# Potential Therapeutic Candidates against *Chlamydia pneumonia* Discovered and Developed In Silico Using Core Proteomics and Molecular Docking and Simulation-Based Approaches

**DOI:** 10.3390/ijerph19127306

**Published:** 2022-06-15

**Authors:** Roqayah H. Kadi, Khadijah A. Altammar, Mohamed M. Hassan, Abdullah F. Shater, Fayez M. Saleh, Hattan Gattan, Bassam M. Al-ahmadi, Qwait AlGabbani, Zuhair M. Mohammedsaleh

**Affiliations:** 1 Department of Biology, College of Science, University of Jeddah, Jeddah 21959, Saudi Arabia; rhkadi@uj.edu.sa; 2 Department of Biology, College of Science, University of Hafr Al Batin, P.O. Box 1803, Hafr Al Batin 31991, Saudi Arabia; khadijahaa@uhb.edu.sa; 3 Department of Biology, College of Science, Taif University, P.O. Box 11099, Taif 21944, Saudi Arabia; m.khyate@tu.edu.sa; 4 Department of Medical Laboratory Technology, Faculty of Applied Medical Sciences, University of Tabuk, Tabuk 71491, Saudi Arabia; ashater@ut.edu.sa (A.F.S.); zuhair.saleh966@gmail.com (Z.M.M.); 5 Department of Medical Microbiology, Faculty of Medicine, University of Tabuk, Tabuk 71491, Saudi Arabia; fsaleh@ut.edu.sa; 6 Department of Medical Laboratory Sciences, Faculty of Applied Medical Sciences, King Abdulaziz University, Jeddah 22254, Saudi Arabia; hsqattan@kau.edu.sa; 7 Special Infectious Agents Unit, King Fahad Medical Research Center, Jeddah 22252, Saudi Arabia; 8 Department of Biology, Faculty of Science, Taibah University, Medina 42353, Saudi Arabia; bmsahmadi@taibahu.edu.sa; 9 Department of Biology, College of Sciences and Humanities, Prince Sattam Bin Abdulaziz University, Al-Kharj 16278, Saudi Arabia

**Keywords:** phytochemicals, molecular docking, drug candidates, molecular dynamic simulation

## Abstract

*Chlamydia pneumonia,* a species of the *family* Chlamydiacea, is a leading cause of pneumonia. Failure to eradicate *C. pneumoniae* can lead to chronic infection, which is why it is also considered responsible for chronic inflammatory disorders such as asthma, arthritis, etc. There is an urgent need to tackle the major concerns arising due to persistent infections caused by *C. pneumoniae* as no FDA-approved drug is available against this chronic infection. In the present study, an approach named subtractive proteomics was employed to the core proteomes of five strains of *C. pneumonia* using various bioinformatic tools, servers, and software. However, 958 non-redundant proteins were predicted from the 4754 core proteins of the core proteome. BLASTp was used to analyze the non-redundant genes against the proteome of humans, and the number of potential genes was reduced to 681. Furthermore, based on subcellular localization prediction, 313 proteins with cytoplasmic localization were selected for metabolic pathway analysis. Upon subsequent analysis, only three cytoplasmic proteins, namely 30S ribosomal protein S4, 4-hydroxybenzoate decarboxylase subunit C, and oligopeptide binding protein, were identified, which have the potential to be novel drug target candidates. The Swiss Model server was used to predict the target proteins’ three-dimensional (3D) structure. The molecular docking technique was employed using MOE software for the virtual screening of a library of 15,000 phytochemicals against the interacting residues of the target proteins. Molecular docking experiments were also evaluated using molecular dynamics simulations and the widely used MM-GBSA and MM-PBSA binding free energy techniques. The findings revealed a promising candidate as a novel target against C. *pneumonia* infections.

## 1. Introduction

*Chlamydia* *pneumoniae* is an established cause of acute lower respiratory tract infections in all age groups [1]. *C. pneumonia* can cause chronic infection and is associated with a range of chronic lung diseases including asthma, chronic bronchitis, and chronic obstructive pulmonary disease (COPD) [2]. Chronic diseases are described as illnesses that last a year or longer and necessitate continuing medical attention, impede everyday activities, or both [3,4]. *C. pneumoniae* has a unique ability to spread from the lungs to pulmonary disease tissues such as arteries, joints, bones, and the central nervous system via peripheral blood mononuclear cells [5]. Chronic infections of the respiratory system account for 70% of bacterial infections, but acute lung illnesses affect only 30% of patients. Indeed, *C. pneumonia* has also long been linked to several serious chronic inflammatory disorders, including atherosclerosis, Alzheimer’s disease, and inflammatory arthritis [6].

Coughing or sneezing can spread *C. pneumoniae* because it produces minute respiratory droplets that contain the bacterium [7]. Other people subsequently inhale the bacteria and droplets. People can also become ill if they come into contact with something that a sick person has deposited droplets on and then touch their mouth or nose. In most cases, *C. pneumonia* infections have a protracted incubation period (the time between breathing in the bacteria and developing symptoms) [8]. It usually infects people while they are school-aged children or young adults for the first time. Reinfection, on the other hand, is more common among the elderly [9]. *C. pneumoniae* can infect a range of different cell types. In regard to respiratory infection, *C. pneumoniae* initially infects lung epithelial cells and alveolar macrophages. Infection can then spread to infiltrating immune cells such as monocytes, macrophages, monocyte-derived dendritic cells (DCs), lymphocytes, and neutrophils. Failure to eradicate *C. pneumoniae* can lead to chronic infection, during which *C. pneumoniae* enters a state of quiescence with intermittent periods of replication [10].

To avoid the challenges that today’s healthcare societies are experiencing, it is critical to diagnose and treat infections as soon as possible [11]. Multiple drugs are available on the market but are not FDA-approved and have side effects [12]. That is why there is a dire need to work on the treatment of chronic *C. pneumoniae* infection. Bioinformatics is increasingly being used in life sciences [13,14]. The emergence of widely acknowledged and highly efficient big data analysis tools has opened up new paths for uncovering more intriguing and promising diagnostic and therapeutic approaches [15]. Recent advancements in technologies have allowed researchers to make tremendous progress in the field of drug discovery with the introduction of high-throughput computational techniques [16]. In the modern postgenomic period, the probabilities of selecting suitable targets through computational methods and the integration of “omics” data, such as proteomics, metabolomics, and genomics, have been expanding continuously [17]. In silico approaches such as subtractive and comparative proteomics are now being used extensively for the identification, as well as the prediction, of drug targets for several pathogenic bacteria. Compared to traditional methods, these techniques are time-saving and cost-effective in drug-designing processes. In recent years, potential drug targets have been designed for several pathogenic bacteria using the subtractive proteomics approach [18,19].

In the current study, the core proteomes of five strains of *C. pneumonia* were analyzed to employ various subtractive proteomics approaches. The essential proteins required for bacterial survival were identified using a variety of computational software tools. To prevent potential drug cross-reactivity with host and bacterial proteins, we analyzed both metabolic and host non-homology pathways, as well as bacterial protein involvement in several host metabolic processes. The study was expanded to model the 3D structures of the likely drug targets using the SWISS-MODEL to identify a selective and potent inhibitor using docking studies. Furthermore, ADMET profiling of compounds was performed using the SwissADME server to determine the pharmacokinetics of compounds. The in silico approach of molecular dynamics simulation is also used to pre-screen the top compounds. The findings of this study could help in the development of effective drug targets against the chronic infection of *C. pneumonia*.

## 2. Materials and Methods

### 2.1. Collection of Data and Core Proteomics Analysis

Genomics data of all available strains of *C. pneumonia* were retrieved from the National Center for Biotechnology Information (NCBI). NCBI is a hub of biomedical and genomics information. The genomics data obtained from NCBI were then further analyzed by Perl script to generate a core proteome that comprises only those proteins that are shared by all pathogenic strains of *C. pneumonia* [20]. A further USEARCH algorithm was used to cluster proteome sequences due to the focus on highly conserved proteins. In this regard, only those proteins that met the precise criteria of ≤50% sequence identity were selected. These conserved proteins are captivating candidates for all-inclusive protein prediction [21].

### 2.2. Identification and Removal of Paralogs

The occurrence of duplicative proteins was then evaluated in the core proteome. Duplicate proteins are the result of duplication events that occurred during the evolution process. CD-Hit analysis [22] was performed to eliminate redundant sequences from the core proteome, as the redundant proteins are not necessary and a single copy of each protein is enough. In this regard, the core proteome was submitted to the CD-HIT suite for the identification of redundant proteins. Meanwhile, the threshold value was set at 80%.

### 2.3. Identification of Non-Homologous Proteins

The protein set obtained from CD-HIT was compared with a human host through Blastp to eliminate the homologs proteins. Host homologous proteins were excluded from the study because this is important to find druggable proteins, which may be considered for therapeutics development. Moreover, merely the proteins of pathogens were retained to minimize the accidental therapeutic blockage by the host involved in the metabolism of the host. The non-homologous proteins were screened by setting >70 query coverage and ≤30% identity parameters of Blastp [23].

### 2.4. Prioritization of Putative Drug Targets

Predicting protein subcellular localization is an important part of drug design. Therefore, it is critically significant to forecast the function of the specific protein in the design of potential drug targets. Subcellular localization is used to determine the proper functioning of specific proteins. In the framework of the current study, the CELLO server combined with SVM was used for the prediction of subcellular localization of homologous/non-host proteins [24].

**Figure 1:**
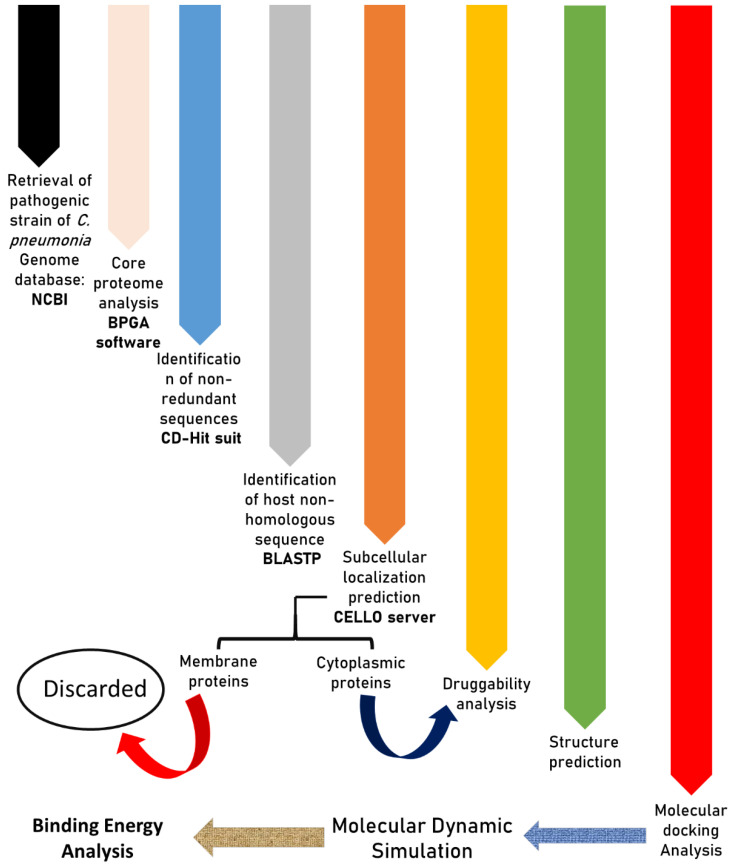
Graphical synopsis representing the overall methodology used in the current analysis.

The bacteria engage in a series of processes that have an impact on the host. Therefore, predicted proteins were screened for comparative analysis of metabolic pathways. The selected proteins were analyzed to determine which metabolic pathways they were linked to. This research is being conducted to find therapeutic targets based on unique and common pathways of bacteria with humans. Therefore, only those proteins in the study’s final list, which are unique to *C. pneumonia*, were included. Lastly, a druggability analysis was performed on predicted cytoplasmic proteins. In this regard, the Drug Bank [25] was employed to locate proteins that are targeted by a wide spectrum of drugs. The Drug Bank is a user-friendly tool in chemoinformatic that combines quantitative drug data with an in-depth understanding of targeted therapies, using a BLAST search with an e-value of 10^−5^. Figure 1 represents the overall methodology used in the current analysis.

### 2.5. Structure Prediction and Structure Evaluation

After the sequence analysis and assessment were completed, the target proteins were submitted to structure prediction. The structure prediction was performed using the SWISS-MODEL program [26]. The Expasy web server accessed the SWISS-MODEL, a fully functional protein structure homology modeling service. One of the most important aspects of computational structure prediction is the accurate evaluation of the 3D model [27]. As a result of novel sequencing methods that have arisen in recent years, scientists have made ground-breaking discoveries in computational structural biology [28]. The introduction of widely accepted and extremely efficient techniques for structure evaluation has opened up new avenues for qualitatively estimating protein structures. In this study, four separate tools, ProCheck, Verify 3D, ERRAT, and ProsA-web, were used to determine the quality of improved pharmacological targets [29].

### 2.6. Refinement of Protein Receptors

UCSF chimera was used for the refinement of the protein structures; firstly, the non-standard residues were removed from the receptor, and after that, at 1000 decent steps, the energy minimization was performed [30]. The resulting structure was improved using the Protonate3D tool in the molecular operating environment (MOE) to add partial charges at a temperature of 310 K and a pH of 7 [31].

### 2.7. Library Preparation

All of the phytochemicals were chosen from a variety of databases, such as Zinc, MDP3 PubChem, and Zinc, using in silico methods to assess their potential inhibitory impact on 30S ribosomal protein S4, 4-hydroxybenzoate decarboxylase subunit C, and Oligopeptide Binding Protein [32]. The plant-based phytochemicals were chosen based on their medicinal potential, according to the literature review. Alkaloids and sterols were the most common phytochemicals chosen. The MOE program was used to produce a ready-to-dock library of the phytochemicals that were chosen [31]. ChemDraw was used to sketch the two-dimensional (2D) chemical structure of the selected ligands [33]. Before using the MOE ligand database, the ligands were refined with Protonate3D, and the energy was decreased.

### 2.8. Molecular Docking Studies

The docking procedure was confirmed by docking co-crystallized ligands into the protein structure using MOE (Molecular Operating Environment) [31]. Molecular docking studies are useful in determining the conformations and interactions that a ligand can have with a protein of interest [34,35]. MOE found the active pocket on the receptor protein molecule. The MOE tool was used to screen a library of 15,000 phytochemicals against the 30S ribosomal protein S4, 4-hydroxybenzoate decarboxylase subunit C, and Oligopeptide Binding Protein. The MOE software used the “Triangular Matcher” technique to verify correct ligand confirmation before using it as the default ligand insertion approach [31]. The London dG scoring algorithm in MOE was utilized to rescore simulated poses. Based on their RMSD values and S-score binding affinity, the phytochemicals with the best conformations were determined after docking was completed. Two-dimensional plots of ligand–protein interactions were analyzed and interpreted using the MOE LigX tool. Three-dimensional pictures of protein–inhibitor complexes were also created using MOE.

### 2.9. Drug Toxicity Prediction and Pharmacological Evaluation

The absence of toxicity of the chosen compound is regarded as a significant element in the selection of a component as a potential therapeutic [36]. The current study examined screened compounds’ toxicity, including carcinogenicity, cytotoxicity, and mutagenicity. The Protox tool was used to assess the compounds’ toxicity [37,38]. The assessment of the pharmacological features of the finalized compounds is the most critical and significant step in the in-silico study process. The Lipinski parameter was used to investigate the compounds. The selected components met the Lipinski parameter’s requirements. So, they were tested for adsorption. The Lipinski characteristics of the selected components were assessed using the SwissADME database at http://www.swissadme.ch/index.php (accessed on 25 December 2021) [39].

### 2.10. Molecular Dynamics Simulation

MD simulation is a successful in silico approach for studying the dynamic behavior and stability of protein–ligand complexes under various conditions [40]. Molecular dynamics (MD) simulation of the best ligand poses was performed using the Desmond v3.6 Program to verify the docking performance, as mentioned earlier [41]. In a nutshell, the TIP3P solvent model was used in conjunction with an orthorhombic designed boundary box. By introducing Na + salt, the OPLS-2005 force field was used to counter the process. A hybrid algorithm of gradient descent and LBFGS algorithms was used to decrease the protein–ligand system [42,43]. After docking, the MD simulation was run at 100 ns on Desmond for early confirmation of the protein–ligand complexes.

### 2.11. Binding Energy Analysis (MMGBSA/MMPBSA)

The binding free energy of drugs towards receptors was assessed to ensure the compounds’ binding stability. This was accomplished using the molecular mechanics generalized Poisson Boltzmann surface area (MMPBSA) method. This method is a well-known, dependable, and powerful analytical tool. The Amber tool 20 MMPBSA script (py) was used to calculate the binding free energy of chosen MD snapshots [44].

## 3. Results

### 3.1. Analysis of Core Proteome

Five significant *C. pneumonia* pathogenic strains were evaluated for the identification of novel inhibitors against *C. pneumonia* infections. The overall protein count of the five pathogenic strains was 81,485, which was reduced to 4745 after performing core proteome analysis (Supplementary File S1). As core proteins are found across all pathogenic strains, using these core proteins in drug designing could provide resistance against *C. pneumonia* infections.

### 3.2. Prediction of Target Proteins

After core proteome analysis, a total of 4745 core proteins were obtained, which were then submitted to the CD-Hit server for the removal of redundant proteins. After CD-Hit analysis, 958 nonredundant proteins were left, which met the precise criteria of 80%. The removal of redundant proteins is necessary because redundant proteins are not essential for an organism’s existence and might produce false outcomes.

The prediction of proteins that are not homologous to the proteins is essential since these proteins are required for pathogen survival as well as preventing drug cross-binding with host proteins. Further, non-homologous proteins were submitted to BlastP to determine the interactivity of the drugs with the proteins of humans. After running BlastP, only 681 proteins were found that are non-homologous to humans. Later, subcellular localization prediction was used to determine how these significant proteins performed their functions. In total, 352 target proteins were projected as membrane proteins and therefore omitted for subsequent analysis. On the other hand, a total of 313 proteins were found to be cytoplasmic proteins, which were then analyzed for therapeutic targets (Supplementary File S2). Finally, a comparative analysis of these 313 proteins revealed that a total of 14 proteins were found to be engaged in various metabolic pathways (Table 1). The names and amino acid sequences of the 14 proteins are presented in Supplementary File S3. From these 14 proteins, only 3 proteins (30S ribosomal protein S4, 4-hydroxybenzoate decarboxylase subunit C, and oligopeptide binding protein) were designated as pathogen-specific because these proteins do not share any pathway similarity with humans.

### 3.3. Draggability Analysis

Druggability is also another important feature for the identification of promising therapeutic targets. Druggability is the likelihood that a small drug molecule may influence the functioning of the target protein. The druggability analysis of predicted proteins was determined by comparing the similarity of proteins to their corresponding drug targets in the Drug Bank database. After analysis, it was noted that three proteins (30S ribosomal protein S4, 4-hydroxybenzoate decarboxylase subunit C, and oligopeptide binding protein) have a strong resemblance to FDA-approved drugs. The sequences of the above three proteins are highlighted in yellow in Supplementary File S3.

### 3.4. Structure Prediction and Structure Evaluation

**Figure 2:**
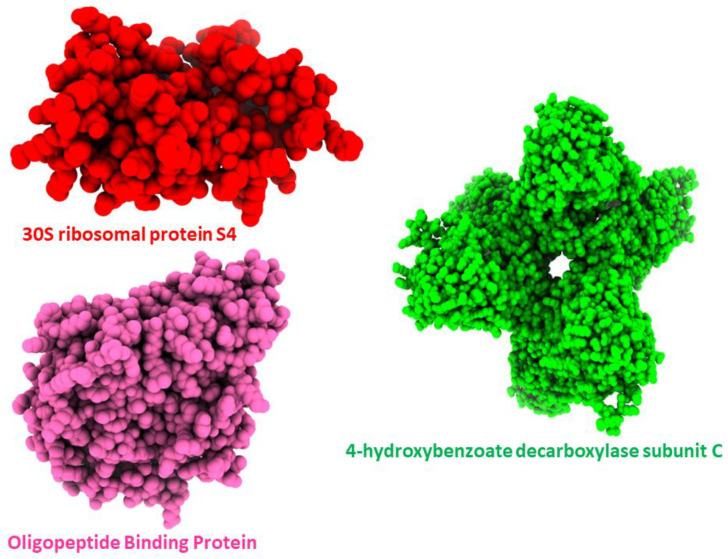
Graphical representation of 3D Structures of the Proteins.

The Swiss Model evaluated the optimal 3D crystal structure for all proteins based on QMEAN and GMQE values. Based on GMQE (global model quality estimate), the confidence level of the model was determined based on the target’s template, coverage, and organization. It uses target–template alignment features in conjunction with a template search to calculate quality. The GMQE score increases in proportion to the model’s quality. It is usually approximated to be between 0 and 1. The GMQE and Q mean for 30S ribosomal protein S4, 4-hydroxybenzoate decarboxylase subunit C, and Oligopeptide Binding Protein indicated that the structures were of excellent quality as shown in Figure 2.

The quality of the protein structures was further assessed using refined pharmacological targets. The 3D models were validated using a variety of ways. To begin, the PROCHECK server was utilized to assess the structural quality of the modelled structure. Finally, an evaluation of the 3D protein models revealed that approximately 90% of residues were found in the favored regions, indicating that all projected models are high quality. The 30S ribosomal protein S4 protein had 94.7% percent of its residues in favorable regions according to the predicted model, while the 4-hydroxybenzoate decarboxylase subunit C and Oligopeptide Binding Protein had over 85 percent of the residues in favored regions. VERIFY 3D projected a good compatibility score of residues, with an average 3D–1D score of ≥0.2. The greater the score, the higher the 3D model’s quality. These results demonstrate that the proposed model is of excellent quality. The ProSA-webserver was used to verify the quality of the 3D models. Z-scores are a parametric quantity that represents the model’s overall quality as shown in Table 2.

### 3.5. Molecular Docking Analysis

The results of docking the receptor protein structures with the phytochemicals library using MOE software are presented in this section. Active sites of the targeted proteins were predicted using the site finder tool in MOE. For each target compound, ten distinct conformations were obtained. The conformations of these compounds were sorted using binding affinity, RMSD values, and bonding interactions with the proteins’ active sites, as shown in Table 3.

**Figure 3:**
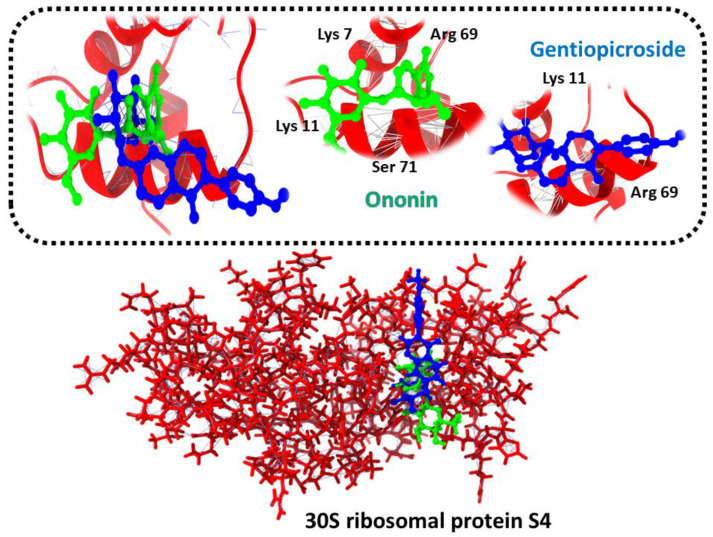
Three-dimensional representation of molecular docking analysis and the interaction of Ononin and Gentiopicroside inhibitors with 30S ribosomal protein S4.

Ononin and Gentiopicroside were the top two drug candidates against 30S ribosomal protein S4. Both compounds indicated a good binding affinity score and showed strong binding interactions with the receptor protein, as shown in Figure 3.

**Figure 4:**
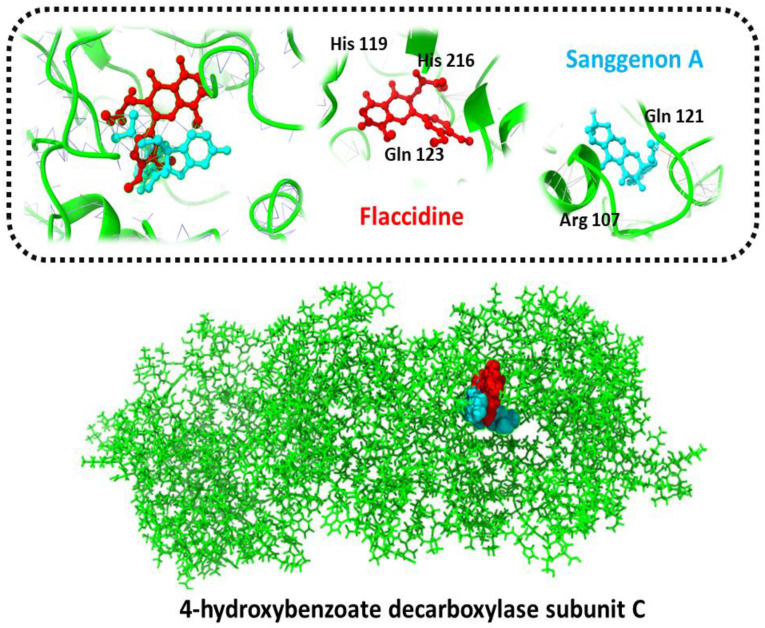
Three-dimensional representation of molecular docking analysis and the interaction of Sanggenon A and Flaccidine inhibitors with 4-hydroxybenzoate decarboxylase subunit C.

The 4-hydroxybenzoate decarboxylase subunit C second targeted protein in complex, with its top drug candidates Sanggenon A and Flaccidine, was discovered to be attached to a score of (−12.49, −12.19) KJ/mol, forming hydrogen bonds with the side-chain/backbone of His 119, His 216, Gln 123, Arg 107, and Gln 121, as shown in Figure 4.

**Figure 5:**
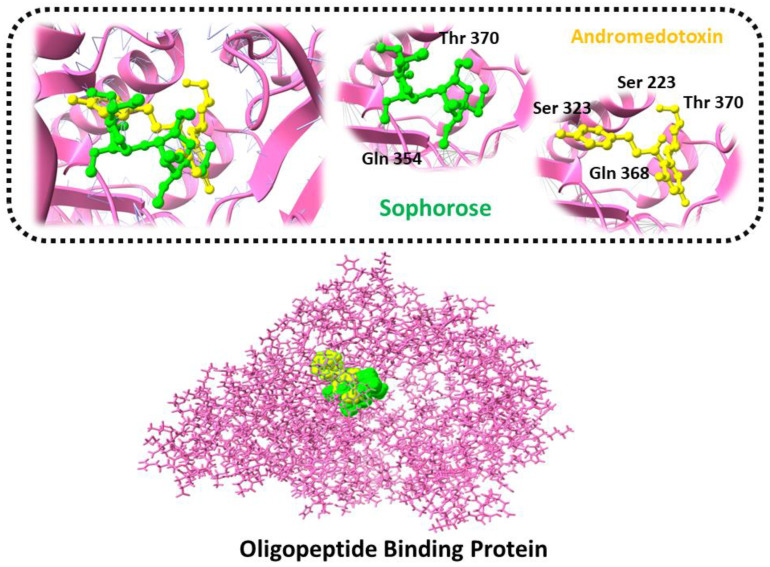
Three-dimensional representation of molecular docking analysis and the interaction of Andromedotoxin and Sophorose inhibitors with Oligopeptide Binding Protein.

Andromedotoxin and Sophorose, in complex with the Oligopeptide Binding Protein, showed the lowest docking score along with a strong hydrogen bond interaction. The LigX tool was used to predict the interacting residues of the receptor and ligand, which indicate that Andromedotoxin and Sophorose showed strong bindings with the side chains of Ser 321, Ser 323, Gln 368, Ser223, Thr 370, and Gln 354, as shown in Figure 5.

### 3.6. Drug Likeness

Lipinski’s rule of five (RO5) was utilized to undertake computational screening of the physicochemical properties of the strongest ligands for the receptor in order to determine its drug-like property. The molecular weight must be ≥500 g/mol, there must be fewer than or equal to five hydrogen bond donors and fewer than or equal to ten hydrogen bond acceptors, and the miLog *p*-value must be <5. It is acceptable to approve a drug candidate that has broken one of these rules. Table 2 shows the top phytochemicals as well as the reference compound’s anticipated drug-likeness characteristics. All of the ligands disclosed demonstrated good drug-like properties, as shown in Table 4.

### 3.7. ADMET Profiling

Several pharmacokinetic factors were evaluated using SwissADME and ADMETsar. Pharmacokinetic parameters can be used to estimate the ADME and toxicity of the top therapeutic candidate drugs. The ADMET characteristics of leading phytochemicals for targets are shown in Table 5. Many drugs do not use this mechanism in their development due to poor pharmacokinetic properties and toxicity. To identify the active best compounds, early drug discovery relies on high-performance and rapid ADMET profiling studies. According to ADMET profiling, none of the candidate compounds had an unfavorable absorption effect. The ADMET properties of putative medications were linked to positive results, indicating that the compounds have therapeutic possibilities. Table 5 shows the most likely drug candidates for the target protein’s pharmacokinetic properties.

### 3.8. MD Simulation

**Figure 6:**
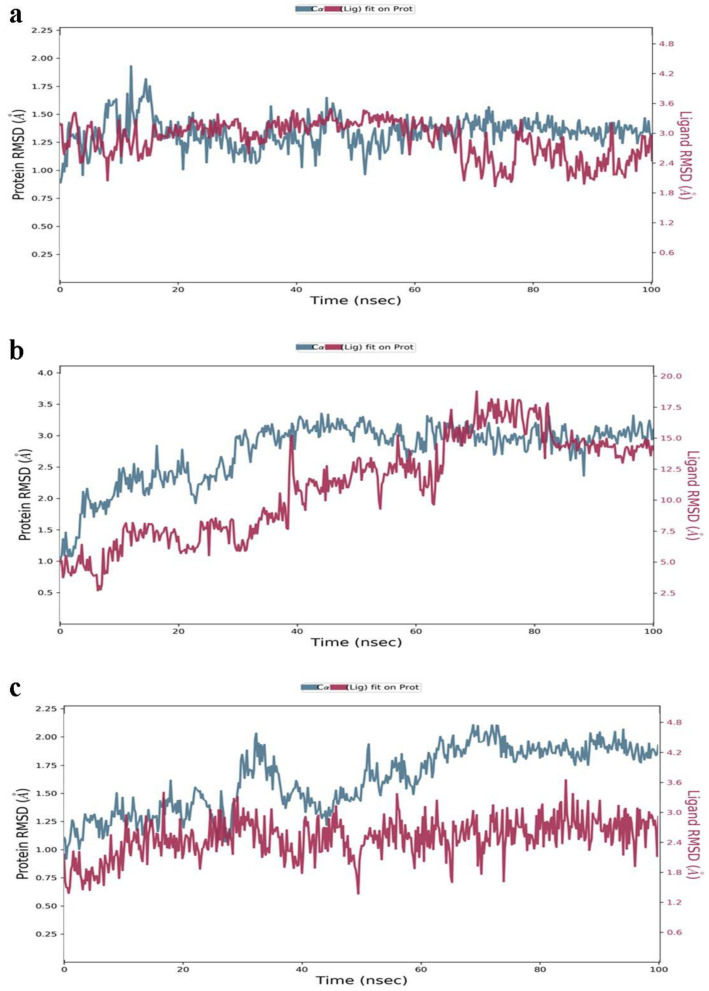
(**a**–**c**) Statistical investigation of the intermolecular stability and dynamics of the complexes based on molecular dynamics simulations.

A 100-ns molecular dynamic simulation was performed for better insight into the molecular mechanisms involved in the top drug candidates’ binding. A compound with the least binding affinity, ranked as the top compound from each target’s top compound, was selected to further understand the stability of the drug candidates with the receptor’s proteins. Root mean square deviation (RMSD) analysis against the Ononin/30S ribosomal protein S4 complex indicates good stability throughout 100 ns of between 1.0 Å and 1.25 Å, as shown in Figure 6a. Although the second complex Sanggenon A/4-hydroxybenzoate decarboxylase subunit C showed a minor deviation up to the period of 55 ns, after that, it remains stable, as represented in Figure 6b. The third complex remains stable up to the period of 45 ns, and after that, it showed a minor deviation of 0.25 Å as indicated in Figure 6c.

**Figure 7:**
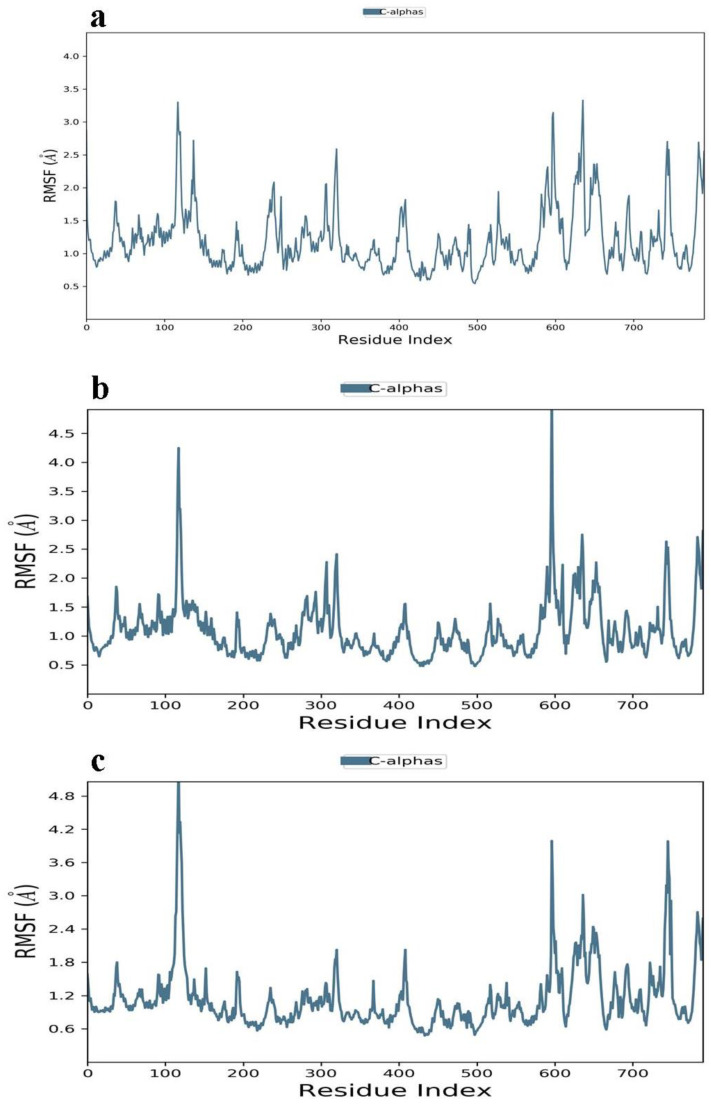
(**a**–**c**) RMSF plot of compounds showing stability index. (**a**) Ononin/30S ribosomal protein S4 complex; (**b**) Sanggenon A/4-hydroxybenzoate decarboxylase subunit C; (**c**) Andromedotoxin/Oligopeptide Binding Protein.

Root mean square fluctuation trajectories indicate that the Ononin/30S ribosomal protein S4 complex showed stability of up to 700 residues with no more than 1 Å fluctuation (Figure 7a). Meanwhile, the second complex Sanggenon A/4-hydroxybenzoate decarboxylase subunit C trajectories showed a minor jump at the residue numbers 120 and 600 (Figure 7b). Although the third complex Andromedotoxin/Oligopeptide Binding Protein indicates a minor deviation at residue number 110, after that, it remains stable up to 700 (Figure 7c).

### 3.9. Binding Energy Analysis (MMGBSA/MMPBSA)

The MMGBSA method was used to calculate the binding energies of docked compounds, as shown in Table 6. The results showed that gas-phase energy, specifically electrostatic energy and van der Waals energy, dominated molecule recognition. The polar solubilization energy appeared to play a reduced role in molecule-targeted protein interactions. In the creation of complexes, non-polar energy played only a minor role. Target 30S ribosomal protein S4 total binding energies were −27.04 kcal mol^−1^. However, the targets 4-hydroxybenzoate decarboxylase subunit C and Oligopeptide Binding Protein total binding energies were −22.43 kcal mol^−1^. and −24.58 kcal mol^−1^, respectively.

## 4. Discussion

*C. Pneumonia* is a pathogen that causes various symptoms, such as pharyngitis, impetigo, necrotizing fasciitis, sepsis, or toxic shock [45]. Antibiotics against C. pneumonia should be discovered as soon as feasible to combat these life-threatening situations. In this research, we employed in silico core proteomics and docking techniques to explore possible medications against *C. pneumonia*. These methods are used to find targets in pathogenic organisms using essential proteins [46]. Recent advances in the bioinformatics domain and computational biology have resulted in various drug design and computational analysis methodologies, reducing the time and expense of drug development via trial and error [47].

After core proteome analysis, a total of 4745 core proteins were obtained, which were then submitted to the CD-Hit server for the removal of redundant proteins. Bacterial life needs the presence of essential proteins. If these crucial proteins are altered or destroyed, bacteria will die. By focusing on these proteins, we can destroy bacteria and cure diseases. In the development of antibacterial drugs and vaccines, essential proteins are the primary targets. As a result, After CD-Hit analysis, 958 non-redundant proteins were left that met the precise criteria of 80% [48]. These genes are likely to be related to those identified in humans.

Such targeting may have fatal consequences and disrupt metabolism. Negative outcomes and cross-reactivity can be reduced by selecting non-homologous proteins that are not found in Homo sapiens [49]. We evaluated 681 non-homologous proteins for toxicity and unfavorable conditions. The best way to uncover novel medicines may be to target non-homologous sequences. Researchers found that 681 pathogen-specific metabolic pathways exist using the KEGG database, while pathogens and hosts share 14 pathways [18,50]. Only three of these fourteen proteins (30S ribosomal protein S4, 4-hydroxybenzoate decarboxylase subunit C, and oligopeptide binding protein) were identified as pathogen-specific.

The Swiss model was used to create 3D models of the target proteins. Predicting the three-dimensional structure is particularly valuable in understanding protein dynamics, functions, ligand interactions, and other protein components [51]. According to Ramachandran’s analysis, 90% of residues were within acceptable, favored areas, and only a few residues were located in prohibited areas. Overall, the model’s quality was satisfactory. Based on the results of various evaluation tools, our models were of high quality.

A low RMSD score and several residues interacting with the target protein 4-hydroxybenzoate decarboxylase subunit C, Flaccidine, Oligopeptide Binding Protein Andromedotoxin, and Sophorose were chosen for the 30S ribosomal protein S4 protein docking. The lowest binding energies of these phytochemicals ranged from approximately −8.12 kcal/mol to −12.49 kcal/mol for the 30S ribosomal protein S4, 4-hydroxybenzoate decarboxylase subunit C, and Oligopeptide Binding protein. The six compounds were evaluated for their drug likelihood using Lipinski’s rule of five. Following that, the compounds were tested for BBB penetration and HIA (human intestinal absorption), as well as monitored using AMES. The behavior, toxicity, and fate of a drug candidate in the human body are all influenced by ADMET properties. A candidate’s toxicity is determined by absorption, metabolism, blood–brain barrier crossing, and subcellular localization [52]. The cytochrome P450 superfamily isoforms CYP3A4, CYP2D6, CYP2C9, CYP1A2, CYP2C19, CYP2A6, and CYP2E1 have been demonstrated to be involved in drug metabolism and elimination [53].

Inhibiting cytochrome P450 isoforms can create drug–drug interactions, limiting subsequent drug metabolism and causing hazardous accumulation levels [54]. According to their ADMET profile, these compounds have no detrimental impacts on absorption. Moreover, none of the compounds were harmful or mutagenic compared to the AMES test. The top six most promising compounds were submitted to a series of toxicity testing modules following the virtual screening. During the toxicity screening, no chemical was hepatotoxic, mutagenic, or cytotoxic. Our study revealed six drug-leading inhibitors that have the potential to be therapeutic inhibitors of apoptosis targeting and inhibition.

The best-docked complexes with the top inhibitors per protein were used for MD simulations and free energy estimates. These compounds exhibited essential drug-target properties such as pathogen metabolic pathway participation, non-homology with the human host, and appropriate size. Ononin, Sanggenon A, and Andromedotoxin are three enzymes that have been identified as novel therapeutic targets against the disease [55]. However, only three pharmacological targets were discovered in this investigation based on a distinct metabolic pathway. Drug targets and drug-like compounds prioritized in this study could be useful in developing new strategies to eradicate the chronic infection of *C. pneumonia*.

## 5. Conclusions

The long-term chronic infection of *C. pneumonia* has prompted the need to investigate novel drug targets that could aid in the development of new therapeutic agents. In *C. pneumonia*, the current investigation discovered three new targets. The current study explored developing drugs against them because those targets are engaged in pathogen-specific metabolic pathways and have been successfully targeted in other bacteria. As a result, this research marks a big step forward in the development of new, effective anti- *C. pneumonia* chemicals. These targets should be tested experimentally in future studies to determine their role in *C. pneumonia* survival and virulence.

## Figures and Tables

**Table 1:** Metabolic pathways of predicted proteins.

Protein Name	Common Pathways within Human	Unique Pathways
**Penicillin-binding protein**	Metabolic pathways	Beta-Lactam resistance Peptidoglycan biosynthesis
**Probable ATP-dependent 6-phosphofructokinase**	Metabolic pathways Biosynthesis of amino acids Carbon metabolism Glycolysis/Gluconeogenesis Pentose phosphate pathway Fructose and mannose metabolism RNA degradation	Microbial metabolism in diverse environments Biosynthesis of secondary metabolites Methane metabolism
**UDP-N-acetylglucosamine 1-carboxyvinyltransferase**	Metabolic pathways Biosynthesis of nucleotide sugars Amino sugar and nucleotide sugar metabolism	Peptidoglycan biosynthesis
**D-Ala-D-Ala Carboxypeptidase**	Metabolic pathways	Peptidoglycan biosynthesis
**NifS protein, putative**	Sulfur relay system Biosynthesis of cofactors Metabolic pathways Thiamine metabolism	
**3-oxoacyl-[acyl-carrier-protein] synthase 3**	Fatty acid biosynthesis Fatty acid metabolism Metabolic pathways	
**Delta-aminolevulinic acid dehydratase**	Metabolic pathways	Microbial metabolism in diverse environments Biosynthesis of secondary metabolites
**Acetyl-coenzyme A carboxylase carboxyl transferase subunit beta**	Fatty acid biosynthesis Fatty acid metabolism Metabolic pathways Carbon metabolism Pyruvate metabolism Propanoate metabolism	Microbial metabolism in diverse environments Biosynthesis of secondary metabolites
**Lipoate--protein ligase A**	Lipoic acid metabolism Metabolic pathways Biosynthesis of cofactors	
**30S ribosomal protein S4**		Two-component system Flagellar assembly
**Acyl carrier protein**	Metabolic pathways	Biosynthesis of secondary metabolites
**4-hydroxybenzoate decarboxylase subunit C**		Microbial metabolism in diverse environments
**Oligopeptide Binding Protein**		Quorum sensing

**Table 2:** Depicting the quality and the refinement of the predicted structure using different computational tools.

Receptors	30S Ribosomal Protein S4	4-hydroxybenzoate Decarboxylase Subunit C	Oligopeptide Binding Protein
**GMQE**	0.75	0.51	0.68
**Q means**	0.71	0.60	0.66
**Ramachandran plot statistics (%)**			
**Core**	94.7%	87.1%	89.0%
**Allowed**	4.5%	11.1%	9.0%
**General**	0.8%	1.2%	1.2%
**Disallowed**	0.0%	0.6%	0.7%
**Verify 3D**			
**Compatibility Score**	79.59%	79.97%	87.74%
**ERRAT**			
**Quality Factor**	97.1223	78.3249	78.1182
**ProSA**			
**z-Score**	−5.18	−5.64	−8.26

**Table 3:** Top drug candidates' binding affinity along with interacting residues.

Target Receptors	Compounds I’D	Compounds Name	Compounds Structure	Binding Affinity	RMSD	Interacting Residues
**30S ribosomal protein S4**	442813	Ononin	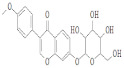	−9.10 kj/mol	1.31	Lys 7 Lys 11 Arg 69 Ser 71
	88708	Gentiopicroside	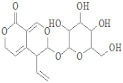	−8.12 KJ/mol	1.08	Lys 11 Arg 69
**4-hydroxybenzoate decarboxylase subunit C**	156707	Sanggenon A	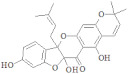	−12.49 KJ/mol	1.65	Arg 107 Gln 121
	44260021	Flaccidine	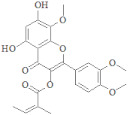	−12.19 KJ/mol	1.72	His 119 His 216 Gln 123
**Oligopeptide Binding Protein**	6072	Andromedotoxin	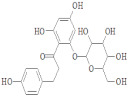	−11.33 kj/mol	0.99	Ser 321 Ser 323 Gln 368 Ser223 Thr 370
	6427838	Sophorose	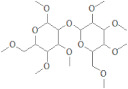	−10.39 KJ/mol	1.50	Gln 354 Thr 370

**Table 4:** The top six phytochemicals were determined following Lipinski’s Rule of Five molecular properties and drug-likeness.

Table.	Compounds I’D	Molecular Weight	Hydrogen Bond Donner	Hydrogen Bond Acceptor	MiLogP
**30S ribosomal protein S4**	442813	428.39	2	9	−1.48
	88708	354.31	2	9	−3.68
**4-hydroxybenzoate decarboxylase subunit C**	156707	436.46	7	3	4.60
	44260021	442.42	9	2	3.32
**Oligopeptide Binding Protein**	6072	434.40	5	10	−2.38
	6427838	454.51	10	0	−0.29
**Target Protein**	**Compounds I’D**	**Molecular Weight**	**Hydrogen Bond Donner**	**Hydrogen Bond Acceptor**	**MiLogP**
**30S ribosomal protein S4**	442813	428.39	2	9	−1.48
	88708	354.31	2	9	−3.68
**4-hydroxybenzoate decarboxylase subunit C**	156707	436.46	7	3	4.60
	44260021	442.42	9	2	3.32
**Oligopeptide Binding Protein**	6072	434.40	5	10	−2.38
	6427838	454.51	10	0	−0.29

**Table 5:** The top anticipated drug candidates for the *C. pneumonia* protein’s pharmacokinetic characteristics.

Compounds	442813	88708	156707	44260021	6072	6427838
**GI absorption**	Low	Low	Low	Low	Low	Low
**BBB permeant**	No	No	No	No	No	No
**P-GP substrate**	No	Yes	No	No	No	No
**CYP1A2 Inhibitor**	No	No	No	No	No	No
**CYP2C19 Inhibitor**	No	No	No	No	No	No
**CYP2C9 Inhibitor**	No	No	No	No	No	No
**CYP2D6 Inhibitor**	No	No	No	No	No	No
**CYP3A4 Inhibitor**	Yes	No	No	No	No	Yes
**Toxicity**						
**Carcinogens**	Non-Toxic	Non-Toxic	Non-Toxic	Non-Toxic	Non-Toxic	Non-Toxic
**Cytotoxicity**	Non-Cytotoxic	Non-Cytotoxic	Non-Cytotoxic	Non-Cytotoxic	Non-Cytotoxic	Non-Cytotoxic
**Mutagenicity**	No	No	No	No	No	No

**Table 6:** Target proteins’ binding energies calculations.

Energy Parameters	30S Ribosomal Protein S4/Ononin	4-hydroxybenzoate Decarboxylase Subunit C/Sanggenon A	Oligopeptide Binding Protein/Andromedotoxin
**MM-PBSA**			
**VDWAALS**	−32.45 kcal mol^−1^	−23.87 kcal mol^−1^	−30.72 kcal mol^−1^
**Delta G gas**	−37.34 kcal mol^−1^	−33.75 kcal mol^−1^	−25.37 kcal mol^−1^
**Delta G solv**	10.20 kcal mol^−1^	9.70 kcal mol^−1^	15.09 kcal mol^−1^
**Delta Total**	−27.04 kcal mol^−1^	−22.43 kcal mol^−1^	−24.58 kcal mol^−1^
**MM-PBSA**			
**VDWAALS**	−32.45 kcal mol^−1^	−23.87 kcal mol^−1^	−30.72 kcal mol^−1^
**Delta G gas**	−37.34 kcal mol^−1^	−33.75 kcal mol^−1^	−25.37 kcal mol^−1^
**Delta G solv**	7.23 kcal mol^−1^	4.26 kcal mol^−1^	8.67 kcal mol^−1^
**Delta Total**	−29.46 kcal mol^−1^	−30.39 kcal mol^−1^	−26.08 kcal mol^−1^

## Data Availability

Not applicable.

## Supplementary Materials

The following supporting information can be downloaded at: https://www.mdpi.com/article/10.3390/ijerph19127306/s1, File S1: Complete detail of *Waddlia chondrophila* proteome; File S2: Sequence of cytoplasmic proteins; File S3: Amino Acid sequences of final proteins.
