# Supplementary material for: Potential Therapeutic Candidates against Chlamydia pneumonia Discovered and Developed In Silico Using Core Proteomics and Molecular Docking and Simulation-Based Approaches
Source: Int J Environ Res Public Health. 2022 Jun 15;19(12):7306. doi: 10.3390/ijerph19127306 (PMC9223490; doi:10.3390/ijerph19127306)
Supplement: Supplementary file 1 [file ijerph-19-07306-s001.zip › file S1.pdf]

Complete detail of the core proteome of *C. pneumonia*

>core/0/1/Org1_Gene58

MKSLPVYVSGIKVRNLKNVSIHFNSEEIVLLTGVSGSGKSSIAFDTLYAAGRKRYISTLPTFFATTITTLPNPKV
EEIHGLSPTIAIKQNHFSHYSHATVGSTTELFSHLALLFTLEGQARDPKTKEVLDLYSKEKVLSTIMELSEGVQI
SILAPLLRKDIAAIHEYAQQGFTKVRCNGTIHPIYSFLTSGIPEDCSVDIVIDTLIKSENNIARLKVSLFTALEFGE
GHCSVLSDEELMTFSTKQQIDDVTYTPLTQQLFSPHALESRCSLCQGSGIFISIDNPLLIDENLSIKENCCSFAGN
CSSYLYHTIYQALADALNFNLETPWKDLSPEIQNIFLRGKNNLVLPVRLFDQTLGKKNLTYKVWRGVLNDIG
DKVRYTTKPSRYLSKGMSAHSCSLCKGTGLGDYASVATWEGKTFTEFQQMSLNNWHVFFSKVKSPSLSIQEI
LQGLKQRLSFLIDLGLGYLTPNRALATLSGGEQERTAIAKHLGGELFGITYILDEPSIGLHPQDTEKLIGVIKKL
RDQGNTVILVEHEERMISLADRIIDIGPGAGIFGGEVLFNGKPEDFLMNSSSLTAKYLRQELTIPIPESREAPTS
WLLLTEATIHNLKNLSIRLPLARLIGVTGVSGSGKSSLINNTLVPAIESFLKQENPKNLHFEWGCIGRLIHITRDL
PGRSQRSIPLTYIKAFDDIRELFASQPRSLRQGLTKAHFSFNQPQGACIQCQGLGTMTISDDDTPIPCSECQGKR
YHSEVLEILYEGKNIADILDMTAYEAEKFFISHPKIHEKIHALCSLRLDYLPLGRPLSTLSGGEIQRLKLAHELL
FASPKQTLYVLDEPTTGLHTHDIQALIEVLLSLTYLGHTVLVIEHNMHVVKVCDYVLELGPEGGDLGGYLLA
SCTPKDLIQLNTPTAKALAPYIEGSLDIPVVKSEPPSSPKSCDILIKDAYQNNLKHIDLALPRNSLIAIAGPGASG
KHSLVFDILYASGNIAYAELFPPYIRQGLLKETPLPSVGEVKGLSPVISVRKCSSSNRSYHTIASALGLSNGLEK
LFAILGEPFSPLTEEKLSKTTPQTIIDSLLKSYKDDYVTITSPIPLGSDLEIFLQEKQKEGFIKLYSEGNLYDLDER
LPLNLIEPAIVIQHTKVSPKNSSSLLSAISVAFSLSSEIWIYISQKKQRKLSYSLGWKDKKGRLYPEITHQLLSSD
HPEGRCLTCGGRGEILKISLEEHKEKIAHYTPLEFFSLFFPKSYMKPVQKLLKDENASQPLKLLTTKEFLNFCR
GSSEFPGMNALLMEQLDTESDSPLIKPLLALTSCPACKGSGLNDYANYVRINNTSLLDIYQEDATFLESFLNTI
GTDDTRSIIQDLMNRLTFISKVGLSYITLGQRQDTLSDGENYRLHLAKKISINLTNIVYLFEEPLSGLHPQDLPTI
VQLLKELVANNNTVIATDRSCSLIPHADHAIFLGPGSGPQGGFLMDSDTEVCPSVDLHANVPQTEVCPKAPLS
ISKANHTRGSDRTLKVNLSIHHIQNLKVSAPLHALVAIGGVSGSGKTSLLLEGFKKQAELLIAKGTTTFSDLVV
IDSHPIASSQRSDISTYFDIAPSLRAFYASLTQAKALNISSTMFSTNTKQGQCSDCQGLGYQWIDRAFYALEKR
PCPTCSGFRIQPLAQEVLYEGKHFGELLHTPIETVALRFPFIKKIQKPLKALLDIGLGYLPIGQKLSSLSVSEKTA
LKTAYFLYQTPETPTLFLIDELFSSLDPIKKQHLPEKLRSLINSGHSVIYIDHDVKLLKSADYLIEIGPGSGKQGG
KLLFSGSPKDIYASKDSLLKKYICNEELDS

>core/1/1/Org1_Gene389

MKWLPATAVFAAVLPALTAFGDPASVEISTSHTGSGDPTSDAALTGFTQSSTETDGTTYTIVGDITFSTFTNIP
VPVVTPDANDSSSNSSKGGSSSSGATSLIRSSNLHSDFDFTKDSVLDLYHLFFPSASNTLNPALLSSSSSGGSSS
SSSSSSSGSASAVVAADPKGGAAFYSNEANGTLTFTTDSGNPGSLTLQNLKMTGDGAAIYSKGPLVFTGLKN
LTFTGNESQKSGGAAYTEGALTTQAIVEAVTFTGNTSAGQGGAIYVKEATLFNALDSLKFEKNTSGQAGGGI
YTESTLTISNITKSIEFISNKASVPAPAPEPTSPAPSSLINSTTIDTSTLQTRAASATPAVAPVAAVTPTPISTQETA
GNGGAIYAKQGISISTFKDLTFKSNSASVDATLTVDSSTIGESGGAIFAADSIQIQQCTGTTLFSGNTANKSGGG
IYAVGQVTLEDIANLKMTNNTCKGEGGAIYTKKALTINNGAILTTFSGNTSTDNGGAIFAVGGITLSDLVEVR
FSKNKTGNYSAPITKAASNTAPVVSSSTTAASPAVPAAAAAPVTNAAKGGALYSTEGLTVSGITSILSFENNEC
QNQGGGAYVTKTFQCSDSHRLQFTSNKAADEGGGLYCGDDVTLTNLTGKTLFQENSSEKHGGGLSLASGKS
LTMTSLESFCLNANTAKENGGGANVPENIVLTFTYTPTPNEPAPVQQPVYGEALVTGNTATKSGGGIYTKNA
AFSNLSSVTFDQNTSSENGGALLTQKAADKTDCSFTYITNVNITNNTATGNGGGIAGGKAHFDRIDNLTVQSN

QAKKGGGVYLEDALILEKVITGSVSQNTATESGGGIYAKDIQLQALPGSFTITDNKVETSLTTSTNLYGGGIYS
SGAVTLTNISGTFGITGNSVINTATSQDADIQGGGIYATTSLSINQCNTPILFSNNSAATKKTSTTKQIAGGAIFS
AAVTIENNSQPIIFLNNSAKSEATTAATAGNKDSCGGAIAANSVTLTNNPEITFKGNYAETGGAIGCIDLTNGS
PPRKVSIADNGSVLFQDNSALNRGGAIYGETIDISRTGATFIGNSSKHDGSAICCSTALTLAPNSQLIFENNKVT
ETTATTKASINNLGAAIYGNNETSDVTISLSAENGSIFFKNNLCTATNKYCSIAGNVKFTAIEASAGKAISFYDA
VNVSTKETNAQELKLNEKATSTGTILFSGELHENKSYIPQKVTFAHGNLILGKNAELSVVSFTQSPGTTITMGP
GSVLSNHSKEAGGIAINNVIIDFSEIVPTKDNATVAPPTLKLVSRTNADSKDKIDITGTVTLLDPNGNLYQNSY
LGEDRDITLFNIDNSASGAVTATNVTLQGNLGAKKGYLGTWNLDPNSSGSKIILKWTFDKYLRWPYIPRDNH
FYINSIWGAQNSLVTVKQGILGNMLNNARFEDPAFNNFWASAIGSFLRKEVSRNSDSFTYHGRGYTAAVDAK
PRQEFILGAAFSQVFGHAESEYHLDNYKHKGSGHSTQASLYAGNIFYFPAIRSRPILFQGVATYGYMQHDTTT
YYPSIEEKNMANWDSIAWLFDLRFSVDLKEPQPHSTARLTFYTEAEYTRIRQEKFTELDYDPRSFSACSYGNL
AIPTGFSVDGALAWREIILYNKVSAAYLPVILRNNPKATYEVLSTKEKGNVVNVLPTRNAARAEVSSQIYLGS
YWTLYGTYTIDASMNTLVQMANGGIRFVF

>core/2/1/Org1_Gene739

MVAKKTVRSYRSSFSHSVIVAILSAGIAFEAHSLHSSELDLGVFNKQFEEHSAHVEEAQTSVLKGSDPVNPSQ
KESEKVLYTQVPLTQGSSGESLDLADANFLEHFQHLFEETTVFGIDQKLVWSDLDTRNFSQPTQEPDTSNAVS
EKISSDTKENRKDLETEDPSKKSGLKEVSSDLPKSPETAVAAISEDLEISENISARDPLQGLAFFYKNTSSQSISE
KDSSFQGIIFSGSGANSGLGFENLKAPKSGAAVYSDRDIVFENLVKGLSFISCESLEDGSAAGVNIVVTHCGDV
TLTDCATGLDLEALRLVKDFSRGGAVFTARNHEVQNNLAGGILSVVGNKGAIVVEKNSAEKSNGGAFACGS
FVYSNNENTALWKENQALSGGAISSASDIDIQGNCSAIEFSGNQSLIALGEHIGLTDFVGGGALAAQGTLTLR
NNAVVQCVKNTSKTHGGAILAGTVDLNETISEVAFKQNTAALTGGALSANDKVIIANNFGEILFEQNEVRNH
GGAIYCGCRSNPKLEQKDSGENINIIGNSGAITFLKNKASVLEVMTQAEDYAGGGALWGHNVLLDSNSGNIQ
FIGNIGGSTFWIGEYVGGGAILSTDRVTISNNSGDVVFKGNKGQCLAQKYVAPQETAPVESDASSTNKDEKSL
NACSHGDHYPPKTVEEEVPPSLLEEHPVVSSTDIRGGGAILAQHIFITDNTGNLRFSGNLGGGEESSTVGDLAI
VGGGALLSTNEVNVCSNQNVVFSDNVTSNGCDSGGAILAKKVDISANHSVEFVSNGSGKFGGAVCALNESV
NITDNGSAVSFSKNRTRLGGAGVAAPQGSVTICGNQGNIAFKENFVFGSENQRSGGGAIIANSSVNIQDNAGD
ILFVSNSTGSYGGAIFVGSLVASEGSNPRTLTITGNSGDILFAKNSTQTAASLSEKDSFGGGAIYTQNLKIVKNA
GNVSFYGNRAPSGAGVQIADGGTVCLEAFGGDILFEGNINFDGSFNAIHLCGNDSKIVELSAVQDKNIIFQDAI
TYEENTIRGLPDKDVSPLSAPSLIFNSKPQDDSAQHHEGTIRFSRGVSKIPQIAAIQEGTLALSQNAELWLAGLK
QETGSSIVLSAGSILRIFDSQVDSSAPLPTENKEETLVSAGVQINMSSPTPNKDKAVDTPVLADIISITVDLSSFV
PEQDGTLPLPPEIIIPKGTKLHSNAIDLKIIDPTNVGYENHALLSSHKDIPLISLKTAEGMTGTPTADASLSNIKID
VSLPSITPATYGHTGVWSESKMEDGRLVVGWQPTGYKLNPEKQGALVLNNLWSHYTDLRALKQEIFAHHTI
AQRMELDFSTNVWGSGLGVVEDCQNIGEFDGFKHHLTGYALGLDTQLVEDFLIGGCFSQFFGKTESQSYKA
KNDVKSYMGAAYAGILAGPWLIKGAFVYGNINNDLTTDYGTLGISTGSWIGKGFIAGTSIDYRYIVNPRRFIS
AIVSTVVPFVEAEYVRIDLPEISEQGKEVRTFQKTRFENVAIPFGFALEHAYSRGSRAEVNSVQLAYVFDVYR
KGPVSLITLKDAAYSWKSYGVDIPCKAWKARLSNNTEWNSYLSTYLAFNYEWREDLIAYDFNGGIRIIF

>core/3/1/Org1_Gene886

MASSSNNSTKQDGIPSWVNPNVQWNRASQVGDQEANSLTPEAQTSRSWFSDRKHFLEVLDVSLEEMENNDL
KKYSRYKTIILIATLVTVAITCIVPISMVFGIPMWVPCLILFGAGLSSAFLSHRLQSKCKEIHLRYRAYQIYRQQL

LSQYPDLRKSTLYKYSITHVKPKKGFVGKLVENLRPDLHKNKDDGGAAADSRLDFAGYGVKHYQTDALLG
VSGVNSVEWQRLASLIMSVKNDILNDVGSREPIDKAQRSALVVSGKDIGGEIQPGGILDISRDILAICGYGMNV
GVEAKKAIDQYKKWYLNSSTFIAWNPQLPAIAQSYLLEQQRHLDYAAKIFQDLSALTTAHGTGQALEDLDSL
LCYYDQLIESKGVGEKIIASIHQKHLDLAMQDSCDQEHLKKWSNLYHVFSITIKEFTEGKLEQNEVVSRIQRL
RGKLEKSKCSILGNCRTNAEYATKSEKKLADYLLQIGDREPFLTGMHKAIATGKAIQGKVEGVISQHPEKQIM
MLRCSIERLEGMLRREDWGAILQKNEDEVLALKSTMEAQLQGFKDLVGTWEGKYQEFKKNKLSKVLVYDF
TKSYSNLLNRLEVLHAESSTDDLVLHVDRMSEDLKKTIEEIDGNLFQVTPEELSLLAREYQGLMNELPLIVQE
GNRLQEAISSEGVSQGLMLLNSLLNRDEKINKNIESSRKNLVAIAKQARSDARNIDSQGLAPLIQRNRASLDNI
LQNMYLFNGSIRNIHALDTETLVATSSNMFSAMHTFDWNIYTNLLDVLEIQSKPAPAPMENPDLPGALPEEVQ
DAVAEDVSGTHRLHHQVLKRRCADLKNMISQLQKSINKWGMAKAIVLGIVAVLFCVLSAIFIGQNILSLLILS
CVGLLLTQVCPLIFDRISKSKEFEKQVLETAQSLIPATKILPSEFNNKDLNRLAKLQDNLNLEGFGPTWARNIV
SDLEGIPTKEKSLKDLTKEFRKDSKNLNKRIKRRFKEGLGQEAPVVRPTIPQDIRGAEVFAELHRELEHLQKQ
KEEISIRGDALVQERMGLCLEKSKYDNEKAHAAAMTKKVGKLQNIDRLQKNNETYVRIQNFFRTLIQEKLGR
DTVQEIDVVKEAKELHELAAIIYGNTSGKSQKQRAKKQFKENVLHIAGKGQLELLEAYLNVTASQGLCRHQ
MQASFRERILLNPDGAKHGEAERTLASREEMLKTLGLSYLTPFVRFSSPESTQSGYNQILKVREQLFDIEQRLQ
NQETVSPEDYAAVQAALAAYVRKHESLIVSTYGLGAQEGQTSSKVTTLMRDLHAVEELVEMGVETYRLNRS
DQILHRVHSVLHSHLRDSDSSGNGIIDVVKKLFELLNNNGNNPNDPECQKYMQILLDAPVSLLYGAFKSFKNE
FLLNFTELNIANSTKAAEEEAKRYVEEKGRGFETYWEEAKQRLEAIAAELDDLRNQETLLEQEIRLANLKISIF
SDLNLREKVSVEKAALEEEIQGIQEQYAEMQGIEDLELKQKFEDLQKKLEALEERLLQIGRRIDSSVDKQKEL
LGLLGREEAA

>core/4/1/Org1_Gene326

MKYSLPWLLTSSALVFSLHPLMAANTDLSSSDNYENGSSGSAAFTAKETSDASGTTYTLTSDVSITNVSAITP
ADKSCFTNTGGALSFVGADHSLVLQTIALTHDGAAINNTNTALSFSGFSSLLIDSAPATGTSGGKGAICVTNTE
GGTATFTDNASVTLQKNTSEKDGAAVSAYSIDLAKTTTAALLDQNTSTKNGGALCSTANTTVQGNSGTVTFS
SNTATDKGGGIYSKEKDSTLDANTGVVTFKSNTAKTGGAWSSDDNLALTGNTQVLFQENKTTGSAAQANNP
EGCGGAICCYLATATDKTGLAISQNQEMSFTSNTTTANGGAIYATKCTLDGNTTLTFDQNTATAGCGGAIYT
ETEDFSLKGSTGTVTFSTNTAKTGGALYSKGNSSLTGNTNLLFSGNKATGPSNSSANQEGCGGAILAFIDSGS
VSDKTGLSIANNQEVSLTSNAATVSGGAIYATKCTLTGNGSLTFDGNTAGTSGGAIYTETEDFTLTGSTGTVT
FSTNTAKTGGALYSKGNNSLSGNTNLLFSGNKATGPSNSSANQEGCGGAILSFLESASVSTKKGLWIEDNENV
SLSGNTATVSGGAIYATKCALHGNTTLTFDGNTAETAGGAIYTETEDFTLTGSTGTVTFSTNTAKTAGALHTK
GNTSFTKNKALVFSGNSATATATTTDQEGCGGAILCNISESDIATKSLTLTENESLSFINNTAKRSGGGIYAPK
CVISGSESINFDGNTAETSGGAIYSKNLSITANGPVSFTNNSGGKGGAIYIADSGELSLEAIDGDITFSGNRATE
GTSTPNSIHLGAGAKITKLAAAPGHTIYFYDPITMEAPASGGTIEELVINPVVKAIVPPPQPKNGPIASVPVVPV
APANPNTGTIVFSSGKLPSQDASIPANTTTILNQKINLAGGNVVLKEGATLQVYSFTQQPDSTVFMDAGTTLE
TTTTNNTDGSIDLKNLSVNLDALDGKRMITIAVNSTSGGLKISGDLKFHNNEGSFYDNPGLKANLNLPFLDLS
STSGTVNLDDFNPIPSSMAAPDYGYQGSWTLVPKVGAGGKVTLVAEWQALGYTPKPELRATLVPNSLWNA
YVNIHSIQQEIATAMSDAPSHPGIWIGGIGNAFHQDKQKENAGFRLISRGYIVGGSMTTPQEYTFAVAFSQLFG
KSKDYVVSDIKSQVYAGSLCAQSSYVIPLHSSLRRHVLSKVLPELPGETPLVLHGQVSYGRNHHNMTTKLAN
NTQGKSDWDSHSFAVEVGGSLPVDLNYRYLTSYSPYVKLQVVSVNQKGFQEVAADPRIFDASHLVNVSIPM

GLTFKHESAKPPSALLLTLGYAVDAYRDHPHCLTSLTNGTSWSTFATNLSRQAFFAEASGHLKLLHGLDCFA
SGSCELRSSSRSYNANCGTRYSF

>core/5/1/Org1_Gene410

MKQKVKRNFAIIICVFALALYYVLPTCLYYAKPLDKKIDGNEAEHIIKSFTKQAQQVRKDVIPRVSAILSSLHL
RGHIQQHPAIPDIVSVRFKRGEDAEDFIGNLVHGEPNVPIKSARLHVVGYSREHDDHVIQVASSINTSLVESDF
SFVSYSSENEQEMASSILQRVYSACTFPKQKDCSCSYPSIWETAPKEQLLQYAKNLSSGFEVFSSRLSAFCQQS
FSSNQDRLAFLSRLSSLSNDAAIDVEDQKLLKSVYETLSQTACIRSLDCPYIEGLRLDCSESSLFFSSIEYCPKER
KIFLTLHSDLLAQRTSLSKEQRLDFDSRLAVEKQKLSKNLTVQVEDYNNGFSFQWMDKDTQGKIILQGERLL
QGIAEHLTALTLHRPAAESCDLIPENFPVFCRQPRESEAFGCYIFSPNTDCKHFSKGSVYILLKGLRSIVAKYQQ
GGGKELQSFEKDLQNLYNCFSHTEAISWTLGEDQVLEIRHPLQQFLDVWGEGFVIGKEGCAFLEVKDIQDRL
ATVNQIEKNRQSDLVRWHEQYRHAKCSMDLQERLSAPIPYQNLFLENMKLNMRKFSRGENILRLGIDFVGG
RQLLLSFKDHQGKQLTDKEDILKVSDELCARLNKLGVSEIELRREGDYIHLSVPGSSTISSSEILGTSKMSFHVV
NERFSSYSASRYEVQRFLDYLWFTSQAQGKTSPEEINTFASALFNEEVDVPPSVHEAITKLKSEGLAFSPSGCE
TPSTDLDTTFSMIAIGKDAEQKANPLVIVFRNYALDGASLKDIRPEFAAGEGYVLNFSVKDTSPKKMAEKLSP
TESFHTWTSAYCQEGISGTANGQYSANRGWRMAVVIDGYMVSSPILNVPLKNHASVSGKFTHREVSKLASD
LKSGAMSFVPEVLSEETISSDLGKKQCTQGIISACCGLAMLIVLMSVYYRFGGVIASGAVLLNLLLIWAALQY
LDAPLTLSGLAGIVLAMGMAVDANVLVFERIREEFLLSQSLKKSVEKGYTKAFGAIFDSNLTTVLASALLFFL
DTGPIKGFALTLILGIFSSMFTALFMTKFFFMLWMNKTQHTQLHMMNKFVGIKHDFLRGCKKLWAVSGSVF
LLGCVALGFGAWNSVLGMDFKGGYAFTFNPKEHGISDVAQMRGKVVHKLQEAGLSSRDFRIQTFGSSEKIKI
YFSDKALSYTKADTSLSPKINDHELALAVGLLSETGLDFSTETLNETQNFWSKVSSKLSKKMRYQATIGLLGA
LAIILLYVSLRFEWQYAFSAVCALIHDLLATCAVLFIAHFFLKKIQIDLQAIGALMTVLGYSLNNTLIIFDRIRED
RQANLFTPMHVLVNDALQKTFSRTVMTTATTLSVLLMLLFIGGSSVFNFAFIMTIGILLGTLSSLYIAPPLLLF
MVRKENRSK

>core/6/1/Org1_Gene885

MFGENSRDIGVLSKEGLFDKLEIGIASDITIRDKWSCGEIKKPETINYRTFKPEKGGLFCEKIFGPTKDWECCCG
KYKKIKHKGIVCDRCGVEVTLSKVRRERMAHIELAVPIVHIWFFKTTPSRIGNVLGMTASDLERVIYYEEYVV
IDPGKTDLTKKQLLNDAQYREVVEKWGKDAFVAKMGGEAIYDLLKSEDLQSLLKDLKERLRKTKSQQARM
KLAKRLKIIEGFVSSSNHPEWMVLKNIPVVPPDLRPLVPLDGGRFATSDLNDLYRRVINRNNRLKAILRLKTPE
VIVRNEKRMLQEAVDALFDNGRHGHPVMGAGNRPLKSLSEMLKGKNGRFRQNLLGKRVDYSGRSVIIVGPE
LKFNQCGLPKEMALELFEPFIIKRLKDQGSVYTIRSAKKMIQRGAPEVWDVLEEIIKGHPVLLNRAPTLHRLGI
QAFEPVLIEGKAIRIHPLVCAAFNADFDGDQMAVHVPLSVEAQLEAKVLMMAPDNIFLPSSGKPVAIPSKDM
TLGLYYLMADPTYFPEEHGGKTKIFKDEIEVLRALNNGGFIDDVFGDRRDETGRGIHIHEKIKVRIDGQIIETTP
GRVLFNRIVPKELGFQNYSMPSKRISELILQCYKKVGLEATVRFLDDLKDLGFIQATKAAISMGLKDVRIPDIK
SHILKDAYDKVAIVKKQYDDGIITEGERHSKTISIWTEVSEQLSDALYVEISKQTRSKHNPLFLMIDSGARGNK
SQLKQLGALRGLMAKPNGAIIESPITSNFREGLTVLEYSISSHGARKGLADTALKTADSGYLTRRLVDVAQDV
IITEKDCGTLNHIEISAIGQGSEELLPLKDRIYGRTVAEDVYQPGDKSRLLAQSGDVLNSVQAEAIDDAGIETIK
IRSTLTCESPRGVCAKCYGLNLANGRLIGMGEAVGIIAAQSIGEPGTQLTMRTFHLGGIAATSSTPEIITNSDGIL
VYMDLRVVLGQEGHNLVLNKKGALHVVGDEGRTLNEYKKLLSTKSIESLEVFPVELGVKILVADGTPVSQG
QRIAEVELHNIPIICDKPGFIKYEDLVEGISTEKVVNKNTGLVELIVKQHRGELHPQIAIYDDADLSELVGTYAI

PSGAIISVEEGQRVDPGMLLARLPRGAIKTKDITGGLPRVAELVEARKPEDAADIAKIDGVVDFKGIQKNKRIL
VVCDEMTGMEEEHLIPLTKHLIVQRGDSVIKGQQLTDGLVVPHEILEICGVRELQKYLVNEVQEVYRLQGVD
INDKHIEIIVRQMLQKVRITDPGDTTLLFGEDVNKKEFYEENRRTEEDGGKPAQAVPVLLGITKASLGTESFIS
AASFQDTTRVLTDAACCSKTDYLLGFKENVIMGHMIPGGTGFETHKRIKQYLEKEQEDLVFDFVSETECVC

>core/7/1/Org1_Gene46

MLKCPERVSVKKKEDIPDLPNLIEIQIKSYKQFLQIGKLAEERENIGLEEVFREIFPIKSYNEATVLEYLSYNLGV
PKYSPEECIRRGITYSVTLKVRFRLTDETGIKEEEVYMGTIPLMTDKGTFIINGAERVVVSQVHRSPGINFEQEK
HSKGNILFSFRIIPYRGSWLEAIFDINDLIYIHIDRKKRRRKILAITFIRALGYSSDADIIEEFFTIGESSLRSEKDFA
LLVGRILADNIIDEASSLVYGKAGEKLSTAMLKRMLDAGIASVKIAVDADENHPIIKMLAKDPTDSYEAALKD
FYRRLRPGEPATLANARSTIMRLFFDPKRYNLGRVGRYKLNRKLGFSIDDEALSQVTLRKEDVIGALKYLIRL
KMGDEKACVDDIDHLANRRVRSVGELIQNQCRSGLARMEKIVRERMNLFDFSSDTLTPGKVVSAKGLASVL
KDFFGRSQLSQFMDQTNPVAELTHKRRLSALGPGGLNRERAGFEVRDVHASHYGRICPIETPEGPNIGLITSLS
SFAKINEFGFIETPYRIVRDGIVTDEIEYMTADVEEECVIAQASASLDEYNMFTEPVCWVRYAGEAFEADTSTV
THMDVSPKQLVSIVTGLIPFLEHDDANRALMGSNMQRQAVPLLKTEAPVVGTGLECRAAKDSGAIVVAEED
GVVDFVDGYKVVVAAKHNPTIKRTYHLKKFLRSNSGTCINQQPLCAVGDVITKGDVIADGPATDRGELALG
KNVLVAFMPWYGYNFEDAIIISEKLIREDAYTSIYIEEFELTARDTKLGKEEITRDIPNVSDEVLANLGEDGIIRI
GAEVKPGDILVGKITPKSETELAPEERLLRAIFGEKAADVKDASLTVPPGTEGVVMDVKVFSRKDRLSKSDDE
LVEEAVHLKDLQKGYKNQVATLKTEYREKLGALLLNEKAPAAIIHRRTAEIVVHEGLLFDQETIERIEQEDLV
DLLMPNCEMYEVLKGLLSDYETALQRLEINYKTEVEHIREGDADLDHGVIRQVKVYVASKRKLQVGDKMA
GRHGNKGVVSKIVPEADMPYLSNGETVQMILNPLGVPSRMNLGQVLETHLGYAAKTAGIYVKTPVFEGFPE
QRIWDMMIEQGLPEDGKSFLYDGKTGERFDNKVVIGYIYMLKLSHLIADKIHARSIGPYSLVTQQPLGGKAQ
MGGQRFGEMEVWALEAYGVAHMLQEILTVKSDDVSGRTRIYESIVKGENLLRSGTPESFNVLIKEMQGLGL
DVRPMVVDA

>core/8/1/Org1_Gene500

MTWIPLHCHSQYSVLDAMSSIKDFVAKGQEFGIPALALTDHGNLYGAVDFYKECTQKGIQPIIGCECYIAPGS
RFDKKKEKRSRAAHHLILLCKNEQGYRNLCILTSLAFTEGFYYFPRIDKDLLRQYSEGLICLSGCLSSSVSDAA
LKSPEALLLELQWFQDLFKDDYFTEVQLHKMSEESIAGFKEEWLKQEYYSLIEKQIKVNTAVLEASKRLGIPT
VATNDIHYINANDWQAHEILLNVQSGETVRIAKQNTHIPNPKRKVYRSREYYFKSPAQMAELFKDIPEVISNT
LEVAKRCDFTFDFSKKHYPIYVPESLKTLNSYTEEDRYQASAVFLKQLAEEALPKKYSSEVLAHIAKKFPHRD
PIDIVKERMDMEMAIIIPKGMCDYLLIVWDIIHWAKANGIPVGPGRGSGAGSVLLFLLGITEIEPIRFDLFFERFI
NPERLSYPDIDIDICMAGRERVINYAIERHGKDNVAQIITFGTMKAKMAVKDVGRTLDMALSKVNHIAKHIPD
LNTTLSKALETDPDLHQLYINDAESAQVIDMALCLEGSIRNTGVHAAGVIICGDQLTNHIPICISKDSTMITTQY
SMKPVESVGMLKVDLLGLKTLTSINIAMSAIEKKTGQSLAMATLPLDDATTFSLLHQGKTMGIFQMESKGMQ
ELAKNLRPDLFEEIIAMGALYRPGPMDMIPSFINRKHGKEIIEYDHPLMESILKETYGIMVYQEQVMQIAGALA
SYSLGEGDVLRRAMGKKDFQQMEQEREKFCKRACNNGIDPELATVIFDKMEKFAAYGFNKSHAAAYGLITY
TTAYLKANYPKEWLAALLTCDSDDIEKIGKLIREAQSMGIPILPPHINVSSNHFVATDEGIRFAMGAIKGIGRG
LIESIVEERDHHGPYESIRDFIQRSDLKKVSKKSIESLIDAGCFDCFDSNRDLLLASVEPLYEAIAKDKKEAASG
VMTFFTLGAMDRKNEVPICLPKDIPTRSKKELLKKEKELLGIYLTEHPMDTVRDHLSRLSVVLAGEFENLPHG
SVVRTVFIIDKVTTKISSKAQKKFAVLRVSDGIDSYELPIWPDMYEEQQELLEEDRLIYAILVLDKRSDSLRISC

RWMKDLSIVNENIIYECDQAFDRIKNQVQKMSFTMSTSGKETKAKGNKPNENGHTQALAPVTLSLDLNELR
HSHLCILKKIVQKHPGSRTLVLVFTQDNERVASMSPDDAYFVCEDIEELRQELVTADLPVRVITV

>core/9/1/Org1_Gene638

MVLEALAIFRQDAMQHLLKHRKEIVVDFCEDSYTIRIPDEEAPEGYWLSTLKLQDIDRLTFASCSCPDGECCL
HLMTAYFAVYDALGLHPLHDKFRHSFWYAVFSHFFLDSIPLQAQGEMVYTLESPHITLTIECLSEEVFQDWLR
TIHASEEPTVFTNKTFLKSALYRTAKKFFFLNEEGAELTIGENSQGFPSHFSLQWQGLVFKAEILDFPTLEDIFP
KLELAHTSLENVSHDISITNVTVCAEEAKVNFTLSPVIHKKDRENHPKTRIGSVEYVAKTHEMITGPKAIALPI
YAIPLLADKFKDQLLSLLCYDSLEYRLRYDIRLLRDASFSFSAYLVTPGDLDNGSLIYPNYCYSPTKGLMQVV
GMLSPKQAFIVKSEQVEDFLNERGHLIQEPGFQTFINERPEGHLTYNVTEQGVLLFHYDVGDPSSTEIRFGTWT
YYTNQGFFLEKKNDLPIQDGLIVEPQDIPAFIVKNDAALRRLPNFFSSPPNLKDLLIEVHRQSRGKGLDLKPILV
GLGESRCWLFGVFLYREDIGFSLIPTPLQGLCFLPRVIPPENVPQFLTQYAQHERILFPNPQTRPPESYELVIQSI
HRPHPASPLHLQLELKTNLGSVPIGIALQGLKSKHTFLFTQAGFLDLKQNLFQFLKQFLSTQKCVIAENTVIANI
TDVFKLDALAPLSVTDDTIANPEDLQFFSQLKAACLPPIPQNLFSSDHQLRPYQNSGLLWMWFLYNHRLSGLL
CDEMGLGKTHQATALLDIVFQSSQPSARPKFLIVCPTSVLPHWEHILSNHLPGVSIFSFHGPNKPSELPPADILL
TSYGTLRQNYDKFYKIAFTIVVFDEIHMAKNKSSQIHKILCRIDAQMKLGLTGTPIENNLLEFKGLLDIILPNYL
PSDALFKKLFTKRCSSEELEEIIPSQDLLLKLTRPFILRRTKKLVLPELPDKVESIIACSLSPDQEKLYMATLQRE
KSHIQKLETPEEPATNFLHIFALLNHLKQICDHPAVFFKDPDQYKNYESGKWNAFVKLLKESLNAGYKVVVF
SQYIHMIRIITLYLEEIGIKYASIQGKSLNRKEEIETFTTDPNCQVFVGSLLAAGTGINLTAGNVVIMYDRWWN
PAKENQALDRVHRIGQKNTVFIYKLITEDTLEERIHYLIEKKIRLLDKVIASQDSNILHMLNREDLLTILSYKDE
HGTSDSEESPVDAPVEDDTGVLPPEDS

>core/10/1/Org1_Gene649

MLNFRKLRRDFSANILQDGKKLFEQGAVIDAKILSMNGETVCISAQVRGLYDNIYECEIEVDRSESDTVDSNC
DCSYNYDCQHIVALLFYLEQYFNEMVVAYARSADLETDHEINEEVKKELKETFVAAATKEEERKDREHQKEI
LREYVHAANALSANPFFLPLEYLEKDSAELAVLFVSVNEDTFAPANQPIEFQLVLRLPCRSKPFYISNIRTFLEG
VLYQEPIVLNGRRFFFTMQSFNASDRKLIDLLIRYVRYPNHTTEEKLLKSAYLMPPALGVILAKMFEHQLADR
GGGSLGEKESFSGLFCGNLEEPLCWSLTPAKMKFNLDFFDMPYKALLMTPVILVDDDEVQPEQTMLLESDAP
GIIHHFVYHRFSPQIKRAHLRSFSRLRDIAIPEALFGSFRENALPVFQEYAEIANVHLLNSFVTLPYVDEVRAIC
DMSYLDGELEAKLHFLYGSLRVPAASLALQYQDVRAFISDEGILARNLVEERKMLEEVFSGFIYDERDGAFR
VKSEKKIVEFMTETIPANQHRITFNCPENLSGQFIYDETIFELSFREGSDINYYEADLKVHGLLKGVPLDLLWD
CISAKKRFLELPKAGQQSKGTRRGKVNSGKLPCILVLDLEKIAPVVQIFNEIGFKVLDDLVQKCPLWSLTGISL
DQFEALPVNFSMSERLIEIQKQIRGEIEFDFQDVPQQIQATLRSYQTEGVHWLERLRKMHLNGILADDMGLGK
TLQAIIAVTQSKLEKGSGCSLIVCPTSLVYNWKEEFRKFNPEFRTLVIDGVPSQRRKQLTALADRDVAITSYNL
LQKDVELYKSFRFDYVVLDEAHHIKNRTTRNAKSVKMIQSDHRLILTGTPIENSLEELWSLFDFLMPGLLSSY
DRFVGKYIRTGNYMGNKADNMVALKKKVSPFILRRMKEDVLKDLPPVSEILYHCHLTESQKELYQSYAASA
KQELSRLVKQEGFERIHIHVLATLTRLKQICCHPAIFAKDAPEPGDSAKYDMLMDLLSSLVDSGHKTVVFSQY
TKMLGIIKKDLESRGIPFVYLDGSTKNRLDLVNQFNEDPSLLVFLISLKAGGTGLNLVGADTVIHYDMWWNP
AVENQATDRVHRIGQSRSVSSYKLVTLNTIEEKILTLQNRKKSLVKKVINSDDEVVSKLTWEEVLELLQI

>core/12/1/Org1_Gene955

MKRPKKFPIYLSIAQKTNRLLSGIVIAFAVIALRLWYLAVVEHEQKLEEAYKPQIRVLPQYVERATICDRFGKT
LAVNQLQYDVSVAYGAIRDLPTRAWRVDEHGHKQLIPVRKHYIMCLSELLSQELHLDREAIEDAIHAKASVL
GSVPYLVAANVSERTYLKLKMLSKDWPGLHVEAVVRRHYPQESVASDILGYVGPISLQEYKRVTQELSQLR
ECVRAYEEGEDPKLPEGLASIDQVRALLESVESNAYSLNALVGKMGVEACWDSKLRGKIGKKPILVDRRGNF
IQEMEGAVPEAPGTKLQLTLSAELQAYADALLLEYEKTETFRSAKSLKKREKLPPLFPWIKGGAIIALDPNNG
EILAMASSPRYRNNDFVNAKVAEDSKAVRSSIYRWLENKEHIAEIYDRKVPLIRERRNPLTGLCYEEILPLTFD
CFLDFLFPENSVIKLQLKRNSFVGQAIEVQNLVTRLLSLFPYEEGTCPCSAIFDAVFPNEEGHILIQEVISLREQK
WIMECLNQHKADIEELKEALDQVFNELPANYDKILYTDILRLIVDPERFSPVLPSEVHRLSLSEFTELQGRYVV
LRSAFSTILEDAFIEVHFKSWRKSEFLQYLAAKRQEEALRKQRYPTPYVDYLEEEKTRQYKMFCQEHLDTFL
AYLFSKTPYKEGLEPYYDILDLWINELDNGAHRALSWHEHYLFLKERVSHLSEHLPALFSTFREFNELQRPLL
GKYPISIVRNKRQTEQDLAASFYPVYGYGYLRPHAYGQAATLGSIFKLVSAYSVLSQRILWGHNEEPANPLVI
IDKNSFGYRSSKPHVGFFKDGTPIPTFFRGGSLPGNDFMGRGFIDLVSALEMSSNPYFSLLVGEGLGDPEDLAD
AASLFGFGEKTGLGLPGEYAGRVPHDLAYNRSGLYATAIGQHTLVVTPLQTAVMLASLVNGGVVYVPKLLL
GEWEGEHVSYLSSKKKRTIFMPDAVVEVLKTGMRNVIWGQYGTARAIQSQFPPQLLSRIIGKTSTAESIMRVG
LDREYGTMKMKDIWFAAVGFSDQDLSLPTIVVIVYLRLGEFGRDAAPMAVKMIDMWEKIQQRESFLRG

>core/13/1/Org1_Gene1027

MDFNPVNLDFSISKEFKEETLPLLLENIHPGATAFLAAKMFHDCRASVIMITTPARLDDLFENLRTFLDQAPVE
FPSSEIDLSPKLVNIDAVGKRDHLLYSLNQHRAPIFCVTTLKALLEKTRSPQATSQQHLDLAVGDVLDPEATTE
LCKSLGYSQVMLTSEKGEFSCRGGIVDIFPLSSPEPFRIEFWGEKIISIRSYNPSDQLSTGKVSKISISPAYTEEAS
GGNYSHSLLDYFSTPPLYLFDNLEILEDDFADISGTLSSLPDRFFSIGTLYDRISTSNQVYFSETPFPNVKNLKEN
RVIIEAFHRNMEASRQAIPILYPEQIIQNDENPLLAFLQHLQEYMPPHGKPLKLAIYSTKTKSLKEARALAETV
ARGDVEIYEKTGNLTSSFALVNEAFAAISLSEFASTKVLRRQKQRTHFSVTTEEVFVPIPGETVVHIHNGIGKFL
GIEKKPNHLNIETDYLVLEYADKARLYVPSNQAYLISRYVGTSDKAADLHHLNSSKWKRSRDLTEKSLIVYA
EKLLQLEAQRSTTPAFVYPPHGESVIKFAETFPYEETPDQLKTIDQIYNDMMSPKLMDRLICGDAGFGKTEVI
MRAAVKAVCDGHRQVIVMVPTTILATQHYETFKERMAGLPIEIAVLSRFSQAKVQKLICEQVASGQIDIIIGTH
KLINKSLEFKNPGLLIIDEEQRFGVKVKDNLKERYPMIDCLTVSATPIPRTLHMSLSGARDLSVIAMPPLDRLP
VSTFVMEHNTETLTAALRHELLRGGQAYVIHNRIESIYTLAETIRNLIPEARIGVAHGQMGAEDLSNIFTKFKN
QKTDILVATALIENGIDIPNANTILIDHADKFGMADLYQMKGRVGRWNKKAYCYFLVPHLDRLSGPAAKRL
AALNKQEYGGGMKIALHDLEIRGAGNILGTDQSGHIGTIGFNLYCKLLKKAVSALKKHTSPLLFNDDVKIEFP
YNSRIPDTYIETGSMRIEFYQKIGNAESSEELTAIQEEMRDRFGPLPQEICWLFALAEIRLFALQHGISSIKGTAN
ALYVQKCLSKSEQTKKTLPYALSPTPELLVKEVIESIERGFLINAS

>core/14/1/Org1_Gene994

MIKKRAIFERMFPIPPPHCPPNNKNNFYHLTTDTKDPLLLRILRTIGYVLLHIITLGLLLLIHYYKHHRVVRKEG
LPTPPTLPKGPEPKTIEIAKQPPKDGEDKKPDVPKPGTPPPEDTPPPPPKAPSPASPKVPKQPADKKPTPPPEAPP
PPVRVATPMPLRPSSQGYWQCLNRMVSMVLRRAPLPLPAMQVDPILGDFNPHFVASYPNRIDNEPMYFQIKQ
FKKIAQNPDLPQQHRRLAQLSLEQALYLNDNYYLVNVPGDGNCFYRAYAVGWLSALYEESSRNDIVFEQEA
TRLLDLPFASSSPANANLCAEMAELLQLCSTYCSFIDLYDGVILSQKHTATLIAFLRKLSAYAIRQQIAASSNEE
TARALFISDMQDDLLPSVLEFLAANRPYSELFQNLINHSALPYMQSRDKLFLLLEHLPALFLTDAELQKMSPE
DQQLRKQYEREIREAFAKLSRRIADSGWDTERFNAIVKDHLPEAIRCQYSRFLATIENRRSGDLPWSPALSFFA

FLCTCPSVRFHKLCATFYKSLEDIIIASAPPQRSIQEILQISNASLSYLNEDLDSSWQREVISSNIMTILTTHESLTL
ESSMPQLETLHKRIANLLKNVISTSFETPPLSNQPDLLSNLVNKLLVAIHSKLELKEHFNTVCSARSLRLTRDE
GSGLSQEQDLLYTQAVQLLFFILQHPQVNNRPETKDAVKELKMLLLPFLQYAFKKVENEKKLQKLLRSILGS
LVLKPPARYPSTPSNKDKETFCKFWSRHPEVMVLDPILEKNCMQFLRATFPNYQLETEAILLEKEIESTFRNG
WNVFLTRLNLFGSKLGSPSSPTALSDQFSKSFLIFCFLNNYPKLLQKKTPLAARLDAFQREASHRFTQVKDKL
LLSLKYGFPLATATINQYSRARDQLICNLLKNTVTASDGFCRSGFRQSLIGYLHSLSSNELGDILDDVKEQAEA
NDVAAMTTVPLQPFAVCLIMSDRDTVSEENIENFVAMHGFLNTISPERDARIFLIRFPNHYGCLLPRNPRTEDQ
NSKPDSSNP

>core/15/1/Org1_Gene890

MKPFNIFDSNSSIQGKFFLEASAGTGKTFTIEQIVLRALIEGSLTHVEHALAITFTNASTNELKVRIKDNLAQTL
RELKAVLNSQPASLPTYLDINCNVKQIYMQVRNALATLDQMSLFTIHGFCNFVLEQYFPKTRLIHKNPALTHS
QLVLHHITNYLKQDLWKNVLFQEQFHLLAVRYNVTSKHTSSLVDKLLASYTQPISSYFSSRVERLEQISLWHQ
QIYNSLLEIPKQVFLDQLTAHISGFKKQPFSILDDLHHFVDLLYTSETHSSLFSFFKIAETFNFKHRLARYKPCA
AFTVLENMSWVERTLEFCNLDRIFNTLLVDLQEYLKQNYTPWLSPDESVFALEKLLSSSEAQPVVQALREQY
QLVLIDEFQDTDKQQWSIFSNLFISPKFTGSLFLIGDPKQSIYEWRSADLPTYLTAKSSFSEDKQLQLVNNYRST
PKLMEAINQIFGKISPFLEIPGYLPIEYHALNPQSSETFENPPHAPIHFFFYETIKDQALWIFSEALRLQKEQKIPL
GNMVVLVSDSNQAFELISYATIPVSFSKNKSIFHLTETHILTTALLEAILHPENYEKISKILFSSLFGLSLDEVTTK
KEDFTIYFQSLHSYISHHGLLATFYRVMTTQGNVLFSSPRGDLIFQEMEKLCGYLDTISSYPYHQLLHLKNFSE
TGRWEEELAISSYSEDLETLKITTIHSSKGLEYDIVFCPGIEKSKKNKSSSELLREMYVACTRAKKQLYLPISTQ
PPSLQRSSALTNYVKLEGTQSSAYDLAIHLHQEHPDLFSYSLPKDHGHATTVLNLPLLETFALKVTPPKTIFSFS
STKFLLDTHKDSQSIPYSKLPISKQQLPLGEKTGILIHKILESIQFSLLQDTEYLMSTIMRFIKHTHLEGFEETILK
LLSKTFFSPLTFSSQTFSLSQVLPNKIFRETSFLFLENQELWQGVIDLFFEHEGKYYIIDWKTSFLGETNSDYSKS
NLSIYIKQEKLDYQGRIYVKAVRKFLNQFEIDDDVELGVIFIRGIDTQGNGFFALNSSEDIPNFNPKAIQKCQAY
H

>core/16/1/Org1_Gene753

MVEVEEKHYTIVKRNGMFVPFNQDRIFQALEAAFRDTRSLETSSPLPKDLEESIAQITHKVVKEVLAKISEGQ
VVTVERIQDLVESQLYISGLQDVARDYIVYRDQRKAERGNSSSIIAIIRRDGGSAKFNPMKISAALEKAFRATL
QINGMTPPATLSEINDLTLRIVEDVLSLHGEEAINLEEIQDIVEKQLMVAGYYDVAKNYILYREARARARANK
DQDGQEEFVPQEETYVVQKEDGTTYLLRKTDLEKRFSWACKRFPKTTDSQLLADMAFMNLYSGIKEDEVTT
ACIMAARANIEREPDYAFIAAELLTSSLYEETLGCSSQDPNLSEIHKKHFKEYILNGEEYRLNPQLKDYDLDAL
SEVLDLSRDQQFSYMGVQNLYDRYFNLHEGRRLETAQIFWMRVSMGLALNEGEQKNFWAITFYNLLSTFRY
TPATPTLFNSGMRHSQLSSCYLSTVKDDLSHIYKVISDNALLSKWAGGIGNDWTDVRATGAVIKGTNGKSQG
VIPFIKVANDTAIAVNQGGKRKGAMCVYLENWHLDYEDFLELRKNTGDERRRTHDINTASWIPDLFFKRLEK
KGMWTLFSPDDVPGLHEAYGLEFEKLYEEYERKVESGEIRLYKKVEAEVLWRKMLSMLYETGHPWITFKDP
SNIRSNQDHVGVVRCSNLCTEILLNCSESETAVCNLGSINLVEHIRNDKLDEEKLKETISIAIRILDNVIDLNFYP
TPEAKQANLTHRAVGLGVMGFQDVLYELNISYASQEAVEFSDECSEIIAYYAILASSLLAKERGTYASYSGSK
WDRGYLPLDTIELLKETRGEHNVLVDTSSKKDWTPVRDTIQKYGMRNSQVMAIAPTATISNIIGVTQSIEPMY
KHLFVKSNLSGEFTIPNTYLIKKLKELGLWDAEMLDDLKYFDGSLLEIERIPNHLKKLFLTAFEIEPEWIIECTS

RRQKWIDMGVSLNLYLAEPDGKKLSNMYLTAWKKGLKTTYYLRSQAATSVEKSFIDINKRGIQPRWMKNK
SASTSIVVERKTTPVCSMEEGCESCQ

>core/17/1/Org1_Gene910

MTADEVGKNSFAKKEEQVLKFWKDNQIFEKSLQNRQGKTLYSFYDGPPFATGLPHYGHLLASTIKDVVGRY
ATMDGYYVPRRFGWDCHGVPVEYEVEKSLSLTAPGAIEDFGIASFNEECRKIVFRYVHEWEYYINRIGRWVD
FSSTWKTMDASFMESVWWVFQSLYNQGLVYEGTKVVPFSTALGTPLSNFEASQNYKEVDDPSLVVRMPLQ
NDSASLLVWTTTPWTLPSNMAIAVGETLVYVRIQDKKSGEQWILSQGCVSRWFSNPEEFVILESFSGKDLVG
RTYEPPFTFFQSKREEGAFRVIAASFVEESEGTGVVHMAPAFGEGDFLVCKENHVPLVCPVDAHGSFTEEIPQ
YQGQYIKHADKEIIKFLKKEGRIFYHGTVKHRYPFCWRTDTPLIYKAVNSWFVAVEKIKDKMLRANSSIHWV
PEHIQEGRFGKWLEGARDWAISRNRYWGTPIPIWKSADGEILVVGSIRELEELTGTQITDIHRHFIDDLNIVKD
GKPFHRIPYVFDCWFDSGAMPYAQNHYPFENQKETEEAFPADFIAEGLDQTRGWFYTLTVISAILFDRPAFRN
AIVNGIILAEDGNKMSKRLNNYPSPKYVLDTYGADALRLYLLHSVVVKAEDLRFSDKGIEGVLKQILLPLTNV
LSFFNTYAELYGFDPKSQDIEPAYTEIDQWILSNLYSVVGKVRESMSQYHLNFAVEPFVTFIDDLTNWYIRRC
RRRFWEAEDTPDRRAAFSTLYEVLTVFCKVIAPFVPFLAEDIYQKLKLEKEPESVHLCDFPQVEMDKILPDLE
KHMHDIREIVGLGHSLRKEHKLKVRQPLANFYVVGSKDRLSLLKTFEGLIAEELNVKNVIFYEEAPSFIYTTV
KPNFRMLGKKVGSKMKEVQKALSELPNNAIDKLIQEETWVLTIDDREIALDGDDVVICRHTDPGYIARSSALF
SVILDCQLREPLIVEGIARELVNKINTMRRNQQLHVSDRIALRIKTTEAVHRAFLDYENYICEETLIIAYDFTQD
SDFQGENWDINGHATQIEITVSSIDS

>core/18/1/Org1_Gene561

MNATKHCRASFSNSPRHLLAQLAEDITSTHQKPFTKRWILVANATTGHWIKNQLVHVLSDHIFMGSTIFTASD
SIVKHLFLGSGCSQPNIPDYLTLPLLINNILEEISKASKFENGREFLSPPTYETTKKLAAAFKQFHTFSQRPTKNA
SHYQELFQILESHFSSYEEMFTTILNNRTQEEDCSLHIFGYAHLPKHLAEFFINLSTYFPVYFYCFSPCREYFGD
LLSDRAIDFFWNQLPDSPIKNAWEHYVLSDRQALLANLAHKSQSSQNFFLDREIDYQEMFLPSKHDSSLGVIQ
NSILDLKPTSPQDFSQTKQTICIYRALNIPREVQEVFCKVTELLHRGVSPEEIFILSSHIESYKVHLNAIFNPHVPI
YFTDEVDPRAEDLRNKILLLSSILQTQGDLHYILQLLTHPQLQQPIDQNKVPYLIKKLSSEWGKISSKDRASGQ
QMKALGDLILEEYPFHQEGGRVSQVEVWETTVPLIYFIQERINLYLSSSQHSYEDLFQNVFSCLEKIFVLSPEET
SFITTLRNSLFPTFATSSCSLLFFTDFCLDFLLHFHKPSPLYDKPGPYIGSLSSLSLIPKGYVFILGANKTTSSDIFD
LLNRTTTHEELAFSSTEDEENFHFLQILVSTKHELHISYISSAAQFNLPSPFLNHIKETLDLPVETLPTQPYLSAFF
KNKACLHTSQEYNYSLAHAFYSKKALLPSLFIPTVKQVNLPQHLSLNEIIKGIFSPLDLFLKTNYNLRISYPEHL
KKQQKLFPTKHQIEDFWNECFVDKEHDLIPSISPHAEELFTYYREKTILLRNGLDKDPKHSPYTVTFSSSIFEER
PYHESYLFPPLSLSFQGNPVQIHGTIHGVCNEGLYLCSIDPRDSLKKTTRTLGSLPETSSEQKQLLERYVALAV
LQMSQHLSSDSALIKLTSFNTKENHHPPFSDPEGYLRKVLEVYHLMSSQPIPLLSPLCWKTLDDEEKFHQAVL
SAISEEAKNPSLPIFWQFHNRNIEEILNHVGASERLKILSLFRGPCEAV

>core/19/1/Org1_Gene866

MSEHPLTLQSMIATILRFWSEQGCVIHQGYDLEVGAGTFNPATFLRALGPEPYKAAYVEPSRRPQDGRYGVH
PNRLQNYHQLQVILKPVPENFLSLYTESLRAIGLDLRDHDIRFIHDDWENPTIGAWGLGWEVWLNGMEITQL
TYFQAIGSKPLDTISGEITYGIERIAMYLQKKTSIYDVLWNDTLTYGQITQASEKAWSEYNFDYANTEMWFKH
FEDFAEEALRTLKNGLSVPAYDFVIKASHAFNILDARGTISVTERTRYIARIRQLTRLVADSYVEWRASLNYPL

LSLSSTSEPKETSESVVPMISSTEDLLLEIGSEELPATFVPIGIQQLESLARQVLTDHNIVYEGLEVLGSPRRLAL
LVKNVAPEVVQKAFEKKGPMLTSLFSPDGDVSPQGQQFFASQGVDISHYQDLSRHASLAIRTVNGSEYLFLL
HPEIRLRTADILMQELPLLIQRMKFPKKMVWDNSGVEYARPIRWLVALYGEHILPITLGTIIASRNSFGHRQLD
PRKISISSPQDYVETLRQACVVVSQKERRMIIEQGLRAHSSDTISAIPLPRLIEEATFLSEHPFVSCGQFSEQFCA
LPKELLIAEMVNHQKYFPTHETSSGAISNFFIVVCDNSPNDTIIEGNEKALTPRLTDGEFLFKQDLQTPLTTFIEK
LKSVTYFEALGSLYDKVERLKAHQRVFSTFSSLAASEDLDIAIQYCKADLVSAVVNEFPELQGIMGEYYLKH
ANLPTASAVAVGEHLRHITMGQKLSTIGTLLSLLDRLDNLLACFILGLKPTSSHDPYALRRQSLEVLTLVSASR
LPIDLASLLDRLADHFPSTIEEKVWDKSKTIHEILEFIWGRLKTFMGSLEFRKDEIAAVLIDSATKNPIEILDTAE
ALQLLKEEHTEKLAVITTTHNRLKKILSSLKLSMTSSPIEVLGDRESNFKQVLDAFPGFPKETSAHAFLEYFLSL
ADLSNDIQDFLNTVHIANDDGAIRNLRISLLLTAMDKFSLCHWESVAV

>core/20/1/Org1_Gene998

MSRLDIFVFDSLIANQDQNLLEEIFCSEDTVLFKAYRTTALQSPLAAKNLNIARKVANYILADNGEIDTVKLVE
AIHHLSQCTYPLGPHRHNEAQDREHLLKMLKALKENPKLKESIKTLFVPSYSTIQNLIRHTLALNPQTILSTIHV
RQAALTALFTYLRQDVGSCFATAPAILIHQEYPERFLKDLNDLISSGKLSRIVNQREIAVPINLSGCIGELFKPLR
ILDLYPDPLVKLSSSPGLKKAFSAANLIETLGDSEAQIQQLLSHQYLMQKLQNVHETLTANDIIKSTLLHYYQL
QESTVRAIFFKEGLFSKEQVAFSTQHPRELSEIQRVYHYLHAYEEAKSAFIHDTQNPLLKAWEYTLATLADAS
QSTISNHIRLALGWKSEDPHSLVSLVTHFVEEEVENIRILVQQCEQTYHEARSQLEYIEGRMRNPLNNQDSQIL
TMDHMRFRQELNKALYEWDSAQEKAKKFLHLPEFLLSFYTKQIPLYFRSSYDAFIQEFAHLYANAPAGFRILF
THGRTHPNTWSPIYSINEFIRFLSEFFTSTESELLGKHAVINLEKETSRLVHNITAMLHTDVFQEALLTRILEAY
QLPVPPSILNHLDQLSQTPWVYVSGGTVDTLLLDYFESSEPLTLTEKHPENPHELAAFYADALKDLPTGIKSYL
EEGSHSLLSSSPTHVFSIIAGSPLFREAWDNDWYSYTWLRDVWVKQHQDFLQDTILPQLSIYAFIENFCNKYA
LQHVVHDFHDFCSDHSLTLPELYDKGSRFLSSLFTKDKTVALIYIRRLLYLMVREVPYVSEQQLPEVLDNVSS
YLGISSRITYEKFRSLIEETIPKMTLLSSADLRHIYKGLLMQSYQKIYTEEDTYLRLTTAMRHHNLAYPAPLLFA
DSNWPSIYFGFILNPGTTEIDLWKFNYAGLQGQPLDNIQELFATSRPWTLYANPIDYGMPPPPGYRSRLPKEFF

>core/21/1/Org1_Gene333

MPLSFKSSSFCLLACLCSASCAFAETRLGGNFVPPITNQGEEILLTSDFVCSNFLGASFSSSFINSSSNLSLLGKG
LSLTFTSCQAPTNSNYALLSAAETLTFKNFSSINFTGNQSTGLGGLIYGKDIVFQSIKDLIFTTNRVAYSPASVT
TSATPAITTVTTGASALQPTDSLTVENISQSIKFFGNLANFGSAISSSPTAVVKFINNTATMSFSHNFTSSGGGVI
YGGSSLLFENNSGCIIFTANSCVNSLKGVTPSSGTYALGSGGAICIPTGTFELKNNQGKCTFSYNGTPNDAGAI
YAETCNIVGNQGALLLDSNTAARNGGAICAKVLNIQGRGPIEFSRNRAEKGGAIFIGPSVGDPAKQTSTLTILA
SEGDIAFQGNMLNTKPGIRNAITVEAGGEIVSLSAQGGSRLVFYDPITHSLPTTSPSNKDITINANGASGSVVFT
SKGLSSTELLLPANTTTILLGTVKIASGELKITDNAVVNVLGFATQGSGQLTLGSGGTLGLATPTGAPAAVDFT
IGKLAFDPFSFLKRDFVSASVNAGTKNVTLTGALVLDEHDVTDLYDMVSLQSPVAIPIAVFKGATVTKTGFPD
GEIATPSHYGYQGKWSYTWSRPLLIPAPDGGFPGGPSPSANTLYAVWNSDTLVRSTYILDPERYGEIVSNSLW
ISFLGNQAFSDILQDVLLIDHPGLSITAKALGAYVEHTPRQGHEGFSGRYGGYQAALSMNYTDHTTLGLSFGQ
LYGKTNANPYDSRCSEQMYLLSFFGQFPIVTQKSEALISWKAAYGYSKNHLNTTYLRPDKAPKSQGQWHNN
SYYVLISAEHPFLNWCLLTRPLAQAWDLSGFISAEFLGGWQSKFTETGDLQRSFSRGKGYNVSLPIGCSSQWF
TPFKKAPSTLTIKLAYKPDIYRVNPHNIVTVVSNQESTSISGANLRRHGLFVQIHDVVDLTEDTQAFLNYTFDG
KNGFTNHRVSTGLKSTF

>core/22/1/Org1_Gene750

MKAGDTYRNFIIKSCKDLPEIESKLLEAEHKPTGASIMMIVNNDEENVFNICFRTCPQTSNGVAHVLEHMVLC
GSENYPVRDPFFSMTRRSLNTFINAFTGPDFTCYPAASQIPEDFYNLLSVYIDAVFHPLLTKQSFLQEAWRYEF
NSENHLCYTGVVFNEMKGAMMSGEARLSEALNAAIFPSVTYGVNSGGEPREIVTLSHEDVRAFHQSQYSINR
CLFYFYGNIKPSRHLDFLEEKLLRQATKLEKQAVSVPLQKRFKEPVRNILTYPVDHQEEDKVLFGISWLTCSIL
EQQELLALHVLEIILMGTDASPLKSRLLKSGFCKQTEMSIENDIREIPMTLVCKGCSPAGAQKLEALIFASLEEII
REGISENIVEGAVHQLELSRKEITGYSLPYGLSLFFRSGLLKQHGGSAEDGLRIHSLFSELRNSLKNSDYLAKLI
RKYFLDNPHFARVILLPDTELVAKDNKDEQQLLLSVSEKLTDENKEKIQQNVRELTESQEQKEDLNGILPNLA
LDKVPTSGKEFPLIKEGLSQGEVLHHECFTNDIVFIDVVLDIPPLSGEELPWLRLLVFLMLQLGCGGRSYKEHL
EFLLEHTGGVDVSYDFSPHANKNSFLSPSVSIRGKALSSKSEKLCGIVSDMLTSVDFTDIPRIRELLMQHNEAL
TNSVRNSPMSYAVSMACSGNSITGAMSYLTTGLPYVKKIRELTKNFDQNIDEAVVILQRLYTKCFSGKRQIVI
SGSAHNYQQLKDNKFYGLLDYLIVIPEPWENPSINLYVTSRGLHIPARAAFNALAFPIGDIAYDHPDAAALTV
AAEILDNVVLHTKIREQGGAYGSGAAANLSRGSFYCYSYRDPEIATTYKTFLKGVSEIASGNFTKEDIYEGAL
GVVQGLDMPVAPGSRASVAFYRLKSGRIPVLRQAFRRSVLEVTKEHICMVMDKYLESTVQETTLISFAGEEM
LRNNVLTLDKDFPIVPAI

>core/23/1/Org1_Gene332

MKTSIRKFLISTTLAPCFASTAFTVEVIMPSENFDGSSGKIFPYTTLSDPRGTLCIFSGDLYIANLDNAISRTSSSC
FSNRAGALQILGKGGVFSFLNIRSSADGAAISSVITQNPELCPLSFSGFSQMIFDNCESLTSDTSASNVIPHASAI
YATTPMLFTNNDSILFQYNRSAGFGAAIRGTSITIENTKKSLLFNGNGSISNGGALTGSAAINLINNSAPVIFSTN
ATGIYGGAIYLTGGSMLTSGNLSGVLFVNNSSRSGGAIYANGNVTFSNNSDLTFQNNTASPQNSLPAPTPPPTP
PAVTPLLGYGGAIFCTPPATPPPTGVSLTISGENSVTFLENIASEQGGALYGKKISIDSNKSTIFLGNTAGKGGAI
AIPESGELSLSANQGDILFNKNLSITSGTPTRNSIHFGKDAKFATLGATQGYTLYFYDPITSDDLSAASAAATV
VVNPKASADGAYSGTIVFSGETLTATEAATPANATSTLNQKLELEGGTLALRNGATLNVHNFTQDEKSVVIM
DAGTTLATTNGANNTDGAITLNKLVINLDSLDGTKAAVVNVQSTNGALTISGTLGLVKNSQDCCDNHGMFN
KDLQQVPILELKATSNTVTTTDFSLGTNGYQQSPYGYQGTWEFTIDTTTHTVTGNWKKTGYLPHPERLAPLIP
NSLWANVIDLRAVSQASAADGEDVPGKQLSITGITNFFHANHTGDARSYRHMGGGYLINTYTRITPDAALSL
GFGQLFTKSKDYLVGHGHSNVYFATVYSNITKSLFGSSRFFSGGTSRVTYSRSNEKVKTSYTKLPKGRCSWS
NNCWLGELEGNLPITLSSRILNLKQIIPFVKAEVAYATHGGIQENTPEGRIFGHGHLLNVAVPVGVRFGKNSH
NRPDFYTIIVAYAPDVYRHNPDCDTTLPINGATWTSIGNNLTRSTLLVQASSHTSVNDVLEIFGHCGCDIRRTS
RQYTLDIGSKLRF

>core/24/1/Org1_Gene643

MLGFLKRFFGSSQERILKKFQKLVDKVNIYDEMLTPLSDDELRNKTAELKQRYQNGESLDSMLPEAYGVVK
NVCRRLAGTPVEVSGYHQRWDMVPYDVQILGAIAMHKGFITEMQTGEGKTLTAVMPLYLNALTGKPVHLV
TVNDYLAQRDCEWVGSVLRWLGLTTGVLVSGTLLEKRKKIYQCDVVYGTASEFGFDYLRDNSIATRLEEQV
GRGYYFAIIDEVDSILIDEARTPLIISGPGEKHNPVYFELKEKVASLVYLQKELCSRIALEARRGLDSFLDVDILP
KDKKVLEGISEFCRSLWLVSKGMPLNRVLRRVREHPDLRAMIDKWDVYYHAEQNKEESLERLSELYIIVDEH
NNDFELTDKGMQQWVEYAGGSTEEFVMMDMGHEYALIENDETLSPADKINKKIAISEEDTLRKARAHGLRQ
LLRAQLLMERDVDYIVRDDQIVIIDEHTGRPQPGRRFSEGLHQAIEAKEHVTIRKESQTLATVTLQNFFRLYEK

LAGMTGTAITESREFKEIYNLYVLQVPTFKPCLRIDHNDEFYMTEREKYHAIVNEIATIHGKGNPILVGTESVE
VSEKLSRILRQNRIEHTVLNAKNHAQEAEIIAGAGKLGAVTVATNMAGRGTDIKLDNEAVIVGGLHVIGTTR
HQSRRIDRQLRGRCARLGDPGAAKFFLSFEDRLMRLFASPKLNTLIRHFRPPEGEAMSDPMFNRLIETAQKRV
EGRNYTIRKHTLEYDDVMNKQRQAIYAFRHDVLHAESVFDLAKEILCHVSLMVASLVMSDRQFKGWTLPNL
EEWITSSFPIALNIEELRQLKDTDSIAEKIAAELIQEFQVRFDHMVEGLSKAGGEELDASAICRDVVRSVMVMH
IDEQWRIHLVDMDLLRSEVGLRTVGQKDPLLEFKHESFLLFESLIRDIRITIARHLFRLELTVEPNPRVNNVIPT
VATSFHNNVNYGPLELTVVTDSEDQD

>core/26/1/Org1_Gene340

MSLNLERISPLLYLLDVTAKKIDTSNLIVEAMNLDEHYGYQGIWSPYWMETTTTTSSTVPEQTNTNHRQLYV
DWTPVGYRPNPERHGEFIANTLWQSAYNALLGIRILPPQNLKEHDLEASLQGLGLLINQHNREGRKGFRNHT
TGYAATTSAKTAARHSFSLGFAQMFSKTRERQSPSTTSSHNYFAGLRFDSLLFRDFISTGLSLGYSYGDHHML
CHYTEILKGSSKAFFNNHTLVASLDCTFLPARITRTLELQPFISAIALRCSQASFQETGDHIRKFHPKHPLTDLSS
PIGFRSEWKTSHHIPMLWTTEISYVPTLYRKNPEMFTTLLISNGTWTTQATPVSYNSVAAKIKNTSQLFSRVTL
SLDYSAQVSSSTVGQYLKAESHCTF

>core/27/1/Org1_Gene388

MKQMRLWGFLFLSSFCQVSYLRANDVLLPLSGIHSGEDLELFTLRSSSPTKTTYSLRKDFIVCDFAGNSIHKPG
AAFLNLKGDLFFINSTPLAALTFKNIHLGARGAGLFSESNVTFKGLHSLVLENNESWGGVLTTSGDLSFINNTS
VLCQNNISYGPGGALLLQGRKSKALFFRDNRGTILFLKNKAVNQDESHPGYGGAVSSISPGSPITFADNQEILF
QENEGELGGAIYNDQGAITFENNFQTTSFFSNKASFGGAVYSRYCNLYSQWGDTLFTKNAAAKVGGAIHAD
YVHIRDCKGSIVFEENSATAGGAIAVNAVCDINAQGPVRFINNSALGLNGGAIYMQATGSILRLHANQGDIEF
CGNKVRSQFHSHINSTSNFTNNAITIQGAPREFSLSANEGHRICFYDPIISATENYNSLYINHQRLLEAGGAVIFS
GARLSPEHKKENKNKTSIINQPVRLCSGVLSIEGGAILAVRSFYQEGGLLALGPGSKLTTQGKNSEKDKIVITN
LGFNLENLDSSDPAEIRATEKASIEISGVPRVYGHTESFYENHEYASKPYTTSIILSAKKLVTAPSRPEKDIQNLII
AESEYMGYGYQGSWEFSWSPNDTKEKKTIIASWTPTGEFSLDPKRRGSFIPTTLWSTFSGLNIASNIVNNNYL
NNSEVIPLQHLCVFGGPVYQIMEQNPKQSSNNLLVQHAGHNVGARIPFSFNTILSAALTQLFSSSSQQNVADK
SHAQILIGTVSLNKSWQALSLRSSFSYTEDSQVMKHVFPYKGTSRGSWRNYGWSGSVGMSYAYPKGIRYLK
MTPFVDLQYTKLVQNPFVETGYDPRYFSSSEMTNLSLPIGIALEMRFIGSRSSLFLQVSTSYIKDLRRVNPQSSA
SLVLNHYTWDIQGVPLGKEALNITLNSTIKYKIVTAYMGISSTQREGSNLSANAHAGLSLSF

>core/28/1/Org1_Gene831

MQNNRSLSKSSFFVGALILGKTTILLNATPLSDYFDNQANQLTTLFPLIDTLTNMTPYSHRATLFGVRDDTNQ
DIVLDHQNSIESWFENFSQDGGALSCKSLAITNTKNQILFLNSFAIKRAGAMYVNGNFDLSENHGSIIFSGNLSF
PNASNFADTCTGGAVLCSKNVTISKNQGTAYFINNKAKSSGGAIQAAIINIKDNTGPCLFFNNAAGGTAGGAL
FANACRIENNSQPIYFLNNQSGLGGAIRVHQECILTKNTGSVIFNNNFAMEADISANHSSGGAIYCISCSIKDNP
GIAAFDNNTAARDGGAICTQSLTIQDSGPVYFTNNQGTWGGAIMLRQDGACTLFADQGDIIFYNNRHFKDTF
SNHVSVNCTRNVSLTVGASQGHSATFYDPILQRYTIQNSIQKFNPNPEHLGTILFSSAYIPDTSTSRDDFISHFR
NHIGLYNGTLALEDRAEWKVYKFDQFGGTLRLGSRAVFSTTDEEQSSSSVGSVININNLAINLPSILGNRVAPK
LWIRPTGSSAPYSEDNNPIINLSGPLSLLDDENLDPYDTADLAQPIAEVPLLYLLDVTAKHINTDNFYPEGLNT
TQHYGYQGVWSPYWIETITTSDTSSEDTVNTLHRQLYGDWTPTGYKVNPENKGDIALSAFWQSFHNLFATL

RYQTQQGQIAPTASGEATRLFVHQNSNNDAKGFHMEATGYSLGTTSNTASNHSFGVNFSQLFSNLYESHSDN
SVASHTTTVALQINNPWLQERFSTSASLAYSYSNHHIKASGYSGKIQTEGKCYSTTLGAALSCSLSLQWRSRP
LHFTPFIQAIAVRSNQTAFQESGDKARKFSVHKPLYNLTVPLGIQSAWESKFRLPTYWNIELAYQPVLYQQNP
EVNVSLESSGSSWLLSGTTLARNAIAFKGRNQIFIFPKLSVFLDYQGSVSSSTTTHYLHAGTTFKF

>core/29/1/Org1_Gene734

MFWKLLCPILICTSLSITSCEQQFKVVPNQCPLQVSTPAAADQKIEKIICSNGLPLLIISDPNLPTSGAALLVKTG
NNADPEEYPGMAHFTEHCVFLGNEKYPEVSGFPGFLSENNGVHNAFTYPNKTVFVFSVEHSAFSDALDQFVH
LFINPKFRQEDLDREKYAVHQEFAAHPLSDGRRVHRIQQLVAPQGHPCARFGCGNASTLTPVTTEKMAEWF
KLHYSPENMCAIAYTSAPLSKAKKQFSKIFSQIPRSKNYERQEPFLPSGDTSSLKNLYINQAIQPTSNLEIYWHI
YESSHPIPLGCYKALAEVLRNESKNSLVSLLKNEQLITDLDVEFFRSSLNTGEFYISYELTEKGDKHYSQVIDST
FQYLRYIQEHGIPNYTLEEISTINALNYCYSSKSPLFDLLCKQIVSLGNEDLSTYPYHSLVYPKYSSEDESALLN
LVSDPEQARFVLSSKNSEHWEEATQLHDPIFDMTYYVKALDGVQDYGKVQSLKPIALPKPNLFIPKEVTLPG
VHLLKKQEFPFAPALSYQDDKLTLYHCEDHYYTAPKLSSQIRIRSPQISRSSPQFLVATELYCLAVNDQLLREY
YPATQAGLSFTSALGGDGIDLRVSGYTTTVPALLNSILTSLPNLEISYETFLVYKKQLLELYQGALLNCPVRSG
LDELASQVMKETYSNTTKLSALEKLSFSEFQAFASNLFNSVHLEVMVLGNLSEQQKKDYLEMLQVFTASRSS
HATKPFYYELQSQEISEIHHDYPLTANGMLLLLQDKSSPSIQGKVCAEMLFEWLHHITFEELRTQQQLGYMV
GARYREFASRPFGFLYIRSDAYSPEELLAKTSLFLNKVSASPEKFGISQEKFANIRKAYINKILEPEHSLDMMNS
ALFSLAFERPFVEFSTPDLKIAIAETLTYEEFLKYCQCFLSNELGTQTSVYIRGTQKTS

>core/30/1/Org1_Gene56

MTTEDFPKAYNFQDTEPELYVFWEKNGMFKAEASSDKPPYSVIMPPPNVTGVLHMGHALVNTLQDVLVRY
KRMSGFEVCWIPGTDHAGIATQAVVERHLQASEGKRRTDYSREDFLKHIWAWKEKSEKVVLSQLRQLGCSC
DWDRKRFTMEPLANRAVKKAFKTLFENGYIYRGYYLVNWDPVLQTALADDEVEYEEKDGWLYYIRYRMV
GSQESIVVATTRPETSLGDTGIAVSPNDERYASWIGASVEVPFVNRQIPIIGDASVDPTFGTGAVKVTPAHDKD
DYLMGTNHHLPMINILTPSGGINENGGPFAGMAKEKAREEILIALEEQGLFVRKEPYKLRVGVSYRSGAVIEP
YLSKQWFVSVSEFRGALREFVESQDIKIFPKDFVKNYLSWVNHLRDWCISRQLWWGHRIPVWYHKNDDERV
LCYDGEGIPEEVAQDPDSWYQDPDVLDTWFSSGLWPLTCLGWPDENSPDLKKFYPTALLVTGHDILFFWVT
RMVLLCSSMSGEKPFSEVFLHGLIFGKSYKRYNDFGEWSYISGKEKLAYDMGEALPDGVVAKWEKLSKSKG
NVIDPLEMIATYGTDAVRLTLCSCANRGEQIDLDYRLFEEYKHFANKVWNGARFIFGHISDLQGKDLLAGIDE
DSLGLEDFYILDGFNQLIHQLEEAYATYAFDKVATLAYEFFRNDLCSTYIEIIKPTLFGKQGNEASQSTKRTLL
AVLLINVLGVLHPVAPFITESLFLRIQDTLGALPEGDGDAFTGHALRMLRSRACMEAPYPKAFDVKIPQDLRE
SFTLAQRLVYTIRNIRGEMQLDPRLHLKAFVVCSDTTEIQSCIPILQALGGLESIQLLDKEPEKGLYSFGVVDTI
RLGIFVPEEHLLKEKGRLEKERVRLERAVENLERLLGDESFCQKANPNLVVAKQEALKNNRIELQGILDKLAS
FA

>core/31/1/Org1_Gene993

MRFFCFGMLLPFTFVLANEGLQLPLETYITLSPEYQAAPQVGFTHNQNQDLAIVGNHNDFILDYKYYRSNGG
ALTCKNLLISENIGNVFFEKNVCPNSGGAIYAAQNCTISKNQNYAFTTNLVSDNPTATAGSLLGGALFAINCSI
TNNLGQGTFVDNLALNKGGALYTETNLSIKDNKGPIIIKQNRALNSDSLGGGIYSGNSLNIEGNSGAIQITSNSS
GSGGGIFSTQTLTISSNKKLIEISENSAFANNYGSNFNPGGGGLTTTFCTILNNREGVLFNNNQSQSNGGAIHAK

SIIIKENGPVYFLNNTATRGGALLNLSAGAGNGSFILSADNGDIIFNNNTASKHALNPPYRNAIHSTPNMNLQI
GARPGYRVLFYDPIEHELPSSFPILFNFETGHTGTVLFSGEHVHQNFTDEMNFFSYLRNTSELRQGVLAVEDG
AGLACYKFFQRGGTLLLGQGAVITTAGTIPTPSSTPTTVGSTITLNHIAIDLPSILSFQAQAPKIWIYPTKTGSTY
TEDSNPTITISGTLTLRNSNNEDPYDSLDLSHSLEKVPLLYIVDVAAQKINSSQLDLSTLNSGEHYGYQGIWST
YWVETTTITNPTSLLGANTKHKLLYANWSPLGYRPHPERRGEFITNALWQSAYTALAGLHSLSSWDEEKGH
AASLQGIGLLVHQKDKNGFKGFRSHMTGYSATTEATSSQSPNFSLGFAQFFSKAKEHESQNSTSSHHYFSGM
CIENTLFKEWIRLSVSLAYMFTSEHTHTMYQGLLEGNSQGSFHNHTLAGALSCVFLPQPHGESLQIYPFITALA
IRGNLAAFQESGDHAREFSLHRPLTDVSLPVGIRASWKNHHRVPLVWLTEISYRSTLYRQDPELHSKLLISQG
TWTTQATPVTYNALGIKVKNTMQVFPKVTLSLDYSADISSSTLSHYLNVASRMRF

>core/32/1/Org1_Gene327

MKSSVSWLFFSSIPLFSSLSIVAAEVTLDSSNNSYDGSNGTTFTVFSTTDAAAGTTYSLLSDVSFQNAGALGIPL
ASGCFLEAGGDLTFQGNQHALKFAFINAGSSAGTVASTSAADKNLLFNDFSRLSIISCPSLLLSPTGQCALKSV
GNLSLTGNSQIIFTQNFSSDNGGVINTKNFLLSGTSQFASFSRNQAFTGKQGGVVYATGTITIENSPGIVSFSQN
LAKGSGGALYSTDNCSITDNFQVIFDGNSAWEAAQAQGGAICCTTTDKTVTLTGNKNLSFTNNTALTYGGAI
SGLKVSISAGGPTLFQSNISGSSAGQGGGGAINIASAGELALSATSGDITFNNNQVTNGSTSTRNAINIIDTAKV
TSIRAATGQSIYFYDPITNPGTAASTDTLNLNLADANSEIEYGGAIVFSGEKLSPTEKAIAANVTSTIRQPAVLA
RGDLVLRDGVTVTFKDLTQSPGSRILMDGGTTLSAKEANLSLNGLAVNLSSLDGTNKAALKTEAADKNISLS
GTIALIDTEGSFYENHNLKSASTYPLLELTTAGANGTITLGALSTLTLQEPETHYGYQGNWQLSWANATSSKI
GSINWTRTGYIPSPERKSNLPLNSLWGNFIDIRSINQLIETKSSGEPFERELWLSGIANFFYRDSMPTRHGFRHIS
GGYALGITATTPAEDQLTFAFCQLFARDRNHITGKNHGDTYGASLYFHHTEGLFDIANFLWGKATRAPWVLS
EISQIIPLSFDAKFSYLHTDNHMKTYYTDNSIIKGSWRNDAFCADLGASLPFVISVPYLLKEVEPFVKVQYIYA
HQQDFYERHAEGRAFNKSELINVEIPIGVTFERDSKSEKGTYDLTLMYILDAYRRNPKCQTSLIASDANWMA
YGTNLARQGFSVRAANHFQVNPHMEIFGQFAFEVRSSSRNYNTNLGSKFCF

>core/33/1/Org1_Gene339

MFGMTPAVYSLQTDSLEKFALERDEEFRTSFPLLDSLSTLTGFSPITTFVGNRHNSSQDIVLSNYKSIDNILLLW
TSAGGAVSCNNFLLSNVEDHAFFSKNLAIGTGGAIACQGACTITKNRGPLIFFSNRGLNNASTGGETRGGAIA
CNGDFTISQNQGTFYFVNNSVNNWGGALSTNGHCRIQSNRAPLLFFNNTAPSGGGALRSENTTISDNTRPIYF
KNNCGNNGGAIQTSVTVAIKNNSGSVIFNNNTALSGSINSGNGSGGAIYTTNLSIDDNPGTILFNNNYCIRDGG
AICTQFLTIKNSGHVYFTNNQGNWGGALMLLQDSTCLLFAEQGNIAFQNNEVFLTTFGRYNAIHCTPNSNLQ
LGANKGYTTAFFDPIEHQHPTTNPLIFNPNANHQGTILFSSAYIPEASDYENNFISSSKNTSELRNGVLSIEDRA
GWQFYKFTQKGGILKLGHAASIATTANSETPSTSVGSQVIINNLAINLPSILAKGKAPTLWIRPLQSSAPFTEDN
NPTITLSGPLTLLNEENRDPYDSIDLSEPLQNIHLLSLSDVTARHINTDNFHPESLNATEHYGYQGIWSPYWVE
TITTTNNASIETANTLYRALYANWTPLGYKVNPEYQGDLATTPLWQSFHTMFSLLRSYNRTGDSDIERPFLEI
QGIADGLFVHQNSIPGAPGFRIQSTGYSLQASSETSLHQKISLGFAQFFTRTKEIGSSNNVSAHNTVSSLYVELP
WFQEAFATSTVLAYGYGDHHLHSLHPSHQEQAEGTCYSHTLAAAIGCSFPWQQKSYLHLSPFVQAIAIRSHQ
TAFEEIGDNPRKFVSQKPFYNLTLPLGIQGKWQSKFHVPTEWTLELSYQPVLYQQNPQIGVTLLASGGSWDIL
GHNYVRNALGYKVHNQTALFRSLDLFLDYQGSVSSSTSTHHLQAGSTLKF

>core/34/1/Org1_Gene57

MERYDIVRIIGKGGMGEVYLAYDPVCSRKVALKKIREDLAENPLLKRRFLREARIAADLIHPGVVPVYTIYSE
KDPVYYTMPYIEGYTLKTLLKSVWQKESLSKELAEKTSVGAFLSIFHKICCTIEYVHSRGILHRDLKPDNILLG
LFSEAVILDWGAAVACGEEEDLLDIDVSKEEVLSSRMTIPGRIVGTPDYMAPERLLGHPASKSTDIYALGVVL
YQMLTLSFPYRRKKGKKIVLDGQRIPSPQEVAPYREIPPFLSAVVMRMLAVDPQERYSSVTELKEDIESHLKG
SPKWTLTTALPPKKSSSWKLNEPILLSKYFPMLEVSPASWYSLAISNIESFSEMRLEYTLSKKGLNEGFGILLPT
SENALGGDFYQGYGFWLHIKERTLSVSLVKNSLEIQRCSQDLESDKETFLIALEQHNHSLSLFVDGTTWLIHM
NYLPSRSGRVAIIVRDMEDILEDIGIFESSGSLRVSCLAVPDAFLAEKLYDRALVLYRRIAESFPGRKEGYEARF
RAGITVLEKASTDNNEQEFALAIEEFSKLHDGVAAPLEYLGKALVYQRLQEYNEEIKSLLLALKRYSQHPEIF
RLKDHVVYRLHESFYKRDRLALVFMILVLEIAPQAITPGQEEKILVWLKDKSRATLFCLLDPTVLELRSSKME
LFLSYWSGFIPHLNSLFHRAWDQSDVRALIEIFYVACDLHKWQFLSSCIDIFKESLEDQKATEEIVEFSFEDLGA
FLFAIQSIFNKEDAEKIFVSNDQLSPILLVYIFDLFANRALLESQGEAIFQALDLIRSKVPENFYHDYLRNHEIRA
HLWCRNEKALSTIFENYTEKQLKDEQHELFVLYGCYLALIQGAEAAKQHFDVCREDRIFPASLLARNYNRLG
LPKDALSYQERRLLLRQKFLYFHCLGNHDERDLCQTMYHLLTEEFQL

>core/35/1/Org1_Gene832

MKIPLHKLLISSTLVTPILLSIATYGADASLSPTDSFDGAGGSTFTPKSTADANGTNYVLSGNVYINDAGKGTA
LTGCCFTETTGDLTFTGKGYSFSFNTVDAGSNAGAAASTTADKALTFTGFSNLSFIAAPGTTVASGKSTLSSA
GALNLTDNGTILFSQNVSNEANNNGGAITAKTLSISGNTSSITFTSNSAKKLGGAIYSSAAASISGNTGQLVFM
NNKGETGGGALGFEASSSITQNSSLFFSGNTATDAAGKGGAIYCEKTGETPTLTISGNKSLTFAENSSVTQGG
AICAHGLDLSAAGPTLFSNNRCGNTAAGKGGAIAIADSGSLSLSANQGDITFLGNTLTSTSAPTSTRNAIYLGS
SAKITNLRAAQGQSIYFYDPIASNTTGASDVLTINQPDSNSPLDYSGTIVFSGEKLSADEAKAADNFTSILKQPL
ALASGTLALKGNVELDVNGFTQTEGSTLLMQPGTKLKADTEAISLTKLVVDLSALEGNKSVSIETAGANKTIT
LTSPLVFQDSSGNFYESHTINQAFTQPLVVFTAATAASDIYIDALLTSPVQTPEPHYGYQGHWEATWADTSTA
KSGTMTWVTTGYNPNPERRASVVPDSLWASFTDIRTLQQIMTSQANSIYQQRGLWASGTANFFHKDKSGTN
QAFRHKSYGYIVGGSAEDFSENIFSVAFCQLFGKDKDLFIVENTSHNYLASLYLQHRAFLGGLPMPSFGSITD
MLKDIPLILNAQLSYSYTKNDMDTRYTSYPEAQGSWTNNSGALELGGSLALYLPKEAPFFQGYFPFLKFQAV
YSRQQNFKESGAEARAFDDGDLVNCSIPVGIRLEKISEDEKNNFEISLAYIGDVYRKNPRSRTSLMVSGASWT
SLCKNLARQAFLASAGSHLTLSPHVELSGEAAYELRGSAHIYNVDCGLRYSF

>core/37/1/Org1_Gene328

MKSSLHWFLISSSLALPLSLNFSAFAAVVEINLGPTNSFSGPGTYTPPAQTTNADGTIYNLTGDVSITNAGSPTA
LTASCFKETTGNLSFQGHGYQFLLQNIDAGANCTFTNTAANKLLSFSGFSYLSLIQTTNATTGTGAIKSTGACS
IQSNYSCYFGQNFSNDNGGALQGSSISLSLNPNLTFAKNKATQKGGALYSTGGITINNTLNSASFSENTAANN
GGAIYTEASSFISSNKAISFINNSVTATSATGGAIYCSSTSAPKPVLTLSDNGELNFIGNTAITSGGAIYTDNLVL
SSGGPTLFKNNSAIDTAAPLGGAIAIADSGSLSLSALGGDITFEGNTVVKGASSSQTTTRNSINIGNTNAKIVQL
RASQGNTIYFYDPITTSITAALSDALNLNGPDLAGNPAYQGTIVFSGEKLSEAEAAEADNLKSTIQQPLTLAGG
QLSLKSGVTLVAKSFSQSPGSTLLMDAGTTLETADGITINNLVLNVDSLKETKKATLKATQASQTVTLSGSLS
LVDPSGNVYEDVSWNNPQVFSCLTLTADDPANIHITDLAADPLEKNPIHWGYQGNWALSWQEDTATKSKAA
TLTWTKTGYNPNPERRGTLVANTLWGSFVDVRSIQQLVATKVRQSQETRGIWCEGISNFFHKDSTKINKGFR
HISAGYVVGATTTLASDNLITAAFCQLFGKDRDHFINKNRASAYAASLHLQHLATLSSPSLLRYLPGSESEQP
VLFDAQISYIYSKNTMKTYYTQAPKGESSWYNDGCALELASSLPHTALSHEGLFHAYFPFIKVEASYIHQDSF

KERNTTLVRSFDSGDLINVSVPIGITFERFSRNERASYEATVIYVADVYRKNPDCTTALLINNTSWKTTGTNLS
RQAGIGRAGIFYAFSPNLEVTSNLSMEIRGSSRSYNADLGGKFQF

>core/38/1/Org1_Gene330

MKTSIPWVLVSSVLAFSCHLQSLANEELLSPDDSFNGNIDSGTFTPKTSATTYSLTGDVFFYEPGKGTPLSDSC
FKQTTDNLTFLGNGHSLTFGFIDAGTHAGAAASTTANKNLTFSGFSLLSFDSSPSTTVTTGQGTLSSAGGVNL
ENIRKLVVAGNFSTADGGAIKGASFLLTGTSGDALFSNNSSSTKGGAIATTAGARIANNTGYVRFLSNIASTSG
GAIDDEGTSILSNNKFLYFEGNAAKTTGGAICNTKASGSPELIISNNKTLIFASNVAETSGGAIHAKKLALSSGG
FTEFLRNNVSSATPKGGAISIDASGELSLSAETGNITFVRNTLTTTGSTDTPKRNAINIGSNGKFTELRAAKNHT
IFFYDPITSEGTSSDVLKINNGSAGALNPYQGTILFSGETLTADELKVADNLKSSFTQPVSLSGGKLLLQKGVT
LESTSFSQEAGSLLGMDSGTTLSTTAGSITITNLGINVDSLGLKQPVSLTAKGASNKVIVSGKLNLIDIEGNIYES
HMFSHDQLFSLLKITVDADVDTNVDISSLIPVPAEDPNSEYGFQGQWNVNWTTDTATNTKEATATWTKTGF
VPSPERKSALVCNTLWGVFTDIRSLQQLVEIGATGMEHKQGFWVSSMTNFLHKTGDENRKGFRHTSGGYVI
GGSAHTPKDDLFTFAFCHLFARDKDCFIAHNNSRTYGGTLFFKHSHTLQPQNYLRLGRAKFSESAIEKFPREIP
LALDVQVSFSHSDNRMETHYTSLPESEGSWSNECIAGGIGLDLPFVLSNPHPLFKTFIPQMKVEMVYVSQNSF
FESSSDGRGFSIGRLLNLSIPVGAKFVQGDIGDSYTYDLSGFFVSDVYRNNPQSTATLVMSPDSWKIRGGNLSR
QAFLLRGSNNYVYNSNCELFGHYAMELRGSSRNYNVDVGTKLRF

>core/39/1/Org1_Gene841

MQRVLRLLFNLHHGEEKRAFLFFLLGLVWGIGCYGTLSLAEGLFIEKLGSAELPKIYLGSSLILCVLSSLILYNL
FKKHISATALFLIPVSLSILCNFYLILSSIFAIDPPRSPLFFYRIVIWSLTILSYTSFWGFVDQFFNLQDGKRHFCIF
NAIIFLGDAIGSGIIASLVHTIGIQGILILFTAALVLTFPIVFYVSKSLKSLSDDHDLFIDTGHPPPLSKALKLCFYD
KYTFYLLCFYFLMQLLAIATEFNYLKIFEIQFASKEEFELVAHIGKCSLWISLGNMCFALFAYSRIVKRLGVNNI
ILFAPLCFLSLFLFWTFKTTLSIAVLAMVVREGVTYALDDNNLQLLIYGVPNKIRNQIRIVVESFIEPIGMLVWS
LVCFLSSQQYVFCLIISLIATILVCLVRSYYAKAILKNLSAQALQLTRSMQDWIKSMTVKQKRQVELFLLAHL
KHPSERHQTFAFQHLLNLASRSVLPSLLAHMNKLSLPNKLKTIEMVKSSLWAKDFLTLELLKRWTSIFPHPAI
ASAIHLYFAEHDLLHITHIAEDLYDTVGDRLLAAILTVRRQEAYGPYRDLADKRLKELLNSDQPEDIVMGLTI
LKLEKNPQNFPILLDFLNTKNEDILIVTCKALHTSVRANHKPYCPELLKRLRQCSHNDEASQYLLKTISIALDIS
FVKDLLMTTSQLKNTSRKYAEAMIGELDKEVAPAFLQVLTDEGTHNRCRILAAKALCKIDNWLLKKHAYKI
VKSKASKALFYSYHGHYIQKKYPTYNLSLLANTLNSNYYAEVNFMLSLLGILGSMEHSGVLIRALTSKNQKI
KAQALESLEKNCDSHLFSLLEPFVNQPGMCYSEKYYFKCGVIPLTLKELLNMMENSPSSLNKLTAQQLKEEL
SYCDPDFQSVNTIYNQEHEDFRTEESETLISFLSI

>core/40/1/Org1_Gene893

MRFSLCGFPLVFSFTLLSVFDTSLSATTISLTPEDSFHGDSQNAERSYNVQAGDVYSLTGDVSISNVDNSALNK
ACFNVTSGSVTFAGNHHGLYFNNISSGTTKEGAVLCCQDPQATARFSGFSTLSFIQSPGDIKEQGCLYSKNAL
MLLNNYVVRFEQNQSKTKGGAISGANVTIVGNYDSVSFYQNAATFGGAIHSSGPLQIAVNQAEIRFAQNTAK
NGSGGALYSDGDIDIDQNAYVLFRENEALTTPIGKGGAVCCLPTSGSSTPVPIVTFSDNKQLVFERNHSIMGG
GAIYARKLSISSGGPTLFINNISYANSQNLGGAIAIDTGGEISLSAEKGTITFQGNRTSLPFLNGIHLLQNAKFLK
LQARNGYSIEFYDPITSEADGSTQLNINGDPKNKEYTGTILFSGEKSLANDPRDFKSTIPQNVNLSAGYLVIKE
GAEVTVSKFTQSPGSHLVLDLGTKLIASKEDIAITGLAIDIDSLSSSSTAAVIKANTANKQISVTDSIELISPTGN

AYEDLRMRNSQTFPLLSLEPGAGGSVTVTAGDFLPVSPHYGFQGNWKLAWTGTGNKVGEFFWDKINYKPRP
EKEGNLVPNILWGNAVDVRSLMQVQETHASSLQTDRGLWIDGIGNFFHVSASEDNIRYRHNSGGYVLSVNN
EITPKHYTSMAFSQLFSRDKDYAVSNNEYRMYLGSYLYQYTTSLGNIFRYASRNPNVNVGILSRRFLQNPLMI
FHFLCAYGHATNDMKTDYANFPMVKNSWRNNCWAIECGGSMPLLVFENGRLFQGAIPFMKLQLVYAYQG
DFKETTADGRRFSNGSLTSISVPLGIRFEKLALPQDVLYDFSFSYIPDIFRKDPSCEAALVISGDSWLVPAAHVS
RHAFVGSGTGRYHFNDYTELLCRGSIECRPHARNYNINCGSKFRF

>core/41/1/Org1_Gene531

MKTVILNIGRKILQGIKKKKKKIGILSGLFFLDLVLLGVSSQRPTETSANVKHNLRDEKLAACPKNSAASLSAK
KSHTKKTTPGSIPSKVFSKFDATQDKTFQKTSGSAFPAKPTTLKELEERKKPRPERRTTADVKRSPRFLPTQEV
EEPVPAASKEQLDSIQVWEEKQNYARRAVNAINLSIKKQLEEQTSTVTEKDVQPKTQATPHASKKNVASPST
SMPGIEKAATTVAVPQDKSEEEKVKERLTKRELTCEDLKDNGYTVNFEDISILELLQFVSKISGTNFVFDSNDL
QFNVTIVSHDPTSVDDLSTILLQVLKMHDLKVVEQGNNVLIYRNPHLSKLSTVVTDSSLKETCEAVVVTRVFR
LYSVSPSAAVNIIQPLLSHDAIVSASEATRHVIISDIAGNVDKVSDLLAALDCPGTSVDMTEYEVKYANPAALV
SYCQDVLGTLAEDDAFQMFIQPGTNKIFVVSSPRLANKAEQLLKSLDVPEMAHTLDDPASTALALGGTGTTS
PKSLRFFMYKLKYQNGEVIANALQDIGYNLYVTTAMDEDFINTLNSIQWLEVNNSIVIIGNQGNVDRVIGLLN
GLDLPPKQVYIEVLILDTSLEKSWDFGVQWVALGDEQSKVAYASGLLNNTGIATPTKATVPPGTPNPGSIPLP
TPGQLTGFSDMLNSSSAFGLGIIGNVLSHKGKSFLTLGGLLSALDQDGDTVIVLNPRIMAQDTQQASFFVGQT
VPYQTTNTIIQETGTVTQNIDYEDIGVNLVVTSTVAPNNVVTLQIEQTISELHSASGSLTPVTDKTYAATRLQIP
DGCFLVMSGHIRDKTTKVVSGVPLLNSIPLIRGLFSRTIDQRQKRNIMMFIKPKVISSFEEGTRVTNKEGYRYN
WEADEGSMQVAPRHAPECQGPPSLQAESDFKIIEIEAQ

>core/43/1/Org1_Gene766

MSKDKKMKPEPKKNFPTVFFFLLFGVVFGVVAFQNFLAGKKARVGFSHQIEHLVNLRLIVPEDSHKIALNDN
LVSFGGRFRDVQTQEGQLRYHYLELIDQGHRLDLDLQETSKSLTTLGKEVTNSILWFSAISGSPIPEQGYAISY
PSEVSGSVLTEPLVVTGPATPQLINLHSLQERYPTLSRSPEALRTYGSDLYELIGKYLSPVLGIGSETLKRELKD
LYQQVEVSLTQETDTEAAYTLYGQVLSTLNRISSSLVVSEGGERFSQLRSVRLYREEWNKYHKLVEARDLNQ
AQLEKLRGELSQTVWYFNNQELSSRSLEKQDPEVFGHWFAGAKEEWTAFKFNHSLSFKAPDQPRNLVLEKT
FKSQEPSPHYLGYLFTFLPIILVLLFVYLVFSRQMRGMSGSAMSFGKSPARMLLKGQNKVTFADVAGIEEAKE
ELIEIVDFLKNPNKFTSLGGRIPKGVLLIGPPGTGKTLIAKAVSGEADRPFFSIAGSDFVEMFVGVGASRIRDMF
EQAKRNAPCIIFIDEIDAVGRHRGAGIGGGHDEREQTLNQLLVEMDGFGTNEGVILMAATNRPDVLDKALLR
PGRFDRRVVMNLPDIKGRFEILMVHAKRIKLDPTVDLMAVARSTPGASGADLENLLNEAALLAARKDRTAV
TAVDVAEARDKVLYGKERRSLEMDAEERKTTAYHESGHAVVGLCVQHGDPVDKVTIIPRGLSLGATHFLPE
KNKLSYWKKELYDQLAVLMGGRAAEEIFLGDISSGAQQDISQATKLVRSMVCEWGMSPQLGNVTYDERSD
GLTGYGGYHEKSYSEETAKTIDTELRMLLDAAYQRALDIINEHKAEIELMTQMLIEFETLDSKDVKEIMDHT
WDPEKKRARLKEEGMLFKKSSDDLPPPPPKEDTLPGLGFNAT

>core/44/1/Org1_Gene273

MDSEFVGQVYSSDMDWIESMYQRFMNHETLDPSWKYFFEGYQLGQAASPSEASTKISGNETIAMLQEQKSQ
FLCTIYRYYGYLQSQISTLAPTTDSRFIQEKIAKIDLDEQVPSAGLLPKAQVSVRELIEALKKCYCGSLTLETLT
CTPELQEFVWNLMEKRQVERFAEQLLRSYKDLCKATFFEEFLQIKFTGQKRFSLEGGETLVPMLEHLVHYGS

ALGISNYVLGMAHRGRLNVLTNVLGKPYRYVFMEFEDDPAARGLESVGDVKYHKGYVLKSHQKDRETTFV
MLPNASHLESVDPIVEGVVAALQHQGHAGKEQSSLAILVHGDAAFSGQGVVYETLQLSRVPGYSTEGTLHIV
VNNYIGFTAVPRESRSTPYCTDIAKMLGIPVFRVNSEDVVACIEAIEYALQVRERFSCDVIIDLCCYRKYGHNE
SDDPSVTAPLLYDQIKRKKSIRELFRQYLLEGQFADISEETLASIEKEIQESLNREFQVLKGTDPEPFPKKECHH
CDRLNNGELILHDCDVSLDRETLFHMSSRLCGFPDNFHPHPKIKTLLEKRMKMAEGGVGYDWAMAEELAFA
SLLIEGYNLRLSGQDSIRGTFSQRHLVWSDTVTGDTYSPLYHLSAEQGSVEMYNSPLSEYAILGFEYGYAQQA
LKTLVLWEAQFGDFANGAQIIFDQYISSGIQKWDLHSDIVLLLPHGYEGQGPEHSSSRIERYLQLAANWNFQV
VLPSTPVQYFRILREHAKRDLSLPLVIFTPKLLLRYPQCVSSIEEFTEPGGFRAILEDADPNYDASILVLCSGKIY
YDYAEMLPQDRRKDFSCLRIESLYPLALEDLVSLIDKYSHLKHFVWLQEESKNMGAYDYMFMALQDILPEK
LLYIGRPRSSSTASGSAKLSRQELVTCMETLFSLR

>core/45/1/Org1_Gene230

MEKVKLTKNLKLKIKNAQLTKAAGLDKLKQKLAQAGSSEAKSSSEKPSAKEKSVKVALAATSTPTASAEQA
SPESTSRRIRAKNRSSFSSSEEESSAHIPVDTSEPAPVSIADPEPELEVVDEVCDESPEVHPVAEVLPEQPVLPET
PPQEKELEPKPVKPAEPKSVVMIKSKFGPTGKHINHLLAKTFKAPAKEEKVVAGSKSTKPVASDKTGKPGTSE
GGEQNNREKQFNPANRSPASGPKRDAGKKNLTDFRDRSKKSDESLKAFTGRDRYGLNEGGEEDRWRKKRV
YKPKKHYDEASIQRPTHIKISLPITVKDLAAEMKLKASEVIQKLFIHGMTYVVNDILDSETAVQFIGLEFGCTID
IDYSEQDKLCLSNDTVRDEIQSTDPSKLVIRSPIVAFMGHVDHGKTTLIDSLRKSNVAATEAGAITQHMGAFC
CSTPVGDITILDTPGHEAFSAMRARGAEVCDIVVLVVAGDEGIKEQTLEAIEHAKAADIAIVVAINKCDKPNF
NSETIYRQLSEINLLPEAWGGSTVTVNTSAKTGEGLSELLEMLALQAEVLELKADPSARARGLVIESELHKGL
GPVATVLIQNGSLKLGEALVFNDCYGKVKTMHNEHNELMKEAGPSIPVLITGLSDIPKAGDPFFVVKNEKTA
RDIIEARSAGQQRFALQQKKRPNFDSMLQNKKTLKLMIKADVQGSIEALVSSISKIKSEKVDVEILTNSVGEIS
ESDIRLAAASKAVLIGFHTGIESHAEPLIKSLGVRVELFTVIYHAIDAIKEIMTSLLDPIAEEKDEGSAEIKEIFRS
SQVGSIYGCIVTEGIMTRNHKVRVLRNKEILWKGTLSSLKRVKEDVKEVRKGLECGILLEGYQQAQIGDVLQ
CYEVIYHPQKL

>core/46/1/Org1_Gene682

MLSNTIRSNFLKFYANRHHTILPSSPVFPHNDPSILFTNAGMNQFKDIFLNKEKVSYSRATTSQKCIRAGGKHN
DLDNVGHTSRHLTFFEMLGNFSFGDYFKAEAIAFAWEVSLSVFNFNPEGIYATVHEKDDEAFALWEAYLPTD
RIFRLTDKDNFWSMANTGPCGYCSELLFDRGPSFGNASSPLDDTDGERFLEYWNLVFMEFNRTSEGSLLALP
NKHVDTGAGLERLVSLIAGTHTVFEADVLRELIAKTEQLSGKVYHPDDSGAAFRVIADHVRSLSFAIADGLLP
GNTERGYVLRKILRRSVNYGRRLGFRNPFLAEIVPSLADAMGEAYPELKNSLSQIQKVLTLEEESFFKTLDRG
GNLLQQVLKSSSSSSCISGEDAFKLKDTYGMPIDEISLLAKDYDYSVDMDTFHKLEQEAKERSRKNVVQSQG
TSESIYNELHLTSEFIGYDHLSCDTFIEAIISKDHIVSSLQEKQEGAIVLKVSPFYAEKGGQVGDSGEIFCSEGTFI
VTHTTSPKAGLIVHHGRISQGSLTVEAAVTAQVNRYRRKRIANNHTACHLLHKALEITLGDHIRQAGSYVDD
TKIRLDFTHPQAISPEDLLCIETLVNESIRENEPVDIREALYSDVMNSSEIKQFFGDKYSDVVRVVSAGHSHELC
GGTHAEATGDIGFFRITKEHAVAMGIRRIEAVTGEKAEATVHQQSEVLEEIATLLQVPRDQIVSRLTATLDER
KQQDKRLNELENSLIQTKLDKLIHNCHQRQGITCLVHHLAEHENHRLQQYAQCLHQRIPEKLISLWTTEKNG
KYIVLSRVSDDLITQGVHAQDLLKAVLTPCGGRWGGKDQSAQGSAPALPATEVLNETLWQWISTQLI

>core/47/1/Org1_Gene553

MSTFSIQNRLRTISGESTRIIKLDHKYSGFDPRSVPAINLEELNSGIYALRHLMNALQSENTNVAALLNPNNTIF
PTTSWTDYKHSRPQASSPRAPSSQTPTDIVSAAALALVLVIDGGLAELVASVTEIDLGALSTISTVRQLMASYL
GLTTLTAEQEKVVFSSSYVPSEKNLLEHVKQEKAAEIQAKQEEIKAVLEAKGVSTEEIEAILKEYPDIYAADFF
KEFIEEPLHTYRAKVGAPIQEMNENAIQLLPTPPAITPDNVNEVNGMNTLSTILQAIDDAIKQAPALGGDQEIIT
ILQTLVPLVDKTTFTKAEFDLIYTATQLPNTASLKLYLTDRQIAEYRGKITKVYQNSIQNLSETKRVVENNRSM
LETQLSMFQQAQNCFVTWISQANALNIAITNKYISAVLTTSMEMYGGLLCLSYMYERLADDEKAIFDKSVNE
YLPIHIVVGGSWVNGWIAKMAAYQELAEYSLGTAVTSQDQIKAYLQTRGNEFKATRHFFHNIGDQMYQFAN
ETVFGNCLTTANGAIQPDLGGFIREAMTNVGTVEADYVSNAQRILNEFNTAATAHVLQLQLQIAELQKKADD
LDPGKASFTENRKFAVAAWITSESLGDALISMILNSQLPKQEAFLKPLIEEINFNNLAANALNSLLQITNEFSTT
SVYYSLSSYLVQSKTGQNLFAGDYYETLLAAAREREYIYRDTARCKQAINLVNGLLQKINSLPGATSAQKQE
MLNATTYYQYSLSVTLNQLTVLESLLAGLKMTLQTTSNNKYDKSVFKIESFDDWIPTLAALESFLTSGFPNIS
ATGGLGPLFTQVQSDQQTYTSQGQTQQLNLQNQMTTIQQEWTLVSTSMQVLNGILSQLAGAIYSN

>core/48/1/Org1_Gene586

MKKSLIIVESPAKIKTLQKLLGSEFVFASSIGHIVDLPAKEFGIDVDHDFEPQYQVLPDKQEVINHIRKLAAKCE
KVYLSPDPDREGEAIAWHIANQLPDSPLIQRVSFNAITKNAVTEALKHPRTIDMALVNAQQARRLLDRIVGYK
ISPILSRKLQQRSGISAGRVQSVALKLVVDREKAIDAFVPVEYWNLRVLMQDPKTTKTFWAHLYAVQGKKW
EKEIPEGKTENDVLLINSEEKARHYAELLEKSSYTITRVEAKAKRRFAPPPFITSTLQQEASRHFRFSASRTMSI
AQTLYEGVDLDSEDSTGLITYMRTDSVRVDPEALTTVREYIQQTFGKEYLPEKANVYTTKKMTQDAHEAIRP
TDINLTPDKLKNKLSDDQFKVYNLIWKRFVASQITPAIYDTLAVQITTDTEIDLRASGSLLKFKGFLAVYEEKQ
DDENDQEEDHPLPPLHAQDALIKEEVSQEQAFTKPLPRFTEASLVKELEKSGIGRPSTYATIMNKIQSREYTTK
ENQRLRPTELGKIISQFLETNFPRIMDIGFTALMEDELELIADNKKPWKLLLQEFWTTFLPVVITAEKEAVIPRI
LTNIECSKCHKGKLVKIWSKNSYFYGCSEYPECDYRTSEEELAFNKEDYAEDTPWDSPCPLCGGVMKVRHG
RYGTFLGCEKYPECRGTISIHKKGEEIEQEEPIPCPAIGCNGKIFKKRSRYNKIFYSCSEYPECSVIGNSIDAVITK
YSGTEKIPYKKKTPTKKKSSAKTTKAAKTPSKKGKAKSSVKKSSEKKTGPLFLPSPDLAKMIGNEPVSRGEAT
KKIWDYIKEHQLQAPENKKLLVPDNNLATIIGPNPIDMFQLSKHLSQHLTKVSNDESSASS

>core/49/1/Org1_Gene949

MKKLFVLDASGFIFRAYFALPEMKNHQGQATQAVFGFIRSLNKLIKEFSPEYMISVFDGPNNKQSRQAIYADY
KSNRQKKFEDIPPQIALVKEYCSLIGLAYLEKESVEADDVIASIAKKAREENYKVYVCTADKDLLQLVNDHV
VAWNPWADQGVVGISEVIERYGIPPGNIPDYLALVGDSSDNIPGLPGCGPKKAAALLKQFGSVEGLLENLDA
VKGLSQTMLSERQETLKLSKRLALLDSNIPIPVPIESLTFPQHPVDEEKLIHFYIQQGFKTLVPSKQTEAATVDV
QIIKDAESLTNILNLVQGGDIAFAVAYTGNHLLSLKLEGLALTQGSGVFFIALEEEGTKILPILKDFFLREDLTF
YGYNLKRDCHALLNAGIVIREISYDLALAEHLTNGGGKISFQSLLVNHGFTETAHRFAKEWGNSGLPIGRLPE
QPEQYFGEFVAYLPIIKDAILEEINRKNLNHILSDIEMPLEKVLFSMERAGVPLDVEELAILEALFETELAVLTE
EIYDLSGGPFNIKSPKQLSDILYNELGLRPIDKAKSTRAEVLEALRSEHPIIEKLLAFRTIEKLLSTYVKALPKQV
DSHTQRIHPSFDQTGAVTGRLACRDPNLQNIPIRSERGILLRKAFRLSEKNSYFLSADYSQIELRFLAHLSQDKS
LKFAFESGEDIHAFTASQVFHVPLEQVSKEQRMQAKTVNFGIVYGQQAFGLAKVLKISIGEAQELIQAYFSRY
PEIAHFVEETIQQAAKDLRVTTMLGRERIIDSWNEFPGSRAASGRFAVNTRIQGSAAELIKLAMLDISQAIKQQ
QMKSRMLLQIHDELLFEVPEEEIEEMQRLVREKMESAMTLSVPIVVNILIGKNWAEC

>core/50/1/Org1_Gene93

MEKFSDAVSEALEKAFELAKSSKHTYVTENHLLLALLENTESLFYLVIKDIHGNPGLLNTAVKDALSREPTVV
EGEVDPKPSPGLQTLLRDAKQEAKTLGDEYISGDHLLLAFWSSNKEPFNSWKQTTKVSFKDLKNLITKIRRGN
RMDSPSAESNFQGLEKYCKNLTALAREGKLDPVIGRDEEIRRTIQVLSRRTKNNPMLIGEPGVGKTAIAEGLA
LRLIQGDVPESLKGKQLYVLDMGALIAGAKYRGEFEERLKSVLKDVESGDGEHIIFIDEVHTLVGAGATDGA
MDAANLLKPALARGTLHCIGATTLNEYQKYIEKDAALERRFQPIFVTEPSLEDAVFILRGLREKYEIFHGVRIT
EGALNAAVLLSYRYIPDRFLPDKAIDLIDEAASLIRMQIGSLPLPIDEKERELAALIVKQEAIKREQSPSYQEEA
DAMQKSIDALREELASLRLGWDEEKKLISGLKEKKNSLESMKFSEEEAERVADYNRVAELRYSLIPQLEEEIK
QDEASLNQRDNRLLQEEVDERLIAQVVANWTGIPVQKMLEGEAEKLLILEESLEERVVGQPFAVSAVSDSIR
AARVGLNDPQRPLGVFLFLGPTGVGKTELAKALADLLFNKEEAMVRFDMSEYMEKHSISKLIGSSPGYVGYE
EGGSLSEALRRRPYSVVLFDEIEKADKEVLNILLQVFDDGILTDGKKRKVNCKNALFIMTSNIGSPELADYCS
KKGSELTKEAILSVVSPVLKRYLSPEFMNRIDEILPFVPLTKEDIVKIVGIQMRRIAQRLKARRINLSWDDSVIL
FLSEQGYDSAFGARPLKRLIQQKVVILLSKALLKGDIKPDTSIELTMAKEVLVFKKVETPS

>core/51/1/Org1_Gene957

MAVRLIVDEGPLSGVIFVLEDGISWSIGRDSSANDIPIEDPKLGASQAIINKTDGSYYITNLDDTIPIVVNGVAIQ
ETTQLKNEDTILLGSNQYSFLSDEFDPQDLVYDFDIPEDNFSNDSGDLSDSNEQGKDLEPRQTSETNHSPKPKE
KLTKDQGSSDPITSGDQELADAFLASAKAEKNQPRAKVAKKGLKESSNESLNPKEQNAKDSPKGEERTNKPQ
NAIMEDNGASPRQDPQPKSAEPSLKNTARDETPLKENKPVEEKANKKATPDSPEKKDQPEEGSKIEATPLDSQ
KESEDKEAEEAFVQEEEENLTEDNKEDSDSAADANDDTASDHTAEDNKETPKKVENEKSAVLSPFHVQDLF
RFDQTIFPAEIDDIAKKNISVDLTQPSRFLLKVLAGANIGAEFHLDSGKTYILGTDPTTCDIVFNDLSVSHQHAK
ITVGNDGGILIEDLDSKNGVIVEGRKIDKTSTLSSNQVVALGTTLFLLIDHHAPADTIVASLSPDDYSLFGRQQ
DAEALERQEAQEEEEKQKRATLPAGSFILTLFVGGLAILFGIGTASLFHTKEVVPLENIDYQEDLAQVINQFPT
VRYTFNKTNSQLFLIGHVKNSTDKSELLYKVDALSFVKSVDDNVIDDEAVWQEMNILLSKRPEFKGISMHSP
EPGKFIITGYVKTEEQAACLVDYLNIHFNYLSLLENKVVVETQMLKAIAGHLLQGGFANIHVAFVNGEVILTG
YVNNDDAEKFRAVVQELSGIPGVRLVKNFAVLLPAEEGIIDLNLRYPNRYRVTGYSRYGEISINVVVNGRILT
RGDVIDGMTVTSIQPNAIFLEKEGLKYKIDYNK

>core/52/1/Org1_Gene833

MFEKFTNRAKQVIKLAKKEAQRLNHNYLGTEHILLGLLKLGQGVAVNVLRNLGIDFDTARQEVERLIGYGPE
IQVYGDPALTGRVKKSFESANEEASLLEHNYVGTEHLLLGILHQSDSVALQVLENLHIDPREVRKEILKELETF
NLQLPPSSSSSSSSSRSNPSSSKSPLGHSLGSDKNEKLSALKAYGYDLTEMVRESKLDPVIGRSSEVERLILILCR
RRKNNPVLIGEAGVGKTAIVEGLAQKIILNEVPDALRKKRLITLDLALMIAGTKYRGQFEERIKAVMDEVRKH
GNILLFIDELHTIVGAGAAEGAIDASNILKPALARGEIQCIGATTIDEYRKHIEKDAALERRFQKIVVHPPSVDE
TIEILRGLKKKYEEHHNVFITEEALKAAATLSDQYVHGRFLPDKAIDLLDEAGARVRVNTMGQPTDLMKLEA
EIENTKLAKEQAIGTQEYEKAAGLRDEEKKLRERLQSMKQEWENHKEEHQVPVDEEAVAQVVSLQTGIPSA
RLTEAESEKLLKLEDTLRRKVIGQNDAVTSICRAIRRSRTGIKDPNRPTGSFLFLGPTGVGKSLLAQQIAIEMFG
GEDALIQVDMSEYMEKFAATKMMGSPPGYVGHEEGGHLTEQVRRRPYCVVLFDEIEKAHPDIMDLMLQILE
QGRLTDSFGRKVDFRHAIIIMTSNLGADLIRKSGEIGFGLKSHMDYKVIQEKIEHAMKKHLKPEFINRLDESVI

FRPLEKESLSEIIHLEINKLDSRLKNYQMALNIPDSVISFLVTKGHSPEMGARPLRRVIEQYLEDPLAELLLKES
CRQEARKLRATLVENRVAFEREEEEQEAALPSPHLES

>core/53/1/Org1_Gene882

MKIPLRFLLISLVPTLSMSNLLGAATTEELSASNSFDGTTSTTSFSSKTSSATDGTNYVFKDSVVIENVPKTGET
QSTSCFKNDAAAGDLNFLGGGFSFTFSNIDATTASGAAIGSEAANKTVTLSGFSALSFLKSPASTVTNGLGAIN
VKGNLSLLDNDKVLIQDNFSTGDGGAINCAGSLKIANNKSLSFIGNSSSTRGGAIHTKNLTLSSGGETLFQGNT
APTAAGKGGAIAIADSGTLSISGDSGDIIFEGNTIGATGTVSHSAIDLGTSAKITALRAAQGHTIYFYDPITVTGS
TSVADALNINSPDTGDNKEYTGTIVFSGEKLTEAEAKDEKNRTSKLLQNVAFKNGTVVLKGDVVLSANGFSQ
DANSKLIMDLGTSLVANTESIELTNLEINIDSLRNGKKIKLSAATAQKDIRIDRPVVLAISDESFYQNGFLNEDH
SYDGILELDAGKDIVISADSRSIDAVQSPYGYQGKWTINWSTDDKKATVSWAKQSFNPTAEQEAPLVPNLLW
GSFIDVRSFQNFIELGTEGAPYEKRFWVAGISNVLHRSGRENQRKFRHVSGGAVVGASTRMPGGDTLSLGFA
QLFARDKDYFMNTNFAKTYAGSLRLQHDASLYSVVSILLGEGGLREILLPYVSKTLPCSFYGQLSYGHTDHR
MKTESLPPPPRRSRRIILLGEDMSGLESWELELLLKIPAAEDFSKSTLHL

>core/54/1/Org1_Gene842

MFNKDEIIVPKNLEEEMKESYLRYSMSVIISRALPDIRDGLKPSQRRVLYAMKQLSLSPGAKHRKCAKICGDT
SGDYHPHGESVIYPTLVRMAQNWAMRYPLVDGQGNFGSIDGDPPAAMRYTEARLTHSAMYLMEDLDKDT
VDIVPNYDETKHEPVVFPSKFPNLLCNGSSGIAVGMATNIPPHNLGELIEATLLLLANPQASVDEILQVMPGPD
FPTGGIICGSEGIRSTYTTGRGKIKVRARLHVEENEDKHRESIIITEMPYNVNKSRLIEQIANLVNEKTLAGISDV
RDESDKDGIRVVLEIKKGESSEIIINRLYKFTDVQVTFGANMLALDKNLPRTMSIHRMISAWIRHRKEVIRRRT
RYELNKAETRAHVLEGYLKALSCLDALVKTIRESGNKEHAKERIIESFGFTEPQALAILELRLYQLTGLEAEKI
QKEYEELLNKIAYYKQVLSDEGLVKDIIRNELQDLLKHHKVARRTTIEFDADDIRDIEDIITNESVIITISGDDY
VKRMPVKVFKEQRRGGHGVTGFDMKKGAGFLKAVYSAFTKDYLLIFTNFGQCYWLKVWQLPEGERRAKG
KPIINFLEGIRPGEELAAILNIKNFDNAGFLFLATKRGVVKKVSLDAFSNPRKKGIRALEIDEGDELIAACHIVS
DEEKVMLFTHLGMAVRFPHEKVRPMGRTARGVRGVSLKNEEDKVVSCQIVTENQSVLIVCDQGFGKRSLVE
DFRETNRGGVGVRSILINERNGNVLGAIPVTDHDSILLMSSQGQAIRINMQDVRVMGRSTQGVRLVHLKEGD
ALVSMEKLSSNENDDEVLSGSEEECSDTVSLR

>core/55/1/Org1_Gene958

MIYSTSISTFYKKLSLVSSMHSFAQRHRESLEHIANYEKTTAERDILKRLIEVLDQRASERYRSAVEKLHKYEV
ERATVAKSIPVAAIHEKPLSSTHASVQVTASTPAATGSGVGAYYNAVKQKWAQDLIVELNTVMTTIMASVNS
KNPANKDVFDKLNTELQALVAAGNNLTEENFQTLYNFPEEIFTAIQRADTFTGGMKTDFTNQLAGKYGNQA
TLTQTFADGRVEGFKDILTAVQGVLTPEQFTIFAEIATELQALADHVGNFDEAGLQRIEDAGEKLAAVINSSD
LTRNDKIMFCQHITDLYSDQVAALGSFDTVLDASIYVNQHQGTMFSNLSSFVGSLIGTFAPIDLSSSQGDISSA
ALAGALQTARGLNSRFNELTAEQQKLINECIKSLVTFKCGEHLGAIWAYFTASTVVALNPTATMDHVKAAIL
EEAKELDNSSFQLASSIKSAMTSIVNSSGSFSVTVNSSTLQYTIYSEKNGKVEINQILLNYGSIGFLPEITKLAKT
NAESTARSYFRFKALAAVESENVQNKIEDLQSQLQQFTNMKTELFDGQLLSQASELRALPLPSAVASVLIDRY
MPKEVDYLNEIYKKLYYSNLGSSVGNSIIDAISQYVNGATYFNFASYVGQQPAVGAGGANAFPGSQESAQAK
LDQERKQAALYLQETRGALTVIEEQRARVLKDDKITNEQRSTILDSLRNYEDNINSISGSLVLLQNYLQPLSIA

GGSVAGTFEVKEGQEQWQARLQILEEALVSGLVGNMINGGMFPLQSTIQSDQQSFADMGQNFQLDLQMHLT
SMQQEWTVVATSLQLLNQMYLSLARSLTG

>core/56/1/Org1_Gene867

MTEKKPTPMMEQWHQCKEKAGDSVLLFRMGDFYEAFYDDAVLLSQHLELTLTQRQGIPMSGIPVSTVDTYV
DRLIGKGFKVAVAEQFGEPAKEKESKKIGPMARDIQRFVTPGTLLSSTLLQEKFNNYIVAINRIGSLFGFACLD
LSTGSFFIEECENTKELVDEICRLAPSEVLSCNKFYNKETAIVMQLQQHLKLTLSTYADWAFEHKFASQKLTT
HFQVASLDGFGLKGLVPAINAAGGLLSYIQDKLLLPTKHIAIPQTRGKQQKLLIDTASQVNLELLAPLNDPQG
KNSLLRIMDHTSTPMGGRLLRQILISPFYNPKEILVRQDAVEFFLRQVTLRKNIKTYLCQVRDIERLMTKVTTG
LAGPRDIGTLRDSFSAGAQIYEQLASATLPEFFIDKCSLDTKLASLIALLSKSLNGDLPLRVSDGNIFVDEFHND
LKRLRHNQEHSQEWIWEYQERIRKETGIKKLKICFAQALGYYIEVSSEFAPQLPKDFIRRQSRLHAERFTTIEL
QQFQDDMSNISEKLQTLETQFFKDLCSHILQLRTEILALSQSLADLDYIISLADLAHAQGYCRPRVDMSDTLCI
YRGCHPVAKTLVDTGKFIPNDTEMRGSQTRMILLTGPNMAGKSTYIRQIALLVIMAQMGSYIPAKSAHIGVID
KIFTRIGAGDNLSKGMSTFMVEMAETANILHNATDRSLVILDEVGRGTSTYDGLAIAQAVVEYLLFTDKKKA
KTLFATHYKELTTLEDHCPHVENFHAGVKDKAGQPVFLYEILKGHSQKSFGIHVARLAGFPLCVVSRAQQIL
RQLEGPESITRPAQDKMQQLTLF

>core/57/1/Org1_Gene1020

MKRAILDRLYLSVVQSPESASPRDIFTAVAKTVMEWLAKGWLKTQNGYYKNDVKRVYYLSMEFLLGRSLK
SNLLNLGILDLVRKALKTLNYDFDHLVEMESDAGLGNGGLGRLAACYLDSMATLAVPAYGYGIRYDYGIFD
QRIVNGYQEEAPDEWLRYGNPWEICRGEYLYPVRFYGRVIHYTDSRGKQVADLVDTQEVLAMAYDIPIPGY
GNDTVNSLRLWQAQSPRGFEFSYFNHGNYIQAIEDIALIENISRVLYPNDSITEGQELRLKQEYFLVSATIQDIIR
RYTKTHICLDNLADKVVVQLNDTHPALGIAEMMHILVDREELPWDKAWEMTTVIFNYTNHTILPEALERWP
LDLFSKLLPRHLEIIYEINSRWLEKVGSRYPKNDDKRRSLSIVEEGYQKRINMANLAVVGSAKVNGVSSFHSQ
LIKDTLFKEFYEFFPEKFINVTNGVTPRRWIALCNPRLSKLLNETIGDRYIIDLSHLSLIRSFAEDSGFRDHWKG
VKLKNKQDLTSRIYNEVGEIVDPNSLFDCHIKRIHEYKRQLMNILRVIYVYNDLKENPNQDVVPTTVIFSGKA
APGYVMAKLIIKLINSVADVVNQDSRVNDKLKVLFLPNYRVSMAEHIIPGTDLSEQISTAGMEASGTGNMKF
ALNGALTIGTMDGANIEMAEHIGKENMFIFGLLEEQIVQLRREYCPQTICDKNPKIRQVLDLLEQGFFNSNDK
DLFKPIVHRLLHEGDPFFVLADLESYIAAHENVNKLFKEPDSWTKISIYNTAGMGFFSSDRAIQDYARDIWHV
PTKSCSGEGN

>core/58/1/Org1_Gene679

MKKLYHPTLFLRPLIRLSLIFALSLTLISGNFPQQKSFGHCCADMHSALISGKNCEELFADFIERVLADRETLTA
RDWGTVVVLVREYLLKCIRKGDCDYGVKILQKLLALRLPKDARKDLQILWHRLNPEQAPLRDVVDQLFTIG
CHESLQDHLLFELYTVTLHSGYENRKQDMLLAKEQGDYKKAIELAKELVAALEKGSCSPHPEIVQIEKTFLQ
KTLLALQIKVAQEAQESCDALLTPYCLSEIAYTEAMDALVLRIARGEVSRTNEVDSVLLSHALQHLPFAREKA
IPELEVLIDHGAYLESTLLYYAYFSLLELYHQNKDFASLERLLEKGDAVFVPEHPYFPEYGFFLGAYFYAKGK
YESAEKVFLQIIDPAVKLGATFARAYEYLGCIAYVQNHYEKAEEYFLRAYKSWGREESGIGLFLAYAVQKKK
TACEDMLYHPKFSFTYRHLLDSLCSLSYPHGENKGSSAIQRVHRAVPELSEIYSRCIYDMIKYRNVTYTHPIIE
LAYNQVRNLEKRNLEEICRDAQDPEYDKALAFWGALQSGASVPRSLIESSDVDEARITIRCYEALYFHNPDAI
AMLPQAFSEECNSWQTALRLVWTLVRPKGAPNHAKYWDHLVLRPHGDSLYFFGYDLQEYLIGKEDALKHL

SVFAELFPKSSLLSLVYYLQGYSESSALRKVGWFVKALEEFTEISWSGEHMKTWAYIYYMVKLDLADTYISL
GNFSQAVHILEEVKEDWQVASHPKLHFLKGEDCYLAMELRWVEGLAYAYFQLHETAHLSNHLLEHVEKNLI
SPRSYRDYYGESLQRTLGLCQRFLGV

>core/59/1/Org1_Gene98

MRYDPNLIEKKWQQFWKEHRSFQANEDEDKVKYYVLDMFPYPSGAGLHVGHLIGYTATDIVARYKRARGF
SVLHPMGWDSFGLPAEQYAIRTGTHPKVTTQKNIANFKKQLSAMGFSYDEGREFATSDPDYYHWTQKLFLF
LYDQGLAYMADMAVNYCPELGTVLSNEEVENGFSIEGGYPVERKMLRQWILKITAYADKLLEGLDALDWP
ENVKQLQKNWIGKSEGALVTFHLTQEGSLEAFTTRLDTLLGVSFLVIAPEHPDLDSIVSEEQRDEVTAYVQES
LRKSERDRISSVKTKTGVFTGNYAKHPITGNLLPVWISDYVVLGYGTGVVMGVPAHDERDREFAEMFSLPIH
EVIDDNGVCIHSNYNDFCLNGLSGQEAKDYVINYLEMRSLGRAKTMYRLRDWLFSRQRYWGEPIPIIHFEDG
THRPLEDDELPLLPPNIDDYRPEGFGQGPLAKAQDWVHIYDEKTGRPGCRETYTMPQWAGSCWYYLRFCDA
HNSQLPWSKEKESYWMPVDLYIGGAEHAVLHLLYSRFWHRVFYDAGLVSTPEPFKKLINQGLVLASSYRIPG
KGYVSIEDVREENGTWISTCGEIVEVRQEKMSKSKLNGVDPQVLIEEYGADALRMYAMFSGPLDKNKTWSN
EGVGGCRRFLNRFYDLVTSSEVQDIEDRDGLVLAHKLVFRITEHIEKMSLNTIPSSFMEFLNDFSKLPVYSKRA
LSMAVRVLEPIAPHISEELWVILGNPPGIDQAAWPQIDESYLVAQTVTFVVQVNGKLRGRLEVAKEAPKEEVL
SLSRSVVAKYLENAQIRKEIYVPNKLVNFVL

>core/60/1/Org1_Gene12

MDSTTNSDSPILDPNPEDVEKLLDESEEESEDQSTERLLPSELFILPLNKRPFFPGMAAPILIESGPYYEVLKVLA
KSSQKYIGLVLTKKENADILKVSFNQLHKTGVAARILRIMPIEGGSAQVLLSIEERIRIIEPIKDKYLKARVSYH
ADNKELTEELKAYSISIVSVIKDLLKLNPLFKEELQIFLGHSDFTEPGKLADFSVALTTATREELQEVLETTNM
HDRIDKALILLKKELDLSRLQSSINQKIEATITKSQKEFFLKEQLKTIKKELGLEKEDRAIDIEKFSERLRKRHVP
DYAMEVIQDEIEKLQTLETSSAEYTVCRNYLDWLTIIPWGIQSKEYHDLKKAEIVLNKDHYGLDEIKQRILELI
SVGKLSKGLKGSIICLVGPPGVGKTSIGRSIAKVLHRKFFRFSVGGMRDEAEIKGHRRTYIGAMPGKMVQALK
QSQAMNPVIMIDEVDKIGASYHGDPASALLEVLDPEQNKDFLDHYLDVRVDLSNVLFILTANVLDTIPDPLLD
RMEILRLSGYILEEKLQIAKKYLVPKARKEIGLTASEVNFQPEALKYMINNYAREAGVRTLNGNIKKVLRKVA
LKIVQNQEKPKSKKITFKISSKNLQTYLGKPIFSSDRFYESTPVGVATGLAWTSLGGATLYIESVQVSSLKTDM
HLTGQAGEVMKESSQIAWTYLHSALHRYAPGYTFFPKSQVHIHIPEGATPKDGPSAGITMVTSLLSLLLETPV
VNNLGMTGEITLTGRVLGVGGIREKLIAARRSRLNILIFPEDNRRDYEELPAYLKTGLKIHFVSHYDDVLKVA
FPKLK

>core/61/1/Org1_Gene972

MKGTPQYHFIGIGGIGMSALAHILLDRGYEVSGSDLYESYTIESLKAKGARCFSGHDSSHVPHDAVVVYSSSI
APDNVEYLTAIQRSSRLLHRAELLSQLMEGYESILVSGSHGKTGTSSLIRAIFQEAQKDPSYAIGGLAANCLNG
YSGSSKIFVAEADESDGSLKHYTPRAVVITNIDNEHLNNYAGNLDNLVQVIQDFSRKVTDLNKVFYNGDCPIL
KGNVQGISYGYSPECQLHIVSYNQKAWQSHFSFTFLGQEYQDIELNLPGQHNAANAAAACGVALTFGIDINII
RKALKKFSGVHRRLERKNISESFLFLEDYAHHPVEVAHTLRSVRDAVGLRRVIAIFQPHRFSRLEECLQTFPKA
FQEADEVILTDVYSAGESPRESIILSDLAEQIRKSSYVHCCYVPHGDIVDYLRNYIRIHDVCVSLGAGNIYTIGE
ALKDFNPKKLSIGLVCGGKSCEHDISLLSAQHVSKYISPEFYDVSYFIINRQGLWRTGKDFPHLIEETQGDSPLS
SEIASALAKVDCLFPVLHGPFGEDGTIQGFFEILGKPYAGPSPSLAATAMDKLLTKRIASAVGVPVVPYQPLNL

CFWKRNPELCIQNLIETFSFPMIVKTAHLGSSIGIFLVRDKEELQEKISEAFLYDTDVFVEESRLGSREIEVSCIG
HSSSWYCMAGPNERCGASGFIDYQEKYGFDGIDCAKISFDLQLSQESLDCVRELAERVYRAMQGKGSARIDF
FLDEEGNYWLSEVNPIPGMTAASPFLQAFVHAGWTQEQIVDHFIIDALHKFDKQQTIEQAFTKEQDLVKR

>core/62/1/Org1_Gene675

MIRERKKSRHPRLPTLPLAAKASLYLFFACFSGLSLWSFHRDQPCTQNWIGLLGWSFSSFLLYFFGAAAFFIPL
YFLWLSFLYFRRTPRPLFFYKAAAFLSLPFCSAILLSMLSPVGTLPALLDTRLPKFILGNNPPVSYVGGIPFYLF
YEGQSFCLKHLIGSVGTALIFGFVMLFSVLYLCGGIALLKKKTFQDGVKKAFCSFFQTCFKNLKKLINRRNYL
PKPSVPFVSKNPFSCTKSQPSPRRVSETIILDGSISPLPQEEIPGSKKESFFLTPHPCKRFLTKFVEPQENKAKEGK
TIALSSTPTVVRESKGKERAALPKLKSLAVPENDLPQYHLLSKNREARPESLQAELERKALILKQTLTSFGIDA
DLGNICSGPTLAAFEVLPHSGVKVQKIKSLENDIALKLQASSIRIIAPIPGKAAVGIEIPTPFPQAVNFRDLLEDY
QKTNRKLQIPLLLGKKANGDNLWADLATMPHLIIAGTTGSGKSVCINTIVMSMIMTTLPSEIKLVIIDPKKVEL
TGYSQLPHMLSPVITESREVYNALVWLVKEMESRYEILRYLGLRNIQAFNSRTRNKTIEASYDREIRETMPFM
VGIIDELSDLLLSSSQDIETPIIRLAQMARAVGIHLILATQRPSREVITGLIKANFPSRISFKVSNKVNSQIIIDEPG
AENLMGNGDMLVLLPSVFGTIRAQGAYICDEDINKVIQDLCSRFPTQYVIPSFHAFDDSDSDNSGEKDPLFAQ
AKTLILQTGNASTTFLQRKLKIGYARAASLIDQLEEARIIGPSEGAKPRQILIQNPLEG

>core/63/1/Org1_Gene194

MDPKEKNYDASAITVLEGLQAVRERPGMYIGDTGITGLHHLVYEVVDNSIDEAMAGYCSRIDVRILEDGGIVI
VDNGRGIPIEVHERESAKQGREVSALEVVLTVLHAGGKFDKDSYKVSGGLHGVGVSCVNALSEKLVATVFK
DKKCYQMEFSRGIPVTPLQYVSVSDRQGTEIVFYPDPKIFSTCTFDRSILMKRLRELAFLNRGITIVFEDDRDVS
FDKVTFFYEGGIQSFVSYLNQNKESLFSEPIYICGTRVGDDGEIEFEAALQWNSGYSELVYSYANNIPTRQGGT
HLTGFSTALTRVINTYIKAHNLAKNNKLALTGEDIREGLTAVISVKVPNPQFEGQTKQKLGNSDVSSVAQQV
VGEALTIFFEENPQIARMIVDKVFVAAQAREAAKKARELTLRKSALDSARLPGKLIDCLEKDPEKCEMYIVEG
DSAGGSAKQGRDRRFQAILPIRGKILNVEKARLQKIFQNQEIGTIIAALGCGIGADNFNLSKLRYRRIIIMTDAD
VDGSHIRTLLLTFFYRHMTALIENECVYIAQPPLYKVSKKKDFRYILSEKEMDSYLLMLGTNESSILFKSTERE
LRGEALESFINVILDVESFINTLEKKAIPFSEFLEMYKEGIGYPLYYLAPATGMQGGRYLYSDEEKEEALAQEE
THKFKIIELYKVAVFVDIQNQLKEYGLDISSYLIPQKNEIVIGNEDSPSCNYSCYTLEEVINYLKNLGRKGIEIQR
YKGLGEMNADQLWDTTMNPEQRTLIHVSLKDAVEADHIFTMLMGEEVPPRREFIESHALSIRINNLDI

>core/64/1/Org1_Gene840

MNKRTLLFVSLIGIAFVGCQIFFGYNEFRSCKNLAEKQRKISEQTLAAVESVGLSVASWDTDVNGEEHKNNY
AVRVGDKLFLLHNGEAAQSVYSSGESWSFVDHKCGFDNIHLALYRQQGSSFNPTNTGKVFLPTNHEGLPVL
VVEFRNNKEPLVFLGEYAQGRISNKDSTIFGTALVFWRSGSDYIPLGLYDSREEKLVSLDLPITRAVIFGNDQD
SAKSSDTANHYVLFNDYMQIIVSEESGSIEGINLPFASTNNKSIVNEIGFDRDLASEKSPEALFPGLSSKLPDGQ
QAKNSIGGYYPLLRRGLLSDSKKLLPLEYHALNVVSGRELATPVALRYRVLSYTPHSIQLESLDRSVQKVYKL
PENPEEKPYVFETAITLTKETEDVWVTSGVPEVEIMSNASAPTIKYRVIKKNKGSLDKVKLPKVKEPLAVRRG
VYPQWILNSNGYFGIILTPLSEIASGYGSLYISGSTAPTRLSAISPKNQLYPVSKYPGYETLLPLPKDAGTHRFL
VYAGPLAEPTLKVLDKTITQEKGENPEYLDSISFRGVFAFITAPFAALLFIIMKFFKLVTGSWGISIILLTVFLKL
LLYPLNAWSIRSMRRMQILSPYIQQIQQKYKNEPKRAQMEIMGLYKTNKVNPITGCLPLLIQLPFLIAMFDLLK

SSFLLRGASFIPGWIDNLTAPDVLFSWQTSIWFIGNEFHLLPILLGIVMFLQQKVTSLHKKGPVTDQQKQQQV
MGNMMAILFTAMFYNFPSGLNIYWLSSMILGVVQQWITNKILDSKHLKNEVVLNNKKHR

>core/65/1/Org1_Gene438

MRIPITLLQTYFSEPLSTKEILEACDHIGIEAEIENTTLYSFASVITAKILHTIPHPNADKLRVATLTDGEKEHQV
VCGAPNCEAGLIVALALPGAKLFDSEGQAYTIKKSKLRGVESQGMCCGADELGLDELQIQERALLELPEATP
LGEDLATVLGNTSLEISLTPNLGHCASFLGLAREICHVTQANLVIPKEFSFENLPTTALDMGNDPDICPFFSYV
VITGISAQPSPIKLQESLQALKQKPINAIVDITNYIMLSLGQPLHAYDASHVALDSLRVEKLSTPESLTLLNGET
VLLPSGVPVVRDDHSLLGLGGVMGAKAPSFQETTTTTVIKAAYFLPEALRASQKLLPIPSESAYRFTRGIDPQN
VVPALQAAIHYILEIFPEATISPIYSSGEICRELKEVALRPKTLQRILGKSFSIEILSQKLQSLGFSTTPQETSLLVK
VPSYRHDINEEIDLVEEICRTESWNIETQNPVSCYTPIYKLKRETAGFLANAGLQEFFTPDLLDPETVALTRKE
KEEISLQGSKHTTVLRSSLLPGLLKSAATNLNRQAPSVQAFEIGTVYAKHGEQYQETQTLAILLTEDGESRSW
LPKPSLSFYSLKGWVERLLYHHHLSIDALTLESSALCEFHPYQQGVLRIHKQSFATLGQVHPELAKKAQIKHP
VFFAELNLDLLCKMLKKTTKLYKPYAIYPSSFRDLTLTVPEDIPANLLRQKLLHEGSKWLESVTIISIYQDKSL
ETRNKNVSLRLVFQDYERTLSNQDIEEEYCRLVALLNELLTDTKGTINS

>core/66/1/Org1_Gene988

MLIMRNKVILQISILALIQTPLTLFSTEKVKEGHVVVDSITIITEGENASNKHPLPKLKTRSGALFSQLDFDEDLR
ILAKEYDSVEPKVEFSEGKTNIALHLIAKPSIRNIHISGNQVVPEHKILKTLQIYRNDLFEREKFLKGLDDLRTY
YLKRGYFASSVDYSLEHNQEKGHIDVLIKINEGPCGKIKQLTFSGISRSEKSDIQEFIQTKQHSTTTSWFTGSGL
YHPDIVEQDSLAITNYLHNNGYADAIVNSHYDLDDKGNILLYMDIDRGSRYTLGHVHIQGFEVLPKRLIEKQS
QVGPNDLYCPDKIWDGAHKIKQTYAKYGYINTNVDVLFIPHATRPIYDVTYEVSEGSPYKVGLIKITGNTHTK
SDVILHETSLFPGDTFNRLKLEDTEQRLRNTGYFQSVSVYTVRSQLDPMGNADQYRDIFVEVKETTTGNLGLF
LGFSSLDNLFGGIELSESNFDLFGARNIFSKGFRCLRGGGEHLFLKANFGDKVTDYTLKWTKPHFLNTPWILGI
ELDKSINRALSKDYAVQTYGGNVSTTYILNEHLKYGLFYRGSQTSLHEKRKFLLGPNIDSNKGFVSAAGVNL
NYDSVDSPRTPTTGIRGGVTFEVSGLGGTYHFTKLSLNSSIYRKLTRKGILKIKGEAQFIKPYSNTTAEGVPVSE
RFFLGGETTVRGYKSFIIGPKYSATEPQGGLSSLLISEEFQYPLIRQPNISAFVFLDSGFVGLQEYKISLKDLRSS
AGFGLRFDVMNNVPVMLGFGWPFRPTETLNGEKIDVSQRFFFALGGMF

>core/67/1/Org1_Gene856

MLLLISGALFLTLGIPGLTAGVSFGLGIGLSALGGVLVVSGLLCLLVKREVSKVCPEEIPAVQPEETPEGVPVTP
FEKPALDEAQKEQKTQKILDQLPQELDQLDRYIQEVFACLGPLKDLKYEDQGFLQDVKEEFQVFDFVQKDMI
AEFVELQQILCQEGRLLEFVINQTRYIGRDLFKREDSLYKLWEWLGYLPSGDVRGERLKKSAREVVDRFMRT
TCNIRKIAMTFDRHVYSVAKTAFEKAFGALETCVYESMRESYREAFCEYEKAKLLGDEEKSAHAEQRFQDIK
NRWEDVKDAFFWVKEDGKIEIDDAIGNSCKWSERYEEHRITRARWYKVAEHQLFNATMRVKDSLREHNEA
RVAFEKERSKENQRQVQKKKEKRLRDLKELHDQELPRAQERLRELQALYPEIAVSVVEARREVASDLEKAH
ESIDKHYQSCVREQELY

>core/68/1/Org1_Gene830

MASGIGGSSGLGKIPPKDNGDRSRSPSPKGELGSHEISLPPQEHGEEGASGSSHIHSSSSFLPEDQESQSSSSAAS
SPGFFSRVRSGVDRALKSFGNFFSAESTSQARETRQAFVRLSKTITADERRDVDSSSAAATEARVAEDASVSG

ENPSQGVPETSSGPEPQRLFSLPSVKKQSGLGRLVQTVRDRIVLPSGAPPTDSEPLSLYELNLRLSSLRQELSDI
QSNDQLTPEEKAEATVTIQQLIQITEFQCGYMEATQSSVSLAEARFKGVETSDEINSLCSELTDPELQELMSDG
DSLQNLLDETADDLEAALSHARLSFSLDDNPTPIDNNPTLISQEEPIYEEIGGAADPQRTRENWSTRLWNQIRE
ALVSLLGMILSILGSILHRLRIARHAAAEAVGRCCTCRGEECTSSEEDSMSVGSPSEIDETERTGSPHDVPRRN
GSPREDSPLMNALVGWAHKHGAKTKESSESSTPEISISAPIVRGWSQDSSVSFIVMEDDHIFYDVPRRKDGIYD
VPSSPRWSPARELEEDVFGDYEVPITSAEPSKDKNIYMTPRLATPAIYDLPSRPGSSGSSRSPSSDRVRSSSPNR
RGVPLPPVPSPAMSEEGSIYEDMSGASGAGESDYEDMSRSPSPRGDLDEPIYANTPEDNPFTQRNIDRILQERS
GGASASPVEPIYDEIPWIHGRPPATLPRPENTLTNVSLRVSPGFGPEVRAALLSESVSAVMVEAESIVPPTEPGD
GESEYLEPLGGLVATTKILLQKGWPRGESNA

>core/69/1/Org1_Gene1003

MLLLISGALFLTLGIPGLSAAISFGLGIGLSALGGVLMISGLLCLLVKREIPTVRPEEIPEGVSLAPSEEPALQAA
QKTLAQLPKELDQLDTDIQEVFACLRKLKDSKYESRSFLNDAKKELRVFDFVVEDTLSEIFELRQIVAQEGWD
LNFLINGGRSLMMTAESESLDLFHVSKRLGYLPSGDVRGEGLKKSAKEIVARLMSLHCEIHKVAVAFDRNSY
AMAEKAFAKALGALEESVYRSLTQSYRDKFLESERAKIPWNGHITWLRDDAKSGCAEKKLRDAEERWKKF
RKAVFWVEEDGGFDINNLLGDWGTVLDPYRQERMDEITFHELYEKTTFLKRLHRKCALAKTTFEKKRSKKN
LQAVEEANARRLKYVRDWYDQEFQKAGERLEKLHALYPEVSVSIRENKIQETRSNLEKAYEAIEENYRCCVR
EQEDYWKEEEKREAEFRERGNKILSPEELESSLEQFDHGLKNFSEKLMELEGHILKLQKEATAEVENKILSDA
ESRLEIVFEDVKEMPCRIEEIEKTLRMAELPLLPTKKAFEKACSQYNSCAEMLEKVKPYCKESLAYVTSKERL
VSLDEDLRRAYTECQKRFQGDSGLESEVRACREQLRERIQEFETQGLDLVEKELLCVSSRLRNTECDCVSGV
KKEAPPGKKFYAQYYDEIYRVRVQSRWMTMSERLREGVQACNKMLKAGLSEEDKVLKEEEYWLYREERK
NKEKRLVGTKIVATQQRVAAFESIEVPEIPEAPEEKPSLLDKARSLFTREDHT

>core/70/1/Org1_Gene883

MLFSKNFSTDNGGAITAKTLSLTGTTMSALFSENTSSKKGGAIQTSDALTITGNQGEVSFSDNTSSDSGAAIFT
EASVTISNNAKVSFIDNKVTGASSSTTGDMSGGAICAYKTSTDTKVTLTGNQMLLFSNNTSTTAGGAIYVKKL
ELASGGLTLFSRNSVNGGTAPKGGAIAIEDSGELSLSADSGDIVFLGNTVTSTTPGTNRSSIDLGTSAKMTALR
SAAGRAIYFYDPITTGSSTTVTDVLKVNETPADSALQYTGNIIFTGEKLSETEAADSKNLTSKLLQPVTLSGGT
LSLKHGVTLQTQAFTQQADSRLEMDVGTTLEPADTSTINNLVINISSIDGAKKAKIETKATSKNLTLSGTITLL
DPTGTFYENHSLRNPQSYDILELKASGTVTSTAVTPDPIMGEKFHYGYQGTWGPIVWGTGASTTATFNWTKT
GYIPNPERIGSLVPNSLWNAFIDISSLHYLMETANEGLQGDRAFWCAGLSNFFHKDSTKTRRGFRHLSGGYVI
GGNLHTCSDKILSAAFCQLFGRDRDYFVAKNQGTVYGGTLYYQHNETYISLPCKLRPCSLSYVPTEIPVLFSG
NLSYTHTDNDLKTKYTTYPTVKGSWGNDSFALEFGGRAPICLDESALFEQYMPFMKLQFVYAHQEGFKEQG
TEAREFGSSRLVNLALPIGIRFDKESDCQDATYNLTLGYTVDLVRSNPDCTTTLRISGDSWKTFGTNLARQAL
VLRAGNHFCFNSNFEAFSQFSFELRGSSRNYNVDLGAKYQF

>core/71/1/Org1_Gene651

MTLQPSYINFTPNVTTALSGGKIDTSAIELSCSALFFQELQDKAQGLKHALGLVQELSAEALRPAQVQTSISYL
PTEESSRPGISAGIIDRTMPTFTDDEVKAILQNPNFETSKIFVEGLDKVFKSYLDSVTPPEGIDPSNPESAIILNYIT
LLNNLKPKFAAGSTPTDADYNALYALPGDFVKEIEALKAADAPPKSKVHAFWQEIMTIYNNMQVLSYPVTD
YLNVQIADLSLNITAAQEVQQYLKNFYSILKDILNPGWTDPQATHYPADAEYNARDAGVIQSLLNLSGNYRQ

LTENMLPNTDTSLPQEIIAQIRSFQNGVNGTIIASNTLLPTTMRLDTLLGVIYTYQCCATIFGMSYGTSTPAKQN
YIDAINQEKSYWQARANGFDVTSDQVFDQFATNIQSGTSYRGIDLFKNNKVNEINPIFLSQAASFLRYPYNLM
SRSMYQTIEDAANRSITALDGLISGWSTQIATFQTQKNSLDPSLLKYFDTMKANKESFVTTAPLQMVYSSLML
DKYLPTQQNVIASLGIQMTYSNKAAKYLNELIKEITTFQSADIYYSLSIYLKQMNLQAVADPIGKAVGVLNDE
KTRAMADITRCNKIKAAIDKMLVEIKADAELSKSQIRELVDTLTNFKSQSDDLIRNLSCLLGFLSGLTLKAVN
DPNATYEAFTAEIFTEPFNNWKRQLATFESFVIQGGQNGITPGGQQQLLQAMESSQQDFSTFNQNQQLALQLE
SSAMQQEWTLVSAALALLNQMVSKIARRIKS

>core/75/1/Org1_Gene620

MVFFRNSLLHLVALSGMLCCSSGVALTIAEKMASLEHSGRGADDYEGMASFNANMREYSLQLSKLYEEARK
LRASGTEDEALWKDLIRRIGEVRGYLREIEELWAAEIREKGGNLEDYALWNHPETTIYNLVTDYGTEDSIYLI
PQEIGAIKIATLSKFVVPKESFEDCLTQILSRLGIGVRQVNSWIKELYMMRKEGCSVAGVFSSRKDLEALPETA
YIGFVLNSNVDAHTNQHVLKKFINPETTHVDVIAGRVWIFGSAGEVGELLKIYNFVQSESIRQEYRVIPLTKID
PGEMISILNAAFREDLTKDVSEESLGLRVVPLQYQGRSLFLSGTAALVQQALTLIRELEEGIENPTDKTVFWYN
VKHSDPQELAALLSQVHDVFSGENKASVGAADGCGSQLNASIQIDTTVSSSAKDGSVKYGNFIADSKTGTLI
MVVEKEVLPRIQMLLKKLDVPKKMVRIEVLLFERKLAHEQKSGLNLLRLGEEVCKKGCSPSVSWAGGTGILE
FLFKGSTGSSIVPGYDLAYQFLMAQEDVRINASPSVVTMNQTPARIAVVDEMSIAVSSDKDKAQYNRAQYGI
MIKMLPVINVGEEDGKSYITLETDITFDTTGKNHDDRPDVTRRNITNKVRIADGETVIIGGLRCKQMSDSHDGI
PFLGDIPGIGKLFGMSSTSDSLTEMFVFITPKILENPVEQQERKEEALLSSRPGEREEYYQALAASEAAARAAH
KKLEMFPASGVSLSQVERQEYDGC

>core/76/1/Org1_Gene708

MGYIESSTFRLYAEVIVGSNINKVLDYGVPENLEHITKGTAVTISLRGGKKVGVIYQIKTTTQCKKILPILGLSD
SEIVLPQDLLDLLFWISQYYFAPLGKTLKLFLPAISSNVIQPKQHYRVVLKQSKAKTKEILAKLEVLHPSQGAV
LKILLQHASPPGLSSLMETAKVSQSPIHSLEKLGILDIVDAAQLELQEDLLTFFPPAPKDLHPEQQSAIDKIFSSL
KTSQFHTHLLFGITGSGKTEIYLRATSEALKQGKSTILLVPEIALTVQTVSLFKARFGKDVGVLHHKLSDSDKS
RTWRQASEGSLRILIGPRSALFCPMKNLGLIIVDEEHDPAYKQTESPPCYHARDVAVMRGKLAHATVVLGSA
TPSLESYTNALSGKYVLSRLSSRAAAAHPAKISLINMNLEREKSKTKILFSQPVLKKIAERLEVGEQVLIFFNRR
GYHTNVSCTVCKHTLKCPHCDMVLTFHKYANVLLCHLCNSSPKDLPQSCPKCLGTMTLQYRGSGTEKIEKIL
QQIFPQIRTIRIDSDTTKFKGSHETLLRQFATGKADVLIGTQMIAKGMNFSAVTLAVILNGDSGLYIPDFRASEQ
VFQLITQVAGRSGRSHLPGEILIQSFLPDHPTIHSAMRQDYSAFYSQEITGRELCEYPPFIRLIRCIFMGKCPKQT
WEEAHRVHNILKEQLESTNPLMPVTPCGHFKIKDTFRYQFLIKSAYVIPVNKKLHHALMLAKLSPKVKFMID
VDPMTTFF

>core/77/1/Org1_Gene78

MEKICGYLEQILVENKDSGDITAYIKIPNKTTPILIKGKLPQPLELGSPIQIYGVWSHSPSNTKYFQIHSYDSPLL
YEYRGVFHYLTSKLIKGIGPKIAEKIIEKFQEKTCYVLDITPERLSEVSGISETRCVSICKQLCEQKILRKTLLFLQ
EYNIPIHYGVRIFKKYQEKSIEKICEDPFLLAREMEGIGFKTADFIAMKLGVPRNSESRLCAGIQHSLEELQEEG
HTCYPIELLIDVVAKLLNQDVFDTPITLEEIDTQILNMQKRKLLHIQDISGTLHVWTRYLHLAEKTIVSDLKRIL
FSSRRIRSIDGEKAIAWVEENLSIDLAEQQREAIKACFSEKLLIITGGPGTGKSTITQAILKIFEQVTHKIILAAPT
GKAAKRMTEITQKHSVTIHALLQYDFKTKSFRKNHDNPIDCDLIIVDESGMMDTHLLHHFLKALPDYTTLVFI

GDIHQLPSVGPGNILKDLITSNKMTVIRLNKIFRQVHDSGIVTNAHRVNEGELPILYSETGRRDFLFFQKDDQE
EALNHIIHLVTKFVPQKYHIYPQDIQVLAPMKKGTLGIYNLNKALKHALNPKKANLHGRFQSYAVGDKVMQI
RNNYNKEVFNGDIGYVSTINFEDKAVVVRMEGKHVGYSFSELDDLVLAYATSVHKYQGSESPCIIIPIHTSHF
MMLYRNLLYTAITRGKKLVILVGTKKAIAIATRNNRVQHRCTGLAEVLKELDTKKNYADL

>core/78/1/Org1_Gene930

MLSFFYKHQKKFIGIVIAVVCVSGTGVGWGRFSRKGSAESTSRRTVFTTASGKRYVEKDFMAMKKFFAHEA
YPFTGNPRAWNFINEGLLTDYFLTTRVGEKLFLKVYHPGEKIFSKEKAYQPYRRFDAPFISSEEVWKSSAPQL
LEILKVFQQIENPISKEGFLARAKLFLEERRFPHYVLRQMLEYRRQMFALPPDEALSRGKDLRLFGYQTIQDW
FGDAYLSAAVELLIRFIDEQKKVLPRPSKQEARDDFYDKAKHAYTKISKNKEFSLGFEEFVNSYFQFLEISESE
FFNMYRDILLCKRALLLLQGGVSFDFQPLTTFFVQGKDSIQVEFFRLPKEYSFKTKQELKAFEVYLKLVSLPKS
DSLDVPNEILPIATIKAKEPRLVGRRFSIDYKRVALQDLAATVPMVEVLHWQQNSEHFQEILQQFPDVETCQS
YKDFQHLKPALRDKISLFTRKEILRARPERILQSLQQVPKQSQEVLLSAGKNSALPGISDGQQLAKVLLENEVL
DLYSQDAETYYTIIVNSSFEKEEVLPYREVLKRDLASQLLTSHGHLVDMERLESALRTRYPGEEGASLWQRR
LWKVVENHRLGRHLEGSFSWSLDRSLKTFSRGDKELPQEFDRIFSMKVGDYSSVFMSPNEGPCYYQCLSHLL
YDRPASVDKLFLAKSQLDEELLGSYMERFIEQGVVR

>core/79/1/Org1_Gene564

MDYLEKLQVLIEEGQSANFLSLWEEYCFNDVVRGRELVEILEKVKSSSLASLFGKIVDTVVPLWEKIPEGKDK
DRVLQLILDLQTSNSQMFFDIATEYVNKKYSGEENFNEALRVVGLRDGRDFQFSLSRFDFLMHMHKGNFVFH
QGGWGVGEVMGVSFLQQKVLIEFEGIMSAKDISFETAFKSLTPLSGDHFLSRRFGDPDGFEAFAKENPIEVVEI
LLRDLGPKTAKEIKDELVDLVIPEADWNRWWQSAKTKIKKGTRIISPDNPKEPYVLSDAGCSHMGQLERKLG
LSLNSAEKISLIYHFIRDLHSELKNIEIRKSLVKALQDLDVEEGNKSLILQRELLLSEYLGIKDASIDKEYITSLSE
DDTSRLLENMPIVALQKSFLSLVRKYSSFWQQVFMQILLYTTSPTMRDFVYKTIKNDPSSVEVLKKRLLDSAH
QPMMFPELFVWFFLKLGNHEDGLFDPEDKEVLRLFLESALNFMYQVASTPHKELGKKLHHYLVGQRYLAVR
QMIEGASLPFLKELLLLSTKCPQFSSSDLNVLQSLAEVVQPTLKKHKSNVEEENVLWSTSESFSRMKAKLQSL
VGKEMVDNAKEIEDARSLGDLRENSEYKFALEKRARLQEEIRVLSEEINRARILTKDLVFTDKVGVGCKVTL
KGDAGEVVEYTILGPWDADPDSCILSLQSKLAQNMLGKKLNDVVILQGKEYKISRIQSIWEEHGA

>core/80/1/Org1_Gene342

MVDKLIHPWDLDLLVSGRQKDPHKLLGILASEDSSDHIVIFRPGAHTVAIELLGELHHAVAYRSGLFFLSVPK
GIGHGDYRVYHQNGLLAHDPYAFPPLWGEIDSFLFHRGTHYRIYERMGAIPMEVQGISGVLFVLWAPHAQR
VSVVGDFNFWHGLVNPLRKISDQGIWELFVPGLGEGIRYKWEIVTQSGNVIVKTDPYGKSFDPPPQGTARVA
DSESYSWSDHRWMERRSKQSEGPVTIYEVHLGSWQWQEGRPLSYSEMAHRLASYCKEMHYTHVELLPITEH
PLNESWGYQVTGYYAPTSRYGTLQEFQYFVDYLHKENIGIILDWVPGHFPVDAFALASFDGEPLYEYTGHSQ
ALHPHWNTFTFDYSRHEVTNFLLGSALFWLDKMHIDGLRVDAVASMLYRDYGREDGEWTPNIYGGKENLE
SIEFLKHLNSVIHKEFSGVLTFAEESTAFPGVTKDVDQGGLGFDYKWNLGWMHDTFHYFMKDPMYRKYHQ
KDLTFSLWYAFQESFILPLSHDEVVHGKGSLVNKLPGDTWTRFAQMRVLLSYQICLPGKKLLFMGGEFGQY
GEWSPDRPLDWELLNHHYHKTLRNCVSALNALYIHQPYLWMQESSQECFHWVDFHDIENNVIAYYRFAGS
NRSSALLCVHHFSASTFPSYVLRCEGVKHCELLLNTDDESFGGSGKGNRAPVVCQDQGVAWGLDIELPPLAT
VIYLVTFF

>core/81/1/Org1_Gene879

MNKFKTYLQTALIAPFFSFPALSGSFSSIQAEEITQQVNHPGAELLSEGSYIPGLQTFRLGIKITASKGSHIYWKN
PGEIGSPLKISWQLPKGFVVEEEHWPTPKVFEEEGTTFFGYEDSALIVADVRAPEGYTPGQEVELRAQVEWLA
CGDSCLPGNVDLKLTLPYEEKEPSLYPDTHAEFTKTLHAQPRVLENDHSVQVAQGKGNEIILNISKKINATKA
WFVSEKADKLFAYAETSYSGGTGTAWRLKVKNLSGVQKNEKLHGILLLADHTGRPVESLTIHSEVLGQTGS
AVAGLSQYITILIMAFLGGVLLNIMPCVLPLVTLKVYGLIKSAGEHRSSVIANGLWFTLGVVGCFWGLAGVAF
ILKVLGHNIGWGFQLQEPMFVATLIIVFFLFALSSLGLFEMGTMFANLGGKLQSSEMKSSNNKAVGAFFNGIL
ATLVTTPCTGPFLGSVLGLVMSLSFLQQLLIFTAIGLGMASPYLVFSVFPKMLSVLPKPGGWMSTFKQLTGFM
LLVTVTWLVWIFGSETSTTSVVVLLGGLWLAGLGAWILGRWGTPVSPKKQRVCASLLFFAFLGGAISVSGLA
SHYFAEPQQTVSVNEDSLWQPFSLEKLAQLRAQGRPVFVNFTAKWCLTCQMNKPVLYGDAVQKMFETHGI
VTLEADWTRKDPGITEELARLGRASVPSYVYYPGDNSAPVVLPEKITQNLLEDVVSRFVR

>core/82/1/Org1_Gene205

MIRSPLPFISSKRALNMLGLQDEFSCPEDVVDFLFSEIELLAQQDEPSEGYLALSRSLLMMTHNHPKVVKRVIF
YGVSYGLKHKSMSIFIDVLTYIDFLFEKLGISASDRLSLCSARTCINFELYSQTGEMKFLSEVVDNFRLIEQLLK
MHPQLKNRLGWEHFRIGAKQEEVSLVASASVYQAVGRSFIELYHKHLELSDLACGMKCLALALDLSPNNAH
IHADYAKGLVVLGTRQGKSLLIERGMEHFSKAIFLSFSRDGDTLAYQNYRYSYALASVKLFDLTYKKEHFDQ
AMNILYQTVQAFPNLSGLWMVWGELLIRSGWLNSNMKYIEVGLEKLASLQKKTNDPIALSGLLATGIAILGL
YLEEPNLFKDSRHRLISAMRTFPGNSALVHALGVVQLCSALYFNEDSHFASAISCFQSCLEWDLDATGMWQK
LFDAYFSWGIKKKSARLLRKAVDVASRLCSLRPEAFLFWSDRGLALKCLAEATIDEAYKEIFLSESLLHYQRA
WDLSGRLEILELWGQSHYLLAELQQSLFHYDEAYTLLTKVDLTLSSSRVKLILAAVLLGKGRLLQDTDPAEE
AREILEPLVEVYLEDENFLLLLGKVYLFLFWKNKNVCLGKLARTYLEKATSLGCPEAYYTLGKFYAVIKDVN
KAWGMVIRSAQYGVRITEAKWLNDPYLANLREIHAFREVVENQKGRLWLGNKTEMKRN

>core/83/1/Org1_Gene235

MNKLLNFVSRTLGGDTALNMINKSSDLILALWMMGVVLMIIIPLPPPIVDLMITINLSISVFLLMVALYIPSALQ
LSVFPSLLLITTMFRLGINISSSRQILLKAYAGHVIQAFGDFVVGGNYVVGFIIFLIITIIQFIVVTKGAERVAEVA
ARFRLDAMPGKQMAIDADLRAGMIDATQARDKRAQIQKESELYGAMDGAMKFIKGDVIAGIVISLINIVGGL
TIGVAMHGMDLAQAAHVYTLLSIGDGLVSQIPSLLIALTAGIVTTRVSSDKNTNLGKEISTQLVKEPRALLLA
GAATLGVGFFKGFPLWSFSILALIFVALGILLLTKKSAAGKKGGGSGASTTVGAAGDGAATVGDNPDDYSLT
LPVILELGKDLSKLIQHKTKSGQSFVDDMIPKMRQALYQDIGIRYPGIHVRTDSPSLEGYDYMILLNEVPYVR
GKIPPHHVLTNEVEDNLSRYNLPFITYKNAAGLPSAWVSEDAKAILEKAAIKYWTPLEVIILHLSYFFHKSSQE
FLGIQEVRSMIEFMERSFPDLVKEVTRLIPLQKLTEIFKRLVQEQISIKDLRTILESLSEWAQTEKDTVLLTEYVR
SSLKLYISFKFSQGQSAISVYLLDPEIEEMIRGAIKQTSAGSYLALDPDSVNLILKSMRNTITPTPAGGQPPVLLT
AIDVRRYVRKLIETEFPDIAVISYQEILPEIRIQPLGRIQIF

>core/84/1/Org1_Gene997

MYKRCVLDKILKGIVAGSLILLYWSSDLLERDIKSIKGNVRDIQEDIREISRVVKQQQTSQAIPAAPGVMLAPK
LVRDEAFALLFGDPSYPNLLSLDPYKQQTLPELLGTNFHPHGILRTAHVGKPENLSPFNGFDYVVGFYDLCIPS
LASPHVGKYEEFSPDLAVKIEEHLVEDGSGDKEFHIYLRPNVFWRPIDPKALPKHVQLDEVFQRPHPVTAHDI

KFFYDAVMNPYVATMRAVALRSCYEDVVSVSVENDLKLVVRWKAHTVINEEGKEERKVLYSAFSNTLSLQ
PLPRFVYQYFANGEKIIEDENIDTYRTNSIWAQNFTMHWANNYIVSCGAYYFAGMDDEKIVFSRNPDFYDPL
AALIDKRFVYFKESTDSLFQDFKTGKIDISYLPPNQRDNFYSFMKSSAYNKQVAKGGAVRETVSADRAYTYI
GWNCFSLFFQSRQVRCAMNMAIDRERIIEQCLDGQGYTISGPFASSSPSYNKQIEGWHYSPEEAARLLEEEGW
IDTDGDGIREKVIDGVTVPFRFRLCYYVKSVTAHTIADYVATACKEIGIECSLLGLDMADLSQAFDEKNFDAL
LMGWCLGIPPEDPRALWHSEGAMEKGSANVVGFHNEEADKIIDRLSYEYDLKERNRLYHRFHEIIHEEAPYA
FLFSRHCSLLYKDYVKNIFVPTHRTDLIPEAQDETVNVTMVWLEKKEDPCLSTS

>core/85/1/Org1_Gene903

MKELRHESYNRALHKLSHQWVRYFLYTFVSCSFIVAIFTFAWLKVLYVPEYKAGEISRISLTAPMDFSLSWSA
HKFYKRTAHISEAFGKVYHLTLSPGSLLSKEGNADENTDYWFKKAADFLLSTNFVDSSTQKCLKDLCIYPPLL
GKEKKTLEININSNKGNVIAQCFCHLKIFLIQENCPQPCFDAIMDILKIANFEVAVDKEMSGCVKGELLGKRCI
EKITKGTPILEKYQRIDDRDAKILKQLRAQLLSVHTLFSCRSLWGAIFVVLLILLWGYGALKALCPEMLKSPQ
RFMLYIAILTLSLLWCRGTEIFCAYWVSYLSYPPILPFTAVLLGYFLGLPIAGFSCTFLALLYTLGSDLWNNSW
FLSINLLCSWRILVSLHRVSRLSSVFWACMKLGGVAMGSLLMFRIFTNTISREALYADGIESFVYSLITAISVVA
LIPVFEASFGASTNFSLLTYLSPENALLKRLFKEAPGTYQHSVLVGSLAEAAAQAIGADSLYCLVAAHYHDIG
KLINPGFFSENQKILQQSGHSLSPLECAKMIMRHIPEGVNLARQAGLPESFIQVIEEHHGTSVIRSAYYSHMVE
NPSTGSFDEELFRYSGNKPSSKETTIIMIADSFEAASRSLKNASLPDLQRLIDQIIQGKLQDGQFSCSPVTLDELA
LISKSMVQTLYGALHSRMKYPEISYQISMDSCPKPSIGGT

>core/86/1/Org1_Gene981

MPEPLYTNKLITEKSPYLLLYAHTPVNWYPWGAEAFHIAAIENKPVFLSIGCKHSRWCQVMLQESYTNPEIAA
MLNEYFVNVKVDKEELPYVAKLYGDLAQMLAVSGDHQETVSWPLNVFLTPDLVPFFSVNYLGNEGKLGLP
SFPQIIDKLHFMWEDAEEREALVEQAMKVLEIASFLEGCVRKEILDESSLKRTVAALYQDIDPHYGGVKAFPK
RLPGLLLQFFLRYSLEYQESRGLFFVDRSLSMVALGGVRDHIGGGVYSYTIDDKWLIPAFEKRLIDNALMALN
YLEAWACLGKEEYRGIGKQILSYILSELYSPEVGAFYSSEQAENWGAGGQNFYTWSVEEISNALGEDAEIFCD
YYGISREGFFNGRNILHIPVHREIEELSEKYHRSIEAIEDIVDRSRDILKGIRAQRSHRSKDDLSLTFNNGWMIYT
FAYAGRLLGEVEYIEIGKKCGEFVRNSLYKHHELYRRWREGEAKYRASLEDYGALILGVLALYESGCGSFWL
SFAEELMQEVVLSFRSEEGGFYSVDGRDSTLLIKQSPLSDGETISGNALICQCLLSLHLITEKKHYLTYAEDILQ
IAQAYAHTHKFSSLGLLIASQNYFSRKHVKVLIALGDQEDRSPVLKCLSGLFLPYLSLIWMTQENQEHLETVL
PEYEHCLIPKGDCTATTIYVLEVDQCKRFKDLELFRRYLISL

>core/88/1/Org1_Gene6

MKRCFLFLASFVLMGSSADALTHQEAVKKKNSYLSHFKSVSGIVTIEDGVLNIHNNLRIQANKVYVENTVGQ
SLKLVAHGNVMVNYRAKTLVCDYLEYYEDTDSCLLTNGRFAMYPWFLGGSMITLTPETIVIRKGYISTSEGP
KKDLCLSGDYLEYSSDSLLSIGKTTLRVCRIPILFLPPFSIMPMEIPKPPINFRGGTGGFLGSYLGMSYSPISRKHF
SSTFFLDSFFKHGVGMGFNLHCSQKQVPENVFNMKSYYAHRLAIDMAEAHDRYRLHGDFCFTHKHVNFSGE
YHLSDSWETVADIFPNNFMLKNTGPTRVDCTWNDNYFEGYLTSSVKVNSFQNANQELPYLTLRQYPISIYNT
GVYLENIVECGYLNFAFSDHIVGENFSSLRLAARPKLHKTVPLPIGTLSSTLGSSLIYYSDVPEISSRHSQLSAKL
QLDYRFLLHKSYIQRRHIIEPFVTFITETRPLAKNEDHYIFSIQDAFHSLNLLKAGIDTSVLSKTNPRFPRIHAKL
WTTHILSNTESKPTFPKTACELSLPFGKKNTVSLDAEWIWKKHCWDHMNIRWEWIGNDNVAMTLESLHRSK

YSLIKCDRENFILDVSRPIDQLLDSPLSDHRNLILGKLFVRPHPCWNYRLSLRYGWHRQDTPNYLEYQMILGT
KIFEHWQLYGVYERREADSRFFFFLKLDKPKKPPF

>core/89/1/Org1_Gene977

MNFQTISINLTEGKILVFETGKIARQANGAVLVRSGETCVFASACAVDLDDKVDFLPLRVDYQEKFSSTGKTL
GGFIKREGRPSEKEILVSRLIDRSLRPSFPYRLMQDVQVLSYVWSYDGQVLPDPLAICAASAALAISDIPQSNIV
AGVRIGCIDNQWVINPTKTELASSTLDLVLAGTENAILMIEGHCDFFTEEQVLDAIEFGHKHIVTICKRLQLWQ
EEIGKSKNLSAVYPLPAEVLTAVKECAQDKFTELFNIKDKKVHAATAHEIEENILEKLQREDDDLFSSFNIKAA
CKTLKSDTMRALIRDREIRADGRSLTTVRPITIETSYLPRTHGSCLFTRGETQTLAVCTLGSEAMAQRYEDLNG
EGLSKFYLQYFFPPFSVGEVGRIGSPGRREIGHGKLAEKALSHALPDSATFPYTIRIESNITESNGSSSMASVCG
GCLALMDAGVPISSPIAGIAMGLILDDQGAIILSDISGLEDHLGDMDFKIAGSGKGITAFQMDIKVEGITPAIMK
KALSQAKQGCNDILNIMNEALSAPKADLSQYAPRIETMQIKPTKIASVIGPGGKQIRQIIEETGVQIDVNDLGV
VSISASSASAINKAKEIIEGLVGEVEVGKTYRGRVTSVVAFGAFVEVLPGKEGLCHISECSRQRIENISDVVKEG
DIIDVKLLSINEKGQLKLSHKATLE

>core/90/1/Org1_Gene399

MSNQEFDLSAIRNIGIMAHIDAGKTTTTERILFYAGRTHKIGEVHEGGATMDWMAQEQERGITITSAATTVFW
LGAKINIIDTPGHVDFTIEVERSLRVLDGAVAVFDAVSGVEPQSETVWRQADKYGVPRIAFVNKMDRMGAD
YFAAVESMKEKLGANAFPVHCPIGSESQFVGMVDLISQKALYFLDDTLGAKWEEKEISEDLKERCAELRANL
LEELATIDESNEAFMMKVLEDPDSITEDEIHQVMRKGVIENKINPVLCGTAFKNKGVQQLLNVIVKWLPSPLD
RGNIRGINLKTDQEISLEPRRDGPLAALAFKIMTDPYVGRITFIRIYSGTLKKGSAILNSTKDKKERISRLLEMH
ANERTDRDEFTVGDIGACVGLKFSVTGDTLCDDNQEIVLERIEFPDPVIDMAIEPKSKGDREKLAQALSSLSEE
DPTFRVSTNEETGQTIISGMGELHLDILRDRMIREFKVEANVGKPQVSYKETITVSGNSETKYVKQSGGRGQY
AHVCLEIEPNEPGKGNEVVSKIVGGVIPKEYIPAVIKGIEEGLNTGVLAGYGLVDVKVSIVFGSYHEVDSSEM
AFKICGSMAVKDACRKAKPVILEPIMKVAVITPEDHLGDVIGDLNRRRGKILGQESSRGMAQVNAEVPLSEM
FGYTTSLRSLTSGRATSTMEPAFFAKVPQKIQEEIVKK

>core/92/1/Org1_Gene351

MYNLLHAHHDAASPDGRLVSHLKKLSPHIYEGEVLIENIPAYFLGFHLPQQCIQVNLKSSLAQLGVEAVLNHL
ELNKARKEARLHVLFMSQDPIATAMLELLEPGSFVCKLFAADDRRLVRSPCYLNRMFTHTDRTGSPLLRFGK
KLEHFITLEIINDRLVVFLPILPGTICYEETIYGFLPLMSKSLTRPHLKIRKFLPLYQMVTDRPPVPEDHKILLIKT
EPLHIRTVFARVVQDLLPQGLRHTAADILEPTTQESGDIYEFYGSTSEPIERIPLEFFTLEPYKEHSFFFYRDMLQ
ETLESPQEVFRVFESIPEGEDQAAMFISKGSELLELSQDSWIIKPRISPSDERHAREIQKHIEDQPCFPFLKAMET
DHITSQGVLFSRYFPSASLKGMFLSNYSRYYLQHIYFQIPSPTSGEFFSNRDRSFLLDLYFAGISVFWADLESKR
LLQYIKRRNKDVGMFVPKHQAEQFAQSYFIGIHGSCLIAGDYDEFLRELLTGMHTLSQQFTIPEFPPQTPLAIL
TGGGSGAMELANRVATELSILSCGNLISLDTTNAYVEAKMSYAIPDLLERQADFHVDLAVFVIGGMGTDFEL
LLELISLKTGKKALVPVFLIGPVDYWKSKITALYNSNHAVGTIRGSEWVHNCLFCLSSAKAGIAIFRRYLNHTL
PIGPEHPVPEDGFVIV

>core/93/1/Org1_Gene763

MKRSRRNFEQALENLEKLKEISLATSNDSYLNNPARFNQRKQTGSSVMEMKEALKNVENYLLEISCVSKSHA
DKALKESDFLIAGVQNVFSFLENQEDLYKSLLDEYSEVTKAYDEVKKNLKEVPTYDLSTDEETEEHKEPECFL
NNLVEVKRDRSYELFYMLDEQDKRFYNDALVQIIYKQNKLHETVNEGDPLTKTLLWNSEEVKNIASSLVIVN
DMPLRLFYQRALSHLDIEAVVKVHNAVMALFFSRYEATMVFKSPKKHNIWYFNDFLLFLREAWKDLNNNVI
DSQERKQTKLLASALSLGIFESKLVFEEASRYLYFNIQTKLENANGKKPLSPGQYLTDAYEELHRLISKYPNGP
LFKAMDRVLEHESRPYDPMILGILPSLEGTLKLHGKSIDIIRSPSPVTQSSILYANCNEEFLGFLNAKAHRSEVT
LVLNIQNRISRKERARSRVIEEALEQEEHAPYVHAFSFPEPEELLQNLESIHGDIETFADFFSILQEEFHKPLLAS
SFFLTKELKEFVGSFLKEKLTALKDIFFAKKKILFRNDKLLLLHLLSYLIVFKLIERTNPNSIVVVSKDGLDYVS
VFIAGFAFFSREAFWDEHSLKLLLTNVLSPTLVARDRLVFVSHIELLSKFVNCLKKNRQGFSSLKSFFKDDIEG
WEFTGYLHELTEVSHKHNL

>core/94/1/Org1_Gene607

MQPCLNMSIVRNSALPLPCLSRSETFKKVRSHMKFMKVLTPWIYRKDLWVTAFLLTAIPGSFAHTLVDIAGEP
RHAAQATGVSGDGKIVIGMKVPDDPFAITVGFQYIDGHLQPLEAVRPQCSVYPNGITPDGTVIVGTNYAIGM
GSVAVKWVNGKVSELPMLPDTLDSVASAVSADGRVIGGNRNINLGASVAVKWEDDVITQLPSLPDAMNAC
VNGISSDGSIIVGTMVDVSWRNTAVQWIGDQLSVIGTLGGTTSVASAISTDGTVIVGGSENADSQTHAYAYK
NGVMSDIGTLGGFYSLAHAVSSDGSVIVGVSTNSEHRYHAFQYADGQMVDLGTLGGPESYAQGVSGDGKVI
VGRAQVPSGDWHAFLCPFQAPSPAPVHGGSTVVTSQNPRGMVDINATYSSLKNSQQQLQRLLIQHSAKVESV
SSGAPSFTSVKGAISKQSPAVQNDVQKGTFLSYRSQVHGNVQNQQLLTGAFMDWKLASAPKCGFKVALHY
GSQDALVERAALPYTEQGLGSSVLSGFGGQVQGRYDFNLGETVVLQPFMGIQVLHLSREGYSEKNVRFPVSY
DSVAYSAATSFMGAHVFASLSPKMSTAATLGVERDLNSHIDEFKGSVSAMGNFVLENSTVSVLRPFASLAMY
YDVRQQQLVTLSVVMNQQPLTGTLSLVSQSSYNLSF

>core/95/1/Org1_Gene17

MGVVQNQVISSIRDVLKLVWELRFAEHKMLLLSRQSGSGGTFQLSCAGHELAGVLAGKSLIPGKDWSFPYY
RDQGFPIGLGCDLSEIFASFLARTTPNHSSARMMPYHYSHKKLRICCQSSVVGTQFLQAAGRAWAVKHSSAD
EVVYVSGGDGATSQGEFHEMLNFVALHQLPLITVIQNNHWAISVPFEDQCGADLASLGRCHQGLAVYEVDG
GNYTSLTETFSHAVDQARQHSVPALILIDVVRLSSHSNSDNQEKYRSALDLKLSMDKDPLILLEKEAINVFGLS
PFEIEEIKAEAQEEVRKSCEIAEALPFPSKGSTSHEVFSPYTETLIDYENSESAQNLRNSEPKVMRDAISEALVEE
MTRDSGVIVFGEDVAGDKGGVFGVTRNLTEKFGPQRCFNSPLAEATIIGTAIGMALDGIHKPVVEIQFADYIW
PGINQLFSEASSIYYRSAGEWEVPLVIRAPSGGYIQGGPYHSQSIEGFLAHCPGIKVAYPSNAADAKALLKAAI
RDPNPVVFLEHKALYQRRIFSACPVFSHDYVLPFGKAAIVHPGKDLTIVSWGMPLVLSLEVAQELASRGISIEV
IDLRTMVPCDFATVLKSLEKTGRLLVIHEASEFCGFGSELVATMSEQGYAYLDAPIRRLGGLHAPVPYSKVLE
NEVLPHKESILQAAKSLAEF

>core/96/1/Org1_Gene362

MLKKPKRKPGRRTYGKSLKIFIPGTLFVHARKGFGFVSPDNPEEYPFDIFVPARDLRGALDGDHVIVSVLPYPR
DGQKLKGTISEVLARGKTTLVGTITSLVSPTSALAYTSMSGSQSLIPVELLPGRTYKIGDRILLSTPPWVDKPQE
GASPALQMLEFIGHITNAKADFQAIQAEYNLAEEFPPEVIEEASLFSQKHITQVLHSRKDLRDLLCFTIDSSTAR
DFDDAISLTYDHNNNYILGVHIADVSHYVTPHSHLDKEAAKRCNSTYFPGKVIPMLPSALSDNLCSLKPNVDR
LAVSVFMTFTKSGHLSDYQIFRSVIRSKYRMTYDEVDNIIEKKHSHPLSKILNEMATLSKKFSDIREERGCIRFV

LPSVTMSLDNLQEPVALIENHQTFSHKLIEEFMLKANEVVAYHISHQGVSLPFRSHEPPNDENLLAFQELAKN
MGFDITFTPTQEPDYQYLLQTTSAGHPLEQVLHSQFVRSMKTASYSTENKGHYGLKLDYYTHFTSPIRRYIDL
IVHRLLFNPLSIDQTHLEIIVRACSTKERVSAKAENSFENLKKTRFINKFLQEQPKTTYHAYIITANHEGLSFVV
TEFCHEGFIAAAELPKEYSLKKNALPESIPDKMKPGASIKVTIDSVNLLTQKIVWSIATTTEDKPKKIKKTPSKK
KGTKKRAS

>core/98/1/Org1_Gene1000

MINKELDIGILGKIAGAIKQISIESIQKASSGHPGLPLGCAELAAYLYGYVLRQNPRDPHWINRDRFVLSAGHG
SVLLYSCLHLAGFDVSLEDLQEFRQLHSRTPGHPEYGETVGVEATTGPLGQGLGNAVGMALSMKMLESRFN
RPGHEIFNGKIYCLAGDGCFMEGVSHEVCSFAGSLNLNNLVVIYDYNNVVLDGYLNEISVEDTKKRFEAYG
WDVYEIDGYDFTHIHETFSSIKRGQERPVLVIAHTIIGHGSPKEGTNKAHGSPLGVEGTHETKQFWHLPEEKFF
VPPAVKNFFAHKIQEDRKAQEQWLDEVRVWSKQFPELHEEFVALTSHKLPKNLESLVQSVEMPDSIAGRAAS
NKLIQVLVQHIPYLIGGSADLSSSDGTWIANEKVIHTYDFSGRNIKYGVREFGMATIMNGLAYSQVFRPFGGT
FLVFSDYMRNAIRLAALSKLPVIYQFTHDSIFVGEDGPTHQPVEQLMSLRAIPGLYVIRPADANEVRGAWIAG
LKHTGPTVIVLSRQALPTLPAAHRPFKDGVGRGAYIVLKESGEKPDYTLFATGSEVSLALSVAKELEHLDKQV
RVVSFPCWELFEAQDVDYKQSIVGGDLGIRVSIEAGSALGWYKYIGSEGLAIAMDRFGYSGASDDVSEECGF
TTEQILQRILSQ

>core/99/1/Org1_Gene282

MEKVSSYPSVPLPLGASKISPNRYRFALYASQATEVILALTDENSEVIEVPLYPDTHRTGAIWHIEIEGISDQSS
YAFRVHGPKKHGMQYSFKEYLADPYAKNIHSPQSFGSRKKQGDYAFCYLKEEPFPWDGDQPLHLPKEEMIIY
EMHVRSFTQSSSSRVHAPGTFLGIIEKIDHLHKLGINAVELLPIFEFDETAHPFRNSKFPYLCNYWGYAPLNFFS
PCRRYAYASDPCAPSREFKTLVKTLHQEGIEVILDVVFNHTGLQGTTCSLPWIDTPSYYILDAQGHFTNYSGC
GNTLNTNRAPTTQWILDILRYWVEEMHVDGFRFDLASVFSRGPSGSPLQFAPVLEAISFDPLLASTKIIAEPWD
AGGLYQVGYFPTLSPRWSEWNGPYRDNVKAFLNGDQNLIGTFASRISGSQDIYPHGSPTNSINYVSCHDGFTL
CDTVTYNHKHNEANGEDNRDGTDANYSYNFGTEGKTEDPGILEVRERQLRNFFLTLMVSQGIPMIQSGDEY
AHTAEGNNNRWALDSNANYFLWDQLTAKPTLMHFLCDLIAFRKKYKTLFNRGFLSNKEISWVDAMGNPMT
WRPGNFLAFKIKSPKAHVYVAFHVGAQDQLATLPKASSNFLPYQIVAESQQGFVPQNVATPTVSLQPHTTLIA
ISHAKEVT

>core/100/1/Org1_Gene96

MKEENSQAHYLALCRELEDHDYSYYVLHRPRISDYEYDMKLRKLLEIERSHPEWKVLWSPSTRLGDRPSGTF
SVVSHKEPMLSIANSYSKEELSEFFSRVEKSLGTSPRYTVELKIDGIAVAIRYEDRVLVQALSRGNGKQGEDIT
SNIRTIRSLPLRLPEDAPEFIEVRGEVFFSYSTFQIINEKQQQLEKTIFANPRNAAGGTLKLLSPQEVAKRKLEISI
YNLIAPGDNDSHYENLQRCLEWGFPVSGKPRLCSTPEEVISVLKTIETERASLPMEIDGAVIKVDSLASQRVLG
ATGKHYRWALAYKYAPEEAETLLEDILVQVGRTGVLTPVAKLTPVLLSGSLVSRASLYNEDEIHRKDIRIGDT
VCVAKGGEVIPKVVRVCREKRPEGSEVWNMPEFCPVCHSHVVREEDRVSVRCVNPECVAGAIEKIRFFVGR
GALNIDHLGVKVITKLFELGLVHTCADLFQLTTEDLMQIPGIRERSARNILESIEQAKHVDLDRFLVALGIPLIG
IGVATVLAGHFETLDRVISATFEELLSLEGIGEKVAHAIAEYFSDSTHLNEIKKMQDLGVCISPYHKSGSTCFG
KAFVITGTLEGMSRLDAETAIRNCGGKVGSSVSKQTDYVVMGNNPGSKLEKARKLGVSILDQEAFTNLIHLE

>core/101/1/Org1_Gene53

MRLNIHKYLFIGRNKADFFSASRELGVVEFISKKCFITTEQGHRFVECLKVFDHLEAEYSLEALEFVKDESVSV
EDIVSEVLTLNKEIKGLLETVKALRKEIVRVKPLGAFSSSEIAELSRKTGISLRFFYRTHKDNEDLEEDSPNVFY
LSTAYNFDYYLVLGVVDLPRDRYTEIEAPRSVNELQVDLANLQREIRNRSDRLCDLYAYRREVLRGLCNYDN
EQRLHQAKECCEDLFDGKVFAVAGWVIVDRIKELQSLCNRYQIYMERVPVDPDETIPTYLENKGVGMMGED
LVQIYDTPAYSDKDPSTWVFFAFVLFFSMIVNDAGYGLLFLMSSLLFSWKFRRKMKFSKHLSRMLKMTAILG
LGCICWGTTTTSFFGMSFSKTSVFREYSMTHVLALKKAEYYLQMRPKAYKELTNEYPSLKAIRDPKAFLLAT
EIGSAGIESRYVVYDKFIDNILMELALFIGVVHLSLGMLRYLRYRYSGIGWILFMVSAYLYVPIYLGTVSLIHY
LFHVPYELGGQIGYYGMFGGIGLAVVLAMIQRSWRGVEEIISVIQVFSDVLSYLRIYALGLAGAMMGATFNQ
MGARLPMLLGSIVILLGHSVNIILSIMGGVIHGLRLNFIEWYHYSFDGGGRPLRPLRKIVCSEDAEASGIHLDN
NSIV

>core/102/1/Org1_Gene361

MSEHKKSSKIIGIDLGTTNSCVSVMEGGQAKVITSSEGTRTTPSIVAFKGNEKLVGIPAKRQAVTNPEKTLGST
KRFIGRKYSEVASEIQTVPYTVTSGSKGDAVFEVDGKQYTPEEIGAQILMKMKETAEAYLGETVTEAVITVPA
YFNDSQRASTKDAGRIAGLDVKRIIPEPTAAALAYGIDKVGDKKIAVFDLGGGTFDISILEIGDGVFEVLSTNG
DTLLGGDDFDEVIIKWMIEEFKKQEGIDLSKDNMALQRLKDAAEKAKIELSGVSSTEINQPFITMDAQGPKHL
ALTLTRAQFEKLAASLIERTKSPCIKALSDAKLSAKDIDDVLLVGGMSRMPAVQETVKELFGKEPNKGVNPD
EVVAIGAAIQGGVLGGEVKDVLLLDVIPLSLGIETLGGVMTTLVERNTTIPTQKKQIFSTAADNQPAVTIVVLQ
GERPMAKDNKEIGRFDLTDIPPAPRGHPQIEVSFDIDANGIFHVSAKDVASGKEQKIRIEASSGLQEDEIQRMV
RDAEINKEEDKKRREASDAKNEADSMIFRAEKAIKDYKEQIPETLVKEIEERIENVRNALKDDAPIEKIKEVTE
DLSKHMQKIGESMQSQSASAAASSAANAKGGPNINTEDLKKHSFSTKPPSNNGSSEDHIEEADVEIIDNDDK

>core/103/1/Org1_Gene929

MKLLLKAVLRHKNHLVILGCSLLAILGLTFSSQMEIFSLGMIAKTGPDAFLLFGRKESGKLVKVSELSQKDILE
NWQAISKDSETLTVSDATTYIAEHGKSTASLTSKLSKFVRNYIDVSRFRGLAIFLICVAIFKAVTLFFQRFLGQV
VAIRVSRDLRQDYFKALQQLPMTFFHDHDIGNLSNRVMTDSASIALAVNSLMINYIQAPITFILTLGVCLSISW
KFSILVCVAFPIFILPIVVIARKIKNLAKRIQKSQDSFSSVLYDFLAGVMTVKVFRTEKFAFTKYCEHNNKISAL
EEKSAAYGLLPRPLLHTIASLFFAFVVVIGIYKFAIPPEELIVFCGLLYLIYDPIKKFGDENTSIMRGCAAAERFY
EVLNHPDLHSQKEREIEFLGLSNTITFENVSFGYQEDKHILKNLSFTLHKGEALGIVGPTGSGKTTLVKLLPRL
YEVSQGKILIDSLPITEYNKGSLRNHIACVLQNPFLFYDTVWNNLTCGKDMEEEAVLEALKRAYADEFILKLP
KGVHSVLEESGKNLSGGQQQRLAIARALLKNASILILDEATSALDAISENYIKNIIGELKGQCTQIIIAHKLTTLE
HVDRVLYIENGQKIAEGTKEELLQTCPEFLKMWELSGTKEYNRVFVPDHKLVANPTDMAITT

>core/104/1/Org1_Gene664

MFSRLFFTSFSAEVVNTFFESGMSEDTSPLLSKQNRKLSHNLPLKSAYLSLGTYLIALLSFWLHAKNLSNLFVV
FTFFLAGTPALIKSLDNICQKVVNIDILMTSAAFGSIFIGGALEGALLLVLFAISEALGQMVSGKAKSTLVSLKQ
LAPTTGWLVLEDGNLQKVAINKIEVGNILRIKSGEVVPLDGEILHGSSSINLMHLTGEKVPKSCHPGSIVPAGA
HNMEGSFDLRVLRTGSDSTIAHIINLVIQAQNSKPRLQQRLDKYSSVYALSIFAIACGIALLVPLFTSIPLLGPQS
AFYRALAFLIAASPCALIIAIPIAYLSAINACAKHGVLLKGGVILDRLVSCNSVVMDKTGTLTTGELTCIGCDYF

GSKNETFFPSVLALEQSSSHPIAEAIVSYLMEQKVSSLPADRYLTVPGEGVRGYFNEQEAFVGRVETGLGKVP
SEYLEDIEQKIYQAKQHGEICSLAYVGNSFALFYFRDIPRPQAKEIIQDLKDLGYPVSMLTGDHKVSAENTAEI
LGISEVFFDLTPEDKLAKIRELATQRQIMMVGDGINDAPALAQATVGIAMGEAGSATAIEAADIVLLHDSLSS
LPWIIQKAKQTKKVVSQNLALALAIILLVSWPASLGIIPLWLAVILHEGSTVIVGLNALRLLKS

>core/105/1/Org1_Gene609

MTFQLHAPFAPCGDQPEAIARLSAGVRNQVKSQVLLGTTGSGKTFTIANVVANVNLPTLVLAHNKTLAAQL
YQEFREFFPNNAVEYFISYYDYYQPEAYIARSDTYIEKSLLINDEIDKLRLSATRSILERRDTLIVSSVSCIYGIGS
PENYTSMALVLEVGKEYPRNILTAQLVKMHYQASPIPQRSAFRERGSVIDIFPAYESELALRLEFLNDTLTSIE
YSDPLTMIPKESVPSATLYPGSHYVIPEAIREQAIRTIQEELEERMAFFDDRPIEKDRIFHRTTHDIEMIKEIGFCK
GIENYSRHFTGAPPGAPPTCLLDYFPEDFLLIIDESHQTLPQIRAMYRGDQSRKQSLVEYGFRLPSAFDNRPLT
YEEAQKYFRKVIYVSATPGDTEVQESSGHIVQQIIRPTGIPDPMPEIRPATGQVDDLLEEIRLRLSQKHEKILVIS
ITKKLAEDMAGFLSELEIPAAYLHSGIETAERTQILTDLRSGVIDVLIGVNLLREGLDLPEVSLVAILDADKEGF
LRSTSSLIQFCGRAARNINGKVIFYADQKTRSIEETLRETERRRQIQLDYNKEHNIVPKPIIKAIFANPILQTSKDS
ESPKESQRPLSKEDLEEQIKKYEALMQRAAKEFRFNEAAKYRDAMQACKEQLLYLF

>core/106/1/Org1_Gene430

MATPAQKSPTFQDPSFVRELGSNHPVFSPLTLEERGEMAIARVQQCGWNHTIVKVSLIILALLTILGGGLLVGL
LPAVPMFIGTGLIALGAVIFALALILCLYDSQGLPEELPPVPEPQQIQIEDLRNETREVLEGTLLEVLLKDRDAK
DPAVPQVVVDCEKRLGMLDRKLRREEEILYRSTAHLKDEERYEFLLELLEMRSLVADRLEFNRRSYERFVQG
IMTVRSEEGEKEISRLQDLISLQQQTVQDLRSRIDDEQKRCWTALQRINQSQKDIQRAHDREASQRACEGTEM
DCAERQQLEKDLRRQLKSMQEWIEMRGTIHQQEKAWRKQNAKLERLQEDLRLTGIAFDEQSLFYREYKEKY
LSQKLDMQKILQEVNAEKSEKACLESLVHDYEKQLEQKDANLKKAAAVWEEELGKQQQEDYEQTQEIRRL
STFILEYQDSLREAEKVEKDFQELQQRYSRLQEEKQVKEKILEESMNHFADLFEKAQKENMAYKKKLADLE
GAAAPTEIGEDDDWVLTDSASLSQKKIRELVEENQELLKALAFKSNELTQLVADAVEAEKEISKLREHIEEQK
EGLRALDKMHAQAIKDCEAAQRKCCDLESLLSPVREDAGMRFELEVELQRLQEENAQLRAEVERLEQEQFQ
G

>core/107/1/Org1_Gene306

MSYRKRSTLIVLGVFALYALLVLRYYKIQICEGDHWAAEALGQHEFCVRDPFRRGTFFANTTVRKGDKDLQ
QPFAVDITKFHLCADPLAIPECHRDEIIQGILQFIEGQTYDDLSLKLDKKSRYCKLYPLLDVSVHDRLSLWWK
GYATKHRLPTNALFFITDYQRSYPFGKLLGQVLHTLREIKDEKTGKAFPTGGMEAYFNHILEGDVGERKLLRS
PLNRLDTNRVIKLPKDGSDIYLTINPVIQTIAEEELERGVLEAKAQGGRLILMNSQTGEILALAQYPFFDPTNYK
EYFNNKERIEHTKVSFVSDVFEPGSIMKPLTVAIALQANEEASLKSQKKIFDPEEPIDVTRTLFPGRKGSPLKDI
SRNSQLNMYMAIQKSSNVYVAQLADRIIQSLGVAWYQQKLLALGFGRKTGIELPSEASGLVPSPHRFHINGSL
EWSLSTPYSLAMGYNILATGIQMVQAYAILANGGYAVRPTLVKKIVSASGEEYHLPTKEKTRLFSEEITREVV
RAMRFTTLPGGSGFRASPKHHSSAGKTGTTEKMIHGKYDKRRHIASFIGFTPVESSEGNFPPLVMLVSIDDPEY
GLRADGTKNYMGGRCAAPIFSRVADRTLLYLGILPDKKLRNCDEEAAALKRLYEEWNRSPKQGGTR

>core/108/1/Org1_Gene554

MVNPIGPGPIDETERTPPADLSAQGLEASAANKSAEAQRIAGAEAKPKESKTDSVERWSILRSAVNALMSLAD
KLGIASSNSSSSTSRSADVDSTTATAPTPPPPTFDDYKTQAQTAYDTIFTSTSLADIQAALVSLQDAVTNIKDTA
ATDEETAIAAEWETKNADAVKVGAQITELAKYASDNQAILDSLGKLTSFDLLQAALLQSVANNNKAAELLK
EMQDNPVVPGKTPAIAQSLVDQTDATATQIEKDGNAIRDAYFAGQNASGAVENAKSNNSISNIDSAKAAIAT
AKTQIAEAQKKFPDSPILQEAEQMVIQAEKDLKNIKPADGSDVPNPGTTVGGSKQQGSSIGSIRVSMLLDDAE
NETASILMSGFRQMIHMFNTENPDSQAAQQELAAQARAAKAAGDDSAAAALADAQKALEAALGKAGQQQ
GILNALGQIASAAVVSAGVPPAAASSIGSSVKQLYKTSKSTGSDYKTQISAGYDAYKSINDAYGRARNDATR
DVINNVSTPALTRSVPRARTEARGPEKTDQALARVISGNSRTLGDVYSQVSALQSVMQIIQSNPQANNEEIRQ
KLTSAVTKPPQFGYPYVQLSNDSTQKFIAKLESLFAEGSRTAAEIKALSFETNSLFIQQVLVNIGSLYSGYLQ

>core/109/1/Org1_Gene946

MKKLVRLCVVLLSLLPNVLFSSDLLREEGIKKMMDKLIEYHVDAQEVSTDILSRSLSSYIQSFDPHKSYLSNQE
VAVFLQSPETKKRLLKNYKAGNFAIYRNINQLIHESILRARQWRNEWVKNPKELVLEASSYQISKQPMQWSK
SLDEVKQRQRALLLSYLSLHLAGASSSRYEGKEEQLAALCLRQIENHENVYLGINDHGVAMDRDEEAYQFHI
RVVKALAHSLDAHTAYFSKDEALAMRIQLEKGMCGIGVVLKEDIDGVVVREIIPGGPAAKSGDLQLGDIIYR
VDGKDIEHLSFRGVLDCLRGGHGSTVVLDIHRGESDHTITLRREKILLEDRRVDVSYEPYGDGVIGKVTLHSF
YEGENQVSSEQDLRRAIQGLKEKNLLGLVLDIRENTGGFLSQAIKVSGLFMTNGVVVVSRYADGTMKCYRT
VSPKKFYDGPLAILVSKSSASAAEIVAQTLQDYGVALVVGDEQTYGKGTIQHQTITGDASQDDCFKVTVGKY
YSPSGKSTQLQGVKSDILIPSLYAEDRLGERFLEHPLPADCCDNVLHDPLTDLDTQTRPWFQKYYLPNLQKQE
TLWREMLPQLTKNSEQRLSENSNFQAFLSQIKSSEKTDLSYGSNDLQLEESINILKDMILLQQCRK

>core/110/1/Org1_Gene811

MTSSSCPLLDLILSPADLKKLSISQLPGLAEEIRYRIISVLSQTGGHLSSNLGIVELTIALHYVFSSPKDKFIFDVG
HQTYPHKLLTGRNNEGFDHIRNDNGLSGFTNPTESDHDLFFSGHAGTALSLALGMAQTTPLESRTHVIPILGD
AAFSCGLTLEALNNISTDLSKFVVILNDNNMSISKNVGAMSRIFSRWLHHPATNKLTKQVEKWLAKIPRYGD
SLAKHSRRLSQCVKNLFCPTPLFEQFGLAYVGPIDGHNVKKLIPILQSVRNLPFPILVHVCTTKGKGLDQAQN
NPAKYHGVRANFNKRESAKHLPAIKPKPSFPDIFGQTLCELGEVSSRLHVVTPAMSIGSRLEGFKQKFPERFFD
VGIAEGHAVTFSAGIAKAGNPVICSIYSTFLHRALDNVFHDVCMQDLPVIFAIDRAGLAYGDGRSHHGIYDMS
FLRAMPQMIICQPRSQVVFQQLLYSSLHWSSPSAIRYPNIPAPHGDPLTGDPNFLRSPGNAETLSQGEDVLIIAL
GTLCFTALSIKHQLLAYGISATVVDPIFIKPFDNDLFSLLLMSHSKVITIEEHSIRGGLASEFNNFVATFNFKVDI
LNFAIPDTFLSHGSKEALTKSIGLDESSMTNRILTHFNFRSKKQTVGDVRV

>core/111/1/Org1_Gene964

MTCISELNEAQRKAVTAPLNPVLVLAGAGAGKTRVVTYRILHLINQGIAPREILAVTFTNKAARELKERIVNQ
CASTNEFDVPMVCTFHSLGVFILRRSINLLNRENNFTIYDQSDAEKLIKHALQQHNLKPNLASKIQAHVSQAK
NRLLFPEDLDPNDYIDPVVSIYQEYQKKLIEANALDFDDLLFLTVRLLRESPEAQELYNQLWKALLIDEYQDT
NHAQYTLMQLLSKQHRNVFAVGDPDQSIYSWRGANIHNILNFENDYPNAKVLCLEENYRSYGNILNAANALI
KNNASRLEKELRSVKGPGEKIRLFLGSTDREEADFVAAEILQLHRVGNIKLRDICIFYRTNSQSRTFEDALLRR
RIPYEIIGGLSFYKRKEIQDILAFLRIFISKSDIVAFDRTVNLPKRGIGSTTIFALTQYAIAQGLPILKACQQALDT
KDVKLSKKQQEGLQEYLALFPQIEHAYNTLSLRDFIESVVRITGYLEILKEDADTFKDRKSNLEELYHKALESE

QQNPKTHLELFLDDLALKGSDDDLNLTADRVNLMTLHNGKGLEFRVSFLVGLEEQLLPHANSLGGTYENIEE
ERRLCYVGITRAQDLLYLTTAQVRSLWGTVRMMKPSRFLKEIPKDYMIQVR

>core/112/1/Org1_Gene67

MKQHYSLNKSRHILRSTYKLLKSKKLAHSPADKKQLQELLEQLEEAIFEHDQETASDLAQQALAFSNRYPNS
FGRKTYELIKALLFAGVVAFLVRQFWFELYEVPTGSMRPTILEQDRILVSKTTFGLHCPFAKKPLAFNPESVTR
GGLVVFTVGDLPIPDADTKYFGLIPGKKRYIKRCMGRPGDFLYFYGGKIYGLDDAGKRIEFPSVHGLENLYH
VPYISFDGTTSSHTEGQKTIIDFKQFNQSYGRLIFPQTSMYGQFFDHKEWHQDEPNKLKDPHLSPVSYADLFG
MGNYAMVRILTEHQARTSHLLPNPGSPTKVYLEICHTANLSYPKPLLRHYEHQLSPAIQPMKTLLPLRKEHLH
LIRNNLTTSRFIVAQGCAYKYHQFKINTSGIAKAYAILLPKVPDGCYEYSKGEAYQIGFGEIRYKLKSSHPLTQ
LNDKQVIELFNCGINFSSIYNPVNPLQAPLPNRYAFFNQGNLYIMDSPVFIKNDPTLQKFVTSETEKQEGSSET
QPYIAFVDKGLPPEDFKEFVEFIHNFGIQVPKGHVLVLGDNYPMSADSREFGFVPMENLLGSPLCTFWPIGRM
GRLTGVSAPTTLSGYLVSGIALATGLSLIGYVYYQKRRRLFPKKEEKNHKK

>core/113/1/Org1_Gene604

MIPFTKTIGFRLWLACAVAIIAPLGINIVWLNLDQYRTIVSAISTALKENAAFKANTLTQIVPLNVDVLSLFSDV
LDLDAGIPETPNVLLSNEMQKVFQGIYNEISLIKVFPNGDKIVVASSIPEHLGENYNHKIDIPKNTPFLAALKQS
PKNQEVFSVMQANVFDAKTQELQGILYTTFSAESLLKDLLINKQSYLTVKTAILSKYGVILKASDPALHLHTV
YPDMTKEKFCQVFLNDDPCPIDSELGPLTLSPLDIGENFYSFKIKDTEIWGCIENVPSIDIAVLSYAKKEESFAPL
WRRARMYTAYFFCILLGSLIAFIVARRLSLPIRKLATAMIESRKNKNCLYTDDSLGFEINRLGHIFNAMVENLH
KQQHLAKTNFEMKENAQNALHLGEQAQQRLLPNTLPSYPHIELAKAYIPAITVGGDFFDVFVVGEGSKARLF
LIVADASGKGVNACGYSLFLKNMLRTFLSRSSSLQQAIQETSRLFYNNTKNSGMFVTLCVYCYHQTSNTMEY
YSCGHPPACYLDPDGETSWLFHPGMALGFLPEVANITSKLFHPKPGSLFVLYSDGITEAHNNNNDMFGEERL
QAAIQGLTGKSAADAVHRLMLSVKTFVGNSHQHDDITLLILKVLES

>core/114/1/Org1_Gene614

MIQVTCDQKNYEVLEGTTAAELAKQLKNSHQFIGVLINERPRDLSTHLNEGDTLVFLTSEDPEGREIFLHTSA
HLLAQAVLRLWPDAIPTIGPVIDHGFYYDFANLSISESDFPLIEDTVKQIVDEKLAISRFTYGDKQQALAQFPQ
NPFKTELIRELPENEEISAYSQGEFFDLCRGPHLPSTAHVKAFKVLRTSAAYWRGDPSRESLVRIYGTSFPTSKE
LRAHLEQIEEAKKRDHRVLGAKLDLFSQQESSPGMPFFHPRGMIVWDALIRYWKQLHTAAGYKEILTPQLM
NRQLWEVSGHWDNYKANMYTLQIDDEDYAIKPMNCPGCMLYYKTRLHSYKEFPLRVAEVGHVHRQEASG
ALSGLMRVRAFHQDDAHVFLTPEQVEEETLNILQLVSTLYGTFGLEYHLELSTRPEKDTIGDDSLWELATDAL
NRALVQSGTPFIVRPGEGAFYGPKIDIHVKDAIQRTWQCGTIQLDMFLPERFELEYTTAQGTKSVPVMLHRAL
FGSIERFLGILIENFKGRFPLWLSPEQVRIITVADRHIPRAKELEEAWKRLGLVVTLDDSSESVSKKIRNAQNM
QVNYMITLGDHEINENVLAVRTRDNRVINDVSVERFLNTILEEKNSLSLTALL

>core/117/1/Org1_Gene602

MDENRKVIVVGGGLAGLSAAMQLANLGIIVELVSLTKVKRSHSVCAQGGINAALNLKPEEEDSPYVHAYDTI
KGGDFLADQPPVLEMCLAAPRIIKMLDNFGCPFNRGPSGNLDVRRFGGTLYHRTVFCGASTGQQLMYTLDE
QVRRREHAGRVIKRENHEFVRLVTDHSGRACGIILMNLFNNRLEILRGDAVIIATGGPGVIFKMSTNSTFCTGA
ANGRLFLQGMAYANPEFIQIHPTAIPGRDKLRLISESVRGEGGRVWVPGDSSKRIVFPDGSERPCGETGAPWY

FLEDMYPAYGNLVSRDVGARAILRVCEAGLGIDGRMEAYLDVTHLPEKTRHKLEVVLDIYKKFTGEDPNTV
PMRIFPAVHYSMGGAWVDWPAADDPDRDSRFRQMTNIPGCFNCGESDFQYHGANRLGANSLLSCLFAGLVS
GDEASRFIEAFGASQATSSDFDRALQQEKEENARLLSASGKENIFVLHEEIAKIMVRNVTVKRNNRDLQETM
DKLKEFRERLKNVSVLDSSPFANKSFHFVRQMGPMLELALAITKGALLRNEFRGSHYKPEFPERDDEHWLKT
TVAVYAPEEPEISYLPVDTRHVAPTLRDYTKSSTGKIELTNIPDNIRLPI

>core/118/1/Org1_Gene838

MTIIYFILAALALGILVLIHELGHLVVAKAVGMAVESFSIGFGPALFKKRIGGIEYRIGCIPFGGYVRIRGMERT
KEKGEKGKIDSVYDIPQGFFSKSPWKRILVLVAGPLANILLAVLAFSILYMNGGRSKNYSDCSKVVGWVHPV
LQAEGLLPGDEILTCNGKPYVGDKDMLTTSLLEGHLNLEIKRPGYLTVPSKEFAIDVEFDPTKFGVPCSGASY
LLYGNQVPLTKNSPMENSELRPNDRFVWMDGTLLFSMAQISQILNESYAFVKVARNDKIFFSRQPRVLASVL
HYTPYLRNELIDTQYEAGLKGKWSSLYTLPYVINSYGYIEGELTAIDPESPLPQPQERLQLGDRILAIDGTPVSG
SVDILRLVQNHRVSIIVQQMSPQELEEVNSRDADKRFIASYHSEDLLQILNHLGESHPVEVAGPYRLLDPVQPR
PWIDVYSSESLDKQLEVAKKIKNKDKQRYYLERLDAEKQKPSLGISLKDLKVRYNPSPVVMLSNITKESLITL
KALVTGHLSPQWLSGPVGIVQVLHTGWSVGFSEVLFWIGLISMNLAVLNLLPIPVLDGGYILLCLWEIVTRRR
LNMKIVERILVPFTFLLIIFFIFLTFQDLFRFFG

>core/119/1/Org1_Gene369

MRRSVCYVNPSIARAGQISTWKFLYSLATPLPAGTKCKFDLAGSGKPTDWEAPATDLSQTRNVIYAEMPEGE
IIEATAIPVKDNPVPQFEFTLPYELQVGETLTIVMGASPNHPQVDDAGNGAQLFAQRRKPFYLYIDPTGEGNY
DEPDVFSMDIRGNVLKKIEIFTPSYVVKNKRFDITVRFEDEFGNLTNFSPEETRIELSYEHLRENLNWQLFIPET
GFVILPNLYFNEPGIYRIQLKNLSTQEIFISAPIKCFADSAPNLMWGLLHGESERVDSEENIETCMRYFRDDRAL
NFYASSSFENQENLSPDIWKLINQTVSDFNEEDRFITLSGFQYSGEPHLEGVRHILHTKETKSHSKHKEYKHIPL
AKLYKSTVNHDMISIPSFTASKEHGFDFENFYPEFERVVEIYNAWGSSETTAALNNPFPIQGKDSEDPRGTVIE
GLKKNLRFGFVAGGLDDRGIYKDYFDSPQVQYSPGLTAIICNKYTRESLVEALFARHCYATTGPRIVLSFNITS
APMGSELSTGSKPGLNVNRHISGHVAGTALLKTVEIIRNGEVLHTFFPDSNNLDYEYDDMVPLSSVTLKDPNG
KAPFVFYYLRVTQADNAMAWSSPIWVDLN

>core/120/1/Org1_Gene913

MESEKDIGAKFLGDYRILYRKGQSLWSEDLLAEHRFIKKRYLIRLLLPDLGSSQPFMEAFHDVVVKLAKLNHP
GILSIENVSESEGRCFLVTQEQDIPILSLTQYLKSIPRKLTELEIVDIVSQLASLLDYVHSEGLAQEEWNLDSVYI
HILNGVPKVILPDLGFASLIKERILDGFISDEENRESKIKERVLLHTSEGKQGREDTYAFGAITYYLLFGFLPQGI
FPMPSKVFSDFIYDWDFLISSCLSCFMEERAKELFPLIRKKTLGEELQNVVTNCIESSLREVPDPLESSQNLPQA
VLKVGETKVSHQQKESAEHLEFVLVEACSIDEAMDTAIESESSSGVEEEGYSLALQSLLVREPVVSRYVEAEK
EEPKPQPILTEMVLIEGGEFSRGSVEGQRDELPVHKVILHSFFLDVHPVTNEQFIRYLECCGSEQDKYYNELIRL
RDSRIQRRSGRLVIEPGYAKHPVVGVTWYGASGYAEWIGKRLPTEAEWEIAASGGVAALRYPCGEEVEKSR
ANFFTADTTTVMSYPPNPYGLYDMAGNVYEWCQDWYGYDFYEISAQEPESPQGPAQGVYRVLRGGCWKSL
KDDLRCAHRHRNNPGAVNSTYGFRCAKNIN

>core/121/1/Org1_Gene779

MKKGKLGAIVFGLLFTSSVAGFSKDLTKDNAYQDLNVIEHLISLKYAPLPWKELLFGWDLSQQTQQARLQLV
LEEKPTTNYCQKVLSNYVRSLNDYHAGITFYRTESAYIPYVLKLSEDGHVFVVDVQTSQGDIYLGDEILEVDG
MGIREAIESLRFGRGSATDYSAAVRSLTSRSAAFGDAVPSGIAMLKLRRPSGLIRSTPVRWRYTPEHIGDFSLV
APLIPEHKPQLPTQSCVLFRSGVNSQSSSSSLFSSYMVPYFWEELRVQNKQRFDSNHHIGSRNGFLPTFGPILW
EQDKGPYRSYIFKAKDSQGNPHRIGFLRISSYVWTDLEGLEEDHKDSPWELFGEIIDHLEKETDALIIDQTHNP
GGSVFYLYSLLSMLTDHPLDTPKHRMIFTQDEVSSALHWQDLLEDVFTDEQAVAVLGETMEGYCMDMHAV
ASLQNFSQSVLSSWVSGDINLSKPMPLLGFAQVRPHPKHQYTKPLFMLIDEDDFSCGDLAPAILKDNGRATLI
GKPTAGAGGFVFQVTFPNRSGIKGLSLTGSLAVRKDGEFIENLGVAPHIDLGFTSRDLQTSRFTDYVEAVKTIV
LTSLSENAKKSEEQTSPQETPEVIRVSYPTTTSAS

>core/122/1/Org1_Gene853

MFGTLVSTLCCPANSERDWEDHEVNCIYIASTSDTQLEAVQGGMHITELRGEPVRVLYETGHLYAFARENTC
HSRLEVSHTVRAMTYFWDRFFSRHWNVGRRFLVFYQGNGGAYVQAALDSSMHTQDIYVLGLSPTVYIRGN
YHVQHYRVRGFWPSCLDSLAACAENTSVLPYGESSDGIFYPSLFSHTFDNAIRYGERCLLVCSEGMGMLPET
QQQTSPLTSLEGGHEVALVLNPQQNPEALSIASRLMHEERGGRLESNYMPGRSSNPFMTSMYVLVRLNTLAQ
IYLMSPYYSFQSNDIVCLIFISSAAVETVSYIFLTVTDSTCGRRYLRVPRLVCTGLRNLALPTTLLELLILSYPRS
VEGVPFNVRFILGYMCTTRVVFFAWNLILHWPFRCLRHGIQLFVHRSIIGHTLGARITDLTLASMRYAIVFPSI
VSSCLLTALAHANTNILALDPYRLIESGDLRRPAFNDDEMQQADNPWDAYSIGLVINTCIYMLILFANLIFMV
YSVRRYHRSRR

>core/123/1/Org1_Gene268

MTLITPAINSSRRKTHTVRIGNLYIGSDHSIKTQSMTTTLTTDIDSTVEQIYALAEHNCDIVRVTVQGIKEAQAC
EKIKERLIALGLNIPLVADIHFFPQAAMLVADFADKVRINPGNYIDKRNMFKGTKIYTEASYAQSLLRLEEKFA
PLVEKCKRLGKAMRIGVNHGSLSERIMQKYGDTIEGMVASAIEYIAVCEKLNYRDVVFSMKSSNPKIMVTAY
RQLAKDLDARGWLYPLHLGVTEAGMGVDGIIKSAVGIGTLLAEGLGDTIRCSLTGCPTTEIPVCDSLLRHTKI
YLDLPEKKNPFSLQHSENFVSAAEKPAKTTLWGDVYGVFLKLYPHHLTDFTPEELLEHLGVNPVTKEKAFTT
PEGVVVPPELKDAPITDVLREHFLVFHHHQVPCLYEHNEEIWDSPAVHQAPFVHFHASDPFIHTSRDFFEKQG
HQGKPTKLVFSRDFDNKEEAAISIATEFGALLLDGLGEAVVLDLPNLPLQDVLKIAFGTLQNAGVRLVKTEYI
SCPMCGRTLFDLEEVTTRIRKRTQHLPGLKIAIMGCIVNGPGEMADADFGFVGSKTGMIDLYVKHTCVKAHIP
MEDAEEELIRLLQEHGVWKDPEETKLTV

>core/124/1/Org1_Gene457

MWTHPIAYDVIVVGAGHAGCEAAYCSAKMGVSVLMLTSNLDTIAKLSCNPAVGGIGKGHIVREIDALGGIM
AEVTDQSGIQFRILNQTKGPAVRAPRAQVDKQLYHIHMKRLLENTPGLHIMQATVESLLDKEGVISGVTTKE
GWMFSGKTVVLSSGTFMRGLIHIGDRNFSGGRLGDPSSQGLSEDLKKRGFPISRLKTGTPPRLLASSINFSCME
EQPGDLGVGFVHRTEPFQPPLPQLSCFITHTMEKTKAIISANLHRSALYGGCIEGVGPRYCPSIEDKIVKFSDKE
RHHVFLEPEGLHTQEIYANGLSTSMPFDVQYDMIRSVLGLENAIITRPAYAIEYDYIHGNVIHPTLESKLIEGLF
LCGQINGTTGYEEAAAQGLIAGINAVNKVFNRPPFIPSRQESYIGVMLDDLTTQILDEPYRMFTGRAEHRLLLR
QDNACARLSHYGYELGLLSEERYELVKKQNQLLEEEKVRLQKTFRQYGQSVVSLAKALSRPEVSYDMLREA
FPNDIRDLGAVLNASLEMEIKYSGYIDRQKILIQSLEKAESLLIPEDLDYKQITALSLEAQEKLAKFTPRTLGSA
SRISGIASADIQVLMIALKKHAHH

>core/125/1/Org1_Gene87

MTTELKTEALPTRTQVDPKHCWDTTLMYANREEWKKDFDLCSSGKDRSPIWPEFSPSHYQIDNPESLLELLS
KKFSVERKLDQLYIYAHLIHDQDITNPEGESDYQSIVYLYTLFSQEISWIQPALIALSEEKVAALLSSSVLAPYR
FYLEKIFRLSPHTGTANEEKILASSFAALNVSNKAFSSLSDAEIPFGIAKDSNGEEHPLSHALASLYMQSPDQEL
RRTAYLAQFQRYYDYRNTFANLLNGKVQAHLFEAKARNYPSCLEASLFQHNIPTTVYINLINETKKHTSLINR
YFNLKKEALNLKEFHFYDVYAPISQTTSKNYSYEEGVDLVCKSLLPLGTHYVEILRNGLLSNRWVDRYENKH
KRSGAYSSGCYDSAPYILLNYTNTLYDVSVIAHEAGHSMHSYFSREAQPYHDAQYPLFLAEIASTFNEMLLM
EALSKSDQSKEDKIVIITKTLDTIFATLFRQTFFAAFEYEIHSAAEQGTPLTEEFLSATYGNLQKEFYGGVVTSD
SLSALEWARIPHFYYNFYVYQYATGIIAALSFAEKILTQEPGALELYLKFLKSGRSDFPLNILKKSGLDMTTSA
PLDKAFAFITKKIDLLSSLLSED

>core/126/1/Org1_Gene572

MFRCILFGIFLLTCFSSGGVLYYLFCSHDFSIGPKEKSRSVWIEEEKEFTDSVLHHLPSQHQHLHILCFQGFLLQ
KQQKFSQAEKIFSKVYDEAQDGPFLFKEEILGSRLINSFFLEKTDVMETILCLLNQRCPNSPYYHLFKALVCYK
QKLYREVIEQLAYWQEEKTRALAPLLNISIEQLLTDFLLDYISAHSLIEQKMFPEGRVILNRNINRLLKHECEW
NAKTYDRIAILLSRSYFLELVESKSADIYFDYYEMVLFYLKKIYILEQCPYAELLPEEELVSLIMEHVFILPKDK
LYPLIQLLEMWQKHYVHPNSSLVVQILVDRFSTHMEGAIRFCEALVSFSGLEELHQQIITTFEELLSNKVQQIK
TEEAKQCVALLHILDPSISISEKLALSSDTLQNIVSGDDEQHTKLRNYLDLWEAIQSYDIDRQQLVHHLVYGA
KDLWKKGGNDEKALNLLQLVLRFTSYDIECESVVFLFIKQAYKQALSSHAIARLLKLEKFISEANIPSIVISEAE
KANFLADAEYLFAHEDYDKCYLYSMWLTKVAPSPQSYRLAGLCLMENKRYDEALEFLCMLSPNDSINDYK
TQKALAFCQKHQSKDRAAS

>core/127/1/Org1_Gene864

MCGIFGYLGNQDGVSIVLEGLAKLEYRGYDSAGLAAVVEQELFIRKTVGRVQELSNLFQEREIPTASVIGHTR
WATHGVPTEINAHPHVDEGRSCAVVHNGIIENFKELRRELTAQGISFASDTDSEIIVQLFSLYYQESQDLVFSF
CQTLAQLRGSVACALIHKDHPHTILCASQESPLILGLGKEETFIASDSRAFFKYTRHSQALASGEFAIVSQGKEP
EVYNLELKKIHKDVRQITCSEDASDKSGYGYYMLKEIYDQPEVLEGLIQKHMDEEGHILSEFLSDVPIKSFKEI
TIVACGSSYHAGYLAKYIIESLVSIPVHIEVASEFRYRRPYIGKDTLGILISQSGETADTLAALKELRRRNIAYLL
GICNVPESAIALGVDHCLFLEAGVEIGVATTKAFTSQLLLLVFLGLKLANVHGALTHAEQCSFGQGLQSLPDL
CQKLLANESLHSWAQPYSYEDKFLFLGRRLMYPVVMEAALKLKEIAYIEANAYPGGEMKHGPIALISKGTPV
IAFCGDDIVYEKMIGNMMEVKARHAHVIAIAPESREDIAAVSDQQIFVPDCHFLAAPVLYTIVGQVMAYAMA
LAKGMEIDCPRNLAKSVTVE

>core/128/1/Org1_Gene741

MIPSGLVYLLYPLGFLASLFFGSAFSIQWWLSKKRKEVYAPRSFWILSSIGATLMIVHGTIQSQFPVTVLHVINL
IIYLRNLNITSSRPISFRATLVLMALSVVFVTLPFLYVNMEWMASPNIFHLPLPPAQLSWHLIGCLGLAIFSGRF
LIQWFYIESNNTKDFPLLFWKIGLLGGLLALVYFIRIGDPINILCYGCGLFPSIANLRLFYKEQRSTPYLDTHCFL
SAGEASGDILGGKLIQSIKSLYPNIRFWGVGGPAMRQEGLQPILNMEEFQVSGFAEVLGSLFRLYRNYRKILKT
ILKHKPATLIFIDFPDFHLLLIKKLRKHGYRGKIIHYVCPSIWAWRPKRKRILEQHLDMLLLILPFEEGLFKNTSL
ETVYLGHPLVEEISDYKEQASWKEKFLNSDRPIVAAFPGSRRGDISRNLRIQVQAFLNSSLSQTHQFVVSSSSA

KYDEIIEDTLKAEGCQHSQIIPMNFRYELMRSCDCALAKCGTIVLETALNQTPTIVMCRLRPFDTFLAKYIFKIL
LPAYSLPNIIMNSVIFPEFIGGKKDFHPEEIATALDLLNQHGSKEKQKEDCRKLCKVMTTGQIASEEFLKRIFDT
LPAV

>core/129/1/Org1_Gene603

MKHTFTKRVLFFFFLVIPIPLLLNLMVVGFFSFSAAKANLVQVLHTRATNLSIEFEKKLTIHKLFLDRLANTLA
LKSYASPSAEPYAQAYNEMMALSNTDFSLCLIDPFDGSVRTKNPGDPFIRYLKQHPEMKKKLSAAVGKAFLL
TIPGKPLLHYLILVEDVASWDSTTTSGLLVSFYPMSFLQKDLFQSLHITKGNICLVNKYGEVLFCAQDSESSFV
FSLDLPNLPQFQARSPSAIEIEKASGILGGENLITVSINKKRYLGLVLNKIPIQGTYTLSLVPVSDLIQSALKVPL
NICFFYVLAFLLMWWIFSKINTKLNKPLQELTFCMEAAWRGNHNVRFEPQPYGYEFNELGNIFNCTLLLLLNS
IEKADIDYHSGEKLQKELGILSSLQSALLSPDFPTFPKVTFSSQHLRRRQLSGHFNGWTVQDGGDTLLGIIGLA
GDIGLPSYLYALSARSLFLAYASSDVSLQKISKDTADSFSKTTEGNEAVVAMTFIKYVEKDRSLELLSLSEGAP
TMFLQRGESFVRLPLETHQALQPGDRLICLTGGEDILKYFSQLPIEELLKDPLNPLNTENLIDSLTMMLNNETE
HSADGTLTILSFS

>core/130/1/Org1_Gene722

MRIEDFSLKLIPSSPGVYLMKDVHDQVLYIGKAKNLKNRLASYFHEKGDSRERIPFLMKKTASIETIVVSNETE
ALLLENNLIKQHHPKYNVLLKDDKTFFCLAISLSHSWPKVEAIRTKAITSSQRQLIFGPYVSAEACHTLLEVISQ
WFPLRTCSDREFALRKRPCILYDMKRCLAPCVGYCTPEEYQGTLDKAILFLKGKIEEVVKDLEKVIQKASDNL
EFEQAANYYRTLSLIKQAMAKQQVEKFHFQNIDALGLYRHKQRTILTLLTVRSGKLLGARHFSFFENAQEDQ
DLLSSFILQYYVSQPYIPKEILTPLPLEFPTLSYVLNAESPPRLRSPKTGYGKELLDLAYRNAKAYAATTLPSST
LPYQDFQNILRMSQYPYRIECYDNAHMQGAHATGVYIVFENNGFDPKQYRTFSIDSEKTQNDLALLEEVLLR
RFHSLTTALPDMIVVDGGKTHYNKTKKIIQTLNLTGIQVVTIAKEKSNHSRGLNKEKIFCETFPEGFSLPPTSNL
LQFFQILRDEAHRFAISKHRKKRGKALFEQEKIPGIGEVKRKRLLQKFKSWKQVMLSSQEELEAIPGLTKKDIA
VLLARQKDFNKSD

>core/131/1/Org1_Gene256

MKEYKIENIRNFSIIAHIDHGKSTIADRLLESTSTVEEREMREQLLDSMDLERERGITIKAHPVTMTYLYEGEV
YQLNLIDTPGHVDFSYEVSRSLSACEGALLIVDAAQGVQAQSLANVYLALERDLEIIPVLNKIDLPAADPVRIA
QQIEDYIGLDTTNIIACSAKTGQGIPAILKAIIDLVPPPKAPAETELKALVFDSHYDPYVGIMVYVRIISGELKKG
DRITFMAAKGSSFEVLGIGAFLPKATFIEGSLRPGQVGFFIANLKKVKDVKIGDTVTKTKHPAKTPLEGFKEIN
PVVFAGIYPIDSSDFDTLKDALGRLQLNDSALTIEQESSHSLGFGFRCGFLGLLHLEIIFERIIREFDLDIIATAPS
VIYKVVLKNGKVLDIDNPSGYPDPAIIEHVEEPWVHVNIITPQEYLSNIMNLCLDKRGICVKTEMLDQHRLVL
AYELPLNEIVSDFNDKLKSVTKGYGSFDYRLGDYRKGSIIKLEVLINEEPIDAFSCLVHRDKAESRGRSICEKL
VDVIPQQLFKIPIQAAINKKVIARETIRALSKNVTAKCYGGDITRKRKLWEKQKKGKKRMKEFGKVSIPNTAFI
EVLKLD

>core/132/1/Org1_Gene1021

MVMVWSTNIKHEGLKSWIDEVAKLTTPKDIRLCDGSDTEYDELCTLMESTGTMIRLNPEFHPNCFLVRSSAD
DVARVEQFTFICTSTEAEAGPTNNWRDPQEMRRELHQLFRGCMQGRTLYIVPFCMGPLDSPFSIVGVELTDSP
YVVCSMKIMTRMGDDVLRSLGTSGKFLKCLHSVGKPLSPGEADVSWPCNPKSMRIVHFQDDSSVMSFGSGY

GGNALLGKKCVALRLASYMAKSQGWLAEHMLIIGITNPEGKKKYFSASFPSACGKTNLAMLMPKLPGWKIE
CIGDDIAWIRPGRDGRLYAVNPEYGFFGVAPGTSERTNPNALATCRSNSIFTNVALTADGDVWWEGLTEQPP
EPLTDWLGKPWKPGGSPAAHPNSRFTAPLRQCPSLDPEWNSPQGVPLDAIIFGGRRSETIPLVYEALSWEHGV
TIGAGMSSTTTAAIVGQLGKLRHDPFAMLPFCGYNMAYYFQHWLSFAENRSLKLPKIFGVNWFRKNNQGEF
LWPGFSENLRVLEWIFQRTDGLEDIAERTPIGYLPNIQKFNLNGLNLDLQTVQELFSVDAEGWLAEVENIGEY
LKIFGSDCPQQITDELLRIKSELKEK

>core/133/1/Org1_Gene542

MAAYTEASILSLASLDHIRLRAGMYIGRLGNGSQKEDGIYTLFKEVVDNGIDEFIMGHGKSLKISASDKQISIQ
DQGRGIPLGKLIDCVSKINTGAKYTQDVFHFSVGLNGVGLKAVNALSEIFSVRSVRKKKYHLATFHRGVLQE
SKQGSTKDPDGTFVSFTPDPSIFPEFTFNHDFLKDKIRQYTYLHSGLEIRFNDEVFISHNGLKDLFDAEITEPPLY
SPLFFQNEDLTFIFSHLEGNTERYFSFVNGQETLDGGTHLTAFKEAIVKGVNEFFGKTFVSNDIREGIVGCIAIKI
ASPIFESQTKNKLGNTQIRSSLIKDVKEAIVQALRKDKVAPELLLEKIKFNEKTRKNIQFIKQDLKSKQKKVHY
KIPKLRDCKFHYNDRSLYGEASSIFLTEGESASASILASRNPLTQAVFSLRGKPMNVFSLEETKMYKNDELFYL
ATALGITQNEIQHLRYNKVILATDADVDGMHIRNLLITFFLKTLLPLVENNHLFILETPLFKVRNKTTTLYYYS
EQEKMQALQQFGKKDSSLEITRFKGLGEISPKEFAAFIGPEIRLTPVTITSLESISSILQFYMGKNTKERKQFIMD
NLITDF

>core/134/1/Org1_Gene26

MKEVEQRIRSLYDAVTAENICRWLSNDCTQQDAKTILGWLDTDPAQLEDLFGATLTFGTGGLRSLMGIGTNR
INLFTIRRTTQGLVQVLRAHLPHPGDPMRVVVGCDTRHNSIEFAQETAKVLAGNGCEVFLFQYPEPLALVSFT
VRYERAIGGVMITASHNPPNYNGYKVYMASGGQVLPPLDQEIVAACSAVNEILSVPSIDHPNIHLIGKEYEAL
YRDTLKQLQLYPEANRISGRSLSISYSPLHGTGISLVPHVLKDWGFLSVHLVEKQAIGDGDFPTVQLPNPEDPE
ALTLGIEQMLANDDDLFIATDPDADRVGVVCLEDGQPYRFNGNQMASLLADHILGAWSKTRHLGEHDKLV
KSLVTTEMLSAIAKHYHVDLINVGTGFKYIGEKIESWRNSTNKFVFGAEESYGCLYGTHVEDKDAIIASALIA
EAALQQKLQGKTLCDALLSLYETYGYFANKTESVVFSAKTDEQEIRKKLSHLEEISSANFFSGKYQVEKFENY
KQGIGFNLLSKDSYALTLPKTSMLCYYFSGGGRVIIRPSGTEPKIKFYFEMSTHYPERVTDKEIQKQREAESFQ
HLDDFIFDFKEKFSNL

>core/135/1/Org1_Gene50

MVTVSEQTAQGHVIEAYGNLLRVRFDGYVRQGEVAYVNVDNTWLKAEVIEVADQEVKVQVFEDTQGACR
GALVTFSGHLLEAELGPGLLQGIFDGLQNRLEVLAEDSSFLQRGKHVNAISDHNLWNYTPVASVGDTLRRGD
LLGTVPEGRFTHKIMVPFSCFQEVTLTWVISEGTYNAHTVVAKARDAQGKECAFTMVQRWPIKQAFIEGEKI
PAHKIMDVGLRILDTQIPVLKGGTFCTPGPFGAGKTVLQHHLSKYAAVDIVILCACGERAGEVVEVLQEFPHL
IDPHTGKSLMHRTCIICNTSSMPVAARESSIYLGVTIAEYYRQMGLDILLLADSTSRWAQALREISGRLEEIPGE
EAFPAYLSSRIAAFYERGGAITTKDGSEGSLTICGAVSPAGGNFEEPVTQSTLAVVGAFCGLSKARADARRYP
SIDPLISWSKYLNQVGQILEEKVSGWGGAVKKAAQFLEKGSEIGKRMEVVGEEGVSMEDMEIYLKAELYDF
CYLQQNAFDPVDCYCPFERQIELFSLISRIFDAKFVFDSPDDARSFFLELQSKIKTLNGLKFLSEEYHESKEVIV
RLLEKTMVQMA

>core/136/1/Org1_Gene276

MTAPTESRSSPPTLLEETEPLSPNPIPADIQIPRITISPPSLDVSTVASSAEDISVFIAGGPRSSSSASVASDVYELV
CLCGGDEDPEPPDSEVRTLYVNGSWQTHQEAVQELLYISEVRGEAVRLLYNDGSGMSPWPISPCRTLPTLDH
PLCQALLTVWEQFFSAPENQNREFLVIFYGDASPYIQQALTQSRHSPRIVVVGISPTVFIQGDFRVHNYRVSGD
FFSSLDCRGTRAENTTILPYSSGLEGVFLPSIRCPSFTWAVRFGEQCLVANRGEDVEDRGGLSQDAERSQLPHS
ERDLAVVIDSTDPSSMSRLVEWLNQGSPSSDMEINPYPQRCPDVALSALYAISRVSGLAQEWILASVHEGLDL
QICYSLILMHTTFAVRYFFLLFTNYPQSRERFRTARIVAQSLYLPSILVLVFDCGNVLRKLWMPQEILRAIFISA
STISGSIVFVECTRWMGRGLRHRVQQFVQQRVIGSGLPVGTVRASYRDRAGFIIGFLQTVHGGLYLPVSIMVL
NQIAIQVPRILVRPNNTAVYDLHNKSAEENWSSGDVLAVGQTLNFILCAFVLFVNLWFFVKSVLRHSRRRRR

>core/137/1/Org1_Gene978

MKKKFIFYFVIVFSLLFLWEMTSRHRPTFSFFCPPPSSIASSTLQSLPLLLTSAWHTLKAILGGFFLAITLSIVLAT
IMLSYKSAKDLLQPLFILLQCTPMFALAPLIVLWFGWGIGAVIVPTALTIFFPLTLTIYQGILSTPEELIEQFVLC
GATKFQLLIKLRIPHALPHIFSGLKIAIGSAGFAAIAGEWVASQSGLGILMLESRRNYEMELAFAGLATLSILTL
SLFQITLLIEKLIFSLFRVKRMSLKHKSVAKKALSVLALIPIMLIPWKGNSKSPPDKKNLTSLTLLLDWTPNPNH
IPLYAGVAKGYFKQHGLDLQLQKNTDSSSAVPHVLFEQVDMALYHALGIMKTSIKGMPIQIVGRLIDSSLQGF
LYRSQDPIYKFEDLNGKVLGFCLNNSRDLNRLLETLNRNGVVPSEVKNVSSDLISPMLLNKIDFLYGAFYNIE
GVKLQTLGMPVKCFLSDTCDLPTGPQLIVFTKKGTKASEPEIVEAFQKALQESIIFSKDHPEDAFKLYAKETKS
IPKNLYQEYLQWEETFPLLAQSQDPLSKDLVDKLLETIIKQYPELASEVAKFSLNDLYNPSLPEEQSV

>core/138/1/Org1_Gene723

MYTEESLDNLRHSIDIVDVLSEHIHLKRSGATYKACCPFHTEKTPSFIVNPAGAHYHCFGCGAHGDAIGFLMQ
HLGYSFTEAILVLSKKFQVDLVLQPKDSGYTPPQGLKEELRHINSEAETFFRYCLYHLPEARHALQYLYHRGF
SPDTIDRFHLGYGPEQSLFLQAMEERKISQEQLHTAGFFGNKWFLFARRIIFPVHDALGHTIGFSARKFLENSQ
GGKYVNTPETPIFKKSRILFGLNFSRRRIAKEKKVILVEGQADCLQMIDSGFNCTVAAQGTAFTEEHVKELSK
LGVLKVFLLFDSDEAGNKAALRVGDLCQTAQMSVFVCKLPQGHDPDSFLMQRGSSGLIALLEQSQDYLTFLI
SEKMSSYPKFGPREKALLVEEAIRQIKHWGSPILVYEHLKQLASLMMVPEDMVLSLANPQVTAEPQNIPIKQK
VPKIHPHIVMETDILRCMLFCGSNTKILYTAQFYFVPEDFKHPECRKLFAFMISYYEKYRKNVPFDEACQVLS
DSQILQLLTKRRLNTEALDTIFVQSLQKMADRRWREQCKPLSLNQNIQDKKLEILEDYVQLRKDRTIITLLDPE
SELIP

>core/139/1/Org1_Gene341

MSTSPISNDPRYLSLSNATEKTSLLANSRSLSPVPNSLVPSNPEDTGLRKSIFTHSVTLFAGLVVLLVAVSVVV
VALTVLAPGVPQAILLGIAISGVGIGGFSIMKSLVYMVRDYMSPRMQESSRIKSALAVGTGFTVMGLVMKVG
ANFVPGGYGGLVGSLGSSAYSRGSQTTLASFSHYIYTKFFRSEKVAKGEKLTEAETIKEAKKLHYITLSIATIG
VGLAVLGILLAIAGTVLLGGAPATIAIILAPPLISIGLTTVLQTILHSSIGKWRAFLLTQEKKDLFVDTSLKDIRLE
KLPPSEVEESETSQSVIEVPDSEGIAETRISAEEIDTRLSLTTRQKVIFALATLLLLASIAAFIVTGFGGLTVMQVL
LVASVGSAVASVTLPMVSSGFSYVAYQLKARLNISKLRWKEAKNKKRVRQFLIESGVIASDREFNQMWKTV
YKKQIQKTDAAIREEVRNFEKGGEVNSALVGGILLGVGTGIMLLALVPAFAPIVPGILALGGSTLGIAGSILMR
KFVNWLYDELVKLYERRRNRRELLYGPESKMRSIATDLVVEALAASHDHLFDLDGPVDFIDVDVDIDGAA

>core/140/1/Org1_Gene409

MTNSDNASAAGLLWAHPKEDPAFLGMIIKEFHLPPTVAQIFISRGFQTIQEIHKFLYSHLSSLYDPGLFLDMSK
AVERLLLARDRKEHVMIYGDSDVDGMTGVALLVEFLRDIDVHVSYFFLGAILKQHGETSTLIAKLKEEGITLL
ITVDCGITAGKEVSDITRQGIDVIITDHHMPTGKIPHCVATLNPKLRDHTYPNRELTGVGVAFKLARGVLNALI
SRNLVPKSQGSLKKLLDLVTLGTITDVGVLLGENRVMVRYGIKEIARGARPGLNKLCALCGVEKSEVTSTDIV
LKIAPKLNSLGRLDDPAKGVELLLTQDDERVDALIMELDNINRERQRIEAEVFQDVQEILNSNPEILKQAAIVL
SSTAWHARVIPIISARLAKTYNKPVVIIAIQRGIGKGSARTIGSFPLLGVLKKCSSLLLSYGGHDFAAGVIMKED
KVEDFKKKFVHLVNSSLKKGDTLPHLEIDAYADFDAIDYDLLASMELFEPFGKGNLMPIFYSKVRQVRYPKV
LPGNHLKLYLSQKERNLEGVAFGLGRHADALKASWHYPLEIAYTPRLSQTSGSGVIHLLVRDFRISSEPRFSD

>core/141/1/Org1_Gene240

MSFLRRHISLFRSQKQLIDVFAPVSPNLELAEIHRRVIEDQGPALLFHNVIGSSFPVLTNLFGTKHRVDQLFSQA
PDNLIARVAHLISSTPKLSSLWKSRDLLKRISSLGLKKARFRRFPFVSMSSVNLDHLPLLTSWPEDGGAFLTLP
LVYTESPTLTTPNLGMYRVQRFNQNTMGLHFQIQKGGGMHLYEAEQKKQNLPVSVFLSGNPFLTLSAIAPLP
ENVSELLFATFLQGAKLLYKKTNDHPHPLLYDAEFILVGESPAGKRRPEGPFGDHFGYYSLQHDFPEFHCHKI
YHRKDAIYPATVVGKPYQEDFYIGNKLQEYLSPLFPLVMPGVRRLKSYGESGFHALTAAVVKERYWRESLTT
ALRILGEGQLSLTKFLMVTDQEVPLDRFSVVLETILERLQPDRDLIIFSETANDTLDYTGPSLNKGSKGIFMGIG
KAIRDLPHGYQGGKIHGVQDIAPFCRGCLVLETSLEDRCIKSLLHHPDLKSWPLIILADNLRETIQSEKDFLWR
TFTRCAPANDLHALHSHFATHRPNYNFPFVIDALMKPSYPKEVEVDPSTKQKVSERWHAYFPNKETFYI

>core/142/1/Org1_Gene496

MKYRTHRCNELTSNHIGENVQLAGWVHRYRNHGGVVFIDLRDRFGITQIVCREDEQPELHQRLDAVRSEWV
LSVRGKVCPRLAGMENPNLATGHIEVEVASFEVLSKSQNLPFSIADDHINVNEELRLEYRYLDMRRGDIIEKL
LCRHQVMLACRNFMDAQGFTEIVTPVLGKSTPEGARDYLVPSRIYPGKFYALPQSPQLFKQLLMVGGLDRYF
QIATCFRDEDLRADRQPEFAQIDIEMSFGDTQDLLPIIEQLVATLFATQGIEIPLPLAKMTYQEAKDSYGTDKP
DLRFDLKLKDCRDYAKRSSFSIFLDQLAHGGTIKGFCVPGGATMSRKQLDGYTEFVKRYGAMGLVWIKNQE
GKVASNIAKFMDEEVFHELFAYFDAKDQDILLLIAAPESVANQSLDHLRRLIAKERELYSDNQYNFVWITDFP
LFSLEDGKIVAEHHPFTAPLEEDIPLLETDPLAVRSSSYDLVLNGYEIASGSQRIHNPDLQSQIFTILKISPESIQE
KFGFFIKALSFGTPPHLGIALGLDRLVMVLTAAESIREVIAFPKTQKASDLMMNAPSEIMSSQLKELSIKVAF

>core/143/1/Org1_Gene259

MSGKKDGVRGMIFVPLSILVLIFLPLPQILLDFGLCISFALSLLTVCWVFTLNSSNSAKLFPPFFLYLCLLRLGLN
LASTRWIVSSGTASSLIVSLGSFFSLGSLWAATFACLLLFFVNFLMVSKGSERIAEVRSRFFLEALPAKQMALD
SDLVSGRASYKAVKKQKNALIEEGDFFSAMEGVFRFVKGDAIISCILLLVNVVSVTCLYYTSGYALEQMWFT
VLGDALVSQVPALLTSCAAATLISKIDKEESLLNYLFEYYKQLRQHFRVVSLLIFSLCCIPSSPKFPIVLLASLL
WLAYRKEEPASEDSCIERAFSYVEGACPKEQESQFYQVYRAASEEVFEDLGVRLPVLTSLRIEERPWLRVFGQ
NVYLDEMTPEAVLPFLRNIAHEALNAEVVQKYLEESERVFGIAVEDIVPKKISLSSLVVLSRLLVRERVSLKLF
PKILEAVAVYQNSGDSLEILAEKVRKSLGYWIGRSLWDQKQTLEVITIDFHVEELINSSYSKSNPVMQENVIRR
VDSLLERSVFKDFRAIVTSCETRFEMKKMLDPHFPDLLVLSHDELPKEIPISFLGIVSDEVLVP

>core/144/1/Org1_Gene228

MPKQAEYTWGSKKILDNIECLTEDVAEFKDLLYTAHRITSSEEESDNEIQPGAILKGTVVDINKDFVVVDVGL
KSEGVIPMSEFIDSSEGLVLGAEVEVYLDQAEDEEGKVVLSREKATRQRQWEYILAHCEEGSIVKGQITRKVK
GGLIVDIGMEAFLPGSQIDNKKIKNLDDYVGKVCEFKILKINVERRNIVVSRRELLEAERISKKAELIEQISIGEY
RKGVVKNITDFGVFLDLDGIDGLLHITDMTWKRIRHPSEMVELNQELEVIILSVDKEKGRVALGLKQKEHNP
WEDIEKKYPPGKRVLGKIVKLLPYGAFIEIEEGIEGLIHISEMSWVKNIVDPSEVVNKGDEVEAIVLSIQKDEGK
ISLGLKQTERNPWDNIEEKYPIGLHVNAEIKNLTNYGAFVELEPGIEGLIHISDMSWIKKVSHPSELFKKGNSV
EAVILSVDKESKKITLGVKQLSSNPWNEIEAMFPAGTVISGVVTKITAFGAFVELQNGIEGLIHVSELSDKPFA
KIEDIISIGENVSAKVIKLDPDHKKVSLSVKEYLADNAYDQDSRTELDFKDSQGPKERKKKGK

>core/145/1/Org1_Gene618

MSTRRPIQLLDPLTINQIAAGEVIENSVSVVKELIENSLDAGADEIEIETLGGGQGAIIIRDNGCGFRAEDIPIALQ
RHATSKIREFSDIFSLNSFGFRGEALPSIASISKMEIQSSIEGDEGVRTVIHGGDIVSCEPCARQLGTTVIVNSLFY
NVPVRRGFQKSMQSDRLGIRKLIENRILSTANIGWSWISEGHHEIQIAKQQGFQERVAYVMGDHFMQDALTI
DKEANGVRIVGVLGSPSFHRPTRQGQKIFINDRPIESLFISKKVGDAYALLLPLHRYPVFVLKLYLPSSWCDFN
VHPQKIEARILKEELVGDCIKEAIVETLACPPGILCRTHQEIEESDSVPLPMFRMLETSDVQEEESVEFDQNLFA
YSSEDVSLEKQEYTSRGPKSQMDWIYSSDVRFLTSLGRVVLAEDLEGVHIIFTAAARKHLFFLSLMQENSRM
YQSQALLIPLRLQVTPEEAFFFSHHGRTLCDLGIEISQVGPCVFSIESTPTVIGEEELKEWLLLLAARGSTDINSE
ALTALMKETLTQATFSKHQHVFDVSWLKLLWSVGKPEKGFDGARIRRLILDSDFMEG

>core/146/1/Org1_Gene242

MAVSGGGGVQPSSDPGKWNPALQGEQAEGPSPLKESIFSETKQASSAAKQESLVRSGSTGMYATESQINKAK
YRKAQDRSSTSPKSKLKGTFSKMRASVQGFMSGFGSRASRVSAKRASDSGEGTSLLPTEMDVALKKGNRISP
EMQGFFLDASGMGGSSSDISQLSLEALKSSAFSGARSLSLSSSESSSVASFGSFQKAIEPMSEEKVNAWTVARL
GGEMVSSLLDPNVETSSLVRRAMATGNEGMIDLSDLGQEEVSTAMTSPRAVEGKVKVSSSDSPEANPTGIPN
SNTLERAEKEAEKQESREQLSEDQMMLARAMAGLLTGAAPQEVLSNSVWSGPSTVFPPPKFSGTLPTQRSGD
KSKHKSPGIEKSTNHTNFSPLREGTVKSAEVKSLPHPESMYRFPKDSIVSREEPEAVVKESTAFKNPENSSQNF
LPIAVESVFPKESGTGGALGSDAVSSSYHFLAQRGVSLLAPLPRATDDYKEKLEAHKGPGGPPDPLIYQYRNV
AVEPPIVLRSPQPFSGSSRLSVQGKPEAASVHDDGGGGNSGGFSGDQRRGSSGQKASRQEKKGKKLSTDI

>core/147/1/Org1_Gene441

MQKHPSFYQRFLSAYYKNLLASLSWKFFISVALIGIYAPLFASSKPLLVTWHGEIFFPLLRYLFFPGYYTKPVD
LFFNVLMVTFPFFILSFKLTRGWLRRWLLGLCIISQCMIFAWAYSGKVQDPALAENLKKMRAEKVRENISKV
NSEMVMLLPKDTRTWEMERRYMSTYEQLGILIKAKYRKKQEASVKKYQVAFEEKRQSPMPTLRHLEMKNE
GICLKRLQQRVDKMQRPYEMAQQAWNRATDNYRPFLMALTRIEHELRLADYNNWGQPEDLCIAYANVEKR
AEPYKKSLLEIRQVLEDYAKLRSAISFIQDKRLWIEKESEDLRILINPFFSSFHWEDDAGGSREMNKYVPWWQ
LSRVTRKDLLAALVFGIRIALVVAGIGITIALAIGIMIGLVSGYFGGTVDMILSRFTEIWETMPVLFILMLVISIT
QQKSLLLNTVLLGCFSWTGFSRYVRIEVLKQRDRGYVLAATNLGYSHYYIMVHQILPNAIVPVISLVPFAMM
AMISCEAGLTFLGLGEESSASWGNLMREGVTGFPAESAVLWPPAIILTMLLIAIALIGDGVRDALDPRLQDS

>core/148/1/Org1_Gene986

MTDFPTHFKGPKLNPIKVNPNFFERNPKVARVLQITAVVLGIIALLSGIVLIIGTPLGAPISMILGGCLLASGGAL
FVGGTIATILQARNSYKKAVNQKKLSEPLMERPELKALDYSLDLKEVWDLHHSVVKHLKKLDLNLSETQRE
VLNQIKIDDEGPSLGECAAMISENYDACLKMLAYREELLKEQTQYQETRFNQNLTHRNKVLLSILSRITDNIS
KAGGVFSLKFSTLSSRMSRIHTTTTVILALSAVVSVMVVAALIPGGILALPILLAVAISAGVIVTGLSYLVRQIL
SNTKRNRQDFYKDFVKNVDIELLNQTVTLQRFLFEMLKGVLKEEEEVSLEGQDWYTQYITNAPIEKRLIEEIR
VTYKEIDAQTKKMKTDLEFLENEVRSGRLSVASPSEDPSETPIFTQGKEFAKLRRQTSQNISTIYGPDNENIDPE
FSLPWVPKKEEEIDHSLEPVTKLEPGSREELLLVEGVNPTLRELNMRIALLQQQLSSVRKWRHPRGEHYGNVI
YSDTELDRIQMLEGAFYNHLREAQEEITQSLGDLVDIQNRILGIIVEGDSDSRTEEEPQE

>core/149/1/Org1_Gene33

MANPTQSRPPSPEISIEELELQELAGSSNTETISNTPPPSCAATAEEVSLFIEGGRRNSEDEEGPLGSCEVYDVVC
ITNQGDPEVRDHEVRVMYINGSGRTQHEGILDAMNICDLRGEPVRFIHNSGYGLGSCFLGIRNRIPPRDNVISQ
AIQARWNEFFIFAENANRDYIVLFSGNGGLYLQVALDNSIYSHHILCVGIGSSYYIQGNYRVHNYRVTGDWTT
LLDRRGATAVNTTTLPYADSAEGLFLPSVRCPSYQWALRCGEQCLIMDNNQQVGFRPQDSSSEIALVVNLNQ
DHSTWTRLIEWIDRGDSQAVLELNPQPSHCRDIALTALYATTRISSLLQECLMISVTYAPEVFVTYAIVTGYSI
MTLRYFILLLTNRPGCRRHFRVLRLAALGLQSLGFLTVLLDHINVTRRVNRRPPLISVIFCTASFATGSFIYVDL
TRMFFTSLRSRLQLFVQRRLTGRGLPLRRVFVNHLDSLRFSQNALITFHGGLFMPLIIGFFNQLVIQVPRVVIRP
NTTAVYDLNQTSQEAWDSGDVLAIGQTINFLLCMILLVINTFFFVRSVRRNLHRRPHR

>core/150/1/Org1_Gene576

MFMNTQNSQATEVSSEEESQKKLEELVALAKEQGFITYEEINEILPMSFDTPEQIDQVLIFLTGMDIQVLNQID
VERQKEKKKEAKELEGLARRTEGTPDDPVRMYLKEMGTVPLLTREEEVEISKRIEKAQVQIERIILRFRYSAK
EAISIAHYLISGKERFDKIISEKEVEDKTHFLKLLPKLITLLKEEDTYLENLLLSLKQPDLSKQEAAKLNDSLEK
CRIRTQAYLRCFHCRHNVTEDFGEVVFKAYDSFLHLEQQINDLKVRAERNKFAAAKLAAAKRKLYKREVAA
GRTLEEFKKDVRMLQRWMDKSQEAKKEMVESNLRLVISIAKKYTNRGLSFLDLIQEGNMGLMKAVEKFEY
RRGYKFSTYATWWIRQAVTRAIADQARTIRIPVHMIETINKVLRGAKKLMMETGKEPTPEELAEELGLTPDR
VREIYKIAQHPISLQAEVGEGSESSFGDFLEDTAVESPAEATGYSMLKDKMKEVLKTLTDRERFVLIHRFGLL
DGKPKTLEEVGSAFNVTRERIRQIEAKALRKMRHPIRSKQLRAFLDLLEEEKTGTSKVKSLKSK

>core/151/1/Org1_Gene901

MDTQSSIGNEEWRIAGTSIVSGMALGKVFFLGTSPLHVRELTLPQEEVEHEIHRYYKALNRSKSDIVALEQEV
TGQQGLQEVSSILQAHLEIMKDPLLTEEVVNTIRKDRKNAEYVFSSVMGKIEESLTAVRGMPSVVDRVQDIH
DISNRVIGHLCCQHKSSLGESDQNLIIFSEELTPSEVASANSAYIRGFVSLVGAATSHTAIVSRAKSIPYLANISE
ELWNIAKRYNGKLVLIDGYRGELIFNPKPATLQSCYKKELSVVAHTSQRLVRKSLHPIVSSHAGSDKDVEDLL
ENFPQTSIGLFRSEFLAVILGRLPTLREQVDLYEKLARFPGDSPSVLRLFDFGEDKPCPGIKNKKERSIRWLLDY
SVILEDQLQAIAKASLQGSIKVLIPGVSDVSEIIEVKKKWETIQTRFPKGHKVSWGTMIEFPSAVWMIEEILPEC
DFLSIGTNDLVQYTLGISRESALPKHLNVTLPPAVIRMIHHVLQAAKQNQVPVSICGEAAGQLSLTPLFIGLGV
QELSVAMPVINRLRNHIALLELNSCLEITEALLQAKTCSEVEELLNRNNKITS

>core/152/1/Org1_Gene1022

MVKVPWAFKNFIPKLYTSIKEGYSFNTFKKDFQAGITVGILAFPFAIAIAIGVGVSPIQGLLASIIGGLLASAMG
GSNVLISGPSSAFISILYCLSAKYGAEALFTVTLLAGVFLIAFGLTGLGTFIKYMPYPVVTGLTTGLAIIIFSSQIK
DFLGLQMGANIPADFLPKWIAYWDHLWTWDSKSFAVGLFTLLIMIYFRNYKPRYPGVMIAIVTATTLVWLL
KIDIPTIGSRYGTLPTAIPLPKIPQLSITKILQLMPDALTIAVLSGLETLLSAVVADGMTGWRHQSNCQLVAQGV
ANIGTSLFSGIPVTGSLSRTAASIKSEATTPIAGIVHSIFICFILLLLAPLTVKIPLTCLAAVLILIAWNMSEIHHFIH
LFTAPKKDIVVLLTVFILTVMTTITAAVQVGMMLAAFLFMKQMSDLSDVISTAKYFDKDSDFLSKAEVPQNT
EIYEINGPFFFGIADRLKNLLNDIEKPPKIFILCMTRVPTIDASAMHALEEFFLECDRQGTLLLLAGVKKTPLAD
LKRYHLDELIGVDHIFSNIKSALLFAQALTNLESKTSTRHLV

>core/153/1/Org1_Gene899

MGLFHLTLFGLLLCSLPISLVAKFPESVGHKILYISTQSTQQALATYLEALDAYGDHDFFVLRKIGEDYLKQSI
HSSDPQTRKSTVIGAGLAGSSEALDVLSQAMETADPLQQLLVLSAVSGHLGKTSDDLLFKALASPYPVIRLEA
AYRLANLKNTKVIDHLHSFIHKLPEEIQCLSAAIFLRLETEESDAYIRDLLAAKKSAIRSATALQIGEYQQKRFL
PTLRNLLTSASPQDQEAILYALGKLKDGQSYYNIKKQLQKPDVDVTLAAAQALIALGKEEDALPVIKKQALE
ERPRALYALRHLPSEIGIPIALPIFLKTKNSEAKLNVALALLELGCDTPKLLEYITERLVQPHYNETLALSFSKG
RTLQNWKRVNIIVPQDPQERERLLSTTRGLEEQILTFLFRLPKEAYLPCIYKLLASQKTQLATTAISFLSHTSHQ
EALDLLFQAAKLPGEPIIRAYADLAIYNLTKDPEKKRSLHDYAKKLIQETLLFVDTENQRPHPSMPYLRYQVT
PESRTKLMLDILETLATSKSSEDIRLLIQLMTEGDAKNFPVLAGLLIKIVE

>core/154/1/Org1_Gene358

MKTSQLFYKTSKNANKSAAVLSNELLEKAGYLFKVSKGVYTYTPLLWRVVSKMMNIIREELNAIGGQELLLP
LLHNAELWQHTGRWEAFTSEGLLYTLKDREGKSHCLAPTHEEVICSFVAQWLSSKRQLPLHLYQIATKFRDE
IRPRFGLIRSRELLMEDSYTFSDSPEQMNEQYEKLRSAYSKIFDRLGLAYVIVTADGGKIGKGKSEEFQVLCSL
GEDTICVSGSYGANIEAAVSIPPQHAYDREFLPVEEVATPGITTIEALANFFSIPLHKILKTLVVKLSYSNEEKFI
AIGMRGDRQVNLVKVASKLNADDIALASDEEIERVLGTEKGFIGPLNCPIDFFADETTSPMTNFVCAGNAKD
KHYVNVNWDRDLLPPQYGDFLLAEEGDTCPENPGHPYRIYQGIEVAHIFNLGTRYTDSFEVNFQDEHGQTQQ
CWMGTYGIGVGRTLAACVEQLADDRGIVWPKALAPFSITIAFNGGDTVSQELAETIYHELQSQGYEPLLDDR
DERLGFKLKDSDLIGIPYKLILGKSYQSSGIFEIESRSGEKYTVSPEAFPTWCQNHLA

>core/155/1/Org1_Gene560

MTVSVKKKSFRALVTTHFLTIINDNLYKFLLAFFLLEGKTLTENAKILSCVSFFFALPFLLLAPLAGSLADRFQ
KRNIILATRFIEILCTILGTYFFFIQSVVGGYVVLILMACHTTIFGPAKLGILPEMLPSEQLSQANGIMTAATYTG
SILGSCLAPLLVDVTHRLGVNSYVWPTLMCVIVSIISTLISFCIRPSNVKNVKQKITLVSFKDLWKVLKDTRMI
HYLTVSIFLGSFFLLIGAYTQLEIIPFVEFTLKYPKHYGAYLFPIVALGVGTGSYITGKISGKDIKIGYVPLAAIGL
ALVFMGLYAFACSILFVLFFLLALGFLGGVYQVPLHAYVQYASPEHKRGQILAANNFLDFFGVLVAAGVIRV
LGSNLGLSPETSFFYIGWFVLAVSIWTLWIWREHVYRLLLGIILRRQLGYYLKIHQSSSPKCYFVAVQSYREIR
RVLAALTKTVRSRVIILDQKLVPGWRAWLLSWCVPTVVSSVRDNDSEAQDAWAVLQANHLKTSLKKFPDV
SVVCLGLPKNVERFTSILQEQGIDLHPIQLVQKEGKKRVIYTLVFPHA

>core/156/1/Org1_Gene416

MSTLLSILSVICSQAIAKAFPNLEDWAPEITPSTKEHFGHYQCNDAMKLARVLKKAPRAIAEAIVAELPQEPFS
LIEIAGAGFINFTFSPVFLNQQLEHFKDALKLGFQVSQPKKIIIDFSSPNIAKDMHVGHLRSTIIGDSLARIFSYVG
HDVLRLNHIGDWGTAFGMLITYLQENPCDYSDLEDLTSLYKKAYVCFTNDEEFKKRSQQNVVALQAKDPQA
IAIWEKICETSEKAFQKIYDILDIVVEKRGESFYNPFLPEIIEDLEKKGLLTVSNDAKCVFHEAFSIPFMVQKSDG
GYNYATTDLAAMRYRIEEDHADKIIIVTDLGQSLHFQLLEATAIAAGYLQPGIFSHVGFGLVLDPQGKKLKTR
SGENVKLRELLDTAIEKAEEALREHRPELTDEAIQERAPVIGINAIKYSDLSSHRTSDYVFSFEKMLRFEGNTA
MFLLYAYVRIQGIKRRLGISQLSLEGPPEIQEPAEELLALTLLRFPEALESTIKELCPHFLTDYLYNLTHKFNGFF
RDSHIQDSPYAKSRLFLCALAEQVLATGMHLLGLKTLERL

>core/157/1/Org1_Gene947

MSKLIRRVVTVLALTSMASCFASGGIEAAVAESLITKIVASAETKPAPVPMTAKKVRLVRRNKQPVEQKSRG
AFCDKEFYPCEEGRCQPVEAQQESCYGRLYSVKVNDDCNVEICQSVPEYATVGSPYPIEILAIGKKDCVDVVI
TQQLPCEAEFVSSDPETTPTSDGKLVWKIDRLGAGDKCKITVWVKPLKEGCCFTAATVCACPELRSYTKCGQ
PAICIKQEGPDCACLRCPVCYKIEVVNTGSAIARNVTVDNPVPDGYSHASGQRVLSFNLGDMRPGDKKVFTV
EFCPQRRGQITNVATVTYCGGHKCSANVTTVVNEPCVQVNISGADWSYVCKPVEYSISVSNPGDLVLHDVVI
QDTLPSGVTVLEAPGGEICCNKVVWRIKEMCPGETLQFKLVVKAQVPGRFINQVAVTSESNCGTCTSCAETT
THWKGLAATHMCVLDTNDPICVGENTVYRICVTNRGSAEDTNVSLILKFSKELQPIASSGPTKGTISGNTVVF
DALPKLGSKESVEFSVTLKGIAPGDARGEAILSSDTLTSPVSDTENTHVY

>core/158/1/Org1_Gene847

MELLSLNKSYFEIQRLRYRPEILTLLETIRSKHIQETSSPPSPPPELQKHIPNLCRIPEVSIYTEQETSSKPLKIGVL
LSGGQAPGGHNVVIGLFDALRVFNPKTRLFGFIKGPLGLTRGLYKDLDISVIYDYYNMGGFDMLSSSREKIKT
EEQKKNILNTVKQLKLDGLLIIGGNNSNTDTAMLAEYFLAHNCKTSVIGVPKTIDGDLKNCWIETSLGFHTSC
RTYSEMIGNLAKDALSAKKYHHFIRLMGQQASYTTLECGLQTLPNIALISELIATRKISLKQLSEQLALGLVRR
YKSGKNYSTVLIPEGLIEHIFDTRKLIDELNVLLANGDSSIEKILSKLSPETLKTFHLFPKDIANQLLLARDSHGN
VRVSKIATEELLAVMVKKEIEKIKPHMEFHSVSHFFGYEARAGFPSNFDCNYGIALGIISALFLVRQKTGYMIT
INNLAQSYTEWQGGATPLYKMMHLENRCGTETPVIKTDSVDPKSPAVQHLLQQSDSCLVEDLYRFPGPLQYF
GKEELIDQRPLTLLWENQTHSPLL

>core/159/1/Org1_Gene813

MSSPPQAVASLTERIKTLLESNFCQIIVKGELSNVSLQPSGHLYFGIKDSQAFLNGAFFHFKSKYYDRKPKDGD
AVIIHGKLAVYAPRGQYQIVAHALVYAGEGDLLQKFEETKRRLTAEGYFATEKKKPLPFAPQCIGVITSPTGA
VIQDILRVLSRRARNYKILVYPVTVQGNSAAHEISKAIEVMNAENLADVLIIARGGGSIEDLWAFNEEILVKAI
HASTIPIVSAVGHETDYTLCDFASDVRAPTPSAAAEIVCKSSEEQVQVFEGYLRHLLSHSRQLLTSKKQQLLP
WRRFLDRAEFYTTAQQQLDSIEIAIQKGVQGKIHESKQRYDNISRWLQGDLVSRMTCRLQSLKKMLSQALSH
KALSLQVRCHQLKKSLTYPRQIQQASQKLSPWRQQLDTLISRRLHYQKEEYFHKHTRLKHAHNVLEQQLRS
HVQKLELLGRRLSRGCELNLQNQKIAYANVKETLATILERRYENSVARYSALKEQLHSLNPKNVLKRGYAM
LFDFNENSAMISVDSLQENARVRIQLQDGEAILTVTNIEICKLIKG

>core/160/1/Org1_Gene973

MDNSDNSFHTLETEQGSFLNDELAVEEVASTESTEISDATLCFADEIQELPSPEKKVAFILNKMREALTGSSQG
SDLRLFWDLRKQCLPLFNEIEDTAKRADHWRCYIELTKEGRHLKGLQDEEGSFVVGQIDLAITCLEKDILKFQ
EGTEDKIFKDREDNFLESQALDKHQAFYKQHHTSLLWLSSFSSKIIDLRKELINVGMRMRLKSKFFQRLSNLG
NQVFPKRKELIEKVSQTFAEDVDAFVAKYFIGSDKETLKKTVFFLRKEIKNLQHAAKRLFVSSHVFAETRLKL
SKCWDQLKGMEKEIRQEQGRLRVVSAENSKEVRQMLAEVSSLLIEGNDLSKVRKDLEGISKKIRALDLTHDD
VISLKKEMQQLFDQLREKQDAAEHSYQEQLAKDKQVKKEAARSLAERITTFSKTCSEGNITSESREEWQTLK
ELLGKMSFLPPPEKISLDNQLNLALQTIVNFFEEQLLSSPDSREKLVNMRQVLKQRRERRQELKDKLEQDKKL
LGSSGLDFDRAMQYSALVEEDKRALEELDASILELKQQIQQLL

>core/161/1/Org1_Gene911

MPQKVLITSALPYANGPLHFGHIAGVYLPADVYARFRRLLGDDVLYICGSDEFGIAITLNADREGLGYQEYV
DMYHKLHKDTFEKLGFALDFFSRTTNPFHAELVQDFYSQLKASGLIENRISEQLYSEQEQRFLADRYVEGTCP
RCGFDHARGDECQSCGADYEAIDLIDPKSKISGVELVKKETEHSYFLLDRMKDALLSFIQGCYLPDHVRKFV
VDYIEHVRSRAITRDLSWGIPVPDFPGKVFYVWFDAPIGYISGTMEWAASQGNPDEWKRFWLEDGVEYVQFI
GKDNLPFHSVVFPAMELGQKLDYKKVDALVVSEFYLLEGRQFSKSEGNYVDMDKFLSSYSLDKLRYVLAAT
APETSDSEFTFLDFKTRCNSELVGKFGNFINRVLAFAEKNHYDKLSYHSVVLEDSDRAFLEEARQLVRDAEK
CYREYSLRKATSVIMSLAALGNVYFNQQAPWKLLKEGTRERVEAILFCACYCQKLLALISYPIIPESAVAIWE
MISPKSLENCNLDTMYARDLWKEEILDVINEEFHLKSPRLLFTTVE

>core/162/1/Org1_Gene140

MHPLYVDLDTIISSYSPPLPKEFQEAASLIAVPDTSHSKPVVPGVKTLFPQTYHLPYLKFVQGENVVHTPLKVG
VMFSGGPAPGGHNVIQGLFNSLKDFHPDSSLVGFVNNGDGLTNNKSIDITEEFLSKFRNSGGFNCIGTGRKKIV
TPEAKEACLKTAEALDLDGLVIIGGDGSNTATAILAEYFAKRRPKTSIVGVPKTIDGDLQHTFLDLTFGFDTAT
KFYSSIISNISRDALSCKAHYHFIKLMGRSASHIALECALQTHPNIALIGEEIAEKNLPLKTIIHKICSVIADRAAM
EKYYGVILIPEGIIEFIPEIINLITEIESLSEYEDKISRLSPESQRLLKSFPAPIIEQILNDRDAHGNVYVSKISVDKLL
IHLVSNHLQQYFPNVPFNAISHFLGYEGRSGLPTKFDNTYGYSLGYGAGILVRNHCNGYLSTIESLACPFMKW
KLRAIPVVKMFTVKQQADGTLQPKIKKYLVDIGSTAFRKFKLYRKIWALEDSYRFLGPLQIETPPEMHSDNFP
PLTLLLNHNFWQRHQGCIEIPDTTY

>core/163/1/Org1_Gene555

MSRKDNEVSLARSIFNILSGTFCSRITGIFREIAMATYFGADPIVAAFWLGFRTVFFLRKILGGLILEQAFIPHFE
FLRAQSLDRAAFFFRRFSRLIKGSTIIFTLLIEAVLWVVLQYVEEGTYDMILLTMILLPCGIFLMMYNVNGALL
HCENKFFGVGLAPVVVNIIWIFFVIAARHSDPRERIIGLSVALVIGFFFEWLITVPGVWKFLLEAKSPPQEHDSV
RALLAPLSLGILTSSIFQLNLLSDICLARYVHEIGPLYLMYSLKIYQLPIHLFGFGVFTVLLPAISRCVQREDHER
GLKLMKFVLTLTMSVMIIMTAGLLLLALPGVRVLYEHGLFPQSAVYAIVRVLRGYGASIIPMALAPLVSVLFY
AQRQYAVPLFIGIGTALANIVLSLVLGRWVLKDVSGISYATSITAWVQLYFLWYYSSKRLPMYSKLLWESIRR
SIKVMGTTMLACMITLGLNILTQTTYVIFLNPLTPLAWPLSSITAQAIAFLSESCIFLAFLFGFAKLLRVEDLINL
ASFEYWRGQRGLLQRQHVMQDTQN

>core/164/1/Org1_Gene85

MAAKNIKYNEEARKKIHKGVKTLAEAVKVTLGPKGRHVVIDKSFGSPQVTKDGVTVAKEIELEDKHENMGA
QMVKEVASKTADKAGDGTTTATVLAEAIYSEGLRNVTAGANPMDLKRGIDKAVKVVVDELKKISKPVQHH
KEIAQVATISANNDSEIGNLIAEAMEKVGKNGSITVEEAKGFETVLDVVEGMNFNRGYLSSYFSTNPETQECV
LEDALILIYDKKISGIKDFLPVLQQVAESGRPLLIIAEEIEGEALATLVVNRLRAGFRVCAVKAPGFGDRRKAM
LEDIAILTGGQLVSEELGMKLENTTLAMLGKAKKVIVTKEDTTIVEGLGNKPDIQARCDNIKKQIEDSTSDYD
KEKLQERLAKLSGGVAVIRVGAATEIEMKEKKDRVDDAQHATIAAVEEGILPGGGTALVRCIPTLEAFLPML
ANEDEAIGTRIILKALTAPLKQIASNAGKEGAIICQQVLARSANEGYDALRDAYTDMIDAGILDPTKVTRSALE
SAASIAGLLLTTEALIADIPEEKSSSAPAMPSAGMDY

>core/165/1/Org1_Gene349

MFGSESLRYQLLIQDFAKVSEEGIGLLESKEYSLLQAKLVLRALAQNSSFDDWFRSFKKCQISYPELAHDRDV
LEEFGIQVLREGIENPSVTVRAVSVLAIGLARDFRLVPLLLQSCNDDSAIVRSLALQVAVNYGSESLKKAIVEL
ARNDDSIHVRITAYQVVALLQIEELLPFLRERAENKLVDSVERREAWKACLELSSQFLETGVAKDDIDQALFT
CEVLRNGMLPETTEIFTELLSVEHPEVQESLLLSALAWSHQLQNHKEFLSKVRHVMCTSPFAKVRFQAAALL
HLHGDPLGRDSLVEGLRSPQPLVCEAASAALCSLGIHGVPLAKEHLESLSSRKAAANLSILLLVSREDIERAGD
VIARYLSNPEMCWAIEYFLWDAQWNLRGDTFPLYSDMIKREIGRKLIRLLAVARYSQAKAVTATFLSGQQA
QGWSFFSGMFWEEGDVKTSEDLVTDACFAAKLEGALASLCQKKDQASLQRVSQLYNDSRWQDKLAILESV
AFSENLDAVPFLLDCCHHEAPSLRSAAAGALFSIFK

>core/166/1/Org1_Gene488

MLRIFCFVISWCLIAFAQPDLSGFVSILGAACGYGFFWYSLEPLKKPSLPLRTLFVSCFFWIFTIEGIHFSWMLS
DQYIGKLIYLVWLTLITILSVLFSGFSCLLVAIVRQKRTAFLWSLPGVWVAIEMLRFYGIFSGMSFDYLGWPM
TASAYGRQFGGFLGWAGQSFAVIAVNMSFYCLLLKKPHAKMLWVLTLLLPYTFGAIHYEYLKHAFQQDKR
ALRVAVVQPAHPPIRPKLKSPIVVWEQLLQLVSPIQQPIDLLIFPEVVVPFGKHRQVYPYESCAHLLSSFAPLPE
GKAFLSNSDCATALSQHFQCPVIIGLERWVKKENVLYWYNSAEVISHKGISVGYDKRILVPGGEYIPGGKFGS
LICRQLFPKYALGCKRLPGRRSGVVQVRGLPRIGITICYEETFGYRLQSYKRQGAELLVNLTNDGWYPESRLP
KVHFLHGMLRNQEFGMPCVRACQTGVTAAVDSLGRILKILPYDTRETKAPSGVLETSLPLFNYKTLYGYCGD
YPMILIAFCAVSYLGGGFLGYRLLAKKEIR

>core/167/1/Org1_Gene455

MQSSEVKPFSRLRAYLCPIYKSEFSKFVPLFLLAFFVGFNYCLLKNMKDTLVIVGSDAGAEVIPFLKVWGIVP
GAVIVTMVYGWLGSRYPRDTVFYCFMAAFLGFFFLFAVIIYPVGDSLHLNSLADKLQELLPQGLRGFIVMVR
YWSYSIYYVMSELWSSVVLSMLFWGLANQITTITEAGRFYALINTGLNLSSICAGEISYWMGKQTFVAYSFA
CDSWHSVMLNLTMLITCSGLIMIWLYRRIHHLTIDTSIPPSRRVLAEEGAATANLKEKKKPKAKARNLFLHLI
QSRYLLGLAIIVLSYNLVIHLFEVVWKDQVSQIYSSHVEFNGYMSRITTLIGVVSVLAAVLLTGQCIRKWGWT
VGALVTPLVMLVSGLLFFGTIFAAKRDISIFGGVLGMTPLALAAWTGGMQNVLSRGTKFTFFDQTKEMAFIP
LSPEDKNHGKAAIDGVVSRIGKSGGSLIYQGLLVIFSSVAASLNVIALVLLIIMVVWIAVVAYIGKEYYSRAAD
AVATLKQPKEPSSSIVREAQESVEQEEMAVL

>core/168/1/Org1_Gene706

MHDQRNRGHNNHNLRLRPGSTLLEAFLILCSEHEEGIACFDEHLGSLSYRELRNAIIAVAIKVSKFSEDRVGV
MMPASIGAFIAYFGILLAGKTPVMMNWSQGLRELRACTKTVEVRRVLTSQQFIKHLTEVQGFVEYPFDLMY
MEDVRKRLSWWEKCRIGLYSKCSVPWLLRIFGVSGVESDDTAVILFTSGTEKLPKAVPLTHKNLMENQEACL
KFFDPNTQDVMLAFLPPFHAYGFNSCGLFPLLMGVHVVFASNPLNPKKLVEFIDDKKVTFFGSTPVFFDYILK
TAKKQNSCLESLRLVVIGGDALKDTLYEETKKLQPQIALYQGYGATECSPVISITTKESPRKSECVGMPIEGM
DVLIISKETHIPVSSGEQGLIVVRGNSVFSGYLGNHEHQSFVSLGGDQWYLTGDLGHIGPSGDLFLEGRLSRFV
KIGGEMVSLEALESILHEHFTENQNEDAGSLVVCGIPGDKVRLCLFTTLATTIHEVNDILKSAETSSIVKISYVH
QVESIPILGIGKPDYVSLNALAVSLFG

>core/169/1/Org1_Gene166

MPFKCIFLTGGVVSSLGKGLTAASLALILERQRLNVAMLKLDPYLNVDPGTMNPFEHGEIYVTDDGVETDLD
LGHYHRFSSAALSRHSSATSGQIYARVIKREREGDYLGSTVQVIPHITNEIIQVILDAAKEHSPDVLIVEIGGTIG
DIESLPFLEAIRQFRYDHSEDCLNIHMTYVPYLQAADEVKSKPTQHSVQTLRGIGIIPDAILCRSEKPLTQEVKS
KISLFCNVPNRAVFNVIDVKHTIYEMPLMLAQEKIANFIGEKLKLATVPENLDDWKVLVNQLSQDLPKVKIG
VVGKYVQHRDAYKSIFEALTHAALRLGHAAEIIPIDAEDENLTMELSQCDACLVPGGFGVRGWEGKIAAAKF
CREQGIPYFGICLGMQVLVVEYARNVLNLDQANSLEMDPNTPHPIVYVMEGQDPLVATGGTMRLGAYPCLL
KPGSKAHKAYNESSLIQERHRHRYEVNPDYIQSLEDHGLRIVGTCPPQGLCEIIEVSDHPWMIGVQFHPEFVSK
LISPHPLFIAFIEAALVYSKDASHV

>core/171/1/Org1_Gene131

MRKISVGICITILLSLSVVLQGCKESSHSSTSRGELAINIRDEPRSLDPRQVRLLSEISLVKHIYEGLVQENNLSG
NIEPALAEDYSLSSDGLTYTFKLKSAFWSNGDPLTAEDFIESWKQVATQEVSGIYAFALNPIKNVRKIQEGHLS
IDHFGVHSPNESTLVVTLESPTSHFLKLLALPVFFPVHKSQRTLQSKSLPIASGAFYPKNIKQKQWIKLSKNPH
YYNQSQVETKTITIHFIPDANTAAKLFNQGKLNWQGPPWGERIPQETLSNLQSKGHLHSFDVAGTSWLTFNIN
KFPLNNMKLREALASALDKEALVSTIFLGRAKTADHLLPTNIHSYPEHQKQEMAQRQAYAKKLFKEALEELQ
ITAKDLEHLNLIFPVSSSASSLLVQLIREQWKESLGFAIPIVGKEFALLQADLSSGNFSLATGGWFADFADPMA
FLTIFAYPSGVPPYAINHKDFLEILQNIEQEQDHQKRSELVSQASLYLETFHIIEPIYHDAFQFAMNKKLSNLGV
SPTGVVDFRYAKEN

>core/172/1/Org1_Gene568

MLGKEEEFTCKQKQCLSHFVTNLTSDVFALKNLPEVVKGALFSKYSRSVLGLRALLLKEFLSNEEDGDVCDE
AYDFETDVQKAADFYQRVLDNFGDDSVGELGGAHLAMENVSILAAKVLEDARIGGSPLEKSTRYVYFDQKV
RGEYLYYRDPILMTSAFKDMFLGTCDFLFDTYSALIPQVRAYFEKLYPKDSKTPASAYATSLRAKVLDCIRGL
LPAATLTNLGFFGNGRFWQNLIHKLQGHNLAELRRLGDESLTELMKVIPSFVSRAEPHHHHHQAMMQYRRA
LKEQLKGLAEQATFSEEMSSSPSVQLVYGDPDGIYKVAAGFLFPYSNRSLTDLIDYCKKMPHEDLVQILESSV
SARENRRHKSPRGLECVEFGFDILADFGAYRDLQRHRTLTQERQLLSTHHGYNFPVELLDTPMEKSYREAME
RANETYNEIVQEFPEEAQYMVPMAYNIRWFFHVNARALQWICELRSQPQGHQNYRTIATGLVREVVKFNPM
YELFFKFVDYSDIDLGRLNQEMRKEPTT

>core/173/1/Org1_Gene849

MFVGGLVSFLLPIPDLECANNVTKTYDKKASVISRDLKLQEDCQKFWNLDPYKLESLCAYQVLYHDDYSSK
RIRELFPQIQKDEVPIFATMILTLGKVDRGFSPEEISLIQKLSYPGLSLASLRGSTEIDPNTDLARALVVSEFSGD
LGKNRADYYSNCLDILALRIHAERQRYLDQSPCVPGTSEFHKATIEAINTILFYEEAVRYPSKKEMFSDEFSFL
SSVTDRKFGVCLGVSSLYFSLSQRLDLPLEAVTPPGHIYLRYQGGEVNIETTAGGRHLPTASYCDCLDLEDLQ
VRTPEEMIGLTFMNQGSFALQKKKYKEAEEAYKKAQEYLGDEELQELLGFVQILGGKKKEGKSLIGKSPRAS
QKGSVAYDYLKGRINIPTLALLFSYPGSNYEEIASYEEELKKAMKSSMPCCEGQRRLASVAFHLGKTAEAVA
LLEKCVEDIPNDLSLHLRLCKILCDRHEYTKALKYFIIAERLMEDQGFLKKDNRSFALFYEVKKIISKVAPQKA
NTLLLMESER

>core/174/1/Org1_Gene8

MSIVLDKIGKSLGTRILFDDVSVVFNPGNCYGLTGPNGAGKSTLLKIIMGMIEPTRGSISLPKKVGILRQNIDSF
HDTTVLDCVIMGNTRLWEALQRRDNLYLQEFTDAIGMELGEIEEIIGEENGYRADSEAEELLTGIGIPNEMFD
KKMAMIPIDLQFRVLLCQALFGHPEALLLDEPTNHLDLYSINWLGNFLKDYEGTVIVVSHDRHFLNTITTHIA
DIDYDTIIIYPGNYDDMVEMKTASREQEKADIKSKEKKISQLKEFVAKFGAGSRASQVQSRLREIKKLQPQEL
KKSNIQRPYIRFPLSDKSSGKVVLSLEAITKDYGDHQVIHPFSLEIYQGDKLGIIGNNGLGKTTLMKLLAGVEA
PSSGSIKLGHQAICSYFPQNHSDVLADCGQETLFEWLRNRKTGINDQEIRSVLGKMLFGGDDAFKQIQALSGG
ETARLLMAGMMLENHNVLILDEANNHLDLESVSALSWAINDYKGTAIFVSHDRGLIQDCATKLLIFDKDKIT
FFDGTMVDYTAGHKQLL

>core/175/1/Org1_Gene845

MKMHRLKPTLKSLIPNLLFLLLTLSSCSKQKQEPLGKHLVIAMSHDLADLDPRNAYLSRDASLAKALYEGLT
RETDQGIALALAESYTLSKDHKVYTFKLRPSVWSDGTPLTAYDFEKSIKQLYFEEFSPSIHTLLGVIKNSSAIHN
AQKSLETLGIQAKDDLTLVITLEQPFPYFLTLIARPVFSPVHHTLRESYKKGTPPSTYISNGPFVLKKHEHQNYL
ILEKNPHYYDHESVKLDRVTLKIIPDASTATKLFKSKSIDWIGSPWSAPISNEDQKVLSQEKILTYSVSSTTLLIY
NLQKPLIQNKALRKAIAHAIDRKSILRLVPSGQEAVTLVPPNLSQLNLQKEISTEERQTKARAYFQEAKETLSE
KELAELSILYPIDSSNSSIIAQEIQRQLKDTLGLKIKIQGMEYHCFLKKRRQGDFFIATGGWIAEYVSPVAFLSIL
GNPRDLTQWRNSDYEKTLEKLYLPHAYKENLKRAEMIIEEETPIIPLYHGKYIYAIHPKIQNTFGSLLGHTDLK
NIDILS

>core/176/1/Org1_Gene861

MPGSVSSPPLSPVIVRERVPSSSGSDLIQPHAVLKISILIFALVTILGIVLVVSSALGALPSLVLTVSGCIAIAVGLI
GLGILVTRLILSTIRKVDAMGYDAAVKEEQYLSRIRELESENREIRDRNRAVEDQCAHLSEENKDLRDPEYLH
GMTERLIASLEIENQALVAENILLKDWNASLSRDFRAYKQKFPLGALEPWKEDIACIMEQNLFLKPECIAMVK
SLPLETQRLFLYPKGFQSLVNRFAPRSRFFQTPKYEYNSRNENEDGKVAAVCARLKKEFFSAVLGACSYEELG
GICERAVALKETLPLPEAVYDTLVQEFPNLLTAESLWKEWCFYSYPYLRPYLSVDYCKRLFVQLFEELCLKLF
TTGSPEDQALVRLFSYYRNHIPAVLASFGLPPPETGGSVFVLLPKQENLLWSQIEVLATRYLKDTFVRNSEWT
GSFEMMFSYNEMCKEISEGRIRFAEDYETRHSEEFPPSPLSEEGEGEEFLPPCSEEEVSVLERPDLDVDSMWV
WHPPVPKGPL

>core/177/1/Org1_Gene714

MTARAEYLDHEDFLYRSHKLQELSELGVVLYPYEFPGVFSCEDIKKTFASQELGNSEAAMSRSTPRVRFAGR
LVLFRAMGKNAFGQILDHNQTIQVMFNREFTSVHGLSEDAEITPIKFIEKKLDLGDILGIDGYLFFTHSGELTV
LVETVTLLCKSLLSLPDKHAGLSDKEVRYRKRWLDLISSREVSDTFVKRSYIIKLIRNYMDAHGFLEVETPILQ
NIYGGAEAKPFTTTMEALHSEMFLRISLEIALKKILVGGAPRIYELGKVFRNEGIDRTHNPEFTMIEAYAAYMD
YKEVMVFVENLVEHLVRAVNHDNTSLVYSYWKHGPQEVDFKAPWIRMTMKESIATYAGIDVDVHSDQKLK
EILKKKTTFPETAFATASRGMLIAALFDELVSDNLIAPHHITDHPVETTPLCKTLRSGDTAFVERFESFCLGKEL
CNAYSELNDPIRQRELLEQQHTKKELLPDSECHPIDEEFLEALCQGMPPAGGFGIGVDRLVMILTNAASIRDVL
YFPVMRRFDAEKTN

>core/178/1/Org1_Gene132

MLRFFAVFISTLWLITSGCSPSQSSKGIFVVNMKEMPRSLDPGKTRLIADQTLMRHLYEGLVEEHSQNGEIKPA
LAESYTISEDGTRYTFKIKNILWSNGDPLTAQDFVSSWKEILKEDASSVYLYAFLPIKNARAIFDDTESPENLG
VRALDKRHLEIQLETPCAHFLHFLTLPIFFPVHETLRNYSTSFEEMPITCGAFRPVSLEKGLRLHLEKNPMYHN
KSRVKLHKIIVQFISNANTAAILFKHKKLDWQGPPWGEPIPPEISASLHQDDQLFSLPGASTTWLLFNIQKKPW
NNAKLRKALSLAIDKDMLTKVVYQGLAEPTDHILHPRLYPGTYPERKRQNERILEAQQLFEEALDELQMTRE
DLEKETLTFSTFSFSYGRICQMLREQWKKVLKFTIPIVGQEFFTIQKNFLEGNYSLTVNQWTAAFIDPMSYLMI
FANPGGISPYHLQDSHFQTLLIKITQEHKKHLRNQLIIEALDYLEHCHILEPLCHPNLRIALNKNIKNFNLFVRR
TSDFRFIEKL

>core/179/1/Org1_Gene238

MNVLKYTKHSPSAHAWKLIGTSPKHGIYLPLFSIHTKNSCGIGEFLDLIPLISWCQKQGFSVIQLLPLNDTGEDT
SPYNSISSVALNPLFLSLSSLPNIDTIPEVAKKLQDMHELCSTPSVSYTQVKEKKWAFLREYYQKCCKSSLEGN
SNFSEFLESERYWLYPYGTFRAIKHHMHGEPINNWPKSLTDQENFPDLTKKFHDEVLFFSYLQFLCYQQLCEV
KAYADQHHVLLKGDLPILISKDSCDVWYFRDYFSSSRSVGAPPDLYNSEGQNWHLPIYNFSQLAKDDYIWW
KERLRYAQNFYSVYRLDHIIGFFRLWIWDSSGRGRFIPDNPKDYIKQGTEILSTMLGASSMLPIGEDLGIIPQDV
KTTLTHLGICGTRIPRWERNWESDSAFIPLKDYNPLSVTTLSTHDSDTFAQWWLNSPKEAKQFAKFLHLPFQK
TLTTETQIDILKLSHESASIFHINLFNDYLALCPDLVSKNLQRERINTPGTISKKNWSYRVRPSLEELAIHKKFN
GYIEKILTGL

>core/180/1/Org1_Gene980

MERKRFIDCDSTKILQELALNPLDLTAPGVLSAERIKKFSLLGGGFTFSFATERLDDAILAALISLAEERGLHES
MLAMQQGQVVNYIEGFPSEMRPALHTATRAWVTDSSFTGEAEDIAVRSRVEAQRLKDFLTKVRSQFTTIVQI
GIGGSELGPKALYRALRAYCPTDKHVHFISNIDPDNGAEVLDTIDCAKALVVVVSKSGTTIETAVNEAFFADY
FAKKGLSFKDHFIAVTCEGSPMDDTGKYLEVFHLWESIGGRFSSTSMVGGVVLGFAYGFEVFLQLLQGASA
MDQIALQPNARENLPMLSALISIWNRNFLGYPTEAVIPYSSGLEFFPAHLQQCCMESNGKSIAQDGRRVGFST
SPVIWGEPGTNGQHSFFQCLHQGTDIIPVEFIGFEKSQKGEDISFQGTTSSQKLFANMIAQAIALACGSENTNPN
KNFDGNRPSSVLVSSQLNPYSLGELLSYYENKIVFQGFCWGINSFDQEGVSLGKALANRVLELLEGADASNFP
EAASLLTLFNIKFR

>core/181/1/Org1_Gene252

MTKTEEKPFGKLRSFLWPIHTHELKKVLPMFLMFFCITFNYTVLRDTKDTLIVGAPGSGAEAIPFIKFWLVVPC
AIIFMLIYAKLSNILSKQALFYAVGTPFLIFFALFPTVIYPLRDVLHPTEFADRLQAILPPGLLGLVAILRNWTFA
AFYVLAELWGSVMLSLMFWGFANEITKIHEAKRFYALFGIGANISLLASGRAIVWASKLRASVSEGVDPWGI
SLRLLMAMTIVSGLVLMASYWWINKNVLTDPRFYNPEEMQKGKKGAKPKMNMKDSFLYLARSPYILLLAL
LVIAYGICINLIEVTWKSQLKLQYPNMNDYSEFMGNFSFWTGVVSVLIMLFVGGNVIRKFGWLTGALVTPVM
VLLTGIVFFALVIFRNQASGLVAMFGTTPLMLAVVVGAIQNILSKSTKYALFDSTKEMAYIPLDQEQKVKGK
AAIDVVAARFGKSGGALIQQGLLVICGSIGAMTPYLAVILLFIIAIWLVSATKLNKLFLAQSALKEQEVAQEDS
APASS

>core/182/1/Org1_Gene592

MVWVFKSQFEGLSALKRGVHALTKAVTPAFGPRGYNVVIKKGKAPIVLTKNGIRIAKEIILQDAFESLGVKLA
KEALLKVVEQTGDGSTTALVVIDALFTQGLKGIAAGLDPQEIKAGILLSVEMVYQQLQRQAIELQSPKDVLH
VAMVAANHDVTLGTVVATVISQADLKGVFSSKDSGISKTRGLGKRVKSGYLSPYFVTRPETMDVVWEEALV
LILSHSLVSLSEELIRYLELISEQNTHPLVIIAEDFDQNVLRTLILNKLRNGLPVCAVKAPGSRELRQVVLEDLAI
LTGATLIGQESENCEIPVSLDVLGRVKQVMITKETFTFLEGGGDAEIIQARKQELCLAIARSTSESECQELEERL
AIFIGSIPQVQITADTDTEQRERQFQLESALRATKAAMKGGIVPGGGVAFLRAAHAIEVPANLSSGMTFGFETL
LQAVRTPLKVLAQNCGRSSEEVIHTILSHENPRFGYNGMTDTFEDLVDAGICDPLIVTTSSLKCAVSVSCLLLT
SSFFISSRTKT

>core/183/1/Org1_Gene5

MYQENLRLLERLLYNSVQKSYADRLFSYEKTKMVHDTPLIPWEEDKEKCAEAEKAFLEQQKILLDYGKSIFW
LNENDEINLNDPWSWGLNTVRTRKVFQEVDDSERWNHKVLIQKLEDDYEKLLEESSKESTEANKKLLSDLV
DRLEDAKTKFFLKKQEEVETRVKDLRARYGGTVDPKQDTEAKKKVELEASLETFLDSIESELVQCLEDQDIY
WKEQDVKDLARTQELEEQDIEAKREEAAEDLRSLNERLKKSKTMLDRAKWHIENAEDSITWWTSQIEMKD
MKARLKILKEDITSVLPEIDEIETCLSLEELPLLTTRELLTKSYLKFKICSETLLKMTSVFENNIYVQEYEVQLQ
NLGFKLQGISQRFGKKQDDFANLEEQVALQKKRLRELTQNFEIQGFNFMKEDFKAAAKDLYIRSTAEQKMN
FDVPCMELFRRYHEEVNKPLLELMYNCADSYRDAKKKLCSLRLDEKELLQKEIKKEEFYQKKQQRHADRSR
HTTYQKLRIAEELALELKKKI

>core/184/1/Org1_Gene996

MVARGLCDFPTLVPNERLPIGPFFVPQHTSGAKGKEFAKRNFSIISGLDDILKLCILQRRPFALQWDNLSVKSD
YEEAGPAIGIRSLEPQVSQISPAHGRLCSTLVQWAPILGSEEQLVWLEETMKRLKFPKSLGSKDAVIVDSEMVP
VNANPTQEIPAASETVESSPVAPGNTTDTMPAASGTTDTTSGVSEAAAAEATVDSTPGTEEEPSFSLRYALVV
QNVPYPEPPKEPEVMFTDEEKSLILEATRARRMELDLYNGYLADYELSKDEIQKHVPDLPENWRTNWRWSE
RLYKFFFKTKKEGLEEIFLNKELGNMILARGLAATQSQARIKVFNSLVAWLLQSFNVGRSCTAKPLPTSKLDL
FKSEFESKPKNNILTEFLVASDEEILFKGLRVLEPGIEGWYDHPDQAGEIRSVLEGLVQAGRISGYWENQPFGR
FVLRGVGERRTELVELLESLVASGEIMQFFESSDEEGAFIIDNEPSKTAMLKQRFKSCVRTKLVGSFADESLPR
GRFTILV

>core/185/1/Org1_Gene826

MVSLLHKFLENASGKKGQSLASTAYLAALDHLLNAFPSIGERIIDELKSQRSHLKMIASENYSSLSVQLAMGN
LLTDKYCEGSPFKRFYSCCENVDAIEWECVETAKELFAADCACVQPHSGADANLLAVMAILTHKVQGPAVS
KLGYKTVNELTEEEYTLLKAEMSSCVCLGPSLNSGGHLTHGNVRLNVMSKLMRCFPYDVNPDTECFDYAEI
SRLAKEYKPKVLIAGYSSYSRRLNFAVLKQIAEDCGSVLWVDMAHFAGLVAGGVFVDEENPIPYADIVTTTT
HKTLRGPRGGLVLATREYESTLNKACPLMMGGPLPHVIAAKTVALKEALSVDFKKYAHQVVNNARRLAER
FLSHGLRLLTGGTDNHMMVIDLGSLGISGKIAEDILSSVGIAVNRNSLPSDAIGKWDTSGIRLGTPALTTLGMG
IDEMEEVADIIVKVLRNIRLSCHVEGSSKKNKGELPEAIAQEARDRVRNLLLRFPLYPEIDLEALV

>core/186/1/Org1_Gene686

MSEQEKLSNYNADKKLFSGIDKLFQIVKGSYGPKQSLSPTSFFKERGFYAISQTELSNSYENLGVDFAKAMVN
KIHKEHSDGATTGLILLHAILQESYAALEKGISTHKLIASLKLQGEKLQEALQQQSWPIKDALKVRNIIFSSLH
MPTIADHFYNAFSVVGPEGLISITKERENDKTSMDVFQGFKIPAGYASTYFVSDTASRLTRIAHPLILITDRKIS
MIHSLLPLLQEISEQNQHLIIFCEDIDPDVLATLVVNKLQGLLQVTVVTIPQLSTTNQELAEDIALFTGTHICPCQ
EASHVLAPEMVTLGSCLSIEISESQTTLIGGLHIPEVLTLKTRQLAEEIRTTSCLETKKRLIKSTNRLQSSVAILPT
DEDNEPLYTLALKIMESALSRGYVPGGGVALFYASLTLGTPKDDADENSIAISLLQKACCAPLKLLATNADLD
GDAVIAKLSSLGTTSLGISVFSREIEDLIAGGILDSLATTSTILAQALDTAILVLSSKILILENQYEISTL

>core/187/1/Org1_Gene736

MENEILLNIESKEIRYAHLKNGQLFDLTIERKKVRQLKGNIYRGRVTNILRNIQSAFINIDERENGFIHISDILENS
KKFEQMFDMDVDALPEEASEAPLLSSEEAPIEEFLKLDSPVLVQVVKEPIGSKGARLTSNISIPGRYLVLLPNSP
HRGVSRKIEDPHMREQLKQLIRSFEMPQDMGLICRTASTTASTEALINEAHDLLLTWKTILEKFYSTEQPCLLY
SETDILKKAVITCIDKNYKRLLIDDYATYQKCKHMLKKYSPDASIKIEYYRDSIPMFERFNIEKEIDKATRRKI
WLSSGGYLFFDKTEAMHTIDVNSGRSTQLESGVEETLVQINLEAAEEIARQLRLRNVGGLVIIDFIDMKSRKN
QRRVLERLKEHMKYDAARCTILSMSEFGLVEMTRQRNRESLMQTLFTLCPYCSGNAIIKTPESVVIEIERDLK
KVINHKEHSHLCLVVHPEIASYMKQENDDNEMINLAKQLKAKLQINTSDSVHLNHYQFFSLITGESIDL

>core/188/1/Org1_Gene203

MFNLFFFTANKETTASHELIYRKNQSFSLSPVTILCLLAISVLLLLGVVFALVGCHVLAAPLGLLVWGCAASV
CSMMAIVSLMCLYKGGKPLIEPSNEEKIDPTKDLEIKDPESLKPVPVEGQSLPKERKTVSFKAKIPSIVEDDFKP
YVIQSTFYHQNKVYSKPIAERMQSLEKEITTLIVDFPRALEESSKSSGSLLRGVISEIKNLFLPRFLSRKVKYSLT
ACLRRLGSIVEEYASSDLLILLLTKPEPLNMVTQQLIAHLNSLKTEKRKLTPHMQKLVLSINFWFYGWSLEEK
CIEKIVAYDPNLLTDELKAHLEAGNIVQFLLSFQSSEMQREFRALFPSDAQELPSAKDGSNYVPAINSSEYMY
DFKDLSVLKKSLSERLAFCEKIPSPSSWNFTSSVASHYKDFSLLFTFFSNQQSVILQNPFLLIELLHENPKCQTFL
KGLLEKAMPMSNWAALFRPMLMGMLCSGIARKKELKIIAEHLGVPFKEITQAIASGKILDLLLQHLFDF

>core/189/1/Org1_Gene331

MTILRNFLTCSALFLALPAAAQVVYLHESDGYNGAINNKSLEPKITCYPEGTSYIFLDDVRISNVKHDQEDAG
VFINRSGNLFFMGNRCNFTFHNLMTEGFGAAISNRVGDTTLTLSNFSYLAFTSAPLLPQGQGAIYSLGSVMIEN
SEEVTFCGNYSSWSGAAIYTPYLLGSKASRPSVNLSGNRYLVFRDNVSQGYGGAISTHNLTLTTRGPSCFENN
HAYHDVNSNGGAIAIAPGGSISISVKSGDLIFKGNTASQDGNTIHNSIHLQSGAQFKNLRAVSESGVYFYDPIS
HSESHKITDLVINAPEGKETYEGTISFSGLCLDDHEVCAENLTSTILQDVTLAGGTLSLSDGVTLQLHSFKQEA

SSTLTMSPGTTLLCSGDARVQNLHILIEDTDNFVPVRIRAEDKDALVSLEKLKVAFEAYWSVYDFPQFKEAFT
IPLLELLGPSFDSLLLGETTLERTQVTTENDAVRGFWSLSWEEYPPSLDKDRRITPTKKTVFLTWNPEITSTP

>core/190/1/Org1_Gene168

MTNVVQETIGGLNSPRTCPPCILVIFGATGDLTARKLLPALYHLTKEGRLSDQFVCVGFARREKSNELFRQEM
KQAVIQFSPSELDIKVWEDFQQRLFYHRSEFDNNMGYTSLKDSLEDLDKTYGTRGNRLFYLSTPPQYFSRIIEN
LNKHKLFYKNQDQGKPWSRVIIEKPFGRDLDSAKQLQQCINENLNENSVYHIDHYLGKETVQNILTTRFANTI
FESCWNSQYIDHVQISLSETIGIGSRGNFFEKSGMLRDMVQNHMMQLLCLLTMEPPTTFDADEIRKEKIKILQR
ISPFSEGSSIVRGQYGPGTVQGVSVLGYREEENVDKDSRVETYVALKTVINNPRWLGVPFYLRAGKRLAKKS
TDISIIFKKSPYNLFAAEECSRCPIENDLLIIRIQPDEGVALKFNCKVPGTNNIVRPVKMDFRYDSYFQTTTPEAY
ERLLCDCIIGDRTLFTGGDEVMASWKLFTPVLEEWDQDSSPSFPNYPAGSSGPKEADALIERDGRSWRPL

>core/191/1/Org1_Gene782

MSSWLSQASEVLLNQDPYIPDAPRSQESSVPKISYSITVAPQEAQKSLPKFFTQKFQSQCKSEPPITHHKTFIIAT
PRERILRFGSSFESQLHNTSQAQTSSPWNLFSQKNSTEASKALMQELTMPKSPEKTSEKALDKNLSSKQEGSC
KNFDTLHLQQHLKLFGTVDSLYSQSLDSEQQELLQSRREERSETYANQQSSEKKIETKVQIKDLCKDLFSQDQ
DSNQKQKKSPFQQDTSRKNRIAKAAQAVPVIPPPSIGVFTLSYLLTKQGILSDFSSYGCHKDSVESTQRELDAL
HEKRIETIKVSIEKEKRERLWGSLSDIIGWLAPFVSIGIGIVAILSGGGIFAFAGFFAGLISLVIKCLEKLKFWDW
LEKHLPIKNEELRRKIITIIQWVVVYLTPVILSICTLKVENLGFSPIIEGAIKGIQPAIESTMAALRCAILFSQAEIYK
LKGKLTKIQLDIELKSFDRDDHYERSQELLDNMESSFEALSRILNYMRELDQVYLHSLRG

>core/192/1/Org1_Gene938

MAVGGVGGSRSPSPIPPNRRNSEDGKVSPKDNLGEHTVSSSDSSLASQGPTIEERKAQLGGTDKIPLPSVKEPG
DSQTSGRSGVLQRIWKGVKGVFKKTPQARPEVSSPRLPSHVQHGQRLPGLEGFRDRIQKRSENPEADLGKMK
RSYSDGDLDRVGHDSNEDSTEDSRSEGGEPSSKSSSFLSGVRGATSTVQGALGDAKEKVSAFGEQAAGAIRS
APGNIRTRFQRSSSEGDLSNVNKAAKHLRKALENLEKVAPEQVSPEVASRVQSLLARMEQLTHQEPPTVEDLI
TFVESNVGSDSVEYASIVPQDGSQAPAETAEAPETGGVEGSAAQGAWKALRDFVVSIFQAVASFFRAIASRLS
SARRESAVDDLASESNTQWFVEQEGVSNPSAAPSLSFAEEIARRAAEMSNRNAQSLEKLESGNVTDPVIQQG
LGLARSFAPEGQ

>core/193/1/Org1_Gene97

MADILVIGANPTGLILANMLIQHGISVKVIDHRASPEDPSFLDCRKLPVILSCSSLELLHNSEMLGDFIQANHKI
FGARYHWKKRTLLFKFSQATDSPVPFSLSTTYQSLEQHLIDEFLKRGGVIDWSTRPVTLVDNSIFIESTKVSQN
FENREIYNPKWIIACEADNNLDIRDLVKSQLRARRINREVIFINCDEGEPFEEDHIHLLPITKNFLNFVFYNPQEK
TKQLCLPQGTHSISPKLKQKLLYTYNLVISDENFHIKTSHHAFPPEHGNVLFLGSLSNTLLLSYLNGINTNIHAA
FNLAWKLLPVLKKAALKHLVITKEQEDGNILPYISPTTEKRAKKLPFSRFYTPALMYYFLKGCRKFNTTGEEY
YYPPHQALKYRSSDIIKMSPQDKEIHGPGPGMRAIDARLENGSFLLDPLKSSKHLLIFFKDIPDLKEALQEEYG
EWIEICNVKEPRILNLYHANPNSLFIIRPDRYIGYRTHTFKLHELISYLLRIFASEKTS

>core/194/1/Org1_Gene406

MNWENVRVRVAPSPTGDPHVGTAYMALFNEIFAKRFKGKMILRIEDTDRTRSRQDYEENIFSALRWCGIQW
DEGPDVGGPYGPYRQSERTKIYQGYVETLLKTDCAYKCFATPQELAEMRAVASTLGYRGGYDRRYRYLSPE
EVASREAAGQPYTIRLKVPLSGECVFEDYSKGRVVFPWADVDDQVLVKSDGFPTYHFANVIDDHLMGITHV
LRGEEWLSSTPKHLLLYEAFGWEPPVFLHMPLLLNPDGTKLSKRKNPTSIFYYRDSGYVKEAFVNFLTLMGY
SMEGDEEVYSLERIIETFNPRRIGKSGAVFDIQKLDWMNKHYLNHEGSPECLLKELQGWLLNDEFFLKILPLC
QSRITTLAEFINLTSFFFSGLLEYRVEELLPQALSPEKAAILLYSYVKYLEKTDQWTKETCYLGSKWLAQAFN
VHHKKAIIPLLYVAITGKKQGLPLFDSIEILGKPRARARLVYAEKLLGGVPKKLAATVDKFMQREDFEEATFD
L

>core/195/1/Org1_Gene956

MDCRGGIPLPEPQVIGGYHVKKILSKKLRSRVVHGLHPETRHSTVIKVFSPSPSFTSRSVYNFLKEAQSLHQIT
HPNIVKFHRYGKWQDCLYIAMEYIEGISLREYILAQFISLPQAIDIIFDIAQALEHLHSRNILHKDIKPENILITPQ
GKIKLIDFGLADWDTEIQRAHPSVIGTPYYMSPEQRQGESHSPASDIYALGLLAYELILGHLSLGRVFLSLVPE
RISKILAKALQPSPNNRYSSTREFIQDIHHYRMSGDMQEDLRIKDHTVALYEQLQTQRFWLAPETLRFPDFISG
VLYHQGYPLYPHAYDTLLEGDVFNLWLGYSPISNATIALSVVKSLVCQQDLQRPLLDRVCEINECLIRMKIPI
DEMGISILCLEISKENKELSWVACGKTVFWIKRQGRVVQDFESFSPGLGKITSLQIRETKVAWEIGDEAVVCTL
ELEESVASLKTLSLAELQDRRQKAIFCPIESIHGGIQSRQHGSNSPSTLISLKRIR

>core/196/1/Org1_Gene314

MLKKFINSLWKLCQQDKYQRFTPIVDAIDTFCYEPIETPSKPPFIRDSVDVKRWMMLVVIALFPATFVAIWNS
GLQSIVYSSGNPVLMEQFLHISGFGSYLSFVYKEIHIVPILWEGLKIFIPLLTISYVVGGTCEVLFAVVRGHKIAE
GLLVTGILYPLTLPPTIPYWMAALGIAFGIVVSKELFGGTGMNILNPALSGRAFLFFTFPAKMSGDVWVGSNP
GVIKDSLMKMNSSTGKVLIDGFSQSTCLQTLNSTPPSVKRLHVDAIAANMLHIPHVPTQDVIHSQFSLWTETH
PGWVLDNLTLTQLQTFVTAPVAEGGLGLLPTQFDSAYAITDVIYGIGKFSAGNLFWGNIIGSLGETSTFACLLG
AIFLIVTGIASWRTMAAFGIGAFLTGWLFKFISVLIVGQNGAWAPARFFIPAYRQLFLGGLAFGLVFMATDPVS
SPTMKLGKWIYGFFIGFMTIVIRLINPAYPEGVMLAILLGNVFAPLIDYFAVRKYRKRGV

>core/197/1/Org1_Gene917

MKFEFSVALKYLIPGRGRLYSAIVSLFSVGIISLVVWLSIVFISVIHGLEQRWIEDLSQLHSPITILPSDTYYSSYY
YQIDKHSSLSNYTTKTLGEKIASPQVDPYDPESDYLLPETFPLKDCDLGGQQKDPVKMTLESLGPYLQSQHGK
VIEFEQGVGYLDIKTSLKLQKPQPRNLTHFLTYPSKLSYEDKVLPYDETDYTSAELNPFNRSPSGWQQDFHHL
EELYRGASIILPSTYKDSGYKVGDTGVFSTYSIENEKETQYTVHVIGFYNPGLSPLGGRTVFIDPDLARSIRSQS
EGLGMSNGFHLFFPDTKRIVFVKKQIENILTSLGVDDYWEISSLHDYDYFQPILDQLQSDQVLFLFVCILILIVA
CSNIVTMSMLLVNNKKKEIGILKAMGTSSRSLKIIFACCGAFSGACGVVIGTIFAIITLKNLQFIVKALNYLQGR
ETFNTAFFGQNLPNSVHPQAIYFLGLGTLLLAAVSGALPARKVAKMHVSEILKAD

>core/198/1/Org1_Gene279

MVLFHAQASGRNRVKADAIVLPFWHFKDAKNAASFEAEFEPSYLPALENFQGKTGEIELLYSSPKAKEKRIV
LLGLGKNEELTSDVVFQTYATLTRVLRKAKCSTVNIILPTISELRLSAEEFLVGLSSGILSLNYDYPRYNKVDR
NLETPLSKVTVIGIVPKMADAIFRKEAAIFEGVYLTRDLVNRNADEITPKKLAEVALNLGKEFPSIDTKVLGKD
AIAKEKMGLLLAVSKGSCVDPHFIVVRYQGRPKSKDHTVLIGKGVTFDSGGLDLKPGKSMLTMKEDMAGG

ATVLGILSALAVLELPINVTGIIPATENAIDGASYKMGDVYVGMSGLSVEICSTDAEGRLILADAITYALKYCK
PTRIIDFATLTGAMVVSLGEEVAGFFSNNDVLAEDLLEASAETSEPLWRLPLVKKYDKTLHSDIADMKNLGS
NRAGAITAALFLQRFLEESSVAWAHLDIAGTAYHEKEEDRYPKYASGFGVRSILYYLENSLSK

>core/199/1/Org1_Gene781

MTVSYQSISTPPPEGEFDIFVDGNATEEAVVAAEVQVALPAGEQYAMLRATSELCFGILTQSECALTQALPPK
EKPLQEEQFLVKNGILMRSTSLPNLKPGQSQQTSLASHRNPLAQQSTSSNSTGKASTETTSSSFPFFSCKAPEG
DSSVDKTFTVSVQTPKAQEQQEASASQSQAQFHVRSYSSSTIKEHSAKEKVSQSTKSAETQKHTQTKSDATLS
PMSLYSTLHKEVPQALSSTKSQQKDEEHRDQRQQEGYEQEQEQEEGKKKTPWCTVESLQQTSSSNQVYESY
TPIIPDPIVEFALSESQLSVLAGKRVTNLDVLRICTELMKLMLKSRANDTMTRLEERELMEREAHELAASYSR
QAKYARWLGIATATLGILGAIAPMVGEISGDSILGFVQRISGRFKDATAKTFFKGIGKVFTSLSQLTEAASKVH
ELSESAVRAVAEYRKEVFRMRQDEVTRTIEEVKDNWKSMDNFLLNILQTEHDAARSLYQ

>core/200/1/Org1_Gene621

MAASILSQELLDILPYTFLKKHCLLPIEESSEAITIAHATATSVIAQDEVKLLIKKPVRFVLKEESEILQRLQQLY
SNREGNVSDMLLTMKEEDGTTISEEEDLLETTDTIPVVRLLNWILKEAIEERASDIHFEPCEDSMRIRYRIDGVL
HDRHSPPSHLRSALTTRLKVLAKMDIAEHRLPQDGRIKIHIGGQEVDMRVSTVPVIYGERVVLRILDKRNVIL
DIAGLHMPKGTEILFKDTITAPEGILLVTGPTGSGKTTTLYSVLQELKGPLTNIMTIEDPPEYKLPGIAQIAVKP
KIGLTFARGLRHLLRQDPDILMVGEIRDQETAEIAIQAALTGHLVVSTLHTNDAISAIPRLLDMGIESYLLSATL
VGVVAQRLVRTICPYCKVAYTPENQEKSFLASLGKDTEMPLYRGQGCVHCFRSGYKGRQGIYEFLRPNTLFR
SEVASNRPYHILRETAEQNGFLPILEHGIALAVSGETTLAEVLRVTKRCD

>core/201/1/Org1_Gene207

MNKKHASFSSRLGFIFSMIGIAVGAGNIWRFPRVAAQNGGGAFLILWLCFLFLWSIPLIIIELSIGKLTKKAPIGA
LIKTAGKKFAWAGGFITLVTTCILAYYSTIVGWGLSYFYYAVSGKIHLGNDFAKLWTSHYQSSIPLWAHLTSL
GLAYLVIRKGIVHGIEKCNKILIPAFFLCTIALLLRAVTLPGAVQGIKQLFSCDKSCFSNYKVWIEALTQNAWD
TGAGWGLLLVYAGFASKKTGVVSNGALTAICNNLVSLIMGIIIFSTCASLDILGTTQLQDGAGASSIGITFIYLP
ELFTRLPGGIYLTTLFSSIFFLAFSMAALSSMISMLFLLSQTLAEFGIKPYISETLATIIAFVLGIPSALSLTFFSNQ
DTVWGVALIVNGLIFIYAALVYGFPKLKKEVINAAPGDLRLNKAFDYIIKYLLPIEGILLLGWYFYEGLFPENG
QWWNPISLYSLGSLVLQWSLGLIILWKFNKQLYLRFSRYNHEIL

>core/203/1/Org1_Gene442

MLKYILKRLVLIPLTLFAIVSINFVILNAAPGDVLEEKSRDALGEAGKSDKMRSYKGPDRYLQFREHYGLTLPI
FFNTRPKITHKKIQTALQELANANNTTPSAKNAAKSLVYWGDCAKFVMPALLFEADDASRDDKYRHIAADL
FIRGGVLQGFVGPNLSPEQRAQNKEIAESNAFLVRQLNEEDLDTKVEALKGWFQDHGGTEVFCYSSKQFWK
TFFLETRFARYMSRVLRLDFGTLRNDAHKTVISEVIKRLRCSLVLSILPMIVGFVLCQIFGMIMALKRNRWIDH
SLNFIFLILFSIPVFVAVPWILDNFVINKTIPFTTIPMPYSGLRSPPEVFNELSTLGRIFDLVSHGFLPFCAVSYGAL
AAQSRLSRSIFLEVLSQDFICAAKARGLRWFDILYKHVGKNAAVSIVTSLASSLGTLLGGALVVETLFNIDGFG
NFFYQAILNRDHNVVLFSVLVGSALSLVGYLLGDICYVLLDPRVQLEGRRI

>core/204/1/Org1_Gene881

MHTEFAPFLEDLVHQQVISPLDIAFASKHISSDFEESFVFLAVSSALWRYGHPFLSLEENRIRPSLGGISETDLY
RGFHNLPKEVRDKLFVVVSGRLYLRSLYTIRSKLLDKLSLLCSATPNYFPPSIDSSILSEEQNFIFNKITQGCFSI
VSGGPGTGKTFLAAQLILSLVKQQPKLRIAIVSPTGKATSHIRQILMKYNIFDDMVLMQTVHHFLQEYAYRRY
NSIDVLLVDEGSMVTFDLLYSLVQTLQGYEKDKKLYTSSLIILGDTNQLPPIGIGVGNPLQDLIGYFHENTFFL
KTSHRAKTGVVDQLTQSVLRGEMISFSPLPSISSAIEVLKNRFVKSLRQSEARLCVLTPMRHGPWGVLNLNTM
IHQRLARSDPDLRIPIMVTSRYETWGLFNGDTGLLCLKTQKLHFPQHEPIDSRALSQYVVYNYVMSVHKSQGSE
YDEVIVIIPKGSEVFGVSILYTAITRAKYRVSVWGDPETLHKIIKKSNY

>core/205/1/Org1_Gene1009

MNLDSKHFDINSANFLEEFAKFISFPSISADSDHLQDCENCAHFLVDHVNKIFDVELWETPGHPPIIYASYKSE
DPLSPTLMLYNHYDVQPAQLSDGWKGDPFILREENGNLYARGASDNKGQCFYTLKALQHYYESQGNFPLNII
WLIEGEEESGSLALFTWLEKKKEALRADYLLIVDGGFLSEKHPYVSIGARGIVSMKISLEEGNKDMHSGVLGG
IAYNTNRALSEILSSLHHPDNSIAIEGFYDDLALPSDSDRPDLPKSDTLRECEENLGFRPQGYEASYSPEESALR
PTVEINGISGGYTGPGFKTVIPYRATAYLSCRLVPNQDPDKAAHQVIHHLKQQVPSSLKFSYEILPGGSRGWRS
SANLPIVKVLQEIYSDLYNEECLRLVMPATIPIGPLLGEAAQTSPIICGTSYLSDDIHAAEEHFSMDQLKKGFLSI
CQLLDKLPKIKE

>core/206/1/Org1_Gene877

MSISSSSGPDNQKNIMSQVLTSTPQGVPQQDKLSGNETKQIQQTRQGKNTEMESDATIAGASGKDKTSSTTKT
ETAPQQGVAAGKESSESQKAGADTGVSGAAATTASNTATKIAMQTSIEEASKSMESTLESLQSLSAAQMKEV
EAVVVAALSGKSSGSAKLETPELPKPGVTPRSEVIEIGLALAKAIQTLGEATKSALSNYASTQAQADQTNKLG
LEKQAIKIDKEREEYQEMKAAEQKSKDLEGTMDTVNTVMIAVSVAITVISIVAAIFTCGAGLAGLAAGAAVG
AAAAGGAAGAAAATTVATQITVQAVVQAVKQAVITAVRQAITAAIKAAVKSGIKAFIKTLVKAIAKAISKGI
SKVFAKGTQMIAKNFPKLSKVISSLTSKWVTVGVGVVVAAPALGKGIMQMQLSEMQQNVAQFQKEVGKLQ
AAADMISMFTQFWQQASKIASKQTGESNEMTQKATKLGAQILKAYAAISGAIAGAA

>core/207/1/Org1_Gene892

MYRYSALELAKAVTLGELTATGVTQHFFHRIEEAEGQVGAFISLCKEQALEQAELIDKKRSRGEPLGKLAGV
PVGIKDNIHVTGLKTTCASRVLENYQPPFDATVVERIKKEDGIILGKLNMDEFAMGSTTLYSAFHPTHNPWDL
SRVPGGSSGGSAAAVSARFCPVALGSDTGGSIRQPAAFCGVVGFKPSYGAVSRYGLVAFASSLDQIGPLANT
VEDVALMMDVFSGRDPKDATSREFFRDSFMSKLSTEVPKVIGVPRTFLEGLRDDIRENFFSSLAIFEGEGTHLV
DVELDILSHAVSIYYILASAEAATNLARFDGVRYGYRSPQAHTISQLYDLSRGEGFGKEVMRRILLGNYVLSA
ERQNVYYKKATAVRAKIVKAFRTAFEKCEILAVPVCSSPAFEIGEILDPVTLYLQDIYTVAMNLAYLPAIAVPS
GFSKEGLPLGLQIIGQQGQDQQVCQVGYSFQEHAQIKQLFSKRYAKSVVLGGQS

>core/208/1/Org1_Gene1011

MNGCWEDLKQTIFWVGEHDCTDIETVRKSCMWLDRYADKFILREKEEKMERHELFHATMVRKASGHAYA
KAKAAFEKERSNENQRKVKDVEKWLSKGLAEFRNQESRRARERLRELQTLYPEVSVEERVLERQRTKKVNL
ENLYADIEKKYHHCVREQEHYWKEVENKEAEYRENGEKVLSAEEVSECLQRLEDCLETWSKKLTKAEESVF
EMKFDATEKLGNKVLSDVTNRLEILCEDAEEMIFRIEEIEMTLRMVELPLLFMKNTFEKASLQYNSCKEMLA
KVEPQCKESPTYRSSQERLERLNQDLQTAYTNCQERLQGFSDLESKVRTCRDHLREQMKHFEVQGLNFINEE

LLWVGAELFTQARLDLVATVPYMEFYLQYHNIKREKVRSQWMAKTERYREIRQAFQGVMKEDLLAEDTIL
KEEDYWLLRDDWLLRDERKNRQRRLICNKIAAAQQRVKGF

>core/209/1/Org1_Gene79

MLGILLIASGIIFLAVAIPGLSSAVALGLGCGMTALGTVLLITGLVLLIRSEKLALEQVEIKQARTRVNNELDQL
SQYVFYTENVLDNLKRWSYRDLGFVRQAQEEVTNLEQDIEEIFLTLRDIRNALDNEEFFMTHAKQCLAQVGE
SLFQDASIDEFINLAHLSEIRQHLDINDPRWSMITKKVKGTVVRFIYVSTMYKQIKSNFEKSDFGQLRKMLLN
NYKTIEEVLYQSFQKGYNRAALLSEKTRIIHTSSLLHWEKDEDKHLNIKNECASRLENFKKFRTLFLGLSEED
VIDFTGASGWDCSKLPRKEVPLDGGKKKLRFKRTFADEQVGDWDRTTSLEHMTPQEEDPLDRLMDQVEQE
ATSVLKDQDRYWKEIETSEAKFRSLPREDDFEKQSQIDSYIRDLDDHLSVWANQLSAAEDALIEVTDVQEHG
NREMLKNIQQGLELIEDAVKATLPRVDFIQELLEKEELPLVAARMSLENS

>core/210/1/Org1_Gene817

MTFPCGNCNCYYRETPPPNPGGEDIPLQEGGQSGSQGGRVITQQPGTGGREMGISLGSDNVLGMVEQAGSLL
NNLLDSARMQRLGHYCYRTGTPWCREHCPGFLQWIWGGCCACCLETVDDPDNPSAQFLQQLIQQYGPICVG
MSFQQLPHCTQKIEQGEPLGDGDKQEVENGCKLHRELLKAAQPRCMGESLVKLLQNNGLGEDMQQTPPWS
LILQAVSEGALSFVTSSDNPPTCWILQPEQQPCPPPPTDEEQLQGAVGGAPAPQQKKHPAQECRVTCKLNFRT
LLQKLSRLEVLSLESGYKGPLGQAAKQIVDLIKKSLKRLVASDLATFLGPGIGLSLESQVFEVLVLLCLLSKGY
LPLDPLHPEQTVLDPRVQGPWQRILRKVLVTTTAGENIWRQTQGEAPRQAPPPPDPWDDDEIERDGIVTGGG
FGIPCQCLRCWRKLPTEKRPNRWL

>core/211/1/Org1_Gene740

MHMSNPISLFSPAELIAKYNLIPKTSPIYPRRTELIILEENACQTRLTNVAQVLHPSSLFSMSKKILNPCGCSGGP
LCWVILNILAFIITSVLFIILLPVNLIVAGLRLFMPLPPKKIVEDLSEPTTEETNEVIQPFIFALQALLFEDNKLRSF
KIVEQSVGKAPLPNPFLNRLVAISPQESQEAMRKIPDLCSQLKKVLKSLGVLTPEWKHMLKYFEGLKNEHDS
NPDKKTFPILIKLLIEALTGKSSLPKTPSTKEKMQAALFIASSCKTCKPTWGEVITRSLNRLYSIANEGDNQLLI
WVQEFKERELMSIQDGDDAEEYRFAAQQHGERYTEAIEQVLRNESAAKLQWHVINTMKFFHGKNLGLVTE
HLQDTLGALTLRQTTVDTHQGREDADLSAALFLNKYLNSGNQLVNSVFKSMQKADPETKALIREFALDILYA
SLRLPQTSAHTEVFSTLLMDPETYEPNKACIAYLLYVLKIIEL

>core/212/1/Org1_Gene3

MSAVYADWESVIGLEVHVELNTASKLFSSALNRFGDEPNTNISTVCTGLPGSLPVLNQSAVEKAVLFGCAVE
GEISLLSRFDRKSYFYPDSPRNFQITQFEHPIIRGGRIKAIVQGEERYFELAQTHIEDDAGMLKHFGEFAGVDYN
RAGVPLIEIVSKPCMFCPEDAVAYATSLVSLLDYIGISDCNMEEGSIRFDVNVSVRPKGSPELRNKVEIKNMNS
FAFMAQALEAEKQRQIDEYLNQPNKDPKLVIPAATYRWDPEKKKTVLMRLKESAEDYKYFPEPDLPTLQLTE
SYIERIRKTLPELPYDKYHRYIQEYGLSEDIASILISDKNIATFFEVACKDCKNFRSLSNWVTVEFGGRCKTLGV
KLPSSGIFPEGVAQLVNAIDQGVITGKIAKEIADLMMESPGKNPEEILKEKPELLPMSDEGELQKIIAEVVLANP
ESIVDYKNGKTKALGFLVGQIMKRTAGKAPPKRVNELLLLELDKG

>core/213/1/Org1_Gene1002

MITKQLRSWLAVLVGSGLLALPLSGQAVGKKESRVSELPQDVLLKEISGGFSKVATKATPAVVYIESFPKSQA
VTHPSPGRRGPYENPFDYFNDEFFNRFFGLPSQREKPQSKEAVRGTGFLVSPDGYIVTNNHVVEDTGKIHVTL
HDGQKYPATVIGLDPKTDLAVIKIKSQNLPYLSFGNSDHLKVGDWAIAIGNPFGLQATVTVGVISAKGRNQL
HIADFEDFIQTDAAINPGNSGGPLLNIDGQVIGVNTAIVSGSGGYIGIGFAIPSLMANRIIDQLIRDGQVTRGFLG
VTLQPIDAELAACYKLEKVYGALVTDVVKGSPADKAGLKQEDVIIAYNGKEVDSLSMFRNAVSLMNPDTRI
VLKVVREGKVIEIPVTVSQAPKEDGMSALQRVGIRVQNLTPETAKKLGIAPETKGILIISVEPGSVAASSGIAPG
QLILAVNRQKVSSIEDLNRTLKDSNNENILLMVSQGDVIRFIALKPEE

>core/214/1/Org1_Gene18

MWNRCQVFSSFFFRYPISSWLIRLRASCECFQQRHPIFLCGLYWLAGITSRGHPECSALILIFLGMFLPRNPKQ
WLPLASAWIISLMLTPAPFLHDGPISGTFVIHHAGGQGTYYGEALCIQTPCGKRAHHLSCQILSESRLELKKVY
ELEGTLHHTSQIVFKSNACYKEIPRSRFYIMKEKCRESSCHFLNHRFPSSEVGPFASSLLLGTPLPQNLRDLFRQ
KGLSHLFAISGWHFSLCATTLWMLCALLPLKIKKILSFIVLTSLACIFPMSLSVWRSWISVTLLCFSWCFSGSCS
GLNRLGAGFILCSIFFSPFSPTFVLSFLATLGILLFFPKIFSFLYTPWTQFLSPFWLYPIRYLAMTLAISLSAQLFIV
LPIMQYFGSLPLEGLLYNLIVPFTILPIIVFLIATIILPCCSPITEALIQGFLSHPWLHNPNILKTLSFAPVPPWMLTL
ASLILFFIGILRTNVSPYASISATSYRFIETL

>core/215/1/Org1_Gene644

MLKIAILGRPNVGKSSLFNRLCKRSLAIVNSQEGTTRDRLYGELHAFGVPAQVIDTGGVDHNSEDYFQKHIYN
QALTGAKEADVLLLVIDIRCGITEEDAHLAKLLLPLKKPLILVANKADSRQEELQIHETYKLGIRDIVVTSTAH
DKHIDTLLQRIKLVANLPEPREEEEEGLEELSVDEHEESEAALPSNTFPDFSEVFTEGFSPEEPCTIPESPQQAPK
TLKIALIGRPNVGKSSIINGLLNEERCIIDNTPGTTRDNIDILYSHKDRQYLFIDTAGLRKMKSVKNSIEWISSSR
TEKAISRADICLLVIDATQKLSSYEKRILSLISKRKKPHIILINKWDLLEEVRMEHYCKDLRATDPYLGQAKML
CISATTKRNLKKIFSAIDELHHVVSNKVPTPIVNKTLASALHRNHPQVIQGRRLRIYYAIQKTTTPLQFLLFINA
KSLLTKHYEYYLKNTLKSSFNLYGIPFDLEFKEKPKRHN

>core/216/1/Org1_Gene784

MNMPVPSAVPSANITLKEDSSTVSTASGILKTATGEVLVSCTALEGSSSTDALISLALGQIILATQQELLLQSTN
VHQLLFLPPEVVELEIQVVDLLVQLEHAETITSEPQETQTQSRSEQTLPQQSSSKQSALSPRSLKPEISDSKQQQ
ALQTPKDSAVRKHSEAPSPETQARASLSQASSSSQRSLPPQESAPERTLLEQQKASSFSPLSQFSAEKQKEALT
TSKSHELYKERDQDRQQREQHDRKHDQEEDAESKKKKKKRGLGVEAVAEEPGENLDIAALIFSDQMRPPAE
ETSKKETTFKKKLPSPMSVFSRFIPSKNPLSVGSSIHGPIQTPKVENVFLRFMKLMARILGQAEAEANELYMRV
KQRTDDVDTLTVLISKINNEKKDIDWSENEEMKALLNRAKEIGVTIDKEKYTWTEEEKRLLKENVQMRKEN
MEKITQMERTDMQRHLQEISQCHQARSNVLKLLKELMDTFIYNLRP

>core/217/1/Org1_Gene859

MTSRTKSSKNLGTIALAGMVVSSIIGGGIFSLPQNMAATAGAGAVILSWILTGFGMFFIANTFRILSTIRPDLKE
GIYMYSREGFGPYIGFTIGWGYWLCQIFGNVGYAVITMDALNYFFPPYFQGGNTLPAILGGSILIWVFNFIVLK
GIRQASIINVIGTIFKIIPLIIFIILTAFFFKLAVFKTDFWGHAVTKAQPSLGSVSSQLKGTMLVTLWAFIGIEGAV
VMSGRAKNPLSVGQATVLGFLGCLTIYILFSLLPFGSLFQHQLANIPNPSTAGVLDILVGKWGEVLMNVGLIIA
VLSSWLSWTIIVAEIPFSAAKNGTFPEIFTIENKEKSPSVSLYITSSVMQLAMLLVYFSSNAWNTMLSITGVMV

LPAYLASAAFLFKLSKSKTYPKKGSIKAPLAMITGILGVVYSLWLIYAGGLKYLFMALVLLALGIPFYIDAGK
KKKNAKTFFAKKEIVGMTFIGLLALTAIFLFSTGRIKI

>core/218/1/Org1_Gene59

MITRTKIICTIGPATNSPEMLAKLLDAGMNVARLNFSHGSHETHGQAIGFLKELREQKRVPLAIMLDTKGPEIR
LGNIPQPISVSQGQKLRLVSSDIDGSAEGGVSLYPKGIFPFVPEGADVLIDDGYIHAVVVSSEADSLELEFMNS
GLLKSHKSLSIRGVDVALPFMTEKDIADLKFGVEQNMDVVAASFVRYGEDIETMRKCLADLGNPKMPIIAKI
ENRLGVENFSKIAKLADGIMIARGDLGIELSVVEVPNLQKMMAKVSRETGHFCVTATQMLESMIRNVLPTRA
EVSDIANAIYDGSSAVMLSGETASGAHPVAAVKIMRSVILETEKNLSHDSFLKLDDSNSALQVSPYLSAIGLA
GIQIAERADAKALIVYTESGSSPMFLSKYRPKFPIIAVTPSTSVYYRLALEWGVYPMLTQESDRAVWRHQACI
YGIEQGILSNYDRILVLSRGACMEETNNLTLTIVNDILTGSEFPET

>core/219/1/Org1_Gene522

MGESVKVFLEEREDYPYGFVTPIESQGLTRGLSEETIEEIAALRNEPQFIIDFRLQAYRYWKQLHEPAWARLH
YGPIAYDDIVYFSSPKQKKPLGRLEDADPEILDTFKKLGIPLDEQKRLLNVENVAVDLVFDSVSIGTTFKEALE
KAGVIFCSLGEAIQEHPNLVKKYLGSVVSHRDNFFAALNAAVFSDGSFVYVPKGVKCPMDISTYFRINNKEA
GQFERTLIVVEDGGYASYLEGCTAPAYSSNQLHAAVVELVAHEHAVIRYSTVQNWYAGDKKTGKGGIYNFV
TKRGLCAGYRSKISWSQVEVGAAITWKYPSCILKGDESVGEFYSVALTSGKMQADTGTKMLHVGKRTTSTV
ISKGISSDESKNTFRSLVSLGKKAEHSSNYTQCDSMLIGKASGAYTDPKIVVENSTSSIEHEATTSKLREDQLLY
LRSRGLSPEEAVSLVIHGFCREIIEQLPLEFAQEASKLLLIKLENSVG

>core/220/1/Org1_Gene305

MDLKELLHGVQAKIYGKVRPLEVRNLTRDSRCVSVGDIFIAHKGQRYDGNDFAVDALANGAIAIASSLYNPF
LSVVQIITPNLEELEAELSAKYYEYPSSKLHTIGVTGTNGKTTVTCLIKALLDSYQKPSGLLGTIEHILGEGVIK
DGFTTPTPALLQKYLATMVRQNRDAVVMEVSSIGLASGRVAYTNFDTAVLTNITLDHLDFHGTFETYVAAK
AKLFSLVPPSGMVVINTDSPYASQCIESAKAPVITYGIESAADYRATDIQLSSSGTKYTLVYGDQKIACSSSFIG
KYNVYNLLAAISTVHASLRCDLEDLLEKIGLCQPPPGRLDPVLMGPCPVYIDYAHTPDALDNVLTGLHELLPE
GGRLIVVFGCGGDRDRSKRKLMAQVVERYGFAVVTSDNPRSEPPEDIVNEICDGFYSKNYFIEIDRKQAITYA
LSIASDRDIVLIAGKGHEAYQIFKHQTVAFDDKQTVCEVLASYV

>core/222/1/Org1_Gene257

MQTNIGLIGLAVMGKNLVLNMIDHGFSVSVYNRTPEKTRDFLKEYPNHRELVGFESLEDFVNSLERPRKIML
MIQAGKPVDQSIHALLPFLEPGDVIIDGGNSYFKDSERRCKELQEKGILFLGVGISGGEEGARHGPSIMPGGNP
EAWPLVAPIFQSIAAKVQGRPCCSWVGTGGAGHYVKAVHNGIEYGDIQLICEAYGILRDFLKLSATAVATILK
EWNTLELESYLIRIASEVLALKDPEGIPVIDTILDVVGQKGTGKWTAIDALNSGVPLSLIIGAVLARFLSSWKEI
REQAARNYPGTPLIFEMPHDPSVFIQDVFHALYASKIISYAQGFMLLGEASKEYNWGLDLGEIALMWRGGCII
QSAFLDVIHKGFAANPENTSLIFQEYFRGALRHAEMGWRRTVVTAIGAGLPIPCLAAAITFYDGYRTASSSMS
LAQGLRDYFGAHTYERNDRPRGEFYHTDWVHTKTTERVK

>core/223/1/Org1_Gene543

MRDVSELFRTHFMHYASYVILERAIPHILDGLKPVQRRLLWTLFLMDDGKMHKVANIAGRTMALHPHGDAP
IVEALVVLANKGYLIDTQGNFGNPLTGDPHAAARYIEARLSPLARETLFNTDLIAFHDSYDGREKEPDILPAKL
PVLLLHGVDGIAVGMTTKIFPHNFAELLKAQIAILNDKKFTVFPDFPSGGLMDPSEYQDGLGSITLRASIDIIND
KTLVVKQICPQSTTETLIRSIENAAKRGTIKIDTIQDFSTDVPHIEIKLPKGSRAKEMLPLLFEHTECQVILYSKPT
VIYENKPVECSISEILKLHTTALQGYLEKELLLLQEQLTLDHYHKTLEYIFIKHKLYDSVREVLAINKKISADDL
HQAVLHALEPWLHELATPVTKQDTSQLASLTIKKILCFNEEACTKELLAIEKKQAAIQKDLGRIKEVTVKYLK
GLLERHGHLGERKTQITNFKTAKTSILKQQTLI

>core/224/1/Org1_Gene792

MLCATVSGPSFCEAKQQILKSLHLVDIIELRLDLINELDDQELHTLITTAQNPILTFRQHKEMSTALWIQKLYSL
AKLEPKWMDIDVSLPKTALQTIRKSHPKIKLILSYHTDKNEDLDAIYNEMLATPAEIYKIVLSPENSSEALNYI
KKARLLPKPSTVLCMGTHGLPSRVLSPLISNAMNYAAGISAPQVAPGQPKLEELLSYNYSKLSEKSHIYGLIG
DPVDRSISHLSHNFLLSKLSLNATYIKFPVTIGEVVTFFSAIRDLPFSGLSVTMPLKTAIFDHVDALDASAQLCE
SINTLVFRNQKILGYNTDGEGVAKLLKQKNISVNNKHIAIVGAGGAAKAIAATLAMQGANLHIFNRTLSSAA
ALATCCKGKAYPLGSLENFKTIDIIINCLPPEVTFPWRFPPIVMDINTKPHPSPYLERAQKHGSLIIHGYEMFIEQ
ALLQFALWFPDFLTPESCDSFRNYVKNFMAKV

>core/225/1/Org1_Gene321

MMSRLRFRLAALGIFFILLVPNSVSAKTIVASDKEKVGVLVYDNSVEAFQQILDCIDHANFYVELCPCMTGGR
TLKEMVDHLEARMDLVPELCSYIIIQPTFTDAEDQKLLKALKERHPNRFFYVFTGCPPSTSILAPNVIEMHIKLS
IIDGKYCILGGTNFEEFMCTPGDEVPEKVDNPRLFVSGVRRPLAFRDQDIMLRSTAFGLQLREEYHKQFAMW
DYYAHHMWFIDNPEQFAGACPPLTLEQAEETVFPGFDKHEDLVLVDSSKIRIVLGGPHDKQPNPVTQEYLKLI
QGARSSVKLAHMYFIPKDELLNALVDVSHNHGVHLSLITNGCHELSPAITGPYAWGNRINYFALLYGKRYPL
WKKWFCEKLKPYERVSIYEFAIWETQLHKKCMIIDDEIFVIGSYNFGKKSDAFDYESIVVIESPEVAAKANKV
FNKDIGLSIPVSHGDIFSWYFHSVHHTLGHLQLTYMPA

>core/226/1/Org1_Gene728

MRIVQVAVEFTPIVKVGGLGDAVASLSKELAKQNDVEVLLPHYPLISKFSSSQVLSERSFYYEFLGKQQASAIS
YSYEGLTLTIITLDSQIELFSTTSVYSENNVVRFSAFAAAAAAYLQEADPADIVHLHDWHVGLLAGLLKNPLN
PVHSKIVFTIHNFGYRGYCSTQLLAASQIDDFHLSHYQLFRDPQTSVLMKGALYCSDYITTVSLTYVQEIINDY
SDYELHDAILARNSVFSGIINGIDEDVWNPKTDPALAVQYDASLLSEPDVLFTKKEENRAVLYEKLGISSDYFP
LICVISRIVEEKGPEFMKEIILHAMEHSYAFILIGTSQNEVLLNEFRNLQDCLASSPNIRLILDFNDPLARLTYAA
ADMICIPSHREACGLTQLIAMRYGTVPLVRKTGGLADTVIPGVNGFTFFDTNNFNEFRAMLSNAVTTYRQEP
DVWLNLIESGMLRASGLDAMAKHYVNLYQSLLS

>core/227/1/Org1_Gene974

MAFSHIEGLYFYNTASQKKELFFPNHTPVRLYTCGPTVYDYAHIGNFRTYVFEDILKRTLVFFGYSVTHVMNI
TDVEDKTIAGASKKNIPLQEYTQPYTEAFFEDLDTLNIARADFYPHATHYIPQMIQAITKLLEQGIAYIGQDAS
VYFSLNRFPNYGKLSHLDLSSLRCCSRISADEYDKENPSDFVLWKAYNPERDGVIYWESPFGKGRPGWHLEC
SIMAMELLGDSLDIHAGGVDNIFPHHENEIAQSEALSGKPFARYWLHSEHLLIDGKKMSKSLGNFLTLRDLLH
QEFTGQEVRYMLLQSHYRTQLNFTEEALLACRHALRRLKDFVSRLEGVDLPGESPLPRTLDSSSQFIEAFSRA

LANDLNVSTGFASLFDFVHEINTLIDQGHFSKADSLYILDTLKKVDTVLGVLPLTTSVCIPETVMQLVAEREEA
RKTKNWAMADTLRDEILAAGFLVEDSKSGPRVKPL

>core/228/1/Org1_Gene939

MDTIDTPGEQGSQSFGNSLGARFDLPRKEQDPSQALAVASYQNKTDSQVVEEHLDELISLADSCGISVLETRS
WILKTPSASTYINVGKLEEIEEILKEFPSIGTLIIDEEITPSQQRNLEKRLGLVVLDRTELILEIFSSRALTAEANIQ
VQLAQARYLLPRLKRLWGHLSRQKSGGGSGGFVKGEGEKQIELDRRMVRERIHKLSAQLKAVIKQRAERRK
VKSRRGIPTFALIGYTNSGKSTLLNLLTAADTYVEDKLFATLDPKTRKCVLPGGRHVLLTDTVGFIRKLPHTL
VAAFKSTLEAAFHEDVLLHVVDASHPLALEHVQTTYDLFQELKIEKPRIITVLNKVDRLPQGSIPMKLRLLSPL
PVLISAKTGEGIQNLLSLMTEIIQEKSLHVTLNFPYTEYGKFTELCDAGVVASSRYQEDFLVVDAYLPKELQK
KFRPFISYVFPEDCGDDEGRGPVLESSFGD

>core/229/1/Org1_Gene204

MDSRTSHLDDELSFKLEKAFTCLSTDIHSHDLSKIVIEYNPIDLAYAVSCLPSESRAILYKNLSCITAKVAFIINT
DSASRWAIFRRLSDSEVCALIEQMPPDEAVWVLDDIPDRRYRRILELIDSKKALKIRDLQKHGRNTAGRLMTN
EFFAFLMETTVKDVSACIRSNPGIDLTRLVFVLDFKGELQGVVTDRSLIINPPEMSLKQIMNQIEHKVLPDATR
EEVVDLVERYKIAALPVVDEENFLIGAITYEDVVEAIEDIADETIARMAGTTEDVGYQTCHVVQRFLLRAPWL
LVTLFAGLISASVMAYFQKISPALLALIIFFIPLINGMSGNVGVQCSTILVRSMATGTLSFGRRRETIFKEMSIGL
LTGVVLGILCGLVVYLMGFLGLNIFSGGGIQLGVTVATGVLGASLTATTLGVLSPFFFAKLGVDPALASGPIV
TALNDIMSMIIFFLIAGGINFLFFN

>core/230/1/Org1_Gene844

MNKKKRFLSLLFLTAVLLGIWFSPHPASINSNAWQLFAIFTTTIMGIIFQPVPMGAIAIIGISTLLLTQTLTLEQG
LSGFHNPIAWLVFLSFSIAKGIIKTGLGERIAYFFVSALGKSPLGLSYGLVITDFFLAPAIPSVTARAGGILYPVV
TSLSDSFGSSAEKGTQDLIGSFLIKVAYQSSVITSAMFLTAMAGNPLVAALAGHVGVSLSWVLWAKAAIIPGL
LSLFLMPIILYKLYPPKITSCEEAIRSAKLRLKEMGPLKKEEKTILMIFFLLVVLWTFGDLLGISATTAALIGLSL
LILTNILDWQKDVIANTTAWETFIWFGALIMMASFLNQLGFIPLVGDSAAALVSGLSWKIGFPLLFLIYFYSHY
LFASNTAHIGAMYPIFLAVSISLGTNPIFAALTLAFASNLFGGLTHYGSGPAPLYFGSHLVTVQEWWRSGFALS
IVNIVIWIGIGSLWWKALGLI

>core/231/1/Org1_Gene1006

MTTKSLGSFNSVISKNKIHFISLGCSRNLVDSEVMLGILLKAGYESTNEIEDADYLILNTCAFLKSARDEAKDY
LDHLIDVKKENAKIIVTGCMTSNHKDELKPWMSHIHYLLGSGDVENILSAIESRESGEKISAKSYIEMGEVPRQ
LSTPKHYAYLKVAEGCRKRCAFCIIPSIKGKLRSKPLDQILKEFRILVNKSVKEIILIAQDLGDYGKDLSTDRSS
QLESLLHELLKEPGDYWLRMLYLYPDEVSDGIIDLMQSNPKLLPYVDIPLQHINDRILKQMRRTTSREQILGFL
EKLRAKVPQVYIRSSVIVGFPGETQEEFQELADFIGEGWIDNLGIFLYSQEANTPAAELPDQIPEKVKESRLKIL
SQIQKRNVDKHNQKLIGEKIEAVIDNYHPETNLLLTARFYGQAPEVDPCIIVNEAKLVSHFGERCFIEITGTAG
YDLVGRVVKKSQNQALLKTSKA

>core/232/1/Org1_Gene200

MHSHSKPTKPLGTFTVGMLSLAVVISLRNLPLTAKHGLSTLFFYGLAVICFMIPYALISAELASFKPQGIYIWA
RDALGKWWGFFAIWMQWFHNMTWYPAVLAFIASTIVYKINPELAHNKVYIATVILAGFWILTFFNFLGITSS
ALFSSICVIIGTLIPGVILVSLALFWIFSGNPIAISLSWGNLLPNFSNVSSLVLLAGMLLALCGLEANANLASDM
VNPRKNYPKAVFIGAIATLTILVLGSLSIAIVIPKEEISLVSGLVKTFTLFFDKYNLSWMTGIVVVMTIAGSLGE
LNAWMFAGTKGLFISTQNDCLPRLFKKVNSKNVPTNLMLFQGIVVTIFTLLFLCLDSADLVYWILTALSVQM
YLAMYICLFLAGPILRIKEPRAQRLYSVPGKFLGICTMSILGILSCAFALWVSFLPPRELAQISEGSKIGYTTFLL
LAFSLNCLIPFGIYFTHKRLSKKS

>core/233/1/Org1_Gene824

MDKSTGVPLPSPPHSKESEMIVLGCMLTGVHYLNLAANQLYEEDFYYLEHKIIFRVLQDAFKQDKPIDVHLA
GEELKRHNQITVIGGPSYLITLAEFAGTAAYLEEYVDIIRSKSILRKMISTAKEIEKRALEQPKNVAEALDEAQN
SFFKISQSTSVSQYTLVADKLRGLTTTTDKPYLVQLQERQELFLQNAQGDNKSFFTGIPTHFIDLDQLIHGFSPS
NLMILAARPAMGKTALALNIAENLCFQNRLPIGIFSLEMTVDQLIHRMICSRSEVDSKKISIGDLSGHDFQRIVS
VINEMQEHTLLIDDQPGLKVSDLRARARRMKESYDIQFLIIDYLQLLSGSGTLRATESRQTEISEISRMLKTLAR
ELNIPILCLSQLSRKVEDRANHRPMMSDLRESGSIEQDSDLVMFLLRREYYDPNDKPGTAELIIAKNRHGSIGS
VPLVFEKELARFRNYSAFECIS

>core/234/1/Org1_Gene566

MKITVNRGLDLSLQGSPKESGFYNKIDPEFVSIDLRPFQPLSLKLKVEQGDAVCSGAPIAEYKHFPNTYITSHV
SGVVTAIRRGNKRSLLDVIIKKTPGPTSTEYTYDLQTLSRSDLSEIFKENGLFALIKQRPFDIPAIPTQTPRDVFIN
LADNRPFTPSPEKHLALFSSREEGFYVFVVGVRAIAKLFGLRPHIVFRDRLTLPTQELKTIAHLHTVSGPFPSGS
PSIHIHSVAPITNEKEVVFTLSFQDVLTIGHLFLKGRILHEQVTALAGTALKSSLRRYVITTKGASFSSLINLNDI
SDNDTLISGDPLTGRLCKKEEEPFLGFRDHSISVLHNPTKRELFSFLRIGFNKPTFTKTYLSGFFKKKRTYTNPD
TNLHGETRPIIDTDIYDKVMPMRIPVVPLIKAVITKNFDLANELGFLEVCGEDFALPTLIDPSKTEMLTIVKESLI
EYAKESGILTPHQD

>core/236/1/Org1_Gene452

MKEERSSEILPRVKETKKHAYVSMQEKSCVGECAVVASESEEAESVTVTKIAKLQRMGIEELNILARQYGVK
NIGSLTKSQVVFEIVKAKSERPDELLIGEGVLEVLPDGFGFLRSPTYNYLPSAEDIYVSPAQIRRFDLKKGDTIIG
TIRSPKEKEKYFALLKVDKINGSTPDKAKERVLFENLTPLYPNQRIVMEMGKDHLAERVLDLTAPIGKGQRG
LIVAPPRSGKTVILQSIAHAIAVNNPDIVLIVLLIDERPEEVTDMIRQVRGEVVASTFDEQPERHIQVAEMVIEK
ARRLVEHGNDVVILLDSITRLARAYNTVQPHSGKILTGGVDASALHKPKRFFGAARNIEGGGSLTILATALIDT
GSRMDEVIFEEFKGTGNMELVLDRRLSDRRTYPAIDLIKSGTRKEELLYHPSELERVYLFRQAIADLTTIDAM
HLLLGRLKKTNSNAEFLLSLKE

>core/237/1/Org1_Gene963

MNFKLPVYHIGLTKAENNTIKIAILQKTCKGWIVCHCEQIPEGKTWSLPKKYFAAPTTFSLQGSDILVKSSSSS
LKNRKNILKVALTNLEASLALPWESLIVQPQLGKPTDRGETPLTLWIAQKNTLKKELSFLSQAQIFPDKLSCRA
ADIFFLAEQSPLKSLPAYLLIYGGSEEVTCIFVKNHAIAVARSFSNHSTKKSCDDIHATLQYIQETFPQTVLPAIH
VAQISPNLQKILEQKLSLPLVVCQSMTYGVEDEDWEIYGDTIAAAHHGASRRPLTFPYDATSVSPAAQKHWL
LRSSLLIGKYALMATVVVSLGSVLKLKSLSSSASNHFAFACPEEGVLPRSLRAAEKTVKAIGKKNSASNYPLL

PTIPTSEQTLKFLLALGKSSPSIKFSYFSYTMTSYPSKDNPSLPYSALVEVKGQGQPEDIPQFLKKISSHPKLQHV
SESLEDQRSFKLQFTLSS

>core/238/1/Org1_Gene655

MTESVYSPSAMHVNSLADKLKAINQEHILDIWPSLSPKQQQRLFQQLTSVDIDFFRKQQQLLSSPTAILKDFHP
ITSFASSGEDPERAHAGTTLLKEKKVACVVLAGGQGSRLKCDGPKGLFPVSPIKKKPLFQLVAEKVRAASKL
AGQPLPLAFMTSPLNTRQTRSFFESNDYFHLDPNQVDFFCQPLWPLLTLSGDLFLEDMDTLALGPNGNGCIAT
LLYTSGVWEKWKNAGIEMVSVIPIDNPLALPFDVELCGFHAMSNNEVTIKAALRQTAIEDVGILVKSHDSGK
TSVIEYSEIPQNERFALNEDGKLKYCLANIGLYCLSMDFIRHAAYQQLPLYKVHKHAKQLGHTSLNEKNAWK
FEEFIFDLFCYSDHCQTLVYPRQECFAPLKNLEGNHSPDTVRQALSDRERQLFHKVTGKKLSPNTTFELEADF
YYPSTSTSLHWENKAFFEEPFFEAS

>core/239/1/Org1_Gene969

MTQEFDCVVIGAGPSGYVAAITAAQSKLRTALIEEDQAGGTCLNRGCIPSKALIAGANVVSHITHAEQFGIHV
DGYTIDYPAMAKRKNTVVQGIRQGLEGLIRSNKITVLKGTGSLVSSTEVKVIGQDTTIIKANHIILATGSEPRPF
PGVPFSSRILSSTGILELEVLPKKLAIIGGGVIGCEFASLFHTLGVEITVIEALDHILAVNNKEVSQTVTNKFTKQ
GIRILTKASISAIEESQNQVRITVNDQVEEFDYVLVAIGRQFNTASIGLDNAGVIRDDRGVIPVDETMRTNVPNI
YAIGDITGKWLLAHVASHQGVIAAKNISGHHEVMDYSAIPSVIFTHPEIAMVGLSLQEAEQQNLPAKLTKFPF
KAIGKAVALGASDGFAAIVSHEITQQILGAYVIGPHASSLIGEMTLAIRNELTLPCIYETVHAHPTLSEVWAEG
ALLATNHPLHFPPKS

>core/240/1/Org1_Gene31

MMSSKRTSKIAVLSILLTFTHSIGFANANSSVGLGTVYITSEVVKKPQKGSERKQAKKEPRARKGYLVPSSRT
LSARAQKMKNSSRKESSGGCNEISANSTPRSVKLRRNKRAEQKAAKQGFSAFSNLTLKSLLPKLPSKQKTSIH
EREKATSRFVNESQLSSARKRYCTPSSAAPSLFLETEIVRAPVERTKELQDNEIHIPVVQVQTNPKEQNTKTTK
QLASQASIQQSEGTEQSLRELAQGASLPVLVRSNPEVSVQRQKEELLKELVAERRQCKRKSVRQALEARSLT
KKVARGGSVTSTLRYDPEKAAEIKSRRNCKVSPEAREQKYSSCKRDARANGKQDKTTPSEDASQEEQQTGA
GLVRKTPKSQVASNAQNFYRNSKNTNIDSYLTANQYSCSSEETDWPCSSCVSKRRTHNSISVCTMVVTVIAMI
VGALIIANATESQTTSDPTPPTPTP

>core/241/1/Org1_Gene223

MRAWEEFLLLQEKEIGTNTVDKWLRSLKVLCFDACNLYLEAQDSFQITWFEEHIRHKVKSGLVNNNNKPIRV
HVTSVDKAAPFYKEKQMQQEKTAYFTMHYGSVNPEMTFSNFLVTPENDLPFRVLQEFTKSPDENGGVTFNPI
YLFGPEGSGKTHLMQSAISVLRESGGKILYVSSDLFTEHLVSAIRSGEMQKFRSFYRNIDALFIEDIEVFSGKSA
TQEEFFHTFNSLHSEGKLIVVSSSYAPVDLVAVEDRLISRFEWGVAIPIHPLVQEGLRSFLMRQVERLSIRIQET
ALDFLIYALSSNVKTLLHALNLLAKRVMYKKLSHQLLYEDDVKTLLKDVLEAAGSVRLTPLKIIRNVAQYYG
VSQESILGRSQSREYVLPRQVAMYFCRQKLSLSYVRIGDVFSRDHSTVISSIRLIEQKIEENSHDIHMAIQDISKN
LNSLHKSLEFFPSEEMII

>core/242/1/Org1_Gene777

MRQEKDSLGIVEVPEDKLYGAQTMRSRNFFSWGPELMPYEVIRALVWIKKCAAQANQDLGFLDSKHCDMIV
AAADEILEGGFEEHFPLKVWQTGSGTQSNMNVNEVIANLAIRHHGGVLGSKDPIHPNDHVNKSQSSNDVFPT
AMHIAAVISLKNKLIPALDHMIRVLDAKVEEFRHDVKIGRTHLMDAVPMTLGQEFSGYSSQLRHCLESIAFSL
AHLYELAIGATAVGTGLNVPEGFVEKIIHYLRKETDEPFIPASNYFSALSCHDALVDAHGSLATLACALTKIAT
DLSFLGSGPRCGLGELFFPENEPGSSIMPGKVNPTQCEALQMVCAQVLGNNQTVIIGGSRGNFELNVMKPVII
YNFLQSVDLLSEGMRAFSEFFVKGLKVNKARLQDNINNSLMLVTALAPVLGYDKCSKAALKAFHESISLKEA
CLALGYLSEKEFDRLVVPENMVGNH

>core/243/1/Org1_Gene940

MNFSLFLFFLIAIQGICLYVGRRGSKKVEDRESYFLAGRSLKIFPLMMTFIATQIGGGVLLGAAEEAFCYGYGG
ILYPLGVALGLIFLGMGPGKRLAEGSLTTVVSIFEVFYGSKKLRKIAFLLSAGSLFFILVAQVIALDRLFSSFPFG
KYVTVAFWIVLASYTSTGGFRGVVRTDVIQAGFLLIAVLVCGVSVWLSVPKSLSVLDPFQSLPCAKLSNWIF
MPMLFMLVEQDMVQRCVAASSPKRLQWAAVGAGLVLLLFNFIPLFLGSLGAKAGLKAGCPLIDTIAYFCNPS
LAAVMAAAIGVAILSTADSLMNAVSQLIAEEYPTLKAPYYRYLVLGLAVAAPLVAIGFTNIVDVLILSYSLSV
CCLSVPVGFYLLAPKGRRVSGAAAWAGVLVGALGYGWVQIVSLGMFGELLAWIGSLVAFSFVGFIEITWKN
KVKTQT

>core/244/1/Org1_Gene743

MQQSVRKLFGTDGVRGRANFEPMTVETTVLLGKAVARVLREGRSGKHRVVVGKDTRLSGYMFENALIAGL
NSMGIETLVLGPIPTPGVAFITRAYRADAGIMISASHNPYRDNGIKIFSLEGFKISDVLEQRIETMVSEADFGPLP
EDHAVGKNKRVIDAMGRYVEFVKATFPKGRTLKGLKIVLDCAHGASYKVAPSVFEELDAEVICYGCEPTGIN
INEHCGALFPQVIQKAVIEHQAHLGIALDGDGDRIIMVDEKGHIVDGDMILSICAGDLKKRSALPHNRVVATI
MTNFGVLKYLEGLGLQVFTSPVGDRHVLHAMLEHEVTLGGEQSGHMIFLDYNTTGDGIVSALQVLRIMIESE
SMLSDLTAPIVKSPQTLINVAVREKIPLETIPLIERTLRDVQDALGPSGRILLRYSGTENICRVMVEGHKKHQV
DCLAKALADVIDAELGTGSRE

>core/245/1/Org1_Gene417

MQIAQVFGCGRLNGEVKVSGAKNAATKLLVASLLSDQKCTLRNVPDIGDVSLTVELCKSLGAHVSWDKETE
VLEIYTPEIQCTRVPPTFSNVNRIPILLLGALLGRCPEGVYVPTVGGDAIGERTLNFHFEGLKQLGVQISSDSSG
YYAKAPRGLKGNYIHLPYPSVGATENLILAAIHAKGRTVIKNVALEAEILDLVLFLQKAGADITTDNDRTIDIF
GTGGLGSVDHTILPDKIEAASFGMAAVVSGGRVFVRNAKQELLIPFLKMLRSIGGGFLVSESGIEFFQERPLVG
GVVLETDVHPGFLTDWQQPFAVLLSQAQGSSVIHETVHENRLGYLHGLQHMGAECQLFHQCLSTKACRYAI
GNFPHSAVIHGATPLWASHLVIPDLRAGFAYVMAALIAEGGGSIIENTHLLDRGYTNWVGKLRSLGAKIQIFD
MEQEELTTSPKSLALRDASL

>core/246/1/Org1_Gene672

MLYFIEQLNKLSTSFCVFPMILLLGGFLTWKLRGLQFHGLKLGFNLMLQNKLDDSSSKANEVSSYEAVAGIL
AGNFGTGNIAGMAVALACGGPGALVWVWLAALLGAIVQYAGSYLGSKYRKPEGNTGEFIGGPIACLAFGM
RKKILAGFFALFTIMTAFCAGNCVQVSCIVPLCAEGTPGKLLVGILLALVVIPVLAGGNNRILRFSARVIPFIAG
FYCISCGIILFQHASAILPAIKLICSSAFGIKAGLAGIGGYTLSQVISTGINRAVMATDCGSGMVSILQANTKSKN
PVVDGLVTLVPPVIVMVVCSITMLVLIVSGAYSSGAQGTLMVMSAFKNSLGSLGSVIVILAMALFGYTTILTW

FACAEKSLQYMIPGRRANLWLKAIYVLIIPLGGVIDMRMIWALSDTGFSGMVILNCIALIALLKDVLSTNRDV
ALLKERECSVADPVRNLDA

>core/247/1/Org1_Gene680

MFNVNFKFLEGLHQPAPRYTSYPTALEWEPSDAAPALLAFQRIRENPQPLSLYFHIPFCQSMCLYCGCSVVLN
RREDIVEAYINTLIQEMKLVVETIGFRPQVSRIHFGGGTPSRLSRELFTLLFDHIHKLFDLSHAEEIAIEVDPRSL
RNDMEKADFFQNVGFNRVSLGVQDTQADVQEAVRRRQSHEESLKAYEKFKELAFQSINIDLIYGLPKQTKES
FSKTIQDILAMYPDRLALFSFASVPWIKPHQKAMKASDMPSMEEKFAIYSQSRHLLTKAGYQAIGMDHFSLP
HDPLTLAFKNKTLIRNFQGYSLPPEEDLLGLGMTSTSFIRGIYLQNAKTLEEYHNTVLRGTFATVKSKILTEDD
RIRKWAIHKLMCTFTINKEEFFNLFGYEFDTYFIESRDRLISMETTGLIHNSPGSLKVTPLGELFVRVIATAFDH
YFLNKVSKKECFSASI

>core/248/1/Org1_Gene468

MTTLRQFFLITELRQKLFYTFALLTACRVGVFIPVPGINGELAVAYFKQLLGSGQNLFQLADIFSGGAFAQMT
VIALGVVPYISASIIVQLFLVFMPALQREMRESSDQGKRRIGRLTRLFTVALAVIQSLLFAKFALRMNLTIPGIV
LPTLLSSKLFGVPWIFYITTVVVMTTGTLLLMWIGEQISDKGIGNGISLIIALGILSSFPSVLGSIVNKLNLGSQD
SSDLGLISILILALVFVFVLITTILIIEGVRKIPVQYARRVIGRREVPGGGSYLPLKVNYAGVIPVIFASSLLMFPA
TIGQFIASESSWMKRIAALLAPGSLVYSICYVLLIIFFTYFWTATQFHPEQIASEMKKNNAFIPGIRQGKPTQHY
LEYTMNRVTLLGALFLAAIAILPSLLGCLLRVDSNVSYFLGGTAMLIVVGVVLDTMKQVDAFLLMRRYDSVL
KTDRTKGRH

>core/250/1/Org1_Gene499

MNVWTKFFQPPKHIKEIEDQEVVKKKYKYWRIRIFYSMFIGYIFYYFTRKSFTFAMPTLIADLGFDKAQLGIIG
STLYFSYGISKFVSGVMSDQSNPRYFMAIGLMITGLTNIFFGMSSSIVLFALWWGLNGWFQGWGWPPCARLL
THWYAKSERGTWWSVWSTSHNIGGALIPILTGFIIDYSGWRGAMYVPGILCIGMGLVLINRLRDTPQSLGLPPI
EKYKRDPHHAHHEGKSASEGTEEIERELSTREILFTYVLTNQWLWFLAAASFFIYIVRMAVNDWSALFLIETK
HYAAVKANFCVSLFEIGGLFGMLVAGWLSDKISKGNRGPMNVLFSLGLLFAILGMWFSRSHNQWWVDGTL
LFVIGFFLYGPQMMIGLAAAELSHKKAAGTASGFTGWFAYFGATFAGYPLGKVTDVWGWKGFFIALLACAS
IALLLFLPTWNATEKNTRSKA

>core/251/1/Org1_Gene119

MKKVLIANRGEIAVRIIRACHDLGLSTVAVYSLADQEALHVLLADEAICIGEPQAAKSYLKISNILAACEITGA
DAVHPGYGFLSENANFASICESCGLTFIGPSSESIAMMGDKIAAKSLAKKIKCPVIPGSEGIIEDESEGLKIAEKI
GFPIVIKAVAGGGGRGIRIVKEKDEFYRAFSAARAEAEAGFNNPNVYIEKFIENPRHLEIQVIGDTHGNYVHLG
ERDCTIQRRRQKLIEETPSPILNAEIRVKVGKVAVDLARSAGYFSVGTVEFLLDKDKKFYFMEMNTRIQVEHT
ITEEVTGIDLVKEQIHVAMGNKLPWKQKNIEFSGHIIQCRINAEDPTNNFSPSPGRLDYYLPPAGPSIRVDGAC
YSGYAIPPYYDSMIAKVIAKGKNREEAIAIMKRALKEFHIGGVQSTIPFHQFMLDNPKFLESNYDINYIDNLLA
QGNSFFKEF

>core/252/1/Org1_Gene24

MATKTKTQWTCNQCGATAPKWLGQCPGCHNWNSLVEEYVPQARSGTSSRSSTSAIALSSIELENESRIFIDHA
GWDRILGGGVVRGSLTLLGGDPGIGKSTLLLQTAERLASQKYKVLYVCGEESVTQTSLRAKRLNISSPLIYLF
PETNLDNIKQQIATLEPDILIIDSIQIIFNPTLNSAPGSVAQVREVTYELMQIAKSAQITTFIIGHVTKSGEIAGPRV
LEHLVDTVLYFEGNSHANYRMIRSVKNRFGPTNELLILSMHADGLKEVSNPSGLFLQEKTGPTTGSMIIPIIEG
SGALLIELQALVSSSPFANPVRKTAGFDPNRFSLLLAVLEKRAQVKLFTMDVFLSITGGLKIIEPAADLGALLA
VASSLYNRLLPNNSIVIGEVGLGGEIRHVAHLERRIKEGKLMGFEGAILPEGQISSLPKEIRENFRLQGVKTIKD
AIRLLL

>core/253/1/Org1_Gene670

MLKILKIKVLVFPLALLMGCNSIGYAGPQGSLQTNSQTKVKIGSEVWIEQKLRQYPELLWLTESGGAPLLTST
PIDMAYSEKLFNKKVPALDIAIRSMIHLHLLIQGSRQSYMQLSQILPSEEGGMTFKQFQTAHKQLLFFLNSPKS
FDNTLRILETAIVLRHVGCSAKAVTTFKPYFTDSCPQSFYAKALHVLRTFPELCPSYARLSPEQQEVLLSLRRL
GNYDSLLNLTEVPSAQLLSAWRTRRSLAILDLYLYCLDTCGDKNCSQEFYINFAPLLSMLQQHATIEEAFSRY
FTYRANRLGFEGTSRTDMTLVRLATLMNLSPSEASTLAWSFKNLPSDEAENLVNSFYTVQGEHIPLTFRGLPS
LVAGLSVATHGSTVSPENRLRQLYSTMLSLLVKSLRSHREMLNKQLLPQGTVLDFSETTLSSGGLDVFAESIA
VRIHLNGAVSINL

>core/254/1/Org1_Gene126

MRKRHSFDSTSTKKEAVSKAIQKIIKIMETTDPSLNVETPNAEIESILQEIKEIKQKLSKQAEDLGLLEKYCSQE
TLSNLENTNASLKLSIGSVIEELASLKQLVEESIEESLGQQDQLIQSVLIEISDKFLSSIGETLSGNLDMNQNVIQ
GLLIKENPEKSEAASVGYVQTLLEPLSKRIGETHKKVATHDVNISSLQFHMMSVAGGRFRGHIDMNGYRVLG
LGEPKNGEDAVSKDYLERYVSSQLTIDKVEDKPITKPNKGKLLYSQGTSPKLESPLPLGLLTSGISGFTWKSAS
KSNDGSFPFSALRHKETESDTDCFQITSTTLSGNQAGTYTWSLSLKVLVPSIFQIEKPEVQLSLVYSYEDWLPID
NIFNMSQPRTIPLALLGQTMLAGQKYDILELAAHQTNQTLMISPNCSRFSLQLKQTNQFENSPVDFYIVHAAH
SCHWSGF

>core/255/1/Org1_Gene311

MLTCNECTTWEQFLNYVKTRCSKTAFENWISPIQVLEETQEKIRLEVPNIFVQNYLLDNYKRDLCSFVPLDVH
GEPALEFVVAEHKKPSAPVASQKESNEGISEVFEETKDFELKLNLSYRFDNFIEGPSNQFVKSAAVGIAGKPGR
SYNPLFIHGGVGLGKTHLLHAVGHYVREHHKNLRIHCITTEAFINDLVYHLKSKSVDKMKNFYRSLDLLLVD
DIQFLQNRQNFEEEFCNTFETLINLSKQIVITSDKPPSQLKLSERIIARMEWGLVAHVGIPDLETRVAILQHKAE
QKGLLIPNEMAFYIADHIYGNVRQLEGAINKLTAYCRLFGKSLTETTVRETLKELFRSPTKQKISVETILKSVA
TVFQVKLNDLKGNSRSKDLVLARQIAMYLAKTLITDSLVAIGAAFGKTHSTVLYACKTIEHKLQNDETLKRQ
VNLCKNHIVG

>core/256/1/Org1_Gene733

MILPPYSYSLKIGAAVLFFCSILHTFLTPWLYTLCQSYEHKKLVFPECWKRYARLSELFRILSRVEIVFFLWAVP
LFFWFLYTEGYRISMAYFNSRNYGFAVFIMVILILLESRPIVYFAELVLSSIAKLGKTSPKSWWWTLMIAPPLL
SCLLKETGAMIIGATLLMRHFYVFSPSRRFAYATMGLLFSNISIGGLTSYVSSRALFLIFPALKWEHSFFLSHFA
WKAIVAILISTTIYYFIFRKEFKKFPDIPSDKDPSVEKVPWWIICVNIIFVGSIILSRSTPLFMGALLLFYLGFQKFT
IFYQDPINLSKVCYVGLFYAGLVVFGDLQEWWVLNLMQGLSDFGYMTVSYTLSIFLDNALVNYLVHNLSVA

TDCYHYLVVAGCMAAGGLTLVSNIPNIVGYLILRSAFPSSTIHMGWLFLGALGPSIISLGVFWLLKNVPEFLYC
FFR

>core/257/1/Org1_Gene578

MSEPRFVCLSLGSNLGNRFKNLQIARTLLGEQAVLGLRSSVILETEALLLPGSPPEWDLPYFNSVLVGETTLSL
RELLVTIKQIEKVVGRAEESPPWSPRTIDVDILLYGDESFCCDHTEITIPLSNLLSRPFLIALIASLCPYRRFCTQG
SPYHNFTFGELAHHLPSPPGMIRRSLSPDTMLMGVVNVTNDSMSDGGMFLDPEKAVAQAEKLFTEGAAVIDF
GAQATNPKVKQFLSVDQEWERLEPVLRLLKETWSNRKQYPIISLDTFYPEIILRAMDIYPIQWINDVSGGSQS
MAEVARDCELSLVMNHSSSLPVDPKNILSFSVPIGEQLLSWGEKQLKMFSDVGLNANQVIFDPGIGFGKGAA
QSLATLYEIAKFKRLGCPILIGHSRKSFLSLFGNHDPKDRDWETVGLSILLQQQGVDYLRVHNVAAHQKALS
VAACEACAPI

>core/258/1/Org1_Gene385

MNRLLSLLSVFDDFFWSYVAFILIIVLGVSFSWKSRFFQFTKFSQFCKLFRYYSQNPQERETKQGVHPLKVFFA
SAGGNIGIGNVVGIVTAACIGGPGALFWVWIAGIFGSIVKYSEVYLGIKFRKLDRDGVYQGGPMYFLIKAFKT
PVVSVIVAILLCIYGVEIYQFSVITDSLAHCWNLPKVYPMLGLLFLVFYAIRGGLQRIGKICSIVLPFFMLLYCA
LSLYILVKEFHTLPHLLSTVFSSAFKGQSALGGFAGCTVATTIHQGISRAAYSGDIGIGFDSIIQSESSAKDPSTQ
AQLSIVGIAIDNLICTLSLLMVLASGSWSLGLENASQVVEHTLASYFPMVKFFLPTFFFVTGYTTIISYFLVGKK
CAKFLYGNTGAKIYTLYGLLILPLFCFLSQNTALLIMSVSGALLLCFNLLGVFILRKEVIFPARAASLTETSLST
E

>core/259/1/Org1_Gene248

MLSCVFSDTIFLSSFLAVTLICMTTALWGTILLISKQPLLSESLSHASYPGLLVGALMAQYVFSLQASIFWIVLF
GCAASVFGYGIIVFLGKVCKLHKDSALCFVLVVFFAIGVILASYVKESSPTLYNRINAYLYGQAATLGFLEAT
LAAIVFCASLFALWWWYRQIVVTTFDKDFAVTCGLKTVLYEALSLIFISLVIVSGVRSVGIVLISAMFVAPSLG
ARQLSDRLSTILILSAFFGGISGALGSYISVAFTCRAIIGQQAVPVTLPTGPLVVICAGLLAGLCLLFSPKSGWVI
RFVRRKHFSFSKDQEHLLKVFWHISHNRLENISVRDFVCSYKYQEYFGPKPFPRWRVQILEWRGYVKKEQDY
YRLTKKGRSEALRLVRAHRLWESYLVNSLDFSKESVHELAEEIEHVLTEELDHTLTEILNDPCYDPHRQIIPNK
KKEV

>core/260/1/Org1_Gene852

MINSLSQKLSSIFSFLVSSRRINEENISESIREVRLALLDADVNYHVVKDFISKVKEKILGEEIWKHVSPGQQFIR
CLHEELVAFLSDGREEFTIQKTPSIILLCGLQGAGKTTTAAKLADYVIKNKKAKKVLVVPCDLKRFAAVDQL
KILVAQTKAEFYQSQENKPIDVVVKALAYAKENGHDFVILDTAGRLNIDNELMEELTAIQKVSQANERLFVM
NVAMGQDVLATVQAFDQSLDLTGVILSMTDGDARAGAVFSIKHVLGKPIKFEGCGERIQDLRSFDPQSMAER
ILGMGDTINFVKEMREYISEEEDAELGKKLVTAAFTYEDYYKQMKAFRRMGPLRKLLGMMPGFNNAKPSQ
KEIEDSEQQMKRTEAIILSMTPEERKELVELDMSRMKRIASGCGLTLGDVNQFRKQMSQSKKFFKGMSKGK
MEQVRKKMSGGNQWR

>core/261/1/Org1_Gene62

MDALILSRIQFGLFITFHYLFVPLSMGLSMMLVIMEGLYLVTKKQIYKQMTWFWVGIFALTFVLGVVTGIMQI
FSFGSNWANFSEYTGNIFGTLLGSEGVFAFFLESGFLGILLFGRHKVSKKMHFFSTCMVALGAHMSAFWIICA
NSWMQTPSGYEMVMHKGKLIPALTSFWGVVFSPTTIDRFIHAVLGTWLSGVFLVISVSAYYLWKKRHHEFA
KQGMKIGTICAVIVLVLQLWSADVTARGVAKNQPAKLAAFEGIFKTEEYTPIWAFGYVDMEKERVIGLPIPG
ALSFLVHRNIKTPVTGLDQIPRDEWPNVQAVFQLYHLMIMLWGVMVALTLISWSAYKGWRWALKPFFLVIL
TFSVLLPEICNECGWCAAEMGRQPWVVQGLLKTKDAVSPIVQANQIVQSLVIFSLVFIALLTLFITVLCKKIKH
GPEEENDLTEFEVK

>core/262/1/Org1_Gene687

MRAMLLEDWVSLMLSDVSCPKCDKKITGFAIDSQQVQPGDLFFALPGNATDGHQFLKHAATAGAVAAVVS
HDYQGDSFGLELIRVDDTKSALQEAGSNQCNLFQGTLVGITGSVGKTTTKEFSKTILSSIYKTHASPKSYNSQL
TVPLSLLMAEGDEDVMILEMGVSEPGNMQDLLRIVQPEIAVITHINDQHAMHFPQGIQEILKEKSYILQKSKL
QLLPKDSPYYLDLRSCSPTAEKFSFSFNDPLADFCYKAISGDSVVIQTPEENYCLPIAFSYKPAYTNLLIAVALS
WILEVPEEGVIRSLPELKLPPMRFEHSMRNGMQVINDAYNACPEAMIAALDALPLPSDGGKIILILGHMAELG
RYSEEGHALVAEKAASRGDMIFFIGEKWIPVQSVLKSYSCEVSFFSSAQDVKDILKQVARYGDVILLKGSRAL
ALESLLACF

>core/263/1/Org1_Gene253

MSNITSPVIQNNRSCNYYFELKNSTTIHIVISAILLCGALIAFLCVAAPVSYILSGALLGLGLLIALIGVILGIKKIT
PMISSKEQVFPQELVNRIRAHYPKFVSDFVSEAKPNLKDLISFIDLLNQLHSEVGSSTNYNVSEELQQKIDTFEG
IARLKNEVRTASLKRLESAASSRPLFPSLPKILQKVFPFFWLGEFISAGSKVVELHRVKKIGGSLEEDLSDYIKP
EMLPTYWLIPLDFRPTNSSILNLHTLVLARVLTRDVFQHLKYAALNGEWNLNHSDLNTMKQQLFAKYHAAY
QSYKHLSQPSLQEDEFYNLLLCIFKHRYSWKQMSLIKTVPADLWENLCCLTLDHTGRPQDMEFASLIGTLYT
QGLIHKESEAFLSSLTLLSLDQFKTIRRQSTNIAMFLENLATHNSTFRSLPPITVHPLKRSVFSQPEEDESSLLIG

>core/264/1/Org1_Gene898

MVITNLSINADTIYGKNPINIVASAANKNITLTGTLALVNADGAFYENHTLQDSQDYSFVKLSPGAGGTIITQD
ASQKPLEVAPSRPHYGYQGHWNVQVIPGTGTQPSQANLEWVRTGYLPNPERQGSLVPNSLWGSFVDQRAIQ
EIMVNSSQILCQERGVWGAGIANFLHRDKINEHGYRHSGVGYLVGVGTHAFSDATINAAFCQLFSRDKDYV
VSKNHGTSYSGVVFLEDTLEFRSPQGFYTDSSSEACCNQVVTIDMQLSYSHRNNDMKTKYTTYPEAQGSWA
NDVFGLEFGATTYYYPNSTFLFDYYSPFLRLQCIYAHQEDFKETGGEVRHFTSGDLFNLAVPIGVKFERFSDC
KRGSYELTFAYVPDVIRKDPKSTATLASGATWSTHGNNLSRQGLQLRLGNHCLINPGIEVFSHGAIELRGSSR
NYNINLGGKYRF

>core/265/1/Org1_Gene795

MLTYKVSPSSVYGNAFIPSSKSHTLRAILWASVAEGKSIIYNYLDSPDTEAMICACKQMGASIKKFPQILEIVG
NPLAIFPKYTLIDAGNSGIVLRFMTALACVFSKEITVTGSSQLQRRPMAPLLQALRNFGASFHFSSDKSVLPFT
MSGPLRSAYSDVEGSDSQFASALAVACSLAEGPCSFTIIEPKERPWFDLSLWWLEKLHLPYSCSDTTYSFPGSS
HPQGFSYHVTGDFSSAAFIAAAALLSKSLQPIRLRNLDILDIQGDKIFFSLMQNLGASIQYDNEEILVFPSSFSGG
SIDMDGCIDALPILTVLCCFADSPSHLYNARSAKDKESDRILAITEELQKMGACIQPTHDGLLVNPSPLYGAVL

DSHDDHRIAMALTIAALYASGDSRIHNTACVRKTFPNFVQTLNIMEARIEECHDNYSMWSTHKRKVFARESF
G

>core/266/1/Org1_Gene615

MTSGVSGSSSQDPTLAAQLAQSSQKAGNAQSGHDTKNVTKQGAQAEVAAGGFEDLIQDASAQSTGKKEATS
STTKSSKGEKSEKSGKSKSSTSVASASETATAQAVQGPKGLRQNNYDSPSLPTPEAQTINGIVLKKGMGTLAL
LGLVMTLMANAAGESWKASFQSQNQAIRSQVESAPAIGEAIKRQANHQASATEAQAKQSLISGIVNIVGFTV
SVGAGIFSAAKGATSALKSASFAKETGASAAGGAASKALTSASSSVQQTMASTAKAATTAASSAGSAATKA
AANLTDDMAAAASKMASDGASKASGGLFGEVLNKPNWSEKVSRGMNVVKTQGARVASFAGNALSSSMQ
MSQLMHGLTAAVEGLSAGQTGIEVAHHQRLAGQAEAQAEVLKQMSSVYGQQAGQAGQLQEQAMQSFNTA
LQTLQNIADSQTQTTSAIFN

>core/267/1/Org1_Gene22

MTLQPYQASSRKYRPQIFREILGQSSVVAVLKNALVFNRAAHAYLFSGIRGTGKTTLARILAKALNCVHLSED
GEPCNQCFSCKEIASGSSLDVLEIDGASHRGIEDIRQINETVLFTPVKAKFKIYIIDEVHMLTKEAFNALLKTLE
EPPQHVKFFFATTEIHKIPGTILSRCQKMHLQRIPEKTILEKLSLMAQDDHIEASQEALAPIARAAQGSLRDAES
LYDYVISLFPKSLSPDTVAQALGFASQDSLRTLDNAILQRDYATALGIVTDFLNSGVAPVTFLHDLTLFYRNLL
LTNSTTSKFSSQYKTEQLLEIIDFLGESAKHLQNTIFEQTFLETVIIHIIRIYQRPVLSELISSIKSRQFEGLRNIKEP
TLTQQVSAPQPQPTYKEQSFLEKKNQPAAEGKIISVEVKSSASIKSAAVDTLLQFAVVEFSGILRQ

>core/268/1/Org1_Gene640

MLKHDTIAAIATPPGEGSIAVVRLSGPQAIVIADRIFSGSVASFASHTIHLGQVIFEETLIDQALLLLMRSPRSFT
GEDVVEFQCHGGFFACSQILDALIALGARPALPGEFSQRAFLNGKIDLVQAEAIQNLIVAENIDAFRIAQTHFQ
GNFSKKIQEIHTLIIEALAFLEVLADFPEEEQPDLLVPQEKIQNALHIVEDFISSFDEGQRLAQGTSLILAGKPNV
GKSSLLNALLQKNRAIVTHIPGTTRDILEEQWLLQGKRIRLLDTAGQRTTDNDIEKEGIERALSAMEEADGIL
WVIDATQPLEDLPKILFTKPSFLLWNKADLTPPPFLDTSLPQFAISAKTGEGLTQVKQALIQWMQKQEAGKTS
KVFLVSSRHHMILQEVARCLKEAQQNLYLQPPEIIALELREALHSIGMLSGKEVTESILGEIFSKFCIGK

>core/269/1/Org1_Gene648

MPRSLSNEQFSVDLEESPGCIVSALVKVSPEVLNKLNKQALKKIKKEITLPGFRKGKAPDDVIASRYPTNVRK
ELGELVTQDAYHALSTVGDRRPLSPKAVRSNSITQFDLQEGAKVEFSYEAFPAISDLPWENLSLPQEEAASEIS
DSDIEKGLTNIGMFFATKTPVERPSQEGDFISISLHVSKSNDENASSAAIFENKYFKLSEEEMTDAFKEKFLGIS
TGHRVVETITSPEIQSFLRGDTLTFTVNAVIEVSIPEIDDEKARQLQAESLDDLKAKLRIQLEKQAKDKQLQKR
FSEAEDALAMLVDFELPTSLLEERISLITREKLLNARLIQYCSDEELEKRKSELIKEAEEDATKALKLLFLTHKI
FSDEKLTISREELQYMMDVCSRERFGQQPPKDISNDTLQELVMSARDRLTYSKAIEHVLRKAELLASTPSA

>core/270/1/Org1_Gene535

MDQLTTDFDTLMSQLGDVNLTTVVGRITEVVGMLIKAVVPNVRVGEVCLVKRNGMEPLVTEVVGFTQSFAF
LSPLGELSGVSPSSEVIPTGLPLHIRAGNGLLGRVLNGLGEPIDVETKGPLQNVDQTFPIFRAPPDPLHRAKLRQ
ILSTGVRCIDGMLTVARGQRIGIFAGAGVGKSSLLGMIARNAEEADVNVIALIGERGREVREFIEGDLGEEGM
KRSVIVVSTSDQSSQLRLNAAYVGTAIAEYFRDQGKTVVLMMDSVTRFARALREVGLAAGEPPARAGYTPS

VFSTLPRLLERSGASDKGTITAFYTVLVAGDDMNEPVADEVKSILDGHIVLSNALAQAYHYPAIDVLASISRL
LTAIVPEEQRRIIGKAREVLAKYKANEMLIRIGEYRRGSDREIDFAIDHIDKLNRFLKQDIHEKTNYEEAAQQL
RAIFR

>core/271/1/Org1_Gene642

MWLVILWALAASLAIALVAKGYYRFVYFRRYAVQVIREVRLSMELKEWALAEQQLLPILKKRSYRRQCLFE
YMRILRKMQRFEESEKLLAEAKKLGLRGPYFFLEIAYKAYRFGAFKECAQAFASVPQDLFEEEDAAKYASAL
VRLGDLDAACSLIEPWISPLSHQETFVTMGHIYFTSKRYKDAIDFYNRANALGVCPVEVTYNLAQAYRITSSY
AKAGKLFRKLLSNPVYKEEALFNIGLCEQKLGRPGKALLIYQSSDLWSRGDALLMKYAAMAAMDQRDYVL
AEPCWELALRCSTFAKDYKCGLGYGFSLCRLRKYGDAERVYCNLIQNFPECLTACKALAWLCGVGYATLLG
SEEGLMYAKKAVELDHSCETLELLSACEARCGNFDAAYEIQSFLSSRDTSLQEKQRRSQILRILRKKLPLNDH
HIVEVDALLAA

>core/272/1/Org1_Gene449

MIENDFPEASNFESSHFYRDKVGVIILCGGEGKRLSPLTNCRCKPTVSFGGRYKLIDIPISHAISAGFSKIFVIGQ
YLTYTLQQHLFKTYFYHGVLQDQIHLLAPEARQGDQIWYQGTADAIRKNLLYFEDTEIEYFLILSGDQLYNM
DFRSIVDTAIRTHVDMVLVAQPIPEKDAYRMGVLDIDSEGKLIDFYEKPQEKEVLKRFQLSSEDRRIHKLTEDS
GDFLGSMGIYLFRRDSLFSLLREEEGNDFGKHLIQAQMKRGQVQTLLYNGYWADIGTIESYYEANIALTQKP
HAEKRGLNCYDDNGMIYSKNHHLPGAIITDSMISSSLLCEGCVINTSHVSRSVLGIRSKIGENSVVDQSIIMGN
ARYGSPSMPSLGIGKDCEIRKAIIDENCCIGNGVKLQNLKGYIKYDSPDKKLFVRDNIIIVPQGTHIPDNYIF

>core/273/1/Org1_Gene89

MLNCSNQKHTVTFEEACQVFPGGVNSPVRACRSVGVTPPIVSSAQGDIFLDTHGREFIDFCGGWGALIHGHSH
PKIVKAIQKTALKGTSYGLTSEEEILFATMLLSSLKLKEHKIRFVSSGTEATMTAVRLARGITNRSIIIKFIGGYH
GHADTLLGGISTTEETIDNLTSLIHTPSPHSLLISLPYNNSQILHHVMEALGPQVAGIIFEPICANMGIVLPKAEF
LDDIIELCKRFGSLSIMDEVVTGFRVAFQGAQDIFNLSPDITIYGKILGGGLPAAALVGHRSILDHLMPEGTIFQ
AGTMSGNFLAMATGHAAIQLCQSEGFYDHLSQLEALFYSPIEEEIRSQGFPVSLVHQGTMFSLFFTESAPTNFD
EAKNSDVEKFQTFYSEVFDNGVYLSPSPLEANFISSAHTEENLTYAQNIIIDSLIKIFDSSAQRFF

>core/274/1/Org1_Gene588

MFQQKQKLSLKYLPSLRMQQGLQMLQSPLTELSSYVVQEIIDNPFFDLSSLEEEEWSPCYRPTNSTFSYLNQTP
GPQESLYTRLLPQIEEAFSTAEERFIAHQIAGNLSDEGLFLRNPEDFAQELELPLEKIHKVWDTIQNLSPEGIASP
SLQSYWMKLLRNSSHQQAYSIVRDCYPLMTNCEFAPIMKKFSLSLSELRNILKKALGSIPWCPAAACTVKPM
VSTPLPDIYLFYSSGSWKIEVSTRGLPSIKLNKETFHFYEHLPKEEQKNLSQQILSAKWLIKNLRKREQTLLQV
METLLPKQEDFLLGKIPAPYPLGIKDLAEDLSFHESTIFRAIENKAVAAPIGIFPLKHLFPRGIHQDSSHSKENVL
QWIRQWIATEQTPLSDSVISDRITAKGIPCARRTVAKYRAQLKILPANKRKKLFYIRSSNSHFRDRQF

>core/275/1/Org1_Gene804

MSKIVYKFGGTSLATAENICLVCDIICKDKPSFVVVSAIAGVTDLLVDFCSSSLREREEVLRKIEGKHEEIVKNL
AIPFPVSTWTSRLLPYLQHLEISDLDFARILSLGEDISASLVRAVCSTRGWDLGFLEARSVILTDDSYRRASPNL
DLMKAHWHQLELNQPSYIIQGFIGSNGLGETVLLGRGGSDYSATLIAELARATEVRIYTDVNGIYTMDPKVIS

DAQRIPELSFEEMQNLASFGAKVLYPPMLFPCMRAGIPIFVTSTFDPEKGGTWVYAVDKSVSYEPRIKALSLS
QYQSFCSVDYTVLGCGGLEEILGILESHGIDPELMIAQNNVVGFVMDDDIISQEAQEHLVDVLSLSSVTRLHHS
VALITMIGDNLSSPKVVSTITEKLRGFQGPVFCFCQSSMALSFVVASELAEGIIEELHNDYVKQKAIVAT

>core/276/1/Org1_Gene51

MQTIYTKITDIKGNLITVEAEGARLGELATITRSDGRSSYASVLRFDLKKVTLQVFGGTSGLSTGDHVTFLGRP
MEVTFGSSLLGRRLNGIGKPIDNEGECFGEPIEIATPTFNPVCRIVPRSMVRTNIPMIDVFNCLVKSQKIPIFSSSG
EHHNALLMRIAAQTDADIVVIGGMGLTFVDYSFFVEESKKLGFADKCVMFIHKAVDAPVECVLVPDMALAC
AEKFAVEEKKNVLVLLTDMTAFADALKEISITMDQIPANRGYPGSLYSDLALRYEKAVEIADGGSITLITVTT
MPSDDITHPVPDNTGYITEGQFYLRNNRIDPFGSLSRLKQLVIGKVTREDHGDLANALIRLYADSRKATERMA
MGFKLSNWDKKLLAFSELFETRLMSLEVNIPLEEALDIGWKILAQSFTSEEVGIKAQLINKYWPKACLSK

>core/277/1/Org1_Gene1010

MISFRFLLLSGLCALGISSYAETPKETTGHYHRYKARIQKKHPESIKESAPSETPHHNSLLSPVTNIFCSHPWKD
GVSVSNLLTSVEKATNTQISLDFSILPQWFYPHKALGQTQALEIPSWQFYFSPSTTWTLYDSPTAGQGIVDFSY
TLIHYWQTNGVDANQAAGTASSMNDYSNRENNLAQLTFSQTFPGDFLTLAIGQYSLYAIDGTLYDNDQYSG
FISYALSQNASATYSLGSTGAYLQFTPNSEIKVQLGFQDSYNIDGTNFSIYNLTKSKYNFYGYASWTPKPSCGD
GQYSVLLYSTRKVPEQNSQVTGWSLNAAQHIHEKLYLFGRINGATGTALPINRSYVLGLVSENPLNRHSQDL
LGIGFATNKVNAKAISNVNKLRRYESVMEAFATIGFGPYISLTPDFQLYIHPALRPERRTSQVYGLRANLSL

>core/278/1/Org1_Gene99

MMLRGVHRIFKCFYDVVLVCAFVIALPKLLYKMLVYGKYKKSLAVRFGLKKPHVPGEGPLVWFHGASVGE
VRLLLPVLEKFCEEFPGWRCLVTSCTELGVQVASQVFIPMGATVSILPLDFSIIIKSVVAKLRPSLVVFSEGDC
WLNFIEEAKRIGATTLVINGRISIDSSKRFKFLKRLGKNYFSPVDGFLLQDEVQKQRFLSLGIPEHKLQVTGNIK
TYVAAQTALHLERETWRDRLRLPTDSKLVILGSMHRSDAGKWLPVVQKLIKEGVSVLWVPRHVEKTKDVE
ESLHRLHIPYGLWSRGANFSYVPVVVVDEIGLLKQLYVAGDLAFVGGTFDPKIGGHNLLEPLQCEVPLIFGPH
ITSQSELAQRLLLSGAGLCLDEIEPIIDTVSFLLNNQEVREAYVQKGKVFVKAETASFDRTWRALKSYIPLYKN
S

>core/279/1/Org1_Gene506

MKRPFFTYLCIIFYGSCASLSLHAGLSFPEVRGATAAVVHADSGKVFYDKDIDAVIYPASMTKIATALFILKHY
PTVLDTLIKVKQDAIASITPQAKKQSGYRSPPHWLETDGSTIQLHLREELLGWDLFHALLVCSANDAANVLA
MACCGSVEKFMDKLNFFLKEEIGCTHTHFNNPHGLHHPNHYTTTRDLISIMRCALKEPPFRGVISTTSYKIGAT
NLHGERILSPTNKLLLPGSTYHYPPALGGKTGTTKTAGKNLIMAAEKNNRLLVTIATGYSGPVSDLYQDVIAL
CETVFNEPLLRKELVPPSDCLQLEIANLGKLSCPLPEGLYYDFYASEDREPLSVSFIAHADAFPIEQGDLLGHW
VFYDDEGKKISSQPFYAPCRFERTIKPWKLYMKRVFTSYRTYMSITMLLMYFRIRKHRKYKNLKHYSKI

>core/280/1/Org1_Gene971

MKRAIIIGAGISGLAAGWWLHKKFPQAEILVLDKEAYAGGFVRTESPQGFSFDLGPKGFLTRGDGEYTLKLIH
ELGLQNSLIFSDRAAKNRFVYYRGKAHKISTWTLLRKGLLPSLIKDFRAPCYTQDSSVQDFLKRHSSQNFTSYI
LDPLITAIRAGHSSILSTHMAFPELAKREASSGSLLRSYLKNRSPKKSKTDRYLASLSPSMGTLITTIQEKLPAT

WKFSTSVTHIDCSPKEACVTTPSETFFADMVIYTGPLQQLPVLLPNYGIENLSKRVLPWNLSSISLGWHHANFS
LPKGYGMLFADELPLLGIVWNSQIFPQATPGKTVLSLLIEGKWRESEAHAFAIAALSEYLNINQKPDAFALFSS
QDGIPQHAVGFLERKERILPHLPGNLKIVGQNIAGPGLNRCIASAYHAICDLHTEETLAQPQSSL

>core/281/1/Org1_Gene133

MFSRWITLFLLFISLTGCSSYSSKHKQSLIIPIHDDPVAFSPEQAKRAMDLSIAQLLFDGLTRETHRESNDLELAI
ASRYTVSEDFCSYTFFIKDSALWSDGTPITSEDIRNAWEYAQENSPHIQIFQGLNFSTPSSNAITIHLDSPNPDFP
KLLAFPAFAIFKPENPKLFSGPYTLVEYFPGHNIHLKKNPNYYDYHCVSINSIKLLIIPDIYTAIHLLNRGKVDW
VGQPWHQGIPWELHKQSQYHYYTYPVEGAFWLCLNTKSPHLNDLQNRHRLATCIDKRSIIEEALQGTQQPAE
TLSRGAPQPNQYKKQKPLTPQEKLVLTYPSDILRCQRIAEILKEQWKAAGIDLILEGLEYHLFVNKRKVQDYA
IATQTGVAYYPGANLISEEDKLLQNFEIIPIYYLSYDYLTQDFIEGVIYNASGAVDLKYTYFP

>core/282/1/Org1_Gene229

MNKNLVAIFDYMEKEKGIQRSTIIGAIESALKIAAKKTLRDDANISVNINSRTGDIEVFCEKEIVEICQNPSKEIP
LDKAREYDPDCQIGQYMDVPFVSDNFGRIAAHAARQIIGQKLRHAERDVIYEEYRHRVNETLSGVVKRFAKG
SNLIIDLGKVEAILPTRFYPKTEKHKIGDKIYALLYEVQESENGGAEVILSRSHAEFVKQLFIQEVPELEEGSVEI
VKIAREAGYRTKLAVRSSDPKTDPVGAFVGMRGSRVKNIIRELNDEKIDIVNYSPVSTELLQNLLYPIEIQKIAI
LEDDKVIAIVVNDADYATVIGKRGINARLISHILDYELEVQRMSEYNKLLEIQRLQLAEFDSPHLDQPLEMEGI
SKLVIQNLEHAGYDTIRRVLLASANDLASVPGISLELAYKILEQVSKYGESKVDEKPEIED

>core/283/1/Org1_Gene925

MSMTIVPHALFKNHCECHSTFPLSSRTIVRIAIASLFCIGALAALGCLAPPVSYIVGSVLAFIAFVILSLVILALIF
GEKKLPPTPRIIPDRFTHVIDEAYGLSISAFVREQQVTLAEFRQFSTALLCNISPEEKIKQLPSELRSKVESFGISR
LAGDLEKNNWPIFEDLLSQTCPLYWLQKFISAGDPQVCRDLGVPRECYGYYWLGPLGYSTAKATIFCKETHH
ILQQLTKEDVLLLKNKALQEKWDTGEVKAIVERIYTTYTARGTLKTEAGGLTKETISKELLLLSLHGYSFDQL
QLITQLPRDAWDWLCFVDNSTAYNLQLCALVGALSSQNLLDESSIDFDVNLGLYVIQDLKEAVQAFSASDEP
KKELGKFLLRHLSSVSKRLESVLRQGLHRIALEHGNARARVYDVNFVTGARIHRKTSIFFKD

>core/284/1/Org1_Gene657

MNHLNKEKLHIHNWQPYRACGLLSKVSGNLIEVDGLSACLGELCKISSTKDPNLLAEVIGFHNHTTLLMSLSP
LHSVALGTEVLPLRRPPSLHLSDHLLGRVLDAFGNPIDKKEDLPKTHRKPLLSLPPSPMMRQPIDQIFPTGIKAI
DAFLTLGKGQRIGVFSEPGSGKSSLLSAIALGSKSTINVIALIGERGREVREYIEKHSNALKQQRTIIIAAPAHET
APTKVIAGRAAMTIAEYFREQGHEVLFIMDSLSRWIAALQEVALARGETLSAHQYAASVFHHVSEFTERAGN
NDKGSITALYAILYYPKHPDIFTDYLKSLLDGHFFLTSQGKALASPPIDILSSLSRSAQALALPHHYAAAERLRS
LLKVYNEALDIIHLGAYTPGQDEELDKAVKLLPSIKAFLAQPLSSYCYLDNTLKQLEALADS

>core/285/1/Org1_Gene774

MLKPMYVLSKRLYRWVNQLIKLGDLVKNSRSFSVEWVFISALLLIFGCLGCASVVKVSLVPFLLLFSFLAFPLI
LCFRGKGYALLLGVFVTLYVAKYVVGETLYVSFWLSGLGVSFLLAFGLFLQGVWLAQEEEMVKGKEQLRL
SEDLDAQRSAYEDLLLTKSQEKEFLDARAQGLDRELTECQELLKAAYQKQEYLTIDLKILADQKNSWLEDY
AELHNKYIELVSKNGDVVFPWVAEPSVGESQGSERVDVSRWVSALQEKEESLERLRNEILVEKQRCSDYEHR

CQELGLLLQNFTALERRCEELQNLLNQKETQINELHQLVCKSEEKVSVEPSAHAETSCVEEKQYKGLYSQLQ
EQFLEKSETLSLVRKKLFAVQEKYLTLKKKEELTKQDISFDDISMIQGLLERIEILEEEVSHLEELVSRSLSL

>core/286/1/Org1_Gene597

MLRQLCFQVFFFCFASLVYAEELEVVVRSEHITLPIEVSCQTDTKDPKIQKYLSSLTEIFCKDIALGDCLQPTAA
SKESSSPLAISLRLHVPQLSVVLLQSSKTPQTLCSFTISQNLSVDRQKIHHAADTVHYALTGIPGISAGKIVFALS
SLGKDQKLKQGELWTTDYDGKNLAPLTTECSLSITPKWVGVGSNFPYLYVSYKYGVPKIFLGSLENTEGKKV
LPLKGNQLMPTFSPRKKLLAFVADTYGNPDLFIQPFSLTSGPMGRPRRLLNENFGTQGNPSFNPEGSQLVFISN
KDGRPRLYIMSLDPEPQAPRLLTKKYRNSSCPAWSPDGKKIAFCSVIKGVRQICIYDLSSGEDYQLTTSPTNKE
SPSWAIDSRHLVFSAGNAEESELYLISLVTKKTNKIAIGVGEKRFPSWGAFPQQPIKRTL

>core/287/1/Org1_Gene676

MTWLSGLYFICIASLIFCAIGVILAGVILLSRKLFIKVHPCKLKINDNEELTKTVESGQTLLVSLLSSGIPIPSPCG
GKATCKQCKVRVVKNADEPLETDRSTFSKRQLEEGWRLSCQCKVQHDMSLEIEERYLNASSWEGTVISNDN
VATFIKELVVAVDPNKPIPFKPGGYLQITVPSYKTNSSDWKQTMAPEYYSDWEHFHLFDQVIDNSQLPADSA
NKAYSLASYPAELPTIKFNIRIATPPFINGKPNSEIPWGVCSSYVFSLKPGDKITVSGPYGESFMKDDDRPLIFLI
GGAGSSFGRSHILDLLLNKHSKREIDLWYGARSLKENIYQEEYENLERQFPNFHYHLVLSEPLPEDIAAGWDK
DDPTKTNFLFRAFNLGQLSRLDNPEDYLYYVCGPPLHNSSILKLLGDYGVERSSIILDDFGS

>core/288/1/Org1_Gene497

MTVTLPKGVFDIFPYLADAKQLWRHTSLWHSVEKAIHTVCMLYGFCEIRTPIFEKSEVFLHVGEESDVVKKE
VYSFLDRKGRSMTLRPEGTAAVVRSFLEHGASHRSDNKFYYILPMFRYERQQAGRYRQHHQFGVEAIGVRH
PLRDAEVLALLWDFYSRVGLQHMQIQLNFLGGSETRFRYDKVLRAYLKESMGELSALSQQRFSTNVLRILDS
KEPEDQEIIRQAPPILDYVSDEDLKYFNEILDALRVLEIPYAINPRLVRGLDYYSDLVFEATTTFQEVSYALGGG
GRYDGLISAFGGASLPACGFGVGLERAIQTLLAQKRIEPQFPHKLRLIPMEPDADQFCLEWSQHLRRLGIPTEV
DWSHKKVKGALKAASTEQVSFVCLIGERELISQQLVIKNMSLRKEFFGTKEEVEQRLLYEIQNTPL

>core/289/1/Org1_Gene125

MFRKLFPFSKKKTGQKQRLRNNGLLQAIIQSIKVLLHNEASKEACVLSYYGLLTCVPILVFFLRLSQHLFTNLN
WKEWLIIKFPDYKKPIVAIVEAAYHATESNIGLVLVGSFFVFCWAGILMLLSLEDGLNKIFRTSWTPISLKRLV
SYFVITLVSPMIFIIVCGSWIYITQIMPIQYAKLFSLSHSMTALYFISRFVPYLLLYLALFCCYAFLPRVAIQKTSA
LISTLIIGSVWIVFQKAFFSLQVSIFNYSFTYGALVALPSFLLLLYIYTMIYLFGGALTFIIQNRGCTFIFLGDKILP
SCYLQLITSTYILALTTRQFNEGLSPLTAQFIAKQSKVPIGEVSQCLDVLEKEGFLFPYNNGYQPVFNFSELTIK
DIADKLLHREIFKKFNPDLGITFIENSFQNIFNQASKNKENLTLSEIARRIK

>core/290/1/Org1_Gene742

MVCENNILSGRGLELLKKKSNITLTPTIYSVSNHNIKLKDFSPHALSVIKTLRKAGYIAYIVGGCIRDLLLNTTP
KDFDISTSAKPEEIKAIFKNCILVGKRFRLAHIRFSKQIIEVSTFRSGSTDEDVLITKDNLWGTPEEDVLRRDFTI
NGLFYDPEHEEIIDYTGGVNDLRNRYLRTIGDPFTRFKQDPVRMLRLLKILSRSPFTVETQTQEALIACRQELIK
SSQARVFEELIKMLNSGAAKNFFQLLIENHLLEILFPYMDKAFRLNRALEEQTATYLKALDDKILKKEAEYDR

HQLMAIFLFPLVNFNVRYKHQKHPYLSLTSVFDYIKNFLEQFFADSFTSCSKKNFILTALILQMQYRLTPLIPTK
KALFFNKKLLHHTRFLEALSLLEIRSIVYPKLDKVYVAWIRHHQTLKCKKDSHSQK

>core/291/1/Org1_Gene221

MISLLKMPKLSPTMEVGTIVKWHKKSNDQVSFGDVIVEISTDKAILEHTANEDGWIREILRHEGEKIVIGTPIA
VLSTEANEPFNLEELLPKTEPSNLEASPKGSSEEVSPATTPQAASATFTAVTFKPEPPLSSPLVFKHVGTTNNLS
PLARQLAKEKNIDVSSIQGSGPGGRIVKKDLEKAPPKSIAGFGYPESPEVPPGSYHEENLSPIREVIAARLQAAK
ISIPHFYVRQQVYASPLLNLLKELQAQGIKLSINDCIVRACALALKEFPSINSGFNSVDNKIVRFDTIDISIAVAIP
DGIITPIIRCADRKNLGMISAEIKSLALKARNQSLQDTEYKGGSFCVSNLGMTGITEFTAIVNPPQAAILAVGSV
TEQALVLDGEITIGSTCNLTLSVDHRVIDGYPAAMFMKRLQKILEAPAVLLLN

>core/293/1/Org1_Gene968

MFEAVIADIQAREILDSRGYPTLHVKVTTSTGSVGEARVPSGASTGKKEALEFRDTDSPRYQGKGVLQAVKN
VKEILFPLVKGCSVYEQSLIDSLMMDSDGSPNKETLGANAILGVSLATAHAAAATLRRPLYRYLGGCFACSLP
CPMMNLINGGMHADNGLEFQEFMIRPIGASSIKEAVNMGADVFHTLKKLLHERGLSTGVGDEGGFAPNLAS
NEEALELLLLAIEKAGFTPGKDISLALDCAASSFYNVKTGTYDGRHYEEQIAILSNLCDRYPIDSIEDGLAEED
YDGWALLTEVLGEKVQIVGDDLFVTNPELILEGISNGLANSVLIKPNQIGTLTETVYAIKLAQMAGYTTIISHR
SGETTDTTIADLAVAFNAGQIKTGSLSRSERVAKYNRLMEIEEELGSEAIFTDSNVFAYEDSEE

>core/294/1/Org1_Gene962

MDIKKLFCLFLCSSLIAMSPIYGKTGDYEKLTLTGINIIDRNGLSETICSKEKLKKYTKVDFLAPQPYQKVMRM
YKNKRGDNVSCLTAYHTNGQIKQYLECLNNRAYGRYREWHVNGNIKIQAEVIGGIADLHPSAESGWLFDQT
TFAYNDEGILEAAIVYEKGLLEGSSVYYHTNGNIWKECPYHKGVPQGKFLTYTSSGKLLKEQNYQQGKRHG
LSIRYSEDSEEDVLAWEEYHEGRLLKAEYLDPQTHEIYATIHEGNGIQAIYGKYAVIETRAFYRGKPYGKVTR
FDNSGTQIVQTYNLLQGAKHGEEFFFYPETGKPKLLLNWHEGILNGIVKTWYPGGTLESCKELVNNKKSGLL
TIYYPEGQIMATEEYDNDLLIKGEYFRPGDRHPYSKIDRGCGTAVFFSSAGTITKKIPYQDGKPLLN

>core/295/1/Org1_Gene909

MKKTMVIDTSVFIYDPEALFSFENTRIIIPFPVIEELEAFGKFRDESAKNASRALSNIRLLLENAKTKVTDGVLLP
SGSELRIEVAPLSNDDRRGKLLTLELLKIIAKREPMVFVTKSLGRRVRAEALQIESRDYESKRFSFRSLYRGFRE
LQVSQEDIENFYKNGYLDLPLDVVPSPNEYFFMSAGENHFALGRYYVSEGKIIALKAMDKSVWGIKPLNTEQ
RCALDLLLRDDVKLVTLIGQAGSGKTILALAAAMHKVFDKETYNKVLVSRPIVPMGRDIGFLPGLKEDKLM
HWMQPIYDNMEVLFSINQMGNSSEALQALMDAKKLEMEALTYIRGRSLPKAFIIIDEAQNLTPHEIKTIISRAG
KGTKIVLTGDPTQIDSLYFDENSNGLTYLVGKFHHLALYGHMFMTRTERSELAAAAATIL

>core/296/1/Org1_Gene666

MLDIKIIRKTPEECETRLRKKDPKISLEPVLSLDKEVRQLKTDSETLQAQRRLLSQDIHKAKTQGVDATNLIQE
VETLAADLEKIEQHLDQKNAQLHELLSHLPNYPADDIPVSEDKAGNQVIKSVGDLPIFSFPPKHHLELNQELDI
LDFQAAAKTTGSGWPAYKNRGVLLEWALLTYMLQKQAAHGFQLWLPPLLVKKEILFGSGQIPKFDGQYYR
VEDGEQYLYLIPTAEVVLNGFRSQDILTEKELPLYYAACTPCFRREAGAAGAQERGLVRVHQFHKVEMFAFT

TPNQDDIAYEKMLSIVEEMLTELKLPYRLSLLSTGDMSFTASKTIDAEVWLPGQKAFYEVSSISQCTDFQSRRS
GTRYKDSQGKLQFVHTLNGSGLATPRLLVAILENNQQADGSVVIPEVLRPYLGGLEILLPKDQ

>core/297/1/Org1_Gene512

MLPLIIFVLLCGFYTSWNIGANDVANAVGPSVGSGVLTLRQAVVIAAIFEFFGALLLGDRVAGTIESSIVSVTN
PMIASGDYMYGMTAALLATGVWLQLASFFGWPVSTTHSIVGAVIGFGLVLGKGTIIYWNSVGIILISWILSPF
MGGCVAYLIFSFIRRHIFYKNDPVLAMVRVAPFLAALVIMTLGTVMISGGVILKVSSTPWAVSGVLVCGLLSY
IITFYYVHTKHCSYISDTPKKGSLTYRLKERGGNYGRKYLVVERIFAYLQIIVACFMAFAHGSNDVANAIAPV
AGVLRQAYPASYTSYTLIRLMAFGGIGLVIGLAIWGWRVIETVGCKITELTPSRGFSVGMGSALTIALASILGL
PISTTHVVVGAVLGIGLARGIRAINLNIIKDIVLSWFITLPAGALLSILFFFALRALFH

>core/298/1/Org1_Gene1025

MIMGLQSRLQHCIEVSQNSNFDSQVKQFIYACQDKTLRQSVLKIFRYHPLLKIHDIARAVYLLMALEEGEDLG
LSFLNVQQYPSGAVELFSCGGFPWKGLPYPAEHAEFGLLLLQIAEFYEESQAYVSKMSHFQQALFDHQGSVF
PSLWSQENSRLLKEKTTLSQSFLFQLGMQIHPEYSLEDPALGFWMQRTRSSSAFVAASGCQSSLGAYSSGDV
GVIAYGPCSGDISDCYYFGCCGIAKEFVCQKSHQTTEISFLTSTGKPHPRNTGFSYLRDSYVHLPIRCKITISDK
QYRVHAALAEATSAMTFSIFCKGKNCQVVDGPRLRSCSLDSYKGPGNDIMILGENDAINIVSASPYMEIFALQ
GKEKFWNADFLINIPYKEEGVMLIFEKKVTSEKGRFFTKMN

>core/299/1/Org1_Gene797

MDKQSSGNSGCIWHPFTQSALDSTPIKIVRGEGAYLYAESGTRYLDAISSWWCNLHGHGHPYITKKLCEQAQ
KLEHVIFANFTHEPALELVSKLAPLLPEGLERFFFSDNGSTSIEIAMKIAVQYYYNQNKAKSHFVGLSNAYHG
DTFGAMSIAGTSPTTVPFHDLFLPSSTIAAPYYGKEELAIAQAKTVFSESNIAAFIYEPLLQGAGGMLMYNPEG
LKEILKLAKHYGVLCIADEILTGFGRTGPLFASEFTDIPPDIICLSKGLTGGYLPLALTVTTKEIHDAFVSQDRM
KALLHGHTFTGNPLGCSAALASLDLTLSPECLQQRQMIERCHQEFQEAHGSLWQRCEVLGTVLALDYPAEAT
GYFSQYRDHLNRFFLERGVLLRPLGNTLYVLPPYCIQEEDLRIIYSHLQDALCLQPQ

>core/300/1/Org1_Gene900

MAEISTPSLPDSSIVSQKTPPVPDPDSSPDHIPTIPTQAPFKPQRKKETPSSIVNAIAFAILAFLSCLGGVFAICLGC
SLEITVPLFILTAVFIAFTLLYFIHYLEKPKIPEPLPTPPPSPTLRAPTLTPEIPAPAPGIPLPPTLPKVDRTKLTCNP
DIHYPSTYDPKACFSLLKQLFSLDPETRPEDRKYSNKLASILLRSKEKSGFRFHCFKGHFSHDKILNKKSGAVV
ISSHSSMDFSTTLGRAFAVTTCLQRSCWEKIKNNIPTPEKHLPIGSCVSGPWDVEEGAQLYTSHLIVINPPTLET
LIKEKMRRAITLKDFSMKEAFTNLVLAYLQCFDICIEHNLESVQLEVFGLNNLSADQEEFTTWESCCHLALLE
SVRILLASKEEYALSNVSVNSISQVPLQTACRALFLN

>core/301/1/Org1_Gene959

MKKQVYQWLASVVLLALTISGYAELPLSEQKVKSHTYTTLDEVKDYLSKRGFVETRKQDGVLRIAGDVRAR
WLYFREDIKNPSDKDKYNPLPVNRYRSEFYLYIDYRAERNWLSSKMNWTAIAGGENTAAGVDINRAFLGYR
FYKNPETRTDFFMEIGRSGLGDLFESEVQFQRNFDGLHIYWTRELSKDYPYQVIVHGGPFVVNMTKKHYAW
VVEGILNRLPKQFFVKCSVVDWNTFVPSETSTTEKAATNAMKYKYCVWQWLVGKRSQVPWINGQKKPLYL

YGAFLMNPLAKATKTTLNGKENLAWFIGGTLGGLRKAGDWSATVRYEYVEALSVPEIDVSGIGRGNLLKFW
FAQAIAANYDPKEANGFTNYKGFSALYMYGITDSLSFRAYGAYSKPANDKLGSDFTFRKFDLGIISAF

>core/302/1/Org1_Gene837

MQSWLQSLQERNILENFTAGLESVEGPIAAYLGFDPTAPALHIGHWIGICFLKRLAALGITPIALVGGATGMV
GDPSGKQSERSLLQTSEVFDNSQKITACLQRYLPGVTLVNNADWLQEISLIDFLRDIGKHFRLGQMLVKDTIK
QRVHSDEGISYTEFSYLILQSYDFYHLFKNYGTILQCGGSDQWGNITSGIDFIRRKGLGQAYGLTYPLLTNAQ
GKKIGKTESGTVWLDSDLTSPFELYQYLLRLPDDTIPKIARTLTLLSNEEIQDIDRRVQTDPVAVKEFVAQDILS
AIHGDLGLEEALSVTRSMHPGNLSSLSEKDFHELFAGGMGASLDKSEVLGKRWLDLFLVLGLCKSKGEIRRLI
EQKGVYINNVPIANEHSVCEEQDICYGHYVLLAQGKKRKLVLYLN

>core/303/1/Org1_Gene829

MTVAEVKGTFKLVCLGCRVNQYEVQAYRDQLTILGYQEVLDSEIPADLCIINTCAVTASAESSGRHAVRQLC
RQNPTAHIVVTGCLGESDKEFFASLDRQCTLVSNKEKSRLIEKIFSYDTTFPEFKIHSFEGKSRAFIKVQDGCNS
FCSYCIIPYLRGRSVSRPAEKILAEIAGVVDQGYREVVIAGINVGDYCDGERSLASLIEQVDQIPGIERIRISSIDP
DDITEDLHRAITSSRHTCPSSHLVLQSGSNSILKRMNRKYSRGDFLDCVEKFRASDPRYAFTTDVIVGFPGESD
QDFEDTLRIIEDVGFIKVHSFPFSARRRTKAYTFDNQIPNQVIYERKKYLAEVAKRVGQKEMMKRLGETTEVL
VEKVTGQVATGHSPYFEKVSFPVVGTVAINTLVSVRLDRVEEEGLIGEIV

>core/304/1/Org1_Gene646

MNKKNLTICSFCGRSEKDVEKLIAGPSVYICDYCIKLCSGILDKKPSSTISSAPVSETPSQPSDLRVLTPKEIKKH
IDEYVIGQERAKKTIAVAVYNHYKRIRALLHNKQVSYGKSNVLLLGPTGSGKTLIAKTLAKILDVPFTIADAT
TLTEAGYVGEDVENIVLRLLQAADYDVARAERGIIYIDEIDKIGRTTANVSITRDVSGEGVQQALLKIVEGTTA
NVPPKGGRKHPNQEYIRVNTENILFIVGGAFVNLDKIIAKRLGKTTIGFSDDQADLSQKTRDHLLAKVETEDLI
AFGMIPEFVGRFNCIVNCEELSLDELVAILTEPTNAIVKQYMELFAEENVKLVFKKEALYAIAKKAKQAKTGA
RALGMILENLLRDLMFEIPSDPTVEAIHIQEDTIAENKAPIIIRRTPEAIA

>core/305/1/Org1_Gene60

MIKFLSQLFIRHWPRKVVSLGFAIIIWILVGQSVTITRTLTNVPVRIVDLHPDQTVLGLQKSGFLNKKVSLTITG
NKNTVQDLRPSNLEVVISAANHTESWIATIDKHNLVSVDHEINIRKDIHSVDANDIFVRLTQYVTEDILLTITKP
IGSPPKGYEYLDVWPKYLNQKVSGPKEYINALKEQGLELTFNLNKISFEELERNRIAQGSHDEIIFPIPKEWKKI
LIPFENTFMDLNDPQADFLRLLFLKRECIPLNLNLPVFLFFPVTFIQTMNPLEYSLDPVPPIILNHGIHQINIPLYV
KDVSRQFLDVVKNNMVLTIVMPSPQDPSSINWAIEFLDEKTLENTFLQTIIAQEHGILHDIALIDEAGIRHRFRE
YLRKLALFTADGEPLNLIAEIKNNKVVIQTKTKETTKLYKKEW

>core/306/1/Org1_Gene778

MLKLQLCALFLFGYLAIVFEHIVRVNKSAIALAMGGLMWLVCFSHIPMADHMILVEEIADMSQVIFFLFSAM
AIVELIDAHKGFSVIVKFCRIQSRTLLLWALIGLSFFLSAALDNLTSIIIIISILKRLVKAREDRLLLGAICVIAVNA
GGAWTPLGDVTTTMLWINNKITSWGIIRALFVPSLVCVLVAGFCGQFFLRKRGSTLIAKDVELQSAPPKSLWII
FIGLGSLLMVPVWKACLGLPPFMGALLGLGLVWLTSDWIHSPHGEDRYHLRVPHILTKIDISSITFFIGILLAVN

ALSFANLLTDFSLWMDKIFSRNVVAIVIGLLSSVLDNVPLVAATMGMYTLPLDDTLWKLIAYAAGTGGSILII
GSAAGVAFMGLEKVDFLWYFKRISWIALASYFGGLFSYFVLESLNFFI

>core/307/1/Org1_Gene146

MSSAIARDCFPSPSPQPSSTLGVHPPKYKSLILSVSLIVLGVLLLCVGMLLLVNAIFSFSVLTVGLGGAGVFLGS
LLLILGLIFFVSYHRKLSEATRSLEQKITLEYQPWADLRKELNEVQEWSNFLLDEWEDFKEVVAQHKSQFATF
EGDLLLFGREVEKYETIWKELDGRDVALLTELKNIWGPLEFLRKKGDRLQCEIDKLRKEVMKVGKSGLKLA
CELTKFKSALKDVKIEQECYRDKRKVEKLEVFPEGYRRELLEVLKTRLSVEKEIQLFEEVVSAFEEKLASLHR
TVFSEEELQEALDKAKAELLDIQVRKSVVEDLSCEPTLIQYHLLRLYEVQCRIVEQFLTQTFSSEQEKVLEEYE
ALKARIRKTLRVKLDQVRANVAFVASTTDLLSESESLDGNDSVFEDAHDDFLD

>core/309/1/Org1_Gene668

MIETREEVGSANFVSLERAIEDLRAGKFVIVVDEASREDEGDLIIAGEKITVEKMTFLLQHTTGVVCAALSQER
LLSLDLPPMVKDNRCRFKTPFTVSVDAAHGVTTGVSAADRTKVVQLLADPKSKPEDFISPGHFFPLASSPGGV
LKRAGHTESTVDLMELAGLQPCGVLAELVNEDYSMMRLPQILEFARKHNIAVIPVTSIIAHRMLSDRLVSKIS
SARLPTIYGDFTIHVYESLLEGMQHLALVKGNVAGKSNVLVRVHSECVTGDILGSKRCDCGEQLSSAMSYIA
EKGTGVLVYLRGQEGRGIGLGHKVRAYALQDNGYDTVDANLAMGFPVDSREYGIGAQILVDLKLTTIKLIT
HNPQKYFGLQGFGLSITERVPLPVRISEDNEQYLRTKQERMGHWLDLPCCNNRVQ

>core/310/1/Org1_Gene876

MLCYSPNYVTDLYRISLSAEESLGGIRAFPQAESLLGGACALNFPDLEERLPDLRKELLFLGSNDRPDACGGK
FSLQLASSKECYIAALKERVYLNVTNSSRGPVYSFSPKGVPTELWIECFSVSVDGRVEVKVRLQGLHKELISKP
RDCETLFLNPPANKLDCWEIAGFRVDASFPVKQKIRRIGVDKFLLMHGGAEYADKATKERVDFVSSDEENYS
RYLAVGDVLLWDGNCWQTCGEFQGASSRAPLFEVKRIDDKVMIADLWNVGGTQRQTISLVKGVPSPIEINE
VIREIEFTGMRSWSKPIVLVGGQRLILSPDDWILRTAKGWEKLSRADQIQDYVTGKVTGPLLVFEKLEKDLRG
FVLRGHMFNAQRTLVETISLPLKQGFEPAVASQEVSSNTRSAAAHPGATNRGGS

>core/311/1/Org1_Gene931

MSQPPINPLGQPQVPAAASPSGQPSVVKRLKTSSTGLFKRFITVPDKYPKMRYVYDTGIIALAAIAILSILLTAS
GNSLVLYALAPALALGALGVTLLISDILDSPKAKKIGEAITAIVVPIIVLAIAAGLIAGAFVASSGTMLVFANPM
FVMGLITVGLYFMSLNKLTLDYFRREHLLRMEKKTQETAEPILVTPSADDAKKIAVEKKKDLSASARMEEHE
ASQRQDARHRRIGREAQGSFFYSSRNPEHRRSFGSLSRFKTKPSDAASTRPASISPPFKDDFQPYHFKDLRSSSF
GSGASSAFTPIMPASSRSPNFSTGTVLHPEPVYPKGGKEPSIPRVSSSSRRSPRDRQDKQQQQNQDEEQKQQS
KKKSGKSNQSLKTPPPDGKSTANLSPSNPFSDGYDEREKRKHRKNK

>core/312/1/Org1_Gene689

MCQRILILGTGITGKSVARFLYQQGHYLIGADNSLESLISVDHLHDRLLMGASEFPENIDLVIRSPGIKPYHPW
VEQAVSLKIPVVTDIQVALKTPEFQRYPSFGITGSNGKTTTTLFLTHLLNTLGIPAIAMGNIGLPILDHMGQPGV
RVVEISSFQLATQEEHIPALSGSVFLNFSRNHLDYHRNLDAYFDAKLRIQKCLRQDKTFWVWEECSLGNSYQI
YSEEIEEILDKGDALKPIYLHDRDNYCAAYALANEVGWVSPEGFLKAIRTFEKPAHRLEYLGKKDGVHYIND

SKATTVTAVEKALMAVGKDVIVILGGKDKGGDFPALASVLSQTTKHVIAMGECRQTIADALSEKIPLTLSKD
LQEAVSIAQTIAQEGDTVLLSPGCASFDQFQSFKERGAYFKLLIREMQAVR

>core/313/1/Org1_Gene700

MSKKRVVVTGFGVVSCLGNEVDTFYDNLLAGVSGVRPITSFPCEDYATRFAGWIPEFNPEPYVDKKQARRV
DPFITYAMVAAKKAIAMSRWDKDHLPSDPVRCGVIVGSGMGGLSTLDQGMERLLVIHKKLSPFFIPYIITNMA
PALIAMDFGLMGPNYSISTACATGNYCIDAAYQHLVSGRADMIICGGTEAAVNRIGLEGFIANRALSERNDAP
DQASRPWDRDRDGFVLGEGAGILVLETLESALRRDAPIFAEMLGSYVTCDAFHITAPRDDGEGITACVLGAL
NSAGIPKERVNYVNAHGTSTPLGDLSEVLAVKKAFGSHVRNLRMNSTKSLIGHCLGAAGGVEAVVAIQAILT
GKLHPTINLDNPIAEIEDFDVVANKAQDWDIDVAMSNSFGFGGHNSTILFSRYVP

>core/314/1/Org1_Gene520

MLVSIETFSSIASGSPVQKAAEACYTQYSKQPSSKEVLSSFSWIQELSLFPDRYNLATGASELIKQHWLHNNHS
LAFECILINGKYEPSLSQLPEGVIVCGIDEARGSLSSFMQGFDVNKHPLAFLNAVCSEDRGVVIYIPEEMQTSDP
IFVRHISFPTVSDHDVIFSPRIVVILGQRASAQIQISHDVDLEMVGSSKTIVNGVTELFVGEGADLTVFMVPGYS
EEDTLSWSTIATVEKDAICRMTQNLLESCQGFGWFDNTSYIVGKKGHAESLVLVQSPRKTWVNNLMSHDAE
ETVSRQNIKSILYSGHFLFEGTISISSQGDLSDANQKHDTLLLSSEARVSTFPRLEIETDEVKASHGATVGPLDP
QQIFYMRSRGMTEAEAQEKLIHGFLKQGLVSDTFLGSSFQLNQTS

>core/315/1/Org1_Gene872

MRYHKYFRYVNSWVFLVVLTLMLLSVVVISSMDPTAMLVTSSKGLLTNKSIMQLRHFALGWVVFFICAYFD
YHLFKRWAWVLYFFMICALVGLFFVPSVQNVHRWYRIPFIHMSVQPSEYGKLVIVIMLSYILESRKADITSKT
TAFLACLVVALPFFLILKEPDLGTALVLCPVTLTIFYLSNVHSLLVKFCTVVATIGIIGSLLIFSGIVSHQKVKPY
ALKVIKEYQYERLSPSNHHQRASLISIGLGGIRGRGWKTGEFAGRGWLPYGYTDSVFSALGEEFGLLGLLFTL
GLFYCLICFGCRTVAVATDDFGKLLAAGITVYLAMHVLINISMMCGLLPITGVPLILISYGGSSVISTMASLGV
LQSIYSHRFAKY

>core/316/1/Org1_Gene377

MKLWMKIFIGLFVGVTLGLVLEDKAIFFKPIGDIFLNLLSMVVYPLVFCSMVLGIASISDMKKLGRIGIKSVGL
YLGTTALAIVIGLCFAWIFSPGNGCDFAQAQSMDSAVTVIDSNKTAAYFLSIIAQVFPSNPVRSFAEGNILQIIIF
AIFLGIALRLSGERGRPVERFIDGFSEIMLRMVNMIMSFAPYGVGASMAWISGNHGLGVLWQLGKFIIAYYLA
CLFHATLVFGGLVRFGCKMSFSKFLSSMMDAISCAVSTASSSATLPVTMRCVSKNLGVSAEVSGFVLPLGAT
VNMNGTAIFQGMAAVFIAQAYNCPLSLSSLLLLVVTATFSAVGSAGVPGGGMITLGSVLASVGLPIQGIAILA
GIDRLRDIVGTPMNILGDAVVATYVASGEGELSPYESIKQESVETT

>core/317/1/Org1_Gene591

MKKIFYSFVLLSCIFPYVGCAQVFVGLDRIFSEGEYTRCIQGKKIALISHSAAINSRGQDALSVFYSRKHDCTVE
ILCTLEHGYYGATPTETVGNQPSRYPNLRSVSLYGVKEVPKEVAEHCDVFVYDVQDIGVRSYSFVTVLMQIV
KASERYGKQLIVLDRPNPMGGRIVDGPLPNPTTSGSLAIPYCYGMTPGELALFFKKTYAPNANVVVIPMKGW
NRSMTFDETGLIWMPTSPQMPDPQSPFFYAATGILGALSVASIGVGYTLPFKVLGAPWMDGEKVADELNRM

KLPGVLFLPFFYEPFFGKYKMEMCSGVLLVLQDPKIFYPVETQCTIWGVLKALYPKQVEQTLKSIERIPARRSS
ICNLFGGDEFLSISHKERYIVWPLRRLCKESRESFHQLRSSCLLSEYAES

>core/318/1/Org1_Gene874

MKKNASHKTNDKKSLSIWSIGGSIFAMFFGAGNIVFPLALGYHYNAHPWSAYFGMMLTAVCVPLLGLVSML
FYSGDYQKFFFSIGRIPGMIFITAIILLIGPFGGIPRAIAVSHATLISLSEHKSAFIPSLPIFSAICCVLIYIFSCKLSRL
IQWLGSVFFPIMLVTLLWVIIRSFMIPTHPMVQEFIPNARQAWLAGFIEGFNTMDLLAAFFFCSIVLISLRQLVA
EEKHPTEEEIPLSFQGISKKNKRSLALGFFLAAILLGMTYLGFVLSAARHAGLLVNVSKGHILGRISAIALGPNS
ILAGVSVFIACLTTEIALVGIVADFLARVVSFKKLNYASAVICTLIPTYLISILNFETISHLLLPLLQLSYPALIVLA
CGNIAYKLWNFRYSPVLFYLTLSLTIVLKLVN

>core/319/1/Org1_Gene160

MDKETLENIYRHFRYRFLKLNILPAFLGLLLLCSPNTLNYTQVDVIFSDRLCSCLLIFLAIASLTKRSLLWLGAP
LGIWVTLFACVAGRSPTIFANDTLIGFAILAVVCISPTRPEALEVGPTLPEGFSYNPSAGGRRAAVLFLSLLGWL
EARYLTASSLGITSSQSSNFLLLYSSIMTVYSLLVVLSLAGSERRWHTRPKIVIATALALTGVIILTLLPIILHQLR
YDCWLCLCLTIEPALAVVFAYDETRATLRYISQFLGDKRALTRASFFGSEYYKHTLSWEERTVLPLRKAYKQ
AFEGISFPINQLLAILVATVFVKVNSSMGLPTFPRNFLNICCWFIIVLFILAFAESLRHLRWMNLIFSAAILFSPVL
FHIPVESPMFLPIIVTGLILIILSIGKRRRTKRKL

>core/320/1/Org1_Gene287

MIPTMLMFFIICFTLCSGFISLSQIALFSLPTSLISHYKRSKSKKQQRVATLLLHPHHLLITLIFCDIGLNIAIQNCF
AILFGDAASWWFTVGLPLAITLILGEILPKAVALPFNTQIASSVAPLILCVTKIFKPLLHWGIVGINYVVQWILS
KQQIDIIQPQELKEVLQSCKDFGVVNQEESRLLYGYLSLSDCSVKERMQPRQDILFYDIQTPLENLYLLFSKQH
CSRVPICNDNLQNLLGICTARSLLLHDKPLQSSDDLLPLLKKPYYMPETISAKMALCQMAAEDETLGMIIDEY
GSIEGLITQEDLFEIVAGEIVDQRDNKILYTTSGADVIIASGTLELREFSEIFDINLPTNNNIATIGGWLIEQIGTIP
TTGMKLSWNNLLFQVLDAAPNRIRRVYIRKLYD

>core/321/1/Org1_Gene846

MDESDGEEASKDSAFSASFSYEFVKSSTRESKNTVTHSTASRTLYILRQDCSYDPRALKVDDEFRYWVEKRL
DAKNPDSLNAFVKEVGTHYVASVTYGGIGFQVLKMSYLQVEELEKEKISISVAAASSLLKSKTSNATEKGYS
SYQSESSAQTVFLGGTVLPDLQQDKLDFKDWSESIPNEPIPLAISVSSITDLIIPELFPSEDAQVLSQKKSALGQV
ILNYLESHKPKEEGPKPVQITSGFNSSSSVFTLQAAKAPKTVSFPYIDYWSTIPYLFPTLKETSGAQPLSFYLRF
DDIFEQQNLVHNTSYILASTSVRLGYFGDSYRDYDALSFYGSWPQAYFDWAGYKDRCTWTLEKLNTTGDLF
IRSGDEIRLKHNTSGKYLATTSMSDGYQTLTCTTQTSDSVFIITV

>core/322/1/Org1_Gene751

MNLPVSLACLLLSGCVFFLGVFVSSSLYARKKRAFLEKIQKLEHENQLLQTSLNLSRHQEQLIEDFSNRLALSS
HKLIKDMKEEAQNYFGDTSKSFQSILSPIQTTLTTFKQSLETFETKHAEDRGRLKEQISQLLAVEKKLEHETHV
LTDILKHPGSRGRWGEIQLERILELAGMLKYCDYDSQTTSAQGAFRADIIIRLPQDRCLIIDAKAPISDSYFSVE
EIDKGDLVDKIKEHIKTLKSKSYWEKFHQSPEYVILFLPGESLFNDAIRLAPELMEIGASSNVILSSPLTLLALLK

TIAYMWKQENLQKQIQEVSLLGKELHRRLQVVFTHFQKIGKNLNQTVQSYNDMTSSFQYRVLPTLRKFEGLE
TSSSHQIEEPTPIESLATSFPHTCDIDTNLAVIESLEKQD

>core/323/1/Org1_Gene828

MKTAFHSCYSWFCWLFSFLVLFVGGIAGGEPLCPDCKYETKSVLRSDQLPDHLWNYENDCYLTGYVQSLLD
MHFLDSRTQVVIEKNRAYLFSLPVDSSLSEAITNFVRDLPFICAVEICERPYGECITRSSAERPLLPKEKTLGMPI
FCGKEGVWLPQNTILFSPLIADPRQVTNSAGIRFNEKVVGNRVGATIFGGDFILLRLFDVSRFHVDCDFGIQGG
VFSVFDLDHPESCMVNSDFFVAGLWSGAIDKWSFRFRLWHLSSHLGDEFILTHPNFPRFNLSDEGVDLFISFR
YTPQIRLYGGCGYIVSRDLTFPERPFYCEWGAELRPFGLREGNLHAQPIFAMHFRCWEEQKFGLDQSYILGME
WAKFQEIGRKIRAVLEYHQGFSKEGQFIREPCNYYGFRLTYGF

>core/324/1/Org1_Gene645

MTTIAIEAAKKVLIKLRNAGYQAYFVGGCVRDMLMNRPLEDIDIATNASPTIVSTIFPDVISIGVAFGIIVVKQD
GRLFEVATFRSDGEYKDGRHPDRIIFSSMREDALRRDFTVNGMYYDPFEDKVFDFVEGTRDIEKKVIRAIGHP
RLRFSEDKLRILRAIRFSSSLGFTLDPTTERAIIKEAPALVNSVSPERIWQELKKMLKRQPYGALSLLLKLKVLIF
IFPELRDIPYSLLRTTIEFARKFNPTHFPEILFLLPLFQGVSEEAATVAFGRLRISNKELKLIESWYEALPHFQNQS
GNRVFWAHFLASPTAPLFLELFSALQKDPSRQQHFISRVQELESRLEQFILRIKTSSPVVSAPDLIAKGISPGRLL
GDLLREAEILSIENECLDKEKILLLLQEKGFWK

>core/325/1/Org1_Gene519

MKNLKEDFPIFAAKAKENEPFIYLDSAATTQKPQQVIDAVANFYTSSYATVNRAIYSSSRNVTEAYAAVREK
VRKWVSAASDSEIVFTRGTTAGLNLLAISVNDLWIPKGGVVLVSEAEHHANVLSWEIACRRRGSLVKKIRVH
DSGLIDLDDLEKLLNEGAQFVSIPHVSNVTGCVQPLQQVAELVHRYDAYLAVDGAQGAPHLPIDVQLWDVD
FYVFSSHKIYGPTGIGVLYGKKDLLDQLPPVEGGGDMVAIYDHQNPEYLPAPMKFEAGTPNIAGVLGLGAAL
DYLDGLSAKFIYDKEIALTTYLHKELLEIPGVEILGPSIEEPRGALIGMTIDGAHPLDLGFLLDLRGIAVRTGHQ
CAQPAMERWNVGHVLRVSLGIYNDEDDIDQFILVLQDSLDKIRR

>core/326/1/Org1_Gene145

MVVVALFILGIFFLSGSLAFLVHTSCGVLLGAALPILCIGLVLLAVALIVFLCHKHKTRQDLDYYDQDLDSLVI
HKKEIPNDISELRVTFEKLQNLFQFHTKDFSDLSQELQGKFINCMEKWLTLEDEVTKFLIVRDRFLETRRNFTT
FGEQVKGIQSNIFDLHEEKSSLYLELYRLRKDLQVLLNFFLLPPGILKVDYDEIEAIKGLFIRLTSRLDKLDVKA
QERKKFINEMSREFKEVEKAFDIVDRATKKLMDRAKKESPARLFMGRTESLLEMKKNEEALKNQGLDPENL
SHPELFSPYQQLLILNYLNSEIVLHHYEFLISGTVTSGLTLEECENRMRAASTGLNALLVRKLQFRGAIKSAYF
EKLTEIEKELRSLQDVIKSLELELIHKIKDIVTEET

>core/327/1/Org1_Gene264

MTDSNPLPSYTDASLYRTPAKHSYPIRLPLNRTDRIEKILKIVTLTLALACALGFSIAAGILAMPIFSAVVVITLA
IAAVSLYSLLKKPKLYEILPQIEPESEQSSLSPSPQPPEQQDLPLQIDPLPDPESLPEVSLADLTTPPEELTAITVTP
GYEALLEQNWDLLPSLAAVDPSFTTETPQQPCFIWKLKDSKLIFISTSGDIAVPRIKTQGRVMIVNAANENISR
EGGGTNKALSLATSLQCWNASRLPRAHSRSGSQLQPGECRSAKWENSDHTSNDHVPGKAHFLAQLLGPEAA

KCNNDPKQAFEVSKKAFHNLFQEAEIIGVDVIQLPLIGCNLFAPSRLLNLGKTRAEWIEAIKLALITSLQDFGW
EQDNQEEQKIIILTDKDQPPIIPPRFDLTTP

>core/328/1/Org1_Gene921

MKKQRSHYTKNNLLLLLSILVGLGLGSVQSPWIVYSAECIANTFLKFLRLLSIPLVFCALGSTITSIQNFNTMVT
LGKRILYYTLLTTVIAASIGLLLFFLLRPQMITQDALATTTKCNPLGYLDVLSDTLPENIFKPFLQGNVISAACL
AVLLGTASLFLQEKEKHFVNQFFNSFFSIFLNLARGGLKLLPIAMLGFSVILFKELKDQSNLTMFAKYLLCVIG
ANLAQGFIVLPILLKINKVSPLKVAKAMSPALVTAFFSKSSAATLPLTMELAEDDLKINKNLSRFSFPLCSVIN
MNGCAAFILITVLFVATSNGMIISPLMSLGWIFIATLAAIGNAGVPMGCYFLTLSLLTSMNVPLSILGLILPFYT
VIDMIETSLNVWSDCCVVSLAN

>core/329/1/Org1_Gene288

MTNSALFWIGVNIICIVLQGFYSMMEMACVSFNRVRLQYYLTKDHKKARYINFLIRRPYRLFGTVMLGVNIA
LQVGSESSRNCYRALGITPDYAPFTQIFIVVIFAELLPLTISRKIPEKLALWGAPILYYSHYIFYPLIQLIGSLTEGL
YYLLNIRKEKLNSTLSRDEFQKALETHHEEQDFNTIATNIFSLSATCADQVCQPLEQVTMLPSSANVKDFCRTI
KNTDINFIPVYHKARKNVIGIAHPKDFVNKALDEPLINNLHSPWFITAKSKLIRILKEFRDNRSSVAVVLNASG
EPIGILSLNAIFKILFNTTNIAHLKPKTISVIERTFPGNSRIKDLQKELDIQFPQYPVETLAQLVLQLLDSPAEVGT
SVIINNLLLEVKEMSLSGIKTVSIKNLLS

>core/330/1/Org1_Gene511

MDKLTVQDLSPEEKKVLVRVDFNVPMQDGKILDDIRIRSAMPTINYLLKKHAAVILMSHLGRPKGQGFQEEY
SLQPVVDVLEGYLGHHVPLAPDCVGEVARQAVAQLSPGRVLLLENLRFHIGEEHPEKDPTFAAELSSYGDFY
VNDAFGTSHRKHASVYVVPQAFPGRAAAGLLMEKELEFLGRHLLTSPKRPFTAILGGAKISSKIGVIEALLNQ
VDYLLLAGGMGFTFLQALGKSLGNSLVEKSALDLARNVLKIAKSRNVTIVLPSDVKAAENLQSKEYSVISIDQ
GIPPHLQGFDIGPRTTEEFIRIINQSATVFWNGPVGVYEVPPFDSGSIAIANALGNHPSAVTVVGGGDAAAVVA
LAGCSTKVSHVSTGGGASLEFLEQGFLPGTEVLSPSKS

>core/331/1/Org1_Gene870

MSTMQNCPHFGVCGGCSFPQSNYSDSLKKKEELLHQLFAPLVPSDMIAPIIPCSPSLRGRNKMEFSFFQTYEGE
KSLGFISSTKPKKGIPVTTCLLIHEQTMDILKLTREWWDKHPELMAYFPPKNKGSLCTLTVRTGSPQQNFMVI
LTTSGTPEYRVNEACIDEWKEILLSSSLNIASIYWEEKVAARGISTYYETKLLYGAPSIQQKLSLPSDGNSASFS
LRPRSFFQPQITQAAKIIETAKEFINPEGSETLLDLYCGAGTIGIMLSPYVKNVIGVEIIPDAVASAQENIKANNK
EDCVEVYLEDAKAFCKRNENCKAPDVIIIDPPRCGMQSKVLKYILRIGSPKIVYISCNPKTQFQECADLISGGY
RIKKMQPIDQFPYSTHLENIILLEREIDL

>core/332/1/Org1_Gene575

MLLVRKWLHTCFKYWIYFLPVVTLLLPLVCYPFLSISQKIYGYFVFTTISSLGWFFALRRRENQLKTAAVQLL
QTKIRKLTENNEGLRQIRESLKEHQQESAQLQIQSQKLKNSLFHLQGLLVKTKGEGQKLETLLLHRTEENRCL
KMQVDSLIQECGEKTEEVQTLNRELAETLAYQQALNDEYQATFSEQRNMLDKRQIYIGKLENKVQDLMYEI
RNLLQLESDIAENIPSQESNAVTGNISLQLSSELKKIAFKAENIEAASSLTASRYLHTDTSVHNYSLECRQLFDS

LREENLGMLFVYARQSQRAVFANALFKTWTGYCAEDFLKFGSDIVISGGKQWMEDLHSSREECSGRLVIKT
KSRGHLPFRYCLMALNKGPLCYHVLGVLYPLHKEVLQS

>core/333/1/Org1_Gene64

MRMLQISMLLLALGTAINSPAIYAADSQSVSFPEQLPSSFTGEIKGNHVRMRLAPHTDGTIIREFSKGDLVAVI
GESKDYYVISAPPGITGYVFRSFVLDNVVEGEQVNVRLEPSTSAPVLVRLSRGTQIQPASQEPHGKWLEVVLP
SQCVFYVAKNFVANKGPIELYTQREGQKKIAMDLINSALNFAHIELEKSLNEIDLEAIYKKINLVQSEEFKDVP
GIQGLIQKALEEIQDAYLSKSLESQNTSIASSQCSTPKVSSSEVTTSLLSRHIRKQTALKTAPLTQGRENLEYSLF
RIWASMQQGNDHSEALTQEAFYRAEQKKKQVLAGVLEVYPHVVKNNPGDYLLKAQENTIAFLYGTSINLEQ
WLGKRVTVECLPRPNNHFAFPAYYVVGIKEAS

>core/334/1/Org1_Gene236

MAASGGTGGLGGTQGVNLAAVEAAAAKADAAEVVASQEGSEMNMIQQSQDLTNPAAATRTKKKEEKFQT
LESRKKGEAGKAEKKSESTEEKPDTDLADKYASGNSEISGQELRGLRDAIGDDASPEDILALVQEKIKDPALQ
STALDYLVQTTPPSQGKLKEALIQARNTHTEQFGRTAIGAKNILFASQEYADQLNVSPSGLRSLYLEVTGDTH
TCDQLLSMLQDRYTYQDMAIVSSFLMKGMATELKRQGPYVPSAQLQVLMTETRNLQAVLTSYDYFESRVPI
LLDSLKAEGIQTPSDLNFVKVAESYHKIINDKFPTASKVEREVRNLIGDDVDSVTGVLNLFFSALRQTSSRLFS
SADKRQQLGAMIANALDAVNINNEDYPKASDFPKPYPWS

>core/335/1/Org1_Gene843

MVYFMVFSPSSESVVKANSVVRSNFCYFLENKFVSPSESTEVMFSEIMKGRVPDIESLFDRPTDMMMTGFKA
AQNLGNLFNSFGILIMCFSQCKSCQTPEKETSAIVLGATLLFFVIALILGPTLGALVYCAYKVYTLGKMIYSLN
KAKAKVLRHPAQNVFHRAAGVATIRSSEEAVKACKLYKSAMIGSLVVSLIASLALIALTAGIVLVLFFVAPGA
APVITAAMMGCCAAGGGALLISLLGLWIAIVRKAKHQEACVGHLTNVVLHTAVSEALLHDPSHFQTNALAR
DLFLTDCLSHYGHLFSNEEVAQLVQGGAPGGGSRPSQHYGGSSDYQNRRGGNGNFGGSHFGGGGGFAGSHF
GAGYPTAPTMPSAPPPFPPPAYDTIYG

>core/336/1/Org1_Gene359

MLSVTIVLVGLEMARSKVSKRDSKILDILFATTELYLKTGQPVGSKTLKESFCSDLSTATIRNYFAELEAEGFL
KKNHTSGGRIPTDLALRHYVDHQEECPEAEISAPIFDKISQLPSESRNIIKDLQKATELLGEILDLPTFFSSPRFEN
DSVTNIQITQVDKQRAVTILSTEFGQIFTDTLWLPEACDTLSIKRIEKFLQNYIRKLPTNEELSKKEEHLSMSLY
NEVVVRYLTRYCNFSEEDLYQTGMSKLLKYEAFKDPEVLALGLSLFENRRQMCELLNIGMHKGRATAFIGK
ELSDILGTSNPGCSVITIPYYMNRSPLGALGILGPINLPYKEALPLLKLFANKINETLTQSFYKFKLSFRRPLTSN
CKLSNEPILRTEYSSIKLLPSKETL

>core/337/1/Org1_Gene354

MRRNPHFSLLKPQYLFSEISKKLAQFRKENPEISVIDLSIGDTTQPLCRSITQAIKEFCVSQEKQETYRGYGPET
GLEKLRTKIASEVYENRISPEEIFISDGAKPDIFRLFSFFGSEKTLGLQDPVYPAYRDIAHITGIRDIIPLACRKET
GFIPELPNQQSLDILCLCYPNNPTGTVLTFQQLQALVNYANQHGTVLIFDAAYSAFVSDPSLPKSIFEIPEAKYC
AIEINSFSKSLGFTGMRLAWNVIPKELTYDNNEPMINDWKRLFATTFNGASLLMQEAGYYGLDLFPTPPAISL

YLTNAQKLKKSLETAGFSVHGGDHAPYLWVELPEGISDEEAFDFFLHQYHIAVTPGHGFGSCGQGFVRFSAL
TQPQNIALACDRLCTASLKETMVLA

>core/338/1/Org1_Gene744

MSNKVLGGSLLIAGSAIGAGVLAVPVLTAKGGFFPATFLYIVSWLFSMASGLCLLEVMTWMKESKNPVNML
SMAESILGHVGKISICLVYLFLFYSLLIAYFCEGGNILCRVFNCQNLGISWIRHLGPLGFAILMGPIIMAGTKVID
YCNRFFMFGLTVAFGIFCALGFLKIQPSFLVRSSWLTTINAFPVFFLAFGFQSIIPTLYYYMDKKVGDVKKAILI
GTLIPLVLYVLWEVVVLGAVSLPILSQAKIGGYTAVEALKQAHRSWAFYIAGELFGFFALVSSFVGVALGVM
DFLADGLKWNKKSHPFSIFFLTFIIPLAWAVCYPEIVLTCLKYAGGFGAAVIIGVFPTLIVWKGRYGKQHHRE
KQLVPGGKFALFLMFLLIVINVVSIYHEL

>core/339/1/Org1_Gene255

MVNIQPVYRNTQVNYSQATQFSVCQPALSLIIVSVVAAVLAIVALVCSQSLLSIELGTALVLVSLILFASAMFM
IYKMRQEPKELLIPKKIMELIQEHYPSIVVDFIRDQEVSIYEIHHLISILNKTNVFDKAPVYLQEKLLQFGIEKFK
DVHPSKLPNFEEILLQHCPLHWLGRLVYPMVSDVTPGTYGYYWCGPLGLYENAPSLFERRSLLLLKKISFGEF
ALLEDGLKKNTWSSSELVQIRQNLFTRYYADKEEVDEAELNADYEQFDSLLHLIFSHKLS

>core/340/1/Org1_Gene563

MSFFNHIPTFSPDAILGLQNVFFADKRPEKVNLVIGVYEHPQKRYGGLSCIRKAQTVILEEEQNKSYLPISGLQI
FLDEMRELVFGAVDPSAIVGFQSLGGTGALHLGARLLSVAKGSGKVYVPEQTWSNHIRIFSQEGLEVIRYPYY
SKEQKQLLFEPLIAFLKEVEKNSVILLHGCCHNPTGVDFTEDMWKELAILMKERELIPFFDTAYQGFAHGIEL
DRKPIEIFISEGNTVLVAASSSKNFALYGERVGYFAVHSTFTDELVKIHSFLEEKIRGEYSSPQRWGVEIVSTILS
NPYLKEEWQSELNFIRESLGKMRTRFVQALRKVAGHTFDFLLSQHGFFAYPGFSDKQVLFLREQHAVYTTAG
GRMNLNGITEKNIDHVVQSFIQAYEL

>core/341/1/Org1_Gene144

MVVSIYSEILSFSELTSCKHSLFPFGPIETASIRIHHVFNVVIVCLIILGTLFVCLGMVFLGVFSTYLLGMSSMILG
LLLISIGLALLKFKERYGLEPKELFGVEGGFDKKLPSEIIQMQDQIADLARELDLEQKKDTLIRGFSARLDVLE
GSKTEKKQILKIGVPRNLSEIQERAQEQNSILEQCKEALLFRRKSAQEIFKKLYDRKAAFWRSYREDLWCYSEI
HVSKKALSNLYIGDVFEGTAPHFLMEAYAMCRTAKNLRNYVKVCVEDMRVNEEKKRAKQLSVSELLCCCT
EIETDLENETNLFTSDSEDVLEEYQIHCIRVTMLHALWAIYNDEVVSRKPIDTLDRVRARMAVEDCIETFEELQ
MCVVHTKTLELEIAQLYVDILLEA

>core/343/1/Org1_Gene39

MSKETFQRNKPHINIGTIGHVDHGKTTLTAAITRALSGDGLASFRDYSSIDNTPEEKARGITINASHVEYETPN
RHYAHVDCPGHADYVKNMITGAAQMDGAILVVSATDGAMPQTKEHILLARQVGVPYIVVFLNKVDMISQE
DAELIDLVEMELSELLEEKGYKGCPIIRGSALKALEGDANYIEKVRELMQAVDDNIPTPEREIDKPFLMPIEDV
FSISGRGTVVTGRIERGIVKVSDKVQLVGLGETKETIVTGVEMFRKELPEGRAGENVGLLLRGIGKNDVERG
MVVCQPNSVKPHTKFKSAVYVLQKEEGGRHKPFFSGYRPQFFFRTTDVTGVVTLPEGTEMVMPGDNVELDV
ELIGTVALEEGMRFAIREGGRTIGAGTISKINA

>core/344/1/Org1_Gene1007

MFEFRFPKIGETSSGGSIVRWLKNLGDHVARDEPLIEVSTDKIATELPSPKAGRLVRFCVNEGDEVASGDVLG
LIELEEISEADDESTSCPLTSCETKSEAGSSSSSVWFSPAVLSLAQREGIGLDNLQKIAGTGKGGRVTRQDLEA
YISESQQVSIPEIFQGEVNRIPMSPLRRAIASSLSKSSDEVPHASLVVDVDVTDLMHLISGERQRFLDTHGVKLT
ITSFIVQCLAQTLRQFPLLNGSLDGTTIVMKKSVNVGVAVNLNKEGVVVPVIHNCQDRGLVSIAKALADLSSR
ARLNKLDPSEVQDGSVTVTNFGMTGALIGMPIIRYPEVAILGIGTIQKRVVVRDDDSLAVRKMVYVTLTFDH
RVLDGIYGSEFLTSLKNRLESVTMG

>core/345/1/Org1_Gene16

MDYYSILGISKTASAEEIKKAYRKLAVKYHPDKNPGDAAAEKRFKEVSEAYEVLSDPQKRDSYDRFGKDGPF
AGAGGFGGAGGMGNMEDALRTFMGAFGGEFGGGSFFDGLFGGLGEAFGMRSDPAGARQGASKKVHINLTF
EEAAHGVEKELVVSGYKSCETCSGQGAVNPQGIKSCERCKGSGQVVQSRGFFSMASTCPECGGEGRIITDPCS
SCRGQGRVKDKRSVHVHIPAGVDSGMRLKMEGYGDAGQNGAPSGDLYVFIDVESHPVFERRGDDLILELPIG
FVDAALGMKKEIPTLLKTEGSCRLTVPEGIQSGTILKVRNQGFPNVHGKGRGDLLVRISVETPQNLSEEQKEL
LRTFASTEKAENFPKKRSFLDKIKGFFSDFTV

>core/346/1/Org1_Gene123

MSSPVNNTPSAPNIPIPAPTTPGIPTTKPRSSFIEKVIIVAKYILFAIAATSGALGTILGLSGALTPGIGIALLVIFFV
SMVLLGLILKDSISGGEERRLREEVSRFTSENQRLTVITTTLETEVKDLKAAKDQLTLEIEAFRNENGNLKTTA
EDLEEQVSKLSEQLEALERINQLIQANAGDAQEISSELKKLISGWDSKVVEQINTSIQALKVLLGQEWVQEAQ
THVKAMQEQIQALQAEILGMHNQSTALQKSVENLLVQDQALTRVVGELLESENKLSQACSALRQEIEKLAQ
HETSLQQRIDAMLAQEQNLAEQVTALEKMKQEAQKAESEFIACVRDRTFGRRETPPPTTPVVEGDESQEEDE
GGTPPVSQPSSPVDRATGDGQ

>core/347/1/Org1_Gene622

MPRYRYTYLDPKERRKRGYLEALHIQEAREKLAQENIQVLDIREVALRRMSIKSTELIVFTKQLLLLLRSGLPL
YESLVSLRDQYHEQKMGLLLTSFMETLRSGGSLSQAMAAHPNIFDHFYCSGVAAGESVGNLEGCLQNIIVVL
EERAQITKKMVGALSYPCVLLVFSFAVMLFFLLGVIPSLKETFENMEVKGLTKIVFGVSDCLSAYRYLFLGFA
SALITVGILMRHRIPWKKILEKLLFALPGTKKFVVKVAVNRFCSVASAILKGGGTLIEGLDLGCDAIPYDRLKT
DMRDIVQAVIGGGSLSQELAQRSWVPKLAIGMIALGEESGDLADVLGYVAHIYNEDTQKTLASITSWCQPVI
LIFLGGLIGVIMLAILIPLTSNIQTL

>core/348/1/Org1_Gene652

MHPKIEKRNSLPLTAVAPVFEESYHPSVATTVDYVDATTLSRHLTVLKDVIKEARNLDLGKAFLTSMKQGFI
NTGTELAIIQASLADQSSRESRKKEEKIFHQHLGKAAPQAATATSGVQPTADPVADKMPLQSAFAYVLLDKYI
PAQEEALYALGRELNLSGYAQNLFSPLLDMIKSFNSAPINYNLGSYISQTSGTANFAYGYEMILSRYNNEVSQ
CRLDIASTVKAKAALANMSASVKANVSLTDAQKKQIEDIIASYTKSLDVIHTQLTDVMTNLASITFVPGLNKY
DPSYRIVGGDLSIIALQNDEKVLVDGKVDITTAVNEGGLLNFFTTVLTDVQNYGDLAQTQQLMLDLELKAM
QQQWSLVSASLKLLNGMYTTVISGFKN

>core/349/1/Org1_Gene524

MKKLLKSALLSAAFAGSVGSLQALPVGNPSDPSLLIDGTIWEGAAGDPCDPCATWCDAISLRAGFYGDYVFD
RILKVDAPKTFSMGAKPTGSAAANYTTAVDRPNPAYNKHLHDAEWFTNAGFIALNIWDRFDVFCTLGASNG
YIRGNSTAFNLVGLFGVKGTTVNANELPNVSLSNGVVELYTDTSFSWSVGARGALWECGCATLGAEFQYAQ
SKPKVEELNVICNVSQFSVNKPKGYKGVAFPLPTDAGVATATGTKSATINYHEWQVGASLSYRLNSLVPYIG
VQWSRATFDADNIRIAQPKLPTAVLNLTAWNPSLLGNATALSTTDSFSDFMQIVSCQINKFKSRKACGVTVG
ATLVDADKWSLTAEARLINERAAHVSGQFRF

>core/350/1/Org1_Gene992

MMKQGVGQDAKELYTFLSRGNEHYQPCLWFSLEEELGFLFDAKMLCAPLSEDHYCHSYLVDLVDQHLKDL
ILSMFLDPQNISAGELLKVSINVGDSFSPLQQKDFLSMVLRDETGKNVVVVFKGVLSLPATQVCKLVEELNSK
DYSYLNIFSCHGDSSPQLLFRKELEGTSGRYFTVICALYLGDTDMRSLQLASERIMVSREFDLVDAYAARCKL
LKIDHTNWRPGTFSRHADFADAVDVSAGFNSREFKLITQANQGILESGELPLPSKTFWEGFLAFCDRVTVTRH
FIPMLDAAIKQAVWTHKHPSLIDKECEALDLKTQCLPSIVSYLEYVTNSHEKTSKGPFIQKEIIADCSPLKEALF
PGSDEDVPSTSEDPSDDHPSDLEDS

>core/351/1/Org1_Gene509

MGTPISGNDGDRNTISDPLEESAAEEGDSDLEDRVSESATQVIETIADTGIPEATPSEGTNSDLNSDLVDRVEY
EARGSLLTTMLARIRKAVSQIWMHVKTKRHPKEQGVRSLGDIPCDLLKATRLPKETAEPPYFYALETALASC
RSFFFHVFLRLFTLLRRQHPEAPLDLCGTDPISPEAAVAFALILRSCCKWVATDAVQEGLPLEVIEEAGMYNA
FSLEATTTVEEVSKRLSELLYSDKRIDGLANVRGITKIITSPYLGAGQCVSVVDNLKTYDLGRNYTQVLACAS
QIDEFADKGENEALVMKDILYLVRQDRSKELGDFLMMWSEEHASEVNYDVVLAILEVNLPILEEDYRSHPLA
YQKKLNYVICQFFCSERLTSIEPKD

>core/352/1/Org1_Gene367

MLHILLAIFCILLFLAFGLTQPSCHGSSKFLKTLNQRFFKDKGREYPPFPSAPTILATLLCILYGALGTKLYTLLP
PKTAHKDLLFWPLYSLSALIAYGFLPPWISTKVPKETTAHLRFLASVFQLGLFPLQLLFYRRRPNQQVRSSTSF
QSQLSEALSAFDNLIVREVMIPKVDIFALPEETTLQEALVLVSEEGYSRVPVYKKNLDNITGILLVKDLLLLYT
SSHDLSQPISSVAKPPFYAPEIKKASSLLQEFRQKHRHLAIIVNEYGFTEGIATMEDIIEEIIGEIADEHDVQENTP
YKKIGSSWIVDGRMNISDAEEYFNLKIDHENSYDTLGGHVFHKVGAVPQKGMRIHHENFDIEIITCTERNVGK
LKITPRKRKFNIS

>core/353/1/Org1_Gene431

MAIKNILVVDDEPLLRDFLSELLTSQGFIPDTAENLRNALQMIRSRDYDLVISDMSMPDGSGLDLIKIIKQSSPH
TPVLVVTAYGSIENAVEAMHQGAFNYLTKPFSSEALFAFISKAEELKNLVHENLFLHSQTTPDSHPLIAESKA
MKDLLAIAKKAASSSANIFIHGESGCGKEVLSFFIHHNSPRANHPYIKVNCAAIPETLLESELFGHEKGAFTGA
TTKKAGRFELAHKGTLLLDEITEVPVNLQAKLLRAIQEKEIEHLGGTKTLSVDVRILATSNRKLKEAIDDKSFR
QDLYYRLNVIPLHLPPLRDRQDDILPLANYFLNKFCRMNNTPLKTLSPKAQELLLNYPWPGNIRELSNVLERV
VILENTSLLTEDMLALA

>core/354/1/Org1_Gene746

MHLHEYQAKDLLASYDVPIPPYWVVSSEEEGELLITKSGLDSAVVKVQVHAGGRGKHGGVIVAKSSAGILQ
AVAKLLGMHFTSNQTADGFLPVEKVLISPLVAIQREYYVAVIMDRKHRCPVLMLSKAGGMDIEEVAHSSPEQ
ILTLPLTSYGHIYSYQLRQATKFMEWEGEVMHQGVQLIKKLAKCFYENDVSLLEINPLVLTLEGELLVLDSKI
TIDDNALYRHPNLEVLYDPSQENVRDVLAKQIGLSYIALSGNIGCIVNGAGLAMSTLDILKLHGGNAANFLDV
GGGASQKQIQEAVSLVLSDESVKVLFINIFGGIMDCSVVASGLVAVMETRDQVVPTVIRLEGTNVELGKEIVQ
QSGIPCQFVSSMEEGARRAVELSM

>core/355/1/Org1_Gene691

MKWFVISCLLGIFSLGLIMVFDTSSAEVLDRSLECSTHKALIRQVTYLILGLGVASLLYMMEWRDFLKISPVLL
SGAALALICVFIPGLGICRNGARRWLGFGQLTIQPSEFVKYLVPIVALYFLTFSSLYQKQLKMFLKLTAILFIPIL
LIAIEPDNGSAAVISASLIPVFIMTSVRLRYWLLPLLCVLIAGGALAYRMPYVRYRLNVYLHPELDIKGRGHQP
YQAKIAAGSGKLLGKGPGASLQKLTYLPEAQNDYIAAIYAEEFGFLGMLVLILLYMCFVYGGYAIAIKASSLE
GAALAMVITLIISMQAFMNLGVVSGLLPSKGVNLPFFSQGGSSLIANMCGVTLLLKVYDEENSKSSLGCRRFR
RPHCPSSLGKGSFFS

>core/356/1/Org1_Gene827

MSSVNQSSGTPNPEEVTSPESTEENKNVVSSDEAQATHAVALPIVTQLSLPEGVGTSSEETASNPKVDEIVAEV
SSSRAVADQISSLVERVGELLDDLKGAQSLFTSFQSELKNCLPAWKSSTRRLETRGAGDNADIARLELFRSDY
EAVLGHANQFHGKAHLILSKLTDVHHKLQGLSREDLSLAFDNNDRVLEHLGSLGLDVDAEGNWSLSCERGI
PRLVLTADSMLVQIKKVNLPTVEELRTLQGTTESSSDPRVEESLSCCERLLNELRRLWANFVGFISSCYDNIVF
VLMWIVRRINLLPGLGCLPFHNPDASQEDQRSSSGERSTRRERLSRRSDLSEEEMIVRAEGESIHPESPHGDGR
NQPSRGDKQDSDSEEETEL

>core/357/1/Org1_Gene660

MEKPQNRKAPRIFWLNNQVAIPPSERVKESYALHSDIFSLPPGSALKLAEKTEESIRQLVGLKDSHIFRFVPHFP
HVVHIVLAALVENLSMFQGRNHIILPAHDQQLLINSLCRHQGLGTTYDWVTVNHEGRIVEEQLIETLSPRSLLF
SLSAAHGLTGVIQPLDPLLSLCKDRRILLHLDISDILGRAPLTPEILNADIITFSSAALGGMGSIGGIFIRKSLERV
FSSWFPPHTSASLCFSAVAAMQTACEERISALPLFTFHTSNLCKKLIQELQSVLPSIQLAFSEVQNRLPNIVVAA
IPDIPAESLAFHLHQQGIYPSLGYERFQPLAQVLQNCGISPFLCHSALHFSLTERSKDLEFSKLARAMHDAIKHL
TPLLGSSS

>core/358/1/Org1_Gene508

MILDFQFSIGYYLRVLELAIRDGTRILAYDRKRLLLDAWPVNDPLPTNYDTSVSTIRQVIHELFSWSAISYSISS
RLLAIIELRLHEEKPQTGWLYRLFFPSKYHIKKAIVDKLCMFKSLILFESKRPVDKIVQAANKVFSKGKSNFSS
WEDFTHEVTVSEVQTPLAGEVQRRLAADASLQMIIEALTTLLEGHTAYLPLSLELLNQFIGEKAQPLKTLSEK
SYVLLRELIQLFSLSAEDFQTIIMSIISDSLSEVLANSLIGNQPLTFHGKTFVGLWQETALASPEDSKLALGFLAE
VLRKVIVEKKLHVSKSDNTTPEEVGNIYSIRDQNPALWDKMITMLLMRWLLDYDRDIGIALRKAAEYYNPHP
SFWRQFLRLWQRRP

>core/360/1/Org1_Gene170

MSAMISLSSSHEASIASNTQVRDVLVSLAMDEFVEHNTEILPIKVFLARGTLSSTAIIDDLKDVVETEGEHHFQ
VYSNISLKMIYQRFFEKIFGIGCCPLLLVTDSHHTDPCGALITGIFAAVLFTVLAIVFGPTLGILCYSAYKIYQLT
KKISSLSRTHTEVINSVQKSDPFIHRSGAVAAAAASQSTIKACKVFRQSTLIFFVLGLIITISLAALIVGLVFALFF
LDPGAPAVMTAAMIGCCAAGGTGILLSVIGFLLASVYSVQKSQEGVHHMHTALLRCIVSNTIIQMPYLPITPG
TKKVLTQSIRRYQQFFSDDEYRDIESEVPLNRQTTPPPSYETLFHEEGSDGSSNVIPRESPPAYSTIDSSNSPFPSS
SPPPYYR

>core/361/1/Org1_Gene1028

MSETIITTPHVVKLISNFFQKKLFSSISTAYPLVIITDVSVQQHLLGPILDHIKMLGYQVIVLTFPPGEPNKTWET
FISLQYQLVDQNISPKSSIIGIGGGTVLDMTGFLAATYCRGLPLYLIPTTITAMVDTSIGGKNGINLRGIKNRLGT
FYLPKEVWMCPQFLSTLPREEWYHGIAEAIKHGFIADAYLWEFLNSHSKMLFSSSQILHEFIKRNCQIKAAIVA
EDPYDRSLRKILNFGHSIAHAIETLAKGTVNHGQAVSVGMMIETRISLAEGVMKTPQLIDQLERLLKRFNLPST
LKDLQSIVPEHLHNSLYSPENIIYTLGYDKKNLSQHELKMIMIEHLGRAAPFNGTYCASPNMEILYDILWSECH
VMRHC

>core/362/1/Org1_Gene799

MLCQQFLIEALARRKSKHTYRSLSLNSHLIDFTSNDYLGFASSPELRKEYITKLHAIESLGATGSRLLTGHSQL
CQRIEEQLAAYHNFESCLIFNTGYTANLGLLYALATDQDRILHDLYIHASIYDGIRLSKAQSFPFNHNDLNHLE
KRLASSHLGRTFVCVESVYSLHGSVAPLQAISELCERYSAYLIVDEAHAVGVFGDQGEGLVSALGLQDKVLA
TVYTFGKALGTHGAAIAGSSILKDYLINFCRPFIYTTAQPPHALTAIELAYEHNQRAFNQREHLSALIHHFREK
AQNLGLQLMKDNTTTPIQSICVSGSHRARQAALQIQNSGYDVRPIVSPTVKQREELLRICLHAFNTKNEIDHLL
HTLEQIFLCNVSSL

>core/363/1/Org1_Gene206

MSCFNLTSTNESLRPISPKASFPKQGWQSYFRSALRKHRSDTLSVSVCKVNKYDANLFVRLTVIALAVVGVLI
LFSIMLASIQGTLVITSWPLVTAAILIPTILLTGGMYILHRLGKKVDVISGVCIPPFSRRCWVPISSSHTLEKFDEK
HVSACSYLDISTLSADGSGIAAVYQCPPLLFRAFPCFGIPCAMPFVALLRMIYNLIRFLVVPFYIIFRMIYEHFFC
KHLPEDDRFIYKDVAREMGRSLAAFLKAPFYASACMIGAFYSLLDPLAGRVLMGSVERDWNDNVILARSVS
LANEAHSLFRFEGGGGRKGLGQHAFYLMLCCQPQSVFLFDKGEIVSGAHPSIQLPERRGLDTSGRYPHISVIPD
SGNDSAKNFIV

>core/364/1/Org1_Gene954

MGINPSGNRSPDDVWVRGAQGDSSSTQGTGATNSNLGAHNVTTSTSQPQVASKAKQLWQTVREFFLGKKSP
DSSQGASGPAMQSPSGPTIRPTRPAPPPPTTGGANAKRPATHGKGRAPQPPTAGSSSGSEQPTAMSSEVAKLV
SELKDAVHSHAESQKVLKKVSQELQTKWTDWENNRGPDYLLHGYRVIARALQQTYTEQSMLIEGTLSTGPV
PQAVTVAKDAVTQTVRGAIKNLENPKPGNDPDGVLMQVVISLGIEGPTLDPGESIQNFLETRVSDFGGDDSDI
DYTSDIARLGSALDRVRENHPNEMPRIWIALARELGAAVHSHATSVRIANAGKNHTRDVVRMANESSRLLQ
GMKVLSVGAWANTMTVLIGDLFE

>core/365/1/Org1_Gene711

MIPSPTPINFRDDTILETDPKPSLIMFSSKKTEIASERRKAHPTLFKVLGTIWNIVKFIISIILFLPLALLWVLKKTC
QFFILPSSIISQSMSKTAVAIRRMTFLSHIKQLLSLKEISAADRVVIQYDDLVVDSLAIKIPHALPHRWILYSQGN
SGLMENLFDRGDSSLHQLAKATGSNLLVFNYPGIMSSKGEAKRENLVKSYQACVRYLRDEETGPKANQIIAF
GYSLGTSVQAAALDREVTDGSDGTSWIVVKDRGPRSLADVANQICKPIASAIIKLVGWNIDSVKPSERLRCPEI
FIYNSNHDQELISDGLFERENCVATPFLELPEVKTSGTKIPIPERDLLHLNPLSPNVVDRLAAVISNYLDSENRK
SQQPD

>core/366/1/Org1_Gene924

MKHLAVLGSTGSIGRQTLEIVRRYPSEFKIISMASYGNNLRLFFQQLEEFAPLAAAVYNEEVYNEACQRFPHM
QFFLGQEGLTQLCIMDTVTTVVAASSGIEALPAILESMKKGKALALANKEILVCAGELVSKTAKENGIKVLPI
DSEHNALYQCLEGRTIEGIKKLILTASGGPLLNKSLEELSCVTKQDVLNHPIWNMGSKVTVDSSTLVNKGLEII
EAYWLFGLENIEILAVIHPQSLIHGMVEFLDGSVISIMNPPDMLFPIQYALTAPERFASPRDGMDFSKKQTLEFF
PVDEERFPSIRLAQQVLEKQGSSGSFFNAANEVLVRRFLCEEISWCDILRKLTTLMECHKVYACHSLEDILEVD
GEARALAQEI

>core/367/1/Org1_Gene606

MKDLGTLGGTSSTAKTVSPDGKVIMGRSQIADGSWHAFMCHTDFSSNNVLFDLDNTYKTLRENGRQLNSIF
NLQNMMLQRASDHEFTEFGRSNIALGAGLYVNALQNLPSNLAAQYFGIAYKIRPKYRLGVFLDHNFSSHVPN
NFNVSHNRLWMGAFIGWQDSDALGSSVKVSFGYGKQKATITREQLENTEAGSGESHFEGVAAQIEGRYGKS
LGGHVRVQPFLGLQFVHITRKEYTENAVQFPVHYDPIDYSTGVVYLGIGSHIALVDSLHVGTRMGMEQNFAA
HTDRFSGSIASIGNFVFEKLDVTHTRAFAEMRVNYELPYLQSLNLILRVNQQPLQGVMGFSSDLRYALGF

>core/368/1/Org1_Gene667

MEDFSEQQLFFMRRAIEIGEKGRITAPPNPWVGCVVVQENRIIGEGFHAYAGGPHAEELAIQNASMPISGSDV
YVSLEPCSHFGSCPPCANLLIKHKVSRVFVALVDPDPKVAGQGIAMLRQAGIQVYVGIGESEAQASLQPYLY
QRTHNFPWTILKSAASVDGQVADSQGKSQWITCPEARHDVGKLRAESQAILVGSRTVLSDDPWLTARQPQG
MLYPKQPLRVVLDSRGSVPPTSKVFDKTSPTLYVTTERCPENYIKVLDSLDVPVLLTESTPSGVDLHKVYEYL
AQKKILQVLVEGGTTLHTSLLKERFVNSLVLYSGPMILGDQKRPLVGVLGNLLESASPLTLKSSQILGNSLKV
VWEISPQVFEPIRN

>core/369/1/Org1_Gene712

MNLSNRSDILSGIFSNPHPVSYFSSTHAKQLSDFSKKHPILTKIVTIIVKIFKLLIGLIIPPLGIYWLCQLVCSLALF
PRSSMLYSVLKTCFKKYRLEQEIQDYFVKNLDPSFKDPAVSESKRITIQQDHLTIDTLAIHFSTARPKRWLLISL
GSGDFLEDMIGLKDSLFLSWKELAKLLGANILIYNYPGVKSSTGKLNLENLATAHNLCAKYLQDKIQGPGAN
EIITYGYSLGGVVQSAALQKNPFTNSETSWVAVKDRAPHSLPAAANSFFGPIGKLIAVLARWKMDAEKNSRE
LPCPEILVYSADRFRPSEVGDDTALLPEFTLAHAIKRTPFARSKKFIGEVNLLHSSPLKHPTIQKLAEAILESLSR
KN

>core/370/1/Org1_Gene92

MNTSLKRPLKSHFDVVGSFLRPEHLKKTRESLKEGSISLDQLMQIEDIAIQDLIKKQKAAGLSFITDGEFRRAT
WHYDFMWGFHGVGHHRATEGVFFDGERAMIDDTYLTDKISVSHHPFVDHFKFVKALEDEFTTAKQTLPAPA

QFLKQMIFPNNIEVTRKFYPTNQELIEDIVAGYRKVIRDLYDAGCRYLQLDDCTRGGLVDPRVCSWYGIDEK
GLQDLIQQYLLINNLVIADRPDDLVVNLHVCRGNYHSKFFASGSYDFIAKPLFEQTNVDGYYLEFDHERSGDF
SPLTFISGEKTVCLGLVTSKTPTLENKDEVIARIHQAADYLPLERLSLSPQCGFASCEIGNKLTEEEQWAKVAL
VKEISEEVWK

>core/371/1/Org1_Gene465

MSDNAHNLLYDKFELPEAVKMLPVEGLPIDKHARFIAEPLERGMGHTLGNALRRALLIGLEAPAIISFAMTGV
LHEYMAIEGVIEDVTNIILNLKGALLKKYPMQDSSLGRTTQVLKASISIDASDLAAANGQKEVTLQDLLQEGD
FEAVNPDQVIFTVTQPIQLEVVLRIAFGRGYTPSERIVLEDKGVYEIVLDAAFSPVTLVNYFVEDTRVGQDTDF
DRLVLIVETDGRVTPKEALAFSTQILTKHFSIFENMDEKKIVFEEAISIEKENKDDILHKLILGINEIELSVRSTNC
LSNANIETIGELVIMPEPRLLQFRNFGKKSLCEIKNKLKEMKLELGMDLTQFGVGLDNVKEKMKWYAEKIRA
KNTKG

>core/372/1/Org1_Gene275

MNGKAPLALYIHIPFCTKKCRYCSFYTIPYKSESVSLYCNAVIQEGLRKLAPIQETHFIETVFFGGGTPSLVSPL
DLKRILKELAPHAREITLEANPENLTVSYLRQLQETPINRISVGVQTFDDSILQLLGRTHSSSAAITALQECQNH
GFSNLSIDLIYGLPTQSLEIFLSDLHQALTLPITHISLYNLTIDPHTSFYKHRKILVPTIAQEEILAEMSLLAENLLL
SQGFQRYELASYAKPDYPAKHNLYYWTDRPFLGLGVSASQYLHGERSKNYSHISHYLRAVRKNLPTQETSEI
LPKKERIKEALALRLRLLEGADLAEFPSTLISMLTQDVKLQNLFSVHGQCLALNRQGRLFHDTIAEEIMGYSF

>core/373/1/Org1_Gene408

MSIAIAREQYAAILDMHPKPSIAMFSSEQARTSWEKRQAHPYLYRLLEIIWGVVKFLLGLIFFIPLGLFWVLQK
ICQNFILLGAGGWIFRPICRDSNLLRQAYAARLFSASFQDHVSSVRRVCLQYDEVFIDGLELRLPNAKPDRWM
LISNGNSDCLEYRTVLQGEKDWIFRIAEESQSNILIFNYPGVMKSQGNITRNNVVKSYQACVRYLRDEPAGPQ
ARQIVAYGYSLGASVQAEALSKEIADGSDSVRWFVVKDRGARSTGAVAKQFIGSLGVWLANLTHWNINSEK
RSKDLHCPELFIYGKDSQGNLIGDGLFKKETCFAAPFLDPKNLEECSGKKIPVAQTGLRHDHILSDDVIKEVAG
HIQRHFDN

>core/374/1/Org1_Gene150

MALKFHLIHQSKKSQARVGQIETSHGVIDTPAFVPVATHGALKGVIDHSDIPLLFCNTYHLLLHPGPEAVAKL
GGLHQFMGRQAPIITDSGGFQIFSLAYGSVAEEIKSCGKKKGMSSLVKITDEGAWFKSYRDGRKLFLSPELSV
QAQKDLGADIIIPLDELLPFHTDQEYFLTSCSRTYVWEKRSLEYHRKDPRHQSMYGVIHGGLDPEQRRIGVRF
VEDEPFDGSAIGGSLGRNLQEMSEVVKITTSFLSKERPVHLLGIGDLPSIYAMVGFGIDSFDSSYPTKAARHGLI
LSKAGPIKIGQQKYSQDSSTIDPSCSCLTCLSGISRAYLRHLFKVREPNAAIWASIHNLHHMQQVMKEIREAIL
KDEI

>core/375/1/Org1_Gene713

MAPIHGSNAFVEDILHSHPSPQATYFSSTRAQKLHEFKDRHPVLTRIASVIIKIFKVLIGLIILPLGIYWLCQTLCT
NSILPSKNLLKIFKKQPNTKTLKTNYLHALQDYSSKNRVASMRRVPILQDNVLIDTLEICLSQAPTNRWMLISL
GSDCSLEEIACKEIFDSWQRFAKLIGANILVYNYPGVMSSTGSSSLKDLASAHNICTRYLKDKEQGPGAKEIIT

YGYSLGGLIQAEALRDQKIVANDDTTWIAVKDRCPLFISPEGFHSCRRIGKLVARLFGWGTKAVERSQDLPCL
EIFLYPTDSLRRSTVRQNKLLAPELTLAHAIKNSPYVQNKEFIEVRLSSDIDPIDSKTRVALATPILKKLS

>core/376/1/Org1_Gene265

MSTTEPNLTNVNLTMLISSESMPTQLASHKLKGLDLVAFILIIGIAVSSGTAAIILGIPLLFILTALAVLAFSILLY
FLLREPKSPISVTHQPTPIIKDTDLPPVPPLALTPVPTEAVLEEPPLPSPRTHQTLLQENWDRIPDLQANTDMPFI
AADNQTGYAWHLKNSNLTLISTLGPIEKPRYKTQGIVMIVNAATPNMANNVKGTSLALAKATSVRCWENSK
KSPDPLRSKQPLQLGECRSAKWENLNGTTNAGKAGLPQFLGQLLGPKASDYNYNPNDAFTFCRQAYLNCLN
EAKRRKTTVVQLPLLSSHFPGSPKDEETTSLRLQWIDGVKLALIDALQTFGSEAENQNQPWVIILTTLARHPLI
TP

>core/377/1/Org1_Gene532

MAVAADSSASWLKSRNNFLSSLGKTEEQVAAPEFPKELCQHKIREKFRLEDVQVSIKFRGSITAVEATKEFGV
HLLIQPMVVQPWEVENLLFLTSEEDLQELMVAVFDDASLASYFYEKDKLLGFHYYFVAEACKLFEELQWVP
SLSAKVGGDAIFTATSLQGSFQVVDISLRLDGKNVRCRLLLPEDTFQSCQKFFSGLHDESDLHNIDQTQQISLS
VEVGYSQLTQEEWHQVVPGSFIMLDSCLYDPETEESGALLTVQKHQFFGGRFLTPSSGEFKITSYPNLTHEDP
PLPENPQASAAPLPGYSRLVVEVARYSLAVSEFIKLNLGSILSLGNHPAYGVDIILDGAKVGRGEIIALGDVLGI
RVLEV

>core/378/1/Org1_Gene289

MIYLDNNAMTPPERGLLEFLQKTFLIEGTYANPSSVHQLGKKSRQLVLEASHWMQKVLSFQGRVLYTSGATE
SLNLAIASLPKDSHVITSGSEHPAILEPLKHSSLSVSYLNPEEGRCVLTIEQIERAVTPKTSAIILGWVNSETGAK
ADIAAIAHFAQERQLQFIVDATANVGKERIVLPSGVTMAAFSGHKFHALSGIGALLVSPGVKLHPQLWGGGQ
QGGLRAGTENLWGIASLLYIFKYLDLHQERISQEILTHRNGFEKAIKARIPDVHIHCADQPRANNVSAIAFPPL
EGEVLQIALDIEGVACGYGSACSSGATAPFKSLVSMGVDEELTLATLRFSFSHLLLQEDVERAVGIIEKVVERL
KNS

>core/379/1/Org1_Gene707

MTTSDVIDFVTNDFLGFARSPTIYCEVSKRFQIHCQQFPHEKLGIRGSRLMVGPSSVIDDLESKIASYHGAPNA
FIVNSGYMANLGLCHHVSRSTDVLLWDEEVHMSVVHSLSAISGQHHTFHHNNLEHLESLLQCYRISSKGRIFI
FVSSVYSFRGTLAPLEQIIALSKKYHAHLIVDEAHAMGIFGDDGKGLCHALGYENFYAVLVTYGKALGTMG
ASLLTSSEVKYDLMQNSPPLRYSTSLSPHTLISIGTAYDFLASEGEIARKQVFKLKEHFHECFDSHAPGCVQPIF
LPHTCLEEAISVLETTGIHVGVVAFAKHPFLRVNLHAYNTVDEVNLLAQVMKPYLEKSSHRVHINHEFHLWR
ELCQH

>core/380/1/Org1_Gene891

MMLVYCFDPSVPTSPEHRLMAALDRWFFLGGHRVRILTLEGNHYRAFQENMSISTVEKILKLISYLLIPIVLIA
LLIRCFLHSRFKCNWKCDSLSDARVPHDVQPFNDFQLFNNQERLNIWKNRRYVSGIDVLMVPVDYLRSQFPG
FKEIPEAIRCENYVSDGQFSEESKTSYLRAMLTDIVGYILSLDETYWTNVILKIRAMCITFESFPGKEADPNYSP
RVTHHYFDESWKALARHVLGEGNMVNRLDEALIRTEKPGKEGECITKQFLKDYCKKHLEVMSCPDFIESLV

DEKIREFRCPSILNSAVCDVIDRKCQEHLLKAIINEANRRLPGMKNSSFTMRGNQVLFYTIFSPPKLPPAASSVYF

>core/381/1/Org1_Gene370

MTTCLPQPPKTSPLYSIFEKLDAQERLSSEDALHLLLLTNKEDQRTLWNFADQVRKQRVGDTVYYSSTLYLYPTNFCDFSCKFCSFYAKPGDPKGWLYSPDDLLQQIQNIKTPITEVHIVGGCFPSCNLQYYSDLFTKIKEYDPQIHIKALTAIEYAYLSDLDNLSIRDVLLTLKDAGLDSIPGGGAEILVDKIRNFLAPKRLSSSDFLNIHKMAHQLGIHSNITMLCYHKEGPEDLVTHMVKVRDLQDETQGFKNFILLKFAQENNVLGKRLRKSGQGHAIPLKSLMAVARIFLDNFSNMKALWNYLGIEAALDLLSCGANDLSSTHMGEKVFQMASSKEPIKMDAEGMAALITQQGRTPCLTNSSHV

>core/382/1/Org1_Gene1013

MRRQVREIMQQTVIVAMSGGVDSSVVAYLFKKFTNYKVIGLFMKNWEEDSEGGLCSSTKDYEDVERVCLQLDIPYYTVSFAKEYRERVFARFLKEYSLGYTPNPDILCNREIKFDLLQKKVQELGGDYLATGHYCRLNTELQETQLLRGCDPQKDQSYFLSGTPKSALHNVLFPLGEMNKTEVRAIAAQAALPTAEKKDSTGICFIGKRPFKEFLEKFLPNKTGNVIDWDTKEIVGQHQGAHYYTIGQRRGLDLGGSEKPCYVVGKNIEENSIYIVRGEDHPQLYLRELTARELNWFTPPKSGCHCSAKVRYRSPDEACTIDYSSGDEVKVRFSQPVKAVTPGQTIAFYQGDTCLGSGVIDVPMIPSEG

>core/383/1/Org1_Gene293

MTKIAFSEKAKNFPVEALKKWFEKNKRSLPWRDNPTPYSVWVSEVMLQQTRAEVVIDYFNQWMERFPTIESLAAAKEEDVIKLWEGLGYYSRARHLLEGARMVMEEFHGKIPDDAISLAQIRGVGPYTVHAILAFAFKRRAAAVDGNVLRVLSRIFLIETSIDLESTRTWVSRIAQALLPHKSPEVIAEALIELGACICKKVPQCHRCPVRQACGAWRENKQFVLPVRHARKKVIFLHRLVAIVLYDGSLVVEKRRPKEMMAGLYEFPYIEVEPEEGLQDIEGFTKKMELSLESPLEFLGNLKEQRHAFTNHKVHLCPIIFKATSLPQFGELHLLSDIDHLAFSSGHKKIKDALLIYLGDVRSRESIGV

>core/384/1/Org1_Gene422

MQENLDKRLEALRTEISLAARSLFDLDKKQKELQVLEEESSEENFWQDSVHAGKISEQIVSLRRQIQEYQELKSKIDAIEFFLEDADALEDPAICEDLEKEFLFCEKKLAVWETQRLLSGEADKNSCFLTINAGAGGTESCDWVEMLFRMYSRWATKHQWALEVVDRLDGEVVGIKHVTVKFSGMYAYGYAKAERGVHRLVRISPFDSNGKRHTSFASVDVFPEIDDQIKIEIRPNDLRIDTFRSSGAGGQHVNVTESAVRITHLPSGVVVSCQNERSQIQNRESCMKMLQAKLYQQVLQERLEKQSLDRKDKKEIAWGSQIRNYVFQPYTLVKDVRTGHETGNVQAMLDGELLDEFIKAYLAEFGEVS

>core/385/1/Org1_Gene34

MAVEGRVNSSQALNQDCQEVLANKQSKGLLRCRILSIVVAVITFIAGVVLIALTLASILTSVPYLALGVFLLIVTLGCIIFALCSEKIKKVPPTPISHKEEIIAWFEERKNIDMEKEKEDPEHFGRTATDIPMRSALDQFNHSCHHIHESPALTETYRSHQDVLLFKDWCPVTLPDVTSEEEVLIRSVVGSYLLMEACVPKVSMLIDELHNKLKSPSERECLFIDKKTLQRKASFLFTQKDLATFFLAYTRVNDGHLAPFRAGAKWILIHYVRLRRQHNQNDFFTPGHSCYYARL

AFNQTQRLYHQLFNVEKLRSIYANMDKDPLCHPWAFIPIYDLLKTEDHGDGFLEQQEDREYPSRAAQDQFW
G

>core/386/1/Org1_Gene650

MSPHRNLFKLKNFSNRLYNRALGRFDKVFNFFSGNVGIDLGTANTLVYVRGRGIVLSEPSVVAVDAQTHAVL
AVGHKAKAMLGKTPRKIMAVRPMKDGVIADFEIAEGMLKALIKRVTPSRSVFRPRILIAVPSGITGVEKRAVE
DSALHAGAQEVILIEEPMAAAIGVDLPVHEPAASMIIDIGGGTTEIAIISLGGIVESRSLRIAGDEFDECIINYMR
RTYNLMIGPRTAEEIKITIGSAYPLGDQELEMEVRGRDQVAGLPITKRINSVEIRECLAEPIQQIIECVRLTLEKC
PPELSADLVERGMVLAGGGALIKGLDKALSKNTGLSVITAPHPLLAVCLGTGKALEHLDQFKKRKGNLV

>core/387/1/Org1_Gene888

MKFVVSRNELGNLIKKIQSVVPQNTPIPVLTHVLIETYNDELVFTATDLTVSTRCVTKAKVYEKGAISIPSKRF
FQLVKELTEANLEISSSAGEMAQITSGSSCFRLLSMEKEDFPMLPDIQNALRFSLPAEQLKTMLQRTSFAVSRE
ESRYVLTGVLLAIANGVATIVGTDGKRLAKIDAEVTLDKSFSGEYIIPIKAVEEIIKMCSDEGEATIFLDQDKIA
VECDNTLLITKLLSGEFPDFSPVISTESNVKLDLHREELITLLKQVALFTNESSHSVKFSFLPGELTLTANCTKV
GEGKVSMAVNYSGELLEIAFNPFFFLDILKHSKDELVSLGISDSYNPGIITDSASGLFVIMPMRLHDD

>core/388/1/Org1_Gene764

MLIWKRHLLTRFWFALTSLLVLALIFYASIHHSLHTLKGASTAASGASVKLSILYYLAQISLKAEFLMPQLVA
VATTSTLFAMQNKREIILLQASGLSLKSLMHPLLLSGAVIMMVLYANFQWLHPICEKISITKENMDRGTTDKE
QGKIPALYLKDQTVLLYSSIEPKTLTLNNVFWIKDPKTIYTMEKLAFTTLSLPIGLNVTQFFANDSENLELKEFF
DMKEFPEIEFNFYENPFSKLFSAGNKNRLSEFFKAIPWNATGLGLSTQVPQRILSLLAQFYYVLISPLACMAAII
LSAYLCLRFSRTPTVTLAYLIPLGTVNIFFVFLKAGIVLASSSVLPTLPVMAFPLIVLFLLTNYAYAKLQ

>core/389/1/Org1_Gene411

MKFPRISISDLIPTQMVIWWRGGGNVHYVPNAQNLPKKILGGVLACFGLALLGCAAFAAGVCQTIFPCIGLMI
LGLVLLGFAYLQYSKGWSRFERPLFRETKVFEKPINWLGCLSLLQSWKKIRPGCYYHPGCPQVEICEGSQEIV
TKIFQKKSDRNTSIFLIQEMDQIALRQGIEKSSLSRKTFAIDPSVVSSLLSEIQREEQQYLDPKVISWSSEDQASD
RTHPKSAIYVNISDAAQEPQGRCYIDAYTKAFFTVLDQIGDPNIVKKHTIYVLTPILGVPDALPKEEQENLKLL
SQAAFLYSAEQVAKRMREEKQDSIRIKFIFTDPTSPTSLYFSPHHSSTPHSVTPISLSGFVGEQESYTFA

>core/390/1/Org1_Gene878

MSKKIKVLGHLTLCTLFRGVLCAAALSNIGYASTSQESPYQKSIEDWKGYTFTDLELLSKEGWSEAHAISGNG
SRIVGASGAGQGSVTAVIWESHLIKHLGTLGGEASSAEGISKDGEVVVGWSDTREGYTHAFVFDGRDMKDL
GTLGATYSVARGVSGDGSIIVGVSATARGEDYGWQVGVKWEKGKIKQLKLLPQGLWSEANAISEDGTVIVG
RGEISRNHIVAVKWNKNAVYSLGTLGGSVASAEAISANGKVIVGWSTTNNGETHAFMHKDETMHDLGTLG
GGFSVATGVSADGRAIVGFSAVKTGEIHAFYYAEGEMEDLTTLGGEEARVFDISSEGNDIIGSIKTDAGAERA
YLFHIHK

>core/391/1/Org1_Gene378

MKKRFPSTLFLFYRRVTIAISLEGILGWGWLGSLLSKVFAFLVACWNRFSWSTPYRARSTVISVGNIVVGGAG
KTPTVLWLAEALRLRGYSCGVLSRGYKSQSSRQKKLTVVDSKVHSASYVGDEPLLMAEKLPEGSVWVHKD
RRISAARAAEKFGILLLDDGLQYRKLHKDVEIAVVNGQDPLGGRAFFPKGRLRDFPLRLKTVDAIIVNGGGKE
AGTVVKRVSNAPQIFVKPTIASVVWTHNGERIPKEALRELRVGVFCGLGFPQGFLNMLREEGIHILGKYLLPD
HAAITKKELNYFCQQMAMRQGQGLLCTEKDSVKLPRLSGEVSLLPIAKVEMRLSVNQDDTLSLLNMIEQIHK
NRGN

>core/392/1/Org1_Gene429

MNVPDSKNLHPPAYELLEIKARITQSYKEASAILTAIPDGILLLSETGHFLICNSQAREILGIDENLEILNRSFTD
VLPDTCLGFSIQEALESLKVPKTLRLSLCKESKEKEVELFIRKNEISGYLFIQIRDRSDYKQLENAIERYKNIAEL
GKMTATLAHEIRNPLSGIVGFASILKKEISSPRHQRMLSSIISGTRSLNNLVSSMLEYTKSQPLNLKIINLQDFFS
SLIPLLSVSFPNCKFVREGAQPLFRSIDPDRMNSVVWNLVKNAVETGNSPITLTLHTSGDISVTNPGTIPSEIMD
KLFTPFFTTKREGNGLGLAEAQKIIRLHGGDIQLKTSDSAVSFFIIIPELLAALPKERAAS

>core/393/1/Org1_Gene233

MSHTECGIVGLPNVGKSGLFNALTGAQVASCNYPFCTIDPNVGIVPVIDERLEALAKISNSQKIIYADMKFVDI
AGLVKGASDGAGLGNRFLSHIRETHAIAHVVRCFDDPDVTHVSGKVNPVEDIEVINLELIFSDFSSAKNIHSKL
EKLAKGKREVGALLPLFDTIIAHLEKGLPLRTLELTPEQIVALKPYPFLTMKPMFYIANVDESSLPDMDNDYV
AAVREVAAKENSKVVPICVRIEEEIVSLPIEERLEFLMSLGLEKSGLHRLVRAAYDTLGLISYFTTGPQESRAW
TVVRGSSAWEAAGEIHTDIQKGFIRAEVITFEDMIECQGRAAARELGKLHIEGRDYIVQDGDTMLFLHN

>core/394/1/Org1_Gene272

MTTEVRIPNIAESISEVTVASLLVTEGALIQENQGLLEIESDKVNQLIYAPVSGRIFWEVSEGDVVPVGGVVGKI
EPAGEGEELGDSQSKETIEAEIICFPQSGVRQSPPENKTFIPLRDQMDQGSQGLSAGDRGETRERMTSIRKTISR
RLLSALHESAMLTTFNEVYMTPLFHLRKEKQEEFLSRYGVKLGFMSFFVKAVLEALKAYPRVNAYIDGEEIV
YRHYYDISIAVGIDRGLVVPVIRDCDKLSNGEIEQKLADLALRAREGLLAIAELEGGGFTITNGGVYGSLLSTPI
INPPQVGILGMHKIEKRPVVLDNEIVIADMMYVALSYDHRLIDGKEAVGFLVKVKEGLENPASLLDL

>core/395/1/Org1_Gene507

MVPFRQHHAYQLLKQLHTSAISEADRVSYYFKQNRSLGSKDRQWIQNIIFNILRHRRLLETLILDSGEQVTPEA
LVAKVNEGVLENLDSYSAIPWPVRYSISDDLAHFLVQDYGEEQAEEIAKIWLTEAPITIRVNTDKISVKELQEK
LEYPSSPGELPEALHFSKRHPLQSTEAFRRGFFEIQDENSQRISQGISLTDKDIVLDFCAGAGGKSLIFAQKAKH
VVINDSRKAILQTAKHRLLRAGARNFSLADQLRLGSFSVVIVDAPCSGTGVFRRHPEHKWQFSKKLLLNYVR
VQKSILKQASAYVGPRGRLVYITCSLLKEENEAHVAYMHSLGWKEVHRKTLPLQVGKGDAFFTSHFQKI

>core/396/1/Org1_Gene810

MKLYQTLRGIVLVSTGCIFLGMHGGYAAEVPVTSSGYENLLESKEQDPSGLAIHDRILFKVDEENVVTALDVI
HKLNLLFYNSYPHLIDSFPARSQYYTAMWPVVLESVIDEFLMVADAKAKRIATDPTAVNQEIEEMFGRDLSP
LYAHFEMSPNDIFNVIDRTLTAQRVMGMMVRSKVMLKVTPGKIREYYRKLEEEASRKVIWKYRVLTIKANT
ESLASQIADKVRARLNEAKTWDKDRLTALVISQGGQLVCSEEFSRENSELSQSHKQELDLIGYPKELCGLPKA

HKSGYKLYMLLDKTSGSIEPLDVMESKIKQHLFALEAESVEKQYKDRLRKRYGYDASMIAKLLSEEAPPLFSLL

>core/397/1/Org1_Gene437

MAFKRKTRWLWQVLILSVGLNMLFLLLFYSAIFRKDIYKLHLFSGPLIAKSSRKVYLSEDFLNEISQASLDDLISLFKDERYMYGRPIKLWALSVAIASHHIDITPVLSKPLTYTELKGSSVRWLLPNIDLKDFPVILDYLRCHKYPYTSKGLFLLIEKMVQEGWVDEDCLYHFCSTPEFLYLRTLLVGADVQASSVASLARMVIRCGSERFFHFCNEESRTSMISATQRQKVLKSYLDCEESLAALLLLVHDSDVVLHEFCDEDLEKVIRLMPQESPYSQNFFSRLQHSPRRELACMSTQRVEAPRVQEDQDEEYVVQDGDSLWLIAKRFGIPMDKIIQKNGLNHHRLFPGKVLKLPAKQS

>core/398/1/Org1_Gene1023

MAVVSTPFFNRNLWYRLLSSRFSLWKSYCPRFFLDYLDAFGLLSDFLDHQAVIKFFELETHFSYYPVSGFVAPHQYLSLLQDRYFPIASVMRTLDKDNFSLTPDLIHDLLGHVPWLLHPSFSEFFINMGRLFTKVIEKVQALPSKKQRIQTLQSNLIAIVRCFWFTVESGLIENHEGRKAYGAVLISSPQELGHAFIDNVRVLPLELDQIIRLPFNTSTPQETLFSIRHFDELVELTSKLEWMLDQGLLESIPLYNQEKYLSGFEVLCQ

>core/400/1/Org1_Gene73

MKIDILSLFPGYFDGPLQTSILGRAIKQRLLDVQLTNLRDFGLGKWKQVDDTPFSGGGMLLMAEPVTSAIRSVRKENSKVIYLSPQGALLTAEKSRELAAASHLILLCGHYEGIDERAIESEVDEEISIGDYVLTNGGIAALVLIDAVSRFIPGVLGNQESAERDSLENGLLEGPQYTRPREFEGKEVPEVLLQGDHKAISQWRLEQSERRTYERRPDLYLNYLYKRSIDHKFDEETTTNRDHFKCDKISVVLEVNKLKRAKNFYCKVFGLDAMSCENKFCLPHEGKTIFWLREVQAEKKNIVTLSLSLDCACEEDFCYLLRRWELFGGKLLEKQADEHAVWALAQDLDGHAWIFSWHRMK

>core/401/1/Org1_Gene234

MGEKTEKATPKRLRDARKKGQVAKSQDFPSAVTFIVSMFTAFSLSTFFFKHLGGFLVSMLSQAPTRHDPVITLFYLKNCLMLILTASLPLLGAVAVVGVIVGFLIVGPTFSTEVFKPDIKKFNPIENIKQKFKIKTLIELIKSILKIFGAALILYITLKSKVSLIIETAGVSPIITAQIFKEIFYKAVTSIGIFFLIVAILDLVYQRHNFAKELKMEKFEVKQEFKDTEGNPEIKGRRRQIAQEIAYEDSSSQVKHASTVVSNPKDIAVAIGYMPEKYKAPWIIAMGINLRAKRILDEAEKYGIPIMRNVPLAHQLLDEGKELKFIPESTYEAIGEILLYITSLNAQNPNNKNTNQPDHL

>core/402/1/Org1_Gene217

MSEAPVYTLKQLAELLQVEVQGNIETPISGVEDISQAQPHHIAFLDNEKYSSFLKNTKAGAIILSRSQAMQHAHLKKNFLITNESPSLTFQKCIELFIEPVTSGFPGIHPTAVIHPTARIEKNVTIEPYVVISQHAHIGSDTYIGAGSVIGAHSVLGANCLIHPKVVIRERVLMGNRVVVQPGAVLGSCGFGYITNAFGHHKPLKHLGYVIVGDDVEIGANTTIDRGRFKNTVIHEGTKIDNQVQVAHHVEIGKHSIIVAQAGIAGSTKIGEHVIIGGQTGITGHISIADHVIMIAQTGVTKSITSPGIYGGAPARPYQETHRLIAKIRNLPKTEERLSKLEKQVRDLSTPSLAEIPSEI

>core/403/1/Org1_Gene944

MSGPSRTESSQVSVLSYVPRDKEIAPKKQFTIAKISTLAILASLALGALVAGISLTIVLGNPVFLALLITTALFSVVTFLVYHQMTSKVSSNWQKVLEQNFKPLGKAWQEKNVDCYSNEMQFYNNHLNPKFKVAIQTDASQPFQPTFLTGLRVIEKNQSTGIIFNPVGPTNLIDNTATNLSTILYSTLKDKSVWDTCKQREGDPAKGEDPFSPTEVRVVK

LPNEALDQTFNLNLSSAEKKSILPTFLGHVCGPKSEELPNQQEYYRQALLAYENCLKAAIESHAAIVALPLFTS
VYEVPPEEILPKEGTFYWDNQTQAFCKRALLDAIQNTALRYPQRSLLVILQDPFNTIESQSRSEE

>core/404/1/Org1_Gene793

MKNSFGSLFSFTTWGESHGPSIGVVIDGCPAGLELHESDFVPAMKRRRPGNPGTSSRKENDIVQILSGVYKGK
TTGTPLSLQILNTDVDSSPYENSERLYRPGHSQYTYEKKFGIVDPNGGGRSSARETACRVAAGVVAEKFLAN
QNIFTLAYLSSLGSLTLPHYLKISPELIHKIHTSPFYSPLPNEKIQEILTSLHDDSDSLGGVISFITSPIHDFLGEPLF
GKVHALLASALMSIPAAKGFEIGKGFASAQMRGSQYTDPFVMEGENITLKSNNCGGTLGGITIGVPIEGRIAF
KPTSSIKRPCATVTKTKKETTYRTPQTGRHDPCVAIRAVPVVEAMINLVLADLVLYQRCSKL

>core/405/1/Org1_Gene530

MTLPNDLLETLVKRKESPQANKVWPVTTFSLARNLSVSKFLPCLSKEQKLEILQFITSHFNHIEGFGEFIVLPLK
DTPLWQKEFLLEHFLLPYDLVGNPEGEALVVSRSGDFLAAINFQDHLVLHGIDFQGNVEKTLDQLVQLDSYL
HSKLSFAFSSEFGFLTTNPKNCGTGLKSQCFLHIPALLYSKEFTNLIDEEVEIITSSLLLGVTGFPGNIVVLSNRC
SLGLTEELLLSSLRITASKLSVAEVAAKKRLSEENSGDLKNLILRSLGLLTHSCQLELKETLDALSWIQLGIDLG
LIKVTENHPLWNPLFWQIRRAHLALQKQAEDSRDLQKDTISHLRASVLKELTKGLSPESF

>core/406/1/Org1_Gene692

MMKKIRKVALAVGGSGGHIVPALSVKEAFSREGIDVLLLGKGLKNHPSLQQGISYREIPSGLPTVLNPIKIMSR
TLSLCSGYLKARKELKIFDPDLVIGFGSYHSLPVLLAGLSHKIPLFLHEQNLVPGKVNQLFSRYARGIGVNFSP
VTKHFRCPAEEVFLPKRSFSLGSPMMKRCTNHTPTICVVGGSQGAQILNTCVPQALVKLVNKYPNMYVHHIV
GPKSDVMKVQHVYNRGEVLCCVKPFEEQLLDVLLAADLVISRAGATILEEILWAKVPGILIPYPGAYGHQEV
NAKFFVDVLEGGTMILEKELTEKLLVEKVTFALDSHNREKQRNSLAAYSQQRSTKTFHAFICECL

>core/407/1/Org1_Gene70

MKKKVAEYLNRLAEVEIKISNPEIFSNSKEYSALSKEHSYLLELKNAYDKILNLEKVLADDKQALAIEKDPEM
VVMLEEGINENKVELEKLNKILESLLVPPDPDDDLNVIMELRAGTGGEEAALFVGDCVRMYHLYASSKGWK
YEVLSASESDLKGYKEYVMGISGTGVKRLLQYEAGTHRVQRVPETETQGRVHTSAITIAVLPEPSEEDTELLI
NEKDLKIDTFRASGAGGQHVNVTDSAVRITHLPTGVVVTCQDERSQHKNKDKAMRILKARIRDAEMQKRHN
EASAMRSAQVGSGDRSERIRTYNFSQNRVTDHRIGLTLYNLDKVMEGDLDPITTAMVSHAYHQLLEHGN

>core/408/1/Org1_Gene976

MPILWKVLIFRYLKTAAFCTLSLICISIISSLQEIVAYIAKDVPYDTVLRLMAYQIPYLLPFILPGSCFVSAFSLFR
KLSDNNHMTFLRASGASQSIIMFPVLMVSGAICCLNFYTCSELASICRYQTCKEIANMAMTSPALLLQTLQKK
ENNRIFIAVDHCAKSKFDNVIVALKGNNEISHVGIIKSIIPDTTKDTVKAKDVVFISKLPDSLTESSSPSSQRFYIE
TLDELLIPKITSTLFAGKSYLKTRTDYLPWKQLVKQSLKHPHLPETLRRVAIGFLCITLTYAGMILGIHKPRFRK
SIALYFIFPILDLILLIVGKNTKNLPLAFMLFVFPQLVSWVVFAARAYRESRGYA

>core/409/1/Org1_Gene294

MSLLSGHRLEGFPPIAEVMAACDRCSMDFCEILKSQSMDLWADAASCVDGLLQDPFWSTAIASGIAKSSLQE
TEFECESKVMVLSSWGEQGAQVCSPFNLERICMSFPSLKVFSLKKNGCENMGIQLSASCMNLLMSIFFVATN

GGSTPIWITKENLMALVALVLSHYQCYFVPATGDPQRGNILGNPEVNAILARGMGMRVDLERKRGGESSSSR
YLELAARCFENSLTKTSLLSDANNVQERDKCLLQMSTSLMHTAGLNLQRPPVPTPSGVTAHPQPQPDPVVTS
QPSLLGARERSPVSSRGRFPVVLPLSVISPRSHPGRVERRDLEDEEEEVMF

>core/410/1/Org1_Gene619

MSHDRILRAQRALSEHNLDAILVEKSEDLAYFLHDEAIAGILLIGQQEVMFFVYRMDKDLYSHIQRVPLTFLT
QDVVADLSLYVQKQRYQKIGFDSASTVYHKFAQRQVLPCLWEPLECFTEKIRSIKSEEEIRRMQEAAALGSA
GYDYVLTLLREGITEKEVVRQLRAFWAEAGAEGPSFPPIIAFGEHSAFPHSIPTDRPLKKGDIVLIDIGVLLNGY
CSDMTRMTALGTPHPKLLESYPVVVEAQKRAMALCKEGVLWGDIDAEAVRVLREHHLDTYFIHGIGHGVG
RHIHEYPCSPRGSQVKLESGMTITVEPGVYFPGIGGIRIEDTLCIDKNKNFSLTARPVISELVCL

>core/411/1/Org1_Gene923

MNKRQKDKLKICVIISTLILVGIFARAPRGDTFKTFLKSEEAIIYSNQCNKDMRKILCDAIEHADEEIFLRIYNLS
EPKIQQSLTRQAQAKNKVTIYYQKFKIPQILKQASNVTLVEQPPAGRKLMHQKALSIDKKDAWLGSANYTNL
SLRLDNNLILGMHSSELCDLIITNTSGDFSIKDQTGKYFVLPQDRKIAIQAVLEKIQTAQKTIQVAMFALTHSEII
QALHQAKQRGIHVDIIIDRSHSKLTFKQLRQLNINKDFVSINTAPCTLHHKFAVIDNKTLLAGSINWSKGRFSL
NDESLIILENLTKQQNQKLRMIWKDLAKHSEHPTVDDEEKEIIEKSLPVEEQEAA

>core/412/1/Org1_Gene961

MNLPDRKKALEAAVAYIEKQFGAGSIMSLGRHSATHEISTIKTGALSLDLALGIHGVPKGRVIEIFGPESSGKT
TLATHIVANAQKMGGVAAYIDAEHALDPSYASLIGVNIDDLMISQPDCGEDALSIAELLARSGAVDVIVIDSV
AALVPKSELEGDIGDVHVGLQARMMSQALRKLTATLSRSQTCAVFINQIREKIGVSFGNPETTTGGRALKFYS
SIRLDIRRIGSIKGSDNSDIGNRIKVKVAKNKLAPPFRIAEFDILFNEGISSAGCILDLAVEYNIIEKKGSWFNYQE
KKLGQGREFVREELKRNRKLFKEIEKRIYDVIAANKTPSVHANETPQEVPAQTVEA

>core/413/1/Org1_Gene697

MNLCKRISFEEGLELFVSSPIERLQERADAIRKERYPSNEVTYVLDANPNYTNICKIDCTFCAFYRKPKSPDAY
LLSFDEVRSLLQRYVSSGVKTVLLQGGVHPGLGIDYLEELVRITVQEFPSIHPHFFSAVEIEHACRVSGISIEQG
LQRLWDAGQRTIPGGGAEILSERVRKIISPKKMQPGGWINLHKLAHLMGFRTTATMMFGHVENPEDILIHLQ
TLRDAQDSCPGFYSFIPWSYKPGNTALRRNVPQQASIETYYRILALGRIFLDNFDHVAASWFGEGKSLGAKAL
HYGADDFGGVILDESVHKATGWSIQSSEEEICNIIRSEGFIPVERNTFYQHISCTVSSL

>core/414/1/Org1_Gene703

MKYSLNFKEIKIDDYERVIEVTCSKVRLHAIIAIHQTAVGPALGGVRASLYSSFEDACTDALRLARGMTYKAII
SNTGTGGGKSVIILPQDAPSLTEDMLRAFGQAVNALEGTYICAEDLGVSINDISIVAEETPYVCGIADVSGDPSI
YTAHGGFLCIKETAKYLWGSSSLRGKKIAIQGIGSVGRRLLQSLFFEGAELYVADVLERAVQDAARLYGATIV
PTEEIHALECDIFSPCARGNVIRKDNLADLNCKAIVGVANNQLEDSSAGMMLHERGILYGPDYLVNAGGLLN
VAAAIEGRVYAPKEVLLKVEELPIVLSKLYNQSKTTGKDLVALSDSFVEDKLLAYTS

>core/417/1/Org1_Gene199

MLNIHDILGNDDENLLSYQCKHITKDKLTLPSHDFVDKVFGLSDRNNRVLRSLQTMFSHGRLANSGYLSILPV
DQGIEHSAGASFAINPIYFDPENIVKLAIESGCSAVASTYGTLSLLSRKYAHKIPFMLKLNHNELLSYPTKYHQI
FFTQVEAAYSMGAVAVGATVYFGSETSNEEIVAVSNAFAKARSLGLATVLWCYLRNPAFVANGVDYHTAA
DLTGQADHLGATLGADIVKQKLPTCQGGFKAINFGKTDERVYSELSSNHPIDLCRYQVLNSYCGKVGLINSG
GPSGKNDFTEAARTAVINKRAGGMGLILGRKAFQRPLSEGIQLLNLVQDIYLDPNITIA

>core/418/1/Org1_Gene688

MIPLIPMFLKQSLFFSLALTGMTTLVLTVALGVPVMKWLKRKNYRDYIHKEYCEKLEMLHKDKAEVPTGGG
VLLFISLIASLLVWLPWGKFSTWFFIILLTCYAGLGWYDDRIKIKRKQGHGLKAKHKFMVQIAIAAFTLIALPY
IYGSTEPLWTLKIPFMEGMLSLPFWLGKVFCLGLALVAIIGTSNAVNLTDGLDGLAAGTMSFAALGFIFVALR
SSTIPIAQDVAYVLAALVGACIGFLWYNGFPAQLFMGDTGSLLLGGLLGSCAVMLRAECILVVIGGVFVAEA
GSVILQVLSCRLRKKRLFLCSPLHHHYEYQGLPETKIVMRFWIFSFVCAGLGIAAVLWR

>core/419/1/Org1_Gene789

MRIAVLGAGYAGLSVTWHLLLHSQGTATIDLFDPIPLGEGASGMSSGLLHAFTGKKALKPPLADQGINATHA
LITEASKALNVPIVISQGILRPAIDEDQAQLFTERVEEFPKEVEWWEKARCEISIPSMVIPPNLGALFIKSGVTLN
NDLYIQGLADACMKLGTQFYDELIEDLADIEEFYDHIIVTPGANASILPELKDMPVNKVKGQLLEISWPKDLA
MLSFSINAHKYMVANTQKNTCILGATFEHNQPEETPDPAIAYQEIMPPVLSLFPGLKDAQVLHCYAGMRSSS
KSRLPVISRIREKLWFLGGLGSKGLLYHGITGDMLAQAVLRKSTAYIAKEFLFTI

>core/420/1/Org1_Gene715

MILLQNIKRCSLKQLKVLATLLLSLSLPTLEAAENRDSDSIVWHLDYQEALQKSKEAELPLLVIFSGSDWNGP
CMKIRKEVLESPEFIKRVQGKFVCVEVEYLKHRPQVENIRQQNLALKSKFKINELPCMILLSHEEREIYRIGSFG
NETGSNLGDSLCHIVESDSLLRRAFPMMTSLSLSELQRYYRLAEELSHKEFLKHALELGVRSDDYFFLSEKFR
LLVEVGKMDSEECQRIKKRLLNKDPKNEKQTHFTVALIEFQELAKRSRAGVRQDASQVIAPLESYISQFGQQD
KDNLWRVEMMIAQFYLDSDQWHHALQHAEVAFEAAPNEVRSHISRSLEYIRHQS

>core/421/1/Org1_Gene397

MYLQEKFKAQQVPLVLRELLSCSDSINDSDPIYRMVFDSNDTTISYKVGDALGVLPENSKEVSEHVLQLLGYS
PTTLVNVKKTSEKVSAQKFIQGYVDLDKIPAKLNSFFPDKDPKITLYDAIQEYRPQIPIELFAESVFPLLPRFYSI
ASSPDLHPKSIELLVKHVSYPGKYQKRFGVCSSFLCSELQVNDSAYIFVQPTKHFTLSTQTEGKPLVMIGAGT
GIAPYKAFLEERLFNKDPGNNLLFFGERKEKVNFYYREFWNHAEEEGKLKLFLAFSRERDQKVYVQDLLRIQ
KDEVRKAYEEGGFFFVCGRKVLGIEVKHALEEILGKDTLASLRKEHRYVVDVY

>core/422/1/Org1_Gene218

MDVSRKINRHTQFYVDSIDGVIKNFDHKPSEDKSRDHEELEEKLLTITKRIVASAQEFQNRKTDSKNYYLKKT
QWLPFKNEELEQTKELFAMLTSMDKKIAQLFFYSPGCSSDWVEFTEVICHLNDSIGLGGVLLCCGLFEQQCEH
VVTVNKKLDLPLLLGTTVVNSLRYYLTYRNISLLNCQSMSELGKELGDVLKQHGVAFTLIFKEIVDIDLLNYV
KLIQGLKRSGNIQARIYDNDVPTLPSVSSSPIALRYSLANTIRGLALHVDFSSLKFISPSILSNTEHTAKALNSGG
ECFIFSNLDEFNLGMKIVMQLLRTGKISPEILNKNIMKILMIKRRVRSLYI

>core/423/1/Org1_Gene754

MEADILDGKLKRVEVSKKGLVNCNQVDVNQLVPIKYKWAWEHYLNGCANNWLPTEVPMARDIELWKSDE
LSEDERRVILLNLGFFSTAESLVGNNIVLAIFKHITNPEARQYLLRQAFEEAVHTHTFLYICESLGLDEGEVFNA
YNERASIRAKDDFQMTLTVDVLDPNFSVQSSEGLGQFIKNLVGYYIIMEGIFFYSGFVMILSFHRQNKMTGIGE
QYQYILRDETIHLNFGIDLINGIKEENPEVWTTELQEEIVALIEKAVELEIEYAKDCLPRGILGLRSSMFIDYVRH
IADRRLERIGLKPIYHSRNPFPWMSETMDLNKEKNFFETRVTEYQTAGNLSW

>core/426/1/Org1_Gene63

MELSLTSLLPLAWYVILGVAVFAYSFGDGFDLGLGAVYLKAKEDKERRILLNSIGPVWDGNEVWLVIIVGGL
FAGFPACYATLLSIFYMPIWTLVLLYIFRGCSLEFRSKSESVSWKIFWDIIFICSGTAISFFLGTIVGNLILGLPLSP
DTSYASLSWILFFRPYAALCGAVVASAFAIHGSCFALMKTSDSLNARIAQQFPYILSSFLVFYVLFLGASLISIP
KRFDAFPTYPLLILLIALTSCCCVAAKTSVSKKRYGYAFIYSTLNLLSLILSAATLTFPNILLSTVDPQYSYTIYN
SAVETKTLKSLLIIVLIGLPFIITYTCYIYRVFRGKTNFPSIY

>core/427/1/Org1_Gene653

MNSKMLKHLRLATLSFSMFFGIVSSPAVYALGAGNPAAPVLPGVNPEQTGWCAFQLCNSYDLFAALAGSLK
FGFYGDYVFSESAHITNVPVITSVTTSGTGTTPTITSTTKNVDFDLNNSSISSSCVFATIALQETSPAAIPLLDIAF
TARVGGLKQYYRLPLNAYRDFTSNPLNAESEVTDGLIEVQSDYGIVWGLSLQKVLWKDGVSFVGVSADYRH
GSSPINYIIVYNKANPEIYFDATDGNLSYKEWSASIGISTYLNDYVLPYASVSIGNTSRKAPSDSFTELEKQFTN
FKFKIRKITNFDRVNFCFGTTCCISNNFYYSVEGRWGYQRAINITSGLQF

>core/428/1/Org1_Gene748

MMEVYSFSPSVRTSFQHRVMAALDNWFFLGGRRLKVVSLDSCNSGQACEEYVPISTTEKVLKILSYLLIPIVII
ALLIRYLLHSNFTAKVSQKPWLKTLQLGIDIKSFILPGSHVNTMDSATLFKAIRLEGKRVDVEYHRLHSSDKV
VFYIPAQKLPDDLRLTHWLPEKETRKTEYVRHMLAHVMGYLTSQGKERLQQVVQDSRSSTSLGAEKVLQYR
FIDHPQSQGEFQRLLNENITTKGSEDKEVVQSDLFDMAFQCWWPQFISVIQSPTFSEELVHEMSQKLDLDCIYP
EDDEFEQKFLNTLLKAVLHHGFEGISVASMGVIFLICPDSLALQIPFLRNQK

>core/429/1/Org1_Gene610

MNKKKRVLTGDRPTGKLHLGHWVGSIKNRLELQNSPEYDCFFIIADLHTLTTKIRKEEVLDVDNHIYEVLAD
WLSVGIDPTKSIIYLQSAIPEIYELHLLFSMLISINRVMGIPSLKDMARNASIEEGSLSYGLIGYPILQSADILLAK
AQFVPVGKDNEAHVELTRDIARNFNRLYGQVFPEPEVLQGELTSLVGIDGQGKMSKSANNAIYLSDSDATITE
KVRKMYTDPNRIRATTPGRVEGNPLFIYHDIFNPHKDEVEEFKARYRQGCIKDIEVKARLAEELIHFLKPIKER
RSEFLSKPLALQNVLEDGTHKMREVAKVTMEEVHDKFGFSHKWRSLLK

>core/430/1/Org1_Gene130

MLTLGLESSCDETACAIVNEDKQILANIIASQDIHASYGGVVPELASRAHLHIFPQVINKALQQANLLIEDMDL
IAVTQTPGLIGSLSVGVHFGKGIAIGAKKSLIGVNHVEAHLYAAYMAAQNVQFPALGLVVSGAHTAAFFIENP
TSYKLIGKTRDDAIGETFDKVGRFLGLPYPAGPLIEKLALEGSEDSYPFSPAKVPNYDFSFSGLKTAVLYAIKG

NNSSPRSPAPEISLEKQRDIAASFQKAACTTIAQKLPTIIKEFSCRSILIGGGVAINEYFRSAIQTACNLPVYFPPA
KLCSDNAAMIAGLGGENFQKNSSIPEIRICARYQWESVSPFSLASP

>core/431/1/Org1_Gene83

MENAMSSSFVYNGPSWILKTSVAQEVFKKHGKGIQVLLSTSVMLFIGLGVCAFIFPQYLIVFVLTIALLMLAIS
LVLFLLIRSVRSSMVDRLWCSEKGYALHQHENGPFLDVKRVQQILLRSPYIKVRALWPSGDIPEDPSQAAVLL
LSPWTFFSSVDVEALLPSPQEKEGKYIDPVLPKLSRIERVSLLVFLSAFTLDDLNEQGVNPLMNNEEFLFFINK
KAREHGIQDLKHEIMSSLEKTGVPLDPSMSFQVSQAMFSVYRYLRQRDLTTSELRCFHLLSCFKGDVVHCLA
SFENPKDLADSDFLEACKNVEWGEFISACEKALLKNPQGISIKDLKQFLVR

>core/432/1/Org1_Gene219

MDSSAPYNIASQGTEKSTVERILDLYGPASCIKFLKQMVLIREFEARGEEAYLEGLVGGFYHSYAGQEAVATA
AIANTGLDPWVFSSYRCHALAILLNIPLQEIAAELLGKETGCALGRGGSMHMCGPNFPGGFGIVGGQIPLAAG
AAFTIKYQEQKNRVSLCFIGDGAVAQGVFHETLNFVSLHQLPLMLIIENNGWSMGTSLNRAVAKQPIAESQG
SSYDIRAVTVNGFDLFNSLLGFREAYRYMVDTESPVLVECLCSRFRGHSISDPNLYRSKEEMQCLFKKDPIVL
AKDWLIRLEVLTEEEFQNIRQECKTAVLEAFSNAKLSSDPSVTTLEEGVYA

>core/433/1/Org1_Gene105

MKLYSISSDVDTPWIFQLMSKVDSYLFLGGNRIKVVSIVMQEPNLIIGKVENVRISTIVKILKILSFLIFPLILIAL
ALHYFLHAKYANHLLVSKILERAPQYVPIPGRSGDTASHYKLTTLVPVSQKNLQAMGSNPLEVEAALRTTKP
SFFCVPAKYRQIIISSHGIRFSLDLEQLADDINLDSVSWPTEYLNSTMDFCSKADKRVIQNVQNLRTGTYINSV
GKRSLLKFMLQHLFIDGITQENPEALPNNTSGRLTLFPSVRYIYSHFTPQNPTIWPQVFFRQGPLDEDRGGGFEI
LEQLQELGVRFPICPSQGPDNPNFQGFQGIRIYWEDSYQPNKEV

>core/434/1/Org1_Gene696

MLPFEFEFNTTSSPECDVCLDPQKLFVKLFKRTIVLLSGPTGSGKTDVSLALAPMIDGEIVSVDSMQVYQGMD
IGTAKVSLKARQEIPHHLIDIRHVQEPFNVVDFYYEAIQACQNILSRNKVPILVGGSGFYFHAFLSGPPKGPAA
DPQIREQLEAIAEEHGVSALYEDLLLKDPEYAQTITKNDKNKIIRGLEIIQLTGKKVSDHEWDIVPKASREYCC
RAWFLSPETEFLKNNIQMRCEAMLQEGLLEEVRGLLNQGIRENPSAFKAIGYREWIEFLDNGEKLEEYEETKR
KFVSNSWHYTKKQKTWFKRYSIFRELPTLGLSSDAIAQKIAKDYLLYS

>core/435/1/Org1_Gene659

MFFQNLAKKLTALGISPLGCLLIGGVVSCAILFGRSSNPSLAPTQVKTEKTSGNWLKLTQMGNPKLIESLTKK
EQLEKDLTSFHPIASAKVAIALSTEDDVMSPLHLSVILTLRKEESLTPSLLFSITDYLCSSLPGLKREHISLSDNL
GNLYIPESITVNSLFIHTLENYLGKIFPKEHFALAYHAKAEKPTLQLTLNENYIAHLTKEESEKIVAHTKHYLY
QNYDDSYDIVIETLPFARLQNKKSFPAKVLIGSMILVISLMIVALASFYLARHAYERVSPEPRKIKRGINISKLLE
IIQKESPEKIALILSYLDPKKAEALLNRLPEDLKHQVLKYKL

>core/436/1/Org1_Gene920

MSEQHSPIISVQDVSKKLGDHILLSKVSFSVYPGEVFGIVGHSGSGKTTLLRCLDFLDMPTSGSISVAGFDNSLP
TQKFSRRNFSKNVAYISQNYGLFSSKTVFENIAYPLRIHHSEMSKSEVEEQVYDTLNFLNLYHRHDAYPGNLS

GGQKQKVAIARAIVCQPEVVLCDEITSALDPKSTENIIERLLQLNQERGITLVLVSHEIDVVKKICSHVLVMHQ
GAVEELGTTEELFLNSENSITNELFHEDINIAALSSCYFAEDREEVLRLNFSKELAIQGIISKVIQTGLVSINILSG
NINLFRKSPMGFLIIVLEGEVEQRKKAKELLIELGVVIKEFY

>core/437/1/Org1_Gene355

MTILRKLSQYLFFFSLFCSFIYVATCGSQPDSVSSPKIAIFLSFPHPLLEDCSKSCIETLKDFENLPEIVVLNAEDSI
VKARKIARSLHTDKNVVAIVTLGTIATKVMSHIETQKPVIYAAVPDRESLTLPKNTMNIYGVNDTLDINQYCF
AIQAVATNAQSIVYLKPSEPFPSDLQKEIVKKLHASGIEVIEISITSSTFKTRIRQAIDKRPSAIFIPLSPLSHKEGT
AFLQEILKEKIPIITDDTSLISEGACIACSVDYKKSGKQIAKIVHHLLYNNHDVDSLRKIIAQRLSPTTTFNEDIIK
YLGIKLHKTERNQFLSFKSKKLEKSEKGKNVAVS

>core/438/1/Org1_Gene762

MEMKEEIEAVKQQFHSELDQVNSSQALADLKVRYLGKKGIFRSFSEKLKQCTDKAKLGSLINDFKTYVEDLL
QEKSLVLLASEQAEAFSKEKIDSSLPGDSQPSGGRHILKSILDDVVDIFVHLGFCVREAPNIESEANNFTLLNFT
EDHPARQMHDTFYLNATTVLRTHTSNVQARELKKQQPPIKVVAPGLCFRNEDISARSHVLFHQVEAFYVDH
NVTFSDLTAILSAFYHSFFQRKTELRFRHSYFPFVEPGIEVDVSCECCGKGCALCKHTGWLEVAGAGMIHPQV
LRNGNVDPEIYSGYAVGMGIERLAMLKYGVSDIRLFSENDLRFLQQFS

>core/439/1/Org1_Gene261

MATISPISLTVDHPLVDTKKKSCSNFDKIQSRILLITAIFAVLVTIGTLLIGLLLNIPVIYFLTGISFIAVVLSNFILY
KRATTLLKPRACGKHKEIKPKRVSTNLQYSSISIAINRSKENWEHQPKDLQNLPAPSALLTDNPYEIWKAKHS
LFSLVSLLPGGNPEHLLISASENLGKTLLIEETSQNAPISSYVDTTPSPKSLLNEAIQETRVEINTELPAGDSGER
LYWQPDFRGRVFLPQIPTTPEAIYQYYYALYVTYIQTAINTNTQIIQIPLYSLREHLYSRELPPQSRMQQSLAMI
TAVKYMAELHPEYPLTIACVERSLAQLPQESIEDLS

>core/440/1/Org1_Gene523

MEEAAKHLAKEFLCSGINLFLSGEYEQAEKRLKETLELDSTAALAYCYLGIIALETGRVSEALNWCSKGLASE
PGDSYLRYCYGVALDRGNQYEAAIEQYSAYVALHPDDVECWFSLGSVYHRLKRLQEALDCFDKILALDPW
NPQSLYNKAVILSEMDDEAESIRLLEVAVAKNPLYWKAWVKLGFLLSRSKRWDKATEAYERVVQLRPDLSD
GHYNLGLCYLTLDKTRLALKAFQEALFLNAEDADAHFYVGLAHLDLKQMREAYEAFNSALSINLEHERAHY
LLGYLHHMQGETDKATKELLFLQKKDSTFAPLLQKTVVSDPSSMQFERRLDTIS

>core/441/1/Org1_Gene541

MVLGVVGISYREAALKERERAIQYLQSFEKNLFLAQRFLGKGGAFIPLLTCHRAELYYYSESPEIAQAALLSEL
TSQGIRPYRHRGLSCFTHLFQVTSGIDSLIFGETEIQGQVKRAYLKGSKERELPFDLHFLFQKALKEGKEYRSRI
GFPDHQVTIESVVQEILLSYDKSIYTNFLFVGYSDINRKVAAYLYQHGYHRITFCSRQQVTAPYRTLSRETLSF
RQPYDVIFFGSSESASQFSDLSCESLASIPKRIVFDFNVPRTFLWKETPTGFVYLDIDFISECVQKRLQCTKEGV
NKAKLLLTCAAKKQWEIYEKKSSHITQRQISSPRIPSVLSY

>core/442/1/Org1_Gene81

MFPSANQESRTRNVPLGIFHGLVACLYWGIVFVIPNFLGSFGDLDIVLTRYTIFGIFSLIACAIKNPSVIKKTPLYI
WRKSLLWTLLINPVYYFGITLGIRYVGSAITVVIASLAPTAVLYHSNTKQKELPYSLLFAISSVIITGVILTHLSA
LNLPTAASPLYSILGVIAVILSTSLWVIYVIRNQSLLEKHPNLTPDTWSYLIGISALIICLPMIIILDLCGITHVTHN
LISHTPGSERLLFLLLCSAMGIFSSAKALIAWNKASLNLSPALLGAILIFEPIFGLVLTYLYSQSLPSLQEGIGIFL
MLGGSLLCLVLFGRKVQKSLENSQVSSSNE

>core/443/1/Org1_Gene967

MKKTCCQNYRSIGVVFSVVLFVLTTQTLFAGHFIDIGTSGLYSWARGVSGDGRVVVGYEGGNAFKYVDGEK
FLLEGLVPRSEALVFKASYDGSVIIGISDQDPSCRAVKWVNGALVDLGIFSEGMQSFAEGVSSDGKTIVGCLY
SDDTETNFAVKWDETGMVVLPNLPEDRHSCAWDASEGGSVIVGDAMGSEEIAKAVYWKDGEQHLLSNIPG
AKRSSAHAVSKDGSFIVGEFISEENEVHAFVYHNGVIKDIGTLGGDYSVATGVSRDGKVIVGHSTRTDGEYR
AFKYVDGRMIDLGTLGGSASFAFGVSDDGKTIVGKFETELGECHAFIYLDD

>core/444/1/Org1_Gene284

MTHQVAVLHQDKKFDVSLRPKGLEEFYGQHHLKERLDLFLCAALQRGEVPGHCLFFGPPGLGKTSLAHIVA
YTVGKGLVLASGPQLIKPSDLLGLLTSLQEGDVFFIDEIHRMGKVAEEYLYSAMEDFKVDITIDSGPGARSVR
VDLAPFTLVGATTRSGMLSEPLRARFAFSARLSYYSDQDLKEILVRSSHLLGIEADSSALLEIAKRSRGTPRLA
NHLLRWVRDFAQIREGNCINGDVAEKALAMLLIDDWGLNEIDIKLLTTIIDYYQGGPVGIKTLSVAVGEDIKT
LEDVYEPFLILKGFIKKTPRGRMVTQLAYDHLKRHAKNLLSLGEGQ

>core/445/1/Org1_Gene562

MSYSLRNKKTKICVYIIIALGILSFRSIPQEVYDKIRSSFVSLHVKFFPKIKQAPSSHLANLELENLVLKERVASL
EEKLKLYEVSNHTPPLFPEILTPYFHKLVEGKVVYRDYTHWSSSCWVNVGKTHGIKKNSPVLSGNVLVGLVD
YVGEHQSRIRLITDVGMKPSVVAMRGDIQSWWIKHSLRELIRQVEQISHAYILEKDKYEKISQLQELDSLIQGE
GENQALLRGILSGVGGALWKEGSLCLEGEGFYFSEGKTLLPGDILVTTGLDGVFPPGLLVARVTKVKAPRDG
ACTFKIEAQSLEEKLMELDQLFILPPLEFNPNDRPDIFGLLWD

>core/446/1/Org1_Gene463

MKVVINGFGRIGRLVLRQILKRNSSVEVLAINDLVPGDALTYLFKFDSTHGRFPEDVRCEADHLIVGKRKIQF
LSERNVQNLPWKDLGVDLVIECTGLFTKKEDAEKHIQAGAKRVLISAPGKGDIPTFVMGVNHKTFNPEKDFV
ISNASCTTNCLAPIAKVLLDNFGITEGLMTTVHAATATQLVVDGPSKKDWRGGRGCLQNIIPASTGAAKAVT
LCLPELKGKLTGMAFRVPIEDVSVVDLTVRLDKSTTYDDICKAMKQASETDLKGILDYTDEQVVSSDFIGSEY
SSIFDALAGIALNDRFFKLVAWYDNETGYATRIVDLLEYVEKNSK

>core/447/1/Org1_Gene393

MFVDQITLELRAGKGGNGVVAWRKEKYLPKGGPYGGNGGNGGSVIIEATTSVYSFEAYRNIRFLKAPDGQS
GATNNRTGRSGKDLIVSVPTGTLLRDAETGEILHDFTVDGERLLVSQGGKGGKGNTFFKTSVNRAPTKATPG
KPGEIRQVELELKLIADIGLVGFPNAGKSTLFNTLAHTEVKVGAYPFTTLAPSLGLVLCKDRLYQKPWIIADIP
GIIEGAHQNKGLGLDFLRHIERTLLLLFVIDVSKRERNSPEEDLETLIHELHSHQPDFEKKDMLVALNKIDDLL
PDEQEECLQSFQKRFPSYTFVLISGLTGEGVDGLYRFFTQRLAV

>core/448/1/Org1_Gene214

MWFSVNKNKKAAIWATGSYLPEKVLSNADLEKMVDTSDEWIVTRTGIKERRIAGPQEYTSLMGAIAAEKAI
ANAGLSKDQIDCIIFSTAAPDYIFPSSGALAQAHLGIEDVPTFDCQAACTGYLYGLSVAKAYVESGTYNHVLLI
AADKLSSFVDYTDRNTCVLFGDGGAACVIGESRPGSLEINRLSLGADGKLGELLSLPAGGSRCPASKETLQSG
KHFIAMEGKEVFKHAVRRMETAAKHSIALAGIQEEDIDWFVPHQANERIIDALAKRFEIDESRVFKSVHKYGN
TAASSVGIALDELVHTESIKLDDYLLLVAFGGGLSWGAVVLKQV

>core/449/1/Org1_Gene585

MAAPIFIKNILLRSSIVYAPLAGFSDYPYRCMSALYQPGLMFCEMVKVEGILYAPERTSKLLDYNENMRPIGA
QLCGSNPETSGEAAKILEGLGFDLIDLNCGCPTDKITKDGSGSGLLKTPELIGRILDKIINSVSIPVTVKIRSGWD
MEHINVEDTVRIIRDAGASAVFVHGRTRAQGYHGPSKQEYISRAKAAAGKEFPVFGNGDIFSPEAAQAMLTT
GCDGVLVARGTLGAPWIGKQIQDYLTTGSYEKIPFIKRKAAFLEHMRLVEDYYQSETKFLSETRKLCGHYLIS
AAKVRFLRSSLAKATSYQEVYQLVNDYEEADDSSLETFVKC

>core/450/1/Org1_Gene37

MSTPLSSGGISPSDQYVPQELFCDRLSSSRSNSPDSNASGDSPIVSPPISALVALTDLKLVPYNQNSFSWTTRLK
NAVEKIGLFLQRNWKYILLYILAWALILVCHHTVALTLTIWLGVGLGIGVVFGIFTATCLDKENKHRHVNSL
WNLINHGILQLDPNGTRQILLATMIASISALIYAVPQAVGLVIGFSIGNQLSINTVYGARLGDEATYAIDRKAH
KKRIENIEQAINQHQIIKHQMINQKQLNALIEINRNNQTDPATANLLASLKLNLNQPMPYCFSMPECGVTSSYL
DLNNNSPDDIIARADQCIMTLSQTLQQIKKEPDRIIESNH

>core/451/1/Org1_Gene632

MVRRSISFCLFFLMTLLCCTSCNSRSLIVHGLPGREANEIVVLLVSKGVAAQKLPQAAAATAGAATEQMWDI
AVPSAQITEALAILNQAGLPRMKGTSLLDLFAKQGLVPSELQEKIRYQEGLSEQMASTIRKMDGVVDASVQIS
FTTENEDNLPLTASVYIKHRGVLDNPNSIMVSKIKRLIASAVPGLVPENVSVVSDRAAYSDITINGPWGLTEEI
DYVSVWGIILAKSSLTKFRLIFYVLILILFVISCGLLWVIWKTHTLIMTMGGTKGFFNPTPYTKNALEAKKAEG
AAADKEKKEDADSQGESKNAETSDKDSSDKDAPEGSNEIEGA

>core/452/1/Org1_Gene654

MKQHIGYLGMGIWGFCLASLLANKGYPVVAWSRNPDLIKQLQEERRHPLAPNVVISPNLSFTTDMKEAIHNA
FMIVEGVTSAGIRPVAEQLKQITDLSVPFVITSKGIEQNTGLLLSEIMLEVLGDSVTPYLGYLSGPSIAKEVLNG
SPCSVVVSAYDSQTLKQIHEAFSLPTFRVYPNTDIKGAALGGALKNVIAIACGIAEGLSFGNNAKAGLVTRGL
HEMRKLAAIMDCKPETLNGLAGLGDLCVTCFSESSRNLRFGHLLAQGLTFEQAKAKIGMVVEGAYTALSAY
QVAKHHKIDMPITTGIYRVLYENLDLKEGIALLLQRNTKEEFL

>core/453/1/Org1_Gene444

MKPLGFQENLEALCNKTSRQLLKYLIKQILFVCGASLLIALEFSFFLYFFLFSGKTVIPAFCLACFFLTLFVCLV
TRLYLLSGKGDFFEDLASEYLQGAVPPNKRSQNIVEEQSHLAAAATKLSINLQNQEYSLLSEIFKFLPKHDLIR
KFSCFCFWKDYFLFRECLLQKAIEAYIKVVQAIPVDLSAHVSLADAYVALSGLYADPRKYPEFDANYWIPSG

RYSAEIQEKFFATARRAIEEFQILNEYAPGNAWVHAQLAYSYHDLQMPMEEIQEYEIVLKLKPNDVETMSKL
GILYFQQGMNAKGLRIYEEIKKRDYKKSQKLIKFYGVEYKY

>core/455/1/Org1_Gene625

MQPFIFTLLCLTSLVSLVAFDAANARKRCACAQTIERGENFFSIKRSACAEIEYQEKSRHASAIERISKDKGKV
TPKQIAKVATKKKQRYRLLQVPFSRPPNNSRYNLYALLSEPPECYSDTASWYAIFIRLLRRAYVDTGNVPPGS
EYAIANALISNKQEILERGAQLGPDVIETLTLPEEQAEIFYKMLKGSSNSQSLLNFLHYEEKSLGHCKLNLIFM
DPLLLEAVLDHPDAYRETSLLRDGIWEAVKRQEHAIQEHGQAAALELFKTRTDFRLELRDKMQLLLSRYDLL
PLLNKKMFDYTLGSAGDYLFLVDPDTKAISRCRCPSKSIKL

>core/456/1/Org1_Gene952

MATNAIRSTGSAASKMLLPVAKEPAAVSSFAQKGIYCIQQFFTNPGNKLAKFVGATKSLDKCFKLSKAVSDC
VVGSLEEAGCTGDALTSARNAQGMLKTTREVVALANVLNGAVPSIVNSTQRCYQYTRQAFELGSKTKERKT
PGEYSKMLLTRGDYLLAASREACTAVGATTYSATFGVLRPLMLINKLTAKPFLDKATVGNFGTAVAGIMTIN
HMAGVAGAVGGIALEQKLFKRAKESLYNERCALENQQSQLSGDVILSAERALRKEHVATLKRNVLTLLEKA
LELVVDGVKLIPLPITVACSAAISGALTAASAGIGLYSIWQKTKSGK

>core/457/1/Org1_Gene803

MRIAVLGVTGLVGQKFVALLHKWYRDWVIAEVVASNSKYGQSYGDACIWQEPIGPMPEMVRDLPIRKIEEV
QSDIVVSFLPSSAESMEAYCLSQGKVVFSNASTYRMHSSVPIIIPEVNSDHFQLLEEQPYPGKIITSPNCCVSGIT
LALAPLRKFSLDHVHIVTLQSASGAGYPGVPSLDLLANTVPHIVGEEEKILRETVKILGSSKQPLPCKLSVTVH
RVPVAYGHTLSLHVTFSKDVDLDEILYSYQEKNKEFPNTYQLYDNPWSPQARKHLSHDDMRVHLGPITYGG
DFRTIKMNVLIHNLVRGAAGTLLASMENYFFDYLKREMCLR

>core/458/1/Org1_Gene454

MKTLWHFVSKAFLSIVGLCCGVVLAFVVIFALIASSLGNGDATFVSLPDAQGEVKDLGKTAPIIAVIEMKDVI
ASSKNTAKTIQNILEGFEKAPLKDRVKGIVIDMDCPGGEVFEIDRIYSMLRFWKERKGFPIYIYVNGLCASGGY
YVSCAATKIYATSSSLIGSIGVRSGPFFNVKEGLNRYGVESDLLTAGKDKAPMNPYTPWTSHDREERQATLDF
LYGQFVDIVTQNRPLLTKEKLVHTLGARIFSPEKAKQEGYIDVVGATKEQVLQDIVAVCKIEDNYRVIGSGGD
GWWKRVASAAASSPLVTGMIKHDILPLSHDAAYIPPYLAL

>core/460/1/Org1_Gene567

MSSLTLSRRPRRNRKTAAIRDLLAETHLSPKDLIAPFFVKYGNNIKEEIPSLPGVFRWSLDLLLKEIERLCTYGL
RAVMLFPIIPDDLKDAYGSYSSNPKNILCHSIHEIKNAFPHLCLISDIALDPYTTHGHDGIFLNGEVLNDESVRIF
GNIATLHAEMGADIVAPSDMMDGRIGYIRSKLDQSGYSKTSIMSYSVKYASCLYSPFRDALSSHVTSGDKKQ
YQMNPKNVLEALLESSLDEEEGADILMVKPAGLYLDVIYRIRQNTCLPLAAYQVSGEYAMILSAFQQGWLD
KETLFHESLIAIKRAGADMIISYSAPFILELLHQGFEF

>core/461/1/Org1_Gene101

MNIYQFSPGASPNWQASLMAQLNSYFCLGGETVTRIISLRPSGLILAKKEKAVVSTAEKILKILSFILFPLVLIAL
AIRYLLYNKFNKDLDRAVFFIPTEITKAEELIIAKNPALVKEAALTVSPLFYSLPKKYQLMKVETP

>core/462/1/Org1_Gene102

MLLEDLDTDSIPWPKLYLSEDFDFAYYPESKAIIDTVAKLEKNNPGEEFCLESKKILARYLLEQLFKLETGLNF
PTSTIDGGRESFLIEFSHETKKPTVWAFIYFYYYHSNGPKLEKDFKQAGCEVHNRLLNLGLKYRPQAGAQND
GRNGGPYGPIGFLIVWEENYGSVLKDHGFIKDN

>core/463/1/Org1_Gene735

MQFSRYLRYAFDNQYLPEPLYQKFSVFHQNYIDAATKKAAADQAEVLCLQWVKVIIEDLKNPFIFPPYHKKI
RAPIDLFRLSIDFFSLVIDDKNSRILNLHRLKEIEEYIARGDNVVLLANHQTECDPQLMYYALGKTHPELMEN
MIFVAGDRVTSDPLARPFSMGCDLLCIYSKRHIATPPELREEKLLHNQKSMQILKTLLNEGGKFIYVAPAGGR
DRKNAEGRLYPSEFSPESIEVFRLLAKASNQTTHFYPFALKTYDILPPPPKIENAIGEQRAIFFAPVFFNFGAELF
FDALCSKEELIHCDKHAQRTLRAEKVFSIVKNLYEEL

>core/464/1/Org1_Gene800

MREETVSWSLEDIREIYHTPVFELIHKANAILRSNFLHSELQTCYLISIKTGGCVEDCAYCAQSSRYHTHVTPEP
MMKIVDVVERAKRAVELGATRVCLGAAWRNAKDDRYFDRVLAMVKSITDLGAEVCCALGMLSEEQAKKL
YDAGLYAYNHNLDSSPEFYETIITTRSYEDRLNTLDVVNKSGISTCCGGIVGMGESEEDRIKLLHVLATRDHIP
ESVPVNLLWPIDGTPLQDQPPISFWEVLRTIATARVVFPRSMVRLAAGRAFLTVEQQTLCFLAGANSIFYGDK
LLTVENNDIDEDAEMIKLLGLIPRPSFGIERGNPCYANNS

>core/465/1/Org1_Gene343

MIDIMQHFKPYTMVPGQKLPIPGSLLYAQVFPTLWRLFSSKHEILNEQTLQVQGPLKRFAVFQDLHRGGLAV
TSERYKYYLLPSGECTQSIKGKLPSAAQAGPLLSLGVHKHADWQKVRCRRDLKEILPLWFRFAAMAPKGSY
RDLETTAIGSLVKTAHQRVLHRETTEIAPALLSIALAGFSECFLPRSYDEEFQGILPQDGDPEGGVPFELLSYSF
GMIQDIFLRHQGQLVEILPALPPEFPCGRLIHVALPNLGTLSIVWTKKTIRQVELHAEYSGEVFLKFCSSLCSAR
LREWSERRLSGSKRLSLGETLEIKAGTTYLWDCFHK

>core/466/1/Org1_Gene681

MSAFFDLLKSQTASHPPIWLLRQVGRYMPPYQELKGSQSLKTFFHNTEAIVEATLLGPSLLHVDAAILFADILS
ILDGFAVTYDFAPGPRIQFSPEQPFTFTSDPQTIFSYLLDAIRTLKQKLPVPLIVFAASPFTLACYLIDGGASKDF
SKTMSFLYVYPEKFDQLISTIIEGTAIYLKTQMDAGAAAVQLFESSSLRLPSALFTRYVTEPNRRLIAKLKEQAI
PVSLFCRCFEENFYTLQATQADTLHPDYHVDLHRIQKNLMLSLQGNLDPAIFLLPQEKLLHYVEAFLVPLRTY
PNFIFNSGHGILPETPLENVQLVVSYVQRQL

>core/467/1/Org1_Gene376

MPSPMISTDVCQDILGKQKEAVDFFFQAFQPKEAMQLAEKILGHSGWVFFSGVGKSGCVARKLVATLQSLSE
RALFFSPVDLLHGDLGLVSPGDIVCLFSKSGETQELLDTVPHLKSRRAILVAITSMPYSNLAALSDLVVILPSV
AELDPFNLIPTNSTTCQMIFGDFLAMLLFHSRGVSLSTYGKNHPSGQVGMKANGKVKDFMFPKTEVPFCHLG
DKVSFSLEVFSAYGCGCVCIVDPQFRLMGIFTDGDLRRSLASYGGEVLSLSLEKVMTANPRCITEDSDIAIALQ
LMESSSPVAVLPVLDNEENRHVTGLLHMHTLAKAGLL

>core/468/1/Org1_Gene788

MAFKEVVRVAVTGGKGQIAYNFLFALAHGDVFGVDRGVDLRIYDVPGTERALSGVRMELDDGAYPLLHRL
RVTTSLNDAFDGIDAAFLIGAVPRGPGMERGDLLKQNGQIFSLQGAALNTAAKRDAKIFVVGNPVNTNCWIA
MKHAPRLHRKNFHAMLRLDQNRMHSMLAHRAEVPLEEVSRVVIWGNHSAKQVPDFTQARISGKPAAEVIG
DRDWLENILVHSVQNRGSAVIEARGKSSAASASRALAEAARSIFCPKSDEWFSSGVCSDHNPYGIPEDLIFGFP
CRMLPSGDYEIIPGLPWEPFIRNKIQISLDEIAQEKASVSSL

>core/469/1/Org1_Gene220

MPKHKTLEIREALREAIDEEMSRDPNVCILGEEVGDYNGAYKVTKGLLDKWGPKRVIDAPISEAAFSGIGIGA
ALSGLRPIIEFMSWNFSFVALDQIISHAAKMHFMTGGKFSVPIVFRGPNGAAAQVSCQHSHCVESLYANIPGLI
IIAPSNPYDAKGLLKSAIRNNNPVLFLENELEYNLKGEVPTEEYLVPIGKAHRVQEGNDLTIITYSRMVSITKEA
CSLAKKRWGLSIEIIDLRTIKPLDISTILSSVRKTSRCIVIEEGHYFAGISSEIIALITEHVFDSLDAPPLRVCQKETP
MPYSKILEQATLPNVNRILDTIEKVMR

>core/470/1/Org1_Gene47

MSNQFDQLKKLSTIVCDSGDPELVKASGSQDATTNPSLILKVAQEPKFQELLNEAVVWGIRQNGDDLQTLSFI
LDKIQVNFALEIIKNIPGRISLEIDARLSFNVEAMVQRAVFLSQLFEAMGGDKKRLLVKIPGTWEGIRAVEFLE
AKGIACNVTLIFNLVQAIAAAKAKATLISPFVGRIYDWWIAAYGDEGYSIDADPGVASVSNIYAYYKKFGIPT
QIMAASFRTKEQVLALAGCDLLTISPKLLDELKKSQHPVKKELDPAEAKKLDVQPIELTESFFRFLMNEDAMA
TEKLAEGIRIFAGDTQILETAITEFIKQIAAEGA

>core/471/1/Org1_Gene445

MTTPAYLLANFGGPRHAKDLQEFLISLLTDRDVTGTFLPRVLHRHLFTFIAKKRVPKVLPQYQSLQNWSPIYF
DTETLAKTLSEILRAPVIPFHRYLPSTHEKTLLALRTLHTRHVIGIPLFPHFTYSVTGSIVRFFMKHVPEIPISWIP
QFGSDSKFVSLITCHIRDFLQKLGILEKECCFLFSVHGLPVRYISQGDPYSKQCYESFSAITTNFKQSENFLCFQS
KFGPGKWLSPSTAQLCQNIDTDKPNVIVVPFGFISDHLETLYEIERDYLPLLRSRGYRALRIPAIYSSPLWVSTL
VDIVKENSTVVAEELIKSGKKHTGIR

>core/472/1/Org1_Gene250

MDAKMGYIFKVMRWIFCFVACGITFGCTNSGFQNANSRPCILSMNRMIHDCVERVVGNRLATAVLIKGSLDP
HAYEMVKGDKDKIAGSAVIFCNGLGLEHTLSLRKHLENNPNSVKLGERLIARGAFVPLEEDGICDPHIWMDL
SIWKEAVIEITEVLIEKFPEWSAEFKANSEELVCEMSILDSWAKQCLSTIPENLRYLVSGHNAFSYFTRRYLATP
EEVASGAWRSRCISPEGLSPEAQISVRDIMAVVDYINEHDVSVVFPEDTLNQDALKKIVSSLKKSHLVRLAQK
PLYSDNVDDNYFSTFKHNVCLITEELGGVALECQR

>core/473/1/Org1_Gene372

MMTYPVPQNPLLLRILRLMDAFSKSDDERDFYLDRVEGFILYIDLDKDQEDLNKIYQELEENAERYCLIPKLT
FYEVKKIMETFINEKIYDIDTKEKFLEILQSKNAREQFLEFIYDHEAELEKWQQFYVERSRIRIIEWLRNNKFHF
VFEEDLDFTKNVLEQLKIHLFDAKVGKEITQARQLLSNKAKIYYSNEALNPRPKRGRPPKQSAKVETETTISSD
IYTKVPQAARRFLFLPEITSPSSITFSEKFDTEEEFLANLRGSTRVEDQLNLTNLSERFASLKELSAKLGYDSLST
GDFFGDDDEKVVTKTKGSKRGRKKSS

>core/474/1/Org1_Gene704

MHSELPNYQNIVESVVTEITTQLLNYRSEHRLVPFWEKSDGSFITAADYGSQYYLKQQLAKAFPNIPFIGEETL
YPDQDNEKIPEILKFTRLLTSSVSRDDLISTLVPPPSPTSLFWLVDPIDGTAGFIRHRAFAVAISLIYEYRPILSVM
ACPAYNQTFKLYSAAKGHGLSIVHSQNLDRRFVYADRKQTKQFCEASLAALNQQHHATRKLSLGLPNTPSP
RRVESQYKYALVAEGAVDFFIRYPFIDSPARAWDHVPGAFLVEEAGGRVTDALGAPLEYRKESLVLNNHAVI
LASGDQETHETTLAALQNQLNVVPTDKLIAL

>core/475/1/Org1_Gene546

MKTVTSFTVCKENSGRLDKYLTEVHPKYSRAFYQEHILSGLVQINGQINTRVATRLNCGDIVTIDIQEKEELLE
LLPEAIPLDKVYEDGMILVINKPRDMVVHPAPGHFHGTLVHALLHEIGERLKEEFPEEPWRPGIVHRLDKDTS
GLIITAKTRQAKKVFSELFSTKRLKKSYLAVCIGKPRSTTIHTHISRHQNKRKEMTVSSQGKEAVTHCQVLAF
NGKLSFVALSPETGRTHQLRVHMKHLGTPILGDPVYGIPSMNSSYGLDKQQLHAYSVDFTHPETRQFCSLKA
GLPEDMRSLLIKEFRNETTILNKNLLESILKEQ

>core/476/1/Org1_Gene190

MTIDMHCDLLSHPHFCRKDPAVRCSPEQLLSGGVRQQVCAIFVPHSRGEPNCDKQNSLFFSLPNQYPDIGLLS
YEEEENGSSSQKKSLSLIRSIENASALGDDTAPLGTLLAKLIHLTKQGPLAYLGIVWKGDNRFGGGTEAPKRL
SNDGKVLLDIMYELGVPIDLSHCSDKLAEDILDYTADKLPNLAVIASHSNFRSVLDHRRNLVDAHAKEIVRRK
GVIGLNLVRSYVGDSLGDLEKHVLHAENLGILSSIVLGSDFFYANEDENFFFNECSSAEAHPVLNQLIHRIFSK
GKAESILSSRAEKFLKQVIVEQVNPKITDVKL

>core/478/1/Org1_Gene850

MIEFAFVPHTSVTADRIEDRMACRMNKLSTLAITSLCVLISSVCIMIGILCISGTVGTYAFVVGIIFSVLALVACV
FFLYFFYFSSEEFKCASSQEFRFLPIPAVVSALRSYEYISQDAINDVIKDTMQLSTLSSLLDPEAFFLEFPYFNSLI
VNHSMKEADRLSREAFLILLGEITWKDCETKILPWLKDPNITPDDFWKLLKDHFDLKDFKKRIATWIRKAYPE
IRLPKKHCLDKSIYKGCCKFLLLAENDVQYQRLLHKVCYFSGEFPAMVLGLGSEVPMVLGLPKVPKDLTWE
MFMENMPVLLQSKREGHWKISLEDVASL

>core/479/1/Org1_Gene302

MELLPHEKQVVEYEKAIAEFKEKNKKNSLLSSSEIQKLEKRLDKLKEKIYSDLTPWERVQICRHPSRPRTVNYI
EGMCEEFVELCGDRTFRDDPAVVGGFVKIQGQRFVLIGQEKGCDTASRLHRNFGMLCPEGFRKALRLGKLA
EKFGLPVVFLVDTPGAYPGLTAEERGQGWAIAKNLFELSRLATPVIIVVIGEGCSGGALGMAVGDSVAMLEH
SYYSVISPEGCASILWKDPKKNSEAASMLKMHGENLKQFGIIDTVIKEPIGGAHHDPALVYSNVREFIIQEWLR
LKDLAIEELLEKRYEKFRSIGLYETTSESGPEA

>core/480/1/Org1_Gene514

MASNPILQIEDLSITLAKQRQQYPIVQSLSFTINEGQTLAIIGESGSGKSVSAHAILRLLPCPPFSVSGQVNFQGH
NLLTASRSIQKKIIGTEISMIFQNPQASLNPVFTIEQQFREIIHTHLALTAEVAKEKMLYALEETGFHDPRLCLNL
YPHQLSGGMLQRICIAMALLCSPKLLIADEPTTALDVSVQYQILQLLKTLQKKTGMSLLIITHNMGVVAETAD

DVLVLYAGRMVECAPAVQMFHNPSHPYTRDLLASRPSLQPQQLGSFNPIPGQPPHYTAFPSGCRYHPRCSKIL
NRCSAEAPEIYPVREGHKVRCWLYDD

>core/481/1/Org1_Gene247

MALGPSPYYGVSFFQFFSVFFSRLFSGSLFTGSLYIDDIQIIVFLAISCSGAFAGTFLVLRKMAMYANAVSHTVL
FGLVCVCLFTHQLTTLSLGTLTLAAMATAMLTGFLIYFIRNTFKVSEESSTALVFSLLFSLSLVLLVFMTKNAH
IGTELVLGNADSLTKEDIFPVTIVILANAVITIFAFRSLVCSSFDSVFASSLGIPIRLVDYLIIFQLSACLVGAFKA
VGVLMALAFLIIPSLIAKVIAKSIRSLMAWSLVFSIGTAFLAPASSRAILSAYDLGLSTSGISVVFLTMMYIVVK
FISYFRGYFSKNFEKISEKSSQY

>core/482/1/Org1_Gene515

MTTNFPQPLIQATSLTKHYYKRSFWFQGKTIASRPVDDVSFSLYSRRAVGLIGESGSGKSTLALALAGLLPLTS
GFLTFNGTPIKLHSKHGRHQLRSQVRLVFQNPQASLNPRKTILDSLGHSLLYHKLVPKEKVLATVREYLELVG
LSEEYFYRYPHQLSGGQQQRVSIARALLGVPQLIICDEIVSALDLSIQAQILNMLAELQKKLSLTYLFISHDLAV
VRSFCTEVFIMYKGQIVEKGNTKRIFSDPQHPYTRMLLNAQLPETPDQRQSKPIFQEYHKDSEESCSTGCYFY
NRCPQKQEACKSEIIPNQGDAHHTYRCIH

>core/483/1/Org1_Gene558

MEKKYYALAYYYITRVDNPHEEIALHKKFLEDLDVSCRIYISEQGINGQFSGYEPHAELYMQWLKERPNFSKI
KFKIHHIKENIFPRITVKYRKELAALGCEVDLSKQAKHISPQEWHEKLQENRCLILDVRNNYEWKIGHFDNAT
LPDIQTFREFPEYAEKLAQECDPETTPVMMYCTGGIRCELYSPVLLEKGFKEVYQLDGGVIAYGQQVGTGKW
LGKLFVFDDRLAIPIDESDPDVAPIAECCHCQTPSDAYYNCANTDCNALFLCCDECIHQHQGCCGEECSQSPR
VRKFDSSRGNKPFRRAHLCEISENSESASCCLI

>core/484/1/Org1_Gene439

MKQLLFCVCVFAMSCSAYASPRRQDPSVMKETFRNNYGIIVSGQEWVKRGSDGTITKVLKNGATLHEVYSG
GLLHGEITLTFPHTTALDVVQIYDQGRLVSRKTFFVNGLPSQEELFNEDGTFVLTRWPDNNDSDTITKPYFIET
TYQGHVIEGSYTSFNGKYSSSIHNGEGVRSVFSSNNILLSEETFNEGVMVKYTTFYPNRDPESITHYQNGQPHG
LRLTYLQGGIPNTIEEWRYGFQDGTTIVFKNGCKTSEIAYVKGVKEGLELRYNEQEIVAEEVSWRNDFLHGER
KIYAGGIQKHEWYYRGRSVSKAKFERLNAAG

>core/486/1/Org1_Gene484

MNLKVVYFGTPTFAATVLQDLLHHKIQITAVVTRVDKPQKRSAQLIPSPVKTIALTHGLPLLQPSKASDPQFIE
ELRAFNADVFIVVAYGAILRQIVLDIPRYGCYNLHAGLLPAYRGAAPIQRCIMEGATESGNTVIRMDAGMDT
GDMANITRVPIGPDMTSGELADALASQGAEVLIKTLQQIESGQLQLVSQDAALATIAPKLSKEEGQVPWDKP
AKEAYAHIRGVTPAPGAWTLFSFSEKAPKRLMIRKASLLAEAGRYGAPGTVVVTDRQELAIACSEGAICLHE
VQVEGKGSTNSKSFLNGYPAKKLKIVFTLNN

>core/488/1/Org1_Gene315

MSKGSSKHTVRINQTWYIVSFILGLSLFAGVLLSTIYYVLSPIQEQAATFDRNKQMLLAAHILDFKGRFQIQEK
KEWVPATFDKKTQLLEVATKKVSEVSYPELELYAERFVRPLLTDAQGKVFSFEEKNLNPIEFFEKYQESPPCQ

QSPLPFYVILENTSRTENMSGADVAKDLSTVQALIFPISGFGLWGPIHGYLGVKNDGDTVLGTAWYQQGETP
GLGANITNPEWQEQFYGKKIFLQDSSGTTNFATTDLGLEVVKGSVRTTLGDSPKALSAIDGISGATLTCNGVT
EAYVQSLACYRQLLINFSNLTHEKKTGE

>core/489/1/Org1_Gene462

MRLLSILKLHLFSLRSSSSLSPHYYHSCSRSMLHLLCRWKDADIMEWQQICNILSGVCSRMSGKLVSLQKETQ
DSCHQEHERIHLQYREQLSALEEEYRRREEAKNQDLEKLQQENTWLQNRLAEKLQQIRHQSDIIDEIKKELLQ
SVQRTEISEGRRLCYEHKIKQLEEQLQRYVSQHGAPSIEIEEDKSSAAYAEINRLKKSLIDLQQEKDIYIKTYHS
EIAKLREKLQRQEGAQTSSEVCSIEKLTEVQTDLAEKKKAIALLQDIVEDQYCQLRDLHKEKGMAMPSNTKL
DHLKGLLGKEPESEVDVVFSESKSLGS

>core/490/1/Org1_Gene738

MEVQIGIDLMGGDHSPLVVWQVLVDVLKSQSSTIPFAFTLFASEEIRKQIQEEFISDLPQEKFPKIISAENFVAM
EDSPLAAIRKKSSSMALGLDYLQEDKLDAFISTGNTGALVTLARAKIPLFPAVSRPALLVCVPTMRGHAVILD
VGANISVKPEEMVGFARMGLAYRQCLGDSKIPTIGLLNIGSEERKGTEAHRQTFRMLRETFGEAFLGNIESGA
VFDGAADIVVTDGFTGNIFLKTAEGVFEFLQRILGDKLEADIQRRLDYTFYPGSVVCGLSKLVIKCHGKACGS
SLFHGILGSINLAQARLCKRILSNLI

>core/491/1/Org1_Gene424

MLISISLATLPILAFSWASFIEPNWLRTTAIPWRLPKKHAHLHGLRIAQISDLHFHKRVPEKFLNKVSKSIKNFSP
DLIVFCGDLLCRARLEDKERLETFLNTLEAPLGVFAILGNHDYSSYISRNTKGEITCIPEEKSRPIQRAIIAVMQG
LFSSPSYRYDPNLTPQEPHPDLLKLLKNTPLTLLHNTTHVIPNTLNIVGLGDLFARQFHPEQAFKNYDPSLPGL
LLSHNPDGITRLQQYPGDFVLSGHSHGPQVTLSWPKFARKFFERLSGLENPYLARGYFVTKEGKQLYVNRGL
GGLKRIRFCSPPEICYITCSYD

>core/492/1/Org1_Gene765

MVLSSDLLRDDKQLDLFFASLDVKKRYLLALSGGSDSLFLFYLLKERGVSFTAVHIDHGWRSTSAQEAKELE
ELCAREGVPFVLYTLTAEEQGDKDLENQARKKRYAFLYESYRQLDAGGIFLAHHANDQAETVLKRLLESAH
LTNLKAMAERSYVEDVLLLRPLLHIPKSSLKEALDARGISYLQDPSNEDERYLRARMRKKLFPWLEEVFGKNI
TFPLLTLGEESAELSEYLEKQAQPFFSAATHQDSQGELPCPDCLIQQAFLCKWVMKKFFNNAGIAVSRHFLQ
MVYDHLSRSSCATLRMRNKIVIIKPGVVVID

>core/493/1/Org1_Gene502

MKFLLYVPLLLVLVSTGCDAKPVSFEPFSGKLSTQRFEPQHSAEEYFSQGQEFLKKGNFRKALLCFGIITHHFP
RDILRNQAQYLIGVCYFTQDHPDLADKAFASYLQLPDAEYSEELFQMKYAIAQRFAQGKRKRICRLEGFPKL
MNADEDALRIYDEILTAFPSKDLGAQALYSKAALLIVKNDLTEATKTLKKLTLQFPLHILSSEAFVRLSEIYLQ
QAKKEPHNLQYLHFAKLNEEAMKKQHPNHPLNEVVSANVGAMREHYARGLYATGRFYEKKKKAEAANIY
YRTAITNYPDTLLVAKCQKRLDRISKHTS

>core/494/1/Org1_Gene357

MNRRKARWVVALFAMTALISVGCCPWSQAKSRCSIDKYIPVVNRLLEVCGLPEAENVEDLIESSSAWVLTPE
ERFSGELVSICQVKDEHAFYNDLSLLHMTQAVPSYSATYDCAVVFGGPLPALRQRLDFLVREWQRGVRFKKI
VFLCGERGRYQSIEEQEHFFDSRYNPFPTEENWESGNRVTPSSEEEIAKFVWMQMLLPRAWRDSTSGVRVTF
LLAKPEENRVVANRKDTLLLFRSYQEAFPGRVLFVSSQPFIGLDACRVGQFFKGESYDLAGPGFAQGVLKYH
WAPRICLHTLAEWLKETNGCLNISEGCFG

>core/495/1/Org1_Gene848

MRKVAFLVSCLFSVAIGASAAPVRVPGFPQIPEDLVQIKTEVCPKQEVCLAVTIKCDDHNLIGVLHLPNTPTPE
GGFPTVVLFHGFRGTKFGGLTGAYRKLGRKFAAAGIATLRVDMAGCGDSEGVAEEVPIETYLRDAQTILETV
QEHPDLNAYRLGISGFSLGCHIAFELAKIYNPRDLNIKALSVWAPIADGGILLKELYENFSKHGEGDIISVGKDF
GFGPPPIIVCSGDVDLLIRIQDHVTANSLPTKPYILHQQGIDDTLVSRTQQTLFKNTAPGRMTFISYPNTGHNLA
TAPDLDMILDQIVSHFQRTL

>core/496/1/Org1_Gene324

MFKLLFHIAAFAGHVLSTPIFIVQDACGIDEEACKNPPPRPFSAQVQYLKVNDAKFKKLPHQTIGYRQYDGTF
LCTLPITEHSGLLFSTGYIGADIQWKSSLPISETDPNGLGWATFQDTSFYNYVLLSLGAYTLSLKNWQWSIILS
GLVDPKNIEMGYGLYQGVLSGKYQATEKLSAIFGVINETGLHQEKAWPLVGVSYKATDQLTLNCIYPVNFSI
DYRSTSVCNLGLAYRLTRFRKKLHKNHLISSRGIFEYQGREIEANVKLTPWPGSFIKGFYGWSIGNDISIADDH
NNNKTSHTFKTSAFFGGSAVMNF

>core/497/1/Org1_Gene142

MLVELEALKREFAHLKDQKPTSDQEITSLYQCLDHLEFVLLGLGQDKFLKATEDEDVLFESQKAIDAWNALL
TKARDVLGLGDIGAIYQTIEFLGAYLSKVNRRAFCIASEIHFLKTAIRDLNAYYLLDFRWPLCKIEEFVDWGN
DCVEIAKRKLCTFEKETKELNESLLREEHAMEKCSIQDLQRKLSDIIIELHDVSLFCFSKTPSQEEYQKDCLYQS
RLRYLLLLYEYTLLCKTSTDFQEQARAKEEFIREKFSLLELEKGIKQTKELEFAIAKSKLERGCLVMRKYEAA
AKHSLDSMFEEETVKSPRKDTE

>core/498/1/Org1_Gene277

MTLPMQKSLTSFDDFSQAYAEKVPAIALIGSALEDDKDALIELLVSESFKELGGQGLMPATLMSWTETFALFQ
EHETLGIIHAEKFPLATKEFLSRYARNPQPHLTILIFTTKQECFRELSKALPSALSLSLFGEWPADRQKRIIRLLL
QRAERVGISCSQSLASLFLRALASTSLPDILSEFDKLLCSVGKKTSLDHSDIKELVVKKEKASLWKFRDSLLKR
DPVEGHQQLHFLLEDGEDPLGIITFLRTQCLYGLRSIEEGSKENKHRMFVLYGKERLHQALNSLFYAETLIKN
NVQDPIVAVETLVIRMVNL

>core/499/1/Org1_Gene134

MFSYIKNRILFNLLSLWIVLTLTFLVMKTIPGDPFNDEGCNVLSEEVLQTLKSRYGLDKPLYQQYTQYLHSIAK
LDFGNSLVYKDRKVTNIISTAFPISAILGLQSLFLSIGGGIALGTIAALKKKKQRRYILGASILQISIPAFIFATLLQ
YVFAVKIPLLPIACWGSFTHTILPTLALAVTPMAFIIQLTYSSVSAALNKDYVLLAYAKGLSPLKVVIKHILPYA
IFPTISYSAFLTTTVITGTFAIENIFCIPGLGKWFICSIKQRDYPVALGLSVFYGTLFMLSSLLSDLIQSIIDPQIRYA
HGKEKKRK

>core/500/1/Org1_Gene246

MAMLPKFFLVLLCLGLCSCSQKTTTIEGEQMTIFYRIVLGTSLSAKEKASLSQQIDRCFHKIDSIYNNWNPYSE
LSIINRAPADVPITLSVELSEFLDQVDTLYKLSEGRFDPTVGPLKTLWLLHLKSQTLPPKDVWEQHYKDMGW
QHLEFQSNTKTLIKKNPHVQIDLCGVVKGYAVDCLNEICNTFCPNNYVEWGGEIKTSGHHPSGRPWRIFSEA
AGTILDIDDMAIATSGNHIQKWCVEGKIYTHILDTRTGKPLELSSYPIQSVSVVHPSCAYADAIATVLMTFDSK
IEAKQWAEEHHILTYINDGASS

>core/501/1/Org1_Gene945

MWRVVLRFLIIFILGRVVFPLRASESFSWETSTCLTVLGIPFIDIILTTNEDFVAQCGLQIGTISSTNNAKIKEIFLI
YKEKFPEASISFKRKEPLNLSQSHLSDLGILCMRNGETYAEGMANKENGPALKQPKDLRLVLRCPNQPDTLL
YSEKEAEKGIETNTCLCNQGYTLLDGQLILYGDSIEKFLKETKRKNNHTLVDLCDSQVVTTFLGRFWSLLNY
VQVLFLSEDSAKILAGIPDLAQATQLLSHTVPLLFIYTNDSIHIIEQGKESSFTYNQDLTEPILGFLFGYINRGSM
EYCFNCAQSSLGET

>core/502/1/Org1_Gene9

MIASIYSFLDYLKMVKSASPHTLRNYCLDLNGLKIFLEERGNLAPSSPLQLATEKRKVSELPFSLFTKEHVRM
YIAKLIENGKAKRTIKRCLSSIKSFAHYCVIQKILLENPAETIHGPRLPKELPSPMTYAQVEVLMATPDISKYHG
LRDRCLMELFYSSGLRISEIVAVNKQDFDLSTHLIRIRGKGKKERIIPVTSNAIQWIQIYLNHPDRKRLEKDPQA
IFLNRFGRRISTRSIDRSFQEYLRRSGLSGHITPHTIRHTIATHWLESGMDLKTIQALLGHSSLETTTVYTQVSV
KLKKQTHQEAHPHA

>core/503/1/Org1_Gene227

MIHSRLIIIGSGPSGYTAAIYASRALLHPLLFEGFFSGISGGQLMTTTEVENFPGFPEGILGPKLMNNMKEQAVR
FGTKTLAQDIISVDFSVRPFILKSKEETYSCDACIIATGASAKRLEIPGAGNDEFWQKGVTACAVCDGASPIFK
NKDLYVIGGGDSALEEALYLTRYGSHVYVVHRRDKLRASKAMEARAQNNEKITFLWNSEIVKISGDSIVRSV
DIKNVQTQEITTREAAGVFFAIGHKPNTDFLGGQLTLDESGYIVTEKGTSKTSVPGVFAAGDVQDKYYRQAV
TSAGSGCIAALDAERFLG

>core/504/1/Org1_Gene780

MRKLILCNPRGFCSGVVRAIQVVEVALEKWGAPIYVKHEIVHNRHVVNALRAKGAIFVEELVDVPEGERVIY
SAHGIPPSVRAEAKARKLIDIDATCGLVTKVHSAAKLYASKGYKIILIGHKKHVEVIGIVGEVPEHITVVEKVA
DVEALPFSSDTPLFYITQTTLSLDDVQEISSALLKRYPSIITLPSSSICYATTNRQKALRSVLSRVNYVYVVGDV
NSSNSNRLREVALRRGVPADLINNPEDIDTNIVNHSGDIAMTAGASTPEDVVQACIRKLSSLIPGLQVENDIFA
VEDVVFQLPKELRCS

>core/505/1/Org1_Gene636

MKCRPTLNTDQPRVRKKLPERFPKWLQRPLPQGSAFHATDATIKRSGMPTVCEEALCPNRAECWSRKTATY
LALGDVCTRSCGFCNIGHSKTPPALDPTEPERIALSAKELGLKHVVITMVARDDLEDGGAQGLVDIIQKLREE
LPQATTEVLASDFQGNVSALHTLLDSGITIYNHNVETVARLSPLVRHKATYARSMFMLEQAANYLPDLKIKS

GIMVGLGEMEGEVKQTLQDLASIGVRIVTIGQYLRPSRKHLQVKSYVTPETFDYYRRVGEAMGLFVYAGPFV
RSSFNADMILASVQDKASA

>core/507/1/Org1_Gene213

MKKRYAFLFPGQGSQYVGMGQDLYMEYPEVRELFDFANERLGFSLTSIMFEGPEDLLMETVHSQLAIYLHS
MAVVKVLSQRSSIQPSLVSGLSLGEYTALVASDRISVLDGLELVRKRGQLMNEACNQSPGAMAALLGLPSEV
IEENITSLGQGIWIANYNAPKQLVVAGIAEKVDQAIELFRDLGCKKAVRLKVSGAFHTPLMQVAQDGLAPDI
YALCMKDSSLPLVSHVVGKSLVNTEEMRECLARQMTSPTLWYQSCYHIESEVDEFLELGPGKVLAGLNRSIG
ISKPITSLGTFAQIEKFLSEV

>core/508/1/Org1_Gene413

MLNSNKFKSKTGAYGDLFQRVVVHSLVLTFLVLLLYSSLFPLTSFALGFITATCGAVGTYEYSSMAKAKMHY
PLSTFSAIGSFLFLALSFLSIRWGHSLPGFFDALPWTLLIVWVVWSIFRVRKSTIGALQLSGVTLFSILYVGIPIRL
FLHVLYSFIHTQEPYLGIWWASFLIATTKGADIFGYFFGKAFGNKKIAPQISPNKTVVGFVAGCLGATLISFIFF
LQIPTRFASYFPMPAILIPLGLALGITGFFGDIIESIFKRDAHLKNSNKLKAVGGMLDTLDSLLLSTPIAYLFLLIT
QSKEFIG

>core/509/1/Org1_Gene28

MRLFSYDKPKIKVQKIKADGFSGWLKCNHCHEMIHANELGQNYNCCPKCSYHYRITAIERVKLLADKDSWR
PLYTDLKSQDPLEFIDTDTYANRLEKARKNTTESEGVIVGICTIGLHPVALAVMDFNFMAGSMGAVVGEKLT
RLIEEAIETRLPVIIVSASGGARMQESVFSLMQMVKTSAALAKLHEAGLPYISVLTNPTSGGVTASFAALGDIII
AEPKALICFAGPRVVAQVIGEDLPEGAQKSEFLLEHGMIDKIVERKELKTTLQTLLDYFLAQEYTGGKSKAPR
DLSKRLKEIFLLTDDSE

>core/510/1/Org1_Gene922

MEIAYSLTSSFSVDSVTVGFFDGCHLGHSNLLSILTSYSGSSGVITFDSHPQTVLSLNHTKLINTKEERLQLLQT
FPIDWLGVLTFDLNFANQSAEEFLTLLHRNLKCKRLILGYDSCIGKEQQSNTEALDTIGKPLGIEVIKIPPYRMD
NIVVSSKAIRQFLSAGNLECAHRFLGHPYAISGKITEGSGIGGSLGFATINLPREESLIPLGVYACEIRYDSTTCQ
GVMNLGTAPTFGRESLYAEAHIFSFAENLYGKEVSIIPRKFLREEKKFQSKETLIRAIEKDILDAQDWFAKGSF
NYEGTA

>core/511/1/Org1_Gene10

MSSRELIILGCSSQQPTRTRNQGAYLFRWNGEGLLFDPGEGTQRQFIFANIAPTTVNRIFVSHFHGDHCLGLGS
MLMRLNLDKVSHPIHCYYPASGKKYFDRLRYGTIYHETIQVVEHPISEEGIVEDFGSFRIEAQRLQHQVDTLG
WRITEPDTIKFLPKELESRGIRGLIIQDLIRDQEISIGGSTVYLSDVSYVRKGDSIAIIADTLPCQAAIDLAKNSCM
MLCESTYLEQHRHLAESHFHMTAKQAATLAKRAATQKLILTHFSARYLNLDDFYKEASAVFPNVSVAQEYR
SYPFPKNPLLNK

>core/512/1/Org1_Gene629

MRSIFRFSLCFFTLSVSCCFADASLYENSCPSRCQPTPPPSNSNPLNVVQQPVAASSVPSYMPPLNADDVLPRD
HLSDGSFSDTYPDITTQAIILIFLALSPFLVMLLTSYLKIIITLVLLRNALGVQQTPPSQVLNGIALILSIYVMFPT

GVAMYKDARKEIEANTIPQSLFTAEGAETVFVALNKSKEPLRSFLIRNTPKAQIQSFYKISQKTFPSEIRAHLTA
SDFVIIIPAFIMGQIKNAFEIGVLIYLPFFVIDLVTANVLVAMQMMMLSPLSISLPLKLLLIVMVDGWTLLLQGL
MISFK

>core/513/1/Org1_Gene757

MKEAAPMHFPFPVRRSVWLNRYSTFRIGGPANYFKAIHTIEEAREVIRFLHSINYPFLIIGKGSNCLFDDRGFD
GFVLYNAIYGKQFLEDARIKAYSGLSFAALGKATAYNGYSGLEFAAGIPGSVGGAIFMNAGTNESDISSVVRN
VETINSEGELCSYSVEELELSYRSSRFHRQQEFILSATFQLSKKQVSADHSKSILQHRLMTQPYTQPSAGCIFRN
PEGTSAGKLIDAAGLKGLAIGGAQISPLHANFIINTGKATSDEVKQLIAIIQSTLKTQGIDLEHEIRIIPYQPKIHS
PVSEK

>core/514/1/Org1_Gene999

MSLFLVFLTAFIWSSSFALSKLVMNASAPIFATGARMVIAGAILALAAWFRGGFVGISKKIFLYIVLLALTGFY
LTNIFEFIGLQSLSSSKTCFIYGLSPLMSALFSYIQLKEKVTLKKVLGLSLGLVSYICYLTFGGGGDDSQPWTW
QIGLPELLILGAASLASFGWTLLRQIEKQSTLSVTAINAYAMLIAGMLSIMHSAVVEPWRPLPVQDISQFLYAT
LALVVISNLICYNLYAKLLRKYSSTFLSFCSLVMPLYSGFYGWILLGEKGVSLGLVLAVAFMVAGCRLIYHEE
FRQGYIVS

>core/515/1/Org1_Gene641

MQKPQYIDRITKKKVIEPIFYEKTMLFLYNSKLGKKLSVFLSTHPIFSRIYGWLQRCSWTRRQIRPFMNRYKIS
EKELTKPVADFTSFNDFFTRKLKPEARPIVGGKEVFITPVDGRYLVYPNVSEFDKFIVKSKAFSLPKLLGDHEL
TKLYAHGSIVFARLAPFDYHRFHFPCDCLPQKTRCVNGALFSVHPLAVKDNFILFCENKRTVTVLETEQFGNV
LYLEVGAMNVGSIVQTFSPNQTYAKGDEKGFFAFGGSTVILLFLPNAIRFDNDLLKNSRMGFETRCLMGQSL
GRSQREEI

>core/516/1/Org1_Gene786

MTMPSTQFHTTILEQFSLFLSVDRGLCQQSIAAYRQDISSFLTISAISSPQDISQNSVYIFAEELYRRKEAETTLA
RRLIALKVFFLFLKDQQLLPYPPIIEHPKIWKRLPSVLTPQEVDALLAVPLQMEKNPRHLAFRDTAILHTLYST
GVRVSELCDLRLGHVSDDCIRVTGKGSKTRLVPLGSRAREAIDAYLCPFRDQYQKKNPHEDHLFLSTRGHKL
ERSCVWRRIHNYAKQVTSKPVSPHSLRHAFATHLLDNKADLRVIQEMLGHARIASTEVYTHVAADSLIEKFL
AHHPRNL

>core/517/1/Org1_Gene296

MLKIDLTGKVAFVAGIGDDQGYGWGIAKLLAEAGATIIVGTWVPIYKIFSQSWELGKFNESRKLSNGTLLEIA
KIYPMDASFDSPEDVPEDIAENKRYKGITGFTISEVAEQVKKDFGHIDILVHSLANSPEISKSLLETSRKGYLAA
LSASSYSFVSLLSHFGSIMNRGGSTISLTYLASMRAVPGYGGGMSSAKAALESDTKTLAWEAGRRWGIRVNT
ISAGPLASRAGKAIGFIERMVDYYQEWAPIPEAMNAEQVGAVAAFLASPLASAITGETLYVDHGANVMGIGP
EMFPKDS

>core/518/1/Org1_Gene815

MPPPFVVTLTTSAQNNLRDQLKEKNFIFSQPQNTVFQARSNTVTCTLYPSGKLVIQGKGSEEFIEFFLEPEILHT
FTHARVEQDLRPRLGVDESGKGDFFGPLCIAAVYASNAEILKKLYENKVQDSKNLKDTKIASLARIIRSLCVC
DVIILYPEKYNELYGKFQNLNTLLAWAHATVINNLAPKPAGDVFAISDQFAASEYTLLKALQKKETDITLIQK
PRAEQDVVVAAASILARDAFVQSIQKLEEQYQVQLPKGAGFNVKAAGREIAKQRGKELLAKISKTHFKTFDE
ICSGK

>core/519/1/Org1_Gene192

MHLEEENQGWEALLRKVYHQEVPPAILLHGFTLPVLQDKAEQLASEILLSSSPGSEHKVSQKIHPDIYQFFPEG
KGRLHSIDLPRGIKKQIYISPFEANYKIYIIHEADRMTLAAISAFLKVFEEPPKHAVIILTTAKVQRLPKTIISRSLS
IFIERGEKILCSKETFSYLFRYAQCEIPVTEVSQIIKESSETDKQVLRDKVQRFMEVLLELYRDRYTLNLGLKAS
ALNYPEHVKEILQLPLLPLDKVLLIVESACRSLNNSSSAASVLEWVAIQLVSLQYKEKELVSVSPGQDLSN

>core/520/1/Org1_Gene186

MRLNYFLNLVNFKYSIFSILFLSASTVFALSINEISQNLSFKEGFKISVFGAIAFVFARTTGIVVNQCIDRFIDKKN
TRTSKRVLPANLVSLNFAWVLSLFCSFLFLFLCKILRIFSLGIASLTLMIVYPYMKRVTFFCHWGLGLVYTVAI
LMNFCAFAESGLSMRLCFLALLWGGSVGMVIAANDIIYAIEDTEFDREEGLRSVPAHYGEKKAVEIAKVNLW
VSYLAYIFSGFVGSLDKEFYFTAIIPLVVILKVVRMYSNYSKKDQEGESKFFLANIAIALSFLVSMTLFWSLSR

>core/521/1/Org1_Gene308

MSERAHIPVLVEECLALFAQRPPQTFRDVTLGAGGHAYAFLEAYPSLTCYDGSDRDLQALAIAEKRLETFQD
RVSFSHASFEDLANQPTPRLYDGVLADLGVSSMQLDTLSRGFSFQGEKEELDMRMDQTQELSASDVLNSLKE
EELGRIFREYGEEPQWKSAAKAVVHFRKHKKILSIQDVKEALLGVFPHYRFHRKIHPLTLIFQALRVYVNGED
RQLKSLLTSAISWLAPQGRLVIISFCSSEDRPVKWFFKEAEASGLGKVITKKVIQPTYQEVRRNPRSRSAKLRC
FEKASQ

>core/522/1/Org1_Gene965

MSRHEICPEVSHKKGKYYSTFIFRCIHSLAGIAFTFFLCEHLFTNMLASSYFSQGKGFVAMVNGFHKIPGLKIIE
VAGLVLPFLCHAIIGIVYLFQGKSNCYSGDGSRPHLRYAKNYSYTWQRWTAWILLFGIAFHVVHLRFIRYPVH
VDIHGTTYYAVDIQPSRYDVIVRGTKGFLTLNLPNTEASSIEVSRHDLGGADAALLSERNSYLLTPSAGTAFLY
VVRDALGSLFIALLYTILVIAAAFHGFNGLWTFCCRWGVVVSLRMQGVLRIVCYLAMTVVTFMGVSAVWN
LYSVA

>core/524/1/Org1_Gene163

MRRFLFLILSSLPLVAFSADNFTILEEKQSPLSRVSIIFALPGVTPVSFDGNCPIPWFSHSKKTLEGQRIYYSGDSF
GKYFVVSALWPNKVSSAVVACNMILKHRVDLILIIGSCYSRSQDSRFGSVLVSKGYINYDADVRPFFERFEIPD
IKKSVFATSEVHREAILRGGEEFISTHKQEIEELLKTHGYLKSTTKTEHTLMEGLVATGESFAMSRNYFLSLQK
LYPEIHGFDSVSGAVSQVCYEYSIPCLGVNILLPHPLESRSNEDWKHLQSEASKIYMDTLLKSVLKELCSSH

>core/525/1/Org1_Gene184

MSHGPRPTKFSFPLYFSKTLSWFILGGFLAACGVQMVLVPNELIDGGIVGLSIIASHFLGHKALPFCLVLFNLPF
VFLAFKQIGKYFVIQMLTAVIIFSCSLWLIDQLPSWLGMSPFVFKGSEMETVVLGGAIIGVGCGLIIRHGGSTD

GTEILGIINKKKGYTVGQIILFVNFFIFALSGIVYKNWHTAFVSFLTYGIATKVMDMVILGLEDTKSVTIITSSP
RKLGHILMETLGIGLTYIHAEGGYSGEPRNLLYVVVERLQLSQLKEIVHREDPSAFIAIENLHEVINGRRT

>core/526/1/Org1_Gene685

MQEKPRHVHRIIHISDVHFHVLPVNPVHCFNKRLKGLLRKVFGLVHFQATTIGQRFPKVVRSLGADSVCITGD
FSLTAMDGEFLLAKHFVETLAKHSSVYLLPGNHDVYTLKSLAQQTFYTHFPNDQLQQNKVSFHKITDHWWL
ILLDCSCLNGWFSANGVVHLAQISAIETFLLSLSPEENVIIANHYPLLSSQNPSHDLINNTHLQNVLKKYPKVRL
YLHGHEHQAAVYNCADTSPSYILNSGSISLPTNSRFHVIDLYPEKYQVHTMILKNLLDFDAPLEIANEATWDC
QKL

>core/527/1/Org1_Gene297

MEKLLVTDIDGTITHQSHHLDKKVYERLYALHQAGWKLFFLTGRYYKYAARLFSDFDAPYLLGCQNGASV
WSSTSSNLLYSKSLPSDLLCILQDCMEGATALFSVESGAPYGDHYYRFSPTPIAQDLHEYVDPRYFPNAKEREI
LFETRSLKDDYAFPSFAAAKVFGLRDEVIRIQKELERQEALTSVATMTLMRWPFDFRYAILFLTDKSVSKGKA
LDRVVNILYDGKKPFVMASGDDANDLDLIERGDFKIVMSSAPEEMHVHADFLAPPADKNGILSAWEAGVRY
YDDLMSL

>core/528/1/Org1_Gene501

MKKLILYFAAFVASLFCGVFLWDRVPCAQKIMRLAADHSSEVFSKSCRFVRKISGFEELQVFERHVSPEQALA
LFPEYRDGKSFVELAFIPHTLMHVRFSKEEPVKKHIISQEGEILWSLVNGEMVLHTGTWTCSKGFRECLLLHA
GKQDMRVIQTLATLGGTTSRESLAQALALKNIRAERVIKECQKKKLIFASGNQIGTHFQQFQPIRGCTTTLNN
NPVWLQKPRHAAVFPAQYSEDRVRHLVKMIFGDNFLIVRSSMVYVPVYKISLVSADNSVRVEYINAVTGKSF
QDL

>core/529/1/Org1_Gene747

MFHSLSKNTPIITQGITGKAGSFHTEQCLAYGTNFVGGVTPGKGGTLWLDLPVYDSVLEAKQATGCRATMIF
VPPPYAAEAILEAEEAGIELIVCITEGIPVRDMLEVARVMDNSTSQLIGPNCPGIIKPGECKIGIMPGYIHLPGNI
GVVSRSGTLTYEAVWQLTQLKIGQSICVGIGGDPLNGTSFIDVLQALEEDPYTELILMIGEIGGSAEEEAAAWI
QAHCTKPVVAFIAGVTAPKGKRMGHAGAIISGNSGDAKSKIQVLRESGVTVVESPAHIGKTVDAVLRAKEL

>core/530/1/Org1_Gene683

MSKHTSESRIAQDMLERYSGSSVKQFCPYLLLTNFSYYIQTFAKLHGVPVFEGSMFSAAHAPHLKTSILDFKL
GSPGAALTIDLCSFLPDLKAALMLGMCGGLRSHYQVGDYFVPVASIRGEGTSDAYFPPEVPALANFVVQKAT
TEVLEDKKANYHIGITHTTNIRFWEFNKKFRKKLYETKAQSAEMECATLFAAGYRRNLPIGALLLISDLPLRK
EGIKTKSSGNFIFNTYTEDHILTGQEVIENLEKVMLKRAASDHKKDQQYRGLPHMEVGEADDTMASGSETSD
SDY

>core/531/1/Org1_Gene556

MKVLPPPSIPLLGAHTSTAGGLKNAIYEGRDIGASTVQIFTANQRQWQRRALKEEVIEDFKAALKETDLSYIM
SHAGYLINPGAPDPVILEKSRIGIYQEILDCITLGISFVNFHPGAALKSSKEDCMNKIVSSFSQSAPLFDSSPPLV

VLLETTAGQGTLIGSNFEELGYLVQNLKNQIPIGVCVDTCHIFAAGYDITSPQGWEDVLNEFDEYVGLSYLRA
FHLNDSMFPLGANKDRHAPLGEGYIGKESFKFLMTDERTRKIPKYLETPGGPENWQKEIGELLKFSKNRDS

>core/532/1/Org1_Gene392

MLSSLIRDSFPLLILLPTFLAALGASVAGGVMGTYIVVKRIVSISGSISHAILGGIGLTLWIQYKLHLSFFPMYGA
IVGAIFLALCIGKIHLKYQEREDSLIAMIWSVGMAIGIIFISRLPTFNGELINFLFGNILWVTPSDLYSLGIFDLLV
LGIVVLCHTRFLALCFDERYTALNHCSVQLWYFLLLVLTAITIVMLIYVMGTILMLSMLVLPVAIACRFSYKM
TRIMFISVLLNILCSFSGICIAYCLDFPVGPTISLLMGLGYTASLCVKKRYNPSTPSPVSPEINTNV

>core/533/1/Org1_Gene775

MKRNDPCWCGSGRKWKQCHYPQPPKMSPEALKQHYASQYNILLKTPEQKAKIYNACQITARILDELCKASQ
KGVTTNELDELSQELHKKYDAIAAPFHYGSPPFPKTICTSLNEVICHGIPNDIPLKDGDIMNIDVSCIVDGYYG
DCSRMVMIGEVPEIKKKICQAALECLNDSIAILKPGIPLCEIGEAIEARADTYGFSVVDQFVGHGVGIEFHENPY
VPHYRNRSMIPLAPGMIFTIEPMINVGKKEGVVDPKNQWEARTCDNQPSAQWEHTIAITETGYEILTLLND

>core/534/1/Org1_Gene805

MHLLTATVTPFFPNGTIDFASLERLLSFQDAVGNGVVLLGSTGEGLSLTKKEKQALICFACDLQLKVPLFVGT
SGTLLEEVLDWIHFCNDLPISGFLMTTPIYTKPKLCGQILWFEAVLNAAKHPAILYNIPSRAATPLYLDTVKAL
AHHPQFLGIKDSGGSVEEFQSYKSIAPHIQLYCGDDVFWSEMAACGAHGLISVLSNAWPEEAREYVLNPQEQ
DYRSLWMETCRWVYTTTNPIGIKAILAYKKAITHAQLRLPLSIEDFDLENVSPAVESMLAWPKLRTSVFSYS

>core/535/1/Org1_Gene1008

MFKFFRNKLQSLFKKNISLDLIEDAESLFYEADFGTELTEELCARLRWTKKADASTIKDLITVLLRESLEGLPS
QASQSSQTRPIVSLLLGTNGSGKTTTAAKLAHYYKERSESVMLVATDTFRAAGMDQARLWANELGCGFVSG
QPGGDAAAIAFDGIQSAIARGYSRVIIDTSGRLHVHGNLMKELSKIVSVCGKALEGAPHEIFMTVDSTLGNNAI
EQVRVFHDVVPLSGLIFTKVDGSAKGGTLFQIAKRLKIPTKFIGYGESLKDLNEFDLDLFLNKLFPEVEKI

>core/536/1/Org1_Gene180

MKRRNLQKILPNASTPSTNVAENTGIKDQNLFLDQATLNVDGNVDIENFLETRDLKVADTITSPCEFTVGGGL
SAESSQFKATTLSKGLEITSEDQDGRVPKFTNVSDPQSPRDALTYNYYRNTGCQALNLYTYYSSSQPTTVGKP
IETVCQNPNPETYRISASAKIYDAVTRFPYIQFKAPGIYQVTIQIRRESGQHSGLDNPNLYLNLMIGNNKTLLCA
SDTRGYSGGHRTSIAVTGTFTLTEIVATPPHDYPWLFLETTIGLDIKSMSTCVIWFPFQANFAEVD

>core/537/1/Org1_Gene889

MQIPRSIGTHDGSFHADEVTACALLIIFDLVDENKIIRSRDPVVLSKCEYVCDVGGVYSIENKRFDHHQVSYDG
SWSSAGMILHYLKEFGYMDCEEYHFLNNTLVHGVDEQDNGRFFSKEGFCSFSDIIKIYNPREEEETNSDADFS
CALHFTIDFLCRLRKKFQYDRVCRGIVREAMETEDMCLYFDRPLAWQENFFFLGGEKHPAAFVCFPSCDQWI
LRGIPPNLDRRMEVRVPFPENWAGLLGKELSKVSGIPGAVFCHKGLFLSVWTNRESCQRALRLTLQDRGII

>core/538/1/Org1_Gene627

MGISLPELFSNLGSAYLDYIFQHPPAYVWSVFLLLLARLLPIFAVAPFLGAKLFPSPIKIGISLSWLAIIFPKVLA
DTQITNYMDNNLFYVLLVKEMIIGIVIGFVLAFPFYAAQSAGSFITNQQGIQGLEGATSLISIEQTSPHGILYHYF
VTIIFWLVGGHRIVISLLLQTLEVIPIHSFFPAEMMSLSAPIWITMIKMCQLCLVMTIQLSAPAALAMLMSDLFL
GIINRMAPQVQVIYLLSALKAFMGLLFLTLAWWFIIKQIDYFTLAWFKEVPIMLLGSNPQVL

>core/539/1/Org1_Gene569

MLHALDTYRPSIESAIEKALEGFGPIGHPIRSPVEYALQGGGKRLRPGLVCMMAQGLGLNHDVMDSALAVEF
VHTSTLIADDLPCMDNDDERRGRPTVHKAFDEATALLASYALIPAAYSHLRLNAKKLKEQGCDPREIDIAYNI
IGDITDKNIGCSGVLGGQYDDMFFSNRGQEHVQSIMIKKTGSLFEIACISGWLFGGGDPQFAPIITSFSNNFGLL
FQIKDDFSDLQKDSQQIGLNYALLFGEKAALELLARSQNNCLELLDRLSAGGLKNSSEFETIISSLGSF

>core/540/1/Org1_Gene179

MSNPTPKTKISIPTFVRFNIQSINLTEDQKKTTFTVGGKVTTENTVVRGDLTCTDGGLTCQSDLTIQKDINIRPT
STNSMVFDGRLNLSNSPLSYKNSQGQDITDYEKMSSGKPQEYVPFGYYKRTQIMMAQRAAHSSGYVGGGSV
PSGSYVPWNKFDQTSTQKTSGTEIYIDPNDSTKLVFEVNNKVPKLFRISVIMAKHGSWLDNGTGADILLAANE
YEQGGGRINVTDLAMTTSRGSSYYETRPLQVVCVTYYAQNNGYFTFQNRAGGGLRVSFFSWNIVALPYVE

>core/541/1/Org1_Gene819

MLKIQKKRMCVSVVITVGAIVGFFNSADAAPKKKKIPIQILYSFTKVSSYLKNEDASTIFCVDVDRGLLQHRY
LGSPGWQETRRRQLFKSLENQSYGNERLGEETLAIDIFRNKECLESEIPEQMEAILANSSALVLGISSFGITGIPA
TLHSLLRQNLSFQKRSIASESFLLKIDSAPSDASVFYKGVLFRGETAIVDALSQLFAQLDLSPKKIIFLGEDPEV
VQAVGSACIGWGMNFLGLVYYPAQESLFSYVHPYSTATELQEAQGLQVISDEVAQLTLNALPKMN

>core/542/1/Org1_Gene11

MSHLIPSLRNSVTSYFHKPQPIKQAAPSKSIRDICNIAYLIIICVLVVVVLVGAMLCMFIPSVGIPLCLSSLALLV
LLSIFNPCLINWISTKKTKEIAPKDASESQPTKSASRKGSPQLSPHHDHEPKNFIRTQLEKGVNYVTNKFKSGEE
SPHISDEHHSPRQSKRSSEIESSDESSPELHRKAKGKAPHTATTKESKTSTTESSKKKKKTKHSLHRTTSSIHKR
SAPKPMVPSKKRKPVLLKKTVPLPIEDLEHQSSGNESSDSSSPPPVQRKAILPWFCKQPTDP

>core/543/1/Org1_Gene71

MEIKKAIQEGTAYLDYYGVPLSDCEALYILMDLLEVSSRAKLFDLVGISETMLMEYRKRLALRGQRCPTAYL
NGAVSFLGLRLRVDSRVLIPRTETELLAEYIINYLLSHSEIQTFYDICCGSGCLGLAIKKSCPHVEVVLSDVCPQ
AVAVANENAKSNGLDVKILLGDLSAPYTRPADAFVCNPPYLSFNEIIHIDPEVRCYEPWKALVGGSTGLEFYQ
RIAQELPKIVTSTGVGWLEIGSSQGESIKNIFSKHGIYGRLHQDLSGRDRIFFLEMDGRDPVSSGAYS

>core/544/1/Org1_Gene224

MAVIYWDRSKIVWSFEPWSLRLTWYGVFFTVGIFLACLSARYLALSYYGLKDHLSFSKSQLRVALENFFIYSI
LFIVPGARLAYVIFYGWSFYLQHPEEIIQIWHGGLSSHGGVLGFLLWAAIFSWIYKKKISKLTFLFLTDLCGSVF
GIAAFFIRLGNFWNQEIVGTPTSLPWGVVFSDPMQGVQGVPVHPVQLYEGISYLVVSGILYFLSYKRYLHLGK
GYVTSIACISVAFIRFFAEYVKSHQGKVLAEDCLLTIGQILSIPLFLFGVALLIICSLKARRHRSHI

>core/545/1/Org1_Gene245

MLLRGIPAAEKILQRLKEEISQSPTSPGLAVVLIGNDPASEVYVGMKVKKATEIGIISKAHKLPSDSTLSSVLKL
IERLNQDPSIHGILVQLPLPKHLDSEVILQAISPDKDVDGLHPVNMGKLLLGNFDGLLPCTPAGIIELLNYYEIP
LRGRHAAIVGRSNIVGKPLAALMMQKHPQTNCTVTVLHSQSENLPEILKTADIIIAALGAPLFIKETMVAPHA
VIVDVGTTRVPADNAKGYTLLGDVDFNNVVTKCAAITPVPGGVGPMTVAMLMSNTWRCYQNFS

>core/546/1/Org1_Gene516

MTEEISKDTIIEVAIDDIRVSPFQPRRVFSNEELQELIASIKAVGLIHPPVVREICTGDRVLYYELIAGERRWRAM
QLAGATTIPVILKHVIADGTAAEATLIENIQRVNLNPIEMAEAFKRLIHVFGLTQDKVAYKVGKKRSTVANYL
RLLALSKTIQESLLQGQITLGHAKVILTLEDPILREKLNEIIIQEHLAVREAELIAKQLISEEGSSIELKPTPLDMA
ESSKQHEELQQRLSDLCGYKVQIKTRGSKATVSFHLQNTQDLQKLEAWLSSHGTLSESLS

>core/547/1/Org1_Gene919

MPDKKAQITFSLPEVMSAIHQGKIVALPTDTVYGFVLSLYAPEAEERLYALKDREPSKAFALYVNSIEDIENIS
GYPLSPTAKKLAQLFPGAITLVVKHRNPRFPKETLAFRIVDHSVVREIVDHCGTLIGTSANLSEFPSALTAQEIF
ADFADHDLCIFDGPCSHGLESTVVASDPLYIYREGLISRSVIENIAGTEAKIFHRTSHAFSKHIKIYTVKNQEQL
VSFLSGSLDFKGVVCEHPKPKNFYTRLREALKKKTPSIVFIYDINTSDYPELFPFLSPYYIE

>core/548/1/Org1_Gene918

MKNNINNNECYFKLDSTVDGDLLAANLKTFDTQAQGISSTETFSVQGNATFKDQVSATGLTSGTTYNLNAQ
NFTSSQISIDFKNNRLSNCALPKEDCDPVPANYVRSPEYFFCSKPLIGDFDFNSGESYLPLTGSEYTLYQSRNVN
SIFRFIGWKQSTRELTVGGNTAIQFLAAGTYIVSFTVGKRWGWNNRWGGAIYINNGLGQVQCESTIYSGGGY
ATIGTLGTSIYRASVDVAPNPNDPNASDRYRAGIFYLSNGGSSAGIGNYSFSLLYYPDDRG

>core/549/1/Org1_Gene347

MHEVLILTFTYPLPRTLKQHPDEVHTVPISPNLSFGEGSPILIAGPCTLESYEHTVSSALTVKEAGAQVFRGSIR
KPRTSPFSFQGWEKECVLWHKEAQSIHGLPTETEVLDVRDVEITAEHVDILRIGAKNMHNTPLLQEVSKSHRP
IILKRSPAATLEEWLCAAEYILASSPSCPGVILCERGIRTFEHSTRYTLDLNTVALLKEISSLPVIVDPSHAAGKR
SLVLPLASAGLSVGADGLMIEVHAHPEKALCDAKQQITPEELHLFAKKHFCPSESRAHAIS

>core/550/1/Org1_Gene173

MKHYLSFSPSADFFSKQGAIETQVLFGERVLVKGSTCYAYSQLFHNELLWKPYPGHSFRSTLVPCTPEFHIHP
NVSVVSVDAFLDPWGIPLPFGTLLHVNSQNTVIFPKDILNHMNTIWGSGTPQCDPRHLRRLNYNFFAELLIKD
ADLLLNFPYVWGGRSVHESLEKPGVDCSGFINILYQAQGYNVPRNAADQYADCHWISSFENLPSGGLIFLYP
KEEKRISHVMLKQDSSTLIHASGGGKKVEYFILEQDGKFLDSTYLFFRNNQRGRAFFGIPRKRKAFL

>core/551/1/Org1_Gene136

MDNYLLNIKDLTITSTNPKRTLIENLSLQLKENRNLALVGESGSGKTTITKAILGFLPENCLIKTGSILFEDIDIT
KLSPKELHKIRGQKIATILQNAMGSLTPSMRIGMQIIETLRQHHKMNKEEAYNKAMQLLTDVCIPNPKYSFSQ
YPFELSGGMRQRVVIAIALASQPKLILADEPTTALDSMSQAQVLRILRNIQQQKQATILLVTHNLSLVKELCND
ICIIKDGKLIETGTVEEIFLSPKHPYTLKLLNAVSKIPIKKTSSPILKNKFQPLMSMQGGL

>core/552/1/Org1_Gene135

MENLSSAPSRSIWKSIIQNKMLVLGLTTLIILMLGALLLPWFYQDYEQTSLKDILVSPCSRFPFGTDTLGRCMF
ARTLRGLRLSLLIATIATLIDVCVGLLWATVAISGGKKIDFLMMRTTEILFSLPRIPIIILLLVIFHHGLLPLILAM
TITGWIPISRIIYGQFLLLKNKPFVLSAKAMHASTFHILKKHLLPNTLAPIISTLIFTIPNAIYTEAFISFLGLGIQPP
QASLGTLVKEGINAIDYYPWLFFFPSLIMIALSISFNLIGEGAKTLCLEEGSHG

>core/553/1/Org1_Gene115

MKQPMSLIFSSVCLGLGLGSLSSCNQKPSWNYHNTSTSEEFFVHGNKSVSQLPHYPSAFRTTQIFSEEHNDPY
VVAKTDEESRKIWREIHKNLKIKGSYIPISTYGSLMHPKSAALTLKTYRPHPIWINGYERSFNIDTGKYLKNGS
RRRTSHDGPKNRAVLNLIKSSGRRCNAIGLEMTEEDFVIARRREGVYSLYPVEVCSYPQGNPFVIAYAWIADE
SACSKEVLPVKGYYSLVWESVSSSDSLNAFGDSFAEDYLRSTFLANGTSILCVHESYKKVPPQP

>core/554/1/Org1_Gene480

MFKKFKPVTPGTRQLVLPAFDELTTRGELRGTKSKRSLRPNKKLSFFKKSSGGRDNLGHISCRHRGGGAKQL
YRVVDFKRNKDGITAKVVTVEYDPNRSAYIALLSYEDGEKRYILAPKGIQRGDVVVSGEGSPFKPGCCMTLK
SIPLGLSVHNIEMRPSSGGKLVRSAGLAAQVIAKSPGYVTLKMPSGEFRMLNEGCRATIGEVSNADHNLRVD
GKAGRRRWMGVRPTVRGTAMNPVDHPHGGGEGRHNGYIPRTPWGKVTKGLKTRDKNKSNKWIVKDRRK

>core/556/1/Org1_Gene485

MASIHPTAIIEPGAKIGKDVVIEPYVVIKATVTLCDNVVVKSYAYIDGNTTIGKGTTIWPSAMIGNKPQDLKYQ
GEKTYVTIGENCEIREFAIITSSTFEGTTVSIGNNCLIMPWAHVAHNCTIGNNVVLSNHAQLAGHVQVGDYAIL
GGMVGVHQFVRIGAHAMVGALSGIRRDVPPYTIGSGNPYQLAGINKVGLQRRQVPFATRLALIKAFKKIYRA
DGCFFESLEETLEEYGDIPEVKNFIEFCQSPSKRGIERSIDKQALEEESADKEGVLIES

>core/557/1/Org1_Gene526

MSDFSMETLKTLRQQTGVGLTKCKEALEACGGNLEEAVVYLRKLGLASAGKKEHRETKEGIIAAKTDANGT
ALIEVNVETDFVANNAVFREFVSNLLNDILKYKVDTVEALSQAASSQDPSLSVDELRAVTMQTVGENIRISRV
AYFPKATNSTVGIYSHGNGKTVALTMLSGSSTADSLAKDIAMHVVAAQPQFLSKESVPAEAIAKEKEVIASQI
QGKPQEVIEKIVTGKLNTFFQEACLLEQPFIKNADLSIQSLIDDFSKTSGSSVAIEQFILWKIGA

>core/558/1/Org1_Gene487

MLERTQRTLKREVRYSGVGIHLGKSSTLHLQPAQTNTGIVFQRQSASGNYENVPALLDHVYTTGRSTTLSRG
SAVIATVEHLMAALRSNNIDNLIIQCSGEEIPIGDGSSNVFVELIDQAGICEQEDKVSIARLTRPVYYQHQDIFL
AAFPSDELKISYTLHYPQSSTIGTQYKSLVINEESFRQEIAPCRTFALYNELCFLMEKGLIGGGCLDNAVVFKD
DGIISRGQLRFADEPVRHKILDLIGDLSLVGRPFVAHVLAVGSGHSSNIAFGKKILEALEL

>core/559/1/Org1_Gene573

MATAHLGRQALLHLRSWTPAIRASGNLFRQQSMSLHNNVLFAGDIVGAIKNSTAISRHALGSSHYAHAALQK
TEGFLGAADGVNTAVAGAMLWGQLLNGSMIFETDEETGELRRCNEADAEGCMTQKLQRRSALTITGKVAR
LASKTLGTATFLHEMDVVSLGANANKIGCKVTSCLNLVATGCSLTESSISLYRILSTRPETISDPENRNKPSAEF
AARSKAIRNAFIAWLGDVVDLVCDALGTLSLFLPAILGVHAVLIMAILGLISCVINFVKDYAKIG

>core/560/1/Org1_Gene183

MVLMNKRLKIILTNDDGITAKGMSCLVSALLEANIGDIYIAAPQAEQSGKSMAISLNQVVCASPYAYPQPVKE
AWAVGGSPTDCVRLGLRTLFESVSPDLVISGINCGNNICKNAWYSGTIGAAKQALVDGIPSMALSQDNHISFF
QQDKAPEILKALVIYLLSQPFPCLTGLNINFPTSPGGSSWEGMRLVPPGDEFFYEEPQYLGSVNKNQYYVGKIS
GVRIGEHPSEELACMLENHISVSPIFSQNSPIGLMTLEEFQKTQENFNASLLSSELTTKIF

>core/561/1/Org1_Gene533

MELKKTAESLYSAKTDNHTVYQNSPEPRDSRDVKVFSLEGKQTRQEKTTSSKGNTRTESRKFADEEKRVDD
EIAEVGSKEEEQESQEFCLAENAFAGMSLIDIAAAGSAEAVVEVAPIAVSSIDTQWIENIILSTVESMVISEINGE
QLVELVLDASSSVPEAFVGANLTLVQSGQDLSVKFSSFVDATQMAEAADLVTNNPSQLSSLVSALKGHQLTL
KEFSVGNLLVQLPKIEEVQTPLHMIASTIRHREEKDQRDQNQKQKQDDKEQDSYKIEEARL

>core/563/1/Org1_Gene860

MRQSFDELSQNAFKNIFNKQRFCFIFCSLCCFGFVFALFLKLCSRLAPEISLSTLGLGAFFCAFSVICASAIIVQF
LLHKESQGETSKLCCAIKNTWSSLWLSLLVSMPFFIAMVAVVTVAMLSSFLGSLPWVGKLFHTVLIFIPYLSA
TALILLFLGSFSCLFFCIPVLHNQESIDYRKLLECFRGNILRQFIGVVIALVPLALCSWLALDSFYLMTHLVEIAD
IHTWSFLAQMFVLIVPIALILTPAVSFFFNFSFSFYLAKQEEEKALVK

>core/564/1/Org1_Gene390

MHKVIVFIFLTLYSLKSYGNDVIDKPHVLVSIAPYKFLVEQIAEETCFVYAIVTNHYDPHTYELPPQQIKELRQ
GDLWFRIGEAFEKTCERNLTCQQVDLSQNVSLIQGKPCCNQHTTNYDTHTWLSPKNLKVQVETIVTTLSKKY
PQHATLYQSNGEKLLLALDQLNEEILTITSKAKQRHILVSHGAFGYFCRDYNFSQHTIEKSSHVEPSPKDVAR
VFRDIEQYKISSVILLEYSGRRSSAMLADRFHMHTVNLDPYAENVLVNLKTIATTFSSL

>core/565/1/Org1_Gene950

MYFTRDPIIETVITSREGYKLSVRNTKHFSQDPFMVEAIEVISLGNICFFRNCDHSKPFLVPAGDYEVMEVRDT
KINLKAVGLDRGVKIAGGREALIKLTKSTPLPVIDEKPLADSPEEGTEPTSPSKKEKKEARKDSFKGEKWKEK
KKLSRRRNHKEIAEVTGASQEILDTVKEELWEESQENEIVEQKKFSLLPPPAKLISEVISQTVVDPVVTSADLN
ESLQALVRESSDLINALLSADDAIHFPETEEEPTSASFEESSAMFFPETSSATEEE

>core/566/1/Org1_Gene982

MTRSSPAQLSRFLSEIQNKPKKSLSQNFLVDQNIVKKIVATSEVIPQDWVLEIGPGFGALTEELIAAGAQVIAIE
KDPMFAPSLEELPIRLEIIDACKYPLDQLQEYTTLGKGRVVANLPYHITTPLLTKLFLEAPDFWKTVTVMVQD
EVARRIVAQPGGKDYGSLTIFLQFFADIHYAFKVSASCFYPKPQVQSAVIHMKVKETLPLSDEEIPVFFTLTRT
AFQQRRKVLANTLKGLYPKEQVEQALKELGLLLNVRPEVLSLNDYLALFHKMQAG

>core/567/1/Org1_Gene525

MESQSCKLTIKDLMSAGAHFGHQTRRWNPKMKLYIFEEKNGLYIINLAKTLQQLRNALPHIRKVIQDNKTVL
FVGTKKQAKCVIREAAIEAGEFFIAERWLGGMLTNMTTIRNSIKTLDKIEKDLSRNQAYLTKKEAALLAKRH
QKLLRNLEGIRYMKKAPGLLVVVDPSYEKIAVAEAKKLGIPVLALVDTNCDPTPIDHVIPCNDDSLKSIRLIIN
VIKENIIEAKHKLGIEIVSPVKSLEVPDLSAFESSQDDESDEENREEDLLAKKFDGEAN

>core/569/1/Org1_Gene631

MTANTFGTLDILMKHSKEDDLSRFLPKNLLVESPHPEEIPLKSLSFTMSWLPTIHPSWITIAMKEFPPEIQGQLL AWLPEPLVQEILPLLPGISIAPHRCAPFGAFYLLDMLSKKIRPCGITEEIFLPASSANAILYYTGPVKIALINCLGL YSIAKELKHILDKVVIERVKNALSPTEKLFLTYCQSHPMKHLETTNFLSSWTTDAELRQFVHKQGLEFLGKAL TKENASFLWYFLRRLDVGRAYIVEQTLKTWYDHPYVDYFKSRLEQCMKVLVK

>core/570/1/Org1_Gene380

MDYKLLDSGDGNKLECFGPVTLIRPSSIAVWPKSRPELWSQAQLQYVREGERGAWKNFKRLPEEWEVAFSD VRCLLKRTPFGHLGVFPEHMGFWPALKQAIEKHKERQVLNLFAYTGAGSIFAAKCGARVTHVDASQAAVR WAQRNVEKNAFPERRIFWVIEDVISFLKKEIRRNKKYQVILLDPPSYGRGPDGEVFKIDKDLFPLLSLCSKLLA DDASYFLLTSHTPGHTPEFLRAIARRSVPTLVSEAWSCGESFCGEGVGALPSGSFVQWIA

>core/571/1/Org1_Gene104

MITGVVLEKHEQRTMFSLTLLNNFTTFGLLHTPLHYNPPYPIVILLHGLASDKTGSKRSHVRLAQELTRLGIAA LRVDLLGHGDCEGELMDFSLENYKQNIREIIEYTHSLLHIDQERLAIFGSSLGGTLALQTLPFFNKIKALAVWA PTISGELMAAEAQKNAPEVITMSQKGAITYAGMTLNPDFYTQFLKIDIVKELMPSARNLPPILYMQGEQDLLV SINHRTLFTEAFANQDKPITILTYPDVDHAFPFAESSALSDLTQWLKRELTSGE

>core/572/1/Org1_Gene551

MLLGFLCDCPCASWQCAAVANCYDSVFMSRPEHKPNIPYITKATRRGLRMKTLAYLASLKDARQLAYDFLK DPGSLARLAKALIAPKEALQEGNLFFYGCSNIEDILEEMRRPHRILLLGFSYCQKPKACPEGRFNDACRYDPSH PTCASCSIGTMMRLNARRYTTVIIPTFIDIAKHLHTLKKRYPGYQILFAVTACELSLKMFGDYASVMNLKGVG IRLTGRICNTFKAFKLAERGVKPGVTILEEDGFEVLARILTEYSSAPFPRDFCEIH

>core/573/1/Org1_Gene820

MRRYLFMVLALCLYRAAPLEAVVIKITDAQAVLKFAREKTLVCFNIEDTVVFPKQMVGQSAWLYNRELDLK TTLSEEQAREQAFLEWMGISFLVDYELVSANLRNVLTGLSLKRSWVLGISQRPVHLIKNTLRILRSFNIDFTSC PAICEDGWLSHPTKDTTFDQAMAIEKNILFVGSLKNGQPMDAALEVLLSGISSPPSQIIYVDQDAERLRSIGAF CKKANIYFIGMLYTPAKQRVESYNPKLTAIQWSQIRKNLSDEYYESLLSYVKSKG

>core/574/1/Org1_Gene1005

MKVKINDQLICIPPFISARWSQIAFIESQEGENKDQGTLRLHLIDGKIISIPNLDQSIIDIAFQEHLLYLETSQSGKE DSRDDDKLGVGVLMNVLQQITKGNDIQVLPKNLISPLFSGTNPIEAILQHTPEHKDHPDAPTDVLEKMADVIR VLSGNNATLLPRPEPHCNCMHCQIGRVMNEEDTLAVSDKDLTFRTWDIMQSGDKLYIVTNPLNPSDQFSVYL GPPIGCTCGEPNCEHIKAVLYT

>core/575/1/Org1_Gene283

MKLLKNVLLGLFFSMSISGFSEVKVSDTFVKQDTVVEPKIRVLLSNESTTALIEAKGPYRIYGDNVLLDTAIQG QRCVVHALYEGIRWGEFYPGLQCLKIEPVDDTASLFFNGIQYQGSLYVHRKDNHCIMVSNEVTIEDYLKSVL SIKYLEELDKEALSACIILERTALYEKLLARNPQNFWHVKAEEEGYAGFGVTKQFYGVEEAIDWTARLVVDS PQGLIIDAQGLLQSNVDRLAIEGFNARQILEKFYKDVDFVVIESWNEELDGEIR

>core/576/1/Org1_Gene197

MKKKLSLLVGLIFVLSSCHKEDAQNKIRIVASPTPHAELLESLQEEAKDLGIKLKILPVDDYRIPNRLLLDKQV
DANYFQHQAFLDDECERYDCKGELVVIAKVHLEPQAIYSKKHSSLERLKSQKKLTIAIPVDRTNAQRALHLL
EECGLIVCKGPANLNMTAKDVCGKENRSINILEVSAPLLVGSLPDVDAAVIPGNFAIAANLSPKKDSLCLEDL
SVSKYTNLVVIRSEDVGSPKMIKLQKLFQSPSVQHFFDTKYHGNILTMTQDNG

>core/577/1/Org1_Gene344

MVRDIQSESIGKLVFLGTGNPEGIPVPFCSCRVCQNTGIHRLRSSVLIQYQNKTLVIDAGPDFRTQMLVAGVSE
LDGVFLTHPHYDHIGGIDDLRAWYIVTQRSLPLVLSASTYRFLNKAKEYLFATPNVESSLPAVLEFTILNEDCG
QEEFQGIPYTYVSYYQKSCHVTGFRFGNLAYLTDLCSYDAKIFSYLDNVETLILSAGPSETPIPFQGHKSSHLT
VEEAKAFANHAGIKNLIITHISHCLEAERDQHPEVTFAYDGMEVLWTL

>core/578/1/Org1_Gene868

MIRRFFKTLFPPGPQYSLCYASILIVLSSLVCVPTFCWLFLPELSLSKFNPSPIRNLFLVSSTLSKVPPTAIAEHLR
LSADAPTYLHEFSIKEAESSLHALGIFSSLVIEKSPDNKGITIFYTLQTPIAYVGNRSNTLCNLEGSCFLGQPYFP
SLNLPQIFFSQEDLKMQKLPKEKMLFTKILLKELAMESPKIIDLSLSDAYPGEIIVTLSSGSLLRLPIKTLDRALD
LYKHMKKSPVIESEKQYVYDLRFPNFLLLKAL

>core/579/1/Org1_Gene548

MFNNKMILIAGPCVIEGEDITLEIAGKLQSILAPYSDRIQWFFKSSYDKANRSSLNSFRGPGLTEGLRILAKVKE
TFGVGILTDVHTPQDAYAAAEVCNILQVPAFLCRQTDLLVATAETGAIVNLKKGQFLSPWDMEGPINKVLST
GNNKILLTERGCSFGYNNLVSDMRSIPVLSRSGFPVIFDATHSVQLPGALSTESGGLTEFVPTLSRAALAAGAH
GLFIETHTNPKIAKSDAASMLSLEEFAALLPTWDQLFTCVSSFDMVSA

>core/580/1/Org1_Gene426

MTKVALLIAYQGTAYSGWQQQPNDLSIQEVIESSLKKITKTRTPLIASGRTDAGVHAYGQVAHFRAPDHPLF
ANANLTKKALNAILPKDIVIRDVALFDDNFHARYLAIAKEYRYSLSRLAKPLPWQRHFCYTPRHPFSTELMQE
GANLLIGTHDFASFANHGRDYNSTVRTIYTLDIVDKGDSLSIICRGNGFLYKMVRNLVGALLDVGKGAYPPE
HLLDILEQKNRREGPSAAPAYGLSLHHVCYSSPYNNFCCEQCSVSTSNEG

>core/582/1/Org1_Gene906

MTQYYFLSSFLPTQLPESVPLFSISDLDDLLYLNLSENDLCNYGLLKRFFDFENFAFFWAGKPIPFSFGEVTQE
NIERMLSSQQWSDDNDFEDFFKDFLMNHKSSQDRLNHFSDLFREFLSYHQTNSSKFLQDYFRFQQQLRVVLA
GFRARVLNMDVSYVLRDEDSSDPVVLEVLMQKDSPNYELPEEFSDLQGVLDDYGLLPHTLNRALALYQFHK
LEGFCSDSYFDGNVILARCATYMFAIRNSLASVEKGREIINHIEKAIKW

>core/583/1/Org1_Gene124

MHSKFLSRRKKNSSHKEETSWDCIASSYNKIVQDKGHYYHRETILPQLLPSLTLGSKSSVLDIGCGQGFLERA
LPKECRYLGIDISSRLIALAKKMRSVNSHQFKVADLSKRLEFVEPTLFSHAVAILSLQNMEFPGEAIRNTATLL
EPLGQFFIVLNHPCFRIPRASSWHYDENKKAISRHIDRYLSPMKIPIMAHPGQKDSPSTLSFHFPLSYWFKELSS
HGFLVSGLEEWTSSKTSTGKRAKAENLCRKEFPLFLMISCIKIK

>core/584/1/Org1_Gene970

MTLPLEPMIFWSSLSAKVMKKFLTPHCAGTFSEEDAEAKEAHLVTGKQGHRLMGNCVTFYWLVDKKNGVI
LDAKFQYFGHPYLIPLAEAVCNLVCGKSYGEAYKMTLDDIDKSLRVHAHQPALPEDSISLYHFVIDALDTAV
EQCLEIPLEDGSLPLQNSPMNLDFEDANPYSQSDWEALTHEQKLYALRATIAEKIGPYIAMDGGEVTVESLEN
FIVTIAYSGNCSGCPSSLGSTLNSIGQLLRAYIYPELQVKVDESSLNLSHP

>core/585/1/Org1_Gene674

MQGFFPLASGSKGNSAYLGTDSCKILIDLGVSKQVVTRELLSMNIDPEDIQAIFVTHEHSDHISGIKSFVKAYN
TPIVCNLETARALCHLLDSHPEFKIFSTGSSFCFQDLEVQTFNVPHDAVDPVAFIFHYREEKLGFCTDLGWVTS
WITHELYDCDYLLIESNHSPELVRQSQRPDVYKKRVLSKLGHISNQECGQLLQKIITPKLKKLYLAHLSTECNT
AELALSTVSESIASITSIAPEIALAQGITSPIYFSRLEVACPR

>core/586/1/Org1_Gene752

MAGLDLEARGKRRVVTPNAITAFGLCCGLFIIFKSVLRTSSSVELFHRLQGLSLLLISAMIADFSDGAIARIMKA
ESAFGAQFDSLSDAVTFGIAPPLIAIKSLDGIYVGNFFSSLLLITSIIYSLCGVLRLVRYNLFSQKTVDVSKPYCFI
GLPIPAAAASIVSLALFLASDFFPDLPAQLRVGLLSFALLFIGGLMISPWKFPGVKHFRFNVSSFLLVVTIGLAA
CLFFSGLVDHFVEVFFLVSWLYTLVGFPIFSIIYRKKS

>core/587/1/Org1_Gene19

MKKPDNDSTFDVRSFFPFDVLCIEQLRKEMSWEVVSAKIPRLPRGWYELMGLSKEDRIDFCLDFWCSVLGIE
HKESPSICRFFSLLETIEVYIYRLEKEPYQLKMFYVFRDGRCGFQGEPPLLDFLGHHRLPPLGDRHYEKFFSIHN
GFGKWEDEGIFPMRSLAKVQQKLRQQLVVMNKMQAEDNCYSLGIFPFYGYEEPFAYQSFFFDPEIRRDLPSP
NVLLNEESLEHRSLETIELLHLSKSYYPSFLSWLENYLHSEEVYNE

>core/590/1/Org1_Gene379

MDCIGKHNPLVKEALALKRSRCRKSSWFLVEGAREIQKALRTGYLCQHVFCSTHLSEKEKEFLYELKRNSTK
ILYCLDSTLAQLSFKEHHDSFVAVIQKRVWNKEDFLIQRKNAQPFYLIIEQVEKPGNVGAILRIADGAGVDGVI
LCNPIVDLYNPNVVRSSLGAVFSLPILSISREEGKELFKQEGWTVFVTSPRAETMYFSKNYLGPTALVFGSEKD
GLTEDWFSEDFSEIALPMLGESDSLNLATSVAAVAYEVVRQRWVN

>core/591/1/Org1_Gene802

MGSSMHVGVIGCSGRTGKVIVSALEQSSEYTLGPGFSRSSALTLFQVIAHNDVLVDFSHPLLTKEVVAHLLISP
KPLIIGTTGFPGKCKEAHDSLEELTHIVPVVVCPNASLGAYIHKRLVMLLSQLCNPQFDIRIRETHHRYKKDSL
SGTAQDLLDTIQQVKQEDWGEEYEVGQRDSSKKTIEVQSSRVGDIPGEHEVAFISSGEQILVRHTVFSRNVFG
RGILSILDWLKTLNPQPGLYSLGDTLELVLRNEHCLLKKTTDH

>core/592/1/Org1_Gene587

MEKLEFVTSLSSPDDDLITFNKQGLIAGPEEEKVAFLVRSNAMLDAGPETPASFPESLREQFDIFPEYVEVLYS
NEGLDVWEAGCTWILNNEVTIQLRKHHRKASRWLGMYSRDEVLAHEAVHAVRMKFHEPVFEEVLAYQTSR
WGWRRFFGPLFRSPGESYLLLFFTILGLGISLWYPAGILIMLVLPMYFLMRLCMAQSYLYRAMKKIRKMLGV
PPLWVLLRLTDKEIKMFAKEPIPVLEHYARKRKLENVRWKQIYQSYFV

>core/593/1/Org1_Gene371

MSNQLQPCISLGCVSYINSFPLSLQLIKRNDIRCVLAPPADLLNLLIEGKLDVALTSSLGAISHNLGYVPGFGIA
ANQRILSVNLYAAPTFFNSPQPRIAATLESRSSIGLLKVLCRHLWRIPTPHILRFITTKVLRQTPENYDGLLLIGD
AALQHPVLPGFVTYDLASGWYDLTKLPFVFALLLHSTSWKEHPLPNLAMEEALQQFESSPEEVLKEAHQHTG
LPPSLLQEYYALCQYRLGEEHYESFEKFREYYGTLYQQARL

>core/594/1/Org1_Gene304

MKLTKYLNTKQLRSMISRLFVRYSLPMSKQLSFFALCVLGSHPIFAQTPNPPQRVRRSEVIFIDPGHGGKDQGT
ASKELHYEEKSLTLSLALTVQSYLKRMGYKPQLTRSSDVYVDLGKRVALSNRGQGDVFISIHCNHSSNAAAF
GTEVYFYNGKVGSPTRNRMSEVLGKNILAAMEKNGILKSRGLKTANFVVIRDTSMPAVLVETGFLSNSRERA
ALQDARYRMHVAKGIAEGVHNFLSGPSFQKPKQNIAKIRKPQIQAN

>core/597/1/Org1_Gene61

MPFDITYYTTPLLEIILIWVMLNYLLKFFWGTRAMDVVFGLLAFLFLFVLADKLHLPIIRRLMLHVVNIAAIVV
FIIFQPEIRLALSRIRFHGKKFFIDTQEQFVEQLAASIYQLSERQIGALVVLENKDSFDEYLSFSSVKINATFSEEL
LETIFEPSSPLHDGAVILRGDILAYARVVLPLAHDTTQLSRSMGTRHRAALGASQRSDALIITVSEENGSVSLSR
DGLLTRGVKIDRFKAVLRSILSPKEHKRKPLFSWIWKR

>core/599/1/Org1_Gene137

MTTLLSIKDLSLTIRGKKILNHINLNLIKGSYLTIVGPSGSGKSSLALTILDLLKPTTGTITFHMDPKIPRARKVQ
VIWQDIDSSLNPCMSIKGIISEPLNIIGTYSKAEQNKEIYNVLDLVNLPKSVLHLKPYKLSGGQKQRIAIAKALV
SKPELLICDEPLSSLDTLNQSLILDLFQTIKKEYQNTLLFITHDMSAAYYIADTIAVMDQGSLVEHACREKIFST
PKHTTTQDLLDAIPIFSLISTEMEPSEEYELQVASK

>core/600/1/Org1_Gene791

MKTWLFFTFLFSCSSFYASCRYAEVRSIHEVAGDILYDEENFWLILDLDDTLLQGGEALSHSIWKSKAIQGLQ
KQGTPEQEAWEAVVPFWIEIQEMGTVQPIESAIFLLIEKIQKQGKTTFVYTERPKTAKDLTLKQLHMLNVSLE
DTAPQPQAPLPKNLLYTSGILFSGDYHKGPGLDLFLEICTPLPAKIIYIDNQKENVLRIGDLCQKYGIAYFGITY
KAQELHPPIYFDNIAQVQYNYSKKLLSNEAAALLLRHQMHE

>core/601/1/Org1_Gene598

MMKYLPYIAITACIHGGILLLVFASPLPKKRLQPKAFQEKLVTIQPKPPVPTPSVVVDPAKTIRPSVATQPQKQ
AKCSPPQENVQKALQKPIPKVIKTEPPKPSPAPTVAKKTTATEKPPPSTTKKNTQLSKTQLQTLSEVAQALSLH
VDKIEKSETSLKNISWPSTAQLTMHSELKATQEDELCELFRTHIALPSKGYVRIKLVLSPNGEIQECSFLSEVSA
ADKQLLTQRIQALPFQKFLEKYKVSKNISFHIKLVSNES

>core/602/1/Org1_Gene601

MDLADAHVHLSDDAFEEDINSVLQRAQDSGVSLVVNVTTTEKELNRSFAYAERFPKIRFCHVGGTPPQDVDQ
DIEEDYRNFHAAAHSKKLAAIGEVGLDYCFATEEGIARQKEVLQRYLALSLECELPLVVHCRGAFNDFFRML
DQYYHNDPRSRPGMLHCFTGTLEEAQELISRGWFISISGIVTFKNAQDLRDLVVELPLEHLLIETDAPFLAPVP
YRGKKNEPAHVLHTINAVANVKGMFPQELAALAYKNVLRFLHG

>core/603/1/Org1_Gene13

MFLQFFHPIVFSDQSLSFLPYLGKSSGIIEKCSNIVEHYLHLGGDTSVIITGVSGATFLSVDHALPISKSEKIIKILS
YILILPLILALFIKIVLRIILFFKYRGLILDVKKEDLKKTLTPDQENLSLPLPSPTTLKKIHALHILVRSGKTYNELI
QEGFSFTKITDLGQAPSPKQDIGFSYNSLLPNFYFHSLVSVPNISGEERALNYHKEQQEEMAVKLKTMQACSF
VFRSLHLPSMQTKDKKAGFGLLTFFPWKIYPL

>core/605/1/Org1_Gene300

MILTAAFSPCPNDIFLFRSFLKDPQFRPLLNQVTIADIETLNTLALQRRLSLMKMSAALFPLVSDYYNLMDVG
NTLGYNSGPIVLSLDPECSLDTLATPGEMTTAHALCKLYYPKAKLIPMPYDKILSAILQGKVDGGALIHEERFS
YDLQLTLRADFGELWRRKTIFPLPLGCLAIAKYVPMATVDALTAALRKSLICSLKDPITAGAKAVEYSKNKN
VTVIHRFIGTYINKETFQLSKTGKKALHMLWKANECCQYT

>core/606/1/Org1_Gene346

MIKQIGRFFRAFIFIMPLSLTSCESKIDRNRIWIVGTNATYPPFEYVDAQGEVVGFDIDLAKAISEKLGKQLEVR
EFAFDALILNLKKHRIDAILAGMSITPSRQKEIALLPYYGDEVQELMVVSKRSLETPVLPLTQYSSVAVQTGTF
QEHYLLSQPGICVRSFDSTLEVIMEVRYGKSPVAVLEPSVGRVVLKDFPNLVATRLELPPECWVLGCGLGVA
KDRPEEIQTIQQAITDLKSEGVIQSLTKKWQLSEVAYE

>core/607/1/Org1_Gene82

MLRNQVLVYCSEGVSPYYLRHTIRFLKYYSTQEGAFDILRVDGNFLIKNPFWEETTRLLVFPGGADRPYHRV
LHGLGTARIFQYVSEGGNFLGICAGAYFGSKMIYFYEPEGAPLQGARDLGFFPGTAKGPAYRGNFSYVSPSGV
RVSPQLFSDFGLGYAMFNGGCFFEGSEGYPGVNIESRYDDLPGKPASIVSRIVSKGLAVLSGPHIEYLPHYCR
MVKENVQKTREFLQRERTTLDRYCQNLVQRLRQPAFSKADC

>core/608/1/Org1_Gene249

MNVKDETFWSVHNLCVNYEHAAVLYHISFSLGKGSLTAILGPNGAGKSTLLKASLGLIKPSSGTVYFFNQKF
KKVRQRIAYMPQRASVDWDFPMTVLDLALMGCYSYKGMWGRISSDDRREAFHILERVGLESVADRQIGQLS
GGQQQRAFLARALMQKADLYLMDELFSAIDMASFKTSVGVLQELRDQGKTIVVVHHDLSHVRQLFDHVVL
LNKRLICCGPTDECLNGDTIFQTYGCEIELLEQTLKLSRGKQFGSC

>core/609/1/Org1_Gene495

MNRRWNLVLATVALALSVASCDVRSKDKDKDQGSLVEYKDNKDTNDIELSDNQKLSRTFGHLLARQLRKS
EDMFFDIAEVAKGLQAELVCKSAPLTETEYEEKMAEVQKLVFEKKSKENLSLAEKFLKENSKNAGVVEVQP
SKLQYKIIKEGAGKAISGKPSALLHYKGSFINGQVFSSSEGNNEPILLPLGQTIPGFALGMQGMKEGETRVLYI
HPDLAYGTAGQLPPNSLLIFEINLIQASADEVAAVPQEGNQGE

>core/611/1/Org1_Gene966

MENLETFILKIYRGVPGKQYWESFELPLHPGENVISALMEIEKRPVNILGEKVNPVVWEQGCLEEVCGSCSILV
NGVPRQACTALIQEYIDATQSREIVLAPLTKFPLIRDLIVDRSIMFDNLERIQGWVAADIEGETFGPQVTQEQQ
ELLYALSQCMTCGCCTEACPQIDNKSDFIGPAAISQARYFNTYPGDKQSKKRWRALMGKGGIEGCGQAHNC
VRVCPKKLPLTESISAMGREISKFSLRSLFSALFKKKK

>core/613/1/Org1_Gene927

MSKFILLLLSLGVAALASKNFFIWPAPSGKTPLKLRQVLFGGALLVFSSLVALSVSSQTAELLSTMTGISLAFAF
LFYLLFLPKDITRAILFSGERPVKTSWRALGSAIRMWIIIIPVTQLIGIMMSKFLTLVLPMQEIHTQEVTQEVQNS
LPITGHYISMILNLGVLTPFGEEVFFRGILQTFLKNKMTRIAAVLCSSIIFSFIHIEHSLGSWVFVPVLFVFSLSAG
FLYEKDRHILSPIALHGLFNLTSLLFLGIK

>core/614/1/Org1_Gene373

MAFYSPSTISKYFIYSGAGNRFLLGETLPEVEDVRFLCQETRVDGFLYLKPSSCADAQLIIFNSDGSRPTMCGN
GLRCAIAHLASQKGKSDISVSTDSGLYSGYFYSWDRVLVDMTLADWRASVHRLESRPDPLPKEVVCIHTGVP
HAVVILPEISTLDLSILGPFLRYHQTFSPDGVNVNFVQILGHCQLRVRTYERGVEGETAACGTGALASALVVS
NSYGWKESIQIHTWGGELMTVSQNRGRVYLQGSVTRDL

>core/615/1/Org1_Gene84

MFKLLKNLFLIGCCIVGYFWMRKESIVEQWLSNRLHTQVTVGRVSIRTSGIKIRHICIHNPLASERFPYAAEIEY
ADVRFSSISMLLTKQLEISELIIHGANFTIFPYDSHGTKTNWSLVWKNFHPQKETPSNLWIDRAPVLIRRCLFLN
TRLYGLRANHKDIPHLSVPSLEFHSHTSSAKELPKLSEALPSLLYLALEESLYHLNLPGDIIKPLSQQAHKHFYS
SYPQFQDRLNDINTPGTPTEEIIGFIRGLFFH

>core/616/1/Org1_Gene65

MVSPLSLFHKMLLENWTPVEEPFPWPPAEKNQKIFAWALNQSKLIFVSTSGNIAQPRLVTDSMSMMIVNAAN
RTMSRDGAGTNQVLSAAVSVDSWGLSQRPLNPERQGTPLNEGECRAGMWRNADGSNHTGKQGKPHYLAQ
LLGPKAVDHHNKSQAAFDRCKNAYLNCFSLAQTLGVTFLQIPLISSGIYAPPENRKKPNSEENKVRMRWIHA
VKCALVAAMQEFGNEPGNTDRRMLIVLTDLKTPAITDPKKKSHL

>core/617/1/Org1_Gene258

MKTQQTQNIIEVWNFYWETQEIEYRDSLIEFYLPLVKSVVHRLISGMPSHVKTEDLYASGVEGLVRAVERYN
PERSRRFEGYAVFLIKAAIIDDLRKQDWVPRSVHQKANKLSGAMDSLRQSLGKEPTDLELCEYLNISQQELSG
WFVSARPALIVSLNEEWPSQSDEGAGMALEERIPDERAETGYDVVDKQEFSLCLANAIQELEEKERKVMALY
YYEELVLKEIGKVLGVSESRVSQIHSKALLKLRAALSAFR

>core/618/1/Org1_Gene317

MWLGAYTWLNVFGILLQAAFIQNILLANFLGMCSYLACSTRVSTANGLGMSVALVLTVTGSINWFVHAFITG
PKALTWISPSLASVNLGFLELIIFIVVIAAFTQILELLLEKVSRNLYLSLGIFLPLIAVNCAILGGVLFGITRSYPFIP
MMIFSLGAGCGWWLAIVILATIKEKLAYSDIPKNLQGMGISFITTGLIAMAFMSLTGIDISKPSAKIQRAPLETE
VVENTTNPLKESSSKHQPSISKARTQRRSL

>core/619/1/Org1_Gene521

MLKIKHLHASCNDVKILDDFNLNIQPGTMHVIMGPNGAGKSTLAKILAGDESVLVSSGEIALQEQNLLSMLPE
ERSRAGLFVGFQMPPEIPGVNNKMFLRDAYNARRRANQEGDISIDEFNTLLSTVLETYEYNATTDLFLDRNV
NEGFSGGERKRNEICQMLVLEPEMVLLDEPDSGLDVDALRLICRVLEKYRELHPTSSLCIVTHNPKLGNLIRP
DVVHLLLDGRVALSGDVSLMHELEAKSYQEVTKRVAWR

>core/621/1/Org1_Gene613

MKTIAVNSFKGGTAKTSTTLHLGAALAQYHQARVLLIDFDAQANLTSGLGLDPDCYDSLAVVLQGEKEIQE
VIRPIQDTQLDLIPADTWLERIEVSGNLAADRYSHERLKYVLGSVQDKYDYVIIDTPPSLCWLTESALIAADYA
LICATPEFYSVKGLERLAGFIQGISARHPLTILGVALSFWNCRGKNNSAFAELIHKTFPGKLLNTKIRRDITVSE
AAIHGKPVFATSPSARASEDYFNLTKELLILLRDI

>core/622/1/Org1_Gene857

MTRQSYVLGNWKMHKTIQEAKEYVQTLASLLQGEPLSCTIGIASPFTSLRAIHEMINTTGAFLWLGAQNVHP
ELSGAFTGEISLPMLKEVGVEFVLVGHSERRHIFGESDAFIASKVKSVAQAGLVPVLCVGESLEVREEGKAHQ
VIKKQLLLGLEQMDNGSEFLIAYEPVWAIGTGKVAEASDVQDIHMFCREVVAERFSEATAEEISILYGGSVKV
DNAQRFGQCSDVDGLLVGGASLEGQSFFEVAKNFNV

>core/623/1/Org1_Gene375

MHDALLSILAIQELDIKMIRLMRVKKEHQKELAKVQSLKSDIRRKVQEKELEMENLKTQIRDGENRIQEISEQI
NKLENQQAAVKKMDEFNALTQEMTTANKERRSLEHQLSDLMDKQAGGEDLIVSLKESLASTENSSSVIEKEI
FESIKKINEEGKALLEQRTELKHATNPELLSIYERLLNNKKDRVVVPIENRVCSGCHIVLTPQHENLVRKKDRL
IFCEHCSRILYWQESQVNAQENSTAKRRRRRAAV

>core/624/1/Org1_Gene165

MKPEESECLCIGVLPARWNSSRYPGKPLAKIHGKSLIQRTYENASQSSLLDKIVVATDDQHIIDHVTDFGGYA
VMTSPTCSNGTERTGEVARKYFPKAEIIVNIQGDEPCLNSEVVDALVQKLRSSPEAELVTPVALTTDREEILTE
KKVKCVFDSEGRALYFSRSPIPFILKKATPVYLHIGVYAFKREALFRYLQHSSTPLSDAEDLEQLRFLEHGGKI
HVCIVDAKSPSVDYPEDIAKVEQYITCLSNAYF

>core/625/1/Org1_Gene705

MLIKLWRATYEGMYTFLVGALLKLRYRMQVEGWDTLNINPKQGCLFLANHVAEVDPIILEYLFWSRFHVRP
MAVEYLFHSRVVQWFLNSVRSIPIPQLVPGKESKRSLERMNVCYEEASRALNRGESLLLYPSGRLSRTGKEEI
VNQYSAYVLLHRVMECNVVLVRVSGLWGSAFSRYKQNSTPKLGPAFKEAFRALLRRGIFFMPKRFVKITLC
QVDHLFLKQFPTKQDLNTFLASWFNQGDDNLPIEVPYA

>core/626/1/Org1_Gene434

MQICVTGVVLRSRPLGKNHTLTTLFTPEGLFTFFAKQGQTLQCDYRETLVPISLGKYTLHRNGSRLPKLTHGD
ILNAFEAIKQTYALLEASGKMIQALLASQWKEKPSHKLFSLFLNFLHRIPESSNPEFFAAIFVLKLLQYEGILDL
TPACSLCKASLPYACYRYQGHKLCKKHQHKQAISIEKEEEQILQAIIHAKQFSELLAIAEFPIAIAEKIFYLFDSL
QEEKKSERNSSEDPYHEILRLSKVVHPY

>core/627/1/Org1_Gene518

MLIVLAFRQVFFSHSRSQLDRLKNYLRLLKQNFAITLPKERTSKGHSLMLTFDFASFDFYTNIFPFLEEQKIPAV
VGVASRYIPSNAAQDLHPSHRLKPSETLAFQDEIFSNYMPFCCQNELIEMAKSPYIQLASSGFAIRNLMNNPPY
LTTEILLSRHHIETITGAKPLAFLFPFGKSDPTSRKLAADHYPYSFLLGNTINRKLKTHNIYRLDIKPMQYVCPS
LFQSSRYLKNWIKEKSKQLYLKKQLPKR

>core/628/1/Org1_Gene139

MLMDISHILEDLAYDEGILPREAIEAAIVKQMQITPYLLHILHDATQRVPEIVNDGSYQGHLYAMYLLAQFRE
SRALPLIIKLFAFEDDTPHAIAGDVLTEDLPRILASVCNDDSLIKELIETPKINPYVKAAAISGLVTLVGAGKIPR
DKVIRYFAELLNYRLEKQPSFAWDNLIAGICTLYPGELFYPISKAFDGGLVDTSFISMEDVENIIHEETVESCIH
TLCSSTELINDTLEEMEKWLEDFPIEP

>core/629/1/Org1_Gene88

MNVADLLSHLETLLSSKIFQDYGPNGLQVGDPQTPVKKIAVAVTADLETIKQAVAAEANVLIVHHGIFWKG
MPYPITGMIHKRIQLLIEHNIQLIAYHLPLDAHPTLGNNWRVALDLNWHDLKPFGSSLPYLGVQGSFSPIDIDS
FIDLLSQYYQAPLKGSALGGPSRVSSAALISGGAYRELSSAATSQVDCFITGNFDEPAWSTALESNINFLAFGH
TATEKVGPKSLAEHLKSEFPISTTFIDTANPF

>core/630/1/Org1_Gene68

MKKQGKTLFLFLFLSFLFSTAFSGLFASQTSSLRTIQENIFLAKTGDYTVLSRGSQRTFVLVKSTTPKTVWIEIIH
FPCIAHKERPSLEQASWKTVIHQLESPSQVFVVSLSSEGSQFFSLNTRTKSLEPVGKSTTVPAFLQIFDLPLSPAP
ANVIKTKGKENKPWSPKVSFEGAPLTSISVNAWQGLWPKDRGPLSETGILMYFTQPDISVFPLWVSIETPKGT
SIVRAVDIGHGATSPYVYSLPDSKTQ

>core/632/1/Org1_Gene412

MSLATNNAESKFPSLQRLPNHVAIIMDGNRRWYKKHREECGHTHTSGHYYGAKVLPNILNAVLDLGIKVLT
LYTFSTENFGRPKEEIQEIFNIFYTQLDKQLPYLMENEICLRCIGDLSKLPKGIQTKINHVSRMTASFSRLELVLA
VNYGGKDELVRAFKKLHVDILNKKISSDDLSESLISSYLDTSGLTDPDLLIRTGGEMRVSNFLLWQIAYTELYI
TDTLWPDFTPQDLFEAINVYQQRSRRGGK

>core/633/1/Org1_Gene612

MGNLKTLLESRFKKNTPTKMEALARKRMEGDPSPLAVRLSNPTLSSKEKEQLRHLLQHYNFREQIEEPDLTQ
LCTLSAEVKQIHHQSVLLHGERITKVRDLLKSYREGAFSSWLLLTYGNRQTPYNFLVYYELFTLLPEPLKIEM
EKMPRQAVYTLASRQGPQEKKEEIIRNYRGERKSELLDRIRKEFPLVETDCRKTSPVKQALAMLTKGSQILTK
CTSLSSDEQIILEKLIKKLEKVKSNLFPDTKV

>core/634/1/Org1_Gene446

MKIKFSWKVNFLICLLAVGLIFFGCSRVKREVLVGRDATWFPKQFGIYTSDTNAFLNDLVSEINYKENLNINIV
NQDWVHLFENLDDKKTQGAFTSVLPTLEMLEHYQFSDPILLTGPVLVVAQDSPYQSIEDLKGRLIGVYKFDSS
VLVAQNIPDAVISLYQHVPIALEALTSNCYDALLAPVIEVTALIETAYKGRLKIISKPLNADGLRLAILKGTNG
DLLEGFNAGLVKTRRSGKYDAIKQRYRLP

>core/635/1/Org1_Gene490

MSLLKDTVFTCLDCEMTGLDVKKDRIIEIAAVRFTFDSVISSIEFLINPERVVSAESQRVHHISNAMLRDQPKIA
EVFPQIKAFFKEGDYIVGHSVGFDLQVLAQEMERIGETFLSKYTIIDTLRLAKEYGDSPNNSLESLAVHFNVPY
DGNHRAMKDVEININIFKHLCKRFRTLEQLKQVLAKPIKMKYMPLGKHKGRCFSEIPLAYLQWASKMDFDS
DLLFSIRHEIKHRQKGTGFSQVNNPFMEL

>core/636/1/Org1_Gene212

MDITLVGKKVIVTGGSRGIGLGIVKLFLENGADVEIWGLNEERGQAVIESLTGLGGEVSFARVDVSHNGGVK
DCVQKFLDKHNKIDILVNNAGITRDNLLMRMSEDDWQSVISTNLTSLYYTCSSVIRHMIKARSGSIINVASIVA
KIGSAGQTNYAAAKAGIIAFTKSLAKEVAARNIRVNCLAPGFIETDMTSVLNDNLKAEWLKSIPLGRAGTPED
VARVALFLASQLSSYMTAQTLVVDGGLTY

>core/637/1/Org1_Gene665

MTQDPHDHFKSRTPEDHIKHVRDKHRVCKGEPHTTFKGFFYHLANNALSTGVFIFFIRTLFFLIPTNRALQVKS
LISLGVGWTFYHGCLKARKAWAYMELSHRSMLEEKNEIEENFEQEKIELRILFENQGFKDPLLQEMVEYVCS
DSTLLLDTMIREELYIRKEDLPHPLIQGGSRILGGLCGLAIFLPLVLCISYTLAGVFSALMVLVLSFLKAKILKN
DKISEMVWVLGIFITSASIISSLMKLL

>core/638/1/Org1_Gene269

MTLSFHTHPLNYWTFEEFDGLPIRHGVFSKQKDAEGTVFAAKNPEIASALQSPKYCDLHQRHGTSVRCVTPT
SPTYQPADGLCTQSPLLSLHIRHSDCQAAIFYDREHHAIANVHSGWRGLLGNIYAVTVGTMKKLFHTKPQDL
FVAIGPSIGPDYAIYPDYATLFPRSFLPFMNPKNHFDLRAIARKQLTNLGISKDRIFISDLCTYTEHDAFFSSRYL
AHHPDPNLTGQHSKNRNNVTAVLLLPRD

>core/639/1/Org1_Gene527

MAKQTRRVLFKISGEALSKDSSNRIDEMRLSRLVSELRAVRNNDIEIALVIGGGNILRGLAEQKELQINRVSAD
QMGMLATLINGMAVADALKAEDIPCLLTSTLSCPQLADLYTPQKSIEALDQGKILICTTGAGSPYLTTDTGAA
LRACELNVDVLIKATMHVDGVYDKDPRLFPDAVKYDFVSYKDFLSNQLGVMDASAISLCMDSHIPIRVFSFL
QHSLEKALFDPTIGTLVSEDVNHVCSPRH

>core/640/1/Org1_Gene48

MKFFILFILIVAQFPAFSAQPRTQVSASHSKQAKARRTSRIRSSAATNASVSRYKTRAAARKKIGKFEKKPSLSP
VQWVRYSGKNYSIQTPSLWQCIDDKTQLPEKLDVLLIGKGKGNLTPTINIAQEITSKSSKEYIEEILAYHKANE
MTLESGIFTQIQSPSGEFTIIKTEKNSSWGRVFCLQATTVIDHTAYIFTSTATLDDYAELSFTFLKVVSSFQIRGG
KEATSGDAILEKALEALQNENK

>core/641/1/Org1_Gene448

MHIYGLADLHLALGVPEKTMEVFGDPWIGYHQKICSEWQAVVHPEDIVLLPGDISWAMNLSEAHKDFAFIG
DLPGTKYMIRGNHDYWSSASTSKILQALPPSLYYLNQGFALLTPHLAVVGVRLWDSPTICVKKENFLTPSTQE
QSYTEQDEKIFLRELGRLKRAFAALPKEVTEVIVMTHYPPISSDGTPGPISEFLEADGRVSLCLFGHIHKVQRPI
DGFGNIRGIHYILVAADYVNFVPQEVM

>core/642/1/Org1_Gene580

MKITTVKTPKIYPYDDLYSILESSLPKLNERSIVVITSKIVSLCEGAVVELEKVSKDELIKQEADAYVFVEKYGI
YLTKKWGILIPSAGIDESNVEGYFVLYPRDFLLSVNTLGDWLRNFYHLEHCGIIISDSHTTPLRRGTMGLGLC
WNGFFPLYNYVGKPDCFGRALKMTYSNLLDGLSAAAVLCMGEGDEQTPIAIIEEAPKITFHSSPTTLQDMSTL
AIAEDEDLYGPLLQSMAWETPAPTS

>core/643/1/Org1_Gene834

MDFDYFGLSDIGRVRARNEDFWQVNLMSQVVAIADGVGGRLGGDIASQEAVTSLMELIDEQQSKLMGYED
DQYKETLKKILLEVNGVVYEHGQMEEHLQGMGTTLSFIQFRKDRAWLFHVGDSRIYRIREGELRRLTEDHSL
ENQLKNRYGLPKQSDKVYSYRHILTNVLGSRPYVMPDIRNLPCEKEDLYCLCSDGLTNMVPDIDIRDILNQPA
TLEERGNALISLANTRGGDDNATVVLVRIQ

>core/644/1/Org1_Gene391

MTIRILAEGLAFRYGSKGPNIIHDVSFSVYDGDFIGIIGPNGGGKSTLTMLILGLLTPTFGSLKTFPSHSAGKQTH
SMIGWVPQHFSYDPCFPISVKDVVLSGRLSQLSWHGKYKKKDFEAVDHALDLVGLSDHHHHCFAHLSGGQI
QRVLLARALASYPEILILDEPTTNIDPDNQQRILSILKKLNRTCTILMVTHDLHHTTNYFNKVFYMNKTLTSLA
DTSTLTDQFCCHPYKNQEFSCSPH

>core/646/1/Org1_Gene979

MSYFNYQKNSVVLRSLGLLAKFFSRLLYRVFFSFREGIYLFSSLYLKYPRLFFYDLGKYVYSLRHCPYAKLGR
LPGASLLKEGNVYGETPWSVLAKICQAFDITSQDVLYDLGCGLGKVCFWFSHVVRCQVIGIDNQPHFIRFSSN
MHRKLSSGFALFDTEEFKNVVLSQASYVYFYGSSFSRRLLNEIILKLSEMAPGSVVISISFPLDSFSRGKECFFT
EKSCSVRFPWGKTIAYKNIRKGS

>core/647/1/Org1_Gene182

MSTLLLNPPWMKAGKRIESLVRKALYTHTMLANHRKIVVALSGGKDSLTLLLMLKAISGRGFPDLDLHAVNI
GGKYSCGAEVNKPYLTRICDQLCIPFRTIPSPYAPETPECYPCSQARRRLLFQAAKEIGASAIAFGHHRDDLVQ
TALLNLLHKAEFAGMLPVLDMVHFGVTILRPLIFTPEFWIRKFAKENGFARVTCRCPVVSLRSKAEQSLKLLE
EVFPLARHNIALAIQEHGSSKSQKI

>core/648/1/Org1_Gene191

MTDYSFFRRKIGNIEAIECPGNPQDPIIILCHGYGSLADNLTFFPSICSFSKLRPTWIFPNGILPLENDFRGSRACF
PLNVLLLQELSRLYANGVGNLQEKYDELFDVDLETPKEALEELILNLNRPYNEIIIGGFSQGAILATHLVLTSQ
NPYAGALIFAGARLFNQGWEEGLKQCAQVPFLQSHGYEDEILPYHLGAHLNDLLLTKLNGQFVSFHGGHEIP
SVVFQKMQVTVPNWIDPARG

>core/650/1/Org1_Gene550

MPILSVCNLVKKYNKKPVTNDVSFQINPGEIVGLLGPNGAGKTTAFYLTVGLIRPDSGKIIFKNVDVTKKTMD
HRARLGIGYLAQEPTIFKELTVQDNLICILEIIYKARKQQSHLLNTLVDDLQLGSCLHKKAGTLSGGERRRLEI
ACVLALNPSVLLLDEPFANVDPLVIQNVKYLIKILAGRGIGILITDHNAKELLSIADRCYLIIDGKIFFEGSSSQM
ISNPMVKQHYLGDSFSY

>core/651/1/Org1_Gene322

MKVRIVDSGKSSAASHMAKDRDLLESLQDGELILHLYEWENPCSLTYGHFMRPEKFLLSNYADLGLDAAVR
PTGGGFVFHKGDYAFSVLMSATHPSYSSSVLENYHTVNSFVAKVLEKVFRIQGMLAPEDENSSSRDSGNFCM
AKTSKYDVLFGDKKIGGAAQRKVQQGFLHQGSLFLSGSSSEFYQRFLKPEVLEEIIEQIQIHAFFPLGLEAADE
VLQEARQQVKEAFIKLFCGEGL

>core/652/1/Org1_Gene835

MTLYLLPNTLGTRAVETLPSVIGELVHRLDGLIVESDRGGRAFLSLWKIPEVHKFPLAILSKHARLPKAWDFY
LEPIVKHGENWGLISDAGLPCIADPGASLVRRARALGIPVQAFSGPCSITLALMLSGLPSQSFTFLGYLPQSPKE
RVKSIKKAATSKEVSTSVCIETPYRNVYTFESLLDTLPSYAELCVASDLSGPSELVLTRQVQSWRTTEDLGSV
KQSITKVPTIFLFHIPN

>core/653/1/Org1_Gene419

MAGHSKWANTKHRKERADHKKGKIFSRIIKELISAVKLGGADPKSNARLRMVIQKAKENNIPNENIERNLKK
ATSAEQKNFEEVTYELYGHGGVGIIVEAMTDNKNRTASDMRIAINKRGGSLVEPGSVLYNFARKGACTVAK
SSIDEEVIFSYAIEAGAEDLDTEDEENFLVICAPSELASVKEKLISQGATCSEDRLIYLPLRLVDCDEKDGEANL
ALIDWLEQIEDVDDVYHNMS

>core/654/1/Org1_Gene492

MDISGAVKQKLLQFLGKQKKPELLATYLFYLEQALSLRPVVFVRDKIIFKTPEDAVRILEQDKKIWRETEIQIS
SEKPQVNENTKRIYICPFTGKVFADNVYANPQDAIYDWLSSCPQNMEKQGGVRIKRFLVSEDPDVIKEYAVPP
KEPIIKTVFASAITGKLFHSLPPLLEDFISSYLRPMTLEEVQNQTKFQLESSFLSLLQDALVEDKIAAFIESLADD
TAFHVYISQWVDTEE

>core/655/1/Org1_Gene745

MGLYDRDYIQDSRVQGTFASRVYGWMTAGLIVTSCVALGLYFSGLYRSLFSFWWVWCFATLGVSFFINSKI
QTLSVSAVGGLFLLYSTLEGMFFGTLLPVYAAQYGGGVIWAAFGSAALVFGLAAVYGAFTKSDLTKISKIMT
FALIGLLLVTLVFAVVSMFVSMPLIYLLICYLGLVIFVGLTAADAQAIRRISSTIGDNNTLSYKLSLMFALKMY
CNVIMVFWYLLQIFSSSGNRD

>core/656/1/Org1_Gene904

MHPPIDITAIEAKLNFTFTQPKLLEIALTHPSYKNESAVQIEDSERLEFLGDAVLGLIVTEHLFLLFPSMDEGTLS
TARASLVNAKACCRYTTVLGIGDYLLIGKGEKIQSERGRLSAYANLFESILGAVYLDGGLSPARKLTVPLLPP
REEILPLMSGNPKNLLQQFTQKQFRVLPVYQSTAVTDAQGNVSYQIQVLVNQEVWGEGNASSKKEAEKIAA
QQALDTYGNKNQNTMDV

>core/657/1/Org1_Gene995

MEPSTNKPDCKKIFDSIASKYDRTNTILSLGMHHFWNRSLIQILGSGYSLLDLCAGTGKVAKRYIAAHPQASV
TLVDFSSAMLDIAKQHLPQGSCSFIHSDINQLPLENHSYPLAAMAYGLRNLSDPHKALQEISRVLMPSGKLGIL
ELTPPKKTHPTYSAHKLYLRAVVPWIGKSVSKDPDAYSYLSKSIQQLPKDHDLEDLFSKSGFYIAKKKKLFLG
AATIWLLEKQ

>core/658/1/Org1_Gene928

MDVLIFYDTETTGTQIERDRIIEIAAYNSVTDESFLTYVNPEIPIPDEASKIHGITTDAVLSAPKFPEAYEGFRKF
CGEDSILVAHNNDGFDFPLLGKECRRHSLEPLTNRTIDSLKWAQKYRPDLPKHNLQYLRQVYGFAENQAHR
ALDDVVILHKVFTSLIGDLPPQQVLDLLQQSYHPKVFKMPFGKYKGQPLVDIPKSYFEWLENQGALDKPENK
DIKAIALLHQPT

>core/659/1/Org1_Gene589

MQNATIDQLPVSWQEQLPLCWREQLKEEWSKPYMQQLLIFLKQEYKEHTVYPEENCVFSALRSTPFDQVRV
VILGQDPYPGKGQAHGLSFSVPEGQRLPPSLINIFRELKTDLGIENHKGCLQSWANQGILLLNTVLTVRAGEPF
SHAGKGWELFTDAIVTKLIQERTHIIFVLWGAAARKKCELLFNSKHQHAVLSSPHPSPLAAHRGFFGCSHFSKI
NYLLNKLNKPMINWKLP

>core/660/1/Org1_Gene232

MDLAVELKEGILLVDKPQGRTSFSLIRALTKLIGVKKIGHAGTLDPFATGVMVMLIGRKFTRLSDILLFEDKEY
EAIAHLGTTTDSYDCDGKVVGRSKKIPSLEEVLSAAEYFQGEIQQLPPMFSAKKVQGKKLYEYARKGLSIERH
HSTVQVHLQITKYEYPLLHFVVSCSKGTYIRSIAHELGTMLGCGAYLEQLRRLRSGRFSIDECIDGNLLDHPDF
DISPYLRDAHGNSL

>core/661/1/Org1_Gene571

MIGDKIILFVTEDLSLSSQLKDLASQRSDYQILVSPVFPTSFESVAIFCEYLLLPEQIFSPGIFPEEDLIVLFDTFQE
EAITKVLNQGATGYLLRPITAKVLDAVIRAFLRQHEVLEHSIPDTMTFGDHTFRVLNLVIESPEGSVYLTPSEA
GILKKLLINRGHLCLRKNLLAEIKGNTKEIIARNVDVHIASLRKKLGPYGSKIVTIRGVGYLFSDDDSIPLQNHD
NTAHPNEE

>core/662/1/Org1_Gene873

MTKVRLNKFLASAGVASRRKCDEIIFSGSVTVNGRVAEGPFVLVDPEDKVQVGGTSVHLTKKVYFMVHKAI
GYLCSSEKKFPGTKLVIDLFAHLPYRVFTVGRLDKETSGLILVTNDGEFANKIIHPSSGITKEYLLKVSRDVSA
KDLGKLMEGTFIDGKHVRPVSVTKIRRGTVKIVVSEGKKHEIRLFADAAGLPILELKRIRIGSLVLGGLRYGEY
RELTDAELGTYMKLSD

>core/663/1/Org1_Gene162

MLQAHRLCYSCDNQVILKDASFQASPGTITIILGSSGVGKTTLFRLLAGFLPLQEGELLWNGSPLNRKDVAYM
QQKEALLPWRTALKNMTLSTELGINTSHNALSNERLEEIIHNFDLGQLLDRYPDELSGGQRQRIALAAQCLSL
KPILLLDEPFSSLDVLLKEQLYQDIVALAKKENKTVLLVTHDFHDVSCLGDVLYVIKNKTLTPVPLDPSMRPL
NNGLCFIKDLKKHLYT

>core/664/1/Org1_Gene770

MRVIFPDKHNNFPNLSKLLKKLPSVILVTSCIAPFFSYIINKFFGIPGLLEILALSVKGIQKHHFWQFLTYPLITAD
SLSLNKDQSFEITQRLLLRNVLDFFLFYKAIQHLIRKLGAFSVLVVISGQALIIGAVLWGFMALIHSSQSFFGPE
SIICGVLTVQIFLDPEKRFTIGPTPLSVSIKWGFLFVLGFYCCILIFSGAFLLLLASMLAIVLAILFCKKEKIPNPYT
TSLRF

>core/665/1/Org1_Gene458

MPTTNCIFLDLRGHSILHQLQIEEALLRVANQNFCIINSGAKDSIVLGISRNLNQDVHISRAQADHIPIIRRYSGG
GTVFIDSNTLMVSWIMNSSEASAQPQELLAWTYGIYSPLLPNTFSIRENDYVLGHKKIGGNAQYIQRHRWVH
HTTFLWDIDLDKLSYYLPIPQQQPTYRNQRSHEEFLTTLRPWFPSRDDFLERIKASGSLLFTWEEFLDNELEEIL
AQPHRKATTVLN

>core/666/1/Org1_Gene23

MLSVCYSDPCLSDFCQGKRPLRIASRNSNLAKAQVHECISLLRSWYPKLWFQLSTTETTGDREKKIPLHLVEN
SYFFTDGVDALVHKGVCDLAIHSAKDLPETPSLPVVAITRCLHPADLLVYADHYVHEPLPLSPRLGSSSLRRS
AVLKQLFPQGQILDIRGTIEERLDQLHRGHYDAIVLAKAASLRLHLHHAYSIELPPPYHALQGSLAITAKDHA
GKWKQLFTPIHCHSS

>core/667/1/Org1_Gene630

MKFFSLIFKDDDVSPNKKVLSPEAFSAFLDAKELLEKTKADSEAYVAETEQKCAQIRQEAKDQGFKEGSESW
SKQIAFLEEETKNLRIRVREALVPLAIASVRKIIGKELELHPETIVSIISQALKELTQNKHIIISVNPKDLPLVEKSR
PELKNIVEYADSLILTAKPDVTPGGCIIETEAGIINAQLDVQLDALEKAFSTILKAKNPVDEPSETSSSTDSSSLS
NDQDKKE

>core/668/1/Org1_Gene690

MNRRDMVITAVVVNAILLVALFVTSKRIGVKDYDEGFRNFASSKVTQAVVSEEKVIEKPVVAEVPSRPIAKET
LAAQFIESKPVIVTTPPVPVVSETPEVPTVAVPPQPVRETVKEEQAPYATVVVKKGDFLERIARANHTTVAKL
MQINDLTTTQLKIGQVIKVPTSQDVSNEKTPQTQTANPENYYIVQEGDSPWTIALRNHIRLDDLLKMNDLDEY
KARRLKPGDQLRIR

>core/669/1/Org1_Gene1026

MFFIVCFGFLIHKKHTILPPKAHIPTNAKHFPTIGNPYAPINITVFEEPSCSACAEFTTEVFPLLKKHYIDTGEISF
TLIPVCFIRGSKPAAQALLCIYHHDPRQADIDAYMEYFHRILTYPKEEGSHWATPEVLTKLAEGLKINSGRSVN
PKGLEQCIASGQYNEQIKKNNLYGSQVLGGQLATPTAVVGDYLIEDPTFHEIERAIQHIRQLQAVEGDHDD

>core/670/1/Org1_Gene600

MVHFSHNPIIQAYTEADFFGKSIFFCLLILSVCTWTVLHQKLAIQKNFLKAGKSLKDFLIKNRHAPLSLDIHPEL
SPFADLYFTIKRGTLELLDKNRQSAPDRGPILSSEDIQSLETLLGAIMPKYKALLHKNSFIPATTISLAPFLGLLG
TVWGILVAFTHISSGSSGNSAIMEGLATALGTTIIGLFVAIPSLIAFNYLKAHSSELISEIEQTAYLLLNSIEVKYR
NTNL

>core/671/1/Org1_Gene43

MTKHGKRIRGILKNYDFSKSYSLREAIDILKQCPPVRFDQTVDVSIKLGIDPKKSDQQIRGAVFLPNGTGKTLR
ILVFASGNKVKEAVEAGADFMGSDDLVEKIKSGWLEFDVAVATPDMMREVGKLGKVLGPRNLMPTPKTGT
VTTDVAKAISELRKGKIEFKADRAGVCNVGVGKLSFESSQIKENIEALSSALIKAKPPAAKGQYLVSFTISSTM
GPGISIDTRELMAS

>core/672/1/Org1_Gene91

MEKDLHLHEKKCLAHEAATQVTSGMILGLGSGSTAKEFIFALAHRIQTESLAVHAIASSQNSYALAKQLAIPL
LNPEKFSSLDLTVDGADEVDPQLRMIKGGGGAIFREKILLRAAKRSIILVDESKLVPVLGKFRVPLEISRFGRSA
IIEEIRHLGYEGEWRLQDTGDLFITDSSNYIYDIFSPNSYPNPEKDLLKLIQIHGVIEVGFVIEKVEVWSSNSQGL
ISKKYSV

>core/673/1/Org1_Gene164

MLQSCKKALLSIVVSILAFHPIPGMGVEAKSGFLGKVKGWFSKKEIQEEARILPVKDSLSWKRYDYTSSSGFS
VEFPGEPDHSGQIVEVPQSEITIRYDTYVTETHPDNTVYVVSVWEYPEKVDISRPELNLQEGFSGMMQALPES
QVLFMQARQIQGHKALEFWIVCEDVYFRGMLISVNHTLYQVFMVYKNKNPQALDKEYEAFSQSFKITKIREP
RTIPSSVKKKVSL

>core/674/1/Org1_Gene187

MALDEINNQNNPSQQIASSTSQTSKINQDRKTFACTVTLLVVATLMILSGIVLLFTIGSLGLSVPLSGILGTFAV
TVGAVLFITGLTILVRKSLGIEQKNEDLNFLKIKTPTPPARPLMSKFSVTCSTTSIVLGMALLIGAVVSVFFLTG
YLQLGLCAGLVGLGTALFVAGLARMSPRSLADQEGSGSADSQSNIVGIGEPKAAQEQKWYKMAVVRGEDGI
PTAIRLTPEK

>core/675/1/Org1_Gene617

MSKPSPRNANQPQKPSASFNKKTRSRLAELAAQKKAKADDLEQVHPVPTEEEIKKALGNIFEGLSNGLDLQQI
LGLSDYLLEEIYTVAYTFYSQGKYNEAVGLFQLLAAAQPQNYKYMLGLSSCYHQLHLYNEAAFGFFLAFDA
QPDNPIPPYYIADSLLKLQQPEESNNFLDVTMDICGNNPEFKILKERCQIMKQSIEKQMAGETKKAPTKKPAG
KSKTTTNKKSGKKR

>core/676/1/Org1_Gene427

MYLEDYDVFFFDLDGLLVDTEPCFYRAFLQACAEFSLEVHWDFSTYYSHTTLGTEIFSKKFIEQYPQAQEYM
AEIFAKRLQIYYKSLEHAGPALMPGVEAFIELVLSLNKTFGVVTNSPRDATHTLRTMYPILNKFLFWVTRENY
ARPKPYGDSYDYAYRTFAREGMKVIGFEDSVKGLRALSKIPATLVCINSMAEITPEDYPELKGKEFFSYPSFD
VLTEHCSQQKLL

>core/677/1/Org1_Gene801

MNTSHRKTLVFSYLSSTFTLLLVLSNLVLSSKLIPTTFFNFIIPGGLILYPLTFLISDVVNEIFGPKKARVMIFSAFI
ANLLASSIVQIFMFFPVASPEMQTAWHCLFDLSPLRFLASLLAFIVSQQLDIVLYTFFKNRTPNSSLWLRSNGS
TWISQIPDTFIVDTCILYFGMGLSFPQTLNIMFYSYIYKITFCVLTTPLFYLAVNTIRKFLGMPSTKIANTVPLIN
QP

>core/678/1/Org1_Gene122

MKKQESVLVGPSIMGADLTCLGVEAKKLEQAGSDFIHIDIMDGHFVPNLTFGPGIIAAINRSTDLFLEVHAMIY
NPFEFIESFVRSGADRIIVHFEASEDIKELLSYIKKCGVQAGLAFSPDTSIEFLPSFLPFCDVVVLMSVYPGFTGQ
SFLPNTIEKIAFARHAIKTLGLKDSCLIEVDGGIDQQSAPLCRDAGADILVTASYLFEADSLAMEDKILLLRGEN
YGVK

>core/679/1/Org1_Gene148

MKGFLSVNELIFGFQTFSVVVLGVFFASRGKAWLTGWLSLLSSIMNVFVLKQIHLWGFEVTSADVYVIGLLT
CLNYAREHYEKNDINDAMLCSWVISIAFLVLTQLHLFLIPSPNDSSQEHFLALFSSTPRIVVASLVTLIFVQIVDI
KLFTFLQRVFSKKYFAMRSTISLLFSQLIDTIIFSFLGLYGLVSNLCDVMIFAMLVKGIVITLAIPTLTVTKAVLD
RRSS

>core/680/1/Org1_Gene432

MRPHRKHVSSKSLALKQSASTHVEITTKAFRLSMPLKQLILEKSDHLPPMETIRVVLTSHKDKLGTEVHVVAS
HGKEILQTKVHNANPYTAVINAFKKIRTMANKHSNKRKDRTKHDLGLAAKEERIAIQEEQEDRLSNEWLPVE
GLDAWDSLKTLGYVPASAKKKISKKKMSIRMLSQDEAIRQLESAAENFLIFLNEQEHKIQCIYKKHDGNYVLI
EPSLKPGFCI

>core/681/1/Org1_Gene661

MALLILLRHGQSVWNEKNLFSGWVDIPLSQQGIEEAFSAGRAIQNLPIDCIFTSTLVRSLMTALLAMTNHHSK
KIPYIVHEDPKAKEMSRIYSAEEENNMIPLYQSSALNERMYGELQGKNKKQTAEQFGEERVKLWRRSYKTAP
PQGESLYDTKQRTLPYFEKNILPQLQNGKNVFVSAHGNSLRSLIMDLEKLSEEEVLSLELPTGKPVVYQWKN
HKIEKHPEFFG

>core/682/1/Org1_Gene161

MSSQPLVTTSSSLSRYVVLTGEEKVACYKKAFNHIWHGAPAIILAAALLMFCIFGFVLGSILLGAPLEGASILY
DVILPWLLPSILVFVLLVLPLNIYAYSHHKQVLALHERITQSNYKEIYDHCEKEKKTPNKKALSLYIESQVLVP
EYSKRFSSMILGKTLKIIPKKDSPESLKHDELIQKALERAKENIYMNKNQREKRDEREAKKEAKNASKTNPLW
EGLGT

>core/683/1/Org1_Gene176

MSLLIEAKNLSKTIQQQNQNISILTDVSLSLHAGETISITGASGNGKTTLLHLLGTLDVPSSGSLRFFDKDLKNQ
DLANFRNQHIGFVFQNFYLLEDDTVLKNVLMPALIARKNISKGSPVYTRALELLDLVNLEDKVRTRCSKLSG
GEKQRVAIARALINEPAILLADEPSGNLDEETSEQIHNLLLEQASALCGILIVTHNKHLASRCSREGVLSNGKLF
FHNS

>core/684/1/Org1_Gene127

MTIRVRNLAYSVNKKKILDGVTFSLERGHITLFVGKSGSGKTMILRALAGLVQPTQGDIWIEGEAPALVFQQP
ELFSHMTVLGNCTHPQIHIKGRSTEEAREKAFELLHLLDIEEVAKNYPDQLSGGQKQRVAIVRSLCMDKHTLL
FDEPTSALDPFATASFRHLLETLRDQELTVGLTTHDMQFVHSCLDRIYLIDQGTVAGVYDKRDGELDSGHPLS
KYIHSAQ

>core/685/1/Org1_Gene854

MDLKLDEVASLLDVSEHTVLQWLKEGAIPSYSMNNEYRFSREEIEDWLLHNQALMIQERGEDKEALKDLSL
KYSLYKAIHRGGVLCDVVVHSKEEALQYASKYIAQKFQLDESVLFEMLSHRENLMSTGIGEGIALPHAKDFLI
NAYYDIVVPMFLAEPIEYGALDGKPVGILFFLFACQDKSHLNLVNKIVHLGMSLNARSFFKNYPNKDQLLAY
VKEWESQTH

>core/686/1/Org1_Gene948

MILRISTVSLLTSCSFSKNSRTCFVTPERITSQKDCPVLLHPKSTTISPPLYDWIPPNREVITAYSFYCRGQGNSII
TPEGVLYDCDGLHHSITKEEFRYIHPRLIEVVRLLQQDHPKVSIIEAFCCPKHFHFLEASGISLSQLHLQGTAAT
FALDPPLPMEKLLATIKKLYKKNSDPSLSNFIVTEATLTNPELRLTQQDLGSHTEITVEILDNLQNKETLSSA

>core/687/1/Org1_Gene709

MENSQNFHDTLCQLLDRYSEELYPTLASLLNVTLPNTAISASVSSIPEKAVEVPNAEPQPITPPPPTNLSQEKTK
PSDWKCVPLHPDLSQNAILKEKYPALKDCSLPAPKIPCSIFVYEENNEEVLFFNRLAKILTQQLFPTKLTLIHAK
TNIFVNNPNFFLALAPLNVIRYKIPTTDYHQSLTQNGCIFLPLYSSLEYEKDSQLKRNLWAILNRLPFAYTPKSS

>core/688/1/Org1_Gene755

MKPQDLSPPFLWKERRPCIQDGVLYVPRHYFEHQNFSTSYHQEFFQNHTSIACELCSGNGDWVVAQAQKDP
QVLWIAVEQRFDRVRKIWSKMINHQIQNLRIVCGTAETFFQYYVPDQFLQRLVVNFPDPWPKMRHRKHRLL
QPSFVQEISRSLQDSAVFALATDDKTYLLESIEALQTHLAPRMETPYYIKMTDTYGNSWFENLWRTKGQEIFY
TEFIKKAGI

>core/689/1/Org1_Gene536

MIDPVECFPNLDGDAEAQSITQNSGTPLASELKKDISPFALGSYAAPKDTTLVQGFKPNPMAMMQDQNSNLI
DPELQEALESEELQEQINNLKGRLWDFRSTFEDSQTTAQFADEHFQAVGVIIDLINEDLNTIAEHTQQDARKE
DKEEGSVTRKIIDWVSSGEEVLNRALLYFSDRDGNRESLANFLKVQYAVQRATQRAELFASIVGTSVSSVKTI
MTTQLG

>core/690/1/Org1_Gene581

MTSWIELLDKQIEDQHMLKHEFYQRWSEGKLEKQQLQAYAKDYYLHIKAFPCYLSALHARCDDLQIRRQIL
ENLMDEEAGNPNHIDLWRQFALSLGVSEEELANHEFSQAAQDMVATFRRLCDMPQLAVGLGALYTYEIQIP
QVCVEKIRGLKEYFGVSARGYAYFTVHQEADIKHASEEKEMLQTLVGRENPDAVLQGSQEVLDTLWNFLSS
FINSTEPCSCK

>core/691/1/Org1_Gene482

MVLLSKFDFSGNKIGEVEVADSLFADEGDGLQLIKDYIVAIRANKRQWSACTRNRSEVSHSTKKPFKQKGTG
NARQGCLASPQFRGGGIVFGPKPKFNQHVRINRKERKAAIRLLLAQKIQTNKLTVVDDTVFVDALTAPKTQS
ALRFLKDCNVECRSILFIDHLDHVEKNENLRLSLRNLTAVKGFVYGININGYDLASAHNIVISKKALQELVERL
VSETKD

>core/692/1/Org1_Gene513

MQTLARLFGQSPFAPLQAHLEMVVSCVEYMLPIFTALRDGRYEELLEMAKLVSDKEYQADCIKNDMRNHLP
AGLFMPISRAGILEIISIQDSIADTAEDVAILLTIRRLNFYPSMETLFFRFLEKNLEAFELTMTLLHEFNQLLESSF
GGRKADKARLLVGRVAKSEHESDVLQRELMQIFFSDDFIIPEKEFYLWLQVIRRTAGISDSSEKLAHRINMTLE
EK

>core/693/1/Org1_Gene796

MSTTTVKHFIHTASRWEPVLKEIVASNYWHAQWINTLSFLENSGAKKISASEHPTEVKEEVLKHAAEEFRHG
HYLKTQISRISETSLPDYTSKNLLGGLLTKYYLHLLDLRTCRVLENEYSLSGQTLKTAAYILVTYAIELRASEL
YPLYHDILKEAQSKITVKSIILEEQGHLQEMERELKDLPHGEELLGYACQFEGELCLQFVERLEQMIFDPSSTF
TKF

>core/694/1/Org1_Gene156

MTKNAINSQTTTPQPNLTDAEPIASRAQCKSIAVIISLFALGMLLLCLGIILISIPIPGLAAQVALGLGIVSLILGIA
LANIGFLCLLLRCKQVPQKPDTLPSESSKQPSEGSTPTALPWQAGEFLEKVQVSATPILLPKNKDEELSAKVM
KEGAEAASSIKQAVLESTEKLIDARKQEESRREARKKIVAEEAEASRKRIQQQMAADQEALRKRKEEVAKRK

>core/695/1/Org1_Gene364

MKKYFITGLVILLPLAITIAIVTMIMNFLTQPFVGLASEFFEKFSFYTKHRALLKFVLQIILLFGLFFATVLLGFL
TRIMIFKSLLSIYDKILHRIPIIKTVYKAAQQVMTTIFGSKSGSFKQVVMVPFPNANVQCIGLVAGDAPTVCCT
GEKEDDPLVTVFIPTTPNPTSGFLTLFRKSDIVFLDMKIEDAFKYIISCGVLSTPMACPSSPLPDELHQDQGS

>core/696/1/Org1_Gene823

MGQKGCPIGFRTGVTKKWRSLWYGNKQEFGKFLIEDVRIRQFLRKKPSCQGAAGFVVRRMSGKIEVTIQTAR
PGLVIGKKGAEVDLLKEELRALTGKEVWLEIAEIKRPELNAKLVADNIARQIERRVSFRRAMKKAMQSVMD
AGAVGVKIQVSGRLAGAEIARSEWYKNGRVPLHTLRADIDYATACAETTYGIIGIKVWINLGENSSSTTPNNP
AAPSAAA

>core/697/1/Org1_Gene942

MTLYLGLNQKTARKYQAHYLPILTLFPYAKSTPQNKRALQFLPQATHVILTSPSSTHLFLSRMTSLLSKATLK
TKTYLCIGESTKERLLSFLGQVKYVVATQEIAEGIFPLLQALPSSARILYPHSSLARPVIREFLYNRFTFFSYPHY
TVKPRKLKKNILSKYKKIILTSPSTVRAFAKIFPRFPEKTYWCQGRMTLQEFQKFSSQKQVSLLETLGKSRTSP

>core/698/1/Org1_Gene559

MLMMLMMIIGITGGSGAGKTTLTQNIKEIFGEDVSVICQDNYYKDRSHYTPEERANLIWDHPDAFDNDLLISD
IKRLKNNEIVQAPVFDFVLGNRSKTEIETIYPSKVILVEGILVFENQELRDLMDIRIFVDTDADERILRRMVRDV
QEQGDSVDCIMSRYLSMVKPMHEKFIEPTRKYADIIVHGNYRQNVVTNILSQKIKNHLENALESDETYYMVN
SK

>core/699/1/Org1_Gene198

MQSDLIQILLKETVNTLYMVSTAFFFSCAIGGMLGLGLFCTSPKSLNPKKSLYATISMILSFLTAIPFAILIVILFP
ITRWIVGTSLGPTASIVPLTIGAIPFVVTIVVDAFRNSALNYLESAVALGIPKRNILFGILLPESYPQLIFSLKSLV
VHLISCSTLAGFVGGGGLGQLLLQYGYYRFEWSVTTSVLVITLVLIESVRILGDFWGRRVLKYRGIL

>core/700/1/Org1_Gene871

MSTVTTEPCSSIHISLNNDWRDSQPYSLDRASELLHFRFLPSLVFSNWKVEQQIETLCHKSEKRRLISPLAKWL
GKLHKQDLLCPPAPPVSVCWINAHVGYGVFARDEIAPWTYIGEYTGILRHRQAIWMDENDYCFRYPMPLFTL
RYFTIDSGKQGNVTRFINHSEQPNAEAIGVFSEGLFHVIIRTIAPIYAGQEICYHYGPLYWKHRKKREEFIPEEE

>core/701/1/Org1_Gene483

MRSHISVMGKKEGMIHIFDKDGSLVACSVIRVEPNVVTQIKTKESDGYFSLQIGAEEMNAPAHTITKRVSKPK
LGHLRKAGGRVFRFLKEVRGSEEALNGVSLGDAFGLEVFEDVSSVDVRGISKGKGFQGVMKKFGFRGGPGS
HGSGFHRHAGSIGMRSTPGRCFPGSKRPSHMGAENVTVKNLEVIKVDLEKKVLLVKGAIPGARGSIVIVKHSS
RT

>core/702/1/Org1_Gene798

MQRIIIVGIDTGVGKTIVSAILARALNAEYWKPIQAGNLENSDSNIVHELSGAYCHPEAYRLHKPLSPHKAAQI
DNVSIEESHICAPKTTSNLIIETSGGFLSPCTSKRLQGDVFSSWSCSWILVSQAYLGSINHTCLTVEAMRSRNLN
ILGMVVNGYPEDEEHWLTQEIKLPIIGTLAKEKEITKTIISCYAEQWKEVWTSNHQGIQGVSGTPSLNLH

>core/703/1/Org1_Gene301

MNAVNTPKKILCIVADYREISPLIEQLDFTQINEHLYSYRCTDYHLDLYIVHVWGSTAVLNALQSYCQAYTDY
DLWINPGFVGACSPEIPLGQCYTIEKIANLTTDTPPVLSEDPPYIFDALPDSLPKSSLVTSPVLYHYGFHKTFKL
LDMEGYAIASQAAEHHIPCSFLKITSDYTVPGDCPFSRLEEVSQKLTQTLVELLPELMERAIPPKLLLPCP

>core/704/1/Org1_Gene345

MLGSLPCYPGAGNIEEYKNRYFYCQLCAEVVSPYVVPVIVVDVQGAPPTGILQVLRCKQHKFQGLPVHGPIT
SLWALEPVGKGAPQLESAMYELCSQVRNFDICSIVSWVFGGLCIFAGLIVGVMVEAPLIAGLSAWVIPCIIGGV
GAILCLFAILMAYLGRGRVREWLNLSHEYITQCHCRQIQAHSQNYSVITEYPATCALSQPITKLPNGSRRDN

>core/705/1/Org1_Gene128

MDHWLAIARLLLRGCGYTLCVSGIGILCGSILGLLIGTVTSLYFPSKLTKLLANSYVTVIRGTPLFIQILIIYFGLP
EVLPIEPTPLVAGIIALSMNSAAYLAENIRGGINSLSIGQWESAMVLGYKKYQIFVYIIYPQVFKNILPSLTNEFV
SLIKESSILMVVGVPELTKVTKDIVSRELNPMEMYLICAGLYFLMTTSFSCISRLSEKRRSYDN

>core/706/1/Org1_Gene671

MKRVIYKTIFCGLTLLTSLSSCSLDPKGYNLETKNSRDLNQESVILKENRETPSLVKRLSRRSRRLFARRDQTQ
KDTLQVQANFKTYAEKISEQDERDLSFVVSSAAEKSSISLALSQGEIKDALYRIREVHPLALIEALAENPALIEG
MKKMQGRDWIWNLFLTQLSEVFSQAWSQGVISEEDIAAFASTLGLDSGTVASIVQGERWPELVDIVIT

>core/707/1/Org1_Gene517

MQKLVHNIWKKFYSFSSAIAICIVLASFLSLKIVSNTYKHSQAKRNSILLLTRAAEVAVSQGFLPSKSALSSLEQ
AYHLGGESMKPYAGFLASCFYIHNEPLRGAYYAGLAYNNSQALQLPHPIQKLLKEISEAQADQLYDVALSKS
YQLLQTANSSPEYPTLSFLTLLRVIELKELLHQDVSQDFAALKSSPLFHQFERMYSDGEWTLSKRFGKKG

>core/708/1/Org1_Gene414

MIITIDGPSGTGKSTTAKALADHLHFNYCNTGKMYRTLAYARLQSPWATLPLTKFLEEPPFSFTFATGQPLESF
FNGHLLTSELTTQEVANAASELSQLPEVRAFMQDLQRRYAQLGNCVFEGRDMGSKVFPNADLKIFLTSSPEV
RAQRRLKDLPEGTLSPEQLQAELVKRDAADAQRAHDPLVIPENGIVIDSSDLTIRQVLEKILALLFRNEL

>core/709/1/Org1_Gene291

MRKMLVLLASLGLLSPTLSSCTHLGSSGSYHPKLYTSGSKTKGVIAMLPVFHRPGKSLEPLPWNLQGEFTEEI
SKRFYASEKVFLIKHNASPQTVSQFYAPIANRLPETIIEQFLPAEFIVATELLEQKTGKEAGVDSVTASVRVRVF
DIRHHKIALIYQEIIECSQPLTTLVNDYHRYGWNSKHFDSTPMGLMHSRLFREVVARVEGYVCANYS

>core/710/1/Org1_Gene702

MSKKPLYVAHPWHSPTLTQDNYESLCCYIEITPYDSVKFELDKATGLLKVDRPQKFSNFCPCLYGLLPQTYC
GTASGNYSGEQTRREGIQGDKDPLDVCVLTEKNIHHGNILLQARPIGGLRIIDSGEADDKIIAVLEDDLVFAEIE
DISDCPGTVLDMIQHYFLTYKATPNHLIKGSPAKIEIVGIYGKKEAQKVIQLAHEDYLSYIGDTAEVN

>core/711/1/Org1_Gene510

MSVNPSGNSKNDLWITGAHDQHPDVKESGVTSANLGSHRVTASGGRQGLLARIKEAVTGFFSRMSFFRSGAP
RGSQQPSAPSADTVRSPLPGGDARATEGAGRNLIKKGYQPGMKVTIPQVPGGGAQRSSGSTTLKPTRPAPPPP
KTGGTNAKRPATHGKGPAPQPPKTGGTNAKRAATHGKGPAPQPPKGILKQPGQSGTSGKKRVSWSDED

>core/712/1/Org1_Gene75

MNTSISEIQRFLSMIAFEKELVSEDFSVVAGIDEAGRGPLAGPVVASACILPKGKVFPGVNDSKKLSPKQRAQV
RDALMQDPEVCFGIGVISVERIDQVNILEATKEAMLQAISSLPISPDILLVDGLYLPHDIPCKKIIQGDAKSASIA
AASILAKEHRDDLMLQLHRLYPEYGFDRHKGYGTSLHVEAIRRYGPSPCHRKSFSPIKQMCAIV

>core/713/1/Org1_Gene172

MTKGSVFIIMGPPGSGKGTQSQYLANRIGLPHISTGDLLRAIIREGTPNGLKAKAYLDKGAFVPSDFVWEILKE
KLQSQACSKGCIIDGFPRTLDQAHLLDSFLMDVHSNYTVIFLEISEDEILKRVCSRFLCPSCSRIYNTSQGHTEC
PDCHVPLIRRSDDTPEIIKERLTKYQERTAPVIAYYDSLGKLCRVSSENKEDLVFEDILKCIYK

>core/714/1/Org1_Gene316

MTSKKSYKSYFFDPLWSNNQILIAILGICSALAVTTTVQTAITMGIAVSIVTGCSSFFVSLLRKFTPDSVRMITQ
LIIISLFVIVIDQFLKAFFFDISKTLSVFVGLIITNCIVMGRSESLARHVTPIPAFLDGFASGLGYGWVLLVIGVIRE
LFGFGTLMGFRIIPQFVYASETHPDGYQNLSLMVLAPSAFFLLGIMIWLVNIRDSKKRKR

>core/715/1/Org1_Gene14

MYFYKYVIIDTSGYYPFLACVDNQQVLEHWSLPVGPDLGIVLEFLFKSKNLSFQGVAVALGPGNFSATRIGIS
FAQGLAMAKNVPLLGYSSLEGYLLSKDEKKALMLPLGKRGGVLTLSSEIPEEGLNEKRRGVGPGALLSYEEA
SDYCVAHGYYHVISPNPQLFASSFSDKITVEEVAPSVEQIRRHVISQFMFVEYDKQLSPDYRSYSCIF

>core/716/1/Org1_Gene323

MATSVAPSPVPESSPLSHATEVLNLPNAYITQPHPIPAAPWETFRSKLSTKHTLCFALTLLLTLGGTISAGYAG
YTGNWIICGIGLGIIVLTLILALLLAIPLKNKQTGTKLIDEISQDISSIGSGFVQRYGLMFSTIKSVHLPELTTQNQ
EKTRILNEIEAKKESIQNLELKITECQNKLAQKQPKRKSSQKSFMRSIKHLSKNPVILFDC

>core/717/1/Org1_Gene415

MIFRICKFFTWVAFSLFYKLKVYGVKKNFIKGPAIIAVNHNSFLDPIALHMCVHECIYHLARASLFNIPWLWK
QWGCFPVRQDEGNSAAFKIASRLFNKRKKLVIYPEGARSPDGQLQPGKVGIGMMAAKSRVPIIPVYIRGTFEA
FNRHQKIPHVWKTITCVFGTPMYFDDIIQNPEIKNKETYQIITNQTMNKIAELKAWYESGCKGDVP

>core/718/1/Org1_Gene425

MIKSSLILLSGGQGTRFGSKIPKQYLPLNGTPLVLHSLKILSSLPQIAEVIVVCDPSYQETFQEYPVSFAIPGERR
QDSVFSGLQQVSYPWVIIHDGARPFIYPDEIHDLLETAEKIGATALASPIPYTIKQRNPVRTLDRDNLAIIHTPQ
CIKTEILREGLALAKEKQLTLVDDIEAAEIIGKPSQLVFNKHPQIKISYPEDLTIAQALL

>core/719/1/Org1_Gene450

MMNYEDAKLRGQAVAILYQIGAIKFGKHILASGEETPLYVDMRLVISSPEVLQTVATLIWRLRPSFNSSLLCG
VPYTALTLATSISLKYNIPMVLRRKELQNVDPSDAIKVEGLFTPGQTCLVINDMVSSGKSIIETAVALEENGLV
VREALVFLDRRKEACQPLGPQGIKVSSVFTVPTLIKALIAYGKLSSGDLTLANKISEILEIES

>core/720/1/Org1_Gene557

MARYCGPKNRVARRFGANIFGRSRNPLLKKPHPPGQHGMQRKKKSDYGLQLEEKQKLKACYGMIMEKQLV
KAFKEVIHKQGNVAQMFLERFECRLDNMVYRMGFAKTIFAAQQLVAHGHILVNGRRVDRRSFFLRPGMQIS
LKEKSKRLQSVKDALESKDESSLPSYISLDKTGFKGELLVSPEQDQIEAQLPLPINISVVCEFLSHRT

>core/721/1/Org1_Gene639

MKQFILRTLNALFPNPKPSLEGWSSPFQLLIAILLSGNSTDKAVNSVTPQLFAKAPDAQSILDLPPGKLYQLIAP
CGLGERKSAYIYQLSQILVRDFHGEPPNDMALLTQLPGVGRKTASVFLGIAYGKPTFPVDTHILRLAQRWKIS
EKKSPSAAEKDLARFFGHENTPKLHLQLIYYARQYCPALHHKIDNCPICSYLAKEANSTRT

>core/722/1/Org1_Gene52

MSVQVKLTKNSFRLEKQKLARLQTYLPTLKLKKALLQAEVQNAVKDAAECDKDYVQAYERIYAFAELFSIP
LCTDCVEKSFEIQSIDNDFENIAGVEVPIVREVTLFPASYSLLGTPIWLDTMLSASKELVVKKVMAEVSKERLK
ILEEELRAVSIRVNLFEKKLIPETTKILKKIAVFLSDRSITDVGQVKMAKKKIELRKARGDECV

>core/723/1/Org1_Gene49

MANLNADGKLKQICDALRLDTLKPAEDEAAALLHNAKEQAKRIIQEAQEEARKILETAEERAHQKIKQGEVA
LSQAGKRALEALKQAVENKIFRESLVEWLEHVTTDPEVSTKLIQALVQALEAQGVSGNLTAYIGKHVSPRAV
NELLGKAVTTKLRKKSVVVGSFVGGVQLKVEEKNWVLDLSSSALLEIFTRYLQKDFREMIFQGS

>core/724/1/Org1_Gene570

MTYLASSIFSPEDFLYPEIISKAHYTWDILDLMDQMLENHVFSGIHGTVESGVTLKNIEKIEIAEDAYVESGAYI
VGPCILGSQTEVRHGAYLRGNVITGSRCVVGHCTEIKNSYLGHHTKAAHFAYLGDSVLSSEVNLGAGVRCA
NFRLDGRNIYVRSTSDKSKKIDTGRRKLGAFLGKGVAIGCNVVINPGQHILPHTRIRPGQVI

>core/726/1/Org1_Gene27

MSFVPYSLPELPYDYDALEPVISSEIMILHHQKHHQIYINNLNAALKRLDAAETQQNLNELIALEPALRFNGGG
HINHSLFWETLAPIDQGGGQPPKHELLSLIERFWGTMDNFLKKLIEVAAGVQGSGWAWLGFCPAKQELVLQ
ATANQDPLEPLTGKLPLLGVDVWEHAYYLQYKNVRMDYLKAFPQIINWGHIENRFSEIISSK

>core/727/1/Org1_Gene460

MYDYIRGTLTYVHTGAIVIECQGIGYHIAITERWAIECIRALHQDFLVFTHVIFRETEHLLYGFHSREERECFRI
LISFSGIGPKLALAILNALPLKVLCSVVRSEDIRALASVSGIGKKTAEKLMVELKQKLPDLLPLDSRVETSQTHT
TSSCLEEGIQALAALGYSKIAAERMIAEAIKDLPEGSSLTDILPIALKKNFSGVNKD

>core/728/1/Org1_Gene880

MKIVIASSHGYKIRETKTFLKRLGDFDIFSLSDFPDYKLPQEQEDSITANALTKGIHAANHLGCWVIADDTMLR
VPALNGLPGPLSANFAGVGAYDKDHRKKLLDLMSSLESLVDRSAYFECCVVLVSPNQEIFKTYGICEGYISHQ
EKGSSGFGYDPIFVKYDYKQTFAELSEDVKNQVSHRAKALQKLAPHLQSLFEKHLLTRD

>core/729/1/Org1_Gene727

MGLPNYITFSRLFITPIFMILYLKGKWFGITPVVLPYVLLALLAISELTDAIDGYVARKFSQVTDLGKLLDPMA
DSIYRISIYLTFTQPPVNLPLLLVFIFLARDSVISTLRTVCAFRGRVVAARASGKLKAILQGVSFFLILLVMIPHSL
GLLSQNGLEIFASVTVSIIAVYSIASGIEYFWMNKNFLSQRAKTKDSEKNHESKD

>core/730/1/Org1_Gene193

MFIVIEGGEGSGKSSLAKALGDQLVAQDRKVLLTREPGGCLIGERLRDLILEPPHLELSRCCELFLFLGSRAQH
IQEVIIPALRDGYIVICERFHDSTIVYQGIAEGLGADFVADLCSKVVGPTPFLPNFVLLLDIPADIGLQRKHRQK
VFDKFEKKPLSYHNRIREGFLSLASADPSRYLVLDARESLASLIDKVMLHTQLGLCT

>core/731/1/Org1_Gene76

MNKILVDSPFSPDHQKCCPKLFTISAPAGVGKTTLVRMLEQEFSSAFAETISVTTRKPREGEVPGKDYHFVSHE
EFQRLLDRQALLEWVFLFGECYGTSMLEIERIWSLGKHAVAVIDIQGALFIRSRMPSVSIFIAPPSQEELERRLA
SRGSEEGSQRKERLEHSLIELAAANQFDYVIINDDLNQAYRVLKSIFIAEEHRNIL

>core/732/1/Org1_Gene595

MHAKLSFFILLSLLFSGIDCSRLHAAGRSPSLQGVLAEIEDISAKLASHEVEIVMLSERLDEQDSKCQKWTAAK
PETLAQKIRELESDQKALAKTLAVLTTSVKDLQTNLQSKLQEIQKDHRALAQDLRLVRRSLLALVDSSSPGA
YADFSDPVPENIYIVREGDSLSKIAKKYKLSVTELKKINKLDSDAIYAGQRLCLQRNKQ

>core/733/1/Org1_Gene278

MIGAQKKQSGKKTASRAVRKPAKKVAAKRTVKKATVRKTAVKKPAVRKTAAKKTVAKKTTAKRTVRKTV
AKKPAVKKVAAKRVVKKTVAKKTTAKRAVRKTVAKKPVARKTTVAKGSPKKAAACALACHKNHKHTSSC
KRVCSSTATRKHGSKSRVRTAHGWRHQLIKMMSR

>core/734/1/Org1_Gene209

MTSPIPFQSSGDASFLAEQPQQLPSTSESQLVTQLLTMMKHTQALSETVLQQQRDRLPTASIILQVGGAPTGG
AGAPFQPGPADDHHHPIPPPVVPAQIETEITTIRSELQLMRSTLQQSTKGARTGVLVVTAILMTISLLAIIIIILAV
LGFTGVLPQVALLMQGETNLIWAMVSGSIICFIALIGTLGLILTNKNTPLPAS

>core/735/1/Org1_Gene647

MTLVPYVVEDTGRGERAMDIYSRLLKDRIVMIGQEITEPLANTVIAQLLFLMSEDPKKDIQIFINSPGGYITAG
LAIYDTIRFLGCDVNTYCIGQAASMGALLLSAGTKGKRHALPHSRMMIHQPSGGIIGTSADIQLQAAEILTLKK
HLANILSECTGQPVEKIIEDSERDFFMGAEEAISYGLIDKVVTSAKETNKDTSST

>core/736/1/Org1_Gene453

MLKLLKVSITGDLSSGKTEACQVFQELGAYVVSADEISHSFLIPHTRIGRRVIDLLGSDVVVDGAFDAQAIAA
KVFYNSVLLQGLEAILHPEVCRIIEEQYHQSIQDGNYPLFVAEVPLLYEIHYAKWFDSVILVMANEDIRRERFM
KKTGRSSEDFDQRCSRFLNVEEKLAQADVVVENNGTKKELHQKIEEYFYALKGAL

>core/737/1/Org1_Gene776

MLILLNLSLLFYVLFDSPGSIPVFVALLKNFSRKKQQRVILRECLFALGALILFVTFGRSFFQFLDISLYAFQIIG
GFLLFTVSIKMMLAPMPEKAKDDTSKTEPIFFPLAFPVITGPAVITALLSYMEEGIYSREIIFTAMIIAWAFSLFT
LLCSSFFDRLFGNFGLLALERLFGIALLLMSVNLMLKGISIAFNIGFYIG

>core/738/1/Org1_Gene381

MFSGIIQELGEVCFFEAQGNGLSLGIKSTPLFVTPLVTGDSVAVDGVCLTLTSCNESKIFFDVIPETLACTTLGE
KRCSDQVNLEAALKMGDSIGGHLLSGHVFGTAEIFLIKENRYYFRGSKELSQYLFEKGFIAIDGISLTLVSVDS
DTFSVGLIPETLQRTTLGKKREGERVNIEIDMSTKIQVDTVKRILASSGKD

>core/739/1/Org1_Gene756

MFAYRTLLTHNVVQVSHEIFKTTVVPGDTVIDATCGNGNDSLFLARLLQGEGRLVVYDIQKEALSNALLLFE
THLSEQERSVIEMKEQSHEHILEKDVKLIHYNLGYLPKGNKEITTLARTTEISLEYALNIVRPDGLITVVCYPGH
PEGEKETHSVESLAQRLHPKEWCVSSFYVANRCRAPRLFIFQRQGSESSVDKG

>core/740/1/Org1_Gene215

MTRYPDYLSKLIFFLRKLPGIGFKTAEKLAFELISWDSEQLKILGNAFHNVASERSHCPLCFTLKESKEADCHF
CREERDNQSLCIVASPKDVFFLERSKVFKGRYHVLGSLLSPITGKHIENERLSILKSRIETLCPKEIILAIDATLEG
DATALFLKQELQHFSVNISRLALGLPIGLSFDYVDSGTLARAFSGRHSY

>core/741/1/Org1_Gene435

MGFACRYLFFFIVLFASGSFGNQLLSVPCWLSEEESFYTHRFDFSKSYPDMENMEIQAQRKKRVEFNLTGEFP
KLETLNYQGSFGHLRAKCRGVYPVLYALNFSCSSCKMDMDFRGKWNRSSTITISNQKESINLKLPKDVGVIV
NTKTSLKGNVCPGSTFIKQGWGVWNKIYHNDLVGFSEVTLIFNVSSEGGTITFS

>core/742/1/Org1_Gene503

MRLFSLGTIYLFFSLALSSCCGYSILNSPYHLSSLGKSLLQERIFIAPIKEDPHGQLCSALTYELSKRSFAISGRSS
CAGYTLKVELLNGIDKNIGFTYAPNKLGDKTHRHFIVSNEGRLSLSAKVQLINNDTQEVLIDQCVARESVDFD
FEPDLGTANAHEFALGQFEMHSEAIKSARRILSIRLAETIAQQVYYDLF

>core/743/1/Org1_Gene915

MNWVPKTIDHVDPESEIDIRKVVSCYKLIKECQPEFRSLISELLGVIRCGLRLLKRSKYKEQARTVSDEDAPLF
CLTRSYYQDGYLTPLRAGPRDLINHYIHLRRRENPKHFFSPKHPCYYARLAFNESVCVYRELFDIERLTKMYV
EGDYSKEQEKNLQAILSFVKTLDEGKDFLIEHKDTDLIGRGFTDVFCT

>core/744/1/Org1_Gene726

MRIALSLLSLLMIFPIFGEESRPGSEDGNSNTQEIVGSQDTQVCLYHSYEQGLQASRIEGKPLVIVVLCNSGDD
GQACTIGLSETCEEVLSVLSGSIFSELANFVVLVPSGVNPLIYPPIEDPILAEIVKFKELFKDESFPTGLSIIVVGV
TPEGPGDIIEVSPVSLTVEEEETLPSEQTTEVESTSELQSEDPAIA

>core/745/1/Org1_Gene403

MSSNLHPVGGTGTGAAAPESVLNIVEEIAASGSVTAGLQAITSSPGMVNLLIGWAKTKFIQPIRESKLFQSRAC
QITLLVLGILLVVAGLACMFIFHSQLGANAFWLIIPAAIGLIKLLVTSLCFDEACTSEKLMVFQKWAGVLEDQL
DDGILNNSNKIFGHVKTEGNTSRATTPVLNDGRGTPVLSPLVSKIARV

>core/746/1/Org1_Gene7

MSLPLVLGSSSPRRKFILEKFRVPFTVIPSNFDESKVSYSGDPIAYTQELAAQKAYAVSELHSPCDCIILTGDTIV
SYDGRIFTKPQDKADAIQMLKTLRNQTHDVVTSIAVLHKGKLLTGSETSQISLTMIPDHRIESYIDTVGTLNNC
GAYDVCHGGLILKKVHGCVYNVQGLPIQTLKYLLEELNIDLWDYSI

>core/747/1/Org1_Gene363

MLQEHFFLSEDVITLAQQLLGHKLITTHEGLITSGYIVETEAYRGPDDKACHAYNYRKTQRNRAMYLKGGSA
YLYRCYGMHHLLNVVTGPEDIPHAVLIRAILPDQGKELMIQRRQWRDKPPHLLTNGPGKVCQALGISLENNR
QRLNTPALYISKEKISGTLTATARIGIDYAQEYRDVPWRFLLSPEDSGKVLS

>core/748/1/Org1_Gene593

MTLSLVGKEAPDFVAQAVVNGETCTVSLKDYLGKYVVLFFYPKDFTYVCPTELHAFQDALGEFHTRGAEVI
GCSVDDIATHQQWLATKKKQGGIEGITYPLLSDEDKVISRSYHVLKPEEELSFRGVFLIDKGGIIRHLVVNDLP
LGRSIEEELRTLDALIFFETNGLVCPANWHEGERAMAPNEEGLQNYFGTID

>core/749/1/Org1_Gene312

MFRRTGKGPFEDVQTLYEEETSSPSSYSPYSRSERPETPPSLFDNPKASEARPLNHNLTEESSLPQWSSTPRTES
LLPLEEPETTLGEGVTFKGELAFERLLRIDGTFEGILVSKGKIIIGPKGVVKADIQLQEAIIEGVVEGNITVSGKV
ELRGGAIIKGDIQANTLCVDEGVRILGYLAIAGITDHSERERDL

>core/750/1/Org1_Gene790

MAYGTRYPTLAFHTGGIGESDDGMPPQPFETFCYDSALLQAKIENFNIVPYTSVLPKELFGNIVPVDTCVKSFK
HGAVLEVIMAGRGAALSDGTHAIATGIGICWGKDKNGELIGGWAAEYVEFFPTWINDEIAETHAKMWLKKS
LQHELDLRSIAKHSEFQFFHNYINIKQKFGFCLTALGFLNFENAEPAKVN

>core/751/1/Org1_Gene583

MKKWISILILSFLSLLSILPVLAITINHVKISQRWSDLNSQILTLKVIRDHEDQVIKHNARISKDRNNLSIESLNAS
CKQLRPLSKERERLNKLNSNSLLAQSKEVWERKRALEKSNHQLVWNCEQMHNDFAFVRLEQATEMDNEDI
ESLFSLFNPENPVAPLVFFTCWKMTKQTTPLGNEVWLTHAEAISRWI

>core/752/1/Org1_Gene596

MNIHSLWKLCTLLALLALPACSLSPNYGWEDSCNTCHHTRRKKPSSFGFVPLYTEEDFNPNFTFGEYDSKEEK
QYKSSQVAAFRNITFATDSYTIKGEENLAILTNLVHYMKKNPKATLYIEGHTDERGAASYNLALGARRANAI
KEHLRKQGISADRLSTISYGKEHPLNSGHNELAWQQNRRTEFKIHAR

>core/753/1/Org1_Gene313

MVLFSLLFPKLCYGCQAPGAYFCSNCLEKLLVEDREGRCLHCFRYLGSSETRLCSQCSPSSQLQAFSLYLPSQ
TALSVYARACEGKRPALQFFSKSIAFELASLDETPSCIAYITSTISRKIVVEVAKLEKLLRIPLWPWLPKKRQIE
KLPKGEGICFLSAYPLSQKWMQTIVGGSASPLVSISLFLSQNDQ

>core/754/1/Org1_Gene185

MKRYVVGISGASGVILAVKLIKELVNAKHQVEVIISPSGRKTLYYELGCQSFDALFSEENLEYIHTHSIQAIESS
LASGSCPVEATIIIPCSMTTVAAISIGLADNLLRRVADVALKERRPLILVPRETPLHTIHLENLLKLSKSGATIFPP
MPMWYFKPQSVEDLENALVGKILAYLNIPSDLTKQWSNPE

>core/755/1/Org1_Gene663

MKVIYYEIEEIPSTNTMAKSYMHLWDPYALTVISTKCQTAGTGKFGKSWKSSKGDLLNTFCFFITDLHIDVSR
LFRLGTEAVVALCKDLGITEAKIKWPNDVLVHGEKLCGVLPETLPVEGLLGVVLGIGLNGNTTKQALKDVG
QPATSLQEILGHPIDLETTRELLIHHLLGVLQENLPDSLATKSNRGNI

>core/757/1/Org1_Gene374

MADGEVHKLRDIIEKELLEARRVFFSEPVTEKSASDAIKKLWYLELKDPGKPIVFVINSPGGSVDAGFAVWDQ
IKMLTSPVTTVVTGLAASMGSVLSLCAAPGRRFATPHSRIMIHQPSIGGPITGQATDLDIHAREILKTKARIIDV
YVEATNQPRDIIEKAIDRDMWMTANEAKDFGLLDGILFSFNDL

>core/758/1/Org1_Gene35

MIKKFFIYSLIFSCSFSAPLKGICNEDVSSQSRIEEDPEVLITQLNELIETPIEEGKEIRNELQAISDGQKSSEEIEES
CGTSDSEGLSEKTDKESSNEYVLDFFDSMVQRLEGISKMCQSGQVAQIIDCFNREFDIRNRELELKNRELELRE
KDLEFKKSILDWNKEKVSRELAFQREQDIKQTLMLLKK

>core/759/1/Org1_Gene285

MSIKEDKWIREMALNADMIHPFVNGQVNVNEETGEKLISYGLSSYGYDLRLSREFKVFTNVYNSVVDPKCFT
EDIFISITDDVCIVPPNSFALARSVEYFRIPRNVLTMCIGKSTYARCGIIVNVTPFEPEWEGHVTIEISNTTPLPAK
IYANEGIAQVLFFESSTTCEVSYADRKGKYQKQQGITVPCV

>core/760/1/Org1_Gene684

MVRVSTSEFRVGLRIEIDGQPYLILQNDFVKPGKGQAFNRIKVKNFLTGRVIERTYKSGESVETADIVERSMRL
LYTDQEGATFMDDETFEQEVVFWEKLENIRQWLLEDTIYTLVLYNGDVVAVEPPIFMELSIAETAPGVRGDT
ASGRVLKPAVTNTGAKIMVPIFIDEGELVKVDTRTGSYESRVSK

>core/762/1/Org1_Gene987

MMKIPYARLEKGSLLVASPDINQGVFARSVILLCEHSLNGSFGLILNKTLGFEISDDIFTFEKVSNHNIRFCMGG
PLQANQMMLLHSCSEIPEQTLEICPSVYLGGDLPFLQEIASSESGPEINLCFGYSGWQAGQLEKEFLSNDWFLA
PGNKDYVFYSEPEDLWALVLKDLGGKYASLSTVPDNLLLN

>core/763/1/Org1_Gene814

MIRRLEYYGSPILRKKSSPIAEITDEIRNLVSDMCDTMEAHRGVGLAAPQVGKNVSLFVMCVDRETEDGELIF
SESPRVFINPVLSDPSETPIIGKEGCLSIPGLRGEVFRPQKITVTAMDLNGKIFTEHLEGFTARIIMHETDHLNGV
LYIDLMEEPKDPKKFKASLEKIKRRYNTHLSKEELVS

>core/764/1/Org1_Gene759

MALNFKINRQIRAPKVRLIGSAGEQLGILAIKDALDLAREAGLDLVEVASNSEPPVCKIMDYGKYRYGLTKKE
KDSKKAQHQVRIKEVKLKPNIDENDFSTKLKQARTFVEKGNKVKITCMFRGRELAYPEHGFKVVQKMSQGL
EDIGFVEAEPKLAGRSLICVVAPGTVKTKKKQEKSHAQDENQ

>core/766/1/Org1_Gene447

MRILAGKYKGKSLKTFSNPHIRPTSGLVKEAFFSICREDIEGAAFLDLFAGMGAIGFEALSRGAASVVFVDISIK
AIQLIHTNSALLGEQLPVVIFRQDAQSAIQRLIKQKRSFDLIYIDPPYELCNCYVETLLQKIVSGNILNPEGTLFL
ENASDEEIACEGLTLRRRRKLGKTYLAEYIVEKDP

>core/768/1/Org1_Gene865

MELVVTSRETGKKSFLKKIRQQGGIPAVVYSAGKSLANITVDALVFKKFLSNLESGALSSTVFSLSYEGRIIKA
LVKDIQYQITTYDVIHLDFEELVEDRPVKLNIPIRCINAVDCIGVKLGGSLRQVIRAVRVVCKPKDIVPFLELDV
RSVGLSQTRKLSDIKIPAGIETITPLKEVAITVSRR

>core/770/1/Org1_Gene121

MVLSSQLSVGMFISTKDGLYKVTSVSKVAGPKGESFIKVALQAADSDVVIERNFKATQEVKEAQFETRTLEY
LYLEDESYLFLDLGNYEKLFIPQEIMKDNFLFLKAGVTVSAMVYDNVVFSVELPHFLELMVSKTDFPGDSLSL
SGGVKKALLETGIEVMVPPFVEIGDVIKIDTRTCEYIQRV

>core/771/1/Org1_Gene360

MTDTPPENEEQHESNVQNENEVEHLQQEIVTLKTELKEKNDKYLMALAESENSRKRLQKERQELMQYALEN
TLIDFLNPIESMEKALGFATQMSDDVKNWALGFNMILNQFKQIFEEKGIIEYSSIGQKFNPFLHEAVQTEETSE
VPEGTILEEFAKGYKIGERPIRVAKVKVAKAPTPKENKE

>core/772/1/Org1_Gene611

MAAKTKTLELEDNVFLLLEGNLKRIFATPIGYTTFREFQNVVFNCANGQQEIANFFFEMLINGKLTQELAPQQ
KQAAHSLIAEFMMPIRVAKDIHERGEFINFITSDMLTQQERCIFLNRLARVDGQEFLLMTDVQNTCHLIRHLL
ARLLEAQKNPVGEKNLQEIQEEITSLKNHFDELTKALQ

>core/773/1/Org1_Gene420

MVETVLHNFQRYLSKYLYRVFRFPCRKKTFLSSHRVLARPSFPVDYCPGKIYDLQEIYEELNAQLFQGALRLQ
IGWFGRKATRKGKSVVLGLFHENEQLIRIHRSLDRQEIPRFFMEYLVYHEMVHSVVPREYSLSGRSIFHGKKF
KEYEQRFPLYDRAVAWEKANAYLLRGYKKRVGGGYGRA

>core/775/1/Org1_Gene807

MNFAKIDHNHLYLTCLGDLGVACPILSTDCLPNYSEKASHEVLVYSKFRCISGEPSRLATSGNDTYYSIVSLPI
GLRYEVTSPSGRHDFNIDMHVAPKIGAVLSHGTREAKEIPGSSKDYAFFSLTARESLMISEKLAMTFQVSEVIQ
NCYSQCTKVTKTNLKEQYRHLSHNTGFELSVKSAF

>core/776/1/Org1_Gene472

MSRKAREPILLPQGVEVSIQDDKIIVKGPKGSLTQKSVKEVEITLKDNSIFVHAAPHVVDRPSCMQGLYWALIS
NMVQGVHLGFEKRLEMIGVGFRASVQGAFLDLSIGVSHPTKIPIPSTLQVSVEKNTLISVKGLDKQLVGEFAA
SIRAKRPPEPYKGKGIRYENEYVRRKAGKAAKTGKK

>core/777/1/Org1_Gene41

MYKWYVVQVFTAQEKKVKKALEDFKESSGMTDFIQEIILPIENVMEVKKGEHKVVEKYIWPGYLLVKMHLT
DESWLYVKSTAGIVEFLGGGVPVALSEDEVRSILTDIEEKKSGVVQKHQFEVGSRVKINDGVFVNFIGMVSEV
FHDKGRLSVMVSIFGRETRVDDLEFWQVEEVAPGQESE

>core/778/1/Org1_Gene433

MSLLNLPSSQDSASEDSTSQSQIFDPIRNRELVSTPEEKVRQRLLSFLMHKLNYPKKLIIIEKELKTLFPLLMRK
GTLIPKRRPDILIITPPTYTDAQGNTHNLGDPKPLLLIECKALAVNQNALKQLLSYNYSIGATCIAMAGKHSQV
SALFNPKTQTLDFYPGLPEYSQLLNYFISLNL

>core/779/1/Org1_Gene309

MVEIFNYSTSIYEQHASNNRIVSDFRKEIQMEGISIRDVAKHAQILDMNPKPSALTSLLQTNQKSHWACFSPPN
NFYKQRFSTPYLAPSLGSPDQQDEDIEKISSFLKVLTRGKFSYRSQITPFLSYKDKEEEEDEDPEEDDDDPRVQ
QGKVLLKALDLGVKSTNVMIDYVISRIFQFVQG

>core/780/1/Org1_Gene151

MSKESIRSYSEISTPTPIFRETPSKEGVAYKLQLRSPAKDCILRNRVSLKGALLRSIPFYGSFLGAKRIHSAWSA
KDAPCTTRVYHYLVGGLELLGLGVVVLACKVLATALKFLFSKASSKIKQMKWREKARNLAAKDTVQSIKEF
CSVDLTSCFTRCFRLRNRVVEEGASENQTVREIIV

>core/781/1/Org1_Gene582

MTDPKIEKSALRKLFISIRRDLSEERKHEASSAVASFVRSFSKESVVLSFVSFNHEIDMQEANRILIQKCTLALP
KIDQENLYPVLIPSIDDLISVVHPKDPFSKQTPISSDKITHVLVPGLAFDQQGYRLGYGHGFYDRWLAQHPYPS
IRTIGIGYCEQKIDRLPQESHDIPLSQIYLC

>core/782/1/Org1_Gene474

MSRLKKFYTEEIRKSLFEKFGYANKMQIPVLKKIVLSMGLAEAAKDKNLFQAHLEELTMISGQKPLVTKARN
SIAGFKLREGQGIGAKVTLRGIRMYDFMDRFCNIVSPRIRDFRGFSNKGDGRGCYSVGLDDQQIFPEINLDRV
KRTQGLNITWVTTAQTDDECTTLLELMGLRFKKAQ

>core/784/1/Org1_Gene729

MAKLIVAIGNPRHGYANTRHNAGFLLADRLVEELQGPPFKPLSKCHALMTLVESSSGPLVFIKPTTFVNLSGK
AVVLAKKYFNVALSHILVLADDVNRSFGKLRLCFNGGSGGHNGLKSITASLGSNEYWQLRFGVGRPLEEGV
ELSNFVLGKFSEEENLQLGSIFVEASTLFTEWCSKF

>core/785/1/Org1_Gene528

MSVLQDTEKKMAAALDFFHKEVKSFRTGKAHPALVETVVVDVYGTTMRLSDIASISVADLRQLVISPYDGN
NASAIAKGIIAANLNLQPEVEGSIIRIKVPEPTADYRQEMIKQLRRKCEEAKINVRNIRREANDKLKKDSALTE
DVVKGNEKKIQELTDKFCKQLDELTKQKEAEIASI

>core/786/1/Org1_Gene658

MTTPQSPGSLSQSHLPHPHDPWDTEPTSLPEDPNDKASQELHSLVHLFRKLSIHLLSEVEKTVQQLKPDLLELA
LLICEKFLYKKLENPQELALLLSTALQRHTTLRSLTPIKVFLHPEDLKTLTDWISTHELPMIKHAEFFPDTSCRR
SGFKIETPNGILRQEISEELDHLLSVLTA

>core/787/1/Org1_Gene407

MACEQHEGCYELEEREEIEDIKDSDTKWVSITQAAKLHNVTRQAIYVAIKQKKLKASKETRWEIDIKDLEEY
KRNRYSRKKSLYQGELVFDNGKGCYSINQVAQILGIPVQKVYYATRTGTIRGERKGAAWVIHVSEIERYKNE
YLSKQAAKKLKGAEPKEHQAPNFEPPTEIFPESN

>core/789/1/Org1_Gene794

MTIILCGLPTSGKSSLGKALAKFLNLPFYDLDDLIVSNYSSALYSSSAEIYKAYGDQKFSECEARILETLPPEDA
LISLGGGTLMYEASYRAIQTRGALVFLSVELPLIYERLEKRGLPERLKEAMKTKPLSEILTERIDRMKEIADYIF
PVDHVDHSSKSSLEQASQDLITLLKS

>core/790/1/Org1_Gene208

MSAPIPTPQELSDQITCLNVQYQQVSELARENKGDIEGLKTLTAALTADAGIQPSADEIYSLQTAAALILSASE
KPGSGPSGSTEGSVTVQSPCKFKKVLAVVLTIIALIAIAVLIACIIAACGGFPLLLSALNLYTIGACVSLPIIASTS
VALICLCTFVANSLIKPVITVRTTR

>core/791/1/Org1_Gene396

MDRDNEVPLPKPKWIYRTGIGQDSHRFLPESSTKPCILGGIIFDHCPGFQANSDGDIIFHAICNAISSVTNKIILG
KVADELLQTRGITDSGIYLEEALKSLKPNQKISHVAITIEGSRPKFLCKLSALRQNIAQVMNLTPTDIGITATSG
EGLSDFGCGDGVQCFCVLTVMEYCD

>core/793/1/Org1_Gene662

MGYVFYVIAGSIFLGISLGAYCQLYYSVKSVLFSWYLLTVYALEKRHALLALSQLVGEEDAQSQKEIDFLSQC
DKLSWRAFLKNSYEIIPTFKEMEDLLSERVQGFLESIETIAEHDRAILCIENFWASKNLFDFEIAAYEEAVEKYL
KLRQRAPLRLASKLFRFLDVPSIRFSS

>core/794/1/Org1_Gene975

MDCVDNLKLYIFRLKLPGDTERISYSISPEYIREKGEEELLNSPIEVEGSLGRIDSDQWILSLSLKTQLGLCCPVC
NNFFSHSVCLPDLQRVISHDEVGSGVFDCRPLIRQELLLESDCFEECSGQGCPERKNILKFLEDRKKHEGNNPF
EYL

>core/795/1/Org1_Gene991

MSSTLNGVFPSSLPEESADLFITNKEIVALGEKGNVFLTHSIPMHIAAITILVIVAPAGIAIICLGCYSQSILLIAVG
IVLTILTLLCLQALVGFIKFIRQLPQQLHTTVQFIREKIRPESSLQLVTNAQRKTTQDTLKLYEELCDLSQKEFK
LQSTLYQKRFELSHKNEKTNQN

>core/796/1/Org1_Gene771

MSIQPVSNTTTKADKVIPDSTKVISDSITINKQSAFYFCISVMLRLSESTTEYGKSILAVLEDNTIVQQQRVKELI
NLPLLKVPDLQKKDGSDDEYKNQNEIQAYQSSNQQISANRQMIQQELSSAQQRAQANQKSVNSTTIESMQIL
QATSSMLSTLKELTIKANLTNSPSD

>core/797/1/Org1_Gene772

MWIIDPLSAKKPLQAAINVPGTPITGGPNTATADDIIAKFSKDSNPLIVTVYYVYQSVLVAQDNLSIIAQELQA
NSSAQTYLNNQEALYQYVSIPKNKLNDNSSSYLQNIQSDNQAIGASRQAIQNQISSLGNAAQVISSNLNTNNNI
IQQSLQVGQALIQTFSQIVSLIANI

>core/798/1/Org1_Gene440

MADDTLIPKLMKNSLSQACSEGLLIAKYPPLQVIVHFDNNLVVKTHLSVAPVFSCLFLGPAAHKAMQEIVLW
CSRYANKEHPPFSSHFAKDLIPSQYLEILNCVAEIPFGEQQTYAEIAKKTDTHPRTVGAACKQNPFLLFFPCHR
VVGSHGERNYVLGPVIHEILLKFENSY

>core/799/1/Org1_Gene325

MGFKNICKQGSQLYLNGIFPERILARKLKNCAKSYPRTALTIEVLVSSVLGALKVILIPCASTYAALTLPLRALF
NAIKTKSCQHLASYAMAWLLHILTIAVIIGLVFSLVFIPPPVVFISLGLLMSVTTSVTLFQVHKNLFPPYEPPPSR
PHTPPPFADEYVPLISESYFD

>core/800/1/Org1_Gene783

MSHLNYLLEKIAASSKEDFPFPDDLESYLEGYVPDKNIALDTYQKIFKISSEDLEKVYKEGYHAYLDKDYAKS ITVFRWLVFFNPFVSKFWFSLGASLHMSEQYSQALHAYGVTAVLRDKDPYPHYYAYICYTLTNEHEEAEKAL EMAWVRAQHKPLYNELKEEILDIRKHK

>core/802/1/Org1_Gene244

MSVHITPRKCFILCILSMFTLPTLFPKAHLILFSPYIVLCFYCFSKDKGLVLALGCGVLSDLALGSRGVFLLLYP LTALITHKAHLIFSKESKAALVIVNMIFYGVFLLLTIPMCALFGHEVRWSIDVLMIPLKCSFLDNLIFTSVIYILP CAINSGIHKMISFFRRLVCY

>core/803/1/Org1_Gene529

MVHSPTHQCYHCQQPATICYTEIDKDKVIRSYVCATCPCPSHYYNNEHLSLSKGVGVLTLECGNCKTVWHS KQDDEQLLGCHQCYTNFKNQITSKLKSERVVSSSFTMEKGQGSLHIGRAPGEASNTNPLLKLIALNEALQDTL EREDYEQAAVIRDQINHLKTKNPDDPS

>core/804/1/Org1_Gene421

MTAEKQNTGILGLEIRYTLPSDATYMLKWLNDPKILRGFPIQTEAEIRETVNFWVGFYRYHSSLTAVYNGNV AGVATLVLNPYVKVSHHALISIIVGEEFRNKGIGTALLNNLIHLAKTRFKLEVLYLEVYEGNPALHLYQRFGF VEVGRQNRFYKDEIGYLAKTTMEKDL

>core/805/1/Org1_Gene216

MKKLLFSTFLLVLGSTSAAHANLGYVNLKRCLEESDLGKKETEELEAMKQQFVKNAEKIEEELTSIYNKLQD EDYMESLSDSASEELRKKFEDLSGEYNAYQSQYYQSINQSNVKRIQKLIQEVKIAAESVRSKEKLEAILNEEA VLAIAPGTDKTTEIIAILNESFKKQN

>core/806/1/Org1_Gene80

MSEVKPLFLKNDSFDLATQRFQNLINMLQEQAEIYNEYEEKNARVQNEIKEQKDFVKRCIEDFEARGLGVLK EELASLTRDFHDKAKAETSMLIECPCIGFYYSIHQEEQRQRQERLQKMAERYRDCKQVLEAVQVEQKDMISS RVVVDDSYFEEEKEEQKVDNRKKEQD

>core/807/1/Org1_Gene579

MCKNRGVRGIVACDPRGVIGLEGKLPWHYPEDLQFFSETIQKFPIVMGRKTWETLPRKYFVDRAVVVFSHEK RQGVHGEIWVTSLEEFLLLDLSSPTFLIGGGELYSLFLENQIVRDFFISHIKKEYAGDTFFPLSLLETWTKTVLR DTQKITTCYYENHHSQNTKNISL

>core/808/1/Org1_Gene44

MKQEKTLLLQEVEDKISAAQGFILLRYLRFTAAYSREFRNSLSGVSAEFEVLKKRIFFKAIEAAGLEVDCSDTD GHLGVVFSCGDPVSAAKQVLDFNKQHKDSLVFLAGRMDNASLSGAEVEAVAKLPSLKELRQQVVGLFAAP MSQVVGIMNSVLSGVISCVDQKAGKN

>core/809/1/Org1_Gene55

MQRLGLSNLFHCLLLFLRYYYSKLVFGLTVLLAAISVICLLGCSEPSLSSFTEYVGPEYSAAAQLSIEQSCHDE
VYGQQVVVTWSLPSRMRKCLPVTLYLWVYYGNGKVEKLTYEVNQSAGYRVYCLKGLEYKELQGIISYRVA
LCSGNQEIVSRRHHLWMEVISLDSP

>core/810/1/Org1_Gene732

MKQQLLLLEDVDGLGRSGDLITARPGYVRNYLIPKKKAVIAGAGTLRLQAKLKEQRLIQAAADKADSERIAQ
ALKDIVLEFQVRVDPDNNMYGSVTIADIIAEAAKKNIFLVRKNFPHAHYAIKNLGKKNIPLKLKEEVTATLLV
EVTSDNEYVTVLAQGKQTEENQEG

>core/811/1/Org1_Gene196

MIINVRAPAFGITSVQQFSTNFQAAIPILNIVIGCSRISSTYAEDIEEVAQEKLEKSTHSKSSTSVNLWAHRVRGV
VEILGGGIVILALEITALVLQVIIKLIKCLIDVLCVCLFGLGVCVVAIIGAIAFCVVVVVKYLGFCSQGEELEPIE
VKTLISPDKPYPTVVYV

>core/812/1/Org1_Gene461

MSELIIGVDPGTIVAGYAIIAVEQRYQLRPYSYGAIRLSSDMPLPMRYKTLFEQLSGVLDDTQPNAMVLETQF
VNKNPQSTMKLAMARGIVLLAAAQRDILIFEYAPNVAKKAVVGKGHASKRQVQVMVSKILNVPEVLHPSNE
DIADAFALAICHTHVARSPLCGVR

>core/813/1/Org1_Gene456

MRQFCNLLSLSRLWLALYFCQEKLHIRLLAIVGAMLSDVLDGYLARRYKATSRLGSILDPITDKVFVFVCITV
LYMEGSLSIAHLFFICARDLFLIIFVCYLSLVKGWKGYDYGSLFWGKIFTVVQFIILLGVTAGGEIPWTGLVPL
VALGFLYFLERIMDYKKQFLR

>core/814/1/Org1_Gene384

MATRFRSTLLVITLFVLIDWVTKLVVLLQYKDLQILTHPTLYTHSWGRFSFSIAPVFNEGAAFGLFSNYKYFLF
LLRIFVILGLLAYLFFKKKSIQSTTQTALVLLCAGAIGNVGDIIFYGHIVDFISFNYKQWAFPTFNVADVLISLGT
LLLVYKFYFPTKQTEKKR

>core/815/1/Org1_Gene534

MAKYPLEPVLAIKKDRVDRAEKVVKEKRRLLEIEQEKLREKEAERDKVKNHYMQKIQQLRDLLDEGTTSDA
VLQIKSYIKVVAVQLSEEEEKVNKQKEVVLAASKELEKAEVNLAKRRKEEEKTRLHKEEWMKEALKEEARA
EEKEQDEMGQLLFQLRQKKKRESGGS

>core/816/1/Org1_Gene251

MAVEQSHIKEEIEKLIGKAIKRVCGNKENDLCRYLPGPSGGYMHHFTLKKMKSAAPEQLLKMLKTFILESETP
RTINPKPRAPRGSKKRRDFINFTKTDIERVLELARQVGDKDLLARFSPKKPLTSLKRELIRSIRNGIVSVELWNA
YVEAVKAVSSPNLEVTSPFV

>core/817/1/Org1_Gene120

MDLKQIEKLMIAMGRNGMKRFAIKREGLELELERDTREGNRQEPVFYDSRLFSGFSQERPIPTDPKKDTIKET
TTENSETSTTTSSGDFISSPLVGTFYGSPAPDSPSFVKPGDIVSEDTIVCIVEAMKVMNEVKAGMSGRVLEVLIT
NGDPVQFGSKLFRIAKDAS

>core/819/1/Org1_Gene181

MSKKINRNDLCPCGSNKKYKQCCLKKEEQTARYTTEGKFKFSAEVLSASEQGEAGDNCTKLFQRLSQSLTSE
QKAAVGKFHQITKNKEVMSKKALKKAQAKEEKLVTEKLQQHNFEILNTGENLAPPMESTATLNQDTNFVCE
DFIPTQEDFRISENSQKPPVEED

>core/820/1/Org1_Gene281

MSRQNAEENLKNFAKELKLPDVAFDQNNTCILFVDGEFSLHLTYEEHSDRLYVYAPLLDGLPDNTQRKLALY
EKLLEGSMLGGQMAGGGVGVATKEQLILMHCVLDMKYAETNLLKAFAQLFIETVVKWRTVCADICAGREP
SVDTMPQMPQGGGGMQPPPTGIRA

>core/821/1/Org1_Gene710

MKFWLQGCAFVGCLLLTLPCCAARRRASGENLQQTRPIAAANLQWESYAEALEHSKQDHKPICLFFTGSDW
CMWCIKMQDQILQSSEFKHFAGVHLHMVEVDFPQKNHQPEEQRQKNQELKAQYKVTGFPELVFIDAEGKQL
ARMGFEPGGGAAYVSKVKSALKLR

>core/822/1/Org1_Gene400

MSRRHSAEKRDIPGDPIYGSVILEKFINKVMMHGKKSVARKIVYSALERFGKKLNLENVLEGFGEALENAKPI
LEVRSRRVGGATYQVPVEVASERRNCLAMQWIIKHARSKPGKSMEVGLATELIDCFNKQGATIKKREDTHR
MAEANKAFAHYKW

>core/823/1/Org1_Gene470

MSLSKNSHKEDQLEEKVLVVNRCSKVVKGGRKFSFSALILVGDGKGRLGYGFAKANELTDAIRKGGEAAKK
NLMKIEALEDGSIPHEVLVHHDGAQLLLKPAKPGTGIVAGSRIRLILEMAGIKDIVAKSFGSNNPMNQVKAAF
KALTGLSPRKDLLRRGAAIND

>core/826/1/Org1_Gene549

MTKFLYCGLFYSLGLLVLAFGTMVAIIQVDQICDVSCMNKHFQESPPFLKIKKVNVSKQICSPEERFFHCKIDK
SCMELHFPQSSYSCKEYLTRISGHILTQNFEKQMQFRGNSGLLNYQDGSLHVYDCRFQVDPVPGYGSPDKED
SSSGGMKTLYLSLFRN

>core/827/1/Org1_Gene725

MMHRYFIPLLALLIFSPSLVRAELQPSENRKGGWPTQLSCAEGSQLFCKFEAAYNNAIEEGKPGILVFFSERPT
PEFADLTNGSFSLSTPIAKGFNVVVLCPGLISPLDFFHKMDPVILYMGSFLEMFPEVEAVSGPRLCYILIDEQGG
AQCQAVLPLETKN

>core/828/1/Org1_Gene773

MSITTLGTLPTVNTINSSRPPLEPLNTPKIGAVLFSIYELLLQAIEIRQQTVLTQSQQLNDNTNIQQQLNQETNQI
KYAIVSAGAKEDEITRVQNQNQNYSAQRSNIQDELVTTRQNGQIILSHASTNINIIQQQSSQDSSFIKTTNSIGST
VNQLNKPLG

>core/829/1/Org1_Gene299

MTTWTLNQNNLTKFLKSSDEEPFLERESGLTYINIQANGNELPLFFVIRSEGEILQLICYLPYQLHESHKASTAR
LLHLLNRDIDIPGFGMDEEQGLIFYRLVLPCLNGEIHDTLLRIYIDTIKLVCDSFSHAIGLISSGNMNLDELRRQ
ALQEQQEKRNE

>core/831/1/Org1_Gene758

MATLSPEKFSGSPISISKEFPQQKMREIILQMLYALDMAPSAEDSLVPLLMSQTAVSQKHVLVALNQTKSILEK
SQELDLIIGNALKNKSFDSLDLVEKNVLRLTLFEHFYSPPINKAILIAEAIRLVKKFSYSEACPFIQAILNDIFTDS
SLNENSLSI

>core/832/1/Org1_Gene280

MMFGHFAGYLGADPEERMTSKGKRVITLRLGVKTRVGMKDETVWCKCNIWHNRYDKMLPYLKKGSGVIV
AGDISVESYMSKDGSPQSSLVISVDSLKFSPFGRNEGSRSPSLEDNHQQVGYESVSVGFEGEALDAEAIKDKD
MYAGYGQEQQYVCEDVPF

>core/834/1/Org1_Gene149

MSKKVFFESYEDFANVASSWPKSLRALVQGRYFVDSELKETPYRIHDFKKTPIHHRLYRSLPIISTIGGIIRLIEA
HSGPIHPRDKMKYRFEVLQAVIEILGLGVLILVFDIIGCFLAFLVAIILSLLLYCNSTFTCVQNLSFTERMLEGIG
EAVNFLA

>core/835/1/Org1_Gene298

MFFNLFSLVFKLSDELALAETIQEPISVHEMFPGSMKLEMFKMLGSLILLLTIFGFGVWAFKKFVRSRSHGFGG
SSQIKILERRSLTPKTSIYLIRVVNKTLVIAETPEKITLLTEFPPDTDINHLLQENNKQSSSSATSDFLSKAIQKIQK
KQQTNQD

>core/837/1/Org1_Gene30

MPSYCQNQQDFSLFSLLSPRLVMFLGKHSRDEILQDLTDLVDAAGLLEDKQAFFDALVRRENIMSTGIGMGV
AIPHGKLESCSNFFIAIGIHTQGILWDAIDGALVRLVFLIGGPENAQAEYLKLLSTLTLSLREESRRQQLLQVNT
IEEVMNVFVGM

>core/838/1/Org1_Gene626

MADETPKENSSKESSSQFDSLKRKVKDLHSNPKVGKWKKFLSHRACEAIGGCLVLVGIIADFISWAGGLFIAC
GVVLGFHVEIRKMLSNLQSYSIANGPIKNAILCGLILFFVLNIPSFAVSFIVLCVILSFITTAPSCSTCSKDHCDKH
QDTSNKPS

>core/839/1/Org1_Gene366

MTQEKIKIHVSNEQTCIPIHLVSVEKLVLTLLEHLKVTTNEIFIYFLEDKALAELHDKVFADPSLTDTITLPIDAP
GDPAYPHVLGEAFISPQAALRFLENTSPNQEDIYEEISRYLVHSILHMLGYDDTSSEEKRKMRVKENQILCML
RKKHALLTA

>core/841/1/Org1_Gene720

MLPISILLFYVILGCLSAYIADKKKRNVIGWFFAGAFFGFIGLVVLLLLPSRRNALEKPQNDPFDNSDLFDDLK
KSLAGNDEIPSSGDLQEIVIDTEKWFYLNKDRENVGPISFEELVVLLKGKTYPEEIWVWKKGMKDWQRVKD
VPSLQQALKEASK

>core/842/1/Org1_Gene241

MRIIRFDPYGALSAQSIAKDSRQNSPLVEKISEEIATNEAIRLALLAIGDREQEEKKQRHRYKLLGQKQAKVLL
SQLRHVHLDFKKLYCDSKKKEDQEKDEKNKQKRSIKVTKKKKGISLGAAASQAIAAAAEAWVIARNKGVLE
TASTLFYQKDEEA

>core/843/1/Org1_Gene721

MEKQNLKLDVKEIEFPETVFSRDIETRVIQVIILHCLAKINGVSLLGGNLIDALFGRDIERMKGIYVEQDSKNHL
VKVRVEVNVDYGVSIPEKTEEIQGCIVSEISEYTGLHVAAVHVIIKGLTQPKDRIDEEIEEEVSVQDLPSPEDFL
LENSEG

>core/844/1/Org1_Gene113

MIMTTISNSPSPALNPELSLIPPPTLVSSGTQTSLAYTIPAQGRRSTLRIILDIFIIILGLATIISTFIVIFFLNGLNLLS
TPSIISSSCLIIVGLLFLIMGLYFMISSLDQGLVGLLQKELSQAEEREEEYIQEIEALRGAPRAESPTESPSTWL

>core/845/1/Org1_Gene494

MRVVLHCPDIPQNTGNIGRTCVALGAELILVRPLGFSLADKFVKRAGMDYWDKLQLTVVDSIEEALHDVPED
QIFCLSTKGSASYTEFSLPSSGTYVFGSESKGLPKEILKKYYKNCLRIPMQQDIRSLNLATSVGIVLYEVVRQKT
VALQKNPTV

>core/846/1/Org1_Gene768

MEKDIFFMQQAFKEARKAYDQDEVPVGCVIVKDDKIIARAHNSVEKLKDATAHAEILCIGSAAQDLDNWRL
LDTVLYCTLEPCLMCAGAIQLARIPRIVWAAPDVRLGAGGSWVNIFTEEHPFHTVSCTGGVCSEEAEHLMKK
FFVEKRREKSEK

>core/847/1/Org1_Gene262

MGYLPVSATDVLFESPAAPLINSANTQNQKLIELKGKQQAESSPRTITSVILEVLLVIGCCLIVLSLLAIRPALQF
TLETGHPAAIAVLAVSGTILLVAVIILFCFLAAVPFAAKKTYKYVKTVDDYASWHSHQQTPTLGTIFSGIVYAE
SQAQL

>core/848/1/Org1_Gene489

MLKKKPVSFSCIDGHIYKIFPNDLNANNTVFGGLLMSLLDRLALVVAERHTESVCVTAFVDALRFYAPAYMG
ENLICKAAVNRTWRTSLEVGVKVWAENIYKQERRHITSAYFTFVAVNEDNQPIPVHQIVPETPEEKRRYNEA
DRRRQARLELK

>core/849/1/Org1_Gene669

MKTLKGHLSAKNLRIAIVGSCFNQAMADALVSGTQETFLKFGGSEDGLMTIRVPGAFEIPCTIKKLLSSERKF
DAIVACGVLIQGETDHYNQIVNQVAAGIGALSLEFCLPITLSIVAAPSAEIAWQRSGIKGRHLGVSGMTTAIEM
ATLFTQI

>core/850/1/Org1_Gene637

MPFAKETEMQRTCWKCEGSVSMHVPQCPYCSAFLQDPPVASGGFSSCHISFPEGASKEEAEDLFAVSSEDWE
AVLGDQNPTQETNKQVIPEWTWLQSWPLAALFLGIGLLAFAFLILLFSTDSGLVLTWPKNRAYFYGIIGAAVA
YRGYRKLPL

>core/851/1/Org1_Gene25

MRSALHLQHLRHFHNHGSILFENLLTIKDCFLLETKLQNFIAKASKTIDTVRWRENIFRSMPEIYTVVRKRRLD
FFAAELVHRPKLSLVRDLWVFPGEEILEGEEDCMLFLLLSGDRAGSGIFFTGPYPSDLYELEKGTTGLLLAFSS
VGIPVI

>core/852/1/Org1_Gene486

MNQPSVIKLRELLDLLPHRYPFLLVDKVLSYDIEARSITAQKNVTINEPFFMGHFPNAPIMPGVLILEALAQAA
GVLIGLVLENDRNKRIALFLGIQKAKFRQAVRPGDVLTLQADFSLISSKGGKAWAQARVDSQLVTEAELSFA
LVDKESI

>core/853/1/Org1_Gene382

MQCPFCNHGELKVIDSRNAPEANAIKRRRECLKCSQRFTTFETVELTLQVLKRDGRYENFQESKLIHGLNAAS
SHTRIGQDQVHAIASNVKSELLGKQNREISTKEIGELVMKYLKKADMIAYIRFACVYRRFKDVGELMEVLLS
ATPDMEK

>core/854/1/Org1_Gene989

MAQKEIVSNRKALRNYEVIETLEAGIVLTGTEIKSLRDHGGNLGDAYVIVSKGEGWLLNASIAPYRFGNIYNH
EERRKRKLLLHRYELRKLEGKIAQKGMTLIPLGMLLSRGYVKVRLGCCRGKKAYDKRRTIIEREKEREVAAA
MKRRHH

>core/855/1/Org1_Gene225

MARNIKYFLILFPGILWISAGMKLLLKATAIALDPLSSFFTYCLLSMVSWGLASLKHRYLLSKTIRKQLSLSSEF
FSQKITWIAYIKQTFISRRFLIMVIMIAFSLVLRRYISNPQALFVIRATVGYALIKTAIAYFSKLQNALMENPEGN

>core/856/1/Org1_Gene167

MSKPSSCKAYLGIDYGKKRIGLAYAAEPLLLTLPIGNIEAGKNLKLSAEALHKIILSRNITCVVLGNPLPMQKG
LYSSLQEEVSLLAEELKKLSTVEIILWDERLSSVQAERMLKQDCGLSRKDRKGKTDSLAATLILTSFLDSLPKK
LTL

>core/857/1/Org1_Gene701

MMKTKYEYSFGVIPIKFFGTPDKNTLKACFICHTRGKHWGFPKGHSEDKEGPQEAAERELVEETGLSVVNFF
PKVLIEQYSFNNEEQVFVRKEVTYFLAEVRGDIHADPMEICDSQWLSLQEGLRLLSFPELRDLTVEADKFINN
YLFSS

>core/858/1/Org1_Gene673

MQLLSPAFAYGAPIPKKYTCQGAGISPPLTFVDVPGAAQSLALIVEDPDVPKEIRSDGLWIHWIVYNLSTTITN
LAEGAEIFAVQGLNTSGKPVYEGPCPPDKQHRYFFTLFALDVVLPEEENVTRDQLYEAMEFHIIEQAELMGTY
EKS

>core/859/1/Org1_Gene175

MEKRKDTKTTIVKSSETTKSWYVVDAAGKTLGRLSSEVAKILRGKHKVTYTPHVAMGDGVIVINAEKVRLT
GAKKGQKIYRYYTGYISGMREIPFENMMARKPNYIIEHAIKGMMPRTRLGKKQLKSLRIVKGDSYETFESQK
PILLDI

>core/860/1/Org1_Gene36

MEFICPLQHARCLKKQHKIIEELFPEPFQKDHLYLKLMENSSSRDAFDKKRMLKENLVVGCQSDLYLYEVYQ
DGILFFFTYTKALMSSGIASLFTEVYSGETPSTILTCKPIFFQRLTPYLSFGRLNGGESLYMRMKQIAVQYLKPP
QT

>core/861/1/Org1_Gene537

MADLEVFQADFALLFEAGLLAIKQGDEDSARKLFQSLHILNPNHYGHDLGLALISLHKMDLFDAEERLSALIK
GNEDNWSIKAFLSLTHMLIVLHQGSSFEVRRESLESCLKFADQVIANCKIESTRALAQSVLDWHDTLVAKSA
GPLG

>core/863/1/Org1_Gene274

MKSERLKKLESELHDLTQWMQLGLVPKKEISRHQEEIRILEHKIYEEKERLQLLKENGEIEEYVTPRRSPAKTV
YPDGPSMSDIEFVEPTETEIDIDPGETVELELTDEGREDGAVEVDYSHEDDEDPFSDRNRWRRGGIIDPDANE
W

>core/864/1/Org1_Gene271

MKTKMNSRKKAGQWAIFNSPTPGVSSTLVLAWTPWGYYDKDVQDILERKDPMSSSLSEKDSKEFLKNLFVD
LLENGFTSVHIHAEEAFTPLDHTGKPHFKRDNVYLPGKLLGALNEAAVQANVSADTQFTLFLTQDECNPFHD
KKRG

>core/865/1/Org1_Gene875

MGSRRKLKRSFLLIEVLMALSLVCAVLLPCIRFYYAIHRSFEEDIFNLQLPALIDHCFLSVEEKMRQQMAEGTV
LTSGKGQTVSLAYTSQGIGYRIPYGYNVDIRQEVRGDNLKMKVCLADVVVELFPDQKQAVSVQRCLCVTL

>core/866/1/Org1_Gene129

MKKKVTIDEALKEILRLEGAATQEELCAKLLAQGFATTQSSVSRWLRKIQAVKVAGERGARYSLPSSTEKTT
TRHLVLSIRHNASLIVIRTVPGSASWIAALLDQGLKDEILGTLAGDDTIFVTPIDEGRLPLLMVSIANLLQVFLD

>core/867/1/Org1_Gene237

MQNQYEQLLESLAPLLNTTLAPDKNNSCLIRFSDTHVPVQIEEDGNSGDLAVSTLLGTLPENVFRERIFKAALS
VNGSFQSSIKGILGYGEVTQQLYLSDILSMNYLNGEKLFEYLKLFSLHAKIWMESLRTGNLPDLHVLGIYYVA

>core/868/1/Org1_Gene693

MEEFVAYIVKNLVTNPEAVEIRSIEDEDNESIKLEIRVAAEDIGKIIGRRGNTIHALRTILRRVCSRLKKKVQIDL
VQPENGTDVIADQDYICDNDSSNSTEDTFGESDTCCSGHCHYDEDLNQEEQEEGNMHHSCECSNHH

>core/869/1/Org1_Gene90

MSLEKELLEETPLVLLNFYKLVSFCNYAGMILGTEEKKFAIYGHVSMGQAFQGADTEGHSPQRPFAHDLLNF
VFSGFDIQVLRVVINDYKDNVFYTRLFLEQKDREFLYVVDVDARPSDSIPLALTHKIPILCVKSVFDAVVPYEE

>core/870/1/Org1_Gene147

MNPVTFDRIQVDFIPEDTSLRINSYIVAGGLLILGVVLSILSVICLDIGLVGLSAGAAFTLGLGCLIFALFLFSFSL
ILLLSQEKRVPDVLSLYLEKEVPQYETPLYKEDLESERDMSAISERLGIIEEKLRIAEKFRYSDSVFV

>core/871/1/Org1_Gene29

MTVFCELDSGGELPEYTTPGAAGADLRANIEEPIALLPGQRALIPTGIKAEIPEGYELQVRPRSGLALKHGITVL
NSPGTIDSDYRGEIRVILINFGDSTFIIEPKMRIAQVVLSPVVQATFVVKQESLAETARGSGGFGHTGAS

>core/872/1/Org1_Gene504

MTFFEGETVFPAVLSELHSMLDLIKRAGKQSKCPQEKLLKLELACEELLVNIISYAYQGENSPGTIAISCISHRG
DLEVVIKDHGPSFNPLAVSINIQEDLPLEQRKLGGLGIFLAKSSVDEFLYAREDHCNIVHLKMLNGQHS

>core/873/1/Org1_Gene459

MEQTLSIIKPDSVSKAHIGEILSIFEQSGLRIAAMKMMHLSQTEAEGFYFVHRERPFFQELVDFMVSGPVVVLV
LEGANAVSRNRELMGATNPAEAASGTIRAKFGESIGVNAVHGSDTLENAAVEIAYFFSKIEVVNASKPLV

>core/874/1/Org1_Gene469

MIKLESLFDISERKRRKKLLGRGPSSGHGKTSGRGHKGDGSRSGYKRRFGYEGGGVPLYRRVPTRGFSHKRF
DKCVEEITTGRLAELFQEGEAITLDALKAKKAIARQAVRVKVILKGDLEKTFVWQDTAVVLSQGVQNLLGIT

>core/875/1/Org1_Gene171

MACSIFFRMSQGDYDDEPLSKKTACLVVDTMLYPVIAVVCAVVSVVLLILKVLFLLLSFPFKLCSASSALPGE
RVSLGSHFKCLYGGGLPYLLACLLIVPVIGTAIHGFIISHRTSEDARLSSAIVFMQAPILQLAGMSGLIKP

>core/876/1/Org1_Gene816

MQEHIHKELLHLGEIFRSSRESQSLSLKDVEAATSIRYSCLEAIEQGCLGKLISPVYAQGFIKKYATYLGLDGD
SILQEHPYVMKIFKEFSDHNMEMLLDLESMGGRNSPERAIHSWSNLWWAGLIIIGGIMVWWLGSLFSIF

>core/877/1/Org1_Gene505

MSLDFFEEFYHQSILNTGTSFPEGYLNIAEILSYPHCTDANTDFLCSQSDNDFIIAESKDKLTLFNADFAIWLVP
ELVQGQAVTRGYIAVSQGEGNYEPEMAFEASGQYNQSSLILEALQLYLKDIKDTENALRSFRFNNDH

>core/878/1/Org1_Gene464

MQHARKKFRVGRTSSHNRCMLANMLKSLIHYERIETTLPKAKELRRHADKMITLAKKNSLAARRIAIGRLM
VRYNKLTSKEARQAKGGDTSVYNVDRLVVNKLFDELGNRFVERKGGYTRILKLQNRIGDNAQKCIIEFLAS

>core/879/1/Org1_Gene565

MNSKSAQKIIDSIKQILTIYNIDFDPSFGSSLSSDSDADYEYLITKTQEKIQELDKRAQEILTQTGMSKEQMEVF
ANNPDNFSPEEWLALEKVRSSCDEYRKETENLINEITLDLHPTKESKRPKQKLSSTKKNKKKNWIPL

>core/880/1/Org1_Gene310

MLDNEWKAILGWGDDELEELRISGYSFLRQGHYSKAILFFEALVILDPLSIYDHQTLGGLYLQIGENSQALAV
LDQALRMQGDHLPTLLNKTKALFCLGRIEEATAIATYLSSCPIPAIANDAEALLMSYSKATKKNAALVR

>core/881/1/Org1_Gene491

MGRYRRVSHSSQETLLLGTELGQVLVPGAVLLLFGDYGAGKTEFVRGIVSGYLGDTIAEEVASPSFSILHVYG
NEPKRLCHYDLYRIDQKNQEYIFQDAEEDDVLCIEWADRLPKPRFCDTINIYITMQTNMEREIIIEKR

>core/882/1/Org1_Gene42

MSVKKVIKIIKLQIPGGKANPAPPIGPALGAAGVNIMGFCKEFNAATQDKPGDLLPVVITVYADKTFTFITKQP
PVSSLIKKTLNLESGSKIPNRNKVGKLTQAQVEAIAEQKMKDMDIVLLESAKRMVEGTARSMGIDVE

>core/883/1/Org1_Gene54

MIDMSVVGPALVLGLAMIGSAIGCGMAGVASHAVMSRIDEGHGKLIGMSAMPSSQSIYGFILMLLMQAAIKN
GTLSPVGGIAIGLSVGAALLVSSVMQGKCCVSGIQAYARSSSIYGKCYAAIGIVESFSLFAVVFALLLL

>core/885/1/Org1_Gene716

MHPLTLPKQSRVLKRKQFLYITRSGFCCRGSQATFYVVPSRHPGTCRMGITVSKKFGKAHERNSFKRVVREV
FRHVRHQLPNCQIVVFPKGHKQRPVFSKLLQDFINQIPEGLHRLGKTKATTGGECTPKSEKCVTAPR

>core/886/1/Org1_Gene478

MLMPKRTKFRKQQKGQFAGLSKGATFVDFGEYAMQTLERGWVTSRQIEACRVAINRYLKRRGKVWIRIFPD
KSVTKKPAETRMGKGKGAPDHWVAVVRPGRILFEVANVSKEDAQDALRRAAAKLGIKTRFVKRVERV

>core/887/1/Org1_Gene210

MNLIDRAFLLKKTIIFQSLDMDLLLTIADKTETIIFKPGSNVFSIGQPGFSFYIIVEGYITISKEKLESPLNLKPLDC
FGEESLFNNKPREYNASANTQVRMLVLSKGQILNIVEECPSVALSFLELYAKQIKFREP

>core/888/1/Org1_Gene152

MVNRYKSSAEFSADHYYDDNLVRMGYKRNLRGLAPVENEVCLFEENNLLESVMASIPIMGSILGLGRLHSV
WSTQDPKDSKISIIFHTALGILETLGLGIIVLLIKITITILLILFTPCLLCYFMYSAAYSDFHPI

>core/889/1/Org1_Gene158

MINFIRSYALYFAWAISCAGTLISIFYSYILNVEPCILCYYQRICLFPLTVILGISAYREDSSIKLYILPQAVLGLGI
SIYQVFLQEIPGMQLDICGRVSCSTKIFLFSYVTIPMASVVAFGAIVCLLVLTKKYRG

>core/890/1/Org1_Gene599

MKYRFTEEIEEEPLVNLTPLIDIVFVILMAFIVAVPLIKLDSIALAPGTQEQEVLSSENDSIAVIKVFADHSLTLN
EHPITLQELTVRLTLLHKAYPEKTPLLLQDGETSFRTYQNVKNAIEAAGFHELHVALQN

>core/891/1/Org1_Gene174

MAKSTIQESVATGRRKQAVSSVRLRPGSGKIDVNGKSFEDYFPLEIQRTTILSPLKKITEDQSQYDLIIRVSGGG
IQGQVIATRLGLARALLKENEENRQDLKSCGFLTRDPRKKERKKYGHKKARKSFQFSKR

>core/892/1/Org1_Gene577

MIAIERYQLIISKFRMWLFLGCSVEERHFKQPVLISVTFSYNEVPSACLSDKLSDACCYLEVTSLIEEIANTKPY
ALIEHLANELFDSLVISFGDKASKIDLEVEKERPPVPNLLNPIKFTISKELCPSPVLSA

>core/893/1/Org1_Gene157

MPYYANTLEFIQGTQSLCPLFKYGFVRHHYKGQLEIEDASHDWDFLEPPSTWKRTLLAAIPILGSVIGLGRLFS
IWSIREPQDSQEYKSIFWHTLCAVLEILGLGIVALILKILATFIMAMPGLKRVATFLFYS

>core/894/1/Org1_Gene473

MGMTSDSIADLLTRIRNALMAEHLYVDVEHSKMREAIVKILKHKGFVAHYLVKEENRKRAMRVFLQYSDD
RKPVIHQLKRVSKPSRRVYVSAAKIPYVFGNMGISVLSTSQGVMEGSLARSKNIGGELLCLVW

>core/895/1/Org1_Gene466

MVKNQAQAKKSVKRKQLKNIPSGVVHVKATFNNTIVSITDPAGNVISWASAGKVGYSGSRKSSAFAATVAA
QDAAKTAMNSGLKEVEVCLKGTGAGRESAVRALISAGLVVSVIRDETPVPHNGCRPRKRRRV

>core/896/1/Org1_Gene698

MNFVSTLTGSDFYAPVLEKLEEAFADTTGQVILFSSSPDFIVHPIAQQLGISSWYASCYRDQSAEQTIYKKCLT
GDKKAQILSYIKKINQARSHTFSDHILDLPFLMLGEEKTVVRPQGRLKKMAKKYYWNIV

>core/898/1/Org1_Gene189

MSQCQSSSTSTWEWMKSFVPNWKNPTPPLSPIPSEDEFILAYEPFVLPKTDPENAQANPPGTSTPNVENGIDDL
NPLLGQPNEQNNANNPGTSGSNPTSLPAPERLPETEENSQEEEQGSQNNEDLIG

>core/899/1/Org1_Gene540

MLEKLIKNFATYMGITSTLELDADGAYVLPISEVVKVRAQQNADNEIVLSASLGALPPSADTAKLYLQMMIG
NLFGRETGGSALGLDSEGNVVMVRRFSGDTTYDDFVRHVESFMNFSETWLSDLGLGKQ

>core/900/1/Org1_Gene292

MRDHAFSKLIGTVRAMVVEGRCPWSLQQSLVSMVEHILGECQEFHEAVLQGKTVQEVGSEAGDVLTLVLIL
CFLLEREGVLASEDVANEAMEKLRRRAPYIFAEDYKPVSIEEADRLWELAKHREKNEST

>core/901/1/Org1_Gene905

MDYKSQLVFSCPCCCKGNVCFSVFNLDVILTCNVCSSTYTFDSVIRNEIRQFVALCKRIHDANSILGNATVSVS
VEDNQMDIPFQLLFSRFPVVLNLSLDGKKIAIRFLFDALNTSILHQESDLIS

>core/902/1/Org1_Gene335

MREEGSVSFREYFRAYMCDKIVAQKNFLFTLDAVIKQAGWRSQEKLNLFYVESQALGREIKVSLEEYIQSMV
GILGSQRTKKSFKFSVDFTPLEQALQERCSSDDDEDATATSTATGATASPTDMHEDE

>core/903/1/Org1_Gene45

MTTESLETLVEKLSNLTVLELSQLKKLLEEKWDVTASAPVVAVAAGGGGEAPVAAEPTEFAVTLEDVPADK
KIGVLKVVREVTGLALKEAKEMTEGLPKTVKEKTSKSDAEDTVKKLQDAGAKASFKGL

>core/904/1/Org1_Gene594

MKFTVALFGEAEKGSYDTAYFCRSLVDLHNYLGDVSSPGITLAIKTLLSDYNVVYFRVREEGYCVDSYFFGL
HFLNTQTTLKNIIAIGLPGVGNQHIIEASRSLCQKHNSLLLFFDHDLYDLLTFNQPF

>core/905/1/Org1_Gene914

MRAGGSLVTTYPKEGQRLRSPEQLRVLDDLVQSYPNHLHAIELDCGAIPQDLIGATYIITFADFSAYILSLRSY
QANSPSDDTWGIWFGSIDDPVQAVISFLKDHGFALPSTLAQDPLLCTNK

>core/906/1/Org1_Gene154

MLIGRYSSDDQFTEATKNTPTIIKLGFVRDNLEGLTNPISEIVSETSSSIKDSVLRSLPILGSILGCARLYSTLSTN
DPLDETQEKIWHTIFGALETLGLGILILLFKIIFVILHCIFHLVIGFCK

>core/908/1/Org1_Gene383

MPLSDDEIEQFKKRLLEMKAKLSHTLEGNAQEVKKPNEATGYSQHQADQGTDTFDRTISLEVTTKEYELLRQINRALEKINESSYGICDVSGEEIPLARLIAIPYATMTVKAQEQFEKGLLSGN

>core/909/1/Org1_Gene471

MESSLCKKSLMKRRRALRVRKVLKGSPTKPRLSVVKTNKHIYVQLIDDSIGKTLASVSTLSKLNKSQGLTKKNQEVAKVLGTQIAELGKNLQLDRVVFDRGPFKYHGIVSMVADGAREGGLQF

>core/910/1/Org1_Gene401

MPTINQLIRKRRKSSLARKKSPALQKCPQKRGVCLQVKTKTPKKPNSALRKVAWVRLSNGQEVIAYIGGEGHNLQEHSIVLIQGGRVKDLPGVRYHIVRGTLDCAAVKNRKQSRSRYGAKRPK

>core/911/1/Org1_Gene678

MALKDTAKKMKDLLDSIQHDLAKAEKGNKAAAQRVRTDSIKLEKVAKLYRKESIKAEKSGLLKRKPSTKAPAKVKKTAEKKAPKKSSAAAAKTSKAVKASKPASKKTAAKKVKKPSKARGFRK

>core/912/1/Org1_Gene623

MKRQKRKQSITLIEMMVVITLIGIIGGALAFNMRGSIHKGKVFQSEQNCAKVYDILMMEYATGGSSLKEIIAHKETVVEEASWCKEGRKLLKDAWGEDLIVQLNDKGDDLVIFSKRVQSSNKK

>core/913/1/Org1_Gene656

MRDRLGSLSLILKVKIHKYLDTLHNQKRLALTVSRNIQATNKRIADLHLERYEHFISRDNIKHYDILLEYLKTLQSSLYKQQSESLRFLEIHHQQLQELINRRKIIEKIKNNKYSKDQEIGT

>core/914/1/Org1_Gene467

MPRIIGIDIPAKKKLKISLTYIYGIGSARSDEIIKKLKLDPEARASELTEEEVGRLNSLLQSEYTVEGDLRRRVQSDIKRLIAIHSYRGQRHRLSLPVRGQRTKTNSRTRKGKRKTVAGKKK

>core/915/1/Org1_Gene476

MIQQESQLKVADNTGAKKVKCFKVLGGSRRRYATVGDVIVCSVRDVEPNSSIKKGDVIKAVIVRTRRHITRKDGSTLKFDTNSCVIIDDKGNPKGTRIFGPVAREIRDRGFIKISSLAPEVI

>core/917/1/Org1_Gene226

MEIIHIGTDIIEISRIREAIATHGNRLLNRIFTEAEQKYCLEKTDPIPSFAGRFAGKEAVAKALGTGIGSVVAWKDIEVFKVSHGPEVLLPSHVYAKIGISKVILSISHCKEYATATAIALA

>core/918/1/Org1_Gene74

MVNLLKELEQEQCRNDLPEFHVGDTIRLATKISEGGKERVQVFQGTVMARRGGGSGETVSLHRVAYGEGMEKSFLLNSPRIVSIEIVKRGKVARARLYYLRGKTGKAAKVKEFVGPRSSKK

>core/919/1/Org1_Gene761

MVRATGSVASRRRRKRILKQAKGFWGDRKGHIRQSRSSVMRAMAFNYMHRKDRKGDFRSLWIARLNVASR
IHSLSYSRLINGLKCANISLNRKMLSEIAIHNPEGFAEIANQAKKALEATV

>core/920/1/Org1_Gene153

MRELNAFELTQPEEYRNRWVLMPCLKCRFCRTQHAKVWSYRCVHEASLYEKNCFLTLTYDDKHLPQYGSL
VKLHLQLFLKRLRKMISPHKIRYFECGAYGTKLQRPHYHLLLS

>core/921/1/Org1_Gene222

MATVAQTPQTTQPQPSVSHKATHRYCSWVFFKPILVSLGLLLASLTTLGLVIASGVTLSLGIGIVLAIQIVLAGI
ALVLAFNHIRQFKQARTAELNSMKMISAPAAATVQKQKLEDRYSSK

>core/922/1/Org1_Gene231

MTENRRIKRVNALLQEAIAKVILKDVKHPKISNLWITVTRVSLSKDLHSARVYYVSVMPHENTKEEALEALKV
SAGFIAHRASKNVVLKYFPELHFYLDDIFSPQDYIENLLWQIQEKEKS

>core/923/1/Org1_Gene616

MNKKPKKTKKAVQSKAAPVKRVPEESQEAAIQQLELAVSDLYKELPLAQTFASLTDKNQINSIIAALSGTLES
LHLEELTQGLFPSAQEDANFAKELSSVVHGLKNLTTVVNKQMVKGAE

>core/924/1/Org1_Gene386

MDNYLLGSLIFCCVLLSIGMCTIFVMTICFLRQLNKILKNIHRVTTILNFEAKILAPLMLGKKLLCGWLKKRKN
RGSLSEDIDELLDEKKQRSWKKNLDQGIKWCAALVLIWKVFRNKD

>core/925/1/Org1_Gene699

MDSFCFDLLKVAAKAIDDKKGNNLVVLDVRTISEFTDYFVFVEGSVNVHVKALANTIVEELKKQKVSPLHVE
GITDGNWVVIDYGFIVVHVFVSEIRGKYRLEELWKDGFIVTSKLLAS

>core/926/1/Org1_Gene266

MPVSSAPLPTSHRPSSGNLGLMEPNSKALKAKHQDKTTKTIKLLVKILVAILVIEVLGIIAAFFIPGTPPICLIILG
GLILTTVLCVLLLVIKLALVNKTEGTTAEQQIKRKLSSKSIS

>core/927/1/Org1_Gene72

MALKIRLRQQGRRNHVVYRLVLADVESPRDGKYIELLGWYDPHSSINYQLKSERIFYWLERGAQLSSKAEAL
VKQGAPGVYSALLSKQEARKLVVRKKRRAYRQRRSTQREEAAKDATK

>core/928/1/Org1_Gene1004

MTVLFYAFLFIFLFLCVILCGLILVQESKSMGLGSSFGVDSGDSVFGVSTPDILKKVTSWCAVAFCIGCLLLSFS
TNLLGKKLDAKEFLLPAAEESDTQASSESVEADES

>core/929/1/Org1_Gene677

MLSRIVTCFLFLLSSLPLFAEEEAAQSKNTFVQPAVMLAIAILFFYFILWRPEQKRRKAMEKRKNDLAKGDKV
TAMGIIGTVDDIREHTVILNIASGKVEVLKGAISEILKPNDNKS

>core/930/1/Org1_Gene368

MSDIQKEEHGSTTIFHLHGKLDGISSPEVQENISQSLAAGSKNIILDCAHLDYMSSAGIRVLLQSYHQVGQHSG
KIVLTTVPKTIEQTLYVTGFLSYFKIFNTVDEAIQTLNKDGD

>core/932/1/Org1_Gene195

MFLKRKKRGGSQVQNKRTASPIKHAKHYLHNYLQELQKIMAARPHDAIDAWNQVFRDKYKGMSQAIGFRD
HILLVKVYNSSLYALLKQTPQNDLIMSLYQVASHVQIREIQFLLG

>core/933/1/Org1_Gene286

MSKVSVRKKNWGFRLLEEVMIKSWWVIFSILIGGFVYDRAIQELRTEELRLQSKVSSLCQDILSAQEKQRQLQ
LHLQHWQDSAAIEAALIQRLGLIPKGYKKLCVSPKQQSENKD

>core/934/1/Org1_Gene320

MWYSDYHVWILPVHERVVRLGLTEKMQKNLGAILHVDLPSVGSLCKEGEVLVILESSKSAIEVLSPVSGEVID
INLDLVDNPQKINEAPEGEGWLAVVRLDQDWDPSNLSLMDEE

>core/936/1/Org1_Gene730

MGKKENQLYEGAYVFSVTLSEEARRKALDKVISGITNYGGEIHKIHDQGRKKLAYTIRGAREGYYYFIYFSVS
PGAITELWKEYHLNEDLLRFMTLRADSVKEVLEFASLPE

>core/937/1/Org1_Gene694

MTAVLILTSFPSEESARSLARHLITERLASCVHVFPKGTSTYLWEGKLCESEEHHIQIKSIDIRFSEICLAIQEFSG
YEVPEVLLFPIENGDPRYLNWLTILSYPEKPPLSD

>core/938/1/Org1_Gene479

MFKATARYIRVQPRKARLAAGLMRNLSVQEAEEQLGFSQLKAGRCLKKVLNSAVANAELHENIKRENLSVT
EVRVDAGPVYKRSKSKSRGGRSPILKRTSHLTVIVGEKER

>core/939/1/Org1_Gene475

MKKQNIRVGDKVFILAGNDKGKEGKVLSLTEDKVVVEGVNVRIKNIKRSQQNPKGKRISIEAPIHISNVRLTIA
GEPAKLSVKVTEQGRELWQRRPDGTSQLYRLVRGKKG

>core/940/1/Org1_Gene318

MTTLPKYVPRSRQNPDTLTFLKRYSSVLLHSENSLSYRIFAKVLAILLTSLAVAFAVTLFSCEGSQLRLCALYI
GIALAICVLLTIVVYCIASKIATACKKPPSISRIEIV

>core/943/1/Org1_Gene481

MKDPYDVIKRHYVTEKAKMLEHLSAGTGEGKKKGSFCKDPKFVFIVSHDATKPLIAQALEAIYVDKNVKVKSVNTINVKPQPARMFRGRRKGKTSGFKKAIVTFYQGHSVG

>core/945/1/Org1_Gene350

MTVFKQIIDGLIDCEKVFENENFIAIKDRFPQAPVHLLIIPKKPIPRFQDIPGDEMILMAEAGKIVQELAAEFGIADGYRVVINNGAEGGQAVFHLHIHLLGGRPLGAIA

>core/946/1/Org1_Gene695

MNLSAKEYGDIIVIYLQGSLDAVSVPSVQEYLEQFIQKKHLKIALNFTDVSYISSAGIRLLLSNFKLVQSLGGKMCLCCVKESVTEVMRIAGLDQLILLCQSEQECLSKL

>core/947/1/Org1_Gene943

MASSATPGFDGTAPSLFPLATRPRYNFKLALFVTIAIALVWIALIATTIAIGLCIHPLCSFIFLTAIPLYFISRYICSHYARNVYIALDVVPDHSKLQDMRSHSPIFSDR

>core/948/1/Org1_Gene69

MKKNTHPEYRQVLFVDSSTGYKFVCGSTYQSEKTEVFEGKEYPVCYVSVSSSSHPFFTGSKKFVDAEGRVDKFLKRYSNVRQPAQQPQPEEDALPAAKGKKKVVTKKKK

>core/949/1/Org1_Gene20

MNEPTRTYLESEKDTQDQIEELQATCIVKNAAGIHVRPAGVIVRLFDGEPCDVHFTYAGKTINAKSIMSILMLGAPQGGEILVTIRSKEAHRILQKIQDAFSSGFGEL

>core/950/1/Org1_Gene836

MSTSPIGVPSMLNAATSLNATTSKAPLPTSTLAERIKEWLPRILLLIVGAIFTIAGCIVMALTKQILYGLLCVVGGLLLALGLLLKPENCIYRNAESAARSLSNALE

>core/952/1/Org1_Gene443

MDEITPNYPLLRQDSLWNRVRVSWRADLSVSSRYEIASAIAILGLLVAFCASAAVSIIFTANPLAQVFIDGCLALGLLPIPLVIGLLIIGIIVLLYGIYLFPQQRE

>core/954/1/Org1_Gene395

MEPYAVIQTGSKQYQVRSGDVIDVELLGEVASDKEVIFQDVLFVFDGTKASLGSPTIANAQVKAEYLSHVKGEKVVAYKYKKRKNYHRKHGHRQKYLRVKIREILI

>core/955/1/Org1_Gene545

MSFTYFLALPVDRLMQERFLCSPKRWAPFINSPLYLTLIADHDTPYLAKNLDKFPLPVEQWEKTVLHVSSLLKSIFLCSDLSSLRLLACTKFEILTLNDLYCAQNI

>core/956/1/Org1_Gene428

MNERTLLLLLKKKKGLFLAILDLTQTESSLTTPELEKVLKQKKIFLSCIDRVDLQIKEFRHAFSSELPQDIQEEL
EEIRDVIIRILDTDKRNYAQKKKEFGIYERP

>core/957/1/Org1_Gene398

MKQQKQKIRIRLKGFDQGQLDRSTADIVETAKRTGARVVGPIPLPTKREVYTVLRSPHVDKKSREQFEIRTHK
RLVDILDPTGKTIDALKMLALPAGVDIKIKAA

>core/958/1/Org1_Gene267

MSSPVVTGTSSASPVEQTKLGEFLERLSGSGRCIKIAFAASTALLLLNTFVSGIVAIAMIFVATSVGAYFTVIGP
LFLLSLILLAIMLISMYKITHPSQNTPISN

>core/959/1/Org1_Gene436

MSFKRFLQQIPVRICLLIIYLYQWLISPLLGSCCRFFPSCSHYAEQALKSHGFLMGCWLSIKRIGKCGPWHPGGI
DMVPKTALQEVLEPYQEIDGGDSSHFSE

>core/961/1/Org1_Gene493

MVKIISSENFDSFIASGLVLVDFFAEWCGPCRMLTPILENLAAELPHVTIGKINIDENSKPAETYEVSSIPTLILFK
DGNEVARVVGLKDKEFLTNLINKHA

>core/962/1/Org1_Gene86

MSDQATTLRIKPLGDRILVKREEEEATARGGIILPDTAKKKQDRAEVLVLGTGKRTDDGTLLPFEVQVGDIIL
MDKYAGQEITIDDEEYVILQSSEIMAVLK

>core/963/1/Org1_Gene319

MDPASPVAPHVLQDHVQLSSEELSALSSGVSRVKKLTIAIMVLSLIAISLVACGLFLTGSAPLQLSIWIAASCIT
LSMLVCACWRYKISNALEKTKVAHES

>core/964/1/Org1_Gene719

MAKKSSVAREAKRRRLVEANFKKRSDLRKIVKSLSVSEEEKENARISLNKMKRDTSPTRLHNRCLLTGRPRG
YLRKFAISRICFRQMASMGEIPGVIKASW

>core/965/1/Org1_Gene2

MEQFHLDREEILLLAKASALQLSEELIQEYQTSLSAVITSMKEALAIEIDDADSCESLFMHVVNVEDLREDSVT
SDFNREEFLRNVPESLGGLVKVPAVIK

>core/967/1/Org1_Gene387

MFRNNHKPKKTKCKRFRWLRGVLFGGFIATLLTCLFTPKSGVQLRKKILKVKNSGAKKSRVFFKNSKQHTKS
FVKQAKLLAKNISHELQDFKKGILDDKD

>core/968/1/Org1_Gene303

MATMTKKKLISTISQDHKIHPNHVRTVIQNFLDKMTDALVKGDRLEFRDFGVLQVVERKPKVGRNPKNAAV
PIHIPARRAVKFTPGKRMKRLIETPNKHS

>core/969/1/Org1_Gene574

MAPKKPNKKNVIQRRPSAEKRILTAQKRELINHSFKSKVKTIVKKFEASLKLDDTQATLSNLQSVYSVVDKA
VKRGIFKDNKAARIKSKATLKVNARAS

>core/970/1/Org1_Gene785

MKKVVTLSIIFFATYCASELSAVTVVAVPLSEAPGKIQVRPVVGLQFQEEQGSVPYSFYYPYDYGYYYPETYG
YTKNTGQESRECYTRFEDGTIFYECD

>core/971/1/Org1_Gene544

MEPRHIYIRKPETPKAPDVEKPGVPEYMTMANTPTFEGPVKTLDQLRRALIEQRGAEEGQKMYDNFIQSILIST
FGLVHKDMDRAQKASKRMRSVYKEQ

>core/972/1/Org1_Gene908

MCLIDCLGQGFEAAINTVCCCSDSSESKANVATVSAGLLALTAIVSFILIILICTGVLGASGMTFGMSNVAAVL
VLVVTILLSMFLSGACFAAKREILR

>core/974/1/Org1_Gene584

MLSYLLRTAINVYSFLILAYIFASWVPDCQSARWYQLVSKCVDPFLNFFRRFVPRIGFIDPSPFVGLLCLGILPF
VILRVLRFIILNIFHSPWLLQYL

>core/975/1/Org1_Gene243

MGKPKKSRTDRALAQEIQKKSTEVLKKPARIKAKNRRKFLIAKEQKTLKHRAQEYDQLVRSLLDSQKKDTD
KVLIFNYENGFVFTDKDHFSKYSIRL

>core/976/1/Org1_Gene77

MIKKDRFTNEKLNKLFDSPFSLVNYAIKQAKIKIAKGDVRSSNVAIETLVLLDREGIQPEFTEEIVVTASPTVER
KRSEHTNSRKKDPSAYTWSDVK

>core/977/1/Org1_Gene402

MYEGKSRMASPTPGQLHLQQKVESKAYDYSRSLAMIATALLFFIVALILSGLSLLPQVFLPFSGAYFIIGSFLAF
IALGILLINCVCDLKQYLTSS

>core/978/1/Org1_Gene21

MGSGYAKKKKEAKIMEQQFLEMEASLLEKRYEGQAGNGLVSVVINGKCDLISVKVQPTCLDPEDPEVIEDLF
RAAFKLAKEQMDQEMSLMRSTMPF

>core/979/1/Org1_Gene307

MNKSRFLRLCCCLCFCGSLFYFYINKQNSLTKLRLEIPCLSVRLRQLEQQNISLRFLIDKIERPDHLMEIAALPE
YQYLEYPSEESISLLSYELP

>core/980/1/Org1_Gene628

MLAFFATSFKSVLFEYSYQSLLLILIVSAPPIILASIVGIMVAIFQAATQIQEQTFAFAVKLVVIFGTLMISGGWL
SNMILRFAGQIFQNFYKWK

>core/982/1/Org1_Gene451

MLIRLFLGISLPKGFPLYLEPPLVLATFQGTQFVGTYSEATNPLYIDNLNLNYHYTQELLYKAVPCNYKSIYRE
IPLIIFPEVLIGSTPTQSTE

>core/983/1/Org1_Gene1

MLGKIIRGLSSLIVILCALNVGLIGITHNKLNIIAKLCGGVSTPATQITYIIIGIAGVICLLSFCPFCSKKSRHSHGD
SCSSGGCHSHHSDKN

>core/988/1/Org1_Gene260

MAKLVITSDDEQQEFELEDNSEIAEPCESMGIPFACTEGVCGTCVIEVLEGRENLSEFTEPEYDFLGEPEDSNER
LACQCRIKGGCVKVTF

>core/989/1/Org1_Gene356

MDDSWILEVKVTPKAKENKIVGFDGQALKVRVTEPPEKGKANDAVISLLAKALSLPKRDVTLIAGETSRKKK
FLLPNRVQDIIFSLHIDV

>core/990/1/Org1_Gene404

MKKAVLIAAMFCGVVSLSSCCRIVDCCFEDPCAPSSCNPCEVIRKKERSCGGNACGSYVPSCSNPCGSTECNS
QSPQVKGCTSPDGRCKQ

>core/991/1/Org1_Gene767

MSLDKGTKEEITKKFQLHEKDTGSADVQIAILTEHIAELKEHLKRSPKDQNSRLALLKLVGQRRKLLEYLNST
DTERYKNLITRLNLRK

>core/992/1/Org1_Gene239

MSRKCPLTGKRPRRGYSYTLRGIAKKKKGIGLKVTGKTKRRFFPNMLTKRLWSTEENRFLKLKISASALRHID
KLGLEKVLERAKSKNF

>core/993/1/Org1_Gene822

MSRSLRKGPFVDHHLLKKVRAMNIEEKKTPIKTWSRRSMITPEMIGHTFEVHNGKKFLTVFVSETMVGHKLG
EFSPTRIFKSHPVKKG

>core/994/1/Org1_Gene116

MKEYLDFLVQRNVERDPQTKRHCTVSQKFGGESIDAKTTTGQLFHIAGKTEPGHGKLCLGESILKQLLALGII
TGYENREREVWVYLD

>core/996/1/Org1_Gene423

MSQKNKNSAFMHPVNISTDLAVIVGKGPMPRTEIVKKVWEYIKKHNCQDQKNKRNILPDANLAKVFGSSDPI
DMFQMTKALSKHIVK

>core/997/1/Org1_Gene724

MKSFKFLLPFLSVILCCGNLLSSPRSRAISVTESIGMSAVKTLVLSEKAHEFLEGIGYGVGASSILRDWQTQQW
LEIESLLAQNEVM

>core/998/1/Org1_Gene477

MASEPRGSRKVKIGVVVSAKMEKTVVVRVERIFSHPQYLKVVRSSKKYYAHTELKVSEGDKVKIQETRPLSK
LKRWRVIEHVGVVS

>core/999/1/Org1_Gene812

MVEIHHKDPSLKKLFALQQSLETLNSLSDIVATYEAMFSLIYEGLNKALRKDQLCYLLSVNSKGELLKSPSGD
PIVQTFPIHPHH

>core/1000/1/Org1_Gene538

MATNKSCTAFDFNKMLDGVCTYVKGVQQYLTELETSTQGTVDLGTMFNLQFRMQILSQYMESVSNILTAVN
TEMITMARAVKGS

>core/1001/1/Org1_Gene394

MAHKKGQGASRNGRDSKSKRLGVKVGAGQKVSTGSILVRQRGTRWNPAQNVGRGRDDTLFALVDGIVVM
KKTNRTYISVVPEQL

>core/1003/1/Org1_Gene40

MKQQHNRKALSRKIGTVKKQAKFAGSFLDEIKKIEWVSKHDLKKYIKVVLISIFGFGFAIYFVDLVLRKSITCL
DGITTFLFG

>core/1004/1/Org1_Gene539

MFNMENTAKEEKNSQPLLDLEQDMQDHDRAQELKASVQDKVHKLHALLREGSDKESFGQQQSLLAGYVA
LQKVLGRINRKMI

>core/1005/1/Org1_Gene731

MNKPVHNNEHRRKRFNKKCPFVSAGWKTIDYKDVETLKKFITERGKVLPRRITGVSSRFQGVLSQAIKRARH
LGLLPFVGED

>core/1007/1/Org1_Gene32

MTKIQCSAQYYRSRPAERAQTPPQPFLARDRADFWERHPRFSACCRVLLLVAWVVLALLFLFVMLLPLAAGSYLLAF

>core/1009/1/Org1_Gene211

MSLEDDVIAIIVEQLGVDPKEVNENSSFIEDLNADSLDLTELIMTLEEKFAFEISEEDAEKLRTVGDVFTYIKKRQAEQ

>core/1012/1/Org1_Gene547

MKEFLAYIIKNLVDRPEEVRIKEVQGTHTIIYELSVAKPDIGKIIGKEGRTIKAIRTLLVSVASRNNVRVSLEIMEEK

>core/1013/1/Org1_Gene1015

MMTLFLVICCAAVLLGLGMGILLVGSHLLGRPLSKGCQKPDCCQKKTCDKTEHCATKSRENSTSKCSSNDDVPPTAP

>core/1014/1/Org1_Gene590

MNEGIHSVCFQKTPRLTAKSVVSMEMLLTTQQLPSAEGMPSVANLEADFLRAEALLAEMREIRGCLEQSLRTLVPSE

>core/1015/1/Org1_Gene769

MKNKIVTLLDQLYEDQESRLQKLGEEIVPNLTPEDLLQPMDFPQLEGNPAFRFEEGVLSGIGEVRAAILAALSQEN

>core/1016/1/Org1_Gene869

MEAKKIKELSKEAQLLKKLREKSRVLDEKNKRKAWVAKLVAMPESIREIEKEERVETPQLFQAIAEKILEEGV

>core/1018/1/Org1_Gene552

MFFAPLLYESLRRGLMHPTSHMQQQLARLEFINDQLTTELEHVNELLCSLGFPEGLTTIKAIAEEVLSDDEPLLD

>core/1019/1/Org1_Gene858

MEEVPFENAMQRLEEIVDLMNQPTTSLDASLALYEEADALMRICESRIRQVEQRVRELAEKRHESSLFEEQAVVR

>core/1021/1/Org1_Gene38

MAKKEDTLVLEGKVEELLPGMHFRVILENGMPVTAHLCGKMRMSNIRLLVGDRVTVEMSAYDLTKARVVYRHR

>core/1022/1/Org1_Gene862

MSGNECNDYPEVFKDDVSAYVLVTCGQMSSEGKIQVEMTYEGDPAVISYLLTKARDSLDES

>core/1023/1/Org1_Gene985

MSYKITLPKADETTAKKVTKISEASTLIFSVLKEKASLGNVHGFCQAENSLSVEANKIISVAENTLAGCFCK

>core/1025/1/Org1_Gene951

MAAKKDLLTQLRGKGDDDLDAYVHENKKALFALRAENLLQNKVVKVHMFSTHKKNIARALTVKQERKGK
VHG

>core/1027/1/Org1_Gene821

MMFDFMLARAFRAANTIVNTKQKPFHIHGMATLRPNATSILNIKEDALSLGTVGSFASTASIKIDSH

>core/1028/1/Org1_Gene498

MKKLIALIGIFLVPIKGNTNKEHDAHATVLKAARAKYNLFFVQDVFPVHEVIEPISPDCLVHYEGWV

>core/1031/1/Org1_Gene825

MKKLMLALLLALVLPVAGSAFGYESCSAPQDEAQGSEDEGRDESSDSDTQTEKDEEGGSEENTAE

>core/1033/1/Org1_Gene760

MPKMKTNKSVSARFKLTASGQLKRTRPGKRHKLSKKSSQEKRNLSKQPLVDKGQVGMYKRMMLV

>core/1034/1/Org1_Gene348

MIKQACKFYLLQCLLCALYWLLKYCRKLLKGTLHHSEETLYQALLSSLIDLLYQLKQLPAPTNE

>core/1037/1/Org1_Gene405

MKLFYFCNIIFLTSLFSVSCTHTPPPRSYILAQGRTPLVNKAHSLEFFLARSVFNTCYNTNL

>core/1045/1/Org1_Gene737

MAVPRNRHSNARKNIRRSHDAKKACHAAKCSNCKHALLPHTICPSCGFYNGKAVMTVEKK

>core/1046/1/Org1_Gene953

MSIALNREEVWDNPRHLMFILMQFQQFSGEQDRFGSFLEATIRDRVSFLVLQEKIATLK

>core/1049/1/Org1_Gene983

MRFFCLFFLGFLGSFHCVAEDKGVDLFGVWDDNQITECDDSYMTEGREEVEKVVDA

>core/1050/1/Org1_Gene15

MPSVKVRVGEPVDRALRILKKKIDKEGILKAAKSHRFYDKPSVKKRAKSKAAAKYRSR

>core/1054/1/Org1_Gene290

MQIENSSILFAEVVMKWFIFSVISAPVVFLPGCTLIPKEKVTKVPSQLWSESLSQP

>core/1056/1/Org1_Gene932

MIDAENQNPKDGGSFTSLHTDPKNLFDEEGMPSPSDTLQCDLNNVFIFIKSMFF

>core/1058/1/Org1_Gene365

MSRHRSYGKSVKGVTKRNVLKRFERVEVLRKLGRWNDSTAKKVTGLPKTPILK

>core/1059/1/Org1_Gene177

MASKNREIIKLKSSESSDMYWTVKNKRKTTGRLELKKYDRKLRRHVIFKEAR

>core/1067/1/Org1_Gene787

MFFIAVRSRGFLDIHGILAARKGKQVVKSTAGAWIGSRGAVFYSLVS

>core/1068/1/Org1_Gene984

MSESLEIPELTEVLSEQPSLSTPDSPPKVITGTLTLYFQEDIDPAS

>core/1069/1/Org1_Gene718

MKVSSSVKADPSKGDKLVRRKGRLYVINKKDPNRKQRQAGPARKK

>core/1070/1/Org1_Gene717

MKRTYQPSKRKRRNSVGFRTRMATRNGRKLLNRRRRHGRHSLVDL

>core/1073/1/Org1_Gene1014

MSSVKKKRRLKIAKHKRKKRRRRDRHKNK

>core/0/2/Org2_Gene73

MKSLPVYVSGIKVRNLKNVSIHFNSEEIVLLTGVSGSGKSSIAFDTLYAAGRKRYISTLPTFFATTITTLPNPKV
EEIHGLSPTIAIKQNHFSHYSHATVGSTTELFSHLALLFTLEGQARDPKTKEVLDLYSKEKVLSTIMELSEGVQI
SILAPLLRKDIAAIHEYAQQGFTKVRCNGTIHPIYSFLTSGIPEDCSVDIVIDTLIKSENNIARLKVSLFTALEFGE
GHCSVLSDEELMTFSTKQQIDDVTYTPLTQQLFSPHALESRCSLCQGSGIFISIDNPLLIDENLSIKENCCSFAGN
CSSYLYHTIYQALADALNFNLETPWKDLSPEIQNIFLRGKNNLVLPVRLFDQTLGKKNLTYKVWRGVLNDIG
DKVRYTTKPSRYLSKGMSAHSCSLCKGTGLGDYASVATWEGKTFTEFQQMSLNNWHVFFSKVKSPSLSIQEI
LQGLKQRLSFLIDLGLGYLTPNRALATLSGGEQERTAIAKHLGGELFGITYILDEPSIGLHPQDTEKLIGVIKKL
RDQGNTVILVEHEERMISLADRIIDIGPGAGIFGGEVLFNGKPEDFLMNSSSLTAKYLRQELTIPIPESREAPTS
WLLLTEATIHNLKNLSIRLPLARLIGVTGVSGSGKSSLINNTLVPAIESFLKQENPKNLHFEWGCIGRLIHITRDL
PGRSQRSIPLTYIKAFDDIRELFASQPRSLRQGLTKAHFSFNQPQGACIQCQGLGTMTISDDDTPIPCSECQGKR
YHSEVLEILYEGKNIADILDMTAYEAEKFFISHPKIHEKIHALCSLRLDYLPLGRPLSTLSGGEIQRLKLAHELL
FASPKQTLYVLDEPTTGLHTHDIQALIEVLLSLTYLGHTVLVIEHNMHVVKVCDYVLELGPEGGDLGGYLLA
SCTPKDLIQLNTPTAKALAPYIEGSLDIPVVKSEPPSSPKSCDILIKDAYQNNLKHIDLALPRNSLIAIAGPGASG
KHSLVFDILYASGNIAYAELFPPYIRQGLLKETPLPSVGEVKGLSPVISVRKCSSSNRSYHTIASALGLSNGLEK
LFAILGEPFSPLTEEKLSKTTPQTIIDSLLKSYKDDYVTITSPIPLGSDLEIFLQEKQKEGFIKLYSEGNLYDLDER
LPLNLIEPAIVIQHTKVSPKNSSSLLSAISVAFSLSSEIWIYISQKKQRKLSYSLGWKDKKGRLYPEITHQLLSSD
HPEGRCLTCGGRGEILKISLEEHKEKIAHYTPLEFFSLFFPKSYMKPVQKLLKDENASQPLKLLTTKEFLNFCR
GSSEFPGMNALLMEQLDTESDSPLIKPLLALTSCPACKGSGLNDYANYVRINNTSLLDIYQEDATFLESFLNTI
GTDDTRSIIQDLMNRLTFISKVGLSYITLGQRQDTLSDGENYRLHLAKKISINLTNIVYLFEEPLSGLHPQDLPTI

VQLLKELVANNNTVIATDRSCSLIPHADHAIFLGPGSGPQGGFLMDSDTEVCPSVDLHANVPQTEVCPKAPLS
ISKANHTRGSDRTLKVNLSIHHIQNLKVSAPLHALVAIGGVSGSGKTSLLLEGFKKQAELLIAKGTTTFSDLVV
IDSHPIASSQRSDISTYFDIAPSLRAFYASLTQAKALNISSTMFSTNTKQGQCSDCQGLGYQWIDRAFYALEKR
PCPTCSGFRIQPLAQEVLYEGKHFGELLHTPIETVALRFPFIKKIQKPLKALLDIGLGYLPIGQKLSSLSVSEKTA
LKTAYFLYQTPETPTLFLIDELFSSLDPIKKQHLPEKLRSLINSGHSVIYIDHDVKLLKSADYLIEIGPGSGKQGG
KLLFSGSPKDIYASKDSLLKKYICNEELDS

>core/1/2/Org2_Gene462

MKWLPATAVFAAVLPALTAFGDPASVEISTSHTGSGDPTSDAALTGFTQSSTETDGTTYTIVGDITFSTFTNIP
VPVVTPDANDSSSNSSKGGSSSSGATSLIRSSNLHSDFDFTKDSVLDLYHLFFPSASNTLNPALLSSSSSGGSSS
SSSSSSSGSASAVVAADPKGGAAFYSNEANGTLTFTTDSGNPGSLTLQNLKMTGDGAAIYSKGPLVFTGLKN
LTFTGNESQKSGGAAYTEGALTTQAIVEAVTFTGNTSAGQGGAIYVKEATLFNALDSLKFEKNTSGQAGGGI
YTESTLTISNITKSIEFISNKASVPAPAPEPTSPAPSSLINSTTIDTSTLQTRAASATPAVAPVAAVTPTPISTQETA
GNGGAIYAKQGISISTFKDLTFKSNSASVDATLTVDSSTIGESGGAIFAADSIQIQQCTGTTLFSGNTANKSGGG
IYAVGQVTLEDIANLKMTNNTCKGEGGAIYTKKALTINNGAILTTFSGNTSTDNGGAIFAVGGITLSDLVEVR
FSKNKTGNYSAPITKAASNTAPVVSSSTTAASPAVPAAAAAPVTNAAKGGALYSTEGLTVSGITSILSFENNEC
QNQGGGAYVTKTFQCSDSHRLQFTSNKAADEGGGLYCGDDVTLTNLTGKTLFQENSSEKHGGGLSLASGKS
LTMTSLESFCLNANTAKENGGGANVPENIVLTFTYTPTPNEPAPVQQPVYGEALVTGNTATKSGGGIYTKNA
AFSNLSSVTFDQNTSSENGGALLTQKAADKTDCSFTYITNVNITNNTATGNGGGIAGGKAHFDRIDNLTVQSN
QAKKGGGVYLEDALILEKVITGSVSQNTATESGGGIYAKDIQLQALPGSFTITDNKVETSLTTSTNLYGGGIYS
SGAVTLTNISGTFGITGNSVINTATSQDADIQGGGIYATTSLSINQCNTPILFSNNSAATKKTSTTKQIAGGAIFS
AAVTIENNSQPIIFLNNSAKSEATTAATAGNKDSCGGAIAANSVTLTNNPEITFKGNYAETGGAIGCIDLTNGS
PPRKVSIADNGSVLFQDNSALNRGGAIYGETIDISRTGATFIGNSSKHDGSAICCSTALTLAPNSQLIFENNKVT
ETTATTKASINNLGAAIYGNNETSDVTISLSAENGSIFFKNNLCTATNKYCSIAGNVKFTAIEASAGKAISFYDA
VNVSTKETNAQELKLNEKATSTGTILFSGELHENKSYIPQKVTFAHGNLILGKNAELSVVSFTQSPGTTITMGP
GSVLSNHSKEAGGIAINNVIIDFSEIVPTKDNATVAPPTLKLVSRTNADSKDKIDITGTVTLLDPNGNLYQNSY
LGEDRDITLFNIDNSASGAVTATNVTLQGNLGAKKGYLGTWNLDPNSSGSKIILKWTFDKYLRWPYIPRDNH
FYINSIWGAQNSLVTVKQGILGNMLNNARFEDPAFNNFWASAIGSFLRKEVSRNSDSFTYHGRGYTAAVDAK
PRQEFILGAAFSQVFGHAESEYHLDNYKHKGSGHSTQASLYAGNIFYFPAIRSRPILFQGVATYGYMQHDTTT
YYPSIEEKNMANWDSIAWLFDLRFSVDLKEPQPHSTARLTFYTEAEYTRIRQEKFTELDYDPRSFSACSYGNL
AIPTGFSVDGALAWREIILYNKVSAAYLPVILRNNPKATYEVLSTKEKGNVVNVLPTRNAARAEVSSQIYLGS
YWTLYGTYTIDASMNTLVQMANGGIRFVF

>core/2/2/Org2_Gene856

MVAKKTVRSYRSSFSHSVIVAILSAGIAFEAHSLHSSELDLGVFNKQFEEHSAHVEEAQTSVLKGSDPVNPSQ
KESEKVLYTQVPLTQGSSGESLDLADANFLEHFQHLFEETTVFGIDQKLVWSDLDTRNFSQPTQEPDTSNAVS
EKISSDTKENRKDLETEDPSKKSGLKEVSSDLPKSPETAVAAISEDLEISENISARDPLQGLAFFYKNTSSQSISE
KDSSFQGIIFSGSGANSGLGFENLKAPKSGAAVYSDRDIVFENLVKGLSFISCESLEDGSAAGVNIVVTHCGDV
TLTDCATGLDLEALRLVKDFSRGGAVFTARNHEVQNNLAGGILSVVGNKGAIVVEKNSAEKSNGGAFACGS
FVYSNNENTALWKENQALSGGAISSASDIDIQGNCSAIEFSGNQSLIALGEHIGLTDFVGGGALAAQGTLTLR
NNAVVQCVKNTSKTHGGAILAGTVDLNETISEVAFKQNTAALTGGALSANDKVIIANNFGEILFEQNEVRNH

GGAIYCGCRSNPKLEQKDSGENINIIGNSGAITFLKNKASVLEVMTQAEDYAGGGALWGHNVLLDSNSGNIQ
FIGNIGGSTFWIGEYVGGGAILSTDRVTISNNSGDVVFKGNKGQCLAQKYVAPQETAPVESDASSTNKDEKSL
NACSHGDHYPPKTVEEEVPPSLLEEHPVVSSTDIRGGGAILAQHIFITDNTGNLRFSGNLGGGEESSTVGDLAI
VGGGALLSTNEVNVCSNQNVVFSDNVTSNGCDSGGAILAKKVDISANHSVEFVSNGSGKFGGAVCALNESV
NITDNGSAVSFSKNRTRLGGAGVAAPQGSVTICGNQGNIAFKENFVFGSENQRSGGGAIIANSSVNIQDNAGD
ILFVSNSTGSYGGAIFVGSLVASEGSNPRTLTITGNSGDILFAKNSTQTAASLSEKDSFGGGAIYTQNLKIVKNA
GNVSFYGNRAPSGAGVQIADGGTVCLEAFGGDILFEGNINFDGSFNAIHLCGNDSKIVELSAVQDKNIIFQDAI
TYEENTIRGLPDKDVSPLSAPSLIFNSKPQDDSAQHHEGTIRFSRGVSKIPQIAAIQEGTLALSQNAELWLAGLK
QETGSSIVLSAGSILRIFDSQVDSSAPLPTENKEETLVSAGVQINMSSPTPNKDKAVDTPVLADIISITVDLSSFV
PEQDGTLPLPPEIIIPKGTKLHSNAIDLKIIDPTNVGYENHALLSSHKDIPLISLKTAEGMTGTPTADASLSNIKID
VSLPSITPATYGHTGVWSESKMEDGRLVVGWQPTGYKLNPEKQGALVLNNLWSHYTDLRALKQEIFAHHTI
AQRMELDFSTNVWGSGLGVVEDCQNIGEFDGFKHHLTGYALGLDTQLVEDFLIGGCFSQFFGKTESQSYKA
KNDVKSYMGAAYAGILAGPWLIKGAFVYGNINNDLTTDYGTLGISTGSWIGKGFIAGTSIDYRYIVNPRRFIS
AIVSTVVPFVEAEYVRIDLPEISEQGKEVRTFQKTRFENVAIPFGFALEHAYSRGSRAEVNSVQLAYVFDVYR
KGPVSLITLKDAAYSWKSYGVDIPCKAWKARLSNNTEWNSYLSTYLAFNYEWREDLIAYDFNGGIRIIF

>core/3/2/Org2_Gene120

MASSSNNSTKQDGIPSWVNPNVQWNRASQVGDQEANSLTPEAQTSRSWFSDRKHFLEVLDVSLEEMENNDL
KKYSRYKTIILIATLVTVAITCIVPISMVFGIPMWVPCLILFGAGLSSAFLSHRLQSKCKEIHLRYRAYQIYRQQL
LSQYPDLRKSTLYKYSITHVKPKKGFVGKLVENLRPDLHKNKDDGGAAADSRLDFAGYGVKHYQTDALLG
VSGVNSVEWQRLASLIMSVKNDILNDVGSREPIDKAQRSALVVSGKDIGGEIQPGGILDISRDILAICGYGMNV
GVEAKKAIDQYKKWYLNSSTFIAWNPQLPAIAQSYLLEQQRHLDYAAKIFQDLSALTTAHGTGQALEDLDSL
LCYYDQLIESKGVGEKIIASIHQKHLDLAMQDSCDQEHLKKWSNLYHVFSITIKEFTEGKLEQNEVVSRIQRL
RGKLEKSKCSILGNCRTNAEYATKSEKKLADYLLQIGDREPFLTGMHKAIATGKAIQGKVEGVISQHPEKQIM
MLRCSIERLEGMLRREDWGAILQKNEDEVLALKSTMEAQLQGFKDLVGTWEGKYQEFKKNKLSKVLVYDF
TKSYSNLLNRLEVLHAESSTDDLVLHVDRMSEDLKKTIEEIDGNLFQVTPEELSLLAREYQGLMNELPLIVQE
GNRLQEAISSEGVSQGLMLLNSLLNRDEKINKNIESSRKNLVAIAKQARSDARNIDSQGLAPLIQRNRASLDNI
LQNMYLFNGSIRNIHALDTETLVATSSNMFSAMHTFDWNIYTNLLDVLEIQSKPAPAPMENPDLPGALPEEVQ
DAVAEDVSGTHRLHHQVLKRRCADLKNMISQLQKSINKWGMAKAIVLGIVAVLFCVLSAIFIGQNILSLLILS
CVGLLLTQVCPLIFDRISKSKEFEKQVLETAQSLIPATKILPSEFNNKDLNRLAKLQDNLNLEGFGPTWARNIV
SDLEGIPTKEKSLKDLTKEFRKDSKNLNKRIKRRFKEGLGQEAPVVRPTIPQDIRGAEVFAELHRELEHLQKQ
KEEISIRGDALVQERMGLCLEKSKYDNEKAHAAAMTKKVGKLQNIDRLQKNNETYVRIQNFFRTLIQEKLGR
DTVQEIDVVKEAKELHELAAIIYGNTSGKSQKQRAKKQFKENVLHIAGKGQLELLEAYLNVTASQGLCRHQ
MQASFRERILLNPDGAKHGEAERTLASREEMLKTLGLSYLTPFVRFSSPESTQSGYNQILKVREQLFDIEQRLQ
NQETVSPEDYAAVQAALAAYVRKHESLIVSTYGLGAQEGQTSSKVTTLMRDLHAVEELVEMGVETYRLNRS
DQILHRVHSVLHSHLRDCDSSGNGIIDVVKKLFELLNNNGNNPNDPECQKYMQILLDAPVSLLYGAFKSFKN
EFLLNFTELNIANSTKAAEEEAKRYVEEKGRGFETYWEEAKQRLEAIAAELDDLRNQETLLEQEIRLANLKISI
FSDLNLREKVSVEKAALEEEIQGIQEQYAEMQGIEDLELKQKFEDLQKKLEALEERLLQIGRRIDSSVDKQKE
LLGLLGREEAA

>core/4/2/Org2_Gene382

MKYSLPWLLTSSALVFSLHPLMAANTDLSSSDNYENGSSGSAAFTAKETSDASGTTYTLTSDVSITNVSAITP
ADKSCFTNTGGALSFVGADHSLVLQTIALTHDGAAINNTNTALSFSGFSSLLIDSAPATGTSGGKGAICVTNTE
GGTATFTDNASVTLQKNTSEKDGAAVSAYSIDLAKTTTAALLDQNTSTKNGGALCSTANTTVQGNSGTVTFS
SNTATDKGGGIYSKEKDSTLDANTGVVTFKSNTAKTGGAWSSDDNLALTGNTQVLFQENKTTGSAAQANNP
EGCGGAICCYLATATDKTGLAISQNQEMSFTSNTTTANGGAIYATKCTLDGNTTLTFDQNTATAGCGGAIYT
ETEDFSLKGSTGTVTFSTNTAKTGGALYSKGNSSLTGNTNLLFSGNKATGPSNSSANQEGCGGAILAFIDSGS
VSDKTGLSIANNQEVSLTSNAATVSGGAIYATKCTLTGNGSLTFDGNTAGTSGGAIYTETEDFTLTGSTGTVT
FSTNTAKTGGALYSKGNNSLSGNTNLLFSGNKATGPSNSSANQEGCGGAILSFLESASVSTKKGLWIEDNENV
SLSGNTATVSGGAIYATKCALHGNTTLTFDGNTAETAGGAIYTETEDFTLTGSTGTVTFSTNTAKTAGALHTK
GNTSFTKNKALVFSGNSATATATTTTDQEGCGGAILCNISESDIATKSLTLTENESLSFINNTAKRSGGGIYAPK
CVISGSESINFDGNTAETSGGAIYSKNLSITANGPVSFTNNSGGKGGAIYIADSGELSLEAIDGDITFSGNRATE
GTSTPNSIHLGAGAKITKLAAAPGHTIYFYDPITMEAPASGGTIEELVINPVVKAIVPPPQPKNGPIASVPVVPV
APANPNTGTIVFSSGKLPSQDASIPANTTTILNQKINLAGGNVVLKEGATLQVYSFTQQPDSTVFMDAGTTLE
TTTTNNTDGSIDLKNLSVNLDALDGKRMITIAVNSTSGGLKISGDLKFHNNEGSFYDNPGLKANLNLPFLDLS
STSGTVNLDDFNPIPSSMAAPDYGYQGSWTLVPKVGAGGKVTLVAEWQALGYTPKPELRATLVPNSLWNA
YVNIHSIQQEIATAMSDAPSHPGIWIGGIGNAFHQDKQKENAGFRLISRGYIVGGSMTTPQEYTFAVAFSQLFG
KSKDYVVSDIKSQVYAGSLCAQSSYVIPLHSSLRRHVLSKVLPELPGETPLVLHGQVSYGRNHHNMTTKLAN
NTQGKSDWDSHSFAVEVGGSLPVDLNYRYLTSYSPYVKLQVVSVNQKGFQEVAADPRIFDASHLVNVSIPM
GLTFKHESAKPPSALLLTLGYAVDAYRDHPHCLTSLTNGTSWSTFATNLSRQAFFAEASGHLKLLHGLDCFA
SGSCELRSSSRSYNANCGTRYSF

>core/5/2/Org2_Gene485

MKQKVKRNFAIIICVFALALYYVLPTCLYYAKPLDKKIDGNEAEHIIKSFTKQAQQVRKDVIPRVSAILSSLHL
RGHIQQHPAIPDIVSVRFKRGEDAEDFIGNLVHGEPNVPIKSARLHVVGYSREHDDHVIQVASSINTSLVESDF
SFVSYSSENEQEMASSILQRVYSACTFPKQKDCSCSYPSIWETAPKEQLLQYAKNLSSGFEVFSSRLSAFCQQS
FSSNQDRLAFLSRLSSLSNDAAIDVEDQKLLKSVYETLSQTACIRSLDCPYIEGLRLDCSESSLFFSSIEYCPKER
KIFLTLHSDLLAQRTSLSKEQRLDFDSRLAVEKQKLSKNLTVQVEDYNNGFSFQWMDKDTQGKIILQGERLL
QGIAEHLTALTLHRPAAESCDLIPENFPVFCRQPRESEAFGCYIFSPNTDCKHFSKGSVYILLKGLRSIVAKYQQ
GGGKELQSFEKDLQNLYNCFSHTEAISWTLGEDQVLEIRHPLQQFLDVWGEGFVIGKEGCAFLEVKDIQDRL
ATVNQIEKNRQSDLVRWHEQYRHAKCSMDLQERLSAPIPYQNLFLENMKLNMRKFSRGENILRLGIDFVGG
RQLLLSFKDHQGKQLTDKEDILKVSDELCARLNKLGVSEIELRREGDYIHLSVPGSSTISSSEILGTSKMSFHVV
NERFSSYSASRYEVQRFLDYLWFTSQAQGKTSPEEINTFASALFNEEVDVPPSVHEAITKLKSEGLAFSPSGCE
TPSTDLDTTFSMIAIGKDAEQKANPLVIVFRNYALDGASLKDIRPEFAAGEGYVLNFSVKDTSPKKMAEKLSP
TESFHTWTSAYCQEGISGTANGQYSANRGWRMAVVIDGYMVSSPILNVPLKNHASVSGKFTHREVSKLASD
LKSGAMSFVPEVLSEETISSDLGKKQCTQGIISACCGLAMLIVLMSVYYRFGGVIASGAVLLNLLLIWAALQY
LDAPLTLSGLAGIVLAMGMAVDANVLVFERIREEFLLSQSLKKSVEKGYTKAFGAIFDSNLTTVLASALLFFL
DTGPIKGFALTLILGIFSSMFTALFMTKFFFMLWMNKTQHTQLHMMNKFVGIKHDFLRGCKKLWAVSGSVF
LLGCVALGFGAWNSVLGMDFKGGYAFTFNPKEHGISDVAQMRGKVVHKLQEAGLSSRDFRIQTFGSSEKIKI
YFSDKALSYTKADTSLSPKINDHELALAVGLLSETGLDFSTETLNETQNFWSKVSSKLSKKMRYQATIGLLGA
LAIILLYVSLRFEWQYAFSAVCALIHDLLATCAVLFIAHFFLKKIQIDLQAIGALMTVLGYSLNNTLIIFDRIRED

RQANLFTPMHVLVNDALQKTFSRTVMTTATTLSVLLMLLFIGGSSVFNFAFIMTIGILLGTLSSLYIAPPLLLF
MVRKENRSK

>core/6/2/Org2_Gene60

MFGENSRDIGVLSKEGLFDKLEIGIASDITIRDKWSCGEIKKPETINYRTFKPEKGGLFCEKIFGPTKDWECCCG
KYKKIKHKGIVCDRCGVEVTLSKVRRERMAHIELAVPIVHIWFFKTTPSRIGNVLGMTASDLERVIYYEEYVV
IDPGKTDLTKKQLLNDAQYREVVEKWGKDAFVAKMGGEAIYDLLKSEDLQSLLKDLKERLRKTKSQQARM
KLAKRLKIIEGFVSSSNHPEWMVLKNIPVVPPDLRPLVPLDGGRFATSDLNDLYRRVINRNNRLKAILRLKTPE
VIVRNEKRMLQEAVDALFDNGRHGHPVMGAGNRPLKSLSEMLKGKNGRFRQNLLGKRVDYSGRSVIIVGPE
LKFNQCGLPKEMALELFEPFIIKRLKDQGSVYTIRSAKKMIQRGAPEVWDVLEEIIKGHPVLLNRAPTLHRLGI
QAFEPVLIEGKAIRIHPLVCAAFNADFDGDQMAVHVPLSVEAQLEAKVLMMAPDNIFLPSSGKPVAIPSKDM
TLGLYYLMADPTYFPEEHGGKTKIFKDEIEVLRALNNGGFIDDVFGDRRDETGRGIHIHEKIKVRIDGQIIETTP
GRVLFNRIVPKELGFQNYSMPSKRISELILQCYKKVGLEATVRFLDDLKDLGFIQATKAAISMGLKDVRIPDIK
SHILKDAYDKVAIVKKQYDDGIITEGERHSKTISIWTEVSEQLSDALYVEISKQTRSKHNPLFLMIDSGARGNK
SQLKQLGALRGLMAKPNGAIIESPITSNFREGLTVLEYSISSHGARKGLADTALKTADSGYLTRRLVDVAQDV
IITEKDCGTLNHIEISAIGQGSEELLPLKDRIYGRTVAEDVYQPGDKSRLLAQSGDVLNSVQAEAIDDAGIETIK
IRSTLTCESPRGVCAKCYGLNLANGRLIGMGEAVGIIAAQSIGEPGTQLTMRTFHLGGIAATSSTPEIITNSDGIL
VYMDLRVVLGQEGHNLVLNKKGALHVVGDEGRTLNEYKKLLSTKSIESLEVFPVELGVKILVADGTPVSQG
QRIGEVELHNIPIICDKPGFIKYEDLVEGISTEKVVNKNTGLVELIVKQHRGELHPQIAIYDDADLSELVGTYAI
PSGAIISVEEGQRVDPGMLLARLPRGAIKTKDITGGLPRVAELVEARKPEDAADIAKIDGVVDFKGIQKNKRIL
VVCDEMTGMEEEHLIPLTKHLIVQRGDSVIKGQQLTDGLVVPHEILEICGVRELQKYLVNEVQEVYRLQGVD
INDKHIEIIVRQMLQKVRITDPGDTTLLFGEDVNKKEFYEENRRTEEDGGKPAQAVPVLLGITKASLGTESFIS
AASFQDTTRVLTDAACCSKTDYLLGFKENVIMGHMIPGGTGFETHKRIKQYLEKEQEDLVFDFVSETECVC

>core/7/2/Org2_Gene59

MLKCPERVSVKKKEDIPDLPNLIEIQIKSYKQFLQIGKLAEERENIGLEEVFREIFPIKSYNEATVLEYLSYNLGV
PKYSPEECIRRGITYSVTLKVRFRLTDETGIKEEEVYMGTIPLMTDKGTFIINGAERVVVSQVHRSPGINFEQEK
HSKGNILFSFRIIPYRGSWLEAIFDINDLIYIHIDRKKRRRKILAITFIRALGYSSDADIIEEFFTIGESSLRSEKDFA
LLVGRILADNIIDEASSLVYGKAGEKLSTAMLKRMLDAGIASVKIAVDADENHPIIKMLAKDPTDSYEAALKD
FYRRLRPGEPATLANARSTIMRLFFDPKRYNLGRVGRYKLNRKLGFSIDDEALSQVTLRKEDVIGALKYLIRL
KMGDEKACVDDIDHLANRRVRSVGELIQNQCRSGLARMEKIVRERMNLFDFSSDTLTPGKVVSAKGLASVL
KDFFGRSQLSQFMDQTNPVAELTHKRRLSALGPGGLNRERAGFEVRDVHASHYGRICPIETPEGPNIGLITSLS
SFAKINEFGFIETPYRIVRDGIVTDEIEYMTADVEEECVIAQASASLDEYNMFTEPVCWVRYAGEAFEADTSTV
THMDVSPKQLVSIVTGLIPFLEHDDANRALMGSNMQRQAVPLLKTEAPVVGTGLECRAAKDSGAIVVAEED
GVVDFVDGYKVVVAAKHNPTIKRTYHLKKFLRSNSGTCINQQPLCAVGDVITKGDVIADGPATDRGELALG
KNVLVAFMPWYGYNFEDAIIISEKLIREDAYTSIYIEEFELTARDTKLGKEEITRDIPNVSDEVLANLGEDGIIRI
GAEVKPGDILVGKITPKSETELAPEERLLRAIFGEKAADVKDASLTVPPGTEGVVMDVKVFSRKDRLSKSDDE
LVEEAVHLKDLQKGYKNQVATLKTEYREKLGALLLNEKAPAAIIHRRTAEIVVHEGLLFDQETIERIEQEDLV
DLLMPNCEMYEVLKGLLSDYETALQRLEINYKTEVEHIREGDADLDHGVIRQVKVYVASKRKLQVGDKMA
GRHGNKGVVSKIVPEADMPYLSNGETVQMILNPLGVPSRMNLGQVLETHLGYAAKTAGIYVKTPVFEGFPE
QRIWDMMIEQGLPEDGKSFLYDGKTGERFDNKVVIGYIYMLKLSHLIADKIHARSIGPYSLVTQQPLGGKAQ

MGGQRFGEMEVWALEAYGVAHMLQEILTVKSDDVSGRTRIYESIVKGENLLRSGTPESFNVLIKEMQGLGL
DVRPMVVDA

>core/8/2/Org2_Gene583

MTWIPLHCHSQYSVLDAMSSIKDFVAKGQEFGIPALALTDHGNLYGAVDFYKECTQKGIQPIIGCECYIAPGS
RFDKKKEKRSRAAHHLILLCKNEQGYRNLCILTSLAFTEGFYYFPRIDKDLLRQYSEGLICLSGCLSSSVSDAA
LKSPEALLLELQWFQDLFKDDYFTEVQLHKMSEESIAGFKEEWLKQEYYSLIEKQIKVNTAVLEASKRLGIPT
VATNDIHYINANDWQAHEILLNVQSGETVRIAKQNTHIPNPKRKVYRSREYYFKSPAQMAELFKDIPEVISNT
LEVAKRCDFTFDFSKKHYPIYVPESLKTLNSYTEEDRYQASAVFLKQLAEEALPKKYSSEVLAHIAKKFPHRD
PIDIVKERMDMEMAIIIPKGMCDYLLIVWDIIHWAKANGIPVGPGRGSGAGSVLLFLLGITEIEPIRFDLFFERFI
NPERLSYPDIDIDICMAGRERVINYAIERHGKDNVAQIITFGTMKAKMAVKDVGRTLDMALSKVNHIAKHIPD
LNTTLSKALETDPDLHQLYINDAESAQVIDMALCLEGSIRNTGVHAAGVIICGDQLTNHIPICISKDSTMITTQY
SMKPVESVGMLKVDLLGLKTLTSINIAMSAIEKKTGQSLAMATLPLDDATTFSLLHQGKTMGIFQMESKGMQ
ELAKNLRPDLFEEIIAMGALYRPGPMDMIPSFINRKHGKEIIEYDHPLMESILKETYGIMVYQEQVMQIAGALA
SYSLGEGDVLRRAMGKKDFQQMEQEREKFCKRACNNGIDPELATVIFDKMEKFAAYGFNKSHAAAYGLITY
TTAYLKANYPKEWLAALLTCDSDDIEKIGKLIREAQSMGIPILPPHINVSSNHFVATDEGIRFAMGAIKGIGRG
LIESIVEERDHHGPYESIRDFIQRSDLKKVSKKSIESLIDAGCFDCFDSNRDLLLASVEPLYEAIAKDKKEAASG
VMTFFTLGAMDRKNEVPICLPKDIPTRSKKELLKKEKELLGIYLTEHPMDTVRDHLSRLSVVLAGEFENLPHG
SVVRTVFIIDKVTTKISSKAQKKFAVLRVSDGIDSYELPIWPDMYEEQQELLEEDRLIYAILVLDKRSDSLRISC
RWMKDLSIVNENIIYECDQAFDRIKNQVQKMSFTMSTSGKETKAKGNKPNENGHTQALAPVTLSLDLNELR
HSHLCILKKIVQKHPGSRTLVLVFTQDNERVASMSPDDAYFVCEDIEELRQELVTADLPVRVITV

>core/9/2/Org2_Gene743

MVLEALAIFRQDAMQHLLKHRKEIVVDFCEDSYTIRIPDEEAPEGYWLSTLKLQDIDRLTFASCSCPDGECCL
HLMTAYFAVYDALGLHPLHDKFRHSFWYAVFSHFFLDSIPLQAQGEMVYTLESPHITLTIECLSEEVFQDWLR
TIHASEEPTVFTNKTFLKSALYRTAKKFFFLNEEGAELTIGENSQGFPSHFSLQWQGLVFKAEILDFPTLEDIFP
KLELAHTSLENVSHDISITNVTVCAEEAKVNFTLSPVIHKKDRENHPKTRIGSVEYVAKTHEMITGPKAIALPI
YAIPLLADKFKDQLLSLLCYDSLEYRLRYDIRLLRDASFSFSAYLVTPGDLDNGSLIYPNYCYSPTKGLMQVV
GMLSPKQAFIVKSEQVEDFLNERGHLIQEPGFQTFINERPEGHLTYNVTEQGVLLFHYDVGDPSSTEIRFGTWT
YYTNQGFFLEKKNDLPIQDGLIVEPQDIPAFIVKNDAALRRLPNFFSSPPNLKDLLIEVHRQSRGKGLDLKPILV
GLGESRCWLFGVFLYREDIGFSLIPTPLQGLCFLPRVIPPENVPQFLTQYAQHERILFPNPQTRPPESYELVIQSI
HRPHPASPLHLQLELKTNLGSVPIGIALQGLKSKHTFLFTQAGFLDLKQNLFQFLKQFLSTQKCVIAENTVIANI
TDVFKLDALAPLSVTDDTIANPEDLQFFSQLKAACLPPIPQNLFSSDHQLRPYQNSGLLWMWFLYNHRLSGLL
CDEMGLGKTHQATALLDIVFQSSQPSARPKFLIVCPTSVLPHWEHILSNHLPGVSIFSFHGPNKPSELPPADILL
TSYGTLRQNYDKFYKIAFTIVVFDEIHMAKNKSSQIHKILCRIDAQMKLGLTGTPIENNLLEFKGLLDIILPNYL
PSDALFKKLFTKRCSSEELEEIIPSQDLLLKLTRPFILRRTKKLVLPELPDKVESIIACSLSPDQEKLYMATLQRE
KSHIQKLETPEEPATNFLHIFALLNHLKQICDHPAVFFKDPDQYKNYESGKWNAFVKLLKESLNAGYKVVVF
SQYIHMIRIITLYLEEIGIKYASIQGKSLNRKEEIETFTTDPNCQVFVGSLLAAGTGINLTAGNVVIMYDRWWN
PAKENQALDRVHRIGQKNTVFIYKLITEDTLEERIHYLIEKKIRLLDKVIASQDSNILHMLNREDLLTILSYKDE
HGTSDSEESPVDAPVEDDTGVLPPEDS

>core/10/2/Org2_Gene754

MLNFRKLRRDFSANILQDGKKLFEQGAVIDAKILSMNGETVCISAQVRGLYDNIYECEIEVDRSESDTVDSNC
DCSYNYDCQHIVALLFYLEQYFNEMVVAYARSADLETDHEINEEVKKELKETFVAAATKEEERKDREHQKEI
LREYVHAANALSANPFFLPLEYLEKDSAELAVLFVSVNEDTFAPANQPIEFQLVLRLPCRSKPFYISNIRTFLEG
VLYQEPIVLNGRRFFFTMQSFNASDRKLIDLLIRYVRYPNHTTEEKLLKSAYLMPPALGVILAKMFEHQLADR
GGGSLGEKESFSGLFCGNLEEPLCWSLTPAKMKFNLDFFDMPYKALLMTPVILVDDDEVQPEQTMLLESDAP
GIIHHFVYHRFSPQIKRAHLRSFSRLRDIAIPEALFGSFRENALPVFQEYAEIANVHLLNSFVTLPYVDEVRAIC
DMSYLDGELEAKLHFLYGSLRVPAASLALQYQDVRAFISDEGILARNLVEERKMLEEVFSGFIYDERDGAFR
VKSEKKIVEFMTETIPANQHRITFNCPENLSGQFIYDETIFELSFREGSDINYYEADLKVHGLLKGVPLDLLWD
CISAKKRFLELPKAGQQSKGTRRGKVNSGKLPCILVLDLEKIAPVVQIFNEIGFKVLDDLVQKCPLWSLTGISL
DQFEALPVNFSMSERLIEIQKQIRGEIEFDFQDVPQQIQATLRSYQTEGVHWLERLRKMHLNGILADDMGLGK
TLQAIIAVTQSKLEKGSGCSLIVCPTSLVYNWKEEFRKFNPEFRTLVIDGVPSQRRKQLTALADRDVAITSYNL
LQKDVELYKSFRFDYVVLDEAHHIKNRTTRNAKSVKMIQSDHRLILTGTPIENSLEELWSLFDFLMPGLLSSY
DRFVGKYIRTGNYMGNKADNMVALKKKVSPFILRRMKEDVLKDLPPVSEILYHCHLTESQKELYQSYAASA
KQELSRLVKQEGFERIHIHVLATLTRLKQICCHPAIFAKDAPEPGDSAKYDMLMDLLSSLVDSGHKTVVFSQY
TKMLGIIKKDLESRGIPFVYLDGSTKNRLDLVNQFNEDPSLLVFLISLKAGGTGLNLVGADTVIHYDMWWNP
AVENQATDRVHRIGQSRSVSSYKLVTLNTIEEKILTLQNRKKSLVKKVINSDDEVVSKLTWEEVLELLQI

>core/12/2/Org2_Gene609

MKRPKKFPIYLSIAQKTNRLLSGIVIAFAVIALRLWYLAVVEHEQKLEEAYKPQIRVLPQYVERATICDRFGKT
LAVNQLQYDVSVAYGAIRDLPTRAWRVDEHGHKQLIPVRKHYIMCLSELLSQELHLDREAIEDAIHAKASVL
GSVPYLVAANVSERTYLKLKMLSKDWPGLHVEAVVRRHYPQESVASDILGYVGPISLQEYKRVTQELSQLR
ECVRAYEEGEDPKLPEGLASIDQVRALLESVESNAYSLNALVGKMGVEACWDSKLRGKIGKKPILVDRRGNF
IQEMEGAVPEAPGTKLQLTLSAELQAYADALLLEYEKTETFRSAKSLKKREKLPPLFPWIKGGAIIALDPNNG
EILAMASSPRYRNNDFVNAKVAEDSKAVRSSIYRWLENKEHIAEIYDRKVPLIRERRNPLTGLCYEEILPLTFD
CFLDFLFPENSVIKLQLKRNSFVGQAIEVQNLVTRLLSLFPYEEGTCPCSAIFDAVFPNEEGHILIQEVISLQEQK
WIMECLNQHKADIEELKEALDQVFNELPANYDKILYTDILRLIVDPERFSPVLPSEVHRLSLSEFTELQGRYVV
LRSAFSTILEDAFIEVHFKSWRKSEFLQYLAAKRQEEALRKQRYPTPYVDYLEEEKTRQYKMFCQEHLDTFL
AYLFSKTPYKEGLEPYYDILDLWINELDNGAHRALSWHEHYLFLKERVSHLSEHLPALFSTFREFNELQRPLL
GKYPISIVRNKRQTEQDLAASFYPVYGYGYLRPHAYGQAATLGSIFKLVSAYSVLSQRILWGHNEEPANPLVI
IDKNSFGYRSSKPHVGFFKDGTPIPTFFRGGSLPGNDFMGRGFIDLVSALEMSSNPYFSLLVGEGLGDPEDLAD
AASLFGFGEKTGLGLPGEYAGRVPHDLAYNRSGLYATAIGQHTLVVTPLQTAVMLASLVNGGVVYVPKLLL
GEWEGEHVSYLSSKKKRTIFMPDAVVEVLKTGMRNVIWGQYGTARAIQSQFPPQLLSRIIGKTSTAESIMRVG
LDREYGTMKMKDIWFAAVGFSDQDLSLPTIVVIVYLRLGEFGRDAAPMAVKMIDMWEKIQQRESFLRG

>core/13/2/Org2_Gene1020

MDFNPVNLDFSISKEFKEETLPLLLENIHPGATAFLAAKMFHDCRASVIMITTPARLDDLFENLRTFLDQAPVE
FPSSEIDLSPKLVNIDAVGKRDHLLYSLNQHRAPIFCVTTLKALLEKTRSPQATSQQHLDLAVGDVLDPEATTE
LCKSLGYSQVMLTSEKGEFSCRGGIVDIFPLSSPEPFRIEFWGEKIISIRSYNPSDQLSTGKVSKISISPAYTEEAS
GGNYSHSLLDYFSTPPLYLFDNLEILEDDFADISGTLSSLPDRFFSIGTLYDRISTSNQVYFSETPFPNVKNLKEN

RVIIEAFHRNMEASRQAIPILYPEQIIQNDENPLLAFLQHLQEYMPPHGKPLKLAIYSTKTKSLKEARALAETV
ARGDVEIYEKTGNLTSSFALVNEAFAAISLSEFASTKVLRRQKQRTHFSVTTEEVFVPIPGETVVHIHNGIGKFL
GIEKKPNHLNIETDYLVLEYADKARLYVPSNQAYLISRYVGTSDKAADLHHLNSSKWKRSRDLTEKSLIVYA
EKLLQLEAQRSTTPAFVYPPHGESVIKFAETFPYEETPDQLKTIDQIYNDMMSPKLMDRLICGDAGFGKTEVI
MRAAVKAVCDGHRQVIVMVPTTILATQHYETFKERMAGLPIEIAVLSRFSQAKVQKLICEQVASGQIDIIIGTH
KLINKSLEFKNPGLLIIDEEQRFGVKVKDNLKERYPMIDCLTVSATPIPRTLHMSLSGARDLSVIAMPPLDRLP
VSTFVMEHNTETLTAALRHELLRGGQAYVIHNRIESIYTLAETIRNLIPEARIGVAHGQMGAEDLSNIFTKFKN
QKTDILVATALIENGIDIPNANTILIDHADKFGMADLYQMKGRVGRWNKKAYCYFLVPHLDRLSGPAAKRL
AALNKQEYGGGMKIALHDLEIRGAGNILGTDQSGHIGTIGFNLYCKLLKKAVSALKKHTSPLLFNDDVKIEFP
YNSRIPDTYIETGSMRIEFYQKIGNAESSEELTAIQEEMRDRFGPLPQEICWLFALAEIRLFALQHGISSIKGTAN
ALYVQKCLSKSEQTKKTLPYALSPTPELLVKEVIESIERGFLINAS

>core/14/2/Org2_Gene413

MIKKRAIFERMFPIPPPHCPPNNKNNFYHLTTDTKDPLLLRILRTIGYVLLHIITLGLLLLIHYYKHHRVVRKEG
LPTPPTLPKGPEPKTIEIAKQPPKDGEDKKPDVPKPGTPPPEDTPPPPPKAPSPASPKVPKQPADKKPTPPPEAPP
PPVRVATPMPLRPSSQGYWQCLNRMVSMVLRRAPLPLPAMQVDPILGDFNPHFVASYPNRIDNEPMYFQIKQ
FKKIAQNPDLPQQHRRLAQLSLEQALYLNDNYYLVNVPGDGNCFYRAYAVGWLSALYEESSRNDIVFEQEA
TRLLDLPFASSSPANANLCAEMAELLQLCSTYCSFIDLYDGVILSQKHTATLIAFLRKLSAYAIRQQIAASSNEE
TARALFISDMQDDLLPSVLEFLAANRPYSELFQNLIDHSALPYMQSRDKLFLLLEHLPALFLTDAELQKMSPE
DQQLRKQYEREIREAFAKLSRRIADSGWDTERFNAIVKDHLPEAIRCQYSRFLATIENRRSGDLPWSPALSFFA
FLCTCPSVRFHKLCATFYKSLEDIIIASAPPQRSIQEILQISNASLSYLNEDLDSSWQREVISSNIMTILTTHESLTL
ESSMPQLETLHKRIANLLKNVISTSFETPPLSNQPDLLSNLVNKLLVAIHSKLELKEHFNTVCSARSLRLTRDE
GSGLSQEQDLLYTQAVQLLFFILQHPQVNNRPETKDAVKELKMLLLPFLQYAFKKVENEKKLQKLLRSILGS
LVLKPPARYPSTPSNKDKETFCKFWSRHPEVMVLDPILEKNCMQFLRATFPNYQLETEAILLEKEIESTFRNG
WNVFLTRLNLFGSKLGSPSSPTALSDQFSKSFLIFCFLNNYPKLLQKKTPLAARLDAFQREASHRFTQVKDKL
LLSLKYGFPLATATINQYSRARDQLICNLLKNTVTASDGFCRSGFRQSLIGYLHSLSSNELGDILDDVKEQAEA
NDVAAMTTVPLQPFAVCLIMSDRDTVSEENIENFVAMHGFLNTISPERDARIFLIRFPNHYGCLLPRNPRTEDQ
NSKPDSSNP

>core/15/2/Org2_Gene652

MKPFNIFDSNSSIQGKFFLEASAGTGKTFTIEQIVLRALIEGSLTHVEHALAITFTNASTNELKVRIKDNLAQTL
RELKAVLNSQPASLPTYLDINCNVKQIYMQVRNALATLDQMSLFTIHGFCNFVLEQYFPKTRLIHKNPALTHS
QLVLHHITNYLKQDLWKNVLFQEQFHLLAVRYNITSKHTSSLVDKLLASYTQPISSYFSSRVERLEQISLWHQ
QIYNSLLEIPKQVFLDQLTAHISGFKKQPFSILDDLHHFVDLLYTSETHSSLFSFFKIAETFNFKHRLARYKPCA
AFTVLENMSWVERTLEFCNLDRIFNTLLVDLQEYLKQNYTPWLSPDESVFALEKLLSSSEAQPVVQALREQY
QLVLIDEFQDTDKQQWSIFSNLFISPKFTGSLFLIGDPKQSIYEWRSADLPTYLTAKSSFSEDKQLQLVNNYRST
PKLMEAINQIFGKISPFLEIPGYLPIEYHALNPQSSETFENPPHAPIHFFFYETIKDQALWIFSEALRLQKEQKIPL
GNMVVLVSDSNQAFELISYATIPVSFSKNKSIFHLTETHILTTALLEAILHPENYEKISKILFSSLFGLSLDEVTTK
KEDFTIYFQSLHSYISHHGLLATFYRVMTTQGNVLFSSPRGDLIFQEMEKLCGYLDTISSYPYHQLLHLKNFSE
TGRWEEELAISSYSEDLETLKITTIHSSKGLEYDIVFCPGIEKSKKNKSSSELLREMYVACTRAKKQLYLPISTQ
PPSLQRSSALTNYVKLEGTQSSAYDLAIHLHQEHPDLFSYSLPKDHGHATTVLNLPLLETFALKVTPPKTIFSFS

STKFLLDTHKDSQSIPYSKLPISKQQLPLGEKTGILIHKILESIQFSLLQDTEYLMSTIMRFIKHTHLEGFEETILK
LLSKTFFSPLTFSSQTFSLSQVLPNKIFRETSFLFLENQELWQGVIDLFFEHEGKYYIIDWKTSFLGETNSDYSKS
NLSIYIKQEKLDYQGRIYVKAVRKFLNQFEIDDDVELGVIFIRGIDTQGNGFFALNSSEDIPNFNPKAIQKCQAY
H

>core/16/2/Org2_Gene874

MVEVEEKHYTIVKRNGMFVPFNQDRIFQALEAAFRDTRSLETSSPLPKDLEESIAQITHKVVKEVLAKISEGQ
VVTVERIQDLVESQLYISGLQDVARDYIVYRDQRKAERGNSSSIIAIIRRDGGSAKFNPMKISAALEKAFRATL
QINGMTPPATLSEINDLTLRIVEDVLSLHGEEAINLEEIQDIVEKQLMVAGYYDVAKNYILYREARARARANK
DQDGQEEFVPQEETYVVQKEDGTTYLLRKTDLEKRFSWACKRFPKTTDSQLLADMAFMNLYSGIKEDEVTT
ACIMAARANIEREPDYAFIAAELLTSSLYEETLGCSSQDPNLSEIHKKHFKEYILNGEEYRLNPQLKDYDLDAL
SEVLDLSRDQQFSYMGVQNLYDRYFNLHEGRRLETAQIFWMRVSMGLALNEGEQKNFWAITFYNLLSTFRY
TPATPTLFNSGMRHSQLSSCYLSTVKDDLSHIYKVISDNALLSKWAGGIGNDWTDVRATGAVIKGTNGKSQG
VIPFIKVANDTAIAVNQGGKRKGAMCVYLENWHLDYEDFLELRKNTGDERRRTHDINTASWIPDLFFKRLEK
KGMWTLFSPDDVPGLHEAYGLEFEKLYEEYERKVESGEIRLYKKVEAEVLWRKMLSMLYETGHPWITFKDP
SNIRSNQDHVGVVRCSNLCTEILLNCSESETAVCNLGSINLVEHIRNDKLDEEKLKETISIAIRILDNVIDLNFYP
TPEAKQANLTHRAVGLGVMGFQDVLYELNISYASQEAVEFSDECSEIIAYYAILASSLLAKERGTYASYSGSK
WDRGYLPLDTIELLKETRGEHNVLVDTSSKKDWTPVRDTIQKYGMRNSQVMAIAPTATISNIIGVTQSIEPMY
KHLFVKSNLSGEFTIPNTYLIKKLKELGLWDAEMLDDLKYFDGSLLEIERIPNHLKKLFLTAFEIEPEWIIECTS
RRQKWIDMGVSLNLYLAEPDGKKLSNMYLTAWKKGLKTTYYLRSQAATSVEKSFIDINKRGIQPRWMKNK
SASTSIVVERKTTPVCSMEEGCESCQ

>core/17/2/Org2_Gene84

MTADEVGKNSFAKKEEQVLKFWKDNQIFEKSLQNRQGKTLYSFYDGPPFATGLPHYGHLLASTIKDVVGRY
ATMDGYYVPRRFGWDCHGVPVEYEVEKSLSLTAPGAIEDFGIASFNEECRKIVFRYVHEWEYYINRIGRWVD
FSSTWKTMDASFMESVWWVFQSLYNQGLVYEGTKVVPFSTALGTPLSNFEASQNYKEVDDPSLVVRMPLQ
NDSASLLVWTTTPWTLPSNMAIAVGETLVYVRIQDKKSGEQWILSQGCVSRWFSNPEEFVILESFSGKDLVG
RTYEPPFTFFQSKREEGAFRVIAASFVEESEGTGVVHMAPAFGEGDFLVCKENHVPLVCPVDAHGSFTEEIPQ
YQGQYIKHADKEIIKFLKKEGRIFYHGTVKHRYPFCWRTDTPLIYKAVNSWFVAVEKIKDKMLRANSSIHWV
PEHIQEGRFGKWLEGARDWAISRNRYWGTPIPIWKSADGEILVVGSIRELEELTGTQITDIHRHFIDDLNIVKD
GKPFHRIPYVFDCWFDSGAMPYAQNHYPFENQKETEEAFPADFIAEGLDQTRGWFYTLTVISAILFDRPAFRN
AIVNGIILAEDGNKMSKRLNNYPSPKYVLDTYGADALRLYLLHSVVVKAEDLRFSDKGIEGVLKQILLPLTNV
LSFFNTYAELYGFDPKSQDIEPAYTEIDQWILSNLYSVVGKVRESMSQYHLNFAVEPFVTFIDDLTNWYIRRC
RRRFWEAEDTPDRRAAFSTLYEVLTVFCKVIAPFVPFLAEDIYQKLKLEKEPESVHLCDFPQVEMDKILPDLE
KRMHDIREIVGLGHSLRKEHKLKVRQPLANFYVVGSKDRLSLLKTFEGLIAEELNVKNVIFYEEAPSFIYTTV
KPNFRMLGKKVGSKMKEVQKALSELPNNAIDKLIQEETWVLTIDDREIALDGDDVVICRHTDPGYIARSSALF
SVILDCQLREPLIVEGIARELVNKINTMRRNQQLHVSDRIALRIKTTEAVHRAFLDYENYICEETLIIAYDFTQD
SDFQGENWDINGHATQIEITVSSIDS

>core/18/2/Org2_Gene651

MNATKHCRASFSNSPRHLLAQLAEDITSTHQKPFTKRWILVANATTGHWIKNQLVHVLSDHIFMGSTIFTASD
SIVKHLFLGSGCSQPNIPDYLTLPLLINNILEEISKASKFENGREFLSPPTYETTKKLAAAFKQFHTFSQRPTKNA
SHYQELFQILESHFSSYEEMFTTILNNRTQEEDCSLHIFGYAHLPKHLAEFFINLSTYFPVYFYCFSPCREYFGD
LLSDRAIDFFWNQLPDSPIKNAWEHYVLSDRQALLANLAHKSQSSQNFFLDREIDYQEMFLPSKHDSSLGVIQ
NSILDLKPTSPQDFSQTKQTICIYRALNIPREVQEVFCKVTELLHRGVSPEEIFILSSHIESYKVHLNAIFNPHVPI
YFTDEVDPRAEDLRNKILLLSSILQTQGDLHYILQLLTHPQLQQPIDQNKVPYLIKKLSSEWGKISSKDRASGQ
QMKALGDLILEEYPFHQEGGRVSQVEVWETTVPLIYFIQERINLYLSSSQHSYEDLFQNVFSCLEKIFVLSPEET
SFITTLRNSLFPTFATSSCSLLFFTDFCLDFLLHFHKPSPLYDKPGPYIGSLSSLSLIPKGYVFILGANKTTSSDIFD
LLNRTTTHEELAFSSTEDEENFHFLQILVSTKHELHISYISSAAQFNLPSPFLNHIKETLDLPVETLPTQPYLSAFF
KNKACLHTSQEYNYSLAHAFYSKKALLPSLFIPTVKQVNLPQHLSLNEIIKGIFSPLDLFLKTNYNLRISYPEHL
KKQQKLFPTKHQIEDFWNECFVDKEHDLIPSISPHAEELFTYYREKTILLRNGLDKDPKHSPYTVTFSSSIFEER
PYHESYLFPPLSLSFQGNPVQIHGTIHGVCNEGLYLCSIDPRDSLKKTTRTLGSLPETSSEQKQLLERYVALAV
LQMSQHLSSDSALIKLTSFNTKENHHPPFSDPEGYLRKVLEVYHLMSSQPIPLLSPLCWKTLDDEEKFHQAVL
SAISEEAKNPSLPIFWQFHNRNIEEILNHVGASERLKILSLFRGPCEAV

>core/19/2/Org2_Gene1007

MSEHPLTLQSMIATILRFWSEQGCVIHQGYDLEVGAGTFNPATFLRALGPEPYKAAYVEPSRRPQDGRYGVH
PNRLQNYHQLQVILKPVPENFLSLYTESLRAIGLDLRDHDIRFIHDDWENPTIGAWGLGWEVWLNGMEITQL
TYFQAIGSKPLDTISGEITYGIERIAMYLQKKISIYDVLWNDTLTYGQITQASEKAWSEYNFDYANTEMWFKH
FEDFAEEALRTLKNGLSVPAYDFVIKASHAFNILDARGTISVTERTRYIARIRQLTRLVADSYVEWRASLNYPL
LSLSSTSEPKETSESVVPMISSTEDLLLEIGSEELPATFVPIGIQQLESLARQVLTDHNIVYEGLEVLGSPRRLAL
LVKNVAPEVVQKAFEKKGPMLTSLFSPDGDVSPQGQQFFASQGVDISHYQDLSRHASLAIRTVNGSEYLFLL
HPEIRLRTADILMQELPLLIQRMKFPKKMVWDNSGVEYARPIRWLVALYGEHILPITLGTIIASRNSFGHRQLD
PRKISISSPQDYVETLRQACVVVSQKERRMIIEQGLRAHSSDTISAIPLPRLIEEATFLSEHPFVSCGQFSEQFCA
LPKELLIAEMVNHQKYFPTHETSSGAISNFFIVVCDNSPNDTIIEGNEKALTPRLTDGEFLFKQDLQTPLTTFIEK
LKSVTYFEALGSLYDKVERLKAHQRVFSTFSSLAASEDLDIAIQYCKADLVSAVVNEFPELQGIMGEYYLKH
ANLPTASAVAVGEHLRHITMGQKLSTIGTLLSLLDRLDNLLACFILGLKPTSSHDPYALRRQSLEVLTLVSASR
LPIDLASLLDRLADHFPSTIEEKVWDKSKTIHEILEFIWGRLKTFMGSLEFRKDEIAAVLIDSATKNPIEILDTAE
ALQLLKEEHTEKLAVITTTHNRLKKILSSLKLSMTSSPIEVLGDRESNFKQVLDAFPGFPKETSAHAFLEYFLSL
ADLSNDIQDFLNTVHIANDDGAIRNLRISLLLTAMDKFSLCHWESVAV

>core/20/2/Org2_Gene997

MSRLDIFVFDSLIANQDQNLLEEIFCSEDTVLFKAYRTTALQSPLAAKNLNIARKVANYILADNGEIDTVKLVE
AIHHLSQCTYPLGPHRHNEAQDREHLLKMLKALKENPKLKESIKTLFVPSYSTIQNLIRHTLALNPQTILSTIHV
RQAALTALFTYLRQDVGSCFATAPAILIHQEYPERFLKDLNDLISSGKLSRIVNQREIAVPINLSGCIGELFKPLR
ILDLYPDPLVKLSSSPGLKKAFSAANLIETLGDSEAQIQQLLSHQYLMQKLQNVHETLTANDIIKSTLLHYYQL
QESTVRAIFFKEGLFSKEQVAFSTQHPRELSEIQRVYHYLHAYEEAKSAFIHDTQNPLLKAWEYTLATLADAS
QPTISNHIRLALGWKSEDPHSLVSLVTHFVEEEVENIRILVQQCEQTYHEARSQLEYIEGRMRNPLNNQDSQIL
TMDHMRFRQELNKALYEWDSAQEKAKKFLHLPEFLLSFYTKQIPLYFRSSYDAFIQEFAHLYANAPAGFRILF
THGRTHPNTWSPIYSINEFIRFLSEFFTSTESELLGKHAVINLEKETSRLVHNITAMLHTDVFQEALLTRILEAY
QLPVPPSILNHLDQLSQTPWVYVSGGTVDTLLLDYFESSEPLTLTEKHPENPHELAAFYADALKDLPTGIKSYL

EEGSHSLLSSSPTHVFSIIAGSPLFREAWDNDWYSYTWLRDVWVKQHQDFLQDTILPQLSIYAFIENFCNKYA
LQHVVHDFHDFCSDHSLTLPELYDKGSRFLSSLFTKDKTVALIYIRRLLYLMVREVPYVSEQQLPEVLDNVSS
YLGISSRITYEKFRSLIEETIPKMTLLSSADLRHIYKGLLMQSYQKIYTEEDTYLRLTTAMRHHNLAYPAPLLFA
DSNWPSIYFGFILNPGTTEIDLWKFNYAGLQGQPLDNIQELFATSRPWTLYANPIDYGMPPPPGYRSRLPKEFF

>core/21/2/Org2_Gene390

MPLSFKSSSFCLLACLCSASCAFAETRLGGNFVPPITNQGEEILLTSDFVCSNFLGASFSSSFINSSSNLSLLGKG
LSLTFTSCQAPTNSNYALLSAAETLTFKNFSSINFTGNQSTGLGGLIYGKDIVFQSIKDLIFTTNRVAYSPASVT
TSATPAITTVTTGASALQPTDSLTVENISQSIKFFGNLANFGSAISSSPTAVVKFINNTATMSFSHNFTSSGGGVI
YGGSSLLFENNSGCIIFTANSCVNSLKGVTPSSGTYALGSGGAICIPTGTFELKNNQGKCTFSYNGTPNDAGAI
YAETCNIVGNQGALLLDSNTAARNGGAICAKVLNIQGRGPIEFSRNRAEKGGAIFIGPSVGDPAKQTSTLTILA
SEGDIAFQGNMLNTKPGIRNAITVEAGGEIVSLSAQGGSRLVFYDPITHSLPTTSPSNKDITINANGASGSVVFT
SKGLSSTELLLPANTTTILLGTVKIASGELKITDNAVVNVLGFATQGSGQLTLGSGGTLGLATPTGAPAAVDFT
IGKLAFDPFSFLKRDFVSASVNAGTKNVTLTGALVLDEHDVTDLYDMVSLQSPVAIPIAVFKGATVTKTGFPD
GEIATPSHYGYQGKWSYTWSRPLLIPAPDGGFPGGPSPSANTLYAVWNSDTLVRSTYILDPERYGEIVSNSLW
ISFLGNQAFSDILQDVLLIDHPGLSITAKALGAYVEHTPRQGHEGFSGRYGGYQAALSMNYTDHTTLGLSFGQ
LYGKTNANPYDSRCSEQMYLLSFFGQFPIVTQKSEALISWKAAYGYSKNHLNTTYLRPDKAPKSQGQWHNN
SYYVLISAEHPFLNWCLLTRPLAQAWDLSGFISAEFLGGWQSKFTETGDLQRSFSRGKGYNVSLPIGCSSQWF
TPFKKAPSTLTIKLAYKPDIYRVNPHNIVTVVSNQESTSISGANLRRHGLFVQIHDVVDLTEDTQAFLNYTFDG
KNGFTNHRVSTGLKSTF

>core/22/2/Org2_Gene871

MKAGDTYRNFIIKSCKDLPEIESKLLEAEHKPTGASIMMIVNNDEENVFNICFRTCPQTSNGVAHVLEHMVLC
GSENYPVRDPFFSMTRRSLNTFINAFTGPDFTCYPAASQIPEDFYNLLSVYIDAVFHPLLTKQSFLQEAWRYEF
NSENHLCYTGVVFNEMKGAMMSGEARLSEALNAAIFPSVTYGVNSGGEPREIVTLSHEDVRAFHQSQYSINR
CLFYFYGNIKPSRHLDFLEEKLLRQATKLEKQAVSVPLQKRFKEPVRNILTYPVDHQEEDKVLFGISWLTCSIL
EQQELLALHVLEIILMGTDASPLKSRLLKSGFCKQTEMSIENDIREIPMTLVCKGCSPAGAQKLEALIFASLEEII
REGISENIVEGAVHQLELSRKEITGYSLPYGLSLFFRSGLLKQHGGSAEDGLRIHSLFSELRNSLKNSDYLAKLI
RKYFLDNPHFARVILLPDTELVAKDNKDEQQLLLSVSEKLTDENKEKIQQNVRELTESQEQKEDLNGILPNLA
LDKVPTSGKEFPLIKEGLSQGEVLHHECFTNDIVFIDVVLDIPPLSGEELPWLRLLVFLMLQLGCGGRSYKEHL
EFLLEHTGGVDVSYDFSPHANKNSFLSPSVSIRGKALSSKSEKLCGIVSDMLTSVDFTDIPRIRELLMQHNEAL
TNSVRNSPMSYAVSMACSGNSITGAMSYLTTGLPYVKKIRELTKNFDQNIDEAVVILQRLYTKCFSGKRQIVI
SGSAHNYQQLKDNKFYGLLDYLIVIPEPWENPSINLYVTSRGLHIPARAAFNALAFPIGDIAYDHPDAAALTV
AAEILDNVVLHTKIREQGGAYGSGAAANLSRGSFYCYSYRDPEIATTYKTFLKGVSEIASGNFTKEDIYEGAL
GVVQGLDMPVAPGSRASVAFYRLKSGRIPVLRQAFRRSVLEVTKEHICMVMDKYLESTVQETTLISFAGEEM
LRNNVLTLDKDFPIVPAI

>core/23/2/Org2_Gene389

MKTSIRKFLISTTLAPCFASTAFTVEVIMPSENFDGSSGKIFPYTTLSDPRGTLCIFSGDLYIANLDNAISRTSSSC
FSNRAGALQILGKGGVFSFLNIRSSADGAAISSVITQNPELCPLSFSGFSQMIFDNCESLTSDTSASNVIPHASAI
YATTPMLFTNNDSILFQYNRSAGFGAAIRGTSITIENTKKSLLFNGNGSISNGGALTGSAAINLINNSAPVIFSTN

ATGIYGGAIYLTGGSMLTSGNLSGVLFVNNSSRSGGAIYANGNVTFSNNSDLTFQNNTASPQNSLPAPTPPPTP
PAVTPLLGYGGAIFCTPPATPPPTGVSLTISGENSVTFLENIASEQGGALYGKKISIDSNKSTIFLGNTAGKGGAI
AIPESGELSLSANQGDILFNKNLSITSGTPTRNSIHFGKDAKFATLGATQGYTLYFYDPITSDDLSAASAAATV
VVNPKASADGAYSGTIVFSGETLTATEAATPANATSTLNQKLELEGGTLALRNGATLNVHNFTQDEKSVVIM
DAGTTLATTNGANNTDGAITLNKLVINLDSLDGTKAAVVNVQSTNGALTISGTLGLVKNSQDCCDNHGMFN
KDLQQVPILELKATSNTVTTTDFSLGTNGYQQSPYGYQGTWEFTIDTTTHTVTGNWKKTGYLPHPERLAPLIP
NSLWANVIDLRAVSQASAADGEDVPGKQLSITGITNFFHANHTGDARSYRHMGGGYLINTYTRITPDAALSL
GFGQLFTKSKDYLVGHGHSNVYFATVYSNITKSLFGSSRFFSGGTSRVTYSRSNEKVKTSYTKLPKGRCSWS
NNCWLGELEGNLPITLSSRILNLKQIIPFVKAEVAYATHGGIQENTPEGRIFGHGHLLNVAVPVGVRFGKNSH
NRPDFYTIIVAYAPDVYRHNPDCDTTLPINGATWTSIGNNLTRSTLLVQASSHTSVNDVLEIFGHCGCDIRRTS
RQYTLDIGSKLRF

>core/24/2/Org2_Gene748

MLGFLKRFFGSSQERILKKFQKLVDKVNIYDEMLTPLSDDELRNKTAELKQRYQNGESLDSMLPEAYGVVK
NVCRRLAGTPVEVSGYHQRWDMVPYDVQILGAIAMHKGFITEMQTGEGKTLTAVMPLYLNALTGKPVHLV
TVNDYLAQRDCEWVGSVLRWLGLTTGVLVSGTLLEKRKKIYQCDVVYGTASEFGFDYLRDNSIATRLEEQV
GRGYYFAIIDEVDSILIDEARTPLIISGPGEKHNPVYFELKEKVASLVYLQKELCSRIALEARRGLDSFLDVDILP
KDKKVLEGISEFCRSLWLVSKGMPLNRVLRRVREHPDLRAMIDKWDVYYHAEQNKEESLERLSELYIIVDEH
NNDFELTDKGMQQWVEYAGGSTEEFVMMDMGHEYALIENDETLSPADKINKKIAISEEDTLRKARAHGLRQ
LLRAQLLMERDVDYIVRDDQIVIIDEHTGRPQPGRRFSEGLHQAIEAKEHVTIRKESQTLATVTLQNFFRLYEK
LAGMTGTAITESREFKEIYNLYVLQVPTFKPCLRIDHNDEFYMTEREKYHAIVNEIATIHGKGNPILVGTESVE
VSEKLSRILRQNRIEHTVLNAKNHAQEAEIIAGAGKLGAVTVATNMAGRGTDIKLDNEAVIVGGLHVIGTTR
HQSRRIDRQLRGRCARLGDPGAAKFFLSFEDRLMRLFASPKLNTLIRHFRPPEGEAMSDPMFNRLIETAQKRV
EGRNYTIRKHTLEYDDVMNKQRQAIYAFRHDVLHAESVFDLAKEILCHVSLMVASLVMSDRQFKGWTLPNL
EEWITSSFPIALNIEELRQLKDTDSIAEKIAAELIQEFQVRFDHMVEGLSKAGGEELDASAICRDVVRSVMVMH
IDEQWRIHLVDMDLLRSEVGLRTVGQKDPLLEFKHESFLLFESLIRDIRITIARHLFRLELTVEPNPRVNNVIPT
VATSFHNNVNYGPLELTVVTDSEDQD

>core/26/2/Org2_Gene400

MSLNLERISPLLYLLDVTAKKIDTSNLIVEAMNLDEHYGYQGIWSPYWMETTTTTSSTVPEQTNTNHRQLYV
DWTPVGYRPNPERHGEFIANTLWQSAYNALLGIRILPPQNLKEHDLEASLQGLGLLINQHNREGRKGFRNHT
TGYAATTSAKTAARHSFSLGFAQMFSKTRERQSPSTTSSHNYFAGLRFDSLLFRDFISTGLSLGYSYGDHHML
CHYTEILKGSSKAFFNNHTLVASLDCTFLPARITRTLELQPFISAIALRCSQASFQETGDHIRKFHPKHPLTDLSS
PIGFRSEWKTSHHIPMLWTTEISYVPTLYRKNPEMFTTLLISNGTWTTQATPVSYNSVAAKIKNTSQLFSRVTL
SLDYSAQVSSSTVGQYLKAESHCTF

>core/27/2/Org2_Gene461

MKQMRLWGFLFLSSFCQVSYLRANDVLLPLSGIHSGEDLELFTLRSSSPTKTTYSLRKDFIVCDFAGNSIHKPG
AAFLNLKGDLFFINSTPLAALTFKNIHLGARGAGLFSESNVTFKGLHSLVLENNESWGGVLTTSGDLSFINNTS
VLCQNNISYGPGGALLLQGRKSKALFFRDNRGTILFLKNKAVNQDESHPGYGGAVSSISPGSPITFADNQEILF
QENEGELGGAIYNDQGAITFENNFQTTSFFSNKASFGGAVYSRYCNLYSQWGDTLFTKNAAAKVGGAIHAD

YVHIRDCKGSIVFEENSATAGGAIAVNAVCDINAQGPVRFINNSALGLNGGAIYMQATGSILRLHANQGDIEF
CGNKVRSQFHSHINSTSNFTNNAITIQGAPREFSLSANEGHRICFYDPIISATENYNSLYINHQRLLEAGGAVIFS
GARLSPEHKKENKNKTSIINQPVRLCSGVLSIEGGAILAVRSFYQEGGLLALGPGSKLTTQGKNSEKDKIVITN
LGFNLENLDSSDPAEIRATEKASIEISGVPRVYGHTESFYENHEYASKPYTTSIILSAKKLVTAPSRPEKDIQNLII
AESEYMGYGYQGSWEFSWSPNDTKEKKTIIASWTPTGEFSLDPKRRGSFIPTTLWSTFSGLNIASNIVNNNYL
NNSEVIPLQHLCVFGGPVYQIMEQNPKQSSNNLLVQHAGHNVGARIPFSFNTILSAALTQLFSSSSQQNVADK
SHAQILIGTVSLNKSWQALSLRSSFSYTEDSQVMKHVFPYKGTSRGSWRNYGWSGSVGMSYAYPKGIRYLK
MTPFVDLQYTKLVQNPFVETGYDPRYFSSSEMTNLSLPIGIALEMRFIGSRSSLFLQVSTSYIKDLRRVNPQSSA
SLVLNHYTWDIQGVPLGKEALNITLNSTIKYKIVTAYMGISSTQREGSNLSANAHAGLSLSF

>core/28/2/Org2_Gene401

MQNNRSLSKSSFFVGALILGKTTILLNATPLSDYFDNQANQLTTLFPLIDTLTNMTPYSHRATLFGVRDDTNQ
DIVLDHQNSIESWFENFSQDGGALSCKSLAITNTKNQILFLNSFAIKRAGAMYVNGNFDLSENHGSIIFSGNLSF
PNASNFADTCTGGAVLCSKNVTISKNQGTAYFINNKAKSSGGAIQAAIINIKDNTGPCLFFNNAAGGTAGGAL
FANACRIENNSQPIYFLNNQSGLGGAIRVHQECILTKNTGSVIFNNNFAMEADISANHSSGGAIYCISCSIKDNP
GIAAFDNNTAARDGGAICTQSLTIQDSGPVYFTNNQGTWGGAIMLRQDGACTLFADQGDIIFYNNRHFKDTF
SNHVSVNCTRNVSLTVGASQGHSATFYDPILQRYTIQNSIQKFNPNPEHLGTILFSSTYIPDTSTSRDDFISHFRN
HIGLYNGTLALEDRAEWKVYKFDQFGGTLRLGSRAVFSTTDEEQSSSSVGSVININNLAINLPSILGNRVAPKL
WIRPTGSSAPYSEDNNPIINLSGPLSLLDDENLDPYDTADLAQPIAEVPLLYLLDVTAKHINTDNFYPEGLNTT
QHYGYQGVWSPYWIETITTSDTSSEDTVNTLHRQLYGDWTPTGYKVNPENKGDIALSAFWQSFHNLFATLR
YQTQQGQIAPTASGEATRLFVHQNSNNDAKGFHMEATGYSLGTTSNTASNHSFGVNFSQLFSNLYESHSDNS
VASHTTTVALQINNPWLQERFSTSASLAYSYSNHHIKASGYSGKIQTEGKCYSTTLGAALSCSLSLQWRSRPL
HFTPFIQAIAVRSNQTAFQESGDKARKFSVHKPLYNLTVPLGIQSAWESKFRLPTYWNIELAYQPVLYQQNPEI
NVSLESSGSSWLLSGTTLARNAIAFKGRNQIFIFPKLSVFLDYQGSVSSSTTTHYLHAGTTFKF

>core/29/2/Org2_Gene851

MFWKLLCPILICTSLSITSCEQQFKVVPNQCPLQVSTPAAADQKIEKIICSNGLPLLIISDPNLPTSGAALLVKTG
NNADPEEYPGMAHFTEHCVFLGNEKYPEVSGFPGFLSENNGVHNAFTYPNKTVFVFSVEHSAFSDALDQFVH
LFINPKFRQEDLDREKYAVHQEFAAHPLSDGRRVHRIQQLVAPQGHPCARFGCGNASTLTPVTTEKMAEWF
KLHYSPENMCAIAYTSAPLSKAKKQFSKIFSQIPRSKNYERQEPFLPSGDTSSLKNLYINQAIQPTSNLEIYWHI
YESSHPIPLGCYKALAEVLRNESKNSLVSLLKNEQLITDLDVEFFRSSLNTGEFYISYELTEKGDKHYSQVIDST
FQYLRYIQEHGIPNYTLEEISTINALNYCYSSKSPLFDLLCKQIVSLGNEDLSTYPYHSLVYPKYSSEDESALLN
LVSDPEQARFVLSSKNSEHWEEATQLHDPIFDMTYYVKALDGVQDYGKVQSLKPIALPKPNLFIPKEVTLPG
VHLLKKQEFPFAPALSYQDDKLTLYHCEDHYYTAPKLSSQIRIRSPQISRSSPQFLVATELYCLAVNDQLLREY
YPATQAGLSFTSALGGDGIDLRVSGYTTTVPALLNSILTSLPNLEISYETFLVYKKQLLELYQGALLNCPVRSG
LDELASQVMKETYSNTTKLSALEKLSFSEFQAFASNLFNSVHLEVMVLGNLSEQQKKDYLEMLQVFTASRSS
HATKPFYYELQSQEISEIHHDYPLTANGMLLLLQDKSSPSIQGKVCAEMLFEWLHHITFEELRTQQQLGYMV
GARYREFASRPFGFLYIRSDAYSPEELLAKTSLFLNKVSASPEKFGISQEKFANIRKAYINKILEPEHSLDMMNS
ALFSLAFERPFVEFSTPDLKIAIAETLTYEEFLKYCQCFLSNELGTQTSVYIRGTQKTS

>core/30/2/Org2_Gene71

MTTEDFPKAYNFQDTEPELYVFWEKNGMFKAEASSDKPPYSVIMPPPNVTGVLHMGHALVNTLQDVLVRY
KRMSGFEVCWIPGTDHAGIATQAVVERHLQASEGKRRTDYSREDFLKHIWAWKEKSEKVVLSQLRQLGCSC
DWDRKRFTMEPLANRAVKKAFKTLFENGYIYRGYYLVNWDPVLQTALADDEVEYEEKDGWLYYIRYRMV
GSQESIVVATTRPETSLGDTGIAVSPNDERYASWIGASVEVPFVNRQIPIIGDASVDPTFGTGAVKVTPAHDKD
DYLMGTNHHLPMINILTPSGGINENGGPFAGMAKEKAREEILIALEEQGLFVRKEPYKLRVGVSYRSGAVIEP
YLSKQWFVSVSEFRGALREFVESQDIKIFPKDFVKNYLSWVNHLRDWCISRQLWWGHRIPVWYHKNDDERV
LCYDGEGIPEEVAQDPDSWYQDPDVLDTWFSSGLWPLTCLGWPDENSPDLKKFYPTALLVTGHDILFFWVT
RMVLLCSSMSGEKPFSEVFLHGLIFGKSYKRYNDFGEWSYISGKEKLAYDMGEALPDGVVAKWEKLSKSKG
NVIDPLEMIATYGTDAVRLTLCSCANRGEQIDLDYRLFEEYKHFANKVWNGARFIFGHISDLQGKDLLAGIDE
DSLGLEDFYILDGFNQLIHQLEEAYATYAFDKVATLAYEFFRNDLCSTYIEIIKPTLFGKQGNEASQSTKRTLL
AVLLINVLGVLHPVAPFITESLFLRIQDTLGALPEGDGDAFTGHALRMLRSRACMEAPYPKAFDVKIPQDLRE
SFTLAQRLVYTIRNIRGEMQLDPRLHLKAFVVCSDTTEIQSCIPILQALGGLESIQLLDKEPEKGLYSFGVVDTI
RLGIFVPEEHLLKEKGRLEKERVRLERAVENLERLLGDESFCQKANPNLVVAKQEALKNNRIELQGILDKLAS
FA

>core/31/2/Org2_Gene398

MRFFCFGMLLPFTFVLANEGLQLPLETYITLSPEYQAAPQVGFTHNQNQDLAIVGNHNDFILDYKYYRSNGG
ALTCKNLLISENIGNVFFEKNVCPNSGGAIYAAQNCTISKNQNYAFTTNLVSDNPTATAGSLLGGALFAINCSI
TNNLGQGTFVDNLALNKGGALYTETNLSIKDNKGPIIIKQNRALNSDSLGGGIYSGNSLNIEGNSGAIQITSNSS
GSGGGIFSTQTLTISSNKKLIEISENSAFANNYGSNFNPGGGGLTTTFCTILNNREGVLFNNNQSQSNGGAIHAK
SIIIKENGPVYFLNNTATRGGALLNLSAGSGNGSFILSADNGDIIFNNNTASKHALNPPYRNAIHSTPNMNLQIG
ARPGYRVLFYDPIEHELPSSFPILFNFETGHTGTVLFSGEHVHQNFTDEMNFFSYLRNTSELRQGVLAVEDGA
GLACYKFFQRGGTLLLGQGAVITTAGTIPTPSSTPTTVGSTITLNHIAIDLPSILSFQAQAPKIWIYPTKTGSTYT
EDSNPTITISGTLTLRNSNNEDPYDSLDLSHSLEKVPLLYIVDVAAQKINSSQLDLSTLNSGEHYGYQGIWSTY
WVETTTITNPTSLLGANTKHKLLYANWSPLGYRPHPERRGEFITNALWQSAYTALAGLHSLSSWDEEKGHA
ASLQGIGLLVHQKDKNGFKGFRSHMTGYSATTEATSSQSPNFSLGFAQFFSKAKEHESQNSTSSHHYFSGMCI
ENTLFKEWIRLSVSLAYMFTSEHTHTMYQGLLEGNSQGSFHNHTLAGALSCVFLPQPHGESLQIYPFITALAIR
GNLAAFQESGDHAREFSLHRPLTDVSLPVGIRASWKNHHRVPLVWLTEISYRSTLYRQDPELHSKLLISQGTW
TTQATPVTYNALGIKVKNTMQVFPKVTLSLDYSADISSSTLSHYLNVASRMRF

>core/32/2/Org2_Gene383

MKSSVSWLFFSSIPLFSSLSIVAAEVTLDSSNNSYDGSNGTTFTVFSTTDAAAGTTYSLLSDVSFQNAGALGIPL
ASGCFLEAGGDLTFQGNQHALKFAFINAGSSAGTVASTSAADKNLLFNDFSRLSIISCPSLLLSPTGQCALKSV
GNLSLTGNSQIIFTQNFSSDNGGVINTKNFLLSGTSQFASFSRNQAFTGKQGGVVYATGTITIENSPGIVSFSQN
LAKGSGGALYSTDNCSITDNFQVIFDGNSAWEAAQAQGGAICCTTTDKTVTLTGNKNLSFTNNTALTYGGAI
SGLKVSISAGGPTLFQSNISGSSAGQGGGGAINIASAGELALSATSGDITFNNNQVTNGSTSTRNAINIIDTAKV
TSIRAATGQSIYFYDPITNPGTAASTDTLNLNLADANSEIEYGGAIVFSGEKLSPTEKAIAANVTSTIRQPAVLA
RGDLVLRDGVTVTFKDLTQSPGSRILMDGGTTLSAKEANLSLNGLAVNLSSLDGTNKAALKTEAADKNISLS
GTIALIDTEGSFYENHNLKSASTYPLLELTTAGANGTITLGALSTLTLQEPETHYGYQGNWQLSWANATSSKI
GSINWTRTGYIPSPERKSNLPLNSLWGNFIDIRSINQLIETKSSGEPFERELWLSGIANFFYRDSMPTRHGFRHIS
GGYALGITATTPAEDQLTFAFCQLFARDRNHITGKNHGDTYGASLYFHHTEGLFDIANFLWGKATRAPWVLS

EISQIIPLSFDAKFSYLHTDNHMKTYYTDNSIIKGSWRNDAFCADLGASLPFVISVPYLLKEVEPFVKVQYIYA
HQQDFYERHAEGRAFNKSELINVEIPIGVTFERDSKSEKGTYDLTLMYILDAYRRNPKCQTSLIASDANWMA
YGTNLARQGFSVRAANHFQVNPHMEIFGQFAFEVRSSSRNYNTNLGSKFCF

>core/33/2/Org2_Gene399

MFGMTPAVYSLQTDSLEKFALERDEEFRTSFPLLDSLSTLTGFSPITTFVGNRHNSSQDIVLSNYKSIDNILLLW
TSAGGAVSCNNFLLSNVEDHAFFSKNLAIGTGGAIACQGACTITKNRGPLIFFSNRGLNNASTGGETRGGAIA
CNGDFTISQNQGTFYFVNNSVNNWGGALSTNGHCRIQSNRAPLLFFNNTAPSGGGALRSENTTISDNTRPIYF
KNNCGNNGGAIQTSVTVAIKNNSGSVIFNNNTALSGSINSGNGSGGAIYTTNLSIDDNPGTILFNNNYCIRDGG
AICTQFLTIKNSGHVYFTNNQGNWGGALMLLQDSTCLLFAEQGNIAFQNNEVFLTTFGRYNAIHCTPNSNLQ
LGANKGYTTAFFDPIEHQHPTTNPLIFNPNANHQGTILFSSAYIPEASDYENNFISSSKNTSELRNGVLSIEDRA
GWQFYKFTQKGGILKLGHAASIATTANSETPSTSVGSQVIINNLAINLPSILAKGKAPTLWIRPLQSSAPFTEDN
NPTITLSGPLTLLNEENRDPYDSIDLSEPLQNIHLLSLSDVTARHINTDNFHPESLNATEHYGYQGIWSPYWVE
TITTTNNASIETANTLYRALYANWTPLGYKVNPEYQGDLATTPLWQSFHTMFSLLRSYNRTGDSDIERPFLEI
QGIADGLFVHQNSIPGAPGFRIQSTGYSLQASSETSLHQKISLGFAQFFTRTKEIGSSNNVSAHNTVSSLYVELP
WFQEAFATSTVLAYGYGDHHLHSLHPSHQEQAEGTCYSHTLAAAIGCSFPWQQKSYLHLSPFVQAIAIRSHQ
TAFEEIGDNPRKFVSQKPFYNLTLPLGIQGKWQSKFHVPTEWTLELSYQPVLYQQNPQIGVTLLASGGSWDIL
GHNYVRNALGYKVHNQTALFRSLDLFLDYQGSVSSSTSTHHLQAGSTLKF

>core/34/2/Org2_Gene72

MERYDIVRIIGKGGMGEVYLAYDPVCSRKVALKKIREDLAENPLLKRRFLREARIAADLIHPGVVPVYTIYSE
KDPVYYTMPYIEGYTLKTLLKSVWQKESLSKELAEKTSVGAFLSIFHKICCTIEYVHSRGILHRDLKPDNILLG
LFSEAVILDWGAAVACGEEEDLLDIDVSKEEVLSSRMTIPGRIVGTPDYMAPERLLGHPASKSTDIYALGVVL
YQMLTLSFPYRRKKGKKIVLDGQRIPSPQEVAPYREIPPFLSAVVMRMLAVDPQERYSSVTELKEDIESHLKG
SPKWTLTTALPPKKSSSWKLNEPILLSKYFPMLEVSPASWYSLAISNIESFSEMRLEYTLSKKGLNEGFGILLPT
SENALGGDFYQGYGFWLHIKERTLSVSLVKNSLEIQRCSQDLESDKETFLIALEQHNHSLSLFVDGTTWLIHM
NYLPSRSGRVAIIVRDMEDILEDIGIFESSGSLRVSCLAVPDAFLAEKLYDRALVLYRRIAESFPGRKEGYEARF
RAGITVLEKASTDNNEQEFALAIEEFSKLHDGVAAPLEYLGKALVYQRLQEYNEEIKSLLLALKRYSQHPEIF
RLKDHVVYRLHESFYKRDRLALVFMILVLEIAPQAITPGQEEKILVWLKDKSRATLFCLLDPTVLELRSSKME
LFLSYWSGFIPHLNSLFHRAWDQSDVRALIEIFYVACDLHKWQFLSSCIDIFKESLEDQKATEEIVEFSFEDLGA
FLFAIQSIFNKEDAEKIFVSNDQLSPILLVYIFDLFANRALLESQGEAIFQALDLIRSKVPENFYHDYLRNHEIRA
HLWCRNEKALSTIFENYTEKQLKDEQHELFVLYGCYLALIQGAEAAKQHFDVCREDRIFPASLLARNYNRLG
LPKDALSYQERRLLLRQKFLYFHCLGNHDERDLCQTMYHLLTEEFQL

>core/35/2/Org2_Gene384

MKIPLHKLLISSTLVTPILLSIATYGADASLSPTDSFDGAGGSTFTPKSTADANGTNYVLSGNVYINDAGKGTA
LTGCCFTETTGDLTFTGKGYSFSFNTVDAGSNAGAAASTTADKALTFTGFSNLSFIAAPGTTVASGKSTLSSA
GALNLTDNGTILFSQNVSNEANNNGGAITTKTLSISGNTSSITFTSNSAKKLGGAIYSSAAASISGNTGQLVFM
NNKGETGGGALGFEASSSITQNSSLFFSGNTATDAAGKGGAIYCEKTGETPTLTISGNKSLTFAENSSVTQGG
AICAHGLDLSAAGPTLFSNNRCGNTAAGKGGAIAIADSGSLSLSANQGDITFLGNTLTSTSAPTSTRNAIYLGS
SAKITNLRAAQGQSIYFYDPIASNTTGASDVLTINQPDSNSPLDYSGTIVFSGEKLSADEAKAADNFTSILKQPL

ALASGTLALKGNVELDVNGFTQTEGSTLLMQPGTKLKADTEAISLTKLVVDLSALEGNKSVSIETAGANKTIT
LTSPLVFQDSSGNFYESHTINQAFTQPLVVFTAATAASDIYIDALLTSPVQTPEPHYGYQGHWEATWADTSTA
KSGTMTWVTTGYNPNPERRASVVPDSLWASFTDIRTLQQIMTSQANSIYQQRGLWASGTANFFHKDKSGTN
QAFRHKSYGYIVGGSAEDFSENIFSVAFCQLFGKDKDLFIVENTSHNYLASLYLQHRAFLGGLPMPSFGSITD
MLKDIPLILNAQLSYSYTKNDMDTRYTSYPEAQGSWTNNSGALELGGSLALYLPKEAPFFQGYFPFLKFQAV
YSRQQNFKESGAEARAFDDGDLVNCSIPVGIRLEKISEDEKNNFEISLAYIGDVYRKNPRSRTSLMVSGASWT
SLCKNLARQAFLASAGSHLTLSPHVELSGEAAYELRGSAHIYNVDCGLRYSF

>core/37/2/Org2_Gene385

MKSSLHWFLISSSLALPLSLNFSAFAAVVEINLGPTNSFSGPGTYTPPAQTTNADGTIYNLTGDVSITNAGSPTA
LTASCFKETTGNLSFQGHGYQFLLQNIDAGANCTFTNTAANKLLSFSGFSYLSLIQTTNATTGTGAIKSTGACS
IQSNYSCYFGQNFSNDNGGALQGSSISLSLNPNLTFAKNKATQKGGALYSTGGITINNTLNSASFSENTAANN
GGAIYTEASSFISSNKAISFINNSVTATSATGGAIYCSSTSAPKPVLTLSDNGELNFIGNTAITSGGAIYTDNLVL
SSGGPTLFKNNSAIDTAAPLGGAIAIADSGSLSLSALGGDITFEGNTVVKGASSSQTTTRNSINIGNTNAKIVQL
RASQGNTIYFYDPITTSITAALSDALNLNGPDLAGNPAYQGTIVFSGEKLSEAEAAEADNLKSTIQQPLTLAGG
QLSLKSGVTLVAKSFSQSPGSTLLMDAGTTLETADGITINNLVLNVDSLKETKKATLKATQASQTVTLSGSLS
LVDPSGNVYEDVSWNNPQVFSCLTLTADDPANIHITDLAADPLEKNPIHWGYQGNWALSWQEDTATKSKAA
TLTWTKTGYNPNPERRGTLVANTLWGSFVDVRSIQQLVATKVRQSQETRGIWCEGISNFFHKDSTKINKGFR
HISAGYVVGATTTLASDNLITAAFCQLFGKDRDHFINKNRASAYAASLHLQHLATLSSPSLLRYLPGSESEQP
VLFDAQISYIYSKNTMKTYYTQAPKGESSWYNDGCALELASSLPHTALSHEGLFHAYFPFIKVEASYIHQDSF
KERNTTLVRSFDSGDLINVSVPIGITFERFSRNERASYEATVIYVADVYRKNPDCTTALLINNTSWKTTGTNLS
RQAGIGRAGIFYAFSPNLEVTSNLSMEIRGSSRSYNADLGGKFQF

>core/38/2/Org2_Gene387

MKTSIPWVLVSSVLAFSCHLQSLANEELLSPDDSFNGNIDSGTFTPKTSATTYSLTGDVFFYEPGKGTPLSDSC
FKQTTDNLTFLGNGHSLTFGFIDAGTHAGAAASTTANKNLTFSGFSLLSFDSSPSTTVTTGQGTLSSAGGVNL
ENIRKLVVAGNFSTADGGAIKGASFLLTGTSGDALFSNNSSSTKGGAIATTAGARIANNTGYVRFLSNIASTSG
GAIDDEGTSILSNNKFLYFEGNAAKTTGGAICNTKASGSPELIISNNKTLIFASNVAETSGGAIHAKKLALSSGG
FTEFLRNNVSSATPKGGAISIDASGELSLSAETGNITFVRNTLTTTGSTDTPKRNAINIGSNGKFTELRAAKNHT
IFFYDPITSEGTSSDVLKINNGSAGALNPYQGTILFSGETLTADELKVADNLKSSFTQPVSLSGGKLLLQKGVT
LESTSFSQEAGSLLGMDSGTTLSTTAGSITITNLGINVDSLGLKQPVSLTAKGASNKVIVSGKLNLIDIEGNIYES
HMFSHDQLFSLLKITVDADVDTNVDISSLIPVPAEDPNSEYGFQGQWNVNWTTDTATNTKEATATWTKTGF
VPSPERKSALVCNTLWGVFTDIRSLQQLVEIGATGMEHKQGFWVSSMTNFLHKTGDENRKGFRHTSGGYVI
GGSAHTPKDDLFTFAFCHLFARDKDCFIAHNNSRTYGGTLFFKHSHTLQPQNYLRLGRAKFSESAIEKFPREIP
LALDVQVSFSHSDNRMETHYTSLPESEGSWSNECIAGGIGLDLPFVLSNPHPLFKTFIPQMKVEMVYVSQNSF
FESSSDGRGFSIGRLLNLSIPVGAKFVQGDIGDSYTYDLSGFFVSDVYRNNPQSTATLVMSPDSWKIRGGNLSR
QAFLLRGSNNYVYNSNCELFGHYAMELRGSSRNYNVDVGTKLRF

>core/39/2/Org2_Gene248

MQRVLRLLFNLHHGEEKRAFLFFLLGLVWGIGCYGTLSLAEGLFIEKLGSAELPKIYLGSSLILCVLSSLILYNL
FKKHISATALFLIPVSLSILCNFYLILSSIFAIDPPRSPLFFYRIVIWSLTILSYTSFWGFVDQFFNLQDGKRHFCIF

NAIIFLGDAIGSGIIASLVHTIGIQGILILFTAALVLTFPIVFYVSKSLKSLSDDHDLFIDTGHPPPLSKALKLCFYD
KYTFYLLCFYFLMQLLAIATEFNYLKIFEIQFASKEEFELVAHIGKCSLWISLGNMCFALFAYSRIVKRLGVNNI
ILFAPLCFLSLFLFWTFKTTLSIAVLAMVVREGVTYALDDNNLQLLIYGVPNKIRNQIRIVVESFIEPIGMLVWS
LICFLSSQQYVFCLIISLIATILVCLVRSYYAKAILKNLSAQALQLTRSMQDWIKSMTVKQKRQVELFLLAHLK
HPSERHQTFAFQHLLNLASRSVLPSLLAHMNKLSLPNKLKTIEMVKSSLWAKDFLTLELLKRWTSIFPHPAIAS
AIHLYFAEHDLLHITHIAEDLYDTVGDRLLAAILTVRRQEAYGPYRDLADKRLKELLNSDQPEDIVMGLTILK
LEKNPQNFPILLDFLNTKNEDILIVTCKALHTSVRANHKPYCPELLKRLRQCSHNDEASQYLLKTISIALDISFV
KDLLMTTSQLKNTSRKYAEAMIGELDKEVAPAFLQVLTDEGTHNRCRILAAKALCKIDNWLLKKHAYKIVK
SKASKALFYSYHGHYIQKKYPTYNLSLLANTLNSNYYAEVNFMLSLLGILGSMEHSGVLIRALTSKNQKIKA
QALESLEKNCDSHLFSLLEPFVNQPGMCYSEKYYFKCGVIPLTLKELLNMMENSPSSLNKLTAQQLKEELSYC
DPDFQSVNTIYNQEHEDFRTEESETLISFLSI

>core/40/2/Org2_Gene5

MRFSLCGFPLVFSFTLLSVFDTSLSATTISLTPEDSFHGDSQNAERSYNVQAGDVYSLTGDVSISNVDNSALNK
ACFNVTSGSVTFAGNHHGLYFNNISSGTTKEGAVLCCQDPQATARFSGFSTLSFIQSPGDIKEQGCLYSKNAL
MLLNNYVVRFEQNQSKTKGGAISGANVTIVGNYDSVSFYQNAATFGGAIHSSGPLQIAVNQAEIRFAQNTAK
NGSGGALYSDGDIDIDQNAYVLFRENEALTTAIGKGGAVCCLPTSGSSTPVPIVTFSDNKQLVFERNHSIMGG
GAIYARKLSISSGGPTLFINNISYANSQNLGGAIAIDTGGEISLSAEKGTITFQGNRTSLPFLNGIHLLQNAKFLK
LQARNGYSIEFYDPITSEADGSTQLNINGDPKNKEYTGTILFSGEKSLANDPRDFKSTIPQNVNLSAGYLVIKE
GAEVTVSKFTQSPGSHLVLDLGTKLIASKEDIAITGLAIDIDSLSSSSTAAVIKANTANKQISVTDSIELISPTGN
AYEDLRMRNSQTFPLLSLEPGAGGSVTVTAGDFLPVSPHYGFQGNWKLAWTGTGNKVGEFFWDKINYKPRP
EKEGNLVPNILWGNAVDVRSLMQVQETHASSLQTDRGLWIDGIGNFFHVSASEDNIRYRHNSGGYVLSVNN
EITPKHYTSMAFSQLFSRDKDYAVSNNEYRMYLGSYLYQYTTSLGNIFRYASRNPNVNVGILSRRFLQNPLMI
FHFLCAYGHATNDMKTDYANFPMVKNSWRNNCWAIECGGSMPLLVFENGRLFQGAIPFMKLQLVYAYQG
DFKETTADGRRFSNGSLTSISVPLGIRFEKLALSQDVLYDFSFSYIPDIFRKDPSCEAALVISGDSWLVPAAHVS
RHAFVGSGTGRYHFNDYTELLCRGSIECRPHARNYNINCGSKFRF

>core/41/2/Org2_Gene617

MKTVILNIGRKILQGIKKKKKKIGILSGLFFLDLVLLGVSSQRPTETSANVKHNLRDEKLAACPKNSAASLSAK
KSHTKKTTPGSIPSKVFSKFDATQDKTFQKTSGSAFPAKPTTLKELEERKKPRPERRTTADVKRSPRFLPTQEV
EEPVPAASKEQLDSIQVWEEKQNYARRAVNAINLSIKKQLEEQTSTVTEKDVQPKTQATPHASKKNVASPST
SMPGIEKAATTVAVPQDKSEEEKVKERLTKRELTCEDLKDNGYTVNFEDISILELLQFVSKISGTNFVFDSNDL
QFNVTIVSHDPTSVDDLSTILLQVLKMHDLKVVEQGNNVLIYRNPHLSKLSTVVTDSSLKETCEAVVVTRVFR
LYSVSPSAAVNIIQPLLSHDAIVSASEATRHVIISDIAGNVDKVSDLLAALDCPGTSVDMTEYEVKYANPAALV
SYCQDVLGTLAEDDAFQMFIQPGTNKIFVVSSPRLANKAEQLLKSLDVPEMAHTLDDPASTALALGGTGTTS
PKSLRFFMYKLKYQNGEVIANALQDIGYNLYVTTAMDEDFINTLNSIQWLEVNNSIVIIGNQGNVDRVIGLLN
GLDLPPKQVYIEVLILDTSLEKSWDFGVQWVALGDEQSKVAYASGLLNNTGIATPTKATVPPGTPNPGSIPLP
TPGQLTGFSDMLNSSSAFGLGIIGNVLSHKGKSFLTLGGLLSALDQDGDTVIVLNPRIMAQDTQQASFFVGQT
VPYQTTNTIIQETGTVTQNIDYEDIGVNLVVTSTVAPNNVVTLQIEQTISELHSASGSLTPVTDKTYAATRLQIP
DGCFLVMSGHIRDKTTKVVSGVPLLNSIPLIRGLFSRTIDQRQKRNIMMFIKPKVISSFEEGTRVTNKEGYRYN
WEADEGSMQVAPRHAPECQGPPSLQAESDFKIIEIEAQ

>core/43/2/Org2_Gene888

MSKDKKMKPEPKKNFPTVFFFLLFGVVFGVVAFQNFLAGKKARVGFSHQIEHLVNLRLIVPEDSHKIALNDN
LVSFGGRFRDVQTQEGQLRYHYLELIDQGHRLDLDLQETSKSLTTLGKEVTNSILWFSAISGSPIPEQGYAISY
PSEVSGSVLTEPLVVTGPATPQLINLHSLQERYPTLSRSPEALRTYGSDLYELIGKYLSPVLGIGSETLKRELKD
LYQQVEVSLTQETDTEAAYTLYGQVLSTLNRISSSLVVSEGGERFSQLRSVRLYREEWNKYHKLVEARDLNQ
AQLEKLRGELSQTVWYFNNQELSSRSLEKQDPEVFGHWFAGAKEEWTAFKFNHSLSFKAPDQPRNLVLEKT
FKSQEPSPHYLGYLFTFLPIILVLLFVYLVFSRQMRGMSGSAMSFGKSPARMLLKGQNKVTFADVAGIEEAKE
ELIEIVDFLKNPNKFTSLGGRIPKGVLLIGPPGTGKTLIAKAVSGEADRPFFSIAGSDFVEMFVGVGASRIRDMF
EQAKRNAPCIIFIDEIDAVGRHRGAGIGGGHDEREQTLNQLLVEMDGFGTNEGVILMAATNRPDVLDKALLR
PGRFDRRVVMNLPDIKGRFEILMVHAKRIKLDPTVDLMAVARSTPGASGADLENLLNEAALLAARKDRTAV
TAVDVAEARDKVLYGKERRSLEMDAEERKTTAYHESGHAVVGLCVQHGDPVDKVTIIPRGLSLGATHFLPE
KNKLSYWKKELYDQLAVLMGGRAAEEIFLGDISSGAQQDISQATKLVRSMVCEWGMSPQLGNVTYDERSD
GLTGYGGYHEKSYSEETAKTIDTELRMLLDAAYQRALDIINEHKAEIELMTQMLIEFETLDSKDVKEIMDHT
WDPEKKRARLKEEGMLFKKSSDDLPPPPPKEDTLPGLGFNAT

>core/44/2/Org2_Gene322

MDSEFVGQVYSSDMDWIESMYQRFMNHETLDPSWKYFFEGYQLGQAASPSEASTKISGNETIAMLQEQKSQ
FLCTIYRYYGYLQSQISTLAPTTDSRFIQEKIAKIDLDEQVPSAGLLPKAQVSVRELIEALKKCYCGSLTLETLT
CTPELQEFVWNLMEKRQVERFAEQLLRSYKDLCKATFFEEFLQIKFTGQKRFSLEGGETLVPMLEHLVHYGS
ALGISNYVLGMAHRGRLNVLTNVLGKPYRYVFMEFEDDPAARGLESVGDVKYHKGYVLKSHQKDRETTFV
MLPNASHLESVDPIVEGVVAALQHQGHAGKEQSSLAILVHGDAAFSGQGVVYETLQLSRVPGYSTEGTLHIV
VNNYIGFTAVPRESRSTPYCTDIAKMLGIPVFRVNSEDVVACIEAIEYALQVRERFSCDVIIDLCCYRKYGHNE
SDDPSVTAPLLYDQIKRKKSIRELFRQYLLEGQFADISEETLASIEKEIQESLNREFQVLKGTDPEPFPKKECHH
CDRLNNGELILHDCDVSLDRETLFHMSSRLCGFPDNFHPHPKIKTLLEKRMKMAEGGVGYDWAMAEELAFA
SLLIEGYNLRLSGQDSIRGTFSQRHLVWSDTVTGDTYSPLYHLSAEQGSVEMYNSPLSEYAILGFEYGYAQQA
LKTLVLWEAQFGDFANGAQIIFDQYISSGIQKWDLHSDIVLLLPHGYEGQGPEHSSSRIERYLQLAANWNFQV
VLPSTPVQYFRILREHAKRDLSLPLVIFTPKLLLRYPQCVSSIEEFTEPGGFRAILEDADPNYDASILVLCSGKIY
YDYAEMLPQDRRKDFSCLRIESLYPLALEDLVSLIDKYSHLKHFVWLQEESKNMGAYDYMFMALQDILPEK
LLYIGRPRSSSTASGSAKLSRQELVTCMETLFSLR

>core/45/2/Org2_Gene271

MEKVKLTKNLKLKIKNAQLTKAAGLDKLKQKLAQAGSSEAKSSSEKPSAKEKSVKVALAATSTPTASAEQA
SPESTSRRIRAKNRSSFSSSEEESSAHIPVDTSEPAPVSIADPEPELEVVDEVCDESPEVHPVAEVLPEQPVLPET
PPQEKELEPKPVKPAEPKSVVMIKSKFGPTGKHINHLLAKTFKAPAKEEKVVAGSKSTKPVASDKTGKPGTSE
GGEQNNREKQFNPANRSPASGPKRDAGKKNLTDFRDRSKKSDESLKAFTGRDRYGLNEGGEEDRWRKKRV
YKPKKHYDEASIQRPTHIKISLPITVKDLAAEMKLKASEVIQKLFIHGMTYVVNDILDSETAVQFIGLEFGCTID
IDYSEQDKLCLSNDTVRDEIQSTDPSKLVIRSPIVAFMGHVDHGKTTLIDSLRKSNVAATEAGAITQHMGAFC
CSTPVGDITILDTPGHEAFSAMRARGAEVCDIVVLVVAGDEGIKEQTLEAIEHAKAADIAIVVAINKCDKPNF
NSETIYRQLSEINLLPEAWGGSTVTVNTSAKTGEGLSELLEMLALQAEVLELKADPSARARGLVIESELHKGL
GPVATVLIQNGSLKLGEALVFNDCYGKVKTMHNEHNELMKEAGPSIPVLITGLSDIPKAGDPFFVVKNEKTA

RDIIEARSAGQQRFALQQKKRPNFDSMLQNKKTLKLMIKADVQGSIEALVSSISKIKSEKVDVEILTNSVGEIS
ESDIRLAAASKAVLIGFHTGIESHAEPLIKSLGVRVELFTVIYHAIDAIKEIMTSLLDPIAEEKDEGSAEIKEIFRS
SQVGSIYGCIVTEGIMTRNHKVRVLRNKEILWKGTLSSLKRVKEDVKEVRKGLECGILLEGYQQAQIGDVLQ
CYEVIYHPQKL

>core/46/2/Org2_Gene792

MLSNTIRSNFLKFYANRHHTILPSSPVFPHNDPSILFTNAGMNQFKDIFLNKEKVSYSRATTSQKCIRAGGKHN
DLDNVGHTSRHLTFFEMLGNFSFGDYFKAEAIAFAWEVSLSVFNFNPEGIYATVHEKDDEAFALWEAYLPTD
RIFRLTDKDNFWSMANTGPCGYCSELLFDRGPSFGNASSPLDDTDGERFLEYWNLVFMEFNRTSEGSLLALP
NKHVDTGAGLERLVSLIAGTHTVFEADVLRELIAKTEQLSGKVYHPDDSGAAFRVIADHVRSLSFAIADGLLP
GNTERGYVLRKILRRSVNYGRRLGFRNPFLAEIVPSLADAMGEAYPELKNSLSQIQKVLTLEEESFFKTLDRG
GNLLQQVLKSSSSSSCISGEDAFKLKDTYGMPIDEISLLAKDYDYSVDMDTFHKLEQEAKERSRKNVVQSQG
TSESIYNELHLTSEFIGYDHLSCDTFIEAIISKDHIVSSLQEKQEGAIVLKVSPFYAEKGGQVGDSGEIFCSEGTFI
VTHTTSPKAGLIVHHGRISQGSLTVEAAVTAQVNRYRRKRIANNHTACHLLHKALEITLGDHIRQAGSYVDD
TKIRLDFTHPQAISPEDLLCIETLVNESIRENEPVDIREALYSDVMNSSEIKQFFGDKYSDVVRVVSAGHSHELC
GGTHAEATGDIGFFRITKEHAVAMGIRRIEAVTGEKAEATVHQQSEVLEEIATLLQVPRDQIVSRLTATLDER
KQQDKRLNELENSLIQTKLDKLIHNCHQRQGITCLVHHLAEHENHRLQQYAQCLHQRIPEKLISLWTTEKNG
KYIVLSRVSDDLITQGVHAQDLLKAVLTPCGGRWGGKDQSAQGSAPALPATEVLNETLWQWISTQLI

>core/47/2/Org2_Gene642

MSTFSIQNRLRTISGESTRIIKLDHKYSGFDPRSVPAINLEELNSGIYALRHLMNALQSENTNVAALLNPNNTIF
PTTSWTDYKHSRPQASSPRAPSSQTPTDIVSAAALALVLVIDGGLAELVASVTEIDLGALSTISTVRQLMASYL
GLTTLTAEQEKVVFSSSYVPSEKNLLEHVKQEKAAEIQAKQEEIKAVLEAKGVSTEEIEAILKEYPDIYAADFF
KEFIEEPLHTYRAKVGAPIQEMNENAIQLLPTPPAITPDNVNEVNGMNTLSTILQAIDDAIKQAPALGGDQEIIT
ILQTLVPLVDKTTFTKAEFDLIYTATQLPNTASLKLYLTDRQIAEYRGKITKVYQNSIQNLSETKRVVENNRSM
LETQLSMFQQAQNCFVTWISQANALNIAITNKYISAVLTTSMEMYGGLLCLSYMYERLADDEKAIFDKSVNE
YLPIHIVVGGSWVNGWIAKMAAYQELAEYSLGTAVTSQDQIKAYLQTRGNEFKATRHFFHNIGDQMYQFAN
ETVFGNCLTTANGAIQPDLGGFIREAMTNVGTVEADYVSNAQRILNEFNTAATAHVLQLQLQIAELQKKADD
LDPGKASFTENRKFAVAAWITSESLGDALISMILNSQLPKQEAFLKPLIEEINFNNLAANALNSLLQITNEFSTT
SVYYSLSSYLVQSKTGQNLFAGDYYETLLAAAREREYIYRDTARCKQAINLVNGLLQKINSLPGATSAQKQE
MLNATTYYQYSLSVTLNQLTVLESLLAGLKMTLQTTSNNKYDKSVFKIESFDDWIPTLAALESFLTSGFPNIS
ATGGLGPLFTQVQSDQQTYTSQGQTQQLNLQNQMTTIQQEWTLVSTSMQVLNGILSQLAGAIYSN

>core/48/2/Org2_Gene681

MKKSLIIVESPAKIKTLQKLLGSEFVFASSIGHIVDLPAKEFGIDVDHDFEPQYQVLPDKQEVINHIRKLAAKCE
KVYLSPDPDREGEAIAWHIANQLPDSPLIQRVSFNAITKNAVTEALKHPRTIDMALVNAQQARRLLDRIVGYK
ISPILSRKLQQRSGISAGRVQSVALKLVVDREKAIDAFVPVEYWNLRVLMQDPKTTKTFWAHLYAVQGKKW
EKEIPEGKTENDVLLINSEEKARHYAELLEKSSYTITRVEAKAKRRFAPPPFITSTLQQEASRHFRFSASRTMSI
AQTLYEGVDLDSEDSTGLITYMRTDSVRVDPEALTTVREYIQQTFGKEYLPEKANVYTTKKMTQDAHEAIRP
TDINLTPDKLKNKLSDDQFKVYNLIWKRFVASQITPAIYDTLAVQITTDTEIDLRASGSLLKFKGFLAVYEEKQ
DDENDQEEDHPLPPLHAQDALIKEEVSQEQAFTKPLPRFTEASLVKELEKSGIGRPSTYATIMNKIQSREYTTK

ENQRLRPTELGKIISQFLETNFPRIMDIGFTALMEDELELIADNKKPWKLLLQEFWTTFLPVVITAEKEAVIPRI
LTNIECSKCHKGKLVKIWSKNSYFYGCSEYPECDYRTSEEELAFNKEDYAEDTPWDSPCPLCGGVMKVRHG
RYGTFLGCEKYPECRGTISIHKKGEEIEQEEPIPCPAIGCNGKIFKKRSRYNKIFYSCSEYPECSVIGNSIDAVITK
YSGTEKIPYKKKTPTKKKSSAKTTKAAKTPSKKGKAKSSVKKSSEKKTGPLFLPSPDLAKMIGNEPVSRGEAT
KKIWDYIKEHQLQAPENKKLLVPDNNLATIIGPNPIDMFQLSKHLSQHLTKVSNDESSASS

>core/49/2/Org2_Gene531

MKKLFVLDASGFIFRAYFALPEMKNHQGQATQAVFGFIRSLNKLIKEFSPEYMISVFDGPNNKQSRQAIYADY
KSNRQKKFEDIPPQIALVKEYCSLIGLAYLEKESVEADDVIASIAKKAREENYKVYVCTADKDLLQLVNDHV
VAWNPWADQGVVGISEVIERYGIPPGNIPDYLALVGDSSDNIPGLPGCGPKKAAALLKQFGSVEGLLENLDA
VKGLSQTMLSERQETLKLSKRLALLDSNIPIPVPIESLTFPQHPVDEEKLIHFYIQQGFKTLVPSKQTEAATVDV
QIIKDAESLTNILNLVQGGDIAFAVAYTGNHLLSLKLEGLALTQGSGVFFIALEEEGTKILPILKDFFLREDLTF
YGYNLKRDCHALLNAGIVIREISYDLALAEHLTNGGGKISFQSLLVNHGFTETAHRFAKEWGNSGLPIGRLPE
QPEQYFGEFVAYLPIIKDAILEEINRKNLNHILSDIEMPLEKVLFSMERAGVPLDVEELAILEALFETELAVLTE
EIYDLSGRPFNIKSPKQLSDILYNELGLRPIDKAKSTRAEVLEALRSEHPIIEKLLAFRTIEKLLSTYVKALPKQV
DSHTQRIHPSFDQTGAVTGRLACRDPNLQNIPIRSERGILLRKAFRLSEKNSYFLSADYSQIELRFLAHLSQDKS
LKFAFESGEDIHAFTASQVFHVPLEQVSKEQRMQAKTVNFGIVYGQQAFGLAKVLKISIGEAQELIQAYFSRY
PEIAHFVEETIQQAAKDLRVTTMLGRERIIDSWNEFPGSRAASGRFAVNTRIQGSAAELIKLAMLDISQAIKQQ
QMKSRMLLQIHDELLFEVPEEEIEEMQRLVREKMESAMTLSVPIVVNILIGKNWAEC

>core/50/2/Org2_Gene115

MEKFSDAVSEALEKAFELAKSSKHTYVTENHLLLALLENTESLFYLVIKDIHGNPGLLNTAVKDALSREPTVV
EGEVDPKPSPGLQTLLRDAKQEAKTLGDEYISGDHLLLAFWSSNKEPFNSWKQTTKVSFKDLKNLITKIRRGN
RMDSPSAESNFQGLEKYCKNLTALAREGKLDPVIGRDEEIRRTIQVLSRRTKNNPMLIGEPGVGKTAIAEGLA
LRLIQGDVPESLKGKQLYVLDMGALIAGAKYRGEFEERLKSVLKDVESGDGEHIIFIDEVHTLVGAGATDGA
MDAANLLKPALARGTLHCIGATTLNEYQKYIEKDAALERRFQPIFVTEPSLEDAVFILRGLREKYEIFHGVRIT
EGALNAAVLLSYRYIPDRFLPDKAIDLIDEAASLIRMQIGSLPLPIDEKERELAALIVKQEAIKREQSPSYQEEA
DAMQKSIDALREELASLRLGWDEEKKLISGLKEKKNSLESMKFSEEEAERVADYNRVAELRYSLIPQLEEEIK
QDEASLNQRDNRLLQEEVDERLIAQVVANWTGIPVQKMLEGEAEKLLILEESLEERVVGQPFAVSAVSDSIR
AARVGLNDPQRPLGVFLFLGPTGVGKTELAKALADLLFNKEEAMVRFDMSEYMEKHSISKLIGSSPGYVGYE
EGGSLSEALRRRPYSVVLFDEIEKADKEVLNILLQVFDDGILTDGKKRKVNCKNALFIMTSNIGSPELADYCS
KKGSELTKEAILSVVSPVLKRYLSPEFMNRIDEILPFVPLTKEDIVKIVGIQMRRIAQRLKARRINLSWDDSVIL
FLSEQGYDSAFGARPLKRLIQQKVVILLSKALLKGDIKPDTSIELTMAKEVLVFKKVETPS

>core/51/2/Org2_Gene627

MAVRLIVDEGPLSGVIFVLEDGISWSIGRDSSANDIPIEDPKLGASQAIINKTDGSYYITNLDDTIPIVVNGVAIQ
ETTQLKNEDTILLGSNQYSFLSDEFDPQDLVYDFDIPEENFSNDSGDLSDSNEQGKDLEPRQTSETNHSPKPKE
KLTKDQGSSDPITSGDQELADAFLASAKAEKNQPRAKVAKKGLKESSNESLNPKEQNAKDSPKGEERTNKPQ
NAIMEDNGASPRQDPQPKSAEPSLKNTARDETPLKENKPVEEKANKKATPDSPEKKDQPEEGSKKEGSKIEA
TPLDSQKESEDKEAEEAFVQEEEENLTEDNKEDSDSAADANDDTASDHTAEDNKETPKKVENEKSAVLSPFH
VQDLFRFDQTIFPAEIDDIAKKNISVDLTQPSRFLLKVLAGANIGAEFHLDSGKTYILGTDPTTCDIVFNDLSVS

HQHAKITVGNDGGILIEDLDSKNGVIVEGRKIDKTSTLSSNQVVALGTTLFLLIDHHAPADTIVASLSPDDYSL
FGRQQDAEALERQEAQEEEEKQKRATLPAGSFILTLFVGGLAILFGIGTASLFHTKEVVPLENIDYQEDLAQVI
NQFPTVRYTFNKTNSQLFLIGHVKNSTDKSELLYKVDALSFVKSVDDNVIDDEAVWQEMNILLSKRPEFKGIS
MHSPEPGKFIITGYVKTEEQAACLVDYLNIHFNYLSLLENKVVVETQMLKAIAGHLLQGGFANIHVAFVNGE
VILTGYVNNDDAEKFRAVVQELSGIPGVRLVKNFAVLLPAEEGIIDLNLRYPNRYRVTGYSRYGEISINVVVN
GRILTRGDVIDGMTVTSIQPNAIFLEKEGLKYKIDYNK

>core/52/2/Org2_Gene376

MFEKFTNRAKQVIKLAKKEAQRLNHNYLGTEHILLGLLKLGQGVAVNVLRNLGIDFDTARQEVERLIGYGPE
IQVYGDPALTGRVKKSFESANEEASLLEHNYVGTEHLLLGILHQSDSVALQVLENLHIDPREVRKEILRELETF
NLQLPPSSSSSSSSRSNPSSSKSPLGHSLGSDKNEKLSALKAYGYDLTEMVRESKLDPVIGRSSEVERLILILCR
RRKNNPVLIGEAGVGKTAIVEGLAQKIILNEVPDALRKKRLITLDLALMIAGTKYRGQFEERIKAVMDEVRKH
GNILLFIDELHTIVGAGAAEGAIDASNILKPALARGEIQCIGATTIDEYRKHIEKDAALERRFQKIVVHPPSVDE
TIEILRGLKKKYEEHHNVFITEEALKAAATLSDQYVHGRFLPDKAIDLLDEAGARVRVNTMGQPTDLMKLEA
EIENTKLAKEQAIGTQEYEKAAGLRDEEKKLRERLQSMKQEWENHKEEHQVPVDEEAVAQVVSLQTGIPSA
RLTEAESEKLLKLEDTLRRKVIGQNDAVTSICRAIRRSRTGIKDPNRPTGSFLFLGPTGVGKSLLAQQIAIEMFG
GEDALIQVDMSEYMEKFAATKMMGSPPGYVGHEEGGHLTEQVRRRPYCVVLFDEIEKAHPDIMDLMLQILE
QGRLTDSFGRKVDFRHAIIIMTSNLGADLIRKSGEIGFGLKSHMDYKVIQEKIEHAMKKHLKPEFINRLDESVI
FRPLEKESLSEIIHLEINKLDSRLKNYQMALNIPDSVISFLVTKGHSPEMGARPLRRVIEQYLEDPLAELLLKES
CRQEARKLRATLVENRVAFEREEEEQEAALPSPHLES

>core/53/2/Org2_Gene9

MKIPLRFLLISLVPTLSMSNLLGAATTEELSASNSFDGTTSTTSFSSKTSSATDGTNYVFKDSVVIENVPKTGET
QSTSCFKNDAAAGDLNFLGGGFSFTFSNIDATTASGAAIGSEAANKTVTLSGFSALSFLKSPASTVTNGLGAIN
VKGNLSLLDNDKVLIQDNFSTGDGGAINCAGSLKIANNKSLSFIGNSSSTRGGAIHTKNLTLSSGGETLFQGNT
APTAAGKGGAIAIADSGTLSISGDSGDIIFEGNTIGATGTVSHSAIDLGTSAKITALRAAQGHTIYFYDPITVTGS
TSVADALNINSPDTGDNKEYTGTIVFSGEKLTEAEAKDEKNRTSKLLQNVAFKNGTVVLKGDVVLSANGFSQ
DANSKLIMDLGTSLVANTESIELTNLEINIDSLRNGKKIKLSAATAQKDIRIDRPVVLAISDESFYQNGFLNEDH
SYDGILELDAGKDIVISADSRSIDAVQSPYGYQGKWTINWSTDDKKATVSWAKQSFNPTAEQEAPLVPNLLW
GSFIDVRSFQNFIELGTEGAPYEKRFWVAGISNVLHRSGRENQRKFRHVSGGAVVGASTRMPGGDTLSLGFA
QLFARDKDYFMNTNFAKTYAGSLRLQHDASLYSVVSILLGEGGLREILLPYVSKTLPCSFYGQLSYGHTDHR
MKTESLPPPPPTLSTDHTSWGGYVWAGELGTRVAVENTSGRGFFQEYTPFVKVQAVYARQDSFVELGAISRD
FSDSHLYNLAIPLGIKLEKRFAEQYYHVVAMYSPDVCRSNPKCTTTLLSNQGSWKTKGSNLARQAGIVQASG
FRSLGAAAELFGNFGFEWRGSSRSYNVDAGSKIKF

>core/54/2/Org2_Gene229

MFNKDEIIVPKNLEEEMKESYLRYSMSVIISRALPDIRDGLKPSQRRVLYAMKQLSLSPGAKHRKCAKICGDT
SGDYHPHGESVIYPTLVRMAQNWAMRYPLVDGQGNFGSIDGDPPAAMRYTEARLTHSAMYLMEDLDKDT
VDIVPNYDETKHEPVVFPSKFPNLLCNGSSGIAVGMATNIPPHNLGELIEATLLLLANPQASVDEILQVMPGPD
FPTGGIICGSEGIRSAYTTGRGKIKVRARLHVEENEDKHRESIIITEMPYNVNKSRLIEQIANLVNEKTLAGISDV
RDESDKDGIRVVLEIKKGESSEIIINRLYKFTDVQVTFGANMLALDKNLPRTMSIHRMISAWIRHRKEVIRRRT

RYELNKAETRAHVLEGYLKALSCLDALVKTIRESGNKEHAKERIIESFGFTEPQALAILELRLYQLTGLEAEKI
QKEYEELLNKIAYYKQVLSDEGLVKDIIRNELQDLLKHHKVARRTTIEFDADDIRDIEDIITNESVIITISGDDY
VKRMPVKVFKEQRRGGHGVTGFDMKKGAGFLKAVYSAFTKDYLLIFTNFGQCYWLKVWQLPEGERRAKG
KPIINFLEGIRPGEELAAILNIKNFDNAGFLFLATKRGVVKKVSLDAFSNPRKKGIRALEIDEGDELIAACHIVS
DEEKVMLFTHLGMAVRFPHEKVRPMGRTARGVRGVSLKNEEDKVVSCQIVTENQSVLIVCDQGFGKRSLVE
DFRETNRGGVGVRSILINERNGNVLGAIPVTDHDSILLMSSQGQAIRINMQDVRVMGRSTQGVRLVHLKEGD
ALVSMEKLSSNENDDEVLSGSEEECSDTVSLR

>core/55/2/Org2_Gene641

MIYSTSISTFYKKLSLVSSMHSFAQRHRESLEHIANYEKTTAERDILKRLIEVLDQRASERYRSAVEKLHKYEV
ERATVAKSIPVAAIHEKPLSSTHASVQVTASTPAATGSGVGAYYNAVKQKWAQDLIVELNTVMTTIMASVNS
KNPANKDVFDKLNTELQALVAAGNNLTEENFQTLYNFPEEIFTAIQRADTFTGGMKTDFTNQLAGKYGNQA
TLTQTFADGRVEGFKDILTAVQGVLTPEQFTIFAEIATELQALADHVGNFDEAGLQRIEDAGEKLAAVINSSD
LTRNDKIMFCQHITDLYSDQVAALGSFDTVLDASIYVNQHQGTMFSNLSSFVGSLIGTFAPIDLSSSQGDISSA
ALAGALQTARGLNSRFNELTAEQQKLINECIKSLVTFKCGEHLGAIWAYFTASTVVALNPTATMDHVKAAIL
EEAKELDNSSFQLASSIKSAMTSIVNSSGSFSVTVNSSTLQYTIYSEKNGKVEINQILLNYGSTGFLPEITKLAKT
NAESTARSYFRFKALAAVESENVQNKIEDLQSQLQQFTNMKTELFDGQLLSQASELRALPLPSAVASVLIDRY
MPKEVDYLNEIYKKLYYSNLGSSIGNSIIDAISQYVNGATYFNFASYVGQQPAVGAGGANAFPGSQESAQAK
LDQERKQAALYLQETRGALTVIEEQRARVLKDDKITNEQRSTILDSLRNYEDNINSISGSLVLLQNYLQPLSIA
GGSVAGTFEVKEGQEQWQARLQILEEALVSGLVGNMINGGMFPLQSTIQSDQQSFADMGQNFQLDLQMHLT
SMQQEWTVVATSLQLLNQMYLSLARSLTG

>core/56/2/Org2_Gene838

MTEKKPTPMMEQWHQCKEKAGDSVLLFRMGDFYEAFYDDAVLLSQHLELTLTQRQGIPMSGIPVSTVDTYV
DRLIGKGFKVAVAEQFGEPAKEKESKKIGPMARDIQRFVTPGTLLSSTLLQEKFNNYIVAINRIGSLFGFACLD
LSTGSFFIEECENTKELVDEICRLAPSEVLSCNKFYNKETAIVMQLQQHLKLTLSTYADWAFEHKFASQKLTT
HFQVASLDGFGLKGLVPAINAAGGLLSYIQDKLLLPTKHIAIPQTRGKQQKLLIDTASQVNLELLAPLNDPQG
KNSLLRIMDHTSTPMGGRLLRQILISPFYNPKEILVRQDAVEFFLRQVTLRKNIKTYLCQVRDIERLMTKVTTG
LAGPRDIGTLRDSFSAGAQIYEQLASATLPEFFIDKCSLDTKLASLIALLSKSLNGDLPLRVSDGNIFVDEFHND
LKRLRHNQEHSQEWIWEYQERIRKETGIKKLKICFAQALGYYIEVSSEFAPQLPKDFIRRQSRLHAERFTTIEL
QQFQDDMSNISEKLQTLETQFFKDLCSHILQLRTEILALSQSLADLDYIISLADLAHAQGYCRPHVDMSDTLCI
YRGCHPVAKTLVDTGKFIPNDTEMRGSQTRMILLTGPNMAGKSTYIRQIALLVIMAQMGSYIPAKSAHIGVID
KIFTRIGAGDNLSKGMSTFMVEMAETANILHNATDRSLVILDEVGRGTSTYDGLAIAQAVVEYLLFTDKKKA
KTLFATHYKELTTLEDHCPHVENFHAGVKDKAGQPVFLYEILKGHSQKSFGIHVARLAGFPLCVVSRAQQIL
RQLEGPESITRPAQDKMQQLTLF

>core/57/2/Org2_Gene1013

MKRAILDRLYLSVVQSPESASPRDIFTAVAKTVMEWLAKGWLKTQNGYYKNDVKRVYYLSMEFLLGRSLK
SNLLNLGILDLVRKALKTLNYDFDHLVEMESDAGLGNGGLGRLAACYLDSMATLAVPAYGYGIRYDYGIFD
QRIVNGYQEEAPDEWLRYGNPWEICRGEYLYPVRFYGRVIHYTDSRGKQVADLVDTQEVLAMAYDIPIPGY
GNDTVNSLRLWQAQSPRGFEFSYFNHGNYIQAIEDIALIENISRVLYPNDSITEGQELRLKQEYFLVSATIQDIIR

RYTKTHICLDNLADKVVVQLNDTHPALGIAEMMHILVDREELPWDKAWEMTTVIFNYTNHTILPEALERWP
LDLFSKLLPRHLEIIYEINSRWLEKVGSRYPKNDDKRRSLSIVEEGYQKRINMANLAVVGSAKVNGVSSFHSQ
LIKDTLFKEFYEFFPEKFINVTNGVTPRRWIALCNPRLSKLLNETIGDRYIIDLSHLSLIRSFAEDSGFRDHWKG
VKLKNKQDLTSRIYNEVGEIVDPNSLFDCHIKRIHEYKRQLMNILRVIYVYNDLKENPNQDVVPTTVIFSGKA
APGYVMAKLIIKLINSVADVVNQDSRVNDKLKVLFLPNYRVSMAEHIIPGTDLSEQISTAGMEASGTGNMKF
ALNGALTIGTMDGANIEMAEHIGKENMFIFGLLEEQIVQLRREYCPQTICDKNPKIRQVLDLLEQGFFNSNDK
DLFKPIVHRLLHEGDPFFVLADLESYIAAHENVNKLFKEPDSWTKISIYNTAGMGFFSSDRAIQDYARDIWHV
PTKSCSGEGN

>core/58/2/Org2_Gene788

MKKLYHPTLFLRPLIRLSLIFALSLTLISGNFPQQKSFGHCCADMHSALISGKNCEELFADFIERVLADRETLTA
RDWGTVVVLVREYLLKCIRKGDCDYGVKILQKLLALRLPKDARKDLQILWHRLNPEQAPLRDVVDQLFTIG
CHESLQDHLLFELYTVTLHSGYENRKQDMLLAKEQGDYKKAIELAKELVAALEKGSCSPHPEIVQIEKTFLQ
KTLLALQIKVAQEAQESCDALLTPYCLSEIAYTEAMDALVLRIARGEVSRTNEVDSVLLSHALQHLPFAREKA
IPELEVLIDHGAYLESTLLYYAYFSLLELYHQNKDFASLERLLEKGDAVFVPEHPYFPEYGFFLGAYFYAKGK
YESAEKVFLQIIDPAVKLGATFARAYEYLGCIAYVQNHYEKAEEYFLRAYKSWGREESGIGLFLAYAVQKKK
TACEDMLYHPKFSFTYRHLLDSLCSLSYPHGENKGSSAIQRVHRAVPELSEIYSRCIYDMIKYRNVTYTHPIIE
LAYNQVRNLEKRNLEEICRDAQDPEYDKALAFWGALQSGASVPRSLIESSDVDEARITIRCYEALYFHNPDAI
AMLPQAFSEECNSWQTALRLVWTLVRPKGAPNHAKYWDHLVLRPHGDSLYFFGYDLQEYLIGKEDALKHL
SVFAELFPKSSLLSLVYYLQGYSESSALRKVGWFVKALEEFTEISWSGEHMKTWAYIYYMVKLDLADTYISL
GNFSQAVHILEEVKEDWQVASHPKLHFLKGEDCYLAMELRWVEGLAYAYFQLHETAHLSNHLLEHVEKNLI
SPRSYRDYYGESLQRTLGLCQRFLGV

>core/59/2/Org2_Gene123

MRYDPNLIEKKWQQFWKEHRSFQANEDEDKVKYYVLDMFPYPSGAGLHVGHLIGYTATDIVARYKRARGF
SVLHPMGWDSFGLPAEQYAIRTGTHPKVTTQKNIANFKKQLSAMGFSYDEGREFATSDPDYYHWTQKLFLF
LYDQGLAYMADMAVNYCPELGTVLSNEEVENGFSIEGGYPVERKMLRQWILKITAYADKLLEGLDALDWP
ENVKQLQKNWIGKSEGALVTFHLTQEGSLEAFTTRLDTLLGVSFLVIAPEHPDLDSIVSEEQRDEVTAYVQES
LRKSERDRISSVKTKTGVFTGNYAKHPITGNLLPVWISDYVVLGYGTGVVMGVPAHDERDREFAEMFSLPIH
EVIDDNGVCIHSNYNDFCLNGLSGQEAKDYVINYLEMRSLGRAKTMYRLRDWLFSRQRYWGEPIPIIHFEDG
THRPLEDDELPLLPPNIDDYRPEGFGQGPLAKAQDWVHIYDEKTGRPGCRETYTMPQWAGSCWYYLRFCDA
HNSQLPWSKEKESYWMPVDLYIGGAEHAVLHLLYSRFWHRVFYDAGLVSTPEPFKKLINQGLVLASSYRIPG
KGYVSIEDVREENGTWISTCGEIVEVRQEKMSKSKLNGVDPQVLIEEYGADALRMYAMFSGPLDKNKTWSN
EGVGGCRRFLNRFYDLVTSSEVQDIEDRDGLVLAHKLVFRITEHIEKMSLNTIPSSFMEFLNDFSKLPVYSKRA
LSMAVRVLEPIAPHISEELWVILGNPPGIDQAAWPQIDESYLVAQTVTFVVQVNGKLRGRLEVAKEAPKEEVL
SLSRSVVAKYLENAQIRKEIYVPNKLVNFVL

>core/60/2/Org2_Gene17

MDSTTNSDSPILDPNPEDVEKLLDESEEESEDQSTERLLPSELFILPLNKRPFFPGMAAPILIESGPYYEVLKVLA
KSSQKYIGLVLTKKENADILKVSFNQLHKTGVAARILRIMPIEGGSAQVLLSIEERIRIIEPIKDKYLKARVSYH
ADNKELTEELKAYSISIVSVIKDLLKLNPLFKEELQIFLGHSDFTEPGKLADFSVALTTATREELQEVLETTNM

HDRIDKALILLKKELDLSRLQSSINQKIEATITKSQKEFFLKEQLKTIKKELGLEKEDRAIDIEKFSERLRKRHVP
DYAMEVIQDEIEKLQTLETSSAEYTVCRNYLDWLTIIPWGIQSKEYHDLKKAEIVLNKDHYGLDEIKQRILELI
SVGKLSKGLKGSIICLVGPPGVGKTSIGRSIAKVLHRKFFRFSVGGMRDEAEIKGHRRTYIGAMPGKMVQALK
QSQAMNPVIMIDEVDKIGASYHGDPASALLEVLDPEQNKDFLDHYLDVRVDLSNVLFILTANVLDTIPDPLLD
RMEILRLSGYILEEKLQIAKKYLVPKARKEIGLTASEVNFQPEALKYMINNYAREAGVRTLNGNIKKVLRKVA
LKIVQNQEKPKSKKITFKISSKNLQTYLGKPIFSSDRFYESTPVGVATGLAWTSLGGATLYIESVQVSSLKTDM
HLTGQAGEVMKESSQIAWTYLHSALHRYAPGYTFFPKSQVHIHIPEGATPKDGPSAGITMVTSLLSLLLETPV
VNNLGMTGEITLTGRVLGVGGIREKLIAARRSRLNILIFPEDNRRDYEELPAYLKTGLKIHFVSHYDDVLKVA
FPKLK

>core/61/2/Org2_Gene804

MKGTPQYHFIGIGGIGMSALAHILLDRGYEVSGSDLYESYTIESLKAKGARCFSGHDSSHVPHDAVVVYSSSI
APDNVEYLTAIQRSSRLLHRAELLSQLMEGYESILVSGSHGKTGTSSLIRAIFQEAQKDPSYAIGGLAANCLNG
YSGSSKIFVAEADESDGSLKHYTPRAVVITNIDNEHLNNYAGNLDNLVQVIQDFSRKVTDLNKVFYNGDCPIL
KGNVQGISYGYSPECQLHIVSYNQKAWQSHFSFTFLGQEYQDIELNLPGQHNAANAAAACGVALTFGIDINII
RKALKKFSGVHRRLERKNISESFLFLEDYAHHPVEVAHTLRSVRDAVGLRRVIAIFQPHRFSRLEECLQTFPKA
FQEADEVILTDVYSAGESPRESIILSDLAEQIRKSSYVHCCYVPHGDIVDYLRNYIRIHDVCVSLGAGNIYTIGE
ALKDFNPKKLSIGLVCGGKSCEHDISLLSAQHVSKYISPEFYDVSYFIINRQGLWRTGKDFPHLIEETQGDSPLS
SEIASALAKVDCLFPVLHGPFGEDGTIQGFFEILGKPYAGPSLSLAATAMDKLLTKRIASAVGVPVVPYQPLNL
CFWKRNPELCIQNLIETFSFPMIVKTAHLGSSIGIFLVRDKEELQEKISEAFLYDTDVFVEESRLGSREIEVSCIG
HSSSWYCMAGPNERCGASGFIDYQEKYGFDGIDCAKISFDLQLSQESLDCVRELAERVYRAMQGKGSARIDF
FLDEEGNYWLSEVNPIPGMTAASPFLQAFVHAGWTQEQIVDHFIIDALHKFDKQQTIEQAFTKEQDLVKR

>core/62/2/Org2_Gene783

MIRERKKSRHPRLPTLPLAAKASLYLFFACFSGLSLWSFHRDQPCTQNWIGLLGWSFSSFLLYFFGAAAFFIPL
YFLWLSFLYFRRTPRPLFFYKAAAFLSLPFCSAILLSMLSPVGTLPALLDTRLPKFILGNNPPVSYVGGIPFYLF
YEGQSFCLKHLIGSVGTALIFGFVMLFSVLYLCGGIALLKKKTFQDGVKKAFCSFFQTCFKNLKKLINRRNYL
PKPSVPFVSKNPFSCTKSQPSPRRVSETIILDGSISPLPQEEIPGSKKESFFLTPHPCKRFLTKFVEPQENKAKEGK
TIALSSTPTVVRESKGKERAALPKLKSLAVPENDLPQYHLLSKNREARPESLQAELERKALILKQTLTSFGIDA
DLGNICSGPTLAAFEVLPHSGVKVQKIKSLENDIALKLQASSIRIIAPIPGKAAVGIEIPTPFPQAVNFRDLLEDY
QKTNRKLQIPLLLGKKANGDNLWADLATMPHLIIAGTTGSGKSVCINTIVMSMIMTTLPSEIKLVIIDPKKVEL
TGYSQLPHMLSPVITESREVYNALVWLVKEMESRYEILRYLGLRNIQAFNSRTRNKTIEASYDREIRETMPFM
VGIIDELSDLLLSSSQDIETPIIRLAQMARAVGIHLILATQRPSREVITGLIKANFPSRISFKVSNKVNSQIIIDEPG
AENLMGNGDMLVLLPSVFGTIRAQGAYICDEDINKVIQDLCSRFPTQYVIPSFHAFDDSDSDNSGEKDPLFAQ
AKTLILQTGNASTTFLQRKLKIGYARAASLIDQLEEARIIGPSEGAKPRQILIQNPLEG

>core/63/2/Org2_Gene230

MDPKEKNYDASAITVLEGLQAVRERPGMYIGDTGITGLHHLVYEVVDNSIDEAMAGYCSRIDVRILEDGGIVI
VDNGRGIPIEVHERESAKQGREVSALEVVLTVLHAGGKFDKDSYKVSGGLHGVGVSCVNALSEKLVATVFK
DKKCYQMEFSRGIPVTPLQYVSVSDRQGTEIVFYPDPKIFSTCTFDRSILMKRLRELAFLNRGITIVFEDDRDVS
FDKVTFFYEGGIQSFVSYLNQNKESLFSEPIYICGTRVGDDGEIEFEAALQWNSGYSELVYSYANNIPTRQGGT

HLTGFSTALTRVINTYIKAHNLAKNNKLALTGEDIREGLTAVISVKVPNPQFEGQTKQKLGNSDVSSVAQQV
VGEALTIFFEENPQIARMIVDKVFVAAQAREAAKKARELTLRKSALDSARLPGKLIDCLEKDPEKCEMYIVEG
DSAGGSAKQGRDRRFQAILPIRGKILNVEKARLQKIFQNQEIGTIIAALGCGIGADNFNLSKLRYRRIIIMTDAD
VDGSHIRTLLLTFFYRHMTALIENECVYIAQPPLYKVSKKKDFRYILSEKEMDSYLLMLGTNESSILFKSTERE
LRGEALESFINVILDVESFINTLEKKAIPFSEFLEMYKEGIGYPLYYLAPATGMQGGRYLYSDEEKEEALAQEE
THKFKIIELYKVAVFVDIQNQLKEYGLDISSYLIPQKNEIVIGNEDSPSCNYSCYTLEEVINYLKNLGRKGIEIQR
YKGLGEMNADQLWDTTMNPEQRTLIHVSLKDAVEADHIFTMLMGEEVPPRREFIESHALSIRINNLDI

>core/64/2/Org2_Gene264

MNKRTLLFVSLIGIAFVGCQIFFGYNEFRSCKNLAEKQRKISEQTLAAVESVGLSVASWDTDVNGEEHKNNY
AVRVGDKLFLLHNGEAAQSVYSSGESWSFVDHKCGFDNIHLALYRQQGSSFNPTNTGKVFLPTNHEGLPVL
VVEFRNNKEPLVFLGEYAQGRISNKDSTIFGTALVFWRSGSDYIPLGLYDSREEKLVSLDLPITRAVIFGNDQD
SAKSSDTANHYVLFNDYMQIIVSEESGSIEGINLPFASTNNKSIVNEIGFDRDLASEKSPEALFPGLSSKLPDGQ
QAKNSIGGYYPLLRRGLLSDSKKLLPLEYHALNVVSGRELATPVALRYRVLSYTPHSIQLESLDRSVQKVYKL
PENPEEKPYVFETAITLTKETEDVWVTSGVPEVEIMSNASAPTIKYRVIKKNKGSLDKVKLPKVKEPLAIRRG
VYPQWILNSNGYFGIILTPLSEIASGYGSLYISGSTAPTRLSAISPKNQLYPVSKYPGYETLLPLPKDAGTHRFL
VYAGPLAEPTLKVLDKTITQEKGENPEYLDSISFRGVFAFITAPFAALLFIIMKFFKLVTGSWGISIILLTVFLKL
LLYPLNAWSIRSMRRMQILSPYIQQIQQKYKNEPKRAQMEIMGLYKTNKVNPITGCLPLLIQLPFLIAMFDLLK
SSFLLRGASFIPGWIDNLTAPDVLFSWQTSIWFIGNEFHLLPILLGIVMFLQQKVTSLHKKGPVTDQQKQQQV
MGNMMAILFTAMFYNFPSGLNIYWLSSMILGVVQQWITNKILDSKHLKNEVVLNNKKHR

>core/65/2/Org2_Gene514

MRIPITLLQTYFSEPLSTKEILEACDHIGIEAEIENTTLYSFASVITAKILHTIPHPNADKLRVATLTDGEKEHQV
VCGAPNCEAGLIVALALPGAKLFDSEGQAYTIKKSKLRGVESQGMCCGADELGLDELQIQERALLELPEATP
LGEDLATVLGNTSLEISLTPNLGHCASFLGLAREICHVTQANLVIPKEFSFENLPTTALDMGNDPDICPFFSYV
VITGISAQPSPIKLQESLQALKQKPINAIVDITNYIMLSLGQPLHAYDASHVALDSLRVEKLSTPESLTLLNGET
VLLPSGVPVVRDDHSLLGLGGVMGAKAPSFQETTTTTVIKAAYFLPEALRASQKLLPIPSESAYRFTRGIDPQN
VVPALQAAIHYILEIFPEATISPIYSSGEICRELKEVALRPKTLQRILGKSFSIEILSQKLQSLGFSTTPQETSLLVK
VPSYRHDINEEIDLVEEICRTESWNIETQNPVSCYTPIYKLKRETAGFLANAGLQEFFTPDLLDPETVALTRKE
KEEISLQGSKHTTVLRSSLLPGLLKSAATNLNRQAPSVQAFEIGTVYAKHGEQYQETQTLAILLTEDGESRSW
LPKPSLSFYSLKGWVERLLYHHHLSIDALTLESSALCEFHPYQQGVLRIHKQSFATLGQVHPELAKKAQIKHP
VFFAELNLDLLCKMLKKTTKLYKPYAIYPSSFRDLTLTVPEDIPANLLRQKLLHEGSKWLESVTIISIYQDKSL
ETRNKNVSLRLVFQDYERTLSNQDIEEEYCRLVALLNELLTDTKGTINS

>core/66/2/Org2_Gene255

MLIMRNKVILQISILALIQTPLTLFSTEKVKEGHVVVDSITIITEGENASNKHPLPKLKTRSGALFSQLDFDEDLR
ILAKEYDSVEPKVEFSEGKTNIALHLIAKPSIRNIHISGNQVVPEHKILKTLQIYRNDLFEREKFLKGLDDLRTY
YLKRGYFASSVDYSLEHNQEKGHIDVLIKINEGPCGKIKQLTFSGISRSEKSDIQEFIQTKQHSTTTSWFTGAGL
YHPDIVEQDSLAITNYLHNNGYADAIVNSHYDLDDKGNILLYMDIDRGSRYTLGHVHIQGFEVLPKRLIEKQS
QVGPNDLYCPDKIWDGAHKIKQTYAKYGYINTNVDVLFIPHATRPIYDVTYEVSEGSPYKVGLIKITGNTHTK
SDVILHETSLFPGDTFNRLKLEDTEQRLRNTGYFQSVSVYTVRSQLDPMGNADQYRDIFVEVKETTTGNLGLF

LGFSSLDNLFGGIELSESNFDLFGARNIFSKGFRCLRGGGEHLFLKANFGDKVTDYTLKWTKPHFLNTPWILGI
ELDKSINRALSKDYAVQTYGGNVSTTYILNEHLKYGLFYRGSQTSLHEKRKFLLGPNIDSNKGFVSAAGVNL
NYDSVDSPRTPTTGIRGGVTFEVSGLGGTYHFTKLSLNSSIYRKLTRKGILKIKGEAQFIKPYSNTTAEGVPVSE
RFFLGGETTVRGYKSFIIGPKYSATEPQGGLSSLLISEEFQYPLIRQPNISAFVFLDSGFVGLQEYKISLKDLRSS
AGFGLRFDVMNNVPVMLGFGWPFRPTETLNGEKIDVSQRFFFALGGMF

>core/67/2/Org2_Gene30

MLLLISGALFLTLGIPGLTAGVSFGLGIGLSALGGVLVVSGLLCLLVKREVSKVCPEEIPAVQPEETPEGVPVTP
FEKPALDEAQKEQKTQKILDQLPQELDQLDRYIQEAFACLGPLKDLKYEDQGFLQDVKEEFQVFDFVQKDMI
AEFVELQQILCQEGRLLEFVINQTRYIGRDLFKREDSLYKLWEWLGYLPSGDVRGERLKKSAREVVDRFMRT
TCNIRKIAMTFDRHVYSVAKTAFEKAFGALETCVYESMRESYREAFCEYEKAKLLGDEEKSAHAEQRFQDIK
NRWEDVKDAFFWVKEDGKIEIDDAIGNSCKWSERYEEHRITRARWYKVAEHQLFNATMRVKDSLREHNEA
RVAFEKERSKENQRQVQKKKEKRLRDLKELHDQELPRAQERLRELQALYPEIAVSVVEARREVASDLEKAH
ESIDKHYQSCVREQELY

>core/68/2/Org2_Gene402

MASGIGGSSGLGKIPPKDNGDRSRSPSPKGELGSHEISLPPQEHGEEGASGSSHIHSSSSFLPEDQESQSSSSAAS
SPGFFSRVRSGVDRALKSFGNFFSAESTSQARETRQAFVRLSKTITADERRDVDSSSAAATEARVAEDASVSG
ENPSQGVPETSSGPEPQRLFSLPSVKKQSGLGRLVQTVRDRIVLPSGAPPTDSEPLSLYELNLRLSSLRQELSDI
QSNDQLTPEEKAEATVTIQQLIQITEFQCGYMEATQSSVSLAEARFKGVETSDEINSLCSELTDPELQELMSDG
DSLQNLLDETADDLEAALSHTRLSFSLDDNPTPIDNNPTLISQEEPIYEEIGGAADPQRTRENWSTRLWNQIRE
ALVSLLGMILSILGSILHRLRIARHAAAEAVGRCCTCRGEECTSSEEDSMSVGSPSEIDETERTGSPHDVPRRN
GSPREDSPLMNALVGWAHKHGAKTKESSESSTPEISISAPIVRGWSQDSSVSFIVMEDDHIFYDVPRRKDGIYD
VPSSPRWSPARELEEDVFGDYEVPITSAEPSKDKNIYMTPRLATPAIYDLPSRPGSSGSSRSPSSDRVRSSSPNR
RGVPLPPVPSPAMSEEGSIYEDMSGASGAGESDYEDMSRSPSPRGDLDEPIYANTPEDNPFTQRNIDRILQERS
GGASASPVEPIYDEIPWIHGRPPATLPRPENTLTNVSLRVSPGFGPEVRAALLSESVSAVMVEAESIVPPTEPGD
GESEYLEPLGGLVATTKILLQKGWPRGESNA

>core/69/2/Org2_Gene990

MLLLISGALFLTLGIPGLSAAISFGLGIGLSALGGVLMISGLLCLLVKREIPTVRPEEIPEGVSLAPSEEPALQAA
QKTLAQLPKELDQLDTDIQEVFACLRKLKDSKYESRSFLNDAKKELRVFDFVVEDTLSEIFELRQIVAQEGWD
LNFLINGGRSLMMTAESESLDLFHVSKRLGYLPSGDVRGEGLKKSAKEIVARLMSLHCEIHKVAVAFDRNSY
AMAEKAFAKALGALEESVYRSLTQSYRDKFLESERAKIPWNGHITWLRDDAKSGCAEKKLRDAEERWKKF
RKAVFWVEEDGGFDINNLLGDWGTVLDPYRQERMDEITFHELYEKTTFLKRLHRKCALAKTTFEKKRSKKN
LQAVEEANARRLKYVRDWYDQEFQKAGERLEKLHALYPEVSVSIRENKIQETRSNLEKAYEAIEENYRCCVR
EQEDYWKEEEKREAEFRERGNKILSPEELESSLEQFDHGLKNFSEKLMELEGHILKLQKEATAEVENKILSDA
ESRLEIVFEDVKEMPCRIEEIEKTLRMAELPLLPTKKAFEKACSQYNSCAEMLEKVKPYCKESLAYVTSKERL
VSLDEDLRRAYTECQKRFQGDSGLESEVRACREQLRERIQEFETQGLDLVEKELLCVSSRLRNTECDCVSGV
KKEAPPGKKFYAQYYDEIYRVRVQSRWMTMSERLREGVQACNKMLKAGLSEEDKVLKEEEYWLYREERK
NKEKRLVGTKIVATQQRVAAFESIEVPEIPEAPEEKPSLLDKARSLFTREDHT

>core/70/2/Org2_Gene974

MLFSKNFSTDNGGAITAKTLSLTGTTMSALFSENTSSKKGGAIQTSDALTITGNQGEVSFSDNTSSDSGAAIFT
EASVTISNNAKVSFIDNKVTGASSSTTGDMSGGAICAYKTSTDTKVTLTGNQMLLFSNNTSTTAGGAIYVKKL
ELASGGLTLFSRNSVNGGTAPKGGAIAIEDSGELSLSADSGDIVFLGNTVTSTTPGTNRSSIDLGTSAKMTALR
SAAGRAIYFYDPITTGSSTTVTDVLKVNETPADSALQYTGNIIFTGEKLSETEAADSKNLTSKLLQPVTLSGGT
LSLKHGVTLQTQAFTQQADSRLEMDVGTTLEPADTSTINNLVINISSIDGAKKAKIETKATSKNLTLSGTITLL
DPTGTFYENHSLRNPQSYDILELKASGTVTSTAVTPDPIMGEKFHYGYQGTWGPIVWGTGASTTATFNWTKT
GYIPNPERIGSLVPNSLWNAFIDISSLHYLMETANEGLQGDRAFWCAGLSNFFHKDSTKTRRGFRHLSGGYVI
GGNLHTCSDKILSAAFCQLFGRDRDYFVAKNQGTVYGGTLYYQHNETYISLPCKLRPCSLSYVPTEIPVLFSG
NLSYTHTDNDLKTKYTTYPTVKGSWGNDSFALEFGGRAPICLDESALFEQYMPFMKLQFVYAHQEGFKEQG
TEAREFGSSRLVNLALPIGIRFDKESDCQDATYNLTLGYTVDLVRSNPDCTTTLRISGDSWKTFGTNLARQAL
VLRAGNHFCFNSNFEAFSQFSFELRGSSRNYNVDLGAKYQF

>core/71/2/Org2_Gene756

MTLQPSYINFTPNVTTALSGGKIDTSAIELSCSALFFQELQDKAQGLKHALGLVQELSAEALRPAQVQTSISYL
PTEESSRPGISAGIIDRTMPTFTDDEVKAILQNPNFETSKIFVEGLDKVFKSYLDSVTPPEGIDPSNPESAIILNYIT
LLNNLKPKFAAGSTPTDADYNALYALPGDFVKEIEALKAADAPPKSKVHAFWQEIMTIYNNMQVLSYPVTD
YLNVQIADLSLNITAAQEVQQYLKNFYSILKDILNPGWTDPQATHYPADAEYNARDAGVIQSLLNLSGNYRQ
LTENMLPNTDTSLPQEIIAQIRSFQNGVNGTIIASNTLLPTTMRLDTLLGVIYTYQCCATIFGMSYGTSTPAKQN
YIDAINQEKSYWQARANGFDVTSDQVFDQFATNIQSGTSYRGIDLFKNNKVNEINPIFLSQAASFLRYPYNLM
SRSMYQTIEDAANRSITALDGLISGWSTQIATFQTQKNSLDPSLLKYFDTMKANKESFVTTAPLQMVYSSLML
DKYLPTQQNVIASLGIQMTYSNKAAKYLNELIKEITTFQSADIYYSLSIYLKQMNLQAVADPIGKAVGVLNDE
KTRAMADITRCNKIKAAIDKMLVEIKADAELSKSQIRELVDTLTNFKSQSDDLIRNLSCLLGFLSGLTLKAVN
DPNATYEAFTAEIFTEPFNNWKRQLATFESFVIQGGQNGITPGGQQQLLQAMESSQQDFSTFNQNQQLALQLE
SSAMQQEWTLVSAALALLNQMVSKIARRIKS

>core/75/2/Org2_Gene724

MVFFRNSLLHLVALSGMLCCSSGVALTIAEKMASLEHSGRGADDYEGMASFNANMREYSLQLSKLYEEARK
LRASGTEDEALWKDLIRRIGEVRGYLREIEELWAAEIREKGGNLEDYALWNHPETTIYNLVTDYGTEDSIYLI
PQEIGAIKIATLSKFVVPKESFEDCLTQILSRLGIGVRQVNSWIKELYMMRKEGCSVAGVFSSRKDLEALPETA
YIGFVLNSNVDAHTNQHVLKKFINPETTHVDVIAGRVWIFGSAGEVGELLKIYNFVQSESIRQEYRVIPLTKID
PGEMISILNAAFREDLTKDVSEESLGLRVVPLQYQGRSLFLSGTAALVQQALTLIRELEEGIENPTDKTVFWYN
VKHSDPQELAALLSQVHDVFSGENKASVGAADGCGSQLNASIQIDTTVSSSAKDGSVKYGNFIADSKTGTLI
MVVEKEVLPRIQMLLKKLDVPKKMVRIEVLLFERKLAHEQKSGLNLLRLGEEVCKKGCSPSVSWAGGTGILE
FLFKGSTGSSIVPGYDLAYQFLMAQEDVRINASPSVVTMNQTPARIAVVDEMSIAVSSDKDKAQYNRAQYGI
MIKMLPVINVGEEDGKSYITLETDITFDTTGKNHDDRPDVTRRNITNKVRIADGETVIIGGLRCKQMSDSHDGI
PFLGDIPGIGKLFGMSSTSDSLTEMFVFITPKILENPVEQQERKEEALLSSRPGEREEYYQALAASEAAARAAH
KKLEMFPASGVSLSQVERQEYDGC

>core/76/2/Org2_Gene821

MGYIESSTFRLYAEVIVGSNINKVLDYGVPENLEHITKGTAVTISLRGGKKVGVIYQIKTTTQCKKILPILGLSD
SEIVLPQDLLDLLFWISQYYFAPLGKTLKLFLPAISSNVIQPKQHYRVVLKQSKAKTKEILAKLEVLHPSQGAV
LKILLQHASPPGLSSLMETAKVSQSPIHSLEKLGILDIVDAAQLELQEDLLTFFPPAPKDLHPEQQSAIDKIFSSL
KTSQFHTHLLFGITGSGKTEIYLRATSEALKQGKSTILLVPEIALTVQTVSLFKARFGKDVGVLHHKLSDSDKS
RTWRQASEGSLRILIGPRSALFCPMKNLGLIIVDEEHDPAYKQTESPPCYHARDVAVMRGKLAHATVVLGSA
TPSLESYTNALSGKYVLSRLSSRAAAAHPAKISLINMNLEREKSKTKILFSQPVLKKIAERLEVGEQVLIFFNRR
GYHTNVSCTVCKHTLKCPHCDMVLTFHKYANVLLCHLCNSSPKDLPQSCPKCLGTMTLQYRGSGTEKIEKIL
QQIFPQIRTIRIDSDTTKFKGSHETLLRQFATGKADVLIGTQMIAKGMNFSAVTLAVILNGDSGLYIPDFRASEQ
VFQLITQVAGRSGRSHLPGEILIQSFLPDHPTIHSAMRQDYSAFYSQEITGRELCEYPPFIRLIRCIFMGKCPKQT
WEEAHRVHNILKEQLESTNPLMPVTPCGHFKIKDTFRYQFLIKSAYVIPVNKKLHHALMLAKLSPKVKFMID
VDPMTTFF

>core/77/2/Org2_Gene98

MEKICGYLEQILVENKDSGDITAYIKIPNKTTPILIKGKLPQPLELGSPIQIYGVWSHSPSNTKYFQIHSYDSPLL
YEYRGVFHYLTSKLIKGIGPKIAEKIIEKFQEKTCYVLDITPERLSEVSGISETRCVSICKQLCEQKILRKTLLFLQ
EYNIPIHYGVRIFKKYQEKSIEKICEDPFLLAREMEGIGFKTADFIAMKLGVPRNSESRLCAGIQHSLEELQEEG
HTCYPIELLIDVVAKLLNQDVFDTPITLEEIDTQILNMQKRKLLHIQDISGTLHVWTRYLHLAEKTIVSDLKRIL
FSSRRIRSIDGEKAIAWVEENLSIDLAEQQREAIKACFSEKLLIITGGPGTGKSTITQAILKIFEQVTHKIILAAPT
GKAAKRMTEITQKHSVTIHALLQYDFKTKSFRKNHDNPIDCDLIIVDESGMMDTHLLHHFLKALPDYTTLVFI
GDIHQLPSVGPGNILKDLITSNKMTVIRLNKIFRQVHDSGIVTNAHRVNEGELPILYSETGRRDFLFFQKDDQE
EALNHIIHLVTKFVPQKYHIYPQDIQVLAPMKKGTLGIYNLNKALKHALNPKKANLHGRFQSYAVGDKVMQI
RNNYNKEVFNGDIGYVSTINFEDKAVVVRMEGKHVGYSFSELDDLVLAYATSVHKYQGSESPCIIIPIHTSHF
MMLYRNLLYTAITRGKKLVILVGTKKAIAIATRNNRVQHRCTGLAEVLKELDTKKNYADL

>core/78/2/Org2_Gene995

MLSFFYKHQKKFIGIVIAVVCVSGIGVGWGRFSRKGSAESTSRRTVFTTASGKRYVEKDFMAMKKFFAHEAY
PFTGNPRAWNFINEGLLTDYFLTTRVGEKLFLKVYHPGEKIFSKEKAYQPYRRFDAPFISSEEVWKSSAPQLLE
ILKVFQQIENPISKEGFLARAKLFLEERRFPHYVLRQMLEYRRQMFALPPDEALSRGKDLRLFGYQTIQDWFG
DAYLSAAVELLIRFIDEQKKVLPRPSKQEARDDFYDKAKHAYTKISKNKEFSLGFEEFVNSYFQFLEISESEFF
NMYRDILLCKRALLLLQGGVSFDFQPLTTFFVQGKDSIQVEFFRLPKEYSFKTKQELKAFEVYLKLVSLPKSD
SLDVPNEILPIATIKAKEPRLVGRRFSIDYKRVALQDLAATVPMVEVLHWQQNSEHFQEILQQFPDVETCQSY
KDFQHLKPALRDKISLFTRKEILRARPERILQSLQQVPKQSQEVLLSAGKNSALPGISDGQQLAKVLLENEVLD
LYSQDAETYYTIIVNSSFEKEEVLPYREVLKRDLASQLLTSHGHLVDMERLESALRTRYPGEEGASLWQRRL
WKVVENHRLGRHLEGSFSWSLDRSLKTFSRGDKELPQEFDRIFSMKVGDYSSVFMSPNEGPCYYQCLSHLLY
DRPASVDKLFLAKSQLDEELLGSYMERFIEQGVVR

>core/79/2/Org2_Gene655

MDYLEKLQVLIEEGQSANFLSLWEEYCFNDVVRGRELVEILEKVKSSSLASLFGKIVDTVVPLWEKIPEGKDK
DRVLQLILDLQTSNSQMFFDIATEYVNKKYSGEENFNEALRVVGLRDGRDFQFSLSRFDFLMHMHKGNFVFH
QGGWGVGEVMGVSFLQQKVLIEFEGIMSAKDISFETAFKSLTPLSGDHFLSRRFGDPDGFEAFAKENPIEVVEI
LLRDLGPKTAKEIKDELVDLVIPEADWNRWWQSAKTKIKKGTRIISPDNPKEPYVLSDAGCSHMGQLERKLG

LSLNSAEKISLIYHFIRDLHSELKNIEIRKSLVKALQDLDVEEGNKSLILQRELLLSEYLGIKDASIDKEYITSLSE
DDTSRLLENMPIVALQKSFLSLVRKYSSFWQQVFMQILLYTTSPTMRDFVYKTIKNDPSSVEVLKKRLLDSAH
QPMMFPELFVWFFLKLGNHEDGLFDPEDKEVLRLFLESALNFMYQVASTPHKELGKKLHHYLVGQRYLAVR
QMIEGASLPFLKELLLLSTKCPQFSSSDLNVLQSLAEVVQPTLKKHKSNVEEENVLWSTSESFSRMKAKLQSL
VGKEMVDNAKEIEDARSLGDLRENSEYKFALEKRARLQEEIRVLSEEINRARILTKDLVFTDKVGVGCKVTL
KGDAGEVVEYTILGPWDADPDSCILSLQSKLAQNMLGKKLNDVVILQGKEYKISRIQSIWEEHGA

>core/80/2/Org2_Gene405

MVDKLIHPWDLDLLVSGRQKDPHKLLGILASEDSSDHIVIFRPGAHTVAIELLGELHHAVAYRSGLFFLSVPK
GIGHGDYRVYHQNGLLAHDPYAFPPLWGEIDSFLFHRGTHYRIYERMGAIPMEVQGISGVLFVLWAPHAQR
VSVVGDFNFWHGLVNPLRKISDQGIWELFVPGLGEGIRYKWEIVTQSGNVIVKTDPYGKSFDPPPQGTARVA
DSESYSWSDHRWMERRSKQSEGPVTIYEVHLGSWQWQEGRPLSYSEMAHRLASYCKEMHYTHVELLPITEH
PLNESWGYQVTGYYAPTSRYGTLQEFQYFVDYLHKENIGIILDWVPGHFPVDAFALASFDGEPLYEYTGHSQ
ALHPHWNTFTFDYSRHEVTNFLLGSALFWLDKMHIDGLRVDAVASMLYRDYGREDGEWTPNIYGGKENLE
SIEFLKHLNSVIHKEFSGVLTFAEESTAFPGVTKDVDQGGLGFDYKWNLGWMHDTFHYFMKDPMYRKYHQ
KDLTFSLWYAFQESFILPLSHDEVVHGKGSLVNKLPGDTWTRFAQMRVLLSYQICLPGKKLLFMGGEFGQY
GEWSPDRPLDWELLNHHYHKTLRNCVSALNALYIHQPYLWMQESSQECFHWVDFHDIENNVIAYYRFAGS
NRSSALLCVHHFSASTFPSYVLRCEGVKHCELLLNTDDESFGGSGKGNRAPVVCQDQGVAWGLDIELPPLAT
VIYLVTFF

>core/81/2/Org2_Gene973

MNKFKTYLQTALIAPFFSFPALSGSFSSIQAEEITQQVNHPGAELLSEGSYIPGLQTFRLGIKITASKGSHIYWKN
PGEIGSPLKISWQLPKGFVVEEEHWPTPKVFEEEGTTFFGYEDSALIVADVRAPEGYTPGQEVELRAQVEWLA
CGDSCLPGNVDLKLTLPYEEKEPSLYPDTHAEFTKTLHAQPRVLENDHSVQVAQGKGNEIILNISKKINATKA
WFVSEKADKLFAYAETSYSGGTGTAWRLKVKNLSGVQKNEKLHGILLLADHTGRPVESLTIHSEVLGQTGS
AVAGLSQYITILIMAFLGGVLLNIMPCVLPLVTLKVYGLIKSAGEHRSSVIANGLWFTLGVVGCFWGLAGVAF
ILKVLGHNIGWGFQLQEPMFVATLIIVFFLFALSSLGLFEMGTMFANLGGKLQSSEMKSSNNKAVGAFFNGIL
ATLVTTPCTGPFLGSVLGLVMSLSFLQQLLIFTAIGLGMASPYLVFSVFPKMLSVLPKPGGWMSTFKQLTGFM
LLVTVTWLVWIFGSETSTTSVVVLLGGLWLAGLGAWILGRWGTPVSPKKQRVCASLLFFAFLGGAISVSGLA
SHYFAEPQQTVSVNEDSLWQPFSLEKLAQLRAQGRPVFVNFTAKWCLTCQMNKPVLYGDAVQKMFETHGI
VTLEADWTRKDPGITEELARLGRASVPSYVYYPGDNSAPVVLPEKITQNLLEDVVSRFVR

>core/82/2/Org2_Gene242

MIRSPLPFISSKRALNMLGLQDEFSCPEDVVDFLFSEIELLAQQDEPSEGYLALSRSLLMMTHNHPKVVKRVIF
YGVSYGLKHKSMSIFIDVLTYIDFLFEKLGISASDRLSLCSARTCINFELYSQTGEMKFLSEVVDNFRLIEQLLK
MHPQLKNRLGWEHFRIGAKQEEVSLVASASVYQAVGRSFIELYHKHLELSDLACGMKCLALALDLSPNNAH
IHADYAKGLVVLGTRQGKSLLIERGMEHFSKAIFLSFSRDGDTLAYQNYRYSYALASVKLFDLTYKKEHFDQ
AMNILYQTVQAFPNLSGLWMVWGELLIRSGWLNSNMKYIEVGLEKLASLQKKTNDPIALSGLLATGIAILGL
YLEEPNLFKDSRHRLISAMRTFPGNSALVHALGVVQLCSALYFNEDSHFASAISCFQSCLEWDLDATGMWQK
LFDAYFSWGIKKKSARLLRKAVDVASRLCSLRPEAFLFWSDRGLALKCLAEATIDEAYKEIFLSESLLHYQRA
WDLSGRLEILELWGQSHYLLAELQQSLFHYDEAYTLLTKVDLTLSSSRVKLILAAVLLGKGRLLQDTDPAEE

AREILEPLVEVYLEDENFLLLLGKVYLFLFWKNKNVCLGKLARTYLEKATSLGCPEAYYTLGKFYAVIKDVN
KAWGMVIRSAQYGVRITEAKWLNDPYLANLREIHAFREVVENQKGRLWLGNKTEMKRN

>core/83/2/Org2_Gene276

MNKLLNFVSRTLGGDTALNMINKSSDLILALWMMGVVLMIIIPLPPPIVDLMITINLSISVFLLMVALYIPSALQ
LSVFPSLLLITTMFRLGINISSSRQILLKAYAGHVIQAFGDFVVGGNYVVGFIIFLIITIIQFIVVTKGAERVAEVA
ARFRLDAMPGKQMAIDADLRAGMIDATQARDKRAQIQKESELYGAMDGAMKFIKGDVIAGIVISLINIVGGL
TIGVAMHGMDLAQAAHVYTLLSIGDGLVSQIPSLLIALTAGIVTTRVSSDKNTNLGKEISTQLVKEPRALLLA
GAATLGVGFFKGFPLWSFSILALIFVALGILLLTKKSAAGKKGGGSGASTTVGAAGDGAATVGDNPDDYSLT
LPVILELGKDLSKLIQHKTKSGQSFVDDMIPKMRQALYQDIGIRYPGIHVRTDSPSLEGYDYMILLNEVPYVR
GKIPPHHVLTNEVEDNLSRYNLPFITYKNAAGLPSAWVSEDAKAILEKAAIKYWTPLEVIILHLSYFFHKSSQE
FLGIQEVRSMIEFMERSFPDLVKEVTRLIPLQKLTEIFKRLVQEQISIKDLRTILESLSEWAQTEKDTVLLTEYVR
SSLKLYISFKFSQGQSAISVYLLDPEIEEMIRGAIKQTSAGSYLALDPDSVNLILKSMRNTITPTPAGGQPPVLLT
AIDVRRYVRKLIETEFPDIAVISYQEILPEIRIQPLGRIQIF

>core/84/2/Org2_Gene519

MYKRCVLDKILKGIVAGSLILLYWSSDLLERDIKSIKGNVRDIQEDIREISRVVKQQQTSQAIPAAPGVMLAPK
LVRDEAFALLFGDPSYPNLLSLDPYKQQTLPELLGTNFHPHGILRTAHVGKPENLSPFNGFDYVVGFYDLCIPS
LASPHVGKYEEFSPDLAVKIEEHLVEDGSGDKEFHIYLRPNVFWRPIDPKALPKHVQLDEVFQRPHPVTAHDI
KFFYDAVMNPYVATMRAVALRSCYEDVVSVSVENDLKLVVRWKAHTVINEEGKEERKVLYSAFSNTLSLQ
PLPRFVYQYFANGEKIIEDENIDTYRTNSIWAQNFTMHWANNYIVSCGAYYFAGMDDEKIVFSRNPDFYDPL
AALIDKRFVYFKESTDSLFQDFKTGKIDISYLPPNQRDNFYSFMKSSAYNKQVAKGGAVRETVSADRAYTYI
GWNCFSLFFQSRQVRCAMNMAIDRERIIEQCLDGQGYTISGPFASSSPSYNKQIEGWHYSPEEAARLLEEEGW
IDTDGDGIREKVIDGVIVPFRFRLCYYVKSVTAHTIADYVATACKEIGIECSLLGLDMADLSQAFDEKNFDALL
MGWCLGIPPEDPRALWHSEGAMEKGSANVVGFHNEEADKIIDRLSYEYDLKERNRLYHRFHEIIHEEAPYAF
LFSRHCSLLYKDYVKNIFVPTHRTDLIPEAQDETVNVTMVWLEKKEDPCLSTS

>core/85/2/Org2_Gene31

MKELRHESYNRALHKLSHQWVRYFLYTFVSCSFIVAIFTFAWLKVLYVPEYKAGEISRISLTAPMDFSLSWSA
HKFYKRTAHISEAFGKVYHLTLSPGSLLSKEGNADENTDYWFKKAADFLLSTNFVDSSTQKCLKDLCIYPPLL
GKEKKTLEININSNKGNVIAQCFCHLKIFLIQENCPQPCFDAIMDILKIANFEVAVDKEMSGCVKGELLGKRCI
EKITKGTPILEKYQRIDDRDAKILKQLRAQLLSVHTLFSCRSLWGAIFVVLLILLWGYGALKALCPEMLKSPQ
RFMLYIAILTLSLLWCRGTEIFCAYWVSYLSYPPILPFTAVLLGYFLGLPIAGFSCTFLALLYTLGSDLWNNSW
FLSINLLCSWRILVSLHRVSRLSSVFWACMKLGGVAMGSLLMFRIFTNTISREALYADGIESFVYSLITAISVVA
LIPVFEASFGASTNFSLLTYLSPENALLKRLFKEAPGTYQHSVLVGSLAEAAAQAIGADSLYCLVAAHYHDIG
KLINPGFFSENQKILQQSGHSLSPLECAKMIMRHIPEGVNLARQAGLPESFIQVIEEHHGTSVIRSAYYSHMVE
NPSTGSFDEELFRYSGNKPSSKETTIIMIADSFEAASRSLKNASLPDLQRLIDQIIQGKLQDGQFSCSPITLDELA
LISKSMVQTLYGALHSRMKYPEISYQISMDSCPKPSIGGT

>core/86/2/Org2_Gene939

MPEPLYTNKLITEKSPYLLLYAHTPVNWYPWGAEAFHIAAIENKPVFLSIGCKHSRWCQVMLQESYTNPEIAA
MLNEYFVNVKVDKEELPYVAKLYGDLAQMLAVSGDHQETVSWPLNVFLTPDLVPFFSVNYLGNEGKLGLP
SFPQIIDKLHFMWEDAEEREALVEQAMKVLEIASFLEGCVRKEILDESSLKRTVAALYQDIDPHYGGVKAFPK
RLPGLLLQFFLRYSLEYQESRGLFFVDRSLSMVALGGVRDHIGGGVYSYTIDDKWLIPAFEKRLIDNALMALN
YLEAWACLGKEEYRGIGKQILSYILSELYSPEVGAFYSSEQAENWGAGGQNFYTWSVEEISNALGEDAEIFCD
YYGISREGFFNGRNILHIPVHREIEELSEKYHRSIEAIEDIVDRSRDILKGIRAQRSHRSKDDLSLTFNNGWMIYT
FAYAGRLLGEVEYIEIGKKCGEFVRNSLYKHHELYRRWREGEAKYRASLEDYGALILGVLALYESGCGSFWL
SFAEELMQEVVLSFRSEEGGFYSVDGRDSTLLIKQSPLSDGETISGNALICQCLLSLHLITEKKHYLTYAEDILQ
IAQACAHTHKFSSLGLLIASQNYFSRKHVKVLIALGDQEDRSPVLKCLSGLFLPYLSLIWMTQENQEHLETVL
PEYEHCLIPKGDCTATTIYVLEVDQCKRFKDLELFRRYLISL

>core/88/2/Org2_Gene10

MKRCFLFLASFVLMGSSADALTHQEAVKKKNSYLSHFKSVSGIVTIEDGVLNIHNNLRIQANKVYVENTVGQ
SLKLVAHGNVMVNYRAKTLVCDYLEYYEDTDSCLLTNGRFAMYPWFLGGSMITLTPETIVIRKGYISTSEGP
KKDLCLSGDYLEYSSDSLLSIGKTTLRVCRIPILFLPPFSIMPMEIPKPPINFRGGTGGFLGSYLGMSYSPISRKHF
SSTFFLDSFFKHGVGMGFNLHCSQKQVPENVFNMKSYYAHRLAIDMAEAHDRYRLHGDFCFTHKHVNFSGE
YHLSDSWETVADIFPNNFMLKNTGPTRVDCTWNDNYFEGYLTSSVKVNSFQNANQELPYLTLRQYPISIYNT
GVYLENIVECGYLNFAFSDHIVGENFSSLRLAARPKLHKTVPLPIGTLSSTLGSSLIYYSDVPEISSRHSQLSAKL
QLDYRFLLHKSYIQRRHIIEPFVTFITETRPLAKNEDHYIFSIQDAFHSLNLLKAGIDTSVLSKTNPRFPRIHAKL
WTTHILSNTESKPTFPKTACELSLPFGKKNTVSLDAEWIWKKHCWDHMNIRWEWIGNDNVAMTLESLHRSK
YSLIKCDRENFILDVSRPIDQLLDSPLSDHRNLILGKLFVRPHPCWNYRLSLRYGWHRQDTPNYLEYQMILGT
KIFEHWQLYGVYERREADSRFFFFLKLDKPKKPPF

>core/89/2/Org2_Gene889

MNFQTISINLTEGKILVFETGKIARQANGAVLVRSGETCVFASACAVDLDDKVDFLPLRVDYQEKFSSTGKTL
GGFIKREGRPSEKEILVSRLIDRSLRPSFPYRLMQDVQVLSYVWSYDGQVLPDPLAICAASAALAISDIPQSNIV
AGVRIGCIDNQWVINPTKTELASSTLDLVLAGTENAILMIEGHCDFFTEEQVLDAIEFGHKHIVTICKRLQLWQ
EEVGKSKNLSAVYPLPAEVLTAVKECAQDKFTELFNIKDKKVHAATAHEIEENILEKLQREDDDLFSSFNIKA
ACKTLKSDTMRALIRDREIRADGRSLTTVRPITIETSYLPRTHGSCLFTRGETQTLAVCTLGSEAMAQRYEDLN
GEGLSKFYLQYFFPPFSVGEVGRIGSPGRREIGHGKLAEKALSHALPDSATFPYTIRIESNITESNGSSSMASVC
GGCLALMDAGVPISSPIAGIAMGLILDDQGAIILSDISGLEDHLGDMDFKIAGSGKGITAFQMDIKVEGITPAIM
KKALSQAKQGCNDILNIMNEALSAPKADLSQYAPRIETMQIKPTKIASVIGPGGKQIRQIIEETGVQIDVNDLG
VVSISASSASAINKAKEIIEGLVGEVEVGKTYRGRVTSVVAFGAFVEVLPGKEGLCHISECSRQRIENISDVVKE
GDIIDVKLLSINEKGQLKLSHKATLE

>core/90/2/Org2_Gene472

MSNQEFDLSAIRNIGIMAHIDAGKTTTTERILFYAGRTHKIGEVHEGGATMDWMAQEQERGITITSAATTVFW
LGAKINIIDTPGHVDFTIEVERSLRVLDGAVAVFDAVSGVEPQSETVWRQADKYGVPRIAFVNKMDRMGAD
YFAAVESMKEKLGANAFPVHCPIGSESQFVGMVDLISQKALYFLDDTLGAKWEEKEISEDLKERCAELRANL
LEELATIDESNEAFMMKVLEDPDSITEDEIHQVMRKGVIENKINPVLCGTAFKNKGVQQLLNVIVKWLPSPLD
RGNIRGINLKTDQEISLEPRRDGPLAALAFKIMTDPYVGRITFIRIYSGTLKKGSAILNSTKDKKERISRLLEMH

ANERTDRDEFTVGDIGACVGLKFSVTGDTLCDDNQEIVLERIEFPDPVIDMAIEPKSKGDREKLAQALSSLSEE
DPTFRVSTNEETGQTIISGMGELHLDILRDRMIREFKVEANVGKPQVSYKETITVSGNSETKYVKQSGGRGQY
AHVCLEIEPNEPGKGNEVVSKIVGGVIPKEYIPAVIKGIEEGLNTGVLAGYGLVDVKVSIVFGSYHEVDSSEM
AFKICGSMAVKDACRKAKPVILEPIMKVAVITPEDHLGDVIGDLNRRRGKILGQESSRGMAQVNAEVPLSEM
FGYTTSLRSLTSGRATSTMEPAFFAKVPQKIQEEIVKK

>core/92/2/Org2_Gene419

MYNLLHAHHDAASPDGRLVSHLKKLSPHIYEGEVLIENIPAYFLGFHLPQQCIQVNLKSSLAQLGVEAVLNHL
ELNKARKEARLHVLFMSQDPIATAMLELLEPGSFVCKLFAADDRRLVRSPCYLNRMFTHTDRTGSPLLRFGK
KLEHFITLEIINDRLVVFLPILPGTICYEETIYGFLPLMSKSLTRPHLKIRKFLPLYQMVTDRPPVPEDHKILLIKT
EPLHIRTVFARVVQDLLPQGLRHTAADILEPTTQESGDIYEFYGSTSEPIERIPLEFFTLEPYKEHSFFFYRDMLQ
ETLESPQEVFRVFESIPEGEDQAAMFISKGSELLELSQDSWIIKPRISPSDERHAREIQKHIEDQPCFPFLKAMET
DHITSQGVLFSRYFPSASLKGMFLSNYSRYYLQHIYFQIPSPTSGEFFSNRDRSFLLDLYFAGISVFWADLESKR
LLQYIKRRNKDVGMFVPKHQAEQFAQSYFIGIHGSCLIAGDYDEFLRELLTGMHTLSQQFTIPEFPPQTPLAIL
TGGGSGAMELANRVATELSILSCGNLISLDTTNAYVEAKMSYAIPDLLERQADFHVDLAVFVIGGMGTDFEL
LLELISLKTGKKALVPVFLIGPVDYWKSKITALYNSNHAVGTIRGSEWVHNCLFCLSSAKAGIAIFRRYLNHTL
PIGPEHPVPEDGFVIV

>core/93/2/Org2_Gene884

MKRSRRNFEQALENLEKLKEISLATSNDSYLNNPARFNQRKQTGSSVMEMKEALKNVENYLLEISCVSKSHA
DKALKESDFLIAGVQNVFSFLENQEDLYKSLLDEYSEVTKAYDEVKKNLKEVPTYDLSTDEETEEHKEPECFL
NNLVEVKRDRSYELFYMLDEQDKRFYNDALVQIIYKQNKLHETVNEGDPLTKTLLWNSEEVKNIASSLVIVN
DMPLRLFYQRALSHLDIEAVVKVHNAVMALFFSRYEATMVFKSPKKHNIWYFNDFLLFLREAWKDLNNNVI
DSQERKQTKLLASALSLGIFESKLVFEEASRYLYFNIQTKLENANGKKPLSPGQYLTDAYEELHRLISKYPNGP
LFKAMDRVLEHESRPYDPMILGILPSLEGTLKLHGKSIDIIRSPSPVTQSSILYANCNEEFLGFLNAKAHRSEVT
LVLNIQNRISRKERARSRVIEEALEQEEHAPYVHAFSFPEPEELLQNLESIHGDIETFADFFSILQEEFHKPLLAS
SFFLTKELKEFVGSFLKEKLTALKDIFFAKKKILFRNDKLLLLHLLSYLIVFKLIERTNPNSIVVVSKDGLDYVS
VFIAGFAFFSREAFWDEHSLKLLLTNVLSPTLVARDRLVFVSHIELLSKFVNCLKKNRQGFSSLKSFFKDDIEG
WEFTGYLHELTEVSHKHNL

>core/94/2/Org2_Gene705

MQPCLNMSIVRNSALPLPCLSRSETFKKVRSHMKFMKVLTPWIYRKDLWVTAFLLTAIPGSFAHTLVDIAGEP
RHAAQATGVSGDGKIVIGMKVPDDPFAITVGFQYIDGHLQPLEAVRPQCSVYPNGITPDGTVIVGTNYAIGM
GSVAVKWVNGKVSELPMLPDTLDSVASAVSADGRVIGGNRNINLGASVAVKWEDDVITQLPSLPDAMNAC
VNGISSDGSIIVGTMVDVSWRNTAVQWIGDQLSVIGTLGGTTSVASAISTDGTVIVGGSENADSQTHAYAYK
NGVMSDIGTLGGFYSLAHAVSSDGSVIVGVSTNSEHRYHAFQYADGQMVDLGTLGGPESYAQGVSGDGKVI
VGRAQVPSGDWHAFLCPFQAPSPAPVHGGSTVVTSQNPRGMVDINATYSSLKNSQQQLQRLLIQHSAKVESV
SSGAPSFTSVKGAISKQSPAVQNDVQKGTFLSYRSQVHGNVQNQQLLTGAFMDWKLASAPKCGFKVALHY
GSQDALVERAALPYTEQGLGSSVLSGFGGQVQGRYDFNLGETVVLQPFMGIQVLHLSREGYSEKNVRFPVSY
DSVAYSAATSFMGAHVFASLSPKMSTAATLGVERDLNSHIDEFKGSVSAMGNFVLENSTVSVLRPFASLAMY
YDVRQQQLVTLSVVMNQQPLTGTLSLVSQSSYNLSF

>core/95/2/Org2_Gene22

MGVVQNQVISSIRDVLKLVWELRFAEHKMLLLSRQSGSGGTFQLSCAGHELAGVLAGKSLIPGKDWSFPYY
RDQGFPIGLGCDLSEIFASFLARTTPNHSSARMMPYHYSHKKLRICCQSSVVGTQFLQAAGRAWAVKHSSAD
EVVYVSGGDGATSQGEFHEMLNFVALHQLPLITVIQNNHWAISVPFEDQCGADLASLGRCHQGLAVYEVDG
GNYTSLTETFSHAVDQARQHSVPALILIDVVRLSSHSNSDNQEKYRSALDLKLSMDKDPLILLEKEAINVFGLS
PFEIEEIKAEAQEEVRKSCEIAEALPFPSKGSTSHEVFSPYTETLIDYENSESAQNLRNSEPKVMRDAISEALVEE
MTRDSGVIVFGEDVAGDKGGVFGVTRNLTEKFGPQRCFNSPLAEATIIGTAIGMALDGIHKPVVEIQFADYIW
PGINQLFSEASSIYYRSAGEWEVPLVIRAPSGGYIQGGPYHSQSIEGFLAHCPGIKVAYPSNAADAKALLKAAI
RDPNPVVFLEHKALYQRRIFSACPVFSHDYVLPFGKAAIVHPGKDLTIVSWGMPLVLSLEVAQELASRGISIEV
IDLRTMVPCDFATVLKSLEKTGRLLVIHEASEFCGFGSELVATMSEQGYAYLDAPIRRLGGLHAPVPYSKVLE
NEVLPHKESILQAAKSLAEF

>core/96/2/Org2_Gene430

MLKKPKRKPGRRTYGKSLKIFIPGTLFVHARKGFGFVSPDNPEEYPFDIFVPARDLRGALDGDHVIVSVLPYPR
DGQKLKGTISEVLARGKTTLVGTITSLVSPTSALAYTSMSGSQSLIPVELLPGRTYKIGDRILLSTPPWVDKPQE
GASPALQMLEFIGHITNAKADFQAIQAEYNLAEEFPPEVIEEASLFSQKHITQVLHSRKDLRDLLCFTIDSSTAR
DFDDAISLTYDHNNNYILGVHIADVSHYVTPHSHLDKEAAKRCNSTYFPGKVIPMLPSALSDNLCSLKPNVDR
LAVSVFMTFTKSGHLSDYQIFRSVIRSKYRMTYDEVDNIIEKKHSHPLSKILNEMATLSKKFSDIREERGCIRFV
LPSVTMSLDNLQEPVALIENHQTFSHKLIEEFMLKANEVVAYHISHQGVSLPFRSHEPPNDENLLAFQELAKN
MGFDITFTPTQEPDYQYLLQTTSAGHPLEQVLHSQFVRSMKTASYSTENKGHYGLKLDYYTHFTSPIRRYIDL
IVHRLLFNPLSIDQTHLEIIVRACSTKERVSAKAENSFENLKKTRFINKFLQEQPKTTYHAYIITANHEGLSFVV
TEFCHEGFIAAAELPKEYSLKKNALPESIPDKMKPGASIKVTIDSVNLLTQKIVWSIATTTEDKPKKIKKTPSKK
KGTKKRAS

>core/98/2/Org2_Gene793

MINKELDIGILGKIAGAIKQISIESIQKASSGHPGLPLGCAELAAYLYGYVLRQNPRDPHWINRDRFVLSAGHG
SALLYSCLHLAGFDVSLEDLQEFRQLHSRTPGHPEYGETVGVEATTGPLGQGLGNAVGMALSMKMLESRFN
RPGHEIFNGKIYCLAGDGCFMEGVSHEVCSFAGSLNLNNLVVIYDYNNVVLDGYLNEISVEDTKKRFEAYG
WDVYEIDGYDFTHIHETFSSIKRGQERPVLVIAHTIIGHGSPKEGTNKAHGSPLGVEGTHETKQFWHLPEEKFF
VPPAVKNFFAHKIQEDRKAQEQWLDEVRVWSKQFPELHEEFVALTSHKLPKNLESLVQSVEMPDSIAGRAAS
NKLIQVLVQHIPYLIGGSADLSSSDGTWIANEKVIHTYDFSGRNIKYGVREFGMATIMNGLAYSQVFRPFGGT
FLVFSDYMRNAIRLAALSKLPVIYQFTHDSIFVGEDGPTHQPVEQLMSLRAIPGLYVIRPADANEVRGAWIAG
LKHTGPTVIVLSRQALPTLPAAHRPFKDGIGRGAYIVLKESGEKPDYTLFATGSEVSLALSVAKELEHLDKQV
RVVSFPCWELFEAQDVDYKQSIVGGDLGIRVSIEAGSALGWYKYIGSEGLAIAMDRFGYSGASDDVSEECGF
TTEQILQRILSQ

>core/99/2/Org2_Gene332

MEKVSSYPSVPLPLGASKISPNRYRFALYASQATEVILALTDENSEVIEVPLYPDTHRTGAIWHIEIEGISDQSS
YAFRVHGPKKHGMQYSFKEYLADPYAKNIHSPQSFGSRKKQGDYAFCYLKEEPFPWDGDQPLHLPKEEMIIY
EMHVRSFTQSSSSRVHAPGTFLGIIEKIDHLHKLGINAVELLPIFEFDETAHPFRNSKFPYLCNYWGYAPLNFFS

PCRRYAYASDPCAPSREFKTLVKTLHQEGIEVILDVVFNHTGLQGTTCSLPWIDTPSYYILDAQGHFTNYSGC
GNTLNTNRAPTTQWILDILRYWVEEMHVDGFRFDLASVFSRGPSGSPLQFAPVLEAISFDPLLASTKIIAEPWD
AGGLYQVGYFPTLSPRWSEWNGPYRDNVKAFLNGDQNLIGTFASRISGSQDIYPHGSPTNSINYVSCHDGFTL
CDTVTYNHKHNEANGEDNRDGTDANYSYNFGTEGKTEDPGILEVRERQLRNFFLTLMVSQGIPMIQSGDEY
AHTAEGNNNRWALDSNANYFLWDQLTAKPTLMHFLCDLIAFRKKYKTLFNRGFLSNKEISWVDAMGNPMT
WRPGNFLAFKIKSPKAHVYVAFHVGAQDQLATLPKASSNFLPYQIVAESQQGFVPQNVATPTVSLQPHTTLIA
ISHAKEVT

>core/100/2/Org2_Gene119

MKEENSQAHYLALCRELEDHDYSYYVLHRPRISDYEYDMKLRKLLEIERSHPEWKVLWSPSTRLGDRPSGTF
SVVSHKEPMLSIANSYSKEELSEFFSRVEKSLGTSPRYTVELKIDGIAVAIRYEDRVLVQALSRGNGKQGEDIT
SNIRTIRSLPLRLPEDAPEFIEVRGEVFFSYSTFQIINEKQQQLEKTIFANPRNAAGGTLKLLSPQEVAKRKLEISI
YNLIAPGDNDSHYENLQRCLEWGFPVSGKPRLCSTPEEVISVLKTIETERASLPMEIDGAVIKVDSLASQRVLG
ATGKHYRWALAYKYAPEEAETLLEDILVQVGRTGVLTPVAKLTPVLLSGSLVSRASLYNEDEIHRKDIRIGDT
VCVAKGGEVIPKVVRVCREKRPEGSEVWNMPEFCPVCHSHVVREEDRVSVRCVNPECVAGAIEKIRFFVGR
GALNIDHLGVKVITKLFELGLVHTCADLFQLTTEDLMQIPGIRERSARNILESIEQAKHVDLDRFLVALGIPLIG
IGVATVLAGHFETLDRVISATFEELLSLEGIGEKVAHAIAEYFSDSTHLNEIKKMQDLGVCISPYHKSGSTCFG
KAFVITGTLEGMSRLDAETAIRNCGGKVGSSVSKQTDYVVMGNNPGSKLEKARKLGVSILDQEAFTNLIHLE

>core/101/2/Org2_Gene68

MRLNIHKYLFIGRNKADFFSASRELGVVEFISKKCFITTEQGHRFVECLKVFDHLEAEYSLEALEFVKDESVSV
EDIVSEVLTLNKEIKGLLETVKALRKEIVRVKPLGAFSSSEIAELSRKTGISLRFFYRTHKDNEDLEEDSPNVFY
LSTAYNFDYYLVLGVVDLPRDRYTEIEAPRSVNELQVDLANLQREIRNRSDRLCDLYAYRREVLRGLCNYDN
EQRLHQAKECCEDLFDGKVFAVAGWVIVDRIKELQSLCNRYQIYMERVPVDPDETIPTYLENKGVGMMGED
LVQIYDTPAYSDKDPSTWVFFAFVLFFSMIVNDAGYGLLFLMSSLLFSWKFRRKMKFSKHLSRMLKMTAILG
LGCICWGTTTTSFFGMSFSKTSVFREYSMTHVLALKKAEYYLQMRPKAYKELTNEYPSLKAIRDPKAFLLAT
EIGSAGIESRYVVYDKFIDNILMELALFIGVVHLSLGMLRYLRYRYSGIGWILFMVSAYLYVPIYLGTVSLIHY
LFHVPYELGGQIGYYGMFGGIGLAVVLAMIQRSWRGVEEIISVIQVFSDVLSYLRIYALGLAGAMMGATFNQ
MGARLPMLLGSIVILLGHSVNIILSIMGGVIHGLRLNFIEWYHYSFDGGGRPLRPLRKIVCSEDAEASGIHLDN
NSIV

>core/102/2/Org2_Gene429

MSEHKKSSKIIGIDLGTTNSCVSVMEGGQAKVITSSEGTRTTPSIVAFKGNEKLVGIPAKRQAVTNPEKTLGST
KRFIGRKYSEVASEIQTVPYTVTSGSKGDAVFEVDGKQYTPEEIGAQILMKMKETAEAYLGETVTEAVITVPA
YFNDSQRASTKDAGRIAGLDVKRIIPEPTAAALAYGIDKVGDKKIAVFDLGGGTFDISILEIGDGVFEVLSTNG
DTLLGGDDFDEVIIKWMIEEFKKQEGIDLSKDNMALQRLKDAAEKAKIELSGVSSTEINQPFITMDAQGPKHL
ALTLTRAQFEKLAASLIERTKSPCIKALSDAKLSAKDIDDVLLVGGMSRMPAVQETVKELFGKEPNKGVNPD
EVVAIGAAIQGGVLGGEVKDVLLLDVIPLSLGIETLGGVMTTLVERNTTIPTQKKQIFSTAADNQPAVTIVVLQ
GERPMAKDNKEIGRFDLTDIPPAPRGHPQIEVSFDIDANGIFHVSAKDVASGKEQKIRIEASSGLQEDEIQRMV
RDAEINKEEDKKRREASDAKNEADSMIFRAEKAIKDYKEQIPETLVKEIEERIENVRNALKDDAPIEKIKEVTE
DLSKHMQKIGESMQSQSASAAASSAANAKGGPNINTEDLKKHSFSTKPPSNNGSSEDHIEEADVEIIDNDDK

>core/103/2/Org2_Gene354

MKLLLKAVLRHKNHLVILGCSLLAILGLTFSSQMEIFSLGMIAKTGPDAFLLFGRKESGKLVKVSELSQKDILE
NWQAISKDSETLTVSDATTYIAEHGKSTASLTSKLSKFVRNYIDVSRFRGLAIFLICVAIFKAVTLFFQRFLGQV
VAIRVSRDLRQDYFKALQQLPMTFFHDHDIGNLSNRVMTDSASIALAVNSLMINYIQAPITFILTLGVCLSISW
KFSILICVAFPIFILPIVVIARKIKNLAKRIQKSQDSFSSVLYDFLAGVMTVKVFRTEKFAFTKYCEHNNKISALE
EKSAAYGLLPRPLLHTIASLFFAFVVVIGIYKFAIPPEELIVFCGLLYLIYDPIKKFGDENTSIMRGCAAAERFYE
VLNHPDLHSQKEREIEFLGLSNTITFENVSFGYQEDKHILKNLSFTLHKGEALGIVGPTGSGKTTLVKLLPRLY
EVSQGKILIDSLPITEYNKGSLRNHIACVLQNPFLFYDTVWNNLTCGKDMEEEAVLEALKRAYADEFILKLPK
GVHSVLEESGKNLSGGQQQRLAIARALLKNASILILDEATSALDAISENYIKNIIGELKGQCTQIIIAHKLTTLEH
VDRVLYIENGQKIAEGTKEELLQTCPEFLKMWELSGTKEYNRVFVPDHKLVANPTDMAITT

>core/104/2/Org2_Gene771

MFSRLFFTSFSAEVVNTFFESGMSEDTSPLLSKQNRKLSHNLPLKSAYLSLGTYLIALLSFWLHAKNLSNLFVV
FTFFLAGTPALIKSLDNICQKVVNIDILMTSAAFGSIFIGGALEGALLLVLFAISEALGQMVSGKAKSTLVSLKQ
LAPTTGWLVLEDGNLQKVAINKIEVGNILRIKSGEVVPLDGEILHGSSSINLMHLTGEKVPKSCHPGSIVPAGA
HNMEGSFDLRVLRTGSDSTIAHIINLVIQAQNSKPRLQQRLDKYSSVYALSIFAIACGIALLVPLFTSIPLLGPQS
AFYRALAFLIAASPCALIIAIPIAYLSAINACAKHGVLLKGGVILDRLVSCNSVVMDKTGTLTTGELTCIGCDYF
GSKNETFFPSVLALEQSSSHPIAEAIVSYLMEQKVSSLPADRYLTVPGEGVRGYFNEQEAFVGRVETGLGKVP
SEYLEDIEQKIYQAKQHGEICSLAYVGNSFALFYFRDIPRPQAKEIIQDLKDLGYPVSMLTGDHKVSAENTAEI
LGISEVFFDLTPEDKLAKIRELATQRQIMMVGDGINDAPALAQATVGIAMGEAGSATAIEAADIVLLHDSLSS
LPWIIQKAKQTKKVVSQNLALALAIILLVSWPASLGIIPLWLAVILHEGSTVIVGLNALRLLKS

>core/105/2/Org2_Gene710

MTFQLHAPFAPCGDQPEAIARLSAGVRNQVKSQVLLGTTGSGKTFTIANVVANVNLPTLVLAHNKTLAAQL
YQEFREFFPNNAVEYFISYYDYYQPEAYIARSDTYIEKSLLINDEIDKLRLSATRSILERRDTLIVSSVSCIYGIGS
PENYTSMALVLEVGKEYPRNILTAQLVKMHYQASPIPQRSAFRERGSVIDIFPAYESELALRLEFLNDTLTSIE
YSDPLTMIPKESVPSATLYPGSHYVIPEAIREQAIRTIQEELEERMAFFDDRPIEKDRIFHRTTHDIEMIKEIGFCK
GIENYSRHFTGAPPGAPPTCLLDYFPEDFLLIIDESHQTLPQIRAMYRGDQSRKQSLVEYGFRLPSAFDNRPLT
YEEAQKYFRKVIYVSATPGDTEVQESSGHIVQQIIRPTGIPDPMPEIRPATGQVDDLLEEIRLRLSQKHEKILVIS
ITKKLAEDMAGFLSELEIPAAYLHSGIETAERTQILTDLRSGVIDVLIGVNLLREGLDLPEVSLVAILDADKEGF
LRSTSSLIQFCGRAARNINGKVIFYADQKTRSIEETLRETERRRQIQLDYNKEHNIVPKPIIKAIFANPILQTSKDS
ESPKESQRPLSKEDLEEQIKKYEALMQRAAKEFRFNEAAKYRDAMQACKEQLLYLF

>core/106/2/Org2_Gene506

MATPAQKSPTFQDPSFVRELGSNHPVFSPLTLEERGEMAIARVQQCGWNHTIVKVSLIILALLTILGGGLLVGL
LPAVPMFIGTGLIALGAVIFALALILCLYDSQGLPEELPPVPEPQQIQIEDLRNETREVLEGTLLEVLLKDRDAK
DPAVPQVVVDCEKRLGMLDRKLRREEEILYRSTAHLKDEERYEFLLELLEMRSLVADRLEFNRRSYERFVQG
IMTVRSEEGEKEISRLQDLISLQQQTVQDLRSRIDDEQKRCWTALQRINQSQKDIQRAHDREASQRACEGTEM
DCAERQQLEKDLRRQLKSMQEWIEMRGTIHQQEKAWRKQNAKLERLQEDLRLTGIAFDEQSLFYREYKEKY
LSQKLDMQKILQEVNAEKSEKACLESLVHDYEKQLEQKDANLKKAAAVWEEELGKQQQEDYEQTQEIRRL

STFILEYQDSLREAEKVEKDFQELQQRYSRLQEEKQVKEKILEESMNHFADLFEKAQKENMAYKKKLADLE
GAAAPTEIGEDDDWVLTDSASLSQKKIRELVEENQELLKALAFKSNELTQLVADAVEAEKEISKLREHIEEQK
EGLRALDKMHAQAIKDCEAAQRKCCDLESLLSPVREDAGMRFELEVELQRLQEENAQLRAEVERLEQEQFQ
G

>core/107/2/Org2_Gene359

MSYRKRSTLIVLGVFALYALLVLRYYKIQICEGDHWAAEALGQHEFCVRDPFRRGTFFANTTVRKGDKDLQ
QPFAVDITKFHLCADPLAIPECHRDEIIQGILQFIEGQTYDDLSLKLDKKSRYCKLYPLLDVSVHDRLSLWWK
GYATKHRLPTNALFFITDYQRSYPFGKLLGQVLHTLREIKDEKTGKAFPTGGMEAYFNHILEGDVGERKLLRS
PLNRLDTNRVIKLPKDGSDIYLTINPVIQTIAEEELERGVLEAKAQGGRLILMNSQTGEILALAQYPFFDPTNYK
EYFNNKERIEHTKVSFVSDVFEPGSIMKPLTVAIALQANEEASLKSQKKIFDPEEPIDVTRTLFPGRKGSPLKDI
SRNSQLNMYMAIQKSSNVYVAQLADRIIQSLGVAWYQQKLLALGFGRKTGIELPSEASGLVPSPHRFHINGSL
EWSLSTPYSLAMGYNILATGIQMVQAYAILANGGYAVRPTLVKKIVSASGEEYHLPTKEKTRLFSEEITREVV
RAMRFTTLPGGSGFRASPKHHSSAGKTGTTEKMIHGKYDKRRHIASFIGFTPVESSEGNFPPLVMLVSIDDPEY
GLRADGTKNYMGGRCAAPIFSRVADRTLLYLGILPDKKLRNCDEEAAALKRLYEEWNRSPKQGGTR

>core/108/2/Org2_Gene643

MVNPIGPGPIDETERTPPADLSAQGLEASAANKSAEAQRIAGAEAKPKESKTDSVERWSILRSAVNALMSLAD
KLGIASSNSSSSTSRSADVDSTTATAPTPPPPTFDDYKTQAQTAYDTIFTSTSLADIQAALVSLQDAVTNIKDTA
ATDEETAIAAEWETKNADAVKVGAQITELAKYASDNQAILDSLGKLTSFDLLQAALLQSVANNNKAAELLK
EMQDNPVVPGKTPAIAQSLVDQTDATATQIEKDGNAIRDAYFAGQNASGAVENAKSNNSISNIDSAKAAIAT
AKTQIAEAQKKFPDSPILQEAEQMVIQAEKDLKNIKPADGSDVPNPGTTVGGSKQQGSSIGSIRVSMLLDDAE
NETASILMSGFRQMIHMFNTENPDSQAAQQELAAQARAAKAAGDDSAAAALADAQKALEAALGKAGQQQ
GILNALGQIASAAVVSAGVPPAAASSIGSSVKQLYKTSKSTGSDYKTQISAGYDAYKSINDAYGRARNDATR
DVINNVSTPALTRSVPRARTEARGPEKTDQALARVISGNSRTLGDVYSQVSALQSVMQIIQSNPQANNEEIRQ
KLTSAVTKPPQFGYPYVQLSNDSTQKFIAKLESLFAEGSRTAAEIKALSFETNSLFIQQVLVNIGSLYSGYLQ

>core/109/2/Org2_Gene996

MKKLVRLCVVLLSLLPNVLFSSDLLREEGIKKMMDKLIEYHVDAQEVSTDILSRSLSSYIQSFDPHKSYLSNQE
VAVFLQSPETKKRLLKNYKAGNFAIYRNINQLIHESILRARQWRNEWVKNPKELVLEASSYQISKQPMQWSK
SLDEVKQRQRALLLSYLSLHLAGASSSRYEGKEEQLAALCLRQIENHENVYLGINDHGVAMDRDEEAYQFHI
RVVKALAHSLDAHTAYFSKDEALAMRIQLEKGMCGIGVVLKEDIDGVVVREIIPGGPAAKSGDLQLGDIIYR
VDGKDIEHLSFRGVLDCLRGGHGSTVVLDIHRGESDHTIALRREKILLEDRRVDVSYEPYGDGVIGKVTLHSF
YEGENQVSSEQDLRRAIQGLKEKNLLGLVLDIRENTGGFLSQAIKVSGLFMTNGVVVVSRYADGTMKCYRT
VSPKKFYDGPLAILVSKSSASAAEIVAQTLQDYGVALVVGDEQTYGKGTIQHQTITGDASQDDCFKVTVGKY
YSPSGKSTQLQGVKSDILIPSLYAEDRLGERFLEHPLPADCCDNVLHDPLTDLDTQTRPWFQKYYLPNLQKQE
TLWREMLPQLTKNSEQRLSENSNFQAFLSQIKSSEKTDLSYGSNDLQLEESINILKDMILLQQCRK

>core/110/2/Org2_Gene942

MTSSSCPLLDLILSPADLKKLSISQLPGLAEEIRYRIISVLSQTGGHLSSNLGIVELTIALHYVFSSPKDKFIFDVG
HQTYPHKLLTGRNNEGFDHIRNDNGLSGFTNPTESDHDLFFSGHAGTALSLALGMAQTTPLESRTHVIPILGD

AAFSCGLTLEALNNISTDLSKFVVILNDNNMSISKNVGAMSRIFSRWLHHPATNKLTKQVEKWLAKIPRYGD
SLAKHSRRLSQCVKNLFCPTPLFEQFGLAYVGPIDGHNVKKLIPILQSVRNLPFPILVHVCTTKGKGLDQAQN
NPAKYHGVRANFNKRESAKHLPAIKPKPSFPDIFGQTLCELGEVSSRLHVVTPAMSIGSRLEGFKQKFPERFFD
VGIAEGHAVTFSAGIAKAGNPVICSIYSTFLHRALDNVFHDVCMQDLPVIFAIDRAGLAYGDGRSHHGIYDMS
FLRAMPQMIICQPRSQVVFQQLLYSSLHWSSPSAIRYPNIPAPHGDPLTGDPNFLRSPGNAETLSQGEDVLIIAL
GTLCFTALSIKHQLLAYGISATVVDPIFIKPFDNDLFSLLLMSHSKVITIEEHSIRGGLASEFNNFVATFNFKVDI
LNFAIPDTFLSHGSKEALTKSIGLDESSMTNRILTHFNFRSKKQTVGDVRV

>core/111/2/Org2_Gene684

MTCISELNEAQRKAVTAPLNPVLVLAGAGAGKTRVVTYRILHLINQGIAPREILAVTFTNKAARELKERIVNQ
CASTNEFDVPMVCTFHSLGVFILRRSINLLNRENNFTIYDQSDAEKLIKHALQQHNLKPNLASKIQAHVSQAK
NRLLFPEDLDPNDYIDPVVSIYQEYQKKLIEANALDFDDLLFLTVRLLRESPEAQELYNQLWKALLIDEYQDT
NHAQYTLMQLLSKQHRNVFAVGDPDQSIYSWRGANIHNILNFENDYPNAKVLCLEENYRSYGNILNAANALI
KNNASRLEKELRSVKGPGEKIRLFLGSTDREEADFVAAEILQLHRVGNIKLRDICIFYRTNSQSRTFEDALLRR
RIPYEIIGGLSFYKRKEIQDILAFLRIFISKSDIVAFDRTVNLPKRGIGSTTIFALTQYAIAQGLPILKACQQALDT
KDVKLSKKQQEGLQEYLALFPQIEHAYNTLSLRDFIESVVRITGYLEILKEDADTFKDRKSNLEELYHKALESE
QQNPKTHLELFLDDLALKGSDDDLNLTADRVNLMTLHNGKGLEFRVSFLVGLEEQLLPHANSLGGTYENIEE
ERRLCYVGITRAQDLLYLTAAQVRSLWGTVRMMKPSRFLKEIPKDYMIQVR

>core/112/2/Org2_Gene85

MKQHYSLNKSRHILRSTYKLLKSKKLAHSPADKKQLQELLEQLEEAIFEHDQETASDLAQQALAFSNRYPNS
FGRKTYELIKALLFAGVVAFLVRQFWFELYEVPTGSMRPTILEQDRILVSKTTFGLHCPFAKKPLAFNPESVTR
GGLVVFTVGDLPIPDADTKYFGLIPGKKRYIKRCMGRPGDFLYFYGGKIYGLDDAGKRIEFPSVHGLENLYH
VPYISFDGTTSSHTEGQKTIIDFKQFNQSYGRLIFPQTSMYGQFFDHKEWHQDEPNKLKDPHLSPVSYADLFG
MGNYAMVRILTEHQARTSHLLPNPGSPTKVYLEICHTANLSYPKPLLRHYEHQLSPAIQPMKTLLPLRKEHLH
LIRNNLTTSRFIVAQGCAYKYHQFKINTSGIAKAYAILLPKVPDGCYEYSKGEAYQIGFGEIRYKLKSSHPLTQ
LNDKQVIELFNCGINFSSIYNPVNPLQAPLPNRYAFFNQGNLYIMDSPVFIKNDPTLQKFVTSETEKQEGSSET
QPYIAFVDKGLPPEDFKEFVEFIHNFGIQVPKGHVLVLGDNYPMSADSREFGFVPMENLLGSPLCTFWPIGRM
GRLTGVSAPTTLSGYLVSGIALATGLSLIGYVVYYQKRRRLFPKKEEKNHKK

>core/113/2/Org2_Gene702

MIPFTKTIGFRLWLACAVAIIAPLGINIVWLNLDQYRTIVSAISTALKENAAFKANTLTQIVPLNVDVLSLFSDV
LDLDAGIPETPNVLLSNEMQKVFQGIYNEISLIKVFPNGDKIVVASSIPEHLGENYNHKIDIPKNTPFLAALKQS
PKNQEVFSVMQANVFDAKTQELQGILYTTFSAESLLKDLLINKQSYLTVKTAILSKYGVILKASDPALHLHTV
YPDMTKEKFCQVFLNDDPCPIDSELGPLTLSPLDIGENFYSFKIKDTEIWGCIENVPSIDIAVLSYAKKEESFAPL
WRRARMYTAYFFCILLGSLIAFIVARRLSLPIRKLATAMIESRKNKNCLYTDDSLGFEINRLGHIFNAMVENLH
KQQHLAKTNFEMKENAQNALHLGEQAQQRLLPNTLPSYPHIELAKAYIPAITVGGDFFDVFVVGEGSKARLF
LIVADASGKGVNACGYSLFLKNMLRTFLSRSSSLQQAIQETSRLFYNNTKNSGMFVTLCVYCYHQTSNTMEY
YSCGHPPACYLDPDGETSWLFHPGMALGFLPEVANITSKLFHPKPGSLFVLYSDGITEAHNNNNDMFGEERL
QAAIQGLTGKSAADAVHRLMLSVKTFVGNSHQHDDITLLILKVLES

>core/114/2/Org2_Gene715

MIQVTCDQKNYEVLEGTTAAELAKQLKNSHQFIGVLINERPRDLSTHLNEGDTLVFLTSEDPEGREIFLHTSA
HLLAQAVLRLWPDAIPTIGPVIDHGFYYDFANLSISESDFPLIEDTVKQIVDEKLAISRFTYGDKQQALAQFPQ
NPFKTELIRELPENEEISAYSQGEFFDLCRGPHLPSTAHVKAFKVLRTSAAYWRGDPSRESLVRIYGTSFPTSKE
LRAHLEQIEEAKKRDHRVLGAKLDLFSQQESSPGMPFFHPRGMIVWDALIRYWKQLHTAAGYKEILTPQLM
NRQLWEVSGHWDNYKANMYTLQIDDEDYAIKPMNCPGCMLYYKTRLHSYKEFPLRVAEVGHVHRQEASG
ALSGLMRVRAFHQDDAHVFLTPEQVEEETLNILQLVSTLYGTFGLEYHLELSTRPEKDTIGDDSLWELATDAL
NRALVQSGTPFIVRPGEGAFYGPKIDIHVKDAIQRTWQCGTIQLDMFLPERFELEYTTAQGTKSVPVMLHRAL
FGSIERFLGILIENFKGRFPLWLSPEQVRIITVADRHIPRAKELEEAWKRLGLVVTLDDSSESVSKKIRNAQNM
QVNYMITLGDHEINENVLAVRTRDNRVINDVSVERFLNTILEEKNSLSLTALL

>core/117/2/Org2_Gene700

MDENRKVIVVGGGLAGLSAAMQLANLGIIVELVSLTKVKRSHSVCAQGGINAALNLKPEEEDSPYVHAYDTI
KGGDFLADQPPVLEMCLAAPRIIKMLDNFGCPFNRGPSGNLDVRRFGGTLYHRTVFCGASTGQQLMYTLDE
QVRRREHAGRVIKRENHEFVRLVTDHSGRACGIILMNLFNNRLEILRGDAVIIATGGPGVIFKMSTNSTFCTGA
ANGRLFLQGMAYANPEFIQIHPTAIPGRDKLRLISESVRGEGGRVWVPGDSSKRIVFPDGSERPCGETGAPWY
FLEDMYPAYGNLVSRDVGARAILRVCEAGLGIDGRMEAYLDVTHLPEKTRHKLEVVLDIYKKFTGEDPNTV
PMRIFPAVHYSMGGAWVDWPAADDPDRDSRFRQMTNIPGCFNCGESDFQYHGANRLGANSLLSCLFAGLVS
GDEASRFIEAFGASQATSSDFDRALQQEKEENARLLSASGKENIFVLHEEIAKIMVRNVTVKRNNRDLQETM
DKLKEFRERLKNVSVLDSSPFANKSFHFVRQMGPMLELALAITKGALLRNEFRGSHYKPEFPERDDEHWLKT
TVAVYAPEEPEISYLPVDTRHVAPTLRDYTKSSTGKIELTNIPDNIRLPI

>core/118/2/Org2_Gene291

MTIIYFILAALALGILVLIHELGHLVVAKAVGMAVESFSIGFGPALFKKRIGGIEYRIGCIPFGGYVRIRGMERT
KEKGEKGKIDSVYDIPQGFFSKSPWKRILVLVAGPLANILLAVLAFSILYMNGGRSKNYSDCSKVVGWVHPV
LQAEGLLPGDEILTCNGKPYVGDKDMLTTSLLEGHLNLEIKRPGYLTVPSKEFAIDVEFDPTKFGVPCSGASY
LLYSNQVPLTKNSPMENSELRPNDRFVWMDGTLLFSMAQISQILNESYAFVKVARNDKIFFSRQPRVLASVL
HYTPYLRNELIDTQYEAGLKGKWSSLYTLPYVINSYGYIEGELTAIDPESPLPQPQERLQLGDRILAIDGTPVSG
SVDILRLVQNHRVSIIVQQMSPQELEEVNSRDADKRFIASYHSEDLLQILNHLGESHPVEVAGPYRLLDPVQPR
PWIDVYSSESLDKQLEVAKKIKNKDKQRYYLERLDAEKQKPSLGISLKDLKVRYNPSPVVMLSNITKESLITL
KALVTGHLSPQWLSGPVGIVQVLHTGWSVGFSEVLFWIGLISMNLAVLNLLPIPVLDGGYILLCLWEIVKRRR
LNMKIVERILVPFTFLLIIFFIFLTFQDLFRFFG

>core/119/2/Org2_Gene437

MRRSVCYVNPSIARAGQISTWKFLYSLATPLPAGTKCKFDLAGSGKPTDWEAPATDLSQTRNVIYAEMPEGE
IIEATAIPVKDNPVPQFEFTLPYELQVGETLTIVMGASPNHPQVDDAGNGAQLFAQRRKPFYLYIDPTGEGNY
DEPDVFSMDIRGNVLKKIEIFTPSYVVKNKRFDITVRFEDEFGNLTNFSPEETRIELSYEHLRENLNWQLFIPET
GFVILPNLYFNEPGIYRIQLKNLSTQEIFISAPIKCFADSAPNLMWGLLHGESERVDSEENIETCMRYFRDDRAL
NFYASSSFENQENLSPDIWKLINQTVSDFNEEDRFITLSGFQYSGEPHLEGVRHILHTKETKSHSKHKEYKHIPL
AKLYKSTVNHDMISIPSFTASKEHGFDFENFYPEFERVVEIYNAWGSSETTAALNNPFPIQGKDSEDPRGTVIE

GLKKNLRFGFVAGGLDDRGIYKDYFDSPQVQYSPGLTAIICNKYTRESLVEALFARHCYATTGPRIVLSFNITS
APMGSELSTGSKPGLNVNRHISGHVAGTALLKTVEIIRNGEVLHTFFPDSNNLDYEYDDMVPLSSVTLKDPNG
KAPFVFYYLRVTQADNAMAWSSPIWVDLN

>core/120/2/Org2_Gene118

MESEKDIGAKFLGDYRILYRKGQSLWSEDLLAEHRFIKKRYLIRLLLPDLGSSQPFMEAFHDVVVKLAKLNHP
GILSIENVSESEGRCFLVTQEQDIPILSLTQYLKSIPRKLTELEIVDIVSQLASLLDYVHSEGLAQEEWNLDSVYI
HILNGVPKVILPDLGFASLIKERILDGFISDEENRESKIKERVLLHTSEGKQGREDTYAFGAITYYLLFGFLPQGI
FPMPSKVFSDFIYDWDFLISSCLSCFMEERAKELFPLIRKKTLGEELQNVVTNCIESSLREVPDPLESSQNLPQA
VLKVGETKVSHQQKESAEHLEFVLVEACSIDEAMDTAIESESSSGVEEEGYSLALQSLLVREPVVSRYVEAEK
EEPKPQPILTEMVLIEGGEFSRGSVEGQRDELPVHKVILHSFFLDVHPVTNEQFIRYLECCGSEQDKYYNELIRL
RDSRIQRRSGRLVIEPGYAKHPVVGVTWYGASGYAEWIGKRLPTEAEWEIAASGGVAALRYPCGEEIEKSRA
NFFTADTTTVMSYPPNPYGLYDMAGNVYEWCQDWYGYDFYEISAQEPESPQGPAQGVYRVLRGGCWKSLK
DDLRCAHRHRNNPGAVNSTYGFRCAKNIN

>core/121/2/Org2_Gene903

MKKGKLGAIVFGLLFTSSVAGFSKDLTKDNAYQDLNVIEHLISLKYAPLPWKELLFGWDLSQQTQQARLQLV
LEEKPTTNYCQKVLSNYVRSLNDYHAGITFYRTESAYIPYVLKLSEDGHVFVVDVQTSQGDIYLGDEILEVDG
MGIREAIESLRFGRGSATDYSAAVRSLTSRSAAFGDAVPSGIAMLKLRRPSGLIRSTPVRWRYTPEHIGDFSLV
APLIPEHKPQLPTQSCVLFRSGVNSQSSSSSLFSSYMVPYFWEELRVQNKQRFDSNHHIGSRNGFLPTFGPILW
EQDKGPYRSYIFKAKDSQGNPHRIGFLRISSYVWTDLEGLEEDHKDSPWELFGEIIDHLEKETDALIIDQTHNP
GGSVFYLYSLLSMLTDHPLDTPKHRMIFTQDEVSSALHWQDLLEDVFTDEQAVAVLGETMEGYCMDMHAV
ASLQNFSQSVLSSWVSGDINLSKPMPLLGFAQVRPHPKHQYTKPLFMLIDEDDFSCGDLAPAILKDNGRATLI
GKPTAGAGGFVFQVTFPNRSGIKGLSLTGSLAVRKDGEFIENLGVAPHIDLGFTSRDLQTSRFTDYVEAVKTIV
LTSLSENAKKSEEQTSPQETPEVIRVSYPTTTSAS

>core/122/2/Org2_Gene48

MTTPPPSRSSSPPPYDWIELQDLGNTNNNSSRATPPPPEVGGELPPYFSASNFVVIERGAPSLPSPQQLLSLPEYS
RQPPPGYFDETASITSRTSEEMFGTLVSTLCCPANSERDWEDHEVNCIYIASTSDTQLEAVQGGMHITELRGEP
VRVLYETGHLYAFARENTCHSRLEVSHTVRAMTYFWDRFFSRHWNVGRRFLVFYQGNGGAYVQAALDSS
MHTQDIYVLGLSPTVYIRGNYHVQHYRVRGFWPSCLDSLAACAENTSVLPYGESSDGIFYPSLFSHTFDNAIR
YGERCLLVCSEGMGMLPETQQQTSPLTSLEGGHEVALVLNPQQNPEALSIASRLMHEERGGRLESNYMPGRS
SNPFMTSMYVLVRLNTLAQIYLMSPYYSFQSNDIVCLIFISSAAVETVSYIFLTVTDSTCGRRYLRVPRLVCTG
LRNLALPTTLLELLILSYPRSVEGVPFNVRFILGYMCTTRVVFFAWNLILHWPFRCLRHGIQLFVHRSIIGHTLG
ARITDLTLASMRYAIVFPSIVSSCLLTALAHANTNILALDPYRLIESGDLRRPAFNDDEMQQADNPWDAYSIGL
VINTCIYMLILFANLIFMVYSVRRYHRSRR

>core/123/2/Org2_Gene317

MTLITPAINSSRRKTHTVRIGNLYIGSDHSIKTQSMTTTLTTDIDSTVEQIYALAEHNCDIVRVTVQGIKEAQAC
EKIKERLIALGLNIPLVADIHFFPQAAMLVADFADKVRINPGNYIDKRNMFKGTKIYTEASYAQSLLRLEEKFA
PLVEKCKRLGKAMRIGVNHGSLSERIMQKYGDTIEGMVASAIEYIAVCEKLNYRDVVFSMKSSNPKIMVTAY

RQLAKDLDARGWLYPLHLGVTEAGMGVDGIIKSAVGIGTLLAEGLGDTIRCSLTGCPTTEIPVCDSLLRHTKI
YLDLPEKKNPFSLQHSENFVSAAEKPAKTTLWGDVYGVFLKLYPHHLTDFTPEELLEHLGVNPVTKEKAFTT
PEGVVVPPELKDAPITDVLREHFLVFHHHQVPCLYEHNEEIWDSPAVHQAPFVHFHASDPFIHTSRDFFEKQG
HQGKPTKLVFSRDFDNKEEAAISIATEFGALLLDGLGEAVVLDLPNLPLQDVLKIAFGTLQNAGVRLVKTEYI
SCPMCGRTLFDLEEVTTRIRKRTQHLPGLKIAIMGCIVNGPGEMADADFGFVGSKTGMIDLYVKHTCVKAHIP
MEDAEEELIRLLQEHGVWKDPEETKLTV

>core/124/2/Org2_Gene535

MWTHPIAYDVIVVGAGHAGCEAAYCSAKMGVSVLMLTSNLDTIAKLSCNPAVGGIGKGHIVREIDALGGIM
AEVTDQSGIQFRILNQTKGPAVRAPRAQVDKQLYHIHMKRLLENTPGLHIMQATVESLLDKEGVISGVTTKE
GWMFSGKTVVLSSGTFMRGLIHIGDRNFSGGRLGDPSSQGLSEDLKKRGFPISRLKTGTPPRLLASSINFSCME
EQPGDLGVGFVHRTEPFQPPLPQLSCFITHTMEKTKAIISANLHRSALYGGCIEGVGPRYCPSIEDKIVKFSDKE
RHHVFLEPEGLHTQEIYANGLSTSMPFDVQYDMIRSVLGLENAIITRPAYAIEYDYIHGNVIHPTLESKLIEGLF
LCGQINGTTGYEEAAAQGLIAGINAVNKVFNRPPFIPSRQESYIGVMLDDLTTQILDEPYRMFTGRAEHRLLLR
QDNACARLSHYGYELGLLSEERYELVKKQNQLLEEEKVRLQKTFRQYGQSVVSLAKALSRPEVSYDMLREA
FPNDIRDLGAVLNASLEMEIKYSGYIDRQKILIQSLEKAESLLIPEDLDYKQITALSLEAQEKLAKFTPRTLGSA
SRISGIASADIQVLMIALKKHAHH

>core/125/2/Org2_Gene109

MTTELKTEALPTRTQVDPKHCWDTTLMYANREEWKKDFDLCSSGKDRSPIWPEFSPSHYQIDNPESLLELLS
KKFSVERKLDQLYIYAHLIHDQDITNPEGESDYQSIVYLYTLFSQEISWIQPALIALSEEKVAALLSSSVLAPYR
FYLEKIFRLSPHTGTANEEKILASSFAALNVSNKAFSSLSDAEIPFGIAKDSNGEEHPLSHALASLYMQSPDQEL
RRTAYLAQFQRYYDYRNTFANLLNGKVQAHLFEAKARNYPSCLEASLFQHNIPTTVYINLINETKKHTSLINR
YFNLKKEALNLKEFHFYDVYAPISQTTSKNYSYEEGVDLVCKSLLPLGTHYVEILRNGLLSNRWVDRYENKH
KRSGAYSSGCYDSAPYILLNYTNTLYDVSVIAHEAGHSMHSYFSREAQPYHDAQYPLFLAEIASTFNEMLLM
EALSKSDQSKEDKIVIITKTLDTIFATLFRQTFFAAFEYEIHSAAEQGTPLTEEFLSATYGNLQKEFYGGVVTSD
SLSALEWARIPHFYYNFYVYQYATGIIAALSFAEKILTQEPGALELYLKFLKSGRSDFPLNILKKSGLDMTTSA
PLDKAFAFITKKIDLLSSLLSED

>core/126/2/Org2_Gene663

MFRCILFGIFLLTCFSSGGVLYYLFCSHDFSIGPKEKSRSVWIEEEKEFTDSVLHHLPSQHQHLHILCFQGFLLQ
KQQKFSQAEKIFSKVYDEAQDGPFLFKEEILGSRLINSFFLEKTDVMETILCLLNQRCPNSPYYHLFKALVCYK
QKLYREVIEQLAYWQEEKTRALAPLLNISIEQLLTDFLLDYISAHSLIEQKMFPEGRVILNRNINRLLKHECEW
NAKTYDRIAILLSRSYFLELVESKSADIYFDYYEMVLFYLKKIYILEQCPYAELLPEEELVSLIMEHVFILPKDK
LYPLIQLLEMWQKHYVHPNSSLVVQILVDRFSTHMEGAIRFCEALVSFSGLEELHQQIITTFEELLSNKVQQIK
TEEAKQCVALLHILDPSISISEKLALSSDTLQNIVSGDDEQHTKLRNYLDLWEAIQSYDIDRQQLVHHLVYGA
KDLWKKGGNDEKALNLLQLVLRFTSYDIECESVVFLFIKQAYKQALSSHAIARLLKLEKFISEANIPSIVISEAE
KANFLADAEYLFAHEDYDKCYLYSMWLTKVAPSPQSYRLAGLCLMENKRYDEALEFLCMLSPNDSINDYK
TQKALAFCQKHQSKDRAAS

>core/127/2/Org2_Gene861

MCGIFGYLGNQDGVSIVLEGLAKLEYRGYDSAGLAAVVEQELFIRKTVGRVQELSNLFQEREIPTASVIGHTR
WATHGVPTEINAHPHVDEGRSCAVVHNGIIENFKELRRELTAQGISFASDTDSEIIVQLFSLYYQESQDLVFSF
CQTLAQLRGSVACALIHKDHPHTILCASQESPLILGLGKEETFIASDSRAFFKYTRHSQALASGEFAIVSQGKEP
EVYNLELKKIHKDVRQITCSEDASDKSGYGYYMLKEIYDQPEVLEGLIQKHMDEEGHILSEFLSDVPIKSFKEI
TIVACGSSYHAGYLAKYIIESLVSTPVHIEVASEFRYRRPYIGKDTLGILISQSGETADTLAALKELRRRNIAYL
LGICNVPESAIALGVDHCLFLEAGVEIGVATTKAFTSQLLLLVFLGLKLANVHGALTHAEQCSFGQGLQSLPD
LCQKLLANESLHSWAQPYSYEDKFLFLGRRLMYPVVMEAALKLKEIAYIEANAYPGGEMKHGPIALISKGTP
VIAFCGDDIVYEKMIGNMMEVKARHAHVIAIAPESREDIAAVSDQQIFVPDCHFLAAPVLYTIVGQVMAYAM
ALAKGMEIDCPRNLAKSVTVE

>core/128/2/Org2_Gene858

MIPSGLVYLLYPLGFLASLFFGSAFSIQWWLSKKRKEVYAPRSFWILSSIGATLMIVHGTIQSQFPVTVLHVINL
IIYLRNLNITSSRPISFRATLVLMALSVVFVTLPFLYVNMEWMASPNIFHLPLPPAQLSWHLIGCLGLAIFSGRF
LIQWFYIESNNTKDFPLLFWKIGLLGGLLALVYFIRIGDPINILCYGCGLFPSIANLRLFYKEQRSTPYLDTHCFL
SAGEASGDILGGKLIQSIKSLYPNIRFWGVGGPAMRQEGLQPILNMEEFQVSGFAEVLGSLFRLYRNYRKILKT
ILKHKPATLIFIDFPDFHLLLIKKLRKHGYRGKIIHYVCPSIWAWRPKRKRILEQHLDMLLLILPFEEGLFKNTSL
ETVYLGHPLVEEISDYKEQASWKEKFLNSDRPIVAAFPGSRRGDISRNLRIQVQAFLNSSLSQTHQFVVSSSSA
KYDEIIEDTLKAEGCQHSQIIPMNFRYELMRSCDCALAKCGTIVLETALNQTPTIVMCRLRPFDTFLAKYIFKIL
LPAYSLPNIIMNSVIFPEFIGGKKDFHPEEIATALDLLNQHGSKEKQKEDCRKLCKVMTTGQIASEEFLKRIFDT
LPAV

>core/129/2/Org2_Gene701

MKHTFTKRVLFFFFLVIPIPLLLNLMVVGFFSFSAAKANLVQVLHTRATNLSIEFEKKLTIHKLFLDRLANTLA
LKSYASPSAEPYAQAYNEMMALSNTDFSLCLIDPFDGSVRTKNPGDPFIRYLKQHPEMKKKLSAAVGKAFLL
TIPGKPLLHYLILVEDVASWDSTTTSGLLVSFYPMSFLQKDLFQSLHITKGNICLVNKYGEVLFCAQDSESSFV
FSLDLPNLPQFQARSPSAIEIEKASGILGGENLITVSINKKRYLGLVLNKIPIQGTYTLSLVPVSDLIQSALKVPL
NICFFYVLAFLLMWWIFSKINTKLNKPLQELTFCMEAAWRGNHNVRFEPQPYGYEFNELGNIFNCTLLLLLNS
IEKADIDYHSGEKLQKELGILSSLQSALLSPDFPTFPKVTFSSQHLRRRQLSGHFNGWTVQDGGDTLLGIIGLA
GDIGLPSYLYALSARSLFLAYASSDVSLQKISKDTADSFSKTTEGNEAVVAMTFIKYVEKDRSLELLSLSEGAP
TMFLQRGESFVRLPLETHQALQPGDRLICLTGGEDILKYFSQLPIEELLKDPLNPLNTENLIDSLTMMLNNETE
HSADGTLTILSFS

>core/130/2/Org2_Gene837

MRIEDFSLKLIPSSPGVYLMKDVHDQVLYIGKAKNLKNRLASYFHEKGDSRERIPFLMKKTASIETIVVSNETE
ALLLENNLIKQHHPKYNVLLKDDKTFFCLAISLSHSWPKVEAIRTKAITSSQRQLIFGPYVSAEACHTLLEVISQ
WFPLRTCSDREFALRKRPCILYDMKRCLAPCVGYCTPEEYQGTLDKAILFLKGKIEEVVKDLEKVIQKASDNL
EFEQAANYYRTLSLIKQAMAKQQVEKFHFQNIDALGLYRHKQRTILTLLTVRSGKLLGARHFSFFENAQEDQ
DLLSSFILQYYVSQPYIPKEILTPLPLEFPTLSYVLNAESPPRLRSPKTGYGKELLDLAYRNAKAYAATTLPSST
LPYQDFQNILRMSQYPYRIECYDNAHMQGAHATGVYIVFENNGFDPKQYRTFSIDSEKTQNDLALLEEVLLR
RFHSLTTALPDMIVVDGGKTHYNKTKKIIQTLNLTGIQVVTIAKEKSNHSRGLNKEKIFCETFPEGFSLPPTSNL

LQFFQILRDEAHRFAISKHRKKRGKALFEQEKIPGIGEVKRKRLLQKFKSWKQVMLSSQEELEAIPGLTKKDIAVLLARQKDFNKSD

>core/131/2/Org2_Gene304

MKEYKIENIRNFSIIAHIDHGKSTIADRLLESTSTVEEREMREQLLDSMDLERERGITIKAHPVTMTYLYEGEVYQLNLIDTPGHVDFSYEVSRSLSACEGALLIVDAAQGVQAQSLANVYLALERDLEIIPVLNKIDLPAADPVRIAQQIEDYIGLDTTNIIACSAKTGQGIPAILKAIIDLVPPPKAPAETELKALVFDSHYDPYVGIMVYVRIISGELKKGDRITFMAAKGSSFEVLGIGAFLPKATFIEGSLRPGQVGFFIANLKKVKDVKIGDTVTKTKHPAKTPLEGFKEINPVVFAGIYPIDSSDFDTLKDALGRLQLNDSALTIEQESSHSLGFGFRCGFLGLLHLEIIFERIIREFDLDIIATAPSVIYKVVLKNGKVLDIDNPSGYPDPAIIEHVEEPWVHVNIITPQEYLSNIMNLCLDKRGICVKTEMLDQHRLVLAYELPLNEIVSDFNDKLKSVTKGYGSFDYRLGDYRKGSIIKLEVLINEEPIDAFSCLVHRDKAESRGRSICEKLVDVIPQQLFKIPIQAAINKKVIARETIRALSKNVTAKCYGGDITRKRKLWEKQKKGKKRMKEFGKVSIPNTAFIEVLKLD

>core/132/2/Org2_Gene1014

MVMVWSTNIKHEGLKSWIDEVAKLTTPKDIRLCDGSDTEYDELCTLMESTGTMIRLNPEFHPNCFLVRSSADDVARVEQFTFICTSTEAEAGPTNNWRDPQEMRRELHQLFRGCMQGRTLYIVPFCMGPLDSPFSIVGVELTDSPYVVCSMKIMTRMGDDVLRSLGTSGKFLKCLHSVGKPLSPGEADVSWPCNPKSMRIVHFQDDSSVMSFGSGYGGNALLGKKCVALRLASYMAKSQGWLAEHMLIIGITNPEGKKKYFSASFPSACGKTNLAMLMPKLPGWKIECIGDDIAWIRPGRDGRLYAVNPEYGFFGVAPGTSERTNPNALATCRSNSIFTNVALTADGDVWWEGLTEQPPEPLTDWLGKPWKPGGSPAAHPNSRFTAPLRQCPSLDPEWNSPQGVPLDAIIFGGRRSETIPLVYEALSWEHGVTIGAGMSSTTTAAIVGQLGKLRHDPFAMLPFCGYNMAYYFQHWLSFAENRSLKLPKIFGVNWFRKNNQGEFLWPGFSENLRVLEWIFQRTDGLEDIAERTPIGYLPNIQKFNLNGLNLDLQTVQELFSVDAEGWLAEVENIGEYLKIFGSDCPQQITDELLRIKSELKEK

>core/133/2/Org2_Gene630

MAAYTEASILSLASLDHIRLRAGMYIGRLGNGSQKEDGIYTLFKEVVDNGIDEFIMGHGKSLKISASDKQISIQDQGRGIPLGKLIDCVSKINTGAKYTQDVFHFSVGLNGVGLKAVNALSEIFSVRSVRKKKYHLATFHRGVLQESKQGSTKDPDGTFVSFTPDPSIFPEFTFNHDFLKDKIRQYTYLHSGLEIRFNDEVFISHNGLKDLFDAEITEPPLYSPLFFQNEDLTFIFSHLEGNTERYFSFVNGQETLDGGTHLTAFKEAIVKGVNEFFGKTFVSNDIREGIVGCIAIKIASPIFESQTKNKLGNTQIRSSLIKDVKEAIVQALRKDKVAPELLLEKIKFNEKTRKNIQFIKQDLKSKQKKVHYKIPKLRDCKFHYNDRSLYGEASSIFLTEGESASASILASRNPLTQAVFSLRGKPMNVFSLEETKMYKNDELFYLATALGITQNEIQHLRYNKVILATDADVDGMHIRNLLITFFLKTLLPLVENNHLFILETPLFKVRNKTTTLYYYSEQEKMQALQQFGKKDSSLEITRFKGLGEISPKEFAAFIGPEIRLTPVTITSLESISSILQFYMGKNTKERKQFIMDNLITDF

>core/134/2/Org2_Gene36

MKEVEQRIRSLYDAVTAENICRWLSNDCTQQDAKTILGWLDTDPAQLEDLFGATLTFGTGGLRSLMGIGTNRINLFTIRRTTQGLVQVLRAHLPHPGDPMRVVVGCDTRHNSIEFAQETAKVLAGNGCEVFLFQYPEPLALVSFTVRYERAIGGVMITASHNPPNYNGYKVYMASGGQVLPPLDQEIVAACSAVNEILSVPSIDHPNIHLIGKEYEALYRDTLKQLQLYPEANRISGRSLSISYSPLHGTGISLVPHVLKDWGFLSVHLVEKQAIGDGDFPTVQLPNPEDPE

ALTLGIEQMLANDDDLFIATDPDADRVGVVCLEDGQPYRFNGNQMASLLADHILGAWSKTRHLGEHDKLV
KSLVTTEMLSAIAKHYHVDLINVGTGFKYIGEKIESWRNSTNKFVFGAEESYGCLYGTHVEDKDAIIASALIA
EAALQQKLQGKTLCDALLSLYETYGYFANKTESVVFSAKTDEQEIRKKLSHLEEISSANFFSGKYQVEKFENY
KQGIGFNLLSKDSYALTLPKTSMLCYYFSGGGRVIIRPSGTEPKIKFYFEMSTHYPERVTDKEIQKQREAESFQ
HLDDFIFDFKEKFSNL

>core/135/2/Org2_Gene65

MVTVSEQTAQGHVIEAYGNLLRVRFDGYVRQGEVAYVNVDNTWLKAEVIEVADQEVKVQVFEDTQGACR
GALVTFSGHLLEAELGPGLLQGIFDGLQNRLEVLAEDSSFLQRGKHVNAISDHNLWNYTPVASVGDTLRRGD
LLGTVPEGRFTHKIMVPFSCFQEVTLTWVISEGTYNAHTVVAKARDAQGKECAFTMVQRWPIKQAFIEGEKI
PAHKIMDVGLRILDTQIPVLKGGTFCTPGPFGAGKTVLQHHLSKYAAVDIVILCACGERAGEVVEVLQEFPHL
IDPHTGKSLMHRTCIICNTSSMPVAARESSIYLGVTIAEYYRQMGLDILLLADSTSRWAQALREISGRLEEIPGE
EAFPAYLSSRIAAFYERGGAITTKDGSEGSLTICGAVSPAGGNFEEPVTQSTLAVVGAFCGLSKARADARRYP
SIDPLISWSKYLNQVGQILEEKVSGWGGAVKKAAQFLEKGSEIGKRMEVVGEEGVSMEDMEIYLKAELYDF
CYLQQNAFDPVDCYCPFERQIELFSLISRIFDAKFVFDSPDDARSFFLELQSKIKTLNGLKFLSEEYHESKEVIV
RLLEKTMVQMA

>core/136/2/Org2_Gene325

MTAPTESRSSPPTLLEETEPLSPNPIPADIQIPRITISPPSLDVSTVASSAEDISVFIAGGPRSSSSASVASDVYELV
CLCGGDEDPEPPDSEVRTLYVNGSWQTHQEAVQELLYISEVRGEAVRLLYNDGSGMSPWPISPCRTLPTLDH
PLCQALLTVWEQFFSAPENQNREFLVIFYGDASPYIQQALTQSRHSPRIVVVGISPTVFIQGDFRVHNYRVSGD
FFSSLDCRGTRAENTTILPYSSGLEGVFLPSIRCPSFTWAVRFGEQCLVANRGEDVEDRGGLSQDAERSQLPHS
ERDLAVVIDSTDPSSMSRLVEWLNQGSPSSDMEINPYPQRCPDVALSALYAISRVSGLAQEWILASVHEGLDL
QICYSLILMHTTFAVRYFFLLFTNYPQSRERFRTARIVAQSLYLPSILVLVFDCGNVLRKLWMPQEILRAIFISA
STISGSIVFVECTRWMGRGLRHRVQQFVQQRVIGSGLPVGTVRASYRDRAGFIIGFLQTVHGGLYLPVSIMVL
NQIAIQVPRILVRPNNTAVYDLHNKSAEENWSSGDVLAVGQTLNFILCAFVLFVNLWFFVKSVLRHSRRRRR

>core/137/2/Org2_Gene900

MKKKFIFYFVIVFSLLFLWEMTSRHRPTFSFFCPPPSSIASSTLQSLPLLLTSAWHTLKAILGGFFLAITLSIVLAT
IMLSYKSAKDLLQPLFILLQCTPMFALAPLIVLWFGWGIGAVIVPTALTIFFPLTLTIYQGILSTPEELIEQFVLC
GATKFQLLIKLRIPHALPHIFSGLKIAIGSAGFAAIAGEWVASQSGLGILMLESRRNYEMELAFAGLATLSILTL
SLFQITLLIEKLIFSLFRVKRMSLKHKSVAKKALSVLALIPIMLIPWKGNSKSPPDKKNLTSLTLLLDWTPNPNH
IPLYAGVAKGYFKQHGLDLQLQKNTDSSSAVPHVLFEQVDMALYHALGIMKTSIKGMPIQIVGRLIDSSLQGF
LYRSQDPIYKFEDLNGKVLGFCLNNSRDLNRLLETLNRNGVVPSEVKNVSSDLISPMLLNKIDFLYGAFYNIE
GVKLQTLGMPVKCFLSDTCDLPTGPQLIVFTKKGTKASEPEIVEAFQKALQESIIFSKDHPEDAFKLYAKETKS
IPKNLYQEYLQWEETFPLLAQSQDPLSKDLVDKLLETIIKRYPELASEVAKFSLNDLYNPSLPEEQSV

>core/138/2/Org2_Gene839

MYTEESLDNLRHSIDIVDVLSEHIHLKRSGATYKACCPFHTEKTPSFIVNPAGAHYHCFGCGAHGDAIGFLMQ
HLGYSFTEAILVLSKKFQVDLVLQPKDSGYTPPQGLKEELRHINSEAETFFRYCLYHLPEARHALQYLYHRGF
SPDTIDRFHLGYGPEQSLFLQAMEERKISQEQLHTAGFFGNKWFLFARRIIFPVHDALGHTIGFSARKFLENSQ

GGKYVNTPETPIFKKSRILFGLNFSRRRIAKEKKVILVEGQADCLQMIDSGFNCTVAAQGTAFTEEHVKELSK
LGVLKVFLLFDSDEAGNKAALRVGDLCQTAQMSVFVCKLPQGHDPDSFLMQRGSSGLIALLEQSQDYLTFLI
SEKMSSYPKFGPREKALLVEEAIRQIKHWGSPILVYEHLKQLASLMMVPEDMVLSLANPQVTAEPQNIPIKQK
VPKIHPHIVMETDILRCMLFCGSNTKILYTAQFYFVPEDFKHPECRKLFAFMISYYEKYRKNVPFDEACQVLS
DSQILQLLTKRRLNTEALDTIFVQSLQKMADRRWREQCKPLSLNQNIQDKKLEILEDYVQLRKDRTIITLLDPE
SELIP

>core/139/2/Org2_Gene404

MSTSPISNDPRYLSLSNATEKTSLLANSRSLSPVPNSLVPSNPEDTGLRKSIFTHSVTLFAGLVVLLVAVSVVV
VALTVLAPGVPQAILLGIAISGVGIGGFSIMKSLVYMVRDYMSPRMQESSRIKSALAVGTGFTVMGLVMKVG
ANFVPGGYGGLVGSLGSSAYSRGSQTTLASFSHYIYTKFFRSEKVAKGEKLTEAETIKEAKKLHYITLSIATIG
VGLAVLGILLAIAGTVLLGGAPATIAIILAPPLISIGLTTVLQTILHSSIGKWRAFLLTQEKKDLFVDTSLKDIRLE
KLPPSEVEESETSQSVIEVPDSEGIAETRISAEEIDTRLSLTTRQKVIFALATLLLLASIAAFIVTGFGGLTVMQVL
LVASVGSAVASVTLPMVSSGFSYVAYQLKARLNISKLRWKEAKNKKRVRQFLIESGVIASDREFNQMWKTV
YKKQIQKTDAAIREEVRNFEKGGEVNSALVGGILLGVGTGIMLLALVPAFAPIVPGILALGGSTLGIAGSILMR
KFVNWLYDELVKLYERRRNRRELLYGPESKMRSIATDLVVEALAASHDHLFDLDGPVDFIDVDVDIDGAA

>core/140/2/Org2_Gene484

MTNSDNASAAGLLWAHPKEDPAFLGMIIKEFHLPPTVAQIFISRGFQTIQEIHKFLYSHLSSLYDPGLFLDMSK
AVERLLLARDRKEHVMIYGDSDVDGMTGVALLVEFLRDIDVHVSYFFLGAILKQHGETSTLIAKLKEEGITLL
ITVDCGITAGKEVSDITRQGIDVIITDHHMPTGKIPHCVATLNPKLRDHTYPNRELTGVGVAFKLARGVLNALI
SRNLVPKSQGSLKKLLDLVTLGTITDVGVLLGENRVMVRYGIKEIARGARPGLNKLCALCGVEKSEVTSTDIV
LKIAPKLNSLGRLDDPAKGVELLLTQDDERVDALIMELDNINRERQRIEAEVFQDVQEILNSNPEILKQAAIVL
SSTAWHARVIPIISARLAKTYNKPVVIAIQRGIGKGSARTIGSFPLLGVLKKCSSLLLSYGGHDFAAGVIMKED
KVEDFKKKFVHLVNSSLKKGDTLPHLEIDAYADFDAIDYDLLASMELFEPFGKGNLMPIFYSKVRQVRYPKV
LPGNHLKLYLSQKERNLEGVAFGLGRHADALKASWHYPLEIAYTPRLSQTSGSGVIHLLVRDFRISSEPRFSD

>core/141/2/Org2_Gene281

MSFLRRHISLFRSQKQLIDVFAPVSPNLELAEIHRRVIEDQGPALLFHNVIGSSFPVLTNLFGTKHRVDQLFSQA
PDNLIARVAHLISSTPKLSSLWKSRDLLKRISSLGLKKARFRRFPFVSMSSVNLDHLPLLTSWPEDGGAFLTLP
LVYTESPTLTTPNLGMYRVQRFNQNTMGLHFQIQKGGGMHLYEAEQKKQNLPVSVFLSGNPFLTLSAIAPLP
ENVSELLFATFLQGAKLLYKKTNDHPHPLLYDAEFILVGESPAGKRRPEGPFGDHFGYYSLQHDFPEFHCHKI
YHRKDAIYPATVVGKPYQEDFYIGNKLQEYLSPLFPLVMPGVRRLKSYGESGFHALTAAVVKERYWRESLTT
ALRILGEGQLSLTKFLMVTDQEVPLDRFSVVLETILERLQPDRDLIIFSETANDTLDYTGPSLNKGSKGIFMGIG
KAIRDLPHGYQGGKIHGVQDIAPFCRGCLVLETSLEDRCIKSLLHHPDLKSWPLIILADNLRETIQSEKDFLWR
TFTRCAPANDLHALHSHFATHRPNYNFPFVIDALMKPSYPKEVEVDPSTKQKVSERWHAYFPNKETFYI

>core/142/2/Org2_Gene579

MKYRTHRCNELTSNHIGENVQLAGWVHRYRNHGGVVFIDLRDRFGITQIVCREDEQPELHQRLDAVRSEWV
LSVRGKVCPRLAGMENPNLATGHIEVEVASFEVLSKSQNLPFSIADDHINVNEELRLEYRYLDMRRGDIIEKL
LCRHQVMLACRNFMDAQGFTEIVTPVLGKSTPEGARDYLVPSRIYPGKFYALPQSPQLFKQLLMVGGLDRYF

QIATCFRDEDLRADRQPEFAQIDIEMSFGDTQDLLPIIEQLVATLFATQGIEIPLPLAKMTYQEAKDSYGTDKP
DLRFDLKLKDCRDYAKRSSFSIFLDQLAHGGTIKGFCVPGGATMSRKQLDGYTEFVKRYGAMGLVWIKNQE
GKVASNIAKFMDEEVFHELFAYFDAKDQDILLLIAAPESVANQSLDHLRRLIAKERELYSDNQYNFVWITDFP
LFSLEDGKIVAEHHPFTAPLEEDIPLLETDPLAVRSSSYDLVLNGYEIASGSQRIHNPDLQSQIFTILKISPESIQE
KFGFFIKALSFGTPPHLGIALGLDRLVMVLTAAESIREVIAFPKTQKASDLMMNAPSEIMSSQLKELSIKVAF

>core/143/2/Org2_Gene307

MSGKKDGVRGMIFVPLSILVLIFLPLPQILLDFGLCISFALSLLTVCWVFTLNSSNSAKLFPPFFLYLCLLRLGLN
LASTRWIVSSGTASSLIVSLGSFFSLGSLWAATFACLLLFFVNFLMVSKGSERIAEVRSRFFLEALPAKQMALD
SDLVSGRASYKAVKKQKNALIEEGDFFSAMEGVFRFVKGDAIISCILLLVNVVSVTCLYYTSGYALEQMWFT
VLGDALVSQVPALLTSCAAATLISKIDKEESLLNYLFEYYKQLRQHFRVVSLLIFSLCCIPSSPKFPIVLLASLL
WLAYRKEEPASEDSCIERAFSYVEGACPKEQESQFYQVYRAASEEVFEDLGVRLPVLTSLRIEERPWLRVFGQ
NVYLDEMTPEAVLPFLRNIAHEALNAEVVQKYLEESERVFGIAVEDIVPKKISLSSLVVLSRLLVRERVSLKLF
PKILEAVAVYQNSGDSLEILAEKVRKSLGYWIGRSLWDQKQTLEVITIDFHVEELINSSYSKSNPVMQENVIRR
VDSLLERSVFKDFRAIVTSCETRFEMKKMLDPHFPDLLVLSHDELPKEIPISFLGIVSDEVLVP

>core/144/2/Org2_Gene269

MPKQAEYTWGSKKILDNIECLTEDVAEFKDLLYTAHRITSSEEESDNEIQPGAILKGTVVDINKDFVVVDVGL
KSEGVIPMSEFIDSSEGLVLGAEVEVYLDQAEDEEGKVVLSREKATRQRQWEYILAHCEEGSIVKGQITRKVK
GGLIVDIGMEAFLPGSQIDNKKIKNLDDYVGKVCEFKILKINVERRNIVVSRRELLEAERISKKAELIEQISIGEY
RKGVVKNITDFGVFLDLDGIDGLLHITDMTWKRIRHPSEMVELNQELEVIILSVDKEKGRVALGLKQKEHNP
WEDIEKKYPPGKRVLGKIVKLLPYGAFIEIEEGIEGLIHISEMSWVKNIVDPSEVVNKGDEVEAIVLSIQKDEGK
ISLGLKQTERNPWDNIEEKYPIGLHVNAEIKNLTNYGAFVELEPGIEGLIHISDMSWIKKVSHPSELFKKGNSV
EAVILSVDKESKKITLGVKQLSSNPWNEIEAMFPAGTVISGVVTKITAFGAFVELQNGIEGLIHVSELSDKPFA
KIEDIISIGENVSAKVIKLDPDHKKVSLSVKEYLADNAYDQDSRTELDFKDSQGPKERKKKGK

>core/145/2/Org2_Gene721

MSTRRPIQLLDPLTINQIAAGEVIENSVSVVKELIENSLDAGADEIEIETLGGGQGAIIIRDNGCGFRAEDIPIALQ
RHATSKIREFSDIFSLNSFGFRGEALPSIASISKMEIQSSIEGDEGVRTVIHGGDIVSCEPCARQLGTTVIVNSLFY
NVPVRRGFQKSMQSDRLGIRKLIENRILSTANIGWSWISEGHHEIQIAKQQGFQERVAYVMGDHFMQDALTI
DKEANGVRIVGVLGSPSFHRPTRQGQKIFINDRPIESLFISKKVGDAYALLLPLHRYPVFVLKLYLPSSWCDFN
VHPQKIEARILKEELVGDCIKEAIVETLACPPGILCRTHQEIEESDSVPLPMFRMLETSDVQEEESVEFDQNLFA
YSSEDVSLEKQEYTSRGPKSQMDWIYSSDVRFLTSLGRVVLAEDLEGVHIIFTAAARKHLFFLSLMQENSRM
YQSQALLIPLRLQVTPEEAFFFSHHGRTLCDLGIEISQVGPCVFSIESTPTVIGEEELKEWLLLLAARGSTDINSE
ALTALMKETLTQATFSKHQHVFDVSWLKLLWSVGKPEKGFDGARIRRLILDSDFMEG

>core/146/2/Org2_Gene284

MAVSGGGGVQPSSDPGKWNPALQGEQAEGPSPLKESIFSETKQASSAAKQESLVRSGSTGMYATESQINKAK
YRKAQDRSSTSPKSKLKGTFSKMRASVQGFMSGFGSRASRVSAKRASDSGEGTSLLPTEMDVALKKGNRISP
EMQGFFLDASGMGGSSSDISQLSLEALKSSAFSGARSLSLSSSESSSVASFGSFQKAIEPMSEEKVNAWTVARL
GGEMVSSLLDPNVETSSLVRRAMATGNEGMIDLSDLGQEEVSTAMTSPRAVEGKVKVSSSDSPEANPTGIPN

SNTLERAEKEAEKQESREQLSEDQMMLARAMAGLLTGAAPQEVLSNSVWSGPSTVFPPPKFSGTLPTQRSGD
KSKHKSPGIEKSTNHTNFSPLREGTVKSAEVKSLPHPESMYRFPKDSIVSREEPEAVVKESTAFKNPENSSQNF
LPIAVESVFPKESGTGGALGSDAVSSSYHFLAQRGVSLLAPLPRATDDYKEKLEAHKGPGGPPDPLIYQYRNV
AVEPPIVLRSPQPFSGSSRLSVQGKPEAASVHDDGGGGNSGGFSGDQRRGSSGQKASRQEKKGKKLSTDI

>core/147/2/Org2_Gene517

MQKHPSFYQRFLSAYYKNLLASLSWKFFISVALIGIYAPLFASSKPLLVTWHGEIFFPLLRYLFFPGYYTKPVD
LFFNVLMVTFPFFILSFKLTRGWLRRWLLGLCIISQCMIFAWAYSGKVQDPALAENLKKMRAEKVRENISKV
NSEMVMLLPKDTRTWEMERRYMSTYEQLGILIKAKYRKKQEASVKKYQVAFEEKRQSPMPTLRHLEMKNE
GICLKRLQQRVDKMQRPYEMAQQAWNRATDNYRPFLMALTRIEHELRLADYNNWGQPEDLCIAYANVEKR
AEPYKKSLLEIRQVLEDYAKLRSAISFIQDKRLWIEKESEDLRILINPFFSSFHWEDDAGGSREMNKYVPWWQ
LSRVTRKDLLAALVFGIRIALVVAGIGITIALAIGIMIGLVSGYFGGTVDMILSRFTEIWETMPVLFILMLVISIT
QQKSLLLNTVLLGCFSWTGFSRYVRIEVLKQRDRGYVLAATNLGYSHYYIMVHQILPNAIVPVISLVPFAMM
AMISCEAGLTFLGLGEESSASWGNLMREGVTGFPAESAVLWPPAIILTMLLIAIALIGDGVRDALDPRLQDS

>core/148/2/Org2_Gene44

MTDFPTHFKGPKLNPIKVNPNFFERNPKVARVLQITAVVLGIIALLSGIVLIIGTPLGAPISMILGGCLLASGGAL
FVGGTIATILQARNSYKKAVNQKKLSEPLMERPELKALDYSLDLKEVWDLHHSVVKHLKKLDLNLSKTQRE
VLNQIKIDDEGPSLGECAAMISENYDACLKMLAYREELLKEQTQYQETRFNQNLTHRNKVLLSILSRITDNIS
KAGGVFSLKFSTLSSRMSRIHTTTTVILALSAVVSVMVVAALIPGGILALPILLAVAISAGVIVTGLSYLVRQIL
SNTKRNRQDFYKDFVKNVDIELLNQTVTLQRFLFEMLKGVLKEEEEVSLEGQDWYTQYITNAPIEKRLIEEIR
VTYKEIDAQTKKMKTDLEFLENEVRSGRLSVASPSEDPSETPIFTQGKEFAKLRRQTSQNISTIYGPDNENIDPE
FSLPWMPKKEEEIDHSLEPVTKLEPGSREELLLVEGVNPTLRELNMRIALLQQQLSSVRKWRHPRGEHYGNVI
YSDTELDRIQMLEGAFYNHLREAQEEITQSLGDLVDIQNRILGIIVEGDSDSRTEEEPQE

>core/149/2/Org2_Gene45

MANPTQSRPPSPEISIEELELQELAGSSNTETISNTPPPSCAATAEEVSLFIEGGRRNSEDEEGPLGSCEVYDVVC
ITNQGDPEVRDHEVRVMYINGSGRTQHEGILDAMNICDLRGEPVRFIHNSGYGLGSCFLGIRNRIPPRDNVISQ
AIQARWNEFFIFAENANRDYIVLFSGNGGLYLQVALDNSIYSHHILCVGIGSSYYIQGNYRVHNYRVTGDWTT
LLDRRGATAVNTTTLPYADSAEGLFLPSVRCPSYQWALRCGEQCLIMDNNQQVGFRPQDSSSEIALVVNLNQ
DHSTWTRLIEWIDRGDSQAVLELNPQPSHCRDIALTALYATTRISSLLQECLMISVTYAPEVFVTYAIVTGYSI
MTLRYFILLLTNRPGCRRHFRVLRLAALGLQSLGFLTVLLDHINVTRRVNRRPPLISVIFCTASFATGSFIYVDL
TRMFFTSLRSRLQLFVQRRLTGRGLPLRRVFVNHLDSLRFSQNALITFHGGLFMPLIIGFFNQLVIQVPRVVIRP
NTTAVYDLNQTSQEAWDSGDVLAIGQTINFLLCMILLVINTFFFVRSVRRNLHRRPHR

>core/150/2/Org2_Gene668

MFMNTQNSQATEVSSEEESQKKLEELVALAKEQGFITYEEINEILPMSFDTPEQIDQVLIFLTGMDIQVLNQID
VERQKEKKKEAKELEGLARRTEGTPDDPVRMYLKEMGTVPLLTREEEVEISKRIEKAQVQIERIILRFRYSAK
EAISIAHYLISGKERFDKIISEKEVEDKTHFLKLLPKLITLLKEEDTYLENLLLSLKQPDLSKQEAAKLNDSLEK
CRIRTQAYLRCFHCRHNVTEDFGEVVFKAYDSFLHLEQQINDLKVRAERNKFAAAKLAAAKRKLYKREVAA
GRTLEEFKKDVRMLQRWMDKSQEAKKEMVESNLRLVISIAKKYTNRGLSFLDLIQEGNMGLMKAVEKFEY

RRGYKFSTYATWWIRQAVTRAIADQARTIRIPVHMIETINKVLRGAKKLMMETGKEPTPEELAEELGLTPDR
VREIYKIAQHPISLQAEVGEGSESSFGDFLEDTAVESPAEATGYSMLKDKMKEVLKTLTDRERFVLIHRFGLL
DGKPKTLEEVGSAFNVTRERIRQIEAKALRKMRHPIRSKQLRAFLDLLEEEKTGTSKVKSLKSK

>core/151/2/Org2_Gene27

MDTQSSTGNEEWRIAGTSIVSGMALGKVFFLGTSPLHVRELTLPQEEVEHEIHRYYKALNRSKSDIVALEQEV
TGQQGLQEVSSILQAHLEIMKDPLLTEEVVNTIRKDRKNAEYVFSSVMGKIEESLTAVRGMPSVVDRVQDIH
DISNRVIGHLCCQHKSSLGESDQNLIIFSEELTPSEVASANSAYIRGFVSLVGAATSHTAIVSRAKSIPYLANISE
ELWNIAKRYNGKLVLIDGYRGELIFNPKPATLQSCYKKELSVVAHTSQRLVRKSLHPIVSSHAGSDKDVEDLL
ENFPQTSIGLFRSEFLAVILGRLPTLREQVDLYEKLARFPGDSPSVLRLFDFGEDKPCPGIKNKKERSIRWLLDY
SVILEDQLQAIAKASLQGSIKVLIPGVSDVSEIIEVKKKWETIRTRFPKGHKVSWGTMIEFPSAVWMIEEILPEC
DFLSIGTNDLVQYTLGISRESALPKHLNVTLPPAVIRMIHHVLQAAKQNQVPVSICGEAAGQLSLTPLFIGLGV
QELSVAMPVINRLRNHIALLELNSCLEITEALLQAKTCSEVEELLNRNNKITS

>core/152/2/Org2_Gene1015

MVKVPWAFKNFIPKLYTSIKEGYSFNTFKKDFQAGITVGILAFPFAIAIAIGVGVSPIQGLLASIIGGLLASAMG
GSNVLISGPSSAFISILYCLSAKYGAEALFTVTLLAGVFLIAFGLTGLGTFIKYMPYPVVTGLTTGLAIIIFSSQIK
DFLGLQMGANIPADFLPKWIAYWDHLWTWDSKSFAVGLFTLLIMIYFRNYKPRYPGVMIAIVTATTLVWLL
KIDIPTIGSRYGTLPTAIPLPKIPQLSITKILQLMPDALTIAVLSGLETLLSAVVADGMTGWRHQSNCQLVAQGV
ANIGTSLFSGIPVTGSLSRTAASIKSEATTPIAGIVHSIFICFILLLLAPLTVKIPLTCLAAVLILIAWNMSEIHHFIH
LFTAPKKDIVVLLTVFILTVMTTITAAVQVGMMLAAFLFMKQMSDLSDVISTAKYFDKDSDFLSKAEVPQNT
EIYEINGPFFFGIADRLKNLLNDIEKPPKIFILCMTRVPTIDASAMHALEEFFLECDRQGTLLLLAGVKKTPLAD
LKRYHLDELIGVDHIFSNIKSALLFAQALTNLESKTSTRHLV

>core/153/2/Org2_Gene11

MGLFHLTLFGLLLCSLPISLVAKFPESVGHKILYISTQSTQQALATYLEALDAYGDHDFFVLRKIGEDYLKQSI
HSSDPQTRKSTIIGAGLAGSSEALDVLSQAMETADPLQQLLVLSAVSGHLGKTSDDLLFKALASPYPVIRLEA
AYRLANLKNTKVIDHLHSFIHKLPEEIQCLSAAIFLRLETEESDAYIRDLLAAKKSAIRSATALQIGEYQQKRFL
PTLRNLLTSASPQDQEAILYALGKLKDGQSYYNIKKQLQKPDVDVTLAAAQALIALGKEEDALPVIKKQALE
ERPRALYALRHLPSEIGIPIALPIFLKTKNSEAKLNVALALLELGCDTPKLLEYITERLVQPHYNETLALSFSKG
RTLQNWKRVNIIVPQDPQERERLLSTTRGLEEQILTFLFRLPKEAYLPCIYKLLASQKTQLATTAISFLSHTSHQ
EALDLLFQAAKLPGEPIIRAYADLAIYNLTKDPEKKRSLHDYAKKLIQETLLFVDTENQRPHPSMPYLRYQVT
PESRTKLMLDILETLATSKSSEDIRLLIQLMTEGDAKNFPVLAGLLIKIVE

>core/154/2/Org2_Gene426

MKTSQLFYKTSKNANKSAAVLSNELLEKAGYLFKVSKGVYTYTPLLWRVVSKMMNIIREELNAIGGQELLLP
LLHNAELWQHTGRWEAFTSEGLLYTLKDREGKSHCLAPTHEEVICSFVAQWLSSKRQLPLHLYQIATKFRDE
IRPRFGLIRSRELLMEDSYTFSDSPEQMNEQYEKLRSAYSKIFDRLGLAYVIVTADGGKIGKGKSEEFQVLCSL
GEDTICVSGSYGANIEAAVSIPPQHAYDREFLPVEEVATPGITTIEALANFFSIPLHKILKTLVVKLSYSNEEKFI
AIGMRGDRQVNLVKVASKLNADDIALASDEEIERVLGTEKGFIGPLNCPIDFFADETTSPMTNFVCAGNAKD
KHYVNVNWDRDLLPPQYGDFLLAEEGDTCPENPGHPYRIYQGIEVAHIFNLGTRYTDSFEVNFQDEHGQTQQ

CWMGTYGIGVGRTLAACVEQLADDRGIVWPKALAPFSITIAFNGGDTVSQELAETIYHELQSQGYEPLLDDR
DERLGFKLKDSDLIGIPYKLILGKSYQSSGIFEIESRSGEKYTVSPEAFPTWCQNHLA

>core/155/2/Org2_Gene650

MTVSVKKKSFRALVTTHFLTIINDNLYKFLLAFFLLEGKTLTENAKILSCVSFFFALPFLLLAPLAGSLADRFQ
KRNIILATRFIEILCTILGTYFFFIQSVVGGYVVLILMACHTTIFGPAKLGILPEMLPSEQLSQANGIMTAATYTG
SILGSCLAPLLVDVTHRLGVNSYVWPTLMCVIVSIISTLISFCIRPSNVKNVKQKITLVSFKDLWKVLKDTRMI
HYLTVSIFLGSFFLLIGAYTQLEIIPFVEFTLKYPKHYGAYLFPIVALGVGTGSYITGKISGKDIKIGYVPLAAIGL
ALVFMGLYAFACSILFVLFFLLALGFLGGVYQVPLHAYVQYASPEHKRGQILAANNFLDFFGVLVAAGVIRV
LGSNLGLSPETSFFYIGWFVLAVSIWTLWIWREHVYRLLLGIILRRQLGYYLKIHQSSSPKCYFVAVQSYREIR
RVLAALTKTVRSRVIILDQKLVPGWRAWLLSWCVPTVVSSVRDNDSEAQDAWAVLQANHLKTSLKKFPDV
SVVCLGLPKNVERFTSILQEQGIDLHPIQLVQKEGKKRVIYTLVFPHA

>core/156/2/Org2_Gene491

MSTLLSILSVICSQAIAKAFPNLEDWAPEITPSTKEHFGHYQCNDAMKLARVLKKAPRAIAEAIVAELPQEPFS
LIEIAGAGFINFTFSPVFLNQQLEHFKDALKLGFQVSQPKKIIIDFSSPNIAKDMHVGHLRSTIIGDSLARIFSYVG
HDVLRLNHIGDWGTAFGMLITYLQENPCDYSDLEDLTSLYKKAYVCFTNDEEFKKRSQQNVVALQAKDPQA
IAIWEKICETSEKAFQKIYDILDIVVEKRGESFYNPFLPEIIEDLEKKGLLTVSNDAKCVFHEAFSIPFMVQKSDG
GYNYATTDLAAMRYRIEEDHADKIIIVTDLGQSLHFQLLEATAIAAGYLQPGIFSHVGFGLVLDPQGKKLKTR
SGENVKLRELLDTAIEKAEEALREHRPELTDEAIQERAPVIGINAIKYSDLSSHRTSDYVFSFEKMLRFEGNTA
MFLLYAYVRIQGIKRRLGISQLSLEGPPEIQEPAEELLALTLLRFPEALESTIKELCPHFLTDYLYNLTHKFNGFF
RDSHIQDSPYAKSRLFLCALAEQVLATGMHLLGLKTLERL

>core/157/2/Org2_Gene478

MSKLIRRVVTVLALTSMASCFASGGIEAAVAESLITKIVASAETKPAPVPMTAKKVRLVRRNKQPVEQKSRG
AFCDKEFYPCEEGRCQPVEAQQESCYGRLYSVKVNDDCNVEICQSVPEYATVGSPYPIEILAIGKKDCVDVVI
TQQLPCEAEFVSSDPETTPTSDGKLVWKIDRLGAGDKCKITVWVKPLKEGCCFTAATVCACPELRSYTKCGQ
PAICIKQEGPDCACLRCPVCYKIEVVNTGSAIARNVTVDNPVPDGYSHASGQRVLSFNLGDMRPGDKKVFTV
EFCPQRRGQITNVATVTYCGGHKCSANVTTVVNEPCVQVNISGADWSYVCKPVEYSISVSNPGDLVLHDVVI
QDTLPSGVTVLEAPGGEICCNKVVWRIKEMCPGETLQFKLVVKAQVPGRFTNQVAVTSESNCGTCTSCAETT
THWKGLAATHMCVLDTNDPICVGENTVYRICVTNRGSAEDTNVSLILKFSKELQPIASSGPTKGTISGNTVVF
DALPKLGSKESVEFSVTLKGIAPGDARGEAILSSDTLTSPVSDTENTHVY

>core/158/2/Org2_Gene129

MELLSLNKSYFEIQRLRYRPEILTLLETIRSKHIQETSSPPSPPPELQKHIPNLCRIPEVSIYTEQETSSKPLKIGVL
LSGGQAPGGHNVVIGLFDALRVFNPKTRLFGFIKGPLGLTRGLYKDLDISVIYDYYNMGGFDMLSSSREKIKT
EEQKKNILNTVKQLKLDGLLIIGGNNSNTDTAMLAEYFLAHNCKTSVIGVPKTIDGDLKNCWIETSLGFHTSC
RTYSEMIGNLAKDALSAKKYHHFIRLMGQQASYTTLECGLQTLPNIALISELIATRKISLKQLSEQLALGLVRR
YKSGKNYSTVLIPEGLIEHIFDTRKLIDELNVLLANGDSSIEKILSKLSPETLKTFHLFPKDIANQLLLARDSHGN
VRVSKIATEELLAVMVKKEIEKIKPHMEFHSVSHFFGYEARAGFPSNFDCNYGIALGIISALFLVRQKTGYMIT

INNLAQSYTEWQGGATPLYKMMHLENRCGTETPVIKTDSVDPKSPAVQHLLQQSDSCLVEDLYRFPGPLQYF
GKEELIDQRPLTLLWENQTHSPFQALYSTSGKRSL

>core/159/2/Org2_Gene944

MSSPPQAVASLTERIKTLLESNFCQIIVKGELSNVSLQPSGHLYFGIKDSQAFLNGAFFHFKSKYYDRKPKDGD
AVIIHGKLAVYAPRGQYQIVAHALVYAGEGDLLQKFEETKRRLTAEGYFATEKKKPLPFAPQCIGVITSPTGA
VIQDILRVLSRRARNYKILVYPVTVQGNSAAHEISKAIEVMNAENLADVLIIARGGGSIEDLWAFNEEILVKAI
HASTIPIVSAVGHETDYTLCDFASDVRAPTPSAAAEIVCKSSEEQVQVFEGYLRHLLSHSRQLLTSKKQQLLP
WRRFLDRAEFYTTAQQQLDSIEIAIQKGVQGKIHESKQRYDNISRWLQGDLVSRMTCRLQSLKKMLSQALSH
KALSLQVRCHQLKKSLTYPRQIQQASQKLSPWRQQLDTLISRRLHYQKEEYFHKHTRLKHAHNVLEQQLRS
HVQKLELLGRRLSRGCELNLQNQKIAYANVKETLATILERRYENSVARYSALKEQLHSLNPKNVLKRGYAM
LFDFNENSAMISVDSLQENARVRIQLQDGEAILTVTNIEICKLIKG

>core/160/2/Org2_Gene810

MDNSDNSFHTLETEQGSFLNDELAVEEVASTESTEISDATLCFAEKKVAFILNKMREALTGSSQGSDLRLFWD
LRKQCLPLFNEIEDTAKRADHWRCYIELTKEGRHLKGLQDEEGSFVVGQIDLAITCLEKDILKFQEGTEDKIFK
DREDNFLESQALDKHQAFYKQHHTSLLWLSSFSSKIIDLRKELINVGMRMRLKSKFFQRLSNLGNQVFPKRK
ELIEKVSQTFAEDVDAFVAKYFIGSDKETLKKTVFFLRKEIKNLQHAAKRLFVSSHVFAETRLKLSKCWDQLK
GMEKEIRQEQGRLRVVSAENSKEVRQMLAEVSSLLIEGNDLSKVRKDLEGISKKIRALDLTHDDVISLKKEM
QQLFDQLREKQDAAEHSYQEQLAKDKQVKKEAARSLAERITTFSKTCSEGNITSESREEWQTLKELLGKMSF
LPPPEKISLDNQLNLALQTIVNFFEEQLLSSPDSREKLVNMRQVLKQRRERRQELKDKLEQDKKLLGSSGLDF
DRAMQYSALVEEDKRALEELDASILELKQQIQQLL

>core/161/2/Org2_Gene97

MPQKVLITSALPYANGPLHFGHIAGVYLPADVYARFRRLLGDDVLYICGSDEFGIAITLNADREGLGYQEYV
DMYHKLHKDTFEKLGFALDFFSRTTNPFHAELVQDFYSQLKASGLIENRISEQLYSEQEQRFLADRYVEGTCP
RCGFDHARGDECQSCGADYEAIDLIGPKSKISGVELVKKETEHSYFLLDRMKDALLSFIQGCYLPDHVRKFV
VDYIEHVRSRAITRDLSWGIPVPDFPGKVFYVWFDAPIGYISGTMEWAASQGNPDEWKRFWLEDGVEYVQFI
GKDNLPFHSVVFPAMELGQKLDYKKVDALVVSEFYLLEGRQFSKSEGNYVDMDKFLSSYSLDKLRYVLAAT
APETSDSEFTFLDFKTRCNSELVGKFGNFINRVLAFAEKNHYDKLSYHSVVLEDSDRAFLEEARQLVRDAEK
CYREYSLRKATSVIMSLAALGNVYFNQQAPWKLLKEGTRERVEAILFCACYCQKLLALISYPIIPESAVAIWE
MISPKSLENCNLDTMYARDLWKEEILDVINEEFHLKSPRLLFTTVE

>core/162/2/Org2_Gene171

MHPLYVDLDTIISSYSPPLPKEFQEAASLIAVPDTSHSKPVVPGVKTLFPQTYHLPYLKFVQGENVVHTPLKVG
VMFSGGPAPGGHNVIQGLFNSLKDFHPDSSLVGFVNNGDGLTNNKSIDITEEFLSKFRNSGGFNCIGTGRKKIV
TPEAKEACLKTAEALDLDGLVIIGGDGSNTATAILAEYFAKRRPKTSIVGVPKTIDGDLQHTFLDLTFGFDTAT
KFYSSIISNISRDALSCKAHYHFIKLMGRSASHIALECALQTHPNIALIGEEIAEKNLPLKTIIHKICSVIADRAAM
EKYYGVILIPEGIIEFIPEIINLITEIESLSEYEDKISRLSPESQRLLKSFPAPIIEQILNDRDAHGNVYVSKISVDKLL
IHLVSNHLQQYFPNVPFNAISHFLGYEGRSGLPTKFDNTYGYSLGYGAGILVRNHCNGYLSTIESLACPFMKW

KLRAIPVVKMFTVKQQADGTLQPKIKKYLVDIGSTAFRKFKLYRKIWALEDSYRFLGPLQIETPPEMHSDNFP
PLTLLLNHNFWQRHQGCIEIPDTTY

>core/163/2/Org2_Gene645

MSRKDNEVSLARSIFNILSGTFCSRITGIFREIAMATYFGADPIVAAFWLGFRTVFFLRKILGGLILEQAFIPHFE
FLRAQSLDRAAFFFRRFSRLIKGSTIIFTLLIEAVLWVVLQYVEEGTYDMILLTMILLPCGIFLMMYNVNGALL
HCENKFFGVGLAPVVVNIIWIFFVIAARHSDPRERIIGLSVALVIGFFFEWLITVPGVWKFLLEAKSPPQEHDSV
RALLAPLSLGILTSSIFQLNLLSDICLARYVHEIGPLYLMYSLKIYQLPIHLFGFGVFTVLLPAISRCVQREDHER
GLKLMKFVLTLTMSVMIIMTAGLLLLALPGVRVLYEHGLFPQSAVYAIVRVLRGYGASIIPMALAPLVSVLFY
AQRQYAVPLFIGIGTALANIVLSLVLGRWVLKDVSGISYATSITAWVQLYFLWYYSSKRLPMYSKLLWESIRR
SIKVMGTTMLACMITLGLNILTQTTYVIFLNPLTPLAWPLSSITAQAIAFLSESCIFLAFLFGFAKLLRVEDLINL
ASFEYWRGQRGLLQRQHVMQDTQN

>core/164/2/Org2_Gene107

MAAKNIKYNEEARKKIHKGVKTLAEAVKVTLGPKGRHVVIDKSFGSPQVTKDGVTVAKEIELEDKHENMGA
QMVKEVASKTADKAGDGTTTATVLAEAIYSEGLRNVTAGANPMDLKRGIDKAVKVVVDELKKISKPVQHH
KEIAQVATISANNDSEIGNLIAEAMEKVGKNGSITVEEAKGFETVLDVVEGMNFNRGYLSSYFSTNPETQECV
LEDALILIYDKKISGIKDFLPVLQQVAESGRPLLIIAEEIEGEALATLVVNRLRAGFRVCAVKAPGFGDRRKAM
LEDIAILTGGQLVSEELGMKLENTTLAMLGKAKKVIVTKEDTTIVEGLGNKPDIQARCDNIKKQIEDSTSDYD
KEKLQERLAKLSGGVAVIRVGAATEIEMKEKKDRVDDAQHATIAAVEEGILPGGGTALVRCIPTLEAFLPML
ANEDEAIGTRIILKALTAPLKQIASNAGKEGAIICQQVLARSANEGYDALRDAYTDMIDAGILDPTKVTRSALE
SAASIAGLLLTTEALIADIPEEKSSSAPAMPSAGMDY

>core/165/2/Org2_Gene416

MFGSESLRYQLLIQDFAKVSEEGIGLLESKEYSLLQAKLVLRALAQNSSFDDWFRSFKKCQISYPELAHDRDV
LEEFGIQVLREGIENPSVTVRAVSVLAIGLARDFRLVPLLLQSCNDDSAIVRSLALQVAVNYGSESLKKAIVEL
ARNDDSIHVRITAYQVVALLQIEELLPFLRERAENKLVDSVERREAWKACLELSSQFLETGVAKDDIDQALFT
CEVLRNGMLPETTEIFTELLSVEHPEVQESLLLSALAWSHQLQNHKEFLSKVRHVMCTSPFAKVRFQAAALL
HLHGDPLGRDSLVEGLRSPQPLVCEAASAALCSLGIHGVPLAKEHLESLSSRKAAANLSILLLVSREDIERAGD
VIARYLSNPEMCWAIEYFLWDAQWNLRGDTFPLYSDMIKREIGRKLIRLLAVARYSQAKAVTATFLSGQQA
QGWSFFSGMFWEEGDVKTSEDLVTDACFAAKLEGALASLCQKKDQASLQRVSQLYNDSRWQDKLAILESV
AFSENLDAVPFLLDCCHHEAPSLRSAAAGALFSIFK

>core/166/2/Org2_Gene571

MLRIFCFVISWCLIAFAQPDLSGFVSILGAACGYGFFWYSLEPLKKPSLPLRTLFVSCFFWIFTIEGIHFSWMLS
DQYIGKLIYLVWLTLITILSVLFSGFSCLLVAIVRQKRTAFLWSLPGVWVAIEMLRFYGIFSGMSFDYLGWPM
TASAYGRQFGGFLGWAGQSFAVIAVNMSFYCLLLKKPHAKMLWVLTLLLPYTFGAIHYEYLKHAFQQDKR
ALRVAVVQPAHPPIRPKLKSPIVVWEQLLQLVSPIQQPIDLLIFPEVVVPFGKHRQVYPYESCAHLLSSFAPLPE
GKAFLSNSDCATALSQHFQCPVIIGLERWVKKENVLYWYNSAEVISHKGISVGYDKRILVPGGEYIPGGKFGS
LICRQLFPKYALGCKRLPGRRSGVVQVRGLPRIGITICYEETFGYRLQSYKRQGAELLVNLTNDGWYPESRLP

KVHFLHGMLRNQEFGMPCVRACQTGVTAAVDSLGRILKILPYDTRETKAPSGVLETSLPLFNYKTLYGYCGD
YPMILIAFCAVSYLGGGFLGYRLLAKKEIR

>core/167/2/Org2_Gene533

MQSSEVKPFSRLRAYLCPIYKSEFSKFVPLFLLAFFVGFNYCLLKNMKDTLVIVGSDAGAEVIPFLKVWGIVP
GAVIVTMVYGWLGSRYPRDTVFYCFMAAFLGFFFLFAVIIYPVGDSLHLNSLADKLQELLPQGLRGFIVMVR
YWSYSIYYVMSELWSSVVLSMLFWGLANQITTITEAGRFYALINTGLNLSSICAGEISYWMGKQTFVAYSFA
CDSWHSVMLNLTMLITCSGLIMIWLYRRIHHLTIDTSIPPSRRVLAEEGAATANLKEKKKPKAKARNLFLHLI
QSRYLLGLAIIVLSYNLVIHLFEVVWKDQVSQIYSSHVEFNGYMSRITTLIGVVSVLAAVLLTGQCIRKWGWT
VGALVTPLVMLVSGLLFFGTIFAAKRDISIFGGVLGMTPLALAAWTGGMQNVLSRGTKFTFFDQTKEMAFIP
LSPEDKNHGKAAIDGVVSRIGKSGGSLIYQGLLVIFSSVAASLNVIALVLLIIMVVWIAVVAYIGKEYYSRAAD
AVATLKQPKEPSSSIVREAQESVEQEEMAVL

>core/168/2/Org2_Gene819

MHDQRNRGHNNHNLRLRPGSTLLEAFLILCSEHEEGIACFDEHLGSLSYRELRNAIIAVAIKVSKFSEDRVGV
MMPASIGAFIAYFGILLAGKTPVMMNWSQGLRELRACTKTVEVRRVLTSQQFIKHLTEVQGFVEYPFDLMY
MEDVRKRLSWWEKCRIGLYSKCSVPWLLRIFGVSGVESDDTAVILFTSGTEKLPKAVPLTHKNLMENQEACL
KFFDPNTQDVMLAFLPPFHAYGFNSCGLFPLLMGVHVVFASNPLNPKKLVEFIDDKKVTFFGSTPVFFDYILK
TAKKQNSCLESLRLVVIGGDALKDTLYEETKKLQPQIALYQGYGATECSPVISITTKESPRKSECVGMPIEGM
DVLIISKETHIPVSSGEQGLIVVRGNSVFSGYLGNHEHQSFVSLGGDQWYLTGDLGHIGPSGDLFLEGRLSRFV
KIGGEMVSLEALESILHEHFTENQNEDAGSLVVCGIPGDKVRLCLFTTLATTIHEVNDILKSAETSSIVKISYVH
QVESIPILGIGKPDYVSLNALAVSLFG

>core/169/2/Org2_Gene197

MPFKCIFLTGGVVSSLGKGLTAASLALILERQRLNVAMLKLDPYLNVDPGTMNPFEHGEIYVTDDGVETDLD
LGHYHRFSSAALSRHSSATSGQIYARVIKREREGDYLGSTVQVIPHITNEIIQVILDAAKEHSPDVLIVEIGGTIG
DIESLPFLEAIRQFRYDHSEDCLNIHMTYVPYLQAADEVKSKPTQHSVQTLRGIGIIPDAILCRSEKPLTQEVKS
KISLFCNVPNRAVFNVIDVKHTIYEMPLMLAQEKIANFIGEKLKLATVPENLDDWKVLVNQLSQDLPKVKIG
VVGKYVQHRDAYKSIFEALTHAALRLGHAAEIIPIDAEDENLTMELSQCDACLVPGGFGVRGWEGKIAAAKF
CREQGIPYFGICLGMQVLVVEYARNVLNLDQANSLEMDPNTPHPIVYVMEGQDPLVATGGTMRLGAYPCLL
KPGSKAHKAYNESSLIQERHRHRYEVNPDYIQSLEDHGLRIVGTCPPQGLCEIIEVSDHPWMIGVQFHPEFVSK
LISPHPLFIAFIEAALVYSKDASHV

>core/171/2/Org2_Gene161

MRKISVGICITILLSLSVVLQGCKESSHSSTSRGELAINIRDEPRSLDPRQVRLLSEISLVKHIYEGLVQENNLSG
NIEPALAEDYSLSSDGLTYTFKLKSAFWSNGDPLTAEDFIESWKQVATQEVSGIYAFALNPIKNVRKIQEGHLS
IDHFGVHSPNESTLVVTLESPTSHFLKLLALPVFFPVHKSQRTLQSKSLPIASGAFYPKNIKQKQWIKLSKNPH
YYNQSQVETKTITIHFIPDANTAAKLFNQGKLNWQGPPWGERIPQETLSNLQSKGHLHSFDVAGTSWLTFNIN
KFPLNNMKLREALASALDKEALVSTIFLGRAKTADHLLPTNIHSYPEHQKQEMAQRQAYAKKLFKEALEELQ
ITAKDLEHLNLIFPVSSSASSLLVQLIREQWKESLGFAIPIVGKEFALLQADLSSGNFSLATGGWFADFADPMA

FLTIFAYPSGVPPYAINHKDFLEILQNIEQEQDHQKRSELVSQASLYLETFHIIEPIYHDAFQFAMNKKLSNLGV
SPTGVVDFRYAKEN

>core/172/2/Org2_Gene659

MLGKEEEFTCKQKQCLSHFVTNLTSDVFALKNLPEVVKGALFSKYSRSVLGLRALLLKEFLSNEEDGDVCDE
AYDFETDVQKAADFYQRVLDNFGDDSVGELGGAHLAMENVSILAAKVLEDARIGGSPLEKSTRYVYFDQKV
RGEYLYYRDPILMTSAFKDMFLGTCDFLFDTYSALIPQVRAYFEKLYPKDSKTPASAYATSLRAKVLDCIRGL
LPAATLTNLGFFGNGRFWQNLIHKLQGHNLAELRRLGDESLTELMKVIPSFVSRAEPHHHHHQAMMQYRRA
LKEQLKGLAEQATFSEEMSSSPSVQLVYGDPDGIYKVAAGFLFPYSNRSLTDLIDYCKKMPHEDLVQILESSV
SARENRRHKSPRGLECVEFGFDILADFGAYRDLQRHRTLTQERQLLSTHHGYNFPVELLDTPMEKSYREAME
RANETYNEIVQEFPEEAQYMVPMAYNIRWFFHVNARALQWICELRSQPQGHQNYRTIATGLVREVVKFNPM
YELFFKFVDYSDIDLGRLNQEMRKEPTT

>core/173/2/Org2_Gene962

MFVGGLVSFLLPIPDLECANNVTKTYDKKASVISRDLKLQEDCQKFWNLDPYKLESLCAYQVLYHDDYSSK
RIRELFPQIQKDEVPIFATMILTLGKVDRGFSPEEISLIQKLSYPGLSLASLRGSTEIDPNTDLARALVVSEFSGD
LGKNRADYYSNCLDILALRIHAERQRYLDQSPCVPGTSEFHKATIEAINTILFYEEAVRYPSKKEMFSDEFSFL
SSVTDRKFGVCLGVSSLYFSLSQRLDLPLEAVTPPGHIYLRYQGGEVNIETTAGGRHLPTASYCDCLDLEDLQ
VRTPEEMIGLTFMNQGSFALQKKKYKEAEEAYKKAQEYLGDEELQELLGFVQILGGKKKEGKSLIGKSPRAS
QKGSVAYDYLKGRINIPTLALLFSYPGSNYEEIASYEEELKKAMKSSMPCCEGQRRLASVAFHLGKTAEAVA
LLEKCVEDIPNDLSLHLRLCKILCDRHEYTKALKYFIIAERLMEDQGFLKKDNRSFALFYEVKKIISKVAPQKA
NTLLLMESER

>core/174/2/Org2_Gene13

MSIVLDKIGKSLGTRILFDDVSVVFNPGNCYGLTGPNGAGKSTLLKIIMGMIEPTRGSISLPKKVGILRQNIDSF
HDTTVLDCVIMGNTRLWEALQRRDNLYLQEFTDAIGMELGEIEEIIGEENGYRADSEAEELLTGIGIPNEMFD
KKMAMIPIDLQFRVLLCQALFGHPEALLLDEPTNHLDLYSINWLGNFLKDYEGTVIVVSHDRHFLNTITTHIA
DIDYDTIIIYPGNYDDMVEMKTASREQEKADIKSKEKKISQLKEFVAKFGAGSRASQVQSRLREIKKLQPQEL
KKSNIQRPYIRFPLSDKSSGKVVLSLEAITKDYGDHQVIHPFSLEIYQGDKLGIIGNNGLGKTTLMKLLAGVEA
PSSGSIKLGHQAICSYFPQNHSDVLADCGQETLFEWLRNRKTGINDQEIRSVLGKMLFGGDDAFKQIQALSGG
ETARLLMAGMMLENHNVLILDEANNHLDLESVSALSWAINDYKGTAIFVSHDRGLIQDCATKLLIFDKDKIT
FFDGTMVDYTAGHKQLL

>core/175/2/Org2_Gene961

MKMHRLKPTLKSLIPNLLFLLLTLSSCSKQKQEPLGKHLVIAMSHDLADLDPRNAYLSRDASLAKALYEGLT
RETDQGIALALAESYTLSKDHKVYTFKLRPSVWSDGTPLTAYDFEKSIKQLYFEEFSPSIHTLLGVIKNSSAIHN
AQKSLETLGIQAKDDLTLVITLEQPFPYFLTLIARPVFSPVHHTLRESYKKGTPPSTYISNGPFVLKKHEHQNYL
ILEKNPHYYDHESVKLDRVTLKIIPDASTATKLFKSKSIDWIGSPWSAPISNEDQKVLSQEKILTYSVSSTTLLIY
NLQKPLIQNKALRKAIAHAIDRKSILRLVPSGQEAVTLVPPNLSQLNLQKEISTEERQTKARAYFQEAKETLSE
KELAELSILYPIDSSNSSIIAQEIQRQLKDTLGLKIKIQGMEYHCFLKKRRQGDFFIATGGWIAEYVSPVAFLSIL

GNPRDLTQWRNSDYEKTLEKLYLPHAYKENLKRAEMIIEEETPIIPLYHGKYIYAIHPKIQNTFGSLLGHTDLKNIDILS

>core/176/2/Org2_Gene914

MPGSVSSPPLSPVIVRERVPSSSGSDLIQPHAVLKISILIFALVTILGIVLVVLSSALGALPSLVLTVSGCIAIAVGLIGLGILVTRLILSTIRKVDAMGYDAAVKEEQYLSRIRELESENREIRDRNRAVEDQCAHLSEENKDLRDPEYLHGMTERLIASLEIENQALVAENILLKDWNASLSRDFRAYKQKFPLGALEPWKEDIACIMEQNLFLKPECIAMVKSLPLETQRLFLYPKGFQSLVNRFAPRSRFFQTPKYEYNSRNENEDGKVAAVCARLKKEFFSAVLGACSYEELGGICERAVALKETLPLPEAVYDTLVQEFPNLLTAESLWKEWCFYSYPYLRPYLSVDYCKRLFVQLFEELCLKLFTTGSPEDQALVRLFSYYRNHIPAVLASFGLPPPETGGSVFVLLPKQENLLWSQIEVLATRYLKDTFVRNSEWTGSFEMMFSYNEMCKEISEGRIRFAEDYETRHSEEFPPSPLSEEGEGEEFLPPCSEEEVSVLERPDLDVDSMWVWHPPVPKGPL

>core/177/2/Org2_Gene828

MTARAEYLDHEDFLYRSHKLQELSELGVVLYPYEFPGVFSCEDIKKTFASQELGNSEAAMSRSTPRVRFAGRLVLFRAMGKNAFGQILDHNQTIQVMFNREFTSVHGLSEDAEITPIKFIEKKLDLGDILGIDGYLFFTHSGELTVLVETVTLLCKSLLSLPDKHAGLSDKEVRYRKRWLDLISSREVSDTFVKRSYIIKLIRNYMDAHGFLEVETPILQNIYGGAEAKPFTTTMEALHSEMFLRISLEIALKKILVGGAPRIYELGKVFRNEGIDRTHNPEFTMIEAYAAYMDYKEVMVFVENLVEHLVRAVNHDNTSLVYSYWKHGPQEVDFKAPWIRMTMKESIATYAGIDVDVHSDQKLKEILKKKTTFPETAFATASRGMLIAALFDELVSDNLIAPHHITDHPVETTPLCKTLRSGDTAFVERFESFCLGKELCNAYSELNDPIRQRELLEQQHTKKELLPDSECHPIDEEFLEALCQGMPPAGGFGIGVDRLVMILTNAASIRDVLYFPVMRRFDAEKTN

>core/178/2/Org2_Gene162

MLRFFAVFISTLWLITSGCSPSQSSKGIFVVNMKEMPRSLDPGKTRLIADQTLMRHLYEGLVEEHSQNGEIKPALAESYTISEDGTRYTFKIKNILWSNGDPLTAQDFVSSWKEILKEDASSVYLYAFLPIKNARAIFDDTESPENLGVRALDKRHLEIQLETPCAHFLHFLTLPIFFPVHETLRNYSTSFEEMPITCGAFRPVSLEKGLRLHLEKNPMYHNKSRVKLHKIIVQFISNANTAAILFKHKKLDWQGPPWGEPIPPEISASLHQDDQLFSLPGASTTWLLFNIQKKPWNNAKLRKALSLAIDKDMLTKVVYQGLAEPTDHILHPRLYPGTYPERKRQNERILEAQQLFEEALDELQMTREDLEKETLTFSTFSFSYGRICQMLREQWKKVLKFTIPIVGQEFFTIQKNFLEGNYSLTVNQWTAAFIDPMSYLMIFANPGGISPYHLQDSHFQTLLIKITQEHKKHLRNQLIIEALDYLEHCHILEPLCHPNLRIALNKNIKNFNLFVRRTSDFRFIEKL

>core/179/2/Org2_Gene279

MNVLKYTKHSPSAHAWKLIGTSPKHGIYLPLFSIHTKNSCGIGEFLDLIPLISWCQKQGFSVIQLLPLNDTGEDTSPYNSISSVALNPLFLSLSSLPNIDTIPEVAKKLQDMHELCSTPSVSYTQVKEKKWAFLREYYQKCCKSSLEGNSNFSEFLESERYWLYPYGTFRAIKHHMHGEPINNWPKSLTDQENFPDLTKKFHDEVLFFSYLQFLCYQQLCEVKAYADQHHVLLKGDLPILISKDSCDVWYFRDYFSSSRSVGAPPDLYNSEGQNWHLPIYNFSQLAKDDYIWWKERLRYAQNFYSVYRLDHIIGFFRLWIWDSSGRGRFIPDNPKDYIKQGTEILSTMLGASSMLPIGEDLGIIPQDVKTTLTHLGICGTRIPRWERNWESDSAFIPLKDYNPLSVTTLSTHDSDTFAQWWLNSPKEAKQFAKFLHLPFQK

TLTTETQIDILKLSHESASIFHINLFNDYLALCPDLVSKNLQRERINTPGTISKKNWSYRVRPSLEELAIHKKFN
GYIEKILTGL

>core/180/2/Org2_Gene912

MERKRFIDCDSTKILQELALNPLDLTAPGVLSAERIKKFSLLGGGFTFSFATERLDDAILAALISLAEERGLHES
MLAMQQGQVVNYIEGFPSEMRPALHTATRAWVTDSSFTGEAEDIAVRSRVEAQRLKDFLTKVRSQFTTIVQI
GIGGSELGPKALYRALRAYCPTDKHVHFISNIDPDNGAEVLDTIDCAKALVVVVSKSGTTIETAVNEAFFADY
FAKKGLSFKDHFIAVTCEGSPMDDTGKYLEVFHLWESIGGRFSSTSMVGGVVLGFAYGFEVFLQLLQGASA
MDQIALQPNARENLPMLSALISIWNRNFLGYPTEAVIPYSSGLEFFPAHLQQCCMESNGKSIAQDGRRVGFST
SPVIWGEPGTNGQHSFFQCLHQGTDIIPVEFIGFEKSQKGEDISFQGTTSSQKLFANMIAQAIALACGSENTNPN
KNFDGNRPSSVLVSSQLNPYSLGELLSYYENKIVFQGLCWGINSFDQEGVSLGKALANRVLELLEGADASNFP
EAASLLTLFNIKFR

>core/181/2/Org2_Gene298

MTKTEEKPFGKLRSFLWPIHTHELKKVLPMFLMFFCITFNYTVLRDTKDTLIVGAPGSGAEAIPFIKFWLVVPC
AIIFMLIYAKLSNILSKQALFYAVGTPFLIFFALFPTVIYPLRDVLHPTEFADRLQAILPPGLLGLVAILRNWTFA
AFYVLAELWGSVMLSLMFWGFANEITKIHEAKRFYALFGIGANISLLASGRAIVWASKLRASVSEGVDPWGI
SLRLLMAMTIVSGLVLMASYWWINKNVLTDPRFYNPEEMQKGKKGAKPKMNMKDSFLYLARSPYILLLAL
LVIAYGICINLIEVTWKSQLKLQYPNMNDYSEFMGNFSFWTGVVSVLIMLFVGGNVIRKFGWLTGALVTPVM
VLLTGIVFFALVIFRNQASGLVAMFGTTPLMLAVVVGAIQNILSKSTKYALFDSTKEMAYIPLDQEQKVKGK
AAIDVVAARFGKSGGALIQQGLLVICGSIGAMTPYLAVILLFIIAIWLVSATKLNKLFLAQSALKEQEVAQEDS
APASS

>core/182/2/Org2_Gene689

MVWVFKSQFEGLSALKRGVHALTKAVTPAFGPRGYNVVIKKGKAPIVLTKNGIRIAKEIILQDAFESLGVKLA
KEALLKVVEQTGDGSTTALVVIDALFTQGLKGIAAGLDPQEIKAGILLSVEMVYQQLQRQAIELQSPKDVLH
VAMVAANHDVTLGTVVATVISQADLKGVFSSKDSGISKTRGLGKRVKSGYLSPYFVTRPETMDVVWEEALV
LILSHSLVSLSEELIRYLELISEQNTHPLVIIAEDFDQNVLRTLILNKLRNGLPVCAVKAPGSRELRQVVLEDLAI
LTGATLIGQESENCEIPVSLDVLGRVKQVMITKETFTFLEGGGDAEIIQARKQELCLAIARSTSESECQELEERL
AIFIGSIPQVQITADTDTEQRERQFQLESALRATKAAMKGGIVPGGGVAFLRAAHAIEVPANLSSGMTFGFETL
LQAVRTPLKVLAQNCGRSSEEVIHTILSHENPRFGYNGMTDTFEDLVDAGICDPLIVTTSSLKCAVSVSCLLLT
SSFFISSRTKT

>core/183/2/Org2_Gene8

MYQENLRLLERLLYNSVQKSYADRLFSYEKTKMVHDTPLIPWEEDKEKCAEAEKAFLEQQKILLDYGKSIFW
LNENDEINLNDPWSWGLNTVRTRKVFQEVDDSERWNHKVLIQKLEDDYEKLLEESSKESTEANKKLLSDLV
DRLEDAKTKFFLKKQEEVETRVKDLRARYGGTVDPKQDTEAKKKVELEASLETFLDSIESELVQCLEDQDIY
WKEQDVKDLARTQELEEQDIEAKREEAAEDLRSLNERLKKSKTMLDRAKWHIENAEDSITWWTSQIEMKD
MKARLKILKEDITSVLPEIDEIETCLSLEELPLLTTRELLTKSYLKFKICSETLLKMTSVFENNIYVQEYEVQLQ
NLGFKLQGISQRFGKKQDDFANLEEQVALQKKRLRELTQNFEIQGFNFMKEDFKAAAKDLYIRSTAEQKMN

FDVPCMELFRRYHEEVNKPLLELMYNCADSYRDAKKKLCSLRLDEKELLQKEIKKEEFYQKKQQRHADRSR
HTTYQKLRIAEELALELKKKI

>core/184/2/Org2_Gene440

MVARGLCDFPTLVPNERLPIGPFFVPQHTSGAKGKEFAKRNFSIISGLDDILKLCILQRRPFALQWDNLSVKSD
YEEAGPAIGIRSLEPQVSQISPAHGRLCSTLVQWAPILGSEEQLVWLEETMKRLKFPKSLGSKDAVIVDSEMVP
VNANPTQEIPAASETVESSPVAPGNTTDTMPAASGTTDTTSGVSEAAAAEAAVDSTPGTEEEPSFSLRYALVV
QNVPYPEPPKEPEVMFTDEEKSLILEATRARRMELDLYNGYLADYELSKDEIQKHVPDLPENWRTNWRWSE
RLYKFFFKTKKEGLEEIFLNKELGNMILARGLAATQSQARIKVFNSLVAWLLQSFNVGRSCTAKPLPTSKLDL
FKSEFESKPKNNILTEFLVASDEEILFKGLRVLEPGIEGWYDHPDQAGEIRSVLEGLVQAGRISGYWENQPFGR
FVLRGVGERRTELVELLESLVASGEIMQFFESSDEEGAFIIDNEPSKTAMLKQRFKSCVRTKLVGSFADESLPR
GRFTILV

>core/185/2/Org2_Gene956

MVSLLHKFLENASGKKGQSLASTAYLAALDHLLNAFPSIGERIIDELKSQRSHLKMIASENYSSLSVQLAMGN
LLTDKYCEGSPFKRFYSCCENVDAIEWECVETAKELFAADCACVQPHSGADANLLAVMAILTHKVQGPAVS
KLGYKTVNELTEEEYTLLKAEMSSCVCLGPSLNSGGHLTHGNVRLNVMSKLMRCFPYDVNPDTECFDYAEI
SRLAKEYKPKVLIAGYSSYSRRLNFAVLKQIAEDCGSVLWVDMAHFAGLVAGGVFVDEENPIPYADIVTTTT
HKTLRGPRGGLVLATREYESTLNKACPLMMGGPLPHVIAAKTVALKEALSVDFKKYAHQVVNNARRLAER
FLSHGLRLLTGGTDNHMMVIDLGSLGISGKIAEDILSSVGIAVNRNSLPSDAIGKWDTSGIRLGTPALTTLGMG
IDEMEEVADIIVKVLRNIRLSCHVEGSSKKNKGELPEAIAQEARDRVRNLLLRFPLYPEIDLEALV

>core/186/2/Org2_Gene797

MSEQEKLSNYNADKKLFSGIDKLFQIVKGSYGPKQSLSPTSFFKERGFYAISQTELSNSYENLGVDFAKAMVN
KIHKEHSDGATTGLILLHAILQESYAALEKGISTHKLIASLKLQGEKLQEALQQQSWPIKDALKVRNIIFSSLH
MPTIADHFYNAFSVVGPEGLISITKERENDKTSMDVFQGFKIPAGYASTYFVSDTASRLTRIAHPLILITDRKIS
MIHSLLPLLQEISEQNQHLIIFCEDIDPDVLATLVVNKLQGLLQVTVVTIPQLSTTNQELAEDIALFTGTHICPCQ
EASHVLAPEMVTLGSCLSIEISESQTTLIGGLHIPEVLTLKTRQLAEEIRTTSCLETKKRLIKSTNRLQSSVAILPT
DEDNEPLYTLALKIMESALSRGYVPGGGVALFYASLTLGTPKDDADENSIAISLLQKACCAPLKLLATNADLD
GDAVIAKLSSLGTTSLGISVFSREIEDLIAGGILDSLATTSTILAQALDTAILVLSSKILILENQYEISTL

>core/187/2/Org2_Gene853

MENEILLNIESKEIRYAHLKNGQLFDLTIERKKVRQLKGNIYRGRVTNILRNIQSAFINIDERENGFIHISDILENS
KKFEQMFDMDVDALPEEASEAPLLSSEEAPIEEFLKLDSPVLVQVVKEPIGSKGARLTSNISIPGRYLVLLPNSP
HRGVSRKIEDPHMREQLKQLIRSFEMPQDMGLICRTASTTASTEALINEAHDLLLTWKTILEKFYSTEQPCLLY
SETDILKKAVITCIDKNYKRLLIDDYATYQKCKHMLKKYSPDASIKIEYYRDSIPMFERFNIEKEIDKATRRKI
WLSSGGYLFFDKTEAMHTIDVNSGRSTQLESGVEETLVQINLEAAEEIARQLRLRNVGGLVIIDFIDMKSRKN
QRRVLERLKEHMKYDAARCTILSMSEFGLVEMTRQRNRESLMQTLFTLCPYCSGNAIIKTPESVVIEIERDLK
KVINHKEHSHLCLVVHPEIASYMKQENDDNEMINLAKQLKAKLQINTSDSVHLNHYQFFSLITGESIDL

>core/188/2/Org2_Gene240

MFNLFFFTANKETTASHELIYRKNQSFSLSPVTILCLLAISVLLLLGVVFALVGCHVLAAPLGLLVWGCAASV
CSMMAIVSLMCLYKGGKPLIEPSNEEKIDPTKDLEIKDPESLKPVPVEGQSLPKERKTVSFKAKIPSIVEDDFKP
YVIQSTFYHQNKVYSKPIAERMQSLEKEITTLIVDFPRALEESSKSSGSLLRGVISEIKNLFLPRFLSRKVKYSLT
ACLRRLGSIVEEYASSDLLILLLTKPEPLNMVTQQLIAHLNSLKTEKRKLTPHMQKLVLSINFWFYGWSLEEK
CIEKIVAYDPNLLTDELKAHLEAGNIVQFLLSFQSSEMQREFRALFPSDAQELPSAKDGSNYVPAINSSEYMY
DFKDLSVLKKSLSERLAFCEKIPSPSSWNFTSSVASHYKDFSLLFTFFSNQQSVILQNPFLLIELLHENPKCQTFL
KGLLEKAMPMSNWAALFRPMLMGMLCSGIARKKELKIIAEHLGVPFKEITQAIASGKILDLLLQHLFDF

>core/189/2/Org2_Gene388

MTILRNFLTCSALFLALPAAAQVVYLHESDGYNGAINNKSLEPKITCYPEGTSYIFLDDVRISNVKHDQEDAG
VFINRSGNLFFMGNRCNFTFHNLMTEGFGAAISNRVGDTTLTLSNFSYLAFTSAPLLPQGQGAIYSLGSVMIEN
SEEVTFCGNYSSWSGAAIYTPYLLGSKASRPSVNLSGNRYLVFRDNVSQGYGGAISTHNLTLTTRGPSCFENN
HAYHDVNSNGGAIAIAPGGSISISVKSGDLIFKGNTASQDGNTIHNSIHLQSGAQFKNLRAVSESGVYFYDPIS
HSESHKITDLVINAPEGKETYEGTISFSGLCLDDHEVCAENLTSTILQDVTLAGGTLSLSDGVTLQLHSFKQEA
SSTLTMSPGTTLLCSGDARVQNLHILIEDTDNFVPVRIRAEDKDALVSLEKLKVAFEAYWSVYDFPQFKEAFT
IPLLELLGPSFDSLLLGETTLERTQVTTENDAVRGFWSLSWEEYPPSLDKDRRITPTKKTVFLTWNPEITSTP

>core/190/2/Org2_Gene199

MTNVVQETIGGLNSPRTCPPCILVIFGATGDLTARKLLPALYHLTKEGRLSDQFVCVGFARREKSNELFRQEM
KQAVIQFSPSELDIKVWEDFQQRLFYHRSEFDNNMGYTSLKDSLEDLDKTYGTRGNRLFYLSTPPQYFSRIIEN
LNKHKLFYKNQDQGKPWSRVIIEKPFGRDLDSAKQLQQCINENLNENSVYHIDHYLGKETVQNILTTRFANTI
FESCWNSQYIDHVQISLSETIGIGSRGNFFEKSGMLRDMVQNHMMQLLCLLTMEPPTTFDADEIRKEKIKILQR
ISPFSEGSSIVRGQYGPGTVQGVSVLGYREEENVDKDSRVETYVALKTVINNPRWLGVPFYLRAGKRLAKKS
TDISIIFKKSPYNLFAAEECSRCPIENDLLIIRIQPDEGVALKFNCKVPGTNNIVRPVKMDFRYDSYFQTTTPEAY
ERLLCDCIIGDRTLFTGGDEVMASWKLFTPVLEEWDQDSSPSFPNYPAGSSGPKEADALIERDGRSWRPL

>core/191/2/Org2_Gene907

MSSWLSQASEVLLNQDPYIPDAPRSQESSVPKISYSITVAPQEAQKSLPKFFTQKFQSQCKSEPPITHHKTFIIAT
PRERILRFGSSFESQLHNTSQAQTSSPWNLFSQKNSTEASKALMQELTMPKSPEKTSEKALDKNLSSKQEGSC
KNFDTLHLQQHLKLFGTVDSLYSQSLDSEQQELLQSRREERSETYANQQSSEKKIETKVQIKDLCKDLFSQDQ
DSNQKQKKSPFQQDTSRKNRIAKAAQAVPVIPPPSIGVFTLSYLLTKQGILSDFSSYGCHKDSVESTQRELDAL
HEKRIETIKVSIEKEKRERLWGSLSDIIGWLAPFVSIGIGIVAILSGGGIFAFAGFFAGLISLVIKCLEKLKFWDW
LEKHLPIKNEELRRKIITIIQWVVYLTPVILSICTLKVENLGFSPIIEGAIKGIQPAIESTMAALRCAILFSQAEIYK
LKGKLTKIQLDIELKSFDRDDHYERSQELLDNMESSFEALSRILNYMRELDQVYLHSLRG

>core/192/2/Org2_Gene403

MAVGGVGGSRSPSPIPPNRRNSEDGKVSPKDNLGEHTVSSSDSSLASQGPTIEERKAQLGGTDKIPLPSVKEPG
DSQTSGRSGVLQRIWKGVKGVFKKTPQARPEVSSPRLPSHVQHGQRLPGLEGFRDRIQKRSENPEADLGKMK
RSYSDGDLDRVGHDSNEDSTEDSRSEGGEPSSKSSSFLSGVRGAVSKVHGALGDIKGKFQRSASEDDLTTQG
EDSAGDTVKERRSEEAEASSKSSSFLSGVRGATSTVQGALGDAKEKVSAFGEQAAGAIRSAPGNIRTRFQRSS
SEGDLSNVNKAAKHLRKALENLEKVAPEQVSPEVASRVQSLLARMEQLTHQEPPTVEDLITFVESNVGSDSV

EYASIVPQDGSQAPAETAEAPETGGVEGSAAQGAWKALRDFVVSIFQAVASFFRAIASRLSSARRESAVDDL
ASESNTQWFVEQEGVSNPSAAPSLSFAEEIARRAAEMSNRNAQSLEKLESGNVTDPVIQQGLGLARSFAPEGQ

>core/193/2/Org2_Gene121

MADILVIGANPTGLILANMLIQHGISVKVIDHRASPEDPSFLDCRKLPVILSCSSLELLHNSEMLGDFIQANHKI
FGARYHWKKRTLLFKFSQATDSPVPFSLSTTYQSLEQHLIDEFLKRGGVIDWSTRPVTLVDNSIFIESTKVSQN
FENREIYNPKWIIACEADNNLDIRDLVKSQLRARRINREVIFINCDEGEPFEEDHIHLLPITKNFLNFVFYNPQEK
TKQLCLPQGTHSISPKLKQKLLYTYNLVISDENFHIKTSHHAFPPEHGNVLFLGSLSNTLLLSYLNGINTNIHAA
FNLAWKLLPVLKKAALKHLVITKEQEDGNILPYISPTTEKRAKKLPFSRFYTPALMYYFLKGCRKFNTTGEEY
YYPPHQALKYRSSDIIKMSPQDKEIHGPGPGMRAIDARLENGSFLLDPLKSSKHLLIFFKDIPDLKEALQEEYG
EWIEICNVKEPRILNLYHANPNSLFIIRPDRYIGYRTHTFKLHELISYLLRIFASEKTS

>core/194/2/Org2_Gene481

MNWENVRVRVAPSPTGDPHVGTAYMALFNEIFAKRFKGKMILRIEDTDRTRSRQDYEENIFSALRWCGIQW
DEGPDVGGPYGPYRQSERTKIYQGYVETLLKTDCAYKCFATPQELAEMRAVASTLGYRGGYDRRYRYLSPE
EVASREAAGQPYTIRLKVPLSGECVFEDYSKGRVVFPWADVDDQVLVKSDGFPTYHFANVIDDHLMGITHV
LRGEEWLSSTPKHLLLYEAFGWEPPVFLHMPLLLNPDGTKLSKRKNPTSIFYYRDSGYVKEAFVNFLTLMGY
SMEGDEEVYSLERIIETFNPRRIGKSGAVFDIQKLDWMNKHYLNHEGSPECLLKELQGWLLNDEFFLKILPLC
QSRITTLAEFINLTSFFFSGLLEYRVEELLPQALSPEKAAILLYSYVKYLEKTDQWTKETCYLGSKWLAQAFN
VHHKKAIIPLLYVAITGKKQGLPLFDSIEILGKPRARARLVYAEKLLGGVPKKLAATVDKFMQREDFEEATFD
L

>core/195/2/Org2_Gene618

MDCRGGIPLPEPQVIGGYHVKKILSKKLRSRVVHGLHPETRHSTVIKVFSPSPSFTSRSVYNFLKEAQSLHQIT
HPNIVKFHRYGKWQDCLYIAMEYIEGISLREYILAQFISLPQAIDIIFDIAQALEHLHSRNILHKDIKPENILITPQ
GKIKLIDFGLADWDTEIQRAHPSVIGTPYYMSPEQRQGESHSPASDIYALGLLAYELILGHLSLGRVFLSLVPE
RISKILAKALQPSPNNRYSSTREFIQDIHHYRMSGDMQEDLRIKDHTVALYEQLQTQRFWLAPETLRFPDFISG
VLYHQGYPLYPHAYDTLLEGDVFNLWLGYSPISNATIALSVVKSLVCQQDLQRPLLDRVCEINECLIRMKIPI
DEMGISILCLEISKENKELSWIACGKTVFWIKRQGRVVQDFESFSPGLGKITSLQIRETKVAWEIGDEAVVCTL
ELEESVASLKTLSLAELQDRRQKAIFCPIESIHGGIQSRQHGSNSPSTLISLKRIR

>core/196/2/Org2_Gene367

MLKKFINSLWKLCQQDKYQRFTPIVDAIDTFCYEPIETPSKPPFIRDSVDVKRWMMLVVIALFPATFVAIWNS
GLQSIVYSSGNPVLMEQFLHISGFGSYLSFVYKEIHIVPILWEGLKIFIPLLTISYVVGGTCEVLFAVVRGHKIAE
GLLVTGILYPLTLPPTIPYWMAALGIAFGIVVSKELFGGTGMNILNPALSGRAFLFFTFPAKMSGDVWVGSNP
GVIKDSLMKMNSSTGKVLIDGFSQSTCLQTLNSTPPSVKRLHVDAIAANMLHIPHVPTQDVIHSQFSLWTETH
PGWVLDNLTLTQLQTFVTAPVAEGGLGLLPTQFDSAYAITDVIYGIGKFSAGNLFWGNIIGSLGETSTFACLLG
AIFLIVTGIASWRTMAAFGIGAFLTGWLFKFISVLIVGQNGAWAPARFFIPAYRQLFLGGLAFGLVFMATDPVS
SPTMKLGKWIYGFFIGFMTIVIRLINPAYPEGVMLAILLGNVFAPLIDYFAVRKYRKRGV

>core/197/2/Org2_Gene209

MKFEFSVALKYLIPGRGRLYSAIVSLFSVGIISLVVWLSIVFISVIHGLEQRWIEDLSQLHSPITILPSDTYYSSYY
YQIDKHSSLSNYTTKTLGEKIASPQVDPYDPESDYLLPETFPLKDCDLGGQQKDPVKMTLESLGPYLQSQHGK
VIEFEQGVGYLDIKTSLKLQKPQPRNLTHFLTYPSKLSYEDKVLPYDETDYTSAELNPFNRSPSGWQQDFHHL
EELYRGASIILPSTYKDSGYKVGDTGVFSTYSIENEKETQYTVHVIGFYNPGLSPLGGRTVFIDPDLARSIRSQS
EGLGMSNGFHLFFPNTKRIVFVKKQIENILTSLGVDDYWEISSLHDYDYFQPILDQLQSDQVLFLFVCILILIVA
CSNIVTMSMLLVNNKKKEIGILKAMGTSSRSLKIIFACCGAFSGACGVVIGTIFAIITLKNLQFIVKALNYLQGR
ETFNTAFFGQNLPNSVHPQAIYFLGLGTLLLAAVSGALPARKVAKMHVSEILKAD

>core/198/2/Org2_Gene329

MVLFHAQASGRNRVKADAIVLPFWHFKDAKNAASFEAEFEPSYLPALENFQGKTGEIELLYSSPKAKEKRIV
LLGLGKNEELTSDVVFQTYATLTRVLRKAKCSTVNIILPTISELRLSAEEFLVGLSSGILSLNYDYPRYNKVDR
NLETPLSKVTVIGIVPKMADAIFRKEAAIFEGVYLTRDLVNRNADEITPKKLAEVALNLGKEFPSIDTKVLGKD
AIAKEKMGLLLAVSKGSCVDPHFIVVRYQGRPKSKDHTVLIGKGVTFDSGGLDLKPGKSMLTMKEDMAGG
ATVLGILSALAVLELPINVTGIIPATENAIDGASYKMGDVYVGMSGLSVEICSTDAEGRLILADAITYALKYCK
PTRIIDFATLTGAMVVSLGEEVAGFFSNNDVLAEDLLEASAETSEPLWRLPLVKKYDKTLHSDIADMKNLGS
NRAGAITAALFLQRFLEESSVAWAHLDIAGTAYHEKEEDRYPKYASGFGVRSILYYLENSLSK

>core/199/2/Org2_Gene906

MTVSYQSISTPPPEGEFDIFVDGNATEEAVVAAEVQVALPAGEQYAMLRATSELCFGILTQSECALTQALPPK
EKPLQEEQFLVKNGILMRSTSLPNLKPGQSQQTSLASHRNPLAQQSTSSNSTGKASTETTSSSFPFFSCKAPEG
DSSVDKTFTVSVQTPKAQEQQEASASQSQAQFHVRSYSSSTIKEHSAKEKVSQSTKSAETQKHTQTKSDATLS
PMSLYSTLHKEVPQALSSTKSQQKDEEHRDQRQQEGYEQEQEQEEGKKKTPWCTVESLQQTSSSNQVYESY
TPIIPDPIVEFALSESQLSVLAGKRVTNLDVLRICTELMKLMLKSRANDTMTRLEERELMEREAHELAASYSR
QAKYARWLGIATATLGILGAIAPMVGEISGDSILGFVQRISGRFKDATAKTFFKGIGKVFTSLSQLTEAASKVH
ELSESAVRAVAEYRKEVFRMRQDEVTRTIEEVKDNWKSMDNFLLNILQTEHDAARSLYQ

>core/200/2/Org2_Gene725

MAASILSQELLDILPYTFLKKHCLLPIEESSEAITIAHATATSVIAQDEVKLLIKKPVRFVLKEESEILQRLQQLY
SNREGNVSDMLLTMKEEDGTTISEEEDLLETTDTIPVVRLLNWILKEAIEERASDIHFEPCEDSMRIRYRIDGVL
HDRHSPPSHLRSALTTRLKVLAKMDIAEHRLPQDGRIKIHIGGQEVDMRVSTVPVIYGERVVLRILDKRNVIL
DIAGLHMPKGTEILFKDTITAPEGILLVTGPTGSGKTTTLYSVLQELKGPLTNIMTIEDPPEYKLPGIAQIAVKP
KIGLTFARGLRHLLRQDPDILMVGEIRDQETAEIAIQAALTGHLVVSTLHTNDAISAIPRLLDMGIESYLLSATL
VGVVAQRLVRTICPYCKVAYTPENQEKSFLASLGKDTEMPLYRGQGCVHCFRSGYKGRQGIYEFLRPNTLFR
SEVASNRPYHILRETAEQNGFLPILEHGIALAVSGETTLAEVLRVTKRCD

>core/201/2/Org2_Gene245

MNKKHASFSSRLGFIFSMIGIAVGAGNIWRFPRVAAQNGGGAFLILWLCFLFLWSIPLIIIELSIGKLTKKAPIGA
LIKTAGKKFAWAGGFITLVTTCILAYYSTIVGWGLSYFYYAVSGKIHLGNDFAKLWTSHYQSSIPLWAHLTSL
GLAYLVIRKGIVHGIEKCNKILIPAFFLCTIALLLRAVTLPGAVQGIKQLFSCDKSCFSNYKVWIEALTQNAWD
TGAGWGLLLVYAGFASKKTGVVSNGALTAICNNLVSLIMGIIIFSTCASLDILGTTQLQDGAGASSIGITFIYLP
ELFTRLPGGIYLTTLFSSIFFLAFSMAALSSMISMLFLLSQTLAEFGIKPYISETLATIIAFVLGIPSALSLTFFSNQ

DTVWGVALIVNGLIFIYAALVYGFPKLKKEVINAAPGDLRLNKAFDYIIKYLLPIEGILLLGWYFYEGLFPENG
QWWNPISLYSLGSLVLQWSLGLIILWKFNKQLYLRFSRYNHEIL

>core/203/2/Org2_Gene518

MLKYILKRLVLIPLTLFAIVSINFVILNAAPGDVLEEKSRDALGEAGKSDKMRSYKGPDRYLQFREHYGLTLPI
FFNTRPKITHKKIQTALQELANANNTTPSAKNAAKSLVYWGDCAKFVMPALLFEADDASRDDKYRHIAADL
FIRGGVLQGFVGPNLSPEQRAQNKEIAESNAFLVRQLNEEDLDTKVEALKGWFQDHGGTEVFCYSSKQFWK
TFFLETRFARYMSRVLRLDFGTLRNDAHKTVISEVIKRLRCSLVLSILPMIVGFVLCQIFGMIMALKRNRWIDH
SLNFIFLILFSIPVFVAVPWILDNFVINKTIPFTTIPMPYSGLRSPPEVFNELSTLGRIFDLVSHGFLPFCAVSYGAL
AAQSRLSRSIFLEVLSQDFICAAKARGLRWFDILYKHVGKNAAVSIVTSLASSLGTLLGGALVVETLFNIDGFG
NFFYQAILNRDHNVVLFSVLVGSALSLVGYLLGDICYVLLDPRVQLEGRRI

>core/204/2/Org2_Gene664

MHTEFAPFLEDLVHQQVISPLDIAFASKHISSDFEESFVFLAVSSALWRYGHPFLSLEENRIRPSLGGISETDLY
RGFHNLPKEARDKLFVVVSGRLYLRSLYTIRSKLLDKLSLLCSATPNYFPPSIDSSILSEEQNFIFNKITQGCFSI
VSGGPGTGKTFLAAQLILSLVKQQPKLRIAIVSPTGKATSHIRQILMKYNIFDDMVLMQTVHHFLQEYAYRRY
NSIDVLLVDEGSMVTFDLLYSLVQTLQGYEKDKKLYTSSLIILGDTNQLPPIGIGVGNPLQDLIGYFHENTFFL
KTSHRAKTGVVDQLTQSVLRGEMISFSPLPSISSAIEVLKNRFVKSLRQSEARLCVLTPMRHGPWGVLNLNTM
IHQRLARSDPDLRIPIMVTSRYETWGLFNGDTGLLCLKTQKLHFPQHEPIDSRALSQYVVYNYVMSVHKSQGSE
YDEVIVIIPKGSEVFGVSILYTAITRAKYRVSVWGDPETLHKIIKKSNY

>core/205/2/Org2_Gene994

MNLDSKHFDINSANFLEEFAKFISFPSISADSDHLQDCENCAHFLVDHVNKIFDVELWETPGHPPIIYASYKSE
DPLSPTLMLYNHYDVQPAQLSDGWKGDPFILREENGNLYARGASDNKGQCFYTLKALQHYYESQGNFPLNII
WLIEGEEESGSLALFTWLEKKKEALRADYLLIVDGGFLSEKHPYVSIGARGIVSMKISLEEGNKDMHSGVLGG
IAYNTNRALSEILSSLHHPDNSIAIEGFYDDLALPSDSDRPDLPKSDTLRECEENLGFRPQGYEASYSPEESALR
PTVEINGISGGYTGPGFKTVIPYRATAYLSCRLVPNQDPDKAAHQVIHHLKQQVPSSLKFSYEILPGGSRGWRS
SANLPIVKVLQEIYSDLYNEECLRLVMPATIPIGPLLGEAAQTSPIICGTSYLSDDIHAAEEHFSMDQLKKGFLSI
CQLLDKLPKIKE

>core/206/2/Org2_Gene718

MSISSSSGPDNQKNIMSQVLTSTPQGVPQQDKLSGNETKQIQQTRQGKNTEMESDATIAGASGKDKTSSTTKT
ETAPQQGVAAGKESSESQKAGADTGVSGAAATTASNTATKIAMQTSIEEASKSMESTLESLQSLSAAQMKEV
EAVVVAALSGKSSGSAKLETPELPKPGVTPRSEVIEIGLALAKAIQTLGEATKSALSNYASTQAQADQTNKLG
LEKQAIKIDKEREEYQEMKAAEQKSKDLEGTMDTVNTVMIAVSVAITVISIVAAIFTCGAGLAGLAAGAAVG
AAAAGGAAGAAAATTVATQITVQAVVQAVKQAVITAVRQAITAAIKAAVKSGIKAFIKTLVKAIAKAISKGI
SKVFAKGTQMIAKNFPKLSKVISSLTSKWVTVGVGVVVAAPALGKGIMQMQLSEMQQNVAQFQKEVGKLQ
AAADMISMFTQFWQQASKIASKQTGESNEMTQKATKLGAQILKAYAAISGAIAGAHKTNNF

>core/207/2/Org2_Gene3

MYRYSALELAKAVTLGELTATGVTQHFFHRIEEAEGQVGAFISLCKEQALEQAELIDKKRSRGEPLGKLAGV
PVGIKDNIHVTGLKTTCASRVLENYQPPFDATVVERIKKEDGIILGKLNMDEFAMGSTTLYSAFHPTHNPWDL
SRVPGGSSGGSAAAVSARFCPVALGSDTGGSIRQPAAFCGVVGFKPSYGAVSRYGLVAFASSLDQIGPLANT
VEDVALMMDVFSGRDPKDATSREFFRDSFMSKLSTEVPKVIGVPRTFLEGLRDDIRENFFSSLAIFEGEGTHLV
DVELDILSHAVSIYYILASAEAATNLARFDGVRYGYRSPQAHTISQLYDLSRGEGFGKEVMRRILLGNYVLSA
ERQNVYYKKATAVRAKIVKAFRTAFEKCEILAMPVCSSPAFEIGEILDPVTLYLQDIYTVAMNLAYLPAIAVP
SGFSKEGLPLGLQIIGQQGQDQQVCQVGYSFQEHAQIKQLFSKRYAKSVVLGGQS

>core/208/2/Org2_Gene1004

MNGCWEDLKQTIFWVGEHDCTDIETVRKSCMWLDRYADKFILREKEEKMERHELFHATMVRKASGHAYA
KAKAAFEKERSNENQRKVKDVEKWLSKGLAEFRNQESRRARERLRELQTLYPEVSVEERVLERQRTKKVNL
ENLYADIEKKYHHCVREQEHYWKEVENKEAEYRENGEKVLSAEEVSECLQRLEDCLETWSKKLTKAEESVF
EMKFDATEKLGNKVLSDVTNRLEILCEDAEEMIFRIEEIEMTLRMVELPLLFMKNTFEKASLQYNSCKEMLA
KVEPQCKESPTYRSSQERLERLNQDLQTAYTNCQERLQGFSDLESKVRTCRDHLREQMKHFEVQGLNFINEE
LLWVGAELFTQARLDLVATVPYMEFYLQYHNIKREKVRSQWMAKTERYREIRQAFQGVMKEDLLAEDTIL
KEEDYWLLRDDWLLRDERKNRQRRLICNKIAAAQQRVKGF

>core/209/2/Org2_Gene99

MLGILLIASGIIFLAVAIPGLSSAVALGLGCGMTALGTVLLITGLVLLIRSEKLALEQVEIKQARTRVNNELDQL
SQYVFYTENVLDNLKRWSYRDLGFVRQAQEEVTNLEQDIEEIFLTLRDIRNALDNEEFFMTHAKQCLAQVGE
SLFQDASIDEFINLAHLSEIRQHLDINDPRWSMITKKVKGTVVRFIYVSTMYKQIKSNFEKSDFGQLRKMLLN
NYKTIEEVLYQSFQKGYNRAALLSEKTRIIHTSSLLHWEKDEDKHLNIKNECASRLENFKKFRTLFLGLSEED
VIDFTGASGWDCSKLPRKEVPLDGGKKKLRFKRTFADEQVGDWDRTTSLEHMTPQEEDPLDRLMDQVEQE
ATSVLKDQDRYWKEIETSEAKFRSLPREDDFEKQSQIDSYIRDLDDHLSVWANQLSAAEDALIEVTDVQEHG
NREMLKNIQQGLELIEDAVKATLPRVDFIQELLEKEELPLVAARMSLENS

>core/210/2/Org2_Gene949

MTFPCGNCNCYYRETPPPNPGGEDIPLQEGGQSGSQGGRVITQQPGTGGREMGISLGSDNVLGMVEQAGSLL
NNLLDSARMQRLGHYCYRTGTPWCREHCPGFLQWIWGGCCACCLETVDDPDNPSAQFLQQLIQQYGPICVG
MSFQQLPHCTQKIEQGEPLGDGDKQEVENGCKLHRELLKAAQPRCMGESLVKLLQNNGLGEDMQQTPPWS
LILQAVSEGALSFVTSSDNPPTCWILQPEQQPCPPPPTDEEQLQGAVGGAPAPQQKKHPAQECRVTCKLNFRT
LLQKLSRLEVLSLESGYKGPLGQAAKQIVDLIKKSLKRLVASDLATFLGPGIGLSLESQVFEVLVLLCLLSKGY
LPLDPLHPEQTVLDPRVQGPWQRILRKVLVTTTAGENIWRQTQGEAPRQAPPPPDPWDDDEIERDGIVTGGG
FGIPCQCLRCWRKLPTEKRPNRWL

>core/211/2/Org2_Gene857

MHMSNPISLFSPAELIAKYNLIPKTSPIYPRRTELIILEENACQTRLTNVAQVLHPSSLFSMSKKILNPCGCSGGP
LCWVILNILAFIITSVLFIILLPVNLIVAGLRLFMPLPPKKIVEDLSEPTTEETNEVIQPFIFALQALLFEDNKLRSF
KIVEQSVGKAPLPNPFLNRLVAISPQESQEAMRKIPDLCSQLKKVLKSLGVLTPEWKHMLKYFEGLKNEHDS
NPDKKTFPILIKLLIEALTGKSSLPKTPSTKEKMQAALFIASSCKTCKPTWGEVITRSLNRLYSIANEGDNQLLI
WVQEFKERELMSIQDGDDAEEYRFAAQQHGERYTEAIEQVLRNESAAKLQWHVINTMKFFHGKNLGLVTE

HLQDTLGALTLRQTTVDTHQGREDADLSAALFLNKYLNSGNQLVNSVFKSMQKADPETKALIREFALDILYA
SLRLPQTSAHTEVFSTLLMDPETYEPNKACIAYLLYVLKIIEL

>core/212/2/Org2_Gene4

MSAVYADWESVIGLEVHVELNTASKLFSSALNRFGDEPNTNISTVCTGLPGSLPVLNQSAVEKAVLFGCAVE
GEISLLSRFDRKSYFYPDSPRNFQITQFEHPIIRGGRIKAIVQGEERYFELAQTHIEDDAGMLKHFGEFAGVDYN
RAGVPLIEIVSKPCMFCPEDAVAYATSLVSLLDYIGISDCNMEEGSIRFDVNVSVRPKGSPELRNKVEIKNMNS
FAFMAQALEAEKQRQIDEYLNQPNKDPKLVIPAATYRWDPEKKKTVLMRLKESAEDYKYFPEPDLPTLQLTE
SYIERIRKTLPELPYDKYHRYIQEYGLSEDIASILISDKNIATFFEVACKDCKNFRSLSNWVTVEFGGRCKTLGV
KLPSSGIFPEGVAQLVNAIDQGVITGKIAKEIADLMMESPGKNPEEILKEKPELLPMSDEGELQKIIAEVVLANP
ESIVDYKNGKTKALGFLVGQIMKRTAGKAPPKRVNELLLLELDKG

>core/213/2/Org2_Gene870

MITKQLRSWLAVLVGSSLLALPLSGQAVGKKESRVSELPQDVLLKEISGGFSKVATKATPAVVYIESFPKSQA
VTHPSPGRRGPYENPFDYFNDEFFNRFFGLPSQREKPQSKEAVRGTGFLVSPDGYIVTNNHVVEDTGKIHVTL
HDGQKYPATVIGLDPKTDLAVIKIKSQNLPYLSFGNSDHLKVGDWAIAIGNPFGLQATVTVGVISAKGRNQL
HIADFEDFIQTDAAINPGNSGGPLLNIDGQVIGVNTAIVSGSGGYIGIGFAIPSLMANRIIDQLIRDGQVTRGFLG
VTLQPIDAELAACYKLEKVYGALVTDVVKGSPADKAGLKQEDVIIAYNGKEVDSLSMFRNAVSLMNPDTRI
VLKVVREGKVIEIPVTVSQAPKEDGMSALQRVGIRVQNLTPETAKKLGIAPETKGILIISVEPGSVAASSGIAPG
QLILAVNRQKVSSIEDLNRTLKDSNNENILLMVSQGDVIRFIALKPEE

>core/214/2/Org2_Gene24

MWNRCQVFSSFFFRYPISSWLIRLRASCECFQQRHPIFLCGLYWLAGITSRGHPECSALILIFLGMFLPRNPKQ
WLPLASAWIISLMLTPAPFLHDGPISGTFVIHHAGGQGTYYGEALCIQTPCGKRAHHLSCQILSESRLELKKVY
ELEGTLHHTSQIVFKSNACYKEIPRSRFYIMKEKCRESSCHFLNHRFPSSEVGPFASSLLLGTPLPQNLRDLFRQ
KGLSHLFAISGWHFSLCATTLWMLCALLPLKIKKILSFIVLTSLACIFPMSLSVWRSWISVTLLCFSWCFSGSCS
GLNRLGAGFILCSIFFSPFSPTFVLSFLATLGILLFFPKIFSFLYTPWTQFLSPFWLYPIRYLAMTLAISLSAQLFIV
LPIMQYFGSLPLEGLLYNLIVPFTILPIIVFLIATIILPCCSPITEALIQGFLSHPWLHNPNILKTLSFAPVPPWMLTL
ASLILFFIGILRTNVSPYASISATSYRFIETL

>core/215/2/Org2_Gene749

MLKIAILGRPNVGKSSLFNRLCKRSLAIVNSQEGTTRDRLYGELHAFGVPAQVIDTGGVDHNSEDYFQKHIYN
QALTGAKEADVLLLVIDIRCGITEEDAHLAKLLLPLKKPLILVANKADSRQEELQIHETYKLGIRDIVVTSTAH
DKHIDTLLQRIKLVANLPEPREEEEEGLEELSVDEHEESEAALPSNTFPDFSEVFTEGFSPEEPCTIPESPQQAPK
TLKIALIGRPNVGKSSIINGLLNEERCIIDNTPGTTRDNIDILYSHKDRQYLFIDTAGLRKMKSVKNSIEWISSSR
TEKAISRADICLLVIDATQKLSSYEKRILSLISKRKKPHIILINKWDLLEEVRMEHYCKDLRATDPYLGQAKML
CISATTKRNLKKIFSAIDELHHVVSNKVPTPIVNKTLASALHRNHPQVIQGRRLRIYYAIQKTTTPLQFLLFINA
KSLLTKHYEYYLKNTLKSSFNLYGIPFDLEFKEKPKRHN

>core/216/2/Org2_Gene909

MNMPVPSAVPSANITLKEDSSTVSTASGILKTATGEVLVSCTALEGSSSTDALISLALGQIILATQQELLLQSTN
VHQLLFLPPEVVELEIQVVDLLVQLEHAETITSEPQETQTQSRSEQTLPQQSSSKQSALSPRSLKPEISDSKQQQ
ALQTPKDSAVRKHSEAPSPETQARASLSQASSSSQRSLPPQESAPERTLLEQQKASSFSPLSQFSAEKQKEALT
TSKSHELYKERDQDRQQREQHDRKHDQEEDAESKKKKKKRGLGVEAVAEEPGENLDIAALIFSDQMRPPAE
ETSKKETTFKKKLPSPMSVFSRFIPSKNPLSVGSSIHGPIQTPKVENVFLRFMKLMARILGQAEAEANELYMRV
KQRTDDVDTLTVLISKINNEKKDIDWSENEEMKALLNRAKEIGVTIDKEKYTWTEEEKRLLKENVQMRKEN
MEKITQMERTDMQRHLQEISQCHQARSNVLKLLKELMDTFIYNLRP

>core/217/2/Org2_Gene917

MTSRTKSSKNLGTIALAGMVVSSIIGGGIFSLPQNMAATAGAGAVILSWILTGFGMFFIANTFRILSTIRPDLKE
GIYMYSREGFGPYIGFTIGWGYWLCQIFGNVGYAVITMDALNYFFPPYFQGGNTLPAILGGSILIWVFNFIVLK
GIRQASIINVIGTIFKIIPLIIFIILTAFFFKLAVFKTDFWGHAVTKAQPSLGSVSSQLKGTMLVTLWAFIGIEGAV
VMSGRAKNPLSVGQATVLGFLGCLTIYILFSLLPFGSLFQHQLANIPNPSTAGVLDILVGKWGEVLMNVGLIIA
VLSSWLSWTIIVAEIPFSAAKNGTFPEIFTIENKEKSPSVSLYITSSVMQLAMLLVYFSSNAWNTMLSITGVMV
LPAYLASAAFLFKLSKSKTYPKKGSIKAPLAMITGILGVVYSLWLIYAGGLKYLFMALVLLALGIPFYIDAGK
KKKNAKTFFAKKEIVGMTFIGLLALTAIFLFLTGRIKI

>core/218/2/Org2_Gene74

MITRTKIICTIGPATNSPEMLAKLLDAGMNVARLNFSHGSHETHGQAIGFLKELREQKRVPLAIMLDTKGPEIR
LGNIPQPISVSQGQKLRLVSSDIDGSAEGGVSLYPKGIFPFVPEGADVLIDDGYIHAVVVSSEADSLELEFMNS
GLLKSHKSLSIRGVDVALPFMTEKDIADLKFGVEQNMDVVAASFVRYGEDIETMRKCLADLGNPKMPIIAKI
ENRLGVENFSKIAKLADGIMIARGDLGIELSVVEVPNLQKMMAKVSRETGHFCVTATQMLESMIRNVLPTRA
EVSDIANAIYDGSSAVMLSGETASGAHPVAAVKIMRSVILETEKNLSHDSFLKLDDSNSALQVSPYLSAIGLA
GIQIAERADAKALIVYTESGSSPMFLSKYRPKFPIIAVTPSTSVYYRLALEWGVYPMLTQESDRAVWRHQACI
YGIEQGILSNYDRILVLSRGACMEETNNLTLTIVNDILTGSEFPET

>core/219/2/Org2_Gene607

MGESVKVFLEEREDYPYGFVTPIESQGLTRGLSEETIEEIAALRNEPQFIIDFRLQAYRYWKQLHEPAWARLH
YGPIAYDDIVYFSSPKQKKPLGRLEDADPEILDTFKKLGIPLDEQKRLLNVENVAVDLVFDSVSIGTTFKEALE
KAGVIFCSLGEAIQEHPNLVKKYLGSVVSHRDNFFAALNAAVFSDGSFVYVPKGVKCPMDISTYFRINNKEA
GQFERTLIVVEDGGYASYLEGCTAPAYSSNQLHAAVVELVAHEHAVIRYSTVQNWYAGDKKTGKGGIYNFV
TKRGLCAGYRSKISWSQVEVGAAITWKYPSCILKGDESVGEFYSVALTSGKMQADTGTKMLHVGKRTTSTV
ISKGISSDESKNTFRSLVSLGKKAEHSSNYTQCDSMLIGKASGAYTDPKIVVENSTSSIEHEATTSKLREDQLLY
LRSRGLSPEEAVSLVIHGFCREIIEQLPLEFAQEASKLLLIKLENSVG

>core/220/2/Org2_Gene358

MDLKELLHGVQAKIYGKVRPLEVRNLTRDSRCVSVGDIFIAHKGQRYDGNDFAVDALANGAIAIASSLYNPF
LSVVQIITPNLEELEAELSAKYYEYPSSKLHTIGVTGTNGKTTVTCLIKALLDSYQKPSGLLGTIEHILGEGVIK
DGFTTPTPALLQKYLATMVRQNRDAVVMEVSSIGLASGRVAYTNFDTAVLTNITLDHLDFHGTFETYVAAK
AKLFSLVPPSGMVVINTDSPYASQCIESAKAPVITYGIESAADYRATDIQLSSSGTKYTLVYGDQKIACSSSFIG
KYNVYNLLAAISTVHASLRCDLEDLLEKIGLCQPPPGRLDPVLMGPCPVYIDYAHTPDALDNVLTGLHELLPE

GGRLIVVFGCGGDRDRSKRKLMAQVVERYGFAVVTSDNPRSEPPEDIVNEICDGFYSKNYFIEIDRKQAITYA
LSIASDRDIVLIAGKGHEAYQIFKHQTVAFDDKQTVCEVLASYV

>core/222/2/Org2_Gene305

MQTNIGLIGLAVMGKNLVLNMIDHGFSVSVYNRTPEKTRDFLKEYPNHRELVGFESLEDFVNSLERPRKIML
MIQAGKPVDQSIHALLPFLEPGDVIIDGGNSYFKDSERRCKELQEKGILFLGVGISGGEEGARHGPSIMPGGNP
EAWPLVAPIFQSIAAKVQGRPCCSWVGTGGAGHYVKAVHNGIEYGDIQLICEAYGILRDFLKLSATAVATILK
EWNTLELESYLIRIASEVLALKDPEGIPVIDTILDVVGQKGTGKWTAIDALNSGVPLSLIIGAVLARFLSSWKEI
REQAARNYPGTPLIFEMPHDPSVFIQDVFHALYASKIISYAQGFMLLGEASKEYNWGLDLGEIALMWRGGCII
QSAFLDVIHKGFAANPENTSLIFQEYFRGALRHAEMGWRRTVVTAIGAGLPIPCLAAAITFYDGYRTASSSMS
LAQGLRDYFGAHTYERNDRPRGEFYHTDWVHTKTTERVK

>core/223/2/Org2_Gene631

MRDVSELFRTHFMHYASYVILERAIPHILDGLKPVQRRLLWTLFLMDDGKMHKVANIAGRTMALHPHGDAP
IVEALVVLANKGYLIDTQGNFGNPLTGDPHAAARYIEARLSPLARETLFNTDLIAFHDSYDGREKEPDILPAKL
PVLLLHGVDGIAVGMTTKIFPHNFAELLKAQIAILNDKKFTVFPDFPSGGLMDPSEYQDGLGSITLRASIDIIND
KTLVVKQICPQSTTETLIRSIENAAKRGTIKIDTIQDFSTDVPHIEIKLPKGSRAKEMLPLLFEHTECQVILYSKPT
VIYENKPVECSISEILKLHTTALQGYLEKELLLLQEQLTLDHYHKTLEYIFIKHKLYDSVREVLAINKKISADDL
HQAVLHALEPWLHELATPVTKQDTSQLASLTIKKILCFNEEACTKELLAIEKKQAAIQKDLGRIKEVTVKYLK
GLLERHGHLGERKTQITNFKTAKTSILKQQTLI

>core/224/2/Org2_Gene921

MLCATVSGPSFCEAKQQILKSLHLVDIIELRLDLINELDDQELHTLITTAQNPILTFRQHKEMSTALWIQKLYSL
AKLEPKWMDIDVSLPKTALQTIRKSHPKIKLILSYHTDKNEDLDAIYNEMLATPAEIYKIVLSPENSSEALNYI
KKARLLPKPSTVLCMGTHGLPSRVLSPLISNAMNYAAGISAPQVAPGQPKLEELLSYNYSKLSEKSHIYGLIG
DPVDRSISHLSHNFLLSKLSLNATYIKFPVTIGEVVTFFSAIRDLPFSGLSVTMPLKTAIFDHVDALDASAQLCE
SINTLVFRNQKILGYNTDGEGVAKLLKQKNISVNNKHIAIVGAGGAAKAIAATLAMQGANLHIFNRTLSSAA
ALATCCKGKAYPLGSLENFKTIDIIINCLPPEVTFPWRFPPIVMDINTKPHPSPYLERAQKHGSLIIHGYEMFIEQ
ALLQFALWFPDFLTPESCDSFRNYVKNFMAKV

>core/225/2/Org2_Gene374

MMSRLRFRLAALGIFFILLVPNSVSAKTIVASDKEKVGVLVYDNSVEAFQQILDCIDHANFYVELCPCMTGGR
TLKEMVDHLEARMDLVPELCSYIIIQPTFTDAEDQKLLKALKERHPNRFFYVFTGCPPSTSILAPNVIEMHIKLS
IIDGKYCILGGTNFEEFMCTPGDEVPEKVDNPRLFVSGVRRPLAFRDQDIMLRSTAFGLQLREEYHKQFAMW
DYYAHHMWFIDNPEQFAGACPPLTLEQAEETVFPGFDKHEDLVLVDSSKIRIVLGGPHDKQPNPVTQEYLKLI
QGARSSVKLAHMYFIPKDELLNALVDVSHNHGVHLSLITNGCHELSPAITGPYAWGNRINYFALLYGKRYPL
WKKWFCEKLKPYERVSIYEFAIWETQLHKKCMIIDDEIFVIGSYNFGKKSDAFDYESIVVIESPEVAAKANKV
FNKDIGLSIPVSHGDIFSWYFHSVHHTLGHLQLTYMPA

>core/226/2/Org2_Gene844

MRIVQVAVEFTPIVKVGGLGDAVASLSKELAKQNDVEVLLPHYPLISKFSSSQVLSERSFYYEFLGKQQASAIS
YSYEGLTLTIITLDSQIELFSTTSVYSENNVVRFSAFAAAAAAYLQEADPADIVHLHDWHVGLLAGLLKNPLN
PVHSKIVFTIHNFGYRGYCSTQLLAASQIDDFHLSHYQLFRDPQTSVLMKGALYCSDYITTVSLTYVQEIINDY
SDYELHDAILARNSVFSGIINGIDEDVWNPKTDPALAVQYDASLLSEPDVLFTKKEENRAVLYEKLGISSDYFP
LICVISRIVEEKGPEFMKEIILHAMEHSYAFILIGTSQNEVLLNEFRNLQDCLASSPNIRLILDFNDPLARLTYAA
ADMICIPSHREACGLTQLIAMRYGTVPLVRKTGGLADTVIPGVNGFTFFDTNNFNEFRAMLSNAVTTYRQEP
DVWLNLIESGMLRASGLDAMAKHYVNLYQSLLS

>core/227/2/Org2_Gene829

MAFSHIEGLYFYNTASQKKELFFPNHTPVRLYTCGPTVYDYAHIGNFRTYVFEDILKRTLVFFGYSVTHVMNI
TDVEDKTIAGASKKNIPLQEYTQPYTEAFFEDLDTLNIARADFYPHATHYIPQMIQAITKLLEQGIAYIGQDAS
VYFSLNRFPNYGKLSHLDLSSLRCCSRISADEYDKENPSDFVLWKAYNPERDGVIYWESPFGKGRPGWHLEC
SIMAMELLGDSLDIHAGGVDNIFPHHENEIAQSEALSGKPFARYWLHSEHLLIDGKKMSKSLGNFLTLRDLLH
QEFTGQEVRYMLLQSHYRTQLNFTEEALLACRHALRRLKDFVSRLEGVDLPGESPLPRTLDSSSQFIEAFSRA
LANDLNVSTGFASLFDFVHEINTLIDQGHFSKADSLYILDTLKKVDTVLGVLPLTTSVCIPETVMQLVAEREEA
RKTKNWAMADTLRDEILAAGFLVEDSKSGPKVKPL

>core/228/2/Org2_Gene408

MDTIDTPGEQGSQSFGNSLGARFDLPRKEQDPSQALAVASYQNKTDSQVVEEHLDELISLADSCGISVLETRS
WILKTPSASTYINVGKLEEIEEILKEFPSIGTLIIDEEITPSQQRNLEKRLGLVVLDRTELILEIFSSRALTAEANIQ
VQLAQARYLLPRLKRLWGHLSRQKSGGGSGGFVKGEGEKQIELDRRMVRERIHKLSAQLKAVIKQRAERRK
VKSRRGIPTFALIGYTNSGKSTLLNLLTAADTYVEDKLFATLDPKTRKCVLPGGRHVLLTDTVGFIRKLPHTL
VAAFKSTLEAAFHEDVLLHVVDASHPLALEHVQTTYDLFQELKIEKPRIITVLNKVDRLPQGSIPMKLRLLSPL
PVLISAKTGEGIQNLLSLMTEIIQEKSLHVTLNFPYTEYGKFTELCDAGVVASSRYQEDFLVVEAYLPKELQKK
FRPFISYVFPEDCGDDEGRGPVLESSFGD

>core/229/2/Org2_Gene241

MDSRTSHLDDELSFKLEKAFTCLSTDIHSHDLSKIVIEYNPIDLAYAVSCLPSESRAILYKNLSCITAKVAFIINT
DSASRWAIFRRLSDSEVCALIEQMPPDEAVWVLDDIPDRRYRRILELIDSKKALKIRDLQKHGRNTAGRLMTN
EFFAFLMETTVKDVSACIRSNPGIDLTRLVFVLDFKGELQGVVTDRSLIINPPEMSLKQIMNQIEHKVLPDATR
EEVVDLVERYKIAALPVVDEENFLIGAITYEDVVEAIEDIADETIARMAGTTEDVGYQTCHVVQRFLLRAPWL
LVTLFAGLISASVMAYFQKISPALLALIIFFIPLINGMSGNVGVQCSTILVRSMATGTLSFGRRRETIFKEMSIGL
LTGVVLGILCGLVVYLMGFLGLNIFSGGGIQLGVTVATGVLGASLTATTLGVLSPFFFAKLGVDPALASGPIV
TALNDIMSMIIFFLIAGGINFLFFN

>core/230/2/Org2_Gene170

MNKKKRFLSLLFLTAVLLGIWFSPHPASINSNAWQLFAIFTTTIMGIIFQPVPMGAIAIIGISTLLLTQTLTLEQG
LSGFHNPIAWLVFLSFSIAKGIIKTGLGERIAYFFVSALGKSPLGLSYGLVITDFFLAPAIPSVTARAGGILYPVV
TSLSDSFGSSAEKGTQDLIGSFLIKVAYQSSVITSAMFLTAMAGNPLVAALAGHVGVSLSWVLWAKAAIIPGL
PSLFLMPIILYKLYPPKITSCEEAIRSAKLRLKEMGPLKKEEKTILMIFFLLVVLWTFGDLLGISATTAALIGLSL
LILTNILDWQKDVIANTTAWETFIWFGALIMMASFLNQLGFIPLVGDSAAALVSGLSWKIGFPLLFLIYFYSHY

LFASNTAHIGAMYPIFLAVSISLGTNPIFAALTLAFASNLFGGLTHYGSGPAPLYFGSHLVTVQEWWRSGFALS
IVNIVIWIGIGSLWWKALGLI

>core/231/2/Org2_Gene993

MTTKSLGSFNSVISKNKIHFISLGCSRNLVDSEVMLGILLKAGYESTNEIEDADYLILNTCAFLKSARDEAKDY
LDHLIDVKKENAKIIVTGCMTSNHKDELKPWMSHIHYLLGSGDVENILSAIESRESGEKISAKSYIEMGEVPRQ
LSTPKHYAYLKVAEGCRKRCAFCIIPSIKGKLRSKPLDQILKEFRILVNKSVKEIILIAQDLGDYGKDLSTDRSS
QLESLLHELLKEPGDYWLRMLYLYPDEVSDGIIDLMQSNPKLLPYVDIPLQHINDRILKQMRRTTSREQILGFL
EKLRAKVPQVYIRSSVIVGFPGETQEEFQELADFIGEGWIDNLGIFLYSQEANTPAAELPDQIPEKVKESRLKIL
SQIQKRNVDKHNQKLIGEKIEAVIDNYHPETNLLLTARFYGQAPEVDPCIIVNEAKLVSHFGERCFIEITGTAG
YDLVGRVVKKSQNQALLKTSKA

>core/232/2/Org2_Gene237

MHSHSKPTKPLGTFTVGMLSLAVVISLRNLPLTAKHGLSTLFFYGLAVICFMIPYALISAELASFKPQGIYIWA
RDALGKWWGFFAIWMQWFHNMTWYPAVLAFIASTIVYKINPELAHNKVYIATVILAGFWILTFFNFLGITSS
ALFSSICVIIGTLIPGVILVSLALFWIFSGNPIAISLSWGNLLPNFSNVSSLVLLAGMLLALCGLEANANLASDM
VNPRKNYPKAVFIGAIATLTILVLGSLSIAIVIPKEEISLVSGLVKTFTLFFDKYNLSWMTGIVVVMTIAGSLGE
LNAWMFAGTKGLFISTQNDCLPRLFKKVNSKNVPTNLMLFQGIVVTIFTLLFLCLDSADLVYWILTALSVQM
YLAMYICLFLAGPILRIKEPRAQRLYSVPGKFLGICTMSILGILSCAFALWVSFLPPRELAQISEGSKIGYTTFLL
LAFSLNCLIPFGIYFTHKRLSKKS

>core/233/2/Org2_Gene954

MDKSTGVPLPSPPHSKESEMIVLGCMLTGVHYLNLAANQLYEEDFYYLEHKIIFRVLQDAFKQDKPIDVHLA
GEELKRHNQITVIGGPSYLITLAEFAGTAAYLEEYVDIIRSKSILRKMISTAKEIEKRALEQPKNVAEALDEAQN
SFFKISQSTSVSQYTLVADKLRGLTTTTDKPYLVQLQERQELFLQNAQGDNKSFFTGIPTHFIDLDQLIHGFSPS
NLMILAARPAMGKTALALNIAENLCFQNRLPIGIFSLEMTVDQLIHRMICSRSEVDSKKISIGDLSGHDFQRIVS
VINEMQEHTLLIDDQPGLKVSDLRARARRMKESYDIQFLIIDYLQLLSGSGTLRATESRQTEISEISRMLKTLAR
ELNIPILCLSQLSRKVEDRANHRPMMSDLRESGSIEQDSDLVMFLLRREYYDPNDKPGTAELIIAKNRHGSIGS
VPLVFEKELARFRNYSAFECIS

>core/234/2/Org2_Gene657

MKITVNRGLDLSLQGSPKESGFYNKIDPEFVSIDLRPFQPLSLKLKVEQGDAVCSGAPIAEYKHFPNTYITSHV
SGVVTAIRRGNKRSLLDVIIKKTPGPTSTEYTYDLQTLSRSDLSEIFKENGLFALIKQRPFDIPAIPTQTPRDVFIN
LADNRPFTPSPEKHLALFSSREEGFYVFVVGVRAIAKLFGLRPHIVFRDRLTLPTQELKTIAHLHTVSGPFPSGS
PSIHIHSVAPITNEKEVVFTLSFQDVLTIGHLFLKGRILHEQVTALAGTALKSSLRRYVITTKGASFSSLINLNDI
SDNDTLISGDPLTGRLCKKEEEPFLGFRDHSISVLHNPTKRELFSFLRIGFNKPTFTKTYLSGFFKKKRTYTNPD
TNLHGETRPIIDTDIYDKVMPMRIPVVPLIKAVITKNFDLANELGFLEVCGEDFALPTLIDPSKTEMLTIVKESLI
EYAKESGILTPHQD

>core/236/2/Org2_Gene529

MKEERSSEILPRVKETKKHAYVSMQEKSCVGECAVVASESEEAESVTVTKIAKLQRMGIEELNILARQYGVK
NIGSLTKSQVVFEIVKAKSERPDELLIGEGVLEVLPDGFGFLRSPTYNYLPSAEDIYVSPAQIRRFDLKKGDTIIG
TIRSPKEKEKYFALLKVDKINGSTPDKAKERVLFENLTPLYPNQRIVMEMGKDHLAERVLDLTAPIGKGQRG
LIVAPPRSGKTVILQSIAHAIAVNNPDIVLIVLLIDERPEEVTDMIRQVRGEVVASTFDEQPERHIQVAEMVIEK
ARRLVEHGNDVVILLDSITRLARAYNTVQPHSGKILTGGVDASALHKPKRFFGAARNIEGGGSLTILATALIDT
GSRMDEVIFEEFKGTGNMELVLDRRLSDRRTYPAIDLIKSGTRKEELLYHPSELERVYLFRQAIADLTTIDAM
HLLLGRLKKTNSNAEFLLSLKE

>core/237/2/Org2_Gene678

MNFKLPVYHIGLTKAENNTIKIAILQKTCKGWIVCHCEQIPEGKTWSLPKKYFAAPTTFSLQGSDILVKSSSSS
LKNRKNILKVALTNLEASLALPWESLIVQPQLGKPTDRGETPLTLWIAQKNTLKKELSFLSQAQIFPDKLSCRA
ADIFFLAEQSPLKSLPAYLLIYGGSEEVTCIFVKNHAIAVARSFSNHSTKKSCDDIHATLQYIQETFPQTVLPAIH
VAQISPNLQKILEQKLSLPLVVCQSMTYGVEDEDWEIYGDTIAAAHHGASRRPLTFPYDATSVSPAAQKHWL
LRSSLLIGKYALMATVVVSLGSVLKLKSLSSSASNHFAFACPEEGVLPRSLKAAEKTVKAIGKKNSASNYPLL
PTIPTSEQTLKFLLALGKSSPSIKFSYFSYTMTSYPSKDNPSLPYSALVEVKGQGQPEDIPQFLKKISSHPKLQHV
SESLEDQRSFKLQFTLSS

>core/238/2/Org2_Gene760

MTESVYSPSAMHVNSLADKLKAINQEHILDIWPSLSPKQQQRLFQQLTSVDIDFFRKQQQLLSSPTAILKDFHP
ITSFASSGEDPERAHAGTTLLKEKKVACVVLAGGQGSRLKCDGPKGLFPVSPIKKKPLFQLVAEKVRAASKL
AGQPLPLAFMTSPLNTRQTRSFFESNDYFHLDPNQVDFFCQPLWPLLTLSGDLFLEDMDTLALGPNGNGCIAT
LLYTSGVWEKWKNAGIEMVSVIPIDNPLALPFDVELCGFHAMSNNEVTIKAALRQTAIEDVGILVKSHDSGK
TSVIEYSEIPQNERFALNEDGKLKYCLANIGLYCLSMDFIRHAAYQQLPLYKVHKHAKQLGHTSLNEKNAWK
FEEFIFDLFCYSDHCQTLVYPRQECFAPLKNLEGNHSPDTVRQALSDRERQLFHKVTGKKLSPNTTFELEADF
YYPSTSTSLHWENKAFFEEPFFEAS

>core/239/2/Org2_Gene741

MTQEFDCVVIGAGPSGYVAAITAAQSKLRTALIEEDQAGGTCLNRGCIPSKALIAGANVVSHIKHAEQFGIHV
DGYTIDYPAMAKRKNTVVQGIRQGLEGLIRSNKITVLKGTGSLVSSTEVKVIGQDTTIIKANHIILATGSEPRPF
PGVPFSSRILSSTGILELEVLPKKLAIIGGGVIGCEFASLFHTLGVEITVIEALDHILAVNNKEVSQTVTNKFTKQ
GIRILTKASISAIEESQNQVRITVNDQVEEFDYVLVAIGRQFNTASIGLDNAGVIRDDRGVIPVDETMRTNVPNI
YAIGDITGKWLLAHVASHQGVIAAKNISGHHEVMDYSAIPSVIFTHPEIAMVGLSLQEAEQQNLPAKLTKFPF
KAIGKAVALGASDGFAAIVSHEITQQILGAYVIGPHASSLIGEMTLAIRNELTLPCIYETVHAHPTLSEVWAEG
ALLATNHPLHFPPKS

>core/240/2/Org2_Gene42

MMSSKRTSKIAVLSILLTFTHSIGFANANSSVGLGTVYITSEVVKKPQKGSERKQAKKEPRARKGYLVPSSRT
LSARAQKMKNSSRKESSGGCNEISANSTPRSVKLRRNKRAEQKAAKQGFSAFSNLTLKSLLPKLPSKQKTSIH
EREKATSRFVNESQLSSARKRYCTPSSAAPSLFLETEIVRAPVERTKELQDNEIHIPVVQVQTNPKEQNTKTTK
QLASQASIQQSEGTEQSLRELAQGASLPVLVRSNPEVSVQRQKEELLKELVAERRQCKRKSVRQALEARSLT
KKVARGGSVTSTLRYDPEKAAEIKSRRNCKVSPEAREQKYSSCKRDARANGKQDKTTPSEDASQEEQQTGA

GLVRKTPKSQVASNAQNFYRNSKNTNIDSYLTANQYSCSSEETDWPCSSCVSKRRTHNSISVCTMVVTVIAMI
VGALIIANATESQTTSDPTPPTPTP

>core/241/2/Org2_Gene263

MRAWEEFLLLQEKEIGTNTVDKWLRSLKVLCFDACNLYLEAQDSFQITWFEEHIRHKVKSGLVNNNNKPIRV
HVTSVDKAAPFYKEKQMQQEKTAYFTMHYGSVNPEMTFSNFLVTPENDLPFRVLQEFTKSPDENGGVTFNPI
YLFGPEGSGKTHLMQSAISVLRESGGKILYVSSDLFTEHLVSAIRSGEMQKFRSFYRNIDALFIEDIEVFSGKSA
TQEEFFHTFNSLHSEGKLIVVSSSYAPVDLVAVEDRLISRFEWGVAIPIHPLVQEGLRSFLMRQVERLSIRIQET
ALDFLIYALSSNVKTLLHALNLLAKRVMYKKLSHQLLYEDDVKTLLKDVLEAAGSVRLTPLKIIRNVAQYYG
VSQESILGRSQSREYVLPRQVAMYFCRQKLSLSYVRIGDVFSRDHSTVISSIRLIEQKIEENSHDIHMAIQDISKN
LNSLHKSLEFFPSEEMII

>core/242/2/Org2_Gene901

MRQEKDSLGIVEVPEDKLYGAQTMRSRNFFSWGPELMPYEVIRALVWIKKCAAQANQDLGFLDSKHCDMIV
AAADEILEGGFEEHFPLKVWQTGSGTQSNMNVNEVIANLAIRHHGGVLGSKDPIHPNDHVNKSQSSNDVFPT
AMHIAAVISLKNKLIPALDHMIRVLDAKVEEFRHDVKIGRTHLMDAVPMTLGQEFSGYSSQLRHCLESIAFSL
AHLYELAIGATAVGTGLNVPEGFVEKIIHYLRKETDEPFIPASNYFSALSCHDALVDAHGSLATLACALTKIAT
DLSFLGSGPRCGLGELFFPENEPGSSIMPGKVNPTQCEALQMVCAQVLGNNQTVIIGGSRGNFELNVMKPVII
YNFLQSVDLLSEGMRAFSEFFVKGLKVNKARLQDNINNSLMLVTALAPVLGYDKCSKAALKAFHESISLKEA
CLALGYLSEKEFDRLVVPENMVGNH

>core/243/2/Org2_Gene959

MNFSLFLFFLIAIQGICLYVGRRGSKKVEDRESYFLAGRSLKIFPLMMTFIATQIGGGVLLGAAEEAFCYGYGG
ILYPLGVALGLIFLGMGPGKRLAEGSLTTVVSIFEVFYGSKKLRKIAFLLSAGSLFFILVAQVIALDRLFSSFPFG
KYVTVAFWIVLASYTSTGGFRGVVRTDVIQAGFLLIAVLVCGVSVWLSVPKSLSVLDPFQSLPCAKLSNWIF
MPMLFMLVEQDMVQRCVAASSPKRLQWAAVGAGLVLLLFNFIPLFLGSLGAKAGLKAGCPLIDTIAYFCNPS
LAAVMAAAIGVAILSTADSLMNAVSQLIAEEYPTLKAPYYRYLVLGLAVAAPLVAIGFTNIVDVLILSYSLSV
CCLSVPVGFYLLAPKGRRVSGAAAWAGVLVGALGYGWVQIVSLGMFGELLAWVGSLVAFSFVGFIEITWK
NKVKTQT

>core/244/2/Org2_Gene860

MQQSVRKLFGTDGVRGRANFEPMTVETTVLLGKAVARVLREGRSGKHRVVVGKDTRLSGYMFENALIAGL
NSMGIETLVLGPIPTPGVAFITRAYRADAGIMISASHNPYRDNGIKIFSLEGFKISDVLEQRIETMVSEADFGPLP
EDHAVGKNKRVIDAMGRYVEFVKATFPKGRTLKGLKIVLDCAHGASYKVAPSVFEELDAEVICYGCEPTGIN
INEHCGALFPQVIQKAVIEHQAHLGIALDGDGDRIIMVDEKGHIVDGDMILSICAGDLKKRSALPHNRVVATI
MTNFGVLKYLEGLGLQVFTSPVGDRHVLHAMLEHEVTLGGEQSGHMIFLDYNTTGDGIVSALQVLRIMIESE
SMLSDLTAPIVKSPQTLINVAVREKIPLETIPLIERTLRDVQDALGPSGRILLRYSGTENICRVMVEGHKKHQV
DCLAKALADVIDAELGTGSRE

>core/245/2/Org2_Gene492

MQIAQVFGCGRLNGEVKVSGAKNAATKLLVASLLSDQKCTLRNVPDIGDVSLTVELCKSLGAHVSWDKETE
VLEIYTPEIQCTRVPPTFSNVNRIPILLLGALLGRCPEGVYVPTVGGDAIGERTLNFHFEGLKQLGVQISSDSSG
YYAKAPRGLKGNYIHLPYPSVGATENLILAAIHAKGRTVIKNVALEAEILDLVLFLQKAGADITTDNDRTIDIF
GTGGLGSVDHTILPDKIEAASFGMAAVVSGGRVFVRNAKQELLIPFLKMLRSIGGGFLVSESGIEFFQERPLVG
GVVLETDVHPGFLTDWQQPFAVLLSQAQGSSVIHETVHENRLGYLHGLQHMGAECQLFHQCLSTKACRYAI
GNFPHSAVIHGATPLWASHLVIPDLRAGFAYVMAALIAEGGGSIIENTHLLDRGYTNWVGKLRSLGAKIQIFD
MEQEELTTSPKSLALRDASL

>core/246/2/Org2_Gene779

MLYFIEQLNKLSTSFCVFPMILLLGGFLTWKLRGLQFHGLKLGFNLMLQNKLDDSSSKANEVSSYEAVAGIL
AGNFGTGNIAGMAVALACGGPGALVWVWLAALLGAIVQYAGSYLGSKYRKPEGNTGEFIGGPIACLAFGM
RKKILAGFFALFTIMTAFCAGNCVQVSCIVPLCAEGTPGKLLVGILLALVVIPVLAGGNNRILRFSARVIPFIAG
FYCISCGIILFQHASAILPAIKLICSSAFGIKAGLAGIGGYTLSQVISTGINRAVMATDCGSGMVSILQANTKSKN
PVVDGLVTLVPPVIVMVVCSITMLVLIVSGAYSSGAQGTLMVMSAFKNSLGSLGSVIVILAMALFGYTTILTW
FACAEKSLQYMIPGRRANLWLKAIYVLIIPLGGVIDMRMIWALSDTGFSGMVILNCIALIALLKDVLSTNRDV
ALLKERECSVADPVRNLDA

>core/247/2/Org2_Gene790

MFNVNFKFLEGLHQPAPRYTSYPTALEWEPSDAAPALLAFQRIRENPQPLSLYFHIPFCQSMCLYCGCSVVLN
RREDIVEAYINTLIQEMKLVVETIGFRPQVSRIHFGGGTPSRLSRELFTLLFDHIHKLFDLSHAEEIAIEVDPRSL
RNDMEKADFFQNVGFNRVSLGVQDTQADVQEAVRRRQSHEESLKAYEKFKELAFQSINIDLIYGLPKQTKES
FSKTIQDILAMYPDRLALFSFASVPWIKPHQKAMKASDMPSMEEKFAIYSQSRHLLTKAGYQAIGMDHFSLP
HDPLTLAFKNKTLIRNFQGYSLPPEEDLLGLGMTSTSFIRGIYLQNAKTLEEYHNTVLRGTFATVKSKILTEDD
RIRKWAIHKLMCTFTINKEEFFNLFGYEFDTYFIESRDRLISMETTGLIHNSPGSLKVTPLGELFVRVIATAFDH
YFLNKVSKKECFSASI

>core/248/2/Org2_Gene547

MTTLRQFFLITELRQKLFYTFALLTACRVGVFIPVPGINGELAVAYFKQLLGSGQNLFQLADIFSGGAFAQMT
VIALGVVPYISASIIVQLFLVFMPALQREMRESSDQGKRRIGRLTRLFTVALAVIQSLLFAKFALRMNLTIPGIV
LPTLLSSKLFGVPWIFYITTVVVMTTGTLLLMWIGEQISDKGIGNGISLIIALGILSSFPSVLGSIVNKLNLGSQD
SSDLGLISILILALVFVFVLITTILIIEGVRKIPVQYARRVIGRREVPGGGSYLPLKVNYAGVIPVIFASSLLMFPA
TIGQFIASESSWMKRIAALLAPGSLVYSICYVLLIIFFTYFWTATQFHPEQIASEMKKNNAFIPGIRQGKPTQHY
LEYTMNRVTLLGALFLAAIAILPSLLGCLLRVDSNVSYFLGGTAMLIVVGVVLDTMKQVDAFLLMRRYDSVL
KTDRTKGRH

>core/250/2/Org2_Gene582

MNVWTKFFQPPKHIKEIEDQEVVKKKYKYWRIRIFYSMFIGYIFYYFTRKSFTFAMPTLIADLGFDKAQLGIIG
STLYFSYGISKFVSGVMSDQSNPRYFMAIGLMITGLTNIFFGMSSSIVLFALWWGLNGWFQGWGWPPCARLL
THWYAKSERGTWWSVWSTSHNIGGALIPILTGFIIDYSGWRGAMYVPGILCIGMGLVLINRLRDTPQSLGLPPI
EKYKRDPHHAHHEGKSASEGTEEIERELSTREILFTYVLTNQWLWFLAAASFFIYIVRMAVNDWSALFLIETK
HYAAVKANFCVSLFEIGGLFGMLVAGWLSDKISKGNRGPMNVLFSLGLLFAILGMWFSRSHNQWWVDGTL

LFVIGFFLYGPQMMIGLAAAELSHKKAAGTASGFTGWFAYFGATFAGYPLGKVTDVWGWKGFFIALLACAS
IALLLFLPTWNATEKNTRSKA

>core/251/2/Org2_Gene148

MKKVLIANRGEIAVRIIRACHDLGLSTVAVYSLADQEALHVLLADEAICIGEPQAAKSYLKISNILAACEITGA
DAVHPGYGFLSENANFASICESCGLTFIGPSSESIAMMGDKIAAKSLAKKIKCPVIPGSEGIIEDESEGLKIAEKI
GFPIVIKAVAGGGGRGIRIVKEKDEFYRAFSAARAEAEAGFNNPNVYIEKFIENPRHLEIQVIGDTHGNYVHLG
ERDCTIQRRRQKLIEETPSPILNAEIRVKVGKVAVDLARSAGYFSVGTVEFLLDKDKKFYFMEMNTRIQVEHT
ITEEVTGIDLVKEQIHVAMGNKLPWKQKNIEFSGHIIQCRINAEDPTNNFSPSPGRLDYYLPPAGPSIRVDGAC
YSGYAIPPYYDSMIAKVIAKGKNREEAIAIMKRALKEFHIGGVQSTIPFHQFMLDNPKFLESNYDINYIDNLLA
QGNSFFKEF

>core/252/2/Org2_Gene33

MATKTKTQWTCNQCGATAPKWLGQCPGCHNWNSLVEEYVPQARSGTSSRSSTSAIALSSIELENESRIFIDHA
GWDRILGGGVVRGSLTLLGGDPGIGKSTLLLQTAERLASQKYKVLYVCGEESVTQTSLRAKRLNISSPLIYLF
PETNLDNIKQQIATLEPDILIIDSIQIIFNPTLNSAPGSVAQVREVTYELMQIAKSAQITTFIIGHVTKSGEIAGPRV
LEHLVDTVLYFEGNSHANYRMIRSVKNRFGPTNELLILSMHADGLKEVSNPSGLFLQEKTGPTTGSMIIPIIEG
SGALLIELQALVSSSPFANPVRKTAGFDPNRFSLLLAVLEKRAQVKLFTMDVFLSITGGLKIIEPAADLGALLA
VASSLYNRLLPNNSIVIGEVGLGGEIRHVAHLERRIKEGKLMGFEGAILPEGQISSLPKEIRENFRLQGVKTIKD
AIRLLL

>core/253/2/Org2_Gene777

MLKILKIKVLVFPLALLMGCNSIGYAGPQGSLQTNSQTKVKIGSEVWIEQKLRQYPELLWLTESGGAPLLTST
PIDMAYSEKLFNKKVPALDIAIRSMIHLHLLIQGSRQSYMQLSQILPSEEGGMTFKQFQTAHKQLLFFLNSPKS
FDNTLRILETAIVLRHVGCSAKAVTTFKPYFTDSCPQSFYAKALHVLRTFPELCPSYARLSPEQQEVLLSLRRL
GNYDSLLNLTEVPSAQLLSAWRTRRSLAILDLYLYCLDTCGDKNCSQEFYINFAPLLSMLQQHATIEEAFSRY
FTYRANRLGFEGTSRTDMTLVRLATLMNLSPSEASTLAWSFKNLPSDEAENLVNSFYTVQGEHIPLTFRGLPS
LVAGLSVATHGSTVSPENRLRQLYSTMLSLLVKSLRSHREMLNKQLLPQGTVLDFSETTLSSGGLDVFAESIA
VRIHLNGAVSINL

>core/254/2/Org2_Gene156

MRKRHSFDSTSTKKEAVSKAIQKIIKIMETTDPSLNVETPNAEIESILQEIKEIKQKLSKQAEDLGLLEKYCSQE
TLSNLENTNASLKLSIGSVIEELASLKQLVEESIEESLGQQDQLIQSVLIEISDKFLSSIGETLSGNLDMNQNVIQ
GLLIKENPEKSEAASVGYVQTLLEPLSKRIGETHKKVATHDVNISSLQFHMMSVAGGRFRGHIDMNGYRVLG
LGEPKNGEDAVSKDYLERYVSSQLTIDKVEDKPITKPNKGKLLYSQGTSPKLESPLPLGLLTSGISGFTWKSAS
KSNDGSFPFSALRHKETESDTDCFQITSTTLSGNQAGTYTWSLSLKVLVPSIFQIEKPEVQLSLVYSYEDWLPID
NIFNMSQPRTIPLALLGQTMLAGQKYDILELAAHQTNQTLMISPNCSRFSLQLKQTNQFENSPVDFYIVHAAH
SCHWSGF

>core/255/2/Org2_Gene364

MLTCNECTTWEQFLNYVKTRCSKTAFENWISPIQVLEETQEKIRLEVPNIFVQNYLLDNYKRDLCSFVPLDVH
GEPALEFVVAEHKKPSAPVASQKESNEGISEVFEETKDFELKLNLSYRFDNFIEGPSNQFVKSAAVGIAGKPGR
SYNPLFIHGGVGLGKTHLLHAVGHYVREHHKNLRIHCITTEAFINDLVYHLKSKSVDKMKNFYRSLDLLLVD
DIQFLQNRQNFEEEFCNTFETLINLSKQIVITSDKPPSQLKLSERIIARMEWGLVAHVGIPDLETRVAILQHKAE
QKGLLIPNEMAFYIADHIYGNVRQLEGAINKLTAYCRLFGKSLTETTVRETLKELFRSPTKQKISVETILKSVA
TVFQVKLNDLKGNSRSKDLVLARQIAMYLAKTLITDSLVAIGAAFGKTHSTVLYACKTIEHKLQNDETLKRQ
VNLCKNHIVG

>core/256/2/Org2_Gene850

MILPPYSYSLKIGAAVLFFCSILHTFLTPWLYTLCQSYEHKKLVFPECWKRYARLSELFRILSRVEIVFFLWAVP
LFFWFLYTEGYRISMAYFNSRNYGFAVFIMVILILLESRPIVYFAELVLSSIAKLGKTSPKSWWWTLMIAPPLL
SCLLKETGAMIIGATLLMRHFYVFSPSRRFAYATMGLLFSNISIGGLTSYVSSRALFLIFPALKWEHSFFLSHFA
WKAIVAILISTTIYYFIFRKEFKKFPDIPSDKDPSVEKVPWWIICVNIIFVGSIILSRSTPLFMGALLLFYLGFQKFT
IFYQDPINLSKVCYVGLFYAGLVVFGDLQEWWVLNLMQGLSDFGYMTVSYTLSIFLDNALVNYLVHNLSVA
TDCYHYLVVAGCMAAGGLTLVSNIPNIVGYLILRSAFPSSTIHMGWLFLGALGPSIISLGVFWLLKNVPEFLYC
FFR

>core/257/2/Org2_Gene670

MSEPRFVCLSLGSNLGNRFKNLQIARTLLGEQAVLGLRSSVILETEALLLPGSPPEWDLPYFNSVLVGETTLSL
RELLVTIKQIEKVVGRAEESPPWSPRTIDVDILLYGDESFCCDHTEITIPLSNLLSRPFLIALIASLCPYRRFCTQG
SPYHNFTFGELAHHLPSPPGMIRRSLSPDTMLMGVVNVTNDSMSDGGMFLDPEKAVAQAEKLFTEGAAVIDF
GAQATNPKVKQFLSVDQEWERLEPVLRLLKETWSNRKQYPIISLDTFYPEIILRAMDIYPIQWINDVSGGSQS
MAEVARDCELSLVMNHSSSLPVDPKNILSFSVPIGEQLLSWGEKQLKMFSDVGLNANQVIFDPGIGFGKGAA
QSLATLYEIAKFKRLGCPILIGHSRKSFLSLFGNHDPKDRDWETVGLSILLQQQGVDYLRVHNVAAHQKALS
VAACEACAPI

>core/258/2/Org2_Gene458

MNRLLSLLSVFDDFFWSYVAFILIIVLGVSFSWKSRFFQFTKFSQFCKLFRYYSQNPQERETKQGVHPLKVFFA
SAGGNIGIGNVVGIVTAACIGGPGALFWVWIAGIFGSIVKYSEVYLGIKFRKLDRDGVYQGGPMYFLIKAFKT
PVVSVIVAILLCIYGVEIYQFSVITDSLAHCWNLPKVYPMLGLLFLVFYAIRGGLQRIGKICSIVLPFFMLLYCA
LSLYILVKEFHTLPHLLSTVFSSAFKGQSALGGFAGCTVATTIHQGISRAAYSGDIGIGFDSIIQSESSAKDPSTQ
AQLSIVGIAIDNLICTLSLLMVLASGSWSLGLENASQVVEHTLASYFPMVKFFLPTFFFVTGYTTIISYFLVGKK
CAKFLYGNTGAKIYTLYGLLILPLFCFLSQNTALLIMSVSGALLLCFNLLGVFILRKEVIFPARAASLTETSLST
E

>core/259/2/Org2_Gene294

MLSCVFSDTIFLSSFLAVTLICMTTALWGTILLISKQPLLSESLSHASYPGLLVGALMAQYVFSLQASIFWIVLF
GCAASVFGYGIIVFLGKVCKLHKDSALCFVLVVFFAIGVILASYVKESSPTLYNRINAYLYGQAATLGFLEAT
LAAIVFCASLFALWWWYRQIVVTTFDKDFAVTCGLKTVLYEALSLIFISLVIVSGVRSVGIVLISAMFVAPSLG
ARQLSDRLSTILILSAFFGGISGALGSYISVAFTCRAIIGQQAVPVTLPTGPLVVICAGLLAGLCLLFSPKSGWVI
RFVRRKHFSFSKDQEHLLKVFWHISHNRLENISVRDFVCSYKYQEYFGPKPFPRWRVQILEWRGYVKKEQDY

YRLTKKGRSEALRLVRAHRLWESYLVNSLDFSKESVHELAEEIEHVLTEELDHTLTEILNDPCYDPHRQIIPNKKKEV

>core/260/2/Org2_Gene90

MINSLSQKLSSIFSFLVSSRRINEENISESIREVRLALLDADVNYHVVKDFISKVKEKILGEEIWKHVSPGQQFIRCLHEELVAFLSDGREEFTIQKTPSIILLCGLQGAGKTTTAAKLADYVIKNKKAKKVLVVPCDLKRFAAVDQLKILVAQTKAEFYQSQENKPINVVVKALAYAKENGHDFVILDTAGRLNIDNELMEELTAIQKVSQANERLFVMNVAMGQDVLATVQAFDQSLDLTGVILSMTDGDARAGAVFSIKHVLGKPIKFEGCGERIQDLRSFDPQSMAERILGMGDTINFVKEMREYISEEEDAELGKKLVTAAFTYEDYYKQMKAFRRMGPLRKLLGMMPGFNNAKPSQKEIEDSEQQMKRTEAIILSMTPEERKELVELDMSRMKRIASGCGLTLGDVNQFRKQMSQSKKFFKGMSKGKMEQVRKKMSGGNQWR

>core/261/2/Org2_Gene78

MDALILSRIQFGLFITFHYLFVPLSMGLSMMLVIMEGLYLVTKKQIYKQMTWFWVGIFALTFVLGVVTGIMQIFSFGSNWANFSEYTGNIFGTLLGSEGVFAFFLESGFLGILLFGRHKVSKKMHFFSTCMVALGAHMSAFWIICANSWMQTPSGYEMVMHKGKLIPALTSFWGVVFSPTTIDRFIHAVLGTWLSGVFLVISVSAYYLWKKRHHEFAKQGMKIGTICAVIVLVLQLWSADVTARGVAKNQPAKLAAFEGIFKTEEYTPIWAFGYVDMEKERVIGLPIPGALSFLVHRNIKTPVTGLDQIPRDEWPNVQAVFQLYHLMIMLWGVMVALTLISWSAYKGWRWALKPFFLVILTFSVLLPEICNECGWCAAEMGRQPWVVQGLLKTKDAVSPIVQANQIVQSLVIFSLVFIALLTLFITVLCKKIKHGPEEENDLTEFEVK

>core/262/2/Org2_Gene798

MRAMLLEDWVSLMLSDVSCPKCDKKITGFAIDSQQVQPGDLFFALPGNATDGHQFLKHAATAGAVAAVVSHDYQGDSFGLELIRVDDTKSALQEAGSNQCNLFQGTLVGITGSVGKTTTKEFSKTILSSIYKTHASPKSYNSQLTVPLSLLMAEGDEDVMILEMGVSEPGNMQDLLRIVQPEIAVITHINDQHAMHFPQGIQEILKEKSYILQKSKLQLLPKDSPYYLDLRSCSPTAEKFSFSFNDPLADFCYKAISGDSVVIQTPEENYCLPIAFSYKPAYTNLLIAVALSWILEVPEEGVIRSLPELKLPPMRFEHSMRNGMQVINDAYNACPEAMIAALDALPLPSDGGKIILILGHMAELGRYSEEGHALVAEKAASRGDMIFFIGEKWIPVQSVLKSYSCEVSFFSSAQDVKDILKQVARYGDVILLKGSRALALESLLACF

>core/263/2/Org2_Gene300

MSNITSPVIQNNRSCNYYFELKNSTTIHIVISAILLCGALIAFLCVAAPVSYILSGALLGLGLLIALIGVILGIKKITPMISSKEQVFPQELVNRIRAHYPKFVSDFVSEAKPNLKDLISFIDLLNQLHSEVGSSTNYNVSEELQQKIDTFEGIARLKNEVRTASLKRLESAASSRPLFPSLPKILQKVFPFFWLGEFISAGSKVVELHRVKKIGGSLEEDLSDYIKPEMLPTYWLIPLDFRPTNSSILNLHTLVLARVLTRDVFQHLKYAALNGEWNLNHSDLNTMKQQLFAKYHAAYQSYKHLSQPSLQEDEFYNLLLCIFKHRYSWKQMSLIKTVPADLWENLCCLTLDHTGRPQDMEFASLIGTLYTQGLIHKESEAFLSSLTLLSLDQFKTIRRQSTNIAMFLENLATHNSTFRSLPPITVHPLKRSVFSQPEEDESSLLIG

>core/264/2/Org2_Gene998

MVITNLSINADTIYGKNPINIVASAANKNITLTGTLALVNADGAFYENHTLQDSQDYSFVKLSPGAGGTIITQDASQKPLEVAPSRPHYGYQGHWNVQVIPGTGTQPSQANLEWVRTGYLPNPERQGSLVPNSLWGSFVDQRAIQ

EIMVNSSQILCQERGVWGAGIANFLHRDKINEHGYRHSGVGYLVGVGTHAFSDATINAAFCQLFSRDKDYV
VSKNHGTSYSGVVFLEDTLEFRSPQGFYTDSSSEACCNQVVTIDMQLSYSHRNNDMKTKYTTYPEAQGSWA
NDVFGLEFGATTYYYPNSTFLFDYYSPFLRLQCTYAHQEDFKETGGEVRHFTSGDLFNLAVPIGVKFERFSDC
KRGSYELTLAYVPDVIRKDPKSTATLASGATWSTHGNNLSRQGLQLRLGNHCLINPGIEVFSHGAIELRGSSR
NYNINLGGKYRF

>core/265/2/Org2_Gene924

MLTYKVSPSSVYGNAFIPSSKSHTLRAILWASVAEGKSIIYNYLDSPDTEAMICACKQMGASIKKFPQILEIVG
NPLAIFPKYTLIDAGNSGIVLRFMTALACVFSKEITVTGSSQLQRRPMAPLLQALRNFGASFHFSSDKSVLPFT
MSGPLRSAYSDVEGSDSQFASALAVACSLAEGPCSFTIIEPKERPWFDLSLWWLEKLHLPYSCSDTTYSFPGSS
HPQGFSYHVTGDFSSAAFIAAAALLSKSLQPIRLRNLDILDIQGDKIFFSLMQNLGASIQYDNEEILVFPSSFSGG
SIDMDGCIDALPILTVLCCFADSPSHLYNARSAKDKESDRILAITEELQKMGACIQPTHDGLLVNPSPLYGAVL
DSHDDHRIAMALTIAALYASGDSRIHNTACVRKTFPNFVQTLNIMEARIEECHDNYSMWSTHKRKVFARESF
G

>core/266/2/Org2_Gene717

MTSGVSGSSSQDPTLAAQLAQSSQKAGNAQSGHDTKNVTKQGAQAEVAAGGFEDLIQDASAQSTGKKEATS
STTKSSKGEKSEKSGKSKSSTSVASASETATAQAVQGPKGLRQNNYDSPSLPTPEAQTINGIVLKKGMGTLAL
LGLVMTLMANAAGESWKASFQSQNQAIRSQVESAPAIGEAIKRQANHQASATEAQAKQSLISGIVNIVGFTV
SVGAGIFSAAKGATSALKSASFAKETGASAAGGAASKALTSASSSVQQTMASTAKAATTAASSAGSAATKA
AANLTDDMAAAASKMASDGASKASGGLFGEVLNKPNWSEKVSRGMNVVKTQGARVASFAGNALSSSMQ
MSQLMHGLTAAVEGLSAGQTGIEVAHHQRLAGQAEAQAEVLKQMSSVYGQQAGQAGQLQEQAMQSFNTA
LQTLQNIADSQTQTTSAIFN

>core/267/2/Org2_Gene29

MTLQPYQASSRKYRPQIFREILGQSSVVAVLKNALVFNRAAHAYLFSGIRGTGKTTLARILAKALNCVHLSED
GEPCNQCFSCKEIASGSSLDVLEIDGASHRGIEDIRQINETVLFTPVKAKFKIYIIDEVHMLTKEAFNALLKTLE
EPPQHVKFFFATTEIHKIPGTILSRCQKMHLQRIPEKTILEKLSLMAQDDHIEASQEALAPIARAAQGSLRDAES
LYDYVISLFPKSLSPDTVAQALGFASQDSLRTLDNAILQRDYATALGIVTDFLNSGVAPVTFLHDLTLFYRNLL
LTNSTTSKFSSQYKTEQLLEIIDFLGESAKHLQNTIFEQTFLETVIIHIIRIYQRPVLSELISSIKSRQFEGLRNIKEP
TLTQQVSAPQPQPTYKEQSFLEKKNQPAAEGKIISVEVKSSASIKSAAVDTLLQFAVVEFSGILRQ

>core/268/2/Org2_Gene745

MLKHDTIAAIATPPGEGSIAVVRLSGPQAIVIADRIFSGSVASFASHTIHLGQVIFEETLIDQALLLLMRSPRSFT
GEDVVEFQCHGGFFACSQILDALIALGARPALPGEFSQRAFLNGKIDLVQAEAIQNLIVAENIDAFRIAQTHFQ
GNFSKKIQEIHTLIIEALAFLEVLADFPEEEQPDLLVPQEKIQNALHIVEDFISSFDEGQRLAQGTSLILAGKPNV
GKSSLLNALLQKNRAIVTHIPGTTRDILEEQWLLQGKRIRLLDTAGQRTTDNDIEKEGIERALSAMEEADGIL
WVIDATQPLEDLPKILFTKPSFLLWNKADLTPPPFLDTSLPQFAISAKTGEGLTQVKQALIQWMQKQEAGKTS
KVFLVSSRHHMILQEVARCLKEAQQNLYLQPPEIIALELREALHSIGMLSGKEVTESILGEIFSKFCIGK

>core/269/2/Org2_Gene753

MPRSLSNEQFSVDLEESPGCIVSALVKVSPEVLNKLNKQALKKIKKEITLPGFRKGKAPDDVIASRYPTNVRK
ELGELVTQDAYHALSTVGDRRPLSPKAVRSNSITQFDLQEGAKVEFSYEAFPAISDLPWENLSLPQEEAASEIS
DSDIEKGLTNIGMFFATKTPVERPSQEGDFISISLHVSKSNDENASSAAIFENKYFKLSEEEMTDAFKEKFLGIS
TGHRVVETITSPEIQSFLRGDTLTFTVNAVIEVSIPEIDDEKARQLQAESLDDLKAKLRIQLEKQAKDKQLQKR
FSEAEDALAMLVDFELPTSLLEERISLITREKLLNARLIQYCSDEELEKRKSELIKEAEEDATKALKLLFLTHKI
FSDEKLTISREELQYMMDVCSRERFGQQPPKDISNDTLQELVMSARDRLTYSKAIEHVLRKAELLASTPSA

>core/270/2/Org2_Gene622

MDQLTTDFDTLMSQLGDVNLTTVVGRITEVVGMLIKAVVPNVRVGEVCLVKRNGMEPLVTEVVGFTQSFAF
LSPLGELSGVSPSSEVIPTGLPLHIRAGNGLLGRVLNGLGEPIDVETKGPLQNVDQTFPIFRAPPDPLHRAKLRQ
ILSTGVRCIDGMLTVARGQRIGIFAGAGVGKSSLLGMIARNAEEADVNVIALIGERGREVREFIEGDLGEEGM
KRSVIVVSTSDQSSQLRLNAAYVGTAIAEYFRDQGKTVVLMMDSVTRFARALREVGLAAGEPPARAGYTPS
VFSTLPRLLERSGASDKGTITAFYTVLVAGDDMNEPVADEVKSILDGHIVLSNALAQAYHYPAIDVLASISRL
LTAIVPEEQRRIIGKAREVLAKYKANEMLIRIGEYRRGSDREIDFAIDHIDKLNRFLKQDIHEKTNYEEAAQQL
RAIFR

>core/271/2/Org2_Gene747

MWLVILWALAASLAIALVAKGYYRFVYFRRYAVQVIREVRLSMELKEWALAEQQLLPILKKRSYRRQCLFE
YMRILRKMQRFEESEKLLAEAKKLGLRGPYFFLEIAYKAYRFGAFKECAQAFASVPQDLFEEEDAAKYASAL
VRLGDLDAACSLIEPWISPLSHQETFVTMGHIYFTSKRYKDAIDFYNRANALGVCPVEVTYNLAQAYRITSSY
AKAGKLFRKLLSNPVYKEEALFNIGLCEQKLGRPGKALLIYQSSDLWSRGDALLMKYAAMAAMDQRDYVL
AEPCWELALRCSTFAKDYKCGLGYGFSLCRLRKYGDAERVYCNLIQNFPECLTACKALAWLCGVGYATLLG
SEEGLMYAKKAVELDHSCETLELLSACEARCGNFDAAYEIQSFLSSRDTSLQEKQRRSQILRILRKKLPLNDH
HIVEVDALLAA

>core/272/2/Org2_Gene526

MIENDFPEASNFESSHFYRDKVGVIILCGGEGKRLSPLTNCRCKPTVSFGGRYKLIDIPISHAISAGFSKIFVIGQ
YLTYTLQQHLFKTYFYHGVLQDQIHLLAPEARQGDQIWYQGTADAIRKNLLYFEDTEIEYFLILSGDQLYNM
DFRSIVDTAIRTHVDMVLVAQPIPEKDAYRMGVLDIDSEGKLIDFYEKPQEKEVLKRFQLSSEDRRIHKLTEDS
GDFLGSMGIYLFRRDSLFSLLREEEGNDFGKHLIQAQMKRGQVQTLLYNGYWADIGTIESYYEANIALTQKP
HAEKRGLNCYDDNGMIYSKNHHLPGAIITDSMISSSLLCEGCVINTSHVSRSVLGIRSKIGENSVVDQSIIMGN
ARYGSPSMPSLGIGKDCEIRKAIIDENCCIGNGVKLQNLKGYIKYDSPDKKLFVRDNIIIVPQGTHIPDNYIF

>core/273/2/Org2_Gene111

MLNCSNQKHTVTFEEACQVFPGGVNSPVRACRSVGVTPPIVSSAQGDIFLDTHGREFIDFCGGWGALIHGHSH
PKIVKAIQKTALKGTSYGLTSEEEILFATMLLSSLKLKEHKIRFVSSGTEATMTAVRLARGITNRSIIIKFIGGYH
GHADTLLGGISTTEETIDNLTSLIHTPSPHSLLISLPYNNSQILHHVMEALGPQVAGIIFEPICANMGIVLPKAEF
LDDIIELCKRFGSLSIMDEVVTGFRVAFQGAQDIFNLSPDITIYGKILGGGLPAAALVGHRSILDHLMPEGTIFQ
AGTMSGNFLAMATGHAAIQLCQSEGFYDHLSQLEALFYSPIEEEIRSQGFPVSLVHQGTMFSLFFTESAPTNFD
EAKNSDVEKFQTFYSEVFDNGVYLSPSPLEANFISSAHTEENLTYAQNIIIDSLIKIFDSSAQRFF

>core/274/2/Org2_Gene683

MFQQKQKLSLKYLPSLRMQQGLQMLQSPLTELSSYVVQEIIDNPFFDLSSLEEEEWSPCYRPTNSTFSYLNQTP
GPQESLYTRLLPQIEEAFSTAEERFIAHQIAGNLSDEGLFLRNPEDFAQELELPLEKIHKVWDTIQNLSPEGIASP
SLQSYWMKLLRNSSHQQAYSIVRDCYPLMTNCEFAPIMKKFSLSLSELRNILKKALGSIPWCPAAACTVKPM
VSTPLPDIYLFYSSGSWKIEVSTRGLPSIKLNKETFHFYEHLPKEEQKNLSQQILSAKWLIKNLRKREQTLLQV
METLLPKQEDFLLGKIPAPYPLGIKDLAEDLSFHESTIFRAIENKAVAAPIGIFPLKHLFPRGIHQDSSHSKENVL
QWIRQWIATEQTPLSDSVISDRITAKGIPCARRTVAKYRAQLKILPANKRKKLFYIRSSNSHFRDRQF

>core/275/2/Org2_Gene933

MSKIVYKFGGTSLATAENICLVCDIICKDKPSFVVVSAIAGVTDLLVDFCSSSLREREEVLRKIEGKHEEIVKNL
AIPFPVSTWTSRLLPYLQHLEISDLDFARILSLGEDISASLVRAVCSTRGWDLGFLEARSVILTDDSYRRASPNL
DLMKAHWHQLELNQPSYIIQGFIGSNGLGETVLLGRGGSDYSATLIAELARATEVRIYTDVNGIYTMDPKVIS
DAQRIPELSFEEMQNLASFGAKVLYPPMLFPCMRAGIPIFVTSTFDPEKGGTWVYAVDKSVSYEPRIKALSLS
QYQSFCSVDYTVLGCGGLEEILGILESHGIDPELMIAQNNVVGFVMDDDIISQEAQEHLVDVLSLSSVTRLHHS
VALITMIGDNLSSPKVVSTITEKLRGFQGPVFCFCQSSMALSFVVASELAEGIIEELHNDYVKQKAIVAT

>core/276/2/Org2_Gene66

MQTIYTKITDIKGNLITVEAEGARLGELATITRSDGRSSYASVLRFDLKKVTLQVFGGTSGLSTGDHVTFLGRP
MEVTFGSSLLGRRLNGIGKPIDNEGECFGEPIEIATPTFNPVCRIVPRSMVRTNIPMIDVFNCLVKSQKIPIFSSSG
EHHNALLMRIAAQTDADIVVIGGMGLTFVDYSFFVEESKKLGFADKCVMFIHKAVDAPVECVLVPDMALAC
AEKFAVEEKKNVLVLLTDMTAFADALKEISITMDQIPANRGYPGSLYSDLALRYEKAVEIADGGSITLITVTT
MPSDDITHPVPDNTGYITEGQFYLRNNRIDPFGSLSRLKQLVIGKVTREDHGDLANALIRLYADSRKATERMA
MGFKLSNWDKKLLAFSELFETRLMSLEVNIPLEEALDIGWKILAQSFTSEEVGIKAQLINKYWPKACLSK

>core/277/2/Org2_Gene919

MISFRFLLLSGLCALGISSYAETPKETTGHYHRYKARIQKKHPESIKESAPSETPHHNSLLSPVTNIFCSHPWKD
GISVSNLLTSVEKATNTQISLDFSILPQWFYPHKALGQTQALEIPSWQFYFSPSTTWTLYDSPTAGQGIVDFSYT
LIHYWQTNGVDANQAAGTASSMNDYSNRENNLAQLTFSQTFPGDFLTLAIGQYSLYAIDGTLYDNDQYSGFI
SYALSQNASATYSLGSTGAYLQFTPNSEIKVQLGFQDSYNIDGTNFSIYNLTKSKYNFYGYASWTPKPSCGDG
QYSVLLYSTRKVPEQNSQVTGWSLNAAQHIHEKLYLFGRINGATGTALPINRSYVLGLVSENPLNRHSQDLL
GIGFATNKVNAKAISNVNKLRRYESVMEAFATIGFGPYISLTPDFQLYIHPALRPERRTSQVYGLRANLSL

>core/278/2/Org2_Gene124

MMLRGVHRIFKCFYDVVLVCAFVIALPKLLYKMLVYGKYKKSLAVRFGLKKPHVPGEGPLVWFHGASVGE
VRLLLPVLEKFCEEFPGWRCLVTSCTELGVQVASQVFIPMGATVSILPLDFSIIIKSVVAKLRPSLVVFSEGDC
WLNFIEEAKRIGATTLVINGRISIDSSKRFKFLKRLGKNYFSPVDGFLLQDEVQKQRFLSLGIPEHKLQVTGNIK
TYVAAQTALHLERETWRDRLRLPTDSKLVILGSMHRSDAGKWLPVVQKLIKEGVSVLWVPRHVEKTKDVE
ESLHRLHIPYGLWSRGANFSYVPVVVVDEIGLLKQLYVAGDLAFVGGTFDPKIGGHNLLEPLQCEVPLIFGPH
ITSQSELAQRLLLSGAGLCLDEIEPIIDTVSFLLNNQEVREAYVQKGKVFVKAETASFDRTWRALKSYIPLYKN
S

>core/279/2/Org2_Gene589

MKRPFFTYLCIIFYGSCASLSLHAGLSFPEVRGATAAVVHADSGKVFYDKDIDAVIYPASMTKIATALFILKHY
PTVLDTLIKVKQDAIASITPQAKKQSGYRSPPHWLETDGSTIQLHLREELLGWDLFHALLVCSANDAANVLA
MACCGSVEKFMDKLNFFLKEEIGCTHTHFNNPHGLHHPNHYTTTRDLISIMRCALKEPPFRGVISTTSYKIGAT
NLHGERILSPTNKLLLPGSTYHYPPALGGKTGTTKTAGKNLIMAAEKNNRLLVTIATGYSGPVSDLYQDVIAL
CETVFNEPLLRKELVPPSDCLQLEIANLGKLSCPLPEGLYYDFYASEDREPLSVSFIAHADAFPIEQGDLLGHW
VFYDDEGKKISSQPFYAPCRFERTIKPWKLYMKRVFTSYRTYMSITMLLMYFRIRKHRKYKNLKHYSKI

>core/280/2/Org2_Gene789

MKRAIIIGAGISGLAAGWWLHKKFPQAEILVLDKEAYAGGFVRTESPQGFSFDLGPKGFLTRGDGEYTLKLIH
ELGLQNSLIFSDRAAKNRFVYYRGKAHKISTWTLLRKGLLPSLIKDFRAPCYTQDSSVQDFLKRHSSQNFTSYI
LDPLITAIRAGHSSILSTHMAFPELAKREASSGSLLRSYLKNRSPKKSKTDRYLASLSPSMGTLITTIQEKLPAT
WKFSTSVTHIDCSPKEACVTTPSETFFADMVIYTGPLQQLPVLLPNYGIENLSKRVLPWNLSSISLGWHHANFS
LPKGYGMLFADELPLLGIVWNSQIFPQATPGKTVLSLLIEGKWRESEAHAFAIAALSEYLNINQKPDAFALFSS
QDGMPQHAVGFLERKERILPHLPGNLKIVGQNIAGPGLNRCIASAYHAICDLHTEETLAQPQSSL

>core/281/2/Org2_Gene163

MFSRWITLFLLFISLTGCSSYSSKHKQSLIIPIHDDPVAFSPEQAKRAMDLSIAQLLFDGLTRETHRESNDLELAI
ASRYTVSEDFCSYTFFIKDSALWSDGTPITSEDIRNAWEYAQENSPHIQIFQGLNFSTPSSNAITIHLDSPNPDFP
KLLAFPAFAIFKPENPKLFSGPYTLVEYFPGHNIHLKKNPNYYDYHCVSINSIKLLIIPDIYTAIHLLNRGKVDW
VGQPWHQGIPWELHKQSQYHYYTYPVEGAFWLCLNTKSPHLNDLQNRHRLATCIDKRSIIEEALQGTQQPAE
TLSRGAPQPNQYKKQKPLTPQEKLVLTYPSDILRCQRIAEILKEQWKAAGIDLILEGLEYHLFVNKRKVQDYA
IATQTGVAYYPGANLISEEDKLLQNFEIIPIYYLSYDYLTQDFIEGVIYNASGAVDLKYTYFP

>core/282/2/Org2_Gene270

MNKNLVAIFDYMEKEKGIQRSTIIGAIESALKIAAKKTLRDDANISVNINSRTGDIEVFCEKEIVEICQNPSKEIP
LDKAREYDPDCQIGQYMDVPFVSDNFGRIAAHAARQIIGQKLRHAERDVIYEEYRHRVNETLSGVVKRFAKG
SNLIIDLGKVEAILPTRFYPKTEKHKIGDKIYALLYEVQESENGGAEVILSRSHAEFVKQLFIQEVPELEEGSVEI
VKIAREAGYRTKLAVRSSDPKTDPVGAFVGMRGSRVKNIIRELNDEKIDIVNYSPVSTELLQNLLYPIEIQKIAI
LEDDKVIAIVVNDADYATVIGKRGINARLISHILDYELEVQRMSEYNKLLEIQRLQLAEFDSPHLDQPLEMEGI
SKLVIQNLEHAGYDTIRRVLLASANDLASVPGISLELAYKILEQVSKYGESKVDEKPEIED

>core/283/2/Org2_Gene301

MSMTIVPHALFKNHCECHSTFPLSSRTIVRIAIASLFCIGALAALGCLAPPVSYIVGSVLAFIAFVILSLVILALIF
GEKKLPPTPRIIPDRFTHVIDEAYGLSISAFVREQQVTLAEFRQFSTALLCNISPEEKIKQLPSELRSKVESFGISR
LAGDLEKNNWPIFEDLLSQTCPLYWLQKFISAGDPQVCRDLGVPRECYGYYWLGPLGYSTAKATIFCKETHH
ILQQLTKEDVLLLKNKALQEKWDTDEVKAIVERIYTTYTARGTLKTEAGGLTKETISKELLLLSLHGYSFDQL
QLITQLPRDAWDWLCFVDNSTAYNLQLCALVGALSSQNLLDESSIDFDVNLGLYVIQDLKEAVQAFSASDEP
KKELGKFLLRHLSSVSKRLESVLRQGLHRIALEHGNARARVYDVNFVTGARIHRKTSIFFKD

>core/284/2/Org2_Gene762

MNHLNKEKLHIHNWQPYRACGLLSKVSGNLIEVDGLSACLGELCKISSTKDPNLLAEVIGFHNHTTLLMSLSP
LHSVALGTEVLPLRRPPSLHLSDHLLGRVLDAFGNPIDKKEDLPKTHRKPLLSLPPSPMMRQPIDQIFPTGIKAI
DAFLTLGKGQRIGVFSEPGSGKSSLLSAIALGSKSTINVIALIGERGREVREYIEKHSNALKQQRTIIIAAPAHET
APTKVIAGRAAMTIAEYFREQGHEVLFIMDSLSRWIAALQEVALARGETLSAHQYAASVFHHVSEFTERAGN
NDKGSITALYAILYYPKHPDIFTDYLKSLLDGHFFLTSQGKALASPPIDILSSLSRSAQALALPHHYAAAERLRS
LLKVYNEALDIIHLGAYTPGQDEELDKAVKLLPSIKAFLAQPLSSYCYLDNTLKQLEALADS

>core/285/2/Org2_Gene897

MLKPMYVLSKRLYRWVNQLIKLGDLVKNSRSFSVEWVFISALLLIFGCLGCASVVKVSLVPFLLLFSFLAFPLI
LCFRGKGYALLLGVFVTLYVAKYVVGETLYVSFWLSGLGVSFLLAFGLFLQGVWLAQEEEMVKGKEQLRL
SEDLDAQRSAYEDLLLTKSQEKEFLDARAQGLDRELTECQELLKAAYQKQEYLTIDLKILADQKNSWLEDY
AELHNKYIELVSKNGDVVFPWVAEPSVGESQGSERVDVSRWVSALQEKEESLERLRNEILVEKQRCSDYEHR
CQELGLLLQNFTALERRCEELQNLLNQKETQINELHQLVCKSEEKVSVEPSAHAETSCVEEKQYKGLYSQLQ
EQFLEKSETLSLVRKKLFAVQEKYLTLKKKEELTKQDISFDDISMIQGLLERIEILEEEVSHLEELVSRSLSL

>core/286/2/Org2_Gene694

MLRQLCFQVFFFCFASLVYAEELEVVVRSEHITLPIEVSCQTDTKDPKIQKYLSSLTEIFCKDIALGDCLQPTAA
SKESSSPLAISLRLHVPQLSVVLLQSSKTPQTLCSFTISQNLSVDRQKIHHAADTVHYALTGIPGISAGKIVFALS
SLGKDQKLKQGELWTTDYDGKNLAPLTTECSLSITPKWVGVGSNFPYLYVSYKYGVPKIFLGSLENTEGKKV
LPLKGNQLMPTFSPRKKLLAFVADTYGNPDLFIQPFSLTSGPMGRPRRLLNENFGTQGNPSFNPEGSQLVFISN
KDGRPRLYIMSLDPEPQAPRLLTKKYRNSSCPAWSPDGKKIAFCSVIKGVRQICIYDLSSGEDYQLTTSPTNKE
SPSWAIDSRHLVFSAGNAEESELYLISLVTKKTNKIAIGVGEKRFPSWGAFPQQPIKRTL

>core/287/2/Org2_Gene784

MTWLSGLYFICIASLIFCAIGVILAGVILLSRKLFIKVHPCKLKINDNEELTKTVESGQTLLVSLLSSGIPIPSPCG
GKATCKQCKVRVVKNADEPLETDRSTFSKRQLEEGWRLSCQCKVQHDMSLEIEERYLNASSWEGTVISNDN
VATFIKELVVAVDPNKPIPFKPGGYLQITVPSYKTNSSDWKQTMAPEYYSDWEHFHLFDQVIDNSQLPADSA
NKAYSLASYPAELPTIKFNIRIATPPFINGKPNSEIPWGVCSSYVFSLKPGDKITVSGPYGESFMKDDDRPLIFLI
GGAGSSFGRSHILDLLLNKHSKREIDLWYGARSLKENIYQEEYENLERQFPNFHYHLVLSEPLPEDIAAGWDK
DDPTKTNFLFRAFNLGQLSRLDNPEDYLYYVCGPPLHNSSILKLLGDYGVERSSIILDDFGS

>core/288/2/Org2_Gene580

MTVTLPKGVFDIFPYLADAKQLWRHTSLWHSVEKAIHTVCMLYGFCEIRTPIFEKSEVFLHVGEESDVVKKE
VYSFLDRKGRSMTLRPEGTAAVVRSFLEHGASHRSDNKFYYILPMFRYERQQAGRYRQHHQFGVEAIGVRH
PLRDAEVLALLWDFYSRVGLQHMQIQLNFLGGSETRFRYDKVLRAYLKESMGELSALSQQRFSTNVLRILDS
KEPEDQEIIRQAPPILDYVSDEDLKYFNEILDALRVLEIPYAINPRLVRGLDYYSDLVFEATTTFQEVSYALGGG
GRYDGLISAFGGASLPACGFGVGLERAIQTLLAQKRIEPQFPHKLRLIPMEPDADQFCLEWSQHLRRLGIPTEV
DWSHKKVKGALKAASTEQVSFVCLIGERELISQQLVIKNMSLRKEFFGTKEEVEQRLLYEIQNTPL

>core/289/2/Org2_Gene154

MFRKLFPFSKKKTGQKQRLRNNGLLQAIIQSIKVLLHNEASKEACVLSYYGLLTCVPILVFFLRLSQHLFTNLN
WKEWLIIKFPDYKKPIVAIVEAAYHATESNIGLVLVGSFFVFCWAGILMLLSLEDGLNKIFRTSWTPISLKRLV
SYFVITLVSPMIFIIVCGSWIYITQIMPIQYAKLFSLSHSMTALYFISRFVPYLLLYLALFCCYAFLPRVAIQKTSA
LISTLIIGSVWIVFQKAFFSLQVSIFNYSFTYGALVALPSFLLLLYIYTMIYLFGGALTFIIQNRGCTFIFLGDKILP
SCYLQLITSTYILALTTRQFNEGLSPLTAQFIAKQSKVPIGEVSQCLDVLEKEGFLFPYNNGYQPVFNFSELTIK
DIADKLLHREIFKKFNPDLGITFIENSFQNIFNQASKNKENLTLSEIARRIK

>core/290/2/Org2_Gene859

MVCENNILSGRGLELLKKKSNITLTPTIYSVSNHNIKLKDFSPHALSVIKTLRKAGYIAYIVGGCIRDLLLNTTP
KDFDISTSAKPEEIKAIFKNCILVGKRFRLAHIRFSKQIIEVSTFRSGSTDEDVLITKDNLWGTPEEDVLRRDFTI
NGLFYDPEHEEIIDYTGGVNDLRNRYLRTIGDPFTRFKQDPVRMLRLLKILSRSPFTVETQTQEALIACRQELIK
SSQARVFEELIKMLNSGAAKNFFQLLIENHLLEILFPYMDKAFRLNRALEEQTATYLKALDDKILKKEAEYDR
HQLMAIFLFPLVNFNVRYKHQKHPYLSLTSVFDYIKNFLEQFFADSFTSCSKKNFILTALILQMQYRLTPLIPTK
KALFFNKKLLHHTRFLEALSLLEIRSIVYPKLDKVYVAWIRHHQTLKCKKDSHSQK

>core/291/2/Org2_Gene261

MISLLKMPKLSPTMEVGTIVKWHKKSNDQVSFGDVIVEISTDKAILEHTANEDGWIREILRHEGEKIVIGTPIA
VLSTEANEPFNLEELLPKTEPSNLEASPKGSSEEVSPATTPQAASATFTAVTFKPEPPLSSPLVFKHVGTTNNLS
PLARQLAKEKNIDVSSIQGSGPGGRIVKKDLEKAPPKSIAGFGYPESPEVPPGSYHEENLSPIREVIAARLQAAK
ISIPHFYVRQQVYASPLLNLLKELQAQGIKLSINDCIVRACALALKEFPSINSGFNSVDNKIVRFDTIDISIAVAIP
DGIITPIIRCADRKNLGMISAEIKSLALKARNQSLQDTEYKGGSFCVSNLGMTGITEFTAIVNPPQAAILAVGSV
TEQALVLDGEITIGSTCNLTLSVDHRVIDGYPAAMFMKRLQKILEAPAVLLLN

>core/293/2/Org2_Gene709

MFEAVIADIQAREILDSRGYPTLHVKVTTSTGSVGEARVPSGASTGKKEALEFRDTDSPRYQGKGVLQAVKN
VKEILFPLVKGCSVYEQSLIDSLMMDSDGSPNKETLGANAILGVSLATAHAAAATLRRPLYRYLGGCFACSLP
CPMMNLINGGMHADNGLEFQEFMIRPIGASSIKEAVNMGADVFHTLKKLLHERGLSTGVGDEGGFAPNLAS
NEEALELLLLAIEKAGFTPGKDISLALDCAASSFYNVKTGTYDGRHYEEQIAILSNLCDRYPIDSIEDGLAEED
YDGWALLTEVLGEKVQIVGDDLFVTNPELILEGISNGLANSVLIKPNQIGTLTETVYAIKLAQMAGYTTIISHR
SGETTDTTIADLAVAFNAGQIKTGSLSRSERVAKYNRLMEIEEELGSEAIFTDSNVFSYEDSEE

>core/294/2/Org2_Gene676

MDIKKLFCLFLCSSLIAMSPIYGKTGDYEKLTLTGINIIDRNGLSETICSKEKLKKYTKVDFLAPQPYQKVMRM
YKNKRGDNVSCLTAYHTNGQIKQYLECLNNRAYGRYREWHVNGNIKIQAEVIGGIADLHPSAESGWLFDQT
TFAYNDEGILEAAIVYEKGLLEGSSVYYHTNGNIWKECPYHKGVPQGKFLTYTSSGKLLKEQNYQQGKRHG
LSIRYSEDSEEDVLAWEEYHEGRLLKAEYLDPQTHEIYATIHEGNGIQAIYGKYAVIETRAFYRGEPYGKVTR
FDNSGTQIVQTYNLLQGAKHGEEFFFYPETGKPKLLLNWHEGILNGIVKTWYPGGTLESCKELVNNKKSGLL
TIYYPEGQIMATEEYDNDLLIKGEYFRPGDRHPYSKIDRGCGTAVFFSSAGTITKKIPYQDGKPLLN

>core/295/2/Org2_Gene81

MKKTMVIDTSVFIYDPEALFSFENTRIIIPFPVIEELEAFGKFRDESAKNASRALSNIRLLLENAKTKVTDGVLLP
SGSELRIEVAPLSNDDRRGKLLTLELLKIIAKREPMVFVTKSLGRRVRAEALQIESRDYESKRFSFRSLYRGFRE
LQVSQEDIENFYKNGYLDLPLDVVSSPNEYFFMSAGENHFALGRYYVSEGKIIALKAMDKSVWGIKPLNTEQ
RCALDLLLRDDVKLVTLIGQAGSGKTILALAAAMHKVFDKETYNKVLVSRPIVPMGRDIGFLPGLKEDKLM
HWMQPIYDNMEVLFSINQMGNSSEALQALMDAKKLEMEALTYIRGRSLPKAFIIIDEAQNLTPHEIKTIISRAG
KGTKIVLTGDPTQIDSLYFDENSNGLTYLVGKFHHLALYGHMFMTRTERSELAAAAATIL

>core/296/2/Org2_Gene773

MLDIKIIRKTPEECETRLRKKDPKISLEPVLSLDKEVRQLKTDSETLQAQRRLLSQDIHKAKTQGVDATNLIQE
VETLAADLEKIEQHLDQKNAQLHELLSHLPNYPADDIPVSEDKAGNQVIKSVGDLPIFSFPPKHHLELNQELDI
LDFQAAAKTTGSGWPAYKNRGVLLEWALLTYMLQKQAAHGFQLWLPPLLVKKEILFGSGQIPKFDGQYYR
VEDGEQYLYLIPTAEVVLNGFRSQDILTEKELPLYYAACTPCFRREAGAAGAQERGLVRVHQFHKVEMFAFT
TPNQDDIAYEKMLSIVEEMLTELKLPYRLSLLSTGDMSFTASKTIDAEVWLPGQKAFYEVSSISQCTDFQSRRS
GTRYKDSQGKLQFVHTLNGSGLATPRLLVAILENNQQADGSVVIPEVLRPYLGGLEILLPKDQ

>core/297/2/Org2_Gene597

MLPLIIFVLLCGFYTSWNIGANDVANAVGPSVGSGVLTLRQAVVIAAIFEFFGALLLGDRVAGTIESSIVSVTN
PMIASGDYMYGMTAALLATGVWLQLASFFGWPVSTTHSIVGAVIGFGLVLGKGTIIYWNSVGIILISWILSPF
MGGCVAYLIFSFIRRHIFYKNDPVLAMVRVAPFLAALVIMTLGTVMISGGVILKVSSTPWAVSGVLVCGLLSY
IITFYYVHTKHCSYISDTPKKGSLTYRLKERGGNYGRKYLVVERIFAYLQIIVACFMAFAHGSNDVANAIAPV
AGVLRQAYPASYTSYTLIRLMAFGGIGLVIGLAIWGWRVIETVGCKITELTPSRGFSVGMGSALTIALASILGL
PISTTHVVVGAVLGIGLARGIRAINLNIIKDIVLSWFITLPAGALLSILFFFALRALFH

>core/298/2/Org2_Gene1018

MIMGLQSRLQHCIEVSQNSNFDSQVKQFIYACQDKTLRQSVLKIFRYHPLLKIHDIARAVYLLMALEEGEDLG
LSFLNVQQYPSGAVELFSCGGFPWKGLPYPAEHAEFGLLLLQIAEFYEESQAYVSKMSHFQQALFDHQGSVF
PSLWSQENSRLLKEKTTLSQSFLFQLGMQIHPEYSLEDPALGFWMQRTRSSSAFVAASGCQSSLGAYSSGDV
GVIAYGPCSGDISDCYYFGCCGIAKEFVCQKSHQTTEISFLTSTGKPHPRNTGFSYLRDSYVHLPIRCKITISDK
QYRVHAALAEATSAMTFSIFCKGKNCQVVDGPRLRSCSLDSYKGPGNDIMILGENDAINIVSASPYMEIFALQ
GKEKFWNADFLINIPYKEEGVMLIFEKKVTSEKGRFFTKMN

>core/299/2/Org2_Gene926

MDKQSSGNSGCIWHPFTQSALDSTPIKIVRGEGAYLYAESGTRYLDAISSWWCNLHGHGHPYITKKLCEQAQ
KLEHVIFANFTHEPALELVSKLAPLLPEGLERFFFSDNGSTSIEIAMKIAVQYYYNQNKAKSHFVGLSNAYHG
DTFGAMSIAGTSPTTVPFHDLFLPSSTIAAPYYGKEELAIAQAKTVFSESNIAAFIYEPLLQGAGGMLMYNPEG
LKEILKLAKHYGVLCIADEILTGFGRTGPLFASEFTDIPPDIICLSKGLTGGYLPLALTVTTKEIHDAFVSQDRM
KALLHGHTFTGNPLGCSAALASLDLTLSPECLQQRQMIERCHQEFQEAHGSLWQRCEVLGTVLALDYPAEAT
GYFSQYRDHLNRFFLERGVLLRPLGNTLYVLPPYCIQEEDLRIIYSHLQDALCLQPQ

>core/300/2/Org2_Gene23

MAEISTPSLPDSSIVSQKTPPVPDPDSSPDHIPTIPTQAPFKPQRKKETPSSIVNAIAFAILAFLSCLGGVFAICLGC
SLEITMPLFILTAVFIAFTLLYFIHYLEKPKIPEPLPTPPPSPTLRAPTLTPEIPAPAPGIPLPPTLPKVDRTKLTCNP
DIHYPSTYDPKACFSLLKQLFSLDPETRPEDRKYSNKLASILLRSKEKSGFRFHCFKGHFSHDKILNKKSGAVV
ISSHSSMDFSTTLGRAFAVTTCLQRSCWEKIKNNIPTPEKHLPIGSCVSGPWDVEEGAQLYTSHLIVINPPTLET
LIKEKMRRAITLKDFSMKEAFTNLVLAYLQCFDICIEHNLESVQLEVFGLNNLSADQEEFTTWESCCHLALLE
SVRILLASKEEYALSNVSVNSISQVPLQTACRALFLN

>core/301/2/Org2_Gene644

MKKQVYQWLASVVLLALTISGYAELPLSEQKVKSHTYTTLDEVKDYLSKRGFVETRKQDGVLRIAGDVRAR
WLYFREDIKNPSDKDKYNPLPVNRYRSEFYLYIDYRAERNWLSSKMNWTAIAGGENTAAGVDINRAFLGYR
FYKNPETRTDFFMEIGRSGLGDLFESEVQFQSNFDGLHIYWTRELSKDYPYQVIVHGGPFVVNMTKKHYAW
VVEGILNRLPKQFFVKCSVVDWNTFVPSETSTTEKAATNAMKYKYCVWQWLVGKHSQVPWINGQKKPLYL
YGAFLMNPLAKATKTTLNGKENLAWFIGGTLGGLRKAGDWSATVRYEYVEALSVPEIDVSGIGRGNLLKFW
FAQAIAANYDPKEANGFTNYKGFSALYMYGITDSLSFRAYGAYSKPANDKLGSDFTFRKFDLGIISAF

>core/302/2/Org2_Gene1000

MQSWLQSLQERNILENFTAGLESVEGPIAAYLGFDPTAPALHIGHWIGICFLKRLAALGITPIALVGGATGMV
GDPSGKQSERSLLQTSEVFDNSQKITACLQRYLPGVTLVNNADWLQEISLIDFLGDIGKHFRLGQMLVKDTIK
QRVHSDEGISYTEFSYLILQSYDFYHLFKNYGTILQCGGSDQWGNITSGIDFIRRKGLGQAYGLTYPLLTNAQ
GKKIGKTESGTVWLDSDLTSPFELYQYLLRLPDDTIPKIARTLTLLSNEEIQDIDRRVQTDPVAVKEFVAQDILS
AIHGDLGLEEALSVTRSMHPGNLSSLSEKDFHELFAGGMGASLDKSEVLGKRWLDLFLVLGLCKSKGEIRRLI
EQKGVYINNVPIANEHSVCEEQDICYGHYVLLAQGKKRKLVLYLN

>core/303/2/Org2_Gene407

MTVAEVKGTFKLVCLGCRVNQYEVQAYRDQLTILGYQEVLDSEIPADLCIINTCAVTASAESSGRHAVRQLC
RQNPTAHIVVTGCLGESDKEFFASLDRQCTLVSNKEKSRLIEKIFSYDTTFPEFKIHSFEGKSRAFIKVQDGCNS
FCSYCIIPYLRGRSVSRPAEKILAEIAGVVDQGYREVVIAGINVGDYCDGERSLASLIEQVDRIPGIERIRISSIDP
DDITEDLHRAITSSRHTCPSSHLVLQSGSNSILKRMNRKYSRGDFLDCVEKFRASDPRYAFTTDVIVGFPGESD
QDFEDTLRIIEDVGFIKVHSFPFSARRRTKAYTFDNQIPNQVIYERKKYLAEVAKRVGQKEMMKRLGETTEVL
VEKVTGQVATGHSPYFEKVSFPVVGTVAINTLVSVRLDRVEEEGLIGEIV

>core/304/2/Org2_Gene751

MNKKNLTICSFCGRSEKDVEKLIAGPSVYICDYCIKLCSGILDKKPSSTISSAPVSETPSQPSDLRVLTPKEIKKH
IDEYVIGQERAKKTIAVAVYNHYKRIRALLHNKQVSYGKSNVLLLGPTGSGKTLIAKTLAKILDVPFTIADAT
TLTEAGYVGEDVENIVLRLLQAADYDVARAERGIIYIDEIDKIGRTTANVSITRDVSGEGVQQALLKIVEGTTA
NVPPKGGRKHPNQEYIRVNTENILFIVGGAFVNLDKIIAKRLGKTTIGFSDDQADLSQKTRDHLLAKVETEDLI
AFGMIPEFVGRFNCIVNCEELSLDELVAILTEPTNAIVKQYMELFAEENVKLVFKKEALYAIAKKAKQAKTGA
RALGMILENLLRDLMFEIPSDPTVEAIHIQEDTIAENKAPIIIRRTPEAIA

>core/305/2/Org2_Gene76

MIKFLSQLFIRHWPRKVVSLGFAIIIWILVGQSVTITRTLTNVPVRIVDLHPDQTVLGLQKSGFLNKKVSLTITG
NKNTVQDLRPSNLEVVISAANHTESWIATIDKHNLVSVDHEINIRKDIHSVDANDIFVRLTQYVTEDILLTITKP
IGSPPKGYEYLDVWPKYLNQKVSGPKEYINALKEQGLELTFNLNKISFEELERNRIAQGSHDEIIFPIPKEWKKI
LIPFENTFMDLNDPQADFLRLLFLKRECIPLNLNLPVFLFFPVTFIQTMNPLEYSLDPVPPIILNHGIHQINIPLYV
KDVSRQFLDVVKNNMVLTIVMPSPQDPSSINWAIEFLDEKTLENTFLQTIIAQEHGILHDIALIDEAGIRHRFRE
YLRKLALFTADGEPLNLIAEIKNNKVVIQTKTKETTKLYKKEW

>core/306/2/Org2_Gene902

MLKLQLCALFLFGYLAIVFEHIVRVNKSAIALAMGGLMWLVCFSHIPMADHMILVEEIADMSQVIFFLFSAM
AIVELIDAHKGFSVIVKFCRIQSRTLLLWALIGLSFFLSAALDNLTSIIIIISILKRLVKAREDRLLLGAICVIAVNA
GGAWTPLGDVTTTMLWINNKITSWGIIRALFVPSLVCVLVAGFCGQFFLRKRGSTLIAKDVELQSAPPKSLWII
FIGLGSLLMVPVWKACLGLPPFMGALLGLGLVWLTSDWIHSPHGEDRYHLRVPHILTKIDISSITFFIGILLAVN
ALSFANLLTDFSLWMDKIFSRNVVAIVIGLLSSVLDNVPLVAATMGMYTLPLDDTLWKLIAYAAGTGGSILII
GSAAGVAFMGLEKVDFLWYFKRISWIALASYFGGLFSYFVLESLNFFI

>core/307/2/Org2_Gene177

MSSAIARDCFPSPSPQPSSTLGVHPPKYKSLILSVSLIVLGVLLLCVGMLLLVNAIFSFSVLTVGLGGAGVFLGS
LLLILGLIFFVSYHRKLSEATRSLEQKITLEYQPWADLRKELNEVQEWSNFLLDEWEDFKEVVAQHKSQFATF
EGDLLLFGREVEKYETIWKELDGRDVALLTELKNIWGPLEFLRKKGDRLQCEIDKLRKEVMKVGKSGLKLA
CELTKFKSALKDVKIEQECYRDKRKVEKLEVFPEGYRRELLEVLKTRLSVEKEIQLFEEVVSAFEEKLASLHR
TVFSEEELQEALDKAKAELLDIQVRKSVVEDLSCEPTLIQYHLLRLYEVQCRIVEQFLTQTFSSEQEKVLEEYE
ALKARIRKTLRVKLDQVRANVAFVASTTDLLSESESLDGNDSVFEDAHDDFLD

>core/309/2/Org2_Gene775

MIETREEVGSANFVSLERAIEDLRAGKFVIVVDEASREDEGDLIIAGEKITVEKMTFLLQHTTGVVCAALSQER
LLSLDLPPMVKDNRCRFKTPFTVSVDAAHGVTTGVSAADRTKVVQLLADPKSKPEDFISPGHFFPLASSPGGV
LKRAGHTESTVDLMELAGLQPCGVLAELVNEDYSMMRLPQILEFARKHNIAVIPVTSIIAHRMLSDRLVSKIS
SARLPTIYGDFTIHVYESLLEGMQHLALVKGNVAGKSNVLVRVHSECVTGDILGSKRCDCGEQLSSAMSYIA
EKGTGVLVYLRGQEGRGIGLGHKVRAYALQDNGYDTVDANLAMGFPVDSREYGIGAQILVDLKLTTIKLIT
HNPQKYFGLQGFGLSITERVPLPVRISEDNEQYLRTKQERMGHWLDLPCCNNRVQ

>core/310/2/Org2_Gene723

MLCYSPNYVTDLYRISLSAEESLGGIRAFPQAESLLGGACALNFPDLEERLPDLRKELLFLGSNDRPDACGGK
FSLQLASSKECYIAALKERVYLNVTNSSRGPVYSFSPKGVPTELWIECFSVSVDGRVEVKVRLQGLHKELISKP
RDCETLFLNPPANKLDCWEIAGFRVDASFPVKQKIRRIGVDKFLLMHGGAEYADKATKERVDFVSSDEENYS
RYLAVGDVLLWDGNCWQTCGEFQGASSRAPLFEVKRIDDKVMIADLWNVGGTQRQTISLVKGVPSPIEINE
VIREIEFTGMRSWSKPIVLVGGQRLILSPDDWVLRTAKGWEKLSRADQIQDYVTGKVTGPLLVFEKLEKDLR
GFVLRGHMFNAQRTLVETISLPLKQGFEPAVASQEVSSNTRSAAAHPGATNRGGS

>core/311/2/Org2_Gene381

MSQPPINPLGQPQVPAAASPSGQPSVVKRLKTSSTGLFKRFITIPDKYPKMRYVYDTGIIALAAIAILSILLTASG
NSLMLYALAPALALGALGVTLLISDILDSPKAKKIGEAITAIVVPIIVLAIAAGLIAGAFVASSGTMLVFANPMF
VMGLITVGLYFMSLNKLTLDYFRREHLLRMEKKTQETAEPILVTPSADDAKKIAVEKKKDLSASARMEEHEA
SQRQDARHRRIGREAQGSFFYSSRNPEHRRSFGSLSRFKTKPSDAASTRPASISPPFKDDFQPYHFKDLRSSSFG
SGASSAFTPIMPASSRSPNFSTGTVLHPEPVYPKGGKEPSIPRVSSSSRRSPRDRQDKQQQQQNQDEEQKQQSK
KKSGKSNQSLKTPPPDGKSTANLSPSNPFSDGYDEREKRKHRKNK

>core/312/2/Org2_Gene800

MCQRILILGTGITGKSVARFLYQQGHYLIGADNSLESLISVDHLHDRLLMGASEFPENIDLVIRSPGIKPYHPW
VEQAVSLKIPVVTDIQVALKTPEFQRYPSFGITGSNGKTTTTLFLTHLLNTLGIPAIAMGNIGLPILDHMGQPGV
RVVEISSFQLATQEEHIPALSGSVFLNFSRNHLDYHRNLDAYFDAKLRIQKCLRQDKTFWVWEECSLGNSYQI
YSEEIEEILDKGDALKPIYLHDRDNYCAAYALANEVGWVSPEGFLKAIRTFEKPAHRLEYLGKKDGVHYIND
SKATTVTAVEKALMAVGKDVIVILGGKDKGGDFPALASVLSQTTKHVIAMGECRQTIADALSEKIPLTLSKD
LQEAVSIAQTIAQEGDTVLLSPGCASFDQFQSFKERGAYFKLLIREMQAVR

>core/313/2/Org2_Gene813

MSKKRVVVTGFGVVSCLGNEVDTFYDNLLAGVSGVRPITSFPCEDYATRFAGWIPEFNPEPYVDKKQARRV
DPFITYAMVAAKKAIAMSRWDKDHLPSDPVRCGVIVGSGMGGLSTLDQGMERLLVIHKKLSPFFIPYIITNMA
PALIAMDFGLMGPNYSISTACATGNYCIDAAYQHLVSGRADMIICGGTEAAVNRIGLEGFIANRALSERNDAP
DQASRPWDRDRDGFVLGEGAGILVLETLESALRRDAPIFAEMLGSYVTCDAFHITAPRDDGEGITACVLGAL
NSAGIPKERVNYVNAHGTSTPLGDLSEVLAVKKAFGSHVRNLRMNSTKSLIGHCLGAAGGVEAVVAIQAILT
GKLHPTINLDNPIAEIEDFDVVANKAQDWDIDVAMSNSFGFGGHNSTILFSRYVP

>core/314/2/Org2_Gene605

MLVSIETFSSIASGSPVQKAAEACYTQYSKQPSSKEVLSSFSWIQELSLFPDRYNLATGASELIKQHWLHNNHS
LAFECILINGKYEPSLSQLPEGVIVCGIDEARGSLSSFMQGFDVNKHPLAFLNAVCSEDRGVVIYIPEEMQTSDP
IFVRHISFPTVSDHDVIFSPRIVVILGQRASAQIQISHDVDLEMVGSSKTIVNGVTELFVGEGADLTVFMVPGYS
EEDTLSWSTIATVEKDAICRMTQNLLESCQGFGWFDNTSYIVGKKGHAESLVLVQSPRKTWVNNLMSHDAE
ETVSRQNIKSILYSGHFLFEGTISISSQGDLSDANQKHDTLLLSSEARVSTFPRLEIETDEVKASHGATVGPLDP
QQIFYMRSRGMTEAEAQEKLIHGFLKQGLVSDTFLGSSFQLNQTS

>core/315/2/Org2_Gene970

MRYHKYFRYVNSWVFLVVLTLMLLSVVVISSMDPTAMLVTSSKGLLTNKSIMQLRHFALGWVVFFICAYFD
YHLFKRWAWVLYFFMICALVGLFFVPSVQNVHRWYRIPFIHMSVQPSEYGKLVIVIMLSYILESRKADITSKT
TAFLACLVVALPFFLILKEPDLGTALVLCPVTLTIFYLSNVHSLLVKFCTVVATIGIIGSLLIFSGIVSHQKVKPY
ALKVIKEYQYERLSPSNHHQRASLISIGLGGIRGRGWKTGEFAGRGWLPYGYTDSVFSALGEEFGLLGLLFTL
GLFYCLICFGCRTVAVATDDFGKLLAAGITVYLAMHVLINISMMCGLLPITGVPLILISYGGSSVISTMASLGV
LQSIYSHRFAKY

>core/316/2/Org2_Gene450

MKLWMKIFIGLFVGVTLGLVLEDKAIFFKPIGDIFLNLLSMVVYPLVFCSMVLGIASISDMKKLGRIGIKSVGL
YLGTTALAIVIGLCFAWIFSPGNGCDFAQAQSMDSAVTVIDSNKTAAYFLSIIAQVFPSNPVRSFAEGNILQIIIF
AIFLGIALRLSGERGRPVERFIDGFSEIMLRMVNMIMSFAPYGVGASMAWISGNHGLGVLWQLGKFIIAYYLA
CLFHATLVFGGLVRFGCKMSFSKFLSSMMDAISCAVSTASSSATLPVTMRCVSKNLGVSAEVSGFVLPLGAT
VNMNGTAIFQGMAAVFIAQAYNCPLSLSSLLLLVVTATFSAVGSAGVPGGGMITLGSVLASVGLPIQGIAILA
GIDRLRDIVGTPMNILGDAVVATYVASGEGELSPYESIKQESVETT

>core/317/2/Org2_Gene688

MKKIFYSFVLLSCIFPYVGCAQVFVGLDRIFSEGEYTRCIQGKKIALISHSAAINSRGQDALSVFYSRKHDCTVE
ILCTLEHGYYGATPTETVGNQPSRYPNLRSVSLYGVKEVPKEVAEHCDVFVYDVQDIGVRSYSFVTVLMQIV
KASERYGKQLIVLDRPNPMGGRIVDGPLPNPTTSGSLAIPYCYGMTPGELALFFKKTYAPNANVVVIPMKGW
NRSMTFDETGLIWMPTSPQMPDPQSPFFYAATGILGALSVASIGVGYTLPFKVLGAPWMDGEKVADELNRM
KLPGVLFLPFFYEPFFGKYKMEMCSGVLLVLQDPKIFYPVETQCTIWGVLKALYPKQVEQTLKSIERIPARRSS
ICNLFGGDEFLSISHKERYIVWPLRRLCKESRESFHQLRSSCLLSEYAES

>core/318/2/Org2_Gene971

MKKNASHKTNDKKSLSIWSIGGSIFAMFFGAGNIVFPLALGYHYNAHPWSAYFGMMLTAVCVPLLGLVSML
FYSGDYQKFFFSIGRIPGMIFITAIILLIGPFGGIPRAIAVSHATLISLSEHKSAFIPSLPIFSAICCVLIYIFSCKLSRL
IQWLGSVFFPIMLVTLLWVIIRSFMIPTHPMVQEFIPNARQAWLAGFIEGFNTMDLLAAFFFCSIVLISLRQLVA
EEKHPTEEEIPLSFQGISKKNKRSLALGFFLAAILLGMTYLGFVLSAARHAGLLVNVSKGHILGRISAIALGPNS
ILAGVSVFIACLTTEIALVGIVADFLARVVSFKKLNYASAVICTLIPTYLISILNFETISHLLLPLLQLSYPALIVLA
CGNIAYKLWNFRYSPVLFYLTLSLTIVLKLVN

>core/319/2/Org2_Gene191

MDKETLENIYRHFRYRFLKLNILPAFLGLLLLCSPNTLNYTQVDVIFSDRLCSCLLIFLAIASLTKRSLLWLGAP
LGIWVTLFACVAGRSPTIFANDTLIGFAILAVVCISPTRPEALEVGPTLPEGFSYNPSAGGRRAAVLFLSLLGWL
EARYLTASSLGITSSQSSNFLLLYSSIMTVYSLLVVLSLAGSERRWHTRPKIVIATALALTGVIILTLLPIILHQLR
YDCWLCLCLTIEPALAVVFAYDETRATLRYISQFLGDKRALTRASFFGSEYYKHTLSWEERTVLPLRKAYKQ
AFEGISFPINQLLAILVATVFVKVNSSMGLPTFPRNFLNICCWFIIVLFILAFAESLRHLRWMNLIFSAAILFSPVL
FHIPVESPMFLPIIVTGLILIILSIGKRRRTKRKL

>core/320/2/Org2_Gene337

MIPTMLMFFIICFTLCSGFISLSQIALFSLPTSLISHYKRSKSKKQQRVATLLLHPHHLLITLIFCDIGLNIAIQNCF
AILFGDAASWWFTVGLPLAITLILGEILPKAVALPFNTQIASSVAPLILCVTKIFKPLLHWGIVGINYVVQWILS
KQQIDIIQPQELKEVLQSCKDFGVVNQEESRLLYGYLSLSDCSVKERMQPRQDILFYDIQTPLENLYLLFSKQH
CSRVPICNDNLQNLLGICTARSLLLHDKPLQSSDDLLPLLKKPYYMPETISAKMALCQMAAEDETLGMIIDEY
GSIEGLITQEDLFEIVAGEIVDQRDNKILYTTSGADVIIASGTLELREFSEIFDINLPTNNNIATIGGWLIEQIGTIP
TTGMKLSWNNLLFQVLDAAPNRIRRVYIRKLYD

>core/321/2/Org2_Gene143

MDESDGEEASKDSAFSASFSYEFVKSSTRESKNTVTHSTASRTLYILRQDCSYDPRALKVDDEFRYWVEKRL
DAKNPDSLNAFVKEVGTHYVASVTYGGIGFQVLKMSYLQVEELEKEKISISVAAASSLLKSKTSNATEKGYS
SYQSESSAQTVFLGGTVLPDLQQDKLDFKDWSESIPNEPIPLAISVSSITDLIIPELFPSEDAQVLSQKKSALGQV
ILNYLESHKPKEEGPKPVQITSGFNSSSSVFTLQAAKAPKTVSFPYIDYWSTIPYLFPTLKETSGAQPLSFYLRF
DDIFEQQNLVHNTSYILASTSVRLGYFGDSYRDYDALSFYGSWPQAYFDWAGYKDRCTWTLEKLNTTGDLF
IRSGDEIRLKHNTSGKYLATTSMSDGYQKLTCTTQTSDSVFIITV

>core/322/2/Org2_Gene872

MNLPVSLACLLLSGCVFFLGVFVSSSLYARKKRAFLEKIQKLEHENQLLQTSLNLSRHQEQLIEDFSNRLALSS
HKLIKDMKEEAQNYFGDTSKSFQSILSPIQTTLTTFKQSLETFETKHAEDRGRLKEQISQLLAVEKKLEHETHV
LTDILKHPGSRGRWGEIQLERILELAGMLKYCDYDSQTTSAQGAFRADIIIRLPQDRCLIIDAKAPISDSYFSVE
EIDKGDLVDKIKEHIKTLKSKSYWEKFHQSPEYVILFLPGESLFNDAIRLAPELMEIGASSNVILSSPLTLLALLK
TIAYMWKQENLQKQIQEVSLLGKELHRRLQVVFTHFQKIGKNLNQTVQSYNDMTSSFQYRVLPTLRKFEGLE
TSSSHQIEEPTPIESLATSFPHTCDIDTNLAVIESLEKQD

>core/323/2/Org2_Gene958

MKTAFHSCYSWFCWLFSFLVLFVGGIAGGEPLCPDCKYETKSVLRSDQLPDHLWNYENDCYLTGYVQSLLD
MHFLDSRTQVVIEKNRAYLFSLPVDSSLSEAITNFVRDLPFICAVEICERPYGECITRSSAERPLLPKEKTLGMPI
FCGKEGVWLPQNTILFSPLIADPRQVTNSAGIRFNEKVVGNRVGATIFGGDFILLRLFDVSRFHVDCDFGIQGG
VFSVFDLDHPESCMVNSDFFVAGLWSGAIDKWSFRFRLWHLSSHLGDEFILTHPNFPRFNLSDEGVDLFISFR
YTPQIRLYGGCGYIVSRDLTFPERPFYCEWGAELRPFGLREGNLHAQPIFAMHFRCWEEQKFGLDQSYILGME
WAKFQEIGRKIRAVLEYHQGFSKEGQFIREPCNYYGFRLTYGF

>core/324/2/Org2_Gene750

MTTIAIEAAKKVLIKLRNAGYQAYFVGGCVRDMLMNRPLEDIDIATNASPTIVSTIFPDVISIGVAFGIIVVKQD
GRLFEVATFRSDGEYKDGRHPDRIIFSSMREDALRRDFTVNGMYYDPFEDKVFDFVEGTRDIEKKVIRAIGHP
RLRFSEDKLRILRAIRFSSSLGFTLDPTTERAIIKEAPALVNSVSPERIWQELKKMLKRQPYGALSLLLKLKVLIF
IFPELRDIPYSLLRTTIEFARKFNPTHFPEILFLLPLFQGVSEEAATVAFGRLRISNKELKLIESWYEALPHFQNQS
GNRVFWAHFLASPTAPLFLELFSALQKDPSRQQHFISRVQELESRLEQFILRIKTSSPVVSAPDLIAKGISPGRLL
GDLLREAEILSIENECLDKEKILLLLQEKGFWK

>core/325/2/Org2_Gene604

MKNLKEDFPIFAAKAKENEPFIYLDSAATTQKPQQVIDAVANFYTSSYATVNRAIYSSSRNVTEAYAAVREK
VRKWVSAASDSEIVFTRGTTAGLNLLAISVNDLWIPKGGVVLVSEAEHHANVLSWEIACRRRGSLVKKIRVH
DSGLIDLDDLEKLLNEGAQFVSIPHVSNVTGCVQPLQQVAELVHRYDAYLAVDGAQGAPHLPIDVQLWDVD
FYVFSSHKIYGPTGIGVLYGKKDLLDQLPPVEGGGDMVAIYDHQNPEYLPAPMKFEAGTPNIAGVLGLGAAL
DYLDGLSAKFIYDKEIALTTYLHKELLEIPGVEILGPSIEEPRGALIGMTIDGAHPLDLGFLLDLRGIAVRTGHQ
CAQPAMERWNVGHVLRVSLGIYNDEDDIDQFILVLQDSLDKIRR

>core/326/2/Org2_Gene176

MVVVALFILGIFFLSGSLAFLVHTSCGVLLGAALPILCIGLVLLAVALIVFLCHKHKTRQDLDYYDQDLDSLVI
HKKEIPNDISELRVTFEKLQNLFQFHTKDFSDLSQELQGKFINCMEKWLTLEDEVTKFLIVRDRFLETRRNFTT
FGEQVKGIQSNIFDLHEEKSSLYLELYRLRKDLQVLLNFFLLPPGILKVDYDEIEAIKGLFIRLTSRLDKLDVKA
QERKKFINEMSREFKEVEKAFDIVDRATKKLMDRAKKESPARLFMGRTESLLEMKKNEEALKNQGLDPENL
SHPELFSPYQQLLILNYLNSEIVLHHYEFLISGTVTSGLTLEECENRMRAASTGLNALLVRKLQFRGAIKSAYF
EKLTEIEKELRSLQDVIKSLELELIHKIKDIVTEET

>core/327/2/Org2_Gene313

MTDSNPLPSYTDASLYRTPAKHSYPIRLPLNRTDRIEKILKIVTLTLALACALGFSIAAGILAMPIFSAVVVITLA
IAAVSLYSLLKKPKLYEILPQIEPESEQSSLSPSPQPPEQQDLPLQIDPLPDPESLPEVSLADLTTPPEELTAITVTP
GYEALLEQNWDLLPSLAAVDPSFTTETPQQPCFIWKLKDSKLIFISTSGDIAVPRIKTQGRVMIVNAANENISR
EGGGTNKALSLATSLQCWNASRLPRAHSRSGSQLQPGECRSAKWENSDHTSNDHVPGKAHFLAQLLGPEAA
KCNNDPKQAFEVSKKAFHNLFQEAEIIGVDVIQLPLIGCNLFAPSRLLNLGKTRAEWIEAIKLALITSLQDFGW
EQDNQEEQKIIILTDKDQPPIIPPRFDLTTP

>core/328/2/Org2_Gene244

MKKQRSHYKKNNLLLLLSILVGLGLGSVQSPWIVYSAECIANTFLKFLRLLSIPLVFCALGSTITSIQNFNTMV
TLGKRILYYTLLTTVIAASIGLLLFFLLRPQMITQDALATTTKCNPLGYLDVLSDTLPENIFKPFLQGNVISAAC
LAVLLGTASLFLQEKEKHFVNQFFNSFFSIFLNLARGGLKLLPIAMLGFSVILFKELKDQSNLTMFAEYLLCVI
GANLAQGFIVLPILLKINKVSPLKVAKAMSPALVTAFFSKSSAATLPLTMELAEDDLKINKNLSRFSFPLCSVIN
MNGCAAFILITVLFVATSNGMIISPLMSLGWIFIATLAAIGNAGVPMGCYFLTLSLLTSMNVPLSILGLILPFYT
VIDMIETSLNVWSDCCVVSLAN

>core/329/2/Org2_Gene338

MTNSALFWIGVNIICIVLQGFYSMMEMACVSFNRVRLQYYLTKDHKKARYINFLIRRPYRLFGTVMLGVNIA
LQVGSESSRNCYRALGITPDYAPFTQIFIVVIFAELLPLTISRKIPEKLALWGAPILYYSHYIFYPLIQLIGSLTEGL
YYLLNIRKEKLNSTLSRDEFQKALETHHEEQDFNTIATNIFSLSATCADQVCQPLEQVTMLPSSANVKDFCRTI
KNTDINFIPVYHKARKNVIGIAHPKDFVNKALDEPLINNLHSPWFITAKSKLIRILKEFRDNRSSVAVVLNASG
EPIGILSLNAIFKILFNTTNIAHLKPKTISVIERTFPGNSRIKDLQKELDIQFPQYPVETLAQLVLQLLDSPAEVGT
SVIINNLLLEVKEMSLSGIKTVSIKNLLS

>core/330/2/Org2_Gene596

MDKLTVQDLSPEEKKVLVRVDFNVPMQDGKILDDIRIRSAMPTINYLLKKHAAVILMSHLGRPKGQGFQEEY
SLQPVVDVLEGYLGHHVPLAPDCVGEVARQAVAQLSPGRVLLLENLRFHIGEEHPEKDPTFAAELSSYGDFY
VNDAFGTSHRKHASVYVVPQAFPGRAAAGLLMEKELEFLGRHLLTSPKRPFTAILGGAKISSKIGVIEALLNQ
VDYLLLAGGMGFTFLQALGKSLGNSLVEKSALDLARNVLKIAKSRNVTIVLPSDVKAAENLQSKEYSVISIDQ
GIPPHLQGFDIGPRTTEEFIRIINQSATVFWNGPVGVYEVPPFDSGSIAIANALGNHPSAVTVVGGGDAAAVVA
LAGCSTKVSHVSTGGGASLEFLEQGFLPGTEVLSPSKS

>core/331/2/Org2_Gene786

MSTMQNCPHFGVCGGCSFPQSNYSDSLKKKEELLHQLFAPLVPSDMIAPIIPCSPSLRGRNKMEFSFFQTYEGE
KSLGFISSTKPKKGIPVTTCLLIHEQTMDILKLTREWWDKHPELMAYFPPKNKGSLCTLTVRTGSPQQNFMVI
LTTSGTPEYRVNEACIDEWKEILLSSSLNIASIYWEEKVAARGISTYYETKLLYGAPSIQQKLSLPSDGNSASFS
LRPRSFFQPQITQAAKIIETAKEFINPEGSETLLDLYCGAGTIGIMLSPYVKNVIGVEIIPDAVASAQENIKANNK
EDCVEVYLEDAKAFCKRNENCKAPDVIIIDPPRCGMQSKVLKYILRIGSPKIVYISCNPKTQFQECADLISGGY
RIKKMQPIDQFPYSTHLENIILLEREIDP

>core/332/2/Org2_Gene667

MLLVRKWLHTCFKYWIYFLPVVTLLLPLVCYPFLSISQKIYGYFVFTTISSLGWFFALRRRENQLKTAAVQLL
QTKIRKLTENNEGLRQIRESLKEHQQESAQLQIQSQKLKNSLFHLQGLLVKTKGEGQKLETLLLHRTEENRCL
KMQVDSLIQECGEKTEEVQTLNRELAETLAYQQALNDEYQATFSEQRNMLDKRQIYIGKLENKVQDLMYEI
RNLLQLESDIAENIPSQESNAVTGNISLQLSSELKKIAFKAENIEAASSLTASRYLHTDTSVHNYSLECRQLFDS
LREENLGMLFVYARQSQRAVFANALFKTWTGYCAEDFLKFGSDIVISGGKQWMEDLHSSREECSGRLVIKT
KSRGHLPFRYCLMALNKGPLCYHVLGVLYPLHKEVLQS

>core/333/2/Org2_Gene80

MRMLQISMLLLALGTAINSPAIYAADSQSVSFPEQLPSSFTGEIKGNHVRMRLAPHTDGTIIREFSKGDLVAVI
GESKDYYVISAPPGITGYVFRSFVLDNVVEGEQVNVRLEPSTSAPVLVRLSRGTQIQPASQEPHGKWLEVVLP
SQCVFYVAKNFVANKGPIELYTQREGQKKIAMDLINSALNFAHIELEKSLNEIDLEAIYKKINLVQSEEFKDVP
GIQGLIQKALEEIQDAYLSKSLESQNTSIASSQCSTPKVSSSEVTTSLLSRHIRKQTALKTAPLTQGRENLEYSLF
RIWASMQQGNDHSEALTQEAFYRAEQKKKQVLAGVLEVYPHVVKNNPGDYLLKAQENTIAFLYGTSINLEQ
WLGKRVTVECLPRPNNHFAFPAYYVVGIKEAS

>core/334/2/Org2_Gene277

MAASGGTGGLGGTQGVNLAAVEAAAAKADAAEVVASQEGSEMNMIQQSQDLTNPAAATRTKKKEEKFQT
LESRKKGEAGKAEKKSESTEEKPDTDLADKYASGNSEISGQELRGLRDAIGDDASPEDILALVQEKIKDPALQ
STALDYLVQTTPPSQGKLKEALIQARNTHTEQFGRTAIGAKNILFASQEYADQLNVSPSGLRSLYLEVTGDTH
TCDQLLSMLQDRYTYQDMAIVSSFLMKGMATELKRQGPYVPSAQLQVLMTETRNLQAVLTSYDYFESRVPI
LLDSLKAEGIQTPSDLNFVKVAESYHKIINDKFPTASKVEREVRNLIGDDVDSVTGVLNLFFSALRQTSSRLFS
SADKRQQLGAMIANALDAVNINNEDYPKASDFPKPYPWS

>core/335/2/Org2_Gene201

MVYFMVFSPSSESVVKANSVVRSNFCYFLENKFVSPSESTEVMFSEIMKGRVPDIESLFDRPTDMMMTGFKA
AQNLGNLFNSFGILIMCFSQCKSCQTPEKETSAIVLGATLLFFVVALILGPTLGALVYCAYKVYTLGKMIYSLN
KAKAKVLRHPAQNVFHRAAGVATIRSSEEAVKACKLYKSAMIGSLVVSLIASLALIALTAGIVLVLFFVAPGA
APVITAAMMGCCAAGGGALLISLLGLWIAIVRKAKHQEACVGHLTNVVLHTAVSEALLHDPSHFQTNALAR
DLFLTDCLSHYGHLFSNEEVAQLVQGGAPGGGSRPSQHYGGSSDYQNRRGGNGNFGGSHFGGGGGFAGSHF
GAGYPTAPTMPSAPPPFPPPAYDTIYG

>core/336/2/Org2_Gene427

MLSVTIVLVGLEMARSKVSKRDSKILDILFATTELYLKTGQPVGSKTLKESFCSDLSTATIRNYFAELEAEGFL
KKNHTSGGRIPTDLALRHYVDHQEECPEAEISAPIFDKISQLPSESRNIIKDLQKATELLGEILDLPTFFSSPRFEN
DSVTNIQITQVDKQRAVTILSTEFGQIFTDTLWLPEACDTLSIKRIEKFLQNYIRKLPTNEELSKKEEHLSMSLY
NEVVVRYLTRYCNFSEEDLYQTGMSKLLKYEAFKDPEVLALGLSLFENRRQMCELLNIGMHKGRATAFIGK
ELSDILGTSNPGCSVITIPYYMNRSPLGALGILGPINLPYKEALPLLKLFANKINETLTQSFYKFKLSFRRPLTSN
CKLSNEPILRTEYSSIKLLPSKETL

>core/337/2/Org2_Gene421

MRRNPHFSLLKPQYLFSEISKKLAQFRKENPEISVIDLSIGDTTQPLCRSITQAIKEFCVSQEKQETYRGYGPET
GLEKLRTKIASEVYENRISPEEIFISDGAKPDIFRLFSFFGSEKTLGLQDPVYPAYRDIAHITGIRDIIPLACRKET
GFIPELPNQQSLDILCLCYPNNPTGTVLTFQQLQALVNYANQHGTVLIFDAAYSAFVSDPSLPKSIFEIPEAKYC
AIEINSFSKSLGFTGMRLAWNVIPKELTYDNNEPMINDWKRLFATTFNGASLLMQEAGYYGLDLFPTPPAISL
YLTNAQKLKKSLETAGFSVHGGDHAPYLWVELPEGISDEEAFDFFLHQYHIAVTPGHGFGSCGQGFVRFSAL
TQPQNIALACDRLCTASLKETMVLA

>core/338/2/Org2_Gene862

MSNKVLGGSLLIAGSAIGAGVLAVPVLTAKGGFFPATFLYIVSWLFSMASGLCLLEVMTWMKESKNPVNML
SMAESILGHVGKISICLVYLFLFYSLLIAYFCEGGNILCRVFNCQNLGISWIRHLGPLGFAILMGPIIMAGTKVID
YCNRFFMFGLTVAFGIFCALGFLKIQPSFLVRSSWLTTINAFPVFFLAFGFQSIIPTLYYYMDKKVGDVKKAILI
GTLIPLVLYVLWEVVVLGAVSLPILSQAKIGGYTAVEALKQAHRSWAFYIAGELFGFFALVSSFVGVALGVM
DFLADGLKWNKKSHPFSIFFLTFIIPLAWAVCYPEIVLTCLKYAGGFGAAVIIGVFPTLIVWKGRYGKQHHRE
KQLVPGGKFALFLMFLLIVINVVSIYHEL

>core/339/2/Org2_Gene303

MVNIQPVYRNTQVNYSQATQFSVCQPALSLIIVSVVAAVLAIVALVCSQSLLSIELGTALVLVSLILFASAMFM
IYKMRQEPKELLIPKKIMELIQEHYPSIVVDFIRDQEVSIYEIHHLISILNKTNVFDKAPVYLQEKLLQFGIEKFK
DVHPSKLPNFEEILLQHCPLHWLGRLVYPMVSDVTPGTYGYYWCGPLGLYENAPSLFERRSLLLLKKISFGEF
ALLEDGLKKNTWSSSELVQIRQNLFTRYYADKEEVDEAELNADYEQFDSLLHLIFSHKLS

>core/340/2/Org2_Gene654

MSFFNHIPTFSPDAILGLQNVFFADKRPEKVNLVIGVYEHPQKRYGGLSCIRKAQTVILEEEQNKSYLPISGLQI
FLDEMRELVFGAVDPSAIVGFQSLGGTGALHLGARLLSVAKGSGKVYVPEQTWSNHIRIFSQEGLEVIRYPYY
SKEQKQLLFEPLIAFLKEVEKNSVILLHGCCHNPTGVDFTEDMWKELAILMKERELIPFFDTAYQGFAHGIEL
DRKPIEIFISEGNTVLVAASSSKNFALYGERVGYFAVHSTFTDELVKIHSFLEEKIRGEYSSPQRWGVEIVSTILS
NPYLKEEWQSELNFIRESLGKMRTRFVQALRKVAGHTFDFLLSQHGFFAYPGFSDKQVLFLREQHAVYTTAG
GRMNLNGITEKNIDHVVQSFIQAYEL

>core/341/2/Org2_Gene175

MVVSIYSEILSFSELTSCKHSLFPFGPIETASIRIHHVFNVVIVCLIILGTLFVCLGMVFLGVFSTYLLGMSSMILG
LLLISIGLALLKFKERYGLEPKELFGVEGGFDKKLPSEIIQMQDQIADLARELDLEQKKDTLIRGFSARLDVLE
GSKTEKKQILKIGVPRNLSEIQERAQEQNSILEQCKEALLFRRKSAQEIFKKLYDRKAAFWRSYREDLWCYSEI

HVSKKALSNLYIGDVFEGTAPHFLMEAYAMCRTAKNLRNYVKVCVEDMRVNEEKKRAKQLSVSELLCCCT
EIETDLENETNLFTSDSEDVLEEYQIHCIRVTMLHALWAIYNDEVVSRKPIDTLDRVRARMAVEDCIETFEELQ
MCVVHTKTLELEIAQLYVDILLEA

>core/343/2/Org2_Gene52

MSKETFQRNKPHINIGTIGHVDHGKTTLTAAITRALSGDGLASFRDYSSIDNTPEEKARGITINASHVEYETPN
RHYAHVDCPGHADYVKNMITGAAQMDGAILVVSATDGAMPQTKEHILLARQVGVPYIVVFLNKVDMISQE
DAELIDLVEMELSELLEEKGYKGCPIIRGSALKALEGDANYIEKVRELMQAVDDNIPTPEREIDKPFLMPIEDV
FSISGRGTVVTGRIERGIVKVSDKVQLVGLGETKETIVTGVEMFRKELPEGRAGENVGLLLRGIGKNDVERG
MVVCQPNSVKPHTKFKSAVYVLQKEEGGRHKPFFSGYRPQFFFRTTDVTGVVTLPEGTEMVMPGDNVELDV
ELIGTVALEEGMRFAIREGGRTIGAGTISKINA

>core/344/2/Org2_Gene1003

MFEFRFPKIGETSSGGSIVRWLKNLGDHVARDEPLIEVSTDKIATELPSPKAGRLVRFCVNEGDEVASGDVLG
LIELEEISEADDESTSCPPTSCETKSEAGSSSSSVWFSPAVLSLAQREGIGLDNLQKIAGTGKGGRVTRQDLEAY
ISESQQVSIPEIFQGEVNRIPMSPLRRAIASSLSKSSDEVPHASLVVDVDVTDLMNLISGERQRFLDTHGVKLTI
TSFIVQCLAQTLRQFPLLNGSLDGTTIVMKKSVNVGVAVNLNKEGVVVPVIHNCQDRGLVSIAKALADLSSR
ARLNKLDPSEVQDGSVTVTNFGMTGALIGMPIIRYPEVAILGIGTIQKRVVVRDDDSLAIRKMVYVTLTFDHR
VLDGIYGSEFLTSLKNRLESVTMG

>core/345/2/Org2_Gene21

MDYYSILGISKTASAEEIKKAYRKLAVKYHPDKNPGDAAAEKRFKEVSEAYEVLSDPQKRDSYDRFGKDGPF
AGAGGFGGAGGMGNMEDALRTFMGAFGGEFGGGSFFDGLFGGLGEAFGMRSDPAGARQGASKKVHINLTF
EEAAHGVEKELVVSGYKSCETCSGQGAVNPQGIKSCERCKGSGQVVQSRGFFSMASTCPECGGEGRIITDPCS
SCRGQGRVKDKRSVHVHIPAGVDSGMRLKMEGYGDAGQNGAPSGDLYVFIDVESHPVFERRGDDLILELPIG
FVDAALGMKKEIPTLLKTEGSCRLTVPEGIQSGTILKVRNQGFPNVHGKGRGDLLVRISVETPQNLSEEQKEL
LRTFASTEKAENFPKKRSFLDKIKGFFSDFTV

>core/346/2/Org2_Gene152

MSSPVNNTPSAPNIPIPAPTTPGIPTTKPRSSFIEKVIIVAKYILFAIAATSGALGTILGLSGALTPGIGIALLVIFFV
SMVLLGLILKDSISGGEERRLREEVSRFTSENQRLTVITTTLETEVKDLKAAKDQLTLEIEAFRNENGNLKTTA
EDLEEQVSKLSEQLEALERINQLIQANAGDAQEISSELKKLISGWDSKVVEQINTSIQALKVLLGQEWVQEAQ
THVKAMQEQIQALQAEILGMHNQSTALQKSVENLLVQDQALTRVVGELLESENKLSQACSALRQEIEKLAQ
HETSLQQRIDAMLAQEQNLAEQVTALEKMKQEAQKAESEFIACVRDRTFGRRETPPPTTPVVEGDESQEEDE
GGTPPVSQPSSPVDRATGDGQ

>core/347/2/Org2_Gene726

MPRYRYTYLDPKERRKRGYLEALHIQEAREKLAQENIQVLDIREVALRRMSIKSTELIVFTKQLLLLLRSGLPL
YESLVSLRDQYHEQKMGLLLTSFMETLRSGGSLSQAMAAHPNIFDHFYCSGVAAGESVGNLEGCLQNIIVVL
EERAQITKKMVGALSYPCVLLVFSFAVMLFFLLGVIPSLKETFENMEVKGLTKIVFGVSDCLSAYRYLFLGFA
SALITVGILMRHRIPWKKILEKLLFALPGTKKFVVKVAVNRFCSVASAILKGGGTLIEGLDLGCDAIPYDRLKT

DMRDIVQAVIGGGSLSQELAQRSWVPKLAIGMIALGEESGDLADVLGYVAHIYNEDTQKTLASITSWCQPVI
LIFLGGLIGVIMLAILIPLTSNIQTL

>core/348/2/Org2_Gene757

MHPKIEKRNSLPLTAVAPVFEESYHPSVATTVDYVDATTLSRHLTVLKDVIKEARNLDLGKAFLTSMKQGFI
NTGTELAIIQASLADQSSRESRKKEEKIFHQHLGKAAPQAATATSGVQPTADPVADKMPLQSAFAYVLLDKYI
PAQEEALYALGRELNLSGYAQNLFSPLLDMIKSFNSAPINYNLGSYISQTSGTANFAYGYEMILSRYNNEVSQ
CRLDIASTVKAKAALANMSASVKANVSLTDAQKKQIEDIIASYTKSLDVIHTQLTDVMTNLASITFVPGLNKY
DPSYRIVGGDLSIIALQNDEKVLVDGKVDITTAVNEGGLLNFFTTVLTDVQNYGDLAQTQQLMLDLELKAM
QQQWSLVSASLKLLNGMYTTVISGFKN

>core/349/2/Org2_Gene610

MKKLLKSALLSAAFAGSVGSLQALPVGNPSDPSLLIDGTIWEGAAGDPCDPCATWCDAISLRAGFYGDYVFD
RILKVDAPKTFSMGAKPTGSAAANYTTAVDRPNPAYNKHLHDAEWFTNAGFIALNIWDRFDVFCTLGASNG
YIRGNSTAFNLVGLFGVKGTTVNANELPNVSLSNGVVELYTDTSFSWSVGARGALWECGCATLGAEFQYAQ
SKPKVEELNVICNVSQFSVNKPKGYKGVAFPLPTDAGVATATGTKSATINYHEWQVGASLSYRLNSLVPYIG
VQWSRATFDADNIRIAQPKLPTAVLNLTAWNPSLLGNATALSTTDSFSDFMQIVSCQINKFKSRKACGVTVG
ATLVDADKWSLTAEARLINERAAHVSGQFRF

>core/350/2/Org2_Gene395

MMKQGVGQDAKELYTFLSRGNEHYQPCLWFSLEEELGFLFDEKMLCAPLSEDHYCHSYLVDLVDQHLKDLI
LSMFLDPQNISAGELLKVSINVGDSFSPLQQKDFLSMVLRDETGKNVVVVFKGVLSLPATQVCKLVEELNSK
DYSYLNIFSCHGDSSPQLLFRKELEGTSGRYFTVICALYLGDTDMRSLQLASERIMVSREFDLVDAYAARCKL
LKIDHTNWRPGTFSRHADFADAVDVSAGFNSREFKLITQANQGILESGELPLPSKTFWEGFLAFCDRVTVTRH
FIPMLDAAIKQAVWTHKHPSLIDKECEALDLKTQCLPSIVSYLEYVTNSHEKTSKGPFIQKEIIADCSPLKEALF
PGSDEDVPSTSEDPSDDHPSDLEDS

>core/351/2/Org2_Gene593

MGTPISGNDGDRNTISDPLEESAAEEGDSDLEDRVSESATQVIETIADTGIPEATPSEGTNSDLNSDLVDRVEY
EARGSLLTTMLARIRKAVSQIWMHVKTKRHPKEQGVRSLGDIPCDLLKATRLPKETAEPPYFYALETALASC
RSFFFHVFLRLFTLLRRQHPEAPLDLCGTDPISPEAAVAFALILRSCCKWVATDAVQEGLPLEVIEEAGMYNA
FSLEATTTVEEVSKRLSELLYSDKRIDGLANVRGITKIITSPYLGAGQCVSVVDNLKTYDLGRNYTQVLACAS
QIDEFADKGENEALVMKDILYLVRQDRSKELGDFLMMWSEEHASEVNYDVVLAILEVNLPILEEDYRSHPLA
YQKKLNYVICQFFCSERLTSIEPKD

>core/352/2/Org2_Gene435

MLHILLAIFCILLFLAFGLTQPSCHGSSKFLKTLNQRFFKDKGREYPPFPSAPTILATLLCILYGALGTKLYTLLP
PKTAHKDLLFWPLYSLSALIAYGFLPPWISTKVPKETTAHLRFLASVFQLGLFPLQLLFYRRRPNQQVRSSTSF
QSQLSEALSAFDNLIVREVMIPKVDIFALPEETTLQEALVLVSEEGYSRVPVYKKNLDNITGILLVKDLLLLYT
SSHDLSQPISSVAKPPFYAPEIKKASSLLQEFRQKHRHLAIIVNEYGFTEGIATMEDIIEEIIGEIADEHDVQENTP

YKKIGSSWIVDGRMNISDAEEYFNLKIDHENSYDTLGGHVFHKVGAVPQKGMRIHHENFDIEIITCTERNVGK
LKITPRKRKFNIS

>core/353/2/Org2_Gene507

MAIKNILVVDDEPLLRDFLSELLTSQGFIPDTAENLRNALQMIRSRDYDLVISDMSMPDGSGLDLIKIIKQSSPH
TPVLVVTAYGSIENAVEAMHQGAFNYLTKPFSSEALFAFISKAEELKNLVHENLFLHSQTTPDSHPLIAESKA
MKDLLAIAKKAASSSANIFIHGESGCGKEVLSFFIHHNSPRANHPYIKVNCAAIPETLLESELFGHEKGAFTGA
TTKKAGRFELAHKGTLLLDEITEVPVNLQAKLLRAIQEKEIEHLGGTKTLSVDVRILATSNRKLKEAIDDKSFR
QDLYYRLNVIPLHLPPLRDRQDDILPLANYFLNKFCRMNNTPLKTLSPKAQELLLNYPWPGNIRELSNVLERV
VILENTSLLTEDMLALA

>core/354/2/Org2_Gene865

MHLHEYQAKDLLASYDVPIPPYWVVSSEEEGELLITKSGLDSAVVKVQVHAGGRGKHGGVIVAKSSAGILQ
AVAKLLGMHFTSNQTADGFLPVEKVLISPLVAIQREYYVAVIMDRKHRCPVLMLSKAGGMDIEEVAHSSPEQ
ILTLPLTSYGHIYSYQLRQATKFMEWEGEVMHQGVQLIKKLAKCFYENDVSLLEINPLVLTLEGELLVLDSKI
TIDDNALYRHPNLEVLYDPSQENVRDVLAKQIGLSYIALSGNIGCIVNGAGLAMSTLDILKLHGGNAANFLDV
GGGASQKQIQEAVSLVLSDESVKVLFINIFGGIMDCSVVASGLVAVMETRDQVVPTVIRLEGTNVELGKEIVQ
QSGIPCQFVSSMEEGARRAVELSM

>core/355/2/Org2_Gene802

MKWFVISCLLGIFSLGLIMVFDTSSAEVLDRSLECSTHKALIRQVTYLILGLGVASLLYMMEWRDFLKISPVLL
SGAALALICVFIPGLGICRNGARRWLGFGQLTIQPSEFVKYLVPIVALYFLTFSSLYQKQLKMFLKLTAILFIPIL
LIAIEPDNGSAAVISASLIPVFIMTSVRLRYWLLPLLCVLIAGGALAYRMPYVRYRLNVYLHPELDIKGRGHQP
YQAKIAAGSGKLLGKGPGASLQKLTYLPEAQNDYIAAIYAEEFGFLGMLVLILLYMCFVYGGYAIAIKASSLE
GAALAMVITLIISMQAFMNLGVVSGLLPSKGVNLPFFSQGGSSLIANMCGVTLLLKVYDEENSKSSLGCRRFR
RPHCPSSLGKGSFFS

>core/356/2/Org2_Gene425

MSSVNQSSGTPNPEEVTSPESTEENKNVVSSDEAQATHAVALPIVTQLSLPEGVGTSSEETASNPRVDEIVAEV
SSSRAVADQISSLVERVGELLDDLKGAQSLFTSFQSELKNCLPAWKSSTRRLETRGAGDNADIARLELFRSDY
EAVLGHANQFHGKAHLILSKLTDVHHKLQGLSREDLSLAFDNNDRVLEHLGSLGLDVDAEGNWSLSCERGI
PRLVLTADSMLVQIKKVNLPTVEELRTLQGTTESSSDPRVEESLSCCERLLNELRRLWANFVGFISSCYDNIVF
VLMWIVRRINLLPGLGCLPFHNPDASQEDQRSSSGERSTRRERLSRRSDLSEEEMIVRAEGESIHPESPHGDGR
NQPSRGDKQDSDSEEETEL

>core/357/2/Org2_Gene766

MEKPQNRKAPRIFWLNNQVAIPPSERVKESYALHSDIFSLPPGSALKLAEKTEESIRQLVGLKDSHIFRFVPHFP
HVVHIVLAALVENLSMFQGRNHIILPAHDQQLLINSLCRHQGLGTTYDWVTVNHEGRIVEEQLIETLSPRSLLF
SLSAAHGLTGVIQPLDPLLSLCKDRRILLHLDISDILGRAPLTPEILNADIITFSSAALGGMGSIGGIFIRKSLERV
FSSWFPPHTSASLCFSAVAAMQTACEERISALPLFTFHTSNLCKKLIQELQSVLPSIQLAFSEVQNRLPNIVVAA

IPDIPAESLAFHLHQQGIYPSLGYERFQPLAQVLQNCGISPFLCHSALHFSLTERSKDLEFSKLARAMHDAIKHL
TPLLGSSS

>core/358/2/Org2_Gene592

MILDFQFSIGYYLRVLELAIRDGTRILAYDRKRLLLDAWPVNDPLPTNYDTSVSTIRQVIHELFSWSAISYSISS
RLLAIIELRLHEEKPQTGWLYRLFFPSKYHIKKAIVDKLCMFKSLILFESKRPVDKIVQAANKVFSKGKSNFSS
WEDFTHEVTVSEVQTPLAGEVQRRLAADASLQMIIEALTTLLEGHTAYLPLSLELLNQFIGEKAQPLKTLSEK
SYVLLRELIQLFSLSAEDFQTIIMSIISDSLSEVLANSLIGNQPLTFHGKTFVGLWQETALASPEDSKLALGFLAE
VLRKVIVEKKLHVSKSDNTTPEEVGNIYSIRDQNPALWDKMITMLLMRWLLDYDRDIGIALRKAAEYYNPHP
SFWRQFLRLWQRRP

>core/360/2/Org2_Gene202

MSAMISLSSSHEASIASNTQVRDVLVSLAMDEFVEHNTEILPIKVFLARGTLSSTAIIDDLKDVVETEGEHHFQ
VYSNISLKMIYQRFFEKIFGIGCCPLLLVTDSHHTDPCGALITGIFAAVLFTVLAIVFGPTLGILCYSAYKIYQLT
KKISSLSRTHTEVINSVQKSDPFIHRSGAVAAAAASQSTIKACKVFRQSTLIFFVLGLIITISLAALIVGLVFALFF
LDPGAPAVMTAAMIGCCAAGGTGILLSVIGFLLASVYSVQKSQEGVHHMHTALLRCIVSNTIIQMPYLPITPG
TKKVLTQSIRRYQQFFSDDEYRDIESEVPLNRQTTPPPSYETLFHEEGSDGSSNVIPRESPPAYSTIDSSNSPFPSS
SPPPYYR

>core/361/2/Org2_Gene1021

MSETIITTPHVVKLISNFFQKKLFSSISTAYPLVIITDVSVQQHLLGPILDHIKMLGYQVIVLTFPPGEPNKTWET
FISLQYQLVDQNISPKSSIIGIGGGTVLDMTGFLAATYCRGLPLYLIPTTITAMVDTSIGGKNGINLRGIKNRLGT
FYLPKEVWMCPQFLSTLPREEWYHGIAEAIKHGFIADAYLWEFLNSHSKMLFSSSQILHEFIKRNCQIKAAIVA
EDPYDRSLRKILNFGHSIAHAIETLAKGTVNHGQAVSVGMMIETRISLAEGVMKTPQLIDQLERLLKRFNLPST
LKDLQSIVPEHLHNSLYSPENIIYTLGYDKKNLSQHELKMIMIEHLGRAAPFNGTYCASPNMEILYDILWSECH
VMRHC

>core/362/2/Org2_Gene928

MLCQQFLIEALARRKSKHTYRSLSLNSHLIDFTSNDYLGFASSPELRKEYITKLHAIESLGATGSRLLTGHSQL
CQRIEEQLAAYHNFESCLIFNTGYTANLGLLYALATDQDRILHDLYIHASIYDGIRLSKAQSFPFNHNDLNHLE
KRLASSHLGRTFVCVESVYSLHGSVAPLQAISELCERYSAYLIVDEAHAVGVFGDQGEGLVSALGLQDKVLA
TVYTFGKALGTHGAAIAGSSILKDYLINFCRPFIYTTAQPPHALTAIELAYEHNQRAFNQREHLSALIHHFREK
AQNLGLQLMKDNTTTPIQSICVSGSHRARQAALQIQNSGYDVRPIVSPTVKQREELLRICLHAFNTKNEIDHLL
HTLEQIFLCNVSSL

>core/363/2/Org2_Gene243

MSCFNLTSTNESLRPISPKASFPKQGWQSYFRSALRKHRSDTLSVSVCKVNKYDANLFVRLTVIALAVVGVLI
LFSIMLASIQGTLVITSWPLVTAAILIPTILLTGGMYILHRLGKKVDVISGVCIPPFSRRCWVPISSSHTLEKFDEK
HVSACSYLDISTLSADGSGIAAVYQCPPLLFRAFPCFGIPCAMPFVALLRMIYNLIRFLVVPFYIIFRMIYEHFFC
KHLPEDDRFIYKDVAREMGRSLAAFLKAPFYASACMIGAFYSLLDPLAGRVLMGSVERDWNDNVILARSVS

LANEAHSLFRFEGGGGRKGLGQHAFYLMLCCQPQSVFLFDKGEIVSGAHPSIQLPERRGLDTSGRYPHISVIPDSGNDSAKNFIV

>core/364/2/Org2_Gene594

MGINPSGNRSPDDVWVRGAQGDSSSTQGTGATNSNLGAHNVTTSTSQPQVASKAKQLWQTVREFFLGKKSPDSSQGASGPAMQSPSGPTIRPTRPAPPPPTTGGANAKRPATHGKGRAPQPPTAGSSSGSEQPTAMSSEVAKLVSELKDAVHSHAESQKVLKKVSQELQTKWTDWENNRGPDYLLHGYRVIARALQQTYTEQSMLIEGTSSTGPVPQAVTVAKDAVTQTVRGAIKNLENPKPGNDPDGVLMQVVISLGIEGPTLDPGESIQNFLETRVSDFGGDDSDIDYTSDIARLGSALDRVRENHPNEMPRIWIALARELGAAVHSHATSVRIANAGKNHTRDVVRMANESSRLLQGMKVLSVGAWANTMTVLIGDLFE

>core/365/2/Org2_Gene824

MIPSPTPINFRDDTILETDPKPSLIMFSSKKTEIASERRKAHPTLFKVLGTIWNIVKFIISIILFLPLALLWVLKKTCQFFILPSSIISQSMSKTAVAIRRMTFLSHIKQLLSLKEISAADRVVIQYDDLVVDSLAIKIPHALPHRWILYSQGNSGLMENLFDRGDSSLHQLAKATGSNLLVFNYPGIMSSKGEAKRENLVKSYQACVRYLRDEETGPKANQIIAFGYSLGTSVQAAALDREVTDGSDGTSWIVVKDRGPRSLADVANQICKPIASAIIKLVGWNIDSVKPSERLRCPEIFIYNSNHDQELISDGLFERENCVATPFLELPEVKTSGTKIPIPERDLLHLNPLSPNVVDRLAAVISNYLDSENRKSQQPD

>core/366/2/Org2_Gene292

MKHLAVLGSTGSIGRQTLEIVRRYPSEFKIISMASYGNNLRLFFQQLEEFAPLAAAVYNEEVYNEACQRFPHMQFFLGQEGLTQLCIMDTVTTVVAASSGIEALPAILESMKKGKALALANKEILVCAGELVSKTAKENGIKVLPIDSEHNALYQCLEGRTIEGIKKLILTASGGPLLNKSLEELSCVTKQDVLNHPIWNMGSKVTVDSSTLVNKGLEIIEAYWLFGLENVEILAVIHPQSLIHGMVEFLDGSVISIMNPPDMLFPIQYALTAPERFASPRDGMDFSKKQTLEFFPVDEERFPSIRLAQQVLEKQGSSGSFFNAANEVLVRRFLCEEISWCDILRKLTTLMECHKVYACHSLEDILEVDGEARALAQEI

>core/367/2/Org2_Gene704

MKDLGTLGGTSSTAKTVSPDGKVIMGRSQIADGSWHAFMCHTDFSSNNVLFDLDNTYKTLRENGRQLNSIFNLQNMMLQRASDHEFTEFGRSNIALGAGLYVNALQNLPSNLAAQYFGIAYKIRPKYRLGVFLDHNFSSHVPNNFNVSHNRLWMGAFIGWQDSDALGSSVKVSFGYGKQKATITREQLENTEAGSGESHFEGVAAQIEGRYGKSLGGHVRVQPFLGLQFVHITRKEYTENAVQFPVHYDPIDYSTGVVYLGIGSHIALVDSLHVGTRMGMEQNFAAHTDRFSGSIASIGNFVFEKLDVTHTRAFAEMRVNYELPYLQSLNLILRVNQQPLQGVMGFSSDLRYALGF

>core/368/2/Org2_Gene774

MEDFSEQQLFFMRRAIEIGEKGRITAPPNPWVGCVVVQENRIIGEGFHAYAGGPHAEELAIQNASMPISGSDVYVSLEPCSHFGSCPPCANLLIKHKVSRVFVALVDPDPKVAGQGIAMLRQAGIQVYVGIGESEAQASLQPYLYQRTHNFPWTILKSAASVDGQVADSQGKSQWITCPEARHDVGKLRAESQAILVGSRTVLSDDPWLTARQPQGMLYPKQPLRVVLDSRGSVPPTSKVFDKTSPTLYVTTERCPENYIKVLDSLDVPVLLTESTPSGVDLHKVYEYLAQKKILQVLVEGGTTLHTSLLKERFVNSLVLYSGPMILGDQKRPLVGVLGNLLESASPLTLKSSQILGNSLKVVWEISPQVFEPIRN

>core/369/2/Org2_Gene825

MNLSNRSDILSGIFSNPHPVSYFSSTHAKQLSDFSKKHPILTKIVTIIVKIFKLLIGLIIPPLGIYWLCQLVCSLALF
PRSSMLYSVLKTCFKKYRLEQEIQDYFVKNLDPSFKDPAVSESKRITIQQDHLTIDTLAIHFSTARPKRWLLISL
GSGDFLEDMIGLKDSLFLSWKELAKLLGANILIYNYPGVKSSTGKLNLENLATAHNLCAKYLQDKIQGPGAN
EIITYGYSLGGVVQSAALQKNPFTNSETSWVAVKDRAPHSLPAAANSFFGPIGKLIAVLARWKMDAEKNSRE
LPCPEILVYSADRFRPSEVGDDTALLPEFTLAHAIKRTPFARSKKFIGEVNLLHSSPLKHPTIQKLAEAILESLSR
KN

>core/370/2/Org2_Gene114

MNTSLKRPLKSHFDVVGSFLRPEHLKKTRESLKEGSISLDQLMQIEDIAIQDLIKKQKAAGLSFITDGEFRRAT
WHYDFMWGFHGVGHHRATEGVFFDGERAMIDDTYLTDKISVSHHPFVDHFKFVKALEDEFTTAKQTLPAPA
QFLKQMIFPNNIEVTRKFYPTNQELIEDIVAGYRKVIRDLYDAGCRYLQLDDCTRGGLVDPRVCSWYGIDEK
GLQDLIQQYLLINNLVIADRPDDLVVNLHVCRGNYHSKFFASGSYDFIAKPLFEQTNVDGYYLEFDHERSGDF
SPLTFISGEKTVCLGLVTSKTPTLENKDEVIARIHQAADYLPLERLSLSPQCGFASCEIGNKLTEEEQWAKVAL
VKEISEEVWK

>core/371/2/Org2_Gene544

MSDNAHNLLYDKFELPEAVKMLPVEGLPIDKHARFIAEPLERGMGHTLGNALRRALLIGLEAPAIISFAMTGV
LHEYMAIEGVIEDVTNIILNLKGALLKKYPMQDSSLGRTTQVLKASISIDASDLAAANGQKEVTLQDLLQEGD
FEAVNPDQVIFTVTQPIQLEVVLRIAFGRGYTPSERIVLEDKGVYEIVLDAAFSPVTLVNYFVEDTRVGQDTDF
DRLVLIVETDGRVTPKEALAFSTQILTKHFSIFENMDEKKIVFEEAISIEKENKDDILHKLILGINEIELSVRSTNC
LSNANIETIGELVIMPEPRLLQFRNFGKKSLCEIKNKLKEMKLELGMDLTQFGVGLDNVKEKMKWYAEKIRA
KNTKG

>core/372/2/Org2_Gene324

MNGKAPLALYIHIPFCTKKCRYCSFYTIPYKSESVSLYCNAVIQEGLRKLAPIQETHFIETVFFGGGTPSLVSPL
DLKRILKELAPHAREITLEANPENLTVSYLRQLQETPINRISVGVQTFDDSILQLLGRTHSSSAAITALQECQNH
GFSNLSIDLIYGLPTQSLEIFLSDLHQALTLPITHISLYNLTIDPHTSFYKHRKILVPTIAQEEILAEMSLLAENLLL
SQGFQRYELASYAKPDYPAKHNLYYWTDRPFLGLGVSASQYLHGERSKNYSHISHYLRAVRKNLPTQETSEI
LPKKERIKEALALRLRLLEGADLAEFPSTLISMLTQDVKLQNLFSVHGQCLALNRQGRLFHDTIAEEIMGYSF

>core/373/2/Org2_Gene483

MSIAIAREQYAAILDMHPKPSIAMFSSEQARTSWEKRQAHPYLYRLLEIIWGVVKFLLGLIFFIPLGLFWVLQK
ICQNFILLGAGGWIFRPICRDSNLLRQAYAARLFSASFQDHVSSVRRVCLQYDEVFIDGLELRLPNAKPDRWM
LISNGNSDCLEYRTVLQGEKDWIFRIAEESQSNILIFNYPGVMKSQGNITRNNVVKSYQACVRYLRDEPAGPQ
ARQIVAYGYSLGASVQAEALSKEIADGSDSVRWFVVKDRGARSTGAVAKQFIGSLGVWLANLTHWNINSEK
RSKDLHCPELFIYGKDSQGNLIGDGLFKKETCFAAPFLDPKNLEECSGKKIPVAQTGLRHDHILSDDVIKEVAG
HIQRHFDN

>core/374/2/Org2_Gene181

MALKFHLIHQSKKSQARVGQIETSHGVIDTPAFVPVATHGALKGVIDHSDIPLLFCNTYHLLLHPGPEAVAKL
GGLHQFMGRQAPIITDSGGFQIFSLAYGSVAEEIKSCGKKKGMSSLVKITDEGAWFKSYRDGRKLFLSPELSV
QAQKDLGADIIIPLDELLPFHTDQEYFLTSCSRTYVWEKRSLEYHRKDPRHQSMYGVIHGGLDPEQRRIGVRF
VEDEPFDGSAIGGSLGRNLQEMSEVVKITTSFLSKERPVHLLGIGDLPSIYAMVGFGIDSFDSSYPTKAARHGLI
LSKAGPIKIGQQKYSQDSSTIDPSCSCLTCLSGISRAYLRHLFKVREPNAAIWASIHNLHHMQQVMKEIREAIL
KDEI

>core/375/2/Org2_Gene826

MAPIHGSNAFVEDILHSHPSPQATYFSSTRAQKLHEFKDRHPVLTRIASVIIKIFKVLIGLIILPLGIYWLCQTLCT
NSILPSKNLLKIFKKQPNTKTLKTNYLHALQDYSSKNRVASMRRVPILQDNVLIDTLEICLSQAPTNRWMLISL
GSDCSLEEIACKEIFDSWQRFAKLIGANILVYNYPGVMSSTGSSSLKDLASAHNICTRYLKDKEQGPGAKEIIT
YGYSLGGLIQAEALRDQKIVANDDTTWIAVKDRCPLFISPEGFHSCRRIGKLVARLFGWGTKAVERSQDLPCL
EIFLYPTDSLRRSTVRQNKLLAPELTLAHAIKNSPYVQNKEFIEVRLSSDIDPIDSKTRVALATPILKKLS

>core/376/2/Org2_Gene314

MSTTEPNLTNVNLTMLISSESMPTQLASHKLKGLDLVAFILIIGIAVSSGTAAIILGIPLLFILTALAVLAFSILLY
FLLREPKSPISVTHQPTPIIKDTDLPPVPPLALTPVPTEAVLEEPPLPSPRTHQTLLQENWDRIPDLQANTDMPFI
AADNQTGYAWHLKNSNLTLISTLGPIEKPRYKTQGIVMIVNAATPNMANNVKGTSLALAKATSVRCWENSK
KSPDPLRSKQPLQLGECRSAKWENLNGTTNAGKAGLPQFLGQLLGPKASDYNYNPNDAFTFCRQAYLNCLN
EAKRRKTTVVQLPLLSSHFPGSPKDEETTSLRLQWIDGVKLALIDALQTFGSEAENQNQPWVIILTTLARHPLI
TP

>core/377/2/Org2_Gene619

MAVAADSSASWLKSRNNFLSSLGKTEEQVAAPEFPKELCQHKIREKFRLEDVQVSIKFRGSITAVEATKEFGV
HLLIQPMVVQPWEVENLLFLTSEEDLQELMVAVFDDASLASYFYEKDKLLGFHYYFVAEACKLFEELQWVP
SLSAKVGGDAIFTATSLQGSFQVVDISLRLDGKNVRCRLLLPEDTFQSCQKFFSGLHDESDLHNIDQTQQISLS
VEVGYSQLTQEEWHQVVPGSFIMLDSCLYDPETEESGALLTVQKHQFFGGRFLTPSSGEFKITSYPNLTHEDP
PLPENPQASAAPLPGYSRLVVEVARYSLAVSEFIKLNLGSILSLGNHPAYGVDIILDGAKVGRGEIIALGDVLGI
RVLEV

>core/378/2/Org2_Gene339

MIYLDNNAMTPPERGLLEFLQKTFLIEGTYANPSSVHQLGKKSRQLVLEASHWMQKVLSFQGRVLYTSGATE
SLNLAIASLPKDSHVITSGSEHPAILEPLKHSSLSVSYLNPEEGRCVLTIEQIERAVTPKTSAIILGWVNSETGAK
ADIAAIAHFAQERQLQFIVDATANVGKERIVLPSGVTMAAFSGHKFHALSGIGALLVSPGVKLHPQLWGGGQ
QGGLRAGTENLWGIASLLYIFKYLDLHQERISQEILTHRNGFEKAIKARIPDVHIHCADQPRANNVSAIAFPPL
EGEVLQIALDIEGVACGYGSACSSGATAPFKSLVSMGVDEELTLATLRFSFSHLLLQEDVERAVGIIEKVVERL
KNS

>core/379/2/Org2_Gene820

MTTSDVIDFVTNDFLGFARSPTIYCEVSKRFQIHCQQFPHEKLGIRGSRLMVGPSSVIDDLESKIASYHGAPNA
FIVNSGYMANLGLCHHVSRSTDVLLWDEEVHMSVVHSLSAISGQHHTFHHNNLEHLESLLQCYRISSKGRIFI

FVSSVYSFRGTLAPLEQIIALSKKYHAHLIVDEAHAMGIFGDDGKGLCHALGYENFYAVLVTYGKALGTMG
ASLLTSSEVKYDLMQNSPPLRYSTSLSPHTLISIGTAYDFLASEGEIARKQVFKLKEHFHECFDSHAPGCVQPIF
LPHTCLEEAISVLETTGIHVGVVAFAKHPFLRVNLHAYNTVDEVNLLAQVMKPYLEKSSHRVHINHEFHLWR
ELCQH

>core/380/2/Org2_Gene867

MMLVYCFDPSVPTSPEHRLMAALDRWFFLGGHRARILTLEGNHYRAFQENMSISTVEKILKLISYLLIPIVLIA
LLIRCFLHSRFKCNWKCDSLSDARVPHDVQPFNDFQLFNNQERLNIWKNRRYVSGIDVLMVPVDYLRSQFPG
FKEIPEAIRCENYVSDGQFSEESKTSYLRAMLTDIVGYILSLDETYWTNVILKIRAMCITFESFPGKEADPNYSP
RVTHHYFDESWKALARHVLGEGNMVNRLDEALIRTEKPGKEGECITKQFLKDYCKKHLEVMSCPDFIESLV
DEKIREFRCPSILNSAVCDVIDRKCQEHLLKAIINEANRRLPGMKNSSFTMRGNQVLFYTIFSPPKLPPAASSVY
F

>core/381/2/Org2_Gene438

MTTCLPQPPKTSPLYSIFEKLDAQERLSSEDALHLLLLTNKEDQRTLWNFADQVRKQRVGDTVYYSSTLYLY
PTNFCDFSCKFCSFYAKPGDPKGWLYSPDDLLQQIQNIKTPITEVHIVGGCFPSCNLQYYSDLFTKIKEYDPQIH
IKALTAIEYAYLSDLDNLSIRDVLLTLKDAGLDSIPGGGAEILVDKIRNFLAPKRLSSSDFLNIHKMAHQLGIHS
NITMLCYHKEGPEDLVTHMVKVRDLQDETQGFKNFILLKFAQENNVLGKRLRKSGQGHAIPLKSLMAVARIF
LDNFSNMKALWNYLGIEAALDLLSCGANDLSSTHMGEKVFQMASSKEPIKMDAEGMAALITQQGRTPCLTN
SSHV

>core/382/2/Org2_Gene1006

MRRQVREIMQQTVIVAMSGGVDSSVVAYLFKKFTNYKVIGLFMKNWEEDSEGGLCSSTKDYEDVERVCLQ
LDIPYYTVSFAKEYRERVFARFLKEYSLGYTPNPDILCNREIKFDLLQKKVQELGGDYLATGHYCRLNTELQE
TQLLRGCDPQKDQSYFLSGTPKSALHNVLFPLGEMNKTEVRAIAAQAALPTAEKKDSTGICFIGKRPFKEFLE
KFLPNKTGNVIDWDTKEIVGQHQGAHYYTIGQRRGLDLGGSEKPCYVVGKNIEENSIYIVRGEDHPQLYLRE
LTARELNWFTPPKSGCHCSAKVRYRSPDEACTIDYSSGDEVKVRFSQPVKAVTPGQTIAFYQGDTCLGSGVID
VPMIPSEG

>core/383/2/Org2_Gene345

MTKIAFSEKAKNFPVEALKKWFEKNKRSLPWRDNPTPYSVWVSEVMLQQTRAEVVIDYFNQWMERFPTIES
LAAAKEEDVIKLWEGLGYYSRARHLLEGARMVMEEFHGKIPDDAISLAQIRGVGPYTVHAILAFAFKRRAAA
VDGNVLRVLSRIFLIETSIDLESTRTWVSRIAQALLPHKSPEVIAEALIELGACICKKVPQCHRCPVRQACGAW
RENKQFVLPVRHARKKVIFLHRLVAIVLYDGSLVVEKRRPKEMMAGLYEFPYIEVEPEEGLQDIEGFTKKME
LSLESPLEFLGNLKEQRHAFTNHKVHLCPIIFKATSLPQFGELHLLSDIDHLAFSSGHKKIKDALLIYLGDVRSR
ESIGV

>core/384/2/Org2_Gene497

MQENLDKRLEALRTEISLAARSLFDLDKKQKELQVLEEESSEENFWQDSVHAGKISEQIVSLRRQIQEYQELK
SKIDAIEFFLEDADALEDPAICEDLEKEFLFCEKKLAVWETQRLLSGEADKNSCFLTINAGAGGTESCDWVEM
LFRMYSRWATKHQWALEVVDRLDGEVVGIKHVTVKFSGMYAYGYAKAERGVHRLVRISPFDSNGKRHTSF

ASVDVFPEIDDQIKIEIRPNDLRIDTFRSSGAGGQHVNVTESAVRITHLPSGVVVSCQNERSQIQNRESCMKML
QAKLYQQVLQERLEKQSLDRKDKKEIAWGSQIRNYVFQPYTLVKDVRTGHETGNVQAMLDGELLDEFIKAY
LAEFGEVS

>core/385/2/Org2_Gene46

MAVEGRVNSSQALNQDCQEVLANKQSKGLLRCRILSIVVAVITFIAGVVLIALTLASILTSVPYLALGVFLLIV
TLGCIIFALCSEKIKKVPPTPISHKEEIIAWFEERKNIDMEKEKEDPEHFGRTATDIPMRSALDQFNHSCHHIHES
PALTETYRSHQDVLLFKDWCPVTLPDVTSEEEVLIRSVVGSYLLMEACVPKVSMLIDELHNKLKSPSERECLF
IDKKTLQRKASFLFTQKDLATFFLAYTRVNDGHLAPFRAGAKWILIHYVRLRRQHNQNDFFTPGHSCYYARL
AFNQTQRLYHQLFNVEKLRSIYANMDKDPLCHPWAFIPIYDLLKTEDHGDGFLEQQEDREYPSRAAQDQFW
G

>core/386/2/Org2_Gene755

MSPHRNLFKLKNFSNRLYNRALGRFDKVFNFFSGNVGIDLGTANTLVYVRGRGIVLSEPSVVAVDAQTHAVL
AVGHKAKAMLGKTPRKIMAVRPMKDGVIADFEIAEGMLKALIKRVTPSRSVFRPRILIAVPSGITGVEKRAVE
DSALHAGAQEVILIEEPMAAAIGVDLPVHEPAASMIIDIGGGTTEIAIISLGGIVESRSLRIAGDEFDECIINYMR
RTYNLMIGPRTAEEIKITIGSAYPLGDQELEMEVRGRDQVAGLPITKRINSVEIRECLAEPIQQIIECVRLTLEKC
PPELSADLVERGMVLAGGGALIKGLDKALSKNTGLSVITAPHPLLAVCLGTGKALEHLDQFKKRKGNLV

>core/387/2/Org2_Gene290

MKFVVSRNELGNLIKKIQSVVPQNTPIPVLTHVLIETYNDELVFTATDLTVSTRCVTKAKVYEKGAISIPSKRF
FQLVKELTEANLEISSSAGEMAQITSGSSCFRLLSMEKEDFPMLPDIQNALRFSLPAEQLKTMLQRTSFAVSRE
ESRYVLTGVLLAIANGVATIVGTDGKRLAKIDAEVTLDKSFSGEYIIPIKAVEEIIKMCSDEGEAAIFLDQDKIA
VECDNTLLITKLLSGEFPDFSPVISTESNVKLDLHREELITLLKQVALFTNESSHSVKFSFLPGELTLTANCTKV
GEGKVSMAVNYSGELLEIAFNPFFFLDILKHSKDELVSLGISDSYNPGIITDSASGLFVIMPMRLHDD

>core/388/2/Org2_Gene885

MLIWKRHLLTRFWFALTSLLVLALIFYASIHHSLHTLKGASTAASGASVKLSILYYLAQISLKAEFLMPQLVA
VATTSTLFAMQNKREIILLQASGLSLKSLMHPLLLSGAVIMMVLYANFQWLHPICEKISITKENMDRGTTDKE
QGKIPALYLKDQTVLLYSSIEPKTLTLNNVFWIKDPKTIYTMEKLAFTTLSLPIGLNVTQFFANDSENLELKEFF
DMKEFPEIEFNFYENPFSKLFSAGNKNRLSEFFKAIPWNATGLGLSTQVPQRILSLLAQFYYVLISPLACMAAII
LSAYLCLRFSRTPTVTLAYLIPLGTVNIFFVFLKAGIVLASSSVLPTLPVMAFPLIVLFLLTNYAYAKLQ

>core/389/2/Org2_Gene486

MKFPRISISDLIPTQMVIWWRGGGNVHYVPNAQNLPKKILGGVLACFGLALLGCAAFAAGVCQTIFPCIGLMI
LGLVLLGFAYLQYSKGWSRFERPLFRETKVFEKPINWLGCLSLLQSWKKIRPGCYYHPGCPQVEICEGSQEIV
TKIFQKKSDRNTSIFLIQEMDQIALRQGIEKSSLSRKTFAIDPSVVSSLLSEIQREEQQYLDPKVISWSSEDQASD
RTHPKSAIYVNISDAAQEPQGRCYIDAYTKAFFTVLDQIGDPNIVKKHTIYVLTPILGVPDALPKEEQENLKLL
SQAAFLYSAEQVAKRMREEKQDSIRIKFIFTDPTSPTSLYFSPHHSSTPHSVTPISLSGFVGEQESYTFA

>core/390/2/Org2_Gene706

MSKKIKVLGHLTLCTLFRGVLCAAALSNIGYASTSQESPYQKSIEDWKGYTFTDLELLSKEGWSEAHAVSGN
GSRIVGASGAGQGSVTAVIWESHLIKHLGTLGGEASSAEGISKDGEVVVGWSDTREGYTHAFVFDGRDMKD
LGTLGATYSVARGVSGDGSIIVGVSATARGEDYGWQVGVKWEKGKIKQLKLLPQGLWSEANAISEDGTVIV
GRGEISRNHIVAVKWNKNAVYSLGTLGGSVASAEAISANGKVIVGWSTTNNGETHAFMHKDETMHDLGTL
GGGFSVATGVSADGRAIVGFSAVKTGEIHAFYYAEGEMEDLTTLGGEEARVFDISSEGNDIIGSIKTDAGAER
AYLFHIHK

>core/391/2/Org2_Gene451

MKKRFPSTLFLFYRRVTIAISLEGILGWGWLGSLLSKVFAFLVACWNRFSWSTPYRARSTVISVGNIVVGGAG
KTPTVLWLAEALRLRGYSCGVLSRGYKSQSSRQKKLTVVDSKVHSASYVGDEPLLMAEKLPEGSVWVHKD
RRISAARAAEKFGILLLDDGLQYRKLHKDVEIAVVNGQDPLGGRAFFPKGRLRDFPLRLKTVDAIIVNGGGKE
AGTVVKRVSNAPQIFVKPTIASVVWTHNGERIPKEALRELRVGVFCGLGFPQGFLNMLREEGIHILGKYLLPD
HAAITKKELNYFCQQMAMRQGQGLLCTEKDSVKLPRLSGEVSLLPIAKVEMRLSVNQDDTLSLLNMIEQIHK
NRGN

>core/392/2/Org2_Gene505

MNVPDSKNLHPPAYELLEIKARITQSYKEASAILTAIPDGILLLSETGHFLICNSQAREILGIDENLEILNRSFTD
VLPDTCLGFSIQEALESLKVPKTLRLSLCKESKEKEVELFIRKNEISGYLFIQIRDRSDYKQLENAIERYKNIAEL
GKMTATLAHEIRNPLSGIVGFASILKKEISSPRHQRMLSSIISGTRSLNNLVSSMLEYTKSQPLNLKIINLQDFFS
SLIPLLSVSFPNCKFVREGAQPLFRSIDPDRMNSVVWNLVKNAVETGNSPITLTLHTSGDISVTNPGTIPSEIMD
KLFTPFFTTKREGNGLGLAEAQKIIRLHGGDIQLKTSDSAVSFFIIIPELLAALPKERAAS

>core/393/2/Org2_Gene274

MSHTECGIVGLPNVGKSGLFNALTGAQVASCNYPFCTIDPNVGIVPVIDERLEALAKISNSQKIIYADMKFVDI
AGLVKGASDGAGLGNRFLSHIRETHAIAHVVRCFDDPDVTHVSGKVNPVEDIEVINLELIFSDFSSAKNIHSKL
EKLAKGKREVGALLPLFDTIIAHLEKGLPLRTLELTPEQIVALKPYPFLTMKPMFYIANVDESSLPDMDNDYV
AAVREVAAKENSKVVPICVRIEEEIVSLPIEERLEFLMSLGLEKSGLHRLVRAAYDTLGLISYFTTGPQESRAW
TVVRGSSAWEAAGEIHTDIQKGFIRAEVITFEDMIECQGRAAARELGKLHIEGRDYIVQDGDTMLFLHN

>core/394/2/Org2_Gene321

MTTEVRIPNIAESISEVTVASLLVTEGALIQENQGLLEIESDKVNQLIYAPVSGRIFWEVSEGDVVPVGGVVGKI
EPAGEGEELGDSQSKETIEAEIICFPQSGVRQSPPENKTFIPLRDQMDQGSQGLSAGDRGETRERMTSIRKTISR
RLLSALHESAMLTTFNEVYMTPLFHLRKEKQEEFLSRYGVKLGFMSFFVKAVLEALKAYPRVNAYIDGEEIV
YRHYYDISIAVGIDRGLVVPVIRDCDKLSNGEIEQKLADLALRAREGLLAIAELEGGGFTITNGGVYGSLLSTPI
INPPQVGILGMHKIEKRPVVLDNEIVIADMMYVALSYDHRLIDGKEAVGFLVKVKEGLENPASLLDL

>core/395/2/Org2_Gene591

MVPFRQHHAYQLLKQLHTSAISEADRVSYYFKQNRSLGSKDRQWIQNIIFNILRHRRLLETLILDSGEQVTPEA
LVAKVNEGVLENLDSYSAIPWPVRYSISDDLAHFLVQDYGEEQAEEIAKIWLTEAPITIRVNTDKISVKELQEK
LEYPSSPGELPEALHFSKRHPLQSTEAFRRGFFEIQDENSQRISQGISLTDKDIVLDFCAGAGGKSLIFAQKAKH

VVINDSRKAILQTAKHRLLRAGARNFSLADQLRLGSFSVVIVDAPCSGTGVFRRHPEHKWQFSKKLLLNYVR
VQKSILKQASAYVGPRGRLVYITCSLLKEENEAHVAYMHSLGWKEVHRKTLPLQVGKGDAFFTSHFQKI

>core/396/2/Org2_Gene940

MKLYQTLRGIVLVSTGCIFLGMHGGYAAEVPVTSSGYENLLESKEQDPSGLAIHDRILFKVDEENVVTALDVI
HKLNLLFYNSYPHLIDSFPARSQYYTAMWPVVLESVIDEFLMVADAKAKRIATDPTAVNQEIEEMFGRDLSP
LYAHFEMSPNDIFNVIDRTLTAQRVMGMMVRSKVMLKVTPGKIREYYRKLEEEASRKVIWKYRVLTIKANT
ESLASQIADKVRARLNEAKTWDKDRLTALVISQGGQLVCSEEFSRENSELSQSHKQELDLIGYPKELCGLPKA
HKSGYKLYMLLDKTSGSIEPLDVMESKIKQHLFALEAESVEKQYKDRLRKRYGYDASMIAKLLSEEAPPLFS
LL

>core/397/2/Org2_Gene513

MAFKRKTRWLWQVLILSVGLNMLFLLLFYSAIFRKDIYKLHLFSGPLIAKSSRKVYLSEDFLNEISQASLDDLI
SLFKDERYMYGRPIKLWALSVAIASHHIDITPVLSKPLTYTELKGSSVRWLLPNIDLKDFPVILDYLRCHKYPY
TSKGLFLLIEKMVQEGWVDEDCLYHFCSTPEFLYLRTLLVGADVQASSVASLARMVIRCGSERFFHFCNEESR
TSMISATQRQKVLKSYLDCEESLAALLLLVHDSDVVLHEFCDEDLEKVIRLMPQESPYSQNFFSRLQHSPRRE
LACMSTQRVEAPRVQEDQDEEYVVQDGDSLWLIAKRFGIPMDKIIQKNGLNHHRLFPGKVLKLPAKQS

>core/398/2/Org2_Gene1016

MAVVSTPFFNRNLWYRLLSSRFSLWKSYCPRFFLDYLEAFGLLSDFLDHQAVIKFFELETHFSYYPVSGFVAP
HQYLSLLQDRYFPIASVMRTLDKDNFSLTPDLIHDLLGHVPWLLHPSFSEFFINMGRLFTKVIEKVQALPSKKQ
RIQTLQSNLIAIVRCFWFTVESGLIENHEGRKAYGAVLISSPQELGHAFIDNVRVLPLELDQIIRLPFNTSTPQET
LFSIRHFDELVELTSKLEWMLDQGLLESIPLYNQEKYLSGFEVLCQ

>core/400/2/Org2_Gene92

MKIDILSLFPGYFDGPLQTSILGRAIKQRLLDVQLTNLRDFGLGKWKQVDDTPFSGGGMLLMAEPVTSAIRSV
RKENSKVIYLSPQGALLTAEKSRELAAASHLILLCGHYEGIDERAIESEVDEEISIGDYVLTNGGIAALVLIDAV
SRFIPGVLGNQESAERDSLENGLLEGPQYTRPREFEGKEVPEVLLQGDHKAISQWRLEQSERRTYERRPDLYL
NYLYKRSIDHKFDEETTTNRDHFKCDKISVVLEVNKLKRAKNFYCKVFGLDAMSCENKFCLPHEGKTIFWLR
EVQAEKKNIVTLSLSLDCACEEDFCYLLRRWELFGGKLLEKQADEHAVWALAQDLDGHAWIFSWHRMK

>core/401/2/Org2_Gene275

MGEKTEKATPKRLRDARKKGQVAKSQDFPSAVTFIVSMFTAFSLSTFFFKHLGGFLVSMLSQAPTRHDPVITL
FYLKNCLMLILTASLPLLGAVAVVGVIVGFLIVGPTFSTEVFKPDIKKFNPIENIKQKFKIKTLIELIKSILKIFGA
ALILYITLKSKVSLIIETAGVSPIITAQIFKEIFYKAVTSIGIFFLIVAILDLVYQRHNFAKELKMEKFEVKQEFKD
TEGNPEIKGRRRQIAQEIAYEDSSSQVKHASTVVSNPKDIAVAIGYMPEKYKAPWIIAMGINLRAKRILDEAEK
YGIPIMRNVPLAHQLLDEGKELKFIPESTYEAIGEILLYITSLNAQNPNNKNTNQPDHL

>core/402/2/Org2_Gene257

MSEAPVYTLKQLAELLQVEVQGNIETPISGVEDISQAQPHHIAFLDNEKYSSFLKNTKAGAIILSRSQAMQHA
HLKKNFLITNESPSLTFQKCIELFIEPVTSGFPGIHPTAVIHPTARIEKNVTIEPYVVISQHAHIGSDTYIGAGSVIG

AHSVLGANCLIHPKVVIRERVLMGNRVVVQPGAVLGSCGFGYITNAFGHHKPLKHLGYVIVGDDVEIGANTT
IDRGRFKNTVIHEGTKIDNQVQVAHHVEIGKHSIIVAQAGIAGSTKIGEHVIIGGQTGITGHISIADHVIMIAQTG
VTKSITSPGIYGGAPARPYQETHRLIAKIRNLPKTEERLSKLEKQVRDLSTPSLAEIPSEI

>core/403/2/Org2_Gene447

MSGPSRTESSQVSVLSYVPRDKEIAPKKQFTIAKISTLAILASLALGALVAGISLTIVLGNPVFLALLITTALFSV
VTFLVYHQMTSKVSSNWQKVLEQNFKPLGKAWQEKNVDCYSNEMQFYNNHLNPKFKVAIQTDASQPFQPT
FLTGLRVIEKNQSTGIIFNPVGPTNLIDNTATNLSTILYSTLKDKSVWDTCKQREGGPAKGEDPFSPTEVRVVK
LPNEALDQTFNLNLSSAEKKSILPTFLGHVCGPKSEELPNQQEYYRQALLAYENCLKAAIESHAAIVALPLFTS
VYEVPPEEILPKEGTFYWDNQTQAFCKRALLDAIQNTALRYPQRSLLVILQDPFNTIESQSRSEE

>core/404/2/Org2_Gene922

MKNSFGSLFSFTTWGESHGPSIGVVIDGCPAGLELHESDFVPAMKRRRPGNPGTSSRKENDIVQILSGVYKGK
TTGTPLSLQILNTDVDSSPYENSERLYRPGHSQYTYEKKFGIVDPNGGGRSSARETACRVAAGVVAEKFLAN
QNIFTLAYLSSLGSLTLPHYLKISPELIHKIHTSPFYSPLPNEKIQEILTSLHDDSDSLGGVISFITSPIHDFLGEPLF
GKVHALLASALMSIPAAKGFEIGKGFASAQMRGSQYTDPFVMEGENITLKSNNCGGTLGGITIGVPIEGRIAF
KPTSSIKRPCATVTKTKKETTYRTPQTGRHDPCVAIRAVPVVEAMINLVLADLVLYQRCSKL

>core/405/2/Org2_Gene616

MTLPNDLLETLVKRKESPQANKVWPVTTFSLARNLSVSKFLPCLSKEQKLEILQFITSHFNHIEGFGEFIVLPLK
DTPLWQKEFLLEHFLLPYDLVGNPEGEALVVSRSGDFLAAINFQDHLVLHGIDFQGNVEKTLDQLVQLDSYL
HSKLSFAFSSEFGFLTTNPKNCGTGLKSQCFLHIPALLYSKEFTNLIDEEVEIITSSLLLGVTGFPGNIVVLSNRC
SLGLTEELLLSSLRITASKLSVAEVAAKKRLSEENSGDLKNLILRSLGLLTHSCQLELKETLDALSWIQLGIDLG
LIKVTENHPLWNPLFWQIRRAHLALQKQAEDSRDLQKDTISHLRASVLKELTKGLSPESF

>core/406/2/Org2_Gene803

MMKKIRKVALAVGGSGGHIVPALSVKEAFSREGIDVLLLGKGLKNHPSLQQGISYREIPSGLPTVLNPIKIMSR
TLSLCSGYLKARKELKIFDPDLVIGFGSYHSLPVLLAGLSHKIPLFLHEQNLVPGKVNQLFSRYARGIGVNFSP
VTKHFRCPAEEVFLPKRSFSLGSPMMKRCTNHTPTICVVGGSQGAQILNTCVPQALVKLVNKYPNMYVHHIV
GPKSDVMKVQHVYNRGEVLCCVKPFEEQLLDVLLAADLVISRAGATILEEILWAKVPGILIPYPGAYGHQEV
NAKFFVDVLEGGTMILEKELTEKLLVEKVTFALDSHNREKQRNSLAAYSQQRSTKTFHAFICECL

>core/407/2/Org2_Gene88

MKKKVAEYLNRLAEVEIKISNPEIFSNSKEYSALSKEHSYLLELKNAYDKILNLEKVLADDKQALAIEKDPEM
VVMLEEGINENKVELEKLNKILESLLVPPDPDDDLNVIMELRAGTGGEEAALFVGDCVRMYHLYASSKGWK
YEVLSASESDLKGYKEYVMGISGTGVKRLLQYEAGTHRVQRVPETETQGRVHTSAITIAVLPEPSEEDTELLI
NEKDLKIDTFRASGAGGQHVNVTDSAVRITHLPTGVVVTCQDERSQHKNKDKAMRILKARIRDAEMQKRHN
EASAMRSAQVGSGDRSERIRTYNFSQNRVTDHRIGLTLYNLDKVMEGDLDPITTAMVSHAYHQLLEHGN

>core/408/2/Org2_Gene886

MPILWKVLIFRYLKTAAFCTLSLICISIISSLQEIVAYIAKDVPYDTVLRLMAYQIPYLLPFILPGSCFVSAFSLFR
KLSDNNHMTFLRASGASQSIIMFPVLMVSGAICCLNFYTCSELASICRYQTCKEIANMAMTSPALLLQTLQKK
ENNRIFIAVDHCAKSKFDNVIVALKGNNEISHVGIIKSIIPDTTKDTVKAKDVVFISKLPDSLTESSSPSSQRFYIE
TLDELLIPKITSTLFAGKSYLKTRTDYLPWKQLVKQSLKHSHLPETLRRVAIGFLCITLTYAGMILGIHKPRFRK
SIALYFIFPILDLILLIVGKNTKNLPLAFMLFVFPQLVSWVVFAARAYRESRGYA

>core/409/2/Org2_Gene346

MSLLSGHRLEGFPPIAEVMAACDRCSMDFCEILKSQSMDLWADAASCVDGLLQDPFWSTAIASGIAKSSLQE
TEFECESKVMVLSSWGEQGAQVCSPFNLERICMSFPSLKVFSLKKNGCENMGIQLSASCMNLLMSIFFVATN
GGSTPIWITKENLMALVALVLSHYQCYFVPATGDPQRGNILGNPEVNAILARGMGMRVDLERKRGGESSSSR
YLELAARCFENSLTKTSLLSDANNVQERDKCLLQMSTSLMHTAGLNLQRPPVPTPSGVTAHPQPQPDPVVTS
QPSLLGARERSPVSSRGRFPVVLPLSVISPRSHPGRVERRDLEDEEEEVMF

>core/410/2/Org2_Gene722

MSHDRILRAQRALSEHNLDAILVEKSEDLAYFLHDEAIAGILLIGQQEVMFFVYRMDKDLYSHIQRVPLTFLT
QDVVADLSLYVQKQRYQKIGFDSASTVYHKFAQRQVLPCLWEPLECFTEKIRSIKSEEEIRRMQEAAALGSA
GYDYVLTLLREGITEKEVVRQLRAFWAEAGAEGPSFPPIIAFGEHSAFPHSIPTDRPLKKGDIVLIDIGVLLNGY
CSDMTRMTALGTPHPKLLESYPVVVEAQKRAMALCKEGVLWGDIDAEAVRVLREHHLDTYFIHGIGHGVG
RHIHEYPCSPRGSQVKLESGMTITVEPGVYFPGIGGIRIEDTLCIDKNKNFSLTARPVISELVCL

>core/411/2/Org2_Gene282

MNKRQKDKLKICVIISTLILVGIFARAPRGDTFKTFLKSEEAIIYSNQCNEDMRKILCDAIEHADEEIFLRIYNLS
EPKIQQSLTRQAQAKNKVTIYYQKFKIPQILKQASNVTLVEQPPAGRKLMHQKALSIDKKDAWLGSANYTNL
SLRLDNNLILGMHSSELCDLIITNTSGDFSIKDQTGKYFVLPQDRKIAIQAVLEKIQTAQKTIQVAMFALTHSEII
QALHQAKQRGIHVDIIIDRSHSKLTFKQLRQLNINKDFVSINTAPCTLHHKFAVIDNKTLLAGSINWSKGRFSL
NDESLIILENLTKQQNQKLRMIWKDLAKHSEHPTVDDEEKEIIEKSLPVEEQEAA

>core/412/2/Org2_Gene674

MNLPDRKKALEAAVAYIEKQFGAGSIMSLGRHSATHEISTIKTGALSLDLALGIHGVPKGRVIEIFGPESSGKT
TLATHIVANAQKMGGVAAYIDAEHALDPSYASLIGVNIDDLMISQPDCGEDALSIAELLARSGAVDVIVIDSV
AALVPKSELEGDIGDVHVGLQARMMSQALRKLTATLSRSQTCAVFINQIREKIGVSFGNPETTTGGRALKFYS
SIRLDIRRIGSIKGSDNSDIGNRIKVKVAKNKLAPPFRIAEFDILFNEGISSAGCILDLAVEYNIIEKKGSWFNYQE
KKLGQGREFVREELKRNRKLFEEIEKRIYDVIAANKTPSVHANETPQEVPAQTVEA

>core/413/2/Org2_Gene809

MNLCKRISFEEGLELFVSSPIERLQERADAIRKERYPSNEVTYVLDANPNYTNICKIDCTFCAFYRKPKSPDAY
LLSFDEVRSLLQRYVSSGVKTVLLQGGVHPGLGIDYLEELVRITVQEFPSIHPHFFSAVEIEHACRVSGISIEQG
LQRLWDAGQRTIPGGGAEILSERVRKIISPKKMQPGGWINLHKLAHLMGFRTTATMMFGHVENPEDILIHLQ
TLRDAQDSCPGFYSFIPWSYKPGNTALRRNVPQQASIETYYRILALGRIFLDNFDHVAASWFGEGKSLGAKAL
HYGADDFGGVILDESVHKATGWSIQSSEEEICNIIRSEGFIPVERNTFYQHISCTVSSL

>core/414/2/Org2_Gene816

MKYSLNFKEIKIDDYERVIEVTCSKVRLHAIIAIHQTAVGPALGGVRASLYSSFEDACTDALRLARGMTYKAII
SNTGTGGGKSVIILPQDAPSLTEDMLRAFGQAVNALEGTYICAEDLGVSINDISIVAEETPYVCGIADVSGDPSI
YTAHGGFLCIKETAKYLWGSSSLRGKKIAIQGIGSVGRRLLQSLFFEGAELYVADVLERAVQDAARLYGATIV
PTEEIHALECDIFSPCARGNVIRKDNLADLNCKAIVGVANNQLEDSSAGMMLHERGILYGPDYLVNAGGLLN
VAAAIEGRVYAPKEVLLKVEELPIVLSKLYNQSKTTGKDLVALSDSFVEDKLLAYTS

>core/417/2/Org2_Gene236

MLNIHDILGNDDENLLSYQCKHITKDKLTLPSHDFVDKVFGLSDRNNRVLRSLQTMFSHGRLANSGYLSILPV
DQGIEHSAGASFAINPIYFDPENIVKLAIESGCSAVASTYGTLSLLSRKYAHKIPFMLKLNHNELLSYPTKYHQI
FFTQVEAAYSMGAVAVGATVYFGSETSNEEIVAVSNAFAKARSLGLATVLWCYLRNPAFVANGVDYHTAA
DLTGQADHLGATLGADIVKQKLPTCQGGFKAINFGKTDERVYSELSSNHPIDLCRYQVLNSYCGKVGLINSG
GPSGKNDFTEAARTAVINKRAGGMGLILGRKAFQRPLSEGIQLLNLVQDIYLDPNITIA

>core/418/2/Org2_Gene799

MIPLIPMFLKQSLFFSLALTGMTTLVLTVALGVPVMKWLKRKNYRDYIHKEYCEKLEMLHKDKAEVPTGGG
VLLFISLIASLLVWLPWGKFSTWFFIILLTCYAGLGWYDDRIKIKRKQGHGLKAKHKFMVQIAIAAFTLIALPY
IYGSTEPLWTLKIPFMEGMLSLPFWLGKVFCLGLALVAIIGTSNAVNLTDGLDGLAAGTMSFAALGFIFVALR
SSTIPIAQDVAYVLAALVGACIGFLWYNGFPAQLFMGDTGSLLLGGLLGSCAVMLRAECILVVIGGVFVAEA
GSVILQVLSCRLRKKRLFLCSPLHHHYEYQGLPETKIVMRFWIFSFVCAGLGIAAVLWR

>core/419/2/Org2_Gene916

MRIAVLGAGYAGLSVTWHLLLHSQGTATIDLFDPIPLGEGASGMSSGLLHAFTGKKALKPPLADQGINATHA
LITEASKALNVPIVISQGILRPAIDEDQAQLFTERVEEFPKEVEWWEKARCEISIPSMVIPPNLGALFIKSGVTLN
NDLYIQGLADACMKLGTQFYDELIEDLADIEEFYDHIIVTPGANASILPELKDMPVNKVKGQLLEISWPKDLA
MLSFSINAHKYMVANTQKNTCILGATFEHNQPEETPDPAIAYQEIMPPVLSLFPGLKDAQVLHCYAGMRSSS
KSRLPVISRIREKLWFLGGLGSKGLLYHGITGDMLAQAVLRKSTAYIAKEFLFTI

>core/420/2/Org2_Gene830

MILLQNIKRCSLKQLKVLATLLLSLSLPTLEAAENRDSDSIVWHLDYQEALQKSKEAELPLLVIFSGSDWNGP
CMKIRKEVLESPEFIKRVQGKFVCVEVEYLKHRPQVENIRQQNLALKSKFKINELPCMILLSHEEREIYRIGSFG
NETGSNLGDSLCHIVESDSLLRRAFPMMTSLSLSELQRYYRLAEELSHKEFLKHALELGVRSDDYFFLSEKFR
LLVEVGKMDSEECQRIKKRLLNKDPKNEKQTHFTVALIEFQELAKRSRAGVRQDASQVIAPLESYISQFGQQD
KDNLWRVEMMIAQFYLDSDQWHHALQHAEVAFEAAPNEVRSHISRSLEYIRHQS

>core/421/2/Org2_Gene470

MYLQEKFKAQQVPLVLRELLSCSDSINDSDPIYRMVFDSNDTTISYKVGDALGVLPENSKEVSEHVLQLLGYS
PTTLVNVKKTSEKVSAQKFIQGYVDLDKIPAKLNSFFPDKDPKITLYDAIQEYRPQIPIELFAESVFPLLPRFYSI
ASSPDLHPKSIELLVKHVSYPGKYQKRFGVCSSFLCSELQVNDSAYIFVQPTKHFTLSTQTEGKPLVMIGAGT

GIAPYKAFLEERLFNKDPGNNLLFFGERKEKVNFYYREFWNHAEEEGKLKLFLAFSRERDQKVYVQDLLRIQ
KDEVRKAYEEGGFFFVCGRKVLGIEVKHALEEILGKDTLASLRKEHRYVVDVY

>core/422/2/Org2_Gene258

MDVSRKINRHTQFYVDSIDGVIKNFDHKPSEDKSRDHEELEEKLLTITKRIVASAQEFQNRKTDSKNYYLKKT
QWLPFKNEELEQTKELFAMLTSMDKKIAQLFFYSPGCSSDWVEFTEVICHLNDSIGLGGVLLCCGLFEQQCEH
VVTVNKKLDLPLLLGTTVVNSLRYYLTYRNISLLNCQSMSELGKELGDVLKQHGVAFTLIFKEIVDIDLLNYV
KLIQGLKRSGNIQARIYDNDVPTLPSVSSSPIALRYSLANTIRGLALHVDFSSLKFISPSILSNTEHTAKALNSGG
ECFIFSNLDEFNLGMKIVMQLLRTGKISPEILNKNIMKILMIKRRVRSLYI

>core/423/2/Org2_Gene875

MEADILDGKLKRVEVSKKGLVNCNQVDVNQLVPIKYKWAWEHYLNGCANNWLPTEVPMARDIELWKSDE
LSEDERRVILLNLGFFSTAESLVGNNIVLAIFKHITNPEARQYLLRQAFEEAVHTHTFLYICESLGLDEGEVFNA
YNERASIRAKDDFQMTLTVDVLDPNFSVQSSEGLGQFIKNLVGYYIIMEGIFFYSGFVMILSFHRQNKMTGIGE
QYQYILRDETIHLNFGIDLINGIKEENPEVWTTELQEEIVALIEKAVELEIEYAKDCLPRGILGLRSSMFIDYVRH
IADRRLERIGLKPIYHSRNPFPWMSETMDLNKEKNFFETRVTEYQTAGNLSW

>core/426/2/Org2_Gene79

MELSLTSLLPLAWYVILGVAVFAYSFGDGFDLGLGAVYLKAKEDKERRILLNSIGPVWDGNEVWLVIIVGGL
FAGFPACYATLLSIFYMPIWTLVLLYIFRGCSLEFRSKSESVSWKIFWDIIFICSGTAISFFLGTIVGNLILGLPLSP
DTSYASLSWILFFRPYAALCGAVVASAFAIHGSCFALMKTSDSLNARIAQQFPYILSSFLVFYVLFLGASLISIP
KRFDAFPTYPLLILLIALTSCCCVAAKTSVSKKRYGYAFIYSTLNLLSLILSAATLTFPNILLSTVDPQYSYTIYN
SAVETKTLKSLLIIVLIGLPFIITYTCYIYRVFRGKTNFPSIY

>core/427/2/Org2_Gene758

MNSKMLKHLRLATLSFSMFFGIVSSPAVYALGAGNPAAPVLPGVNPEQTGWCAFQLCNSYDLFAALAGSLK
FGFYGDYVFSESAHITNVPVITSVTTSGTGTTPTITSTTKNVDFDLNNSSISSSCVFATIALQETSPAAIPLLDIAF
TARVGGLKQYYRLPLNAYRDFTSNPLNAESEVTDGLIEVQSDYGIVWGLSLQKVLWKDGVSFVGVSADYRH
GSSPINYIIVYNKANPEIYFDATDGNLSYKEWSASIGISTYLNDYVLPYASVSIGNTSRKAPSDSFTELEKQFTN
FKFKIRKITNFDRVNFCFGTTCCISNNFYYSVEGRWGYQRAINITSGLQF

>core/428/2/Org2_Gene868

MMEVYSFSPSVRTSFQHRVMAALDNWFFLGGRRLKVVSLDSCNSGQACEEYVPISTTEKVLKILSYLLIPIVII
ALLIRYLLHSNFTAKVSQKPWLKTLQLGIDIKSFILPGSHVNTMDSATLFKAIRLEGKRVDVEYHRLHSSDKV
VFYIPAQKLPDDLRLTHWLPEKETRKTEYVRHMLAHVMGYLTSQGKERLQQVVQDSRSSTSLGAEKVLQYR
FIDHPQSQGEFQRLLNENITTKGSEDKEVVQSDLFDMAFQCWWPQFISVIQSPTFSEELVHEMSQKLDLDCIYP
EDDEFEQKFLNTLLKAVLHHGFEGISVASMGVIFLICPDSLALQIPFLRNQK

>core/429/2/Org2_Gene711

MNKKKRVLTGDRPTGKLHLGHWVGSIKNRLELQNSPEYDCFFIIADLHTLTTKIRKEEVLDVDNHIYEVLAD
WLSVGIDPTKSIIYLQSAIPEIYELHLLFSMLISINRVMGIPSLKDMARNASIEEGSLSYGLIGYPILQSADILLAK

AQFVPVGKDNEAHVELTRDIARNFNRLYGQVFPEPEVLQGELTSLVGIDGQGKMSKSANNAIYLSDSDATITE
KVRKMYTDPNRIRATTPGRVEGNPLFIYHDIFNPHKDEVEEFKARYRQGCIKDIEVKARLAEELIHFLKPIKER
RSEFLSKPLALQNVLEDGTHKMREVAKVTMEEVHDKFGFSHKWRSLLK

>core/430/2/Org2_Gene160

MLTLGLESSCDETACAIVNEDKQILANIIASQDIHASYGGVVPELASRAHLHIFPQVINKALQQANLLIEDMDL
IAVTQTPGLIGSLSVGVHFGKGIAIGAKKSLIGVNHVEAHLYAAYMAAQNVQFPALGLVVSGAHTAAFFIENP
TSYKLIGKTRDDAIGETFDKVGRFLGLPYPAGPLIEKLALEGSEDSYPFSPAKVPNYDFSFSGLKTAVLYAIKG
NNSSPRSPAPEISLEKQRDIAASFQKAACTTIAQKLPTIIKEFSCRSILIGGGVAINEYFRSAIQTACNLPVYFPPA
KLCSDNAAMIAGLGGENFQKNSSIPEIRICARYQWESVSPFSLASP

>core/431/2/Org2_Gene104

MENAMSSSFVYNGPSWILKTSVAQEVFKKHGKGIQVLLSTSVMLFIGLGVCAFIFPQYLIVFVLTIALLMLAIS
LVLFLLIRSVRSSMVDRLWCSEKGYALHQHENGPFLDVKRVQQILLRSPYIKVRALWPSGDIPEDPSQAAVLL
LSPWTFFSSVDVEALLPSPQEKEGKYIDPVLPKLSRIERVSLLVFLSAFTLDDLNEQGVNPLMNNEEFLFFINK
KAREHGIQDLKHEIMSSLEKTGVPLDPSMSFQVSQAMFSVYRYLRQRDLTTSELRCFHLLSCFKGDVVHCLA
SFENPKDLADSDFLEACKNVEWGEFISACEKALLKNPQGISIKDLKQFLVR

>core/432/2/Org2_Gene259

MDSSAPYNIASQGTEKSTVERILDLYGPASCIKFLKQMVLIREFEARGEEAYLEGLVGGFYHSYAGQEAVATA
AIANTGLDPWVFSSYRCHALAILLNIPLQEIAAELLGKETGCALGRGGSMHMCGPNFPGGFGIVGGQIPLAAG
AAFTIKYQEQKNRVSLCFIGDGAVAQGVFHETLNFVSLHQLPLMLIIENNGWSMGTSLNRAVAKQPIAESQG
SSYDIRAVTVNGFDLFNSLLGFREAYRYMVDTESPVLVECLCSRFRGHSISDPNLYRSKEEMQCLFKKDPIVL
AKDWLIRLEVLTEEEFQNIRQECKTAVLEAFSNAKLSSDPSVTTLEEGVYA

>core/433/2/Org2_Gene131

MKLYSISSDVDTPWIFQLMSKVDSYLFLGGNRIKVVSIVMQEPNLIIGKVENVRISTIVKILKILSFLIFPLILIAL
ALHYFLHAKYANHLLVSKILERAPQYVPIPGRSGDTASHYKLTTLVPVSQKNLQAMGSNPLEVEAALRTTKP
SFFCVPAKYRQIIISSHGIRFSLDLEQLADDINLDSVSWPTEYLNSTMDFCSKADKRVIQNVQNLRTGTYINSV
GKRSLLKFMLQHLFIDGITQENPEALPNNTSGRLTLFPSVRYIYSHFTPQNPTIWPQVFFRQGPLDEDRGGGFEI
LEQLQELGVRFPICPSQGPDNPNFQGFQGIRIYWEDSYQPNKEV

>core/434/2/Org2_Gene808

MLPFEFEFNTTSSPECDVCLDPQKLFVKLFKRTIVLLSGPTGSGKTDVSLALAPMIDGEIVSVDSMQVYQGMD
IGTAKVSLKARQEIPHHLIDIRHVQEPFNVVDFYYEAIQACQNILSRNKVPILVGGSGFYFHAFLSGPPKGPAA
DPQIREQLEAIAEEHGVSALYEDLLLKDPEYAQTITKNDKNKIIRGLEIIQLTGKKVSDHEWDIVPKASREYCC
RAWFLSPETEFLKNNIQMRCEAMLQEGLLEEVRGLLNQGIRENPSAFKAIGYREWIEFLDNGEKLEEYEETKR
KFVSNSWHYTKKQKTWFKRYSIFRELPTLGLSSDAIAQKIAKDYLLYS

>core/435/2/Org2_Gene764

MFFQNLAKKLTALGISPLGCLLIGGVVSCAILFGRSSNPSLAPTQVKTEKTSGNWLKLTQMGNPKLIESLTKK
EQLEKDLTSFHPIASAKVAIALSTEDDVMSPLHLSVILTLRKEESLTPSLLFSITDYLCSSLPGLKREHISLSDNL
GNLYIPESITVNSLFIHTLENYLGKIFPKEHFALAYHAKAEKPTLQLTLNENYIAHLTKEESEKIVAHTKHYLY
QNYDDSYDIVIETLPFARLQNKKSFPAKVLIGSMILVISLMIVALASFYLARHAYERVSPEPRKIKRGINISKLLE
IIQKESPEKIALILSYLDPKKAEALLNRLPEDLKHQVLKYKL

>core/436/2/Org2_Gene235

MSEQHSPIISVQDVSKKLGDHILLSKVSFSVYPGEVFGIVGHSGSGKTTLLRCLDFLDMPTSGSISVAGFDNSLP
TQKFSRRNFSKKVAYISQNYGLFSSKTVFENIAYPLRIHHSEMSKSEVEEQVYDTLNFLNLYHRHDAYPGNLS
GGQKQKVAIARAIVCQPEVVLCDEITSALDPKSTENIIERLLQLNQERGITLVLVSHEIDVVKKICSHVLVMHQ
GAVEELGTTEELFLNSENSITNELFHEDINIAALSSCYFAEDREEVLRLNFSKELAIQGIISKVIQTGLVSINILSG
NINLFRKSPMGFLIIVLEGEVEQRKKAKELLIELGVVIKEFY

>core/437/2/Org2_Gene422

MTILRKLSQYLFFFSLFCSFIYVATCGSQPDSVSSPKIAIFLSFPHPLLEDCSKSCIETLKDFENLPEIVVLNAEDSI
VKARKIARSLHTDKNVVAIVTLGTIATKVMSHIETQKPVIYAAVPDRESLTLPKNTMNIYGVNDTLDINQYCF
AIQAVATNAQSIVYLKPSEPFPSDLQKEIVKKLHASGIEVIEISITSSTFKTRIRQAIDKRPSAIFIPLSPLSHKEGT
AFLQEILKEKIPIITDDTSLISEGACIACSVDYKKSGKQIAKIVHHLLYNNHDVDSLRKIIAQRLSPTTTFNEDIIK
YLGIKLHKTERNQFLSFKSKKLEKSEKGKNVAVS

>core/438/2/Org2_Gene883

MEMKEEIEAVKQQFHSELDQVNSSQALADLKVRYLGKKGIFRSFSEKLKQCTDKAKLGSLINDFKTYVEDLL
QEKSLVLLASEQAEAFSKEKIDSSLPGDSQPSGGRHILKSILDDVVDIFVHLGFCVREAPNIESEANNFTLLNFT
EDHPARQMHDTFYLNATTVLRTHTSNVQARELKKQQPPIKVVAPGLCFRNEDISARSHVLFHQVEAFYVDH
NVTFSDLTAILSAFYHSFFQRKTELRFRHSYFPFVEPGIEVDVSCECCGKGCALCKHTGWLEVAGAGMIHPQV
LRNGNVDPEIYSGYAVGMGIERLAMLKYGVSDIRLFSENDLRFLQQFS

>core/439/2/Org2_Gene309

MATISPISLTVDHPLVDTKKKSCSNFDKIQSRILLITAIFAVLVTIGTLLIGLLLNIPVIYFLTGISFIAVVLSNFILY
KRATTLLKPRACGKHKEIKPKRVSTNLQYSSISIAINRSKENWEHQPKDLQNLPAPSALLTDNPYEIWKAKHS
LFSLVSLLPGGNPEHLLISASENLGKTLLIEETSQNAPISSYVDTTPSPKSLLNEAIQETRVEINTELPAGDSGER
LYWQPDFRGRVFLPQIPTTPEAIYQYYYALYVTYIQTAINTNTQIIQIPLYSLREHLYSRELPPQSRMQQSLAMI
TAVKYMAELHPEYPLTIACVERSLAQLPQESIEDLS

>core/440/2/Org2_Gene608

MEEAAKHLAKEFLCSGINLFLSGEYEQAEKRLKETLELDSTAALAYCYLGIIALETGRVSEALNWCSKGLASE
PGDSYLRYCYGVALDRGNQYEAAIEQYSAYVALHPDDVECWFSLGSVYHRLKRLQEALDCFDKILALDPW
NPQSLYNKAVILSEMDDEAESIRLLEVAVAKNPLYWKAWVKLGFLLSRSKRWDKATEAYERVVQLRPDLSD
GHYNLGLCYLTLDKTRLALKAFQEALFLNAEDADAHFYVGLAHLDLKQMREAYEAFNSALSINLEHERAHY
LLGYLHHMQGETDKATKELLFLQKKDSTFAPLLQKTVVSDPSSMQFERRLDTIS

>core/441/2/Org2_Gene629

MVLGVVGISYREAALKERERAIQYLQSFEKNLFLAQRFLGKGGAFIPLLTCHRAELYYYSESPEIAQAALLSEL
TSQGIRPYRHRGLSCFTHLFQVTSGIDSLIFGETEIQGQVKRAYLKGSKERELPFDLHFLFQKALKEGKEYRSRI
GFPDHQVTIESVVQEILLSYDKSIYTNFLFVGYSDINRKVAAYLYQHGYHRITFCSRQQVTAPYRTLSRETLSF
RQPYDVIFFGSSESASQFSDLSCESLASIPKRIVFDFNVPRTFLWKETPTGFVYLDIDFISECVQKRLQCTKEGV
NKAKLLLTCAAKKQWEIYEKKSSHITQRQISSPRIPSVLSY

>core/442/2/Org2_Gene102

MFPSANQESRTRNVPLGIFHGLVACLYWGIVFVIPNFLGSFGDLDIVLTRYTIFGIFSLIACAIKNPSVIKKTPLYI
WRKSLLWTLLINPVYYFGITLGIRYVGSAITVVIASLAPTAVLYHSNTKQKELPYSLLFAISSVIITGVILTHLSA
LNLPTAASPLYSILGVIAVILSTSLWVIYVIRNQSLLEKHPNLTPDTWSYLIGISALIICLPMIIILDLCGITHVTHN
LISHTPGSERLLFLLLCSAMGIFSSAKALIAWNKASLNLSPALLGAILIFEPIFGLVLTYLYSQSLPSLQEGIGIFL
MLGGSLLCLVLFGRKVQKSLENSQVSSSNE

>core/443/2/Org2_Gene707

MKKTCCQNYRSIGVVFSVVLFVLTTQTLFAGHFIDIGTSGLYSWARGVSGDGRVVVGYEGGNAFKYVDGEK
FLLEGLVPRSEALVFKASYDGSVIIGISDQDPSCRAVKWVNGALVDLGIFSEGMQSFAEGVSSDGKTIVGCLY
SDDTETNFAVKWDETGMVVLPNLPEDRHSCAWDASEDGSVIVGDAMGSEEIAKAVYWKDGEQHLLSNIPG
AKRSSAHAVSKDGSFIVGEFISEENEVHAFVYHNGVIKDIGTLGGDYSVATGVSRDGKVIVGHSTRTDGEYR
AFKYVDGRMIDLGTLGGSASFAFGVSDDGKTIVGKFETELGECHAFIYLDD

>core/444/2/Org2_Gene334

MTHQVAVLHQDKKFDVSLRPKGLEEFYGQHHLKERLDLFLCAALQRGEVPGHCLFFGPPGLGKTSLAHIVA
YTVGKGLVLASGPQLIKPSDLLGLLTSLQEGDVFFIDEIHRMGKVAEEYLYSAMEDFKVDITIDSGPGARSVR
VDLAPFTLVGATTRSGMLSEPLRARFAFSARLSYYSDQDLKEILVRSSHLLGIEADSSALLEIAKRSRGTPRLA
NHLLRWVRDFAQIREGNCINGDVAEKALAMLLIDDWGLNEIDIKLLTTIIDYYQGGPVGIKTLSVAVGEDIKT
LEDVYEPFLILKGFIKKTPRGRMVTQLAYDHLKRHAKNLLSLGEGQ

>core/445/2/Org2_Gene653

MSYSLRNKKTKICVYIIIALGILSFRSIPQEVYDKIRSSFVSLHVKFFPKIKQAPSSHLANLELENLVLKERVASL
EEKLKLYEVSNHTPPLFPEILTPYFHKLVEGKVVYRDYTHWSSSCWVNVGKTHGIKKNSPVLSGNVLVGLVD
YVGEHQSRIRLITDVGMKPSVVAMRGDIQSWWIKHSLRELIRQVEQISHAYILEKDKYEKISQLQELDSLIQGE
GENQALLRGILSGVGGALWKEGSLCLEGEGFYFSEGKTLLPGDILVTTGLDGVFPPGLLVARVTKVKAPRDG
ACTFKIEAQSLEEKLMELDQLFILPPLEFNPNDRPDIFGLLWD

>core/446/2/Org2_Gene542

MKVVINGFGRIGRLVLRQILKRNSSVEVLAINDLVPGDALTYLFKFDSTHGRFPEDVRCEADHLIVGKRKIQF
LSERNVQNLPWKDLGVDLVIECTGLFTKKEDAEKHIQAGAKRVLISAPGKGDIPTFVMGVNHKTFNPEKDFV
ISNASCTTNCLAPIAKVLLDNFGITEGLMTTVHAATATQLVVDGPSKKDWRGGRGCLQNIIPASTGAAKAVT

LCLPELKGKLTGMAFRVPIEDVSVVDLTVRLDKSTTYDDICKAMKQASETDLKGILDYTDEQVVSSDFIGSEY
SSIFDALAGIALNDRFFKLVAWYDNETGYATRIVDLLEYVEKNSK

>core/447/2/Org2_Gene466

MFVDQITLELRAGKGGNGVVAWRKEKYLPKGGPYGGNGGNGGSVIIEATTSVYSFEAYRNIRFLKAPDGQS
GATNNRTGRSGKDLIVSVPTGTLLRDAETGEILHDFTVDGERLLVSQGGKGGKGNTFFKTSVNRAPTKATPG
KPGEIRQVELELKLIADIGLVGFPNAGKSTLFNTLAHTEVKVGAYPFTTLAPSLGLVLCKDRLYQKPWIIADIP
GIIEGAHQNKGLGLDFLRHIERTLLLLFVIDVSKRERNSPEEDLETLIHELHSHQPDFEKKDMLVALNKIDDLL
PDEQEECLQSFQKRFPSYTFVLISGLTGEGVDGLYRFFTQRLAV

>core/448/2/Org2_Gene253

MWFSVNKNKKAAIWATGSYLPEKVLSNADLEKMVDTSDEWIVTRTGIKERRIAGPQEYTSLMGAIAAEKAI
ANAGLSKDQIDCIIFSTAAPDYIFPSSGALAQAHLGIEDVPTFDCQAACTGYLYGLSVAKAYVESGTYNHVLLI
AADKLSSFVDYTDRNTCVLFGDGGAACVIGESRPGSLEINRLSLGADGKLGELLSLPAGGSRCPASKETLQSG
KHFIAMEGKEVFKHAVRRMETAAKHSIALAGIQEEDIDWFVPHQANERIIDALAKRFEIDESRVFKSVHKYGN
TAASSVGIALDELVHTESIKLDDYLLLVAFGGGLSWGAVVLKQV

>core/449/2/Org2_Gene680

MAAPIFIKNILLRSSIVYAPLAGFSDYPYRCMSALYQPGLMFCEMVKVEGILYAPERTSKLLDYNENMRPIGA
QLCGSNPETSGEAAKILEGLGFDLIDLNCGCPTDKITKDGSGSGLLKTPELIGRILDKIINSVSIPVTVKIRSGWD
MEHINVEDTVRIIRDAGASAVFVHGRTRAQGYHGPSKQEYISRAKAAAGKEFPVFGNGDIFSPEAAQAMLTT
GCDGVLVARGTLGAPWIGKQIQDYLTTGSYEKIPFIKRKAAFLEHMRLVEDYYQSETKFLSETRKLCGHYLIS
AAKVRFLRSSLAKATSYQEVYQLVNDYEEADDSSLETFVKC

>core/450/2/Org2_Gene50

MSTPLSSGGISPSDQYVPQELFCDRLSSSRSNSPDSNASGDSPIVSPPISALVALTDLKLVPYNQNSFSWTTRLK
NAVEKIGLFLQRNWKYILLYILAWALILVCHHTVALTLTIWLGVGLGIGVVFGIFTATCLDKENKHRHVNSL
WNLINHGILQLDPNGTRQILLATMIASISALIYAVPQAVGLVIGFSIGNQLSINTVYGARLGDEATYAIDRKAH
KKRIENIEQAINQHQIIKHQMINQKQLNALIEINRNNQTDPATANLLASLKLNLNQPMPYCFSMPECGVTSSYL
DLNNNSPDDIIARADQCIMTLSQTLQQIKKEPDRIIESNH

>core/451/2/Org2_Gene736

MVRRSISFCLFFLMTLLCCTSCNSRSLIVHGLPGREANEIVVLLVSKGVAAQKLPQAAAATAGAATEQMWDI
AVPSAQITEALAILNQAGLPRMKGTSLLDLFAKQGLVPSELQEKIRYQEGLSEQMASTIRKMDGVVDASVQIS
FTTENEDNLPLTASVYIKHRGVLDNPNSIMVSKIKRLIASAVPGLVPENVSVVSDRAAYSDITINGPWGLTEEI
DYVSVWGIILAKSSLTKFRLIFYVLILILFVISCGLLWVIWKTHTLIMTMGGTKGFFNPTPYTKNALEAKKAEG
AAADKEKKEDADSQGESKNAETSDKDSSDKDAPEGSNEIEGA

>core/452/2/Org2_Gene759

MKQHIGYLGMGIWGFCLASLLANKGYPVVAWSRNPDLIKQLQEERRHPLAPNVVISPNLSFTTDMKEAIHNA
FMIVEGVTSAGIRPVAEQLKQITDLSVPFVITSKGIEQNTGLLLSEIMLEVLGDSVTPYLGYLSGPSIAKEVLNG

SPCSVVVSAYDSQTLKQIHEAFSLPTFRVYPNTDIKGAALGGALKNVIAIACGIAEGLSFGNNAKAGLVTRGL
HEMRKLAAIMDCKPETLNGLAGLGDLCVTCFSESSRNLRFGHLLAQGLTFEQAKAKIGMVVEGAYTALSAY
QVAKHHKIDMPITTGIYRVLYENLDLKEGIALLLQRNTKEEFL

>core/453/2/Org2_Gene521

MKPLGFQENLEALCNKTSRQLLKYLIKQILFVCGASLLIALEFSFFLYFFLFSGKTVIPAFCLACFFLTLFVCLV
TRLYLLSGKGDFFEDLASEYLQGAVPPNKRSQNIVEEQSHLAAAATKLSINLQNQEYSLLSEIFKFLPKHDLIR
KFSCFCFWKDYFLFRECLLQKAIEAYIKVVQAIPVDLSAHVSLADAYVALSGLYADPRKYPEFDANYWIPSG
RYSAEIQEKFFATARRAIEEFQILNEYAPGNAWVHAQLAYSYHDLQMPMEEIQEYEIVLKLKPNDVETMSKL
GILYFQQGMNAKGLRIYEEIKKRDYKKSQKLIKFYGVEYKY

>core/455/2/Org2_Gene729

MQPFIFTLLCLTSLVSLVAFDAANARKRCACAQTIERGENFFSIKRSACAEIEYQEKSRHASAIERISKDKGKV
TPKQIAKVATKKKQRYRLLQVPFSRPPNNSRYNLYALLSEPPECYSDTASWYAIFIRLLRRAYVDTGNVPPGS
EYAIANALISNKQEILERGAQLGPDVIETLTLPEEQAEIFYKMLKGSSNSQSLLNFLHYEEKSLGHCKLNLIFM
DPLLLEAVLDHPDAYRETSLLRDGIWEAVKRQEHAIQEHGQAAALELFKTRTDFRLELRDKMQLLLSRYDLL
PLLNKKMFDYTLGSAGDYLFLVDPDTKAISRCRCPSKSIKL

>core/456/2/Org2_Gene566

MATNAIRSAGSAASKMLLPVAKEPAAVSSFAQKGIYCIQQFFTNPGNKLAKFVGATKSLDKCFKLSKAVSDC
VVGSLEEAGCTGDALTSARNAQGMLKTTREVVALANVLNGAVPSIVNSTQRCYQYTRQAFELGSKTKERKT
PGEYSKMLLTRGDYLLAASREACTAVGATTYSATFGVLRPLMLINKLTAKPFLDKATVGNFGTAVAGIMTIN
HMAGVAGAVGGIALEQKLFKRAKESLYNERCALENQQSQLSGDVILSAERALRKEHVATLKRNVLTLLEKA
LELVVDGVKLIPLPITVACSAAISGALTAASAGIGLYSIWQKTKSGK

>core/457/2/Org2_Gene932

MRIAVLGVTGLVGQKFVALLHKWYRDWVIAEVVASNSKYGQSYGDACIWQEPIGPMPEMVRDLPIRKIEEV
QSDIVVSFLPSSAESMEAYCLSQGKVVFSNASTYRMHSSVPIIIPEVNSDHFQLLEEQPYPGKIITSPNCCVSGIT
LALAPLRKFSLDHVHIVTLQSASGAGYPGVPSLDLLANTVPHIVGEEEKILRETVKILGSSKQPLPCKLSVTVH
RVPVAYGHTLSLHVTFSKDVDLDEILYSYQEKNKEFPNTYQLYDNPWSPQARKHLSHDDMRVHLGPITYGG
DFRTIKMNVLIHNLVRGAAGTLLASMENYFFDYLKREMCLR

>core/458/2/Org2_Gene532

MKTLWHFVSKAFLSIVGLCCGVVLAFVVIFALIASSLGNGDATFVSLPDAQGEVKDLGKTAPIIAVIEMKDVI
ASSKNTAKTIQNILEGFEKAPLKDRVKGIVIDMDCPGGEVFEIDRIYSMLRFWKERKGFPIYIYVNGLCASGGY
YVSCAATKIYATSSSLIGSIGVRSGPFFNVKEGLNRYGVESDLLTAGKDKAPMNPYTPWTSHDREERQATLDF
LYGQFVDIVTQNRPLLTKEKLVHTLGARIFSPEKAKQEGYIDVVGATKEQVLQDIVAVCKIEDNYRVIGSGGD
GWWKRVASAAASSPLVTGMIKHDILPLSHDAAYIPPYLAL

>core/460/2/Org2_Gene658

MSSLTLSRRPRRNRKTAAIRDLLAETHLSPKDLIAPFFVKYGNNIKEEIPSLPGVFRWSLDLLLKEIERLCTYGL
RAVMLFPIIPDDLKDAYGSYSSNPKNILCHSIHEIKNAFPHLCLISDIALDPYTTHGHDGIFLNGEVLNDESVRIF
GNIATLHAEMGADIVAPSDMMDGRIGYIRSKLDQSGYSKTSIMSYSVKYASCLYSPFRDALSSHVTSGDKKQ
YQMNPKNVLEALLESSLDEEEGADILMVKPAGLYLDVIYRIRQNTCLPLAAYQVSGEYAMILSAFQQGWLD
KETLFHESLIAIKRAGADMIISYSAPFILELLHQGFEF

>core/461/2/Org2_Gene126

MNIYQFSPGASPNWQASLMAQLNSYFCLGGETVTRIISLRPSGLILAKKEKAVVSTAEKILKILSFILFPLVLIAL
AIRYLLYNKFNKDLDRAVFFIPTEITKAEELIIAKNPALVKEAALTVSPLFYSLPKKYQLMKVETP

>core/462/2/Org2_Gene127

MLLEDLDTDSIPWPKLYLSEDFDFAYYPESKAIIDTVAKLEKNNPGEEFCLESKKILARYLLEQLFKLETGLNF
PTSTIDGGRESFLIEFSHETKKPTVWAFIYFYYYHSNGPKLEKDFKQAGCEVHNRLLNLGLKYRPQAGAQND
GRNGGPYGPIGFLIVWEENYGSVLKDHGFIKDN

>core/463/2/Org2_Gene852

MQFSRYLRYAFDNQYLPEPLYQKFSVFHQNYIDAATKKAAADQAEVLCLQWVKVIIEDLKNPFIFPPYHKKI
RAPIDLFRLSIDFFSLVIDDKNSRILNLHRLKEIEEYIARGDNVVLLANHQTECDPQLMYYALGKTHPELMEN
MIFVAGDRVTSDPLARPFSMGCDLLCIYSKRHIATPPELREEKLLHNQKSMQILKTLLNEGGKFIYVAPAGGR
DRKNAEGRLYPSEFSPESIEVFRLLAKASNQTTHFYPFALKTYDILPPPPKIENAIGEQRAIFFAPVFFNFGAELF
FDALCSKEELIHCDKHAQRTLRAEKVFSIVKNLYEEL

>core/464/2/Org2_Gene929

MREETVSWSLEDIREIYHTPVFELIHKANAILRSNFLHSELQTCYLISIKTGGCVEDCAYCAQSSRYHTHVTPEP
MMKIVDVVERAKRAVELGATRVCLGAAWRNAKDDRYFDRVLAMVKSITDLGAEVCCALGMLSEEQAKKL
YDAGLYAYNHNLDSSPEFYETIITTRSYEDRLNTLDVVNKSGISTCCGGIVGMGESEEDRIKLLHVLATRDHIP
ESVPVNLLWPIDGTPLQDQPPISFWEVLRTIATARVVFPRSMVRLAAGRAFLTVEQQTLCFLAGANSIFYGDK
LLTVENNDIDEDAEMIKLLGLIPRPSFGIERGNPCYANNS

>core/465/2/Org2_Gene406

MIDIMQHFKPYTMVPGQKLPIPGSLLYAQVFPTLWRLFSSKHEILNEQTLQVQGPLKRFAVFQDLHRGGLAV
TSERYKYYLLPSGECTQSIKGKLPSAAQAGPLLSLGVHKHADWQKVRCRRDLKEILPLWFRFAAMAPKGSY
RDLETTAIGSLVKTAHQRVLHRETTEIAPALLSIALAGFSECFLPRSYDEEFQGILPQDGDPEGGVPFELLSYSF
GMIQDIFLRHQGQLVEILPALPPEFPCGRLIHVALPNLGTLSIVWTKKTIRQVELHAEYSGEVFLKFCSSLCSAR
LREWSERRLSGSKRLSLGETLEIKAGTTYLWDCFHK

>core/466/2/Org2_Gene791

MSAFFDLLKSQTASHPPIWLLRQVGRYMPPYQELKGSQSLKTFFHNTEAIVEATLLGPSLLHVDAAILFADILS
ILDGFAVTYDFAPGPRIQFSPEQPFTFTSDPQTIFSYLLDAIRTLKQKLPVPLIVFAASPFTLACYLIDGGASKDF
SKTMSFLYVYPEKFDQLISTIIEGTAIYLKTQMDAGAAAVQLFESSSLRLPSALFTRYVTEPNRRLIAKLKEQAI

PVSLFCRCFEENFYTLQATQADTLHPDYHVDLHRIQKNLMLSLQGNLDPAIFLLPQEKLLHYVEAFLVPLRTY PNFIFNSGHGILPETPLENVQLVVSYVQRQL

>core/467/2/Org2_Gene449

MPSPMISTDVCQDILGKQKEAVDFFFQAFQPKEAMQLAEKILGHSGWVFFSGVGKSGCVARKLVATLQSLSE RALFFSPVDLLHGDLGLVSPGDIVCLFSKSGETQELLDTVPHLKSRRAILVAITSMPYSNLAALSDLVVILPSV AELDPFNLIPTNSTTCQMIFGDFLAMLLFHSRGVSLSTYGKNHPSGQVGMKANGKVKDFMFPKTEVPFCHLG DKVSFSLEVFSAYGCGCVCIVDPQFRLMGIFTDGDLRRSLASYGGEVLSLSLEKVMTANPRCITEDSDIAIALQ LMESSSPVAVLPVLDNEENRHVTGLLHMHTLAKAGLL

>core/468/2/Org2_Gene915

MAFKEVVRVAVTGGKGQIAYNFLFALAHGDVFGVDRGVDLRIYDVPGTERALSGVRMELDDGAYPLLHRL RVTTSLNDAFDGIDAAFLIGAVPRGPGMERGDLLKQNGQIFSLQGAALNTAAKRDAKIFVVGNPVNTNCWIA MKHAPRLHRKNFHAMLRLDQNRMHSMLAHRAEVPLEEVSRVVIWGNHSAKQVPDFTQARISGKPAAEVIG DRDWLENILVHSVQNRGSAVIEARGKSSAASASRALAEAARSIFCPKSDEWFSSGVCSDHNPYGIPEDLIFGFP CRMLPSGDYEIIPGLPWEPFIRNKIQISLDEIAQEKASVSSL

>core/469/2/Org2_Gene260

MPKHKTLEIREALREAIDEEMSRDPNVCILGEEVGDYNGAYKVTKGLLDKWGPKRVIDAPISEAAFSGIGIGA ALSGLRPIIEFMSWNFSFVALDQIISHAAKMHFMTGGKFSVPIVFRGPNGAAAQVSCQHSHCVESLYANIPGLI IIAPSNPYDAKGLLKSAIRNNNPVLFLENELEYNLKGEVPTEEYLVPIGKAHRVQEGNDLTIITYSRMVSITKEA CSLAKKRWGLSIEIIDLRTIKPLDISTILSSVRKTSRCIVIEEGHYFAGISSEIIALITEHVFDSLDAPPLRVCQKETP MPYSKILEQATLPNVNRILDTIEKVMR

>core/470/2/Org2_Gene61

MSNQFDQLKKLSTIVCDSGDPELVKASGSQDATTNPSLILKVAQEPKFQELLNEAVVWGIRQNGDDLQTLSFI LDKIQVNFALEIIKNIPGRISLEIDARLSFNVEAMVQRAVFLSQLFEAMGGDKKRLLVKIPGTWEGIRAVEFLE AKGIACNVTLIFNLVQAIAAAKAKATLISPFVGRIYDWWIAAYGDEGYSIDADPGVASVSNIYAYYKKFGIPT QIMAASFRTKEQVLALAGCDLLTISPKLLDELKKSQHPVKKELDPAEAKKLDVQPIELTESFFRFLMNEDAMA TEKLAEGIRIFAGDTQILETAITEFIKQIAAEGA

>core/471/2/Org2_Gene522

MTTPAYLLANFGGPRHAKDLQEFLISLLTDRDVTGTFLPRVLHRHLFTFIAKKRVPKVLPQYQSLQNWSPIYF DTETLAKTLSEILRAPVIPFHRYLPSTHEKTLLALRTLHTRHVIGIPLFPHFTYSVTGSIVRFFMKHVPEIPISWIP QFGSDSKFVSLITCHIRDFLQKLGILEKECCFLFSVHGLPVRYISQGDPYSKQCYESFSAITTNFKQSENFLCFQS KFGPGKWLSPSTAQLCQNIDTDKPNVIVVPFGFISDHLETLYEIERDYLPLLRSRGYRALRIPAIYSSPLWVSTL VDIVKENSTVVAEELIKSGKKHTGIR

>core/472/2/Org2_Gene296

MDAKMGYIFKVMRWIFCFVACGITFGCTNSGFQNANSRPCILSMNRMIHDCVERVVGNRLATAVLIKGSLDP HAYEMVKGDKDKIAGSAVIFCNGLGLEHTLSLRKHLENNPNSVKLGERLIARGAFVPLEEDGICDPHIWMDL

SIWKEAVIEITEVLIEKFPEWSAEFKANSEELVCEMSILDSWAKQCLSTIPENLRYLVSGHNAFSYFTRRYLATP
EEVASGAWRSRCISPEGLSPEAQISVRDIMAVVDYINEHDVSVVFPEDTLNQDALKKIVSSLKKSHLVRLAQK
PLYSDNVDDNYFSTFKHNVCLITEELGGVALECQR

>core/473/2/Org2_Gene442

MMTYPVPQNPLLLRILRLMDAFSKSDDERDFYLDRVEGFILYIDLDKDQEDLNKIYQELEENAERYCLIPKLT
FYEVKKIMETFINEKIYDIDTKEKFLEILQSKNAREQFLEFIYDHEAELEKWQQFYVERSRIRIIEWLRNNKFHF
VFEEDLDFTKNVLEQLKIHLFDAKVGKEITQARQLLSNKAKIYYSNEALNPRPKRGRPPKQSAKVETETTISSD
IYTKVPQAARRFLFLPEITSPSSITFSEKFDTEEEFLANLRGSTRVEDQLNLTNLSERFASLKELSAKLGYDSLST
GDFFGDDDEKVVTKTKGSKRGRKKSS

>core/474/2/Org2_Gene817

MHSELPNYQNIVESVVTEITTQLLNYRSEHRLVPFWEKSDGSFITAADYGSQYYLKQQLAKAFPNIPFIGEETL
YPDQDNEKIPEILKFTRLLTSSVSRDDLISTLVPPPSPTSLFWLVDPIDGTAGFIRHRAFAVAISLIYEYRPILSVM
ACPAYNQTFKLYSAAKGHGLSIVHSQNLDRRFVYADRKQTKQFCEASLAALNQQHHATRKLSLGLPNTPSP
RRVESQYKYALVAEGAVDFFIRYPFIDSPARAWDHVPGAFLVEEAGGRVTDALGAPLEYRKESLVLNNHAVI
LASGDQETHETTLAALQNQLNVVPTDKLIAL

>core/475/2/Org2_Gene634

MKTVTSFTVCKENSGRLDKYLTEVHPKYSRAFYQEHILSGLVQINGQINTRVATRLNCGDIVTIDIQEKEELLE
LLPEAIPLDKVYEDGMILVINKPRDMVVHPAPGHFHGTLVHALLHEIGERLKEEFPEEPWRPGIVHRLDKDTS
GLIITAKTRQAKKVFSELFSTKRLKKSYLAVCIGKPRSTTIHTHISRHQNKRKEMTVSSQGKEAVTHCQVLAF
NGKLSFVALSPETGRTHQLRVHMKHLGTPILGDPVYGIPSMNSSYGLDKQQLHAYSVDFTHPETRQFCSLKA
GLPEDMRSLLIKEFRNETTILNKNLLESILKEQ

>core/476/2/Org2_Gene224

MTIDMHCDLLSHPHFCRKDPAVRCSPEQLLSGGVRQQVCAIFVPHSRGEPNCDKQNSLFFSLPNQYPDIGLLS
YEEEENGSSSQKKSLSLIRSIENASALGDDTAPLGTLLAKLIHLTKQGPLAYLGIVWKGDNRFGGGTEAPKRL
SNDGKVLLDIMYELGVPIDLSHCSDKLAEDILDYTADKLPNLAVIASHSNFRSVLDHRRNLVDAHAKEIVRRK
GVIGLNLVRSYVGDSLGDLEKHVLHAENLGILSSIVLGSDFFYANEDENFFFNECSSAEAHPVLNQLIHRIFSK
GKAESILSSRAEKFLKQVIVEQVNPKITDVKL

>core/478/2/Org2_Gene105

MIEFAFVPHTSVTADRIEDRMACRMNKLSTLAITSLCVLISSVCIMIGILCISGTVGTYAFVVGIIFSVLALVACV
FFLYFFYFSSEEFKCASSQEFRFLPIPAVVSALRSYEYISQDAINDVIKDTMQLSTLSSLLDPEAFFLEFPYFNSLI
VNHSMKEADRLSREAFLILLGEITWKDCETKILPWLKDPNITPDDFWKLLKDHFDLKDFKKRIATWIRKAYPE
IRLPKKHCLDKSIYKGCCKFLLLSENDVQYQRLLHKVCYFSGEFPAMVLGLGSEVPMVLGLPKVPKDLTWE
MFMENMPVLLQSKREGHWKISLEDVASL

>core/479/2/Org2_Gene355

MELLPHEKQVVEYEKAIAEFKEKNKKNSLLSSSEIQKLEKRLDKLKEKIYSDLTPWERVQICRHPSRPRTVNYI
EGMCEEFVELCGDRTFRDDPAVVGGFVKIQGQRFVLIGQEKGCDTASRLHRNFGMLCPEGFRKALRLGKLA
EKFGLPVVFLVDTPGAYPGLTAEERGQGWAIAKNLFELSRLATPVIIVVIGEGCSGGALGMAVGDSVAMLEH
SYYSVISPEGCASILWKDPKKNSEAASMLKMHGENLKQFGIIDTVIKEPIGGAHHDPALVYSNVREFIIQEWLR
LKDLAIEELLEKRYEKFRSIGLYETTSESGPEA

>core/480/2/Org2_Gene599

MASNPILQIEDLSITLAKQRQQYPIVQSLSFTINEGQTLAIIGESGSGKSVSAHAILRLLPCPPFSVSGQVNFQGH
NLLTASRSIQKKIIGTEISMIFQNPQASLNPVFTIEQQFREIIHTHLALTAEVAKEKMLYALEETGFHDPRLCLNL
YPHQLSGGMLQRICIAMALLCSPKLLIADEPTTALDVSVQYQILQLLKTLQKKTGMSLLIITHNMGVVAETAD
DVLVLYAGRMVECAPAVQMFHNPSHPYTRDLLASRPSLQPQQLGSFNPIPGQPPHYTAFPSGCRYHPRCSKIL
NRCSAEAPEIYPVREGHKVRCWLYDD

>core/481/2/Org2_Gene293

MALGPSPYYGVSFFQFFSVFFSRLFSGSLFTGSLYIDDIQIIVFLAISCSGAFAGTFLVLRKMAMYANAVSHTVL
FGLVCVCLFTHQLTTLSLGTLTLAAMATAMLTGFLIYFIRNTFKVSEESSTALVFSLLFSLSLVLLVFMTKNAH
IGTELVLGNADSLTKEDIFPVTIVILANAVITIFAFRSLVCSSFDSVFASSLGIPIRLVDYLIIFQLSACLVGAFKA
VGVLMALAFLIIPSLIAKVIAKSIRSLMAWSLVFSIGTAFLAPASSRAILSAYDLGLSTSGISVVFLTMMYIVVK
FISYFRGYFSKNFEKISEKSSQY

>core/482/2/Org2_Gene600

MTTNFPQPLIQATSLTKHYYKRSFWFQGKTIASRPVDDVSFSLYSRRAVGLIGESGSGKSTLALALAGLLPLTS
GFLTFNGTPIKLHSKHGRHQLRSQVRLVFQNPQASLNPRKTILDSLGHSLLYHKLVPKEKVLATVREYLELVG
LSEEYFYRYPHQLSGGQQQRVSIARALLGVPQLIICDEIVSALDLSIQAQILNMLAELQKKLSLTYLFISHDLAV
VRSFCTEVFIMYKGQIVEKGNTKRIFSDPQHPYTRMLLNAQLPETPDQRQSKPIFQEYHKDSEESCSTGCYFY
NRCPQKQEACKSEIIPNQGDAHHTYRCIH

>core/483/2/Org2_Gene648

MEKKYYALAYYYITRVDNPHEEIALHKKFLEDLDVSCRIYISEQGINGQFSGYEPHAELYMQWLKERPNFSKI
KFKIHHIKENIFPRITVKYRKELAALGCEVDLSKQAKHISPQEWHEKLQENRCLILDVRNNYEWKIGHFDNAT
LPDIQTFREFPEYAEKLAQECDPETTPVMMYCTGGIRCELYSPVLLEKGFKEVYQLDGGVIAYGQQVGTGKW
LGKLFVFDDRLAIPIDESDPDVAPIAECCHCQTPSDAYYNCANTDCNALFLCCDECIHQHQGCCGEECSQSPR
VRKFDSSRGNKPFRRAHLCEISENSESASCCLI

>core/484/2/Org2_Gene515

MKQLLFCVCVFAMSCSAYASPRRQDPSVMKETFRNNYGIIVSGQEWVKRGSDGTITKVLKNGATLHEVYSG
GLLHGEITLTFPHTTALDVVQIYDQGRLVSRKTFFVNGLPSQEELFNEDGTFVLTRWPDNNDSDTITKPYFIET
TYQGHVIEGSYTSFNGKYSSSIHNGEGVRSVFSSNNILLSEETFNEGVMVKYTTFYPNRDPESITHYQNGQPHG
LRLTYLQGGIPNTIEEWRYGFQDGTTIVFKNGCKTSEIAYVKGVKEGLELRYNEQEIVAEEVSWRNDFLHGER
KIYAGGIQKHEWYYRGRSVSKAKFERLNAAG

>core/486/2/Org2_Gene567

MNLKVVYFGTPTFAATVLQDLLHHKIQITAVVTRVDKPQKRSAQLIPSPVKTIALTHGLPLLQPSKASDPQFIE
ELRAFNADVFIVVAYGAILRQIVLDIPRYGCYNLHAGLLPAYRGAAPIQRCIMEGATESGNTVIRMDAGMDT
GDMANITRVPIGPDMTSGELADALASQGAEVLIKTLQQIESGQLQLVSQDAALATIAPKLSKEEGQVPWDKP
AKEAYAHIRGVTPAPGAWTLFSFSEKAPKRLMIRKASLLAEAGRYGAPGTVVVTDRQELAIACSEGAICLHE
VQVEGKGSTNSKSFLNGYPAKKLKIVFTLNN

>core/488/2/Org2_Gene368

MSKGSSKHTVRINQTWYIVSFILGLSLFAGVLLSTIYYVLSPIQEQAATFDRNKQMLLAAHILDFKGRFQIQEK
KEWVPATFDKKTQLLEVATKKVSEVSYPELELYAERFVRPLLTDAQGKVFSFEEKNLNPIEFFEKYQESPPCQ
QSPLPFYVILENTSRTENMSGADVAKDLSTVQALIFPISGFGLWGPIHGYLGVKNDGDTVLGTAWYQQGETP
GLGANITNPEWQEQFYGKKIFLQDSSGTTNFATTDLGLEVVKGSVRTTLGDSPKALSAIDGISGATLTCNGVT
EAYVQSLACYRQLLINFSNLTHEKKTGE

>core/489/2/Org2_Gene540

MRLLSILKLHLFSLRSSSSLSPHYYHSCSRSMLHLLCRWKDADIMEWQQICNILSGVCSRMSGKLVSLQKETQ
DSCHQEHERIHLQYREQLSALEEEYRRREEAKNQDLEKLQQENTWLQNRLAEKLQQIRHQSDIIDEIKKELLQ
SVQRTEISEGRRLCYEHKIKQLEEQLQRYVSQHGAPSIEIEEDKSSAAYAEINRLKKSLIDLQQEKDIYIKTYHS
EIAKLREKLQRQEGAQTSSEVCSIEKLTEVQTDLAEKKKAIALLQDIVEDQYCQLRDLHKEKGMAMPSNTKL
DHLKGLLGKEPESEVDVVFSESKSLGS

>core/490/2/Org2_Gene855

MEVQIGIDLMGGDHSPLVVWQVLVDVLKSQSSTIPFAFTLFASEEIRKQIQEEFISDLPQEKFPKIISAENFVAM
EDSPLAAIRKKSSSMALGLDYLQEDKLDAFISTGNTGALVTLARAKIPLFPAVSRPALLVCVPTMRGHAVILD
VGANISVKPEEMVGFARMGLAYRQCLGDSKIPTIGLLNIGSEERKGTEAHRQTFRMLRETFGEAFLGNIESGA
VFDGAADIVVTDGFTGNIFLKTAEGVFEFLQRILGDKLEADIQRRLDYTFYPGSVVCGLSKLVIKCHGKACGS
SLFHGILGSINLAQARLCKRILSNLI

>core/491/2/Org2_Gene499

MLISISLATLPILAFSWASFIEPNWLRTTAIPWRLPKKHAHLHGLRIAQISDLHFHKRVPEKFLNKVSKSIKNFSP
DLIVFCGDLLCRARLEDKERLETFLNTLEAPLGVFAILGNHDYSSYISRNTKGEITCIPEEKSRPIQRAIIAVMQG
LFSSPSYRYDPNLTPQEPHPDLLKLLKNTPLTLLHNTTHVIPNTLNIVGLGDLFARQFHPEQAFKNYDPSLPGL
LLSHNPDGITRLQQYPGDFVLSGHSHGPQVTLSWPKFARKFFERLSGLENPYLARGYFVTKEGKQLYVNRGL
GGLKRIRFCSPPEICYITCSYD

>core/492/2/Org2_Gene887

MVLSSDLLRDDKQLDLFFASLDVKKRYLLALSGGSDSLFLFYLLKERGVSFTAVHIDHGWRSTSAQEAKELE
ELCAREGVPFVLYTLTAEEQGDKDLENQARKKRYAFLYESYRQLDAGGIFLAHHANDQAETVLKRLLESAH
LTNLKAMAERSYVEDVLLLRPLLHIPKSSLKEALDARGISYLQDPSNEDERYLRARMRKKLFPWLEEVFGKNI

TFPLLTLGEESAELSEYLEKQAQPFFSAATHQDSQGELPCPDCLIQQAFLCKWVMKKFFNNAGIAVSRHFLQ
MVYDHLSRSSCATLRMRNKIVIIKPGVVVID

>core/493/2/Org2_Gene585

MKFLLYVPLLLVLVSTGCDAKPVSFEPFSGKLSTQRFEPQHSAEEYFSQGQEFLKKGNFRKALLCFGIITHHFP
RDILRNQAQYLIGVCYFTQDHPDLADKAFASYLQLPDAEYSEELFQMKYAIAQRFAQGKRKRICRLEGFPKL
MNADEDALRIYDEILTAFPSKDLGAQALYSKAALLIVKNDLTEATKTLKKLTLQFPLHILSSEAFVRLSEIYLQ
QAKKEPHNLQYLHFAKLNEEAMKKQHPNHPLNEVVSANVGAMREHYARGLYATGRFYEKKKKAEAANIY
YRTAITNYPDTLLVAKCQKRLDRISKHTS

>core/494/2/Org2_Gene424

MNRRKARWVVALFAMTALISVGCCPWSQAKSRCSIDKYIPVVNRLLEVCGLPEAENVEDLIESSSAWVLTPE
ERFSGELVSICQVKDEHAFYNDLSLLHMTQAVPSYSATYDCAVVFGGPLPALRQRLDFLVREWQRGVRFKKI
VFLCGERGRYQSIEEQEHFFDSRYNPFPTEENWESGNRVTPSSEEEIAKFVWMQMLLPRAWRDSTSGVRVTF
LLAKPEENRVVANRKDTLLLFRSYQEAFPGRVLFVSSQPFIGLDACRVGQFFKGESYDLAGPGFAQGVLKYH
WAPRICLHTLAEWLKETNGCLNISEGCFG

>core/495/2/Org2_Gene122

MRKVAFLVSCLFSVAIGASAAPVRVPGFPQIPEDLVQIKTEVCPKQEVCLAVTIKCDDHNLIGVLHLPNTPTPE
GGFPTVVLFHGFRGTKFGGLTGAYRKLGRKFAAVGIATLRVDMAGCGDSEGVAEEVPIETYLRDAQTILETV
QEHPDLNAYRLGISGFSLGCHIAFELAKIYNPRDLNIKALSVWAPIADGGILLKELYENFSKHGEGDIISVGKDF
GFGPPPIIVCSGDVDLLIRIQDHVTANSLPTKPYILHQQGIDDTLVSRTQQTLFKNTAPGRMTFISYPNTGHNLA
TAPDLDMILDQIVSHFQRTL

>core/496/2/Org2_Gene379

MFKLLFHIAAFAGHVLSTPIFIVQDACGIDEEACKNPPPRPFSAQVQYLKVNDAKFKKLPHQTIGYRQYDGTF
LCTLPITEHSGLLFSTGYIGADIQWKSSLPISETDPNGLGWATFQDTSFYNYVLLSLGAYTLSLKNWQWSIILS
GLVDPKNIEMGYGLYQGVLSGKYQATEKLSAIFGVINETGLHQEKAWPLVGVSYKATDQLTLNCIYPVNFSI
DYRSTSVCNLGLAYRLTRFRKKLHKNHLISSRGIFEYQGREIEANVKLTPWPGSFIKGFYGWSIGNDISIADDH
NNNKTSHTFKTSAFFGGSAVMNF

>core/497/2/Org2_Gene173

MLVELEALKREFAHLKDQKPTSDQEITSLYQCLDHLEFVLLGLGQDKFLKATEDEDVLFESQKAIDAWNALL
TKARDVLGLGDIGAIYQTIEFLGAYLSKVNRRAFCIASEIHFLKTAIRDLNAYYLLDFRWPLCKIEEFVDWGN
DCVEIAKRKLCTFEKETKELNESLLREEHAMEKCSIQDLQRKLSDIIIELHDVSLFCFSKTPSQEEYQKDCLYQS
RLRYLLLLYEYTLLCKTSTDFQEQARAKEEFIREKFSLLELEKGIKQTKELEFAIAKSKLERGCLVMRKYEAA
AKHSLDSMFEEETVKSPRKDTE

>core/498/2/Org2_Gene327

MTLPMQKSLTSFDDFSQAYAEKVPAIALIGSALEDDKDALIELLVSESFKELGGQGLMPATLMSWTETFALFQ
EHETLGIIHAEKFPLATKEFLSRYARNPQPHLTILIFTTKQECFRELSKALPSALSLSLFGEWPADRQKRIIRLLL

QRAERVGISCSQSLASLFLRALASTSLPDILSEFDKLLCSVGKKTSLDHSDIKELVVKKEKASLWKFRDSLLKR
DPVEGHQQLHFLLEDGEDPLGIITFLRTQCLYGLRSIEEGSKENKHRMFVLYGKERLHQALNSLFYAETLIKN
NVQDPIVAVETLVIRMVNL

>core/499/2/Org2_Gene164

MFSYIKNRILFNLLSLWIVLTLTFLVMKTIPGDPFNDEGCNVLSEEVLQTLKSRYGLDKPLYQQYTQYLHSIAK
LDFGNSLVYKDRKVTNIISTAFPISAILGLQSLFLSIGGGIALGTIAALKKKKQRRYILGASILQISIPAFIFATLLQ
YVFAVKIPLLPIACWGSFTHTILPTLALAVTPMAFIIQLTYSSVSAALNKDYVLLAYAKGLSPLKVVIKHILPYA
IFPTISYSAFLTTTVITGTFAIENIFCIPGLGKWFICSIKQRDYPVALGLSVFYGTLFMLSSLLSDLIQSIIDPQIRYA
HGKEKKRK

>core/500/2/Org2_Gene288

MAMLPKFFLVLLCLGLCSCSQKTTTIEGEQMTIFYRIVLGTSLSAKEKASLSQQIDRCFHKIDSIYNNWNPYSE
LSIINRAPADVPITLSVELSEFLDQVDTLYKLSEGRFDPTVGPLKTLWLLHLKSQTLPPKDVWEQHYKDMGW
QHLEFQSNTKTLIKKNPHVQIDLCGVVKGYAVDCLNEICNTFCPNNYVEWGGEIKTSGHHPSGRPWRIFSEA
AGTILDIDDMAIATSGNHIQKWCVEGKIYTHILDTRTGKPLELSSYPIQSVSVVHPSCAYADAIATVLMTFDSK
IEAKQWAEEHHILTYINDGASS

>core/501/2/Org2_Gene475

MWRVVLRFLIIFILGRAVFPLRASESFSWETSTCLTVLGIPFIDIILTTNEDFVAQCGLQIGTISSTNNAKIKEIFLI
YKEKFPEASISFKRKEPLNLSQSHLSDLGILCMRNGETYAEGMANKENGPALKQPKDLRLVLRCPNQPDTLL
YSEKEAEKGIETNTCLCNQGYTLLDGQLILYGDSIEKFLKETKRKNNHTLVDLCDSQVVTTFLGRFWSLLNY
VQVLFLSEDSAKILAGIPDLAQATQLLSHTVPLLFIYTNDSIHIIEQGKESSFTYNQDLTEPILGFLFGYINRGSM
EYCFNCAQSSLGET

>core/502/2/Org2_Gene14

MIASIYSFLDYLKMVKSASPHTLRNYCLDLNGLKIFLEERGNLAPSSPLQLATEKRKVSELPFSLFTKEHVRM
YIAKLIENGKAKRTIKRCLSSIKSFAHYCVIQKILLENPAETIHGPRLPKELPSPMTYAQVEVLMATPDISKYHG
LRDRCLMELFYSSGLRISEIVAVNKQDFDLSTHLIRIRGKGKKERIIPVTSNAIQWIQIYLNHPDRKRLEKDPQA
IFLNRFGRRISTRSIDRSFQEYLRRSGLSGHITPHTIRHTIATHWLESGMDLKTIQALLGHSSLETTTVYTQVSV
KLKKQTHQEAHPHA

>core/503/2/Org2_Gene268

MIHSRLIIIGSGPSGYTAAIYASRALLHPLLFEGFFSGISGGQLMTTTEVENFPGFPEGILGPKLMNNMKEQAVR
FGTKTLAQDIISVDFSVRPFILKSKEETYSCDACIIATGASAKRLEIPGAGNDEFWQKGVTACAVCDGASPIFK
NKDLYVIGGGDSALEEALYLTRYGSHVYVVHRRDKLRASKAMEARAQNNEKITFLWNSEIVKISGDSIVRSV
DIKNVQTQEITTREAAGVFFAIGHKPNTDFLGGQLTLDESGYIVTEKGTSKTSVPGVFAAGDVQDKYYRQAV
TSAGSGCIAALDAERFLG

>core/504/2/Org2_Gene904

MRKLILCNPRGFCSGVVRAIQVVEVALEKWGAPIYVKHEIVHNRHVVNALRAKGAIFVEELVDVPEGERVIY
SAHGIPPSVRAEAKARKLIDIDATCGLVTKVHSAAKLYASKGYKIILIGHKKHVEVIGIVGEVPEHITVVEKVA
DVEALPFSSDTPLFYITQTTLSLDDVQEISSALLKRYPSIITLPSSSICYATTNRQKALRSVLSRVNYVYVVGDV
NSSNSNRLREVALRRGVPADLINNPEDIDTNIVNHSGDIAMTAGASTPEDVVQACIRKLSSLIPGLQVENDIFA
VEDVVFQLPKELRCS

>core/505/2/Org2_Gene740

MKCRPTLNTDQPRVRKKLPERFPKWLQRPLPQGSAFHATDATIKRSGMPTVCEEALCPNRAECWSRKTATY
LALGDVCTRSCGFCNIGHSKTPPALDPTEPERIALSAKELGLKHVVITMVARDDLEDGGAQGLVDIIQKLREE
LPQATTEVLASDFQGNVSALHTLLDSGITIYNHNVETVARLSPLVRHKATYARSMFMLEQAANYLPDLKIKS
GIMVGLGEMEGEVKQTLQDLASIGVRIVTIGQYLRPSRKHLQVKSYVTPETFDYYRRVGEAMGLFVYAGPFV
RSSFNADMILASVQDKASA

>core/507/2/Org2_Gene252

MKKRYAFLFPGQGSQYVGMGQDLYMEYPEVRELFDFANERLGFSLTSIMFEGPEDLLMETVHSQLAIYLHS
MAVVKVLSQRSSIQPSLVSGLSLGEYTALVASDRISVLDGLELVRKRGQLMNEACNQSPGAMAALLGLPSEV
IEENITSLGQGIWIANYNAPKQLVVAGIAEKVDQAIELFRDLGCKKAVRLKVSGAFHTPLMQVAQDGLAPDI
YALCMKDSSLPLVSHVVGKSLVNTEEMRECLARQMTSPTLWYQSCYHIESEVDEFLELGPGKVLAGLNRSIG
ISKPITSLGTFAQIEKFLSEV

>core/508/2/Org2_Gene488

MLNSNKFKSKTGAYGDLFQRVVVHSLVLTFLVLLLYSSLFPLTSFALGFITATCGAVGTYEYSSMAKAKMHY
PLSTFSAIGSFLFLALSFLSIRWGHSLPGFFDALPWTLLIVWVVWSIFRVRKSTIGALQLSGVTLFSILYVGIPIRL
FLHVLYSFIHTQEPYLGIWWASFLIATTKGADIFGYFFGKAFGNKKIAPQISPNKTVVGFVAGCLGATLISFIFF
LQIPTRFASYFPMPAILIPLGLALGITGFFGDIIESIFKRDAHLKNSNKLKAVGGMLDTLDSLLLSTPIAYLFLLIT
QSKEFIG

>core/509/2/Org2_Gene38

MRLFSYDKPKIKVQKIKADGFSGWLKCNHCHEMIHANELGQNYNCCPKCSYHYRITAIERVKLLADKDSWR
PLYTDLKSQDPLEFIDTDTYANRLEKARKNTTESEGVIVGICTIGLHPVALAVMDFNFMAGSMGAVVGEKLT
RLIEEAIETRLPVIIVSASGGARMQESVFSLMQMVKTSAALAKLHEAGLPYISVLTNPTSGGVTASFAALGDIII
AEPKALICFAGPRVVAQVIGEDLPEGAQKSEFLLEHGMIDKIVERKELKTTLQTLLDYFLAQEYTGGKSKAPR
DLSKRLKEIFLLTDDSE

>core/510/2/Org2_Gene979

MEIAYSLTSSFSVDSVTVGFFDGCHLGHSNLLSILTSYSGSSGVITFDSHPQTVLSLNHTKLINTKEERLQLLQT
FPIDWLGVLTFDLNFANQSAEEFLTLLHRNLKCKRLILGYDSCIGKEQQSNTEALDTIGKPLGIEVIKIPPYRMD
NIVVSSKAIRQFLSAGNLECAHRFLGHPYAISGKITEGSGIGGSLGFATINLPREESLIPLGVYACEIRYDSTTCQ
GVMNLGTAPTFGRESLYAEAHIFSFAENLYGKEVSIIPRKFLREEKKFQSKETLIRAIEKDILDAQDWFAKGSF
NYEGTA

>core/511/2/Org2_Gene15

MSSRELIILGCSSQQPTRTRNQGAYLFRWNGEGLLFDPGEGTQRQFIFANIAPTTVNRIFVSHFHGDHCLGLGS
MLMRLNLDKVSHPIHCYYPASGKKYFDRLRYGTIYHETIQVVEHPISEEGIVEDFGSFRIEAQRLQHQVDTLG
WRITEPDTIKFLPKELESRGIRGLIIQDLIRDQEISIGGSTVYLSDVSYVRKGDSIAIIADTLPCQAAIDLAKNSCM
MLCESTYLEQHRHLAESHFHMTAKQAATLAKRAATQKLILTHFSARYLNLDDFYKEASAVFPNVSVAQEYR
SYPFPKNPLLNK

>core/512/2/Org2_Gene733

MRSIFRFSLCFFTLSVSCCFADASLYENSCPSRCQPTPPPSNSNPLNVVQQPVAASSVPSYMPPLNADDVLPRD
HLSDGSFSDTYPDITTQAIILIFLALSPFLVMLLTSYLKIIITLVLLRNALGVQQTPPSQVLNGIALILSIYVMFPT
GVAMYKDARKEIEANTIPQSLFTAEGAETVFVALNKSKEPLRSFLIRNTPKAQIQSFYKISQKTFPSEIRAHLTA
SDFVIIIPAFIMGQIKNAFEIGVLIYLPFFVIDLVTANVLVAMQMMMLSPLSISLPLKLLLIVMVDGWTLLLQGL
MISFK

>core/513/2/Org2_Gene878

MKEAAPMHFPFPVRRSVWLNRYSTFRIGGPANYFKAIHTIEEAREVIRFLHSINYPFLIIGKGSNCLFDDRGFD
GFVLYNAIYGKQFLEDARIKAYSGLSFAALGKATAYNGYSGLEFAAGIPGSVGGAIFMNAGTNESDISSVVRN
VETINSEGELCSYSVEELELSYRSSRFHRQQEFILSATFQLSKKQVSADHSKSILQHRLMTQPYTQPSAGCIFRN
PEGTSAGKLIDAAGLKGLAIGGAQISPLHANFIINTGKATSDEVKQLIAIIQSTLKTQGIDLEHEIRIIPYQPKIHS
PVSEK

>core/514/2/Org2_Gene716

MSLFLVFLTAFIWSSSFALSKLVMNASAPIFATGARMVIAGAILALAAWFRGGFVGISKKIFLYIVLLALTGFY
LTNIFEFIGLQSLSSSKTCFIYGLSPLMSALFSYIQLKEKVTLKKVLGLSLGLVSYICYLTFGGGGDDSQPWTW
QIGLPELLILGAASLASFGWTLLRQIEKQSTLSVTAINAYAMLIAGMLSIMHSAVVEPWRPLPVQDISQFLYAT
LALVVISNLICYNLYAKLLRKYSSTFLSFCNLVMPLYSGFYGWILLGEKGVSLGLVLAVAFMVAGCRLIYHEE
FRQGYIVS

>core/515/2/Org2_Gene746

MQKPQYIDRITKKKVIEPIFYEKTMLFLYNSKLGKKLSVFLSTHPIFSRIYGWLQRCSWTRRQIRPFMNRYKIS
EKELTKPVADFTSFNDFFTRKLKPEARPIVGGKEVFITPVDGRYLVYPNVSEFDKFIVKSKAFSLPKLLGDHEL
TKLYAHGSIVFARLAPFDYHRFHFPCDCLPQKTRCVNGALFSVHPLAVKDNFILFCENKRTVTVLETEQFGNV
LYLEVGAMNVGSIVQTFSPNQTYAKGDEKGFFAFGGSTVILLFLPNAIRFDNDLLKNSRMGFETRCLMGQSL
GRSQREEI

>core/516/2/Org2_Gene911

MTMPSTQFHTTILEQFSLFLSVDRGLCQQSIAAYRQDISSFLTISAISSPQDISQNSVYIFAEELYRRKEAETTLA
RRLIALKVFFLFLKDQQLLPYPPIIEHPKIWKRLPSVLTPQEVDALLAVPLQMEKNPRHLAFRDTAILHTLYST
GVRVSELCDLRLGHVSDDCIRVTGKGSKTRLVPLGSRAREAIDAYLCPFRDQYQKKNPHEDHLFLSTRGHKL

ERSCVWRRIHNYAKQVTSKPVSPHSLRHAFATHLLDNKADLRVIQEMLGHARIASTEVYTHVAADSLIEKFL
AHHPRNL

>core/517/2/Org2_Gene348

MLKIDLTGKVAFVAGIGDDQGYGWGIAKLLAEAGATIIVGTWVPIYKIFSQSWELGKFNESRKLSNGTLLEIA
KIYPMDASFDSPEDVPEDIAENKRYKGITGFTISEVAEQVKKDFGHIDILVHSLANSPEISKSLLETSRKGYLAA
LSASSYSFVSLLSHFGSIMNRGGSTISLTYLASMRAVPGYGGGMSSAKAALESDTKTLAWEAGRRWGIRVNT
ISAGPLASRAGKAIGFIERMVDYYQEWAPIPEAMNAEQVGAVAAFLASPLASAITGETLYVDHGANVMGIGP
EMFPKDS

>core/518/2/Org2_Gene947

MPPPFVVTLTTSAQNNLRDQLKEKNFIFSQPQNTVFQARSNTVTCTLYPSGKLVIQGKGSEEFIEFFLEPEILHT
FTHARVEQDLRPRLGVDESGKGDFFGPLCIAAVYASNAEILKKLYENKVQDSKNLKDTKIASLARIIRSLCVC
DVIILYPEKYNELYGKFQNLNTLLAWAHATVINNLAPKPAGDVFAISDQFAASEYTLLKALQKKETDITLIQK
PRAEQDVVVAAASILARDAFVQSIQKLEEQYQVQLPKGAGFNVKAAGREIAKQRGKELLAKISKTHFKTFDE
ICSGK

>core/519/2/Org2_Gene227

MHLEEENQGWEALLRKVYHQEVPPAILLHGFTLPVLQDKAEQLASEILLSSSPGSEHKVSQKIHPDIYQFFPEG
KGRLHSIDLPRGIKKQIYISPFEANYKIYIIHEADRMTLAAISAFLKVFEEPPKHAVIILTTAKVQRLPKTIISRSLS
IFIERGEKILCSKETFSYLFRYAQCEIPVTEVSQIIKESSETDKQVLRDKVQRFMEVLLELYRDRYTLNLGLKAS
ALNYPEHVKEILQLPLLPLDKVLLIVESACRSLNNSSSAASVLEWVAIQLVSLQYKEKELVSVSPGQDLSN

>core/520/2/Org2_Gene220

MRLNYFLNLVNFKYSIFSILFLSASTVFALSINEISQNLSFKEGFKISVFGAIAFVFARTTGIVVNQCIDRFIDKKN
TRTSKRVLPANLVSLNFAWVLSLFCSFLFLFLCKILRIFSLGIASLTLMIVYPYMKRVTFFCHWGLGLVYTVAI
LMNFCAFAESGLSMRLCFLALLWGGSVGMVIAANDIIYAIEDTEFDREEGLRSVPAHYGEKKAVEIAKVNLW
VSYLAYIFSGFVGSLDKEFYFTAIIPLVVILKVVRMYSNYSKKDQEGESKFFLANIAIALSFLVSMTLFWSLSR

>core/521/2/Org2_Gene361

MSERAHIPVLVEECLALFAQRPPQTFRDVTLGAGGHAYAFLEAYPSLTCYDGSDRDLQALAIAEKRLETFQD
RVSFSHASFEDLANQPTPRLYDGVLADLGVSSMQLDTLSRGFSFQGEKEELDMRMDQTQELSASDVLNSLKE
EELGRIFREYGEEPQWKSAAKAVVHFRKHKKILSIQDVKEALLGVFPHYRFHRKIHPLTLIFQALRVYVNGED
RQLKSLLTSAISWLAPQGRLVIISFCSSEDRPVKWFFKEAEASGLGKVITKKVIQPTYQEVRRNPRSRSAKLRC
FEKASQ

>core/522/2/Org2_Gene699

MSRHEICPEVSHKKGKYYSTFIFRCIHSLAGIAFTFFLCEHLFTNMLASSYFSQGKGFVAMVNGFHKIPGLKIIE
VAGLVLPFLCHAIIGIVYLFQGKSNCYSGDGSRPHLRYAKNYSYTWQRWTAWILLFGIAFHVVHLRFIRYPVH
VDIHGTTYYAVDIQPSRYDVIVRGTKGFLTLNLPNTEASSIEVSRHDLGGADAALLSERNSYLLTPSAGTAFLY

VVRDALGSLFIALLYTILVIAAAFHGFNGLWTFCCRWGVVVSLRMQGVLRIVCYLAMIVVTFMGVSAVWNLYSVA

>core/524/2/Org2_Gene194

MRRFLFLILSSLPLVAFSADNFTILEEKQSPLSRVSIIFALPGVTPVSFDGNCPIPWFSHSKKTLEGQRIYYSGDSFGKYFVVSALWPNKVSSAVVACNMILKHRVDLILIIGSCYSRSQDSRFGSVLVSKGYINYDADVRPFFERFEIPDIKKSVFATSEVHREAILRGGEEFISTHKQEIEELLKTHGYLKSTTKTEHTLMEGLVATGESFAMSRNYFLSLQKLYPEIHGFDSVSGAVSQVCYEYSIPCLGVNILLPHPLESRSNEDWKHLQSEASKIYMDTLLKSVLKELCSSH

>core/525/2/Org2_Gene218

MSHGPRPTKFSFPLYFSKTLSWFILGGFLAACGVQMVLVPNELIDGGIVGLSIIASHFLGHKALPFCLVLFNLPFVFLAFKQIGKYFVIQMLTAVIIFSCSLWLIDQLPSWLGMSPFVFKGSEMETVVLGGAIIGVGCGLIIRHGGSTDGTEILGIIINKKKGYTVGQIILFVNFFIFALSGIVYKNWHTAFVSFLTYGIATKVMDMVILGLEDTKSVTIITSSPRKLGHILMETLGIGLTYIHAEGGYSGEPRNLLYVVVERLQLSQLKEIVHREDPSAFIAIENLHEVINGRRT

>core/526/2/Org2_Gene796

MQEKPRHVHRIIHISDVHFHVLPVNPVHCFNKRLKGLLRKVFGLVHFQATTIGQRFPKVVRSLGADSVCITGDFSLTAMDGEFLLAKHFVETLAKHSSVYLLPGNHDVYTLKSLAQQTFYTHFPNDQLQQNKVSFHKITDHWWLILLDCSCLNGWFSANGVVHLAQISAIETFLLSLSPEENVIIANHYPLLSSQNPSHDLINNTHLQNVLKKYPKVRLYLHGHEHQAAVYNCADTSPSYILNSGSISLPTNSRFHVIDLYPEKYQVHTMILKNLLDFDAPLEIANEATWDCQKL

>core/527/2/Org2_Gene349

MEKLLVTDIDGTITHQSHHLDKKVYERLYALHQAGWKLFFLTGRYYKYAARLFSDFDAPYLLGCQNGASVWSSTSSNLLYSKSLPSDLLCILQDCMEGATALFSVESGAPYGDHYYRFSPTPIAQDLHEYVDPRYFPNAKEREILFETRSLKDDYAFPSFAAAKVFGLRDEVIRIQKELERQEALTSVATMTLMRWPFDFRYAILFLTDKSVSKGKALDRVVNILYDGKKPFVMASGDDANDLDLIERGDFKIVMSSAPEEMHVHADFLAPPADKNGILSAWEAGVRYYDDLMSL

>core/528/2/Org2_Gene584

MKKLILYFAAFVASLFCGVFLWDRVPCAQKIMRLAADHSSEVFSKSCRFVRKISGFEELQVFERHVSPEQALALFPEYRDGKSFVELAFIPHTLMHVRFSKEEPVKKHIISQEGEILWSLVNGEMVLHTGTWTCSKGFRECLLLHAGKQDMRVIQTLATLGGTTSRESLAQALALKNIRAERVIKECQKKKLIFASGNQIGTHFQQFQPIRGCTTTLNNNPVWLQKPRHAAVFPAQYSEDRVRHLVKMIFGDNFLIVRSSMVYVPVYKISLVSADNSVRVEYINAVTGKSFQDL

>core/529/2/Org2_Gene866

MFHSLSKNTPIITQGITGKAGSFHTEQCLAYGTNFVGGVTPGKGGTLWLDLPVYDSVLEAKQATGCRATMIFVPPPYAAEAILEAEEAGIELIVCITEGIPVRDMLEVARVMDNSTSQLIGPNCPGIIKPGECKIGIMPGYIHLPGNIGVVSRSGTLTYEAVWQLTQLKIGQSICVGIGGDPLNGTSFIDVLQALEEDPYTELILMIGEIGGSAEEEAAAWIQAHCTKPVVAFIAGVTAPKGKRMGHAGAIISGNSGDAKSKIQVLRESGVTVVESPAHIGKTVDAVLRAKEL

>core/530/2/Org2_Gene794

MSKHTSESRIAQDMLERYSGSSVKQFCPYLLLTNFSYYIQTFAKLHGVPVFEGSMFSAAHAPHLKTSILDFKL
GSPGAALTIDLCSFLPDLKAALMLGMCGGLRSHYQVGDYFVPVASIRGEGTSDAYFPPEVPALANFVVQKAT
TEVLEDKKANYHIGITHTTNIRFWEFNKKFRKKLYETKAQSAEMECATLFAAGYRRNLPIGALLLISDLPLRK
EGIKTKSSGNFIFNTYTEDHILTGQEVIENLEKVMLKRAASDHKKDQQYRGLPHMEVGEADDTMASGSETSD
SDY

>core/531/2/Org2_Gene646

MKVLPPPSIPLLGAHTSTAGGLKNAIYEGRDIGASTVQIFTANQRQWQRRALKEEVIEDFKAALKETDLSYIM
SHAGYLINPGAPDPVILEKSRIGIYQEILDCITLGISFVNFHPGAALKSSKEDCMNKIVSSFSQSAPLFDSSPPLV
VLLETTAGQGTLIGSNFEELGYLVQNLKNQIPIGVCVDTCHIFAAGYDITSPQGWEDVLNEFDEYVGLSYLRA
FHLNDSMFPLGANKDRHAPLGEGYIGKESFKFLMTDERTRKIPKYLETPGGPENWQKEIGELLKFSKNRDS

>core/532/2/Org2_Gene465

MLSSLIRDSFPLLILLPTFLAALGASVAGGVMGTYIVVKRIVSISGSISHAILGGIGLTLWIQYKLHLSFFPMYGA
IVGAIFLALCIGKIHLKYQEREDSLIAMIWSVGMAIGIIFISRLPTFNGELINFLFGNILWVTPSDLYSLGIFDLLV
LGIVVLCHTRFLALCFDERYTALNHCSVQLWYFLLLVLTAITIVMLIYVMGTILMLSMLVLPVAIACRFSYKM
TRIMFISVLLNILCSFSGICIAYCLDFPVGPTISLLMGLGYTASLCVKKRYNPSTPSPVSPEINTNV

>core/533/2/Org2_Gene898

MKRNDPCWCGSGRKWKQCHYPQPPKMSPEALKQHYASQYNILLKTPEQKAKIYNACQITARILDELCKASQ
KGVTTNELDELSQELHKKYDAIAAPFHYGSPPFPKTICTSLNEVICHGIPNDIPLKDGDIMNIDVSCIVDGYYG
DCSRMVMIGEVPEIKKKICQAALECLNDSIAILKPGIPLCEIGEAIEARADTYGFSVVDQFVGHGVGIEFHENPY
VPHYRNRSMIPLAPGMIFTIEPMINVGKKEGVVDPKNQWEARTCDNQPSAQWEHTIAITETGYEILTLLND

>core/534/2/Org2_Gene934

MHLLTATVTPFFPNGTIDFASLERLLSFQDAVGNGVVLLGSTGEGLSLTKKEKQALICFACDLQLKVPLFVGT
SGTLLEEVLDWIHFCNDLPISGFLMTTPIYTKPKLCGQILWFEAVLNAAKHPAILYNIPSRAATPLYLDTVKAL
AHHPQFLGIKDSGGSVEEFQSYKSIAPHIQLYCGDDVFWSEMAACGAHGLISVLSNAWPEEAREYVLNPQEQ
DYRSLWMETCRWVYTTTNPIGIKAILAYKKAITHAQLRLPLSIEDFDLENVSPAVESMLAWPKLRTSVFSYS

>core/535/2/Org2_Gene864

MFKFFRNKLQSLFKKNISLDLIEDAESLFYEADFGTELTEELCARLRRTKKADASTIKDLITVLLRESLEGLPSQ
ASQSSQTRPIVSLLLGTNGSGKTTTAAKLAHYYKERSESVMLVATDTFRAAGMDQARLWANELGCGFVSGQ
PGGDAAAIAFDGIQSAIARGYSRVIIDTSGRLHVHGNLMKELSKIVSVCGKALEGAPHEIFMTVDSTLGNNAIE
QVRVFHDVVPLSGLIFTKVDGSAKGGTLFQIAKRLKIPTKFIGYGESLKDLNEFDLDLFLNKLFPEVEKI

>core/536/2/Org2_Gene214

MKRRNLQKILPNASTPSTNVAENTGIKDQNLFLDQATLNVDGNVDIENFLETRDLKVADTITSPCEFTVGGGL
SAESSQFKATTLSKGLEITSEDQDGRVPKFTNVSDPQSPRDALTYNYYRNTGCQALNLYTYYSSSQPTTVGKP

IETVCQNPNPETYRISASAKIYDAVTRFPYIQFKAPGIYQVTIQIRRESGQHSGLDNPNLYLNLMIGNNKTLLCA
SDTRGYSGGHRTSIAVTGTFTLTEIVATPPHDYPWLFLETTIGLDIKSMSTCVIWFPFQANFAEVD

>core/537/2/Org2_Gene418

MQIPRSIGTHDGSFHADEVTACALLIIFDLVDENKIIRSRDPVVLSKCEYVCDVGGVYSIENKRFDHHQVSYDG
SWSSAGMILHYLKEFGYMDCEEYHFLNNTLVHGVDEQDNGRFFSKEGFCSFSDIIKIYNPREEEETNSDADFS
CALHFTIDFLCRLRKKFQYDRVCRGIVREAMETEDMCLYFDRPLAWQENFFFLGGEKHPAAFVCFPSCDQWI
LRGIPPNLDRRMDVRVPFPENWAGLLGKELSKVSGIPGAVFCHKGLFLSVWTNRESCQRALRLTLQDRGII

>core/538/2/Org2_Gene731

MGISLPELFSNLGSAYLDYIFQHPPAYVWSVFLLLLARLLPIFAVAPFLGAKLFPSPIKIGISLSWLAIIFPKVLA
DTQITNYMDNNLFYVLLVKEMIIGIVIGFVLAFPFYAAQSAGSFITNQQGIQGLEGATSLISIEQTSPHGILYHYF
VTIIFWLVGGHRIVISLLLQTLEVIPIHSFFPAEMMSLSAPIWITMIKMCQLCLVMTIQLSAPAALAMLMSDLFL
GIINRMAPQVQVIYLLSALKAFMGLLFLTLAWWFIIKQIDYFTLAWFKEVPIMLLGSNPQVL

>core/539/2/Org2_Gene660

MLHALDTYRPSIESAIEKALEGFGPIGHPIRSPVEYALQGGGKRLRPGLVCMMAQGLGLNHDVMDSALAVEF
VHTSTLIADDLPCMDNDDERRGRPTVHKAFDEATALLASYALIPAAYSHLRLNAKKLKEQGCDPREIDIAYNI
IGDITDKNIGCSGVLGGQYDDMFFSNRGQEHVQSIMIKKTGSLFEIACISGWLFGGGDPQFAPIITSFSNNFGLL
FQIKDDFSDLQKDSQQIGLNYALLFGEKAALELLARSQNNCLELLDRLSAGGLKNSSEFETIISSLGSF

>core/540/2/Org2_Gene213

MSNPTPKTKISIPTFVRFNIQSINLTEDQKKTTFTVGGKVTTENTVVRGDLTCTDGGLTCQSDLTIQKDINIRPT
STNSMVFDGRLNLSNSPLSYKNSQGQDITDYEKMSSGKPQEYVPFGYYKRTQIMMAQRAAHSSGYVGGGSV
PSGSYVPWNKFDQTSTQKTSGTEIYIDPNDSTKLVFEVNNKVPKLFRISVIMAKHGSWLDNGTGADILLAANE
YEQGGGRINVTDLAMTTSRGSSYYETRPLQVVCVTYYAQNNGYFTFQNRAGGGLRVSFFSWNIVALPYVE

>core/541/2/Org2_Gene951

MLKIQKKRMCVSVVITVGAIVGFFNSADAAPKKKKIPIQILYSFTKVSSYLKNEDASTIFCVDVDRGLLQHRY
LGSPGWQETRRRQLFKSLENQSYGNERLGEETLAIDIFRNKECLESEIPEQMEAILANSSALVLGISSFGITGIPA
TLHSLLRQNLSFQKRSIASESFLLKIDSAPSDASVFYKGVLFRGETAIVDALSQLFAQLDLSPKKIIFLGEDPEV
VQAVGSACIGWGMNFLGLVYYPAQESLFSYVHPYSTATELQEAQGLQVISDEVAQLTLNALPKMN

>core/542/2/Org2_Gene16

MSHLIPSLRNSVTSYFHKPQPIKQAAPSKSIRDICNIAYLIIICVLVVVVLVGAMLCMFIPSVGIPLCLSSLALLV
LLSIFNPCLINWISTKKTKEIAPKDASESQPTKSASRKGSPQLSPHHDHEPKNFIRTQLEKGVNYVTNKFKSGEE
SPHISDEHHSPRQSKRSSEIESSDESSPELHRKAKGKAPHTATTKESKTSTTESSKKKKKTKHSLHRTTSSIHKR
SAPKPMVPSKKRKPVLLKKTVPLPIEDLEHQSSGNESSDSSSPPPVQRKAILPWFCKQPTDP

>core/543/2/Org2_Gene89

MEIKKAIQEGTAYLDYYGVPLSDCEALYILMDLLEVSSRAKLFDLVGISETMLMEYRKRLALRGQRCPTAYL
NGAVSFLGLRLRVDSRVLIPRTETELLAEYIINYLLSHSEIQTFYDICCGSGCLGLAIKKSCPHVEVVLSDVCPQ
AVAVANENAKSNGLDVKILLGDLSAPYTRPADAFVCNPPYLSFNEIIHIDPEVRCYEPWKALVGGSTGLEFYQ
RIAQELPKIVTSTGVGWLEIGSSQGESIKNIFSKHGIYGRLHQDLSGRDRIFFLEMDGRDPVSSGAYS

>core/544/2/Org2_Gene265

MAVIYWDRSKIVWSFEPWSLRLTWYGVFFTVGIFLACLSARYLALSYYGLKDHLSFSKSQLRVALENFFIYSI
LFIVPGARLAYVIFYGWSFYLQHPEEIIQIWHGGLSSHGGVLGFLLWAAIFSWIYKKKISKLTFLFLTDLCGSVF
GIAAFFIRLGNFWNQEIVGTPTSLPWGVVFSDPMQGVQGVPVHPVQLYEGISYLVVSGILYFLSYKRYLHLGK
GYVTSIACISVAFIRFFAEYVKSHQGKVLAEDCLLTIGQILSIPLFLFGVALLIICSLKARRHRSHI

>core/545/2/Org2_Gene287

MLLRGIPAAEKILQRLKEEISQSPTSPGLAVVLIGNDPASEVYVGMKVKKATEIGIISKAHKLPSDSTLSSVLKL
IERLNQDPSIHGILVQLPLPKHLDSEVILQAISPDKDVDGLHPVNMGKLLLGNFDGLLPCTPAGIIELLNYYEIP
LRGRHAAIVGRSNIVGKPLAALMMQKHPQTNCTVTVLHSQSENLPEILKTADIIIAALGAPLFIKETMVAPHA
VIVDVGTTRVPADNAKGYTLLGDVDFNNVVTKCAAITPVPGGVGPMTVAMLMSNTWRCYQNFS

>core/546/2/Org2_Gene601

MTEEISKDTIIEVAIDDIRVSPFQPRRVFSNEELQELIASIKAVGLIHPPVVREICTGDRVLYYELIAGERRWRAM
QLAGATTIPVILKHVIADGTAAEATLIENIQRVNLNPIEMAEAFKRLIHVFGLTQDKVAYKVGKKRSTVANYL
RLLALSKTIQESLLQGQITLGHAKVILTLEDPILREKLNEIIIQEHLAVREAELIAKQLISEEGSSIELKPTPLDMA
ESSKQHEELQQRLSDLCGYKVQIKTRGSKATVSFHLQNTQDLQKLEAWLSSHGTLSESLS

>core/547/2/Org2_Gene225

MPDKKAQITFSLPEVMSAIHQGKIVALPTDTVYGFVLSLYASEAEERLYALKDREPSKAFALYVNSIEDIENIS
GYPLSPTAKKLAQLFPGAITLVVKHRNPRFPKETLAFRIVDHSVVREIVDHCGTLIGTSANLSEFPSALTAQEIF
ADFADHDLCIFDGPCSHGLESTVVASDPLYIYREGLISRSVIENIAGTEAKIFHRTSHAFSKHIKIYTVKNQEQL
VSFLSGSLDFKGVVCEHPKPKNFYTRLREALKKKTPSIVFIYDINTSDYPELFPFLSPYYIE

>core/548/2/Org2_Gene212

MKNNINNNECYFKLDSTVDGDLLAANLKTFDTQAQGISSTETFSVQGNATFKDQVSATGLTSGTTYNLNAQ
NFTSSQISIDFKNNRLSNCALPKEDCDPVPANYVRSPEYFFCSKPLIGDFDFNSGESYLPLTGSEYTLYQSRNVN
SIFRFIGWKQSTRELTVGGNTAIQFLAAGTYIVSFTVGKRWGWNNGWGGAIYINNGLGQVQCESTIYSGGGY
ATIGTLGTSIYRASVDVAPNPNDPNASDRYRAGIFYLSNGGSSAGIGNYSFSLLYYPDDRG

>core/549/2/Org2_Gene414

MHEVLILTFTYPLPRTLKQHPDEVHTVPISPNLSFGEGSPILIAGPCTLESYEHTVSSALTVKEAGAQVFRGSIR
KPRTSPFSFQGWEKECVLWHKEAQSIHGLPTETEVLDVRDVEITAEHVDILRIGAKNMHNTPLLQEVSKSHRP
IILKRSPAATLEEWLCAAEYILASSPSCPGVILCERGIRTFEHSTRYTLDLNTVALLKEISSLPVIVDPSHAAGKR
SLVLPLASAGLSVGADGLMIEVHAHPEKALCDAKQQITPEELHLFAKKHFCPSESRAHAIS

>core/550/2/Org2_Gene205

MKHYLSFSPSADFFSKQGAIETQVLFGERVLVKGSTCYAYSQLFHNELLWKPYPGHSFRSTLVPCTPEFHIHP
NVSVVSVDAFLDPWGIPLPFGTLLHVNSQNTVIFPKDILNHMNTIWGSGTPQCDPRHLRRLNYNFFAELLIKD
ADLLLNFPYVWGGRSVHESLEKPGVDCSGFINILYQAQGYNVPRNAADQYADCHWISSFENLPSGGLIFLYP
KEEKRISHVMLKQDSSTLIHASGGGKKVEYFILEQDGKFLDSTYLFFRNNQRGRAFFGIPRKRKAFL

>core/551/2/Org2_Gene166

MDNYLLNIKDLTITSTNPKRTLIENLSLQLKENRNLALVGESGSGKTTITKAILGFLPENCLIKTGSILFEDIDIT
KLSPKELHKIRGQKIATILQNAMGSLTPSMRIGMQIIETLRQHHKMNKEEAYNKAMQLLTDVCIPNPKYSFSQ
YPFELSGGMRQRVVIAIALASQPKLILADEPTTALDSMSQAQVLRILRNIQQQKQATILLVTHNLSLVKELCND
ICIIKDGKLIETGTVEEIFLSPKHPYTLKLLNAVSKIPIKKTSSPILKNKFQPLMSMQGGL

>core/552/2/Org2_Gene165

MENLSSAPSRSIWKSIIQNKMLVLGLTTLIILMLGALLLPWFYQDYEQTSLKDILVSPCSRFPFGTDTLGRCMF
ARTLRGLRLSLLIATIATLIDVCVGLLWATVAISGGKKIDFLMMRTTEILFSLPRIPIIILLLVIFHHGLLPLILAM
TITGWIPISRIIYGQFLLLKNKPFVLSAKAMHASTFHILKKHLLPNTLAPIISTLIFTIPNAIYTEAFISFLGLGIQPP
QASLGTLVKEGINAIDYYPWLFFFPSLIMIALSISFNLIGEGAKTLCLEEGSHG

>core/553/2/Org2_Gene144

MKQPMSLIFSSVCLGLGLGSLSSCNQKPSWNYHNTSTSEEFFVHGNKSVSQLPHYPSAFRTTQIFSEEHNDPY
VVAKTDEESRKIWREIHKNLKIKGSYIPISTYGSLMHPKSAALTLKTYRPHPIWINGYERSFNIDTGKYLKNGS
RRRTSHDGPKNRAVLNLIKSSGRRCNAIGLEMTEEDFVIARRREGVYSLYPVEVCSYPQGNPFVIAYAWIADE
SACSKEVLPVKGYYSLVWESVSSSDSLNAFGDSFAEDYLRSTFLANGTSILCVHESYKKVPPQP

>core/554/2/Org2_Gene562

MFKKFKPVTPGTRQLVLPAFDELTTRGELRGTKSKRSLRPNKKLSFFKKSSGGRDNLGHISCRHRGGGAKQL
YRVVDFKRNKDGITAKVVTVEYDPNRSAYIALLSYEDGEKRYILAPKGIQRGDVVVSGEGSPFKPGCCMTLK
SIPLGLSVHNIEMRPSSGGKLVRSAGLAAQVIAKSPGYVTLKMPSGEFRMLNEGCRATIGEVSNADHNLRVD
GKAGRRRWMGVRPTVRGTAMNPVDHPHGGGEGRHNGYIPRTPWGKVTKGLKTRDKNKSNKWIVKDRRK

>core/556/2/Org2_Gene568

MASIHPTAIIEPGAKIGKDVVIEPYVVIKATVTLCDNVVVKSYAYIDGNTTIGKGTTIWPSAMIGNKPQDLKYQ
GEKTYVTIGENCEIREFAIITSSTFEGTTVSIGNNCLIMPWAHVAHNCTIGNNVVLSNHAQLAGHVQVGDYAIL
GGMVGVHQFVRIGAHAMVGALSGIRRDVPPYTIGSGNPYQLAGINKVGLQRRQVPFATRLALIKAFKKIYRA
DGCFFESLEETLEEYGDIPEVKNFIEFCQSPSKRGIERSIDKQALEEESADKEGVLIES

>core/557/2/Org2_Gene612

MSDFSMETLKTLRQQTGVGLTKCKEALEACGGNLEEAVVYLRKLGLASAGKKEHRETKEGIIAAKTDANGT
ALIEVNVETDFVANNAVFREFVSNLLNDILKYKVDTVEALSQAASSQDPSLSVDELRAVTMQTVGENIRISRV
AYFPKATNSTVGIYSHGNGKTVALTMLSGSSTADSLAKDIAMHVVAAQPQFLSKESVPAEAIAKEKEVIASQI
QGKPQEVIEKIVTGKLNTFFQEACLLEQPFIKNADLSIQSLIDDFSKTSGSSVAIEQFILWKIGA

>core/558/2/Org2_Gene570

MLERTQRTLKREVRYSGVGIHLGKSSTLHLQPAQTNTGIVFQRQSASGNYENVPALLDHVYTTGRSTTLSRG
SAVIATVEHLMAALRSNNIDNLIIQCSGEEIPIGDGSSNVFVELIDQAGICEQEDKVSIARLTRPVYYQHQDIFL
AAFPSDELKISYTLHYPQSSTIGTQYKSLVINEESFRQEIAPCRTFALYNELCFLMEKGLIGGGCLDNAVVFKD
DGIISRGQLRFADEPVRHKILDLIGDLSLVGRPFVAHVLAVGSGHSSNIAFGKKILEALEL

>core/559/2/Org2_Gene665

MATAHLGRQALLHLRSWTPAIRASGNLFRQQSMSLHNNVLFAGDIVGAIKNSTAISRHALGSSHYAHAALQK
TEGFLGAADGVNTAVAGAMLWGQLLNGSMIFETDEETGELRRCNEADAEGCMTQKLQRRSALTITGKVAR
LASKTLGTATFLHEMDVVSLGANANKIGCKVTSCLNLVATGCSLTESSISLYRILSTRPETISDPENRNKPSAEF
AARSKAIRNAFIAWLGDVVDLVCDALGTLSLFLPAILGVHAVLIMAILGLISCVINFVKDYAKIG

>core/560/2/Org2_Gene217

MVLMNKRLKIILTNDDGITAKGMSCLVSALLEANIGDIYIAAPQAEQSGKSMAISLNQVVCASPYAYPQPVKE
AWAVGGSPTDCVRLGLRTLFESVSPDLVISGINCGNNICKNAWYSGTIGAAKQALVDGIPSMALSQDNHISFF
QQDKAPEILKALVIYLLSQPFPCLTGLNINFPTSPGGSSWEGMRLVPPGDEFFYEEPQYLGSVNKNQYYVGKIS
GVRIGEHPSEELACMLENHISVSPIFSQNSPIGLMTLEEFQKTQENFNASLLSSELTTKIF

>core/561/2/Org2_Gene620

MELKKTAESLYSAKTDNHTVYQNSPEPRDSRDVKVFSLEGKQTRQEKTTSSKGNTRTESRKFADEEKRVDD
EIAEVGSKEEEQESQEFCLAENAFAGMSLIDIAAAGSAEAVVEVAPIAVSSIDTQWIENIILSTVESMVISEINGE
QLVELVLDASSSVPEAFVGANLTLVQSGQDLSVKFSSFVDATQMAEAADLVTNNPSQLSSLVSALKGHQLTL
KEFSVGNLLVQLPKIEEVQTPLHMIASTIRHREEKDQRDQNQKQKQDDKEQDSYKIEEARL

>core/563/2/Org2_Gene965

MRQSFDELSQNAFKNIFNKQRFCFIFCSLCCFGFVFALFLKLCSRLAPEISLSTLGLGAFFCAFSVICASAIIVQF
LLHKESQGETSKLCCAIKNTWSSLWLSLLVSMPFFIAMVAVVTVAMLSSFLGSLPWVGKLFHTVLIFIPYLSA
TALILLFLGSFSCLFFCIPVLHNQESIDYRKLLECFRGNILRQFIGVVIALVPLALCSWLALDSFYLMTHLVEIAD
IHTWSFLAQMFVLIVPIALILTPAVSFFFNFSFSFYLAKQEEEKALVK

>core/564/2/Org2_Gene463

MHKVIVFIFLTLYSLKSYGNDVIDKPHVLVSIAPYKFLVEQIAEETCFVYAIVTNHYDPHTYELPPQQIKELRQ
GDLWFRIGEAFEKTCERNLTCQQVDLSQNVSLIQGKPCCNQHTTNYDTHTWLSPKNLKVQVETIVTTLSKKY
PQHATLYQSNGEKLLLALDQLNEEILTITSKAKQRHILVSHGAFGYFCRDYNFSQHTIEKSSHVEPSPKDVAR
VFRDIEQYKISSVILLEYSGRRSSAMLADRFHMHTVNLDPYAENVLVNLKTIATTFSSL

>core/565/2/Org2_Gene541

MYFTRDPVIETVITSREGYKLSVRNTKHFSQDPFMVEAIEVISLGNICFFRNCDHSKPFLVPAGDYEVMEVRDT
KINLKAVGLDRGVKIAGGREALIKLTKSTPLPVIDEKPLADSPEEGTEPTSPSKKEKKEARKDSFKGEKWKEK
KKLSRRRNHKEIAEVTGASQEILDTVKEELWEESQENEIVEQKKFSLLPPPAKLISEVISQTVVDPVVTSADLN
ESLQALVRESSDLINALLSADDAIHFPETEEEPTSASFEESSAMFFPETSSATEEE

>core/566/2/Org2_Gene941

MTRSSPAQLSRFLSEIQNKPKKSLSQNFLVDQNIVKKIVATSEVIPQDWVLEIGPGFGALTEELIAAGAQVIAIE
KDPMFAPSLEELPIRLEIIDACKYPLDQLQEYKTLGKGRVVANLPYHITTPLLTKLFLEAPDFWKTVTVMVQD
EVARRIVAQPGGRDYGSLTIFLQFFADIHYAFKVSASCFYPKPQVQSAVIHMKVKETLPLSDEEIPVFFTLTRT
AFQQRRKVLANTLKGLYPKEQVEQALKELGLLLNVRPEVLSLNDYLALFHKMQAG

>core/567/2/Org2_Gene611

MESQSCKLTIKDLMSAGAHFGHQTRRWNPKMKLYIFEEKNGLYIINLAKTLQQLRNALPHIRKVIQDNKTVL
FVGTKKQAKCVIREAAIEAGEFFIAERWLGGMLTNMTTIRNSIKTLDKIEKDLSRNQAYLTKKEAALLAKRH
QKLLRNLEGIRYMKKAPGLLVVVDPSYEKIAVAEAKKLGIPVLALVDTNCDPTPIDHVIPCNDDSLKSIRLIIN
VIKENIIEAKHKLGIEIVSPVKSLEVPDLSAFESSQDDESDEENREEDLLAKKFDGEAN

>core/569/2/Org2_Gene735

MTANTFGTLDILMKHSKEDDLSRFLPKNLLVESPHPEEIPLKSLSFTMSWLPTIHPSWITIAMKEFPPEIQGQLL
AWLPEPLVQEILPLLPGISIAPHRCAPFGAFYLLDMLSKKIRPCGITEEIFLPASSANAILYYTGPVKIALINCLGL
YSIAKELKHILDKVVIERVKNALSPTEKLFLTYCQSHPMKHLETTNFLSSWTTDAELRQFVHKQGLEFLGKAL
TKENASFLWYFLRRLDVGRAYIVEQTLKTWYDHPYVDYFKSRLEQCMKVLVK

>core/570/2/Org2_Gene453

MDYKLLDSGDGNKLECFGPVTLIRPSSIAVWPKSRPELWSQAQLQYVREGERGAWKNFKRLPEEWEVAFSD
VRCLLKRTPFGHLGVFPEHMGFWPALKQAIEKHKERQVLNLFAYTGAGSIFAAKCGARVTHVDASQAAVR
WAQRNVEKNAFPERRIFWVIEDVISFLKKEIRRNKKYQVILLDPPSYGRGPDGEVFKIDKDLFPLLSLCSKLLA
DDASYFLLTSHTPGHTPEFLRAIARRSVPTLVSEAWSCGESFCGEGVGALPSGSFVQWIA

>core/571/2/Org2_Gene130

MITGVVLEKHEQRTMFSLTLLNNFTTFGLLHTPLHYNPPYPIVILLHGLASDKTGSKRSHVRLAQELTRLGIAA
LRVDLLGHGDCEGELMDFSLENYKQNIREIIEYTHSLLHIDQERLAIFGSSLGGTLALQTLPFFNKIKALAVWA
PTISGELMAAEAQKNAPEVITMSQKGAITYAGMTLNPDFYTQFLKIDIVKELMPSARNLPPILYMQGEQDLLV
SINHRTLFTEAFANQDKPITILTYPDVDHAFPFAESSALSDLTQWLKRELTSGE

>core/572/2/Org2_Gene639

MLLGFLCDCPCASWQCAAVANCYDSVFMSRPEHKPNIPYITKATRRGLRMKTLAYLASLKDARQLAYDFLK
DPGSLARLAKALIAPKEALQEGNLFFYGCSNIEDILEEMRRPHRILLLGFSYCQKPKACPEGRFNDACRYDPSH
PTCASCSIGTMMRLNARRYTTVIIPTFIDIAKHLHTLKKRYPGYQILFAVTACELSLKMFGDYASVMNLKGVG
IRLTGRICNTFKAFKLAERGVKPGVTILEEDGFEVLARILTEYSSAPFPRDFCEIH

>core/573/2/Org2_Gene952

MRRYLFMVLALCLYRAAPLEAVVIKITDAQAVLKFAREKTLVCFNIEDTVVFPKQMVGQSAWLYNRELDLK
TTLSEEQAREQAFLEWMGISFLVDYELVSANLRNVLTGLSLKRSWVLGISQRPVHLIKNTLRILRSFNIDFTSC
PAICEDGWLSHPTKDTTFDQAMAIEKNILFVGSLKNGQPMDAALEVLLSGISSPPSQIIYVDQDAERLRSIGAF
CKKANIYFIGMLYTPAKQRVESYNPKLTAIQWSQIRKNLSDEYYESLLSYVKSKG

>core/574/2/Org2_Gene992

MKVKINDQLICIPPFISARWSQIAFIESQEGENKDQGTLRLHLIDGKIISIPNLDQSIIDIAFQEHLLYLETSQSGKE
DSRDDDKLGVGVLMNVLQQITKGNDIQVLPKNLISPLFSGTNPIEAILQHTPEHKDHPDAPTDVLEKMADVIR
VLSGNNATLLPRPEPHCNCMHCQIGRVMNEEDTLAVSDKDLTFRTWDIMQSGDKLYIVTNPLNPSDQFSVYL
GPPIGCTCGEPNCEHIKAVLYT

>core/575/2/Org2_Gene333

MKLLKNVLLGLFFSMSISGFSEVKVSDTFVKQDTVVEPKIRVLLSNESTTALIEAKGPYRIYGDNVLLDTAIQG
QRCVVHALYEGIRWGEFYPGLQCLKIEPVDDTASLFFNGIQYQGSLYVHRKDNHCIMVSNEVTIEDYLKSVL
SIKYLEELDKEALSACIILERTALYEKLLARNPQNFWHVKAEEEGYAGFGVTKQFYGVEEAIDWTARLVVDS
PQGLIIDAQGLLQSNVDRLAIEGFNARQILEKFYKDVDFVVIESWNEELDGEIR

>core/576/2/Org2_Gene233

MKKKLSLLVGLIFVLSSCHKEDAQNKIRIVASPTPHAELLESLQEEAKDLGIKLKILPVDDYRIPNRLLLDKQV
DANYFQHQAFLDDECERYDCKGELVVIAKVHLEPQAIYSKKHSSLERLKSQKKLTIAIPVDRTNAQRALHLL
EECGLIVCKGPANLNMTAKDVCGKENRSINILEVSAPLLVGSLPDVDAAVIPGNFAIAANLSPKKDSLCLEDL
SVSKYTNLVVIRSEDVGSPKMIKLQKLFQSPSVQHFFDTKYHGNILTMTQDNG

>core/577/2/Org2_Gene409

MVRDIQSESIGKLVFLGTGNPEGIPVPFCSCRVCQNTGIHRLRSSVLIQYQNKTLVIDAGPDFRTQMLVAGVSE
LDGVFLTHPHYDHIGGIDDLRAWYIVTQRSLPLVLSASTYRFLNKAKEYLFATPNVESSLPAVLEFTILNEDCG
QEEFQGIPYTYVSYYQKSCHVTGFRFGNLAYLTDLCSYDAKIFSYLDNVETLILSAGPSETPIPFQGHKSSHLT
VEEAKAFANHAGIKNLIITHISHCLEAERDQHPEVTFAYDGMEVLWTL

>core/578/2/Org2_Gene968

MIRRFFKTLFPPGPQYSLCYASILIVLSSLVCVPTFCWLFLPELSLSKFNPSPIRNLFLVSSTLSKVPPTAIAEHLR
LSADAPTYLHEFSIKEAESSLHALGIFSSLVIEKSPDNKGITIFYTLQTPIAYVGNRSNTLCNLEGSCFLGQPYFP
SLNLPQIFFSQEDLKMQKLPKEKMLFTKILLKELAMESPKIIDLSLSDAYPGEIIVTLSSGSLLRLPIKTLDRALD
LYKHMKKSPVIESEKQYVYDLRFPNFLLLKAL

>core/579/2/Org2_Gene636

MFNNKMILIAGPCVIEGEDITLEIAGKLQSILAPYSDRIQWFFKSSYDKANRSSLNSFRGPGLTEGLRILAKVKE
TFGVGILTDVHTPQDAYAAAEVCNILQVPAFLCRQTDLLVATAETGAIVNLKKGQFLSPWDMEGPINKVLST
GNNKILLTERGCSFGYNNLVSDMRSIPVLSRSGFPVIFDATHSVQLPGALSTESGGLTEFVPTLSRAALAAGAH
GLFIETHTNPKIAKSDAASMLSLEEFAALLPTWDQLFTCVSSFDMVSA

>core/580/2/Org2_Gene501

MTKVALLIAYQGTAYSGWQQQPNDLSIQEVIESSLKKITKTRTPLIASGRTDAGVHAYGQVAHFRAPDHPLF
ANANLTKKALNAILPKDIVIRDVALFDDNFHARYLAIAKEYRYSLSRLAKPLPWQRHFCYTPRHPFSTELMQE
GANLLIGTHDFASFANHGRDYNSTVRTIYTLDIVDKGDSLSIICRGNGFLYKMVRNLVGALLDVGKGAYPPE
HLLDILEQKNRREGPSAAPAYGLSLHHVCYSSPYNNFCCEQCSVSTSNEG

>core/582/2/Org2_Gene64

MTQYYFLSSFLPTQLPESVPLFSISDLDDLLYLNLSENDLCNYGLLKRFFDFENFAFFWAGKPIPFSFGEVTQE
NVERMLSSQQWSDDNDFEDFFKDFLMNHKSSQDRLNHFSDLFREFLSYHQTNSSKFLQDYFRFQQQLRVVL
AGFRARVLNMDVSYVLRDEDSSDPVVLEVLMQKDSPNYELPEEFSDLQGVLDDYGLLPHTLNRALALYQFH
KLEGFCSDSYFDGNVILARCATYMFAIRNSLASVEKGREIINHIEKAIKW

>core/583/2/Org2_Gene153

MHSKFLSRRKKNSSHKEETSWDCIASSYNKIVQDKGHYYHRETILPQLLPSLTLGSKSSVLDIGCGQGFLERA
LPKECRYLGIDISSRLIALAKKMRSVNSHQFKVADLSKRLEFVEPTLFSHAVAILSLQNMEFPGEAIRNTATLL
EPLGQFFIVLNHPCFRIPRASSWHYDENKKAISRHIDRYLSPMKIPIMAHPGQKDSPSTLSFHFPLSYWFKELSS
HGFLVSGLEEWTSSKTSTGKRAKAENLCRKEFPLFLMISCIKIK

>core/584/2/Org2_Gene765

MTLPLEPMIFWSSLSAKVMKKFLTPHCAGTFSEEDAEAKEAHLVTGKQGHRLMGNCVTFYWLVDKKNGVI
LDAKFQYFGHPYLIPLAEAVCNLVCGKSYSEAYKMTLDDIDKSLRVHAHQPALPEDSISLYHFVIDALDTAVE
QCLEIPLEDGSLPLQNSPMNLDFEDANPYSQSDWEALTHEQKLYALRATIAEKIGPYIAMDGGEVTVESLENF
IVTIAYSGNCSGCPSSLGSTLNSIGQLLRAYIYPELQVKVDESSLNLSHP

>core/585/2/Org2_Gene782

MQGFFPLASGSKGNSAYLGTDSCKILIDLGVSKQVVTRELLSMNIDPEDIQAIFVTHEHSDHISGIKSFVKAYN
TPIVCNLETARALCHLLDSHPEFKIFSTGSSFCFQDLEVQTFNVPHDAVDPVAFIFHYREEKLGFCTDLGWVTS
WITHELYDCDYLLIESNHSPELVRQSQRPDVYKKRVLSKLGHISNQECGQLLQKIITPKLKKLYLAHLSTECNT
AELALSTVSESIASITSIAPEIALAQGITSPIYFSRLEVACPR

>core/586/2/Org2_Gene873

MAGLDLEARGKRRVVTPNAITAFGLCCGLFIIFKSVLRTSSSVELFHRLQGLSLLLISAMIADFSDGAIARIMKA
ESAFGAQFDSLSDAVTFGIAPPLIAIKSLDGIYVGNFFSSLLLITSIIYSLCGVLRLVRYNLFSQKTVDVSKPYCFI
GLPIPAAAASIVSLALFLASDFFPDLPAQLRVGLLSFALLFIGGLMISPWKFPGVKHFRFNVSSFLLVVTIGLAA
CLFFSGLVDHFVEVFFLVSWLYTLVGFPIFSIIYRKKS

>core/587/2/Org2_Gene25

MKKPDNDSTFDVRSFFPFDVLCIEQLRKEMSWEVVSAKIPRLPRGWYELMGLSKEDRIDFCLDFWCSVLGIE
HKESPSICRFFSLLETIEVYIYRLEKEPYQLKMFYVFRDGRCGFQGEPPLLDFLGHHRLPPLGDRHYEKFFSIHN
GFGKWEDEGIFPMRSLAKVQQKLRQQLVVMNKMQAEDNCYSLGIFPFYGYEEPFAYQSFFFDPEIRRDLPSP
NVLLNEESLEHRSLETIELLHLSKSYYPSFLSWLENYLHSEEVYNE

>core/590/2/Org2_Gene452

MDCIGKHNPLVKEALALKRSRCRKSSWFLVEGAREIQKALRTGYLCQHVFCSTHLSEKEKEFLYELKRNSTK
ILYCLDSTLAQLSFKEHHDSFVAVIQKRVWNKEDFLIQRKNAQPFYLIIEQVEKPGNVGAILRIADGAGVDGVI
LCNPIVDLYNPNVVRSSLGAVFSLPILSISREEGKELFKQEGWTVFVTSPRAETMYFSKNYLGPTALVFGSEKD
GLTEDWFSEDFSEIALPMLGESDSLNLATSVAAVAYEVVRQRWVN

>core/591/2/Org2_Gene931

MGSSMHVGVIGCSGRTGKVIVSALEQSSEYTLGPGFSRSSALTLFQVIAHNDVLVDFSHPLLTKEVVAHLLISP
KPLIIGTTGFPGKCKEAHDSLEELTHIVPVVVCPNASLGAYIHKRLVMLLSQLCNPQFDIRIRETHHRYKKDSL
SGTAQDLLDTIQQVKQEDWGEEYEVGQRDSSKKTIEVQSSRVGDIPGEHEVAFISSGEQILVRHTVFSRNVFG
RGILSILDWLKTLNPQPGLYSLGDTLELVLRNEHCLLKKTTDH

>core/592/2/Org2_Gene682

MEKLEFVTSLSSPDDDLITFNKQGLIAGPEEEKVAFLVRSNAMLDAGPETPASFPESLREQFDIFPEYVEVLYS
NEGLDVWEAGCTWILNNEVTIQLRKHHRKASRWLGMYSRDEVLAHEAVHAVRMKFHEPVFEEVLAYQTSR
WGWRRFFGPLFRSPGESYLLLFFTILGLGISLWYPAGILIMLVLPMYFLMRLCMAQSYLYRAMKKIRKMLGV
PPLWVLLRLTDKEIKMFAKEPIPVLEHYARKRKLENVRWKQIYQSYFV

>core/593/2/Org2_Gene439

MSNQLQPCISLGCVSYINSFPLSLQLIKRNDIRCVLAPPADLLNLLIEGKLDVALTSSLGAISHNLGYVPGFGIA
ANQRILSVNLYAAPTFFNSPQPRIAATLESRSSIGLLKVLCRHLWRIPTPHILRFITTKVLRQTPENYDGLLLIGD
AALQHPVLPGFVTYDLASGWYDLTKLPFVFALLLHSTSWKEHPLPNLAMEEALQQFESSPEEVLKEAHQHTG
LPPSLLQEYYALCQYRLGEEHYESFEKFREYYGTLYQQARL

>core/594/2/Org2_Gene357

MKLTKYLNTKQLRSMISRLFVRYSLPMSKQLSFFALCVLGSHPIFAQTPNPPQRVRRSEVIFIDPGHGGKDQGT
ASKELHYEEKSLTLSLALTVQSYLKRMGYKPQLTRSSDVYVDLGKRVALSNRGQGDVFISIHCNHSSNAAAF
GTEVYFYNGKVGSPTRNRMSEVLGKNILAAMEKNGILKSRGLKTANFVVIRDTSMPAVLVETGFLSNSRERA
ALQDARYRMHVAKGIAEGVHNFLSGPSFQKPKQNIAKIRKPQIQAN

>core/597/2/Org2_Gene77

MPFDITYYTTPLLEIILIWVMLNYLLKFFWGTRAMDVVFGLLAFLFLFVLADKLHLPIIRRLMLHVVNIAAIVV
FIIFQPEIRLALSRIRFHGKKFFIDTQEQFVEQLAASIYQLSERQIGALVVLENKDSFDEYLSFSSVKINATFSEEL
LETIFEPSSPLHDGAVILRGDILAYARVVLPLAHDTTQLSRSMGTRHRAALGASQRSDALIITVSEENGSVSLSR
DGLLTRGVKIDRFKAVLRSILSPKEHKRKPLFSWIWKR

>core/599/2/Org2_Gene167

MTTLLSIKDLSLTIRGKKILNHINLNLIKGSYLTIVGPSGSGKSSLALTILDLLKPTTGTITFHMDPKIPRARKVQ
VIWQDIDSSLNPCMSIKGIISEPLNIIGTYSKAEQNKEIYNVLDLVNLPKSVLHLKPYKLSGGQKQRIAIAKALV
SKPELLICDEPLSSLDTLNQSLILDLFQTIKKEYQNTLLFITHDMSAAYYIADTIAVMDQGSLVEHACREKIFST
PKHTTTQDLLDAIPIFSLISTEMEPSEEYELQVASK

>core/600/2/Org2_Gene920

MKTWLFFTFLFSCSSFYASCRYAEVRSIHEVAGDILYDEENFWLILDLDDTLLQGGEALSHSIWKSKAIQGLQ
KQGTPEQEAWEAVVPFWIEIQEMGTVQPIESAIFLLIEKIQKQGKTTFVYTERPKTAKDLTLKQLHMLNVSLE
DTAPQPQAPLPKNLLYTSGILFSGDYHKGPGLDLFLEICTPLPAKIIYIDNQKENVLRIGDLCQKYGIAYFGITY
KAQELHPPIYFDNIAQVQYNYSKKLLSNEAAALLLRHQMHE

>core/601/2/Org2_Gene695

MMKYLPYIAITACIHGGILLLVFASPLPKKRLQPKAFQEKLVTIQPKPPVPTPSVVVDPAKTIRPSVATQPQKQ
AKCSPPQENVQKALQKPIPKVIKTEPPKPSPAPTVAKKTTATEKPPPSTTKKNTQLSKTQLQTLSEVAQALSLH
VDKIEKSETSLKNISWPSTAQLTMHSELKATQEDELCELFRTHIALPSKGYVRIKLVLSPNGEIQECSFLSEVSA
ADKQLLTQRIQALPFQKFLEKYKVSKNISFHIKLVSNES

>core/602/2/Org2_Gene698

MDLADAHVHLSDDAFEEDINSVLQRAQDSGVSLVVNVTTTEKELNRSFAYAERFPKIRFCHVGGTPPQDVDQ
DIEEDYRNFHAAAHSKKLAAIGEVGLDYCFATEEGIARQKEVLQRYLALSLECELPLVVHCRGAFNDFFRML
DQYYHNDPRSRPGMLHCFTGTLEEAQELISRGWFISISGIVTFKNAQDLRDLVVELPLEHLLIETDAPFLAPVP
YRGKKNEPAHVLHTINAVANVKGMFPQELAALAYKNVLRFLHG

>core/603/2/Org2_Gene18

MFLQFFHPIVFSDQSLSFLPYLGKSSGIIEKCSNIVEHYLHLGGDTSVIITGVSGATFLSVDHALPISKSEKIIKILS
YILILPLILALFIKIVLRIILFFKYRGLILDVKKEDLKKTLTPDQENLSLPLPSPTTLKKIHALHILVRSGKTYNELI
QEGFSFTKITDLGQAPSPKQDIGFSYNSLLPNFYFHSLVSVPNISGEERALNYHKEQQEEMAVKLKTMQACSF
VFRSLHLPSMQTKDKKAGFGLLTFFPWKIYPL

>core/605/2/Org2_Gene352

MILTAAFSPCPNDIFLFRSFLKDPQFRPLLNQVTIADIETLNTLALQRRLSLMKMSAALFPLVSDYYNLMDVG
NTLGYNSGPIVLSLDPECSLDTLATPGEMTTAHALCKLYYPKAKLIPMPYDKILSAILQGKVDGGALIHEERFS
YDLQLTLRADFGELWRRKTIFPLPLGCLAIAKYVPMATVDALTAALRKSLICSLKDPITAGAKAVEYSKNKN
VTVIHRFIGTYINKETFQLSKTGKKALHMLWKANECCQYT

>core/606/2/Org2_Gene412

MIKQIGRFFRAFIFIMPLSLTSCESKIDRNRIWIVGTNATYPPFEYVDAQGEVVGFDIDLAKAISEKLGKQLEVR
EFAFDALILNLKKHRIDAILAGMSITPSRQKEIALLPYYGDEVQELMVVSKRSLETPVLPLTQYSSVAVQTGTF
QEHYLLSQPGICVRSFDSTLEVIMEVRYGKSPVAVLEPSVGRVVLKDFPNLVATRLELPPECWVLGCGLGVA
KDRPEEIQTIQQAITDLKSEGVIQSLTKKWQLSEVAYE

>core/607/2/Org2_Gene103

MLRNQVLVYCSEGVSPYYLRHTIRFLKYYSTQEGAFDILRVDGNFLIKNPFWEETTRLLVFPGGADRPYHRV
LHGLGTARIFQYVSEGGNFLGICAGAYFGSKMIYFYEPEGAPLQGARDLGFFPGTAKGPAYRGNFSYVSPSGV
RVSPQLFSDFGLGYAMFNGGCFFEGSEGYPGVNIESRYDDLPGKPASIVSRIVSKGLAVLSGPHIEYLPHYCR
MVKENVQKTREFLQRERTTLDRYCQNLVQRLRQPAFSKADC

>core/608/2/Org2_Gene295

MNVKDETFWSVHNLCVNYEHAAVLYHISFSLGKGSLTAILGPNGAGKSTLLKASLGLIKPSSGTVYFFNQKF
KKVRQRIAYMPQRASVDWDFPMTVLDLALMGCYSYKGMWGRISSDDRREAFHILERVGLESVADRQIGQLS
GGQQQRAFLARALMQKADLYLMDELFSAIDMASFKTSVGVLQELRDQGKTIVVVHHDLSHVRQLFDHVVL
LNKRLICCGPTDECLNGDTIFQTYGCEIELLEQTLKLSRGKQFGSC

>core/609/2/Org2_Gene578

MNRRWNLVLATVALALSVASCDVRSKDKDKDQGSLVEYKDNKDTNDIELSDNQKLSRTFGHLLARQLRKS
EDMFFDIAEVAKGLQAELVCKSAPLTETEYEEKMAEVQKLVFEKKSKENLSLAEKFLKENSKNAGVVEVQP
SKLQYKIIKEGAGKAISGKPSALLHYKGSFINGQVFSSSEGNNEPILLPLGQTIPGFALGMQGMKEGETRVLYI
HPDLAYGTAGQLPPNSLLIFEINLIQASADEVAAVPQEGNQGE

>core/611/2/Org2_Gene1001

MENLETFILKIYRGVPGKQYWESFELPLHPGENVISALMEIEKRPVNILGEKVNPVVWEQGCLEEVCGSCSILV
NGVPRQACTALIQEYIDATQSREIVLAPLTKFPLIRDLIVDRSIMFDNLERIQGWVAADIEGETFGPQVTQEQQ
ELLYALSQCMTCGCCTEACPQIDNKSDFIGPAAISQARYFNTYPGDKRSKKRWRALMGKGGIEGCGQAHNC
VRVCPKKLPLTESISAVGREISKFSLRSLFSALFKKKK

>core/613/2/Org2_Gene343

MSKFILLLSLGVAALASKNFFIWPAPSGKTPLKLRQVLFGGALLVFSSLVALSVSSQTAELLSTMTGISLAFAF
LFYLLFLPKDITRAILFSGERPVKTSWRALGSAIRMWIIIIPVTQLIGIMMSKFLTLVLPTQEIHTQEVTQEVQNS
LPITGHYISMILNLGVLTPFGEEVFFRGILQTFLKNKMTRIAAVLCSSIIFSFIHIEHSLGSWVFVPVLFVFSLSAG
FLYEKDRHILSPIALHGLFNLTSLLFLGIK

>core/614/2/Org2_Gene443

MAFYSPSTISKYFIYSGAGNRFLLGETLPEVEDVRFLCQETRVDGFLYLKPSSCADAQLIIFNSDGSRPTMCGN
GLRCAIAHLASQKGKSDISVSTDSGLYSGYFYSWDRVLVDMTLADWRASVHRLESRPDPLPKEVVCIHTGVP
HAVVILPEISTLDLSILGPFLRYHQTFSPDGVNVNFVQILGHCQLRVRTYERGVEGETAACGTGALASALVVS
NSYGWKESIQIHTWGGELMTVSQNRGRVYLQGSVTRDL

>core/615/2/Org2_Gene106

MFKLLKNLFLIGCCIVGYFWMRKESIVEQWLSNRLHTQVTVGRVSIRTSGIKIRHICIHNPLASERFPYAAEIEY
ADVRFSSISMLLTKQLEISELIIHGANFTIFPYDSHGTKTNWSLVWKNFHPQKETPSNLWIDRAPVLIRRCLFLN
TRLYGLRANHKDIPHLSVPSLEFHSHTSSAKELPKLSEALPSLLYLALEESLYHLNLPGDIIKPLSQQAHKHFYS
SYPQFQDRLNDINTPGTPTEEIIGFIRGLFFH

>core/616/2/Org2_Gene82

MVSPLSLFHKMLLENWTPVEEPFPWPPAEKNQKIFAWALNQSKLIFVSTSGNIAQPRLVTDSMSMMIVNAAN
RTMSRDGAGTNQVLSAAVSVDSWGLSQRPLNPERQGTPLNEGECRAGMWRNADGSNHTGKQGKPHYLAQ
LLGPKAVDHHNKSQAAFDRCKNAYLNCFSLAQTLGVTFLQIPLISSGIYAPPENRKKPNSEENKVRMRWIHA
VKCALVAAMQEFGNEPGNTDRRMLIVLTDLKTPAITDPKKKSHL

>core/617/2/Org2_Gene306

MKTQQTQNIIEVWNFYWETQEIEYRDSLIEFYLPLVKSVVHRLISGMPSHVKTEDLYASGVEGLVRAVERYN
PERSRRFEGYAVFLIKAAIIDDLRKQDWVPRSVHQKANKLSGAMDSLRQSLGKEPTDLELCEYLNISQQELSG
WFVSARPALIVSLNEEWPSQSDEGAGMALEERIPDERAETGYDVVDKQEFSLCLANAIQELEEKERKVMALY
YYEELVLKEIGKVLGVSESRVSQIHSKALLKLRAALSAFR

>core/618/2/Org2_Gene370

MWLGAYTWLNVFGILLQAAFIQNILLANFLGMCSYLACSTRVSTANGLGMSVALVLTVTGSINWFVHAFITG
PKALTWISPSLASVNLGFLELIIFIVVIAAFTQILELLLEKVSRNLYLSLGIFLPLIAVNCAILGGVLFGITRSYPFIP
MMIFSLGAGCGWWLAIVILATIKEKLAYSDIPKNLQGMGISFITTGLIAMAFMSLTGIDISKPSAKIQRAPLETE
VVENTTNPLKESSSKHQPSISKARTQRRSL

>core/619/2/Org2_Gene606

MLKIKHLHASCNDVKILDDFNLNIQPGTMHVIMGPNGAGKSTLAKILAGDESVLVSSGEIALQEQNLLSMLPE
ERSRAGLFVGFQMPPEIPGVNNKMFLRDAYNARRANQEGDISIDEFNTLLSTVLETYEYNATTDLFLDRNV
NEGFSGGERKRNEICQMLVLEPEMVLLDEPDSGLDVDALRLICRVLEKYRELHPTSSLCIVTHNPKLGNLIRP
DVVHLLLDGRVALSGDVSLMHELEAKSYQEVTKRVAWR

>core/621/2/Org2_Gene714

MKTIAVNSFKGGTAKTSTTLHLGAALAQYHQARVLLIDFDAQANLTSGLGLDPDCYDSLAVVLQGEKEIQE
VIRPIQDTQLDLIPADTWLERIEVSGNLAADRYSHERLKYVLGSVQDKYDYVIIDTPPSLCWLTESALIAADYA
LICATPEFYSVKGLERLAGFIQGISARHPLTILGVALSFWNCRGKNNSAFAELIHKTFPGKLLNTKIRRDITVSE
AAIHGKPVFATSPSARASEDYFNLTKELLILLRDI

>core/622/2/Org2_Gene945

MTRQSYVLGNWKMHKTIQEAKEYVQTLASLLQGEPLSCTIGIASPFTSLRAIHEVINTTGAFLWLGAQNVHPE
LSGAFTGEISLPMLKEVGVEFVLVGHSERRHIFGESDAFIASKVKSVAQAGLVPVLCVGESLEVREEGKAHQV
IKKQLLLGLEQMDNGSEFLIAYEPVWAIGTGKVAEASDVQDIHMFCREVVAERFSEATAEEISILYGGSVKVD
NAQRFGQCSDVDGLLVGGASLEGQSFFEVAKNFNV

>core/623/2/Org2_Gene448

MHDALLSILAIQELDIKMIRLMRVKKEHQKELAKVQSLKSDIRRKVQEKELEMENLKTQIRDGENRIQEISEQI
NKLENQQAAVKKMDEFNALTQEMTTANKERRSLEHQLSDLMDKQAGGEDLIVSLKESLASTENSSSVIEKEI
FESIKKINEEGKALLEQRTELKHATNPELLSIYERLLNNKKDRVVVPIENRVCSGCHIVLTPQHENLVRKKDRL
IFCEHCSRILYWQESQVNAQENSTAKRRRRRAAV

>core/624/2/Org2_Gene196

MKPEESECLCIGVLPARWNSSRYPGKPLAKIHGKSLIQRTYENASQSSLLDKIVVATDDQHIIDHVTDFGGYA
VMTSPTCSNGTERTGEVARKYFPKAEIIVNIQGDEPCLNSEVVDALVQKLRSSPEAELVTPVALTTDREEILTE
KKVKCVFDSEGRALYFSRSPIPFILKKATPVYLHIGVYAFKREALFRYLQHSSTPLSDAEDLEQLRFLEHGGKI
HVCIVDAKSPSVDYPEDIAKVEQYITCLSNAYF

>core/625/2/Org2_Gene818

MLIKLWRATYEGMYTFLVGALLKLRYRMQVEGWDTLNINPKQGCLFLANHVAEVDPIILEYLFWSRFHVRP
MAVEYLFHSRVVQWFLNSVRSIPIPQLVPGKESKRSLERMNVCYEEASRALNRGESLLLYPSGRLSRTGKEEI
VNQYSAYVLLHRVMECNVVLVRVSGLWGSAFSRYKQNSTPKLGPAFKEAFRALLRRGIFFMPKRFVKITLC
QVDHLFLKQFPTKQDLNTFLASWFNQGDDNLPIEVPYA

>core/626/2/Org2_Gene510

MQICVTGVVLRSRPLGKNHTLTTLFTPEGLFTFFAKQGQTLQCDYRETLVPISLGKYTLHRNGSRLPKLTHGD
ILNAFEAIKQTYALLEASGKMIQALLASQWKEKPSHKLFSLFLNFLHRIPESSNPEFFAAIFVLKLLQYEGILDL
TPACSLCKASLPYACYRYQGHKLCKKHQHKQAISIEKEEEQILQAIIHAKQFSELLAIAEFPIAIAEKIFYLFDSL
QEEKKSERNSSEDPYHEILRLSKVVHPY

>core/627/2/Org2_Gene603

MLIVLAFRQVFFSHSRSQLDRLKNYLRLLKQNFAITLPKERTSKGHSLMLTFDFASFDFYTNIFPFLEEQKIPAV
VGVASRYIPSNAAQDLHPSHRLKPSETLAFQDEIFSNYMPFCCQNELIEMAKSPYIQLASSGFAIRNLMNNPPY
LTTEILLSRHHIETITGAKPLAFLFPFGKSDPTSRKLAADHYPYSFLLGNTINRKLKTHNIYRLDIKPMQYVCPS
LFQSSRYLKNWIKEKSKQLYLKKQLPKR

>core/628/2/Org2_Gene169

MLMDISHILEDLAYDEGILPREAIEAAIVKQMQITPYLLHILHDATQRVPEIVNDGSYQGHLYAMYLLAQFRE
SRALPLIIKLFAFEDDTPHAIAGDVLTEDLPRILASVCNDDSLIKELIETPKINPYVKAAAISGLVTLVGAGKIPR
DKVIRYFAELLNYRLEKQPSFAWDNLIAGICTLYPGELFYPISKAFDGGLVDTSFISMEDVENIIHEETVESCIH
TLCSSTELINDTLEEMEKWLEDFPIEP

>core/629/2/Org2_Gene110

MNVADLLSHLETLLSSKIFQDYGPNGLQVGDPQTPVKKIAVAVTADLETIKQAVAAEANVLIVHHGIFWKG
MPYPITGMIHKRIQLLIEHNIQLIAYHLPLDAHPTLGNNWRVALDLNWHDLKPFGSSLPYLGVQGSFSPIDIDS
FIDLLSQYYQAPLKGSALGGPSRVSSAALISGGAYRELSSAATSQVDCFITGNFDEPAWSTALESNINFLAFGH
TATEKVGPKSLAEHLKSEFPISTTFIDTANPF

>core/630/2/Org2_Gene86

MKKQGKTLFLFLFLSFLFSTAFSGLFASQTSSLRTIQENIFLAKTGDYTVLSRGSQRTFVLVKSTTPKTVWIEIIH
FPCIAHKERPSLEQASWKTVIHQLESPSQVFVVSLSSEGSQFFSLNTRTKSLEPVGKSTTVPAFLQIFDLPLSPAP
ANVIKTKGKENKPWSPKVSFEGAPLTSISVNAWQGLWPKDRGPLSETGILMYFTQPDISVFPLWVSIETPKGT
SIVRAVDIGHGATSPYVYSLPDSKTQ

>core/632/2/Org2_Gene487

MSLATNNAESKFPSLQRLPNHVAIIMDGNRRWYKKHREECGHTHTSGHYYGAKVLPNILNAVLDLGIKVLT
LYTFSTENFGRPKEEIQEIFNIFYTQLDKQLPYLMENEICLRCIGDLSKLPKGIQTKINHVSRMTASFSRLELVLA
VNYGGKDELVRAFKKLHVDILNKKISSDDLSESLISSYLDTSGLTDPDLLIRTGGEMRVSNFLLWQIAYTELYI
TDTLWPDFTPQDLFEAINVYQQRSRRGGK

>core/633/2/Org2_Gene713

MGNLKTLLESRFKKNTPTKMEALARKRMEGDPSPLAVRLSNPTLSSKEKEQLRHLLQHYNFREQIEEPDLTQ
LCTLSAEVKQIHHQSVLLHGERITKVRDLLKSYREGAFSSWLLLTYGNRQTPYNFLVYYELFTLLPEPLKIEM
EKMPRQAVYTLASRQGPQEKKEEIIRNYRGERKSELLDRIRKEFPLVETDCRKTSPVKQALAMLTKGSQILTK
CTSLSSDEQIILEKLIKKLEKVKSNLFPDTKV

>core/634/2/Org2_Gene523

MKIKFSWKVNFLICLLAVGLIFFGCSRVKREVLVGRDATWFPKQFGIYTSDTNAFLNDLVSEINYKENLNINIV
NQDWVHLFENLDDKKTQGAFTSVLPTLEMLEHYQFSDPILLTGPVLVVAQDSPYQSIEDLKGRLIGVYKFDSS
VLVAQNIPDAVISLYQHVPIALEALTSNCYDALLAPVIEVTALIETAYKGRLKIISKPLNADGLRLAILKGTNG
DLLEGFNAGLVKTRRSGKYDAIKQRYRLP

>core/635/2/Org2_Gene573

MSLLKDTVFTCLDCEMTGLDVKKDRIIEIAAVRFTFDSVISSIEFLINPERVVSAESQRVHHISNAMLRDQPKIA
EVFPQIKAFFKEGDYIVGHSVGFDLQVLAQEMERIGETFLSKYTIIDTLRLAKEYGDSPNNSLESLAVHFNVPY
DGNHRAMKDVEININIFKHLCKRFRTLEQLKQVLAKPIKMKYMPLGKHKGRCFSEIPLAYLQWASKMDFDS
DLLFSIRHEIKHRQKGTGFSQVNNPFMEL

>core/636/2/Org2_Gene251

MDITLVGKKVIVTGGSRGIGLGIVKLFLENGADVEIWGLNEERGQAVIESLTGLGGEVSFARVDVSHNGGVK
DCVQKFLDKHNKIDILVNNAGITRDNLLMRMSEDDWQSVISTNLTSLYYTCSSVIRHMIKARSGSIINVASIVA
KIGSAGQTNYAAAKAGIIAFTKSLAKEVAARNIRVNCLAPGFIETDMTSVLNDNLKAEWLKSIPLGRAGTPED
VARVALFLASQLSSYMTAQTLVVDGGLTY

>core/637/2/Org2_Gene772

MTQDPHDHFKSRTPEDHIKHVRDKHRVCKGEPHTTFKGFFYHLANNALSTGVFIFFIRTLFFLIPTNRALQVKS
LISLGVGWTFYHGCLKARKAWAYMELSHRSMLEEKNEIEENFEQEKIELRILFENQGFKDPLLQEMVEYVCS
DSTLLLDTMIREELYIRKEDLPHPLIQGGSRILGGLCGLAIFLPLVLCISYTLAGVFSALMVLVLSFLKAKILKN
DKISEMVWVLGIFITSASIISSLMKLL

>core/638/2/Org2_Gene318

MTLSFHTHPLNYWTFEEFDGLPIRHGVFSKQKDAEGTVFAAKNPEIASALQSPKYCDLHQRHGTSVRCVTPT
SPTYQPADGLCTQSPLLSLHIRHSDCQAAIFYDREHHAIANVHSGWRGLLGNIYAVTVGTMKKLFHTKPQDL
FVAIGPSIGPDYAIYPDYATLFPRSFLPFMNPKNHFDLRAIARKQLTNLGISKDRIFISDLCTYTEHDAFFSSRYL
AHHPDPNLTGQHSKNRNNVTAVLLLPRD

>core/639/2/Org2_Gene613

MAKQTRRVLFKISGEALSKDSSNRIDEMRLSRLVSELRAVRNNDIEIALVIGGGNILRGLAEQKELQINRVSAD
QMGMLATLINGMAVADALKAEDIPCLLTSTLSCPQLADLYTPQKSIEALDQGKILICTTGAGSPYLTTDTGAA
LRACELNVDVLIKATMHVDGVYDKDPRLFPDAVKYDFVSYKDFLSNQLGVMDASAISLCMDSHIPIRVFSFL
QHSLEKALFDPTIGTLVSEDVNHVCSPRH

>core/640/2/Org2_Gene62

MKFFILFILIVAQFPAFSAQPRTQVSASHSKQAKARRTSRIRSSAATNASVSRYKTRAAARKKIGKFEKKPSLSP
VQWVRYSGKNYSIQTPSLWQCIDDKTQLPEKLDVLLIGKGKGNLTPTINIAQEITSKSSKEYIEEILAYHKANE
MTLESGIFTQIQSPSGEFTIIKTEKNSSWGRVFCLQATTVIDHTAYIFTSTATLDDYAELSFTFLKVVSSFQIRGG
KEATSGDAILEKALEALQNENK

>core/641/2/Org2_Gene525

MHIYGLADLHLALGVPEKTMEVFGDPWIGYHQKICSEWQAVVHPEDIVLLPGDISWAMNLSEAHKDFAFIG
DLPGTKYMIRGNHDYWSSASTSKILQALPPSLYYLNQGFALLTPHLAVVGVRLWDSPTICVKKENFLTPSTQE
QSYTEQDEKIFLRELGRLKRAFAALPKEVTEVIVMTHYPPISSDGTPGPISEFLEADGRVSLCLFGHIHKVQRPI
DGFGNIRGIHYILVAADYVNFVPQEVM

>core/642/2/Org2_Gene672

MKITTVKTPKIYPYDDLYSILESSLPKLNERSIVVITSKIVSLCEGAVVELEKVSKDELIKQEADAYVFVEKYGI
YLTKKWGILIPSAGIDESNVEGYFVLYPRDFLLSVNTLGDWLRNFYHLEHCGIIISDSHTTPLRRGTMGLGLC
WNGFFPLYNYVGKPDCFGRALKMTYSNLLDGLSAAAVLCMGEGDEQTPIAIIEEAPKITFHSSPTTLQDMSTL
AIAEDEDLYGPLLQSMAWETPAPTS

>core/643/2/Org2_Gene340

MDFDYFGLSDIGRVRARNEDFWQVNLMSQVVAIADGVGGRLGGDIASQEAVTSLMELIDEQQSKLMGYGD
DQYKETLKKILLEVNGVVYEHGQMEEHLQGMGTTLSFIQFRKDRAWLFHVGDSRIYRIREGELRRLTEDHSL
ENQLKNRYGLPKQSDKVYSYRHILTNVLGSRPYVMPDIRNLPCEKEDLYCLCSDGLTNMVPDIDIRDILNQPA
TLEERGNALISLANTRGGDDNATVVLVRIQ

>core/644/2/Org2_Gene464

MTIRILAEGLAFRYGSKGPNIIHDVSFSVYDGDFIGIIGPNGGGKSTLTMLILGLLTPTFGSLKTFPSHSAGKQTH
SMIGWVPQHFSYDPCFPISVKDVVLSGRLSQLSWHGKYKKKDFEAVDHALDLVGLSDHHHHCFAHLSGGQI
QRVLLARALASYPEILILDEPTTNIDPDNQQRILSILKKLNRTCTILMVTHDLHHTTNYFNKVFYMNKTLTSLA
DTSTLTDQFCCHPYKNQEFSCSPH

>core/646/2/Org2_Gene905

MSYFNYQKNSVVLRSLGLLAKFFSRLLYRVFFSFREGIYLFSSLYLKYPRLFFYDLGKYVYSLRHCPYAKLGR
LPGASLLKEGNVYGETPWSVLAKICQAFDITSQDILYDLGCGLGKVCFWFSHVVRCQVIGIDNQPHFIRFSSN
MHRKLSSGFALFDTEEFKNVVLSQASYVYFYGSSFSRRLLNEIILKLSEMAPGSVVISISFPLDSFSRGKECFFT
EKSCSVRFPWGKTIAYKNIRKGS

>core/647/2/Org2_Gene216

MSTLLLNPPWMKAGKRIESLVRKALYTHTMLANHRKIVVALSGGKDSLTLLLMLKAISGRGFPDLDLHAVNI
GGKYSCGAEVNKPYLTRICDQLCIPFRTIPSPYAPETPECYPCSQARRRLLFQAAKEIGASAIAFGHHRDDLVQ
TALLNLLHKAEFAGMLPVLDMVHFGVTILRPLIFTPEFWIRKFAKENGFARVTCRCPVVSLRSKAEQSLKLLE
EVFPLARHNIALAIQEHGSSKSQKI

>core/648/2/Org2_Gene226

MTDYSFFRRKIGNIEAIECPGNPQDPIIILCHGYGSLADNLTFFPSICSFSKLRPTWIFPNGILPLENDFRGSRACF
PLNVLLLQELSRLYANGVGNLQEKYDELFDVDLETPKEALEELILNLNRPYNEIIIGGFSQGAILATHLVLTSQ
NPYAGALIFAGARLFNQGWEEGLKQCAQVPFLQSHGYEDEILPYHLGAHLNDLLLTKLNGQFVSFHGGHEIP
SVVFQKMQVTVPNWIDPARG

>core/650/2/Org2_Gene638

MPILSVCNLVKKYNKKPVTNDVSFQINPGEIVGLLGPNGAGKTTAFYLTVGLIRPDSGKIIFKNVDVTKKTMD
HRARLGIGYLAQEPTIFKELTVQDNLICILEIIYKARKQQSHLLNTLVDDLQLGSCLHKKAGTLSGGERRRLEI
ACVLALNPSVLLLDEPFANVDPLVIQNVKYLIKILAGRGIGILITDHNAKELLSIADRCYLIIDGKIFFEGSSSQM
ISNPMVKQHYLGDSFSY

>core/651/2/Org2_Gene375

MKVRIVDSGKSSAASHMAKDRDLLESLQDGELILHLYEWENPCSLTYGHFMRPEKFLLSNYADLGLDAAVR
PTGGGFVFHKGDYAFSVLMSATHPSYSSSVLENYHTVNSFVAKVLEKVFRIQGMLAPEDENSSSRDSGNFCM
AKTSKYDVLFGDKKIGGAAQRKVQQGFLHQGSLFLSGSSSEFYQRFLKPEVLEEIIEQIQIHAFFPLGLEAADE
VLQEARQQVKEAFIKLFCGEGL

>core/652/2/Org2_Gene326

MTLYLLPNTLGTRAVETLPSVIGELVHRLDGLIVESDRGGRAFLSLWKIPEVHKFPLAILSKHARLPKAWDFY
LEPIVKHGENWGLISDAGLPCIADPGASLVRRARALGIPVQAFSGPCSITLALMLSGLPSQSFTFLGYLPQSPKE
RVKSIKKAATSKEVSTSVCIETSYRNVYTFESLLDTLPSYAELCVASDLSGPSELVLTRQVQSWRTTEDLGSV
KQSITKVPTIFLFHIPN

>core/653/2/Org2_Gene494

MAGHSKWANTKHRKERADHKKGKIFSRIIKELISAVKLGGADPKSNARLRMVIQKAKENNIPNENIERNLKK
ATSAEQKNFEEVTYELYGHGGVGIIVEAMTDNKNRTASDMRIAINKRGGSLVEPGSVLYNFARKGACTVAK
SSIDEEVIFSYAIEAGAEDLDTEDEENFLVICAPSELASVKEKLISQGATCSEDRLIYLPLRLVDCDEKDGEANL
ALIDWLEQIEDVDDVYHNMS

>core/654/2/Org2_Gene575

MDISGAVKQKLLQFLGKQKKPELLATYLFYLEQALSLRPVVFVRDKIIFKTPEDAVRILEQDKKIWRETEIQIS
SEKPQVNENTKRIYICPFTGKVFADNVYANPQDAIYDWLSSCPQNMEKQGGVRIKRFLVSEDPDVIKEYAVPP
KEPIIKTVFASAITGKLFHSLPPLLEDFISSYLRPMTLEEVQNQTKFQLESSFLSLLQDALVEDKIAAFIESLADD
TAFHVYISQWVDTEE

>core/655/2/Org2_Gene863

MGLYDRDYIQDSRVQGTFASRVYGWMTAGLIVTSCVALGLYFSGLYRSLFSFWWVWCFATLGVSFFINSKI
QTLSVSAVGGLFLLYSTLEGMFFGTLLPVYAAQYGGGVIWAAFGSAALVFGLAAVYGAFTKSDLTKISKIMT
FALIGLLLVTLVFAVVSMFVSMPLIYLLICYLGLVIFVGLTAADAQAIRRISSTIGDNNTLSYKLSLMFALKMY
CNVIMVFWYLLQIFSSSGNRD

>core/656/2/Org2_Gene34

MHPPIDITAIEAKLNFTFTQPKLLEIALTHPSYKNESAVQIEDSERLEFLGDAVLGLIVTEHLFLLFPSMDEGTLS
TARASLVNAKACCRYTTMLGIGDYLLIGKGEKIQSERGRLSAYANLFESILGAVYLDGGLSPARKLTVPLLPP
REEILPLMSGNPKNLLQQFTQKQFRVLPVYQSTAVTDAQGNVSYQIQVLVNQEVWGEGNASSKKEAEKIAA
QQALDTYGNKNQNTMDV

>core/657/2/Org2_Gene989

MEPSTNKPDCKKIFDSIASKYDRTNTILSLGMHHFWNRSLIQILGSGYSLLDLCAGTGKVAKRYIAAHPQASV
TLVDFSSAMLDIAKQHLPQGSCSFIHSDINQLPLENHSYPLAAMAYGLRNLSDPHKALQEISRVLMPSGKLGIL
ELTPPKKTHPTYSAHKLYLRAVVPWIGKSVSKDPDAYSYLSKSIQQLPKDHDLEDLFSKSGFYIAKKKKLFLG
AATIWLLEKQ

>core/658/2/Org2_Gene980

MDVLIFYDTETTGTQIERDRIIEIAAYNSVTDESFLTYVNPEIPIPDEASKIHGITTDAVLSAPKFPEAYEGFRKF
CGEDSILVAHNNDGFDFPLLGKECRRHSLEPLTNRTIDSLKWAQKYRPDLPKHNLQYLRQVYGFAENQAHR
ALDDVVILHKVFTSLIGDLPPQQVLDLLQQSYHPKVFKMPFGKYKGQPLVDIPKSYFEWLENQGALDKPENK
DIKAAIALLHQPT

>core/659/2/Org2_Gene685

MQNATIDQLPVSWQEQLPLCWREQLKEEWSKPYMQQLLIFLKQEYKEHTVYPEENCVFSALRSTPFDQVRV
VILGQDPYPGKGQAHGLSFSVPEGQRLPPSLINIFRELKTDLGIENHKGCLQSWANQGILLLNTVLTVRAGEPF
SHAGKGWELFTDAIVTKLIQERTHIIFVLWGAAARKKCELLFNSKHQHAVLSSPHPSPLAAHRGFFGCSHFSKI
NYLLNKLNKPMINWKLP

>core/660/2/Org2_Gene273

MDLAVELKEGILLVDKPQGRTSFSLIRALTKLIGVKKIGHAGTLDPFATGVMVMLIGRKFTRLSDILLFEDKEY
EAIAHLGTTTDSYDCDGKVVGRSKKIPSLEEVLSAAEYFQGEIQQLPPMFSAKKVQGKKLYEYARKGLSIERH
HSTVQVHLQITKYEYPLLHFVVSCSKGTYIRSIAHELGTMLGCGAYLEQLRRLRSGRFSIDECIDGNLLDHPDF
DISPYLRDAHGNSL

>core/661/2/Org2_Gene662

MIGDKIILFVTEDLSLSSQLKDLASQRSDYQILVSPVFPTSFESVAIFCEYLLLPEQIFSPGIFPEEDLIVLFDTFQE
EAITKVLNQGATGYLLRPITAKVLDAVIRAFLRQHEVLEHSIPDTMTFGDHTFRVLNLVIESPEGSVYLTPSEA
GILKKLLINRGHLCLRKNLLAEIKGNTKEIIARNVDVHIASLRKKLGPYGSKIVTIRGVGYLFSDDDSIPLQNHD
NTAHPNEE

>core/662/2/Org2_Gene768

MTKVRLNKFLASAGVASRRKCDEIIFSGSVTVNGRVAEGPFVLVDPEDKVQVGGTSVHLTKKVYFMVHKAI
GYLCSSEKKFPGTKLVIDLFAHLPYRVFTVGRLDKETSGLILVTNDGEFANKIIHPSSGITKEYLLKVSRDVSA
KDLGKLMEGTFIDGKHVRPVSVTKIRRGTVKIVVSEGKKHEIRLFADAAGFPILELKRIRIGSLVLGGLRYGEY
RELTDAELGTYMKLSD

>core/663/2/Org2_Gene193

MLQAHRLCYSCDNQVILKDASFQASPGTITIILGSSGVGKTTLFRLLAGFLPLQEGELLWNGSPLNRKDVAYM
QQKEALLPWRTALKNMTLSTELGINTSHNALSNERLEEIIHNFDLGQLLDRYPDELSGGQRQRIALAAQCLSL
KPILLLDEPFSSLDVLLKEQLYQDIVALAKKENKTVLLVTHDFHDVSCLGDVLYVIKNKTLTPVPLDPSMRPL
NNGLCFIKDLKKHLYT

>core/664/2/Org2_Gene893

MRVIFPDKHNNFPNLSKLLKKLPSVILVTSCIAPFFSYIINKFFGIPGLLEILALSVKGIQKHHFWQFLTYPLITAD
SLSLNKDQSFEITQRLLLRNVLDFFLFYKAIQHLIRKLGAFSVLVVISGQALIIGAVLWGFMALIHSSQSFFGPE
SIICGVLTVQIFLDPEKRFTIGPTPLSVSIKWGFLFVLGFYCCILIFSGAFLLLLASMLAIVLAILFCKKEKIPNPYT
TSLRF

>core/665/2/Org2_Gene536

MPTTNCIFLDLRGHSILHQLQIEEALLRVANQNFCIINSGAKDSIVLGISRNLNQDVHISRAQADHIPIIRRYSGG
GTVFIDSNTLMVSWIMNSSEASAQPQELLAWTYGIYSPLLPNTFSIRENDYVLGHKKIGGNAQYIQRHRWVH
HTTFLWDIDLDKLSYYLPIPQQQPTYRNQRSHEEFLTTLRPWFPSRDDFLERIKASGSLLFTWEEFLDNELEEIL
AQPHRKATTVLN

>core/666/2/Org2_Gene32

MLSVCYSDPCLSDFCQGKRPLRIASRNSNLAKAQVHECISLLRSWYPKLWFQLSTTETTGDREKKIPLHLVEN
SYFFTDGVDALVHKGVCDLAIHSAKDLPETPSLPVVAITRCLHPADLLVYADHYVHEPLPLSPRLGSSSLRRS
AVLKQLFPQGQILDIRGTIEERLDQLHRGHYDAIVLAKAASLRLHLHHAYSIELPPPYHALQGSLAITAKDHA
GKWKQLFTPIHCHSS

>core/667/2/Org2_Gene734

MKFFSLIFKDDDVSPNKKVLSPEAFSAFLDAKELLEKTKADSEAYVAETEQKCAQIRQEAKDQGFKEGSESW
SKQIAFLEEETKNLRIRVREALVPLAIASVRKIIGKELELHPETIVSIISQALKELTQNKHIIISVNPKDLPLVEKSR
PELKNIVEYADSLILTAKPDVTPGGCIIETEAGIINAQLDVQLDALEKAFSTILKAKNPVDEPSETSSSTDSSSLS
NDQDKKE

>core/668/2/Org2_Gene801

MNRRDMVITAVVVNAILLVALFVTSKRIGVKDYDEGFRNFASSKVTQAVVSEEKVIEKPVVAEVPSRPIAKET
LAAQFIESKPVIVTTPPVPVVSETPEVPTVAVPPQPVRETVKEEQAPYATVVVKKGDFLERIARANHTTVAKL
MQINDLTTTQLKIGQVIKVPTSQDVSNEKTPQTQTANPENYYIVQEGDSPWTIALRNHIRLDDLLKMNDLDEY
KARRLKPGDQLRIR

>core/669/2/Org2_Gene1019

MFFIVCFGFLIHKKHTILPPKAHIPTNAKHFPTIGNPYAPINITVFEEPSCSACAEFTTEVFPLLKKHYIDTGEISF
TLIPVCFIRGSKPAAQALLCIYHHDPRQADIDAYMEYFHRILTYPKEEGSHWATPEVLTKLAEGLKINSGRSVN
PKGLEQCIASGQYNEQIKKNNLYGSQVLGGQLATPTAVVGDYLIEDPTFHEIERAIQHIRQLQAVEGDHDD

>core/670/2/Org2_Gene697

MVHFSHNPIIQAYTEADFFGKSIFFCLLILSVCTWTVLHQKLAIQKNFLKAGKSLKDFLIKNRHAPLSLDIHPEL
SPFADLYFTIKRGTLELLDKNRQSAPDRGPILSSEDIQSLETLLGAIMPKYKALLHKNSFIPATTISLAPFLGLLG
TVWGILVAFTHISSGSSGNSAIMEGLATALGTTIIGLFVAIPSLIAFNYLKAHSSELISEIEQTAYLLLLNSIEVKYR
NTNL

>core/671/2/Org2_Gene56

MTKHGKRIRGILKNYDFSKSYSLREAIDILKQCPPVRFDQTVDVSIKLGIDPKKSDQQIRGAVFLPNGTGKTLR
ILVFASGNKVKEAVEAGADFMGSDDLVEKIKSGWLEFDVAVATPDMMREVGKLGKVLGPRNLMPTPKTGT
VTTDVAKAISELRKGKIEFKADRAGVCNVGVGKLSFESSQIKENIEALSSALIKAKPPAAKGQYLVSFTISSTM
GPGISIDTRELMAS

>core/672/2/Org2_Gene113

MEKDLHLHEKKCLAHEAATQVTSGMILGLGSGSTAKEFIFALAHRIQTESLAVHAIASSQNSYALAKQLAIPL
LNPEKFSSLDLTVDGADEVDPQLRMIKGGGGAIFREKILLRAAKRSIILVDESKLVPVLGKFRVPLEISRFGRSA
IIEEIRHLGYEGEWRLQDTGDLFITDSSNYIYDIFSPNSYPNPEKDLLKLIQIHGVIEVGFVIEKVEVWSSNSQGL
ISKKYSV

>core/673/2/Org2_Gene195

MLQSCKKALLSIVVSILAFHPIPGMGVEAKSGFLGKVKGWFSKKEIQEEARILPVKDSLSWKRYDYTSSSGFS
VEFPGEPDHSGQIVEVPQSEITIRYDTYVTETHPDNTVYVVSVWEYPEKVDISRPELNLQEGFSGMMQALPES
QVLFMQARQIQGHKALEFWIVCEDVYFRGMLISVNHTLYQVFMVYKNKNPQALDKEYEAFSQSFKITKIREP
RTIPSSVKKKVSL

>core/674/2/Org2_Gene221

MALDEINNQNNPSQQIASSTSQTSKINQDRKTFACTVTLLVVATLMILSGIVLLFTIGSLGLSVPLSGILGTFAV
TVGAVLFITGLTILVRKSLGIEQKNEDLNFLKIKTPTPPARPLMSKFSVTCSTTSIVLGMALLIGAVVSVFFLTG
YLQLGLCAGLVGLGTALFVAGLARMSPRSLADQEGSGSADSQSNIVGIGEPKAAQEQKWYKMAVVRGEDGI
PTAIRLTPEK

>core/675/2/Org2_Gene720

MSKPSPRNANQPQKPSASFNKKTRSRLAELAAQKKAKADDLEQVHPVPTEEEIKKALGNIFEGLSNGLDLQQI
LGLSDYLLEEIYTVAYTFYSQGKYNEAVGLFQLLAAAQPQNYKYMLGLSSCYHQLHLYNEAAFGFFLAFDA
QPDNPIPPYYIADSLLKLQQPEESNNFLDVTMDICGNNPEFKILKERCQIMKQSIEKQMAGETKKAPTKKPAG
KSKTTTNKKSGKKR

>core/676/2/Org2_Gene502

MYLEDYDVFFFDLDGLLVDTEPCFYRAFLQACAEFSLEVHWDFSTYYSHTTLGTEIFSKKFIEQYPQAQEYM
AEIFAKRLQIYYKSLEHAGPALMPGVEAFIELVLSLNKTFGVVTNSPRDATHTLRTMYPILNKFLFWVTRENY
ARPKPYGDSYDYAYRTFAREGMKVIGFEDSVKGLRALSKIPATLVCINSMAEITPEDYPELKGKEFFSYPSFD
VLTEHCSQQKLL

>core/677/2/Org2_Gene930

MNTSHRKTLVFSYLSSTFTLLLVLSNLVLSSKLIPTTFFNFIIPGGLILYPLTFLISDVVNEIFGPKKARVMIFSAFI
ANLLASSIVQIFMFFPVASPEMQTAWHCLFDLSPLRFLASLLAFIVSQQLDIVLYTFFKNRTPNSSLWLRSNGS
TWISQIPDTFIVDTCILYFGMGLSFPQTLNIMFYSYIYKITFCVLTTPLFYLAVNTIRKFLGMPSTKIANTVPLIN
QP

>core/678/2/Org2_Gene151

MKKQESVLVGPSIMGADLTCLGVEAKKLEQAGSDFIHIDIMDGHFVPNLTFGPGIIAAINRSTDLFLEVHAMIY
NPFEFIESFVRSGADRIIVHFEASEDIKELLSYIKKCGVQAGLAFSPDTSIEFLPSFLPFCDVVVLMSVYPGFTGQ
SFLPNTIEKIAFARHAIKTLGLKDSCLIEVDGGIDQQSAPLCRDAGADILVTASYLFEADSLAMEDKILLLRGEN
YGVK

>core/679/2/Org2_Gene179

MKGFLSVNELIFGFQTFSVVVLGVFFASRGKAWLTGWLSLLSSIMNVFVLKQIHLWGFEVTSADVYVIGLLT
CLNYAREHYEKNDINDAMLCSWVISIAFLVLTQLHLFLIPSPNDSSQEHFLALFSSTPRIVVASLVTLIFVQIVDI
KLFTFLQRVFSKKYFAMRSTISLLFSQLIDTIIFSFLGLYGLVSNLCDVMIFAMLVKGIVITLAIPTLTVTKAVLD
RRSS

>core/680/2/Org2_Gene508

MRPHRKHVSSKSLALKQSASTHVEITTKAFRLSMPLKQLILEKSDHLPPMETIRVVLTSHKDKLGTEVHVVAS
HGKEILQTKVHNANPYTAVINAFKKIRTMANKHSNKRKDRTKHDLGLAAKEERIAIQEEQEDRLSNEWLPVE
GLDAWDSLKTLGYVPASAKKKISKKKMSIRMLSQDEAIRQLESAAENFLIFLNEQEHKIQCIYKKHDGNYVLI
EPSLKPGFCI

>core/681/2/Org2_Gene767

MALLILLRHGQSVWNEKNLFSGWVDIPLSQQGIEEAFSAGRAIQNLPIDCIFTSTLVRSLMTALLAMTNHHSK
KIPYIVHEDPKAKEMSRIYSAEEENNMIPLYQSSALNERMYGELQGKNKKQTAEQFGEERVKLWRRSYKTAP
PQGESLYDTKQRTLPYFEKNILPQLQNGKNVFVSAHGNSLRSLIMDLEKLSEEEVLSLELPTGKPVVYQWKN
HKIEKHPEFFG

>core/682/2/Org2_Gene192

MSSQPLVTTSSSLSRYVVLTGEEKVACYKKAFNHIWHGAPAIILAAALLMFCIFGFVLGSILLGAPLEGASILY
DVILPWLLPSILVFVLLVLPLNIYAYSHHKQVLALHERITQSNYKEIYDHCEKEKKTPNKKALSLYIESQVLVP
EYSKRFSSMILGKTLKIIPKKDSPESLKHDELIQKALERAKENIYMNKNQREKRDEREAKKEAKNASKTNPLW
EGLGT

>core/683/2/Org2_Gene208

MSLLIEAKNLSKTIQQQNQNISILTDVSLSLHAGETISITGASGNGKTTLLHLLGTLDVPSSGSLRFFDKDLKNQ
DLANFRNQHIGFVFQNFYLLEDDTVLKNVLMPALIARKNISKGSPVYTRALELLDLVNLEDKVRTRCSKLSG
GEKQRVAIARALINEPAILLADEPSGNLDEETSEQIHNLLLEQASALCGILIVTHNKHLASRCSREGVLSNGKLF
FHNS

>core/684/2/Org2_Gene157

MTIRVRNLAYSVNKKKILDGVTFSLERGHITLFVGKSGSGKTMILRALAGLVQPTQGDIWIEGEAPALVFQQP
ELFSHMTVLGNCTHPQIHIKGRSTEEAREKAFELLHLLDIEEVAKNYPDQLSGGQKQRVAIVRSLCMDKHTLL
FDEPTSALDPFATASFRHLLETLRDQELTVGLTTHDMQFVHSCLDRIYLIDQGTVAGVYDKRDGELDSGHPLS
KYIHSAQ

>core/685/2/Org2_Gene41

MDLKLDEVASLLDVSEHTVLQWLKEGAIPSYSMNNEYRFSREEIENWLLHNQALMIQERGEDKEALKDLSL
KYSLYKAIHRGGVLCDVVVHSKEEALQYASKYIAQKFQLDESVLFEMLSHRENLMSTGIGEGIALPHAKDFLI
NAYYDIVVPMFLAEPIEYGALDGKPVGILFFLFACQDKSHLNLVNKIVHLGMSLNARSFFKNYPNKDQLLAY
VKEWESQTH

>core/686/2/Org2_Gene503

MILRISTVSLLTSCSFSKNSRTCFVTPERITSQKDCPVLLHPKSTTISPPLYDWISPNREVITAYSFYCRGQGNSII
TPEGVLYDCDGLHHSITKEEFRYIHPRLIEVVRLLQQDHPKVSIIEAFCCPKHFHFLEASGISLSQLHLQGTAAT
FALDPPLPMEKLLATIKKLYKKNSDPSLSNFIVTEATLTNPELRLTQQDLGSHTEITVEILDNLQNKEALSSA

>core/687/2/Org2_Gene822

MENSQNFHDTLCQLLDRYSEELYPTLASLLNVTLPNTAISASVSSIPEKAVEVPNAEPQPITPPPPTNLSQEKTK
PSDWKCVPLHPDLSQNAILKEKYPALKDCSLPAPKIPCSIFVYEENNEEVLFFNRLAKILTQQLFPTKLTLIHAK
TNIFVNNPNFFLALAPLNVIRYKIPTTDYHQSLTQNGCIFLPLYSSLEYEKDSQLKRNLWAILNRLPFAYTPKSS

>core/688/2/Org2_Gene876

MKPQDLSPPFLWKERRPCIQDGVLYVPRHYFEHQNFSTSYHQEFFQNHTSIACELCSGNGDWVVAQAQKDP
QVLWIAVEQRFDRVRKIWSKMINHQIQNLRIVCGTAETFFQYYVPDQFLQRLVVNFPDPWPKMRHRKHRLL
QPSFVQEISRSLQDSAVFALATDDKTYLLESIEALQTHLAPRMETPYYIKMTDTYGNSWFENLWRTKGQEIFY
TEFIKKAGI

>core/689/2/Org2_Gene623

MIDPVECFPNLDGDAEAQSITQNSGTPLASELKKDISPFALGSYAAPKDTTLVQGFKPNPMAMMQDQNSNLI
DPELQEALESEELQEQINNLKGRLWDFRSTFEDSQTTAQFADEHFQAVGVIIDLINEDLNTIAEHTQQDARKE
DKEEGSVTRKIIDWVSSGEEVLNRALLYFSDRDGNRESLANFLKVQYAVQRATQRAELFASIVGTSVSSVKTI
MTTQLG

>core/690/2/Org2_Gene673

MTSWIELLDKQIEDQHMLKHEFYQRWSEGKLEKQQLQAYAKDYYLHIKAFPCYLSALHARCDDLQIRRQIL
ENLMDEEAGNPNHIDLWRQFALSLGVSEEELANHEFSQAAQDMVATFRRLCDMPQLAVGLGALYTYEIQIP
QVCVEKIRGLKEYFGVSARGYAYFTVHQEADIKHASEEKEMLQTLVGRENPDAVLQGSQEVLDTLWNFLSS
FINSTEPCSCK

>core/691/2/Org2_Gene564

MVLLSKFDFSGNKIGEVEVADSLFADEGDGLQLIKDYIVAIRANKRQWSACTRNRSEVSHSTKKPFKQKGTG
NARQGCLASPQFRGGGIVFGPKPKFNQHVRINRKERKAAIRLLLAQKIQTNKLTVVDDTVFVDALTAPKTQS
ALRFLKDCNVECRSILFIDHLDHVEKNENLRLSLRNLTAVKGFVYGININGYDLASAHNIVISKKALQELVERL
VSETKD

>core/692/2/Org2_Gene598

MQTLARLFGQSPFAPLQAHLEMVVSCVEYMLPIFTALRDGRYEELLEMAKLVSDKEYQADCIKNDMRNHLP
AGLFMPISRAGILEIISIQDSIADTAEDVAILLTIRRLNFYPSMETLFFRFLEKNLEAFELTMTLLHEFNQLLESSF
GGRKADKARLLVGRVAKSEHESDVLQRELMQIFFSDDFIIPEKEFYLWLQVIRRTAGISDSSEKLAHRINMTLE
EK

>core/693/2/Org2_Gene925

MSTTTVKHFIHTASRWEPVLKEIVASNYWHAQWINTLSFLENSGAKKISASEHPTEVKEEVLKHAAEEFRHG
HYLKTQISRISETSLPDYTSKNLLGGLLTKYYLHLLDLRTCRVLENEYSLSGQTLKTAAYILVTYAIELRASEL
YPLYHDILKEAQSKITVKSIILEEQGHLQEMERELKDLPHGEELLGYACQFEGELCLQFVERLEQMIFDPSSTF
TKF

>core/694/2/Org2_Gene187

MTKNAINSQTTTPQPNLTDAEPIASRAQCKSIAVIISLFALGMLLLCLGIILISIPIPGLAAQVALGLGIVSLILGIA
LANIGFLCLLLRCKQVPQKPDTLPSESSKQPSEGSTPTALPWQAGEFLEKVQVSATPILLPKNKDEELSAKVM
KEGAEAASSIKQAVLESTEKLIDARKQEESRREARKKIVAEEAEASRKRIQQQMAADQEALRKRKEEVAKRK

>core/695/2/Org2_Gene432

MKKYFITGLVILLPLAITIAIVTMIMNFLTQPFVGLASEFFEKFSFYTKHRALLKFVLQIILLFGLFFATVLLGFL
TRIMIFKSLLSIYDKILHRIPIIKTVYKAAQQVMTTIFGSKSGSFKQVVMVPFPNANVQCIGLVAGDAPTVCCT
GEKEDDPLVTVFIPTTPNPTSGFLTLFRKSDIVFLDMKIEDAFKYIISCGVLSTPMACPSSPLPDELHQDQGS

>core/696/2/Org2_Gene559

MGQKGCPIGFRTGVTKRWRSLWYGNKQEFGKFLIEDVRIRQFLRKKPSCQGAAGFVVRRMSGKIEVTIQTAR
PGLVIGKKGAEVDLLKEELRALTGKEVWLEIAEIKRPELNAKLVADNIARQIERRVSFRRAMKKAMQSVMD
AGAVGVKIQVSGRLAGAEIARSEWYKNGRVPLHTLRADIDYATACAETTYGIIGIKVWINLGENSSSTTPNNP
AAPSAAA

>core/697/2/Org2_Gene445

MTLYLGLNQKTARKYQAHYLPILTLFPYAKSTPQNKRALQFLPQATHVILTSPSSTHLFLSRMTSLLSKATLK
TKTYLCIGESTKERLLSFLGQVKYVVATQEIAEGIFPLLQALPSSARILYPHSSLARPVIREFLYNRFTFFSYPHY
TVKPRKLKKNILSKYKKIIFTSPSTVRAFAKIFPRFPEKTYWCQGRMTLQEFQKFSSQKQVSLLETLGKSRTSP

>core/698/2/Org2_Gene649

MLMMLMMIIGITGGSGAGKTTLTQNIKEIFGEDVSVICQDNYYKDRSHYTPEERANLIWDHPDAFDNDLLISD
IKRLKNNEIVQAPVFDFVLGNRSKTEIETIYPSKVILVEGILVFENQELRDLMDIRIFVDTDADERILRRMVRDV
QEQGDSVDCIMSRYLSMVKPMHEKFIEPTRKYADIIVHGNYRQNVVTNILSQKIKNHLENALESDETYYMVN
SK

>core/699/2/Org2_Gene234

MQSDLIQILLKETVNTLYMVSTAFFFSCAIGGMLGLGLFCTSPKSLNPKKSLYATISMILSFLTAIPFAILIVILFP
ITRWIVGTSLGPTASIVPLTIGAIPFVVTIVVDAFRNSALNYLESAVALGIPKRNILFGILLPESYPQLIFSLKSLV
VHLISCSTLAGFVGGGGLGQLLLQYGYYRFEWSVTTSVLVITLVLIESVRILGDFWGRRVLKYRGIL

>core/700/2/Org2_Gene781

MSTVTTEPCSSIHISLNNDWRDSQPYSLDRASELLHFRFLPSLVFSNWKVEQQIETLCHKSEKRRLISPLAKWL
GKLHKQDLLCPPAPPVSVCWINAHVGYGVFARDEIAPWTYIGEYTGILRHRQAIWMDENDYCFRYPMPLFTL
RYFTIDSGKQGNVTRFINHSEQPNAEAIGVFSEGLFHVIIRTVAPIYAGQEICYHYGPLYWKHRKKREEFIPEEE

>core/701/2/Org2_Gene565

MRSHISVMGKKEGMIHIFDKDGSLVACSVIRVEPNVVTQIKTKESDGYFSLQIGAEEMNAPAHTITKRVSKPK
LGHLRKAGGRVFRFLKEVRGSEEALNGVSLGDAFGLEVFEDVSSVDVRGISKGKGFQGVMKKFGFRGGPGS
HGSGFHRHAGSIGMRSTPGRCFPGSKRPSHMGAENVTVKNLEVIKVDLEKKVLLVKGAIPGARGSIVIVKHSS
RT

>core/702/2/Org2_Gene927

MQRIIIVGIDTGVGKTIVSAILARALNAEYWKPIQAGNLENSDSNIVHELSGAYCHPEAYRLHKPLSPHKAAQI
DNVSIEESHICAPKTTSNLIIETSGGFLSPCTSKRLQGDVFSSWSCSWILVSQAYLGSINHTCLTVEAMRSRNLN
ILGMVVNGYPEDEEHWLTQEIKLPIIGTLAKEKEITKTIISCYAEQWKEVWTSNHQGIQGVSGTPSLNLH

>core/703/2/Org2_Gene353

MNAVNTPKKILCIVADYREISPLIEQLDFTQINEHLYSYRCTDYHLDLYIVHVWGSTAVLNALQSYCQAYTDY
DLWINPGFVGACSPEIPLGQCYTIEKIANLTTDTPPVLSEDPPYIFDALPDSLPKSSLVTSPVLYHYGFHKTFKL
LDMEGYAIASQAAEHHIPCSFLKITSDYTVPGDCPFSRLEEVSQKLTQTLVELLPELMERAIPPKLLLPCP

>core/704/2/Org2_Gene410

MLGSLPCYPGAGNIEEYKNRYFYCQLCAEVVSPYVVPVIVVDVQGAPPTGILQVLRCKQHKFQGLPVHGPIT
SLWALEPVGKGAPQLESAMYELCSQVRNFDICSIVSWVFGGLCIFAGLIVGVMVEAPLIAGLSAWVIPCIIGGV
GAILCLFAILMAYLGRGRVREWLNLSHEYITQCHCRQIQAHSQNYSVITEYPATCALSQPITKLPNGSRRDN

>core/705/2/Org2_Gene158

MDHWLAIARLLLRGCGYTLCVSGIGILCGSILGLLIGTVTSLYFPSKLTKLLANSYVTVIRGTPLFIQILIIYFGLP
EVLPIEPTPLVAGIIALSMNSAAYLAENIRGGINSLSIGQWESAMVLGYKKYQIFVYIIYPQVFKNILPSLTNEFV
SLIKESSILMVVGVPELTKVTKDIVSRELNPMEMYLICAGLYFLMTTSFSCISRLSEKRRSYDN

>core/706/2/Org2_Gene778

MKRVIYKTIFCGLTLLTSLSSCSLDPKGYNLETKNSRDLNQESVILKENRETPSLVKRLSRRSRRLFARRDQTQ
KDTLQVQANFKTYAEKISEQDERDLSFVVSSAAEKSSISLALSQGEIKDALYRIREVHPLALIEALAENPALIEG
MKKMQGRDWIWNLFLTQLSEVFSQAWSQGVISEEDIAAFASTLGLDSGTVASIVQGERWPELVDIVIT

>core/707/2/Org2_Gene602

MQKLVHNIWKKFYSFSSAIAICIVLASFLSLKIVSNTYKHSQAKRNSILLLTRAAEVAVSQGFLPSKSALSSLEQ
AYHLGGESMKPYAGFLASCFYIHNEPLRGAYYAGLAYNNSQALQLPHPIQKLLKEISEAQADQLYDVALSKS
YQLLQTANSSPEYPTLSFLTLLRVIELKELLHQDVSQDFAALKSSPLFHQFERMYSDGEWTLSKRFGKKG

>core/708/2/Org2_Gene489

MIITIDGPSGTGKSTTAKALADHLHFNYCNTGKMYRTLAYARLQSPWATLPLTKFLEEPPFSFTFATGQPLESF
FNGHLLTSELTTQEVANAASELSQLPEVRAFMQDLQRRYAQLGNCVFEGRDMGSKVFPNADLKIFLTSSPEV
RAQRRLKDLPEGTLSPEQLQAELVKRDAADAQRAHDPLVIPENGIVIDSSDLTIRQVLEKILALLFRNEL

>core/709/2/Org2_Gene342

MRKMLVLLASLGLLSPTLSSCTHLGSSGSYHPKLYTSGSKTKGVIAMLPVFHRPGKSLEPLPWNLQGEFTEEI
SKRFYASEKVFLIKHNASPQTVSQFYAPIANRLPETIIEQFLPAEFIVATELLEQKTGKEAGVDSVTASVRVRVF
DIRHHKIALIYQEIIECSQPLTTLVNDYHRYGWNSKHFDSTPMGLMHSRLFREVVARVEGYVCANYS

>core/710/2/Org2_Gene815

MSKKPLYVAHPWHSPTLTQDNYESLCCYIEITPYDSVKFELDKATGLLKVDRPQKFSNFCPCLYGLLPQTYC
GTASGNYSGEQTRREGIQGDKDPLDVCVLTEKNIHHGNILLQARPIGGLRIIDSGEADDKIIAVLEDDLVFAEIE
DISDCPGTVLDMIQHYFLTYKATPNHLIKGSPAKIEIVGIYGKKEAQKVIQLAHEDYLSYIGDTAEVN

>core/711/2/Org2_Gene595

MSVNPSGNSKNDLWITGAHDQHPDVKESGVTSANLGSHRVTASGGRQGLLARIKEAVTGFFSRMSFFRSGAP
RGSQQPSAPSADTVRSPLPGGDARATEGAGRNLIKKGYQPGMKVTIPQVPGGGAQRSSGSTTLKPTRPAPPPP
KTGGTNAKRPATHGKGPAPQPPKTGGTNAKRAATHGKGPAPQPPKGILKQPGQSGTSGKKRVSWSDED

>core/712/2/Org2_Gene94

MNTSISEIQRFLSMIAFEKELVSEDFSVVAGIDEAGRGPLAGPVVASACILPKGKVFPGVNDSKKLSPKQRAQV
RDALMQDPEVCFGIGVISVERIDQVNILEATKEAMLQAISSLPISPDILLVDGLYLPHDIPCKKIIQGDAKSASIA
AASILAKEHRDDLMLQLHRLYPEYGFDRHKGYGTSLHVEAIRRYGPSPCHRKSFSPIKQMCAIV

>core/713/2/Org2_Gene204

MTKGSVFIIMGPPGSGKGTQSQYLANRIGLPHISTGDLLRAIIREGTPNGLKAKAYLDKGAFVPSDFVWEILKE
KLQSQACSKGCIIDGFPRTLDQAHLLDSFLMDVHSNYTVIFLEISEDEILKRVCSRFLCPSCSRIYNTSQGHTEC
PDCHVPLIRRSDDTPEIIKERLTKYQERTAPVIAYYDSLGKLCRVSSENKEDLVFEDILKCIYK

>core/714/2/Org2_Gene369

MTSKKSYKSYFFDPLWSNNQILIAILGICSALAVTTTVQTAITMGIAVSIVTGCSSFFVSLLRKFTPDSVRMITQ
LIIISLFVIVIDQFLKAFFFDISKTLSVFVGLIITNCIVMGRSESLARHVTPIPAFLDGFASGLGYGWVLLVIGVIRE
LFGFGTLMGFRIIPQFVYASETHPDGYQNLSLMVLAPSAFFLLGIMIWLVNIRDSKKRKR

>core/715/2/Org2_Gene19

MYFYKYVIIDTSGYYPFLACVDNQQVLEHWSLPVGPDLGIVLEFLFKSKNLSFQGVAVALGPGNFSATRIGIS
FAQGLAMAKNVPLLGYSSLEGYLLSKDEKKALMLPLGKRGGVLTLSSEIPEEGLNEKRRGVGPGALLSYEEA
SDYCVAHGYYHVISPNPQLFASSFSDKITVEEVAPSVEQIRRHVISQFMFVEYDKQLSPDYRSYSCIF

>core/716/2/Org2_Gene378

MATSVAPSPVPESSPLSHATEVLNLPNAYITQPHPIPAAPWETFRSKLSTKHTLCFALTLLLTLGGTISAGYAG
YTGNWIICGIGLGIIVLTLILALLLAIPLKNKQTGTKLIDEISQDISSIGSGFVQRYGLMFSTIKSVHLPELTTQNQ
EKTRILNEIEAKKESIQNLELKITECQNKLAQKQPKRKSSQKSFMRSIKHLSKNPVILFDC

>core/717/2/Org2_Gene490

MIFRICKFFTWVAFSLFYKLKVYGVKKNFIKGPAIIAVNHNSFLDPIALHMCVHECIYHLARASLFNIPWLWK
QWGCFPVRQDEGNSAAFKIASRLFNKRKKLVIYPEGARSPDGQLQPGKVGIGMMAAKSRVPIIPVYIRGTFEA
FNRHQKIPHVWKTITCVFGTPMYFDDIIQNPEIKNKETYQIITNQTMNKIAELKAWYESGCKGDVP

>core/718/2/Org2_Gene500

MIKSSLILLSGGQGTRFGSKIPKQYLPLNGTPLVLHSLKILSSLPQIAEVIVVCDPSYQETFQEYPVSFAIPGERR
QDSVFSGLQQVSYPWVIIHDGARPFIYPDEIHDLLETAEKIGATALASPIPYTIKQRNPVRTLDRDNLAIIHTPQ
CIKTEILREGLALAKEKQLTLVDDIEAAEIIGKPSQLVFNKHPQIKISYPEDLTIAQALL

>core/719/2/Org2_Gene527

MMNYEDAKLRGQAVAILYQIGAIKFGKHILASGEETPLYVDMRLVISSPEVLQTVATLIWRLRPSFNSSLLCG
VPYTALTLATSISLKYNIPMVLRRKELQNVDPSDAIKVEGLFTPGQTCLVINDMVSSGKSIIETAVALEENGLV
VREALVFLDRRKEACQPLGPQGIKVSSVFTVPTLIKALIAYGKLSSGDLTLANKISEILEIES

>core/720/2/Org2_Gene647

MARYCGPKNRVARRFGANIFGRSRNPLLKKPHPPGQHGMQRKKKSDYGLQLEEKQKLKACYGMIMEKQLV
KAFKEVIHKQGNVAQMFLERFECRLDNMVYRMGFAKTIFAAQQLVAHGHILVNGRRVDRRSFFLRPGMQIS
LKEKSKRLQSVKDALESKDESSLPSYISLDKTGFKGELLVSPEQDQIEAQLPLPINISVVCEFLSHRT

>core/721/2/Org2_Gene744

MKQFILRTLNALFPNPKPSLEGWSSPFQLLIAILLSGNSTDKAVNSVTPQLFAKAPDAQSILDLPPGKLYQLIAP
CGLGERKSAYIYQLSQILVRDFHGEPPNDMALLTQLPGVGRKTASVFLGIAYGKPTFPVDTHILRLAQRWKIS
EKKSPSAAEKDLARFFGHENTPKLHLQLIYYARQYCPALHHKIDNCPICSYLAKEANSTRT

>core/722/2/Org2_Gene67

MSVQVKLTKNSFRLEKQKLARLQTYLPTLKLKKALLQAEVQNAVKDAAECDKDYVQAYERIYAFAELFSIP
LCTDCVEKSFEIQSIDNDFENIAGVEVPIVREVTLFPASYSLLGTPIWLDTMLSASKELVVKKVMAEVSKERLK
ILEEELRAVSIRVNLFEKKLIPETTKILKKIAVFLSDRSITDVGQVKMAKKKIELRKARGDECV

>core/723/2/Org2_Gene63

MANLNADGKLKQICDALRLDTLKPAEDEAAALLHNAKEQAKRIIQEAQEEARKILETAEERAHQKIKQGEVA
LSQAGKRALEALKQAVENKIFRESLVEWLEHVTTDPEVSTKLIQALVQALEAQGVSGNLTAYIGKHVSPRAV
NELLGKAVTTKLRKKSVVVGSFVGGVQLKVEEKNWVLDLSSSALLEIFTRYLQKDFREMIFQGS

>core/724/2/Org2_Gene661

MTYLASSIFSPEDFLYPEIISKAHYTWDILDLMDQMLENHVFSGIHGTVESGVTLKNIEKIEIAEDAYVESGAYI
VGPCILGSQTEVRHGAYLRGNVITGSRCVVGHCTEIKNSYLGHHTKAAHFAYLGDSVLSSEVNLGAGVRCA
NFRLDGRNIYVRSTSDKSKKIDTGRRKLGAFLGKGVAIGCNVVINPGQHILPHTRIRPGQVI

>core/726/2/Org2_Gene37

MSFVPYSLPELPYDYDALEPVISSEIMILHHQKHHQIYINNLNAALKRLDAAETQQNLNELIALEPALRFNGGG
HINHSLFWETLAPIDQGGGQPPKHELLSLIERFWGTMDNFLKKLIEVAAGVQGSGWAWLGFCPAKQELVLQ
ATANQDPLEPLTGKLPLLGVDVWEHAYYLQYKNVRMDYLKAFPQIINWGHIENRFSEIISSK

>core/727/2/Org2_Gene538

MYDYIRGTLTYVHTGAIVIECQGIGYHIAITERWAIECIRALHQDFLVFTHVIFRETEHLLYGFHSREERECFRI
LISFSGIGPKLALAILNALPLKVLCSVVRSEDIRALASVSGIGKKTAEKLMVELKQKLPDLLPLDSRVETSQTHT
TSSCLEEGIQALAALGYSKIAAERMIAEAIKDLPEGSSLTDILPIALKKNFSGVNKD

>core/728/2/Org2_Gene687

MKIVIASSHGYKIRETKTFLKRLGDFDIFSLSDFPDYKLPQEQGDSITANALTKGIHAANHLGCWVIADDTML
RVPALNGLPGPLSANFAGVGAYDKDHRKKLLDLMSSLESLVDRSAYFECCVVLVSPNQEIFKTYGICEGYISH
QEKGSSGFGYDPIFVKYDYKQTFAELSEDVKNQVSHRAKALQKLAPHLQSLFEKHLLTRD

>core/729/2/Org2_Gene843

MGLPNYITFSRLFITPIFMILYLKGKWFGITPVVLPYVLLALLAISELTDAIDGYVARKFSQVTDLGKLLDPMA
DSIYRISIYLTFTQPPVNLPLLLVFIFLARDSVISTLRTVCAFRGRVVAARASGKLKAILQGVSFFLILLVMIPHSL
GLLSQNGLEIFASVTVSIIAVYSIASGIEYFWMNKNFLSQRAKTKDSEKNHESKD

>core/730/2/Org2_Gene228

MFIVIEGGEGSGKSSLAKALGDQLVAQDRKVLLTREPGGCLIGERLRDLILEPPHLELSRCCELFLFLGSRAQH
IQEVIIPALRDGYIVICERFHDSTIVYQGIAEGLGADFVADLCSKVVGPTPFLPNFVLLLDIPADIGLQRKHRQK
VFDKFEKKPLSYHNRIREGFLSLASADPSRYLVLDARESLASLIDKVMLHTQLGLCT

>core/731/2/Org2_Gene95

MNKILVDSPFSPDHQKCCPKLFTISAPAGVGKTTLVRMLEQEFSSAFAETISVTTRKPREGEVPGKDYHFVSHE
EFQRLLDRQALLEWVFLFGECYGTSMLEIERIWSLGKHAVAVIDIQGALFIRSRMPSVSIFIAPPSQEELERRLA
SRGSEEGSQRKERLEHSLIELAAANQFDYVIINDDLNQAYRVLKSIFIAEEHRNIL

>core/732/2/Org2_Gene692

MHAKLSFFILLSLLFSGIDCSRLHAAGRSPSLQGVLAEIEDISAKLASHEVEIVMLSERLDEQDSKCQKWTAAK
PETLAQKIRELESDQKALAKTLAVLTTSVKDLQTNLQSKLQEIQKDHRALAQDLRLVRRSLLALVDSSSPGA
YADFSDPVPENIYIVREGDSLSKIAKKYKLSVTELKKINKLDSDAIYAGQRLCLQRNKQ

>core/733/2/Org2_Gene328

MIGAQKKQSGKKTASRAVRKPAKKVAAKRTVKKATVRKTAVKKPAVRKTAAKKTVAKKTTAKRTVRKTV
AKKPAVKKVAAKRVVKKTVAKKTTAKRAVRKTVAKKPVARKTTVAKGSPKKAAACALACHKNHKHTSSC
KRVCSSTATRKHGSKSRVRTAHGWRHQLIKMMSR

>core/734/2/Org2_Gene247

MTSPIPFQSSGDASFLAEQPQQLPSTSESQLVTQLLTMMKHTQALSETVLQQQRDRLPTASIILQVGGAPTGG
AGAPFQPGPADDHHHPIPPPVVPAQIETEITTIRSELQLMRSTLQQSTKGARTGVLVVTAILMTISLLAIIIIILAV
LGFTGVLPQVALLMQGETNLIWAMVSGSIICFIALIGTLGLILTNKNTPLPAS

>core/735/2/Org2_Gene752

MTLVPYVVEDTGRGERAMDIYSRLLKDRIVMIGQEITEPLANTVIAQLLFLMSEDPKKDIQIFINSPGGYITAG
LAIYDTIRFLGCDVNTYCIGQAASMGALLLSAGTKGKRHALPHSRMMIHQPSGGIIGTSADIQLQAAEILTLKK
HLANILSECTGQPVEKIIEDSERDFFMGAEEAISYGLIDKVVTSAKETNKDTSST

>core/736/2/Org2_Gene530

MLKLLKVSITGDLSSGKTEACQVFQELGAYVVSADEISHSFLIPHTRIGRRVIDLLGSDVVVDGAFDAQAIAA
KVFYNSVLLQGLEAILHPEVCRIIEEQYHQSIQDGNYPLFVAEVPLLYEIHYAKWFDSVILVMANEDIRRERFM
KKTGRSSEDFDQRCSRFLNVEEKLAQADVVVENNGTKKELHQKIEEYFYALKGAL

>core/737/2/Org2_Gene899

MLILLNLSLLFYVLFDSPGSIPVFVALLKNFSRKKQQRVILRECLFALGALILFVTFGRSFFQFLDISLYAFQIIG
GFLLFTVSIKMMLAPMPEKAKDDTSKTEPIFFPLAFPVITGPAVITALLSYMEEGIYSREIIFTAMIIAWAFSLFT
LLCSSFFDRLFGNFGLLALERLFGIALLLMSVNLMLKGISIAFNIGFYIG

>core/738/2/Org2_Gene454

MFSGIIQELGEVCFFEAQGNGLSLGIKSTPLFVTPLVTGDSVAVDGVCLTLTSCNESKIFFDVIPETLACTTLGE
KRCSDQVNLEAALKMGDSIGGHLLSGHVFGTAEIFLIKENRYYFRGSKELSQYLFEKGFIAIDGISLTLVSVDS
DTFSVGLIPETLQRTTLGKKREGERVNIEIDMSTKIQVDTVKRILASSGKD

>core/739/2/Org2_Gene877

MFAYRTLLTHNVVQVSHEIFKTTVVPGDTVIDATCGNGNDSLFLARLLQGEGRLVVYDIQKEALSNALLLFE
THLSEQERSVIEMKEQSHEHILEKDVKLIHYNLGYLPKGNKEITTLARTTEISLEYALNIVRPDGLITVVCYPGH
PEGEKETHSVESLAQRLHPKEWCVSSFYVANRCRAPRLFIFQRQGSESSVDKG

>core/740/2/Org2_Gene254

MTRYPDYLSKLIFFLRKLPGIGFKTAEKLAFELISWDSEQLKILGNAFHNVASERSHCPLCFTLKESKEADCHF
CREERDNQSLCIVASPKDVFFLERSKVFKGRYHVLGSLLSPITGKHIENERLSILKSRIETLCPKEIILAIDATLEG
DATALFLKQELQHFSVNISRLALGLPIGLSFDYVDSGTLARAFSGRHSY

>core/741/2/Org2_Gene511

MGFACRYLFFFIVLFASGSFGNQLLSVPCWLSEEESFYTHRFDFSKSYPDMENMEIQAQRKKRVEFNLTGEFP
KLETLNYQGSFGHLRAKCRGVYPVLYALNFSCSSCKMDMDFRGKWNRSSTITISNQKESINLKLPKDVGVIV
NTKTSLKGNVCPGSTFIKQGWGVWNKIYHNDLVGFSEVTLIFNVSSEGGTITFS

>core/742/2/Org2_Gene586

MRLFSLGTIYLFFSLALSSCCGYSILNSPYHLSSLGKSLLQERIFIAPIKEDPHGQLCSALTYELSKRSFAISGRSS
CAGYTLKVELLNGIDKNIGFTYAPNKLGDKTHRHFIVSNEGRLSLSAKVQLINNDTQEVLIDQCVARESVDFD
FEPDLGTANAHEFALGQFEMHSEAIKSARRILSIRLAETIAQQVYYDLF

>core/743/2/Org2_Gene134

MNWVPKTIDHVDPESEIDIRKVVSCYKLIKECQPEFRSLISELLGVIRCGLRLLKRSKYQEQARTVSDEDAPLF
CLTRSYYQDGYLTPLRAGPRDLINHYIHLRRRENPKHFFSPKHPCYYARLAFNESVCVYRELFDIERLTKMYV
EGDYSKEQEKNLQAILSFVKTLDEGKDFLIEHKDTDLIGRGFTDVFCT

>core/744/2/Org2_Gene842

MRIALSLLSLLMIFPIFGEESRPGSEDGNSNTQEIVGSQDTQVCLYHSYEQGLQASRIEGKPLVIVVLCNSGDD
GQACTIGLSETCEEVLSVLSGSIFSELANFVVLVPSGVNPLIYPPIEDPILAEIVKFKELFKDESFPTGLSIIVVGV
TPEGPGDIIEVSPVSLTVEEEETLPSEQTTEVESTSELQSEDPAIA

>core/745/2/Org2_Gene477

MSSNLHPVGGTGTGAAAPESVLNIVEEIAASGSVTAGLQAITSSPGMVNLLIGWAKTKFIQPIRESKLFQSRAC
QITLLVLGILLVVAGLACMFIFHSQLGANAFWLIIPAAIGLIKLLVTSLCFDEACTSEKLMVFQKWAGVLEDQL
DDGILNNSNKIFGHVKTEGNTSRATTPVLNDGRGTPVLSPLVSKIARV

>core/746/2/Org2_Gene12

MSLPLVLGSSSPRRKFILEKFRVPFTVIPSNFDESKVSYSGDPIAYTQELAAQKAYAVSELHSPCDCIILTGDTIV
SYDGRIFTKPQDKADAIQMLKTLRNQTHDVVTSIAVLHKGKLLTGSETSQISLTMIPDHRIESYIDTVGTLNNC
GAYDVCHGGLILKKVHGCVYNVQGLPIQTLKYLLEELNIDLWDYSI

>core/747/2/Org2_Gene431

MLQEHFFLSEDVITLAQQLLGHKLITTHEGLITSGYIVETEAYRGPDDKACHAYNYRKTQRNRAMYLKGGSA
YLYRCYGMHHLLNVVTGPEDIPHAVLIRAILPDQGKELMIQRRQWRDKPPHLLTNGPGKVCQALGISLENNR
QRLNTPALYISKEKISGTLTATARIGIDYAQEYRDVPWRFLLSPEDSGKVLS

>core/748/2/Org2_Gene690

MTLSLVGKEAPDFVAQAVVNGETCTVSLKDYLGKYVVLFFYPKDFTYVCPTELHAFQDALGEFHTRGAEVI
GCSVDDIATHQQWLATKKKQGGIEGITYPLLSDEDKVISRSYHVLKPEEELSFRGVFLIDKGGIIRHLVVNDLP
LGRSIEEELRTLDALIFFETNGLVCPANWHEGERAMAPNEEGLQNYFGTID

>core/749/2/Org2_Gene365

MFRRTGKGPFEDVQTLYEEETSSPSSYSPYSRSERPETPPSLFDNPKASEARPLNHNLTEESSLPQWSSTPRTES
LLPLEEPETTLGEGVTFKGELAFERLLRIDGTFEGILVSKGKIIGPKGVVKADIQLQEAIIEGVVEGNITVSGKV
ELRGGAIIKGDIQANTLCVDEGVRILGYLAIAGITDHSERERDL

>core/750/2/Org2_Gene918

MAYGTRYPTLAFHTGGIGESDDGMPPQPFETFCYDSALLQAKIENFNIVPYTSVLPKELFGNIVPVDTCVKSFK
HGAVLEVIMAGRGAALSDGTHAIATGIGICWGKDKNGELIGGWAAEYVEFFPTWINDEIAETHAKMWLKKS
LQHELDLRSIAKHSEFQFFHNYINIKQKFGFCLTALGFLNFENAEPAKVN

>core/751/2/Org2_Gene677

MKKWISILILSFLSLLSILPVLAITINHVKISQRWSDLNSQILTLKVIRDHEDQVIKHNARISKDRNNLSIESLNAS
CKQLRPLSKERERLNKLNSNSLLAQSKEVWERKRALEKSNHQLVWNCEQMHNDFAFVRLEQATEMDNEDI
ESLFSLFNPENPVAPLVFFTCWKMTKQTTPLGNEVWLTHAEAISRWI

>core/752/2/Org2_Gene693

MNIHSLWKLCTLLALLALPACSLSPNYGWEDSCNTCHHTRRKKPSSFGFVPLYTEEDFNPNFTFGEYDSKEEK
QYKSSQVAAFRNITFATDSYTIKGEENLAILTNLVHYMKKNPKATLYIEGHTDERGAASYNLALGARRANAI
KEHLRKQGISADRLSTISYGKEHPLNSGHNELAWQQNRRTEFKIHAR

>core/753/2/Org2_Gene366

MVLFSLLFPKLCYGCQAPGAYFCSNCLEKLLVEDREGRCLHCFRYLGSSETRLCSQCSPSSQLQAFSLYLPSQ
TALSVYARACEGKRPALQFFSKSIAFELASLDETPSCIAYITSTISRKIVVEVAKLEKLLRIPLWPWLPKKRQIE
KLPKGEGICFLSAYPLSQKWMQTIVGGSASPLVSISLFLSQNDQ

>core/754/2/Org2_Gene219

MKRYVVGISGASGVILAVKLIKELVNAKHQVEVIISPSGRKTLYYELGCQSFDALFSEENLEYIHTHSIQAIESS
LASGSCPVEATIIIPCSMTTVAAISIGLADNLLRRVADVALKERRPLILVPRETPLHTIHLENLLKLSKSGATIFPP
MPMWYFKPQSVEDLENALVGKILAYLNIPSDLTKQWSNPE

>core/755/2/Org2_Gene770

MKVIYYEIEEIPSTNTMAKSYMHLWDPYALTVISTKCQTAGTGKFGKSWKSSKGDLLNTFCFFITDLHIDVSR
LFRLGTEAVVALCKDLGITEAKIKWPNDVLVHGEKLCGVLPETLPVEGLLGVVLGIGLNGNTTKQALKDVG
QPATSLQEILGHPIDLETTRELLIHHLLGVLQENLPDSLATKSNRGNI

>core/757/2/Org2_Gene444

MADGEVHKLRDIIEKELLEARRVFFSEPVTEKSASDAIKKLWYLELKDPGKPIVFVINSPGGSVDAGFAVWDQIKMLTSPVTTVVTGLAASMGSVLSLCAAPGRRFATPHSRIMIHQPSIGGPITGQATDLDIHAREILKTKARIIDVYVEATNQPRDIIEKAIDRDMWMTANEAKDFGLLDGILFSFNDL

>core/758/2/Org2_Gene47

MIKKFFIYSLIFSCSFSAPLKGICNEDVSSQSRIEEDPEVLITQLNELIETPIEEGKEIRNELQAISDGQKSSEEIEESCGTSDSEGLSEKTDKESSNEYVLDFFDSMVQRLEGISKMCQSGQVAQIIDCFNREFDIRNRELELKNRELELREKDLEFKKSILDWNKEKVSRELAFQREQDIKQTLMLLKK

>core/759/2/Org2_Gene335

MSIKEDKWIREMALNADMIHPFVNGQVNVNEETGEKLISYGLSSYGYDLRLSREFKVFTNVYNSVVDPKCFTEDIFISITDDVCIVPPNSFALARSVEYFRIPRNVLTMCIGKSTYARCGIIVNVTPFEPEWEGHVTIEISNTTPLPAKIYANEGIAQVLFFESSTTCEVSYADRKGKYQKQQGITVPCV

>core/760/2/Org2_Gene795

MVRVSTSEFRVGLRIEIDGQPYLILQNDFVKPGKGQAFNRIKVKNFLTGRVIERTYKSGESVETADIVERSMRLLYTDQEGATFMDDETFEQEVVFWEKLENIRQWLLEDTIYTLVLYNGDVVAVEPPIFMELSIAETAPGVRGDTASGRVLKPAVTNTGAKIMVPIFIDEGELVKVDTRTGSYESRVSK

>core/762/2/Org2_Gene988

MMKIPYARLEKGSLLVASPDINQGVFARSVILLCEHSLNGSFGLILNKTLGFEISDDIFTFEKVSNHNIRFCMGGPLQANQMMLLHSCSEIPEQTLEICPSVYLGGDLPFLQEIASSESGPEINLCFGYSGWQAGQLEKEFLSNDWFLAPGNKDYVFYSEPEDLWALVLKDLGGKYASLSTVPDNLLLN

>core/763/2/Org2_Gene946

MIRRLEYYGSPILRKKSSPIAEITDEIRNLVSDMCDTMEAHRGVGLAAPQVGKNVSLFVMCVDRETEDGELIFSESPRVFINPVLSDPSETPIIGKEGCLSIPGLRGEVFRPQKITVTAMDLNGKIFTEHLEGFTARIIMHETDHLNGVLYIDLMEEPKDPKKFKASLEKIKRRYNTHLSKEELVS

>core/764/2/Org2_Gene880

MALNFKINRQIRAPKVRLIGSAGEQLGILAIKDALDLAREAGLDLVEVASNSEPPVCKIMDYGKYRYGLTKKEKDSKKAQHQVRIKEVKLKPNIDENDFSTKLKQARTFVEKGNKVKITCMFRGRELAYPEHGFKVVQKMSQGLEDIGFVEAEPKLAGRSLICVVAPGTVKTKKKQEKSHAQDENQ

>core/766/2/Org2_Gene524

MRILAGKYKGKSLKTFSNPHIRPTSGLVKEAFFSICREDIEGAAFLDLFAGMGAIGFEALSRGAASVVFVDISIKAIQLIHTNSALLGEQLPVVIFRQDAQSAIQRLIKQKRSFDLIYIDPPYELCNCYVETLLQKIVSGNILNPEGTLFLENASDEEIACEGLTLRRRRKLGKTYLAEYIVEKDP

>core/768/2/Org2_Gene845

MELVVTSRETGKKSFLKKIRQQGGIPAVVYSAGKSLANITVDALVFKKFLSNLESGALSSTVFSLSYEGRIIKA
LVKDIQYQITTYDVIHLDFEELVEDRPIKLNIPIRCINAVDCIGVKLGGSLRQVIRAVRVVCKPKDIVPFLELDV
RSVGLSQTRKLSDIKIPAGIETITPLKEVAITVSRR

>core/770/2/Org2_Gene150

MVLSSQLSVGMFISTKDGLYKVTSVSKVAGPKGESFIKVALQAADSDVVIERNFKATQEVKEAQFETRTLEY
LYLEDESYLFLDLGNYEKLFIPQEIMKDNFLFLKAGVTVSAMVYDNVVFSVELPHFLELMVSKTDFPGDSLSL
SGGVKKALLETGIEVMVPPFVEIGDVIKIDTRTCEYIQRV

>core/771/2/Org2_Gene428

MTDTPPENEEQHESNVQNENEVEHLQQEIVTLKTELKEKNDKYLMALAESENSRKRLQKERQELMQYALEN
TLIDFLNPIESMEKALGFATQMSDDVKNWALGFNMILNQFKQIFEEKGIIEYSSIGQKFNPFLHEAVQTEETSE
VPEGTILEEFAKGYKIGERPIRVAKVKVAKAPTPKENKE

>core/772/2/Org2_Gene712

MAAKTKTLELEDNVFLLLEGNLKRIFATPIGYTTFREFQNVVFNCANGQQEIANFFFEMLINGKLTQELAPQQ
KQAAHSLIAEFMMPIRVAKDIHERGEFINFITSDMLTQQERCIFLNRLARVDGQEFLLMTDVQNTCHLIRHLL
ARLLEAQKNPVGEKNLQEIQEEITSLKNHFDELTKALQ

>core/773/2/Org2_Gene495

MVETVLHNFQRYLSKYLYRVFRFPCRKKTFLSSHRVLARPSFPVDYCPGKIYDLQEIYEELNAQLFQGALRLQ
IGWFGRKATRKGKSVVLGLFHENEQLIRIHRSLDRQEIPRFFMEYLVYHEMVHSVVPREYSLSGRSIFHGKKF
KEYEQRFPLYDRAVAWEKANAYLLRGYKKRVGGGYGRA

>core/775/2/Org2_Gene936

MNFAKIDHNHLYLTCLGDLGVACPILSTDCLPNYSEKASHEVLVYSKFRCISGEPSRLATSGNDTYYSIVSLPI
GLRYEVTSPSGRHDFNIDMHVAPKIGAVLSHGTREAKEIPGSSKDYAFFSLTARESLMISEKLAMTFQVSEVIQ
NCYSQCTKVTKTNLKEQYRHLSHNTGFELSVKSAF

>core/776/2/Org2_Gene551

MSRKAREPILLPQGVEVSIQDDKIIVKGPKGSLTQKSVKEVEITLKDNSIFVHAAPHVVDRPSCMQGLYWALIS
NMVQGVHLGFEKRLEMIGVGFRASVQGAFLDLSIGVSHPTKIPIPSTLQVSVEKNTLISVKGLDKQLVGEFAA
SIRAKRPPEPYKGKGIRYENEYVRRKAGKAAKTGKK

>core/777/2/Org2_Gene54

MYKWYVVQVFTAQEKKVKKALEDFKESSGMTDFIQEIILPIENVMEVKKGEHKVVEKYIWPGYLLVKMHLT
DESWLYVKSTAGIVEFLGGGVPVALSEDEVRSILTDIEEKKSGVVQKHQFEVGSRVKINDGVFVNFIGMVSEV
FHDKGRLSVMVSIFGRETRVDDLEFWQVEEVAPGQESE

>core/778/2/Org2_Gene509

MSLLNLPSSQDSASEDSTSQSQIFDPIRNRELVSTPEEKVRQRLLSFLMHKLNYPKKLIIIEKELKTLFPLLMRK GTLIPKRRPDILIITPPTYTDAQGNTHNLGDPKPLLLIECKALAVNQNALKQLLSYNYSIGATCIAMAGKHSQV SALFNPKTQTLDFYPGLPEYSQLLNYFISLNL

>core/779/2/Org2_Gene362

MVEIFNYSTSIYEQHASNNRIVSDFRKEIQMEGISIRDVAKHAQILDMNPKPSALTSLLQTNQKSHWACFSPPN NFYKQRFSTPYLAPSLGSPDQQDEDIEKISSFLKVLTRGKFSYRSQITPFLSYKDKEEEEDEDPEEDDDDPRVQ QGKVLLKALDLGVKSTNVMIDYVISRIFQFVQG

>core/780/2/Org2_Gene182

MSKESIRSYSEISTPTPIFRETPSKEGVAYKLQLRSPAKDCILRNRVSLKGALLRSIPFYGSFLGAKRIHSAWSA KDAPCTTRVYHYLVGGLELLGLGVVVLACKVLATALKFLFSKASSKIKQMKWREKARNLAAKDTVQSIKEF CSVDLTSCFTRCFRLRNRVVEEGASENQTVREIIV

>core/781/2/Org2_Gene675

MTDPKIEKSALRKLFISIRRDLSEERKHEASSAVASFVRSFSKESVVLSFVSFNHEIDMQEANRILIQKCTLALP KIDQENLYPVLIPSIDDLISVVHPKDPFSKQTPISSDKITHVLVPGLAFDQQGYRLGYGHGFYDRWLAQHPYPS IRTIGIGYCEQKIDRLPQESHDIPLSQIYLC

>core/782/2/Org2_Gene553

MSRLKKFYTEEIRKSLFEKFGYANKMQIPVLKKIVLSMGLAEAAKDKNLFQAHLEELTMISGQKPLVTKARN SIAGFKLREGQGIGAKVTLRGIRMYDFMDRFCNIVSPRIRDFRGFSNKGDGRGCYSVGLDDQQIFPEINLDRV KRTQGLNITWVTTAQTDDECTTLLELMGLRFKKAQ

>core/784/2/Org2_Gene846

MAKLIVAIGNPRHGYANTRHNAGFLLADRLVEELQGPPFKPLSKCHALMTLVESSSGPLVFIKPTTFVNLSGK AVVLAKKYFNVALSHILVLADDVNRSFGKLRLCFNGGSGGHNGLKSITASLGSNEYWQLRFGVGRPLEEGV ELSNFVLGKFSEEENLQLGSIFVEASTLFTEWCSKF

>core/785/2/Org2_Gene614

MSVLQDTEKKMAAALDFFHKEVKSFRTGKAHPALVETVVVDVYGTTMRLSDIASISVADLRQLVISPYDGN NASAIAKGIIAANLNLQPEVEGSIIRIKVPEPTADYRQEMIKQLRRKCEEAKINVRNIRREANDKLKKDSALTE DVVKGNEKKIQELTDKFCKQLDELTKQKEAEIASI

>core/786/2/Org2_Gene763

MTTPQSPGSLSQSHLPHPHDPWDTEPTSLPEDPNDKASQELHSLVHLFRKLSIHLLSEVEKTVQQLKPDLLELA LLICEKFLYKKLENPQELALLLSTALQRHTTLRSLTPIKVFLHPEDLKTLTDWISTHELPMIKHAEFFPDTSCRR SGFKIETPNGILRQEISEELDHLLSVLTA

>core/787/2/Org2_Gene482

MACEQHEGCYELEEREEIEDIKDSDTKWVSITQAAKLHNVTRQAIYVAIKQKKLKASKETRWEIDIKDLEEY
KRNRYSRKKSLYQGELVFDNGKGCYSINQVAQILGIPVQKVYYATRTGTIRGERKGAAWVIHVSEIERYKNE
YLSKQAAKKLKGAEPKEHQAPNFEPPTEIFPESN

>core/789/2/Org2_Gene923

MTIILCGLPTSGKSSLGKALAKFLNLPFYDLDDLIVSNYSSALYSSSAEIYKAYGDQKFSECEARILETLPPEDA
LISLGGGTLMYEASYRAIQTRGALVFLSVELPLIYERLEKRGLPERLKEAMKTKPLSEILTERIDRMKEIADYIF
PVDHVDHSSKSSLEQASQDLITLLKS

>core/790/2/Org2_Gene246

MSAPIPTPQELSDQITCLNVQYQQVSELARENKGDIEGLKTLTAALTADAGIQPSADEIYSLQTAAALILSASE
KPGSGPSGSTEGSVTVQSPCKFKKVLAVVLTIIALIAIAVLIACIIAACGGFPLLLSALNLYTIGACVSLPIIASTS
VALICLCTFVANSLIKPVITVRTTR

>core/791/2/Org2_Gene469

MDRDNEVPLPKPKWIYRTGIGQDSHRFLPESSTKPCILGGIIFDHCPGFQANSDGDIIFHAICNAISSVTNKIILG
KVADELLQTRGITDSGIYLEEALKSLKPNQKISHVAITIEGSRPKFLCKLSALRQNIAQVMNLTPTDIGITATSG
EGLSDFGCGDGVQCFCVLTVMEYCD

>core/793/2/Org2_Gene769

MGYVFYVIAGSIFLGISLGAYCQLYYSVKSVLFSWYLLTVYALEKRHALLALSQLVGEEDAQSQKEIDFLSQC
DKLSWRAFLKNSYEIIPTFKEMEDLLSERVQGFLESIETIAEHDRAILCIENFWASKNLFDFEIAAYEEAVEKYL
KLRQRAPLRLASKLFRFLDVPSIRFSS

>core/794/2/Org2_Gene1002

MDCVDNLKLYIFRLKLPGDTERISYSISPEYIREKGEEELLNSPIEVEGSLGRIDSDQWILSLSLKTQLGLCCPVC
NNFFSHSVCLPDLQRVISHDEVGSGVFDCRPLIRQELLLESDCFEECSGQGCPERKNILKFLEDRKKHEGNSPF
EYL

>core/795/2/Org2_Gene377

MSSTLNGVFPSSLPEESADLFITNKEIVALGEKGNVFLTHSIPMHIAAITILVIVALAGIAIICLGCYSQSILLIAVG
IVLTILTLLCLQALVGFIKFIRQLPQQLHTTVQFIREKIRPESSLQLVTNAQRKTTQDTLKLYEELCDLSQKEFK
LQSTLYQKRFELSHKNEKTNQN

>core/796/2/Org2_Gene894

MSIQPVSNTTTKADKVIPDSTKVISDSITINKQSAFYFCISVMLRLSESTTEYGKSILAVLEDNTIVQQQRVKELI
NLPLLKVPDLQKKDGSDDEYKNQNEIQAYQSSNQQISANRQMIQQELSSAQQRAQANQKSVNSTTIESMQIL
QATSSMLSTLKELTIKANLTNSPSD

>core/797/2/Org2_Gene895

MWIIDPLSAKKPLQAAINVPGTPITGGPNTATADDIIAKFSKDSNPLIVTVYYVYQSVLVAQDNLSIIAQELQA
NSSAQTYLNNQEALYQYVSIPKNKLNDNSSSYLQNIQSDNQAIGASRQAIQNQISSLGNAAQVISSNLNTNNNI
IQQSLQVGQALIQTFSQIVSLIANI

>core/798/2/Org2_Gene516

MADDTLIPKLMKNSLSQACSEGLLIAKYPPLQVIVHFDNNLVVKTHLSVAPVFSCLFLGPAAHKAMQEIVLW
CSRYANKEHPPFSSHFAKDLIPSQYLEILNCVAEIPFGEQQTYAEIAKKTDTHPRTVGAACKQNPFLLFFPCHR
VVGSHGERNYVLGPVIHEILLKFENSY

>core/799/2/Org2_Gene380

MGFKNICKQGSQLYLNGIFPERILARKLKNCAKSYPRTALTIEVLVSSVLGALKVILIPCASTYAALTLPLRALF
NAIKTKSCQHLASYAMAWLLHILTIAVIIGLVFSLVFIPPPVVFISLGLLMSVTTSVTLFQVHKNLFPPYEPPPSR
PHTPPPFADEYVPLISESYFD

>core/800/2/Org2_Gene908

MSHLNYLLEKIAASSKEDFPFPDDLESYLEGYVPDKNIALDTYQKIFKISSEDLEKVYKEGYHAYLDKDYAKS
ITVFRWLVFFNPFVSKFWFSLGASLHMSEQYSQALHAYGVTAVLRDKDPYPHYYAYICYTLTNEHEEAEKAL
EMAWVRAQHKPLYNELKEEILDIRKHK

>core/802/2/Org2_Gene286

MSVHITPRKCFILCILSMFTLPTLFPKAHLILFSPYIVLCFYCFSKDKGLVLALGCGVLSDLALGSRGVFLLLYP
LTALITHKAHLIFSKESKAALVIVNMIFYGVFLLLTIPMCALFGHEVRWSIDVLMIPLKCSFLDNLIFTSVIYILP
CAINSGIHKMISFFRRLVCY

>core/803/2/Org2_Gene615

MVHSPTHQCYHCQQPATICYTEIDKDKVIRSYVCATCPCPSHYYNNEHLSLSKGVGVLTLECGNCKTVWHS
KQDDEQLLGCHQCYTNFKNQITSKLKSERVVSSSFTMEKGQGSLHIGRAPGEASNTNPLLKLIALNEALQDTL
EREDYEQAAVIRDQINHLKTKNPDDPS

>core/804/2/Org2_Gene496

MTAEKQNTGILGLEIRYTLPSDATYMLKWLNDPKILRGFPIQTEAEIRETVNFWVGFYRYHSSLTAVYNGNV
AGVATLVLNPYVKVSHHALISIIVGEEFRNKGIGTALLNNLIHLAKTRFKLEVLYLEVYEGNPALHLYQRFGF
VEVGRQNRFYKDEIGYLAKTTMEKDL

>core/805/2/Org2_Gene256

MKKLLFSTFLLVLGSTSAAHANLGYVNLKRCLEESDLGKKETEELEAMKQQFVKNAEKIEEELTSIYNKLQD
EDYMESLSDSASEELRKKFEDLSGEYNAYQSQYYQSINQSNVKRIQKLIQEVKIAAESVRSKEKLEAILNEEA
VLAIAPGTDKTTEIIAILNESFKKQN

>core/806/2/Org2_Gene100

MSEVKPLFLKNDSFDLATQRFQNLINMLQEQAEIYNEYEEKNARVQNEIKEQKDFVKRCIEDFEARGLGVLK
EELASLTRDFHDKAKAETSMLIECPCIGFYYSIHQEEQRQRQERLQKMAERYRDCKQVLEAVQVEQKDMISS
RVVVDDSYFEEEKEEQKVDNRKKEQD

>core/807/2/Org2_Gene671

MCKNRGVRGIVACDPRGVIGLEGKLPWHYPEDLQFFSETIQKFPIVMGRKTWETLPRKYFVDRAVVVFSHEK
RQGVHGEIWVTSLEEFLLLDLSSPTFLIGGGELYSLFLENQIVRDFFISHIKKEYAGDTFFPLSLLETWTKTVLR
DTQKITTCYYENHHSQNTKNISL

>core/808/2/Org2_Gene57

MKQEKTLLLQEVEDKISAAQGFILLRYLRFTAAYSREFRNSLSGVSAEFEVLKKRIFFKAIEAAGLEVDCSDTD
GHLGVVFSCGDPVSAAKQVLDFNKQHKDSLVFLAGRMDNASLSGAEVEAVAKLPSLKELRQQVVGLFAAP
MSQVVGIMNSVLSGVISCVDQKAGKN

>core/809/2/Org2_Gene70

MQRLGLSNLFHCLLLFLRYYYSKLVFGLTVLLAAISVICLLGCSEPSLSSFTEYVGPEYSAAAQLSIEQSCHDE
VYGQQVVVTWSLPSRMRKCLPVTLYLWVYYGNGKVEKLTYEVNQSAGYRVYCLKGLEYKELQGIISYRVA
LCSGNQEIVSRRHHLWMEVISLDSP

>core/810/2/Org2_Gene849

MKQQLLLLEDVDGLGRSGDLITARPGYVRNYLIPKKKAVIAGAGTLRLQAKLKEQRLIQAAADKADSERIAQ
ALKDIVLEFQVRVDPDNNMYGSVTIADIIAEAAKKNIFLVRKNFPHAHYAIKNLGKKNIPLKLKEEVTATLLV
EVTSDNEYVTVLAQGKQTEENQEG

>core/811/2/Org2_Gene232

MIINVRAPAFGITSVQQFSTNFQAAIPILNIVIGCSRISSTYAEDIEEVAQEKLEKSTHSKSSTSVNLWAHRVRGV
VEILGGGIVILALEITALVLQVIIKLIKCLIDVLCVCLFGLGVCVVAIIGAIAFCVVVVVVKYLGFCSQGEELEPIE
VKTLISPDKPYPTVVYV

>core/812/2/Org2_Gene539

MSELIIGVDPGTIVAGYAIIAVEQRYQLRPYSYGAIRLSSDMPLPMRYKTLFEQLSGVLDDTQPNAMVLETQF
VNKNPQSTMKLAMARGIVLLAAAQRDILIFEYAPNVAKKAVVGKGHASKRQVQVMVSKILNVPEVLHPSNE
DIADAFALAICHTHVARSPLCGVR

>core/813/2/Org2_Gene534

MRQFCNLLSLSRLWLALYFCQEKLHIRLLAIVGAMLSDVLDGYLARRYKATSRLGSILDPITDKVFVFVCITV
LYMEGSLSIAHLFFICARDLFLIIFVCYLSLVKGWKGYDYGSLFWGKIFTVVQFIILLGVTAGGEIPWTGLVPL
VALGFLYFLERIMDYKKQFLR

>core/814/2/Org2_Gene457

MATRFRSTLLVITLFVLIDWVTKLVVLLQYKDLQILTHPTLYTHSWGRFSFSIAPVFNEGAAFGLFSNYKYFLF
LLRIFVILGLLAYLFFKKKSIQSTTQTALVLLCAGAIGNVGDIIFYGHIVDFISFNYKQWAFPTFNVADVLISLGT
LLLVYKFYFPTKQTEKKR

>core/815/2/Org2_Gene621

MAKYPLEPVLAIKKDRVDRAEKVVKEKRRLLEIEQEKLREKEAERDKVKNHYMQKIQQLRDLLDEGTTSDA
VLQIKSYIKVVAVQLSEEEEKVNKQKEVVLAASKELEKAEVNLAKRRKEEEKTRLHKEEWMKEALKEEARA
EEKEQDEMGQLLFQLRQKKKRESGGS

>core/816/2/Org2_Gene297

MAVEQSHIKEEIEKLIGKAIKRVCGNKENDLCRYLPGPSGGYMHHFTLKKMKSAAPEQLLKMLKTFILESETP
RTINPKPRAPRGSKKRRDFINFTKTDIERVLELARQVGDKDLLARFSPKKPLTSLKRELIRSIRNGIVSVELWNA
YVEAVKAVSSPNLEVTSPFV

>core/817/2/Org2_Gene149

MDLKQIEKLMIAMGRNGMKRFAIKREGLELELERDTREGNRQEPVFYDSRLFSGFSQERPIPTDPKKDTIKET
TTENSETSTTTSSGDFISSPLVGTFYGSPAPDSPSFVKPGDIVSEDTIVCIVEAMKVMNEVKAGMSGRVLEVLIT
NGDPVQFGSKLFRIAKDAS

>core/819/2/Org2_Gene215

MSKKINRNDLCPCGSNKKYKQCCLKKEEQTARYTTEGKFKFSAEVLSASEQGEAGDNCTKLFQRLSQSLTSE
QKAAVGKFHQITKNKEVMSKKALKKAQAKEEKLVTEKLQQHNFEILNTGENLAPPMESTATLNQDTNFVCE
DFIPTQEDFRISENSQKPPVEED

>core/820/2/Org2_Gene331

MSRQNAEENLKNFAKELKLPDVAFDQNNTCILFVDGEFSLHLTYEEHSDRLYVVYAPLLDGLPDNTQRKLALY
EKLLEGSMLGGQMAGGGVGVATKEQLILMHCVLDMKYAETNLLKAFAQLFIETVVKWRTVCADICAGREP
SVDTMPQMPQGGGGMQPPPTGIRA

>core/821/2/Org2_Gene823

MKFWLQGCAFVGCLLLTLPCCAARRRASGENLQQTRPIAAANLQWESYAEALEHSKQDHKPICLFFTGSDW
CMWCIKMQDQILQSSEFKHFAGVHLHMVEVDFPQKNHQPEEQRQKNQELKAQYKVTGFPELVFIDAEGKQL
ARMGFEPGGGAAYVSKVKSALKLR

>core/822/2/Org2_Gene473

MSRRHSAEKRDIPGDPIYGSVILEKFINKVMMHGKKSVARKIVYSALERFGKKLNLENVLEGFGEALENAKPI
LEVRSRRVGGATYQVPVEVASERRNCLAMQWIIKHARSKPGKSMEVGLATELIDCFNKQGATIKKREDTHR
MAEANKAFAHYKW

>core/823/2/Org2_Gene549

MSLSKNSHKEDQLEEKVLVVNRCSKVVKGGRKFSFSALILVGDGKGRLGYGFAKANELTDAIRKGGEAAKKNLMKIEALEDGSIPHEVLVHHDGAQLLLKPAKPGTGIVAGSRIRLILEMAGIKDIVAKSFGSNNPMNQVKAAFKALTGLSPRKDLLRRGAAIND

>core/826/2/Org2_Gene637

MTKFLYCGLFYSLGLLVLAFGTMVAIIQVDQICDVSCMNKHFQESPPFLKIKKVNVSKQICSPEERFFHCKIDKSCMELHFPQSSYSCKEYLTRISGHILTQNFEKQMQFRGNSGLLNYQDGSLHVYDCRFQVDPVPGYGSPDKEDSSSGGMKTLYLSLFRN

>core/827/2/Org2_Gene841

MMHRYFIPLLALLIFSPSLVRAELQPSENRKGGWPTQLSCAEGSQLFCKFEAAYNNAIEEGKPGILVFFSERPTPEFADLTNGSFSLSTPIAKGFNVVVLCPGLISPLDFFHKMDPVILYMGSFLEMFPEVEAVSGPRLCYILIDEQGGAQCQAVLPLETKN

>core/828/2/Org2_Gene896

MSITTLGTLPTVNTINSSRPPLEPLNTPKIGAVLFSIYELLLQAIEIRQQTVLTQSQQLNDNTNIQQQLNQETNQIKYAIVSAGAKEDEITRVQNQNQNYSAQRSNIQDELVTTRQNGQIILSHASTNINIIQQQSSQDSSFIKTTNSIGSTVNQLNKPLG

>core/829/2/Org2_Gene351

MTTWTLNQNNLTKFLKSSDEEPFLERESGLTYINIQANGNELPLFFVIRSEGEILQLICYLPYQLHESHKASTARLLHLLNRDIDIPGFGMDEEQGLIFYRLVLPCLNGEIHDTLLRIYIDTIKLVCDSFSHAIGLISSGNMNLDELRRQALQEQQEKRNE

>core/831/2/Org2_Gene879

MATLSPEKFSGSPISISKEFPQQKMREIILQMLYALDMAPSAEDSLVPLLMSQTAVSQKHVLVALNQTKSILEKSQELDLIIGNALKNKSFDSLDLVEKNVLRLTLFEHFYSPPINKAILIAEAIRLVKKFSYSEACPFIQAILNDIFTDSSLNENSLSI

>core/832/2/Org2_Gene330

MMFGHFAGYLGADPEERMTSKGKRVITLRLGVKTRVGMKDETVWCKCNIWHNRYDKMLPYLKKGSGVIVAGDISVESYMSKDGSPQSSLVISVDSLKFSPFGRNEGSRSPSLEDNHQQVGYESVSVGFEGEALDAEAIKDKDMYAGYGQEQQYVCEDVPF

>core/834/2/Org2_Gene180

MSKKVFFESYEDFANVASSWPKSLRALVQGRYFVDSELKETPYRIHDFKKTPIHHRLYRSLPIISTIGGIIRLIEAHSGPIHPRDKMKYRFEVLQAVIEILGLGVLILVFDIIGCFLAFLVAIILSLLLYCNSTFTCVQNLSFTERMLEGIGEAVNFLA

>core/835/2/Org2_Gene350

MFFNLFSLVFKLSDELALAETIQEPISVHEMFPGSMKLEMFKMLGSLILLLTIFGFGVWAFKKFVRSRSHGFGG
SSQIKILERRSLTPKTSIYLIRVVNKTLVIAETPEKITLLTEFPPDTDINHLLQENNKQSSSSATSDFLSKAIQKIQK
KQQTNQD

>core/837/2/Org2_Gene40

MPSYCQNQQDFSLFSLLSPRLVMFLGKHSRDEILQDLTDLVDAAGLLEDKQAFFDALVRRENIMSTGIGMGV
AIPHGKLESCSNFFIAIGIHTQGILWDAIDGALVRLVFLIGGPENAQAEYLKLLSTLTLSLREESRRQQLLQVNT
IEEVMNVFVGM

>core/838/2/Org2_Gene730

MADETPKENSSKESSSQFDSLKRKVKDLHSNPKVGKWKKFLSHRACEAIGGCLVLVGIIADFISWAGGLFIAC
GVVLGFHVEIRKMLSNLQSYSIANGPIKNAILCGLILFFVLNIPSFAVSFIVLCVILSFITTAPSCSTCSKDHCDKH
QDTSNKPS

>core/839/2/Org2_Gene434

MTQEKIKIHVSNEQTCIPIHLVSVEKLVLTLLEHLKVTTNEIFIYFLEDKALAELHDKVFADPSLTDTITLPIDAP
GDPAYPHVLGEAFISPQAALRFLENTSPNQEDIYEEISRYLVHSILHMLGYDDTSSEEKRKMRVKENQILCML
RKKHALLTA

>core/841/2/Org2_Gene835

MLPISILLFYVILGCLSAYIADKKKRNVIGWFFAGAFFGFIGLVVLLLLPSRRNALEKPQNDPFDNSDLFDDLK
KSLAGNDEIPSSGDLQEIVIDTEKWFYLNKDRENVGPISFEELVVLLKGKTYPEEIWVWKKGMKDWQRVKD
VPSLQQALKEASK

>core/842/2/Org2_Gene283

MRIIRFDPYGALSAQSIAKDSRQNSPLVEKISEEIATNEAIRLALLAIGDREQEEKKQRHRYKLLGQKQAKVLL
SQLRHVHLDFKKLYCDSKKKEDQEKDEKNKQKRSIKVTKKKKGISLGAAASQAIAAAAEAWVIARNKGVLE
TASTLFYQKDEEA

>core/843/2/Org2_Gene836

MEKQNLKLDVKEIEFPETVFSRDIETRVIQVIILHCLAKINGVSLLGGNLIDALFGRDIERMKGIYVEQDSKNHL
VKVRVEVNVDYGVSIPEKTEEIQGCIVSEISEYTGLHVAAVHVIIKGLTQPKDRIDEEIEEEVSVQDLPSPEDFL
LENSEG

>core/844/2/Org2_Gene141

MIMTTISNSPSPALNPELSLIPPPTLVSSGTQTSLAYTIPAQGRRSTLRIILDIFIIILGLATIISTFIVIFFLNGLNLLS
TPSIISSSCLIIVGLLFLIMGLYFMISSLDQGLVGLLQKELSQAEEREEEYIQEIEALRGAPRAESPTESPSTWL

>core/845/2/Org2_Gene577

MRVVLHCPDIPQNTGNIGRTCVALGAELILVRPLGFSLADKFVKRAGMDYWDKLQLTVVDSIEEALHDVPED
QIFCLSTKGSASYTEFSLPSSGTYVFGSESKGLPKEILKKYYKNCLRIPMQQDIRSLNLATSVGIVLYEVVRQKT
VALQKNPTV

>core/846/2/Org2_Gene891

MEKDIFFMQQAFKEARKAYDQDEVPVGCVIVKDDKIIARAHNSVEKLKDATAHAEILCIGSAAQDLDNWRL
LDTVLYCTLEPCLMCAGAIQLARIPRIVWAAPDVRLGAGGSWVNIFTEEHPFHTVSCTGGVCSEEAEHLMKK
FFVEKRREKSEK

>core/847/2/Org2_Gene310

MGYLPVSATDVLFESPAAPLINSANTQNQKLIELKGKQQAESSPRTITSVILEVLLVIGCCLIVLSLLAIRPALQF
TLETGHPAAIAVLAVSGTILLVAVIILFCFLAAVPFAAKKTYKYVKTVDDYASWHSHQQTPTLGTIFSGIVYAE
SQAQL

>core/848/2/Org2_Gene572

MLKKKPVSFSCIDGHIYKIFPNDLNANNTVFGGLLMSLLDRLALVVAERHTESVCVTAFVDALRFYAPAYMG
ENLICKAAVNRTWRTSLEVGVKVWAENIYKQERRHITSAYFTFVAVNEDNQPIPVHQIVPETPEEKRRYNEA
DRRRQARLELK

>core/849/2/Org2_Gene776

MKTLKGHLSAKNLRIAIVGSCFNQAMADALVSGTQETFLKFGGSEDGLMTIRVPGAFEIPCTIKKLLSSERKF
DAIVACGVLIQGETDHYNQIVNQVAAGIGALSLEFCLPITLSIVAAPSAEIAWQRSGIKGRHLGVSGMTTAIEM
ATLFTQI

>core/850/2/Org2_Gene742

MPFAKETEMQRTCWKCEGSVSMHVPQCPYCSAFLQDPPVASGGFSSCHISFPEGASKEEAEDLFAVSSEDWE
AVLGDQNPTQETNKQVIPEWTWLQSWPLAALFLGIGLLAFAFLILLFSTDSGLVLTWPKNRAYFYGIIGAAVA
YRGYRKLPL

>core/851/2/Org2_Gene35

MRSALHLQHLRHFHNHGSILFENLLTIKDCFLLETKLQNFIAKASKTIDTVRWRENIFRSMPEIYTVVRKRRLD
FFAAELVHRPKLSLVRDLWVFPGEEILEGEEDCMLFLLLSGDRAGSGIFFTGPYPSDLYELEKGTTGLLLAFSS
VGIPVI

>core/852/2/Org2_Gene569

MNQPSVIKLRELLDLLPHRYPFLLVDKVLSYDIEARSITAQKNVTINEPFFMGHFPNAPIMPGVLILEALAQAA
GVLIGLVLENDRNKRIALFLGIQKAKFRQAVRPGDVLTLQADFSLISSKGGKAWAQARVDSQLVTEAELSFA
LVDKESI

>core/853/2/Org2_Gene455

MQCPFCNHGELKVIDSRNAPEANAIKRRRECLKCSQRFTTFETVELTLQVLKRDGRYENFQESKLIHGLNAAS
SHTRIGQDQVHAIASNVKSELLGKQNREISTKEIGELVMKYLKKADMIAYIRFACVYRRFKDVGELMEVLLS
ATPDMEK

>core/854/2/Org2_Gene289

MAQKEIVSNRKALRNYEVIETLEAGIVLTGTEIKSLRDHGGNLGDAYVIVSKGEGWLLNASIAPYRFGNIYNH
EERRKRKLLLHRYELRKLEGKIAQKGMTLIPLGMFLSRGYVKVRLGCCRGKKAYDKRRTIIEREKEREVAAA
MKRRHH

>core/855/2/Org2_Gene266

MARNIKYFLILFPGILWISAGMKLLLKATAIALDPLSSFFTYCLLSMVSWGLASLKHRYLLSKTIRKQLSLSSEF
FSQKITWIAYIKQTFISRRFLIMVIMIAFSLVLRRYISNPQALFVIRATVGYALIKTAIAYFSKLQNALMENPEGN

>core/856/2/Org2_Gene198

MSKPSSCKAYLGIDYGKKRIGLAYAAEPLLLTLPIGNIEAGKNLKLSAEALHKIILSRNITCVVLGNPLPMQKG
LYSSLQEEVSLLAEELKKLSTVEIILWDERLSSVQAERMLKQDCGLSRKDRKGKTDSLAATLILTSFLDSLPKK
LTL

>core/857/2/Org2_Gene814

MMKTKYEYSFGVIPIKFFGTPDKNTLKACFICHTRGKHWGFPKGHSEDKEGPQEAAERELVEETGLSVVNFF
PKVLIEQYSFNNEEQVFVRKEVTYFLAEVRGDIHADPMEICDSQWLSLQEGLRLLSFPELRDLTVEADKFINN
YLFSS

>core/858/2/Org2_Gene780

MQLLSPAFAYGAPIPKKYTCQGAGISPPLTFVDVPGAAQSLALIVEDPDVPKEIRSDGLWIHWIVYNLSTTITN
LAEGAEIFAVQGLNTSGKPVYEGPCPPDKQHRYFFTLFALDVVLPEEENVTRDQLYEAMEFHIIEQAELMGTY
EKS

>core/859/2/Org2_Gene207

MEKRKDTKTTIVKSSETTKSWYVVDAAGKTLGRLSSEVAKILRGKHKVTYTPHVAMGDGVIVINAEKVRLT
GAKKGQKIYRYYTGYISGMREIPFENMMARKPNYIIEHAIKGMMPRTRLGKKQLKSLRIVKGDSYETFESQK
PILLDI

>core/860/2/Org2_Gene49

MEFICPLQHARCLKKQHKIIEELFPEPFQKDHLYLKLMENSSSRDAFDKKRMLKENLVVGCQSDLYLYEVYQ
DGILFFFTYTKALMSSGIASLFTEVYSGETPSTILTCKPIFFQRLTPYLSFGRLNGGESLYMRMKQIAVQYLKPP
QT

>core/861/2/Org2_Gene624

MADLEVFQADFALLFEAGLLAIKQGDEDSARKLFQSLHILNPNHYGHDLGLALISLHKMDLFDAEERLSALIK
GNEDNWSIKAFLSLTHMLIVLHQGSSFEVRRESLESCLKFADQVIANCKIESTRALAQSVLDWHDTLVAKSA
GPLG

>core/863/2/Org2_Gene323

MKSERLKKLESELHDLTQWMQLGLVPKKEISRHQEEIRILEHKIYEEKERLQLLKENGEIEEYVTPRRSPAKTV
YPDGPSMSDIEFVEPTETEIDIDPGETVELELTDEGREDGAVEVDYSHEDDEDPFSDRNRWRRGGIIDPDANE
W

>core/864/2/Org2_Gene320

MKTKMNSRKKAGQWAIFNSPTPGVSSTLVLAWTPWGYYDKDVQDILERKDPMSSSLSEKDSKEFLKNLFVD
LLENGFTSVHIHAEEAFTPLDHTGKPHFKRDNVYLPGKLLGALNEAAVQANVSADTQFTLFLTQDECNPFHD
KKRG

>core/865/2/Org2_Gene972

MGSRRKLKRSFLLIEVLMALSLVCAVLLPCIRFYYAIHRSFEEDIFNLQLPALIDHCFLSVEEKMRQQMAEGTV
LTSGKGQTVSLAYTSQGIGYRIPYGYNVDIRQEVRGDNLKMKVCLADVVVELFPDQKQAVSVQRCLCVTL

>core/866/2/Org2_Gene159

MKKKVTIDEALKEILRLEGAATQEELCAKLLAQGFATTQSSVSRWLRKIQAVKVAGERGARYSLPSSTEKTT
TRHLVLSIRHNASLIVIRTVPGSASWIAALLDQGLKDEILGTLAGDDTIFVTPIDEGRLPLLMVSIANLLQVFLD

>core/867/2/Org2_Gene278

MQNQYEQLLESLAPLLNTTLAPDKNNSCLIRFSDTHVPVQIEEDGNSGDLAVSTLLGTLPENVFRERIFKAALS
VNGSFQSSIKGILGYGEVTQQLYLSDILSMNYLNGEKLFEYLKLFSLHAKIWMESLRTGNLPDLHVLGIYYVA

>core/868/2/Org2_Gene805

MEEFVAYIVKNLVTNPEAVEIRSIEDEDNESIKLEIRVAAEDIGKIIGRRGNTIHALRTILRRVCSRLKKKVQIDL
VQPENGTDVIADQDYICDNDSSNSTEDTFGESDTCCSGHCHYDEDLNQEEQEEGNMHHSCECSNHH

>core/869/2/Org2_Gene112

MSLEKELLEETPLVLLNFYKLVSFCNYAGMILGTEEKKFAIYGHVSMGQAFQGADTEGHSPQRPFAHDLLNF
VFSGFDIQVLRVVINDYKDNVFYTRLFLEQKDREFLYVVDVDARPSDSIPLALTHKIPILCVKSVFDAVVPYEE

>core/870/2/Org2_Gene178

MNPVTFDRIQVDFIPEDTSLRINSYIVAGGLLILGVVLSILSVICLDIGLVGLSAGAAFTLGLGCLIFALFLFSFSL
ILLLSQEKRVPDVLSLYLEKEVPQYETPLYKEDLESERDMSAISERLGIIEEKLRIAEKFRYSDSVFV

>core/871/2/Org2_Gene39

MTVFCELDSGGELPEYTTPGAAGADLRANIEEPIALLPGQRALIPTGIKAEIPEGYELQVRPRSGLALKHGITVL
NSPGTIDSDYRGEIRVILINFGDSTFIIEPKMRIAQVVLSPVVQATFVVKQESLAETARGSGGFGHTGAS

>core/872/2/Org2_Gene587

MTFFEGETVFPAVLSELHSMLDLIKRAGKQSKCPQEKLLKLELACEELLVNIISYAYQGENSPGTIAISCISHRG
DLEVVIKDHGPSFNPLAVSINIQEDLPLEQRKLGGLGIFLAKSSVDEFLYAREDHCNIVHLKMLNGQHS

>core/873/2/Org2_Gene537

MEQTLSIIKPDSVSKAHIGEILSIFEQSGLRIAAMKMMHLSQTEAEGFYFVHRERPFFQELVDFMVSGPVVVLV
LEGANAVSRNRELMGATNPAEAASGTIRAKFGESIGVNAVHGSDTLENAAVEIAYFFSKIEVVNASKPLV

>core/874/2/Org2_Gene548

MIKLESLFDISERKRRKKLLGRGPSSGHGKTSGRGHKGDGSRSGYKRRFGYEGGGVPLYRRVPTRGFSHKRF
DKCVEEITTGRLAELFQEGEAITLDALKAKKAIARQAVRVKVILKGDLEKTFVWQDTAVVLSQGVQNLLGIT

>core/875/2/Org2_Gene203

MACSIFFRMSQGDYDDEPLSKKTACLVVDTMLYPVIAVVCAVVSVVLLILKVLFLLLSFPFKLCSASSALPGE
RVSLGSHFKCLYGGGLPYLLACLLIVPVIGTAIHGFIISHRTSEDARLSSAIVFMQAPILQLAGMSGLIKP

>core/876/2/Org2_Gene948

MQEHIHKELLHLGEIFRSSRESQSLSLKDVEAATSIRYSCLEAIEQGCLGKLISPVYAQGFIKKYATYLGLDGD
SILQEHPYVMKIFKEFSDHNMEMLLDLESMGGRNSPERAIHSWSNLWWAGLIIIGGIMVWWLGSLFSIF

>core/877/2/Org2_Gene588

MSLDFFEEFYHQSILNTGTSFPEGYLNIAEILSYPHCTDANTDFLCSQSDNDFIIAESKDKLTLFNADFAIWLVP
ELVQGQAVTRGYIAVSQGEGNYEPEMAFEASGQYNQSSLILEALQLYLKDIKDTENALRSFRFNNDH

>core/878/2/Org2_Gene543

MQHARKKFRVGRTSSHNRCMLANMLKSLIHYERIETTLPKAKELRRHADKMITLAKKNSLAARRIAIGRLM
VRYNKLTSKEARQAKGGDTSVYNVDRLVVNKLFDELGNRFVERKGGYTRILKLQNRIGDNAQKCIIEFLAS

>core/879/2/Org2_Gene656

MNSKSAQKIIDSIKQILTIYNIDFDPSFGSSLSSDSDADYEYLITKTQEKIQELDKRAQEILTQTGMSKEQMEVF
ANNPDNFSPEEWLALEKVRSSCDEYRKETENLINEITLDLHPTKESKRPKQKLSSTKKNKKKNWIPL

>core/880/2/Org2_Gene363

MLDNEWKAILGWGDDELEELRISGYSFLRQGHYSKAILFFEALVILDPLSIYDHQTLGGLYLQIGENSQALAV
LDQALRMQGDHLPTLLNKTKALFCLGRIEEATAIATYLSSCPIPAIANDAEALLMSYSKATKKNAALVR

>core/881/2/Org2_Gene574

MGRYRRVSHSSQETLLLGTELGQVLVPGAVLLLFGDYGAGKTEFVRGIVSGYLGDTIAEEVASPSFSILHVYG
NEPKRLCHYDLYRIDQKNQEYIFQDAEEDDVLCIEWADRLPKPRFCDTINIYITMQTNMEREIIIEKR

>core/882/2/Org2_Gene55

MSVKKVIKIIKLQIPGGKANPAPPIGPALGAAGVNIMGFCKEFNAATQDKPGDLLPVVITVYADKTFTFITKQP
PVSSLIKKTLNLESGSKIPNRNKVGKLTQAQVEAIAEQKMKDMDIVLLESAKRMVEGTARSMGIDVE

>core/883/2/Org2_Gene69

MIDMSVVGPALVLGLAMIGSAIGCGMAGVASHAVMSRIDEGHGKLIGMSAMPSSQSIYGFILMLLMQAAIKN
GTLSPVGGIAIGLSVGAALLVSSVMQGKCCVSGIQAYARSSSIYGKCYAAIGIVESFSLFAVVFALLLL

>core/885/2/Org2_Gene831

MHPLTLPKQSRVLKRKQFLYITRSGFCCRGSQATFYVVPSRHPGTCRMGITVSKKFGKAHERNSFKRVVREV
FRHVRHQLPNCQIVVFPKGHKQRPVFSKLLQDFINQIPEGLHRLGKTKATTGGECTPKSEKCVTAPR

>core/886/2/Org2_Gene558

MLMPKRTKFRKQQKGQFAGLSKGATFVDFGEYAMQTLERGWVTSRQIEACRVAINRYLKRRGKVWIRIFPD
KSVTKKPAETRMGKGKGAPDHWVAVVRPGRILFEVANVSKEDAQDALRRAAAKLGIKTRFVKRVERV

>core/887/2/Org2_Gene249

MNLIDRAFLLKKTIIFQSLDMDLLLTIADKTETIIFKPGSNVFSIGQPGFSFYIIVEGYITISKEKLESPLNLKPLDC
FGEESLFNNKPREYNASANTQVRMLVLSKGQILNIVEECPSVALSFLELYAKQIKFREP

>core/888/2/Org2_Gene183

MVNRYKSSAEFSADHYYDDNLVRMGYKRNLRGLAPVENEVCLFEENNLLESVMASIPIMGSILGLGRLHSV
WSTQDPKDSKISIIFHTALGILETLGLGIIVLLIKITITILLILFTPCLLCYFMYSAAYSDFHPI

>core/889/2/Org2_Gene189

MINFIRSYALYFAWAISCAGTLISIFYSYILNVEPCILCYYQRICLFPLTVILGISAYREDSSIKLYILPQAVLGLGI
SIYQVFLQEIPGMQLDICGRVSCSTKIFLFSYVTIPMASVVAFGAIVCLLVLTKKYRG

>core/890/2/Org2_Gene696

MKYRFTEEIEEEPLVNLTPLIDIVFVILMAFIVAVPLIKLDSIALAPGTQEQEVLSSENDSIAVIKVFADHSLTLN
EHPITLQELTVRLTLLHKAYPEKTPLLLQDGETSFRTYQNVKNAIEAAGFHELHVALQN

>core/891/2/Org2_Gene206

MAKSTIQESVATGRRKQAVSSVRLRPGSGKIDVNGKSFEDYFPLEIQRTTILSPLKKITEDQSQYDLIIRVSGGG
IQGQVIATRLGLARALLKENEENRQDLKSCGFLTRDPRKKERKKYGHKKARKSFQFSKR

>core/892/2/Org2_Gene669

MIAIERYQLIISKFRMWLFLGCSVEERHFKQPVLISVTFSYNEVPSACLSDKLSDACCYLEVTSLIEEIANTKPY
ALIEHLANELFDSLVISFGDKASKIDLEVEKERPPVPNLLNPIKFTISKELCPSPVLSA

>core/893/2/Org2_Gene188

MPYYANTLEFIQGTQSLCPLFKYGFVRHHYKGQLEIEDASHDWDFLEPPSTWKRTLLAAIPILGSVIGLGRLFS
IWSIREPQDSQEYKSIFWHTLCAVLEILGLGIVALILKILATFIMAMPGLKRVATFLFYS

>core/894/2/Org2_Gene552

MGMTSDSIADLLTRIRNALMAEHLYVDVEHSKMREAIVKILKHKGFVAHYLVKEENRKRAMRVFLQYSDD
RKPVIHQLKRVSKPSRRVYVSAAKIPYVFGNMGISVLSTSQGVMEGSLARSKNIGGELLCLVW

>core/895/2/Org2_Gene545

MVKNQAQAKKSVKRKQLKNIPSGVVHVKATFNNTIVSITDPAGNVISWASAGKVGYSGSRKSSAFAATVAA
QDAAKTAMNSGLKEVEVCLKGTGAGRESAVRALISAGLVVSVIRDETPVPHNGCRPRKRRRV

>core/896/2/Org2_Gene811

MNFVSTLTGSDFYAPVLEKLEEAFADTTGQVILFSSSPDFIVHPIAQQLGISSWYASCYRDQSAEQTIYKKCLT
GDKKAQILSYIKKINQARSHTFSDHILDLPFLMLGEEKTVVRPQGRLKKMAKKYYWNIV

>core/898/2/Org2_Gene223

MSQCQSSSTSTWEWMKSFVPNWKNPTPPLSPIPSEDEFILAYEPFVLPKTDPENAQANPPGTSTPNVENGIDDL
NPLLGQPNEQNNANNPGTSGSNPTSLPAPERLPETEENSQEEEQGSQNNEDLIG

>core/899/2/Org2_Gene628

MLEKLIKNFATYMGITSTLELDADGAYVLPISEVVKVRAQQNADNEIVLSASLGALPPSADTAKLYLQMMIG
NLFGRETGGSALGLDSEGNVVMVRRFSGDTTYDDFVRHVESFMNFSETWLSDLGLGKQ

>core/900/2/Org2_Gene344

MRDHAFSKLIGTVRAMVVEGRCPWSLQQSLVSMVEHILGECQEFHEAVLQGKTVQEVGSEAGDVLTLVLIL
CFLLEREGVLASEDVANEAMEKLRRRAPYIFAEDYKPVSIEEADRLWELAKHREKNEST

>core/901/2/Org2_Gene978

MDYKSQLVFSCPCCCKGNVCFSVFNLDVILTCNVCSSTYTFDSVIRNEIRQFVALCKRIHDANSILGNATVSVS
VEDNQMDIPFQLLFSRFPVVLNLSLDGKKIAIRFLFDALNTSILHQESDLIS

>core/902/2/Org2_Gene392

MREEGSVSFREYFRAYMCDKIVAQKNFLFTLDAVIKQAGWRSQEKLNLFYVESQALGREIKVSLEEYIQSMV
GILGSQRTKKSFKFSVDFTPLEQALQERCSSDDDEDATATSTATGATASPTDMHEDE

>core/903/2/Org2_Gene58

MTTESLETLVEKLSNLTVLELSQLKKLLEEKWDVTASAPVVAVAAGGGGEAPVAAEPTEFAVTLEDVPADK
KIGVLKVVREVTGLALKEAKEMTEGLPKTVKEKTSKSDAEDTVKKLQDAGAKASFKGL

>core/904/2/Org2_Gene691

MKFTVALFGEAEKGSYDTAYFCRSLVDLHNYLGDVSSPGITLAIKTLLSDYNVVYFRVREEGYCVDSYFFGL
HFLNTQTTLKNIIAIGLPGVGNQHIIEASRSLCQKHNSLLLFFDHDLYDLLTFNQPF

>core/905/2/Org2_Gene132

MRAGGSLVTTYPKEGQRLRSPEQLRVLDDLVQSYPNHLHAIELDCGAIPQDLIGATYIITFADFSTYILSLRSY
QANSPSDDTWGIWFGSIDDPVQAVISFLKDHGFALPSTLAQDPLLCTNK

>core/906/2/Org2_Gene185

MLIGRYSSDDQFTEATKNTPTIIKLGFVRDNLEGLTNPISEIVSETSSSIKDSVLRSLPILGSILGCARLYSTLSTN
DPLDETQEKIWHTIFGALETLGLGILILLFKIIFVILHCIFHLVIGFCK

>core/908/2/Org2_Gene456

MPLSDDEIEQFKKRLLEMKAKLSHTLEGNAQEVKKPNEATGYSQHQADQGTDTFDRTISLEVTTKEYELLRQ
INRALEKINESSYGICDVSGEEIPLARLIAIPYATMTVKAQEQFEKGLLSGN

>core/909/2/Org2_Gene550

MESSLCKKSLMKRRRALRVRKVLKGSPTKPRLSVVKTNKHIYVQLIDDSIGKTLASVSTLSKLNKSQGLTKK
NQEVAKVLGTQIAELGKNLQLDRVVFDRGPFKYHGIVSMVADGAREGGLQF

>core/910/2/Org2_Gene474

MPTINQLIRKRRKSSLARKKSPALQKCPQKRGVCLQVKTKTPKKPNSALRKVAWVRLSNGQEVIAYIGGEGH
NLQEHSIVLIQGGRVKDLPGVRYHIVRGTLDCAAVKNRKQSRSRYGAKRPK

>core/911/2/Org2_Gene787

MALKDTAKKMKDLLDSIQHDLAKAEKGNKAAAQRVRTDSIKLEKVAKLYRKESIKAEKSGLLKRKPSTKAP
AKVKKTAEKKAPKKSSAAAAKTSKAVKASKPASKKTAAKKVKKPSKARGFRK

>core/912/2/Org2_Gene727

MKRQKRKQSITLIEMMVVITLIGIIGGALAFNMRGSIHKGKVFQSEQNCAKVYDILMMEYATGGSSLKEIIAH
KETVVEEASWCKEGRKLLKDAWGEDLIVQLNDKGDDLVIFSKRVQSSNKK

>core/913/2/Org2_Gene761

MRDRLGSLSLILKVKIHKYLDTLHNQKRLALTVSRNIQATNKRIADLHLERYEHFISRDNIKHYDILLEYLKTL
QSSLYKQQSESLRFLEIHHQQLQELINRRKIIEKIKNNKYSKDQEIGT

>core/914/2/Org2_Gene546

MPRIIGIDIPAKKKLKISLTYIYGIGSARSDEIIKKLKLDPEARASELTEEEVGRLNSLLQSEYTVEGDLRRRVQS
DIKRLIAIHSYRGQRHRLSLPVRGQRTKTNSRTRKGKRKTVAGKKK

>core/915/2/Org2_Gene555

MIQQESQLKVADNTGAKKVKCFKVLGGSRRRYATVGDVIVCSVRDVEPNSSIKKGDVIKAVIVRTRRHITRK
DGSTLKFDTNSCVIIDDKGNPKGTRIFGPVAREIRDRGFIKISSLAPEVI

>core/917/2/Org2_Gene267

MEIIHIGTDIIEISRIREAIATHGNRLLNRIFTEAEQKYCLEKTDPIPSFAGRFAGKEAVAKALGTGIGSVVAWKD
IEVFKVSHGPEVLLPSHVYAKIGISKVILSISHCKEYATATAIALA

>core/918/2/Org2_Gene93

MVNLLKELEQEQCRNDLPEFHVGDTIRLATKISEGGKERVQVFQGTVMARRGGGSGETVSLHRVAYGEGME
KSFLLNSPRIVSIEIVKRGKVARARLYYLRGKTGKAAKVKEFVGPRSSKK

>core/919/2/Org2_Gene882

MVRATGSVASRRRRKRILKQAKGFWGDRKGHIRQSRSSVMRAMAFNYMHRKDRKGDFRSLWIARLNVASR
IHSLSYSRLINGLKCANISLNRKMLSEIAIHNPEGFAEIANQAKKALEATV

>core/920/2/Org2_Gene184

MRELNAFELTQPEEYRNRWVLMPCLKCRFCRTQHAKVWSYRCVHEASLYEKNCFLTLTYDDKHLPQYGSL
VKLHLQLFLKRLRKMISPHKIRYFECGAYGTKLQRPHYHLLLS

>core/921/2/Org2_Gene262

MATVAQTPQTTQPQPSVSHKATHRYCSWVFFKPILVSLGLLLASLTTLGLVIASGVTLSLGIGIVLAIQIVLAGI
ALVLAFNHIRQFKQARTAELNSMKMISAPAAATVQKQKLEDRYSSK

>core/922/2/Org2_Gene272

MTENRRIKRVNALLQEAIAKVILKDVKHPKISNLWITVTRVSLSKDLHSARVYYVSVMPHENTKEEALEALKV
SAGFIAHRASKNVVLKYFPELHFYLDDIFSPQDYIENLLWQIQEKEKS

>core/923/2/Org2_Gene719

MNKKPKKTKKAVQSKAAPVKRVPEESQEAAIQQLELAVSDLYKELPLAQTFASLTDKNQINSIIAALSGTLES
LHLEELTQGLFPSAQEDANFAKELSSVVHGLKNLTTVVNKQMVKGAE

>core/924/2/Org2_Gene459

MDNYLLGSLIFCCVLLSIGMCTIFVMTICFLRQLNKILKNIHRVTTILNFEAKILAPLMLGKKLLCGWLKKRKN
RGSLSEDIDELLDEKKQRSWKKNLDQGIKWCAALVLIWKVFRNKD

>core/925/2/Org2_Gene812

MDSFCFDLLKVAAKAIDDKKGNNLVVLDVRTISEFTDYFVFVEGSVNVHVKALANTIVEELKKQKVSPLHVE
GITDGNWVVIDYGFIVVHVFVSEIRGKYRLEELWKDGFIVTSKLLAS

>core/926/2/Org2_Gene315

MPVSSAPLPTSHRPSSGNLGLMEPNSKALKAKHQDKTTKTIKLLVKILVAILVIEVLGIIAAFFIPGTPPICLIILG GLILTTVLCVLLLVIKLALVNKTEGTTAEQQIKRKLSSKSIS

>core/927/2/Org2_Gene91

MALKIRLRQQGRRNHVVYRLVLADVESPRDGKYIELLGWYDPHSSINYQLKSERIFYWLERGAQLSSKAEAL VKQGAPGVYSALLSKQEARKLVVRKKRRAYRQRRSTQREEAAKDATK

>core/928/2/Org2_Gene991

MTVLFYAFLFIFLFLCVILCGLILVQESKSMGLGSSFGVDSGDSVFGVSTPDILKKVTSWCAVAFCIGCLLLSFS TNLLGKKLDAKEFLLPAAEESDTQASSESVEADES

>core/929/2/Org2_Gene785

MLSRIVTCFLFLLSSLPLFAEEEAAQSKNTFVQPAVMLAIAILFFYFILWRPEQKRRKAMEKRKNDLAKGDKV TAMGIIGTVDDIREHTVILNIASGKVEVLKGAISEILKPNDNKS

>core/930/2/Org2_Gene436

MSDIQKEEHGSTTIFHLHGKLDGISSPEVQENISQSLAAGSKNIILDCAHLDYMSSAGIRVLLQSYHQVGQHSG KIVLTTVPKTIEQTLYVTGFLSYFKIFNTVDEAIQTLNKDGD

>core/932/2/Org2_Gene231

MFLKRKKRGGSQVQNKRTASPIKHAKHYLHNYLQELQKIMAARPHDAIDAWNQVFRDKYKGMSQAIGFRD HILLVKVYNSSLYALLKQTPQNDLIMSLYQVASHVQIREIQFLLG

>core/933/2/Org2_Gene336

MSKVSVRKKNWGFRLLEEVMIKSWWVIFSILIGGFVYDRAIQELRTEELRLQSKVSSLCQDILSAQEKQRQLQ LHLQHWQDSAAIEAALIQRLGLIPKGYKKLCVSPKQQSENKD

>core/934/2/Org2_Gene373

MWYSDYHVWILPVHERVVRLGLTEKMQKNLGAILHVDLPSVGSLCKEGEVLVILESSKSAIEVLSPVSGEVID INLDLVDNPQKINEAPEGEGWLAVVRLDQDWDPSNLSLMDEE

>core/936/2/Org2_Gene847

MGKKENQLYEGAYVFSVTLSEEARRKALDKVISGITNYGGEIHKIHDQGRKKLAYTIRGAREGYYYFIYFSVS PGAITELWKEYHLNEDLLRFMTLRADSVKEVLEFASLPE

>core/937/2/Org2_Gene806

MTAVLILTSFPSEESARSLARHLITERLASCVHVFPKGTSTYLWEGKLCESEEHHIQIKSIDIRFSEICLAIQEFSG YEVPEVLLFPIENGDPRYLNWLTILSYPEKPPLSD

>core/938/2/Org2_Gene560

MFKATARYIRVQPRKARLAAGLMRNLSVQEAEEQLGFSQLKAGRCLKKVLNSAVANAELHENIKRENLSVT
EVRVDAGPVYKRSKSKSRGGRSPILKRTSHLTVIVGEKER

>core/939/2/Org2_Gene554

MKKQNIRVGDKVFILAGNDKGKEGKVLSLTEDKVVVEGVNVRIKNIKRSQQNPKGKRISIEAPIHISNVRLTIA
GEPAKLSVKVTEQGRELWQRRPDGTSQLYRLVRGKKG

>core/940/2/Org2_Gene371

MTTLPKYVPRSRQNPDTLTFLKRYSSVLLHSENSLSYRIFAKVLAILLTSLAVAFAVTLFSCEGSQLRLCALYI
GIALAICVLLTIVVYCIASKIATACKKPPSISRIEIV

>core/943/2/Org2_Gene563

MKDPYDVIKRHYVTEKAKMLEHLSAGTGEGKKKGSFCKDPKFVFIVSHDATKPLIAQALEAIYVDKNVKVK
SVNTINVKPQPARMFRGRRKGKTSGFKKAIVTFYQGHSVG

>core/945/2/Org2_Gene417

MTVFKQIIDGLIDCEKVFENENFIAIKDRFPQAPVHLLIIPKKPIPRFQDIPGDEMILMAEAGKIVQELAAEFGIA
DGYRVVINNGAEGGQAVFHLHIHLLGGRPLGAIA

>core/946/2/Org2_Gene807

MNLSAKEYGDIIVIYLQGSLDAVSVPSVQEYLEQFIQKKHLKIALNFTDVSYISSAGIRLLLSNFKLVQSLGGK
MCLCCVKESVTEVMRIAGLDQLILLCQSEQECLSKL

>core/947/2/Org2_Gene446

MASSATPGFDGTAPSLFPPATRPRYNFKLALFVTIAIALVWIALIATTIAIGLCIHPLCSFIFLTAIPLYFISRYICS
HYARNVYIALDVVPDHSKLQDMRSHSPIFSDR

>core/948/2/Org2_Gene87

MKKNTHPEYRQVLFVDSSTGYKFVCGSTYQSEKTEVFEGKEYPVCYVSVSSSSHPFFTGSKKFVDAEGRVDK
FLKRYSNVRQPAQQPQPEEDALPAAKGKKKVVTKKKK

>core/949/2/Org2_Gene26

MNEPTRTYLESEKDTQDQIEELQATCIVKNAAGIHVRPAGVIVRLFDGEPCDVHFTYAGKTINAKSIMSILML
GAPQGGEILVTIRSKEAHRILQKIQDAFSSGFGEL

>core/950/2/Org2_Gene960

MSTSPIGVPSMLNAATSLNATTSKAPLPTSTLAERIKEWLPRILLLIVGAIFTIAGCIVMALTKQILYGLLCVVG
GLLLALGLLLKPENCIYRNAESAARSLSNALE

>core/952/2/Org2_Gene520

MDEITPNYPLLRQDSLWNRVRVSWRADLSVSSRYEIASAIAILGLLVAFCASAAVSIIFTANPLAQVFIDGCLALGLLPIPLVIGLLIIGIIVLLYGIYLFPQQRE

>core/954/2/Org2_Gene468

MEPYAVIQTGSKQYQVRSGDVIDVELLGEVASDKEVIFQDVLFVFDGTKASLGSPTIANAQVKAEYLSHVKGEKVVAYKYKKRKNYHRKHGHRQKYLRVKIREILI

>core/955/2/Org2_Gene633

MSFTYFLALPVDRLMQERFLCSPKRWAPFINSPLYLTLIADHDTPYLAKNLDKFPLPVEQWEKTVLHVSSLLKSIFLCSDLSSLRLLACTKFEILTLNDLYCAQNI

>core/956/2/Org2_Gene504

MNERTLLLLLKKKKGLFLAILDLTQTESSLTTPELEKVLKQKKIFLSCIDRVDLQIKEFRHAFSSELPQDIQEELEEIRDVIIRILDTDKRNYAQKKKEFGIYERP

>core/957/2/Org2_Gene471

MKQQKQKIRIRLKGFDQGQLDRSTADIVETAKRTGARVVGPIPLPTKREVYTVLRSPHVDKKSREQFEIRTHKRLVDILDPTGKTIDALKMLALPAGVDIKIKAA

>core/958/2/Org2_Gene316

MSSPVVTGTSSASPVEQTKLGEFLERLSGSGRCIKIAFAASTALLLLNTFVSGIVAIAMIFVATSVGAYFTVIGPLFLLSLILLAIMLISMYKITHPSQNTPISN

>core/959/2/Org2_Gene512

MSFKRFLQQIPVRICLLIIYLYQWLISPLLGSCCRFFPSCSHYAEQALKSHGFLMGCWLSIKRIGKCGPWHPGGIDMVPKTALQEVLEPYQEIDGGDSSHFSE

>core/961/2/Org2_Gene576

MVKIISSENFDSFIASGLVLVDFFAEWCGPCRMLTPILENLAAELPHVTIGKINIDENSKPAETYEVSSIPTLILFKDGNEVARVVGLKDKEFLTNLINKHA

>core/962/2/Org2_Gene108

MSDQATTLRIKPLGDRILVKREEEEATARGGIILPDTAKKKQDRAEVLVLGTGKRTDDGTLLPFEVQVGDIILMDKYAGQEITIDDEEYVILQSSEIMAVLK

>core/963/2/Org2_Gene372

MDPASPVAPHVLQDHVQLSSEELSALSSGVSRVKKLTIAIMVLSLIAISLVACGLFLTGSAPLQLSIWIAASCITLSMLVCACWRYKISNALEKTKVAHES

>core/964/2/Org2_Gene834

MAKKSSVAREAKRRRLVEANFKKRSDLRKIVKSLSVSEEEKENARISLNKMKRDTSPTRLHNRCLLTGRPRG
YLRKFAISRICFRQMASMGEIPGVIKASW

>core/965/2/Org2_Gene2

MEQFHLDREEILLLAKASALQLSEELIQEYQTSLSAVITSMKEALAIEIDDADSCESLFMHVVNVEDLREDSVT
SDFNREEFLRNVPESLGGLVKVPAVIK

>core/967/2/Org2_Gene460

MFRNNHKPKKTKCKRFRWLRGVLFGGFIATLLTCLFTPKSGVQLRKKILKVKNSGAKKSRVFFKNSKQHTKS
FVKQAKLLAKNISHELQDFKKGILDDKD

>core/968/2/Org2_Gene356

MATMTKKKLISTISQDHKIHPNHVRTVIQNFLDKMTDALVKGDRLEFRDFGVLQVVERKPKVGRNPKNAAV
PIHIPARRAVKFTPGKRMKRLIETPNKHS

>core/969/2/Org2_Gene666

MAPKKPNKKNVIQRRPSAEKRILTAQKRELINHSFKSKVKTIVKKFEASLKLDDTQATLSNLQSVYSVVDKA
VKRGIFKDNKAARIKSKATLKVNARAS

>core/970/2/Org2_Gene910

MKKVVTLSIIFFATYCASELSAVTVVAVPLSEAPGKIQVRPVVGLQFQEEQGSVPYSFYYPYDYGYYYPETYG
YTKNTGQESRECYTRFEDGTIFYECD

>core/971/2/Org2_Gene632

MEPRHIYIRKPETPKAPDVEKPGVPEYMTMANTPTFEGPVKTLDQLRRALIEQRGAEEGQKMYDNFIQSILIST
FGLVHKDMDRAQKASKRMRSVYKEQ

>core/972/2/Org2_Gene987

MCLIDCLGRGFEAAINTVCCCSDSSESKANVATVSAGLLALTAIVSFILIILICTGVLGASGMTFGMSNVAAVL
VLVVTILLSMFLSGACFAAKREILR

>core/974/2/Org2_Gene679

MLSYLLRTAINVYSFLILAYIFASWVPDCQSARWYQLVSKCVDPFLNFFRRFVPRIGFIDPSPFVGLLCLGILPF
VILRVLRFIILNIFHSPWLLQYL

>core/975/2/Org2_Gene285

MGKPKKSRTDRALAQEIQKKSTEVLKKPARIKAKNRRKFLIAKEQKTLKHRAQEYDQLVRSLLDSQKKDTD
KVLIFNYENGFVFTDKDHFSKYSIRL

>core/976/2/Org2_Gene96

MIKKDRFTNEKLNKLFDSPFSLVNYAIKQAKIKIAKGDVRSSNVAIETLVLLDREGIQPEFTEEIVVTASPTVERKRSEHTNSRKKDPSAYTWSDVK

>core/977/2/Org2_Gene476

MYEGKSRMASPTPGQLHLQQKVESKAYDYSRSLAMIATALLFFIVALILSGLSLLPQVFLPFSGAYFIIGSFLAFIALGILLINCVCDLKQYLTSS

>core/978/2/Org2_Gene28

MGSGYAKKKKEAKIMEQQFLEMEASLLEKRYEGQAGNGLVSVVINGKCDLISVKVQPTCLDPEDPEVIEDLFRAAFKLAKEQMDQEMSLMRSTMPF

>core/979/2/Org2_Gene360

MNKSRFLRLCCCLCFCGSLFYFYINKQNSLTKLRLEIPCLSVRLRQLEQQNISLRFLIDKIERPDHLMEIAALPEYQYLEYPSEESISLLSYELP

>core/980/2/Org2_Gene732

MLAFFATSFKSVLFEYSYQSLLLILIVSAPPIILASIVGIMVAIFQAATQIQEQTFAFAVKLVVIFGTLMISGGWLSNMILRFAGQIFQNFYKWK

>core/982/2/Org2_Gene528

MLIRLFLGISLPKGFPLYLEPPLVLATFQGTQFVGTYSEATNPLYIDNLNLNYHYTQELLYKAVPCNYKSIYREIPLIIFPEVLIGSTPTQSTE

>core/983/2/Org2_Gene1

MLGKIIRGLSSLIVILCALNVGLIGITHNKLNIIAKLCGGVSTPATQITYIIIGIAGVICLLSFCPFCSKKSRHSHGDSCSSGGCHSHHSDKN

>core/988/2/Org2_Gene308

MAKLVITSDDEQQEFELEDNSEIAEPCESMGIPFACTEGVCGTCVIEVLEGRENLSEFTEPEYDFLGEPEDSNERLACQCRIKGGCVKVTF

>core/989/2/Org2_Gene423

MDDSWILEVKVTPKAKENKIVGFDGQALKVRVTEPPEKGKANDAVISLLAKALSLPKRDVTLIAGETSRKKKFLLPNRVQDIIFSLHIDV

>core/990/2/Org2_Gene479

MKKAVLIAAMFCGVVSLSSCCRIVDCCFEDPCAPSSCNPCEVIRKKERSCGGNACGSYVPSCSNPCGSTECNSQSPQVKGCTSPDGRCKQ

>core/991/2/Org2_Gene890

MSLDKGTKEEITKKFQLHEKDTGSADVQIAILTEHIAELKEHLKRSPKDQNSRLALLKLVGQRRKLLEYLNST
DTERYKNLITRLNLRK

>core/992/2/Org2_Gene280

MSRKCPLTGKRPRRGYSYTLRGIAKKKKGIGLKVTGKTKRRFFPNMLTKRLWSTEENRFLKLKISASALRHID
KLGLEKVLERAKSKNF

>core/993/2/Org2_Gene561

MGRSLRKGPFVDHHLLKKVRAMNIEEKKTPIKTWSRRSMITPEMIGHTFEVHNGKKFLTVFVSETMVGHKL
GEFSPTRIFKSHPVKKG

>core/994/2/Org2_Gene145

MKEYLDFLVQRNVERDPQTKRHCTVSQKFGGESIDAKTTTGQLFHIAGKTEPGHGKLCLGESILKQLLALGII
TGYENREREVWVYLD

>core/996/2/Org2_Gene498

MSQKNKNSAFMHPVNISTDLAVIVGKGPMPRTEIVKKVWEYIKKHNCQDQKNKRNILPDANLAKVFGSSDPI
DMFQMTKALSKHIVK

>core/997/2/Org2_Gene840

MKSFKFLLPFLSVILCCGNLLSSPRSRAISVTESIGMSAVKTLVLSEKAHEFLEGIGYGVGASSILRDWQTQQW
LEIESLLAQNEVM

>core/998/2/Org2_Gene556

MASEPRGSRKVKIGVVVSAKMEKTVVVRVERIFSHPQYLKVVRSSKKYYAHTELKVSEGDKVKIQETRPLSK
LKRWRVIEHVGVVS

>core/999/2/Org2_Gene943

MVEIHHKDPSLKKLFALQQSLETLNSLSDIVATYEAMFSLIYEGLNKALRKDQLCYLLSVNSKGELLKSPSGD
PIVQTFPIHPHH

>core/1000/2/Org2_Gene625

MATNKSCTAFDFNKMLDGVCTYVKGVQQYLTELETSTQGTVDLGTMFNLQFRMQILSQYMESVSNILTAVN
TEMITMARAVKGS

>core/1001/2/Org2_Gene467

MAHKKGQGASRNGRDSKSKRLGVKVGAGQKVSTGSILVRQRGTRWNPAQNVGRGRDDTLFALVDGIVVM
KKTNRTYISVVPEQL

>core/1003/2/Org2_Gene53

MKQQHNRKALSRKIGTVKKQAKFAGSFLDEIKKIEWVSKHDLKKYIKVVLISIFGFGFAIYFVDLVLRKSITCLDGITTFLFG

>core/1004/2/Org2_Gene626

MFNMENTAKEEKNSQPLLDLEQDMQDHDRAQELKASVQDKVHKLHALLREGSDKESFGQQQSLLAGYVALQKVLGRINRKMI

>core/1005/2/Org2_Gene848

MNKPVHNNEHRRKRFNKKCPFVSAGWKTIDYKDVETLKKFITERGKVLPRRITGVSSRFQGVLSQAIKRARHLGLLPFVGED

>core/1007/2/Org2_Gene43

MTKIQCSAQYYRSRPAERAQTPPQPFLARDRADFWERHPRFSACCRVLLLVAWVVLALLFLFVMLLPLAAGSYLLAF

>core/1009/2/Org2_Gene250

MSLEDDVIAIIVEQLGVDPKEVNENSSFIEDLNADSLDLTELIMTLEEKFAFEISEEDAEKLRTVGDVFTYIKKRQAEQ

>core/1012/2/Org2_Gene635

MKEFLAYIIKNLVDRPEEVRIKEVQGTHTIIYELSVAKPDIGKIIGKEGRTIKAIRTLLVSVASRNNVRVSLEIMEEK

>core/1013/2/Org2_Gene957

MMTLFLVICCATVLLGLGMGILLVGSHLLGRPLSKGCQKPDCCQKKTCDKTEHCATKSRENSTSKCSSNDDVPPTAP

>core/1014/2/Org2_Gene686

MNEGIHSVCFQKTPRLTAKSVVSMEMLLTTQQLPSAEGMPSVANLEADFLRAEALLAEMREIRGCLEQSLRTLVPSE

>core/1015/2/Org2_Gene892

MKNKIVTLLDQLYEDQESRLQKLGEEIVPNLTPEDLLQPMDFPQLEGNPAFRFEEGVLSGIGEVRAAILAALSQEN

>core/1016/2/Org2_Gene969

MEAKKIKELSKEAQLLKKLREKSRVLDEKNKRKAWVAKLVAMPESIREIEKEERVETPQLFQAIAEKILEEGV

>core/1018/2/Org2_Gene640

MFFAPLLYESLRRGLMHPTSHMQQQLARLEFINDQLTTELEHVNELLCSLGFPEGLTTIKAIAEEVLSDDEPLLD

>core/1019/2/Org2_Gene964

MEEVPFENAMQRLEEIVDLMNQPTTSLDASLALYEEADALMRICESRIRQVEQRVRELAEKRHESSLFEEQA
VVR

>core/1021/2/Org2_Gene51

MAKKEDTLVLEGKVEELLPGMHFRVILENGMPVTAHLCGKMRMSNIRLLVGDRVTVEMSAYDLTKARVVY
RHR

>core/1022/2/Org2_Gene966

MSGNECNDYPEVFKDDVSAYVLVTCGQMSSEGKIQVEMTYEGDPAVISYLLTKARDSLDES

>core/1023/2/Org2_Gene986

MSYKITLPKADETTAKKVTKISEASTLIFSVLKEKASLGNVHGFCQAENSLSVEANKIISVAENTLAGCFCK

>core/1025/2/Org2_Gene557

MAAKKDLLTQLRGKSDDDLDAYVHENKKALFALRAENLLQNKVVKVHMFSTHKKNIARALTVKQERKGK
VHG

>core/1027/2/Org2_Gene953

MMFDFMLARAFRAANTIVNTKQKPFHIHGMATLRPNATSILNIKEDALSLGTVGSFASTASIKIDSH

>core/1028/2/Org2_Gene581

MKKLIALIGIFLVPIKGNTNKEHDAHATVLKAARAKYNLFFVQDVFPVHEVIEPISPDCLVHYEGWV

>core/1031/2/Org2_Gene955

MKKLMLALLLALVLPVAGSAFGYESCSAPQDEAQGSEDEGRDESSDSDTQTEKDEEGGSEENTAE

>core/1033/2/Org2_Gene881

MPKMKTNKSVSARFKLTASGQLKRTRPGKRHKLSKKSSQEKRNLSKQPLVDKGQVGMYKRMMLV

>core/1034/2/Org2_Gene415

MIKQACKFYLLQCLLCALYWLLKYCRKLLKGTLHHSEETLYQALLSSLIDLLYQLKQLPAPTNE

>core/1037/2/Org2_Gene480

MKLFYFCNIIFLTSLFSVSCTHTPPPRSYILAQGRTPLVNKAHSLEFFLARSVFNTCYNTNL

>core/1045/2/Org2_Gene854

MAVPRNRHSNARKNIRRSHDAKKACHAAKCSNCKHALLPHTICPSCGFYNGKAVMTVEKK

>core/1046/2/Org2_Gene590

MSIALNREEVWDNPHHLMFILMQFQQFSGEQDRFGSFLEATIRDRVSFLVLQEKIATLK

>core/1049/2/Org2_Gene984

MRFFCLFFLGFLGSFHCVAEDKGVDLFGVWDDNQITECDDSYMTEGREEVEKVVDA

>core/1050/2/Org2_Gene20

MPSVKVRVGEPVDRALRILKKKIDKEGILKAAKSHRFYDKPSVKKRAKSKAAAKYRSR

>core/1054/2/Org2_Gene341

MQIENSSILFAEVVMKWFIFSVISAPVVFLPGCTLIPKEKVTKVPSQLWSESLSQP

>core/1056/2/Org2_Gene981

MIDAENQNPKDGGSFTSLHTDPKNLFDEEGMPSPSDTLQCDLNNVFIFIKSMFF

>core/1058/2/Org2_Gene433

MSRHRSYGKSVKGVTKRNVLKRFERVEVLRKLGRWNDSTAKKVTGLPKTPILK

>core/1059/2/Org2_Gene210

MASKNREIIKLKSSESSDMYWTVKNKRKTTGRLELKKYDRKLRRHVIFKEAR

>core/1067/2/Org2_Gene913

MFFIAVRSRGFLDIHGILAARKGKQVVKSTAGAWIGSRGAVFYSLVS

>core/1068/2/Org2_Gene985

MSESLEIPELTEVLSEQPSLSTPDSPPKVITGTLTLYFQEDIDPAS

>core/1069/2/Org2_Gene833

MKVSSSVKADPSKGDKLVRRKGRLYVINKKDPNRKQRQAGPARKK

>core/1070/2/Org2_Gene832

MKRTYQPSKRKRRNSVGFRTRMATRNGRKLLNRRRRHGRHSLVDL

>core/1073/2/Org2_Gene1008

MSSVKKKRRLKIAKHKRKKRRRRDRHKNK

>core/0/3/Org3_Gene880

MKSLPVYVSGIKVRNLKNVSIHFNSEEIVLLTGVSGSGKSSIAFDTLYAAGRKRYISTLPTFFATTITTLPNPKV
EEIHGLSPTIAIKQNHFSHYSHATVGSTTELFSHLALLFTLEGQARDPKTKEVLDLYSKEKVLSTIMELSEGVQI
SILAPLLRKDIAAIHEYAQQGFTKVRCNGTIHPIYSFLTSGIPEDCSVDIVIDTLIKSENNIARLKVSLFTALEFGE
GHCSVLSDEELMTFSTKQQIDDVTYTPLTQQLFSPHALESRCSLCQGSGIFISIDNPLLIDENLSIKENCCSFAGN
CSSYLYHTIYQALADALNFNLETPWKDLSPEIQNIFLRGKNNLVLPVRLFDQTLGKKNLTYKVWRGVLNDIG
DKVRYTTKPSRYLSKGMSAHSCSLCKGTGLGDYASVATWEGKTFTEFQQMSLNNWHVFFSKVKSPSLSIQEI
LQGLKQRLSFLIDLGLGYLTPNRALATLSGGEQERTAIAKHLGGELFGITYILDEPSIGLHPQDTEKLIGVIKKL

RDQGNTVILVEHEERMISLADRIIDIGPGAGIFGGEGLFNGKPEDFLMNSSSLTAKYLRQELTIPIPESREAPTS
WLLLTEATIHNLKNLSIRLPLARLIGVTGVSGSGKSSLINNTLVPAIESFLKQENPKNLHFEWGCIGRLIHITRDL
PGRSQRSIPLTYIKAFDDIRELFASQPRSLRQGLTKAHFSFNQPQGACIQCQGLGTMTISDDDTPIPCSECQGKR
YHSEVLEILYEGKNIADILDMTAYEAEKFFISHPKIHEKIHALCSLRLDYLPLGRPLSTLSGGEIQRLKLAHELL
FASPKQTLYVLDEPTTGLHTHDIQALIEVLLSLTYLGHTVLVIEHNMHVVKVCDYVLELGPEGGDLGGYLLA
SCTPKDLIQLNTPTAKALAPYIEGSLDIPVVKSEPPSSPKSCDILIKDAYQNNLKHIDLALPRNSLIAIAGPGASG
KHSLVFDILYASGNIAYAELFPPYIRQGLLKETPLPSVGEVKGLSPVISVRKCSSSNRSYHTIASALGLSNGLEE
LFAILGEPFSPLTEEKLSKIHLRPSSIATQSYNDELTSLLTSPIPLGADLEIFLQEKQKEGFIKLYSEGNLYDLDER
LPLNLIEPAIVIQHTKVSPKNSSSLLSAISVAFSLSSEIWIYISQKKQRKLSYSLGWKDKKGRLYPEITHQLLSSD
HPEGRCLTCGGRGEILKISLEEHKEKIAHYTPLEFFSLFFPKSYMKPVQKLLKDENASQPLKLLTTKEFLNFCR
GSSEFPGMNALLMEQLDTESDSPLIKPLLALTSCPACKGSGLNDYANYVRINNTSLLDIYQEDATFLESFLNTI
GTDDTRSIIQDLMNRLTFISKVGLSYITLGQRQDTLSDGENYRLHLAKKISINLTNIVYLFEEPLSGLHPQDLPTI
VQLLKELVANNNTVIATDRSCSLIPHADHAIFLGPGSGPQGGFLMDSDTEVCPSVDLHANVPQTEVCPKAPLS
ISKANHTRGSDRTLKVNLSIHHIQNLKVSAPLHALVAIGGVSGSGKTSLLLEGFKKQAELLIAKGTTTFSDLVV
IDSHPIASSQRSDISTYFDIAPSLRAFYASLTQAKALNISSTMFSTNTKQGQCSDCQGLGYQWIDRAFYALEKR
PCPTCSGFRIQPLAQEVLYEGKHFGELLHTPIETVALRFPFIKKIQKPLKALLDIGLGYLPIGQKLSSLSVSEKTA
LKTAYFLYQTPETPTLFLIDELFSSLDPIKKQHLPEKLRSLINSGHSVIYIDHDVKLLKSADYLIEIGPGSGKQGG
KLLFSGSPKDIYASKDSLLKKYICNEELDS

>core/1/3/Org3_Gene918

MKWLPATAVFAAVLPALTAFGDPASVEISTSHTGSGDPTSDAALTGFTQSSTETDGTTYTIVGDITFSTFTNIP
VPVVTPDANDSSSNSSKGGSSSSGATSLIRSSNLHSDFDFTKDSVLDLYHLFFPSASNTLNPALLSSSSSGGSSS
SSSSSSSGSASAVVAADPKGGAAFYSNEANGTLTFTTDSGNPGSLTLQNLKMTGDGAAIYSKGPLVFTGLKN
LTFTGNESQKSGGAAYTEGALTTQAIVEAVTFTGNTSAGQGGAIYVKEATLFNALDSLKFEKNTSGQAGGGI
YTESTLTISNITKSIEFISNKASVPAPAPEPTSPAPSSLINSTTIDTSTLQTRAASATPAVAPVAAVTPTPISTQETA
GNGGAIYAKQGISISTFKDLTFKSNSASVDATLTVDSSTIGESGGAIFAADSIQIQQCTGTTLFSGNTANKSGGG
IYAVGQVTLEDIANLKMTNNTCKGEGGAIYTKKALTINNGAILTTFSGNTSTDNGGAIFAVGGITLSDLVEVR
FSKNKTGNYSAPITKAASNTAPVVSSSTTAASPAVPAAAAAPVTNAAKGGALYSTEGLTVSGITSILSFENNEC
QNQGGGAYVTKTFQCSDSHRLQFTSNKAADEGGGLYCGDDVTLTNLTGKTLFQENSSEKHGGGLSLASGKS
LTMTSLESFCLNANTAKENGGGANVPENIVLTFTYTPTPNEPAPVQQPVYGEALVTGNTATKSGGGIYTKNA
AFSNLSSVTFDQNTSSENGGALLTQKAADKTDCSFTYITNVNITNNTATGNGGGIAGGKAHFDRIDNLTVQSN
QAKKGGGVYLEDALILEKVITGSVSQNTATESGGGIYAKDIQLQALPGSFTITDNKVETSLTTSTNLYGGGIYS
SGAVTLTNISGTFGITGNSVINTATSQDADIQGGGIYATTSLSINQCNTPILFSNNSAATKKTSTTKQIAGGAIFS
AAVTIENNSQPIIFLNNSAKSEATTAATAGNKDSCGGAIAANSVTLTNNPEITFKGNYAETGGAIGCIDLTNGS
PPRKVSIADNGSVLFQDNSALNRGGAIYGETIDISRTGATFIGNSSKHDGSAICCSTALTLAPNSQLIFENNKVT
ETTATTKASINNLGAAIYGNNETSDITISLSAENGSIFFKNNLCTATNKYCSIAGNVKFTAIEASAGKAISFYDA
VNVSTKETNAQELKLNEKATSTGTILFSGELHENKSYIPQKVTFAHGNLILGKNAELSVVSFTQSPGTTITMGP
GSVLSNHSKEAGGIAINNVIIDFSEIVPTKDNATVAPPTLKLVSRTNADSKDKIDITGTVTLLDPNGNLYQNSY
LGEDRDITLFNIDNSASGAVTATNVTLQGNLGAKKGYLGTWNLDPNSSGSKIILKWTFDKYLRWPYIPRDNH
FYINSIWGAQNSLVTVKQGILGNMLNNARFEDPAFNNFWASAIGSFLRKEVSRNSDSFTYHGRGYTAAVDAK
PRQEFILGAAFSQVFGHAESEYHLDNYKHKGSGHSTQASLYAGNIFYFPAIRSRPILFQGVATYGYMQHDTTT

YYPSIEEKNMANWDSIAWLFDLRFSVDLKEPQPHSTARLTFYTEAEYTRIRQEKFTELDYDPRSFSACSYGNL
AIPTGFSVDGALAWREIILYNKVSAAYLPVILRNNPKATYEVLSTKEKGNVVNVLPTRNAARAEVSSQIYLGS
YWTLYGTYTIDASMNTLVQMANGGIRFVF

>core/2/3/Org3_Gene968

MVAKKTVRSYRSSFSHSVIVAILSAGIAFEAHSLHSSELDLGVFNKQFEEHSAHVEEAQTSVLKGSDPVNPSQ
KESEKVLYTQVPLTQGSSGESLDLADANFLEHFQHLFEETTVFGIDQKLVWSDLDTRNFSQPTQEPDTSNAVS
EKISSDTKENRKDLETEDPSKKSGLKEVSSDLPKSPETAVAAISEDLEISENISARDPLQGLAFFYKNTSSQSISE
KDSSFQGIIFSGSGANSGLGFENLKAPKSGAAVYSDRDIVFENLVKGLSFISCESLEDGSAAGVNIVVTHCGDV
TLTDCATGLDLEALRLVKDFSRGGAVFTARNHEVQNNLAGGILSVVGNKGAIVVEKNSAEKSNGGAFACGS
FVYSNNENTALWKENQALSGGAISSASDIDIQGNCSAIEFSGNQSLIALGEHMGLTDFVGGGALAAQGTLTLR
NNAVVQCVKNTSKTHGGAILAGTVDLNETISEVAFKQNTAALTGGALSANDKVIIANNFGEILFEQNEVRNH
GGAIYCGCRSNPKLEQKDSGENINIIGNSGAITFLKNKASVLEVMTQAEDYAGGGALWGHNVLLDSNSGNIQ
FIGNIGGSTFWIGEYVGGGAILSTDRVTISNNSGDVVFKGNKGQCLAQKYVAPQETAPVESDASSTNKDEKSL
NACSHGDHYPPKTVEEEVPPSLLEEHPVVSSTDIRGGGAILAQHIFITDNTGNLRFSGNLGGGEESSTVGDLAI
VGGGALLSTNEVNVCSNQNVVFSDNVTSNGCDSGGAILAKKVDISANHSVEFVSNGSGKFGGAVCALNESV
NITDNGSAVSFSKNRTRLGGAGVAAPQGSVTICGNQGNIAFKENFVFGSENQRSGGGAIIANSSVNIQDNAGD
ILFVSNSTGSYGGAIFVGSLVASEGSNPRTLTITGNSGDILFAKNSTQTAASLSEKDSFGGGAIYTQNLKIVKNA
GNVSFYGNRAPSGAGVQIADGGTVCLEAFGGDILFEGNINFDGSFNAIHLCGNDSKIVELSAVQDKNIIFQDAI
TYEENTIRGLPDKDVSPLSAPSLIFNSKPQDDSAQHHEGTIRFSRGVSKIPQIAAIQEGTLALSQNAELWLAGLK
QETGSSIVLSAGSILRIFDSQVDSSAPLPTENKEETLVSAGVQINMSSPTPNKDKAVDTPVLADIISITVDLSSFV
PEQDGTLPLPPEIIIPKGTKLHSNAIDLKIIDPTNVGYENHALLSSHKDIPLISLKTAEGMTGTPTADASLSNIKID
VSLPSITPATYGHTGVWSESKMEDGRLVVGWQPTGYKLNPEKQGALVLNNLWSHYTDLRALKQEIFAHHTI
AQRMELDFSTNVWGSGLGVVEDCQNIGEFDGFKHHLTGYALGLDTQLVEDFLIGGCFSQFFGKTESQSYKA
KNDVKSYMGAAYAGILAGPWLIKGAFVYGNINNDLTTDYGTLGISTGSWIGKGFIAGTSIDYRYIVNPRRFIS
AIVSTVVPFVEAEYVRIDLPEISEQGKEVRTFQKTRFENVAIPFGFALEHAYSRGSRAEVNSVQLAYVFDVYR
KGPVSLITLKDAAYSWKSYGVDIPCKAWKARLSNNTEWNSYLSTYLAFNYEWREDLIAYDFNGGIRIIF

>core/3/3/Org3_Gene885

MASSSNNSTKQDGIPSWVNPNVQWNRASQVGDQEANSLTPEAQTSRSWFSDRKHFLEVLDVSLEEMENNDL
KKYSRYKTIILIATLVTVAITCIVPISMVFGIPMWVPCLILFGAGLSSAFLSHRLQSKCKEIHLRYRAYQIYRQQL
LSQYPDLRKSTLYKYSITHVKPKKGFVGKLVENLRPDLHKNKDDGGAAADSRLDFAGYGVKHYQTDALLG
VSGVNSVEWQRLASLIMSVKNDILNDVGSREPIDKAQRSALVVSGKDIGGEIQPGGILDISRDILAICGYGMNV
GVEAKKAIDQYKKWYLNSSTFIAWNPQLPAIAQSYLLEQQRHLDYAAKIFQDLSALTTAHGTGQALEDLDSL
LCYYDQLIESKGVGEKIIASIHQKHLDLAMQDSCDQEHLKKWSNLYHVFSITIKEFTEGKLEQNEVVSRIQRL
RGKLEKSKCSILGNCRTNAEYATKSEKKLADYLLQIGDREPFLTGMHKAIATGKAIQGKVEGVISQHPEKQIM
MLRCSIERLEGMLRREDWGAILQKNEDEVLALKSTMEAQLQGFKDLVGTWEGKYQEFKKNKLSKVLVYDF
TKSYSNLLNRLEVLHAESSTDDLVLHVDRMSEDLKKTIEEIDGNLFQVTPEELSLLAREYQGLMNELPLIVQE
GNRLQEAISSEGVSQGLMLLNSLLNRDEKINKNIESSRKNLVAIAKQARSDARNIDSQGLAPLIQRNRASLDNI
LQNMYLFNGSIRNIHALDTETLVATSSNMFSAMHTFDWNIYTNLLDVLEIQSKPAPAPMENPDLPGALPEEVQ
DAVAEDVSGTHRLHHQVLKRRCADLKNMISQLQKSINKWGMAKAIVLGIVAVLFCVLSAIFIGQNILSLLILS

CVGLLLTQVCPLIFDRISKSKEFEKQVLETAQSLIPATKILPSEFNNKDLNRLAKLQDNLNLEGFGPTWARNIV
SDLEGIPTKEKSLKDLTKEFRKDSKNLNKRIKRRFKEGLGQEAPVVRPTIPQDIRGAEVFAELHRELEHLQKQ
KEEISIRGDALVQERMGLCLEKSKYDNEKAHAAAMTKKVGKLQNIDRLQKNNETYVRIQNFFRTLIQEKLGR
DTVQEIDVVKEAKELHELAAIIYGNTSGKSQKQRAKKQFKENVLHIAGKGQLELLEAYLNVTASQGLCRHQ
MQASFRERILLNPDGAKHGEAERTLASREEMLKTLGLSYLTPFVRFSSPESTQSGYNQILKVREQLFDIEQRLQ
NQETVSPEDYAAVQAALAAYVRKHESLIVSTYGLGAQEGQTSSKVTTLMRDLHAVEELVEMGVETYRLNRS
DQILHRVHSVLHSHLRDSDSSGNGIIDVVKKLFELLNNNGNNPNDPECQKYMQILLDAPVSLLYGAFKSFKNE
FLLNFTELNIANSTKAAEEEAKRYVEEKGRGFETYWEEAKQRLEAIAAELDDLRNQETLLEQEIRLANLKISIF
SDLNLREKVSVEKAALEEEIQGIQEQYAEMQGIEDLELKQKFEDLQKKLEALEERLLQIGRRIDSSVDKQKEL
LGLLGREEAA

>core/4/3/Org3_Gene784

MKYSLPWLLTSSALVFSLHPLMAANTDLSSSDNYENGSSGSAAFTAKETSDASGTTYTLTSDVSITNVSAITP
ADKSCFTNTGGALSFVGADHSLVLQTIALTHDGAAINNTNTALSFSGFSSLLIDSAPATGTSGGKGAICVTNTE
GGTATFTDNASVTLQKNTSEKDGAAVSAYSIDLAKTTTAALLDQNTSTKNGGALCSTANTTVQGNSGTVTFS
SNTATDKGGGIYSKEKDSTLDANTGVVTFKSNTAKTGGAWSSDDNLALTGNTQVLFQENKTTGSAAQANNP
EGCGGAICCYLATATDKTGLAISQNQEMSFTSNTTTANGGAIYATKCTLDGNTTLTFDQNTATAGCGGAIYT
ETEDFSLKGSTGTVTFSTNTAKTGGALYSKGNSSLTGNTNLLFSGNKATGPSNSSANQEGCGGAILSFLESAS
VSTKKGLWIEDNENVSLSGNTATVSGGAIYATKCALHGNTTLTFDGNTAETAGGAIYTETEDFTLTGSTGTV
TFSTNTAKTAGALHTKGNTSFTKNKALVFSGNSATATATTTTDQEGCGGAILCNISESDIATKSLTLTENESLS
FINNTAKRSGGGIYAPKCVISGSESINFDGNTAETSGGAIYSKNLSITANGPVSFTNNSGGKGGAIYIADSGELS
LEAIDGDITFSGNRATEGTSTPNSIHLGAGAKITKLAAAPGHTIYFYDPITMEAPASGGTIEELVINPVVKAIVPP
PQPKNGPIASVPVVPVAPANPNTGTIVFSSGKLPSQDASIPANTTTILNQKINLAGGNVVLKEGATLQVYSFTQ
QPDSTVFMDAGTTLETTTTNNTDGSIDLKNLSVNLDALDGKRMITIAVNSTSGGLKISGDLKFHNNEGSFYDN
PGLKANLNLPFLDLSSTSGTVNLDDFNPIPSSMAAPDYGYQGSWTLVPKVGAGGKVTLVAEWQALGYTPKP
ELRATLVPNSLWNAYVNIHSIQQEIATAMSDAPSHPGIWIGGIGNAFHQDKQKENAGFRLISRGYIVGGSMTT
PQEYTFAVAFSQLFGKSKDYVVSDIKSQVYAGSLCAQSSYVIPLHSSLRRHVLSKVLPELPGETPLVLHGQVS
YGRNHHNMTTKLANNTQGKSDWDSHSFAVEVGGSLPVDLNYRYLTSYSPYVKLQVVSVNQKGFQEVAAD
PRIFDASHLVNVSIPMGLTFKHESAKPPSALLLTLGYAVDAYRDHPHCLTSLTNGTSWSTFATNLSRQAFFAE
ASGHLKLLHGLDCFASGSCELRSSSRSYNANCGTRYSF

>core/5/3/Org3_Gene379

MKQKVKRNFAIIICVFALALYYVLPTCLYYAKPLDKKIDGNEAEHIIKSFTKQAQQVRKDVIPRVSAILSSLHL
RGHIQQHPAIPDIVSVRFKRGEDAEDFIGNLVHGEPNVPIKSARLHVVGYSREHDDHVIQVASSINTSLVESDF
SFVSYSSENEQEMASSILQRVYSACTFPKQKDCSCSYPSIWETAPKEQLLQYAKNLSSGFEVFSSRLSAFCQQS
FSSNQDRLAFLSRLSSLSNDAAIDVEDQKLLKSVYETLSQTACIRSLDCPYIEGLRLDCSESSLFFSSIEYCPKER
KIFLTLHSDLLAQRTSLSKEQRLDFDSRLAVEKQKLSKNLTVQVEDYNNGFSFQWMDKDTQGKIILQGERLL
QGIAEHLTALTLHRPAAESCDLIPENFPVFCRQPRESEAFGCYIFSPNTDCKHFSKGSVYILLKGLRSIVAKYQQ
GGGKELQSFEKDLQNLYNCFSHTEAISWTLGEDQVLEIRHPLQQFLDVWGEGFVIGKEGCAFLEVKDIQDRL
ATVNQIEKNRQSDLVRWHEQYRHAKCSMDLQERLSAPIPYQNLFLENMKLNMRKFSRGENILRLGIDFVGG
RQLLLSFKDHQGKQLTDKEDILKVSDELCARLNKLGVSEIELRREGDYIHLSVPGSSTISSSEILGTSKMSFHVV

NERFSSYSASRYEVQRFLDYLWFTSQAQGKTSPEEINTFASALFNEEVDVPPSVHEAITKLKSEGLAFSPSGCE
TPSTDLDTTFSMIAIGKDAEQKANPLVIVFRNYALDGASLKDIRPEFAAGEGYVLNFSVKDTSPKKMAEKLSP
TESFHTWTSAYCQEGISGTANGQYSANRGWRMAVVIDGYMVSSPILNVPLKNHASVSGKFTHREVSKLASD
LKSGAMSFVPEVLSEETISSDLGKKQCTQGIISACCGLAMLIVLMSVYYRFGGVIASGAVLLNLLLIWAALQY
LDAPLTLSGLAGIVLAMGMAVDANVLVFERIREEFLLSQSLKKSVEKGYTKAFGAIFDSNLTTVLASALLFFL
DTGPIKGFALTLILGIFSSMFTALFMTKFFFMLWMNKTQHTQLHMMNKFVGIKHDFLRGCKKLWAVSGSVF
LLGCVALGFGAWNSVLGMDFKGGYAFTFNPKEHGISDVAQMRGKVVHKLQEAGLSSRDFRIQTFGSSEKIKI
YFSDKALSYTKADTSLSPKINDHELALAVGLLSETGLDFSTETLNETQNFWSKVSSKLSKKMRYQATIGLLGA
LAIILLYVSLRFEWQYAFSAVCALIHDLLATCAVLFIAHFFLKKIQIDLQAIGALMTVLGYSLNNTLIIFDRIRED
RQANLFTPMHVLVNDALQKTFSRTVMTTATTLSVLLMLLFIGGSSVFNFAFIMTIGILLGTLSSLYIAPPLLLF
MVRKENRSK

>core/6/3/Org3_Gene878

MFGENSRDIGVLSKEGLFDKLEIGIASDITIRDKWSCGEIKKPETINYRTFKPEKGGLFCEKIFGPTKDWECCCG
KYKKIKHKGIVCDRCGVEVTLSKVRRERMAHIELAVPIVHIWFFKTTPSRIGNVLGMTASDLERVIYYEEYVV
IDPGKTDLTKKQLLNDAQYREVVEKWGKDAFVAKMGGEAIYDLLKSEDLQSLLKDLKERLRKTKSQQARM
KLAKRLKIIEGFVSSSNHPEWMVLKNIPVVPPDLRPLVPLDGGRFATSDLNDLYRRVINRNNRLKAILRLKTPE
VIVRNEKRMLQEAVDALFDNGRHGHPVMGAGNRPLKSLSEMLKGKNGRFRQNLLGKRVDYSGRSVIIVGPE
LKFNQCGLPKEMALELFEPFIIKRLKDQGSVYTIRSAKKMIQRGAPEVWDVLEEIIKGHPVLLNRAPTLHRLGI
QAFEPVLIEGKAIRIHPLVCAAFNADFDGDQMAVHVPLSVEAQLEAKVLMMAPDNIFLPSSGKPVAIPSKDM
TLGLYYLMADPTYFPEEHGGKTKIFKDEIEVLRALNNGGFIDDVFGDRRDETGRGIHIHEKIKVRIDGQIIETTP
GRVLFNRIVPKELGFQNYSMPSKRISELILQCYKKVGLEATVRFLDDLKDLGFIQATKAAISMGLKDVRIPDIK
SHILKDAYDKVAIVKKQYDDGIITEGERHSKTISIWTEVSEQLSDALYVEISKQTRSKHNPLFLMIDSGARGNK
SQLKQLGALRGLMAKPNGAIIESPITSNFREGLTVLEYSISSHGARKGLADTALKTADSGYLTRRLVDVAQDV
IITEKDCGTLNHIEISAIGQGSEELLPLKDRIYGRTVAEDVYQPGDKSRLLAQSGDVLNSVQAEAIDDAGIETIK
IRSTLTCESPRGVCAKCYGLNLANGRLIGMGEAVGIIAAQSIGEPGTQLTMRTFHLGGIAATSSTPEIITNSDGIL
VYMDLRVVLGQEGHNLVLNKKGALHVVGDEGRTLNEYKKLLSTKSIESLEVFPVELGVKILVADGTPVSQG
QRIAEVELHNIPIICDKPGFIKYEDLVEGISTEKVVNKNTGLVELIVKQHRGELHPQIAIYDDADLSELVGTYAI
PSGAIISVEEGQRVDPGMLLARLPRGAIKTKDITGGLPRVAELVEARKPEDAADIAKIDGVVDFKGIQKNKRIL
VVCDEMTGMEEEHLIPLTKHLIVQRGDSVIKGQQLTDGLVVPHEILEICGVRELQKYLVNEVQEVYRLQGVD
INDKHIEIIVRQMLQKVRITDPGDTTLLFGEDVNKKEFYEENRRTEEDGGKPAQAVPVLLGITKASLGTESFIS
AASFQDTTRVLTDAACCSKTDYLLGFKENVIMGHMIPGGTGFETHKRIKQYLEKEQEDLVFDFVSETECVC

>core/7/3/Org3_Gene877

MLKCPERVSVKKKEDIPDLPNLIEIQIKSYKQFLQIGKLAEERENIGLEEVFREIFPIKSYNEATVLEYLSYNLGV
PKYSPEECIRRGITYSVTLKVRFRLTDETGIKEEEVYMGTIPLMTDKGTFIINGAERVVVSQVHRSPGINFEQEK
HSKGNILFSFRIIPYRGSWLEAIFDINDLIYIHIDRKKRRRKILAITFIRALGYSSDADIIEEFFTIGESSLRSEKDFA
LLVGRILADNIIDEACSLVYGKAGEKLSIAMLKRMLDAGIASVKIAVDADENHPIIKMLAKDPTDSYEAALKD
FYRRLRPGEPATLANARSTIMRLFFDPKRYNLGRVGRYKLNRKLGFSIDDEALSQVTLRKEDVIGALKYLIRL
KMGDEKACVDDIDHLANRRVRSVGELIQNQCRSGLARMEKIVRERMNLFDFSSDTLTPGKVVSAKGLASVL
KDFFGRSQLSQFMDQTNPVAELTHKRRLSALGPGGLNRERAGFEVRDVHASHYGRICPIETPEGPNIGLITSLS

SFAKINEFGFIETPYRIVRDGIVTDEIEYMTADVEEECVIAQASASLDEYNMFTEPVCWVRYAGEAFEADTSTV
THMDVSPKQLVSIVTGLIPFLEHDDANRALMGSNMQRQAVPLLKTEAPVVGTGLECRAAKDSGAIVVAEED
GVVDFVDGYKVVVAAKHNPTIKRTYHLKKFLRSNSGTCINQQPLCAVGDVITKGDVIADGPATDRGELALG
KNVLVAFMPWYGYNFEDAIIISEKLIREDAYTSIYIEEFELTARDTKLGKEEITRDIPNVSDEVLANLGEDGIIRI
GAEVKPGDILVGKITPKSETELAPEERLLRAIFGEKAADVKDASLTVPPGTEGVVMDVKVFSRKDRLSKSDDE
LVEEAVHLKDLQKGYKNQVATLKTEYREKLGALLLNEKAPAAIIHRRTAEIVVHEGLLFDQETIERIEQEDLV
DLLMPNCEMYEVLKGLLSDYETALQRLEINYKTEVEHIREGDADLDHGVIRQVKVYVASKRKLQVGDKMA
GRHGNKGVVSKIVPEADMPYLSNGETVQMILNPLGVPSRMNLGQVLETHLGYAAKTAGIYVKTPVFEGFPE
QRIWDMMIEQGLPEDGKSFLYDGKTGERFDNKVVIGYIYMLKLSHLIADKIHARSIGPYSLVTQQPLGGKAQ
MGGQRFGEMEVWALEAYGVAHMLQEILTVKSDDVSGRTRIYESIVKGENLLRSGTPESFNVLIKEMQGLGL
DVRPMVVDA

>core/8/3/Org3_Gene462

MTWIPLHCHSQYSVLDAMSSIKDFVAKGQEFGIPALALTDHGNLYGAVDFYKECTQKGIQPIIGCECYIAPGS
RFDKKKEKRSRAAHHLILLCKNEQGYRNLCILTSLAFTEGFYYFPRIDKDLLRQYSEGLICLSGCLSSSVSDAA
LKSPEALLLELQWFQDLFKDDYFTEVQLHKMSEESIAGFKEEWLKQEYYSLIEKQIKVNTAVLEASKRLGIPT
VATNDIHYINANDWQAHEILLNVQSGETVRIAKQNTHIPNPKRKVYRSREYYFKSPAQMAELFKDIPEVISNT
LEVAKRCDFTFDFSKKHYPIYVPESLKTLNSYTEEDRYQASAVFLKQLAEEALPKKYSSEVLAHIAKKFPHRD
PIDIVKERMDMEMAIIIPKGMCDYLLIVWDIIHWAKANGIPVGPGRGSGAGSVLLFLLGITEIEPIRFDLFFERFI
NPERLSYPDIDIDICMAGRERVINYAIERHGKDNVAQIITFGTMKAKMAVKDVGRTLDMALSKVNHIAKHIPD
LNTTLSKALETDPDLHQLYINDAESAQVIDMALCLEGSIRNTGVHAAGVIICGDQLTNHIPICISKDSTMITTQY
SMKPVESVGMLKVDLLGLKTLTSINIAMSAIEKKTGQSLAMATLPLDDATTFSLLHQGKTMGIFQMESKGMQ
ELAKNLRPDLFEEIIAMGALYRPGPMDMIPSFINRKHGKEIIEYDHPLMESILKETYGIMVYQEQVMQIAGALA
SYSLGEGDVLRRAMGKKDFQQMEQEREKFCKRACNNGIDPELATVIFDKMEKFAAYGFNKSHAAAYGLITY
TTAYLKANYPKEWLAALLTCDSDDIEKIGKLIREAQSMGIPILPPHINVSSNHFVATDEGIRFAMGAIKGIGRG
LIESIVEERDHHGPYESIRDFIQRSDLKKVSKKSIESLIDAGCFDCFDSNRDLLLASVEPLYEAIAKDKKEAASG
VMTFFTLGAMDRKNEVPICLPKDIPTRSKKELLKKEKELLGIYLTEHPMDTVRDHLSRLSVVLAGEFENLPHG
SVVRTVFIIDKVTTKISSKAQKKFAVLRVSDGIDSYELPIWPDMYEEQQELLEEDRLIYAILVLDKRSDSLRISC
RWMKDLSIVNENIIYECDQAFDRIKNQVQKMSFTMSTSGKETKAKGNKPNENGHTQALAPVTLSLDLNELR
HSHLCILKKIVQKHPGSRTLVLVFTQDNERVASMSPDDAYFVCEDIEELRQELVTADLPVRVITV

>core/9/3/Org3_Gene955

MVLEALAIFRQDAMQHLLKHRKEIVVDFCEDSYTIRIPDEEAPEGYWLSTLKLQDIDRLTFASCSCPDGECCL
HLMTAYFAVYDALGLHPLHDKFRHSFWYAVFSHFFLDSIPLQAQGEMVYTLESPHITLTIECLSEEVFQDWLR
TIHASEEPTVFTNKTFLKSALYRTAKKFFFLNEEGAELTIGENSQGFPSHFSLQWQGLVFKAEILDFPTLEDIFP
KLELAHTSLENVSHDISITNVTVCAEEAKVNFTLSPVIHKKDRENHPKTRIGSVEYVAKTHEMITGPKAIALPI
YAIPLLADKFKDQLLSLLCYDSLEYRLRYDIRLLRDASFSFSAYLVTPGDLDNGSLIYPNYCYSPTKGLMQVV
GMLSPKQAFIVKSEQVEDFLNERGHLIQEPGFQTFINERPEGHLTYNVTEQGVLLFHYDVGDPSSTEIRFGTWT
YYTNQGFFLEKKNDLPIQDGLIVEPQDIPAFIVKNDAALRRLPNFFSSPPNLKDLLIEVHRQSRGKGLDLKPILV
GLGESRCWLFGVFLYREDIGFSLIPTPLQGLCFLPRVIPPENVPQFLTQYAQHERILFPNPQTRPPESYELVIQSI
HRPHPASPLHLQLELKTNLGSVPIGIALQGLKSKHTFLFTQAGFLDLKQNLFQFLKQFLSTQKCVIAENTVIANI

TDVFKLDALAPLSVTDDTIANPEDLQFFSQLKAACLPPIPQNLFSSDHQLRPYQNSGLLWMWFLYNHRLSGLL
CDEMGLGKTHQATALLDIVFQSSQPSARPKFLIVCPTSVLPHWEHILSNHLPGVSIFSFHGPNKPSELPPADILL
TSYGTLRQNYDKFYKIAFTIVVFDEIHMAKNKSSQIHKILCRIDAQMKLGLTGTPIENNLLEFKGLLDIILPNYL
PSDALFKKLFTKRCSSEELEEIIPSQDLLLKLTRPFILRRTKKLVLPELPDKVESIIACSLSPDQEKLYMATLQRE
KSHIQKLETPEEPATNFLHIFALLNHLKQICDHPAVFFKDPDQYKNYESGKWNAFVKLLKESLNAGYKVVVF
SQYIHMIRIITLYLEEIGIKYASIQGKSLNRKEEIETFTTDPNCQVFVGSLLAAGTGINLTAGNVVIMYDRWWN
PAKEIQALDRVHRIGQKNTVFIYKLITEDTLEERIHYLIEKKIRLLDKVIASQDSNILHMLNREDLLTILSYKDE
HGTSDSEESPVDAPVEDDTGVLPPEDS

>core/10/3/Org3_Gene956

MLNFRKLRRDFSANILQDGKKLFEQGAVIDAKILSMNGETVCISAQVRGLYDNIYECEIEVDRSESDTVDSNC
DCSYNYDCQHIVALLFYLEQYFNEMVVAYARSADLETDHEINEEVKKELKETFVAAATKEEERKDREHQKEI
LREYVHAANALSANPFFLPLEYLEKDSAELAVLFVSVNEDTFAPANQPIEFQLVLRLPCRSKPFYISNIRTFLEG
VLYQEPIVLNGRRFFFTMQSFNASDRKLIDLLIRYVRYPNHTTEEKLLKSAYLMPPALGVILAKMFEHQLADR
GGGSLGEKESFSGLFCGNLEEPLCWSLTPAKMKFNLDFFDMPYKALLMTPVILVDDDEVQPEQTMLLESDAP
GIIHHFVYHRFSPQIKRAHLRSFSRLRDIAIPEALFGSFRENALPVFQEYAEIANVHLLNSFVTLPYVDEVRAIC
DMSYLDGELEAKLHFLYGSLRVPAASLALQYQDVRAFISDEGILARNLVEERKMLEEVFSGFIYDERDGAFR
VKSEKKIVEFMTETIPANQHRITFNCPENLSGQFIYDETIFELSFREGSDINYYEADLKVHGLLKGVPLDLLWD
CISAKKRFLELPKAGQQSKGTRRGKVNSGKLPCILVLDLEKIAPVVQIFNEIGFKVLDDLVQKCPLWSLTGISL
DQFEALPVNFSMSERLIEIQKQIRGEIEFDFQDVPQQIQATLRSYQTEGVHWLERLRKMHLNGILADDMGLGK
TLQAIIAVTQSKLEKGSGCSLIVCPTSLVYNWKEEFRKFNPEFRTLVIDGVPSQRRKQLTALADRDVAITSYNL
LQKDVELYKSFRLDYVVLDEAHHIKNRTTRNAKSVKMIQSDHRLILTGTPIENSLEELWSLFDFLMPGLLSSY
DRFVGKYIRTGNYMGNKADNMVALKKKVSPFILRRMKEDVLKDLPPVSEILYHCHLTESQKELYQSYAASA
KQELSRLVKQEGFERIHIHVLATLTRLKQICCHPAIFAKDAPEPGDSAKYDMLMDLLSSLVDSGHKTVVFSQY
TKMLGIIKKDLESRGIPFVYLDGSTKNRLDLVNQFNEDPSLLVFLISLKAGGTGLNLVGADTVIHYDMWWNP
AVENQATDRVHRIGQSRSVSSYKLVTLNTIEEKILTLQNRKKSLVKKVINSDDEVVSKLTWEEVLELLQI

>core/12/3/Org3_Gene935

MKRPKKFPIYLSIAQKTNRLLSGIVIAFAVIALRLWYLAVVEHEQKLEEAYKPQIRVLPQYVERATICDRFGKT
LAVNQLQYDVSVAYGAIRDLPTRAWRVDEHGHKQLIPVRKHYIMCLSELLSQELHLDREAIEDAIHAKASVL
GSVPYLVAANVSERTYLKLKMLSKDWPGLHVEAVVRRHYPQESVASDILGYVGPISLQEYKRVTQELSQLR
ECVRAYEEGEDPKLPEGLASIDQVRALLESVESNAYSLNALVGKMGVEACWDSKLRGKIGKKPILVDRRGNF
IQEMEGAVPEAPGTKLQLTLSAELQAYADALLLEYEKTETFRSAKSLKKREKLPPLFPWIKGGAIIALDPNNG
EILAMASSPRYRNNDFVNAKVAEDSKAVRSSIYRWLENKEHIAEIYDRKVPLIRERRNPLTGLCYEEILPLTFD
CFLDFLFPENSVIKLQLKRNSFVGQAIEVQNLVTRLLSLFPYEEGTCPCSAIFDAVFPNEEGHILIQEVISLQEQK
WIMECLNQHKADIEELKEALDQVFNELPANYDKILYTDILRLIVDPERFSPVLPSEVHRLSLSEFTELQGRYVV
LRSAFSTILEDAFIEVHFKSWRKSEFLQYLAAKRQEEALRKQRYPTPYVDYLEEEKTRQYKMFCQEHLDTFL
AYLFSKTPYKEGLEPYYDILDLWINELDNGAHRALSWNEHYLFLKERVSHLSEHLPALFSTFREFNELQRPLL
GKYPISIVRNKRQTEQDLAASFYPVYGYGYLRPHAYGQAATLGSIFKLVSAYSVLSQRILWGHNEEPANPLVI
IDKNSFGYRSSKPHVGFFKDGTPIPTFFRGGSLPGNDFMGRGFIDLVSALEMSSNPYFSLLVGEGLGDPEDLAD
AASLFGFGEKTGLGLPGEYAGRVPHDLAYNRSGLYATAIGQHTLVVTPLQTAVMLASLVNGGVVYVPKLLL

GEWEGEHVSYLSSKKKRTIFMPDAVVEVLKTGMRNVIWGQYGTARAIQSQFPPQLLSRIIGKTSTAESIMRVG
LDREYGTMKMKDIWFAAVGFSDQDLSLPTIVVIVYLRLGEFGRDAAPMAVKMIDMWEKIQQRESFLRG

>core/13/3/Org3_Gene1019

MDFNPVNLDFSISKEFKEETLPLLLENIHPGATAFLAAKMFHDCRASVIMITTPARLDDLFENLRTFLDQAPVE
FPSSEIDLSPKLVNIDAVGKRDHLLYSLNQHRAPIFCVTTLKALLEKTRSPQATSQQHLDLAVGDVLDPEATTE
LCKSLGYSQVMLTSEKGEFSCRGGIVDIFPLSSPEPFRIEFWGEKIISIRSYNPSDQLSTGKVSKISISPAYTEEAS
GGNYSHSLLDYFSTPPLYLFDNLEILEDDFADISGTLSSLPDRFFSIGTLYDRISTSNQVYFSETPFPNVKNLKEN
RVIIEAFHRNMEASRQAIPILYPEQIIQNDENPLLAFLQHLQEYMPPHGKPLKLAIYSTKTKSLKEARALAETV
ARGDVEIYEKTGNLTSSFALVNEAFAAISLSEFASTKVLRRQKQRTHFSVTTEEVFVPIPGETVVHIHNGIGKFL
GIEKKPNHLNIETDYLVLEYADKARLYVPSNQAYLISRYVGTSDKAADLHHLNSSKWKRSRDLTEKSLIVYA
EKLLQLEAQRSTTPAFVYPPHGESVIKFAETFPYEETPDQLKTIDQIYNDMMSPKLMDRLICGDAGFGKTEVI
MRAAVKAVCDGHRQVIVMVPTTILATQHYETFKERMAGLPIEIAVLSRFSQAKVQKLICEQVASGQIDIIIGTH
KLINKSLEFKNPGLLIIDEEQRFGVKVKDNLKERYPMIDCLTVSATPIPRTLHMSLSGARDLSVIAMPPLDRLP
VSTFVMEHNTETLTAALRHELLRGGQAYVIHNRIESIYTLAETIRNLIPEARIGVAHGQMGAEDLSNIFTKFKN
QKTDILVATALIENGIDIPNANTILIDHADKFGMADLYQMKGRVGRWNKKAYCYFLVPHLDRLSGPAAKRL
AALNKQEYGGGMKIALHDLEIRGAGNILGTDQSGHIGTIGFNLYCKLLKKAVSALKKHTSPLLFNDDVKIEFP
YNSRIPDTYIETGSMRIEFYQKIGNAESSEELTAIQEEMRDRFGPLPQEICWLFALAEIRLFALQHGISSIKGTAN
ALYVQKCLSKSEQTKKTLPYALSPTPELLVKEVIESIERGFLINAS

>core/14/3/Org3_Gene914

MIKKRAIFERMFPIPPPHCPPNNKNNFYHLTTDTKDPLLLRILRTIGYVLLHIITLGLLLLIHYYKHHRVVRKEG
LPTPPTLPKGPEPKTIEIAKQPPKDGEDKKPDVPKPGTPPPEDTPPPPPKAPSPASPKVPKQPADKKPTPPPEAPP
PPVRVATPMPLRPSSQGYWQCLNRMVSMVLRRAPLPLPAMQVDPILGDFNPHFVASYPNRIDNEPMYFQIKQ
FKKIAQNPDLPQQHRRLAQLSLEQALYLNDNYYLVNVPGDGNCFYRAYAVGWLSALYEESSRNDIVFEQEA
TRLLDLPFASSSPANANLCAEMAELLQLCSTYCSFIDLYDGVILSQKHTATLIAFLRKLSAYAIRQQIAASSNEE
TARALFISDMQDDLLPSVLEFLAANRPYSELFQNLIDHSALPYMQSRDKLFLLLEHLPALFLTDAELQKMSPE
DQQLRKQYEREIREAFAKLSRRIADSGWDTERFNAIVKDYLPEAIRCQYSRFLATIENRRSGDLPWSPALSFFA
FLCTCPSVRFHKLCATFYKSLEDIIIASAPPQRSIQEILQISNASLSYLNEDLDSSWQREVISSNIMTILTTHESLTL
ESSMPQLETLHKRIANLLKNVISTSFETPPLSNQPDLLSNLVNKLLVAIHSKLELKEHFNTVCSARSLRLTRDE
GSGLSQEQDLLYTQAVQLLFFILQHPQVNNRPETKDAVKELKMLLLPFLQYAFKKVENEKKLQKLLRSILGS
LVLKPPARYPSTPSNKDKETFCKFWSRHPEVMVLDPILEKNCMQFLRATFPNYQLETEAILLEKEIESTFRNG
WNVFLTRLNLFGSKLGSPSSPTALSDQFSKSFLIFCFLNNYPKLLQKKTPLAARLDAFQREASHRFTQVKDKL
LLSLKYGFPLATATINQYSRARDQLICNLLKNTVTASDGFCRSGFRQSLIGYLHSLSSNELGDILDDVKEQAEA
NDVAAMTTVPLQPFAVCLIMSDRDTVSEENIENFVAMHGFLNTISPERDARIFLIRFPNHYGCLLPRNPRTEDQ
NSKPDSSNP

>core/15/3/Org3_Gene945

MKPFNIFDSNSSIQGKFFLEASAGTGKTFTIEQIVLRALIEGSLTHVEHALAITFTNASTNELKVRIKDNLAQTL
RELKAVLNSQPASLPTYLDINCNVKQIYMQVRNALATLDQMSLFTIHGFCNFVLEQYFPKTRLIHKNPALTHS
QLVLHHITNYLKQDLWKNVLFQEQFHLLAVRYNVTSKHTSSLVDKLLASYTQPISSYFSSRVERLEQISLWHQ

QIYNSLLEIPKQVFLDQLTAHISGFKKQPFSILDDLHHFVDLLYTSETHSSLFSFFKIAETFNFKHRLARYKPCA
AFTVLENMSWVERTLEFCNLDRIFNTLLVDLQEYLKQNYTPWLSPDESVFALEKLLSSSEAQPVVQALREQY
QLVLIDEFQDTDKQQWSIFSNLFISPKFTGSLFLIGDPKQSIYEWRSADLPTYLTAKSSFSEDKQLQLVNNYRST
PKLMEAINQIFGKISPFLEIPGYLPIEYHALNPQSSETFENPPHAPIHFFFYETIKDQALWIFSEALRLQKEQKIPL
GNMVVLVSDSNQAFELISYATIPVSFSKNKSIFHLTETHILTTALLEAILHPENYEKISKILFSSLFGLSLDEVTTK
KEDFTIYFQSLHSYISHHGLLATFYRVMTTQGNVLFSSPRGDLIFQEMEKLCGYLDTISSYPYHQLLHLKNFSE
TGRWEEELAISSYSEDLETLKITTIHSSKGLEYDIVFCPGIEKSKKNKSSSELLREMYVACTRAKKQLYLPISTQ
PPSLQRSSALTNYVKLEGTQSSAYDLAIHLHQEHPDLFSYSLPKDHGHATTVLNLPLLETFALKVTPPKTIFSFS
STKFLLDTHKDSQSIPYSKLPISKQQLPLGEKTGILIHKILESIQFSLLQDTEYLMSTIMRFIKHTHLEGFEETILK
LLSKTFFSPLTFSSQTFSLSQVLPNKIFRETSFLFLENQELWQGVIDLFFEHEGKYYIIDWKTSFLGETNSDYSKS
NLSIYIKQEKLDYQGRIYVKAVRKFLNQFEIDDDVELGVIFIRGIDTQGNGFFALNSSEDIPNFNPKAIQKCQAY
H

>core/16/3/Org3_Gene690

MVEVEEKHYTIVKRNGMFVPFNQDRIFQALEAAFRDTRSLETSSPLPKDLEESIAQITHKVVKEVLAKISEGQ
VVTVERIQDLVESQLYISGLQDVARDYIVYRDQRKAERGNSSSIIAIIRRDGGSAKFNPMKISAALEKAFRATL
QINGMTPPATLSEINDLTLRIVEDVLSLHGEEAINLEEIQDIVEKQLMVAGYYDVAKNYILYREARARARANK
DQDGQEEFVPQEETYVVQKEDGTTYLLRKTDLEKRFSWACKRFPKTTDSQLLADMAFMNLYSGIKEDEVTT
ACIMAARANIEREPDYAFIAAELLTSSLYEETLGCSSQDPNLSEIHKKHFKEYILNGEEYRLNPQLKDYDLDAL
SEVLDLSRDQQFSYMGVQNLYDRYFNLHEGRRLETAQIFWMRVSMGLALNEGEQKNFWAITFYNLLSTFRY
TPATPTLFNSGMRHSQLSSCYLSTVKDDLSHIYKVISDNALLSKWAGGIGNDWTDVRATGAVIKGTNGKSQG
VIPFIKVANDTAIAVNQGGKRKGAMCVYLENWHLDYEDFLELRKNTGDERRRTHDINTASWIPDLFFKRLEK
KGMWTLFSPDDVPGLHEAYGLEFEKLYEEYERKVESGEIRLYKKVEAEVLWRKMLSMLYETGHPWITFKDP
SNIRSNQDHVGVVRCSNLCTEILLNCSESETAVCNLGSINLVEHIRNDKLDEEKLKETISIAIRILDNVIDLNFYP
TPEAKQANLTHRAVGLGVMGFQDVLYELNISYASQEAVEFSDECSEIIAYYAILASSLLAKERGTYASYSGSK
WDRGYLPLDTIELLKETRGEHNVLVDTSSKKDWTPVRDTIQKYGMRNSQVMAIAPTATISNIIGVTQSIEPMY
KHLFVKSNLSGEFTIPNTYLIKKLKELGLWDAEMLDDLKYFDGSLLEIERIPNHLKKLFLTAFEIEPEWIIECTS
RRQKWIDMGVSLNLYLAEPDGKKLSNMYLTAWKKGLKTTYYLRSQAATSVEKSFIDINKRGIQPRWMKNK
SASTSIVVERKTTPVCSMEEGCESCQ

>core/17/3/Org3_Gene63

MTADEVGKNSFAKKEEQVLKFWKDNQIFEKSLQNRQGKTLYSFYDGPPFATGLPHYGHLLASTIKDVVGRY
ATMDGYYVPRRFGWDCHGVPVEYEVEKSLSLTAPGAIEDFGIASFNEECRKIVFRYVHEWEYYINRIGRWVD
FSSTWKTMDASFMESVWWVFQSLYNQGLVYEGTKVVPFSTALGTPLSNFEASQNYKEVDDPSLVVRMPLQ
NDSASLLVWTTTPWTLPSNMAIAVGETLVYVRIQDKKSGEQWILSQGCVSRWFSNPEEFVILESFSGKDLVG
RTYEPPFTFFQSKREEGAFRVIAASFVEESEGTGVVHMAPAFGEGDFLVCKENHVPLVCPVDAHGSFTEEIPQ
YQGQYIKHADKEIIKFLKKEGRIFYHGTVKHRYPFCWRTDTPLIYKAVNSWFVAVEKIKDKMLRANSSIHWV
PEHIQEGRFGKWLEGARDWAISRNRYWGTPIPIWKSADGEILVVGSIRELEELTGTQITDIHRHFIDDLNIVKD
GKPFHRIPYVFDCWFDSGAMPYAQNHYPFENQKETEEAFPADFIAEGLDQTRGWFYTLTVISAILFDRPAFRN
AIVNGIILAEDGNKMSKRLNNYPSPKYVLDTYGADALRLYLLHSVVVKAEDLRFSDKGIEGVLKQILLPLTNV
LSFFNTYAELYGFDPKSQDIEPAYTEIDQWILSNLYSVVGKVRESMSQYHLNFAVEPFVTFIDDLTNWYIRRC

RRRFWEAEDTPDRRAAFSTLYEVLTVFCKVIAPFVPFLAEDIYQKLKLEKEPESVHLCDFPQVEMDKILPDLE
KRMHDIREIVGLGHSLRKEHKLKVRQPLANFYVVGSKDRLSLLKTFEGLIAEELNVKNVIFYEEAPSFIYTTV
KPNFRMLGKKVGSKMKEVQKALSELPNNAIDKLIQEETWVLTIDDREIALDGDDVVICRHTDPGYIARSSALF
SVILDCQLREPLIVEGIARELVNKINTMRRNQQLHVSDRIALRIKTTEAVHRAFLDYENYICEETLIIAYDFTQD
SDFQGENWDINGHATQIEITVSSIDS

>core/18/3/Org3_Gene944

MNATKHCRASFSNSPRHLLAQLAEDITSTHQKPFTKRWILVANATTGHWIKNQLVHVLSDHIFMGSTIFTASD
SIVKHLFLGSGCSQPNIPDYLTLPLLINNILEEISKASKFENGREFLSPPTYETTKKLAAAFKQFHTFSQRPTKNA
SHYQELFQILESHFSSYEEMFTTILNNRTQEEDCSLHIFGYAHLPKHLAEFFINLSTYFPVYFYCFSPCREYFGD
LLSDRAIDFFWNQLPDSPIKNAWEHYVLSDRQALLANLAHKSQSSQNFFLDREIDYQEMFLPSKHDSSLGVIQ
NSILDLKPTSPQDFSQTKQTICIYRALNIPREVQEVFCKVTELLHRGVSPEEIFILSSHIESYKVHLNAIFNPHVPI
YFTDEVDPRAEDLRNKILLLSSILQTQGDLHYILQLLTHPQLQQPIDQNKVPYLIKKLSSEWGKISSKDRASGQ
QMKALGDLILEEYPFHQEGGRVSQVEVWETTVPLIYFIQERINLYLSSSQHSYEDLFQNVFSCLEKIFVLSPEET
SFITTLRNSLFPTFATSSCSLLFFTDFCLDFLLHFHKPSPLYDKPGPYIGSLSSLSLIPKGYVFILGANKTTSSDIFD
LLNRTTTHEELAFSSTEDEENFHFLQILVSTKHELHISYISSAAQFNLPSPFLNHIKETLDLPVETLPTQPYLSAFF
KNKACLHTSQEYNYSLAHAFYSKKALLPSLFIPTVKQVNLPQHLSLNEIIKGIFSPLDLFLKTNYNLRISYPEHL
KKQQKLFPTKHQIEDFWNECFVDKEHDLIPSISPHAEELFTYYREKTILLRNGLDKDPKHSPYTVTFSSSIFEER
PYHESYLFPPLSLSFQGNPVQIHGTIHGVCNEGLYLCSIDPGDSLKKTTRTLGSLPETSSEQKQLLERYVALAV
LQMSQHLSSDSALIKLTSFNTKENHHPPFSDPEGYLRKVLEVYHLMSSQPIPLLSPLCWKTLDDEEKFHQAVL
SAISEEAKNPSLPIFWQFHNRNIEEILNHVGASERLKILSLFRGPCEAV

>core/19/3/Org3_Gene841

MSEHPLTLQSMIATILRFWSEQGCVIHQGYDLEVGAGTFNPATFLRALGPEPYKAAYVEPSRRPQDGRYGVH
PNRLQNYHQLQVILKPVPENFLSLYTESLRAIGLDLRDHDIRFIHDDWENPTIGAWGLGWEVWLNGMEITQL
TYFQAIGSKPLDTISGEITYGIERIAMYLQKKTSIYDVLWNDTLTYGQITQASEKAWSEYNFDYANTEMWFKH
FEDFAEEALRTLKNGLSVPAYDFVIKASHAFNILDARGTISVTERTRYIARIRQLTRLVADSYVEWRASLNYPL
LSLSSTSEPKETSESVVPMISSTEDLLLEIGSEELPATFVPIGIQQLESLARQVLTDHNIVYEGLEVLGSPRRLAL
LVKNVAPEVVQKAFEKKGPMLTSLFSPDGDVSPQGQQFFASQGVDISHYQDLSRHASLAIRTVNGSEYLFLL
HPEIRLRTADILMQELPLLIQRMKFPKKMVWDNSGVEYARPIRWLVALYGEHILPITLGTIIASRNSFGHRQLD
PRKISISSPQDYVETLRQACVVVSQKERRMIIEQGLRAHSSDTISAIPLPRLIEEATFLSEHPFVSCGQFSEQFCA
LPKELLIAEMVNHQKYFPTHETSSGAISNFFIVVCDNSPNDTIIEGNEKALTPRLTDGEFLFKQDLQTPLTTFIEK
LKSVTYFEALGSLYDKVERLKAHQRVFSTFSSLAASEDLDIAIQYCKADLVSAVVNEFPELQGIMGEYYLKH
ANLPTASAVAVGEHLRHITMGQKLSTIGTLLSLLDRLDNLLACFILGLKPTSSHDPYALRRQSLEVLTLVSASR
LPIDLASLLDRLADHFPSTIEEKVWDKSKTIHEILEFIWGRLKTFMGSLEFRKDEIAAVLIDSATKNPIEILDTAE
ALQLLKEEHTEKLAVITTTHNRLKKILSSLKLSMTSSPIEVLGDRESNFKQVLDAFPGFPKETSAHAFLEYFLSL
ADLSNDIQDFLNTVHIANDDGAIRNLRISLLLTAMDKFSLCHWESVAV

>core/20/3/Org3_Gene1003

MSRLDIFVFDSLIANQDQNLLEEIFCSEDTVLFKAYRTTALQSPLAAKNLNIARKVANYILADNGEIDTVKLVE
AIHHLSQCTYPLGPHRHNEAQDREHLLKMLKALKENPKLKESIKTLFVPSYSTIQNLIRHTLALNPQTILSTIHV

RQAALTALFTYLRQDVGSCFATAPAILIHQEYPERFLKDLNDLISSGKLSRIVNQREIAVPINLSGCIGELFKPLR
ILDLYPDPLVKLSSSPGLKKAFSAANLIETLGDSEAQIQQLLSHQYLMQKLQNVHETLTANDIIKSTLLHYYQL
QESTVRAIFFKEGLFSKEQVAFSTQHPRELSEIQRVYHYLHAYEEAKSAFIHDTQNPLLKAWEYTLATLADAS
QPTISNHIRLALGWKSEDPHSLVSLVTHFVEEEVENIRILVQQCEQTYHEARSQLEYIEGRMRNPLNNQDSQIL
TMDHMRFRQELNKALYEWDSAQEKAKKFLHLPEFLLSFYTKQIPLYFRSSYDAFIQEFAHLYANAPAGFRILF
THGRTHPNTWSPIYSINEFIRFLSEFFTSTESELLGKHAVINLEKETSRLVHNITAMLHTDVFQEALLTRILEAY
QLPVPPSILNHLDQLSQTPWVYVSGGTVDTLLLDYFESSEPLTLTEKHPENPHELAAFYADALKDLPTGIKSYL
EEGSHSLLSSSPTHVFSIIAGSPLFREAWDNDWYSYTWLRDVWVKQHQDFLQDTILPQLSIYAFIENFCNKYA
LQHVVHDFHDFCSDHSLTLPELYDKGSRFLSSLFTKDKTVALIYIRRLLYLMVREVPYVSEQQLPEVLDNVSS
YLGISSRITYEKFRSLIEETIPKMTLLSSADLRHIYKGLLMQSYQKIYTEEDTYLRLTTAMRHHNLAYPAPLLFA
DSNWPSIYFGFILNPGTTEIDLWKFNYAGLQGQPLDNIQELFATSRPWTLYANPIDYGMPPPPGYRSRLPKEFF

>core/21/3/Org3_Gene780

MPLSFKSSSFCLLACLCSASCAFAETRLGGNFVPPITNQGEEILLTSDFVCSNFLGASFSSSFINSSSNLSLLGKG
LSLTFTSCQAPTNSNYALLSAAETLTFKNFSSINFTGNQSTGLGGLIYGKDIVFQSIKDLIFTTNRVAYSPASVT
TSATPAITTVTTGASALQPTDSLTVENISQSIKFFGNLANFGSAISSSPTAVVKFINNTATMSFSHNFTSSGGGVI
YGGSSLLFENNSGCIIFTANSCVNSLKGVTPSSGTYALGSGGAICIPTGTFELKNNQGKCTFSYNGTPNDAGAI
YAETCNIVGNQGALLLDSNTAARNGGAICAKVLNIQGRGPIEFSRNRAEKGGAIFIGPSVGDPAKQTSTLTILA
SEGNIAFQGNMLNTKPGIRNAITVEAGGEIVSLSAQGGSRLVFYDPITHSLPTTSPSNKDITINANGASGSVVFT
SKGLSSTELLLPANTTTILLGTVKIASGELKITDNAVVNVLGFATQGSGQLTLGSGGTLGLATPTGAPAAVDFT
IGKLAFDPFSFLKRDFVSASVNAGTKNVTLTGALVLDEHDVTDLYDMVSLQSPVAIPIAVFKGATVTKTGFPD
GEIATPSHYGYQGKWSYTWSRPLLIPAPDGGFPGGPSPSANTLYAVWNSDTLVRSTYILDPERYGEIVSNSLW
ISFLGNQAFSDILQDVLLIDHPGLSITAKALGAYVEHTPRQGHEGFSGRYGGYQAALSMNYTDHTTLGLSFGQ
LYGKTNANPYDSRCSEQMYLLSFFGQFPIVTQKSEALISWKAAYGYSKNHLNTTYLRPDKAPKSQGQWHNN
SYYVLISAEHPFLNWCLLTRPLAQAWDLSGFISAEFLGGWQSKFTETGDLQRSFSRGKGYNVSLPIGCSSQWF
TPFKKAPSTLTIKLAYKPDIYRVNPHNIVTVVSNQESTSISGANLRRHGLFVQIHDVVDLTEDTQAFLNYTFDG
KNGFTNHRVSTGLKSTF

>core/22/3/Org3_Gene972

MKAGDTYRNFIIKSCKDLPEIESKLLEAEHKPTGASIMMIVNNDEENVFNICFRTCPQTSNGVAHVLEHMVLC
GSENYPVRDPFFSMTRRSLNTFINAFTGPDFTCYPAASQIPEDFYNLLSVYIDAVFHPLLTKQSFLQEAWRYEF
NSENHLCYTGVVFNEMKGAMMSGEARLSEALNAAIFPSVTYGVNSGGEPREIVTLSHEDVRAFHQSQYSINR
CLFYFYGNIKPSRHLDFLEEKLLRQATKLEKQAVSVPLQKRFKDPVRNILTYPVDHQEEDKVLFGISWLTCSIL
EQQELLALHVLEIILMGTDASPLKSRLLKSGFCKQTEMSIENDIREIPMTLVCKGCSPAGAQKLEALIFASLEEII
REGISENIVEGAVHQLELSRKEITGYSLPYGLSLFFRSGLLKQHGGSAEDGLRIHSLFSELRNSLKNSDYLAKLI
RKYFLDNPHFARVILLPDTELVAKDNKDEQQLLLSVSEKLTDENKEKIQQNVRELTESQEQKEDLNGILPNLA
LDKVPTSGKEFPLIKEGLSQGEVLHHECFTNDIVFIDVVLDIPPLSGEELPWLRLLVFLMLQLGCGGRSYKEHL
EFLLEHTGGVDVSYDFSPHANKNSFLSPSVSIRGKALSSKSEKLCGIVSDMLTSVDFTDIPRIRELLMQHNEAL
TNSVRNSPMSYAVSMACSGNSITGAMSYLTTGLPYVKKIRELTKNFDQNIDEAVVILQRLYTKCFSGKRQIVI
SGSAHNYQQLKDNKFYGLLDYLIVIPEPWENPSINLYVTSRGLHIPARAAFNALAFPIGDIAYDHPDAAALTV
AAEILDNVVLHTKIREQGGAYGSGAAANLSRGSFYCYSYRDPEIATTYKTFLKGVSEIASGNFTKEDIYEGAL

GVVQGLDMPVAPGSRASVAFYRLKSGRIPVLRQAFRRSVLEVTKEHICMVMDKYLESTVQETTLISFAGEEM
LRNNVLTLDKDFPIVPAI

>core/23/3/Org3_Gene305

MKTSIRKFLISTTLAPCFASTAFTVEVIMPSENFDGSSGKIFPYTTLSDPRGTLCIFSGDLYIANLDNAISRTSSSC
FSNRAGALQILGKGGVFSFLNIRSSADGAAISSVITQNPELCPLSFSGFSQMIFDNCESLTSDTSASNVIPHASAI
YATTPMLFTNNDSILFQYNRSAGFGAAIRGTSITIENTKKSLLFNGNGSISNGGALTGSAAINLINNSAPVIFSTN
ATGIYGGAIYLTGGSMLTSGNLSGVLFVNNSSRSGGAIYANGNVTFSNNSDLTFQNNTASPQNSLPAPTPPPTP
PAVTPLLGYGGAIFCTPPATPPPTGVSLTISGENSVTFLENIASEQGGALYGKKISIDSNKSTIFLGNTAGKGGAI
AIPESGELSLSANQGDILFNKNLSITSGTPTRNSIHFGKDAKFATLGATQGYTLYFYDPITSDDLSAASAAATV
VVNPKASADGAYSGTIVFSGETLTATEAATPANATSTLNQKLELEGGTLALRNGATLNVHNFTQDEKSVVIM
DAGTTLATTNGANNTDGAITLNKLVINLDSLDGTKAAVVNVQSTNGALTISGTLGLVKNSQDCCDNHGMFN
KDLQQVPILELKATSNTVTTTDFSLGTNGYQQSPYGYQGTWEFTIDTTTHTVTGNWKKTGYLPHPERLAPLIP
NSLWANVIDLRAVSQASAADGEDVPGKQLSITGITNFFHANHTGDARSYRHMGGGYLINTYTRITPDAALSL
GFGQLFTKSKDYLVGHGHSNVYFATVYSNITKSLFGSSRFFSGGTSRVTYSRSNEKVKTSYTKLPKGRCSWS
NNCWLGELEGNLPITLSSRILNLKQIIPFVKAEVAYATHGGIQENTPEGRIFGHGHLLNVAVPVGVRFGKNSH
NRPDFYTIIVAYAPDVYRHNPDCDTTLPINGATWTSIGNNLTRSTLLVQASSHTSVNDVLEIFGHCGCDIRRTS
RQYTLDIGSKLRF

>core/24/3/Org3_Gene595

MLGFLKRFFGSSQERILKKFQKLVDKVNIYDEMLTPLSDDELRNKTAELKQRYQNGESLDSMLPEAYGVVK
NVCRRLAGTPVEVSGYHQRWDMVPYDVQILGAIAMHKGFITEMQTGEGKTLTAVMPLYLNALTGKPVHLV
TVNDYLAQRDCEWVGSVLRWLGLTTGVLVSGTLLEKRKKIYQCDVVYGTASEFGFDYLRDNSIATRLEEQV
GRGYYFAIIDEVDSILIDEARTPLIISGPGEKHNPVYFELKEKVASLVYLQKELCSRIALEARRGLDSFLDVDILP
KDKKVLEGISEFCRSLWLVSKGMPLNRVLRRVREHPDLRAMIDKWDVYYHAEQNKEESLERLSELYIIVDEH
NNDFELTDKGMQQWVEYAGGSTEEFVMMDMGHEYALIENDETLSPADKINKKIAISEEDTLRKARAHGLRQ
LLRAQLLMERDVDYIVRDDQIVIIDEHTGRPQPGRRFSEGLHQAIEAKEHVTIRKESQTLATVTLQNFFRLYEK
LAGMTGTAITESREFKEIYNLYVLQVPTFKPCLRIDHNDEFYMTEREKYHAIVNEIATIHGKGNPILVGTESVE
VSEKLSRILRQNRIEHTVLNAKNHAQEAEIIAGAGKLGAVTVATNMAGRGTDIKLDNEAVIVGGLHVIGTTR
HQSRRIDRQLRGRCARLGDPGAAKFFLSFEDRLMRLFASPKLNTLIRHFRPPEGEAMSDPMFNRLIETAQKRV
EGRNYTIRKHTLEYDDVMNKQRQAIYAFRHDVLHAESVFDLAKEILCHVSLMVASLVMSDRQFKGWTLPNL
EEWITSSFPIALNIEELRQLKDTDSIAEKIAAELIQEFQVRFDHMVEGLSKAGGEELDASAICRDVVRSVMVMH
IDEQWRIHLVDMDLLRSEVGLRTVGQKDPLLEFKHESFLLFESLIRDIRITIARHLFRLELTVEPNPRVNNVIPT
VATSFHNNVNYGPLELTVVTDSEDQD

>core/26/3/Org3_Gene312

MSLNLERISPLLYLLDVTAKKIDTSNLIVEAMNLDEHYGYQGIWSPYWMETTTTTSSTVPEQTNTNHRQLYV
DWTPVGYRPNPERHGEFIANTLWQSAYNALLGIRILPPQNLKEHDLEASLQGLGLLINQHNREGRKGFRNHT
TGYAATTSAKTAARHSFSLGFAQMFSKTRERQSPSTTSSHNYFAGLRFDSLLFRDFISTGLSLGYSYGDHHML
CHYTEILKGSSKAFFNNHTLVASLDCTFLPARITRTLELQPFISAIALRCSQASFQETGDHIRKFHPKHPLTDLSS

PIGFRSEWKTSHHIPMLWTTEISYVPTLYRKNPEMFTTLLISNGTWTTQATPVSYNSVAAKIKNTSQLFSRVTL
SLDYSAQVSSSTVGQYLKAESHCTF

>core/27/3/Org3_Gene917

MKQMRLWGFLFLSSFCQVSYLRANDVLLPLSGIHSGEDLELFTLRSSSPTKTTYSLRKDFIVCDFAGNSIHKPG
AAFLNLKGDLFFINSTPLAALTFKNIHLGARGAGLFSESNVTFKGLHSLVLENNESWGGVLTTSGDLSFINNTS
VLCQNNISYGPGGALLLQGRKSKALFFRDNRGTILFLKNKAVNQDESHPGYGGAVSSISPGSPITFADNQEILF
QENEGELGGAIYNDQGAITFENNFQTTSFFSNKASFGGAVYSRYCNLYSQWGDTLFTKNAAAKVGGAIHAD
YVHIRDCKGSIVFEENSATAGGAIAVNAVCDINAQGPVRFINNSALGLNGGAIYMQATGSILRLHANQGDIEF
CGNKVRSQFHSHINSTSNFTNNAITIQGAPREFSLSANEGHRICFYDPIISATENYNSLYINHQRLLEAGGAVIFS
GARLSPEHKKDNKNKTSIINQPVRLCSGVLSIEGGAILAVRSFYQEGGLLALGPGSKLTTQGKNSEKDKIVITN
LGFNLENLDSSDPAEIRATEKASIEISGVPRVYGHTESFYENHEYASKPYTTSIILSAKKLVTAPSRPEKDIQNLII
AESEYMGYGYQGSWEFSWSPNDTKEKKTIIASWTPTGEFSLDPKRRGSFIPTTLWSTFSGLNIASNIVNNNYL
NNSEVIPLQHLCVFGGPVYQIMEQNPKQSSNNLLVQHAGHNVGARIPFSFNTILSAALTQLFSSSSQQNVADK
SHAQILIGTVSLNKSWQALSLRSSFSYTEDSQVMKHVFPYKGTSRGSWRNYGWSGSVGMSYAYPKGIRYLK
MTPFVDLQYTKLVQNPFVETGYDPRYFSSSEMTNLSLPIGIALEMRFIGSRSSLFLQVSTSYIKDLRRVNPQSSA
SLVLNHYTWDIQGVPLGKEALNITLNSTIKYKIVTAYMGISSTQREGSNLSANAHAGLSLSF

>core/28/3/Org3_Gene776

MQNNRSLSKSSFFVGALILGKTTILLNATPLSDYFDNQANQLTTLFPLIDTLTNMTPYSHRATLFGVRDDTNQ
DIVLDHQNSIESWFENFSQDGGALSCKSLAITNTKNQILFLNSFAIKRAGAMYVNGNFDLSENHGSIIFSGNLSF
PNASNFADTCTGGAVLCSKNVTISKNQGTAYFINNKAKSSGGAIQAAIINIKDNTGPCLFFNNAAGGTAGGAL
FANACRIENNSQPIYFLNNQSGLGGAIRVHQECILTKNTGSVIFNNNFAMEADISANHSSGGAIYCISCSIKDNP
GIAAFDNNTAARDGGAICTQSLTIQDSGPVYFTNNQGTWGGAIMLRQDGACTLFADQGDIIFYNNRHFKDTF
SNHVSVNCTRNVSLTVGASQGHSATFYDPILQRYTIQNSIQKFNPNPEHLGTILFSSAYIPDTSTSRDDFISHFR
NHIGLYNGTLALEDRAEWKVYKFDQFGGTLRLGSRAVFSTTDEEQSSSSVGSVININNLAINLPSILGNRVAPK
LWIRPTGSSAPYSEDNNPIINLSGPLSLLDDENLDPYDTADLAQPIAEVPLLYLLDVTAKHINTDNFYPEGLNT
TQHYGYQGVWSPYWIETITTSDTSSEDTVNTLHRQLYGDWTPTGYKVNPENKGDIALSAFWQSFHNLFATL
RYQTQQGQIAPTASGEATRLFVHQNSNNDAKGFHMEATGYSLGTTSNTASNHSFGVNFSQLFSNLYESHSDN
SVASHTTTVALQINNPWLQERFSTSASLAYSYSNHHIKASGYSGKIQTEGKCYSTTLGAALSCSLSLQWRSRP
LHFTPFIQAIAVRSNQTAFQESGDKARKFSVHKPLYNLTVPLGIQSAWESKFRLPTYWNIELAYQPVLYQQNP
EVNVSLESSGSSWLLSGTTLARNAIAFKGRNQIFIFPKLSVFLDYQGSVSSSTTTHYLHAGTTFKF

>core/29/3/Org3_Gene676

MFWKLLCPILICTSLSITSCEQQFKVVPNQCPLQVSTPAAADQKIEKIICSNGLPLLIISDPNLPTSGAALLVKTG
NNADPEEYPGMAHFTEHCVFLGNEKYPEVSGFPGFLSENNGVHNAFTYPNKTVFVFSVEHSAFSDALDQFVH
LFINPKFRQEDLDREKYAVHQEFAAHPLSDGRRVHRIQQLVAPQGHPCARFGCGNASTLTPVTTEKMAEWF
KLHYSPENMCAIAYTSAPLSKAKKQFSKIFSQIPRSKNYERQEPFLPSGDTSSLKNLYINQAIQPTSNLEIYWHI
YESSHPIPLGCYKALAEVLRNESKNSLVSLLKNEQLITDLDVEFFRSSLNTGEFYISYELTEKGDKHYSQVIDST
FQYLRYIQEHGIPNYTLEEISTINALNYCYSSKSPLFDLLCKQIVSLGNEDLSTYPYHSLVYPKYSSEDESALLN
LVSDPEQARFVLSSKNSEHWEEATQLHDPIFDMTYYVKALDGVQDYGKVQSLKPIALPKPNLFIPKEVTLPG

VHLLKKQEFPFAPALSYQDDKLTLYHCEDHYYTAPKLSSQIRIRSPQISRSSPQFLVATELYCLAVNDQLLREY
YPATQAGLSFTSALGGDGIDLRVSGYTTTVPALLNSILTSLPNLEISYETFLVYKKQLLELYQGALLNCPVRSG
LDELASQVMKETYSNTTKLSALEKLSFSEFQAFASNLFNSVHLEVMVLGNLSEQQKKDYLEMLQVFTASRSS
HATKPFYYELQSQEISEIHHDYPLTANGMLLLLQDKSSPSIQGKVCAEMLFEWLHHITFEELRTQQQLGYMV
GARYREFASRPFGFLYIRSDAYSPEELLAKTSLFLNKVSASPEKFGISQEKFANIRKAYINKILEPEHSLDMMNS
ALFSLAFERPFVEFSTPDLKIAIAETLTYEEFLKYCQCFLSNELGTQTSVYIRGTQKTS

>core/30/3/Org3_Gene53

MTTEDFPKAYNFQDTEPELYVFWEKNGMFKAEASSDKPPYSVIMPPPNVTGVLHMGHALVNTLQDVLVRY
KRMSGFEVCWIPGTDHAGIATQAVVERHLQASEGKRRTDYSREDFLKHIWAWKEKSEKVVLSQLRQLGCSC
DWDRKRFTMEPLANRAVKKAFKTLFENGYIYRGYYLVNWDPVLQTALADDEVEYEEKDGWLYYIRYRMV
GSQESIVVATTRPETSLGDTGIAVSPNDERYASWIGASVEVPFVNRQIPIIGDASVDPTFGTGAVKVTPAHDKD
DYLMGTNHHLPMINILTPSGGINENGGPFAGMAKEKAREEILIALEEQGLFVRKEPYKLRVGVSYRSGAVIEP
YLSKQWFVSVSEFRGALREFVESQDIKIFPKDFVKNYLSWVNHLRDWCISRQLWWGHRIPVWYHKNDDERV
LCYDGEGIPEEVAQDPDSWYQDPDVLDTWFSSGLWPLTCLGWPDENSPDLKKFYPTALLVTGHDILFFWVT
RMVLLCSSMSGEKPFSEVFLHGLIFGKSYKRYNDFGEWSYISGKEKLAYDMGEALPDGVVAKWEKLSKSKG
NVIDPLEMIATYGTDAVRLTLCSCANRGEQIDLDYRLFEEYKHFANKVWNGARFIFGHISDLQGKDLLAGIDE
DSLGLEDFYILDGFNQLIHQLEEAYATYAFDKVATLAYEFFRNDLCSTYIEIIKPTLFGKQGNEASQSTKRTLL
AVLLINVLGVLHPVAPFITESLFLRIQDTLGALPEGDGDAFTGHALRMLRSRACMEAPYPKAFDVKIPQDLRE
SFTLAQRLVYTIRNIRGEMQLDPRLHLKAFVVCSDTTEIQSCIPILQALGGLESIQLLDKEPEKGLYSFGVVDTI
RLGIFVPEEHLLKEKGRLEKERVRLERAVENLERLLGDESFCQKANPNLVVAKQEALKNNRIELQGILDKLAS
FA

>core/31/3/Org3_Gene311

MRFFCFGMLLPFTFVLANEGLQLPLETYITLSPEYQAAPQVGFTHNQNQDLAIVGNHNDFILDYKYYRSNGG
ALTCKNLLISENIGNVFFEKNVCPNSGGAIYAAQNCTISKNQNYAFTTNLVSDNPTATAGSLLGGALFAINCSI
TNNLGQGTFVDNLALNKGGALYTETNLSIKDNKGPIIIKQNRALNSDSLGGGIYSGNSLNIEGNSGAIQITSNSS
GSGGGIFSTQTLTISSNKKLIEISENSAFANNYGSNFNPGGGGLTTTFCTILNNREGVLFNNNQSQSNGGAIHAK
SIIIKENGPVYFLNNTATRGGALLNLSAGSGNGSFILSADNGDIIFNNNTASKHALNPPYRNAIHSTPNMNLQIG
ARPGYRVLFYDPIEHELPSSFPILFNFETGHTGTVLFSGEHVHQNFTDEMNFFSYLRNTSELRQGVLAVEDGA
GLACYKFFQRGGTLLLGQGAVITTAGTIPTPSSTPTTVGSTITLNHIAIDLPSILSFQAQAPKIWIYPTKTGSTYT
EDSNPTITISGTLTLRNSNNEDPYDSLDLSHSLEKVPLLYIVDVAAQKINSSQLDLSTLNSGEHYGYQGIWSTY
WVETTTITNPTSLLGANTKHKLLYANWSPLGYRPHPERRGEFITNALWQSAYTALAGLHSLSSWDEEKGHA
ASLQGIGLLVHQKDKNGFKGFRSHMTGYSATTEATSSQSPNFSLGFAQFFSKAKEHESQNSTSSHHYFSGMCI
ENTLFKEWIRLSVSLAYMFTSEHTHTMYQGLLEGNSQGSFHNHTLAGALSCVFLPQPHGESLQIYPFITALAIR
GNLAAFQESGDHAREFSLHRPLTDVSLPVGIRASWKNHHRVPLVWLTEISYRSTLYRQDPELHSKLLISQGTW
TTQATPVTYNALGIKVKNTMQVFPKVTLSLDYSADISSSTLSHYLNVASRMRF

>core/32/3/Org3_Gene783

MKSSVSWLFFSSIPLFSSLSIVAAEVTLDSSNNSYDGSNGTTFTVFSTTDAAAGTTYSLLSDVSFQNAGALGIPL
ASGCFLEAGGDLTFQGNQHALKFAFINAGSSAGTVASTSAADKNLLFNDFSRLSIISCPSLLLSPTGQCALKSV

GNLSLTGNSQIIFTQNFSSDNGGVINTKNFLLSGTSQFASFSRNQAFTGKQGGVVYATGTITIENSPGIVSFSQN
LAKGSGGALYSTDNCSITDNFQVIFDGNSAWEAAQAQGGAICCTTTDKTVTLTGNKNLSFTNNTALTYGGAI
SGLKVSISAGGPTLFQSNISGSSAGQGGGGAINIASAGELALSATSGDITFNNNQVTNGSTSTRNAINIIDTAKV
TSIRAATGQSIYFYDPITNPGTAASTDTLNLNLADANSEIEYGGAIVFSGEKLSPTEKAIAANVTSTIRQPAVLA
RGDLVLRDGVTVTFKDLTQSPGSRILMDGGTTLSAKEANLSLNGLAVNLSSLDGTNKAALKTEAADKNISLS
GTIALIDTEGSFYENHNLKSASTYPLLELTTAGANGTITLGALSTLTLQEPETHYGYQGNWQLSWANATSSKI
GSINWTRTGYIPSPERKSNLPLNSLWGNFIDIRSINQLIETKSSGEPFERELWLSGIANFFYRDSMPTRHGFRHIS
GGYALGITATTPAEDQLTFAFCQLFARDRNHITGKNHGDTYGASLYFHHTEGLFDIANFLWGKATRAPWVLS
EISQIIPLSFDAKFSYLHTDNHMKTYYTDNSIIKGSWRNDAFCADLGASLPFVISVPYLLKEVEPFVKVQYIYA
HQQDFYERYAEGRAFNKSELINVEIPIGVTFERDSKSEKGTYDLTLMYILDAYRRNPKCQTSLIASDANWMA
YGTNLARQGFSVRAANHFQVNPHMEIFGQFAFEVRSSSRNYNTNLGSKFCF

>core/33/3/Org3_Gene913

MFGMTPAVYSLQTDSLEKFALERDEEFRTSFPLLDSLSTLTGFSPITTFVGNRHNSSQDIVLSNYKSIDNILLLW
TSAGGAVSCNNFLLSNVEDHAFFSKNLAIGTAGAIACQGACTITKNRGPLIFFSNRGLNNASTGGETRGGAIA
CNGDFTISQNQGTFYFVNNSVNNWGGALSTNGHCRIQSNRAPLLFFNNTAPSGGGALRSENTTISDNTRPIYF
KNNCGNNGGAIQTSVTVAIKNNSGSVIFNNNTALSGSINSGNGSGGAIYTTNLSIDDNPGTILFNNNYCIRDGG
AICTQFLTIKNSGHVYFTNNQGNWGGALMLLQDSTCLLFAEQGNIAFQNNEVFLTTFGRYNAIHCTPNSNLQ
LGANKGYTTAFFDPIEHQHPTTNPLIFNPNANHQGTILFSSAYIPEASDYENNFISSSKNTSELRNGVLSIEDRA
GWQFYKFTQKGGILKLGHAASIATTANSETPSTSVGSQVIINNLAINLPSILAKGKAPTLWIRPLQSSAPFTEDN
NPTITLSGPLTLLNEENRDPYDSIDLSEPLQNIHLLSLSDVTARHINTDNFHPESLNATEHYGYQGIWSPYWVE
TITTTNNASIETANTLYRALYANWTPLGYKVNPEYQGDLATTPLWQSFHTMFSLLRSYNRTGDSDIERPFLEI
QGIADGLFVHQNSIPGAPGFRIQSTGYSLQASSETSLHQKISLGFAQFFTRTKEIGSSNNVSAHNTVSSLYVELP
WFQEAFATSTVLAYGYGDHHLHSLHPSHQEQAEGTCYSHTLAAAIGCSFPWQQKSYLHLSPFVQAIAIRSHQ
TAFEEIGDNPRKFVSQKPFYNLTLPLGIQGKWQSKFHVPTEWTLELSYQPVLYQQNPQIGVTLLASGGSWDIL
GHNYVRNALGYKVHNQTALFRSLDLFLDYQGSVSSSTSTHHLQAGSTLKF

>core/34/3/Org3_Gene54

MERYDIVRIIGKGGMGEVYLAYDPVCSRKVALKKIREDLAENPLLKRRFLREARIAADLIHPGVVPVYTIYSE
KDPVYYTMPYIEGYTLKTLLKSVWQKESLSKELAEKTSVGAFLSIFHKICCTIEYVHSRGILHRDLKPDNILLG
LFSEAVILDWGAAVACGEEEDLLDIDVSKEEVLSSRMTIPGRIVGTPDYMAPERLLGHPASKSTDIYALGVVL
YQMLTLSFPYRRKKGKKIVLDGQRIPSPQEVAPYREIPPFLSAVVMRMLAVDPQERYSSVTELKEDIESHLKG
SPKWTLTTALPPKKSSSWKLNEPILLSKYFPMLEVSPASWYSLAISNIESFSEMRLEYTLSKKGLNEGFGILLPT
SENALGGDFYQGYGFWLHIKERTLSVSLVKNSLEIQRCSQDLESDKETFLIALEQHNHSLSLFVDGTTWLIHM
NYLPSRSGRVAIIVRDMEDILEDIGIFESSGSLRVSCLAVPDAFLAEKLYDRALVLYRRIAESFPGRKEGYEARF
RAGITVLEKASTDNNEQEFALAIEEFSKLHDGVAAPLEYLGKALVYQRLQEYNEEIKSLLLALKRYSQHPEIF
RLKDHVVYRLHESFYKRDRLALVFMILVLEIAPQAITPGQEEKILVWLKDKSRATLFCLLDPTVLELRSSKME
LFLSYWSGFIPHLNSLFHRAWDQSDVRALIEIFYVACDLHKWQFLSSCIDIFKESLEDQKATEEIVEFSFEDLGA
FLFAIQSIFNKEDAEKIFVSNDQLSPILLVYIFDLFANRALLESQGEAIFQALDLIRSKVPENFYHDYLRNHEIRA
HLWCRNEKALSTIFENYTEKQLKDEQHELFVLYGCYLALIQGAEAAKQHFDVCREDRIFPASLLARNYNRLG
LPKDALSYQERRLLLRQKFLYFHCLGNHDERDLCQTMYHLLTEEFQL

>core/35/3/Org3_Gene782

MKIPLHKLLISSTLVTPILLSIATYGADASLSPTDSFDGAGGSTFTPKSTADANGTNYVLSGNVYINDAGKGTA
LTGCCFTETTGDLTFTGKGYSFSFNTVDAGSNAGAAASTTADKALTFTGFSNLSFIAAPGTTVASGKSTLSSA
GALNLTDNGTILFSQNVSNEANNNGGAITAKTLSISGNTSSITFTSNSAKKLGGAIYSSAAASISGNTGQLVFM
NNKGETGGGALGFEASSSITQNSSLFFSGNTATDAAGKGGAIYCEKTGETPTLTISGNKSLTFAENSSVTQGG
AICAHGLDLSAAGPTLFSNNRCGNTAAGKGGAIAIADSGSLSLSANQGDITFLGNTLTSTSAPTSTRNAIYLGS
SAKITNLRAAQGQSIYFYDPIASNTTGASDVLTINQPDSNSPLDYSGTIVFSGEKLSADEAKAADNFTSILKQPL
ALASGTLALKGNVELDVNGFTQTEGSTLLMQPGTKLKADTEAISLTKLVVDLSALEGNKSVSIETAGANKTIT
LTSPLVFQDSSGNFYESHTINQAFTQPLVVFTAATAASDIYIDALLTSPVQTPEPHYGYQGHWEATWADTSTA
KSGTMTWVTTGYNPNPERRASVVPDSLWASFTDIRTLQQIMTSQANSIYQQRGLWASGTANFFHKDKSGTN
QAFRHKSYGYIVGGSAEDFSENIFSVAFCQLFGKDKDLFIVENTSHNYLASLYLQHRAFLGGLPMPSFGSITD
MLKDIPLILNAQLSYSYTKNDMDTRYTSYPEAQGSWTNNSGALELGGSLALYLPKEAPFFQGYFPFLKFQAV
YSRQQNFKESGAEARAFDDGDLVNCSIPVGIRLEKISEDEKNNFEISLAYIGDVYRKNPRSRTSLMVSGASWT
SLCKNLARQAFLASAGSHLTLSPHVELSGEAAYELRGSAHIYNVDCGLRYSF

>core/37/3/Org3_Gene301

MKSSLHWFLISSSLALPLSLNFSAFAAVVEINLGPTNSFSGPGTYTPPAQTTNADGTIYNLTGDVSITNAGSPTA
LTASCFKETTGNLSFQGHGYQFLLQNIDAGANCTFTNTAANKLLSFSGFSYLSLIQTTNATTGTGAIKSTGACS
IQSNYSCYFGQNFSNDNGGALQGSSISLSLNPNLTFAKNKATQKGGALYSTGGITINNTLNSASFSENTAANN
GGAIYTEASSFISSNKAISFINNSVTATSATGGAIYCSSTSAPKPVLTLSDNGELNFIGNTAITSGGAIYTDNLVL
SSGGPTLFKNNSAIDTAAPLGGAIAIADSGSLSLSALGGDITFEGNTVVKGASSSQTTTRNSINIGNTNAKIVQL
RASQGNTIYFYDPITTSITAALSDALNLNGPDLAGNPAYQGTIVFSGEKLSEAEAAEADNLKSTIQQPLTLAGG
QLSLKSGVTLVAKSFSQSPGSTLLMDAGTTLETADGITINNLVLNVDSLKETKKATLKATQASQTVTLSGSLS
LVDPSGNVYEDVSWNNPQVFSCLTLTADDPANIHITDLAADPLEKNPIHWGYQGNWALSWQEDTATKSKAA
TLTWTKTGYNPNPERRGTLVANTLWGSFVDVRSIQQLVATKVRQSQETRGIWCEGISNFFHKDSTKINKGFR
HISAGYVVGATTTLASDNLITAAFCQLFGKDRDHFINKNRASAYAASLHLQHLATLSSPSLLRYLPGSESEQP
VLFDAQISYIYSKNTMKTYYTQAPKGESSWYNDGCALELASSLPHTALSHEGLFHAYFPFIKVEASYIHQDSF
KERNTTLVRSFDSGDLINVSVPIGITFERFSRNERASYEATVIYVADVYRKNPDCTTALLINNTSWKTTGTNLS
RQAGIGRAGIFYAFSPNLEVTSNLSMEIRGSSRSYNADLGGKFQF

>core/38/3/Org3_Gene303

MKTSIPWVLVSSVLAFSCHLQSLANEELLSPDDSFNGNIDSGTFTPKTSATTYSLTGDVFFYEPGKGTPLSDSC
FKQTTDNLTFLGNGHSLTFGFIDAGTHAGAAASTTANKNLTFSGFSLLSFDSSPSTTVTTGQGTLSSAGGVNL
ENIRKLVVAGNFSTADGGAIKGASFLLTGTSGDALFSNNSSSTKGGAIATTAGARIANNTGYVRFLSNIASTSG
GAIDDEGTSILSNNKFLYFEGNAAKTTGGAICNTKASGSPELIISNNKTLIFASNVAETSGGAIHAKKLALSSGG
FTEFLRNNVSSATPKGGAISIDASGELSLSAETGNITFVRNTLTTTGSTDTPKRNAINIGSNGKFTELRAAKNHT
IFFYDPITSEGTSSDVLKINNGSAGALNPYQGTILFSGETLTADELKVADNLKSSFTQPVSLSGGKLLLQKGVT
LESTSFSQEAGSLLGMDSGTTLSTTAGSITITNLGINVDSLGLKQPVSLTAKGASNKVIVSGKLNLIDIEGNIYES
HMFSHDQLFSLLKITVDADVDTNVDISSLIPVPAEDPNSEYGFQGQWNVNWTTDTATNTKEATATWTKTGF
VPSPERKSALVCNTLWGVFTDIRSLQQLVEIGATGMEHKQGFWVSSMTNFLHKTGDENRKGFRHTSGGYVI

GGSAHTPKDDLFTFAFCHLFARDKDCFIAHNNSRTYGGTLFFKHSHTLQPQNYLRLGRAKFSESAIEKFPREIP
LALDVQVSFSHSDNRMETHYTSLPESEGSWSNECIAGGIGLDLPFVLSNPHPLFKTFIPQMKVEMVYVSQNSF
FESSSDGRGFSIGRLLNLSIPVGAKFVQGDIGDSYTYDLSGFFVSDVYRNNPQSTATLVMSPDSWKIRGGNLSR
QAFLLRGSNNYVYNSNCELFGHYAMELRGSSRNYNVDVGTKLRF

>core/39/3/Org3_Gene796

MQRVLRLLFNLHHGEEKRAFLFFLLGLVWGIGCYGTLSLAEGLFIEKLGSAELPKIYLGSSLILCVLSSLILYNL
FKKHISATALFLIPVSLSILCNFYLILSSIFAIDPPRSPLFFYRIVIWSLTILSYTSFWGFVDQFFNLQDGKRHFCIF
NAIIFLGDAIGSGIIASLVHTIGIQGILILFTAALVLTFPIVFYVSKSLKSLSDDHDLFIDTGHPPPLSKALKLCFYD
KYTFYLLCFYFLMQLLAIATEFNYLKIFEIQFASKEEFELVAHIGKCSLWISLGNMCFALFAYSRIVKRLGVNNI
ILFAPLCFLSLFLFWTFKTTLSIAVLAMVVREGVTYALDDNNLQLLIYGVPNKIRNQIRIVVESFIEPIGMLVWS
LVCFLSSQQYVFCLIISLIATILVCLVRSYYAKAILKNLSAQALQLTRSMQDWIKSMTVKQKRQVELFLLAHL
KHPSERHQTFAFQHLLNLASRSVLPSLLAHMNKLSLPNKLKTIEMVKSSLWAKDFLTLELLKRWTSIFPHPAI
ASAIHLYFAEHDLLHITHIAEDLYDTVGDRLLAAILTVRRQEAYGPYRDLADKRLKELLNSDQPEDIVMGLTI
LKLEKNPQNFPILLDFLNTKNEDILIVTCKALHTSVRANHKPYCPELLKRLRQCSHNDEASQYLLKTISIALDIS
FVKDLLMTTSQLKNTSRKYAEAMIGELDKEVAPAFLQVLTDEGTHNRCRILAAKALCKIDNWLLKKHAYKI
VKSKASKALFYSYHGHYIQKKYPTYNLSLLANTLNSNYYAEVNFMLSLLGILGSMEHSGVLIRALTSKNQKI
KAQALESLEKNCDSHLFSLLEPFVNQPGMCYSEKYYFKCGVIPLTLKELLNMMENSPSSLNKLTAQQLKEEL
SYCDPDFQSVNTIYNQEHEDFRTEESETLISFLSI

>core/40/3/Org3_Gene4

MRFSLCGFPLVFSFTLLSVFDTSLSATTISLTPEDSFHGDSQNAERSYNVQAGDVYSLTGDVSISNVDNSALNK
ACFNVTSGSVTFAGNHHGLYFNNISSGTTKEGAVLCCQDPQATARFSGFSTLSFIQSPGDIKEQGCLYSKNAL
MLLNNYVVRFEQNQSKTKGGAISGANVTIVGNYDSVSFYQNAATFGGAIHSSGPLQIAVNQAEIRFAQNTAK
NGSGGALYSDGDIDIDQNAYVLFRENEALTTAIGKGGAVCCLPTSGSSTPVPIVTFSDNKQLVFERNHSIMGG
GAIYARKLSISSGGPTLFINNISYANSQNLGGAIAIDTGGEISLSAEKGTITFQGNRTSLPFLNGIHLLQNAKFLK
LQARNGYSIEFYDPITSEADGSTQLNINGDPKNKEYTGTILFSGEKSLANDPRDFKSTIPQNVNLSAGYLVIKE
GAEVTVSKFTQSPGSHLVLDLGTKLIASKEDIAITGLAIDIDSLSSSSTAAVIKANTANKQISVTDSIELISPTGN
AYEDLRMRNSQTFPLLSLEPGAGGSVTVTAGDFLPVSPHYGFQGNWKLAWTGTGNKVGEFFWDKINYKPRP
EKEGNLVPNILWGNAVDVRSLMQVQETHASSLQTDRGLWIDGIGNFFHVSASEDNIRYRHNSGGYVLSVNN
EITPKHYTSMAFSQLFSRDKDYAVSNNEYRMYLGSYLYQYTTSLGNIFRYASRNPNVNVGILSRRFLQNPLMI
FHFLCAYGHATNDMKTDYANFPMVKNSWRNNCWAIECGGSMPLLVFENGRLFQGAIPFMKLQLVYAYQG
DFKETTADGRRFSNGSLTSISVPLGIRFEKLALSQDVLYDFSFSYIPDIFRKDPSCEAALVISGDSWLVPAAHVS
RHAFVGSGTGRYHFNDYTELLCRGSIECRPHARNYNINCGSKFRF

>core/41/3/Org3_Gene491

MKTVILNIGRKILQGIKKKKKKIGILSGLFFLDLVLLGVSSQRPTETSANVKHNLRDEKLAACPKNSAASLSAK
KSHTKKTTPGSIPSKVFSKFDATQDKTFQKTSGSAFPAKPTTLKELEERKKPRPERRTTADVKRSPRFLPTQEV
EEPVPAASKEQLDSIQVWEEKQNYARRAVNAINLSIKKQLEEQTSTVTEKDVQPKTQATPHASKKNVASPST
SMPGIEKAATTVAVPQDKSEEEKVKERLTKRELTCEDLKDNGYTVNFEDISILELLQFVSKISGTNFVFDSNDL
QFNVTIVSHDPTSVDDLSTILLQVLKMHDLKVVEQGNNVLIYRNPHLSKLSTVVTDSSLKETCEAVVVTRVFR

LYSVSPSAAVNIIQPLLSHDAIVSASEATRHVIISDIAGNVDKVSDLLAALDCPGTSVDMTEYEVKYANPAALV
SYCQDVLGTLAEDDAFQMFIQPGTNKIFVVSSPRLANKAEQLLKSLDVPEMAHTLDDPASTALALGGTGTTS
PKSLRFFMYKLKYQNGEVIANALQDIGYNLYVTTAMDEDFINTLNSIQWLEVNNSIVIIGNQGNVDRVIGLLN
GLDLPPKQVYIEVLILDTSLEKSWDFGVQWVALGDEQSKVAYASGLLNNTGIATPTKATVPPGTPNPGSIPLP
TPGQLTGFSDMLNSSSAFGLGIIGNVLSHKGKSFLTLGGLLSALDQDGDTVIVLNPRIMAQDTQQASFFVGQT
VPYQTTNTIIQETGTVTQNIDYEDIGVNLVVTSTVAPNNVVTLQIEQTISELHSASGSLTPVTDKTYAATRLQIP
DGCFLVMSGHIRDKTTKVVSGVPLLNSIPLIRGLFSRTIDQRQKRNIMMFIKPKVISSFEEGTRVTNKEGYRYN
WEADEGSMQVAPRHAPECQGPPSLQAESDFKIIEIEAQ

>core/43/3/Org3_Gene703

MSKDKKMKPEPKKNFPTVFFFLLFGVVFGVVAFQNFLAGKKARVGFSHQIEHLVNLRLIVPEDSHKIALNDN
LVSFGGRFRDVQTQEGQLRYHYLELIDQGHRLDLDLQETSKSLTTLGKEVTNSILWFSAISGSPIPEQGYAISY
PSEVSGSVLTEPLVVTGPATPQLINLHSLQERYPTLSRSPEALRTYGSDLYELIGKYLSPVLGIGSETLKRELKD
LYQQVEVSLTQETDTEAAYTLYGQVLSTLNRISSSLVVSEGGERFSQLRSVRLYREEWNKYHKLVEARDLNQ
AQLEKLRGELSQTVWYFNNQELSSRSLEKQDPEVFGHWFAGAKEEWTAFKFNHSLSFKAPDQPRNLVLEKT
FKSQEPSPHYLGYLFTFLPIILVLLFVYLVFSRQMRGMSGSAMSFGKSPARMLLKGQNKVTFADVAGIEEAKE
ELIEIVDFLKNPNKFTSLGGRIPKGVLLIGPPGTGKTLIAKAVSGEADRPFFSIAGSDFVEMFVGVGASRIRDMF
EQAKRNAPCIIFIDEIDAVGRHRGAGIGGGHDEREQTLNQLLVEMDGFGTNEGVILMAATNRPDVLDKALLR
PGRFDRRVVMNLPDIKGRFEILMVHAKRIKLDPTVDLMAVARSTPGASGADLENLLNEAALLAARKDRTAV
TAVDVAEARDKVLYGKERRSLEMDAEERKTTAYHESGHAVVGLCVQHGDPVDKVTIIPRGLSLGATHFLPE
KNKLSYWKKELYDQLAVLMGGRAAEEIFLGDISSGAQQDISQATKLVRSMVCEWGMSPQLGNVTYDERSD
GLTGYGGYHEKSYSEETAKTIDTELRMLLDAAYQRALDIINEHKAEIELMTQMLIEFETLDSKDVKEIMDHT
WDPEKKRARLKEEGMLFKKSSDDLPPPPPKEDTLPGLGFNAT

>core/44/3/Org3_Gene253

MDSEFVGQVYSSDMDWIESMYQRFMNHETLDPSWKYFFEGYQLGQAASPSEASTKISGNETIAMLQEQKSQ
FLCTIYRYYGYLQSQISTLAPTTDSRFIQEKIAKIDLDEQVPSAGLLPKAQVSVRELIEALKKCYCGSLTLETLT
CTPELQEFVWNLMEKRQVERFAEQLLRSYKDLCKATFFEEFLQIKFTGQKRFSLEGGETLVPMLEHLVHYGS
ALGISNYVLGMAHRGRLNVLTNVLGKPYRYVFMEFEDDPAARGLESVGDVKYHKGYVLKSHQKDRETTFV
MLPNASHLESVDPIVEGVVAALQHQGHAGKEQSSLAILVHGDAAFSGQGVVYETLQLSRVPGYSTEGTLHIV
VNNYIGFTAVPRESRSTPYCTDIAKMLGIPVFRVNSEDVVACIEAIEYALQVRERFSCDVIIDLCCYRKYGHNE
SDDPSVTAPLLYDQIKRKKSIRELFRQYLLEGQFADISEETLASIEKEIQESLNREFQVLKGTDPEPFPKKECHH
CDRLNNGELILHDCDVSLDRETLFHMSSRLCGFPDNFHPHPKIKTLLEKRMKMAEGGVGYDWAMAEELAFA
SLLIEGYNLRLSGQDSIRGTFSQRHLVWSDTVTGDTYSPLYHLSAEQGSVEMYNSPLSEYAILGFEYGYAQQA
LKTLVLWEAQFGDFANGAQIIFDQYISSGIQKWDLHSDIVLLLPHGYEGQGPEHSSSRIERYLQLAANWNFQV
VLPSTPVQYFRILREHAKRDLSLPLVIFTPKLLLRYPQCVSSIEEFTEPGGFRAILEDADPNYDASILVLCSGKIY
YDYAEMLPQDRRKDFSCLRIESLYPLALEDLVSLIDKYSHLKHFVWLQEESKNMGAYDYMFMALQDILPEK
LLYIGRPRSSSTASGSAKLSRQELVTCMETLFSLR

>core/45/3/Org3_Gene794

MEKVKLTKNLKLKIKNAQLTKAAGLDKLKQKLAQAGSSEAKSSSEKPSAKEKSVKVALAATSTPTASAEQA
SPESTSRRIRAKNRSSFSSSEEESSAHIPVDTSEPAPVSIADPEPELEVVDEVCDESPEVHPVAEVLPEQPVLPET
PPQEKELEPKPVKPAEPKSVVMIKSKFGPTGKHINHLLAKTFKAPAKEEKVVAGSKSTKPVASDKTGKPGTSE
GGEQNNREKQFNPANRSPASGPKRDAGKKNLTDFRDRSKKSDESLKAFTGRDRYGLNEGGEEDRWRKKRV
YKPKKHYDEASIQRPTHIKISLPITVKDLATEMKLKASEVIQKLFIHGMTYVVNDILDSETAVQFIGLEFGCTID
IDYSEQDKLCLSNDTVRDEIQSTDPSKLVIRSPIVAFMGHVDHGKTTLIDSLRKSNVAATEAGAITQHMGAFC
CSTPVGDITILDTPGHEAFSAMRARGAEVCDIVVLVVAGDEGIKEQTLEAIEHAKAADIAIVVAINKCDKPNF
NSETIYRQLSEINLLPEAWGGSTVTVNTSAKTGEGLSELLEMLALQAEVLELKADPSARARGLVIESELHKGL
GPVATVLIQNGSLKLGEALVFNDCYGKVKTMHNEHNELMKEAGPSIPVLITGLSDIPKAGDPFFVVKNEKTA
RDIIEARSAGQQRFALQQKKRPNFDSMLQNKKTLKLMIKADVQGSIEALVSSISKIKSEKVDVEILTNSVGEIS
ESDIRLAAASKAVLIGFHTGIESHAEPLIKSLGVRVELFTVIYHAIDAIKEIMTSLLDPIAEEKDEGSAEIKEIFRS
SQVGSIYGCIVTEGIMTRNHKVRVLRNKEILWKGTLSSLKRVKEDVKEVRKGLECGILLEGYQQAQIGDVLQ
CYEVIYHPQKL

>core/46/3/Org3_Gene630

MLSNTIRSNFLKFYANRHHTILPSSPVFPHNDPSILFTNAGMNQFKDIFLNKEKVSYSRATTSQKCIRAGGKHN
DLDNVGHTSRHLTFFEMLGNFSFGDYFKAEAIAFAWEVSLSVFNFNPEGIYATVHEKDDEAFALWEAYLPTD
RIFRLTDKDNFWSMANTGPCGYCSELLFDRGPSFGNASSPLDDTDGERFLEYWNLVFMEFNRTSEGSLLALP
NKHVDTGAGLERLVSLIAGTHTVFEADVLRELIAKTEQLSGKVYHPDDSGAAFRVIADHVRSLSFAIADGLLP
GNTERGYVLRKILRRSVNYGRRLGFRNPFLAEIVPSLADAMGEAYPELKNSLSQIQKVLTLEEESFFKTLDRG
GNLLQQVLKSSSSSSCISGEDAFKLKDTYGMPIDEISLLAKDYDYSVDMDTFHKLEQEAKERSRKNVVQSQG
TSESIYNELHLTSEFIGYDHLSCDTFIEAIISKDHIVSSLQEKQEGAIVLKVSPFYAEKGGQVGDSGEIFCSEGTFI
VTHTTSPKAGLIVHHGRISQGSLTVEAAVTAQVNRYRRKRIANNHTACHLLHKALEITLGDHIRQAGSYVDD
TKIRLDFTHPQAISPEDLLCIETLVNESIRENEPVDIREALYSDVMNSSEIKQFFGDKYSDVVRVVSAGHSHELC
GGTHAEATGDIGFFRITKEHAVAMGIRRIEAVTGEKAEATVHQQSEVLEEIATLLQVPRDQIVSRLTATLDER
KQQDKRLNELENSLIQTKLDKLIHNCHQRQGITCLVHHLAEHENHRLQQYAQCLHQRIPEKLISLWTTEKNG
KYIVLSRVSDDLITQGVHAQDLLKAVLTPCGGRWGGKDQSAQGSAPALPATEVLNETLWQWISTQLI

>core/47/3/Org3_Gene942

MSTFSIQNRLRTISGESTRIIKLDHKYSGFDPRSVPAINLEELNSGIYALRHLMNALQSENTNVAALLNPNNTIF
PTTSWTDYKHSRPQASSPRAPSSQTPTDIVSAAALALVLVIDGGLAELVASVTEIDLGALSTISTVRQLMASYL
GLTTLTAEQEKVVFSSSYVPSEKNLLEHVKQEKAAEIQAKQEEIKAVLEAKGVSTEEIEAILKEYPDIYAADFF
KEFIEEPLHTYRAKVGAPIQEMNENAIQLLPTPPAITPDNVNEVNGMNTLSTILQAIDDAIKQAPALGGDQEIIT
ILQTLVPLVDKTTFTKAEFDLIYTATQLPNTASLKLYLTDRQIAEYRGKITKVYQNSIQNLSETKRVVENNRSM
LETQLSMFQQAQNCFVTWISQANALNIAITNKYISAVLTTSMEMYGGLLCLSYMYERLADDEKAIFDKSVNE
YLPIHIVVGGSWVNGWIAKMAAYQELAEYSLGTAVTSQDQIKAYLQTRGNEFKATRHFFHNIGDQMYQFAN
ETVFGNCLTTANGAIQPDLGGFIREAMTNVGTVEADYVSNAQRILNEFNTAATAHVLQLQLQIAELQKKADD
LDPGKASFTENRKFAVAAWITSESLGDALISMILNSQLPKQEAFLKPLIEEINFNNLAANALNSLLQITNEFSTT
SVYYSLSSYLVQSKTGQNLFAGDYYETLLAAAREREYIYRDTARCKQAINLVNGLLQKINSLPGATSAHKQE
MLNATTYYQYSLSVTLNQLTVLESLLAGLKMTLQTTSNNKYDKSVFKIESFDDWIPTLAALESFLTSGFPNIS
ATGGLGPLFTQVQSDQQTYTSQGQTQQLNLQNQMTTIQQEWTLVSTSMQVLNGILSQLAGAIYSN

>core/48/3/Org3_Gene542

MKKSLIIVESPAKIKTLQKLLGSEFVFASSIGHIVDLPAKEFGIDVDHDFEPQYQVLPDKQEVINHIRKLAAKCE
KVYLSPDPDREGEAIAWHIANQLPDSPLIQRVSFNAITKNAVTEALKHPRTIDMALVNAQQARRLLDRIVGYK
ISPILSRKLQQRSGISAGRVQSVALKLVVDREKAIDAFVPVEYWNLRVLMQDPKTTKTFWAHLYAVQGKKW
EKEIPEGKTENDVLLINSEEKARHYAELLEKSSYTITRVEAKAKRRFAPPPFITSTLQQEASRHFRFSASRTMSI
AQTLYEGVDLDSEDSTGLITYMRTDSVRVDPEALTTVREYIQQTFGKEYLPEKANVYTTKKMTQDAHEAIRP
TDINLTPDKLKNKLSDDQFKVYNLIWKRFVASQITPAIYDTLAVQITTDTEIDLRASGSLLKFKGFLAVYEEKQ
DDENDQEEDHPLPPLHAQDALIKEEVSQEQAFTKPLPRFTEASLVKELEKSGIGRPSTYATIMNKIQSREYTTK
ENQRLRPTELGKIISQFLETNFPRIMDIGFTALMEDELELIADNKKPWKLLLQEFWTTFLPVVITAEKEAVIPRI
LTNIECSKCHKGKLVKIWSKNSYFYGCSEYPECDYRTSEEELAFNKEDYAEDTPWDSPCPLCGGVMKVRHG
RYGTFLGCEKYPECRGTISIHKKGEEIEQEEPIPCPAIGCNGKIFKKRSRYNKIFYSCSEYPECSVIGNSIDAVITK
YSGTEKIPYKKKTPTKKKSSAKTTKAAKTPSKKGKAKSSVKKSSEKKTGPLFLPSPDLAKMIGNEPVSRGEAT
KKIWDYIKEHQLQAPENKKLLVPDNNLATIIGPNPIDMFQLSKHLSQHLTKVSNDESSASS

>core/49/3/Org3_Gene416

MKKLFVLDASGFIFRAYFALPEMKNHQGQATQAVFGFIRSLNKLIKEFSPEYMISVFDGPNNKQSRQAIYADY
KSNRQKKFEDIPPQIALVKEYCSLIGLAYLEKESVEADDVIASIAKKAREENYKVYVCTADKDLLQLVNDHV
VAWNPWADQGVVGISEVIERYGIPPGNIPDYLALVGDSSDNIPGLPGCGPKKAAALLKQFGSVEGLLENLDA
VKGLSQTMLSERQETLKLSKRLALLDSNIPIPVPIESLTFPQHPVDEEKLIHFYIQQGFKTLVPSKQTEAATVDV
QIIKDAESLTNILNLVQGGDIAFAVAYTGNHLLSLKLEGLALTQGSGVFFIALEEEGTKILPILKDFFLREDLTF
YGYNLKRDCHALLNAGIVIREISYDLALAEHLTNGGGKISFQSLLVNHGFTETAHRFAKEWGNSGLPIGRLPE
QPEQYFGEFVAYLPIIKDAILEEINRKNLNHILSDIEMPLEKVLFSMERAGVPLDVEELAILEALFETELAVLTE
EIYDLSGRPFNIKSPKQLSDILYNELGLRPIDKAKSTRAEVLEALRSEHPIIEKLLAFRTIEKLLSTYVKALPKQV
DSHTQRIHPSFDQTGAVTGRLACRDPNLQNIPIRSERGILLRKAFRLSEKNSYFLSADYSQIELRFLAHLSQDKS
LKFAFESGEDIHAFTASQVFHVPLEQVSKEQRMQAKTVNFGIVYGQQAFGLAKVLKISIGEAQELIQAYFSRY
PEIAHFVEETIQQAAKDLRVTTMLGRERIIDSWNEFPGSRAASGRFAVNTRIQGSAAELIKLAMLDISQAIKQQ
QMKSRMLLQIHDELLFEVPEEEIEEMQRLVREKMESAMTLSVPIVVNILIGKNWAEC

>core/50/3/Org3_Gene86

MEKFSDAVSEALEKAFELAKSSKHTYVTENHLLLALLENTESLFYLVIKDIHGNPGLLNTAVKDALSREPTVV
EGEVDPKPSPGLQTLLRDAKQEAKTLGDEYISGDHLLLAFWSSNKEPFNSWKQTTKVSFKDLKNLITKIRRGN
RMDSPSAESNFQGLEKYCKNLTALAREGKLDPVIGRDEEIRRTIQVLSRRTKNNPMLIGEPGVGKTAIAEGLA
LRLIQGDVPESLKGKQLYVLDMGALIAGAKYRGEFEERLKSVLKDVESGDGEHIIFIDEVHTLVGAGATDGA
MDAANLLKPALARGTLHCIGATTLNEYQKYIEKDAALERRFQPIFVTEPSLEDAVFILRGLREKYEIFHGVRIT
EGALNAAVLLSYRYIPDRFLPDKAIDLIDEAASLIRMQIGSLPLPIDEKERELAALIVKQEAIKREQSPSYQEEA
DAMQKSIDALREELASLRLGWDEEKKLISGLKEKKNSLESMKFSEEEAERVADYNRVAELRYSLIPQLEEEIK
QDEASLNQRDNRLLQEEVDERLIAQVVANWTGIPVQKMLEGEAEKLLILEESLEERVVGQPFAVSAVSDSIR
AARVGLNDPQRPLGVFLFLGPTGVGKTELAKALADLLFNKEEAMVRFDMSEYMEKHSISKLIGSSPGYVGYE
EGGSLSEALRRRPYSVVLFDEIEKADKEVLNILLQVFDDGILTDGKKRKVNCKNALFIMTSNIGSPELADYCS

KKGSELTKEAILSVVSPVLKRYLSPEFMNRIDEILPFVPLTKEDIVKIVGIQMRRIAQRLKARRINLSWDDSVIL
FLSEQGYDSAFGARPLKRLIQQKVVILLSKALLKGDIKPDTSIELTMAKEVLVFKKVETPS

>core/51/3/Org3_Gene501

MAVRLIVDEGPLSGVIFVLEDGISWSIGRDSSANDIPIEDPKLGASQAIINKTDGSYYITNLDDTIPIVVNGVAIQ
ETTQLKNEDTILLGSNQYSFLSDEFDPQDLVYDFDIPEENFSNDSGDLSDSNEQGKDLEPRQTSETNHSPKPKE
KLTKDQGSSDPITSGDQELADAFLASAKAEKNQPRAKVAKKGLKESSNESLNPKEQNAKDSPKGEERTNKPQ
NAIMEDNGASPRQDPQPKSAEPSLKNTARDETPLKENKPVEEKANKKATPDSPEKKDQPEEGSKKEGSKIEA
TPLDSQKESEDKEAEEAFVQEEEENLTEDNKEDSDSAADANDDTASDHTAEDNKETPKKVENEKSAVLSPFH
VQDLFRFDQTIFPAEIDDIAKKNISVDLTQPSRFLLKVLAGANIGAEFHLDSGKTYILGTDPTTCDIVFNDLSVS
HQHAKITVGNDGGILIEDLDSKNGVIVEGRKIDKTSTLSSNQVVALGTTLFLLIDHHAPADTIVASLSPDDYSL
FGRQQDAEALERQEAQEEEEKQKRATLPAGSFILTLFVGGLAILFGIGTASLFHTKEVVPLENIDYQEDLAQVI
NQFPTVRYTFNKTNSQLFLIGHVKNSTDKSELLYKVDALSFVKSVDDNVIDDEAVWQEMNILLSKRPEFKGIS
MHSPEPGKFIITGYVKTEEQAACLVDYLNIHFNYLSLLENKVVVETQMLKAIAGHLLQGGFANIHVAFVNGE
VILTGYVNNDDAEKFRAVVQELSGIPGVRLVKNFAVLLPAEEGIIDLNLRYPNRYRVTGYSRYGEISINVVVN
GRILTRGDVIDGMTVTSIQPNAIFLEKEGLKYKIDYNK

>core/52/3/Org3_Gene911

MFETFTNRAKQVIKLAKKEAQRLNHNYLGTEHILLGLLKLGQGVAVNVLRNLGIDFDTARQEVERLIGYGPE
IQVYGDPALTGRVKKSFESANEEASLLEHNYVGTEHLLLGILHQSDSVALQVLENLHIDPREVRKEILKELETF
NLQLPPSSSSSSSSRSNPSSSKSPLGHSLGSDKNEKLSALKAYGYDLTEMVRESKLDPVIGRSSEVERLILILCR
RRKNNPVLIGEAGVGKTAIVEGLAQKIILNEVPDALRKKRLITLDLALMIAGTKYRGQFEERIKAVMDEVRKH
GNILLFIDELHTIVGAGAAEGAIDASNILKPALARGEIQCIGATTIDEYRKHIEKDAALERRFQKIVVHPPSVDE
TIEILRGLKKKYEEHHNVFITEEALKAAATLSDQYVHGRFLPDKAIDLLDEAGARVRVNTMGQPTDLMKLEA
EIENTKLAKEQAIGTQEYEKAAGLRDEEKKLRERLQSMKQEWENHKEEHQVPVDEEAVAQVVSLQTGIPSA
RLTEAESEKLLKLEDTLRRKVIGQNDAVTSICRAIRRSRTGIKDPNRPTGSFLFLGPTGVGKSLLAQQIAIEMFG
GEDALIQVDMSEYMEKFAATKMMGSPPGYVGHEEGGHLTEQVRRRPYCVVLFDEIEKAHPDIMDLMLQILE
QGRLTDSFGRKVDFRHAIIIMTSNLGADLIRKSGEIGFGLKSHMDYKVIQEKIEHAMKKHLKPEFINRLDESVI
FRPLEKESLSEIIHLEINKLDSRLKNYQMALNIPDSVISFLVTKGHSPEMGARPLRRVIEQYLEDPLAELLLKES
CRQEARKLRATLVENRVAFEREEEEQEAALPSPHLES

>core/53/3/Org3_Gene868

MKIPLRFLLISLVPTLSMSNLLGAATTEELSASNSFDGTTSTTSFSSKTSSATDGTNYVFKDSVVIENVPKTGET
QSTSCFKNDAAAGDLNFLGGGFSFTFSNIDATTASGAAIGSEAANKTVTLSGFSALSFLKSPASTVTNGLGAIN
VKGNLSLLDNDKVLIQDNFSTGDGGAINCAGSLKIANNKSLSFIGNSSSTRGGAIHTKNLTLSSGGETLFQGNT
APTAAGKGGAIAIADSGTLSISGDSGDIIFEGNTIGATGTVSHSAIDLGTSAKITALRAAQGHTIYFYDPITVTGS
TSVADALNINSPDTGDNKEYTGTIVFSGEKLTEAEAKDEKNRTSKLLQNVAFKNGTVVLKGDVVLSANGFSQ
DANSKLIMDLGTSLVANTESIELTNLEINIDSLRNGKKIKLSAATAQKDIRIDRPVVLAISDESFYQNGFLNEDH
SYDGILELDAGKDIVISADSRSIDAVQSPYGYQGKWTINWSTDDKKATVSWAKQSFNPTAEQEAPLVPNLLW
GSFIDVRSFQNFIELGTEGAPYEKRFWVAGISNVLHRSGRENQRKFRHVSGGAVVGASTRMPGGDTLSLGFA

QLFARDKDYFMNTNFAKTYAGSLRLQHDASLYSVVSILLGEGGLREILLPYVSKTLPCSFYGQLSYGHTDHR
MKTESLPPPPRRSRRIILLGEDMSGLESWELELLLKIPAAEDFSKSTLHL

>core/54/3/Org3_Gene799

MFNKDEIIVPKNLEEEMKESYLRYSMSVIISRALPDIRDGLKPSQRRVLYAMKQLSLSPGAKHRKCAKICGDT
SGDYHPHGESVIYPTLVRMAQNWAMRYPLVDGQGNFGSIDGDPPAAMRYTEARLTHSAMYLMEDLDKDT
VDIVPNYDETKHEPVVFPSKFPNLLCNGSSGIAVGMATNIPPHNLGELIEATLLLLANPQASVDEILQVMPGPD
FPTGGIICGSEGIRSTYTTGRGKIKVRARLHVEENEDKHRESIIITEMPYNVNKSRLIEQIANLVNEKTLAGISDV
RDESDKDGIRVVLEIKKGESSEIIINRLYKFTDVQVTFGANMLALDKNLPRTMSIHRMISAWIRHRKEVIRRRT
RYELNKAETRAHVLEGYLKALSCLDALVKTIRESGNKEHAKERIIESFGFTEPQALAILELRLYQLTGLEAEKI
QKEYEELLNKIAYYKQVLSDEGLVKDIIRNELQDLLKHHKVARRTTIEFDADDIRDIEDIITNESVIITISGDDY
VKRMPVKVFKEQRRGGHGVTGFDMKKGAGFLKAVYSAFTKDYLLIFTNFGQCYWLKVWQLPEGERRAKG
KPIINFLEGIRPGEELAAILNIKNFDNAGFLFLATKRGVVKKVSLDAFSNPRKKGIRALEIDEGDELIAACHIVS
DEEKVMLFTHLGMAVRFPHEKVRPMGRTARGVRGVSLKNEEDKVVSCQIVTENQSVLIVCDQGFGKRSLVE
DFRETNRGGVGVRSILINERNGNVLGAIPVTDHDSILLMSSQGQAIRINMQDVRVMGRSTQGVRLVHLKEGD
ALVSMEKLSSNENDDEVLSGSEEECSDTVSLR

>core/55/3/Org3_Gene941

MIYSTSISTFYKKLSLVSSMHSFAQRHRESLEHIANYEKTTAERDILKRLIEVLDQRASERYRSAVEKLHKYEV
ERATVAKSIPVAAIHEKPLSSTHASVQVTASTPAATGSGVGAYYNAVKQKWAQDLIVELNTVMTTIMASVNS
KNPANKDVFDKLNTELQALVAAGNNLTEENFQTLYNFPEEIFTAIQRADTFTGGMKTDFTNQLAGKYGNQA
TLTQTFADGRVEGFKDILTAVQGVLTPEQFTIFAEIATELQALADHVGNFDEAGLQRIEDAGEKLAAVINSSD
LTRNDKIMFCQHITDLYSDQVAALGSFDTVLDASIYVNQHQGTMFSNLSSFVGSLIGTFAPIDLSSSQGDISSA
ALAGALQTARGLNSRFNELTAEQQKLINECIKSLVTFKCGEHLGAIWAYFTASTVVALNPTATMDHVKAAIL
EEAKELDNSSFQLASSIKSAMTSIVNSSGSFSVTVNSSTLQYTIYSEKNGKVEINQILLNYGSTGFLPEITKLAKT
NAESTARSYFRFKALAAVESENVQNKIEDLQSQLQQFTNMKTELFDGQLLSQASELRALPLPSAVASVLIDRY
MPKEVDYLNEIYKKLYYSNLGSSVGNSIIDAISQYVNGATYFNFASYVGQQPAVGAGGANAFPGSQESAQAK
LDQERKQAALYLQETRGALTVIEEQRARVLKDDKITNEQRSTILDSLRNYEDNINSISGSLVLLQNYLQPLSIA
GGSVAGTFEVKEGQEQWQARLQILEEALVSGLVGNMINGGMFPLQSTIQSDQQSFADMGQNFQLDLQMHLT
SMQQEWTVVATSLQLLNQMYLSLARSLTG

>core/56/3/Org3_Gene842

MTEKKPTPMMEQWHQCKEKAGDSVLLFRMGDFYEAFYDDAVLLSQHLELTLTQRQGIPMSGIPVSTVDTYV
DRLIGKGFKVAVAEQFGEPAKEKESKKIGPMARDIQRFVTPGTLLSSTLLQEKFNNYIVAINRIGSLFGFACLD
LSTGSFFIEECENTKELVDEICRLAPSEVLSCNKFYNKETAIVMQLQQHLKLTLSTYADWAFEHKFASQKLTT
HFQVASLDGFGLKGLVPAINAAGGLLSYIQDKLLLPTKHIAIPQTRGKQQKLLIDTASQVNLELLAPLNDPQG
KNSLLRIMDHTSTPMGGRLLRQILISPFYNPKEILVRQDAVEFFLRQVTLRKNIKTYLCQVRDIERLMTKVTTG
LAGPRDIGTLRDSFSAGAQIYEQLASATLPEFFIDKCSLDTKLASLIALLSKSLNGDLPLRVSDGNIFVDEFHND
LKRLRHNQEHSQEWIWEYQERIRKETGIKKLKICFAQALGYYIEVSSEFAPQLPKDFIRRQSRLHAERFTTIEL
QQFQDDMSNISEKLQTLETQFFKDLCSHILQLRTEILALSQSLADLDYIISLADLAHAQGYCRPRVDMSDTLCI
YRGCHPVAKTLVDTGKFIPNDTEMRGSQTRMILLTGPNMAGKSTYIRQIALLVIMAQMGSYIPAKSAHIGVID

KIFTRIGAGDNLSKGMSTFMVEMAETANILHNATDRSLVILDEVGRGTSTYDGLAIAQAVVEYLLFTDKKKA
KTLFATHYKELTTLEDHCPHVENFHAGVKDKAGQPVFLYEILKGHSQKSFGIHVARLAGFPLCVVSRAQQIL
RQLEGPESITRPAQDKMQQLTLF

>core/57/3/Org3_Gene1013

MKRAILDRLYLSVVQSPESASPRDIFTAVAKTVMEWLAKGWLKTQNGYYKNDVKRVYYLSMEFLLGRSLK
SNLLNLGILDLVRKALKTLNYDFDHLVEMESDAGLGNGGLGRLAACYLDSMATLAVPAYGYGIRYDYGIFD
QRIVNGYQEEAPDEWLRYGNPWEICRGEYLYPVRFYGRVIHYTDSRGKQVADLVDTQEVLAMAYDIPIPGY
GNDTVNSLRLWQAQSPRGFEFSYFNHGNYIQAIEDIALIENISRVLYPNDSITEGQELRLKQEYFLVSATIQDIIR
RYTKTHICLDNLADKVVVQLNDTHPALGIAEMMHILVDREELPWDKAWEMTTVIFNYTNHTILPEALERWP
LDLFSKLLPRHLEIIYEINSRWLEKVGSRYPKNDDKRRSLSIVEEGYQKRINMANLAVVGSAKVNGVSSFHSQ
LIKDTLFKEFYEFFPEKFINVTNGVTPRRWIALCNPRLSKLLNETIGDRYIIDLSHLSLIRSFAEDSGFRDHWKG
VKLKNKQDLTSRIYNEVGEIVDPNSLFDCHIKRIHEYKRQLMNILRVIYVYNDLKENPNQDVVPTTVIFSGKA
APGYVMAKLIIKLINSVADVVNQDSRVNDKLKVLFLPNYRVSMAEHIIPGTDLSEQISTAGMEASGTGNMKF
ALNGALTIGTMDGANIEMAEHIGKENMFIFGLLEEQIVQLRREYCPQTICDKNPKIRQVLDLLEQGFFNSNDK
DLFKPIVHRLLHEGDPFFVLADLESYIAAHENVNKLFKEPDSWTKISIYNTAGMGFFSSDRAIQDYARDIWHV
PTKSCSGEGN

>core/58/3/Org3_Gene960

MKKLYHPTLFLRPLIRLSLIFALSLTLISGNFPQQKSFGHCCADMHSALISGKNCEELFADFIERVLADRETLTA
RDWGTVVVLVREYLLKCIRKGDCDYGVKILQKLLALRLPKDARKDLQILWHRLNPEQAPLRDVVDQLFTIG
CHESLQDHLLFELYTVTLHSGYANRKQDMLLAKEQGDYKKAIELAKELVAALEKGSCSPHPEIVQIEKTFLQ
KTLLALQIKVAQEAQESCDALLTPYCLSEIAYTEAMDALVLRIARGEVSRTNEVDSVLLSHALQHLPFAREKA
IPELEVLIDHGAYLESTLLYYAYFSLLELYHQNKDFASLERLLEKGDAVFVPEHPYFPEYGFFLGAYFYAKGK
YESAEKVFLQIIDPAVKLGATFARAYEYLGCIAYVQNHYEKAEEYFLRAYKSWGREESGIGLFLAYAVQKKK
TACEDMLYHPKFSFTYRHLLDSLCSLSYPHGENKGSSAIQRVHRAVPELSEIYSRCIYDMIKYRNVTYTHPIIE
LAYNQVRNLEKRNLEEICRDAQDPEYDKALAFWGALQSGASVPRSLIESSDVDEAGITIRCYEALYFHNPDAI
AMLPQAFSEECNSWQTALRLVWTLVRPKGAPNHAKYWDHLVLRPHGDSLYFFGYDLQEYLIGKEDALKHL
SVFAELFPKSSLLSLVYYLQGYSESSALRKVGWFVKALEEFTEISWSGEHMKTWAYIYYMVKLDLADTYISL
GNFSQAVHILEEVKEDWQVASHPKLHFLKGEDCYLAMELRWVEGLAYAYFQLHETAHLSNHLLEHVEKNLI
SPRSYRDYYGESLQRTLGLCQRFLGV

>core/59/3/Org3_Gene92

MRYDPNLIEKKWQQFWKEHRSFQANEDEDKVKYYVLDMFPYPSGAGLHVGHLIGYTATDIVARYKRARGF
SVLHPMGWDSFGLPAEQYAIRTGTHPKVTTQKNIANFKKQLSAMGFSYDEGREFATSDPDYYHWTQKLFLF
LYDQGLAYMADMAVNYCPELGTVLSNEEVENGFSIEGGYPVERKMLRQWILKITAYADKLLEGLDALDWP
ENVKQLQKNWIGKSEGALVTFHLTQEGSLEAFTTRLDTLLGVSFLVIAPEHPDLDSIVSEEQRDEVTAYVQES
LRKSERDRISSVKTKTGVFTGNYAKHPITGNLLPVWISDYVVLGYGTGVVMGVPAHDERDREFAEMFSLPIH
EVIDDNGVCIHSNYNDFCLNGLSGQEAKDYVINYLEMRSLGRAKTMYRLRDWLFSRQRYWGEPIPIIHFEDG
THRPLEDDELPLLPPNIDDYRPEGFGQGPLAKAQDWVHIYDEKTGRPGCRETYTMPQWAGSCWYYLRFCDA
HNSQLPWSKEKESYWMPVDLYIGGAEHAVLHLLYSRFWHRVFYDAGLVSTPEPFKKLINQGLVLASSYRIPG

KGYVSIEDVREENGTWISTCGEIVEVRQEKMSKSKLNGVDPQVLIEEYGADALRMYAMFSGPLDKNKTWSN
EGVGGCRRFLNRFYDLVTSSEVQDIEDRDGLVLAHKLVFRITEHIEKMSLNTIPSSFMEFLNDFSKLPVYSKRA
LSMAVRVLEPIAPHISEELWVILGNPPGIDQAAWPQIDESYLVAQTVTFVVQVNGKLRGRLEVAKEAPKEEVL
SLSRSVVAKYLENAQIRKEIYVPNKLVNFVL

>core/60/3/Org3_Gene13

MDSTTNSDSPILDPNPEDVEKLLDESEEESEDQSTERLLPSELFILPLNKRPFFPGMAAPILIESGPYYEVLKVLA
KSSQKYIGLVLTKKENADILKVSFNQLHKTGVAARILRIMPIEGGSAQVLLSIEERIRIIEPIKDKYLKARVSYH
ADNKELTEELKAYSISIVSVIKDLLKLNPLFKEELQIFLGHSDFTEPGKLADFSVALTTATREELQEVLETTNM
HDRIDKALILLKKELDLSRLQSSINQKIEATITKSQKEFFLKEQLKTIKKELGLEKEDRAIDIEKFSERLRKRHVP
DYAMEVIQDEIEKLQTLETSSAEYTVCRNYLDWLTIIPWGIQSKEYHDLKKAEIVLNKDHYGLDEIKQRILELI
SVGKLSKGLKGSIICLVGPPGVGKTSIGRSIAKVLHRKFFRFSVGGMRDEAEIKGHRRTYIGAMPGKMVQALK
QSQAMNPVIMIDEVDKIGASYHGDPASALLEVLDPEQNKDFLDHYLDVRVDLSNVLFILTANVLDTIPDPLLD
RMEILRLSGYILEEKLQIAKKYLVPKARKEIGLTASEVNFQPEALKYMINNYAREAGVRTLNGNIKKVLRKVA
LKIVQNQEKPKSKKITFKISSKNLQTYLGKPIFSSDRFYESTPVGVATGLAWTSLGGATLYIESVQVSSLKTDM
HLTGQAGEVMKESSQIAWTYLHSALHRYAPGYTFFPKSQVHIHIPEGATPKDGPSAGITMVTSLLSLLLETPV
VNNLGMTGEITLTGRVLGVGGIREKLIAARRSRLNILIFPEDNRRDYEELPAYLKTGLKIHFVSHYDDVLKVA
FPKLK

>core/61/3/Org3_Gene638

MKGTPQYHFIGIGGIGMSALAHILLDRGYEVSGSDLYESYTIESLKAKGARCFSGHDSSHVPHDAVVVYSSSI
APDNVEYLTAIQRSSRLLHRAELLSQLMEGYESILVSGSHGKTGTSSLIRAIFQEAQKDPSYAIGGLAANCLNG
YSGSSKIFVAEADESDGSLKHYTPRAVVITNIDNEHLNNYAGNLDNLVQVIQDFSRKVTDLNKVFYNGDCPIL
KGNVQGISYGYSPECQLHIVSYNQKAWQSHFSFTFLGQEYQDIELNLPGQHNAANAAAACGVALTFGIDINII
RKALKKFSGVHRRLERKNISESFLFLEDYAHHPVEVAHTLRSVRDAVGLRRVIAIFQPHRFSRLEECLQTFPKA
FQEADEVILTDVYSAGESPRESIILSDLAEQIRKSSYVHCCYVPHGDIVDYLRNYIRIHDVCVSLGAGNIYTIGE
ALKDFNPKKLSIGLVCGGKSCEHDISLLSAQHVSKYISPEFYDVSYFIINRQGLWRTGKDFPHLIEETQGDSPLS
SEIASALAKVDCLFPVLHGPFGEDGTIQGFFEILGKPYAGPSLSLAATAMDKLLTKRIASAVGVPVVPYQPLNL
CFWKRNPELCIQNLIETFSFPMIVKTAHLGSSIGIFLVRDKEELQEKISEAFLYDTDVFVEESRLGSREIEVSCIG
HSSSWYCMAGPNERCGASGFIDYQEKYGFDGIDCAKISFDLQLSQESLDCVRELAERVYRAMQGKGSARIDF
FLDEEGNYWLSEVNPIPGMTAASPFLQAFVHAGWTQEQIVDHFIIDALHKFDKQQTIEQAFTKEQDLVKR

>core/62/3/Org3_Gene625

MIRERKKSRHPRLPTLPLAAKASLYLFFACFSGLSLWSFHRDQPCTQNWIGLLGWSFSSFLLYFFGAAAFFIPL
YFLWLSFLYFRRTPRPLFFYKAAAFLSLPFCSAILLSMLSPVGTLPALLDTRLPKFILGNNPPVSYVGGIPFYLF
YEGQSFCLKHLIGSVGTALIFGFVMLFSVLYLCGGIALLKKKTFQDGVKKAFCSFFQTCFKNLKKLINRRNYL
PKPSVPFVSKNPFSCTKSQPSPRRVSETIILDGSISPLPQEEIPGSKKESFFLTPHPCKRFLTKFVEPQENKAKEGK
TIALSSTPTVVRESKGKERAALPKLKSLAVPENDLPQYHLLSKNREARPESLQAELERKALILKQTLTSFGIDA
DLGNICSGPTLAAFEVLPHSGVKVQKIKSLENDIALKLQASSIRIIAPIPGKAAVGIEIPTPFPQAVNFRDLLEDY
QKTNRKLQIPLLLGKKANGDNLWADLATMPHLIIAGTTGSGKSVCINTIVMSMIMTTLPSEIKLVIIDPKKVEL
TGYSQLPHMLSPVITESREVYNALVWLVKEMESRYEILRYLGLRNIQAFNSRTRNKTIEASYDREIRETMPFM

VGIIDELSDLLLSSSQDIETPIIRLAQMARAVGIHLILATQRPSREVITGLIKANFPSRISFKVSNKVNSQIIIDEPG
AENLMGNGDMLVLLPSVFGTIRAQGAYICDEDINKVIQDLCSRFPTQYVIPSFHAFDDSDSDNSGEKDPLFAQ
AKTLILQTGNASTTFLQRKLKIGYARAASLIDQLEEARIIGPSEGAKPRQILIQNPLEG

>core/63/3/Org3_Gene176

MDPKEKNYDASAITVLEGLQAVRERPGMYIGDTGITGLHHLVYEVVDNSIDEAMAGYCSRIDVRILEDGGIVI
VDNGRGIPIEVHERESAKQGREVSALEVVLTVLHAGGKFDKDSYKVSGGLHGVGVSCVNALSEKLVATVFK
DKKCYQMEFSRGIPVTPLQYVSVSDRQGTEIVFYPDPKIFSTCTFDRSILMKRLRELAFLNRGITIVFEDDRDVS
FDKVTFFYEGGIQSFVSYLNQNKESLFSEPIYICGTRVGDDGEIEFEAALQWNSGYSELVYSYANNIPTRQGGT
HLTGFSTALTRVINTYIKAHNLAKNNKLALTGEDIREGLTAVISVKVPNPQFEGQTKQKLGNSDVSSVAQQV
VGEALTIFFEENPQIARMIVDKVFVAAQAREAAKKARELTLRKSALDSARLPGKLIDCLEKDPEKCEMYIVEG
DSAGGSAKQGRDRRFQAILPIRGKILNVEKARLQKIFQNQEIGTIIAALGCGIGADNFNLSKLRYRRIIIMTDAD
VDGSHIRTLLLTFFYRHMTALIENECVYIAQPPLYKVSKKKDFRYILSEKEMDSYLLMLGTNESSILFKSTERE
LRGEALESFINVILDVESFINTLEKKAIPFSEFLEMYKEGIGYPLYYLAPATGMQGGRYLYSDEEKEEALAQEE
THKFKIIELYKVAVFVDIQNQLKEYGLDISSYLIPQKNEIVIGNEDSPSCNYSCYTLEEVINYLKNLGRKGIEIQR
YKGLGEMNADQLWDTTMNPEQRTLIHVSLKDAVEADHIFTMLMGEEVPPRREFIESHALSIRINNLDI

>core/64/3/Org3_Gene795

MNKRTLLFVSLIGIAFVGCQIFFGYNEFRSCKNLAEKQRKISEQTLAAVESVGLSVASWDTDVNGEEHKNNY
AVRVGDKLFLLHNGEAAQSVYSSGESWSFVDHKCGFDNIHLALYRQQGSSFNPTNTGKVFLPTNHEGLPVL
VVEFRNNKEPLVFLGEYAQGRISNKDSTIFGTALVFWRSGSDYIPLGLYDSREEKLVSLDLPITRAVIFGNDQD
SAKSSDTANHYVLFNDYMQIIVSEESGSIEGINLPFASTNNKSIVNEIGFDRDLASEKSPEALFPGLSSKLPDGQ
QAKNSIGGYYPLLRRGLLSDSKKLLPLEYHALNVVSGRELATPVALRYRVLSYTPHSIQLESLDRSVQKVYKL
PENPEEKPYVFETAITLTKETEDVWVTSGVPEVEIMSNASAPTIKYRVIKKNKGSLDKVKLPKVKEPLAVRRG
VYPQWILNSNGYFGIILTPLSEIASGYGSLYISGSTAPTRLSAISPKNQLYPVSKYPGYETLLPLPKDAGTHRFL
VYAGPLAEPTLKVLDKTITQEKGENPEYLDSISFRGVFAFITAPFAALLFIIMKFFKLVTGSWGISIILLTVFLKL
LLYPLNAWSIRSMRRMQILSPYIQQIQQKYKNEPKRAQMEIMGLYKTNKVNPITGCLPLLIQLPFLIAMFDLLK
SSFLLRGASFIPGWIDNLTAPDVLFSWQTSIWFIGNEFHLLPILLGIVMFLQQKVTSLHKKGPVTDQQKQQQV
MGNMMAILFTAMFYNFPSGLNIYWLSSMILGVVQQWITNKILDSKHLKNEVVLNNKKHR

>core/65/3/Org3_Gene924

MRIPITLLQTYFSEPLSTKEILEACDHIGIEAEIENTTLYSFASVITAKILHTIPHPNADKLRVATLTDGEKEHQV
VCGAPNCEAGLIVALALPGAKLFDSEGQAYTIKKSKLRGVESQGMCCGADELGLDELQIQERALLELPEATP
LGEDLATVLGNTSLEISLTPNLAHCASFLGLAREICHVTQANLVIPKEFSFENLPTTALDMGNDPDICPFFSYV
VITGISAQPSPIKLQESLQALKQKPINAIVDITNYIMLSLGQPLHAYDASHVALDSLRVEKLSTPESLTLLNGET
VLLPSGVPVVRDDHSLLGLGGVMGAKAPSFQETTTTTVIKAAYFLPEALRASQKLLPIPSESAYRFTRGIDPQN
VVPALQAAIHYILEIFPEATISPIYSSGEICRELKEVALRPKTLQRILGKSFSIEILSQKLQSLGFSTTPQETSLLVK
VPSYRHDINEEIDLVEEICRTESWNIETQNPVSCYTPIYKLKRETAGFLANAGLQEFFTPDLLDPETVALTRKE
KEEISLQGSKHTTVLRSSLLPGLLKSAATNLNRQAPSVQAFEIGTVYAKHGEQCQETQTLAILLTEDGESRSW
LPKPSLSFYSLKGWVERLLYHHHLSIDALTLESSALCEFHPYQQGVLRIHKQSFATLGQVHPELAKKAQIKHP

VFFAELNLDLLCKMLKKTTKLYKPYAIYPSSFRDLTLTVPEDIPANLLRQKLLHEGSKWLESVTIISIYQDKSL
ETRNKNVSLRLVFQDYERTLSNQDIEEEYCRLVALLNELLTDTKGTINS

>core/66/3/Org3_Gene196

MLIMRNKVILQISILALIQTPLTLFSTEKVKEGHVVVDSITIITEGENASNKHPLPKLKTRSGALFSQLDFDEDLR
ILAKEYDSVEPKVEFSEGKTNIALHLIAKPSIRNIHISGNQVVPEHKILKTLQIYRNDLFEREKFLKGLDDLRTY
YLKRGYFASSVDYSLEHNQEKGHIDVLIKINEGPCGKIKQLTFSGISRSEKSDIQEFIQTKQHSTTTSWFTGAGL
YHPDIVEQDSLAITNYLHNNGYADAIVNSHYDLDDKGNILLYMDIDRGSRYTLGHVHIQGFEVLPKRLIEKQS
QVGPNDLYCPDKIWDGAHKIKQTYAKYGYINTNVDVLFIPHATRPIYDVTYEVSEGSPYKVGLIKITGNTHTK
SDVILHETSLFPGDTFNRLKLEDTEQRLRNTGYFQSVSVYTVRSQLDPMGNADQYRDIFVEVKETTTGNLGLF
LGFSSLDNLFGGIELSESNFDLFGARNIFSKGFRCLRGGGEHLFLKANFGDKVTDYTLKWTKPHFLNTPWILGI
ELDKSINRALSKDYAVQTYGGNVSTTYILNEHLKYGLFYRGSQTSLHEKRKFLLGPNIDSNKGFVSAAGVNL
NYDSVDSPRTPTTGIRGGVTFEVSGLGGTYHFTKLSLNSSIYRKLTRKGILKIKGEAQFIKPYSNTTAEGVPVSE
RFFLGGETTVRGYKSFIIGPKYSATEPQGGLSSLLISEEFQYPLIRQPNISAFVFLDSGFVGLQEYKISLKDLRSS
AGFGLRFDVMNNVPVMLGFGWPFRPTETLNGEKIDVSQRFFFALGGMF

>core/67/3/Org3_Gene825

MLLLISGALFLTLGIPGLTAGVSFGLGIGLSALGGVLVVSGLLCLLVKREVSKVCPEEIPAVQPEETPEGVPVTP
FEKPALDEAQKEQKTQKILDQLPQELDQLDRYIQEVFACLGPLKDLKYEDQGFLQDVKEEFQVFDFVQKDMI
AEFVELQQILCQEGRLLEFVINQTRYIGRDLFKREDSLYKLWEWLGYLPSGDVRGERLKKSAREVVDRFMRT
TCNIRKIAMTFDRHVYSVAKTAFEKAFGALETCVYESMRESYREAFCEYEKAKLLGDEEKSAHAEQRFQDIK
NRWEDVKDAFFWVKEDGKIEIDDAIGNSCKWSERYEEHRITRARWYKVAEHQLFNATMRVKDSLREHNEA
RVAFEKERSKENQRQVQKKKEKRLRDLKELHDQELPRAQERLRELQALYPEIAVSVVEARREVASDLEKAH
ESIDKHYQSCVREQELY

>core/68/3/Org3_Gene775

MASGIGGSSGLGKIPPKDNGDRSRSPSPKGELGSHEISLPPQEHGEEGASGSSHIHSSSSFLPEDQESQSSSSAAS
SPGFFSRVRSGVDRALKSFGNFFSAESTSQARETRQAFVRLSKTITADERRDVDSSSAAATEARVAEDASVSG
ENPSQGVPETSSGPEPQRLFSLPSVKKQSGLGRLVQTVRDRIVLPSGAPPTDSEPLSLYELNLRLSSLRQELSDI
QSNDQLTPEEKAEATVTIQQLIQITEFQCGYMEATQSSVSLAEARFKGVETSDEINSLCSELTDPELQELMSDG
DSLQNLLDETADDLEAALSHARLSFSLDDNPTPIDNNPTLISQEEPIYEEIGGAADPQRTRENWSTRLWNQIRE
ALVSLLGMILSILGSILHRLRIARHAAAEAVGRCCTCRGEECTSSEEDSMSVGSPSEIDETERTGSPHDVPRRN
GSPREDSPLMNALVGWAHKHGAKTKESSESSTPEISISAPIVRGWSQDSSVSFIVMEDDHIFYDVPRRKDGIYD
VPSSPRWSPARELEEDVFGDYEVPITSAEPSKDKNIYMTPRLATPAIYDLPSRPGSSGSSRSPSSDRVRSSSPNR
RGVPLPPVPSPAMSEEGSIYEDMSGASGAGESDYEDMSRSPSPRGDLDEPIYANTPEDNPFTQRNIDRILQERS
GGASASPVEPIYDEIPWIHGRPPATLPRPENTLTNVSLRVSPGFGPEVRAALLSESVSAVMVEAESIVPPTEPGD
GESEYLEPLGGLVATTKILLQKGWPRGESNA

>core/69/3/Org3_Gene867

MLLLISGALFLTLGIPGLSAAISFGLGIGLSALGGVLMISGLLCLLVKREIPTVRPEEIPEGVSLAPSEEPALQAA
QKTLAQLPKELDQLDTDIQEVFACLRKLKDSKYESRSFLNDAKKELRVFDFVVEDTLSEIFELRQIVAQEGWD

LNFLINGGRSLMMTAESESLDLFHVSKRLGYLPSGDVRGEGLKKSAKEIVARLMSLHCEIHKVAVAFDRNSY
AMAEKAFAKALGALEESVYRSLTQSYRDKFLESERAKIPWNGHITWLRDDAKSGCAEKKLRDAEERWKKF
RKAVFWVEEDGGFDINNLLGDWGTVLDPYRQERMDEITFHELYEKTTFLKRLHRKCALAKTTFEKKRSKKN
LQAVEEANARRLKYVRDWYDQEFQKAGERLEKLHALYPEVSVSIRENKIQETRSNLEKAYEAIEENYRCCVR
EQEDYWKEEEKREAEFRERGNKILSPEELESSLEQFDHGLKNFSEKLMELEGHILKLQKEATAEVENKILSDA
ESRLEIVFEDVKEMPCRIEEIEKTLRMAELPLLPTKKAFEKACSQYNSCAEMLEKVKPYCKESLAYVTSKERL
VSLDEDLRRAYTECQKRFQGDSGLESEVRACREQLRERIQEFETQGLDLVEKELLCVSSRLRNTECDCVSGV
KKEAPPGKKFYAQYYDEIYRVRVQSRWMTMSERLREGVQACNKMLKAGLSEEDKVLKEEEYWLYREERK
NKEKRLVGTKIVATQQRVAAFESIEVPEIPEAPEEKPSLLDKARSLFTREDHS

>core/70/3/Org3_Gene870

MLFSKNFSTDNGGAITAKTLSLTGTTMSALFSENTSSKKGGAIQTSDALTITGNQGEVSFSDNTSSDSGAAIFT
EASVTISNNAKVSFIDNKVTGASSSTTGDMSGGAICAYKTSTDTKVTLTGNQMLLFSNNTSTTAGGAIYVKKL
ELASGGLTLFSRNSVNGGTAPKGGAIAIEDSGELSLSADSGDIVFLGNTVTSTTPGTNRSSIDLGTSAKMTALR
SAAGRAIYFYDPITTGSSTTVTDVLKVNETPADSALQYTGNIIFTGEKLSETEAADSKNLTSKLLQPVTLSGGT
LSLKHGVTLQTQAFTQQADSRLEMDVGTTLEPADTSTINNLVINISSIDGAKKAKIETKATSKNLTLSGTITLL
DPTGTFYENHSLRNPQSYDILELKASGTVTSTAVTPDPIMGEKFHYGYQGTWGPIVWGTGASTTATFNWTKT
GYIPNPERIGSLVPNSLWNAFIDISSLHYLMETANEGLQGDRAFWCAGLSNFFHKDSTKTRRGFRHLSGGYVI
GGNLHTCSDKILSAAFCQLFGRDRDYFVAKNQGTVYGGTLYYQHNETYISLPCKLRPCSLSYVPTEIPVLFSG
NLSYTHTDNDLKTKYTTYPTVKGSWGNDSFALEFGGRAPICLDESALFEQYMPFMKLQFVYAHQEGFKEQG
TEAREFGSSRLVNLALPIGIRFDKESDCQDATYNLTLGYTVDLVRSNPDCTTTLRISGDSWKTFGTNLARQAL
VLRAGNHFCFNSNFEAFSQFSFELRGSSRNYNVDLGAKYQF

>core/71/3/Org3_Gene602

MTLQPSYINFTPNVTTALSGGKIDTSAIELSCSALFFQELQDKAQGLKHALGLVQELSAEALRPAQVQTSISYL
PTEESSRPGISAGIIDRTMPTFTDDEVKAILQNPNFETSKIFVEGLDKVFKSYLDSVTPPEGIDPSNPESAIILNYIT
LLNNLKPKFAAGSTPTDADYNALYALPGDFVKEIEALKAADAPPKSKVHAFWQEIMTIYNNMQVLSYPVTD
YLNVQIADLSLNITAAQEVQQYLKNFYSILKDILNPGWTDPQATHYPADAEYNARDAGVIQSLLNLSGNYRQ
LTENMLPNTDTSLPQEIIAQIRSFQNGVNGTIIASNTLLPTTMRLDTLLGVIYTYQCCATIFGMSYGTSTPAKQN
YIDAINQEKSYWQARANGFDVTSDQVFDQFATNIQSGTSYRGIDLFKNNKVNEINPIFLSQAASFLRYPYNLM
SRSMYQTIEDAANRSITALDGLISGWSTQIATFQTQKNSLDPSLLKYFDTMKANKESFVTTAPLQMVYSSLML
DKYLPTQQNVIASLGIQMTYSNKAAKYLNELIKEITTFQSADIYYSLSIYLKQMNLQAVADPIGKAVGVLNDE
KTRAMADITRCNKIKAAIDKMLVEIKADAELSKSQIRELVDTLTNFKSQSDDLIRNLSCLLGFLSGLTLKAVN
DPNATYEAFTAEIFTEPFNNWKRQLATFESFVIQGGQNGITPGGQQQLLQAMESSQQDFSTFNQNQQLALQLE
SSAMQQEWTLVSAALALLNQMVSKIARRIKS

>core/75/3/Org3_Gene953

MVFFRNSLLHLVALSGMLCCSSGVALTIAEKMASLEHSGRGADDYEGMASFNANMREYSLQLSKLYEEARK
LRASGTEDEALWKDLIRRIGEVRGYLREIEELWAAEIREKGGNLEDYALWNHPETTIYNLVTDYGTEDSIYLI
PQEIGAIKIATLSKFVVPKESFEDCLTQILSRLGIGVRQVNSWIKELYMMRKEGCSVAGVFSSRKDLEALPETA
YIGFVLNSNVDAHTNQHVLKKFINPETTHVDVIAGRVWIFGSAGEVGELLKIYNFVQSESIRQEYRVIPLTKID

PGEMISILNAAFREDLTKDVSEESLGLRVVPLQYQGRSLFLSAIAALVQQALTLIRELEEGIENPTDKTVFWYN
VKHSDPQELAALLSQVHDVFSGENKASVGAADGCGSQLNASIQIDTTVSSSAKDGSVKYGNFIADSKTGTLI
MVVEKEVLPRIQMLLKKLDVPKKMVRIEVLLFERKLAHEQKSGLNLLRLGEEVCKKGCSPSVSWAGGTGILE
FLFKGSTGSSIVPGYDLAYQFLMAQEDVRINASPSVVTMNQTPARIAVVDEMSIAVSSDKDKAQYNRAQYGI
MIKMLPVINVGEEDGKSYITLETDITFDTTGKNHDDRPDVTRRNITNKVRIADGETVIIGGLRCKQMSDSHDGI
PFLGDIPGIGKLFGMSSTSDSLTEMFVFITPKILENPVEQQERKEEALLSSRPGEREEYYQALAASEAAARAAH
KKLEMFPASGVSLSQVERQEYDGC

>core/76/3/Org3_Gene650

MGYIESSTFRLYAEVIVGSNINKVLDYGVPENLEHITKGTAVTISLRGGKKVGVIYQIKTTTQCKKILPILGLSD
SEIVLPQDLLDLLFWISQYYFAPLGKTLKLFLPAISSNVIQPKQHYRVVLKQSKAKTKEILAKLEVLHPSQGAV
LKILLQHASPPGLSSLMETAKVSQSPIHSLEKLGILDIVDAAQLELQEDLLTFFPPAPKDLHPEQQSAIDKIFSSL
KTSQFHTHLLFGITGSGKTEIYLRATSEALKQGKSTILLVPEIALTVQTVSLFKARFGKDVGVLHHKLSDSDKS
RTWRQASEGSLRILIGPRSALFCPMKNLGLIIVDEEHDPAYKQTESPPCYHARDVAVMRGKLAHATVVLGSA
TPSLESYTNALSGKYVLSRLSSRAAAAHPAKISLINMNLEREKSKTKILFSQPVLKKIAERLEVGEQVLIFFNRR
GYHTNVSCTVCKHTLKCPHCDMVLTFHKYANVLLCHLCNSSPKDLPQSCPKCLGTMTLQYRGSGTEKIEKIL
QQIFPQIRTIRIDSDTTKFKGSHETLLRQFATGKADVLIGTQMIAKGMNFSAVTLAVILNGDSGLYIPDFRASEQ
VFQLITQVAGRSGRSHLPGEILIQSFLPDHPTIHSAMRQDYSAFYSQEITGRELCEYPPFIRLIRCIFMGKCPKQT
WEEAHRVHNILKEQLESTNPLMPVTPCGHFKIKDTFRYQFLIKSAYVIPVNKKLHHALMLAKLSPKVKFMID
VDPMTTFF

>core/77/3/Org3_Gene883

MEKICGYLEQILVENKDSGDITAYIKIPNKTTPILIKGKLPQPLELGSPIQIYGVWSHSPSNTKYFQIHSYDSPLL
YEYRGVFHYLTSKLIKGIGPKIAEKIIEKFQEKTCYVLDITPERLSEVSGISETRCVSICKQLCEQKILRKTLLFLQ
EYNIPIHYGVRIFKKYHETSIEKICEDPFLLAREMEGIGFKTADFIAMKLGVPRNSESRLCAGIQHSLEELQEEG
HTCYPIELLIDVVAKLLNQDVFDTPITLEEIDTQILNMQKRKLLHIQDISGTLHVWTRYLHLAEKTIVSDLKRIL
FSSRRIRSIDGEKAIAWVEENLSIDLAEQQREAIKACFSEKLLIITGGPGTGKSTITQAILKIFEQVTHKIILAAPT
GKAAKRMTEITQKHSVTIHALLQYDFKTKSFRKNHDNPIDCDLIIVDESGMMDTHLLHHFLKALPDYTTLVFI
GDIHQLPSVGPGNILKDLITSNKMTVIRLNKIFRQVHDSGIVTNAHRVNEGELPILYSETGRRDFLFFQKDDQE
EALNHIIHLVTKFVPQKYHIYPQDIQVLAPMKKGTLGIYNLNKALKHALNPKKANLHGRFQSYAVGDKVMQI
RNNYNKEVFNGDIGYVSTINFEDKAVVVRMEGKHVGYSFSELDDLVLAYATSVHKYQGSESPCIIIPIHTSHF
MMLYRNLLYTAITRGKKLVILVGTKKAIAIATRNNRVQHRCTGLAEVLKELDTKKNYADL

>core/78/3/Org3_Gene999

MLSFFYKHQKKFIGIVIAVVCVSGIGVGWGRFSRKGSAESTSRRTVFTTASGKRYVEKDFMAMKKFFAHEAY
PFTGNPRAWNFINEGLLTDYFLTTRVGEKLFLKVYHPGEKIFSKEKAYQPYRRFDAPFISSEEVWKSSAPQLLE
ILKVFQQIENPISKEGFLARAKLFLEERRFPHYVLRQMLEYRRQMFALPPDEALSRGKDLRLFGYQTIQDWFG
DAYLSAAVELLIRFIDEQKKVLPRPSKQEARDDFYDKAKHAYTKISKNKEFSLGFEEFVNSYFQFLEISESEFF
NMYRDILLCKRALLLLQGGVSFDFQPLTTFFVQGKDSIQVEFFRLPKEYSFKTKQELKAFEVYLKLVSLPKSD
SLDVPNEILPIATIKAKEPRLVGRRFSIDYKRVALQDLAATVPMVEVLHWQQNSEHFQEILQQFPDVETCQSY
KDFQHLKPALRDKISLFTRKEILRARPERILQSLQQVPKQSQEVLLSAGKNSALPGISDGQQLAKVLLENEVLD

LYSQDAETYYTIIVNSSFEKEEVLPYREVLKRDLASQLLTSHGHLVDMERLESALRTRYPGEEGASLWQRRL
WKVVENHRLGRHLEGSFSWSLDRSLKTFSRGDKELPQEFDRIFSMKVGDYSSVFMSPNEGPCYYQCLSHLLY
DRPASVDKLFLAKSQLDEELLGSYMERFIEQGVVR

>core/79/3/Org3_Gene520

MDYLEKLQVLIEEGQSANFLSLWEEYCFNDVVRGRELVEILEKVKSSSLASLFGKIVDTVVPLWEKIPEGKDK
DRVLQLILDLQTSNSQMFFDIATEYVNKKYSGEENFNEALRVVGLRDGRDFQFSLSRFDFLMHMHKGNFVFH
QGGWGVGEVMGVSFLQQKVLIEFEGIMSAKDISFETAFKSLTPLSGDHFLSRRFGDPDGFEAFAKENPIEVVEI
LLRDLGPKTAKEIKDELVDLVIPEADWNRWWQSAKTKIKKGTRIISPDNPKEPYVLSDAGCSHMGQLERKLG
LSLNSAEKISLIYHFIRDLHSELKNIEIRKSLVKALQDLDVEEGNKSLILQRELLLSEYLGIKDASIDKEYITSLSE
DDTSRLLENMPIVALQKSFLSLVRKYSSFWQQVFMQILLYTTSPTMRDFVYKTIKNDPSSVEVLKKRLLDSAH
QPMMFPELFVWFFLKLGNHEDGLFDPEDKEVLRLFLESALNFMYQVASTPHKELGKKLHHYLVGQRYLAVR
QMIEGASLPFLKELLLLSTKCPQFSSSDLNVLQSLAEVVQPTLKKHKSNVEEENVLWSTSESFSRMKAKLQSL
VGKEMVDNAKEIEDARSLGDLRENSEYKFALEKRARLQEEIRVLSEEINRARILTKDLVFTDKVGVGCKVTL
KGDAGEVVEYTILGPWDADPDSCILSLQSKLAQNMLGKKLNDVVILQGKEYKISRIQSIWEEHGA

>core/80/3/Org3_Gene314

MVDKLIHPWDLDLLVSGRQKDPHKLLGILASEDSSDHIVIFRPGAHTVAIELLGELHHAVAYRSGLFFLSVPK
GIGHGDYRVYHQNGLLAHDPYAFPPLWGEIDSFLFHRGTHYRIYERMGAIPMEVQGISGVLFVLWAPHAQR
VSVVGDFNFWHGLVNPLRKISDQGIWELFVPGLGEGIRYKWEIVTQSGNVIVKTDPYGKSFDPPPQGTARVA
DSESYSWSDHRWMERRSKQSEGPVTIYEVHLGSWQWQEGRPLSYSEMAHRLASYCKEMHYTHVELLPITEH
PLNESWGYQVTGYYAPTSRYGTLQEFQYFVDYLHKENIGIILDWVPGHFPVDAFALASFDGEPLYEYTGHSQ
ALHPHWNTFTFDYSRHEVTNFLLGSALFWLDKMHIDGLRVDAVASMLYRDYGREDGEWTPNIYGGKENLE
SIEFLKHLNSVIHKEFSGVLTFAEESTAFPGVTKDVDQGGLGFDYKWNLGWMHDTFHYFMKDPMYRKYHQ
KDLTFSLWYAFQESFILPLSHDEVVHGKGSLVNKLPGDTWTRFAQMRVLLSYQICLPGKKLLFMGGEFGQY
GEWSPDRPLDWELLNHHYHKTLRNCVSALNALYIHQPYLWMQESSQECFHWVDFHDIENNVIAYYRFAGS
NRSSALLCVHHFSASTFPSYVLRCEGVKHCELLLNTDDESFGGSGKGNRAPVVCQDQGVAWGLDIELPPLAT
VIYLVTFF

>core/81/3/Org3_Gene861

MNKFKTYLQTALIAPFFSFPALSGSFSSIQAEEITQQVNHPGAELLSEGSYIPGLQTFRLGIKITASKGSHIYWKN
PGEIGSPLKISWQLPKGFVVEEEHWPTPKVFEEEGTTFFGYEDSALIVADVRAPEGYTPGQEVELRAQVEWLA
CGDSCLPGNVDLKLTLPYEEKEPSLYPDTHAEFTKTLHAQPRVLENDHSVQVAQGKGNEIILNISKKINATKA
WFVSEKADKLFAYAETSYSGGTGTAWRLKVKNLSGVQKNEKLHGILLLADHTGRPVESLTIHSEVLGQTGS
AVAGLSQYITILIMAFLGGVLLNIMPCVLPLVTLKVYGLIKSAGEHRSSVIANGLWFTLGVVGCFWGLAGVAF
ILKVLGHNIGWGFQLQEPMFVATLIIVFFLFALSSLGLFEMGTMFANLGGKLQSSEMKSSNNKAVGAFFNGIL
ATLVTTPCTGPFLGSVLGLVMSLSFLQQLLIFTAIGLGMASPYLVFSVFPKMLSVLPKPGGWMSTFKQLTGFM
LLVTVTWLVWIFGSETSTTSVVVLLGGLWLAGLGAWILGRWGTPVSPKKQRVCASLLFFAFLGGAISVSGLA
SHYFAEPQQTVSVNEDSLWQPFSLEKLAQLRAQGRPVFVNFTAKWCLTCQMNKPVLYGDAVQKMFETHGI
VTLEADWTRKDPGITEELARLGRASVPSYVYYPGDNSAPVVLPEKITQNLLEDVVSRFVR

>core/82/3/Org3_Gene187

MIRSPLPFISSKRALNMLGLQDEFSCPEDVVDFLFSEIELLAQQDEPSEGYLALSRSLLMMTHNHPKVVKRVIF
YGVSYGLKHKSMSIFIDVLTYIDFLFEKLGISASDRLSLCSARTCINFELYSQTGEMKFLSEVVDNFRLIEQLLK
MHPQLKNRLGWEHFRIGAKQEEVSLVASASVYQAVGRSFIELYHKHLELSDLACGMKCLALALDLSPNNAH
IHADYAKGLVVLGTRQGKSLLIERGMEHFSKAIFLSFSRDGDTLAYQNYRYSYALASVKLFDLTYKKEHFDQ
AMNILYQTVQAFPNLSGLWMVWGELLIRSGWLNSNMKYIEVGLEKLASLQKKTNDPIALSGLLATGIAILGL
YLEEPNLFKDSRHRLISAMRTFPGNSALVHALGVVQLCSALYFNEDSHFASAISCFQSCLEWDLDATGMWQK
LFDAYFSWGIKKKSARLLRKAVDVASRLCSLRPEAFLFWSDRGLALKCLAEATIDEAYKEIFLSESLLHYQRA
WDLSGRLEILELWGQSHYLLAELQQSLFHYDEAYTLLTKVDLTLSSSRVKLILAAVLLGKGRLLQDTDPAEE
AREILEPLVEVYLEDENFLLLLGKVYLFLFWKNKNVCLGKLARTYLEKATSLGCPEAYYTLGKFYAVIKDVN
KAWGMVIRSAQYGVRITEAKWLNDPYLANLREIHAFREVVENQKGRLWLGNKTEMKRN

>core/83/3/Org3_Gene214

MNKLLNFVSRTLGGDTALNMINKSSDLILALWMMGVVLMIIIPLPPPIVDLMITINLSISVFLLMVALYIPSALQ
LSVFPSLLLITTMFRLGINISSSRQILLKAYAGHVIQAFGDFVVGGNYVVGFIIFLIITIIQFIVVTKGAERVAEVA
ARFRLDAMPGKQMAIDADLRAGMIDATQARDKRAQIQKESELYGAMDGAMKFIKGDVIAGIVISLINIVGGL
TIGVAMHGMDLAQAAHVYTLLSIGDGLVSQIPSLLIALTAGIVTTRVSSDKNTNLGKEISTQLVKEPRALLLA
GAATLGVGFFKGFPLWSFSILALIFVALGILLLTKKSAAGKKGGGSGASTTVGAAGDGAATVGDNPDDYSLT
LPVILELGKDLSKLIQHKTKSGQSFVDDMIPKMRQALYQDIGIRYPGIHVRTDSPSLEGYDYMILLNEVPYVR
GKIPPHHVLTNEVEDNLSRYNLPFITYKNAAGLPSAWVSEDAKAILEKAAIKYWTPLEVIILHLSYFFHKSSQE
FLGIQEVRSMIEFMERSFPDLVKEVTRLIPLQKLTEIFKRLVQEQISIKDLRTILESLSEWAQTEKDTVLLTEYVR
SSLKLYISFKFSQGQSAISVYLLDPEIEEMIRGAIKQTSAGSYLALDPDSVNLILKSMRNTITPTPAGGQPPVLLT
AIDVRRYVRKLIETEFPDIAVISYQEILPEIRIQPLGRIQIF

>core/84/3/Org3_Gene407

MYKRCVLDKILKGIVAGSLILLYWSSDLLERDIKSIKGNVRDIQEDIREISRVVKQQQTSQAIPAAPGVMLAPK
LVRDEAFALLFGDPSYPNLLSLDPYKQQTLPELLGTNFHPHGILRTAHVGKPENLSPFNGFDYVVGFYDLCIPS
LASPHVGKYEEFSPDLAVKIEEHLVEDGSGDKEFHIYLRPNVFWRPIDPKALPKHVQLDEVFQRPHPVTAHDI
KFFYDAVMNPYVATMRAVALRSCYEDVVSVSVENDLKLVVRWKAHTVINEEGKEERKVLYSAFSNTLSLQ
PLPRFVYQYFANGEKIIEDENIDTYRTNSIWAQNFTMHWANNYIVSCGAYYFAGMDDEKIVFSRNPDFYDPL
AALIDKRFVVYFKESTDSLFQDFKTGKIDISYLPPNQRDNFYSFMKSSAYNKQVAKGGAVRETVSADRAYTYI
GWNCFSLFFQSRQVRCAMNMAIDRERIIEQCLDGQGYTISGPFASSSPSYNKQIEGWHYSPEEAARLLEEEGW
IDTDGDGIREKVIDGVIVPFRFRLCYYVKSVTAHTIADYVATACKEIGIECSLLGLDMADLSQAFDEKNFDALL
MGWCLGIPPEDPRALWHSEGAMEKGSANVVGFHNEEADKIIDRLSYEYDLKERNRLYHRFHEIIHEEAPYAF
LFSRHCSLLYKDYVKNIFVPTHRTDLIPEAQDETVNVTMVWLEKKEDPCLSTS

>core/85/3/Org3_Gene23

MKELRHESYNRALHKLSHQWVRYFLYTFVSCSFIVAIFTFAWLKVLYVPEYKAGEISRISLTAPMDFSLSWSA
HKFYKRTAHISEAFGKVYHLTLSPGSLLSKEGNADENTDYWFKKAADFLLSTNFVDSSTQKCLKDLCIYPPLL
GKEKKTLEININSNKGNVIAQCFCHLKIFLIQENCPQPCFDAIMDILKIANFEVAVDKEMSGCVKGELLGKRCI

EKITKGTPILEKYQRIDDRDAKILKQLRAQLLSVHTLFSCRSLWGAIFVVLLILLWGYGALKALCPEMLKSPQ
RFMLYIAILTLSLLWCRGTEIFCAYWVSYLSYPPILPFTAVLLGYFLGLPIAGFSCTFLALLYTLGSDLWNNSW
FLSINLLCSWRILVSLHRVSRLSSVFWACMKLGGVAMGSLLMFRIFTNTISREALYADGIESFVYSLITAISVVA
LIPVFEASFGASTNFSLLTYLSPENALLKRLFKEAPGTYQHSVLVGSLAEAAAQAIGADSLYCLVAAHYHDIG
KLINPGFFSENQKILQQSGHSLSPLECAKMIMRHIPEGVNLARQAGLPESFIQVIEEHHGTSVIRSAYYSHMVE
NPSTGSFDEELFRYSGNKPSSKETTIIMIADSFEAASRSLKNASLPDLQRLIDQIIQGKLQDGQFSCSPITLDELA
LISKSMVQTLYGALHSRMKYPEISYQISMDSCPKPSIGGT

>core/86/3/Org3_Gene745

MPEPLYTNKLITEKSPYLLLYAHTPVNWYPWGAEAFHIAAIENKPVFLSIGCKHSRWCQVMLQESYTNPEIAA
MLNEYFVNVKVDKEELPYVAKLYGDLAQMLAVSGDHQETVSWPLNVFLTPDLVPFFSVNYLGNEGKLGLP
SFPQIIDKLHFMWEDAEEREALVEQAMKVLEIASFLEGCVRKEILDESSLKRTVAALYQDIDPHYGGVKAFPK
RLPGLLLQFFLRYSLEYQESRGLFFVDRSLSMVALGGVRDHIGGGVYSYTIDDKWLIPAFEKRLIDNALMALN
YLEAWACLGKEEYRGIGKQILSYILSELYSPEVGAFYSSEQAENWGAGGQNFYTWSVEEISNALGEDAEIFCD
YYGISREGFFNGRNILHIPVHREIEELSEKYHRSIEAIEDIVDRSRDILKGIRAQRSHRSKDDLSLTFNNGWMIYT
FAYAGRLLGEVEYIEIGKKCGEFVRNSLYKHHELYRRWREGEAKYRASLEDYGALILGVLALYESGCGSFWL
SFAEELMQEVVLSFRSEEGGFYSVDGRDSTLLIKQSPLSDGETISGNALICQCLLSLHLITEKKHYLTYAEDILQ
IAQACAHTHKFSSLGLLIASQNYFSRKHVKVLIALGDQEDRSPVLKCLSGLFLPYLSLIWMTQENQEHLETVL
PEYEHCLIPKGDCTATTIYVLEVDQCKRFKDLELFRRYLISL

>core/88/3/Org3_Gene7

MKRCFLFLASFVLMGSSADALTHQEAVKKKNSYLSHFKSVSGIVTIEDGVLNIHNNLRIQANKVYVENTVGQ
SLKLVAHGNVMVNYRAKTLVCDYLEYYEDTDSCLLTNGRFAMYPWFLGGSMITLTPETIVIRKGYISTSEGP
KKDLCLSGDYLEYSSDSLLSIGKTTLRVCRIPILFLPPFSIMPMEIPKPPINFRGGTGGFLGSYLGMSYSPISRKHF
SSTFFLDSFFKHGVGMGFNLHCSQKQVPENVFNMKSYYAHRLAIDMAEAHDRYRLHGDFCFTHKHVNFSGE
YHLSDSWETVADIFPNNFMLKNTGPTRVDCTWNDNYFEGYLTSSVKVNSFQNANQELPYLTLRQYPISIYNT
GVYLENIVECGYLNFAFSDHIVGENFSSLRLAARPKLHKTVPLPIGTLSSTLGSSLIYYSDVPEISSRHSQLSAKL
QLDYRFLLHKSYIQRRHIIEPFVTFITETRPLAKNEDHYIFSIQDAFHSLNLLKAGIDTSVLSKTNPRFPRIHAKL
WTTHILSNTESKPTFPKTACELSLPFGKKNTVSLDAEWIWKKHCWDHMNIRWEWIGNDNVAMTLESLHRSK
YSLIKCDRENFILDVSRPIDQLLDSPLSDHRNLILGKLFVRPHPCWNYRLSLRYGWHRQDTPNYLEYQMILGT
KIFEHWQLYGVYERREADSRFFFFLKLDKPKKPPF

>core/89/3/Org3_Gene704

MNFQTISINLTEGKILVFETGKIARQANGAVLVRSGETCVFASACAVDLDDKVDFLPLRVDYQEKFSSTGKTL
GGFIKREGRPSEKEILVSRLIDRSLRPSFPYRLMQDVQVLSYVWSYDGQVLPDPLAICAASAALAISDIPQSNIV
AGVRIGCIDNQWVINPTKTELASSTLDLVLAGTENAILMIEGHCDFFTEEQVLDAIEFGHKHIVTICKRLQLWQ
EEVGKSKNLSAVYPLPAEVLTAVKECAQDKFTELFNIKDKKVHAATAHEIEENILEKLQREDDDLFSSFNIKA
ACKTLKSDTMRALIRDREIRADGRSLTTVRPITIETSYLPRTHGSCLFTRGETQTLAVCTLGSEAMAQRYEDLN
GEGLSKFYLQYFFPPFSVGEVGRIGSPGRREIGHGKLAEKALSHALPDSATFPYTIRIESNITESNGSSSMASVC
GGCLALMDAGVPISSPIAGIAMGLILDDQGAIILSDISGLEDHLGDMDFKIAGSGKGITAFQMDIKVEGITPAIM
KKALSQAKQGCNDILNIMNEALSAPKADLSQYAPRIETMQIKPTKIASVIGPGGKQIRQIIEETGVQIDVNDLG

VVSISASSASAINKAKEIIEGLVGEVEVGKTYRGRVTSVVAFGAFVEVLPGKEGLCHISECSRQRIENISDVVKE
GDIIDVKLLSINEKGQLKLSHKATLE

>core/90/3/Org3_Gene920

MSNQEFDLSAIRNIGIMAHIDAGKTTTTERILFYAGRTHKIGEVHEGGATMDWMAQEQERGITITSAATTVFW
LGAKINIIDTPGHVDFTIEVERSLRVLDGAVAVFDAVSGVEPQSETVWRQADKYGVPRIAFVNKMDRMGAD
YFAAVESMKEKLGANAFPVHCPIGSESQFVGMVDLISQKALYFLDDTLGAKWEEKEISEDLKERCAELRANL
LEELATIDESNEAFMMKVLEDPDSITEDEIHQVMRKGVIENKINPVLCGTAFKNKGVQQLLNVIVKWLPSPLD
RGNIRGINLKTDQEISLEPRRDGPLAALAFKIMTDPYVGRITFIRIYSGTLKKGSAILNSTKDKKERISRLLEMH
ANERTDRDEFTVGDIGACVGLKFSVTGDTLCDDNQEIVLERIEFPDPVIDMAIEPKSKGDREKLAQALSSLSEE
DPTFRVSTNEETGQTIISGMGELHLDILRDRMIREFKVEANVGKPQVSYKETITVSGNSETKYVKQSGGRGQY
AHVCLEIEPNEPGKGNEVVSKIVGGVIPKEYIPAVIKGIEEGLKKGVLAGYGLVDVKVSIVFGSYHEVDSSEM
AFKICGSMAVKDACRKAKPVILEPIMKVAVITPEDHLGDVIGDLNRRRGKILGQESSRGMAQVNAEVPLSEM
FGYTTSLRSLTSGRATSTMEPAFFAKVPQKIQEEIVKK

>core/92/3/Org3_Gene324

MYNLLHAHHDAASPDGRLVSHLKKLSPHIYEGEVLIENIPAYFLGFHLPQQCIQVNLKSSLAQLGVEAVLNHL
ELNKARKEARLHVLFMSQDPIATAMLELLEPGSFVCKLFAADDRRLVRSPCYLNRMFTHTDRTGSPLLRFGK
KLEHFITLEIINDRLVVFLPILPGTICYEETIYGFLPLMSKSLTRPHLKIRKFLPLYQMVTDRPPVPEDHKILLIKT
EPLHIRTVFARVVQDLLPQGLRHTAADILEPTTQESGDIYEFYGSTSEPIERIPLEFFTLEPYKEHSFFFYRDMLQ
ETLESPQEVFRVFESIPEGEDQAAMFISKGSELLELSQDSWIIKPRISPSDERHAREIQKHIEDQPCFPFLKAMET
DHITSQGVLFSRYFPSASLKGMFLSNYSRYYLQHIYFQIPSPTSGEFFSNRDRSFLLDLYFAGISVFWADLESKR
LLQYIKRRNKDVGMFVPKHQAEQFAQSYFIGIHGSCLIAGDYDEFLRELLTGMHTLSQQFTIPEFPPQTPLAIL
TGGGSGAMELANRVATELSILSCGNLISLDTTNAYVEAKMSYAIPDLLERQADFHVDLAVFVIGGMGTDFEL
LLELISLKTGKKALVPVFLIGPVDYWKSKITALYNSNHAVGTIRGSEWVHNCLFCLSSAKAGIAIFRRYLNHTL
PIGPEHPVPEDGFVIV

>core/93/3/Org3_Gene699

MKRSRRNFEQALENLEKLKEISLATSNDSYLNNPARFNQRKQTGSSVMEMKEALKNVENYLLEISCVSKSHA
DKALKESDFLIAGVQNVFSFLENQEDLYKSLLDEYSEVTKAYDEVKKNLKEVPTYDLSTDEETEEHKEPECFL
NNLVEVKRDRSYELFYMLDEQDKRFYNDALVQIIYKQNKLHETVNEGDPLTKTLLWNSEEVKNIASSLVIVN
DMPLRLFYQRALSHLDIEAVVKVHNAVMALFFSRYEATMVFKSPKKHNIWYFNDFLLFLREAWKDLNNNVI
DSQERKQTKLLASALSLGIFESKLVFEEASRYLYFNIQTKLENANGKKPLSPGQYLTDAYEELHRLISKYPNGP
LFKAMDRVLEHESRPYDPMILGILPSLEGTLKLHGKSIDIIRSPSPVTQSSILYANCNEEFLGFLNAKAHRSEVT
LVLNIQNRISRKERARSRVIEEALEQEEHAPYVHAFSFPEPEELLQNLESIHGDIETFADFFSILQEEFHKPLLAS
SFFLTKELKEFVGSFLKEKLTALKDIFFAKKKILFRNDKLLLLHLLSYLIVFKLIERTNPNSIVVVSKDGLDYVS
VFIAGFAFFSREAFWDEHSLKLLLTNVLSPTLVARDRLVFVSHIELLSKFVNCLKKNRQGFSSLKSFFKDDIEG
WEFTGYLHELTEVSHKHNL

>core/94/3/Org3_Gene560

MQPCLNMSIVRNSALPLPCLSRSETFKKVRSHMKFMKVLTPWIYRKDLWVTAFLLTAIPGSFAHTLVDIAGEP
RHAAQATGVSGDGKIVIGMKVPDDPFAITVGFQYIDGHLQPLEAVRPQCSVYPNGITPDGTVIVGTNYAIGM
GSVAVKWVNGKVSELPMLPDTLDSVASAVSADGRVIGGNRNINLGASVAVKWEDDVITQLPSLPDAMNAC
VNGISSDGSIIVGTMVDVSWRNTAVQWIGDQLSVIGTLGGTTSVASAISTDGTVIVGGSENADSQTHAYAYK
NGVMSDIGTLGGFYSLAHAVSSDGSVIVGVSTNSEHRYHAFQYADGQMVDLGTLGGPESYAQGVSGDGKVI
VGRAQVPSGDWHAFLCPFQAPSPAPVHGGSTVVTSQNPRGMVDINATYSSLKNSQQQLQRLLIQHSAKVESV
SSGAPSFTSVKGAISKQSPAVQNDVQKGTFLSYRSQVHGNVQNQQLLTGAFMDWKLASAPKCGFKVALHY
GSQDALVERAALPYTEQGLGSSVLSGFGGQVQGRYDFNLGETVVLQPFMGIQVLHLSREGYSEKNVRFPVSY
DSVAYSAATSFMGAHVFASLSPKMSTAATLGVERDLNSHIDEFKGSVSAMGNFVLENSTVSVLRPFASLAMY
YDVRQQQLVTLSVVMNQQPLTGTLSLVSQSSYNLSF

>core/95/3/Org3_Gene18

MGVVQNQVISSIRDVLKLVWELRFAEHKMLLLSRQSGSGGTFQLSCAGHELAGVLAGKSLIPGKDWSFPYY
RDQGFPIGLGCDLSEIFASFLARTTPNHSSARMMPYHYSHKKLRICCQSSVVGTQFLQAAGRAWAVKHSSAD
EVVYVSGGDGATSQGEFHEMLNFVALHQLPLITVIQNNHWAISVPFEDQCGADLASLGRCHQGLAVYEVDG
GNYTSLTETFSHAVDQARQHSVPALILIDVVRLSSHSNSDNQEKYRSALDLKLSMDKDPLILLEKEAINVFGLS
PFEIEEIKAEAQEEVRKSCEIAEALPFPSKGSTSHEVFSPYTETLIDYENSESAQNLRNSEPKVMRDAISEALVEE
MTRDSGVIVFGEDVAGDKGGVFGVTRNLTEKFGPQRCFNSPLAEATIIGTAIGMALDGIHKPVVEIQFADYIW
PGINQLFSEASSIYYRSAGEWEVPLVIRAPSGGYIQGGPYHSQSIEGFLAHCPGIKVAYPSNAADAKALLKAAI
RDPNPVVFLEHKALYQRRIFSACPVFSHDYVLPFGKAAIVHPGKDLTIVSWGMPLVLSLEVAQELASRGISIEV
IDLRTMVPCDFATVLKSLEKTGRLLVIHEASEFCGFGSELVATMSEQGYAYLDAPIRRLGGLHAPVPYSKVLE
NEVLPHKESILQAAKSLAEF

>core/96/3/Org3_Gene332

MLKKPKRKPGRRTYGKSLKIFIPGTLFVHARKGFGFVSPDNPEEYPFDIFVPARDLRGALDGDHVIVSVLPYPR
DGQKLKGTISEVLARGKTTLVGTITSLVSPTSALAYTSMSGSQSLIPVELLPGRTYKIGDRILLSTPPWVDKPQE
GASPALQMLEFIGHITNAKADFQAIQAEYNLAEEFPPEVIEEASLFSQKHITQVLHSRKDLRDLLCFTIDSSTAR
DFDDAISLTYDHNNNYILGVHIADVSHYVTPHSHLDKEAAKRCNSTYFPGKVIPMLPSALSDNLCSLKPNVDR
LAVSVFMTFTKSGHLSDYQIFRSVIRSKYRMTYDEVDNIIEKKHSHPLSKILNEMATLSKKFSDIREERGCIRFV
LPSVTMSLDNLQEPVALIENHQTFSHKLIEEFMLKANEVVAYHISHQGVSLPFRSHEPPNDENLLAFQELAKN
MGFDITFTPTQEPDYQYLLQTTSAGHPLEQVLHSQFVRSMKTASYSTENKGHYGLKLDYYTHFTSPIRRYIDL
IVHRLLFNPLSIDQTHLEIIVRACSTKERVSAKAENSFENLKKTRFINKFLQEQPKTTYHAYIITANHEGLSFVV
TEFCHEGFIAAAELPKEYSLKKNALPESIPDKMKPGASIKVTIDSVNLLTQKIVWSIATTTEDKPKKIKKTPSKK
KGTKKRAS

>core/98/3/Org3_Gene961

MINKELDIGILGKIAGAIKQISIESIQKASSGHPGLPLGCAELAAYLYGYVLRQNPRDPHWINRDRFVLSAGHG
SALLYSCLHLAGFDVSLEDLQEFRQLHSRTPGHPEYGETVGVEATTGPLGQGLGNAVGMALSMKMLESRFN
RPGHEIFNGKIYCLAGDGCFMEGVSHEVCSFAGSLNLNNLVVIYDYNNVVLDGYLNEISVEDTKKRFEAYG
WDVYEIDGYDFTHIHETFSSIKRGQERPVLVIAHTIIGHGSPKEGTNKAHGSPLGVEGTHETKQFWHLPEEKFF
VPPAVKNFFAHKIQEDRKAQEQWLDEVRVWSKQFPELHEEFVALTSHKLPKNLESLVQSVEMPDSIAGRAAS

NKLIQVLVQHIPYLIGGSADLSSSDGTWIANEKVIHTYDFSGRNIKYGVREFGMATIMNGLAYSQVFRPFGGT
FLVFSDYMRNAIRLAALSKLPVIYQFTHDSIFVGEDGPTHQPVEQLMSLRAIPGLYVIRPADANEVRGAWIAG
LKHTGPTVIVLSRQALPTLPAAHRPFKDGVGRGAYIVLKESGEKPDYTLFATGSEVSLALSVAKELEHLDKQV
RVVSFPCWELFEAQDVDYKQSIVGGDLGIRVSIEAGSALGWYKYIGSEGLAIAMDRFGYSGASDDVSEECGF
TTEQILQRILSQ

>core/99/3/Org3_Gene261

MEKVSSYPSVPLPLGASKISPNRYRFALYASQATEVILALTDENSEVIEVPLYPDTHRTGAIWHIEIEGISDQSS
YAFRVHGPKKHGMQYSFKEYLADPYAKNIHSPQSFGSRKKQGDYAFCYLKEEPFPWDGDQPLHLPKEEMIIY
EMHVRSFTQSSSSRVHAPGTFLGIIEKIDHLHKLGINAVELLPIFEFDETAHPFRNSKFPYLCNYWGYAPLNFFS
PCRRYAYASDPCAPSREFKTLVKTLHQEGIEVILDVVFNHTGLQGTTCSLPWIDTPSYYILDAQGHFTNYSGC
GNTLNTNRAPTTQWILDILRYWVEEMHVDGFRFDLASVFSRGPSGSPLQFAPVLEAISFDPLLASTKIIAEPWD
AGGLYQVGYFPTLSPRWSEWNGPYRDNVKAFLNGDQNLIGTFASRISGSQDIYPHGSPTNSINYVSCHDGFTL
CDTVTYNHKHNEANGEDNRDGTDANYSYNFGTEGKTEDPGILEVRERQLRNFFLTLMVSQGIPMIQSGDEY
AHTAEGNNNRWALDSNANYFLWDQLTAKPTLMHFLCDLIAFRKKYKTLFNRGFLSNKEISWVDAMGNPMT
WRPGNFLAFKIKSPKAHVYVAFHVGAQDQLATLPKASSNFLPYQIVAESQQGFVPQNVATPTVSLQPHTTLIA
ISHAKEVT

>core/100/3/Org3_Gene90

MKEENSQAHYLALCRELEDHDYSYYVLHRPRISDYEYDMKLRKLLEIERSHPEWKVLWSPSTRLGDRPSGTF
SVVSHKEPMLSIANSYSKEELSEFFSRVEKSLGTSPRYTVELKIDGIAVAIRYEDRVLVQALSRGNGKQGEDIT
SNIRTIRSLPLRLPEDAPEFIEVRGEVFFSYSTFQIINEKQQQLEKTIFANPRNAAGGTLKLLSPQEVAKRKLEISI
YNLIAPGDNDSHYENLQRCLEWGFPVSGKPRLCSTPEEVISVLKTIETERASLPMEIDGAVIKVDSLASQRVLG
ATGKHYRWALAYKYAPEEAETLLEDILVQVGRTGVLTPVAKLTPVLLSGSLVSRASLYNEDEIHRKDIRIGDT
VCVAKGGEVIPKVVRVCREKRPEGSEVWNMPEFCPVCHSHVVREEDRVSVRCVNPECVAGAIEKIRFFVGR
GALNIDHLGVKVITKLFELGLVHTCADLFQLTTEDLMQIPGIRERSARNILESIEQAKHVDLDRFLVALGIPLIG
IGVATVLAGHFETLDRVISATFEELLSLEGIGEKVAHAIAEYFSDSTHLNEIKKMQDLGVCISPYHKSGSTCFG
KAFVITGTLEGMSRLDAETAIRNCGGKVGSSVSKQTDYVVMGNNPGSKLEKARKLGVSILDQEAFTNLIHLE

>core/101/3/Org3_Gene879

MRLNIHKYLFIGRNKADFFSASRELGVVEFISKKCFITTEQGHRFVECLKVFDHLEAEYSLEALEFVKDESVSV
EDIVSEVLTLNKEIKGLLETVKALRKEIVRVKPLGAFSSSEIAELSRKTGISLRFFYRTHKDNEDLEEDSPNVFY
LSTAYNFDYYLVLGVVDLPRDRYTEIEAPRSVNELQVDLANLQREIRNRSDRLCDLYAYRREVLRGLCNYDN
EQRLHQAKECCEDLFDGKVFAVAGWVIVDRIKELQSLCNRYQIYMERVPVDPDETIPTYLENKGVGVMGED
LVQIYDTPAYSDKDPSTWVFFAFVLFFSMIVNDAGYGLLFLMSSLLFSWKFRRKMKFSKHLSRMLKMTAILG
LGCICWGTTTTSFFGMSFSKTSVFREYSMTHVLALKKAEYYLQMRPKAYKELTNEYPSLKAIRDPKAFLLAT
EIGSAGIESRYVVYDKFIDNILMELALFIGVVHLSLGMLRYLRYRYSGIGWILFMVSAYLYVPIYLGTVSLIHY
LFHVPYELGGQIGYYGMFGGIGLAVVLAMIQRSWRGVEEIISVIQVFSDVLSYLRIYALGLAGAMMGATFNQ
MGARLPMLLGSIVILLGHSVNIILSIMGGVIHGLRLNFIEWYHYSFDGGGRPLRPLRKIVCSEDAEASGIHLDN
NSIV

>core/102/3/Org3_Gene331

MSEHKKSSKIIGIDLGTTNSCVSVMEGGQAKVITSSEGTRTTPSIVAFKGNEKLVGIPAKRQAVTNPEKTLGST
KRFIGRKYSEVASEIQTVPYTVTSGSKGDAVFEVDGKQYTPEEIGAQILMKMKETAEAYLGETVTEAVITVPA
YFNDSQRASTKDAGRIAGLDVKRIIPEPTAAALAYGIDKVGDKKIAVFDLGGGTFDISILEIGDGVFEVLSTNG
DTLLGGDDFDEVIIKWMIEEFKKQEGIDLSKDNMALQRLKDAAEKAKIELSGVSSTEINQPFITMDAQGPKHL
ALTLTRAQFEKLAASLIERTKSPCIKALSDAKLSAKDIDDVLLVGGMSRMPAVQETVKELFGKEPNKGVNPD
EVVAIGAAIQGGVLGGEVKDVLLLDVIPLSLGIETLGGVMTTLVERNTTIPTQKKQIFSTAADNQPAVTIVVLQ
GERPMAKDNKEIGRFDLTDIPPAPRGHPQIEVSFDIDANGIFHVSAKDVASGKEQKIRIEASSGLQEDEIQRMV
RDAEINKEEDKKRREASDAKNEADSMIFRAEKAIKDYKEQIPETLVKEIEERIENVRNALKDDAPIEKIKEVTE
DLSKHMQKIGESMQSQSASAAASSAANAKGGPNINTEDLKKHSFSTKPPSNNGSSEDHIEEADVEIIDNDDK

>core/103/3/Org3_Gene788

MKLLLKAVLRHKNHLVILGCSLLAILGLTFSSQMEIFSLGMIAKTGPDAFLLFGRKESGKLVKVSELSQKDILE
NWQAISKDSETLKVSDATTYIAEHGKSTASLTSKLSKFVRNYIDVSRFRGLAIFLICVAIFKAVTLFFQRFLGQ
VVAIRVSRDLRQDYFKALQQLPMTFFHDHDIGNLSNRVMTDSASIALAVNSLMINYIQAPITFILTLGVCLSIS
WKFSILICVAFPIFILPIVVIARKIKNLAKRIQKSQDSFSSVLYDFLAGVMTVKVFRTEKFAFTKYCEHNNKISA
LEEKSAAYGLLPRPLLHTIASLFFAFVVVIGIYKFAIPPEELIVFCGLLYLIYDPIKKFGDENTSIMRGCAAAERF
YEVLNHPDLHSQKEREIEFLGLSNTITFENVSFGYQEDKHILKNLSFTLHKGEALGIVGPTGSGKTTLVKLLPR
LYEVSQGKILIDSLPITEYNKGSLRNHIACVLQNPFLFYDTVWNNLTCGKDMEEEAVLEALKRAYADEFILKL
PKGVHSVLEESGKNLSGGQQQRLAIARALLKNASILILDEATSALDAISENYIKNIIGELKGQCTQIIIAHKLTTL
EHVDRVLYIENGQKIAEGTKEELLQTCPEFLKMWELSGTKEYNRVFVPDHKLVANPTDMAITT

>core/104/3/Org3_Gene614

MFSRLFFTSFSAEVVNTFFESGMSEDTSPLLSKQNRKLSHNLPLKSAYLSLGTYLIALLSFWLHAKNLSNLFVV
FTFFLAGTPALIKSLDNICQKVVNIDILMTSAAFGSIFIGGALEGALLLVLFAISEALGQMVSGKAKSTLVSLKQ
LAPTTGWLVLEDGNLQKVAINKIEVGNILRIKSGEVVPLDGEILHGSSSINLMHLTGEKVPKSCHPGSIVPAGA
HNMEGSFDLRVLRTGSDSTIAHIINLVIQAQNSKPRLQQRLDKYSSVYALSIFAIACGIALLVPLFTSIPLLGPQS
AFYRALAFLIAASPCALIIAIPIAYLSAINACAKHGVLLKGGVILDRLVSCNSVVMDKTGTLTTGELTCIGCDYF
GSKNETFFPSVLALEQSSSHPIAEAIVSYLMEQKVSSLPADRYLTVPGEGVRGYFNEQEAFVGRVETGLGKVP
SEYLEDIEQKIYQAKQHGEICSLAYVGNSFALFYFRDIPRPQAKEIIQDLKDLGYPVSMLTGDHKVSAENTAEI
LGISEVFFDLTPEDKLAKIRELATQRQIMMVGDGINDAPALAQATVGIAMGEAGSATAIEAADIVLLHDSLSS
LPWIIQKAKQTKKVVSQNLALALAIILLVSWPASLGIIPLWLAVILHEGSTVIVGLNALRLLKS

>core/105/3/Org3_Gene563

MTFQLHAPFAPCGDQPEAIARLSAGVRNQVKSQVLLGTTGSGKTFTIANVVANVNLPTLVLAHNKTLAAQL
YQEFREFFPNNAVEYFISYYDYYQPEAYIARSDTYIEKSLLINDEIDKLRLSATRSILERRDTLIVSSVSCIYGIGS
PENYTSMALVLEVGKEYPRNILTAQLVKMHYQASPIPQRSAFRERGSVIDIFPAYESELALRLEFLNDTLTSIE
YSDPLTMIPKESVPSATLYPGSHYVIPEAIREQAIRTIQEELEERMAFFDDRPIEKDRIFHRTTHDIEMIKEIGFCK
GIENYSRHFTGAPPGAPPTCLLDYFPEDFLLIIDESHQTLPQIRAMYRGDQSRKQSLVEYGFRLPSAFDNRPLT
YEEAQKYFRKVIYVSATPGDTEVQESSGHIVQQIIRPTGIPDPMPEIRPATGQVDDLLEEIRLRLSQKHEKILVIS

ITKKLAEDMAGFLSELEIPAAYLHSGIETAERTQILTDLRSGVIDVLIGVNLLREGLDLPEVSLVAILDADKEGF
LRSTSSLIQFCGRAARNINGKVIFYADQKTRSIEETLRETERRRQIQLDYNKEHNIVPKPIIKAIFANPILQTSKDS
ESPKESQRPLSKEDLEEQIKKYEALMQRAAKEFRFNEAAKYRDAMQACKEQLLYLF

>core/106/3/Org3_Gene397

MATPAQKSPTFQDPSFVRELGSNHPVFSPLTLEERGEMAIARVQQCGWNHTIVKVSLIILALLTILGGGLLVGL
LPAVPMFIGTGLIALGAVIFALALILCLYDSQGLPEELPPVPEPQQIQIEDLRNETREVLEGTLLEVLLKDRDAK
DPAVPQVVVDCEKRLGMLDRKLRREEEILYRSTAHLKDEERYEFLLELLEMRSLVADRLEFNRRSYERFVQG
IMTVRSEEGEKEISRLQDLISLQQQTVQDLRSRIDDEQKRCWTALQRINQSQKDIQRAHDREASQRACEGTEM
DCAERQQLEKDLRRQLKSMQEWIEMRGTIHQQEKAWRKQNAKLERLQEDLRLTGIAFDEQSLFYREYKEKY
LSQKLDMQKILQEVNAEKSEKACLESLVHDYEKQLEQKDANLKKAAAVWEEELGKQQQEDYEQTQEIRRL
STFILEYQDSLREAEKVEKDFQELQQRYSRLQEEKQVKEKILEESMNHFADLFEKAQKENMAYKKKLADLE
GAAAPTEIGEDDDWVLTDSASLSQKKIRELVEENQELLKALAFKSNELTQLVADAVEAEKEISKLREHIEEQK
EGLRALDKMHAQAIKDCEAAQRKCCDLESLLSPVREDAGMRFELEVELQRLQEENAQLRAEVERLEQEQFQ
G

>core/107/3/Org3_Gene910

MSYRKRSTLIVLGVFALYALLVLRYYKIQICEGDHWAAEALGQHEFCVRDPFRRGTFFANTTVRKGDKDLQ
QPFAVDITKFHLCADPLAIPECHRDEIIQGILQFIEGQTYDDLSLKLDKKSRYCKLYPLLDVSVHDRLSLWWK
GYATKHRLPTNALFFITDYQRSYPFGKLLGQVLHTLREIKDEKTGKAFPTGGMEAYFNHILEGDVGERKLLRS
PLNRLDTNRVIKLPKDGSDIYLTINPVIQTIAEEELERGVLEAKAQGGRLILMNSQTGEILALAQYPFFDPTNYK
EYFNNKERIEHTKVSFVSDVFEPGSIMKPLTVAIALQANEEASLKSQKKIFDPEEPIDVTRTLCPGRKGSPLKDI
SRNSQLNMYMAIQKSSNVYVAQLADRIIQSLGVAWYQQKLLALGFGRKTGIELPSEASGLVPSPHRFHINGSL
EWSLSTPYSLAMGYNILATGIQMVQAYAILANGGYAVRPTLVKKIVSASGEEYHLPTKEKTRLFSEEITREVV
RAMRFTTLPGGSGFRASPKHHSSAGKTGTTEKMIHGKYDKRRHIASFIGFTPVESSEGNFPPLVMLVSIDDPEY
GLRADGTKNYMGGRCAAPIFSRVADRTLLYLGILPDKKLRNCDEEAAALKRLYEEWNRSPKQGGTR

>core/108/3/Org3_Gene512

MVNPIGPGPIDETERTPPADLSAQGLEASAANKSAEAQRIAGAEAKPKESKTDSVERWSILRSAVNALMSLAD
KLGIASSNSSSSTSRSADVDSTTATAPTPPPPTFDDYKTQAQTAYDTIFTSTSLADIQAALVSLQDAVTNIKDTA
ATDEETAIAAEWETKNADAVKVGAQITELAKYASDNQAILDSLGKLTSFDLLQAALLQSVANNNKAAELLK
EMQDNPVVPGKTPAIAQSLVDQTDATATQIEKDGNAIRDAYFAGQNASGAVENAKSNNSISNIDSAKAAIAT
AKTQIAEAQKKFPDSPILQEAEQMVIQAEKDLKNIKPADGSDVPNPGTTVGGSKQQGSSIGSIRVSMLLDDAE
NETASILMSGFRQMIHMFNTENPDSQAAQQELAAQARAAKAAGDDSAAAALADAQKALEAALGKAGQQQ
GILNALGQIASAAVVSAGVPPAAASSIGSSVKQLYKTSKSTGSDYKTQISAGYDAYKSINDAYGRARNDATR
DVINNVSTPALTRSVPRARTEARGPEKTDQALARVISGNSRTLGDVYSQVSALQSVMQIIQSNPQANNEEIRQ
KLTSAVTKPPQFGYPYVQLSNDSTQKFIAKLESLFAEGSRTAAEIKALSFETNSLFIQQVLVNIGSLYSGYLQ

>core/109/3/Org3_Gene1001

MKKLVRLCVVLLSLLPNVLFSSDLLREEGIKKMMDKLIEYHVDAQEVSTDILSRSLSSYIQSFDPHKSYLSNQE
VAVFLQSPETKKRLLKNYKAGNFAIYRNINQLIHESILRARQWRNEWVKNPKELVLEASSYQISKQPMQWSK

SLDEVKQRQRALLLSYLSLHLAGASSSRYEGKEEQLAALCLRQIENHENVYLGINDHGVAMDRDEEAYQFHIRVVKALAHSLDAHTAYFSKDEALAMRIQLEKGMCGIGVVLKEDIDGVVVREIIPGGPAAKSGDLQLGDIIYRVDGKDIEHLSFRGVLDCLRGGHGSTVVLDIHRGESDHTIALRREKILLEDRRVDVSYEPYGDGVIGKVTLHSFYEGENQVSSEQDLRRAIQGLKEKNLLGLVLDIRENTGGFLSQAIKVSGLFMTNGVVVVSRYADGTMKCYRTVSPKKFYDGPLAILVSKSSASAAEIVAQTLQDYGVALVVGDEQTYGKGTIQHQTITGDASQDDCFKVTVGKYYSPSGKSTQLQGVKSDILIPSLYAEDRLGERFLEHPLPADCCDNVLHDPLTDLDTQTRPWFQKYYLPNLQKQETLWREMLPQLTKNSEQRLSENSNFQAFLSQIKSSEKTDLSYGSNDLQLEESINILKDMILLQQCRK

>core/110/3/Org3_Gene748

MTSSSCPLLDLILSPADLKKLSISQLPGLAEEIRYRIISVLSQTGGHLSSNLGIVELTIALHYVFSSPKDKFIFDVGHQTYPHKLLTGRNNEGFDHIRNDNGLSGFTNPTESDHDLFFSGHAGTALSLALGMAQTTPLESRTHVIPILGDAAFSCGLTLEALNNISTDLSKFVVILNDNNMSISKNVGAMSRIFSRWLHHPATNKLTKQVEKWLAKIPRYGDSLAKHSRRLSQCVKNLFCPTPLFEQFGLAYVGPIDGHNVKKLIPILQSVRNLPFPILVHVCTTKGKGLDQAQNNPAKYHGVRANFNKRESAKHLPAIKPKPSFPDIFGQTLCELGEVSSRLHVVTPAMSIGSRLEGFKQKFPERFFDVGIAEGHAVTFSAGIAKAGNPVICSIYSTFLHRALDNVFHDVCMQDLPVIFAIDRAGLAYGDGRSHHGIYDMSFLRAMPQMIICQPRSQVVFQQLLYSSLHWSSPSAIRYPNIPAPHGDPLTGDPNFLRSPGNAETLSQGEDVLIIALGTLCFTALSIKHQLLAYGISATVVDPIFIKPFDNDLFSLLLMSHSKVITIEEHSIRGGLASEFNNFVATFNFKVDILNFAIPDTFLSHGSKEALTKSIGLDESSMTNRILTHFNFRSKKQTVGDVRV

>core/111/3/Org3_Gene545

MTCISELNEAQRKAVTAPLNPVLVLAGAGAGKTRVVTYRILHLINQGIAPREILAVTFTNKAARELKERIVNQCASTNEFDVPMVCTFHSLGVFILRRSINLLNRENNFTIYDQSDAEKLIKHALQQHNLKPNLASKIQAHVSQAKNRLLFPEDLDPNDYIDPVVSIYQEYQKKLIEANALDFDDLLFLTVRLLRESPEAQELYNQLWKALLIDEYQDTNHAQYTLMQLLSKQHRNVFAVGDPDQSIYSWRGANIHNILNFENDYPNAKVLCLEENYRSYGNILNAANALIKNNASRLEKELRSVKGPGEKIRLFLGSTDREEADFVAAEILQLHRVGNIKLRDICIFYRTNSQSRTFEDALLRRRIPYEIIGGLSFYKRKEIQDILAFLRIFISKSDIVAFDRTVNLPKRGIGSTTIFALTQYAIAQGLPILKACQQALDTKDVKLSKKQQEGLQEYLALFPQIEHAYNTLSLRDFIESVVRITGYLEILKEDADTFKDRKSNLEELYHKALESEQQNPKTHLELFLDDLALKGSDDDLNLTADRVNLMTLHNGKGLEFRVSFLVGLEEQLLPHANSLGGTYENIEEERRLCYVGITRAQDLLYLTAAQVRSLWGTVRMMKPSRFLKEIPKDYMIQVR

>core/112/3/Org3_Gene882

MKQHYSLNKSRHILRSTYKLLKSKKLAHSPADKKQLQELLEQLEEAIFEHDQETASDLAQQALAFSNRYPNSFGRKTYELIKALLFAGVVAFLVRQFWFELYEVPTGSMRPTILEQDRILVSKTTFGLHCPFAKKPLAFNPESVTRGGLVVFTVGDLPIPDADTKYFGLIPGKKRYIKRCMGRPRDFLYFYGGKIYGLDDAGKRIEFPSVHGLENLYHVPYISFDGTTSSHTEGQKTIIDFKQFNQSYGRLIFPQTSMYGQFFDHKEWHQDEPNKLKDPHLSPVSYADLFGMGNYAMVRILTEHQARTSHLLPNPGSPTKVYLEICHTANLSYPKPLLRHYEHQLSPAIQPMKTLLPLRKEHLHLIRNNLTTSRFIVAQGCAYKYHQFKINTSGIAKAYAILLPKVPDGCYEYSKGEAYQIGFGEIRYKLKSSHPLTQLNDKQVIELFNCGINFSSIYNPVNPLQAPLPNRYAFFNQGNLYIMDSPVFIKNDPTLQKFVTSETEKQEGSSETQPYIAFVDKGLPPEDFKEFVEFIHNFGIQVPKGHVLVLGDNYPMSADSREFGFVPMENLLGSPLCTFWPIGRMGRLTGVSAPTTLSGYLVSGIALATGLSLIGYVYYQKRRRLFPKKEEKNHKK

>core/113/3/Org3_Gene951

MIPFTKTIGFRLWLACAVAIIAPLGINIVWLNLDQYRTIVSAISTALKENAAFKANTLTQIVPLNVDVLSLFSDV
LDLDAGIPETPNVLLSNEMQKVFQGIYNEISLIKVFPNGDKIVVASSIPEHLGENYNHKIDIPKNTPFLAALKQS
PKNQEVFSVMQANVFDAKTQELQGILYTTFSAESLLKDLLINKQSYLTVKTAILSKYGVILKASDPALHLHTV
YPDMTKEKFCQVFLNDDPCPIDSELGPLTLSPLDIGENFYSFKIKDTEIWGCIENVPSIDIAVLSYAKKEESFAPL
WRRARMYTAYFFCILLGSLIAFIVARRLSLPIRKLATAMIESRKNKNCLYTDDSLGFEINRLGHIFNAMVENLH
KQQHLAKTNFEMKENAQNALHLGEQAQQRLLPNTLPSYPHIELAKAYIPAITVGGDFFDVFVVGEGSKARLF
LIVADASGKGVNACGYSLFLKNMLRTFLSRSSSLQQAIQETSRLFYNNTKNSGMFVTLCVYCYHQTSNTMEY
YSCGHPPACYLDPDGETSWLFHPGMALGFLPEVANITSKLFHPKPGSLFVLYSDGITEAHNNNNDMFGEERL
QAAIQGLTGKSAADAVHRLMLSVKPFVGNSHQHDDITLLILKVLES

>core/114/3/Org3_Gene568

MIQVTCDQKNYEVLEGTTAAELAKQLKNSHQFIGVLINERPRDLSTHLNEGDTLVFLTSEDPEGREIFLHTSA
HLLAQAVLRLWPDAIPTIGPVIDHGFYYDFANLSISESDFPLIEDTVKQIVDEKLAISRFTYGDKQQALAQFPQ
NPFKTELIRELPENEEISAYSQGEFFDLCRGPHLPSTAHVKAFKVLRTSAAYWRGDPSRESLVRIYGTSFPTSKE
LRAHLEQIEEAKKRDHRVLGAKLDLFSQQESSPGMPFFHPRGMIVWDALIRYWKQLHTAAGYKEILTPQLM
NRQLWEVSGHWDNYKANMYTLQIDDEDYAIKPMNCPGCMLYYKTRLHSYKEFPLRVAEVGHVHRQEASG
ALSGLMRVRAFHQDDAHVFLTPEQVEEETLNILQLVSTLYGTFGLEYHLELSTRPEKDTIGDDSLWELATDAL
NRALVQSGTPFIVRPGEGAFYGPKIDIHVKDAIQRTWQCGTIQLDMFLPERFELEYTTAQGTKSVPVMLHRAL
FGSIERFLGILIENFKGRFPLWLSPEQVRIITVADRHIPRAKELEEAWKRLGLVVTLDDSSESVSKKIRNAQNM
QVNYMITLGDHEINENVLAVRTRDNRVINDVSVERFLNTILEEKNSLSLTALL

>core/117/3/Org3_Gene557

MDENRKVIVVGGGLAGLSAAMQLANLGIIVELVSLTKVKRSHSVCAQGGINAALNLKPEEEDSPYVHAYDTI
KGGDFLADQPPVLEMCLAAPRIIKMLDNFGCPFNRGPSGNLDVRRFGGTLYHRTVFCGASTGQQLMYTLDE
QVRRREHAGRVIKRENHEFVRLVTDHSGRACGIILMNLFNNRLEILRGDAVIIATGGPGVIFKMSTNSTFCTGA
ANGRLFLQGMAYANPEFIQIHPTAIPGRDKLRLISESVRGEGGRVWVPGDSSKRIVFPDGSERPCGETGAPWY
FLEDMYPAYGNLVSRDVGARAILRVCEAGLGIDGRMEAYLDVTHLPEKTRHKLEVVLDIYKKFTGEDPNTV
PMRIFPAVHYSMGGAWVDWPAADDPDRDSRFRQMTNIPGCFNCGESDFQYHGANRLGANSLLSCLFAGLVS
GDEASRFIEAFGASQATSSDFDRALQQEKEENARLLSASGKENIFVLHEEIAKIMVRNVTVKRNNRDLQETM
DKLKEFRERLKNVSVLDSSPFANKSFHFVRQMGPMLELALAITKGALLRNEFRGSHYKPEFPERDDEHWLKT
TVAVYAPEEPEISYLPVDTRHVAPTLRDYTKSSTGKIELTNIPDNIRLPI

>core/118/3/Org3_Gene901

MTIIYFILAALALGILVLIHELGHLVVAKAVGMAVESFSIGFGPALFKKRIGGIEYRIGCIPFGGYVRIRGMERT
KEKGEKGKIDSVYDIPQGFFSKSPWKRILVLVAGPLANILLAVLAFSILYMNGGRSKNYSDCSKVVGWVHPV
LQAEGLLPGDEILTCNGKPYVGDKDMLTTSLLEGHLNLEIKRPGYLTVPSKEFAIDVEFDPTKFGVPCSGASY
LLYGNQVPLTKNSPMENSELRPNDRFVWMDGTLLFSMAQISQILNESYAFVKVARNDKIFFSRQPRVLASVL
HYTPYLRNELIDTQYEAGLKGKWSSLYTLPYVINSYGYIEGELTAIDPESPLPQPQERLQLGDRILAIDGTPVSG
SVDILRLVQNHRVSIIVQQMSPQELEEVNSRDADKRFIASYHSEDLLQILNHLGESHPVEVAGPYRLLDPVQPR

PWIDVYSSESLDKQLEVAKKIKNKDKQRYYLERLDAEKQKPSLGISLKDLKVRYNPSPVVMLSNITKESLITL
KALVTGHLSPQWLSGPVGIVQVLHTGWSVGFSEVLFWIGLISMNLAVLNLLPIPVLDGGYILLCLWEIVTRRR
LNMKIVERILDPFTFLLIIFFIFLTFQDLFRFFG

>core/119/3/Org3_Gene339

MRRSVCYVNPSIARAGQISTWKFLYSLATPLPAGTKCKFDLAGSGKPTDWEAPATDLSQTRNVIYAEMPEGE
IIEATAIPVKDNPVPQFEFTLPYELQVGETLTIVMGASPNHPQVDDAGNGAQLFAQRRKPFYLYIDPTGEGNY
DEPDVFSMDIRGNVLKKIEIFTPSYVVKNKRFDITVRFEDEFGNLTNFSPEETRIELSYEHLRENLNWQLFIPET
GFVILPNLYFNEPGIYRIQLKNLSTQEIFISAPIKCFADSAPNLMWGLLHGESERVDSEENIETCMRYFRDDRAL
NFYASSSFENQENLSPDIWKLINQTVSDFNEEDRFITLSGFQYSGEPHLEGVRHILHTKETKSHSKHKEYKHIPL
AKLYKSTVNHDMISIPSFTASKEHGFDFENFYPEFERVVEIYNAWGSSETTAALNNPFPIQGKDSEDPRGTVIE
GLKKNLRFGFVAGGLDDRGIYKDYFDSPQVQYSPGLTAIICNKYTRESLVEALFARHCYATTGPRIVLSFNITS
APMGSELSTGSKPGLNVNRHISGHVAGTALLKTVEIIRNGEVLHTFFPDSNNLDYEYDDMVPLSSVTLKDPNG
KAPFVFYYLRVTQADNAMAWSSPIWVDLN

>core/120/3/Org3_Gene89

MESEKDIGAKFLGDYRILYRKGQSLWSEDLLAEHRFIKKRYLIRLLLPDLGSSQPFMEAFHDVVVKLAKLNHP
GILSIENVSESEGRCFLVTQEQDIPILSLTQYLKSIPRKLTELEIVDIVSQLASLLDYVHSEGLAQEEWNLDSVYI
HILNGVPKVILPDLGFASLIKERILDGFISDEENRESKIKERVLLHTSEGKQGREDTYAFGAITYYLLFGFLPQGI
FPMPSKVFSDFIYDWDFLISSCLSCFMEERAKELFPLIRKKTLGEELQNVVTNCIESSLREVPDPLESSQNLPQA
VLKVGETKVSHQQKESAEHLEFVLVEACSIDEAMDTAIESESSSGVEEEGYSLALQSLLVREPVVSRYVEAEK
EEPKPQPILTEMVLIEGGEFSRGSVEGQRDELPVHKVILHSFFLDVHPVTNEQFIRYLECCGSEQDKYYNELIRL
RDSRIQRRSGRLVIEPGYAKHPVVGVTWYGASGYAEWIGKRLPTEAEWEIAASGGVAALRYPCGEEIEKSRA
NFFTADTTTVMSYPPNPYGLYDMAGNVYEWCQDWYGYDFYEISAQEPESPQGPAQGVYRVLRGGCWKSLK
DDLRCAHRHRNNPGAVNSTYGFRCAKNIN

>core/121/3/Org3_Gene833

MKKGKLGAIVFGLLFTSSVAGFSKDLTKDNAYQDLNVIEHLISLKYAPLPWKELLFGWDLSQQTQQARLQLV
LEEKPTTNYCQKVLSNYVRSLNDYHAGITFYRTESAYIPYVLKLSEDGHVFVVDVQTSQGDIYLGDEILEVDG
MGIREAIESLRFGRGSATDYSAAVRSLTSRSAAFGDAVPSGIAMLKLRRPSGLIRSTPVRWRYTPEHIGDFSLV
APLIPEHKPQLPTQSCVLFRSGVNSQSSSSSLFSSYMVPYFWEELRVQNKQRFDSNHHIGSRNGFLPTFGPILW
EQDKGPYRSYIFKAKDSQGNPHRIGFLRISSYVWTDLEGLEEDHKDSPWELFGEIIDHLEKETDALIIDQTHNP
GGSVFYLYSLLSMLTDHPLDTPKHRMIFTQDEVSSALHWQDLLEDVFTDEQAVAVLGETMEGYCMDMHAV
ASLQNFSQSVLSSWVSGDINLSKPMPLLGFAQVRPHPKHQYTKPLFMLIDEDDFSCGDLAPAILKDNGRATLI
GKPTAGAGGFVFQVTFPNRSGIKGLSLTGSLAVRKDGEFIENLGVAPHIDLGFTSRDLQTSRFTDYVEAVKTIV
LTSLSENAKKSEEQTSPQETPEVIRVSYPTTTSAL

>core/122/3/Org3_Gene821

MFGTLVSTLCCPANSERDWEDHEVNCIYIASTSDTQLEAVQGGMHITELRGEPVRVLYETGHLYAFARENTC
HSRLEVSHTVRAMTYFWDRFFSRHWNVGRRFLVFYQGNGGAYVQAALDSSMHTQDIYVLGLSPTVYIRGN
YHVQHYRVRGFWPSCLDSLAACAENTSVLPYGESSDGIFYPSLFSHTFDNAIRYGERCLLVCSEGMGMLPET

QQQTSPLTSLEGGHEVALVLNPQQNPEALSIASRLMHEERGGRLESNYMPGRSSNPFMTSMYVLVRLNTLAQ
IYLMSPYYSFQSNDIVCLIFISSAAVETVSYIFLTVTDSTCGRRYLRVPRLVCTGLRNLALPTTLLELLILSYPRS
VEGVPFNVRFILGYMCTTRVVFFAWNLILHWPFRCLRHGIQLFVHRSIIGHTLGARITDLTLASMRYAIVFPSI
VSSCLLTALAHANTNILALDPYRLIESGDLRRPAFNDDEMQQADNPWDAYSIGLVINTCIYMLILFANLIFMV
YSVRRYHRSRR

>core/123/3/Org3_Gene249

MTLITPAINSSRRKTHTVRIGNLYIGSDHSIKTQSMTTTLTTDIDSTVEQIYALAEHNCDIVRVTVQGIKEAQAC
EKIKERLIALGLNIPLVADIHFFPQAAMLVADFADKVRINPGNYIDKRNMFKGTKIYTEASYAQSLLRLEEKFA
PLVEKCKRLGKAMRIGVNHGSLSERIMQKYGDTIEGMVASAIEYIAVCEKLNYRDVVFSMKSSNPKIMVTAY
RQLAKDLDARGWLYPLHLGVTEAGMGVDGIIKSAVGIGTLLAEGLGDTIRCSLTGCPTTEIPVCDSLLRHTKI
YLDLPEKKNPFSLQHSENFVSAAEKPAKTTLWGDVYGVFLKLYPHHLTDFTPEELLEHLGVNPVTKEKAFTT
PEGVVVPPELKDAPITDVLREHFLVFHHHQVPCLYEHNEEIWDSPAVHQAPFVHFHASDPFIHTSRDFFEKQG
HQGKPTKLVFSRDFDNKEEAAISIATEFGALLLDGLGEAVVLDLPNLPLQDVLKIAFGTLQNAGVRLVKTEYI
SCPMCGRTLFDLEEVTTRIRKRTQHLPGLKIAIMGCIVNGPGEMADADFGFVGSKTGMIDLYVKHTCVKAHIP
MEDAEEELIRLLQEHGVWKDPEETKLTV

>core/124/3/Org3_Gene420

MWTHPIAYDVIVVGAGHAGCEAAYCSAKMGVSVLMLTSNLDTIAKLSCNPAVGGIGKGHIVREIDALGGIM
AEVTDQSGIQFRILNQTKGPAVRAPRAQVDKQLYHIHMKRLLENTPGLHIMQATVESLLDKEGVISGVTTKE
GWMFSGKTVVLSSGTFMRGLIHIGDRNFSGGRLGDPSSQGLSEDLKKRGFPISRLKTGTPPRLLASSINFSCME
EQPGDLGVGFVHRTEPFQPPLPQLSCFITHTMEKTKAIISANLHRSALYGGCIEGVGPRYCPSIEDKIVKFSDKE
RHHVFLEPEGLHTQEIYANGLSTSMPFDVQYDMIRSVLGLENAIITRPAYAIEYDYIHGNVIHPTLESKLIEGLF
LCGQINGTTGYEEAAAQGLIAGINAVNKVFNRPPFIPSRQESYIGVMLDDLTTQILDEPYRMFTGRAEHRLLLR
QDNACARLSHYGYELGLLSEERYELVKKQNQLLEEEKVRLQKTFRQYGQSVVSLAKALSRPEVSYDMLREA
FPNDIRDLGAVLNASLEMEIKYSGYIDRQKILIQSLEKAESLLIPEDLDYKQITALSLEAQEKLAKFTPRTLGSA
SRISGIASADIQVLMIALKKHAHH

>core/125/3/Org3_Gene81

MTTELKTEALPTRTQVDPKHCWDTTLMYANREEWKKDFDLCSSGKDRSPIWPEFSPSHYQIDNPESLLELLS
KKFSVERKLDQLYIYAHLIHDQDITNPEGESDYQSIVYLYTLFSQEISWIQPALIALSEEKVAALLSSSVLAPYR
FYLEKIFRLSPHTGTANEEKILASSFAALNVSNKAFSSLSDAEIPFGIAKDSNGEEHPLSHALASLYMQSPDQEL
RRTAYLAQFQRYYDYRNTFANLLNGKVQAHLFEAKARNYPSCLEASLFQHNIPTTVYINLINETKKHTSLINR
YFNLKKEALNLKEFHFYDVYAPISQTTSKNYSYEEGVDLVCKSLLPLGTHYVEILRNGLLSNRWVDRYENKH
KRSGAYSSGCYDSAPYILLNYTNTLYDVSVIAHEAGHSMHSYFSREAQPYHDAQYPLFLAEIASTFNEMLLM
EALSKSDQSKEDKIVIITKTLDTIFATLFRQTFFAAFEYEIHSAAEQGTPLTEEFLSATYGNLQKEFYGGVVTSD
SLSALEWARIPHFYYNFYVYQYATGIIAALSFAEKILTQEPGALELYLKFLKSGRSDFPLNILKKSGLDMTTSA
PLDKAFAFITKKIDLLSSLLSED

>core/126/3/Org3_Gene947

MFRCILFGIFLLTCFSSGGVLYYLFCSHDFSIGPKEKSRSVWIEEEKEFTDSVLHHLPSQHQHLHILCFQGFLLQ
KQQKFSQAEKIFSKVYDEAQDGPFLFKEEILGSRLINSFFLEKTDVMETILCLLNQRCPNSPYYHLFKALVCYK
QKLYREVIEQLAYWQEEKTRALAPLLNISIEQLLTDFLLDYISAHSLIEQKMFPEGRVILNRNINRLLKHECEW
NAKTYDRIAILLSRSYFLELVESKSADIYFDYYEMVLFYLKKIYILEQCPYAELLPEEELVSLIMEHVFILPKDK
LYPLIQLLEMWQKHYVHPNSSLVVQILVDRFSTHMEGAIRFCEALVSFSGLEELHQQIITTFEELLSNKVQQIK
TEEAKQCVALLHILDPSISISEKLALSSDTLQNIVSGDDEQHTKLRNYLDLWEAIQSYDIDRQQLVHHLVYGA
KDLWKKGGNDEKALNLLQLVLRFTSYDIECESVVFLFIKQAYKQALSSHAIARLLKLEKFISEANIPSIVISEAE
KANFLADAEYLFAHEDYDKCYLYSMWLTKVAPSPQSYRLAGLCLMENKRYDEALEFLCMLSPNNSINDYK
TQKALAFCQKHQSKDRVAS

>core/127/3/Org3_Gene838

MCGIFGYLGNQDGVSIVLEGLAKLEYRGYDSAGLAAVVEQELFIRKTVGRVQELSNLFQEREIPTASVIGHTR
WATHGVPTEINAHPHVDEGRSCAVVHNGIIENFKELRRELTAQGISFASDTDSEIIVQLFSLYYQESQDLVFSF
CQTLAQLRGSVACALIHKDHPHTILCASQESPLILGLGKEETFIASDSRAFFKYTRHSQALASGEFAIVSQGKEP
EVYNLELKKIHKDVRQITCSEDASDKSGYGYYMLKEIYDQPEVLEGLIQKHMDEEGHILSEFLSDVPIKSFKEI
TIVACGSSYHAGYLAKYIIESLVSIPVHIEVASEFRYRRPYIGKDTLGILISQSGETADTLAALKELRRRNIAYLL
GICNVPESAIALGVDHCLFLEAGVEIGVATTKAFTSQLLLLVFLGLKLANVHGALTHAEQCSFGQGLQSLPDL
CQKLLANESLHSWAQPYSYEDKFLFLGRRLMYPVVMEAALKLKEIAYIEANAYPGGEMKHGPIALISKGTPV
IAFCGDDIVYEKMIGNMMEVKARHAHVIAIAPESREDIAAVSDQQIFVPDCHFLAAPVLYTIVGQVMAYAMA
LAKGMEIDCPRNLAKSVTVE

>core/128/3/Org3_Gene680

MIPSGLVYLLYPLGFLASLFFGSAFSIQWWLSKKRKEVYAPRSFWILSSIGATLMIVHGTIQSQFPVTVLHVINL
IIYLRNLNITSSRPISFRATLVLMALSVVFVTLPFLYVNMEWMASPNIFHLPLPPAQLSWHLIGCLGLAIFSGRF
LIQWFYIESNNTKDFPLLFWKIGLLGGLLALVYFIRIGDPINILCYGCGLFPSIANLRLFYKEQRSTPYLDTHCFL
SAGEASGDILGGKLIQSIKSLYPNIRFWGVGGPAMRQEGLQPILNMEEFQVSGFAEVLGSLFRLYRNYRKILKT
ILKHKPATLIFIDFPDFHLLLIKKLRKHGYRGKIIHYVCPSIWAWRPKRKRILEQHLDMLLLILPFEEGLFKNTSL
ETVYLGHPLVEEISDYKEQASWKEKFLNSDRPIVAAFPGSRRGDISRNLRIQVQAFLNSSLSQTHQFVVSSSSA
KYDEIIEDTLKAEGCQHSQIIPMNFRYELMRSCDCALAKCGTIVLETALNQTPTIVMCRLRPFDTFLAKYIFKIL
LPAYSLPNIIMNSVIFPEFIGGKKDFHPEEIATALDLLNQHGSKEKQKEDCRKLCKVMTTGQIASEEFLKRIFDT
LPAV

>core/129/3/Org3_Gene558

MKHTFTKRVLFFFFLVIPIPLLLNLMVVGFFSFSAAKANLVQVLHTRATNLSIEFEKKLTIHKLFLDRLANTLA
LKSYASPSAEPYAQAYNEMMALSNTDFSLCLIDPFDGSVRTKNPGDPFIRYLKQHPEMKKKLSAAVGKAFLL
TIPGKPLLHYLILVEDVASWDSTTTSGLLVSFYPMSFLQKDLFQSLHITKGNICLVNKYGEVLFCAQDSESSFV
FSLDLPNLPQFQARSPSAIEIEKASGILGGENLITVSINKKRYLGLVLNKIPIQGTYTLSLVPVSDLIQSALKVPL
NICFFYVLAFLLMWWIFSKINTKLNKPLQELTFCMEAAWRGNHNVRFEPQPYGYEFNELGNIFNCTLLLLLNS
IEKADIDYHSGEKLQKELGILSSLQSALLSPDFPTFPKVTFSSQHLRRRQLSGHFNGWTVQDGGDTLLGIIGLA
GDIGLPSYLYALSARSLFLAYASSDVSLQKISKDTADSFSKTTEGNEAVVAMTFIKYVEKDRSLELLSLSEGAP

TMFLQRGESFVRLPLETHQALQPGDRLICLTGGEDILKYFSQLPIEELLKDPLNPLNTENLIDSLTMMLNNETE
HSADGTLTILSFS

>core/130/3/Org3_Gene664

MRIEDFSLKLIPSSPGVYLMKDVHDQVLYIGKAKNLKNRLASYFHEKGDSRERIPFLMKKTASIETIVVSNETE
ALLLENNLIKQHHPKYNVLLKDDKTFFCLAISLSHSWPKVEAIRTKAITSSQRQLIFGPYVSAEACHTLLEVISQ
WFPLRTCSDREFALRKRPCILYDMKRCLAPCVGYCTPEEYQGTLDKAILFLKGKIEEVVKDLEKVIQKASDNL
EFEQAANYYRTLSLIKQAMAKQQVEKFHFQNIDALGLYRHKQRTILTLLTVRSGKLLGARHFSFFENAQEDQ
DLLSSFILQYYVSQPYIPKEILTPLPLEFPTLSYVLNAESPPRLRSPKTGYGKELLDLAYRNAKAYAATTLPSST
LPYQDFQNILRMSQYPYRIECYDNAHMQGAHATGVYIVFENNGFDPKQYRTFSIDSEKTQNDLALLEEVLLR
RFHSLTTALPDMIVVDGGKTHYNKTKKIIQTLNLTGIQVVTIAKEKSNHSRGLNKEKIFCETFPEGFSLPPTSNL
LQFFQILRDEAHRFAISKHRKKRGKALFEQEKIPGIGEVKRKRLLQKFKSWKQVMLSSQEELEAIPGLTKKDIA
VLLARQKDFNKSD

>core/131/3/Org3_Gene237

MKEYKIENIRNFSIIAHIDHGKSTIADRLLESTSTVEEREMREQLLDSMDLERERGITIKAHPVTMTYLYEGEV
YQLNLIDTPGHVDFSYEVSRSLSACEGALLIVDAAQGVQAQSLANVYLALERDLEIIPVLNKIDLPAADPVRIA
QQIEDYIGLDTTNIIACSAKTGQGIPAILKAIIDLVPPPKAPAETELKALVFDSHYDPYVGIMVYVRIISGELKKG
DRITFMAAKGSSFEVLGIGAFLPKATFIEGSLRPGQVGFFIANLKKVKDVKIGDTVTKTKHPAKTPLEGFKEIN
PVVFAGIYPIDSSDFDTLKDALGRLQLNDSALTIEQESSHSLGFGFRCGFLGLLHLEIIFERIIREFDLDIIATAPS
VIYKVVLKNGKVLDIDNPSGYPDPAIIEHVEEPWVHVNIITPQEYLSNIMNLCLDKRGICVKTEMLDQHRLVL
AYELPLNEIVSDFNDKLKSVTKGYGSFDYRLGDYRKGSIIKLEVLINEEPIDAFSCLVHRDKAESRGRSICEKL
VDVIPQQLFKIPIQAAINKKVIARETIRALSKNVTAKCYGGDITRKRKLWEKQKKGKKRMKEFGKVSIPNTAFI
EVLKLD

>core/132/3/Org3_Gene1014

MVMVWSTNIKHEGLKSWIDEVAKLTTPKDIRLCDGSDTEYDELCTLMESTGTMIRLNPEFHPNCFLVRSSAD
DVARVEQFTFICTSTEAEAGPTNNWRDPQEMRRELHQLFRGCMQGRTLYIVPFCMGPLDSPFSIVGVELTDSP
YVVCSMKIMTRMGDDVLRSLGTSGKFLKCLHSVGKPLSPGEADVSWPCNPKSMRIVHFQDDSSVMSFGSGY
GGNALLGKKCVALRLASYMAKSQGWLAEHMLIIGITNPEGKKKYFSASFPSACGKTNLAMLMPKLPGWKIE
CIGDDIAWIRPGRDGRLYAVNPEYGFFGVAPGTSERTNPNALATCRSNSIFTNVALTADGDVWWEGLTEQPP
EPLTDWLGKPWKPGGSPAAHPNSRFTAPLRQCPSLDPEWNSPQGVPLDAIIFGGRRSETIPLVYEALSWEHGV
TIGAGMSSTTTAAIVGQLGKLRHDPFAMLPFCGYNMAYYFQHWLSFAENRSLKLPKIFGVNWFRKNNQGEF
LWPGFSENLRVLEWIFQRTDGLEDIAERTPIGYLPNIQKFNLNGLNLDLQTVQELFSVDAEGWLAEVENIGEY
LKIFGSDCPQQITDELLRIKSELKEK

>core/133/3/Org3_Gene504

MAAYTEASILSLASLDHIRLRAGMYIGRLGNGSQKEDGIYTLFKEVVDNGIDEFIMGHGKSLKISASDKQISIQ
DQGRGIPLGKLIDCVSKINTGAKYTQDVFHFSVGLNGVGLKAVNALSEIFSVRSVRKKKYHLATFHRGVLQE
SKQGSTKDPDGTFVSFTPDPSIFPEFTFNHDFLKDKIRQYTYLHSGLEIRFNDEVFISHNGLKDLFDAEITEPPLY
SPLFFQNEDLTFIFSHLEGNTERYFSFVNGQETLDGGTHLTAFKEAIVKGVNEFFGKTFVSNDIREGIVGCIAIKI

ASPIFESQTKNKLGNTQIRSSLIKDVKEAIVQALRKDKVAPELLLEKIKFNEKTRKNIQFIKQDLKSKQKKVHY
KIPKLRDCKFHYNDRSLYGEASSIFLTEGESASASILASRNPLTQAVFSLRGKPMNVFSLEETKMYKNDELFYL
ATALGITQNEIQHLRYNKVILATDADVDGMHIRNLLITFFLKTLLPLVENNHLFILETPLFKVRNKTTTLYYYS
EQEKMQALQQFGKKDSSLEITRFKGLGEISPKEFAAFIGPEIRLTPVTITSLESISSILQFYMGKNTKERKQFIMD
NLITDF

>core/134/3/Org3_Gene823

MKEVEQRIRSLYDAVTAENICRWLSNDCTQQDAKTILGWLDTDPAQLEDLFGATLTFGTGGLRSLMGIGTNR
INLFTIRRTTQGLVQVLRAHLPHPGDPMRVVVGCDTRHNSIEFAQETAKVLAGNGCEVFLFQYPEPLALVSFT
VRYERAIGGVMITASHNPPNYNGYKVYMASGGQVLPPLDQEIVAACSAVNEILSVPSIDHPNIHLIGKEYEAL
YRDTLKQLQLYPEANRISGRSLSISYSPLHGTGISLVPHVLKDWGFLSVHLVEKQAIGDGDFPTVQLPNPEDPE
ALTLGTEQMLANDDDLFIATDPDADRVGVVCLEDGQPYRFNGNQMASLLADHILGAWSKTRHLGEHDKLV
KSLVTTEMLSAIAKHYHVDLINVGTGFKYIGEKIESWRNSTNKFVFGAEESYGCLYGTHVEDKDAIIASALIA
EAALQQKLQGKTLCDALLSLYETYGYFANKTESVVFSAKTDEQEIRKKLSHLEEISSANFFSGKYQVEKFENY
KQGIGFNLLSKDSYALTLPKTSMLCYYFSGGGRVIIRPSGTEPKIKFYFEMSTHYPERVTDKEIQKQREAESFQ
HLDDFIFDFKEKFSNL

>core/135/3/Org3_Gene48

MVTVSEQTAQGHVIEAYGNLLRVRFDGYVRQGEVAYVNVDNTWLKAEVIEVADQEVKVQVFEDTQGACR
GALVTFSGHLLEAELGPGLLQGIFDGLQNRLEVLAEDSSFLQRGKHVNAISDHNLWNYTPVASVGDTLRRGD
LLGTVPEGRFTHKIMVPFSCFQEVTLTWVISEGTYNAHTVVAKARDAQGKECAFTMVQRWPIKQAFIEGEKI
PAHKIMDVGLRILDTQIPVLKGGTFCTPGPFGAGKTVLQHHLSKYAAVDIVILCACGERAGEVVEVLQEFPHL
IDPHTGKSLMHRTCIICNTSSMPVAARESSIYLGVTIAEYYRQMGLDILLLADSTSRWAQALREISGRLEEIPGE
EAFPAYLSSRIAAFYERGGAITTKDGSEGSLTICGAVSPAGGNFEEPVTQSTLAVVGAFCGLSKARADARRYP
SIDPLISWSKYLNQVGQILEEKVSGWGGAVKKAAQFLEKGSEIGKRMEVVGEEGVSMEDMEIYLKAELYDF
CYLQQNAFDPVDCYCPFERQIELFSLISRIFDAKFVFDSPDDARSFFLELQSKIKTLNGLKFLSEEYHESKEVIV
RLLEKTMVQMA

>core/136/3/Org3_Gene907

MTAPTESRSSPPTLLEETEPLSPNPIPADIQIPRITISPPSLDVSTVASSAEDISVFIAGGPRSSSSASVASDVYELV
CLCGGDEDPEPPDSEVRTLYVNGSWQTHQEAVQELLYISEVRGEAVRLLYNDGSGMSPWPISPCRTLPTLDH
PLCQALLTVWEQFFSAPENQHREFLVIFYGDASPYIQQALTQSRHSPRIVVVGISPTVFIQGDFRVHNYRVSGD
FFSSLDCRGTRAENTTILPYSSGLEGVFLPSIRCPSFTWAVRFGEQCLVANRGEDVEDRGGLSQDAERSQLPHS
ERDLAVVIDSTDPSSMSRLVEWLNQGSPSSDMEINPYPQRCPDVALSALYAISRVSGLAQEWILASVHEGLDL
QICYSLILMHTTFAVRYFFLLFTNYPQSRERFRTARIVAQSLYLPSILVLVFDCGNVLRKLWMPQEILRAIFISA
STISGSIVFVECTRWMGRGLRHRVQQFVQQRVIGSGLPVGTVRASYRDRAGFIIGFLQTVHGGLYLPVSIMVL
NQIAIQVPRILVRPNNTAVYDLHNKSAEENWSSGDVLAVGQTLNFILCAFVLFVNLWFFVKSVLRHSRRRRR

>core/137/3/Org3_Gene714

MKKKFIFYFVIVFSLLFLWEMTSRHRPTFSFFCPPPSSIASSTLQSLPLLLTSAWHTLKAILGGFFLAITLSIVLAT
IMLSYKSAKDLLQPLFILLQCTPMFALAPLIVLWFGWGIGAVIVPTALTIFFPLTLTIYQGILSTPEELIEQFVLC

GATKFQLLIKLRIPHALPHIFSGLKIAIGSAGFAAIAGEWVASQSGLGILMLESRRNYEMELAFAGLATLSILTL
SLFQITLLIEKLIFSLFRVKRMSLKHKSVAKKALSVLALIPIMLIPWKGNSKSPPDKKNLTSLTLLLDWTPNPNH
IPLYAGVAKGYFKQHGLDLQLQKNTDSSSAVPHVLFEQVDMALYHALGIMKTSIKGMPIQIVGRLIDSSLQGF
LYRSQDPIYKFEDLNGKVLGFCLNNSRDLNRLLETLNRNGVVPSEVKNVSSDLISPMLLNKIDFLYGAFYNIE
GVKLQTLGMPVKCFLSDTCDLPTGPQLIVFTKKGTKASEPEIVEAFQKALQESIIFSKDHPEDAFKLYAKETKS
IPKNLYQEYLQWEETFPLLAQSQDPLSKDLVDKLLETIIKRYPELASEVAKFSLNDLYNPSLPEEQSV

>core/138/3/Org3_Gene665

MYTEESLDNLRHSIDIVDVLSEHIHLKRSGATYKACCPFHTEKTPSFIVNPAGAHYHCFGCGAHGDAIGFLMQ
HLGYSFTEAILVLSKKFQVDLVLQPKDSGYTPPQGLKEELRHINSEAETFFRYCLYHLPEARHALQYLYHRGF
SPDTIDRFHLGYGPEQSLFLQAMEERKISQEQLHTAGFFGNKWFLFARRIIFPVHDALGHTIGFSARKFLENSQ
GGKYVNTPETPIFKKSRILFGLNFSRRRIAKEKKVILVEGQADCLQMIDSGFNCTVAAQGTAFTEEHVKELSK
LGVLKVFLLFDSDEAGNKAALRVGDLCQTAQMSVFVCKLPQGHDPDSFLMQRGSSGLIALLEQSQDYLTFLI
SEKMSSYPKFGPREKALLVEEAIRQIKHWGSPILVYEHLKQLASLMMVPEDMVLSLANPQVTAEPQNIPIKQK
VPKIHPHIVMETDILRCMLFCGSNTKILYTAQFYFVPEDFKHPECRKLFAFMISYYEKYRKNVPFDEACQVLS
DSQILQLLTKRRLNTEALDTIFVQSLQKMADRRWREQCKPLSLNQNIQDKKLEILEDYVQLRKDRTIITLLDPE
SELIP

>core/139/3/Org3_Gene313

MSTSPISNDPRYLSLSNATEKTSLLANSRSLSPVPNSLVPSNPEDTGLRKSIFTHSVTLFAGLVVLLVAVSVVV
VALTVLAPGVPQAILLGIAISGVGIGGFSIMKSLVYMVRDYMSPRMQESSRIKSALAVGTGFTVMGLVMKVG
ANFVPGGYGGLVGSLGSSAYSRGSQTTLASFSHYIYTKFFRSEKVAKGEKLTEAETIKEAKKLHYITLSIATIG
VGLAVLGILLAIAGTVLLGGAPATIAIILAPPLISIGLTTVLQTILHSSIGKWRAFLLTQEKKDLFVDTSLKDIRLE
KLPPSEVEESETSQSVIEVPDSEGIAETRISAEEIDTRLSLTTRQKVIFALATLLLLASIAAFIVTGFGGLTVMQVL
LVASVGSAVASVTLPMVSSGFSYVAYQLKARLNISKLRWKEAKNKKRVRQFLIESGVIASDREFNQMWKTV
YKKQIQKTDAAIREEVRNFEKGGEVNSALVGGILLGVGTGIMLLALVPAFAPIVPGILALGGSTLGIAGSILMR
KFVNWLYDELVKLYERRRNRRELLYGPESKMRSIATDLVVEALAASHDHLFDLDGPVDFIDVDVDIDGAA

>core/140/3/Org3_Gene378

MTNSDNASAAGLLWAHPKEDPAFLGMIIKEFHLPPTVAQIFISRGFQTIQEIHKFLYSHLSSLYDPGLFLDMSK
AVERLLLARDRKEHVMIYGDSDVDGMTGVALLVEFLRDIDVHVSYFFLGAILKQHGETSTLIAKLKEEGITLL
ITVDCGITAGKEVSDITRQGIDVIITDHHMPTGKIPHCVATLNPKLRDHTYPNRELTGVGVAFKLARGVLNALI
SRNLVPKSQGSLKKLLDLVTLGTITDVGVLLGENRVMVRYGIKEIARGARPGLNKLCALCGVEKSEVTSTDIV
LKIAPKLNSLGRLDDPAKGVELLLTQDDERVDALIMELDNINRERQRIEAEVFQDVQEILNSNPEILKQAAIVL
SSTAWHARVIPIISARLAKTYNKPVVIIAIQRGIGKGSARTIGSFPLLGVLKKCSSLLLSYGGHDFAAGVIMKED
KVEDFKKKFVHLVNSSLKKGDTLPHLEIDAYADFDAIDYDLLASMELFEPFGKGNLMPIFYSKVRQVRYPKV
LPGNHLKLYLSQKERNLEGVAFGLGRHADALKASWHYPLEIAYTPRLSQTSGSGVIHLLVRDFRISSEPRFSD

>core/141/3/Org3_Gene219

MSFLRRHISLFRSQKQLIDVFAPVSPNLELAEIHRRVIEDQGPALLFHNVIGSSFPVLTNLFGTKHRVDQLFSQA
PDNLIARVAHLISSTPKLSSLWKSRDLLKRISSLGLKKARFRRFPFVSMSSVNLDHLPLLTSWPEDGGAFLTLP

LVYTESPTLTTPNLGMYRVQRFNQNTMGLHFQIQKGGGMHLYEAEQKKQNLPVSVFLSGNPFLTLSAIAPLP
ENVSELLFATFLQGAKLLYKKTNDHPHPLLYDAEFILVGESPAGKRRPEGPFGDHFGYYSLQHDFPEFHCHKI
YHRKDAIYPATVVGKPYQEDFYIGNKLQEYLSPLFPLVMPGVRRLKSYGESGFHALTAAVVKERYWRESLTT
ALRILGEGQLSLTKFLMVTDQEVPLDRFSVVLETILERLQPDRDLIIFSETANDTLDYTGPSLNKGSKGIFMGIG
KAIRDLPHGYQGGKIHGVQDIAPFCRGCLVLETSLEDRCIKSLLHHPDLKSWPLIILADNLRETIQSEKDFLWR
TFTRCAPANDLHALHSHFATHRPNYNFPFVIDALMKPSYPKEVEVDPSTKQKVSERWHAYFPNKETFYI

>core/142/3/Org3_Gene459

MKYRTHRCNELTSNHIGENVQLAGWVHRYRNHGGVVFIDLRDRFGITQIVCREDEQPELHQRLDAVRSEWV
LSVRGKVCPRLAGMENPNLATGHIEVEVASFEVLSKSQNLPFSIADDHINVNEELRLEYRYLDMRRGDIIEKL
LCRHQVMLACRNFMDAQGFTEIVTPVLGKSTPEGARDYLVPSRIYPGKFYALPQSPQLFKQLLMVGGLDRYF
QIATCFRDEDLRADRQPEFAQIDIEMSFGDTQDLLPIIEQLVATLFATQGIEIPLPLAKMTYQEAKDSYGTDKP
DLRFDLKLKDCRDYAKRSSFSIFLDQLAHGGTIKGFCVPGGATMSRKQLDGYTEFVKRYGAMGLVWIKNQE
GKVASNIAKFMDEEVFHELFAYFDAKDQDILLLIAAPESVANQSLDHLRRLIAKERELYSDNQYNFVWITDFP
LFSLEDGKIVAEHHPFTAPLEEDIPLLETDPLAVRSSSYDLVLNGYEIASGSQRIHNPDLQSQIFTILKISPESIQE
KFGFFIKALSFGTPPHLGIALGLDRLVMVLTAAESIREVIAFPKTQKASDLMMNAPSEIMSSQLKELSIKVAF

>core/143/3/Org3_Gene240

MSGKKDGVRGMIFVPLSILVLIFLPLPQILLDFGLCISFALSLLTVCWVFTLNSSNSAKLFPPFFLYLCLLRLGLN
LASTRWIVSSGTASSLIVSLGSFFSLGSLWAATFACLLLFFVNFLMVSKGSERIAEVRSRFFLEALPAKQMALD
SDLVSGRASYKAVKKQKNALIEEGDFFSAMEGVFRFVKGDAIISCILLLVNVVSVTCLYYTSGYALEQMWFT
VLGDALVSQVPALLTSCAAATLISKIDKEESLLNYLFEYYKQLRQHFRVVSLLIFSLCCIPSSPKFPIVLLASLL
WLAYRKEEPASEDSCIERAFSYVEGACPKEQESQFYQVYRAASEEVFEDLGVRLPVLTSLRIEERPWLRVFGQ
NVYLDEMTPEAVLPFLRNIAHEALNAEVVQKYLEESERVFGIAVEDIVPKKISLSSLVVLSRLLVRERVSLKLF
PKILEAVAVYQNSGDSLEILAEKVRKSLGYWIGRSLWDQKQTLEVITIDFHVEELINSSYSKSNPVMQENVIRR
VDSLLERSVFKDFRAIVTSCETRFEMKKMLDPHFPDLLVLSHDELPKEIPISFLGIVSDEVLVP

>core/144/3/Org3_Gene208

MPKQAEYTWGSKKILDNIECLTEDVAEFKDLLYTAHRITSSEEESDNEIQPGAILKGTVVDINKDFVVVDVGL
KSEGVIPMSEFIDSSEGLVLGAEVEVYLDQAEDEEGKVVLSREKATRQRQWEYILAHCEEGSIVKGQITRKVK
GGLIVDIGMEAFLPGSQIDNKKIKNLDDYVGKVCEFKILKINVERRNIVVSRRELLEAERISKKAELIEQISIGEY
RKGVVKNITDFGVFLDLDGIDGLLHITDMTWKRIRHPSEMVELNQELEVIILSVDKEKGRVALGLKQKEHNP
WEDIEKKYPPGKRVLGKIVKLLPYGAFIEIEEGIEGLIHISEMSWVKNIVDPSEVVNKGDEVEAIVLSIQKDEGK
ISLGLKQTERNPWDNIEEKYPIGLHVNAEIKNLTNYGAFVELEPGIEGLIHISDMSWIKKVSHPSELFKKGNSV
EAVILSVDKESKKITLGVKQLSSNPWNEIEAMFPAGTVISGVVTKITAFGAFVELQNGIEGLIHVSELSDKPFA
KIEDIISIGENVSAKVIKLDPDHKKVSLSVKEYLADNAYDQDSRTELDFKDSQGPKERKKKGK

>core/145/3/Org3_Gene573

MSTRRPIQLLDPLTINQIAAGEVIENSVSVVKELIENSLDAGADEIEIETLGGGQGAIIIRDNGCGFRAEDIPIALQ
RHATSKIREFSDIFSLNSFGFRGEALPSIASISKMEIQSSIEGDEGVRTVIHGGDIVSCEPCARQLGTTVIVNSLFY
NVPVRRGFQKSMQSDRLGIRKLIENRILSTANIGWSWISEGHHEIQIAKQQGFQERVAYVMGDHFMQDALTI

DKEANGVRIVGVLGSPSFHRPTRQGQKIFINDRPIESLFISKKVGDAYALLLPLHRYPVFVLKLYLPSSWCDFN
VHPQKIEARILKEELVGDCIKEAIVETLACPPGILCRTHQEIEESDSVPLPMFRMLETSDVQEEESVEFDQNLFA
YSSEDVSLEKQEYTSRGPKSQMDWIYSSDVRFLTSLGRVVLAEDLEGVHIIFTAAARKHLFFLSLMQENSRM
YQSQALLIPLRLQVTPEEAFFFSHHGRTLCDLGIEISQVGPCVFSIESTPTVIGEEELKEWLLLLAARGSTDINSE
ALTALMKETLTQATFSKHQHVFDVSWLKLLWSVGKPEKGFDGARIRRLILDSDFMEG

>core/146/3/Org3_Gene222

MAVSGGGGVQPSSDPGKWNPALQGEQAEGPSPLKESIFSETKQASSAAKQESLVRSGSTGMYATESQINKAK
YRKAQDRSSTSPKSKLKGTFSKMRASVQGFMSGFGSRASRVSAKRASDSGEGTSLLPTEMDVALKKGNRISP
EMQGFFLDASGMGGSSSDISQLSLEALKSSAFSGARSLSLSSSESSSVASFGSFQKAIEPMSEEKVNAWTVARL
GGEMVSSLLDPNVETSSLVRRAMATGNEGMIDLSDLGQEEVSTAMTSPRAVEGKVKVSSSDSPEANPTGIPN
SNTLERAEKEAEKQESREQLSEDQMMLARAMAGLLTGAAPQEVLSNSVWSGPSTVFPPPKFSGTLPTQRSGD
KSKHKSPGIEKSTNHTNFSPLREGTVKSAEVKSLPHPESMYRFPKDSIVSREEPEAVVKESTAFKNPENSSQNF
LPIAVESVFPKESGTGGALGSDAVSSSYHFLAQRGVSLLAPLPRATDDYKEKLEAHKGPGGPPDPLIYQYRNV
AVEPPIVLRSPQPFSGSSRLSVQGKPEAASVHDDGGGGNSGGFSGDQRRGSSGQKASRQEKKGKKLSTDI

>core/147/3/Org3_Gene406

MQKHPSFYQRFLSAYYKNLLASLSWKFFISVALIGIYAPLFASSKPLLVTWHGEIFFPLLRYLFFPGYYTKPVD
LFFNVLMVTFPFFILSFKLTRGWLRRWLLGLCIISQCMIFAWAYSGKVQDPALAENLKKMRAEKVRENISKV
NSEMVMLLPKDTRTWEMERRYMSTYEQLGILIKAKYRKKQEASVKKYQVAFEEKRQSPMPTLRHLEMKNE
GICLKRLQQRVDKMQRPYEMAQQAWNRATDNYRPFLMALTRIEHELRLADYNNWGQPEDLCIAYANVEKR
AEPYKKSLLEIRQVLEDYAKLRSAISFIQDKRLWIEKESEDLRILINPFFSSFHWEDDAGGSREMNKYVPWWQ
LSRVTRKDLLAALVFGIRIALVVAGIGITIALAIGIMIGLVSGYFGGTVDMILSRFTEIWETMPVLFILMLVISIT
QQKSLLLNTVLLGCFSWTGFSRYVRIEVLKQRDRGYVLAATNLGYSHYYIMVHQILPNAIVPVISLVPFAMM
AMISCEAGLTFLGLGEESSASWGNLMREGVTGFPAESAVLWPPAIILTMLLIAIALIGDGVRDALDPRLQDS

>core/148/3/Org3_Gene875

MTDFPTHFKGPKLNPIKVNPNFFERNPKVARVLQITAVVLGIIALLSGIVLIIGTPLGAPISMILGGCLLASGGAL
FVGGTIATILQARNSYKKAVNQKKLSEPLMERPELKALDYSLDLKEVWDLHHSVVKHLKKLDLNLSETQRE
VLNQIKIDDEGPSLGECAAMISENYDACLKMLAYREELLKEQTQYQETRFNQNLTHRNKVLLSILSRITDNIS
KAGGVFSLKFSTLSSRMSRIHTTTTVILALSAVVSVMVVAALIPGGILALPILLAVAISAGVIVTGLSYLVRQIL
SNTKRNRQDFYKDFVKNVDIELLNQTVTLQRFLFEMLKGVLKEEEEVSLEGQDWYTQYITNAPIEKRLIEEIR
VTYKEIDAQTKKMKTDLEFLENEVRSGRLSVASPSEDPSETPIFTQGKEFAKLRRQTSQNISTIYGPDNENIDPE
FSLPWMPKKEEEIDHSLEPVTKLEPGSREELLLVEGVNPTLRELNMRIALLQQQLSSVRKWRHPRGEHYGNVI
YSDTELDRIQMLEGAFYNHLREAQEEITQSLGDLVDIQNRILGIIVEGDSDSRTEEEPQE

>core/149/3/Org3_Gene34

MANPTQSRPPSPEISIEELELQELAGSSNTETISNTPPPSCAATAEEVSLFIEGGRRNSEDEEGPLGSCEVYDVVC
ITNQGDPEVRDHEVRVMYINGSGRTQHEGILDAMNICDLRGEPVRFIHNSGYGLGSCFLGIRNRIPPRDNVISQ
AIQARWNEFFIFAENANRDYIVLFSGNGGLYLQVALDNSIYSHHILCVGIGSSYYIQGNYRVHNYRVTGDWTT
LLDRRGATAVNTTTLPYADSAEGLFLPSVRCPSYQWALRCGEQCLIMDNNQQVGFRPQDSSSEIALVVNLNQ

DHSTWTRLIEWIDRGDSQAVLELNPQPSHCRDIALTALYATTRISSLLQECLMISVTYAPEVFVTYAIVTGYSI
MTLRYFILLLTNRPGCRRHFRVLRLAALGLQSLGFLTVLLDHINVTRRVNRRPPLISVIFCTASFATGSFIYVDL
TRMFFTSLRSRLQLFVQRRLTGRGLPLRRVFVNHLDSLRFSQNALITFHGGLFMPLIIGFFNQLVIQVPRVVIRP
NTTAVYDLNQTSQEAWDSGDVLAIGQTINFLLCMILLVINTFFFVRSVRRNLHRRPHR

>core/150/3/Org3_Gene531

MFMNTQNSQATEVSSEEESQKKLEELVALAKEQGFITYEEINEILPMSFDTPEQIDQVLIFLTGMDIQVLNQID
VERQKEKKKEAKELEGLARRTEGTPDDPVRMYLKEMGTVPLLTREEEVEISKRIEKAQVQIERIILRFRYSAK
EAISIAHYLISGKERFDKIISEKEVEDKTHFLKLLPKLITLLKEEDTYLENLLLSLKQPDLSKQEAAKLNDSLEK
CRIRTQAYLRCFHCRHNVTEDFGEVVFKAYDSFLHLEQQINDLKVRAERNKFAAAKLAAAKRKLYKREVAA
GRTLEEFKKDVRMLQRWMDKSQEAKKEMVESNLRLVISIAKKYTNRGLSFLDLIQEGNMGLMKAVEKFEY
RRGYKFSTYATWWIRQAVTRAIADQARTIRIPVHMIETINKVLRGAKKLMMETGKEPTPEELAEELGLTPDR
VREIYKIAQHPISLQAEVGEGSESSFGDFLEDTAVESPAEATGYSMLKDKMKEVLKTLTDRERFVLIHRFGLL
DGKPKTLEEVGSAFNVTRERIRQIEAKALRKMRHPIRSKQLRAFLDLLEEEKTGTSKVKSLKSK

>core/151/3/Org3_Gene826

MDTQSSIGNEEWRIAGTSVVSGMALGKVFFLGTSPLHVRELTLPQEEVEHEIHRYYKALNRSKSDIVALEQEV
TGQQGLQEVSSILQAHLEIMKDPLLTEEVVNTIRKDRKNAEYVFSSVMGKIEESLTAVRGMPSVVDRVQDIH
DISNRVIGHLCCQHKSSLGESDQNLIIFSEELTPSEVASANSAYIRGFVSLVGAATSHTAIVSRAKSIPYLANISE
ELWNIAKRYNGKLVLIDGYRGELIFNPKPATLQSCYKKELSVVAHTSQRLVRKSLHPIVSSHAGSDKDVEDLL
ENFPQTSIGLFRSEFLAVILGRLPTLREQVDLYEKLARFPGDSPSVLRLFDFGEDKPCPGIKNKKERSIRWLLDY
SVILEDQLQAIAKASLQGSIKVLIPGVSDVSEIIEVKKKWETIQTRFPKGHKVSWGTMIEFPSAVWMIEEILPEC
DFLSIGTNDLVQYTLGISRESALPKHLNVTLPPAVIRMIHHVLQAAKQNQVPVSICGEAAGQLSLTPLFIGLGV
QELSVAMPVINRLRNHIALLELNSCLEITEALLQAKTCSEVEELLNRNNKITS

>core/152/3/Org3_Gene1021

MVKVPWAFKNFIPKLYTSIKEGYSFNTFKKDFQAGITVGILAFPFAIAIAIGVGVSPIQGLLASIIGGLLASAMG
GSNVLISGPSSAFISILYCLSAKYGAEALFTVTLLAGVFLIAFGLTGLGTFIKYMPYPVVTGLTTGLAIIIFSSQIK
DFLGLQMGANIPADFLPKWIAYWDHLWTWDSKSFAVGLFTLLIMIYFRNYKPRYPGVMIAIVTATTLVWLL
KIDIPTIGSRYGTLPTAIPLPKIPQLSITKILQLMPDALTIAVLSGLETLLSAVVADGMTGWRHQSNCQLVAQGV
ANIGTSLFSGIPVTGSLSRTAASIKSEATTPIAGIVHSIFICFILLLLAPLTVKIPLTCLAAVLILIAWNMSEIHHWI
HLLTAPKKDIVVLLTVFILTVMTTITAAVQVGMMLAAFLFMKQMSDLSDVISTAKYFDKDSDFLSKAEVPQN
TEIYEINGPFFFGIADRLKNLLNDIEKPPKIFILCMTRVPTIDASAMHALEEFFLECDRQGTLLLLAGVKKTPLA
DLKRYHLDELIGVDHIFSNIKSALLFAQALTNLESKTSTRHLV

>core/153/3/Org3_Gene8

MGLFHLTLFGLLLCSLPISLVAKFPESVGHKILYISTQSTQQALATYLEALDAYGDHDFFVLRKIGEDYLKQSI
HSSDPQTRKSTIIGAGLAGSSEALDVLSQAMETADPLQQLLVLSAVSGHLGKTSDDLLFKALASPYPVIRLEA
AYRLANLKNTKVIDHLHSFIHKLPEEIQCLSAAIFLRLETEESDAYIRDLLAAKKSAIRSATALQIGEYQQKRFL
PTLRNLLTSASPQDQEAILYALGKLKDGQSYYNIKKQLQKPDVDVTLAAAQALIALGKEEDALPVIKKQALE
ERPRALYALRHLPSEIGIPIALPIFLKTKNSEAKLNVALALLELGCDTPKLLEYITERLVQPHYNETLALSFSKG

RTLQNWKRVNIIVPQDPQERERLLSTTRGLEEQILTFLFRLPKEAYLPCIYKLLASQKTQLATTAISFLSHTSHQ
EALDLLFQAAKLPGEPIIRAYADLAIYNLTKDPEKKRSLHDYAKKLIQETLLFVDTENQRPHPSMPYLRYQVT
PESRTKLMLDILETLATSKSSEDIRLLIQLMTEGDAKNFPVLAGLLIKIVE

>core/154/3/Org3_Gene916

MKTSQLFYKTSKNANKSAAVLSNELLEKAGYLFKVSKGVYTYTPLLWRVVSKMMNIIREELNAIGGQELLLP
LLHNAELWQHTGRWEAFTSEGLLYTLKDREGKSHCLAPTHEEVICSFVAQWLSSKRQLPLHLYQIATKFRDE
IRPRFGLIRSRELLMEDSYTFSDSPEQMNEQYEKLRSAYSKIFDRLGLAYVIVTADGGKIGKGKSEEFQVLCSL
GEDTICVSGSYGANIEAAVSIPPQHAYDREFLPVEEVATPGITTIEALANFFSIPLHKILKTLVVKLSYSNEEKFI
AIGMRGDRQVNLVKVASKLNADDIALASDEEIERVLGTEKGFIGPLNCPIDFFADETTSPMTNFVCAGNAKD
KHYVNVNWDRDLLPPQYGDFLLAEEGDTCPENPGHPYRIYQGIEVAHIFNLGTRYTDSFEVNFLDEHGQTQQ
CWMGTYGIGVGRTLAACVEQLPDDRGIVWPKALAPFSITIAFNGGDTVSQELAETIYHELQSQGYEPLLDDR
DERLGFKLKDSDLIGIPYKLILGKSYQSSGIFEIESRSGEKYTVSPEAFPTWCQNHLA

>core/155/3/Org3_Gene518

MTVSVKKKSFRALVTTHFLTIINDNLYKFLLAFFLLEGKTLTENAKILSCVSFFFALPFLLLAPLAGSLADRFQ
KRNIILATRFIEILCTILGTYFFFIQSVVGGYVVLILMACHTTIFGPAKLGILPEMLPSEQLSQANGIMTAATYTG
SILGSCLAPLLVDVTHRLGVNSYVWPTLMCVIVSIISTLISFCIRPSNVKNVKQKITLVSFKDLWKVLKDTRMI
HYLTVSIFLGSFFLLIGAYTQLEIIPFVEFTLKYPKHYGAYLFPIVALGVGTGSYITGKISGKDIKIGYVPLAAIGL
ALVFMGLYAFACSILFVLFFLLALGFLGGVYQVPLHAYVQYASPEHKRGQILAANNFLDFFGVLVAAGVIRV
LGSNLGLSPETSFFYIGWFVLAVSIWTLWIWREHVYRLLLGIILRRQLGYYLKIHQSSSPKCYFVAVQSYREIR
RVLAALTKTVRSRVIILDQKLVPGWRAWLLSWCVPTVVSSVRDNDSEAQDAWAVLQANHLKTSLKKFPDV
SVVCLGLPKNVERFTSILQEQGIDLHPIQLVQKEGKKRVIYTLVFPHA

>core/156/3/Org3_Gene385

MSTLLSILSVICSQAIAKAFPNLEDWAPEITPSTKEHFGHYQCNDAMKLARVLKKAPRAIAEAIVAELPQEPFS
LIEIAGAGFINFTFSPVFLNQQLEHFKDALKLGFQVSQPKKIIIDFSSPNIAKDMHVGHLRSTIIGDSLARIFSYVG
HDVLRLNHIGDWGTAFGMLITYLQENPCDYSDLEDLTSLYKKAYVCFTNDEEFKKRSQQNVVALQAKDPQA
IAIWEKICETSEKAFQKIYDILDIVVEKRGESFYNPFLPEIIEDLEKKGLLTVSNDAKCVFHEAFSIPFMVQKSDG
GYNYATTDLAAMRYRIEEDHADKIIIVTDLGQSLHFQLLEATAIAAGYLQPGIFSHVGFGLVLDPQGKKLKTR
SGENVKLRELLDTAIEKAEEALREHRPELTDEAIQERAPVIGINAIKYSDLSSHRTSDYVFSFEKMLRFEGNTA
MFLLYAYVRIQGIKRRLGISQLSLEGPPEIQEPAEELLALTLLRFPEALESTIKELCPHFLTDYLYNLTHKFNGFF
RDSHIQDSPYAKSRLFLCALAEQVLATGMHLLGLKTLERL

>core/157/3/Org3_Gene373

MSKLIRRVVTVLALTSMASCFASGGIEAAVAESLITKIVASAETKPAPVPMTAKKVRLVRRNKQPVEQKSRG
AFCDKEFYPCEEGRCQPVEAQQESCYGRLYSVKVNDDCNVEICQSVPEYATVGSPYPIEILAIGKKDCVDVVI
TQQLPCEAEFVSSDPETTPTSDGKLVWKIDRLGAGDKCKITVWVKPLKEGCCFTAATVCACPELRSYTKCGQ
PAICIKQEGPDCACLRCPVCYKIEVVNTGSAIARNVTVDNPVPDGYSHASGQRVLSFNLGDMRPGDKKVFTV
EFCPQRRGQITNVATVTYCGGHKCSANVTTVVNEPCVQVNISGADWSYVCKPVEYSISVSNPGDLVLHDVVI
QDTLPSGVTVLEAPGGEICCNKVVWRIKEMCPGETLQFKLVVKAQVPGRFTNQVAVTSESNCGTCTSCAETT

THWKGLAATHMCVLDTNDPICVGENTVYRICVTNRGSAEDTNVSLILKFSKELQPIASSGPTKGTISGNTVVF
DALPKLGSKESVEFSVTLKGIAPGDARGEAILSSDTLTSPVSDTENTHVY

>core/158/3/Org3_Gene808

MELLSLNKSYFEIQRLRYRPEILTLLETIRSKHIQETSSPPSPPPELQKHIPNLCRIPEVSIYTEQETSSKPLKIGVL
LSGGQAPGGHNVVIGLFDALRVFNPKTRLFGFIKGPLGLTRGLYKDLDISVIYDYYNMGGFDMLSSSREKIKT
EEQKKNILNTVKQLKLDGLLIIGGNNSNTDTAMLAEYFLAHNCKTSVIGVPKTIDGDLKNCWIETSLGFHTSC
RTYSEMIGNLAKDALSAKKYHHFIRLMGQQASYTTLECGLQTLPNIALISELIATRKISLKQLSEQLALGLVRR
YKSGKNYSTVLIPEGLIEHIFDTRKLIDELNVLLANGDSSIEKILSKLSPETLKTFHLFPKDIANQLLLARDSHGN
VRVSKIATEELLAVMVKKEIEKIKPHMEFHSVSHFFGYEARAGFPSNFDCNYGIALGIISALFLVRQKTGYMIT
INNLAQSYTEWQGGATPLYKMMHLENRCGTETPVIKTDSVDPKSPAVQHLLQQSDSCLVEDLYRFPGPLQYF
GKEELIDQRPLTLLWENQTHSPLL

>core/159/3/Org3_Gene749

MSSPPQAVASLTERIKTLLESNFCQIIVKGELSNVSLQPSGHLYFGIKDSQAFLNGAFFHFKSKYYDRKPKDGD
AVIIHGKLAVYAPRGQYQIVAHALVYAGEGDLLQKFEETKRRLTAEGYFATEKKKPLPFAPQCIGVITSPTGA
VIQDILRVLSRRARNYKILVYPVTVQGNSAAHEISKAIEVMNAENLADVLIIARGGGSIEDLWAFNEEILVKAI
HASTIPIVSAVGHETDYTLCDFASDVRAPTPSAAAEIVCKSSEEQVQVFEGYLRHLLSHSRQLLTSKKQQLLP
WRRFLDRAEFYTTAQQQLDSIEIAIQKGVQGKIHESKQRYDNISRWLQGDLVSRMTCRLQSLKKMLSQALSH
KALSLQVRCHQLKKSLTYPRQIQQASQKLSPWRQQLDTLISRRLHYQKEEYFHKHTRLKHAHNVLEQQLRS
HVQKLELLGRRLSRGCELNLQNQKIAYANVKETLATILERRYENSVARYSALKEQLHSLNPKNVLKRGYAM
LFDFNENSAMISVDSLQENARVRIQLQDGEAILTVTNIEICKLIKG

>core/160/3/Org3_Gene845

MDNSDNSFHTLETEQGSFLNDELAVEEVAYTESTEISDATLCFADEIQELPSPEKKVAFILNKMREALTGSSQG
SDLRLFWDLRKQCLPLFNEIEDTAKRADHWRCYIELTKEGRHLKGLQDEEGSFVVGQIDLAITCLEKDILKFQ
EGTEDKIFKDREDNFLESQALDKHQAFYKQHHTSLLWLSSFSSKIIDLRKELINVGMRMRLKSKFFQRLSNLG
NQVFPKRKELIEKVSQTFAEDVDAFVAKYFIGSDKETLKKTVFFLRKEIKNLQHAAKRLFVSSHVFAETRLKL
SKCWDQLKGMEKEIRQEQGRLRVVSAENSKEVRQMLAEVSSLLIEGNDLSKVRKDLEGISKKIRALDLTHDD
VISLKKEMQQLFDQLREKQDAAEHSYQEQLAKDKQVKKEAARSLAERITTFSKTCSEGNITSESREEWQTLK
ELLGKMSFLPPPEKISLDNQLNLALQTIVNFFEEQLLSSPDSREKLVNMRQVLKQRRERRQELKDKLEQDKKL
LGSSGLDFDRAMQYSALVEEDKRALEELDASILELKQQIQQLL

>core/161/3/Org3_Gene816

MPQKVLITSALPYANGPLHFGHIAGVYLPADVYARFRRLLGDDVLYICGSDEFGIAITLNADREGLGYQEYV
DMYHKLHKDTFEKLGFALDFFSRTTNPFHAELVQDFYSQLKASGLIENRISEQLYSEQEQRFLADRYVEGTCP
RCGFDHARGDECQSCGADYEAIDLIDPKSKISGVELVKKETEHSYFLLDRMKDALLSFIQGCYLPDHVRKFV
VDYIEHVRSRAITRDLSWGIPVPDFPGKVFYVWFDAPIGYISGTMEWAASQGNPDEWKRFWLEDGVEYVQFI
GKDNLPFHSVVFPAMELGQKLDYKKVDALVVSEFYLLEGRQFSKSEGNYVDMDKFLSSYSLDKLRYVLAAT
APETSDSEFTFLDFKTRCNSELVGKFGNFINRVLAFAEKNHYDKLSYHSVVLEDSDRAFLEEVRQLVRDAEK

CYREYSLRKATSVIMSLAALGNVYFNQQAPWKLLKEGTRERVEAILFCACYCQKLLALISYPIIPESAVAIWE
MISPKSLENCNLDTMYARDLWKEEILDVINEEFHLKSPRLLFTTVE

>core/162/3/Org3_Gene127

MHPLYVDLDTIISSYSPPLPKEFQEAASLIAVPDTSHSKPVVPGVKTLFPQTYHLPYLKFVQGENVVHTPLKVG
VMFSGGPAPGGHNVIQGLFNSLKDFHPDSSLVGFVNNGDGLTNNKSIDITEEFLSKFRNSGGFNCIGTGRKKIV
TPEAKEACLKTAEALDLDGLVIIGGDGSNTATAILAEYFAKRRPKTSIVGVPKTIDGDLQHTFLDLTFGFDTAT
KFYSSIISNISRDALSCKAHYHFIKLMGRSASHIALECALQTHPNIALIGEEIAEKNLPLKTIIHKICSVIADRAAM
EKYYGVILIPEGIIEFIPEIINLITEIESLSEYEDKISRLSPESQRLLKSFPAPIIEQILNDRDAHGNVYVSKISVDKLL
IHLVSNHLQQYFPNVPFNAISHFLGYEGRSGLPTKFDNTYGYSLGYGAGILVRNHCNGYLSTIESLACPFMKW
KLRAIPVVKMFTVKQQADGTLQPKIKKYLVDIGSTAFRKFKLYRKIWALEDSYRFLGPLQIETPPEMHSDNFP
PLTLLLNHNFWQRHQGCIEIPDTTY

>core/163/3/Org3_Gene513

MSRKDNEVSLARSIFNILSGTFCSRITGIFREIAMATYFGADPIVAAFWLGFRTVFFLRKILGGLILEQAFIPHFE
FLRAQSLDRAAFFFRRFSRLIKGSTIIFTLLIEAVLWVVLQYVEEGTYDMILLTMILLPCGIFLMMYNVNGALL
HCENKFFGVGLAPVVVNIIWIFFVIAARHSDPRERIIGLSVALVIGFFFEWLITVPGVWKFLLEAKSPPQEHDSV
RALLAPLSLGILTSSIFQLNLLSDICLARYVHEIGPLYLMYSLKIYQLPIHLFGFGVFTVLLPAISRCVQREDHER
GLKLMKFVLTLTMSVMIIMTAGLLLLALPGVRVLYEHGLFPQSAVYAIVRVLRGYGASIIPMALAPLVSVLFY
AQRQYAVPLFIGIGTALANIVLSLVLGRWVLKDVSGISYATSITAWVQLYFLWYYSSKRLPMYSKLLWESIRR
SIKVMGTTMLACMITLGLNILTQTTYVIFLNPLTPLAWPLSSITAQAIAFLSESCIFLAFLFGFAKLLRVEDLINL
ASFEYWRGQRGLLQRQHVMQDTQN

>core/164/3/Org3_Gene79

MAAKNIKYNEEARKKIHKGVKTLAEAVKVTLGPKGRHVVIDKSFGSPQVTKDGVTVAKEIELEDKHENMGA
QMVKEVASKTADKAGDGTTTATVLAEAIYSEGLRNVTAGANPMDLKRGIDKAVKVVVDELKKISKPVQHH
KEIAQVATISANNDSEIGNLIAEAMEKVGKNGSITVEEAKGFETVLDVVEGMNFNRGYLSSYFSTNPETQECV
LEDALILIYDKKISGIKDFLPVLQQVAESGRPLLIIAEEIEGEALATLVVNRLRAGFRVCAVKAPGFGDRRKAM
LEDIAILTGGQLVSEELGMKLENTTLAMLGKAKKVIVTKEDTTIVEGLGNKPDIQARCDNIKKQIEDSTSDYD
KEKLQERLAKLSGGVAVIRVGAATEIEMKEKKDRVDDAQHATIAAVEEGILPGGGTALVRCIPTLEAFLPML
ANEDEAIGTRIILKALTAPLKQIASNAGKEGAIICQQVLARSANEGYDALRDAYTDMIDAGILDPTKVTRSALE
SAASIAGLLLTTEALIADIPEEKSSSAPAMPSAGMDY

>core/165/3/Org3_Gene322

MFGSESLRYQLLIQDFAKVSEEGIGLLESKEYSLLQAKLVLRALAQNSSFDDWFRSFKKCQISYPELAHDRDV
LEEFGIQVLREGIENPSVTVRAVSVLAIGLARDFRLVPLLLQSCNDDSAIVRSLALQVAVNYGSESLKKAIVEL
ARNDDSIHVRITAYQVVALLQIEELLPFLRERAENKLVDSVERREAWKACLELSSQFLETGVAKDDIDQALFT
CEVLRNGMLPETTEIFTELLSVEHPEVQESLLLSALAWSHQLQNHKEFLSKVRHVMCTSPFAKVRFQAAALL
HLHGDPLGRDSLVEGLRSPQPLVCEAASAALCSLGIHGVPLAKEHLESLSSRKAAANLSILLLVSREDIERAGD
VIARYLSNPEMCWAIEYFLWDAQWNLRGDTFPLYSDMIKREIGRKLIRLLAVARYSQAKAVTATFLSGQQA

QGWSFFSGMFWEEGDVKTSEDLVTDACFAAKLEGALASLCQKKDQASLQRVSQLYNDSRWQDKLAILESV
AFSENLDAVPFLLDCCHHEAPSLRSAAAGALFSIFK

>core/166/3/Org3_Gene451

MLRIFCFVISWCLIAFAQPDLSGFVSILGAACGYGFFWYSLEPLKKPSLPLRTLFVSCFFWIFTIEGIHFSWMLS
DQYIGKLIYLVWLTLITILSVLFSGFSCLLVAIVRQKRTAFLWSLPGVWVAIEMLRFYGIFSGMSFDYLGWPM
TASAYGRQFGGFLGWAGQSFAVIAVNMSFYCLLLKKPHAKMLWVLTLLLPYTFGAIHYEYLKHAFQQDKR
ALRVAVVQPAHPPIRPKLKSPIVVWEQLLQLVSPIQQPIDLLIFPEVVVPFGKHRQVYPYESCAHLLSSFAPLPE
GKAFLSNSDCATALSQHFQCPVIIGLERWVKKENVLYWYNSAEVISHKGISVGYDKRILVPGGEYIPGGKFGS
LICRQLFPKYALGCKRLPGRRSGVVQVRGLPRIGITICYEETFGYRLQSYKRQGAELLVNLTNDGWYPESRLP
KVHFLHGMLRNQEFGMPCVRACQTGVTAAVDSLGRILKILPYDTRETKAPSGVLETSLPLFNYKTLYGYCGD
YPMILIAFCAVSYLGGGFLGYRLLAKKEIR

>core/167/3/Org3_Gene418

MQSSEVKPFSRLRAYLCPIYKSEFSKFVPLFLLAFFVGFNYCLLKNMKDTLVIVGSDAGAEVIPFLKVWGIVP
GAVIVTMVYGWLGSRYPRDTVFYCFMAAFLGFFFLFAVIIYPVGDSLHLNSLADKLQELLPQGLRGFIVMVR
YWSYSIYYVMSELWSSVVLSMLFWGLANQITTITEAGRFYALINTGLNLSSICAGEISYWMGKQTFVAYSFA
CDSWHSVMLNLTMLITCSGLIMIWLYRRIHHLTIDTSIPPSRRVLAEEGAATANLKEKKKPKAKARNLFLHLI
QSRYLLGLAIIVLSYNLVIHLFEVVWKDQVSQIYSSHVEFNGYMSRITTLIGVVSVLAAVLLTGQCIRKWGWT
VGALVTPLVMLVSGLLFFGTIFAAKRDISIFGGVLGMTPLALAAWTGGMQNVLSRGTKFTFFDQTKEMAFIP
LSPEDKNHGKAAIDGVVSRIGKSGGSLIYQGLLVIFSSVAASLNVIALVLLIIMVVWIAVVAYIGKEYYSRAAD
AVATLKQPKEPSSSIVREAQESVEQEEMAVL

>core/168/3/Org3_Gene648

MHDQRNRGHNNHNLRLRPGSTLLEAFLILCSEHEEGIACFDEHLGSLSYRELRNAIIAVAIKVSKFSEDRVGV
MMPASIGAFIAYFGILLAGKTPVMMNWSQGLRELRACTKTVEVRRVLTSQQFIKHLTEVQGFVEYPFDLMY
MEDVRKRLSWWEKCRIGLYSKCSVPWLLRIFGVSGVESDDTAVILFTSGTEKLPKAVPLTHKNLMENQEACL
KFFDPNTQDVMLAFLPPFHAYGFNSCGLFPLLMGVHVVFASNPLNPKKLVEFIDDKKVTFFGSTPVFFDYILK
TAKKQNSCLESLRLVVIGGDALKDTLYEETKKLQPQIALYQGYGATECSPVISITTKESPRKSECVGMPIEGM
DVLIISKETHIPVSSGEQGLIVVRGNSVFSGYLGNHEHQSFVSLGGDQWYLTGDLGHIGPSGDLFLEGRLSRFV
KIGGEMVSLEALESILHEHFTENQNEDAGSLVVCGIPGDKVRLCLFTTLATTIHEVNDILKSAETSSIVKISYVH
QVESIPILGIGKPDYVSLNALAVSLFG

>core/169/3/Org3_Gene801

MPFKCIFLTGGVVSSLGKGLTAASLALILERQRLNVAMLKLDPYLNVDPGTMNPFEHGEIYVTDDGVETDLD
LGHYHRFSSAALSRHSSATSGQIYARVIKREREGDYLGSTVQVIPHITNEIIQVILDAAKEHSPDVLIVEIGGTIG
DIESLPFLEAIRQFRYDHSEDCLNIHMTYVPYLQAADEVKSKPTQHSVQTLRGIGIIPDAILCRSEKPLTQEVKS
KISLFCNVPNRAVFNVIDVKHTIYEMPLMLAQEKIANFIGEKLKLATVPENLDDWRVLVNQLSQDLPKVKIG
VVGKYVQHRDAYKSIFEALTHAALRLGHAAEIIPIDAEDENLTMELSQCDACLVPGGFGVRGWEGKIAAAKF
CREQGIPYFGICLGMQVLVVEYARNVLNLDQANSLEMDPNTPHPIVYVMEGQDPLVATGGTMRLGAYPCLL

KPGSKAHKAYNESSLIQERHRHRYEVNPDYIQSLEDHGLRIVGTCPPQGLCEIIEVSDHPWMIGVQFHPEFVSK
LISPHPLFIAFIEAALVYSKDASHV

>core/171/3/Org3_Gene119

MRKISVGICITILLSLSVVLQGCKESSHSSTSRGELAINIRDEPRSLDPRQVRLLSEISLVKHIYEGLVQENNLSG
NIEPALAEDYSLSSDGLTYTFKLKSAFWSNGDPLTAEDFIESWKQVATQEVSGIYAFALNPIKNVRKIQEGHLS
IDHFGVHSPNESTLVVTLESPTSHFLKLLALPVFFPVHKSQRTLQSKSLPIASGAFYPKNIKQKQWIKLSKNPH
YYNQSQVETKTITIHFIPDANTAAKLFNQGKLNWQGPPWGERIPQETLSNLQSKGHLHSFDVAGTSWLTFNIN
KFPLNNMKLREALASALDKEALVSTIFLGRAKTADHLLPTNIHSYPEHQKQEMAQRQAYAKKLFKEALEELQ
ITAKDLEHLNLIFPVSSSASSLLVQLIREQWKESLGFAIPIVGKEFALLQADLSSGNFSLATGGWFADFADPMA
FLTIFAYPSGVPPYAINHKDFLEILQNIEQEQDHQKRSELVSQASLYLETFHIIEPIYHDAFQFAMNKKLSNLGV
SPTGVVDFRYAKEN

>core/172/3/Org3_Gene524

MLGKEEEFTCKQKQCLSHFVTNLTSDVFALKNLPEVVKGALFSKYSRSVLGLRALLLKEFLSNEEDGDVCDE
AYDFETDVQKAADFYQRVLDNFGDDSVGELGGAHLAMENVSILAAKVLEDARIGGSPLEKSTRYVYFDQKV
RGEYLYYRDPILMTSAFKDMFLGTCDFLFDTYSALIPQVRAYFEKLYPKDSKTPASAYATSLRAKVLDCIRGL
LPAATLTNLGFFGNGRFWQNLIHKLQGHNLAELRRLGDESLTELMKVIPSFVSRAEPHHHHHQAMMQYRRA
LKEQLKGLAEQATFSEEMSSSPSVQLVYGDPDGIYKVAAGFLFPYSNRSLTDLIDYCKKMPHEDLVQILESSV
SARENRRHKSPRGLECVEFGFDILADFGAYRDLQRHRTLTQERQLLSTHHGYNFPVELLDTPMEKSYREAME
RANETYNEIVQEFPEEAQYMVPMAYNIRWFFHVNARALQWICELRSQPQGHQNYRTIATGLVREVVKFNPM
YELFFKFVDYSDIDLGRLNQEMRKEPTT

>core/173/3/Org3_Gene811

MFVGGLVSFLLPIPDLECANNVTKTYDKKASVISRDLKLQEDCQKFWNLDPYKLESLCAYQVLYHDDYSSK
RIRELFPQIQKDEVPIFATMILTLGKVDRGFSPEEISLIQKLSYPGLSLASLRGSTEIDPNTDLARALVVSEFSGD
LGKNRADYYSNCLDILALRIHAERQRYLDQSPCVPGTSEFHKATIEAINTILFYEEAVRYPSKKEMFSDEFSFL
SSVTDRKFGVCLGVSSLYFSLSQRLDLPLEAVTPPGHIYLRYQGGEVNIETTAGGRHLPTASYCDCLDLEDLQ
VRTPEEMIGLTFMNQGSFALQKKKYKEAEEAYKKAQEYLGDEELQELLGFVQILGGKKKEGKSLIGKSPRAS
QKGSVAYDYLKGRINIPTLALLFSYPGSNYEEIASYEEELKKAMKSSMPCCEGQRRLASVAFHLGKTAEAVA
LLEKCVEDIPNDLSLHLRLCKILCDRHEYTKALKYFIIAERLMEDQGFLKKDNRSFALFYEVKKIISKVAPQKA
NTLLLMESER

>core/174/3/Org3_Gene10

MSIVLDKIGKSLGTRILFDDVSVVFNPGNCYGLTGPNGAGKSTLLKIIMGMIEPTRGSISLPKKVGILRQNIDSF
HDTTVLDCVIMGNTRLWEALQRRDNLYLQEFTDAIGMELGEIEEIIGEENGYRADSEAEELLTGIGIPNEMFD
KKMAMIPIDLQFRVLLCQALFGHPEALLLDEPTNHLDLYSINWLGNFLKDYEGTVIVVSHDRHFLNTITTHIA
DIDYDTIIIYPGNYDDMVEMKTASREQEKADIKSKEKKISQLKEFVAKFGAGSRASQVQSRLREIKKLQPQEL
KKSNIQRPYIRFPLSDKSSGKVVLSLEAITKDYGDHQVIHPFSLEIYQGDKLGIIGNNGLGKTTLMKLLAGVEA
PSSGSIKLGHQAICSYFPQNHSDVLADCGQETLFEWLRNRKTGINDQEIRSVLGKMLFGGDDAFKQIQALSGG

ETARLLMAGMMLENHNVLILDEANNHLDLESVSALSWAINDYKGTAIFVSHDRGLIQDCATKLLIFDKDKIT
FFDGTMVDYTAGHKQLL

>core/175/3/Org3_Gene804

MKMHRLKPTLKSLIPNLLFLLLTLSSCSKQKQEPLGKHLVIAMSHDLADLDPRNAYLSRDASLAKALYEGLT
RETDQGIALALAESYTLSKDHKVYTFKLRPSVWSDGTPLTAYDFEKSIKQLYFEEFSPSIHTLLGVIKNSSAIHN
AQKSLETLGIQAKDDLTLVITLEQPFPYFLTLIARPVFSPVHHTLRESYKKGTPPSTYISNGPFVLKKHEHQNYL
ILEKNPHYYDHESVKLDRVTLKIIPDASTATKLFKSKSIDWIGSPWSAPISNEDQKVLSQEKILTYSVSSTTLLIY
NLQKPLIQNKALRKAIAHAIDRKSILRLVPSGQEAVTLVPPNLSQLNLQKEISTEERQTKARAYFQEAKETLSE
KELAELSILYPIDSSNSSIIAQEIQRQLKDTLGLKIKIQGMEYHCFLKKRRQGDFFIATGGWIAEYVSPVAFLSIL
GNPRDLTQWRNSDYEKTLEKLYLPHAYKENLKRAEMIIEEETPIIPLYHGKYIYAIHPKIQNTFGSLLGHTDLK
NIDILS

>core/176/3/Org3_Gene831

MPGSVSSPPLSPVIVRERVPSSSGSDLIQPHAVLKISILIFALVTILGIVLVVSSALGALPSLVLTVSGCIAIAVGLI
GLGILVTRLILSTIRKVDAMGYDAAVKEEQYLSRIRELESENREIRDRNRAVEDQCAHLSEENKDLRDPEYLH
GMTERLIASLEIENQALVAENILLKDWNASLSRDFRAYKQKFPLGALEPWKEDIACIMEQNLFLKPECIAMVK
SLPLETQRLFLYPKGFQSLVNRFAPRSRFFQTPKYEYNSRNENEDGKVAAVCARLKKEFFSAVLGACSYEELG
GICERAVALKETLPLPEAVYDTLVQEFPNLLTAESLWKEWCFYSYPYLRPYLSVDYCKRLFVQLFEELCLKLF
TTGSPEDQALVRLFSYYRNHIPAVLASFGLPPPETGGSVFVLLPKQENLLWSQIEVLATRYLKDTFVRNSEWT
GSFEMMFSYNEMCKEISEGRIRFAEDYETRHSEEFPPSPLSEEGEGEEFLPPCSEEEVSVLERPDLDVDSMWV
WHPPVPKGPL

>core/177/3/Org3_Gene966

MTARAEYLDHEDFLYRSHKLQELSELGVVLYPYEFPGVFSCEDIKKTFASQELGNSEAAMSRSTPRVRFAGR
LVLFRAMGKNAFGQILDHNQTIQVMFNREFTSVHGLSEDAEITPIKFIEKKLDLGDILGIDGYLFFTHSGELTV
LVETVTLLCKSLLSLPDKHAGLSDKEVRYRKRWLDLISSREVSDTFVKRSYIIKLIRNYMDAHGFLEVETPILQ
NIYGGAEAKPFTTTMEALHSETFLRISLEIALKKILVGGAPRIYELGKVFRNEGIDRTHNPEFTMIEAYAAYMD
YKEVMVFVENLVEHLVRAVNHDNTSLVYSYWKHGPQEVDFKAPWIRMTMKESIATYAGIDVDVHSDQKLK
EILKKKTTFPETAFATASRGMLIAALFDELVSDNLIAPHHITDHPVETTPLCKTLRSGDTAFVERFESFCLGKEL
CNAYSELNDPIRQRELLEQQHTKKELLPDSECHPIDEEFLEALCQGMPPAGGFGIGVDRLVMILTNAASIRDVL
YFPVMRRFDAEKTN

>core/178/3/Org3_Gene120

MLRFFAVFISTLWLITSGCSPSQSSKGIFVVNMKEMPRSLDPGKTRLIADQTLMRHLYEGLVEEHSQNGEIKPA
LAESYTISEDGTRYTFKIKNILWSNGDPLTAQDFVSSWKEILKEDASSVYLYAFLPIKNARAIFDDTESPENLG
VRALDKRHLEIQLETPCAHFLHFLTLPIFFPVHETLRNYSTSFEEMPITCGAFRPVSLEKGLRLHLEKNPMYHN
KSRVKLHKIIVQFISNANTAAILFKHKKLDWQGPPWGEPIPPEISASLHQDDQLFSLPGASTTWLLFNIQKKPW
NNAKLRKALSLAIDKDMLTKVVYQGLAEPTDHILHPRLYPGTYPERKRQNERILEAQQLFEEALDELQMTRE
DLEKETLTFSTFSFSYGRICQMLREQWKKVLKFTIPIVGQEFFTIQKNFLEGNYSLTVNQWTAAFIDPMSYLMI

FANPGGISPYHLQDSHFQTLLIKITQEHKKHLRNQLIIEALDYLEHCHILEPLCHPNLRIALNKNIKNFNLFVRR
TSDFRFIEKL

>core/179/3/Org3_Gene217

MNVLKYTKHSPSAHAWKLIGTSPKHGIYLPLFSIHTKNSCGIGEFLDLIPLISWCQKQGFSVIQLLPLNDTGEDT
SPYNSISSVALNPLFLSLSSLPNIDTIPEVAKKLQDMHELCSTPSVSYTQVKEKKWAFLREYYQKCCKSSLEGN
SNFSEFLESERYWLYPYGTFRAIKHHMHGEPINNWPKSLTDQENFPDLTKKFHDEVLFFSYLQFLCYQQLCEV
KAYADQHHVLLKGDLPILISKDSCDVWYFRDYFSSSRSVGAPPDLYNSEGQNWHLPIYNFSQLAKDDYIWW
KERLRYAQNFYSVYRLDHIIGFFRLWIWDSSGRGRFIPDNPKDYIKQGTEILSTMLGASSMLPIGEDLGIIPQDV
KTTLTHLGICGTRIPRWERNWESDSAFIPLKDYNPLSVTTLSTHDSDTFAQWWLNSPKEAKQFAKFLHLPFQK
TLTTETQIDILKLSHESASIFHINLFNDYLALCPDLVSKNLQRERINTPGTISKKNWSYRVRPSLEELAIHKKFN
GYIEKILTGL

>core/180/3/Org3_Gene974

MERKRFIDCDSTKILQELALNPLDLTAPGVLSAERIKKFSLLGGGFTFSFATERLDDAILAALISLAEERGLHES
MLAMQQGQVVNYIEGFPSEMRPALHTATRAWVTDSSFTGEAEDIAVRSRVEAQRLKDFLTKVRSQFTTIVQI
GIGGSELGPKALYRALRAYCPTDKHVHFISNIDPDNGAEVLDTIDCAKALVVVVSKSGTTIETAVNEAFFADY
FAKKGLSFKDHFIAVTCEGSPMDDTGKYLEVFHLWESIGGRFSSTSMVGGVVLGFAYGFEVFLQLLQGASA
MDQIALQPNARENLPMLSALISIWNRNFLGYPTEAVIPYSSGLEFFPAHLQQCCMESNGKSIVQDGRRVGFST
SPVIWGEPGTNGQHSFFQCLHQGTDIIPVEFIGFEKSQKGEDISFQGTTSSQKLFANMIAQAIALACGSENTNPN
KNFDGNRPSSVLVSSQLNPYSLGELLSYYENKIVFQGFCWGINSFDQEGVSLGKALANRVLELLEGADASNFP
EAASLLTLFNIKFR

>core/181/3/Org3_Gene903

MTKTEEKPFGKLRSFLWPIHTHELKKVLPMFLMFFCITFNYTVLRDTKDTLIVGAPGSGAEAIPFIKFWLVVPC
AIIFMLIYAKLSNILSKQALFYAVGTPFLIFFALFPTVIYPLRDVLHPTEFADRLQAILPPGLLGLDAILRNWTFA
AFYVLAELWGSVMLSLMFWGFANEITKIHEAKRFYALFGIGANISLLASGRAIVWASKLRASVSEGVDPWGI
SLRLLMAMTIVSGLVLMASYWWINKNVLTDPRFYNPEEMQKGKKGAKPKMNMKDSFLYLARSPYILLLAL
LVIAYGICINLIEVTWKSQLKLQYPNMNDYSEFMGNFSFWTGVVSVLIMLFVGGNVIRKFGWLTGALVTPVM
VLLTGIVFFALVIFRNQASGLVAMFGTTPLMLAVVVGAIQNILSKSTKYALFDSTKEMAYIPLDQEQKVKGK
AAIDVVAARFGKSGGALIQQGLLVICGSIGAMTPYLAVILLFIIAIWLVSATKLNKLFLAQSALKEQEVAQEDS
APASS

>core/182/3/Org3_Gene863

MVWVFKSQFEGLSALKRGVHALTKAVTPAFGPRGYNVVIKKGKAPIVLTKNGIRIAKEIILQDAFESLGVKLA
KEALLKVVEQTGDGSTTALVVIDALFTQGLKGIAAGLDPQEIKAGILLSVEMVYQQLQRQAIELQSPKDVLH
VAMVAANHDVTLGTVVATVISQADLKGVFSSKDSGISKTRGLGKRVKSGYLSPYFVTRPETMDVVWEEALV
LILSHSLVSLSEELIRYLELISEQNTHPLVIIAEDFDQNVLRTLILNKLRNGLPVCAVKAPGSRELRQVVLEDLAI
LTGATLIGQESENCEIPVSLDVLGRVKQVMITKETFTFLEGGGDAEIIQARKQELCLAIAGSTSESECQELEERL
AIFIGSIPQVQITADTDTEQRERQFQLESALRATKAAMKGGIVPGGGVAFLRAAHAIEVPANLSSGMTFGFETL

LQAVRTPLKVLAQNCGRSSEEVIHTILSHENPRFGYNGMTDTFEDLVDAGICDPLIVTTSSLKCAVSVSCLLLT
SSFFISSRTKT

>core/183/3/Org3_Gene6

MYQENLRLLERLLYNSVQKSYADRLFSYEKTKMVHDTPLIPWEEDKEKCAEAEKAFLEQQKILLDYGKSIFW
LNENDEINLNDPWSWGLNTVRTRKVFQEVDDSERWNHKVLIQKLEDDYEKLLEESSKESTEANKKLLSDLV
DRLEDAKTKFFLKKQEEVETRVKDLRARYGGTVDPKQDTEAKKKVELEASLETFLDSIESELVQCLEDQDIY
WKEQDVKDLARTQELEEQDIEAKREEAAEDLRSLNERLKKSKTMLDRAKWHIENAEDSITWWTSQIEMKD
MKARLKILKEDITSVLPEIDEIETCLSLEELPLLTTRELLTKSYLKFKICSETLLKMTSVFENNIYVQEYEVQLQ
NLGFKLQGISQRFGKKQDDFANLEEQVALQKKRLRELTQNFEIQGFNFMKEDFKAAAKDLYIRSTAEQKMN
FDVPCMELFRRYHEEVNKPLLELMYNCADSYRDAKKKLCSLRLDEKELLQKEIKKEEFYQKKQQRHADRSR
HTTYQKLRIAEELALELKKKI

>core/184/3/Org3_Gene765

MVARGLCDFPTLVPNERLPIGPFFVPQHTSGAKGKEFAKRNFSIISGLDDILKLCILQRRPFALQWDNLSVKSD
YEEAGPAIGIRSLEPQVSQISPAHGRLCSTLVQWAPILGSEERLVWLEETMKRLKFPKSLGSKDAVIVDSEMVP
VNANPTQEIPAASETVESSPVAPGNTTDTMPAASGTTDTTSGVSEAAAAEATVDSTPGTEEEPSFSLRYALVV
QNVPYPEPPKEPEVMFTDEEKSLILEATRARRMELDLYNGYLADYELSKDEIQKHVPDLPENWRTNWRWSE
RLYKFFFKTKKEGLEEIFLNKELGNMILARGLAATQSQARIKVFNSLVAWLLQSFNVGRSCTAKPLPTSKLDL
FKSEFESKPKNNILTEFLVASDEEILFKGLRVLEPGIEGWYDHPDQAGEIRSVLEGLVQAGRISGYWENQPFGR
FVLRGVGERRTELVELLESLVASGEIMQFFESSDEEGAFIIDNEPSKTAMLKQRFKSCVRTKLVGSFADESLPR
GRFTILV

>core/185/3/Org3_Gene764

MVSLLHKFLENASGKKGQSLASTAYLAALDHLLNAFPSIGERIIDELKSQRSHLKMIASENYSSLSVQLAMGN
LLTDKYCEGSPFKRFYSCCENVDAIEWECVETAKELFAADCACVQPHSGADANLLAVMAILTHKVQGPAVS
KLGYKTVNELTEEEYTLLKAEMSSCVCLGPSLNSGGHLTHGNVRLNVMSKLMRCFPYDVNPDTECFDYAEI
SRLAKEYKPKVLIAGYSSYSRRLNFAVLKQIAEDCGSVLWVDMAHFAGLVAGGVFVDEENPIPYADIVTTTT
HKTLRGPRGGLVLATREYESTLNKACPLMMGGPLPHVIAAKTVALKEALSVDFKKYAHQVVNNARRLAER
FLSHGLRLLTGGTDNHMMVIDLGSLGISGKIAEDILSSVGIAVNRNSLPSDAIGKWDTSGIRLGTPALTTLGMG
IDEMEEVADIIVKVLRNIRLSCHVEGSSKKNKGELPEAIAQEARDRVRNLLLRFPLYPEIDLEALV

>core/186/3/Org3_Gene634

MSEQEKLSNYNADKKLFSGIDKLFQIVKGSYGPKQSLSPTSFFKERGFYAISQTELSNSYENLGVDFAKAMVN
KIHKEHSDGATTGLILLHAILQESYAALEKGISTHKLIASLKLQGEKLQEALQQQSWPIKDALKVRNIIFSSLH
MPTIADHFYNAFSVVGPEGLISITKERENDKTSMDVFQGFKIPAGYASTYFVSDTASRLTRIAHPLILITDRKIS
MIHSLLPLLQEISEQNQHLIIFCEDIDPDVLATLVVNKLQGLLQVTVVTIPQLSTTNQELAEDIALFTGTHICPCQ
EASHVLAPEMVTLGSCLSIEISESQTTLIGGLHIPEVLTLKTRQLAEEIRTTSCLETKKRLIKSTNRLQSSVAILPT
DEDNEPLYTLALKIMESALSRGYVPGGGVALFYASLTLGTPKDDADENSIAISLLQKACCAPLKLLATNADLD
GDAVIAKLSSLGTTSLGISVFSREIEDLIAGGILDSLATTSTILAQALDTAILVLSSKILILENQYEISTL

>core/187/3/Org3_Gene967

MENEILLNIESKEIRYAHLKNGQLFDLTIERKKVRQLKGNIYRGRVTNILRNIQSAFINIDERENGFIHISDILENS
KKFEQMFDMDVDALPEEASEAPLLSSEEAPIEEFLKLDSPVLVQVVKEPIGSKGARLTSNISIPGRYLVLLPNSP
HRGVSRKIEDPHMREQLKQLIRSFEMPQDMGLICRTASTTASTEALINEAHDLLLTWKTILEKFYSTEQPCLLY
SETDILKKAVITCIDKNYKRLLIDDYATYQKCKHMLKKYSPDASIKIEYYRDSIPMFERFNIEKEIDKATRRKI
WLSSGGYLFFDKTEAMHTIDVNSGRSTQLESGVEETLVQINLEAAEEIARQLRLRNVGGLVIIDFIDMKSRKN
QRRVLERLKEHMKYDAARCTILSMSEFGLVEMTRQRNRESLMQTLFTLCRYCSGNAIIKTPESVVIEIERDLK
KVINHKEHSHLCLVVHPEIASYMKQENDDNEMINLAKQLKAKLQINTSDSVHLNHYQFFSLITGESIDL

>core/188/3/Org3_Gene185

MFNLFFFTANKETTASHELIYRKNQSFSLSPVTILCLLAISVLLLLGVVFALVGCHVLAAPLGLLVWGCAASV
CSMMAIVSLMCLYKGGKPLIEPSNEEKIDPTKDLEIKDPESLKPVPVEGQSLPKERKTVSFKAKIPSIVEDDFKP
YVIQSTFYHQNKVYSKPIAERMQSLEKEITTLIVDFPRALEESSKSSGSLLRGVISEIKNLFLPRFLSRKVKYSLT
ACLRRLGSIVEEYASSDLLILLLTKPEPLNMVTQQLIAHLNSLKTEKRKLTPHMQKLVLSINFWFYGWSLEEK
CIEKIVAYDPNLLTDELKAHLEAGNIVQFLLSFQSSEMQREFRALFPSDAQELPSAKDGSNYVPAINSSEYMY
DFKDLSVLKKSLSERLAFCEKIPSPSSWNFTSSVASHYKDFSLLFTFFSNQQSVILQNPFLLIELLHENPKCQTFL
KGLLEKAMPMSNWAALFRPMLMGMLCSGIARKKELKIIAEHLGVPFKEITQAIASGKILDLLLQHLFDF

>core/189/3/Org3_Gene304

MTILRNFLTCSALFLALPAAAQVVYLHESDGYNGAINNKSLEPKITCYPEGTSYIFLDDVRISNVKHDQEDAG
VFINRSGNLFFMGNRCNFTFHNLMTEGFGAAISNRVGDTTLTLSNFSYLAFTSAPLLPQGQGAIYSLGSVMIEN
SEEVTFCGNYSSWSGAAIYTPYLLGSKASRPSVNLSGNRYLVFRDNVSQGYGGAISTHNLTLTTRGPSCFENN
HAYHDVNSNGGAIAIAPGGSISISVKSGDLIFKGNTASQDGNTIHNSIHLQSGAQFKNLRAVSESGVYFYDPIS
HSESHKITDLVINAPEGKETYEGTISFSGLCLDDHEVCAENLTSTILQDVTLAGGTLSLSDGVTLQLHSFKQEA
SSTLTMSPGTTLLCSGDARVQNLHILIEDTDNFVPVRIRAEDKDALVSLEKLKVAFEAYWSVYDFPQFKEAFT
IPLLELLGPSFDSLLLGETTLERTQVTTENDAVRGFWSLSWEEYPPSLDKDRRITPTKKTVFLTWNPEITSTP

>core/190/3/Org3_Gene150

MTNVVQETIGGLNSPRTCPPCILVIFGATGDLTARKLLPALYHLTKEGRLSDQFVCVGFARREKSNELFRQEM
KQAVIQFSPSELDIKVWEDFQQRLFYHRSEFDNNMGYTSLKDSLEDLDKTYGTRGNRLFYLSTPPQYFSRIIEN
LNKHKLFYKNQDQGKPWSRVIIEKPFGRDLDSAKQLQQCINENLNENSVYHIDHYLGKETVQNILTTRFANTI
FESCWNSQYIDHVQISLSETIGIGSRGNFFEKSGMLRDMVQNHMMQLLCLLTMEPPTTFDADEIRKEKIKILQR
ISPFSEGSSIVRGQYGPGTVQGVSVLGYREEENVDKDSRVETYVALKTVINNPRWLGVPFYLRAGKRLAKKS
TDISIIFKKSPYNLFAAEECSRCPIENDLLIIRIQPDEGVALKFNCKVPGTNNIVRPVKMDFRYDSYFQTTTPEAY
ERLLCDCIIGDRTLFTGGDEVMASWKLFTPVLEEWDQDSSPSFPNYPAGSSGPKEADALIERDGRSWRPL

>core/191/3/Org3_Gene973

MSSWLSQASEVLLNQDPYIPDAPRSQESSVPKISYSITVAPQEAQKSLPKFFTQKFQSQCKSEPPITHHKTFIIAT
PRERILRFGSSFESQLHNTSQAQTSSPWNLFSQKNSTEASKALMQELTMPKSPEKTSEKALDKNLSSKQEGSC
KNFDTLHLQQHLKLFGTVDSLYSQSLDSEQQELLQSRREERSETYANQQSSEKKIETKVQIKDLCKDLFSQDQ

DSNQKQKKSPFQQDTSRKNRIAKAAQAVPVIPPPSIGVFTLSYLLTKQGILSDFSSYGCHKDSVESTQRELDAL
HEKRIETIKVSIEKEKRERLWGSLSDIIGWLAPFVSIGIGIVAILSGGGIFAFAGFFAGLISLVIKCLEKLKFWDW
LEKHLPIKNEELRRKIITIIPWVVYLTPVILSICTLKVENLGFSPIIEGAIKGIQPAIESTMAALRCAILFSQAEIYKL
KGKLTKIQLDIELKSFDRDDHYERSQELLDNMESSFEALSRILNYMRELDQVYLHSLRG

>core/192/3/Org3_Gene774

MAVGGVGGSRSPSPIPPNRRNSEDGKVSPKDNLGEHTVSSSDSSLASQGPTIEERKAQLGGTDKIPLPSVKEPG
DSPTSGRSGVLQRIWKGVKGVFKKTPQARPEVSSPRLPSHVQHGQRLPGLEGFRDRIQKRSENPEADLGKMK
RSYSDGDLDRVGHDSNEDSTEDSRSEGGEPSSKSSSFLSGVRGAVSKVHGALGDIKGKFQRSASEDDLTTQG
EDSAGDTVKERRSEEAEASSKSSSFLSGVRGATSTVQGALGDAKEKVSAFGEQAAGAIRSAPGNIRTRFQRSS
SEGDLSNVNKAAKHLRKALENLEKVAPEQVSPEVASRVQSLLARMEQLTHQEPPTVEDLITFVESNVGSDSV
EYASIVPQDGSQAPAETAEAPETGGVEGSAAQGAWKALRDFVVSIFQAVASFFRAIASRLSSARRESAVDDL
ASESNTQWFVEQEGVSNPSAAPSLSFAEEIARRAAEMSNRNAQSLEKLESGNVTDPVIQQGLGLARSFAPEGQ

>core/193/3/Org3_Gene91

MADILVIGANPTGLILANMLIQHGISVKVIDHRASPEDPSFLDCRKLPVILSCSSLELLHNSEMLGDFIQANHKI
FGARYHWKKRTLLFKFSQATDSPVPFSLSTTYQSLEQHLIDEFLKRGGVIDWSTRPVTLVDNSIFIESTKVSQN
FENREIYNPKWIIACEADNNLDIRDLVKSQLRARRINREVIFINCDEGEPFEEDHIHLLPITKNFLNFVFYNPQEK
TKQLCLPQGTHSISPKLKQKLLYTYNLVISDENFHIKTSHHAFPPEHGNVLFLGSLSNTLLLSYLNGINTNIHAA
FNLAWKLLPVLKKAALKHLVITKEQEDGNILPYISPTTEKRAKKLPFSRFYTPALMYYFLKGCRKFNTTGEEY
YYPPHQALKYRSSDIIKMSPQDKEIHGPGPGMRAIDARLENGSFLLDPLKSSKHLLIFFKDIPDLKEALQEEYG
EWIEICNVKEPRILNLYHANPNSLFIIRPDRYIGYRTHTFKLHELISYLLRIFASEKTS

>core/194/3/Org3_Gene376

MNWENVRVRVAPSPTGDPHVGTAYMALFNEIFAKRFKGKMILRIEDTDRTRSRQDYEENIFSALRWCGIQW
DEGPDVGGPYGPYRQSERTKIYQGYVETLLKTDCAYKCFATPQELAEMRAVASTLGYRGGYDRRYRYLSPE
EVASREAAGQPYTIRLKVPLSGECVFEDYSKGRVVFPWADVDDQVLVKSDGFPTYHFANVIDDHLMGITHV
LRGEEWLSSTPKHLLLYEAFGWEPPVFLHMPLLLNPDGTKLSKRKNPTSIFYYRDSGYVKEAFVNFLTLMGY
SMEGDEEVYSLERIIETFNPRRIGKSGAVFDIQKLDWMNKHYLNHEGSPECLLKELQGWLLNDEFFLKILPLC
QSRITTLAEFINLTSFFFSGLLEYRVEELLPQALSPEKAAILLYSYVKYLEKTDQWTKETCYLGSKWLAQAFN
VHHKKAIIPLLYVAITGKKQGLPLFDSIEILGKPRARARLVYAEKLLGGVPKKLAATVDKFMQREDFEEATFD
L

>core/195/3/Org3_Gene492

MDCRGGIPLPEPQVIGGYHVKKILSKKLRSRVVHGLHPETRHSTVIKVFSPSPSFTSRSVYNFLKEAQSLHQIT
HPNIVKFHRYGKWQDCLYIAMEYIEGISLREYILAQFISLPQAIDIIFDIAQALEHLHSRNILHKDIKPENILITPQ
GKIKLIDFGLADWDTEIQRAHPSVIGTPYYMSPEQRQGESHSPASDIYALGLLAYELILGHLSLGRVFLSLVPE
RISKILAKALQPSPNNRYSSTREFIQDIHHYRMSGDMQEDLRIKDHTVALYEQLQTQRFWLAPETLRFPDFISG
VLYHQGYPLYPHAYDTLLEGDVFNLWLGYSPISNATIALSVVKSLVCQQDLQRPLLDRVCEINECLIRMKIPI
DEMGISILCLEISKENKELSWIACGKTVFWIKRQGRVVQDFESFSPGLGKITSLQIRETKVAWEIGDEAVVCTL
ELEESVASLKTLSLAELQDRRQKAIFCPIESIHGGIQSRQHGSNSPSTLISLKRIR

>core/196/3/Org3_Gene290

MLKKFINSLWKLCQQDKYQRFTPIVDAIDTFCYEPIETPSKPPFIRDSVDVKRWMMLVVIALFPATFVAIWNS
GLQSIVYSSGNPVLMEQFLHISGFGSYLSFVYKEIHIVPILWEGLKIFIPLLTISYVVGGTCEVLFAVVRGHKIAE
GLLVTGILYPLTLPPTIPYWMAALGIAFGIVVSKELFGGTGMNILNPALSGRAFLFFTFPAKMSGDVWVGSNP
GVIKDSLMKMNSSTGKVLIDGFSQSTCLQTLNSTPPSVKRLHVDAIAANMLHIPHVPTQDVIHSQFSLWTETH
PGWVLDNLTLTQLQTFVTAPVAEGGLGLLPTQFDSAYAITDVIYGIGKFSAGNLFWGNIIGSLGETSTFACLLG
AIFLIVTGIASWRTMAAFGIGAFLTGWLFKFISVLIVGQNGAWAPARFFIPAYRQLFLGGLAFGLVFMATDPVS
SPTMKLGKWIYGFFIGFMTIVIRLINPAYPEGVMLAILLGNVFAPLIDYFAVRKYRKRGV

>core/197/3/Org3_Gene156

MKFEFSVALKYLIPGRGRLYSAIVSLFSVGIISLVVWLSIVFISVIHGLEQRWIEDLSQLHSPITILPSDTYYSSYY
YQIDKHSSLSNYTTKTLGEKIASPQVDPYDPESDYLLPETFPLKDCDLGGQQKDPVKMTLESLGPYLQSQHGK
VIEFEQGVGYLDIKTSLKLQKPQPRNLTHFLTYPSKLSYEDKVLPYDETDYTSAELNPFNRSPSGWQQDFHHL
EELYRGASIILPSTYKDSGYKVGDTGVFSTYSIENEKETQYTVHVIGFYNPGLSPLGGRTVFIDPDLARSIRSQS
EGLGMSNGFHLFFPNTKRIVFVKKQIENILTSLGVDDYWEISSLHDYDYFQPILDQLQSDQVLFLFVCILILIVA
CSNIVTMSMLLVNNKKKEIGILKAMGTSSRSLKIIFACCGAFSGACGVVIGTIFAIITLKNLQFIVKALNYLQGR
ETFNTAFFGQNLPNSVHPQAIYFLGLGTLLLAAVSGALPARKVAKMHVSEILKAD

>core/198/3/Org3_Gene258

MVLFHAQASGRNRVKADAIVLPFWHFKDAKNAASFEAEFEPSYLPALENFQGKTGEIELLYSSPKAKEKRIV
LLGLGKNEELTSDVVFQTYATLTRVLRKAKCSTVNIILPTISELRLSAEEFLVGLSSGILSLNYDYPRYNKVDR
NLETPLSKVTVIGIVPKMADAIFRKEAAIFEGVYLTRDLVNRNADEITPKKLAEVALNLGKEFPSIDTKVLGKD
AIAKEKMGLLLAVSKGSCVDPHFIVVRYQGRPKSKDHTVLIGKGVTFDSGGLDLKPGKSMLTMKEDMAGG
ATVLGILSALAVLELPINVTGIIPATENAIDGASYKMGDVYVGMSGLSVEICSTDAEGRLILADAITYALKYCK
PTRIIDFATLTGAMVVSLGEEVAGFFSNNDVLAEDLLEASAETSEPLWRLPLVKKYDKTLHSDIADMKNLGS
NRAGAITAALFLQRFLEESSVAWAHLDIAGTAYHEKEEDRYPKYASGFGVRSILYYLENSLSK

>core/199/3/Org3_Gene719

MTVSYQSISTPPPEGEFDIFVDGNATEEAVVAAEVQVALPAGEQYAMLRATSELCFGILTQSECALTQALPPK
EKPLQEEQFLVKNGILMRSTSLPNLKPGQSQQTSLASHRNPLAQQSTSSNSTGKASTETTSSSFPFFSCKAPEG
DSSVDKTFTVSVQTPKAQEQQEASASQSQAQFHVRSYSSSTIKEHSAKEKVSQSTKSAETQKHTQTKSDATLS
PMSLYSTLHKEVPQALSSTKSQQKDEEHRDQRQQEGYEQEQEQEEGKKKTPWCTVESLQQTSSSNQVYESY
TPIIPDPIVEFALSESQLSVLAGKRVTNLDVLRICTELMKLMLKSRANDTMTRLEERELMEREAHELAASYSR
QAKYARWLGIATATLGILGAIAPMVGEISGDSILGFVQRISGRFKDATAKTFFKGIGKVFTSLSQLTEAASKVH
ELSESAVRAVAEYRKEVFRMRQDEVTRTIEEVKDNWKSMDNFLLNILQTEHDAARSLYQ

>core/200/3/Org3_Gene575

MAASILSQELLDILPYTFLKKHCLLPIEESSEAITIAHATATSVIAQDEVKLLIKKPVRFVLKEESEILQRLQQLY
SNREGNVSDMLLTMKEEDGTTISEEEDLLETTDTIPVVRLLNWILKEAIEERASDIHFEPCEDSMRIRYRIDGVL
HDRHSPPSHLRSALTTRLKVLAKMDIAEHRLPQDGRIKIHIGGQEVDMRVSTVPVIYGERVVLRILDKRNVIL

DIAGLHMPKGTEILFKDTITAPEGILLVTGPTGSGKTTTLYSVLQELKGPLTNIMTIEDPPEYKLPGIAQIAVKP
KIGLTFARGLRHLLRQDPDILMVGEIRDQETAEIAIQAALTGHLVVSTLHTNDAISAIPRLLDMGIESYLLSATL
VGVVAQRLVRTICPYCKVAYTPENQEKSFLASLGKDTEMPLYRGQGCVHCFRSGYKGRQGIYEFLRPNTLFR
SEVASNRPYHILRETAEQNGFLPILEHGIALAVSGETTLAEVLRVTKRCD

>core/201/3/Org3_Gene797

MNKKHASFSSRLGFIFSMIGIAVGAGNIWRFPRVAAQNGGGAFLILWLCFLFLWSIPLIIIELSIGKLTKKAPIGA
LIKTAGKKFAWAGGFITLVTTCILAYYSTIVGWGLSYFYYAVSGKIHLGNDFAKLWTSHYQSSIPLWAHLTSL
GLAYLVIRKGIVHGIEKCNKILIPAFFLCTIALLLRAVTLPGAVQGIKQLFSCDKSCFSNYKVWIEALTQNAWD
TGAGWGLLLVYAGFASKKTGVVSNGALTAICNNLVSLIMGIIIFSTCASLDILGTTQLQDGAGASSIGITFIYLP
ELFTRLPGGIYLTTLFSSIFFLAFSMAALSSMISMLFLLSQTLAEFGIKPYISETLATIIAFVLGIPSALSLTFFSNQ
DTVWGVALIVNGLIFIYAALVYGFPKLKKEVINAAPGDLRLNKAFDYIIKYLLLIEGILLLGWYFYEGLFPENG
QWWNPISLYSLGSLVLQWSLGLIILWKFNKQLYLRFSRYNHEIL

>core/203/3/Org3_Gene925

MLKYILKRLVLIPLTLFAIVSINFVILNAAPGDVLEEKSRYALGEAGKSDKMRSYKGRDRYLQFREAYGLNAP
HFFNTRPKITHKKIQTALQELANANNTTPSAKNAAKSLVYWGDCAKFVMPALLFEADDASRDDKYRHIAAD
LFIRGGVLQGFVGPNLSPEQRAQNKEIAESNAFLVRQLNEEDLDTKVEALKGWFQDHGGTEVFCYSSKQFW
KTFFLETRFARYMSRVLRLDFGTLRNDAHKTVISEVIKRLRCSLVLSILPMIVGFVLCQIFGMIMALKRNRWID
HSLNFIFLILFSIPVFVAVPWILDNFVINKTIPFTTIPMPYSGLRSPPEVFNELSTLGRIFDLVSHGFLPFCAVSYG
ALAAQSRLSRSIFLEVLSQDFICAAKARGLRWFDILYKHVGKNAAVSIVTSLASSLGTLLGGALVVETLFNIDG
FGNFFYQAILNRDHNVVLFSVLVGSALSLVGYLLGDICYVLLDPRVQLEGRRI

>core/204/3/Org3_Gene865

MHTEFAPFLEDLVHQQVISPLDIAFASKHISSDFEESFVFLAVSSALWRYGHPFLSLEENRIRPSLGGISETDLY
RGFHNLPKEVRDKLFVVVSGRLYLRSLYTIRSKLLDKLSLLCSATPNYFPPSIDSSILSEEQNFIFNKITQGCFSI
VSGGPGTGKTFLAAQLILSLVKQQPKLRIAIVSPTGKATSHIRQILMKYNIFDDMVLMQTVHHFLQEYAYRRY
NSIDVLLVDEGSMVTFDLLYSLVQTLQGYEKDKKLYTSSLIILGDTNQLPPIGIGVGNPLQDLIGYFHENTFFL
KTSHRAKTGVVDQLTQSVLRGEMISFSPLPSISSAIEVLKNRFVKSLRQSEARLCVLTPMRHGPWGVLNLNTM
IHQRLARSDPDLRIPIMVTSRYETWGLFNGDTGLLCLKTQKLHFPQHEPIDSRALSQYVVYNYVMSVHKSQGSE
YDEVIVIIPKGSEVFGVSILYTAITRAKYRVSVWGDPETLHKIIKKSNY

>core/205/3/Org3_Gene996

MNLDSKHFDINSANFLEEFAKFISFPSISADSDHLQDCENCAHFLVDHVNKIFDVELWETPGHPPIIYASYKSE
DPLSPTLMLYNHYDVQPAQLSDGWKGDPFILREENGNLYARGASDNKGQCFYTLKALQHYYESQGNFPLNII
WLIEGEEESGSLALFTWLEKKKEALRADYLLIVDGGFLSEKHPYVSIGARGIVSMKISLEEGNKDMHSGVLGG
IAYNTNRALSEILSSLHHPDNSIAIEGFYDDLALPSDSDRPDLPKSDTLRECEENLGFRPQGYEASYSPEESALR
PTVEINGISGGYTGPGFKTVIPYRATAYLSCRLVPNQDPDKAAHQVIHHLKQQVPSSLKFSYEILPGGSRGWRS
SANLPIVKVLQEIYSDLYNEECLRLVMPATIPIGPLLGEAAQTSPIICGTSYLSDDIHAAEEHFSMDQLKKGFLSI
CQLLDKLPKIKE

>core/206/3/Org3_Gene858

MSISSSSGPDNQKNIMSQVLTSTPQGVPQQDKLSGNETKQIQQTRQGKNTEMESDATIAGASGKDKTSSTTKT
ETAPQQGVAAGKESSESQKAGADTGVSGAAATTASNTATKIAMQTSIEEASKSMESTLESLQSLSAAQMKEV
EAVVVAALSGKSSGSAKLETPELPKPGVTPRSEVIEIGLALAKAIQTLGEATKSALSNYASTQAQADQTNKLG
LEKQAIKIDKEREEYQEMKAAEQKSKDLEGTMDTVNTVMIAVSVAITVISIVAAIFTCGAGLAGLAAGAAVG
AAAAGGAAGAAAATTVATQITVQAVVQAVKQAVITAVRQAITAAIKAAVKSGIKAFIKTLVKAIAKAISKGI
SKVFAKGTQMIAKNFPKLSKVISSLTSKWVTVGVGVVVAAPALGKGIMQMQLSEMQQNVAQFQKEVGKLQ
AAADMISMFTQFWQQASKIASKQTGESNEMTQKATKLGAQILKAYAAISGAIAGAA

>core/207/3/Org3_Gene3

MYRYSALELAKAVTLGELTATGVTQHFFHRIEEAEGQVGAFISLCKEQALEQAELIDKKRSRGEPLGKLAGV
PVGIKDNIHVTGLKTTCASRVLENYQPPFDATVVERIKKEDGIILGKLNMDEFAMGSTTLYSAFHPTHNPWDL
SRVPGGSSGGSAAAVSARFCPVALGSDTGGSIRQPAAFCGVVGFKPSYGAVSRYGLVAFASSLDQIGPLANT
VEDVALMMDVFSGRDPKDATSREFFRDSFMSKLSTEVPKVIGVPRTFLEGLRDDIRENFFSSLAIFEGEGTHLV
DVELDILSHAVSIYYILASAEAATNLARFDGVRYGYRSPQAHTISQLYDLSRGEGFGKEVMRRILLGNYVLSA
ERQNVYYKKATAVRAKIVKAFRTAFEKCEILAMPVCSSPAFEIGEILDPVTLYLQDIYTVAMNLAYLPAIAVP
SGFSKEGLPLGLQIIGQQGQDQQVCQVGYSFQEHAQIKQLFSKRYAKSVVLGGQS

>core/208/3/Org3_Gene1005

MNGCWEDLKQTIFWVGEHDCTDIETVRKSCMWLDRYADKFILREKEEKMERHELFHATMVRKASGHAYA
KAKAAFEKERSNENQRKVKDVEKWLSKGLAEFRNQESRRARERLRELQTLYPEVSVEERVLERQRTKKVNL
ENLYADIEKKYHHCVREQEHYWKEVENKEAEYRENGEKVLSAEEVSECLQRLEDCLETWSKKLTKAEESVF
EMKFDATEKLGNKVLSDVTNRLEILCEDAEEMIFRIEEIEMTLRMVELPLLFMKNTFEKASLQYNSCKEMLA
KVEPQCKESPTYRSSQERLERLNQDLQTAYTNCQERLQGFSDLESKVRTCRDHLREQMKHFEVQGLNFINEE
LLWVGAELFTQARLDLVATVPYMEFYLQYHNIKREKVRSQWMAKTERYREIRQAFQGVMKEDLLAEDTIL
KEEDYWLLRDDWLLRDERKNRQRRLICNKIAAAQQRVKGF

>core/209/3/Org3_Gene815

MLGILLIASGIIFLAVAIPGLSSAVALGLGCGMTALGTVLLITGLVLLIRSEKLALEQVEIKQARTRVNNELDQL
SQYVFYTENVLDNLKRWSYRDLGFVRQAQEEVTNLEQDIEEIFLTLRDIRNALDNEEFFMTHAKQCLAQVGE
SLFQDASIDEFINLAHLSEIRQHLDINDPRWSMITKKVKGTVVRFIYVSTMYKQIKSNFEKSDFGQLRKMLLN
NYKTIEEVLYQSFQRGYNRAALLSEKTRIIHTSSLLHWEKDEDKHLNIKNECASRLENFKKFRTLFLGLSEEDV
IDFTGASGWDCSKLPRKEVPLDGGKKKLRFKRTFADEQVGDWDRTTSLEHMTPQEEDPLDRLMDQVEQEAT
SVLKDQDRYWKEIETSEAKFRSLPREDDFEKQSQIDSYIRDLDDHLSVWANQLSAAEDALIEVTDVQEHGNR
EMLKNIQQGLELIEDAVKATLPRVDFIQELLEKEELPLVAARMSLENS

>core/210/3/Org3_Gene753

MTFPCGNCNCYYRETPPPNPGGEDIPLQEGGQSGSQGGRVITQQPGTGGREMGISLGSDNVLGMVEQAGSLL
NNLLDSARMQRLGHYCYRTGTPWCREHCPGFLQWIWGGCCACCLETVDDPDNPSAQFLQQLIQQYGPICVG
MSFQQLPHCTQKIEQGEPLGDGDKQEVENGCKLHRELLKAAQPRCMGESLVKLLQNNGLGEDMQQTPPWS

LILQAVSEGALSFVTSSDNPPTCWILQPEQQPCPPPPTDEEQLQGAVGGAPAPQQKKHPAQECRVTCKLNFRT
LLQKLSRLEVLSLESGYKGPLGQAAKQIVDLIKKSLKRLVASDLATFLGPGIGLSLESQVFEVLVLLCLLSKGY
LPLDPLHPEQTVLDPRVQGPWQRILRKVLVTTTAGENIWRQTQGEAPRQAPPPPDPWDDDEIERDGIVTGGG
FGIPCQCLRCWRKLPTEKRPNRWL

>core/211/3/Org3_Gene969

MHMSNPISLFSPAELIAKYNLIPKTSPIYPRRTELIILEENACQTRLTNVAQVLHPSSLFSMSKKILNPCGCSGGP
LCWVILNILAFIITSVLFIILLPVNLIVAGLRLFMPLPPKKIVEDLSEPTTEETNEVIQPFIFALQALLFEDNKLRSF
KIVEQSVGKAPLPNPFLKRLVAISPQESQEAMRKIPDLCSQLKKVLKSLGVLTPEWKHMLKYFEGLKNEHDS
NPDKKTFPILIKLLIEALTGKSSLPKTPSTKEKMQAALFIASSCKTCKPTWGEVITRSLNRLYSIANEGDNQLLI
WVQEFKERELMSIQDGDDAEEYRFAAQQHGERYTEAIEQVLRNESAAKLQWHVINTMKFFHGKNLGLVTE
HLQDTLGALTLRQTTVDTHQGREDADLSAALFLNKYLNSGNQLVNSVFKSMQKADPETKALIREFALDILYA
SLRLPQTSAHTEVFSTLLMDPETYEPNKACIAYLLYVLKIIEL

>core/212/3/Org3_Gene866

MSAVYADWESVIGLEVHVELNTASKLFSSALNRFGDEPNTNISTVCTGLPGSLPVLNQSAVEKAVLFGCAVE
GEISLLSRFDRKSYFYPDSPRNFQITQFEHPIIRGGRIKAIVQGEERYFELAQTHIEDDAGMLKHFGEFAGVDYN
RAGVPLIEIVSKPCMFCPEDAVAYATSLVSLLDYIGISDCNMEEGSIRFDVNVSVRPKGSPELRNKVEIKNMNS
FAFMAQALEAEKQRQIDEYLNQPNKDPKLVIPAATYRWDPEKKKTVLMRLKESAEDYKYFPEPDLPTLQLTE
SYIERIRKTLPELPYDKYHRYIQEYGLSEDIASILISDKNIATFFEVACKDCKNFRSLSNWVSVEFGGRCKTLGV
KLPSSGIFPEGVAQLVNAIDQGVITGKIAKEIADLMMESPGKNPEEILKEKPELLPMSDEGELQKIIAEVVLANP
ESIVDYKNGKTKALGFLVGQIMKRTAGKAPPKRVNELLLLELDKG

>core/213/3/Org3_Gene687

MITKQLRSWLAVLVGSSLLALPLSGQAVGKKESRVSELPQDVLLKEISGGFSKVATKATPAVVYIESFPKSQA
VTHPSPGRRGPYENPFDYFNDEFFNRFFGLPSQREKPQSKEAVRGTGFLVSPDGYIVTNNHVVEDTGKIHVTL
HDGQKYPATVIGLDPKTDLAVIKIKSQNLPYLSFGNSDHLKVGDWAIAIGNPFGLQATVTVGVISAKGRNQL
HIADFEDFIQTDAAINPGNSGGPLLNIDGQVIGVNTAIVSGSGGYIGIGFAIPSLMANRIIDQLIRDGQVTRGFLG
VTLQPIDAELAACYKLEKVYGALVTDVVKGSPADKAGLKQEDVIIAYNGKEVDSLSMFRNAVSLMNPDTRI
VLKVVREGKVIEIPVTVSQAPKEDGMSALQRVGIRVQNLTPETAKKLGIAPETKGILIISVEPGSVAASSGIAPG
QLILAVNRQKVSSIEDLNRTLKDSNNENILLMVSQGDVIRFIALKPEE

>core/214/3/Org3_Gene827

MWNRCQVFSSFFFRYPISSWLIRLRASCECFQQRHPIFLCGLYWLAGITSRGYPECSALILIFLGMFLPRNPKQ
WLPLASAWIISLMLTPAPFLHDGPISGTFVIHHAGGQGTYYGEALCIQTPCGKRAHHLSCQILSESRLELKKVY
ELEGTLHHTSQIVFKSNACYKEIPRSRFYIMKEKCRESSCHFLNHRFPSSEVGPFASSLLLGTPLPQNLRDLFRQ
KGLSHLFAISGWHFSLCATTLWMLCALLPLKIKKILSFIVLTSLACIFPMSLSVWRSWISVTLLCFSWCFSGSCS
GLNRLGAGFILCSIFFSPFSPTFVLSFLATLGILLFFPKIFSFLYTPWTQFLSPFWLYPIRYLAMTLAISLSAQLFIV
LPIMQYFGSLPLEGLLYNLIVPFTILPIIVFLIATIILPCCSPITEALIQGFLSHPWLHNPNILKTLSFAPVPPWMLTL
ASLILFFIGILRTNVSPYASTSATSYRFIETL

>core/215/3/Org3_Gene596

MLKIAILGRPNVGKSSLFNRLCKRSLAIVNSQEGTTRDRLYGELHAFGVPAQVIDTGGVDHNSEDYFQKHIYN
QALTGAKEADVLLLVIDIRCGITEEDAHLAKLLLPLKKPLILVANKADSRQEELQIHETYKLGIRDIVVTSTAH
DKHIDTLLQRIKLVANLPEPREEEEEGLEELSVDEHEESEAALPSNTFPDFSEVFTEGFSPEEPCTIPESPQQAPK
TLKIALIGRPNVGKSSIINGLLNEERCIIDNTPGTTRDNIDILYSHKDRQYLFIDTAGLRKMKSVKNSIEWISSSR
TEKAISRADICLLVIDATQKLSSYEKRILSLISKRKKPHIILINKWDLLEEVRMEHYCKDLRATDPYLGQAKML
CISATTKRNLKKIFSAIDELHHVVSNKVPTPIVNKTLASALHRNHPQVIQGRRLRIYYAIQKTTTPLQFLLFINA
KSLLTKHYEYYLKNTLKSSFNLYGIPFDLEFKEKPKRHN

>core/216/3/Org3_Gene832

MNMPVPSAVPSANITLKEDSSTVSTASGILKTATGEVLVSCTALEGSSSTDALISLALGQIILATQQELLLQSTN
VHQLLFLPPEVVELEIQVVDLLVQLEHAETITSEPQETQTQSRSEQTLPQQSSSKQSALSPRSLKPEISDSKQQQ
ALQTPKGSAVRKHSEAPSPETQARASLSQASSSSQRSLPPQESAPERTLLEQQKASSFSPLSQFSAEKQKEALT
TSKSHELYKERDQDRQQREQHDRKHDQEEDAESKKKKKKRGLGVEAVAEEPGENLDIAALIFSDQMRPPAE
ETSKKETTFKKKLPSPMSVFSRFIPSKNPLSVGSSIHGPIQTPKVENVFLRFMKLMARILGQAEAEANELYMRV
KQRTDDVDTLTVLISKINNEKKDIDWSENEEMKALLNRAKEIGVTIDKEKYTWTEEEKRLLKENVQMRKEN
MEKITQMERTDMQRHLQEISQCHQARSNVLKLLKELMDTFIYNLRP

>core/217/3/Org3_Gene975

MTSRTKSSKNLGTIALAGMVVSSIIGGGIFSLPQNMAATAGAGAVILSWILTGFGMFFIANTFRILSTIRPDLKE
GIYMYSREGFGPYIGFTIGWGYWLCQIFGNVGYAVITMDALNYFFPPYFQGGNTLPAILGGSILIWVFNFIVLK
GIRQASIINVIGTIFKIIPLIIFIILTAFFFKLAVFKTDFWGHAVTKAQPSLGSVSSQLKGTMLVTLWAFIGIEGAV
VMSGRAKNPLSVGQATVLGFLGCLTIYILFSLLPFGSLFQHQLANIPNPSTAGVLDILVGKWGEVLMNVGLIIA
VLSSWLSWTIIVAEIPFSAAKNGTFPEIFTIENKEKSPSVSLYITSSVMQLAMLLVYFSSNAWNTMLSITGVMV
LPAYLASAAFLFKLSKSKTYPKKGSIKAPLAMITGILGVVYSLWLIYAGGLKYLFMALVLLALGIPFYIDAGK
KKKNAKTFFAKKEIVGMTFIGLLALTAIFLFSTG

>core/218/3/Org3_Gene881

MITRTKIICTIGPATNSPEMLAKLLDAGMNVARLNFSHGSHETHGQAIGFLKELREQKRVPLAIMLDTKGPEIR
LGNIPQPISVSQGQKLRLVSSDIDGSAEGGVSLYPKGIFPFVPEGADVLIDDGYIHAVVVSSEADSLELEFMNS
GLLKSHKSLSIRGVDVALPFMTEKDIADLKFGVEQNMDVNAASFVRYGEDIETMRKCLADLGNPKMPIIAKI
ENRLGVENFSKIAKLADGIMIARGDLGIELSVVEVPNLQKMMAKVSRETGHFCVTATQMLESMIRNVLPTRA
EVSDIANAIYDGSSAVMLSGETASGAHPVAAVKIMRSVILETEKNLSHDSFLKLDDSNSALQVSPYLSAIGLA
GIQIAERADAKALIVYTESGSSPMFLSKYRPKFPIIAVTPSTSVYYRLALEWGVYPMLTQESDRAVWRHQACI
YGIEQGILSNYDRILVLSRGACMEETNNLTLTIVNDILTGSEFPET

>core/219/3/Org3_Gene484

MGESVKVFLEEREDYPYGFVTPIESQGLTRGLSEETIEEIAALRNEPQFIIDFRLQAYRYWKQLHEPAWARLH
YGPIAYDDIVYFSSPKQKKPLGRLEDADPEILDTFKKLGIPLDEQKRLLNVENVAVDLVFDSVSIGTTFKEALE
KAGVIFCSLGEAIQEHPNLVKKYLGSVVSHRDNFFAALNAAVFSDGSFVYVPKGVKCPMDISTYFRINNKEA

GQFERTLIVVEDGGYASYLEGCTAPAYSSNQLHAAVVELVAHEHAVIRYSTVQNWYAGDKKTGKGGIYNFV
TKRGLCAGYRSKISWSQVEVGAAITWKYPSCILKGDESVGEFYSVALTSGKMQADTGTKMLHVGKRTTSTV
ISKGISSDESKNTFRSLVSLGKKAEHSSNYTQCDSMLIGKASGAYTDPKIVVENSTSSIEHEATTSKLREDQLLY
LRSRGLSPEEAVSLVIHGFCREIIEQLPLEFAQEASKLLLIKLENSVG

>core/220/3/Org3_Gene282

MDLKELLHGVQAKIYGKVRPLEVRNLTRDSRCVSVGDIFIAHKGQRYDGNDFAVDALANGAIAIASSLYNPF
LSVVQIITPNLEELEAELSAKYYEYPSSKLHTIGVTGTNGKTTVTCLIKALLDSYQKPSGLLGTIEHILGEGVIK
DGFTTPTPALLQKYLATMVRQNRDAVVMEVSSIGLASGRVAYTNFDTAVLTNITLDHLDFHGTFETYVAAK
AKLFSLVPPSGMVVINTDSPYASQCIESAKAPVITYGIESAADYRATDIQLSSSGTKYTLVYGDQKIACSSSFIG
KYNVYNLLAAISTVHASLRCDLEDLLEKIGLCQPPPGRLDPVLMGPCPVYIDYAHTPDALDNVLTGLHELLPE
GGRLIVVFGCGGDRDRSKRKLMAQVVERYGFAVVTSDNPRSEPPEDIVNEICDGFYSKNYFIEIDRKQAITYA
LSIASDRDIVLIAGKGHEAYQIFKHQTVAFDDKQTVCEVLASYV

>core/222/3/Org3_Gene238

MQTNIGLIGLAVMGKNLVLNMIDHGFSVSVYNRTPEKTRDFLKEYPNHRELVGFESLEDFVNSLERPRKIML
MIQAGKPVDQSIHALLPFLEPGDVIIDGGNSYFKDSERRCKELQEKGILFLGVGISGGEEGARHGPSIMPGGNP
EAWPLVAPIFQSIAAKVQGRPCCSWVGTGGAGHYVKAVHNGIEYGDIQLICEAYGILRDFLKLSATAVATILK
EWNTLELESYLIRIASEVLALKDPEGIPVIDTILDVVGQKGTGKWTAIDALNSGVPLSLIIGAVLARFLSSWKEI
REQAARNYPGTPLIFEMPHDPSVFIQDVFHALYASKIISYAQGFMLLGEASKEYNWGLDLGEIALMWRGGCII
QSAFLDVIHKGFAANPENTSLIFQEYFRGALRHAEMGWRRTVVTAIGAGLPIPCLAAAITFYDGYRTASSSMS
LAQGLRDYFGAHTYERNDRPRGEFYHTDWVHTKTTERVK

>core/223/3/Org3_Gene938

MRDVSELFRTHFMHYASYVILERAIPHILDGLKPVQRRLLWTLFLMDDGKMHKVANIAGRTMALHPHGDAP
IVEALVVLANKGYLIDTQGNFGNPLTGDPHAAARYIEARLSPLARETLFNTDLIAFHDSYDGREKEPDILPAKL
PVLLLHGVDGIAVGMTTKIFPHNFAELLKAQIAILNDKKFTVFPDFPSGGLMDPSEYQDGLGSITLRASIDIIND
KTLVVKQICPQSTTETLIRSIENAAKRGTIKIDTIQDFSTDVPHIEIKLPKGSRAKEMLPLLFEHTECQVILYSKPT
VIYENKPVECSISEILKLHTTALQGYLEKELLLLQEQLTLDHYHKTLEYIFIKHKLYDSVREVLAINKKISADDL
HQAVLHALEPWLHELATPVTKQDTSQLASLTIKKILCFNEEACTKELLAIEKKQAAIQKDLGRLKEVTVKYL
KGLLERHGHLGERKTQITNFKTAKTSILKQQTLI

>core/224/3/Org3_Gene729

MLCATVSGPSFCEAKQQILKSLHLVDIIELRLDLINELDDQELHTLITTAQNPILTFRQHKEMSTALWIQKLYSL
AKLEPKWMDIDVSLPKTALQTIRKSHPKIKLILSYHTDKNEDLDAIYNEMLATPAEIYKIVLSPENSSEALNYI
KKARLLPKPSTVLCMGTHGLPSRVLSPLISNAMNYAAGISAPQVAPGQPKLEELLSYNYSKLSEKSHIYGLIG
DPVDRSISHLSHNFLLSKLSLNATYIKFPVTIGEVVTFFSAIRDLPFSGLSVTMPLKTAIFDHVDALDASAQLCE
SINTLVFRNQKILGYNTDGEGVAKLLKQKNISVNNKHIAIVGAGGAAKAIAATLAMQGANLHIFNRTLSSAA
ALATCCKGKAYPLGSLENFKTIDIIINCLPPEVTFPWRFPPIVMDINTKPHPSPYLERAQKHGSLIIHGYEMFIEQ
ALLQFALWFPDFLTPESCDSFRNYVKNFMAKV

>core/225/3/Org3_Gene297

MMSRLRFRLAALGIFFILLVPNSVSAKTIVASDKEKVGVLVYDNSVEAFQQILDCIDHANFYVELCPCMTGGR
TLKEMVDHLEARMDLVPELCSYIIIQPTFTDAEDQKLLKALKERHPNRFFYVFTGCPPSTSILAPNVIEMHIKLS
IIDGKYCILGGTNFEEFMCTPGDEVPEKVDNPRLFVSGVRRPLAFRDQDIMLRSTAFGLQLREEYHKQFAMW
DYYAHHMWFIDNPEQFAGACPPLTLEQAEETVFPGFDKHEDLVLVDSSKIRIVLGGPHDKQPNPVTQEYLKLI
QGARSSVKLAHMYFIPKDELLNALVDVSHNHGVHLSLITNGCHELSPAITGPYAWGNRINYFALLYGKRYPL
WKKWFCEKLKPYERVSIYEFAIWETQLHKKCMIIDDEIFVIGSYNFGKKSDAFDYESIVVIESPEVAAKANKV
FNKDIGLSIPVSHGDIFSWYFHSVHHTLGHLQLTYMPA

>core/226/3/Org3_Gene670

MRIVQVAVEFTPIVKVGGLGDAVASLSKELAKQNDVEVLLPHYPLISKFSSSQVLSERSFYYEFLGKQQASAIS
YSYEGLTLTIITLDSQIELFSTTSVYSENNVVRFSAFAAAAAAYLQEADPADIVHLHDWHVGLLAGLLKNPLN
PVHSKIVFTIHNFGYRGYCSTQLLAASQIDDFHLSHYQLFRDPQTSVLMKGALYCSDYITTVSLTYVQEIINDY
SDYELHDAILARNSVFSGIINGIDEDVWNPKTDPALAVQYDASLLSEPDVLFTKKEENRAVLYEKLGISSDYFP
LICVISRIVEEKGPEFMKEIILHAMEHSYAFILIGTSQNEVLLNEFRNLQDCLASSPNIRLILDFNDPLARLTYAA
ADMICIPSHREACGLTQLIAMRYGTVPLVRKTGGLADTVIPGVNGFTFFDTNNFNEFRAMLSNAVTTYRQEP
DVWLNLIESGMLRASGLDAMAKHYVNLYQSLLS

>core/227/3/Org3_Gene656

MAFSHIEGLYFYNTASQKKELFFPNHTPVRLYTCGPTVYDYAHIGNFRTYVFEDILKRTLVFFGYSVTHVMNI
TDVEDKTIAGASKKNIPLQEYTQPYTEAFFEDLDTLNIARADFYPHATHYIPQMIQAITKLLEQGIAYIGQDAS
VYFSLNRFPNYGKLSHLDLSSLRCCSRISADEYDKENPSDFVLWKAYNPERDGVIYWESPFGKGRPGWHLEC
SIMAMELLGDSLDIHAGGVDNIFPHHENEIAQSEALSGKPFARYWLHSEHLLIDGKKMSKSLGNFLTLRDLLH
QEFTGQEVRYMLLQSHYRTQLNFTEEALLACRHALRRLKDFVSRLEGVDLPGESPLPRTLDSSSQFIEAFSRA
LANDLNVSTGFASLFDFVHEINTLIDQGHFSKADSLYILDTLKKVDTVLGVLPLTTSVCIPETVMQLVAEREEA
RKTKNWAMADTLRDEILAAGFLVEDSKSGPKVKPL

>core/228/3/Org3_Gene316

MDTIDTPGEQGSQSFGNSLGARFDLPRKEQDPSQALAVASYQNKTDSQVVEEHLDELISLADSCGISVLETRS
WILKTPSASTYINVGKLEEIEEILKEFPSIGTLIIDEEITPSQQRNLEKRLGLVVLDRTELILEIFSSRALTAEANIQ
VQLAQARYLLPRLKRLWGHLSRQKSGGGSGGFVKGEGEKQIELDRRMVRERIHKLSAQLKAVIKQRAERRK
VKSRRGIPTFALIGYTNSGKSTLLNLLTAADTYVEDKLFATLDPKTRKCVLPGGRHVLLTDTVGFIRKLPHTL
VAAFKSTLEAAFHEDVLLHVVDASHPLALEHVQTTYDLFQELKIEKPRIITVLNKVDRLPQGSIPMKLRLLSPL
PVLISAKTGEGIQNLLSLMTEIIQEKSLHVTLNFPYTEYGKFTELCDAGVVASSRYQEDFLVVEAYLPKELQKK
FRPFISYVFPEDCGDDEGRGPVLESSFGD

>core/229/3/Org3_Gene186

MDSRTSHLDDELSFKLEKAFTCLSTDIHSHDLSKIVIEYNPIDLAYAVSCLPSESRAILYKNLSCITAKVAFIINT
DSASRWAIFRRLSDSEVCALIEQMPPDEAVWVLDDIPDRRYRRILELIDSKKALKIRDLQKHGRNTAGRLMTN
EFFAFLMETTVKDVSACIRSNPGIDLTRLVFVLDFKGELQGVVTDRSLIINPPEMSLKQIMNQIEHKVLPDATR

EEVVDLVERYKIAALPVVDEENFLIGAITYEDVVEAIEDIADETIARMAGTTEDVGYQTCHVVQRFLLRAPWL
LVTLFAGLISASVMAYFQKISPALLALIIFFIPLINGMSGNVGVQCSTILVRSMATGTLSFGRRRETIFKEMSIGL
LTGVVLGILCGLVVYLMGFLGLNIFSGGGIQLGVTVATGVLGASLTATTLGVLSPFFFAKLGVDPALASGPIV
TALNDIMSMIIFFLIAGGINFLFFN

>core/230/3/Org3_Gene803

MNKKKRFLSLLFLTAVLLGIWFSPHPASINSNAWQLFAIFTTTIMGIIFQPVPMGAIAIIGISTLLLTQTLTLEQG
LSGFHNPIAWLVFLSFSIAKGIIKTGLGERIAYFFVSALGKSPLGLSYGLVITDFFLAPAIPSVTARAGGILYPVV
TSLSDSFGSSAEKGTQDLIGSFLIKVAYQSSVITSAMFLTAMAGNPLVAALAGHVGVSLSWVLWAKAAIIPGL
LSLFLMPIILYKLYPPKITSCEEAIRSAKLRLKEMGPLKKEEKTILMIFFLLVVLWTFGDLLGISATTAALIGLSL
LILTNILDWQKDVIANTTAWETFIWFGALIMMASFLNQLGFIPLVGDSAAALVSGLSWKIGFPLLFLIYFYSHY
LFASNTAHIGAMYPIFLAVSISLGTNPIFAALTLAFASNLFGGLTHYGSGPAPLYFGSHLVTVQEWWRSGFALS
IVNIVIWIGIGSLWWKALGLI

>core/231/3/Org3_Gene997

MTTKSLDSFNSVISKNKIHFISLGCSRNLVDSEVMLGILLKAGYESTNEIEDADYLILNTCAFLKSARDEAKDY
LDHLIDVKKENAKIIVTGCMTSNHKDELKPWMSHIHYLLGSGDVENILSAIESRESGEKISAKSYIEMGEVPRQ
LSTPKHYAYLKVAEGCRKRCAFCIIPSIKGKLRSKPLDQILKEFRILVNKSVKEIILIAQDLGDYGKDLSTDRSS
QLESLLHELLKEPGDYWLRMLYLYPDEVSDGIIDLMQSNPKLLPYVDIPLQHINDRILKQMRRTTSREQILGFL
EKLRAKVPQVYIRSSVIVGFPGETQEEFQELADFIGEGWIDNLGIFLYSQEANTPAAELPDQIPEKVKESRLKIL
SQIQKRNVDKHNQKLIGEKIEAVIDNYHPETNLLLTARFYGQAPEVDPCIIVNEAKLVSHFGERCFIEITGTAG
YDLVGRVVKKSQNQALLKTSKA

>core/232/3/Org3_Gene183

MHSHSKPTKPLGTFTVGMLSLAVVISLRNLPLTAKHGLSTLFFYGLAVICFMIPYALISAELASFKPQGIYIWA
RDALGKWWGFFAIWMQWFHNMTWYPAVLAFIASTIVYKINPELAHNKVYIATVILAGFWILTFFNFLGITSS
ALFSSICVIIGTLIPGVILVSLALFWIFSGNPIAISLSWGNLLPNFSNVSSLVLLAGMLLALCGLEANANLASDM
VNPRKNYPKAVFIGAIATLTILVLGSLSIAIVIPKEEISLVSGLVKTFTLFFDKYNLSWMTGIVVVMTIAGSLGE
LNAWMFAGTKGLFISTQNDCLPRLFKKVNSKNVPTNLMLFQGIVVTIFTLLFLCLDSADLVYWILTALSVQM
YLAMYICLFLAGPILRIKEPRAQRLYSVPGKFLGICTMSILGILSCAFALWVSFLPPRELAQISEGSKIGYTTFLL
LAFSLNCLIPFGIYFTHKRLSKKS

>core/233/3/Org3_Gene760

MDKSTGVPLPSPPHSKESEMIVLGCMLTGVHYLNLAANQLYEEDFYYLEHKIIFRVLQDAFKQDKPIDVHLA
GEELKRHNQITVIGGPSYLITLAEFAGTAAYLEEYVDIIRSKSILRKMISTAKEIEKRALEQPKNVAEALDEAQN
SFFKISQSTSVSQYTLVADKLRGLTTTTDKPYLVQLQERQELFLQNAQGDNKSFFTGIPTHFIDLDQLIHGFSPS
NLMILAARPAMGKTALALNIAENLCFQNRLPIGIFSLEMTVDQLIHRMICSRSEVDSKKISIGDLSGHDFQRIVS
VINEMQEHTLLIDDQPGLKVSDLRARARRMKESYDIQFLIIDYLQLLSGSGTLRATESRQTEISEISRMLKTLAR
ELNIPILCLSQLSRKVEDRANHRPMMSDLRESGSIEQDSDLVMFLLRREYYDPNDKPGTAELIIAKNRHGSIGS
VPLVFEKELARFRNYSAFECIS

>core/234/3/Org3_Gene522

MKITVNRGLDLSLQGSPKESGFYNKIDPEFVSIDLRPFQPLSLKLKVEQGDAVCSGAPIAEYKHFPNTYITSHV
SGVVTAIRRGNKRSLLDVIIKKTPGPTSTEYTYDLQTLSRSDLSEIFKENGLFALIKQRPFDIPAIPTQTPRDVFIN
LADNRPFTPSPEKHLALFSSREEGFYVFVVGVRAIAKLFGLRPHIVFRDRLTLPTQELKTIAHLHTVSGPFPSGS
PSIHIHSVAPITNEKEVVFTLSFQDVLTIGHLFLKGRILHEQVTALAGTALKSSLRRYVITTKGASFSSLINLNDI
SDNDTLISGDPLTGRLCKKEEEPFLGFRDHSISVLHNPTKRELFSFLRIGFNKPTFTKTYLSGFFKKKRTYTNPD
TNLHGETRPIIDTDIYDKVMPMRIPVVPLIKAVITKNFDLANELGFLEVCGEDFALPTLIDPSKTEMLTIVKESLI
EYAKESGILTPHQD

>core/236/3/Org3_Gene415

MKEERSSEILPRVKETKKHAYVSMQEKSCVGECAVVASESEEAESVTVTKIAKLQRMGIEELNILARQYGVK
NIGSLTKSQVVFEIVKAKSERPDELLIGEGVLEVLPDGFGFLRSPTYNYLPSAEDIYVSPAQIRRFDLKKGDTIIG
TIRSPKEKEKYFALLKVDKINGSTPDKAKERVLFENLTPLYPNQRIVMEMGKDHLAERVLDLTAPIGKGQRG
LIVAPPRSGKTVILQSIAHAIAVNNPDIVLIVLLIDERPEEVTDMIRQVRGEVVASTFDEQPERHIQVAEMVIEK
ARRLVEHGNDVVILLDSITRLARAYNTVQPHSGKILTGGVDASALHKPKRFFGAARNIEGGGSLTILATALIDT
GSRMDEVIFEEFKGTGNMELVLDRRLSDRRTYPAIDLIKSGTRKEELLYHPSELERVYLFRQAIADLTTIDAM
HLLLGRLKKTNSNAEFLLSLKE

>core/237/3/Org3_Gene539

MNFKLPVYHIGLTKAENNTIKIAILQKTCKGWIVCHCEQIPEGKTWSLPKKYFAAPTTFSLQGSDILVKSSSSS
LKNRKNILKVALTNLEASLALPWESLIVQPQLGKPTDRGETPLTLWIAQKNTLKKELSFLSQAQIFPDKLSCRA
ADIFFLAEQSPLKSLPAYLLIYGGSEEVTCIFVKNHAIAVARSFSNHSTKKSCDDIHATLQYIQETFPQTVLPAIH
VAQISPNLQKILEQKLSLPLVVCQSMTYGVEDEDWEIYGDTIAAAHHGASRRPLTFPYDATSVSPAAQKHWL
LRSSLLIGKYALMATVVVSLGSVLKLKSLSSSASNHFAFACPEEGVLPRSLKAAEKTVKAIGKKNSASNYPLL
PTIPTSEQTLKFLLALGKSSPSIKFSYFSYTMTSYPSKDNPSLPYSALVEVKGQGQPEDIPQFLKKISSHPKLQHV
SESLEDQRSFKLQFTLSS

>core/238/3/Org3_Gene606

MTESVYSPSAMHVNSLADKLKAINQEHILDIWPSLSPKQQQRLFQQLTSVDIDFFRKQQQLLSSPTAILKDFHP
ITSFASSGEDPERAHAGTTLLKEKKVACVVLAGGQGSRLKCDGPKGLFPVSPIKKKPLFQLVAEKVRAASKL
AGQPLPLAFMTSPLNTRQTRSFFESNDYFHLDPNQVDFFCQPLWPLLTLSGDLFLEDMDTLALGPNGNGCIAT
LLYTSGVWEKWKNAGIEMVSVIPIDNPLALPFDVELCGFHAMSNNEVTIKAALRQTAIEDVGILVKSHDSGK
TSVIEYSEIPQNERFALNEDGKLKYCLANIGLYCLSMDFIRHAAYQQLPLYKVHKHAKQLGHTSLNEKNAWK
FEEFIFDLFCYSDHCQTLVYPRQECFAPLKNLEGNHSPDTVRQALSDRERQLFHKVTGKKLSPNTTFELEADF
YYPSTSTSLHWENKAFFEEPFFEAS

>core/239/3/Org3_Gene589

MTQEFDCVVIGAGPSGYVAAITAAQSKLRTALIEEDQAGGTCLNRGCIPSKALIAGANVVSHIKHAEQFGIHV
DGYTIDYPAMAKRKNTVVQGIRQGLEGLIRSNKITVLKGTGSLVSSTEVKVIGQDTTIIKANHIILATGSEPRPF
PGVPFSSRILSSTGILELEVLPKKLAIIGGGVIGCEFASLFHTLGVEITVIEALDHILAVNNKEVSQTVTNKFTKQ

GIRILTKASISAIEESQNQVRITVNDQVEEFDYVLVAIGRQFNTASIGLDNAGVIRDDRGVIPVDETMRTNVPNI
YAIGDITGKWLLAHVASHQGVIAAKNISGHHEVMDYSAIPSVIFTHPEIAMVGLSLQEAEQQNLPAKLTKFPF
KAIGKAVALGASDGFAAIVSHEITQQILGAYVIGPHASSLIGEMTLAIRNELTLPCIYETVHAHPTLSEVWAEG
ALLATNHPLHFPPKS

>core/240/3/Org3_Gene32

MMSSKRTSKIAVLSILLTFTHSIGFANANSSVGLGTVYITSEVVKKPQKGSERKQAKKEPRARKGYLVPSSRT
LSARAQKMKNSSRKESSGGCNEISANSTPRSVKLRRNKRAEQKAAKQGFSAFSNLTLKSLLPKLPSKQKTSIH
EREKATSRFVNESQLSSARKRYCTPSSAAPSLFLETEIVRAPVERTKELQDNEIHIPVVQVQTNPKEQNTKTTK
QLASQASIQQSEGTEQSLRELAQGASLPVLVRSNPEVSVQRQKEELLKELVAERRQCKRKSVRQALEARSLT
KKVARGGSVTSTLRYDPEKAAEIKSRRNCKVSPEAREQKYSSCKRDARANGKQDKTTPSEDASQEEQQTGA
GLVRKTPKSQVASNAQNFYRNSKNTNIDSYLTANQYSCSSEETDWPCSSCVSKRRTHNSISVCTMVVTVIAMI
VGALIIANATESQTTSDPTPPTPTP

>core/241/3/Org3_Gene899

MRAWEEFLLLQEKEIGTNTVDKWLRSLKVLCFDACNLYLEAQDSFQITWFEEHIRHKVKSGLVNNNNKPIRV
HVTSVDKAAPFYKEKQMQQEKTAYFTMHYGSVNPEMTFSNFLVTPENDLPFRVLQEFTKSPDENGGVTFNPI
YLFGPEGSGKTHLMQSAISVLRESGGKILYVSSDLFTEHLVSAIRSGEMQKFRSFYRNIDALFIEDIEVFSGKSA
TQEEFFHTFNSLHSEGKLIVVSSSYAPVDLVAVEDRLISRFEWGVAIPIHPLVQEGLRSFLMRQVERLSIRIQET
ALDFLIYALSSNVKTLLHALNLLAKRVMYKKLSHQLLYEDDVKTLLKDVLEAAGSVRLTPLKIIRNVAQYYG
VSQESILGRSQSREYVLPRQVAMYFCRQKLSLSYVRIGDVFSRDHSTVISSIRLIEQKIEENSHDIHMAIQDISKN
LNSLHKSLEFFXSEEMII

>core/242/3/Org3_Gene715

MRQEKDSLGIVEVPEDKLYGAQTMRSRNFFSWGPELMPYEVIRALVWIKKCAAQANQDLGFLDSKHCDMIV
AAADEILEGGFEEHFPLKVWQTGSGTQSNMNVNEVIANLAIRHHGGVLGSKDPIHPNDHVNKSQSSNDVFPT
AMHIAAVISLKNKLIPALDHMIRVLDAKVEEFRHDVKIGRTHLMDAVPMTLGQEFSGYSSQLRHCLESIAFSL
AHLYELAIGATAVGTGLNVPEGFVEKIIHYLRKETDEPFIPASNYFSALSCHDALVDAHGSLATLACALTKIAT
DLSFLGSGPRCGLGELFFPENEPGSSIMPGKVNPTQCEALQMVCAQVLGNNQTVIIGGSRGNFELNVMKPVII
YNFLQSVDLLSEGMRAFSEFFVKGLKVNKARLQDNINNSLMLVTALAPVLGYDKCSKAALKAFHESISLKEA
CLALGYLSEKEFDRLVVPENMVGNH

>core/243/3/Org3_Gene771

MNFSLFLFFLIAIQGICLYVGRRGSKKVEDRESYFLAGRSLKIFPLMMTFIATQIGGGVLLGAAEEAFCYGYGG
ILYPLGVALGLIFLGMGPGKRLAEGSLTTVVSIFEVFYGSKKLRKIAFLLSAGSLFFILVAQVIALDRLFSSFPFG
KYVTVAFWIVLASYTSTGGFRGVVRTDVIQAGFLLIAVLVCGVSVWLSVPKSLSVLDPFQSLPCAKLSNWIF
MPMLFMLVEQDMVQRCVAASSPKRLQWAAVGAGLVLLLFNFIPLFLGSLGAKAGLKAGCPLIDTIAYFCNPS
LAAVMAAAIGVAILSTADSLMNAVSQLIAEEYPTLKAPYYRYLVLGLAVAAPLVAIGFTNIVDVLILSYSLSV
CCLSVPVGFYLLAPKGRRVSGAAAWAGVLVGALGYGWVQIVSLGMFGELLAWVGSLVAFSFVGFIEITWK
NKVKTQT

>core/244/3/Org3_Gene681

MQQSVRKLFGTDGVRGRANFEPMTVETTVLLGKAVARVLREGRSGKHRVVVGKDTRLSGYMFENALIAGL
NSMGIETLVLGPIPTPGVAFITRAYRADAGIMISASHNPYRDNGIKIFSLEGFKISDVLEQRIETMVSEADFGPLP
EDHAVGKNKRVIDAMGRYVEFVKATFPKGRTLKGLKIVLDCAHGASYKVAPSVFEELDAEVICYGCEPTGIN
INEHCGALFPQVIQKAVIEHQAHLGIALDGDGDRIIMVDEKGHIVDGDMILSICAGDLKKRSALPHNRVVATI
MTNFGVLKYLEGLGLQVFTSPVGDRHVLHAMLEHEVTLGGEQSGHMIFLDYNTTGDGIVSALQVLRIMIESE
SMLSDLTAPIVKSPQTLINVAVREKIPLETIPLIERTLRDVQDALGPSGRILLRYSGTENICRVMVEGHKKHQV
DCLAKALADVIDAELGTGSRE

>core/245/3/Org3_Gene386

MQIAQVFGCGRLNGEVKVSGAKNAATKLLVASLLSDQKCTLRNVPDIGDVSLTVELCKSLGAHVSWDKETE
VLEIYTPEIQCTRVPPTFSNVNRIPILLLGALLGRCPEGVYVPTVGGDAIGERTLNFHFEGLKQLGVQISSDSSG
YYAKAPRGLKGNYIHLPYPSVGATENLILAAIHAKGRTVIKNVALEAEILDLVLFLQKAGADITTDNDRTIDIF
GTGGLGSVDHTILPDKIEAASFGMAAVVSGGRVFVRNAKQELLIPFLKMLRSIGGGFLVSESGIEFFQERPLVG
GVVLETDVHPGFLTDWQQPFAVLLSQAQGSSVIHETVHENRLGYLHGLQHMGAECQLFHQCLSTKACRYAI
GNFPHSAVIHGATPLWASHLVIPDLRAGFAYVMAALIAEGGGSIIENTHLLDRGYTNWVGKLRSLGAKIQIFD
MEQEELTTSPKSLALRDASL

>core/246/3/Org3_Gene622

MLYFIEQLNKLSTSFCVFPMILLLGGFLTWKLRGLQFHGLKLGFNLMLQNKLDDSSSKANEVSSYEAVAGIL
AGNFGTGNIAGMAVALACGGPGALVWVWLAALLGAIVQYAGSYLGSKYRKPEGNTGEFIGGPIACLAFGM
RKKILAGFFALFTIMTAFCAGNCVQVSCIVPLCAEGTPGKLLVGILLALVVIPVLAGGNNRILRFSARVIPFIAG
FYCISCGIILFQHASAILPAIKLICSSAFGIKAGLAGIGGYTLSQVISTGINRAVMATDCGSGMVSILQANTKSKN
PVVDGLVTLVPPVIVMVVCSITMLVLIVSGAYSSGAQGTLMVMSAFKNSLGSLGSVIVILAMALFGYTTILTW
FACAEKSLQYMIPGRRANLWLKAIYVLIIPLGGVIDMRMIWALSDTGFSGMVILNCIALIALLKDVLSTNRDV
ALLKERECSVADPVRNLDA

>core/247/3/Org3_Gene628

MFNVNFKFLEGLHQPAPRYTSYPTALEWEPSDAAPALLAFQRIRENPQPLSLYFHIPFCQSMCLYCGCSVVLN
RREDIVEAYINTLIQEMKLVVETIGFRPQVSRIHFGGGTPSRLSRELFTLLFDHIHKLFDLSHAEEIAIEVDPRSL
RNDMEKADFFQNVGFNRVSLGVQDTQADVQEAVRRRQSHEESLKAYEKFKELAFQSINIDLIYGLPKQTKES
FSKTIQDILAMYPDRLALFSFASVPWIKPHQKAMKASDMPSMEEKFAIYSQSRHLLTKAGYQAIGMDHFSLP
HDPLTLAFKNKTLIRNFQGYSLPPEEDLLGLGMTSTSFIRGIYLQNAKTLEEYHNTVLRGTFATVKSKILTEDD
RIRKWAIHKLMCTFTINKEEFFNLFGYEFDTYFIESRDRLISMETTGLIHNSPGSLKVTPLGELFVRVIATAFDH
YFLNKVSKKECFSASI

>core/248/3/Org3_Gene431

MTTLRQFFLITELRQKLFYTFALLTACRVGVFIPVPGINGELAVAYFKQLLGSGQNLFQLADIFSGGAFAQMT
VIALGVVPYISASIIVQLFLVFMPALQREMRESSDQGKRRIGRLTRLFTVALAVIQSLLFAKFALRMNLTIPGIV
LPTLLSSKLFGVPWIFYITTVVVMTTGTLLLMWIGEQISDKGIGNGISLIIALGILSSFPSVLGSIVNKLNLGSQD

SSDLGLISILILALVFVFVLITTILIIEGVRKIPVQYARRVIGRREVPGGGSYLPLKVNYAGVIPVIFASSLLMFPA
TIGQFIASESSWMKRIAALLAPGSLVYSICYVLLIIFFTYFWTATQFHPEQIASEMKKNNAFIPGIRQGKPTQHY
LEYTMNRVTLLGALFLAAIAILPSLLGCLLRVDSNVSYFLGGTAMLIVVGVVLDTMKQVDAFLLMRRYDSVL
KTDRTKGRH

>core/250/3/Org3_Gene461

MNVWTKFFQPPKHIKEIEDQEVVKKKYKYWRIRIFYSMFIGYIFYYFTRKSFTFAMPTLIADLGFDKAQLGIIG
STLYFSYGISKFVSGVMSDQSNPRYFMAIGLMITGLTNIFFGMSSSIVLFALWWGLNGWFQGWGWPPCARLL
THWYAKSERGTWWSVWSTSHNIGGALIPILTGFIIDYSGWRGAMYVPGILCIGMGLVLINRLRDTPQSLGLPPI
EKYKRDPHHAHHEGKSASEGTEEIERELSTREILFTYVLTNQWLWFLAAASFFIYIVRMAVNDWSALFLIETK
HYAAVKANFCVSLFEIGGLFGMLVAGWLSDKISKGNRGPMNVLFSLGLLFAILGMWFSRSHNQWWVDGTL
LFVIGFFLYGPQMMIGLAAAELSHKKAAGTASGFTGWFAYFGATFAGYPLGKVTDVWGWKGFFIALLACAS
IALLLFLPTWNATEKNTRSKA

>core/251/3/Org3_Gene806

MKKVLIANRGEIAVRIIRACHDLGLSTVAVYSLADQEALHVLLADEAICIGEPQAAKSYLKISNILAACEITGA
DAVHPGYGFLSENANFASICESCGLTFIGPSSESIAMMGDKIAAKSLAKKIKCPVIPGSEGIIEDESEGLKIAEKI
GFPIVIKAVAGGGGRGIRIVKEKDEFYRAFSAARAEAEAGFNNPNVYIEKFIENPRHLEIQVIGDTHGNYVHLG
ERDCTIQRRRQKLIEETPSPILNAEIRVKVGKIAVDLARSAGYFSVGTVEFLLDKDKKFYFMEMNTRIQVEHTI
TEEVTGIDLVKEQIHVAMGNKLPWKQKNIEFSGHIIQCRINAEDPTNNFSPSPGRLDYYLPPAGPSIRVDGACY
SGYAIPPYYDSMIAKVIAKGKNREEAIAIMKRALKEFHIGGVQSTIPFHQFMLDNPKFLESNYDINYIDNLLAQ
GNSFFKEF

>core/252/3/Org3_Gene25

MATKTKTQWTCNQCGATAPKWLGQCPGCHNWNSLVEEYVPQARSGTSSRSSTSAIALSSIELENESRIFIDHA
GWDRILGGGVVRGSLTLLGGDPGIGKSTLLLQTAERLASQKYKVLYVCGEESVTQTSLRAKRLNISSPLIYLF
PETNLDNIKQQIATLEPDILIIDSIQIIFNPTLNSAPGSVAQVREVTYELMQIAKSAQITTFIIGHVTKSGEIAGPRV
LEHLVDTVLYFEGNSHANYRMIRSVKNRFGPTNELLILSMHADGLKEVSNPSGLFLQEKTGPTTGSMIIPIIEG
SGALLIELQALVSSSPFANPVRKTAGFDPNRFSLLLAVLEKRAQVKLFTMDVFLSITGGLKIIEPAADLGALLA
VASSLYNRLLPNNSIVIGEVGLGGEIRHVAHLERRIKEGKLMGFEGAILPEGQISSLPKEIRENFRLQGVKTIKD
AIRLLL

>core/253/3/Org3_Gene620

MLKILKIKVLVFPLALLMGCNSIGYAGPQGSLQTNSQTKVKIGSEVWIEQKLRQYPELLWLTESGGAPLLTST
PIDMAYSEKLFNKKVPALDIAIRSMIHLHLLIQGSRQSYMQLSQILPSEEGGMTFKQFQTAHKQLLFFLNSPKS
FDNTLRILETAIVLRHVGCSAKAVTTFKPYFTDSCPQSFYAKALHVLRTFPELCPSYARLSPEQQEVLLSLRRL
GNYDSLLNLTEVPSAQLLSAWRTRRSLAILDLYLYCLDTCGDKNCSQEFYINFAPLLSMLQQHATIEEAFSRY
FTYRANRLGFEGTSRTDMTLVRLATLMNLSPSEASTLAWSFKNLPSDEAENLVNSFYTVQGEHIPLTFRGLPS
LVAGLSVATHGSTVSPENRLRQLYSTMLSLLVKSLRSHREMLNKQLLPQGTVLDFSETTLSSGGLDVFAESIA
VRIHLNGAVSINL

>core/254/3/Org3_Gene805

MRKRHSFDSTSTKKEAVSKAIQKIIKIMETTDPSLNVETPNAEIESILQEIKEIKQKLSKQAEDLGLLEKYCSQE
TLSNLENTNASLKLSIGSVIEELASLKQLVEESIEESLGQQDQLIQSVLIEISDKFLSSIGETLSGNLDMNQNVIQ
GLLIKENPEKSEAASVGYVQTLLEPLSKRIGETHKKVATHDVNISSLQFHMMSVAGGRFRGHIDMNGYRVLG
LGEPKNGEDAVSKDYLERYVSSQLTIDKVEDKPITKPNKGKLLYSQGTSPKLEGPLPLGLLTSGISGFTWKSAS
KSNDGSFPFSALRHKETESDTDCFQITSTTLSGNQAGTYTWSLSLKVLVPSIFQIEKPEVQLSLVYSYEDWLPID
NIFNMSQPRTIPLALLGQTMLAGQKYDILELAAHQTNQTLMISPNCSRFSLQLKQTNQFENSPVDFYIVHAAH
SCHWSGF

>core/255/3/Org3_Gene287

MLTCNECTTWEQFLNYVKTRCSKTAFENWISPIQVLEETQEKIRLEVPNIFVQNYLLDNYKRDLCSFVPLDVH
GEPALEFVVAEHKKPSAPVASQKESNEGISEVFEETKDFELKLNLSYRFDNFIEGPSNQFVKSAAVGIAGKPGR
SYNPLFIHGGVGLGKTHLLHAVGHYVREHHKNLRIHCITTEAFINDLVYHLKSKSVDKMKNFYRSLDLLLVD
DIQFLQNRQNFEEEFCNTFETLINLSKQIVITSDKPPSQLKLSERIIARMEWGLVAHVGIPDLETRVAILQHKAE
QKGLLIPNEMAFYIADHIYGNVRQLEGAINKLTAYCRLFGKSLTETTVRETLKELFRSPTKQKISVETILKSVA
TVFQVKLNDLKGNSRSKDLVLARQIAMYLAKTLITDSLVAIGAAFGKTHSTVLYACKTIEHKLQNDETLKRQ
VNLCKNHIVG

>core/256/3/Org3_Gene675

MILPPYSYSLKIGAAVLFFCSILHTFLTPWLYTLCQSYEHKKLVFPECWKRYARLSELFRILSRVEIVFFLWAVP
LFFWFLYTEGYRISMAYFNSRNYGFAVFIMVILILLESRPIVYFAELVLSSIAKLGKTSPKSWWWTLMIAPPLL
SCLLKETGAMIIGATLLMRHFYVFSPSRRFAYATMGLLFSNISIGGLTSYVSSRALFLIFPALKWEHSFFLSHFA
WKAIVAILISTTIYYFIFRKEFKKFPDIPSDKDPSVEKVPWWIICVNIIFVGSIILSRSTPLFMGALLLFYLGFQKFT
IFYQDPINLSKVCYVGLFYAGLVVFGDLQEWWVLNLMQGLSDFGYMTVSYTLSIFLDNALVNYLVHNLSVA
TDCYHYLVVAGCMAAGGLTLVSNIPNIVGYLILRSAFPSSTIHMGWLFLGALGPSIISLGVFWLLKNVPEFLYC
FFR

>core/257/3/Org3_Gene533

MSEPRFVCLSLGSNLGNRFKNLQIARTLLGEQAVLGLRSSVILETEALLLPGSPPEWDLPYFNSVLVGETTLSL
RELLVTIKQIEKVVGRAEESPPWSPRTIDVDILLYGDESFCCDHTEITIPLSNLLSRPFLIALIASLCPYRRFCTQG
SPYHNFTFGELAHHLPSPPGMIRRSLSPDTMLMGVVNVTNDSMSDGGMFLDPEKAVAQAEKLFTEGAAVIDF
GAQATNPKVKQFLSVDQEWERLEPVLRLLKETWSNRKQYPIISLDTFYPEIILRAMDIYPIQWINDVSGGSQS
MAEVARDCELSLVMNHSSSLPVDPKNILSFSVPIGEQLLSWGEKQLKMFSDVGLNANQVIFDPGIGFGKGAA
QSLATLYEIAKFKRLGCPILIGHSRKSFLSLFGNHDPKDRDWETVGLSILLQQQGVDYLRVHNVAAHQKALS
VAACEACAPI

>core/258/3/Org3_Gene358

MNRLLSLLSVFDDFFWSYVAFILIIVLGVSFSWKSRFFQFTKFSQFCKLFRYYSQNPQERETKQGVHPLKVFFA
SAGGNIGIGNVVGIVTAACIGGPGALFWVWIAGIFGSIVKYSEVYLGIKFRKLDRDGVYQGGPMYFLIKAFKT
PVVSVIVAILLCIYGVEIYQFSVITDSLAHCWNLPKVYPMLGLLFLVFYAIRGGLQRIGKICSIVLPFFMLLYCA

LSLYILVKEFHTLPHLLSTVFSSAFKGQSALGGFAGCTVATTIHQGISRAAYSGDIGIGFDSIIQSESSAKDPSTQ
AQLSIVGIAIDNLICTLSLLMVLASGSWSLGLENASQVVEHTLASYFPMVKFFLPTFFFVTGYTTIISYFLVGKK
CAKFLYGNTGAKIYTLYGLLILPLFCFLSQNTALLIMSVSGALLLCFNLLGVFILRKEVIFPARAASLTETSLST
E

>core/259/3/Org3_Gene229

MLSCVFSDTIFLSSFLAVTLICMTTALWGTILLISKQPLLSESLSHASYPGLLVGALMAQYVFSLQASIFWIVLF
GCAASVFGYGIIVFLGKVCKLHKDSALCFVLVVFFAIGVILASYVKESSPTLYNRINAYLYGQAATLGFLEAT
LAAIVFCASLFALWWWYRQIVVTTFDKDFAVTCGLKTVLYEALSLIFISLVIVSGVRSVGIVLISAMFVAPSLG
ARQLSDRLSTILILSAFFGGISGALGSYISVAFTCRAIIGQQAVPVTLPTGPLVVICAGLLAGLCLLFSPKSGWVI
RFVRRKHFSFSKDQEHLLKVFWHISHNRLENISVRDFVCSYKYQEYFGPKPFPRWRVQILEWRGYVKKEQDY
YRLTKKGRSEALRLVRAHRLWESYLVNSLDFSKESVHELAEEIEHVLTEELDHTLTEILNDPCYDPHRQIIPNK
KKEV

>core/260/3/Org3_Gene817

MINSLSQKLSSIFSFLVSSRRINEENISESIREVRLALLDADVNYHVVKDFISKVKEKILGEEIWKHVSPGQQFIR
CLHEELVAFLSDGREEFTIQKTPSIILLCGLQGAGKTTTAAKLADYVIKNKKAKKVLVVPCDLKRFAAVDQL
KILVAQTKAEFYQSQENKPIDVVVKALAYAKENGHDFVILDTAGRLNIDNELMEELTAIQKVSQANERLFVM
NVAMGQDVLATVQAFDQSLDLTGVILSMTDGDARAGAVFSIKHVLGKPIKFEGCGERIQDLRSFDPQSMAER
ILGMGDTINFVKEMREYISEEEDAELGKKLVTAAFTYEDYYKQMKAFRRMGPLRKLLGMMPGFNNAKPSQ
KEIEDSEQQMKRTEAIILSMTPEERKELVELDMSRMKRIASGCGLTLGDVNQFRKQMSQSKKFFKGMSKGK
MEQVRKKMSGGNQWR

>core/261/3/Org3_Gene57

MDALILSRIQFGLFITFHYLFVPLSMGLSMMLVIMEGLYLVTKKQIYKQMTWFWVGIFALTFVLGVVTGIMQI
FSFGSNWANFSEYTGNIFGTLLGSEGVFAFFLESGFLGILLFGRHKVSKKMHFFSTCMVALGAHMSAFWIICA
NSWMQTPSGYEMVMHKGKLIPALTSFWGVVFSPTTIDRFIHAVLGTWLSGVFLVISVSAYYLWKKRHHEFA
KQGMKIGTICAVIVLVLQLWSADVTARGVAKNQPAKLAAFEGIFKTEEYTPIWAFGYVDMEKERVIGLPIPG
ALSFLVHRNIKTPVTGLDQIPRDEWPNVQAVFQLYHLMIMLWGVMVALTLISWSAYKGWRWALKPFFLVIL
TFSVLLPEICNECGWCAAEMGRQPWVVQGLLKTKDAVSPIVQANQIVQSLVIFSLVFIALLTLFITVLCKKIKH
GPEEENDLTEFEVK

>core/262/3/Org3_Gene848

MRAMLLEDWVSLMLSDVSCPKCDKKITGFAIDSQKVQPGDLFFALPGNATDGHQFLKHAATAGAVAAVVS
HDYQGDSFGLELIRVDDTKSALQEAGSNQCNLFQGTLVGITGSVGKTTTKEFSKTILSSIYKTHASPKSYNSQL
TVPLSLLMAEGDEDVMILEMGVSEPGNMQDLLRIVQPEIAVITHINDQHAMHFPQGIQEILKEKSYILQKSKL
QLLPKDSPYYLDLRSCSPTAEKFSFSFNDPLADFCYKAISGDSVVIQTPEENYCLPIAFSYKPAYTNLLIAVALS
WILEVPEEGVIRSLPELKLPPMRFEHSMRNGMQVINDAYNACPEAMIAALDALPLPSDGGKIILILGHMAELG
RYSEEGHALVAEKAASRGDMIFFIGEKWIPVQSVLKSYSCEVSFFSSAQDVKDILKQVARYGDVILLKGSRAL
ALESLLACF

>core/263/3/Org3_Gene234

MSNITSPVIQNNRSCNYYFELKNSTTIHIVISAILLCGALIAFLCVAAPVSYILSGALLGLGLLIALIGVILGIKKIT
PMISSKEQVFPQELVNRIRAHYPKFVSDFVSEAKPNLKDLISFIDLLNQLHSEVGSSTNYNVSEELQQKIDTFEG
IARLKNEVRTASLKRLESAASSRPLFPSLPKILQKVFPFFWLGEFISAGSKVVELHRVKKIGGSLEEDLSDYIKP
EMLPTYWLIPLDFRPTNSSILNLHTLVLARVLTRDVFQHLKYAALNGEWNLNHSDLNTMKQQLFAKYHAAY
QSYKHLSQPSLQEDEFYNLLLCIFKHRYSWKQMSLIKTVPADLWENLCCLTLDHTGRPQDMEFASLIGTLYT
QGLIHKESEAFLSSLTLLSLDQFKTIRRQSTNIAMFLENLATHNSTFRSLPPITVHPLKRSVFSQPEEDESSLLIG

>core/264/3/Org3_Gene872

MVITNLSINADTIYGKNPINIVASAANKNITLTGTLALVNADGAFYENHTLQDSQDYSFVKLSPGAGGTIITQD
ASQKPLEVAPSRPHYGYQGHWNVQVIPGTGTQPSQANLEWVRTGYLPNPERQGSLVPNSLWGSFVDQRAIQ
EIMVNSSQILCQERGVWGAGIANFLHRDKINEHGYRHSGVGYLVGVGTHAFSDATINAAFCQLFSRDKDYV
VSKNHGTSYSGVVFLEDTLEFRSPQGFYTDSSSEACCNQVVTIDMQLSYSHRNNDMKTKYTTYPEAQGSWA
NDVFGLEFGATTYYYPNSTFLFDYYSPFLRLQCTYAHQEDFKETGGEVRHFTSGDLFNLAVPIGVKFERFSDC
KRGSYELTFAYVPDVIRKDPKSTATLASGATWSTHGNNLSRQGLQLRLGNHCLINPGIEVFSHGAIELRGSSR
NYNINLGGKYRF

>core/265/3/Org3_Gene731

MLTYKVSPSSVYGNAFIPSSKSHTLRAILWASVAEGKSIIYNYLDSPDTEAMICACKQMGASIKKFPQILEIVG
NPLAIFPKYTLIDAGNSGIVLRFMTALACVFSKEITVTGSSQLQRRPMAPLLQALRNFGASFHFSSDKSVLPFT
MSGPLRSAYSDVEGSDSQFASALAVACSLAEGPCSFTIIEPKERPWFDLSLWWLEKLHLPYSCSDTTYSFPGSS
HPQGFSYHVTGDFSSAAFIAAAALLSKSLQPIRLRNLDILDIQGDKIFFSLMQNLGASIQYDNEEILVFPSSFSGG
SIDMDGCIDALPILTVLCCFADSPSHLYNARSAKDKESDRILAITEELQKMGACIQPTHDGLLVNPSPLYGAVL
DSHDDHRIAMALTIAALYASGDSRIHNTACVRKTFPNFVQTLNIMEARIEECHDNYSMWSTHKRKVFARESF
G

>core/266/3/Org3_Gene570

MTSGVSGSSSQDPTLAAQLAQSSQKAGNAQSGHDTKNVTKQGAQAEVAAGGFEDLIQDASAQSTGKKEATS
STTKSSKGEKSEKSGKSKSSTSVASASETATAQAVQGPKGLRQNNYDSPSLPTPEAQTINGIVLKKGMGTLAL
LGLVMTLMANAAGESWKASFQSQNQAIRSQVESAPAIGEAIKRQANHQASATEAQAKQSLISGIVNIVGFTV
SVGAGIFSAAKGATSALKSASFAKETGASAAGGAASKALTSASSSVQQTMASTAKAATTAASSAGSAATKA
AANLTDDMAAAASKMASDGASKASGGLFGEVLNKPNWSEKVSRGMNVVKTQGARVASFAGNALSSSMQ
MSQLMHGLTAAVEGLSAGQTGIEVAHHQRLAGQAEAQAEVLKQMSSVYGQQAGQAGQLQEQAMQSFNTA
LQTLQNIADSQTQTTSAIFN

>core/267/3/Org3_Gene873

MTLQPYQASSRKYRPQIFREILGQSSVVAVLKNALVFNRAAHAYLFSGIRGTGKTTLARILAKALNCVHLSED
GEPCNQCFSCKEIASGSSLDVLEIDGASHRGIEDIRQINETVLFTPVKAKFKIYIIDEVHMLTKEAFNALLKTLE
EPPQHVKFFFATTEIHRIPGTILSRCQKMHLQRIPEKTILEKLSLMAQDDHIEASQEALAPIARAAQGSLRDAES
LYDYVISLFPKSLSPDTVAQALGFASQDSLRTLDNAILQRDYATALGIVTDFLNSGVAPVTFLHDLTLFYRNLL

LTNSTTSKFSSQYKTEQLLEIIDFLGESAKHLQNTIFEQTFLETVIIHIIRIYQRPVLSELISSIKSRQFEGLRNIKEP
TLTQQVSAPQPQPTYKEQSFLEKKNQPAAEGKIISVEVKSSASIKSAAVDTLLQFAVVEFSGILRQ

>core/268/3/Org3_Gene592

MLKHDTIAAIATPPGEGSIAVVRLSGPQAIVIADRIFSGSVASFASHTIHLGQVIFEETLIDQALLLLMRSPRSFT
GEDVVEFQCHGGFFACSQILDALIALGARPALPGEFSQRAFLNGKIDLVQAEAIQNLIVAENIDAFRIAQTHFQ
GNFSKKIQEIHTLIIEALAFLEVLADFPEEEQPDLLVPQEKIQNALHIVEDFISSFDEGQRLAQGTSLILAGKPNV
GKSSLLNALLQKNRAIVTHIPGTTRDILEEQWLLQGKRIRLLDTAGQRTTDNDIEKEGIERALSAMEEADGIL
WVIDATQPLEDLPKILFTKPSFLLWNKADLTPPPFLDTSLPQFAISAKTGEGLTQVKQALIQWMQKQEAGKTS
KVFLVSSRHHMILQEVARCLKEAQQNLYLQPPEIIALELREALHSIGMLSGKEVTESILGEIFSKFCIGK

>core/269/3/Org3_Gene600

MPRSLSNEQFSVDLEESPGCIVSALVKVSPEVLNKLNKQALKKIKKEITLPGFRKGKAPDDVIASRYPTNVRK
ELGELVTQDAYHALSTVGDRRPLSPKAVRSNSITQFDLQEGAKVEFSYEAFPAISDLPWENLSLPQEEAASEIS
DSDIEKGLTNIGMFFATKTPVERPSQEGDFISISLHVSKSNDENASSAAIFENKYFKLSEEEMTDAFKEKFLGIS
TGHRVVETITSPEIQSFLRGDTLTFTVNAVIEVSIPEIDDEKARQLQAESLDDLKAKLRIQLEKQAKDKQLQKR
FSEAEDALAMLVDFELPTSLLEERISLITREKLLNARLIQYCSDEELEKRKSELIKEAEEDATKALKLLFLTHKI
FSDEKLTISREELQYMMDVCSRERFGQQPPKDISNDTLQELVMSARDRLTYSKAIEHVLRKAELLASTPSA

>core/270/3/Org3_Gene496

MDQLTTDFDTLMSQLGDVNLTTVVGRITEVVGMLIKAVVPNVRVGEVCLVKRNGMEPLVTEVVGFTQSFAF
LSPLGELSGVSPSSEVIPTGLPLHIRAGNGLLGRVLNGLGEPIDVETKGPLQNVDQTFPIFRAPPDPLHRAKLRQ
ILSTGVRCIDGMLTVARGQRIGIFAGAGVGKSSLLGMIARNAEEADVNVIALIGERGREVREFIEGDLGEEGM
KRSVIVVSTSDQSSQLRLNAAYVGTAIAEYFRDQGKTVVLMMDSVTRFARALREVGLAAGEPPARAGYTPS
VFSTLPRLLERSGASDKGTITAFYTVLVAGDDMNEPVADEVKSILDGHIVLSNALAQAYHYPAIDVLASISRL
LTAIVPEEQRRIIGKAREVLAKYKANEMLIRIGEYRRGSDREIDFAIDHIDKLNRFLKQDIHEKTNYEEAAQQL
RAIFR

>core/271/3/Org3_Gene594

MWLVILWALAASLAIALVAKGYYRFVYFRRYAVQVIREVRLSMELKEWALAEQQLLPILKKRSYRRQCLFE
YMRILRKMQRFEESEKLLAEAKKLGLRGPYFFLEIAYKAYRFGAFKECAQAFASVPQDLFEEEDAAKYASAL
VRLGDLDAACSLIEPWISPLSHQETFVTMGHIYFTSKRYKDAIDFYNRANALGVCPVEVTYNLAQAYRITSSY
AKAGKLFRKLLSNPVYKEEALFNIGLCEQKLGRPGKALLIYQSSDLWSRGDALLMKYAAMAAMDQRDYVL
AEPCWELALRCSTFAKDYKCGLGYGFSLCRLRKYGDAERVYCNLIQNFPECLTACKALAWLCGVGYATLLG
SEEGLMYAKKAVELDHSCETLELLSACEARCGNFDAAYEIQSFLSSRDTSLQEKQRRSQILRILRKKLPLNDH
HIVEVDALLAA

>core/272/3/Org3_Gene413

MIENDFPEASNFESSHFYRDKVGVIILCGGEGKRLSPLTNCRCKPTVSFGGRYKLIDIPISHAISAGFSKIFVIGQ
YLTYTLQQHLFKTYFYHGVLQDQIHLLAPEARQGDQIWYQGTADAIRKNLLYFEDTEIEYFLILSGDQLYNM
DFRSIVDTAIRTHVDMVLVAQPIPEKDAYRMGVLDIDSEGKLIDFYEKPQEKEVLKRFQLSSEDRRIHKLTEDS

GDFLGSMGIYLFRRDSLFSLLREEEGNDFGKHLIQAQMKRGQVQTLLYNGYWADIGTIESYYEANIALTQKP
HAEKRGLNCYDDNGMIYSKNHHLPGAIITDSMISSSLLCEGCVINTSHVSRSVLGIRSKIGENSVVDQSIIMGN
ARYGSPSMPSLGIGKDCEIRKAIIDENCCIGNGVKLQNLKGYIKYDSPDKKLFVRDNIIIVPQGTHIPDNYIF

>core/273/3/Org3_Gene812

MLNCSNQKHTVTFEEACQVFPGGVNSPVRACRSVGVTPPIVSSAQGDIFLDTHGREFIDFCGGWGALIHGHSH
PKIVKAIQKTALKGTSYGLTSEEEILFATMLLSSLKLKEHKIRFVSSGTEATMTAVRLARGITNRSIIIKFIGGYH
GHADTLLGGISTTEETIDNLTSLIHTPSPHSLLISLPYNNSQILHHVMEALGPQVAGIIFEPICANMGIVLPKAEF
LDDIIELCKRFGSLSIMDEVVTGFRVAFQGAKDIFNLSPDITIYGKILGGGLPAAALVGHRSILDHLMPEGTIFQ
AGTMSGNFLAMATGHAAIQLCQSEGFYDHLSQLEALFYSPIEEEIRSQGFPVSLVHQGTMFSLFFTESAPTNFD
EAKNSDVEKFQTFYSEVFDNGVYLSPSPLEANFISSAHTEENLTYAQNIIIDSLIKIFDSSAQRFF

>core/274/3/Org3_Gene544

MFQQKQKLSLKYLPSLRMQQGLQMLQSPLTELSSYVVQEIIDNPFFDLSSLEEEEWSPCYRPTNSTFSYLNQTP
GPQESLYTRLLPQIEEAFSTAEERFIAHQIAGNLSDEGLFLRNPEDFAQELELPLEKIHKVWDTIQNLSPEGIASP
SLQSYWMKLLRNSSHQQAYSIVRDCYPLMTNCEFAPIMKKFSLSLSELRNILKKALGSIPWCPAAACTVKPM
VSTPLPDIYLFYSSGSWKIEVSTRGLPSIKLNKETFHFYEHLPKEEQKNLSQQILSAKWLIKNLRKREQTLLQV
METLLPKQEDFLLGKIPAPYPLGIKDLAEDLSFHESTIFRAIENKAVAAPIGIFPLKHLFPRGIHQDSSHSKENVL
QWIRQWIATEQTPLSDSVISDRITAKGIPCARRTVAKYRAQLKILPANKRKKLFYIRSSNSHFRDRQF

>core/275/3/Org3_Gene739

MSKIVYKFGGTSLATAENICLVCDIICKDKPSFVVVSAIAGVTDLLVDFCSSSLREREEVLRKIEGKHEEIVKNL
AIPFPVSTWTSRLLPYLQHLEISDLDFARILSLGEDISASLVRAVCSTRGWDLGFLEARSVILTDDSYRRASPNL
DLMKAHWHQLELNQPSYIIQGFIGSNGLGETVLLGRGGSDYSATLIAELARATEVRIYTDVNGIYTMDPKVIS
DAQRIPELSFEEMQNLASFGAKVLYPPMLFPCMRAGIPIFVTSTFDPEKGGTWVYAVDKSVSYEPRIKALSLS
QYQSFCSVDYTVLGCGGLEEILGILESHGIDPELMIAQNNVVGFVMDDDIISQEAQEHLVDVLSLSSVTRLHHS
VALITMIGDNLSSPKVVSTITEKLRGFQGPVFCFCQSSMALSFVVASELAEGIIEELHNDYVKQKAIVAT

>core/276/3/Org3_Gene49

MQTIYTKITDIKGNLITVEAEGARLGELATITRSDGRSSYASVLRFDLKKVTLQVFGGTSGLSTGDHVTFLGRP
MEVTFGSSLLGRRLNGIGKPIDNEGECFGEPIEIATPTFNPVCRIVPRSMVRTNIPMIDVFNCLVKSQKIPIFSSSG
EHHNALLMRIAAQTDADIVVIGGMGLTFVDYSFFVEESKKLGFADKCVMFIHKAVDAPVECVLVPDMALAC
AEKFAVEEKKNVLVLLTDMTAFADALKEISITMDQIPANRGYPGSLYSDLALRYEKAVEIADGGSITLITVTT
MPSDDITHPVPDNTGYITEGQFYLRNNRIDPFGSLSRLKQLVIGKVTREDHGDLANALIRLYADSRKATERMA
MGFKLSNWDKKLLAFSELFETRLMSLEVNIPLEEALDIGWKILAQSFTSEEVGIKAQLINKYWPKACLSK

>core/277/3/Org3_Gene727

MISFRFLLLSGLCALGISSYAETPKETTGHYHRYKARIQKKHPESIKESAPSETPHHNSLLSPVTNIFCSHPWKD
GISVSNLLTSVEKATNTQISLDFSILPQWFYPHKALGQTQALEIPSWQFYFSPSTTWTLYDSPTAGQGIVDFSYT
LIHYWQTNGVDANQAAGTASSMNDYSNRENNLAQLTFSQTFPGDFLTLAIGQYSLYAIDGTLYDNDQYSGFI
SYALSQNASATYSLGSTGAYLQFTPNSEIKVQLGFQDSYNIDGTNFSIYNLTKSKYNFYGYASWTPKPSCGDG

QYSVLLYSTRKVPEQNSQVTGWSLNAAQHIHEKLYLFGRINGATGTALPINRSYVLGLVSENPLNRHSQDLL
GIGFATNKVNAKAISNVNKLRRYESVMEAFATIGFGPYISLTPDFQLYIHPALRPERRTSQVYGLRANLSL

>core/278/3/Org3_Gene809

MMLRGVHRIFKCFYDVVLVCAFVIALPKLLYKMLVYGKYKKSLAVRFGLKKPHVPGEGPLVWFHGASVGE
VRLLLPVLEKFCEEFPGWRCLVTSCTELGVQVASQVFIPMGATVSILPLDFSIIIKSVVAKLRPSLAVFSEGDC
WLNFIEEAKRIGATTLVINGRISIDSSKRFKFLKRLGKNYFSPVDGFLLQDEVQKQRFLSLGIPEHKLQVTGNIK
TYVAAQTALHLERETWRDRLRLPTDSKLVILGSMHRSDAGKWLPVVQKLIKEGVSVLWVPRHVEKTKDVE
ESLHRLHIPYGLWSRGANFSYVPVVVVDEIGLLKQLYVAGDLAFVGGTFDPKIGGHNLLEPLQCEVPLIFGPH
ITSQSELAQRLLLSGAGLCLDEIEPIIDTVSFLLNNQEVREAYVQKGKVFVKAETASFDRTWRALKSYIPLYKN
S

>core/279/3/Org3_Gene468

MKRPFFTYLCIIFYGSCASLSLHAGLSFPEVRGATAAVVHADSGKVFYDKDIDAVIYPASMTKIATALFILKHY
PTVLDTLIKVKQDAIASITPQAKKQSGYRSPPHWLETDGSTIQLHLREELLGWDLFHALLVCSANDAANVLA
MACCGSVEKFMDKLNFFLKEEIGCTHTHFNNPHGLHHPNHYTTTRDLISIMRCALKEPPFRGVISTTSYKIGAT
NLHGERILSPTNKLLLPGSTYHYPPALGGKTGTTKTAGKNLIMAAEKNNRLLVTIATGYSGPVSDLYQDVIAL
CETVFNEPLLRKELVPPSDCLQLEIANLGKLSCPLPEGLYYDFYASEDREPLSVSFIAHADAFPIEQGDLLGHW
VFYDDEGKKISSQPFYAPCRFERTIKPWKLYMKRVFTSYRTYMSITMLLMYFRIRKHRKYKNLKHYSKI

>core/280/3/Org3_Gene850

MKRAIIIGAGISGLAAGWWLHKKFPQAEILVLDKEAYAGGFVRTESPQGFSFDLGPKGFLTRGDGEYTLKLIH
ELGLQNSLIFSDRAAKNRFVYYRGKARKISTWTLLRKGLLPSLIKDFRAPCYTQDSSVQDFLKRHSSQNFTSYI
LDPLITAIRAGHSSILSTHMAFPELAKREASSGSLLRSYLKNRSPKKSKTDRYLASLSPSMGTLITTIQEKLPAT
WKFSTSVTHIDCSPKEACVTTPSETFFADMVIYTGPLQQLPVLLPNYGIENLSKRVLPWNLSSISLGWHHANFS
LPKGYGMLFADELPLLGIVWNSQIFPQATPGKTVLSLLIEGKWRESEAHAFAIAALSEYLNINQKPDAFALFSS
QDGMPQHAVGFLERKERILPHLPGNLKIVGQNIAGPGLNRCIASAYHAICDLHTEETLAQPQSSL

>core/281/3/Org3_Gene121

MFSRWITLFLLFISLTGCSSYSSKHKQSLIIPIHDDPVAFSPEQAKRAMDLSIAQLLFDGLTRETHRESNDLELAI
ASRYTVSEDFCSYTFFIKDSALWSDGTPITSEDIRNAWEYAQENSPHIQIFQGLNFSTPSSNAITIHLDSPNPDFP
KLLAFPAFAIFKPENPKLFSGPYTLVEYFPGHNIHLKKNPNYYDYHCVSINSIKLLIIPDIYTAIHLLNRGKVDW
VGQPWHQGIPWELHKQSQYHYYTYPVEGAFWLCLNTKSPHLNDLQNRHRLATCIDKRSIIEEALQGTQQPAE
TLSRGAPQPNQYKKQKPLTPQEKLVLTYPSDILRCQRIAEILKEQWKAAGIDLILEGLEYHLFVNKRKVQDYA
IATQTGVAYYPGANLISEEDKLLQNFEIIPIYYLSYDYLTQDFIEGVIYNASGAVDLKYTYFP

>core/282/3/Org3_Gene209

MNKNLVAIFDYMEKEKGIQRSTIIGAIESALKIAAKKTLRDDANISVNINSRTGDIEVFCEKEIVEICQNPSKEIP
LDKAREYDPDCQIGQYMDVPFVSDNFGRIAAHAARQIIGQKLRHAERDVIYEEYRHRVNETLSGVVKRFAKG
SNLIIDLGKVEAILPTRFYPKTEKHKIGDKIYALLYEVQESENGGAEVILSRSHAEFVKQLFIQEVPELEEGSVEI
VKIAREAGYRTKLAVRSSDPKTDPVGAFVGMRGSRVKNIIRELNDEKIDIVNYSPVSTELLQNLLYPIEIQKIAI

LEDDKVIAIVVNDADYATVIGKRGINARLISHILDYELEVQRMSEYNKLLEIQRLQLAEFDSPHLDQPLEMEGI
SKLVIQNLEHAGYDTIRRVLLASANDLASVPGISLELAYKILEQVSKYGESKVDEKPEIED

>core/283/3/Org3_Gene904

MSMTIVPHALFKNHCECHSTFPLSSRTIVRIAIASLFCIGALAALGCLAPPVSYIVGSVLAFIAFVILSLVILALIF
GEKKLPPTPRIIPDRFTHVIDEAYGLSISAFVREQQVTLAEFRQFSTALLCNISPEEKIKQLPSELRSKVESFGISR
LAGDLEKNNWPIFEDLLSQTCPLYWLQTFISAGDPQVCRDLGVPRECYGYYWLGPLGYSTAKATIFCKETHH
ILQQLTKEDVLLLKNKALQEKWDTDEVKAIVERIYTTYTARGTLKTEAGGLTKETISKELLLLSLHGYSFDQL
QLITQLPRDAWDWLCFVDNSTAYNLQLCALVGALSSQNLLDESSIDFDVNLGLYVIQDLKEAVQAFSASDEP
KKELGKFLLRHLSSVSKRLESVLRQGLHRIALEHGNARARVYDVNFVTGARIHRKTSIFFKD

>core/284/3/Org3_Gene957

MNHLNKEKLHIHNWQPYRACGLLSKVSGNLIEVDGLSACLGELCKISSTKDPNLLAEVIGFHNHTTLLMSLSP
LHSVALGTEVLPLRRPPSLHLSDHLLGRVLDAFGNPIDKKEDLPKTHRKPLLSLPPSPMMRQPIDQIFPTGIKAI
DAFLTLGKGQRIGVFSEPGSGKSSLLSAIALGSKSTINVIALIGERRREVREHIEKHSNALKQQRTIIIAAPAHET
APTKVIAGRAAMTIAEYFREQGHEVLFIMDSLSRWIAALQEVALARGETLSAHQYAASVFHHVSEFTERAGN
NDKGSITALYAILYYPKHPDIFTDYLKSLLDGHFFLTSQGKALASPPIDILSSLSRSAQALALPHHYAAAERLRS
LLKVYNEALDIIHLGAYTPGQDEELDKAVKLLPSIKAFLAQPLSSYCYLDNTLKQLEALADS

>core/285/3/Org3_Gene712

MLKPMYVLSKRLYRWVNQLIKLGDLVKNSRSFSVEWVFISALLLIFGCLGCASVVKVSLVPFLLLFSFLAFPLI
LCFRGKGYALLLGVFVTLYVAKYVVGETLYVSFWLSGLGVSFLLAFGLFLQGVWLAQEEEMVKGKEQLRL
SEDLDAQRSAYEDLLLTKSQEKEFLDARAQGLDRELTECQELLKAAYQKQEYLTIDLKILADQKNSWLEDY
AELHNKYIELVSKNGDVVFPWVAEPSVGESQGSERVDVSRWVSALQEKEESLERLRNEILVEKQRCSDYEHR
CQELGLLLQNFTALERRCEELQNLLNQKETQINELHQLVCKSEEKVSVEPSAHAETSCVEEKQYKGLYSQLQ
EQFLEKSETLSLVRKKLFAVQEKYLTLKKKEELTKQDISFDDISMIQGLLERIEILEEEVSHLEELVSRSLSL

>core/286/3/Org3_Gene552

MLRQLCFQVFFFCFASLVYAEELEVVVRSEHITLPIEVSCQTDTKDPKIQKYLSSLTEIFCKDIALGDCLQPTAA
SKESSSPLAISLRLHVPQLSVVLLQSSKTPQTLCSFTISQNLSVDRQKIHHAADTVHYALTGIPGISAGKIVFALS
SLGKDQKLKQGELWTTDYDGKNLAPLTTECSLSITPKWVGVGSNFPYLYVSYKYGVPKIFLGSLENTEGKKV
LPLKGNQLMPTFSPRKKLLAFVADTYGNPDLFIQPFSLTSGPMGRPRRLLNENFGTQGNPSFNPEGSQLVFISN
KDGRPRLYIMSLDPEPQAPRLLTKKYRNSSCPAWSPDGKKIAFCSVIKGVRQICIYDLSSGEDYQLTTSPTNKE
SPSWAIDSRHLVFSAGNAEESELYLISLVTKKTNKIAIGVGEKRFPSWGAFPQQPIKRTL

>core/287/3/Org3_Gene959

MTWPSGLYFICIASLIFCAIGVILAGVILLSRKLFIKVHPCKLKINDNEELTKTVESGQTLLVSLLSSGIPIPSPCG
GKATCKQCKVRVVKNADEPLETDRSTFSKRQLEEGWRLSCQCKVQHDMSLEIEERYLNASSWEGTVISNDN
VATFIKELVVAVDPNKPIPFKPGGYLQITVPSYKTNSSDWKQTMAPEYYSDWEHFHLFDQVIDNSQLPADSA
NKAYSLASYPAELPTIKFNIRIATPPFINGKPNSEIPWGVCSSYVFSLKPGDKITVSGPYGESFMKDDDRPLIFLI

GGAGSSFGRSHILDLLLNKHSKREIDLWYGARSLKENIYQEEYENLERQFPNFHYHLVLSEPLPEDIAAGWDK
DDPTKTNFLFRAFNLGQLSRLDNPEDYLYYVCGPPLHNSSILKLLGDYGVERSSIILDDFGS

>core/288/3/Org3_Gene932

MTVTLPKGVFDIFPYLADAKQLWRHTSLWHSVEKAIHTVCMLYGFCEIRTPIFEKSEVFLHVGEESDVVKKE
VYSVLDRKGRSMTLRPERTAAVVSAFLEHGASHRSDIKFYYILPMFRYERQQAGRYRQHHQFGVEAIGVRHP
LRDAEVLALLWDFYSRVGLQHMQIQLNFLGGSETRFRYDKVLRAYLKESMGELSALSQQRFSTNVLRILDSK
EPEDQEIIRQAPPILDYVSDEDLKYFNEILDALRVLEIPYAINPRLVRGLDYYSDLVFEATTTFQEVSYALGGGG
RYDGLISAFGGASLPACGFGVGLERAIQTLLAQKRIEPQFPHKLRLIPMEPDADQFCLEWSQHLRRLGIPTEVD
WSHKKVKGALKAASTEQVSFVCLIGERELISQQLVIKNMSLRKEFFGTKEEVEQRLLYEIQNTPL

>core/289/3/Org3_Gene889

MFRKLFPFSKKKTGQKQRLRNNGLLQAIIQSIKVLLHNEASKEACVLSYYGLLTCVPILVFFLRLSQHLFTNLN
WKEWLIIKFPDYKKPIVAIVEAAYHATESNIGLVLVGSFYVFCWAGILMLLSLEDGLNKIFRDSWTPISLKRLV
SYFVITLVSPMIFIIVCGSWIYITQIMPIQYAKLFSLSHSMTALYFISRFVPYLLLYLALFCCYAFLPRVAIQKTSA
LISTLIIGSVWIVFQKAFFSLQVSIFNYSFTYGALVALPSFLLLLYIYTMIYLFGGALTFIIQNRGCTFIFLGDKILP
SCYLQLITSTYILALTTRQFNEGLSPLTAQFIAKQSKVPIGEVSQCLDVLEKEGFLFPYNNGYQPVFNFSELTIK
DIADKLLHREIFKKFNPDLGITFIENSFQNIFNQASKNKENLTLSEIARRIK

>core/290/3/Org3_Gene839

MVCENNILSGRGLELLKKKSNITLTPTIYSVSNHNIKLKDFSPHALSVIKTLRKAGYIAYIVGGCIRDLLLNTTP
KDFDISTSAKPEEIKAIFKNCILVGKRFRLAHIRFSKQIIEVSTFRSGSTDEDVLITKDNLWGTPEEDVLRRDFTI
NGLFYDPEHEEIIDYTGGVNDLRNRYLRTIGDPFTRFKQDPVRMLRLLKILSRSPFTVETQTQEALIACRQELIK
SSRARVFEELIKMLNSGAAKNFFQLLIENHLLEILFPYMDKALRLNRALEEQTATYLKALDDKILKKEAEYDR
HQLMAIFLFPLVNFNVRYKHQKHPYLSLTSVFDYIKNFLEQFFADSFTSCSKKNFILTALILQMQYRLTPLIPTK
KALFFNKKLLHHTRFLEALSLLEIRSIVYPKLDKVYVAWIRHHQTLKCKKDSHSQK

>core/291/3/Org3_Gene202

MISLLKMPKLSPTMEVGTIVKWHKKSNDQVSFGDVIVEISTDKAILEHTANEDGWIREILRHEGEKIVIGTPIA
VLSTEANEPFNLEELLPKTEPSNLEASPKGSSEEVSPATTPQAASATFTAVTFKPEPPLSSPLVFKHVGTTNNLS
PLARQLAKEKNIDVSSIQGSGPGGRIVKKDLEKAPPKSIAGFGYPESPEVPPGSYHEENLSPIREVIAARLQAAK
ISIPHFYVRQQVYASPLLNLLKELQAQGIKLSINDCIVRACALALKEFPSINSGFNSVDNKIVRFDTIDISIAVAIP
DGIITPIIRCADRKNLGMISAEIKSLALKARNQSLQDTEYKGGSFCVSNLGMTGITEFTAIVNPPQAAILAVGSV
TEQALVLDGEITIGSTCNLTLSVDHRVIDGYPAAMFMKRLQKILEAPAVLLLN

>core/293/3/Org3_Gene859

MFEAVIADIQAREILDSRGYPTLHVKVTTSTGSVGEARVPSGASTGKKEALEFRDTDSPRYQGKGVLQAVKN
VKEILFPLVKGCSVYEQSLIDSLMMDSDGSPNKETLGANAILGVSLATAHAAAATLRRPLYRYLGGCFACSLP
CPMMNLINGGMHADNGLGFQEFMIRPIGASSIKEAVNMGADVFHTLKKLLHERGLSTGVGDEGGFAPNLAS
NEEALELLLLAIEKAGFTPGKDISLALDCAASSFYNVKTGTYDGRHYEEQIAILSNLCDRYPIDSIEDGLAEED

YDGWALLTEVLGEKVQIVGDDLFVTNPELILEGISNGLANSVLIKPNQIGTLTETVYAIKLAQMAGYTTIISHR
SGETTDTTIADLAVAFNAGQIKTGSLSRSERVAKYNRLMEIEEELGSEAIFTDSNVFSYEDSEE

>core/294/3/Org3_Gene949

MDIKKLFCLFLCSSLIAMSPIYGKTGDYEKLTLTGINIIDRNGLSETICSKEKLKKYTKVDFLAPQPYQKVMRM
YKNKRGDNVSCLTAYHTNGQIKQYLECLNNRAYGRYREWHVNGNIKIQAEVIGGIADLHPSAESGWLFDQT
TFAYNDEGILEAAIVYEKGLLEGSSVYYHTNGNIWKECPYHKGVPQGKFLTYTSSGKLLKEQNYQQGKRHG
LSIRYSEDSEEDVLAWEEYHEGRLLKAEYLDPQTHEIYATIHEGNGIQAIYGKYAVIETRAFYRGEPYGKVTR
FDNSGTQIVQTYNLLQGAKHGEEFFFYPETGKPKLLLNWHEGILNGIVKTWYPGGTLESCKELVNNKKSGLL
TIYYPEGQIMANEEYDNDLLIKGEYFRPGDRHPYSKIDRGCGTAVFFSSAGTITKKIPYQDGKPLLN

>core/295/3/Org3_Gene60

MKKTMVIDTSVFIYDPEALFSFENTRIIIPFPVIEELEAFGKFRDESAKNASRALSNIRLLLENAKTKVTDGVLLP
SGSELRIEVAPLSNDDRRGKLLTLELLKIIAKREPMVFVTKSLGRRVRAEALQIESRDYESKRFSFRSLYRGFRE
LQVSQEDIENFYKNGYLDLPLDVVSSPNEYFFMSAGENHFALGRYYVSEGKIIALKAMDKSVWGIKPLNTEQ
RCALDLLLRDDVKLVTLIGQAGSGKTILALAAAMHKVFDKETYNKVLVSRPIVPMGRDIGFLPGLKEDKLM
HWMQPIYDNMEVLFSINQMGNSSEALQALMDAKKLEMEALTYIRGRSLPKAFIIIDEAQNLTPHEIKTIISRAG
KGTKIVLTGDPTQIDSLYFDENSNGLTYLVGKFHHLALYGHMFMTRTERSELAAAAATIL

>core/296/3/Org3_Gene616

MLDIKIIRKTPEECETRLRKKDPKISLEPVLSLDKEVRQLKTDSETLQAQRRLLSQDIHKAKTQGVDATNLIQE
VETLAADLEKIEQHLDQKNAQLHELLSHLPNYPADDIPVSEDKAGNQVIKSVGDLPIFSFPPKHHLELNQELDI
LDFQAAAKTTGSGWPAYKNRGVLLEWALLTYMLQKQAAHGFQLWLPPLLVKKEILFGSGQIPKFDGQYYR
VEDGEQYLYLIPTAEVVLNGFRSQDILTEKELPLYYAACTPCFRREAGAAGAQERGLVRVHQFHKVEMFAFT
TPNQDDIAYEKMLSIVEEMLTELKLPYRLSLLSTGDMSFTASKTIDAEVWLPGQKAFYEVSSISQCTDFQSRRS
GTRYKDSQGKLQFVHTLNGSGLATPRLLVAILENNQQADGSVVIPEVLRPYLGGLEILLPKDQ

>core/297/3/Org3_Gene933

MLPLIIFVLLCGFYTSWNIGANDVANAVGPSVGSGVLTLRQAVVIAAIFEFFGALLLGDRVAGTIESSIVSVTN
PMIASGDYMYGMTAALLATGVWLQLASFFGVPVSTTHSIVGAVIGFGLVLGKGTIIYWNSVGIILISWILSPFM
GGCVAYLIFSFIRRHIFYKNDPVLAMVRVAPFLAALVIMTLGTVMISGGVILKVSSTPWAVSGVLVCGLLSYII
TFYYVHTKHCSYISDTPKKGSLTYRLKERGGNYGRKYLVVERIFAYLQIIVACFMAFAHGSNDVANAIAPVA
GVLRQAYPASYTSYTLIRLMAFGGIGSVIGLAIWGWRVIETVGCKITELTPSRGFSVGMGSALTIALASILGLPI
STTHVVVGAVLGIGLARGIRAINLNIIKDIVLSWFITLPAGALLSILFFFALRALFH

>core/298/3/Org3_Gene1017

MIMGLQSRLQHCIEVSQNSNFDSQVKQFIYACQDKTLRQSVLKIFRYHPLLKIHDIARAVYLLMALEEGEDLG
LSFLNVQQYPSGAVELFSCGGFPWKGLPYPAEHAEFGLLLLQIAEFYEESQAYVSKMSHFQQALFDHQGSVF
PSLWSQENSRLLKEKTTLSQSFLFQLGMQIHPEYSLEDPALGFWMQRTRSSSAFVAASGCQSSLGAYSSGDV
GVIAYGPCSGDISDCYYFGCCGIAKEFVCQKSHQTTEISFLTSTGKPHPRNTGFSYLRDSYVHLPIRCKITISDK

QYRVHAALAEATSAMTFSIFCKGKNCQVVDGPRLRSCSLDSYKGPGNDIMILGENDAINIVSASPYMEIFALQ
GKEKFWNADFLINIPYKEEGVMLIFEKKVTSEKGRFFTKMN

>core/299/3/Org3_Gene733

MDKQSSGNSGCIWHPFTQSALDSTPIKIVRGEGAYLYAESGTRYLDAISSWWCNLHGHGHPYITKKLCEQAQ
KLEHVIFANFTHEPALELVSKLAPLLPEGLERFFFSDNGSTSIEIAMKIAVQYYYNQNKAKSHFVGLSNAYHG
DTFGAMSIAGTSPTTVPFHDLFLPSSTIAAPYYGKEELAIAQAKTVFSESNIAAFIYEPLLQGAGGMLMYNPEG
LKEILKLAKHYGVLCIADEILTGFGRTGPLFASEFTDIPPDIICLSKGLTGGYLPLALTVTTKEIHDAFVSQDRM
KALLHGHTFTGNPLGCSAALASLDLTLSPECLQQRQMIERCHQEFQEAHGSLWQRCEVLGTVLALDYPAEAT
GYFSQYRDHLNRFFLERGVLLRPLGNTLYVLPPYCIQEEDLRIIYSHLQDALCLQPQ

>core/300/3/Org3_Gene19

MAEISTPSLPDSSIVSQKTPPVPDPDSSPDHIPTIPTQAPFKPQRKKETPSSIVNAIAFAILAFLSCLGGVFAICLGC
SLEITMPLFILTAVFIAFTLLYFIHYLEKPKIPEPLPTPPPSPTLRAPTLTPEIPAPAPGIPLPPTLPKVDRTKLTCNP
DIHYPSTYDPKACFSLLKQLFSLDPETRPEDRKYSNKLASILLRSKEKSGFRFHCFKGHFSHDKILNKKSGAVV
ISSHSSMDFSTTLGRAFAVTTCLQRSCWEKIKNNIPTPEKHLPIGSCVSGPWDVEEGAQLYTSHLIVINPPTLET
LIKEKMRRAITLKDFSMKEAFTNLVLAYLQCFDICIEHNLESVQLEVFGLNNLSADQEEFTTWESCCHLALLE
SVRILLASKEEYALSNVSVNSISQVPLQTACRALFLN

>core/301/3/Org3_Gene943

MKKQVYQWLASVVLLALTISGYAELPLSEQKVKSHTYTTLDEVKDYLSKRGFVKTRKQDGVLRIAGDVRAR
WLYFREDIKNPSDKDKYNPLPVNRYRSEFYLYIDYRAERNWLSSKMNWTAIAGGENTAAGVDINRAFLGYR
FYKNPETRTDFFMEIGRSGLGDLFESEVQFQSNFDGLHIYWTRELSKDYPYQVIVHGGPFVVNMTKKHYAW
VVEGILNRLPKQFFVKCSVVDWNTFVPSETSTTEKAATNAMKYKYCVWQWLVGKHSQVPWINGQKKPLYL
YGAFLMNPLAKATKTTLNGKENLAWFIGGTLGGLRKAGDWSATVRYEYVEALSVPEIDVSGIGRGNLLKFW
FAQAIAANYDPKEANGFTNYKGFSALYMYGITDSLSFRAYGAYSKPANDKLGSDFTFRKFDLGIISAF

>core/302/3/Org3_Gene998

MQSWLQSLQERNILENFTAGLESVEGPIAAYLGFDPTAPALHIGHWIGICFLKRLAALGITPIALVGGATGMV
GDPSGKQSERSLLQTSEVFDNSQKITACLQRYLPGVTLVNNADWLQEISLIDFLRDIGKHFRLGQMLVKDTIK
QRVHSDEGISYTEFSYLILQSYDFYHLFKNYGTILQCGGSDQWGNITSGIDFIRRKGLGQAYGLTYPLLTNAQ
GKKIGKTESGTVWLDSDLTSPFELYQYLLRLPDDTIPKIARTLTLLSNEEIQDIDRRVQTDPVAVKEFVAQDILS
AIHGDLGLEEALSVTRSMHPGNLSSLSEKDFHELFAGGMGASLDKSEVLGKRWLGLFLVLGLCKSKGEIRRLI
EQKGVYINNVPIANEHSVCEEQDICYGHYVLLAQGKKRKLVLYLN

>core/303/3/Org3_Gene773

MTVAEVKGTFKLVCLGCRVNQYEVQAYRDQLTILGYQEVLDSEIPADLCIINTCAVTASAESSGRHAVRQLC
RQNPTAHIVVTGCLGESDKEFFASLDRQCTLVSNKEKSRLIEKIFSYDTTFPEFKIHSFEGKSRAFIKVQDGCNS
FCSYCIIPYLRGRSVSRPAEKILAEIAGVVDQGYREVVIAGINVGDYCDGERSLASLIEQVDQIPGIERIRISSIDP
DDITEDLHRAITSSRHTCPSSHLVLQSGSNSILKRMNRKYSRGDFLDCVEKFRASDPRYAFTTDVIVGFPGESD

QDFEDTLRIIEDVGFIKVHSFPFSARRRTKAYTFDNQIPNQVIYERKKYLAEVAKRVGQKEMMKRLGETTEVL
VEKVTGQVATGHSPYFEKVSFPVVGTVAINTLVSVRLDRVEEEGLIGEIV

>core/304/3/Org3_Gene598

MNKKNLTICSFCGRSEKDVEKLIAGPSVYICDYCIKLCSGILDKKPSSTISSAPVSETPSQPSDLRVLTPKEIKKH
IDEYVIGQERAKKTIAVAVYNHYKRIRALLHNKQVSYGKSNVLLLGPTGSGKTLIAKTLAKILDVPFTIADAT
TLTEAGYVGEDVENIVLRLLQAADYDVARAERGIIYIDEIDKIGRTTANVSITRDVSGEGVQQALLKIVEGTTA
NVPPKGGRKHPNQEYIRVNTENILFIVGGAFVNLDKIIAKRLGKTTIGFSDDQADLSQKTRDHLLAKVETEDLI
AFGMIPEFVGRFNCIVNCEELSLDELVAILTEPTNAIVKQYMELFAEENVKLVFKKEALYAIAKKAKQAKTGA
RALGMILENLLRDLMFEIPSDPTVEAIHIQEDTIAENKAPIIIRRTPEAIA

>core/305/3/Org3_Gene55

MIKFLSQLFIRHWPRKVVSLGFAIIIWILVGQSVTITRTLTNVPVRIVDLHPDQTVLGLQKSGFLNKKVSLTITG
NKNTVQDLRPSNLEVVISAANHTESWIATIDKHNLVSVDHEINIRKDIHSVDANDIFVRLTQYVTEDILLTITKP
IGSPPKGYEYLDVWPKYLNQKVSGPKEYINALKEQGLELTFNLNKISFEELERNRIAQGSHDEIIFPIPKEWKKI
LIPFENTFMDLNDPQADFLRLLFLKRECIPLNLNLPVFLFFPVTFIQTMNPLEYSLDPVPPIILNHGIHQINIPLYV
KDVSRQFLDVVKNNMVLTIVMPSPQDPSSINWAIEFLDEKTLENTFLQTIIAQEHGILHDIALIDEAGIRHRFRE
YLRKLALFTADGEPLNLIAEIKNNKVVIQTKTKETTKLYKKEW

>core/306/3/Org3_Gene716

MLKLQLCALFLFGYLAIVFEHIVRVNKSAIALAMGGLMWLVCFSHIPMADHMILVEEIADMSQVIFFLFSAM
AIVELIDAHKGFSVIVKFCRIQSRTLLLWALIGLSFFLSAALDNLTSIIIIISILKRLVKAREDRLLLGAICVIAVNA
GGAWTPLGDVTTTMLWINNKITSWGIIRALFVPSLVCVLVAGFCGQFFLRKRGSTLIAKDVELQSAPPKSLWII
FIGLGSLLMVPVWKACLGLPPFMGALLGLGLVWLTSDWIHSPHGEDRYHLRVPHILTKIDISSITFFIGILLAVN
ALSFANLLTDFSLWMDKIFSRNVVAIVIGLLSSVLDNVPLVAATMGMYTLPLDDTLWKLIAYAAGTGGSILII
GSAAGVAFMGLEKVDFLWYFKRISWIALASYFGGLFSYFVLESLNFFI

>core/307/3/Org3_Gene893

MSSAIARDCFPSPSPQPSSTLGVHPPKYKSLILSVSLIVLGVLLLCVGMLLLVNAIFSFSVLTVGLGGAGVFLGS
LLLILGLIFFVSYHRKLSEATRSLEQKITLEYHPWADLRKELNEVQEWSNFLLDEWEDFKEVVAQHKSQFATF
EGDLLLFGREVEKYETIWKELDGRDVALLTELKNIWGPLEFLRKKGDRLQCEIDKLRKEVMKVGKSGLKLA
CELTKFKSALKDVKIEQECYRDKRKVEKLEVFPEGYRRELLEVLKTRLSVEKEIQLFEEVVSAFEEKLASLHR
TVFSEEELQEALDKAKAELLDIQVRKSVVEDLSCEPTLIQYHLLRLYEVQCRIVEQFLTQTFSSEQEKVLEEYE
ALKARIRKTLRVKLDQVRANVAFVASTTDLLSESESLDGNDSVFEDAHDDFLD

>core/309/3/Org3_Gene618

MIETREEVGSANFVSLERAIEDLRAGKFVIVVDEASREDEGDLIIAGEKITVEKMTFLLQHTTGVVCAALSQER
LLSLDLPPMVKDNRCRFKTPFTVSVDAAHGVTTGVSAADRTKVVQLLADPKSKPEDFISPGHFFPLASSPGGV
LKRAGHTESTVDLMELAGLQPCGVLAELVNEDYSMMRLPQILEFARKHNIAVIPVTSIIAHRMLSDRLVSKIS
SARLPTIYGDFTIHVYESLLEGMQHLALVKGNVAGKSNVLVRVHSECVTGDILGSKRCDCGEQLSSAMSYIA

EKGTGVLVYLRGQEGRGIGLGHKVRAYALQDNGYDTVDANLAMGFPVDSREYGIGAQILVDLKLTTIKLIT
HNPQKYFGLQGFGLSITERVPLPVRISEDNEQYLRTKQERMGHWLDLPCCNNRVQ

>core/310/3/Org3_Gene857

MLCYSPNYVTDLYRISLSAEESLGGIRAFPQAESLLGGACALNFPDLEERLPDLRKELLFLGSNDRPDACGGK
FSLQLASSKECYIAALKERVYLNVTNSSRGPVYSFSPKGVPTELWIECFSVSVDGRVEVKVRLQGLHKELISKP
RDCETLFLNPPANKLDCWEIAGFRVDASFPVKQKIRRIGVDKFLLMHGGAEYADKATKERVDFVSSDEENYS
RYLAVGDVLLWDGNCWQTCGEFQGASSRAPLFEVKRIDDKVMIADLWNVGGTQRQTISLVKGVPSPIEINE
VIREIEFTGMRSWSKPIVLVGGQRLILSPDDWILRTAKGWEKLSRADQIQDYVTGKVTGPLLVFEKLEKDLRG
FVLRGHMFNAQRTLVETISLPLKQGFEPAVASQEVSSNTRSAAAHPGATNRGGS

>core/311/3/Org3_Gene785

MSQPPINPLGQPQVPAAASPSGQPSVVKRLKTSSTGLFKRFITVPDKYPKMRYVYDTGIIALAAIAILSILLTAS
GNSLMLYALAPALALGALGVTLLISDILDSPKAKKIGEAITAIVVPIIVLAIAAGLIAGAFVASSGTMLVFANPM
FVMGLITVGLYFMSLNKLTLDYFRREHLLRMEKKTQETAEPILVTPSADDAKKIAVEKKKDLSASARMEEHE
ASQRQDARHRRIGREAQGSFFYSSRNPEHRRSFGSLSRFKTKPSDAASTRPASISPPFKDDFQPYHFKDLRSSSF
GSGASSAFTPIMPASSRSPNFSTGTVLHPEPVYPKGGKEPSIPRVSSSSRRSPRDRQDKQQQQQNQDEEQKQQS
KKKSGKSNQSLKTPPPDGKSTANLSPSNPFSDGYDEREKRKHRKNK

>core/312/3/Org3_Gene962

MCQRILILGTGITGKSVARFLYQQGHYLIGADNSLESLISVDHLHDRLLMGASEFPENIDLVIRSPGIKPYHPW
VEQAVSLKIPVVTDIQVALKTPEFQRYPSFGITGSNGKTTTTLFLTHLLNTLGIPAIAMGNIGLPILDHMGQPGV
RVVEISSFQLATQEEHVPALSGSVFLNFSRNHLDYHRNLDAYFDAKLRIQKCLRQDKTFWVWEECSLGNSYQ
IYSEEIEEILDKGDALKPIYLHDRDNYCAAYALANEVGWVSPEGFLKAIRTFEKPAHRLEYLGKKDGVHYIND
SKATTVTAVEKALMAVGKDVIVILGGKDKGGDFPALASVLSQTTKHVIAMGECRQTIADALSEKIPLTLSKD
LQEAVSIAQTIAQEGDTVLLSPGCASFDQFQSFKERGAYFKLLIREMQAVR

>core/313/3/Org3_Gene644

MSKKRVVVTGFGVVSCLGNEVDTFYDNLLAGVSGVRPITSFPCEDYATRFAGWIPEFNPEPYVDKKQARRV
DPFITYAMVAAKKAIAMSRWDKDHLPSDPVRCGVIVGSGMGGLSTLDQGMERLLVIHKKLSPFFIPYIITNMA
PALIAMDFGLMGPNYSISTACATGNYCIDAAYQHLVSGRADMIICGGTEAAVNRIGLEGFIANRALSERNDAP
DQASRPWDRDRDGFVLGEGAGILVLETLESALRRDAPIFAEMLGSYVTCDAFHITAPRDDGEGITACVLGAL
NSAGIPKERVNYVNAHGTSTPLGDLSEVLAVKKAFGSHVRNLRMNSTKSLIGHCLGAAGGVEAVVAIQAILT
GKLHPTINLDNPIAEIEDFDVVANKAQDWDIDVAMSNSFGFGGHNSTILFSRYVP

>core/314/3/Org3_Gene482

MLVSIETFSSIASGSPVQKAAEACYTQYSKQPSSKEVLSSFSWIQELSLFPDRYNLATGASELIKQHWLHNNHS
LAFECILINGKYEPSLSQLPEGVIVCGIDEARGSLSSFMQGFDVNKHPLAFLNAVCSEDRGVVIYIPEEMQTSDP
IFVRHISFPTVSDHDVIFSPRIVVILGQRASAQIQISHDVDLEMVGSSKTIVNGVTELFVGEGADLTVFMVPGYS
EEDTLSWSTIATVEKDAICRMTQNLLESCQGFGWFDNTSYIVGKKGHAESLVLVQSPRKTWVNNLMSHDAE

ETVSRQNIKSILYSGHFLFEGTISISSQGDLSDANQKHDTLLLSSEARVSTFPRLEIETDEVKASHGATVGPLDP
QQIFYMRSRGMTEAEAQEKLIHGFLKQGLVSDTFLGSSFQLNQTS

>core/315/3/Org3_Gene853

MRYHKYFRYVNSWVFLVVLTLMLLSVVVISSMDPTAMLVTSSKGLLTNKSIMQLRHFALGWVVFFICAYFD
YHLFKRWAWVLYFFMICALVGLFFVPSVQNVHRWYRIPFIHMSVQPSEYGKLVIVIMLSYILESRKADITSKT
TAFLACLVVALPFFLILKEPDLGTALVLCPVTLTIFYLSNVHSLLVKFCTVVATIGIIGSLLIFSGIVSHQKVKPY
ALKVIKEYQYERLSPSNHHQRASLISIGLGGIRGRGWKTGEFAGRGWLPYGYTDSVFSALGEEFGLLGLLFTL
GLFYCLICFGCRTVAVATDDFGKLLAAGITVYLAMHVLINISMMCGLLPITGVPLILISYGGSSVISTMASLGV
LQSIYSHRFAKY

>core/316/3/Org3_Gene350

MKLWMKIFIGLFVGVTLGLVLEDKAIFFKPIGDIFLNLLSMVVYPLVFCSMVLGIASISDMKKLGRIGIKSVGL
YLGTTALAIVIGLCFAWIFSPGNGCDFAQAQSMDSAVTVIDSNKTAAYFLSIIAQVFPSNPVRSFAEGNILQIIIF
AIFLGIALRLSGERGRPVERFIDGFSEIMLRMVNMIMSFAPYGVGASMAWISGNHGLGVLWQLGKFIIAYYLA
CLFHATLVFGGLVRFGCKMSFSKFLSSMMDAISCAVSTASSSATLPVTMRCVSKNLGVSAEVSGFVLPLGAT
VNMNGTAIFQGMAAVFIAQAYNCPLSLSSLLLLVVTATFSAVGSAGVPGGGMITLGSVLASVGLPIQGIAILA
GIDRLRDIVGTPMNILGDAVVATYVASGEGELSPYESIKQESVETT

>core/317/3/Org3_Gene548

MKKIFYSFVLLSCIFPYVGCAQVFVGLDRIFSEGEYTRCIQGKKIALISHSAAINSRGQDALSVFYSRKHDCTVE
ILCTLEHGYYGATPTETVGNQPSRYPNLRSVSLYGVKEVPKEVAEHCDVFVYDVQDIGVRSYSFVTVLMQIV
KASERYGKQLIVLDRPNPMGGRIVDGPLPNPTTSGSLAIPYCYGMTPGELALFFKKTYAPNANVVVIPMKGW
NRSMTFDETGLIWMPTSPQMPDPQSPFFYAATGILGALSVASIGVGYTLPFKVLGAPWMDGEKVADELNRM
KLPGVLFLPFFYEPFFGKYKMEMCSGVLLVLQDPKIFYPVETQCTIWGVLKALYPKQVEQTLKSIERIPARRSS
ICNLFGGDEFLSISHKERYIVWPLRRLCKESRESFHQLRSSCLLSEYAES

>core/318/3/Org3_Gene855

MKKNASHKTNDKKSLSIWSIGGSIFAMFFGAGNIVFPLALGYHYNAHPWSAYFGMMLTAVCVPLLGLVSML
FYSGDYQKFFFSIGRIPGMIFITAIILLIGPFGGIPRAIAVSHATLISLSEHKSAFIPSLPIFSAICCVLIYIFSCKLSRL
IQWLGSVFFPIMLVTLLWVIIRSFMIPTHPMVQEFIPNARQAWLAGFIEGFNTMDLLAAFFFCSIVLISLRQLVA
EEKHPTEEEIPLSFQGISKKNKRSLALGFFLAAILLGMTYLGFVLSAARHAGLLVNVSKGHILGRISAIALGPNS
ILAGVSVFIACLTTEIALVGIVADFLARVVSFKKLNYASAVICTLIPTYLISILNFETISHLLLPLLQLSYPALIVLA
CGNIAYKLWNFRYSPVLFYLTLSLTIVLKLVN

>core/319/3/Org3_Gene144

MDKETLENIYRHFRYRFLKLNILPAFLGLLLLCSPNTLNYTQVDVIFSDRLCSCLLIFLAIASLTKRSLLWLGAP
LGIWVTLFACVAGRSPTIFANDTLIGFAILAVVCISPTRPEALEVGPTLPEGFSYNPSAGGRRAAVLFLSLLGWL
EARYLTASSLGITSSQSSNFLLLYSSIMTVYSLLVVLSLAGSERRWHTRPKIVIATALALTGVIILTLLPIILHQLR
YDCWLCLCLTIEPALAVVFAYDETRATLRYISQFLGDKRALTRASFFGSEYYKHTLSWEERTVLPLRKAYKQ

AFEGISFPINQLLAILVATVFVKVNSSMGLPTFPRNFLNICCWFIIVLFILAFAESLRHLRWMNLIFSAAILFSPVL
FHIPVESPMFLPIIVTGLILIILSIGKRRRTKRKL

>core/320/3/Org3_Gene908

MIPTMLMFFIICFTLCSGFISLSQIALFSLPTSLISHYKRSKSKKQQRVATLLLHPHHLLITLIFCDIGLNIAIQNCL
AILFGDAASWWFTVGLPLAITLILGEILPKAVALPFNTQIASSVAPLILCVTKIFKPLLHWGIVGINYVVQWILS
KQQIDIIQPQELKEVLQSCKDFGVVNQEESRLLYGYLSLSDCSVKERMQPRQDILFYDIQTPLENLYLLFSKQH
CSRVPICNDNLQNLLGICTARSLLLHDKPLQSSDDLLPLLKKPYYMPETISAKMALCQMAAEDETLGMIIDEY
GSIEGLITQEDLFEIVAGEIVDQRDNKILYTTSGADVIIASGTLELREFSEIFDINLPTNNNIATIGGWLIEQIGTIP
TTGMKLSWNNLLFQVLDAAPNRIRRVYIRKLYD

>core/321/3/Org3_Gene807

MDESDGEEASKDSAFSASFSYEFVKSSTRESKNTVTHSTASRTLYILRQDCSYDPRALKVDDEFRYWVEKRL
DAKNPDSLNAFVKEVGTHYVASVTYGGIGFQVLKMSYLQVEELEKEKISISVAAASSLLKSKTSNATEKGYS
SYQSESSAQTVFLGGTVLPDLQQDKLDFKDWSESIPNEPIPLAISVSSITDLIIPELFPSEDAQVLSQKKSALGQV
ILNYLESHKPKEEGPKPVQITSGFNSSSSVFTLQAAKAPKTVSFPYIDYWSTIPYLFPTLKETSGAQPLSFYLRF
DDIFEQQNLVHNTSYILASTSVRLGYFGDSYRDYDALSFYGSWPQAYFDWAGYKDRCTWTLEKLNTTGDLF
IRSGDEIRLKHNTSGKYLATTSMSDGYQTLTCTTQTSDSVFIITV

>core/322/3/Org3_Gene688

MNLPVSLACLLLSGCVFFLGVFVSSSLYARKKRAFLEKIQKLEHENQLLQTSLNLSRHQEQLIEDFSNRLALSS
HKLIKDMKEEAQNYFGDTSKSFQSILSPIQTTLTTFKQSLETFETKHAEDRGRLKEQISQLLAVEKKLEHETHV
LTDILKHPGSRGRWGEIQLERILELAGMLKYCDYDSQTTSAQGAFRADIIIRLPQDRCLIIDAKAPISDSYFSVE
EIDKGDLVDKIKEHIKTLKSKSYWEKFHQSPEYVILFLPGESLFNDAIRLAPELMEIGASSNVILSSPLTLLALLK
TIAYMWKQENLQKQIQEVSLLGKELHRRLQVVFTHFQKIGKNLNQTVQSYNDMTSSFQYRVLPTLRKFEGLE
TSSSHQIEEPTPIESLATSFPHTCDIDTNLAVIESLEKQD

>core/323/3/Org3_Gene770

MKTAFHSCYSWFCWLFSFLVLFVGGIAGGEPLCPDCKYETKSVLRSDQLPDHLWNYENDCYLTGYVQSLLD
MHFLDSRTQVVIEKNRAYLFSLPVDSSLSEAITNFVRDLPFICAVEICERPYGECITRSSAERPLLPKEKTLGMPI
FCGKEGVWLPQNTILFSPLIADPRQVTNSAGIRFNEKVVGNRVGATIFGGDFILLRLFDVSRFHVDCDFGIQGG
VFSVFDLDHPESCMVNSDFFVAGLWSGAIDKWSFRFRLWHLSSHLGDEFILTHPNFPRFNLSDEGVDLFISFR
YTPQIRLYGGCGYIVSRDLTFPERPFYCEWGAELRPFGLREGNLHAQPIFAMHFRCWEEQKFGLDQSYILGME
WAKFQEIGRKIRAVLEYHQGFSKEGQFIREPCNYYGFRLTYGF

>core/324/3/Org3_Gene597

MTTIAIEAAKKVLIKLRNAGYQAYFVGGCVRDMLMNRPLEDIDIATNASPTIVSTIFPDVISIGVAFGIIVVKQD
GRLFEVATFRSDGEYKDGRHPDRIIFSSMREDALRRDFTVNGMYYDPFEDKVFDFVEGTRDIEKKVIRAIGHP
RLRFSEDKLRILRAIRFSSSLGFTLDPTTERAIIKEAPALVNSVSPERIWQELKKMLKRQPYGALSLLLKLKVLIF
IFPELRDIPYSLLRTTIEFARKFNPTHFPEILFLLPLFQGVSEEAATVAFGRLRISNKELKLIESWYEALPHFQNQS

GNRVFWAHFLASPTAPLFLELFSALQKDPSRQQHFISRVQELESRLEQFILRIKTSSPVVSAPDLIAKGISPGRLL
GDLLREAEILSIENECLDKEKILLLLQEKGFWK

>core/325/3/Org3_Gene481

MKNLKEDFPIFAAKAKENEPFIYLDSAATTQKPQQVIDAVANFYTSSYATVNRAIYSSSRNVTEAYAAVREK
VRKWVSAASDSEIVFTRGTTAGLNLLAISVNDLWIPKGGVVLVSEAEHHANVLSWEIACRRRGSLVKKIRVH
DSGLIDLDDLEKLLNEGAQFVSIPHVSNVTGCVQPLQQVAELVHRYDAYLAVDGAQGAPHLPIDVQLWDVD
FYVFSSHKIYGPTGIGVLYGKKDLLDQLPPVEGGGDMVAIYDHQNPEYLPAPMKFEAGTPNIAGVLGLGAAL
DYLDGLSAKFIYDKEIALTTYLHKELLEIPGVEILGPSIEEPRGALIGMTIDGAHPLDLGFLLDLRGIAVRTGHQ
CAQPAMERWNVGHVLRVSLGIYNDEDDIDQFILVLQDSLDKIRR

>core/326/3/Org3_Gene131

MVVVALFILGIFFLSGSLAFLVHTSCGVLLGAALPILCIGLVLLAVALIVFLCHKHKTRQDLDYYDQDLDSLVI
HKKEIPNDISELRVTFEKLQNLFQFHTKDFSDLSQELQGKFINCMEKWLTLEDEVTKFLIVRDRFLETRRNFTT
FGEQVKGIQSNIFDLHEEKSSLYLELYRLRKDLQVLLNFFLLPPGILKVDYDEIEAIKGLFIRLTSRLDKLDVKA
QERKKFINEMSREFKEVEKAFDIVDRATKKLMDRAKKESPARLFMGRTESLLEMKKNEEALKNQGLDPENL
SHPELFSPYQQLLILNYLNSEIVLHHYEFLISGTVTSGLTLEECENRMRAASTGLNALLVRKLQFRGAIKSAYF
EKLTEIEKELRSLQDVIKSLELELIHKIKDIVTEET

>core/327/3/Org3_Gene245

MTDSNPLPSYTDASLYRTPAKHSYPIRLPLNRTDRIEKILKIVTLTLALACALGFSIAAGILAMPIFSAVVVITLA
IAAVSLYSLLKKPKLYEILPQIEPESEQSSLSPSPQPPEQQDLPLQIDPLPDPESLPEVSLADLTTPPEELTAITVTP
GYEALLEQNWDLLPSLAAVDPSFTTETPQQPCFIWKLKDSKLIFISTSGDIAVPRIKTQGRVMIVNAANENISR
EGGGTNKALSLATSLQCWNASRLPRAHSRSGSQLQPGECRSAKWENSDHTSNDHVPGKAHFLAQLLGPEAA
KCNNDPKQAFEVSKKAFHNLFQEAEIIGVDVIQLPLIGCNLFAPSRLLNLGKTRAEWIEAIKLALITSLQDFGW
EQDNQEEQKIIILTDKDQPPIIPPRFDLTTP

>core/328/3/Org3_Gene798

MKKQRSHYTKNNLLLLLSILVGLGLGSVQSPWIVYSAECIANTFLKFLRLLSIPLVFCALGSTITSIQNFNTMVT
LGKRILYYTLLTTVIAASIGLLLFFLLRPQMITQDALATTTKCNPLGYLDVLSDTLPENIFKPFLQGNVISAACL
AVLLGTASLFLQEKEKHFVNQFFNSFFSIFLNLARGGLKLLPIAMLGFSVILFKELKDQSNLTMFAEYLLCVIG
ANLAQGFIVLPILLKINKVSPLKVAKAMSPALVTAFFSKSSAATLPLTMELAEDDLKINKNLSRFSFPLCSVIN
MNGCAAFILITVLFVATSNGMIISPLMSLGWIFIATLAAIGNAGVPMGCYFLTLSLLTSMNVPLSILGLILPFYT
VIDMIETSLNVWSDCCVVSLAN

>core/329/3/Org3_Gene265

MTNSALFWIGVNIICIVLQGFYSMMEMACVSFNRVRLQYYLTKDHKKARYINFLIRRPYRLFGTVMLGVNIA
LQVGSESSRNCYRALGITPDYAPFTQIFIVVIFAELLPLTISRKIPEKLALWGAPILYYSHYIFYPLIQLIGSLTEGL
YYLLNIRKEKLNSTLSRDEFQKALETHHEEQDFNTIATNIFSLSATCADQVCQPLEQVTMLPSSANVKDFCRTI
KNTDINFIPVYHKARKNVIGIAHPKDFVNKALDEPLINNLHSPWFITAKSKLIRILKEFRDNRSSVAVVLNASG

EPIGILSLNAIFKILFNTTNIAHLKPKTISVIERTFPGNSRIKDLQKELDIQFPQYPVETLAQLVLQLLDSPAEVGT
SVIINNLLLEVKEMSLSGIKTVSIKNLLS

>core/330/3/Org3_Gene475

MDKLTVQDLSPEEKKVLVRVDFNVPMQDGKILDDIRIRSAMPTINYLLKKHAAVILMSHLGRPKGQGFQEEY
SLQPVVDVLEGYLGHHVPLAPDCVGEVARQAVAQLSPGRVLLLENLRFHIGEEHPEKDPTFAAELSSYGDFY
VNDAFGTSHRKHASVYVVPQAFPGRAAAGLLMEKELEFLGRHLLTSPKRPFTAILGGAKISSKIGVIEALLNQ
VDYLLLAGGMGFTFLQALGKSLGNSLVEKSALDLARNVLKIAKSRNVTIVLPSDVKAAENLQSKEYSVISIDQ
GIPPHLQGFDIGPRTTEEFIRIINQSATVFWNGPVGVYEVPPFDSGSIAIANALGNHPSAVTVVGGGDAAAVVA
LAGCSTKVSHVSTGGGASLEFLEQGFLPGTEVLSPSKS

>core/331/3/Org3_Gene851

MSTMQNCPHFGVCGGCSFPQSNYSDSLKKKEELLHQLFAPLVPSDMIAPIIPCSPSLRGRNKMEFSFFQTYEGE
KSLGFISSTKPKKGIPVTTCLLIHEQTMDILKLTREWWDKHPELMAYFPPKNKGSLCTLTVRTGSPQQNFMVI
LTTSGTPEYRVNEACIDEWKEILLSSSLNIASIYWEEKVAARGISTYYETKLLYGAPSIQQKLSLPSDGNSASFS
LRPRSFFQPQITQAAKIIETAKEFINPEGSETLLDLYCGAGTIGIMLSPYVKNVIGVEIIPDAVASAQENIKANNK
EDCVEVYLEDAKAFCKRNENCKAPDVIIIDPPRCGMQSKVLKYILRIGSPKIVYISCNPKTQFQECADLISGGY
RIKKMQPIDQFPYSTHLENIILLEREIDL

>core/332/3/Org3_Gene530

MLLVRKWLHTCFKYWIYFLPVVTLLLPLVCYPFLSISQKIYGYFVFTTISSLGWFFALRRRENQLKTAAVQLL
QTKIRKLTENNEGLRQIRESLKEHQQESAQLQIQSQKLKNSLFHLQGLLVKTKGEGQKLETLLLHRTEENRCL
KMQVDSLIQECGEKTEEVQTLNRELAETLAYQQALNDEYQATFSEQRNMLDKRQIYIGKLENKVQDLMYEI
RNLLQLESDIAENIPSQESNAVTGNISLQLSSELKKIAFKAENIEAASSLTASRYLHTDTSVHNYSLECRQLFDS
LREENLGMLFVYARQSQRAVFANALFKTWTGYCAEDFLKFGSDIVISGGKQWMEDLHSSREECSGRLVIKT
KSRGHLPFRYCLMALNKGPLCYHVLGVLYPLHKEVLQS

>core/333/3/Org3_Gene59

MRMLQISMLLLALGTAINSPAIYAADSQSVSFPEQLPSSFTGEIKGNHVRMRLAPHTDGTIIREFSKGDLVAVI
GESKDYYVISAPPGITGYVFRSFVLDNVVEGEQVNVRLEPSTSAPVLVRLSRGTQIQPASQEPHGKWLEVVLP
SQCVFYVAKNFVANKGPIELYTQREGQKKIAMDLINSALNFAHIELEKSLNEIDLEAIYKKINLVQSEEFKDVP
GIQGLIQKALEEIQDAYLSKSLESQNTSIASSQCSTPKVSSSEVTTSLLSRHIRKQTALKTAPLTQGRENLEYSLF
RIWASMQQGNDHSEALTQEAFYRAEQKKKQVLAGVLEVYPHVVKNNPGDYLLKAQENTIAFLYGTSINLEQ
WLGKRVTVECLPRPNNHFAFPAYYVVGIKEAS

>core/334/3/Org3_Gene215

MAASGGTGGLGGTQGVNLAAVEAAAAKADAAEVVASQEGSEMNMIQQSQDLTNPAAATRTKKKEEKFQT
LESRKKGEAGKAEKKSESTEEKPDTDLADKYASGNSEISGQELRGLRDAIGDDASPEDILALVQEKIKDPALQ
STALDYLVQTTPPSQGKLKEALIQARNTHTEQFGRTAIGAKNILFASQEYADQLNVSPSGLRSLYLEVTGDTH
TCDQLLSMLQDRYTYQDMAIVSSFLMKGMATELKRQGPYVPSAQLQVLMTETRNLQAVLTSYDYFESRVPI

LLDSLKAEGIQTPSDLNFVKVAESYHKIINDKFPTASKVEREVRNLIGDDVDSVTGVLNLFFSALRQTSSRLFS
SADKRQQLGAMIANALDAVNINNEDYPKASDFPKPYPWS

>core/335/3/Org3_Gene800

MVYFMVFSPSSESVVKANSVVRSNFCYFLENKFVSPSESTEVMFSEIMKGRVPDIESLFDRPTDMMMTGFKA
AQNLGNLFNSFGILIMCFSQCKSCQTPEKETSAIVLGATLLFFVIALILGPTLGALVYCAYKVYTLGKMIYSLN
KAKAKVLRHPAQNVFHRAAGVATIRSSEEAVKACKLYKSAMIGSLVVSLIASLALIALTAGIVLVLFFVAPGA
APVITAAMMGCCAAGGGALLISLLGLWIAIVRKAKHQEACVGHLTNVVLHTAVSEALLHDPSHFQTNALAR
DLFLTDCLSHYGHLFSNEEVAQLVQGGAPGGGSRPSQHYGGSSDYQNRRGGNGNFGGSHFGGGGGFAGSHF
GAGYPTAPTMPSAPPPFPPPAYDTIYG

>core/336/3/Org3_Gene329

MLSVTIVLVGLEMARSKVSKRDSKILDILFATTELYLKTGQPVGSKTLKESFCSDLSTATIRNYFAELEAEGFL
KKNHTSGGRIPTDLALRHYVDHQEECPEAEISAPIFDKISQLPSESRNIIKDLQKATELLGEILDLPTFFSSPRFEN
DSVTNIQITQVDKQRAVTILSTEFGQIFTDTLWLPEACDTLSIKRIEKFLQNYIRKLPTNEELSKKEEHLSMSLY
NEVVVRYLTRYCNFSEEDLYQTGMSKLLKYEAFKDPEVLALGLSLFENRRQMCELLNIGMHKGRATAFIGK
ELSDILGTSNPGCSVITIPYYMNRSPLGALGILGPINLPYKEALPLLKLFANKINETLTQSFYKFKLSFRRPLTSN
CKLSNEPILRTEYSSIKLLPSKETL

>core/337/3/Org3_Gene326

MRRNPHFSLLKPQYLFSEISKKLAQFRKENPEISVIDLSIGDTTQPLCRSITQAIKEFCVSQEKQETYRGYGPET
GLEKLRTKIASEVYENRISPEEIFISDGAKPDIFRLFSFFGSEKTLGLQDPVYPAYRDIAHITGIRDIIPLACRKET
GFIPELPNQQSLDILCLCYPNNPTGTVLTFQQLQALVNYANQHGTVLIFDAAYSAFVSDPSLPKSIFEIPEAKYC
AIEINSFSKSLGFTGMRLAWNVIPKELTYDNNEPMINDWKRLFATTFNGASLLMQEAGYYGLDLFPTPPAISL
YLTNAQKLKKSLETAGFSVHGGDHAPYLWVELPEGISDEEAFDFFLHQYHIAVTPGHGFGSCGQGFVRFSAL
TQPQNIALACDRLCTASLKETMVLA

>core/338/3/Org3_Gene682

MSNKVLGGSLLIAGSAIGAGVLAVPVLTAKGGFFPATFLYIVSWLFSMASGLCLLEVMTWMKESKNPVNML
SMAESILGHVGKISICLVYLFLFYSLLIAYFCEGGNILCRVFNCQNLGISWIRHLGPLGFAILMGPIIMAGTKVID
YCNRFFMFGLTVAFGIFCALGFLKIQPSFLVRSSWLTTINAFPVFFLAFGFQSIIPTLYYYMDKKVGDVKKAILI
GTLIPLVLYVLWEVVVLGAVSLPILSQAKIGGYTAVEALKQAHRSWAFYIAGELFGFFALVSSFVGVALGVM
DFLADGLKWNKKSHPFSIFFLTFIIPLAWAVCYPEIVLTCLKYAGGFGAAVIIGVFPTLIVWKGRYGKQHHRE
KQLVPGGKFALFLMFLLIVINVVSIYHEL

>core/339/3/Org3_Gene236

MVNIQPVYRNTQVNYSQATQFSVCQPALSLIIVSVVAAVLAIVALVCSQSLLSIELGTALVLVSLILFASAMFM
IYKMRQEPKELLIPKKIMELIQEHYPSIVVDFIRDQEVSIYEIHHLISILNKTNVFDKAPVYLQEKLLQFGIEKFK
DVHPSKLPNFEEILLQHCPLHWLGRLVYPMVSDVTPGTYGYYWCGPLGLYENAPSLFERRSLLLLKKISFGEF
ALLEDGLKKNTWSSSELVQIRQNLFTRYYADKEEVDEAELNADYEQFDSLLHLIFSHKLS

>core/340/3/Org3_Gene519

MSFFNHIPTFSPDAILGLQNVFFADKRPEKVNLVIGVYEHPQKRYGGLSCIRKAQTVILEEEQNKSYLPISGLQI
FLDEMRELVFGAVDPSAIVGFQSLGGTGALHLGARLLSVAKGSGKVYVPEQTWSNHIRIFSQEGLEVIRYPYY
SKEQKQLLFEPLIAFLKEVEKNSVILLHGCCHNPTGVDFTEDMWKELAILMKERELIPFFDTAYQGFAHGIEL
DRKPIEIFISEGNTVLVAASSSKNFALYGERVGYFAVHSTFTDELVKIHSFLEEKIRGEYSSPQRWGVEIVSTILS
NPYLKEEWQSELNFIRESLGKMRTRFVQALRKVAGHTFDFLLSQHGFFAYPGFSDKQVLFLREQHAVYTTAG
GRMNLNGITEKNIDHVVQSFIQAYEL

>core/341/3/Org3_Gene130

MVVSIYSEILSFSELTSCKHSLFPFGPIETASIRIHHVFNVVIVCLIILGTLFVCLGMVFLGVFSTYLLGMSSMILG
LLLISIGLALLKFKERYGLEPKELFGVEGGFDKKLPSEIIQMQDQIADLARELDLEQKKDTLIRGFSARLDVLE
GSKTEKKQILKIGVPRNLSEIQERAQEQNSILEQCKEALLFRRKSAQEIFKKLYDRKAAFWRSYREDLWCYSEI
HVSKKALSNLYIGDVFEGTAPHFLMEAYAMCRTAKNLRNYVKVCVEDMRVNEEKKRAKQLSVSELLCCCT
EIETDLENETNLFTSDSEDVLEEYQIHCIRVTMLHALWAIYNDEVVSRKPIDTLDRVRARMAVEDCIETFEELQ
MCVVHTKTLELEIAQLYVDILLEA

>core/343/3/Org3_Gene39

MSKETFQRNKPHINIGTIGHVDHGKTTLTAAITRALSGDGLASFRDYSSIDNTPEEKARGITINASHVEYETPN
RHYAHVDCPGHADYVKNMITGAAQMDGAILVVSATDGAMPQTKEHILLARQVGVPYIVVFLNKVDMISQE
DAELIDLVEMELSELLEEKGYKGCPIIRGSALKALEGDANYIEKVRELMQAVDDNIPTPEREIDKPFLMPIEDV
FSISGRGTVVTGRIERGIVKVSDKVQLVGLGETKETIVTGVEMFRKELPEGRAGENVGLLLRGIGKNDVERG
MVVCQPNSVKPHTKFKSAVYVLQKEEGGRHKPFFSGYRPQFFFRTTDVTGVVTLPEGTEMVMPGDNVELDV
ELIGTVALEEGMRFAIREGGRTIGAGTISKINA

>core/344/3/Org3_Gene1004

MFEFRFPKIGETSSGGSIVRWLKNLGDHVARDEPLIEVSTDKIATELPSPKAGRLVRFCVNEGDEVASGDVLG
LIELEEEISEADDESTSCPLTSCETKSEAGSSSSSVWFSPAVLSLAQREGIGLDNLQKIAGTGKGGRVTRQDLEA
YISESQQVSIPEIFQGEVNRIPMSPLRRAIASSLSKSSDEVPHASLVVDVDVTDLMNLISGERQRFLDTHGVKLT
ITSFIVQCLAQTLRQFPLLNGSLDGTTIVMKKSVNVGVAVNLNKEGVVVPVIHNCQDRGLVSIAKALADLSSR
ARLNKLDPSEVQDGSVTVTNFGMTGALIGMPIIRYPEVAILGIGTIQKRVVVRDDDSLAIRKMVYVTLTFDHR
VLDGIYGSEFLTSLKNRLESVTMG

>core/345/3/Org3_Gene17

MDYYSILGISKTASAEEIKKAYRKLAVKYHPDKNPGDAAAEKRFKEVSEAYEVLSDPQKRDSYDRFGKDGPF
AGAGGFGGAGGMGNMEDALRTFMGAFGGEFGGGSFFDGLFGGLGEAFGMRSDPAGARQGASKKVHINLTF
EEAAHGVEKELVVSGYKSCETCSGQGAVNPQGIKSCERCKGSGQVVQSRGFFSMASTCPECGGEGRIITDPCS
SCRGQGRVKDKRSVHVHIPAGVDSGMRLKMEGYGDAGQNGAPSGDLYVFIDVESHPVFERRGDDLILELPIG
FVDAALGMKKEIPTLLKTEGSCRLTVPEGIQSGTILKVRNQGFPNVHGKGRGDLLVRISVETPQNLSEEQKEL
LRTFASTEKAENFPKKRSFLDKIKGFFSDFTV

>core/346/3/Org3_Gene115

MSSPVNNTPSAPNIPIPAPTTPGIPTTKPRSSFIEKVIIVAKYILFAIAATSGALGTILGLSGALTPGIGIALLVIFFV
SMVLLGLILKDSISGGEERRLREEVSRFTSENQRLTVITTTLETEVKDLKAAKDQLTLEIEAFRNENGNLKTTA
EDLEEQVSKLSEQLEALERINQLIQANAGDAQEISSELKKLISGWDSKVVEQINTSIQALKVLLGQEWVQEAQ
THVKAMQEQIQALQAEILGMHNQSTALQKSVENLLVQDQALTRVVGELLESENKLSQACSALRQEIEKLAQ
HETSLQQRIDAMLAQEQNLAEQVTALEKMKQEAQKAESEFIACVRDRTFGRRETPPPTTPVVEGDESQEEDE
GGTPPVSQPSSPVDRATGDGQ

>core/347/3/Org3_Gene576

MPRYRYTYLDPKERRKRGYLEALHIQEAREKLAQENIQVLDIREVALRRMSIKSTELIVFTKQLLLLLRSGLPL
YESLVSLRDQYHEQKMGLLLTSFMETLRSGGSLSQAMAAHPNIFDHFYCSGVAAGESVGNLEGCLQNIIVVL
EERAQITKKMVGALSYPCVLLVFSFAVMLFFLLGVIPSLKETFENMEVKGLTKIVFGVSDCLSAYRYLFLGFA
SALITVGILMRHRIPWKKILEKLLFALPGTKKFVVKVAVNRFCSVASAILKGGGTLIEGLDLGCDAIPYDRLKT
DMRDIVQAVIGGGSLSQELAQRSWVPKLAIGMIALGEESGDLADVLGYVAHIYNEDTQKTLASITSWCQPVI
LIFLGGLIGVIMLAILIPLTSNIQTL

>core/348/3/Org3_Gene603

MHPKIEKRNSLPLTAVAPVFEESYHPSVATTVDYVDATTLSRHLTVLKDVIKEARNLDLGKAFLTSMKQGFI
NTGTELAIIQASLADQSSRESRKKEEKIFHQHLGKAAPQAATATSGVQPTADPVADKMPLQSAFAYVLLDKYI
PAQEEALYALGRELNLSGYAQNLFSPLLDMIKSFNSAPINYNLGSYISQTSGTANFAYGYEMILSRYNNEVSQ
CRLDIASTVKAKAALANMSASVKANVSLTDAQKKQIEDIIASYTKSLDVIHTQLTDVMTNLASITFVPGLNKY
DPSYRIVGGDLSIIALQNDEKVLVDGKVDITTAVNEGGLLNFFTTVLTDVQNYGDLAQTQQLMLDLELKAM
QQQWSLVSASLKLLNGMYTTVISGFKN

>core/349/3/Org3_Gene486

MKKLLKSALLSAAFAGSVGSLQALPVGNPSDPSLLIDGTIWEGAAGDPCDPCATWCDAISLRAGFYGDYVFD
RILKVDAPKTFSMGAKPTGSAAANYTTAVDRPNPAYNKHLHDAEWFTNAGFIALNIWDRFDVFCTLGASNG
YIRGNSTAFNLVGLFGVKGTTVNANELPNVSLSNGVVELYTDTSFSWSVGARGALWECGCATLGAEFQYAQ
SKPKVEELNVICNVSQFSVNKPKGYKGVAFPLPTDAGVATATGTKSATINYHEWQVGASLSYRLNSLVPYIG
VQWSRATFDADNIRIAQPKLPTAVLNLTAWNPSLLGNATALSTTDSFSDFMQIVSCQINKFKSRKACGVTVG
ATLVDADKWSLTAEARLINERAAHVSGQFRF

>core/350/3/Org3_Gene309

MMKQGVGQDAKELYTFLSRGNEHYQPCLWFSLEEELGFLFDEKMLCAPLSEDHYCHSYLVDLVDQHLKDLI
LSMFLDPQNISAGELLKVSINVGDSFSPLQQKDFLSMVLRDETGKNVVVVFKGVLSLPATQVCKLVEELNSK
DYSYLNIFSCHGDSSPQLLFRKELEGTSGRYFTVICALYLGDTDMRSLQLASERIMVSREFDLVDAYAARCKL
LKIDHTNWRPGTFSRHADFADAVDVSAGFNSREFKLITQANQGILESGELPLPSKTFWEGFLAFCDRVTVTRH
FIPMLDAAIKQAVWTHKHPSLIDKECEALDLKTQCLPSIVSYLEYVTNSHEKTSKGPFIQKEIIADCSPLKEALF
PGSDEDVPSTSEDPSDDHPSDLEDS

>core/351/3/Org3_Gene472

MGTPISGNDGDRNTISDPLEESAAEEGDSDLEDRVSESATQVIETIADTGIPEATPSEGTNSDLNSDLVDRVEY
EARGSLLTTMLARIRKAVSQIWMHVKTKRHPKEQGVRSLGDIPCDLLKATRLPKETAEPPYFYALETALASC
RSFFFHVFLRLFTLLRRQHPEAPLDLCGTDPISPEAAVAFALILRSCCKWVATDAVQEGLPLEVIEEAGMYNA
FSLEATTTVEEVSKRLSELLYSDKRIDGLANVRGITKIITSPYLGAGQCVSVVDNLKTYDLGRNYTQVLACAS
QIDEFADKGENEALVMKDILYLVRQDRSKELGDFLMMWSEEHASEVNYDVVLAILEVNLPILEEDYRSHPLA
YQKKLNYVICQFFCSERLTSIEPKD

>core/352/3/Org3_Gene337

MLHILLAIFCILLFLAFGLTQPSCHGSSKFLKTLNQRFFKDKGREYPPFPSAPTILATLLCILYGALGTKLYTLLP
PKTAHKDLLFWPLYSLSALIAYGFLPPWISTKVPKETTAHLRFLASVFQLGLFPLQLLFYRRRPNQQVRSSTSF
QSQLSEALSAFDNLIVREVMIPKVDIFALPEETTLQEALVLVSEEGYSRVPVYKKNLDNITGILLVKDLLLLYT
SSHDLSQPISSVAKPPFYAPEIKKASSLLQEFRQKHRHLAIIVNEYGFTEGIATMEDIIEEIIGEIADEHDVQENTP
YKKIGSSWIVDGRMNISDAEEYFNLKIDHENSYDTLGGHVFHKVGAVPQKGMRIHHENFDIEIITCTERNVGK
LKITPRKRKFNIS

>core/353/3/Org3_Gene398

MAIKNILVVDDEPLLRDFLSELLTSQGFIPDTAENLRNALQMIRSRDYDLVISDMSMPDGSGLDLIKIIKQSSPH
TPVLVVTAYGSIENAVEAMHQGAFNYLTKPFSSEALFAFISKAEELKNLVHENLFLHSQTTPDSHPLIAESKA
MKDLLAIAKKAASSSANIFIHGESGCGKEVLSFFIHHNSPRANHPYIKVNCAAIPETLLESELFGHEKGAFTGA
TTKKAGRFELAHKGTLLLDEITEVPVNLQAKLLRAIQEKEIEHLGGTKTLSVDVRILATSNRKLKEAIDDKSFR
QDLYYRLNVIPLHLPPLRDRQDDILPLANYFLNKFCRMNNTPLKTLSPKAQELLLNYPWPGNIRELSNVLERV
VILENTSLLTEDMLALA

>core/354/3/Org3_Gene685

MHLHEYQAKDLLASYDVPIPPYWVVSSEEEGELLITKSGLDSAVVKVQVHAGGRGKHGGVIVAKSSAGILQ
AVAKLLGMHFTSNQTADGFLPVEKVLISPLVAIQREYYVAVIMDRKHRCPVLMLSKAGGMDIEEVAHSSPEQ
ILTLPLTSYGHIYSYQLRQATKFMEWEGEVMHQGVQLIKKLAKCFYENDVSLLEINPLVLTLEGELLVLDSKI
TIDDNALYRHPNLEVLYDPSQENVRDVLAKQIGLSYIALSGNIGCIVNGAGLAMSTLDILKLHGGNAANFLDV
GGGASQKQIQEAVSLVLSDESVKVLFINIFGGIMDCSVVASGLVAVMETRDQVVPTVIRLEGTNVELGKEIVQ
QSGIPCQFVSSMEEGARRAVELSM

>core/355/3/Org3_Gene636

MKWFVISCLLGIFSLGLIMVFDTSSAEVLDRSLECSTHKALIRQVTYLILGLGVASLLYMMEWRDFLKISPVLL
SGAALALICVFIPGLGICRNGARRWLGFGQLTIQPSEFVKYLVPIVALYFLTFSSLYQKQLKMFLKLTAILFIPIL
LIAIEPDNGSAAVISASLIPVFIMTSVRLRYWLLPLLCVLIAGGALAYRMPYVRYRLNVYLHPELDIKGRGHQP
YQAKIAAGSGKLLGKGPGASLQKLTYLPEAQNDYIAAIYAEEFGFLGMLVLILLYMCFVYGGYAIAIKASSLE
GAALAMVITLIISMQAFMNLGVVSGLLPSKGVNLPFFSQGGSSLIANMCGVTLLLKVYDEENSKSSLGCRRFR
RPHCPSSLGKGSFFS

>core/356/3/Org3_Gene768

MSSVNQSSGTPNPEEVTSPESTEENKNVVSSDEAQATHAVALPIVTQLSLPEGVGTSSEETASNPKVDEIVAEV
SSSRAVADQISSLVERVGELLDDLKGAQSLFTSFQSELKNCLPAWKSSTRRLETRGAGDNADIARLELFRSDY
EAVLGHANQFHGKAHLILSKLTDVHHKLQGLSREDLSLAFDNNDRVLEHLGSLGLDVDAEGNWSLSCERGI
PRLVLTADSMLVQIKKVNLPTVEELRTLQGTTESSSDPRVEESLSCCERLLNELRRLWANFVGFISSCYDNIVF
VLMWIVRRINLLPGLGCLPFHNPDASQEDQRSSSGERSTRRERLSRRSDLSEEEMIVRAEGESIHPESPHGDGR
NQPSRGDKQDSDSEEETEL

>core/357/3/Org3_Gene611

MEKPQNRKAPRIFWLNNQVAIPPSERVKESYALHSDIFSLPPGSALKLAEKTEESIRQLVGLKDSHIFRFVPHFP
HVVHIVLAALVENLSMFQGRNHIILPAHDQQLLINSLCRHQGLGTTYDWVTVNHEGRIVEEQLIETLSPRSLLF
SLSAAHGLTGVIQPLDPLLSLCKDRRILLHLDISDILGRAPLTPEILNADIITFSSAALGGMGSIGGIFIRKSLERV
FSSWFPPHTSASLCFSAVAAMQTACEERISALPLFTFHTSNLCKKLIQELQSVLPSIQLAFSEVQNRLPNIVVAA
IPDIPAESLAFHLHQQGIYPSLGYERFQPLAQVLQNCGISPFLCHSALHFSLTERSKDLEFSKLARAMHDAIKHL
TPLLGSSS

>core/358/3/Org3_Gene471

MILDFQFSIGYYLRVLELAIRDGTRILAYDRKRLLLDAWPVNDPLPTNYDTSVSTIRQVIHELFSWSAISYSISS
RLLAIIELRLHEEKPQTGWLYRLFFPSKYHIKKAIVDKLCMFKSLILFESKRPVDKIVQAANKVFSKGKSNFSS
WEDFTHEVTVSEVQTPLAGEVQRRLAADASLQMIIEALTTLLEGHTAYLPLSLELLNQFIGEKAQPLKTLSEK
SYVLLRELIQLFSLSAEDFQTIIMSIISDSLSEVLANSLIGNQPLTFHGKTFVGLWQETALASPEDSKLALGFLAE
VLRKVIVEKKLHVSKSDNTTPEEVGNIYSIRDQNPALWDKMITMLLMRWLLDYDRDIGIALRKAAEYYNPHP
SFWRQFLRLWQRRP

>core/360/3/Org3_Gene895

MSAMISLSSSHEASIASNTQVRDVLVSLAMDEFVEHNTEILPIKVFLARGTLSSTAIIDDLKDVVETEGEHHFQ
VYSNISLKMIYQRFFEKIFGIGCCPLLLVTDSHHTDPCGALITGIFAAVLFTVLAIVFGPTLGILCYSAYKIYQLT
KKISSLSRTHTEVINSVQKSDPFIHRSGAVAAAAASQSTIKACKVFRQSTLIFFVLGLIITISLAALIVGLVFALFF
LDPGAPAVMTAAMIGCCAAGGTGILLSVIGFLLASVYSVQKSQEGVHHMHTALLRCIVSNTIIQMPYLPITPG
TKKVLTQSIRRYQQFFSDDEYRDIESEVPLNRQTTPPPSYETLFHEEGSDGSSNVIPRESPPAYSTIDSSNSPFPSS
SPAPYYR

>core/361/3/Org3_Gene1020

MSETIITTPHVVKLISNFFQKKLFSSISTAYPLVIITDVSVQQHLLGPILDHIKMLGYQVIVLTFPPGEPNKTWET
FISLQYQLVDQNISPKSSIIGIGGGTVLDMTGFLAATYCRGLPLYLIPTTITAMVDTSIGGKNGINLRGIKNRLGT
FYLPKEVWMCPQFLSTLPREEWYHGIAEAIKHGFIADAYLWEFLNSHSKMLFSSSQILHEFIKRNCQIKAAIVA
EDPYDRSLRKILNFGHSIAHAIETLAKGTVNHGQAVSVGMMIETRISLAEGVMKTPQLIDQLERLLKRFNLPST
LKDLQSIVPEHLHNSLYSPENIIYTLGYDKKNLSQHELKMIMIEHLGRAAPFNGTYCASPNMEILYDILWSECH
VMRHC

>core/362/3/Org3_Gene735

MLCQQFLIEALARRKSKHTYRSLSLNSHLIDFTSNDYLGFASSPELRKEYITKLHAIESLGATGSRLLTGHSQL
CQRIEEQLAAYHNFESCLIFNTGYTANLGLLYALATDQDRILHDLYIHASIYDGIRLSKAQSFPFNHNDLNHLE
KRLASSHLGRTFVCVESVYSLHGSVAPLQAISELCERYSAYLIVDEAHAVGVFGDQGEGLVSALGLQDKVLA
TVYTFGKALGTHGAAIAGSSILKDYLINFCRPFIYTTAQPPHALTAIELAYEHNQRAFNQREHLSALIHHFREK
AQNLGLQLMKDNTTTPIQSICVSGSHRARQAALQIQNSGYDVRPIVSPTVKQREELLRICLHAFNTKNEIDHLL
HTLEQIFLCNVSSL

>core/363/3/Org3_Gene188

MSCFNLTSTNESLRPISPKASFPKQGWQSYFRSALRKHRSDTLSVSVCKVNKYDANLFVRLTVIALAVVGVLI
LFSIMLASIQGTLVITSWPLVTAAILIPTILLTGGMYILHRLGKKVDVISGVCIPPFSRRCWVPISSSHTLEKFDEK
HVSACSYLDISTLSADGSGIAAVYQCPPLLFRAFPCFGIPCAMPFVALLRMIYNLIRFLVVPFYIIFRMIYEHFFC
KHLPEDDRFIYKDVAREMGRSLAAFLKAPFYASACMIGAFYSLLDPLAGRVLMGSVERDWNDNVILARSVS
LANEAHSLFRFEGGGGRKGLGQHAFYLMLCCQPQSVFLFDKGEIVSGAHPSIQLPERRGLDTSGRYPHISVIPD
SGNDSAKNFIV

>core/364/3/Org3_Gene473

MGINPSGNRSPDDVWVRGAQGDSSSTQGTGATNSNLGAHNVTTSTSQPQVASKAKQLWQTVREFFLGKKSP
DSSQGASGPAMQSPSGPTIRPTRPAPPPPTTGGANAKRPATHGKGRAPQPPTAGSSSGSEQPTAMSSEVAKLV
SELKDAVHSHAESQKVLKKVSQELQTKWTDWENNRGPDYLLHGYRVIARALQQTYTEQSMLIEGTSSTGPV
PQAVTVAKDAVTQTVRGAIKNLENPKPGNDPDGVLMQVVISLGIEGPTLDPGESIQNFLETRVSDFGGDDSDI
DYTSDIARLGSALDRVRENHPNEMPRIWIALARELGAAVHSHATSVRIANAGKNHTRDVVRMANESSRLLQ
GMKVLSVGAWANTMTVLIGDLFE

>core/365/3/Org3_Gene653

MIPSPTPINFRDDTILETDPKPSLIMFSSKKTEIASERRKAHPTLFKVLGTIWNIVKFIISIILFLPLALLWVLKKTC
QFFILPSSIISQSMSKTAVAIRRMTFLSHIKQLLSLKEISAADRVVIQYDDLVVDSLAIKIPHALPHRWILYSQGN
SGLMENLFDRGDSSLHQLAKATGSNLLVFNYPGIMSSKGEAKRENLVKSYQACVRYLRDEETGPKANQIIAF
GYSLGTSVQAAALDREVTDGSDGTSWIVVKDRGPRSLADVANQICKPIASAIIKLVGWNIDSVKPSERLRCPEI
FIYNSNHDQELISDGLFERENCVATPFLELPEVKTSGTKIPIPERDLLHLNPLSPNVVDRLAAVISNYLDSENRK
SQQPD

>core/366/3/Org3_Gene902

MKHLAVLGSTGSIGRQTLEIVRRYPSEFKIISMASYGNNLRLFFQHLEEFAPLAAAVYNEEVYNEACQRFPHM
QFFLGQEGLTQLCIMDTVTTVVAASSGIEALPAILESMKKGKALALANKEILVCAGELVSKTAKENGIKVLPI
DSEHNALYQCLEGRTIEGIKKLILTASGGPLLNKSLEELSCVTKQDVLNHPIWNMGSKVTVDSSTLVNKGLEII
EAYWLFGLENVEILAVIHPQSLIHGMVEFLDGSVISIMNPPDMLFPIQYALTAPERFASPRDGMDFSKKQTLEF
FPVDEERFPSIRLAQQVLEKQGSSGSFFNAANEVLVRRFLCEEISWCDILRKLTTLMECHKVYACHSLEDILEV
DGEARALAQEI

>core/367/3/Org3_Gene952

MKDLGTLGGTSSTAKTVSPDGKVIMGRSQIADGSWHAFMCHTDFSSNNVLFDLDNTYKTLRENGRQLNSIF
NLQNMMLQRASDHEFTEFGRSNIALGAGLYVNALQNLPSNLAAQYFGIAYKIRPKYRLVVFLDHNFSSHVPN
NFNVSHNRLWMGAFIGWQDSDALGSSVKVSFGYGKQKATITREQLENTEAGSGESHFEGVAAQIEGRYGKS
LGGHVRVQPFLGLQFVHITRKEYTENAVQFPVHYDPIDYSTGVVYLGIGSHIALVDSLHVGTRMGMEQNFAA
HTDRFSGSIASIGNFVFEKLDVTHTRAFAEMRVNYELPYLQSLNLILRVNQQPLQGVMGFSSDLRYALGF

>core/368/3/Org3_Gene617

MEDFSEQQLFFMRRAIEIGEKGRITAPPNPWVGCVVVQENRIIGEGFHAYAGGPHAEELAIQNASMPISGSDV
YVSLEPCSHFGSCPPCANLLIKHKVSRVFVALVDPDPKVAGQGIAMLRQAGIQVYVGIGESEAQASLQPYLY
QRTHNFPWTILKSAASVDGQVADSQGKSQWITCPEARHDVGKLRAESQAILVGSRTVLSDDPWLTARQPQG
MLYPKQPLRVVLDSRGSVPPTSKVFDKTSPTLYVTTERCPENYIKVLDSLDVPVLLTESTPSGVDLHKVYEYL
AQKKILQVLVEGGTTLHTSLLKERFVNSLVLYSGPMILGDQKRPLVGVLGNLLESASPLTLKSSQILGNSLKV
VWEISPQVFEPIRN

>core/369/3/Org3_Gene654

MNLSNRSDILSGIFSNPHPVSYFSSTHAKQLSDFSKKHPILTKIVTIIVKIFKLLIGLIIPPLGIYWLCQLVCSLALF
PRSSMLYSVLKTCFKKYRLEQEIQDYFVKNLDPSFKDPAVSESKRITIQQDHLTIDTLAIHFSTARPKRWLLISL
GSGDFLEDMIGLKDSLFLSWKELAKLLGANILIYNYPGVKSSTGKLNLENLATAHNLCAKYLQDKIQGPGAN
EIITYGYSLGGVVQSAALQKNPFTNSETSWVAVKDRAPHSLPAAANSFFGPIGKLIAVLARWKMDAEKNSRE
LPCPEILVYSADRFRPSEVGDDTALLPEFTLAHAIKRTPFARSKKFIGEVNLLHSSPLKHPTIQKLAEAILESLSR
KN

>core/370/3/Org3_Gene85

MNTSLKRPLKSHFDVVGSFLRPEHLKKTRESLKEGSISLDQLMQIEDIAIQDLIKKQKAAGLSFITDGEFRRAT
WHYDFMWGFHGVGHHRATEGVFFDGERAMIDDTYLTDKISVSHHPFVDHFKFVKALEDEFTTAKQTLPAPA
QFLKQMIFPNNIEVTRKFYPTNQELIEDIVAGYRKVIRDLYDAGCRYLQLDDCTRGGLVDPRVCSWYGIDEK
GLQDLIQQYLLINNLVIADRPDDLVVNLHVCRGNYHSKFFASGSYDFIAKPLFEQTNVDGYYLEFDHERSGDF
SPLTFISGEKTVCLGLVTSKTPTLENKDEVIARIHQAADYLPLERLSLSPQCGFASCEIGNKLTEEEQWAKVAL
VKEISEEVWK

>core/371/3/Org3_Gene929

MSDNAHNLLYDKFELPEAVKMLPVEGLPIDKHARFIAEPLERGMGHTLGNALRRALLIGLEAPAIISFAMTGV
LHEYMAIEGVIEDVTNIILNLKGALLKKYPMQDSSLGRTTQVLKASISIDASDLAAANGQKEVTLQDLLQEGD
FEAVNPDQVIFTVTQPIQLEVVLRIAFGRGYTPSERIVLEDKGVYEIVLDAAFSPVTLVNYFVEDTRVGQDTDF
DRLVLIVETDGRVTPKEALAFSTQILTKHFSIFENMDEKKIVFEEAISIEKENKDDILHKLILGINEIELSVRSTNC
LSNANIETIGELVIMPEPRLLQFRNFGKKSLCEIKNKLKEMKLELGMDLTQFGVGLDNVKEKMKWYAEKIRA
KNIKG

>core/372/3/Org3_Gene255

MNGKAPLALYIHIPFCTKKCRYCSFYTIPYKSESVSLYCNAVIQEGLRKLAPIQETHFIETVFFGGGTPSLVSPL
DLKRILKELAPHAREITLEANPENLTVSYLRQLQETPINRISVGVQTFDDSILQLLGRTHSSSAAITALQECQNH

GFSNLSIDLIYGLPTQSLEIFLSDLHQALTLPITHISLYNLTIDPHTSFYKHRKILVPTIAQEEILAEMSLLAENLLL SQGFQRYELASYAKPDYPAKHNLYYWTDRPFLGLGVSASQYLHGERSKNYSHISHYLRAVRKNLPTQETSEI LPKKERIKEALALRLRLLEGADLAEFPSTLISMLTQDVKLQNLFSVHGQCLALNRQGRLFHDTIAEEIMGYSF

>core/373/3/Org3_Gene763

MSIAIAREQHAAILDMHPKPSIAMFSSEQARTSWEKRQAHPYLYRLLEIIWGVVKFLLGLIFFIPLGLFWVLQK ICQNFILLGAGGWIFRPICRDSNLLRQAYAARLFSASFQDHVSSVRRVCLQYDEVFIDGLELRLPNAKPDRWM LISNGNSDCLEYRTVLQGEKDWIFRIAEESQSNILIFNYPGVMKSQGNITRNNVVKSYQACVRYLRDEPAGPQ ARQIVAYGYSLGASVQAEALSKEIADGSDSVRWFVVKDRGARSTGAVAKQFIGSLGVWLANLTHWNINSEK RSKDLHCPELFIYGKDSQGNLIGDGLFKKETCFAAPFLDPKNLEECSGKKIPVAQTGLRHDHILSDDVIKEVAG HIRRHFDN

>core/374/3/Org3_Gene135

MALKFHLIHQSKKSQARVGQIETSHGVIDTPAFVPVATHGALKGVIDHSDIPLLFCNTYHLLLHPGPEAVAKL GGLHQFMGRQAPIITDSGGFQIFSLAYGSVAEEIKSCGKKKGMSSLVKITDEGAWFKSYRDGRKLFLSPELSV QAQKDLGADIIIPLDELLPFHTDQEYFLTSCSRTYVWEKRSLEYHRKDPRHQSMYGVIHGGLDPEQRRIGVRF VEDEPFDGSAIGGSLGRNLQEMSEVVKITTSFLSKERPVHLLGIGDLPSIYAMVGFGIDSFDSSYPTKAARHGLI LSKAGPIKIGQQKYSQDSSTIDPSCSCLTCLSGISRAYLRHLFKVREPNAAIWASIHNLHHMQQVMKEIREAIL KDEI

>core/375/3/Org3_Gene843

MAPIHGSNAFVEDILHSHPSPQATYFSSTRAQKLHEFKDRHPVLTRIASVIIKIFKVLIGLIILPLGIYWLCQTLCT NSILPSKNLLKIFKKQPNTKTLKTNYLRALQDYSSKNRVASMRRVPILQDNVLIDTLEICLSQAPTNRWMLISL GSDCSLEEIACKEIFDSWQRFAKLIGANILVYNYPGVMSSTGSSSLKDLASAHNICTRYLKDKEQGPGAKEIIT YGYSLGGLIQAEALRDQKIVANDDTTWIAVKDRCPLFISPEGFHSCRRIGKLVARLFGWGTKAVERSQDLPCL EIFLYPTDSLRRSTVRQNKLLAPELTLAHAIKNSPYVQNKEFIEVRLSSDIDPIDSKTRVALATPILKKLS

>core/376/3/Org3_Gene246

MSTTEPNLTNVNLTMLISSESMPTQLASHKLKGLDLVAFILIIGIAVSSGTAAIILGIPLLFILTALAVLAFSILLY FLLREPKSPISVTHQPTPIIKDTDLPPVPPLALTPVPTEAVLEEPPLPSPRTHQTLLQENWDRIPDLQANTDMPFI AADNQTGYAWHLKNSNLTLISTLGPIEKPRYKTQGIVMIVNAATPNMANNVKGTSLALAKATSVRCWENSK KSPDPLRSKQPLQLGECRSAKWENLNGTTNAGKAGLPQFLGQLLGPKASDYNYNPNDAFTFCRQAYLNCLN EAKRRKTTVVQLPLLSSHFPGSPKDEETTSLRLQWIDGVKLALIDALQTFGSEAENQNQPWVIILTTLARHPLI TP

>core/377/3/Org3_Gene493

MAVAADSSASWLKSRNNFLSSLGKTEEQVAAPEFPKELCQHKIREKFRLEDVQVSIKFRGSITAVEATKEFGV HLLIQPMVVQPWEVENLLFLTSEEDLQELMVAVFDDASLASYFYEKDKLLGFHYYFVAEACKLFEELQWVP SLSAKVGGDAIFTATSLQGSFQVVDISLRLDGKNVRCRLLLPEDTFQSCQKFFSGLHDESDLHNIDQTQQISLS VEVGYSQLTQEEWHQVVPGSFIMLDSCLYDPETEESGALLTVQKHQFFGGRFLTPSSGEFKITSYPNLTHEDP

PLPENPQASAAPLPGYSRLVVEVARYSLAVSEFIKLNLGSILSLGNHPAYGVDIILDGAKVGRGEIIALGDVLGI
RVLEV

>core/378/3/Org3_Gene266

MIYLDNNAMTPPERGLLEFLQKTFLIEGTYANPSSVHQLGKKSRQLVLEASHWMQKVLSFQGRVLYTSGATE
SLNLAIASLPKDSHVITSGSEHPAILEPLKHSSLSVSYLNPEEGRCVLTIEQIERAVTPKTSAIILGWVNSETGAK
ADIAAIAHFAQERQLQFIVDATANVGKERIVLPSGVTMAAFSGHKFHALSGIGALLVSPGVKLHPQLWGGGQ
QGGLRAGTENLWGIASLLYIFKYLDLHQERISQEILTHRNGFEKAIKARIPDVHIHCADQPRANNVSAIAFPPL
EGEVLQIALDIEGVACGYGSACSSGATAPFKSLVSMGVDEELTLATLRFSFSHLLLQEDVERAVGIIEKVVERL
KNS

>core/379/3/Org3_Gene649

MTTSDVIDFVTNDFLGFARSPTIYCEVSKRFQIHCQQFPHEKLGIRGSRLMVGPSSVIDDLESKIASYHGAPNA
FIVNSGYMANLGLCHHVSRSTDVLLWDEEVHMSVVHSLSAISGQHHTFHHNNLEHLESLLQCYRISSKGRIFI
FVSSVYSFRGTLAPLEQIIALSKKYHAHLIVDEAHAMGIFGDDGKGLCHALGYENFYAVLVTYGKALGTMG
ASLLTSSEVKYDLMQNSPPLRYSTSLSPHTLISIGTAYDFLASEGEIARKQVFKLKEHFHECFDSHAPGCVQPIF
LPHTCLEEAISVLETTGIHVGVVAFAKHPFLRVNLHAYNTVDEVNLLAQVMKPYLEKSSHRVHINHEFHLWR
ELCQH

>core/380/3/Org3_Gene970

MMLVYCFDPSVPTSPEHRLMAALDRWFFLGGHRVRILTLEGNHYRAFQENMSISTVEKILKLISYLLIPIVLIA
LLIRCFLHSRFKCNWKCDSLSDARVPHDVQPFNDFQLFNNQERLNIWKNRRYVSGIDVLMVPVDYLRSQFPG
FKEIPEAIRCENYVSDGQFSEESKTSYLRAMLTDIVGYILSLDETYWTNVILKIRAMCITFESFPGKEADPNYSP
RVTHHYFDESWKALARHVLGEGNMVNRLDEALIRTEKPGKEGECITKQFLKDYCKKHLEVMSCPDFIESLV
DEKIREFRCPSILNSAVCDVIDRKCQEHLLKAIINEANRRLPGMKNSSFTMRGNQVLFYTIFSPPKLPPAASSVY
F

>core/381/3/Org3_Gene340

MTTCLPQPPKTSPLYSIFEKLDAQERLSSEDALHLLLLTNKEDQRTLWNFADQVRKQRVGDTVYYSSTLYLY
PTNFCDFSCKFCSFYAKPGDPKGWLYSPDDLLQQIQNIKTPITEVHIVGGCFPSCNLQYYSDLFTKIKEYDPQIH
IKALTAIEYAYLSDLDNLSIRDVLLTLKDAGLDSIPGGGAEILVDKIRNFLAPKRLSSSDFLNIHKMAHQLGIHS
NITMLCYHKEGPEDLVTHMVKVRDLQDETQGFKNFILLKFAQENNVLGKRLRKSGQGHAIPLKSLMAVARIF
LDNFSNMKALWNYLGIEAALDLLSCGANDLSSTHMGEKVFQMASSKEPIKMDAEGMAALITQQGRTPCLTN
SSHV

>core/382/3/Org3_Gene1007

MRRQVREIMQQTVIVAMSGGVDSSVVAYLFKKFTNYKVIGLFMKNWEEDSEGGLCSSTKDYEDVERVCLQ
LDIPYYTVSFAKEYRERVFARFLKEYSLGYTPNPDILCNREIKFDLLQKKVQELGGDYLATGHYCRLNTELQE
TQLLRGCDPQKDQSYFLSGTPKSALHNVLFPLGEMNKTEVRAIAAQAALPTAEKKDSTGICFIGKRPFKEFLE
KFLPNKTGNVIDWDTKEIVGQHQGAHYYTIGQRRGLDLGGSEKPCYVVGKNIEENSIYIVRGEDHPQLYLRE

LTARELNWFTPPKSGCHCSAKVRYRSPDEACTIDYSSGDEVKVRFSQPVKAVTPGQTIAFYQGDTCLGSGVID
VPMIPSEG

>core/383/3/Org3_Gene271

MTKIAFSEKAKNFPVEALKKWFEKNKRSLPWRDNPTPYSVWVSEVMLQQTRAEVVIDYFNQWMERFPTIES
LAAAKEEDVIKLWEGLGYYSRARHLLEGARMVMEEFHGKIPDDAISLAQIRGVGPYTVHAILAFAFKRRAAA
VDGNVLRVLSRIFLIETSIDLESTRTWVSRIAQALLPHKSPEVIAEALIELGACICKKVPQCHRCPVRQACGAW
RENKQFVLPVRHARKKVIFLHRLVAIVLYDGSLVVEKRRPKEMMAGLYEFPYIEVEPEEGLQDIEGFTKKME
LSLESPLEFLGNLKEQRHAFTNHKVHLCPIIFKATSLPQFGELHLLSDIDHLAFSSGHKKIKDALLIYLGDVRSR
ESIGV

>core/384/3/Org3_Gene390

MQENLDKRLEALRTEISLAARSLFDLDKKQKELQVLEEESSEENFWQDSVHAGKISEQIVSLRRQIQEYQELK
SKIDAIEFFLEDADALEDPAICEDLEKEFLFCEKKLAVWETQRLLSGEADKNSCFLTINAGAGGTESCDWVEM
LFRMYSRWATKHQWALEVVDRLDGEVVGIKHVTVKFSGMYAYGYAKAERGVHRLVRISPFDSNGKRHTSF
ASVDVFPEIDDQIKIEIRPNDLRIDTFRSSGAGGQHVNVTESAVRITHLPSGVVVSCQNERSQIQNRESCMKML
QAKLYQQVLQERLEKQSLDRKDKKEIAWGSQIRNYVFQPYTLVKDVRTGHETGNVQAMLDGELLDEFIKAY
LAEFGEVS

>core/385/3/Org3_Gene35

MAVEGRVNSSQALNQDCQEVLANKQSKGLLRCRILSIVVAVITFIAGVVLIALTLASILTSVPYLALGVFLLIV
TLGCIIFALCSEKIKKVPPTPISHKEEIIAWFEERKNIDMEKEKEDPEHFGRTATDIPMRSALDQFNHSCHHIHES
PALTETYRSHQDVLLFKDWCPVTLPDVTSEEEVLIRSVVGSYLLMEACVPKVSMLIDELHNKLKSPSERECLF
IDKKTLQRKASFLFTQKDLATFFLAYTRVNDGHLAPFRAGAKWILIHYVRLRRQHNQNDFFTPGHSCYYARL
AFNQTQRLYHQLFNVEKLRSIYANMDKDPLCHPWAFIPIYDLLKTEDHGDGFLEQQEDREYPSRAAQDQFW
G

>core/386/3/Org3_Gene601

MSPHRNLFKLKNFSNRLYNRALGRFDKVFNFFSGNVGIDLGTANTLVYVRGRGIVLSEPSVVAVDAQTHAVL
AVGHKAKAMLGKTPRKIMAVRPMKDGVIADFEIAEGMLKALIKRVTPSRSVFRPRILIAVPSGITGVEKRAVE
DSALHAGAQEVILIEEPMAAAIGVDLPVHEPAASMIIDIGGGTTEIAIISLGGIVESRSLRIAGDEFDECIINYMR
RTYNLMIGPRTAEEIKITIGSAYPLGDQELEMEVRGRDQVAGLPITKRINSVEIRECLAEPIQQIIECVRLTLEKC
PPELSADLVERGMVLAGGGALIKGLDKALSKNTGLSVITAPHPLLAVCLGTGKALEHLDQFKKRKGNLV

>core/387/3/Org3_Gene900

MKFVVSRNELGNLIKKIQSVVPQNTPIPVLTHVLIETYNDELVFTATDLTVSTRCVTKAKVYEKGAISIPSKRF
FQLVKELTEANLEISSSAGEMAQITSGSSCFRLLSMEKEDFPMLPDIQNALRFSLPAEQLKTMLQRTSFAVSRE
ESRYVLTGVLLAIANGVATIVGTDGKRLAKIDAEVTLDKSFSGEYIIPIKAVEEIIKMCSDEGEATIFLDQDKIA
VECDNTLLITKLLSGEFPDFSPVISTESNVKLDLHREELITLLKQVALFTNESSHSVKFSFLPGELTLTANCTKV
GEGKVSMAVNYSGELLEIAFNPFFFLDILKHSKDELVSLGISDSYNPGIITDSASGLFVIMPMRLHDD

>core/388/3/Org3_Gene700

MLIWKRHLLTRFWFALTSLLVLALIFYASIHHSLHTLKGASTAASGASVKLSILYYLAQISLKAEFLMPQLVA
VATTSTLFAMQNKREIILLQASGLSLKSLMHPLLLSGAVIMMVLYANFQWLHPICEKISITKENMDRGTTDKE
QGKIPALYLKDQTVLLYSSIEPKTLTLNNVFWIKDPKTIYTMEKLAFTTLSLPIGLNVTQFFANDSENLELKEFF
DMKEFPEIEFNFYENPFSKLFSAGNKNRLSEFFKAIPWNATGLGLSTQVPQRILSLLAQFYYVLISPLACMAAII
LSAYLCLRFSRTPTVTLAYLIPLGTVNIFFVFLKAGIVLASSSVLPTLPVMAFPLIVLFLLTNYAYAKLQ

>core/389/3/Org3_Gene380

MKFPRISISDLIPTQMVIWWRGGGNVHYVPNAQNLPKKILGGVLACFGLALLGCAAFAAGVCQTIFPCIGLMI
LGLVLLGFAYLQYSKGWSRFERPLFRETKVFEKPINWLGCLSLLQSWKKIRPGCYYHPGCPQVEICEGSQEIV
TKIFQKKSDRNTSIFLIQEMDQIALRQGIEKSSLSRKTFAIDPSVVSSLLSEIQREEQQYLDPKVISWSSEDQASD
RTHPKSAIYVNISDAAQEPQGRCYIDAYTKAFFTVLDQIGDPNIVKKHTIYVLTPILGVPDALPKEEQENLKLL
SQAAFLYSAEQVAKRMREEKQDSIRIKFIFTDPTSPTSLYFSPHHSSTPHSVTPISLSGFVGEQESYTFA

>core/390/3/Org3_Gene860

MSKKIKVLGHLTLCTLFRGVLCAAALSNIGYASTSQESPYQKSIEDWKGYTFTDLELLSKEGWSEAHAISGNG
SRIVGASGAGQGSVTAVIWESHLIKHLGTLGGEASSAEGISKDGEVVVGWSDTREGYTHAFVFDGRDMKDL
GTLGATYSVARGVSGDGSIIVGVSATARGEDYGWQVGVKWEKGKIKQLKLLPQGLWSEANAISEDGTVIVG
RGEISRNHIVAVKWNKNAVYSLGTLGGSVASAEAISANGKVIVGWSTTNNGETHAFMHKDETMHDLGTLG
GGFSVATGVSADGRAIVGFSAVKTGEIHAFYYAEGEMEDLTTLGGEEARVFDISSEGNDIIGSIKTDAGAERA
YLFHIHK

>core/391/3/Org3_Gene351

MKKRFPSTLFLFYRRVTIAISLEGILGWGWLGSLLSKVFAFLVACWNRFSWSTPYRARSTVISVGNIVVGGAG
KTPTVLWLAEALRLRGYSCGVLSRGYKSQSSRQKKLTVVDSKVHSASYVGDEPLLMAEKLPEGSVWVHKD
RRISAARAAEKFGILLLDDGLQYRKLHKDVEIAVVNGQDPLGGRAFFPKGRLRDFPLRLKTVDAIIVNGGGKE
AGTVVKRVSNAPQIFVKPTIASVVWTHNGERIPKEALRELRVGVFCGLGFPQGFLNMLREEGIHILGKYLLPD
HAAITKKELNYFCQQMAMRQGQGLLCTEKDSVKLPRLSGEVSLLPIAKVEMRLSVNQDDTLSLLNMIEQIHK
NRGN

>core/392/3/Org3_Gene922

MNVPDSKNLHPPAYELLEIKARITQSYKEASAILTAIPDGILLLSETGHFLICNSQAREILGIDENLEILNRSFTD
VLPDTCLGFSIQEALESLKVPKTLRLSLCKESKEKEVELFIRKNEISGYLFIQIRDRSDYKQLENAIERYRNIAEL
GKMTATLAHEIRNPLSGIVGFASILKKEISSPRHQRMLSSIISGTRSLNNLVSSMLEYTKSQPLNLKIINLQDFFS
SLIPLLSVSFPNCKFVREGAQPLFRSIDPDRMNSVVWNLVKNAVETGNSPITLTLHTSGDISVTNPGTIPSEIMD
KLFTPFFTTKREGNGLGLAEAQKIIRLHGGDIQLKTSDSAVSFFIIIPELLAALPKERAAS

>core/393/3/Org3_Gene212

MSHTECGIVGLPNVGKSGLFNALTGAQVASCNYPFCTIDPNVGIVPVIDERLEALAKISNSQKIIYADMKFVDI
AGLVKGASDGAGLGNRFLSHIRETHAIAHVVRCFDDPDVTHVSGKVNPVEDIEVINLELIFSDFSSAKNIHSKL

EKLAKGKREVGALLPLFDTIIAHLEKGLPLRTLELTPEQIVALKPYPFLTMKPMFYIANVDESSLPDMDNDYV
AAVREVAAKENSKVVPICVRIEEEIVSLPIEERLEFLMSLGLEKSGLHRLVRAAYDTLGLISYFTTGPQESRAW
TVVRGSSAWEAAGEIHTDIQKGFIRAEVITFEDMIECQGRAAARELGKLHIEGRDYIVQDGDTMLFLHN

>core/394/3/Org3_Gene252

MTTEVRIPNIAESISEVTVASLLVTEGALIQENQGLLEIESDKVNQLIYAPVSGRIFWEVSEGDVVPVGGVVGKI
EPAGEGEELGDSQSKETIEAEIICFPQSGVRQSPPENKTFIPLRDQMDQGSQGLSAGDRGETRERMTSIRKTISR
RLLSALHESAMLTTFNEVYMTPLFHLRKEKQEEFLSRYGVKLGFMSFFVKAVLEALKAYPRVNAYIDGEEIV
YRHYYDISIAVGIDRGLVVPVIRDCDKLSNGEIEQKLADLALRAREGLLAIAELEGGGFTITNGGVYGSLLSTPI
INPPQVGILGMHKIEKRPVVLDNEIVIADMMYVALSYDHRLIDGKEAVGFLVKVKEGLENPASLLDL

>core/395/3/Org3_Gene470

MVPFRQHHAYQLLKQLHTSAISEADRVSYYFKQNRSLGSKDRQWIQNIIFNILRHRRLLETLILDSGEQVTPEA
LVAKVNEGVLENLDSYSAIPWPVRYSISDDLAHFLVQDYGEEQAEEIAKIWLTEAPITIRVNTDKISVKELQEK
LEYPSSPGELPEALHFSKRHPLQSTEAFRRGFFEIQDENSQRISQGISLTDKDIVLDFCAGAGGKSLIFAQKAKH
VVINDSRKAILQTAKHRLLRAGARNFSLADQLRLGSFSVVIVDAPCSGTGVFRRHPEHKWQFSKKLLLNYVR
VQKSILKQASAYVGPRGRLVYITCSLLKEENEAHVAYMHSLGWKEVHRKTLPLQVGKGDAFFTSHFQKI

>core/396/3/Org3_Gene746

MKLYQTLRGIVLVSTGCIFLGMHGGYAAEVPVTSSGYENLLESKEQDPSGLAIHDRILFKVDEENVVTALDVI
HKLNLLFYNSYPHLIDSFPARSQYYTAMWPVVLESVIDEFLMVADAKAKRIATDPTAVNQEIEEMFGRDLSP
LYAHFEMSPNDIFNVIDRTLTAQRVMGMMVRSKVMLKVTPGKIREYYRKLEEEASRKVIWKYRVLTIKANT
ESLASQIADKVRARLNEAKTWDKDRLTALVISQGGQLVCSEEFSRENSELSQSHKQELDLIGYPKELCGLPKA
HKSGYKLYMLLDKTSGSIEPLDVMESKIKQHLFALEAESVEKQYKDRLRKRYGYDASMIAKLLSEEAPPLFS
LL

>core/397/3/Org3_Gene923

MAFKRKTRWLWQVLILSVGLNMLFLLLFYSAIFRKDIYKLHLFSGPLIAKSSRKVYLSEDFLNEISQASLDDLI
SLFKDERYMYGRPIKLWALSVAIASHHIDITPVLSKPLTYTELKGSSVRWLLPNIDLKDFPVILDYLRCHKYPY
TSKGLFLLIEKMVQEGWVDEDCLYHFCSTPEFLYLRTLLVGADVQASSVASLARMVIRCGSERFFHFCNEESR
TSMISATQRQKVLKSYLDCEESLAALLLLVHDSDVVLHEFCDEDLEKVIRLMPQESPYSQNFFSRLQHSPRRE
LACMSTRRVEAPRVQEDQDEEYVVQDGDSLWLIAKRFGIPMDKIIQKNGLNHHRLFPGKVLKLPAKQS

>core/398/3/Org3_Gene1015

MAVVSTPFFNRNLWYRLLSSRFSLWKSYCPRFFLDYLEAFGLLSDFLDHQAVIKFFELETHFSYYPVSGFVAP
HQYLSLLQDRYFPIASVMRTLDKDNFSLTPDLIHDLLGHVPWLLHPSFSEFFINMGRLFTKVIEKVQALPSKKQ
RIQTLQSNLIAIVRCFWFTVESGLIENHEGRKAYGAVLISSPQELGHAFIDNVRVLPLELDQIIRLPFNTSTPQET
LFSIRHFDELVELTSKLEWMLDQGLLESIPLYNQEKYLSGFEVLCQ

>core/400/3/Org3_Gene69

MKIDILSLFPGYFDGPLQTSILGRAIKQRLLDVQLTNLRDFGLGKWKQVDDTPFSGGGMLLMAEPVTSAIRSV
RKENSKVIYLSPQGALLTAEKSRELAAASHLILLCGHYEGIDERAIESEVDEEISIGDYVLTNGGIAALVLIDAV
SRFIPGVLGNQESAERDSLENGLLEGPQYTRPREFEGKEVPEVLLQGDHKAISQWRLEQSERRTYERRPDLYL
NYLYKRSIDHKFDEETTTNRDHFKCDKISVVLEVNKLKRAKNFYCKVFGLDAMSCENKFCLPHEGKTIFWLR
EVQAEKKNIVTLSLSLDCACEEDFCYLLRRWELFGGKLLEKQADEHAVWALAQDLDGHAWIFSWHRMK

>core/401/3/Org3_Gene213

MGEKTEKATPKRLRDARKKGQVAKSQDFPSAVTFIVSMFTAFSLSTFFFKHLGGFLVSMLSQAPTRHDPVITL
FYLKNCLMLILTASLPLLGAVAVVGVIVGFLIVGPTFSTEVFKPDIKKFNPIENIKQKFKIKTLIELIKSILKIFGA
ALILYITLKSKVSLIIETAGVSPIITAQIFKEIFYKAVTSIGIFFLIVAILDLVYQRHNFAKELKMEKFEVKQEFKD
TEGNPEIKGRRRQIAQEIAYEDSSSQVKHASTVVSNPKDIAVAIGYMPEKYKAPWIIAMGINLRAKRILDEAEK
YGIPIMRNVPLAHQLLDEGKELKFIPESTYEAIGEILLYITSLNAQNPNNKNTNQPDHL

>core/402/3/Org3_Gene198

MSEAPVYTLKQLAELLQVEVQGNIETPISGVEDISQAQPHHIAFLDNEKYSSFLKNTKAGAIILSRSQAMQHA
HLKKNFLITNESPSLTFQKCIELFIEPVTSGFPGIHPTAVIHPTARIEKNVTIEPYVVISQHAHIGSDTYIGAGSVIG
AHSVLGANCLIHPKVVIRERVLMGNRVVVQPGAVLGSCGFGYITNAFGHHKPLKHLGYVIVGDDVEIGANTT
IDRGRFKNTVIHEGTKIDNQVQVAHHVEIGKHSIIVAQAGIAGSTKIGEHVIIGGQTGITGHISIADHVIMIAQTG
VTKSITSPGIYGGAPARPYQETHRLIAKIRNLPKTEERLSKLEKQVRDLSTPSLAEIPSEI

>core/403/3/Org3_Gene347

MSGPSRTESSQVSVLSYVPRDKEIAPKKQFTIAKISTLAILASLALGALVAGISLTIVLGNPVFLALLITTALFSV
VTFLVYHQMTSKVSSNWQKVLEQNFKPLGKAWQEKNVDCYSNEMQFYNNHLNPKFKVAIQTDASQPFQPT
FLTGLRVIEKNQSTGIIFNPVGPTNLIDNTATNLSTILYSTLKDKSVWDTCKQREGGPAKGEDPFSPTEVRVVK
LPNEALDQTFNLNLSSAEKKSILPTFLGHVCGPKSEELPNQQEYYRQALLAYENCLKAAIESHAAIVALPLFTS
VYEVPPEEILPKEGTFYWDNQTQAFCKRALLDAIQNTALRYPQRSLLVILQDPFNTIESQSRSEE

>core/404/3/Org3_Gene730

MKNSFGSLFSFTTWGESHGPSIGVVIDGCPAGLELHESDFVPAMKRRRPGNPGTSSRKENDIVQILSGVYKGK
TTGTPLSLQILNTDVDSSPYENSERLYRPGHSQYTYEKKFGIVDPNGGGRSSARETACRVAAGVVAEKFLAN
QNIFTLAYLSSLGSLTLPHYLKISPELIHKIHTSPFYSPLPNEKIQEILTSLHDDSDSLGGVISFITSPIHDFLGEPLF
GKVHALLASALMSIPAAKGFEIGKGFASAQMRGSQYTDPFVMEGENITLKSNNCGGTLGGITIGVPIEGRIAF
KPTSSIKRPCATVTKTKKETTYRTPQTGRHDPCVAIRAVPVVEAMINLVLADLVLYQRCSKL

>core/405/3/Org3_Gene937

MTLPNDLLETLVKRKESPQANKVWPVTTFSLARNLSVSKFLPCLSKEQKLEILQFITSHFNHIEGFGEFIVLPLK
DTPLWQKEFLLEHFLLPYDLVGNPEGEALVVSRSGDFLAAINFQDHLVLHGIDFQGNVEKTLDQLVQLDSYL
HSKLSFAFSSEFGFLTTNPKNCGTGLKSQCFLHIPALLYSKEFTNLIDEEVEIITSSLLLGVTGFPGNIVVLSNRC
SLGLTEELLLSSLRITASKLSVAEVAAKKRLSEENSGDLKNLILRSLGLLTHSCQLELKETLDALSWIQLGIDLG
LIKVTENHPLWNPLFWQIRRAHLALQKQAENSRDLQKDTISHLRASVLKELTKGLSPESF

>core/406/3/Org3_Gene637

MMKKIRKVALAVGGSGGHIVPALSVKEAFSREGIDVLLLGKGLKNHPSLQQGISYREIPSGLPTVLNPIKIMSR
TLSLCSGYLKARKELKIFDPDLVIGFGSYHSLPVLLAGLSHKIPLFLHEQNLVPGKVNQLFSRYARGIGVNFSP
VTKHFRCPAEEVFLPKRSFSLGSPMMKRCTNHTPTICVVGGSQGAQILNTCVPQALVKLVNKYPNMYVHHIV
GPKSDVMKVQHVYNRGEVLCCVKPFEEQLLDVLLAADLVISRAGATILEEILWAKVPGILIPYPGAYGHQEV
NAKFFVDVLEGGTMILEKELTEKLLVEKVTFALDSHNREKQRNSLAAYSQQRSTKTFHAFICECL

>core/407/3/Org3_Gene66

MKKKVAEYLNRLAEVEIKISNPEIFSNSKEYSALSKEHSYLLELKNAYDKILNLEKVLADDKQALAIEKDPEM
VVMLEEGINENKVELEKLNKILESLLVPPDPDDDLNVIMELRAGTGGEEAALFVGDCVRMYHLYASSKGWK
YEVLSASESDLKGYKEYVMGISGTGVKRLLQYEAGTHRVQRVPETETQGRVHTSAITIAVLPEPSEEDTELLI
NEKDLKIDTFRASGAGGQHVNVTDSAVRITHLPTGVVVTCQDERSQHKNKDKAMRILKARIRDAEMQKRHN
EASAMRSAQVGSGDRSERIRTYNFSQNRVTDHRIGLTLYNLDKVMEGDLDPITTAMVSHAYHQLLEHGN

>core/408/3/Org3_Gene701

MPILWKVLIFRYLKTAAFCTLSLICISIISSLQEIVAYIAKDVPYDTVLRLMAYQIPYLLPFILPGSCFVSAFSLFR
KLSDNNHMTFLRASGASQSIIMFPVLMVSGAICCLNFYTCSELASICRYQTCKEIANMAMTSPALLLQTLQKK
ENNRIFIAVDHCAKSKFDNVIVALKGNNEISHVGIIKSIIPDTTKDTVKAKDVVFISKLPDSLTESSSPSSQRFYIE
TLDELLIPKITSTLFAGKSYLKTRTDYLPWKQLVKQSLKHSHLPETLRRVAIGFLCITLTYAGMILGIHKPRFRK
SIALYFIFPILDLILLIVGKNTKNLPLAFMLFVFPQLVSWVVFAARAYRESRGYA

>core/409/3/Org3_Gene272

MSLLSGHRLEGFPPIAEVMAACDRCSMDFCEILKSQSMDLWADAASCVDGLLQDPFWSTAIASGIAKSSLQE
TEFECESKVMVLSSWGEQGAQVCSPFNLERICMSFPSLKVFSLKKNGCENMGIQLSASCMNLLMSIFFVATN
GGSTPIWITKENLMALVALVLSHYQCYFVPATGDPQRGNILGNPEVNAILARGMGMRVDLERKRGGESSSSR
YLELAARCFENSLTKTSLLSDANNVQERDKCLLQMSTSLMHTAGLNLQRPPVPTPSGVTAHPQPQPDPVVTS
QPSLLGARERSPVSSRGRFPVVLPLSVISPRSHPGRVERRDLEDEEEEVMF

>core/410/3/Org3_Gene574

MSHDRILRAQRALSEHNLDAILVEKSEDLAYFLHDEAIAGILLIGQQEVMFFVYRMDKDLYSHIQRVPLTFLT
QDVVADLSLYVQKQRYQKIGFDSASTVYHKFAQRQVLPCLWEPLECFTEKIRSIKSEEEIRRMQEAAALGSA
GYDYVLTLLREGITEKEVVRQLRAFWAEAGAEGPSFPPIIAFGEHSAFPHSIPTDRPLKKGDIVLIDIGVLLNGY
CSDMTRMTALGTPHPKLLESYPVVVEAQKRAMALCKEGVLWGDIDAEAVRVLREHHLDTYFIHGIGHGVG
RHIHEYPCSPRGSQVKLESGMTITVEPGVYFPGIGGIRIEDTLCIDKNKNFSLTARPVISELVCL

>core/411/3/Org3_Gene220

MNKRQKDKLKICVIISTLILVGIFARAPRGDTFKTFLKSEEAIIYSNQCNEDMRKILCDAIEHADEEIFLRIYNLS
EPKIQQSLTRQAQAKNKVTIYYQKFKIPQILKQASNVTLVEQPPAGRKLMHQKALSIDKKDAWLGSANYTNL
SLRLDNNLILGMHSSELCDLIITNTSGDFSIKDQTGKYFVLPQDRKIAIQAVLEKIQTAQKTIQVAMFALTHSEII

QALHQAKQRGIHVDIIIDRSHSKLTFKQLRQLNINKDFVSINTAPCTLHHKFAVIDNKTLLAGSINWSKGRFSL
NDESLIILENLTKQQNQKLRMIWKDLAKHSEHPTVDDEEKEIIEKSLPVEEQEAA

>core/412/3/Org3_Gene536

MNLPDRKKALEAAVAYIEKQFGAGSIMSLGRHSATHEISTIKTGALSLDLALGIHGVPKGRVIEIFGPESSGKT
TLATHIVANAQKMGGVAAYIDAEHALDPSYASLIGVNIDDLMISQPDCGEDALSIAELLARSGAVDVIVIDSV
AALVPKSELEGDIGDVHVGLQARMMSQALRKLTATLSRSQTCAVFINQIREKIGVSFGNPETTTGGRALKFYS
SIRLDIRRIGSIKGSDNSDIGNRIKVKVAKNKLAPPFRIAEFDILFNEGISSAGCILDLAVEYNIIEKKGSWFNYQE
KKLGQGREFVREELKRNRKLFEEIEKRIYDVIAANKTPSVHANETPQEVPAQTVEA

>core/413/3/Org3_Gene642

MNLCKRISFEEGLELFVSSPIERLQERADAIRKERYPSNEVTYVLDANPNYTNICKIDCTFCAFYRKPKSPDAY
LLSFDEVRSLLQRYVSSGVKTVLLQGGVHPGLGIDYLEELVRITVQEFPSIHPHFFSAVEIEHACRVSGISIEQG
LQRLWDAGQRTIPGGGAEILSERVRKIISPKKMQPGGWINLHKLAHLMGFRTTATMMFGHVENPEDILIHLQ
TLRDAQDSCPGFYSFIPWSYKPGNTALRRNVPQQASIETYYRILALGRIFLDNFDHVAASWFGEGKSLGAKAL
HYGADDFGGVILDESVHKATGWSIQSSEEEICNIIRSEGFIPVERNTFYQHISCTVSSL

>core/414/3/Org3_Gene647

MKYSLNFKEIKIDDYERVIEVTCSKVRLHAIIAIHQTAVGPALGGVRASLYSSFEDACTDALRLARGMTYKAII
SNTGTGGGKSVIILPQDAPSLTEDMLRAFGQAVNALEGTYICAEDLGVSINDISIVAEETPYVCGIADVSGDPSI
YTAHGGFLCIKETAKYLWGSSSLRGKKIAIQGIGSVGRRLLQSLFFEGAELYVADVLERAVQDAARLYGATIV
PTEEIHALECDIFSPCARGNVIRKDNLADLNCKAIVGVANNQLEDSSAGMMLHERGILYGPDYLVNAGGLLN
VAAAIEGRVYAPKEVLLKVEELPIVLSKLYNQSKTTGKDLVALSDSFVEDKLLAYTS

>core/417/3/Org3_Gene182

MLNIHDILGNDDENLLSYQCKHITKDKLTLPSHDFVDKVFGLSDRNNRVLRSLQTMFSHGRLANSGYLSILPV
DQGIEHSAGASFAINPIYFDPENIVKLAIESGCSAVASTYGTLSLLSRKYAHKIPFMLKLNHNELLSYPTKYHQI
FFTQVEAAYSMGAVAVGATVYFGSETSNEEIVAVSNAFAKARSLGLATVLWCYLRNPAFVANGVDYHTAA
DLTGQADHLGATLGADIVKQKLPTCQGGFKAINFGKTDERVYSELSSNHPIDLCRYQVLNSYCGKVGLINSG
GPSGKNDFTEAARTAVINKRAGGMGLILGRKAFQRPLSEGIQLLNLVQDIYLDPNITIA

>core/418/3/Org3_Gene847

MIPLIPMFLKQSLFFSLALTGMTTLVLTVSLGVPVMKWLKRKNYRDYIHKEYCEKLEMLHKDKAEVPTGGG
VLLFISLIASLLVWLPWGKFSTWFFIILLTCYAGLGWYDDRIKIKRKQGHGLKAKHKFMVQIAIAAFTLIALPY
IYGSTEPLWTLKIPFMEGMLSLPFWLGKVFCLGLALVAIIGTSNAVNLTDGLDGLAAGTMSFAALGFIFVALR
SSTIPIAQDVAYVLAALVGACIGFLWYNGFPAQLFMGDTGSLLLGGLLGSCAVMLRAECILVVIGGVFVAEA
GSVILQVLSCRLRKKRLFLCSPLHHHYEYQGLPETKIVMRFWIFSFVCAGLGIAAVLWR

>core/419/3/Org3_Gene725

MRIAVLGAGYAGLSVTWHLLLHSQGTATIDLFDPIPLGEGASGMSSGLLHAFTGKKALKPPLADQGINATHA
LITEASKALNVPIVISQGILRPAIDEDQAQLFTERVEEFPKEVEWWEKARCEISIPSMVIPPNLGALFIKSGVTLN

NDLYIQGLADACMKLGTQFYDELIEDLADIEEFYDHIIVTPGANASILPELKDMPVNKVKGQLLEISWPKDLA
MLSFSINAHKYMVANTQKNTCILGATFEHNQPEETPDPAIAYQEIMPPVLSLFPGLKDAQVLHCYAGMRSSS
KSRLPVISRIREKLWFLGGLGSKGLLYHGITGDMLAQAVLRKSTAYIAKEFLFTI

>core/420/3/Org3_Gene657

MILLQNIKRCSLKQLKVLATLLLSLSLPTLEAAENRDSDSIVWHLDYQEALQKSKEAELPLLVIFSGSDWNGP
CMKIRKEVLESPEFIKRVQGKFVCVEVEYLKHRPQVENIRQQNLALKSKFKINELPCMILLSHEEREIYRIGSFG
NETGSNLGDSLCHIVESDSLLRRAFPMMTSLSLSELQRYYRLAEELSHKEFLKHALELGVRSDDYFFLSEKFR
LLVEVGKMDSEECQRIKKRLLNKDPKNEKQTHFTVALIEFQELAKRSRAGVRQDASQVIAPLESYISQFGQQD
KDNLWRVEMMIAQFYLDSDQWHHALQHAEVAFEAAPNEVRSHISRSLEYIRHQS

>core/421/3/Org3_Gene367

MYLQEKFKAQQVPLVLRELLSCSDSINDSDPIYRMVFDSNDTTISYKVGDALGVLPENSKEVSEHVLQLLGYS
PTTLVNVKKTSEKVSAQKFIQGYVDLDKIPAKLNSFFPDKDPKITLYDAIQEYRPQIPIELFAESVFPLLPRFYSI
ASSPDLHPKSIELLVKHVSYPGKYQKRFGVCSSFLCSELQVNDSAYIFVQPTKHFTLSTQTEGKPLVMIGAGT
GIAPYKAFLEERLFNKDPGNNLLFFGERKEKVNFYYREFWNHAEEEGKLKLFLAFSRERDQKVYVQDLLRIQ
KDEVRKAYEEGGFFFVCGRKVLGIEVKHALEEILGKDTLASLRKEHRYVVDVY

>core/422/3/Org3_Gene199

MDVSRKINRHTQFYVDSIDGVIKNFDHKPSEDKSRDHEELEEKLLTITKRIVASAQEFQNRKTDSKNYYLKKT
QWLPFKNEELEQTKELFAMLTSMDKKIAQLFFYSPGCSSDWVEFTEVICHLNDSIGLGGVLLCCGLFEQQCEH
VVTVNKKLDLPLLLGTTVVNSLRYYLTYRNISLLNCQSMSELGKELGDVLKQHGVAFTLIFKEIVDIDLLNYV
KLIQGLKRSGNIQARIYDNDVPTLPSVSSSPIALRYSLANTIRGLALHVDFSSLKFISPSILSNTEHTAKALNSGG
ECFIFSNLDEFNLGMKIVMQLLRTGKISPEILNKNIMKILMIKRRVRSLYI

>core/423/3/Org3_Gene691

MEADILDGKLKRVEVSKKGLVNCNQVDVNQLVPIKYKWAWEHYLNGCANNWLPTEVPMARDIELWKSDE
LSEDERRVILLNLGFFSTAESLVGNNIVLAIFKHITNPEARQYLLRQAFEEAVHTHTFLYICESLGLDEGEVFNA
YNERASIRAKDDFQMTLTVDVLDPNFSVQSSEGLGQFIKNLVGYYIIMEGIFFYSGFVMILSFHRQNKMTGIGE
QYQYILRDETIHLNFGIDLINGIKEENPEVWTTELQEEIVALIEKAVELEIEYAKDCLPRGILGLRSSMFIDYVRH
IADRRLERIGLKPIYHSRNPFPWMSETMDLNKEKNFFETRVTEYQTAGNLSW

>core/426/3/Org3_Gene58

MELSLTSLLPLAWYVILGVAVFAYSFGDGFDLGLGAVYLKAKEDKERRILLNSIGPVWDGNEVWLVIIVGGL
FAGFPACYATLLSIFYMPIWTLVLLYIFRGCSLEFRSKSESVSWKIFWDIIFICSGTAISFFLGTIVGNLILGLPLSP
DTSYASLSWILFFRPYAALCGAVVASAFAIHGSCFALMKTSDSLNARIAQQFPYILSSFLVFYVLFLGASLISIP
KRFDAFPTYPLLILLIALTSCCCVAAKTSVSKKRYGYAFIYSTLNLLSLILSAATLTFPNILLSTVDPQYSYTIYN
SAVETKTLKSLLIIVLIGLPFIITYTCYIYRVFRGKTNFPSIY

>core/427/3/Org3_Gene604

MNSKMLKHLRLATLSFSMFFGIVSSPAVYALGAGNPAAPVLPGVNPEQTGWCAFQLCNSYDLFAALAGSLK
FGFYGDYVFSESAHITNVPVITSVTTSGTGTTPTITSTTKNVDFDLNNSSISSSCVFATIALQETSPAAIPLLDIAF
TARVGGLKQYYRLPLNAYRDFTSNPLNAESEVTDGLIEVQSDYGIVWGLSLQKVLWKDGVSFVGVSADYRH
GSSPINYIIVYNKANPEIYFDATDGNLSYKEWSASIGISTYLNDYVLPYASVSIGNTSRKAPSDSFTELEKQFTN
FKFKIRKITNFDRVNFCFGTTCCISNNFYYSVEGRWGYQRAINITSGLQF

>core/428/3/Org3_Gene971

MMEVYSFSPSVRTSFQHRVMAALDNWFFLGGRRLKVVSLDSCNSGQACEEYVPISTTEKVLKILSYLLIPIVII
ALLIRYLLHSNFTAKVSQKPWLKTLQLGIDIKSFILPGSHVNTMDSATLFKAIRLEGKRVDVEYHRLHSSDKV
VFYIPAQKLPDDLRLTHWLPEKETRKTEYVRHMLAHVMGYLTSQGKERLQQVVQDSRSSTSLGAEKVLQYR
FIDHPQSQGEFQRLLNENITTKGSEDKEVVQSDLFDMAFQCWWPQFISVIQSPTFSEELVHEMSQKLDLDCIYP
EDDEFEQKFLNTLLKAVLLHGFEGISVASMGVIFLICPDSLALQIPFLRNQK

>core/429/3/Org3_Gene564

MNKKKRVLTGDRPTGKLHLGHWVGSIKNRLELQNSPEYDCFFIIADLHTLTTKIRKEEVLDVDNHIYEVLAD
WLSVGIDPTKSIIYLQSAIPEIYELHLLFSMLISINRVMGIPSLKDMARNASIEEGSLSYGLIGYPILQSADILLAK
AQFVPVGKDNEAHVELTRDIARNFNRLYGQVFPEPEVLQGELTSLVGIDGQGKMSKSANNAIYLSDSDATITE
KVRKMYTDPNRIRATTPGRVEGNPLFIYHDIFNPHKDEVEEFKARYRQGCIKDIEVKARLAEELIHFLKPIKER
RSEFLSKPLALQNVLEDGTHKMREVAKVTMEEVHDKFGFSHKWRSLLK

>core/430/3/Org3_Gene118

MLTLGLESSCDETACAIVNEDKQILANIIASQDIHASYGGVVPELASRAHLHIFPQVINKALQQANLLIEDMDL
IAVTQTPGLIGSLSVGVHFGKGIAIGAKKSLIGVNHVEAHLYAAYMAAQNVQFPALGLVVSGAHTAAFFIENP
TSYKLIGKTRDDAIGETFDKVGRFLGLPYPAGPLIEKLALEGSEDSYPFSPAKVPNYDFSFSGLKTAVLYAIKG
NNSSPRSPAPEISLEKQRDIAASFQKAACTTIAQKLPTIIKEFSCRSILIGGGVAINEYFRSAIQTACNLPVYFPPA
KLCSDNAAMIAGLGGENFQKNSSIPEIRICARYQWESVSPFSLASP

>core/431/3/Org3_Gene77

MENAMSSSFVYNGPSWILKTSVAQEVFKKHGKGIQVLLSTSVMLFIGLGVCAFIFPQYLIVFVLTIALLMLAIS
LVLFLLIRSVRSSMVDRLWCSEKGYALHQHENGPFLDVKRVQQILLRSPYIKVRALWPSGDIPEDPSQAAVLL
LSPWTFFSSVDVEALLPSPQEKEGKYIDPVLPKLSRIERVSLLVFLSAFTLDDLNEQGVNPLMNNEEFLFFINK
KAREHGIQDLKHEIMSSLEKTGVPLDPSMSFQVSQAMFSVYRYLRQRDLTTSELRCFHLLSCFKGDVVHCLA
SFENPKDLADSDFLEACKNVEWGEFISACEKALLKNPQGISIKDLKQFLVR

>core/432/3/Org3_Gene200

MDSSAPYNIASQGTEKSTVERILDLYGPASCIKFLKQMVLIREFEARGEEAYLEGLVGGFYHSYAGQEAVATA
AIANTGLDPWVFSSYRCHALAILLNIPLQEIAAELLGKETGCALGRGGSMHMCGPNFPGGFGIVGGQIPLAAG
AAFTIKYQEQKNRVSLCFIGDGAVAQGVFHETLNFVSLHQLPLMLIIENNGWSMGTSLNRAVAKQPIAESQG
SSYDIRAVTVNGFDLFNSLLGFREAYRYMVDTESPVLVECLCSRFRGHSISDPNLYRSKEEMQCLFKKDPIVL
AKDWLIRLEVLTEEEFQNIRQECKTAVLEAFSNAKLSSDPSVTTLEEGVYA

>core/433/3/Org3_Gene98

MKLYSISSDVDTPWIFQLMSKVDSYLFLGGNRIKVVSIVMQEPNLIIGKVENVRISTIVKILKILSFLIFPLILIAL
ALHYFLHAKYANHLLVSKILERAPQYVPIPGRSGDTASHYKLTTLVPVSQKNLQAMGSNPLEVEAALRTTKP
SFFCVPAKYRQIIISSHGIRFSLDLEQLADDINLDSVSWPTEYLNSTMDFCSKADKRVIQNVQNLRTGTYINSV
GKRSLLKFMLQHLFIDGITQENPEALPNNTSGRLTLFPSVRYIYSHFTPQNPTIWPQVFFRQGPLDEDRGGGFEI
LEQLQELGVRFPICPSQGPDNPNFQGFQGIRIYWEDSYQPNKEV

>core/434/3/Org3_Gene641

MLPFEFEFNTTSSPECDVCLDPQKLFVKLFKRTIVLLSGPTGSGKTDVSLALAPMIDGEIVSVDSMQVYQGMD
IGTAKVSLKARQEIPHHLIDIRHVQEPFNVVDFYYEAIQACQNILSRNKVPILVGGSGFYFHAFLSGPPKGPAA
DPQIREQLEAIAEEHGVSALYEDLLLKDPEYAQTITKNDKNKIIRGLEIIQLTGKKVSDHEWDIVPKASREYCC
RAWFLSPETEFLKNNIQMRCEAMLQEGLLEEVRGLLNQGIRENPSAFKAIGYREWIEFLDNGEKLEEYEETKR
KFVSNSWHYTKKQKTWFKRYSIFRELPTLGLSSDAIAQKIAKDYLLYS

>core/435/3/Org3_Gene609

MFFQNLAKKLTALGISPLGCLLIGGVVSCAILFGRSSNPSLAPTQVKTEKTSGNWLKLTQMGNPKLIESLTKK
EQLEKDLTSFHPIASAKVAIALSTEDDVMSPLHLSVILTLRKEESLTPSLLFSITDYLCSSLPGLKREHISLSDNL
GNLYIPESITVNSLFIHTLENYLGKIFPKEHFALAYHAKAEKPTLQLTLNENYIAHLTKEESEKIVAHTKHYLY
QNYDDSYDIVIETLPFARLQNKKSFPAKVLIGSMILVISLMIVALASFYLARHAYERVSPEPRKIKRGINISKLLE
IIQKESPEKIALILSYLDPKKAEALLNRLPEDLKHQVLKYKL

>core/436/3/Org3_Gene181

MSEQHSPIISVQDVSKKLGDHILLSKVSFSVYPGEVFGIVGHSGSGKTTLLRCLDFLDMPTSGSISVAGFDNSLP
TQKFSRRNFSKKVAYISQNYGLFSSKTVFENIAYPLRIHHSEMSKSEVEEQVYDTLNFLNLYHRHDAYPGNLS
GGQKQKVAIARAIVCQPEVVLCDEITSALDPKSTENIIERLLQLNQERGITLVLVSHEIDVVKKICSHVLVMHQ
GAVEELGTTEELFLNSENSITNELFHEDINIAALSSCYFAEDREEVLRLNFSKELAIQGIISKVIQTGLVSINILSG
NINLFRKSPMGFLIIVLEGEVEQRKKAKELLIELGVVIKEFY

>core/437/3/Org3_Gene769

MTILRKLSQYLFFFSLFCSFIYVATCGSQPDSVSSPKIAIFLSFPHPLLEDCSKSCIETLKDFENLPEIVVLNAEDSI
VKARKIARSLHTDKNVVAIVTLGTIATKVMSHIETQKPVIYAAVPDRESLTPPKNTMNIYGVNDTLDINQYCF
AIQAVATNAQSIVYLKPSEPFPSDLQKEIVKKLHASGIEVIEISITSSTFKTRIRQAIDKRPSAIFIPLSPLSHKEGT
AFLQEILKEKIPIITDDTSLISEGACIACSVDYKKSGKQIAKIVHHLLYNNHDVDSLRKIIAQRLSPTTTFNEDIIK
YLGIKLHKTERNQFLSFKSKKLEKSEKGKNVAVS

>core/438/3/Org3_Gene698

MEMKEEIEAVKQQFHSELDQVNSSQALADLKVRYLGKKGIFRSFSEKLKQCTDKAKLGSLINDFKTYVEDLL
QEKSLVLLASEQAEAFSKEKIDSSLPGDSQPSGGRHILKSILDDVVDIFVHLGFCVREAPNIESEANNFTLLNFT
EDHPARQMHDTFYLNATTVLRTHTSNVQARELKKQQPPIKVVAPGLCFRNEDISARSHVLFHQVEAFYVDH

NVTFSDLTAILSAFYHSFFQRKTELRFRHSYFPFVEPGIEVDVSCECCGKGCALCKHTGWLEVAGAGMIHPQV
LRNGNVDPEIYSGYAVGMGIERLAMLKYGVSDIRLFSENDLRFLQQFS

>core/439/3/Org3_Gene242

MATISPISLTVDHPLVDTKKKSCSNFDKIQSRILLITAIFAVLVTIGTLLIGLLLNIPVIYFLTGISFIAVVLSNFILY
KRATTLLKPRACGKHKEIKPKRVSTNLQYSSISIAINRSKENWEHQPKDLQNLPAPSALLTDNPYEIWKAKHS
LFSLVSLLPGGNPEHLLISASENLGKTLLIEETSQNAPISSYVDTTPSPKSLLNEAIQETRVEINTELPAGDSGER
LYWQPDFRGRVFLPQIPTTPEAIYQYYYALYVTYIQTAINTNTQIIQIPLYSLREHLYSRELPPQSRMQQSLAMI
TAVKYMAELHPEYPLTIACVERSLAQLPQESIEDLS

>core/440/3/Org3_Gene485

MEEAAKHLAKEFLCSGINLFLSGEYEQAEKRLKETLELDSTAALAYCYLGIIALETGRVSEALNWCSKGLASE
PGDSYLRYCYGVALDRGNQYEAAIEQYSAYVALHPDDVECWFSLGSVYHRLKRLQEALDCFDKILALDPW
NPQSLYNKAVILSEMDDEAESIRLLEVAVAKNPLYWKAWVKLGFLLSRSKRWDKATEAYERVVQLRPDLSD
GHYNLGLCYLTLDKTRLALKAFQEALFLNAEDADAHFYVGLAHLDLKQMREAYEAFNSALSINLEHERAHY
LLGYLHHMQGETDKATKELLFLQKKDSTFAPLLQKTVVSDPSSMQFERRLDTIS

>core/441/3/Org3_Gene503

MVLGVVGISYREAALKERERAIQYLQSFEKNLFLAQRFLGKGGAFIPLLTCHRAELYYYSESPEIAQAALLSEL
TSQGIRPYRHRGLSCFTHLFQVTSGIDSLIFGETEIQGQVKRAYLKGSKERELPFDLHFLFQKALKEGKEYRSRI
GFPDHQVTIESVVQEILLSYDKSIYTNFLFVGYSDINRKVAAYLYQHGYHRITFCSRQQVTAPYRTLSRETLSF
RQPYDVIFFGSSESASQFSDLSCESLASIPKRIVFDFNVPRTFLWKETPTGFVYLDIDFISECVQKRLQCTKEGV
NKAKLLLTCAAKKQWEIYEKKSSHITQRQISSPRIPSVLSY

>core/442/3/Org3_Gene75

MFPSANQESRTRNVPLGIFHGLVACLYWGIVFVIPNFLGSFGDLDIVLTRYTIFGIFSLIACAIKNPSVIKKTPLYI
WRKSLLWTLLINPVYYFGITLGIRYVGSAITVVIASLAPTAVLYHSNTKQKELPYSLLFAISSVIITGVILTHLSA
LNLPTAASPLYSILGVIAVILSTSLWVIYVIRNQSLLEKHPNLTPDTWSYLIGISALIICLPMIIILDLCGITHVTHN
LISHTPGSERLLFLLLCSAMGIFSSAKALIAWNKASLNLSPALLGAILIFEPIFGLVLTYLYSQSLPSLQEGIGIFL
MLGGSLLCLVLFGRKVQKSLENSQVSSSNE

>core/443/3/Org3_Gene561

MKKTCCQNYRSIGVVFSVVLFVLTTQTLFAGHFIDIGTSGLYSWARGVSGDGRVVVGYEGGNAFKYVDGEK
FLLEGLVPRSEALVFKASYDGSVIIGISDQDPSCRAVKWVNGALVDLGIFSEGMQSFAEGVSSDGKTIVGCLY
SDDTETNFAVKWDETGMVVLPNLPEDRHSCAWDASEDGSVIVGDAMGSEEIAKAVYWKDGEQHLLSNIPG
AKRSSAHAVSKDGSFIVGEFISEENEVHAFVYHNGVIKDIGTLGGDYSVATGVSRDGKVIVGHSTRTDGEYR
AFKYVDGRMIDLGTLGGSASFAFGVSDDGKTIVGKFETELGECHAFIYLDD

>core/444/3/Org3_Gene790

MTHQVAVLHQDKKFDVSLRPKGLEEFYGQHHLKERLDLFLCAALQRGEVPGHCLFFGPPGLGKTSLAHIVA
YTVGKGLVLASGPQLIKPSDLLGLLTSLQEGDVFFIDEIHRMGKVAEEYLYSAMEDFKVDITIDSGPGARSVR

VDLAPFTLVGATTRSGMLSEPLRTRFAFSARLSYYSDQDLKEILVRSSHLLGIEADSSALLEIAKRSRGTPRLA
NHLLRWVRDFAQIREGNCINGDVAEKALAMLLIDDWGLNEIDIKLLTTIIDYYQGGPVGIKTLSVAVGEDIKT
LEDVYEPFLILKGFIKKTPRGRMVTQLAYDHLKRHAKNLLSLGEGQ

>core/445/3/Org3_Gene946

MSYSLRNKKTKICVYIIIALGILSFRSIPQEVYDKIRSSFVSLHVKFFPKIRQAPSSHLANLELENLVLKERVASL
EEKLKLYEVSNHTPPLFPEILTPYFHKLVEGKVVYRDYTHWSSSCWVNVGKTHGIKKNSPVLSGNVLVGLVD
YVGEHQSRIRLITDVGMKPSVVAMRGDIQSWWIKHSLRELIRQVEQISHAYILEKDKYEKISQLQELDSLIQGE
GENQALLRGILSGVGGALWKEGSLCLEGEGFYFSEGKTLLPGDILVTTGLDGVFPPGLLVARVTKVKAPRDG
ACTFKIEAQSLEEKLMELDQLFILPPLEFNPNDRPDIFGLLWD

>core/446/3/Org3_Gene427

MKVVINGFGRIGRLVLRQILKRNSSVEVLAINDLVPGDALTYLFKFDSTHGRFPEDVRCEADHLIVGKRKIQF
LSERNVQNLPWKDLGVDLVIECTGLFTKKEDAEKHIQAGAKRVLISAPGKGDIPTFVMGVNHKTFNPEKDFV
ISNASCTTNCLAPIAKVLLDNFGITEGLMTTVHAATATQLVVDGPSKKDWRGGRGCLQNIIPASTGAAKAVT
LCLPELKGKLTGMAFRVPIEDVSVVDLTVRLDKSTTYDDICKAMKQASETDLKGILDYTDEQVVSSDFIGSEY
SSIFDALAGIALNDRFFKLVAWYDNETGYATRIVDLLEYVEKNSK

>core/447/3/Org3_Gene363

MFVDQITLELRAGKGGNGVVAWRKEKYLPKGGPYGGNGGNGGSVIIEATTSVYSFEAYRNIRFLKAPDGQS
GATNNRTGRSGKDLIVSVPTGTLLRDAETGEILHDFTVDGERLLVSQGGKGGKGNTFFKTSVNRAPTKATPG
KPGEIRQVELELKLIADIGLVGFPNAGKSTLFNTLAHTEVKVGAYPFTTLAPSLGLVLCKDRLYQKPWIIADIP
GIIEGAHQNKGLGLDFLRHIERTLLLLFVIDVSKRERNSPEEDLETLIHELHSHQPDFEKKDMLVALNKIDDLL
PDEQEECLQSFQKRFPSYTFVLISGLTGEGVDGLYRFFTQRLAV

>core/448/3/Org3_Gene194

MWFSVNKNKKAAIWATGSYLPEKVLSNADLEKMVDTSDEWIVTRTGIKERRIAGPQEYTSLMGAIAAEKAI
ANAGLSKDQIDCIIFSTAAPDYIFPSSGALAQAHLGIEDVPTFDCQAACTGYLYGLSVAKAYVESGTYNHVLLI
AADKLSSFVDYTDRNTCVLFGDGGAACVIGESRPGSLEINRLSLGADGKLGELLSLPAGGSRCPASKETLQSG
KHFIAMEGKEVFKHAVRRMETAAKHSIALAGIQEEDIDWFVPHQANERIIDALAKRFEIDESRVFKSVHKYGN
TAASSVGIALDELVHTESIKLDDYLLLVAFGGGLSWGAVVLKQV

>core/449/3/Org3_Gene541

MAAPIFIKNILLRSSIVYAPLAGFSDYPYRCMSALYQPGLMFCEMVKVEGILYAPERTSKLLDYNENMRPIGA
QLCGSNPETSGEAAKILEGLGFDLIDLNCGCPTDKITKDGSGSGLLKTPELIGRILDKIINSVSIPVTVKIRSGWD
MEHINVEDTVRIIRDAGASAVFVHGRTRAQGYHGPSKQEYISRAKAAAGKEFPVFGNGDIFSPEAAQAMLTT
GCDGVLVARGTLGAPWIGKQIQDYLTTGSYEKIPFIKRKAAFLEHMRLVEDYYQSETKFLSETRKLCGHYLIS
AAKVRFLRSSLAKATSYQEVYQLVNDYEEADDSSLETFVKC

>core/450/3/Org3_Gene37

MSTPLSSGGISPSDQYVPQELFCDRLSSSRSNSPDSNASGDSPIVSPPISALVALTDLKLVPYNQNSFSWTTRLK
NAVEKIGLFLQRNWKYILLYILAWALILVCHHTVALTLTIWLGVGLGIGVVFGIFTATCLDKENKHRHVNSL
WNLINHGILQLDPNGTRQILLATMIASISALIYAVPQAVGLVIGFSIGNQLSINTVYGARLGDEATYAIDRKAH
KKRIENIEQAINQHQIIKHQMINQKQLNALIEINRNNQTDPATANLLASLKLNLNQPMPYCFSMPECGVTSSYL
DLNNNSPDDIIARADQCIMTLSQTLQQIKKEPDRIIESNH

>core/451/3/Org3_Gene585

MVRRSISFCLFFLMTLLCCTSCNSRSLIVHGLPGREANEIVVLLVSKGVAAQKLPQAAAATAGAATEQMWDI
AVPSAQITEALAILNQAGLPRMKGTSLLDLFAKQGLVPSELQEKIRYQEGLSEQMASTIRKMDGVVDASVQIS
FTTENEDNLPLTASVYIKHRGVLDNPNSIMVSKIKRLIASAVPGLVPENVSVVSDRAAYSDITINGPWGLTEEI
DYVSVWGIILAKSSLTKFRLIFYVLILILFVISCGLLWVIWKTHTLIMTMGGTKGFFNPTPYTKNALEAKKAEG
AAADKEKKEDADSQGESKNAETSDKDSSDKDAPEGSNEIEGA

>core/452/3/Org3_Gene605

MKQHIGYLGMGIWGFCLASLLANKGYPVVAWSRNPDLIKQLQEERRHPLAPNVVISPNLSFTTDMKEAIHNA
FMIVEGVTSAGIRPVAEQLKQITDLSVPFVITSKGIEQNTGLLLSEIMLEVLGDSVTPYLGYLSGPSIAKEVLNG
SPCSVVVSAYDSQTLKQIHEAFSLPTFRVYPNTDIKGAALGGALKNVIAIACGIAEGLSFGNNAKAGLVTRGL
HEMRKLAAIMDCKPETLNGLAGLGDLCVTCFSESSRNLRFGHLLAQGLTFEQAKAKIGMVVEGAYTALSAY
QVAKHHKIDMPITTGIYRVLYENLDLKEGIALLLQRNTKEEFL

>core/453/3/Org3_Gene409

MKPLGFQENLEALCNKTSRQLLKYLIKQILFVCGASLLIALEFSFFLYFFLFSGKTVIPAFCLACFFLTLFVCLV
TRLYLLSGKGDFFEDLASEYLQGAVPPNKRSQNIVEEQSHLAAAATKLSINLQNQEYSLLSEIFKFLPKHDLIR
KFSCFCFWKDYFLFRECLLQKAIEAYIKVVQAIPVDLSAHVSLADAYVALSGLYADPRKYPEFDANYWIPSG
RYSAEIQEKFFATARRAIEEFQILNEYAPGNAWVHAQLAYSYHDLQMPMEEIQEYEIVLKLKPNDVETMSKL
GILYFQQGMNAKGLRIYEEIKKRDYKKSQKLIKFYGVEYKY

>core/455/3/Org3_Gene578

MQPFIFTLLCLTSLVSLVAFDAANARKRCACAQTIERGENFFSIKRSACAEIEYQEKSRHASAIERISKDKGKV
TPKQIAKVATKKKQRYRLLQVPFSRPPNNSRYNLYALLSEPPECYSDTASWYAIFIRLLRRAYVDTGNVPPGS
EYAIANALISNKQEILERGAQLGPDVIETLTLPEEQAEIFYKMLKGSSNSQSLLNFLHYEEKSLGHCKLNLIFM
DPLLLEAVLDHPDAYRETSLLRDGIWEAVKRQEHAIQEHGQAAALELFKTRTDFRLELRDKMQLLLSRYDLL
PLLNKKMFDYTLGSAGDYLFLVDPDTKAISRCRCPSKSIKL

>core/456/3/Org3_Gene446

MATNAIRSAGSAASKMLLPVAKEPAAVSSFAQKGIYCIQQFFTNPGNKLAKFVGATKSLDKCFKLSKAVSDC
VVGSLEEAGCTGDALTSARNAQGMLKTTREVVALANVLNGAVPSIVNSTQRCYQYTRQAFELGSKTKERKT
PGEYSKMLLTRGDYLLAASREACTAVGATTYSATFGVLRPLMLINKLTAKPFLDKATVGNFGTAVAGIMTIN
HMAGVAGAVGGIALEQKLFKRAKESLYNERCALENQQSQLSGDVILSAERALRKEHVATLKRNVLTLLEKA
LELVVDGVKLIPLPITVACSAAISGALTAASAGIGLYSIWQKTKSGK

>core/457/3/Org3_Gene977

MRIAVLGVTGLVGQKFVALLHKWYRDWVIAEVVASNSKYGQSYGDACIWQEPIGPMPEMVRDLPIRKIEEV
QSDIVDSFLPSSAESMEAYCLSQGKVVFSNASTYRMHSSVPIIIPEVNSDHFQLLEEQPYPGKIITSPNCCVSGIT
LALAPLRKFSLDHVHIVTLQSASGAGYPGVPSLDLLANTVPHIVGEEEKILRETVKILGSSKQPLPCKLSVTVH
RVPVAYGHTLSLHVTFSKDVDLDEILYSYQEKNKEFPNTYQLYDNPWSPQARKHLSHDDMRVHLGPITYGG
DFRTIKMNVLIHNLVRGAAGTLLASMENYFFDYLKREMCLR

>core/458/3/Org3_Gene417

MKTLWHFVSKAFLSIVGLCCGVVLAFVVIFALIASSLGNGDATFVSLPDAQGEVKDLGKTAPIIAVIEMKDVI
ASSKNTAKTIQNILEGFEKAPLKDRVKGIVIDMDCPGGEVFEIDRIYSMLRFWKERKGFPIYIYVNGLCASGGY
YVSCAATKIYATSSSLIGSIGVRSGPFFNVKEGLNRYGVESDLLTAGKDKAPMNPYTPWTSHDREERQATLDF
LYGQFVDIVTQNRPLLTKEKLVHTLGARIFSPEKAKQEGYIDVVGATKEQVLQDIVAVCKIEDNYRVIGSGGD
GWWKRVASAAASSPLVTGMIKHDILPLSHDAAYIPPYLAL

>core/460/3/Org3_Gene523

MSSLTLSRRPRRNRKTAAIRDLLAETHLSPKDLIAPFFVKYGNNIKEEIPSLPGVFRWSLDLLLKEIERLCTYGL
RAVMLFPIIPDDLKDAYGSYSSNPKNILCHSIHEIKNAFPHLCLISDIALDPYTTHGHDGIFLNGEVLNDESVRIF
GNIATLHAEMGADIVAPSDMMDGRIGYIRSKLDQSGYSKTSIMSYSVKYASCLYSPFRDALSSHVTSGDKKQ
YQMNPKNVLEALLESSLDEEEGADILMVKPAGLYLDVIYRIRQNTCLPLAAYQVSGEYAMILSAFQQGWLD
KETLFHESLIAIKRAGADMIISYSAPFILELLHQGFEF

>core/461/3/Org3_Gene94

MNIYQFSPGASPNWQASLMAQLNSYFCLGGETVTRIISLRPSGLILAKKEKAVVSTAEKILKILSFILFPLVLIAL
AIRYLLYNKFNKDLDRAVFFIPTEITKAEELIIAKNPALVKEAALTVSPLFYSLPKKYQLMKVETP

>core/462/3/Org3_Gene95

MLLEDLDTDSIPWPKLYLSEDFDFAYYPESKAIIDTVAKLEKNNPGEEFCLESKKILARYLLEQLFKLETGLNF
PTSTIDGGRESFLIEFSHETKKPTVWAFIYFYYYHSNGPKLEKDFKQAGCEVHNRLLNLGLKYRPQAGAQND
GRNGGPYGPIGFLIVWEENYGSVLKDHGFIKDN

>core/463/3/Org3_Gene677

MQFSRYLRYAFDNQYLPEPLYQKFSVFHQNYIDAATKKAAADQAEVLCLQWVKVIIEDLKNPFIFPPYHKKI
RAPIDLFRLSIDFFSLVIDDKNSRILNLHRLKEIEEYIARGDNVVLLANHQTECDPQLMYYALGKTHPELMEN
MIFVAGDRVTSDPLARPFSMGCDLLCIYSKRHIATPPELREEKLLHNQKSMQILKTLLNEGGKFIYVAPAGGR
DRKNAEGRLYPSEFSPESIEVFRLLAKASNQTTHFYPFALKTYDILPPPPKIENAIGEQRAIFFAPVFFNFGAELF
FDALCSKEELIHCDKHAQRTLRAEKVFSIVKNLYEEL

>core/464/3/Org3_Gene736

MREETVSWSLEDIREIYHTPVFELIHKANAILRSNFLHSELQTCYLISIKTGGCVEDCAYCAQSSRYHTHVTPEP
MMKIVDVVERAKRAVELGATRVCLGAAWRNAKDDRYFDRVLAMVKSITDLGAEVCCALGMLSEEQAKKL
YDAGLYAYNHNLDSSPEFYETIITTRSYEDRLNTLDVVNKSGISTCCGGIVGMGESEEDRIKLLHVLATRDHIP

ESVPVNLLWPIDGTPLQDQPPISFWEVLRTIATARVVFPRSMVRLAAGRAFLTVEQQTLCFLAGANSIFYGDK
LLTVENNDIDEDAEMIKLLGLIPRPSFGIERGNPCYANNS

>core/465/3/Org3_Gene315

MIDIMQHFKPYTMVPGQKLPIPGSLLYAQVFPTLWRLFSSKHEILNEQTLQVQGPLKRFAVFQDLHRGGLAV
TSERYKYYLLPSGECTQSIKGKLPSAAQAGPLLSLGVHKHADWQKVRCRRDLKEILPLWFRFAAMAPKGSY
RDLETTAIGSLVKTAHQRVLHRETTEIAPALLSIALAGFSECFLPRSYDEEFQGILPQDGDPEGGVPFELLSYSF
GMIQDIFLRHQGQLVEILPALPPEFPCGRLIHVALPNLGTLSIVWTKKTIRQVELHAEYSGEVFLKFCSSLCSAR
LREWSERRLSGSKRLSLGETLEIKAGTTYLWDCFHK

>core/466/3/Org3_Gene629

MSAFFDLLKSQTASHPPIWLLRQVGRYMPPYQELKGSQSLKTFFHNTEAIVEATLLGPSLLHVDAAILFADILS
ILDGFAVTYDFAPGPRIQFSPEQPFTFTSDPQTIFSYLLDAIRTLKQKLPVPLIVFAASPFTLACYLIDGGASKDF
SKTMSFLYVYPEKFDQLISTIIEGTAIYLKTQMDAGAAAVQLFESSSLRLPSALFTRYVTEPNRRLIAKLKEQAI
PVSLFCRCFEENFYTLQATQADTLHPDYHVDLHRIQKNLMLSLQGNLDPAIFLLPQEKLLHYVEAFLVPLRTY
PNFIFNSGHGILPETPLENVQLVVSYVQRQL

>core/467/3/Org3_Gene349

MPSPMISTDVCQDILGKQKEAVDFFFQAFQPKEAMQLAEKILGHSGWVFFSGVGKSGCVARKLVATLQSLSE
RALFFSPVDLLHGDLGLVSPGDIVCLFSKSGETQELLDTVPHLKSRRAILVAITSMPYSNLAALSDLVVILPSV
AELDPFNLIPTNSTTCQMIFGDFLAMLLFHSRGVSLSTYGKNHPSGQVGMKANGKVKDFMFPKTEVPFCHLG
DKVSFSLEVFSAYGCGCVCIVDPQFRLMGIFTDGDLRRSLASYGGEVLSLSLEKVMTANPRCITEDSDIAIALQ
LMESSSPVAVLPVLDNEENRHVTGLLHMHTLAKAGLL

>core/468/3/Org3_Gene724

MAFKEVVRVAVTGGKGQIAYNFLFALAHGDVFGVDRGVDLRIYDVPGTERALSGVRMELDDGAYPLLHRL
RVTTSLNDAFDGIDAAFLIGAVPRGPGMERGDLLKQNGQIFSLQGAALNTAAKRDAKIFVVGNPVNTNCWIA
MKHAPRLHRKNFHAMLRLDQNRMHSMLAHRAEVPLEEVSRVVIWGNHSAKQVPDFTQARISGKPAAEVIG
DRDWLENILVHSVQNRGSAVIEARGKSSAASASRALAEAARSIFCPKSDEWFSSGVCSDHNPYGIPEDLIFGFP
CRMLPSGDYEIIPGLPWEPFIRNKIQISLDEIAQEKASVSSL

>core/469/3/Org3_Gene201

MPKHKTLEIREALREAIDEEMSRDPNVCILGEEVGDYNGAYKVTKGLLDKWGPKRVIDAPISEAAFSGIGIGA
ALSGLRPIIEFMSWNFSFVALDQIISHAAKMHFMTGGKFSVPIVFRGPNGAAAQVSCQHSHCVESLYANIPGLI
IIAPSNPYDAKGLLKSAIRNNNPVLFLENELEYNLKGEVPTEEYLVPIGKAHRVQEGNDLTIITYSRMVSITKEA
CSLAKKRWGLSIEIIDLRTIKPLDISTILSSVRKTSRCIVIEEGHYFAGISSEIIALITEHVFDSLDAPPLRVCQKETP
MPYSKILEQATLPNVNRILDTIEKVMR

>core/470/3/Org3_Gene45

MSNQFDQLKKLSTIVCDSGDPELVKASGSQDATTNPSLILKVAQEPKFQELLNEAVVWGIRQNGDDLQTLSFI
LDKIQVNFALEIIKNIPGRISLEIDARLSFNVEAMVQRAVFLSQLFEAMGGDKKRLLVKIPGTWEGIRAVEFLE

AKGIACNVTLIFNLVQAIAAAKAKATLISPFVGRIYDWWIAAYGDEGYSIDADPGVASVSNIYAYYKKFGIPT
QIMAASFRTKEQVLALAGCDLLTISPKLLDELKKSQHPVKKELDPAEAKKLDVQPIELTESFFRFLMNEDAMA
TEKLAEGIRIFAGDTQILETAITEFIKQIAAEGA

>core/471/3/Org3_Gene926

MTTPAYLLANFGGPRHAKDLQEFLISLLTDRDVTGTFLPRVLHRHLFTFIAKKRVPKVLPQYQSLQNWSPIYF
DTETLAKTLSEILRAPVIPFHRYLPSTHEKTLLALRTLHTRHVIGIPLFPHFTYSVTGSIVRFFMKHVPEIPISWIP
QFGSDSKFVSLITCHIRDFLQKLGILEKECCFLFSVHGLPVRYISQGDPYSKQCYESFSAITTNFKQSENFLCFQS
KFGPGKWLSPSTAQLCQNIDTDKPNVIAVPFGFISDHLETLYEIERDYLPLLRSRGYRALRIPAIYSSPLWVSTL
VDIVKENSTVVAEELIKSGKKHTGIR

>core/472/3/Org3_Gene231

MDAKMGYIFKVMRWIFCFVACGITFGCTNSGFQNANSRPCILSMNRMIHDCVERVVGNRLATAVLIKGSLDP
HAYEMVKGDKDKIAGSAVIFCNGLGLEHTLSLRKHLENNPNSVKLGERLIARGAFVPLEEDGICDPHIWMDL
SIWKEAVIEITEVLIEKFPEWSAEFKANSEELVCEMSILDSWAKQCLSTIPENLRYLVSGHNAFSYFTRRYLATP
EEVASGAWRSRCISPEGLSPEAQISVRDIMAVVDYINEHDVSVVFPEDTLNQDALKKIVSSLKKSHLVRLAQK
PLYSDNVDDNYFSTFKHNVCLITEELGGVALECQR

>core/473/3/Org3_Gene342

MMTYPVPQNPLLLRILRLMDAFSKSDDERDFYLDRVEGFILYIDLDKDQEDLNKIYQELEENAERYCLIPKLT
FYEVKKIMETFINEKIYDIDTKEKFLEILQSKNAREQFLEFIYDHEAELEKWQQFYVERSRIRIIEWLRNNKFHF
VFEEDLDFTKNVLEQLKIHLFDAKVGKEITQARQLLSNKAKIYYSNEALNPRPKRGRPPKQSAKVETETTISSD
IYTKVPQAARRFLFLPEITSPSSITFSEKFDTEEEFLANLRGSTRVEDQLNLTNLSERFASLKELSAKLGYDSLST
GDFFGDDDEKVVTKTKGSKRGRKKSS

>core/474/3/Org3_Gene964

MHSELPNYQNIVESVVTEITTQLLNYRSEHRLVPFWEKSDGSFITAADYGSQYYLKQQLAKAFPNIPFIGEETL
YPDQDNEKIPEILKFTRLLTSSVSRDDLISTLVPPPSPTSLFWLVDPIDGTAGFIRHRAFAVAISLIYEYRPILSVM
ACPAYNQTFKLYSAAKGHGLSIVHSQNLDRRFVYADRKQTKQFCEASLAALNQQHHATRKLSLGLTNTPSP
RRVESQYKYALVAEGAVDFFIRYPFIDSPARAWDHVPGAFLVEEAGGRVTDALGAPLEYRKESLVLNNHAVI
LASGDQETHETTLAALQNQLNVVPTDKLIAL

>core/475/3/Org3_Gene507

MKTVTSFTVCKENSGRLDKYLTEVHPKYSRAFYQEHILSGLVQINGQINTRVATRLNCGDIVTIDIQEKEELLE
LLPEAIPLDKVYEDGMILVINKPRDMVVHPAPGHFHGTLVHALLHEIGERLKEEFPEEPWRPGIVHRLDKDTS
GLIITAKTRQAKKVFSELFSTKRLKKSYLAVCIGKPRSTTIHTHISRHQNKRKEMTVSSQGKEAVTHCQVLAF
NGKLSFVALSPETGRTHQLRVHMKHLGTPILGDPVYGIPSMNSSYGLDKQQLHAYSVDFTHPETRQFCSLKA
GLPEDMRSLLIKEFRNETTILNKNLLESILKEQ

>core/476/3/Org3_Gene171

MTIDMHCDLLSHPHFCRKDPAVRCSPEQLLSGGVRQQVCAIFVPHSRGEPNCDKQNSLFFSLPNQYPDIGLLS
YEEEENGSSSQKKSLSLIRSIENASALGDDTAPLGTLLAKLIHLTKQGPLAYLGIVWKGDNRFGGGTEAPKRL
SNDGKVLLDIMYELGVPIDLSHCSDKLAEDILDYTADKLPNLAVIASHSNFRSVLDHRRNLVDAHAKEIVRRK
GVIGLNLVRSYVGDSLGDLEKHVLHAENLGILSSIVLGSDFFYANEDENFFFNECSSAEAHPVLNQLIHRIFSK
GKAESILSSRAEKFLKQVIVEQVNPKITDVKL

>core/478/3/Org3_Gene813

MIEFAFVPHTSVTADRIEDRMACRMNKLSTLAITSLCVLISSVCIMIGILCISGTVGTYAFVVGIIFSVLALVACV
FFLYFFYFSSEEFKCASSQEFRFLPIPAVVSALRSYEYISQDAINDVIKDTMQLSTLSSLLDPEAFFLEFPYFNSLI
VNHSMKEADRLSREAFLILLGEITWKDCETKILPWLKDPNITPDDFWKLLKDHFDLKDFKKRIATWIRKAYPE
IRLPKKHCLDKSIYKGCCKFLLLAENDVQYQRLLHKVCYFSGEFPAMVLGLGSEVPMVLGLPKVPKDLTWE
MFMENMPVLLQSKREGHWKISLEDVASL

>core/479/3/Org3_Gene280

MELLPHEKQVVEYEKAIAEFKEKNKKNSLLSSSEIQKLEKRLDKLKEKIYSDLTPWERVQICRHPSRPRTVNYI
EGMCEEFVELCGDRTFRDDPAVVGGFVKIQGQRFVLIGQEKGCDTASRLHRNFGMLCPEGFRKALRLGKLA
EKFGLPVVFLVDTPGAYPGLTAEERGQGWAIAKNLFELSRLATPVIIVVIGEGCSGGALGMAVGDSVAMLEH
SYYSVISPEGCASILWKDPKKNSEAASMLKMHGENLKQFGIIDTVIKEPIGGAHHDPALVYSNVREFIIQEWLR
LKDLAIEELLEKRYEKFRSIGLYETTSESGPEA

>core/480/3/Org3_Gene477

MASNPILQIEDLSITLAKQRQQYPIVQSLSFTINEGQTLAIIGESGSGKSVSAHAILRLLPCPPFSVSGQVNFQGH
NLLTASRSIQKKIIGTEISMIFQNPQASLNPVFTIEQQFREIIHTHLALTAEVAKEKMLYALEETGFHDPRLCLNL
YPHQLSGGMLQRICIAMALLCSPKLLIADEPTTALDVSVQYQILQLLKTLQKKTGMSLLIITHNMGVVAETAD
DVLVLYAGRMVECAPAVQMFHNPSHPYTRDLLASRPSLQPQQLGSFNPIPGQPPHYTAFPSGCRYHPRCSKIL
NRCSAEAPEIYPVREGHKVRCWLYDD

>core/481/3/Org3_Gene228

MALGPSPYYGVSFFQFFSVFFSRLFSGSLFTGSLYIDDIQIIVFLAISCSGAFAGTFLVLRKMAMYANAVSHTVL
FGLVCVCLFTHQLTTLSLGTLTLAAMATAMLTGFLIYFIRNTFKVSEESSTALVFSLLFSLSLVLLVFMTKNAH
IGTELVLGNADSLTKEDIFPVTIVILANAVITIFAFRSLVCSSFDSVFASSLGIPIRLVDYLIIFQLSACLVGAFKA
VGVLMALAFLIIPSLIAKVIAKSIRSLMAWSLVFSIGTAFLAPASSRAILSAYDLGLSTSGISVVFLTMMYIVVK
FISYFRGYFSKNFEKISEKSSQY

>core/482/3/Org3_Gene478

MTTNFPQPLIQATSLTKHYYKRSFWFQGKTIASRPVDDVSFSLYSRRAVGLIGESGSGKSTLALALAGLLPLTS
GFLTFNGTPIKLHSKHGRHQLRSQVRLVFQNPQASLNPRKTILDSLGHSLLYHKLVPKEKVLATVREYLELVG
LSEEYFYRYPHQLSGGQQQRVSIARALLGVPQLIICDEIVSALDLSIQAQILNMLAELQKKLSLTYLFISHDLAV
VRSFCTEVFIMYKGQIVEKGNTKRIFSDPQHPYTRMLLNAQLPETPDQRQSKPIFQEYHKDSEESCSTGCYFY
NRCPQKQEACKSEIIPNQGDAHHTYRCIH

>core/483/3/Org3_Gene516

MEKKYYALAYYYITRVDNPHEEIALHKKFLEDLDVSCRIYISEQGINGQFSGYEPHAELYMQWLKERPNFSKI
KFKIHHIKENIFPRITVKYRKELAALGCEVDLSKQAKHISPQEWHEKLQENRCLILDVRNNYEWKIGHFDNAT
LPDIQTFREFPEYAEKLAQECDPETTPVMMYCTGGIRCELYSPVLLEKGFKEVYQLDGGVIAYGQQVGTGKW
LGKLFVFDDRLAIPIDESDPDVAPIAECCHCQTPSDAYYNCANTDCNALFLCCDECIHQHQGCCGEECSQSPR
VRKFDSSRGNKPFRRAHLCEISENSESASCCLI

>core/484/3/Org3_Gene404

MKQLLFCVCVFAMSCSAYASPRRQDPSVMKETFRNNYGIIVSGQEWVKRGSDGTITKVLKNGATLHEVYSG
GLLHGEITLTFPHTTALDVVQIYDQGRLVSRKTFFVNGLPSQEELFNEDGTFVLTRWPDNNDSDTITKPYFIET
TYQGHVIEGSYTSFNGKYSSSIHNGEGVRSVFSSNNILLSEETFNEGVMVKYTTFYPNRDPESITHYQNGQPHG
LRLTYLQGGIPNTIEEWRYGFQDGTTIVFKNGCKTSEIAYVKGVKEGLELRYNEQEIVAEEVSWRNDFLHGER
KIYAGGIQKHEWYYRGRSVSKAKFERLNAAG

>core/486/3/Org3_Gene447

MNLKVVYFGTPTFAATVLQDLLHHKIQITAVVTRVDKPQKRSAQLIPSPVKTIALTHGLPLLQPSKASDPQFIE
ELRAFNADVFIVVAYGAILRQIVLDIPRYGCYNLHAGLLPAYRGAAPIQRCIMEGATESGNTVIRMDAGMDT
GDMANITRVPIGPDMTSGELADALASQGAEVLIKTLQQIESGQLQLVSQDAALATIAPKLSKEEGQVPWDKP
AKEAYAHIRGVTPAPGAWTLFSFSEKAPKRLMIRKASLLAEAGRYGAPGTVVVTDRQELAIACSEGAICLHE
VQVEGKGSTNSKSFLNGYPAKKLKIVFTLNN

>core/488/3/Org3_Gene291

MSKGSSKHTVRINQTWYIVSFILGLSLFAGVLLSTIYYVLSPIQEQAATFDRNKQMLLAAHILDFKGRFQIQEK
KEWVPATFDKKTQLLEVATKKVSEVSYPELELYAERFVRPLLTDAQGKVFSFEEKNLNPIEFFEKYQESPPCQ
QSPLPFYVILENTSRTENMSGADVAKDLSTVQALIFPISGFGLWGPIHGYLGVKNDGDTVLGTAWYQQGETP
GLGANITNPEWQEQFYGKKIFLQDSSGTTNFATTDLGLEVVKGSVRTTLGDSPKALSAIDGISGATLTCNGVT
EAYVQSLACYRQLLINFSNLTHEKKTGE

>core/489/3/Org3_Gene425

MRLLSILKLHLFSLRSSSSLSPHYYHSCSRSMLHLLCRWKDADIMEWQQICNILSGVCSRMSGKLVSLQKETQ
DSCHQEHERIHLQYREQLSALEEEYRRREEAKNQDLEKLQQENTWLQNRLAEKLQQIRHQSDIIDEIKKELLQ
SVQRTEISEGRRLCYEHKIKQLEEQLQRYVSQHGAPSIEIEEDKSSAAYAEINRLKKSLIDLQQEKDIYIKTYHS
EIAKLREKLQRQEGAQTSSEVCSIEKLTEVQTDLAEKKKAIALLQDIVEDQYCQLRDLHKEKGMAMPSNTKL
DHLKGLLGKEPESEVDVVFSESKSLGS

>core/490/3/Org3_Gene679

MEVQIGIDLMGGDHSPLVVWQVLVDVLKSQSSTIPFAFTLFASEEIRKQIQEEFISDLPQEKFPKIISAENFVAM
EDSPLAAIRKKSSSMALGLDYLQEDKLDAFISTGNTGALVTLARAKIPLFPAVSRPALLVCVPTMRGHAVILD
VGANISVKPEEMVGFARMGLAYRQCLGDSKIPTIGLLNIGSEERKGTEAHRQTFRMLRETFGEAFLGNIESGA

VFDGAADIVVTDGFTGNIFLKTAEGVFEFLQRILGDKLEADIQRRLDYTFYPGSVVCGLSKLVIKCHGKACGS
SLFHGILGSINLAQARLCKRILSNLI

>core/491/3/Org3_Gene392

MLISISLATLPILAFSWASFIEPNWLRTTAIPWRLPKKHAHLHGLRIAQISDLHFHKRVPEKFLNKVSKSIKNFSP
DLIVFCGDLLCRARLEDKERLETFLNTLEAPLGVFAILGNHDYSSYISRNTKGEITCIPEEKSRPIQRAIIAVMQG
LFSSPSYRYDPNLTPQEPHPDLLKLLKNTPLTLLHNTTHVIPNTLNIVGLGDLFARQFHPEQAFKNYDPSLPGL
LLSHNPDGITRLQQYPGDFVLSGHSHGPQVTLSWPKFARKFFERLSGLENPYLARGYFVTKEGKQLYVNRGL
GGLKRIRFCSPPEICYITCSYD

>core/492/3/Org3_Gene702

MVLSSDLLRDDKQLDLFFASLDVKKRYLLALSGGSDSLFLFYLLKERGVSFTAVHIDHGWRSTSAQEAKELE
ELCAREGVPFVLYTLTAEEQGDKDLENQARKKRYAFLYESYRQLDAGGIFLAHHANDQAETVLKRLLESAH
LTNLKAMAERSYVEDVLLLRPLLHIPKSSLKEALDARGISYLQDPSNEDERYLRARMRKKLFPWLEEVFGKNI
TFPLLTLGEESAELSEYLEKQAQPFFSAATHQDSQGELPCPDCLIQQAFLCKWVMKKFFNNAGIAVSRHFLQ
MVYDHLSRSSCATLRMRNKIVIIKPGVVVID

>core/493/3/Org3_Gene464

MKFLLYVPLLLVLVSTGCDAKPVSFEPFSGKLSTQRFEPQHSAEEYFSQGQEFLKKGNFRKALLCFGIITHHFP
RDILRNQAQYLIGVCYFTQDHPDLADKAFASYLQLPDAEYSEELFQMKYAIAQRFAQGKRKRICRLEGFPKL
MNADEDALRIYDEILTAFPSKDLGAQALYSKAALLIVKNDLTEATKTLKKLTLQFPLHILSSEAFVRLSEIYLQ
QAKKEPHNLQYLHFAKLNEEAMKKQHPNHPLNEVVSANVGAMREHYARGLYATGRFYEKKKKAEAANIY
YRTAITNYPDTLLVAKCQKRLDRISKHTS

>core/494/3/Org3_Gene328

MNRRKARWVVALFAMTALISVGCCPWSQAKSRCSIDKYIPVVNRLLEVCGLPEAENVEDLIESSSAWVLTPE
ERFSGELVSICQVKDEHAFYNDLSLLHMTQAVPSYSATYDCAVVFGGPLPALRQRLDFLVREWQRGVRFKKI
VFLCGERGRYQSIEEQEHFFDSRYNPFPTEENWESGNRVTPSSEEEIAKFVWMQMLLPRAWRDSTSGVRVTF
LLAKPEENRVVANRKDTLLLFRSYQEAFPGRVLFVSSQPFIGLDACRVGQFFKGESYDLAGPGFAQGVLKYH
WAPRICLHTLAEWLKETNGCLNISEGCFG

>core/495/3/Org3_Gene810

MRKVAFLVSCLFSVAIGASAAPVRVPGFPQIPEDLVQIKTEVCPKQEVCLAVTIKCDDHNLIGVLHLPNTPTPE
GGFPTVVLFHGFRGTKFGGLTGAYRKLGRKFAAAGIATLRVDMAGCGDSEGVAEEVPIETYLRDAQTILETV
QEHPDLNAYRLGISGFSLGCHIAFELAKIYNPRDLNIKALSVWAPIADGGILLKELYENFSKHGEGDIISVGKDF
GFGPPPIIVCSGDVDLLIRIQDHVTANSLPTKPYILHQQGIDDTLVSRTQQTLFKNTAPGRMTFISYPNTGHNLA
TAPDLDMILDQIVSHFQRTL

>core/496/3/Org3_Gene787

MFKLLFHIAAFAGHVLSTPIFIVQDACGIDEEACKNPPPRPFSAQVQYLKVNDAKFKKLPHQTIGYRQYDGTF
LCTLPITEHSGLLFSTGYIGADIQWKSSLPISETDPNGLGWATFQDTSFYNYVLLSLGAYTLSLKNWQWSIILS

GLVDPKNIEMGYGLYQGVLSGKYQATEKLSAIFGVINETGLHQEKAWPLVGVSYKATDQLTLNCIYPVNFSI
DYRSTSVCNLGLAYRLTRFRKKLYKNHLISSRGIFEYQGREIEANVKLTPWPGSFIKGFYGWSIGNDISIADDH
NNNKTSHTFKTSAFFGGSAVMNF

>core/497/3/Org3_Gene128

MLVELEALKREFAHLKDQKPTSDQEITSLYQCLDHLEFVLLGLGQDKFLKATEDEDVLFESQKAIDAWNALL
TKARDVLGLGDIGAIYQTIEFLGAYLSKVNRRAFCIASEIHFLKTAIRDLNAYYLLDFRWPLCKIEEFVDWGN
DCVEIAKRKLCTFEKETKELNESLLREEHAMEKCSIQDLQRKLSDIIIELHDVSLFCFSKTPSQEEYQKDCLYQS
RLRYLLLLYEYTLLCKTSTDFQEQARAKEEFIREKFSLLELEKGIKQTKELEFAIAKSKLERGCLVMRKYEAA
AKHSLDSMFEEETVKSPRKDTE

>core/498/3/Org3_Gene256

MTLPMQKSLTSFDDFSQAYAEKVPAIALIGSALEDDKDALIELLVSESFKELGGQGLMPATLMSWTETFALFQ
EHETLGIIHAEKFPLATKEFLSRYARNPQPHLTILIFTTKQECFRELSKALPSALSLSLFGEWPADRQKRIIRLLL
QRAERVGISCSQSLASLFLRALASTSLPDILSEFDKLLCSVGKKTSLDHSDIKELVVKKEKASLWKFRDSLLKR
DPVEGHQQLHFLLEDGEDPLGIITFLRTQCLYGLRSIEEGSKENKHRMFVLYGKERLHQALNSLFYAETLIKN
NVQDPIVAVETLVIRMVNL

>core/499/3/Org3_Gene122

MFSYIKNRILFNLLSLWIVLTLTFLVMKTIPGDPFNDEGCNVLSEEVLQTLKSRYGLDKPLYQQYTQYLHSIAK
LDFGNSLVYKDRKVTNIISTAFPISAILGLQSLFLSIGGGIALGTIAALKKKKQRRYILGASILQISIPAFIFATLLQ
YVFAVKIPLLPIACWGSFTHTILPTLALAVTPMAFIIQLTYSSVSAALNKDYVLLAYAKGLSPLKVVIKHILPYA
IFPTISYSAFLTTTVITGTFAIENIFCIPGLGKWFICSIKQRDYPVALGLSVFYGTLFMLSSLLSDLIQSIIDPQIRYA
HGKEKKRK

>core/500/3/Org3_Gene226

MAMLPKFFLVLLCLGLCSCSQKTTTIEGEQMTIFYRIVLGTSLSAKEKASLSQQIDRCFHKIDSIYNNWNPYSE
LSIINRAPADVPITLSVELSEFLDQVDTLYKLSEGRFDPTVGPLKTLWLLHLKSQTLPPKDVWEQHYKDMGW
QHLEFQSNTKTLIKKNPHVQIDLCGVVKGYAVDCLNEICNTFCPNNYVEWGGEIKTSGHHPSGRPWRIFSEA
AGTILDIDDMAIATSGNHIQKWCVEGKIYTHILDTRTGKPLELSSYPIQSVSVVHPSCAYADAIATVLMTFDSK
IEAKQWAEEHHILTYINDGASS

>core/501/3/Org3_Gene370

MWRVVLRFLIIFILGRAVFPLRASESFSWETSTCLTVLGIPFIDIILTTNEDFVAQCGLQIGTISSTNNAKIKEIFLI
YKEKFPEASISFKRKEPLNLSQSHLSDLGILCMRNGETYAEGMANKENGPALKQPKDLRLVLRCPNQPDTLL
YSEKEAEKGIETNTCLCNQGYTLLDGQLILYGDSIEKFLKETKRKNNHTLVDLCDSQVVTTFLGRFWSLLNY
VQVLFLSEDSAKILAGIPDLAQATQLLSHTVPLLFIYTNDSIHIIEQGKESSFTYNQDLTEPILGFLFGYINRGSM
EYCFNCAQSSLGET

>core/502/3/Org3_Gene11

MIASIYSFLDYLKMVKSASPHTLRNYCLDLNGLKIFLEERGNLAPSSPLQLATEKRKVSELPFSLFTKEHVRM
YIAKLIENGKAKRTIKRCLSSIKSFAHYCVIQKILLENPAETIHGPRLPKELPSPMTYAQVEVLMATPDISKYHG
LRDRCLMELFYSSGLRISEIVAVNKQDFDLSTHLIRIRGKGKKERIIPVTSNAIQWIQIYLNHPDRKRLEKDPQA
IFLNRFGRRISTRSIDRSFQEYLRRSGLSGHITPHTIRHTIATHWLESGMDLKTIQALLGHSSLETTTVYTQVSV
KLKKQTHQEAHPHA

>core/503/3/Org3_Gene207

MIHSRLIIIGSGPSGYTAAIYASRALLHPLLFEGFFSGISGGQLMTTTEVENFPGFPEGILGPKLMNNMKEQAVR
FGTKTLAQDIISVDFSVRPFILKSKEETYSCDACIIATGASAKRLEIPGAGNDEFWQKGVTACAVCDGASPIFK
NKDLYVIGGGDSALEEALYLTRYGSHVYVVHRRDKLRASKAMEARAQNNEKITFLWNSEIVKISGDSIVRSV
DIKNVQTQEITTREAAGVFFAIGHKPNTDFLGGQLTLDESGYIVTEKGTSKTSVPGVFAAGDVQDKYYRQAV
TSAGSGCIAALDAERFLG

>core/504/3/Org3_Gene717

MRKLILCNPRGFCSGVVRAIQVVEVALEKWGAPIYVKHEIVHNRHVVNALRAKGAIFVEELVDVPEGERVIY
SAHGIPPSVRAEAKARKLIDIDATCGLVTKVHSAAKLYASKGYKIILIGHKKHVEVIGIVGEVPEHITVVEKVA
DVEALPFSSDTPLFYITQTTLSLDDVQEISSALLKRYPSIITLPSSSICYATTNRQKALRSVLSRVNYVYVVGDV
NSSNSNRLREVALRRGVPADLINNPEDIDTNIVNHSGDIAMTAGASTPEDVVQACIRKLSSLIPGLQVENDIFA
VEDVVFQLPKELRCS

>core/505/3/Org3_Gene588

MKCRPTLNTDQPRVRKKLPERFPKWLQRPLPQGSAFHATDATIKRSGMPTVCEEALCPNRAECWSRKTATY
LALGDVCTRSCGFCNIGHSKTPPALDPTEPERIALSAKELGLKHVVITMVARDDLEDGGAQGLVDIIQKLREE
LPQATTEVLASDFQGNVSALHTLLDSGITIYNHNVETVARLSPLVRHKATYARSMFMLEQAANYLPDLKIKS
GIMVGLGEMEGEVKQTLQDLASIGVRIVTIGQYLRPSRKHLQVKSYVTPETFDYYRRVGEAMGLFVYAGPFV
RSSFNADMILASVQDKASA

>core/507/3/Org3_Gene898

MKKRYAFLFPGQGSQYVGMGQDLYMEYPEVRELVDFANERLGFSLTSIMFEGPEDLLMETVHSQLAIYLHS
MAVVKVLSQRSSIQPSLVSGLSLGEYTALVASDRISVLDGLELVRKRGQLMNEACNQSPGAMAALLGLPSEV
IEENITSLGQGIWIANYNAPKQLVVAGIAEKVDQAIELFRDLGCKKAVRLKVSGAFHTPLMQVAQDGLAPDI
YALCMKDSSLPLVSHVVGKSLVNTEEMRECLARQMTSPTLWYQSCYHIESEVDEFLELGPGKVLAGLNRSIG
ISKPITSLGTFAQIEKFLSEV

>core/508/3/Org3_Gene382

MLNSNKFKSKTGAYGDLFQRVVVHSLVLTFLVLLLYSSLFPLTSFALGFITATCGAVGTYEYSSMAKAKMHY
PLSTFSAIGSFLFLALSFLSIRWGHSLPGFFDALPWTLLIVWVVWSIFRVRKSTIGALQLSGVTLFSILYVGIPIRL
FLHVLYSFIHTQEPYLGIWWASFLIATTKGADIFGYFFGKAFGNKKIAPQISPNKTVVGFVAGCLGATLISFIFF
LQIPTRFASYFPMPAILIPLGLALGITGFFGDIIESIFKRDAHLKNSNKLKAVGGMLDTLDSLLLSTPIAYLFLLIT
QSKEFIG

>core/509/3/Org3_Gene29

MRLFSYDKPKIKVQKIKADGFSGWLKCNHCHEMIHANELGQNYNCCPKCSYHYRITAIERVKLLADKDSWR
PLYTDLKSQDPLEFIDTDTYANRLEKARKNTTESEGVIVGICTIGLHPVALAVMDFNFMAGSMGAVVGEKLT
RLIEEAIETRLPVIIVSASGGARMQESVFSLMQMVKTSAALAKLHEAGLPYISVLTNPTSGGVTASFAALGDIII
AEPKALICFAGPRVVAQVIGEDLPEGAQKSEFLLEHGMIDKIVERKELKTTLQTLLDYFLAQEYTGGKSKAPR
DLSKRLKEIFLLTDDSE

>core/510/3/Org3_Gene983

MEIAYSLTSSFSVDSVTVGFFDGCHLGHSNLLSILTSYSGSSGVITFDSHPQTVLSLNHTKLINTKEERLQLLQT
FPIDWLGVLTFDLNFANQSAEEFLTLLHRNLKCKRLILGYDSCIGKEQQSNTEALDTIGKPLGIEVIKIPPYRMD
NIVVSSKAIRQFLSAGNLECAHRFLGHPYAISGKITEGSGIGGSLGFATINLPREESLIPLGVYACEIRYDSTTCQ
GVMNLGTAPTFGRESLYAEAHIFSFAENLYGKEVSIIPRKFLREEKKFQSKETLIRAIEKDILDAQDWFAKGSF
NYEGTA

>core/511/3/Org3_Gene12

MSSRELIILGCSSQQPTRTRNQGAYLFRWNGEGLLFDPGEGTQRQFIFANIAPTTVNRIFVSHFHGDHCLGLGS
MLMRLNLDKVSHPIHCYYPASGKKYFDRLRYGTIYHETIQVVEHPISEEGIVEDFGSFRIEAQRLQHQVDTLG
WRITEPDTIKFLPKELESRGIRGLIIQDLIRDQEISIGGSTVYLSDVSYVRKGDSIAIIADTLPCQAAIDLAKNSCM
MLCESTYLEQHRHLAESHFHMTAKQAATLAKRAATQKLILTHFSARYLNLDDFYKEASAVFPNVSVAQEYR
SYPFPKNPLLNK

>core/512/3/Org3_Gene582

MRSIFRFSLCFFTLSVSCCFADASLYENSCPSRCQPTPPPSNSNPLNVVQQPVAASSVPSYMPPLNADDVLPRD
HLSDGSFSDTYPDITTQAIILIFLALSPFLVMLLTSYLKIIITLVLLRNALGVQQTPPSQVLNGIALILSIYVMFPT
GVAMYKDARKEIEANTIPQSLFTAEGAETVFVALNKSKEPLRSFLIRNTPKAQIQSFYKISQKTFPSEIRAHLTA
SDFVIIIPAFIMGQIKNAFEIGVLIYLPFFVIDLVTANVLVAMQMMMLSPLSISLPLKLLLIVMVDGWTLLLQGL
MISFK

>core/513/3/Org3_Gene836

MKEAAPMHFPFPVRRSVWLNRYSTFRIGGPANYFKAIHTIEEAREVIRFLHSINYPFLIIGKGSNCLFDDRGFD
GFVLYNAIYGKQFLEDARIKAYSGLSFAALGKATAYNGYSGLEFAAGIPGSVGGAIFMNAGTNESDISSVVRN
VETINSEGELCSYSVEELELSYRSSRFHRQQEFILSATLQLSKKQVSADHSKSILQHRLMTQPYTQPSAGCIFRN
PEGTSAGKLIDAAGLKGLAIGGAQISPLHANFIINTGKATSDEVKQLIAIIQSTLKTQGIDLEHEIRIIPYQPKIHS
PVSEK

>core/514/3/Org3_Gene569

MSLFLVFLTAFIWSSSFALSKLVMNASAPIFATGARMVIAGAILALAAWFRGGFVGISKKIFLYIVLLALTGFY
LTNIFEFIGLQSLSSSKTCFIYGLSPLMSALFSYIQLKEKVTLKKVLGLSLGLVSYICYLTFGGGGDDSQPWTW
QIGLPELLILGAASLASFGWTLLRQIEKQSTLSVTAINAYAMLIAGMLSIMHSAVVEPWRPLPVQDISQFLYAT

LALVVISNLICYNLYAKLLRKYSSTFLSFCNLVMPLYSGFYGWILLGEKGVSLGLVLAVAFMVAGCRLIYHEE
FRQGYIVS

>core/515/3/Org3_Gene593

MQKPQYIDRITKKKVIEPIFYEKTMLFLYNSKLGKKLSVFLSTHPIFSRIYGWLQRCSWTRRQIRPFMNRYKIS
EKELTKPVADFTSFNDFFTRKLKPEARPIVGGKEVFITPVDGRYLVYPNVSEFDKFIVKSKAFSLPKLLGDHEL
TKLYAHGSIVFARLAPFDYHRFHFPCDCLPQKTRCVNGALFSVHPLAVKDNFILFCENKRTVTVLETEQFGNV
LYLEVGAMNVGSIVQTFSPNQTYAKGDEKGFFAFGGSTVILLFLPNAIRFDNDLLKNSRMGFETRCLMGQSL
GRSQREEI

>core/516/3/Org3_Gene722

MTMPSTQFHTTILEQFSLFLSVDRGLCQQSIAAYRQDISSFLTISAISSPQDISQNSVYIFAEELYRRKEAETTLA
RRLIALKVFFLFLKDQQLLPYPPIIEHPKIWKRLPSVLTPQEVDALLAVPLQMEKNPRHLAFRDTAILHTLYST
GVRVSELCDLRLGHVSDDCIRVTGKGSKTRLVPLGSRAREAIDAYLCPFRDQYQKKNPHEDHLFLSTRGHKL
ERSCVWRRIHNYAKQVTSKPVSPHSLRHAFATHLLDNKADLRVIQEMLGHARIASTEVYTHVAADSLIEKFL
AHHPRNL

>core/517/3/Org3_Gene274

MLKIDLTGKVAFVAGIGDDQGYGWGIAKLLAEAGATIIVGTWVPIYKIFSQSWELGKFNESRKLSNGTLLEIA
KIYPMDASFDSPEDVPEDIAENKRYKGITGFTISEVAEQVKKDFGHIDILVHSLANSPEISKSLLETSRKGYLAA
LSASSYSFVSLLSHFGSIMNRGGSTISLTYLASMRAVPGYGGGMSSAKAALESDTKTLAWEAGRRWGIRVNT
ISAGPLASRAGKAIGFIERMVDYYQEWAPIPEAMNAEQVGAVAAFLASPLASAITGETLYVDHGANVMGIGP
EMFPKDS

>core/518/3/Org3_Gene751

MPPPFVVTLTTSAQNNLRDQLKEKNFIFSQPQNTVFQARSNTVTCTLYPSGKLVIQGKGSEEFIEFFLEPEILHT
FTHARVEQDLRPRLGVDESGKGDFFGPLCIAAVYASNAEILKKLYENKVQDSKNLKDTKIASLARIIRSLCVC
DVIILYPEKYNELYGKFQNLNTLLAWAHATVINNLAPKPAGDVFAISDQFAASEYTLLKALQKKETDITLIQK
PRAEQDVVVAAASILARDAFVQSIQKLEEQYQVQLPKGAGFNVKAAGREIAKQRGKELLAKISKTHFKTFDE
ICSGK

>core/519/3/Org3_Gene174

MHLEEENQGWEALLRKVYHQEVPPAILLHGFTLPVLQDKAEQLASEILLSSSPGSEHKVSQKIHPDIYQFFPEG
KGRLHSIDLPRGIKKQIYISPFEANYKIYIIHEADRMTLAAISAFLKVFEEPPKHAVIILTTAKVQRLPKTIISRSLS
IFIERGEKILCSKETFSYLFRYAQCEIPVTEVSQIIKESSETDKQVLRDKVQRFMEVLLELYRDRYTLNLGLKAS
ALNYPEHVKEILQLPLLPLDKVLLIVESACRSLNNSSSAASVLEWVAIQLVSLQYKEKELVSVSPGQDLSN

>core/520/3/Org3_Gene167

MRLNYFLNLVNFKYSIFSILFLSASTVFALSINEISQNLSFKEGFKISVFGAIAFVFARTTGIVVNQCIDRFIDKKN
TRTSKRVLPANLVSLNFAWVLSLFCSFLFLFLCKILRIFSLGIASLTLMIVYPYMKRVTFFCHWGLGLVYTVAI

LMNFCAFAESGLSMRLCFLALLWGGSVGMVIAANDIIYAIEDTEFDREEGLRSVPAHYGEKKAVEIAKVNLW
VSYLAYIFSGFVGSLDKEFYFTAIIPLVVILKVVRMYSNYSKKDQEGESKFFLANIAIALSFLVSMTLFWSLSR

>core/521/3/Org3_Gene284

MSERAHIPVLVEECLALFAQRPPQTFRDVTLGAGGHAYAFLEAYPSLTCYDGSDRDLQALAIAEKRLETFQD
RVSFSHASFEDLANQPTPRLYDGVLADLGVSSMQLDTLSRGFSFQGEKEELDMRMDQTQELSASDVLNSLKE
EELGRIFREYGEEPQWKSAAKAVVHFRKHKKILSIQDVKEALLGVFPHYRFHRKIHPLTLIFQALRVYVNGED
RQLKSLLTSAISWLAPQGRLVIISFCSSEDRPVKWFFKEAEASGLGKVITKKVIQPTYQEVRRNPRSRSAKLRC
FEKASQ

>core/522/3/Org3_Gene556

MSRHEICPEVSHKKGKYYSTFIFRCIHSLAGIAFTFFLCEHLFTNMLASSYFSQGKGFVAMVNGFHKIPGLKIIE
VAGLVLPFLCHAIIGIVYLFQGKSNCYSGDGSRPHLRYAKNYSYTWQRWTAWILLFGIAFHVVHLRFIRYPVH
VDIHGTTYYAVDIQPSRYDVIVRGTKGFLTLNLPNTEASSIEVSRHDLGGADAALLSERNSYLLTPSAGTAFLY
VVRDALGSLFIALLYTILVIAAAFHGFNGLWTFCCRWGVVVSLRMQGVLRIVCYLAMIVVTFMGVSAVWNL
YSVA

>core/524/3/Org3_Gene802

MRRFLFLILSSLPLVAFSADNFTILEEKQSPLSRVSIIFALPGVTPVSFDGNCSIPWFSHSKKTLEGQRIYYSGDSF
GKYFVVSALWPNKVSSAVVACNMILKHRVDLILIIGSCYSRSQDSRFGSVLVSKGYINYDADVRPFFERFEIPD
IKKSVFATSEVHREAILRGGEEFISTHKQEIEELLKTHGYLKSTTKTEHTLMEGLVATGESFAMSRNYFLSLQK
LYPEIHGFDSVSGAVSQVCYEYSIPCLGVNILLPHPLESRSNEDWKHLQSEASKIYMDTLLKSVLKELCSSH

>core/525/3/Org3_Gene165

MSHGPRPTKFSFPLYFSKTLSWFILGGFLAACGVQMVLVPNELIDGGIVGLSIIASHFLGHKALPFCLVLFNLPF
VFLAFKQIGKYFVIQMLTAVIIFSCSLWLIDQLPSWLGMSPFVFKGSEMETVVLGGAIIGVGCGLIIRHGGSTD
GTEILGIIINKKKGYTVGQIILFVNFFIFALSGIVYKNWHTAFVSFLTYGIATKVMDMVILGLEDTKSVTIITSSP
RKLGHILMETLGIGLTYIHAEGGYSGEPRNLLYVVVERLQLSQLKEIVHREDPSAFIAIENLHEVINGRRT

>core/526/3/Org3_Gene633

MQEKPRHVHRIIHISDVHFHVLPVNPVHCFNKRLKGLLRKVFGLVHFQATTIGQRFPKVVRSLGADSVCITGD
FSLTAMDGEFLLAKHFVETLAKHSSVYLLPGNHDVYTLKSLAQQTFYTHFPNDQLQQNKVSFHKITDHWWL
ILLDCSCLNGWFSANGVVHLAQISAIETFLLSLSPEENVIIANHYPLLSSQNPSHDLINNTHLQNVLKKYPKVRL
YLHGHEHQAAVYNCADTSPSYILNSGSISLPTNSRFHVIDLYPEKYQVHTMILKNLLDFDAPLEIANEATWDC
QKL

>core/527/3/Org3_Gene275

MEKLLVTDIDGTITHQSHHLDKKVYERLYALHQAGWKLFFLTGRYYKYAARLFSDFDAPYLLGCQNGASV
WSSTSSNLLYSKSLPSDLLCILQDCMEGATALFSVESGAPYGDHYYRFSPTPIAQDLHEYVDPRYFPNAKEREI
LFETRSLKDDYAFPSFAAAKVFGLRDEVIRIQKELERQEALTSVATMTLMRWPFDFRYAILFLTDKSVSKGKA

LDRVVNILYDGKKPFVMASGDDANDLDLIERGDFKIVMSSAPEEMHVHADFLAPPADKNGILSAWEAGVRYYDDLMSL

>core/528/3/Org3_Gene463

MKKLILYFAAFVASLFCGVFLWDRVPCAQKIMRLAADHSSEVFSKSCRFVRKISGFEELQVFERHVSPEQALALFPEYRDGKSFVELAFIPHTLMHVRFSKEEPVKKHIISQEGEILWSLVNGEMVLHTGTWTCSKGFRECLLLHAGKQDMRVIQTLATLGGTTSRESLAQALALKNIRAERVIKECQKKKLIFASGNQIGTHFQQFQPIRGCTTTLNNNPVWLQKPRHAAVFPAQYSEDRVRHLVKMIFGDNFLIVRSSMVYVPVYKISLVSADNSVRVEYINAVTGKSFQDL

>core/529/3/Org3_Gene686

MFHSLSKNTPIITQGITGKAGSFHTEQCLAYGTNFVGGVTPGKGGTLWLDLPVYDSVLEAKQATGCRATMIFVPPPYAAEAILEAEEAGIELIVCITEGIPVRDMLEVARVMDNSTSQLIGPNCPGIIKPGECKIGIMPGYIHLPGNIGVVSRSGTLTYEAVWQLTQLKIGQSICVGIGGDPLNGTSFIDVLQALEEDPYTELILMIGEIGGSAEEEAAAWIQAHCTKPVVAFIAGVTAPKGKRMGHAGAIISGNSGDAKSKIQVLRESGVTVVESPAHIGKTVDAVLRAKEL

>core/530/3/Org3_Gene631

MSKHTSESRIAQDMLERYSGSSVKQFCPYLLLTNFSYYIQTFAKLHGVPVFEGSMFSAAHAPHLKTSILDFKLGSPGAALTIDLCSFLPDLKAALMLGMCGGLRSHYQVGDYFVPVASIRGEGTSDAYFPPEVPALANFVVQKATTEVLEDKKANYHIGITHTTNIRFWEFNKKFRKKLYETKAQSAEMECATLFAAGYRRNLPIGALLLISDLPLRKEGIKTKSSGNFIFNTYTEDHILTGQEVIENLEKVMLKRAASDHKKDQQYRGLPHMEVGEADDTMASGSETSDSDY

>core/531/3/Org3_Gene514

MKVLPPPSIPLLGAHTSTAGGLKNAIYEGRDIGASTVQIFTANQRQWQRRALKEEVIEDFKAALKETDLSYIMSHAGYLINPGAPDPVILEKSRIGIYQEILDCITLGISFVNFHPGAALKSSKEDCMNKIVSSFSQSAPLFDSSPPLVVLLETTAGQGTLIGSNFEELGYLVQNLKNQIPIGVCVDTCHIFAAGYDITSPQGWEDVLNEFDEYVGLSYLRAFHLNDSMFPLGANKDRHAPLGEGYIGKESFKFLMTDERTRKIPKYLETPGGPENWQKEIGELLKFSKNRDS

>core/532/3/Org3_Gene362

MLSSLIRDSFPLLILLPTFLAALGASVAGGVMGTYIVVKRIVSISGSISHAILGGIGLTLWIQYKLHLSFFPMYGAIVGAIFLALCIGKIHLKYQEREDSLIAMIWSVGMAIGIIFISRLPTFNGELINFLFGNILWVTPSDLYSLGIFDLLVLGIVVLCHTRFLALCFDERYTALNHCSVQLWYFLLLVLTAITIVMLIYVMGTILMLSMLVLPVAIACRFSYKMTRIMFISVLLNILCSFSGICIAYCLDFPVGPTISLLMGLGYTASLCVKKRYNPSTPSPVSPEINTNV

>core/533/3/Org3_Gene713

MKRNDPCWCGSGRKWKQCHYPQPPKMSPEALKQHYASQYNILLKTPEQKAKIYNACQITARILDELCKASQKGVTTNELDELSQELHKKYDAIAAPFHYGSPPFPKTICTSLNEVICHGIPNDIPLKDGDIMNIDVSCIVDGYYGDCSRMVMIGEVPEIKKKICQAALECLNDSIAILKPGIPLCEIGEAIEARADTYGFSVVDQFVGHGVGIEFHENPYVPHYRNRSMIPLAPGMIFTIEPMINVGKKEGVVDPKNQWEARTCDNQPSAQWEHTIAITETGYEILTLLND

>core/534/3/Org3_Gene740

MHLLTATVTPFFPNGTIDFASLERLLSFQDAVGNGVVLLGSTGEGLSLTKKEKQALICFACDLQLKVPLFVGT
SGTLLEEVLDWIHFCNDLPISGFLMTTPIYTKPKLCGQILWFEAVLNAAKHPAILYNIPSRAATPLYLDTVKAL
AHHPQFLGIKDSGGSVEEFQSYKSIAPHIQLYCGDDVFWSEMAACGAHGLISVLSNAWPEEAREYVLNPQEQ
DYRSLWMETCRWVYTTTNPIGIKAILAYKKAITHAQLRLPLSIEDFDLENVSPAVESMLAWPKLRTSVFSYS

>core/535/3/Org3_Gene684

MFKFFRNKLQSLFKKNISLDLIEDAESLFYEADFGTELTEELCARLRRTKKADASTIKDLITVLLRESLEGLPSQ
ASQSSQTRPIVSLLLGTNGSGKTTTAAKLAHYYKERSESVMLVATDTFRAAGMDQARLWANELGCGFVSGQ
PGGDAAAIAFDGIQSAIARGYSRVIIDTSGRLHVHGNLMKELSKIVSVCGKALEGAPHEIFMTVDSTLGNNAIE
QVRVFHDVVPLSGLIFTKVDGSAKGGTLFQIAKRLKIPTKFIGYGESLKDLNEFDLDLFLNKLFPEVEKI

>core/536/3/Org3_Gene161

MKRRNLQKILPNASTPSTNVAENTGIKDQNLFLDQATLNVDGNVDIENFLETRDLKVADTITSPCEFTVGGGL
SAESSQFKATTLSKGLEITSEDQDGRVPKFTNVSDPQSPRDALTYNYYRNTGCQALNLYTYYSSSQPTTVGKP
IETVCQNPNPETYRISASAKIYDAVTRFPYIQFKAPGIYQVTIQIRRESGQHSGLDNPNLYLNLMIGNNKTLLCA
SDTRGYSGGHRTSIAVTGTFTLTEIVATPPHDYPWLFLETTIGLDIKSMSTCVIWFPFQANFAEVD

>core/537/3/Org3_Gene915

MQIPRSIGTHDGSFHADEVTACALLIIFDLVDENKIIRSRDPVVLSKCEYVCDVGGVYSIENKRFDHHQVSYDG
SWSSAGMILHYLKEFGYMDCEEYHFLNNTLVHGVDEQDNGRFFSKEGFCSFSDIIKIYNPREEEETNSDADFS
CALHFTIDFLCRLRKKFQYDRVCRGIVREAMETEDMCLYFDRPLAWQENFFFLGGEKHPAAFVCFPSCDQWI
LRGIPPNLDRRMEVRVPFPENWAGLLGKELSKVSGIPGAVFCHKGLFLSVWTNRESCQRALRLTLQDRGII

>core/538/3/Org3_Gene580

MGISLPELFSNLGSAYLDYIFQHPPAYVWSVFLLLLARLLPIFAVAPFLGAKLFPSPIKIGISLSWLAIIFPKVLA
DTQITNYMDNNLFYVLLVKEMIIGIVIGFVLAFPFYAAQSAGSFITNQQGIQGLEGATSLISIEQTSPHGILYHYF
VTIIFWLVGGHRIVISLLLQTLEVIPIHSFFPAEMMSLSAPIWITMIKMCQLCLVMTIQLSAPAALAMLMSDLFL
GIINRMAPQVQVIYLLSALKAFMGLLFLTLAWWFIIKQIDYFTLAWFKEVPIMLLGSNPQVL

>core/539/3/Org3_Gene525

MLHALDTYRPSIESAIEKALEGFGPIGHPIRSPVEYALQGGGKRLRPGLVCMMAQGLGLNHDVMDSALAVEF
VHTSTLIADDLPCMDNDDERRGRPTVHKAFDEATALLASYALIPAAYSHLRLNAKKLKEQGCDPREIDIAYNI
IGDITDKNIGCSGVLGGQYDDMFFSNRGQEHVQSIMIKKTGSLFEIACISGWLFGGGDPQFAPIITSFSNNFGLL
FQIKDDFSDLQKDSQQIGLNYALLFGEKAALELLARSQNNCLELLDRLSAGGLKNSSEFETIISSLGSF

>core/540/3/Org3_Gene160

MSNPTPKTKISIPTFVRFNIQSINLTEDQKKTTFTVGGKVTTENTVVRGDLTCTDGGLTCQSDLTIQKDINIRPT
STNSMVFDGRLNLSNSPLSYKNSQGQDITDYEKMSSGKPQEYVPFGYYKRTQIMMAQRAAHSSGYVGGGSV
PSGSYVPWNKFDQTSTQKTSGTEIYIDPNDSTKLVFEVNNKVPKLFRISVIMAKHGSWLDNGTGADILLAANE
YEQGGGRINVTDLAMTTSRGSSYYETRPLQVVCVTYYAQNNGYFTFQNRAGGGLRVSFFSWNIVALPYVE

>core/541/3/Org3_Gene755

MLKIQKKRMCVSVVITVGAIVGFFNSADAAPKKKKIPIQILYSFTKVSSYLKNEDASTIFCVDVDRGLLQHRY
LGSPGWQETRRRQLFKSLENQSYGNERLGEETLAIDIFRNKECLESEIPEQMEAILANSSALVLGISSFGITGIPA
TLHSLLRQNLSFQKRSIASESFLLKIDSAPSDASVFYKGVLFRGETAIVDALSQLFAQLDLSPKKIIFLGEDPEV
VQAVGSACIGWGMNFLGLVYYPAQESLFSYVHPYSTATELQEAQGLQVISDEVAQLTLNALPKMN

>core/542/3/Org3_Gene828

MSHLIPSLRNSVTSYFHKPQPIKQAAPSKSIRDICNIAYLIIICVLVVVVLVGAMLCMFIPSVGIPLCLSSLALLV
LLSIFNPCLINWISTKKTKEIAPKDASESQPTKSASRKGSPQLSPHHDHEPKNFIRTQLEKGVNYVTNKFKSGEE
SPHISDEHHSPRQSKRSSEIESSDESSPELHRKAKGKAPHTATTKGSKTSTTESSKKKKKTKHSLHRTTSSIHKR
SAPKPMVPSKKRKPVLLKKTVPLPIEDLEHQSSGNESSDSSSPPPVQRKAILPWFCKQPTDP

>core/543/3/Org3_Gene67

MEIKKAIQEGTAYLDYYGVPLSDCEALYILMDLLEVSSRAKLFDLVGISETMLMEYRKRLALRGQRCPTAYL
NGAVSFLGLRLRVDSRVLIPRTETELLAEYIINYLLSHSEIQTFYDICCGSGCLGLAIKKSCPHVEVVLSDVCPQ
AVAVANENAKSNGLDVKILLGDLSAPYTRPADAFVCNPPYLSFNEIIHIDPEVRCYEPWKALVGGSTGLEFYQ
RIAQELPKIVTSTGVGWLEIGSSQGESIKNIFSKHGIYGRLHQDLSGRDRIFFLEMDGRDPVSSGAYS

>core/544/3/Org3_Gene204

MAVIYWDRSKIVWSFEPWSLRLTWYGVFFTVGIFLACLSARYLALSYYGLKDHLSFSKSQLRVALENFFIYSI
LFIVPGARLAYVIFYGWSFYLQHPEEIIQIWHGGLSSHGGVLGFLLWAAIFSWIYKKKISKLTFLFLTDLCGSVF
GIAAFFIRLGNFWNQEIVGTPTSLPWGVVFSDPMQGVQGVPVHPVQLYEGISYLVVSGILYFLSYKRYLHLGK
GYVTSIACISVAFIRFFAEYVKSHQGKVLAEDCLLTIGQILSIPLFLFGVALLIICSLKARRHRSHI

>core/545/3/Org3_Gene225

MLLRGIPAAEKILQRLKEEISQSPTSPGLAVVLIGNDPASEVYVGMKVKKATEIGIISKAHKLPSDSTLSSVLKL
IERLNQDPSIHGILVQLPLPKHLDSEVILQAISPDKDVDGLHPVNMGKLLLGNFDGLLPCTPAGIIELLNYYEIP
LRGRHAAIVGRSNIVGKPLAALMMQKHPQTNCTVTVLHSQSENLPEILKTADIIIAALGAPLFIKETMVAPHA
VIVDVGTTRVPADNAKGYTLLGDVDFNNVVTKCAAITPVPGGVGPMTVAMLMSNTWRCYQNFS

>core/546/3/Org3_Gene479

MTEEISKDTIIEVAIDDIRVSPFQPRRVFSNEELQELIASIKAVGLIHPPVVREICTGDRVLYYELIAGERRWRAM
QLAGATTIPVILKHVIADGTAAEATLIENIQRVNLNPIEMAEAFKRLIHVFGLTQDKVAYKVGKKRSTVANYL
RLLALSKTIQESLLQGQITLGHAKVILTLEDPILREKLNEIIIQEHLAVREAELIAKQLISEEGSSIELKPTPLDMA
ESSKQHEELQQRLSDLCGYKVQIKTRGSKATVSFHLQNTQDLQKLEAWLSSHGTLSESLS

>core/547/3/Org3_Gene172

MPDKKAQITFSLPEVMSAIHQGKIVALPTDTVYGFVLSLYASEAEERLYALKDREPSKAFALYVNSIEDIENIS
GYPLSPTAKKLAQLFPGAITLVVKHRNPRFPKETLAFRIVDHSVVREIVDHCGTLIGTSANLSEFPSALTAQEIF
ADFADHDLCIFDGPCSHGLESTVVASDPLYIYREGLISRSVIENIAGTEAKIFHRTSHAFSKHIKIYTVKNQEQL
VSFLSGSLDFKGVVCEHPKPKNFYTRLREALKKKTPSIVFIYDINTSDYPELFPFLSPYYIE

>core/548/3/Org3_Gene159

MKNNINNNECYFKLDSTVDGDLLAANLKTFDTQAQGISSTETFSVQGNATFKDQVSATGLTSGTTYNLNAQ
NFTSSQISIDFKNNRLSNCALPKEDCDPVPANYVRSPEYFFCSKPLIGDFDFNSGESYLPLTGSEYTLYQSRNVN
SIFRFIGWKQSTRELTVGGNTAIQFLAAGTYIVSFTVGKRWGWNNGWGGAIYINNGLGQVQCESTIYSGGGY
ATIGTLGTSIYRASVDVAPNPNDPNASDRYRAGIFYLSNGGSSAGIGNYSFSLLYYPDDRG

>core/549/3/Org3_Gene320

MHEVLILTFTYPLPRTLKQHPDEVHTVPISPNLSFGEGSPILIAGPCTLESYEHTVSSALTVKEAGAQVFRGSIR
KPRTSPFSFQGWEKECVLWHKEAQSIHGLPTETEVLDVRDVEITAEHVDILRIGAKNMHNTPLLQEVSKSHRP
IILKRSPAATLEEWLCAAEYILASSPSCPGVILCERGIRTFEHSTRYTLDLNTVALLKEISSLPVIVDPSHAAGKR
SLVLPLASAGLSVGADGLMIEVHAHPEKALCDAKQQITPEELHLFAKKHFCPSESRAHAIS

>core/550/3/Org3_Gene896

MKHYLSFSPSADFFSKQGAIETQVLFGERVLVKGSTCYAYSQLFHNELLWKPYPGHSFRSTLVPCTPEFHIHP
NVSVVSVDAFLDPWGIPLPFGTLLHVNSQNTVIFPKDILNHMNTIWGSGTPQCDPRHLRRLNYNFLAELLIKD
ADLLLNFPYVWGGRSVHESLEKPGVDCSGFINILYQAQGYNVPRNAADQYADCHWISSFENLPSGGLIFLYP
KEEKRISHVMLKQDSSTLIHASGGGKKVEYFILEQDGKFLDSTYLFFRNNQRGRAFFGIPRKRKAFL

>core/551/3/Org3_Gene123

MDNYLLNIKDLTITSTNPKRTLIENLSLQLKENRNLALVGESGSGKTTITKAILGFLPENCLIKTGSILFEDIDIT
KLSPKELHKIRGQKIATILQNAMGSLTPSMRIGMQIIETLRQHHKMNKEEAYNKAMQLLTDVCIPNPKYSFSQ
YPFELSGGMRQRVVIAIALASQPKLILADEPTTALDSMSQAQVLRILRNIQQQKQATILLVTHNLSLVKELCND
ICIIKDGKLIETGTVEEIFLSPKHPYTLKLLNAVSKIPIKKTSSPILKNKFQPLMSMQGGL

>core/552/3/Org3_Gene891

MENLSSAPSRSIWKSIIQNKMLVLGLTTLIILMLGALLLPWFYQDYEQTSLKDILVSPCSRFPFGTDTLGRCMF
ARTLRGLRLSLLIATIATLIDVCVGLLWATVAISGGKKIDFLMMRTTEILFSLPRIPIIILLLVIFHHGLLALILAM
TITGWIPISRIIYGQFLLLKNKPFVLSAKAMHASTFHILKKHLLPNTLAPIISTLIFTIPNAIYTEAFISFLGLGIQPP
QASLGTLVKEGINAIDYYPWLFFFPSLIMIALSISFNLIGEGAKTLCLEEGSHG

>core/553/3/Org3_Gene110

MKQPMSLIFSSVCLGLGLGSLSSCNQKPSWNYHNTSTSEEFFVHGNKSVSQLPHYPSAFRTTQIFSEEHNDPY
VVAKTDEESRKIWREIHKNLKIKGSYIPISTYGSLMHPKSAALTLKTYRPHPIWINGYERSFNIDTGKYLKNGS
RRRTSHDGPKNRAVLNLIKSSGRRCNAIGLEMTEEDFVIARRREGVYSLYPVEVCSYPQGNPFVIAYAWIADE
SACSKEVLPVKGYYSLVWESVSSSDSLNAFGDSFAEDYLRSTFLANGTSILCVHESYKKVPPQP

>core/554/3/Org3_Gene442

MFKKFKPVTPGTRQLVLPAFDELTTRGELRGTKSKRSLRPNKKLSFFKKSSGGRDNLGHISCRHRGGGAKQL
YRVVDFKRNKDGITAKVVTVEYDPNRSAYIALLSYEDGEKRYILAPKGIQRGDVVVSGEGSPFKPGCCMTLK
SIPLGLSVHNIEMRPSSGGKLVRSAGLAAQVIAKSPGYVTLKMPSGEFRMLNEGCRATIGEVSNADHNLRVD
GKAGRRRWMGVRPTVRGTAMNPVDHPHGGGEGRHNGYIPRTPWGKVTKGLKTRDKNKSNKWIVKDRRK

>core/556/3/Org3_Gene448

MASIHPTAIIEPGAKIGKDVVIEPYVVIKATVTLCDNVVVKSYAYIDGNTTIGKGTTIWPSAMIGNKPQDLKYQ
GEKTYVTIGENCEIREFAIITSSTFEGTTVSIGNNCLIMPWAHVAHNCTIGNNVVLSNHAQLAGHVQVGDYAIL
GGMVGVHQFVRIGAHAMVGALSGIRRDVPPYTIGSGNPYQLAGINKVGLQRRQVPFATRLALIKAFKKIYRA
DGCFFESLEETLEEYGDIPEVKNFIEFCQSPSKRGIERSIDKQALEEESADKEGVLIES

>core/557/3/Org3_Gene936

MSDFSMETLKTLRQQTGVGLTKCKEALEACGGNLEEAVVYLRKLGLASAGKKEHRETKEGIIAAKTDANGA
ALIEVNVETDFVANNAVFREFVSNLLNDILKYKVDTVEALSQAASSQDPSLSVDELRAVTMQTVGENIRISRV
AYFPKATNSTVGIYSHGNGKTVALTMLSGSSTADSLAKDIAMHVVAAQPQFLSKESVPAEAIAKEKEVIASQI
QGKPQEVIEKIVTGKLNTFFQEACLLEQPFIKNADLSIQSLIDDFSKTSGSSVAIEQFILWKIGA

>core/558/3/Org3_Gene450

MLERTQRTLKREVRYSGVGIHLGKSSTLHLQPAQTNTGIVFQRQSASGNYENVPALLDHVYTTGRSTTLSRG
SAVIATVEHLMAALRSNNIDNLIIQCSGEEIPIGDGSSNVFVELIDQAGICEQEDKVSIARLTRPVYYQHQDIFL
AAFPSDELKISYTLHYPQSSTIGTQYKSLVINEESFRQEIAPCRTFALYNELCFLMEKGLIGGGCLDNAVVFKD
DGIISRGQLRFADEPVRHKILDLIGDLSLVGRPFVAHVLAVGSGHSSNIAFGKKILEALEL

>core/559/3/Org3_Gene528

MATAHLGRQALLHLRSWTPAIRASGNLFRQQSMSLHNNVLFAGDIVGAIKNSTAISRHALGSSHYAHAALQK
TEGFLGAADGVNTAVAGAMLWGQLLNGSMIFETDEETGELRRCNEADAEGCMTQKLQRRSALTITGKVAR
LASKTLGTATFLHEMDVVSLGANANKIGCKVTSCLNLVATGCSLTESSISLYRILSTRPETISDPENRNKPSAEF
AARSKAIRNAFIAWLGDVVDLVCDALGTLSLFLPAILGVHAVLIMAILGLISCVINFVKDYAKIG

>core/560/3/Org3_Gene164

MVLMNKRLKIILTNDDGITAKGMSCLVSALLEANIGDIYIAAPQAEQSGKSMAISLNQVVCASPYAYPQPVKE
AWAVGGSPTDCVRLGLRTLFESVSPDLVISGINCGNNICKNAWYSGTIGAAKQALVDGIPSMALSQDNHISFF
QQDKAPEILKALVIYLLSQPFPCLTGLNINFPTSPGGSSWEGMRLVPPGDEFFYEEPQYLGSVNKNQYYVGKIS
GVRIGEHPSEELACMLENHISVSPIFSQNSPIGLMTLEEFQKTQENFNASLLSSELTTKIF

>core/561/3/Org3_Gene494

MELKKTAESLYSAKTDNHTVYQNSPEPRDSRDVKVFSLEGKQTRQEKTTSSKGNTRTESRKFADEEKRVDD
EIAEVGSKEEEQESQEFCLAENAFAGMSLIDIAAAGSAEAVVEVAPIAVSSIDTQWIENIILSTVESMVISEINGE
QLVELVLDASSSVPEAFVGANLTLVQSGQDLSVKFSSFVDATQMAEAADLVTNNPSQLSSLVSALKGHQLTL
KEFSVGNLLVQLPKIEEVQTPLHMIASTIRHREEKDQRDQNQKQKQDDKEQDSYKIEEARL

>core/563/3/Org3_Gene830

MRQSFDELSQNAFKNIFNKQRFCFIFCSLCCFGFVFALFLKLCSRLAPEISLSTLGLGAFFCAFSVICASAIIVQF
LLHKESQGETSKLCCAIKNTWSSLWLSLLVSMPFFIAMVAVVTVAMLSSFLGSLPWVGKLFHTVLIFIPYLSA
TALILLFLGSFSCLFFCIPVLHNQESIDYRKLLECFRGNILRQFIGVVIALVPLALCSWLALDSFYLMTHLVEIAD
IHTWSFLAQMFVLIVPIALILTPAVSFFFNFSFSFYLAKQEEEKALVK

>core/564/3/Org3_Gene919

MHKVIVFIFLTLYSLKSYGNDVIDKPHVLVSIAPYKFLVEQIAEETCFVYAIVTNHYDPHTYELPPQQIKELRQ
GDLWFRIGEAFEKTCERNLTCQQVDLSQNVSLIQGKPCCNQHTTNYDTHTWLSPKNLKVQVETIVTTLSKKY
PQHATLYQSNGEKLLLALDQLNEEILTITSKAKQRHILVSHGAFGYFCRDYNFSQHTIEKSSHVEPSPKDVAR
VFRDIEQYKISSVILLEYSGRRSSAMLADRFHMHTVNLDPYAENILVNLKTIATTFSSL

>core/565/3/Org3_Gene426

MYFTRDPVIETVITSREGYKLSVRNTKHFSQDPFMVEAIEVISLGNICFFRNCDHSKPFLVPAGDYEVMEVRDT
KINLKAVGLDRGVKIAGGREALIKLTKSTPLPVIDEKPLADSPEEGTEPTSPSKKEKKEARKDSFKGEKWKEK
KKLSRRRNHKEIAEVTGASQEILDTVKEELWEESQENEIVEQKKFSLLPPPAKLISEVISQTVVDPVVTSADLN
ESLQALVRESSDLINALLSADDAIHFPETEEEPTSASFEESSAMFFPETSSATEEE

>core/566/3/Org3_Gene747

MTRSSPAQLSRFLSEIQNKPKKSLSQNFLVDQNIVKKIVATSEVIPQDWVLEIGPGFGALTEELIAAGAQVIAIE
KDPMFAPSLEELPIRLEIIDACKYPLDQLQEYKTLGKGRVVANLPYHITTPLLTKLFLEAPDFWKTVTVMVQD
EVARRIVAQPGGRDYGSLTIFLQFFADIHYAFKVSASCFYPKPQVQSAVIHMKVKETLPLSDEEIPVFFTLTRT
AFQQRRKVLANTLKGLYPKEQVEQALKELGLLLNVRPEVLSLNDYLALFHKMQAG

>core/567/3/Org3_Gene487

MESQSCKLTIKDLMSAGAHFGHQTRRWNPKMKLYIFEEKNGLYIINLAKTLQQLRNALPHIRKVIQDNKTVL
FVGTKKQAKCVIREAAIEAGEFFIAERWLGGMLTNMTTIRNSIKTLDKIEKDLSRNQAYLTKKEAALLAKRH
QKLLRNLEGIRYMKKAPGLLVVVDPSYEKIAVAEAKKLGIPVLALVDTNCDPTPIDHVIPCNDDSLKSIRLIIN
VIKENIIEAKHKLGIEIVSPVKSLEVPDLSAFESSQDDESDEENREEDLLAKKFDGEAN

>core/569/3/Org3_Gene584

MTANTFGTLDILMKHSKEDDLSRFLPKNLLVESPHPEEIPLKSLSFTMSWLPTIHPSWITIAMKEFPPEIQGQLL
AWLPEPLVQEILPLLPGISIAPHRCAPFGAFYLLDMLSKKIRPCGITEEIFLPASSANAILYYTGPVKIALINCLGL
YSIAKELKHILDKVVIERVKNALSPTEKLFLTYCQSHPMKHLETTNFLSSWTTDAELRQFVHKQGLEFLGKAL
TKENASFLWYFLRRLDVGRAYIVEQTLKTWYDHPYVDYFKSRLEQCMKVLVK

>core/570/3/Org3_Gene353

MDYKLLDSGDGNKLECFGPVTLIRPSSIAVWPKSRPELWSQAQLQYVREGERGAWKNFKRLPEEWEVAFSD
VRCLLKRTPFGHLGVFPEHMGFWPALKQAIEKHKERQVLNLFAYTGAGSIFAAKCGARVTHVDASQAAVR
WAQRNVEKNAFPERRIFWVIEDVISFLKKEIRRNKKYQVILLDPPSYGRGPDGEVFKIDKDLFPLLSLCSKLLA
DDASYFLLTSHTPGHTPEFLRAIARRSVPTLVSEAWSCGESFCGEGVGALPSGSFVQWIA

>core/571/3/Org3_Gene97

MITGVVLEKHEQRTMFSLTLLNNFTTFGLLHTPLHYNPPYPIVILLHGLASDKTGSKRSHVRLAQELTRLGIAA
LRVDLLGHGDCEGELMDFSLENYKQNIREIIEYTHSLLHIDQERLAIFGSSLGGTLALQTLPFFNKIKALAVWA
PTISGELMAAEAQKNAPEVITMSQKGAITYAGMTLNPDFYTQFLKIDIVKELMPSARNLPPILYMQGEQDLLV
SINHRTLFTEAFANQDKPITILTYPDVDHAFPFAESSALSDLTQWLKRELTSGE

>core/572/3/Org3_Gene510

MLLGFLCDCPCASWQCAAVANCYDSVFMSRPEHKPNIPYITKATRRGLRMKTLAYLASLKDARQLAYDFLK
DPGSLARLAKALIAPKEALQEGNLFFYGCSNIEDILEEMRRPHRILLLGFSYCQKPKACPEGRFNDACRYDPSH
PTCASCSIGTMMRLNARRYTTVIIPTFIDIAKHLHTLKKRYPGYQILFAVTACELSLKMFGDYASVMNLKGVG
IRLTGRICNTFKAFKLAERGVKPGVTILEEDGFEVLARILTEYSSAPFPRDFCEIH

>core/573/3/Org3_Gene756

MRRYLFMVLALCLYRAAPLEAVVIKITDAQAVLKFAREKTLVCFNIEDTVVFPKQMVGQSAWLYNRELDLK
TTLSEEQAREQAFLEWMGISFLVDYELVSANLRNVLTGLSLKRSWVLGISQRPVHLIKNTLRILRSFNIDFTSC
PAICEDGWLSHPTKDTTFDQAMAIEKNILFVGSLKNGQPMDAALEVLLSGISSPPSQIIYVDQDAERLRSIGAF
CKKANIYFIGMLYTPAKQRVESYNPKLTAIQWSQIRKNLSDEYYESLLSYVKSKG

>core/574/3/Org3_Gene995

MKVKINDQLICIPPFISARWSQIAFIESQEGENKDQGTLRLHLIDGKIISIPNLDQSIIDIAFQEHLLYLETSQSGKE
DSRDDDKLGVGVLMNVLQQITKGNDIQVLPKNLISPLFSGTNPIEAILQHTPEHKDHPDAPTDVLEKMADVIR
VLSGNNATLLPRPEPHCNCMHCQIGRVMNEEDTLAVSDKDLTFRTWDIMQSGDKLYIVTNPLNPSDQFSVYL
GPPIGCTCGEPNCEHIKAVLYT

>core/575/3/Org3_Gene262

MKLLKNVLLGLFFSMSISGFSEVKVSDTFVKQDTVVEPKIRVLLSNESTTALIEAKGPYRIYGDNVLLDTAIQG
QRCVVHALYEGIRWGEFYPGLQCLKIEPVDDTASLFFNGIQYQGSLYVHRKDNHCIMVSNEVTIEDYLKSVL
SIKYLEELDKEALSACIILERTALYEKLLARNPQNFWHVKAEEEGYAGFGVTKQFYGVEEAIDWTARLVVDS
PQGLIIDAQGLLQSNVDRLAIEGFNARQILEKFYKDVDFVVIESWNEELDGEIR

>core/576/3/Org3_Gene179

MKKKLSLLVGLIFVLSSCHKEDAQNKIRIVASPTPHAELLESLQEEAKDLGIKLKILPVDDYRIPNRLLLDKQV
DANYFQHQAFLDDECERYDCKGELVVIAKVHLEPQAIYSKKHSSLERLKSQKKLTIAIPVDRTNAQRALHLL
EECGLIVCKGPANLNMTAKDVCGKENRSINILEVSAPLLVGSLPDVDAAVIPGNFAIAANLSPKKDSLCLEDL
SVSKYTNLVVIRSEDVGSPKMIKLQKLFQSPSVQHFFDTKYHGNILTMTQDNG

>core/577/3/Org3_Gene317

MVRDIQSESIGKLVFLGTGNPEGIPVPFCSCRVCQNTGIHRLRSSVLIQYQNKTLVIDAGPDFRTQMLVAGVSE
LDGVFLTHPHYDHIGGIDDLRAWYIVTQRSLPLVLSASTYRFLNKAKEYLFATPNVESSLPAVLEFTILNEDCG
QEEFQGIPYTYVSYYQKSCHVTGFRFGNLAYLTDLCSYDAKIFSYLDNVETLILSAGPSETPIPFQGHKSSHLT
VEEAKAFANHAGIKNLIITHISHCLEAERDQHPEVTFAYDGMEVLWTL

>core/578/3/Org3_Gene846

MIRRFFKTLFPPGPQYSLCYASILIVLSSLVCVPTFCWLFLPELSLSKFNPSPIRNLFLVSSTLSKVPPTAIAEHLR
LSADAPTYLHEFSIKEAESSLHALGIFSSLVIEKSPDNKGITIFYTLQTPIAYVGNRSNTLCNLEGSCFLGQPYFP
SLNLPQIFFSQEDLKMQKLPKEKMLFTKILLKELAMESPKIIDLSLSDAYPGEIIVTLSSGSLLRLPIKTLDRALD
LYKHMKKSPVIESEKQYVYDLRFPNFLLLKAL

>core/579/3/Org3_Gene940

MFNNKMILIAGPCVIEGEDITLEIAGKLQSILAPYSDRIQWFFKSSYDKANRSSLNSFRGPGLTEGLRILAKVKE
TFGVGILTDVHTPQDAYAAAEVCNILQVPAFLCRQTDLLVATAETGAIVHLKKGQFLSPWDMEGPINKVLST
GNNKILLTERGCSFGYNNLVSDMRSIPVLSRSGFPVIFDATHSVQLPGALSTESGGLTEFVPTLSRAALAAGAH
GLFIETHTNPKIAKSDAASMLSLEEFAALLPTWDQLFTCVSSFDMVSA

>core/580/3/Org3_Gene762

MTKVALLIAYQGTAYSGWQQQPNDLSIQEVIESSLKKITKTRTPLIASGRTDAGVHAYGQVAHFRAPDHPLF
ANANLTKKALNAILPKDIVIRDVALFDDNFHARYLTIAKEYRYSLSRLAKPLPWQRHFCYTPRHPFSTELMQE
GANLLIGTHDFASFANHGRDYNSTVRTIYTLDIVDKGDSLSIICRGNGFLYKMVRNLVGALLDVGKGAYPPE
HLLDILEQKNRREGPSAAPAYGLSLHHVCYSSPYNNFCCEQCSVSTSNEG

>core/582/3/Org3_Gene819

MTQYYFLSSFLPTQLPESVPLFSISDLDDLLYLNLSENDLCNYGLLKRFFDFENFAFFWAGKPIPFSFGEVTQE
NVERMLSSQQWSDDNDFEDFFKDFLMNHKSSQDRLNHFSDLFREFLSYHQTNSSKFLRDYFRFQQQLRVVL
AGFRARVLNMDVSYVLRDEDSSDPVVLEVLMQKDSPNYELPEEFSDLQGVLDDYGLLPHTLNRALALYQFH
KLEGFCSDSYFDGNVILARCATYMFAIRNSLASVEKGREIINHIEKAIKW

>core/583/3/Org3_Gene888

MHSKFLSRRKKNSSHKEETSWDCIASSYNKIVQDKGHYYHRETILPQLLPSLTLGSKSSVLDIGCGQGFLERA
LPKECRYLGIDISSRLIALAKKMRSVNSHQFKVADLSKRLEFVEPTLFSHAVAILSLQNMEFPGEAIRNTATLL
DPLGQFFIVLSHPCFRIPRASSWHYDENKKAISRHIDRYLSPMKIPIMAHPGQKDSPSTLSFHFPLSYWFKELSS
HGFLVSGLEEWTSSKTSTGKRAKAENLCRKEFPLFLMISCIKIK

>core/584/3/Org3_Gene610

MTLPLEPMIFWSSLSAKVMKKFLTPHCAGTFSEEDAEAKEAHLVTGKQGHRLMGNCVTFYWLVDKKNGVI
LDAKFQYFGHPYLIPLAEAVCNLVCGKSYSEAYKMTLDDIDKSLRVHAHQPALPEDSISLYHFVIDALDTAVE
QCLEIPLEDGSLPLQNSPMNLDFEDANPYSQSDWEALTHEQKLYALRATIAEKIGPYIAMDGGEVTVESLENF
IVTIAYSGNCSGCPSSLGSTLNSIGQLLRAYIYPELQVKVDESSLNLSHP

>core/585/3/Org3_Gene624

MQGFFPLASGSKGNSAYLGTDSCKILIDLGVSKQVVTRELLSMNIDPEDIQAIFVTHEHSDHISGIKSFVKAYN
TPIVCNLETARALCHLLDSHPEFKIFSTGSSFCFQDLEVQTFNVPHDAVDPVAFIFHYREEKLGFCTDLGWVTS
WITHELYDCDYLLIESNHSPELVRQSQRPDVYKKRVLSKLGHISNQECGQLLQKIITPKLKKLYLAHLSTECNT
AELALSTVSESIASITSIAPEIALAQGITSPIYFSRLEVACPR

>core/586/3/Org3_Gene689

MAGLDLEARGKRRVVTPNAITAFGLCCGLFIIFKSVLRTSSSVELFHRLQGLSLLLISAMIADFSDGAIARIMKA
ESAFGAQFDSLSDAVTFGIAPPLIAIKSLDGIYVGNFFSSLLLITSIIYSLCGVLRLVRYNLFSQKTVDVSKPYCFI
GLPIPAAAASIVSLALFLASDFFPDLPAQLRVGLLSFALLFIGGLMISPWKFPGVKHFRFNVSSFLLVVTIGLAA
CLFFSGLVDHFVEVFFLVSWLYTLVGFPIFSIIYRKKS

>core/587/3/Org3_Gene20

MKKPDNDSTFDVRSFFPFDVLCIEQLRKEMSWEVVSAKIPRLPRGWYELMGLSKEDRIDFCLDFWCSVLGIE
HKESPSICRFFSLLETIEVYIYRLEKEPYQLKMFYVFRDGRCGFQGEPPLLDFLGHHRLPPLGDRHYEKFFSIHN
GFGKWEDEGIFPMRSLAKVQQKLRQQLVVMNKMQAEDNCYSLGIFPFYGYEEPFAYQSFFFDPEIRRDLPSP
NVLLNEESLEHRSLETIELLHLSKSYYPSFLSWLENYLHSEEVYNE

>core/590/3/Org3_Gene352

MDCIGKHNPLVKEALALKRSRCRKSSWFLVEGAREIQKALRTGYLCQHVFCSTHLSEKEKEFLYELKRNSTK
ILYCLDSTLAQLSFKEHHDSFVAVIQKRVWNKEDFLIQRKNAQPFYLIIEQVEKPGNVGAILRIADGAGVDGVI
LCNPIVDLYNPNVVRSSLGAVFSLPILSISREEGKELFKQEGWTVFVTSPRAETMYFSKNYLGPTALVFGSEKD
GLTEDWFSEDFSEIALPMLGESDSLNLATSVAAVAYEVVRQRWVN

>core/591/3/Org3_Gene738

MGSSMHVGVIGCSGRTGKVIVSALEQSSEYTLGPGFSRSSALTLFQVIAHNDVLVDFSHPLLTKEVVAHLLISP
KPLIIGTTGFPGKCKEAHDSLEELTHIVPVVVCPNASLGAYIHKRLVMLLSQLCNPQFDIRIRETHHRYKKDSL
SGTAQDLLDTIQQVKQEDWGEEYEVGQRDSSKKTIEVQSSRVGDIPGEHEVAFISSGEQILVRHTVFSRNVFG
RGILSILDWLKTLNPQPGLYSLGDTLELVLRNEHCLLKKTTDH

>core/592/3/Org3_Gene543

MEKLEFVTSLSSPDDDLITFNKQGLIAGPEEEKVAFLVRSNAMLDAGPETPASFPESLREQFDIFPEYVEVLYS
NEGLDVWEAGCTWILNNEVTIQLRKHHRKASRWLGMYSRDEVLAHEAVHAVRMKFHEPVFEEVLAYQTSR
WGWRRFFGPLFRSPGESYLLLFFTILGLGISLWYPAGILIMLVLPMYFLMRLCMAQSYLYRAMKKIRKMLGV
PPLWVLLRLTDKEIKMFAKEPIPVLEHYARKRKLENVRWKQIYQSYFV

>core/593/3/Org3_Gene766

MSNQLQPCISLGCVSYINSFPLSLQLIKRNDIRCVLAPPADLLNLLIEGKLDVALTSSLGAISHNLGYVPGFGIA
ANQRILSANLYAAPTFFNSPQPRIAATLESRSSIGLLKVLCRHLWRIPTPHILRFITTKVLRQTPENYDGLLLIGD
AALQHPVLPGFVTYDLASGWYDLTKLPFVFALLLHSTSWKEHPLPNLAMEEALQQFESSPEEVLKEAHQHTG
LPPSLLQEYYALCQYRLGEEHYESFEKFREYYGTLYQQARL

>core/594/3/Org3_Gene909

MKLTKYLNTKQLRSMISRLFVRYSLPMSKQLSFFALCVLGSHPIFAQTPNPPQRVRRSEVIFIDPGHGGKDQGT
ASKELHYEEKSLTLSLALTVQSYLKRMGYKPQLTRSSDVYVDLGKRVALSNRGQGDVFISIHCNHSSNAAAF
GTEVYFYNGKVGSPTRNRMSEVLGKNILAAMEKNGILKSRGLKTANFVVIRDTSMPAVLVETGFLSNSRERA
DLQDARYRMHVAKGIAEGVHNFLSGPSFQKPKQNIAKIRKPQIQAN

>core/597/3/Org3_Gene56

MPFDITYYTTPLLEIILIWVMLNYLLKFFWGTRAMDVVFGLLAFLFLFVLADKLHLPIIRRLMLHVVNIAAIVV
FIIFQPEIRLALSRIRFHGKKFFIDTQEQFVEQLAASIYQLSERQIGALVVLENKDSFDEYLSFSSVKINATFSEEL
LETIFEPSSPLHDGAVILRGDILAYARVVLPLAHDTTQLSRSMGTRHRAALGASQRSDALIITVSEENGSVSLSR
DGLLTRGVKIDRFKAVLRSILSPKEHKRKPLFSWIWKR

>core/599/3/Org3_Gene124

MTTLLSIKDLSLTIRGKKILNHINLNLIKGSYLTIVGPSGSGKSSLALTILDLLKPTTGTITFHMDPKIPRARKVQ
VIWQDIDSSLNPCMSIKGIISEPLNIIGTYSKAEQNKEIYNVLDLVNLPKSVLHLKPYKLSGGQKQRIAIAKALV
SKPELLICDEPLSSLDTLNQSLILDLFQTIKKEYQNTLLFITHDMSAAYYIADTIAVMDQGSLVEHACREKIFST
PKHTTTQDLLDAIPIFSLISTEMEPSEEYELQVASK

>core/600/3/Org3_Gene728

MKTWLFFTFLFSCSSFYASCRYAEVRSIHEVAGDILYDEENFWLILDLDDTLLQGGEALSHSIWKSKAIQGLQ
KQGTPEQEAWEAVVPFWIEIQEMGTVQPIESAIFLLIEKIQKQGKTTFVYTERPKTAKDLTLKQLHMLNVSLE
DTAPQPQAPLPKNLLYTSGILFSGDYHKGPGLDLFLEICTPLPAKIIYIDNQKENVLRIGDLCQKYGIAYFGITY
KAQELHPPIYFDNIAQVQYNYSKKLLSNEAAALLLRHQMHE

>core/601/3/Org3_Gene862

MMKYLPYIAITACIHGGILLLVFASPLPKKRLQPKAFQEKLVTIQPKPPVPTPSVVVDPAKTIRPSVATQPQKQ
AKCSPPQENVQKALQKPIPKVIKTEPPKPSPAPTVAKKTTATEKPPPSTTKKNTQLSKTQLQTLSEVAQALSLH
VDKIEKSETSLKNISWPSTAQLTMHSELKATQEDELCELFRTHIALPSKGYVRIKLVLSPNGEIQECSFLSEVSA
ADKQLLTQRIHALPFQKFLEKYKVSKNISFHIKLVSNES

>core/602/3/Org3_Gene555

MDLADAHVHLSDDAFEEDINSVLQRAQDSGVSLVVNVTTTEKELNRSFAYAERFPKIRFCHVGGTPPQDVDQ
DIEEDYRNFHAAAHSKKLAAIGEVGLDYCFATEEGIARQKEVLQRYLALSLECELPLVVHCRGAFNDFFRML
DQYYHNDPRSRPGMLHCFTGTLEEAQELISRGWFISISGIVTFKNAQDLRDLVVELPLEHLLIETDAPFLAPVP
YRGKKNEPAHVLHTINAVANVKGMFPQELAALAYKNVLRFLHG

>core/603/3/Org3_Gene14

MFLQFFHPIVFSDQSLSFLPYLGKSSGIIEKCSNIVEHYLHLGGDTSVIITGVSGATFLSVDHALPISKSEKIIKILS
YILILPLILALFIKIVLRIILFFKYRGLILDVKKEDLKKTLTPDQENLSLPLPSPTTLKKIHALHILVRSGKTYNELI
QEGFSFTKITDLGQAPSPKQDIGFSYNSLLPNFYFHSLVSVPNISGEERALNYHKEQQEEMAVKLKTMQACSF
VFRSLHLPSMQTKDKKAGFGLLTFFPWKIYPL

>core/605/3/Org3_Gene278

MILTAAFSPCPNDIFLFRSFLKDPQFRPLLNQVTIADIETLNTLALQRRLSLMKMSAALFPLVSDYYNLMDVG
NTLGYNSGPIVLSLDPECSLDTLATPGEMTTAHALCKLYYPKAKLIPMPYDKILSAILQGKVDGGALIHEERFS
YDLQLTLRADFGELWRRKTIFPLPLGCLAIAKYVPMATVDALTAALRKSLICSLKDPITAGAKAVEYSKNKN
VTVIHRFIGTYINKETFQLSKTGKKALHMLWKANECCQYT

>core/606/3/Org3_Gene772

MIKQIGRFFRAFIFIMPLSLTSCESKIDRNRIWIVGTNATYPPFEYVDAQGEVVGFDIDLAKAISEKLGKQLEVR
EFAFDALILNLKKHRIDAILAGMSITPSRQKEIALLPYYGDEVQELMVVSKRSLETPVLPLTQHSSVAVQTGTF
QEHYLLSQPGICVRSFDSTLEVIMEVRYGKSPVAVLEPSVGRVVLKDFPNLVATRLELPPECWVLGCGLGVA
KDRPEEIQTIQQAITDLKSEGVIQSLTKKWQLSEVAYE

>core/607/3/Org3_Gene76

MLRNQVLVYCSEGVSPYYLRHTIRFLKYYSTQEGAFDILRVDGNFLIKNPFWEETTRLLVFPGGADRPYHRV
LHGLGTARIFQYVSEGGNFLGICAGAYFGSKMIYFYEPEGAPLQGARDLGFFPGTAKGPAYRGNFSYVSPSGV
RVSPQLFSDFGLGYAMFNGGCFFEGSEGYPGVNIESRYDDLPGKPASIVSRIVSKGLAVLSGPHIEYLPHYCR
MVKENVQKTREFLQRERTTLDRYCQNLVQRLRQPAFSKADC

>core/608/3/Org3_Gene230

MNVKDETFWSVHNLCVNYEHAAVLYHISFSLGKGSLTAILGPNGAGKSTLLKASLGLIKPSSGTVYFFNQKF
KKVRQRIAYMPQRASVDWDFPMTVLDLALMGCYSYKGMWGRISSDDRREAFHILERVGLESVADRQIGQLS
GGQQQRAFLARALMQKADLYLMDELFSAIDMASFKTSVGVLQELRDQGKTIVVVHHDLSHVRQLFDHVVL
LNKRLICCGPTDECLNGDTIFQTYGCEIELLEQTLKLSRGKQFGSC

>core/609/3/Org3_Gene458

MNRRWNLVLATVALALSVASCDVRSKDKDKDQGSLVEYKDNKDTNDIELSDNQKLSRTFGHLLARQLRKS
EDMFFDIAEVAKGLQAELVCKSAPLTETEYEEKMAEVQKLVFEKKSKENLSLAEKFLKENSKNAGVVEVQP
SKLQYKIIKEGAGKAISGKPSALLHYKGSFINGQVFSSSEGNNEPILLPLGQTIPGFALGMQGMKEGETRVLYI
HPDLAYGTAGQLPPNSLLIFEINLIQASADEVAAVPQEGNQGE

>core/611/3/Org3_Gene1002

MENLETFILKIYRGVPGKQYWESFELPLHPGENVISALMEIEKRPVNILGEKVNPVVWEQGCLEEVCGSCSILV
NGVPRQACTALIQEYIDATQSREIVLAPLTKFPLIRDLIVDRSIMFDNLERIQGWVAADIEGETFGPQVTQEQQ
ELLYALSQCMTCGCCTEACPQIDNKSDFIGPAAISQARYFNTYPGDKQSKKRWRALMGKGGIEGCGQAHNC
VRVCPKKLPLTESISAVGREISKFSLRSLFSALFKKKK

>core/613/3/Org3_Gene269

MSKFILLLSLGVAALASKNFFIWPAPSGKTPLKLRQVLFGGALLVFSSLVALSVSSQTAELLSTMTGISLAFAF
LFYLLFLPKDITRAILFSGERPVKTSWRALGSAIRMWIIIIPVTQLIGIMMSKFLTLVLPTQEIHTQEVTQEVQNS
LPITGHYISMILNLGVLTPFGEEVFFRGILQTFLKNKMTRIAAVLCSSIIFSFIHIEHSLGSWVFVPVLFVFSLSAG
FLYEKDRHILSPIALHGLFNLTSLLFLGIK

>core/614/3/Org3_Gene343

MAFYSPSTISKYFIYSGAGNRFLLGETLPEVEDVRFLCQETRVDGFLYLKPSSCADAQLIIFNSDGSRPTMCGN
GLRCAIAHLASQKGKSDISVSTDSGLYSGYFYSWDRVLVDMTLADWRASVHRLESRPDPLPKEVVCIHTGVP
HAVVILPEISTLDLSILGPFLRYHQTFSPDGVNVNFVQILGHCQLRVRTYERGVEGETAACGTGALASALVVS
NSYGWKESIQIHTWGGELMTVSQNRGRVYLQGSVTRDL

>core/615/3/Org3_Gene78

MFKLLKNLFLIGCCIVGYFWMRKESIVEQWLSNRLHTQVTVGRVSIRTSGIKIRHICIHNPLASERFPYAAEIEY
ADVRFSSISMLLTKQLEISELIIHGANFTIFPYDSHGTKTNWSLVWKNFHPQKETPSNLWIDRAPVLIRRCLFLN
TRLYGLRANHKDIPHLSVPSLEFHSHTSSAKELPKLSEALPSLLYLALEESLYHLNLPGDIIKPLSQQAHKHFYS
SYPQFQDRLNDINTPGTPTEEIIGFIRGLFFH

>core/616/3/Org3_Gene61

MVSPLSLFHKMLLENWTPVEEPFPWPPAEKNQKIFAWALNQSKLIFVSTSGNIAQPRLVTDSMSMMIVNAAN
RTMSRDGAGTNQVLSAAVSVDSWGLSQRPLNPERQGTPLNEGECRAGMWRNADGSNHTGKQGKPHYLAQ
LLGPKAVDHHNKSQAAFDRCKNAYLNCFSLAQTLGVTFLQIPLISSGIYAPPENRKKPNSEENKVRMRWIHA
VKCALVAAMQEFGNEPGNTDRRMLIVLTDLKTPAITDPKKKSHL

>core/617/3/Org3_Gene239

MKTQQTQNIIEVWNFYWETQEIEYRDSLIEFYLPLVKSVVHRLISGMPSHVKTEDLYASGVEGLVRAVERYN
PERSRRFEGYAVFLIKAAIIDDLRKQDWVPRSVHQKANKLSGAMDSLRQSLGKEPTDLELCEYLNISQQELSG
WFVSARPALIVSLNEEWPSQSDEGAGMALEERIPDERAETGYDVVDKQEFSLCLANAIQELEEKERKVMALY
YYEELVLKEIGKVLGVSESRVSQIHSKALLKLRAALSAFR

>core/618/3/Org3_Gene293

MWLGAYTWLNVFGILLQAAFIQNILLANFLGMCSYLACSTRVSTANGLGMSVALVLTVTGSINWFVHAFITG
PKALTWISPSLASVNLGFLELIIFIVVIAAFTQILELLLEKVSRNLYLSLGIFLPLIAVNCAILGGVLFGITRSYPFIP
MMIFSLGAGCGWWLAIVILATIKEKLAYSDIPKNLQGMGISFITTGLIAMAFMSLTGIDISKPSAKIQRAPLETE
VVENTTNPLKESSSKHQPSISKARTQRRSL

>core/619/3/Org3_Gene483

MLKIKHLHASCNDVKILDDFNLNIQPGTMHVIMGPNGAGKSTLAKILAGDESVLVSSGEIALQEQNLLSMLPE
ERSRAGLFVGFQMPPEIPGVNNKMFLRDAYNARRRANQEGDISIDEFNTLLSTVLETYEYNATTDLFLDRNV
NEGFSGGERKRNEICQMLVLEPEMVLLDEPDSGLDVDALRLICRVLEKYRELHPTSSLCIVTHNPKLGNLIRP
DVVHLLLDGRVALSGDVSLMHELEAKSYQEVTKRVAWR

>core/621/3/Org3_Gene567

MKTIAVNSFKGGTAKTSTTLHLGAALAQYHQARVLLIDFDAQANLTSGLGLDPDCYDSLAVVLQGEKEIQE
VIRPIQDTQLDLIPADTWLERIEVSGNLAADRYSHERLKYVLGSVQDKYDYVIIDTPPSLCWLTESALIAADYA
LICATPEFYSVKGLERLAGFIQGISARHPLTILGVALSFWNCRGKNNSAFAELIHKTFPGKLLNTKIRRDITVSE
AAIHGKPVFATSPSARASEDYFNLTKELLILLRDI

>core/622/3/Org3_Gene979

MTRQSYVLGNWKMHKTIQEAKEYVQTLASLLQGEPLSCTIGIASPFTSLRAIHEMINTTGAFLWLGAQNVHP
ELSGAFTGEISLPMLKEVGVEFVLVGHSERRHIFGESDALIASKVKSVAQAGLVPVLCVGESLEVREEGKAHQ
VIKKQLLLGLEQMDNGSEFLIAYEPVWAIGTGKVAEASDVQDIHMFCREVVAERFSEATAEEISILYGGSVKV
DNAQRFGQCSDVDGLLVGGASLEGQSFFEVAKNFNV

>core/623/3/Org3_Gene348

MHDALLSILAIQELDIKMIRLMRVKKEHQKELAKVQSLKSDIRRKVQEKELEMENLKTQIRDGENRIQEISEQI
NKLENQQAAVKKMDEFNALTQEMTTANKERRSLEHQLSDLMDKQAGGEDLIVSLKESLASTENSSSVIEKEI
FESIKKINEEGKALLEQRTELKHATNPELLSIYERLLNNKKDRVVVPIENRVCSGCHIVLTPQHENLVRKKDRL
IFCEHCSRILYWQESQVNAQENSTAKRRRRRAAV

>core/624/3/Org3_Gene148

MKPEESECLCIGVLPARWNSSRYPGKPLAKIHGKSLIQRTYENASQSSLLDKIVVATDDQHIIDHVTDFGGYA
VMTSPTCSNGTERTGEVARKYFPKAEIIVNIQGDEPCLNSEVVDALVQKLRSSPEAELVTPVALTTDREEILTE
KKVKCVFDSEGRALYFSRSPIPFILKKATPVYLHIGVYAFKREALFRYLQHSSTPLSDAEDLEQLRFLEHGGKI
HVCIVDAKSPSVDYPEDIAKVEQYITCLSNAYF

>core/625/3/Org3_Gene965

MLIKLWRATYEGMYTFLVGALLKLRYRMQVEGWDTLNINPKQGCLFHANHVAEVDPIILEYLFWSRFHVRP
MAAEYLFHSRVVQWFLNSVRSIPIPQLVPGKESKRSLERMNVCYEEASRALNRGESLLLYPSGRLSRTGKEEI
VNQYSAYVLLHRVMECNVVLVRVSGLWGSAFSRYKQNSTPKLGPAFKEAFRALLRRGIFFMPKRFVKITLC
QVDHLFLKQFPTKQDLNTFLASWFNQGDDNLPIEVPYA

>core/626/3/Org3_Gene401

MQICVTGVVLRSRPLGKNHTLTTLFTPEGLFTFFAKQGQTLQCDYRETLVPISLGKYTLHRNGSRLPKLTHGD
ILNAFEAIKQTYALLEASGKMIQALLASQWKEKPSHKLFSLFLNFLHRIPESSNPEFFAAIFVLKLLQYEGILDL
TPACSLCKASLPYACYRYQGHKLCKKHQHKQAISIEKEEEQILQAIIHAKQFSELLAIAEFPIAIAEKIFYLFDSL
QEEKKSERNSSEDPYHEILRLSKVVHPY

>core/627/3/Org3_Gene480

MLIVLAFRQVFFSHSRSQLDRLKNYLRLLKQNFAITLPKERTSKGHSLMLTFDFASFDFYTNIFPFLEEQKIPAV
VGVASRYIPSNAAQDLHPSHRLKPSETLAFQDEIFSNYMPFCCQNELIEMAKSPYIQLASSGFAIRNLMNNPPY
LTTEILLSRHHIETITGAKPLAFLFPFGKSDPTSRKLAADHYPYSFLLGNTINRKLKTHNIYRLDIKPMQYVCPS
LFQSSRYLKNWIKEKSKQLYLKKQLPKR

>core/628/3/Org3_Gene126

MLMDISHILEDLAYDEGILPREAIEAAIVKQMQITPYLLHILHDATQRVPEIVNDGSYQGHLYAMYLLAQFRE
SRALPLIIKLFAFEDDTPHAIAGDVLTEDLPRILASVCNDDSLIKELIETPKINPYVKAAAISGLVTLVGAGKIPR
DKVIRYFAELLNYRLEKQPSFAWDNLIAGICTLYPGELFYPISKAFDGGLVDTSFISMEDVENIIHEETVESCIH
TLCSSTELINDTLEEMEKWLEDFPIEP

>core/629/3/Org3_Gene82

MNVADLLSHLETLLSSKIFQDYGPNGLQVGDPQTPVKKIAVAVTADLETIKQAVAAEANVLIVHHGIFWKG
MPYPITGMIHKRIQLLIEHNIQLIAYHLPLDAHPTLGNNWRVALDLNWHDLKPFGSSLPYLGVQGSFSPIDIDS
FIDLLSQYYQAPLKGSALGGPSRVSSAALISGGAYRELSSAATSQVDCFITGNFDEPAWSTALESNINFLAFGH
TATEKVGPKSLAEHLKSEFPISTTFIDTANPF

>core/630/3/Org3_Gene64

MKKQGKTLFLFLFLSFLFSTAFSGLFASQTSSLRTIQENIFLAKTGDYTVLSRGSQRTFVLVKSTTPKTVWIEIIH
FPCIAHKERPSLEQASWKTVIHQLESPSQVFVVSLSSEGSQFFSLNTRTKSLEPVGKSTTVPAFLQIFDLPLSPAP
ANVIKTKGKENKPWSPKVSFEGAPLTSISVNAWQGLWPKDRGPLSETGILMYFTQPDISVFPLWVSIETPKGT
SIVRAVDIGHGATSPYVYSLPDSKTQ

>core/632/3/Org3_Gene381

MSLATNNAESKFPSLQRLPNHVAIIMDGNRRWYKKHREECGHTHTSGHYYGAKVLPNILNAVLDLGIKVLT
LYTFSTENFGRPKEEIQEIFNIFYTQLDKQLPYLMENEICLRCIGDLSKLPKGIQTKINHVSRMTASFSRLELVLA
VNYGGKDELVRAFKKLHVDILNKKISSDDLSESLISSYLDTSGLTDPDLLIRTGGEMRVSNFLLWQIAYTELYI
TDTLWPDFTPQDLFEAINVYQQRSRRGGK

>core/633/3/Org3_Gene566

MGNLKTLLESRFKKNTPTKMEALARKRMEGDPSPLAVRLSNPTLSSKEKEQLRHLLQHYNFREQIEEPDLTQ
LCTLSAEVKQIHHQSVLLHGERITKVRDLLKSYREGAFSSWLLLTYGNRQTPYNFLVYYELFTLLPEPLKIEM
EKMPRQAVYTLASRQGPQEKKEEIIRNYRGERKSELLDRIRKEFPLVETDCRKTSPVKQALAMLTKGSQILTK
CTSLSSDEQIILEKLIKKLEKVKSNLFPDTKV

>core/634/3/Org3_Gene410

MKIKFSWKVNFLICLLAVGLIFFGCSRVKREVLVGRDATWFPKQFGIYTSDTNAFLNDLVSEINYKENLNINIV
NQDWVHLFENLDDKKTQGAFTSVLPTLEMLEHYQFSDPILLTGPVLVVAQDSPYQSIEDLKGRLIGVYKFDSS
VLVAQNIPDAVISLYQHVPIALEALTSNCYDALLAPVIEVTALIETAYKGRLKIISKPLNADGLRLAILKGTNG
DLLEGFNAGLVKTRRSGKYDAIKQRYRLP

>core/635/3/Org3_Gene453

MSLLKDTVFTCLDCEMTGLDVKKDRIIEIAAVRFTFDSVISSIEFLINPERVVSAESQRVHHISNAMLRDQPKIA
EVFPQIKAFFKEGDYIVGHSVGFDLQVLAQEMERIGETFLSKYTIIDTLRLAKEYGDSPNNSLESLAVHFNVPY
DGNHRAMKDVEININIFKHLCKRFRTLEQLKQVLAKPIKMKYMPLGKHKGRCFSEIPLAYLQWASKMDFDS
DLLFSIRHEIKHRQKGTGFSQVNNPFMEL

>core/636/3/Org3_Gene193

MDITLVGKKVIVTGGSRGIGLGIVKLFLENGADVEIWGLNEERGQAVIESLTGLGGEVSFARVDVSHNGGVK
DCVQKFLDKHNKIDILVNNAGITRDNLLMRMSEDDWQSVISTNLTSLYYTCSSVIRHMIKARSGSIINVASIVA
KIGSAGQTNYAAAKAGIIAFTKSLAKEVAARNIRVNCLAPGFIETDMTSVLNDNLKAEWLKSIPLGRAGTPED
VARVALFLASQLSSYMTAQTLVVDGGLTY

>core/637/3/Org3_Gene615

MTQDPHDHFKSRTPEDHIKHVRDKHRVCKGEPHTTFKGFFYHLANNALSTGVFIFFIRTLFFLIPTNRALQVKS
LISLGVGWTFYHGCLKARKAWAYMELSHRSMLEEKNEIEENFEQEKIELRILFENQGFKDPLLQEMVEYVCS
DSTLLLDTMIREELYIRKEDLPHPLIQGGSRILGGLCGLAIFLPLVLCISYTLAGVFSALMVLVLSFLKAKILKN
DKISEMVWVLGIFITSASIISSLMKLL

>core/638/3/Org3_Gene250

MTLSFHTHPLNYWTFEEFDGLPIRHGVFSKQKDAEGTVFAAKNPEIASALQSPKYCDLHQRHGTSVRCVTPT
SPTYQPADGLCTQSPLLSLHIRHSDCQAAIFYDREHHAIANVHSGWRGLLGNIYAVTVGTMKKLFHTKPQDL
FVAIGPSIGPDYAIYPDYATLFPRSFLPFMNPKNHFDLRAIARKQLTNLGISKDRIFISDLCTYTEHDAFFSSRYL
AHHPDPNLTGQHSKNRNNVTAVLLLPRD

>core/639/3/Org3_Gene488

MAKQTRRVLFKISGEALSKDSSNRIDEMRLSRLVSELRAVRNNDIEIALVIGGGNILRGLAEQKELQINRVSAD
QMGMLATLINGMAVADALKAEDIPCLLTSTLSCPQLADLYTPQKSIEALDQGKILICTTGAGSPYLTTDTGAA
LRACELNVDVLIKATMHVDGVYDKDPRLFPDAVKYDFVSYKDFLSNQLGVMDASAISLCMDSHIPIRVFSFL
QHSLEKALFDPTIGTLVSEDVNHVCSPRH

>core/640/3/Org3_Gene46

MKFFILFILIVAQFPAFSAQPRTQVSASHSKQAKARRTSRIRSSAATNASVSRYKTRAAARKKIGKFEKKPSLSP
VQWVRYSGKNYSIQTPSLWQCIDDKTQLPEKLDVLLIGKGKGNLTPTINIAQEITSKSSKEYIEEILAYHKANE
MTLESGIFTQIQSPSGEFTIIKTEKNSSWGRVFCLQATTVIDHTAYIFTSTATLDDYAELSFTFLKVVSSFQIRGG
KEATSGDAILEKALEALQNENK

>core/641/3/Org3_Gene412

MHIYGLADLHLALGVPEKTMEVFGDPWIGYHQKICSEWQAVVHPEDIVLLPGDISWAMNLSEAHKDFAFIG
DLPGTKYMIRGNHDYWSSASTSKILQALPPSLYYLNQGFALLTPHLAVVGVRLWDSPTICVKKENFLTPSTQE
QSYTEQDEKIFLRELGRLKRAFAALPKEVTEVIVMTHYPPISSDGTPGPISEFLEADGRVSLCLFGHIHKVQRPI
DGFGNIRGIHYILVAADYVNFVPQEVM

>core/642/3/Org3_Gene948

MKITTVKTPKIYPYDDLYSILESSLPKLNERSIVVITSKIVSLCEGAVVELEKVSKDELIKQEADAYVFVEKYGI
YLTKKWGILIPSAGIDESNVEGYFVLYPRDVLLSVNTLGDWLRNFYHLEHCGIIISDSHTTPLRRGTMGLGLC
WNGFFPLYNYVGKPDCFGRALKMTYSNLLDGLSAAAVLCMGEGDEQTPIAIIEEAPKITFHSSPTTLQDMSTL
AIAEDEDLYGPLLQSMAWETPAPTS

>core/643/3/Org3_Gene789

MDFDYFGLSDIGRVRARNEDFWQVNLMSQVVAIADGVGGRLGGDIASQEAVTSLMELIDEQQSKLMGYED
DQYKETLKKILLEVNGVVYEHGQMEEHLQGMGTTLSFIQFRKDRAWLFHVGDSRIYRIREGELRRLTEDHSL
ENQLKNRYGLPKQSDKVYSYRHILTNVLGSRPYVMPDIRNLPCEKEDLYCLCSDGLTNMVPDIDIRDILNQPA
TLEERGNALISLANTRGGDDNATVVLVRIQ

>core/644/3/Org3_Gene361

MTIRILAEGLAFRYGSKGPNIIHDVSFSVYDGDFIGIIGPNGGGKSTLTMLILGLLTPTFGSLKTFPSHSAGKQTH
SMIGWVPQHFSYDPCFPISVKDVVLSGRLSQLSWHGKYKKKDFEAVDHALDLVGLSDHHHHCFAHLSGGQI
QRVLLARALASYPEILILDEPTTNIDPDNQQRILSILKKLNRTCTILMVTHDLHHTTNYFNKVFYMNKTLTSLA
DTSTLTDQFCCHPYKNQEFSCSPH

>core/646/3/Org3_Gene718

MSYFNYQKNSVVLRSLGLLAKFFSRLLYRVFFSFREGIYLFSSLYLKYPRLFFYDLGKYVYSLRHCPYAKLGR
LPGASLLKEGNVYGETPWSVLAKICQAFDITSQDILYDLGCGLGKVCFWFSHVVRCQVIGIDNQPHFIRFSSN
MHRKLSSGFALFDTEEFKNVVLSQASYVYFYGSSFSRRLLNEIILKLSEMAPGSVVISISFPLDSFSRGKECFFT
EKSCSVRFPWGKTIAYKNIRKGS

>core/647/3/Org3_Gene163

MSTLLLNPPWMKAGKRIESLVRKALYTHTMLANHRKIVVALSGGKDSLTLLLMLKAISGRGFPDLDLHAVNI
GGKYSCGAEVNKPYLTRICDQLCIPFRTIPSPYAPETPECYPCSQARRRLLFQAAKEIGASAIAFGHHRDDLVQ
TALLNLLHKAEFAGMLPVLDMVHFGVTILRPLIFTPEFWIRKFAKENGFARVTCRCPVVSLRSKAEQSLKLLE
EVFPLARHNIALAIQEHGSSKSQKI

>core/648/3/Org3_Gene173

MTDYSFFRRKIGNIEAIECPGNPQDPIIILCHGYGSLADNLTFFPSICSFSKLRPTWIFPNGILPLENDFRGSRACF
PLNVLLLQELSRLYANGVGNLQEKYDELFDVDLETPKEALEELILNLNRPYNEIIIGGFSQGAILATHLVLTSQ
NPYAGALIFAGARLFNQGWEEGLKQCAQVPFLQSHGYEDEILPYHLGAHLNDLLLTKLNGQFVSFHGGHEIP
SVVFQKMQVTVPNWIDPARG

>core/650/3/Org3_Gene509

MPILSVCNLVKKYNKKPVTNDVSFQINPGEIVGLLGPNGAGKTTAFYLTVGLIRPDSGKIIFKNVDVTKKTMD
HRARLGIGYLAQEPTIFKELTVQDNLICILEIIYKARKQQSHLLNTLVDDLQLGSCLHKKAGTLSGGERRRLEI
ACVLALNPSVLLLDEPFANVDPLVIQNVKYLIKILAGRGIGILITDHNAKELLSIADRCYLIIDGKIFFEGSSSQM
ISNPMVKQHYLGDSFSY

>core/651/3/Org3_Gene298

MKVRIVDSGKSSAASHMAKDRDLLESLQDGELILHLYEWENPCSLTYGHFMRPEKFLLSNYADLGLDAAVR
PTGGGFVFHKGDYAFSVLMSATHPSYSSSVLENYHTVNSFVAKVLEKVFRIQGMLAPEDENSSSRDSGNFCM
AKTSKYDVLFGDKKIGGAAQRKVQQGFLHQGSLFLSGSSSEFYQRFLKPEVLEEIIEQIQIHAFFPLGLEAADE
VLQEARQQVKEAFIKLFCGEGL

>core/652/3/Org3_Gene791

MTLYLLPNTLGTRAVETLPSVIGELVHRLDGLIVESDRGGRAFLSLWKIPEVHKFPLAILSKHARLPKAWDFY
LEPIVKHGENWGLISDAGLPCIADPGASLVRRARALGIPVQAFSGPCSITLALMLSGLPSQSFTFLGYLPQSPKE
RVKSIKKAATSKEVSTSVCIETPYRNVYTFESLLDTLPSYAELCVASDLSGPSELVLTRQVQSWRTTEDLGSV
KQSITKVPTIFLFHIPN

>core/653/3/Org3_Gene388

MAGHSKWANTKHRKERADHKKGKIFSRIIKELISAVKLGGADPKSNARLRMVIQKAKENNIPNENIERNLKK
ATSAEQKNFEEVTYELYGHGGVGIIVEAMTDNKNRTASDMRIAINKRGGSLVEPGSVLYNFARKGACTVAK
SSIDEEVIFSYAIEAGAEDLDTEDEENFLVICAPSELASVKEKLISQGATCSEDRLIYLPLRLVDCDEKDGEANL
ALIDWLEQIEDVDDVYHNMS

>core/654/3/Org3_Gene455

MDISGAVKQKLLQFLGKQKKPELLATYLFYLEQALSLRPVVFVRDKIIFKTPEDAVRILEQDKKIWRETEIQIS
SEKPQVNENTKRIYICPFTGKVFADNVYANPQDAIYDWLSSCPQNMEKQGGVRIKRFLVSEDPDVIKEYAVPP
KEPIIKTVFASAITGKLFHSLPPLLEDFISSYLRPMTLEEVQNQTKFQLESSFLSLLQDALVEDKIAAFIESLADD
TAFHVYISQWVDTEE

>core/655/3/Org3_Gene683

MGLYDRDYIQDSRVQGTFASRVYGWMTAGLIVTSCVALGLYFSGLYRSLFSFWWVWCFATLGVSFFINSKI
QTLSVSAVGGLFLLYSTLEGMFFGTLLPVYAAQYGGGVIWAAFGSAALVFGLAAVYGAFTKSDLTKISKIMT
FALIGLLLVTLVFAVVSMFVSMPLIYLLICYLGLVIFVGLTAADAQAIRRISSTIGDNNTLSYKLSLMFALKMY
CNVIMVFWYLLQIFSSSGNRD

>core/656/3/Org3_Gene26

MHPPIDITAIEAKLNFTFTQPKLLEIALTHPSYKNESAVQIEDSERLEFLGDAVLGLIVTEHLFLLFPSMDEGTLS
TARASLVNAKACCRYTTMLGIGDYLLIGKGEKIQSERGRLSAYANLFESILGAVYLDGGLSPARKLTVPLLPP
REEILPLMSGNPKNLLQQFTQKQFRVLPVYQSTAVTDAQGNVSYQIQVLVNQEVWGEGNASSKKEAEKIAA
QQALDTYGNKNQNTMDV

>core/657/3/Org3_Gene993

MEPSTNKPDCKKIFDSIASKYDRTNTILSLGMHHFWNRSLIQILGSGYSLLDLCAGTGKVAKRYIAAHPQASV
TLVDFSSAMLDIAKQHLPQGSCSFIHSDINQLPLENHSYPLAAMAYGLRNLSDPHKALQEISRVLMPSGKLGIL
ELTPPKKTHPTYSAHKLYLRAVVPWIGKSVSKDPDAYSYLSKSIQQLPKDHDLEDLFSKSGFYIAKKKKLFLG
AATIWLLEKQ

>core/658/3/Org3_Gene984

MDVLIFYDTETTGTQIERDRIIEIAAYNSVTDESFLTYVNPEIPIPDEASKIHGITTDAVLSAPKFPEAYEGFRKF
CGEDSILVAHNNDGFDFPLLGKECRRHSLEPLTNRTIDSLKWAQKYRPDLPKHNLQYLRQVYGFAENQAHR
ALDDVVILHKVFTSLIGDLPPQQVLDLLQQSYHPKVFKMPFGKYKGQPLVDIPKSYFEWLENQGALDKPENK
DIKAAIALLHQPT

>core/659/3/Org3_Gene546

MQNATIDQLPVSWQEQLPLCWREQLKEEWSKPYMQQLLIFLKQEYKEHTVYPEENCVFSALRSTPFDQVRV
VILGQDPYPGKGQAHGLSFSVPEGQRLPPSLINIFRELKTDLGIENHKGCLQSWANQGILLLNTVLTVRAGEPF
SHAGKGWELFTDAIVTKLIQERTHIIFVLWGAAARKKCELLFNSKHQHAVLSSPHPSPLAAHRGFFGCSHFSKI
NYLLNKLNKPMINWKLP

>core/660/3/Org3_Gene211

MDLAVELKEGILLVDKPQGRTSFSLIRALTKLIGVKKIGHAGTLDPFATGVMVMLIGRKFTRLSDILLFEDKEY
EAIAHLGTTTDSYDCDGKVVGRSKKIPSLEEVLSAAEYFQGEIQQLPPMFSAKKVQGKKLYEYARKGLSIERH
HSTVQVHLQITKYEYPLLHFVVSCSKGTYIRSIAHELGTMLGCGAYLEQLRRLRSGRFSIDECIDGNLLDHPDF
DISPYLRDAHGNSL

>core/661/3/Org3_Gene527

MIGDKIILFVTEDLSLSSQLKDLASQRSDYQILVSPVFPTSFESVAIFCEYLLLPEQIFSPGIFPEEDLIVLFDTFQE
EAITKVLNQGATGYLLRPITAKVLDAVIRAFLRQHEVLEHSIPDTMTFGDHTFRVLNLVIESPEGSVYLTPSEA
GILKKLLINRGHLCLRKNLLAEIKGNTKEIIARNVDVHIASLRKKLGPYGSKIVTIRGVGYLFSDDDSIPLQNHD
NTAHPNEE

>core/662/3/Org3_Gene854

MTKVRLNKFLASAGVASRRKCDEIIFSGSVTVNGRVAEGPFVLVDPEDKVQVGGTSVHLTKKVYFMVHKAI
GYLCSSEKKFPGTKLVIDLFAHLPYRVFTVGRLDKETSGLILVTNDGEFANKIIHPSSGITKEYLLKVSRDVSA
KDLGKLMEGTFIDGKHVRPVSVTKIRRGTVKIVVSEGKKHEIRLFADAAGLPILELKRIRIGSLVLGGLRYGEY
RELTDAELGTYMKLSD

>core/663/3/Org3_Gene146

MLQAHRLCYSCDNQVILKDASFQASPGTITIILGSSGVGKTTLFRLLAGFLPLQEGELLWNGSPLNRKDVAYM
QQKEALLPWRTALKNMTLSTELGINTSHNALSNERLEEIIHNFDLGQLLDRYPDELSGGQRQRIALAAQCLSL
KPILLLDEPFSSLDVLLKEQLYQDIVALAKKENKTVLLVTHDFHDVSCLGDVLYVIKNKTLTPVPLDPSMRPL
NNGLCFIKDLKKHLYT

>core/664/3/Org3_Gene708

MRVIFPDKHNNFPNLSKLLKKLPSVILVTSCIAPFFSYIINKFFGIPGLLEILALSVKGIQKHHFWQFLTYPLITAD
SLSLNKDQSFEITQRLLLRNVLDFFLFYKAIQHLIRKLGAFSVLVVISGQALIIGAVLWGFMALIHSSQSFFGPE
SIICGVLTVQIFLDPEKRFTIGPTPLSVSIKWGFLFVLGFYCCILIFSGAFLLLLASMLAIVLAILFCKKEKIPNPYT
TSLRF

>core/665/3/Org3_Gene421

MPTTNCIFLDLRGHSILHQLQIEEALLRVANQNFCIINSGAKDSIVLGISRNLNQDVHISRAQADHIPIIRRYSGG
GTVFIDSNTLMVSWIMNSSEASAQPQELLAWTYGIYSPLLPNTFSIRENDYVLGHKKIGGNAQYIQRHRWVH
HTTFLWDIDLDKLSYYLPIPQQQPTYRNQRSHEEFLTTLRPWFPSRDDFLERIKASGSLLFTWEEFLDNELEEIL
AQPHRKATTVLN

>core/666/3/Org3_Gene24

MLSVCYSDPCLSDFCQGKRPLRIASRNSNLAKAQVHECISLLRSWYPKLWFQLSTTETTGDREKKIPLHLVEN
SYFFTDGVDALVHKGVCDLAIHSAKDLPETPSLPVVAITRCLHPADLLVYADHYVHEPLPLSPRLGSSSLRRS
AVLKQLFPQGQILDIRGTIEERLDQLHRGHYDAIVLAKAASLRLHLHHAYSIELPPPYHALQGSLAITAKDHA
GKWKQLFTPIHCHSS

>core/667/3/Org3_Gene583

MKFFSLIFKDDDVSPNKKVLSPEAFSAFLDAKELLEKTKADSEAYVAETEQKCAQIRQEAKDQGFKEGSESW
SKQIAFLEEETKNLRIRVREALVPLAIASVRKIIGKELELHPETIVSIISQALKELTQNKHIIISVNPKDLPLVEKSR
PELKNIVEYADSLILTAKPDVTPGGCIIETEAGIINAQLDVQLDALEKAFSTILKAKNPVDEPSETSSSTDSSSLS
NDQDKKE

>core/668/3/Org3_Gene635

MNRRDMVITAVVVNAILLVALFVTSKRIGVKDYDEGFRNFASSKVTQAVVSEEKVIEKPVVAEVPSRPIAKET
LAAQFIESKPVIVTTPPVPVVSETPEVPTVAVPPQPVRETVKEEQAPYATVVVKKGDFLERIARANHTTVAKL
MQINDLTTTQLKIGQVIKVPTSQDVSNEKTPQTQTANPENYYIVQEGDSPWTIALRNHIRLDDLLKMNDLDEY
KARRLKPGDQLRIR

>core/669/3/Org3_Gene1018

MFFIVCFGFLIHKKHTILPPKAHIPTNAKHFPTIGNPYAPINITVFEEPSCSACAEFTTEVFPLLKKHYIDTGEISF
TLIPVCFIRGSKPAAQALLCIYHHDPRQADIDAYMEYFHRILTYPKEEGSHWATPEVLTKLAEGLKINSGRSVN
PKGLEQCIASGQYNEQIKKNNLYGSQVLGGQLATPTAVVGDYLIEDPTFHEIERAIQHIRQLQAVEGDHDD

>core/670/3/Org3_Gene554

MVHFSHNPIIQAYTEADFFGKSIFFCLLILSVCTWTVLHQKLAIQKNFLKAGKSLKDFLIKNRHAPLSLDIHPEL
SPFADLYFTIKRGTLELLDKNRQSAPDRGPILSSEDIQSLETLLGAIMPKYKALLHKNSFIPATTISLAPFLGLLG
TVWGILVAFTHISSGSSGNSAIMEGLATALGTTIIGLFVAIPSLIAFNYLKAHSSELISEIEQTAYLLLNSIEVKYR
NTNL

>core/671/3/Org3_Gene42

MTKHGKRIRGILKNYDFSKSYSLREAIDILKQCPPVRFDQTVDVSIKLGIDPKKSDQQIRGAVFLPNGTGKTLR
ILVFASGNKVKEAVEAGADFMGSDDLVEKIKSGWLEFDVAVATPDMMREVGKLGKVLGPRNLMPTPKTGT
VTTDVAKAISELRKGKIEFKADRAGVCNVGVGKLSFESSQIKENIEALSSALIKAKPPAAKGQYLVSFTISSTM
GPGISIDTRELMAS

>core/672/3/Org3_Gene84

MEKDLHLHEKKCLAHEAATQVTSGMILGLGSGSTAKEFIFALAHRIQTESLAVHAIASSQNSYALAKQLAIPL
LNPEKFSSLDLTVDGADEVDPQLRMIKGGGGAIFREKILLRAAKRSIILVDESKLVPVLGKFRVPLEISRFGRSA
IIEEIRHLGYEGEWRLQDTGDLFITDSSNYIYDIFSPNSYPNPEKDLLKLIQIHGVIEVGFVIEKVEVWSSNSQGL
ISKKYSV

>core/673/3/Org3_Gene147

MLQSCKKALLSIVVSILAFHPIPGMGVEAKSGFLGKVKGWFSKKEIQEEARILPVKDSLSWKRYDYTSSSGFS
VEFPGEPDHSGQIVEVPQSEITIRYDTYVTETHPDNTVYVVSVWEYPEKVDISRPELNLQEGFSGMMQALPES
QVLFMQARQIQGHKALEFWIVCEDVYFRGMLISVNHTLYQVFMVYKNKNPQALDKEYEAFSQSFKITKIREP
RTIPSSVKKKVSL

>core/674/3/Org3_Gene168

MALDEINNQNNPSQQIASSTSQTSKINQDRKTFACTVTLLVVATLMILSGIVLLFTIGSLGLSVPLSGILGTFAV
TVGAVLFITGLTILVRKSLGIEQKNEDLNFLKIKTPTPPARPLMSKFSVTCSTTSIVLGMALLIGAVVSVFFLTG
YLQLGLCAGLVGLGTALFVAGLARMSPRSLADQEGSGSADSQSNIVGIGEPKAAQEQKWYKMAVVRGEDGI
PTAIRLTPEK

>core/675/3/Org3_Gene572

MSKPSPRNANQPQKPSASFNKKTRSRLAELAAQKKAKADDLEQVHPVPTEEEIKKALGNIFEGLSNGLDLQQI
LGLSDYLLEEIYTVAYTFYSQGKYNEAVGLFQLLAAAQPQNYKYMLGLSSCYHQLHLYNEAAFGFFLAFDA
QPDNPIPPYYIADSLLKLQQPEESNNFLDVTMDICGNNPEFKILKERCQIMKQSIEKQMAGETKKAPTKKPAG
KSKTTTNKKSGKKR

>core/676/3/Org3_Gene394

MYLEDYDVFFFDLDGLLVDTEPCFYRAFLQACAEFSLEVHWDFSTYYSHTTLGTEIFSKKFIEQYPQAQEYM
AEIFAKRLQIYYKSLEHAGPALMPGVEAFIELVLSLNKTFGVVTNSPRDATHTLRTMYPILNKFLFWVTRENY
ARPKPYGDSYDYAYRTFAREGMKVIGFEDSVKGLRALSKIPATLVCINSMAEITPEDYPELKGKEFFSYPSFD
VLTEHCSQQKLL

>core/677/3/Org3_Gene737

MNTSHRKTLVFSYLSSTFTLLLVLSNLVLSSKLIPTTFFNFIIPGGLILYPLTFLISDVVNEIFGPKKARVMIFSAFI
ANLLASSIVQIFMFFPVASPEMQTAWHCLFDLSPLRFLASLLAFIVSQQLDIVLYTFFKNRTPNSSLWLRSNGS
TWISQIPDTFIVDTCILYFGMGLSFPQTLNIMFYSYIYKITFCVLTTPLFYLAVNTIRKFLGMPSTKIANTVPLIN
QP

>core/678/3/Org3_Gene887

MKKQESVLVGPSIMGADLTCLGVEAKKLEQAGSDFIHIDIMDGHFVPNLTFGPGIIAAINRSTDLFLEVHAMIY
NPFEFIESFVRSGADRIIVHFEASEDIKELLSYIKKCGVQAGLAFSPDTSIEFLPSFLPFCDVVVLMSVYQGFTVQ
SFLPHTIEKIAFARHAIKTLGLKDSCLIEVDGGIDQQSAPLCRDAGADILVTASYLFEADSLAMEDKILLLRGEN
YGVK

>core/679/3/Org3_Gene133

MKGFLSVNELIFGFQTFSVVVLGVFFASRGKAWLTGWLSLLSSIMNVFVLKQIHLWGFEVTSADVYVIGLLT
CLNYAREHYEKNDINDAMLCSWVISIAFLVLTQLHLFLIPSPNDSSQEHFLALFSSTPRIVVASLVTLIFVQIVDI
KLFTFLQRVFSKKYFAMRSTISLLFSQLIDTIIFSFLGLYGLVSNLCDVMIFAMLVKGIVITLAIPTLTVTKAVLD
RRSS

>core/680/3/Org3_Gene399

MRPHRKHVSSKSLALKQSASTHVEITTKAFRLSMPLKQLILEKSDHLPPMETIRVVLTSHKDKLGTEVHVVAS
HGKEILQTKVHNANPYTAVINAFKKIRTMANKHSNKRKDRTKHDLGLAAKEERIAIQEEQEDRLSNEWLPVE
GLDAWDSLKTLGYVPASAKKKISKKKMSIRMLSQDEAIRQLESAAENFLIFLNEQEHKIQCIYKKHDGNYVLI
EPSLKPGFCI

>core/681/3/Org3_Gene958

MALLILLRHGQSVWNEKNLFSGWVDIPLSQQGIEEAFSAGRAIQNLPIDCIFTSTLVRSLMTALLAMTNHHSK
KIPYIVHEDPKAKEMSRIYSAEEENNMIPLYQSSALNERMYGELQGKNKKQTAEQFGEERVKLWRRSYKTAP
PQGESLYDTKQRTLPYFEKNILPQLQHGKNVFVSAHGNSLRSLIMDLEKLSEEEVLSLELPTGKPVVYQWKN
HKIEKHPEFFG

>core/682/3/Org3_Gene145

MSSQPLVTTSSSLSRYVVLTGEEKVACYKKAFNHIWHGAPAIILAAALLMFCIFGFVLGSILLGAPLEGASILY
DVILPWLLPSILVFVLLVLPLNIYAYSHHKQVLALHERITQSNYKEIYDHCEKEKKTPNKKALSLYIESQVLVP
EYSKRFSSMILGKTLKIIPKKDSPESLKHDELIQKALERAKENIYMNKNQREKRDEREAKKEAKNASKTNPLW
EGLGT

>core/683/3/Org3_Gene155

MSLLIEAKNLSKTIQQQNQNISILTDVSLSLHAGETISITGASGNGKTTLLHLLGTLDVPSSGSLRFFDKDLKNQ
DLANFRNQHIGFVFQNFYLLEDDTVLKNVLMPALIARKNISKGSPVYTRALELLDLVNLEDKVRTRCSKLSG
GEKQRVAIARALINEPAILLADEPSGNLDEETSEQIHNLLLEQASALCGILIVTHNKHLASRCSREGVLSNGKLF
FHNS

>core/684/3/Org3_Gene116

MTIRVRNLAYSVNKKKILDGVTFSLERGHITLFVGKSGSGKTMILRALAGLVQPTQGDIWIEGEAPALVFQQP
ELFSHMTVLGNCTHPQIHIKGRSTEEAREKAFELLHLLDIEEVAKNYPDQLSGGQKQRVAIVRSLCMDKHTLL
FDEPTSALDPFATASFRHLLETLRDQELTVGLTTHDMQFVHSCLDRIYLIDQGTVAGVYDKRDGELDSGHPLS
KYIHSAQ

>core/685/3/Org3_Gene822

MDLKLDEVASLLDVSEHTVLQWLKEGAIPSYSMNNEYRFSREEIEDWLLHNQALMIQERGEDKEALKDLSL
KYSLYKAIHRGGVLCDVVVHSKEEALQYASKYIAQKFQLDESVLFEMLSHRENLMSTGIGEGIALPHAKDFLI
NAYYDIVVPMFLAEPIEYGALDGKPVGILFFLFACQDKSHLNLVNKIVHLGMSLNARSFFKNYPNKDQLLAY
VKEWESQTH

>core/686/3/Org3_Gene395

MILRISTVSLLTSCSFSKNSRTCFVTPERITSQKDCPVLLHPKSTTISPPLYDWISPNREVITAYSFYCRGQGNSII
TPEGVLYDCDGLHHSITKEEFRYIHPRLIEVVRLLQQDHPKVSIIEAFCCPKHFHFLEASGISLSQLHLQGTAAT
FALDPPLPMEKLLATIKKLYKKNSDPSLSNFIVTEATLTNPELRLTQQDLGSHTEITVEILDNLQNKEALSSA

>core/687/3/Org3_Gene651

MENSQNFHDTLCQLLDRYSEELYPTLASLLNVTLPNTAISASVSSIPEKAVEVPNAEPQPITPPPPTNLSQEKTK
PSDWKCVPLHPDLSQNAILKEKYPALKDCSLPAPKIPCSIFVYEENNEEVLFFNRLAKILTQQLFPTKLTLIHAK
TNIFVNNPNFFLALAPLNVIRYKIPTTDYHQSLTQNGCIFLPLYSSLEYEKDSQLKRNLWAILNRLPFAYTPKSS

>core/688/3/Org3_Gene692

MKPQDLSPPFLWKERRPCIQDGVLYVPRHYFEHQNFSTSYHQEFFQNHTSIACELCSGNGDWVVAQAQKDP
QVLWIAVEQRFDRVRKIWSKMINHQIQNLRIVCGTAETFFQYYVPDQFLQRLVVNFPDPWPKMRHRKHRLL
QPSFVQEISRSLQDSAVFALATDDKTYLLESIEALQTHLAPRMETPYYIKMTDTYGNSWFENLWRTKGQEIFY
TEFIKKAGI

>core/689/3/Org3_Gene497

MIDPVECFPNLDGDAEAQSITQNSGTPLASELKKDISPFALGSYAAPKDTTLVQGFKPNPMAMMQDQNSNLI
DPELQEALESEELQEQINNLKGRLWDFRSTFEDSQTTAQFADEHFQAVGVIIDLINEDLNTIAEHTQQDARKE
DKEEGSVTRKIIDWVSSGEEVLNRALLYFSDRDGNRESLANFLKVQYAVQRATQRAELFASIVGTSVSSVKTI
MTTQLG

>core/690/3/Org3_Gene535

MTSWIELLDKQIEDQHMLKHEFYQRWSEGKLEKQQLQAYAKDYYLHIKAFPCYLSALHARCDDLQIRRQIL
ENLMDEEAGNPNHIDLWRQFALSLGVSEEELANHEFSQAAQDMVATFRRLCDMPQLAVGLGALYTYEIQIP
QVCVEKIRGLKEYFGVSARGYAYFTVHQEADIKHASEEKEMLQTLVGRENPDAVLQGSQEVLDTLWNFLSS
FINSTEPCSCK

>core/691/3/Org3_Gene444

MVLLSKFDFSGNKIGEVEVADSLFADEGDGLQLIKDYIVAIRANKRQWSACTRNRSEVSHSTKKPFKQKGTG
NARQGCLASPQFRGGGIVFGPKPKFNQHVRINRKERKAAIRLLLAQKIQTNKLTVVDDTVFVDALTAPKTQS
ALRFLKDCNVECRSILFIDHLDHVEKNENLRLSLRNLTAVKGFVYGININGYDLASAHNIVISKKALQELVERL
VSETKD

>core/692/3/Org3_Gene476

MQTLARLFGQSPFAPLQAHLEMVVSCVEYMLPIFTALRDGRYEELLEMAKLVSDKEYQADCIKNDMRNHLP
AGLFMPISRAGILEIISIQDSIADTAEDVAILLTIRRLNFYPSMETLFFRFLEKNLEAFELTMTLLHEFNQLLESSF
GGRKADKARLLVGRVAKSEHESDVLQRELMQIFFSDDFIIPEKEFYLWLQVIRRTAGISDSSEKLAHRINMTLE
EK

>core/693/3/Org3_Gene732

MSTTTVKHFIHTASRWEPVLKEIVASNYWHAQWINTLSFLENSGAKKISASEHPTEVKEEVLKHAAEEFRHG
HYLKTQISRISETSLPDYTSKNLLGGLLTKYYLHLLDLRTCRVLENEYSLSGQTLKTAAYILVTYAIELRASEL
YPLYHDILKEAQSKITVKSIILEEQGHLQEMERELKDLPHGEELLGYACQFEGELCLQFVERLEQMIFDPSSTF
TKF

>core/694/3/Org3_Gene140

MTKNAINSQTTTPQPNLTDAEPIASRAQCKSIAVIISLFALGMLLLCLGIILISIPIPGLAAQVALGLGIVSLILGIA
LANIGFLCLLLRCKQVPQKPDTLPSESSKQPSEGSTPTALPWQAGEFLEKVQVSATPILLPKNKDEELSAKVM
KEGAEAASSIKQAVLESTEKLIDARKQEESRREARKKIVAEEAEASRKRIQQQMAADQEALRKRKEEVAKRK

>core/695/3/Org3_Gene334

MKKYFITGLVILLPLAITIAIVTMIMNFLTQPFVGLASEFFEKFSFYTKHRALLKFVLQIILLFGLFFATVLLGFL
TRIMIFKSLLSIYDKILHRIPIIKTVYKAAQQVMTTIFGSKSGSFKQVVMVPFPNANVQCIGLVAGDAPTVCCT
GEKEDDPLVTVFIPTTPNPTSGFLTLFRKSDIVFLDMKIEDAFKYIISCGVLSTPMACPSSPLPDELHQDQGS

>core/696/3/Org3_Gene759

MGQKGCPIGFRTGVTKKWRSLWYGNKQEFGKFLIEDVRIRQFLRKKPSCQGAAGFVVRRMSGKIEVTIQTAR
PGLVIGKKGAEVDLLKEELRALTGKEVWLEIAEIKRPELNAKLVADNIARQIERRVSFRRAMKKAMQSVMD
AGAVGVKIQVSGRLAGAEIARSEWYKNGRVPLHTLRADIDYATACAETTYGIIGIKVWINLGENSSSTTPNNP
AAPSAAA

>core/697/3/Org3_Gene345

MTLYLGLNQKTARKYQAHYLPILTLFPYAKSTPQNKRALQFLPQATHVILTSPSSTHLFLSRMTSLLSKATLK
TKTYLCIGESTKERLLSFLGQVKYVVATQEIAEGIFPLLQALPSSARILYPHSSLARPVIREFLYNRFTFFSYPHY
TVKPRKLKKNILSKYKKIIFTSPSTVRAFAKIFPRFPEKTYWCQGRMTLQEFQKFSSQKQVSLLETLGKSRTSP

>core/698/3/Org3_Gene517

MLMMLMMIIGITGGSGAGKTTLTQNIKEIFGEDVSVICQDNYYKDRSHYTPEERANLIWDHPDAFDNDLLISD
IKRLKNNEIVQAPVFDFVLGNRSKTEIETIYPSKVILVEGILVFENQELRDLMDIRIFVDTDADERILRRMVRDV
QEQGDSVDCIMSRYLSMVKPMHEKFIEPTRKYADIIVHGNYRQNVVTNILSQKIKNHLENALESDETYYMVN
SK

>core/699/3/Org3_Gene180

MQSDLIQILLKETVNTLYMVSTAFFFSCAIGGMLGLGLFCTSPKSLNPKKSLYATISMILSFLTAIPFAILIVILFP
ITRWIVGTSLGPTASIVPLTIGAIPFVVTIVVDAFRNSALNYLESAVALGIPKRNILFGILLPESYPQLIFSLKSLV
VHLISCSTLAGFVGGGGLGQLLLQYGYYRFEWSVTTSVLVITLVLIESVRILGDFWGRRVLKYRGIL

>core/700/3/Org3_Gene852

MSTVTTEPCSSIHISLNNDWRDSQPYSLDRASELLHFRFLPSLVFSNWKVEQQIETLCHKSEKRRLISPLAKWL
GKLHKQDLLCPPAPPVSVCWINAHVGYGVFARDEIAPWTYIGEYTGILRHRQAIWMDENDYCFRYPMPLFTL
RYFTIDSGKQGNVTRFINHSEQPNAEAIGVFSEGLFHVIIRTIAPIYAGQEICYHYGPLYWKHRKKREEFIPEEE

>core/701/3/Org3_Gene445

MRSHISVMGKKEGMIHIFDKDGSLVACSVIRVEPNVVTQIKTKESDGYFSLQIGAEEMNAPAHTITKRVSKPK
LGHLRKAGGRVFRFLKEVRGSEEALNGVSLGDAFGLEVFEDVSSVDVRGISKGKGFQGVMKKFGFRGGPGS
HGSGFHRHAGSIGMRSTPGRCFPGSKRPSHMGAENVTVKNLEVIKVDLEKKVLLVKGAIPGARGSIVIVKHSS
RT

>core/702/3/Org3_Gene734

MQRIIIVGIDTGVGKTIVSAILARALNAEYWKPIQAGNLENSDSNIVHELSGAYCHPEAYRLHKPLSPHKAAQI
DNVSIEESHICAPKTTSNLIIETSGGFLSPCTSKRLQGDVFSSWSCSWILVSQAYLGSINHTCLTVEAMRSRNLN
ILGMVVNGYPEDEEHWLTQEIKLPIIGTLAKEKEITKTIISCYAEQWKEVWTSNHQGIQGVSGTPSLNLH

>core/703/3/Org3_Gene279

MNAVNTPKKILCIVADYREISPLIEQLDFTQINEHLYSYRCTDYHLDLYIVHVWGSTAVLNALQSYCQAYTDY
DLWINPGFVGACSPEIPLGQCYTIEKIANLTTDTPPVLSEDPPYIFDALPDSLPKSSLVTSPVLYHYGFHKTFKL
LDMEGYAIASQAAEHHIPCSFLKITSDYTVPGDCPFSRLEEVSQKLTQTLVELLPELMERAIPPKLLLPCP

>core/704/3/Org3_Gene318

MLGSLPCYPGAGNIEEYKNRYFYCQLCAEVVSPYVVPVIVVDVQGAPPTGILQVLRCKQHKFQGLPVHGPIT
SLWALEPVGKGAPQLESAMYELCSQVRNFDICSIVSWVFGGLCIFAGLIVGVMVEAPLIAGLSAWVIPCIIGGV
GAILCLFAILMAYLGRGRVREWLNLSHEYITQCHCRQIQAHSQNYSVITEYPATCALSQPITKLPNGSRRDN

>core/705/3/Org3_Gene890

MDHWLAIARLLLRGCGYTLCVSGIGILCGSILGLLIGTVTSLYFPSKLTKLLANSYVTVIRGTPLFIQILIIYFGLP
EVLPIEPTPLVAGIIALSMNSAAYLAENIRGGINSLSIGQWESAMVLGYKKYQIFVYIIYPQVFKNILPSLTNEFV
SLIKESSILMVVGVPELTKVTKDIVSRELNPMEMYLICAGLYFLMTSSFSCISRLSEKRRSYDN

>core/706/3/Org3_Gene621

MKRVIYKTIFCGLTLLTSLSSCSLDPKGYNLETKNSRDLNQESVILKENRETPSLVKRLSRRSRRLFARRDQTQ
KDTLQVQANFKTYAEKISEQDERDLSFVVSSAAEKSSISLALSQGEIKDALYRIREVHPLALIEALAENPALIEG
MKKMQGRDWIWNLFLTQLSEVFSQAWSQGVISEEDIAAFASTLGLDSGTVASIVQGERWPELVDIVIT

>core/707/3/Org3_Gene934

MQKLVHNIWKKFYSFSSAIAICIVLASFLSLKIVSNTYKHSQAKRNSILLLTRAAEVAVSQGFLPSKSALSSLEQ
AYHLGGESMKPYAGYLASCFYIHNEPLRGAYYAGLAYNNSQALQLPHPIQKLLKEISEAQADQLYDVALSKS
YQLLQTANSSPEYPTLSFLTLLRVIELKELLHQDVSQDFAALKSSPLFHQFERMYSDGEWTLSKRFGKKG

>core/708/3/Org3_Gene383

MIITIDGPSGTGKSTTAKALADHLHFNYCNTGKMYRTLAYARLQSPWATLPLTKFLEEPPFSFTFATGQPLESF
FNGHLLTSELTTQEVANAASELSQLPEVRAFMQDLQRRYAQLGNCVFEGRDMGSKVFPNADLKIFLTSSPEV
RAQRRLKDLPEGTLSPEQLQAELVKRDAADAQRAHDPLVIPENGIVIDSSDLTIRQVLEKILALLFRNEL

>core/709/3/Org3_Gene268

MRKMLVLLASLGLLSPTLSSCTHLGSSGSYHPKLYTSGSKTKGVIAMLPVFHRPGKSLEPLPWNLQGEFTEEI
SKRFYASEKVFLIKHNASPQTVSQFYAPIANRLPETIIEQFLPAEFIVATELLEQKTGKEAGVDSVTASVRVRVF
DIRHHKIALIYQEIIECSQPLTTLVNDYHRYGWNSKHFDSTPMGLMHSRLFREVVARVEGYVCANYS

>core/710/3/Org3_Gene646

MSKKPLYVAHPWHSPTLTQDNYESLCCYIEITPYDSVKFELDKATGLLKVDRPQKFSNFCPCLYGLLPQTYC
GTASGNYSGEQTRREGIQGDKDPLDVCVLTEKNIHHGNILLQARPIGGLRIIDSGEADDKIIAVLEDDLVFAEIE
DISDCPGTVLDMIQHYFLTYKATPNHLIKGSPAKIEIVGIYGKKEAQKVIQLAHEDYLSYIGDTAEVN

>core/711/3/Org3_Gene474

MSVNPSGNSKNDLWITGAHDQHPDVKESGVTSANLGSHRVTASGGRQGLLARIKEAVTGFFSRMSFFRSGAP
RGSQQPSAPSADTVRSPLPGGDARATEGAGRNLIKKGYQPGMKVTIPQVPGGGAQRSSGSTTLKPTRPAPPPP
KTGGTNAKRPATHGKGPAPQPPKTGGTNAKRAATHGKGPAPQPPKGILKQPGQSGTSGKKRVSWSDED

>core/712/3/Org3_Gene71

MNTSISEIQRFLSMIAFEKELVSEDFSVVAGIDEAGRGPLAGPVVASACILPKGKVFPGVNDSKKLSPKQRAQV
RDALMQDPEVCFGIGVISVERIDQVNILEATKEAMLQAISSLPISPDILLVDGLYLPHDIPCKKIIQGDAKSASIA
AASILAKEHRDDLMLQLHRLYPEYGFDRHKGYGTSLHVEAIRRYGPSPCHRKSFSPIKQMCAIV

>core/713/3/Org3_Gene152

MTKGSVFIIMGPPGSGKGTQSQYLANRIGLPHISTGDLLRAIIREGTPNGLKAKAYLDKGAFVPSDFVWEILKE
KLQSQACSKGCIIDGFPRTLDQAHLLDSFLMDVHSNYTVIFLEISEDEILKRVCSRFLCPSCSRIYNTSQGHTEC
PDCHVPLIRRSDDTPEIIKERLTKYQERTAPVIAYYDSLGKLCRVSSENKEDLVFEDILKCIYK

>core/714/3/Org3_Gene292

MTSKKSYKSYFFDPLWSNNQILIAILGICSALAVTTTVQTAITMGIAVSIVTGCSSFFVSLLRKFTPDSVRMITQ
LIIISLFVIVIDQFLKAFFFDISKTLSVFVGLIITNCIVMGRSESLARHVTPIPAFLDGFASGLGYGWVLLVIGVIRE
LFGFGTLMGFRIIPQFVYASETHPDGYQNLSLMVLAPSAFFLLGIMIWLVNIRDSKKRKR

>core/715/3/Org3_Gene15

MYFYKYVIIDTSGYYPFLACVDNQQVLEHWSLPVGPDLGIVLEFLFKSKNLSFQGVAVALGPGNFSATRIGIS
FAQGLAMAKNVPLLGYSSLEGYLLSKDEKKALMLPLGKRGGVLTLSSEIPEEGLNEKRRGVGPGALLSYEEA
SDYCVAHGYYHVISPNPQLFASSFSDKITVEEVAPSVEQIRRHVISQFMFVEYDKQLSPDYRSYSCIF

>core/716/3/Org3_Gene300

MATSVAPSPVPESSPLSHATEVLNLPNAYITQPHPIPAAPWETFRSKLSTKHTLCFALTLLLTLGGTISAGYAG
YTGNWIICGIGLGIIVLTLILALLLAIPLKNKQTGTKLIDEISQDISSIGSGFVQRYGLMFSTIKSVHLPELTTQNQ
EKTRILNEIEAKKESIQNLELKITECQNKLAQKQPKRKSSQKSFMRSIKHLSKNPVILFDC

>core/717/3/Org3_Gene384

MIFRICKFFTWVAFSLFYKLKVYGVKKNFIKGPAIIAVNHNSFLDPIALHMCVHECIYHLARASLFNIPWLWK
QWGCFPVRQDEGNSAAFKIASRLFNKRKKLVIYPEGARSPDGQLQPGKVGIGMMAAKSRVPIIPVYIRGTFEA
FNRHQKIPHVWKTITCVFGTPMYFDDIIQNPEIKNKETYQIITNQTMNKIAELKAWYESGCKGDVP

>core/718/3/Org3_Gene393

MIKSSLILLSGGQGTRFGSKIPKQYLPLNGTPLVLHSLKILSSLPQIAEVIVVCDPSYQETFQEYPVSFAIPGERR
QDSVFSGLQQVSYPWVIIHDGARPFIYPDEIHDLLETAEKIGATALASPIPYTIKQRNPVRTLDRDNLAIIHTPQ
CIKTEILREGLALAKEKQLTLVDDIEAAEIIGKPSQLVFNKHPQIKISYPEDLTIAQALL

>core/719/3/Org3_Gene927

MMNYEDAKLRGQAVAILYQIGAIKFGKHILASGEETPLYVDMRLVISSPEVLQTVATLIWQLRPSFNSSLLCG
VPYTALTLATSISLKYNIPMVLRRKELQNVDPSDAIKVEGLFTPGQTCLVINDMVSSGKSIIETAVALEENGLV
VREALVFLDRRKEACQPLGPQGIKVSSVFTVPTLIKALIAYGKLSSGDLTLANKISEILEIES

>core/720/3/Org3_Gene515

MARYCGPKNRVARRFGANIFGRSRNPLLKKPHPPGQHGMQRKKKSDYGLQLEEKQKLKACYGMIMEKQLV
KAFKEVIHKQGNVAQMFLERFECRLDNMVYRMGFAKTIFAAQQLVAHGHILVNGRRVDRRSFFLRPGMQIS
LKEKSKRLQSVKDALESKDESSLPSYISLDKTGFKGELLVSPEQDQIEAQLPLPINISVVCEFLSHRT

>core/721/3/Org3_Gene591

MKQFILRTLNALFPNPKPSLEGWSSPFQLLIAILLSGNSTDKAVNSVTPQLFAKAPDAQSILDLPPGKLYQLIAP
CGLGERKSAYIYQLSQILVRDFHGEPPNDMALLTQLPGVGRKTASVFLGIAYGKPTFPVDTHILRLAQRWKIS
EKKSPSAAEKDLARFFGHENTPKLHLQLIYYARQYCPALHHKIDNCPICSYLAKEANSTRT

>core/722/3/Org3_Gene50

MSVQVKLTKNSFRLEKQKLARLQTYLPTLKLKKALLQAEVQNAVKDAAECDKDYVQAYERIYAFAELFSIP
LCTDCVEKSFEIQSIDNDFENIAGVEVPIVREVTLFPASYSLLGTPIWLDTMLSASKELVVKKVMAEVSKERLK
ILEEELRAVSIRVNLFEKKLIPETTKILKKIAVFLSDRSITDVGQVKMAKKKIELRKARGDECV

>core/723/3/Org3_Gene47

MANLNADGKLKQICDALRLDTLKPAEDEAAALLHNAKEQAKRIIQEAQEEARKILETAEERAHQKIKQGEVA
LSQAGKRALEALKQAVENKIFRESLVEWLEHVTTDPEVSTKLIQALVQALEAQGVSGNLTAYIGKHVSPRAV
NELLGKAVTTKLRKKSVVVGSFVGGVQLKVEEKNWVLDLSSSALLEIFTRYLQKDFREMIFQGS

>core/724/3/Org3_Gene526

MTYLASSIFSPEDFLYPEIISKAHYTWDILDLMDQMLENHVFSGIHGTVESGVTLKNIEKIEIAEDAYVESGAYI
VGPCILGSQTEVRHGAYLRGNVITGSRCVVGHCTEIKNSYLGHHTKAAHFAYLGDSVLSSEVNLGAGVRCA
NFRLDGRNIYVRSTSDKSKKIDTGRRKLGAFLGKGVAIGCNVVINPGQHILPHTRIRPGQVI

>core/726/3/Org3_Gene28

MSFVPYSLPELPYDYDALEPVISSEIMILHHQKHHQIYINNLNAALKRLDAAETQQNLNELIALEPALRFNGGG
HINHSLFWETLAPIDQGGGQPPKHELLSLIERFWGTMDNFLKKLIEVAAGVQGSGWAWLGFCPAKQELVLQ
ATANQDPLEPLTGKLPLLGVDVWEHAYYLQYKNVRMDYLKAFPQIINWGHIENRFSEIISSK

>core/727/3/Org3_Gene423

MYDYIRGTLTYVHTGAIVIECQGIGYHIAITERWAIECIRALHQDFLVFTHVIFRETEHLLYGFHSREERECFRI
LISFSGIGPKLALAILNALPLKVLCSVVRSEDIRALASVSGIGKKTAEKLMVELKQKLPDLLPLDSRVETSQTHT
TSSCLEEGIQALAALGYSKIAAERMIAEAIKDLPEGSSLTDILPIALKKNFSGVNKD

>core/728/3/Org3_Gene864

MKIVIASSHGYKIRETKTFLKRLGDFDIFSLSDFPDYKLPQEQEDSITANALTKGIHAANHLGCWVIADDTMLR
VPALNGLPGPLSANFAGVGAYDKDHRKKLLDLMSSLESLVDRSAYFECCVVLVSPNQEIFKTYGICEGYISHQ
EKGSSGFGYDPIFVKYDYKQTFAELSEDVKNQVSHRAKALQKLAPHLQSLFEKHLLTRD

>core/729/3/Org3_Gene669

MGLPNYITFSRLFITPIFMILYLKGKWFGITPVVLPYVLLALLAISELTDAIDGYVARKFSQVTDLGKLLDPMA
DSIYRISIYLTFTQPPVNLPLLLVFIFLARDSVISTLRTVCAFRGRVVAARASGKLKAILQGVSFFLILLVMIPHSL
GLLSQNGLEIFASVTVSIIAVYSIASGIEYFWMNKNFLSQRAKTKDSEKNHESKD

>core/730/3/Org3_Gene175

MFIVIEGGEGSGKSSLAKALGDQLVAQDRKVLLTREPGGCLIGERLRDLILEPPHLELSRCCELFLFLGSRAQH
IQEVIIPALRDGYIVICERFHDSTIVYQGIAEGLGADFVADLCSKVVGPTPFLPNFVLLLDIPADIGLQRKHRQK
VFDKFEKKPLSYHNRIREGFLSLASADPSRYLVLDARESLASLIDKVMLHTQLGLCT

>core/731/3/Org3_Gene72

MNKILVDSPFSPDHQKCCPKLFTISAPAGVGKTTLVRMLEQEFSSAFAETISVTTRKPREGEVPGKDYHFVSHE
EFQRLLDRQALLEWVFLFGECYGTSMLEIERIWSLGKHAVAVIDIQGALFIRSRMPSVSIFIAPPSQEELERRLA
SRGSEEGSQRKERLEHSLIELAAANQFDYVIINDDLNQAYRVLKSIFIAEEHRNIL

>core/732/3/Org3_Gene550

MHAKLSFFILLSLLFSGIDCSRLHAAGRSPSLQGVLAEIEDISAKLASHEVEIVMLSERLDEQDSKCQKWTAAK
PETLAQKIRELESDQKALAKTLAVLTTSVKDLQTNLQSKLQEIQKDHRALAQDLRLVRRSLLALVDSSSPGA
YADFSDPVPENIYIVREGDSLSKIAKKYKLSVTELKKINKLDSDAIYAGQRLCLQRNKQ

>core/733/3/Org3_Gene257

MIGAQKKQSGKKTASRAVRKPAKKVAAKRTVKKATVRKTAVKKPAVRKTAAKKTVAKKTTAKRTVRKTV
AKKPAVKKVAAKRVVKKTVAKKTTAKRAVRKTVAKKPVARKTTVAKGSPKKAAACALACHKNHKHTSSC
KRVCSSTATRKHGSKSRVRTAHGWRHQLIKMMSR

>core/734/3/Org3_Gene190

MTSPIPFQSSGDASFLAEQPQQLPSTSESQLVTQLLTMMKHTQALSETVLQQQRDRLPTASIILQVGGAPTGG
AGAPFQPGPADDHHHPIPPPVVPAQIETEITTIRSELQLMRSTLQQSTKGARTGVLVVTAILMTISLLAIIIIILAV
LGFTGVLPQVALLMQGETNLIWAMVSGSIICFIALIGTLGLILTNKNTPLPAS

>core/735/3/Org3_Gene599

MTLVPYVVEDTGRGERAMDIYSRLLKDRIVMIGQEITEPLANTVIAQLLFLMSEDPKKDIQIFINSPGGYITAG
LAIYDTIRFLGCDVNTYCIGQAASMGALLLSAGTKGKRHALPHSRMMIHQPSGGIIGTSADIQLQAAEILTLKK
HLANILSECTGQPVEKIIEDSERDFFMGAEEAISYGLIDKVVTSAKETNKDTSST

>core/736/3/Org3_Gene928

MLKLLKVSITGDLSSGKTEACQVFQELGAYVVSADEISNSFLIPHTRIGRRVIDLLGSDVVVDGAYDAQAIAA
KVFYNSVLLQGLEAILHPEVCRIIEEQYHQSIQDGNYPLFVAEVPLLYEIHYAKWFDSVILVMANEDIRRERFM
KKTGRSSEDFDQRCSRFLNVEEKLAQADVVVENNGTKKELHQKIEEYFYALKGAL

>core/737/3/Org3_Gene834

MLILLNLSLLFYVLFDSPGSIPVFVALLKNFSRKKQQRVILRECLFALGALILFVTFGRSFFQFLDISLYAFQIIG
GFLLFTVSIKMMLAPMPEKAKDDTSKTEPIFFPLAFPVITGPAVITALLSYMEEGIYSREIIFTAMIIAWAFSLFT
LLCSSFFDRLSGNFGLLALERLFGIALLLMSVNLMLKGISIAFNIGFYIG

>core/738/3/Org3_Gene354

MFSGIIQELGEVCFFEAQGNGLSLGIKSTPLFVTPLVTGDSVAVDGVCLTLTSCNESKIFFDVIPETLACTTLGE
KRCSDQVNLEAALKMGDSIGGHLLSGHVFGTAEIFLIKENRYYFRGSKELSQYLFEKGFIAIDGISLTLVSVDS
DTFSVGLIPETLQRTTLGKKREGERVNIEIDMSTKIQVDTVKRILASSGKD

>core/739/3/Org3_Gene693

MFAYRTLLTHNVVQVSHEIFKTTVVPGDTVIDATCGNGNDSLFLARLLQGEGRLVVYDIQKEALSNALLLFE
THLSEQERSVIEMKEQSHEHILEKDVKLIHYNLGYLPKGNKEITTLARTTEISLEYALNIVRPDGLITVVCYPGH
PEGEKETHSVESLAQRLHPKEWCVSSFYVANRCRAPRLFIFQRQGSESSVDKG

>core/740/3/Org3_Gene195

MTRYPDYLSKLIFFLRKLPGIGFKTAEKLAFELISWDSEQLKILGNAFHNVASERSHCPLCFTLKESKEADCHF
CREERDNQSLCIVASPKDVFFLERSKVFKGRYHVLGSLLSPITGKHIENERLSILKSRIETLCPKEIILAIDATLEG
DATALFLKQELQHFSVNISRLALGLPIGLSFDYVDSGTLARAFSGRHSY

>core/741/3/Org3_Gene402

MGFACRYLFFFIVLFASGSFGNQLLSVPCWLSEEESFYTHRFDFSKSYPDMENMEIQAQRKKRVEFNLTGEFP
KLETLNYQGSFGHLRAKCRGVYPVLYALNFSCSSCKMDMDFRGKWNRSSTITISNQKESINLKLPKDVGVIV
NTKTSLKGNVCPGSTFIKQGWGVWNKIYHNDLVGFSEVTLIFNVSSEGGTITFS

>core/742/3/Org3_Gene465

MRLFSLGTIYLFFSLALSSCCGYSILNSPYHLSSLGKSLLQERIFIAPIKEDPHGQLCSALTYELSKRSFAISGRSS
CAGYTLKVELLNGIDKNIGFTYAPNKLGDKTHRHFIVSNEGRLSLSAKVQLINNDTQEVLIDQCVARESVDFD
FEPDLGTANAHEFALGQFEMHSEAIKSARRILSIRLAETIAQQVYYDLF

>core/743/3/Org3_Gene101

MNWVPKTIDHVDPESEIDIRKVVSCYKLIKECQPEFRSLISELLGVIRCGLRLLKRSKYQEQARTVSDEDAPLF
CLTRSYYQDGYLTPLRAGPRDLINHYIHLRRRENPKHFFSPKHPCYYARLAFNESVCVYRELFDIERLTKMYV
EGDYSKEQEKNLQAILSFVKTLDEGKDFLIEHKDTDLIGRGFTDVFCT

>core/744/3/Org3_Gene668

MRIALSLLSLLMIFPIFGEESRPGSEDGNSNTQEIVGSQDTQVCLYHSYEQGLQASRIEGKPLVIVVLCNSGDD
GQACTIGLSETCEEVLSVLSGSIFSELANFVVLVPSGVNPLIYPPIEDPILAEIVKFKELFKDESFPTGLSIIVVGV
TPEGPGDIIEVSPVSLTVEEEETLPSEQTTEVESTSELQSEDPAIA

>core/745/3/Org3_Gene372

MSSNLHPVGGTGTGAAAPESVLNIVEEIAASGSVTAGLQAITSSPGMVNLLIGWAKTKFIQPIRESKLFQSRAC
QITLLVLGILLVVAGLACMFIFHSQLGANAFWLIIPAAIGLIKLLVTSLCFDEACTSEKLMVFQKWAGVLEDQL
DDGILNNSNKIFGHVKTEGNTSRATTPVLNDGRGTPVLSPLVSKIARV

>core/746/3/Org3_Gene9

MSLPLVLGSSSPRRKFILEKFRVPFTVIPSNFDESKVSYSGDPIAYTQELAAQKAYAVSELHSPCDCIILTGDTIV
SYDGRIFTKPQDKADAIQMLKTLRNQTHDVVTSIAVLHKGKLLTGSETSQISLTMIPDHRIESYIDTVGTLNNC
GAYDVCHGGLILKKVHGCVYNVQGLPIQTLKYLLEELNIDLWDYSI

>core/747/3/Org3_Gene333

MLQEHFFLSEDVITLAQQLLGHKLITTHEGLITSGYIVETEAYRGPDDKACHAYNYRKTQRNRAMYLKGGSA
YLYRCYGMHHLLNVVTGPEDIPHAVLIRAILPDQGKELMIQRRQWRDKPPHLLTNGPGKVCQALGISLENNR
QRLNTPALYISKEKISGTLTATARIGIDYAQEYRDVPWRFLLSPEDSGKVLS

>core/748/3/Org3_Gene549

MTLSLVGKEAPDFVAQAVVNGETCTVSLKDYLGKYVVLFFYPKDFTYVCPTELHAFQDALGEFHTRGAEVI
GCSVDDIATHQQWLATKKKQGGIEGITYPLLSDEDKVISRSYHVLKPEEELSFRGVFLIDKGGIIRHLVVNDLP
LGRSIEEELRTLDALIFFETNGLVCPANWHEGERAMAPNEEGLQNYFGTID

>core/749/3/Org3_Gene288

MFRRTGKGPFEDVQTLYEEETSSPSSYSPYSRSERPETPPSLFDNPKASEARPLNHNLTEESSLPQWSSTPRTES
LLPLEEPETTLGEGVTFKGELAFERLLRIDGTFEGILVSKGKIIIGPKGVVKADIQLQEAIIEGVVEGNITVSGKV
ELRGGAIIKGDIQANTLCVDEGVRILGYLAIAGITDHSERERDL

>core/750/3/Org3_Gene726

MAYGTRYPTLAFHTGGIGESDDGMPPQPFETFCYDSALLQAKIENFNIVPYTSVLPKELFGNIVPVDTCVKSFK
HGAVLEVIMAGRGAALSDGTHAIATGIGICWGKDKNGELIGGWAAEYVEFFPTWINDEIAETHAKMWLKKS
LQHELDLRSIAKHSEFQFFHNYINIKQKFGFCLTALGFLNFENAEPAKVN

>core/751/3/Org3_Gene538

MKKWISILILSFLSLLSILPVLAITINHVKISQRWSDLNSQILTLKVIRDHEDQVIKHNARISKDRNNLSIESLNAS
CKQLRPLSKERERLNKLNSNSLLAQSKEVWERKRALEKSNHQLVWNCEQMHNDFAFVRLEQATEMDNEDI
ESLFSLFNPENPVAPLVFFTCWKMTKQTTPLGNEVWLTHAEAISRWI

>core/752/3/Org3_Gene551

MNIHSLWKLCTLLALLALPACSLSPNYGWEDSCNTCHHTRRKKPSSFGFVPLYTEEDFNPNFTFGEYDSKEEK
QYKSSQVAAFRNITFATDSYTIKGEENLAILTNLVHYMKKNPKATLYIEGHTDERGAASYNLALGARRANAI
KEHLRKQGISADRLSTISYGKEHPLNSGHNELAWQQNRRTEFKIHAR

>core/753/3/Org3_Gene289

MVLFSLLFPKLCYGCQAPGAYFCSNCLEKLLVEDREGRCLHCFRYLGSSETRLCSQCSPSSQLQAFSLYLPSQ
TALSVYARACEGKRPALQFFSKSIAFELASLDETPSCIAYITSTISRKIVVEVAKLEKLLRIPLWPWLPKKRQIE
KLPKGEGICFLSAYPLSQKWMQTIVGGSASPLVSISLFLSQNDQ

>core/754/3/Org3_Gene166

MKRYVVGISGASGVILAVKLIKELVNAKHQVEVIISPSGRKTLYYELGCQSFDALFSEENLEYIHTHSIQAIESS LASGSCPVEATIIIPCSMTTVAAISIGLADNLLRRVADVALKERRPLILVPRETPLHTIHLENLLKLSKSGATIFPP MPMWYFKPQSVEDLENALVGKILAYLNIPSDLTKQWSNPE

>core/755/3/Org3_Gene613

MKVIYYEIEEIPSTNTMAKSYMHLWDPYALTVISTKCQTAGTGKFGKSWKSSKGDLLNTFCFFITDLHIDVSR LFRLGTEAVVALCKDLGITEAKIKWPNDVLVHGEKLCGVLPETLPVEGLLGVVLGIGLNGNTTKQALKDVG QPATSLQEILGHPIDLETTRELLIHHLLGVLQENLPDSLATKSNRGNI

>core/757/3/Org3_Gene344

MADGEVHKLRDIIEKELLEARRVFFSEPVTEKSASDAIKKLWYLELKDPGKPIVFVINSPGGSVDAGFAVWDQ IKMLTSPVTTVVTGLAASMGSVLSLCAAPGRRFATPHSRIMIHQPSIGGPITGQATDLDIHAREILKTKARIIDV YVEATNQPRDIIEKAIDRDMWMTANEAKDFGLLDGILFSFNDL

>core/758/3/Org3_Gene36

MIKKFFIYSLIFSCSFSAPLKGICNEDVSSQSRIEEDPEVLITQLNELIETPIEEGKEIRNELQAISDGQKSSEEIEES CGTSDSEGLSEKTDKESSNEYVLDFFDSMVQRLEGISKMCQSGQVAQIIDCFNREFDIRNRELELKNRELELRE KDLEFKKSILDWNKEKVSRELAFQREQDIKQTLMLLKK

>core/759/3/Org3_Gene263

MSIKEDKWIREMALNADMIHPFVNGQVNVNEETGEKLISYGLSSYGYDLRLSREFKVFTNVYNSVVDPKCFT EDIFISITDDVCIVPPNSFALARSVEYFRIPRNVLTMCIGKSTYARCGIIVNVTPFEPEWEGHVTIEISNTTPLPAK IYANEGIAQVLFFESSTTCEVSYADRKGKYQKQQGITVPCV

>core/760/3/Org3_Gene632

MVRVSTSEFRVGLRIEIDGQPYLILQNDFVKPGKGQAFNRIKVKNFLTGRVIERTYKSGESVETADIVERSMRL LYTDQEGATFMDDETFEQEVVFWEKLENIRQWLLEDTIYTLVLYNGDVVAVEPPIFMELSIAETAPGVRGDT ASGRVLKPAVTNTGAKIMVPIFIDEGELVKVDTRTGSYESRVSK

>core/762/3/Org3_Gene992

MMKIPYARLEKGSLLVASPDINQGVFARSVILLCEHSLNGSFGLILNKTLGFEISDDIFTFEKVSNHNIRFCMGG PLQANQMMLLHSCSEIPEQTLEICPSVYLGGDLPFLQEIASSESGPEINLCFGYSGWQAGQLEKEFLSNDWFLA PGNKDYVFYSEPEDLWALVLKDLGGKYASLSTVPDNLLLN

>core/763/3/Org3_Gene750

MIRRLEYYGSPILRKKSSPIAEITDEIRNLVSDMCDTMEAHRGVGLAAPQVGKNVSLFVMCVDRETEDGELIF SESPRVFINPVLSDPSETPIIGKEGCLSIPGLRGEVFRPQKITVTAMDLNGKIFTEHLEGFTARIIMHETDHLNGV LYIDLMEEPKDPKKFKASLEKIKRRYNTHLSKEELVS

>core/764/3/Org3_Gene695

MALNFKINRQIRAPKVRLIGSAGEQLGILAIKDALDLAREAGLDLVEVASNSEPPVCKIMDYGKYRYGLTKKE
KDSKKAQHQVRIKEVKLKPNIDENDFSTKLKQARTFVEKGNKVKITCMFRGRELAYPEHGFKVVQKMSQGL
EDIGFVEAEPKLAGRSLICVVAPGTVKTKKKQEKSHAQDENQ

>core/766/3/Org3_Gene411

MRILAGKYKGKSLKTFSNPHIRPTSGLVKEAFFSICREDIEGAAFLDLFAGMGAIGFEALSRGAASVVFVDISIK
AIQLIHTNSALLGEQLPVVIFRQDAQSAIQRLIKQKRSFDLIYIDPPYELCNCYVETLLQKIVSGNILNPEGTLFL
ENASDEEIACEGLTLRRRRKLGKTYLAEYIVEKDP

>core/768/3/Org3_Gene840

MELVVTSRETGKKSFLKKIRQQGGIPAVVYSAGKSLANITVDALVFKKFLSNLESGALSSTVFSLSYEGRIIKA
LVKDIQYQITTYDVIHLDFEELVEDRPVKLNIPIRCINAVDCIGVKLGGSLRQVIRAVRVVCKPKDIVPFLELDV
RSVGLSQTRKLSDIKIPAGIETITPLKEVAITVSRR

>core/770/3/Org3_Gene114

MVLSSQLSVGMFISTKDGLYKVTSVSKVAGPKGESFIKVALQAADSDVVIERNFKATQEVKEAQFETRTLEY
LYLEDESYLFLDLGNYEKLFIPQEIMKDNFLFLKAGVTVSAMVYDNVVFSVELPHFLELMVSKTDFPGDSLSL
SGGVKKALLETGIEVMVPPFVEIGDVIKIDTRTCEYIQRV

>core/771/3/Org3_Gene330

MTDTPPENEEQHESNVQNENEVEHLQQEIVTLKTELKEKNDKYLMALAESENSRKRLQKERQELMQYALEN
TLIDFLNPIESMEKALGFATQMSDDVKNWALGFNMILNQFKQIFEEKGIIEYSSIGQKFNPFLHEAVQTEETSE
VPEGTILEEFAKGYKIGERPIRVAKVKVAKAPTPKENKE

>core/772/3/Org3_Gene565

MAAKTKTLELEDNVFLLLEGNLKRIFATPIGYTTFREFQNVVFNCANGQQEIANFFFEMLINGKLTQELAPQQ
KQAAHSLIAEFMMPIRVAKDIHERGEFINFITSDMLTQQERCIFLNRLARVDGQEFLLMTDVQNTCHLIRHLL
ARLLEAQKNPVGEKNLQEIQEEITSLKNHFDELTKALQ

>core/773/3/Org3_Gene389

MVETVLHNFQRYLSKYLYRVFRFPCRKKTFLSSHRVLARPSFPVDYCPGKIYDLQEIYEELNAQLFQGALRLQ
IGWFGRKATRKGKSVVLGLFHENEQLIRIHRSLDRQEIPRFFMEYLVYHEMVHSVVPREYSLSGRSIFHGKKF
KEYEQRFPLYDRAVAWEKANAYLLRGYKKRVGGGYGRA

>core/775/3/Org3_Gene742

MNFAKIDHNHLYLTCLGDLGVACPILSTDCLPNYSEKASHEVLVYSKFRCISGEPSRLATSGNDTYYSIVSLPI
GLRYEVTSPSGRHDFNIDMHVAPKIGAVLSHGTREAKEIPGSSKDYAFFSLTARESLMISEKLAMTFQVSEVIQ
NCYSQCTKVTKTNLKEQYRHLSHNTGFELSVKSAF

>core/776/3/Org3_Gene434

MSRKAREPILLPQGVEVSIQDDKIIVKGPKGSLTQKSVKEVEITLKDNSIFVHAAPHVVDRPSCMQGLYWALIS
NMVQGVHLGFEKRLEMIGVGFRASVQGAFLDLSIGVSHPTKIPIPSTLQVSVEKNTLISVKGLDKQLVGEFAA
SIRAKRPPEPYKGKGIRYENEYVRRKAGKAAKTGKK

>core/777/3/Org3_Gene820

MYKWYVVQVFTAQEKKVKKALEDFKESSGMTDFIQEIILPIENVMEVKKGEHKVVEKYIWPGYLLVKMHLT
DESWLYVKSTAGIVEFLGGGVPVALSEDEVRSILTDIEEKKSGVVQKHQFEVGSRVKINDGVFVNFIGTVSEV
FHDKGRLSVMVSIFGRETRVDDLEFWQVEEVAPGQESE

>core/778/3/Org3_Gene400

MSLLNLPSSQDSASEDSTSQSQIFDPIRNRELVSTPEEKVRQRLLSFLMHKLNYPKKLIIIEKELKTLFPLLMRK
GTLIPKRRPDILIITPPTYTDAQGNTHNLGDPKPLLLIECKALAVNQNALKQLLSYNYSIGATCIAMAGKHSQV
SALFNPKTQTLDFYPGLPEYSQLLNYFISLNL

>core/779/3/Org3_Gene285

MVEIFNYSTSIYEQHASNNRIVSDFRKEIQMEGISIRDVAKHAQILDMNPKPSALTSLLQTNQKSHWACFSPPN
NFYKQRFSTPYLAPSLGSPDQQDEDIEKISSFLKVLTRGKFSYRSQITPFLSYKDKEEEEDEDPEEDDDDPRVQ
QGKVLLKALDLGVKSTNVMIDYVISRIFQFVQG

>core/780/3/Org3_Gene136

MSKESIRSYSEISTPTPIFRETPSKEGVAYKLQLRSPAKDCILRNRVSLKGALLRSIPFYGSFLGAKRIHSAWSA
KDAPCTTRVYHYLVGGLELLGLGVVVLACKVLATALKFLFSKASSKIKQMKWREKARNLAAKDTVQSIKEF
CSVDLTSCFTRCFRLRNRVVEEGASENQTVREIIV

>core/781/3/Org3_Gene537

MTDPKIEKSALRKLFISIRRDLSEERKHEASSAVASFVRSFSKESVVLSFVSFNHEIDMQEANRILIQKCTLALP
KIDQENLYPVLIPSIDDLISVVHPKDPFSKQTPISSDKITHVLVPGLAFDQQGYRLGYGHGFYDRWLAQHPYPS
IRTIGIGYCEQKIDRLPQESHDIPLSQIYLC

>core/782/3/Org3_Gene436

MSRLKKFYTEEIRKSLFEKFGYANKMQIPVLKKIVLSMGLAEAAKDKNLFQAHLEELTMISGQKPLVTKARN
SIAGFKLREGQGIGAKVTLRGIRMYDFMDRFCNIVSPRIRDFRGFSNKGDGRGCYSVGLDDQQIFPEINLDRV
KRTQGLNITWVTTAQTDDECTTLLELMGLRFKKAQ

>core/784/3/Org3_Gene671

MAKLIVAIGNPRHGYANTRHNAGFLLADRLVEELQGPPFKPLSKCHALMTLVESSSGPLVFIKPTTFVNLSGK
AVVLAKKYFNVALSHILVLADDVNRSFGKLRLCFNGGSGGHNGLKSITASLGSNEYWQLRFGVGRPLEEGV
ELSNFVLGKFSEEENLQLGSIFVEASTLFTEWCSKF

>core/785/3/Org3_Gene489

MSVLQDTEKKMAAALDFFHKEVKSFRTGKAHPALVETVVVDVYGTTMRLSDIASISVADLRQLVISPYDGN
NASAIAKGIIAANLNLQPEVEGSIIRIKVPEPTADYRQEMIKQLRRKCEEAKINVRNIRREANDKLKKDSALTE
DVVKGNEKKIQELTDKFCKQLDELTKQKEAEIASI

>core/786/3/Org3_Gene608

MTTPQSPGSLSQSHLPHPHDPWDTEPTSLPEDPNDKASQELHSLVHLFRKLSIHLLSEVEKTVQQLKPDLLELA
LLICEKFLYKKLENPQELALLLSTALQRHTTLRSLTPIKVFLHPEDLKTLTDWISTHELPMIKHAEFFPDTSCRR
SGFKIETPNGILRQEISEELDHLLSVLTA

>core/787/3/Org3_Gene377

MACEQHEGCYELEEREEIEDIKDSDTKWVSITQAAKLHNVTRQAIYVAIKQKKLKASKETRWEIDIKDLEEY
KRNRYSRKKSLYQGELVFDNGKGCYSINQVAQILGIPVQKVYYATRTGTIRGERKGAAWVIHVSEIERYKNE
YLSKQAAKKLKGAEPKEHQAPNFEPPTEIFPESN

>core/789/3/Org3_Gene976

MTIILCGLPTSGKSSLGKALAKFLNLPFYDLDDLIVSNYSSALYSSSAEIYKAYGDQKFSECEARILETLPPEDA
LISLGGGTLMYEASYRAIQTRGALVFLSVELPLIYERLEKRGLPERLKEAMKTKPLSEILTERIDRMKEIADYIF
PVDHVDHSSKSSLDQASQDLITLLKS

>core/790/3/Org3_Gene189

MSAPIPTPQELSDQITCLNVQYQQVSELARENKGDIEGLKTLTAALTADAGIQPSADEIYSLQTAAALILSASE
KPGSGPSGSTEGSVTVQSPCKFKKVLAVVLTIIALIAIAVLIACIIAACGGFPLLLSALNLYTIGACVSLPIIASTS
VALICLCTFVANSLIKPVITVRTTR

>core/791/3/Org3_Gene366

MDRDNEVPLPKPKWIYRTGIGQDSHRFLPESSTKPCILGGIIFDHCPGFQANSDGDIIFHAICNAISSVTNKIILG
KVADELLQTRGITDSGIYLEEALKSLKPNQKISHVAITIEGSRPKFLCKLSALRQNIAQVMNLTPTDIGITATSG
EGLSDFGCGDGVQCFCVLTVMEYCD

>core/793/3/Org3_Gene612

MGYVFYVIAGSIFLGISLGAYCQLYYSVKSVLFSWYLLTVYALEKRHALLALSQLVGEEDAQSQKEIDFLSQC
DKLSWRAFLKNSYEIIPTFKEMEDLLSERVQGFLESIETIAEHDRAILCIENFWASKNLFDFEIAAYEEAVEKYL
KLRQRAPLRLASKLFRFLDVPSIRFSS

>core/794/3/Org3_Gene988

MDCVDNLKLYIFRLKLPGDTERISYSISPEYIREKGEEELLNSPIEVEGSLGRIDSDQWILSLSLKTQLGLCCPVC
NNFFSHSVCLPDLQRVISHDEVGSGVFDCRPLIRQELLLESDCFEECSGQGCPERKNILKFLEDRKKHEGNNPF
EYL

>core/795/3/Org3_Gene299

MSSTLNGVFPSSLPEESADLFITNKEIVALGEKGNVFLTHSIPMHIAAITILVIVALAGIAIICLGCYSQSILLIAVG
IVLTILTLLCLQALVGFIKFIRQLPQQLHTTVQFIREKIRPESSLQLVTNAQRKTTQDTLKLYEELCDLSQKEFK
LQSTLYQKRFELSHKNEKTNQN

>core/796/3/Org3_Gene709

MSIQPVSNTTTKADKVIPDSTKVISDSITINKQSAFYFCISVMLRLSESTTEYGKSILAVLEDNTIVQQQRVKELI
NLPLLKVPDLQKKDGSDDEYKNQNEIQAYQSSNQQISANRQMIQQELSSAQQRAQANQKSVNSTTIESMQIL
QATSSMLSTLKELTIKANLTNSPSD

>core/797/3/Org3_Gene710

MWIIDPLSAKKPLQAAINVPGTPITGGPNTATADDIIAKFSKDSNPLIVTVYYVYQSVLVAQDNLSIIAQELQA
NSSAQTYLNNQEALYQYVSIPKNKLNDNSSSYLQNIQSDNQAIGASRQAIQNQISSLGNAAQVISSNLNTNNNI
IQQSLQVGQALIQTFSQIVSLIANI

>core/798/3/Org3_Gene405

MADDTLIPKLMKNSLSQACSEGLLIAKYPPLQVIVHFDNNLVVKTHLSVAPVFSCLFLGPAAHKAMQEIVLW
CSRYANKEHPPFSSHFAKDLIPSQYLEILNCVAEIPFGEQQTYAEIAKKTDTHPRTVGAACKQNPFLLFFPCHR
VVGSHGERNYVLGPVIHEILLKFENSY

>core/799/3/Org3_Gene786

MGFKNICKQGSQLYLNGIFPERILARKLKNCAKSYPRTALTIEVLVSSVLGALKVILIPCASTYAALTLPLRALF
NAIKTKSCQHLASYAMAWLLNILTIAVIIGLVFSLVFIPPPVVFISLGLLMSVTTSVTLFQVHKNLFPPYEPPPSR
PHTPPPFADEYVPLISESYFD

>core/800/3/Org3_Gene720

MSHLNYLLEKIAASSKEDFPFPDDLESYLEGYVPDKNIALDTYQKIFKISSEDLEKVYKEGYHAYLDKDYAKS
ITVFRWLVFFNPFVSKFWFSLGASLHMSEQYSQALHAYGVTAVLRDKDPYPHYYAYICYTLTNEHEEAEKAL
EMAWVRAQHKPLYNELKEEILDIRKHK

>core/802/3/Org3_Gene224

MSVHITPRKCFILCILSMFTLPTLFPKAHLILFSPYIVLCFYCFSKDKGLVLALGCGVLSDLALGSRGVFLLLYP
LTALITHKAHLIFSKESKAALVIVNMIFYGVFLLLTIPMCALFGHEVRWSIDVLMIPLKCSFLDNLIFTSVIYILP
CAINSGIHKMISFFRRLVCY

>core/803/3/Org3_Gene490

MVHSPTHQCYHCQQPATICYTEIDKDKVIRSYVCATCPCPSHYYNNEHLSLSKGVGVLTLECGNCKTVWHS
KQDDEQLLGCHQCYTNFKNQITSKLKSERVVSSSFTMEKGQGSLHIGRAPGEASNTNPLLKLIALNEALQDTL
EREDYEQAAVIRDQINHLKTKNPDDPS

>core/804/3/Org3_Gene921

MTAEKQNTGILGLEIRYTLPSDATYMLKWLNDPKILRGFPIQTEAEIRETVNFWVGFYRYHSSLTAVYNGNV
AGVATLVLNPYVKVSHHALISIIVGEEFRNKGIGTALLNNLIHLAKTRFKLEVLYLEVYEGHPALHLYQRFGF
VEVGRQNRFYKDEIGYLAKTTMEKDL

>core/805/3/Org3_Gene197

MKKLLFSTFLLVLGSTSAAHANLGYVNLKRCLEESDLGKKETEELEAMKQQFVKNAEKIEEELTSIYNKLQD
EDYMESLSDSASEELRKKFEDLSGEYNAYQSQYYQSINQSNVKRIQKLIQEVKIAAESVRSKEKLEAILNEEA
VLAIAPGTDKTTEIIAILNESFKKQN

>core/806/3/Org3_Gene884

MSEVKPLFLKNDSFDLATQRFQNLINMLQEQAEIYNEYEEKNARVQNEIKEQKDFVKRCIEDFEARGLGVLK
EELASLTRDFHDKAKAETSMLIECPCIGFYYSIHQEEQRQRQERLQKMAERYRDCKQVLEAVQVEQKDMISS
RVVVDDSYFEEEKEDQKVDNRKKEQD

>core/807/3/Org3_Gene534

MCKNRGVRGIVACDPRGVIGLEGKLPWHYPEDLQFFSETIQKFPIVMGRKTWETLPRKYFVDRAVVVFSHEK
RQGVHGEIWVTSLEEFLLLDLSSPTFLIGGGELYSLFLENQIVRDFFISHIKKEYAGDTFFPLSLLETWTKTVLR
DTQKITTCYYENHHSQNTKNISL

>core/808/3/Org3_Gene43

MKQEKTLLLQEVEDKISAAQGFILLRYLRFTAAYSREFRNSLSGVSAEFEVLKKRIFFKAIEAAGLEVDCSDTD
GHLGVVFSCGDPVSAAKQVLDFNKQHKDSLVFLAGRMDNASLSGAEVEAVAKLPSLKELRQQVVGLFAAP
MSQVVGIMNSVLSGVISCVDQKAGKN

>core/809/3/Org3_Gene52

MQRLGLSNLFHCLLLFLRYYYSKLVFGLTVLLAAISVICLLGCSEPSLSSFTEYVGPEYSAAAQLSIEQSCHDE
VYGQQVVVTWSLPSRMRKCLPVTLYLWVYYGNGKVEKLTYEVNQSAGYRVYCLKGLEYKELQGIISYRVA
LCSGNQEIVSRRHHLWMEVISLDSP

>core/810/3/Org3_Gene674

MKQQLLLLEDVDGLGRSGDLITARPGYVRNYLIPKKKAVIAGAGTLRLQAKLKEQRLIQAAADKADSERIAQ
ALKDIVLEFQVRVDPDNNMYGSVTIADIIAEAAKKNIFLVRKNFPHAHYAIKNLGKKNIPLKLKEEVTATLLV
EVTSDNEYVTVLAQGKQTEENQEG

>core/811/3/Org3_Gene178

MIINVRAPAFGITSVQQFSTNFQAAIPILNIVIGCSRISSTYAEDIEEVAQEKLEKSTHSKSSTSVNLWAHRVRGV
VEILGGGIVILALEITALVLQVIIKLIKCLIDVLCVCLFGLGVCVVAIIGAIAFCVVVVVKYLGFCSQGEELEPIE
VKTLISPDKPYPTVVYV

>core/812/3/Org3_Gene424

MSELIIGVDPGTIVAGYAIIAVEQRYQLRPYSYGAIRLSSDMPLPMRYKTLFEQLSGVLDDTQPNAMVLETQF
VNKNPQSTMKLAMARGIVLLAAAQRDILIFEYAPNVAKKAVVGKGHASKRQVQVMVSKILNVPEVLHPSNE
DIADAFALAICHTHVARSPLCGVR

>core/813/3/Org3_Gene419

MRQFCNLLSLSRLWLALYFCQEKLHIRLLAIVGAMLSDVLDGYLARRYKATSRLGSILDPITDKVFVFVCITV
LYMEGSLSIAHLFFICARDLFLIIFVCYLSLVKGWKGYDYGSLFWGKIFTVVQFIILLGVTAGGEIPWTGLVPL
VALGFLYFLERIMDYKKQFLR

>core/814/3/Org3_Gene357

MATRFRSTLLVITLFVLIDWVTKLVVLLQYKDLQILTHPTLYTHSWGRFSFSIAPVFNEGAAFGLFSNYKYFLF
LLRIFVILGLLAYLFFKKKSIQSTTQTALVLLCAGAIGNVGDIIFYGHIVDFISFNYKQWAFPTFNVADVLISLGT
LLLVYKFYFPTKQTEKKR

>core/815/3/Org3_Gene495

MAKYPLEPVLAIKKDRVDRAEKVVKEKRRLLEIEQEKLREKEAERDKVKNHYMQKIQQLRDLLDEGTTSDA
VLQIKSYIKVVAVQLSEEEEKVNKQKEVVLAASKELEKAEVNLAKRRKEEEKTRLHKEEWMKEALKEEARA
EEKEQDEMGQLLFQLRQKKKRESGGS

>core/816/3/Org3_Gene232

MAVEQSHIKEEIEKLIGKAIKRVCGNKENDLCRYLPGPSGGYMHHFTLKKMKSAAPEQLLKMLKTFILESETP
RTINPKPRAPRGSKKRRDFINFTKTDIERVLELARQVGDKDLLARFSPKKPLTSLKRELIRSIRNGIVSVELWNA
YVEAVKAVSSPNLEVTSPFV

>core/817/3/Org3_Gene113

MDLKQIEKLMIAMGRNGMKRFAIKREGLELELERDTREGNRQEPVFYDSRLFSGFSQERPIPTDPKKDTIKET
TTENSETSTTTSSGDFISSPLVGTFYGSPAPDSPSFVKPGDIVSEDTIVCIVEAMKVMNEVKAGMSGRVLEVLIT
NGDPVQFGSKLFRIAKDAS

>core/819/3/Org3_Gene162

MSKKINRNDLCPCGSNKKYKQCCLKKEEQTARYTTEGKFKFSAEVLSASEQGEAGDNCTKLFQRLSQSLTSE
QKAAVGKFHQITKNKEVMSKKALKKAQAKEEKLVTEKLQQHNFEILNTGENLAPPMESTATLNQDTNFVCE
DFIPTQEDFRISENSQKPPVEED

>core/820/3/Org3_Gene260

MSRQNAEENLKNFAKELKLPDVAFDQNNTCILFVDGEFSLHLTYEEHSDRLYVVYAPLLDGLPDNTQRKLALY
EKLLEGSMLGGQMAGGGVGVATKEQLILMHCVLDMKYAETNLLKAFAQLFIETVVKWRTVCADICAGREP
SVDTMPQMPQGGGGMQPPPTGIRA

>core/821/3/Org3_Gene652

MKFWLQGCAFVGCLLLTLPCCAARRRASGENLQQTRPIAAANLQWESYAEALEHSKQDHKPICLFFTGSDW
CMWCIKMQDQILQSSEFKHFAGVHLHMVEVDFPQKNHQPEEQRQKNQELKAQYKVTGFPELVFIDAEGKQL
ARMGFEPGGGAAYVSKVKSALKLR

>core/822/3/Org3_Gene1000

MSRRHSAEKRDIPGDPIYGSVILEKFINKVMMHGKKSVARKIVYSALERFGKKLNLENVLEGFGEALENAKPI
LEVRSRRVGGATYQVPVEVASERRNCLAMQWIIKHARSKPGKSMEVGLATELIDCFNKQGATIKKREDTHR
MADAYKAFAHYKWVRLVKLRRK

>core/823/3/Org3_Gene433

MSLSKNSHKEDQLEEKVLVVNRCSKVVKGGRKFSFSALILVGDGKGRLGYGFAKANELTDAIRKGGEAAKK
NLMKIEALEDGSIPHEVLVHHDGAQLLLKPAKPGTGIVAGSRIRLILEMAGIKDIVAKSFGSNNPMNQVKAAF
KALTGLSPRKDLLRRGAAIND

>core/826/3/Org3_Gene508

MTKFLYCGLFYSLGLLVLAFGTMVAIIQVDQICDVSCMNKHFQESPPFLKIKKVNVSKQICSPEERFFHCKIDK
SCMELHFPQSSYSCKEYLTRISGHILTQNFEKQMQFRGNSGLLNYQDGSLHVYDCRFQVDPVPGYGSPDKED
SSSGGMKTLYLSLFRN

>core/827/3/Org3_Gene667

MMHRYFIPLLALLIFSPSLVRAELQPSENRKGGWPTQLSCAEGSQLFCKFEAAYNNAIEEGKPGILVFFSERPT
PEFADLTNGSFSLSTPIAKGFNVVVLCPGLISPLDFFHKMDPVILYMGSFLEMFPEVEAVSGPRLCYILIDEQGG
AQCQAVLPLETKN

>core/828/3/Org3_Gene711

MSITTLGTLPTVNTINSSRPPLEPLNTPKIGAVLFSIYELLLQAIEIRQQTVLTQSQQLNDNTNIQQQLNQETNQI
KYAIVSAGAKEDEITRVQNQNQNYSAQRSNIQDELVTTRQNGQIILSHASTNINIIQQQSSQDSSFIKTTNSIGST
VNQLNKPLG

>core/829/3/Org3_Gene277

MTTWTLNQNNLTKFLKSSDEEPFLERESGLTYINIQANGNELPLFFVIRSEGEILQLICYLPYQLHESHKASTAR
LLHLLNRDIDIPGFGMDEEQGLIFYRLVLPCLNGEIHDTLLRIYIDTIKLVCDSFSHAIGLISSGNMNLDELRRQ
ALQEQQEKRNE

>core/831/3/Org3_Gene694

MATLSPEKFSGSPISISKEFPQQKMREIILQMLYALDMAPSAEDSLVPLLMSQTAVSQKHVLVALNQTKSILEK
SQELDLIIGNALKNKSFDSLDLVEKNVLRLTLFEHFYSPPINKAILIAEAIRLVKKFSYSEACPFIQAILNDIFTDS
SLNENSLSI

>core/832/3/Org3_Gene259

MMFGHFAGYLGADPEERMTSKGKRVITLRLGVKTRVGMKDETVWCKCNIWHNRYDKMLPYLKKGSGVIV
AGDISVESYMSKDGSPQSSLVISVDSLKFSPFGRNEGSRSPSLEDNHQQVGYESVSVGFEGEALDAEAIKDKD
MYAGYGQEQQYVCEDVPF

>core/834/3/Org3_Gene134

MSKKVFFESYEDFANVASSWPKSLRALVQGRYFVDSELKETPYRIHDFKKTPIHHRLYRSLPIISTIGGIIRLIEA
HSGPIHPRDKMKYRFEVLQAVIEILGLGVLILVFDIIGCFLAFLVAIILSLLLYCNSTFTCVQNLSFTERMLEGIG
EAVNFLA

>core/835/3/Org3_Gene276

MFFNLFSLVFKLSDELALAETIQEPISVHEMFPGSMKLEMFKMLGSLILLLTIFGFGVWAFKKFVRSRSHGFGG
SSQIKILERRSLTPKTSIYLIRVVNKTLVIAETPEKITLLTEFPPDTDINHLLQENNKQSSSSATSDFLSKAIQKIQK
KQQTNQD

>core/837/3/Org3_Gene31

MPSYCQNQQDFSLFSLLSPRLVMFLGKHSRDEILQDLTDLVDAAGLLEDKQAFFDALVRRENIMSTGIGMGV
AIPHGKLESCSNFFIAIGIHTQGILWDAIDGALVRLVFLIGGPENAQAEYLKLLSTLTLSLREESRRQQLLQVNT
IEEVMNVFVGM

>core/838/3/Org3_Gene579

MADETPKENSSKESSSQFDSLKRKVKDLHSNPKVGKWKKFLSHRACEAIGGCLVLVGIIADFISWAGGLFIAC
GVVLGFHVEIRKMLSNLQSYSIANGPIKNAILCGLILFFVLNIPSFAVSFIVLCVILSFITTAPSCSTCSKDHCDKH
QDTSNKPS

>core/839/3/Org3_Gene336

MTQEKIKIHVSNEQTCIPIHLVSVEKLVLTLLEHLKVTTNEIFIYFLEDKALAELHDKVFADPSLTDTITLPIDAP
GDPAYPHVLGEAFISPQAALRFLENTSPNQEDIYEEISRYLVHSILHMLGYDDTSSEEKRKMRVKENQILCML
RKKHALLTA

>core/841/3/Org3_Gene662

MLPISILLFYVILGCLSAYIADKKKRNVIGWFFAGAFFGFIGLVVLLLLPSRRNALEKPQNDPFDNSDLFDDLK
KSLAGNDEIPSSGDLQEIVIDTEKWFYLNKDRENVGPISFEELVVLLKGKTYPEEIWVWKKGMKDWQRVKD
VPSLQQALKEASK

>core/842/3/Org3_Gene221

MRIIRFDPYGALSAQSIAKDSRQNSPLVEKISEEIATNEAIRLALLAIGDREQEEKKQRHRYKLLGQKQAKVLL
SQLRHVHLDFKKLYCDSKKKEDQEKDEKNKQKRSIKVTKKKKGISLGAAASQAIAAAAEAWVIARNKGVLE
TASTLFYQKDEEA

>core/843/3/Org3_Gene663

MEKQNLKLDVKEIEFPETVFSRDIETRVIQVIILHCLAKINGVSLLGGNLIDALFGRDIERMKGIYVEQDSKNHL
VKVRVEVNVDYGVSIPEKTEEIQGCIVSEISEYTGLHVAAVHVIIKGLTQPKDRIDEEIEEEVSVQDLPSPEDFL
LENSEG

>core/844/3/Org3_Gene108

MIMTTISNSPSPALNPELSLIPPPTLVSSGTQTSLAYTIPAQGRRSTLRIILDIFIIILGLATIISTFIVIFFLNGLNLLS
TPSIISSSCLIIVGLLFLIMGLYFMISSLDQGLVGLLQKELSQAEEREEEYIQEIEALRGAPRAESPTESPSTWL

>core/845/3/Org3_Gene457

MRVVLHCPDIPQNTGNIGRTCVALGAELILVRPLGFSLADKFVKRAGMDYWDKLQLTVVDSIEEALHDVPED
QIFCLSTKGSASYTEFSLPSSGTYVFGSESKGLPKEILKKYYKNCLRIPMQQDIRSLNLATSVGIVLYEVVRQKT
VALQKNPTV

>core/846/3/Org3_Gene706

MEKDIFFMQQAFKEARKAYDQDEVPVGCVIVKDDKIIARAHNSVEKLKDATAHAEILCIGSAAQDLDNWRL
LDTVLYCTLEPCLMCAGAIQLARIPRIVWAAPDVRLGAGGSWVNIFTEEHPFHTVSCTGGVCSEEAEHLMKK
FFVEKRREKSEK

>core/847/3/Org3_Gene905

MGYLPVSATDVLFESPAAPLINSAYTQNQKLIELKGKQQAESSPRTITSVILEVLLVIGCCLIVLSLLAIRPALQF
TLETGHPAAIAVLAVSGTILLVAVIILFCFLAAVPFAAKKTYKYVKTVDDYASWHSHQQTPTLGTIFSGIVYAE
SQAQL

>core/848/3/Org3_Gene452

MLKKKPVSFSCIDGHIYKIFPNDLNANNTVFGGLLMSLLDRLALVVAERHTESVCVTAFVDALRFYAPAYMG
ENLICKAAVNRTWRTSLEVGVKVWAENIYKQERRHITSAYFTFVAVNEDNQPIPVHQIVPETPEEKRRYNEA
DRRRQARLELK

>core/849/3/Org3_Gene619

MKTLKGHLSAKNLRIAIVGSCFNQAMADALVSGTQETFLKFGGSEDGLMTIRVPGAFEIPCTIKKLLSSERKF
DAIVACGVLIQGETDHYNQIVNQVAAGIGALSLEFCLPITLSIVAAPSAEIAWQRSGIKGRHLGVSGMTTAIEM
ATLFTQI

>core/850/3/Org3_Gene590

MPFAKETEMQRTCWKCEGSVSMHVPQCPYCSAFLQDPPVASGGFSSCHISFPEGASKEEAEDLFAVSSEDWE
AVLGDQNPTQETNKQVIPEWTWLQSWPLAALFLGIGLLAFAFLILLFSTDSGLVLTWPKNRAYFYGIIGAAVA
YRGYRKLPL

>core/851/3/Org3_Gene27

MRSALHLQHLRHFHNHGSILFENLLTIKDCFLLETKLQNFIAKASKTIDTVRWRENIFRSMPEIYTVVRKRRLDFFAAELVHRPKLSLVRDLWVFPGEEILEGEEDCMLFLLLSGDRAGSGIFFTGPYPSDLYELEKGTTGLLLAFSSVGIPVI

>core/852/3/Org3_Gene449

MNQPSVIKLRELLDLLPHRYPFLLVDKVLSYDIEARSITAQKNVTINEPFFMGHFPNAPIMPGVLILEALAQAAGVLIGLVLENDRNKRIALFLGIQKAKFRQAVRPGDVLTLQADFSLISSKGGKAWAQARVDSQLVTEAELSFALVDKESI

>core/853/3/Org3_Gene355

MQCPFCNHGELKVIDSRNAPEANAIKRRRECLKCSQRFTTFETVELTLQVLKRDGRYENFQESKLIHGLNAASSHTRIGQDQVHAIASNVKSELLGKQNREISTKEIGELVMKYLKKADMIAYIRFACVYRRFKDVGELMEVLLSATPDMEK

>core/854/3/Org3_Gene227

MAQKEIVSNRKALRNYEVIETLEAGIVLTGTEIKSLRDHGGNLGDAYVIVSKGEGWLLNASIAPYRFGNIYNHEERRKRKLLLHRYELRKLEGKIAQKGMTLIPLGMFLSRGYVKVRLGCCRGKKAYDKRRTIIEREKEREVAAAMKRRHH

>core/855/3/Org3_Gene205

MARNIKYFLILFPGILWISAGMKLLLKATAIALDPLSSFFTYCLLSMVSWGLASLKHRYLLSKTIRKQLSLSSEFFSQKITWIAYIKQTFISRRFLIMVIMIAFSLVLRRYISNPQALFVIRATVGYALIKTAIAYFSKLQNALMENPEGN

>core/856/3/Org3_Gene149

MSKPSSCKAYLGIDYGKKRIGLAYAAEPLLLTLPIGNIEAGKNLKLSAEALHKIILSRNITCVVLGNPLPMQKGLYSSLQEEVSLLAEELKKLSTVEIILWDERLSSVQAERMLKQDCGLSRKDRKGKTDSLAATLILTSFLDSLPKKLTL

>core/857/3/Org3_Gene645

MMKTKYEYSFGVIPIKFFGTPDKNTLKACFICHTRGKHWGFPKGHSEDKEGPQEAAERELVEETGLSVVNFFPKVLIEQYSFNNEEQVFVRKEVTYFLAEVRGDIHADPMEICDSQWLSLQEGLRLLSFPELRDLTVEADKFINNYLFSS

>core/858/3/Org3_Gene623

MQLLSPAFAYGAPIPKKYTCQGAGISPPLTFVDVPGAAQSLALIVEDPDVPKEIRSDGLWIHWIVYNLSTTITNLAEGAEIFAVQGLNTSGKPVYEGPCPPDKQHRYFFTLFALDVVLPEEENVTRDQLYEAMEFHIIEQAELMGTYEKS

>core/859/3/Org3_Gene154

MEKRKDTKTTIVKSSETTKSWYVVDAAGKTLGRLSSEVAKILRGKHKVTYTPHVAMGDGVIVINAEKVRLT
GAKKGQKIYRYYTGYISGMREIPFENMMARKPNYIIEHAIKGMMPRTRLGKKQLKSLRIVKGDSYETFESQK
PILLDI

>core/860/3/Org3_Gene876

MEFICPLQHARCLKKQHKIIEELFPEPFQKDHLYLKLMENSSSRDAFDKKRVLKENLVVGCQSDLYLYEVYQ
DGILFFFTYTKALMSSGIASLFTEVYSGETPSTILTCKPIFFQRLTPYLSFGRLNGGESLYMRMKQIAVQYLKPP
QT

>core/861/3/Org3_Gene498

MADLEVFQADFALLFEAGLLAIKQGDEDSARKLFQSLHILNPNHYGHDLGLALISLHKMDLFDAEERLSALIK
GNEDNWSIKAFLSLTHMLIVLHQGSSFEVRRESLESCLKFADQVIANCKIESTRALAQSVLDWHDTLVAKSA
GPLG

>core/863/3/Org3_Gene254

MKSERLKKLESELHDLTQWMQLGLVPKKEISRHQEEIRILEHKIYEEKERLQLLKENGEIEEYVTPRRSPAKTV
YPDGPSMSDIEFVEPTETEIDIDPGETVELELTDEGREDGAVEVDYSHEDDEDPFSDRNRWRRGGIIDPDANE
W

>core/864/3/Org3_Gene251

MKTKMNSRKKAGQWAIFNSPTPGVSSTLVLAWTPWGYYDKDVQDILERKDPMSSSLSEKDSKEFLKNLFVD
LLENGFTSVHIHAEEAFTPLDHTGKPHFKRDNVYLPGKLLGALNEAAVQANVSADTQFTLFLTQDECNPFHD
KKRG

>core/865/3/Org3_Gene856

MGSRRKLKRSFLLIEVLMALSLVCAVLLPCIRFYYAIHRSFEEDIFNLQLPALIDHCFLSVEEKMRQQMAEGTV
LTSGKGQTVSLAYTSQGIGYRIPYGYNVDIRQEVRGDNLKMKVCLADVVVELFPDQKQAVSVQRCLCVTL

>core/866/3/Org3_Gene117

MKKKVTIDEALKEILRLEGAATQEELCAKLLAQGFATTQSSVSRWLRKIQAVKVAGERGARYSLPSSTEKTT
TRHLVLSIRHNASLIVIRTVPGSASWIAALLDQGLKDEILGTLAGDDTIFVTPIDEGRLPLLMVSIANLLQVFLD

>core/867/3/Org3_Gene216

MQNQYEQLLESLAPLLNTTLAPDKNNSCLIRFSDTHVPVQIEEDGNSGDLAVSTLLGTLPENVFRERIFKAALS
VNGSFQSSIKGILGYGEVTQQLYLSDILSMNYLNGEKLFEYLKLFSLHAKIWMESLRTGNLPDLHVLGIYYVA

>core/868/3/Org3_Gene639

MEEFVAYIVKNLVTNPEAVEIRSIEDEDNESIKLEIRVAAEDIGKIIGRRGNTIHALRTILRRVCSRLKKKVQIDL
VQPENGTDVIADQDYICDNDSSNSTEDTFGESDTCCSGHCHYDEDLNQEEQEEGNMHHSCECSNHH

>core/869/3/Org3_Gene83

MSLEKELLEETPLVLLNFYKLVSFCNYAGMILGTEEKKFAIYGHVSMGQAFQGADTEGHSPQRPFAHDLLNF
VFSGFDIQVLRVVINDYKDNVFYTRLFLEQKDREFLYVVDVDARPSDSIPLALTHKIPILCVKSVFDAVVPYEE

>core/870/3/Org3_Gene132

MNPVTFDRIQVDFIPEDTSLRINSYIVAGGLLILGVVLSILSVICLDIGLVGLSAGAAFTLGLGCLIFALFLFSFSL
ILLLSQEKRVPDVLSLYLEKEVPQYETPLYKEDLESERDMSAISERLGIIEEKLRIAEKFRYSDSVFV

>core/871/3/Org3_Gene30

MTVFCELDSGGELPEYTTPGAAGADLRANIEEPIALLPGQRALIPTGIKAEIPEGYELQVRPRSGLALKHGITVL
NSPGTIDSDYRGEIRVILINFGDSTFIIEPKMRIAQVVLSPVVQATFVVKQESLAETARGSGGFGHTGAS

>core/872/3/Org3_Gene466

MTFFEGETVFPAVLSELHSMLDLIKRAGKQSKCPQEKLLKLELACEELLVNIISYAYQGENSPGTIAISCISHRG
DLEVVIKDHGPSFNPLAVSINIQEDLPLEQRKLGGLGIFLAKSSVDEFLYAREDHCNIVHLKMLNGQHS

>core/873/3/Org3_Gene422

MEQTLSIIKPDSVSKAHIGEILSIFEQSGLRIAAMKMMHLSQTEAEGFYFVHRERPFFQELVDFMVSGPVVVLV
LEGANAVSRNRELMGATNPAEAASGTIRAKFGESIGVNAVHGSDTLENAAVEIAYFFSKIEVVNASKPLV

>core/874/3/Org3_Gene432

MIKLESLFDISERKRRKKLLGRGPSSGHGKTSGRGHKGDGSRSGYKRRFGYEGGGVPLYRRVPTRGFSHKRF
DKCVEEITTGRLAELFQEGEAITLDALKAKKAIARQAVRVKVILKGDLEKTFVWQDTAVVLSQGVQNLLGIT

>core/875/3/Org3_Gene151

MACSIFFRMSQGDYDDEPLSKKTACLVVDTMLYPVIAVVCAVVSVVLLILKVLFLLLSFPFKLCSASSALPGE
RVSLGSHFKCLYGGGLPYLLACLLIVPVIGTAIHGFIISHRTSEDARLSSAIVFMQAPILQLAGMSGLIKP

>core/876/3/Org3_Gene752

MQEHIHKELLHLGEIFRSSRESQSLSLKDVEAATSIRYSCLEAIEQGCLGKLISPVYAQGFIKKYATYLGLDGD
SILQEHPYVMKIFKEFSDHNMEMLLDLESMGGRNSPERAIHSWSNLWWAGLIIIGGIMVWWLGSLFSIF

>core/877/3/Org3_Gene467

MSLDFFEEFYHQSILNTGTSFPEGYLNIAEILSYPHCTDANTDFLCSQSDNDFIIAESKDKLTLFNADFAIWLVP
ELVQGQAVTRGYIAVSQGEGNYEPEMAFEASGQYNQSSLILEALQLYLKDIKDTENALRSFRFNNDH

>core/878/3/Org3_Gene428

MQHARKKFRVGRTSSHNRCMLANMLKSLIHYERIETTLPKAKELRRHADKMITLAKKNSLAARRIAIGRLM
VRYNKLTSKEARQAKGGDTSVYNVDRLVVNKLFDELGNRFVERKGGYTRILKLQNRIGDNAQKCIIEFLAS

>core/879/3/Org3_Gene521

MNSKSAQKIIDSIKQILTIYNIDFDPSFGSSLSSDSDADYEYLITKTQEKIQELDKRAQEILTQTGMSKEQMEVF
ANNPDNFSPEEWLALEKVRSSCDEYRKETENLINEITLDLHPTKESKRPKQKLSSTKKNKKKNWIPL

>core/880/3/Org3_Gene286

MLDNEWKAILGWGDDELEELRISGYSFLRQGHYSKAILFFEALVILDPLSIYDHQTLGGLYLQIGENSQALAV
LDQALRMQGDHLPTLLNKTKALFCLGRIEEATAIATYLSSCPIPAIANDAEALLMSYSKATKKNAALVR

>core/881/3/Org3_Gene454

MGRYRRVSHSSQETLLLGTELGQVLVPGAVLLLFGDYGAGKTEFVRGIVSGYLGDTIAEEVASPSFSILHVYG
NEPKRLCHYDLYRIDQKNQEYIFQDAEEDDVLCIEWADRLPKPRFCDTINIYITMQTNMEREIIIEKR

>core/882/3/Org3_Gene41

MSVKKVIKIIKLQIPGGKANPAPPIGPALGAAGVNIMGFCKEFNAATQDKPGDLLPVVITVYADKTFTFITKQP
PVSSLIKKTLNLESGSKIPNRNKVGKLTQAQVEAIAEQKMKDMDIVLLESAKRMVEGTARSMGIDVE

>core/883/3/Org3_Gene51

MIDMSVVGPALVLGLAMIGSAIGCGMAGVASHAVMSRIDEGHGKLIGMSAMPSSQSIYGFILMLLMQAAIKN
GTLSPVGGIAIGLSVGAALLVSSVMQGKCCVSGIQAYARSSSIYGKCYAAIGIVESFSLFAVVFALLLL

>core/885/3/Org3_Gene658

MHPLTLPKQSRVLKRKQFLYITRSGFCCRGSQATFYVVPSRHPGTCRMGITVSKKFGKAHERNSFKRVVREV
FRHVRHQLPNCQIVVFPKGHKQRPVFSKLLQDFINQIPEGLHRLGKTKATTGGECTPKSEKCVTAPR

>core/886/3/Org3_Gene440

MLMPKRTKFRKQQKGQFAGLSKGATFVDFGEYAMQTLERGWVTSRQIEACRVAINRYLKRRGKVWIRIFPD
KSVTKKPAETRMGKGKGAPDHWVAVVRPGRILFEVANVSKEDAQDALRRAAAKLGIKTRFVKRVERV

>core/887/3/Org3_Gene191

MNLIDRAFLLKKTIIFQSLDMDLLLTIADKTETIIFKPGSNVFSIGQPGFSFYIIVEGYITISKEKLESPLNLKPLDC
FGEESLFNNKPREYNASANTQVRMLVLSKGQILNIVEECPSVALSFLELYAKQIKFREP

>core/888/3/Org3_Gene137

MVNRYKSSAEFSADHYYDDNLVRMGYKRNLRGLAPVENEVCLFEENNLLESVMASIPIMGSILGLGRLHSV
WSTQDPKDSKISIIFHTALGILETLGLGIIVLLIKITITILLILFTPCLLCYFMYSAAYSDFHPI

>core/889/3/Org3_Gene142

MINFIRSYALYFAWAISCAGTLISIFYSYILNVEPCILCYYQRICLFPLTVILGISAYREDSSIKLYILPQAVLGLGI
SIYQVFLQEIPGMQLDICGRVSCSTKIFLFSYVTIPMASVVAFGAIVCLLVLTKKYRG

>core/890/3/Org3_Gene553

MKYRFTEEIEEEPLVNLTPLIDIVFVILMAFIVAVPLIKLDSIALAPGTQEQEVLSSENDSIAVIKVFADHSLTLN
EHPITLQELTVRLTLLHKAYPEKTPLLLQDGETSFRTYQNVKNAIEAAGFHELHVALQN

>core/891/3/Org3_Gene153

MAKSTIQESVATGRRKQAVSSVRLRPGSGKIDVNGKSFEDYFPLEIQRTTILSPLKKITEDQSQYDLIIRVSGGG
IQGQVIATRLGLARALLKENEENRQDLKSCGFLTRDPRKKERKKYGHKKARKSFQFSKR

>core/892/3/Org3_Gene532

MIAIERYQLIISKFRMWLFLGCSVEERHFKQPVLISVTFSYNEVPSACLSDKLSDACCYLEVTSLIEEIANTKPY
ALIEHLANELFDSLVISFGDKASKIDLEVEKERPPVPNLLNPIKFTISKELCPSPVLSA

>core/893/3/Org3_Gene141

MPYYANTLEFIQGTQSLCPLFKYGFVRHHYKGQLEIEDASHDWDFLEPPSTWKRTLLAAIPILGSVIGLGRLFS
IWSIREPQDSQEYKSIFWHTLCAVLEILGLGIVALILKILATFIMAMPGLKRVATFLFYS

>core/894/3/Org3_Gene435

MGMTSDSIADLLTRIRNALMAEHLYVDVEHSKMREAIVKILKHKGFVAHYLVKEENRKRAMRVFLQYSDD
RKPVIHQLKRVSKPSRRVYVSAAKIPYVFGNMGISVLSTSQGVMEGSLARSKNIGGELLCLVW

>core/895/3/Org3_Gene429

MVKNQAQAKKSVKRKQLKNIPSGVVHVKATFNNTIVSITDPAGNVISWASAGKVGYSGSRKSSAFAATVAA
QDAAKTAMNSGLKEVEVCLKGTGAGRESAVRALISAGLVVSVIRDETPVPHNGCRPRKRRRV

>core/896/3/Org3_Gene844

MNFVSTLTGSDFYAPVLEKLEEAFADTTGQAILFSSSPDFIVHPIAQQLGISSWYASCYRDQSAEQTIYKKCLT
GDKKAQILSYIKKINQARSHTFSDHILDLPFLMLGEEKTVVRPQGRLKKMAKKYYWNIV

>core/898/3/Org3_Gene170

MSQCQSSSTSTWEWMKSFVPNWKNPTPPLSPIPSEDEFILAYEPFVLPKTDPENAQANPPGTSTPNVENGIDDL
NPLLGQPNEQNNANNPGTSGSNPTSLPAPERLPETEENSQEEEQGSQNNEDLIG

>core/899/3/Org3_Gene502

MLEKLIKNFATYMGITSTLELDADGAYVLPISEVVKVRAQQNADNEIVLSASLGALPPSADTAKLYLQMMIG
NLFGRETGGSALGLDSEGNVVMVRRFSGDTTYDDFVRHVESFMNFSETWLSDLGLGKQ

>core/900/3/Org3_Gene270

MRDHAFSKLIGTVRAMVVEGRCPWSLQQSLVSMVEHILGECQEFHEAVLQGKTVQEVGSEAGDVLTLVLIL
CFLLEREGVLASEDVANEAMEKLRRRAPYIFAEDYKPVSIEEADRLWELAKHREKNEST

>core/901/3/Org3_Gene982

MDYKSQLVFSCPCCCKGNVCFSVFNLDVILTCNVCSSTYTFDSVIRNEIRQFVALCKRIHDANSILGNATVSVS
VEDNQMDIPFQLLFSRFPVVLNLSLDGKKIAIRFLFDALNTSILHQESDLIS

>core/902/3/Org3_Gene306

MREEGSVSFREYFRAYMCDKIVAQKNFLFTLDAVIKQAGWRSQEKLNLFYVESQALGREIKVSLEEYIQSMV
GILGSQRTKKSFKFSVDFTPLEQALQERCSSDDDEDATATSTATGATASPTDMHEDE

>core/903/3/Org3_Gene44

MTTESLETLVEKLSNLTVLELSQLKKLLEEKWDVTASAPVVAVAAGGGGEAPVAAEPTEFAVTLEDVPADK
KIGVLKVVREVTGLALKEAKEMTEGLPKTVKEKTSKSDAEDTVKKLQDAGAKASFKGL

>core/904/3/Org3_Gene950

MKFTVALFGEAEKGSYDTAYFCRSLVDLHNYLGDVSSPGITLAIKTLLSDYNVVYFRVREEGYCVDSYFFGL
HFLNTQATLKNIIAIGLPGVGNQHIIEASRSLCQKHNSLLLFFDHDLYDLLTFNQPF

>core/905/3/Org3_Gene99

MRAGGSLVTTYPKEGQRLRSPEQLRVLDDLVQSYPNHLHAIELDCGAIPQDLIGATYIITFADFSTYILSLRSY
QANSPSDDTWGIWFGSIDDPVQAVISFLKDHGFALPSTLAQDPLLCTNK

>core/906/3/Org3_Gene894

MLIGRYSSDDQFTEATKNTPTIIKLGFARDNLEGLTNPISEIVSETSSSIKDSVLRSLPILGSILGCARLYSTLSTN
DPLDETQEKIWHTIFGALETLGLGILILLFKIIFVILHCIFHLVIGFCK

>core/908/3/Org3_Gene356

MPLSDDEIEQFKKRLLEMKAKLSHTLEGNAQEVKKPNEATGYSQHQADQGTDTFDRTISLEVTTKEYELLRQ
INRALEKINESSYGICDVSGEEIPLARLIAIPYATMTVKAQEQFEKGLLSGN

>core/909/3/Org3_Gene930

MESSLCKKSLMKRRRALRVRKVLKGSPTKPRLSVVKTNKHIYVQLIDDSIGKTLASVSTLSKLNKSQGLTKK
NQEVAKVLGTQIAELGKNLQLDRVVFDRGTFKYHGIVSMVADGAREGGLQF

>core/910/3/Org3_Gene369

MPTINQLIRKRRKSSLARKKSPALQKCPQKRGVCLQVKTKTPKKPNSALRKVAWVRLSNGQEVIAYIGGEGH
NLQEHSIVLIQGGRVKDLPGVRYHIVRGTLDCAAVKNRKQSRSRYGAKRPK

>core/911/3/Org3_Gene627

MALKDTAKKMKDLLDSIQHDLAKAEKGNKAAAQRVRTDSIKLEKVAKLYRKESIKAEKSGLLKRKPSTKAP
AKVKKTAEKKAPKKSSAAAAKTSKAVKASKPASKKTAAKKVKKPSKARGFRK

>core/912/3/Org3_Gene577

MKRQKRKQSITLIEMMVVITLIGIIGGALAFNMRGSIHKGKVFQSEQNCAKVYDILMMEYATGGSSLKEIIAH
KETVVEEASWCKEGRKLLKDAWGEDLIVQLNDKGDDLVIFSKRVQSSNKK

>core/913/3/Org3_Gene607

MRDRLGSLSLILKVKIHKYLDTLHNQKRLALTVSRNIQATNKRIADLHLERYEHFISRDNIKHYDILLEYLKTL
QSSLYKQQSESLRFLEIHHQQLQELINRRKIIEKIKNNKYSKDQEIGT

>core/914/3/Org3_Gene430

MPRIIGIDIPAKKKLKISLTYIYGIGSARSDEIIKKLKLDPEARASELTEEEVGRLNSLLQSEYTVEGDLRRRVQS
DIKRLIAIHSYRGQRHRLSLPVRGQRTKTNSRTRKGKRKTVAGKKK

>core/915/3/Org3_Gene931

MIQQESQLKVADNTGAKKVKCFKVLGGSRRRYATVGDVIVCSVRDVEPNSSIKKGDVIKAEIVRTRRHITRK
DGSTLKFDTNSCVIIDDKGNPKGTRIFGPVAREIRDRGFIKISSLAPEVI

>core/917/3/Org3_Gene206

MEIIHIGTDIIEISRIREAIATHGNRLLNRIFTEAEQKYCLEKTDPIPSFAGRFAGKEAVAKALGTGIGSVVAWKD
IEVFKVSHGPEVLLPSHVYAKIGISKVILSISHCKEYATATAIALA

>core/918/3/Org3_Gene70

MVNLLKELEQEQCRNDLPEFHVGDTIRLATKISEGGKERVQVFQGTVMARRGGGSGETVSLHRVAYGEGME
KSFLLNSPRIVSIEIVKRGKVARARLYYLRGKTGKAAKVKEFVGPRSSKK

>core/919/3/Org3_Gene697

MVRATGSVASRRRRKRILKQAKGFWGDRKGHIRQSRSSVMRAMAFNYMHRKDRKGDFRSLWIARLNVASR
IHSLSYSRLINGLKCANISLNRKMLSEIAIHNPEGFAEIANQAKKALEATV

>core/920/3/Org3_Gene138

MRELNAFELTQPEEYRNRWVLMPCLKCRFCRTQHAKVWSYRCVHEASLYEKNCFLTLTYDDKHLPQYGSL
VKLHLQLFLKRLRKMISPHKIRYFECGAYGTKLQRPHYHLLLS

>core/921/3/Org3_Gene203

MATVAQTPQTTQPQPSVSHKATHRYCSWVFFKPILVSLGLLLASLTTLGLVIASGVTLSLGIGIVLAIQIVLAGI
ALVLAFNHIRQFKQARTAELNSMKMISAPAAATVQKQKLEDRYSSK

>core/922/3/Org3_Gene210

MTENRRIKRVNALLQEAIAKVILKDVKHPKISNLWITVTRVSLSKDLHSARVYYVSVMPHENTKEEALEALKV
SAGFIAHRASKNVVLKYFPELHFYLDDIFSPQDYIENLLWQIQEKEKS

>core/923/3/Org3_Gene571

MNKKPKKTKKAVQSKAAPVKRVPEESQEAAIQQLELAVSDLYKELPLAQTFASLTDKNQINSIIAALSGTLES
LHLEELTQGLFPSAQEDANFAKELSSVVHGLKNLTTVVNKQMVKGAE

>core/924/3/Org3_Gene359

MDNYLLGSLIFCCVLLSIGMCTIFVMTICFLRQLNKILKNIHRVTTILNFEAKILAPLMLGKKLLCGWLKKRKN
RGSLSEDIDELLDEKKQRSWKKNLDQGIKWCAALVLIWKVFRNKD

>core/925/3/Org3_Gene643

MDSFCFDLLKVAAKAIDDKKGNNLVVLDVRTISEFTDYFVFVEGSVNVHVKALANTIVEELKKQKVSPLHVE
GITDGNWVVIDYGFIVVHVFVSEIRGKYRLEELWKDGFIVTSKLLAS

>core/926/3/Org3_Gene247

MPVSSAPLPTSHRPSSGNLGLMEPNSKALKAKHQDKTTKTIKLLVKILVAILVIEVLGIIAAFFIPGTPPICLIILG
GLILTTVLCVLLLVIKLALVNKTEGTTAEQQIKRKLSSKSIS

>core/927/3/Org3_Gene68

MALKIRLRQQGRRNHVVYRLVLADVESPRDGKYIELLGWYDPHSSINYQLKSERIFYWLERGAQLSSKAEAL
VKQGAPGVYSALLSKQEARKLVVRKKRRAYRQRRSTQREEAAKDATK

>core/928/3/Org3_Gene994

MTVLFYAFLFIFLFLCVILCGLILVQESKSMGLGSSFGVDSGDSVFGVSTPDILKKVTSWCAVAFCIGCLLLSFS
TNLLGKKLDAKEFLLPAAEESDTQASSESVEADES

>core/929/3/Org3_Gene626

MLSRIVTCFLFLLSSLPLFAEEEAAQSKNTFVQPAVMLAIAILFFYFILWRPEQKRRKAMEKRKNDLAKGDKV
TAMGIIGTVDDIREHTVILNIASGKVEVLKGAISEILKPNDNKS

>core/930/3/Org3_Gene338

MSDIQKEEHGSTTIFHLHGKLDGISSPEVQENISQSLAAGSKNIILDCAHLDYMSSAGIRVLLQSYHQVGQHSG
KIVLTTVPKTIEQTLYVTGFLSYFKIFNTVDEAIQTLNKDGD

>core/932/3/Org3_Gene177

MFLKRKKRGGSQVQNKRTASPIKHAKHYLHNYLQELQKIMAARPHDAIDAWNQVFRDKYKGMSQAIGFRD
HILLVKVYNSSLYALLKQTPQNDLIMSLYQVASHVQIREIQFLLG

>core/933/3/Org3_Gene264

MSKVSVRKKNWGFRLLEEVMIKSWWVIFSILIGGFVYDRAIQELRTEELRLQSKVSSLCQDILSAQEKQRQLQ
LHLQHWQDSAAIEAALIQRLGLIPKGYKKLCVSPKQQSENKD

>core/934/3/Org3_Gene296

MWYSDYHVWILPVHERVVRLGLTEKMQKNLGAILHVDLPSVGSLCKEGEVLVILESSKSAIEVLSPVSGEVIDINLDLVDNPQKINEAPEGEGWLAVVRLDQDWDPSNLSLMDEE

>core/936/3/Org3_Gene672

MGKKENQLYEGAYVFSVTLSEEARRKALDKVISGITNYGGEIHKIHDQGRKKLAYTIRGAREGYYYFIYFSVSPGAITELWKEYHLNEDLLRFMTLRADSVKEVLEFASLPE

>core/937/3/Org3_Gene640

MTAVLILTSFPSEESARSLARHLITERLASCVHVFPKGTSTYLWEGKLCESEEHHIQIKSIDIRFSEICLAIQEFSGYEVPEVLLFPIENGDPRYLNWLTILSYPEKPPLSD

>core/938/3/Org3_Gene441

MFKATARYIRVQPRKARLAAGLMRNLSVQEAEEQLGFSQLKAGRCLKKVLNSAVANAELHENIKRENLSVTEVRVDAGPVYKRSKSKSRGGRSPILKRTSHLTVIVGEKER

>core/939/3/Org3_Gene437

MKKQNIRVGDKVFILAGNDKGKEGKVLSLTEDKVVVEGVNVRIKNIKRSQQNPKGKRISIEAPIHISNVRLTIAGEPAKLSVKVTEQGRELWQRRPDGTSQLYRLVRGKKG

>core/940/3/Org3_Gene294

MTTLPKYVPRSRQNPDTLTFLKRYSSVLLHSENSLSYRIFAKVLAILLTSLAVAFAVTLFSCEGSQLRLCALYIGIALAICVLLTIVVYCIASKIATACKKPPSISRIEIV

>core/943/3/Org3_Gene443

MKDPYDVIKRHYVTEKAKMLEHLSAGTGEGKKKGSFCKDPKFVFIVSHDATKPLIAQALEAIYVDKNVKVKSVNTINVKPQPARMFRGRRKGKTSGFKKAIVTFYQGHSVG

>core/945/3/Org3_Gene323

MTVFKQIIDGLIDCEKVFENENFIAIKDRFPQAPVHLLIIPKKPIPRFQDIPGDEMILMAEAGKIVQELAAEFGIADGYRVVINNGAEGGQAVFHLHIHLLGGRPLGAIA

>core/946/3/Org3_Gene963

MNLSAKEYGDIIVIYLQGSLDAVSVPSVQEYLEQFIQKKHLKIALNVTDVSYISSAGIRLLLSNFKLVQSLGGKMCLCCVKESVTEVMRIAGLDQLILLCQSEQECLSKL

>core/947/3/Org3_Gene346

MASSATPGFDGTAPSLFPPATRPRYNFKLALFVTIAIALVWIALIATTIAIGLCIHPLCSFIFLTAIPLYFISRYICSHYARNVYIALDVVPDHSKLQDMRSHSPIFSDR

>core/948/3/Org3_Gene65

MKKNTHPEYRQVLFVDSSTGYKFVCGSTYQSEKTEVFEGKEYPVCYVSVSSSSHPFFTGSKKFVDAEGRVDKFLKRYSNVRQPAQQPQPEEDALPAAKGKKKVVTKKKK

>core/949/3/Org3_Gene21

MNEPTRTYLESEKDTQDQIEELQATCIVKNAAGIHVRPAGVIVRLFDGEPCDVHFTYAGKTINAKSIMSILMLGAPQGGEILVTIRSKEAHRILQKIQDAFSSGFGEL

>core/950/3/Org3_Gene792

MSTSPIGVPSMLNAATSLNATTSKAPLPTSTLAERIKEWLPRILLLIVGAIFTIAGCIVMALTKQILYGLLCVVGGLLLALGLLLKPENCIYRNAESAARSLSNALE

>core/952/3/Org3_Gene408

MDEITPNYPLLRQDSLWNRVRVSWRADLSVSSRYEIASAIAILGLLVAFCASAAVSIIFTANPLAQVFIDGCLALGLLPIPLVIGLLIIGIIVLLYGIYLFPQQRE

>core/954/3/Org3_Gene365

MEPYAVIQTGSKQYQVRSGDVIDVELLGEVASDKEVIFQDVLFVFDGTKASLGSPTIANAQVKAEYLSHVKGEKVVAYKYKKRKNYHRKHGHRQKYLRVKIREILI

>core/955/3/Org3_Gene506

MSFTYFLALPVDRLMQERFLCSPKRWAPFINSPLYLTLIADHDTPYLAKNLDKFPLPVEQWEKTVLHVSSLLKSIFLCSDLSSLRLLACTKFEILTLNDLYCAQNI

>core/956/3/Org3_Gene396

MNERTLLLLLLKKKKGLFLAILDLTQTESSLTTPELEKVLKQKKIFLSCIDRVDLQIKEFRHAFSSELPQDIQEELEEIRDVIIRILDTDKRNYAQKKKEFGIYERP

>core/957/3/Org3_Gene368

MKQQKQKIRIRLKGFDQGQLDRSTADIVETAKRTGARVVGPIPLPTKREVYTVLRSPHVDKKSREQFEIRTHKRLVDILDPTGKTIDALKMLALPAGVDIKIKAA

>core/958/3/Org3_Gene248

MSSPVVTGTSSASPVEQTKLGEFLERLSGSGRCIKIAFAASTALLLLNTFVSGIVAIAMIFVATSVGAYFTVIGPLFLLSLILLAIMLISMYKITHPSQNTPISN

>core/959/3/Org3_Gene403

MSFKRFLQQIPVRICLLIIYLYQWLISPLLGSCCRFFPSCSHYAEQALKSHGFLMGCWLSIKRIGKCGPWHPGGIDMVPKTALQEVLEPYQEIDGGDSSHFSE

>core/961/3/Org3_Gene456

MVKIISSENFDSFIASGLVLVDFFAEWCGPCRMLTPILENLAAELPHVTIGKINIDENSKPAETYEVSSIPTLILFK
DGNEVARVVGLKDKEFLTNLINKHA

>core/962/3/Org3_Gene80

MSDQATTLRIKPLGDRILVKREEEEATARGGIILPDTAKKKQDRAEVLVLGTGKRTDDGTLLPFEVQVGDIIL
MDKYAGQEITIDDEEYVILQSSEIMAVLK

>core/963/3/Org3_Gene295

MDPASPVAPHVLQDHVQLSSEELSALSSGVSRVKKLTIAIMVLSLIAISLVACGLFLTGSAPLQLSIWIAASCIT
LSMLVCACWRYKISNALEKTKVAHES

>core/964/3/Org3_Gene661

MAKKSSVAREAKRRRLVEANFKKRSDLRKIVKSLSVSEEEKENARISLNKMKRDTSPTRLHNRCLLTGRPRG
YLRKFAISRICFRQMASMGEIPGVIKASW

>core/965/3/Org3_Gene2

MEQFHLDREEILLLAKASALQLSEELIQEYQTSLSAVITSMKEALAIEIDDADSCESLFMHVVNVEDLREDSVT
SDFNREEFLRNVPESLGGLVKVPAVIK

>core/967/3/Org3_Gene360

MFRNNHKPKKTKCKRFRWLRGVLFGGFIATLLTCLFTPKSGVQLRKKILKVKNSGAKKSRVFFKNSKQHTKS
FVKQAKLLAKNISHELQDFKKGILDDKD

>core/968/3/Org3_Gene281

MATMTKKKLISTISQDHKIHPNHVRTVIQNFLDKMTDALVKGDRLEFRDFGVLQVVERKPKVGRNPKNAAV
PIHIPARRAVKFTPGKRMKRLIETPNKHS

>core/969/3/Org3_Gene529

MAPKKPNKKNVIQRRPSAEKRILTAQKRELINHSFKSKVKTIVKKFEASLKLDDTQATLSNLQSVYSVVDKA
VKRGIFKDNKAARIKSKATLKVNARAS

>core/970/3/Org3_Gene721

MKKVVTLSIIFFATYCASELSAVTVVAVPLSEAPGKIQVRPVVGLQFQEEQGSVPYSFYYPYDYGYYYPETYG
YTKNTGQESRECYTRFEDGTIFYECD

>core/971/3/Org3_Gene505

MEPRHIYIRKPETPKAPDVEKPGVPEYMTMANTPTFEGPVKTLDQLRRALIEQRGAEEGQKMYDNFIQSILIST
FGLVHKDMDRAQKASKRMRSVYKEQ

>core/972/3/Org3_Gene818

MCLIDCLGQGFEAAINTVCCCSDSSESKANVATVSAGLLALTAIVSFILIILICTGVLGASGMTFGMSNVAAVL
VLLVTILLSMFLSGACFAAKREILR

>core/974/3/Org3_Gene540

MLSYLLRTAINVYSFLILAYIFASWVPDCQSARWYQLVSKCVDPFLNFFRRFVPRIGFIDPSPFVGLLCLGILPF
VILRVLRFIILNIFHSPWLLQYL

>core/975/3/Org3_Gene223

MGKPKKSRTDRALAQEIQKKSTEVLKKPARIKAKNRRKFLIAKEQKTLKHRAQEYDQLVRSLLDSQKKDTD
KVLIFNYENGFVFTDKDHFSKYSIRL

>core/976/3/Org3_Gene73

MIKKDRFTNEKLNKLFDSPFSLVNYAIKQAKIKIAKGDVRSSNVAIETLVLLDREGIQPEFTEEIVVTASPTVER
KRSEHTNSRKKDPSAYTWSDVK

>core/977/3/Org3_Gene371

MYEGKSRMASPTPGQLHLQQKVESKAYDYSRSLAMIATALLFFIVALILSGLSLLPQVFLPFSGAYFIIGSFLAF
IALGILLINCVCDLKQYLTSS

>core/978/3/Org3_Gene22

MGSGYAKKKKEAKIMEQQFLEMEASLLEKRYEGQAGNGLVSVVINGKCDLISVKVQPTCLDPEDPEVIEDLF
RAAFKLAKEQMDQEMSLMRSTMPF

>core/979/3/Org3_Gene283

MNKSRFLRLCCCLCFCGSLFYFYINKQNSLTKLRLEIPCLSVRLRQLEQQNISLRFLIDKIERPDHLMEIAALPE
YQYLEYPSEESISLLSYELP

>core/980/3/Org3_Gene581

MLAFFATSFKSVLFEYSYQSLLLILIVSAPPIILASIVGIMVAIFQAATQIQEQTFAFAVKLVVIFGTLMISGGWL
SNMILRFAGQIFQNFYKWK

>core/982/3/Org3_Gene414

MLIRLFLGISLPKGFPLYLEPPLVLATFQGTQFVGTYSEATNPLYIDNLNLNYHYTQELLYKAVPCNYKSIYRE
IPLIIFPEVLIGSTPTQSTE

>core/983/3/Org3_Gene1

MLGKIIRGLSSLIVILCALNVGLIGITHNKLNIIAKLCGGVSTPATQITYIIIGIAGVICLLSFCPFCSKKSRHSHGD
SCSSGGCHSHHSDKN

>core/988/3/Org3_Gene241

MAKLVITSDDEQQEFELEDNSEIAEPCESMGIPFACTEGVCGTCVIEVLEGRENLSEFTEPEYDFLGEPEDSNERLACQCRIKGGCVKVTF

>core/989/3/Org3_Gene327

MDDSWILEVKVTPKAKENKIVGFDGQALKVRVTEPPEKGKANDAVISLLAKALSLPKRDVTLIAGETSRKKKFLLPNRVQDIIFSLHIDV

>core/990/3/Org3_Gene374

MKKAVLIAAMFCGVVSLSSCCRIVDCCFEDPCAPSSCNPCEVIRKKERSCGGNACGSYVPSCSNPCGSTECNSQSPQVKGCTSPDGRCKQ

>core/991/3/Org3_Gene705

MSLDKGTKEEITKKFQLHEKDTGSADVQIAILTEHIAELKEHLKRSPKDQNSRLALLKLVGQRRKLLEYLNSTDTERYKNLITRLNLRK

>core/992/3/Org3_Gene218

MSRKCPLTGKRPRRGYSYTLRGIAKKKKGIGLKVTGKTKRRFFPNMLTKRLWSTEENRFLKLKISASALRHIDKLGLEKVLERAKSKNF

>core/993/3/Org3_Gene758

MSRSLRKGPFVDHHLLKKVRAMNIEEKKTPIKTWSRRSMITPEMIGHTFEVHNGKKFLTVFVSETMVGHKLGEFSPTRIFKSHPVKKG

>core/994/3/Org3_Gene111

MKEYLDFLVQRNVERDPQTKRHCTVSQKFGGESIDAKTTTGQLFHIAGKTEPGHGKLCLGESILKQLLALGIITGYENREREVWVYLD

>core/996/3/Org3_Gene391

MSQKNKNSAFMHPVNISTDLAVIVGKGPMPRTEIVKKVWEYIKKHNCQDQKNKRNILPDANLAKVFGSSDPIDMFQMTKALSKHIVK

>core/997/3/Org3_Gene666

MKSFKFLLPFLSVILCCGNLLSSPRSRAISVTESIGMSAVKTLVLSEKAHEFLEGIGYGVGASSILRDWQTQQWLEIESLLAQNEVM

>core/998/3/Org3_Gene438

MASEPRGSRKVKIGVVVSAKMEKTVVVRVERIFSHPQYLKVVRSSKKYYAHTELKVSEGDKVKIQETRPLSKLKRWRVIEHVGVVS

>core/999/3/Org3_Gene978

MVEIHHKDPSLKKLFALQQSLETLNSLSDIVATYEAMFSLIYEGLNKALRKDQLCYLLSGNSKGELLKSPSGD PIVQTFPIHPHH

>core/1000/3/Org3_Gene499

MATNKSCTAFDFNKMLDGVCTYVKGVQQYLTELETSTQGTVDLGTMFNLQFRMQILSQYMESVSNILTAVN TEMITMARAVKGS

>core/1001/3/Org3_Gene364

MAHKKGQGASRNGRDSKSKRLGVKVGAGQKVSTGSILVRQRGTRWNPAQNVGRGRDDTLFALVDGIVVM KKTNRTYISVVPEQL

>core/1003/3/Org3_Gene40

MKQQHNRKALSRKIGTVKKQAKFAGSFLDEIKKIEWVSKHDLKKYIKVVLISIFGFGFAIYFVDLVLRKSITCL DGITTFLFG

>core/1004/3/Org3_Gene500

MFNMENTAKEEKNSQPLLDLEQDMQDHDRAQELKASVQDKVHKLHALLREGSDKESFGQQQSLLAGYVA LQKVLGRINRKMI

>core/1005/3/Org3_Gene673

MNKPVHNNEHRRKRFNKKCPFVSAGWKTIDYKDVETLKKFITERGKVLPRRITGVSSRFQGVLSQAIKRARH LGLLPFVGED

>core/1007/3/Org3_Gene33

MTKIQCSAQYYRSRPAERAQTPPQPFLARDRADFWERHPRFSACCRVLLLVAWVVLALLFLFVMLLPLAAG SYLLAF

>core/1009/3/Org3_Gene192

MSLEDDVIAIIVEQLGVDPKEVNENSSFIEDLNADSLDLTELIMTLEEKFAFEISEEDAEKLRTVGDVFTYIKKR QAEQ

>core/1012/3/Org3_Gene939

MKEFLAYIIKNLVDRPEEVRIKEVQGTHTIIYELSVAKPDIGKIIGKEGRTIKAIRTLLVSVASRTNVRVSLEIME EK

>core/1013/3/Org3_Gene767

MMTLFLVICCATVLLGLGMGILLVGSHLLGRPLSKGCQKPDCCQKKTCDKTEHCATKSRENSTSKCSSNDDV PPTAP

>core/1014/3/Org3_Gene547

MNEGIHSVCFQKTPRLTAKSVVSMEMLLTTQQLPSAEGMPSVANLEADFLRAEALLAEMREIRGCLEQSLRT
LVPSE

>core/1015/3/Org3_Gene707

MKNKIVTLLDQLYEDQESRLQKLGEEIVPNLTPEDLLQPMDFPQLEGNPAFRFEEGVLSGIGEVRAAILAALS
QEN

>core/1016/3/Org3_Gene849

MEAKKIKELSKEAQLLKKLREKSRVLDEKNKRKAWVAKLVAMPESIREIEKEERVETPQLFQAIAEKILEEGV

>core/1018/3/Org3_Gene511

MFFAPLLYESLRRGLMHPTSHMQQQLARLEFINDQLTTELEHVNELLCSLGFPEGLTTIKAIAEEVLSDDEPLL
D

>core/1019/3/Org3_Gene829

MEEVPFENAMQRLEEIVDLMNQPTTSLDASLALYEEADALMRICESRIRQVEQRVRELAEKRHESSLFEEQA
VVR

>core/1021/3/Org3_Gene38

MAKKEDTLVLEGKVEELLPGMHFRVILENGMPVTAHLCGKMRMSNIRLLVGDRVTVEMSAYDLTKARVVY
RHR

>core/1022/3/Org3_Gene835

MSGNECNDYPEVFKDDVSAYVLVTCGQMSSEGKIQVEMTYEGDPAVISYLLTKARDSLDES

>core/1023/3/Org3_Gene991

MSYKITLPKADETTAKKVTKISEASTLIFSVLKEKASLGNVHGFCQAENSLSVEANKIISVAENTLAGCFCK

>core/1025/3/Org3_Gene439

MAAKKDLLTQLRGKSDDDLDAYVHENKKALFALRAENLLQNKVVKVHMFSTHKKNIARALTVKQERKGK
VHG

>core/1027/3/Org3_Gene757

MMFDFMLARAFRAANTIVNTKQKPFHIHGMATLRPNATSILNIKEDALSLGTVGSFASTASIKIDSH

>core/1028/3/Org3_Gene460

MKKLIALIGIFLVPIKGNTNKEHDAHATVLKAARAKYNLFFVQDVFPVHEVIEPISPDCLVHYEGWV

>core/1031/3/Org3_Gene761

MKKLMLALLLALVLPVAGSAFGYESCSAPQDEAQGSEDEGRDESSDSDTQTEKDEEGGSEENTAE

>core/1033/3/Org3_Gene696

MPKMKTNKSVSARFKLTASGQLKRTRPGKRHKLSKKSSQEKRNLSKQPLVDKGQVGMYKRMMLV

>core/1034/3/Org3_Gene321

MIKQACKFYLLQCLLCALYWLLKYCRKLLKGTLHHSEETLYQALLSSLIDLLYQLKQLPAPTNE

>core/1037/3/Org3_Gene375

MKLFYFCNIIFLTSLFSVSCTHTPPPRSYILAQGRTPLVNKAHSLEFFLARSVFNTCYNTNL

>core/1045/3/Org3_Gene678

MAVPRNRHSNARKNIRRSHDAKKACHAAKCSNCKHALLPHTICPSCGFYNGKAVMTVEKK

>core/1046/3/Org3_Gene469

MSIALNREEVWDNPHHLMFILMQFQQFSGEQDRFGSFLEATIRDRVSFLVLQEKIATLK

>core/1049/3/Org3_Gene989

MRFFCLFFLGFLGSFHCVAEDKGVDLFGVWDDNQITECDDSYMTEGREEVEKVVDA

>core/1050/3/Org3_Gene16

MPSVKVRVGEPVDRALRILKKKIDKEGILKAAKSHRFYDKPSVKKRAKSKAAAKYRSR

>core/1054/3/Org3_Gene267

MQIENSSILFAEVVMKWFIFSVISAPVVFLPGCTLIPKEKVTKVPSQLWSESLSQP

>core/1056/3/Org3_Gene985

MIDAENQNPKDGGSFTSLHTDPKNLFDEEGMPSPSDTLQCDLNNVFIFIKSMFF

>core/1058/3/Org3_Gene335

MSRHRSYGKSVKGVTKRNVLKRFERVEVLRKLGRWNDSTAKKVTGLPKTPILK

>core/1059/3/Org3_Gene157

MASKNREIIKLKSSESSDMYWTVKNKRKTTGRLELKKYDRKLRRHVIFKEAR

>core/1067/3/Org3_Gene723

MFFIAVRSRGFLDIHGILAARKGKQVVKSTAGAWIGSRGAVFYSLVS

>core/1068/3/Org3_Gene990

MSESLEIPELTEVLSEQPSLSTPDSPPKVITGTLTLYFQEDIDPAS

>core/1069/3/Org3_Gene660

MKVSSSVKADPSKGDKLVRRKGRLYVINKKDPNRKQRQAGPARKK

>core/1070/3/Org3_Gene659

MKRTYQPSKRKRRNSVGFRTRMATRNGRKLLNRRRRHGRHSLVDL

>core/1073/3/Org3_Gene1008

MSSVKKKRRLKIAKHKRKKRRRRDRHKNK

>core/0/4/Org4_Gene652

MKSLPVYVSGIKVRNLKNVSIHFNSEEIVLLTGVSGSGKSSIAFDTLYAAGRKRYISTLPTFFATTITTLPNPKV
EEIHGLSPTIAIKQNHFSHYSHATVGGTTELFSHLALLFTLEGQARDPKTKEVLDLYSKEKVLSTIMELSEGVQI
SILAPLLRKDIAAIHEYAQQGFTKVRCNGTIHPIYSFLTSGIPEDCSVDIVIDTLIKSENNIARLKVSLFTALEFGE
GHCSVLSDEELMTFSTKQQIDDVTYTPLTQQLFSPHALESRCSLCQGSGIFISIDNPLLIDENLSIKENCCSFAGN
CSSYLYHTIYQALADALNFNLETPWKDLSPEIQNIFLRGKNNLVLPVRLFDQTLGKKNLTYKVWRGVLNDIG
DKVRYTTKPSRYLSKGMSAHSCSLCKGTGLGDYASVATWEGKTFAEFQQMSLNNWHVFFSKVKSPSLSIQEI
LQGLKQRLSFLIDLGLGYLTPNRALATLSGGEQERTAIAKHLGGELFGITYILDEPSIGLHPQDTEKLIGVIKKL
RDQGNTVILVEHEERMISLADRIIDIGPGAGIFGGEVLFNGKPEDFLMNFSSLTAKYLRQELTIPIPESREAPTS
WLLLTEATIHNLKNLSIRLPLARLIGVTGVSGSGKSSLINNTLVPAIESFLKQENPKNLHFEGGCIGRLIHITRDL
PGRSQRSIPLTYIKAFDDIRELFASQPRSLRQGLTKAHFSFNQPQGACIQCQGLGTMTISDDDTPIPCSECQGKR
YHSEVLEVLYEGKNIADILDMTAYEAEKFFISHPKIHEKIHALCSLRLDYLPLGRPLSTLSGGEIQRLKLAHELL
FASPKQTLYVLDEPTTGLHTHDIQALIEVLLSLTCLGHTVLVIEHNMHVVKVCDYVLELGPEGGDLGGYLLA
SCTPKDLIQLNTPTAKALAPYIEGSLDIPVVKSEPPSSPKSCDILIKDAYQNNLKHIDLALPRNSLIAIAGPGASG
KHSLVFDILYASGNIAYAELFPPYIRQGLLKETPLPSVGEVKGLSPVISVRKCSSSNRSYHTIASALGLSNGLEK
LFAILGEPFSPLTEEKLSKTTPQTIIDSLLKSYKDDYVTITSPIPLGTDLEIFLQEKQKEGFIKLYSEGNLYDLDET
LPLSLIEPAIVIQHTKVSPKNSSSLLSAISVAFSLSSEIWIYISQKKQRKLSYSLGWKDKKGRLYPEITHQLLSSD
HPEGRCLTCGGRGEILKISLEEHKEKIAHYTPLEFFSLFFPKSSTKPVQKLLKDENASKPLKLLTTKEFLNFCRG
SSEFPGMNALLMEQLDTESDSPLIKPLLALTSCPACKGSGLNDYANYVRINNTSLLDIYQEDATFLESFLNTIG
TDDTRSIIQDLMNRLTFISKVGLSYITLGQRQDTLSDGENYRLHLAKKISTNLTNIVYLFEEPLSGLHPQDLPTI
VQLLKELVANNNTVIATDRSCSLIPHADHAIFLGPGSGPQGGFLMDSDTEVCPSVDLHANVPQTEVCPKAPLS
ISKANHTRGSDRTLKVNLSIHHIQNLKVSAPLHALVAIGGVSGSGKTSLLLEGFKKQAELLIAKGTTTFSDLVV
IDSHPIASSQRSDISTYFDIAPSLRAFYASLTQAKALNISSTMFSTNTKQGQCSDCQGLGYQWIDRAFYALEKR
PCPTCSGFRIQPLAQEVLYEGKHFGELLHTPIETVALRFPFIKKIQKPLKVLLDIGLGYLPIGQKLSSLSVSEKTA
LKTAYFLYQTPETSTLFLIDELFSSLDPIKKQHLPEKLRSLINSGHSVIYIDHDVKLLKSADYLIEIGPGSGKQGG
KLLFSGSPKDIYASKDSLLKKYICNEELDS

>core/1/4/Org4_Gene389

MKWLPATAVFAAVLPALTAFGDPASVEISTSHTGSGDPTSDAALTGFTQSSTETDGTTYTIVGDITFSTFTNIP
VPVVTPDANDSSSDSSKGGSSSSGATSLIRSSNLNSDFDFTKDSVLDLYHLFFPSASNTLNPALLSSSSSGGSSS
SSSSSSSGSASAVVAADPKGGAAFYSNEANGTLTFTTDSGNPGSLTLQNLKMTGDGAAIYSKGPLVFTGLKN
LTFTGNESQKSGGAAYTEGALTTQAIVEAVTFTGNTSAGQGGAIYVKGATLFNALDSLKFEKNTSGQAGGGI
YTKSTLTISNITKSIEFISNKASVPAPAPEPTPPAPAPSPAPSSLINSTTIDTSTLQTLAASATPAVTPVAAVTPTPI
STQETAGNGGAIYAEQGISISTFKDLTLKSNSASVDATLTVDSSTIGESGGAIFAADSIQIQQCTGTTLFSGNTA
NKSGGGIYAVGQVTLEDIANLKMTNNTCNGEGGAIYTKKALTINNGAILTTFSGNTSTDNGGAIFAVGGITLS
DLVEVRFSKNKTGNYSAPVTKAASNTAPVVSSSTTAASPAVPAAAAAPVTNAAKGGALYSTEGLTVSGITSIL

SFENNECQNQGGGAYVTKTFQCSDSHRLQFTSNKAADEGGGLYCGDDVTLTNLTGKTLFQENSSEKHGGGL
SLASGKSLTMTSLESFCLNANTAKENGGGANVPENVVLTFTYTPTPNEPAPVQQPVYGEALVTGNTAKKSGG
GIYTKSAAFSNLSSVTFDQNTSSENGGGLLTQTIADKTDCSFTYITNVNITNNTATGNGGGIAGQKAHFDRIDN
LTVQSNQAKKGGGVYLGDSLILEKVITGSVSQNTATESGGGIYAKDIQLQALPGSFTITDNKVETSLTTSTNLY
GGGIYSSGAVTLTNISGTFGITGNSVINTATSQDADIQGGGIYATTSLSINQCNTPILFSNNSAATKKTSTTKQIA
GGAIFSAAVTIENNSQPIIFLNNSAKSEATTAATAGNKDSCGGAIAANSVTLTNNPEITFKGNYAETGGAIGCID
LTNGSPPRKVSIADNGSVLFQDNSALNRGGAIYGETIDISRTGATFIGNSSKHDGSAICCSTALTLAPNSQLIFE
NNKVTETTATTKASINNLGAAIYGNNETSDVTISLSAENGSIFFKNNLCTATNKYCSIAGNVKFTAIEASAGKA
ISFYDAVNVSTKETNAQELKLNEKATSTGTILFSGELHENKSYIPQKVTFAHGNLILGKNAELSVVSFTQSPGT
TITMGPGSVLSNHSKEAGGIAINNVIIDFSEIVPTKDNATVAPPTLKLVSRTNTDSKDKIDITGTVTLLDPNGNL
YQNSYLGEDRDITLFNIDNSASGAVTATNVTLQGNLGAKKGYLGTWNLDPNSSGSKIILKWTFDKYLRWPYI
PRDNHFYINSIWGAQNSLVTVKQGILGNMLNNARFEDPAFNNFWASAIGSFLRKEVSRNSDSFTYHGRGYTA
AVDAKPRQEFILGAAFSQVFGHAESEYHLDNYKHKGSGHSTQASLYAGNIFYFPAIRSRPILFQGVATYGYM
QHDTTTYYPSIEEKNMANWDSIAWLFDLRFSVDLKEPQPHSTARLTFYTEAEYTRIRQEKFTELDYDPRSFSA
CSYGNLAIPTGFSVDGALAWREIILYNKVSAAYLPVILRNNPKATYEVLSTKEKGNVVNVLPTRNAARAEVS
SQIYLGSYWTLYGTYTIDASMNTLVQMANGGIRFVF

>core/2/4/Org4_Gene773

MVAKKTVRSYRSSFSHSVIVAILSAGIAFEAHSLHSSELDLGVFNKQFEEHSAHVEEAQTPVLKGSDPVNPSQ
KESEKVLYTQVPLTQGSSGESLDLADANFLEHFQHLFEETTVFGIDQKLVWSDLDTRNFSQPTQEPDTSNAVS
EKISSDTKENRKDLETEDPSKKSGLKEVSSDLPKSPETAVAAISEDLEISENISARDPLQGLAFFYKNTSSQSISE
KDSSFQGIIFSGSGANSGLGFENLKAPKSGAAVYSDRDIVFENLAKGLSFISCESLEDGSAAGVNIVVTHCGDV
TLTDCATGLDLEALRLVKDFSRGGAVFTARNHEVQNNLAGGILSVVGNKGAIVVEKNSAEKSNGGAFACGS
FVYSNNENTALWKENQALSGGAISSASDIDIQGNCSAIEFSGNQSLIALGEHIGLTDFVGGGALAAQGTLTLR
NNAVVQCVKNTSKTHGGAILAGTVDLNETISEVAFKQNTAALTGGALSANDKVIIANNFGEILFEQNEVRNH
GGAIYCGCRSNPKLEQKDSGENINIIGNSGAITFLKNKASVLEVMTQAEDYAGGGALWGHNVLLDSNSGNIQ
FIGNIGGSTFWIGEYVGGGAILSTDRVTISNNSGDVVFKGNKGQCLAQKYVAPQETAPVESDASSTNKDEKSL
NACSHGDHYPPKTVEEEVPPSLLEEHPVVSSTDIRGGGAILAQHIFITDNTGNLRFSGNLGGGEESSTVGDLAI
VGGGALLSTNEVNVCSNQNVVFSDNVTSNGCDSGGAILAKKVDISANHSVEFVSNGSGKFGGAVCALNESV
NITDNGSAVSFSKNRTRLGGAGVAAPQGSVTICGNQGNIAFKENFVFGSENQRSGGGAIIANSSVNIQDNAGD
ILFVSNSTGSYGGAIFVGSLVASEGSNPRTLTITGNSGDILFAKNSTQTAASLSEKDSFGGGAIYTQNLKIVKNA
GNVSFYGNRAPSGAGVQIADGGTVCLEAFGGDILFEGNINFDGSFNAIHLCGNDSKIVELSAVQDKNIIFQDAI
TYEENTIRGLPDKDVSPLSAPSLIFNSKPQDDSAQHHEGTIRFSRGVSKIPQIAAIQEGTLALSQNAELWLAGLK
QETGSSIVLSAGSILRIFDSQVDSSAPLPTENKEETLVSAGVQINMSSPTPNKDKAVDTPVLADIISITVDLSSFV
PEQDGTLPLPPEIIIPKGTKLHSNAIDLKIIDPTNVGYENHALLSSHKDIPLISLKTAEGMTGTPTADASLSNIKID
VSLPSITPATYGHTGVWSESKMEDGRLIVGWQPTGYKLNPEKQGALVLNNLWSHYTDLRALKQEIFAHHTIA
QRMELDFSTNVWGSGLGVVEDCQNIGEFDGFKHHLTGYSLGLDTQLVEDFLIGGCFSQFFGKTESQSYKAKN
DVKSYMGAAYAGILAGPWLIKGAFVYGNINNDLTTDYGTLGISTGSWIGKGFIAGTSIDYRYIVNPRRFISAIV
STVVPFVEAEYVRIDLPEISEQGKEVRTFQKTRFENVAIPFGFALEHAYSRGSRAEVNSVQLAYVFDVYRKRP
VSLITLKDAAYSWKSYGVDIPCKAWKARLSNNTEWNSYLSTYLAFNYEWREDLIAYDFNGGIRIIF

>core/3/4/Org4_Gene620

MASSSNNSTKQDGIPSWVNPNVQWNRASQVGDQEAKSLTPEAQTSRSWFSDRKHFLEVLDVSLEEMENNDL
KKYSRYKTIILIATLVTVAITCIVPISMVFGIPMWVPCLILFGVGLSSAFLSHRLQSKCKEIHLRYRAYQIYRQQL
LSQYPDLRKSTLYKYSITHVKPKKGFVGKLVENLRPDLHKNKDDGGAAADSRLDFAGYGVKHYQTDALLG
VSGVNSVEWQRLASLIMSVKNDILNDVGSREPIDKAQRSALVVSGKDIGGEIQPGGILDISRDILAICGYGMNV
GVEAKKAIDQYKKWYLNSSTFIAWNPQLPAIAQSYLLEQQQHLDYAAKIFQDLSALTTAYGTGQALEDLDSL
LCYYDQLIESKGVGEKIIASIHQKHLDLAMQDSCDQEHLKKWSNLYHVFSLTIKEFTEGKLEQNEVVSRIQRL
RGKLEKSKCSILGNCRTNAEYATKSEKKLADYLLQIGDREPFLTGMHKAIATGKAIQGKVEGVISQHPEKQIM
MLRCSIERLEGMLRREDWGAILQKNEDEVLALKSTMEAQLQGFKDLVGAWEGKYQEFKKNKLSKVLVYDF
TKSYSNLLNRLEVLHAESSTDDLVLHVDRMSEDLKKTIEEIDGNLFQVTPEELSLLAREYQGLMNELPLIVQE
GNRLQEVISSEGVSQGLMLLNSLLNRDEKINKNIESSRKNLVAIAKQARSDARNIDSQGLAPLIQRNRASLDNI
LQNMHLFNGSIRNIHALDTETLVATSSNMFSAMHTFDWNIYTNLLDVLEIQSKPAPAPMENPDLPGALPEEVQ
DAVAEDVSGTHRLHHQVLKRRCADLKNMISQLQKSINKWGMAKAIVLGIVAVLFCVLSTIFIGQNILSLLILS
CVGLLLTQVCPLIFDRISKSKEFEKQVLETAQSLIPATKILPSEFNNKDLNRLAKLQDNLNLEGFGPTWARNIV
SDLEGIPTKEKSLKDLTKEFRKDSKNLNKRIKRRFKEGLGQEAPVVRPTIPQDIRGAEVFAELHRELEHLQKQ
KEEISIRGDALVQERMGLCLEKSKYDNEKAHAAAMTKKVGKLQNIDRLQKNNETYVRIQNFFRTLIQEKLGR
DTVQEIDVVKEAKELHELAAIIYGNTSGKSQKQRAKKQFKENVLHIAGKGQLELLEAYLNVTASQGLCRHQ
MQASFRERILLNPDGAKHGEAERTLASREEMLKTLGLSYLTPFVRFSSPESTQSGYNQILKVREQLFDIEQRLQ
NQETVSPEDYAAVQAALAAYVRKHESLIVSTYGLGAQEGQTSSKVTTLMRDLHAVEELVEMGVETYRLNRS
DQILHRVHSVLHSHLRDSNSSGNGIIDVVKKLFELLNNNGNNPSDPECQKYMQILLDAPVSLLYGAFKSFKNE
FLLNFTELNIANSTKAAEEEAKRYVEEKGRGFETYWEEAKQRLEAIAAELDDLKNQETLLEQEIRLANLKISIF
SDLNLREKVSVEKAALEEEIQGIQEQYAEMQGIEDLELKQKFEDLQKKLEALEERLLQIGRRIDSSVDKQKEL
LGLLGREEAA

>core/4/4/Org4_Gene449

MKHSLPWLLTSSALVFSLHPLMAANTGLSSSDNYENGSSGSAAFTAKETSDASGTTYTLTSDVSITNVSTITPA
DKSCFTNTGGALSFVGADHSLVLQTIALTHDGAAINNTNTALSFSGFSSLLIDSAPATGTSGGKGAICVTNTEG
GTATFTDNASVTLQKNTSEKDGAAVSAYSIDLAKTTTAALLDQNTSTKNGGALCSTANTTVQGNSGTVTFSS
NTATDKGGAIYSKEKDSTLDANTGVVTFKSNTAKTGGAWSSDDNLALTSNTQVLFQENKTTGSAGQANNPE
GCGGAICCYLATATDKTGLAISQNQEMSFTSNTATANGGAIYATKCTLDGNTTLTFDQNTATAGCGGAIYTE
TEDFALTGSTGTVTFSTNTAKTGGALYSKGNSSLTGNTNLLFSGNKATDSASSPANQDGCGGAILAFIDSGSV
SDKTGLSIANNQEVSLTSNTATVSGGAIYATKCILTGNGSLTFDGNTAGTSGGAIYTETEDFTLTGSTGTVTFN
ANTAKTGGALYSKGNNSLSGNTNLIFSGNKSTDSSGSSANQDGCGGAILSFLESASASAKKGLVIEDNENVSL
SGNTATVSGGAIYATKCALHGNTTLTFDGNTAGTSGGAIYTEDQDFTLTGSTGTVTFSTNTAKTAGALHTKG
NTSFTKNKALVFSGNSATATTTTDQEGCGGAILCNISESDIATKSLTLTENESLSFINNTAKRSGGGIYAPKCVI
SGSESINFDGNTAETSGGAIYSKNLSITANGPVSFTNNSGGKGGAIYIADRGELSLEAIDGDITFSGNRATEGTS
TPNSIHLGAGAKITKLAAAPGHTIYFYDPITMEAPASGGTIEELVINPVVKAIVPPPQPKNGPIASVPVVPVAPA
NPNTGTIVFSSGKLSSQDASIPANTTTILNQKINLAGGNVVLKEGATLQVYSFTQQPDSTVFMDAGTTLETTTT
NNTDGSIDLKNLSVNLDALDGKRMITIAVNSTSGGLKISGDLKFHNNEGSFYDNPGLKANLNLPFLDLSSTSG
TVNLDDFNPIPSSMAAPDYGYQGSWTLVPKVGAGGKVTLVAEWQALGYTPKPELRATLVPNSLWNAYVNI

HSIQQEIATAMSDAPSHPGIWIGGIGNAFHQDKQKENAGFRLISGGYIVGGSMTTPQEYTFAVAFSQLFGKSK
DYVVSDIKSQVYAGSLYAQSSYVIPLHSSLRRHVLSKVLPELPGETPLVLHGQVSYGRNHHNMTTKLANNTQ
GKSDWDSHSFAVEVGGSLPVDLNYRYLTSYSPYVKLQVVSVNQKGFQEVAADPRIFDASHLVNVSIPMGLTF
KHESAKLPSALLLTLGYAVDAYRDHPHCLTSLTNGTSWSTFATNLSRQAFFAEASGHLKLLHGLDCFASGSC
ELRSSSRSYNANCGTRYSF

>core/5/4/Org4_Gene372

MKQKVKRNFAIIICVFALALYYVLPTCLYYAKPLDKKIDGNEAEHIIKSFTKQAQQVRKDVIPRVSAILSSLHL
RGHIQQHPAIPDIVSVRFKRGEDAEGFIGNLVHGEPNVPIKSSRLHVVGYSREHDDHVIQVASSINTSLVESDFS
FVSYSFENEQEIASSILQRVYSACTFPKQKDCSCSYPSIWETAPKEQLLQYTKNLSSGFEVFSSRLSAFCQQSFS
SNQDRLTFLSRLSSLSNDAAIDVEDQKLLKSVYETLSQTACIRSLDCPYIEGLRLDCSESSLFFSSIEYCPKERKI
FLTLHSDLLAQRTSLSKEQRLDFDSRLAVAKQKLSKNLTVQVEDYNDGFSFQWMDKDTQGKIILQGERLLQ
GIAEHLTALTLHRPAAESCDLIPENFPVFCRQPRESEAFGCYIFSPNTDCKHFSKGSVYILLKGLRSIVAKYQQG
GGKELQSFEKDLQNLYNCFSHTEAISWTLGEDQVLEIRHPLQQFLDVWGEGFVIGKEGCAFLEVKDIKDRLA
TVNQIEKNRQSDLVRWHEQYRHAKCSMDLQERLSAPIPYQNLFLENMKLNMRKFSRGENILRLGIDFVGGR
QLLLSFKDHQGKQLTDKEDILKVSDELYARLNKLGVSEIELRREGDYIHLSVPGSSTISSSEILRTSKMSFHVV
NERFSSYSASRYEVQRFLDYLWFTSQAQGKTSPEEINTFASALFNKEVDVPPSVHEAITKLKSEGLAFSPSGCE
TPSTDLDTTFSMIAIGKDAEQKANPLVIVFRNYALDGASLKDIRPEFAAGEGYVLNFSVKDTSPKKMAEKLSP
TESFHTWTSAYCQEGISGTANGQYSANRGWRMAVVIDGYMVSSPILNVPLKNHASVSGKFTHREVSKLASD
LKSGAMSFVPEVLSEETISSDLGKKQCTQGIISACCGLAMLIVLMSVYYRFGGVIASGAVLLNLLLIWAALQY
LDAPLTLSGLAGIVLAMGMAVDANVLVFERIREEFLLSQSLKKSVEKGYTKAFGAIFDSNLTTVLASALLFFL
DTGPIKGFALTLILGIFSSMFTALFMTKFFFMLWMNKTQHTQLHMMNKFVGIKHDFLGGCKKLWAVSGSVF
LLGCVALGFGAWNSVLGMDFKGGYAFTFNPKEHGISDVAQMRGKVVHKLQEAGLSSRDFRIQTFGSSEKIKI
YFSDKALSYTKADTSLSPKINDHELALAVGLLSETGLDFSTETLNETQNFWSKVSSKLSKKMRYQATIGLLGA
LAIILLYVSLRFEWQYAFSAVCALIHDLLATCAVLFIAHFFLKKIQIDLQAIGALMTVLGYSLNNTLIIFDRIRED
RQANLFTPMHVLVNDALQKTFSRTVMTTATTLSVLLMLLFIGGSSVFNFAFIMTIGILLGTLSSLYIAPPLLLF
MVRKENRSK

>core/6/4/Org4_Gene663

MFGENSRDIGVLSKEGLFDKLEIGIASDITIRDKWSCGEIKKPETINYRTFKPEKGGLFCEKIFGPTKDWECCCG
KYKKIKHKGIVCDRCGVEVTLSKVRRERMAHIELAVPIVHIWFFKTTPSRIGNVLGMTASDLERVIYYEEYVV
IDPGKTDLTKKQLLNDAQYREVVEKWGKDAFVAKMGGEAIYDLLKSEDLQSLLKDLKERLRKTKSQQARM
KLAKRLKIIEGFVSSSNHPEWMVLKNIPVVPPDLRPLVPLDGGRFATSDLNDLYRRVINRNNRLKAILRLKTPE
VIVRNEKRMLQEAVDALFDNGRHGHPVMGAGNRPLKSLSEMLKGKNGRFRQNLLGKRVDYSGRSVIIVGPE
LKFNQCGLPKEMALELFEPFIIKRLKDQGSVYTIRSAKKMIQRGAPEVWDVLEEIIKGHPVLLNRAPTLHRLGI
QAFEPVLIEGKAIRVHPLVCAAFNADFDGDQMAVHVPLSVEAQLEAKVLMMAPDNIFLPSSGKPVAIPSKDM
TLGLYYLMADPTYFPEEHGGKTKIFKDEIEVLRALNNGGFIDDVFGDRRDETGRGIHIHEKIKVRIDGQIIETTP
GRVLFNRIVPKELGFQNYSMPSKRISELILQCYKKVGLEATVRFLDDLKDLGFIQATKAAISMGLKDVRIPDIK
SHILKDAYDKVAIVKKQYDDGIITEGERHSKTISIWTEVSEQLSDALYVEISKQTRSKHNPLFLMIDSGARGNK
SQLKQLGALRGLMAKPNGAIIESPITSNFREGLTVLEYSISSHGARKGLADTALKTADSGYLTRRLVDVAQDV
IITEKDCGTLNHIEISAICQGSEELLPLKDRIYGRTVAEDVYQPGDKSRLLAQSGDVLNSVQAEAIDDAGIETIKI

RSTLTCESPRGVCAKCYGLNLANGRLIGMGEAVGIIAAQSIGEPGTQLTMRTFHLGGIAATSSTPEIITNSDGIL
VYMDLRVVLGQEGHNLVLNKKGALHVVRDEGRTLNEYKKLLSTKSIESLEVFPVELGVKILVADGTPVSQG
QRIAEVELHNIPIICDKPGFIKYEDLVEGISTEKVVNKNTGLVELIVKQHRGELHPQIAIYDDADLSELVGTYAI
PSGAIISVEEGQRVDPGMLLARLPRGAIKTKDITGGLPRVAELVEARKPEDAADIAKIDGVVDFKGIQKNKRIL
VVCDEITGMEEEHLIPLTKHLIVQRGDSVIKGQQLTDGLVVPHEILEICGVRELQKYLVNEVQEVYRLQGVDI
NDKHIEIIVRQMLQKVRITDPGDTTLLFGEDVNKKEFYEENRRTEEDGGKPAQAVPVLLGITKASLGTESFISA
ASFQDTTRVLTDAACCSKTDYLLGFKENVIMGHMIPGGTGFETHKRIKQYLEKEQEDLVFDFVSETECVC

>core/7/4/Org4_Gene664

MLKCPERVSVKKKEDILDLPNLIEIQIKSYKQFLQIGKLAEERENIGLEEVFREIFPIKSYNEATVLEYLSYNLG
VPKYSPEECIRRGITYSVTLKVRFRLTDETGIKEEEVYMGTIPLMTDKGTFIINGAERVVVSQVHRSPGINFEQE
KHSKGNILFSFRIIPYRGSWLEAIFDINDLIYIHIDRKKRRRKILAITFIRALGYSSDADIIEEFFTIEESSLRSEKDF
ALLVGRILADNIIDEASSLVYGKAGEKLSTAMLKRMLDAGIASVKIAVDADENHPIIKMLAKDPTDSYEAALK
DFYRRLRPGEPATLANARSTIMRLFFDPKRYNLGRVGRYKLNRKLGFSMDDEALSQVTLRKEDVIGALKYLI
RLKMGDEKACVDDIDHLANRRVRSVGELIQNQCRSGLARMEKIVRERMNLFDFSSDTLTPGKVVSAKGLAS
VLKDFFGRSQLSQFMDQTNPVAELTHKRRLSALGPGGLNRERAGFEVRDVHASHYGRICPIETPEGPNIGLITS
LSSFAKINEFGFIETPYRIVRDGIVTDEIEYMTADVEEECVIAQASASLDEYNMFTEPVCWVRYAGEAFEADTS
TVTHMDVSPKQLVSIVTGLIPFLEHDDANRALMGSNMQRQAVPLLKTEAPVVGTGLECRAAKDSGAIVVAE
EDGVVDFVDGYKVVVAAKHNPTIKRTYHLKKFLRSNSGTCINQQPLCAVGDVITKGDVIADGPATDRGELA
LGKNVLVAFMPWYGYNFEDAIIISEKLIREDAYTSIYIEEFELTARDTKLGKEEITRDIPNVSDEVLANLGEDGII
RIGAEVKPGDILVGKITPKSETELAPEERLLRAIFGEKAADVKDASLTVPPGTEGVVMDVKVFSRKDRLSKSD
DELVEEAVHLKDLQKGYKNQVATLKTEYREKLGALLLNEKAPAAIIHRRTAEIVVHEGLLFDQETIERIEQED
LVDLLMPNCEMYEVLKGLLSDYETALQRLEINYKTEVEHIREGDADLDHGVIRQVKVYVASKRKLQVGDK
MAGRHGNKGVVSKIVPEADMPYLSNGETVQMILNPLGVPSRMNLGQVLETHLGYAAKTAGIYVKTPVFEGF
PEQRIWDMMIEQGLPEDGKSFLYDGKTGERFDNKVVIGYIYMLKLSHLIADKIHARSIGPYSLVTQQPLGGKA
QMGGQRFGEMEVWALEAYGVAHMLQEILTVKSDDVSGRTRIYESIVKGENLLRSGTPESFNVLIKEMQGLG
LDVRPMVVDA

>core/8/4/Org4_Gene312

MTWIPLHCHSQYSVLDAMSSIKDFVAKGQEFGIPALALTDHGNLYGAVDFYKECTQKGIQPIIGCECYIAPGS
RFDKKKEKRSRAAHHLILLCKNEQGYRNLCILTSLAFTEGFYYFPRIDKDLLRQYSEGLICLSGCLSSSVSDAA
LKSPEALLLELQWFQDLFKDDYFTEVQLHKMSEESIAGFKEEWLKQEYYSLIEKQIKVNTAVLEASKRLGIPT
VATNDIHYINANDWQAHEILLNVQSGETVRIAKQNTHIPNPKRKVYRSREYYFKSPAQMAELFKDIPEVISNT
LEVAKRCDFTFDFSKKHYPIYVPESLKTLNSYTEEDRYQASAVFLKQLAEEALPKKYSSEVLAHIAKKFPHRD
PIDIVKERMDMEMAIIIPKGMCDYLLIVWDIIHWAKANGIPVGPGRGSGAGSVLLFLLGITEIEPIRFDLFFERFI
NPERLSYPDIDIDICMAGRERVINYAIERHGKDNVAQIITFGTMKAKMAVKDVGRTLDMALSKVNHIAKHIPD
LNTTLSKALETDPDLHQLYINDAESAQVIDMALCLEGSIRNTGVHAAGVIICGDQLTNHIPICISKDSTMITTQY
SMKPVESVGMLKVDLLGLKTLTSINIAMSAIEKKTGQSLAMATLPLDDATTFSLLHQGKTMGIFQMESKGMQ
ELAKNLRPDLFEEIIAMGALYRPGPMDMIPSFINRKHGKEIIEYDHPLMESILKETYGIMVYQEQVMQIAGALA
SYSLGEGDVLRRAMGKKDFQQMEQEREKFCKRACNNGIDPELATVIFDKMEKFAAYGFNKSHAAAYGLITY
TTAYLKANYPKEWLAALLTCDSDDIEKVGKLIREAQSMGIPILPPHINVSSNHFVATDEGIRFAMGAIKGIGHG

LIESIVEERDHHGPYESIRDFIQRSDLKKVSKKSIESLIDAGCFDCFDSNRDLLLASVEPLYEAIAKDKKEAASG
VMTFFTLGAMDRKNEVPICLPKDIPTRSKKELLKKEKELLGIYLTEHPMDTVRDHLSRLSVVLSGEFENLPHG
SVVRTVFIIDKVTTKISSKAQKKFAVLRVSDGIDSYELPIWPDMYEEQQELLEEDRLIYAILVLDKRSDSLRISC
RWMKDLSIVNENIIYECDQAFDRIKNQVQKMSFTMSTSGKETKTKGNKPNENGHTQALAPVTLSLDLNELRH
SHLCILKKIVQKHPGSRTLVLVFTQDNERVASMSPDDAYFVCEDIEELRQELVTADLPVRVITV

>core/9/4/Org4_Gene860

MVLEALAIFRQDAMQHLLKHRKEIVVDFCEDSYTIRIPDEEAPEGYWLSTLKLQDIDRLTFASCSCPDGECCL
HLMTAYFAVYDALGLHPLHDKFRHSFWYAVFSHFFLESIPLQAQGEMVYTLESPHITLTIECLSEEVFQDWLR
TIHASEEPTVFTNKTFLKSALYRAAKKFFFLNEEGAELTIGENSQGFPSDFSLQWQGLVFKAEILDFPTLEDIFP
KLELAHTSLKNVSHDISITNVTVCAEEAKVNFTLSPVIHKKDRENHPKTKIGSVEYVAKTHEMITGPKAIALPI
YAIPLLADKFKDQLLSLLCYDSLEYRLRYDIRLLRDASFSFSAYLVTPGDLDNGSLIYPNYCYSPTKGLMQVV
GMLSPKQAFIVKSEQVEDFLNERGHLIQEPGFQTFINERPEGHLTYNVTEQGVLLFHYDVGDPSSTEIRFGTWT
YYTNQGFFLEKKNDLPIQDGLIVEPQDIPAFIVKNDAALRRLPNFFSSPPNLKDLLIEVHRQSRGKGLDLKPILV
GLGESRCRLFGVFLYREDIGFSLIPTPLQGLCSLPRVIPPENVPQFLTQYAQHERILFPDPQTRPPESYELVIQSIH
RPHPASPLHLQLELKTNLGSVPIGIALQGLKSKHTFLFTQAGFLDLKQNLFQFLQQFLSTQKCVIAENTVIANIT
DVFKLDALAPLSVTDDTIANPEDLQFFSQLKAACLPPIPQNLFSSDHQLRPYQNSGLLWMWFLYNHRLSGLL
CDEMGLGKTHQATALLDIVFQSSQPSARPKFLIVCPTSVLPHWEHILSNHLPGVSIFSFHGPNKPSELPTADILL
TSYGTLRQNYDKFYKIAFTIVVFDEIHMAKNKSSQIHKILCRIDAQMKLGLTGTPIENNLLEFKGLLDIILPNYL
PSDALFKKLFTKRCSSEELEEIIPSQDLLLKLTRPFILRRTKKLVLPELPDKVESIIACSLSPDQEKLYMATLQRE
KSHIQKLETPEEPATNFLHIFALLNHLKQICDHPAVFFKDPDQYKNYESGKWNAFVKLLKESLNAGYKVVVF
SQYIHMIRIITLYLEEIGIKYASIQGKSLNRKEEIETFTTDPNCQVFVGSLLAAGTGINLTAGNVVIMYDRWWN
PAKENQALDRVHRIGQKNTVFIYKLITEDTLEERIHYLIEKKIRLLDKVIASQDSNILHMLNREDLLTILSYKDE
HGTSDSEESPVDTPVEDDTGVLPPEDS

>core/10/4/Org4_Gene849

MLNFRKLRRDFSANILQDGKELFEQGAVIDAKILSMNGETVCISAQIRGLYDNIYECEIEVDRSESDTVDSNCD
CSYNYDCQHIVALLFYLEQYFNEMVVAYARSADLETDHEINEEVKKELKETFVAAATKEEERKDREHQKEIL
REYVHAANALSANPFFLPLEYLEKDSAELAVLFVSVNEDTFAPANQPIEFQLVLRLPCRSKPFYISNIRTFLEG
VLYQEPIVLNGRRFFFTMQSFNASDRKLIDLLIRYVRYPNHTTEEKLLKSAYLMPPALGVILAKMFEHQLADR
GGGSLGEKESFSGLFCGNLEEPLCWSLTPAKMKFNLDFFDMPYKALLMTPVILVDDDEVQPEQTMLLESDAP
GIIHHFVYHRFSPQIKRAHLRSFSRLRDIAIPEALFGSFRENALPVFQEYAEIANVHLLNSFVTLPYVDEVRAIC
DMSYLDGELEAKLHFLYGSLRVPAASLALQYQDVRAFISDEGILARNLVEERKMLEEVFSGFIYDERDGAFH
VKSEKKIVEFMTETIPANQHRITFNCPENLSDQFIYDETIFELSFREGSDINYYEADLKVHGLLKGVPLDLLWD
CISAKKRFLELPKAGQQSKGTRRGKVNSGKLPCILVLDLEKIAPVVQIFNEIGFKVLDDLVQKCPLWSLTGISL
DQFEALPVNFSMSERLIEIQKQIRGEIEFDFQDVPQQIQATLRSYQTEGVHWLERLRKMHLNGILADDMGLGK
TLQAIIAVTQSKLEKGSGCSLIVCPTSLVYNWKEEFRKFNPEFRTLVIDGVPSQRRKQLTALADHDVAITSYNL
LQKDVELYKSFRFDYVVLDEAHHIKNRTTRNAKSVKMIQSDHRLILTGTPIENSLEELWSLFDFLMPGLLSSY
DRFVGKYIRTGNYMGNKADNMVALKKKVSPFILRRMKEDVLKDLPPVSEILYHCHLTESQKELYQSYAASA
KQELSRLVKQEGFERIHIHVLATLTRLKQICCHPAIFAKDAPEPGDSAKYDMLMDLLSSLVDSGHKTAVFSQY

TKMLGIIKKDLESRGIPFVYLDGSTKNRLDLVNQFNEDPSLLVFLISLKAGGTGLNLVGADTVIHYDMWWNP
AVENQATDRVHRIGQSRSVSSYKLVTLNTIEEKILTLQNRKKSLVKKVINSDDEVVSKLTWEEVLELLQI

>core/12/4/Org4_Gene292

MKRPKKFPIYLSIAQKTNRLLSGIVIAFAVIALRLWYLAVVEHEQKLEEAYKPQIRVLPQYVERATICDRFGKT
LAVNQLQYDVSVAYGAIRDLPTRAWRVDEHGHKQLIPVRKHYIMCLSELLSQELHLDREAIEDAIHAKASVL
GSVPYLVAANVSERTYLKLKMLSKDWPGLHVEAVVRRHYPQESVASDILGYVGPISPQEYKRVTQELSQLRE
CVRAYEEGEDPKLPEGLASIDQVRALLESMESNAYSLNALVGKMGVEACWDSKLRGKIGKKPILVDRRGNFI
QEMEGAVPEAPGTKLQLTLSAELQAYADALLLEYEKTETFRSAKSLKKREKLPPLFPWIKGGAIIALDPNNGE
ILAMASSPRYRNNDFVNAKVAEDSKAVRSSIYRWLENKEHIAEIYDRKVPLIRERRNPLTGLCYEEILPLTFDC
FLDFLFPENSVIKLQLKRNSFVGQAIEVQNLVTRLLSLFPYEEGTCPCSAIFDAVFPNEEGHILIQEVISLQEQK
WIMECLNQHKADIEELKEGLDQVFNELPANYDKILYTDILRLIVDPERFSPVLPSEVHRLSLSEFTELQGRYVV
LRSAFSTILEDAFIEVHFKSWRKSEFPQYLAAKRQEEALRKQRYPTPYVDYLEEEKTRQYKMFCQEHLDTFLA
YLFSKTPYKEGLEPYYDILDLWINELDNGAHRALSWHEHYLFLKERVSHLSQHLPALFSTFREFNELQRPLLG
KYPISIVRNKRQTEQDLAASFYPVYGYGYLRPHAYGQAATLGSIFKLVSAYSVLSQRILWGHNEEPANPLVII
DKNSFGYRSSKPHVGFFKDGTPIPTFFRGGSLPGNDFMGRGFIDLVSALEMSSNPYFSLLVGEGLGDPEDLAD
AASLFGFGEKTGLGLPGEYAGRVPHDLAYNRSGLYATAIGQHTLVVTPLQTAVMLASLVNGGVVYVPKLLL
GEWEGEHVSYLSSKKKRTIFMPDAVVEVLKTGMRNVIWGQYGTARAIQSQFPPQLLSRIIGKTSTAESIMRVG
LDREYGTMKMKDIWFAAVGFSDQDLSLPTIVVIVYLRLGEFGRDAAPMAVKMIDMWEKIQQRESFLRG

>core/13/4/Org4_Gene819

MDFNPVNLDFSISKEFKEETLPLLLENIHPGATAFLAAKMFHDCRASVIMITTPARLDDLFENLRTFLDQAPVE
FPSSEIDLSPKLVNIDAVGKRDHLLYSLNQHRAPIFCVTTLKALLEKTRSPQATSQQHLDLAVGDVLDPEATTE
LCKSLGYSQVMLTSEKGEFSCRGGIVDIFPLSSPEPFRIEFWGEKIISIRSYNPSDQLSTGKVSKISISPAYTEEAS
GGNYSHSLLDYFSTPPLYLFDNLEILEDDFADISGTLSSLPDRFFSIGTLYDRISTSNQVYFSETPFPNVKNLKEN
RVIIEAFHRNIEASRQAIPILYPEQIIQNDENPLLAFLQHLQEYMPPHGKPLKLAIYSTKTKSLKEARALAETVA
RGDVEIYEKTGNLTSSFALVNEAFAAISLSEFASTKVLRRQKQRTHFSVTTEEVFVPIPGETVVHIHNGIGKFLG
IEKKPNHLNIETDYLVLEYADKARLYVPSNQAYLISRYVGTSDKAADLHHLNSSKWKRSRDLTEKSLIVYAE
KLLQLEAQRSTTPAFVYPPHGESVIKFAETFPYEETPDQLKTIDQIYNDMMSPKLMDRLICGDAGFGKTEVIM
RAAVKAVCDGHRQVIVMVPTTILATQHYETFKERMAGLPIEIAVLSRFSQAKVQKLIYEQVASGQIDIIIGTHK
LINKSLEFKNPGLLIIDEEQRFGVKVKDNLKERYPMIDCLTVSATPIPRTLHMSLSGARDLSVIAMPPLDRLPVS
TFVMEHNTETLTAALRHELLRGGQAYVIHNRIESIYTLAETIRNLIPEARIGVAHGQMGAEDLSNIFTKFKNQK
TNILVATALIENGIDIPNANTILIDHADKFGMADLYQMKGRVGRWNKKAYCYFLVPHLDRLSGPAAKRLAAL
NKQEYGGGMKIALHDLEIRGAGNILGTDQSGHIGTIGFNLYCKLLKKAVSALKKHTSPLLFNDDVKIEFPYNS
RIPDTYIETASMRIEFYQKIGNAESSEELTAIQEEMRDRFGPLPQEICWLFALAEVRLFALQHGISSIKGTANAL
YVQKCLSKSEQTKKTLPYALSPTPELLVKEVIESIERGFLINAS

>core/14/4/Org4_Gene955

MIKKRAIFERMFPIPPPHCPPNNKNNFYHLTTDTKDPLLLRILRTIGYVLLHIITLGLLLLIHYYKHHRVVRKEG
LPTPPTLPKGPEPKTIEIAKQPPKDGEDKKPDVPKPGTPPPEDTPPPPPKAPSPASPKVPKQPADKKPTPPPEPPP
PVKKPTPPPKAPPPVRVATPMPLRPSSQGYWQCLNRMVSMVLRRAPLPLPAMQIDPILGDFNPHFVASYPNRI

DNEPMCFQIEQFKKIAQNPDLPQQHRRLAQLSLEQALYLNDNYYLVNVPGDGNCFYRAYAVGWLSALYEES
SRNDIVFEQEATRLLDLPFAASSPANANLCAEMAELLQLCSTYCSFIDLYDGVILSQKHTATLIAFLRKLSAYA
IRQQIAASSNEETARALFISDMQDDLLPSVLEFLAANRPYSELFQNLIDHSALPDMQSRDKLFLLLEHLPALFL
TDAELQKMSPEDQQLRKQYEREIREAFAKLSRRIADSGWDTDRFNAIVKDHLPEAIRCQYSRFLATIENRRSG
DLPWSPALSFFAFLCTCPSVRFHKLCATFYKSLEDIIIASAPPQRSIQEILQISNASLSYLNEDLDSSWQREVISSN
IMTILTTHESLTLESSMPQLETLHKRIANLLKNVISTSFETPPLSNQPDLLPNLVNKLLVAIHSKHELKEHFNTV
CSARSLRLTRDEGSGLSQEQDLLYTQAVQLLFFILQHPQVNNRPETKDAVKELKMLLLPFLQYAFKKVENEK
KFQKLLRSILGSLVLKPPARYPSTPSNKDKETFCKFWSRHPEVMVLDPILEKNCMQFLRATFPNYQLETEAILL
EKEIESTFRNGWNVFLTRLNLFGSKLGSPSSPTALSDQFSKSFLIFCFLNNYPKLLQKETPLAARLDAFQREAS
HRFTQVKDKLLLSLKYGFPLATATINQYSRARDQLICNLLKNTVTASDGFCRSGFRQSLIGYLHSLSSNELGDI
LDDVKEQAEANDVTAMTTVSLQPFAVCQIMSDRDTVSEENIENFVAMHGFLNTISPERDARIFLIRFPNHYGC
LLPRNPRTEDQNSKPDSSNP

>core/15/4/Org4_Gene261

MKPFNIFDSNSSIQGKFFLEASAGTGKTFTIEQIVLRALIEGSLTHVEHALAITFTNASTNELKVRIKDNLAQTL
RELKAVLNSQPASLPTYLDINCNVKQIYMQVRNALATLDQMSLFTIHGFCNFVLEQYFPKTRLIHKNPALTHS
QLVLHHITNYLKQDLWKNVLFQEQFHLLAVRYNVTSKHTSSLVDKLLASYTQPISSYFSSRVERLEQISLWHQ
QIYNSLLEIPKQVFLDQLTAHISGFKKQPFFILDDLHHFVDLLYTSETHSSLFSFFKIAETFNFKHRLARYKPCA
AFTVLENMSWVERTLEFCNLDRIFNTLLVDLQEYLKQNYTPWLSPDESVFALEKLLSSSEAQPVVQALREQY
QLVLIDEFQDTDKQQWSIFSNLFISPKFTGSLFLIGDPKQSIYEWRNADLPTYLKAKSSFSEDKQLQLVNNYRS
TPKLMEAINQIFGKISPFLEIPGYLPIEYHALNPQSSETFENPPHAPIHFFFYETIKDQALWIFSEALRLQKEQKIP
LGNMVVLVSDSNQAFELISYATIPVSFSKNKSIFHLTETHILTTALLEAILHPENYEKISKILFSSLFGLSLDEVTT
KKEDFTIYFQSLHSYILHHGLLATFYRVMTTQGNVLFSSPRGDLIFQEMEKLCGYLDTISSYPYHQLLHLKNFS
ETGRWEEELAISSYSEDLETLKITTIHSSKGLEYDIVFCPGIEKSKKNKSSSELLREMYVACTRAKKQLYLPIST
QPPSLQRSSALTNYVKLEGTQSSAYDLAIHLHQEHPDLFSYSLPKDHGHATTVLNLPLLETFALKVTPPKTIFS
FSSTKFLLDTHKDSQSIPYSKLPISKQQLPLGEKTGILIHKILESIQFSLLQDTEYLMSTIMRFIKHTHLEGFEETIL
KLLSKTFFSPLTFSSQTFSLSQVLPNKIFRETSFLFLENQELWQGVIDLFFEHEGKYYIIDWKTSFLGETNSEYSK
SNLSIYIKQEKLDYQGRIYVKAVRKFLNQFEIDDDVELGVIFIRGIDTQGNGFFALNSSEDIPTFNPKAIQKCQA
YH

>core/16/4/Org4_Gene757

MVEVEEKHYTIVKRNGMFVPFNQDRIFQALEAAFRDTRSLETSSPLPKDLEESIAQITHKVVKEVLAKISEGQ
VVTVERIQDLVESQLYISGLQDVARDYIVYRDQRKAERGNSSSIIAIIRRDGGSAKFNPMKISAALEKAFRATL
QINGMTPPATLSEINDLTLRIVEDVLSLHGEGAINLEEIQDIVEKQLMVAGYYDVAKNYILYREARARARANK
DQDGQEEFVPQEETYVVQKEDGTTYLLRKTDLEKRFSWACKRFPKTTDSQLLADMAFMNLYSGIKEEEVTT
ACIMAARANIEREPDYAFIAAELLTSSLYEETLGCNSQDPNLSEIHKKHFKEYILNGEEYRLNPQLKDYDLDAL
SEVLDLSRDQQFSYMGVQNLYDRYFNLHEGRRLETAQIFWMRVSMGLALNEGEQKNFWAITFYNLLSTFRY
TPATPTLFNSGMRHSQLSSCYLSTVKDDLSHIYKVISDNALLSKWAGGIGNDWTDVRATGAVIKGTNGKSQG
VIPFIKVANDTAIAVNQGGKRKGAMCVYLENWHLDYEDFLELRKNTGDERRRTHDINTASWIPDLFFKRLEK
KGMWTLFSPDDVPGLHEAYGLEFEKLYEEYERKVESGEIRLYKKVEAEVLWRKMLSMLYETGHPWITFKDP
SNIRSNQDHVGVVRCSNLCTEILLNCSESETAVCNLGSINLVEHIRNGKLDEEKLKETISIAIRILDNVIDLNFYP

TPEAKQANLTHRAVGLGVMGFQDVLYELNISYASQEAVEFSDECSEIIAYYAILASSLLAKERGTYASYSGSK
WDRGYLPLDTIELLKETRGEHNVLVDTSSKKDWTPVRDTIQKYGMRNSQVMAIAPTATISNIIGVTQSIEPMY
KHLFVKSNLSGEFTIPNTYLIKKLKELGLWDAEMLDDLKYFDGSLLEIERIPNHLKKLFLTAFEIEPEWIIECTS
RRQKWIDMGVSLNLYLAEPDGKKLSNMYLMAWKKGLKTTYYLRSQAATSVEKSFIDINKRGIQPRWMKNK
SASTSIVVERKTTPVCSMEEGCESCQ

>core/17/4/Org4_Gene644

MTADEVGKNSFAKKEEQVLKFWKDNQIFEKSLQNRQGKTLYSFYDGPPFATGLPHYGHLLASTIKDVVGRY
ATMDGYYVPRRFGWDCHGVPVEYEVEKSLSLTAPGAIEDFGIASFNEECRKIVFRYVHEWEYYINRIGRWVD
FSSTWKTMDASFMESVWWVFQSLYNQGLVYEGTKVVPFSTALGTPLSNFEASQNYKEVDDPSLVVRMPLQ
NDSASLLVWTTTPWTLPSNMAIAVGEALVYVRIQDKKSGEQWILSQGCVSRWFSNPEEFVILESFSGKDLVG
RTYEPPFTFFQSKREEGAFRVIAASFVEESEGTGVVHMAPAFGEGDFLVCKENHVPLVCPVDAHGSFTEEIPQ
YQGQYIKHADKEIIKFLKKEGRIFYHGTVKHRYPFCWRTDTPLIYKAVNSWFVAVEKIKDKMLRANSSIHWV
PEHIQEGRFGKWLEGARDWAISRNRYWGTPIPIWKSADGEILVVGSIRELEELTGTQITDIHRHFIDDLNIVKD
GKPFHRIPYVFDCWFDSGAMPYAQNHYPFENQKETEEAFPADFIAEGLDQTRGWFYTLTVISAILFDRPAFRN
AIVNGIILAEDGNKMSKRLNNYPSPKYVLDTYGADALRLYLLHSVVVKAEDLRFSDKGIEGVLKQILLPLTSV
LSFFNTYAELYGFDPKSQDIEPAYTEIDQWILSNLYSVVGKVRESMSQYHLNFAVEPFVTFIDDLTNWYIRRC
RRRFWEAENTPDRRAAFSTLYEVLTVFCKVIAPFVPFLAEDIYQKLKLEKEPESVHLCDFPQVEMDKILPDLE
KRMHDIREIVGLGHSLRKEHKLKVRQPLANFYVVGSKDRLSLLKTFEGLIAEELNVKNVIFYEEAPSFIYTTV
KPNFRMLGKKVGSKMKEVQKALSELPNNAIDKLIQGETWVLTIDDREIALDGDDVVICRHTDPGYIARSSAL
FSVILDCQLTEPLIVEGIARELVNKINTMRRNQQLHVSDRIALRIKTTEAVHRAFLDYENYICEETLIIAYDFTQ
NSDFQGEDWDINGHATQIEITVSSIDS

>core/18/4/Org4_Gene262

MNATKHCRASFSNSPRHLLAQLAEDITSTHQKPFTKRWILVANATTGHWIKNQLVHALSDHIFMGSTIFTASD
SIVKHLFLGSGCSQPNIPDYLTLPLLINNILEEISKASKFENGREFLSPPTYETTKKLAAAFKQFHTFSQRPTKNA
SHYQELFQILESHFSSYEEMFTTILNNRTQEEDCSLHIFGYAHLPKHLAEFFINLSTYFPVYFYCFSPCREYFGD
LLSDRAIDFFWNQLPDSPIKNAWEHYVLSDRQALLANLAHKSQSSQNFFLDREIDYQEMFLPSKHDSSLGVIQ
NSILDLKPTSPQDFSQTKQTICIYRALNIPREVQEVFCKVTELLHRGVSPEEIFILSSHIESYKVHLNAIFNPHVPI
YFTDEVDPQAEDLRNKILLLSSILQTQGDLHYILQLLTHPQLQQPIDQNKVPYLIKKLSLEWGKISSKDRASGQ
QMKALGDLILEEYPFHQEGGRVSQVEVWETTVPLIYFIQERINLYLSSSQHSYEDLFQNVFSCLEKIFVLSPEET
SFITTLRNSLFPTFATSSCSLLFFTDFCLDFLLHFHKPSPLYDKPGPYIGSLSSLSLIPKGYVFILGANKTTSSDIFD
LLNRTTTHEELAFSSTEDEENFHFLQILVSTKHELHISYISSAAQFNLPSPFLNHIKETLDLPVETLPTQPYLSAFF
KNKACLHASQEYNYSLAHAFYSKKALLPSLFIPNVKQVNLPQHLSLNEIIKGIFSPLDLFLKTNYNLRISSPEHL
KKQQKLFPTKHQIEDFWNECFVDKEHDLIPSISPHAEELFTYYREKTILLHNGLDKDPKHSPYTVTFSSSIFEER
PYHESYLFPPLSLSFQGNPVQIHGTIHGVCNEGLYLCSIDPRDSLKKTTRTLGSLPETSSEQKQLLERYVALAV
LQMSQHLSSDSALIKLTSFNTKENHHPPFSDPEGYLRKVLEVYHLMSSQPIPLLSPLCWKTLDDEEKFHQAVL
SAISEEAKNPSLPIFWQFHNRNIEETLNHVGASERLKILSLFRGPCEAV

>core/19/4/Org4_Gene786

MSEHPLTLQSMIATILRFWSEQGCVIHQGYDLEVGAGTFNPATFLRALGPEPYKAAYVEPSRRPQDGRYGMH
PNRLQNYHQLQVILKPVPENFLSLYTESLRAIGLDLRDHDIRFIHDDWENPTIGAWGLGWEVWLNGMEITQL
TYFQAIGSKPLDTISGEITYGIERIAMYLQKKTSIYDVLWNDTLTYGQITQASEKAWSEYNFDYANTEMWFKH
FEDFAEEALRTLKNGLSVPAYDFVIKASHAFNILDARGTISVTERTRYIARIRQLARLVADSYVEWRASLNYPL
LSLSSTSEPKETSESVVPMISSTEDLLLEIGSEELPATFVPIGIQQLESLARQVLTDHNIVYEGLEVLGSPRRLAL
LVKNIAPEVVQKAFEKKGPMLTSLFSPNGDVSPQGQQFFASQGVDISHYQDLSRHASLAIRTVNGSEYLFLLH
PEVRLRTADILMQELPLLIQRMKFPKKMVWDNSGVEYARPIRWLVALYGEHILPITLGTIIASRNSFGHRQLDP
REISISSPQDYVETLRQACVVVSQKERRMIIEQGLRAHSSDTISAIPLPRLIEEATFLSEHPFVSCGQFSEQFCALP
KELLIAEMVNHQKYFPTHETSSGAISNFFIVVCDNSPNDTIIEGNEKALTPRLTDGEFLFKQDLQTPLTTFIEKL
KSVTYFEALGSLYDKVERLKAHQRVFSTFSSLAASKDLDIAIQYCKADLVSAVVNEFPELQGIMGEYYLKHA
NLPTASAVAVGEHLRHITMGQKLSTIGTLLSLLDRLDNLLACFILGLKPTSSHDPYALRRQSLEVLTLVSASRL
PIDLASLLDRLADHFPSTIEEKVWDKSKTIHEILEFIWGRLKTFMGSLEFRKDEIAAVLIDSATKNPIEILDTAEA
LQLLKEEHTEKLAVITTTHNRLKKILSSLKLSMTSSPIEVLGDRESNFKQVLDAFPEFPKETSAHAFLEYFLSLA
DLSNDIQDFLNTVHIASDDGAIRNLRISLLLTAMDKFSLCHWESVAV

>core/20/4/Org4_Gene980

MYSCYSKGISHNYLLHPMSRLDIFVFDSLIANQDQNLLEEIFCSEDTVLFKAYRTTALQSPLAAKNLNIARKV
ANYILADNGEIDTVKLVDAIHHLSQCTYPLGPHRHNEAQDREHLLKMLKALKENPKLKESIKTLFVPSYSTIQ
NLIRHTLALNPQTILSTIHVRQAALTALFTYLRQDVGSCFATAPAILIHQEYPERFLKDLNDLISSGKLSRIVNQ
REIAVPINLSGCIGELFKPLRILDLYPDPVVKLSSSPGLKKAFSAANLIETLGDSEAQIQQLLSHQYLMQKLQNV
HETLTANDIIKSTLLHYYQLQESTVRAIFFKEGLFSKEQVAFSTQHPRELSEIQRVYHYLHAYEEAKSAFIHDT
QNPLLKAWEYTLATLADASQPTISNHIRLALGWKSEDPHSLVSLVTHFVEEEVENIRILVQQCEQTYHEARSQ
LEYIEGRMRNPLNNQDSQILTMDHMRFRQELNKALYEWDSAQEKAKKFLHLPEFLLSFYTKQIPLYFRSSYD
AFIQEFAHLYADAPAGFRILFTHGRTHPNTWSPIYSINEFIRFLSEFFTSTESELLGKHAVINLEKETSRLVHNIT
AMLHTDVFQEALLTRILEAYQLPVPPSILNHLDQLSQTPWVYVSGGTVDTLLLDYFESSEPLTLTEKHPENPH
ELAAFYADALKDLPTGIKSYLEEGSHSLLSSSPTHVFSIIAGSPLFREAWDNDWYSYTWLRDVWVKQHQDFL
QDTILPQPSIYAFIENFCNKYALQHVVHDFHDFCSDHSLTLPELYDKGSRFLSSLFTKDKTVALIYIRRLLYLM
VREVPYVSEQQLPEVLDNVSSYLGISSRITYEKFRSLIEETIPKMTLLSSADLRHIYKGLLMQSYQKIYTEEDM
YLRLTTAMRHHNLAYPAPLLFADSNWPSIYFGFILNPGTTEIDLWKFNYAGLQGQPLDNIQELFATSRPWTLY
ANPIDYGMPPPPGYRSRLPKEFF

>core/21/4/Org4_Gene440

MPLSFKSSSFCLLACLCSASCAFAETRLGGNFVPPITNQGEEILLTSDFVCSNFLGASFSSSFINSSGNLSLLGKG
LSLTFTSCQAPTNSNYALLSAAETLTFKNFSSINFTGNQSTGLGGLIYGKDIVFQSIKDLIFTTNRVAYSPASAT
TTANPAVTTVTTGASALQPTDSLTVENISQSIKFFGNLANFGSAISSSPTAVVKFINNTATMNFSHNFTSSGGG
VIYGGSSLLFENNSGCIIFTANSCVNSLKGLTPSSGTYALGSGGAICIPTGTFELKNNQGKCTFSYNGTPNDAG
AIYAETCNIIGNQGALVLDSNTAAKNGGAICAKVLNIQGRGPIEFAKNRAEKGGAIFVGPSVGDPAKKTSTLTI
LASEGDITFQGNMLNTKPGIRNAITVEAGGEIVSLSAQGGSRLVFYDPITHSLPTTSPSNKDITINANGASGSVV
FTSQGLSSTELLLPANTTTTLLGTVKIASGELKITDNAVVNVLGFATQNSGQLTLGSGGTLGLATTSGTPAAV
NFTIGKLAFDPFSFLKRDFVSASVNAGTKDITLTGALVLDEHDVTDLYDMVSLQSPVAIPIAVFKGANVTKTG
FPDGEIATPSHYGYQGKWSYTWSRPLLIPAPDGGFPGGLSPSANTLYAVWNSDTLMRSTYILDPERYGEIVSN

SLWISFLGNQAFSDILQDVLLIDHPGLSITAKALGAYVEHTPRQGHEGFSGRYGGYQAALSMNYTDHTTLGLS
FGQLYGKTNANPYDSRCSEQMYLLSFFGRFPIVTQKSEAFISWEAAYGYSKNHLNTTYLRPDKAPKSQGQW
HNNSYYVLISAEHPFLNWCLLTRPLAQAWDLSGFISAEFLGGWQSKFTETGDLQRSFSRGKGYNVSLPIGCSS
QWFTPFKKAPSTLTIKLAYKPDIYRVNPHNIVTVVSNQESTSISGANLRRHGLFVQIHDVVDLTEDTQAFLNY
TFDGKNGFTNHRVSTGLKSTF

>core/22/4/Org4_Gene760

MKAGDTYRNFIIKSCKDLPEIESKLLEAEHKPTGASIMMIVNDDEENVFNICFRTCPQTSNGVAHVLEHMVLC
GSENYPVRDPFFSMTRRSLNTFINAFTGPDFTCYPAASQIPEDFYNLLSVYIDAVFHPLLTKQSFLQEAWRYEF
NSENQLCYTGVVFNEMKGAMMSGEARLSEALNAAIFPSVTYGVNSGGEPREIVTLSHEDVRAFHQSQYSINR
CLFYFYGNIKPSRHLDFLEEKLLRQATKLEKQAVSVPLQKRFKEPVRNILTYPVDHQEEDKVLFGISWLTCSIL
EQQELLALHVLEIILMGTDASPLKSRLLKSGFCKQTEMSIENDIREIPMTLVCKGCSPAGAQKLEALIFASLEEII
REGISENIVEGAVHQLELSRKEITGYSLPYGLSLFFRSGLLKQHGGSAEDGLRIHSLFSELRNSLKNSDYLAKLI
RKYFLDNPHFARVILLPDTELVAKENKDEQQLLLSVSEKLTDENKEKIQQNVRELTESQEQKEDLNGILPNLA
LDKVPTSGKEFPLIKEGLSQGEVLHHECFTNDIVFIDVVLDIPPLSGEELPWLRLLVFLMLQLGCGGRSYKEHL
EFLLEHTGGVDVSYDFSPHANKNSFLSPSVSIRGKALSSKSEKLCGIISDMLTSVDFTDIPRIRELLMQHNEALT
NSVRNSPMSYAVSMACSGNSITGAMSYLTTGLPYVKKIRELTKNFDQNIDEAVVILQRLYTKCFSGKRQIVIS
GSAHNYQQLKYNKFYGLLDYLIVIPEPWENPSINLYVTSQGLHIPARAAFNALAFPIGDIAYDHPDAAALTVA
AEILDNVVLHTKIREQGGAYGSGAAANLSRGSFYCYSYRDPEIATTYKTFLKGVSEIASGNFTKEDIYEGALG
VIQGLDMPVAPGSRASVAFYRLKSGRIPVLRQAFRRSVLEVTKEHICMVMDKYLESTVQETTLISFAGEEMLR
NNVLTLDKDFPIVPAI

>core/23/4/Org4_Gene441

MKTSIRKFLISTTLAPCFASTAFTVEVIMPSENFDGSSGKIFPYTTLSDPRGTLCIFSGDLYIANLDNAISRTSSSC
FSNRAGALQILGKGGVFSFLNIRSSADGAAISSVITQNPELCPLSFSGFSQMIFDNCESLTSDTSASNVIPHASAI
YATTPMLFTNNDSILFQYNRSAGFGAAIRGTSITIENTTKSLLFNGNGSISNGGALTGSAAINLINNSAPVIFSTN
ATGIYGGAIYLTGGSMLTSGNLSGVSFVNNSARSGGAIYANGNVTFSNNSDLTFQNNTASPQNSLPAPTPPPT
PPAVTPLLGYGGAIFCTPPATPPPTGVSLTISGENTVTFLENIASEQGGALYGKKISIDSNKSTIFLGNTAGKGG
AIAIPESGELSLSANQGDILFNKNLSITSGASTRNSIHFGKDAKFATLGATQGYTLYFYDPITSDDLSAASAAAT
VVVNPKASADGAYSGTIVFSGETLTATEAATPANATSTLNQKLELEGGTLALRNGATLNVHSFTQDERSVVI
MDAGTTLATTNGAANNTDGAITLNNLVINLDSLDGTKAAVVNVQSTNGALSISGTLGLVKNSQDCCDNHG
MFNKDLQQVPILELNATSTTVTTTDFSVGTNGYQQSPYGYQGTWEFTIDTTNHKVTGDWKKTGYIPHPERLA
PLIPNSLWANVIDLRAVSQASAAGGEDIPGKQLSITGITNFFHANHTNDARSYRHMGGGYLINTYTRITPDAA
LSLGFGQLFTKSKDYLVGHGHSNVYFATVYSNITKSLFGSSRFFSGGTSRVTYSRSNEKVKTSYTKLPKGRCS
WSNNCWLGELEGNLPITLSSRILNLKQIIPFVKAEVAYATHGGIQENTPEGRIFGHGHLLNVAVPVGVRFDKN
SHNRPDFYTIIVAYAPDVYRHNPNCDTTLPINGATWTSIGNNLTRSTLLVQASSHTSVNDVLEIFGHCGCDIRR
TSRQYTLDIGSKLRF

>core/24/4/Org4_Gene183

MLGFLKRFFGSSQERILKKFQKLVDKVNIYDEMLTPLSDDELRNKTAELKQRYQNGESLDSMLPEAYGVVK
NVCRRLAGTPVEVSGYHQRWDMVPYDVQILGAIAMHKGFITEMQTGEGKTLTAVMPLYLNALTGKPVHLV

TVNDYLAQRDCEWVGSVLRWLGLTTGVLVSGTLLEKRKKIYQCDVVYGTASEFGFDYLRDNSIATRLEEQV
GRGYYFAIIDEVDSILIDEARTPLIISGPGEKHNPVYFELKEKVASLVYLQKELCSRIALEARRGLDSFLDVDILP
KDKKVLEGISEFCRSLWLVSKGMPLNRVLRRVREHPDLRAMIDKWDVYYHAEQNKEESLERLSELYIIVDEH
NNDFELTDKGMQQWVEYAGGSTEEFVMMDMGHEYALIENDETLSPADKINKKIAISEEDTLRKARAHGLRQ
LLRAQLLMERDVDYIVRDDQIVIIDEHTGRPQPGRRFSEGLHQAIEAKEHVTIRKESQTLATVTLQNFFRLYEK
LAGMTGTAITESREFKEIYNLYVLQVPTFKPCLRIDHNDEFYMTEREKYHAIVNEIATIHGKGNPILVGTESVE
VSEKLSRILRQNRIEHTVLNAKNHAQEAEIIAGAGKLGAVTVATNMAGRGTDIKLDNEAVIVGGLHVIGTTR
HQSRRIDRQLRGRCARLGDPGAAKFFLSFEDRLMRLFASPKLNTLIRHFRPPEGEAMSDPMFNRLIETAQKRV
EGRNYTIRKHTLEYDDVMNKQRQAIYAFRHDVLHAESVFDLAKEILCHVSLMVASLVMSDRQFKGWTLPNL
EEWITSSFPIALNIEELRQLKDTDSIAEKIAAELIQEFQVRFDHMVEGLSKAGGEELDASAICRDVVRSVMVMH
IDEQWRIHLVDMDLLRSEVGLRTVGQKDPLLEFKHESFLLFESLIRDIRITIARHLFRLELTVEPNPRVNNVIPT
VATSFHNNVNYGPLELTVVTDSEDQD

>core/26/4/Org4_Gene437

MRPICLSILSTALCCSLSGNEVPNLASCQMSRKDISAFHTSPSFRLNVTPEPLVSSFRPSNLLNGFGHDITQDITI
TGNSNNSVIDYNYHYEDGGILACKNLFISENKGNLSFERNSSHSSGGALYSVQKCLISKNQNYSFISNAASLAT
TTTSGFGGALHALDGYITNNLGEGKFLDNVSKNRGGAIYVGVSLSITDNLGPIVIKKNQTLEDSSFGGGIFGRA
INIEGNYQNIQINDNASGQGGGIFSALGISISSNKEIIEISNNSASSVNTASGKLYPGGGGIMCTSLIVENNPKGLI
FNNNTAALSGGAIHTRSFIFQNNGPTAFINNSATSGGALINLSGIGSTPQNFFLSADYGDILFNNNTITSSSPQPG
YRNALYAAPGINLKLGARQGYKILFYDPIDHDQTTTDPIVFNYEPHHLGTVLFSGINVDSNATNPLNFLSKLSN
SSRLERGVLAIEDRAAISCKTLSQTGGTLRLGNAALIRTKGPGSSINFNAVAINLPSILQSEASAPKFWIYPTLTG
SAYSEDTSSTITLSGPLTFLNDENENPYDSLDLSEPRKDIPLLYLLDVTAKKIDTSNLIVEAMNLDEHYGYQGI
WSPYWMETTTTTSSTVPEQTNTNHRQLYVDWTPLGYRPNPERQGEFVANTLWQSAYNALLGIDTLSPQNLT
EHSLEASLQGLGLLINQHNREGRKGFRNHTTGYAATTSAKTTASHKFSLGFAQMFSKTRERQSHNKISSHNY
FAGLRFDSLLFKDFLSTALSIAYSYGDHHLHCHYTKTLKGSSEAFFNNHTLAASLDCTFLSARITRTLQLQPFI
KAIALHSSHASFQETGSHARKFNTEHPLIDLSSPIGFRSEWKTSHQAMLWTTEISYIPTLYRKNPELLTTLIVSN
GTWKTEATPVSYNALSAKVKNTSRLSSRITLSLDYSAQVSSSTVGQYLKAESRYTF

>core/27/4/Org4_Gene390

MKQMRLWGFLFLSSFCQVSYLRANDVLLPLSGIHSGEDLELFTLRSSSPTKTTYSLRKDFIVCDFSGNSIHKPG
AAFLNLKGDLFFINSTPLAALTFKNIHLGARGAGLFSESNVTFKGLRSLVLENNESWGGVLTTSGDLSFINNTS
VLCQNNISYGPGGALLLQGRKSKALFFRDNRGTILFLKNKAVNQDESHPGYGGAVSSISPGSPITFADNQEILF
QENEGELGGAIYNDQGAITFENNFQTTSFFSNKASFGGAVYSRYCNLYSQWGDTLFTKNAAVKVGGAIHAD
YVHIRDCKGSIVFEENSATAGGAIAVNAVCDINAQGPVRFINNSALGLNGGAIYMQATGSILRLHANQGDIEF
CGNKVRSQFHSHINSTSNFTNNAITIQGAPREFSLSANEGHRICFYDPIISATENYNSLYINHQRLLEAGGAVIFS
GARLSPEHKKENKNKTSIINQPVRLCSGVLSIEGGAILAVRSFYQEGGLLALGPGSKLTTQGKNSEKDKIVITN
LGFNLENLDSSDPAEIRATEKASIEISGVPRVYGHTESFYENHEYASKPYTTSIILSAKKLVTAPSRPEKDIQNLII
AESEYMGYGYQGSWEFSWSPNDTKEKKTIIASWTPTGEFSLDPKHRGSFIPTTLWSTFSGLNIASNIVNNNYL
NNSEVIPLQHLCVFGGPVYQIMEQNPKQSSNNLLVQHAGHNVGARIPFSFNTILSAALTQLFSSSSQQNVADK
SHAQMLIGTVSLNKSWQALSLRSSFSYTEDSQVMKHVFPYKGTSRGSWRNYGWSGSVGMSYAYPKGIRYL

KMTPFVDLQYTKLVQNPFVETGYDPRYFSSSEMTNLSLPIGIALEMRFIGSRSSLFLQVSTSYIKDLRRVNPQSS
ASLVLNHYTWDIQGVPLGKEALNITLNSTIKYKIVTAYMGISSTQREGSNLSANAHAGLSLSF

>core/28/4/Org4_Gene990

MQQQPQRKLQHDIIAPTASGEATGLFVHQNSNNDAKGFHMEATGYSLGTTSNTASNHSFGINFSQLFSNLHE
SRSDNSVASHTTTVALQMNNPWLQERFSTSTSLAYSYSNHHIKASRYSGKIQTEGKCYSTTLGAALSCSLPLQ
WRSRPLHFTPFIQAIAVRSNQTAFQESGDKSRKFAVHKPLYNLTVPLGIQSAWESKFRLPTYWNIELAYQPVL
YQQNPEINVSLESSGSSWLLSGTTLARNAIAFKGRNQIFIFPKLSVFLDYQGSVSSSTTTHYLHTGTTFKF

>core/29/4/Org4_Gene777

MFWKLLCPILICTSLSITSCKQQFKVVPNQCPLQVSTPAAADQKIEKIICSNGLPLLIISDPNLPTSGAALLVKTG
NNADPEEYPGMAHFTEHCVFLGNEKYPEVSGFPGFLSENNGVHNAFTYPNKTVFVFSVEHSAFSDALDQFVH
LFINPKFRQEDLDREKYAVHQEFAAHPLSDGRRVHRIQQLVAPQGHPCARFGCGNASTLTPVTTEKMAEWF
KLHYSPENMCAIAYTSAPLSKAKKQFSKIFSQIPRSKNYERQEPFLPSGDTSSLKKLYINQAIQPTSNLEIYWHI
YESSHPIPLGCYKALAEVLRNESKNSLVSLLKNEQLITDLDVEFFRSSLNTGEFYISYELTEKGDKHYSQVIDST
FQYLRYIQEHGIPNYTLEEISTINALNYCYSSKSPLFDLLCKQIVSLGNEDLSTYPYHSLVYPKYSSEDESALLN
LVSDPEQARFVLSSKNSEHWEEATQLYDPIFDMTYYVKALDGVQDYGKVQSLKPIALPKPNLFIPKEVTLPG
VHLLKKQEFPFAPALSYQDDKLTLYHCEDHYYTAPKLSSQIRIRSPQISRSSPQFLVATELYCLAVNDQLLREY
YPATQAGLSFTSALGGDGIDLRVSGYTTTVPTLLNSILTSLPNLEISYETFLVYKKQLLELYQGALLNCPVRSG
LDELASQVMKETYSNTTKLSALEKLSFSEFQAFASNLFNSVHLEVMVLGNLSEQQKKDYLEMLQAFTASRSS
HATKPFYYELQSQEISEIHHDYPLTANGMLLLLQDKSSPSIQGKVCAEMLFEWLHHITFEELRTQQQLGYMV
GARYREFASRPFGFLYIRSDAYSPEELLAKTSLFLNKVSASPEKFGMSQEKFANIRKAYINKILEPEHSLDMMN
SALFSLAFERPFVEFSTPDLKIAIAETLTYEEFLKYCQCFLSNELGTQTSVYIRGTQKTS

>core/30/4/Org4_Gene654

MTTEDFPKAYNFQDTEPELYVFWEKNGMFKAEASSDKPPYSVIMPPPNVTGVLHMGHALVNTLQDVLVRY
KRMSGFEVCWIPGTDHAGIATQTVVERHLQASEGKRRTDYSREDFLKHIWAWKEKSEKVILSQLRQLGCSC
DWDRKRFTMEPLANRAVKKAFKTLFENGYIYRGYYLVNWDPVLQTALADDEVEYEEKDGWLYYIRYRMV
GSQESIVVATTRPETLLGDTAIAVSPNDERYASWIGASVEVPFVNRQIPIIGDASVDPTFGTGAVKVTPAHDKD
DYLMGTNHHLPMINILTPSGGINENGGLFAGMAKEKAREEILIALEEQGLFVRKEPYKLRVGVSYRSGAVIEP
YLSKQWFVSVSEFRGALREFVESQDIKIFPKDFVKNYLSWVNHLRDWCISRQLWWGHRIPVWYHKNDDERV
LCYDGEGIPEEVAQDPDSWYQDPDVLDTWFSSGLWPLTCLGWPDENSPDLKKFYPTALLVTGHDILFFWVT
RMVLLCSSMSGEKPFSEVFLHGLIFGKSYKRYNDFGEWSYISGKEKLAYDMGEALPDGVVAKWEKLSKSKG
NVIDPLEMIATYGTDAVRLTLCSCANRGEQIDLDYRLFEEYKHFANKVWNGARFIFGHISDLQGKDLLAGIDE
DSLGLEDFYILDGFNELIHQLEEAYATYAFDKVATLAYEFFRNDLCSTYIEIIKPTLFGKQGNEASQSTKRTLL
AVLLINVLGVLHPVAPFITESLFLRIQDTLGALPEGDGDAFTGHALRMLRSRACMEAPYPKAFDVKMPQDLR
ESFTLAQRLVYTIRNIRGEMQLDPRLHLKAFVVCSDTTEIQSCIPILQALGGLESIQLLDKEPEKGLYSFGVVDT
IRLGIFVPEEHLLKEKGRLEKERVRLERAVENLERLLGDESFCQKANPNLVVAKQEALKNNRIELQGILDKLA
SFA

>core/31/4/Org4_Gene438

MRFFCFGMLLPSLFTFVLANEGLQLHLETYITLSPEYQAAPQAGLTHNQNQDLAIVGNHNDFILDYKYYQSN
GGALTCKNLLISENIGNIFFEKNVCPTSGGAIYTAQNCTISKNQNYAFATNLVSDNPTVTGGSRFGGALFAINC
SITNNLGQGTFVDNLALNKGGALYTETNLSIKDNKGPIIIKQNRTLNSESLGGGIYSGNSLNIEGNSGAIQISSNS
SGAGAGIFSTQTLTISSNKKLIEISENSAFANNYGSNFNPGGGGLSTNFCTISNNREGVLFNNNQSQRNGGAIH
AKSIIIKENGPVYFLNNTATWGGALLNLSGGSGNGSFVLSADNGDIIFNNNTASKNAPNPPYRNAIHSTPNMN
LQVGARPGYRVLFYDPIEHEHPSSFPLLFNFETGHTGTVLFSGKYVHQNFTEEMNFFSYLRNTSELRQGVLAV
EDGAGLACYKFFQRGGTLLLGKGAVITTAGTIPTPSSTRTTVGSTITLNHIAIDLPSILSFQAQAPKIWIYPTKTG
STYTEDSNPTITISGTLNLLNSNNEDPYDSLDLSHSLEKVPLLYIVDVAAQKINSSQLDLSTLNSSEHYGYQGI
WSTYWVETTTITNPTSLLGANTKHKLLYANWTPLGYRPHPERRGEFITNALWQSAYTALAGLHSLSSWDEE
KGHAASLQGIGLLVHQKDKNGFKGFRSHTTGYSATTEATSSQSPNFSLGFAQFFSKAKEHESQNSTSSHHYFS
GMCIENTLLKEWIRLSMSLAYMFSSEHTHTMYQGLLGGSSQGSFHNHTLAGALSCVFLPQPQGHSLQIYPFIT
ALAIRGKLAAFQESGDHAREFSLHHPLTNVSLPVGIRASWKNHHRVPLVWLTEISYRSTLYRQDPELHSKLLI
SQGTWTTHATPVTYNALAIKVKNTMQVFPKVTLSLDYSADISSSTLSHYLNVASRMRF

>core/32/4/Org4_Gene447

MKSSVSWLFFSSIPLFSSLSIVAAEVTLDSSNSYDGSNGTTFTVFSTTDAAAGTTYSLLSDVSFQNAGALGIPLA
SGCFLEAGGDLTFQGNQHALKFAFINAGSSAGTVASTSVADKNLLFNDFSRLSIISCPSLLLSPTGQCALKSAG
NLSLTGNSQIIFTQNFSSDNGGVINTKNFLLSGTSQFASFSRNQAFTGKQGGVVYATGTVTIENNPGIVSFSQN
LAKGSGGALYSTDNCSITDNFQVIFDGNSAWEAAQAQGGAICCTTTDKTVTLTGNKNLSFTNNTALTYGGAI
SGLKVSISAGGPTLFQSNISGSSAGQGGGGAINIASAGELALSATSGDITFNNNQVTNGSTSTRNAINIIDTAKV
TSIRAATGQSIYFYDPITNPGTTASTDTLNLNLADANSEIEYGGAIVFSGEKLSPTEKAIAANVTSTIRQPAVLA
RGDLVLRDGVTVTFKDLTQSPGSRILMDGGTTLSAKEANLSLNGLAVNLSSLDGTNKAALKTEAADKNISLS
GTIALIDTEGSFYENHNLKSASTYPLLELTTAGANGTITLGALSTLTLQEPETHYGYQGNWQLSWANATSSKI
GSINWTRTGYIPSPERRSNLPLNSLWGNFIDIRSINQLIETKSNGEPFERELWLSGIANFFYRDSMPTRHGFRHIS
GGYALGITATTPAEDQLTFAFCQLFARDRNHITGKNHGDTYGASLYFHHTEGLFDIANFLWGKATRAPWVLS
EISQIIPLSFDAKFSYLHTDNHMKTYYTDNSIIKGSWRNDAFCADLGASLPFVISVPYLLKEVEPFVKVQYIYA
HQQDFYERHAEGRAFNKSELINVEIPIGVTFERDSKSEKGTYDLTLMYILDAYRRNPKCQTSLIASDANWMA
YGTNLARQGFSVRAANHFQVNPHMEIFGQFAFEVRSSSRNYNTNLGSKFCF

>core/33/4/Org4_Gene967

MFGMTPAVYSVQTNSLEKSALERDEEFRTSFPLLDSLSTLTGFSPITTFVGNRHNSPQDIVLSNYKSIDNILLLW
TSAGGALSCNNFLLSNVEDHAFFSKNLAIGTGGAIACQGACTITKNKGPLIFFSNRGLNNASTGGETRGGAIA
CNGDFMISQNQGTFYFVNNSVNNWGGALSTNGHCRIQSNTGPLLFFNNTAPSAGGALRSENTTISDNTRPIYF
KNNCANNGGAIQTNSTITIKNNSGSVIFNNNTALSASIDSGNGSGGAIYTTSLSIDDNPGTIIFNNNYCSRDGGA
ICIQSLTIKNSGHVYFTNNQGNWGGALMLRRDSTCLLFAEQGNIAFQNNEVFLTAFGRYNAIHCTPNSNLQLG
ANKGYTTAFFDPIEHQHPTTNPLIFNPNANHQGTILFSSAYIPEASDYENNFISSSKNTSELRNGVLSIEDRAGW
QFYKFTQKGGILKLGHAASIATTANSETPSTSVGSQVIINNLAINLPSILAKGKAPTLWIRPLQSSAPFTEDNNP
TITLSGPLTLLNEENRDPYDSMDLSEPLQNIHLLSLSDVTARHINTDNFHPESLNTTEHYGYQGIWSPYWVETI
TTTNNASIETANTLYRALYANWTPLGYKVNPEYQGDLATTPLWQSFHTMFSLLRSYNRTGDSDIERPFLEIQG
IADGLFVHQNSIPGTPGFRIQSTGYSLQASSETSLHQKISLGFAQFFTRTKEIGSSNNVSAHNTVSSLYVELPWF
QEAFATSTVLAYGYGDHHLHSLHPSHQEQAEGTCYSHTLAAAIGCSFPWQQKSYLHLSPFVQAIAIRSHQTAF

EEIGDNPRKFVSQKPFYNLTLPLGIQGKWQSKFHVPTEWTLELSYQPVLYQQNPQIGVTLLASGGSWDILGPN
YVRNALGYKVHNQTALFRSLDLFLDYQGSVSSSTSTHHLQAGSTLKF

>core/34/4/Org4_Gene653

MERYDIVRIIGKGGMGEVYLAYDPVCSRKVALKKIREDLAENPLLKRRFLREARIAADLIHPGVVPVYTIYSE
KDPVYYTMPYIEGYTLKTLLKSVWQKESLSKELAEKTSVGAFLSIFHKICCTIEYVHSRGILHRDLKPDNILLG
LFSEAVILDWGAAVACGEEEDLLDIDVSKEEVLSSRMTIPGRIVGTPDYMAPERLLGHPASKSTDIYALGVVL
YQMLTLSFPYRRKKGKKIVLDGQRIPSPQEVAPYREIPPFLSAVVMRTLAVDPQERYSSVTELKEDIESHLKGS
PKWTLTTALPPKKSSSWKLNEPILLSKYFPMLEVSPASWYSLAISNIESFSEMRLEYTLSKKGLNEGFGILLPTS
ENALGGDFYQGYGFWLHIKERTLSVSLVKNSLEIQRCSQDLESDKETFLIALEQHNHSLSLFVDGTTWLIHMN
YLPSRSGRVAIIVRDMEDILEDIGIFESSGSLRVSCLAVPDAFLAEKLYDRALVLYRRIAESFPGRKEGYEARFR
AGITVLEKASTDNNEQEFSLAIEEFSKLHDGVAAPLEYLGKALVYQRLQEYNEEIKSLLLALKRYSQHPEIFRL
KDHVVYRLHESFYKRDRLALVFMILVLEIAPQAITPGQEEKILVWLKDKSRATLFCLLDPTVLELRSSKMELF
LSYWSGFIPHLNSLFHRAWDQSDVRALIEIFYVACDLHKWQFLSSCIDIFKESLEDQKAKEEIVEFSFEDLRAF
LFAIQSIFNKEDAEKIFVSNDQLSPILLVYIFDLFANRALLESQGEAIFQALDLIRSKVPENFYHDYLRNHEIRAH
LWCRNEKALSTIFENYTEKQLKDEQHELFVLYGCYLALIQGAEAAKQHFDVCREDRIFPASLLARNYNRLGL
PKDALSYQERRLLLRQKFLYFHCLGNHDERDLCQTMYHLLTEEFQL

>core/35/4/Org4_Gene446

MKIPLHKLLISSTLVTPILLSIATYGADASLSPTDSFDGAGGSTFTPKSTAEANGTNYILSGNVYINDAGKGTAL
TGCCFTETTGDLTFTGKGYSFSFNTVDAGSNAGAAASTTADKALTFTGFSNLSFIAAPGTTVASGKSTLSSAG
ALNLTDNGTILFSQNVSNEANNNGGAITAKTLSISGNTSSITFTSNSAKKLGGAIYSSDGASISGNTGQLVFMN
NKGETGGGALGFEASSSITQNSSLFFSGNTATDTAGKGGAICCEKTSQTPTLTISGNKSLTFTENSSVAEGGAIC
AHGLDLSAAGPTLFSNNRCGNTAAGKGGAIAIADSGSLSLSANQGDITFLGNTLTSTSAPTSTRNAIYLGSSAK
ITNLRAAQGQSIYFYDPIASNTTGASDVLTINQPDSNSPLDYSGTIVFSGEKLSADEAKAADNFTSTLKQPLAL
ASGTLALKGNVELDVNGFTQTEGSTLLMQPGTKLKADTEAISLTKLVVDLSALEGNKSVSIETAGANKTITLT
SPLVFQDSSGNFYESHTINQAFTQPLVVFTAATAASDIHIDALLTSPVQTPESHYGYQGHWEATWADTSTAKS
GTMTWVTTGYNPNPERRASVVPDSLWASFTDIRTLQQIMTSQANSIYQQRGLWASGTANFFHKDKSGTNQA
FRHKSYGYIVGGSAEDFSENIFSVAFCQLFGKDKDLFIVENTSHNYLASLYLQHRAFLGGLPMPSFGSITDML
KDIPLILNAQLSYSYTKNDMDTRYTSYPEAQGSWTNNSGALELGGSLALYLPKEAPFFQGYFPFLKFQAVYS
RQQNFKESGAEARAFDDGDLVNCSIPVGIRLEKISEDEKNNFEISLAYIGDVYRKNPRSRTSLMVSGASWTSL
CKNLARQAFLASAGSHLTLSPHVELSGEAAYELRGSAHIYNVDCGLRYSF

>core/37/4/Org4_Gene445

MKSSLHWFLISSSLALPLSLNFSAFAAVVEINLGPTNSFSGPGTYTPPAQTTNADGTIYNLTGDVSITNAGSPTA
LTASCFKETTGNLSFQGHGYQFLLQNIDAGANCTFTNTAANKLLSFSGFSYLSLIQTTNATTGTGAIKSTGACS
IQSNYSCYFGQNFSNDNGGALQGSSISLSLNPNLTFAKNKATQKGGALYSTGGITINNTLNSASFSENTAATN
GGAIYTEASSFISSNKAISFLNNSTTAASATGGAIYCSSTSAPKPVLTLSDNGELNFIGNTAITSGGAIYTDNLVL
SSGGPTLFKNNSAIDTAAPLGGAIAIADSGSLSLSALGGDITFEGNTVVKGASSSQTTTRNSINIGNTNAKIVQL
RASQGNTIYFYDPITTSITAALSDALNLNGPDLAGNPAYQGTIVFSGEKLSEAEAAEADNLKSTIQQPLTLAGG
QLSLKSGVTLVAKSFSQSPGSTLLMDAGTTLETADGITINNLVLNVASLKETKKATLKATQASQTVTLSGSLS

LVDPSGNVYEDVSWNNPQVFSCLTLTADDPANIHITDLAADPLEKNPIHWGYQGNWALSWQEDTATKSKAA
TLTWTKTGYNPNPERRGTLVANTLWGSFVDVRSIQQLVATKVRQSQETRGIWCEGISNFFHKDSTKINKGFR
HISAGYVVGATTTLASDNLITAAFCQLFGKDRDHFINKNRASAYAASLHLQHLATLSSPSLLRYLPGSESEQP
VLFDAQISYIYSKNTMKTYYTQAPKGESSWYNDGCALELASSLPHTALSHEGLFHAYSPFIKVEASYIHQDSF
KERDTTLVRSFDSGDLINVSVPIGITFERFSRNERASYEATVIYVADVYRKNPDCTTALLINNTSWKTTGTNLS
RQAGIGRAGIFYAFSPNLEVTSNLSMEIRGSSRSYNADLGGKFQF

>core/38/4/Org4_Gene443

MKTSIPWVLVSSVLAFSCHLQSLANEELLSPDDSFNGNIDSGTFTPKTSATTYSLTGDVFFYEPGKGTPLSDSC
FKQTTDNLTFLGNGHSLTFGFIDAGTNPGAAASTTANKNLTFSGFSLLSFDSSPNTTVTTGQGTLSSAGGVNL
ENIRKLVVAGNFSTADGGAIKGASFLLTGTSGDALFSNNSSSTKGGAIATTAGARIANNTGYVRFLSNIASTSG
GAIDDEGTSILSNNKFLYFEGNAAKTTGGAICNTKASGSPELIISNNETLIFASNVAETSGGAIHAKKLALSSGG
FTEFLRNNVSSATPKGGAISIDASGELSLSAETGNITFVRNTLTTTGSTDTPKRNAINIGSNGKFTELRAAKNHT
IFFYDPITSEGTSSDVLKINNGSAGAPNPYQGTILFSGETLTADELKVADNLKSSFTQPVSLSGGKLLLQKGVTL
ESTSFSQEAGSLLGMDSGTTLSTTAGSITITNLGINVDSLGLKQPVSLTAKGASNKVIVSGKLNLIDIEGNIYES
HMFSHDQLFSLLKITVDADVDTNVDISSLIPVPAEDPNSEYGFQGKWNVNWTTDTTTNTKEATATWTKTGFV
PSPERKSALVCNTLWGVFTDIRSLQQLVEIGATGMEHKQGFWVSSMTNFLHKTGDENRKGFRHTSGGYVIG
GSAHTPKDDLFTFAFCHLFARDKDCFIAHNNSRTYGGTLFFKHSHTLQPQSYLRLGRAKFSESAIEKFPREIPL
ALDVQVSFSHSDNRMETHYTSLPESEGSWSNECIAGGIGLDLPFVLSNPHPLFKTFIPQMKVEMVYVSQNSFF
ESSSDGRGFSSGRLLNLSIPVGAKFVQGDIGDSYTYDLSGFFVSDVYRNNPQSTATLVMSPDSWKIRGGNLSR
QAFLLRGSNNYVYNSNCELFGHYAMELRGSSRNYNVDVGTKLRF

>core/39/4/Org4_Gene534

MQRVLRLLFNLHHGEEKRAFLFFLLGLVWGIGCYGTLSLAEGLFIEKLGSAELPKIYLGSSLILCVLSSLILYNL
FKKHISATALFLIPVSLSILCNFYLILSSIFAIDLPRSPLFFYRIVIWSLTILSYTSFWGFVDQFFNLQDGKRHFCIF
NAIIFLGDAIGSGIIASLVHTIGIQGILILFTAALVLTFPIVFYVSKSLKSLSDDHDLFIDTGHPPPLSKALKLCFYD
KYTFYLLCFYFLMQLLAIATEFNYLKIFEIQFASKEEFELVAHIGKCSLWISLGNMCFALFAYSRIVKRLGVNNI
ILFAPLCFLSLFLFWTFKTTLSIAVLAMVVREGVTYALDDNNLQLLIYGVPNKIRNQIRIVVESFIEPIGMLVWS
LVCFLSSQQYVFCLIISLIATILVCLVRSYYAKAILKNLSAQALQLTRSMQDWIKSMTVKQKRQVELFLLAHL
KHPSERHQTFAFQHLLNLASRSVLPSLLAHMNKLSLPNKLKTIEMVKSSLWAKDFLTLELLKRWTSIFPHPAI
ASAIHLYFAEHDLLHITHIAEDLYDTVGDRLLAAILTVRRQEAYGPYRDLADKRLKELLNSDQPEDIVMGLTI
LKLEKNPQNFPILLDFLNTKNEDILIVTCKALHTSVRANHKPYCPELLKRLRQCSHNDEASQYLLKTISNALDI
SFVKDLLMTTSQLKNTSRKYAEAMIGELDKEVAPAFLQVLTDEGTHNRCRILAAKALCKIDNWLLKKHAYK
IVKSKASKALFYSYHGHYIQKKYPTYNLSLLVNTLNSNYYAEVNFMLSLLGILGSMEHSDVLIRALTSKNQKI
KAQALESLEKNCDSHLFSLLEPFVNQPGMCYSEKYYFKCGVIPLTLKELLNMMENSPSSLNKLTAQQLKEEL
SYCDPDFQSVNTIYNQEHEDFRTEESETLISFLSI

>core/40/4/Org4_Gene700

MRFSLCGFPLVFSFTLLSVFDTSLSATTISLTPEDSFHGDSQNAERSYDVQAGDVYSLTGDVSISNVDNSALNK
ACFNVTSGSVTFAGNHHGLYFNNISSGTTKEGAVLCCQDPQATARFSGFSTLSFIQSPGDIKEQGCLYSKNAL
MLLNNYVVRFEQNQSKTKGGAISGANVTIVGNYDSVSFYQNAATFGGAIHSSGPLQIAVNQAEIRFAQNTAK

NGSGGALYSDGDIDIDQNAYVLFRENEALTTAIGKGGAVCCLPTSGSSTPVPIVTFSDNKQLVFERNHSIMGG
GAIYARKLSISSGGPTLFINNISYANSQNLGGAIAIDTGGEISLSAEKGTITFQGNRTSLPFLNGIHLLQNAKFLK
LQARNGYSIEFYDPITSEADGSTQLNINGDPKNKEYTGTILFSGEKSLANDPRDFKSTIPQNVNLSAGYLVIKE
GAEVTVSKFTQSPGSHLVLDLGTKLIASKEDIAITGLAIDIDSLSSSSTAAVIKANTANKQISVTDSIELISPTGN
AYEDLRMRNSQTFPLLSLEPGAGGSVTVTSGDFLPVSPHYGFQGNWKLAWTGTGNKVGEFFWDKINYKPRP
EKEGNLVPNILWGNAVDVRSLMQVQETHASSLQTDRGLWIDGIGNFFHVSASEDNIRYRHNSGGYVLSVNN
EITPKHYTSMAFSQLFSRDKDYAVSKNEYRMYLGSYLYQYTTSLGNIFRYASRNPNVNVGILSRRFLQNPLMI
FHFLCAYGHATNDMKTDYANFPMVKNSWRNNCWAIECGGSMPLLVFENGRLFQGAIPFMKLQLVYAYQG
DFKETTADGRRFSNGSLTSISVPLGIRFEKLALSQDVLYDFSFSYIPDIFRKDPSCEAALVISGDSWLVPAAHVS
RHAFVGSGTGRYHFNDYTELLCRGSIECRPHARNYNINCGSKFRF

>core/41/4/Org4_Gene286

MKTVTLNIGRKILQGIKKKKKKIGILSGLFFLDLVLLGVSSQRPTETSANVKHNLRDEKLAACPKNSAASLSA
KKSHTKKTTPGSIPSKVFSKFDATQDKTFQKTSGSAFPAKPTTLKELEERKKPRPERRTTADVKRSPRFLPKQE
VEEPVPAASKEKLDSIQVWEEKQNYARRAVNAINLSIKKQLEEQTSTVTEKDVQPKTQATPHAAKKNVASPS
TSMPGIEKAATTVAVPQDKSEEEKVKERLTKRELTCEDLKDNGYTVNFEDISILELLQFVSKISGTNFVFDSND
LQFNVTIVSHDPTSVDDLSTILLQVLKMHDLKVVEQGNNVLIYRNPHLSKLSTVVTDSSLKETCEAVVVTRVF
RLYSVSPSAAVNIIQPLLSHDAIVSASEATRHVIISDIAGNVDKVSDLLAALDCPGTSVDMTEYEVKYANPAAL
VSYCQDVLGTMAEDDAFQMFIQPGTNKIFVVSSPRLANKAEQLLKSLDVPEMAHTLDDPASTALALGGTGT
TSPKSLRFFMYKLKYQNGEVIANALQDIGYNLYVTTAMDEDFINTLNSIQWLEVNNSIVIIGNQGNVDRVVSL
LNGLDLPPKQVYIEVLILDTSLEKSWDFGVQWVALGDEQSKVAYASGLLNNTGIATPTKATVPPGTPNPGSIP
LPTPGQLTGFSDMLNSSSAFGLGIIGNVLSHKGKSFLTLGGLLSALDQDGDTVIVLNPRIMAQDTQQASFFVG
QTVPYQTTNTIIQETGTVTQNIDYEDIGVNLVVTSTVAPNNVVTLQIEQTISELHSASGSLTPVTDKTYAATRL
QIPDGCFLVMSGHIRDKTTKVVSGVPLLNSIPLIRGLFSRTIDQRQKRNIMMFIKPKVISSFEEGTRVTNKEGYR
YNWEADEGSMQVAPRHAPECQGPPSLQAESDFKIIEIEAQ

>core/43/4/Org4_Gene223

MSKDKKMKPEPKKNFPTVFFFLLFGVVFGVVAFQNFLAGKKARVGFSHQIEHLVNLRLIVPEDSHKIALNDN
LVSFGGRFRDVQTQEGQLRYHYLELIDQGHRLDLDLQETSKSLTTLGKEVTNSILWFSAISGSPIPEQGYAISY
PSEVSGSVLTEPLVVTGPATPQLINLHSLQERYPTLSRSPEALRTYGSDLYELIGKYLSPVLGIGSETLKRELKD
LYQQVEVSLTQETDTEAAYTLYGQVLSTLNRISSSLVVSEGGERFSQLRSVRLYREEWNKYHKLVEARDLNQ
AQLEKLRGELSQTVWYFNNQELSSRSLEKQDPEVFGHWFAGAKEEWTAFKFNHSLSFKAPDQPRNLVLEKT
FKSQEPSPHYLGYLFTFLPIILVLLFVYLVFSRQMRGMSGSAMSFGKSPARMLLKGQNKVTFADVAGIEEAKE
ELIEIVDFLKNPNKFTSLGGRIPKGVLLIGPPGTGKTLIAKAVSGEADRPFFSIAGSDFVEMFVGVGASRIRDMF
EQAKRNAPCIIFIDEIDAVGRHRGAGIGGGHDEREQTLNQLLVEMDGFGTNEGVILMAATNRPDVLDKALLR
PGRFDRRVVMNLPDIKGRFEILMVHAKRIKLDPTVDLMAVARSTPGASGADLENLLNEAALLAARKDRTAV
TAVDVAEARDKVLYGKERRSLEMDAEERKTTAYHESGHAVVGLCVQHGDPVDKVTIIPRGLSLGATHFLPE
KNKLSYWKKELYDQLAVLMGGRAAEEIFLGDISSGAQQDISQATKLVRSMVCEWGMSPQLGNVTYDERSD
GLTGYGGYHEKSYSEETAKTIDTELRMLLDAAYQRALDIINEHKAEIELMTQMLIEFETLDSKDVKEIMDHT
WDPEKKRARLKEEGMLFKKSSDDLPPPPPKEDTLPGLGFNAT

>core/44/4/Org4_Gene486

MDSEFVGQVYSSDMDWIESMYQRFMNHETLDPSWKYFFEGYQLGQAASPSEASTKISGNETIAMLQEQKSQ
FLCTIYRYYGYLQSQISTLAPTTDSRFIQEKIAKIDLDEQVPSAGLLPKAQVSVRELIEALKKCYCGSLTVETLT
CTPELQEFVWNLMEKRQVERSAEQLLRSYKDLCKATFFEEFLQIKFTGQKRFSLEGGETLVPMLEHLVHYGS
ALGISNYVLGMAHRGRLNVLTNVLGKPYRYVFMEFEDDPAARGLESVGDVKYHKGYVLKSHQKDRETTFV
MLPNASHLESVDPIVEGVVAALQHQGHAGKEQSSLAILVHGDAAFSGQGVVYETLQLSRVPGYSTEGTLHIV
VNNYIGFTAVPRESRSTPYCTDIAKMLGIPVFRVNSEDVVACIEAIEYALQVRERFSCDVIIDLCCYRKYGHNE
SDDPSVTAPLLYDQIKRKKSIRELFRQHLLEGQFADISEETLASIEKEIQESLNCEFQVLKGTGPEPFPKKECHH
CDRLNNGELILRDCDVSLDRETLFHMSSRLCGFPENFHPHPKIKTLLEKRMKMAEGGVGYDWAMAEELAFA
SLLIEGYNLRLSGQDSIRGTFSQRHLVWSDTVTGDTYSPLYHLSAEQGSVEMYNSPLSEYAILGFEYGYAQQA
LKTLVLWEAQFGDFANGAQIIFDQYISSGIQKWDLHSDIVLLLPHGYEGQGPEHSSSRIERYLQLAANWNFQV
VLPSTPVQYFRILREHAKRDLSLPLVIFTPKLLLRYPQCVSSIEEFTEPGGFRAILEDADPNYDASILVLCSGKIY
YDYAEMLPQDRRKDFSCLRIESLYPLALEDLVSLIDKYSHLKHFVWLQEEPKNMGAYDYMFMALQDILPEK
LLYIGRPRSSSTASGSAKLSRQELVTCMETLFSLR

>core/45/4/Org4_Gene928

MEKVKLTKNLKLKIKNAQLTKAAGLDKLKQKLAQAGSSEAKSSSEKPSAKEKSVKVALAATSTPTASAEQA
SPESTSRRIRAKNRSSFSSSEEESSAHIPVDTSEPAPVSIADPEPELEVVDEVCDESPEVHPVAEVLPEQPVLPET
PPQEKELEPKPVKPAEPKSVVMIKSKFGPTGKHINHLLAKTFKAPAKEEKVVAGSKSTKPVASDKTGKPGTSE
GGEQNNREKQFNPANRSPASGPKRDAGKKNLTDFRDRSKKSDESLKAFTGRDRYGLNEGGEEDRWRKKRV
YKPKKHYDEASIQRPTHIKISLPITVKDLAAEMKLKASEVIQKLFIHGMTYVVNDILDSETAVQFIGLEFGCTID
IDYSEQDKLCLSNDTVRDEIQSTDPSKLVIRSPIVAFMGHVDHGKTTLIDSLRKSNVAATEAGAITQHMGAFC
CSTPVGDITILDTPGHEAFSAMRARGAEVCDIVVLVVAGDEGIKEQTLEAIEHAKAADIAIVVAINKCDKPNF
NSETIYRQLSEINLLPEAWGGSTVTVNTSAKTGEGLSELLEMLALQAEVLELKADPSARARGLVIESELHKGL
GPVATVLIQNGSLKLGEALVFNDCYGKVKTMHNEHNELMKEAGPSIPVLITGLSDIPKAGDPFFVVKNEKTA
RDIIEARSAGQQRFALQQKKRPNFDSMLQNKKTLKLMVKADVQGSIEALVSSISKIKSEKVDVEILTNSVGEIS
ESDIRLAAASKAVLIGFHTGIESHAEPLIKSLGVRVELFTVIYHAIDAIKEIMTSLLDPIAEEKDEGSAEIKEIFRS
SQVGSIYGCIVTEGIMTRNHKVRVLRNKEILWKGTLSSLKRVKEDVKEVRKGLECGILLEGYQQAQIGDVLQ
CYEVIYHPQKL

>core/46/4/Org4_Gene195

MLSNTIRSNFLKFYANRHHTILPSSPVFPHNDPSILFTNAGMNQFKDIFLNKEKVSYSRATTSQKCIRAGGKHN
DLDNVGHTSRHLTFFEMLGNFSFGDYFKAEAIAFAWEVSLSVFNFNPEGIYATVHEKDDEAFALWEAYLPTD
RIFRLTDKDNFWSMANTGPCGYCSELLFDRGPSFGNASSPLDDTDGERFLEYWNLVFMEFNRTSEGSLLALP
NKHVDTGAGLERLVSLIAGTHTVFEADVLRELIAKTEQLSGKVYHPDDSGAAFRVIADHVRSLSFAIADGLLP
GNTERGYVLRKILRRSVNYGRRLGFRNPFLAEIVPSLADAMGEAYPELKNSLSQIQKVLTLEEESFFKTLDRG
GNLLQQVLKSSSSSSCISGEDAFKLKDTYGMPIDEISLLAKDYDYSVDMDTFHKLEQEAKERSRKNVVQSQG
TSESIYNELHLTSEFIGYDHLSCDTFIEAIISKDHIVSSLQEKQEGAIVLKVSPFYAEKGGQVGDSGEIFCSEGTFI
VTHTTSPKAGLIVHHGRISQGSLTVEAAVTAQVNRYRRKRIANNHTACHLLHKALEITLGDHIRQAGSYVDD
TKIRLDFTHPQAISPEDLLCIETLVNESIRENEPVDIREALYSDVMNSSEIKQFFGDKYSDVVRVVSAGHSHELC

GGTHAEATGDIGFFRITKEHAVAMGIRRIEAVTGEKAEATVHQQSEVLEEIATLLQVPRDQIVSRLTATLDER
KQQDKRLNELENSLIQTKLDKLIHNCHQRQGITCLVHHLAEHENHRLQQYAQCLHQRIPEKLISLWTTEKNG
KYIVLSRVSDDLITQGVHAQDLLKAVLTPCGGRWGGKDQSAQGSAPALPATEVLNETLWQWISTQLI

>core/47/4/Org4_Gene270

MSTFSIQNRPRTISGESTRIIKLDHKYSGFDPRSVPAINLEELNSGIYALRHLMNALQSENTNVAALLNPNNTIF
PTTSWTDYNHSRPQASSPRAPSSQTPTDIVSAAALALVPVIDGGLAELVASVTEIDLGALSTISTVRQLMASYL
GLTTLTAEQEKVVFSSSYVPSEKNLLEHVKQEKAAEIQAKQEEIKAELEAKGVSTEEIEAILKEYPDIYAADFF
KEFIEEPLHTYRAKVGAPIQEMNENAIQLLPTPPAITPDNVNEVNGMNTLSTILQAIDDAIKQAPALGGDQEIIT
ILQTLVPLVDKTTFTKAEFDLIYTATQLPNTASLKLYLTDRQIAEYRGKITKVYQNSVQNLSETKRVVENNRS
MLETQLSMFQQAQNCFVTWISQANALNIAITNKYISAVLTTSMEMYGGLLCLSYMYERLADDEKAIFDKSVN
EYLPIHIVVGGSWVNGWIAKMAAYQELAEYSLGTAVTSQDQIKAYLQTRGNEFKATRHFFHDIGDQMYQFA
NETVFGNCLTTANGAIQPNLGGFIREAMTNVGTVEADYVSNAQRILNEFNTAATAHVLQLQLQIAELQKKAD
DLDPGKASFTENRKFAVAAWITSESLGDALISMILNSQLPKQEAFLKPLIEEINFNNLAANALNSLLQITNEFST
TSVYYSLSSYLVQSKTGQNLFAGDYYETLLAAAREREYIYRDTARCKQAINLVNGLLQKINSLPGATSAQKQ
EMLNATTYYQYSLSVTLNQLTVLESLLAGLKMTLQTTSNNKYNKSVFKIESFDDWIPTLAALESFLTSGFPNIS
ATGGLGPLFTQVQSDQQTYTSQGQTQQLNLQNQMTTIQQEWTLVSTSMQVLNGILSQLAGAIYSN

>core/48/4/Org4_Gene894

MKKSLIIVESPAKIKTLQKLLGSEFVFASSIGHIVDLPAKEFGIDVDHDFEPQYQVLPDKQEVINHIRKLAAKCE
KVYLSPDPDREGEAIAWHIANQLPDSPLIQRVSFNAITKNAVTEALKHPRTIDMALVNAQQARRLLDRIVGYK
ISPILSRKLQQRSGISAGRVQSVALKLVVDREKAIDAFVPVEYWNLRVLMQDPKTTKTFWAHLYAVQGKKW
EKEIPEGKTENDVLLINSEEKARHYAELLEKSSYTITRVEAKAKRRFAPPPFITSTLQQEASRHFRFSASRTMSI
AQTLYEGVDLDSEDSTGLITYMRTDSVRVDPEALTTVREYIQQTFGKEYLPEKANVYTTKKMTQDAHEAIRP
TDINLTPDKLKNKLSDDQFKVYNLIWKRFVASQITPAIYDTLAVQITTDTEIDLRASGSLLKFKGFLAVYEEKQ
DDENDQEEDHPLPPLHAQDVLIKEEVSQEQAFTKPLPRFTEASLVKELEKSGIGRPSTYATIMNKIQSREYTTK
ENQRLRPTELGKIISQFLETNFPRIMDIGFTALMEDELELIADNKKPWKLLLQEFWTTFLPVVITAEKEAVIPRI
LTNIECSKCHKGKLVKIWSKNSYFYGCSEYPECDYRTSEEELAFNKEDYAEDTPWDSPCPLCGGVMKVRHG
RYGTFLGCEKYPECRGTISIHKKGEEIEQEEPIPCPAIGCNGKIFKKRSRYNKIFYSCSEYPECSVIGNSIDAVITK
YSGTEKIPYKKKTPIKKKSSAKTTKAAKAPSKKGKAKSSVKKSSEKKTGPLFLPSPDLAKMIGNEPVSRGEAT
KKIWDYIKEHQLQAPENKKLLVPDNNLATIIGPNPIDMFQLSKHLSQHLTKVSNDESSASS

>core/49/4/Org4_Gene340

MKKLFVLDASGFIFRAYFALPEMKNHQGQATQAVFGFIRSLNKLIKEFSPEYMISVFDGPNNKQSRQAIYADY
KSNRQKKFEDIPPQIALVKEYCSLIGLAYLEKESVEADDVIASIAKKAREENYKVYVCTADKDLLQLVNDHV
VAWNPWADQGVVGISEVIERYGIPPGNIPDYLALVGDSSDNIPGVPGCGPKKAAALLKQFGSVEGLLENLDA
VKGLSQTMLSERQETLKLSKRLALLDSNIPIPVPIESLTFPQHPVDEEKLIHFYIQQGFKTLVPSKQTEAATVDV
QIIKDAESLTNILNLVQGGDIAFAAAYTGNHLLSLKLEGLALTQGSGVFFIALEEEGTKILPILKDFFLREDLTF
YGYNLKRDCHALLNAGIVIREISYDLALAEHLTNGGGKISFQSLLVNHGFTETAHRFAKEWGNLGLPIGRLPE
QPEQYFGELVAYLPIIKDAILEEINRKNLNHILSDIEMPLEKVLVSMERAGVPLDVEELAILEALFETELAVLTE
EIYDLSGRPFNIKSPKQLSDILYNELGLRPIDKAKSTRAEVLEALRSEHPIIEKLLAFRTIEKLLSTYVKALPKQV

DSHTQRIHPSFDQTGTVTGRLACRDPNLQNIPIRSERGILLRKAFRLSEKNSYFLSADYSQIELRFLAHLSQDKS
LKFAFESGEDIHAFTASQVFHVPLEQVSKEQRMQAKTVNFGIVYGQQAFGLAKVLKISVGEAQELIQAYFSR
YPEIAHFVEETIQQAAKDLRVTTMLGRERIIDSWNEFPGSRAASGRFAVNTRIQGSAAELIKLAMLDISQAIKQ
QQMKSRMLLQIHDELLFEVPEEEIEEMQRLVREKMESAMTLSVPIAVNILIGKNWAEC

>core/50/4/Org4_Gene34

MEKFSDAVSEALEKAFELAKSSKHTYVTENHLLLALLENTESLFYLVIKDIHGNPGLLNTAVKDALSREPTVV
EGEVDPKPSPGLQTLLRDAKQEAKTLGDEYISGDHLLLAFWSSNKEPFNSWKQTTKVSFKDLKNLITKIRRGN
RMDSPSAESNFQGLEKYCKNLTALAREGKLDPVIGRDEEIRRTIQVLSRRTKNNPMLIGEPGVGKTAIAEGLA
LRLIQGDVPESLKGKQLYVLDMGALIAGAKYRGEFEERLKSVLKDVESGDGEHIIFIDEVHTLVGAGATDGA
MDAANLLKPALARGTLHCIGATTLNEYQKYIEKDAALERRFQPIFVTEPSLEDAVFILRGLREKYEIFHGVRIT
EGALNAAVLLSYRYIPDRFLPDKAIDLIDEAASLIRMQIGSLPLPIDEKERELAALIVKQEAIKREQSPSYQEEA
DAMQKSIDALREELASLRLGWDEEKKLISGLKEKKNSLESMKFSEEEAERVADYNRVAELRYSLIPQLEEEIK
QDEASLNQRDNRLLQEEVDERLIAQVVANWTGIPVQKMLEGEAEKLLILEESLEERVVGQPFAVSAVSDSIR
AARVGLNDPQRPLGVFLFLGPTGVGKTELAKALADLLFNKEEAMVRFDMSEYMEKHSISKLIGSSPGYVGYE
EGGSLSEALRRRPYSVVLFDEIEKADKEVLNILLQVFDDGILTDGKKRKVNCKNALFIMTSNIGSPELADYCS
KKGSELTKEAILSVVSPVLKRYLSPEFMNRIDEILPFVPLTKEDIVKIVGIQMRRIAQRLKARRINLSWDDSVIL
FLSEQGYDSAFGARPLKRLIQQKVVILLSKALLKGDIKPDTSIELTMAKEVLVFKKVETPS

>core/51/4/Org4_Gene280

MAVRLIVDEGPLSGVIFVLEDGISWSIGRDSSANDIPIEDPKLGASQAIINKTDGSYYITNLDDTIPIVVNGVAIQ
ETTQLKNEDTILLGSNQYSFLSDEFDPQDLVYDFDIPEENFSNDSGDLSDSNEQGKDLEPRQTSETNHSPKPKE
KLTKDQGSSDPITSRDQELADAFLASAKAEKNQPRAKVAKKGLKESSNESLNPKEQNAKDSPKGEERTNKPQ
NAIMEDNGASPSQDPQPKSAEPSLENTARDETPLKENKPVEEKANKKATPDSPEKKDQPEEGSKKEGSKIEAT
PADSQKESEDKEAEEAFVQEEEENLTEDNKEDSDSAADANDDTASDHTAEDNKETPKKVENEKSAVLSPFH
VQDLFRFDQTIFPAEIDDIAKKNISVDLTQPSRFLLKVLAGANIGAEFHLDSGKTYILGTDPTTCDIVFNDLSVS
HQHAKITVGNDGGILIEDLDSKNGVIVEGRKIDKTSTLSSNQVVALGTTLFLLIDHHAPADTIVASLSPDDYSL
FGRQQDAEALERQEAQEEEEKQKRATLPAGSFILTLFVGGLAILFGIGTASLFHTKEVVPLENIDYQEDLAQVI
NQFPTVRYTFNKTNSQLFLIGHVKNSTDKSELLYKVDALSFVKSVDDNVIDDEAVWQEMNILLSKRPEFKGIS
MHSPEPGKFIITGYVKTEEQAACLVDYLNIHFNYLSLLENKVVVETQMLKAIAGHLLQGGFANIHVAFVNGE
VILTGYVNNDDAEKFRAVVQELSGIPGVRLVKNFAVLLPAEEGIIDLNLRYPNRYRVTGYSRYGEISINVVVN
GRILTRGDVIDGMTVTSIQPNAIFLEKEGLKYKIDYNK

>core/52/4/Org4_Gene455

MFEKFTNRAKQVIKLAKKEAQRLNHNYLGTEHILLGLLKLGQGVAVNVLRNLGIDFDTARQEVERLIGYGPE
IQVYGDPALTGRVKKSFESANEEASLLEHNYVGTEHLLLGILHQSDSVALQVLENLHIDPREVRKEILKELETF
NLQLPPSSSSSSSSRSNPSSSKSPLGHSLGSDKNEKLSALKAYGYDLTEMFRESKLDPVIGRSSEVERLILILCR
RRKNNPVLIGEAGVGKTAIVEGLAQKIILNEVPDALRKKRLITLDLALMIAGTKYRGQFEERIKAVMDEVRKH
GNILLFIDELHTIVGAGAAEGAIDASNILKPALARGEIQCIGATTIDEYRKHIEKDAALERRFQKIVVHPPSVDE
TIEILRGLKKKYEEHHNVFITEEALKAAATLSDQYVHGRFLPDKAIDLLDEAGARVRVNTMGQPTDLMKLEA
EIENTKLAKEQAIGTQEYEKAAGLRDEEKKLRERLQSMKQEWENHKEEHQVPVDEEAVAQVVSLQTGIPSA

RLTEAESEKLLKLEDTLRRKVIGQNDAVTSICRAIRRSRTGIKDPNRPTGSFLFLGPTGVGKSLLAQQIAIEMFG
GEDALIQVDMSEYMEKFAATKMMGSPPGYVGHEEGGHLTEQVRRRPYCVVLFDEIEKAHPDIMDLMLQILE
QGRLTDSFGRKVDFRHAIIIMTSNLGADLIRKSGEIGFGLKSHMDYKVIQEKIEHAMKKHLKPEFINRLDESVI
FRPLEKESLSEIIHLEINKLDSRLKNYQMALNIPDSVISFLVTKGHSPEMGARPLRRVIEQYLEDPLAELLLKES
CRQEARKLRATLVENRVAFEREEEEQEAALPSPHLES

>core/53/4/Org4_Gene699

MKIPLRFLLISLVPTLSMSNLLGAATTEELSASNSFDGTTSTTSFSSKTSSATDGTNYVFKDSVVIENVPKTGET
QSTSCFKNEAAAGDLNFIGGGFSFTFSNIDATTASGAAIGSEAANKTVTLSGFSALSFLKSPASTVTNGLGAIN
VKGNLSLLDNDKVLIQDNFSTGDGGAINCADSLKIANNKSLSFIGNSSSTSGGAIHTKNLTLSSGGETLFQGNT
APTAAGKGGAIAIADSGTLSISGDSGDIIFEGNTIGATGTVSHSAIDLGTSAKITALRAAQGHTIYFYDPITVTGS
TSVADALNINSPDTGDNKEYTGTIVFSGEKLTEAEAKDEKNRTSKLLQNVAFKNGTVVLKGDVVLSANGFSQ
DANSKLIMDLGTSLVANTESIELTNLEINIDSLRNGKKIKLSAATAQKDIRIDRPVVLAISDESFYQNGFLNEDH
SYDGILELDAGKDIVISADSRSIDAVQSPYGYQGKWTINWSTDDKKATVSWAKQSFNPTAEQEAPLVPNLLW
GSFIDVRSFQNFMELGTEGAPYEKRFWVAGISNVLHRSGRENQRKFRHVSGGAVVGASTRMPGGDTLSLGF
AQLFARDKDYFMNTNFAKTYAGSLRLQHDASLYSVVSILLGEGGLREILLPYVSKTQPCSFYGQLSYGHTDH
RMKTESLPPPPPTLSTDHTSWGGYVWAGELGTRVAVENTSGRGFFQEYTPFVKVQAVYARQDSFVELGAISR
DFSDSHLYNLAIPLGIKLEKRFAEQYYHVVAMYSPDVCRSNPKCTTTLLSNQGSWKTKGSNLARQAGIVQAS
GFRSLGAAAELFGNFGFEWRGSSRSYNVDAGSKIKF

>core/54/4/Org4_Gene545

MFNKDEIIVPKNLEEEMKESYLRYSMSVIISRALPDIRDGLKPSQRRILYAMKQLSLSPGAKHRKCAKICGDTS
GDYHPHGESVIYPTLVRMAQNWAMRYPLVDGQGNFGSIDGDPPAAMRYTEARLTHSAMYLMEDLDKDTV
DIVPNYDETKHEPVVFPSKFPNLLCNGSSGIAVGMATNIPPHNLGELIEATLLLLANPQASVDEILQVMPGPDF
PTGGIICGSEGIRSTYTTGRGKIKVRARLHVEENEDKHRESIIITEMPYNVNKSRLIEQIANLVNEKTLAGISDVR
DESDKDGIRVVLEIKKGESSEIIINRLYKFTDVQVTFGANMLALDKNLPRTMSIHRMISAWIRHRKEVIRRRTR
YELNKAETRAHVLEGYLKALSCLDALVKTIRESGNKEHAKERIIESFGFTEPQALAILELRLYQLTGLEAEKIQ
KEYEELLNKIAYYKQVLSDEGLVKDIIRNELQDLLKHHKVARRTTIEFDADDIRDIEDIITNESVIITISGDDYV
KRMPVKVFKEQRRGGHGVTGFDMKKGAGFLKAVYSAFTKDYLLIFTNFGQCYWLKVWQLPEGERRAKGK
PIINFLEGIRPGEELAAILNIKNFDNAGFLFLATKRGVVKKVSLDAFSNPRKKGIRALEIDEGDELIAACHIVSDE
EKVMLFTHLGMAVRFPHEKVRPMGRTARGVRGVSLKNEEDKVVSCQIVTENQSVLIVCDQGFGKRSLVEDF
RETNRGGVGVRSILINERNGNVLGAIPVTDHDSILLMSSQGQAIRINMQDVRVMGRSTQGVRLVHLKEGDAL
VSMEKLSSNENDDEVLSGSEEECSDTVSLR

>core/55/4/Org4_Gene271

MIYSTSISTFYKKLPLVSSMHSFAQRHRESLEHIANYEKTTAERDILKRLIEVLDQRASERYRSAVEKLHKYEV
ERATVAKSIPVAAIHEKPLSSTHASVQVTASTPAATGSGVGAYYNAVKQKWAQDLIVELNTVMTTIMASVNS
KNPANKDVFDKLNTELQALVAAGNNLTEENFQTLYNFPEEIFTAIQRADTFTGGMKTDFTNQLAGKYGNQA
TLTQTFADGRVEGFKDILTAVQGVLTPEQFTIFAEIATELQALADHVGNFDEAGLPRIEDAGEKLAAVINSSDL
TRNDKIMFCQHITDLYSDQVAALGSFDTVLDASIYVNQHQGTMFSNLSSFVGSLIGTFAPIDLSSSQGDISSAA
LAGALQTARGLNSRFNELTAEQQKLINECVKSLDTFKCGEHLGAIWAYFTASTVVALNPTATMDHVKAILE

EAKELDNSSFQLASSMKSAMTSIVSSNGSFSVTVNSNTLQYTIYSEKSGKVEINQILLNYGSTGFLPEITKLAKT
NAESTARSYFRFKALAAVESENVQNKIEDLQSQLQQFTNMKTELFDGQLLSQASELRALPLPSAVASVLIDRY
MPKEVDYLNEIYKKLYYSNLGSSVGNSIIDAISQYVNGATYFNFASYVGQQPAVGAGGANAFPGSQESAQAK
LDQERKQAALYLQETRGALTVIEEQRARVLKDDKITNEQRSTILDSLRNYEDNINSISGSLVLLQNYLQPLSIA
GGSVAGTFEVKGGQEQWQARLQILEEALVSGLVGNMINGGMFPLQSTIQSDQQSFADMGQNFQLDLQMHL
TSMQQEWTVVATSLQLLNQMYLSLARSLTG

>core/56/4/Org4_Gene789

MTEKKPTPMMEQWHQCKEKAGDSVLLFRMGDFYEAFYDDAVLLSQHLELTLTQRQGIPMSGIPVSTVDTYV
DRLIGKGFKVAVAEQFGEPAKEKESKKIGPMARDIQRFVTPGTLLSSTLLQEKSNNYIVAINRIGSLFGFACLD
LSTGSFFIEECENTKELVDEICRLAPSEVLSCNKFYNKETAIVMQLQQHLKLTLSTYADWAFEHKFASQKLTT
HFQVASLDGFGLKGLVPAINAAGGLLSYIQDKLLLPTKHIAIPQTRGKQQKLLIDTASQVNLELLAPLNDPQG
KNSLLRIMDHTSTPMGGRLLRQILISPFYNPKEILVRQDAVEFFLRQVTLRKNIKTYLCQVRDIERLMTKVTTG
LAGPRDIGTLRDSFSAGAQIYEQLASATLPEFFIDKCSLDTKLASLIALLSKSLNGDLPLRVSDGNIFVDEFHND
LKRLRHNQEHSQEWIWEYQERIRKETGIKKLKICFAQALGYYIEVSSEFAPQLPKDFIRRQSRLHAERFTTIEL
QQFQDDMSNISEKLQTLETQFFKDLCSHILQLRTEILALSQSLADLDYIISLADLAHAQGYCRPRVDMSDTLCI
YRGCHPVAKTLVDTGKFIPNDTEMRGSQTRMILLTGPNMAGKSTYIRQIALLVIMAQMGSYIPAKSAHIGVID
KIFTRIGAGDNLSKGMSTFMVEMAETANILHNATDRSLVILDEVGRGTSTYDGLAIAQAVVEYLLFTDKKKA
KTLFATHYKELTTLEDHCPHVENFHAGVKDKAGQPVFLYEILKGHSQKSFGIHVARLAGFPLCVVSRAQQIL
RQLEGPESITRPAQDKMQQLTLF

>core/57/4/Org4_Gene993

MKRAILDRLYLSVVQSPESASPRDIFTAVAKTVMEWLAKGWLKTQNGYYKNDVKRVYYLSMEFLLGRSLK
SNLLNLGILDLVRKALKTLNYDFDHLVEMESDAGLGNGGLGRLAACYLDSMATLAVPAYGYGIRYDYGIFD
QRIVNGYQEEAPDEWLRYGNPWEICRGEYLYPVRFYGRVIHYTDSRGKQVADLVDTQEVLAMAYDIPIPGY
GNDTVNSLRLWQAQSPRGFEFSYFNHGNYIQAIEDIALIENISRVLYPNDSITEGQELRLKQEYFLVSATIQDIIR
RYTKTHICLDNLADKVVVQLNDTHPALGIAEMMHILVDREELPWDKAWEMTTVIFNYTNHTILPEALERWP
LDLFSKLLPRHLEIIYEINSRWLEKVGSRYPKNDDKRRSLSIVEEGCQKRINMANLAVVGSAKVNGVSSFHSQ
LIKDTLFKEFYEFFPEKFINVTNGVTPRRWIALCNPRLSKLLNETIGDRYIVDLSHLSLIRSFAEDSGFRDHWKE
VKLKNKQDLTSRIYNEVGEIVDPNSLFDCHIKRIHEYKRQLMNILRVIYVYNDLKENPNQDVVPTTVIFSGKA
APGYVMAKLIIKLINSVADVVNQDSRVNDKLKVLFLPNYRVSMAEHIIPGTDLSEQISTAGMEASGTGNMKF
ALNGALTIGTMDGANIEMAEHIGKENMFIFGLLEEQIVQLRREYCPQTICDKNPKIRQVLDLLEQGFFNSNDK
DLFKPIVHRLLHEGDPFFVLADLESYIAAHENVNKLFKEPDSWTKISIYNTAGMGFFSSDRAIQDYARDIWHV
PTKSCSGEGN

>core/58/4/Org4_Gene823

MKKLYHPTLFLRPLIRLSLIFALSLTLISGNFPQQKSFGHCCADMHSALTSGKNCEELFADFIERVLADRETLT
ARDWGTVVVLVREYLLKCIRKGDCDYGVKILQKLLALRLPKDARKDLQILWHRLNPEQAPLRDVVDQLFTI
GCHESLQDHLLFELYTMTLHSGYENRKQDMLLAKEQGDYKKAIELAKELVAALEKGSCSPHPEIVQIEKTFL
QKTLLALQIKVAQEAQESCDALLTPYCLSEIAYTEAMDALVLRIARGEVSRTNEVDSVLLSHALQHLPFAREK
AILELEVLIDHGAYLESTLLYYAYFSLLELYHQNKDFASLERLLEKGDAVFVPEHPYFPEYGFFLGAYFYAKG

KYESAEKVFLQIIDPAVKLGATFARAYEYLGCIAYVQNHYEKAEEYFLRAYKSWGREESGIGLFLAYAVQKK
KTACEDMLYHPKFSFTYRHLLDSLCSLSYPHGENKGSSAIQRVHRAVPELSEIYSRCIYDMIKYRNVTYTHPII
ELAYNQVRNLEKRNLEEICRDAQDPEYDKALAFWGALQSGASVPRSLIESSDVDEARITIRCYEALYFHNPDA
IAMLPQAFSEECNSWQTALRLVWTLVRPKGAPNHAKYWDHLVLRPHGDSLYFFGYDLQEYLIGKEDALKHL
SVFAELFPKSSLLSLVYYLQGYSESSALRKVGWFVKALEEFNEISWSGEHMKTWAYIYYMVKLDLADTYISL
GNFSQAVHILEEVKEDWQVASHPKLHFLKGEDCYLAMELRWVEGLAYAYFQLHETAHLSNHLLEHVEKNLI
SPRSYRDYYGESLQRTLGLCQRFLGV

>core/59/4/Org4_Gene617

MRYDPNLIEKKWQQFWKEHRSFQANEDEDKVKYYVLDMFPYPSGAGLHVGHLIGYTATDIVARYKRARGF
SVLHPMGWDSFGLPAEQYAIRTGTHPKVTTQKNIANFKKQLSAMGFSYDEGREFATSDPDYYHWTQKLFLF
LYDQGLAYMADMAVNYCPELGTVLSNEEVENGFSIEGGYPVERKMLRQWILKITAYADKLLEGLDALDWP
ENVKQLQKNWIGKSEGALVTFHLTQEGSLEAFTTRLDTLLGVSFLVIAPEHPDLDSIVSEEQRDEVTAYVQES
LRKSERDRISSVKTKTGVFTGNYAKHPITGNLLPVWISDYVVLGYGTGVVMGVPAHDERDREFAEKFSLPIH
EVIDDDGVCIHSTYNDFCLNGLSGQEAKDYVINYLEMRSLGRAKTMYRLRDWLFSRQRYWGEPIPIIHFEDG
THRPLEHDELPLLPPNIDDYRPEGFGQGPLAKAQDWVHIYDEKTGRPGCRETYTMPQWAGSCWYYLRFCDA
HNSQLPWSKEKETYWMPVDLYIGGAEHAVLHLLYSRFWHRVFYDAGLVSTPEPFKKLINQGLVLAFSYRIPG
KGYVSIEDVREENGTWISTCGEIVEVRQEKMSKSKLNGVDPQVLIEEYGADALRMYAMFSGPLDKNKTWSN
EGVGGCRRFLNRFYDLVTSSEVQDIEDRDGLVLAHKLVFRITEHIEKMSLNTIPSSFMEFLNDFSKLPVYSKHA
LSMAVRVLEPIAPHISEELWVILGNPPGIDQAAWPQIDESYLVAQTVTFVVQVNGKLRGRIEVAKEAPKEEVL
SLSRSVVAKYLENAQIRKEIYVPNKLVNFVL

>core/60/4/Org4_Gene5

MDSTTNSDSPILDPNPEDVEKLLDESEEESEDQSTERLLPSELFILPLNKRPFFPGMAAPILIESGPYYEVLKVLA
KSSQKYIGLVLTKKENADILKVSFNQLHKTGVAARILRIMPIEGGSAQVLLSIEERIRIIEPIKDKYLKARVSYH
ADNKELTEELKAYSISIVSVIKDLLKLNPLFKEELQIFLGHSDFTEPGKLADFSVALTTATREELQEVLETTNM
HDRIDKALILLKKELDLSRLQSSINQKIEATITKSQKEFFLKEQLKTIKKELGLEKEDRAIDIEKFSERLRKRHVP
DYAMEVIQDEIEKLQTLETSSAEYTVCRNYLDWLTIIPWGIQSKEYHDLKKAEIVLNKDHYGLDEIKQRILELI
SVGKLSKGLKGSIICLVGPPGVGKTSIGRSIAKVLHRKFFRFSVGGMRDEAEIKGHRRTYIGAMPGKMVQALK
QSQAMNPVIMIDEVDKIGASYHGDPASALLEVLDPEQNKDFLDHYLDVRVDLSNVLFILTANVLDTIPDPLLD
RMEILRLSGYILEEKLQIAKKYLVPKARKEIGLTASEVNFQPEALKYMINNYAREAGVRTLNGNIKKVLRKVA
LKIVQNQEKPKSKKITFKISSKNLQTYLGKPIFSSDRFYESTPVGVATGLAWTSLGGATLYIESVQVSSLKTDM
HLTGQAGEVMKESSQIAWTYLHSALHRYAPGYTFFPKSQVHIHIPEGATPKDGPSAGITMVTSLLSLLLETPV
VNNLGMTGEITLTGRVLGVGGIREKLIAARRSRLNILIFPEDNRRDYEELPAYLKTGLKIHFVSHYDDVLKVA
FPKLK

>core/61/4/Org4_Gene810

MKGTPQYHFIGIGGIGMSALAHILLDRGYEVSGSDLYESYTIESLKAKGARCFSGHDSSHVPHDAVVVYSSSI
APDNVEYLTAIQRSSRLLHRAELLSQLMEGYESILVSGSHGKTGTSSLIRAIFQEAQKDPSYAIGGLAANCLNG
YSGSSKIFVAEADESDGSLKHYTPHAVVITNIDNEHLNNYAGNLDNLVQVIQDFSRKVTDLNKVFYNGDCPIL
KGNVQGISYGYSPECQLHIVSYNQKAWQSHFSFTFLGQEYQDIELNLPGQHNAANAAAACGVALTFGIDINII

RKALKKFSGVHRRLERKNISESFLFLEDYAHHPVEVAHTLRSVRDAVGLRRVIAIFQPHRFSRLEECLQTFPKA
FQEADEVILTDVYSAGESPRESIILSDLAEQIRKSSYVHCCYVPHGDIVDYLRNYIRIHDVCVSLGAGNIYTIGE
ALKDFNPKKLSIGLVCGGKSCEHDISLLSAQHVSKYISPEFYDVSYFIINRQGLWRTGKDFPHLIEETQGDSPLS
SEIASALAKVDCLFPVLHGPFGEDGTIQGFFEILGKPYAGPSLSLAATAMDKLLTKRIASAVGVPVVPYQPLNL
CFWKRNPELCIQNLIETFSFPMIVKTAHLGSSIGIFLVRDKEELQEKISEAFLYDTDVFVEESRLGSREIEVSCIG
HSSSWYCMAGPNERCGASGFIDYQEKYGFDGIDCAKISFDLQLSQESLDCVRELAERVYRAMQGKGSARIDF
FLDEEGNYWLSEVNPIPGMTAASPFLQAFVHAGWTQEQVVDHFIIDALHKFDKQQTIEQAFTKEQDLVKR

>core/62/4/Org4_Gene828

MIRERKKSRHPRLPTLPLAAKASLYLFFACFSGLSLWSFHRDQPCTQNWIGLLGWSFSSFLLYFFGAAAFFIPL
YFLWLSFLYFRRTPRPLFFYKAAAFLSLPFCSAILLSMLSPVGTLPGLLDTRLPKFILGNNPPVSYVGGIPFYLF
YEGQSFCLKHLIGSVGTALIFGFVMLFSVLYLCGGIALLKKKTFQDGVKKAFCSFFQTCFKNLKKLINRRNYL
PKPSVPFVSKNPFSCTKSQPSPRRVSETIILDGSISPLPQEEIPGSKKESFFLTPHPCKRFLTKFVEPQENKAKEGK
TIALPSTPTVVRESKGKERAALPKLKSLAVPENDLPQYHLLSKNREARPESLQAELERKALILKQTLTSFGIDA
DLGNICSGPTLAAFEVLPHSGVKVQKIKSLENDIALKLQASSIRIIAPIPGKAAVGIEIPTPFPQAVNFRDLLEDY
QKTNRKLQIPLLLGKKANGDNLWADLATMPHLIIAGTTGSGKSVCINTIVMSMIMTTLPSEIKLVIIDPKKVEL
TGYSQLPHMLSPVITESREVYNALVWLVKEMESRYEILRYLGLRNIQAFNSRTRNKTIEASYDREIPETMPFM
VGIIDELSDLLLSSSQDIETPIIRLAQMARAVGIHLILATQRPSREVITGLIKANFPSRISFKVSNKVNSQIIIDEPG
AENLMGNGDMLVLLPSVFGTIRAQGAYICDEDINKVIQDLCSRFPTQYVIPSFHAFDDSDSDNSGEKDPLFAQ
AKTLILQTGNASTTFLQRKLKIGYARAASLIDQLEEARIIGPSEGAKPRQILIQNPLEG

>core/63/4/Org4_Gene544

MDPKEKNYDASAITVLEGLQAVRERPGMYIGDTGITGLHHLVYEVVDNSIDEAMAGYCSRIDVRILEDGGIVI
VDNGRGIPIEVHERESAKQGREVSALEVVLTVLHAGGKFDKDSYKVSGGLHGVGVSCVNALSEKLVATVFK
DKKCYQMEFSRGIPVTPLEYVSASDRQGTEIVFYPDPKIFSTCTFDRSILMKRLRELAFLNRGITIVFEDDRDVS
FDKVTFFYEGGIQSFVSYLNQNKESLFSEPIYICGTRVGDDGEIEFEAALQWNSGYSELVYSYANNIPTRQGGT
HLTGFATALTRVINTYIKAHNLAKNNKLALTGEDIREGLTAVISVKVPNPQFEGQTKQKLGNSDVSSVAQQV
VGEALTIFFEENPQIARMIVDKVFVAAQAREAAKKARELTLRKSALDSARLPGKLIDCLEKDPEKCEMYIVEG
DSAGGSAKQGRDRRFQAILPIRGKILNVEKARLQKIFQNQEIGTIIAALGCGIGADNFNLSKLRYRRIIIMTDAD
VDGSHIRTLLLTFFYRHMTALIENECVYIAQPPLYKVSKKKDFRYILSEKEMDSYLLMLGTNESSILFKSTERE
LRGEALESFINVILDVESFINALEKKAIPFSEFLEMYKEGIGYPLYYLAPATGMQGGRYLYSDEEKEEALAQEE
THKFKIIELYKVAVFVDIQNQLKEYGLDISSYLIPQKNEIVIGNEDSPSCNYSCYTLEEVINYLKNLGRKGIEIQR
YKGLGEMNADQLWDTTMNPEQRTLIRVSLKDAVEADHIFTMLMGEEVPPRREFIESHALSIRMNNLDI

>core/64/4/Org4_Gene526

MNKRTLLFVSLIGIAFVGCQIFFGYNEFRSCKNLAEKQRKISEQTLAAVESVGLSVASWDTDVNGEEHKNNY
AVRVGDKLFLLHNGEAAQSVYSSGESWSFVDHKCGFDNIHLALYRQQGSSFNPTNTGKVFLPTNHEGLPVL
VVEFRNNKEPLVFLGEYAQGRISNKDSTIFGTALVFWRSGSDYIPLGLYDSREEKLVSLDLPITRAVIFGNDQD
SAKSSDTANHYVLFNDYMQIIVSEESGSIEGINLPFASTNNKSIVNEIGFDRDLASEKSPEALFPGLSSKLPDGQ
QAKNSIGGYYPLLRRGLLSDSKKLLPLEYHALNVVSGRELATPVALRYRVLSYTPHSIQLESLDRSVQKVYKL
PENPEEKPYVFETAITLTKETEDVWVTSGVPEVEIMSNASVPTIKYRVIKKNKGSLDKVKLPKVKEPLAVRRG

VYPQWILNSNGYFGIILTPLSEIASGYGSLYISGSTAPTRLSAISPKNQLYPASKYPGYETLLPLPKDAGTHRFL
VYAGPLAEPTLKVLDKTITQEKGENPEYLDSISFRGVFAFITAPFAALLFIIMKFFKLVTGSWGISIILLTVFLKL
LLYPLNAWSIRSMRRMQILSPYIQQIQQKYKNEPKRAQMEIMGLYKTNKVNPITGCLPLLIQLPFLIAMFDLLK
SSFLLRGASFIPGWIDNLTAPDVLFSWQTSIWFIGNEFHLLPILLGIVMFLQQKVTSLHKKGPVTDQQKQQQV
MGNMMAILFTAMFYNFPSGLNIYWLSSMILGVVQQWITNKILDSKHLKNEVVLNNKKHR

>core/65/4/Org4_Gene354

MRIPITLLQTYFSEPLSTKEILEACDHIGIEAEIENTTLYSFTSVITAKILHTIPHPNADKLRVATLTDGAKEHQV
VCGAPNCEAGLIVALALPGAKLFDSEGKAYTIKKSKLRGVESQGMCCGADELGLDELQIQERALLELPEATP
LGEDLATVLGNTSLEISLTPNLGHCASLLGLAREICHVTQANLVIPKEFSFENLPTTALDMGNDPDICPFFSYV
VITGISAQPSPIKLQESLQALKQKPINAIVDITNYIMLSLGQPLHAYDASHVALDSLRVEKLSTPESLTLLNGET
VLLPSGVPVVRDDHSLLGLGGVMGAKAPSFQETTTTTVIEAAYFLPEALRASQKLLPIPSESAYRFTRGIDPQN
VVPALQAAIHYILEIFPEATISPIYSSGEICRELKEVALRPKTLQRILGKSFSIEILSQKLQSLGFSTTPQETSLLVK
VPSYRHDINEEIDLVEEICRTESWNIETQNPVSCYTPIYKLKRETARFLANAGLQEFFTPDLLDPETAALTRKEK
EEISLQGSKHTTVLRSSLLPGLLKSAATNLNRQAPSVQAFEIGTVYAKHGEQYQETQTLAILLTEDGESRSWLP
KPSLSFYSLKGWVEKLLYHHHLSIDALTLESSALCEFHPYQQGVLRIHKQSFATLGQVHPELAKKAQIKHPVF
FAELNLDLLCKMLKKTTKLYKPYAIYPSSFRDLTLTVPEDMPANLLRQKLLHEGSKWLESVTIISIYQDKSLET
RNKNVSLRLVFQDYERTLSNQDIEEEYCRLVALLNELLTDTKGTINS

>core/66/4/Org4_Gene532

MLIMRNKVILQISILALIQTPLTLFSTEKVKEGHVVVDSITIITEGENASNKHPLPKLKTRSGALFSQLDFDEDLR
ILAKEYDSVEPKVEFSEGKTNIALHLIAKPSIRNIHISGNQVVPEHKILKTLQIYRNDLFEREKFLKGLDDLRTY
YLKRGYFASSVDYSLEHNQEKGHIDVLIKINEGPCGKIKQLTFSGISRSEKSDIQEFIQTKQHSTTTSWFTGAGL
YHPDIVEQDSLAITNYLHNNGYADAIVNSHYDLDDKGNILLYMDIDRGSRYTLGHVHIQGFEVLPKRLIEKQS
QVGPNDLYCPDKIWDGAHKIKQTYAKYGYINTNVDVLFIPHATRPIYDVTYEVSEGSPYKVGLIKITGNTHTK
SDVILHETSLFPGDTFNRLKLEDTEQRLRNTGYFQSVSVYTVRSQLDPMGNADQYRDIFVEVKETTTGNLGLF
LGFSSLDNLFGGIELSESNFDLFGARNIFSKGFRCLRGGGEHLFLKANFGDKVTDYTLKWTKPHFLNTPWILGI
ELDKSINRALSKDYAVQTYGGNVSTTYILNEHLKYGLFYRGSQTSLHEKRKFLLGPNIDSNKGFVSAAGVNL
NYDSVDSPRTPTTGIRGGVTFEVSGLGGTYHFTKLSLNSSIYRKLTRKGVLKIKGEAQFIKPYSNTTAEGVPVS
ERFFLGGETTVRGYKSFIIGPKYSATEPQGGLSSLLISEEFQYPLIRQPNISAFVFLDSGFVGLQEYKISLKDLRSS
AGFGLRFDVMNNVPVMLGFGWPFRPTETLNGEKIDVSQRFFFALGGMF

>core/67/4/Org4_Gene970

MLLLISGALFLTLGISGLSAAISFGLGIGFSALGGVLVVSGLLCLLVKQEVSEERPEEIPAVQPEEIPEGVPVTPF
EKPALDEAQKEQKAQKILEQLPKELDQLDRYIQEVVSCFRKLKDLKCEDQGLLKDAKEKLQVFDFVWKDM
MTEFVELQQIIVQEGWYKELHIQNLDDLGPNLFNDSRLSEHCVSLCYLEELCGYLPSGDARASRLKRSLREVV
ARFMKVVHDTRKVAITFERNNYGVAKKTFEETFRALEDCVCRSLSKSYRDAFYEYEKAKILRSGHIEWLRDD
RNSVLAEQRFQDARDRWEYLKETVFWVKEDGSIDIEDVANPSAWNDRFPVRMFFQDRQDKILGDESWEWW
GGCRKAYHTSHAVLQAAFEKDKSKDNLQKVKKHDERMFTFFQKNCDREYQKALERLRELQALYPEVSVSL
VEAGREENLGSNLERAYENLEEKYQRCVRDQEDYWRKVEKEEAEFRANGTKIRSMEEVLGYIQILEDLIESW
SLKINKFEQSVLGFNFEVTQELDNKILFHATNRLNVLGEDIKDMLSRVEEIEMMLRVIELPLLPIKQALEKAFV

QYNSCKAKLTKVEPCFRESPAYITSEERLQSLDQTLERAYKEYQKRFQEPSRLESEVSGCREHLREQVKQFET
QGLDLIKEELIFVSDVLFRKMVSRLVSTVHVPFMEFYYEYFELHRLRLRAQWMANAEIYSKVRKAFPEMLKE
TLEKAKAPREEEYWLLCEERKSKEKRLILNKIEAAQQRVKDLEPPPIKETGKQKRK

>core/68/4/Org4_Gene436

MASGIGGSSGLGKIPPKDNGDRSRSPSPKGELGSHEISLPPQEHGEEGASGSSHIHSSSSFLPEDQESQSSSSAAS
SPGFFSRVRSGVDRALKSFGNFFSAESTSQARETRQAFVRLSKTITADERQDVDSSSAAATEARVAEDASVSG
ENPSQGVPETSSGPEPQRLFSLPSVKKQSGLGRLVQTVRDRIVLPSGAPPPDSEPLSLYELNLRLSSLRQELSDI
QSNDQLTPEEKAEATVTIQQLIQITEFQCGYMEATQSSVSLAEARFEGVETSDEINSLCSELTDPELQELMSDG
DSLQNLLDETADDLEAALSHVRVSFSLDDNPTPIDNNPTPISQEEPIYEEIGGAADPQRTRENWSTRLWNQIRE
ALVSLLGMILSILGSILHRLRIARHAAAEAVGRCCTCRGEECTSSEGDSMSVGSPSEIDETERTGSPHDVPRRD
GSPREDSPLMNALVGWARKHGAKTKESSESSTPEISISAPIVRGWSQDSSVSFIVMEDDHIIYDVPRRKDGIYD
VPSSPRWSPARELEEDVFGDDEVPTTSVEPSKDENIYMTPRLATPAIYDLPSRPGSSGSSRSPSSDRVRSSSPNR
RGVPLPPVPSPAMSEEGSIYEDMSGASGAGESDYEDMSRSPSPRGDLDEPIYANTPEDNPFTQRNIDRILQERS
GGASASPVEPIYDEIPWIHGRPPATLPRPEHTLTNVSLRVSPGFGPEVRAALLSESVSATMVGAENAVAPTEPG
HGESEYLEPLGELVATTKILLQKGWPSGESNA

>core/69/4/Org4_Gene683

MLLLISGALFLTLGIPGLSGAISFSLGIGLSALGGVLMISGLLCLLVKREVSEERPEEIPTVQPEGVPLAPSEEPA
LQAAQKTLAQLPKELDQLDTDIQEVFACLRKLKDSKYESRSFLQDAKEKLRIFDFVVEDALSEIFELRQIVDQ
EGWDLNFLINGGRSLMVTAEPESLDLFHVWKRLGYLPSGDVRGEELKKSVKEIVARLMRLYCEIHKVAVAF
DRNSYAMAEKAFAKALGALEESVYRSLTQSYRDKFLESERAKILWNGHITWLRDDAKSGCAEKKLRDAEER
WKKFRKAVFWVEEDGRIEITYVIGDRGEVLEPYRQERMNEITFHELYDKITSVKDTHRKYALAKTTFEKKRS
RRNLQAVEEANACRLKYVRDWYDQEFQKAGERLEKLHALYPEVSVSIRENRIQATRSNLEKAYEAIEENYR
CCVREQEDYWKEEEKREAEFREKGTKVYSPEEVSEHLQILEDLLEVCSTQLTIAEVVVLGVGLEATEEIEYTIL
SDAANRLKVLCEDADDMTFRIKEIEMMLRMAELPLLPIKQAFTNAFVQYNSCKENLAKVEPYCQESPAYVNS
NKRLESLDQALQNVYQEYQKRFQGASGLESEVSAYREYLREQITEFETQGLDVIKEELLFVSSTLKSKSSYDP
LIAKVPCMKFYYQYYDGIDKARVQSRWLEKSERYRKAKKGFQEMLKEGLFKEDQALKEEDYRLYREERMN
KESHLICNKIAAARRRVQEFESMEISEIPEEKAGFMDKARSLFTREDRS

>core/70/4/Org4_Gene969

MLFSKNFSTDNGGAITAKTLSLTGTTTSALFSENTSSKKGGAIQTSDALTITGNQGEVSFSDNTSSDSGAAIFTE
ASVTISNNAKVSFIGNKVTGASSTTTGDMSGGAICAYKTSTDTKVALTGNQMLLFSNNTSTTAGGAIYVKKL
ELASGGLTLFSGNSVNGGTAPKGGAIAIEDSGELSLSADSGDIVFLGNTVTSTTPGTNRSSIDLGTSAKMTALR
SAAGRAIYFYDPITTAATTTVADVLKVNETPADSALQYTGNIIFTGEKLSETEAADSKNLTSKLLQPVTLSGGT
LSLKHGVTLQTQAFTQQADSRLEMDVGTTLEPADTSTINNLVINISSIDGAKKAKIETKDTAKNLTLSGTITLL
DPTGTFYENHSLRNPQSYDILELKASGTVTSTAVTPDPIMGEKFHYGYQGTWGPIVWGTGASTTATFNWTKT
GYIPNPERIGSLVPNSLWNAFIDISSLHHLMETANEGLQGDRALWCAGLSNFFHKDSTKTRRGFRHLSGGYVI
GGNLHTCSDKILSAAFCQLFGRDRDYFVAKNQGTVYGGTLYYQHNETYISLPCKLRPCSLSYVPTEIPVLFSG
NLSYTHTDNDLKTKYTTYPTVKGSWGNDSFALEFGGRAPICLDESALFEQYMPFMKLQFVYAHQEGFKEQG

TEAREFGSSRLVNLALPIGIRFDKESDCQDATYNLTLGYTVDLVRSNPDCTTTLRISGDSWKTFGTNLARQAL
VLRAGNHFCFNSNFEAFSQFSFELRGSSRNYNVDLGAKYQF

>core/71/4/Org4_Gene848

MTLQPSYIHFTRNVTTALSGGKIDTSAIELSCSALFFQELQDKAQGLKHALGLVQELSAEALPPAQVQTSISYP
PTEEFSRPGISAGIIDRTMPTFTDDEVKAILQNPNFETSKIFVEGLDKVFKSYLDSVTPPEGIDPSNPESAIILNYIT
LLNNLKPKFAAGATPTDADYNALYALPGDFVKEIEALKAPDAPPKSKVHAFWQEIMTIYNNMQVLSYPVTD
YLNVQIADLSLNITAAQEVQQYLKNFYSILKDILNPGWTDPQATHYPADAEYNARDAGVIQSLLNLSGNYRQ
LTENMLPNTDTSLPQEIIAQIRNFQNGVNGTIISTQTTPSATMRLDTLLGVIYTYQCCATIFGTSYGTSTPAKQN
YIDAINQEKSYWQARANGFDVTSDQVFEQFVTNIQSGTQYRGMDLFKNNKVNEINPIFLSQAASFLRYPYNL
MSRSMYQTIEDAANRSITALDGLISGWSTQIATFQTQKNSLDPSLLKYFDTMKANKESFVTTAPLQMVYSSL
MLDKYLPTQQNVIASLGIQMTYSNKAAKYLNELIKEITTFQSADIYYSLSIYLKQMNLQAVADPIGKAVGVLN
DEKTRAMADITRCNKIKAAIDKMLVEIKADAELSKSQIRELVDTLTNFKSQSDDLIRNLSCLLGFLSGLTLKAV
NDPNATYEAFTAEIFTEPFNNWKRQLATFESFVIQGGQNGITPGGQQQLLQAMESSQQDFSTFNQNQQLALQ
LESSAMQQEWTLVSAALALLNQMVSKIARRIKS

>core/75/4/Org4_Gene871

MVFFRNSLLHLVALSGMLCCSSGVALTIAEKMASLEHSGSGADDYEGMASFNANMREYSLQLSKLYEEARK
LRASGTEDEALWKDLIRRIGEVRGYLREIEELWAAEIREKGGNLEDYALWNHPETTIYNLVTDYGTEDSIYLI
PQEIGAIKIATLSKFVVPKESFEDCLTQILSRLGIGVRQVNSWIKELYMMRKEGCSVAGVFSSRKDLEALPETA
YIGFVLNSNVDAHTNQHVLKKFINPETTHVDVIAGRVWIFGSAGEVGELLKIYNFVQSESIRQEYRVIPLTKID
AGEMISILNAAFREDLTKDVSEESLGLRVVPLQYQGRSLFLSGTSALVQQALTLIRELEEGIENPTDKTVFWYN
VKHSDPQELAALLSQVHDVFSGENKASVGAADGCGSQLNASIQIDTTVSSSAKDGSVKYGNFIADSKTGTLI
MVVEKEVLPRIQMLLKKLDVPKKMVRIEVLLFERKLAHEQKSGLNLLRLGEEVCKKGCSPSVSWAGGTGILE
FLFKGSTGSSIVPGYDLAYQFLMAQEDVRINASPSVVTMNQTPARIAVVDEMSIAVSSDKDKAQYNRAQYGI
MIKMLPVINVGEEDGKSYITLETDITFDTTGKNHDDRPDVTRRNITNKVRIADGETVIIGGLRCKQMSDSHDGI
PFLGDIPGIGKLFGMSSTSDSLTEMFVFITPKILENPVEQQERKEEALLSSRPGEREEYYQALAASEAAARAAH
KKLEVFPASGVSLSQVEGQEYDGC

>core/76/4/Org4_Gene799

MGYIESSTFRLYAEVIVGSNINKVLDYGVPENLEHITKGTAVTISLRGGKKVGVIYQIKTTTQCKKILPILGLSD
SEIVLPQDLLDLLFWISQYYFAPLGKTLKLFLPAISSNVIQPKQHYRVVLKQSKAKTKEILAKLEVLHPSQGAV
LKILLQHASPPGLSSLMETAKVSQSPIHSLEKLGILDIVDAAQLELQEDRLTFFPPAPKDLHPEQQSAIDKIFSSL
KTSQFHTHLLFGITGSGKTEIYLRATSEALKQGKSTILLVPEIALTVQTVSLFKARFGKDVGVLHHKLSDSDKS
RTWRQASEGSLRILIGPRSALFCPMKNLGLIIVDEEHDPAYKQTESPPCYHARDVAVMRGKLAHATVVLGSA
TPSLESYTNALSGKYVLSRLSSRAAAAHPAKISLINMNLEREKSKTKILFSQPVLKKIAERLEVGEQVLMFFNR
RGYHTNVSCTVCKHTLKCPHCDMVLTFHKYANVLLCHLCNSSPKDLPQSCPKCLGTMTLQYRGSGTEKIEKI
LQQIFPQIRTIRIDSDTTKFKGSHETLLRQFATGKADVLIGTQMIAKGMNFSAVTLAVILNGDSGLYIPDFRASE
QVFQLITQVAGRSGRSHLPGEILIQSFLPDHPTIHSAMRQDYSAFYSQEITGRELCEYPPFIRLIRCIFMGKCPKQ
TWEEAHRVHNILKEQLESTNPLMPVTPCGHFKIKDTFRYQFLIKSAYVIPVNKKLHHALMLAKLSPKVKFMID
VDPMTTFF

>core/77/4/Org4_Gene30

MEKICGYLEQILVENKDSGDITAYIKIPNKTTPILIKGKLPQPLELGSPIQIYGVWSHSPSNTKYFQIHSYDSPLL
YEYRGVFHYLTSKLIKGIGPKIAEKIIEKFQEKTCYVLDITPERLSEVSGISETRCVSICKQLCEQKILRKTLLFLQ
EYNIPIHYGVRIFKKYQEKSIEKICEDPFLLAREMEGIGFKTADFIAMKLGVPRNSESRLCAGIQHSLEELQEEG
HTCYPIELLIDVVAKLLNQDVFDTPITLEEIDTQILNMQKRKLLHIQDISGTLHVWTRYLHLAEKTIVSDLKRIL
FSSRRIRSIDGEKAIAWVEENLSIDLAEQQREAIKACFSEKLLIITGGPGTGKSTITQAILKIFEQVTHKIILAAPT
GKAAKRMTEITQKHSVTIHALLQYDFKTKSFRKNHDNPIDCDLIIVDESGMMDTHLLHHFLKALPDYTTLVFI
GDIHQLPSVGPGNILKDLITSNKMTVIRLNKIFRQVHDSGIVTNAHRVNEGELPILYSETGRRDFLFFQKDDQE
EALNHIIHLVTKFVPQKYHIYPQDIQVLAPMKKGTLGIYNLNKALKHALNPKKANLHGRFQSYAVGDKVMQI
RNNYNKEVFNGDIGYVSTINFEDKAVVVRMEGKHVGYSFSELDDLVLAYATSVHKYQGSESPCIIIPIHTSHF
MMLYRNLLYTAITRGKKLVILVGTKKAIAIATRNNRVQHRCTGLAEVLKELDTKKNYADL

>core/78/4/Org4_Gene457

MLSFFYKHQKKFIGIVIAVVCVSGIGVGWGRFSREGSAESTSRRTVFTTASGKRYVEKDFMAMKKFFAHEAY
PFTGNPRAWNFINEGLLTDYFLTTRVGEKLFLKVYHPGEKIFSKEKAYQPYRRFDAPFISSEEVWKSSAPQLLE
ILKVFQQIENPISKEGFLARVKLFLEERRFPHYVLRQMLEYRRQMFALPPDEALSRGKDLRLFGYQTIQDWFG
DAYLSAAVELLIRFIDEQKKVLPRPSKQEARDDFYDKAKHAYTKISKNKEFSLGFEEFVNSYFQFLEISESEFF
NMYRDILLCKRALLLLQGGVSFDFQPLTTFFVQGKDSIQVEFFRLPKEYSFKTKQELKAFEVYLKLVSLPKSD
SLDVPNEILPIATIKAKEPRLVGRRFSIDYKRVALQDLAATVPMVEVLHWQQNSEHFQEILQQFPDVETCQSY
KDFQHLKPALRDKISLFTRKEILRARPERILQSLQQVPKQSQEVLLSAGKNSALPGISDGQQLAKVLLENEVLD
LYSQDAETYYTIIVNSSFEKEEVLPYREVLKRDLASQLLTSHGHLVDMERLESALRTRYPGEEGASLWQRRL
WKVVENHRLGRHLEGSFSWSLDRSLKTFSRGDKELPQEFDRIFSMKVGDYSSVFMSPNEGPCYYQCLSHLLY
DRPASVDKLFLAKSQLDEELLGSYMERFIEQGVVR

>core/79/4/Org4_Gene258

MDYLEKLQVLIEEGQSANFLSLWEEYCFNDVVRGRELVEILEKVKSSSLASLFGKIVDTVVPLWEKIPEGKDK
DRVLQLILDLQTSNSQMFFDIATEYVNKKYSGEENFNEALRVVGLRDGRDFQFSLSRFDFLMHMHKGNFVFH
QGGWGVGEVMGVSFLQQKVLIEFEGIMSAKDISFETAFKSLTPLSGDHFLSRRFGDPDGFEAFAKENPIEVIEI
LLRDLGPKTAKEIKDELVDLVIPEADWNRWWQSAKTKIKKGTRIISPDNPKEPYVLSDAECSHMGQLERKLG
LSLNSAEKISLIYHFIRDLHSELKNIEIRKSLVKALQDLDVEEGNKSLILQRELLLSEYLGIKDASIDKEYITSLSE
DDISRLLENMPIVALQKSFLSLVRKYSSFWQQVFMQILLYTTSPTMRDFVYKTIKNDPSSVEVLKKRLLDSAH
QPMMFPELFVWFFLKLGNHEDGLFDPEDKEVLRLFLESALNFMYQVASTPHKELGKKLHHYLVGQRYLAVR
QMIEGASLPFLKELLLLSTKCPQFSSADLNVLQSLAEVVQPTLKKNKSNVEEENVLWSTSESFSRMKAKLQSL
VGKEMVDNAKEIEDARSLGDLRENSEYKFALEKRARLQEEIRVLSEEINRARILTKDLVFTDKVGVGCKVTL
KGDAGEVVEYTILGPWDADPDSCILSLQSKLAQNMLGKKLNDVVILQGKEYKISRIQSIWEEHGA

>core/80/4/Org4_Gene433

MVDKLIHPWDLDLLVSGRQKDPHKLLGILASEDSSDHIVIFRPGAHTVAIELLGELHHAVAHRSGLFFLSVPK
GIGHGDYRVYHQNGLLAHDPYAFPPLWGEIDSFLFHRGTHYRIYERMGAIPMEVQGISGVLFVLWAPHAQR
VSVVGDFNFWHGLVNPLRKISDQGIWELFVPGLGEGIRYKWEIVTQSGNVIVKTDPYGKSFDPPPQGTARVA

DSESYSWSDHRWMERRSKQSEGPIAIYEVHLGSWQWQEGRPLSYSEMAHRLASYCKEMHYTHVELLPITEH
PLNESWGYQVTGYYAPTSRYGTLQEFQYFVDYLHKENIGVILDWVPGHFPVDAFALASFDGEPLYEYTGHS
QALHPHWNTFTFDYSRHEVTNFLLGSALFWLDKMHIDGLRVDAVASMLYRDYGREDGEWTPNIYGGKENL
ESIEFLKHLNSVIHKEFPGVLTFAEESTAFPGVTKDVDQGGLGFDYKWNLGWMHDTFHYFMKDPMYRKYH
QKDLTFSLWYAFQESFILPLSHDEVVHGKGSLVNKLPGDTWTRFAQMRVLLSYQICLPGKKLLFMGGEFGQ
YGEWSPDRPLDWELLNHHYHKTLRNCVSALNALYMHQPYLWMQESSQECFHWVDFHDIENNVIAYYRFA
GSNRSSALLCVHHFSASTFPSYVLRCEGVKHCELLLNTDDESFGGSGKGNRAPVVCQDHGVAWGLDIELPPL
ATVIYLVTFF

>core/81/4/Org4_Gene953

MNKFKTYLQTALIALFFSFPALSGSFSSIQAEEITQQVNHPGAELLSEGSYIPGLQTFRLGIKITASKGSHIYWK
NPGEIGSPLKISWQLPKGFVVEEEHWPTPKVFEEEGTTFFGYEDSALIVADVRAPEGYTPGQEVELRAQVEWL
ACGDSCLPGNVDLKLTLPYEEKEPSLYPDTHAEFTKTLHAQPRVLENDHSVQVAQGKGNEIILNISKKINATK
AWFVSEKADKLFAYAETSYSEGTGTAWRLKVKNLSGVQKNEKLHGILLLADHTGRPVESLTIHSEVLGQTGS
AVAGLSQYITILIMAFLGGVLLNIMPCVLPLVTLKVYGLIKSAGEHRSSVIANGLWFTLGVVGCFWGLAGVAF
ILKVLGHNIGWGFQLQEPMFVATLIIVFFLFALSSLGLFEMGTMFANLGGKLQSSEMKSSNNKAVGAFFNGIL
ATLVTTPCTGPFLGSVLGLVMSLSFLQQLLIFTAIGLGMASPYLVFSVFPKMLSVLPKPGGWMSTFKQLTGFM
LLATVTWLVWIFGSETSTTSVVVLLGGLWLAGLGAWILGRWGTPVSPKKQRVCASLLFFAFLGGAISVSGLA
SHYFAEPQQTVSVNEDSLWQPFSLEKLAQLRAQGRPVFVNFTAKWCLTCQMNKPVLYGDAVQKMFETHGI
VTLEADWTRKDPGITEELARLGRASVPSYVYYPGDNSAPVVLPEKITQNLLEDVVSRFVR

>core/82/4/Org4_Gene539

MIRSPLPFISSKRALNMLGLQDEFSCPEDVVDFLFSEIELLAQQDEPSEGYLALSRSLLMMTHNHPKVVKRVIF
YGVSYGLKHKSMSIFIDVLIYIDFLFEKLGISASDRLSLCSARTCINFELYSQTGEMKFLSEVVDNFRLIEQLLK
MHPQLKNRLGWEHFRIGAKQEEVSLVASASVYQAVGRSFIELYHKHLELSDLACGMKCLALALDLSPNNAH
IHADYAKGLVVLGTRQGKSLLIERGMEHFSKAIFLSFSRDGDTLAYQNYRYSYALASVKLFDLTYKKEHFDQ
AMNILYQTVQAFPNLSGLWMVWGELLIRSGWLNSNMKYIEVGLEKLASLQKKTNDPIALSGLLATGIAILGL
YLEEPNLFKESRHRLISAMRTFPGNSALVHALGVVQLCSALYFNEDSHFASAISCFQSCLEWDLDATGMWQK
LFDAYFSWGVKKKSARLLRKAVDVASRLCSLRPEAFLFWSDRGLALKCLAEATIDEAYKEIFLSESLLHYQR
AWDLSGRLEILELWGQSHYLLAELQQSLFHYDEAYTLLTKVDLTLSSSRVKLILAAVLLGKGRLLQDTDPAE
EAREILEPLVEVYLEDENFLLLLGKVYLFLFWKNKNVCLGKLARTYLEKATSLGCPEAYYTLGKFYAVIKDV
NKAWGMVIRSAQYGVRITEAKWLNDPYLANLREIHTFREVVENQKGRLWLGNKTEMKRN

>core/83/4/Org4_Gene518

MNKLLNFVSRTLGGDAALNMINKSSDLILALWMMGVVLMIIIPLPPPIVDLMITINLSISVFLLMVALYIPSALQ
LSVFPSLLLITTMFRLGINISSSRQILLKAYAGHVIQAFGDFVVGGNYVVGFIIFLIITIIQFIVVTKGAERVAEVA
ARFRLDAMPGKQMAIDADLRAGMIDATQARDKRAQIQKESELYGAMDGAMKFIKGDVIAGIVISLINIVGGL
TIGVAMHGMDLAQAAHVYTLLSIGDGLVSQIPSLLIALTAGIVTTRVSSDKNTNLGKEISTQLVKEPRALLLA
GAATLGVGFFKGFPLWSFSILALIFVALGILLLTKKSAAGKKGGGSGASTTVGAAGDGAATAGDNPDDYSLT
LPVILELGKDLSKLIQHKTKSGQSFVDDMIPKMRQALYQDIGIRYPGIHVRTDSPSLEGYDYMILLNEVPYVR
GKIPPRHVLTNEVEDNLSRYNLPFITYKNAAGLPSAWVSEDAKAILEKAAIKYWTPLEVIILHLSYFFHKSSQE

FLGIQEVRSMIEFMERSFPDLVKEVTRLIPLQKLTEIFKRLVQEQISIKDLRTILESLSEWAQTEKDTVLLTEYVR
SSLKLYISFKFSQGQSAISVYLLDPEIEEMIRGAIKQTSAGSYLALDPDSVNLILKSMRNTITPTPAGGQPPVLLT
AIDVRRYVRKLIETEFPDIAVISYQEILPEIRIQPLGRIQIF

>core/84/4/Org4_Gene350

MYKRCVLDKILKGIVAGSLILLYWSSDLLERDIKSIKGNVRDIQEDIREISRVVKQQQTSQAIPAAPGVMLAPK
LVRDEAFALLSGDPSYPNLLSLDPYKQQTLPELLGTNFHPHGILRTAHVGKPENLSPFNGFDYVVGFYDLCIPS
LASPHVGKYEEFSPDLAVKIEEHLVEDGSGDKEFHIYLRPNVFWRPIDPKAFPKHVQLDEVFQRPHPVTAHDI
KFFYDAVMNPYVATMRAVALRSCYEDVVSVSVENDLKLVVRWKAHTVINEEGKEERKVLYSAFSNTLSLQ
PLPRFVYQYFANGEKIIEDENIDTYRTNSIWAQNFTMHWANNYIVSCGAYYFAGMDDEKIVFSRNPDFYDPL
AALIDKRFVYFKESTDSLFQDFKTGKIDISYLPPNQRDNFYSFMKSSAYNKQVAKGGAVRETVSADRAYTYI
GWNCFSLFFQSRQVRCAMNMAIDRERIIEQCLDGQGYTISGPFASSSPSYNKQIEGWHYSPEEAARLLEEEGW
IDTDGDGIREKVIDGVIVPFRFRLCYYVKSVTAHTIADYVATACKEIGIECSLLGLDMADLSQAFDEKNFDALL
MGWCLGIPPEDPRALWHSEGAMEKGSANVVGFHNEEADKIIDRLSYEYDLKERNRLYHRFHEIIHEEAPYAF
LFSRHCSLLYKDYVKNIFVPTHRTDLIPEAQDETVNVTMVWLEKKEDPCLSTS

>core/85/4/Org4_Gene680

MKELRHESYNRALHKLSHQWVRYFLYTFVSCSFIVAIFTFAWLKVLYVPEYKAGEISRISLTAPMDFSLSWSA
HKFYKRTAHISEAFGKVYHLTLSPGSLLSKEGNADENTDYWFKKAADFLFSTNFVDSSTQKCLKDLCIYPPLL
GKEKKTLEININSNKGNVIAQCFCHLKIFLIQENCPQPCFDAIMDILKIANFEMAVDKEMSGCVKGELLGKRCI
EKITKGTPILEKYQRIDDRDAKILKQLRAQLLSVHTLFSCRSLWGAIFVVLLILLWGYGALKALCPEMLKSPQ
RFMLYIAILTLSLLWCRGTEIFCAYWVSYLSYPPILPFTAVLLGYFLGLPIAGFSCTFLALLYTLGSDLWNNSW
FLSINLLCSWRILVSLHRVSRLSSVFWACMKLGGVAMGSLLMFRIFTNTISREALYADGIESFVYSLITAISVVA
LIPVFEASFGASTNFSLLTYLSPENALLKRLFKEAPGTYQHSVLVGSLAEAAAQAIGADSLYCLVAAHYHDIG
KLINPGFFSENQKILQQSGHSLSPLECAKMIMRHIPEGVNLARQAGLPESFIQVIEEHHGTSVIRSAYYSHMVE
NPSTGSFDEELFRYSGNKPSSKETTIIMIADSFEAASRSLKNASLPDLQRLIDQIIQGKLQDGQFSCSPVTLDELA
LISKSMVQTLYGALHSRMKYPEISYQISMDSCPKPSIGGT

>core/86/4/Org4_Gene714

MPEPLYTNKLITEKSPYLLLYAHTPVNWYPWGAEAFHIAAIENKPVFLSIGCKHSRWCQVMLQESYTNPEIAA
MLNEYFVNVKVDKEELPYVAKLYGDLAQMLAVSGDHQETVSWPLNVFLTPDLIPFFSVNYLGNEGKLGLPS
FPQIIDKLHFMWEDAEEREALVEQAMKVLEIASFLEGCVRKEILDESSLKRTVAALYQDIDPHYGGVKAFPKR
LPGLLLQFFLRYSLEYQESRGLFFVDRSLSMVALGGVRDHIGGGVYSYTIDDKWLIPAFEKRLIDNALMALNY
LEAWACLGKEEYRGIGKQILSYILSELYSPEVGAFYSSEQAENWGAGGQNFYTWSVEEISNALGEDAEIFCDY
YGISREGFFNGRNILHIPVHREIEELSEKYHRSIEAIEDIVDRSRDILKGIRAQRSHRSKDDLSLTFNNGWMIYTF
AYAGRLLGEVEYIEIGKKCGEFVRNSLYKHHELYRRWREGEAKYRASLEDYGALILGVLALYESGCGSFWLS
FAEELMQEVVLSFRSEEGGFYSVDGRDSTLLIKQSPLSDGETISGNALICQCLLSLHLITEKKHYLTYAEDILQI
AQAYAHTHKFSSLGLLIASQNYFSRKHVKVLIALGDQEDRSPVLKCLSGLFLPYLSLIWMTQENQEHLETVLP
EYEHCLIPKGDCTATTIYVLEVDQCKRFKDLELFRRYLISL

>core/88/4/Org4_Gene697

MKRCFLFLASFVLMGSSADALTHQEAVKKKNSYLSHFKSVSGIVTIEDGVLNIHNNLRIQANKVYVENTVGQ
SLKLVAHGNVMVNYRAKTLVCDYLEYYEDTDSCLLTNGRFAMYPWFLGGSMITLTPETIVIRKGYISTSEGP
KKDLCLSGDYLEYSSDSLLSIGKTTLRVCRIPILFLPPFSIMPMEIPKPPINFRGGTGGFLGSYLGMSYSPISRKHF
SSTFFLDSFFKHGVGMGFNLHCSQKQVPENVFNMKSYYAHRLAIDMAEAHDRYRLHGDFRFTHKHVNFSGE
YHLSDSWETVADIFPNNFMLKNTGPTRVDCTWNDNYFEGYLTSSVKVNSFQNANQELPYLTLRQYPISIYNT
GVYLENIVECGYLNFAFSDHIVGENFSSLRLAARPKLHKTVPLPIGTLSSTLGSSLIYYSDVPVISSRHSQLSAK
LQLDYRFLLHKSYIQRRHIIEPFVTFITETRPLAKNEDHYIFSIQDAFHSLNLLKAGIDTSVLSKTNPRFPRIHAK
LWTTHILSNTESKPTFPKTACELSLPFGKKNTVSLDAEWIWKKHCWDHMNIRWEWIGNDNVAMTLESLHRS
KYSLIKCDRENFILDVSRPIDQLLDSPLSDHRNLILGKLFVRPHPCWNYRLSLRYGWHRQDTPNYLEYQMILG
TKIFEHWQLYGVYERREADSRFFFFLKLDKPKKPPF

>core/89/4/Org4_Gene748

MNFQTISINLTEGKILVFETGKIARQANGAVLVRSGETCVFASACAVDLDDKVDFLPLRVDYQEKFSSTGKTL
GGFIKREGRPSEKEILVSRLIDRSLRPSFPYRLMQDVQVLSYVWSYDGQVLPDPLAICAASAALAISDIPQSNIV
AGVRIGCIDNQWVINPTKTELASSTLDLVLAGTENAILMIEGHCDFFTEEQVLDAIEFGHKHIVTICKRLQLWQ
EEIGKSKNLSAVYPLPTEVLTAVKECAQDKFTELFNIKDKKVHAATAHEIEENILEKLQREDDDLFSSFNIKAA
CKTLKSDTMRALIRDREIRADGRSLTTVRPITIETSYLPRTHGSCLFTRGETQTLAVCTLGSEAMAQRYEDLNG
EGLSKFYLQYFFPPFSVGEVGRIGSPGRREIGHGKLAEKALSHALPDSATFPYTIRIESNITESNGSSSMASVCG
GCLALMDAGVPISSPIAGIAMGLILDDQGAIILSDISGLEDHLGDMDFKIAGSGKGITAFQMDIKVEGITPAIMK
KALSQAKQGCNDILNIMNEALSAPKADLSQYAPRIETMQIKPTKIASVIGPGGKQIRQIIEETGVQIDINDLGVV
SISASSASAINKAKEIIEGLVGEVEVGKTYRGRVTSVVAFGAFVEVLPGKEGLCHISEFSRQRIENISDVVKEGD
IIDVKLLSINEKGQLKLSHKATLE

>core/90/4/Org4_Gene383

MSNQEFDLSAIRNIGIMAHIDAGKTTTTERILFYAGRTHKIGEVHEGGATMDWMAQEQERGITITSAATTVFW
LGAKINIIDTPGHVDFTIEVERSLRVLDGAVAVFDAVSGVEPQSETVWRQADKYGVPRIAFVNKMDRMGAD
YFAAVESMKEKLGANAFPVHCPIGSESQFVGMVDLISQKALYFLDDTLGAKWEEKEIPEDLKERCAELRANL
LEELATIDESNEAFMMKVLEDPDSITEDEIHQVMRKGVIENKINPVLCGTAFKNKGVQQLLNVIVKWLPSPLD
RGNIRGINLKTDQEISLEPRRDGPLAALAFKIMTDPYVGRITFIRIYSGTLKKGAAILNSTKDKKERISRLLEMH
ANERTDRDEFTVGDIGACVGLKFSVTGDTLCDDNQEIVLERIEFPDPVIDMAIEPKSKGDREKLAQALSSLSEE
DPTFRVSTNEETGQTIISGMGELHLDILRDRMIREFKVEANVGKPQVSYKETITVSGNSETKYVKQSGGRGQY
AHVCLEIEPNEPGKGNEVVSKIVGGVIPKEYIPAVIKGVEEGLNTGVLAGYGLVDVKVSIVFGSYHEVDSSEM
AFKICGSMAVKDACRKAKPVILEPIMKVAVITPDDHLGDVIGDLNRRRGKILGQESSRGMAQVNAEVPLSEM
FGYTTSLRSLTSGRATSTMEPAFFAKVPQKIQEEIVKK

>core/92/4/Org4_Gene420

MYNLLHAHHDAASPDGRLVSHLRKLSPHIYEGEVLIENIPAYFLGFHLPQQCIQVNLKSSLAQLGVEAVLNHL
ELNKARKEARLHVLFMSQDPIATAMLELLEPGSFVCKLFAADDRRLVRSPCYLNRMFTHTDRTGSPLLRFGK
KLEHFITLEIINDRLVVFLPILPGTICYEETIYGFLPLMSKSLTRPHLKIRKFLPLYQMVTDRPPVPEDHKILLIKT
EPLHIRTVFARVVQDLLPQGLRHTAADILEPTTQESGDIYEFYGSTSEPIERIPLEFFTLEPYKEHSFFFYRDMLQ
ETLESPQEVFRVFESIPEGEDQAAMFISKGSELLELSQDSWIIKPRISPPDERHGREIQKYIEDQPCFPFLKAMET

DLITSQGVLFSRYFPSAWLKGMFLSNYSRYYLQHIYFQIPSPTSGEFFSNRDRSFLLDLYFAGISVFWADLESK
RLLQYIKRRNKDVGMFVPKYQAEQFAQSYFIGIHGSCLIAGDYDEFLRELLTGMHTLSQQFTIPEFPPQTPLAI
LTGGGSGAMELANRVATELSILSCGNLISLDTTNAYVEAKMSYAIPDLLERQADFHVDLAVFVIGGMGTDFE
LLLELISLKTGKKALVPVFLIGPVDYWKSKITALYNSNHAVGTIRGSEWVHNCLFCLSSAKAGIEIFRRYLNHT
LPIGPEHPVPEDGFVIV

>core/93/4/Org4_Gene221

MKRSRRNFEQALENLEKLKEISLATSNDSYLNNPARFNQRKQTGSSVMEMKEALKNVENYLLEISCVSKSHA
DKALKESDFLIAGVQNVFSFLENQEDLYKSLLDEYSEVTKAYDEVKKNLKEVPTYDLSTDEETEEHKEPECFL
NNLVEVKRDRSYELFYMLDEQDKRFYNDALVQIIYKQNKLHETVNEGDPLTKTLLWNSEEVKNIASSLVIVN
DMPLRLFYQRALSHLDIEAVVKVHNAVMALFFSRYEATMVFKSPKKHNIWYFNDFLLFLREAWKDLNNNVI
DSQERKQTKLLASALSLGIFESKLVFEEASRYLYFNIQTKLENANGKKPLSPGQYLTDAYEELHRLISKYPNGP
LFKAMDRVLEHESRPYDPMILGILPSLEGTLKLHGKSIDIIRSPSPVTQSSILYANCNEEFLGFLNAKAHRSEVT
LVLNIQNRISRKERARSRVIEEALEQEEHAPYVHAFSFPEPEELLQNLESIHGDIETFADFFSILQEEFHKPLLAS
SFFLTKELKEFVGSFLKEKLTALKDIFFAKKKILFRNDKLLLLHLLSYLIVFKLIERTNPNSIVVVSKDGLDYVS
VFIAGFAFFSREAFWDEHSLKLLLTNVLSPTLVARDRLVFVSHIELLSKFVNCLKKNRQGFSSLKSFFKDDIEG
WEFTGYLHELTEVSHKHNL

>core/94/4/Org4_Gene959

MQPCLNMSIVRNSALPLPCLSRSETFKKVRSHMKFMKVLTPWIYRKDLWVTAFLLTAIPGSFAHTLVDIAGEP
RHAAQATGVSGDGKIVIGMKVPDDPFAITVGFQYIDGHLQPLETVRPQCSVYPNGITPDGTVIVGTNYSPMM
GSVAVKWVNGKVFELPILPDTLDSVASAVSADGRVIGGNRNINLGASVAVKWEDDVITQLPSLPDAMNAYV
NGISSDGSIIVGTMVDVSWRNTAVQWIGDQLSVIGTLGGATSVANAISTDGTVIVGGSENADSQTHAYAYKN
GVMSDIGTLGGFYSLAHAVSSDGSVIVGVSTDSKHRYHAFQYADGQMIDLGTLGGSESYAQSVSGDGKVIV
GRAQVPSGDWHAFLCPFQAPSPAPVQGGSTVVTSQNPRGMVDINATYSSLKNSQQQLQRLLTQHSAKVQSV
SSGAPSFISVKDAISRQSPAVQNDVQKGTFLSYRSQVHGNVQNQQLLTGAFMDWKLVSAPRCGFKVALHYG
SQDALVERAALPYTEQGLGSSVLSGFGGQVQGRYDFNLGETVVLQPFMGIQVLHLSREGYSEKNVRFPVSYD
SVAYSAATSFMGAHLFASLSPKMSTAATVGVERDLNSHIDEFKGSVSAMGNFVLENSTVSVLRPFASLAMYY
DLRQQQLVTLSVVMNQQPLTGTLSLISQSSYNLSF

>core/95/4/Org4_Gene689

MGVVQNQVISSIRDVLKLVWELRFAEHKMLLLSRQSGSGGTFQLSCAGHELAGVLAGKSLIPGKDWSFPYY
RDQGFPIGLGCDLSEIFASFLARTTPNHSSARMMPYHYSHKKLRICCQSSVVGTQFLQAAGRAWAVKHSSAD
EVVYVSGGDGATSQGEFHEMLNFVALHQLPLITVIQNNHWAISVPFEDQCGADLASLGRCHQGLAVYEVDG
GNYTSLTETFSHAVDQARQHSVPALILIDVVRLSSHSNSDNQEKYRSALDLKLSMDKDPLILLEKEAINVFGLS
PFEIEEIKAEAQEEVRRSCEIAEAFPFPSKGSTSHEVFSPYTETLIDYENSESAQNLRNSEPKVMRDAISEALVEE
MTRDSGVIVFGEDVAGDKGGVFGVTRNLTEKFGPQRCFNSPLAEATIIGTAIGMALDGIHKPVVEIQFADYIW
PGINQLFSEASSIYYRSAGEWEVPLVIRAPSGGYIQGGPYHSQSIEGFLAHCPGIKVAYPSNAADAKALLKAAI
RDPNPVVFLEHKALYQRRIFSACPVFSHDYVLPFGKAAIVHPGKDLTIVSWGMPLVLSLEVAQELASQGISIEV
IDLRTIVPCDFATVLKSLEKTGRLLVIHEASEFCGFGSELVATMSEQGYAYLDAPIRRLGGLHAPVPYSKVLEN
EVLPQKESILQAAKSLAEF

>core/96/4/Org4_Gene920

MLKKPKRKPGRRTYGKSLKIFIPGTLFVHARKGFGFVSPDNPEEYPFDIFVPARDLRGALDGDHVIVSVLPYPR
DGQKLKGTISEVLARGKTTLVGTITSLVSPTSALAYTSMSGSQSLIPVELLPGRTYKIGDRILLSTPPWVDKPQE
GASPALQMLEFIGHITNAKADFQAIQAEYNLAEEFPPEVIEEASLFSQKHITQVLHSRKDLRDLLCFTIDSSTAR
DFDDAISLTYDHNNNYILGVHIADVSHYVTPHSHLDKEAAKRCNSTYFPGKVIPMLPSALSDNLCSLKPNVDR
LAVSVFMTFTKSGHLSDYQIFRSVIRSKYRMTYDEVDNIIEKKHSHPLSKILNEMATLSKKFSDIREERGCIRFV
LPSVTMSLDNLQEPVALIENHQTFSHKLIEEFMLKANEVVAYHISHQGVSLPFRSHEPPNDENLLAFQELAKN
MGFDITFTPTQEPDYQYLLQTTSAGHPLEQVLHSQFVRSMKTASYSTENKGHYGLKLDYYTHFTSPIRRYIDL
IVHRLLFNPLSIDQTHLEIIVRACSTKERVSAKAENSFENLKKTRFINKFLQEQPKTTYHAYIITANHEGLSFVV
TEFCHEGFIAAAELPKEYSLKKNALPESIPDKMKPGASIKVTIDSVNLLTQKIVWSIVTTTEDKPKKIKKTPSKK
KGTKKRAS

>core/98/4/Org4_Gene818

MINKELDIGILGKIAGAIKQISIESIQKASSGHPGLPLGCAELAAYLYGYVLRQNPRDPHWINRDRFVLSAGHG
SALLYSCLHLAGFDVSLEDLQEFRQLHSRTPGHPEYGETVGVEATTGPLGQGLGNAVGMALSMKMLESRFN
RPGHKIFNGKIYCLAGDGCFMEGVSHEVCSFAGSLNLNNLVVIYDYNNVVLDGYLNEISVEDTKKRFEAYG
WDVYEIDGYDFTHIHETFSSIKRGQERPVLVIAHTIIGHGSPKEGTNKAHGSPLGIEGTHETKQFWHLPEEKFF
VPPAVKNFFAHKIQEDRKAQEQWLDEVRVWSKQFPELHEEFVALTSHKLPKNLESLVQSVEMPDSIAGRAAS
NKLIQVLVQHIPYLIGGSADLSSSDGTWIANEKVIHTYDFSGRNIKYGVREFGMATIMNGLAYSQVFRPFGGT
FLVFSDYMRNAIRLAALSKLPVIYQFTHDSIFVGEDGPTHQPVEQLMSLRAIPGLYVIRPADANEVKGAWIAG
LKHTGPTVIVLSRQALPTLPAAHRPFKDGVGRGAYIVLKESGEKPDYTLFATGSEVSLALSVAKELEHLDKQV
RVVSFPCWELFEAQDVDYKQSIVGGDLGIRVSIEAGSALGWYKYIGSEGLAIAMDRFGYSGAPDDVSEECGF
TTEQILQRILSQ

>core/99/4/Org4_Gene480

MEKVSSYPSVPLPLGASKISPNRYRFALYASQATEVILALTDENSEVIEVPLHPDTDRMGAIWHIEIEGISDQW
SYAFRVHGPKKHGMQYSFKEYLADPYAKNIHSPQSFGSRKKQGDYAFCYLKEEPFPWDGDQPLHLPKEEMII
YEMHVRSFTQSSSSRVHAPGTFLGIIEKIDHLHKLGINAVELLPIFEFDETAHPFRNSKFPYLCNYWGYAPLNFF
SPCRRYAYASDPCAPSREFKTLVKTLHQEGIEVILDVVFNHTGLQRTTCSLPWIDTPSYYILDAQGHFTNYSGC
GNTLNTNRAPTTQWILDILRYWVEEMHVDGFRFDLASVFSRGPSGSPLQFAPVLEAISFDPLLASTKIIAEPWD
AGGLYQVGYFPTLSPRWSEWNGPYRDNVKAFLNGDQNLIGTFASRISGSQDIYPHGSPTNSINYVSCHDGFTL
CDTVTYNHKHNEANGEDNRDGTDANYSYNFGTEGKTEDPGILEVRERQLRNFFLTLMVSQGIPMIQSGDEY
AHTAEGNNNRWALDSNANYFLWDQLTAKPTLMNFLCDLIAFRKKYKTLFNRGFLSDKEISWVDAMGNPMT
WRPGNFLAFKIKSPKAHVYVAFHVGAQDQLATLPKASSNFLPYQIVAESQQGFVPQNVATPTVSLQPHTTLIA
ISHAKEVT

>core/100/4/Org4_Gene621

MKEENSQAHYLALCRELEDHDYSYYVLHRPRISDYEYDMKLRKLLEIERSHPEWKVLWSPSTRLGDRPSGTF
SVVSHKEPMLSIANSYSKEELSEFFSRVEKSLGTSPRYTVELKIDGIAVAIRYEDRVLVQALSRGNGKQGEDIT
LNIRTIRSLPLRLPEDAPEFIEVRGEVFFSYSTFQTINEKQQQLEKTIFANPRNAAGGTLKLLSPQEVAKRKLEIS

IYNLIAPGDNDSHYENLQRCLEWGFPVSGKPRLCSTPEEVISVLKTIETERASLPMEIDGAVIKVDSLASQRVL
GATGKHYRWALAYKYAPEEAETLLEDILVQVGRTGVLTPVAKLTPVLLSGSLVSRASLYNEDEIHRKDIRIG
DTVCVAKGGEVIPKVVRVCREKRPEGSEVWNMPEFCPVCHSHVVREEDRVSVRCVNPECVAGAIEKIRFFVG
RGALNIDHLGVKVITKLFELGLVHTCADLFQLTTEDLMQIPGIRERSARNILESIEQAKHVDLDRFLVALGIPLI
GIGVATVLAGHFETLDRVISATFEELLSLEGVGEKVAHAIAEYFSDSTHLNEIKKMQDLGVCISPYHKSGSTCF
GKAFVITGTLEGMSRLDAETAIRNCGGKVGSSVSKQTDYLVMGNNPGSKLEKARKLGVSILDQEAFTNLIHL
E

>core/101/4/Org4_Gene655

MRLNIHKYLFIGRNKADFFSASRELGVVEFISKKCFITTEQGHRFVECLKVFDHLEAEYSLEALEFVKDESVSV
EDIVSEVLTLNKEIKGLLETVKALRKEIVRVKPLGAFSSSEIAELSRKTGISLRFFYRTHKDNEDLEEDSPNVFY
LSTAYNFDYYLVLGVVDLPRDRYTEIEAPRSVNELQVDLANLQREIRNRSDRLCDLYAYRREVLRGLCDYDN
EQRLHQAKECCEDLFDGKVFAVAGWVIVDRIKELQSLCNRYQIYMERVPVDPDETIPTYLENKGVGMMGED
LVQIYDTPAYSDKDPSTWVFFAFVLFFSMIVNDAGYGLLFLMSSLLFSWKFRRKMKFSKHLSRVLKMTAILG
LGCICWGTTTTSFFGMSFSKTSVFREYSMTHVLALKKAEYYLQMRPKAYKELTNEYPSLKAIRDPKAFLLAT
ETGSAGIESRYVVYDKFIDNILMELALFIGVIHLSLGMLRYLRYRYSGIGWILFMVSAYLYVPIYLGTVSLIHY
LFHVPYELGGQIGYYGMFGGIGLAVVLAMIQRSWRGVEEIISVIQVFSDVLSYLRIYALGLAGAMMGATFNQ
MGARLPMLLGSIVILLGHSVNIILSIMGGVIHGLRLNFIEWYHYSFDGGGRPLRPLRKIVCSEDAEASGIHLDN
NSIV

>core/102/4/Org4_Gene414

MSEHKKSSKIIGIDLGTTNSCVSVMEGGQAKVITSSEGTRTTPSIVAFKGNEKLVGIPAKRQAVTNPEKTLGST
KRFIGRKYSEVASEIQTVPYTVTSGSKGDAVFEVDGKQYTPEEIGAQILMKMKETAEAYLGETVTEAVITVPA
YFNDSQRASTKDAGRIAGLDVKRIIPEPTAAALAYGIDKVGDKKIAVFDLGGGTFDISILEIGDGVFEVLSTNG
DTHLGGDDFDEVIIKWMIEEFKKQEGIDLSKDNMALQRLKDAAEKAKIELSGVSSTEINQPFITMDAQGPKHL
ALTLTRAQFEKLAASLIERTKSPCIKALSDAKLSAKDIDDVLLVGGMSRMPAVQETVKELFGKEPNKGVNPD
EVVAIGAAIQGGVLGGEVKDVLLLDVIPLSLGIETLGGVMTTLVERNTTIPTQKKQIFSTAADNQPAVTIVVLQ
GERPMAKDNKEIGRFDLTDIPPAPRGHPQIEVSFDIDANGILHVSAKDVASGKEQKIRIEASSGLQEDEIQRMV
RDAEINKEEDKKRREASDAKNEADSMIFRAEKAIKDYKEQIPETLVKEIEERIENVRNALKDDAPIEKIKEVTE
DLSKHMQKIGESMQSQSASAAAASAANAKGGPNINTEDLKKHSFSTKPPSNNGSSEDHIEEADVEIIDNDDK

>core/103/4/Org4_Gene466

MKLLLKAVLRHKNHLVILGCSLLAILGLTFSSQMEIFSLGMIAKTGPDAFLLFGRKESGKLVKVSELSQKDILE
NWQAISKDSETLTVSDATTYIAEHGKSTASLTSKLSKFVRNYIDVSRFRGLAIFLICVAIFKAVTLFFQRFLGQV
VAIRVSRDLRQDYFKALQQLPMTFFHDHDIGNLSNRVMTDSASIALAVNSLMINYIQAPITFILTLGVCLSISW
KFSILVCVAFPIFILPIVVIARKIKNLAKRIQKSQDSFSSVLYDFLAGVMTVKVFRTEKFAFTKYCEHNNKISAL
EEKSAAYGLLPRPLLHTIASLFFAFVVVIGIYKFTIPPEELIVFCGLLYLIYDPIKKFGDENTSIMRGCAAAERFY
EVLNHPDLHSQKEREIEFLGLSNTITFENVSFGYQEDKHILKDLSFTLHKGEALGIVGPTGSGKTTLVKLLPRL
YEVSQGKILIDSLPITEYNKGSLRNHIACVLQNPFLFYDTVWNNLTCGKDMEEEAVLEALKRAYADEFILELP
KGVHSVLEESGKNLSGGQQQRLAIARALLKNASILILDEATSALDAISENYIKNIIGELKGQCTQIIIAHKLTTLE
HVDRVLYIENGQKIAEGTKEELLQTCPEFLKMWELSGTKEYDRVFVPDHKLVANPTDMAITT

>core/104/4/Org4_Gene837

MFSRLFFTSFSAEVLNTFFESGMSEDTSPLLSKQNRKLSHNLPLKSAYLSLGTYLIALLSFWLHAKNLSNLFVV
FTFFLAGTPALIKSLDNICQKVVNIDILMTSAAFGSIFIGGALEGALLLVLFAISEALGQMVSGKAKSTLVSLKQ
LAPTTGWLVLEDGNLQKVAINKIEVGNILRIKSGEVVPLDGEILHGSSSINLMHLTGEKVPKSCHPGSIVPAGA
HNMEGSFDLRVLRTGSDSTIAHIINLVIQAQNSKPRLQQRLDKYSSVYALSIFAIASGIALLVPLFTSIPLLGPQS
AFYRALAFLIAASPCALIIAIPIAYLSAINACAKHGVLLKGGVILDRLVSCNSVVMDKTGTLTTGELTCIGCDYF
GPKNETFFPSVLALEQSSSHPIAEAIVSYLMEQKVSSLPADRYLTVPGEGVRGYFNEQEAFVGRVETGLGKVP
SEYLEDIEQKIYQAKQHGEICSLAYVGNSFALFYFRDIPRPQAKEIIQDLKDLGYPVSMLTGDHKVSAENTAEI
LGISEVFFDLTPEDKLAKIRELATQRQIMMVGDGINDAPALAQATVGIAMGEAGSATAIEAADIVLLHDSLSS
LPWIIQKAKQTKKVVSQNLALALAIILLVSWPASLGIIPLWLAVILHEGSTVIVGLNALRLLKS

>core/105/4/Org4_Gene877

MTFQLHAPFAPCGDQPEAIARLSAGVRNQVKSQVLLGTTGSGKTFTIANVVANVNLPTLVLAHNKTLAAQL
YQEFREFFPNNAVEYFISYYDYYQPEAYIARSDTYIEKSLLINDEIDKLRLSATRSILERRDTLIVSSVSCIYGIGS
PENYTSMALVLEVGKEYPRNILTAQLVKMHYQASPIPQRSAFRERGSVIDIFPAYESELALRLEFLNDTLTSIE
YSDPLTMIPKESVSSATLYPGSHYVIPEAIREQAIRTIQEELEERMAFFDDRPIEKDRIFHRTTHDIEMIKEIGFCK
GIENYSRHFTGAPPGAPPTCLLDYFPEDFLLVIDESHQTLPQIRAMYRGDQSRKQSLVEYGFRLPSAFDNRPLT
YEEAQKYFRKVIYVSATPGDTEVQESSGHIVQQIIRPTGIPDPMPEIRPATGQVDDLLEEIRLRLSQKHEKILVIS
ITKKLAEDMAGFLSELEIPAAYLHSGIETAERTQILTDLRSGVIDVLIGVNLLREGLDLPEVSLVAILDADKEGF
LRSTSSLIQFCGRAARNINGKVIFYADQKTRSIEETLRETERRRQIQLDYNKEHNIVPKPIIKAIFANPILQTSKDS
ESPKESQRPLSKEDLEEQIKKYEALMQRAAKEFRFNEAAKYRDAMQACKEQLLYLF

>core/106/4/Org4_Gene361

MATPAQKSPTFQDPSFVRELGSNHPVFSPLTLEERGEMAIAPVQQCGWNHTIVKVSLFILALLTILGGGLLVGL
LPAVPMFIGTGLIALGAVIFALALILCLCDSQGLPEELPPVPEPQQIQIEYLRNETREVLEGTLLEVLLKDRDAK
DPAVPQVVVDCEKRLGMLDRKLRREEEILYRSTAHLKDEARYEFLLELLEMRSLVADRLEFNRRSYERFVQG
IMTVRSEEGEKEISRLQDLIGLQQQTVQDLRSQIDDEQKRCWTSLQRIHQSQKDIQRAHDREASQRACEGTEM
DCAERHQLEKDLRRQLKSMREWIEMRGTIHQQEKAWRKQNAKLERLQEDLRLTKIAFDEQPLFYREYKEKY
LSQKLDMQKILQEVNAEKSEKARLKSLVRDYEKQLEQQDANLKKAKAIWEEELGKQQLKDHEQTQEIKRLN
TFMLEYQDGLREAEQVAEQVRQDLRQLEEKYSHLQEEKQEKEKILEQSVNHFAERFEALQKENVVYKKKLA
DLEGAAAPTEIREEDGWILAGSASLSQKKIRELNEENQELLKLLAFKTRELTQLAADVGEAEKEISKLREQIEE
QKEELRVLDTMHSEAVKDCEAAQRKCRDLEGLLSPVREDAGMRFELEIELQRLREENAQLRLEVERLEQEQL
QG

>core/107/4/Org4_Gene464

MSYRKRSTLIVLGVFALYALLVLRYYKIQICEGDHWAAEALGQHEFCVRDPFRRGTFFANTTVRKGDKDLQ
QPFAVDITKFHLCADPLAIPECHRDEIIQGILQFIEGQTYDDLSLKLDKKSRYCKLYPLLDVSVHDRLSLWWK
GYATKHRLPTNALFFITDYQRSYPFGKLLGQVLHTLREIKDEKTGKAFPTGGMEAYFNHILEGDVGERKLLRS
PLNRLDTNRVIKLPKDGSDIYLTINPVIQTIAEEELERGVLEAKAQGGRLILMNSQTGEILALAQYPFFDPTNYK
EYFNNKERIEHTKVSFVSDVFEPGSIMKPLTVAIALQANKEASLKSQKKIFDPEEPIDVTRTLFPGRKGSPLKDI

SRNSQLNMYMAIQKSSNVYVAQLADRIIQSLGVAWYQQKLLALGFGRKTGIELPSEASGLVPSPHRFHINGSP
EWSLSTPYSLAMGYNILATGIQMVQAYAILANGGYAVRPTLVKKIVSASGEEYHLPTKEKTRLFSEEITREVV
RAMRFTTLPGGSGFRASPKHHSSAGKTGTTEKMIHGKYDKRRHIASFIGFTPVESSEGNFPPLVMLVSIDDPEY
GLRADGTKNYMGGRCAAPIFSRVADRTLLYLGILPDKKLRNCDEEAAALKRLYEEWNRSPK

>core/108/4/Org4_Gene269

MVNPIGPGPIDETERTPPADLSAQGLEASAANKSAEAQRIAGAEAKPKESKTDSVERWSILRSAVNALMSLAD
KLGIASSNSSSSTSRSADVDSTTATAPTPPPPTFDDYKTQAQTAYDTIFTSTSLADIQAALVSLQDAVTNMKDT
AATDEETAIAAEWETKNADAVKVGAQITELAKYASDNQAILDSLGKLTSFDLLQAALLQSVANNNKAAELL
KEMQDNPVVPGKTPAIAQSLVDQTDATATQIEKDGNVIRDAYFAGQNASGAVENAKSNNSISNIDSAKAAIA
TAKTQIAEAQQKFPDSPILQEAEQMVIQAEKDLKNIKPADGSDVPNPGTTVGGSKQQGSSIGSIRVSMLLDDA
ENETASILMSGFRQMIHMFNTENPDSQAAQQELAAQAGAAKAAGDDSAAAALADAQKALEAALGKAGQQ
QGILNALGQIASAAVVSAGVPPAAASSIGSSVKQLYKTSKSTGSDYKTQISAGYDAYKSINDAYGRARNDAT
RDVINNVSTPALTRSVPRARTEARGPEKTDQALARVISGNSRTLGDVYSQVSALQSVMQMIQSNPQANNEEI
RQKLTSAVTKPPQFGYPYVQLSNDSTQKFIAKLESLFAEGSRTAAEIKALSFETNSLFIQQVLVNIGSLYSGYL
Q

>core/109/4/Org4_Gene379

MKKLVRLCVVLLSLLPNVLFSSDLLREEGIKKMMDKLIEYHVDAQEVSTDILSRSLSSYIQSFDPHKSYLSNQE
VAVFLQSPETKKRLLKNYKAGNFAIYRNINQLIHESILRARQWRNEWVKNPKELVLEASSYQISKQPMQWSK
SLDEVKQRQRALLLSYLSLHLAGASSSRYEGKEEQLAALCLRQIENHENVYLGINDHGVAMDRDEEAYQFHI
RVVKALAHSLDAHTAYFSKDEALAMRIQLEKGMCGIGVVLKEDIDGVVVREIIPGGPAAKSGDLQLGDIIYR
VDGKDIEHLSFRGVLDCLRGGHGSTVVLDIHRGESDHTITLRREKILLEDRRVDVSYEPYGDGVIGKVTLHSF
YEGENQVSSEQDLRRAIQGLKEKNLLGLVLDIRENTGGFLSQAIKVSGLFMTNGVVVVSRYADGTMKCYRT
VSPKKFYDGPLAILVSKSSASAAEIVAQTLQDYGVALVVGDEQTYGKGTIQHQTITGDASQDDCFKVTVGKY
YSPSGKSTQIQGVKSDILIPSLYAEDRLGERFLEHPLPADCCDNVLHDPLTDLDTQTRPWFQKYYLPNLQKQE
TLWREMLPQLMKNSEQRLSENSNFQAFLSQIKSSEKMDLSYGSNDLQLEESINILKDMILLQQCKK

>core/110/4/Org4_Gene711

MTSSSFPLLDLILSPADLKKLSISQLPGLAEEIRYRIISVLSQTGGHLSSNLGIVELTIALHYVFSSPKDKFIFDVG
HQTYPHKLLTGRNNEGFDHIRNDNGLSGFTNPTESDHDLFFSGHAGTALSLALGMAQTTPLESRTHVIPILGD
AAFSCGLTLEALNNISTDLSKFVVILNDNNMSISKNVGAMSRIFSRWLHHPATNKLTKQVEKWLAKIPRYGD
SLAKHSRRLSQCVKNLFCPTPLFEQFGLAYVGPIDGHNVKKLIPILQSVRNLPFPILVHVCTTKGKGLDQAQN
NPAKYHGVSANFNKRESAKHLPAIKPKPSFPDIFGQTLCELGEVSSRLHVVTPAMSIGSRLEGFKQKFPERFFD
VGIAEGHAVTFSAGIAKAGNPVICSIYSTFLHRALDNVFHDVCMQDLPVIFAIDRAGLAYGDGRSHHGIYDMS
FLRAMPQMIICQPRSQVVFQQLLYSSLHWSSPSAIRYPNIPAPHGDPLTGDPNFLRSPGNAETLSQGEDVLIIAL
GTLCFTALSIKHQLLAYGISATVVDPIFIKPFDNDLFSLLLMSHSKVITIEEHSIRGGLASEFNNFVATFNFKVDI
LNFAIPDTFLSHGSKEALTKSIGLDESSMTNRILTHFNFRSKKQTVGDVRV

>core/111/4/Org4_Gene891

MTCISELNEAQRKAVTAPLNPVLVLAGAGAGKTRVVTYRILHLINQGIAPREILAVTFTNKAARELKERIVNQ
CASTNEFDVPMVCTFHSLGVFILRRSINLLNRENNFTIYDQSDAEKLIKYALQQHNLKPNLASKIQAHISQAKN
RLLFPEDLDPNDYIDPVVSIYQEYQKKLIEANALDFDDLLFLTVRLLRESPEAQELYNQLWKALLIDEYQDTN
HAQYTLMQLLSKQHRNVFAVGDPDQSIYSWRGANIHNILNFENDYPNAKVLCLEENYRSYGNILNAANALIK
NNASRLEKELRSVKGPGEKIRLFLGSTDREEADFVAAEILQLHRVGNIKLRDICIFYRTNSQSRTFEDALLRRRI
PYEIIGGLSFYKRKEIQDILAFLRIFISKSDIVAFDRTVNLPKRGIGSTTIFALTQYAIAQGLPILKACQQALDTKD
VKLSKKQQEGLQEYLALFPQIEHAYNTLSLRDFIESVVRITGYLEILKEDADTFKDRKSNLEELYHKALESEQQ
NPKTHLELFLDDLALKGSDDDQNLTADRVNLMTLHNGKGLEFRVSFLVGLEEQLLPHANSLGGTYENIEEER
RLCYVGITRAQDLLYLTAAQVRSLWGTVRMMKPSRFLKEIPKDYMIQVR

>core/112/4/Org4_Gene643

MKQHYSLNKSRHILRSTYKLLKSKKLAHSPADKKQLQELLEQLEEAIFEHDQETASDLAQQALAFSNRYPNS
FGRKTYELIKALLFAGVVAFLVRQFWFELYEVPTGSMRPTILEQDRILVSKTTFGLHCPFAKKPLAFNPESVTR
GGLVVFTVGDLPIPDADTKYFGLIPGKKRYIKRCMGRPGDFLYFYGGKIYGLDDAGKRIEFPSVHGLENLYH
VPYISFDGTTSSHTEGQKTIIDFKQFNQSYGRLIFPQTSMYGQFFDHKEWHQDEPNKLKDPHLSLVSYADLFG
MGNYAMVRILTEHQARTSHLLPNPGSPTKVYLEICHTANLSYPKPLLRHYEHQLSPTIQPMKTLLPLRKEHLH
LIRNNLTTSRFIVAQGCAYKYHQFKINTSGIAKAYAILLPKVPDGCYEYSKGEAYQIGFGGIRYKLKSSHPLTQ
LNDKQVIELFNCGINFSSIYNPVNPLQAPLPNRYAFFNQGNLYIMDSPVFIKNDPTLQKFVTSEKEKQEGSSET
QPYIAFVDKGLPPEDFKEFVEFIHNFGIQVPKGHVLVLGDNYPMSADSREFGFVPMENLLGSPLCTFWPIGRM
GRLTGVSAPTTLSGYLVSGIALATGLSLIGYVVYYQKRRRLFPKKEEKNHKK

>core/113/4/Org4_Gene880

MIPFTKTIGFRLWLACAVAIIAPLGINIVWLNLDQYRTIVSAISTALKENAAFKANTLTQIVPLNVDVLSLFSDV
LDLDAGIPETPNVLLSNEMQKVFQGIYNEISLIKVFPNGDKIVVASSIPEHLGENYNHKIDIPENTPFLAALKQS
PKNQEVFSVMQANVFDAKTQELQGILYTTFSAESLLKDLLINKQSYLTVKTAILSKYGVILKASDPALHLHTV
YPDMTKEKFCQVFLNDDPCPIDSELGPLTLSPLDIGENFYSFKIKDTEIWGCIENVPSIDIAVLSYAKKEESFAPL
WRRARMYTAYFFCILLGSLIAFIVARRLSLPIRKLATAMIESRKNKNYLYTDDSLGFEINRLGHIFNAMVENLH
KQQYLAKTNFEMKENAQNALHLGEQAQQRLLPNTLPSYPHIELAKAYIPAITVGGDFFDVFVVGEGSEARLF
LIVADASGKGVNACGYSLFLKNMLRTFLSRSSSLQQAIQETSRLFYNNTKNSGMFVTLCVYCYHQTSNTMEY
YSCGHPPACYLDPDGETSWLFHPGMALGFLPEVANITSKLFHPKPGSLFVLYSDGITEAHNNNNDMFGEERL
QAAIQGLTGKSAADAVHRLMLSVKTFVGNSHQHDDITLLILKVLES

>core/114/4/Org4_Gene875

MIQVTCDQKNYEVLEGTTAAELAKQLKNSHQFIGVLINERPRDLSTHLNEGDTLVFLTSEDPEGREIFLHTSA
HLLAQAVLRLWPDAIPTIGPVIDHGFYYDFANLSISESDFPLIEDTVKQIVDEKLTISRFTYGDKQQALAQFPQN
PFKTELIRELPENEEISAYSQGEFFDLCRGPHLPSTAHVKAFKVLRTSAAYWRGDPSRESLVRIYGTSFPTSKEL
RAHLEQIEEAKKRDHRVLGAKLDLFSQQESSPGMPFFHPRGMIVWDALIRYWKQLHTAAGYKEILTPQLMN
RQLWEVSGHWDNYKANMYTLQIDDEDYAIKPMNCPGCMLYYKTRLHSYKEFPLRVAEIGHVHRQEASGAL
SGLMRVRAFHQDDAHVFLTPEQVEEETLNILQLVSTLYGTFGLEYHLELSTRPEKDTIGDDSLWELATDALN
RALVQSGTPFIVRPGEGAFYGPKIDIHVKDAIQRTWQCGTIQLDMFLPERFELEYTTAQGTKSVPVMLHRALF

GSIERFLGILIENFKGRFPLWLSPEQVRIITVADRHIPGAKELEEAWKRLGLVVTLDDSSESVSKKIRNAQNMQ
VNYMITLGDHEINENVLAVRTRDNRVINDVSVERFLNTILEEKNSLSLTALL

>core/117/4/Org4_Gene882

MDENRKVIVVGGGLAGLSAAMQLANLGIIVELVSLTKVKRSHSVCAQGGINAALNLKPEEEDSPYVHAYDTI
KGGDFLADQPPVLEMCLAAPRIIKMLDNFGCPFNRGPSGNLDVRRFGGTLYHRTVFCGASTGQQLMYTLDE
QVRRREHAGRVIKRENHEFVRLVTDHSGRACGIILMNLFNNRLEILRGDAVIIATGGPGVIFKMSTNSTFCTGA
ANGRLFLQGMAYANPEFIQIHPTAIPGRDKLRLISESVRGEGGRVWVPGDSSKRIVFPDGSERPCGETGAPWY
FLEDMYPAYGNLVSRDVGARAILRVCEAGLGIDGRMEVYLDVTHLPEKTRHKLEVVLDIYKKFTGEDPNTV
PMRIFPAVHYSMGGAWVDWPAADDPDRDSRFRQMTNIPGCFNCGESDFQYHGANRLGANSLLSCLFAGLVS
GDEASRFIEAFGASQATSSDFDRALQQEKEENARLLSASGKENIFVLHEEIAKIMVRNVTVKRNNRDLQETM
DKLKEFRERLKNVSVLDSSPFANKSFHFVRQMGPMLELALAITKGALLRNEFRGSHYKPEFPERDDEHWLKT
TVVVYAPEEPEISYLPVDTRHVAPTLRDYTKSSTGKIELTNIPDNIRLPI

>core/118/4/Org4_Gene242

MTIIYFILAALALGILVLIHELGHLVVAKAVGMAVESFSIGFGPALFKKRIGGIEYRIGCIPFGGYVRIRGMERT
KEKGEKGKIDSVYDIPQGFFSKSPWKRILVLVAGPLANILLAVLAFSILYMNGGRSKNYSDCSKVVGWVHPV
LQAEGLLPGDEILTCNGKPYVGDKDMLTTSLLEGHLNLEIKRPGYLTVPSKEFAIDVEFDPTKFGVPCSGASY
LLYGNQVPLTKNSPMENSELRPNDRFVWMDGTLLFSMAQISQILNESYAFVKVARNDKIFFSRQPRVLASVL
HYTPYLRNELIDTQYEAGLKGKWSSLYTLPYVINSYGYIEGELTAIDPESPLPQPQERLQLGDRILAIDGTPVSG
SVDILRLVQNHRVSIIVQQMSPQELEEVNSRDADKRFIASYHSEDLLQILNHLGESHPVEVAGPYRLLDPVQPR
PWIDVYSSESLDKQLEVAKKIKNKDKQRYYLERLDAEKQKPSLGISLKDLKVRYNPSPVVMLSNITKESLITL
KALVTGHLSPQWLSGPVGIVQVLHTGWSVGFSEVLFWIGLISMNLAVLNLLPIPVLDGGYILLCLWEIVTRRR
LNMKIVERILVPFTFLLIIFFIFLTFQDLFRFFG

>core/119/4/Org4_Gene410

MRRSVCYVNPSIARAGQISTWKFLYSLATPLPAGTKCKFDLAGSGKPTDWEAPATDLSQTRNVIYAEMPEGE
IIEATAIPVKDNPVPQFEFTLPYELQVGETLTIVMGASPNHPQVDDAGNGAQLFAQRRKPFYLYIDPTGEGNY
DEPDVFSMDIRGNVLKKIEIFTPSYVVKNKRFDITVRFEDEFGNLTNFSPEDTRIELSYEHLRENLNWQLFIPET
GFVILPNLYFNEPGIYRIQLKNLSTQEIFISAPIKCFADSAPNLMWGLLHGESERVDSEENIETCMRYFRDDRAL
NFYASSSFENQENLSPDIWKLINQTVSDFNEEDRFITLSGFQYSGEPHLEGVRHILHTKETKSHSKHKEYKHIPL
AKLYKSTVNHDMISIPSFTASKEHGFDFENFYPEFERVVEIYNAWGSSETTAALNNPFPIQGKDSEDPRGTVIE
GLKKNLRFGFVAGGLDDRGIYKDYFDSPQVQYSPGLTAIICNKYTRESLVEALFARHCYATTGPRIVLSFNITS
APMGSELSTGSKPGLNVNRHISGHVAGTALLKTVEIIRNGEVLHTFFPDSNNLDYEYDDMVPLSSVTLKDPNG
KAPFVFYYLRVTQADNAMAWSSPIWVDLN

>core/120/4/Org4_Gene622

MESEKDIGARFLGDYRILYRKGQSLWSEDLLAEHRFIKKRYFIRLLLPNLGSSEPFMEAFHDVVVRLAKLNHP
GVLCIENVSESEGRCFLVTQEQNIPILSLTQYLKSIPRKLTELEIVDIVSQLAVLLDYVHSEGLVQEEWNLDSIYI
HVLNGVPKVVLPDLGFASLIKERVLEGLISDEENRESKIKERVLLHTSEGKQGREDTYAFGAITYYLLFGFLPQ
GIFPMPSKVFSDFIYDWDFLISSCLSCFMEERAKELFPLIRKKTLGEELQNVVTNCIESSLREVPDPLESSQNLPQ

AVLKVGETKVSHQQKESAEHLEFVLVEACSIDEAMDTAIESESSSGVEEEGYSLALQSLLVREPVVSRYVEAE
KEEPKPQPILTEMVLIEGGEFSRGSVEGQRDELPVHKVILHSFFLDVHPVTNEQFIRYLECCGSEQDKYYNELI
RLRDSRIQRRSGRLVIEPGYAKHPVVGVTWYGASGYAEWIGKRLPTEAEWEIAASGGVAALRYPCGEEIEKS
RANFFTADTTTVMSYPPNPYGLYDMAGNVYEWCQDWYGYDFYEISAQEPESPQGPAQGVYRVLRGGCWK
SLKDDLRCAHRHRNNPGAVNSTYGFRCAKNIN

>core/121/4/Org4_Gene738

MKKGKLGAIVFGLLFTSSVAGFSKDLTKDNAYQDLNVIEHLISLKYAPLPWKELLFGWDLSQQTQQARLQLV
LEEKPTTNYCQKVLSNYVRSLNDYHAGITFYRTESAYIPYVLKLSEDGHVFVVDVQTSQGDIYLGDEILEVDG
MGIHEAIESLRFGRGSATDYSAAVRSLTSRSAAFGDAVPSGIAMLKLRRPSGLIRSTPVRWRYTPEHIGDFSLV
APLIPEHKPQLPTQSCVLFRSGVNSQSSSSSLFSSYMVPYFWEELRVQNKQRFDSNHHIGSRNGFLPTFGPILW
EQDKGPYRSYIFKAKDSQGNPHRIGFLRISSYVWTDLEGLEEDHKDSPWELFGEIIDHLEKETDALIIDQTHNP
GGSVFYLYSLLSMLTDHPLDTPKHRMIFTQDEVSSALHWQDLLEDVFTDEQAVAVLGETMEGYCMDMHAV
ASLQNFSQSVLSSWVSGDINLSKPMPLLGFAQVRPHPKHQYTKPLFMLIDEDDFSCGDLAPAILKDNGRATLI
GKPTAGAGGFVFQVTFPNRSGIKGLSLTGSLAVRKDGEFIENLGVAPHIDLGFTSRDLQTSRFTDYVEAVKTIV
LTSLSENAKKSEEQTSPQETPEVIRVSYPTTTSAS

>core/122/4/Org4_Gene670

MTTPPPSRSSSPPPYDWIELQDLGNTNNNSSRATPPPPEVGGELPPYFSASNFVVIERGAPSLPSPQQLLSLPEYS
RQPPPGYFDETASITSRTSEEMFGTLVSTLCCPANSERDWEDHEVNCIYIASTSDTQLEAVQGGMHITELRGEP
VRVLYETGHLYAFARENTCHSRLEVSHTVRAMTYFWDRFFSRHWNVGRRFLVFYQGNGGAYVQAALDSS
MHTQDIYVLGLSPTVYIRGNYHVQHYRVRGFWPSCLDSLAACAENTSVLPYGESSDGIFYPSLFSHTFDNAIR
YGERCLLVCSEGMGMLPETQQQTSPLTSLEGGHEVALVLNPQQNPEALSIASRLMHEERGGRLESNYMPGRS
SNPFMTSMYVLVRLNTLAQIYLMSPYYSFQSNDIVCLIFISSAAVETVSYIFLTVTDSTCGRRYLRVPRLVCTG
LRNLALPTTLLELLILSYPRSVEGVPFNVRFILGYMCTTRVVFFAWNLILHWPFRCLRHGIQLFVHRSIIGHTLG
ARITDLTLASMRYAIVFPSIVSSCLLTALAHANTNILALDPYRLIESGDLRRPAFNDDEMQQADNPWDAYSIGL
VINTCIYMLVLFANLIFMVYSVRRYHRSRR

>core/123/4/Org4_Gene489

MTLITPAINSSRRKTHTVRIGNLYIGSDHSIKTQSMTTTSTTDIDSTVEQIYALAEHNCDIVRVTVQGIKEAQAC
EKIKERLIALGLNIPLVADIHFFPQAAMLVADFADKVRINPGNYIDKRNMFKGTKIYTEASYAQSLLRLEEKFA
PLVEKCKRLGKAMRIGVNHGSLSERIMQKYGDTIEGMVASAIEYIAVCEKLNYRDVVFSMKSSNPKIMVTAY
RQLAKDLDARGWLYPLHLGVTEAGMGMDGIIKSAVGIGTLLAEGLGDTIRCSLTGCPTTEIPVCDSLLRHTKI
YLDLPEKKNPFSLQHSENFVSAAKKPAKTTLWGDVYGVFLKLYPHHLTNFTPEELLEHLGVNPVTKEKAFTT
PEGVVVPPELKDAPITNVLREHFLVFHHHQVPCLYEHNEEIWDSPAVHQAPFVHFHASDPFIHTSRDFFEKQG
HQGKPTKLVFSRDFDNKEEAAISIATEFGALLLDGLGEAVVLDLPNLPLQDVLEIAFGTLQNAGVRLVKTEYI
SCPMCGRTLFDLEEVTTRIRKRTQHLPGLKIAIMGCIVNGPGEMADADFGFVGSKTGMIDLYVKHTCVKAHIP
MEDAEEELIRLLQEHGVWKDPEETKLTV

>core/124/4/Org4_Gene336

MWTHPIAYDVIVVGAGHAGCEAAYCSAKMGASVLMLTSNLDTIAKLSCNPAVGGIGKGHIVREIDALGGIM
AEVTDQSGIQFRILNQTKGPAVRAPRAQVDKQLYHIHMKRLLENTPGLHIMQATVESLLDKEGVISGVTTKE
GWMFSGKTVVLSSGTFMRGLIHIGDRNFSGGRLGDPSSQGLSEDLKKRGFPISRLKTGTPPRLLASSINFSCME
EQPGDLGVGFVHRTEPFQPPLPQLSCFITHTTEKTKEIISANLHRSALYGGCIEGVGPRYCPSIEDKIVKFSDKE
RHHVFLEPEGLHTQEIYANGLSTSMPFDVQYDMIRSVLGLENAIITRPAYAIEYDYIHGNVIHPTLESKLIEGLF
LCGQINGTTGYEEAAAQGLIAGINAVNKVFNKPPFIPSRQESYIGVMLDDLTTQILDEPYRMFTGRAEHRLLL
RQDNACARLSHYGYELGLLSEERYELVKKQNQLLEEEKVRLQKTFRQYGQSVVSLAKALSRPEVSYDMLRE
AFPNDIRDLGTVLNASLEMEIKYSGYIDRQKILIQSLEKAESLLIPEDLDYKQITALSLEAQEKLAKFTPRTLGS
ASRISGIASADIQVLMIALKKHAHH

>core/125/4/Org4_Gene629

MTTELKTEALPTRTQVDPEHCWDTTLMYANRKEWKKDFDLCSSGKDRSPIWPEFSPSHYQIDNPESLLELLS
KKFSVERKLDQLYVYAHLIHDQDITNPEGESDYQSIVYLYTLFSQEISWIQPALIALSEEKVAALLSSSVLALY
RFYLEKIFRLSPHTGTANEEKILASSFAALNVANKAFSSLSDAEIPFGIAKDSNGEEHPLSHALASLYMQSPDQ
ELRRTAYLAQFQRYYDYRNTFANLLNGKVQAHLFEAKARNYPSCLEASLFQHNIPTTVYINLINETKKHTSLI
NRYFNLKKEALNLKEFHFYDVYAPISQTTSKNYSYEEGVDLVCKSLLPLGTHYVEILRNGLLSNRWVDRYEN
KHKRSGAYSSGCYDSAPYILLNYTNTLYDVSVIAHEAGHSMHSYFSREAQPYHDAQYPLFLAEIASTFNEML
LMEALSKSDQSKEDKIVIITKTLDTIFATLFRQTFFAAFEYEIHSAAEQGTPLTEEFLSATYGNLQKEFYGGVVT
SDSLSALEWARIPHFYYNFYVYQYATGIIAALSFAEKILTQEPGALELYLKFLKSGGSDFPLNILKKSGLDMTT
SAPLDKAFAFITKKIDLLSSLLSED

>core/126/4/Org4_Gene905

MFRCILFGIFLLTCFSSGGVLYYLFCSHDFSIGPKEKSRSVWIEEEKEFTDSVLHHLPSQHQHLHILCFQGFLLQ
KQQKFSQAEKIFSKVYDEAQDGPFLFKEEILGARLINSFFLEKTDVMETILCLLNQRCPNSPYYHLFKALVCYK
QKLYREVIEQLAYWQEEKTRALAPLLNISIEQLLTDFLLDYISAHSLIEQKMFPEGRVILNRNINRLLKHECEW
NAKTYDRIAILLSRSYFLELVESKSADIYFDYYEMVLFYLKKIYILEQCPYAELLPEEELVSLIMEHVFILPKDK
LYPLIQLLEMWQKHYVHPNSSLVVQILVDHFSTHMEGAIRFCEALVSFSGLEELHQQIITTFEELLSNKVQQIK
TEEAKQCVALLHILDPSISISEKLALSSDTLQNIVSGDDEQHTKLRNYLDLWEAIQSYDIDRQQLVHHLVYGA
KDLWKKGGNDEKALNLLQLVLRFTSYDIECESVVFLFVKQAYKQALSSHAIARLLKLEKFISEANIPSIVISEA
EKANFLADAEYLFAHEDYDKCYLYSMWLTKVAPSPQSYRLAGLCLMENKRYDEALEFLCMLSPNDSINDY
KTQKALAFCQKHQSKDRAAS

>core/127/4/Org4_Gene770

MCGIFGYLGNQDGVSIVLEGLEKLEYRGYDSAGLAAVVEQELFIRKTVGRVQELSNLFQEREIPTASVIGHTR
WATHGVPTEINAHPHVDEGRSCAVVHNGIIENFKELRRELTAQGISFASDTDSEIIVQLFSLYYQESQDLVFSF
CQTLAQLRGSVACALIHKDHPHTILCASQESPLILGLGKEETFIASDARAFLKYTRHSQALASGEFAIVSQGKE
PEVYNLELKKIHKDVRQITCSEDASDKSGYGYYMLKEIYDQPEVLEGLIQKHMDEEGHILSEFLSDVPIKSFKE
ITIVACGSSYHAGYLAKYIIESLVSIPVHIEVASEFRYRRPYIGKDTLGILISQSGETADTLAALKELRRRNIAYL
LGICNVPESAIALGVDHCLFLEAGVEIGVATTKAFTSQLLLLVFLGLKLANVHGALTHAEQCSFGQGLQSLPD
LCQKLLANESLHSWAQPYSYEDKFLFLGRRLMYPVVMEAALKLKEIAYIEANAYPGGEMKHGPIALISKGTP

VIAFCGDDIVYEKMIGNMMEVKARHAHVIAIAPESREDIAAVSDQQIFVPDCHFLAAPVLYTIVGQVMAYAM
ALAKGTEIDCPRNLAKSVTVE

>core/128/4/Org4_Gene772

MIPSGLVYLLYPLGFLASLFFGSAFSIQWWLSKKRKEVYAPRSFWILSSIGATLMIVHGTIQSQFPVTVLHVINL
IIYLRNLNITSSRPISFRATLVLMALSVVFVTLPFLYVNMEWMASPDIFHLPLPPAQLSWHLIGCLGLAIFSGRF
LIQWFYIESNNTKDFPLLFWKIGLLGGLLALVYFIRIGDPINILCYGCGLFPSIANLRLFYKEQRSTPYLDTHCFL
SAGEASGDILGGKLIQSIKSLYPNIRFWGVGGPAMRQEGLQPILNMEEFQVSGFAEVLGSLFRLYRNYRKILKT
ILKHKPATLIFIDFPDFHLLLIKKLRKHGYRGKIIHYVCPSIWAWRPKRKRILEQHLDMLLLILPFEEGLFKNTSL
ETVYLGHPLVEEISDYKEQASWKEKFLNSDRPIVAAFPGSRRGDISRNLRIQVQAFLNSSLSQTHQFVVSSSSA
KYDEIIEDTLKAEGCQHSQIIPMNFRYELMRSCDCALAKCGTIVLETALNQTPTIVMCRLRPFDTFLAKYIFKIL
LPAYSLPNIIMNSVIFPEFIGGKKDFHPEEIATALDLLNQHGSKEKQKEDCRKLCKVMTTGQIASEEFLKRIFDT
LPAV

>core/129/4/Org4_Gene881

MKHTFTKRVLFFFFLVIPIPLLLNLMVVGFFSFSAAKANLVQVLHTHATNLSIEFEKKLTIHKLFLDRLANTLA
LKSYASPSAEPYAQAYNEMMALSNTDFSLCLIDPFDGSVRTKNPGDPFIRYLKQHPEMKKKLSAAVGKAFLL
TIPGKPLLHYLILVEDVASWDSTTTSGLLVSFYPMSFLQKDLFQSLHITKGNICLVNKYGEVLFCAQDSESSFV
FSLDLPNLPQFQARSSSAIEIEKASGILGGENLITVSINKKRYLGLVLNKIPIQGTYTLSLVPVSDLIQSALKVPL
NICFFYVLAFLLMWWIFSKINTKLNKPLQELTFCMEAAWRGNHNVRFEPQPYGYEFNELGNIFNCTLLLLLNS
IEKANIDYHSGEKLQKELGILSSLQSALLSPDFPTFPKVTFSSQHLRGRQLSGHFNGWTVQDGGDTLLGIIGLA
GDIGLPSYLYALSARSLFLAYASLDVSLQKISKDTADSFSKTTEGNEAVVAMTFIKYVEKDRSLELLSLSEGAP
TMFLQRGESFVRLPLETHQALQPGDRLICLTGGEDILKYFSQLPIEELLKDPLNPLNTENLIDSLTMMLNNETE
HSADGTLTILSFS

>core/130/4/Org4_Gene790

MRIEDFSLKLIPSSPGVYLMKDVHDQVLYIGKAKNLKNRLASYFHEKGDSRERIPFLMKKTASVETIVVSNET
EALLLENNLIKQHHPKYNVLLKDDKTFFCLAISLSHSWPKVEAIRTKAITSSQRQLIFGPYVSAEACHTLLEVIS
QWFPLRTCSDREFALRKRPCILYDMKRCLAPCVGYCTPEEYQGTLDKAILFLKGKIEEVVKDLEKAIQKASD
NLEFEQAANYYRTLSLIKQAMAKQQVEKFHFQNIDALGLYRHKQRTILTLLTVRSGKLLGARHFSFFENAQE
DQDLLSSFILQYYVSQPYIPKEILTPLPLEFPTLSYVLNAESPPRLRSPKTGYGKELLDLAYRNAKAYAATTLPS
STLPYQDFQNILRMSQYPYRIECYDNAHMQGAHATGVYIVFENNGFDPKQYRTFSIDSEKTQNDLALLEEVL
LRRFHSLTTALPDMIVVDGGKTHYNKTKKILQTLNLTGIQVVTIAKEKSNHSRGLNKEKIFCETFPEGFSLPPT
SNLLQFFQILRDEAHRFAISKHRKKRGKALFEQEKIPGIGEVKRKRLLQKFKSWKQVMLSSQEELEAIPGLTK
KDIAVLLARQKDFNKSD

>core/131/4/Org4_Gene497

MKEYKIENIRNFSIIAHIDHGKSTIADRLLESTSTVEEREMREQLLDSMDLERERGITIKAHPVTMTYLHEGEV
YQLNLIDTPGHVDFSYEVSRSLSACEGALLIVDAAQGVQAQSLANVYLALERDLEIIPVLNKIDLPAADPVRIA
QQIEDYIGLDTTNIIACSAKTGQGIPAILKAIIDLVPPPKAPEETELKALVFDSHYDPYVGIMVYVRIISGELKKG
DRITFMAAKCSSFEVLGIGAFLPKATFIEGSLRPGQVGFFIANLKKVKDVKIGDTVTKTKHPAKTPLEGFKEIN

PVVFAGIYPIDSSDFDTLKDALGRLQLNDSALTIEQESSHSLGFGFRCGFLGLLHLEIIFERIIREFDLDIIATAPS
VIYKVVLKNGKVLDIDNPSGYPDPAIIEHVEEPWVHVNIITPQEYLSNIMNLCLDKRGICVKTEMLDQHRLVL
AYELPLNEIVSDFNDKLKSVTKGYGSFDYRLGDYRKGSIIKLEVLINEEPIDAFSCLVHRDKAESRGRSICEKL
VNVIPQQLFKIPIQAAINKKVIARETIRALSKNVTAKCYGGDITRKRKLWEKQKKGKKRMKEFGKVSIPNTAFI
EVLKLD

>core/132/4/Org4_Gene994

MVMVWSTNIKHEGLKSWIDEVAKLTTPKDIRLCDGSDAEYDELCTLMESTGTMIRLNPEFHPNCFLVRSSAD
DVARVEQFTFICTPTEAEAGPTNNWRDPQEMRRELHQLFRGCMQGRTLYIVPFCMGPLDSPFSIVGVELTDSP
YVVCSMKIMTRMGDDVLRSLGTSGKFLKCLHSVGKPLSPGEADVSWPCNPKSMRIVHFQDDSSVMSFGSGY
GGNALLGKKCVALRLASYMAKSQGWLAEHMLIIGITNPEGKKKYFSASFPSACGKTNLAMLMPKLPGWKIE
CIGDDIAWIRPGRDGRLYAVNPEYGFFGVAPGTSERTNPNALATCRSNSIFTNVALTADGDVWWEGLTEQPP
EPLTDWLGKPWKPGGSPAAHPNSRFTAPLRQCPSLDPEWNSPQGVPLDAIIFGGRRSETIPLVYEALSWEHGV
TIGAGMSSTTTAAIVGQLGKLRHDPFAMLPFCGYNMAYYFQHWLSFAENRSLKLPKIFGVNWFRKNNQGEF
LWPGFSENLRVLEWIFQRTDGLEDIAERTPIGYLPNIQKFNLNGLNLDPQTVQELFSVDAEGWLAEVENIGEY
LKIFGSDCPQQITDELLRIKSELKEK

>core/133/4/Org4_Gene278

MAAYTEASILSLASLDHIRLRAGMYIGRLGNGSQKEDGIYTLFKEVVDNGIDEFIMGHGKSLKISASDKQISIQ
DQGRGIPLGKLIDCVSKINTGAKYTQDVFHFSVGLNGVGLKAVNALSEIFSVRSVRKKKYHLATFHRGVLQE
SKQGSTKDPDGTFVSFTPDPSIFPEFTFNHDFLKDKIRQYTYLHSGLEIRFNDEVFISHNGLKDLFDAEITEPPLY
SPLFFQNEDLTFIFSHLEGNTERYFSFVNGQETLDGGTHLTAFKEAIVKGVNEFFGKTFVSNDIREGIVGCIAIKI
ASPIFESQTKNKLGNTQIRSSLIKDVKEAIVQALRKDKVAAELLLEKIKFNEKTRKNIQFIKQDLKSKQKKVHY
KIPKLRDCKFHYNDRSLYGEASSIFLTEGESASASILASRNPLTQAVFSLRGKPMNVFSLEETKMYKNDELFYL
ATALGITQNGIQHLRYNKVILATDADVDGMHIRNLLITFFLKTLLPLVENNHLFILETPLFKVRNKTTTLYCYS
EQEKMQALQQFGKKDSSLEITRFKGLGEISPKEFAAFIGPEIRLTPVTITSLESISSILQFYMGKNTKERKQFIMD
NLITDF

>core/134/4/Org4_Gene677

MKEVEQRIRSLYDAVTAENICRWLSNDCTQQDAKTILGWLDTDPAQLEDLFGATLTFGTGGLRSLMGIGTNR
INLFTIRRTTQGLVQVLRAHLPHPGDPMRVVVGCDTRHNSIEFAQETAKVLAGNGCEVFLFQYPEPLALVSFT
VRYERAIGGVMITASHNPPNYNGYKVYMASGGQVLPPLDQEIVAACSAVNEILSVPSIDHPNIHLIGKEYEAL
YRDTLKQLQLYPEANRISGRSLSISYSPLHGTGISLVPHVLKDWGFLSVHLVEKQAIGDGDFPTVQLPNPEDPE
ALTLGIEQMLANDDDLFIATDPDADRVGVVCLEDGQPYRFNGNQMASLLADHILGAWSKTRHLGEHDKLV
KSLVTTEMLSAIAKHYHVDLINVGTGFKYIGEKIESWRNSTNKFVFGAEESYGCLYGTHVEDKDAIIASALIA
EAALQQKLQGKTLRDALLSLYETYGYFANKTESVVFSAKTDEQEIRKKLSHLEEISSANFFSGKYQVEKFENY
KQGIGFNLLSKDSYALTLPKTSMLCYYFSGGGRVIIRPSGTEPKIKFYFEMSTHYPERVTDKEIQKQREAESFQ
HLDDFIFDFKEKFSNL

>core/135/4/Org4_Gene658

MVTVSEQTAQGHVIEAYGNLLRVRFDGYVRQGEVAYVNVDNTWLKAEVIEVADQEVKVQVFEDTQGACR
GALVTFSGHLLEAELGPGLLQGIFDGLQNRLEVLAEDSSFLQRGKYVNAISDHNLWNYTPVASVGDTLRRGD
LLGTVPEGRFTHKIMVPFSCFQEVTLTWVISEGTYNAHTVVAKARDAQGKEYAFTMVQRWPIKQAFIEGEKI
PAHKIMDVGLRILDTQIPVLKGGTFCTPGPFGAGKTVLQHHLSKYAAVDIVILCACGERAGEVVEVLQEFPHL
IDPHTGESLMHRTCIICNTSSMPVAARESSIYLGVTIAEYYRQMGLDILLLADSTSRWAQALREISGRLEEIPGE
EAFPAYLSSRIAAFYERGGAITTKDGSEGSLTICGAVSPAGGNFEEPVTQSTLAVVGAFCGLSKARADARRYP
SIDPLISWSKYLNQVGQILEEKVSGWGGAVKKAAQFLEKGSEIGKRMEVVGEEGVSMEDMEIYLKAELYDF
CYLQQNAFDPVDCYCPFERQIELFSLISRIFDAKFVFDSPDDARSFFLELQSKIKTLNGLKFLSEEYHESKEVIV
RLLEKTMVQMA

>core/136/4/Org4_Gene484

MTAPTESRSSSPTLLEETEPLSPNPIPADIRIPRITISPPSPDVSTVASSAEDISVLIEGGPRSSSSASVASDVYELVC
LCGGDEDPEPPDSEVRTLYVNGSWQTHQEAVQELLYISEVRGEAVRLLYNDGSGMSPWPISPCRTLPTHDHP
LCQALLMVWEQFFSAPENQNSEFLVIFYGDASPYIQQALTQSMYSQRIVVVGISPTVFIQGDFRVHNYRVSGD
FFSSLDCRGTRAENTTILPYSSGLEGVFLPSIRCPSFTWAVRFGEQCLITNRGEDVEDRGGRPQDIERSQLPHSE
RDLAVVIDSTDPSSMTRLVEWLNQGSPSSDMEINPYPQRCPDVALSALYAISRVSGLVQEWILASVHEGLDLQ
ICHSLILMHTTFAVRYFFLLFTNYPQCRERFRTARIVAQSLYLPSILVLVFDYGNVLRRLWMPQEILRAIFISAS
TISGSIVFVECTRWMGRGLRHRVQQFVQQRVIGSGLPVGTVRASYRDRAGFIIGFLQTVHGGLYLPISIMVLN
QIAIQVPRTLVRPNNTAVYDLHNKSAEDSWNSGDVLAVGQTLNFILCAFVLFVNLWFFVKSVLRHSRRRRR

>core/137/4/Org4_Gene741

MKKKFIFYFVIVFSLLFLWEMTSRHRPTFSFLFPPPSSIASSTLQSLPLLLTSAWHTLKAILGGFFLAITLSIVLAT
IMLSYKSAKDLLQPLFILLQCTPMFALAPLIVLWFGWGIGAVIVPTALTIFFPLTLTIYQGILSTPDELIEQFVLC
GATKFQLLIKLRIPHALPHIFSGLKIAIGSAGFAAIAGEWVASQSGLGILMLESRRNYEMELALAGLATLSILTL
SLFQITLLIEKLIFSLFRVKRMSLKHKSVAKKALSVLALIPIMLIPWKGNSKSPPDKKNLTSLTLLLDWTPNPNH
IPLYAGVAKGYFKQHGLDLQLQKNTDSSSAVPHVLFEQVDMALYHALGIMKTSIKGMPIQIVGRLIDSSLQGF
LYRSQDPIYKFEDLNGKVLGFCLNNSRDLNRLLETLNRNGVVPSEVKNVSSDLISPMLLNKIDFLYGAFYNIE
GVKLQTLGMPVKCFLSDTYDLPTGPQLIVFTKKGTKASEPEIVEAFQKALQESIIFSKDHPEDAFKLYAKETKS
IPKNLYQEYLQWEETFPLLAQSQDPLSKDLVDKLLETIIKRYPELASEVAKFSLNDLYNPSLPEEQSV

>core/138/4/Org4_Gene788

MYTEESLDNLRHSIDIVDVLSEHIHLKRSGATYKACCPFHTEKTPSFIVNPAGAHYHCFGCGAHGDAIGFLMQ
HLGYSFTEAILVLSKKFQVDLVLQPKDSGYTPPQGLKEELRHINSEAETFFRYCLYHLPEARHALQYLYHRGF
SPDTIDRFHLGYGPEQSLFLQAMEERKISQEQLHTAGFFGNKWFLFARRIIFPVHDALGHTIGFSARKFLENSQ
GGKYVNTPETPIFKKSRILFGLNFSRRRIAKEKKVILVEGQADCLQMIDSGFNCTVAAQGTAFTEEHVKELSK
LGVLKVFLLFDSDEAGNKAALRVGDLCQTAQMSVFVCKLPQGHDPDSFLMQRGSSGLIALLEQSQDYLTFLI
SEKMSSYPKFGPREKALLVEEAIRQIKHWGSPILVYEHLKQLASLMMVPEDMVLSLANPQVTAEPQNILIKQK
VPKIHPHIVMETDILRCMLFCGSNTKILYTAQFYFVPEDFKHPECRKLFAFMISYYEKYRKNVPFDEACQVLS
DSQILQLLTKRRLNTEALDTIFVQSLQKMADRRWREQCKPLSLNQNIQDKKLEILEDYVQLRKDRTIITLLDPE
SELIP

>core/139/4/Org4_Gene434

MSTSPISNDPRYLSLSNATEKTSLLANSRSLSPVPNALVPSNPEDTGLRKSIFTHSVTLFAGLVVLLVAVSVVV
VALTVLAPGVPQAILLGIAISGVGIGGFSIMKSLVYMVRDYMSPRMQESSRIKSALAVGTGFTVMGLVMKVG
ANFVPGGYGGLVGSLGSSAYSRGSQTTLASFSHYIYTKFFRSEKVAKGEKLTEAETIKEAKKLHYITLSIATIG
VGLAVLGILLAIAGTVLLGGTPATIAIILAPPLISIGLTTVLQTILHSSIGKWRAFLLTQEKKDLFVDTSLKDIRLE
KLPPSEVEESETSQSVIEVPDSEGIAETRISAEEIDTRLSLTTRQKVIFALATLLLLASIAAFIVTGFGGLTVMQVL
LVASVGSAVASVTLPMVSSGFSYVAYQLKARLNISKLRWKEAKNKKRVRQFLIESGVIASDREFNQMWKTV
YKKQIQKTDAAIREEVRNFEKGGEVNSALVGGILLGVGIGIMLLALVPAFAPIVPGILALGGSTLGIAGSILMR
KFVNWLYDELVKLYERRRNRRELLYGPESKMRSIATDLVVEALAASHDHLFDLDGPVDFIDVDVDIDGAA

>core/140/4/Org4_Gene373

MTNSDNASATGLLWAHPKEDPAFLGMIIKEFHLPPTVAQIFISRGFQTIQEIHKFLYSHLSSLYDPGLFLDMSK
AVERLLLARDRKEHVMIYGDSDVDGMTGVALLVEFLRDIDVHVSYFFLGAILKQHGETSTLIAKLKEEGITLL
ITVDCGITAGKEVSDITRQGIDVIITDHHMPTGKIPHCVATLNPKLRDHTYPNRELTGVGVAFKLARGVLNALI
SRNLVPKSQGSLKKLLDLVTLGTITDVGVLLGENRVMVRYGIKEIARGARLGLNKLCALCGVEKSEVSSTDIV
LKIAPKLNSLGRLDDPAKGVELLLTQDDERVDALIMELDNINKERQRIEADVFQDVQEILNSNPEILNQAAIVL
SSTAWHARVIPIISARLAKTYNKPVVIAIQGGIGKGSARTIGSFPLLGVLKKCSSLLLSYGGHDFAAGVIMKED
KVEDFKKKFVHLVNSSLKKGDTLPHLEIDAYADFDAIDYDLLASMELFEPFGKGNLMPIFYSKVRQVRYPKV
LPGNHLKLYLSQKERNLEGVAFGLGRHADALKARWHYPLEIAYTPRLSQTSGSGVIHLLVRDFRISSEPRFSD

>core/141/4/Org4_Gene515

MSFLRRHISLFRSQKQLIDVFAPVSPNLELAEIHRRVIEDQGPALLFHNVIGSSFPVLTNLFGTKHRVDQLFSQA
PDNLIARVAHLISSTPKLSSLWKSRDLLKRMSSLGLKKARFRRFPFVSMSSVNLDHLPLLTSWPEDGGAFLTL
PLVYTESPTLTTPNLGMYRMQRFNQNTMGLHFQIQKGGGMHLYEAEQKKQNLPVSVFLSGNPFLTLSAIAPL
PENVSELLFATFLQGAKLLYKKTNDHPHPLLYDAEFILVGKSPAGKRRPEGPFGDHFGYYSLQHDFPEFHCHK
IYHRKDAIYPATVVGKPYQEDFYIGNKLQEYLSPLFPLVMPGVRSLKSYGESGFHALTAAVVKERYWRESLT
TALRILGEGQLSLTKFLMVTDQEVPLDRFSVVLETILERLQPDRDLIIFSETANDTLDYTGPSLNKGSKGIFMGI
GKAIRDLPHRYQGGKIHGVQDIAPFCRGCLVLETSLEDRCIKSLLHHPDLKSWPLIILADNLRETIQSEKDFLW
RTFTRCAPANDLHALHSHFATHRPNYNFPFVIDALMKPSYPKEVEVDPSTKQKVSERWHEYFPNKETFYI

>core/142/4/Org4_Gene314

MKYRTHRCNELTSNHIGENVQLAGWVHRYRNHGGVVFIDLRDRFGITQIVCREDEQPELHQRLDAVRSEWV
LSVRGKVCPRLAGMENPNLATGHIEVEVASFEVLSKSQNLPFSIADDHINVNEELRLEYRYLDMRRGDIIEKM
LCRHQVMLACRNFMDAQGFTEIVTPVLGKSTPEGARDYLVPSRIYPGKFYALPQSPQLFKQLLMVGGLDRYF
QIATCFRDEDLRADRQPEFAQIDIEMSFGDTQDLFPIIEQLVATLFATQGIEIPLPLAKMTYQEAKDSYGTDKPD
LRFDLKLKDCRDYAKRSSFSIFLDQLAHGGTIKGFCVPGGATMSRKQLDGYTEFVKRYGAMGLVWIKNQEG
KVASNIAKFMDEEVFHELFAYFDAKDQDILLLIAALESVANQSLDHLRRLIAKERELYSDNQYNFVWITDFPL
FSLEDGKIVAEHHPFTAPLEEDIPLLETDPLAVRSSSYDLVLNGYEIASGSQRIHNPDLQSQIFTILKMSPESIQE
KFGFFIKALSFGTPPHLGIALGLDRLVMVLTAAESIREVIAFPKTQKASDLMMNAPSEIMSSQLKELSIKVAF

>core/143/4/Org4_Gene494

MSGKKDGVRGMIFVPLSILVLIFLPLPQILLDFGLCISFALSLLTVCWVFTLNSSNSAKLFPPFFLYLCLLRLGLN
LASTRWIVSSGTASSLIVSLGSFFSLGSLWAATFACLLLFFVNFLMVSKGSERIAEVRSRFFLEALPAKQMALD
SDLVSGRASYKAVKKQKNALIEEGDFFSAMEGVFRFVKGDAIISCILLLVNVVSVTCLYYTSGYALEQMWFT
VLGDALVSQVPALLTSCAAATLISKIDKEESLLNYLFEYYKQLRQHFRVVSLLIFSLCCIPSSPKFPIILLASLLW
LAYRKEEPASEDSCIERAFSYVEGACPKEQESQFYQVYRAASEEVLEDLGVRLPVLTSLRIEERPWLRVFGQN
VYLDEVTPEAVLPFLRNIAHEALNAEVVQKYLEESERVFGIAVEDIVPKKISLSSLVVLSRLLVRERVSLKLFP
KILEAVAVYQNSGDSLEILAEKVRKSLGYWIGRSLWDQKQTLEVITIDFHVEELINSSYSKSNPVMQENVIRR
VDSLLERSVFKDFRAIVTSSETRFEMKKMLDPHFPDLLVLSHNELPKEIPISFLGIVSDEVLVP

>core/144/4/Org4_Gene522

MPKQAEYTWGSKKILDNIECLTEDVAEFKDLLYTAHRITSSEEESDNEIQPGAILKGTVVDINKDFVVVDVGL
KSEGVIPMSEFIDSSEGLVLGAEVEVYLDQAEDEEGKVVLSREKATRQRQWEYILAHCEEGSIVKGQITRKVK
GGLIVDIGMEAFLPGSQIDNKKIKNLDDYVGKVCEFKILKINVERRNVVVSRRELLEAERISKKAELIEQISIGE
YRKGVVKNITDFGVFLDLDGIDGLLHITDMTWKRIRHPSEMVELNQELEVIILSVDKEKGRVALGLKQKEHN
PWEDIEKKYPPGKRVLGKIVKLLPYGAFIEIEEGIEGLIHVSEMSWVKNIVDPSEVVNKGDEVEAIVLSIQKDE
GKISLGLKQTEHNPWDNIEEKYPIGLHVNAEIKNLTNYGAFVELEPGIEGLIHISDMSWIKKVSHPSELFKKGN
SVEAVILSVDKESKKITLGVKQLSSNPWNEIEAMFPAGTVISGVVTKITAFGAFVELQNGIEGLIHVSELSDKPF
AKIEDIISIGENVSAKVIKLDPDHKKVSLSVKEYLADNAYDQDSRTELDFEDSQGPKERKKKGK

>core/145/4/Org4_Gene873

MSTRRPIQLLDPLTINQIAAGEVIENSVSVVKELIENSLDAGADEIEIETLGGGQGAIIIRDNGCGFRAEDIPIALQ
RHATSKIREFSDIFSLNSFGFRGEALPSIASISKMEIQSSIEGDEGVRTVIHGGDIVSCEPCARQLGTTVIVNSLFY
NVPVRRGFQKSMQSDRLGIRKLIENRILSTANIGWSWISEGHHEIQIAKQQGFQERVAYVMGDHFMQDALTI
DKEANGVRIVGVLGSPSFHRPTRQGQKIFINDRPIESLFISKKVGDAYALLLPLHRYPVFVLKLYLPSSWCDFN
VHPQKIEARILKEELVGDCIKEAIVETLTCPPGILCRTHQEIEESDSVPLPMFRMLETSDVQEEESVEFNQNLFA
YSSEDVSLEKQEYTSRGPKSQMDWMYSSDVRFLTSLGRVVLAEDLEGVHIIFTAAARKHLFFLSLMQENSRM
YQSQALLIPLRLQVTPEEAFFFSHHGRTLCDLGIEISQVGPCVFSIESTPTVIGEEELKEWLLLLAARGSTDINSE
ALTALMKETLTQATFSKHQHVFDVSWLKLLWSVGKPEKGFDGARIRRLILDSDFMEG

>core/146/4/Org4_Gene512

MAVSGGGGIQPSSDPGKWNPALQGEQAEGPSPLKESIFSETKQASSAAKQESLVRSGSTGMYATESQINKAK
YRKAQDRSSTSPKSKLKGTFSKMRASVQGFMSGFGSRASRVSAKRASDSGEGTSLLPTEMDVALKKGNRISP
EMQGFFLDASGMGGSSSDISQLSLEALKSSAFSGARSLSLSSSESSSVASFGSFQKAIEPMSEEKVNAWTVARL
GGEMVSSLLDPNVETSSLVRRAMATGNEGMIDLSDLGQEEVSTAMTSPRAVEGKVKVSSSDSPEANPTGIPN
SNTLERAEKEAEKQESREQLSEDQMMLARAMAGLLTGAAPQEVLSNSVWSGPSTVFPPPKFSGTLPTQRSGD
KSKHKSPGIEKSTNHTNFSPLREGTVKSAEVKSLPHPESMYRFPKDSIVSREEPEAVVKESTAFKNPENSSQNF
LPIAEESVFPKESGTGGALGSDAVSSSYHFLAQRGVSLLAPLPRATDDYKEKLEAHKGPGGPPDPLIYQYRNV
AVEPPIVLRSPQPFSGSSRLSVQGKPEAASVHDDGGGGNSGGFSGDQRRGSSGQKASRQEKKGKKLSRDI

>core/147/4/Org4_Gene352

MQKHPSFYQRFLSAYHKNLLASLSWKFFISVALIGIYAPLFASSKPLLVTWHGEIFFPLLRYLFFPGYYTKPVD
LFFNVLMVTFPFFILSFKLTRGWLRRWLLGLCIISQCMIFAWAYSGKVQDPALAENLKKMRAEKVRENISKV
NSEMVMLLPKDTRTWEMERRYMSTYEQLGILIKAKYRKKQEASVKKYQVAFEEKRQSPMPTLRHLEMKNE
GICLKRLQQRVDKMQRPYEMAQQAWNRATDNYRPFLMALTRIEHELRLADYNNWGQPEDLCIAYANVEKR
AEPYKKSLLEIRQVLEDYAKLRSAISFIQDKRLWIEKESEDLRILINPFFSSFHWEDDAGGSREMNKYVPWWQ
LSRVTRKDLLAALVFGIRIALVVAGIGITIALAIGIMIGLVSGYFGGTVDMILSRFTEIWETMPVLFILMLVISIT
QQKSLLLNTVLLGCFSWTGFSRYVRIEVLKQRDRGYVLAATNLGYSHYYIMVHQILPNAIVPVISLVPFAMM
AMISCEAGLTFLGLGEESSASWGNLMREGVTGFPAESAVLWPPAIILTMLLIAIALIGDGVRDALDPRLQDS

>core/148/4/Org4_Gene941

MTDFPTHFKGPKLNPIKVNPNFFERNPKVARVLQITAVVLGIIALLSGIVLIIGTPLGAPISMILGGCLLASGGAL
FVGGTIATILQARNSYKKAVNQKKLSEPLMERPELKALDYSLDLKEVWDLHHSVVKHLKKLDLNLSETQRE
VLNQIKIDDEGPSLGECAAMISENYDACLKMLAYREELLKEQTQYQETRFNQNLTHRNKVLLSILSRITDNIS
KAGGVFSLKFSTLSSRMSRIHTTITVILALSAVVSVMVVAALIPGGILALPILLAVAISAGVIVTGLSYLVRQILS
NTKRNRQDFYKDFVKNVDIELLNQTVTLQRFLFEMLKGVLKEEEEEEVSLEGQDWYTQYITNAPIEKRLIEEI
RVTYKEIDAQTKKMKTDLEFLENEVRSGRLSVASPSEDPSETPIFTQGKEFAKLRRQTSQNISTIYGPDNENIDP
EFSLPWMPKKEEEIDDSLEPVTKLEPGSREELLLVEGVSPTLRELNMRIALLQQQLSSVRKWRHPRGEHYGNV
IYSDTELDRIQMLEGAFYNPLREAQEEITQSLGDLVDIQNRILGVIVEGDSDSRTEEEPQE

>core/149/4/Org4_Gene673

MANPTQSRPPSPEISIEEIELQELPGSSNTETTSNTPPPSCAATAEEVSLFIEGGRRNSEDEEGLLGPCDVYDVVC
ITNQGDPEVRDHEVRVMYINGSGRTQHEGILDAVNICELRGEPVRFIHNSGYGLANCFLGTQNRIPPRDNVISQ
AIQARWNEFFVSPENANRNYIVLFSGNGGLYLQVALDNSIYSHHILCVGIGSSYYIQGSYRVHNYRVTGDWT
TLLDRRGATAVNTTTLPYADSAEGLFLPSVRCPSYQWALRYGEQCLIMDNNQQGGFRPQDPSSEIALLVNLN
QDQGNWTRLIEWIDQGDSQAVLELNPQPSHCRDIALTALYATTRISSLLQECLMISVTYSPDVFVTYAIVTGY
SIMTLRYFILLLTNRPGCRQHFRVLRLAALGLQPLGFLTVLLDHINVTRRVNRRPPLISVIFCTASFATGSFIYV
DLTRMFFTSLRSRLQLFVQRRLTGRGLPLRRVCVNHLDSLRFSQNALTTFHGGLFMPLIIGFFNQLVIQVPRVV
IRPNTNAVYDLNQTSQEAWDSGDVLAIGQTINFLLCMILLVINTFFFVRSVRRNLHRRPHR

>core/150/4/Org4_Gene965

MFMNTQNSQATEVSSEEESQKKLEELVALAKEQGFITYEEINEILPMSFDTPEQIDQVLIFLTGMDIQVLNQID
VERQKEKKKEAKELEGLARRTEGTPDDPVRMYLKEMGTVPLLTREEEVEISKRIEKAQVQIERIILRFRYSAK
EAISIAHYLISGKERFDKIISEKEVEDKTHFLKLLPKLITLLKEEDTYLENLLLSLKQPDLSKQEAAKLNDSLEK
CRIRTQAYLRCFHCRHNVTEDFGEVVFKAYDSFLHLEQQINDLKVRAERNKFAAAKLAAAKRKLYKREVAA
GRTLEEFKKDVRMLQRWMDKSQEAKKEMVESNLRLVISIAKKYTNRGLSFLDLIQEGNMGLMKAVEKFEY
RRGYKFSTYATWWIRQAVTRAIADQARTIRIPVHMIETINKVLRGAKKLMMETGKEPTPEELAEELGLTPDR
VREIYKIAQHPISLQAEVGEGGESSFGDFLEDTAVESPAEATGYSMLKDKMKEVLKTLTDRERFVLIHRFGLL
DGKPKTLEEVGSAFNVTRERIRQIEAKALRKMRHPIRSKQLRAFLDLLEEEKTGTSKVKSLKSK

>core/151/4/Org4_Gene685

MDTQSSIGNEEWRIAGTSIVSGMALGKVFFLGTSPLHVRELTLPQEEVEHEIHRYYKALNRSKSDIVALEQEV
TGQQGLQEVSSILQAHLEIMKDPLLTEEVVNTIRKDRKNAEYVFSSVMGKIEESLTAVRGMPSVVDRVQDIH
DISNRVIGHLCCQHKSSLGESDQNLIIFSEELTPSEVASANSAYIRGFVSLVGAATSHTAIVSRAKSIPYLANISE
ELWNIAKRYNGKLVLIDGYRGELIFNPKPATLQSCYKKELSVVAHTSQRLVRKSLHPIVSSHAGSDKDVEDLL
ENFPQTSIGLFRSEFLAVILGRLPTLREQVDLYEKLARFPGDSPSVLRLFDFGEDKPCPGIKNKKERSIQWLLDY
SVILEDQLQAIAKASLQGSIKVLIPGVSDVSEIIEVKKKWETIQTRFPKGHKVSWGTMIEFPSAVWMIEEILPEC
DFLSIGTNDLVQYTLGISRESALPKHLNVTLPPAVIRMIHHVLQAAKQNQVPVSICGEAAGQLSLTPLFIGLGV
QELSVAMPVINRLRNHIALLELNSCLEITEALLQAKTCSEVEELLNRNNKITS

>core/152/4/Org4_Gene997

MVKVPWAFKNFIPKLYTSIKEGYSFNTFKKDFQAGITVGILAFPFAIAIAIGVGVSPIQGLLASIIGGLLASAMG
GSNVLISGPSSAFISILYCLSAKYGAEALFTVTLLAGVFLIAFGLTGLGTFIKYMPYPVVTGLTTGLAVIIFSSQI
KDFLGLQMGANIPADFLPKWVAYWDHLWTWDSKSFAVGLFTLLIMIYFRNYKPRYPGVMIAIVTATTLVWL
LKIDIPTIGSRYGTLPTAIPLPKIPQLSITKILQLMPDALTIAVLSGLETLLSAVVADGMTGWRHQSNCQLVAQG
VANIGTSLFSGIPVTGSLSRTAASIKSGATTPIAGIVHSIFICFILLLLAPLTVKIPLTCLAAVLILIAWNMSEIHHFI
HLFTAPKKDIVVLLTVFILTVMTTITAAVQVGMMLAAFLFMKQMSDLSDVISTAKYFDDDNQPKDSDFLSKA
EVPQNTEIYEINGPFFFGIADRLKNLLNDIEKPPKIFILCMTRVPTIDASAMHALEEFFLECDRQGTLLLLAGVK
KTPLADLKRYHLDELIGVDHIFSNIKSALLFAQALTNLESKTSTRHLA

>core/153/4/Org4_Gene696

MGLFHLTLFGLLLCSLPISLVAKFPESVGHKILYISTQSTQQALATYLEALDAYGDHDFFVLRKIGEDYLKQSI
HSSDPQTRKSTIIGAGLAGSSEALDVLSQAMETADPLQQLLVLSAVSGHLGKTSDDLLFKALASPYPVIRLEA
AYRLANLKNTKVIDHLHSFIHKLPEEIQCLSAAIFLRLETEESDAYIRDLLAAKKSAIRSATALQIGEYQQKRFL
PTLRNLLTSASPQDQEAVLYALGKLKDGQSYYNIKKQLQKPDVDVTLAAAQALIALGKEEDALPVIKKQALE
ERPRALYALRHLPSEIGIPIALPIFLKTKNSEAKLNVALALLELGCDTPKLLEYITEWLVQPHYNETLALSFSKG
RTLQNWKRVNIIVPQDPQERERLLSTTRGLEEQILTFLFRLPKEAYLPCIYKLLASQKTQLATTAISFLSHTSHQ
EALDLLFQAAKLPGEPIIRAYADLAIYNLTKDPEKKRSLHDYAKKLIQETLLFVGTENQRPHPSMPYLRYQVT
PESRTKLMLDILETLATSKSSEDIRLLIQLMTEGDAKNFPVLAGLLIKIVE

>core/154/4/Org4_Gene416

MKTSQLFYKTSKNANKSAAVLSNELLEKAGYLFKVSKGVYTYTPLLWRVVSKMMNIIREELNAIGGQELLLP
LLHNAELWQHTGRWEAFTSEGLLYTLKDREGKSHCLAPTHEEVICSFVAQWLSSKRQLPLHLYQIATKFRDE
IRPRFGLIRSRELLMEDSYTFSDSPEQMNEQYEKLRSGYSKIFDRLGLAYAIVTADGGKIGKGKSEEFQVLCSL
GEDTICVSGSYGANIEAAVSIPPQHAYDREFLPVEEVATPGITTIEALANFFSIPLHKILKTLVVKLSYSNEEKFI
AIGMRGDRQVNLVKVASKLNADDIALASDEEIERVLGTEKGFIGPLNCPIDFFADETTSPMTNFVCAGNAKD
KHYVNVNWDRDLPPPQYGDFLLAEEGDTCPENPGHPYRIYQGIEVAHIFNLGTRYTDSFEVNFQDEHGQTQQ
CWMGTYGIGVGRTLAACVEQLADDRGIVWPKALAPFSITIAFNGGDTVSQELAETLYHELQSQGYEPLLDDR
DERLGFKLKDSDLIGIPYKLILGKSYQSSGIFEIESRSGEKYTVSPEAFPTWCQNHLA

>core/155/4/Org4_Gene263

MTVSVKKKSFRALVTTHFLTIINDNLYKFLLAFFLLEGKTLTENAKILSCVSFFFALPFLLLAPLAGSLADRFQ
KRNIILATRFIEILCTILGTYFFFIQSVVGGYVVLILMACHTTIFGPAKLGILPEMLPSEQLSQANGIMTAATYTG
SILGSCLAPLLVDVTHRLGVNSYVWPTLMCVVVSIISTLISFCIRPSNVKNVKQKITLVSFKDLWKVLKDTRMI
HYLTVSIFLGSFFLLIGAYTQLEIIPFVEFTLKYPKHYGAYLFPIVALGVGMGSYITGKISGKDIKIGYVPLAAIG
LALVFMGLYAFACSILFVLFFLLALGFLGGVYQVPLHAYVQYASPEHKRGQILAANNFLDFFGVLVAAGVIR
VLGSNLGLSPETSFFYIGWFVLAVSIWTLWIWREHVYRLLLGIILRRQLGYYLKIHQSSSPKCYFVAVQSYREI
RRVLAALTKTVRSRVIILDQKLVPGWRAWLLSWCVPTVVSSVRDNDSEAQDAWAVLQANHLKTSLKKFPD
VSVVCLGLPKNVERFTSILQEQGIDLHPIQLVQKEGKKRVIYTLVFPHA

>core/156/4/Org4_Gene369

MSTLLSILSVICSQAIAKAFPNLEDWAPEITPSTKEHFGHYQCNDAMKLARVLKKAPRAIAEAIVAELPQEPFS
LIEIAGAGFINFTFSPVFLNQQLEHFRDALKLGFQVSQPKKIIIDFSSPNIAKDMHVGHLRSTIIGDSLARIFSYVG
HDVLRLNHIGDWGTAFGMLISYLQENPCDYSDLEDLTSLYKKAYVCFTNDEEFKKRSQQNVVALQAKDPQA
IAIWEKICETSEKAFQKIYDILDIVVEKRGESFYNPFLPEIIEDLEKKGLLTVSNDAKCVFHEAFSIPFMVQKSDG
GYNYATTDLAAMRYRIEEDHADKIIIVTDLGQSLHFQLLEATAIAAGYLQPGIFSHVGFGLVLDPQGKKLKTR
SGENVKLRELLDTAIEKAEEALREHRPELTDEEIQERAPVIGINAIKYSDLSSHRTSDYVFSFEKMLRFEGNTA
MFLLYAYVRIQGIKRRLGISQLSLEGPPEIQEPAEELLALTLLRFPEALESTIKELCPHFLTDYLYNLTHKFNGFF
RDCHIQDSPYAKSRLFLCALTEQVLATGMHLLGLKTLERL

>core/157/4/Org4_Gene377

MSKLIRRVVTVLALTSMASCFASGGIEAAVAESLITKIVASAETKPAPVPVTAKKVRLVRKNKQPVEQKSRG
AFCDKEFYPCEEGRCQPVEAQQESCYGRLYSVKVNDDCNVEICQSVPEYATVGSPYPIEILAIGKKDCVDVVI
TQQLPCEAEFVSSDPETTPTSDGKLVWKIDRLGAGDKCKITVWVKPLKEGCCFTAATVCACPELRSYTKCGQ
PAICIKQEGPDCACLRCPVCYKIEVVNTGSAIARNVTVDNPVPDGYSHASGQRVLSFNLGDMRPGDKKVFTV
EFCPQRRGQITNVATVTYCGGHKCSANVTTVVNEPCVQVNISGADWSYVCKPVEYSISVSNPGDLVLHDVVI
QDTLPSGVTVLEAPGGEICCNKVVWRIKEMCPGETLQFKLVVKAQVPGRFTNQVAVTSESNCGTCTSCAETT
THWKGLAATHMCVLDTNDPICVGENTVYRICVTNRGSAEDTNVSLILKFSKELQPIASSGPTKGTISGNTVVF
DALPKLGSKESVEFSVTLKGIAPGDARGEAILSSDTLTSPVSDTENTHVY

>core/158/4/Org4_Gene613

MELLSLNKSYFEIQRLRYRPEILTLLETIRSKHIQETSSPLSPPPELQKHIPNLCRIPEVSIYTEQETSSKPLKIGVL
LSGGQAPGGHNVVIGLFDALRVFNPETRLFGFIKGPLGLTRGLYKDLDISVIYDYYNMGGFDMLSSSREKIKT
EEQKKNILNTVKKLKLDGLLIIGGNNSNTDTAMLAEYFLAHNCKTSVIGVPKTIDGDLKNCWIETSLGFHTSC
RTYSEMIGNLAKDALSAKKYHHFIRLMGQQASYTTLECGLQTLPNIALISELIATRKISLKQLSEQLALGLVRR
YKSGKNYSTVLIPEGLIEHIFDTRKLIDELNVLLANGHSAIEKILSRLSPEALKTFHLFPKDIANQLLLARDSHG
NVRVSKIATEELLAVMVKKEIEKIEPHMEFHSVSHFFGYEARAGFPSNFDCNYGIALGIISALFLVRQKTGYMI
TINNLARAYTEWQGGATPLYKMMHLENRCGTETPVIKTDSVDPKSPAVQHLLQQSDSCLVEDLYRFPGPLQ
YFGKEELIDQRPLTFLWENQTHSPLL

>core/159/4/Org4_Gene237

MSSPPQAVASLTERIKTLLESNFCQIIVKGELSNVSLQPSGHLYFGIKDSQAFLNGAFFHFKSKYYDRKPKDGD
AVIIHGKLAVYAPRGQYQIVAHALVYAGEGDLLQKFEETKRRLTAEGYFATEKKKPLPFAPQCIGVITSPTGA
VIQDILRVLSRRARNYKILVYPVTVQGNSAAHEISKAIEVMNAENLADVLIIARGGGSIEDLWAFNEEILVKAI
HASTIPIVSAVGHETDYTLCDFASDVRAPTPSAAAEIVCKSSEEQVQVFEGYLRHLLSHSRQLLTSKKQQLLP
WRRFLDRAEFYTTAQQQLDSIEIAIQKGVQGKIHESKQRYDNISRWLQGDLVSRMTCRLQSLKKMLSQALSH
KALSLQVRCHQLKKSLTYPRQIQQASQKLSPWRQQLDTLISRRLHYQKEEYFHKHTRLKHAHNVLEQQLRS
HVQKLELLGRRLSRGCELNLQNQKIAYANVKETLATILERRYENSVARYSALKEQLHSLNPKNVLKRGYAM
LFDFNENSAMISVDSLQENARVRIQLQDGEAILTVTNIEICKLIKG

>core/160/4/Org4_Gene805

MDNSDNSFHTLETEQGSFLNDELAVEEVASTESTEMSDATLCFADEIQELPSPEKKVAFILNKMREALTGSSQ
GSDLRLFWDLRKQCLPLFNEIEDTAKRADHWRCYIELTKEGRHLKGLQDEEGSFVVGQIDLAITCLEKDILKF
QEGTEDKIFKDREDNFLESQALDKHQAFYKQHHTSLLWLSSFSSKIIDLRKELMNVGMRMRLKSKFFQRLSN
LGNQVFPKRKELIEKVSQTFAEDVDAFVAKYFIGSDKETLKKTVFFLRKEIKNLQHAAKRLFVSSHVFAETRL
KLSKCWDQLKGMEKEIRQEQGRLRVVSAENSKEVRQMLAEVSSLLIEGNDLSKVRKNLEGISKKIRALDLTH
DDVISLKKEMQQLFDQLREKQDAAEHSYQEQLAKDKQVKKEAARSLAERITTFSKTCSEGNITSESREEWQT
LKELLGKMSFLPPPEKISLDNQLNLALQTIVNFFEEQLLSSPDSREKLVNMRQVLKQRRERRQELKDKLEQDK
KLLGSSGLDFDRAMQYSALVEEDKRALEELDASILELKQQIQQLL

>core/161/4/Org4_Gene252

MPQKVLITSALPYANGPLHFGHIAGVYLPADVYARFRRLLGDDVLYICGSDEFGIAITLNADREGLGYQEYV
DMYHKLHKDTFEKLGFALDFFSRTTNPFHAELVQDFYSQLKASGLIENRISEQLYSEQEQRFLADRYVEGTCP
RCGFDHARGDECQSCGADYEAIDLIDPKSKISGVELVKKETEHSYFLLDRMKDALLSFIQGCYLPDHVRKFV
VDYIEHVRSRAITRDLSWGIPVPDFPGKVFYVWFDAPIGYISGTMEWAASQGNPDEWKRFWLEDGVEYVQFI
GKDNLPFHSVVFPAMELGQKLDYKKVDALVVSEFYLLEGRQFSKSEGNYVDMDKFLSSYSLDKLRYVLAAT
APETSDSEFTFLDFKTRCNSELVGKFGNFINRVLAFAEKNHYDKLSYHSVVLEDSDRAFLEEARQLVRDAEK
CYREYSLRKATSVIMSLAALGNVYFNQQAPWKLLKEGTRERVEAILFCACYCQKLLALISYPIIPESAVAIWE
MISPKSLENCNLDTMYARDLWKEEILDVINEEFHLKSPRLLFTTVE

>core/162/4/Org4_Gene587

MHPSYFDLDTIISSYSPPLPKEFQEAASLIAVPDTSHSKPVVPGVKTLFPQTYHLPYLKFVQGENVVHTPLKVG
VMFSGGPAPGGHNVIQGLFNSLKDFHPDSSLVGFINNGDGLTNNKSIDITEEFLSKFRNSGGFNCIGTGRKKIV
TPEAKEACLKTAEALDLDGLVIIGGDGSNTATAILAEYFAKRRPKTSIVGVPKTIDGDLQHTFLDLTFGFDTAT
KFYSSIISNISRDALSCKAHYHFIKLMGRSASHIALECALQTHPNIALIGEEIAEKNLPLKTIIHKICSVIADRAAM
EKYYGVILIPEGIIEFIPEIINLITEIESLSEYEDKISRLSPESQRLLKSFPAPIIEQILNDRDAHGNVVYVSKISVDKLL
IHLVSNHLQQYFPNVPFNAISHFLGYEGRSGLPTKFDNTYGYSLGYGAGILVRNHCNGYLSTIESLACPFMKW
KLRAIPVVKMFTVKQQADGTLQPKIKKYLVDIGSTAFRKFKLYRKIWALEDSYRFLGPLQIETPPEMHSDNFP
PLTLLLNHNFWQRHQGCIEIPDTTY

>core/163/4/Org4_Gene267

MSRKDNEVSLARSIFNILSGTFCSRITGIFREIAMATYFGADPIVAAFWLGFRTVFFLRKILGGLILEQAFIPHFE
FLRAQSLDRAAFFFRRFSRLIKGSAIIFTLLIEAVLWVVLQYVEEGTYDMILLTMILLPCGIFLMMYNVNGALL
HCENKFFGVGLAPVVVNIIWIFFVIAARHSDPRERIIGLSVALVIGFFFEWLITVPGVWKFLLQAKSPPQEHDSV
RALLAPLSLGILTSSIFQLNLLSDICLARYVHEIGPLYLMYSLKIYQLPIHLFGFGVFTVLLPAISRCVQREDHER
GLKLMKFVLTLTMSVMIIMTAGLLLLALPGVRVLYEHGLFPQSAVYAIVRVLRGYGASIIPMALAPLVSVLFY
AQRQYAVPLFIGIGTALANIVLSLVLGRWVLKDVSGISYATSITAWVQLYFLWYYSSKRLPMYSKLLWESIRR
SIKVMGTTMLACMITLGLNILTQTTYVIFLNPLTPLAWPLSSITAQAIAFLSESCIFLAFLFGFAKLLRVEDLINL
ASFEYWRGQRGLLQRQHVMQDTQN

>core/164/4/Org4_Gene630

MAAKNIKYNEEARKKIHKGVKTLAEAVKVTLGPKGRHVVIDKSFGSPQVTKDGVTVAKEIELEDKHENMGA
QMVKEVASKTADKAGDGTTTATVLAEAIYSEGLRNVTAGANPMDLKRGIDKAVKVVVDELKKISKPVQHH
KEIAQVATISANNDSEIGNLIAEAMEKVGKNGSITVEEAKGFETVLDVVEGMNFNRGYLSSYFSTNPETQECV
LEDALILIYDKKISGIKDFLPVLQQVAESGRPLLIIAEEIEGEALATLVVNRLRAGFRVCAVKAPGFGDRRKAM
LEDIAILTGGQLVSEELGMKLENTTLAMLGKAKKVIVTKEDTTIVEGLGNKPDIQARCDNIKKQIEDSTSDYD
KEKLQERLAKLSGGVAVIRVGAATEIEMKEKKDRVDDAQHATIAAVEEGILPGGGTALVRCIPTLEAFLPML
ANEDEAIGARIILKALTAPLKQIASNAGKEGAIICQQVLARSANEGYDALRDAYTDMIDAGILDPTKVTRSAL
ESAASIAGLLLTTEALIADIPEEKSSSAPAMPSAGMDY

>core/165/4/Org4_Gene422

MFGSESLRYQLLIQDFAKVSEEGIDLLESKEYSLLQAKLVLRALAQNSSFDDWFRSFKKCQISYPELAHDRDV
LEEFGIQVLREGIENPSVTVRAVSVLAIGLARDFRLVPLLLQSCNDDSAIVRSLALQVAVNYGSESLKKAIVEL
ARNDDSIHVRITAYQVVALLQIEELLPFLRERAENKLVDGVERREAWKACLELSSQFLETGVAKDDIDQALFT
CEVLHNGMLPETTEIFTELLSVEHPEVQESLLLSALAWSHQLQNHKEFLSKVRHVMRTSPFAKVRFQAAALL
HLHGDSLGRDSLVEGLRSHQPLVCEAASAALCSLGIHGVPLAKEHLESLSSRKAAANLSILLLVSREDIERAG
DVIARYLSNPEMCWAIEYFLWDAQWNLRGDAFPLYSDMIKREIGRKLIRLLAVARYSQAKAVTATFLSGQQ
AQGWSFFSGMFWEEGDVKTSEDLVTDACFAAKLEGALASLCQKKDQASLQRVSQLYNDSRWQDKLAILES
VAFSENLHAVPFLLDCCHHEAPSLRSAAAGALFSIFK

>core/166/4/Org4_Gene318

MLRIFCFVISWCLIAFAQPDLSRFVSILGAACGYGLFWYSLEPLKKTSLPLRTLFVSCFFWIFTIEGIHFSWMLS
DQYIGKLIYLVWLTLITILSVLFSGFSCLLVAIVRQKRTAFLWSLPGVWVAIEMLRFYGIFSGMSFDYLGWPM
TASAYGRQFGGFLGWAGQSFAVIAVNMSFYCLLLKKPHAKMLWVLTLLLPYTFGAIHYEYLKHAFQQDKR
ALRVAVVQPAHPPIRLKLKSPIVVWEQLLQLVSPIQQPIDLLIFPEVVVPFGKHRQVYPYESCAHLLSSFAPLPE
GKAFLSNSDCATALSQHFQCPVIIGLERWVKKENVLYWYNSAEVISHKGISVGYDKRILVPGGEYIPGGKFGS
LICRQLFPKYALGCKRLPGTRSGVVQVPGLPRIGITICYEETFGYRLQSYKRQGAELLVNLTNDGWYPESRLP
KVHFLHGMLRNQEFGMPCVRACQTGVTAAVDSLGRILKILPYDTRETKAPSGVLETSLPLFNYKTLYGYCGD
YPMILIAFCAVSYLGGGFLGYRLLAKKEIR

>core/167/4/Org4_Gene338

MQSSEVKPFSRLRAYLCPIYKSEFSKFVPLFLLAFFVGFNYCLLKNMKDTLVIVGSDAGAEVIPFLKVWGIVP
GAVIVTMVYGWLGSRYPRDTVFYCFMAAFLGFFFLFAVIIYPVGDSLHLNSLADKLQELLPQGLRGFVVMVR
YWSYSIYYVMSELWSSVVLSMLFWGLANQITTITEAGRFYALINTGLNLSSICAGEISYWMGKQTFVAYSFA
CDSWHSVMLNLTMLITCSGLIMIWLYRRIHHLTIDTSIPPSRRVLAEERAATANLKEKKKPKAKARNLFLHLI
QSRYLLGLAIIVLSYNLVIHLFEVVWKDQVSQIYSSHVEFNGYMSRITTLIGVVSVLAAVLLTGQCIRKWGWT
VGALVTPLVMLVSGLLFFGTIFAAKRDISIFGGVLGMTPLALAAWTGGMQNVLSRGTKFTFFDQTKEMAFIP
LSPEDKNHGKAAIDGVVSRIGKSGGSLIYQGLLVIFSSVAASLNVIALVLLIIMVVWIAVVAYIGKEYYSRAAD
AVATLKQPKEPSSSIVREAQESVEQEEMAVL

>core/168/4/Org4_Gene202

MHDQRNRGHNNHNLRLRPGSTLLEAFLILCSEHEEGIACFDEHLGSLSYRELRNAIIAVAIKVSKFSEDRVGV
MMPASIGAFIAYFGILLAGKTPVMMNWSQGLRELRACTKTVEVRRVLTSQQFIKHLTEVQGFVEYPFDLMY
MEDVRKRLSWWEKCRIGLYSKCSVPWLLRIFGVSGVESDDTAVILFTSGTEKLPKAVPLTHKNLMENQEACL
KFFDPNTQDVMLAFLPPFHAYGFNSCGLFPLLMGVHVVFASNPLNPKKLVEFIDDKKVTFFGSTPVFFDYILK
TAKKQNSCLESLRLVVIGGDALKDTLYEETKKLQPQIALYQGYGATECSPVISITTKESPRKSECVGMPIEGM
DVLIISKETHIPVSSGEQGLIVVRGNSVFSGYLGNHEHQSFVSLGGDQWYLTGDLGHIGPSGDLFLEGRLSRFV
KIGGEMVSLEALESILHEHFTENQNEDAGSLVVCGIPGDKVRLCLFTTLATTIHEVNDILKSAETSSIVKISYVH
QVESIPILGIGKPDYVSLNALAVSLFG

>core/169/4/Org4_Gene40

MPFKCIFLTGGVVSSLGKGLTAASLALILERQRLNVAMLKLDPYLNVDPGTMNPFEHGEIYVTDDGVETDLD
LGHYHRFSSAALSRHSSATSGQIYARVIKREREGDYLGSTVQVIPHITNEIIQVILDAAKEHSPDVLIVEIGGTIG
DIESLPFLEAIRQFRYDHSEDCLNIHMTYVPYLQAADEVKSKPTQHSVQTLRGIGIIPDAILCRSEKPLTQEVKS
KISLFCNVPNRAVFNVIDVKHTIYEMPLMLAQEKIANFIGEKLKLATVPENLDDWKVLVNQLSQDLPKVKIG
VVGKYVQHRDAYKSIFEALTHAALRLGHAAEIIPIDAEDENLTMELSQCDACLVPGGFGVRGWEGKIAAAKF
CREQGIPYFGICLGMQVLVVEYARNVLNLDQANSLEMDPNTPHPIVYVMEGQDPLVATGGTMRLGAYPCLL
KPGSKAHKAYNESSLIQERHRHRYEVNPDYIQSLEDHGLRIVGTCPPQGLCEIIEVSDHPWMIGVQFHPEFVSK
LISPHPLFIAFIEAALVYSKDASHV

>core/171/4/Org4_Gene594

MRKISVGICITILLSLSVVLQGCKESSHSSTSRGELAINIRDEPRSLDPRQVRLLSEISLVKHIYEGLVQENNLSG
NIEPALAEDYSLSSDGLTYTFKLKSAFWSNGDPLTAEDFIESWKQVATQEVSGIYAFALNPIKNVRKIQEGHLS
IDHFGVHSPNESTLVVTLESPTSHFLKLLALPVFFPVHKSQRTLQSKSLPIASGAFYPKNIKQKQWIKLSKNPH
YYNQSQVETKTITIHFIPDANTAAKLFNQGKLNWQGPPWGERIPQETLSNLQSKGHLHSFDVAGTSWLTFNIN
KFPLNNMKLREALASALDKEALVSTIFLGRAKTADHLLPTNIHSYPEHQKQEVAQRQAYAKKLFKEALEELQ
ITAKDLEHLNLIFPVSSSASSLLVQLIREQWKESLGFAIPIFGKEFALLQADLSSGNFSLATGGWFADFADPMAF
LTIFAYPSGVPPYAINHKDFLEILQNIEQEQDHQKRSELVSQASLYLETFHIIEPIYHDAFQFAMNKKLSNLGVS
PTGVVDFRYAKEN

>core/172/4/Org4_Gene908

MLGKEEEFTCKQKQCLSHFVTNLTSDVFALKNLPEVVKGALFSKYSRSVLGLRALLLKEFLSNEEDGDVCDE
AYDFETDVQKAADFYQRVLDNFGDDSVGELGGAHLAMENVSILAAKVLEDARIGGSPLEKSTRYVYFDQKV
RGEYLYYRDPILMTSAFKDMFLGTCDFLFDTYSALIPQVRAYFEKLYPKDSKTPASAYATSLRAKVLDCIRGL
LPAATLTNLGFFGNGRFWQNLIHKLQGHNLAELRRLGDESLTELMKVIPSFVSRAEPHHHHHQAMMQYRRA
LKEQLKGLAEQATFSEEMSSSPSVQLVYGDPDGIYKVAAGFLFPYSNRSLTDLIDYCKKMPHEDLVQILESSV
SARENRRHKSPRGLECVEFGFDILADFGAYRDLQRHRTLTQERQLLSTHHGYNFPVELLDTPMEKSYREAME
RANETYNEIVQEFPEEAQYVVPMAYNIRWFFHVNARALQWICELRSQPQGHQNYRTIATGLVREVVKFNPM
YELFFKFVDYSDIDLGRLNQEMRKEPTT

>core/173/4/Org4_Gene624

MFVGGFVSFLLPIPDLECANNVTKTYDKKASVISRDLKLQEDCQKFWNLDPYKLESLCAYQVLYHDDYSSK
RIRELFPQIQKDEVPIFATMILTLGKVDRGFSPEEISLIQKLSYPGLSLASLRGSTEIDPNTDLARALVVSEFSGD
LGKNRADYYSNCLDILALRIHAERQRYLDQSPCVPGTSEFHKATIEAINTILFYEEAVRYPSKKEMFSDEFSFL
SSVTDRKFGVCLGVSSLYFSLSQRLDLPLEAVTPPGHIYLRYQGGEVNIETTAGGRHLPTASYCDCLDLEDLQ
VRTPEEMIGLTFMNQGSFALQKKKYKEAEEAYKKAQEYLGDEELQELLGFVQILGGKKKEGKSLIGKSPRAS
QKGSVAYDYLKGRINIPTLALLFSYPGSNYEEIASYEEELKKAMKSSMPCCEGQRRLASVAFHLGKTAEAVA
LLEKCVEDIPNDLSLHLRLCKILCDRHDYTKALKYFIIAERLMEDQGFLKKDNRSFALFYEVKKIISKVAPQKA
NTLLLMESER

>core/174/4/Org4_Gene695

MSIVLDKIGKSLGTRILFDDVSVVFNPGNCYGLTGPNGAGKSTLLKIIMGMIEPTRGSISLPKKVGILRQNIDSF
HDTTVLDCVIMGNTRLWEALQRRDNLYLQEFTDAIGMELGEIEEIIGEENGYRADSEAEELLTGIGIPNEMFD
KKMAMIPIDLQFRVLLCQALFGHPEALLLDEPTNHLDLYSINWLGNFLKDYEGTVIVVSHDRHFLNTITTHIA
DIDYDTIIIYPGNYDDMVEMKTASREQEKADIKSKEKKISQLKEFVAKFGAGSRASQVQSRLREIKKLQPQEL
KKSNIQRPYIRFPLSDKSSGKVVLSLEAITKDYGDHQVIHPFSLEIYQGDKLGIIGNNGLGKTTLMKLLAGVEA
PSSGSIKLGHQAICSYFPQNHSDVLADCGQETLFEWLRNRKTGINDQEIRSVLGKMLFGGDDAFKQIQALSGG
ETARLLMAGMMLENHNVLILDEANNHLDLESVSALSWAINDYKGTAIFVSHDRGLIQDCATKLLIFDKDKIT
FFDGTMADYTAGHKQLL

>core/175/4/Org4_Gene591

MKMHRLKPTLKSLIPNLLFLLLTLSSCSKQKQEPLGKHLVIAMSHDLADLDPRNAYLSRDASLAKALYEGLT
RETDQGIALALAESYTLSKDHKVYTFKLRPSVWSDGTPLTAYDFEKSIKQLYFEEFSPSIHTLLGVIKNSSAIHN
AQKSLETLGIHAKDDLTLVITLEQPFPYFLTLIARPVFSPVHHTLRESYKKGTPPSTYISNGPFVLKKHEHQNYL
ILEKNPHYYDHESVKLDRVTLKIIPDASTATKLFKSKSIDWIGSPWSAPISNEDQKVLSQEKILTYSVSSTTLLIY
NLQKPLIQNKALRKAIAHAIDRKSILRLVPSGQEAVTLVPPNLSQLNLQKEISTEERQKKARAYFQEAKETLSE
KELAELSILYPIDSSNSSIIAQEIQRQLKDTLGLKIKIQGMEYHCFLKKRRQGDFFIATGGWIAEYVSPVAFLSIL
GNPRDLTQWRNSDYEKTLEKLYLPHAYKENLKRAEMIIEEETPIIPLYHGKYIYAIHPKIQNTFGSLLGHTDLK
NIDILS

>core/176/4/Org4_Gene246

MPGSVSSPPLSPVIVRERVPSSSGSDLIQPHAVLKISILIFALVTILGIVLVVSSALGALPSLVLTVSGCIAIAVGLI
GLGILVTRLILSTIRKVDAMGYDAAVKEEQYLSRIRELESENREIRDRNRAVEDQCAHLSEENKDLRDPEYLH
GMTERLIASLEIENQALVAENILLKDWNASLSRDFRAYKQKFPLGALEPWKEDIACIMEQNLFLKPECIAMVK
SLPLETQRLFLYPKGFQSLVNRFAPRSRFFQTPKYEYNSRNENEDGKVAAVCARLKKEFFSAVLGACSYEELG
GICERAVALKETLPLPEAVYDTLVQEFPNLLTAESLWKEWCFYSYPYLRPYLSVDYCKRLFVQLFEELCLKLF
TTGSPEDQALVRLFSYYRNHIPAVLASFGLPPPETGGSVFVLLPKQENLLWSQIEVLATRYLKDTFVRNSEWT
GSFEMMFSYNEMCKEISEGRIRFAEDYETRHSEEFPPSPLSEEGEGEEFLPPCSEEEVSVLERPDLDVDSMWV
WHPPVPKGPL

>core/177/4/Org4_Gene793

MTARAEYLNHEDFLYRSHKLQELSALGVVPYPYEFPGVLSCEDIKKAFTGQELGNSEAAMSRSTPKVRFAGR
LVLFRAMGKNAFGQILDHNQTIQVMFNSEFTSVHGLSKDAGVVPIKFIEKKLDLGDILGIDGYLFFTHSGELT
VLVETVTLLCKSLLSLPDKHAGLSDKEVRYRKRWLDLISSREVSDTFVKRSYIIKLIRNYMDAHGFLEVETPIL
QNIYGGAEAKPFTTKMEALHSEMFLRISLEIALKKILVGGAPRIYELGKVFRNEGIDRTHNPEFTMIEAYAAY
MDYQEVMVFVENLVEHLVRAVNHDNTSLVYSHWKHGPQEVDFKAPWIRMTMKESIATYAGIDVDVHSDQ
KLKEILKKKTTLPETAFATASRGMLIAALFDELVSDNLIAPHHITDHPVETTPLCKTLRSGDTAFVERFESFCLG
KELCNAYSELNDPIRQRELLEQQHTKKELLPDSECHPIDEEFLEALCQGMPPAGGFGIGVDRLVMILTNAASIR
DVLYFPVMRRFDAEKTN

>core/178/4/Org4_Gene593

MLRFFAVFISTLWLITSGCSPSQSSKGIFVVNMKEMPRSLDPRKTRLIADQTLMRHLYEGLVEEHSQNGEIKPA
LAESYTISEDGTRYTFKIKNILWSNGDPLTAQDFVSSWKEILKEDASSVYLYAFLPIKNARAIFDDTESPENLG
VQALDKRHLEIQLETPCAHFLHFLTLPIFFPVHETLRNYSTSFEEMPITCGAFRPVSLEKGLRLHLEKNPMYHN
KSRVKLHKIIVQFISDPNTAAILFKHKKLDWQGPPWGEPIPPEISASLHQDDQLFSLPGASTTWLLFNIQKKPW
NNAKLRKALSLAIDKDMLTKVVYQGLAEPTDHILHPRLYPGTYPERKRQNERILEAQQLFEEALDELQMTRE
DLEKETLTFSTFSFSYGRICQMLREQWKKVLKFTIPIVGQEFFIIQKNFLEGNYSLTVNQWTAAFIDPMSYLMIF
ANPGGISPYHLQDSHFQTLLIKITQEHTKHLRNQLIIEALDYLEHCHILEPLCHPNLRIALNKNIKNFNLFVRRTS
DFRFIEKL

>core/179/4/Org4_Gene516

MNVLKYTKHSSSAHAWKLIGTSPKHGIYLPLFSIHTKNSCGIGEFLDLVPLISWCQKQGFGVIQLLPLNDTGED
TSPYNSISSVALNPLFLSLSSLPNIDTIPEVAKKLQDMHELCSTPSVSYTQVKEKKWAFLREYYQKCCKSSLEG
NSNFSEFLESERYWLYPYGTFRAIKHHMHGEPINNWPKSLTDQENFPDLTKKFHDEVLFFSYLQFLCYQQLCE
VKAYADQHHVLLKGDLPILISKDSCDVWYFRDYFSSSRSVGAPPDLYNSEGQNWHLPIYNFSQLAKDDYIW
WKERLRYAQNFYSVYRLDHIIGFFRLWIWDSSGRGRFIPDNPKDYIKQGTEILSTMLGASSMLPIGEDLGIIPQ
DVKTTLTHLGICGTRIPRWERNWESDSAFIPLKDYNPLSVTTLSTHDSDTFAQWWLNSPKEAKQFAKFLHLPF
QKTLTTETQIDILKLSHESASIFHINLFNDYLALCPDLVSKNLQRERINTPGTISKKNWSYRVRPSLEELAIHKK
FNGYIEKILTGL

>core/180/4/Org4_Gene733

MERKRFIDCDSTKILQELALNPLDLTAPGVLSAERIKKFSLLGGGFTFSFATERLDDAILAALISLAEERGLHES
MLAMQQGQVVNYIEGFPSEMRPALHTATRAWVTDSSFTGEAEDIAVRSRVEAQRLKDFLTKVRSQFTTIVQI
GIGGSELGPKALYRALRAYCPTDKHVHFISNIDPDNGAEVLDTIDCAKALVVVVSKSGTTIETAVNEAFFADY
FAKKGLSFKDHFIAVTCEGSPMDDTGKYLEVFHLWESIGGRFSSTSMVGGVVLGFAYGFEVFLQLLQGASA
MDQIALQPNARENLPMLSALISIWNRNFLGYPTEAVIPYSSGLEFFPAHLQQCCMESNGKSITQDGRRVGFSTS
PVIWGEPGTNGQHSFFQCLHQGTDIIPVEFIGFEKSQKGEDISFQGTTSSQKLFANMIAQAIALACGSENTNPN
KNFDGNRPSSVLVSSQLNPYSLGELLSYYENKIVFQGFCWGINSFDQEGVSLGKALANRVLELLEGADASNFP
EAASLLTLFNIKFR

>core/181/4/Org4_Gene501

MTKTEEKPFGKLRSFLWPIHTHELKKVLPMFLMFFCITFNYTVLRDTKDTLIVGAPGSGAEAIPFIKFWLVVPC
AIIFMLIYAKLSNILSKQALFYAVGTPFLIFFALFPTVIYPLRDVLHPTEFADRLQAILPPGLLGLVAILRNWTFA
AFYVLAELWGSVMLSLMFWGFANEITKIHEAKRFYALFGIGANISLLASGRAIVWASKLRASVSEGVDPWGI
SLRLLMAMTIVSGLVLMASYWWINKNVLTDPRFYNPEEMQKGKKGAKPKMNMKDSFLYLARSPYILLLAL
LVIAYGICINLIEVTWKSQLKLQYPNMNDYSEFMGNFSFWTGVVSVLIMLFVGGNVIRKFGWLTGALVTPVM
VLLTGIVFFALVIFRNQASGLVAMFGTTPLMLAVVVGAIQNILSKSTKYALFDSTKEMAYIPLDQEQKVKGK
AAIDVVAARFGKSGGALIQQGLLVICGSIGAMTPYLAVILLFIIAIWLVSATKLNKLFLAQSALKEQEVAQEEA
ASVQTRTQEDPAPASS

>core/182/4/Org4_Gene888

MVWVFKSQFEGLSALKRGVHALTKAVTPAFGPRGYNAVIKKGKAPIVLTKNGIRIAKEIILQDAFESLGVKLA
KEALLKVVEQTGAGSTTALVVIDALFTQGLKGIAAGLDPQEIKAGILLSVEMVYQQLQRQAIELQSPKDVLH
VAMVAANHDVTLGTVVATVISQADLKGVFSSKDSGISKTRGLGKRVKSGYLSPYFVTRPETMDVVWEEALV
LILSHSLVSLSEELIRYLELISEQNTHPLVIIAEDFDQNVLRTLILNKLRNGLPVCAVKAPGSRELRQVVLEDLAI
LTGATLIGQESENCEIPVSLDVLGRVKQVMITKETFTFLEGGGDAEIIQARKQELCLAIARSTSESECQELEERL
AIFIGSIPQVQITADTDTEQRERQFQLESALRATKAAMKGGIVPGGGVAFLRAAHAIEVPANLSSGMTFGFETL
LQAVRTPLKVLAQNCGRSSEEVIHTILSHENPRFGYNGMTDTFEDLVDAGICDPLIVTTSSLKCAVSVSCLLLT
SSFFISSRTKT

>core/183/4/Org4_Gene964

MYQENLRLLERLLYNSFQKSYADRLFSYEKTKMVHDTPLIPWEEDKEKCAEAEKAFLEQQKILLDYGKSIFW
LNENDEINLNDPWSWGLNTVRTRKVFQEVDDSERWNHKVLIQKLEDDYEKLLEESSKESTEANKKLLSDLV
DRLEDAKTKFFLKKQEEVETRVKDLRAGYGGTVDPKQDTEAKKKVELEASLETFLDSIESELVQCLEDQDIY
WKEQDVKDLARRQELEEQDVEAKREEAAEDLRSLNERLKKSKTMLDRAKWHIENAEDSITWWTSQIEMKD
MKARLKILKEDITSVLPEIDEIETCLSLEELPLLTTRELLTKSYLKFKICSEKLLKMTSVFENNIYVQEYEVQLQ
NLGFKLQGISQRFGKKQDDFANLEEQVALQKKRLRELTQNFEIQGFNFMKEDFKAAAKDLYIRSTAEQKMN
FDVPCMELFRRYHEEVNKPLLELMYNCADSYRDAKKKLCSLRLDEKELLQKEIKKEEFYQKKQQRHADRSR
HTTYQKLRIAEELALELKKKI

>core/184/4/Org4_Gene966

MGPFFVPKQTSCEGDKDFSARNVSIILGLNDVLKLCLLQRCPFALQWDNISVRRDYEEEEPVIKIRSLEPKVSQ
ISPTHGLLCSTLVQWASILGSEEQLFWLEETIKHLTFPKSLGSKDAVIIDSEEVPLNANPTQDVPMPSGTMKSTP
VVPETGDSLPTASTFAAAEDSTSEAGRESFSQRYALVLQGVPYPEPPKEPLVMFTDQEKSLILEATRARRMEL
DLYNGYLADYELSKDEIQKHVSDLPENWRTNWRWSERLYKFFFKTKKEGLEEIFLNKELGNMILARGLAAT
QSQARIKVFNSLVAWLLQGFNVGRSCTAKPLPTLKLDLFKSEFESKPKNNILTEFLVASDEEILFKGLRVLEPG
IEGWYDHPDQAGEIRSVLEGLVQAGRISGYWENQPCGRFVLRGVGERRTELVELLESLVASGEIMQFFESSDE
EGAFIIDNEPSKTAMLKQRFKSCVRTKLVGSFADESLPRGRFTILV

>core/185/4/Org4_Gene405

MVSLLHKFLENASGKKGQSLASTAYLAALDHLLNAFPSIGERIIDELKSQRSHLKMIASENYSSLSVQLAMGN
LLTDKYCEGSPFKRFYSCCENVDAIEWECVETAKELFAADCACVQPHSGADANLLAVMAILTHKVQGPAVS
KLGYKTVNELTEEEYTLLKAEMSSCVCLGPSLNSGGHLTHGNVRLNVMSKLMRCFPYDVNPDTECFDYAEI
SRLAKEYKPKVLIAGYSSYSRRLNFAVLKQIAEDCGSVLWVDMAHFAGLVAGGVFVDEENPIPYADIVTTTT
HKTLRGPRGGLVLATREYESTLNKACPLMMGGPLPHVIAAKTVALKEALSVDFKKYAHQVVNNARRLAER
FLSHGLRLLTGGTDNHMMVIDLGSLGISGKIAEDILSSVGIAVNRNSLPSDAIGKWDTSGIRLGTPALTTLGMG
IDEMEEVADIIVKVLRNIRLSCHVEGSSKKNKGELPEAIAQEARDRVCNLLLRFPLYPEIDLEALV

>core/186/4/Org4_Gene815

MSEQEKLSNYNADKKLFSGIDKLFQIVKGSYGPKQSLSPTSFFKERGFYAISQTELSNSYENLGVDFAKAMVN
KIHKEHSDGATTGLILLHAILQESYAALEKGISTHKLIASLKLQGEKLQEALQQQSWPIKDALKVRNIIFSSLH
MPTIADHFYNAFSVVGPEGLISITKERENEKTSMDVFQGFKIPAGYASTYFVSDTASRLTRIAHPLILITDRKIS
MIHSLLPLLQEISEQNQHLIIFCEDIDPDALATLVVNKLQGLLQVTVVTIPQLSTTNQELAEDIALFTGTHICPCQ
EASHVLAPEMVTLGSCLSIEISESQTTLIGGLHIPEVLTLKTRQLAEEIRTTSCLETKKRLIKRTNRLQSSVAILPT
DEDNEPLYTLALKIMESALSRGYVPGGGVALFYASLTLGTPKDDADENSIAISLLQKACCAPLKLLATNADLD
GDAVIAKLSSLGTTSLGISVFSREIEDLIAGGILDSLATTSTILAQALDTAILVLSSKILILENQYEISTL

>core/187/4/Org4_Gene775

MENEILLNIESKEIRYAHLKNGQLFDLIIERKKVRQLKGNIYRGRVTNILRNIQSAFINIDERENGFIHISDILENS
KKFEQMFDMDVDALPEEASEAPLLSSEEAPIEEFLKLDSPVLVQVVKEPIGSKGARLTSNISIPGRYLVLLPNSP
HRGVSRKIEDPHMREQLKQLIRSFEMPQDMGLICRTASTTASTEALINEAHDLLLTWKTILEKFYSTEQPCLLY
SETDILKKAVITCIDKNYKRLLIDDYATYQKCKHMLKKYSPDASIKIEYYRDSIPMFERFNIEKEIDKATRRKI
WLSSGGYLFFDKTEAMHTIDVNSGRSTQLESGVEETLVQINLEAAEEIARQLRLRNVGGLVIIDFIDMKSRKN
QRRVLERLKEHMKYDAARCTILSMSEFGLVEMTRQRNRESLMQTLFTLCPYCSGNAIIKTPESVVIEIERDLK
KVINHKEHSHLCLVVHPEIASYMKQENDDNEMINLAKQLKAKLQINTSDSVHLNHYQFFSLITGESIDL

>core/188/4/Org4_Gene929

MFNLFFFTANKETTASHELIYRKNQSFSLSPVTILCLLAISVLLLLGVVFALVGCHVLAAPLGLLVWGCAASV
CSMMAIVSLMCLYRGGKPLIEPSNEEKIDPTKDPESLKPVPVEGQSLPKERKTVSFKAKIPSIVEDDFKPYVIQS
TFYHQNKVYSKPIAERMQSLEKEITTLIVDFPRALEESSKSSGSLLRGVISEIKNLFLPRFLSRKVKYSLTACLR
RLGSIVEEYASSDLLILLLTKPEFLNMVTQQLIAHLNSLKTEKQKLTSHMQKLVLSINFWFYGWSLEEKCIEKI
VAYDPNLLTDELKAHLEAGNIVQFLLSFQSSEMQREFRALFPSDAQELPSAKDGSNYVPAMNSSEYMYDFKD

LSVLKKSLSERLAFCEKIPSPSSWNFTSSVASHYKDFSLLFTFFSNQQSVILQNPFLLIELLHENPKCQTFLKGLL
EKAMPMSNWAALFRPMLMGMLCSGIARKKELKIIAEHLGVPFKEITQAIASGKILDLLLQHLFDF

>core/189/4/Org4_Gene442

MTLLRNFLTCSALFLALPAAAQVVYLHESDGYNGAINNKSLEPRITCDPEGTSYIFLDDVRISNVKHDQEDAG
VFINRSGNLFFMGNRCNFTFHNLMTEGFGAAISNRVGDTTLTLSNFSYLAFTSAPLLPQGRGAIYSLGSVMIEN
SEEVTFCGNYSLWGGAAIYTPYLLGSKASRPSVNLSGNRYLVFRDNVSQGYGGAISTHNLTLTTRGPSCFENN
HAYHDVNSNGGAIAIAPGGSISISVKSGDLIFKGNTASQDGNTIHNSIHLQSGAQFKNLRAVSESGVYFYDPIS
HSESHKITDLVINAPEGKETYEGTISFSGLCLDDHEVCAENLTSTILQDVTLAGGTLSLSDGVTLQLHSFKQEA
NSTLTMFPGTTLFCSGDARVQNLHILIEDTDNFVPVRIRAEDKDALVSLEKLIVTFEAYWSVYDFPQFKEAFTI
PLLELLGPSFDSLLLGETTLERTQVTTENDAVRGFWSLSWEEYPPSLDKDRRITPTKKTVFLTWNPEITSTP

>core/190/4/Org4_Gene568

MTNVVQETTGGLNSPRTCPPCILVIFGATGDLTARKLLPALYHLTKEGRLSDQFVCVGFARREKSNEQFRQE
MKQAVIQFSPSELDIKVWEDFQQRLFYHRSEFDNNTGYTSLKDSLEDLDKTYGTRGNRLFYLSTPPQYFSGIIE
NLNKHKLFYKNQDQGKPWSRVIIEKPFGRDLDSAKQLQQCINENLNENSVYHIDHYLGKETVQNILTTRFAN
TIFESCWNSQYIDHVQISLSETIGIGSRGNFFEKSGMLRDMVQNHMMQLLCLLTMEPPTTFDADEIRKEKIKIL
QRISPFSEGSSIVRGQYGPGTVQGVSVLGYREEENVDKDSRVETYVALKTVINNPRWLGVPFYLRAGKRLAK
KSTDISIIFKKSPYNLFAAEECSRCPIENDLLIIRIQPDEGVALKFNCKVPGTNNIVRPVKMDFRYDSYFQTTTPE
AYERLLCDCIIGDRTLFTGGDEVMASWKLFTPVLEEWDQDSSPSFPNYPAGSSGPKEADALIERDGRSWRPL

>core/191/4/Org4_Gene735

MSSWLSQASEVLLNQDPYIPDAPSSQESSVPKISYSITVAPQEAQKSLPKFFTQKFQSQCKSEPPITHHKTFIIAT
PRERILRFGSSFESQLHNTSQAQTSSPWNLFSQKNSTEASKALMQELTMPKSPEKTSEKALDKNLSSKQEGSC
KNFDTLHLQQHLKLFGTVDSLYSQSLDSEQQELLQSRREERSETYANQQSSEKKIETKVQIKDLCKDLFSQDQ
DSNQKQKKSPFQQDTSRKNRIAKAAQAVPVIPPPSIGVFTLSYLLTKQGILSDFSSYACHKDSVESTQRELDAL
HEKRIETIKVSIEKEKRERLWGSLSDIIGWLAPFVSIGIGIVAILSGGGIFAFAGFFAGLISLVIKCLEKLKFWDW
LEKHLPIKNEELRRKIITIIQWVVVYLTPVILSICTLKVENLGFSPIIEGAIKGIQPAIESTMAALRCAILFSQAEIYK
LKGKLTKIQLDIELKSFDRDDHYERSQELLDNMESSFEALSRILNYMRELDQVYLHSLRG

>core/192/4/Org4_Gene435

MAVGGVGGSRSLSPIPPNRRNSEDGKVSPKDNLGEHTVSSSDSSLASQGPTIEERKAQLGGTDKIPLPSVKEPG
DSQTSGRSGVLQRIWKGVKGVFKKTPQARPEVSSPRLPSHVQHGQRLPGFEGLRDRIQKRSENPEADLGKFQ
RSASEGDLTTQGRDSVGDAAEDSRSEGGEPSSRSSSFLSGVRGAASTVQGALGDAKEKVSAFGEQAPGAIRS
APGNIRTRFQRSSSEGDLSNVNKAAKHLRKALENLEKVAPEQVSPEVASGVQSLLARMEQLTHQEPPTVEDLI
TFVESNVGSDSVEYASLDLFATPGESGVRGAGDSVEYASIVPQDGSQASAETAEAPETGGVERSAAQGAWK
ALRDFVVSIFQAVASFFRAIASRLSSARRESAVDDLASESNTQWFVEQEGVSNPSAAPSLSFAEESARRAAEM
SNRNAQSLEKLESGNVTDPVIQQGLGLARSFAPEGQ

>core/193/4/Org4_Gene619

MADILVIGANPTGLILANMLIQHGISVKVIDHRASPEDPSFLDCRKLPVILSCSSLELLHNSEMLGDFIQANHKI
FGARYHWKKRTLLFKFSQATDSPVPFSLSTTYQSLEQHLIDEFLKRGGVIDWSTRPVTLVDNSIFIESTKVSQN
FENREIYNPKWIIACEADNNLDIRDLVKSQLRARRINREVIFINCDEGEPFEEDHIHLLPITKNFLNFVFYNPQEK
TKQLCLPQGTHSISPKLKQKLLYTYNLVISDENFHIKTSHHAFPPEHGNVLFLGSLSNTLLLSYLNGINTNIHAA
FNLAWKLLPVLKKAALKHLVITKEQEDGNILPYISPTTEKRAKKLPFSRFYTPALMYYFLKGCRKFNTTGEEY
YYPPHQALKYRSSDIIKMSPQDKEIHGPGPGMRAIDARLENGSFLLDPLKSSKHLLIFFKDIPDLKEALQEEYG
EWIEICNVKEPRILKLYHANPNSLFIIRPDRYIGYRTHTFKLHELISYLLRIFASEKTS

>core/194/4/Org4_Gene375

MNWENVRVRVAPSPTGDPHVGTAYMALFNEIFAKRFKGKMILRIEDTDRTRSRRDYEENIFSALRWCGIQW
DEGPDVGGPYGPYRQSERTKIYQGYVETLLKTDCAYKCFATPQELAEMRAVASTLGYRGGYDRRYRYLSPE
EVASREAAGQPYTIRLKVPLSGECVFEDYSKGRVVFPWADVDDQVLVKSDGFPTYHFANVIDDHLMGITHV
LRGEEWLSSTPKHLLLYEAFGWEPPVFLHMPLLLNPDGTKLSKRKNPTSIFYYRDSGYVKEAFVNFLTLMGY
SMEGDEEVYSLERIIETFNPRRIGKSGAVFDIQKLDWMNKHYLNHEGSPERLLKELQGWLLNDEFFLKILPLC
QSRITTLAEFINLTSFFFSGLLEYRVEELLPQVLSPEKAAILLYSYVKYLEKTDQWTKETCYLGSKWLAQAFN
VHHKKAIIPLLYVAITGKKQGLPLFDSIEILGKPRARARLVYAEKLLGGVPKKLAAAVDKFMQREDFEEATFD
L

>core/195/4/Org4_Gene285

MDCRGDIPLPEPQVIGGYHVKKILSKKLRSRVVHGLHPETRHSTVIKVFSPSPSFTSRSVHNFLKEAQSLHQIT
HPNIVKFHRYGKWQDCLYIAMEYIEGISLREYILAQFISLPQAIDIIFDIAQALEHLHSRNILHKDIKPENILITPQ
GKIKLIDFGLADWDTEIQRAHPSVIGTPYYMSPEQRQGESHSPASDIYALGLLAYELILGHLSLGRVFLSLVPE
RISKILAKALQPSPNNRYSSTREFIQDIHHYRMSGDMQEDLRIKDHTVALYEQLQTQRFWLAPETLRFPDFISG
VLYHQGYPLYPHAYYDTLLEGDVFNLWLGYSPISNATIALSVVKSLVCQQDLQRPLLDRVCEINECLIRMKIP
IDEMGISILCLEISKENKELSWIACGKTVFWIKRQGRVVQDFESFSPGLGKITSLQIRETKVAWEIGDEAVVRTL
ELEESVASLKTLSLAELQDRRQKAIFCPIESIHGGIQSRQHGSNSPSTLISLKRIR

>core/196/4/Org4_Gene462

MLKKFINSLWKLCQQDKYQRFTPIVDAIDTFCYEPIETPSKPPFIRDSVDVKRWMMLVVIALFPATFVAIWNS
GLQSIVYSSGNPVLMEQFLHISGFGSYLSFVYKEIHIVPILWEGLKIFIPLLTISYVVGGTCEVLFAVVRGHKIAE
GLLVTGILYPLTLPPTIPYWMAALGIAFGIVVSKELFGGTGMNILNPALSGRAFLFFTFPAKMSGDVWVGSNP
GVIKDSLIKMNSSTGKVLIDGFSQSTCLQTLNSTPPSVKRLHVDAIAANMLHIPHVPTQDVIHSQFSLWTETHP
GWVLDNLTLTQLQTFVTAPVAEGGLGLLPTQFDSAYAITDVIYGIGKFSAGNLFWGNIIGSLGETSTFACLLG
AIFLIVTGIASWRTMAAFGIGAFLTGWLFKFISVLIVGQNGAWAPARFFIPAYRQLFLGGLAFGLVFMATDPVS
SPTMKLGKWIYGFFIGFMTIVIRLINPAYPEGVMLAILLGNVFAPLIDYFAVRKYRKRRV

>core/197/4/Org4_Gene560

MKFEFSVALKYLIPGKGRLSSAIVSLFSVGIISLVVWLSIVFISVIHGLEQRWIEDLSQLHSPITILPSDTYYSSYY
YQIDKHSNLSNYTTKTLGEKIASPQVDPYDPESDYLLPETFPLKDCDLGGQQKDPVKMTLESLGPYLQSQHG
KVIEFEQGVGYLDIKTSLKLQKPQPRNLTHFLTYPSKLSYEDKVLPYDETDYTSAELNPFNRSPSGWQQDFHH
LEELYRGASIILPSTYKDSGYKVGDTGVFSTYSIENQKETQYTVHVIGFYNPGLSPLGGRTVFIDPDLARSIRSQ

SEGLGMSNGFHLFFPNTKRIVFVKKQIENILTSLGIDDYWEISSLYDYDYFQPILDQLQSDQVLFLFVCILILIVA
CSNIVTMSMLLVNNKKKEIGILKAMGTSSRSLKIIFACCGAFSGACGVVIGTIFAIITLKNLQFIVKALNYLQGR
ETFNTAFFGQNLPNSVHPQAIYFLGLGTLFLAAVSGALPARKVAKMHVSEILKAD

>core/198/4/Org4_Gene481

MVLFHAQASGRNRVKADAIVLPFWHFKDAKNAASFEAEFEPSYLPALENFQGKTGEIELLYSSPKAKEKRIV
LLGLGKNEELTSDVVFQTYAKLTRVLRKAKCSTVNIILPTISELRLSAEEFLVGLSSGIFSLNYDYPRYNKVDR
NLETPLSKVTVIGIVPKMADAIFRKEEAIFEGVYLTRDLVNRNADEITPKKLAEVALNLGKEFPSIDTKVLGKD
AIAKEKMGLLLAVSKGSCVDPQFIVVRYQGRPKSKDHTVLIGKGVTFDSGGLDLKPGKSMLTMKEDMAGG
ATVLGILSALAVLELPINVTGIIPATENAIDGASYKMGDVYVGMSGLSVEICSTDAEGRLILADAITYALKYCK
PTRIIDFATLTGAMVVSLGEEVAGFFSNNDVLAEDLLEASAETSEPLWRLPLVKKYDKTLHSDIADMKNLGS
NRAGAITAALFLQRFLEESSVAWAHLDIAGTAYHEKEEDHYPKYASGFGVRSILYYLEKSLSK

>core/199/4/Org4_Gene736

MTVSYQSISTPPPEGEFDIFVDGNATEEAVVAAEVQVALPAGEQYAMLRATSELCFGILTQSECALTQALPPK
EKPLQEEQFLVKNGILMRSTSLPNLKPGQSQQTSLASHRNPLAQQSTSSNSTGKASTETTSSSFPFFSCKAPEG
DSSVDKTFTVSVQTPKAQEQQEASASQSQAQFHVRSYSSSTIKEHSAKEKVSQSTKSAETQKHTQTKSDATLS
PMSLYSTLHKEAPQALSSTKSQQKDEEHRDQGQQEGYEQEQEQEEGKKKTPWCTVESLQQTSSSNQVYESY
TPIIPDPIVEFALSESQLSVLAGKRVTNLDVLRICTELMKLMLKSRANDTMTRLEERELMEREAHELAASYSR
QAKYARWLGIATATLGILGAIAPMVGEISGDSILGFVQRISGRFKDATAKTFFKGIGKVFTSLSQLTEAASKVH
ELSESAVRAVAEYRKEVFRMRQDEVTRTIEEVKDNWKSMDNFLLNILQTEHDAARSLYQ

>core/200/4/Org4_Gene870

MAASILSQELLDILPYTFLKKHCLLPIEESSEAITIAHATATSVIAQDEVKLLIKKPVRFVLKEESEILQRLQQLY
SNREGNVSDMLLTMKEEDGTTISEEEDLLETTDTIPVVRLLNLILKEAIEERASDIHFEPCEDSMRIRYRIDGVL
HDRHSPPSHLRSALTTRLKVLAKMDIAEHRLPQDGRIKIHIGGQEVDMRVSTVPVIYGERVVLRILDKRNVIL
DIAGLHMPKDTEILFKDTITAPEGILLVTGPTGSGKTTTLYSVLQELKGPLTNIMTIEDPPEYKLPGIAQIAVKP
KIGLTFARGLRHLLRQDPDILMVGEIRDQETAEIAIQAALTGHLVVSTLHTNDAISAIPRLLDMGIESYLLSATL
VGVVAQRLVRTICPYCKVAYTPENQEKSFLASLGKDTEMPLYRGQGCVHCFRSGYKGRQGIYEFLRPNTLFR
SEVASNRPYHILRETAEQNGFLPILEHGIALAVSGETTLAEVLRVTKRCD

>core/201/4/Org4_Gene50

MNKKHASFSSRLGFIFSMIGIAVGAGNIWRFPRVAAQNGGGAFLILWLCFLFLWSIPLIIIELSIGKLTKKAPIGA
LIKTAGKKFAWAGGFITLVTTCILAYYSTIVGWGLSYFYYAVSGKIHLGNDFAKLWTSHYQSSIPLWAHLTSL
GLAYLVIRKGIVHGIEKCNKILIPAFFLCTIALLLRAVTLPGAVQGIKQLFSCDKSCFSNYKVWIEALTQNAWD
TGAGWGLLLVYAGFASKKTGVVSNGALTAICNNLVSLIMGIIIFSTCASLDILGTTQLQDGAGASSIGITFIYLP
ELFTRLPGGIYLTTLFSSIFFLAFSMAALSSMISMLFLLSQTLAEFGIKPYISETLATIIAFVLGIPSALSLTFFSNQ
DTVWGVALIVNGLIFIYAALVYGFPKLKKEVINAAPGDLRLNKAFDYIIKYLLPIEGILLLGWYFYEGLFPENG
QWWNPISLYSLGSLVLQWSLGLIILWKFNKQLYLRFSRYNHEIL

>core/203/4/Org4_Gene351

MLKYILKRLVLIPLTLFAIVSINFVILNAAPGDVLEEKSRDALGEAGKSDKMRSYKGPDRYLQFREHYGLTLPI
FFNTRPKITHKKIQTALQELANANNTTPSAKNAAKSLVYWGDCAKFVMPALLFEADDASRDDKYRHIAADL
FIRGGVLQGFVGPNLSPEQRAQNKEIAESNAFLVRQLNEEDLDTKVEALKGWFQDHGGTEVFCYSPKQFWK
TFFLETRFARYMSRVLRLDFGTLRNDAHKTVISEVIKRLRCSLVLSILPMIVGFVLCQIFGMIMALKRNHWIDH
SLNFIFLILFSIPVFVAVPWILDNFVINKTIPFTTIPMPYSGLRSPPEVFNELSTLGRIFDLVSHGFLPFCAVSYGAL
AAQSRLSRSIFLEVLSQDFICAAKARGLRWFDILYKHVGKNAAVSIVTSLASSLGTLLGGALVVETLFNIDGFG
NFFYQAILNRDHNVVLFSVLVGSALSLVGYLLGDICYVLLDPRVQLEGRRI

>core/204/4/Org4_Gene904

MHTEFAPFLEDLVHQQVISLLDIAFASKHISSDFEESFVFLAVSSALWRYGHPFLSLEENRIRPSLGGISETDLY
RGFHNLPKEVRDKLFVVVSGRLYLRSLYTIRSKLLDKLSLLCSATPNYFPPSIDSSILSEEQNFIFNKITQGCFSI
VSGGPGTGKTFLAAQLILSLVKQQPKLRIAIVSPTGKATSHIRQILMKYNIFDDMVLMQTVHHFLQEYAYRRY
NSIDVLLVDEGSMVTFDLLYSLVQTLQGYEKDKKLYTSSLIILGDTNQLPPIGIGVGNPLQDLIGYFHENTFFL
KTSHRAKTGAVDQLTQSVLRGEMIPFSPLPSISSAIEVLKDRFVKSLRQSEARLCVLTPMRHGPWGVLNLNTM
IHQRLARSDPDLRIPIMVTSRYETWGLFNGDTGLLCLKTQKLHFPQHEPIDSRALSQYVYNYVMSVHKSQGSE
YDEVIVIIPKGSEVFGVSILYTAITRAKYRVSVWGDPETLHKIIKKSNY

>core/205/4/Org4_Gene761

MNLDSKHFDINSANFLEEFAKFISFPSISADSDHLQDCENCAHFLVDHVNKIFDVELWETPGHPPIIYASYKSE
DPLSPTLMLYNHYDVQPAQLSDGWKGDPFILREENGNLYARGASDNKGQCFYTLKALQHYYESRGNFPLNII
WLIEGEEESGSLALFTWLEKKKEALHADYLLIVDGGFLSEKHPYVSIGARGIVSMKISLEEGNKDMHSGVLG
GIAYNTNRALSEILSSLHHPDNSIAIEGFYDDLALPSDSDRPDLPKSDTLRECEENLGFRPQGYEASYSPEESAL
RPTVEINGISGGYTGPGFKTVIPYRATAYLSCRLVPNQDPDKAAHQVIHHLKQQVPSSLKFSYEILPGGSRGW
RSSANLPIVKVLQEIYSDLYNEECLRLMMPATIPIGPLLGEAAQTSPIICGTSYLSDDIHAAEEHFSIDQLKKGFL
SICQLLDKLPKIKE

>core/206/4/Org4_Gene249

MSISSSSGPDNQKNIMSQVLTSTPQGVPQQDKLSGNETKQIQQTRQGKNTEMESDATIAGASGKDKTSSTTKT
ETAPQQGVAAGKESSESQKAGADTGVSGAAATTASNTATKIAMQTSIEEASKSMESTLESLQSLSAAQMKEV
EAVVVAALSGKSSGSAKLETPELPKPGVTPRSEVIEIGLALAKAIQTLGEATKSALSNYASTQAQADQTNKLG
LEKQAIKIDKEREEYQEMKAAEQKSKDLEGTMDTVNTVMIAVSVAITVISIVAAIFTCGAGLAGLAAGAAVG
AAAAGGAAGAAAATTVATQITVQAVVQAVKQAVITAVRQAITAAIKAAVKSGIKAFIKTLVKAIAKAISKGI
SKVFAKGTQMIAKNFPKLSKVISSLTSKWVTVGVGVVVAAPALGKGIMQMQLSEMQQNVAQFQKEVGKLQ
AAADMISMFTQFWQQASKIASKQTGESNEMTQKATKLGAQILKAYAAISGAIAGAA

>core/207/4/Org4_Gene702

MYRYSALELAKAVTLGELTATGVTQHFFHRIEEAEGQVGAFISLCKEQALEQAELIDKKRSRGEPLGKLAGV
PVGIKDNIHVTGLKTTCASRMLENYQPPFDATVVERIKKEDGIILGKLNMDEFAMGSTTLYSAFHPTHNPWD
LSRVPGGSSGGSAAAVSARFCPVALGSDTGGSIRQPAAFCGVVGFKPSYGAVSRYGLVAFASSLDQIGPLANT
VEDVALMMDVFSGRDPKDATSREFFRDSFMSKLSTEVPKVIGVPRTFLEGLRDDIRENFFSSLAIFEGEGTHLV
DVELDILSHAVSIYYILASAEAATNLARFDGVRYGYRSPQAHTISQLYDLSRGEGFGKEVMRRILLGNYVLSA

ERQNVYYKKATAVRAKIVKAFRTAFEKCEILAMPVCSSPAFEIGEILDPVTLYLQDIYTVAMNLAYLPAIAVP
SGFSKEGLPLGLQIIGQQGQDQQVCQVGYSFQEHAQIKQLFSKRYAKSVVLGGQS

>core/208/4/Org4_Gene991

MQRDGRFKWLQDEDTSAHAEQRFRDINGCWEDLKQTIFWVGEHDCMDIETVRKSYMMWLDRYADKFILR
EKEEKMKRHELFHATMVRKASGHAYAKAKAAFEKERSNENQRKVKDIEKWLSKGLAEFRNQESRRARERL
RELQTLYPGVSVEERVLERQRTKKVNLENLYADIEKKYHHCVREQEHYWKEVENKEAEFRENREKVLSTEE
VLECLQRLEDCLETWSKQLTKAEGSVFEMMFDATEELGNKVLSDVTNRLEILCEDAEEMIFRIEEIEMTLRM
VELPLLFMKNTFEKASLQYNSCKEMLAKAEPHCKESAIYRSSEERLERLNRDLQTAYTNCHERLQGFSKLESE
VRTCKDHLREQMKHFEVQGLNFINEELLWVGAELFTQARLDLVATVPYMEFYLQYHNIKREKVRSQWMAK
TERYREIRQAFQGVMKEDLLAEDTILKEEDYWLLRDDWLLRDERKNRQRRLICNKIAAAQQRVKGF

>core/209/4/Org4_Gene637

MLGILLIASGIIFLAVAIPGLSSAVALGLGCGMTALGTVLLITGLVSLIRSEQLALEQVEIKQARTRVNNELDQL
SQYVFYTENVLDNLKRWSYRDLGFVRQAQEEVTNLEQDIEEIFLTLRDIRNALDNEEFFMTHAKQCLAQVGE
SLFQDASIDEFINLAHLSEIRQHLDINDPRWSMITKKVKGTVVRFIYVSTMYKQIKSNFEKSDFGQLRKMLLN
NYKTIEEVLYQSFQKGYNRAALLSEKTRIIHTSSLLHWEKDEDKHLNIKNECASRLENFKKFRTLFLGLSEED
VIDFTGASGWDCSKLPRKELPLDGGKKKLRFKRTFADEQVGDWDRTTSLEHMTPQEEDPLDKLMDQVEQE
ATSVLKDQDRYWKEIETSEAKFRSLPQEDDFEKQSQIDSYIRDLDDHLSVWANQLSAAEDALIEVTDVQEHG
NREMLKNIQQGLELIEDAVKATLPRVDFIQELLEKEELPLVAARMSLENSYLKISS

>core/210/4/Org4_Gene705

MSFPCGNCDCYYRETPSLNPGGEDIPLQEGGTPNPQMGHAITNQPGHAAGISFGSDNLLGTVEQAGKMLDNL
LSSVRMQRLGHYCYRTGTPWCRTHCPGFLQWIWGGCCACCLEAVDDPENPSEICLRDLVDRFGPICVGMAF
QQLPRCTSKVEQGDPLDDEDQRNLETMCQEQLKLLKAAQPRCMGLSLIKILKNTDLGEGMRSESPSSLLLQA
VSEGSRPYITSIESVPTCWILQPDQHPSPPPPPTDEEQLQGAVGGAPATQPKKNPAQECRVTCKLDFKVLLQKL
SKLDVYCLSTGYQGPLGRAARPVLDTVKRSLKRLVVREPMIFQAPGIGLSLESKIFELMILLCLLSQGFLPLDP
LNPEQPILDPRAQGPWQDILKEMLFTTMAGENIWRHTHGEPPRHPTPPSGNEIEIDGGGPQAAASGTGRGATR
RRDPTDDENTGRAGNLGMPLSDGDVDDDDEDPWSPDNQRALLSTAFRVCNIFNLG

>core/211/4/Org4_Gene946

MHMSNPISLFSPAELIAKYNLIPKTSPIYPRRTELIILEENACQTRLTNVAQILHPSSLFSMSKKILNPCGCSGGPL
CWVILNILAFIITSVLFIILLPVNLIVAGLRLFMPLPPKKIVEDLSEPTTEETNEVIQPFIFALQALLFEDNKLRSFK
IVEQSVSKAPLPNPFLNRLVATSPQESQEAMRKIPDLCSQLKKVLKSLGVLTPEWKHMLKYFEGLKNEHDSN
PDKKTFPILIKLLIEALTGKSSFFSNLPKTPSTKEKMQAALFIASSCKTCKPTWGEVITRSLNRLYSIANEGDNQ
LLIWVQEFKERELMSIQDGDDAEEYRFAAQQHGERYTEAIEQVLRNESAAKLQWHVINTMKFFHGKNLGLV
TEHLQDTLGALTLRQTTVDTHQGREDADLSAALFLNKYLNSGNQLVNSVFKSMQKADPETKALIREFALDIL
YASLRLPQTSAHTEVFSTLLMDPETYEPNKACIAYLLYVLKIIEL

>core/212/4/Org4_Gene701

MSAVYADWESVIGLEVHVELNTASKLFSSALNRFGDEPNTNISTVCTGLPGSLPVLNQSAVEKAVLFGCAVE
GEISLLSRFDRKSYFYPDSPRNFQITQFEHPIIRGGRIKAIVQGEERYFELAQTHIEDDAGMLKHFGEFAGVDYN
RAGVPLIEIVSKPCMFCPEDAVAYATSLVSLLDYIGISDCNMEEGSIRFDVNVSVRPKGSPELRNKVEIKNMNS
FAFMAQALEAEKQRQIDEYLNQPNKDPKLVIPAATYRWDPEKKKTVLMRLKESAEDYKYFPEPDLPTLQLTE
SYIEGIRKTLPELPYDKYHRYIQEYGLSEDIASILISDKNIATFFEVACKDCKNFRSLSNWVTVEFGGRCKTLGV
KLPSSGIFPEGVAQLVNAIDQGVITGKIAKEIADLMMESPGKNPEEILKEKPELLPMSDEGELQKIIAEVVLANP
ESIVDYKNGKTKALGFLVGQIMKRTAGKAPPKRVNELLLLELDKG

>core/213/4/Org4_Gene762

MITKQLRSWLAVLVGSSLLALPLSGQAVGKKESRVSELPQDVLLKEISGGFSKVATKATPAVVYIESFPKSQA
VTHPSPGRRGPYENPFDYFNDEFFNRFFGLPSQREKPQSKEAVRGTGFLVSPDGYIVTNNHVVEDTGKIHVTL
HDGQKYPATVIGLDPKTDLAVIKIKSQNLPYLSFGNSDHLKVGDWAIAIGNPFGLQATVTVGVISAKGRNQL
HIADFEDFIQTDAAINPGNSGGPLLNIDGQVIGVNTAIVSGSGGYIGIGFAIPSLMANRIIDQLIRDGQVTRGFLG
VTLQPIDAELAACYKLEKVYGALVTDVVKGSPADKAGLKQEDVIIAYNGKEVDSLSTFRNAVSLMNPDTRIV
LKVVREGKVIEIPVTVSQAPKEDGMSALQRVGIRVQNLTPETAKKLGIAPETKGILIISVEPGSVAASSGIAPGQ
LILAVNRQKVSSIEDLNRTLKDSNNENILLMVSQGDVIRFIALKPEE

>core/214/4/Org4_Gene687

MWNRCQVFSSFFFRYPISSWLIRLRASCECFQQRHPIFLCGLYWLAGITSRGHPECSALILIFLGMFLPRNPKQ
WLPLASAWIISLMLTPAPFLHDGPISGTFVIHHAGGQGTYYGEALCIQTPCGKRAHHLSCQILSESRLELKKVY
ELEGTLHHTSQIVFKSNACYKEIPRSRFYIMKEKCRESSCHFLNHRFPSSEVGPFASSLLLGTPLPQNLRDLFRQ
KGLSHLFAISGWHFSLCATTLWMLCALLPLKIKKILSFIVLTSLACIFPMSPSVWRSWISVTLLCFSWCFSGSCS
GLNRLGAGFILCSIFFSPFSPTFVLSFLATLGILLFFPKIFSFLYTPWTQFLSPFWLYPIRYLAMTLAISLSAQLFIV
LPIMQYFGSLPLEGLLYNLIVPFTILPIIVFLIATIILPCCSPITEALIQGFLSHPWLHNPNILKTLSFAPVPPWMLTL
ASLILFFIGILRTNVSPYASISATSYRFIETL

>core/215/4/Org4_Gene853

MLKIAILGRPNVGKSSLFNRLCKRSLAIVNSQEGTTRDRLYGELHAFGVPAQVIDTGGVDHNSEDYFQKHIYN
QALTGAKEADVLLLVIDIRCGITEEDAHLAKLLLPLKKPLILVANKADSRQEELQIHETYKLGIRDIVVTSTAH
DKHIDTLLQRIKLVANLPEPREEEGLEELSVDEHEESEAALPSNTFPDFSEVFTEGFSPEEPCTIPESPQQAPKTL
KIALIGRPNVGKSSIINGLLNEERCIIDNTPGTTRDNIDILYSHKDRQYLFIDTAGLRKMKSVKNSIEWISSSRTE
KAISRADICLLVIDATQKLSSYEKRILSLISKRKKPHIILINKWDLLEEVRMEHYCKDLRATDPYLGQAKMLCIS
ATTKRNLKKIFSAIDELHHVVSNKVPTPIVNKTLASALHRNHPQVIQGRRLRIYYAIQKTTTPLQFLLFINAKSL
LTKHYEYYLKNTLKSSFNLYGIPFDLEFKEKPKRHN

>core/216/4/Org4_Gene734

MNMPVPSAVPSANITLKEDSSTVSTASGILKTATGEVLVSCTALEGSSSTDALISLALGQIILATQQELLLQSTN
VHQLLFLPPEVVELEIQVVDLLVQLEHTETITSEPQETQTQSRSEQTLPQQSSSKQSALSPRSLKPEISDSKQQQ
ALQTPKDSAVRKHSEAPSPETQARASLSQASSSSQRSLPPQESAPERTLLEQQKASSFSPLSQFSAEKQKEALT
TSKSHELYKERDQDRQQREQHDRKHDQEEDAESKKKKKKRGLGVEAVAEEPGETLDIAALIFSDQMRPPAE
ETSKKETTFKKKLPSPMSVFSRFIPSKNPLSVGSSIHGPIQTPKVENVFLRFMKLMARILGQAEAEANELYMRV

KQRTDDVDTLTVLISKINNEKKDIDWSENEEMKALLNRAKEIGVTIDKEKYTWTEEEKRLLKENVQMRKEN
MEKITQMERTDMQRHLQEISQCHQARSNVLKLLKELMDTFIYNLRP

>core/217/4/Org4_Gene729

MTSRTKSSKNLGTIALAGMVVSSMIGGGIFSLPQNMAATAGAGAVILSWILTGFGMFFIANTFRILSTIRPDLK
EGIYMYSREGFGPYIGFTIGWGYWLCQIFGNVGYAVITMDALNYFFPPYFQGGNTLPAILGGSILIWVFNFIVL
KGIRQASIINVIGTIFKIIPLIIFIILTAFFFKLAVFKTDFWGHAVTKAQPSLGSVSSQLKGTMLVTLWAFIGIEGA
VVMSGRAKNPLSVGKATVLGFLGCLTIYILLSLLPFGSLFQHQLANIPNPSTAGVLDILVGKWGEVLMNVGLII
AVLSSWLSWTIIVAEIPFSAAKNGTFPEIFTIENKEKSPSVSLYITSSVMQLAMLLVYFSSNAWNTMLSITGVM
VLPAYLASAAFLFKLSKSKTYPKKGSIKAPLAMITGILGVVYSLWLIYAGGLKYLFMALVLLALGIPFYIDAG
KKKKNAKTFFAKKEIVGMTFIGLLALTAIFLFSTGRIKI

>core/218/4/Org4_Gene651

MITRTKIICTIGPATNSPEMLAKLLDAGMNVARLNFSHGSHETHGQTIGLLKELREQKRVPLAIMLDTKGPEIR
LGNIPQPISVSQGQKLRLVSSDIDGSAEGGVSLYPKGIFPFVPEGADVLIDDGYIHAVVVSSEADSLELEFMNS
GLLKSHKSLSIRGVDVALPFMTEKDIADLKFGVEQNVDVVAASFVRYGEDIETMRKCLADLGNPKMPIIAKIE
NRLGVENFSKIAKLADGIMIARGDLGIELSVVEVPNLQKMMAKVSRETGRFCVTATQMLESMIRNVLPTRAE
VSDIANAIYDGSSAVMLSGETASGAHPVAAVKIMRSVILETEKNLSYDSFLKLDDSNSALQVSPYLSAIGLAGI
QIAERADAKALIVYTESGSSPMFLSKYRPKFPIIAVTPSTSVYYRLALEWGVYPMLTQESDRAVWRHQACIYG
IEQRILSNYDRILVLSRGACMEETNNLTLTIVNDILTGSEFPET

>core/219/4/Org4_Gene294

MGESVKVFLEEREDYPYGFVTPIESQGLTRGLSEETIEEIAALRNEPQFIIDFRLQAYRHWKQLHEPAWARLH
YGPIAYDDIVYFSSPKQKKPLGRLEDADPEILDTFKKLGIPLDEQKRLLNVENVAVDLVFDSVSIGTTFKEALE
KAGVIFCSLGEAIQEHPNLVKKYLGSVVSHRDNFFAALNAAVFSDGSFVYVPKGVKCPMDISTYFRINNKEA
GQFERTLIVVEDGGYASYLEGCTAPAYSSNQLHAAVVELVAHEHAVIRYSTVQNWYAGDKKTGKGGIYNFV
TKRGLCAGYRSKISWSQVEVGAAITWKYPSCILKGDESVGEFYSVALTSGKMQADTGTKMLHVGKRTTSTV
ISKGISSDESKNTFRSLVSLGKKAEHSSNYTQCDSMLIGKASGAYTDPKIVVENSTSSIEHEATTSKLREDQLLY
LRSRGLSPEEAVSLVIHGFCREIIEQLPLEFAQEASKLLLIKLENSVG

>core/220/4/Org4_Gene465

MDLKELLHGVQAKIYGKVRPLEVRNLTRDSRCVGVGDIFIAHKGQRYDGNDFAADALANGAIAIASSLYNPF
LSVVQIITPNLEELEAELSAKYYEYPSSKLHTIGVTGTNGKTTVTCLIKALLDSYQKPSGLLGTIEHILGEGVIK
DGFTTPTPALLQKYLATMVRQNRDAVVMEVSSIGLASGRVAYTNFDTAVLTNITLDHLDFHGTFEAYVAAK
AKLFSLVPPSGMVVINTDSPYASQCIESAKAPVITYGIESAADYRATDIQLSSSGTKYTLVYGDQKIACSSSFIG
KYNVYNLLAAISTVHASLRCDLEDLLEKIGLCQPPPGRLDPVLMGPCPVYIDYAHTPDALDNVLTGLHELLPE
GGRLIVVFGCGGDRDRSKRKLMAQVVERYGFAVVTSDNPRSEPPEDIVNEICDGFYSKNYFIEIDRKQAITYA
LSIASDRDIVLIAGKGHEAYQIFKHQTVAFDDKQTVCEVLASYV

>core/222/4/Org4_Gene496

MQTNIGLIGLAVMGKNLVLNMIDHGFSVSVYNRTPEKTRDFLKEYPNHRELIGFESLEDFVNSLERPRKIMLM
IQAGKPVDQSIHSLLPFLESGDVIIDGGNSYFKDSERRCKELQEKGILFLGIGISGGEEGARHGPSIMPGGNPEA
WPLVAPIFQSIAAKVQGRPCCSWVGTGGAGHYVKAVHNGIEYGDIQLICEAYGILRDFLKLSPTAVATIFKEW
NTLELESYLIRIASEVLALKDPEGIPVIDTILDVVGQKGTGKWTAIDALNSGVPLSLIIEAVLARFLSSWKEIREQ
AARNYPGTPLIFEMPHDPSVFIQDVFHALYASKIISYAQGFMLLGEASKEYNWGLDLGEIALMWRGGCIIQSA
FLDVIHKGFAANPENTSLIFQEYFRGALRHAEMGWRRTVVTAIGAGLPIPCLAAAITFYDGYRTANSSMSLAQ
GLRDYFGAHTYERNDRPRGEFYHTDWVYTKTTERVK

>core/223/4/Org4_Gene277

MRDVSELFRTHFMHYASYVILERAIPHILDGLKPVQRRLLWTLFLMDDGKMHKVANIAGRTMALHPHGDAP
IVEALVVLANKGYLIDTQGNFGNPLTGDPHAAARYIEARLSPLARETLFNTDLIAFHDSYDGREKEPDILPAKL
PVLLLHGVDGIAVGMTTKIFPHNFAELLKAQIAILNDKKFTVFPDFPSGGLMDPSEYQDGLGSITLRASIDIIND
KTLVVKQICPQSTTETLIRSIENAAKRGTIKIDTIQDFSTDVPHIEIKLPKGSRAKEMLPLLFEHTECQVILYSKPT
VIYENKPVECSISEILKLHTTALQGYLEKELLLLQEQLTLDHHHKTLEYIFIKHKLYDSVREALAINKKISADDL
HQAVLHALEPWLHELATPVTKQDTSQLASLTIKKILCFNEEACTKELLAIEKKQAAIQKDLGRIKEVTVKYLK
GLLERHGHLGERKTQITNFKTAKTSILKQQTLI

>core/224/4/Org4_Gene726

MLCATVSGPSFCEAKQQILKSLHLVDIIELRLDLINELDDQELHTLITTAQNPILTFRQHKEMSTALWIQKLYSL
AKLEPKWMDIDVSLPKTALQTIRKSHPKIKLILSYHTDKNEDLDAIYNEMLATPAEIYKIVLSPENSSEALNYI
KKARLLPKPSTVLCMGTHGLPSRVLSPLISNAMNYAAGISAPQVAPGQPKLEELLSYNYSKLSEKSHIYGLIG
DPVDRSISHLSHNFLLSKLSLNATYIKFPVTIGEVVTFFSAIRDLPFSGLSVTMPLKTAIFDHVDALDASAQLCE
SINTLVFRNQKILGYNTDGEGVAKLLKQKNISVNNKHIAIVGAGGAAKAIAATLAMQGATLHIFNRTLSSAA
ALATCCKGKAYPLGSLENFKTIDIIINCLPPEVTFPWRFPPIVMDINTKPHPSPYLERAQKHGSLIIHGYEMFIEQ
ALLQFALWFPDFLTPESCDSFRNYVKNFMAKV

>core/225/4/Org4_Gene923

MMSRLRFRLAALGIFFILLVPNFVSAKTIVASDNEKVGVLVYDNSVEAFQQILDCIDHANSYVELCPCMAGGR
TLKEMVDHLEARMDLVPELCSYIIIQPTFTNAEDQKLLKALKERHPNRFFYVFTGCPPSTSILAPNVIEMHIKLS
IIDGKYCILGGTNFEEFMCTPGDKVPENVDNPRLFVSGVRRPLAFRDQDIMLRSTAFGLQLREEYHKQFAMW
DYYAHHMWFIDNPEQFAGACPPLTLEQAEETVFPGFDKHEDLVLVDSSKIRIVLGGPHDKQPNPVTQEYLKLI
QGARSSVKLAHMYFIPKDELLNALVDVSHNHGVHLSLITNGCHELSPAITGPYAWGNRINYFALLYGKRYPL
WKKWFCEKLKPYERVSIYEFAIWETQLHKKCMIIDDEIFVIGSYNFGKKSDAFDYESIVVIESPEVAAKANKV
FNKDIGLSIPVSHGDIFSWYFHPVHHTLGHLQLTYMPA

>core/226/4/Org4_Gene784

MRIVQVAVEFTPIVKVGGLGDAVASLSKELAKQNDVEVLLPHYPLIFKFSSSQVLSERSFYYEFLGKQQASAIS
YSYEGLTLTIITLDSQIELFSTTSVYSENNVVRFSAFAAAAAAYLQEADPADIVHLHDWHVGLLAGLLKNPLN
PVHSKIVFTIHNFGYRGYCSTQLLAASQIDDFHLSHYQLFRDPQTSVLMKGALYCSDYITTVSLTYVQEIINDY
SDYELHDAILARNSVFSGIINGIDEDVWNPKTDPALAVQYDASLLSEPDVLFTKKEENRAVLYEKLGISSDYFP
LICVISRIVEEKGPEFMKEIILHAMEHSYAFILIGTSQNEVLLNEFRNLQDCLASSPNIRLILDFNDPLARLTYAA

ADMICIPSHREACGLTQLIAMRYGTVPLVRKTGGLADTVIPGVNGFTFFDTNNFNEFRAMLSNAVTTYRQEP
DVWLNLIESGMLRASGLDAMAKHYVNLYHSLLS

>core/227/4/Org4_Gene792

MAFSHIEGLYFYNTASQKKELFFPNHTPVRLYTCGPTVYDYAHIGNFRTYVFEDILKRTLVFFGYSVTHVMNI
TDVEDKTIAGASKKNIPLQEYTQPYTEAFFEDLDTLNIAKADFYPHATHYIPQMIQAITKLLEQGIAYIGQDAS
VYFSLNRFPNYGKLSHLDLSSLRCCSRISADEYDKENPSDFVLWKAYNPERDGVIYWESPFGKGRPGWHLEC
SIMAMELLGDSLDIHAGGVDNIFPHHENEIAQSEALSGKPFVRYWLHSEHLLIDGKKMSKSLGNFLTLRDLLH
QGFTGQEVRYMLLQSHYRTQLNFTEEALLACRHALRRLKDFVSRLEGVDLPGESPLPRTLDSSSQFIEAFSRA
LANDLNVSTGFASLFDFVHEINTLIDQGHFSKADSLYILDTLKKVDTVLGVLPLTTSVCIPETVMQLVAEREEA
RKTKNWAMADTLRDEILAAGFLVEDSKSGPKVKPL

>core/228/4/Org4_Gene430

MDTIDTPGEQGSQSFGNSLGARFDLPRKEQDPSQALAVASYQKKTDSQVVEEHLDELISLADSCGISVLETRS
WILKTPSASTYINVGKLEEIEEILKEFPSIGTLIIDEEITPSQQRNLEKRLGLVVLDRTELILEIFSSRALTAEANIQ
VQLAQARYLLPRLKRLWGHLSRQKSGGGSGGFVKGEGEKQIELDRRMVRERIHKLSAQLKAVIKQRAERRK
VKSRRGIPTFALIGYTNSGKSTLLNLLTAADTYVEDKLFATLDPKTRKCVLPGGRHVLLTDTVGFIRKLPHTL
VAAFKSTLEAAFHEDVLLHVVDASHPLALEHVQTTYDLFQELKIEKPRIITVLNKVDRLPQGSIPMKLRLLSPL
PVLISAKTGEGIQNLLSLMTEIIQEKSLHVTLNFPYTEYGKFTELCDAGVVASSRYQEDFLVVEAYLPKELQKK
FRPFISYVFPEDCGDDEGRGPVLESSFGD

>core/229/4/Org4_Gene49

MDSRTSHLDDELSFKLEKAFTCLSTDIHSHDLSKIVIEYNPIDLAYAVSCLPSESRAILYKNLSCITAKVAFIINT
DSASRWAIFRRLSDSEVCALIEQMPPDEAVWVLDDIPDRRYRRILELIDSKKALKIRDLQKHGRNTAGRLMTN
EFFAFLMETTVKDVSACIRSNPGIDLTRLVFVLDFKGELQGVVTDRSLIINPPEMSLKQIMNQIEHKVLPDATR
EEVVDLVERYKIAALPVVDEENFLIGAITYEDVVEAIEDIADETIARMAGTTEDVGYQTCHVVQRFLLRAPWL
LVTLFAGLISASVMAYFQKISPALLALIIFFIPLINGMSGNVGVQCSTILVRSMATGTLSFGRRRETIFKEMSIGL
LTGVVLGILCGLVVYLMGFLGLNIFSGGGIQLGVTVATGVLGASLTATTLGVLSPFFFAKLGVDPALASGPIV
TALNDIMSMIIFFLIAGGINFLFFN

>core/230/4/Org4_Gene244

MNKKKRFLSLLFLTAVLLGIWFSPHPASINSNAWQLFAIFTTTIMGIIFQPVPMGAIAIIGISTLLLTQTLTLEQG
LSGFHNPIAWLVFLSFSIAKGIIKTGLGERIAYFFVSALGKSPLGLSYGLVITDFFLAPAIPSVTARAGGILYPVV
TSLSDSFGSSAEKGTQDLIGSFLIKVAYQSSVITSAMFLTAMAGNPLVAALAGHVGVSLSWVLWAKAAIIPGL
LSLFLMPIILYKLYPPKITSCEEAIRSAKLRLKEMGPLKKEEKTILMIFFLLVVLWTFGDLLGISATTAALIGLSL
LILTNILDWQKDVIANTTAWETFIWFGALIMMASFLNQLGFIPLVGDSAAALVSGLSWKIGFPLLFLIYFYSHY
LFASNTAHIGAMYPIFLAVSISLGTNPIFAALTLAFASNLFGGLTHYGSGPAPLYFGSHLVTVQEWWRSGFALS
IVNIVIWIGIGSLWWKALGLI

>core/231/4/Org4_Gene559

MTTKSLGSFNSVISKNKIHFISLGCSRNLVDSEVMLGILLKAGYESTNEIEDADYLILNTCAFLKSARDEAKDY
LDHLIDVKKENAKIIVTGCMTSNHKDELKPWMSHIHYLLGSGDVENILSAIESRESGEKISAKSYIEMGEVPRQ
LSTPKHYAYLKVAEGCRKRCAFCIIPSIKGKLRSKPLDQILKEFRILVNNGVKEIILIAQDLGDYGKDLSTDRSS
QLESLLHELLKEPGDYWLRMLYLYPDEVSDGIIDLMRSNPKLLPYVDIPLQHINNRILKQMRRTTSREQILGFL
EKLRAKVPQVYIRSSVIVGFPGETQEEFQELADFIGEGWIDNLGIFLYSQEANTPAAELPDQIPEKVKESRLKIL
SQIQKRNVDKHNQKLIGEKIEAVIDNYHPETNLLLTARFYGQAPEVDPCIIVNEAKLVSHFGERCFIEITGAAG
YDLVGRVVKKSQNQALLKASKA

>core/232/4/Org4_Gene540

MHSHSKPTKPLGTFTVGMLSLAVVISLRNLPLTAKHGLSTLFFYGLAVICFMIPYALISAELASFKPQGIYIWA
RDALGKWWGFFAIWMQWFHNMTWYPAMLAFIASTIVYKINPELAHNKVYIATVILAGFWILTFFNFLGITSS
ALFSSICVIIGTLIPGVILVSLALFWIFSGNPIAISLSWGNLLPDFSNVSSLVLLAGMLLALCGLEANANLASDM
VNPRKNYPKAVFIGAIATLTILVLGSLSIAIVIPKEEISLVSGLVKTFTLFFDKYNLSWMTGIVVVMTIAGSLGE
LNAWMFAGTKGLFISTQNDCLPRLFKKVNSKNVPTNLMLFQGIVVTIFTLLFLCLDSADLVYWILTALSVQM
YLAMYICLFLAGPILRIKEPRAQRLYSVPGKFLGICTMSILGILSCAFALWVSFLPPRELAQISEGSKIGYTTFLL
LAFSLNCLIPFGIYFTHKRLSKKS

>core/233/4/Org4_Gene337

MDKSTAVPLPSPPHSKESEMIVLGCMLTGVHYLNLAANQLYEEDFYYLEHKIIFRVLQDAFKQDKPIDVHLA
GEELKRRNQITVIGGPSYLITLAEFAGTAAYLEEYVDIIRSKSILRKMISTAKEIEKRALEQPKNVAEALDEAQN
SFFKISQSTSVSQYTLVADKLRGLTTTTDKPYLVQLQERQELFLQNAQGDNKSFFTGIPTHFIDLDQLIHGFSPS
NLMILAARPAMGKTALALNIAENLCFQNRLPIGIFSLEMTVDQLIHRMICSRSEVDSKKISIGDLSGHDFQRIVS
VINEMQEHTLLIDDQPGLKVSDLRARARRMKESYDIQFLIIDYLQLLSGSGTLRATESRQTEISEISRMLKTLAR
ELNIPILCLSQLSRKVEDRANHRPMMSDLRESGSIEQDSDLVMFLLRREYYDPNDKPGTAELIIAKNRHGSIGS
VPLVFEKELARFRNYSAFECIS

>core/234/4/Org4_Gene160

MKITVNRGLDLSLQGSPKESGFYNKIDPEFVSIDLRPFQPLSLKLKVEQGDAVCSGAPIAEYKHFPNTYITSHV
SGVVTAIRRGNKRSLLDVIIKKTPGPTSTEYTYDLQTLSRSDLSEIFKENGLFALIKQRPFDIPAIPTQTPRDVFIN
LADNRPFTPSPEKHLALFSSREEGFYVFVVGVRAIAKLFGLRPHIVFRDRLTLPTQELKTIAHLHTVSGPFPSGS
PSIHIHSVAPITNEKEVVFTLSFQDVLTIGHLFLKGRILHEQVTALAGTALKSSLRRYVITTKGASFSSLINLNDI
SDNDTLISGDPLTGRLCKKEEEPFLGFRDHSISVLHNPTKRELFSFLRIGFNKPTFTKTYLSGFFKKKRTYTNPD
TNLHGETRPIIDTDIYDKVMPMRIPVVPLIKAVITKNFDLANELGFLEVCGEDFALPTLIDPSKTEMLTIVKESLI
EYAKESGILTPHQD

>core/236/4/Org4_Gene342

MKEERSSEILPKVKETKKHAYVSMQEKSCVGECAVVASESEEAESVTVTKIAKLQRMGIEELNILARQYGVK
NIGSLTKSQVVFEIVKAKSERPDELLIGEGVLEVLPDGFGFLRSPTYNYLPSAEDIYVSPAQIRRFDLKKGDTIIG
TIRSPKEKEKYFALLKVDKINGSTPDKAKERVLFENLTPLYPNQRIVMEMGKDHLAERVLDLTAPIGKGQRG
LIVAPPRSGKTVILQSIAHAIAVNNPDIVLIVLLIDERPEEVTDMIRQVRGEVVASTFDEQPERHIQVAEMVIEK
ARRLVEHGNDVVILLDSITRLARAYNTVQPHSGKILTGGVDASALHKPKRFFGAARNIEGGGSLTILATALIDT

GSRMDEVIFEEFKGTGNMELVLDRRLSDRRTYPAIDLIKSGTRKEELLYHPSELERVYLFRQAIADLTAIDAM
HLLLGRLKKTNSNAEFLLSLKE

>core/237/4/Org4_Gene896

MNFKLPVYHIGLTKAENNTIKIAILQKTCKGWIVCHCEQIPEGKTWSLPKKYFAAPTTFSLQGSDILVKSSSSS
LKNRKNILKVALTNLEASLALPWESLIVQPQLGKPTDRGETPLTLWIAQKNTLKKELSFLSQAQIFPDKLSCRA
ADIFFLAEQSPLKSLPAYLLIYGGSEEVTCIFVKNHAIAVARSFSNYSTKKSCDDIHATLQYIQETFPQTVLPAIH
VAQISPNLQKILEQKLSLPLVVCQSMTYGVEDEDWEIYGDTIAAAHHGASRRPLTFPYDATSVSPAAQKHWL
LRSSLLIGKYALMATVVVSLGSVLKLKSLSSSASNHFALACPEEGVLPRSLKAAEKTVKAIGMKNSASNYPLL
PTIPTSEQTLKFLLALGKSSPSIKFSYFSYTMTSYPSKDNPSLPYSALVEVKGQGQPEDIPQFLKKISSHPKLQHV
SESLEDQRSFKLQFTLSS

>core/238/4/Org4_Gene186

MTESVYSPSAMHVNSLADKLKAINQEHILDIWPSLSPKQQQRLFQQLTSVDIDFFRKQQQLLSSPTAILKDFHP
ITSFASSGEDPERAHAGTTLLKEKKVACVVLAGGQGSRLKCDGPKGLFPVSPIKKKPLFQLVAEKVRAASKL
AGQPLPLAFMTSPLNTRQTRSFFESNDYFHLDPNQVDFFCQPLWPLLTLSGDLFLEDMDTLALGPNGNGCIAT
LLYTSGVWEKWKNAGIEMVSVIPIDNPLALPFDVELCGFHAMSNNEVTIKAALRQTAIEDVGILVKSHDSGK
TSVIEYSEIPQNERFALNEDGKLKYCLANIGLYCLSMDFIRHAAYQQLPLYKVHKHAKQLGHTSLNEKNAWK
FEEFIFDLFCYSDHCQTLVYPRQECFAPLKNLEGNHSPDTVRQALSDRERQLFHKVTGKKLSPNTTFELEADF
YYPSTSTSLHWENKAFFEEPFFEAS

>core/239/4/Org4_Gene950

MTQEFDCVVIGAGPGGYVAAITAAQSKLRTALIEEDQAGGTCLNRGCIPSKALIAGANVVSHIKHAERFGIHV
DGYTIDYPAMAKRKNTVVQGIRQGLEGLIRSNKITVLKGTGSLISSTEVKVIGQDTTIIKANHIILATGSEPRPFP
GVPFSSRILSSTGILELEVLPKKLAIIGGGVIGCEFASLFHTLGVEITVIEALDHILAVNNKEVSQTVTNKFTKQG
IRILTKASISAIEESQNQVRITVNDQVEEFDYVLVAIGRQFNTASIGLDNAGVIRDDRGVIPVDETMRTNVPNIY
AIGDITGKWLLAHVASHQGVIAAKNISGHHEVMDYSAIPSVIFTHPEIAMVGLSLQEAEQQNLPAKLSKFPFK
AIGKAVALGESDGFAAIVSHEITQQILGAYVIGPHASSLIGEMTLAIRNELTLPCIYETVHAHPTLSEIWAEGAL
LATNHPLHFPPKS

>core/240/4/Org4_Gene674

MMSSKRTSKIAVLSILLTFTHSIGFANANSSVGLGTVYITSEAVKKPQKGSERKQAKKEPRARKGYLVPSSRT
LSARAQKMKNSSRKESSGGCNEISANSTPRSVKLRRNKRAEQKAAKQGFSAFSNLTLKSLLPKLPSKQKTSIH
EREKATSRFVNESQLSSARKRYCTPSSAAPSLFLETEIVRAPVERTKELQDNEIHIPVVQVQTNPKEQNTKTTK
QLASQASIQQSEGTEQSLRELAQGASLPVLVRPNPEVSVQRQKEELLEELVAERRQCKRKSVRQALEARSLTK
KVARDGSVTSTLRYDPEKAAEIKSRRNCKVSPEAREQKYSSCKRDARANGKQDKTTPSEDASQEEQQTGAG
PVRKTPKSQVASNAQNFYRNSKNTNIDSYLTANQYSCSSEETDWPCSSCVSKRRTHNSISVCTMVVTVIAMIV
GALIIANATESQTTPDPTPPTPTP

>core/241/4/Org4_Gene527

MRAWEEFLLLQEKEIGTNTVDKWLRSLKVLCFDACNLYLEAQDSFQITWFEEHIRHKVKSGLVNNNNKPIRV
HVTSVDKAAPFYKEKQMQQEKTAYFTMHYGSVNPEMTFSNFLVTPENDLPFRVLQEFTKSPDENGGVTFNPI
YLFGPEGSGKTHLMQSAISVLRESGGKILYVSSDLFTEHLVSAIRSGEMQKFRSFYRNIDALFIEDIEVFSGKSA
TQEEFFHTFNSLHSEGKLIVVSSSYAPADLVAVEDRLISRFEWGVAIPIHPLVQEGLRSFLMRQVERLSIRIQET
ALDFLIYALSSNVKTLLHALNLLAKRVMYKKLSHQLLYEDDVKTLLKDVLEAAGSVRLTPLKIIRNVAQYYG
VSQESILGRSQSREYVLPRQVAMYFCRQKLSLSYVRIGDVFSRDHSTVISSIRLIEQKIEENSHDIHMAIQDISKN
LNSLHKSLEFFPSEEMII

>core/242/4/Org4_Gene740

MRQEKDSLGIVEVPEDKLYGAQTMRSRNFFSWGPELMPYEVIRALVWIKKCAAKANQDLGFLDSKHCDMIV
AAADEILEGGFEEHFPLKVWQTGSGTQSNMNVNEVIANLAIRHHGGVLGSKDPIHPNDHVNKSQSSNDVFPT
AMHIAAVISLKNKLIPALDHMIRVLDAKVEEFRHDVKIGRTHLMDAVPMTLGQEFSGYSSQLRHCLESIAFSL
AHLYELAIGATAVGTGLNVPEGFVEKIIHYLRKETDEPFIPASNYFSALSCHDALVDAHGSLATLACALTKIAT
DLSFLGSGPRCGLGELFFPENEPGSSIMPGKVNPTQCEALQMVCAQVLGNNQTVIIGGSRGNFELNVMKPVII
YNFLQSVDLLSEGMRAFSEFFVKGLKVNKARLQDNINNSLMLVTALAPVLGYDKCSKAALKAFHESISLKEA
CLALGYLSEKEFDRLVVPENMVGNH

>core/243/4/Org4_Gene423

MNFSLFLFFLIAIQGICLYVGRRGSKKVEDRESYFLAGRSLKIFPLMMTFIATQIGGGVLLGAAEEAFCYGYGV
ILYPLGVALGLIFLGMGPGKRLAEGSLTTVVSIFEVFYGSKKLRKIAFLLSAGSLFFILVAQVIALDRLFSSFPFG
KYVTVAFWIVLASYTSTGGFRGVVRTDVIQAGFLLIAVLVCGVSVWLSVPKSLPVLDPFQSLPCAKLSNWIF
MPMLFMLVEQDMVQRCVAASSPKRLQWAAVGAGLVLLLFNFIPLFLGSLGAKAGLKAGCPLIDTIAYFCNPS
LAAVMAAAIGVAILSTADSLMNAVAQLIAEEYPTLKAPYYRYLVLGLAVAAPLVAIGFTNIVDVLILSYSLSV
CCLSVPVGFYLLAPKGRRVSGAAAWAGVLVGALGYGWVQIVSLGMFGELLAWVGSLVAFSFVGFIEITWK
NKVKTQT

>core/244/4/Org4_Gene771

MQQSVRKLFGTDGVRGRANFEPMTVETTVLLGKAVARVLREGRSGKHRVVVGKDTRLSGYMFENALIAGL
NSMGIETLVLGPIPTPGVAFITRAYRADAGIMISASHNPYRDNGIKIFSSEGFKISDVLEQRIETMVSEADFGPLP
EDHAVGKNKRVIDAMGRYIEFVKATFPKGRTLKGLKIVLDCAHGASYKVAPSVFEELDAEVICYGCEPTGINI
NEHCGALFPQVIQKVVIEHQAHLGIALDGDGDRIIMVDEKGHIVDGDMILSICAGDLKKRSALPHNRVVATIM
TNFGVLKYLEGLGLQVFTSPVGDRHVLHAMLEHEVTLGGEQSGHMIFLDYNTTGDGIVSALQVLRIMIESES
TLSDLTAPIVKSPQTLINVAVREKIPLETIPLIERTLRDVQDALGPSGRILLRYSGTENICRVMVEGHKKHQVDC
LAKALADVIDAELGTGSRE

>core/245/4/Org4_Gene368

MQIAQVFGGGRLNGEVKVSGAKNAATKLLVASLLSDQKCTLRNVPDIGDVSLTVELCKSLGAHVSWDKETE
VLEIYTPEIQCTRVPPTFSNVNRIPILLLGALLGRCPEGVYVPTVGGDAIGERTLNFHFEGLKQLGVQISSDSSG
YYAKAPRGLKGNYIHLPYPSVGATENLILAAIHAKGRTVIKNVALEAEILDLVLFLQKAGADITTDNDRTIDIF
GTGGLGSVDHTILPDKIEAASFGMAAVVSGGRVFVRNAKQELMIPFLKMLRSIGGGFLVSESGIEFFQERPLV
GGVVLETDVHPGFLTDWQQPFAVLLSQAQGSSVIHETVHENRLGYLHGLQHMGAECQLFHQCLSTKACRY

AIGNFPHSAVIHGATPLRASHLVIPDLRAGFAYVMAALIAEGGGSIIENTHLLDRGYTNWVGKLRSLGAKIQIF
DMEQEELTTSPKSLALRDASL

>core/246/4/Org4_Gene191

MLYFIEQLNKLSTSFCVFPMILLLGGFLTWKLRGLQFHGLKLGFNLMLQNKLDDSSSKANEVSSYEAVAGIL
AGNFGTGNIAGMAVALACGGPGALVWVWLAALLGAIVQYAGSYLGSKYRKPEGNTGEFIGGPIACLAFGM
RKKILAGFFALFTIMTAFCAGNCVQVSCIVPLCAEGTPGKLLVGILLALVVIPVLAGGNNRILRFSARVIPFIAG
FYCISCGIILFQHASAILPAIKLICSSAFGIKAGLAGIGGYTLSQVISTGINRAVMATDCGSGMVSILQANTKSKN
PVVDGLVTLVPPVIVMVVCSITMLVLIVSGAYSSGAQGTLMVMSAFKNSLGSLGSVIVILAMALFGYTTILTW
FACAEKSLQYMIPGRRANLWLKAIYVLIIPLGGVIDMRMIWALSDTGFSGMVILNCIALIALLKDVLSTNRDV
ALLKERECSVADPVRNLDA

>core/247/4/Org4_Gene821

MFNVNFKFLEGLHQPAPRYTSYPTALEWEPSDAAPALLAFQRIRENPQPLSLYFHIPFCQSMCLYCGCSVVLN
RREDIVEAYINTLIQEMKLVVETIGFRPQVSRIHFGGGTPSRLSRELFTLLFDHIHKLFDLSHAEEIAIEVDPRSL
RNDMEKADFFQNVGFNRVSLGVQDTQADVQEAVRRRQSHEESLKAYEKFKELGFQSINIDLIYGLPKQTKES
FSKTIQDILAMYPDRLALFSFASVPWIKPHQKAMKASDMPSMEEKFAIYSQSRHLLTKAGYQAIGMDHFSLP
HDPLTLAFKNKTLIRNFQGYSLPPEEDLLGLGMTSTSFIRGIYLQNAKTLEEYHNTVLRGTFATVKSKILTEDD
RIRKWAIHKLMCTFTINKEEFYNLFGYEFDTYFIESRDRLISMETTGLIHNSPGSLKVTPLGELFVRVIATAFDH
YFLNKVSKKECFSASI

>core/248/4/Org4_Gene328

MTTLRQFFLITELRQKLFYTFALLAACRVGVFIPVPGINGELAVAYFKQLLGSGQNLFQLADIFSGGAFAQMT
VIALGVVPYISASIIVQLFLVFMPALQREMRESPDQGKRRIGRLTRLFTVALAVIQSLLFAKFALRMNLTIPGIV
LPTLLSSKLFGVPWIFYITTVVVMTTGTLLLMWIGEQISDKGIGNGISLIIALGILSSFPSVLGSIVNKLNLGSQD
SSDLGLISILILALVFVFVLITTILIIEGVRKIPVQYARRVIGRREVPGGGSYLPLKVNYAGVIPVIFASSLLMFPA
TIGQFIASESSWMKRIAALLAPGSLVYSICYVLLIIFFTYFWTATQFHPEQIASEMKKNNAFIPGIRQGKPTQHY
LEYTMNRVTLLGALFLAAIAILPSLLGCLLRVDSNVSYFLGGTAMLIVVGVVLDTMKQVDAFLLMRRYDSVL
KTDRTKGRH

>core/250/4/Org4_Gene139

MNVWTKFFQPPKHIKEIEDQEVVKKKYKYWRIRIFYSMFIGYIFYYFTRKSFTFAMPTLIADLGFDKAQLGIIG
STLYFSYGISKFVSGVMSDQSNPRYFMAIGLMITGLTNIFFGMSSSIVLFALWWGLNGWFQGWGWPPCARLL
THWYAKSERGTWWSVWSTSHNIGGALIPILTGFIIDYSGWRGAMYVPGILCIGMGLVLINRLRDTPQSLGLPPI
EKYKRDPHHAHHEGKSASEGTEEIERELSTREILFTYVLTNQWLWFLAAASFFIYIVRMAVNDWSALFLIETK
HYAAVKANFCVSLFEIGGLFGMLVAGWLSDKISKGNRGPMNVLFSLGLLFAILGMWFSRSHNQWWVDGTL
LFVIGFFLYGPQMMIGLAAAELSHKKAAGTASGFTGWFAYFGATFAGYPLGKVTDVWGWKGFFIALLACAS
IALLLFLPTWNATEKNTRSKA

>core/251/4/Org4_Gene606

MKKVLIANRGEIAVRIIRACHDLGLSTVAVYSLADQEALHVLLADEAVCIGEPQAAKSYLKISNILAACEITGA
DAVHPGYGFLSENANFASICESCGLTFIGPSSESIAMMGDKIAAKSLAKKIKCPVIPGSEGIIEDESEGLKIAEKI
GFPIVIKAVAGGGGRGIRIVKEKDEFYRAFSAARAEAEAGFNNPNVYIEKFIENPRHLEIQVIGDTHGNYVHLG
ERDCTIQRRRQKLIEETPSPILNAEIRAKVGKVAVDLARSAGYFSVGTVEFLLDKDKKFYFMEMNTRIQVEHT
ITEEVTGIDLVKEQIHVAMGNKLPWKQKNIEFSGHIIQCRINAEDPTNNFSPSPGRLDYYLPPAGPSIRVDGAC
YSGYAIPPYYDSMIAKVIAKGKNREEAIAIMKRALKEFHIGGVQSTIPFHQFMLDNPKFLESNYDINYIDNLLA
QGNSFFKEF

>core/252/4/Org4_Gene10

MATKTKTQWTCNQCGATAPKWLGQCPGCHNWNSLVEEYVPQARSGTSSRSSTSAIALSSIELENESRIFIDHA
GWDRILGGGVVRGSLTLLGGDPGIGKSTLLLQTAERLASQKYKVLYVCGEESVTQTSLRAKRLNISSPLIYLF
PETNLDNIKQQIATLEPDILIIDSIQIIFNPTLNSAPGSVAQVREVTYELMQIAKSAQITTFIIGHVTKSGEIAGPRV
LEHLVDTVLYFEGNSHANYRMIRSVKNRFGPTNELLILSMHADGLKEVSNPSGLFLQEKTGPTTGSMIIPIIEG
SGALLIELQALVSSSPFANPVRKTAGFDPNRFSLLLAVLEKRAQVKLFTMDVFLSITGGLKIIEPAADLGALLA
VASSLYNRLLPNNSIVIGEVGLGGEIRHVAHLERRIKEGKLMGFEGAILPEGQISSLPKEIRENFRLQGVKTIKD
AIRLLL

>core/253/4/Org4_Gene831

MLKILKIKVLVFPLALLMGCNSIGYAGPQGSLQTNSQTKVKIGSEVWIEQKLRQYPELLWLTESGGAPLLTST
PIDMAYSEKLFNKKVPALDIAIRSMIHLHLLIQGSRQSYMQLSQILPSEEGGMTFKQFQTAHKQLLFFLNSPKS
FDNTLRILETAIVLRHVGCSAKAVTTFKPYFTDSCPQSFYAKALHVLRTFPELCPSYARLSPEQQEVLLSLRRL
GNYDSLLNLTEVPSAQLLSAWRTRRSLAILDLYLYCLDTCGDKNCSQEFYVNFAPLLSMLQQHATIEEAFSR
YFTYRANRLGFEGTSRTDMTLVRLATLMNLSPSEASTLAWSFKNLPSDEAENLVNSFYTVQGEHIPLTFRGLP
SLVAGLSVATHGSTVSPENRLRQVYSTMLSLLVKSLRSHREMLNKQLLPQGTVLDFSETTLSSGGLDVFAESI
AVRIHLNGAVSINL

>core/254/4/Org4_Gene598

MRKRHSFDSTSTKKEAVSKAIQKIIKIMETTDPSLNVETPNAEIESILQEIKEIKQKLSKQAEDLGLLEKYCSQE
TLSNLENTNASLKLSIGSVIEELASLKQLVEESIEESLGQQDQLIQSVLIEISDKFLSSIGGTLSGNLDMNQNVIQ
GLLIKENPEKSEAASVGYVQTLLEPLSKRIGETHEKVATHDVNISSLQFHMMSVAGGRFRGHIDMNGYRVLG
LGEPKNGEDAVSKDYLERYVSSQLTIDKVEDKPITKPNKGKLLYSQGTSPKLESPLPLGLLTSGISGFTWKSAS
KSNDGSFPFSALRHKETESDTDCFQITSTTLSGNQAGTYTWSLSLKVLVPSIFQIEKPEVQLSLVYSYEDWLPID
NIFNMSQPRTIPLALLGQTMLAGQKYDILELAAHQTNQTLMISPNCSRFSLQLKQTNQFENSPVDFYIVHAAH
SCLWSGF

>core/255/4/Org4_Gene84

MLTCNECTTWEQFLNYVKTRCSKTAFENWISPIQVLEETQEKIRLEVPNIFVQNYLLDNYKRDLCSFVPLDVH
GEPALEFVVAEHKKPSAPVASQKESNEGISEVFEETKDFELKLNLSYRFDNFIEGPSNQFVKSAAVGIAGKPGR
SYNPLFIHGGVGLGKTHLLHAVGHYVREHHKNLRIHCITTEAFINDLVYHLKSKSVDKMKNFYRSLDLLLVD
DIQFLQNRQNFEEEFCNTFETLINLSKQIVITSDKPPSQLKLSERIIARMEWGLVAHVGIPDLETRVAILQHKAE
QKGLLIPNEMAFYIADHIYGNVRQLEGAINKLTAYCRLFGKSLTETTVRETLKELFRSPTKQKISVETILKSVA

TVFQVKLNDLKGNSRSKDLVLARQIAMYLAKTLITDSLVAIGAAFGKTHSTVLYACKTIEHKLQNDETLKRQVNLCKNHIVG

>core/256/4/Org4_Gene778

MILPPYSYSLKIGAAVLFFCSILHTFLTPWLYTLCQSYEHKKLVFPECWKRYVRLSELFRILSRVEIVFFLWAVPLFFWFLYTEGYRISMAYFNSRNYGFAVFIMVILILLESRPIVYFAELVLSSIAKLGKTSPKSWWWTLMIAPPLLSCLLKETGAMIIGATLLMRHFYVFSPSRRFAYATMGLLFSNISIGGLTSYVSSRALFLIFPALKWEHSFFLSHFAWKAIVAILISTTIYYFIFRKEFKKFPDIPSDKDPSVEKVPWWIICVNIIFVGSIILSRSTPLFMGALLLFYLGFQKFTIFYQDPINLPKVCYVGLFYAGLVVFGDLQEWWVLNLMQGLSDFGYMTVSYTLSIFLDNALVNYLVHNLSVATDCYHYLVVAGCMAAGGLTLVSNIPNIVGYLILRSAFPSSTIHMGWLFLGALGPSIISLGVFWLLKNVPEFLYCFFR

>core/257/4/Org4_Gene901

MSEPRFVCLSLGSNLGNRFKNLQIARTLLGEQAVLGLRSSVILETEALLLPGSPPEWDLPYFNSVLVGETTLSLRELLVTIKQIEKVVGRAEESPPWSPRTIDIDILLYGDESFCCDHTEITIPLSNLLSRPFLIALIASLCPYRRFCTQGSPYHNFTFGELAHHLPSPPGMIRRSLSPDTMLMGVVNVTNDSMSDGGMFLDPEKAVAQAEKLFTEGAAVIDFGAQATNPKVKQFLSVDQEWERLEPVLRLLKETWSNRKQYPIISLDTFYPEIILRAMDIYPIQWINDVSGGSQSMAEVARDCELSLVMNHSSSLPVDPKNILSFSVPIGEQLLSWGEKQLKMFSDVGLNANQVIFDPGIGFGKGAAQSLATLYEIAKFKRLGCPILIGHSRKSFLSLFGNHDPKDRDWETVGLSILLQQQGVDYLRVHNVAAHQKALSVAACEACAPI

>core/258/4/Org4_Gene393

MNRLLSLLSVFDDFFWSYVAFILIIVLGVSFSWKSRFFQFTKFSQFCKLFRYYSQNPQERETKQGVHPLKVFFASAGGNIGIGNVVGIVTAACIGGPGALFWVWIAGIFGSIVKYSEVYLGIKFRKLDRDGVYQGGPMYFLTKAFKTPVVSVIVAILLCIYGVEIYQFSVITDSLAHCWNLPKVYPMLGLLFLVFYAIRGGLQRIGKICSIVLPFFMLLYCALSLYILVKEFHTLPHLLSTVFSSAFKGQSALGGFAGCTVATTIHQGISRAAYSGDIGIGFDSIIQSESSAKDPSTQAQLSIVGIAIDNLICTLSLLMVLASGSWSLGLENASQVVEHTLASYFPMVKFFLPTFFFVTGYTTIISYFLVGKKCAKFLYGNTGAKIYTLYGLLILPLFCFLSQNTALLIMSVSGALLLCFNLLGVFILRKEVIFPARAASLTETSLSTE

>core/259/4/Org4_Gene505

MLSCVFSDTIFLSSFLAVTLICMTTALWGTILLISKQPLLSESLSHASYPGLLVGALMAQYVFSLQASIFWIVLFGCAASVFGYGIIVFLGKVCKLHKDSALCFVLVVFFAIGVILASYVKESSPTLYNRINAYLYGQAATLGFLEATLAAIVFCASLFALWWWYRQIVVTTFDKDFAVTCGLKTVLYEALSLIFISLVIVSGVRSVGIVLISAMFVAPSLGARQLSDRLSTILILSAFFGGISGALGSYISVAFTCRAIIGQQAVPVTLPTGPLVVICAGLLAGLCLLFSPKSGWVIRFVRRKHFSFSKDQEHLLKVFWHISHNRLENISVRDFVCSYKYQEYFGPKPFPRWRVQILEWQGYVKKEQDYYRLTKKGRSEALRLVRAHRLWESYLVNSLDFSKESVHELAEEIEHVLTEELDHTLTEILNDPCYDPHRQIIPNKKKEV

>core/260/4/Org4_Gene641

MINSLSQKLSSIFSSLVSSRRINEENISESIREVRLALLDADVNYHVVKDFISKVKEKILGEEIWKHVSPGQQFIR
CLHEELVAFLSDGREEFTIQKTPSIILLCGLQGAGKTTTAAKLADYVIKNKKAKKVLVVPCDLKRFAAVDQL
KILVAQTKAEFYQSQENKPIDVVVKALAYAKENGHDFVILDTAGRLNIDNELMEELTAIQKVSQANERLFVM
NVAMGQDVLATVQAFDQSLDLTGVILSMTDGDARAGAVFSIKHVLGKPIKFEGCGERIQDLRSFDPQSMAER
ILGMGDTINFVKEMREYISEEEDAELGKKLVTAAFTYEDYYKQMKAFRRMGPLRKLLGMMPGFNNAKPSQ
KEIEDSEKQMKRTEAIILSMTPEERKELVELDMSRMKRIASGCGLTLGDVNQFRKQMSQSKKFFKGMSKGK
MEQVRKKMSGGNQWR

>core/261/4/Org4_Gene648

MDALILSRIQFGLFITFHYLFVPLSMGLSMMLVIMEGLYLVTKKQIYKQMTWFWVGIFALTFVLGVVTGIMQI
FSFGSNWANFSEYTGNIFGTLLGSEGVFAFFLESGFLGILLFGRHKVSKKMHFFSTCMVALGAHMSAFWIICA
NSWMQTPSGYEMVMHKGKLIPALTSFWGVVFSPTTIDRFIHAVLGTWLSGVFLVISVSAYYLWKKRHHEFA
KQGMKIGTICAVIVLVLQLWSADVTARGVAKNQPAKLAAFEGIFKTEEYTPIWAFGYVDMEKERVIGLPIPG
ALSFLVHRNIKTPVTGLDQIPRDEWPNVQAVFQLYHLMIMLWGVMVALTLISWSAYKGWRWALKPFFLVIL
TFSVLLPEICNECGWCAAEMGRQPWVVQGLLKTKDAVSPIVQANQIVQSLVMFSLVFIALLTLFITVLCKKIK
HGPEEENDLTEFEVK

>core/262/4/Org4_Gene814

MRAMLLEDWVSLMLSDVSCPKCDKKITGFAIDSQQVQPGDLFFALPGNATDGHQFLKHAATAGAVAAVVS
HDYQGDSFGLELIRVDDTKSALQEAGSNQCNLFQGTLVGITGSVGKTTTKEFSKTILSSIYKTHASPKSYNSQL
TVPLSLLMAEGDEDVMILEMGVSEPGNMQDLLRIVQPEIAVITHINDQHAMHFPQGIQEILKEKSYILQKSKL
QLLPKDSPYYLDLRSCSPTAEKFSFSFNDPLADFCYKAISRDSVVIQTPEENYCLPIAFSYKPAYTNLLIAVALS
WILEVPEEGVIRSLPELKLPPMRFEHSMRNGMQVINDAYNACPEAMIAALDALPLPSDGGKIILILGHMAELG
RYSEEGHALVAEKAASRGDMIFFIGEKWIPVQSVLKSYSCEVSFFSSAQDVKDILKQVARYGDVILLKGSRAL
ALESLLACF

>core/263/4/Org4_Gene500

MNNITSPVIQNNRSCNYYFELKNSTTIHIVISAILLCGALIAFLCVAAPVSYILSGALLGLGLLIALIGVILGIKKIT
PMISSKEQVFPQELVNRIRAHYPKFVSDFVSEAKPNLKDLISFIDLLNQLHSEVGSSTNYNVSEELQQKIDTFG
GIARLKNEVRTASLKRLESAASSRPLFPSLPKILQKLFPFFSLGEFISAGSKVVELHRVKKIGGSLEEDLSDYIKP
EMLPTYWLIPLDFRPTNSSILNLHTLVLARVLTRDVFQHLKYAALNGEWNLNHSDLNTMKQQLFAKYHAAY
QSYKHLSQPSLQEDEFYNLLLCIFKHRYSWKQMSLIKTVPADLWENLCCLTLDHTGRPQDMEFASLIGTLYT
QGLIHKESEAFLSSLTLLSLDQFKTIRRQSTNIAMFLENLATHNSTFRSLPPITVHPLKRSVFSQPEEDESSLLIG

>core/264/4/Org4_Gene963

MVITNLSINADTIYGKNPINIVASAANKNITLTGTLALVNADGAFYENHTLQDSQDYSFVKLSPGAGGTIITQD
ASQKPLQVAPSRPHYGYQGHWNVQVIPGTGNQPSQPILEWVRTGYLPNPERQGSLVPNSLWGSFVDQRAIQE
IMVNSSQILCQERGVWGAGIANFLHRDKINEHGYRHSGVGYLVGVGTHAFSDATINAAFCQLFSRDKDYVVS
KNHGTSYSGVVFLEDTLEFRSPQGFYTDSSSEACCNQVVTIDMQLSYSHRNNDMKTKYTTYPEAQGSWAND
VFGLEFGATTYYYPNSTFLFDYYSPFLRLQCIYAHQEDFKETGGEVRHFTSGDLFNLAVPIGVKFERFSDCKR

GSYELTFAYVPDVIRKDPKSTATLASGAAWSTHGNNLSRQGLQLRLGNHCLINPGIEVFSHGAIELRGSSRNY
NINLGGKYRF

>core/265/4/Org4_Gene724

MLTYKVSPSSVYGNAFIPSSKSHTLRAILWASVAEGKSIIYNYLDSPDTEAMICACKQMGASIKKFPQILEIVG
NPLAIFPKYTLIDAGNSGIVLRFMTALACVFSKEITVTGSSQLQRRPMAPLLQALRNFGASFHFSSDKSVLPFT
MSGPLRSAYSDVEGSDSQFASALAVACSLAEGPCSFTIIEPKERPWFDLSLWWLEKLHLPYSCSDTTYSFPGSS
HPQGFSYHVTGDFSSAAFIAAAALLSKSLQPIRLRNLDILDIQGDKIFFSLMQSLGASIQYDNEEILVFPSSFSGG
SIDMDGCIDALPILTVLCCFADSPSHLYNARSAKDKESDRILAITEELQKMGACIQPTHDGLLVNPSPLYGAVL
DSHDDHRIAMALTIAALYASGDSRIHNTACVRKTFPNFVQTLNIMEARIEECHDNYSMWSTHKRKVFARESF
G

>core/266/4/Org4_Gene874

MTSGVSGSSSQDPTLAAQLAQSSQKAGNAQSGHDTKNVTKQGTQAEVAAGGFEDLIQDASAQSTGKKEATS
STTKSSKGEKSEKSGKSKSSTSVASASETATAQAVQGPKGLRQNNYDSPSLPTPEAQTINGIVLKKGMGTLAL
LGLVMTLMANAAGESWKASFQSQNQAIQSQVESAPAIGEAIKRQANHQASATEAQAKQSLISGIVNIVGFTV
SVGAGIFSAAKGATSALKSASFAKETGASAAGGAASKALTSASSSVQQTTASTAKAATTAASSAGSAATKAA
ANLTDDMAAAASKMASDGASKASGGLFGEVLNKPNWSEKVSRGMNVVKTQGARVASFAGNALSSSMQMS
QLMHGLTAAVEGLSAGQTGMEVAHHQRLAGQAEAQAEVLKQMSSVYGQQAGQAGQLQEQAMQSFNTAL
QTLQNIADSQTQTTSAIFN

>core/267/4/Org4_Gene684

MTLQPYQASSRKYRPQIFREILGQSSVVAVLKNALIFNRAAHAYLFSGIRGTGKTTLARILAKALNCVHLSED
GEPCNQCFSCKEIASGSSLDVLEIDGASHRGIEDIRQINETVLFTPVKAKFKIYIIDEVHMLTKEAFNALLKTLE
EPPQHVKFFFATTEIHKIPGTILSRCQKMHLQRIPEKTILEKLSLMAQDDHIEASQEALAPIARAAQGSLRDAES
LYDYVISLFPKSLSPDTVAQALGFASQDSLRTLDNAILQRDYATALGIVTDFLNSGVAPVTFLHDLTLFYRNLL
LTNSTTSKFSSQYKTEQLLEIIDFLGESAKHLQNTIFEQTFLETVIIHIIRIYQRPVLSELISSIKSRQFEGLRNIKEP
TLTQQVSAPQPQPTYKEQSFLEKKNQPAAEGKIISVEVKSSASIKSAAVDTLLQFAVVEFSGILRQ

>core/268/4/Org4_Gene857

MLKHDTIAAIATPPGEGSIAVVRLSGPQAIVIADRIFSGSVASFASHTIHLGQVIFEETLIDQALLLLMRSPRSFT
GEDVVEFQCHGGFFACSQILDALIALGARPALPGEFSQRAFLNGKIDLVQAEAIQNLIVAENIDAFRIAQTHFQ
GNFSKKIQEIHTLIIEALAFLEVLADFPEEEQPDLLVPQEKIQNALHIVEDFISSFDEGQRLAQGTSLILAGKPNV
GKSSLLNALLQKNRAIVTHIPGTTRDILEEQWLLQGKRIRLLDTAGQRTTDNDIEKEGIERALSAMEEADGIL
WVIDATQPLEDLPKILFTKPSFLLWNKADLAPPPFLDTSLPQFAISAKTGEGLTQVKQALIQWMQKQEAGKTS
KVFLVSSRHHMILQEVARCLKEAQQNLYLQPPEIIALELREALHSIGMLSGKEVTESILGEIFSKFCIGK

>core/269/4/Org4_Gene850

MPRSLSNEQFSVDLEESPGCIVSALVKVSPEVLNKLNKQALKKIKKEITLPGFRKGKAPDDVIASRYPTNVRK
ELGELVTQDAYHALSTVGDRRPLSPKAVRSNSITQLDLQEGAKVEFSYEAFPAISDLPWENLSLPQEEAASEIS
DSDIEKGLTNIGMFFATKTPVERPSQEGDFISISLHVSKSNDENASSAAIFENKYFKLSEEEMTDAFKEKFLGIS

TGHRVVETITSPEIQSFLRGDTLTFTVNAVIEVSIPEIDDEKARQLQAESLDDLKAKLRIQLEKQAKDKQLQKR
FSEAEDALAMLVDFELPTSLLEERISLITREKLLNARLIQYCSDEELEKRKSELIKEAEEDATKALKLLFLTHKI
FSDEKLTISREELQYMMDVCSRERFGQQPPKDISNDTLQELVMSARDRLTYSKAIEHVLRKAELLASTPSA

>core/270/4/Org4_Gene282

MDQLTTDFDTLMSQLGDVNLTTVVGRITEVVGMLIKAVVPNVRVGEVCLVKRNGMEPLVTEVVGFTQSFAF
LSPLGELSGVSPSSEVIPTGLPLHIRAGNGLLGRVLNGLGEPIDVETKGPLQNVDQTFPIFRAPPDPLHRAKLRQ
ILSTGVRCIDGMLTVARGQRIGIFAGAGVGKSSLLGMIARNAEEADVNVIALIGERGREVREFIEGDLGEEGM
KRSVIVVSTSDQSSQLRLNAAYVGTAIAEYFRDQGKTVVLMMDSVTRFARALREVGLAAGEPPARAGYTPS
VFSTLPRLLERSGASDKGTITAFYTVLVAGDDMNEPVADEVKSILDGHIVLSNALAQAYHYPAIDVLASISRL
LTAIVPEEQRRIIGKAREVLAKYKANEMLIRIGEYRRGSDREVDFAIDHIDKLNRFLKQDIHEKTNYEEAAQQL
RAIFR

>core/271/4/Org4_Gene855

MWLVILWALAASLAIALVAKGYYRFVYFRRYAVQVIREVRLSMELKEWALAEQQLLPILKKRSYRRQCLFE
YMRILRKMQRFEESEKLLAEAKKLGLRGPYFFLEIAYKAYRFGAFKECAQAFASVPQDLFEEEDAAKYASAL
VRLGDLDAACSLIEPWISPLSHQETFVTMGHIYFTSKRYKDAIDFYNRANALGVCPVEVTYNLAQAYRITSSY
AKAGKLFRKLLSNPVYKEEALFNIGLCEQKLGRPGKALLIYQSSDLWSRGDALLMKYAAMAAMDQRDYVL
AERCWELALRCSTFAKDYKCGLGYGFSLCRLRKYGDAERVYCNLIQNFPECLTACKALAWLCGVGYATLL
GSEEGLMYAKKAVELDHSCETLELLSACEARCGNFDAAYEIQSFLSSRDTSLQDKQRRSQILRILRKKLPLND
HHIVEVDALLAA

>core/272/4/Org4_Gene344

MIENDFPEASNFESSHFYRDKVGVIILCGGEGKRLSPLTNCRCKPTVSFGGRYKLIDIPISHAISAGFSKIFVIGQ
YLTYTLQQHLFKTYFYHGVLQDQIHLLAPEARQGDQIWYQGTADAIRKNLLYFEDTEIEYFLILSGDQLYNM
DFRSIVDTAIRTHVDMVLVAQPIPEKDAYRMGVLDIDSKGKLIDFYEKPQEKEVLKRFQLSSEDRRIHKLTED
SGDFLGSMGIYLFRRDSLFSLLQEEEGNDFGKHLIQAQMKRGQVQTLLYNGYWTDIGTIESYYEANIALTQKP
HAEKRGLNCYDDNGMIYSKNHHLPGAIITDSMISSSLLCEGCVIDTSHVSRSVLGIRSKIGENSVVDQSIIMGN
ARYGSRSMPSLGIGKDCEIHKAIIDENCCIGNGVKLQNLKGYIEYDSPDKKLFVRDNIIIVPQGTHIPDNYIF

>core/273/4/Org4_Gene627

MLNCSNQKHTVTFEEACQVFPGGVNSPVRACRSVGVTPPIVSSAQGDIFLDTHGREFIDFCGGWGALIHGHSH
PKIVEAIQKTALKGTSYGLTSEEEILFATMLLSSLKLKEHKIRFVSSGTEATMTAVRLARGITNRSIIIKFIGGYH
GHPDTLLGGISITEETIDNLTSLIHTPSPHSLLISLPYNNSQILHHVMEALGPQVAGIIFEPICANMGIVLPKAEFL
DDIIELCKRFGSLSIMDEVVTGFRVAFQGAQDIFNLSPDITIYGKILGGGLPAAALVGHRSILDHLMPEGTIFQA
GTMSGNFLAMATGHAAIQLCQSEGFYDHLSQLEALFYSPIEEEIRSQGFPVSLVHQGTMFSLFFTESAPTNFDE
AKNSDVEKFQTFYSEVFDNGVYLSPSPLEANFISSAHTEENLTYAQNIIIDSLIKIFDSSAQRFF

>core/274/4/Org4_Gene892

MFQQKQKLSLKYLPSLRMQQGLQMLQSPLTELSSYVVQEIIDNPFFDLSSLEEEWSPCYRPTNSTFSYLNQTP
GPQESLYTRLLPQIEEAFSTAEERFIAHQIAGNLSDEGLFLRNPEDFAQELELPLEKIHKVWDTIQNLSPEGIASP

SLQSYWMKLLRNSSHQQAYSIVRDCYPLMTNCEFAPIMKKFSLSLSELRNILKKALGSIPWCPAAACTVKPM
VSTPLPDIYLFYSSGSWKIEVSTRGLPSIKLNKETFHFYEHLPKEEQKNLSQQILSAKWLIKNLRKREQTLLQV
METLLPKQEDFLLGKIPAPYPLGIKDLAEDLSFHESTIFRAIENKAVAAPIGIFPLKHLFPRGIHQDSSHSKENVL
QWIRQWIATEQTPLSDSVISDRITAKGIPCARRTVAKYRAQLKILPANKRKIQM

>core/275/4/Org4_Gene717

MSKIVYKFGGTSLATAENICLVCDIICKDKPSFVVVSAIAGVTDLLVDFCSSSLREREEVLRKIEGKHEEIVKNL
AIPFPVSTWTSRLLPYLQHLEISDLDFARILSLGEDISASLVRAVCSTRGWDLGFLEARSVILTDDSYRRASPNL
DLMKAHWHQLELNQPSYIIQGFIGSNGLGETVLLGRGGSDYSATLIAELARATEVRIYTDVNGIYTMDPKVIS
DAQRIPELSFEEMQNLASFGAKVLYPPMLFPCMRAGIPIFVTSTFDPEKGGTWVYAVDKSVSYEPRIKALSLS
QHQSFCSVDYTVLGCGGLEEILGILESHGIDPELMIAQNNVVGFVMDDDIISQEAQEHLVDVLSLSSVTRLHHS
VALITMIGDNLSSPKVVSTITEKLRGFQGPVFCFCQSSMALSFVVASELAEGIIEELHNDYVKQKAIVAT

>core/276/4/Org4_Gene657

MQTIYTKITDIKGNLITVEAEGARLGELATITRSDGRSSYASVLRFDLKKVTLQVFGGTSGLSTGDHVTFLGRP
MEVTFGSSLLGRRLNGIGKPIDNEGECFGEPIEIATPTFNPVCRIVPRSMVRTNIPMIDVFNCLVKSQKIPIFSSSG
EHHNALLMRIAAQTDADIVVIGGMGLTFVDYSFFVEESKKLGFADKCVMFIHKAVDAPVECVLVPDMALAC
AEKFAVEEKKNVLVLLTDMTAFADALKEISITMDQIPANRGYPGSLYSDLALRYEKAVEIADGGSITLITVTT
MPGDDITHPVPDNTGYITEGQFYLRNNRIDPFGSLSRLKQLVIGKVTREDHGDLANALIRLYADSRKATERMA
MGFKLSNWDKKLLAFSELFETRLMSLEVNIPLEEALDIGWKILAQSFTSEEVGIKAQLINKYWPKACLSK

>core/277/4/Org4_Gene728

MISFRFLLLSGLCALGISSYAETPKETTGHYHRYKARIQKKHPESIKESAPSETPHHNSLLSPVTNIFCSHPWKD
GISVSNLLTSVEKATNTQISLDFSILPQWFYPHKALGQTQALEIPSWQFYFSPSITWTLYDSPTAGQGIVDFSYT
LIRYWQTNGVDANQAAGTASGMNDYSNRENNLAQLTFSQTFPGDFLTLAIGQYSLYAIDGTLYDNDQYSGFI
SYALSQNASATYSLGSTGAYLQFTPNSEIKVQVGFQDSYNIDGTNFSIYNLTKSKYNFYGYASWTPKPSCGDG
QYSVLLYSTRKVPEQNSQVTGWSLNAAQHIHEKLYLFGRINGATGTALPINRSYVLGLVSENPLNRHSQDLL
GIGFATNKVNAKAISNVNKLRRYESVMEAFATIGFGPYISLTPDFQLYIHPALRPERRTSQVYGLRANLSL

>core/278/4/Org4_Gene616

MMLRGIHRIFKCFYDVVLVCAFVIALPKLLYKMLVYGKYKKSLAVRFGLKKPHVPGEGPLVWFHGASVGEV
RLLLPVLEKFCEEFPGWRCLVTSCTELGVQVASQVFIPMGATVSILPLDFSIIIKSVVAKLRPSLVVFSEGDCWL
NFIEEAKRIGATTLVINGRISIDSSKRFKFLKRLGKNYFSPVDGFLLQDEVQKQRFLSLGIPEHKLQVTGNIKTY
VAAQTALDLEREAWRDRLRLPTDSKLVVLGSMHRSDAGKWLPVVQKLIKEGVSVLWVPRHVEKTKDVEES
LHRLHIPYGLWSRGANFSYTSVVVVDEIGLLKQLYVAGDLAFVGGTFDPKIGGHNLLEPLQCEVPLIFGPHITS
QSELAQRLLLSGAGLCLDEIEPIIDTVSFLLNNQEMREAYVQKGKVFLKAETASFDRTWRALKSYIPLYKNS

>core/279/4/Org4_Gene307

MKRPFFTYLCIIFYGSCASLSLHAGLSFPEVRGATAAVVHADSGKVFYDKDIDAVIYPASMTKIATALFILKHY
PTVLDTLIKVKQDAIASITPQAKKQSGYRSPPHWLETDGSTIQLHLREELLGWDLFHALLVCSANDAANVLA
MACCGSVEKFMDKLNFFLKEEIGCTHTHFNNPHGLHHPNHYTTTRDLISIMRCALKEPPFRGVISTTSYKIGAT

NLHGERILSPTNKLLLPGSTYHYPPALGGKTGTTKTAGKNLIMAAEKNNRLLVTIATGYSGPVSDLYQDVIAL
CETVFNEPLLRKELVPPSDCLQLEIANLGKLSCPLPEGLYYDFYASEDREPLSVSFIAHADAFPIEQGDLLGHW
VFYDDEGKKISSQPFYAPCRFERTIKPWKLYMKRVFTSYRTYISITMLLMYFRIRKHRKYKNLKHYSKI

>core/280/4/Org4_Gene822

MKRAIIIGAGISGLAAGWWLHKKFPQAEILVLDKEAYAGGFVRTESPQGFSFDLGPKGFLTRGDGEYTLKLIH
ELGLQNSLIFSDRAAKNRFVYYRGKAHKISTWTLLRKGLLPSLIKDFRAPCYAQDSSVQDFLKRHSSQNFTSY
ILDPLITAIRAGHSSILSTHMAFPELAKREASSGSLLRSYLKNRSPKKSKTDRYLASLSPSMGTLITTIQEKLPAT
WKFSTSVTHIDCSPKEACVTTPSETFFADMVIYTGPLHQLPVLLPNYGIENLSKRVLPWNLSSISLGWHHANFS
LPKGYGMLFADELPLLGIVWNSQIFPQATPGKTVLSLLIEGKWRESEAHAFAIAALSEYLNINQKPDAFALFSS
QDGMPQHAVGFLERKERILPHLPGNLKIVGQNIAGPGLNRCIASAYHAICDLHTEETLAQPQSSL

>core/281/4/Org4_Gene592

MFSRWITLFLLFISLTGCSSYSSKHKQSLIIPIHDDPVAFSPEQAKRAMDLSIAQLLFDGLTRETHRESNDLELAI
ASRYTVSEDFCSYTFFIKDSALWSDGTPITSEDIRNAWEYAQENSPHIQIFQGLNFSTPSSNAITIHLDSPNPDFP
KLLAFPAFAIFKPENPKLFSGPYTLVEYFPGHNIHFKKNPNYYDYHCVSINSIKLLIIPDIYTAIHLLNRGKVDW
VGQPWHQGIPWELHKQSQYHYYTYPVEGAFWLCLNTKSPHLNDLQNRHRLATCIDKRSIIEEALQGTQQPAE
TLSRGAPQPNQYKKQKPLTPQEKLVLTYPSDILRCQRIAELLKEQWKAAGIDLILEGLEYHLFVNKRKVQDY
AIATQTGVAYYPGANLISEEDKLLQNFEIIPIYYLSYDYLTQDFIEGVIYNASGAVDLKYTYFP

>core/282/4/Org4_Gene521

MNKNLVAIFDYMEKEKGIQRSTIIGAIESALKIAAKKTLRDDANISVNINSRTGDIEVFCEKEIVEICQNPSKEIP
LDKAREYDPDCQIGQYMDVPFVSDNFGRIAAHAARQIIGQKLRHAERDVIYEEYRHRVNEILSGVVKRFAKG
SNLIIDLGKVEAILPTRFYPKTEKHKIGDKIYALLYEVQESENGGAEVILSRSHAEFVKQLFIQEVPELEEGSVEI
VKIAREAGYRTKLAVRSSDPKTDPVGAFVGMRGSRVKNIIRELNDEKIDIVNYSPVSTELLQNLLYPIEIQKIAI
LEDDKVIAIVVNDADYATVIGKRGINARLISQILDYELEVQRMSEYNKLLEIQRLQLAEFDSPHLDQPLEMEGI
SKLVIQNLEHAGYDTIRRVLLASANDLASVPGISLELAYKILEQVSKYGESKVDEKPEIED

>core/283/4/Org4_Gene499

MSMTIVPHALFKNHCECHSTFPLSSRTIVRIAIASLFCIGALAALGCLAPPVSYIVGSVLAFIAFVILSLVILALIF
GEKKLPPTPRIIPDRFTHVIDQAYGLSISAFVREQQVTLAEFRQFSTALLCNISPEEKIKQLPSELRSKVESFGISR
LAGDLEKNNWPIFEDLLSQTCPLYWLQKFISAGDPQVCRDLGVPRECYGYYWLGPLGYSTAKATIFCKETHH
ILQQLTKEDVLLLKNKALQEKWDTDEVKAIVERIYTTYTARGTLKTEAGGLTKETISKELLLLSLHGYSFDQL
QLITQLPRDTWDWLCSVDNSTAYNLQLCALVGALSSQNLLDESSIDFDVNLGLYVIQDLKEAVQAFSASDEP
KKELGKFLLRHLSSVSKRLESVLRQGLHRIALEHGNARARVYDVNFVTGARIHRKTSIFFKD

>core/284/4/Org4_Gene187

MNHLNKEKLHIHNWQPYRACGLLSKVSGNLIEVDGLSACLGELCKISSTKDPNLLAEVIGFHNHTTLLMSLSP
LHSVALGTEVLPLRRPPSLHLSDHLLGRVLDAFGNPIDKKEDLPKTHRKPLLSLPPSPMMRQPIDQIFPTGIKAI
DAFLTLGKGQRIGVFSEPGSGKSSLLSAIALGSKSTINVIALIGERGREVREYIEKHSNALKQQRTIIIAAPAHET
APTKVIAGRAAMTIAEYFREQGHEVLFIMDSLSRWIAALQEVALARGETLSAHQYAASVFHHVSEFTERAGN

NDKGSITALYAILYYPKHPDIFTDYLKSLLDGHFFLTSQGKALASPPIDILSSLSRSAQALALPHHYAAAERLRS
LLKVYNEALDIIHLGAYTPGQDEELDKAVKLLPSIKAFLAQPLSSYCYLDNTLKQLEALADS

>core/285/4/Org4_Gene944

MLKPMYVLSKRLYRWVNQLIKLGDLVKNSRSFSVEWVFISALLLIFGCLGCASVVKVSLVPFLLLFSFLAFPLI
LCFRGKGYALLLGVFVTLYVAKYVVGETLYVSFWLSGLGVSFLLAFGLFLQGVWLAQEEEMVKGKEQLRL
SEDLDAQRSAYEDLLLTKSQEKEFLDARAQGLDRELTECQELLKAAYQKQEYLTIDLKILADQKNSWLEDY
AELHNKYIELVSKNGDVVFPWVAEPSVGESQGSEKVDVSRWVSALQEKEESLERLRNEILVEKQRCSDYEHR
CQELGLLLQNFTALERRCEELQNLLNQKETQINELHQLVCKSEEKVSVEPSAHAETSCVEEKQYKGLYSQLQ
EQFLEKSETLSLVRKKLFAVQEKYLTLKKKEELTKQDMSFDDISMIQGLLERIEILEEEVSHLEELVSRSLSL

>core/286/4/Org4_Gene171

MLRQLCFQVFFFCFASLVYAEELEVVVRSEHITLPIEVSCQTDTKDPKIQKYLSSLTEIFCKDIALGDCLQPTAA
SKESSSPLAISLRLHVPQLSVVLLQSSKTPQTLCSFTISQNLSVDRQKIHHAADTVHYALTGIPGISAGKIVFALS
SLGKDQKLKQGELWTTDYDGKNLAPLTTECSLSITPKWVGVGSNFPYLYVSYKYGVPKIFLGSLENTEGKKV
LPLKGNQLMPTFSPRKKLLAFVADTYGNPDLFIQPFSLTSGPMGRPRRLLNENFGTQGNPSFNPEGSQLVFISN
KDGRPRLYIMSLDPEPQAPRLLTKKYRNSSCPAWSPDGKKIAFCSVIKGVRQICIYDLSSGEDYQLTTSPTNKE
SPSWAIDSRHLVFSAGNAEESELYLISLVTKKTNKIAIGVGEKRFPSWGAFPQQPIKRTL

>core/287/4/Org4_Gene826

MTWLSGLYFICIASLIFCAIGVILAGVILLSRKLFIKVHPCKLKINDNEELTKTVESGQTLLVSLLSSGIPIPSPCG
GKATCKQCKVRVVKNADEPLETDRSTFSKRQLEEGWRLSCQCKVQHDMSLEIEERYLNASSWEGTVVSND
NVATFIKELVVAVDPNKPIPFKPGGYLQITVPSYKTNSSDWKQTMAPEYYSDWEHFHLFDQVIDNSQLPADS
ANKAYSLASYPAELPTIKFNIRIATPPFINGKPNSEIPWGVCSSYVFSLKPGDKITVSGPYGESFMKDDDRPLIFL
IGGAGSSFGRSHILDLLLNKHSKREIDLWYGARSLKENIYQEEYENLERQFPNFHYHLVLSEPLPEDIAAGWD
KDDPTKTNFLFRAFNLGQLSRLDNPEDYLYYVCGPPLHNSSILKLLDDYGVERSSIILDDFGS

>core/288/4/Org4_Gene313

MTVTLPKGVFDIFPYLADAKQLWRHTSLWHSVEKAIHTVCMLYGFCEIRTPIFEKSEVFLHVGEESDVVKKE
VYSFLDRKGRSMTLRPEGTAAVVRSFLEHGASHRSDNKFYYILPMFRYERQQAGRYRQHHQFGVEAIGVRH
PLRDAEVLALLWDFYSRVGLQHMQIQLNFLGGSETRFRYDKVLRAYLKESMGELSALSQQRFSTNVLRILDS
KEPEDQEIIRQAPPILDYVSDEDLKYFNEILDALRVLEIPYAINPRLVRGLDYYSDLVFEATTTFQEVSYALGGG
GRYDGLISAFGGASLPACGFGVGLERVIQTLLAQKRIEPQFPHKLRLIPMEPDADQFCLEWSQHLRRLGIPTEV
DWSHKKVKGALKAASTEQVSFVCLIGERELISQQLVIKNMSLRKEFFGTKEEVEQRLLYEIQNTPL

>core/289/4/Org4_Gene600

MFRKLFPFSKKKTGQKQRLRNNGLLQAIIQSIKVLLHNEASKEACVLSYYGLLTCVPILVFFLRLSQHLFTNLN
WKEWLIIKFPDYKKPIVAIVEAAYHATESNIGLVLVGSFFVFCWAGILMLLSLEDGLNKIFRTSWTPISLKRLV
SYFVITLVSPMIFIIVCGSWIYITQIMPIQYAKLFSLSHSMTALYFISRFVPYLLLYLALFCCYAFLPRVAIQKTSA
LISTLIIGSVWIVFQKAFFSLQVSIFNYSFTYGALVALPSFLLLLYIYAMIYLFGGALTFIIQNRGCTFIFLGDKILP

SCYLQLITSTYILALTTRQFNEGLSPLTAQFIAKQSKVPIGEVSQCLDVLEKEGFLFPYNNGYQPVFNFSELTIK
DIADKLLHREIFKKFNPDLGITFIENSFQNIFNQASKNKENLTLSEIARRIK

>core/290/4/Org4_Gene215

MVCENNILSGRGLELLKKKSNITLTPTIYSVSNHNIKLKDFSPHALSVIKTLRKAGYIAYIVGGCIRDLLLNTTP
KDFDISTSAKPEEIKAIFKNCILVGKRFRLAHIRFSKQIIEVSTFRSGSTDEDVLITKDNLWGTPEEDVLRRDFTI
NGLFYDPEHEEIIDYTGGVNDLRNRYLRTIGDPFTRFKQDPVRMLRLLKILSRSPFTVETQTQEALIACRQELIK
SSQARVFEELIKMLNSGAAKNFFQLLIENHLLEILFPYMDKAFRLNRALEEQTATYLKALDDKILKKEAEYDR
HQLMAIFLFPLVNFNVRYKHQKHPYLSLTSVFDYIKNFLEQFFADSFTSCSKKNFILTALILQMQYRLTPLIPTK
KALFFNKKLLHHTRFLEALSLLEIRSIVYPKLDKVYVAWIRHHQTLKCKKDSHSQK

>core/291/4/Org4_Gene58

MISLLKMPKLSPTMEVGTIVKWHKKSNDQVSFGDVIVEISTDKAILEHTANEDGWIREILRHEGEKIVIGTPIA
VLSTEANEPFNLEELLPKTEPSNLEASPKGSSEEVSPATTPQAASATFTAVTFKPEPPLSSPLVFKHVGTTNNLS
PLARQLAKEKNIDVSSIQGSGPGGRIVKKDLEKAPPKSIAGFGYPESPEVPPGSYHEENLSPIREVIAARLQAAK
ISIPHFYVRQQVYASPLLNLLKELQAQGIKLSINDCIVRACALALKEFPSINSGFNSVDNKIVRFDTIDISIAVAIP
DGIITPIIRCADRKNLGMISAEIKSLALKARNQSLQDTEYKGGSFCVSNLGMTGITEFTAIVNPPQAAILAVGSV
TEQALVLDGEITIGSTCNLTLSVDHRVIDGYPAAMFMKRLQKILEAPAVLLLN

>core/293/4/Org4_Gene255

MFEAVIADIQAREILDSRGYPTLHVKVTTSTGSVGEARVPSGASTGKKEALEFRDTDSPRYQGKGVLQAVKN
VKEILFPLVKGCSVYEQSLIDSLMMDSDGSPNKETLGANAILGVSLATAHAAAATLRRPLYRYLGGCFACSLP
CPMMNLINGGMHADNGLEFQEFMIRPIGASSIKEAVNMGADVFHTLKKLLHERGLSTGVGDEGGFAPNLAS
NEEALELLLLAIEKAGFTPGKDISLALDCAASSFYNVKTGTYDGRHYEEQIAILSNLCDRYPIDSIEDGLAEED
YDGWALLTEVLGEKVQIVGDDLFVTNPELILEGISNGLANSVLIKPNQIGTLTETVYAIKLAQMAGYTTIISHR
SGETTDTTIADLAVAFNAGQIKTGSLSRSERVAKYNRLMEIEEELGSEAIFTDSNVFAYEDSEE

>core/294/4/Org4_Gene897

MDIKKLFCLFLCSSLIAMSPIYGKTGDYEKLTLTGINIIDRNGLSETICSKEKLKKYTKVDFLAPQPYQKVMRM
YKNKRGDNVSCLTAYHTNGQIKQYLECLNNRAYGRYREWHVNGNIKIQAEVIGGIADLHPSAESGWLFDQT
TFAYNDEGILEAAIVYEKGLLEGSSVYYHTNGNIWKECPYHKGVPQGKFLTYTSSGKLLKEQNYQQGKRHG
LSIRYSEDSEEDVLAWEEYHEGRLLKAEYLDPQTHEIYATIHEGNGIQAIYGKYAVIETRAFYRGEPYGKVSR
FDNSGTQIVQTYNLLQGAKHGEEFFFYPETGKPKLLLNWHEGILHGIVKTWYPGGTLESCKELVNNKKSGLL
TIYYPEGQIMATEEYDNDLLIKGEYFRPGDRHPYSKIDRGCGTAVFFSSAGTITKKIPYQDGKPLLN

>core/295/4/Org4_Gene939

MKKTMVIDTSVFIYDPEALFSFENTRIIIPFPVIEELEAFGKFRDESAKNASRALSNIRLLLENAKTKVTDGVLLP
NGSELRIEVAPLSNDDRRGKLLTLELLKIIATREPMVFVTKSLGRRVRAEALQIESRDYESKRFSFRSLYRGFR
ELQVSQEDIENFYKNGYLDLPLDVVPSPNEYFFMSAGENHFALGRYYVSQGKIIALKAMDKSVWGIKPLNTE
QRCALDLLLRDDVKLVTLIGQAGSGKTILALAAAMHKVFDKETYNKVLVSRPIVPMGRDIGFLPGLKEDKL

MHWMQPIYDNMEVLFSINQMGNSSEALQALMDAKKLEMEALTYIRGRSLPKAFIIIDEAQNLTPHEIKTIISRA
GKGTKIVLTGDPTQIDSLYFDENSNGLTYLVGKFHHLALYGHMFMTRTERSELAAAAATIL

>core/296/4/Org4_Gene835

MLDIKIIRKTPEECETRLRKKDPKISLEPVLSLDKEVRQLKTDSETLQAQRRLLSQDIHKAKTQGVDATNLIQE
VETLAADLEKIEQHLDQKNAQLHELLSHLPNYPADDIPVSEDKAGNQVIKSVGDLPTFSFPPKHHLELNQELD
ILDFQAAAKTTGSGWPAYKNRGVLLEWALLTYMLQKQAAHGFQLWLPPLLVKKEILFGSGQIPKFDGQYYR
VEDGEQYLYLIPTAEVVLNGFRSQDILTEKELPLYYAACTPCFRREAGAAGAQERGLVRVHQFHKVEMFAFT
TPNQDDIAYEKMLSIVEEMLTELKLPYRLSLLSTGDMSFTASKTIDAEVWLPGQKAFYEVSSISQCTDFQSRRS
GTRYKDSQGKLQFVHTLNGSGLATPRLLVAILENNQQADGSVVIPEVLRPYLGGLEILLPKDQ

>core/297/4/Org4_Gene301

MLPLIIFVLLCGFYTSWNIGANDVANAVGPSVGSGVLTLRQAVVIAAIFEFFGALLLGDRVAGTIESSIVSVTN
PMIASGDYMYGMTAALLATGVWLQLASFFGWPVSTTHSIVGAVIGFGLVLGKGTIIYWNSVGIILISWILSPF
MGGCVAYLIFSFIRRHIFYKNDPVLAMVRVAPFLAALVIMTLGTVMISGGVILKVSSTPWAVSGVLVCGLLSY
VITFYYVHTKHCSYISDTPKKGSLTYRLKERGGNYGRKYLVVERIFAYLQIIVACFMAFAHGSNDVANAIAPV
AGVLRQAYPASYTSYTLIGLMAFGGIGLVIGLAIWGWRVIETVGCKITELTPSRGFSVGMGSALTIALASILGL
PISTTHVVVGAVLGIGLARGIRAINLNIIKDIVLSWFITLPAGALLSILFFFALRALFH

>core/298/4/Org4_Gene961

MIMGLQSRLQHCIEVSQNSNFDSQVKQFIYACQDKTLRQSVLKIFRYHPLLKIHDIARAVYLLMALEEGEDLG
LSFLNVQQYPSGAVELFSCGGFPWKGLPYPAEHAEFGLLLLQIAEFYEESQAYVSKMSHFQQALFDHQGSVF
PSLWSQENSRLLKEKTTLSQSFLFQLGMQIHPEYSLEDPALGFWMQRTRSSSAFVAASGCQSSLGAYSSGDV
GVIAYGPCSGDISDCYYFGCCGIAKEFVCQKSHQTTEISFLTSTGKPHPRNTGFSYLRDSYVHLPIRCKITISDK
QYRVHAALAETTSAMTFSIFCKGKNCQVVDGPRLRSCSLDSYKGPGNDIMILGENDAINIVSASPYMEIFALQ
GKEKFWNADFLINIPYKEEGVMLIFEKK

>core/299/4/Org4_Gene722

MDKQSSGNSGCIWHPFTQSALDSTPIKIVRGEGAYLYAESGTRYLDAISSWWCNLHGHGHPYITKKLCEQAQ
KLEHVIFANFTHEPALELVSKLAPLLPEGLERFFFSDNGSTSIEIAMKIAVQYYYNQNKAKSHFVGLSNAYHG
DTFGAMSIAGTSPTTVPFHDLFLPSSTIAAPYYGKEELAIAQAKNVFSESNVAAFIYEPLLQGAGGMLMYNPE
GLKEILKLAKHYGVLCIADEILTGFGRTGPLFASEFTDIPPDIICLSKGLTGGYLPLALTVTTKEIHDAFVSQDR
MKALLHGHTFTGNPLGCSAALASLDLTLSPECLQQRQMIERCHQEFQEAHGSLWQRCEVLGTVLALDYPAE
ATGYFSQYRDHLNRFFLERGVLLRPLGNTLYVLPPYCIQEEDLRIIYSHLQDALCLQPQ

>core/300/4/Org4_Gene688

MAEISTPSLPDSSIVSQKTPPVPDPDSSPDHIPTIPTQAPFKPQRKKETPSSIVNAIAFAILAFLSCLGGVFAICLGC
SLEITMPLFILTAVFIAFTLLYFIHYLEKPKIPEPLPTPLPPKLSPPPSPTLRAPTLTPEIPVPAPGIPLPPTLPKVDRT
KLTCNPDIHYPSTYDPKACSSLLKQLFSLDPETRPEDRKYSNKLASILLRSKEKPGFRFHCFKGHFSHDKILNK
KSGAVVISSHSSMDFSTTLGRAFAVTTCLQRSCWEKIKNNIPTPEKHLPIGSCVSGPWDVEEGAQLYTSHLIVI

NPPTLETLIKEKMRRAVTLKDFSMKEAFTNLVLAYLQCFDICIEHNLESVQLEVFGLNNLSADQEEFTTWESC CHLALLESVRILLASKEEYALSNVSVNSIPQVPLQTACRALFLN

>core/301/4/Org4_Gene268

MKKQVYQWLASVVLLALTISGYAELPLSEQKVKSHTYTTLDEVKDYLSKRGFVETRKQDGVLRIAGDVRAR WLYFREDIKNPSDKDKYNPLPVNRYRSEFYLYIDYRAERNWLSSKMNWTAIAGGENTAAGVDINRAFLGYR FYKNPETRTDFFMEIGRSGLGDLFESEVQFQSNFDGLHIYWTRELSKDYPYQVIVHGGPFVVNMTKKHYAW VVEGILNRLPKQFFVKCSVVDWNTFVPSETSTTEKAATNAMKYKYCVWQWLVGKHSQVPWINGQKKPLYL YGAFLMNPLAKATKTTLNGKENLAWFVGGTLGGLRKAGDWSATVRYEYVEALSVPEIDVSGIGRGNLLKF WFAQAIAANYDPKEANGFTNYKGFSALYMYGITDSLSFRAYGAYSKPANDKLGSDFTFRKFDLGIISAF

>core/302/4/Org4_Gene495

MQSWLQSLQERNILENFTAGLDSVEGPIAAYLGFDPTAPALHIGHWIGICFLKRLAALGITPIALVGGATGMV GDPSGKQSERSLLQTSEVFDNSQKITACLQRYLPGVTLVNNADWLQEISLIDFLRDIGKHFRLGQMLAKDTIK QRVHSDEGISYTEFSYLILQSYDFYHLFKNYGTILQCGGSDQWGNITSGIDFIRRKGLGQAYGLTYPLLTNAQ GKKIGKTESGTVWLDSDLTSPFELYQYLLRLPDDTIPKIARTLTLLSNEEIQDIDRRVQTDPVAVKEFVAQDIL RAIHGDLGLEEALSVTRSMHPGNLSSLSEKDFHELFAGGMGASLDKSEVLGKRWLDLFLVLGLCKSKGEIRR LIEQKGVYINNVPIANEHSVCEEQDICYGHYVLLAQGKKRKLVLYLN

>core/303/4/Org4_Gene431

MTVAEVKGTFKLVCLGCRVNQYEVQAYRDQLTILGYQEVLDSEIPADLCIINTCAVTASAESSGRHAVRQLC RQNPTAHIVVTGCLGESDKEFFASLDRQCTLVSNKEKSRLIEKIFSYDTTFPEFKIHSFEGKSRAFIKVQDGCNS FCSYCIIPYLRGRSVSRPAEKILAEIAGVVDQGYREVVIAGINVGDYCDGERSLASLIEQVDQIPGIERIRISSIDP DDITEDLHRAITSSRHTCPSSHLVLQSGSNSILKRMNRKYSRGDFLDCVEKFRASDPRYAFTTDVIVGFPGESD QDFEDTLRIIEDVGFIKVHSFPFSARRRTKAYTFDNQIPNQVIYERKKYLAEVAKRVGQKEMMKRLGETTEVL VEKVTGQVAMGHSPYFEKVSFPVVGTVAINTLVSVRLDRVEEEGLIGEIV

>core/304/4/Org4_Gene851

MNKKNLTICSFCGRSEKDVEKLIAGPSVYICDYCIKLCSGILDKKPSSTISSAPVSETPSQPSDLRVLTPKEIKKH IDEYVIGQERAKKTIAVAVYNHYKRIRALLHNKQVSYGKSNVLLLGPTGSGKTLIAKTLAKILDVPFTIADAT TLTEAGYVGEDVENIVLRLLQAADYDVARAERGIIYIDEIDKIGRTTANVSITRDVSGEGVQQALLKIVEGTTA NVPPKGGRKHPNQEYIRVNTENILFIVGGAFVNLDKIIAKRLGKTTIGFSDDQADLSQKTRDHLLAKVETEDLI AFGMIPEFVGRFNCIVNCEELSLDELVAILTEPTNAIVKQYIELFAEENVKLVFKKEALYAIAKKAKQAKTGA RALGMILENLLRDLMFEIPSDPTVEAIHIQEDTIAENKAPIIIRRTPEAIA

>core/305/4/Org4_Gene975

MIKFLSQLFIRHWPRKVVSLGFAIIIWILVGQSVTITRTLTNVPVRIVDLHPDQTVLGLQKSGFLNKKVSLTITG NKNTVQDLRPSNLEVVISAANHTESWIATIDKHNLVSVDHEINIRKDIHSVDANDIFVRLTQYVTEDILLTITKP IGSPPKGYEYLDVWPKYLNQKVSGPKEYINALKEQGLELTFNLNKISFEELERNRIAQGSHDEIIFPIPKEWKKI LIPFENTFMDLNDPQADFLRLLFLKRECIPLNLNLPVFLFFPVTFIQTMNPLEYSLDPVPPIILNHGIHQINIPLYV

KDVSRQFLDVVKNNMALTIVMPSPQDPSSINWAIEFLDEKTLENTFLQTIIAQEHGILHDIALIDEAGIRHRFRE
YLRKLALFTADGEPLNLIAEIKNNKVVIQTKTKETTKLYKKEW

>core/306/4/Org4_Gene739

MLKLQLCALFLFGYLAIVFEHIVRVNKSAIALAMGGLMWLVCFSHIPMADHMILVEEIADMSQVIFFLFSAM
AIVELIDAHKGFSVIVKFCRIQSRTLLLWALIGLSFFLSAALDNLTSIIIIISILKRLVKAREDRLLLGAICVIAVNA
GGAWTPLGDVTTTMLWINNKITSWGIIRALFVPSLVCVLVAGFCGQFFLRKRGSTLIAKDVELQSAPPKSLWII
FIGLGSLLMVPVWKACLGLPPFMGALLGLGLVWLTSDWIHSPHGEDRYHLRVPHILTKIDISSITFFIGILLAVN
ALSFANLLTDFSLWMDKIFSRNVVAIVIGLLSSVLDNVPLVAATMGMYNLPLDDTLWKLIAYAAGTGGSILII
GSAAGVAFMGLEKVDFLWYFKRISWIALASYFGGLFSYFVLESLNFFI

>core/307/4/Org4_Gene585

MSSAIARDCFPSPSPQPSSTLGVHPPKYKSLILSVSLIVLGALLLCVGMLLLVNAIFSFSVLTVGLGGAGVFLGS
LLLILGLIFFVSYHRKLSEATRSLEQKITLEYQPWADLRKELNEVQEWSNFLLDEWEDFKEVVAQHKSQFATF
EGDLLLFGREVEKYETIWKELDGRDVALLTELKNIWGPLEFLGKKGDRLQCEIDKLRKEVMKVGKSGLKLA
CELTKFKSALKDVKIEQECYRDKRKVEKLEVLPEGYRRELLEVLKTRLSVEKEIQLFEEVVSAFEEKLASLHR
TVFSEEELQEALDKAKAELLDIQVRKSVVEDLSCEPTLIQYHLLRLYEVQCRIVEQFLTQTFSSEQEKVLEEYE
ALKARIRKTLRVKLDQVRANVAFVASTTDLSSASESLDGNDSVFEDAHDDFLD

>core/309/4/Org4_Gene833

MIETREEVGSANFVSLERAIEDLRAGKFVIVVDEASREDEGDLIIAGEKITVEKMTFLLQHTTGVVCAALSQER
LLSLDLPPMVKDNRCRFKTPFTVSVDAAHGVTTGVSAADRTKVVQLLADPKSKPEDFISPGHFFPLASSPGGV
LKRAGHTESTVDLMELAGLQPCGVLAELVNEDYSMMRLPQILEFARKHNIAVIPVTSIIAHRMLSDRLVSKIS
SARLPTIYGDFTIHVYESLLDGMQHLALVKGNVAGKSNVLVRVHSECVTGDILGSKRCDCGEQLSSAMSYIA
EKGTGVLVYLRGQEGRGIGLGHKVRAYALQDNGYDTVDANLAMGFPVDSREYGIGAQILVDLKLTTIKLIT
HNPQKYFGLQGFGLSITERVPLPVRISEDNEQYLRTKQERMGHWLDLPCCNNRVQ

>core/310/4/Org4_Gene951

MLCYSPNYVTDLYSISLSAEESLGGIRAFPQAESLLGGACALNFPDLEERLPDLRKELLFLGSNDRPDACGGKF
SLQLASSKECYIAALKERVYLNVTTSSRGPVYSFSPKGVPTELWIECFSVSMDGRVEVKVRLQGLHKELISKP
RDCETLFLNPPANKLDCWEIAGFRVDASFPVKQKIRRIGVDKFLLMHGGAEYADKATKERVDFVSSDEENYS
RYLAVGDVLLWDGNCWQTCGEFQGASSRAPLFEVKRIDDKVMIADLWNVGGTQRQTISLVKGVPSPIEINE
VIREIEFTGMRSWSKPIVLVGGQRLILSPDDWILRTAKGWEKLSRADQIQDYVTGKVTGPLLVFEKLEKDLRG
FVLRGHMFNAQRTLVETISLPLKQGFEPAVASQEVSSNTRSAAAHPGATNRGGS

>core/311/4/Org4_Gene450

MSQPPINPLGQPQVPAAASPSGQPSVVKRLKTSSTGLFKRFITVPDKYPKMRYVYDIGIIALAAIAILSILLTASG
NSLMLYALAPALALGALGVTLLISDILDSPKAKKIAEAITAIVVPIIVLAIAAGLIAGAFVASGGTMLVFANPMF
VMGLITVGLYFMSLNKLTLNYFRREHLLRMEKKTQETAEPILVTPSADDAKKIAVEKKKDLSASARMEEHEA
SQRQDARHRRIGREAQGSFFYSSRNPEHRRSFGSLSRFKTKPSDAASTRPASISPPFKDDFQPHHFKDLRSSSFG

SGASSAFTPIMPASSRSPNFSTGTVLHPEPVYPKGGKEPSIPRVSSSSRRSSRDRQDKQQQQQNQDEEQKQQSK
KKSGKSNQSRKTPPPDGKSTANLSPSNPFSDGYDEREKRKHRKNK

>core/312/4/Org4_Gene813

MCQRILILGTGITGKSVARFLYQQGHYLIGADNSLESLISVDHLHDRLLMGASEFPENVDLVIRSPGIKPYHPW
VEQAVSLKIPVVTDIQVALKTPEFQRYPSFGITGSNGKTTTTLFLTHLLNTLGIPAIAMGNIGLPILDHMGQPGV
RVVEISSFQLATQEEHIPALSGSVFLNFSRNHLDYHRNLDAYFDAKLRIQKCLRQDKTFWVWEECSLGNSYQI
YSEEIEEILDKGDALKPIYLHDRDNYCAAYALANEVGWVSPEGFLKAIRTFEKPAHRLEYLGEKDGVHYINDS
KATTVTAVEKALMAVGKDVIVILGGKDKGGDFPALASVLSQTTKHVIAMGECRQTIADALSEKIPLTLSKDL
QEAVSIAQTIAQEGDTVLLSPGCASFDQFQSFEERGAYFKLLIREMQAVR

>core/313/4/Org4_Gene804

MSKKRVVVTGFGVVSCLGNEVDTFYDNLLAGVSGVRPITSFPCEDYATRFAGWIPEFNPEPYVDKKQARRV
DPFITYAMVAAKKAIAMSRWDKDHLPSDPVRCGVIVGSGMGGLSTLDQGMERLLVIHKKLSPFFIPYIITNMA
PALIAMDFGLMGPNYSISTACATGNYCIDAAYQHLVSGRADMIICGGTEAAVNRIGLEGFIANRALSERNDAP
DQASRPWDRDRDGFVLGEGAGILVLETLESALRRDAPIFAEMLGSYVTCDAFHITAPRDDGEGITACVLGAL
NSAGIPKERVNYVNAHGTSTPLGDLSEVLAVKKAFGSHVRNLRMNSTKSLIGHCLGAAGGVEAIVAIQAILT
GKLHPTINLDNPIAEIEDFDVVANKAQDWDIDVAMSNSFGFGGHNSTILFSRYVP

>core/314/4/Org4_Gene296

MLVSIETFSSIASGSPVQKAAEACYTQYSKQPSSKEVLSSFSWIQELSLFPDRYNLATGASELIKQHWLHNNHS
LAFECILINGKYEPSLSQLPEGVIVCGIDEARGSLSSFMQGFDVNKHPLAFLNAVCSEDRGVVIYIPEEMQTSDP
IFVRHISFPTVSDHDVIFSPRIVVILGQRASAQIQISHDVDLEMVGSSKTIVNGVTELFVGEGADLTVFMVPRYS
EEDTLSWSTIATVEKDAVCRMTHNLLESCQGFGWFDNTSYIVGKKGHAESLVLVQSPRKTWVNNLMSHDA
EETVSRQNIKSILYSGHFLFEGTISISSQGDLSDAYQKHDTLLLSSEARVSTFPRLEIETDEVKASHGATVGPLD
PQQIFYMRSRGMTEAEAQEKLIHGFLKQGLVSDTFLGSSFQLNQTS

>core/315/4/Org4_Gene838

MRYHKYFRYVNSWVFLVVLTLMLLSVVVISSMDPTAMLVTSSKGLLTNKSIMQLRHFALGWVVFFICAYFD
YHLFKRWAWVLYFFMICALVGLFFVPSVQNVHRWYRIPFIHMSVQPSEYGKLVIVIMLSYILESRKADITSKT
TAFLACLVVALPFFLILKEPDLGTALVLCPVTLTIFYLSNVHSLLVKFCTVVATIGIIGSLLIFSGIVSHQKVKPY
ALKVIKEYQYERLSPSNHHQRASLISIGLGGIRGRGWKTGEFAGRGWLPYGYTDSVFSALGEEFGLLGLLFTL
GLFYCLICFGCRTVAVATDDFGKLLAAGITVYLAMHVLINISMMCGLLPITGVPLILISYGGSSVISTMASLGV
LQSIYSRRFAKY

>core/316/4/Org4_Gene400

MKLWMKIFIGLFVGVTLGLVLEDKAIFFKPIGDIFLNLLSMVVYPLVFCSMVLGIASISDMKKLGRIGIKSVGL
YLGTTALAIVIGLCFAWIFSPGNGCDFAQTQSMDSAVTVIDSNKTASYFLSIIAQVFPSNPVRSFAEGNILQIIIF
AIFLGIALRLSGERGRPVERFIDGFSEIMLRMVNMIMSFAPYGVGASMAWISGNHGLGVLWQLGKFIIAYYLA
CLFHATLVFGGLVRFGCKMSFSKFLSSMMDAISCAVSTASSSATLPVTMRCVSKNLGVSAEVSGFVLPLGAT

VNMNGTAIFQGMAAVFIAQAYNCPLSLSSLLLLVVTATFSAVGSAGVPGGGMITLGSVLASVGLPIQGIAILA
GIDRLRDIVGTPMNILGDAVVATYVASGEGELSPSESIKQESVETT

>core/317/4/Org4_Gene889

MKRIFYSFVLLSCIFPYVGCAQVFVGLDRIFSEGEYTRCIQGKKIALISHSAAINSRGQDALSVFYSRKHDCTVE
ILCTLEHGYYGATPTETVGNQPSRYPNLRSVSLYGVKEVPKEVAEHCDVFVYDVQDIGVRSYSFVTVLMQIV
KASERYGKQLIVLDRPNPMGGRIVDGPLPNPTTSGSLAIPYCYGMTPGELALFFKKTYAPNANVVVIPMKGW
NRSMTFDETGLIWMPTSPQMPDPQSPFFYAATGILGALSVASIGVGYTLPFKVLGAPWMDGEKVADELNRM
KLPGVLFLPFFYEPFFGKYKMEMCSGVLLVLQDPKIFYPVETQCTIWGVLKALYPKQVEQTLKSIERIPARRSS
ICNLFGGDEFLSISHKERYIVWPLRRLCKESRESFHQLRSSCLLSEYAES

>core/318/4/Org4_Gene859

MKKNASHKTNDKKSLSIWSIGGSIFAMFFGAGNIVFPLALGYHYNAHPWSAYFGMMLTAVCVPLLGLVSML
FYSGDYQKFFFSIGRIPGMIFITAIILLIGPFGGIPRAIAVSHATLISLSEHKSAFIPSLPIFSAICCVLIYIFSCKLSRL
IQWLGSVFFPIMLVTLLWVIIRSFMIPTHPMVQEFIPNARQAWLAGFIEGFNTMDLLAAFFFCSIVLISLRQLVA
EEKHPTEEEIPLSFQGISKKNKRSLALGFFLAAILLGVTYLGFVLSAARHAGLLVNVSKGHILGRISAIALGPNSI
LTGVSVFIACLTTEIALVGIVADFLARVVSFKKLNYASAVICTLIPTYLISILNFETISHLLLPLLQLSYPALIVLA
CGNIAYKLWNFRYSPVLFYLTLSLTIVLKLVN

>core/319/4/Org4_Gene573

MDKETLENIYRHFRYRFLKLNILPAFLGLLLLCSPNTLNYTQVDVIFSDRLCGCLLIFLAIASLTKRSLLWLGAP
LGIWVTLFACVAGRSPTIFANDTLIGFAILAVVCISPTRPEALEVGPTLPEGFSYNPSAGGRRAAVLFLSLLGWL
EARYLTASSLGITSSQSSNFLLLYSSIMTVYSLLVVLSLAGSERRWHTRPKIVIATALALTGVIILTLLPIILHQLR
YDCWLCLCLTIEPALAVVFAYDETRATLRYIAQFLGDKRALTRASFFGSEYYKHTLSWEERTVLPLRKAYKQ
AFEGISFPINQLLAILVATVFVKVNSSMGLPTFPRNFLNICCWFIIVLFILAFAESLRHLRWMNLIFSAAILFSPVL
FHIPVESPMFLPIIVTGLILIILSIGKRRRTKRKL

>core/320/4/Org4_Gene477

MIPTMLMFFIICFTLCSGFISLSQIALFSLPTSLISHYKRSKSKKQQRVATLLLHPHHLLITLIFCDIGLNIAIQNCF
AILFGDAASWWFTVGLPLAITLILGEILPKAVALPFNTQIASSVAPLILCVTKIFKPLLHWGIVGINYVVQWILS
KQQIDIIQPQELKEVLQSCKDFGVVNQEESRLLYGYLSLSDCSVKERMQPRQDILFYDIQTPLENLYLLFSKQH
CSRVPVCNDNLQNLLGICTARSLLLHDKPLQSSDDLLPLLKKPYYMPETISAKMALCQMAAEDETLGMIIDE
YGSIEGLITQEDLFEIVAGEIVDQRDNKILYTTSGADVIIASGTLELREFSEIFDINLPTNNNIATIGGWLIEQIGTI
PTTGMKLSWNNLLFQVLDAAPNRIRRVYIRKLYD

>core/321/4/Org4_Gene986

MDESDGEEASKDSAFSASFSYEFVKSSTRESKNTVTHSTASRTLYILRQDCSYDPRALKVDDEFRYWVEKRL
DAKNPDSLNAFVKEVGTHYVTSVTYGGIGFQVLKMSYLQVEELEKEKISISVAAASSLLKSKTSNATEKGYSS
YQSESSAQTVFLGGTVLPDLQQDKLDFKDWSESVPNEPIPLAISVSSITDLIIPELFPSEDAQVLSQKKSALGKVI
LNYLESHKPKEEGPKPVQITSGFNSSSSVFTLQAAKAPKTVSFPYIDYWSTIPYLFPTLKETSGAQPLSFYLRFD

DIFEQQNLVHNTSYILASTSVRLGYFGDSYRDYDALSFYGSWPQAYFDWAGYKDRCTWTLEKLNTTGDLFIRSGDEIRLKHNTSGKYLATTSMSDGYQTLTCTTQTSDSVFIITV

>core/322/4/Org4_Gene759

MNLPVSLACLLLSGCVFFLGVFVSSSLYARKKRAFLEKIQKLEHENQLLQTSLNLSRHQEQLIEDFSNRLALSSHKLIKDMKEEAQNYFGDTSKSFQSILSPIQTTLTTFKQSLETFETKHAEDRGRLKEQISQLLAVEKKLEHETHVLTDILKHPGSRGRWGEIQLERILELAGMLKYCDYDSQTTSAQGAFRADIIIRLPQDRCLIIDAKAPISDSYFSVEEIDKGDLVDKIKEHIKTLKSKSYWEKFHQSPEYVILFLPGESLFNDAIRLAPELMEIGASSNVILSSPLTLLALLKTIAYMWKQENLQKQIQEVGLLGKELHRRLQVVFTHFQKIGKNLNQTVQSYNDMASSFQYRVLPTLRKFEGLETSSSHQIEEPTPIESLATSFPHTCDIDTNLAVIESLEKQD

>core/323/4/Org4_Gene241

MKTAFHSCYSWFCWLFSFLVLFVGGIAGGEPLCPDCKYETKSVLRSDQLPDHLWNYENDCYLTGYVQSLLDMHFLDSRTQVVIEKNRAYLFSLPVDSSLSEAITNFVRDLPFICAVEICERPYGECITRSSAERPLLPKEKTLGMPIFCGKEGVWLPQNTILFSPLIADPRQVTNSAGIRFNEKVVGNRVGATIFGGDFILLRLFDVSRFHVDCDFGIQGGVFSVFDLDHPESCMVNSDFFVAGLWSGAIDKWSFRFRLWHLSSHLGDEFILTHPNFPRFNLSDEGVDLFISFRYTPQIRLYGGCGYIVSRDLTFPERPFYCEWGAELRPFGLREGNLHAQPIFAMHFRCWEEQKFGLDQSYILGMEWAKFQEIGRKIRAVLEYHQGFSKEGQFIREPCNYYGFRLTYGF

>core/324/4/Org4_Gene852

MTTIAIEAAKKVLIKLRNAGYQAYFVGGCVRDMLMNRPLEDIDIATNASPTIVSTIFPDVISIGVAFGIIVVKQDGRLFEVATFRSDGEYKDGRHPDRIIFSSMREDALRRDFTVNGMYYDPFEDKVFDFVEGTRDIEKKVIRAIGHPRLRFSEDKLRILRAIRFSSSLGFTLDPTTERAIIKEAPALVNSVSPERIWQELKKMLKRQPHGALSLLLKLKVLIFIFPELRDIPYSLLRTTIEFARKFNPTHFPEILFLLPLFQGVSEEAATVAFGRLRISNKELKLIESWYEALPHFQNQSSNRVFWAHFLASPTAPLFLELFSALQKDPSRQQHFISRVQELESRLEQFILRIKTSSPVVSAPDLIAKGITPGRLLGDLLREAEILSIENECLDKEKILLLLQEKGFWK

>core/325/4/Org4_Gene297

MKNLKEDFPIFAAKAKENEPFIYLDSAATTQKPQQVIDAVANFYTSSYATVNRAIYSSSRNVTEAYAAVREKVRKWVSAASDSEIVFTRGTTAGLNLLAISVNDLWIPKGGVVLVSEAEHHANVLSWEIACRRRGSLVKKIRVHDSGLIDLDDLEKLLNEGAQFVSIPHVSNVTGCVQPLQQVAELVHRYDAYLAVDGAQGAPHLPIDVQLWDVDFYVFSSHKIYGPTGIGVLYGKKDLLDQLPPVEGGGDMVAIYDHQNPEYLPAPMKFEAGTPNIAGVLGLGAALDYLDGLSAKFIYDKEIALTTYLHKELLEIPGVEILGPSIEEPRGALISMTIDGAHPLDLGFLLDLRGIAVRTGHQCAQPAMERWNVGHVLRVSLGIYNDEDDINQFILVLQDSLDKIRR

>core/326/4/Org4_Gene933

MVVVALFILGIFFLSGSLAFLVHTSCGVLLGAALPILCIGLVLLAVALIVFLCHKHKTRQDLDYYDQDLDSLVIHKKEIPNDISELRVTFEKLQNLFQFHTKDFSDLSQELQGKFINCMEKWLTLEDEVTKFLIVRDRFLETRRNFTTFGEQVKEIQSNIFDLHEEKSSLYLELYRLRKDLQVLLNFFLLPPGILKVDYDEIEAIKGLFIRLTSRLDKLDVKAQERKKFINEMSREFKEVEKAFDIVDRATKKLMDRAKKESPARLFMGRTESLLEMKKNEEALKNQGLDPENL

SHPELFSPYQQLLILNYLNSEIVLHHYEFLISGAVTSGLTLEECENRMRAASTRLNAFLVRKLQFRGAIKSAYF
EKLTEIEKELRSLQDVIKSLELELIHKIKDIVTEET

>core/327/4/Org4_Gene492

MTDSNPLPSYTDASLYRTPAKHSYPIRLPLNRTDRIEKILKIVTLTLALACALGFSIAAGILAMPIFSAVVVITLA
IAAVSLYSLLKKPKLYEILPQIEPESEESSLSPSPQPPEQQDLPLQIDPLPDPESLPEVSLADLTTPPEELTAITVTP
GYEALLQQNWDLLPSLAAVDPSFTTETPQQPCFIWKLKDSKLIFISTSGDIAVPRIKTQGRVMIVNAANENISR
EGGGTNKALSLATSLQCWNASRLPRAHSRSGSQLQPGECRSAKWENSDHTSNDHVPGKAHFLAQLLGPEAA
KCNNDPKQAFEVSKKAFHNLFQEAEIIGVDVIQLPLIGCNLFAPSRLLNLGKTRAEWIEAVKLALITSLQDFG
WEQDNQEEQKIIILTDQDQPPIIPPLFDLTTP

>core/328/4/Org4_Gene537

MKKQRSHYTKNNLLLLLSILVGLGLGSVQSPWIVYSAECIANTFLKFLRLLSIPLVFCALGSTITSIQNFNTMVT
LGKRILYYTLLTTVIAASIGLLLFFLLRPQMITQDALATTTKCNPLGYLDVLSDTLPENIFKPFLQGNVISAACL
AVLLGTASLFLQEKEKHFVNQFFNSFFSIFLNLARGGLKLLPIAMLGFSVILFKELKDQSNLTMFAKYLLCVIG
ANLAQGFIVLPILLKINKVSPLKVAKAMSPALVTAFFSKSSAATLPLTMELAEDDLKINKNLSRFSFPLCSVIN
MNGCAAFILITVLFVATSNGMIISPLMSLGWIFIATLAAIGNAGVPMGCYFLTLSLLTSMNVPLSILGLILPFYT
VIDMIETSLNVWSDCCVVSLANQQLSKKLSL

>core/329/4/Org4_Gene476

MTNSALFWIGVNIICIVLQGFYSMMEMACVSFNRVRLQYYLTKDHKKARYINFLIRRPYRLFGTVMLGVNIA
LQVGSESSRNCYRALGITPDYAPFTQIFIVVIFAELLPLTISRKIPEKLALWGAPILYYSHYIFYPLIQLIGSLTEGL
YYLLNIKKEKLNSTLSRDEFQKALETHHEEQDFNTIATNIFSLSATCADQVCQPLEQVTMLPSSANVKDFCRTI
KNTDINFIPVYHKARKNVIGIAHPKDFVNKALDEPLINNLHSPWFITAKSKLIRILKEFRDNRSSVAVVLNASG
EPIGILSLNAIFKILFNTTNIAHLKPKTISVIERTFPGNSRIKDLQKELDIQFPKYPVETLAQLVLQLLDSPAEVGT
SVIINNLLLEVKEMSLSGIKTVSIKNLLS

>core/330/4/Org4_Gene142

MDKLTVQDLSPEEKKVLVRVDFNVPMQDGKILDDIRIRSAMPTINYLLKKHAAVILMSHLGRPKGQGFQEEY
SLQPVVDVLEGYLGHHVPLAPDCVGEVARQAVAQLSPGRVLLLENLRFHIGEEHPEKDPTFAAELSSYGDFY
VNDAFGTSHRKHASVYVVPQAFPGRAAAGLLMEKELEFLGRHLLTSPKRPFTAILGGAKISSKIGVIEALLNQ
VDYLLLAGGMGFTFLQALGKSLGNSLVEKSALDLARNVLKIAKSRNVTIVLPSDVKAAENLQSKEYSVISIDQ
GIPPHLQGFDIGPRTTEEFIRIINQSATVFWNGPVGVYEVPPFDSGSIAIANALGNHPSAVTVVGGGDAAAVVA
LAGCSTKVSHVSTGGGASLEFLEQGFLPGTEVLSPSKS

>core/331/4/Org4_Gene825

MSTMQNCPHFGVCGGCSFPQSNYSDSLKKKEELLHQLFAPLVPSDMIAPIIPCSPSLRGRNKMEFSFFQTYEGE
KSLGFISSTKPKKGIPVTTCLLIHEQTMDILKLTREWWDKHPELMAYFPPKNKGSLCTLTVRTGSPQQNFMVI
LTTSGTPEYRVNEACIDEWKEILLSSSLNIASIYWEEKVAARGISTYYETKLLYGAPSIQQKLSLPSDDNSASFS
LRPRSFFQPQITQAAKIIETAKEFINPEGSETLLDLYCGAGTIGIMLSPYVKNVIGVEIIPDAVASAQENIKANNK

EDCVEVYLEDAKAFCKRNENCKAPDVIVIDPPRCGMQSKVLKYILRIESPKIVYISCNPKTQFQECADLISGGY
RIKKMQPIDQFPYSTHLENIILLEREIDL

>core/332/4/Org4_Gene163

MLLVRKWLHTCFKYWIYFLPVVTLLLPLVCYPFLSISQKIYGYFVFTTISSLGWFFALRRRENQLKTAAVQLL
QTKIRKLTENNEGLRQIRESLKEHQQESAQLQIQSQKLKNSLFHLQGLLVKTKGEGQKLETLLLHRTEENRCL
KMQVDSLIQECGEKTEEVQTLNRELAETLAYQQALNDEYQATFSEQRNMLDKRQIYIGKLENKVQDLMYEI
RNLLQLESDIAENIPSQESNAVTGNISLQLSSELKKIAFKAENIEAASSLTASRYLHTDTSVHNYSLECRQLFDS
LREENLGMLFVYARQSQRAVFANALFKTWTGYCAEDFLKFGSDIVISGGKQWMEDLHSSREECSGRLVIKT
KSRGHLPFRYCLMALNKGPLCYHVLGVLYPLHKEVLQS

>core/333/4/Org4_Gene646

MRMLQISMLLLALGTAINSPAIYAADSQSVSFPEQLLSSFTGEIKGNHVRMRLAPHTDGTIIREFSKGDLVAVI
GESKDYYVISAPPGITGYVFRSFVLDNVVEGEQVNVRLEPSTSAPVLVRLSRGTQIQPASQEPHGKWLEVVLP
SQCVFYVAKNFVANKGPIELYTQREGQKKIAMDLINSALNFAHIELEKSLNEIDLEAIYKKINLVQSEEFKDVP
GIQGLIQKALEEIQDAYLSKSLESQNTSIASSQCSTPKVSSSSEVTTSLLSRHIRKQTALKTAPLTQGRENLEYSL
FRIWASMQQGNDHSEALTQEAFYRAEQKKKQVLVGVLEVYPHVVKNNPGDYLLKAQENTIAFLYGTSINLE
QWLGKRVTVECLPRPNNHFAFPAYYVVGIKEAS

>core/334/4/Org4_Gene62

MAASGGTGGLGGTQGVNLAAVEAAAAKADAAEVVASQEGSEMNMIQQSQDLTNPAAATRTKKKEEKFQT
LESRKKGEAGKAEKKSESTEEKPDTDLADKYASGNSEISGQELRGLRDAIGDDASPEDILALVQEKIKDPALQ
STALDYLVQTTPPSQGKLKEALIQARNTHTEQFGRTAIGAKNILFASQEYADQLNVSPSGLRSLYLEVTGDTH
TCDQLLSMLQDRYTYQDMAIVSSFLMKGMATELKRQGPYVPSAQLQVLMTETRNLQAVLTSYDYFESRVPI
LLDSLKAEGIQTPSDLNFVKVAESYHKIINDKFPTASKVEREVRNLIGDDVDSVTGVLNLFFSALRQTSSRLFS
SADKRQQLGAMIANALDAVNINNEDYPKASDFPKPYPWS

>core/335/4/Org4_Gene931

MVYFMVFFPSSESVVKANSVVRSNFCYFLENKFVSPSESTEVMFSEIMNGRVPDIESLFDRPTDMMMTGFKA
AQNLGNLFNSFGILIMCFSQCKSCQTPEKETSAIVLGATLLFFVIALILGPTLGALVYCAYKVYTLGKMIYSLN
KAKAKVLRHPAQNVFHRAAGVATIRSSEEAVKACKLYKSAMIGSLVVSLIASLALIALTAGIVLALFFVAPGA
APVITAAMIGCCAAGGGALLISLLGLWIAMVRKAKHQEACVGHLTNVVLHTAVSEALLHDPSHFQTNALAR
DLFLTDCLSHYGHLFSNEEVAQLVQGGAPGGGSRPSQHYGGPSDYQNRRGGNGNFGGSHFGGGGGFAGSHF
GAGYPTAPTMPSAPPPPPYNPAFNPPPFPPPAYDTIYG

>core/336/4/Org4_Gene90

MLSVTIVLVGLEMARSKVSKRDSKILDILFATTELYLKTGQPVGSKTLKESFCSDLSTATIRNYFAELEAEGFL
KKNHTSGGRIPTDLALRHYVDHQEECPEAEISAPIFDKISQLPSESRNIIKDLQKATELLGEILDLPTFFSSPRFEN
DSVTNIQITQVDKQRAVTILSTEFGQIFTDTLWLPEACDTLSIKRIEKFLQNYIRKLPTNEELSKKEEHLSMSLY
NEVVVRYLTRYCNFSEEDLYQTGMSKLLKYEAFKDPEVLALGLSLFENRRQMCELLNIGMHKGRATAFIGK

ELSDILGTSNPGCSVITIPYYMNRSPLGALGILGPINLPYKEALPLLKLFANKINETLTQSFYKFKLSFRRPLTSN
CKLSNEPILRTEYSSIKLLPSKETL

>core/337/4/Org4_Gene419

MRRNPHFSLLKPQYLFSEISKKLAQFRKENPEISVIDLSIGDTTQPLCRSITQAIKEFCVSQEKQETYRGYGPET
GLEKLRTKIASEVYENRISPEEIFISDGAKPDIFRLFSFFGPEKTLGLQDPVYPAYRDIAHITGIRDIIPLACRKET
GFIPELPNQQSLDILCLCYPNNPTGTVLTFQQLQALVNYANQHGTVLIFDAAYSAFVSDPSLPKSIFEIPEAKYC
AIEINSFSKSLGFTGMRLAWNVIPKELTYDNNEPMINDWKRLFATTFNGASLLMQEAGYYGLDLFPTPPAISL
YLTNAQKLKKSLETAGFSVHGGDHAPYLWVELPEGISDEEAFDFFLHQYHIAVTPGHGFGSCGQGFVRFSAL
AQPQNIALACDRLCTASLKETMVLA

>core/338/4/Org4_Gene769

MSNKVLGGSLLIAGSAIGAGVLAVPVLTAKGGFFPATFLYIVSWLFSIASGLCLLEVMTWMKESKNPVNMLS
MAESILGHVGKISICLVYLFFFYSLLIAYFCEGGNILCRVFNCQNLGISWIRHLGPLGFAILMGPIIMAGTKVIDY
CNRFFMFGLTVAFGIFCALGFLKIQPSFLVRSSWLTTINAFPVFFLAFGFQSIIPTLYYYMDKKVGDVKKAILIG
TLIPLVLYVLWEVVVLGAVSLPILSQAKIGGYTAVEALKQAHRSWAFYIAGELFGFFALVSSFVGVALGVMD
FLADGLKWNKKSHPFSIFFLTFIIPLAWAVCYPEIVLTCLKYAGGFGAAVIIGVFPTLIVWKGRYGKQHHREK
QLVPGGKFTLFLMFLLIVINVVSLYHEL

>core/339/4/Org4_Gene498

MVNIQPVYRNTQVNYSKATPFSVCQPALSLIIVSVVAAVLAIVALVCSQSLLSIGLGIALVLVSLILFASAMFMI
YKMRQEPKELLIPKKIMELIQEHYPSIVVDFIRDQEVSIYEIHHLISILNKTSVFDKAPVYLQEKLLQFGIEKFKD
VHPSKLPNFEEILLQHCPLHWLGRLVYPMVPDVTPGTYGYYWCGPLGLYENAPSLFERRSLLLLKKMSFGEF
ALLEDGLKKNTWSSSELVQIRQNLFTRYYADKEEVDEAELNADYEQFNSLLHLIFSHKLSWKQVQLFQYMN
ESGWDWLCDFDSQGEGFQLSRLVGLLHSSWALYEAKEQFYLPEVSLLTWEELIEMQWLSKPAKHGVAKDL
CNVFEKHFQRFRQYLGSLESKSKVRKYLLELS

>core/340/4/Org4_Gene259

MSFFNHIPTFSPDAILGLQNVFFADKRPEKVNLVIGVYEHPQKRYGGLSCIRKAQTVILEEEQNKSYLPISGLQI
FLDEMRELVFGAVDPSAIVGFQSLGGTGALHLGARLLSVAKGSGKVYVPEQTWSNHIRIFSQEGLEVIRYPYY
SKEQKQLLFEPLIAFLKEVEKNSVILLHGCCHNPTGVDFTEDMWKELAILMKERELIPFFDTAYQGFAQGIEL
DRKPIEIFISEGNTVLVAASSSKNFALYGERVGYFAVHSTFTDELVKIRSFLEEKIRGEYSSPQRWGVEIVSTILS
NPYLKEEWQSELNFIRESLGKMRTRFVQALRKVAGHTFDFLLSQHGFFAYPGFSDKQVLFLREQHAVYTTAG
GRMNLNGITEKNIDHVVQSFIQAYEL

>core/341/4/Org4_Gene932

MVVSIYSEILSFSELTSCKHSLFPFGPIETASIRIHHVFNVVIVCLIILGTLFVCLGMVFLGVFSTYLLGMSSMILG
LLLISIGLALLKFKERYGLEPKELFGVEGGFDKKLPSEIIQMQDQIADLARELDLEQKKDTLIRGFSARLDVLE
GSKTEKKQILKIGVPRNLSEIQERAQELNSILEQCKEALLFRRKSAQEIFKKLYDRKAAFWRSYREDLWCYSEI
HVSKKALSNLYIGDVFEGTAPHFLMEAYAMCRTAKNLRNYVKVCVEDMRMRIDEEKKRAKQLSVSELLCC

CTEIETDLENETNLFTSDSEDVLEEYQIHCIRVTMLHALWAIYNDEVVCRKPIDTLDRVRARMAVEDCIETFEE
LQMCVVHTKTLELEIAQLYVDILLEA

>core/343/4/Org4_Gene16

MSKETFQRNKPHINIGTIGHVDHGKTTLTAAITRALSGDGLASFRDYSSIDNTPEEKARGITINASHVEYETPN
RHYAHVDCPGHADYVKNMITGAAQMDGAILVVSATDGAMPQTKEHILLARQVGVPYIVVFLNKVDMISQE
DAELIDLVEMELSELLEEKGYKGCPIIRGSALKALEGDANYIEKVRELMQAVDDNIPTPEREIDKPFLMPIEDV
FSISGRGTVVTGRIERGIVKVSDKVQLVGLGETKETIVTGVEMFRKELPEGRAGENVGLLLRGIGKNDVERG
MVVCQPNSVKPHTKFKSAVYVLQKEEGGRHKPFFSGYRPQFFFRTTDVTGVVTLPEGTEMVMPGDNVELDV
ELIGTVALEEGMRFAIREGGRTIGAGTISKINA

>core/344/4/Org4_Gene960

MFEFRFPKIGETSSGGSIVRWLKNLGDHVARDEPLIEVSTDKIATELPSPKAGRLVRFCVNEGDEVASGDVLG
LIELEEEISEADDESTSCPLTSCETKSEAGSSSSSAWFSPAVLSLAQREGIGLDNLQKIAGTGKGGRVTRQDLEA
YISESQQVSIPETFQGEVNRIPMSPLRRAIASSLSKSSDEVPHASLVVDVDVTDLMNLISGERQRFLDTHGVKL
TITSFIVQCLAQTLRQFPLLNGSLDGTTIVMKKSVNVGVAVNLNKEGVVVPVIHNCQDRGLVSIAKALADLSS
RARLNKLDPSEVQDGSVTVTNFGMTGALIGMPIIRYPEVAILGIGTIQKRVVVRDDDSLAIRKMVYVTLTFDH
RVLDGIYGSEFLTSLKNRLESVTMG

>core/345/4/Org4_Gene690

MDYYSILGISKTASAEEIKKAYRKLAVKYHPDKNPGDAEAEKRFKEVSEAYEVLSDPQKRDSYDRFGKDGPF
AGAGGFGGAGGMGNMEDALRTFMGAFGGEFGGGSFFDGLFGGLGEAFGMRSDPAGARQGASKKVHINLTF
EEAAHGVEKELVVSGYKSCETCSGQGAVNPQGIKSCERCKGSGQVVQSRGFFSMASTCPECGGEGRIITDPCS
SCRGQGRVKDKRSVHVHIPAGVDSGMRLKMEGYGDAGQNGAPSGDLYVFIDVESHPVFERRGDDLILELPIG
FVDAALGMKKEIPTLLKTEGSCRLTVPEGIQSGTILKVRNQGFPNVHGKGRGDLLVRISVETPQNLSEEQKEL
LRTFASTEKAENFPKKRSFLDKIKGFFSDFTV

>core/346/4/Org4_Gene602

MSSPVNNTPSAPNIPIPAPTTPGIPTTKPRSSFIEKVIIVAKYILFAIAATSGALGTILGLSGALTPGIGIALLVIFFV
SMVLLGLILKDSISGGEERRLREEVSRFTSENQRLTVITTTLETEVKDLKAAKDQLTLEIEAFRNENGNLKTTA
EDLQEQVSKLSEQLEALERINQLIQANAGDAQEISSELKKLISDWDPKVVEQINTSIQALKVLLGQEWVQEAQ
THVKAMQEQIQALQSEILEMHNQSTALKKSVENLLVQDQALTRVVGDLLESEKKLSQACSALREEIEKLAQH
ETSLQQRIDMMLAQEQNLAEQVASLEKMKQEAQQAQSDFIACVRDRTFGRRETPPPPTPPVVKGDESQEEDE
GSTPPVSQPSSPVDRATGDGQ

>core/347/4/Org4_Gene869

MPRYRYTYLDPKERRKRGYLEALHIQEAREKLAQENIQVLDIREVALRRMSIKSTELIVFTKQLLLLLRSGLPL
YESLVSLRDQYHGQKMGLLLTSFMETLRSGGSLSQAMAAHPNIFDHFYCSGVAAGESVGNLEGCLQNIIVVL
EERAQITKKMIGALSYPCVLLVFSFAVMLFFLLGVIPALKETFENMEVKGLTKIVFGVSDCLSAYRYLFLGFAS
ALITVGILMRHRIPWKKILEKLLFALPGTKKFVVKVAVNRFCSVASAILKGGGTLIEGLDLGCDAIPYDRLKTD

MRDIVQAVIGGGSLSQELAQRSWVPKLAIGMIALGEESGDLADVLGYVAHIYNEDTQKTLASITSWCQPVILI
FLGGLIGVIMLAILIPLTSNIQTL

>core/348/4/Org4_Gene847

MHPKIEKRNSLPLTAVAPVFEESYHPSVATTVDYVDATTLSRHLTVLKDVIKEARNLDLGKAFLTSMKQGFI
NTGTELAIIQASLADQSSRESRKKEEKIFHQHLGKAAPQASTATSGVQPTADPVADKMPLQSAFAYVLLDKYI
PAQEEALYALGRELNLSGYAQNLFSPLLDMIKSFNSAPINYNLGSYISQTSGTANFAYGYEMILSRYNNEVSQ
CRLDIASTVKAKAALANMSASVKANVSLTDAQKKQIEDIIASYTKSLDVIHTQLTDVMTNLASITFVPGLNKY
DPSYRIVGGDLSIIALQNDEKVLVDGKVDITTAVNEGGLLNFFTTVLTDVQNYGDLAQTQQLMLDLELKAM
QQQWSLVSASLKLLNGMYTTVISGFKN

>core/349/4/Org4_Gene291

MKKLLKSALLSAAFAGSVGSLQALPVGNPSDPSLLIDGTIWEGAAGDPCDPCATWCDAISLRAGFYGDYVFD
RILKVDAPKTFSMGAKPTGSATANYTTAVDRPNPAYNKHLHDAEWFTNAGFIALNIWDRFDVFCTLGASNG
YIKGNSTAFNLVGLFGVKGTSVAANELPNVSLSNGVVELYTDTSFSWSVGARGALWECGCATLGAEFQYAQ
SKPKVEELNVICNVAQFSVNKPKGYKGVAFPLPTDAGVATATGTKSATINYHEWQVGASLSYRLNSLVPYIG
VQWSRATFDADNIRIAQPKLPTAVLNLTAWNPSLLGNTTTLPTSDSFSDFMQIVSCQINKFKSRKACGVTVGA
TLVDADKWSLTAEARLINERAAHVSGQFRF

>core/350/4/Org4_Gene921

MMKQGVGQDAKELYTFLSRGNENYQPCLWFSLEEELGFLFDEKMLCAPLSEDHYCHSYLVDLVDQHLKDLI
LSMFLDPQNISGGELLKVSINIGDSLSPQQQKDFLSMVLRDETEKNVVVVSQGVLSLPATQVCKLVEELNTKD
YSYLNIFSCHADSSPQLLFRKELEGTSGRYFTVICTLYLGGADMRSLQLASERIMVSREFGLVDAYAARCKLL
KIDHTNWRPGTFSRHADFADAVDVSAGFNSKEFKLITQANQGILESGELPLPSKTFWEGFLAFCDRVTVTRHF
VPMLDAAIKQAVWTHKHPSLIDKECKALDLKTQCLPSIVSYLEYVTNSQEKTSKVPFIQKEIIVDCSPLKEALF
PGSDEDVPSTSEDPSDDHPSDLEDS

>core/351/4/Org4_Gene304

MGTPISGDDGDRNTISDPLEESAAEEGDSDLEDRVSESATQVIETIADTGIPEATPSEGTNSDLNSDLVDRVEY
EARGSLLTTMLARIRKAVSQIWMHVKTKRHPKEQGVRSLGDIPCDLLKATRLPKETAEPPYFYALETALASC
RSFFFHVFLRLFTLLRRQHPEAPLDLCGTDPISPEAAVAFALILRSCCKWVATDAVQEGLPLEVIEEAGIYNAF
SLEATTTVEEVSKRLSELLYSDKRIDGLANVRGITKIITSPYLGAGQCVSVVDNLKTYDLGRNYTQVLACASQ
IDEFADKGENEALVMKDILYLVRQDRSKELGDFLMMWSEEHASEVNYDVVLAILEVNLPILEEDYRSHPLAY
QKKLNYVICQFFCSERLTSIEPKD

>core/352/4/Org4_Gene93

MLHILLAIFCILLFLAFGLTQPSCHGSSKFLKTLNQRFFKDKGREYPPFPSAPTILATLLCILYGALGTKLYTLLP
PKTAHKDLLFWPLYSLSALIAYGFLPPWISTKVPKETTAHLRFLASVFQLGLFPLQLLFYRRRPNQQVRSSTSF
QSQLSEALSAFDNLIVREVMIPKVDIFALPEETTLQEALVLVSEEGYSRVPVYKKNLDNITGILLVKDLLLLYT
SSHDLSQPISSVAKPPFYAPEIKKASSLLQEFRQKHRHLAIIVNEYGFTEGIATMEDIIEEIIGEIADEHDVQENTP

YKKIGSSWIVDGRMNISDAEEYFNLKIDHENSYDTLGGHVFHKVGAVPQKGMRIHHENFDIEIITCTERNVGK
LKITPRKRKFNIS

>core/353/4/Org4_Gene360

MAIKNILVVDDEPLLRDFLSELLTSQGFIPDTAENLRNALQMIRTRDYDLVISDMSMPDGSGLDLIKIIKQSSPH
TPVLVVTAYGSIENAVEAMHQGAFNYLTKPFSSEALFAFISKAEELKNLVHENLFLHSQTTPDSHPLIAESKA
MKDLLAIAKKAASSSANIFIHGESGCGKEVLSFFIHHNSPRANHPYIKVNCAAIPETLLESELFGHEKGAFTGA
TTKKAGRFELAHKGTLLLDEITEVPVNLQAKLLRAIQEKEIEHLGGTKTLSVDVRILATSNRNLKEAIDDKSFR
QDLYYRLNVIPLHLPPLRDRQDDILPLANYFLNKFCRMNNTPLKTLSPKAQELLLNYPWPGNIRELSNVLERV
VILENTSLLTEDMLALA

>core/354/4/Org4_Gene767

MHLHEYQAKDLLASYDVPIPPYWVVSSEEEGELLITKSGLDSAVVKVQVHAGGRGKHGGVIVAKSSAGILQ
AVAKLLGMHFTSNQTADGFLPVEKVLISPLVAIQREYYVAVIMDRKHRCPVLMLSKAGGMDVEEVAQSSPE
QILTLPLTSYGHIYSYQLRQATKFMEWEGEVMHQGVQLIKKLAKCFYENDVSLLEINPLVLTLEGELLVLDSK
ITIDDNALYRHPNLEVLYDPSQENVRDVLAKQIGLSYIALSGNIGCIVNGAGLAMSTLDILKLHGGNAANFLD
VGGGASQKQIQEAVSLVLSDESVKVLFINIFGGIMDCSVVASGLVAVMETRDQVVPTVIRLEGTNVELGKEIV
QQSGIPCQFVSSMEEGARRAVELSM

>core/355/4/Org4_Gene811

MKWFVISCLLGIFSLGLIMVFDTSSAEVLDRSLECSTHKALIRQVTYLILGLGVASLLYMMEWRDFLKISPVLL
SGATLALICVFIPGLGICRNGARRWLGFGQLTIQPSEFVKYLVPIVALYFLTFSSLYQKQLKMFLKLTAILFIPIL
LIAIEPDNGSAAVISASLIPVFIMTSVRLRYWLLPLLCVLIAGGALAYRMPYVRYRLNVYLHPELDIKGRGHQP
YQAKIAAGSGKLLGKGPGASLQKLTYLPEAQNDYIAAIYAEEFGFLGMLVLILLYMCFVYGGYAIAIKASSLE
GAALAMVITLIISMQAFMNLGVVSGLLPSKGVNLPFFSQGGSSLIANMCGVTLLLKVYDEENSKSSLGCRRFR
RPHCPSSLGKGSFFS

>core/356/4/Org4_Gene417

MSSVNQSSGTPNPEEVTSPESTEENKNVVSSDEAQATHAVALPIVTQLSLPEGVGTSPEETASNPKVDEIVAEV
SSSRAVVDQISSLVERVGELLDDLQGVQSLFTSFQSELKNCLPAWKSSTRRLETRGAGDNADIARLELFRSDY
EAVLDHVNQFHGDAHLTLSKLTDIHHKLQGNSREDLFLAFDNNDRVLEHLGSLGLDVDAEGNWSLSCERGI
PRLVLTADSMLVQIKKVNLPTVEELRTLQGTTESSSDPRVEESLSCCERLLNELRRLWANFVGFISSCYDNIVF
VLMWIMRRINLLPGQGCLPFHNPDASQEDHRSSSGERSTRRERLSRRSDLSEEEMIVRAEGESIHPESPHGDGR
NQPSRGDKQDSDSEEETEL

>core/357/4/Org4_Gene841

MEKPQNRKAPRIFWLNNQVAIPPSERVKESYALHSDIFSLPPGSALKLAEKTEESIRQLVGLKDSHIFRFVPHFP
HVVHIVLAALVENLSMFQGRNHIILPAHDQQLLINSLCRHQGLGTTYDWVTVNHEGRIVEEQLIETLSPRSLLF
SLSAAHGLTGVIQPLDPLLSLCKDRRILLHLDISDILGRAPLTPEILNADIITFSSVALGGMGSIGGIFIRKSLERV
FSSWFPPHTSASLCFSAVAAMQTACEERISALPLFTFHTSNLCKKLIQELQSVLPSIQLAFSEVQNRLPNIVVAA

IPDIPAESLAFHLHQQGIYPSLGYERFQPLAQVLQNCGISPFLCHSALHFSLTERSKDLEFSKLARAMHDAVKH
LTPLLGSSS

>core/358/4/Org4_Gene305

MILDFQFSIGYYLRVLELAIRDGTRILAYDRKRLLLDAWPVNDPLPTNYDTSVSTIRQVIHELFSWSAISYSISS
RLLAIIELRLHEEKPQTGWLYRLFFPSKYHIKKAIVDKLCMFKSLILFESKRPVDKIVHAANKVFSKGKSNFSS
WEDFTHEVTVSEVQTPLAGEVQGRLAADASLQMIIEALTTLLEGHTAYLPLSLELLNQFIGEKAQPLKTLSEK
SYVLLRELIQLFSWSAEDFQTIIMSIISDSLSEVLANSLIGNQPLTFHGKTFVGLWQETALVSPEDSKLALGFLA
EVLRKVIVEKKLHVSKSDNTTPEEVGNIYSIRDQNPALWDKMITMLLMRWLLDYDRDIGIALRKAAEYYNPH
PSFWRQFLRLWQRRP

>core/360/4/Org4_Gene567

MSAMISLSSSHEASIASNTQVRDVLVSLAMDEFVEHNTEILPIKVFLARGTLSSTAIIDDLKDVVETEGEHHFQ
VYSNISLKMIYQRFFEKIFGIGCCPLLLVTDSHHTDPCGALITGIFAAVLFTVLAIVFGPTLGILCYSAYKIYQLT
KKISSLSRTHTEVINSVQKSDPFIHRSGAVAAAAASQSTIKACKVFRQSTLIFFVLGLIITISLAALIVGLVFALFF
LGPGAPAVMTAAMIGCCAAGGTGILLSVIGFLLASVYSVQKSKEGVHHMHTALLRCMVSNTIIQMPYLPITP
GTKKVLTQSIRRYQQFFSDDEYRDIESEVPLTPPPSYETLFHEEGSDGSSNVIPRESPPAYSTIDSSNSPFPSSSPP
PYYR

>core/361/4/Org4_Gene996

MSETIITTPHVVKLISNFFQKKLFSSISTAYPLVIITDVSVQQHLLGPILDHIKMLGYQVIVLTFPPGEPNKTWET
FISLQYQLVDQNISPKSSIIGIGGGTVLDMTGFLAATYCRGLPLYLIPTTITAMVDASIGGKNGINLRGIKNRLG
TFYLPKEVWMCPQFLSTLPREEWYHGIAEAIKHGFIADAYLWEFLNSHSKMLFSSSQILHEFIKRNCQIKAAIV
AEDPYDRSLRKILNFGHSIAHAIETLAKGTVNHGQAVSVGMMIETRISLAEGVMKTPQLIDQLERLLKRFNLP
STLKDLQSIVPEHLHNSLYSPENIIYTLGYDKKNLSQHELKMIMIEHLGRAAPFNGTYCASPNMEILYDILWSE
CHVMRHC

>core/362/4/Org4_Gene721

MSCQQFLIEALARRKSKHTYRSLSLNSHLIDFTSNDYLGFASSPELRKEYITKLHAIESLGATGSRLLTGHSQLC
QRIEEQLAAYHNFESCLIFNTGYTANLGLLYALATDQDRILHDLYIHASIYDGIRLSKAQSFPFNHNDLNHLEK
RLASSHLGRTFVCVESVYSLHGSVAPLQAISELCERYSAYLIVDEAHAVGVFGDQGEGLVSALGLQDKVLAT
VYTFGKALGTHGAAIAGSSILKDYLINFCRPFIYTTAQPPHALTAIELAYEHNQRAFNQREHLSALIHHFREKA
QNLGLQLMKDNTTTPIQSICVSGSHRARQAALQIQNSGYDVRPIVSPTVKQREELLRICLHAFNTKNEIDHLLH
TLEQIFLCNVSSL

>core/363/4/Org4_Gene538

MSCFNLTSTNESLRPISPKASFPKQGWQSYFRSALRKHRSNTLSVSVCKVNKYDVNLFVRLTVIALAVVGVLI
LFSIMLASIQGTLVITSWPLVTAAILIPTILLTGGMYILHRLGKKVDVISGVCIPPFSRRCWVPISCSHTLEKFDE
KHVSACSYLDISTLSADGSGIAAVYQCPPLLFRAFPCFGIPCAMPFVALLRMIYNLIRFLVVPFYIIFRMIYEHFF
CKHLPEDDRFICKDIAREMGRSLAAFLKAPFYASACMIGAFYSLLDPLAGRVLMGSVERDWNDNVILARSVS

LANEAHSLFRFEGGGGRKGLGQHAFYLMLCCQPQSVFLFDKGEIVSGAHPSIQLPERRGLDTSGRYPHISVIPD
SGNDSAKNFIV

>core/364/4/Org4_Gene303

MGINPSGNRSPDDVWVRGAQGDSSSTQGTGATNSNLGAHNVTTSTSQPQVASRAKQLWQMVKEFFLGKKS
PDSSQGASGPAMQSPSGPTIRPTRPAPPPPTMGGVNAKRPATHGKGPAPQPPTTGSSSGSEQPTAMSPEVVKL
VSELKDAVHSHAESQKVLKKVSQELQTKWSDWENNKGPDYSLHGYRVVTRALQQTYTEQSMLIEGTSSTGP
VPQAVTVAKDAVTQTVREAVKNLENPKPGNDPDGVLMQVVTSLGIEGPTLDPGESIQNFLETRVSDFGGDDS
DIDYTGDIARLGAALDRVRENHPNEMPRIWIALARELGAAVHSHATSVRIANAGKNHTRDVVRMANESSRL
LQGMKVLSVGAWANTMTVLIGDLFE

>core/365/4/Org4_Gene797

MIPSPTPINFRDDTILETDPKPSLIMFSSKKTEIASERRKAHPTLFKVLGTIWNIVKFIISIILFLPLALLWVLKKTC
QFFILPSSIISQSMSKTAVAIRRMTFLSHIKQLLSLKEISAADRVVIQYDDLVVDSLAIKIPHALPHRWILYSQGN
SGLMENLFDRGDSSLHQLAKATRSNLLVFNYPGIMSSKGEVKRENLIKSYQACVRYLRDEETGPKANQIIAFG
YSLGTSVQAAALDREVTDGSDGTSWIVVKDRGPRSLADVANQICKPIASAIIKLVGWNIDSVKPSERLRCPEIF
IYNSNHDQELISDGLFERENCVATPFLELPEVKTSGTKIPIPERDLLHLNPLSPNVVDRLAAVISNYLDSENRKS
QQPD

>core/366/4/Org4_Gene507

MKHLAVLGSTGSIGRQTLEIVRRYPSEFKIISMASYGNNPRLFFQQLEEFAPLAAAVYNEEVYNEACQRFPHM
QFFLGQEGLTQLCIMDTVTTVVAASSGIEALPAILESMKKGKALALANKEILVCAGELVSKTAKENGIKVLPI
DSEHNALYQCLEGRTIEGIKKLILTASGGPLLNKSLEELSCVTKQDVLNHPIWNMGSKVTVDSSTLVNKGLEII
EAYWLFGLENVEILAVIHPQSLIHGMVEFLDGSVISIMNPPDMLFPIQYALTAPERFASPRDGMDFSKKQTLEF
FPVDEERFPSIRLAQQVLEKQGSSGSFFNAANEVLVRRFLCEEISWCDILRKLTTLMECHKVYACHSLEDILEV
DGEARALAQEI

>core/367/4/Org4_Gene954

MKDLGTLGGTSSTAKTVSPDGKVIMGRSQIADGSWHAFMCHTDFSSNNVLFDLDNTYKTLRENGRQLNSIF
NLQNMMLQRASDHEFTEFGRSNIVLGAGLYVNALQNLPSNLAAQYFGIAYKIRPEYRLGVFLDHNFSSHVPN
NFNVSHNRLWMGAFIGWQDSDALGSSVKVSFGYGKQKATITREQLENTEAGSGESHFEGVAAQIEGRYGKS
LGGHVRVQPFLGLQFVHITRKEYTENGVQFPVHYDPIDYSTGVVYLGIGSHIALVDSLHVGTRMGMEQNFAA
HTDRFSGSIASIGNFVFEKLDVTHTRAFAEMRVNYELPYLQSLNLILRVNQQPLQGVMGFSSDLRYALGF

>core/368/4/Org4_Gene834

MEDFSEQQLFFMRRAIEIGEKGRITAPPNPWVGCVVVQENRIIGEGFHAYAGGPHAEELAIQNASMPISGSDV
YVSLEPCSHFGSRPPCANLLIKYKVSRVFVALVDPDPKVAGQGIAMLRQAGIQVYVGIGESEAQASLQPYLY
QRTHNFPWTILKSAASVDGQVADSQGKSQWITCPEARHDVGKLRAESQAILVGSRTVLSDDPWLTARQPQG
MLYPKQPLRVVLDSRGSVPPTSKVFDKTSPTLYVTTERCPKNYIKVLDSLDVPVLLTESTPSGVDLHKVYEYL
AQKKILQVLVEGGTTLHTSLLKERFVNSLVLYSGPMILGDQKRPLVGVLGNLLESASPLTLKSSQILGNSLKV
VWEISPQVFEPIRN

>core/369/4/Org4_Gene796

MNLSNRSDILSGIFSNPHPVSYFSSTHAKQLSDFSKQHPILTKIVTIIVKIFKLLIGLIILPLGIYWLCQSVCSLALF
PRSSMLYSVLKTCFKKYRLEQEIQDYFVKNLDPSFKDPAVSESKRITIQQDHLTIDTLAIHFSTARPKRWLLISL
GSGDFLEDMIGLKDSLFLSWKELAKLLGANILIYNYPGVKSSTGKLNLENLATAHNLCAKYLQDKIQGPGAN
EIITYGYSLGGVVQSAALQKNPFTNSETSWVAVKDRAPHSLPAAANSFFGPIGKLIAVLAGWKMDAEKNSRE
LPCPEILVYSADRFRPSEVGDDIALLPEFTLAHAIKRTPFARSKKFIGEVNLLHSSPLKHPTIQKLAEAILESLSR
KN

>core/370/4/Org4_Gene625

MNTSLKRPLKSHFDVVGSFLRPEHLKKTRESLEEGSISLDQLMQIEDIAIQDLIKKQKAAGLSFITDGEFRRAT
WHYDFMWGFHGVGHHRATEGVFFDGERAMIDDTYLTDKISVSHHPFVDHFKFVKALEDEFTTAKQTLPAPA
QFLKQMIFPNNIEVTRKFYPTNQELIEDIVAGYRKVIRDLYDAGCRYLQLDDCTWGGLVDPRVCSWYGIDEK
GLQDLIQQYLLINNLVIADRPDDLVVNLHVCRGNYHSKFFASGSYDFIAKPLFEQTNVDGYYLEFDDERSGDF
SPLTFISGEKTVCLGLVTSKTPTLENKDEVIARIHQAADYLPLERLSLSPQCGFASCEIGNKLTEEEQWAKVAL
VKEISEEVWK

>core/371/4/Org4_Gene329

MSDNAHNLLYDKFELPEAVKMLPVEGLPIDKHARFIAEPLERGMGHTLGNALRRALLIGLEAPAIISFAMTGV
LHEYMAIEGVIEDVTNIILNLKGALLKKYPMQDSSLGRTTQVLKASISIDASDLAAANGQKEVTLQDLLQEGD
FEAVNPDQVIFTVTQPIQLEVVLRIAFGRGYTPSERIVLEDKGVYEIILDAAFSPVTLVNYFVEDTRVGQDTDF
DRLVLIVETDGRVTPKEALAFSTQILTKHFSIFENMDEKKIVFEEAISIEKENKDDILHKLILGINEIELSVRSTNC
LSNANIETIGELVIMPEPRLLQFRNFGKKSLCEIKNKLKEMKLELGMDLTQFGVGLDNVKEKMKWYAEKIRA
KNTKG

>core/372/4/Org4_Gene485

MNGKSPLALYIHIPFCTKKCRYCSFYTIPYKSESVSLYCNAVIQEGLRKLAPIQETHFVETVFFGGGTPSLVSHL
DLKRILKELAPHAQEITLEANPENLTASYLRQLQETPINRISVGVQTFDDSILQLLGRTHSSSVAITALQECHNH
GFSNLSIDLIYGLPTQSLEIFLSDLHQALTLPITHISLYNLTIDPHTSFYKHRKILVPTIAQEEILAEMSLLAENLLL
SQGFQRYELASYAKPGYPAKHNLYYWTDRPFLGLGVSASQYLHGERSKNYSRISHYLRAVRKNLPTQETSEI
LPEKERIKEALALRLRLLGGAYLAEFPSTLISMLTQDVKLQNLFSVHGQFLTLNRQGRLFHDTIAEEIMGYSF

>core/373/4/Org4_Gene374

MSIAIAREQYAAILDTHPKPSIAMFSSEQARTSWEKRQAHPYLYRLLEIIWIVVKFLLGLVFFIPLGLFWVLQKI
CQNFILPGAGGWIFRPICRDSNLLRQAYAARLFSASFQDHVSSVRRVCLQYDKVFIDGLELRLPNAKPDRWM
LISNGNSDCLEYRTVLQGENDWIFRIAEESQSNILIFNYPGVMKSQGNITRDNVVKSYQACVRYLRDEPAGPQ
ARQIVAYGYSLGASVQAEALSKEITDGSDSVRWFVVKDRGARSTGAVAKQFIGSLGVWLANLTHWNINSEK
RSKDLHCPELFIYGKDSQGNLIGDGLFKKETCFAAPFLDPKNLEECSGKKIPVAQTGLRHDHILSDDVIKEVAG
HIQRHFDN

>core/374/4/Org4_Gene581

MALKFHLIHKSKKSQARVGQIETSHGVIDTPAFVPVATHGALKGVIDHSDIPLLFCNTYHLLLHPGPEAVAKL
GGLHQFMGRQAPIITDSGGFQIFSLAYGSVAEEIKSCGKKKGMSSLVKITDEGAWFKSYRDGRKLFLSPELSV
QAQKDLGADIIIPLDELLPFHTDQEYFLTSCSRTYVWEKRSLEYHRKDPRHQSMYGVIHGGLDPEQRRIGVRF
VEDEPFDGSAIGGSLGRNLQEMSEVVKITTSFLSKERPVHLLGIGDLPSIYAMVGFGIDSFDSSYPTKAARHGLI
LSKAGPIKIGQQKYSQDSSTIDPSCSCLTCLSGISRAYLRHLFKVREPNAAIWASIHNLHHMQQVMKEIREAIL
KDEI

>core/375/4/Org4_Gene795

MAPIHGSNAFVEDILHSHPSPQATYFSSTHAQKLHEFKDRHPVLTRIASVIIKIFKVLIGLIILPLGIYWLCQTLC
TNSILPSKNIENLLTIFKKKPNTKTLRTNYLRALQDYSSKNRVASMRRVPILQDNVLIDTLEICLSQAPTNRWM
LISLGSDCSLEEIACKEIFDSWQRFAKLIGANILVYNYPGVMSSTGSSSLKDLASAHNICTRYLKDKEQGPGAK
EIITYGYSLGGLIQAEALRDQKIVANDDTTWIAVKDRCPLFISPEGFHSCRRIGKLVARLFGWGTKAVERSQDL
PCLEIFLYPTDSLRRSTVRQNKLLAPELTLAHAIKNSPYVQNKEFIEGRLSSDIDPIDSKTRVALATPILKKLS

>core/376/4/Org4_Gene491

MSTTEPNLTNVNLTMPISSKSMPTQLASHKLKGLDLVAFILIIGIAVSSGTAAIILGIPLLFILTALAVLAFSILLY
FLLREPKSPISVTHQPTPIIKDTDLPPVPPLALTPVPTEAMLEEPPLPSPRTHQTLLQENWDRIPDLQANTDMPFI
AADNQTGYAWHLKNSNLTLISTLGPIEKPRYKTQGIVMIVNAATPNMANNVKGTSLALAKATSVRCWENSK
KSPDPLRSKQPLQLGECRSGKWENLNGTTNAGKAGLPQFLGQLLGPKASDYNYNPNDAFTFCRQAYLNCLN
EAKRRKTTVVQLPLLSSHFPSSPKDEETTSLRLQWIDGVKLALIDALQTFGSEAENQNQPWVIILTTLARHPLI
TP

>core/377/4/Org4_Gene284

MAVAADPSASWLKSRNDFLSSLGKTEEQVAAPQFPKELCQHKIREKFRLEDVQVSIKFRGSITAVEATKEFGV
HLLIQPMVVQPWEVENLLFLTSEEDLQELMVAVFDDASLASYFYEKDRLLGFHYYFVAEACKLFEELQWVP
SLSAKVGGDAIFTATSLQGSFQVVDISLRLDGKNVRCRLLLPEDTFQSCQKFFSGLHDESDLHNIDQTQQISLS
VEVGYSQLTQEEWHQVVPGSFIMLDSCLYDPETEESGALLTVQKHQFFGGRFLTPSSGEFKITSYPNLTHEDP
PLPENPQAPAAPLPGYSRLVVEVARYSLAVSEFIKLNLGSILSLGNHPAYGVDIILDGAKVGRGEIIALGDVLGI
RVLEV

>core/378/4/Org4_Gene475

MIYLDNNAMTPPERGLLEFLQKTFLIEGTYANPSSVHQLGKKSRQLVLEASHWMQKVLSFQGRVLYTSGATE
SLNLAIASLPKDSHVITSGSEHPAILEPLKHSSLSVSYLNPEEGRCVLTIEQIERAVTPKTSAIILGWVNSETGAK
ADIAAIAHFAQEQQLQFIVDATAIVGKERIVLPSGVTMAAFSGHKFHALSGIGALLVSPGVKLHPQLWGGGQ
QGGLRAGTENLWGIASLLYIFKYLDLHQERISQEILTHRNGFENAIKARIPDVHIHCADQPRANNVSAIAFPPL
EGEVLQIALDIEGVACGYGSACSSGATAPFKSLVSMGVDEELTLATLRFSFSHLLLQEDVERAVGIIEKVVERL
KNS

>core/379/4/Org4_Gene947

MTTSDVIDFVTNDFLGFARSPTIYCEVSKRFQIHCQQFPHEKLGIRGSRLMVGSSSVIDDLEAKIASYHGAPNA
FIVNSGYMANLGLCHHVSRSTDVLLWDEEVHMSVVHSLSAISGQHHTFHHNDLEHLESLLQCYRISSKGRIFI

FVSSVYSFRGTLAPLEQIIALSKKYHAHLIVDEAHAMGIFGDDGKGLCHALGYENFYAVLVTYGKALGTMG
ASLLTSSEVKYDLMQNSPPLRYSTSLSPHTLISIGTAYDFLASEGEIARKQVFKLKEHFHECFDSHAPGCVQPIF
LPHTCLEEAISVLETTGIHVGVVAFAKHPFLRVNLHAYNTVDEVNLLAQVMKPYLEKSSHRVHINHEFHLWR
ELCQH

>core/380/4/Org4_Gene764

MEVYSFHPAVRTSFQHRVMAGLDNWFFLGGYRLKVVFLDKNNCGWACQKLVSISKTEKILKILSYLLVPIVL
IALLIRCFLHARFKCTWNRDSLIDSRVPSDVRPFHDFRLFNNQERLNIWKNRIYVSGVDVLVASVDYLKSQFS
GFNHIPKAIRCENYVRNGQFSEEGKKSYLRGMLTHIVGYILSVDETYWEDVILKIPAMNILSEAAYSRSVSTDP
NYQHHGVYLFLDKSLQVPARHVLGEGNMLNTAKWMHIRAERPGKEGECIAKQFLKDYCKKHLEVMNCPDF
IESLLDEKIREFHCPSVLNSAVCDIIDHKCQEHLLKAIINEANRRLPGMKNSSFTMCGDQVLFYTVFSPPKLPPA
ASSAYF

>core/381/4/Org4_Gene409

MTTCLPQPPKTSPLYSIFEKLDAQERLSSEDALHLLLLTNKEDQRTLWNFADQVRKQRVGDTVYYSSTLYLY
PTNFCDFSCKFCSFYAKPGDPKGWLYSPNHLLQQIQNIKTPITEVHIVGGCFPSCNLQYYSDLFTKIKEYDPQIH
IKALTAIEYAYLSDLDNLSIRDVLLTLKDAGLDSIPGGGAEILVDKIRNFLAPKRLSSSDFLNIHKMAHQLGIHS
NITMLCYHKEGPEDLVTHMVKVRDLQDETQGFKNFILLKFAQENNVLGKRLRKSGQGHAIPLKSLMAVARIF
LDNFSNMKALWNYLGIEAALDLLSCGANDLSSTHMGEKVFQMASSKEPIKMDAEGMAALITQQGRTPCLTN
SSHV

>core/382/4/Org4_Gene972

MRRQVREIMQRTVIVAMSGGVDSSVVAYLFKKFTNYKVIGLFMKNWEEDSEGGLCSSTKDYEDVERVCLQL
DIPYYTVSFAKEYRERVFARFLKEYSLGYTPNPDILCNREIKFDLLQKKVQELGGDYLATGHYCRLNTELQET
QLLRGCDPQKDQSYFLSGTPKSALHNVLFPLGEMNKTEVRAIAAQAALPTAEKKDSTGICFIGKRPFKEFLEK
FLPNKTGNIIDWDTKEIVGQHQGAHYYTIGQRRGLDLGGSEKPCYVVGKNIEENSIYIVRGEDHPQLYLRELT
ARELNWFTPPKSGCHCSAKVRYRSPDEACTIDYSSDDEVKVRFSQPVKAVTPGQTIAFYQGDTCLGSGVIDVP
MIPSEG

>core/383/4/Org4_Gene470

MTKIAFSEKAKNFPVEALKKWFEKNKRSLPWRDNPTPYSVWVSEVMLQQTRAEVVIDYFNQWMERFPTLES
LAAAKEEDVIKLWEGLGYYSRARHLLEGARMVMEEFHGKIPDDAISLAQIRGVGPYTVHAILAFAFKRRAAA
VDGNVLRVLSRIFLIETSIDLESTRTWVSRIAQALLPHKSPEVIAEALIELGACICKKVPQCHRCPVRQACGAW
RENKQFVLPVRHARKKVIFLHRLVAIVLYDGSLVVEKRRPKEMMAGLYEFPYIEVEPEEGLQDIEGFTKKME
LSLESPLEFLGNLKEQRHAFTNHKVHLCPIIFKATSLPQFGELHRLSDIDHLAFSSGHKKIKDALLIYLGDVRSG
ESIGV

>core/384/4/Org4_Gene984

MQENLDKRLEVLRTEISLAARSLFDLDKKQKELQVLEEESSEENFWQDSVHAGKISEQIVSLRRQIQEYQELK
SKIDAIEFFLEDADALEDPAICEDLEKEFLFCEKKLAVWETQRLLSGEADKNSCFLTINAGAGGTESCDWVEM
LFRMYSRWATKHQWALEVVDRLDGEVAGIKHVTVKFSGMYAYGYAKAERGVHRLVRISPFDSNGKRHTSF

ASVDVFPEIDDQIKIEIRPNDLRIDTFRSSGAGGQHVNVTESAVRITHLPSGVVVSCQNERSQIQNRESCMKML
QAKLYQQVLQERLEKQSLDRKDKKEIAWGSQIRNYVFQPYTLVKDVRTGHETGNVQAMLDGELLDEFIKAY
LAEFGEVS

>core/385/4/Org4_Gene672

MAVEGRVNSSQALNQDCQEVLANKQSKGLLRCRILSIVVAVITFIAGVVLIALTLASILTSVPYLALGVFLLLV
TLGCIIFALCSEKIKKVPPTPISHKEEIIAWFEERKNIDMEKEKEDPEHFGRTATDIPMRSALDQFNHSCHHIHES
PTLTETYRGHQDVLLFKDWCPVTLPDVTSEEEVLIRSVVGSYLLIEACVPKVSMLIDELHNKLKSPSERECLFI
DKKTLQRKASFLFTQKDLATFFLAYTRVNDGHLAPFRAGAKWILIHYVRLRRQHNQNDFFTPGHSCYYARL
AFNQTQRLYHQLFNVEKLRSIYANMEKDPLCHPWAFIPIYDLLKTEDHGDGFLEQQEDREYPSRAAQDQFW
G

>core/386/4/Org4_Gene185

MSPHRNLFKLKNFSNRLYNRALGRFDKVFNFFSGNVGIDLGTANTLVYVRGRGIVLSEPSVVAVDAQTHAVL
AVGHKAKAMLGKTPRKIMAVRPMKDGVIADFEIAEGMLKALIKRVTPSRSVFRPRILIAVPSGITGVEKRAVE
DSALHAGAQEVILIEEPMAAAIGVDLPVHEPAASMIIDIGGGTTEIAIISLGGIVESRSLRIAGDEFDECIINYMR
RTYNLMIGPRTAEEIKITIGSAYPLGDQELEMEVRGRDQVAGLPITKRINSVEIRECLAEPIQQIIECVRLTLEKC
PPELSADLVERGMVLAGGGALIKGLDKALSKNTGLSVITAPHPLLAVCLGTGKALEHLDQFKKRKGNLV

>core/387/4/Org4_Gene508

MKFVVSRNELGNLIKKIQSVVPQNTPIPVLTHVLIETYNDELVFTATDLTVSTRCVTKAKVYEKGAISIPSKRF
FQLVKELTEANLEISSSAGEMAQITSGSSCFRLLSMEKEDFPMLPDIQNALRFSLPAEQLKTMLQRTSFAVSRE
ESRYVLTGVLLAIANGVATIVGTDGKRLAKIDAEVTLDKSFSGEYIIPIKAVEEIIKMCSDEGEATIFLDQDKIA
VECDNTLLITKLLSGEFPDFSPVISTESNVKLDLHREELITLLKQVALFTNESSHSVKFSFLPGELTLTANCTKV
GEGKVSMAVNYSGELLEIAFNPFFFLDILKHSKDELVCLGISDSYNPGIITDSASGLFVIMPMRLHDD

>core/388/4/Org4_Gene222

MLIWKRHLLTRFWFALTSLLVLALIFYASIHHSLHTLKGASTAASGASVKLSILYYLAQISLKAEFLMPQLVA
VATTSTLFAMQNKREIILLQASGLSLKSLMHPLLLSGAVIMMVLYANFQWLHPICEKISITKENMDRGTTDKE
QGKIPALYLKDQTVLLYSSIEPKTLTLNNVFWIKDPKTIYTMEKLAFTTLSLPIGLNVTQFFANDSENLELKEFF
DMKEFPEIEFNFYENPFSKLFSAGNKNRLSEFFKAIPWNATGLGLSTQVPQRILSLLAQFYYVLISPLACMAAII
LSAYLCLRFSRTPTVTLAYLIPLGTVNIFFVFLKAGIVLASSSVLPTLPVMAFPLIVLFLLTNYAYAKLQ

>core/389/4/Org4_Gene104

MKFPRISISDLIPTQMVIWWRGGGNVHYVPNAQNLPKKILGGVLACFGLALLGCAAFAAGVCQTIFPCIGLMI
LGLVLLGFAYLQYSKGWSRFERPLFRETKVFEKPINWLGCLSLLQSWKKIRPGCYYHPGCPQVEICEGSQEIV
TKIFQKKSDRNTSIFLIQEMDQIALRQGIEKSSLSRKTFAIDPSVVSSLLSEIQREEQQYLDPKVISWSSEDQASD
RTHPKSAIYVNISDAAQEPQGRCYIDAYTKAFFTVLDQIGDPNIVKKHTIYVLTPILGVPDALPKEEQENLKLL
SQAAFLYSAEQVAKRMREEKQDSIRIKFIFTDPTSPTSLYFSPHHSSTPHSVTPISLSGFVGEQESYTFA

>core/390/4/Org4_Gene879

MSKKITVLGYLKLRTLFRRVLCAAALSNIGYASTSQESPYQKSIEDWKGHTFTDLELLSKEGWSEAHAISGNG
SSIVGASGANQGSATAVIWESHLIKHLGTLGGEASSAEGISKDGEVVVGWSDTREGHTHAFVFDGRDMKDL
GTLGATYSVARGVSGDGSIIVGVSATAHGEDYGWQVGVKWEKGKIKQLKLLPQGLWSEAHAISEDGTVIVG
RGEISRDHIVAVKWNKNAVYSLGTLGGSVASAEAISANGKVIVGWSTTNNGETHAFMHKDETMHDLGTLG
GSFSVATGVSADGRAIVGFSAVKTGEIHAFYYAEGEMADLTTFGGEEARVFDISSEGNDIIGSIRTDAGAERA
YLFHIHK

>core/391/4/Org4_Gene399

MKKRFPSTLFLFYRRVTIAISLEGILGWGWLGSLLSKVFAFLVACWNRFSWSTPYRARSTVISVGNIVVGGAG
KTPTVLWLAEALRLRGYSCGVLSRGYKSQSSRQKKLTVVDSKVHSASYVGDEPLLMAEKLPEGSVWVHKD
RRISAARAAEKFDILLLDDGLQYRKLHKDVEIAVVNGQDPLGGRAFFPKGRLRDFPLRLKTVDAIIVNGGGKE
AGTVVKRVSNAPQIFVKPTIASVVWTHNGERIPKEALRELRVGVFCGLGFPQGFLNMLREEGIHILGKYLLPD
HAAITKKELNYFCQQMAMRQGQGLLCTEKDSVKLPRLSGEVSLLPIAKVEMRLSVNQDDTLSLLNMIEQIHK
NRGN

>core/392/4/Org4_Gene362

MNVPDSKNLHPPAYELLEIKARITQSYKEASAILTAIPDGILLLSETGHFLICNSQAREILGIDENLEILNRSFTD
VLPDTCLGFSIQEALESLKVPKTLRLSLCKDSKEKEVELFIRKNEISGYLFIQIRDRSDYKQLENAIERYKNIAEL
GKMTATLAHEIRNPLSGIVGFASILKKEISSPRHQRMLSSIISGTRSLNNLVSSMLEYTKSQPLNLKIINLQDFFS
SLIPLLSVSFPNCKFVREGAQPLFRSIDPDRMNSVVWNLVKNAVETGNSPITLTLHTSGDISVTNPGTIPSEIMD
KLFTPFFTTKREGNGLGLAEAQKIIRLHGGDIQLKTSDSAVSFFIIIPELLAALPKERAAG

>core/393/4/Org4_Gene61

MSHTECGIVGLPNVGKSGLFNALTGAQVASCNYPFCTIDPNVGIVPVIDERLEALAKISNSQKIIYADMKFVDI
AGLVKGASDGAGLGNRFLSHIRETHAIAHVVRCFDDPDVTHVSGKVNPVEDIEVINLELIFSDFSSAKNIHSKL
EKLAKGKREVGALLPLFDTIIAHLEKGLPLRTLELTPEQIVALKPYPFLTMKPMFYIANVDESSLPDMDNDYV
AAVREVAAKENSKVVPICVRIEEEIVSLPIEERLEFLMSLGLEKSGLHRLVRAAYDTLGLISYFTTGPQESRAW
TVVRGSSAWEAAGEIHTDIQKGFIRAEVITFEDMIECQGRAAARELGKLHIEGRDYIVQDGDTMLFLHN

>core/394/4/Org4_Gene487

MTTEVRIPNIAESISEVTVASLLVTEGALIQENQGLLEIESDKVNQLIYAPASGRIFWEVSEGDVVPVGGVVGKI
EPAGEGEELGDSQSKETIEAEIICFPQSGVRQSPPENKTFIPLRDQMDQGSQGLSAGDRGETRERMTSIRKTISR
RLLSALHESAMLTTFNEVYMTPLLHLRKEKQEEFLSRYGVKLGFMSFFVKAVLEALKAYPRVNAYIDGEEIV
YRHYYDISIAVGTDRGLVVPVIRDCDKLSNGEIEQKLADLALRAREGLLAIGELEGGGFTITNGGVYGSLLSTP
IINPPQVGILGMHKIEKRPVVLDNEIVIADMMYVALSYDHRLIDGKEAVGFLVKVKEDLENPTSLLDL

>core/395/4/Org4_Gene306

MVPFRQHHAYQLLKQLHTSAISEADRVSYYFKQNRSLGSKDRQWIQNIIFNILRHRRLLETLILDSGEQVTPEA
LVAKVNEGVLENLDSYSAIPWPVRYSISDDLAHFLVQDYGEEQAEEIAKIWLTEAPITIRVNTDKISVKELQEK
LEYPSCPGELPEALHFSKRHPLQSTEAFRRGFFEIQDENSQRISQGISLTDKDIVLDFCAGAGGKSLIFAQKAKH

VVINDSRKAILQTAKHRLLRAGARNFSLADQLRLGSFSVVIVDAPCSGTGVFRRHPEHKWQFSKKLLLNYVR
VQKSILKQASAYVGPRGRLVYITCSLLKEENEAHVAYMHSLGWKEVHRKTLPLQVGKGDAFFTSHFQKI

>core/396/4/Org4_Gene713

MKLYQTLRGIVLVSTGCIFLGMHGGYAAEVPVTSSGYENLLESKEQDPSGLAIHDRILFKVDEENVVTTLDVI
HKLNLLFYNSYPHLIDSFPARSQYYTAMWPVVLESVIDEFLMVADAKAKRIATDPTAVNQEIEEMFGRDLSP
LYAHFEMSPNDIFNVIDRTLTAQRVMGMMVRSKVMLKVTPGKIREYYRKLEEEASRKVIWKYRVLTIKANT
ESLASQIADKVRARLNEAKTWDKDRLTALVISQGGQLVCSEEFSRENSELSQSHKQELDLIGYPKELCGLPKA
HKSGYKLYMLLDKTSGSIEPLDVMESKIKQHLFALEAESVEKQYKDRLRKRYGYDASMIAKLLSEEAPPLFS
LL

>core/397/4/Org4_Gene355

MAFKRKTRWLWQVLILSLGLNMLFLLLFYSAIFRKDIYKLHLFSGPLIAKSSRKVYLSEDFLNEISQASLDDLI
SLFKDERYMYGRPIKLWALSVAIASHHIDITPVLSKPLTYTELKGSSVRWLLPNIDLKDFPVILDYLRCHKYPY
TSKGLFLLIEKMVQEGWVDEDCLYHFCSTPEFLYLRTLLVGADVQASSVASLARMVIRCGSERFFHFCNEESR
TSMISATQRQKVLKSYLDCEESLAALLLLVHDSDVVLHEFCDEDLEKVIRLMPQESPYSQNFFSRLQHSPRRE
LACMSTQRVEAPRVQEDQDEEYVVQDGDSLWLIAKRFGIPMDKIIQKNGLNHHRLFPGKVLKLPAKQS

>core/398/4/Org4_Gene718

MAVVSTPFFNQNLWYRLLSSRFSLWKSYCPRFFLDYLEAFGLLSDFLDHQAVIKFFELETHFSYYPVSGFVAP
HQYLSLLQDRYFPIASVMRTLDKDNFSLTPDLIHDLLGHVPWLLHPSFSEFFINMGRLFTKVIEKVQALPSKKQ
RIQTLQSNLIAIIRCFWFTVESGLIENHEGRKAYGAVLISSPQELRHAFIDNVRVLPLELDQIIRLPFNTSTPQETL
FSIRHFDELVELTSKLEWMLDQGLLESIPLYNQEKYLSGFEVLCQ

>core/400/4/Org4_Gene640

MKIDILSLFPGYFDGPLQTSILGRAIKQRLLDVQLTNLRDFGLGKWKQVDDTPFSGGGMLLMAEPVTSAIRSV
RKENSKVIYLSPQGALLTAEKSRELAAASHLILLCGHYEGIDERAIESEVDEEISIGDYVLTNGGIAALVLIDAV
SRFIPGVLGNQESAERDSLENGLLEGPQYTRPREFEGKEVPEVLLQGDHKAISQWRLEQSERRTYERRPDLYL
NYLCKRSIDHKFDEETTTNRDHFKCDKISVVLEVNKLKRAKNFYCKVFGLDAMSCENKFCLPHEGKTIFWLR
EVQAEKKNIVTLSLSLDCACEEDFCYLLRRWELFGGKLLEKQADEHAVWALAQDLDGHAWIFSWHRMK

>core/401/4/Org4_Gene519

MGEKTEKATPKRLRDARKKGQVAKSQDFPSAVTFIVSMFTAFSLSTFFFKHLGGFLVSMLSQAPTRHDPVITL
FYLKNCLMLILTASLPLLGAVAVVGVIVGFLIVGPTFSTEVFKPDIKKFNPIENIKQKFKIKTLIELIKSILKIFGA
ALILYITLKSKVSLIIETAGVSPIITAQIFKEIFYKAVTSIGIFFLIVAVLDLVYQRHNFAKELKMEKFEVKQEFKD
TEGNPEIKGRRRQIAQEIAYEDSSSQVKHASTVVSNPKDIAVAIGYMPEKYKAPWIIAMGINLRAKRILDEAEK
YGIPIMRNVPLAHQLLDEGKELKFIPESTYEAIGEILLYITSLNAQNPNNKNTNQPDHL

>core/402/4/Org4_Gene57

MSEAPVYTLKQLAELLQVEVQGNIETPISGVEDISQAQPHHIAFLDNEKYSSFLKNTKAGAIILSRSQAMQHA
HLKKNFLITNESPSLTFQKCIELFIEPVTSGFPGIHPTAVIHPTARIEKNVTIEPYVVISQHAHIGSDTYIGAGSVIG

AHSVLGANCLIHPKVVIRERVLMGNRVVVQPGAVLGSCGFGYITNAFGHHKPLKHLGYVIVGDDVEIGANTT
IDRGRFKNTVIHEGTKIDNQVQVAHHVEIGKHSIIVAQAGIAGSTKIGEHVIIGGQTGITGHISIADHVIMIAQTG
VTKSITSPGIYGGAPARPYQETHRLIAKIRNLPKTEERLSKLEKQVRDLSTPSLAEIPSEI

>core/403/4/Org4_Gene402

MSGPSRTESSQVSVLSYVPRDKEIAPKKQFTIAKISTLAILASLALGALVAGISLTIVLGNPVFLALLITTALFSV
VTFLVYHQMTSKVSSNWQKALEQNFKPLGKAWQEKNVDCYSNEMQFYNNHLNPKFKVAIQTDASQPFQPT
FLTGLRVIEKNQSTGIIFNPVGPTNLIDNTATNLSTILYSTLKDKSVWDTCKQREGDPAKGEDPFSPTEVRVVK
LPNEALDQTFNLNLSSAEKKSILPTFLGHVCGPKSEELPNQQEYYRQALLAYENCLKAAIESHAAIVALPLFTS
VYEVPPEEILPKEGTFYWDNQTQAFCKRALLDAIQNTALRYPQRSLLVILQDPFNTIESQSRSEE

>core/404/4/Org4_Gene725

MKNSFGSLFSFTTWGESHGPSIGVVIDGCPAGLELHESDFVPAMKRRRPGNPGTSSRKENDIVQILSGVYKGK
TTGTPLSLQILNTDVDSTPYENSERLYRPGHSQYTYEKKFGIVDPNGGGRSSARETACRVAAGVVAEKFLAN
QNIFTLAYLSSLGSLTLPHYLKISPELIHKIHTSPFYSPLPNEKIQEILTSLHDDSDSLGGVISFITSPIHDFLGEPLF
GKVHALLASALMSIPAAKGFEIGKGFASAQMRGSQYTDPFVMEGENITLKSNNCGGTLGGITIGVPIEGRIAF
KPTSSIKRPCATVTKTKKETTYRTPQTGRHDPCVAIRAVPVVEAMINLVLADLVLYQRCSKL

>core/405/4/Org4_Gene287

MTLPNDLLETLVKRKESPQANKVWPVTTFSLARNLSVSKFLPCLSKEQKLEILQFITSHFNHIEGFGEFIVLPLK
DTPLWQKEFLLEHFLLPYDLVGNPEGEALVVSRSGDFLAAINFQDHLVLHGIDFQGNAEKTLDQLVQLDSYL
HSKLSFAFSSEFGFLTTNPKNCGTGLKSQCFLHIPALLYSKEFTNLIDEEVEIITSSLLPGVTGFPGNIVVLSNRC
SLGLTEEQLLSSLRITASKLSVAEVAAKKRLSEENSGDLKNLILRSLGLLTHSYQLELKETLDALSWIQLGIDL
GLIQVTENHPLWNPLFWQIRRAHLALQKQAEDSRDLQKDTISHLRASVLKELTKGLSPESF

>core/406/4/Org4_Gene948

MMKKIRKVALAVGGSGGHIVPALSVKEAFSREGIDVLLLGKGLENHPSLQQGISYREIPSGLPTVLNPIKIMSR
TLSLCSGYLKARKELKIFDPDLVIGFGSYHSLPVLLAGLSHKIPLFLHEQNLVPGKVNQLFSRYARGVGVNFSP
VTKHFRCPAEEVFLPKRSFSLGSPMMKRCTNHTPTICVVGGSQGAQILNTCVPQALVKLVNKYPNMYVHHIV
GPKSDVMKVQHVYNRGEVLCCVKPFEEQLLDVLLAADLVISRAGATILEEILWAKVPGILIPYPGAYGHQEV
NAKFFVDVLEGGTMILEKELTEKLLVEKVTFALDSHNREKQRNSLAAYSQQRSTKTFHAFICECL

>core/407/4/Org4_Gene25

MKKKVAEYLNRLAEVEIKISNPEIFSNSKEYSALSKEHSYLLELKNAYDKILNLEKVLADDKQALAIEKDPEM
VVMLEEGINENKVELEKLNKILESLLVPPDPDDDLNVIMELRAGTGGEEAALFVGDCVRMYHLYASSKGWK
YEVLSASESDLKGYKEYVMGISGTGVKRLLQYEAGTHRVQRVPETETQGRVHTSAITIAVLPEPSEEDTELLI
NEKDLKIDTFRASGAGGQHVNVTDSAVRITHLPTGVVVTCQDERSQHKNKDKAMRILKARIRDAEMQKRHN
EASAMRSAQVGSGDRSERIRTYNFSQNRVTDHRIGLTLYNLDKVMEGDLDPITTAMVSHAYHQLLEHGN

>core/408/4/Org4_Gene750

MPILWKVLIFRYLKTAAFCTLSLICISIISSLQEIVAYIAKDVPYDTVLRLMAYQIPYLLPFILPGSCFVSAFSLFR
KLSDNNQMTFLRASGASQSIIMFPVLMVSGAICCLNFYTCSELASICRYQTCKEIANMAMTSPALLLQTLQKK
ENNRIFIAVDHCAKSKFDNVIVALKGNNEISHVGIIKSIIPDTTKDTVKAKDVVFISKLPDSLTESSSPSSQRFYIE
TLDELLIPKITSTLFAGKSYLKTRTDYLPWKQLVKQSLKHSHLPETLRRVAIGFLCITLTYAGMILGIHKPRFRK
SIALYFIFPILDLILLIVGKNTKNLPLAFMLFVFPQLVSWVVFAARAYRESRGYA

>core/409/4/Org4_Gene924

MSLLSGHRLEGFPPIAEVMAACDRCSMDFCEILKSQSMDLWADAASRVDGLLQDPFWSTAIASGIAKSSLQE
TEFECESKVMVLSSWGEQGAQVCSPFNLERICMSFPSLKVFSLKKNGCENMGIQLSASCMNLLMSIFFVATN
GGSTPIWITKENLMALVALVLSHYQCYFVPATGDPQRGNILGNPEVNAILARGMGMRVDLERKRGAYRTHK
GKVFAKVHHQERESSSSRYLELAARCFENSLTKTSLLSDANNVQERDKCLLQMSTSLMHTAGLNLQRPPVPT
PSGVTAHPQPQPDPVVTSQPSSLGARERSPVSSRGRFPVVLPLSVISPRSHPGRVERRDLEDEEEEVMF

>core/410/4/Org4_Gene872

MSHDRILRAQRALSEHNLDAILVEKSEDLAYFLHDEAIAGILLIGQQEVMFFVYRMDKDLYSHIQRVPLTFLT
QDVVADLSLYVQKQRYQKIGFDSASTVYHKFAQRQVLPCLWEPLECFTEKIRSIKSEEEIRRMQEAAALGSA
GYDYVLTLLREGITEKEVVRQLRAFWAEAGAEGPSFPPIIAFGEHSAFPHSIPADRPLKKGDIVLIDIGVLLNGY
CSDMTRMTALGTPHPKLLESYPVVVEAQKRAMALCKEGVLWGDIDAEAVRVLREHHLDTYFIHGIGHGVG
RNIHEYPCSPRGSQVKLESGMTITVEPGVYFPGIGGIRIEDTLCIDKNKNFSLTARPVISELVCL

>core/411/4/Org4_Gene514

MNKRQKDKLKICVIISTLILVGIFARAPRGDTFKTFLKSEEAIIYSNQCNEDMRKILCDAIEHADEEIFLRIYNLS
EPKIQQSLTRQAQAKKKVTIYYQKFKIPQILKQASNVTLVEQPAAGRKLMHQKALSIDKKDAWLGSANYTNL
SLRLDNNLILGMHSSELCDLIITNTSGDFSIKDQTGKYFVLPQDRKIAIQAVLEKIQTTQKTIQVAMFALTHSEII
QALHQAKQRGIHVDIIIDRSHSKLTFKQLRQLNINKDFVSINTAPCTLHHKFAVIDNKTLLAGSINWSKGGFSL
NDESLIILENLTKRQNQKLRMIWKDLAKHSEHPTVDDEEKEIIEKSLPVEEQEAA

>core/412/4/Org4_Gene165

MNLPDRKKALEAAVAYIEKQFGAGSIMSLGRHSATHEISTIKTGALSLDLALGIHGVPKGRVIEIFGPESSGKT
TLATHIVANAQKMGGVAAYIDAEHALDPSYASLIGVNIDDLMISQPDCGEDALSIAELLARSGAVDVIVIDSV
AALVPKSELEGDIGDVHVGLQARMMSQALRKLTATLSRSQTCAVFINQIREKIGVSFGNPETTTGGRALKFYS
SIRLDIRRIGSIKGSDNSDIGNRIKVKVAKNKLAPPFRIAEFDILFNEGISSAGCILDLAVEYNIIEKKGSWFNYQE
KKLGQGREFVREELKRNRKLFEEIEKRIYDVIAANKTPSVHANETPQEVPAQTVEA

>core/413/4/Org4_Gene806

MNLCKRISFEEGLELFVSSPIERLQERADAIRKERYPSNEVTYVLDANPNYTNICKIDCTFCAFYRKPKSPDAY
LLSFDEARSLLQRYVSSGVKTVLLQGGVHPRLGIDYLEELVRITVQEFPSIHPHFFSAVEIEHACRVSGISIEQG
LQRLWDAGQRTIPGGGAEILSERVRKIISPKKMQPGGWINLHKLAHLMGFRTTATMMFGHVENPEDILIHLQ
TLRDAQDSCPGFYSFIPWSYKPGNTALRRNVPQQASIETYYRILALGRIFLDNFDHVAASWFGEGKSLGAKAL
HYGADDFGGVILDESVHKATGWSIQSSEEEICNIIRSEGFIPVERNTFYQHISCTVSSL

>core/414/4/Org4_Gene801

MKYSLNFKEIKIDDYERVIEVTCSEVRLHAIIAIHQTAVGPALGGVRASLYSSFEDACTDALRLARGMTYKAII
SNTGTGGGKSVIILPQDAPSLTEDMLRAFGQAVNALEGTYICAEDLGVSINDISIVAEETPYVCGIADVSGDPSI
YTAHGGFLCIKETAKYLWGSSSLRGKKIAIQGIGSVGRRLLQSLFFEGAELYVADVLERAVQDAARLYGATIV
PTEEIHALECDIFSPCARGNVIRKDNLADLNCKAIVGVANNQLEDSSVGMMLHERGILYGPDYLVNAGGLLN
VAAAIEGRVYAPKEVLLKVEELPVVLSKLYNQSKTTGKDLVALSDSFVEDKLLAYTS

>core/417/4/Org4_Gene48

MLNIHDILGNDDENLLSYQCKHITKDKLTLPSHDFVDKVFGLSDRNNRVLRSLQTMFSHGRLANSGYLSILPV
DQGIEHSAGASFAINPIYFDPENIVKLAIESGCSAVASTYGTLSLLSRKYAHKIPFMLKLNHNELLSYPTKYHQI
FFTQVEAAYSMGAVAVGATVYFGSETSNEEIVAVSNAFAKARSLGLATVLWCYLRNPAFVANGVDYHTAA
DLTGQADHLGATLGADIVKQKLPTCQGGFKAINFGKTDERVYSELSSNHPIDLCRYQVLNSYCGKVGLINSG
GPSGKNDFTEAARTAVINKRAGGMGLILGRKAFQRPLSEGIQLLNLVQDIYLDPNITIA

>core/418/4/Org4_Gene197

MIPLIPMFLKQSLFFSLALTGMTTLVLTVALGVPVMKWLKRKNYRDYIHKEYCEKLEMLHKDKAEVPTGGG
VLLFISLIASLLVWLPWGKFSTWFFIILLTCYAGLGWYDDRIKIKRKQGHGLKAKHKFMVQIAIAAFTLIALPY
IYGSTEPLWTLKIPFMEGMLSLPFWLGKVFCLGLALVAIIGTSNAVNLTDGLDGLAAGTMSFAALGFIFVALR
SSTIPIAQDVAYVLAALVGACIGFLWYNGFPAQLFMGDTGSLLLGGLLGSCAVMLRAECILVVIGGVFVAEA
GSVILQVLSCRLRKKRLFLCSPLHHHYEYQGLPETKIVMRFWIFSFVCAGLGIAAVLWR

>core/419/4/Org4_Gene730

MRIAVLGAGYAGLSVTWHLLLHSQGTATIDLFDPIPLGEGASGMSSGLLHAFTGKKALKPPLADQGINATHA
LITEASKALNVPIVISQGILRPAIDEDQAQLFMKRVEEFPKEVEWWEKARCEISIPSMVIPPNLGALFIKSGVTL
NNDLYIQGLADACMKLGTQFYDELIEDLADIEEFYDHIIVTPGANASILPELKDMPVNKVKGQLLEISWPKDL
AMPSFSINAHKYMVANTQKNTCILGATFEHNQPEETPDPAIAYQEIMPPVLSLFPGLKDAQVLHCYAGMRSSS
KSRLPVISRISEKLWFLGGLGSKGLLYHGITGDMLAQAVLRKSTAYIAKEFLFTI

>core/420/4/Org4_Gene204

MILLQNIKRCSLKQLKVLATLLLSLSLPTLEAAENRDSDSIVWHLDYQEALQKSKEAELPLLVIFSGSDWNGP
CMKIRKEVLESPEFIKRVQGKFVCVEVEYLKHRPQVENIRQQNLALKSKFKINELPCMILLSHEEREIYRIGSFG
NETGSNLGDSLCHIVESDSLLRRAFPMMTSLSLSELQRYYRLAEELSHKEFLKHALELGVRSDDYFFLSEKFR
LLVEVGKMDSEECQRIKKRLLNKDPKNEKQTHFTVALIEFQELAKRSRAGVRQDASQVIAPLESYISQFGQQD
KDNLWRVEMMIAQFYLDSDQWHHALQHAEVAFEAAPNEVRSHISRSLEYIRHQS

>core/421/4/Org4_Gene384

MYLQEKFKAQQVPLVLRELLSCSDSINDSDPVYRIVFDSNDTTISYKVGDALGVLPENSKEVSEHVLQLLGYS
PTTLVNVKKTSEKVSAQKFIQGYVDLDKIPAKLNSFFPDKDPKITLYDAIQEYHPQIPIELFAESVFPLLPRFYSI
ASSPDLHPKSIELLVKHVSYPGKYQKRFGVCSSFLCSELQVNDSAYIFVQPTKHFTLSTQIEGKPLVMIGAGTG

IAPYKAFLEERLFNKDPGNNLLFFGERKEKVNFYYREFWNHSEEEGKLKLFLAFSRESDQKVYVQDLLRIQK
DEVRKAYEEGGFFFVCGRKVLGIEVKHALEEILGKDTLASLRKEHRYVVDVY

>core/422/4/Org4_Gene531

MDVSRKINRHTQFYVDSIDGVIKKFDQKPSEDKSRDHEELEEKLLTITKRIVASAQEFQNRKTDSKNYYLKKT
QWLPFKNEELEQTKELFAMLTSMDKKIAQLFFYSPGCSSDWVEFTEVICHLNDSIGLGGVLLCCGLFEQQCEH
VVTVNKKLDLPLLLGTTVVNSLRYYLTYRNISLLNCQSMSELGKELGDVLKQHGVAFTLIFKEIVDIDLLNYA
KLIQGLKRSGNIQARIYDNDVPTLPSVSSSPIALRYSLANTIRGLALHVDFSSLKFISPSILSNTEHTAKALNSGG
ECFIFSNLDEFNLGMKIVMQLLRTGKISPEILNKNIMKILMIKRRVRSLYI

>core/423/4/Org4_Gene218

MEADILDGKLKRVEVSKKGLVNCNQVDVNQLVPIKYKWAWEHYLNGCANNWLPTEVPMARDIELWKSDE
LSEDERRVILLNLGFFSTAESLVGNNIVLAIFKHITNPEARQYLLRQAFEEAVHTHTFLYICESLGLDEGEVFNA
YNERASIRAKDDFQMTLTVDVLDPNFSVQSSEGLGQFIKNLVGYYIIMEGIFFYSGFVMILSFHRQNKMTGIGE
QYQYILRDETIHLNFGIDLINGIKEENPEVWTTELQEEIVALIEKAVELEIEYAKDCLPRGILGLRSSMFIDYVRH
IADRRLERIGLKPIYHSRNPFPWMSETMDLNKEKNFFETRVTEYQTAGNLSW

>core/426/4/Org4_Gene647

MELSLTSLLPLAWYVILGVAVFAYSFGDGFDLGLGAVYLKAKEDKERRILLNSIGPVWDGNEVWLVIIVGGL
FAGFPACYATLLSIFYMPIWTLVLLYIFRGCSLEFRSKSESVSWKTFWDIIFICSGTAISFFLGTIVGNLILGLPLS
PDTSYASLSWILFFRPYAALCGAVVASAFAIHGSCFALMKTSDSLNARIAQQFPYILSSFLVFYVLFLGASLISIP
KRFDAFPTYPLLILLIALTSCCCVAAKTSVSKKRYGYAFIYSTLNLLSLILSAATLTFPNILLSTVDPQYSYTIYN
SAVETKTLKSLLIIVLIGLPFIITYTCYIYRVFRGKTNFPSIY

>core/427/4/Org4_Gene846

MNSKMLKHLRLATLSFSMFLGIVSSPAVYALGAGNPAAPVLPGVNPEQTGWCAFQLCNSYDLFAALAGSLK
FGFYGDYIFSESAHITNVPVITSVTTSGTGTTPTITSTTKNVDFDLNNSSISSSCVFATIALQETSPAAIPLLDIAFT
ARVGGLKQYYRLPLNAYRDFTSNPLNAESEVTDGLIEVQSDYGIVWDLSLQKVLWKDGVSFVGVSADYRH
GSSPINYIIVYNKANPEIYFDATDGNLSYKEWSASIGISTYLNDYVLPYASVSIGNTSRKAPSDSFTELEKQFTN
FKFKIRKITNFDRVNFCFGTTCCISNNFYYSVEGRWGYQRAINITSGLQF

>core/428/4/Org4_Gene978

MMEVYSFSPSVRTSFQHRVMAGLDNWFFLGGHRLKVVSLDSCNSGRVCEEYVPISMTEKILKILSYLLIPIVII
ALLIRYLLHSNFTAKVSPKPWLKTLQLGIDIKSFILPSSHVNTMDSATLFKAIHLEGKRVDVEYHRLHSSDKVV
FYIPAQKLPDDLRLTHWLPEKETRKTEYVRHMLAHVMGYLTSQGKERLQQVVQDSRSSTSLGAEKVLQYRF
IDHPQSQGEFQRLLNENITTKGSEDKEVVQSDLFDMAFQCWWPQFISVIQSPTFSEELVHEMSQKLDLDCIYPE
DDEFEQTFLNTLLKAVLYHGFQGISVASMGVIFLICLDSLELQIPFLRNQK

>core/429/4/Org4_Gene876

MNKKKRVLTGDRPTGKLHLGHWVGSIKNRLELQNSPEYDCFFIIADLHTLTTKIRKEEVLDVDNHIYEVLAD
WLSVGIDPTKSIIYLQSAIPEIYELHLLFSMLISINRVMGIPSLKDMARNASIEEGGLSYGLIGYPILQSADILLAK

AQFVPVGKDNEAHVELTRDIARNFNRLYGQVFPEPEVLQGELTSLVGIDGQGKMSKSANNAIYLSDSDATITE
KVRKMYTDPNRIRATTPGRVEGNPLFIYHDIFNPHKDEVEEFKARYRQGCIKDIEVKARLAEELIHFLKPIKER
RLEFLSKPLALQNVLEDGTHKMREVAKATMEEVHDKFGFSHKWRSLLK

>core/430/4/Org4_Gene595

MLTLGLESSCDETACAIVNEDKQILANIIASQDIHASYGGVVPELASRAHLHIFPQVINKALQQANLLIEDMDL
IAVTQTPGLIGSLSVGVHFGKGIAIGAKKPLIGVNHVEAHLYAAYMAAQNVQFPALGLVVSGAHTAAFFIENP
TSYKLIGKTRDDAIGETFDKVGRFLGLPYPAGPLIEKLALEGSEDSYPFSPAKVPNYDFSFSGLKTAVLYAIKG
NNSSPRSPAPEISLEKQRDIAASFQKAACTTIAQKLPTIIKEFSCRSILIGGGVAINEYFRSAIQTACNLPVYFPPA
KLCSDNAAMIAGLGGENFQKNSIIPEIRICARYQWESVSPFSLASP

>core/431/4/Org4_Gene632

MENAMSSSFVYNGPSWILKTSVAQEVFKKHGRGIQVFLSTSVMLFIGLGVCAFIFPQYLIVFVLTIALLMLAIS
LVLFLLIRSVRSSMVDRLWCSEKGYALHQHENGPFLDLKHVQQVLLRSPYIKVRALWPSGDIPEDPSQAAVL
LLSPWTFFSSVDVEALLPSPQEKEGKYIDPVLPKLSRIERVSLLVFLSAFTLDDLNEQGVNPLMNNEEFLFFINK
KAREHGIQDLKHEIMSSLEKTGVPLDPSMSFQVSQAMFSVYRYLRQRDLTSSELRCFHLLSCFKGDVVHCLA
SFENPKDLADSDFLEACKNVEWGEFISACEKALLKNPQGISIKDLKQFLVR

>core/432/4/Org4_Gene530

MDSSAPYNIASQGIEKSTVERILDLYGPASCIKFLKQMVLIREFEARGEEAYLEGLVGGFYHSYAGQEAVATA
AIANTGLDPWVFSSYRCHALAILLNIPLQEIAAELLGKETGCALGRGGSMHMCGPNFPGGFGIVGGQIPLAAG
AAFTIKYQEQKNRVSLCFIGDGAVAQGVFHETLNFVSLHQLPLMLIIENNGWGMGTSLNRAVAKQPIAESQG
SSYDIRAVTVNGFDLFNSLLGFREAYRYMVDTESPVLVECLCSRFRGHSISDPNLYRSKEEMQCLFKKDPIVL
AKDWLIRLEVLTEEEFQNIRQECKTAVLEAFSNAKLSSDPSVTTLEEGVYA

>core/433/4/Org4_Gene611

MKLYSISSAVDTPWIFQLMSKVDSYLFLGGNRIKVVSIVMQEPNLIIGKVENVQISTIVKILKILSFLIFPLILIAL
ALHYFLHAKYANHLLVSKILERAPQYVPIPGRSGDTTSHYKLTTLVPISQKNLQAMGSNPLEVEAALRTTKPS
FFCVPAKYRQIIISSHGIRFSLDLEQLADDINLDSVSWPTEYLNSTMDFCSKADKRVIQNVRNLQTGTYINSVG
KRSLLKFMLRYLFIDGIIQENPEALPNNTSGRLTLFPGVPYIYSRFTPQNPTIWPQVFFRQGPLDEDRGGGFEIL
EQLQELGVRFPICPSQGPDNPNFKGFQGIRIYWEDSYQPNKEV

>core/434/4/Org4_Gene807

MLPFEFEFNTTSSPECDVCLDPQKLFVKLFKRTIVLLSGPTGSGKTDVSLALAPMIDGEIVSVDSMQVYQGMD
IGTAKVSLKARQEIPHHLIDIRHVQEPFNVVDFYYEAIQACQNILSRNKVPILVGGSGFYFHAFLSGPPKGPAA
DPQIREQLEAIAEEHGVSALYEDLLLKDPEYAQTITKNDKNKIIRGLEIIQLTGKKVSDHEWDIVPKASREYCC
RAWFLSPETEFLKNNIQMRCEAMLQEGLLEEVRGLLNQGIRENPSAFKAIGYREWIEFLDNGEKLEKYEETKR
KFVSNSWHYTKKQKTWFKRYSIFRELPTLGLSSDAIAQKIAKDYLLYS

>core/435/4/Org4_Gene843

MFFQNLAKKLTALGISPLGCLLIGGVVSCAILFGRSSNPSLAPTQVKTEKTSGNWLKLTQMGNPKLIESLTKK
EQLEKDLTSFHPIASAKVAIALSTEDDVMSPLHLSVILTLRKEESLTPSLLFSITDYLCSSLPGLKREHISLSDNL
GNLYIPESITVNSLFIHTLENYLGKIFPKEHFALAYHAKAEKPTLQLTLNENYIAHLAKEESEKIVAHTKHYLY
QNYDDSYDIVIETLPFARLQNKKSFPAKVLIGSMILVISLMIVALASFYLARHAYERVSPEPRKIKRGINISKLLE
IIQKESPEKIALILSYLDPKKAEALLNRLPEDLKHQVLKYKL

>core/436/4/Org4_Gene541

MSEQHSPIISVQDVSKKLGDHILLSKVSFSVYPGEVFGIVGHSGSGKTTLLRCLDFLDMPTSGSISVAGFDNSLP
IQKFSRRNFSKKVAYISQNYGLFSSKTVFENIAYPLRIHHSEMSKSEVEEQVYDTLNFLNLYHRHDAYPGNLS
GGQKQKVAIARAIVCQPEVVLCDEITSALDPKSTEDIIERLLQLNQERGITLVLVSHEIDVVKKICSHVLVMHQ
GAVEELGTTEELFLNSENSITNELFHEDINIAALSSCYFAEDREEVLRLNFSKELAIQGIISKVIQTGLVSINILSG
NINLFRKSPMGFLIIVLEGEVEQRKKAKELLIELGVVIKEFY

>core/437/4/Org4_Gene418

MTILRKLSQYLFFFSLLCSFIYVATCGSQPDSVSSPKIAIFLSFPHPLLEDCSKSCIETLKDFENLPEIVVLNAEDSI
VKARKIARSLHTDKNVVAIVTLGTIATKVMSHIETQKPVIYAAVPDRESLTLPKNTMNIYGVNDTLDINQYCF
AIQAVATNAQSIVYLKPSEPFPSDLQKEIVKKLHASGIEVIEIPITSSTFKTRIRQAIDKRPSAIFIPLSPLSHKEGT
AFLQEILKEKIPIITDDTSLISEGACIACSVDYKKSGKQIAKIVHHLLYNNHDVDSLRKIIAQPLSPTTTFNEDIIK
YLGIKLHKTERNQFLSFKSKKLEKSEKGKNVAVS

>core/438/4/Org4_Gene751

MEMKEEIEAVKQQFHSELDQVNSSQALADLKVRYLGKKGIFRSFSEKLKQCTDKAKLGSLINDFKTYVEDLL
QEKSLVLLASEQAEAFSKEKIDSSLPGDSQHSGGRHILKSILDDVVDIFVHLGFCVREAPNIESEANNFTLLNFT
EDHPARQMHDTFYLNATTVLRTHTSNVQARELKKQQPPIKVVAPGLCFRNEDISARSHVLFHQVEAFYVDH
NVTFSDLTAILSAFYHSFFQRKTELRFRHSYFPFVEPGIEVDVSCECCGKGCALCKHTGWLEVAGAGMIHPQV
LRNGNVDPEIYSGYAVGMGIERLAMLKHGISDIRLFSENDLRFLQQFS

>core/439/4/Org4_Gene957

MATISPISLTVDHPLVDTKKKSCSNFDKIQSRILLITAIFAVLVTIGTLLIGLLLNIPVIYFLTGISFIAVVLSNFILY
KRATTLLKPCACGKHKEIKPKRVSTNLQYSSISIAINRSKENWEHQPKDLQNLPAPSALLTDNPYEIWKAKHS
LFSLVSLPGGNPEHLLISASENLGKTLLIEETSQNAPISSYVDTTPSPKSLLNEAIQETRVEINTELPAGDSGERL
YWQPDFRGRVFLPQIPTTPEAIYQYYYALYVTYIQTAINTNTQIIQIPLYSLREHLYSRELPPQSRMQQSLAMIT
AVKYMAELHPEYPLTIACVERSLAQLPQESIEDLS

>core/440/4/Org4_Gene293

MEEAAKHLAKEFLCSGINLFLSGEYEQAEKRLKETLELDSTAALAYCYLGIIALETGRVPEALNWCSKGLASE
PGDSYLRYCYGVALDRGNQYEAAIEQYSAYVALHPDDVECWFSLGSVYHRLKRLQEALDCFDKILALDPW
NPQSLYNKAVILSEMDDEAESIRLLEVAVAKNPLYWKAWVKLGFLLSRSKRWDKATEAYERVVQLRPDLSD
GHYNLGLCYLTLDKTRLALKAFQEALFLNAEDADAHFYVGLAHMDLKQMREAYEAFNSALSINLEHERAH
YLLGYLHHMQGETDKATKELLFLQKKDSTFAPLLQKTVVSDPSSMQFERRLDTIS

>core/441/4/Org4_Gene279

MVLGVVGISYREAALKERERAIQYLQSFEKNLFLAQRFLGKGGAFIPLLTCHRAELYYYSESPEIAQAALLSEL
TSQGIRPYRHRGLSCFTHLFQVTSGIDSLIFGETEIQGQVKRAYLKGSKERDLPFDLHFLFQKALKEGKEYRSRI
GFPDHQVTIESVVQEILLSYDKSIYTNFLFVGYSDINRKVAAYLYQHGYHRITFCSRQQVTTPYRTLSRETLSF
RQPYDVIFFGSSESASQFSDLSCESLSSIPKRIVFDFNVPRTFLWKETPTGFVYLDIDFISECVQKRLQCTKEGVN
KAKLLLTCAAKKQWEIYEKKSSHITQRQISSPRIPSVLSY

>core/442/4/Org4_Gene635

MFPSANPESRTRNVPLGIFHGLVACLYWGIVFVIPNFLGSFGDLDIVLTRYTIFGIFSLIACAIKNPSVIKKTPLYI
WRKSLLWTLLINPVYYFGITLGIRYVGSAITVVIAGLAPIAVLYHSNTKQKELPYSLLFAISSVIITGVILTHLSA
LNLPTAASPLYSILGVIAVILSTSLWVIYVIRNQSLLEKHPNLTPDTWSYLIGISALIICLPMIIILDLCGITHATHN
LISHTPGSERLLFLLLCSAMGIFSSAKALTAWNKASLNLSPALLGAILIFEPIFGLVLTYLYSQSLPSLQEGIGIFL
MLGGSLLCLVLFGRKVQKSLENSQVSSSNE

>core/443/4/Org4_Gene878

MKKTCFQNYRSIGVVFSVVLFVLTTQTLFAGHFIDIGTSGLYSWARGVSGDGRVVVGYEGGNAFKYVDGEK
FLLEGLVPRSEVLVFKASYDGSVIIGISDQDPSCRAVKWVNGALVDLGIFSEGMQSFAEGVSSDGKTIVGCLY
SDDTETNFAVKWDETGMVVLPNLPEDRHSYAWDASEDGSVIVGDAMGSEEIAKAVYWKDGEQHLLSNIPG
AKRSSAHAVSKDGSFIVGEFISEENEVHAFVYHNGVIKDIGTLGGDYSVATGVSRDGKVIVGHSTRTDGEYR
AFKYVDGRMIDLGTLGGSASFAFGVSDDGKTIVGKSETELGECHAFIYLDD

>core/444/4/Org4_Gene478

MTHQVAVLHQDKKFDVSLRPKGLEEFYGQHHLKERLDLFLCAALQRGEVPGHCLFFGPPGLGKTSLAHIVA
YTVGKGLVLASGPQLIKPSDLLGLLTSLQEGDVFFIDEIHRMGKVAEEYLYSAMEDFKVDITIDSGPGARSVR
VDLAPFTLVGATTRSGMLSEPLRARFAFSARLSYYSDQDLKEILVRSSHLLGIEADSSALLEIAKRSRGTPRLA
NHLLRWVRDFAQIREGNCINGDVAEKALAMLLIDDWGLNEIDIKLLTTIIDYYQGGPVGIKTLSVAVGEDIKT
LEDVYEPFLILKGLIKKTPRGRMVTQLAYDHLKRHAKNLLSLGEGQ

>core/445/4/Org4_Gene260

MSYSLRNKKTKICVYIIIALGILSFRSIPQEVYDKIRSSFVSLHVKFFPKIKQAPSSHLANLELENLVLKERVASL
EEKLKLYEVSNHTPPLFPEILTPYFHKLVEGKVVYRDYTHWSSSCWVNVGKTHGIKKNSPVLSGNVLVGLVD
YVGEHQSRIRLITDVGMKPSVVAMRGDIQSWWIKHSLRELIRQVEQISHAYILEKDKYEKISQLQELDSLIQGE
GENQALLRGILSGVGGALWKEGSLCLEGEGFYFSEGKTLLPGDILVTTGLDGVFPPGLLVARVTKVKAPRDG
ACAFKIEAQSLEEKLMELDQLFILPPLEFNPNDRPDIFGLLWD

>core/446/4/Org4_Gene331

MKVVINGFGRIGRLVLRQILKRNSSVEVLAINDLVPGDALTYLFKFDSTHGRFPEDVRCEADHLIVGKRKIQF
LSERNVQNLPWKDLGVDLVIECTGLFTKKEDAEKHIQAGAKRVLISAPGKGDIPTFVMGVNHKTFNPEKDFV
ISNASCTTNCLAPIAKVLLDNFGITEGLMTTVHAATATQLVVDGPSKKDWRGGRGCLQNIIPASTGAAKAVT

LCLPELKGKLTGMAFRVPIEDVSVVDLTVRLDKSTTYDDICKAMKQASETDLKGILDYTDEQVVSSDFIGSEY
SSIFDALAGIALNDRFFKLVAWYDNEIGYATRIVDLLEYVEKNSK

>core/447/4/Org4_Gene386

MFVDQITLELRAGKGGNGVVAWRKEKYLPKGGPYGGNGGNGGSVIIEATTNVYSFEAYRNIRFLKAPDGQS
GATNNRTGRSGKDLIVSVPTGTLLRDAETGEILHDFTVDGERLLVSQGGKGGKGNTFFKTSVNRAPTKATPG
KPGEIRQVELELKLIADIGLVGFPNAGKSTLFNTLAHTEVKVGAYPFTTLAPSLGLVLCNDRLYQKPWIIADIP
GIIEGAHQNKGLGLDFLRHIERTLLLLFVIDVSKRERNSPEEDLETLIHELHSHQPDFEKKDMLVALNKIDDLL
PDEQEECLQSFQKRFPSYTFVLISGLTGEGVDGLYRFFTQRLAV

>core/448/4/Org4_Gene54

MWFSVNKNKKAAIWATGSYLPEKVLSNADLEKMVDTSDEWIVTRTGIKERRIAGPQEYTSLMGAIAAEKAI
ANAGLSKDQIDCIIFSTAAPDYIFPSSGALAQAHLGIEDVPTFDCQAACTGYLYGLSVAKAYVESGTYNHVLLI
AADKLSSFVDYTDRNTCVLFGDGGAACVIGESRPGSLEINRLSLGADGKLGELLSLPAGGSRCPASKETLQSG
KHFIAMEGKEVFKHAVRRMETAAKHSIALAGIQEEDIDWFVPHQANERIIDALAKRFEIDESRVFKSVHKYGN
TAASSVGIALDELVHTESIKLDDYLLLVAFGGGLSWGAVVLKQV

>core/449/4/Org4_Gene895

MAAPIFIKNILLRSSIVYAPLAGFSDYPYRCMSALYQPGLMFCEMVKVEGMLYAPERTSKLLDYNENMRPIG
AQLCGSNPETSGEAAKILEGLGFDLIDLNCGCPTDKITKDGSGSGLLKTPELIGRILDKIINSVSIPVTVKIRSGW
DMEHINIEDTVRIIRDAGASAVFVHGRTRAQGYHGPSNQEYISRAKAAAGKEFPVFGNGDIFSPEAAQAMLTT
GCDGVLVARGTMGAPWIGKQIQDYLTTGSYEKIPFIKRKAAFLEHMRLVEDYYQSETKFLSETRKLCGHYLI
SAAKVRFLRSSLAKATSYQEVYQLVNDYEEADDSSLETFVKC

>core/450/4/Org4_Gene668

MSTPLSSGGISPSEQYVPQELFCDRLSSSRSNSPDSNASGDSPIVSPPISALVALTDLKLVPYNQNSFSWTTRLK
NAAEKIGLFLQRNWKYILLYILAWALILVCHHTVALTLTIWLGVGLGIGVVFGIFTATCLDKENKHRHVNSL
WNLINHGILQLDPNGTRQILLATMIASISALIYAVPQAVGLVIGFSIGNQLSINTVYGARLGDEATYAIDRKAH
KKRIENIEQAINQHQIIKHQMINQKQLNALIEINRNNQTDPATANLLASLKLNLNQPMPYCFSMPECGVTSSYL
DLNNNSPDDIIARADQCIMTLSQTLQQIKKEPDRIIESNH

>core/451/4/Org4_Gene863

MVRRSVSFCLFFLMTLLCCTGCNSRSLIVHGLPGRDANEIVVLLVSKGVAAQKLPQAAAATAGAATEQMWD
IAVPSAQITEALAILNQAGLPRMKGTSLLDLFAKQGLVPSELQEKIRYQEGLSEQMASTIRKMDGIVDASVQIS
FTTENEDNLPLTASVYIKHRGVLDNPNSIMVSKIKRLIASAVPGLVPENVSVVSDRAAYSDITINGPWGLTEEI
DYVSVWGIILAKSSLTKFRLIFYVLILILFVISCGLLWVIWKTHTLIMTMGGTKGFFNPTPYTKNALEAKKAEG
AAADKEKKEDADSQGESKNAETSDKDSSDKDAPEGSNEIEGA

>core/452/4/Org4_Gene845

MKQHIGYLGMGIWGFCLASLLANKGYPVVAWSRNPDLIKQLQEERRHPLAPNVVISPNLSFTTDIKEAIHNAF
MIVEGVTSAGIRPVAEQLKQITDLSVPFVITSKGIEQNTGLLLSEIMLEVLGDSVTPYLGYLSGPSIAKEVLNGS

PCSVVVSAYDSQTLKQIHEAFSLPTFRVYPNTDIKGAALGGALKNVIAIACGIAEGLSFGNNAKAGLVTRGLH
EMRKLAAIMDCKPETLNGLAGLGDLCVTCFSESSRNLRFGHLLAQGLTFEQAKAKIGMVVEGAYTALSAYQ
VAKHHKIDMPITTGIYRVLYENLDLKEGIALLLQRNTKEEFL

>core/453/4/Org4_Gene348

MKPLGFQENLEALCNKTSRQLLKYLIKQILFVCGASLLIALEFSFFLYFFLFSGETVIPAFCLACFFLTLFVCLVT
RLYLLSGKGDFFEDLASEYLQGAVPPNKRSQNIVEEQSHLAAAATKLSINLQNQEYSLLSEIFKFLPKHDLIRK
FSCFCFWKDYFLFRECLLQKAIEAYIKVVQAIPVDLSAHVSLADAYVALSGLYADPRKYPEFDANYWIPSGR
YSAEIQEKFFATARRAIEEFQILNEYAPGNAWVHAQLAYSYHDLQMPMEEIQEYEIVLKLKPNDVETMSKLGI
LYFQQGMNAKGLRIYEEIKKRDYKKSQKLIKFYGVEYKY

>core/455/4/Org4_Gene180

MQPFIFTLLCLTSLVSLVAFDAANARKRCACAQTIERGENFFSIKRSACAEIEYQEKSRHASAIERISKDKGKV
TPKQIAKVATKKKQRYRLLQVPFSRPPNNSRYNLYALLSEPPECYSDTASWYAIFIRLLRRAYVDTGNVPPGS
EYAIANALISNKQEILERGAQLGPDVIETLTLPEEQAEIFYKMLKGSSNSQSLLNFLHYEEKSLGHCKLNLIFM
DPLLLEAVLDHPDAYRETSLLRDGIWEAVKRQEHAIQEHGQAAALELFKTRTDFRLELRDKMQLLLSRYDLL
PLLNKKMFDYTLGSAGDYLFLVDPDTKAISRCRCPSKSIKL

>core/456/4/Org4_Gene321

MATNAIRLAGSAASKMLLPVAKEPAAVSSFAQKGIYCIQQFFTNPGNKLAKFVGATKSLDKCFKLSKAVSDC
VVGSLEEAGCTGDALTSARNAQGMLKTTREVVALANVLNGAVPSIVNSTQRCYRYTRQAFELGSKTKERET
PGEYSKMLLTRGDYLLAASREACTAVGATTYSATFGVLRPLMLINKLTAKPFLDKATVGNFGTAVAGIMTIN
HMAGVAGAGCGIALEQKLYKRAKESLYNERCALENQQSQLSGDVILSAERALRKEHVATLKRNVLTLLEKA
LELVVDGVKLIPLPITVACSAAISGALTAASAGIGLYSIWQKTKSGK

>core/457/4/Org4_Gene235

MRIAVLGVTGLVGQKFVALLHKWYRDWVIAEVVASNSKYGQSYGDACIWQEPIGPMPEMVRDLPIRKIEEV
QSDIVVSFLPSSAESMEAYCLSQGKVVFSNASTYRMHSSVPIIIPEVNSDHFQLLEEQPYPGKIITSPNCCVSGIT
LALAPLRKFSLDHVHIVTLQSASGAGYPGVPSLDLLANTVPHIVGEEEKILRETVKILGSSKQPLPCKLSVTVH
RVPVAYGHTLSLHVTFSKDVDLDEILYSYQEKNKEFPNTYQLYDNPWSPQARKHLSHDDMRVHLGPITYGG
DFRTIKMNVLIHNLVRGAAGTLLASMENYFFDYLKREMCLR

>core/458/4/Org4_Gene339

MKTLWHFVSKAFLSIVGLCCGVVLAFVVIFALIASSLGNGDATFVSLPDAQGEVKDLGKTAPIIAVIEMKDVI
ASSKNTAKTIQNILEGFEKAPLKDRVKGIVIDMDCPGGEVFEIDRIYSMLRFWKERKGFPIYIYVNGLCASGGY
YVSCAATKIYATSSSLIGSIGVRSGPFFNVKEGLNRYGVESDLLTAGKDKAPMNPYIPWTSHDREERQATLDF
LYGQFVDIVTQNRPLLTKEKLVHTLGARIFSPEKAKQEGYIDVVGATKEQVLQDIVAVCKIEDNYRVIGSGGD
GWWKRVASAAASSPLVTGMIKHDILPLSHDAAYIPPYLAL

>core/460/4/Org4_Gene916

MSSLTLSRRPRRNRKTAAIRDLLAETHLSPKDLIAPFFVKYGNNIKEEIPSLPGVFRWSLDLLLKEIERLCAYGL
RAVMLFPIIPDDLKDAYGSYSSNPKNILCHSIHEIKNAFPHLCLISDIALDPYTTHGHDGIFLNGEVLNDESVRIF
GNIATLHAEMGADIVAPSDMMDGRIGYIRSKLDQSGYSKTSIMSYSVKYASCLYSPFRDALSSHVTSGDKKQ
YQMNPKNVLEALLESSLDEEEGADILMVKPAGLYLDVIYRIRQNTCLPLAAYQVSGEYAMILSAFQQGWLD
KETLFHESLIAIKRAGADMIISYSAPFILELLHQGFEF

>core/461/4/Org4_Gene615

MNIYQFSPGVSPNWQASLMARLDSYFCLGGETVTRIISLRPSGLILAKKEKAVVSIAAKILKILSFILFPLVLTAL
AIRYLLYNKFNKDLDRAVFFIPTEITKTEELIIAKNPALVKEAALNVSPFFYSLPKKYQRMKVETPEGKFYKITF
SVNLDLLVEDLHLETLDWPTELLLKNRPFDFTGHPEEEKLIKDILLKEEGNEYFSLESKKLLARHMMHNIVVL
SEEPGRSAFLGRTAFFPNKYPIAKGGVGIPSTTSNLFTIWYCFYFYRAATPQSDHPDGCGFILLERLKELGAEFF
YCDLRESNTTGFTLLFEGSNKGVLKNHLFIRDE

>core/462/4/Org4_Gene614

MSNIYSFSPGICPNWQAGLMSKLDSLCCFGGETATRIFSMTPSGFSLATEEKVHISTAEKVIKILALIFFPIILIAL
AIRYFLHRKFDRKCFVIPQDTPKELELILAANPQLVEKAAREVHPGFFALPTKYQSMYIQTSKGQPPKITLSINI
DLLLEDLDTDSIPWPKLYLDEDFDFAYYPESKAIIDTVTKLEKNNPGEEFCLESKKILAHYLLEQLFKLETGLN
FPTSTIDGGRESFLIKFSHETKKPTVWAFIYFYYHHSNGPKLEKDFKQAGCEVHNRLLNLGLKYRPQAGAQN
DGCDGGPYGPRGFLIVWEENYGSVLKDHGFIKDN

>core/463/4/Org4_Gene776

MQFSRYLRYAFDNQYLPEPLYQKFSVFHQNYIDAATKKAAADQAEALCLQWVKVIIEDLKNPFIFPPYHKKI
RAPIDLFRLSIDFFSLVIDDKNSRILNLHRLKEIEEYIARGDNVVLLANHQTECDPQLMYYALGKTHPELMEN
MIFVAGDRVTSDPLARPFSMGCDLLCIYSKRHIATPPELREEKLLHNQKSMQILKTLLNEGGKFIYVAPAGGR
DRKNAEGKLYPSEFSPESIEMFRLLAKASNQTTHFYPFALKTYDILPPPPKIENAIGEQRAIFFAPVFFNFGAELF
FDALCSKEELIHCDKHAQRTLRAEKVFSIVKNLYEEL

>core/464/4/Org4_Gene720

MREETVSWSLEDIREIYHTPVFELIHKANAILRSNFPHSELQTCYLISIKTGGCVEDCAYCAQSSRYHTHVTPEP
MMKIVDVVERAKRAVELGATRVCLGAAWRNAKDDRYFDRVLAMVKSITDLGAEVCCALGMLSEEQAKKL
YDAGLYAYNHNLDSSPEFYETIITTRSYEDRLNTLDVVNKSGISTCCGGIVGMGESEEDRIKLLHVLATRDHIP
ESVPVNLLWPIDGTPLQDQPPISFWEVLRTIATARVVFPRSMVRLAAGRAFLTVEQQTLCFLAGANSIFYGDK
LLTVENNDIDEDAEMIKLLGLIPRSSFGIERGNPCHANNS

>core/465/4/Org4_Gene432

MIDIMQHFKPYTMVPGQKLPIPGSLLYAQVFPTLWRLFSSKHEILNEQTLQVQGPLKRFAVFQDLHRGGLAV
TSERYKYYLLPSGECTQSIKGKLPSAAQAGPLLSLGVHKHADWQKVRRRRDLKEILPLWFRFAAMAPKGSY
RDLETTAIGSLVKTAHQSVLHRETTEIAPALLSIALAGFSECFLPRSYDEEFQGILPQDGDPEGGVPFELLSYSF
GMIQDIFLRHQGQLVEILPALPPEFPCGRLIHVALPNLGTLSIVWTKKTIRQVELHAEYSGEVFLKFCSSLCSAR
LREWSERRLSGSKRLSLGETLEIKAGTTYLWDCFHK

>core/466/4/Org4_Gene820

MSAFFDLLKSQTASHPPIWLLRQVGRYMPPYQELKGSQSLKTFFHNTEAIVEATLLGPSLLHVDAAILFADILS
ILDGFAVTYDFAPGPRIQFFPEQPFTFTSDPQTIFSYLLDAIRTLKQKLPVPLIVFAASPFTLACYLIDGGASKDF
SKTMSFLYAYPEKFDQLISTIIEGTAIYLKTQMDAGAAAVQLFESSSLRLPSALFTRYVTEPNRRLIAKLKEQAI
PVSLFCRCFEENFYTLQATQADTLHPDYHVDLHRIQKNLMPSLQGNLDPAIFLLPQEKLLHYVEAFLVPLRTY
PNFIFNSGHGILPETPLENVQLVVSYVQRQL

>core/467/4/Org4_Gene401

MPSPMISTDVCQDILGKQKEAVDFFFQAFQPKEAMQLAEKILGHSGWVFFSGVGKSGCVARKLVATLQSLSE
RALFFSPVDLLHGDLGLVSPGDIVCLFSKSGETQELLDTVPHLKSRGAILVAITSMPYSNLAALSDLVVILPSV
AELDPFNLIPTNSTTCQMIFGDFLAMLLFHSRGVSLSTYGKNHPSGQVGMKANGKVKDFMFPKTEVPFCHLG
DKVSFSLEVFSAYGCGCVCIVDPQFRLMGIFTDGDLRRSLASYGGEVLSLSLDKVMTANPRCITEDSDIAIALQ
LMESSSPVAVLPVLDNEENRHVTGLLHMHTLAKAGLL

>core/468/4/Org4_Gene732

MAFKEVVRVAVTGGTGQIAYNFLFALAHGDVFGVDRGVDLRIYDVPGTERALSGVRMELDDGAYPLLHHL
RVTTSLNDAFDGIDAAFLIGAVPRGPGMERGDLLKQNGQIFSLQGAALNTAAKRDAKIFVVGNPVNTNCWIA
MKHAPRLHRKNFHAMLRLDQNRMHSMLAHRAEIPLEEVSRVVIWGNHSAKQVPDFTQARISGKPAAEVIGD
RDWLENILVHSVQNRGSAVIEARGKSSAASASRALAEAARSIFCPKSDEWFSSGVCSDHNPYGIPEDLIFGFPC
RMLPSGDYEIIPGLPWEPFIRNKIQISLDEIAQEKASVSSL

>core/469/4/Org4_Gene529

MPKHKTLEIREALREAIDEEMSRDPNVCILGEEIGDYNGAYKVTKGLLDKWGPKRVIDTPISEAAFSGIGIGAA
LSGLRPIIEFMSWNFSFVALDQIISHAAKMHFMTGGKFSVPIVFRGPNGAAAQVSCQHSHCVESLYANIPGLIII
APSNPYDAKGLLKSAIRNNNPVLFLENELEYNLKGEVPTEEYLVPIGKAHRVQEGNDLTIITYSRMVSITKEAC
SLAKKRWGLSIEIIDLRTIKPLDISTILSSVRKTSRCIVVEEGHYFAGISSEIIALITEHVFDSLDAPPLRVCQKETP
MPYSKILEQATLPNVNRILDTIEKVMR

>core/470/4/Org4_Gene662

MSNQFDQLKKLSTIVCDSGDPELVKASGSQDATTNPSLILKVAQEPKFQELLNEAVVWGIRQNGDDLQTLSFI
LDKIQVNFALEIIKNIPGRISLEIDARLSFNVEAMVQRAVFLSQLFEAMGGDKKRLLVKIPGTWEGIRAVEFLE
AKGIACNVTLIFNLVQAIAAAKAKATLISPFVGRIYDWWIAAYGDEGYSIDADPGVASVSNIYAYYKKFGIPT
QIMAASFRTKEQVLALAGCDLLTISPKLLDELKKSQDPVKKELDPAEAKKLDVQPIELTESVFRFLMNEDAM
ATEKLAEGIRIFAGDTQILETAITEFIKQIAAEGA

>core/471/4/Org4_Gene115

MTTPAYLLANFGGPRHAKDLQEFLISLLTDRDVTGTFLPRVLHRHLFTFIAKKRVPKVLPQYQSLQNWSPIYF
DTETLAKTLSEILRAPVIPFHRYLPSTHEKTLLALRTLHTRHVIGIPLFPHFTYSVTGSIVRFFMKHVPEIPISWIP
QFGSDSKFVSLITCHIRDFLQKLGILEKECCFLFSVHGLPVRYISQGDPYSKQCYESFSAITTNFKQSENFLCFQS

KFGPGKWLSPSTAQLCQNIDTDKPNVIVVPFGFISDHLETLYEIERDYLPLLRSRGYRALRIPAIYSSPLWVSTL
VDIVKENSTVVAEELIKSGKKHTGIR

>core/472/4/Org4_Gene503

MDAKMGYIFKVMRWIFCFVACGITFGCTNSGFQNANSRPCILSMNRMIHDCVERVVGNRLATAVLIKGSLDP
HAYEMVKGDEDKIAGSAVIFCNGLGLEHTLSLRKHLENNPNSVKLGERLIARGVFVPLEEDGICDPHIWMDL
SIWKEAVIEITEVLIEKFPEWSAEFKANSEELVCEMSILDSWAKQCLSTIPENLRYLVSGHNAFSYFTRRYLATP
EEVASGAWRSRCISPEGLSPEAQISVRDIMAVVDYINEHDVSVVFPEDTLNQDALKKIVSSLKKSHLVRLAQK
PLYSDNVDDNYFSTFKHNVCLITEELGGVALECQR

>core/473/4/Org4_Gene406

MMTYPVPQNPLLLRILRLMDAFSKSDDERDFYLDRVEGFILYIDLDKDQEDLNKIYQELEENAERYCLIPKLT
FYEVKKIMETFINEKIYDIDTKEKFLEILQSKNAREQFLEFIYDHEAELEKWQQFYVERSRIRIIEWLRNNKFHF
VFEEDLDFTKNVLEQLKIHLFDAKVGKEITQARQLLSNKAKIYYSNEALNPRPKRGRPPKQSAKVETETTISSD
IYTKVPQAARRFLFLPEITSPSSITFSEKFDTEEEFLANLRGSTRVEDQLNLTNLSERFASLKELSAKLGYDSLST
GDFFGDDEEEEEVVTKTKGSKRGRKKSS

>core/474/4/Org4_Gene201

MHSELPNYQNIVESVVTEITTQLLNYRSEHRLVPFWEKSDGSFITAADYGSQYYLKQQLAKAFPNIPFIGEETL
YPDQDNEKIPEILKFTRLLTSSVSRDDLISTLVPPPSPTSLFWLVDPIDGTAGFIRHRAFAVAISLIYEYRPILSVM
ACPAYNQTFKLYSAAKGHGLSIVHSQNLDRRFVYADRKQTKQFCEASLAALNQQHHATRKLSLGLPNTPSP
RRVESQYKYALVAEGAVDFFIRYPFIDSPARAWDHVPGAFLVEEAGGRVTDALGAPLEYRKESLVLNNHAVI
LASGDQETHETTLAALQNQLNVVPTDKLIAL

>core/475/4/Org4_Gene155

MKTVTSFTVCKENSGRLDKYLTEVHPKYSRAFYQEHILSGLVQINGQINTRVATRLNCGDIVTIDIQEKEELLE
LLPEAIPLDKVYEDGMILVINKPRDMVVHPAPGHFHGTLVHALLHEIGERLKEEFPEEPWRPGIVHRLDKDTS
GLIITAKTRQAKKVFSELFSTKRLKKSYLAVCIGKPRSTTIHTHISRHQNKRKEMTVSSQGKEAVTHCQVLAF
NGKLSFVALSPETGRTHQLRVHMKHLGTPILGDPVYGIPSMNSSYGLDKQQLHAYSVDFTHPETRQFCSLKA
GLPEDMRSLLIKEFRNETTILNKNLLESILKEQ

>core/476/4/Org4_Gene549

MTIDMHCDLLSHPHFCRKDPAVRCSPEQLLSGGVRQQVCAIFVPHSQGEPHCDKQNSLFFSLPNQYPDIGLLS
YEEEENGSSSQKKSLSLIRSIENASALGDDTAPLGTLLAKLIHLTKQGPLAYLGIVWKGDNRFGGGTEAPKRL
SNDGKVLLDIMYELGVPIDLSHCSDKLAEDILDYTADKLPNLAVIASHSNFRSVLDHRRNLVDAHAKEIVRRK
GVIGLNLVRSYVGDSLGDLEKHVLHAENLGILSSIVLGSDFFYANEDENFFFNECSSAEAHPVLNQLIHRIFSK
GKAESILSSRAEKFLKQVIVEQVNRKITDVKL

>core/478/4/Org4_Gene631

MIEFAFVPHTSVTADRIEDRMACRMNKLSILAITSLCVLISSVCIMIGILCISGTVGTYAFVVGIIFSVLALVACV
FFLYFFYFSSEEFKCASSQEFRFLPIPAVVSALRSYEYISQDAINDVIKDTMQLSTLSSLLDPEAFFLEFPYFNSLI

VNHSMKEADRLSREAFLILLGEITWKNCETKILPWLKDPNITPDDFWKLLKDHFGLKDFKKRIATWIRKAYPE
IRLPKKHCLDKSIYKGCCKFLLPAENDVQYQRLLHKVCYFSGEFPAMVLGLGCEVPMVLGLPKVPKDLTWE
MFMENMPVLLQSKREGHWKISLEDVASL

>core/479/4/Org4_Gene77

MELLPHEKQVVEYEKAIAEFKEKNKKNSLLSSSEIQKLEKRLDKLKEKIYSDLTPWERVQICRHPSRPRTVNYI
EGMCEEFVELCGDRTFRDDPAVVGGFVKIQGQRFVLIGQEKGCDTASRLHRNFGMLCPEGFRKALRLGKLA
EKFGLPVVFLVDTPGAYPGLTAEERGQGWAIAKNLFELSRLATPVIIVVIGEGCSGGALGMAVGDSVAMLEH
SYYSVISPEGCASILWKDPKKNSEAASMLKMHGENLKQFGIIDTVIKEPIGGAHHDPALVYSNVREFIIQEWLR
LKDLAIEELLEKRYEKFRSIGLYETTSESGPEA

>core/480/4/Org4_Gene300

MASNPILQIEDLSITLAKQRQQYPIVQSLSFTINEGQTLAIIGESGSGKSVSAHAILRLLPCPPFSVSGQVNFQGH
NLLTASRSIQKKIIGTEISMIFQNPQASLNPVFTIEQQFREIIHTHLALTAEVAKEKMLYALEETGFHDPRLCLNL
YPHQLSGGMLQRICIAMALLCSPKLLIADEPTTALDVSVQYQILQLLKTLQKKTGMSLLIITHNMGVVAETAD
DVLVLYAGRMVECAPAVQIFHNPSHPYTRDLLASRPSLQPQPLGSFNPIPGQPPHYTAFPSGCRYHPRCSKILN
RCSAEAPEIYPVREGHKVRCWLYDD

>core/481/4/Org4_Gene506

MALGPSPYYGVSFFQFFSVFFSRLFSGSLFTGSLYIDDIQIIVFLAISCSGAFAGTFLVLRKMAMYANAVSHTVL
FGLVCVCLFTHQLTTLSLGTLTLAAMATAMLTGFLIYFIRNTFKVSEESSTALVFSLLFSLSLVLLVFMTKNAH
IGTELVLGNADSLTKEDIFPVTIVILANAVITIFAFRSLVCSSFDSVFASSLGIPIRLVDYLIIFQLSACLVGAFKA
VGVLMALAFLIIPSLIAKVIAKSIRSLMAWSLVFSIGTAFLAPASSRAILSAYDVGLSTSGISVVFLTMMYIVVK
FISYFRGYFSKNFEKISEKSSQY

>core/482/4/Org4_Gene299

MTTNFPQPLIQATSLTKHYYKRSFWFQGKTIASRPVDDVSFSLYSRRAVGLIGESGSGKSTLALALAGLLPLTS
GFLTFNGTPIKLHSKHGRHQLRSQVRLVFQNPQASLNPRKTILDSLGHSLLYHKLVPKEKVLATVREYLELVG
LSEEYFYRYPHQLSGGQQQRVSIARALLGVPQLIICDEIVSALDLSIQAQILNMLAELQKKLSLTYLFISHDLAV
VRSFCTEVFIMYKGQIVEKGNTERIFSDPQHPYTRMLLNAQLPETPDQRQSKPIFQEYHKDSEESCSTGCSFYN
RCPQKQEACKSETSPNQGDAHHTYRCIH

>core/483/4/Org4_Gene265

MEKKYYALAYYYITRVDNPHEEIALHKKFLEDLDVSCRIYISEQGINGQFSGYEPHAELYMQWLKERPNFSKI
KFKIHHIKENIFPRITVKYRKELAALGCEVDLSKQAKHISPQEWHEKLQENRCLILDVRNNYEWKIGHFDNAT
LPDIQTFREFPEYAEKLAQECDPETTPVMMYCTGGIRCELYSPVLLEKGFKEVYQLDGGVIAYGQQVGTGKW
LGKLFVFDDRLAIPIDESDPDVAPIAECCHCQTPSDAYYNCANTDCNALFLCCDECIHQHQGCCGEECSQSPR
VRKFDSSRGNKPFRRAHLCEISENSESTSCCLI

>core/484/4/Org4_Gene113

MKQLLFCVCVFAMSCSAYASPRRQDPSVMKETFRNNYGIIVSGQEWVKRGSDGTITKVLKNGATLHEVYSG
GLLHGEITLTFPHTTALDVVQIYDQGRLVSRKTFFVNGLPSQEELFNEDGTFVLTRWPDNNDSDTITKPYFIET
TYQGHVIEGSYTSFNGKYSSSIHNGEGVRSVFSSNNILLSEETFNEGVMVKYTTFYPNRDPESITHYQNGQPHG
LRLTYLQGGIPNTIEEWRYGFQDGTTIVFKNGCKTSEIAYVKGVKEGLELRYNEQEIVAEEVSWRNDFLHGER
KIYAGGIQKHEWYYRGRSVSKAKFERLNAAG

>core/486/4/Org4_Gene320

MNLKVVYFGTPTFAATVLQDLLHHKIPITAVVTRVDKPQKRSAQLIPSPVKTIALTHGLPLLQPSKASDPQFIE
ELRAFNADVFIVVAYGAILRQIVLDIPRYGCYNLHAGLLPAYRGAAPIQRCIMEGATESGNTVIRMDAGMDT
GDMANITRVPIGPDMTSGELADALASQGAEVLIKTLQQIESGQLQLVSQDAALATIAPKLSKEEGQVPWDKP
AKEAYAHIRGVTPAPGAWTLFSFSEKAPKRLMIRKASLLAEAGRYGAPGTVVVTDRQELAIACSEGAICLHE
VQVEGKGSTNSKSFLNGYPAEKLKIVFTLNN

>core/488/4/Org4_Gene461

MSKGSSKHTVRINQTWYIVSFILGLSLFAGVLLSTIYYVLSPIQEQAATFDRNKQMLLAAHILDFKGRFQIQEK
KEWVAATFDKKTQLLEVATKKVSAVSYPELELYAERFVRPLLTDAQGKVFSFEEKNLNPIEFFEKYQKSPPC
QQSLLPFYVILENTSRTENMSGADVAKDLSTVQALIFPISGFGLWGPIHGYLGVKNDGDTVLGTAWYQQGET
PGLGANITNPEWQEQFYGKKIFLQDSSGTTNFATTDLGLEVIKGSVRTTLGDSPKALSAIDGISGATLTCNGVT
EAYVQSLACYRQLLINFSNLTHEKKTGE

>core/489/4/Org4_Gene333

MRLLSILKLHLFSLRSSSSLSPHYYHSCSRSMLHLLCRWKDADIMEWQQICNILSGVCSRMSGKLVSLQKETQ
DSCHQEHERIHLQYREQLSALEEEYRRREEAKNQDLEKLQQENTWLQNRLAEKLQQIRHQSDIIDEIKKELLQ
SVQRTEISEGRRLCYEHKIKQLEEQLQRYVSQHGAPSIEIEEDKSSAAYAEINRLKKSLIDLQQEKDIYIKTYHS
EIAKLREKLQRQEGAQTSSEVCSIEKLTEVQTDLAEKKKAIALLQDIVEDQYCQLRDLHKEKGMAMPSNTKL
DHLKGLLGKEPECEVDVVFSESKSLGS

>core/490/4/Org4_Gene774

MEVQIGIDLMGGDHSPLVVWQVLVDVLKSQSSTIPFAFTLFASEEIRKQIQEEFISNLPQEKFPKIISAENFVAM
EDSPLAAIRKKSSSMALGLDYLQEDKLDAFISTGNTGALVTLARAKIPLFPAVSRPALLVCVPTMRGHAVILD
VGANISVKPEEMVGFARMGLAYRQCLGDSKIPTIGLLNIGSEERKGTEAHRQTFRMLRDTFGEAFLGNIESGA
VFDGAADIVVTDGFTGNIFLKTAEGVFEFLQRILGDKLEADIQRRLDYTFYPGSVVCGLSKLVIKCHGKACGS
SLFHGILGSINLAQARLCKRILSNLI

>core/491/4/Org4_Gene365

MLISISLATLPILAFSWASFIEPNWLRTTAIPWRLPKKHAHLHGLRIAQISDLHFHKRVPEKFLNKVSKSIKNFSP
DLIVFCGDLLCRARLEDKERLETFLNTLEAPLGAFAILGNHDYSSYISRNTKGEITCIPEEKSRPIQRAIIAVMQG
LFSSPSYRYDPNLTPQEPHPDLLKLLKNTPLTLLHNTTHVIPNTLNIVGLGDLFARQFHPEQAFKNYDPSLPGL
LLSHNPDGITRLQQYPGDFVLSGHSHGPQVTLSWPKFARKFFERLSGLENPYLARGYFVTKEGKQLYVNRGL
GGLKRIRFCSPPEICYITCSYD

>core/492/4/Org4_Gene749

MVLSSDLLRDDKQLDLFFASLDVKKRYLLALSGGSDSLFLFYLLKERGVSFTAVHIDHGWRSTSAQEAKELE
ELCAREGVPFVLYTLTAEEQGDKDLENQARKKRYAFLYESYRQLDAGGIFLAHHANDQAETVLKRLLESAH
LTNLKAMAERSYVEDVLLLRPLLHIPKSSLKEALDARGISYVQDPSNEDERYLRARMRKKLFPWLEEVFGKN
ITFPLLTLGEESAELSEYLEKQAQPFFSAATHQDSQGELPCPDCLIQQAFLCKWVMKKFFNNAGIAVSRHFLQ
MVYDHLSRSSCATLRMRNKIVIIKPGVVMID

>core/493/4/Org4_Gene311

MKFLLYVPLLLVLVSMGCDAKPVSFEPFSGKLSTQRFEPQHSAEEYFSQGQEFLKKGNFRKALLCFGIITHHFP
KDILRNQAQYLIGVCYFKQDHPDLADKAFASYLQLPDAEYSEELFQMKYAIAQRFAQGKRKRICRLEGFPKL
MNADEDALRIYDEILTAFPSKDLGAQALYSKAALLIVKNDLTEAIKTLKKLTLQFPLHILSSEAFVRLSEIYLQ
QAKKEPHNLQYLHFAKLNEEAMKKQHPNHPLNEVVSANVGAMREHYARGLYATGRFYEKKKKAEAANIY
YRTAITNYPDTLLVAKCQKRLDRISKHTS

>core/494/4/Org4_Gene89

MNRRKARWVVALFAMTALISVGCCPWSQAKSRCSIDKYIPVVNRLLEVCGLPEAENVEDLIESSSAWVLTPE
ERFSGELVSICQVKDEHAFYNDLSLLHMTQAVPSYSATYDCAVVFGGPLPALRQRLDFLVREWQRGVRFKKI
VFLCGERGRYQSIEEQEHFFDSRYNPFPTEENWESGNRVTPSSEEEIAKFVWMQMLLPRAWRDSTSGVRVTF
LLAKPEENRVVANRKDTLLLFRSYQEAFPGRVLFVSSQPFIGLDACRVGQFFKGESYDLAGPGFAQGVLKYH
WAPRICLHTLAEWLKETNGCLNISEGCFG

>core/495/4/Org4_Gene618

MRKVAFLVSCLFSVAIGASAAPVRVPGFPQIPEDLVQIKTEVCPKQEVCLAVTIKCDDHNLIGVLHLPNTPTPE
GGFPTVVLFHGFRGTKFGGLTGAYRKLGRKFAAAGIATLRVDMAGCGDSEGVAEEVPIETYLRDAQTILETV
QEHPDLNAYRLGISGFSLGCHIAFELAKIYNPRDLNIKALSVWAPIADGGILLKELYENFSKHGEGDIISVGKDF
GFGPPPLIVCSGDVDLLIRIQDHVTANSLPTKPYILHQQGIDDTLVSRTQQTLFKNTAPGRMTFISYPNTGHNL
ATAPDLDMILDQIVSHFQRTL

>core/496/4/Org4_Gene452

MFKLLFHIAAFAGHVLSTPIFIVQDACGIDEEACKNPPPRPFSAQVQYLKVNDAKFKKLPHQTIGYRQYDGTF
LCTLPITEHSGLLFSTGYIGADIQWKSSLPISETDPNGLGWATFQDTSFYNYVLLSLGAYTLSLKNWQWSIILS
GLVDPKNIEMGYGLYQGVLSGKYQATEKLSAIFGVINETGLHQEKAWPLVGVSYKATDQLTLNCIYPVNFSI
DYRSTSVCNLGLAYRLTRFRKKLHKNHLTSSRGIFEYQGREIEANVKLTPWPGSFIKGFYGWSIGNDISIADDH
NNNKTSHTFKTSAFFGGSAVMNF

>core/497/4/Org4_Gene985

MLVELEALKKEFAHLKDQKPTSDQEITSLYQRLDHLEFVLLELGQDKFLKATEDEDMLFESQKAIDAWNALL
TKARDALGLGDIGAIYQTIEFLGAYLSKVNRRAFCIASEIHFLKTAIRDLNAYYLLDFRWPLCKIEEFVDWGN
DCVELAKRKLCTFEKETKELNESLLREEHAMEKCSIQDLQRKLSDIIIELHDVSLFCFSKTPSQEEYQKDCLYQ

SRLRYLLLLYEYTLLCKTSTDFQEQARAKEEFIREKFSLLELQKGIKQTKELEFAIAKSKLERGCLVMRKYEA
AAKHSLDSMFEEETVKSPRKDTE

>core/498/4/Org4_Gene482

MTLPMQKSLTSFDDFSQAYAEKVPAIALIGSALEDDKDALIELLVSESFKELDGQGLMPATLMSWTETFALFQ
EHETLGIIHAEKFPLATKEFLSRYARNPQPHLTILIFTTKQECFRELSKALPSALSLSLFGEWPADRQKRIIRLLL
QRAERVGISCSQSLASLFLRALASTSLPDILSEFDKLLCSVGKKTSLDHSDIKELVVKKEKASLWKFRDSLLKR
DPVEGHQQLHFLLEDGEDPLGIIAFLRTQCLYGLRSIEEGSKENKHRMFVLYGKERLHQALNSLFYAETLIKN
NVQDPIVAVETLVIRMVNL

>core/499/4/Org4_Gene590

MFSYIKNRILFNLLSLWIVLTLTFLVMKTIPGDPFNDEGCNVLSEEVLQTLKSRYGLDKPLYQQYTQYLHSIAK
LDFGNSLVYKDRKVTNIISTAFPISAILGLQSLFLSIGGGIALGTIAALKKKKQRRYILGASILQISIPAFIFATLLQ
YVFAVKIPLLPIACWGSFTHTILPTLALAVTPMAFIIQLTYSSVSAALNKDYVLLAYAKGLSPLKVVIKHILPYA
IFPTISYSAFLITTVITGTFAIENIFCIPGLGKWFICSIKQRDYPVALGLSVFYGTLFMLSSLLSDLIQSIIDPQIRYA
HGKEKKRK

>core/500/4/Org4_Gene509

MAMLPKFFLVLLCLGLCSCSQKTTTIEGEQMTIFYRIVLGTSLSAKEKASLSQQIDRCFHKIDSIYNNWNPHSE
LSIINRAPADVPITLSVELSEFLDQVDTLYKLSEGRFDPTVGPLKTLWLLHLKSQTLPPKDVWEQHYKDMGW
QHLEFQSNTKTLIKKNPHVQIDLCGVVKGYAVDCLNEICNTFCPNNYVEWGGEIKTSGHHPSGRPWRIFSEA
AGTILDIDDMAIATSGNHIQKWCVEGKIYTHILDTRTGKPLELSSYPIQSVSVVHPSCAYADAIATVLMTFDSK
IEAKQWAEEHHILTYINDGASS

>core/501/4/Org4_Gene381

MWRVVLRFLIIFILGRAVFPLRASESFSWETSTCLTVLGIPFIDIILTTNEDFVAQCGLQIGTISSTNNATIKEIFLI
YKEKFPETSISFKRKEPLNLSQSHLSDLGILCMRNGETYAEGMANKENGPALKQPKDLRLVLRCPNQPDTLLY
SEKEAEKGIETNICLCNQGYTLLDGQLILYGDSIEKFLKETKRKNNHTLVDLCDSQVVTTFLSRFWSLLNYIQV
LFLSEDSAKILAGIPDLAQATQLLSHTVPLLFIYTNDSIHIIEQGKESSFTYNQDLTEPILGFLFGYINRGSMEYCF
NCAQSSLGET

>core/502/4/Org4_Gene694

MIASIYSFLDYLKMVKSASPHTLRNYCLDLNGLKIFLEERGNLAPSSPLQLATEKRKVSELPFSLFTKEHVRM
YIAKLIENGKAKRTIKRCLSSIKSFAHYCVVQKILLENPAETIHGPRLPKELPSPMTYAQVEVLMATPDISKYH
GLRDRCLMELFYSSGLRISEIVAVNKQDFDLSTHLIRIRGKGKKERIIPVTSNAIQWIQTYLNHPDRKRLEKDP
QAIFLNRFGRRISTRSIDRSFQEYLRRSGLSGHITPHTIRHTIATHWLESGMDLKTIQALLGHSSLETTTVYTQV
SVKLKKQTHQEAHPHA

>core/503/4/Org4_Gene523

MIHSRLIIIGSGPSGYTAAIYASRALLHPLLFEGFFSGISGGQLMTTTEVENFPGFPEGILGPKLMDNMKEQAVR
FGTKTLAQDIISVDFSVRPFILKSKEETYSCDACIIATGASAKRLEIPGAGNDEFWQKGVTACAVCDGASPIFK

NKDLYVIGGGDSALEEALYLTRYGSHVYVVHRRDKLRASKAMEARAQNNEKITFLWNSEIVKISGDSIVRSV
DIKNVQTQEITTREAAGVFFAIGHKPNTDFLGGQLTLDESGYIVTEKGTSKTSVPGVFAAGDVQDKYYRQAV
TSAGSGCIAALDAERFLG

>core/504/4/Org4_Gene227

MRKLILCNPRGFCSGVVRAIQVVEVALEKWGAPIYVKHEIVHNRHVVNALRAKGAIFVEELVDVPEGERVIY
SAHGIPPSVRAEAKARKLIDIDATCGLVTKVHSAAKLYASKGYKIILIGHKKHVEVIGIVGEVPEHITVVEKVA
DVEALPFSSDTPLFYITQTTLSLDDVQEISSALLKRYPSIITLPSSSICYATTNRQKALRSVLSRVNYVYVVGDV
NSSNSNRLREVALRRGVPADLINNPEDIDTNIVNHSGDIAMTAGASTPEDVVQACIRKLSSLIPGLQVENDIFA
VEDVVFQLPKELRCS

>core/505/4/Org4_Gene862

MKCRPTLNTDQPRVRKKLPERFPKWLQRPLPQGPAFHATDATIKRSGMPTVCEEALCPNRAECWSRKTATY
LALGDVCTRSCGFCNIGHSKTPPALDPTEPERIALSAKELGLKHVVITMVARDDLEDGGAQGLVDIIRKLGEE
LPQATTEVLASDFQGNVSALHTLLDSGITIYNHNIETIERLTPLVRHKATYARSMFMLEQAANYLPNLKIKSGI
MVGLGETEGEVKQTLQDLASVGVRIVTIGQYLRPSRKHIQVKSYVTPETFDYYRRVGTAMGLFVYAGPFVRS
SFNADMILTSVQDKASAQQT

>core/507/4/Org4_Gene53

MKKRYAFLFPGQGSQYVGMGQDLYMEYPEVRELFDFANERLGFSLTSIMFEGPEDLLMETVHSQLAIYLHS
MAVVKVLSQRSSIQPSLVSGLSLGEYTALVASDRISVLDGLELVRKRGQLMNEACNQSPGAMAALLGLPSEV
IEENITSLGQGIWIANYNAPKQLVVAGIAEKVDQAIELFRDLGCKKAVRLKVSGAFHTPLMQVAQDGLAPDI
YALCMKDSSLPLVSHVVGKSLVNTEEMRECLARQMTSPTLWYQSCYHIESEVDEFLELGPGKVLAGLNRSIG
ISKPITSLGTFAQIEKFLSEV

>core/508/4/Org4_Gene371

MLNSNKFKSKTGAYGDLFQRVVVHSLVLTFLVLLLYSSLFPLTSFALGFITATCGAVGTYEYSSMAKAKMHY
PLSTFSAIGSFLFLALSFLSIRWGHSLPGFFDALPWTLLIVWVVWSIFRVRKSTIGALQLSGVTLFSILYVGIPIRL
FLHVLYGFIHTQEPYLGIWWASFLIATTKGADIFGYFFGKAFGNKKITPQISPNKTVVGFVAGCLGATLISFIFF
LQIPTRFASYFPMPAILIPLGLALGITGFFGDIIESIFKRDAHLKNSNKLKAVGGMLDTLDSLLLSTPIAYLFLLIT
QSKEFIG

>core/509/4/Org4_Gene12

MRLFSYDKPKIKVQKIKADGFSGWLKCNHCHEMIHANELGQNYNCCPKCSYHYRITAIERVKLLADKDSWR
PLYTDLKSQDPLEFIDTDTYANRLEKARKNTTESEGVIVGICTIGLHPVALAVMDFNFMAGSMGAVVGEKLT
RLIEEAIETRLPVIIVSASGGARMQESVFSLMQMVKTSAALAKLHEAGLPYISVLTNPTSGGVTASFAALGDIII
AEPKALICFAGPRVVAQVIGEDLPEGAQKSEFLLEHGMIDKIVERKELKTTLQTLLDYFLAQEYTGGKSKAPR
DLSKRLKEIFLLTDDSE

>core/510/4/Org4_Gene520

MEIAYSLTSSFSVDSVTVGFFDGCHLGHSNLLSILTSYSGSSGVITFDSHPQTVLSLNHTKLINTKEERLQLLQT
FPIDWLGVLTFDLNFANQSAEEFLTLLHRNLKCKRLILGYDSCIGKEQQSNTEALDTIGKPLGIEVIKIPPYRMD
NIVVSSKAIRQFLSAGNLECAHRFLGHPYAISGKITEGSGIGSSLGFATINLPREESLIPLGVYACEIRYDSTTCQ
GVMNLGTAPTFGRESLYAEAHIFSFAENLYGKEVSIIPRKFLREEKKFQSKETLIRAIEKDILDAQDWFAKGSF
NYEGTA

>core/511/4/Org4_Gene693

MSSRELIILGCSSQQPTRTRNQGAYLFRWNGEGLLFDPGEGTQRQFIFANIAPTTVNRIFVSHFHGDHCLGLGS
MLMRLNLDKVSHPIHCYYPASGKKYFDRLRYGTIYHETIQVVEHPISEEGIVEDFGSFRIEARRLQHQVDTLG
WRITEPDTIKFLPKELESRGIRGPIIQDLIRDQEISIGGSTVYLSDVSYVRKGDSIAIIADTLPCQAAIDLAKNSCM
MLCESTYLEQHRHLAESHFHMTAKQAATLAKRAATQKLILTHFSARYLNLDDFYKEASAVFPNVSVAQEYR
SYPFPKNPLLNK

>core/512/4/Org4_Gene866

MRSIFRFFLCFFTLSVSCCFADASLYENSCPSRCQPTPPPSNSNPLNVVQQPVAASSVPSYMPPVNADDVLPRD
HLSDGSFSDTYPDITTQAIILIFLALSPFLVMLLTSYLKIIITLVLLRNALGVQQTPPSQVLNGIALILSIYVMFPT
GVAMYKDARKEIEANTIPQSLFTAEGAETVFVALNKSKEPLRSFLIRNTPKAQIQSFYKISQKTFPSEIREHLTA
SDFVIIIPAFIMGQIKNAFEIGVLIYLPFFVIDLVTANVLVAMQMMMLSPLSISLPLKLLLIVMVDGWTLLLQGL
MISFK

>core/513/4/Org4_Gene755

MKESAPVHFPFPVRRSVWLNRYSTFRIGGPANYFKAIHTIEEAREVIRFLHSINYPFLIIGKGSNCLFDDRGFDG
FVLYNAIYGKQFLEDARIKAYSGLSFAALGKATAYNGYSGLEFAAGIPGSVGGAIFMNAGTNESDISSVVRNV
ETINSEGELCSYSVEELELSYRSSRFHRQQEFILSATFQLSKKQVSADHSKSILQHRLMTQPYTQPSAGCIFRNP
EGTSAGKLIDAAGLKGLAIGGAQISPLHANFIINTGKATSDEVKQLIAIIQSTLKTQGIDLEHEIRIIPYQPKIHSP
VSEK

>core/514/4/Org4_Gene949

MSLFLVFLTAFIWSSSFALSKLVMNASAPIFATGARMVIAGAILALAAWFRGGFVGISKKIFLYIVLLALTGFY
LTNIFEFIGLQSLSSSKTCFIYGLSPLMSALFSYIQLKEKVTLKKVLGLSLGLVSYICYLTFGGGGDDSQPWTW
QIGLPELLILGAASLASFGWTLLRQIEKQSTLSVTAINAYAMLIAGILSIMHSAVVEPWRPLPVQDISQFLYATL
ALVVISNLICYNLYAKLLRKYSSTFLSFCNLVMPLYSGFYGWILLGEKGVSLGLVLAVAFMVAGCRLIYHEEF
RQGYIVS

>core/515/4/Org4_Gene856

MQKPQYIDRITKKKVIEPIFYEKTMLFLYNSKLGKKLSVFLSTHPIFSRIYGWLQRCSWTRRQIRPFMNRYKIS
EKELTKPVADFTSFNDFFTRKLKPEARPIVGGKEVFITPVDGRYLVYPNVSEFDKFIVKSKAFSLPKLLGDHEL
TKLYAHGSIVFARLAPFDYHRFHFPCDCFPQKTRCVNGALFSVHPLAVKDNFILFCENKRTVTVLETEQFGNV
LYLEVGAMNVGSIVQTFSPNQTYPKGDEKGFFAFGGSTVILLFLPNAIRFDNDLLKNSRMGFETRCLMGQSLG
RSQREEI

>core/516/4/Org4_Gene230

MTMPSTQFHTTILEQFSLFLSVDRGLCQQSIAAYRQDISSFLTISAISSPQDISQNSVYIFAEELYRRKEAETTLA
RRLIALKVFFLFLKDQQLLPYPPIIEHPKIWKRLPSVLTPQEVDALLAVPLQMEKNPRHLAFRDTAILHTLYST
GVRVSELCDLRLGHVSDDCIRVTGKGSKTRLVPLGSRAREAIDAYLCPFRDQYQKKNPHEDHLFLSTRGHKL
ERSCVWRRIHNYAKQVTSKPVSPHSLRHAFATHLLDNKADLRVIQEMLGHARIASTEVYTHVAADSLIEKFL
AHHPRNL

>core/517/4/Org4_Gene73

MLKIDLTGKVAFVAGIGDDQGYGWGIAKLLAEAGATIIVGTWVPIYKIFSQSWELGKFNESRKLSNGTLLEIA
KIYPMDASFDSPEDVPEDIAENKRYKGITGFTISEVAEQVKKDFGHIDILVHSLANSPEISKSLLETSRKGYLAA
LSASSYSFVSLLSHFGSIMNRGGSTISLTYLASMRAVPGYGGGMSSAKAALESDTKTLAWEAGRRWGIRVNT
ISAGPLASRAGKAIGFIERMVDYYQEWAPIPEAMNAEQVGAVAAFLASPLASAITGETLYVDHGANVMGIGP
EMFPKDS

>core/518/4/Org4_Gene707

MPPPLVVTLTTSAQNNLRDRLKEKNFLFSQPQNTVFQARSNTVTCTLYPSGKLVIQGKGSEEFIEFFLEPEILHT
FTHARVEQDLRPRLGVDESGKGDFFGPLCIAGVYASNAEILKKLYENKVQDSKNLKDTKIASLARIIRSLCVC
DVIILYPEKYNELYGKFQNLNTLLAWAHATIINNLAPKPAGDVFAISDQFAASEYTLLKALQKKETDITLIQKP
RAEQDVVVAAASILARDAFVQSIQKLEEQYQVQLPKGAGFNVKAAGREIAKQRGKELLAKISKTHFKTFDEI
CSGK

>core/519/4/Org4_Gene546

MHLEEENQGWEALLRKVYHQEMPPAILLHGFTLPVLQDKAEQLASEILLSSSPGSEHKVSQKIHPDIYQFFPE
GKGRLHSIDLPRGIKKQIYISPFEANYKIYIIHEADRMTLAAISAFLKVFEEPPKHAVIILTTAKVQRLPKTIISRS
LSIFIERGEKILCSKETFSYLFRYAQCEIPVTEVSQIIKESSETDKQVLRDKVQRFMEVLLELYRDRYTLNLGLK
ASALNYPEHVKEILQLPLLPLDKVLLIVESACRSLNNSSSAASVLEWVAIQLVSLQYKEKELVSVSPGQDLSN

>core/520/4/Org4_Gene551

MRLNYFLNLVNFKYSIFSILFLSASTVFALSINEISQNLSFKEGFKISVFGATAFVFARTTGIVVNQCIDRFIDKK
NTRTSKRVLPANLVSLNFAWVLSLFCSFLFLFLCKILHIFSLGIASLTLMIVYPYMKRVTFFCHWGLGLVYTVA
ILMNFCAFAESGLSMRLCFLAFLWGGSVGMVIAANDIIYAIQDTEFDREEGLRSVPAHYGEKKAVEIAKVNL
WVSYLAYIFSGFVGSLDKEFYFTAIIPLVVILKVVRMYSNYSKKDQEGESKFFLANIAIALSFLVSMTLFWSLS
R

>core/521/4/Org4_Gene81

MSERAHIPVLVEECLALFAQRPPQTFRDVTLGAGGHAYAFLEAYPSLTCYDGSDRDLQALAIAEKRLETFQD
RVSFSHASFEDLANQPTPRLYDGVLADLGVSSMQLDTLSRGFSFQGEKEELDMRMDQTQELSASDVLNSLKE
EELGRIFREYGEEPQWKSAAKAVVHFRKHKKILSIQDVKEALLGVFPHYRFHRKIHPLTLIFQALRVYVNGED
RQLKSLLTSAISWLAPQGRLVIISFCSSEDRPVKWFFKEAEASGLGKVITKKVIQPTYQEVRRNPRSRSAKLRC
FEKASQ

>core/522/4/Org4_Gene883

MSRREICPEVSHKKGKYYSTFIFRCIHSLAGIAFTFFLCEHLFTNMLASSYFSQGKGFVAMVNGFHKIPGLKIIE
VVGLALPCLCHAIIGIVYLFQGKSNCYSGDGSRPHLRYAKNYSYTWQRWTAWILLFGIAFHVVHLRFIRYPV
HVDIHGTTYYAVDIQPFRYDVIVRGTKGFLTLNLPNTEASSIEVSRHDLGGADAALLSEGNSYLLTPSAGTAF
LYVVRDALGSLFIALLYTILVIAAAFHGFNGLWTFCCRWGVVVSLRMQGVLRIVCYLAMIVVTFMGVSVVW
NLYSVA

>core/524/4/Org4_Gene570

MRRFLFLILSSLPLVAFSADNFTILEEKQSPLSRVGIIFALPEVTPVSFDGNCPIPWFSHSKKTLEGQRIYYSGDS
FGKYFVVSALWPNKVSSAVVACNMILKHRVDLILIIGSCYSRSQDSRFGSVLVSKGYINYDADVRPFFERFEIP
DIKKSVFATSEVHREAILRGGEEFISTHKQEIEELLKTHGYLKSTTKTEHTLMEGLVATGESFAMSRNYFLSLQ
KLYPEIHGFDSVSGAVSQVCYEYSIPCLGVNILLPHPLESRSNEDWKHLQSEASKIYMDTLLKSVLKELCSSH

>core/525/4/Org4_Gene44

MSHGPRPTKFSFPLYFSKTLSWFILGGFLAACGVQMVLVPNELIDGGIVGLSIIASHFLGHKALPFCLVLFNLPF
VFLAFKQIGKYFVIQMLTAVIIFSCSLWLIDQLPSWLGMSPFVFKGSEMETVVLGGAIIGVGCGLIIRHGGSTD
GTEILGIIINKKKGYTVGQIILFVNFFIFALSGIVYKNWHTAFVSFLTYGIATKVMDMVILGLEDTKSVTIITSSP
RKLGHILMETLGIGLTYIHAEGGYSGEPRNLLYVVVERLQLSQLKEIVHREDPSAFIAIENLHEVINGRRT

>core/526/4/Org4_Gene816

MQEKPRHVHRIIHISDVHFHVLPVNPVHCFNKRLKGLLRKVFGLVHFQATTIGQRFPKVVRSLGADSVCITGD
FSLTAMDGEFLLAKHFVETLAKHSSVYLLPGNHDVYTPKSLAQQTFYTHFPNDQLQQNKVSFHKITDHWWL
ILLDCSCLNGWFSANGVVHLAQISAIETFLLSLSPEENVIIANHYPLLSSQNPSHDLINNTHLQNVLKKYPKVRL
YLHGHEHQAAVYNCADTSPSYILNSGSISLPTNSRFHVIDLYPEKYQVHTMILKNLLDFDAPLEIANEATWDC
QKL

>core/527/4/Org4_Gene468

MEKLLVTDIDGTITHQSHHLDKKVYERLYALHQAGWKLFFLTGRYYKYAARLFSDFDAPYLLGCQNGASV
WSSTSSNLLYSKSLPSDLLCILQDCMEGATALFSVESGAPYGDHYYRFSPTPIAQDLHEYVDPRYFPNAKEREI
LFETRSLKDDYAFPSFAAAKVFGLRDEVIRIQKELERQEALTSVATMTLMRWPFDFRYAILFLTDKSVSKGKA
LDRVVNILYDGKKPFVMASGDDANDLDLIERGDFKIVMSSAPEEMHVHADFLAPPADKNGILSAWEAGVRY
YDELMSL

>core/528/4/Org4_Gene140

MKKLILYFAAFVASLFCGVFLWDRVPCAQKIMRLAADHSSEVFSKSCRFVRKISGFEELQVFERHVSPEQALA
LFPEYRDGKSFVELAFIPHTLMHVRFSKEEPVKKHIISQEGEILWSLVNGEMVLHTGTWTCSKGFRECLLLHA
GKQDMRVIQTLATLGGTTSRESLAQALALKNIRAERVIKECQKKKLIFASGNQIGTHFQQFQPIRGCTTTLNN
NPVWLQKPRHAAVFPAQYSEDRVRHLVKMIFGDNFLIVRSSMVYVPVYKISLVSADNSVRVEYINAVTGKSF
QDL

>core/529/4/Org4_Gene217

MFHSLSKNTPIITQGITGKAGSFHTEQCLAYGTNFVGGVTPGKGGTLWLDLPVYDSVLEAKQATGCRATMIF
VPPPYAAEAILEAEEAGIELIVCITEGIPVRDMLEVARVMDNSTSQLIGPNCPGIIKPGECKIGIMPGYIHLPGNI
GVVSRSGTLTYEAVWQLTQLKIGQSICVGIGGDPLNGTSFIDVLQALEEDPYTELILMIGEIGGSAEEEAAAWI
QAHCTKPVVAFIAGVTAPKGKRMGHAGAIISGNSGDAKSKIQVLRESGVTVVESPAHIGKTVDAVLRAKEL

>core/530/4/Org4_Gene817

MSKHTSESRIAQDMLERYSGSNVKQFCPYLLLTNFSYYIQTFAKLHGVPIFEGSMFSAAHAPHLKTSILDFKLG
SPGAALTIDLCSFLPDLKAALMLGMCGGLRSHYQVGDYFVPVASIRGEGTSDAYFPPEVPALANFVVQKATT
EVLEDKKANYHIGITHTTNIRFWEFNKKFRKKLYETKAQSAEMECATLFAAGYRRNLPIGALLLISDLPLRKE
GIKTKSSGNFVFNTYTEDHILTGQEVIENLEKVMLKRAASDHKKDQQYRGLPHMEVGEADDTMASGSETSD
SDY

>core/531/4/Org4_Gene266

MKVLPPPSIPLLGAHTSTAGGLKNAIYEGRDIGASTVQIFTANQRQWQRRALKEEVIEDFKAALKETDLSYIM
SHAGYLINPGAPDPVILEKSRIGIYQEILDCITLGISFVNFHPGAALKSSKEDCMNKIVSSFSQSAPLFDSSPPLV
VLLETTAGQGTLIGSNFEELGYLVQNLKNQIPIGVCVDTCHIFAAGYDITSPQGWEDVLNEFDEYVGLSYLRA
FHLNDSMFPLGANKDRHAPLGEGYIGKESFKFLMADERTRKIPKYLETPGGPENWQKEIGELLKFSKKQR

>core/532/4/Org4_Gene919

MLSSLIRDSFPLLILLPTFLAALGASVAGGVMGTYIVVKRIVSISGSISHAILGGIGLTLWIQYKLHLSFFPMYGA
IVGAIFLALCIGKIHLKYQEREDSLIAMIWSVGMAIGIIFISRLPTFNGELINFLFGNILWVTPSDLYSLGIFDLLV
LGIVVLCHTRFLALCFDERYMALNHCSVQLWYFLLLVLTAITIVMLIYVMGTILMLSMLVLPVAIACRFSYK
MTRIMFISVLLNILCSFSGICIAYCLDFPVGPTISLLMGLGYTASLCVKKRYNPSTPSPVSPEINTNV

>core/533/4/Org4_Gene945

MKRNDPCWCGSGRKWKQCHYPQPPKMSPEALKQHYASQYNILLKTPEQKAKIYNACQVTARILDELCKAS
QKGVTTNELDELSQELHKKYDAIAAPFHYGSPPFPKTICTSLNEVICHGIPNDIPLKDGDIMNIDVSCIVDGYY
GDCSRMVMIGEVPEIKKKICQAALECLNDSIAILKPGIPLCEIGEAIEARADTYGFSVVDQFVGHGVGIEFHENP
YVPHYRNRSMIPLAPGMIFTIEPMINVGKKEGVVDPKNQWEARTCDNQPSAQWEHTIAITETGYEILTLLND

>core/534/4/Org4_Gene716

MHLLTATVTPFFPNGTIDFASLERLLSFQDAVGNGVVLLGSTGEGLSLTKKEKQALICFACDLQLKVPLFVGT
SGTLLEEVLDWIHFCNDLPISGFLMTTPIYTKPKLCGQILWFEAVLNAAKHPAILYNIPSRAATPLYLDTVKAL
AHHPQFLGIKDSGGSVEEFQSYKSIAPHIQLYCGDDVLWSEMAACGAHGLISVLSNAWPEEAREYVLNPQEQ
DYRSLWMETCRWVYTTTNPIGIKAILAYKKAITHAQLRLPLSIEDFDLENVSPAVESMLAWPKLRTSVFSYS

>core/535/4/Org4_Gene216

MFKFFRNKLQSLFKKNISLDLIEDAESLFYEADFGTELTEELCARLRRTKKADASTIKDLITVLLRESLEGLPSQ
ASQSSQTRPIVSLLLGTNGSGKTTTAAKLAHYYKERSESVMLVATDTFRAAGMDQARLWANELGCGFVSGQ
PGGDAAAIAFDGIQSAIARGYSRVIIDTSGRLHVHGNLMKELSKIVSVCGKALEGAPHEIFMTVDSTLGNNAIE
QVRVFHDVVPLSGLIFTKVDGSAKGGTLFQIAKRLKIPTKFIGYGESLKDLNEFDLDLFLNKLFPEVEKI

>core/536/4/Org4_Gene556

MKRRNLQKILPNASTPSTNVAENTGIKDQNLFLDQATLNVDGSVDVENFLETRDLKVADTITSPCEFTVGGA
LSAESSQFKATTLSRGLEITSEDQNGRVPKFINVSDPQSPRDALTYNYYRNTGCQALNMYTFYDGLYSAQPTT
VGKPIEPVSQNPGPTTYHTSASAKIYDAIGTTPYVEFKAPGIYQVTIQVLRDLGQHSGNDYPNLYLNLMIGNN
KTLLCASDTRGYSGGHRTSVAVTGTFTLTEIVTTPPRDYPWLFLETTIGLNIKSMSTCIIWFPFQSNFAEVD

>core/537/4/Org4_Gene251

MQIPRSIGTHDGSFHADEVTACALLIIFDLVDENKIIRSRDPVVLSKCEYVCDVGGVYSIENKRFDHHQVSYDG
SWSSAGMILHYLKEFGYMDCEEYHFLNNTLVHGVDEQDNGRFFSKEGFCSFSDIIKIYNPREEEETNSDADFS
CALHFTIDFLCRLRKKFQYDRVCRGIVREAMETEDMCLYFDRPLAWQENFFFLGGEKHPAAFVCFPSCDQWI
LRGIPPNLDRRMEVRVPFPENWAGLLGKELSKVSGIPGAVFCHKGLFLSVWTNRESCQRALRLTLQDRGII

>core/538/4/Org4_Gene867

MGISLPELFSNLGSAYLDYIFQHPPAYVWSVFLLLLARLLPIFAVAPFLGAKLFPSPIKIGIGLSWLAIIFPKVLA
DTQITNYMDNNLFYVLLVKEMIIGIVIGFVLAFPFYAAQSAGSFITNQQGIQGLEGATSLISIEQTSPHGILYHYF
VTIVFWLVGGHRIVISLLLQTLEVIPIHSFFPAEMMSLSAPIWITMIKMCQLCLVMTIQLSAPAALAMLMSDLF
LGIINRMAPQVQVIYLLSALKAFMGLLFLTLAWWFIIKQIDYFTLAWFKEVPIMLLGSNPQVL

>core/539/4/Org4_Gene161

MLHALDTYRPSIESAIEKALEGFGPIGHPIRSPVEYALQGGGKRLRPGLVCMMAQGLGLNHDVMDSALAVEF
VHTSTLIADDLPCMDNDDERRGRPTVHKAFDEATALLASYALIPAAYSHLRLNAKKLKEQGCDPREIDIAYNI
IGDITDKNIGCSGVLGGQYDDMFFSNRGQEHVQSIMIKKTGSLFEIACISGWLFGGGDPQFAPIITSFSNNFGLL
FQIKDDFSDLQKDSQQIGLNYALLFGEKAALELLARSQNNCLELLDRLSAGGLKNSSEFETIISSLGSF

>core/540/4/Org4_Gene557

MSNPTPKTKISIPTFVRFNVQSINLTEDQKKTALTVGGKVTTENTVVGGDLTCTDGGLTCQSDLTIQKDINIRP
TSTNSMVFDGRLNLSNSPLSYKNSQGQDITDYEKISSGKPQEYVPFGYYKRTQIMMAQRGATSSGHVGSGSV
PSGSYVPWNKFDQTSVQKTSGTEIYIDPNDGTKLVFEVNSKVPKLFRISIIMAKNGSWLDNGTGADVLLAANE
YEQSGGKIKVTELAMTSSRGTSYYETRPLQVVCVTYYAKNNGYFTFQNRAGGGFRVSFFSWNIVSLPYVE

>core/541/4/Org4_Gene704

MLKIQKKRMCVSVVITVGAIVGFFNSADAAPKKKKIPIQILYSFTKVSSYLKNEDASTIFCVDVDRGLLQHRY
LGSPGWQETRRRQLFKSLENQSYGNERLGEETLAIDIFRNKECLESEIPEQMEAILANSSALVLGISSFGITGIPA
TLHSLLRQNLSFQKRSIASESFLLKIDSAPSDASVFYKGVLFRGETAIVDALSQLFAQLDLSPKKIIFLGEDPEV
VRAVGSACIGWGMNFLGLVYYPAQESLFSYVHPYSTATELQEAQGLQVISDEVAQLTLNALPKMN

>core/542/4/Org4_Gene692

MSHLIPSLRNSVTSYFHKPQPIKQAAPSKSIRDICNIAYLIIICVLVVVVLVGAMLCMFIPSVGIPLCLSSLALLV
LLSIFNPCLINWISTKKTKEIAPKDASESQPTKSASRKGSPQLSPHHDHEPKNFIRTQLEKGVNYVTNKFKSGEE
SPHISDEHHSPRQSKRSSEIESSDESSPELHRKAKGKAPHTATTKESKTSTTESSKKKKKTKHSLHRTTSSIHKR
SAPKPMVPSKKRKPVPLKKPVPLPIEDLEHQSSGNESSDSSSPPPVQRKAILPWFCKQPTDP

>core/543/4/Org4_Gene26

MEIKKAIQEGTAYLDYYGVPLSDCEALYILMDLLEVSSRAKLFDLVGISETMLMEYRKRLALRGQRCPTAYL
NGAVSFLGLRLRVDSRVLIPRTETELLAEYIINYLLSHSEIQTFYDICCGSGCLGLAIKKSCPHVEVVLSDVCPQ
AVAVANENAKSNGLDVKILLGDLSAPYTRPADAFVCNPPYLSFNEIIHIDPEVRCYEPWKALVGGSTGLEFYQ
RIAQELPKIVTSTGVGWLEIGSSQGESIKNIFSKHGIYGRLHQDLSGRDRIFFLEMDGRDPVSSGAYS

>core/544/4/Org4_Gene982

MAVIYWDRSKIVWSFEPWSLRLTWYGVFFTVGIFLACLSARYLALSYYGLKDHLSFSKSQLRVALENFFIYSI
LFIVPGARLAYVIFYGWSFYLQHPEEIIQIWHGGLSSHGGILGFLLWAAIFSWIYKKKISKLTFLFLTDLCGSVF
GIAAFFIRLGNFWNQEIVGTPTSLPWGVVFSDPMQGVQGVPVHPVQLYEGISYLVVSGILYFLSYKRYLHLGK
GYVTSIVCISVALIRFLAEYVKSHQGKVLAEDCLLTIGQILSIPLFLFGVALLIICSLKARRHRSHI

>core/545/4/Org4_Gene510

MLLRGIPAAEKILQRLKEEISQSPTSPGLAVVLIGNDPASEVYVGMKVKKAAEIGIISKAHKLPSDSTLSSVLKL
IERLNQDPSIHGILVQLPLPKHLDSEVILQAISPDKDVDGLHPVNMGKLLLGNFDGLLPCTPAGIIELLNYYEIP
LRGRHAAIVGRSNIVGKPLAALMMQKHPQTNCTVTVLHSQSENLPKILKTADIIIAALGAPLFIKETMVAPHA
VIVDVGTTRVPADNAKGYTLLGDVDFNNVVTKCAAITPVPGGVGPMTVAMLMSNTWRCYQNFS

>core/546/4/Org4_Gene144

MTEEISKDTIIEVAIDDIRVSPFQPRRVFSNEELQELIASIKAVGLIHPPVVREICTGDRVLYYELIAGERRWRAM
QLAGATTIPVILKHVIADGTAAEATLIENIQRVNLNPIEMAEAFKRLIHVFGLTQDKVAYKVGKKRSTVANYL
RLLALSKTIQESLLQGQITLGHAKVILTLEDPILREKLNEIIIQEHLAVREAELIAKQLISEEGSSIELKPTPLDMA
ESSKQHEELQQRLSDLCGYKVQIKTRGSKATVSFHLQNTQDLQKLEAWLSSHGTLSESLS

>core/547/4/Org4_Gene548

MPDKKAQITFSLPEVMSAIHQGKIVALPTDTVYGFVLSLYASEAEERLYALKDREPSKAFALYVNSIEDIENIS
GYPLSPTAKKLAQLFPGAITLVVKHRNPRFPKETLAFRIVDHSVVREIVDHCGTLIGTSANLSEFPSALTAQEV
FADFADHDLCIFDGPCSHGLESTVVASDPLYIYREGLISRSVIENIAGTEAKIFHRTSHAFSKHIKIYTVKNQEQ
LVSFLSGSLDFKGVVCEHPKPKNFYTRLREALKKKTPSIVFIYDINTSDYPELFPFLSPYYIE

>core/548/4/Org4_Gene555

MTTTNNQDNNECYFKLDSTVDGDLLASNIQTFDTQAKGISSTETFSVEGNATFKDQVSATGLTSASTYKLNA
TGPAPSSITIDMKNNRLSNPALPKNPCDPVPANYVRSPQYFFCSKPIEGTFTFDGSSRYLPITGDGSNYTLYQSN
KAGDVFRFVSWDQNSKKLNLGGNTAIQLLAAGTYILTFTIGKRWGWNNGWGGSIRLFEGKFTGDGTILCGST
VYSGGGYSTIGYLSTAVYRDHSDVDPDPNNPSNRYMNNFLFVRNGDSSAVIGNYSFTLLYFAGDKV

>core/549/4/Org4_Gene425

MHEVLILTFTYPLPRTLKQHPDEVHTVPISPNLSFGEGSPILIAGPCTLESYEHTVSSALTVKEAGAQVFRGSIR
KPRTSPFSFQGWEKECVLWHKEAQRIHGLPTETEVLDVRDVEITAEHVDILRIGAKNMHNTPLLQEVSKSHRP
IILKRSPAATLEEWLCAAEYILASPSCPGVILCERGIRTFEHSTRYTLDLNTVALLKEISSLPVIVDPSHAAGKRS
LVLPLASAGLSVGADGLMIEVHAHPEKALCDAKQQITPEELRLFAKKHFCPSESRAHAIS

>core/550/4/Org4_Gene563

MKHYLSFSPSADLFSKQGAIETQVLFGERVLVKGSTCYAYSQLFHNELLWKPYPGHSFRSTLVPCTPEFHIHP
NVSVVSVDAFLDPWGIPLPFGTLLHVNSQNTVIFPKDILNHMNTIWGSGTPQCDPRHLRRLNYNFFAELLIKD
ADLLLNFPYVWGGRSVHESLEKPGVDCSGFINILYQAQGYNVPRNAADQYADCHWISSFENLPSGGLIFLYP
KEEKRISHVMLKQDSSTLIHASGGGKKVEYFILEQDGKFLDSTYLFFRNNQRGQAFFGIPRKRKAFL

>core/551/4/Org4_Gene36

MDNYLLNIKDLTITSTNPKRTLIENLSLQLKENRNLALVGESGSGKTTITKAILGFLPENCLIKTGSILFEDIDIT
KLSPKELHKIRGQKIATILQNAMGSLTPSMRIGMQIIETLRQHHKMNKEEAYNKAMQLLTDVCIPNPKYSFSQ
YPFELSGGMRQRVVIAIALASQPKLILADEPTTALDSMSQAQVLRILRNIQQQKQATILLVTHNLSLVKELCND
ICIIKDGKLIETGTVEEIFLSPKHPYTLKLLNAVSKIPIKKTSSPILKNKFQPLMSMQGGL

>core/552/4/Org4_Gene589

MENLSSAPSRSIWKSIIQNKMLVLGLTTLIILILGALLLPWFYQDYEQTSLKDILVSPCSRFPFGTDTLGRCMFA
RTLRGLRLSLLIATIATLIDVCVGLLWATVAISGGKKIDFLMMRTTEILFSLPRIPIIILLLVIFHHGLLPLILAMTI
TGWIPISRIIYGQFLLLKNKPFVLSAKAMHASTFHILKKHLLPNTLAPIISTLIFTIPNAIYTEAFISFLGLGIQPPQ
ASLGTLVKEGINAIDYYPWLFFFPSLIMIALSISFNLIGEGAKTLCLEEGSHG

>core/553/4/Org4_Gene607

MKQPMSLIFSSICLGLGLGSLSSCNQKPSWNYHNTSTSEEFFVHGNKSVSQLPHYPSAFRTTQIFSEEHNDPYV
VAKTDEESRKIWREIHKNLKIKGSYIPISTYGSLMHPKSAALTLKTYRPHPIWINGYERSFNIDTGKYLKNGSR
RRTSHDGPKNRAVLNLIKSSGRRCNAIGLEMTEEDFVIARRREGVYSLYPVEVCSYPQGNPCGIAYAWIADES
ACSKEVLPVKGYYSLVWESVSSSDSLNAFGDSFAEDYLRSTFLANGTSILCVHESYKKVPPQP

>core/554/4/Org4_Gene130

MFKKFKPVTPGTRQLVLPAFDELTTRGELRGTKSKRSLRPNKKLSFFKKSSGGRDNLGHISCRHRGGGAKQL
YRVVDFKRNKDGITAKVVTVEYDPNRSAYIALLSYEDGEKRYILAPKGIQRGDVVVSGEGSPFKPGCCMTLK
SIPLGLSVHNIEMRPSSGGKLVRSAGLAAQVIAKSPGYVTLKMPSGEFRMLNEGCRATIGEVSNADHNLRVD
GKAGRRRWMGVRPTVRGTAMNPVDHPHGGGEGRHNGYIPRTPWGKVTKGLKTRDKNKSNKWIVKDRRK

>core/556/4/Org4_Gene319

MASIHPTAIIEPGAKIGKDVVIEPYVVIKATVTLCDNVVVKSYAYIDGNTTIGKGTTIWPSAMIGNKPQDLKYQ
GEKTYVTIGENCEIREFAIITSSTFEGTTVSIGNNCLIMPWAHVAHNCTIGNNVVLSNHAQLAGHVQVGDYAIL
GGMVGVHQFVRIGAHAMVGALSGIRRDVPPYTIGSGNPYQLAGINKVGLQRRQVPFTTRLALIKAFKKIYRA
DGCFFESLEETLEETLEEYGDIPEVKNFIEFCQSPSKRGIERSIDKQALEEESADKEGVLIES

>core/557/4/Org4_Gene289

MSDFSMETLKTLRQQTGVGLTKCKEALEACGGNLEEAVVYLRKLGLASAGKKEHRETKEGIIAAKTDANGT
ALIEVNVETDFVANNAVFREFVSNLLNDILKYKVDTVEALSQAASSQDPSLSVDELRAVTMQTVGENIRISRV
AYFPKAANSTVGIYSHGNGKTVALTMLSGSSTADSLAKDIAMHVVAAQPQFLSKESVPAEAIAKEKEVIASQI
QGKPQEVIEKIVTGKLNTFFQEACLLEQPFIKNADLSIQSLIDDFSKTSGSSVAIEQFILWKIGA

>core/558/4/Org4_Gene133

MLERTQRTLKREVRYSGVGIHLGKSSTLHLQPAQTNTGIVFQRQSASGNYENVPALLDHVYTTGRSTTLSRG
SAVIATVEHLMAALRSNNIDNLIIQCSGEEIPIGDGSSNVFVELIDQAGICEQEDKVSIARLTRPVYYQHQDIFL
AAFPSDELKISYTLHYPQSSTIGTQYKSLVINEESFRQEIAPCRTFALYNELCFLMEKGLIGGGCLDNAVVFKD
DGIISRGQLRFADEPVRHKILDLIGDLSLVGRPFVAHVLAVGSGHSSNIAFGKKILEALEL

>core/559/4/Org4_Gene903

MATAHLGRQALLHLRSWTPAIRTSGNLFRQQSMSLHNNILFAGDIIGAIKNSTAISRYALGSSHHAHAALQKT
EGFLGAADGVNTAVAGAMLWGQLLNGSMIFETDEETGELRRCNEADAEGCRTQKLQRRSALTITGKVARL
ASKTLGTATFLHEMDVVSLGANANKIGCKVTSCLNLVATGCSLTESSISLYRILSTRPETISDPENRNKPSAEF
AARSKAIRNAFIAWLGDMVDLVCDALGTLSLFLPAILGVHAVLIMAILGLISCVINFVKDYAKIG

>core/560/4/Org4_Gene974

MVLMNKRLKIILTNDDGITAKGMSCLVSALLEANIGDIYIAAPQAEQSGKSMAISLNQVVCASPYAYPQPVKE
AWAVGGSPTDCVRLGLRTLFESVSPDLVISGINCGNNICKNAWYSGTVGAAKQALVDGIPSMALSQDNHISF
FQQYKAPEILKALVIYLLSQPFPCLTGLNINFPTSPGGSSWEGMRLVPPGDEFFYEEPQYLGSVNKNQYYVGKI
SGVRIGEHPSEELACMLENHISVSPIFSQNSPIGLMTLEEFQKTQENFNASLLSSELTTKIF

>core/561/4/Org4_Gene283

MELKKTAESLYSAKTDNHTVHQNSPEPRDSRDVKVFSLEGKQTRQEKTTSSKGNTRTESRKFADEEKRVGD
EIAEVGSKEEEQESQEFCLAENAFAGMSLIDIAAAGSAEAVVEVAPIAVSSMDTQWIENIILSTVESMVISEING
EQLVELVLDASSSVPEAFVGANLTLVQSGQDLSVKFSSFVDATQMAEAADLVTNNPSQLSSLVSALKGHQLT
LKEFSVGNLLVQLPKIEEVQTPLHMIASTIRHREEKDQRDQNQKQKQDDKEQDSYKIEEARL

>core/563/4/Org4_Gene731

MRQSFDELSQNAFKNIFNKQRFCFIFCSLCCFGFVFALFLKLCSRLAPEISLSTLGLGAFFCAFSVICASAIIVQF
LLHKESQGETSKLCCAIKNTWSSLWLSLLVSMPFFIAMVAVVTVVMLSSFLGSLPWVGKLFHTVLIFIPYLSA
TALILLFLGSFSCLFFCIPVLHNQESIDYRKLLDCFRGNILRQFIGVVIALVPLALCSWLALDSFYLMTHLVEIA
DIHTWSFLAQMFVLIVPIALILTPAVSFFFNFSFSFYLAKQEEEKALVK

>core/564/4/Org4_Gene388

MHKVIVFVFLTLYSLKSYGNDVIDKPHVLVSIAPYKFLVEQIAEETCFVYAIVTNHYDPHTYELPPRQIKELRQ
GDLWFRIGEAFEKTCERNLTCQQVDLSQNVSLIQGKPCCNQHTTNYDTHTWLSPKNLKVQVETIVTTLSKKY
PEHATLYQSNGEKLLLALDQLNEEILTITSKAKQRHILVSHGAFGYFCRDYNFSQHTIEKSSHVEPSPKDVARV
FRDIEQYKISSVILLEYSGRRSSAMLADRFHMHTVNLDPYAENVLVNLKTIATTFSSL

>core/565/4/Org4_Gene332

MYFTRDPVIETVITSREGYKLSVRNTKHFSQDPFMVEAIEVISLGNICFFRNCDHSKPFLVPAGDYEVMEVRDT
KINLKAVGLDRGVKIAGGREALIKLTKSTPLPVIDEKPLADSPEEGTEPTSPSKKEKKEARKDSFKGEKWKEK
KKLSRRRNHKEIAEVTGASQEILDTVKEELWEESQENEIVEQKKFSLLPPPAKLISEVISQTVVDPVVTSADLN
ESLQALVRESSDVINALLSADDAIHFPETEEEPTSASFEESSAMFFPETSSATEEE

>core/566/4/Org4_Gene712

MTRSSPAQLSRLLSEIQNKPKKSLSQNFLVDQNIVKKIVATSEVIPQDWVLEIGPGFGALTEELIAAGAQVIAIE
KDPMFAPSLEELPIRLEIIDACKYPLDQLQEYKTLGKGRVVANLPYHITTPLLTKLFLEAPDFWKTVTVMVQD
EVARRIVAQPGGKDYGSLTIFLQFFADIHYAFKVSASCFYPKPQVQSAVIHMKVKETLPLSDEEIPVFFTLTRT
AFQQRRKVLANTLKGLYPKEQVEQALKELGLLLNVRPEVLSLNDYLALFHKMQAG

>core/567/4/Org4_Gene290

MESQSCKLTIKDLMSAGAHFGHQTRRWHPKMKLYIFEEKNGLYIINLAKTLQQLRNALPHIRKVIQDNKTVL
FVGTKKQAKCVIREAAIEAGEFFVAERWLGGMLTNMTTIRNSIKTLDKIEKDLSRNQAYLTKKEAALLAKRH
QKLLRNLEGIRYMKKAPGLLVVVDPSYEKIAVAEAKKLGIPVLALVDTNCDPTPIDHVIPCNDDSLKSIRLIIN
VIKENIIEAKHKLGIEIVSPVKSLEVPDLSAFESSQDDESDEENREEDLLAKKFDSEAN

>core/569/4/Org4_Gene864

MTANTFGTLDILMKHSKEDDLSRFLPKNLLVESPHPEEIPLKSLSFTMSWLPTIHPSWITIAMKEFPPEIQGQLL
AWLPEPLVQEILPLLPGISMAPHRCAPFGAFYLLDMLSKKIRPCGITEEIFLPASSANAILYYTGPVKIALINCLG
LYSIAKELKHILDKVVIERVKNALSPTEKLFLTYCQSHPMKHLETTNFLSSWTTDAELRQFVHKQGLEFLGKA
LTKENASFLWYFLRRLDVGRAYIVEQTLKTWYDHPYVDYFKSRLEQCMKVLVK

>core/570/4/Org4_Gene397

MDYKLLDSGDGNKLECFGPVTLIRPSSIAVWPKSRPELWSQAQLQYVREGERGAWKNFKRLPEEWEVAFSD
VRCLLKRTPFGHLGVFPEHMGFWPALKQAIEKHKECQVLNLFAYTGAGSIFAAKCGARVTHVDASQAAVR
WAQRNVEKNAFPERRIFWVIEDVISFLKKEIRRNKKYQVILLDPPSYGRGPDGDVFKIDKDLFPLLSLCSKLLA
DDASYFLLTSHTPGHTPEFLRAIARRSVPTLVSEAWSCGESFCGEGVGALPSGSFVQWIA

>core/571/4/Org4_Gene612

MITGVVLEKHEQRTMFSLTLLNNFTTFGVLHTPLHYNPPYPIVILLHGLASDKTGSKRSHVRLAQELTRLGIA
ALRVDLLGHGDGEGELMDFSLENYKQNIREIIEYTHSLLHIDQERLAIFGSSLGGTLALQTLPFFDKIKALAVW
APTISGELMAAEAQKNAPEVITMSQKGAITYAGMTLNPDFYTQFLKIDIVKELMPSARNLPPILYMQGEQDHL
VSMNHRTLFTEAFANQDKPITILTYPDVDHAFPFAESSALSDLTQWLKRELTSRE

>core/572/4/Org4_Gene272

MLLGFLCDCPCASWQCAAVANCYDSVFMSRPEHKPNIPYITKATRRGLRMKTLAYLASLKAARQLAYDFLK
DPGSLARLAKALIAPKEALQEGNLFFYGCSNIEDILEEMRRPHRILLLGFSYCQKPKACPEGRFNDACRYDPSH
PTCASCSIGTMMRLNARRYTTVIIPTFIDIAKHLHTLKKRYPGYQILFAVTACELSLKMFGDYASVMNLKGVG
IRLTGRICNTFKAFKLAERGVKPGVTILEEDGFEVLARILTEYSSAPFPRDFCEIH

>core/573/4/Org4_Gene703

MRRYLFMVLALCLYRAAPLEAVVIKITDAQAVLKFAREKTLVCFNIEDTVVFPKQMVGQSAWLYNRELDLK
TTLSEEQAREQAFLEWMGISFLVDYELVSANLRNVLTGLSLKRSWVLGISQRPVHLIKNTLRILRSFNIDFTSC
PAICEDGWLSHPTKDTTFDQAMAIEKNILFVGSLKNGQPMDAALEVLLSGISSAPSQIIYVDQDAERLRSIGAF
CKKANIYFIGMLYTPAKQRVESYNPKLTAIQWSQIRKNLSDEYYESLLSYVKSKG

>core/574/4/Org4_Gene645

MKVKINDQLICIPPFISARWSQIAFIESQEGENKDQGTLRLHLIDGKIISIPNLDQSIIDIAFQEHLLYLETSQSGKE
DSRDDDKLGVGVLMNVLQQITKGNDIQVLPKNLISPLFSGTNPIEAILQHTPEHKDHPDAPTDVLEKMADVIR
VLSGNNATLLPKPEPHCNCMHCQIGRVMNEEDTLAVSDKDLTFRTWDIMQSGDKLYIVTNPLNPSEQFSVYL
GSPIGCTCGEPNCEHIKAVLYT

>core/575/4/Org4_Gene479

MKLLKNVLLGLFFSMSISGFSEVKVSDTFVKQDTVVEPKIRVLLSNESTTALIEAKGPYRIYGDNVLLDTAIQG
QRCVVHALYEGIRWGEFYPGLQCLKIEPVDDTASLFFNGIQYQGSLYVHRKDNHCIMVSNEVTIEDYLKSVL
SIKYLKELDKEALSACIILERTALYEKLLARNPQNFWHVKAEEEGYAGFGVTKQFYGVEEAIDWTARLVVDS
PQGLIIDAQGLLQSNVDRLAIEGFNARQILEKFYKDVDFVVIESWNEELDGEIR

>core/576/4/Org4_Gene542

MKKKLSLLVGLIFVLSSCHKEDAQNKIRIVASPTPHAELLESLQEEAKDLGIKLKILPVDDYRIPNRLLLDKQV
DANYFQHQAFLDDECERYDCKGELVVIAKVHLEPQAIYSKKHSSLESLKSQKKLTIAIPVDRTNAQRALHLLE
ECGLIVCKGPANLNMTAKDVCGKENRSINILEVSAPLLVGSLPDVDAAVIPGNFAIAANLSPKKDSLCLEDLS
VSKYTNLVVIRSEDVGSPKMIKLQKLFQSPSVQHFFDTKYHGNILTMTQDNG

>core/577/4/Org4_Gene429

MVRDIQSESIGKLVFLGTGNPEGIPVPFCSCRVCQNTGIHRLRSSVLIQYQNKTLVIDAGPDFRTQMLVAGVSE
LDGVFLTHPHYDHIGGIDDLRAWYIVTQRSLPLVLSASTYRFLNKAKEYLFATPNVESSLPAVLEFTILNEDYG
QEEFQGIPYTYVSYYQKSCHVTGFRFGNLAYLTDLCSYDAKIFSYLDNVETLILSAGPSETPIAFQGHKSSHLT
VEEAKAFANHAGIKNLIITHISHCLEAERDQHLEVTFAYDGMEVLWTL

>core/578/4/Org4_Gene247

MIRRFFKTLFPPGPQYSLCYASILIVLSSLVCVPTFCWLFLPELSLSKFNPSPIRNLFLVSSTLSKVPPTAIAEHLR
LSADAPTYLHEFSIKEAESSLHALGIFSSLVIEKSPDNKGITIFYTLQTPIAYVGNRSNTLCNLEGSCFLGQPYFP
SLNLPQIFFSQEDLKMQKLPKEKMLFTKILLKELAMESPKIIDLSLSDAYPGEIIVTLSSGSLLRLPIKTLDRALD
LYKHMKKSPVIESEKQYVYDLRFPNFLLLKAL

>core/579/4/Org4_Gene274

MFNNKMILIAGPCVIEGEDITLEIAGKLQSILAPYSDRIQWFFKSSYDKANRSSLNSFRGPGLTEGLRILAKVKE
TFGVGILTDVHTPQDAYAAAEVCDILQVPAFLCRQTDLLIATAETGAIVNLKKGQFLSPWDMEGPINKVLSTG
NNKILLTERGCSFGYNNLVSDMRSIPVLSRSGFPVIFDATHSVQLPGALSTESGGQTEFVPTLSRAALAAGAHG
LFIETHTNPKIAKSDAASMLSLEEFAALLPTWDQLFTCVSSFDMVSA

>core/580/4/Org4_Gene918

MTKVALLIAYQGTAYSGWQQQPNDLSIQEVIESSLKKITKTRTPLIASGRTDAGVHAYGQVAHFRAPDHPLF
ANPNLTKKALNAILPKDIVIRDVALFDDNFHARYLAIAKEYRYSLSRLAKPLPWQRHFCYTPRHPFSTELMQE
GANLLIGTHDFASFANHGRDYNSTVRTIYTLDIVDKGDSLSIICRGNGFLYKMVRNLVGALLDVGKGAYPPE
HLLDILEQKNRREGPSAAPAYGLSLHHVCYSSPYNNFCCEQCSVSTSNEG

>core/582/4/Org4_Gene20

MTQYYFLSSFLPTQLPESVPLFSISDLDDLLYLNLSENDLCNYGLLKRFFDFENFAFFWAGKPIPFSFGEVTQE
NVERMLSSQQWSDDNDFEDFFKDFLMNHKSSQDRLNHFSDLFREFLSYHQTNSSKFLQDYFRFQQQLRVVL
AGFRARVLNMDVSYVLRDEDSSDPVVLEVLMQKDSPNYELPEEFSDLQGVLDDYGLLPHTLNRALALYQFH
KLEGFCSDSYFDGNVILARCATYMFAIRNSLASVEKGREIINHIEKAIKW

>core/583/4/Org4_Gene601

MHSKFLSRRKKNSSHKEETSWDCIASSYNKIVQDKGHYYHRETILPQLLPSLTLNSKSSVLDIGCGQGFLERA
LPKECRYLGIDISSRLIALAKKMRSVNSHQFKVADLSKRLEFVEPTLFSHAVAILSLQNMEFPGEAIRNTATLL
EPLGQFFIVLNHPCFRIPRASSWHYDENKKAISRHIDRYLSPMKIPIMAHPGQKDSPSTLSFHFPLSYWFKELSS
HGFLVSGLEEWTSSKTSTGKRAKAENLCRKEFPLFLMISCIKIK

>core/584/4/Org4_Gene842

MTLPLEPMIFWSSLSAKVMKKFLTPHCAGTFSEEDAEAKEAHLVTGKQGHRLMGNCVTFYWLVDKKNGVI
LDAKFQYFGHPYLIPLAEAVCNLVCGKSYSEAYKMTLDDIDKSLRVHAHQPALPEDSISLYHFVIDALDTAVE
QCLEIPLEDGSLPFQNSPMNLDFEDANPYSQSDWEALTHEQKLYALRATIAEKIGPYIAMDGGEVTVESLENFI
VTIAYSGNCSGCPSSLGSTLNSIGQLLRAYIYPELQVKVDESSLNLSHP

>core/585/4/Org4_Gene193

MQGFFPLASGSKGNSAYLGTDSCKILIDLGVSKQVVTRELLSMNIDPEDIQAIFVTHEHSDHISGIKSFVKAYN
TPIVCNLETARALCHLLDSHPEFKIFSTGSSFCFQDLEVQTFNVPHDAVDPVAFIFHYREEKLGFCTDLGWVTS
WITHELYDCDYLLIESNHSPELVRQSQRPDVYKKRVLSKLGHISNQECGQLLQKIITPKLKKLYLAHLSTECNT
AELALSTVSESIASITSIAPEIALAQGITSPIYFSRLEVACPR

>core/586/4/Org4_Gene758

MAGLDLEARGKRRVVTPNAITAFGLCCGLFIIFKSVLRTSSSVELFHRLQGLSLLLISAMIADFSDGAIARIMKA
ESAFGAQFDSLSDAVTFGIAPPLIAIKSLDGIYVGNFFSSLLLITSIIYSLCGVLRLVRYNLFSQKTVDVSKPYCFI
GLPIPAAAASIVSLALFLASDFFPDLPAQLRVGLLSFALLFIGGLMISPWKFPGVKHFRFNVSSFLLVVTIGLAA
CLFFSGLVDHFVEVFFLVSWLYTLVGFPIFSIIYRKRS

>core/587/4/Org4_Gene686

MKKPDNDSTFDVRSFFPFDVLCIEQLRKEMSWEVVSAKIPRLPRGWYELMGLSKEDRIDFCLDFWCSVLGIE
HKESPSICRFFSLLETIEVYIYRLEKGPYQLKMFYVFRDGRCGFQGEPPLLDFLGHHRLPPLGDRHYEKFFSIHN
GFGKWEDEGIFPMRSLAKVQQKLRQQLVVMNKMQAEDNCYSLGIFPFYGYEEPFAYQSFFFDPEIRRDLPSP
NVLLNEESLEHRSLETIELLHLSKSYYPSFLSWLENYLHSEEVYNE

>core/590/4/Org4_Gene398

MDCIGKHNPLVKEALALKRSRCRKSSWFLVEGAREIQKALRAGYLCQHVFCSTHLSEKEKEFLYELKRNSTK
ILYCLDSTLAQLSFKEHHDSFVAVIQKRVWNKEDFLIQRKNAQPFYLIIEQVEKPGNVGAILRIADGAGVDGVI
LCNPIVDLYNPNVVRSSLGAVFSLPILSISREEGKELFKQEGWTVFVTSPRAETMYFSKNYLGPTALVFGSEKD
GLTEDWFSEDFSEIALPMLGESDSLNLATSVAAVAYEVVRQRWVN

>core/591/4/Org4_Gene977

MGSSMHVGVIGCSGRTGKVIVSALEQSSEYTLGPGFSRSSALTLSQVIAHNDVLVDFSHPLLTKEVVAHLLISP
KPLIIGTTGFPGKCKEAHDSLEELTHIVPVVVCPNASLGAYIHKRLVMLLSQLCNPQFDIRIRETHHRYKKDSL
SGTAQDLLDTIQQVKQEDWGEEYEVGQRDSSKKTIEVQSSRVGDIPGEHEVAFISSGEQILVRHTVFSRNVFG
QGILSILDWLKTLNPQPGLYSLGDDTLELVLRNEHCLLKKTTDH

>core/592/4/Org4_Gene893

MEKLEFVTSLSSPDDDLITFNKQGLIAGPEEEKVAFLVRSNAMLDAGPETPASFPESLREQFDIFPEYVEVLYS
NEGLDVWEAGCTWILNNEVTIQLRKHLRKASRWLGMYSRDEVLAHEAVHAVRMKFHEPVFEEVLAYQTSR
WGWRRFFGPLFRSPGESYLLLFFTILGLGISLWYPAGILIMLVLPMYFLMRLCMAQSYLYRAMKKIRKMLGV
PPLWVLLRLTDKEIKMFAKEPIPVLEHYARKRKLENVRWKQIYQSYFV

>core/593/4/Org4_Gene408

MPNQLQPRISLGCVSYINSFPLSLQLIKRNDILCVLAPPADLLNLLIEGKLDVALTSSLGAISHNLGYVPGFGIA
ANQRILSVNLYAAPTFFNSPQPRIAATLESRSSIGLLKVLCRHLWRIPTPHILRFITTKVLRQTPENYDGLLLIGD
AALQHPVLPGFVTYDLASGWYDLTKLPFVFALLLHSTSWKEHPLPNLAMEEALQQFESSPEEVLKEAHQHTG
LPPSLLQEYYALCQYRLGEEHYESFEKFREYYGTLYQQARL

>core/594/4/Org4_Gene79

MKLTKYLNTKQLRSMISRLFVRYSLPMSKQLSFFALCVLGSHPIFAQTPNPPQRVRRSEVIFIDPGHGGKDQGT
ASKELHYEEKSLTLSLALTVQSYLKRMGYKPQLTRSSDVYVDLGKRVALSNRGQGDVFISIHCNHSSNAAAF
GTEVYFYNGKVGSPTRNRMSEVLGKNILAAMEKNGILKSRGLKTANFVVIRDTSMPAVLVETGFLSNSRERA
ALQDARYRMHVAKGIAEGVHNFLSGPSFQKPKQNIAKIRKPQIQAN

>core/597/4/Org4_Gene23

MPFDITYYTTPLLEIILIWVMLNYLLKFFWGTRAMDVVFGLLAFLFLFVLADKLHLPIIRRLMLHVVNIAAIVV
FIIFQPEIRLALSRIRFHGKKFFIDTQEQFVEQLAASIYQLSERQIGALVVLENKDSFDEYLSFSSVKINATFSEEL
LETIFEPSSPLHDGAVILRGDILAYARVVLPLAHDTTQLSRSMGTRHRAALGASQRSDALIITVSEENGSVSLSR
DGLLTRGVKIDRFKAVLRSILSPKEHKRKPLFSWIWKR

>core/599/4/Org4_Gene588

MTTLLSIKDLSLTIRGKKILNHINLNLIKGSCLTIVGPSGSGKSSLALTILDLLKPTTGTITFHMDPKIPRARKVQ
VIWQDIDSSLNPCMSIKGIISEPLNIIGTYSKAEQNKEIYNVLDLVNLPKSVLHLKPYKLSGGQKQRIAIAKALV
SKPELLICDEPLSSLDTLNQSLILDLFQTIKKEYQNTLLFITHDMSAAYYIADTIAVMDQGSLVEHACREKIFST
PEHTTTQDLLDAIPIFSLISTEMEPSEEYELQVASK

>core/600/4/Org4_Gene727

MKTWLFFTFLFSCSSFYASCRYAEVRSIHEVAGDILYDEENFWLILDLDDTLLQGGEALSHSIWKSKAIQGLQ
KQGIPEQEAWEAVVPFWLEIQEMGTVQPIESAIFLLIEKIQKQGKTTFVYTERPKTAKDLTLKQLHMLNVSLE
DTAPQPQAPLPKNLLYTSGILFSGDYHKGPGLDLFLEICTPLPAKIIYIDNQKENVLRIGDLCQKYGIAYFGITY
KAQELQPPIYFDNIAQVQYNYSKKLLSNEAAALLLRHQMHE

>core/601/4/Org4_Gene885

MMKYLPYIAITACIHGGILLLVFASPLPKKRLQPKAFQEKLVTIQPKPPVPTPSVVVDPAKTIRPSVATQPQQQ
AKCSPPQENVQKALKKPIPKVVKTEPPKPSPAPTVAKKTTATEKPPPSTTKKNTQLSKTQLQTLSEVAQALFL
HVDKIEKSETSLKNISWPSTAQLTMHSELKATQEDELCELFRTHIALPSKGYVRIKLVLSPNGEIQECSFLSEVS
AADKQLLTQRIQALPFQKFLEKYKVSKNISFHIKLVSNES

>core/602/4/Org4_Gene173

MDLADAHVHLSDDAFEEDINSVLQRAQDSGVSLVVNVTTTEKELNRSFAYAERFPKIRFCHVGGTPPQDVDQ
DIEEDYRNFHAAAHSKKLAAIGEVGLDYCFATEEGIARQKEVLQRYLALSLECELPLVVHCRGAFNDFFRML
DQYYHNDPRSRPGMLHCFTGTLEEAQELISRGWFISISGIVTFKNAQDLRDLVVELPLEHLLIETDAPFLAPVP
YRGKKNEPAHVLHTINAVANVKGMFPQELAALAYKNVLRFLHG

>core/603/4/Org4_Gene6

MFLQFFHPIVFSDQSLSFLPYLGKSSGIIEKCSNIVEHYLHLGGDTSVIITGVSGATFLSVDHALPISKSEKIIKILS
YILILPLILALFIKIVLRIILFFKYRGLILDVKKEDLKKTLTPDQENLSLPLPSPTTLKKIHALHILVRSGKTYNELI
QEGFSFTKITDLGQAPSPKQDIGFSYNSLLPNFYFHSLVSVPNISGEERALNYHKEQQEEMAVKLKTMQACSF
VFRSLHLPSMQTKDKKAGFGLLTFFPWKIYPL

>core/605/4/Org4_Gene75

MILTAAFSPCPNDIFLFRSFLKDPQFRPLLNQVTIADIETLNTLALQRRLSLMKMSAALFPLVSDYYNLMDVG
NTLGYNSGPIVLSLDPECSLDTLATPGEMTTAHALCKLYYPKAKLIPMPYDKILSAILQGKVDGGALIHEERFS
YDLQLTLRADFGELWRRKTIFPLPLGCLAIAKYVPMATVDALTAALRKSLICSLKDPITAGAKAVEYSKNKN
VTVIHRFIGTYINKETFQLSKTGKKALHMLWKANECCQYT

>core/606/4/Org4_Gene426

MIKQIGRFFRAFIFIMLLSLTSCESKIDRNRIWIVGTNATYPPFEYVDAQGEVVGFDIDLAKAISEKLGKQLEVR
EFAFDALILNLKKHRIDAILAGMSITPSRQKEIALLPYYGDEVQELMVVSKRSLETPVLPLTQYSSVAVQTGTF
QEHYLLSQPGICVRSFDSTLEVIMEVRYGKSPVAVLEPSVGRVVLKDFPNLVATRLELPPECWVLGCGLGVA
KDRPEEIQTIQQAITDLKSEGVIQSLTKKWQLSEVAYE

>core/607/4/Org4_Gene634

MLRNQVLVYCSEGVSPYYLRHTIRFLKHYSTQEGAFDILRVDGNFLIKNPFWEETTRLLVFPGGADRPYHRV
LHGLGTARIFQYVSEGGNFLGICAGAYFGSKMIYFYEPEGAPLQGARDLGFFPGTAKGPAYRGNFSYVSPSGV
RVSPQLFSDFGLGYAMFNGGRFFEGSEGYPGVNIESRYDDLPGKPASIVSRIVGKGLAVLSGPHIEYLPHYCR
MVEENVQKTREFLQREGTTLDRYCQNLVRRLLQPAFSKVDC

>core/608/4/Org4_Gene504

MNVKDETFWSVHNLCVNYEHAAVLYHISFSLGKGSLTAILGPNGAGKSTLLKASLGLIKPSSGTVYFFNQKF
KKVRQRIAYMPQRASVDWDFPMTVLDLVLMGCYSYKGMWGRISSDDRREAFHILERVGLESVADRQIGQLS
GGQQQRAFLARALMQKADLYLMDELFSAIDMASFKTSVGVLQELRDQGKTIVVVHHDLSHVRQLFDHVVL
LNKRLICCGPTDECLNGDTIFQTYGCEIELLEQTLKLSRGKQFGSC

>core/609/4/Org4_Gene137

MNRRWNLVLATVALALSVASCDVRSKDKDKDQGSLVEYKDNKDTNDIELSDNQKLSRTFGHLLARQLRKS
EDMFFDIAEVAKGLQAELVCKSAPLTETEYEEKMAEVQKLVFEKKSKENLSLAEKFLKENSKNAGVVEVQP
SKLQYKIIKEGAGKAISGKPSALLHYKGSFINGQVFSSSEGNNEPILLPLGQTIPGFALGMQGMKEGETRVLYI
HPDLAYGTAGQLPPNSLLIFEINLIQASADEVAAVPQEGNQGE

>core/611/4/Org4_Gene952

MENLETFILKIYRGIPGKQYWESFELPLHPGENVISALMEIEKRPVNILGEKVNPVVWEQGCLEEVCGSCSILV
NGVPRQACTALIQEYIDATQSREIVLAPLTKFPLIRDLIVDRSIMFDNLERIQGWVAADIEGETFGPQVTQEQQ
ELLYALSQCMTCGCCTEACPQIDNKSDFIGPAAISQARYFNTYPGDKQSKKRWRALMGKGGIEGCGQAHNC
VRVCPKKLPLTESISAMGREISKFSLRSLFSALFKKKK

>core/613/4/Org4_Gene472

MSKFILLLSLGVAALASKNFFIWPAPSGKTPLKLRQVLFGGALLVFSSLVALSVSSQTAELLSTMTGISLAFAF
LFYLLFLPKDITRAILFSGERPIKTSWRALGSAIRMWIIIIPVTQLIGIMMSKFLTLVLPTQEIHTQEVTQEVQNSL
PITGHYISMILNLGVLTPFGEEVFFRGILQTFLKNKMTRIAAVLCSSIIFSFIHIEHSLGSWVFVPVLFVFSLSAGF
LYEKDRHILSPIALHGLFNLTSLLFVGIK

>core/614/4/Org4_Gene94

MAFYSPSTISKYFIYSGAGNRFLLGETLPEVEDVRFLCQETRVDGFLYLKPSSCADAQLIIFNSDGSRPTMCGN
GLRCAIAHLASQKGKSDISVSTDSGLYSGYFYSWDRVLVDMTLADWRASVHRLESRPDPLPKEVVCIHTGVP
HAVVILPEISTLDLSILGPFLRYHQTFSPDGVNVNFVQILGHCQLRVRTYERGVEGETAACGTGALASALVVS
NSYGWKESIQIHTWGGELMTVSQNRGRVYLQGSVTRDL

>core/615/4/Org4_Gene31

MFKLLKNLFLIGCCIVGYFWMRKESIVEQWLSNRLHTQVTVGRVSIRTSGIKIRHICIHNPLASERFPYAAEIEY
ADVRFSSISMLLTKQLEISELIIHGANFTIFPYDSHGTKTNWSLVWKNFHPQKETPSNLWIDRAPVLIRRCLFLN
TRLYGLRANHKDIPHLSVPSLEFHSHTSSAKELPKLSEALPSLLYLALEESLYHLNLPGDIIKPLSQQAHKHFYS
SYPQFQDRLNDINTPGTPTEEIIGFIRGLFFH

>core/616/4/Org4_Gene940

MVSPLSLFHKMLLENWTPVEEPFPWPPAEKNQKIFAWALNQSKLIFVSTSGNIAQPRLVTDSMSMMIVNAAN
RTMSRDGAGTNQVLSAAVSVDSWGLSQQPLNPERRGTPLNEGECRAGMWRNADGSNHTGKQGKPHYLAQ
LLGPKAVDHHNESQAAFDCCKNAYLNCFRLAQTLGVTFLQIPLISSGIYAPPENRKKPNSEENKVRLRWIHAV
KCALVAAMQEFGNEPGNADRKMLIVLTDLKTPAITDPKKKSHL

>core/617/4/Org4_Gene65

MKTQQTQNIIEVWNFYWETQEIEYRDSLIEFYLPLVKSVVHRLISGMPSHVKTEDLYASGVEGLVRAVERYN
PERSRRFEGYAVFLIKAAIIDDLRKQDWVPRSVHQKANKLSGAMDSLRQSLGKEPTDLELCEYLNISQQELSG
WFVSARPALIVSLNEEWPSQSDEGAGMALEERIPDERAETGYDVVDKQEFSLCLANAIQELEEKERKVMALY
YYEELVLKEIGKVLGVSESRVSQIHSKALLKLRAALSAFR

>core/618/4/Org4_Gene86

MWLGAYTWLNVFGILLQAAFIQNILLANFLGMCSYLACSTRVSTANGLGMSVALVLTVTGSINWFVHAFITG
PKALTWISPSLASVNLGFLELIIFIVVIAAFTQILELLLEKVSRNLYLSLGIFLPLIAVNCAILGGVLFGITRSYPFIP
MMIFSLGAGCGWWLAIVILATIKEKLAYSDIPKNLQGMGISFITTGLIAMAFMSLTGIDISKPSAKIQRAPLETE
VVENTTNPLKESSSKHQPSISKARTQRRSL

>core/619/4/Org4_Gene295

MLKIKHLHASCNDVKILDDFNLNIQPGTMHVIMGPNGAGKSTLAKILAGDESVLVSSGEIALQEQNLLSMLPE
ERSRAGLFVGFQMPPEIPGVNNKMFLRDAYNARRANQEGDISIDEFNTLLSTVLETYEYNTTTDLFLDRNVN
EGFSGGERKRNEICQMLVLEPEMVLLDEPDSGLDVDALRLICRVLEKYRELHPTSSLCIVTHNPKLGNLIRPD
VVHLLLDGRVALSGDVSLMHELEAKSYQEVTKRVAWR

>core/621/4/Org4_Gene176

MKTIAVNSFKGGTAKTSTTLHLGAALAQYHQARVLLIDFDAQANLTSGLGLDPDCYDSLAVVLQGEKEIQE
VIRPIQDTQLDLIPADTWLERIEVSGNLAADRYSHERLKYVLGSVQDKYDYVIIDTPPSLCWLTESALIAADYA
LICATPEFYSVKGLERLAGFIQGISARHPLTILGVALSFWNCRGKNNSAFAELIHKTFPGKLLNTKIRRDITVSE
AAIHGKPVFATSPSARASEDYFNLTKELLILLRDI

>core/622/4/Org4_Gene710

MTRQSYVLGNWKMHKTIQEAKEYVQTLASLLQGEPLSCTIGIAPPFTSLRAIHEMINTTGAFLWLGAQNVHP
ELSGAFTGEISLPMLKEVGVEFVLVGHSERRHIFGESDAFIASKVKSVAQAGLVPVLCVGESLEVREEGKAHQ
VIKKQLLLGLEQMDNGSEFLIAYEPVWAIGTGKVAEASDVQDIHMFCREVVAERFSEATAEEISILYGGSVKV
DNAQRFGQCSDVDGLLVGGASLEAQSFFEVAKNFNV

>core/623/4/Org4_Gene96

MHDALLSILAIQELDIKMIRLMRVKKEHQKELAKVQSLKSDIRRKVQEKELEMENLKTQIRDGENRIQEISEQI
NKLENQQAAVKKMDEFNALTQEMTTANKERRSLEHQLSDLMDKQAGGEDLIVSLKESLASTENSSSVIEKEI
FESIKKINEEGKALLEQRTELKHATNPELLSIYERLLNNKKDRVVVPIENRVCSGCHIVLTPQHENLVRKKDRL
IFCEHCSRILYWQESQVNAQENSTAKRRRRRAAV

>core/624/4/Org4_Gene569

MKPEESECLCIGVLPARWNSSRYPGKPLAKIHGKSLIQRTYENASQSSLLDKIVVATDDQRIIDHVTDFGGYA
VMTSPTCSNGTERTGEVATKYFPKTEIIVNIQGDEPCLNSEVVDALVQKLRSSPEAELVTPVALTTDREEILTE
KKVKCVFDSEGRALYFSRSPIPFILKKATPVYLHIGVYAFKREALFRYLQHSSTPLSDAEDLEQLRFLEHGGKI
HVCIVDAKSPSVDYPEDIAKVEQYITCLSNAYF

>core/625/4/Org4_Gene800

MLIKLWRATYEGMYTFLVGALLKLRYRMQVEGWDTLNINPKQGCLFLANHVAEVDPIILEYLFWSRFHVRP
MAVEYLFHSRIVRWFLNSVRSIPIPQLVPGKESKRSLERMNVCYEEASRALNRGESLLLYPSGRLSRTGKEEIV
NQYSAYVLLHRVMECNVVLVRVSGLWGSAFSRYKQNSTPKLGPAFKEAFRALLRRGIFFMPKRFVKITLCQ
VDHLFLKQFPTKQDLNTFLASWFNQGDDNLPIEVPYA

>core/626/4/Org4_Gene357

MQICVTGVVLRSRPLGKNHTLTTLFTPEGLFTFFAKQGQTLQCDYRETLVPISLGKYTLHRNGSRLPKLTHGD
ILNAFEAIKQTYALLEASGKMIQALLASQWKEKPSHKLFSLFLNFLHRIPESNNPEFFAAIFVLKLLQYEGILDL
TPACSLCKASLPYACYRYQGHKLCKKHQHKQAISIEKEEEQILQAIIHAKQFSELLAIAEFPIAIAEKIFYLFDSL
QEEKKSERNSSEDPYHEILRLSKVVHPY

>core/627/4/Org4_Gene146

MLIVLAFRQVFFSHSRSQLDRLKNYLRLLKQNFAITLPKERTSKGHSLMLTFDFASFDFYTNIFPFLEEQKIPAV
VGVASRYIPSNAAQDLHPSHRLKPSETLAFQDEIFSNYMPFCCQNELIEMAKSPYIQLASSGFAIRNLMNNPPY
LTTEILLSRHHIETITGAKPLAFLFPFGKSDPTSRKLAADHYPYSFLLGNTINRKLKTHNIYRLDIKPMQYVCPS
LFQSSRYLKNWIKEKSKQLYLKKQLPKR

>core/628/4/Org4_Gene38

MLMDISHILEDLAYDEGILPREAIEAAIVKQMQITPYLLHILHDATQRVPEIVNDGSYQGHLYAMYLLAQFRE
SRALPLIIKLFAFEDDTPHAIAGDVLTEDLPRILASVCNDDSLIKELIETPKINPYVKAAAISGLVTLVGAGKIPR
DKVIRYFAELLNYRLEKQPSFAWDNLIAGICTLYPGELFYPISKAFDGGLVDTSFISMEDVENIIHEETVESCIH
TLCSSTELINDTLEEMEKWLEDFPIEP

>core/629/4/Org4_Gene628

MNVADLLSHLETLLSSKIFQDYGPNGLQVGDPQTPVKKIAVAVTADLETIKQAVAAEANVLIVHHGIFWKG
MPYPITGMIYKRIQLLIEHNIQLIAYHLPLDAHPTLGNNWRVALDLNWHDLKPFGSSLPYLGVQGSFSPIDIDS
FIDLLSQYYQAPLKGSALGGPSRVSSAALISGGAYRELSSAATSQVDCFITGNFDEPAWSTALESNINFLAFGH
TATEKVGPKSLAEHLKSEFPIPTTFIDTANPF

>core/630/4/Org4_Gene642

MKKQGKTLFLFLFLSFLFSTAFSGLFASQTSSLRTIQENIFLAKTGDYTVLSRGSQRTFVLVKSTTPKTVWIEIIH
FPCIAYKERPSLEQASWKTVIHQLESPSQVFVVSLSSEGSQFFSLNTRTKSLEPVGKSTTVPAFLQIFDLPLSPAP
ANVIKTKGKENKPWSPRVSFEGAPLTSISVNAWQGLWPKDRGPLSETGILMYFTQPDISVFPLWVSIETPKGT
SIVRAVDIGHGATSPYVYSLPHSKTQ

>core/632/4/Org4_Gene105

MSLATNNAESKFPSLQRLPNHVAIIMDGNRRWYKKHREECGHTHTSGHYYGAKVLPNILNAVLDLGIKVLT
LYTFSTENFGRPKEEIQEIFNIFYTQLDKQLPYLMENEICLRCIGDLSKLPKGIQTKINHVSRMTASFSRLELVLA
VNYGGKDELVRAFKKLHVDILNKKISSDDLSESLISSYLDTSGLTDPDLLIRTGGEMRVSNFLLWQIAYTELYI
TDTLWPDFTPQDLFEAINVYQQRSRRGGK

>core/633/4/Org4_Gene175

MGNLKTLLESRFKKNTPTKMEALARKRMEGDPSPLAVRLSNPTLSSKEKEQLRHLLQHYNFREQIEEPDLTQ
LCTLSAEVKQIHHQSVLLHGERITKVRDLLKSYREGAFSSWLLLTYGNRQTPYNFLVYYELFTLLPEPLKIEM
EKMPRQAVYTLASRQGPQEKKEEIIRNYRGERKSELLDRIRKEFPLVETDCRKTSPVKQALAMLTKGSQILTK
CTSLSSDEQIILEKLIKKLEKVKSNLFPDTKV

>core/634/4/Org4_Gene347

MKIKFSWKVNFLICLLAVGLIFFGCSRVKREVLVGRDATWFPKQFGIYTSDINAFLNDLVSEINYKENLNINIV
NQDWAHLFENLDDKKTQGAFTSVLPTLEMLEHYQFSDPILLTGPVLVVAQDSPYQSIEDLKGRLIGVYKFDSS
VLVAQNIPDAVISLYQHVPIALEALTSNCYDALLAPVIEVTALIETAYKGRLKIISKPLNADGLRLAILKGTNG
DLLEGFNAGLVKTRRSGKYDAIKQRYRLP

>core/635/4/Org4_Gene317

MSLLKDTVFTCLDCEMTGLDVKKDRIIEIAAVRFTFDSVISSIEFLINPERVVSAESQRVHHISNAMLRDQPKIA
EVFPQIKAFFKEGDYIVGHSVGFDLQVLSQEMERIGETFLSKYTIIDTLRLAKEYGDSPNNSLESLAVHFNVPY
DGNHRAMKDVEININIFKHLCKRFRTLEQLKQVLAKPIKMKYMPLGKHKGRCFSEIPLAYLQWASKMDFDS
DLLFSIRHEIKHRQKGTGFSQVNNPFMEL

>core/636/4/Org4_Gene533

MDITLVGKKVIVTGGSRGIGLGIVKLFLENGADVEIWGLNEERGQAVIESLTGLGGEVSFARVDVSHNSEVKD
CVQKFLDKHNKIDILVNNAGITRDNLLMRMSEDDWQSVISTNLTSLYYTCSSVIRHMIKARSGSIINVASIVAK
IGSAGQTNYAAAKAGIIAFTKSLAKEVAARNIRVNCLAPGFIETDMTSVLNDNLKAEWLKSIPLGRAGTPEDV
ARVALFLASQLSSYMTAQTLVVDGGLTY

>core/637/4/Org4_Gene836

MTQDPHDHFKSRTPEDHIKHVRDKHRVCKGEPHTTFKGFFYHLANNALSTGVFIFFIRTLFFLIPTNRALQVKS
LISLGVGWTFYHGCLKARKAWAYMELSHRSMLEEKNEIEENFEQEKIELRILFENQGFKDPLLQEMVEYVCS
DSTLLLDTMIREELYIRKEDLPHPLIQGGSRILGGLCGLAIFLPLVLCISYTLAGVFSALMVLVLSFLKAKILEN
DKISEMVWVLGIFITSASIISSLMKLL

>core/638/4/Org4_Gene488

MTLSFHTHPLNYWTFEEFDGLPIRHGVFSKQKDAEGTVFAAKNPEIASALQSPKYCDLHQRHGTSVRCVTPT
SPTYQPADGVCTQSPLLSLHIRHSDCQAAIFYDREHHAIANVHSGWRGLLGNIYAVTVGTMKKLFHTKPQDL
FVASGPSIGPDYAIYPDYATLFPRSFLPFMNPKNHFDLRAIARKQLTNLGISKDRIFISDLCTYTEHDAFFSSRYL
AHHPDPNLTGQHSKNRNNVTAVLLLPRD

>core/639/4/Org4_Gene288

MAKQTRRVLFKISGEALSKDSSNRIDEMRLSRLVSELRAVRNNDIEIALVIGGGNILRGLAEQKELQINRVSAD
QMGMLATLINGMAVADALKAEDIPCLLTSTLSCPQLADLYTPQKSIEALDQGKILICTTGAGSPYLTTDTGAA
LRACELNVDVLIKATMHVDGVYDKDPRLFPDAVKYDFVSYKDFLSNQLGVMDASAISLCMDSHIPIRVFSFL
QHSLEKALFDPTIGTLISEDVNHVCSPRH

>core/640/4/Org4_Gene660

MKFFILFILIVAQFPAFSAQPRTQVSASHSKQAKARRTSRTRSSATTNASVSRYKTRAAARKKTGKFEKKPSLS
PVQWVRYSGKNYSIQTPSLWQCIDDKTQLPEKLDILLIGKGKGNLTPTINIAQEITSKSSKEYIEEILAYHKANE
MTLESGIFTQIQSPSGEFTIIKTEKNSSWGRVFCLQATTVIDHTAYIFTSTATLDDYAELSFTFLKVVSSFQIRGG
KEATSGDAILEKALEALQNENK

>core/641/4/Org4_Gene345

MHIYGLADLHLALGVPEKTMEVFGDPWIGYHQKICSEWQAVVHPEDIVLLPGDISWAMNLSEAHKDFAFIG
DLPGTKYMIRGNHDYWSSASTSKILQALPPSLYYLNQGFALLTPHLAVVGVRLWDSPTICVKKENFLNPSTQ
EQSYTEQDEKIFLRELGRLKRAFAALPKEVSEVIVMTHYPPISSDGTPGPVSEFLEADGRVSLCLFGHIHKVQR
PIDGFGNIRGIHYILVAADYVNFVPQEVM

>core/642/4/Org4_Gene164

MKITTVKTPKIYPYDDLYSILESSLPKLNERSIVVITSKIVSLCEGAVVELEKVSKDELIKQEADAYVFVEKYGI
YLTKKWGILIPSAGIDESNVEGYFVLYPRDFLLSVNTLGDWLRNFYHLEHCGIIISDSHTTPLRRGTMGLGLC
WNGFFPLYNYVGKPDCFGRALKMTYSNLLDGLSAAAVLCMGEGDEQTPIAIIEEAPKITFHSSPTTLQDMSTL
AIAEDEDLYGPLLQSMAWETPAPTS

>core/643/4/Org4_Gene474

MDFDYFGLSDIGRVRARNEDFWQVNLMSQVVAVADGVGGRLGGDIASQEAVTSLMELIDEQQSKLMGYED
DQYKETLKKILLEVNGVVYEHGQMEEHLQGMGTTLSFIQFRKDRAWLFHVGDSRIYRIREGELRCLTEDHSL
ENQLKNRYGLPKQSDKVYSYRHILTNVLGSRPYVMPDIRNLPCEKEDLYCLCSDGLTNMVPDIDIRDILNQPA
TLEERGNALISLANTRGGDDNATVVLVRIQ

>core/644/4/Org4_Gene387

MTIRILAEGLAFRYGSKGPNIIHDVSFSVYDGDFIGIIGPNGGGKSTLTMLILGLLTPTFGSLKTFPSHSAGKQTH
SMIGWVPQHFSYDPCFPISVKDVVLSGRLSQLSWHGKYKKKDFEAVDHALDLVGLADHHHHCFAHLSGGQI
QRVLLARALASYPEILILDEPTTNIDPDNQQRILSILKKLNRTCTILMVTHDLHHTTNYFNKVFYMNKTLTSLA
DTSTLTDQFCCHPYKNQEFSCSPH

>core/646/4/Org4_Gene737

MSYFNYQKNSVVLRSLGLLAKFFSRLLYRVFFSFREGIYLFSSLYLKYPRLFFYDLGKYVYSLRHCPYAKLGR
LPGASLLKEGNVYGETPWSVLAKISQAFDITSQDILYDLGCGLGKVCFWFSHVVRCQVIGIDNQPNFIRFSSN
MHRKLSSGFALFDTEEFKNVVLSQASYVYFYGSSFSRRLLNEIILKLSEMAPGSVVISISFPLDSFSKGKECFFT
EKSCSVRFPWGKTIAYKNIRKGS

>core/647/4/Org4_Gene553

MSTSLLNPPWMKAGKRIESLVRKALYTHTMLANHHKIVVALSGGKDSLTLLLMLKAISGRGFPDLDLHAVNI
GGKYSCGAEVNKPYLTRICDQLCIPFRTIPSPYAPETPECYPCSQARRRLLFQAAKEIGASAIAFGHHRDDLVQ
TALLNLLHKAEFAGMLPVLDMVHFGVTILRPLIFTPEFWIRKFAKENGFARVTCRCPVVSLRSKAEQSLKLLE
EVFPLARHNIALAIQEHGSSKSQKI

>core/648/4/Org4_Gene547

MTDYSFFRRKIGNIEAIECPGNPQDPIIILCHGYGSLADNLTFFPSICSFSKLRPTWIFPNGILPLENDFRGSRAWF
PLNVLLLQELSRLYANGVGNLQEKYDELFDVDLETPKEALEELILNLNRPYNEIIIGGFSQGAILTTHLVLTSQ
NPYAGALIFAGARLFNQGWEEGLKQCAQVPFLQSHGYEDEILPYHLGAHLNDLLLTKLNGQFVSFHGGHEIP
SIVFQKMQVTVPNWIDPARG

>core/650/4/Org4_Gene156

MPILSVCNLVKKYNKKPVTNDVSFQINPGEIVGLLGPNGAGKTTAFYLTVGLIRPDSGKIIFKNVDVTKKTMD
HRARLGIGYLAQEPTIFKELTVQDNLICILEIIYKARKQQSHLLNTLVDDLQLGSCLHKKAGTLSGGERRRLEI
ACVLALNPSVLLLDEPFANVDPLVIQNVKYLIKILAGRGIGILITDHNAKELLSIADRCYLIIDGKIFFEGSSSQM
ISNPMVKQHYLGDSFSY

>core/651/4/Org4_Gene456

MKVRIVDSGKSSVASHMAKDRDLLESLQDGELILHLYEWENPCSLTYGHFMRPEKFLVSNYPDLGLDAAVR
PTGGGFVFHKGDYAFSVLMSATHPSYSSSVLENYHTVNSFVAKVLEKVFRIQGMLAPEDENSSSRDSGNFCM
AKTSKYDVLFGDKKIGGAAQRKVQQGFLHQGSLFLSGSSSEFYQRFLKPEVLEEIIEQIQIHAFFPLGLESADE
VLQEARQQVKEAFIKLFCGERL

>core/652/4/Org4_Gene483

MTLYLLPNTLGTRAVETLPSVIGELVHRLDGLIVESDRGGRAFLSLWKIPEVHKFPLAILSKHARLPKAWDFY
LEPIVKHGENWGLISDAGLPCIADPGASLVRRARALGIPVQAFSGPCSITLALMLSGLPSQSFTFLGYLPQSPKE
RVKSIKKAATSKEVSTSVCIETPYRNVYTFESLLDTLPSYAELCVASDLSAPSELVLTRQVKSWRTNEDLGSV
KQSITKVPTIFLFHIPN

>core/653/4/Org4_Gene367

MAGHSKWANTKHRKERADHKKGKIFSRIIKELISAVKLGGADPKSNARLRMVIQKAKENNIPNENIERNLKK
ATSAEQKNFEEVTYELYGHGGVGIIVEAMTDNKNRTASDMRIAINKRGGSLVEPGSVLYNFARKGACTVAK
SSIDEEVLFSYAIEAGAEDLDTEDEENFLVICAPSELASVKEKLISQGATCSEDRLIYLPLRLVDCDEKDGEANL
ALIDWLEQIEDVDDVYHNMS

>core/654/4/Org4_Gene135

MDISGAVKQKLLQFLGKQKKPELLATYLFYLEQALSLRPVVFVRDKIIFKTPEDAVRILEQDKKIWRETEIQIS
SEKPQVNENTKRIYICPFTGKVFADNVYANPQDAIYDWLSSCPQNMEKQGGVRIKRFLVSEDPDVIKEYAVPP
KEPIIKTVFASAITGKLFHSLPPLLEDFISSYLRPMTLEEVQNQTKFQLESSFLSLLQDALVEDKIAAFIESLADD
TAFHVYISQWVDTEE

>core/655/4/Org4_Gene768

MGLYDRDYIQDSRMQGTFASRVYGWMTAGLIVTSCVALGLYFSGLYRSLFSFWWVWCFATLGVSFFINSKI
QTLSVSAVGGLFLLYSTLEGMFFGTLLPVYAAQYGGGVIWAAFGSAALVFGLAAVYGAFTKSDLTKISKIMT
FALIGLLLVTLVFAVVSMFVSMPLIYLLICYLGLVIFVGLTAADAQAIRRISSTIGDNNTLSYKLSLMFALKMY
CNVIMVFWYLLQIFSSSGNRD

>core/656/4/Org4_Gene678

MHPPIDITAIEAKLNFTFTQPKLLEIALTHPSYKNESAVQIEDSERLEFLGDAVLGLIVTEHLFLLFPSMDEGTLS
TARASLVNAKACCRYTTTLGIGDYLLIGKGEKIQSERGRLSAYANLFESILGAVYLDGGLSPARKLTVPLLPPR
EEILPLMSGNPKNLLQQFTQKQFRVLPVYQSTAVTDAQGNVSYQIQVLVNQEVWGEGNASSKKEAEKIAAQ
QALDTYGNKNQNTMDV

>core/657/4/Org4_Gene407

MEPSTNKPDCKKIFDSIASKYDRINTILSLGMHHFWNRSLIQILGSGHSLLDLCAGTGKVAKRYIAAHPQASVT
LVDFSSAMLDIAKQHLPQGSCSFIHSDINQLPLENHSYPLAAMAYGLRNLSDPHKALQEISRVLMPSGKLGILE
LTPPKKTHPTYSAHKLYLRAVVPWIGKSVSKDPDAYSYLSKSIQQLPKDHDLEDLFSKSGFYIAKKKKLFLGA
ATIWLLEKQ

>core/658/4/Org4_Gene253

MDVLIFYDTETTGTQIERDRIIEIAAYNSVTDESFLTYVNPEIPIPDEASKIHGITTDAVLSAPKFPEAYEGFRKF
CGEDSILVAHNNDGFDFPLLGKECRRHSLEPLTNRTIDSLKWAQKYRPDLPKHNLQYLRQVYGFAENQAHR
ALDDVVILHKVFTSLIGDLPPQQVLDLLQQSYHPKVFKMPFGKYKGQPLVDIPKSYFEWLENQGALDKPENK
DIKAAIALLHQPT

>core/659/4/Org4_Gene890

MQNATIDQLPVSWQEQLPLCWREQLKEEWSKPYMQQLLIFLKQEYKEHTVYPEENCVFSALRSTPFDQVRV
VILGQDPYPGKGQAHGLSFSVPEGQRLPPSLINIFRELKTDLGIENHKGCLQSWANQGVLLLNTVLTVRAGEP
FSHAGKGWELFTDAIVTKLIQERTHIIFVLWGAAARKKCELLFNSKHQHAVLSSPHPSPLAAHRGFFGCSHFS
KINYLLNKLNKPMINWKLP

>core/660/4/Org4_Gene60

MDLAVELKEGILLVDKPQGRTSFSLIRALTKLIGVKKIGHAGTLDPFATGVMVMLIGRKFTRLSDILLFEDKEY
EAIAHLGTTTDSYDCDGKVVGRSKKIPSLEEVLSAAEYFQGEIQQLPPMFSAKKVQGKKLYEYARKGLSIERH
HSTVQVHLQITKYEYPLLHFVVSCSKGTYIRSIAHELGTMLGCGAYLEQLRRLRSGRFSIDECIDGNLLDHPDF
DISPYLRDAHGNSL

>core/661/4/Org4_Gene906

MIGDKIILFVTEDLSLSSQLKDLASQRSDYQILVSPVFPTSFEAVAIFCEYLLLPEQIFSPGIFPEEDLIVLFDTFQE
EAITKVLNQGATGYLLRPITAKVLDAVIRAFLRQHEVLEHSIPDTMTFGDHTFRVLNLVIESPEGSVYLTPSEA
GILKKLLINRGHLCLRKNLLAEIKGNTKEIIARNVDVHIASLRKKLGPYGSKIVTIRGVGYLFSDDDSIPLQNHD
NTAHPNEE

>core/662/4/Org4_Gene839

MTKVRLNKFLASAGVASRRKCDEIIFSGSVTVNGRVAEGPFVLVDPEDKVQVGGTSVHLTKKVYFMVHKAI
GYLCSSEKKFPGTKLVIDLFAHLPYRVFTVGRLDKETSGLILVTNDGEFANKIIHPSSGITKEYLLKVSRDVSA
KDLEKLMEGTFIDGKHVRPVSVTKIRRGTVKIVVSEGKKHEIRLFADAAGLPILELKRIRIGSLVLGGLRYGEY
RELTDAELGTYMKLSD

>core/663/4/Org4_Gene571

MLQAHRLCYSYDNQVILKDASFQASPGTITIILGSSGVGKTTLFRLLAGFLPLQEGELLWNGSPLNCKDVAYM
QQKEALLPWRTALKNMTLSTELGINTSHNALSNERLQEIIHNFDLGQLLDRYPDELSGGQRQRIALAAQCLSL
KPILLLDEPFSSLDVLLKEQLYQDIVALAKKENKTVLLVTHDFHDVSCLGDVLYVIKNKTLTPVPLDPSMRSL
NNGLCFIKDLKKHLYT

>core/664/4/Org4_Gene746

MRVIFPDKHNNFPNLSKLLKKLPSVILVTSCIAPFFSYIINKFFGIPGLLEILALSVKGIQKHHFWQFLTYPLITAD
SLSLNKDQSFEITQRLLLRNVLDFFLFYKAIQHLIRKLGAFSVLVVISGQALIIGAVLWGFMALIHSSQSFFGPE
SIICGVLTVQIFLDPEKRFTIGPTPLSVSRKWGFLFVLGFYCCILIFSGAFLLLLASMLAIVLAILFCKKEKIPNPY
TTSLRF

>core/665/4/Org4_Gene335

MPTTNCIFLDLRGHSILHQLQIEEALLRVANQNFCIINSGVKDSIVLGISRNLNQDVHISRAQADHIPIIRRYSGG
GTVFIDSNTLMVSWIMNSSEASAQPQELLAWTYGIYSPLLPNTFSIRENDYVLGHKKIGGNAQYIQRHRWVH
HTTFLWDIDLDKLSYYLPIPQQQPTYRNQRSHEEFLTTLRPWFPSRDDFLERIKASGSLLFTWEEFLDNELEEIL
AQPHRKATTVLN

>core/666/4/Org4_Gene679

MLSVCYSDPFLSDFCQGKRPLRIASRNSNLAKAQVHECISLLRSWYPKLWFQLSTTETTGDRDKKIPLHLVEN
SYFFTDGVDALVHKGVCDLAIHSAKDLPETPSLPVVAITRCLHPADLLVYADHYVHEPLPLRPRLGSSSLRRS
AVLKQLFPQGQILDIRGTIEERLDQLHRGHYDAIVLAKAASLRLHLHHAYSIELPPPYHALQGSLAITAKDHA
GKWKQLFTPIHCHSS

>core/667/4/Org4_Gene865

MKFFSLIFKDDDVSPNKKVLSPEAFSAFLDAKELLEKTKADSEAYVAETEQKCVQIRQEAKDQGFKEGSESW
SKQIAFLEEETKNLRIRVREALVPLAIASVRKIIGKELELHPETIVSIISQALKELTQNKHIIISVNPKDLPLVEKSR
PELKNIVEYADSLILTAKPDVTPGGCIIETEAGIINAQLDVQLDALEKAFSTILKAKNPVDEPSETSSSTDSASLS
NDQDKKE

>core/668/4/Org4_Gene812

MNRRDMVITAVVVNAILLVALFVTSKRIGVKDYDEGFRNFASSKVTQAVVSEEKVIEKPVVAEVPSRPIAKET
LAAQFIESKPVIVTTPPVPVVSETPEVPTVAVPPQPVRETVKEEQAPYATVVVKKGDFLERIARANHTTVAKL
MQINDLTTTQLKIGQVIKVPTSQDVSNEKTPQTQTANSENYYIVQEGDSPWTIALRNHIRLDDLLKMNDLDEY
KARRLKPGDQLRIR

>core/669/4/Org4_Gene995

MFFIVCFGFLIHKKHTILPPKAHIPTNAKHFPTIGNPYAPINITVFEEPSCSACAEFTTEVFPLLKKHYIDTGEISF
TLIPVCFIRGSKPAAQALLCIYHHDPRQADIDAYMEYFHRILTYPKEEGSHWATPEVLTKLAEGLKTNSGRSIN
PKGLEQCIASGQYNEQIKKNNLYGSQVLGGQLATPTAVVGDYLIEDPTFHEIERAIQHIRQLQAVEGDHDD

>core/670/4/Org4_Gene884

MVHFSHNPIIQAYTEADFFGKSIFFCLLILSVCTWTVLHQKLAIQKKFLKAGKSLKDFLIKNRHAPLSLDIHPEL
SPFADLYFTIKRGTLELLDKNRQSAPDRGPILSSEDIQSLETLLGAIMPKYKALLHKNSFIPATTISLAPFLGLLG
TVWGILVAFTHISSGSSGNSAIMEGLATALGTTIIGLFVAIPSLIAFNYLKAHSSELISEIEQTAYLLLNSIEVKYR
NTNL

>core/671/4/Org4_Gene666

MTKHGKRIRGILKNYDFSKSYSLQEAIDILKQCPPVRFDQTVDVSIKLGIDPKKSDQQIRGAVFLPNGTGKTLR
ILVFASGNKVKEAVEAGADFVGSDDLVEKIKSGWLEFDVAVATPDMMREVGKLGKVLGPRNLMPTPKTGT
VTTDVAKAISELRKGKIEFKADRAGVCNVGVGKLSFESSQIKENIEALSSALIKAKPPAAKGQYLVSFTISSTM
GPGISIDTRELMAS

>core/672/4/Org4_Gene626

MEKDLHLHEKKCLAHEAATQVTSGMILGLGSGSTAKEFIFALAHRIQTESLAVHAIASSQNSYVLAKQLAIPL
LNPEKFSSLDLTVDGADEVDPQLRMIKGGGGAIFREKILLRAAKRSIILVDESKLVPVLGKFRVPLEISRFGRSA
IVEEIRHLGYEGEWRLQDTGDLFITDSGNYIYDIFSPNSYPNPEKDLLKLIQIHGVIEVGFVIEKVEVWSSNSQG
LISKKYSV

>core/673/4/Org4_Gene39

MLQSCKKALLSIVVSILAFHPIPGMGVEAKSGFLGKVKGWFSKKEIQEEARILPVKDSLSWKRYDYTSSSGFS
VEFPGEPDHSGQIVEVPQSEITIRYDTYVTETHPDNTVYVVSVWEYPEKVDISRPELNLQEGFSGMMQALPES
QVLFMQARQIQGHKALEFWIVCEDVYFRGMLISVNHTLYQVFMVYKNKNPQALDKEYEAFSQSFKITKIREP
RTIPSSVKKKVSL

>core/674/4/Org4_Gene550

MALDEINNQNNPSQQIASSTSQTSKINQDRKTFACTVTLLVVATLMILSGIVLLFTIGSLGLSVPLSGILGTFAV
TVGAVLFITGLTILVRKSLGIEQKNEDLNFLKIKTPPPPARPLMSKFSVTCSTISIVLGMALLIGAVVSVFFLTGY
LQLGLCAGLVGLGTALFVAGLARMSPRSLADQEGSGSADSQSNIVGIGEPKAAQEQKWYKMAVVRGEDGIP
TAIRLTPEK

>core/675/4/Org4_Gene178

MSKPSPRNANQPQKPSASFNKKTRSRLAELAAQKKAKADDLEQVHPVPTEEEIKKALGNIFEGLSNGLDLQQI
LGLSDYLLEEIYTVAYTFYSQGKYNEAVGLFQLLAAAQPQNYKYMLGLSSCYHQLHLYNEAAFGFFLAFDA
QPDNPIPPYYIADSLLKLQQPEESNNFLDVTMDICGNNPEFKILKERCQIMKQSIEKQMAGETKKAPTKKPAG
KSKTTTNKKSGKKR

>core/676/4/Org4_Gene109

MYLEDYDVFFFDLDGLLVDTEPCFYRAFLQACAEFSLEVHWDFSTYYSHTTLGTEIFSKKFIEQYPQAQEYM
AEIFAKRLQIYYKSLEHAGPALMPGVEAFIELVLSLNKTFGVVTNSPRDATHTLRTMYPILNKFLFWVTRENY
ARPKPYGDSYDYAYRTFAREGMKVIGFEDSVKGLRALSKIPATLVCINSMAEITPEDYPELKGKEFFSYPSFD
VLTEHCSQQKLL

>core/677/4/Org4_Gene719

MNTSHRKTLVFSYLSSTFTLLLVLSNLVLSSKLIPTTFFNFIIPGGLILYPLTFLISDVVNEIFGPKKARVMIFSAFI
ANLLASSIVQIFMFFPVASPEMQTAWHCLFDLSPLRFLASLLAFIVSQQLDIVLYTFFKNRTPNSSLWLRSNGS
TWISQIPDTFIVDTYILYFGMGLSFPQTLNIMFYSYIYKITFCVLTTPLFYLAVNTIRKFLGMPSTKIANTVPLIN
QP

>core/678/4/Org4_Gene603

MKKQESVLVGPSIMGADLTCLGVEAKKLEQAGSDFIHIDIMDGHFVPNLTFGPGIIAAINRSTDLFLEVHAMIY
NPFEFIESFVRSGADRIIVHFEASEDIKELLSYIKKCGVQAGLAFSPDTSIEFLPSFLPLCDVVVLMSVYPGFTGQ
SFLPNTIEKIAFARHAIKTLGLKDSCLIEVDGGIDQQSAPLCRDAGADILVTASYLFEADSLAMEDKILLLRGEN
YGVK

>core/679/4/Org4_Gene583

MKGFLSVNELIFGFQTFSVVVLGVFFASRGKAWLTGWLSLLSSIMNVFVLKQIHLWGFEVTSADVYVIGLLT
CLNYAREHYEKNDVNDAMLCSWVISIAFLVLTQLHLFLIPSPNDSSQKHFLALFSSTPRIVVASLVTLIFVQIVD
IKLFTFLQRVFSKKYFAMRSTISLLFSQLIDTIIFSFLGLYGLVSNLCDVMIFAMLVKGIVITLAIPTLTVTKAVL
DRRSS

>core/680/4/Org4_Gene359

MRPHRKHVSSKSLASKQSTSTHVEITTKAFRLSMPLKQLILEKSDHLPPMETIRVVLTSHKDKLGTEVHVVAS
HGKEILQTKVHNANPYTAVINAFKKIRTMANKHSNKRKDRTKHDLGLAAKEERIAIQEEQEDRLSNEWLPVE
DLDAWDSLKTLGYVPASAKKKISKKKMSIRMLSQDEAIRQLESAPENFLIFLNEQEHKIQCIYKKHNGNYVLI
EPSLKPGFCI

>core/681/4/Org4_Gene840

MALLILLRHGQSVWNEKNLFSGWVDIPLSQQGIEEAFSAGRAIQNLPIDCIFTSTLVRSLMTALLAMTNHHSK
KIPYIVHEDPKAKEMSRIYSAEEENNMIPLYQSSALNERMYGELQGKNKKQTAEQFGEEQVKLWRRSYKTAP
PQGESLYDTKQRTLPYFEKNILPQLQNRKNVFVSAHGNSLRSLIMDLEKLSEEEVLSLELPTGKPVVYQWKN
HKIEKHPEFFG

>core/682/4/Org4_Gene572

MSSQPLVTTSSSLSRYVVLTGEEKVACYKKAFNHIWHGAPAIILAAALLMFCIFGFVLGSILLGAPLEGASILY
DVILPWLLPSILVFVLLVLPLNIYAYSHHKQVLALHERITQSNYKEIYDHCEKEKKTPNKKALSLYIESQVLVP
EYSKRFSSMILGKTLKIIPKKDSPESLKHDELIQKALERAKENIYMNKNQREKRDEREAKKEAKNAPKTNPLW
EGLGT

>core/683/4/Org4_Gene561

MSLLIEAKNLSKTIQQQNQNISILTDVSLSLHAGETISITGASGNGKTTLLHLLGTLDVPSSGSLRFFDKDLKNQ
DLANFRNQHIGFVFQNFYLLEDDTVLKNVLMPALIARKNISKGSPVYTRALELLDLVNLDDKVQTRCSKLSG
GEKQRVAIARALINEPAILLADEPSGNLDEETSEQIHNLLLEQASALCGILIVTHNKHLASRCSREGVLSNGKLF
FHNS

>core/684/4/Org4_Gene597

MTIRVRNLAYSVNKKKILDGVTFSLERGHITLFVGKSGSGKTTILRTLAGLVQPTQGDIWIEGEAPALVFQQPE
LFSHMTVLGNCTHPQIHIKGRSTEEAREKAFELLHLLDIEEVAKNYPDQLSGGQKQRVAIVRSLCMDKHTLLF
DEPTSALDPFATASFRHLLETLRDQELTVGLTTHDMQFVHSCLDRIYLIDQGTVAGVYDKRDGELDSDHPLS
KYIHSAQ

>core/685/4/Org4_Gene675

MDLKLDEVASLLDVSEHTVLQWLKEGAIPSYSMNNEYRFSREEIEDWLLHNQALMIQERGEDKEALKDLSL
KYSLYKAIHRGGVLCDVVVHSKEEALQYASKYIAQKFQLDESVLFEMLFHRENLMSTGIGEGIALPHAKDFLI
NAYYDIVVPMFLAEPIEYEALDGKPVSILFFLFACQDKSHLNLVNKIVHLGMSLNARSFFKNYPNKDQLLAY
VKEWESQTH

>core/686/4/Org4_Gene363

MILRISTVSLLTSCSFSKNSRTCFVTPERITSQKDCPVLLHPKSTTISPPLYDWISPNREVITAYSFYCRGQGNSII
TPEGVLYDCDGLHHSITKEEFRYIHPRLIEVVRLLQQDHPKVSIIEAFCCPKHFHFLEASGISLSQLHLEGTAAT
FALDPPLPMEKLLATIKKLYTKNSDPSLTNFIVTEATLTNPELRLTQQDLGSHTEITVEILDNLQNKEALSSA

>core/687/4/Org4_Gene798

MENSQNFHDTLCQLLDRYSEELYPTLASLLNVTLPNTAISASVSPIPEKAVEVPNAEPQPITPPPPTNLSQEKTK
PSDWKCVPLHPDLSQNAILKEKYPALKDCSLPAPKIPCSIFVYEENNEEVLFFNRLAKILTQQLFPTKLTLIHAK
TNIFVNNPNFFLALAPLNVIRYKIPTTDYHQSLTQNGCIFLPLYSSLEYEKDSQLKRNLWAILNRLPFAYTPKSS

>core/688/4/Org4_Gene756

MKPQDLSPPFLWKERRPCIQDGVLYVPRHYFEHQNFSTSYHQEFFQNHTSIACELCSGNGDWVVAQAQKDP
QVLWIAVEQRFDRVRKIWSKMINHQIQNLRIVCGTAETFFQYYVPDQFLQRLVVNFPDPWPKMRHRKHRLL
QPSFVQEISRSLQDSAVFALATDDKTYLLESIEALQTRLAPRMETPYYIKMTDTYGNSWFENLWRTKGQEIFY
TEFIKKAGI

>core/689/4/Org4_Gene150

MIDPVECFPNLDGDAEAQSITQNSGTPLASELKKDISPFALGSYAAPKDTTLVQGFKPNPMAMMQDQNSNLI
DPELQEALESEELQEQINNLKGRLWDFRSTFEDSQTTAQFADEHFQAVGVIIDLINEDLNTIAEHTQQDARKE
DKEEGSVTRKIIDWVSSGEEVLNRALLYFSDRDGNRESLANFLKVQYAVQRATQRAELFASIVGTSVSSVKTI
MTTQLG

>core/690/4/Org4_Gene899

MTSWIELLDKQIEDQHMLKHEFYQRWSEGKLEKQQLQAYAKDYYLHIKAFPCYLSALHARCDDLQIRRQIL
ENLMDEEAGNPNHIDLWRQFALSLGVSEEELANHEFSQAAQDMVATFRRLCDMPQLAVGLGALYTYESQIP
QVCVEKIRGLKEYFGVSARGYAYFTVHQEADIKHASEEKEMLQTLVGRENPDAVLQGSQEVLDTLWNFLSS
FINSTEPCSCK

>core/691/4/Org4_Gene323

MVLLSKFDFSGNKIGEVEVADSLFADEGDGLQLIKDYIVAIRANKRQWSACTRNRSEVSHSTKKPFKQKGTG
NARQGCLASPQFRGGGIVFGPKPKFNQHVRINRKERKAAIRLLLAQKIQTNKLTVVDDTVFVDALTAPKTQS
ALKFLKDCNVECRSILFIDHLDHVEKNENLRLSLRNLTAVKGFVYGININGYDLASAHNIVISKKALQELVER
LVSETKD

>core/692/4/Org4_Gene143

MQTLARLFGQSPFAPLQAHLEMVVSCVEYMLPIFTALRDGRYEELLEMAKLVSDKEYQADCIKNDMRNHLP
AGLFMPISRAGILEIISIQDSIADTAEDVAILLTIRRLNFYPSMETLFFRFLEKNLEAFELTMTLLHEFNQLLESSF
GGRKADKARLLVGRVAKSEHESDVLQRELMQIFFSDDFIIPEKEFYLWLQVIRRTAGISDSSEKLAHRINMTLE
EK

>core/693/4/Org4_Gene723

MSTTTVKHFIHTASRWEPVLKEIVASNYWHAQWINTLSFLENSGAKKISASEHPTEVKEEVLKHAAEEFRHA
HYLKTQISRISETSLPDYTSKNLLGGLLTKYYLHLLDLRTCRVLENEYSLSGQTLKTAAYILVTYAIELRASEL
YPLYHDILKEAQSKITVKSIILEEQGHLQEMERELKDLPHGEELLGYACQFEGELCLQFVERLEQMIFDPSSTF
TKF

>core/694/4/Org4_Gene576

MTKNAINSQTTTLQPNLTDAEPIASPAKYKSVVAAISLFALGILLICLGIILISIPIPGLAAQVALGLGIVSLILGIA
LANIGFLCLLLRCKQVPQKPDTLPSESSKQPSEGSTPTALPWQPGEFLEKVQVSATPILLPKNKDEELSAKVMK
EGAEAASSIKQAVLESTEKLIDARKQEESRREARKKIVAEEAEASRKRIQQQMAADQEALRKRKEEVAKRK

>core/695/4/Org4_Gene91

MKKYFITGLVILLPLAITIAIVTMIMNFLTQPFVGLASEFFEKFSFYTKHRALLKFVLQIILLFGLFFATVLLGFL
TRIMIFKSLLSIYDKILHRIPIIKTVYKAAQQVMTTIFGSKSGSFKQVVMVPFPNANVQCIGLVAGDAPTVCCT
GEKEDDPLVTVFIPTTPNPTSGFLTLFRKSDIVFLDMKIEDAFKYIISCGVLSTPMACPSSPLPDELHQDQGS

>core/696/4/Org4_Gene325

MGQKGCPIGFRTGVTKKWRSLWYGNKQEFGKFLIEDVRIREFLRKKPSCQGAAGFVVRRMSGKIEVTIQTAR
PGLVIGKKGAEVDLLKEELRALTGKEVWLEIAEIKRPELNAKLVADNIARQIERRVSFRRAMKKAMQSVMD
AGAVGVKIQVSGRLAGAEIARSEWYKNGRVPLHTLRADIDYATACAETTYGIIGIKVWINLGENSSSTTPNNP
AAPSAAA

>core/697/4/Org4_Gene404

MTLYLGLNQKTARKYQAHYLPILTLFPYAKSTPQNKRALQFLPQATHVILTSPSSTHLFLSRMTSLLSKATLK
TKTYLCIGESTKERLLSFLGQVKYAVATQEIAEGIFPLLQALPSSARILYPHSSLARPVIREFLYNRFTFFSYPHY
TVKPRKLKKNILSKYKEIIFTSPSTVRAFAKIFPRFPEKTYWCQGRMTLQEFQKFYSQKQVSLLETLGKSRTSP

>core/698/4/Org4_Gene264

MLMMLMMIIGITGGSGAGKTTLTQNIKEIFGEDVSVICQDNYYKDRSHYTPEERANLIWDHPDAFDNDLLISD
IKRLKNNEIVQAPVFDFVLGNRSKTEIETIYPSKVILVEGILVFENQELRDLMDIRIFVDTDADERILRRMVRDV
QERGDSVDCIMSRYLSMVKPMHEKFIEPTRKYADIIVHGNYRQNVVTNILSQKIKNHLENALESDETYYMVN
SK

>core/699/4/Org4_Gene47

MQSDLIQILLKETVNTLYMVSTAFFFSCAIGGMLGLGLFCTSPKSLNPKKSLYATISMILSFLTAIPFAILIVILFP
ITRWIVGTSLGPTASIVPLTIGAIPFVVTIVVDAFRNSALNYLESAVALGIPKRNILFGILLPESYPQLIFSLKSLV
VHLISCSTLAGFVGGGGLGQLLLQYGYYRFEWSVTTSVLVITLVLIESVRILGDFWGRRVLKYRGIL

>core/700/4/Org4_Gene829

MSTVTTEPCSSIHISLNNDWRDSLPYSLDRASELLHFRFLPSLVFSNWKVEQQIETLCRKSEKRRLISPLAKWL
GKLHKQDLLCPPAPPVSVCWINAHVGYGVFARDEIAPWTYIGEYTGILRHRQAIWMDENDYCFRYPMPLFTL
RYFTIDSGKQGNVTRFINHSEQPNAEAIGVFCEGLFHVIIRTIAPIYAGQEICYHYGPLYWKHRKKREEFIPEEE

>core/701/4/Org4_Gene322

MRSHISVMGKKEGMIHIFDKDGSLVACSVIRVEPNVVAQIKTKESDGYFSLQMGAEEMNAPAHTITKRVSKP
KLGHLRKAGGRVFRFLKEVRGSEEALNGVSLGDAFGLEVFEDVSSVDVRGISKGKGFQGVMKKFGFRGGPG
SHGSGFHRHAGSIGMRSTPGRCFPGSKRPSHMGAENVTVKNLEVIKVDLEKKVLLVKGAIPGARGSIVIVKHS
SRT

>core/702/4/Org4_Gene234

MQRIIIVGIDTGVGKTIVSAILARALNAEYWKPIQAGNLENSDSNIVHELSGAYCHPEAYRLHKPLSPHKAAQI
DNVSIEESHICAPKTTSNLIIETSGGFLSPCTSKRLQGDVFSSWSCSWILVSQAYLGSINHTCLTVEAMRSRNLN
ILGMVVNGYPEDEEHWLTQEIKLPIIGTLAKEKEITKTIISCYAEQWKEVWTSNHQGIQGVSGTPSLNLH

>core/703/4/Org4_Gene76

MNAVNTPKKILCIVADYREISPLIEQLDFTQINEHLYSYRCTDYHLDLYIVHVWGSTAVLNALQSYCQAYTDY
DLWINPGFVGACSPEIPLGQCYTIEKIANLTTDTPPVLSEDPPYIFDALPDSLPKSSLVTSPVLYHYGFHKTFKL
LDMEGYAIASQAAEHHIPCSFLKITSDYTVPGDCPFSRLEEVSQKLTQTLVELLPELMERAIPPKLLLPCP

>core/704/4/Org4_Gene428

MLGSLPCYPGAGNIEEYKNRYFYCQLCAEVVSPYVVPVVVVDVQGAPPTGILQVLRCKQHKFQGLPVHGPIT
SLWALEPVGKGAPQLESAMYELCSQVRNFDICSIVSWVFGGLCIFAGLIVGVMVEAPLIAGLSAWVIPCIIGGI
GAILCLFAILMAYLGRGRVREWLNLSHEYITQCHCRQIQAHSQNYSVITEYPATCALSQPITKLPNGSRRDN

>core/705/4/Org4_Gene596

MDHWLAIARLLLRGCGYTLCVSGIGILCGSILGLLIGTVTSLYFPSKLTKLLANSYVTVIRGTPLFIQILIVYFGL
PEVLPIEPTPLVAGIIALSMNSAAYLAENIRGGINSLSIGQWESAMVLGYKKYQIFIYIIYPQVFKNILPSLTNEF
VSLIKESSILMVVGVPELTKVTKDIVSRELNPMEMYLICAGLYFLMTTAFSCISRLSEKRRSYDN

>core/706/4/Org4_Gene830

MKRFIYKTIFCGLTLLTSLSSCSLDPKGYNLETKNSGDLNQESVILKENRETPSLVKRLSRRSRRLFARRDQTQ
KDTLQVQANFKTYAEKISEQDERDLSFVVSSAAEKSSISLALSQGEIKDALYRIREVHPLALIEALAENPALIEG
MKKMQGRDWIWNFFLTQLSEVFSQAWSQGVITEEDIAAFASTLGLDSGTVASIVQGERWPELVDIVIT

>core/707/4/Org4_Gene145

MQKLVHNIWKKFYSFSSAIAICIVLASFLSLKIVSNTYKHSQAKRNSILLLTRAAEVAVSQGFLPSKSALSSLEQ
AYHLGGESMKPYAGFLASCFYIHNEPLRGAYYAGLAYNNSQALQLPHPIQKLLKEISEAQADQLYDVALSKS
YQLLQTANSSPEYPTLSFLTLLRVIELKELLHQDVSQDFAALKSSPLFHQFERMYSDGEWTLSKRFGKKG

>core/708/4/Org4_Gene106

MIITIDGPSGTGKSTTAKALADHLHFNYCNTGKMYRTLAYARLQSPWATLPLTKFLEEPPFSFTFATGQPLESF
FNGHLLTSELTTQEVANAASELSQLPEVRAFMQDLQRRYAQLGNCVFEGRDMGSKVFPNADLKIFLTSSPEV
RAQRRLKDLPEGTLSPEQLQAELVKRDAADAQRAHDPLVIPENGIVIDSSDLTIRQVLEKILALLFRNEL

>core/709/4/Org4_Gene473

MRKMLVLLASLGLLSPTLSSCTHLGSSGSYHPKLYTSGSKTKGVIAMLPVFHRPGKSLEPLPWNLQGEFTEEI
SKRFYASEKVFLIKHNASPQTVSQFYAPIANRLPETVIEQFLPAEFIVATELLEQKTGKEAGVDSVTASVRVRV
FDIRHHKIALIYQEIIECSQPLTTLVNDYHRYGWNSKHFDSTPMGLMHSRLFREVVARVEGYVCANYS

>core/710/4/Org4_Gene802

MSKKPLYVAHPWHSPTLTQDNYESLCCYIEITPYDSVKFELDKTTGLLKVDRPQKFSNFCPCLYGLLPQTYCG
TASGNYSGEQTRREGIQGDKDPLDVCVLTEKNIHHGNILLQARPIGGLRIIDSGEADDKIIAVLEDDLVFAEIED
ISDCPGTVLDMIQHYFLTYKATPDHLIKGSPAKIEIVGIYGKKEAQKVIQLAHEDYLSYIGDTAEVN

>core/711/4/Org4_Gene302

MSVNPSGNSKNDLWITGAHDQHPDVKESGVTSANLGSHRVTASGGRQGLLARIKEAVTRFFSRMSFFRSGAP
RGSQQPSAPSADTVHSPLPGGDARATEGAGRNLIKKGYQPGMKVTIPQVPGGGAQRSSGSATLKPTRPAPPPP
KTGGTNAKRPATHGKGPAPQPPKIGGTNAKRAATHGKGPAPQPPKGILKQPGQSGTSGKKRVSWSDEDSM

>core/712/4/Org4_Gene639

MNTSISEIQRFLSMIAFEKELVSEGFSVVAGIDEAGRGPLAGPVVASACILPKGKVFPGVNDSKKLSPKQRAQV
RDALMQDPEVCFGIGVISVERIDQVNILEATKEAMLQAISSLPISPDILLVDGLYLPHDIPCKKIIQGDAKSASIA
AASILAKEHRDDLMLQLHRLYPEYGFDRHKGYGTSLHVEAIRRYGPSPCHRKSFSPIKQMCAIV

>core/713/4/Org4_Gene564

MTKGSVFIIMGPPGSGKGTQSQYLANRIGLPHISTGDLLRAIIQEGTPNGLKAKAYLDKGAFVPSDFVWEILKE
KLQSQACSKGCIIDGFPRTLDQAHLLDSFLMDVHSNYTVIFLEISEDEILKRVCSRFLCPSCSRIYNTSQGHTEC
PDCHVPLIRRSDDTPEIIKERLKKYQERTAPVIAYYDSLGKLCRVSSENKEDLVFEDILKCIYK

>core/714/4/Org4_Gene460

MTSKKSYRSYFFDPLWSNNQILIAILGICSALAVTTTVQTAITMGIAVSIVTGCSSFFVSLLRKFTPDSVRMITQ
LIIISLFVIVIDQFLKAFFFDISKTLSVFVGLIITNCIVMGRSESLARHVTPIPAFLDGFASGLGYGWVLLVIGVIRE
LFGFGTLMGFRIIPQFVYASETHPDGYQNLSLMVLAPSAFFLLGIMIWLVNIRDSKKRKR

>core/715/4/Org4_Gene691

MYFYKYVIIDTSGYYPFLAYVDNQQVLEHWSLPVGPDLGIVLEFLFKSKNLSFQGVAVALGPGNFSATRIGIS
FAQGLAMAKNVPLLGYSSLEGYLLSKDEKKALMLPLGKRGGVLTLSSEIPEEGLNEKRRGVGPGALLSYEEA
SDYCVAHGYYHVISPNPQLFASSFSDKITVEEVAPSVEQIRRHVISQFMFVEYDKQLSPDYRSYSCIF

>core/716/4/Org4_Gene453

MATSVAPSPVPESSPLSHATEVLNLPNAYITQPHPIPAAPWETFRSKLSTKHTLCFALTLLLTLGGTILAGYAG
YTGNWIICGIGLGIIVLTLILALLLAIPLKNRQTGTKLIDEISQDISSIGSGFVQRYGLMFSTIKSVHLPELTTQNQ
EKTRILNEIEAKKESIQNLELKITECQNKLAQKQPKRKSSQKSFMHSIKHRSKNP

>core/717/4/Org4_Gene370

MIFRICKFFTWVAFSLFYKLKVYGVKKNFIKGPAIIAVNHNSFLDPIALHMCVHECIYHLARASLFNIPWLWK
QWGCFPVRQDEGNSAAFKIASRLFNKRKKLVIYPEGARSPDGQLQPGKVGIGMMAAKSRVPIIPVYIGGTFEA
FNRHQKIPHVWKTITCVFGTPMYFDDIIQNPEIKNKETYQIITNQTMNKIAELKAWYESGCKGDVP

>core/718/4/Org4_Gene364

MIKSSLILLSGGQGTRFGSKIPKQYLPLNGTPLVLHSLKILSSLPQIAEVIVVCDPSYQETFQEYPVSFAIPGERR
QDSVFSGLQQVSYPWVIIHDGARPFIYPDEIHDLLETAEKIGAAALASPIPYTIKQRNPVRTLDRDNLAIIHTPQ
CIKTEILREGLALAKEKQLTLVDDIEAAEIIGKPSQLVFNKHPQIKISYPEDLTIAQALL

>core/719/4/Org4_Gene917

MMNYEDAKLRGQAVAILYQIGAIKFGKHILASGEETPLYVDMRLVISSPEVLQTVATLIWRLRPSFNSSLLCG
VPYTALTLATSISLKYNIPMVLRRKELQNADPSDAIKVEGLFTPGQTCLVINDIVSSGKSIIETAAALEENGLVV
REALVFLDRRKEACQPLGPQGIKVSSVFTLPTLIKALIAYGKLSSGDLTLANKISENLEIES

>core/720/4/Org4_Gene158

MARYCGPKNRVARRFGANIFGRSRNPLLKKPHPPGQHGMQRKKKSDYGLQLEEKQKLKACYGMIMEKQLV
KAFKEVIHKQGNVAQMFLERFECRLDNMVYRMGFAKTIFAAQQLVAHGHILVNGRRVDRRSFFLRPGMQIS
LKEKSKRLQSVKDALESKDESSLPSYISLDKTGFKGELLVSPEQDQIEAQLPLPINISVVCEFLSHRT

>core/721/4/Org4_Gene858

MKQFILRTLNTLFPNPKPSLEGWSSPFQLLIAILLSGNSTDKAVNSVTPQLFAKAPDAQSILDLPPGKLYQLIAP
CGLGERKSAYIYQLSQILVRDFHGEPPNDMALLTQLPGVGRKTASVFLGIAYGKPTFPVDTHILRLAQRWKIS
EKKSPSAAEKDLARFFGHENTPKLHLQLIYYARQYCPALHHKIDNCPICSYLAKEANSTRT

>core/722/4/Org4_Gene656

MSVQVKLTKNSFRLEKQKLARLQTYLPTLKLKKALLQAEVQNAVKDAAERDKDYVQAYERIYAFAELFSIP
LCTDCVEKSFEIQSIDKDFENIAGVEVPIVREVTLFPASYSLLGTPIWLDTMLSASKELVVKKVMAEVSKERLK
ILEEELRAVSIRVNLFEKKLIPETTKILKKIAVFLSDRSITDVGQVKMAKKKIELRKARGDECV

>core/723/4/Org4_Gene659

MANLNADDKLKQICDALRLDTLKPAEDEAAALLHNAKEQAKRIIQEAQEEARKILETAEERAHQKIKQGEVA
LSQGGKRALEALKQAVENKIFRESLVEWLEHVTTDPEVSTKLIQALVQALEAQGVSGNLTAYIGKHVSPRAV
NELLGKAVTTKLRKKSVVVGGFVGGVQLKVEEKNWVLDLSSSALLEIFTRYLQKDFREMIFQGS

>core/724/4/Org4_Gene907

MTYLASSIFSPEDFLYPEIISKAHYTWDILDLMDQMLENHVFSGIHGTVESGVTLKNIEKIEIAEGAYVESGAYI
VGPCILGSQTEVRHGAYLRGNVITGSRCVVGHCTEIKNSYLGHHTKAAHFAYLGDSVLSSEVNLGAGVRCA
NFRLDGRNIYVRSTSDKSKKIDTGRRKLGAFLGKGVAIGCNVVINPGQHILPHTRIRPGQVI

>core/726/4/Org4_Gene676

MSFVPYSLPELPYDYDALEPVISSEIMILHHQKHHQTYINNLNAALKRLDAAETQQNLNELIALEPALRFNGG
GHINHSLFWETLAPIDQGGGQPPKHELLSLIERFWGTMDNFLKKLIEVAAGVQGSGWAWLGFCPAKQELVL
QATANQDPLEPLTGKLPLLGVDVWEHAYYLQYKNVRMDYLKAFPQIINWGYIENRFSEIISSK

>core/727/4/Org4_Gene118

MYDYIRGTLTYVHTGAIVIECQGIGYHIAITERWAIECIRALHQDFLVFTHVIFRETEHLLYGFHSREERECFRI
LISFSGIGPKLALAILNALPLKVLCSVVRSEDIRALASVSGIGKKTAEKLMVELKQKLPDLLPLDSRVETSQTHT
TSSCLEEGIQALAALGYSKIAAERMIAEAIKDLPEGSSLTDILPIALKKNFSGVNKD

>core/728/4/Org4_Gene250

MKIVIASSHGYKIRETKTFLKRLGDFDIFSLSDFPDYKLPQEQEDSITANALTKGIHAANHLGCWVIADDTMLR
VPALNGLPGPLSANFAGVGAYDKDHRKKLLDLMSSLESLVDRSAYFECCVVLVSPNQEIFKTYGICEGYISHQ
EKGSSGFGYDPIFVKYDYKQTFAELSEDVKNQVSHRAKALQKLAPHLQSLFEKHLLTRD

>core/729/4/Org4_Gene785

MGLPNYITFSRLFITPIFMILYLKGKWFGITPVVLPYVLLALLAISELTDAIDGYVARKFSQVTDLGKLLDPMA
DSIYRISIYLTFTQPPVNLPLLLVFIFLARDSVISTLRTVCAFRGRVVAARASGKLKAILQGVSFFLILLVMIPHSL
GLLSQNGLEIFASVTVSIIAVYSIASGIEYFWMNKNFLSQRAKTKDSEKNHESKE

>core/730/4/Org4_Gene45

MFIVIEGGEGSGKSSLAKALGDQLVAQDRKVLLTREPGGCLIGERLRDLILEPPHLELSRCCELFLFLGSRAQH
IQEVIIPALRDGYIVICERFHDSTIVYQGIAEGLGADFVADLCSKVVGPTPFLPNFVLLLDIPADIGLQRKHRQK
VFDKFEKKPLSYHNRIREGFLSLASADPSRYLVLDARESLASLIDKVMLHTQLGLCT

>core/731/4/Org4_Gene638

MNKILVDSPFSPDHQKCCPKLFTISAPAGVGKTTLVRMLEQEFSSAFAETISVTTRKPREGEVPGKDYHFVSHE
EFQRLLDRQALLEWVFLFGEYYGTSMLEIERIWSLGKHAVAVIDIQGALFIRSRMPSVSIFIAPPSQEELERRLA
SRGSEEGSQRKERLEHSLIELAAANQFDYVIINDDLNQAYKVLKSIFIAEEHRNIL

>core/732/4/Org4_Gene887

MHAKLSFFILLSLLFSGIDCCRLHAAGRSPSLQGVLAEIEDISAKLASHEVEIVMLSERLDEQDSKCQKWTAAK
PETLAQKIRELESDQKALAKTLAVLTTSVKDLQTNLQSKLQEIQKDHRALAQDLRLVRRSLLALVDSSSPGA
YADFSDPVPENIYIVREGDSLSKIAKKYKLSVTELKKINKLDSDAIYAGQRLCLQRNKQ

>core/733/4/Org4_Gene956

MIGAQKKQSGKKTASRAVRKPAKKVAAKRTVKKATVRKTAVKKPAVRKTAAKKTVAKKTTAKRTVRKTV
AKKPAVKKVAAKRVVKKTVAKKTTAKRTVRKTVAKKPAVKKVAAKRVVKKTVAKKTTAKRTVRKTVAK
KPVARKTTVAKGSPKKAAACALACHKNHKHTSSCKRVCSSTATRKHGSKSRVRTAHGWRHQLIKMMSR

>core/734/4/Org4_Gene535

MMSPIPFQSSGDASFLAEQSQQLPSTSESQLVTQLLTMMKHTQALSETVLQQQRDRLPTAPIILQVGGAPTGG
AGAPFQPGPADDHHPIPPPVVPAQIETEIATIRSELQLMRSTLQQSTKGARTGVLVVTAILMTISLLAIIIIILAVL
GFTGVLPQVALLMQGETNLIWAMVSGSIICFIALIGTLGLILTNKNTPLRAS

>core/735/4/Org4_Gene184

MTLVPYVVEDTGRGERAMDIYSRLLKDRIVMIGQEITEPLANTVIAQLLFLMSEDPKKDIQIFINSPGGYITAG
LAIYDTIRFLGCDVNTYCIGQAASMGALLLSAGTKGKRHALPHSRMMIHQPSGGIIGTSADIQLQAAEILTLKK
HLANILSECTGQPVEKIIEDSERDFFMGAEEAISYGLIDKVVTSAKETNKDTSST

>core/736/4/Org4_Gene341

MLKLLKVSITGDLSSGKTEACQVFQELGAYVVSADEISHSFLIPHTRIGRRVIDLLGSDVVVDGAFDAQAIAA
KVFYNSVLLQGLEAILHPEVCRIIEEQYHQSIQDGDYPLFVAEVPLLYEIHYAKWFDSVILVMANEDIRRERFM
KKTGRSSEDFDQRCSRFLNVEEKLAQADVVVENNGTKKELHQKIEEYFYALKGAL

>core/737/4/Org4_Gene742

MLTLLNLSLLFYVLFDSPGSIPVFVALLKNFSRKKQQRVILRECLFALGALILFVTFGRSFFQFLDISLYAFQIIG
GFLLFTVSIKMMLAPMPEKAKDDTSKTEPIFFPLAFPVITGPAVITALLSYMEEGIYSREIIFTAMIIAWAFSLFT
LLCSSFFDRLFGNFGLLALERLFGIALLLMSVNLMLKGISIAFNIGFYIG

>core/738/4/Org4_Gene396

MFSGIIQELGEVCFFEAQGNGLSLGIKSTPLFVSPLVTGDSVAVDGVCLTLTSCNESKIFFDVIPETLACTTLGE
KRCSDQVNLEAALKMGDSIGGHLLSGHVFGTAEIFLIKENRYYFRGSKELSQYLFEKGFIAIDGISLTLVSVDS
DTFSVGLIPETLQRTTLGKKREGERVNIEIDMSTKIQVDTVKRILASSGKD

>core/739/4/Org4_Gene219

MFAYRTLLTHNVVQVSHEIFKTTVVPGDTVIDATCGNGNDSLFLARLLQGEGRLVVYDIQKEALSNALLLFE
THLSEQERSVIEMKEQSHEHILEKDVKLIHYNLGYLPKGNKEITTLARTTEISLEYALNIVRPDGLITVVCYPGH
PEGEKETHSVESLAQRLHPKEWCVSSFYVANRCRAPRLFIFQRQGSESSVDKG

>core/740/4/Org4_Gene55

MTRYPDYLSKLIFFLRKLPGIGFKTAEKLAFELISWDSEQLKILGNAFHNVASERSHCPLCFTLKESKEADCHF
CREERDNQSLCIVASPKDVFFLERSKVFKGRYHVLGSLLSPITGKHIENERLSILKSRIETLCPKEIILAIDATLEG
DATALFLKQELQHFSVNISRLALGLPIGLSFDYVDSGTLARAFSGRHSY

>core/741/4/Org4_Gene111

MGFACRYLFFFIVLFASGSFGNQLLSVPCWLSEEESFYTHRFDFSKSYPDMENMEIQAQRKKRVEFNLTGEFP
KLETLNYQGSFGHLRAKCRGVYPVLYALNFSCSSCKMDMDFRGKWNRSSTITISNQKESINLKLPKDVGVIV
NTKTSLKGNVCPGSTFIKQGWGVWNKIYHNDLVGFSEVTLIFNVSSEGGTITFS

>core/742/4/Org4_Gene310

MRLFSLGTIYLFFSLALSSCCGYSILNSPYHLSSLGKSLLQERIFIAPIKEDPHGQLCSALTYELSKRSFAISGRSS
CAGYTLKVELLNGIDENIGFTYAPNKLGDKTHRHFIVSNEGRLSLSAKVQLINNDTQEVLIDQCVARESVDFD
FEPDLGTANAHEFALGQFEMHSEAIKSARRILSIRLAETIAQQVYYDLF

>core/743/4/Org4_Gene935

MNWAPKTMDHVDPKSETNIRLVISCYKLIKACQLEFPSLVDEVLLGMKCCAWESFKLVRQYQEQAKTVSAK
NAPLFCLTRSYYRDGHLTPLRAGPRAALSNYLDLRRRENSEKFFNPGHPCYYARLAFNETIQIYRTLFDISKLQ
IMFESGDYEKGQRENIAVILNFVKTLDDNEKVNFLLRHNDTRIPGRESATVFCS

>core/744/4/Org4_Gene211

MRIALSLLSLLMIFPIFGEESRPGSEDGNSNTQEIVGSQDTQVCLYHSYEQGLQASRIEGKPLVIVVLCNSGDD
GQACTIGLSETCEEVLSVLSGSIFSELANFVVLVPSGVNPLIYPPIEDPILAEIVKFKELFKDESFPTGLSIIVVGV
TPEGPGDIIEVSPVSLTVEEEETLPSEQTTEVESTSELQSEDPAIA

>core/745/4/Org4_Gene378

MSSNLHPVGGTGTGAAATESVLNIVEETAASGSVTAGLQAITSSPGMVNLLIGWAKTKFIQPIRESKLFQSRA
CQITLLVLGILLVVAGLACMFIFHSQLGANAFWLIIPATIGLIKLLVTSLCFDEACTSEKLMVFQKWAGVLEDQ
LDDGILNNSNKIFGHVKTEGSNSRATTPVPTSEGRGTPILSPLVSKIARV

>core/746/4/Org4_Gene4

MSLPLVLGSSSPRRKFILEKFRVPFTVIPSNFDESKVSYSGDPIAYTQELAAQKAYAVSELHSPCDCIILTGDTIV
SYDGRIFTKPQDKADAIQMLKTLRNQTHDVVTSIAVLHKGKLLTGSETSQISLTMIPDHRIESYIDTVGTLNNC
GAYDVCHGGLILKKVHGCVYNVQGLPIQTLKYLLEELNIDLWDYSI

>core/747/4/Org4_Gene413

MLQEHFFLSEDVITLAQQLLGHKLITTHEGLITSGYIVETEAYRGPDDKACHAYNYRKTQRNRAMYLKGGSA
YVYRCYGMHHLLNVVTGPEDIPHAVLIRAILPDQGKELMIQRRQWRDKPPHLLTNGPGKVCQALGISLGNN
KQRLNTPALYISKEKISGTLTATARIGIDYAQEYRDVPWRFLLSSEDSGKVLS

>core/748/4/Org4_Gene169

MTLSLVGKEAPDFVAQAVVNGETCTVSLKDYLGKYVVLFFYPKDFTYVCPTELHAFQDALGEFHTRGAEVI
GCSVDDIATHQQWLATKKKQGGIEGITYPLLSDEDKVISRSYHVLKPEEELSFRGVFLIDKGGIIRHLVVNDLP
LGRSIEEELRTLDALIFFETNGLVCPANWHEGERAMAPNEEGLQNYFGTID

>core/749/4/Org4_Gene85

MFRRTGKGPFEDVQTLYEEETSSPSSYSPYSRSERPETPPSLFDNPKASEARPLNHNLTEESSLPQWSSTPRTES
LLPLEEPETTLGEGVTFKGELAFERLLRIDGTFEGILVSKGKIIGPKGVVKADIQLQEAIIEGVVEGNITVSGKV
ELRGGAIIKGDIQANTLCVDEGVRILGYLAIAGITDHSERERDL

>core/750/4/Org4_Gene232

MAYGTRYPTLAFHTGGIGESDDGMPPQPFETFCYDSALLQAKIENFNIVPYTSVLPKELFGNIVPVDTCVKSFK
HGAVLEVIMAGRGAALSDGTHAIATGIGICWGKDKNGELIGGWAAEYVEFFPTWINDEIAETHAKMWLKKS
LQHELDLRSIAKHSEFQFFHNYINIKQKFGFCLTALGFLNFENAEPAKVN

>core/751/4/Org4_Gene166

MKKWISILILSFLSLLSILPVLAITINHVKISQRWSDLNSQILTLKVIRDHEDQVIKHNARISKDRNNLSIESLNAS
CKQLRPLSKERERLNKLNSNSLLAQSKEVWERKRALEKSNHQLVWNCEQMHNDFAFVRLEQATEMDNEDI
ESLFSLFNPENPVAPLVFFTCWKMTKQTTPLGNEVWLTHAEAISRWI

>core/752/4/Org4_Gene886

MNIHSLWKLCILLALLALPACSLSPNYGWEDSCNTCHHTRRKKPSSFGFVPLYTEEDFNPNFTFGEYDSKEEK
QYKSSQVAAFRNITFATDSYTIKGEENLAILTNLVHYMKKNPKATLYIEGHTDERGAASYNLALGARRANAI
KEHLRKQGISADRLPTISYGKEHPLNSGHNELAWQQNRRTEFKIHAR

>core/753/4/Org4_Gene463

MVLFSLLFPKLCYGCQAPGAYFCSNCLEKLLVEDREGRCLHCFRYLGSSETRLCSQCSPSSQLQAFSLYLPSQ
TALSVYARACEGKRPALQFFSKSIAFELASLDETPSCIAYITSTISREIVVEVAKLEKLLRIPLWPWLPKKRQIEK
LPKGECICFLSAYPLSQKWMQAIVGGSASPVVSISLFLSQNDQ

>core/754/4/Org4_Gene552

MKRYVVGISGASGVILAVKLIKELVNAKHQVEVIISPSGRKTLYYELGCQSFDALFSEENLEYIHTHSIQAIESS
LASGSCPVEATIIIPCSMTTVAAISIGLADNLLRRVADVALKERRPLILVPRETPLHTIHLENLLKLSRSGATIFPP
MPMWYFKPQSVEDLENALVGKILAYLNIPSDLTKQWSNPE

>core/755/4/Org4_Gene190

MKVIYYEIEEIPSTNTMAKSYMHLWDPYALTVISTKCQTAGTGKFGKSWKSSKGDLLNTFCFFITDLHIDVSR
LFRLGTEAVVALCKDLGITEAKIKWPNDVLVHGEKLCGVLPETLPVEGLLGVVLGIGLNGNTTKQALKDVG
QPATSLQEILGHPIDLETTRELLIHHLLGVLQENLPDSLATKSNRGNI

>core/757/4/Org4_Gene95

MADGEVHKLRDIIEKELLEARRVFFSEPVTEKSASDAIKKLWYLELKDPGKPIVFVINSPGGSVDAGFAVWDQIKMLTSPVTTVVTGLAASMGSVLSLCAAPGRRFATPHSRIMIHQPSIGGPITGQATDLDIHAREILKTKARIIDVYVEATNQPRDIIEKAIDRDMWMTANEAKDFGLLDGILFSFNDL

>core/758/4/Org4_Gene671

MIKKFFIYSLIFSCSFSAPLKGICNEDVSSQSRIEEDPDVLITQLNGLIETLIEEGKEIRNELQAISDGQKSSEEIEESCGTSDSEGLSEKTDEESSNEYVLDFFDSMVQRLEGISKMCQSGQVAQIIDCFNREFDIRNRELELKNRELELREKDLEFKKSILDWNKEKVSRELAFQREQDIKQTLMLLKK

>core/759/4/Org4_Gene70

MSIKEDKWIREMALNADMIHPFVNGQVNVNEETGEKLISYGLSSYGYDLRLSREFKVFTNVYNSVVDPKCFTEDIFISITDDVCIVPPNSFALARSVEYFRIPRNVLTMCIGKSTYARCGIIVNVTPFEPEWEGHVTIEISNTTPLPAKIYANEGIAQVLFFESSTTCEVSYADRKGKYQKQQGITVPCV

>core/760/4/Org4_Gene196

MVRVSTSEFRVGLRIEIDGQPYLILQNDFVKPGKGQAFNRIKVKNFLTGRVIERTYKSGESVETADIVERSMRLLYTDQEGATFMDDETFEQEVVFWEKLENIRQWLLEDTIYTLVLYNGDVVAVEPPIFMELSIAETAPGVRGDTASGRVLKPAVTNTGAKIMVPIFIDEGELVKVDTRTGSYESRVSK

>core/762/4/Org4_Gene938

MMKIPYARLEKGSLLVASPDMNQGVFARSVILLCEHSLNGSFGLILNKTLGFEISDDIFTFEKVSNHNIRFCMGGPLQANQMMLLHSCSEIPEQTLEICPSVYLGGDLPFLQEIASSESGPEINLCFGYSGWQAGQLEKEFLSNDWFLAPGNKDYVFYSEPEDLWALVLKDLGGKYASLSTVPDNLLLN

>core/763/4/Org4_Gene708

MIRRLEYYGSPILRKKSSPIAEITDEIRNLVSDMCDTMEAHRGVGLAAPQVGKNVSLFVMCVDRETEDGELIFSESPRVFINPVLSDPSETLIIGKEGCLSIPGLRGEVFRPQKITVTAMDLNGKIFTEHLEGFTARIIMHETDHLNGVLYIDLMEEPKDPKKFKASLEKIKRRYNTHLSKEELVS

>core/764/4/Org4_Gene753

MALNFKINRQIRAPKVRLIGSAGEQLGILAIKDALDLAREAGLDLVEVASNSEPPVCKIMDYGKYRYDLTKKEKDSKKAQHQVRIKEVKLKPNIDENDFSTKLKQARTFVEKGNKVKITCMFRGRELAYPEHGFKVVQKMSQGLEDIGFVEAEPKLAGRSLICVVAPGTVKTKKKQEKSHAQDENQ

>core/766/4/Org4_Gene346

MRILAGKYKGKSLKTFSNPHIRPTSGLVKEAFFSICREDIEGAAFLDLFAGMGAIGFEALSRGAACVVFVDISIKAIQLIHTNSALLGEQLPVVIFRQDAQSAIQRLIKQKRSFDLIYIDPPYELCNRYVQTLLQKIVSGNILNPEGTLFLENASDEEIACEGLTLRRRRKLGKTYLAEYIVEKDP

>core/768/4/Org4_Gene783

MELVVTSRETGKKSFLKKIRQQGGIPAVVYSAGKSLANITVDALVFKKFLSNLESGALSSTVFSLSYEGRIIKALVKDIQYQITTYDVIHLDFEELVEDRPVKLNIPIRCINAVDCIGVKLGGSLRQVIRAVRVVCKPKDIVPFLELDVQSVGLSQTRKLSDIKIPAGIETITPLKEVAITVSRR

>core/770/4/Org4_Gene604

MVLSSQLSVGMFISTKDGLYKVTSVSKVAGPKGESFIKVALQAADSDVVVERNFKATQEVKEAQFETRTLEYLYLEDESYLFLDLGNYEKLFIPQEIMKDNFLFLKAGVTVSAMVYDNVVFSVELPHFLELMVSKTDFPGDSLSLSGGVKKALLETGIEVMVPPFVEIGDVIKIDTRTCEYIQRV

>core/771/4/Org4_Gene415

MTDTPPENEEQHESNVQNENEVEHLQQEIVTLKTELKEKNDKYLMALAESENSRKRLQKERQELMQYALENTLIDFLNPIESMEKALGFATQMSDDVKNWALGFNMILNQFKQIFEEKGIIEYSSIGQKFNPFLHEAVETEETSEVPEGTILEEFAKGYKIGERPIRVAKVKVAKAPTPKENKE

>core/772/4/Org4_Gene174

MAAKTKTLELEDNVFLLLEGNLKRIFATPIGYTTFREFQNVVFNCANGQQEIANFFFEMLINGKLTQELAPQQKQAAHSLIAEFMMPIRVAKDIHERGEFINFITSDMLTQQERCIFLNRLARVDGQEFLLMTDVQNTCHLIRHLLARLLEAQKNPVGEKNLQEIQEEITSLKNHFDELTKALQ

>core/773/4/Org4_Gene107

MVETVLHNFQRYLSKYLYRVFRFPCRKKTFLSSHRVLARPSFPVDYCPGKIYDLQEIYEELNAQLFQGALRLQIGWFGRKATRKGKSVVLGLFHENEQLIRIHRSLDRQEIPRFFMEYLVYHEMVHSVVPREYSLSGRSIFHGKKFKEYEQRFPLYDRAVAWEKANAYLLRGYKKRVGGGYGRA

>core/775/4/Org4_Gene943

MNFAKIDHNHLYLTCLGDLGVACPILSTDCLPNYSEKASHEVLVYSKFRCISGEPSRLATSGNDTYYSIVSLPIGLRYEVTSPSGRHDFNIDMHVAPKIGAVLSHGAREAKEIPGSSKDYAFFSLTARESLMLSEKLAMTFQVSEVIQNCYSQCTKVTKTNLKEQYRHLSHNTGFELSVKSAF

>core/776/4/Org4_Gene326

MSRKAREPILLPQGVEVSIQDDKIIVKGPKGSLTQKLVKEVEITLKDNSIFVHAAPHVVDRPSCMQGLYWALISNMVQGVHLGFEKRLEMIGVGFRASVQGAFLDLSIGVSHPTKIPIPSTLQVSVEKNTLISVKGLDKQLVGEFAASIRAKRPPEPYKGKGIRYENEYVRRKAGKAAKTGKK

>core/777/4/Org4_Gene667

MYKWYVVQVFTAQEKKVKKALEDFKESSGMTDFIQEIILPIENVMEVKKGEHKVVEKYIWPGYLLIKMHLTDESWLYVKSTAGVVEFLGGGVPVALSEDEVRSILTDIEEKKSGVVQKHQFEVGSRVKINDGVFVNFIGMVSEVFHDKGRLSVMVSIFGRETRVDDLEFWQVEEVAPGQESE

>core/778/4/Org4_Gene358

MSLLNLPSSQDSASEDSTSQSQIFDPIRNRALVSTPEEKVRQRLLSFLMHKLNYPKKLIIIEKELKTLFPLLMRK
GTLIPKRRPDILIITPPTYTDAQGNTHNLGDPKPLLLIECKALAVNQNALKQLLSYNYSIGATCIAMAGKHSQV
SALFNPKTQTLDFYPGLPEYSQLLNYFISLNL

>core/779/4/Org4_Gene82

MVEIFNYSTSIYEQHASNNRIVSDFRKEIQMEGISIRDVAKHAQILDMNPKPSALTSLLQTNQKSHWACFSPPN
NFYKQRFSTPYLAPSLGSPDQQDEDIEKISSFLKVLTRGKFSYRSQITPFLSYKDKEEEEDEDPEEDDDDPRVQ
QGKVLLKALDLGVKSTNVMIDYVISRIFQFVQG

>core/780/4/Org4_Gene580

MSKESIRSYSEISTPTPIFRETPSKEGVAYKLQLRSPAKDCILRNRVSLKGALLRSIPFYGSFLGAKRIHSAWSA
KDAPCTTRVYHYLVGGLELLGLGVVVLACKVLATALKFLFSKASSKIKQMKWREKARNLAAKDTVQSIKEF
CSVDLTSCFTRCFRLRNRVVEEGESENQTVREIIV

>core/781/4/Org4_Gene898

MTDPKIEKSALRKLFISIRRDLSEERKHEASSAVASFVRSFSKESVVLSFVSFNHEIDMQEANRILIQKCTLALP
KIDQENLYPVLIPSIDDLISVVHPKDPFSKQTPISSDKITHVLVPGLAFDQQGYRLGYGHGFYDRWLAQHPYPS
IRTIGIGYCEQKIDKLPQESHDIPLSQIYLC

>core/782/4/Org4_Gene124

MSRLKKFYTEEIRKSLFEKFGYANKMQIPVLKKIVLSMGLAEAAKDKNLFQAHLEELTMISGQKPLVTKARN
SIAGFKLREGQGIGAKVTLRGIRMYDFMDRFCNIVSPRIRDFRGFSNKGDGRGCYSVGLDDQQIFPEINLDRV
KRTQGLNITWVTTAQTDDECTTLLELMGLRFKKAQ

>core/784/4/Org4_Gene782

MAKLIVAIGNPGHGYANTRHNAGFLLADRLVEELQGPPFKPLSKCHALMTLVESSSGPLVFIKPTTFVNLSGK
AVVLAKKYFNVALSHILVLADDVNRSFGKLRLCFNGGSGGHNGLKSITASLGSNEYWQLRFGVGRPLEEGV
ELSNFVLGKFSEEENLQLGSIFVEASTLFTEWCSKF

>core/785/4/Org4_Gene147

MSVLQDTEKKMAAALDFFHKEVKSFRTGKAHPALVETVVVDVYGTTMRLSDIASISVADLRQLVISPYDGN
NASAIAKGIIAANLNLQPEVEGSIIRIKVPEPTADYRQEMIKQLRRKCEEAKINVRNIRREANDKLKKDSALTE
DVVKGNEKKIQELTDKFCKQLDELTKQKEAEIASI

>core/786/4/Org4_Gene188

MTTPQSPGSLSQSHLPHPHDPWDTEPTSLPEDPNDKASQELHSLVHLFRKLSIHLLSEVEKTVQQLKPDLLELA
LLICEKFLYKKLENPQELALLLSTALQRHTTLRSLTPIKVFLHPEDLKTLTDWISTHELPMIKHAEFFPDTSCRR
SGFKIETPNGILRQEISEELDHLLSVLTA

>core/787/4/Org4_Gene103

MACEQHEGCYELEEREEIEDIKDSDTKWVSITQAAKLHNVTRQAIYVAIKQKKLKASKETRWEIDIKDLEEY
KRNRYSRKKSLYQGELVFDNGKGCYSINQVAQILGIPVQKVYYATRTGTIRGERKGAAWVIHVSEIERYKNE
YLSKQAAKKLKGAEPKEHQAPNFEPPTEIFPESN

>core/789/4/Org4_Gene233

MTIILCGLPTSGKSSLGKALAKFLNLPFYDLDDLIVSNYSSALYSSSAEIYKAYGDQKFSECEARILETLPPEDA
LISLGGGTLMYEASYRAIQTRGALVFLSVELPLIYERLEKRGLPERLKEAMKTKPLSEILTERIDRMKEIADYIF
PVDHVDHSSKSSLEQASQDLITLLKS

>core/790/4/Org4_Gene536

MSAPIPTPQELSDQITCLNVQYQQVSELARENKGDIEGLKTLTAALTAHAGIQPSADEIYSLQTAAALILSASE
KPGSGPSGSTEGSVTVQSPCKFKKVLAVVLTIIALIAIAVLIACIVAACGGFPLLLSALNLYTIGACVSLPIIASAS
VALICLCTFVANSLTKPVITVHTAR

>core/791/4/Org4_Gene99

MDRDNEVPLPKPKWIYRTGIGQDSHRFLPESSTKPCILGGIIFDHCPGFQANSDGDIIFHAICNAISSVTNKIILG
KVADELLQTRGITDSGIYLEEALKSLKPNQKISHVAITIEGSRPKFLCKLSALRQNIAQVMNLTPTDIGITATSG
EGLSDFGCGDGVQCFCVLTVMEYCD

>core/793/4/Org4_Gene189

MGYVFYVIAGSIFLGISLGAYCQLYYSVKSVLFSWYLLTVYALEKRHALLALSQLVGEEDAQSQKEIDFLSQC
DKLSWRAFLKNSYEIIPTFKEMEDLLSERVQGFLESIETIAEHDRAILCIENFWASKNLFDFEIAAYEEAVEKYL
KLRQRAPLRLASKLFRFLDVPSIRFSS

>core/794/4/Org4_Gene256

MDCVDNLKLYIFRLKLPGDTERISYSISPEYIREKGEEELLNSPIEVEGSLGRIDSDQWILSLSLKTQLGLCCPVC
NNFFSHSVCLPDLQRVISHDEVGSGVFDCRPLIRQELLLESDCFEECSGQGCPERKNILKFLEDRKKHEGNNPF
EYL

>core/795/4/Org4_Gene454

MSSTLNGIFPSSLPEESADLFITNKEIVALGEKGNVFLTHSIPMHIAAITILVIVALAGIAIICLGCYSQSILLIAVGI
VLTILTLLCLQALVGFIKFIRQLPQQLHTTVQFIREKIRPESSLQLVTNAQRKTTQDTLKLYEELCDLSQKEFKL
QSTLYQKRFELSHKNEKTNQN

>core/796/4/Org4_Gene745

MSIQPVSNTTTKADKVIPDSTKVISDSITINKQSAFYFCISVMLRLSESTTEYGKSILAVLEDNTIVQQQRVKELI
NLPLLKVPDLQKKDGSDDEYKNQNEIQAYQSSNQQISANRQMIQQELSSAQQRAQANQKSVNSTTTESMQIL
QATSSMLSTLKELTIKANLTNSPSD

>core/797/4/Org4_Gene226

MWIIDPLSAKKPLQAAINVPGTPITGGPNTATADDIIAKFSKDSNPLIVTVYYVYQSVLVAQDNLSIIAQELQA
NSSAQTYLNNQEALYQYVSIPKNKLNDNSSSYLQNIQSDNQAIGASRQAIQNQISSLGNAAQVISSNLNTNNNI
IQQSLQVGQALIQTFSQIVSLIANI

>core/798/4/Org4_Gene353

MADDTLIPKLMKNSLSQACSEGLLIAKYPPLQVIVHFDNNLVVKTHLSVAPVFSCLFLGPAAHKAMQELVLW
CSRYANKEHPPFSSHFAKDLIPSQYLEILNCVAEIPFGEQQTYAEIAKKTDTHPRTVGAACKQNPFLLFFPCHR
VVGSHGERNYVLGPVIHEILLKFENSY

>core/799/4/Org4_Gene451

MGFKNICKQGSQLYLNGIFPERILARKLKNCAKSYPRTALTIEVLVSSVLGALKVILIPCASTYAALTLPLRALF
NAIKTKSCQHLASYAMAWLFHILTIAVIIGLVFSLVFIPPPVVFISLGLLMSVTTSVTLFQVHKNLFPPHEPLPSR
PHTPPPFADEYVPLISESYFD

>core/800/4/Org4_Gene228

MSHLNYLLEKIAASSKEDFPFPDDLESYLEGYVPDKNIALDTYQKIFKISSEDLEKVYKEGYHAYLDKDYAKS
ITVFRWLVFFNPFVSKFWFSLGASLHMSEQYSQALHAYGVTAVLRDKDPYPHYYAYICYTLTNEHEEAEKAL
EMAWVRAQHKPLYNELKEEILDIRKHK

>core/802/4/Org4_Gene511

MPVHTTPRKCFILCILSMFTLPTLFPKAHLILFSPYIVLCFYCFSKDKGLVLALGCGVLSDLALGSRGVFLLLYP
LTALITHKAHLIFSKESKAALVIVNMIFYGVFLLLTIPMCALFGHEVRWSIDVLMIPLKCSFLDNLIFTSVIYILP
CAINSGIHKMMSFFRRLVCY

>core/803/4/Org4_Gene148

MVHSPTHQCYHCQQPATICYTEIDKDKVIRSYVCATCPCPSHYYNNEHLSLSKGVGVLTLECGNCKTVWHS
KQDDEQLLGCHQCYTNFKNQITSKLKSERVVSSSFTMEKGQGSLHIGRAPGEASNTNPLLKLIALNEALQDTL
EREDYEQAAVIRDQINHLKTKNPDDPS

>core/804/4/Org4_Gene366

MTAEKQNTGIPGLEIRYTLPSDATYMLKWLNDPKILRGFPIQTEAEIRETVNFWVGFYRYHSSLTAVYNGNV
AGVATLVLNPYVKVSHHALISIIVGEEFRNKGIGTALLNNLIHLAKTRFKLEVLYLEVYEGNPALHLYQRFGF
VEVGRQNRFYKDEIGYLAKTTMEKDL

>core/805/4/Org4_Gene56

MKKLLFSTFLLVLGSTSAAHANLGYVNLKRCLEESDLGKKETEELEAMKQQFVKNAEKIEEELTSIYNKLQD
EDYMESLSDSASEELRKKFEDLSGEYNAYQSQYYQSINQSNVKRIQKLIQEVKIAAESVRSKEKLEAILNEEA
VLAIAPGTDKTTEIIAILNESFKKQN

>core/806/4/Org4_Gene636

MSEVKPLLLKNDSFDLATQRFQNLINMLQEQAEIYNEYEEKNARVQNEIKEQKDSVKRCIEDFEARGLGVLK
EELASLTRDFHDKAKAETSMLIECPCIGFYYSIHQEEQRQRQERLQKMAERYRDCKQVLEAVQVEQKDMISP
RVVVDDSYFEEEKEEQKVDNRKKEQD

>core/807/4/Org4_Gene900

MCKNRGVRGIVACDPRGVIGLEGKLPWHYPEDLQFFSETIQKFPIVMGRKTWETLPRKYFVDRAVVVFSHEK
RQGVHGEIWVTSLEEFLLLDLSSPTFLIGGGELYSLFLENQIVRDFFISHIKKEYAGDTFFPLSLLETWTKTVLR
DTQEIITCYYENHHSQNTKNISL

>core/808/4/Org4_Gene665

MKQEKTLLLQEVEDKISAAQGFILLRYLGFTAAYSREFRNSLSGVSAEFEVLKKRIFFKAIEAAGLEVDCSDTD
GHLGVVFSYGDPVSAAKQVLDFNKQHKDSLVFLAGRMDNASLSGAEVEAVAKLPSLKELRQQVVGLFAAP
MSQVVGIMNSVLSGVISCVDQKAEKN

>core/809/4/Org4_Gene22

MQRLGLSNLFHCLLLFLRYYYSKLVFGLTVLLAAISVICLLGCSEPSLSSFTEYVGPEYSAAAQLSIEQSCHDE
VYGQQVVVTWSLPSRMRKCLPVTLYLWVYYGNGKVEKLTYEVNQSAGYRVYCLKGLEYKELQGIISYRVA
LCSGNQEIVSRRHHLWMEVISLDSP

>core/810/4/Org4_Gene781

MKQQLLLLEDVDGLGRSGDLITARPGYVRNYLIPKKKAVIAGAGTLRLQAKLKEQRLIQAAADKADSERIAQ
ALKDIVLEFQVRVDSDNNMYGSVTIADIIAEAAKKNIFLVRKNFPHAHYAIKNLGKKNIPLKLKEEVTATLLV
EVTSDNEYVTVLAQGKQTEENQEG

>core/811/4/Org4_Gene543

MIINVRAPAFGITSVQQFSTNFQAAIPILNIVIGCSRISSTYAEDIEEVAQEKSEKSTHSKSSTSVNLWAHRVRGV
VEILGGGIVILALEITALVLQVIIKLIKCLIDVLCVCLFGLGVCVVAIIGAIAFCVVVVVVKYLGFCSQGEELEPIE
VKTLISPDKPYPTVVYV

>core/812/4/Org4_Gene334

MSELIIGVDPGTIVAGYAIIAVEQRYQLRPYSYGAIRLSSDMPLPMRYKTLFEQLSGVLDDTQPNAMVLETQF
VNKNPQSTMKLAMARGIVLLAAAQRDIPIFEYAPNVAKKAVVGKGHASKRQVQVMVSKILNVPEVLHPSNE
DIADAFALAICHTHVARSPLCGVR

>core/813/4/Org4_Gene116

MRQFCNLLSLSRLWLALYFCQEKLHIRLLAIVGAMLSDVLDGYLARRYKATSRLGSILDPITDKVFVFVCITV
LYMEGSLSIAHLFFICARDLFLIIFVCYLSLVKGWKGYDYGSLFWGKIFTVVQFIILLGVTAGGEIPWTGLVPL
VALGFLYFLERIMDYKKQFLR

>core/814/4/Org4_Gene394

MATRFRSTLLVITLFVLIDWVTKLVVLLQYKDLQILTHPTLYTHSWGRFSFSIAPVFNEGAAFGLFSNYKYFLF
LLRIFVILGLLAYLFLKKKSIQSTTQTALVLLCAGAIGNVGDIIFYGHVVDFISFNYKQWAFPTFNVADVLISLG
TLLLVYKFYFPTKQTEKKR

>core/815/4/Org4_Gene149

MAKYPLEPVLAIKKDRVDRAEKVVKEKRRLLEIEQEKLREKEAERDKVKNHYMQKIQQLRDLLDEGTTSDA
VLQIKSYIKVVAVQLSEEEEKVNKQKEVVLAASKELEKAEVNLAKRRKEEEKTRLHKEEWMKEALKEEARA
EEKEQDEMGQLLFQLRQKKKRESGGS

>core/816/4/Org4_Gene502

MAVEQSHIKEEIEKLIGKAIKKVCGNKENDLCRYLPGPSGGYMHHFTLKKMKSAAPEQLLKMLKTFILESETP
RTINPKPRAPRGSKKRRDFINFTKTDIERVLELARQVGDKDLLARFSPKKPLTSLKRELIRSIRNGIVSVELWNA
YVEAVKAVSSPNLEVTSPFV

>core/817/4/Org4_Gene605

MDLKQIEKLMIAMGRNGMKRFAIKREGLELELERDTGEGNRQEPVFYDSRLFSGFSQERPIPTDPKKDTIKET
TTENSETSTTTSSGDFISSPLVGTFYGSPAPDSPAFVKPGDIVSEDTIVCIVEAMKVMNEVKAGMSGRVLEVLI
TNGDPVQFGSKLFRIAKDAS

>core/819/4/Org4_Gene554

MSKKINRNDLCPCGSNKKYKQCCLKKEEQTARYTTEGKFKFSAEVLSSSEQGEAGDNCTKLFQRLSQSLTSE
QKAAVGKFHQITKNKEVTSKKALKKAQAKEEKLVTEKLQQHNFEILNTGENLAPPMESTATLNQDTNFVCE
DFIPTQEDFRISENSQKPPVEED

>core/820/4/Org4_Gene69

MSRQNAEENLKNFAKELKLPDVAFDQNNTCILFVDGEFSLHLTYEEHSDRLYVVYAPLLDGLPDNTQRKLALY
EKLLEGSMLGGQMAGGGVGVATKEQLILMHCVLDMKYAETNLLKAFAQLFIETVVKWRTVCADICAGREP
SVDTMPQMPQGGGGMQPPPTGIRA

>core/821/4/Org4_Gene203

MKFWLQGCAFVGCLLLTLPCCAARRRASGENLQQTRPIAAANLQWESYAEALEHSKQDHKPICLFFTGSDW
CMWCIKMQDQILQSSEFKHFAGVHLHMVEVDFPQKNHQPEEQRQKNQELKAQYKVTGFPELVFIDAEGKQL
ARMGFEPGGGAAYVSKVKSALKLR

>core/822/4/Org4_Gene382

MSRRHSAEKRDIPGDPIYGSVILEKFINKVMMHGKKSVARKIVYSALERFGKKLNLENVLEGFEEALENAKPI
LEVRSRRVGGATYQVPVEVASERRNCLAMQWIIKHARSKPGKSMEVGLATELIDCFNKQGATIKKREDTHR
MAEANKAFAHYKW

>core/823/4/Org4_Gene327

MSLSKNSHKEDQLEEKVLVVNRCSKVVKGGRKFSFSALILVGDGKGRLGYGFAKANELTDAIRKGGEAAKK
NLMKIEALENGSIPHEVLVHHDGAQLLLKPAKPGTGIVAGSRIRLILEMAGIKDIVAKSFGSNNPMNQVKAAF
KALTGLSPRKDLLRRGAAIND

>core/826/4/Org4_Gene273

MTKFLYCGLFYSLGLLVLAFGTMVAIIQVDQICDVSCMNKHFQESPPFLKIKKVNVSKQICSPEERFFHCKIDK
SCMELHFPQSSYSCKEYLTRISGHILTQNFEKQMQFRGNSGLLNYQDGSLHVYDCRFQVDPLPGYGSPDKED
SSSGGMKTLYLSLFRN

>core/827/4/Org4_Gene787

MMHRYFIPLLALLIFSPSLVKAELQPSENRKGGWPTQLSCAEGSQLFCKFEAAYNNAIEEGKPGILVFFSERPT
PEFADLTNGSFSLSTPIAKGFNVVVLCPGLISPLDFFHKMDPVILYMGSFLEMFPEVEAVSGPRLCYILIDEQGG
AQCQAVLPLETKI

>core/828/4/Org4_Gene744

MSITTLGTLPTVNTINSSRPPLEPLNTPKIGAVLFSIYELLLQAIEIRQQTVLTQSQQLNDNTNIQQQLNQETNQI
KYAIVSAGAKEDEITRVQNQNQNYSAQRSNIQDELVTTRQNGQIILSHASTNINIIQQLSSQDSSFIKTTNSIGST
VNQLNKPLG

>core/829/4/Org4_Gene467

MTTWTLNQNNLTKFLKSSDEEPFLERESGLTYINIQANGNELPLFFVIRSEGEILQLICYLPYQLHESHKASTAR
LLHLLNRDIDIPGFGMDEEQGLIFYRLVLPCLNGKIHDTLLRIYIDTIKLVCDSFSHAIGLISSGNMNLDELRRQ
ALQEQQEKRNE

>core/831/4/Org4_Gene754

MATLSPEKFSGSPISISKEFPQQKMREIILQMLYALDMAPSAEDSLVPLLMSQTAVSQKHVLVALNQTKSILEK
SQELDLIIGNALKNKSFDSLDLVEKNVLRLTLFEHFYSPPINKAILIAEAIRLVKKFSYSEACPFIHAILNDIFTDS
SLNENSLSI

>core/832/4/Org4_Gene973

MMFGHFAGYLGADPEERMTSKGKRVITLRLGVKTRVGMKDETVWCKCNIWHNRYDKMLPYLKKGSGVIV
AGDISVESYMSKDGSPQSSLVISVDSLKFSPFGRNEGSRSPSLEDNHQQAGYESVSVGFEGEALDAEAIKDKD
MYAGYGQEQQYVCEDVPF

>core/834/4/Org4_Gene582

MSKKVFFESYEDFANVASSWPKSLRALVQGRYFVDSELKETPYRIHDFKKTPIHHRLYRSLPIISTIGGIIRLIEA
HSGPIHPRDKMKYRFEVLQAVIEILGLGVLILVFDIIRCFLAFLVAIILSLLLYCNSTFTCVQNLSFTERMLEGIG
EAVNFLA

>core/835/4/Org4_Gene74

MFFNLFSLVFKLSDELALAETIQEPISVHEMFPGSMKLEMFKMLGSLILLLTIFGFGVWAFKKFVRSRSHGFGG
SSQIKILERRSLTPKTSIYLIRVVNKTLVIAETPEKITLLTEFPPDTDINHLLQENNKQSSSSATSDFLSKAIQKIQK
KQQTNQD

>core/837/4/Org4_Gene14

MPSYCQNQQDFSLFSLLSPRLVMFLGKHSRDEILQDLTDLVDAAGLLEDKQAFFDALVRRENIMSTGIGMGV
AIPHGKLESCSNFFIAIGIHTQGILWDAIDGALVRLVFLIGGPENAQAEYLKLLSTLTLSLREESRRQQLLQVNT
IEEVMNVFVGM

>core/838/4/Org4_Gene181

MADETPKENSSKESSSQFDSLKRKVKDLHSNPKVGKWKKFLSHRACEAIGGCLVLVGIIADFISWAGGLFIAC
GVVLGFHVEIRKMLSNLQSYSIANGPIKNAILCGLILFFVLNIPSFAVSFIVLCVILSFITTAPSCSTCSKDHCDKH
QDTSNKPS

>core/839/4/Org4_Gene412

MTQEKIKIHVSNEQTCIPIHLISVEKLVLTLLEHLKVTTNEIFIYFLEDKALAELHDKVFADPSLTDTITLPIDAP
GDPAYPHVLGEAFISPQAALRFLENTSPNQEDIYEEISRYLVHSILHMLGYDDTSSEEKRKMRVKENQILRML
RKKHALLTA

>core/841/4/Org4_Gene209

MLPISILLFYVILGCLSAYIADKKKRNVIGWFFAGAFFGFIGLVVLLLLPSRRNALEKPQNDPFDNSDLFDDLK
KSLAGNDEIPSSGDLQEIVIDTEKWFYLNKDRENVGPISFEELVVLLKGKTYPEEIWVWKKGMKDWQRVKD
VPSLQQALKEASK

>core/842/4/Org4_Gene513

MRIIRFDPYGALSAQSIAKDSRQNSPLVEKISEEIATNEAIRLALLAIGDREQEEKKQRHRYKLLGQKQAKVLL
SQLRHVHLDFKKLHCDSKKKEDQEKDEKNKQKRSIKVTKKKKGISLGAAASQAIAAAAEAWVISRNKGVLE
TASTLFYQKDEEA

>core/843/4/Org4_Gene791

MEKQNLKLDVKEIEFPETVFSRDIETRVIQVIILHCLAKINGVSLLGGNLIDALFGRDIERMKGIYVEQDSKNHL
VKVRVEVNVDYGVSIPEKTEEIQGCIVSEISEYTGLHVAAVHVIIKGLTQPKDRIDEEIEEEVPVQDLPSPEDFL
LENSEG

>core/844/4/Org4_Gene608

MTVISNSPSPALNPELSLIPPPTLVSSGTQTSLAYTIPAQGRRSTLRIILDIFIIILGLATIISTFIVIFFLNGLNLLSTP
SIISSSCLIIVGLLFLIMGLYFMISSLDQGLVGLLQKELSQAEEREEEYIQEIEALRGAPRAESPTESPSTWL

>core/845/4/Org4_Gene136

MRVVLHCPDIPQNTGNIGRTCVALGAELILVRPLGFSLADKFVKRAGMDYWDKLQLTVVDSIEEALHDVPED
QIFCLSTKGSASYTEFSLPSSGTYVFGSESKGLPKEILKKYYKNCLRIPMQQDIRSLNLATSVGIVLYEVVRQKT
VALQKNPTV

>core/846/4/Org4_Gene747

MEKDIFFMQQAFKEARKAYDQDEVPVGCVIVKDDKIIARAHNSVEKLKDATAHAEILCIGSAAQDLDNWRL
LDTVLYCTLEPCLMCAGAIQLARIPRIVWAAPDVRLGAGGSWVNIFTEKHPFHTVSCTGGVCSEEAEHLMKK
FFVEKRREKSEK

>core/847/4/Org4_Gene493

MRYLPVSATDVLFESPAAPLINSANTQNQELIELKGKQQAESSPRTITSVILEVLLLIGCCLIVLSLLAIYPALQF
TLETGHPAAIAVLAVSGTILLVAVIILFCFLAAVPFAAKKTYEYVKTVDDYASWHSHKQTPTLGTIFSGIVYAE
SQAQL

>core/848/4/Org4_Gene134

MLKKKPVSFSCIDGHIYKIFPNDLNANNTVFGGLLMSLLDRLALVVAERHTESVCVTAFVDALRFYAPAYMG
ENLICKAAVNRTWRTSLEVGVKVWAENIYKQERRHITSAYFTFVAVNEDNQPIPVHQIVPETPEEKRRYNEA
DRRRQARLELK

>core/849/4/Org4_Gene832

MKTLKGHLSAKNLRIAIVGSCFNQAMADALVSGAQETFLKFGGSEDGLMTIRVPGAFEIPCTIKKLLSSERKF
DAIVACGVLIQGETDHYNQIVNQVAAGIGALSLEFCLPITLSIVAAPSAEIAWQRSGIKGCHLGVSGMTTAIEM
ATLFTQI

>core/850/4/Org4_Gene861

MPFAKETEMQRTCWKCEGSVSMHVPQCPYCSAFLQDPPVASGGFSSCHISFSEGASKEEAEDLFAVSSEDWE
AVLGDQNPTQETNKQVIPEWTWLQSWPLAALFLGIGLLAFAFLILLFSTDSGLVLTWPKNRAYSYGIIGAAVA
YRGYRKLPL

>core/851/4/Org4_Gene11

MRSALHLQHLRHFHNHGSILFENLLTIKDCFLLETKLQNFIAKASKTIDTVRWRENIFRSMPEIYTVVRKRRLD
FFAAELVHRPKLSLVRDLWVFPGEEILEGEEDCMLFLLLSGDRAGSGIFFTGPYPSDLYELEKGTTGLLLAFSS
VGIPVI

>core/852/4/Org4_Gene132

MNQPSVIKLRELLDLLPHRYPFLLVDKVLSYDIEARSITAQKNVTINEPFFMGHFPNAPIMPGVLILEALAQAA
GVLIGLVLENDRNKRIALFLGIQKAKFRQAVRPGDVLTLQADFSLISSKGGKAWAQARVDSQLVTEAELSFA
LVDKESI

>core/853/4/Org4_Gene97

MQCPFCNHGELKVIDSRNAPEANAIKRRRECLKCSQRFTTFETVELTLQVLKRDGRYENFQESKLIHGLNAAS
SHTRIGQDQVHAIASNVKSELLGKQNREISTKEIGELVMKYLKKADMIAYIRFACVYRRFKDVGELMEVLLS
ATPDMEK

>core/854/4/Org4_Gene927

MAQKEIVSNRKALRNYEVIETLEAGIVLTGTEIKSLRDHGGNLGDAYVIVSKGEGWLLNASIAPYRFGNIYNH
EERRKRKLLLHRYELRKLEGKIAQKGMTLIPLGMFLSRGYVKVRLGCCRGKKTYDKHRTIIEREKEREVAAA
MKRRHH

>core/855/4/Org4_Gene525

MARNIKYFLILFPGILWISAGMKLLLKATAIALDPLSSFFTYCLLSMISWGLASLKHRYLLSKTIRKQLSLSSEF
FSQKITWIAYIKQTFISRRFLIMVIMIAFSLVLRRYISNPQALFVIRATVGYALIKTAIAYFSKLQNALMENPEGN

>core/856/4/Org4_Gene41

MSKPSSCKAYLGIDYGKKRIGLAYAAEPLLLTLPIGNIEAGKNLKLSAEALHKIILSRNITCVVLGNPLPMQKG
LYSSLQEEVSLLAEELKKLSTVEIILWDERLSSVQAERMLKQDCGLSRKDRKGKTDSLAATLILTSFLDSLPKK
LTL

>core/857/4/Org4_Gene803

MMKTKYEYSFGVIPIKFFGTPDKSTLKACFICHTRGKHWGFPKGHSEDKEGPQEAARELVEETGLSVVNFFP
KVLIEQYSFNNEEQVFVRKEVTYFLAEVRGDIHADPMEICDSQWLSLQEGLRLLSFPELRDLTVEADKFINNY
LFSS

>core/858/4/Org4_Gene192

MQLLSPAFAYGAPIPKKYTCQGAGISPPLTFVDVPGAAQSLALIVEDPDVPKEIRSDGLWIHWIVYNLSTTITN
LAEGAEIFAVQGLNTSGKPVYEGPCPPDKQHRYFFTLFALDVVLPEEENVTRDQLYEAMEFHIIEQAELMGTY
EKS

>core/859/4/Org4_Gene562

MEKRKDTKTTIVKSSETTKSWYVVDAAGKTLGRLSSEVAKILRGKHKVTYTPHVAMGDGVIVINAEKVRLT
GAKKGQKIYRYYTGYISGMREIPFENMMARRPNYIIEHAIKGMMPRTRLGKKQLKSLRIVKGDSYETFESQKP
ILLDI

>core/860/4/Org4_Gene669

MEFICPLQHARCLKKQHKIIEKLFPEPFQKDHLYLKLMENSSSRDAFDKKRMLKENLVVGCQSDLYLYEVYQ
DGILFFFTYTKALMSSGIASLFTEVYSGETPSTILTCKPIFFQRLTPYLSFGRLNGGESLYMRMKQIAVQYLKPP
QT

>core/861/4/Org4_Gene151

MADLEVFQADFALLFEAGLLAIKQGDEDSARKLFQSLHILNPNHYGHDLGLALISLHKMDLFDAEERLSALIK
GNEDNWSIKAFLSLTHMLIVLHQGSSFEVRRESLESCLKFADQVIANCKIESTRALAQSVLDWHDTLVAKSA
GPLG

>core/863/4/Org4_Gene68

MKSERLKKLESELHDLTQWMQLGLVPKKEISRHQEEIRILEHKIYEEKERLQLLKENGEIEEYVTPRRSPAKTV
YPDGPSMSDIEFVEPTETEIDIDPGETVELELTDEGREDGAVEVDYSHEDDEDPFSDRNRWRRGGIIDPDANE
W

>core/864/4/Org4_Gene925

MKTKMNSRKKAGQWAIFNSPTPGVSSTLVLAWTPWGYYDKDVQDILERKDPMSSSLSDKDSKEFLKNLFVD
LLENGFTSVHIHAEEAFTPLDHTGKPHFKRDNVYLPGKLLGALNEAAVQANVSADTQFTLFLTQDDRNPFHD
KKKRD

>core/865/4/Org4_Gene979

MSNIMGSRRKLKRSFLLIEVLMALSLVCAVLLPCIRFYYAIHRSFEEDIFNLQLPALIDHCFLSVEEKMRQQMA
EGTVFTSGKGQTVSLAYTSQGIGYRIPYGYNVDIRQEVRGDNLKMKVCLADVVVELFPDQKQAVSVQRCLC
VTL

>core/866/4/Org4_Gene35

MKKKVTIDEALKEILRLEGAATQEELCAKLLAQGFATTQSSVSRWLRKIQAVKVAGERGARYSLPSSTEKTT
TRHLVLSIRHNASLIVIRTVPGSASWIAALLDQGLKDEILGTLAGDDTIFVTPIDEGRLPLLMVSIANLLQVFLD

>core/867/4/Org4_Gene517

MQNQYEQLLESLAPLLNTTLAPDKNNSCLIRFSDTQVPVQIEEDGNSGDLAVSTLLGTLPENVFRERIFKAALS
VNGSFQSSIKGILGYGEVTQQLYLSDILSMNYLNGEKLFEYLKLFSLHAKIWMESLRTGNLPDLHVLGIYYVA

>core/868/4/Org4_Gene809

MEEFVAYIVKNLVTNPEAVEIRSIEDEDNESIKLEIRVAAEDIGKIIGRRGNTIHALRTILRRVCSRLKKKVQIDL
IQPENGTDVIADQDYICDNDSSNSTEDTFGESDTCCSGHCHYDEDLNQEELDQEEQEEDNMHHSCECSNHH

>core/869/4/Org4_Gene33

MSLEKELLEETPLVLLNFYKLVSFCNYAGMILGTEEKKFAIYGHVSMGQAFQGADTEGHSPQRPFAHDLLNF
VFSGFDIQVLRVVINDYKDNVFYTRLFLEQKDREFLYVVDVDARPSDSIPLALTHKIPILCVKSVFDAVVPYEE

>core/870/4/Org4_Gene584

MNPVTFDRIQVDFIPEDTSLRINSYIVAGGLLILGVVLSILSVICLDIGLVGLSAGAAFTLGLGCLIFALFLFSFSL
ILLLSQEKRVPDVLCLYLEKEVPQYETPLYKEDLESERDMSAISERLGIIEEKLRIAEKFRYSDSVFV

>core/871/4/Org4_Gene13

MTVFCELDSGGELPEYTTPGAAGADLRANIEEPIALLPGQRALIPTGIKAEIPEGYELQVRPRSGLALKHGITVL
NSPGTIDSDYRGEIRVILINFGDSTFIIEPKMRIAQVVLSPVVQATFVVKQESLAETARGSGGFGHTGAS

>core/872/4/Org4_Gene309

MTFFEGETVFPAVLSELHSMLDLIKRAGKQSKCPQEKLLKLELACEELLVNIISYAYQGGNSPGTIAISCISHRG
DLEVVIKDHGPSFNPLAVSINIQEDLPLEQRKLGGLGIFLAKSSVDEFLYAREDHCNIVHLKILNSQHS

>core/873/4/Org4_Gene117

MEQTLSIIKPDSVSKAHIGEILSIFEQSGLRIAAMKMMHLSQTEAEGFYFVHRERPFFQELVDFMVSGPVVVLV
LEGANAVSRNRELMGATNPAEAASGTIRAKFGESIGVNAVHGSDTLENAAVEIAYFFSKIEVVNASKPLV

>core/874/4/Org4_Gene121

MIKLESLFDISERKRRKKLLGRGPSSGHGKTSGRGHKGDGSRSGYKRRFGYEGGGVPLYRRVPTRGFSHKRF
DKCVEEITTGRLAELFQEGEAITLDALKAKKAIARQAVRVKVILKGDLEKTFVWQDTAVVLSQGVQNLLGIT

>core/875/4/Org4_Gene566

MACSIFFRMSQGDYDDEPLSKKTACLVVDTMLYPVIAVVCAVVSVVLLILKVLFLLLSFPFKLCSASSALPGE
RVSLGSHFKCLYGGGLPDLLACLLIVPVIGTAIHGVIISHRTSEDDRLSSAIVFMQAPILQLAGMSGLIKP

>core/876/4/Org4_Gene706

MQEHVHKELLHLGEIFRSSREFQSLSLKDVEAATSIRYSCLEAIEQGCLGKLISPVYAQGFIKKYATYLGLDGD
SILQEHPYVMKIFKEFSDHNMEMLLDLESMGGRNSPERAIHSWSNLWWAGLIIIGGIVVWWLGSLFSIF

>core/877/4/Org4_Gene308

MSLDFFEEFYHQSILNTGTSFPEGYLNIAEILSHPHCTDANADFLCSQSDNDFIIAESKDKLTLFNADFAIWLVP
ELVQGQAVTRGYIAVSQGEGNYEPEMAFEASGQYNQSSLILEALQLYLKDIKDTENALRSFRFNNDH

>core/878/4/Org4_Gene330

MQHARKKFRVGRTSSHNRCMLANMLKSLIHHERIETTLPKAKELRRHADKMITLAKKNSLAARRIAIGRLM
VRYNKLTSKEARQAKGGDTSVYNVDRLVVNKLFDELGNRFVERKGGYTRILKLQNRIGDNAQKCIIEFLAS

>core/879/4/Org4_Gene159

MNSKSAQKIIDSIKQILTIYNIDFDPSFGSSLSSDSDADYEYLITKTQEKIQELDKRAQEILTQTGMSKEQMEVF
ANNPDNFSPEEWLALEKVRSSCDEYRKETENLINEITLDLHPTKESKRPKQKLSSTKKNKKKNWIPL

>core/880/4/Org4_Gene83

MLDNEWKAILGWGDDELEELRISGYSFLRQGHYSKAILFFEALVILDPLSIYDHQTLGGLYLQIGENSQALAV
LDQALRMQGDHLPTLLNKTKALFCLGRIEEATAIATYLSSCPIPAIANDAEALLMSYSKATKKNAALVR

>core/881/4/Org4_Gene316

MGRYRRVSHSSQETLLLGTELGQVLVPGAVLLLFGDYGAGKTEFVRGIVSGYLGDTIAEEVASPSFSILHVYG
NEPKRLCHYDLYRIDQKNQEYIFQDAEEDDVLCIEWADRLPKPRFCDTINIYIAMLTNMEREIIIEKR

>core/882/4/Org4_Gene18

MSVKKVIKIIKLQIPGGKANPAPPIGPALGAAGVNIMGFCKEFNAATQDKPGDLLPVVITVYADKTFTFITKQP
PVSSLIKKTLNLESGSKIPNRNKVGKLTQAQVEAIAEQKMKDMDIVLLESAKRMVEGTARSMGIDVE

>core/883/4/Org4_Gene21

MIDMSVVGPALVLGLAMIGSAIGCGMAGVASHAVMSRIDEGHGKLIGMSAMPSSQSIYGFILMLLMQAAIKN
GTLSPVGGIAIGLSVGAALLVSSVMQGKCCVSGIQAYARSSSIYGKCYAAIGIVESFSLFAVVFALLLL

>core/885/4/Org4_Gene205

MHPLTLPKQSRVLKRKQFLYITRSGFCCRGSQATFYVVPSRHPGTCRMGITVSKKFGKAHERNSFKRVVREV
FRHVRHQLPNCQIVVFPKGHKQRPVFSKLLQDFINQIPEGLHRLGKTKATTGGECTPKSEKCVTAPR

>core/886/4/Org4_Gene129

MLMPKRTKFRKQQKGQFAGLSKGATFVDFGEYAMQTLERGWVTSRQIEACRVAINRYLKRRGKVWIRIFPD
KSVTKKPAETRMGKGKGAPDHWVAVVRPGRILFEVANVSKEDAQDALRRAAAKLGIKTRFVKRVERV

>core/887/4/Org4_Gene51

MNLIDRAFLLKKTIIFQSLDMDLLLTIADKTETIIFKPGSNVFSIGQPGFSFYIIVEGYITISKEKLESPLNLKPLDC
FGEESLFNNKPREYNASANTQVRMLVLSKGQILNIVEECPSVALSFLELYAKQIKFREP

>core/888/4/Org4_Gene579

MVNRYKSSAEFSADHYYDDNLLRMGYKRNLRGLAPVENEVCLFEENNLLESVMASIPIMGSILGLGRLHSV
WSTQDPKDSKISIIFHTALGILETLGLGIIVLLIKITITILLILFTPCLLCYFMYSAAYSDFHPI

>core/889/4/Org4_Gene574

MINFIRSYALYFAWVISCAGTLISIFYSYILNVEPCILCYYQRICLFPLTVILGISAYREDSSIKLYTLPQAVLGLGI
SIYQVFLQEIPGMQLDICGRVSCSTKIFLFSYVTIPMASVVAFGAIVCLLVLTKKCRG

>core/890/4/Org4_Gene172

MKYRFTEEIEEEPLVNLTPLIDIVFVILMAFIVAVPLIKLDSIALAPGTQEQEVLSSENDSIAVIKVFADHSLTLN
EHPITLQELTVRLTLLHKAYPEKTPLLLQDGETSFRTYQNVKNAIEAAGFHELHVALQN

>core/891/4/Org4_Gene43

MAKSTIQESVATGRRKQAVSSVRLRPGSGKIDVNGKSFEDYFPLEIQRTTILSPLKKITEDQSQYDLIIRVSGGG
IQGQVIATRLGLARALLKENEENRQDLKSCGFLTRDPRKKERKKYGHKKARKSFQFSKR

>core/892/4/Org4_Gene902

MIAIERYQLIISKFRMWLFLGCSVEERHFKQPVLISVTFSYNEVPSACLSDKLSDACCYLEVTSLIEEIVNTKPYALIEHLANELFDSLVISFGDKASKIDLEVEKERPPVPNLLNPIKFTISKELCPSPVLSA

>core/893/4/Org4_Gene575

MPYYANTLEFIQGTQSLCPLFKYGFVRHHNKGQLEIEDASHDWDFLEPPSTWKRTLLAAIPILGSIIGLGRLFSIWSIREPQDSQEYKSIFWHTLCAVLEILGLGIVALILKILATFIMAMPGLKRVATFLFYS

>core/894/4/Org4_Gene123

MGMTSDSIADLLTRIRNALMAEHLYVDVEHSKMREAIVKILKHKGFVAHYLVKEENRKRAMRVFLQYSDDRKPVIHQLKRVSKPSRRVYVSAAKIPYVFGNMGISVLSTSQGVMEGSLARSKNIGGELLCLVW

>core/895/4/Org4_Gene119

MVKNQAQAKKSVKRKQLKNIPSGVVHVKATFNNTIVSITDPAGNVISWASAGKVGYSGSRKSSAFAATVAAQDAAKTAMNSGLKEVEVCLKGTGAGRESAVRALISAGLVVSVIRDETPVPHNGCRPRKRRRV

>core/896/4/Org4_Gene199

MNFVSTLTGSDFYAPVLEKLEEAFADTTGQVILFSSSPDFIVHPIAQQLGISSWYASCYRDQSAEQTIYKKCLTGDKKAQILSYIKKINQARSHTFSDHILDLPFLMLGEEKTVVRPQGRLKKMAKKYYWNIV

>core/898/4/Org4_Gene930

MSQCQSSSISTWEWMKSFVPNWSTEPKYSTPLSSPTPSEDEFTLAYESFVLKGDPENAPANPPGTPTPNSENGIDDLNPPLGQPNEQNNANNPGTSGSNPISLPAPERLPETEENSQEEEQGSQNSEDLIG

>core/899/4/Org4_Gene153

MLEKLIKNFATYMGITSTLELDADGAYVLPISEVVKVRAQQNADNEIVLSASLGALPPSADTAKLYLQMMIGNLFGRETGGSALGLDSEGNVVMVRRFSGDTTYDDFVRHVESFMNFSETWLSDLGLGKQ

>core/900/4/Org4_Gene471

MRDHAFSKLIGTVRAMVVEGRCPWSLQQSLVSMVEHILGECQEFHEAVLQGKTAQEVGSEAGDVLTLVLILCFLLEREGVLASEDVANEAIEKLRRRAPYIFAEDYKPVSIEEADRLWELAKHREKNEST

>core/901/4/Org4_Gene661

MDYKSQLVFSCPCCCKGNVCFSVFNLDVILTCNVCSSTYTFDSIIRNEIRQFVALCKRIHDANSILGNATVSVSVEDNQMDIPFQLLFSRFPVVLNLSLDGKKIAIRFLFDALNTSILHQESDLIS

>core/902/4/Org4_Gene968

MCDKIVAQKNFLFTLDAVIKQAGWRSQEKLNLFYVESQALGREIKVSLEEYIQSMVGILGSQRTKKSFKFSVDFTLLEQALQERCSSDDDEDATATSTATGATASPTDTHEDE

>core/903/4/Org4_Gene19

MTTESLETLVEKLSNLTVLELSQLKKLLEEKWDVTASAPVVAVAAGGGGEAPVAAEPTEFAVTLEDVPADKKIGVLKVVREVTGLALKEAKEMTEGLPKTVKEKTSKSDAEDTVKKLQDAGAKASFKGL

>core/904/4/Org4_Gene170

MKFTVALFGEAEKGSYDTAYFCRSLVDLHNYLGDVSSPGITLAIKTLLSDYNVVYFRVREEGYCVDSYFFGLHFLNTQTTLKNIIAIGLPGVGNQHIIEASRSLCQKHNSLLLFFDHDLYDLLTFNQPF

>core/905/4/Org4_Gene937

MRAGGSLVTTYPKEGRRLRSPEQLRVLDDLVQSYPNHLHAIELDCDAIPQDLIGATYIITFADFSTYILSLRSYQANSPSDDTWGIWFGSIDDPVIADRFQAVISFLKDHGFALPSTLAQDPLLCTNK

>core/906/4/Org4_Gene577

MLIGRYSSDDQFTEATKNTPTIIKLGFVRDNLDGLTNPISEMVSETSSSIKDSVLRSLPILGSILGCARLYSTLSTNDPLDETQEKIWHTIFGALETLGLGILVLLFKIIFVILHCIFHLVIGFCN

>core/908/4/Org4_Gene395

MPLSDDEIEQFKKRLLEMKAKLSHTLEGNAQEVKKPNEATGYSQHQADQGTDTFDRTISLEVTTKEYELLRQINRALEKINESSYGICDVSREEIPLARLIAIPYATMTVKAQEQFEKGLLSGN

>core/909/4/Org4_Gene122

MESSLCKKSLMKRRRALRVRKVLKGSPTKPRLSVVKTNKHIYVQLIDDSIGKTLASVSTLSKLNKSQGLTKKNQEVAKVLGTQIAELGKNLQLDRVVFDRGPFKYHGIVSMVADGAREGGLQF

>core/910/4/Org4_Gene101

MPTINQLIRKRRKSSLARKKSPALQKCPQKRGVCLQVKTKTPKKPNSALRKVAWVRLSNGQEVIAYIGGEGHNLQEHSIVLIQGGRVKDLPGVRYHIVRGTLDCAAVKNRKQSRSRYGAKRPK

>core/911/4/Org4_Gene824

MALKDTAKKMKDLLDSIQHDLAKAEKGNKAAAQRVRTDSIKLEKVAKLYRKESIKAEKSGLLKRKPSTKAPAKVKKTAVKKAPKKSSAAAAKTSKAVKASKPASKKTAAKKVKKPSKARGFRK

>core/912/4/Org4_Gene868

MKRQKRKQSITLIEMMVVITLIGIIGGALAFNMRGSIHKGKVFQSEQNCAKVYDILMMEYATGGSSLKEIIAHKETVVEEASWCKEGRKLLKDAWGEDLIVQLNDKGDDLVIFSKRVQNSNKK

>core/913/4/Org4_Gene844

MRDRLGSLSLILKVKIHKYLDTLHNQKRLALTVSRNIQATNKRIADLHLERYEHFISRDNIKHYDVLLEYLKTLQSSLYKQQSESLRFLEIHHQQLQELINRRKIIEKIKNNKYSKDQEIGT

>core/914/4/Org4_Gene120

MPRIIGIDIPAKKKLKISLTYIYGIGSARSDEIIKKLKLDPEARASELTEEEVGRLNSLLQSEYTVEGDLRRRVQS
DIKRLIAIHSYRGQRHRLSLPVRGQRTKTNSRTRKGKRKTVAGKKK

>core/915/4/Org4_Gene126

MIQQESQLKVADNTGAKKVKCFKVLGGSRRRYATVGDVIVCSVRDVEPNSSIKKGDVIKAVIVRTRRHITRK
DGSTLKFDTNSCVIIDDKGNPKGTRIFGPVAREIRDRGFIKISSLAPEVI

>core/917/4/Org4_Gene524

MEIIHIGTDIIEISRIRKAIATHGNRLLNRIFTEAEQKYCLEKTDPIPSFAGRFAGKEAVAKALGTGIGSVVAWK
DIEVFKVSHRPEVLLPSHVYAKIGISKVILSISHCKEYATATAIALA

>core/918/4/Org4_Gene28

MVNLLKELEQEQCRNDLPEFHVGDTIRLATKISEGGKERVQVFQGTVMARRGGGSGETVSLHRVAYGEGME
KSFLLNSPRIVSIEIVKRGKVARARLYYLRGKTGKAAKVKEFVGPRSSKK

>core/919/4/Org4_Gene752

MVRATGSVASRRRRKRILKQAKGFWGDRKGHIRQSRSSVMRAMAFNYMHRKDRKGDFRSLWIARLNVASR
IHGLSYSRLINGLKCANISLNRKMLSEIAIHNPEGFAEIANQAKKALEATV

>core/920/4/Org4_Gene578

MRELNAFELTQPEEYRNRWVLMPCLKCRFCRTQHAKVWSYRCVHEASLYEKNCFLTLTYDDKHLPQYGSL
VKLHLQLFLKRLRDRISPHKIRYFGCGEYGTKLQRPHYHLLIFNYDSLLDG

>core/921/4/Org4_Gene528

MATVAQTPQTTQPQPSVSHKATHRYCSWAFFKPILVSLGLLLASLTTLGLVIASGVTLSLGIGIVLAIQIVLAGI
ALVLAFNHIRQFKQARTAELNSMKMISAPAAATVRKQKLEDRYSSK

>core/922/4/Org4_Gene59

MTENRRIKRVNALLQEAIAKVILKDVKHPKISNLWITVTRVSLSKDLHSARVYYVSVMPHENTKEEALEALKV
SAGFIAHRASKNVVLKYFPELHFYLDDIFSPQDYIENLLWQIQEKEKS

>core/923/4/Org4_Gene177

MNKKPKKTKKAVQSKAAPVKRVPEESQEAAIQQLELAVSDLYKELPLAQTFASLTDKNQINSIIAALSGTLES
LHLEELTQGLFPSAQEDANFAKELSSVVHGLKNLTTVVNKQMVKGAE

>core/924/4/Org4_Gene392

MDNYLLGILIFCCVLLSIGMCTIFVMTICFLRQLNKILKNIHRVTTILNFEAKILAPLMLGKKLLCGWLKKRKN
RGSLSEDIDELLDEKKQRSWKKNLGQGIKWCAALVLIWKVFRNKD

>core/925/4/Org4_Gene200

MDSFCFDLLKVAAKAIDDKKGNNLVVLDVRTISEFTDYFVFVEGSVNVHVKALANTIVEELKKQKVSPLHVE
GITDGNWVVIDYGFIVVHVFVSEIRGKYRLEELWKDGFIVTSKLLAS

>core/926/4/Org4_Gene67

MPVSSAPLPTSHRPSSGNLGLMEPNSKALKAKHQDKTTKTIKLLVKILVAILVIEVLGIIAAFFIPGTPPICLIILG
GLILTTVLCVLLLVIKLALVNKTEGTTAEQQIKRKLSSKSIS

>core/927/4/Org4_Gene27

MALKIRLRQQGRRNHVVYRLVLADVESPRDGKYIELLGWYDPHSSINYQLKSERIFYWLERGAQLSSKAEAL
VKQGAPGVYSALLSKQEARKLVVRKKRRAYRQRRSTQREEAAKDATK

>core/928/4/Org4_Gene976

MSLNKEIGMTVLFYAFLFIFLFLCVILCGLILVQESKSMGLGSSFGVDSGDSVFGVSTPDILKKVTSWCAVAFC
IGCLLLSFSTNLLGKKLDAKEFLLPAAEESDIQASSESVEADKS

>core/929/4/Org4_Gene194

MLSRIVTCFLFLLSSLPLFAEEEAAQSKNTFVQPAVMLAIAILFFYFILWRPEQKRRKAMEKRKNDLAKGDKV
TAMGIIGTVDDIREHTVILNIASGKVEVLKGAISEILKPNDNKS

>core/930/4/Org4_Gene411

MSDIQKEEHGSTTVFHLHGKLDGISSPEVQENISQSLAAGSKNIILDCAHLDYMSSAGIRVLLQSYHQVGQHS
GKIVLTTVPKTIEQTLYVTGFLSYFKIFNTVDEAIQTLNKDGD

>core/932/4/Org4_Gene46

MFLKRKKRGGSQVQNKRTASPIKHAKHYLHNYLQELQKIMAARPHDAIDAWNQVFRDKYKGMSQAIGFRD
HILLVKVYNSSLYALLKQTPQNDLIMSLYQVASHVQIREIQFLLG

>core/933/4/Org4_Gene71

MSKVSVRKKNWGFRLLEEVMIKSWWVIFSILIGGFVYDRAIQELRTEELRLQSKVSSLCQDILSAQEKQRQLQ
LHLQHWQDSAAIEAALIQRLGLIPKGYKKLCVSPKQQSENKD

>core/934/4/Org4_Gene87

MWYSDYHVWILPVHERVVRLGLTEKMQKNLGAILHVDLPSVGSLCKEGEVLVILESSKSAIEVLSPVSGEVID
INLDLVDNPQKINEAPEGEGWLAVVRLDQDWDPSNLSLMDEE

>core/936/4/Org4_Gene212

MGKKENQLYEGAYVFSVTLSEEARRKALDKVISGITNYGGEIHKIHDQGRKKLAYTIRGAREGYYYFIYFSVS
PGAITELWKEYHLNEDLLRFMTLRADSVKEVLEFASLPE

>core/937/4/Org4_Gene808

MTAVLILTSFPSEESARSLARHLITERLASCVHVFPKGTSTYLWEGKLCESEEHHIQIKSIDIRFSEICLAIQEFSG
YEVPEVLLFPIENGDPRYLNWLTIHSYPEKPPLSD

>core/938/4/Org4_Gene324

MFKATARYIRVQPRKARLAAGLMRNLSVQEAEEQLGFSQLKAGRCLKKVLNSAVANAELHENIKRENLSVI
EVRVDAGPVYKRSKSKSRGGRSPILKRTSHLTVIVGEKER

>core/939/4/Org4_Gene125

MKKQNIRVGDKVFILAGNDKGKEGKVLSLTEDKVVVEGVNVRIKNIKRSQQNPKGKRISIEAPIHISNVRLTIA
GEPAKLSVKVTEQGRELWQRRPDGTSQLYRLVRGKKG

>core/940/4/Org4_Gene459

MTTLPKYVPRSRQNPDTLTFLKRYSSVLLHSENSLSYRIFAKVLAILLTSLAVAFAVTLFSCEGSQLRVCALYI
GIALAICVLLTIVVYCIASKIATACKKPPSISRIEIV

>core/943/4/Org4_Gene131

MKDPYDVIKRHYVTEKAKMLEHLSAGTGEGKKKGSFCKDPKFVFIVSHDATKPLIAQALEAIYVDKNVKVK
SVNTINVKPQPARMFRGRRKGKTSGFKKAIVTFYQGHSVG

>core/945/4/Org4_Gene421

MTVFKQIIDGLIDCEKVFENENFIAIKDRFPQAPIHLLIIPKKPIPRFQDIPGDEMILMAEAGKIVQELAAEFGIAD
GYRVVINNGAEGGQAVFHLHIHLLGGRPLGAIA

>core/946/4/Org4_Gene198

MNLSAKEYGDIIVIYLQGSLDAVSVPSVQEYLEQFIQKKHLKIALNFTDVSYISSAGIRLLLSNFKLVQSLGGK
MCLCCVKESVTEVMRIAGLDQLILLCQSEQECLSKL

>core/947/4/Org4_Gene403

MASSATPGFDGTAPSLFPPATRPRYNFKLALFVTIAIALVWLALIATTIAIGLCIHPLCSSIFLTAIPLYFISRYIFS
HYARNVYIALDVVPDHSKLQDMRSHSPIFSDR

>core/948/4/Org4_Gene24

MKKNTHPEYRQVLFVDSSTGYKFVCGSTYQSEKTEVFEGKEYPVCYVSVSSSSHPFFTGSKKFVDAEGRVDK
FLKRYSNVRQPAQQPQPEEDALPAAKGKKKVVTKKKK

>core/949/4/Org4_Gene8

MNEPTRTYLESEKDTQDQIEELQATCIVKNAAGIHVRPAGVIVRLFDGEPCDVHFTYAGKTINAKSIMSILML
GAPQGGEILVTIRSKEAHRILQKIQDAFSSGFGEL

>core/950/4/Org4_Gene926

MSTSPIGVPSMLNAATSLNATTSKAPLPTSTLSERIKEWLPCILLLIVGAIFTIAGCIVMALTKQILYGLLCVVG
GILLALGLLLKPENCIYRNAESAARSLSNALE

>core/952/4/Org4_Gene114

MDEITPNYPLLRQDSLWNRVRVSWRADLSVSSRYEIASAIAILGLLVAFCASAAVSIIFTANPLAQVFIDGCLA
LGLLPIPLVIGLLIIGIIVLLYGIYLFPQQRE

>core/954/4/Org4_Gene98

MEPYAVIQTGSKQYQVRSGDVIDVELLGEVASDKEVIFQDVLFVFDGTKASLGSPTIANAQVKAEYLSHVKG
EKVVAYKYKKRKNYHRKHGHRQKYLRVKIREILI

>core/955/4/Org4_Gene276

MSFTYFLALPVDRLMQERFLCSPKRWAPFINSPLYLTLIADHDTPYLAKNLDKFPLPVEQWEKTVLHVSSLLK
SIFLCSDLSSLRLLACTKFEILTLNDLYCVQNI

>core/956/4/Org4_Gene110

MNERTLLLLLKKKKGLFLAILDLTQTESSLTTPELEKVLKQKKIFLSCIDRVDLQIKEFRHAFSSELPQDIQEEL
EEIRDVIIRILDTDKRNYAQKKKEFGIYERP

>core/957/4/Org4_Gene100

MKQQKQKIRIRLKGFDQGQLDRSTADIVETAKRTGARVVGPIPLPTKREVYTVLRSPHVDKKSREQFEIRTHK
RLVDILDPTGKTIDALKMLALPAGVDIKIKAA

>core/958/4/Org4_Gene490

MSSPVVTGTSSASPVEQTALGEFLERLSGSGRCIKFAFAASTALLLLNTFVSGIVAIAMIFVATSVGAYFTVIGP
LFLLSLILLAIMLISMYKITHPSQNTPISN

>core/959/4/Org4_Gene112

MSFKRFLQQIPVRICLLIIYLYQWLISPLLGSCCRFFPSCSHYAEQALKSHGFLMGCWLSIKRIGKCGPWHPGGI
DMVPKTALQEVLEPYQEIDGGDSSHFSE

>core/961/4/Org4_Gene315

MVKIISSENFDSFIASGLVLVDFFAEWCGPCRMLTPILENLAAELPHVTIGKINIDENSKPAETYEVSSIPTLILFK
DGNEMARVVGLKDKEFLTNLINKHA

>core/962/4/Org4_Gene32

MSDQATTLRIKPLGDRILVKREEEEATARGGIILPDTAKKKQDRAEVLVLGTGKRTDDGTLLPFEVQVGDIIL
MDKYAGQEITIDDEEYVILQSSEIMAVLK

>core/963/4/Org4_Gene458

MDPASPVAPNVLQDHVQLSSEELSALSSGVSRVKKLTIAIMILSLIAISLVACGLFLTGSAPLQLSIWIAASCITLSMLVCACWRYKISNALEKTKVAHES

>core/964/4/Org4_Gene208

MAKKSSVAREAKRRRLVEANFKKRSDLRKIVKSLSVSEEEKENARISLNKMKRDTSPTRLHNRCLLTGRPRGYLRKFAISRICFRQMASMGEIPGVIKASW

>core/965/4/Org4_Gene3

MEQFHLDREEILLLAKASALQLSEELIQEYQTSLSAVITSMKEALAIEIDDADSCESLFMHVVNVEDLREDSVTSDFNREEFLRNVPESLGGLVKVPAVIK

>core/967/4/Org4_Gene391

MFRSNHKPKKTKCKRFRWLRGVLFGGFIATLLTCLFTPKSGVQLRKKILKVKNSGAKKSRVFFKNSKQHTKSFVKQAKLLAKNISHELQDFKKGILDDKD

>core/968/4/Org4_Gene78

MATMTKKKLISTISQDHKIHPNHVRTVIQNFLDKMTDALVKGDRLEFRDFGVLQVVERKPKVGRNPKNAAVPIHIPARRAVKFTPGKRMKRLIETPNKHS

>core/969/4/Org4_Gene162

MAPKKPNKKNVIQRRPSAEKRILTAQKRELINHSFKSKVKTIVKKFEASLKLDDTQATLSNLQSVYSVVDKAVKRGIFKDNKAARIKSKATLKVNARAS

>core/970/4/Org4_Gene229

MKKVVTLSIIFFATYCASELSAVTVVAVPLSEAPGKIQVRPVVGLQFQEEQGSVPYSFYYPYDYGYYYPETYGYTKNTGQESRECYTRFEDGTIFYECD

>core/971/4/Org4_Gene154

MEPRHIYIRKPETPKAPDVEKPGVPEYMTMANTPTFEGPVKTLDQLRRALIEQRGAEEGQKMYDNFIQSILISTFGLVHKDMDRAQKASKRMRSVYKEQ

>core/972/4/Org4_Gene649

MCLIDCLGQGFEAAINTVCCCSDSSESKANVAKVSAGLLALTAIVSFILIILICTGVLGASGMTFGMSNVAAVLVLVVTILLSMFLSGACFAAKREILR

>core/974/4/Org4_Gene167

MLSYLLRTAINVYSFLILAYIFASWVPDCQSARWYQLVSKCVDPFLNFFRRFVPRIGFIDPSPFVGLLCLGILPFVILRVLRFIILNIFHSPWLLQYL

>core/975/4/Org4_Gene64

MGKPKKSRTDRALAQEIQKKSTEVLKKPARIKAKNRRKFLIAKEQKTLKHRAQEYDQLVRSLLDSQKKDTD
KVLIFNYENGFVFTDKDHFSKYSIRL

>core/976/4/Org4_Gene29

MIKKDRFTNEKLNKLFDSPFSLVNYAIKQAKIKIAKGDVRSSNVAIETLVLLDREGIQPEFTEEIVVTASPTVER
KRSEHTNSRKKDPSAYTWSDVK

>core/977/4/Org4_Gene380

MASPTPGQLHLQQKVESKVYDYSRSLAMIATALLFFIVALILSGLSLLPQVLLPFSGAYFIIGSFLAFIALGILLI
NCVCDLKQYLTSS

>core/978/4/Org4_Gene9

MGSGYAKKKKEAKIMEQQFLEMEASLLEKRYEGQAGNGLVSVVINGKCDLISVKVQPTCLDPEDPEVIEDLF
RAAFKLAKEQMDQEMSLMRSTMPF

>core/979/4/Org4_Gene80

MNKSRFLRLCCCLCFCGSLFYFYINKQNSLTKLRLEIPCLSVRLRQLEQQNISLRFLIDKIERPDHLMEIAALPE
YQYLEYPSEESISLLSYELP

>core/980/4/Org4_Gene182

MLAFFATSFKSVLFEYSYQSLLLILIVSAPPIILASIVGIMVAIFQAATQIQEQTFAFAVKLVVIFGTLMISGGWL
SNMILRFAGQIFQNFYKWK

>core/982/4/Org4_Gene343

MLIRLFLGISLPKGFPLYVEPPLVLATFQGTQFVGTYSEATNPLYIDNLNLNYHYTQELLYKAVPCNYKPIYRE
IPLIIFPEVLIGSTPTQSTE

>core/983/4/Org4_Gene2

MLGKIIRGLSSLIVILCALNVGLIGITHNKLNIIAKLCGGVSTPATQITYIIIGIAGVICLLSFCPFCSKKSRHSHGD
SCSSGGCHSHHSDKN

>core/988/4/Org4_Gene66

MAKLVITSDDEQQEFELEDNSEIAEPCESMGIPFACTEGVCGTCVIEVLEGRENLSEFTEPEYDFLGEPEDSNER
LACQCRIKGGCVKVTF

>core/989/4/Org4_Gene88

MDDSWILEVKVTPKAKENKIVGFDGQALKVRVTEPPEKGKANDAVISLLAKALSLPKRDVTLIAGETSRKKK
FLLPNRVQDIIFSLHIDV

>core/990/4/Org4_Gene102

MKKAVLIAAMFCGVVSLSSCCRIVDCCFEDPCAPSSCNPCEVIRKKERSCGGNACGSYVPSCSNPCGSTECNSQSPQVKGCTSPDGRCKQ

>core/991/4/Org4_Gene224

MSLDKGTKEEITKKFQLHEKDTGSADVQIAILTEHIAELKEHLKRSPKDQNSRLALLKLVGQRRKLLEYLNSTDTERYKNLITRLNLRK

>core/992/4/Org4_Gene63

MSRKCPLTGKRPRRGYSYTLRGIAKKKKGIGLKVTGKTKRRFFPNMLTKRLWSTEENRFLKLKISASALRHIDKLGLEKVLERAKSKNF

>core/993/4/Org4_Gene239

MSRSLRKGPFVDHHLLKKVRAMNIEEKKTPIKTWSRRSMITPEMIGHTFEVHNGKKFLTVFVSETMVGHKLGEFSPTRIFKSHPVKKG

>core/994/4/Org4_Gene934

MKEYLDFLVQRNVERDPQTKRHCTVSQKFGGESIDAKTTTGQIFHIAGKTEPGHGKLCLGESILKQLLALGIITGYENREREVWVYLD

>core/996/4/Org4_Gene108

MSQKNKNSAFMHPVNISTDLAVIVGKGPMPRTEIVKKVWEYIKKHNCQDQKNKRNILPDANLAKVFGSSDPIDMFQMTKALSKHIVK

>core/997/4/Org4_Gene210

MKSFKFLLPFLSVILCCGNLLSSPRSRAISVTESIGMSAVKTLVLSEKAHEFLEGIGYGVGASSILRDWQTQQWLEIESLLAQNEVM

>core/998/4/Org4_Gene127

MASEPRGSRKVKIGVVVSAKMEKTVVVRVERIFSHPQYLKVVRSSKKYYAHTELKVSEGDKVKIQETRPLSKLKRWRVIEHVGVVS

>core/999/4/Org4_Gene236

MVEIHHKDPSLKKLFALQQSLETLNSLSDIVATYEAMFSLIYEGLNKALRKDQLCYLLSVNSKGELLKSPSGDPIVQTFPIHPHH

>core/1000/4/Org4_Gene152

MATNKSCTAFDFNKMLDGVCTYVKGVQQYLTELETSTQGTVDLGTMFNLQFRMQILSQYMESVSNILTAVNTEMITMARAVKGS

>core/1001/4/Org4_Gene385

MAHKKGQGASRNGRDSKSKRLGVKVGAGQKVSTGSILVRQRGTRWNPAQNVGRGRDDTLFALVDGIVVM
KKTKRTYISVVPEQL

>core/1003/4/Org4_Gene17

MKQQHNRKALSRKIGTVKKQAKFAGSFLDEIKKIEWVSKHDLKKYIKVVLISIFGFGFAIYFVDLVLRKSITCL
DGITTFLFG

>core/1004/4/Org4_Gene281

MFNMENTAKEEKNSQPLLDLEQDMQDHNRAQELKASVQDKVHKLHALLREGSDKESFGQQQSLLAGYVA
LQKVLGRINRKMI

>core/1005/4/Org4_Gene213

MNKPVHNNEHRRKRFNKKCPFVSAGWKTIDYKDVETLKKFITERGKVLPRRITGVSSRFQGVLSQAIKRARH
LGLLPFVGED

>core/1007/4/Org4_Gene942

MTKIQCGAQYYRSRPAERAQTPPQPFLARDRADFWERHPRFSACCRVLLFVAWVVLALLFLFVMLLPLAAG
RGPAPIWLLW

>core/1009/4/Org4_Gene52

MSLEDDVIAIIVEQLGVDPKEVNENSSFIEDLNADSLDLTELIMTLEEKFAFEISEEDAEKLRTVGDVFTYIKKR
QAEQ

>core/1012/4/Org4_Gene275

MKEFLAYIIKNLVDRPEEVRIKEVQGTHTIIYELSVAKPDIGKIIGKEGRTIKAIRNLLVSVASRNNVRVSLEIME
EK

>core/1013/4/Org4_Gene240

MMTLFLVICCATVLLGLGMGILLVGSHLLGRPLSKGCQKPDCCQKKTCDKTEHCATKSRENSTSKCSSNDDV
PPTAP

>core/1014/4/Org4_Gene168

MNEGIHSVCFQKTPRLTAKSVVSMEMLLTTQQLPSAEGMPSVANLEADFLRAEALLAEMREIRGCLEQSLRT
LVPSE

>core/1015/4/Org4_Gene225

MKNKIVTLLDQLYEDQESRLQKLGEEIVPNLTPEDLLQPMDFPQLEGNPAFRFEEGVLSGIGEVRAAILAALS
QEN

>core/1016/4/Org4_Gene248

MEAKKIKELSKEAQLLKKLREKSRVLDEKNKRKAWVAKLVAMPESIREIEKEERVETPQLFQAIAEKILEEGV

>core/1018/4/Org4_Gene157

MFFAPLLYESLRRGLMHPTSHMQQQLARLEFINDQLTTELEHVNELLCSLGFPEGLTTIKAIAEEVLSDDEPLL
D

>core/1019/4/Org4_Gene245

MEEVPFENAMQRLEEIVDLMNQPTTSLDASLALYEEADALMRICESRIRQVEQRVRELAEKRHESSLFEEQA
VVR

>core/1021/4/Org4_Gene15

MAKKEDTLVLEGKVEELLPGMHFRVILENGMPVTAHLCGKMRMSNIRLLVGDRVTVEMSAYDLTKARVVY
RHR

>core/1022/4/Org4_Gene743

MSGNECNDYPEVFKDDVSAYVLVTCGQMSSDGKIQVEMTYEGDPAVISYLLTKARDSLDES

>core/1023/4/Org4_Gene779

MSYKITLPKADETTAKKVTKISEASTLIFSVLKEKASLGNVHGFCQAENSLSVEANKIISVAENTLAGCFCK

>core/1025/4/Org4_Gene128

MAAKKDLLTQLRGKSDDDLDAYVHENKKALFALRAENLLQNKVVKVHMFSTHKKNIARALTVKQERKGK
VHG

>core/1027/4/Org4_Gene238

MMFDFMLARAFRAANTIVNTKQKPFHIHGMATLRPNATSILNIKEDALSLGTVGSFASTASIKIDSH

>core/1028/4/Org4_Gene138

MKKLIALIGIFLVPIKGNTNKEHDAHATVLKAARAKYNLFFVQDVFPVHEVIEPISPDCLVHYEGWV

>core/1031/4/Org4_Gene349

MKKLMLAFLLALVLPVAGSAFGYESCSAPQDEAQGSEDEGRDESSDSETQTGKDEEGGSEENTAE

>core/1033/4/Org4_Gene220

MPKMKTNKSVSARFKLTASGQLKRTRPGKRHKLSKKSSQEKRNLSKQPLVDKGQVGMYKRMMLV

>core/1034/4/Org4_Gene424

MIKQACKFYLLQCLLCALYWLLKYCRKLLKGTLHHSEETFYQALLSSLIDLLYQLKQLPAPTNE

>core/1037/4/Org4_Gene376

MKLFYFCNIIFLTSLFSVSCTHTPPPRSYILAQGRTPLVNKAHSLEFFLARAVFNTCYNTNL

>core/1045/4/Org4_Gene214

MAVPRNRHSNARKNIRRSHDAKKACHAAKCSNCKHALLPHTICPSCGFYNGKAVMTVEKK

>core/1046/4/Org4_Gene141

MSIALNREEVWDNPHHLMFILMQFQQFSGEQDRFGSFLEATIRDRVSFLVLQEKIATLK

>core/1049/4/Org4_Gene257

MRFFCLFFLGFLGSFHCVAEDKGVDLFGVWDDNQITECDDSYMTEGREEVEKVVDA

>core/1050/4/Org4_Gene7

MPSVKVRVGEPVDRALRILKKKIDKEGILKAAKSHRFYDKPSVKKRAKSKAAAKYRSR

>core/1054/4/Org4_Gene72

MQIENSSILFAEVVMKWFIFSVISAPVVFLPGCTLIPKEKVTKVPSQLWSESLSQP

>core/1056/4/Org4_Gene448

MIDAENQNPKDGGSFTSLHTDPKNLFDEEGMPSHLILYSAISITYSSL

>core/1058/4/Org4_Gene92

MSRHRSYGKSVKGVTKRNVLKRFERVEVLRKLGRWNDSTAKKVTGLPKTPILK

>core/1059/4/Org4_Gene1

MASKNREIIKLKSSESSDMYWTVKNKRKTTGRLELKKYDRKLRRHVIFKEAK

>core/1067/4/Org4_Gene231

MFFIAVRSRGFLDIHGILAARKGKQVVKSTAGAWIGSRGAVFYSLVS

>core/1068/4/Org4_Gene298

MSESLEIPELTEVLSEQPSLSTPDSPPKVITGTLTLYFQEDIDPAS

>core/1069/4/Org4_Gene207

MKVSSSVKADPSKGDKLVRRKGRLYVINKKDPNRKQRQAGPARKK

>core/1070/4/Org4_Gene206

MKRTYQPSKRKRRNSVGFRTRMATRNGRKLLNRRRRHGRHSLVDL

>core/1073/4/Org4_Gene971

MSSVKKKRRLKIAKHKRKKRRRRDRHKNK

>core/0/5/Org5_Gene64

MKSLPVYVSGIKVRNLKNVSIHFNSEEIVLLTGVSGSGKSSIAFDTLYAAGRKRYISTLPTFFATTITTLPNPKV
EEIHGLSPTIAIKQNHFSHYSHATVGSTTELFSHLALLFTLEGQARDPKTKEVLDLYSKEKVLSTIMELSEGVQI
SILAPLLRKDIAAIHEYAQQGFTKVRCNGTIHPIYSFLTSGIPEDCSVDIVIDTLIKSENNIARLKVSLFTALEFGE

GHCSVLSDEELMTFSTKQQIDDVTYTPLTQQLFSPHALESRCSLCQGSGIFISIDNPLLIDENLSIKENCCSFAGN
CSSYLYHTIYQALADALNFNLETPWKDLSPEIQNIFLRGKNNLVLPVRLFDQTLGKKNLTYKVWRGVLNDIG
DKVRYTTKPSRYLSKGMSAHSCSLCKGTGLGDYASVATWEGKTFTEFQQMSLNNWHVFFSKVKSPSLSIQEI
LQGLKQRLSFLIDLGLGYLTPNRALATLSGGEQERTAIAKHLGGELFGITYILDEPSIGLHPQDTEKLIGVIKKL
RDQGNTVILVEHEERMISLADRIIDIGPGAGIFGGEVLFNGKPEDFLMNSSSLTAKYLRQELTIPIPESREAPTS
WLLLTEATIHNLKNLSIRLPLARLIGVTGVSGSGKSSLINNTLVPAIESFLKQENPKNLHFEWGCIGRLIHITRDL
PGRSQRSIPLTYIKAFDDIRELFASQPRSLRQGLTKAHFSFNQPQGACIQCQGLGTMTISDDDTPIPCSECQGKR
YHSEVLEILYEGKNIADILDMTAYEAEKFFISHPKIHEKIHALCSLRLDYLPLGRPLSTLSGGEIQRLKLAHELL
FASPKQTLYVLDEPTTGLHTHDIQALIEVLLSLTYLGHTVLVIEHNMHVVKVCDYVLELGPEGGDLGGYLLA
SCTPKDLIQLNTPTAKALAPYIEGSLDIPVVKSEPPSSPKSCDILIKDAYQNNLKHIDLALPRNSLIAIAGPGASG
KHSLVFDILYASGNIAYAELFPPYIRQGLLKETPLPSVGEVKGLSPVISVRKCSSSNRSYHTIASALGLSNGLEK
LFAILGEPFSPLTEEKLSKTTPQTIIDSLLKSYKDDYVTITSPIPLGSDLEIFLQEKQKEGFIKLYSEGNLYDLDER
LPLNLIEPAIVIQHTKVSPKNSSSLLSAISVAFSLSSEIWIYISQKKQRKLSYSLGWKDKKGRLYPEITHQLLSSD
HPEGRCLTCGGRGEILKISLEEHKEKIAHYTPLEFFSLFFPKSYMKPVQKLLKDENASQPLKLLTTKEFLNFCR
GSSEFPGMNALLMEQLDTESDSPLIKPLLALTSCPACKGSGLNDYANYVRINNTSLLDIYQEDATFLESFLNTI
GTDDTRSIIQDLMNRLTFISKVGLSYITLGQRQDTLSDGENYRLHLAKKISINLTNIVYLFEEPLSGLHPQDLPTI
VQLLKELVANNNTVIATDRSCSLIPHADHAIFLGPGSGPQGGFLMDSDTEVCPSVDLHANVPQTEVCPKAPLS
ISKANHTRGSDRTLKVNLSIHHIQNLKVSAPLHALVAIGGVSGSGKTSLLLEGFKKQAELLIAKGTTTFSDLVV
IDSHPIASSQRSDISTYFDIAPSLRAFYASLTQAKALNISSTMFSTNTKQGQCSDCQGLGYQWIDRAFYALEKR
PCPTCSGFRIQPLAQEVLYEGKHFGELLHTPIETVALRFPFIKKIQKPLKALLDIGLGYLPIGQKLSSLSVSEKTA
LKTAYFLYQTPETPTLFLIDELFSSLDPIKKQHLPEKLRSLINSGHSVIYIDHDVKLLKSADYLIEIGPGSGKQGG
KLLFSGSPKDIYASKDSLLKKYICNEELDS

>core/1/5/Org5_Gene1033

MKWLPATAVFAAVLPALTAFGDPASVEISTSHTGSGDPTSDAALTGFTQSSTETDGTTYTIVGDITFSTFTNIP
VPVVTPDANDSSSNSSKGGSSSSGATSLIRSSNLHSDFDFTKDSVLDLYHLFFPSASNTLNPALLSSSSSGGSSS
SSSSSSSGSASAVVAADPKGGAAFYSNEANGTLTFTTDSGNPGSLTLQNLKMTGDGAAIYSKGPLVFTGLKN
LTFTGNESQKSGGAAYTEGALTTQAIVEAVTFTGNTSAGQGGAIYVKEATLFNALDSLKFEKNTSGQAGGGI
YTESTLTISNITKSIEFISNKASVPAPAPEPTSPAPSSLINSTTIDTSTLQTRAASATPAVAPVAAVTPTPISTQETA
GNGGAIYAKQGISISTFKDLTFKSNSASVDATLTVDSSTIGESGGAIFAADSIQIQQCTGTTLFSGNTANKSGGG
IYAVGQVTLEDIANLKMTNNTCKGEGGAIYTKKALTINNGAILTTFSGNTSTDNGGAIFAVGGITLSDLVEVR
FSKNKTGNYSAPITKAASNTAPVVSSSTTAASPAVPAAAAAPVTNAAKGGALYSTEGLTVSGITSILSFENNEC
QNQGGGAYVTKTFQCSDSHRLQFTSNKAADEGGGLYCGDDVTLTNLTGKTLFQENSSEKHGGGLSLASGKS
LTMTSLESFCLNANTAKENGGGANVPENIVLTFTYTPTPNEPAPVQQPVYGEALVTGNTATKSGGGIYTKNA
AFSNLSSVTFDQNTSSENGGALLTQKAADKTDCSFTYITNVNITNNTATGNGGGIAGGKAHFDRIDNLTVQSN
QAKKGGGVYLEDALILEKVITGSVSQNTATESGGGIYAKDIQLQALPGSFTITDNKVETSLTTSTNLYGGGIYS
SGAVTLTNISGTFGITGNSVINTATSQDADIQGGGIYATTSLSINQCNTPILFSNNSAATKKTSTTKQIAGGAIFS
AAVTIENNSQPIIFLNNSAKSEATTAATAGNKDSCGGAIAANSVTLTNNPEITFKGNYAETGGAIGCIDLTNGS
PPRKVSIADNGSVLFQDNSALNRGGAIYGETIDISRTGATFIGNSSKHDGSAICCSTALTLAPNSQLIFENNKVT
ETTATTKASINNLGAAIYGNNETSDITISLSAENGSIFFKNNLCTATNKYCSIAGNVKFTAIEASAGKAISFYDA
VNVSTKETNAQELKLNEKATSTGTILFSGELHENKSYIPQKVTFAHGNLILGKNAELSVVSFTQSPGTTITMGP

GSVLSNHSKEAGGIAINNVIIDFSEIVPTKDNATVAPPTLKLVSRTNADSKDKIDITGTVTLLDPNGNLYQNSY
LGEDRDITLFNIDNSASGAVTATNVTLQGNLGAKKGYLGTWNLDPNSSGSKIILKWTFDKYLRWPYIPRDNH
FYINSIWGAQNSLVTVKQGILGNMLNNARFEDPAFNNFWASAIGSFLRKEVSRNSDSFTYHGRGYTAAVDAK
PRQEFILGAAFSQVFGHAESEYHLDNYKHKGSGHSTQASLYAGNIFYFPAIRSRPILFQGVATYGYMQHDTTT
YYPSIEEKNMANWDSIAWLFDLRFSVDLKEPQPHSTARLTFYTEAEYTRIRQEKFTELDYDPRSFSACSYGNL
AIPTGFSVDGALAWREIILYNKVSAAYLPVILRNNPKATYEVLSTKEKGNVVNVLPTRNAARAEVSSQIYLGS
YWTLYGTYTIDASMNTLVQMANGGIRFVF

>core/2/5/Org5_Gene781

MVAKKTVRSYRSSFSHSVIVAILSAGIAFEAHSLHSSELDLGVFNKQFEEHSAHVEEAQTSVLKGSDPVNPSQ
KESEKVLYTQVPLTQGSSGESLDLADANFLEHFQHLFEETTVFGIDQKLVWSDLDTRNFSQPTQEPDTSNAVS
EKISSDTKENRKDLETEDPSKKSGLKEVSSDLPKSPETAVAAISEDLEISENISARDPLQGLAFFYKNTSSQSISE
KDSSFQGIIFSGSGANSGLGFENLKAPKSGAAVYSDRDIVFENLVKGLSFISCESLEDGSAAGVNIVVTHCGDV
TLTDCATGLDLEALRLVKDFSRGGAVFTARNHEVQNNLAGGILSVVGNKGAIVVEKNSAEKSNGGAFACGS
FVYSNNENTALWKENQALSGGAISSASDIDIQGNCSAIEFSGNQSLIALGEHIGLTDFVGGGALAAQGTLTLR
NNAVVQCVKNTSKTHGGAILAGTVDLNETISEVAFKQNTAALTGGALSANDKVIIANNFGEILFEQNEVRNH
GGAIYCGCRSNPKLEQKDSGENINIIGNSGAITFLKNKASVLEVMTQAEDYAGGGALWGHNVLLDSNSGNIQ
FIGNIGGSTFWIGEYVGGGAILSTDRVTISNNSGDVVFKGNKGQCLAQKYVAPQETAPVESDASSTNKDEKSL
NACSHGDHYPPKTVEEEVPPSLLEEHPVVSSTDIRGGGAILAQHIFITDNTGNLRFSGNLGGGEESSTVGDLAI
VGGGALLSTNEVNVCSNQNVVFSDNVTSNGCDSGGAILAKKVDISANHSVEFVSNGSGKFGGAVCALNESV
NITDNGSAVSFSKNRTRLGGAGVAAPQGSVTICGNQGNIAFKENFVFGSENQRSGGGAIIANSSVNIQDNAGD
ILFVSNSTGSYGGAIFVGSLVASEGSNPRTLTITGNSGDILFAKNSTQTAASLSEKDSFGGGAIYTQNLKIVKNA
GNVSFYGNRAPSGAGVQIADGGTVCLEAFGGDILFEGNINFDGSFNAIHLCGNDSKIVELSAVQDKNIIFQDAI
TYEENTIRGLPDKDVSPLSAPSLIFNSKPQDDSAQHHEGTIRFSRGVSKIPQIAAIQEGTLALSQNAELWLAGLK
QETGSSIVLSAGSILRIFDSQVDSSAPLPTENKEETLVSAGVQINMSSPTPNKDKAVDTPVLADIISITVDLSSFV
PEQDGTLPLPPEIIIPKGTKLHSNAIDLKIIDPTNVGYENHALLSSHKDIPLISLKTAEGMTGTPTADASLSNIKID
VSLPSITPATYGHTGVWSESKMEDGRLVVGWQPTGYKLNPEKQGALVLNNLWSHYTDLRALKQEIFAHHTI
AQRMELDFSTNVWGSGLGVVEDCQNIGEFDGFKHHLTGYALGLDTQLVEDFLIGGCFSQFFGKTESQSYKA
KNDVKSYMGAAYAGILAGPWLIKGAFVYGNINNDLTTDYGTLGISTGSWIGKGFIAGTSIDYRYIVNPRRFIS
AIVSTVVPFVEAEYVRIDLPEISEQGKEVRTFQKTRFENVAIPFGFALEHAYSRGSRAEVNSVQLAYVFDVYR
KGPVSLITLKDAAYSWKSYGVDIPCKAWKARLSNNTEWNSYLSTYLAFNYEWREDLIAYDFNGGIRIIF

>core/3/5/Org5_Gene954

MASSSNNSTKQDGIPSWVNPNVQWNRASQVGDQEANSLTPEAQTSRSWFSDRKHFLEVLDVSLEEMENNDL
KKYSRYKTIILIATLVTVAITCIVPISMVFGIPMWVPCLILFGAGLSSAFLSHRLQSKCKEIHLRYRAYQIYRQQL
LSQYPDLRKSTLYKYSITHVKPKKGFVGKLVENLRPDLHKNKDDGGAAADSRLDFAGYGVKHYQTDALLG
VSGVNSVEWQRLASLIMSVKNDILNDVGSREPIDKAQRSALVVSGKDIGGEIQPGGILDISRDILAICGYGMNV
GVEAKKAIDQYKKWYLNSSTFIAWNPQLPAIAQSYLLEQQRHLDYAAKIFQDLSALTTAHGTGQALEDLDSL
LCYYDQLIESKGVGEKIIASIHQKHLDLAMQDSCDQEHLKKWSNLYHVFSITIKEFTEGKLEQNEVVSRIQRL
RGKLEKSKCSILGNCRTNAEYATKSEKKLADYLLQIGDREPFLTGMHKAIATGKAIQGKVEGVISQHPEKQIM
MLRCSIERLEGMLRREDWGAILQKNEDEVLALKSTMEAQLQGFKDLVGTWEGKYQEFKKNKLSKVLVYDF

TKSYSNLLNRLEVLHAESSTDDLVLHVDRMSEDLKKTIEEIDGNLFQVTPEELSLLAREYQGLMNELPLIVQE
GNRLQEAISSEGVSQGLMLLNSLLNRDEKINKNIESSRKNLVAIAKQARSDARNIDSQGLAPLIQRNRASLDNI
LQNMYLFNGSIRNIHALDTETLVATSSNMFSAMHTFDWNIYTNLLDVLEIQSKPAPAPMENPDLPGALPEEVQ
DAVAEDVSGTHRLHHQVLKRRCADLKNMISQLQKSINKWGMAKAIVLGIVAVLFCVLSAIFIGQNILSLLILS
CVGLLLTQVCPLIFDRISKSKEFEKQVLETAQSLIPATKILPSEFNNKDLNRLAKLQDNLNLEGFGPTWARNIV
SDLEGIPTKEKSLKDLTKEFRKDSKNLNKRIKRRFKEGLGQEAPVVRPTIPQDIRGAEVFAELHRELEHLQKQ
KEEISIRGDALVQERMGLCLEKSKYDNEKAHAAAMTKKVGKLQNIDRLQKNNETYVRIQNFFRTLIQEKLGR
DTVQEIDVVKEAKELHELAAIIYGNTSGKSQKQRAKKQFKENVLHIAGKGQLELLEAYLNVTASQGLCRHQ
MQASFRERILLNPDGAKHGEAERTLASREEMLKTLGLSYLTPFVRFSSPESTQSGYNQILKVREQLFDIEQRLQ
NQETVSPEDYAAVQAALAAYVRKHESLIVSTYGLGAQEGQTSSKVTTLMRDLHAVEELVEMGVETYRLNRS
DQILHRVHSVLHSHLRDSDSSGNGIIDVVKKLFELLNNNGNNPNDPECQKYMQILLDAPVSLLYGAFKSFKNE
FLLNFTELNIANSTKAAEGEAKRYVEEKGRGFETYWEEAKQRLEAIAAELDDLRNQETLLEQEIRLANLKISIF
SDLNLREKVSVEKAALEEEIQGIQEQYAEMQGIEDLELKQKFEDLQKKLEALEERLLQIGRRIDSSVDKQKEL
LGLLGREEAA

>core/4/5/Org5_Gene914

MKYSLPWLLTSSALVFSLHPLMAANTDLSSSDNYENGSSGSAAFTAKETSDASGTTYTLTSDVSITNVSAITP
ADKSCFTNTGGALSFVGADHSLVLQTIALTHDGAAINNTNTALSFSGFSSLLIDSAPATGTSGGKGAICVTNTE
GGTATFTDNASVTLQKNTSEKDGAAVSAYSIDLAKTTTAALLDQNTSTKNGGALCSTANTTVQGNSGTVTFS
SNTATDKGGGIYSKEKDSTLDANTGVVTFKSNTAKTGGAWSSDDNLALTGNTQVLFQENKTTGSAAQANNP
EGCGGAICCYLATATDKTGLAISQNQEMSFTSNTTTANGGAIYATKCTLDGNTTLTFDQNTATAGCGGAIYT
ETEDFSLKGSTGTVTFSTNTAKTGGALYSKGNSSLTGNTNLLFSGNKATGPSNSSANQEGCGGAILSFLESAS
VSTKKGLWIEDNENVSLSGNTATVSGGAIYATKCALHGNTTLTFDGNTAETAGGAIYTETEDFTLTGSTGTV
TFSTNTAKTAGALHTKGNTSFTKNKALVFSGNSATATATTTTDQEGCGGAILCNISESDIATKSLTLTENESLS
FINNTAKRSGGGIYAPKCVISGSESINFDGNTAETSGGAIYSKNLSITANGPVSFTNNSGGKGGAIYIADSGELS
LEAIDGDITFSGNRATEGTSTPNSIHLGAGAKITKLAAAPGHTIYFYDPITMEAPASGGTIEELVINPVVKAIVPP
PQPKNGPIASVPVVPVAPANPNTGTIVFSSGKLPSQDASIPANTTTILNQKINLAGGNVVLKEGATLQVYSFTQ
QPDSTVFMDAGTTLETTTTNNTDGSIDLKNLSVNLDALDGKRMITIAVNSTSGGLKISGDLKFHNNEGSFYDN
PGLKANLNLPFLDLSSTSGTVNLDDFNPIPSSMAAPDYGYQGSWTLVPKVGAGGKVTLVAEWQALGYTPKP
ELRATLVPNSLWNAYVNIHSIQQEIATAMSDAPSHPGIWIGGIGNAFHQDKQKENAGFRLISRGYIVGGSMTT
PQEYTFAVAFSQLFGKSKDYVVSDIKSQVYAGSLCAQSSYVIPLHSSLRRHVLSKVLPELPGETPLVLHGQVS
YGRNHHNMTTKLANNTQGKSDWDSHSFAVEVGGSLPVDLNYRYLTSYSPYVKLQVVSVNQKGFQEVAAD
PRIFDASHLVNVSIPMGLTFKHESAKPPSALLLTLGYAVDAYRDHPHCLTSLTNGTSWSTFATNLSRQAFFAE
ASGHLKLLHGLDCFASGSCELRSSSRSYNANCGTRYSF

>core/5/5/Org5_Gene432

MKQKVKRNFAIIICVFALALYYVLPTCLYYAKPLDKKIDGNEAEHIIKSFTKQAQQVRKDVIPRVSAILSSLHL
RGHIQQHPAIPDIVSVRFKRGEDAEDFIGNLVHGEPNVPIKSARLHVVGYSREHDDHVIQVASSINTSLVESDF
SFVSYSSENEQEMASSILQRVYSACTFPKQKDCSCSYPSIWETAPKEQLLQYAKNLSSGFEVFSSRLSAFCQQS
FSSNQDRLAFLSRLSSLSNDAAIDVEDQKLLKSVYETLSQTACIRSLDCPYIEGLRLDCSESSLFFSSIEYCPKER
KIFLTLHSDLLAQRTSLSKEQRLDFDSRLAVEKQKLSKNLTVQVEDYNNGFSFQWMDKDTQGKIILQGERLL

QGIAEHLTALTLHRPAAESCDLIPENFPVFCRQPRESEAFGCYIFSPNTDCKHFSKGSVYILLKGLRSIVAKYQQ
GGGKELQSFEKDLQNLYNCFSHTEAISWTLGEDQVLEIRHPLQQFLDVWGEGFVIGKEGCAFLEVKDIQDRL
ATVNQIEKNRQSDLVRWHEQYRHAKCSMDLQERLSAPIPYQNLFLENMKLNMRKFSRGENILRLGIDFVGG
RQLLLSFKDHQGKQLTDKEDILKVSDELCARLNKLGVSEIELRREGDYIHLSVPGSSTISSSEILGTSKMSFHVV
NERFSSYSASRYEVQRFLDYLWFTSQAQGKTSPEEINTFASALFNEEVDVPPSVHEAITKLKSEGLAFSPSGCE
TPSTDLDTTFSMIAIGKDAEQKANPLVIVFRNYALDGASLKDIRPEFAAGEGYVLNFSVKDTSPKKMAEKLSP
TESFHTWTSAYCQEGISGTANGQYSANRGWRMAVVIDGYMVSSPILNVPLKNHASVSGKFTHREVSKLASD
LKSGAMSFVPEVLSEETISSDLGKKQCTQGIISACCGLAMLIVLMSVYYRFGGVIASGAVLLNLLLIWAALQY
LDAPLTLSGLAGIVLAMGMAVDANVLVFERIREEFLLSQSLKKSVEKGYTKAFGAIFDSNLTTVLASALLFFL
DTGPIKGFALTLILGIFSSMFTALFMTKFFFMLWMNKTQHTQLHMMNKFVGIKHDFLRGCKKLWAVSGSVF
LLGCVALGFGAWNSVLGMDFKGGYAFTFNPKEHGISDVAQMRGKVVHKLQEAGLSSRDFRIQTFGSSEKIKI
YFSDKALSYTKADTSLSPKINDHELALAVGLLSETGLDFSTETLNETQNFWSKVSSKLSKKMRYQATIGLLGA
LAIILLYVSLRFEWQYAFSAVCALIHDLLATCAVLFIAHFFLKKIQIDLQAIGALMTVLGYSLNNTLIIFDRIRED
RQANLFTPMHVLVNDALQKTFSRTVMTTATTLSVLLMLLFIGGSSVFNFAFIMTIGILLGTLSSLYIAPPLLLF
MVRKENRSK

>core/6/5/Org5_Gene1029

MFGENSRDIGVLSKEGLFDKLEIGIASDITIRDKWSCGEIKKPETINYRTFKPEKGGLFCEKIFGPTKDWECCCG
KYKKIKHKGIVCDRCGVEVTLSKVRRERMAHIELAVPIVHIWFFKTTPSRIGNVLGMTASDLERVIYYEEYVV
IDPGKTDLTKKQLLNDAQYREVVEKWGKDAFVAKMGGEAIYDLLKSEDLQSLLKDLKERLRKTKSQQARM
KLAKRLKIIEGFVSSSNHPEWMVLKNIPVVPPDLRPLVPLDGGRFATSDLNDLYRRVINRNNRLKAILRLKTPE
VIVRNEKRMLQEAVDALFDNGRHGHPVMGAGNRPLKSLSEMLKGKNGRFRQNLLGKRVDYSGRSVIIVGPE
LKFNQCGLPKEMALELFEPFIIKRLKDQGSVYTIRSAKKMIQRGAPEVWDVLEEIIKGHPVLLNRAPTLHRLGI
QAFEPVLIEGKAIRIHPLVCAAFNADFDGDQMAVHVPLSVEAQLEAKVLMMAPDNIFLPSSGKPVAIPSKDM
TLGLYYLMADPTYFPEEHGGKTKIFKDEIEVLRALNNGGFIDDVFGDRRDETGRGIHIHEKIKVRIDGQIIETTP
GRVLFNRIVPKELGFQNYSMPSKRISELILQCYKKVGLEATVRFLDDLKDLGFIQATKAAISMGLKDVRIPDIK
SHILKDAYDKVAIVKKQYDDGIITEGERHSKTISIWTEVSEQLSDALYVEISKQTRSKHNPLFLMIDSGARGNK
SQLKQLGALRGLMAKPNGAIIESPITSNFREGLTVLEYSISSHGARKGLADTALKTADSGYLTRRLVDVAQDV
IITEKDCGTLNHIEISAIGQGSEELLPLKDRIYGRTVAEDVYQPGDKSRLLAQSGDVLNSVQAEAIDDAGIETIK
IRSTLTCESPRGVCAKCYGLNLANGRLIGMGEAVGIIAAQSIGEPGTQLTMRTFHLGGIAATSSTPEIITNSDGIL
VYMDLRVVLGQEGHNLVLNKKGALHVVGDEGRTLNEYKKLLSTKSIESLEVFPVELGVKILVADGTPVSQG
QRIAEVELHNIPIICDKPGFIKYEDLVEGISTEKVVNKNTGLVELIVKQHRGELHPQIAIYDDADLSELVGTYAI
PSGAIISVEEGQRVDPGMLLARLPRGAIKTKDITGGLPRVAELVEARKPEDAADIAKIDGVVDFKGIQKNKRIL
VVCDEMTGMEEEHLIPLTKHLIVQRGDSVIKGQQLTDGLVVPHEILEICGVRELQKYLVNEVQEVYRLQGVD
INDKHIEIIVRQMLQKVRITDPGDTTLLFGEDVNKKEFYEENRRTEEDGGKPAQAVPVLLGITKASLGTESFIS
AASFQDTTRVLTDAACCSKTDYLLGFKENVIMGHMIPGGTGFETHKRIKQYLEKEQEDLVFDFVSETECVC

>core/7/5/Org5_Gene52

MLKCPERVSVKKKEDIPDLPNLIEIQIKSYKQFLQIGKLAEERENIGLEEVFREIFPIKSYNEATVLEYLSYNLGV
PKYSPEECIRRGITYSVTLKVRFRLTDETGIKEEEVYMGTIPLMTDKGTFIINGAERVVVSQVHRSPGINFEQEK
HSKGNILFSFRIIPYRGSWLEAIFDINDLIYIHIDRKKRRRKILAITFIRALGYSSDADIIEEFFTIGESSLRSEKDFA

LLVGRILADNIIDEASSLVYGKAGEKLSTAMLKRMLDAGIASVKIAVDADENHPIIKMLAKDPTDSYEAALKD
FYRRLRPGEPATLANARSTIMRLFFDPKRYNLGRVGRYKLNRKLGFSIDDEALSQVTLRKEDVIGALKYLIRL
KMGDEKACVDDIDHLANRRVRSVGELIQNQCRSGLARMEKIVRERMNLFDFSSDTLTPGKVVSAKGLASVL
KDFFGRSQLSQFMDQTNPVAELTHKRRLSALGPGGLNRERAGFEVRDVHASHYGRICPIETPEGPNIGLITSLS
SFAKINEFGFIETPYRIVRDGIVTDEIEYMTADVEEECVIAQASASLDEYNMFTEPVCWVRYAGEAFEADTSTV
THMDVSPKQLVSIVTGLIPFLEHDDANRALMGSNMQRQAVPLLKTEAPVVGTGLECRAAKDSGAIVVAEED
GVVDFVDGYKVVVAAKHNPTIKRTYHLKKFLRSNSGTCINQQPLCAVGDVITKGDVIADGPATDRGELALG
KNVLVAFMPWYGYNFEDAIIISEKLIREDAYTSIYIEEFELTARDTKLGKEEITRDIPNVSDEVLANLGEDGIIRI
GAEVKPGDILVGKITPKSETELAPEERLLRAIFGEKAADVKDASLTVPPGTEGVVMDVKVFSRKDRLSKSDDE
LVEEAVHLKDLQKGYKNQVATLKTEYREKLGALLLNEKAPAAIIHRRTAEIVVHEGLLFDQETIERIEQEDLV
DLLMPNCEMYEVLKGLLSDYETALQRLEINYKTEVEHIREGDADLDHGVIRQVKVYVASKRKLQVGDKMA
GRHGNKGVVSKIVPEADMPYLSNGETVQMILNPLGVPSRMNLGQVLETHLGYAAKTAGIYVKTPVFEGFPE
QRIWDMMIEQGLPEDGKSFLYDGKTGERFDNKVVIGYIYMLKLSHLIADKIHARSIGPYSLVTQQPLGGKAQ
MGGQRFGEMEVWALEAYGVAHMLQEILTVKSDDVSGRTRIYESIVKGENLLRSGTPESFNVLIKEMQGLGL
DVRPMVVDA

>core/8/5/Org5_Gene526

MTWIPLHCHSQYSVLDAMSSIKDFVAKGQEFGIPALALTDHGNLYGAVDFYKECTQKGIQPIIGCECYIAPGS
RFDKKKEKRSRAAHHLILLCKNEQGYRNLCILTSLAFTEGFYYFPRIDKDLLRQYSEGLICLSGCLSSSVSDAA
LKSPEALLLELQWFQDLFKDDYFTEVQLHKMSEESIAGFKEEWLKQEYYSLIEKQIKVNTAVLEASKRLGIPT
VATNDIHYINANDWQAHEILLNVQSGETVRIAKQNTHIPNPKRKVYRSREYYFKSPAQMAELFKDIPEVISNT
LEVAKRCDFTFDFSKKHYPIYVPESLKTLNSYTEEDRYQASAVFLKQLAEEALPKKYSSEVLAHIAKKFPHRD
PIDIVKERMDMEMAIIIPKGMCDYLLIVWDIIHWAKANGIPVGPGRGSGAGSVLLFLLGITEIEPIRFDLFFERFI
NPERLSYPDIDIDICMAGRERVINYAIERHGKDNVAQIITFGTMKAKMAVKDVGRTLDMALSKVNHIAKHIPD
LNTTLSKALETDPDLHQLYINDAESAQVIDMALCLEGSIRNTGVHAAGVIICGDQLTNHIPICISKDSTMITTQY
SMKPVESVGMLKVDLLGLKTLTSINIAMSAIEKKTGQSLAMATLPLDDATTFSLLHQGKTMGIFQMESKGMQ
ELAKNLRPDLFEEIIAMGALYRPGPMDMIPSFINRKHGKEIIEYDHPLMESILKETYGIMVYQEQVMQIAGALA
SYSLGEGDVLRRAMGKKDFQQMEQEREKFCKRACNNGIDPELATVIFDKMEKFAAYGFNKSHAAAYGLITY
TTAYLKANYPKEWLAALLTCDSDDIEKIGKLIREAQSMGIPILPPHINVSSNHFVATDEGIRFAMGAIKGIGRG
LIESIVEERDHHGPYESIRDFIQRSDLKKVSKKSIESLIDAGCFDCFDSNRDLLLASVEPLYEAIAKDKKEAASG
VMTFFTLGAMDRKNEVPICLPKDIPTRSKKELLKKEKELLGIYLTEHPMDTVRDHLSRLSVVLAGEFENLPHG
SVVRTVFIIDKVTTKISSKAQKKFAVLRVSDGIDSYELPIWPDMYEEQQELLEEDRLIYAILVLDKRSDSLRISC
RWMKDLSIVNENIIYECDQAFDRIKNQVQKMSFTMSTSGKETKAKGNKPNENGHTQALAPVTLSLDLNELR
HSHLCILKKIVQKHPGSRTLVLVFTQDNERVASMSPDDAYFVCEDIEELRQELVTADLPVRVITV

>core/9/5/Org5_Gene675

MVLEALAIFRQDAMQHLLKHRKEIVVDFCEDSYTIRIPDEEAPEGYWLSTLKLQDIDRLTFASCSCPDGECCL
HLMTAYFAVYDALGLHPLHDKFRHSFWYAVFSHFFLDSIPLQAQGEMVYTLESPHITLTIECLSEEVFQDWLR
TIHASEEPTVFTNKTFLKSALYRTAKKFFFLNEEGAELTIGENSQGFPSHFSLQWQGLVFKAEILDFPTLEDIFP
KLELAHTSLENVSHDISITNVTVCAEEAKVNFTLSPVIHKKDRENHPKTRIGSVEYVAKTHEMITGPKAIALPI
YAIPLLADKFKDQLLSLLCYDSLEYRLRYDIRLLRDASFSFSAYLVTPGDLDNGSLIYPNYCYSPTKGLMQVV

GMLSPKQAFIVKSEQVEDFLNERGHLIQEPGFQTFINERPEGHLTYNVTEQGVLLFHYDVGDPSSTEIRFGTWT
YYTNQGFFLEKKNDLPIQDGLIVEPQDIPAFIVKNDAALRRLPNFFSSPPNLKDLLIEVHRQSRGKGLDLKPILV
GLGESRCWLFGVFLYREDIGFSLIPTPLQGLCFLPRVIPPENVPQFLTQYAQHERILFPNPQTRPPESYELVIQSI
HRPHPASPLHLQLELKTNLGSVPIGIALQGLKSKHTFLFTQAGFLDLKQNLFQFLKQFLSTQKCVIAENTVIANI
TDVFKLDALAPLSVTDDTIANPEDLQFFSQLKAACLPPIPQNLFSSDHQLRPYQNSGLLWMWFLYNHRLSGLL
CDEMGLGKTHQATALLDIVFQSSQPSARPKFLIVCPTSVLPHWEHILSNHLPGVSIFSFHGPNKPSELPPADILL
TSYGTLRQNYDKFYKIAFTIVVFDEIHMAKNKSSQIHKILCRIDAQMKLGLTGTPIENNLLEFKGLLDIILPNYL
PSDALFKKLFTKRCSSEELEEIIPSQDLLLKLTRPFILRRTKKLVLPELPDKVESIIACSLSPDQEKLYMATLQRE
KSHIQKLETPEEPATNFLHIFALLNHLKQICDHPAVFFKDPDQYKNYESGKWNAFVKLLKESLNAGYKVVVF
SQYIHMIRIITLYLEEIGIKYASIQGKSLNRKEEIETFTTDPNCQVFVGSLLAAGTGINLTAGNVVIMYDRWWN
PAKENQALDRVHRIGQKNTVFIYKLITEDTLEERIHYLIEKKIRLLDKVIASQDSNILHMLNREDLLTILSYKDE
HGTSDSEESPVDAPVEDDTGVLPPEDS

>core/10/5/Org5_Gene686

MLNFRKLRRDFSANILQDGKKLFEQGAVIDAKILSMNGETVCISAQVRGLYDNIYECEIEVDRSESDTVDSNC
DCSYNYDCQHIVALLFYLEQYFNEMVVAYARSADLETDHEINEEVKKELKETFVAAATKEEERKDREHQKEI
LREYVHAANALSANPFFLPLEYLEKDSAELAVLFVSVNEDTFAPANQPIEFQLVLRLPCRSKPFYISNIRTFLEG
VLYQEPIVLNGRRFFFTMQSFNASDRKLIDLLIRYVRYPNHTTEEKLLKSAYLMPPALGVILAKMFEHQLADR
GGGSLGEKESFSGLFCGNLEEPLCWSLTPAKMKFNLDFFDMPYKALLMTPVILVDDDEVQPEQTMLLESDAP
GIIHHFVYHRFSPQIKRAHLRSFSRLRDIAIPEALFGSFRENALPVFQEYAEIANVHLLNSFVTLPYVDEVRAIC
DMSYLDGELEAKLHFLYGSLRVPAASLALQYQDVRAFISDEGILARNLVEERKMLEEVFSGFIYDERDGAFR
VKSEKKIVEFMTETIPANQHRITFNCPENLSGQFIYDETIFELSFREGSDINYYEADLKVHGLLKGVPLDLLWD
CISAKKRFLELPKAGQQSKGTRRGKVNSGKLPCILVLDLEKIAPVVQIFNEIGFKVLDDLVQKCPLWSLTGISL
DQFEALPVNFSMSERLIEIQKQIRGEIEFDFQDVPQQIQATLRSYQTEGVHWLERLRKMHLNGILADDMGLGK
TLQAIIAVTQSKLEKGSGCSLIVCPTSLVYNWKEEFRKFNPEFRTLVIDGVPSQRRKQLTALADRDVAITSYNL
LQKDVELYKSFRFDYVVLDEAHHIKNRTTRNAKSVKMIQSDHRLILTGTPIENSLEELWSLFDFLMPGLLSSY
DRFVGKYIRTGNYMGNKADNMVALKKKVSPFILRRMKEDVLKDLPPVSEILYHCHLTESQKELYQSYAASA
KQELSRLVKQEGFERIHIHVLATLTRLKQICCHPAIFAKDAPEPGDSAKYDMLMDLLSSLVDSGHKTVVFSQY
TKMLGIIKKDLESRGIPFVYLDGSTKNRLDLVNQFNEDPSLLVFLISLKAGGTGLNLVGADTVIHYDMWWNP
AVENQATDRVHRIGQSRSVSSYKLVTLNTIEEKILTLQNRKKSLVKKVINSDDEVVSKLTWEEVLELLQI

>core/12/5/Org5_Gene878

MKRPKKFPIYLSIAQKTNRLLSGIVIAFAVIALRLWYLAVVEHEQKLEEAYKPQIRVLPQYVERATICDRFGKT
LAVNQLQYDVSVAYGAIRDLPTRAWRVDEHGHKQLIPVRKHYIMCLSELLSQELHLDREAIEDAIHAKASVL
GSVPYLVAANVSERTYLKLKMLSKDWPGLHVEAVVRRHYPQESVASDILGYVGPISLQEYKRVTQELSQLR
ECVRAYEEGEDPKLPEGLASIDQVRALLESVESNAYSLNALVGKMGVEACWDSKLRGKIGKKPILVDRRGNF
IQEMEGAVPEAPGTKLQLTLSAELQAYADALLLEYEKTETFRSAKSLKKREKLPPLFPWIKGGAIIALDPNNG
EILAMASSPRYRNNDFVNAKVAEDSKAVRSSIYRWLENKEHIAEIYDRKVPLIRERRNPLTGLCYEEILPLTFD
CFLDFLFPENSVIKLQLKRNSFVGQAIEVQNLVTRLLSLFPYEEGTCPCSAIFDAVFPNEEGHILIQEVISLXEQK
WIMECLNQHKADIEELKEALDQVFNELPANYDKILYTDILRLIVDPERFSPVLPSEVHRLSLSEFTELQGRYVV
LRSAFSTILEDAFIEVHFKSWRKSEFLQYLAAKRQEEALRKQRYPTPYVDYLEEEKTRQYKMFCQEHLDTFL

AYLFSKTPYKEGLEPYYDILDLWINELDNGAHRALSWNEHYLFLKERVSHLSEHLPALFSTFREFNELQRPLL
GKYPISIVRNKRQTEQDLAASFYPVYGYGYLRPHAYGQAATLGSIFKLVSAYSVLSQRILWGHNEEPANPLVI
IDKNSFGYRSSKPHVGFFKDGTPIPTFFRGGSLPGNDFMGRGFIDLVSALEMSSNPYFSLLVGEGLGDPEDLAD
AASLFGFGEKTGLGLPGEYAGRVPHDLAYNRSGLYATAIGQHTLVVTPLQTAVMLASLVNGGVVYVPKLLL
GEWEGEHVSYLSSKKKRTIFMPDAVVEVLKTGMRNVIWGQYGTARAIQSQFPPQLLSRIIGKTSTAESIMRVG
LDREYGTMKMKDIWFAAVGFSDQDLSLPTIVVIVYLRLGEFGRDAAPMAVKMIDMWEKIQQRESFLRG

>core/13/5/Org5_Gene721

MAMDFNPVNLDFSISKEFKEETLPLLLENIHPGATAFLAAKMFHDCRASVIMITTPARLDDLFENLRTFLDQA
PVEFPSSEIDLSPKLVNIDAVGKRDHLLYSLNQHRAPIFCVTTLKALLEKTRSPQATSQQHLDLAVGDVLDPE
ATTELCKSLGYSQVMLTSEKGEFSCRGGIVDIFPLSSPEPFRIEFWGEKIISIRSYNPSDQLSTGKVSKISISPAYT
EEASGGNYSHSLLDYFSTPPLYLFDNLEILEDDFADISGTLSSLPDRFFSIGTLYDRISTSNQVYFSETPFPNVKN
LKENRVIIEAFHRNMEASRQAIPILYPEQIIQNDENPLLAFLQHLQEYMPPHGKPLKLAIYSTKTKSLKEARAL
AETVARGDVEIYEKTGNLTSSFALVNEAFAAISLSEFASTKVLRRQKQRTHFSVTTEEVFVPIPGETVVHIHNG
IGKFLGIEKKPNHLNIETDYLVLEYADKARLYVPSNQAYLISRYVGTSDKAADLHHLNSSKWKRSRDLTEKS
LIVYAEKLLQLEAQRSTTPAFVYPPHGESVIKFAETFPYEETPDQLKTIDQIYNDMMSPKLMDRLICGDAGFG
KTEVIMRAAVKAVCDGHRQVIVMVPTTILATQHYETFKERMAGLPIEIAVLSRFSQAKVQKLICEQVASGQID
IIIGTHKLINKSLEFKNPGLLIIDEEQRFGVKVKDNLKERYPMIDCLTVSATPIPRTLHMSLSGARDLSVIAMPPL
DRLPVSTFVMEHNTETLTAALRHELLRGGQAYVIHNRIESIYTLAETIRNLIPEARIGVAHGQMGAEDLSNIFT
KFKNQKTDILVATALIENGIDIPNANTILIDHADKFGMADLYQMKGRVGRWNKKAYCYFLVPHLDRLSGPA
AKRLAALNKQEYGGGMKIALHDLEIRGAGNILGTDQSGHIGTIGFNLYCKLLKKAVSALKKHTSPLLFNDDV
KIEFPYNSRIPDTYIETGSMRIEFYQKIGNAESSEELTAIQEEMRDRFGPLPQEICWLFALAEIRLFALQHGISSIK
GTANALYVQKCLSKSEQTKKTLPYALSPTPELLVKEVIESIERGFLINAS

>core/14/5/Org5_Gene1031

MIKKRAIFERMFPIPPPHCPPNNKNNFYHLTTDTKDPLLLRILRTIGYVLLHIITLGLLLLIHYYKHHRVVRKEG
LPTPPTLPKGPEPKTIEIAKQPPKDGEDKKPDVPKPGTPPPEDTPPPPPKAPSPASPKVPKQPADKKPTPPPEAPP
PPVRVATPMPLRPSSQGYWQCLNRMVSMVLRRAPLPLPAMQVDPILGDFNPHFVASYPNRIDNEPMYFQIKQ
FKKIAQNPDLPQQHRRLAQLSLEQALYLNDNYYLVNVPGDGNCFYRAYAVGWLSALYEESSRNDIVFEQEA
TRLLDLPFASSSPANANLCAEMAELLQLCSTYCSFIDLYDGVILSQKHTATLIAFLRKLSAYAIRQQIAASSNEE
TARALFISDMQDDLLPSVLEFLAANRPYSELFQNLIDHSALPYMQSRDKLFLLLEHLPALFLTDAELQKMSPE
DQQLRKQYEREIREAFAKLSRRIADSGWDTERFNAIVKDYLPEAIRCQYSRFLATIENRRSGDLPWSPALSFFA
FLCTCPSVRFHKLCATFYKSLEDIIIASAPPQRSIQEILQISNASLSYLNEDLDSSWQREVISSNIMTILTTHESLTL
ESSMPQLETLHKRIANLLKNVISTSFETPPLSNQPDLLSNLVNKLLVAIHSKLELKEHFNTVCSARSLRLTRDE
GSGLSQEQDLLYTQAVQLLFFILQHPQVNNRPETKDAVKELKMLLLPFLQYAFKKVENEKKLQKLLRSILGS
LVLKPPARYPSTPSNKDKETFCKFWSRHPEVMVLDPILEKNCMQFLRATFPNYQLETEAILLEKEIESTFRNG
WNVFLTRLNLFGSKLGSPSSPTALSDQFSKSFLIFCFLNNYPKLLQKKTPLAARLDAFQREASHRFTQVKDKL
LLSLKYGFPLATATINQYSRARDQLICNLLKNTVTASDGFCRSGFRQSLIGYLHSLSSNELGDILDDVKEQAEA
NDVAAMTTVPLQPFAVCLIMSDRDTVSEENIENFVAMHGFLNTISPERDARIFLIRFPNHYGCLLPRNPRTEDQ
NSKPDSSNP

>core/15/5/Org5_Gene875

MKPFNIFDSNSSIQGKFFLEASAGTGKTFTIEQIVLRALIEGSLTHVEHALAITFTNASTNELKVRIKDNLAQTL
RELKAVLNSQPASLPTYLDINCNVKQIYMQVRNALATLDQMSLFTIHGFCNFVLEQYFPKTRLIHKKPALTHS
QLVLHHITNYLKQDLWKNVLFQEQFHLLAVRYNVTSKHTSSLVDKLLASYTQPISSYFSSRVERLEQISLWHQ
QIYNSLLEIPKQVFLDQLTAHISGFKKQPFSILDDLHHFVDLLYTSETHSSLFSFFKIAETFNFKHRLARYKPCA
AFTVLENMSWVERTLEFCNLDRIFNTLLVDLQEYLKQNYTPWLSPDESVFALEKLLSSSEAQPVVQALREQY
QLVLIDEFQDTDKQQWSIFSNLFISPKFTGSLFLIGDPKQSIYEWRSADLPTYLTAKSSFSEDKQLQLVNNYRST
PKLMEAINQIFGKISPFLEIPGYLPIEYHALNPQSSETFENPPHAPIHFFFYETIKDQALWIFSEALRLQKEQKIPL
GNMVVLVSDSNQAFELISYATIPVSFSKNKSIFHLTETHILTTALLEAILHPENYEKISKILFSSLFGLSLDEVTTK
KEDFTIYFQSLHSYISHHGLLATFYRVMTTQGNVLFSSPRGDLIFQEMEKLCGYLDTISSYPYHQLLHLKNFSE
TGRWEEELAISSYSEDLETLKITTIHSSKGLEYDIVFCPGIEKSKKNKSSSELLREMYVACTRAKKQLYLPISTQ
PPSLQRSSALTNYVKLEGTQSSAYDLAIHLHQEHPDLFSYSLPKDHGHATTVLNLPLLETFALKVTPPKTIFSFS
STKFLLDTHKDSQSIPYSKLPISKQQLPLGEKTGILIHKILESIQFSLLQDTEYLMSTIMRFIKHTHLEGFEETILK
LLSKTFFSPLTFSSQTFSLSQVLPNKIFRETSFLFLENQELWQGVIDLFFEHEGKYYIIDWKTSFLGETNSDYSKS
NLSIYIKQEKLDYQGRIYVKAVRKFLNQFEIDDDVELGVIFIRGIDTQGNGFFALNSSEDIPNFNPKAIQKCQAY
H

>core/16/5/Org5_Gene796

MVEVEEKHYTIVKRNGMFVPFNQDRIFQALEAAFRDTRSLETSSPLPKDLEESIAQITHKVVKEVLAKISEGQ
VVTVERIQDLVESQLYISGLQDVARDYIVYRDQRKAERGNSSSIIAIIRRDGGSAKFNPMKISAALEKAFRATL
QINGMTPPATLSEINDLTLRIVEDVLSLHGEEAINLEEIQDIVEKQLMVAGYYDVAKNYILYREARARARANK
DQDGQEEFVPQEETYVVQKEDGTTYLLRKTDLEKRFSWACKRFPKTTDSQLLADMAFMNLYSGIKEDEVTT
ACIMAARANIEREPDYAFIAAELLTSSLYEETLGCSSQDPNLSEIHKKHFKEYILNGEEYRLNPQLKDYDLDAL
SEVLDLSRDQQFSYMGVQNLYDRYFNLHEGRRLETAQIFWMRVSMGLALNEGEQKNFWAITFYNLLSTFRY
TPATPTLFNSGMRHSQLSSCYLSTVKDDLSHIYKVISDNALLSKWAGGIGNDWTDVRATGAVIKGTNGKSQG
VIPFIKVANDTAIAVNQGGKRKGAMCVYLENWHLDYEDFLELRKNTGDERRRTHDINTASWIPDLFFKRLEK
KGMWTLFSPDDVPGLHEAYGLEFEKLYEEYERKVESGEIRLYKKVEAEVLWRKMLSMLYETGHPWITFKDP
SNIRSNQDHVGVVRCSNLCTEILLNCSESETAVCNLGSINLVEHIRNDKLDEEKLKETISIAIRILDNVIDLNFYP
TPEAKQANLTHRAVGLGVMGFQDVLYELNISYASQEAVEFSDECSEIIAYYAILASSLLAKERGTYASYSGSK
WDRGYLPLDTIELLKETRGEHNVLVDTSSKKDWTPVRDTIQKYGMRNSQVMAIAPTATISNIIGVTQSIEPMY
KHLFVKSNLSGEFTIPNTYLIKKLKELGLWDAEMLDDLKYFDGSLLEIERIPNHLKKLFLTAFEIEPEWIIECTS
RRQKWIDMGVSLNLYLAEPDGKKLSNMYLTAWKKGLKTTYYLRSQAATSVEKSFIDINKRGIQPRWMKNK
SASTSIVVERKTTPVCSMEEGCESCQ

>core/17/5/Org5_Gene962

MTADEVGKNSFAKKEEQVLKFWKDNQIFEKSLQNRQGKTLYSFYDGPPFATGLPHYGHLLASTIKDVVGRY
ATMDGYYVPRRFGWDCHGVPVEYEVEKSLSLTAPGAIEDFGIASFNEECRKIVFRYVHEWEYYINRIGRWVD
FSSTWKTMDASFMESVWWVFQSLYNQGLVYEGTKVVPFSTALGTPLSNFEASQNYKEVDDPSLVVRMPLQ
NDSASLLVWTTTPWTLPSNMAIAVGETLVYVRIQDKKSGEQWILSQGCVSRWFSNPEEFVILESFSGKDLVG
RTYEPPFTFFQSKREEGAFRVIAASFVEESEGTGVVHMAPAFGEGDFLVCKENHVPLVCPVDAHGSFTEEIPQ

YQGQYIKHADKEIIKFLKKEGRIFYYGTVKHRYPFCWRTDTPLIYKAVNSWFVAVEKIKDKMLRANSSIHWV
PEHIQEGRFGKWLEGARDWAISRNRYWGTPIPIWKSADGEILVVGSIRELEELTGTQITDIHRHFIDDLNIVKD
GKPFHRIPYVFDCWFDSGAMPYAQNHYPFENQKETEEAFPADFIAEGLDQTRGWFYTLTVISAILFDRPAFRN
AIVNGIILAEDGNKMSKRLNNYPSPKYVLDTYGADALRLYLLHSVVVKAEDLRFSDKGIEGVLKQILLPLTNV
LSFFNTYAELYGFDPKSQDIEPAYTEIDQWILSNLYSVVGKVRESMSQYHLNFAVEPFVTFIDDLTNWYIRRC
RRRFWEAEDTPDRRAAFSTLYEVLTVFCKVIAPFVPFLAEDIYQKLKLEKEPESVHLCDFPQVEMDKILPDLE
KXMHDIREIVGLGHSLRKEHKLKVRQPLANFYVVGSKDRLSLLKTFEGLIAEELNVKNVIFYEEAPSFIYTTV
KPNFRMLGKKVGSKMKEVQKALSELPNNAIDKLIQEETWVLTIDDREIALDGDDVVICRHTDPGYIARSSALF
SVILDCQLREPLIVEGIARELVNKINTMRRNQQLHVSDRIALRIKTTEAVHRAFLDYENYICEETLIIAYDFTQD
SDFQGENWDINGHATQIEITVSSIDS

>core/18/5/Org5_Gene876

MNATKHCRASFSNSPRHLLAQLAEDITSTHQKPFTKRWILVANATTGHWIKNQLVHVLSDHIFMGSTIFTASD
SIVKHLFLGSGCSQPNIPDYLTLPLLINNILEEISKASKFENGREFLSPPTYETTKKLAAAFKQFHTFSQRPTKNA
SHYQELFQILESHFSSYEEMFTTILNNRTQEEDCSLHIFGYAHLPKHLAEFFINLSTYFPVYFYCFSPCREYFGD
LLSDRAIDFFWNQLPDSPIKNAWEHYVLSDRQALLANLAHKSQSSQNFFLDREIDYQEMFLPSKHDSSLGVIQ
NSILDLKPTSPQDFSQTKQTICIYRALNIPREVQEVFCKVTELLHRGVSPEEIFILSSHIESYKVHLNAIFNPHVPI
YFTDEVDPRAEDLRNKILLLSSILQTQGDLHYILQLLTHPQLQQPIDQNKVPYLIKKLSSEWGKISSKDRASGQ
QMKALGDLILEEYPFHQEGGRVSQVEVWETTVPLIYFIQERINLYLSSSQHSYEDLFQNVFSCLEKIFVLSPEET
SFITTLRNSLFPTFATSSCSLLFFTDFCLDFLLHFHKPSPLYDKPGPYIGSLSSLSLIPKGYVFILGANKTTSSDIFD
LLNRTTTHEELAFSSTEDEENFHFLQILVSTKHELHISYISSAAQFNLPSPFLNHIKETLDLPVETLPTQPYLSAFF
KNKACLHTSQEYNYSLAHAFYSKKALLPSLFIPTVKQVNLPQHLSLNEIIKGIFSPLDLFLKTNYNLRISYPEHL
KKQQKLFPTKHQIEDFWNECFVDKEHDLIPSISPHAEELFTYYREKTILLRNGLDKDPKHSPYTVTFSSSIFEER
PYHESYLFPPLSLSFQGNPVQIHGTIHGVCNEGLYLCSIDPGDSLKKTTRTLGSLPETSSEQKQLLERYVALAV
LQMSQHLSSDSALIKLTSFNXKENHHPPFSDPEXYLRKVLEVYHLMSSQPIPLLSPLCWKTLDDEEKFHQAVL
SAISEEAKNPSLPIFWQFHNRNIEEILNHVGASERLKILSLFRGPCEAV

>core/19/5/Org5_Gene1037

MESFVSEHPLTLQSMIATILRFWSEQGCVIHQGYDLEVGAGTFNPATFLRALGPEPYKAAYVEPSRRPQDGRY
GVHPNRLQNYHQLQVILKPVPENFLSLYTESLRAIGLDLRDHDIRFIHDDWENPTIGAWGLGWEVWLNGMEI
TQLTYFQAIGSKPLDTISGEITYGIERIAMYLQKKTSIYDVLWNDTLTYGQITQASEKAWSEYNFDYANTEMW
FKHFEDFAEEALRTLKNGLSVPAYDFVIKASHAFNILDARGTISVTERTRYIARIRQLTRLVADSYVEWRASLN
YPLLSLSSTSEPKETSESVVPMISSTEDLLLEIGSEELPATFVPIGIQQLESLARQVLTDHNIVYEGLEVLGSPRRL
ALLVKNVAPEVVQKAFEKKGPMLTSLFSPDGDVSPQGQQFFASQGVDISHYQDLSRHASLAIRTVNGSEYLF
LLHPEIRLRTADILMQELPLLIQRMKFPKKMVWDNSGVEYARPIRWLVALYGEHILPITLGTIIASRNSFGHRQ
LDPRKISISSPQDYVETLRQACVVVSQKERRMIIEQGLRAHSSDTISAIPLPRLIEEATFLSEHPFVSCGQFSEQF
CALPKELLIAEMVNHQKYFPTHETSSGAISNFFIVVCDNSPNDTIIEGNEKALTPRLTDGEFLFKQDLQTPLTTF
IEKLKSVTYFEALGSLYDKVERLKAHQRVFSTFSSLAASEDLDIAIQYCKADLVSAVVNEFPELQGIMGEYYL
KHANLPTASAVAVGEHLRHITMGQKLSTIGTLLSLLDRLDNLLACFILGLKPTSSHDPYALRRQSLEVLTLVS
ASRLPIDLASLLDRLADHFPSTIEEKVWDKSKTIHEILEFIWGRLKTFMGSLEFRKDEIAAVLIDSATKNPIEILD

TAEALQLLKEEHTEKLAVITTTHNRLKKILSSLKLSMTSSPIEVLGDRESNFKQVLDAFPGFPKETSAHAFLEY
FLSLADLSNDIQDFLNTVHIANDDGAIRNLRISLLLTAMDKFSLCHWESVAV

>core/20/5/Org5_Gene637

MYSCYSKGISHNYLLHPMSRLDIFVFDSLIANQDQNLLEEIFCSEDTVLFKAYRTTALQSPLAAKNLNIARKV
ANYILADNGEIDTVKLVEAIHHLSQCTYPLGPHRHNEAQDREHLLKMLKALKENPKLKESIKTLFVPSYSTIQ
NLIRHTLALNPQTILSTIHVRQAALTALFTYLRQDVGSCFATAPAILIHQEYPERFLKDLNDLISSGKLSRIVNQ
REIAVPINLSGCIGELFKPLRILDLYPDPLVKLSSSPGLKKAFSAANLIETLGDSEAQIQQLLSHQYLMQKLQNV
HETLTANDIIKSTLLHYYQLQESTVRAIFFKEGLFSKEQVAFSTQHPRELSEIQRVYHYLHAYEEAKSAFIHDT
QNPLLKAWEYTLATLADASQPTISNHIRLALGWKSEDPHSLVSLVTHFVEEEVENIRILVQQCEQTYHEARSQ
LEYIEGRMRNPLNNQDSQILTMDHMRFRQELNKALYEWDSAQEKAKKFLHLPEFLLSFYTKQIPLYFRSSYD
AFIQEFAHLYANAPAGFRILFTHGRTHPNTWSPIYSINEFIRFLSEFFTSTESELLGKHAVINLEKETSRLVHNIT
AMLHTDVFQEALLTRILEAYQLPVPPSILNHLDQLSQTPWVYVSGGTVDTLLLDYFESSEPLTLTEKHPENPH
ELAAFYADALKDLPTGIKSYLEEGSHSLLSSSPTHVFSIIAGSPLFREAWDNDWYSYTWLRDVWVKQHQDFL
QDTILPQLSIYAFIENFCNKYALQHVVHDFHDFCSDHSLTLPELYDKGSRFLSSLFTKDKTVALIYIRRLLYLM
VREVPYVSEQQLPEVLDNVSSYLGISSRITYEKFRSLIEETIPKMTLLSSADLRHIYKGLLMQSYQKIYTEEDTY
LRLTTAMRHHNLAYPAPLLFADSNWPSIYFGFILNPGTTEIDLWKFNYAGLQGQPLDNIQELFATSRPWTLYA
NPIDYGMPPPPGYRSRLPKEFF

>core/21/5/Org5_Gene910

MPLSFKSSSFCLLACLCSASCAFAETRLGGNFVPPITNQGEEILLTSDFVCSNFLGASFSSSFINSSSNLSLLGKG
LSLTFTSCQAPTNSNYALLSAAETLTFKNFSSINFTGNQSTGLGGLIYGKDIVFQSIKDLIFTTNRVAYSPASVT
TSATPAITTVTTGASALQPTDSLTVENISQSIKFFGNLANFGSAISSSPTAVVKFINNTATMSFSHNFTSSGGGVI
YGGSSLLFENNSGCIIFTANSCVNSLKGVTPSSGTYALGSGGAICIPTGTFELKNNQGKCTFSYNGTPNDAGAI
YAETCNIVGNQGALLLDSNTAARNGGAICAKVLNIQGRGPIEFSRNRAEKGGAIFIGPSVGDPAKQTSTLTILA
SEGNIAFQGNMLNTKPGIRNAITVEAGGEIVSLSAQGGSRLVFYDPITHSLPTTSPSNKDITINANGASGSVVFT
SKGLSSTELLLPANTTTILLGTVKIASGELKITDNAVVNVLGFATQGSGQLTLGSGGTLGLATPTGAPAAVDFT
IGKLAFDPFSFLKRDFVSASVNAGTKNVTLTGALVLDEHDVTDLYDMVSLQSPVAIPIAVFKGATVTKTGFPD
GEIATPSHYGYQGKWSYTWSRPLLIPAPDGGFPGGPSPSANTLYAVWNSDTLVRSTYILDPERYGEIVSNSLW
ISFLGNQAFSDILQDVLLIDHPGLSITAKALGAYVEHTPRQGHEGFSGRYGGYQAALSMNYTDHTTLGLSFGQ
LYGKTNANPYDSRCSEQMYLLSFFGQFPIVTQKSEALISWKAAYGYSKNHLNTTYLRPDKAPKSQGQWHNN
SYYVLISAEHPFLNWCLLTRPLAQAWDLSGFISAEFLGGWQSKFTETGDLQRSFSRGKGYNVSLPIGCSSQWF
TPFKKAPSTLTIKLAYKPDIYRVNPHNIVTVVSNQESTSISGANLRRHGLFVQIHDVVDLTEDTQAFLNYTFDG
KNGFTNHRVSTGLKSTF

>core/22/5/Org5_Gene793

MKAGDTYRNFIIKSCKDLPEIESKLLEAEHKPTGASIMMIVNNDEENVFNICFRTCPQTSNGVAHVLEHMVLC
GSENYPVRDPFFSMTRRSLNTFINAFTGPDFTCYPAASQIPEDFYNLLSVYIDAVFHPLLTKQSFLQEAWRYEF
NSENHLCYTGVVFNEMKGAMMSGEARLSEALNAAIFPSVTYGVNSGGEPREIVTLSHEDVRAFHQSQYSINR
CLFYFYGNIKPSRHLDFLEEKLLRQATKLEKQAVSVPLQKRFKEPVRNILTYPVDHQEEDKVLFGISWLTCSIL
EQQELLALHVLEIILMGTDASPLKSRLLKSGFCKQTEMSIENDIREIPMTLVCKGCSPAGAQKLEALIFASLEEII

REGISENIVEGAVHQLELSRKEITGYSLPYGLSLFFRSGLLKQHGGSAEDGLRIHSLFSELRNSLKNSDYLAKLI
RKYFLDNPHFARVILLPDTELVAKDNKDEQQLLLSVSEKLTDENKEKIQQNVRELTESQEQKEDLNGILPNLA
LDKVPTSGKEFPLIKEGLSQGEVLHHECFTNDIVFIDVVLDIPPLSGEELPWLRLLVFLMLQLGCGGRSYKEHL
EFLLEHTGGVDVSYDFSPHANKNSFLSPSVSIRGKALSSKSEKLCGIVSDMLTSVDFTDIPRIRELLMQHNEAL
TNSVRNSPMSYAVSMACSGNSITGAMSYLTTGLPYVKKIRELTKNFDQNIDEAVVILQRLYTKCFSGKRQIVI
SGSAHNYQQLKDNKFYGLLDYLIVIPEPWENPSINLYVTSRGLHIPARAAFNALAFPIGDIAYDHPDAAALTV
AAEILDNVVLHTKIREQGGAYGSGAAANLSRGSFYCYSYRDPEIATTYKTFLKGVSEIASGNFTKEDIYEGAL
GVVQGLDMPVAPGSRASVAFYRLKSGRIPVLRQAFRRSVLEVTKEHICMVMDKYLESTVQETTLISFAGEEM
LRNNVLTLDKDFPIVPAI

>core/23/5/Org5_Gene350

MKTSIRKFLISTTLAPCFASTAFTVEVIMPSENFDGSSGKIFPYTTLSDPRGTLCIFSGDLYIANLDNAISRTSSSC
FSNRAGALQILGKGGVFSFLNIRSSADGAAISSVITQNPELCPLSFSGFSQMIFDNCESLTSDTSASNVIPHASAI
YATTPMLFTNNDSILFQYNRSAGFGAAIRGTSITIENTKKSLLFNGNGSISNGGALTGSAAINLINNSAPVIFSTN
ATGIYGGAIYLTGGSMLTSGNLSGVLFVNNSSRSGGAIYANGNVTFSNNSDLTFQNNTASPQNSLPAPTPPPTP
PAVTPLLGYGGAIFCTPPATPPPTGVSLTISGENSVTFLENIASEQGGALYGKKISIDSNKSTIFLGNTAGKGGAI
AIPESGELSLSANQGDILFNKNLSITSGTPTRNSIHFGKDAKFATLGATQGYTLYFYDPITSDDLSAASAAATV
VVNPKASADGAYSGTIVFSGETLTATEAATPANATSTLNQKLELEGGTLALRNGATLNVHNFTQDEKSVVIM
DAGTTLATTNGANNTDGAITLNKLVINLDSLDGTKAAVVNVQSTNGALTISGTLGLVKNSQDCCDNHGMFN
KDLQQVPILELKATSNTVTTTDFSLGTNGYQQSPYGYQGTWEFTIDTTTHTVTGNWKKTGYLPHPERLAPLIP
NSLWANVIDLRAVSQASAADGEDVPGKQLSITGITNFFHANHTGDARSYRHMGGGYLINTYTRITPDAALSL
GFGQLFTKSKDYLVGHGHSNVYFATVYSNITKSLFGSSRFFSGGTSRVTYSRSNEKVKTSYTKLPKGRCSWS
NNCWLGELEGNLPITLSSRILNLKQIIPFVKAEVAYATHGGIQENTPEGRIFGHGHLLNVAVPVGVRFGKNSH
NRPDFYTIIVAYAPDVYRHNPDCDTTLPINGATWTSIGNNLTRSTLLVQASSHTSVNDVLEIFGHCGCDIRRTS
RQYTLDIGSKLRF

>core/24/5/Org5_Gene680

MLGFLKRFFGSSQERILKKFQKLVDKVNIYDEMLTPLSDDELRNKTAELKQRYQNGESLDSMLPEAYGVVK
NVCRRLAGTPVEVSGYHQRWDMVPYDVQILGAIAMHKGFITEMQTGEGKTLTAVMPLYLNALTGKPVHLV
TVNDYLAQRDCEWVGSVLRWLGLTTGVLVSGTLLEKRKKIYQCDVVYGTASEFGFDYLRDNSIATRLEEQV
GRGYYFAIIDEVDSILIDEARTPLIISGPGEKHNPVYFELKEKVASLVYLQKELCSRIALEARRGLDSFLDVDILP
KDKKVLEGISEFCRSLWLVSKGMPLNRVLRRVREHPDLRAMIDKWDVYYHAEQNKEESLERLSELYIIVDEH
NNDFELTDKGMQQWVEYAGGSTEEFVMMDMGHEYALIENDETLSPADKINKKIAISEEDTLRKARAHGLRQ
LLRAQLLMERDVDYIVRDDQIVIIDEHTGRPQPGRRFSEGLHQAIEAKEHVTIRKESQTLATVTLQNFFRLYEK
LAGMTGTAITESREFKEIYNLYVLQVPTFKPCLRIDHNDEFYMTEREKYHAIVNEIATIHGKGNPILVGTESVE
VSEKLSRILRQNRIEHTVLNAKNHAQEAEIIAGAGKLGAVTVATNMAGRGTDIKLDNEAVIVGGLHVIGTTR
HQSRRIDRQLRGRCARLGDPGAAKFFLSFEDRLMRLFASPKLNTLIRHFRPPEGEAMSDPMFNRLIETAQKRV
EGRNYTIRKHTLEYDDVMNKQRQAIYAFRHDVLHAESVFDLAKEILCHVSLMVASLVMSDRQFKGWTLPNL
EEWITSSFPIALNIEELRQLKDTDSIAEKIAAELIQEFQVRFDHMVEGLSKAGGEELDASAICRDVVRSVMVMH
IDEQWRIHLVDMDLLRSEVGLRTVGQKDPLLEFKHESFLLFESLIRDIRITIARHLFRLELTVEPNPRVNNVIPT
VATSFHNNVNYGPLELTVVTDSEDQD

>core/26/5/Org5_Gene357

MSLNLERISPLLYLLDVTAKKIDTSNLIVEAMNLDEHYGYQGIWSPYWMETTTTTSSTVPEQTNTNHRQLYV
DWTPVGYRPNPERHGEFIANTLWQSAYNALLGIRILPPQNLKEHDLEASLQGLGLLINQHNREGRKGFRNHT
TGYAATTSAKTAARHSFSLGFAQMFSKTRERQSPSTTSSHNYFAGLRFDSLLFRDFISTGLSLGYSYGDHHML
CHYTEILKGSSKAFFNNHTLVASLDCTFLPARITRTLELQPFISAIALRCSQASFQETGDHIRKFHPKHPLTDLSS
PIGFRSEWKTSHHIPMLWTTEISYVPTLYRKNPEMFTTLLISNGTWTTQATPVSYNSVAAKIKNTSQLFSRVTL
SLDYSAQVSSSTVGQYLKAESHCTF

>core/27/5/Org5_Gene410

MKQMRLWGFLFLSSFCQVSYLRANDVLLPLSGIHSGEDLELFTLRSSSPTKTTYSLRKDFIVCDFAGNSIHKPG
AAFLNLKGDLFFINSTPLAALTFKNIHLGARGAGLFSESNVTFKGLHSLVLENNESWGGVLTTSGDLSFINNTS
VLCQNNISYGPGGALLLQGRKSKALFFRDNRGTILFLKNKAVNQDESHPGYGGAVSSISPGSPITFADNQEILF
QENEGELGGAIYNDQGAITFENNFQTTSFFSNKASFGGAVYSRYCNLYSQWGDTLFTKNAAAKVGGAIHAD
YVHIRDCKGSIVFEENSATAGGAIAVNAVCDINAQGPVRFINNSALGLNGGAIYMQATGSILRLHANQGDIEF
CGNKVRSQFHSHINSTSNFTNNAITIQGAPREFSLSANEGHRICFYDPIISATENYNSLYINHQRLLEAGGAVIFS
GARLSPEHKKENKNKTSIINQPVRLCSGVLSIEGGAILAVRSFYQEGGLLALGPGSKLTTQGKNSEKDKIVITN
LGFNLENLDSSDPAEIRATEKASIEISGVPRVYGHTESFYENHEYASKPYTTSIILSAKKLVTAPSRPEKDIQNLII
AESEYMGYGYQGSWEFSWSPNDTKEKKTIIASWTPTGEFSLDPKRRGSFIPTTLWSTFSGLNIASNIVNNNYL
NNSEVIPLQHLCVFGGPVYQIMEQNPKQSSNNLLVQHAGHNVGARIPFSFNTILSAALTQLFSSSSQQNVADK
SHAQILIGTVSLNKSWQALSLRSSFSYTEDSQVMKHVFPYKGTSRGSWRNYGWSGSVGMSYAYPKGIRYLK
MTPFVDLQYTKLVQNPFVETGYDPRYFSSSEMTNLSLPIGIALEMRFIGSRSSLFLQVSTSYIKDLRRVNPQSSA
SLVLNHYTWDIQGVPLGKEALNITLNSTIKYKIVTAYMGISSTQREGSNLSANAHAGLSLSF

>core/28/5/Org5_Gene904

MQNNRSLSKSSFFVGALILGKTTILLNATPLSDYFDNQANQLTTLFPLIDTLTNMTPYSHRATLFGVRDDTNQ
DIVLDHQNSIESWFENFSQDGGALSCKSLAITNTKNQILFLNSFAIKRAGAMYVNGNFDLSENHGSIIFSGNLSF
PNASNFADTCTGGAVLCSKNVTISKNQGTAYFINNKAKSSGGAIQAAIINIKDNTGPCLFFNNAAGGTAGGAL
FANACRIENNSQPIYFLNNQSGLGGAIRVHQECILTKNTGSVIFNNNFAMEADISANHSSGGAIYCISCSIKDNP
GIAAFDNNTAARDGGAICTQSLTIQDSGPVYFTNNQGTWGGAIMLRQDGACTLFADQGDIIFYNNRHFKDTF
SNHVSVNCTRNVSLTVGASQGHSATFYDPILQRYTIQNSIQKFNPNPEHLGTILFSSAYIPDTSTSRDDFISHFR
NHIGLYNGTLALEDRAEWKVYKFDQFGGTLRLGSRAVFSTTDEEQSSSSVGSVININNLAINLPSILGNRVAPK
LWIRPTGSSAPYSEDNNPIINLSGPLSLLDDENLDPYDTADLAQPIAEVPLLYLLDVTAKHINTDNFYPEGLNT
TQHYGYQGVWSPYWIETITTSDTSSEDTVNTLHRQLYGDWTPTGYKVNPENKGDIALSAFWQSFHNLFATL
RYQTQQGQIAPTASGEATRLFVHQNSNNDAKGFHMEATGYSLGTTSNTASNHSFGVNFSQLFSNLYESHSDN
SVASHTTTVALQINNPWLQERFSTSASLAYSYSNHHIKASGYSGKIQTEGKCYSTTLGAALSCSLSLQWRSRP
LHFTPFIQAIAVRSNQTAFQESGDKARKFSVHKPLYNLTVPLGIQSAWESKFRLPTYWNIELAYQPVLYQQNP
EVNVSLESSGSSWLLSGTTLARNAIAFKGRNQIFIFPKLSVFLDYQGSVSSSTTTHYLHAGTTFKF

>core/29/5/Org5_Gene776

MFWKLLCPILICTSLSITSCEQQFKVVPNQCPLQVSTPAAADQKIEKIICSNGLPLLIISDPNLPTSGAALLVKTG
NNADPEEYPGMAHFTEHCVFLGNEKYPEVSGFPGFLSENNGVHNAFTYPNKTVFVFSVEHSAFSDALDQFVH
LFINPKFRQEDLDREKYAVHQEFAAHPLSDGRRVHRIQQLVAPQGHPCARFGCGNASTLTPVTTEKMAEWF
KLHYSPENMCAIAYTSAPLSKAKKQFSKIFSQIPRSKNYERQEPFLPSGDTSSLKNLYINQAIQPTSNLEIYWHI
YESSHPIPLGCYKALAEVLRNESKNSLVSLLKNEQLITDLDVEFFRSSLNTGEFYISYELTEKGDKHYSQVIDST
FQYLRYIQEHGIPNYTLEEISTINALNYCYSSKSPLFDLLCKQIVSLGNEDLSTYPYHSLVYPKYSSEDESALLN
LVSDPEQARFVLSSKNSEHWEEATQLHDPIFDMTYYVKALDGVQDYGKVQSLKPIALPKPNLFIPKEVTLPG
VHLLKKQEFPFAPALSYQDDKLTLYHCEDHYYTAPKLSSQIRIRSPQISRSSPQFLVATELYCLAVNDQLLREY
YPATQAGLSFTSALGGDGIDLRVSGYTTTVPALLNSILTSLPNLEISYETFLVYKKQLLELYQGALLNCPVRSG
LDELASQVMKETYSNTTKLSALEKLSFSEFQAFASNLFNSVHLEVMVLGNLSEQQKKDYLEMLQVFTASRSS
HATKPFYYELQSQEISEIHHDYPLTANGMLLLLQDKSSPSIQGKVCAEMLFEWLHHITFEELRTQQQLGYMV
GARYREFASRPFGFLYIRSDAYSPEELLAKTSLFLNKVSASPEKFGISQEKFANIRKAYINKILEPEHSLDMMNS
ALFSLAFERPFVEFSTPDLKIAIAETLTYEEFLKYCQCFLSNELGTQTSVYIRGTQKTS

>core/30/5/Org5_Gene62

MTTEDFPKAYNFQDTEPELYVFWEKNGMFKAEASSDKPPYSVIMPPPNVTGVLHMGHALVNTLQDVLVRY
KRMSGFEVCWIPGTDHAGIATQAVVERHLQASEGKRRTDYSREDFLKHIWAWKEKSEKVVLSQLRQLGCSC
DWDRKRFTMEPLANRAVKKAFKTLFENGYIYRGYYLVNWDPVLQTALADDEVEYEEKDGWLYYIRYRMV
GSQESIVVATTRPETSLGDTGIAVSPNDERYASWIGASVEVPFVNRQIPIIGDASVDPTFGTGAVKVTPAHDKD
DYLMGTNHHLPMINILTPSGGINENGGPFAGMAKEKAREEILIALEEQGLFVRKEPYKLRVGVSYRSGAVIEP
YLSKQWFVSVSEFRGALREFVESQDIKIFPKDFVKNYLSWVNHLRDWCISRQLWWGHRIPVWYHKNDDERV
LCYDGEGIPEEVAQDPDSWYQDPDVLDTWFSSGLWPLTCLGWPDENSPDLKKFYPTALLVTGHDILFFWVT
RMVLLCSSMSGEKPFSEVFLHGLIFGKSYKRYNDFGEWSYISGKEKLAYDMGEALPDGVVAKWEKLSKSKG
NVIDPLEMIATYGTDAVRLTLCSCANRGEQIDLDYRLFEEYKHFANKVWNGARFIFGHISDLQGKDLLAGIDE
DSLGLEDFYILDGFNQLIHQLEEAYATYAFDKVATLAYEFFRNDLCSTYIEIIKPTLFGKQGNEASQSTKRTLL
AVLLINVLGVLHPVAPFITESLFLRIQDTLGALPEGDGDAFTGHALRMLRSRACMEAPYPKAFDVKIPQDLRE
SFTLAQRLVYTIRNIRGEMQLDPRLHLKAFVVCSDTTEIQSCIPILQALGGLESIQLLDKEPEKGLYSFGVVDTI
RLGIFVPEEHLLKEKGRLEKERVRLERAVENLERLLGDESFCQKANPNLVVAKQEALKNNRIELQGILDKLAS
FA

>core/31/5/Org5_Gene355

MRFFCFGMLLPFTFVLANEGLQLPLETYITLSPEYQAAPQVGFTHNQNQDLAIVGNHNDFILDYKYYRSNGG
ALTCKNLLISENIGNVFFEKNVCPNSGGAIYAAQNCTISKNQNYAFTTNLVSDNPTATAGSLLGGALFAINCSI
TNNLGQGTFVDNLALNKGGALYTETNLSIKDNKGPIIIKQNRALNSDSLGGGIYSGNSLNIEGNSGAIQITSNSS
GSGGGIFSTQTLTISSNKKLIEISENSAFANNYGSNFNPGGGGLTTTFCTILNNREGVLFNNNQSQSNGGAIHAK
SIIIKENGPVYFLNNTATRGGALLNLSAGSGNGSFILSADNGDIIFNNNTASKHALNPPYRNAIHSTPNMNLQIG
ARPGYRVLFYDPIEHELPSSFPILFNFETGHTGTVLFSGEHVHQNFTDEMNFFSYLRNTSELRQGVLAVEDGA
GLACYKFFQRGGTLLLGQGAVITTAGTIPTPSSTPTTVGSTITLNHIAIDLPSILSFQAQAPKIWIYPTKTGSTYT
EDSNPTITISGTLTLRNSNNEDPYDSLDLSHSLEKVPLLYIVDVAAQKINSSQLDLSTLNSGEHYGYQGIWSTY
WVETTTITNPTSLLGANTKHKLLYANWSPLGYRPHPERRGEFITNALWQSAYTALAGLHSLSSWDEEKGHA
ASLQGIGLLVHQKDKNGFKGFRSHMTGYSATTEATSSQSPNFSLGFAQFFSKAKEHESQNSTSSHHYFSGMCI

ENTLFKEWIRLSVSLAYMFTSEHTHTMYQGLLEGNSQGSFHNHTLAGALSCVFLPQPHGESLQIYPFITALAIR
GNLAAFQESGDHAREFSLHRPLTDVSLPVGIRASWKNHHRVPLVWLTEISYRSTLYRQDPELHSKLLISQGTW
TTQATPVTYNALGIKVKNTMQVFPKVTLSLDYSADISSSTLSHYLNVASRMRF

>core/32/5/Org5_Gene913

MKSSVSWLFFSSIPLFSSLSIVAAEVTLDSSNNSYDGSNGTTFTVFSTTDAAAGTTYSLLSDVSFQNAGALGIPL
ASGCFLEAGGDLTFQGNQHALKFAFINAGSSAGTVASTSAADKNLLFNDFSRLSIISCPSLLLSPTGQCALKSV
GNLSLTGNSQIIFTQNFSSDNGGVINTKNFLLSGTSQFASFSRNQAFTGKQGGVVYATGTITIENSPGIVSFSQN
LAKGSGGALYSTDNCSITDNFQVIFDGNSAWEAAQAQGGAICCTTTDKTVTLTGNKNLSFTNNTALTYGGAI
SGLKVSISAGGPTLFQSNISGSSAGQGGGGAINIASAGELALSATSGDITFNNNQVTNGSTSTRNAINIIDTAKV
TSIRAATGQSIYFYDPITNPGTAASTDTLNLNLADANSEIEYGGAIVFSGEKLSPTEKAIAANVTSTIRQPAVLA
RGDLVLRDGVTVTFKDLTQSPGSRILMDGGTTLSAKEANLSLNGLAVNLSSLDGTNKAALKTEAADKNISLS
GTIALIDTEGSFYENHNLKSASTYPLLELTTAGANGTITLGALSTLTLQEPETHYGYQGNWQLSWANATSSKI
GSINWTRTGYIPSPERKSNLPLNSLWGNFIDIRSINQLIETKSSGEPFERELWLSGIANFFYRDSMPTRHGFRHIS
GGYALGITATTPAEDQLTFAFCQLFARDRNHITGKNHGDTYGASLYFHHTEGLFDIANFLWGKATRAPWVLS
EISQIIPLSFDAKFSYLHTDNHMKTYYTDNSIIKGSWRNDAFCADLGASLPFVISVPYLLKEVEPFVKVQYIYA
HQQDFYERYAEGRAFNKSELINVEIPIGVTFERDSKSEKGTYDLTLMYILDAYRRNPKCQTSLIASDANWMA
YGTNLARQGFSVRAANHFQVNPHMEIFGQFAFEVRSSSRNYNTNLGSKFCF

>core/33/5/Org5_Gene356

MFGMTPAVYSLQTDSLEKFALERDEEFRTSFPLLDSLSTLTGFSPITTFVGNRHNSSQDIVLSNYKSIDNILLLW
TSAGGAVSCNNFLLSNVEDHAFFSKNLAIGTGGAIACQGACTITKNRGPLIFFSNRGLNNASTGGETRGGAIA
CNGDFTISQNQGTFYFVNNSVNNWGGALSTNGHCRIQSNRAPLLFFNNTAPSGGGALRSENTTISDNTRPIYF
KNNCGNNGGAIQTSVTVAIKNNSGSVIFNNNTALSGSINSGNGSGGAIYTTNLSIDDNPGTILFNNNYCIRDGG
AICTQFLTIKNSGHVYFTNNQGNWGGALMLLQDSTCLLFAEQGNIAFQNNEVFLTTFGRYNAIHCTPNSNLQ
LGANKGYTTAFFDPIEHQHPTTNPLIFNPNANHQGTILFSSAYIPEASDYENNFISSSKNTSELRNGVLSIEDRA
GWQFYKFTQKGGILKLGHAASIATTANSETPSTSVGSQVIINNLAINLPSILAKGKAPTLWIRPLQSSAPFTEDN
NPTITLSGPLTLLNEENRDPYDSIDLSEPLQNIHLLSLSDVTARHINTDNFHPESLNATEHYGYQGIWSPYWVE
TITTTNNASIETANTLYRALYANWTPLGYKVNPEYQGDLATTPLWQSFHTMFSLLRSYNRTGDSDIERPFLEI
QGIADGLFVHQNSIPGAPGFRIQSTGYSLQASSETSLHQKISLGFAQFFTRTKEIGSSNNVSAHNTVSSLYVELP
WFQEAFATSTVLAYGYGDHHLHSLHPSHQEQAEGTCYSHTLAAAIGCSFPWQQKSYLHLSPFVQAIAIRSHQ
TAFEEIGDNPRKFVSQKPFYNLTLPLGIQGKWQSKFHVPTEWTLELSYQPVLYQQNPQIGVTLLASGGSWDIL
GHNYVRNALGYKVHNQTALFRSLDLFLDYQGSVSSSTSTHHLQAGSTLKF

>core/34/5/Org5_Gene63

MERYDIVRIIGKGGMGEVYLAYDPVCSRKVALKKIREDLAENPLLKRRFLREARIAADLIHPGVVPVYTIYSE
KDPVYYTMPYIEGYTLKTLLKSVWQKESLSKELAEKTSVGAFLSIFHKICCTIEYVHSRGILHRDLKPDNILLG
LFSEAVILDWGAAVACGEEEDLLDIDVSKEEVLSSRMTIPGRIVGTPDYMAPERLLGHPASKSTDIYALGVVL
YQMLTLSFPYRRKKGKKIVLDGQRIPSPQEVAPYREIPPFLSAVVMRMLAVDPQERYSSVTELKEDIESHLKG
SPKWTLTTALPPKKSSSWKLNEPILLSKYFPMLEVSPASWYSLAISNIESFSEMRLEYTLSKKGLNEGFGILLPT
SENALGGDFYQGYGFWLHIKERTLSVSLVKNSLEIQRCSQDLESDKETFLIALEQHNHSLSLFVDGTTWLIHM

NYLPSRSGRVAIIVRDMEDILEDIGIFESSGSLRVSCLAVPDAFLAEKLYDRALVLYRRIAESFPGRKEGYEARF
RAGITVLEKASTDNNEQEFALAIEEFSKLHDGVAAPLEYLGKALVYQRLQEYNEEIKSLLLALKRYSQHPEIF
RLKDHVVYRLHESFYKRDRLALVFMILVLEIAPQAITPGQEEKILVWLKDKSRATLFCLLDPTVLELRSSKME
LFLSYWSGFIPHLNSLFHRAWDQSDVRALIEIFYVACDLHKWQFLSSCIDIFKESLEDQKATEEIVEFSFEDLGA
FLFAIQSIFNKEDAEKIFVSNDQLSPILLVYIFDLFANRALLESQGEAIFQALDLIRSKVPENFYHDYLRNHEIRA
HLWCRNEKALSTIFENYTEKQLKDEQHELFVLYGCYLALIQGAEAAKQHFDVCREDRIFPASLLARNYNRLG
LPKDALSYQERRLLLRQKFLYFHCLGNHDERDLCQTMYHLLTEEFQL

>core/35/5/Org5_Gene912

MKIPLHKLLISSTLVTPILLSIATYGADASLSPTDSFDGAGGSTFTPKSTADANGTNYVLSGNVYINDAGKGTA
LTGCCFTETTGDLTFTGKGYSFSFNTVDAGSNAGAAASTTADKALTFTGFSNLSFIAAPGTTVASGKSTLSSA
GALNLTDNGTILFSQNVSNEANNNGGAITAKTLSISGNTSSITFTSNSAKKLGGAIYSSAAASISGNTGQLVFM
NNKGETGGGALGFEASSSITQNSSLFFSGNTATDAAGKGGAIYCEKTGETPTLTISGNKSLTFAENSSVTQGG
AICAHGLDLSAAGPTLFSNNRCGNTAAGKGGAIAIADSGSLSLSANQGDITFLGNTLTSTSAPTSTRNAIYLGS
SAKITNLRAAQGQSIYFYDPIASNTTGASDVLTINQPDSNSPLDYSGTIVFSGEKLSADEAKAADNFTSILKQPL
ALASGTLALKGNVELDVNGFTQTEGSTLLMQPGTKLKADTEAISLTKLVVDLSALEGNKSVSIETAGANKTIT
LTSPLVFQDSSGNFYESHTINQAFTQPLVVFTAATAASDIYIDALLTSPVQTPEPHYGYQGHWEATWADTSTA
KSGTMTWVTTGYNPNPERRASVVPDSLWASFTDIRTLQQIMTSQANSIYQQRGLWASGTANFFHKDKSGTN
QAFRHKSYGYIVGGSAEDFSENIFSVAFCQLFGKDKDLFIVENTSHNYLASLYLQHRAFLGGLPMPSFGSITD
MLKDIPLILNAQLSYSYTKNDMDTRYTSYPEAQGSWTNNSGALELGGSLALYLPKEAPFFQGYFPFLKFQAV
YSRQQNFKESGAEARAFDDGDLVNCSIPVGIRLEKISEDEKNNFEISLAYIGDVYRKNPRSRTSLMVSGASWT
SLCKNLARQAFLASAGSHLTLSPHVELSGEAAYELRGSAHIYNVDCGLRYSF

>core/37/5/Org5_Gene346

MKSSLHWFLISSSLALPLSLNFSAFAAVVEINLGPTNSFSGPGTYTPPAQTTNADGTIYNLTGDVSITNAGSPTA
LTASCFKETTGNLSFQGHGYQFLLQNIDAGANCTFTNTAANKLLSFSGFSYLSLIQTTNATTGTGAIKSTGACS
IQSNYSCYFGQNFSNDNGGALQGSSISLSLNPNLTFAKNKATQKGGALYSTGGITINNTLNSASFSENTAANN
GGAIYTEASSFISSNKAISFINNSVTATSATGGAIYCSSTSAPKPVLTLSDNGELNFIGNTAITSGGAIYTDNLVL
SSGGPTLFKNNSAIDTAAPLGGAIAIADSGSLSLSALGGDITFEGNTVVKGASSSQTTTRNSINIGNTNAKIVQL
RASQGNTIYFYDPITTSITAALSDALNLNGPDLAGNPAYQGTIVFSGEKLSEAEAAEADNLKSTIQQPLTLAGG
QLSLKSGVTLVAKSFSQSPGSTLLMDAGTTLETADGITINNLVLNVDSLKETKKATLKATQASQTVTLSGSLS
LVDPSGNVYEDVSWNNPQVFSCLTLTADDPANIHITDLAADPLEKNPIHWGYQGNWALSWQEDTATKSKAA
TLTWTKTGYNPNPERRGTLVANTLWGSFVDVRSIQQLVATKVRQSQETRGIWCEGISNFFHKDSTKINKGFR
HISAGYVVGATTTLASDNLITAAFCQLFGKDRDHFINKNRASAYAASLHLQHLATLSSPSLLRYLPGSESEQP
VLFDAQISYIYSKNTMKTYYTQAPKGESSWYNDGCALELASSLPHTALSHEGLFHAYFPFIKVEASYIHQDSF
KERNTTLVRSFDSGDLINVSVPIGITFERFSRNERASYEATVIYVADVYRKNPDCTTALLINNTSWKTTGTNLS
RQAGIGRAGIFYAFSPNLEVTSNLSMEIRGSSRSYNADLGGKFQF

>core/38/5/Org5_Gene348

MKTSIPWVLVSSVLAFSCHLQSLANEELLSPDDSFNGNIDSGTFTPKTSATTYSLTGDVFFYEPGKGTPLSDSC
FKQTTDNLTFLGNGHSLTFGFIDAGTHAGAAASTTANKNLTFSGFSLLSFDSSPSTTVTTGQGTLSSAGGVNL

ENIRKLVVAGNFSTADGGAIKGASFLLTGTSGDALFSNNSSSTKGGAIATTAGARIANNTGYVRFLSNIASTSG
GAIDDEGTSILSNNKFLYFEGNAAKTTGGAICNTKASGSPELIISNNKTLIFASNVAETSGGAIHAKKLALSSGG
FTEFLRNNVSSATPKGGAISIDASGELSLSAETGNITFVRNTLTTTGSTDTPKRNAINIGSNGKFTELRAAKNHT
IFFYDPITSEGTSSDVLKINNGSAGALNPYQGTILFSGETLTADELKVADNLKSSFTQPVSLSGGKLLLQKGVT
LESTSFSQEAGSLLGMDSGTTLSTTAGSITITNLGINVDSLGLKQPVSLTAKGASNKVIVSGKLNLIDIEGNIYES
HMFSHDQLFSLLKITVDADVDTNVDISSLIPVPAEDPNSEYGFQGQWNVNWTTDTATNTKEATATWTKTGF
VPSPERKSALVCNTLWGVFTDIRSLQQLVEIGATGMEHKQGFWVSSMTNFLHKTGDENRKGFRHTSGGYVI
GGSAHTPKDDLFTFAFCHLFARDKDCFIAHNNSRTYGGTLFFKHSHTLQPQNYLRLGRAKFSESAIEKFPREIP
LALDVQVSFSHSDNRMETHYTSLPESEGSWSNECIAGGIGLDLPFVLSNPHPLFKTFIPQMKVEMVYVSQNSF
FESSSDGRGFSIGRLLNLSIPVGAKFVQGDIGDSYTYDLSGFFVSDVYRNNPQSTATLVMSPDSWKIRGGNLSR
QAFLLRGSNNYVYNSNCELFGHYAMELRGSSRNYNVDVGTKLRF

>core/39/5/Org5_Gene936

MQRVLRLLFNLHHGEEKRAFLFFLLGLVWGIGCYGTLSLAEGLFIEKLGSAELPKIYLGSSLILCVLSSLILYNL
FKKHISATALFLIPVSLSILCNFYLILSSIFAIDPPRSPLFFYRIVIWSLTILSYTSFWGFVDQFFNLQDGKRHFCIF
NAIIFLGDAIGSGIIASLVHTIGIQGILILFTAALVLTFPIVFYVSKSLKSLSDDHDLFIDTGHPPPLSKALKLCFYD
KYTFYLLCFYFLMQLLAIATEFNYLKIFEIQFASKEEFELVAHIGKCSLWISLGNMCFALFAYSRIVKRLGVNNI
ILFAPLCFLSLFLFWTFKTTLSIAVLAMVVREGVTYALDDNNLQLLIYGVPNKIRNQIRIVVESFIEPIGMLVWS
LVCFLSSQQYVFCLIISLIATILVCLVRSYYAKAILKNLSAQALQLTRSMQDWIKSMTVKQKRQVELFLLAHL
KHPSERHQTFAFQHLLNLASRSVLPSLLAHMNKLSLPNKLKTIEMVKSSLWAKDFLTLELLKRWTSIFPHPAI
ASAIHLYFAEHDLLHITHIAEDLYDTVGDRLLAAILTVRRQEAYGPYRDLADKRLKELLNSDQPEDIVMGLTI
LKLEKNPQNFPILLDFLNTKNEDILIVTCKALHTSVRANHKPYCPELLKRLRQCSHNDEASQYLLKTISIALDIS
FVKDLLMTTSQLKNTSRKYAEAMIGELDKEVAPAFLQVLTDEGTHNRCRILAAKALCKIDNWLLKKHAYKI
VKSKASKALFYSYHGHYIQKKYPTYNLSLLANTLNSNYYAEVNFMLSLLGILGSMEHSGVLIRALTSKNQKI
KAQALESLEKNCDSHLFSLLEPFVNQPGMCYSEKYYFKCGVIPLTLKELLNMMENSPSSLNKLTAQQLKEEL
SYCDPDFQSVNTIYNQEHEDFRTEESETLISFLSI

>core/40/5/Org5_Gene981

MRFSLCGFPLVFSFTLLSVFDTSLSATTISLTPEDSFHGDSQNAERSYNVQAGDVYSLTGDVSISNVDNSALNK
ACFNVTSGSVTFAGNHHGLYFNNISSGTTKEGAVLCCQDPQATARFSGFSTLSFIQSPGDIKEQGCLYSKNAL
MLLNNYVVRFEQNQSKTKGGAISGANVTIVGNYDSVSFYQNAATFGGAIHSSGPLQIAVNQAEIRFAQNTAK
NGSGGALYSDGDIDIDQNAYVLFRENEALTTAIGKGGAVCCLPTSGSSTPVPIVTFSDNKQLVFERNHSIMGG
GAIYARKLSISSGGPTLFINNISYANSQNLGGAIAIDTGGEISLSAEKGTITFQGNRTSLPFLNGIHLLQNAKFLK
LQARNGYSIEFYDPITSEADGSTQLNINGDPKNKEYTGTILFSGEKSLANDPRDFKSTIPQNVNLSAGYLVIKE
GAEVTVSKFTQSPGSHLVLDLGTKLIASKEDIAITGLAIDIDSLSSSSTAAVIKANTANKQISVTDSIELISPTGN
AYEDLRMRNSQTFPLLSLEPGAGGSVTVTAGDFLPVSPHYGFQGNWKLAWTGTGNKVGEFFWDKINYKPRP
EKEGNLVPNILWGNAVDVRSLMQVQETHASSLQTDRGLWIDGIGNFFHVSASEDNIRYRHNSGGYVLSVNN
EITPKHYTSMAFSQLFSRDKDYAVSNNEYRMYLGSYLYQYTTSLGNIFRYASRNPNVNVGILSRRFLQNPLMI
FHFLCAYGHATNDMKTDYANFPMVKNSWRNNCWAIECGGSMPLLVFENGRLFQGAIPFMKLQLVYAYQG
DFKETTADGRRFSNGSLTSISVPLGIRFEKLALXQDVLYDFSFSYIPDIFRKDPSCEAALVISGDSWLVPAAHVS
RHAFVGSGTGRYHFNDYTELLCRGSIECRPHARNYNINCGSKFRF

>core/41/5/Org5_Gene559

MKTVILNIGRKILQGIKKKKKKIGILSGLFFLDLVLLGVSSQRPTETSANVKHNLRDEKLAACPKNSAASLSAK
KSHTKKTTPGSIPSKVFSKFDATQDKTFQKTSGSAFPAKPTTLKELEERKKPRPERRTTADVKRSPRFLPTQEV
EEPVPAASKEQLDSIQVWEEKQNYARRAVNAINLSIKKQLEEQTSTVTEKDVQPKTQATPHASKKNVASPST
SMPGIEKAATTVAVPQDKSEEEKVKERLTKRELTCEDLKDNGYTVNFEDISILELLQFVSKISGTNFVFDSNDL
QFNVTIVSHDPTSVDDLSTILLQVLKMHDLKVVEQGNNVLIYRNPHLSKLSTVVTDSSLKETCEAVVVTRVFR
LYSVSPSAAVNIIQPLLSHDAIVSASEATRHVIISDIAGNVDKVSDLLAALDCPGTSVDMTEYEVKYANPAALV
SYCQDVLGTLAEDDAFQMFIQPGTNKIFVVSSPRLANKAEQLLKSLDVPEMAHTLDDPASTALALGGTGTTS
PKSLRFFMYKLKYQNGEVIANALQDIGYNLYVTTAMDEDFINTLNSIQWLEVNNSIVIIGNQGNVDRVIGLLN
GLDLPPKQVYIEVLILDTSLEKSWDFGVQWVALGDEQSKVAYASGLLNNTGIATPTKATVPPGTPNPGSIPLP
TPGQLTGFSDMLNSSSAFGLGIIGNVLSHKGKSFLTLGGLLSALDQDGDTVIVLNPRIMAQDTQQASFFVGQT
VPYQTTNTIIQETGTVTQNIDYEDIGVNLVVTSTVAPNNVVTLQIEQTISELHSASGSLTPVTDKTYAATRLQIP
DGCFLVMSGHIRDKTTKVVSGVPLLNSIPLIRGLFSRTIDQRQKRNIMMFIKPKVISSFEEGTRVTNKEGYRYN
WEADEGSMQVAPRHAPECQGPPSLQAESDFKIIEIEAQ

>core/43/5/Org5_Gene809

MSKDKKMKPEPKKNFPTVFFFLLFGVVFGVVAFQNFLAGKKARVGFSHQIEHLVNLRLIVPEDSHKIALNDN
LVSFGGRFRDVQTQEGQLRYHYLELIDQGHRLDLDLQETSKSLTTLGKEVTNSILWFSAISGSPIPEQGYAISY
PSEVSGSVLTEPLVVTGPATPQLINLHSLQERYPTLSRSPEALRTYGSDLYELIGKYLSPVLGIGSETLKRELKD
LYQQVEVSLTQETDTEAAYTLYGQVLSTLNRISSSLVVSEGGERFSQLRSVRLYREEWNKYHKLVEARDLNQ
AQLEKLRGELSQTVWYFNNQELSSRSLEKQDPEVFGHWFAGAKEEWTAFKFNHSLSFKAPDQPRNLVLEKT
FKSQEPSPHYLGYLFTFLPIILVLLFVYLVFSRQMRGMSGSAMSFGKSPARMLLKGQNKVTFADVAGIEEAKE
ELIEIVDFLKNPNKFTSLGGRIPKGVLLIGPPGTGKTLIAKAVSGEADRPFFSIAGSDFVEMFVGVGASRIRDMF
EQAKRNAPCIIFIDEIDAVGRHRGAGIGGGHDEREQTLNQLLVEMDGFGTNEGVILMAATNRPDVLDKALLR
PGRFDRRVVMNLPDIKGRFEILMVHAKRIKLDPTVDLMAVARSTPGASGADLENLLNEAALLAARKDRTAV
TAVDVAEARDKVLYGKERRSLEMDAEERKTTAYHESGHAVVGLCVQHGDPVDKVTIIPRGLSLGATHFLPE
KNKLSYWKKELYDQLAVLMGGRAAEEIFLGDISSGAQQDISQATKLVRSMVCEWGMSPQLGNVTYDERSD
GLTGYGGYHEKSYSEETAKTIDTELRMLLDAAYQRALDIINEHKAEIELMTQMLIEFETLDSKDVKEIMDHT
WDPEKKRARLKEEGMLFKKSSDDLPPPPPKEDTLPGLGFNAT

>core/44/5/Org5_Gene289

MDSEFVGQVYSSDMDWIESMYQRFMNHETLDPSWKYFFEGYQLGQAASPSEASTKISGNETIAMLQEQKSQ
FLCTIYRYYGYLQSQISTLAPTTDSRFIQEKIAKIDLDEQVPSAGLLPKAQVSVRELIEALKKCYCGSLTLETLT
CTPELQEFVWNLMEKRQVERFAEQLLRSYKDLCKATFFEEFLQIKFTGQKRFSLEGGETLVPMLEHLVHYGS
ALGISNYVLGMAHRGRLNVLTNVLGKPYRYVFMEFEDDPAARGLESVGDVKYHKGYVLKSHQKDRETTFV
MLPNASHLESVDPIVEGVVAALQHQGHAGKEQSSLAILVHGDAAFSGQGVVYETLQLSRVPGYSTEGTLHIV
VNNYIGFTAVPRESRSTPYCTDIAKMLGIPVFRVNSEDVVACIEAIEYALQVRERFSCDVIIDLCCYRKYGHNE
SDDPSVTAPLLYDQIKRKKSIRELFRQYLLEGQFADISEETLASIEKEIQESLNREFQVLKGTDPEPFPKKECHH
CDRLNNGELILHDCDVSLDRETLFHMSSRLCGFPDNFHPHPKIKTLLEKRMKMAEGGVGYDWAMAEELAFA
SLLIEGYNLRLSGQDSIRGTFSQRHLVWSDTVTGDTYSPLYHLSAEQGSVEMYNSPLSEYAILGFEYGYAQQA

LKTLVLWEAQFGDFANGAQIIFDQYISSGIQKWDLHSDIVLLLPHGYEGQGPEHSSSRIERYLQLAANWNFQV
VLPSTPVQYFRILREHAKRDLSLPLVIFTPKLLLRYPQCVSSIEEFTEPGGFRAILEDADPNYDASILVLCSGKIY
YDYAEMLPQDRRKDFSCLRIESLYPLALEDLVSLIDKYSHLKHFVWLQEESKNMGAYDYMFMALQDILPEK
LLYIGRPRSSSTASGSAKLSRQELVTCMETLFSLR

>core/45/5/Org5_Gene933

MEKVKLTKNLKLKIKNAQLTKAAGLDKLKQKLAQAGSSEAKSSSEKPSAKEKSVKVALAATSTPTASAEQA
SPESTSRRIRAKNRSSFSSSEEESSAHIPVDTSEPAPVSIADPEPELEVVDEVCDESPEVHPVAEVLPEQPVLPET
PPQEKELEPKPVKPAEPKSVVMIKSKFGPTGKHINHLLAKTFKAPAKEEKVVAGSKSTKPVASDKTGKPGTSE
GGEQNNREKQFNPANRSPASGPKRDAGKKNLTDFRDRSKKSDESLKAFTGRDRYGLNEGGEEDRWRKKRV
YKPKKHYDEASIQRPTHIKISLPITVKDLATEMKLKASEVIQKLFIHGMTYVVNDILDSETAVQFIGLEFGCTID
IDYSEQDKLCLSNDTVRDEIQSTDPSKLVIRSPIVAFMGHVDHGKTTLIDSLRKSNVAATEAGAITQHMGAFC
CSTPVGDITILDTPGHEAFSAMRARGAEVCDIVVLVVAGDEGIKEQTLEAIEHAKAADIAIVVAINKCDKPNF
NSETIYRQLSEINLLPEAWGGSTVTVNTSAKTGEGLSELLEMLALQAEVLELKADPSARARGLVIESELHKGL
GPVATVLIQNGSLKLGEALVFNDCYGKVKTMHNEHNELMKEAGPSIPVLITGLSDIPKAGDPFFVVKNEKTA
RDIIEARSAGQQRFALQQKKRPNFDSMLQNKKTLKLMIKADVQGSIEALVSSISKIKSEKVDVEILTNSVGEIS
ESDIRLAAASKAVLIGFHTGIESHAEPLIKSLGVRVELFTVIYHAIDAIKEIMTSLLDPIAEEKDEGSAEIKEIFRS
SQVGSIYGCIVTEGIMTRNHKVRVLRNKEILWKGTLSSLKRVKEDVKEVRKGLECGILLEGYQQAQIGDVLQ
CYEVIYHPQKL

>core/46/5/Org5_Gene722

MLSNTIRSNFLKFYANRHHTILPSSPVFPHNDPSILFTNAGMNQFKDIFLNKEKVSYSRATTSQKCIRAGGKHN
DLDNVGHTSRHLTFFEMLGNFSFGDYFKAEAIAFAWEVSLSVFNFNPEGIYATVHEKDDEAFALWEAYLPTD
RIFRLTDKDNFWSMANTGPCGYCSELLFDRGPSFGNASSPLDDTDGERFLEYWNLVFMEFNRTSEGSLLALP
NKHVDTGAGLERLVSLIAGTHTVFEADVLRELIAKTEQLSGKVYHPDDSGAAFRVIADHVRSLSFAIADGLLP
GNTERGYVLRKILRRSVNYGRRLGFRNPFLAEIVPSLADAMGEAYPELKNSLSQIQKVLTLEEESFFKTLDRG
GNLLQQVLKSSSSSSCISGEDAFKLKDTYGMPIDEISLLAKDYDYSVDMDTFHKLEQEAKERSRKNVVQSQG
TSESIYNELHLTSEFIGYDHLSCDTFIEAIISKDHIVSSLQEKQEGAIVLKVSPFYAEKGGQVGDSGEIFCSEGTFI
VTHTTSPKAGLIVHHGRISQGSLTVEAAVTAQVNRYRRKRIANNHTACHLLHKALEITLGDHIRQAGSYVDD
TKIRLDFTHPQAISPEDLLCIETLVNESIRENEPVDIREALYSDVMNSSEIKQFFGDKYSDVVRVVSAGHSHELC
GGTHAEATGDIGFFRITKEHAVAMGIRRIEAVTGEKAEATVHQQSEVLEEIATLLQVPRDQIVSRLTATLDER
KQQDKRLNELENSLIQTKLDKLIHNCHQRQGITCLVHHLAEHENHRLQQYAQCLHQRIPEKLISLWTTEKNG
KYIVLSRVSDDLITQGVHAQDLLKAVLTPCGGRWGGKDQSAQGSAPALPATEVLNETLWQWISTQLI

>core/47/5/Org5_Gene583

MSTFSIQNRLRTISGESTRIIKLDHKYSGFDPRSVPAINLEELNSGIYALRHLMNALQSENTNVAALLNPNNTIF
PTTSWTDYKHSRPQASSPRAPSSQTPTDIVSAAALALVLVIDGGLAELVASVTEIDLGALSTISTVRQLMASYL
GLTTLTAEQEKVVFSSSYVPSEKNLLEHVKQEKAAEIQAKQEEIKAVLEAKGVSTEEIEAILKEYPDIYAADFF
KEFIEEPLHTYRAKVGAPIQEMNENAIQLLPTPPAITPDNVNEVNGMNTLSTILQAIDDAIKQAPALGGDQEIIT
ILQTLVPLVDKTTFTKAEFDLIYTATQLPNTASLKLYLTDRQIAEYRGKITKVYQNSIQNLSETKRVVENNRSM
LETQLSMFQQAQNCFVTWISQANALNIAITNKYISAVLTTSMEMYGGLLCLSYMYERLADDEKAIFDKSVNE

YLPIHIVVGGSWVNGWIAKMAAYQELAEYSLGTAVTSQDQIKAYLQTRGNEFKATRHFFHNIGDQMYQFAN
ETVFGNCLTTANGAIQPDLGGFIREAMTNVGTVEADYVSNAQRILNEFNTAATAHVLQLQLQIAELQKKADD
LDPGKASFTENRKFAVAAWITSESLGDALISMILNSQLPKQEAFLKPLIEEINFNNLAANALNSLLQITNEFSTT
SVYYSLSSYLVQSKTGQNLFAGDYYETLLAAREREYIYRDTARCKQAINLVNGLLQKINSLPGATSAQKQE
MLNATTYYQYSLSVTLNQLTVLESLLAGLKMTLQTTSNNKYDKSVFKIESFDDWIPTLAALESFLTSGFPNIS
ATGGLGPLFTQVQSDQQTYTSQGQTQQLNLQNQMTTIQQEWTLVSTSMQVLNGILSQLAGAIYSN

>core/48/5/Org5_Gene619

MKKSLIIVESPAKIKTLQKLLGSEFVFASSIGHIVDLPAKEFGIDVDHDFEPQYQVLPDKQEVINHIRKLAAKCE
KVYLSPDPDREGEAIAWHIANQLPDSPLIQRVSFNAITKNAVTEALKHPRTIDMALVNAQQARRLLDRIVGYK
ISPILSRKLQQRSGISAGRVQSVALKLVVDREKAIDAFVPVEYWNLRVLMQDPKTTKTFWAHLYAVQGKKW
EKEIPEGKTENDVLLINSEEKARHYAELLEKSSYTITRVEAKAKRRFAPPPFITSTLQQEASRHFRFSASRTMSI
AQTLYEGVDLDSEDSTGLITYMRTDSVRVDPEALTTVREYIQQTFGKEYLPEKANVYTTKKMTQDAHEAIRP
TDINLTPDKLKNKLSDDQFKVYNLIWKRFVASQITPAIYDTLAVQITTDTEIDLRASGSLLKFKGFLAVYEEKQ
DDENDQEEDHPLPPLHAQDALIKEEVSQEQAFTKPLPRFTEASLVKELEKSGIGRPSTYATIMNKIQSREYTTK
ENQRLRPTELGKIISQFLETNFPRIMDIGFTALMEDELELIADNKKPWKLLLQEFWTTFLPVVITAEKEAVIPRI
LTNIECSKCHKGKLVKIWSKNSYFYGCSEYPECDYRTSEEELAFNKEDYAEDTPWDSPCPLCGGVMKVRHG
RYGTFLGCEKYPECRGTISIHKKGEEIEQEEPIPCPAIGCNGKIFKKRSRYNKIFYSCSEYPECSVIGNSIDAVITK
YSGTEKIPYKKKTPTKKKSSAKTTKAAKTPSKKGKAKSSVKKSSEKKTGPLFLPSPDLAKMIGNEPVSRGEAT
KKIWDYIKEHQLQAPENKKLLVPDNNLATIIGPNPIDMFQLSKHLSQHLTKVSNDESSASS

>core/49/5/Org5_Gene477

MKKLFVLDASGFIFRAYFALPEMKNHQGQATQAVFGFIRSLNKLIKEFSPEYMISVFDGPNNKQSRQAIYADY
KSNRQKKFEDIPPQIALVKEYCSLIGLAYLEKESVEADDVIASIAKKAREENYKVYVCTADKDLLQLVNDHV
VAWNPWADQGVVGISEVIERYGIPPGNIPDYLALVGDSSDNIPGLPGCGPKKAAALLKQFGSVEGLLENLDA
VKGLSQTMLSERQETLKLSKRLALLDSNIPIPVPIESLTFPQHPVDEEKLIHFYIQQGFKTLVPSKQTEAATVDV
QIIKDAESLTNILNLVQGGDIAFAVAYTGNHLLSLKLEGLALTQGSGVFFIALEEEGTKILPILKDFFLREDLTF
YGYNLKRDCHALLNAGIVIREISYDLALAEHLTNGGGKISFQSLLVNHGFTETAHRFAKEWGNSGLPIGRLPE
QPEQYFGEFVAYLPIIKDAILEEINRKNLNHILSDIEMPLEKVLFSMERAGVPLDVEELAILEALFETELAVLTE
EIYDLSGRPFNIKSPKQLSDILYNELGLRPIDKAKSTRAEVLEALRSEHPIIEKLLAFRTIEKLLSTYVKALPKQV
DSHTQRIHPSFDQTGAVTGRLACRDPNLQNIPIRSERGILLRKAFRLSEKNSYFLSADYSQIELRFLAHLSQDKS
LKFAFESGEDIHAFTASQVFHVPLEQVSKEQRMQAKTVNFGIVYGQQAFGLAKVLKISIGEAQELIQAYFSRY
PEIAHFVEETIQQAAKDLRVTTMLGRERIIDSWNEFPGSRAASGRFAVNTRIQGSAAELIKLAMLDISQAIKQQ
QMKSRMLLQIHDELLFEVPEEEIEEMQRLVREKMESAMTLSVPIVVNILIGKNWAEC

>core/50/5/Org5_Gene102

MEKFSDAVSEALEKAFELAKSSKHTYVTENHLLLALLENTESLFYLVIKDIHGNPGLLNTAVKDALSREPTVV
EGEVDPKPSPGLQTLLRDAKQEAKTLGDEYISGDHLLLAFWSSNKEPFNSWKQTTKVSFKDLKNLITKIRRGN
RMDSPSAESNFQGLEKYCKNLTALAREGKLDPVIGRDEEIRRTIQVLSRRTKNNPMLIGEPGVGKTAIAEGLA
LRLIQGDVPESLKGKQLYVLDMGALIAGAKYRGEFEERLKSVLKDVESGDGEHIIFIDEVHTLVGAGATDGA
MDAANLLKPALARGTLHCIGATTLNEYQKYIEKDAALERRFQPIFVTEPSLEDAVFILRGLREKYEIFHGVRIT

EGALNAAVLLSYRYIPDRFLPDKAIDLIDEAASLIRMQIGSLPLPIDEKERELAALIVKQEAIKREQSPSYQEEA
DAMQKSIDALREELASLRLGWDEEKKLISGLKEKKNSLESMKFSEEEAERVADYNRVAELRYSLIPQLEEEIK
QDEASLNQRDNRLLQEEVDERLIAQVVANWTGIPVQKMLEGEAEKLLILEESLEERVVGQPFAVSAVSDSIR
AARVGLNDPQRPLGVFLFLGPTGVGKTELAKALADLLFNKEEAMVRFDMSEYMEKHSISKLIGSSPGYVGYE
EGGSLSEALRRRPYSVVLFDEIEKADKEVLNILLQVFDDGILTDGKKRKVNCKNALFIMTSNIGSPELADYCS
KKGSELTKEAILSVVSPVLKRYLSPEFMNRIDEILPFVPLTKEDIVKIVGIQMRRIAQRLKARRINLSWDDSVIL
FLSEQGYDSAFGARPLKRLIQQKVVILLSKALLKGDIKPDTSIELTMAKEVLVFKKVETPS

>core/51/5/Org5_Gene569

MAVRLIVDEGPLSGVIFVLEDGISWSIGRDSSANDIPIEDPKLGASQAIINKTDGSYYITNLDDTIPIVVNGVAIQ
ETTQLKNEDTILLGSNQYSFLSDEFDPQDLVYDFDIPEENFSNDSGDLSDSNEQGKDLEPRQTSETNHSPKPKE
KLTKDQGSSDPITSGDQELADAFLASAKAEKNQPRAKVAKKGLKESSNESLNPKEQNAKDSPKGEERTNKPQ
NAIMEDNGASPRQDPQPKSAEPSLKNTARDETPLKENKPVEEKANKKATPDSPEKKDQPEEGSKKEGSKIEA
TPLDSQKESEDKEAEEAFVQEEEENLTEDNKEDSDSAADANDDTASDHTAEDNKETPKKVENEKSAVLSPFH
VQDLFRFDQTIFPAEIDDIAKKNISVDLTQPSRFLLKVLAGANIGAEFHLDSGKTYILGTDPTTCDIVFNDLSVS
HQHAKITVGNDGGILIEDLDSKNGVIVEGRKIDKTSTLSSNQVVALGTTLFLLIDHHAPADTIVASLSPDDYSL
FGRQQDAEALERQEAQEEEEKQKRATLPAGSFILTLFVGGLAILFGIGTASLFHTKEVVPLENIDYQEDLAQVI
NQFPTVRYTFNKTNSQLFLIGHVKNSTDKSELLYKVDALSFVKSVDDNVIDDEAVWQEMNILLSKRPEFKGIS
MHSPEPGKFIITGYVKTEEQAACLVDYLNIHFNYLSLLENKVVVETQMLKAIAGHLLQGGFANIHVAFVNGE
VILTGYVNNDDAEKFRAVVQELSGIPGVRLVKNFAVLLPAEEGIIDLNLRYPNRYRVTGYSRYGEISINVVVN
GRILTRGDVIDGMTVTSIQPNAIFLEKEGLKYKIDYNK

>core/52/5/Org5_Gene918

MFEKFTNRAKQVIKLAKKEAQRLNHNYLGTEHILLGLLKLGQGVAVNVLRNLGIDFDTARQEVERLIGYGPE
IQVYGDPALTGRVKKSFESANEEASLLEHNYVGTEHLLLGILHQSDSVALQVLENLHIDPREVRKEILKELETF
NLQLPPSSSSSSSSSRSNPSSSKSPLGHSLGSDKNEKLSALKAYGYDLTEMVRESKLDPVIGRSSEVERLILILCR
RRKNNPVLIGEAGVGKTAIVEGLAQKIILNEVPDALRKKRLITLDLALMIAGTKYRGQFEERIKAVMDEVRKH
GNILLFIDELHTIVGAGAAEGAIDASNILKPALARGEIQCIGATTIDEYRKHIEKDAALERRFQKIVVHPPSVDE
TIEILRGLKKKYEEHHNVFITEEALKAAATLSDQYVHGRFLPDKAIDLLDEAGARVRVNTMGQPTDLMKLEA
EIENTKLAKEQAIGTQEYEKAAGLRDEEKKLRERLQSMKQEWENHKEEHQVPVDEEAVAQVVSLQTGIPSA
RLTEAESEKLLKLEDTLRRKVIGQNDAVTSICRAIRRSRTGIKDPNRPTGSFLFLGPTGVGKSLLAQQIAIEMFG
GEDALIQVDMSEYMEKFAATKMMGSPPGYVGHEEGGHLTEQVRRRPYCVVLFDEIEKAHPDIMDLMLQILE
QGRLTDSFGRKVDFRHAIIIMTSNLGADLIRKSGEIGFGLKSHMDYKVIQEKIEHAMKKHLKPEFINRLDESVI
FRPLEKESLSEIIHLEINKLDSRLKNYQMALNIPDSVISFLVTKGHSPEMGARPLRRVIEQYLEDPLAELLLKES
CRQEARKLRATLVENRVAFEREEEEQEAALPSPHLES

>core/53/5/Org5_Gene8

MKIPLRFLLISLVPTLSMSNLLGAATTEELSASNSFDGTTSTTSFSSKTSSATDGTNYVFKDSVVIENVPKTGET
QSTSCFKNDAAAGDLNFLGGGFSFTFSNIDATTASGAAIGSEAANKTVTLSGFSALSFLKSPASTVTNGLGAIN
VKGNLSLLDNDKVLIQDNFSTGDGGAINCAGSLKIANNKSLSFIGNSSSTRGGAIHTKNLTLSSGGETLFQGNT
APTAAGKGGAIAIADSGTLSISGDSGDIIFEGNTIGATGTVSHSAIDLGTSAKITALRAAQGHTIYFYDPITVTGS

TSVADALNINSPDTGDNKEYTGTIVFSGEKLTEAEAKDEKNRTSKLLQNVAFKNGTVVLKGDVVLSANGFSQ
DANSKLIMDLGTSLVANTESIELTNLEINIDSLRNGKKIKLSAATAQKDIRIDRPVVLAISDESFYQNGFLNEDH
SYDGILELDAGKDIVISADSRSIDAVQSPYGYQGKWTINWSTDDKKATVSWAKQSFNPTAEQEAPLVPNLLW
GSFIDVRSFQNFIELGTEGAPYEKRFWVAGISNVLHRSGRENQRKFRHVSGGAVVGASTRMPGGDTLSLGFA
QLFARDKDYFMNTNFAKTYAGSLRLQHDASLYSVVSILLGEGGLREILLPYVSKTLPCSFYGQLSYGHTDHR
MKTESLPPPPPTLSTDHTSWGGYVWAGELGTRVAVENTSGRGFFQEYTPFVKVQAVYARQDSFVELGAISRD
FSDSHLYNLAIPLGIKLEKRFAEQYYHVVAMYSPDVCRSNPKCTTTLLSNQGSWKTKGSNLARQAGIVQASG
FRSLGAAAELFGNFGFEWRGSSRSYNVDAGSKIKF

>core/54/5/Org5_Gene939

MFNKDEIIVPKNLEEEMKESYLRYSMSVIISRALPDIRDGLKPSQRRVLYAMKQLSLSPGAKHRKCAKICGDT
SGDYHPHGESVIYPTLVRMAQNWAMRYPLVDGQGNFGSIDGDPPAAMRYTEARLTHSAMYLMEDLDKDT
VDIVPNYDETKHEPVVFPSKFPNLLCNGSSGIAVGMATNIPPHNLGELIEATLLLLANPQASVDEILQVMPGPD
FPTGGIICGSEGIRSTYTTGRGKIKVRARLHVEENEDKHRESIIITEMPYNVNKSRLIEQIANLVNEKTLAGISDV
RDESDKDGIRVVLEIKKGESSEIIINRLYKFTDVQVTFGANMLALDKNLPRTMSIHRMISAWIRHRKEVIRRRT
RYELNKAETRAHVLEGYLKALSCLDALVKTIRESGNKEHAKERIIESFGFTEPQALAILELRLYQLTGLEAEKI
QKEYEELLNKIAYYKQVLSDEGLVKDIIRNELQDLLKHHKVARRTTIEFDADDIRDIEDIITNESVIITISGDDY
VKRMPVKVFKEQRRGGHGVTGFDMKKGAGFLKAVYSAFTKDYLLIFTNFGQCYWLKVWQLPEGERRAKG
KPIINFLEGIRPGEELAAILNIKNFDNAGFLFLATKRGVVKKVSLDAFSNPRKKGIRALEIDEGDELIAACHIVS
DEEKVMLFTHLGMAVRFPHEKVRPMGRTARGVRGVSLKNEEDKVVSCQIVTENQSVLIVCDQGFGKRSLVE
DFRETNRGGVGVRSILINERNGNVLGAIPVTDHDSILLMSSQGQAIRINMQDVRVMGRSTQGVRLVHLKEGD
ALVSMEKLSSNENDDEVLSGSEEECSDTVSLR

>core/55/5/Org5_Gene1035

MIYSTSISTFYKKLSLVSSMHSFAQRHRESLEHIANYEKTTAERDILKRLIEVLDQRASERYRSAVEKLHKYEV
ERATVAKSIPVAAIHEKPLSSTHASVQVTASTPAATGSGVGAYYNAVKQKWAQDLIVELNTVMTTIMASVNS
KNPANKDVFDKLNTELQALVAAGNNLTEENFQTLYNFPEEIFTAIQRADTFTGGMKTDFTNQLAGKYGNQA
TLTQTFADGRVEGFKDILTAVQGVLTPEQFTIFAEIATELQALADHVGNFDEAGLQRIEDAGEKLAAVINSSD
LTRNDKIMFCQHITDLYSDQVAALGSFDTVLDASIYVNQHQGTMFSNLSSFVGSLIGTFAPIDLSSSQGDISSA
ALAGALQTARGLNSRFNELTAEQQKLINECIKSLVTFKCGEHLGAIWAYFTASTVVALNPTATMDHVKAAIL
EEAKELDNSSFQLASSIKSAMTSIVNSSGSFSVTVNSSTLQYTIYSEKNGKVEINQILLNYGSTGFLPEITKLAKT
NAESTARSYFRFKALAAVESENVQNKIEDLQSQLQQFTNMKTELFDGQLLSQASELRALPLPSAVASVLIDRY
MPKEVDYLNEIYKKLYYSNLGSSVGNSIIDAISQYVNGATYFNFASYVGQQPAVGAGGANAFPGSQESAQAK
LDQERKQAALYLQETRGALTVIEEQRARVLKDDKITNEQRSTILDSLRNYEDNINSISGSLVLLQNYLQPLSIA
GGSVAGTFEVKEGQEQWQARLQILEEALVSGLVGNMINGGMFPLQSTIQSDQQSFADMGQNFQLDLQMHLT
SMQQEWTVVATSLQLLNQMYLSLARSLTG

>core/56/5/Org5_Gene997

MTEKKPTPMMEQWHQCKEKAGDSVLLFRMGDFYEAFYDDAVLLSQHLELTLTQRQGIPMSGIPVSTVDTYV
DRLIGKGFKVAVAEQFGEPAKEKESKKIGPMARDIQRFVTPGTLLSSTLLQEKFNNYIVAINRIGSLFGFACLD
LSTGSFFIEECENTKELVDEICRLAPSEVLSCNKFYNKETAIVMQLQQHLKLTLSTYADWAFEHKFASQKLTT

HFQVASLDGFGLKGLVPAINAAGGLLSYIQDKLLLPTKHIAIPQTRGKQQKLLIDTASQVNLELLAPLNDPQG
KNSLLRIMDHTSTPMGGRLLRQILISPFYNPKEILVRQDAVEFFLRQVTLRKNIKTYLCQVRDIERLMTKVTTG
LAGPRDIGTLRDSFSAGAQIYEQLASATLPEFFIDKCSLDTKLASLIALLSKSLNGDLPLRVSDGNIFVDEFHND
LKRLRHNQEHSQEWIWEYQERIRKETGIKKLKICFAQALGYYIEVSSEFAPQLPKDFIRRQSRLHAERFTTIEL
QQFQDDMSNISEKLQTLETQFFKDLCSHILQLRTEILALSQSLADLDYIISLADLAHAQGYCRPRVDMSDTLCI
YRGCHPVAKTLVDTGKFIPNDTEMRGSQTRMILLTGPNMAGKSTYIRQIALLVIMAQMGSYIPAKSAHIGVID
KIFTRIGAGDNLSKGMSTFMVEMAETANILHNATDRSLVILDEVGRGTSTYDGLAIAQAVVEYLLFTDKKKA
KTLFATHYKELTTLEDHCPHVENFHAGVKDKAGQPVFLYEILKGHSQKSFGIHVARLAGFPLCVVSRAQQIL
RQLEGPESITRPAQDKMQQLTLF

>core/57/5/Org5_Gene236

MEDFSSFDKNKVSVDSMKRAILDRLYLSVVQSPESASPRDIFTAVAKTVMEWLAKGWLKTQNGYYKNDVK
RVYYLSMEFLLGRSLKSNLLNLGILDLVRKALKTLNYDFDHLVEMESDAGLGNGGLGRLAACYLDSMATLA
VPAYGYGIRYDYGIFDQRIVNGYQEEAPDEWLRYGNPWEICRGEYLYPVRFYGRVIHYTDSRGKQVADLVD
TQEVLAMAYDIPIPGYGNDTVNSLRLWQAQSPRGFEFSYFNHGNYIQAIEDIALIENISRVLYPNDSITEGQELR
LKQEYFLVSATIQDIIRRYTKTHICLDNLADKVVVQLNDTHPALGIAEMMHILVDREELPWDKAWEMTTVIF
NYTNHTILPEALERWPLDLFSKLLPRHLEIIYEINSRWLEKVGSRYPKNDDKRRSLSIVEEGYQKRINMANLAV
VGSAKVNGVSSFHSQLIKDTLFKEFYEFFPEKFINVTNGVTPRRWIALCNPRLSKLLNETIGDRYIIDLSHLSLIR
SFAEDSGFRDHWKGVKLKNKQDLTSRIYNEVGEIVDPNSLFDCHIKRIHEYKRQLMNILRVIYVYNDLKENP
NQDVVPTTVIFSGKAAPGYVMAKLIIKLINSVADVVNQDSRVNDKLKVLFLPNYRVSMAEHIIPGTDLSEQIS
TAGMEASGTGNMKFALNGALTIGTMDGANIEMAEHIGKENMFIFGLLEEQIVQLRREYCPQTICDKNPKIRQ
VLDLLEQGFFNSNDKDLFKPIVHRLLHEGDPFFVLADLESYIAAHENVNKLFKEPDSWTKISIYNTAGMGFFS
SDRAIQDYARDIWHVPTKSCSGEGN

>core/58/5/Org5_Gene1005

MKKLYHPTLFLRPLIRLSLIFALSLTLISGNFPQQKSFGHCCADMHSALISGKNCEELFADFIERVLADRETLTA
RDWGTVVVLVREYLLKCIRKGDCDYGVKILQKLLALRLPKDARKDLQILWHRLNPEQAPLRDVVDQLFTIG
CHESLQDHLLFELYTVTLHSGYENRKQDMLLAKEQGDYKKAIELAKELVAALEKGSCSPHPEIVQIEKTFLQ
KTLLALQIKVAQEAQESCDALLTPYCLSEIAYTEAMDALVLRIARGEVSRTNEVDSVLLSHALQHLPFAREKA
IPELEVLIDHGAYLESTLLYYAYFSLLELYHQNKDFASLERLLEKGDAVFVPEHPYFPEYGFFLGAYFYAKGK
YESAEKVFLQIIDPAVKLGATFARAYEYLGCIAYVQNHYEKAEEYFLRAYKSWGREESGIGLFLAYAVQKKK
TACEDMLYHPKFSFTYRHLLDSLCSLSYPHGENKGSSAIQRVHRAVPELSEIYSRCIYDMIKYRNVTYTHPIIE
LAYNQVRNLEKRNLEEICRDAQDPEYDKALAFWGALQSGASVPRSLIESSDVDEAGITIRCYEALYFHNPDAI
AMLPQAFSEECNSWQTALRLVWTLVRPKGAPNHAKYWDHLVLRPHGDSLYFFGYDLQEYLIGKEDALKHL
SVFAELFPKSSLLSLVYYLQGYSESSALRKVGWFVKALEEFTEISWSGEHMKTWAYIYYMVKLDLADTYISL
GNFSQAVHILEEVKEDWQVASHPKLHFLKGEDCYLAMELRWVEGLAYAYFQLHETAHLSNHLLEHVEKNLI
SPRSYRDYYGESLQRTLGLCQRFLGV

>core/59/5/Org5_Gene108

MRYDPNLIEKKWQQFWKEHRSFQANEDEDKVKYYVLDMFPYPSGAGLHVGHLIGYTATDIVARYKRARGF
SVLHPMGWDSFGLPAEQYAIRTGTHPKVTTQKNIANFKKQLSAMGFSYDEGREFATSDPDYYHWTQKLFLF

LYDQGLAYMADMAVNYCPELGTVLSNEEVENGFSIEGGYPVERKMLRQWILKITAYADKLLEGLDALDWP
ENVKQLQKNWIGKSEGALVTFHLTQEGSLEAFTTRLDTLLGVSFLVIAPEHPDLDSIVSEEQRDEVTAYVQES
LRKSERDRISSVKTKTGVFTGNYAKHPITGNLLPVWISDYVVLGYGTGVVMGVPAHDERDREFAEMFSLPIH
EVIDDNGVCIHSNYNDFCLNGLSGQEAKDYVINYLEMRSLGRAKTMYRLRDWLFSRQRYWGEPIPIIHFEDG
THRPLEDDELPLLPPNIDDYRPEGFGQGPLAKAQDWVHIYDEKTGRPGCRETYTMPQWAGSCWYYLRFCDA
HNSQLPWSKEKESYWMPVDLYIGGAEHAVLHLLYSRFWHRVFYDAGLVSTPEPFKKLINQGLVLASSYRIPG
KGYVSIEDVREENGTWISTCGEIVEVRQEKMSKSKLNGVDPQVLIEEYGADALRMYAMFSGPLDKNKTWSN
EGVGGCRRFLNRFYDLVTSSEVQDIEDRDGLVLAHKLVFRITEHIEKMSLNTIPSSFMEFLNDFSKLPVYSKRA
LSMAVRVLEPIAPHISEELWVILGNPPGIDQAAWPQIDESYLVAQTVTFVVQVNGKLRGRLEVAKEAPKEEVL
SLSRSVVAKYLENAQIRKEIYVPNKLVNFVL

>core/60/5/Org5_Gene15

MDSTTNSDSPILDPNPEDVEKLLDESEEESEDQSTERLLPSELFILPLNKRPFFPGMAAPILIESGPYYEVLKVLA
KSSQKYIGLVLTKKENADILKVSFNQLHKTGVAARILRIMPIEGGSAQVLLSIEERIRIIEPIKDKYLKARVSYH
ADNKELTEELKAYSISIVSVIKDLLKLNPLFKEELQIFLGHSDFTEPGKLADFSVALTTATREELQEVLETTNM
HDRIDKALILLKKELDLSRLQSSINQKIEATITKSQKEFFLKEQLKTIKKELGLEKEDRAIDIEKFSERLRKRHVP
DYAMEVIQDEIEKLQTLETSSAEYTVCRNYLDWLTIIPWGIQSKEYHDLKKAEIVLNKDHYGLDEIKQRILELI
SVGKLSKGLKGSIICLVGPPGVGKTSIGRSIAKVLHRKFFRFSVGGMRDEAEIKGHRRTYIGAMPGKMVQALK
QSQAMNPVIMIDEVDKIGASYHGDPASALLEVLDPEQNKDFLDHYLDVRVDLSNVLFILTANVLDTIPDPLLD
RMEILRLSGYILEEKLQIAKKYLVPKARKEIGLTASEVNFQPEALKYMINNYAREAGVRTLNGNIKKVLRKVA
LKIVQNQEKPKSKKITFKISSKNLQTYLGKPIFSSDRFYESTPVGVATGLAWTSLGGATLYIESVQVSSLKTDM
HLTGQAGEVMKESSQIAWTYLHSALHRYAPGYTFFPKSQVHIHIPEGATPKDGPSAGITMVTSLLSLLLETPV
VNNLGMTGEITLTGRVLGVGGIREKLIAARRSRLNILIFPEDNRRDYEELPAYLKTGLKIHFVSHYDDVLKVA
FPKLK

>core/61/5/Org5_Gene732

MKGTPQYHFIGIGGIGMSALAHILLDRGYEVSGSDLYESYTIESLKAKGARCFSGHDSSHVPHDAVVVYSSSI
APDNVEYLTAIQRSSRLLHRAELLSQLMEGYESILVSGSHGKTGTSSLIRAIFQEAQKDPSYAIGGLAANCLNG
YSGSSKIFVAEADESDGSLKHYTPRAVVITNIDNEHLNNYAGNLDNLVQVIQDFSRKVTDLNKVFYNGDCPIL
KGNVQGISYGYSPECQLHIVSYNQKAWQSHFSFTFLGQEYQDIELNLPGQHNAANAAAACGVALTFGIDINII
RKALKKFSGVHRRLERKNISESFLFLEDYAHHPVEVAHTLRSVRDAVGLRRVIAIFQPHRFSRLEECLQTFPKA
FQEADEVILTDVYSAGESPRESIILSDLAEQIRKSSYVHCCYVPHGDIVDYLRNYIRIHDVCVSLGAGNIYTIGE
ALKDFNPKKLSIGLVCGGKSCEHDISLLSAQHVSKYISPEFYDVSYFIINRQGLWRTGKDFPHLIEETQGDSPLS
SEIASALAKVDCLFPVLHGPFGEDGTIQGFFEILGKPYAGPSLSLAATAMDKLLTKRIASAVGVPVVPYQPLNL
CFWKRNPELCIQNLIETFSFPMIVKTAHLGSSIGIFLVRDKEELQEKISEAFLYDTDVFVEESRLGSREIEVSCIG
HSSSWYCMAGPNERCGASGFIDYQEKYGFDGIDCAKISFDLQLSQESLDCVRELAERVYRAMQGKGSARIDF
FLDEEGNYWLSEVNPIPGMTAASPFLQAFVHAGWTQEQIVDHFIIDALHKFDKQQTIEQAFTKEQDLVKR

>core/62/5/Org5_Gene713

MIRERKKSRHPRLPTLPLAAKASLYLFFACFSGLSLWSFHRDQPCTQNWIGLLGWSFSSFLLYFFGAAAFFIPL
YFLWLSFLYFRRTPRPLFFYKAAAFLSLPFCSAILLSMLSPVGTLPALLDTRLPKFILGNNPPVSYVGGIPFYLF

YEGQSFCLKHLIGSVGTALIFGFVMLFSVLYLCGGIALLKKKTFQDGVKKAFCSFFQTCFKNLKKLINRRNYL
PKPSVPFVSKNPFSCTKSQPSPRRVSETIILDGSISPLPQEEIPGSKKESFFLTPHPCKRFLTKFVEPQENKAKEGK
TIALSSTPTVVRESKGKERAALPKLKSLAVPENDLPQYHLLSKNREARPESLQAELERKALILKQTLTSFGIDA
DLGNICSGPTLAAFEVLPHSGVKVQKIKSLENDIALKLQASSIRIIAPIPGKAAVGIEIPTPFPQAVNFRDLLEDY
QKTNRKLQIPLLLGKKANGDNLWADLATMPHLIIAGTTGSGKSVCINTIVMSMIMTTLPSEIKLVIIDPKKVEL
TGYSQLPHMLSPVITESREVYNALVWLVKEMESRYEILRYLGLRNIQAFNSRTRNKTIEASYDREIRETMPFM
VGIIDELSDLLLSSSQDIETPIIRLAQMARAVGIHLILATQRPSREVITGLIKANFPSRISFKVSNKVNSQIIIDEPG
AENLMGNGDMLVLLPSVFGTIRAQGAYICDEDINKVIQDLCSRFPTQYVIPSFHAFDDSDSDNSGEKDPLFAQ
AKTLILQTGNASTTFLQRKLKIGYARAASLIDQLEEARIIGPSEGAKPRQILIQNPLEG

>core/63/5/Org5_Gene209

MDPKEKNYDASAITVLEGLQAVRERPGMYIGDTGITGLHHLVYEVVDNSIDEAMAGYCSRIDVRILEDGGIVI
VDNGRGIPIEVHERESAKQGREVSALEVVLTVLHAGGKFDKDSYKVSGGLHGVGVSCVNALSEKLVATVFK
DKKCYQMEFSRGIPVTPLQYVSVSDRQGTEIVFYPDPKIFSTCTFDRSILMKRLRELAFLNRGITIVFEDDRDVS
FDKVTFFYEGGIQSFVSYLNQNKESLFSEPIYICGTRVGDDGEIEFEAALQWNSGYSELVYSYANNIPTRQGGT
HLTGFSTALTRVINTYIKAHNLAKNNKLALTGEDIREGLTAVISVKVPNPQFEGQTKQKLGNSDVSSVAQQV
VGEALTIFFEENPQIARMIVDKVFVAAQAREAAKKARELTLRKSALDSARLPGKLIDCLEKDPEKCEMYIVEG
DSAGGSAKQGRDRRFQAILPIRGKILNVEKARLQKIFQNQEIGTIIAALGCGIGADNFNLSKLRYRRIIIMTDAD
VDGSHIRTLLLTFFYRHMTALIENECVYIAQPPLYKVSKKKDFRYILSEKEMDSYLLMLGTNESSILFKSTERE
LRGEALESFINVILDVESFINTLEKKAIPFSEFLEMYKEGIGYPLYYLAPATGMQGGRYLYSDEEKEEALAQEE
THKFKIIELYKVAVFVDIQNQLKEYGLDISSYLIPQKNEIVIGNEDSPSCNYSCYTLEEVINYLKNLGRKGIEIQR
YKGLGEMNADQLWDTTMNPEQRTLIHVSLKDAVEADHIFTMLMGEEVPPRREFIESHALSIRINNLDI

>core/64/5/Org5_Gene934

MNKRTLLFVSLIGIAFVGCQIFFGYNEFRSCKNLAEKQRKISEQTLAAVESVGLSVASWDTDVNGEEHKNNY
AVRVGDKLFLLHNGEAAQSVYSSGESWSFVDHKCGFDNIHLALYRQQGSSFNPTNTGKVFLPTNHEGLPVL
VVEFRNNKEPLVFLGEYAQGRISNKDSTIFGTALVFWRSGSDYIPLGLYDSREEKLVSLDLPITRAVIFGNDQD
SAKSSDTANHYVLFNDYMQIIVSEESGSIEGINLPFASTNNKSIVNEIGFDRDLASEKSPEALFPGLSSKLPDGQ
QAKNSIGGYYPLLRRGLLSDSKKLLPLEYHALNVVSGRELATPVALRYRVLSYTPHSIQLESLDRSVQKVYKL
PENPEEKPYVFETAITLTKETEDVWVTSGVPEVEIMSNASAPTIKYRVIKKNKGSLDKVKLPKVKEPLAVRRG
VYPQWILNSNGYFGIILTPLSEIASGYGSLYISGSTAPTRLSAISPKNQLYPVSKYPGYETLLPLPKDAGTHRFL
VYAGPLAEPTLKVLDKTITQEKGENPEYLDSISFRGVFAFITAPFAALLFIIMKFFKLVTGSWGISIILLTVFLKL
LLYPLNAWSIRSMRRMQILSPYIQQIQQKYKNEPKRAQMEIMGLYKTNKVNPITGCLPLLIQLPFLIAMFDLLK
SSFLLRGASFIPGWIDNLTAPDVLFSWQTSIWFIGNEFHLLPILLGIVMFLQQKVTSLHKKGPVTDQQKQQQV
MGNMMAILFTAMFYNFPSGLNIYWLSSMILGVVQQWITNKILDSKHLKNEVVLNNKKHR

>core/65/5/Org5_Gene886

MRIPITLLQTYFSEPLSTKEILEACDHIGIEAEIENTTLYSFASVITAKILHTIPHPNADKLRVATLTDGEKEHQV
VCGAPNCEAGLIVALALPGAKLFDSEGQAYTIKKSKLRGVESQGMCCGADELGLDELQIQERALLELPEATP
LGEDLATVLGNTSLEISLTPNLGHCASFLGLAREICHVTQANLVIPKEFSFENLPTTALDMGNDPDICPFFSYV
VITGISAQPSPIKLQESLQALKQKPINAIVDITNYIMLSLGQPLHAYDASHVALDSLRVEKLSTPESLTLLNGET

VLLPSGVPVVRDDHSLLGLGGVMGAKAPSFQETTTTTVIKAAYFLPEALRASQKLLPIPSESAYRFTRGIDPQN
VVPALQAAIHYILEIFPEATISPIYSSGEICRELKEVALRPKTLQRILGKSFSIEILSQKLQSLGFSTTPQETSLLVK
VPSYRHDINEEIDLVEEICRTESWNIETQNPVSCYTPIYKLKRETAGFLANAGLQEFFTPDLLDPETVALTRKE
KEEISLQGSKHTTVLRSSLLPGLLKSAATNLNRQAPSVQAFEIGTVYAKHGEQCQETQTLAILLTEDGESRSW
LPKPSLSFYSLKGWVERLLYHHHLSIDALTLESSALCEFHPYQQGVLRIHKQSFATLGQVHPELAKKAQIKHP
VFFAELNLDLLCKMLKKTTKLYKPYAIYPSSFRDLTLTVPEDIPANLLRQKLLHEGSKWLESVTIISIYQDKSL
ETRNKNVSLRLVFQDYERTLSNQDIEEEYCRLVALLNELLTDTKGTINS

>core/66/5/Org5_Gene230

MLIMRNKVILQISILALIQTPLTLFSTEKVKEGHVVVDSITIITEGENASNKHPLPKLKTRSGALFSQLDFDEDLR
ILAKEYDSVEPKVEFSEGKTNIALHLIAKPSIRNIHISGNQVVPEHKILKTLQIYRNDLFEREKFLKGLDDLRTY
YLKRGYFASSVDYSLEHNQEKGHIDVLIKINEGPCGKIKQLTFSGISRSEKSDIQEFIQTKQHSTTTSWFTGAGL
YHPDIVEQDSLAITNYLHNNGYADAIVNSHYDLDDKGNILLYMDIDRGSRYTLGHVHIQGFEVLPKRLIEKQS
QVGPNDLYCPDKIWDGAHKIKQTYAKYGYINTNVDVLFIPHATRPIYDVTYEVSEGSPYKVGLIKITGNTHTK
SDVILHETSLFPGDTFNRLKLEDTEQRLRNTGYFQSVSVYTVRSQLDPMGNADQYRDIFVEVKETTTGNLGLF
LGFSSLDNLFGGIELSESNFDLFGARNIFSKGFRCLRGGGEHLFLKANFGDKVTDYTLKWTKPHFLNTPWILGI
ELDKSINRALSKDYAVQTYGGNVSTTYILNEHLKYGLFYRGSQTSLHEKRKFLLGPNIDSNKGFVSAAGVNL
NYDSVDSPRTPTTGIRGGVTFEVSGLGGTYHFTKLSLNSSIYRKLTRKGILKIKGEAQFIKPYSNTTAEGVPVSE
RFFLGGETTVRGYKSFIIGPKYSATEPQGGLSSLLISEEFQYPLIRQPNISAFVFLDSGFVGLQEYKISLKDLRSS
AGFGLRFDVMNNVPVMLGFGWPFRPTETLNGEKIDVSQRFFFALGGMF

>core/67/5/Org5_Gene974

MLLLISGALFLTLGIPGLTAGVSFGLGIGLSALGGVLVVSGLLCLLVKREVSKVCPEEIPAVQPEETPEGVPVTP
FEKPALDEAQKEQKTQKILDQLPQELDQLDRYIQEVFACLGPLKDLKYEDQGFLQDVKEEFQVFDFVQKDMI
AEFVELQQILCQEGRLLEFVINQTRYIGRDLFKREDSLYKLWEWLGYLPSGDVRGERLKKSAREVVDRFMRT
TCNIRKIAMTFDRHVYSVAKTAFEKAFGALETCVYESMRESYREAFCEYEKAKLLGDEEKSAHAEQRFQDIK
NRWEDVKDAFFWVKEDGKIEIDDAIGNSCKWSERYEEHRITRARWYKVAEHQLFNATMRVKDSLREHNEA
RVAFEKERSKENQRQVQKKKEKRLRDLKELHDQELPRAQERLRELQALYPEIAVSVVEARREVASDLEKAH
ESIDKHYQSCVREQELY

>core/68/5/Org5_Gene903

MASGIGGSSGLGKIPPKDNGDRSRSPSPKGELGSHEISLPPQEHGEEGASGSSHIHSSSSFLPEDQESQSSSSAAS
SPGFFSRVRSGVDRALKSFGNFFSAESTSQARETRQAFVRLSKTITADERRDVDSSSAAATEARVAEDASVSG
ENPSQGVPETSSGPEPQRLFSLPSVKKQSGLGRLVQTVRDRIVLPSGAPPTDSEPLSLYELNLRLSSLRQELSDI
QSNDQLTPEEKAEATVTIQQLIQITEFQCGYMEATQSSVSLAEARFKGVETSDEINSLCSELTDPELQELMSDG
DSLQNLLDETADDLEAALSHARLSFSLDDNPTPIDNNPTLISQEEPIYEEIGGAADPQRTRENWSTRLWNQIRE
ALVSLLGMILSILGSILHRLRIARHAAAEAVGRCCTCRGEECTSSEEDSMSVGSPSEIDETERTGSPHDVPRRN
GSPREDSPLMNALVGWAHKHGAKTKESSESSTPEISISAPIVRGWSQDSSVSFIVMEDDHIFYDVPRRKDGIYD
VPSSPRWSPARELEEDVFGDYEVPITSAEPSKDKNIYMTPRLATPAIYDLPSRPGSSGSSRSPSSDRVRSSSPNR
RGVPLPPVPSPAMSEEGSIYEDMSGASGAGESDYEDMSRSPSPRGDLDEPIYANTPEDNPFTQRNIDRILQERS

GGASASPVEPIYDEIPWIHGRPPATLPRPENTLTNVSLRVSPGFGPEVRAALLSESVSAVMVEAESIVPPTEPGD
GESEYLEPLGGLVATTKILLQKGWPRGESNA

>core/69/5/Org5_Gene980

MLLLISGALFLTLGIPGLSAAISFGLGIGLSALGGVLMISGLLCLLVKREIPTVRPEEIPEGVSLAPSEEPALQAA
QKTLAQLPKELDQLDTDIQEVFACLRKLKDSKYESRSFLNDAKKELRVFDFVVEDTLSEIFELRQIVAQEGWD
LNFLINGGRSLMMTAESESLDLFHVSKRLGYLPSGDVRGEGLKKSAKEIVARLMSLHCEIHKVAVAFDRNSY
AMAEKAFAKALGALEESVYRSLTQSYRDKFLESERAKIPWNGHITWLRDDAKSGCAEKKLRDAEERWKKF
RKAVFWVEEDGGFDINNLLGDWGTVLDPYRQERMDEITFHELYEKTTFLKRLHRKCALAKTTFEKKRSKKN
LQAVEEANARRLKYVRDWYGQEFQKAGERLEKLHALYPEVSVSIRENKIQETRSNLEKAYEAIEENYRCCVR
EQEDYWKEEEKREAEFRERGNKILSPEELESSLEQFDHGLKNFSEKLMELEGHILKLQKEATAEVENKILSDA
ESRLEIVFEDVKEMPCRIEEIEKTLRMAELPLLPTKKAFEKACSQYNSCAEMLEKVKPYCKESLAYVTSKERL
VSLDEDLRRAYTECQKRFQGDSGLESEVRACREQLRERIQEFETQGLDLVEKELLCVSSRLRNTECDCVSGV
KKEAPPGKKFYAQYYDEIYRVRVQSRWMTMSERLREGVQACNKMLKAGLSEEDKVLKEEEYWLYREERK
NKEKRLVGTKIVATQQRVAAFESIEVPEIPEAPEEKPSLLDKARSLFTREDHS

>core/70/5/Org5_Gene1024

MLFSKNFSTDNGGAITAKTLSLTGTTMSALFSENTSSKKGGAIQTSDALTITGNQGEVSFSDNTSSDSGAAIFT
EASVTISNNAKVSFIDNKVTGASSSTTGDMSGGAICAYKTSTDTKVTLTGNQMLLFSNNTSTTAGGAIYVKKL
ELASGGLTLFSRNSVNGGTAPKGGAIAIEDSGELSLSADSGDIVFLGNTVTSTTPGTNRSSIDLGTSAKMTALR
SAAGRAIYFYDPITTGSSTTVTDVLKVNETPADSALQYTGNIIFTGEKLSETEAADSKNLTSKLLQPVTLSGGT
LSLKHGVTLQTQAFTQQADSRLEMDVGTTLEPADTSTINNLVINISSIDGAKKAKIETKATSKNLTLSGTITLL
DPTGTFYENHSLRNPQSYDILELKASGTVTSTAVTPDPIMGEKFHYGYQGTWGPIVWGTGASTTATFNWTKT
GYIPNPERIGSLVPNSLWNAFIDISSLHYLMETANEGLQGDRAFWCAGLSNFFHKDSTKTRRGFRHLSGGYVI
GGNLHTCSDKILSAAFCQLFGRDRDYFVAKNQGTVYGGTLYYQHNETYISLPCKLRPCSLSYVPTEIPVLFSG
NLSYTHTDNDLKTKYTTYPTVKGSWGNDSFALEFGGRAPICLDESALFEQYMPFMKLQFVYAHQEGFKEQG
TEAREFGSSRLVNLALPIGIRFDKESDCQDATYNLTLGYTVDLVRSNPDCTTTLRISGDSWKTFGTNLARQAL
VLRAGNHFCFNSNFEAFSQFSFELRGSSRNYNVDLGAKYQF

>core/71/5/Org5_Gene689

MTLQPSYINFTPNVTTALSGGKIDTSAIELSCSALFFQELQDKAQGLKHALGLVQELSAEALRPAQVQTSISYL
PTEESSRPGISAGIIDRTMPTFTDDEVKAILQNPNFETSKIFVEGLDKVFKSYLDSVTPPEGIDPSNPESAIILNYIT
LLNNLKPKFAAGSTPTDADYNALYALPGDFVKEIEALKAADAPPKSKVHAFWQEIMTIYNNMQVLSYPVTD
YLNVQIADLSLNITAAQEVQQYLKNFYSILKDILNPGWTDPQATHYPADAEYNARDAGVIQSLLNLSGNYRQ
LTENMLPNTDTSLPQEIIAQIRSFQNGVNGTIIASNTLLPTTMRLDTLLGVIYTYQCCATIFGMSYGTSTPAKQN
YIDAINQEKSYWQARANGFDVTSDQVFDQFATNIQSGTSYRGIDLFKNNKVNEINPIFLSQAASFLRYPYNLM
SRSMYQTIEDAANRSITALDGLISGWSTQIATFQTQKNSLDPSLLKYFDTMKANKESFVTTAPLQMVYSSLML
DKYLPTQQNVIASLGIQMTYSNKAAKYLNELIKEITTFQSADIYYSLSIYLKQMNLQAVADPIGKAVGVLNDE
KTRAMADITRCNKIKAAIDKMLVEIKADAELSKSQIRELVDTLTNFKSQSDDLIRNLSCLLGFLSGLTLKAVN
DPNATYEAFTAEIFTEPFNNWKRQLATFESFVIQGGQNGITPGGQQQLLQAMESSQQDFSTFNQNQQLALQLE
SSAMQQEWTLVSAALALLNQMVSKIARRIKS

>core/75/5/Org5_Gene656

MVFFRNSLLHLVALSGMLCCSSGVALTIAEKMASLEHSGRGADDYEGMASFNANMREYSLQLSKLYEEARK
LRASGTEDEALWKDLIRRIGEVRGYLREIEELWAAEIREKGGNLEDYALWNHPETTIYNLVTDYGTEDSIYLI
PQEIGAIKIATLSKFVVPKESFEDCLTQILSRLGIGVRQVNSWIKELYMMRKEGCSVAGVFSSRKDLEALPETA
YIGFVLNSNVDAHTNQHVLKKFINPETTHVDVIAGRVWIFGSAGEVGELLKIYNFVQSESIRQEYRVIPLTKID
PGEMISILNAAFREDLTKDVSEESLGLRVVPLQYQGRSLFLSGTAALVQQALTLIRELEEGIENPTDKTVFWYN
VKHSDPQELAALLSQVHDVFSGENKASVGAADGCGSQLNASIQIDTTVSSSAKDGSVKYGNFIADSKTGTLI
MVVEKEVLPRIQMLLKKLDVPKKMVRIEVLLFERKLAHEQKSGLNLLRLGEEVCKKGCSPSVSWAGGTGILE
FLFKGSTGSSIVPGYDLAYQFLMAQEDVRINASPSVVTMNQTPARIAVVDEMSIAVSSDKDKAQYNRAQYGI
MIKMLPVINVGEEDGKSYITLETDITFDTTGKNHDDRPDVTRRNITNKVRIADGETVIIGGLRCKQMSDSHDGI
PFLGDIPGIGKLFGMSSTSDSLTEMFVFITPKILENPVEQQERKEEALLSSRPGEREEYYQALAASEAAARAAH
KKLEMFPASGVSLSQVERQEYDGC

>core/76/5/Org5_Gene749

MGYIESSTFRLYAEVIVGSNINKVLDYGVPENLEHITKGTAVTISLRGGKKVGVIYQIKTTTQCKKILPILGLSD
SEIVLPQDLLDLLFWISQYYFAPLGKTLKLFLPAISSNVIQPKQHYRVVLKQSKAKTKEILAKLEVLHPSQGAV
LKILLQHASPPGLSSLMETAKVSQSPIHSLEKLGILDIVDAAQLELQEDLLTFFPPAPKDLHPEQQSAIDKIFSSL
KTSQFHTHLLFGITGSGKTEIYLRATSEALKQGKSTILLVPEIALTVQTVSLFKARFGKDVGVLHHKLSDSDKS
RTWRQASEGSLRILIGPRSALFCPMKNLGLIIVDEEHDPAYKQTESPPCYHARDVAVMRGKLAHATVVLGSA
TPSLESYTNALSGKYVLSRLSSRAAAAHPAKISLINMNLEREKSKTKILFSQPVLKKIAERLEVGEQVLIFFNRR
GYHTNVSCTVCKHTLKCPHCDMVLTFHKYANVLLCHLCNSSPKDLPQSCPKCLGTMTLQYRGSGTEKIEKIL
QQIFPQIRTIRIDSDTTKFKGSHETLLRQFATGKADVLIGTQMIAKGMNFSAVTLAVILNGDSGLYIPDFRASEQ
VFQLITQVAGRSGRSHLPGEILIQSFLPDHPTIHSAMRQDYSAFYSQEITGRELCEYPPFIRLIRCIFMGKCPKQT
WEEAHRVHNILKEQLESTNPLMPVTPCGHFKIKDTFRYQFLIKSAYVIPVNKKLHHALMLAKLSPKVKFMID
VDPMTTFF

>core/77/5/Org5_Gene86

MEKICGYLEQILVENKDSGDITAYIKIPNKTTPILIKGKLPQPLELGSPIQIYGVWSHSPSNTKYFQIHSYDSPLL
YEYRGVFHYLTSKLIKGIGPKIAEKIIEKFQEKTCYVLDITPERLSEVSGISETRCVSICKQLCEQKILRKTLLFLQ
EYNIPIHYGVRIFKKYQEKSIEKICEDPFLLAREMEGIGFKTADFIAMKLGVPRNSESRLCAGIQHSLEELQEEG
HTCYPIELLIDVVAKLLNQDVFDTPITLEEIDTQILNMQKRKLLHIQDISGTLHVWTRYLHLAEKTIVSDLKRIL
FSSRRIRSIDGEKAIAWVEENLSIDLAEQQREAIKACFSEKLLIITGGPGTGKSTITQAILKIFEQVTHKIILAAPT
GKAAKRMTEITQKHSVTIHALLQYDFKTKSFRKNHDNPIDCDLIIVDESGMMDTHLLHHFLKALPDYTTLVFI
GDIHQLPSVGPGNILKDLITSNKMTVIRLNKIFRQVHDSGIVTNAHRVNEGELPILYSETGRRDFLFFQKDDQE
EALNHIIHLVTKFVPQKYHIYPQDIQVLAPMKKGTLGIYNLNKALKHALNPKKANLHGRFQSYAVGDKVMQI
RNNYNKEVFNGDIGYVSTINFEDKAVVVRMEGKHVGYSFSELDDLVLAYATSVHKYQGSESPCIIIPIHTSHF
MMLYRNLLYTAITRGKKLVILVGTKKAIAIATRNNRVQHRCTGLAEVLKELDTKKNYADL

>core/78/5/Org5_Gene341

MRIYQQDLFCRLCRDPAWFFSLLSFTLRFYCLGRGWTLLSFFYKHQKKFIGIVIAVVCVSGIGVGWGRFSRKG
SAESTSRRTVFTTASGKRYVEKDFMAMKKFFAHEAYPFTGNPRAWNFINEGLLTDYFLTTRVGEKLFLKVYH
PGEKIFSKEKAYQPYRRFDAPFISSEEVWKSSAPQLLEILKVFQQIENPISKEGFLARAKLFLEERRFPHYVLRQ
MLEYRRQMFALPPDEALSRGKDLRLFGYQTIQDWFGDAYLSAAVELLIRFIDEQKKVLPRPSKQEARDDFYD
KAKHAYTKISKNKEFSLGFEEFVNSYFQFLEISESEFFNMYRDILLCKRALLLLQGGVSFDFQPLTTFFVQGKD
SIQVEFFRLPKEYSFKTKQELKAFEVYLKLVSLPKSDSLDVPNEILPIATIKAKEPRLVGRRFSIDYKRVALQDL
AATVPMVEVLHWQQNSEHFQEILQQFPDVETCQSYKDFQHLKPALRDKISLFTRKEILRARPERILQSLQQVP
KQSQEVLLSAGKNSALPGISDGQQLAKVLLENEVLDLYSQDAETYYTIIVNSSFEKEEVLPYREVLKRDLASQ
LLTSHGHLVDMERLESALRTRYPGEEGASLWQRRLWKVVENHRLGRHLEGSFSWSLDRSLKTFSRGDKELP
QEFDRIFSMKVGDYSSVFMSPNEGPCYYQCLSHLLYDRPASVDKLFLAKSQLDEELLGSYMERFIEQGVVR

>core/79/5/Org5_Gene595

MDYLEKLQVLIEEGQSANFLSLWEEYCFNDVVRGRELVEILEKVKSSSLASLFGKIVDTVVPLWEKIPEGKDK
DRVLQLILDLQTSNSQMFFDIATEYVNKKYSGEENFNEALRVVGLRDGRDFQFSLSRFDFLMHMHKGNFVFH
QGGWGVGEVMGVSFLQQKVLIEFEGIMSAKDISFETAFKSLTPLSGDHFLSRRFGDPDGFEAFAKENPIEVVEI
LLRDLGPKTAKEIKDELVDLVIPEADWNRWWQSAKTKIKKGTRIISPDNPKEPYVLSDAGCSHMGQLERKLG
LSLNSAEKISLIYHFIRDLHSELKNIEIRKSLVKALQDLDVEEGNKSLILQRELLLSEYLGIKDASIDKEYITSLSE
DDTSRLLENMPIVALQKSFLSLVRKYSSFWQQVFMQILLYTTSPTMRDFVYKTIKNDPSSVEVLKKRLLDSAH
QPMMFPELFVWFFLKLGNHEDGLFDPEDKEVLRLFLESALNFMYQVASTPHKELGKKLHHYLVGQRYLAVR
QMIEGASLPFLKELLLLSTKCPQFSSSDLNVLQSLAEVVQPTLKKHKSNVEEENVLWSTSESFSRMKAKLQSL
VGKEMVDNAKEIEDARSLGDLRENSEYKFALEKRARLQEEIRVLSEEINRARILTKDLVFTDKVGVGCKVTL
KGDAGEVVEYTILGPWDADPDSCILSLQSKLAQNMLGKKLNDVVILQGKEYKISRIQSIWEEHGA

>core/80/5/Org5_Gene359

MVDKLIHPWDLDLLVSGRQKDPHKLLGILASEDSSDHIVIFRPGAHTVAIELLGELHHAVAYRSGLFFLSVPK
GIGHGDYRVYHQNGLLAHDPYAFPPLWGEIDSFLFHRGTHYRIYERMGAIPMEVQGISGVLFVLWAPHAQR
VSVVGDFNFWHGLVNPLRKISDQGIWELFVPGLGEGIRYKWEIVTQSGNVIVKTDPYGKSFDPPPQGTARVA
DSESYSWSDHRWMERRSKQSEGPVTIYEVHLGSWQWQEGRPLSYSEMAHRLASYCKEMHYTHVELLPITEH
PLNESWGYQVTGYYAPTSRYGTLQEFQYFVDYLHKENIGIILDWVPGHFPVDAFALASFDGEPLYEYTGHSQ
ALHPHWNTFTFDYSRHEVTNFLLGSALFWLDKMHIDGLRVDAVASMLYRDYGREDGEWTPNIYGGKENLE
SIEFLKHLNSVIHKEFSGVLTFAEESTAFPGVTKDVDQGGLGFDYKWNLGWMHDTFHYFMKDPMYRKYHQ
KDLTFSLWYAFQESFILPLSHDEVVHGKGSLVNKLPGDTWTRFAQMRVLLSYQICLPGKKLLFMGGEFGQY
GEWSPDRPLDWELLNHHYHKTLRNCVSALNALYIHQPYLWMQESSQECFHWVDFHDIENNVIAYYRFAGS
NRSSALLCVHHFSASTFPSYVLRCEGVKHCELLLNTDDESFGGSGKGNRAPVVCQDQGVAWGLDIELPPLAT
VIYLVTFF

>core/81/5/Org5_Gene633

MIAPFFSFPALSGSFSSIQAEEITQQVNHPGAELLSEGSYIPGLQTFRLGIKITASKGSHIYWKNPGEIGSPLKISW
QLPKGFVVEEEHWPTPKVFEEEGTTFFGYEDSALIVADVRAPEGYTPGQEVELRAQVEWLACGDSCLPGNV
DLKLTLPYEEKEPSLYPDTHAEFTKTLHAQPRVLENDHSVQVAQGKGNEIILNISKKINATKAWFVSEKADKL
FAYAETSYSGGTGTAWRLKVKNLSGVQKNEKLHGILLLADHTGRPVESLTIHSEVLGQTGSAVAGLSQYITIL

IMAFLGGVLLNIMPCVLPLVTLKVYGLIKSAGEHRSSVIANGLWFTLGVVGCFWGLAGVAFILKVLGHNIGW
GFQLQEPMFVATLIIVFFLFALSSLGLFEMGTMFANLGGKLQSSEMKSSNNKAVGAFFNGILATLVTTPCTGP
FLGSVLGLVMSLSFLQQLLIFTAIGLGMASPYLVFSVFPKMLSVLPKPGGWMSTFKQLTGFMLLVTVTWLVW
IFGSETSTTSVVVLLGGLWLAGLGAWILGRWGTPVSPKKQRVCASLLFFAFLGGAISVSGLASHYFAEPQQTV
SVNEDSLWQPFSLEKLAQLRAQGRPVFVNFTAKWCLTCQMNKPVLYGDAVQKMFETHGIVTLEADWTRKD
PGITEELARLGRASVPSYVYYPGDNSAPVVLPEKITQNLLEDVVSRFVR

>core/82/5/Org5_Gene220

MIRSPLPFISSKRALNMLGLQDEFSCPEDVVDFLFSEIELLAQQDEPSEGYLALSRSLLMMTHNHPKVVKRVIF
YGVSYGLKHKSMSIFIDVLTYIDFLFEKLGISASDRLSLCSARTCINFELYSQTGEMKFLSEVVDNFRLIEQLLK
MHPQLKNRLGWEHFRIGAKQEEVSLVASASVYQAVGRSFIELYHKHLELSDLACGMKCLALALDLSPNNAH
IHADYAKGLVVLGTRQGKSLLIERGMEHFSKAIFLSFSRDGDTLAYQNYRYSYALASVKLFDLTYKKEHFDQ
AMNILYQTVQAFPNLSGLWMVWGELLIRSGWLNSNMKYIEVGLEKLASLQKKTNDPIALSGLLATGIAILGL
YLEEPNLFKDSRHRLISAMRTFPGNSALVHALGVVQLCSALYFNEDSHFASAISCFQSCLEWDLDATGMWQK
LFDAYFSWGIKKKSARLLRKAVDVASRLCSLRPEAFLFWSDRGLALKCLAEATIDEAYKEIFLSESLLHYQRA
WDLSGRLEILELWGQSHYLLAELQQSLFHYDEAYTLLTKVDLTLSSSRVKLILAAVLLGKGRLLQDTDPAEE
AREILEPLVEVYLEDENFLLLLGKVYLFLFWKNKNVCLGKLARTYLEKATSLGCPEAYYTLGKFYAVIKDVN
KAWGMVIRSAQYGVRITEAKWLNDPYLANLREIHAFREVVENQKGRLWLGNKTEMKRN

>core/83/5/Org5_Gene249

MNKLLNFVSRTLGGDTALNMINKSSDLILALWMMGVVLMIIIPLPPPIVDLMITINLSISVFLLMVALYIPSALQ
LSVFPSLLLITTMFRLGINISSSRQILLKAYAGHVIQAFGDFVVGGNYVVGFIIFLIITIIQFIVVTKGAERVAEVA
ARFRLDAMPGKQMAIDADLRAGMIDATQARDKRAQIQKESELYGAMDGAMKFIKGDVIAGIVISLINIVGGL
TIGVAMHGMDLAQAAHVYTLLSIGDGLVSQIPSLLIALTAGIVTTRVSSDKNTNLGKEISTQLVKEPRALLLA
GAATLGVGFFKGFPLWSFSILALIFVALGILLLTKKSAAGKKGGGSGASTTVGAAGDGAATVGDNPDDYSLT
LPVILELGKDLSKLIQHKTKSGQSFVDDMIPKMRQALYQDIGIRYPGIHVRTDSPSLEGYDYMILLNEVPYVR
GKIPPHHVLTNEVEDNLSRYNLPFITYKNAAGLPSAWVSEDAKAILEKAAIKYWTPLEVIILHLSYFFHKSSQE
FLGIQEVRSMIEFMERSFPDLVKEVTRLIPLQKLTEIFKRLVQEQISIKDLRTILESLSEWAQTEKDTVLLTEYVR
SSLKLYISFKFSQGQSAISVYLLDPEIEEMIRGAIKQTSAGSYLALDPDSVNLILKSMRNTITPTPAGGQPPVLLT
AIDVRRYVRKLIETEFPDIAVISYQEILPEIRIQPLGRIQIF

>core/84/5/Org5_Gene464

MYKRCVLDKILKGIVAGSLILLYWSSDLLERDIKSIKGNVRDIQEDIREISRVVKQQQTSQAIPAAPGVMLAPK
LVRDEAFALLFGDPSYPNLLSLDPYKQQTLPELLGTNFHPHGILRTAHVGKPENLSPFNGFDYVVGFYDLCIPS
LASPHVGKYEEFSPDLAVKIEEHLVEDGSGDKEFHIYLRPNVFWRPIDPKALPKHVQLDEVFQRPHPVTAHDI
KFFYDAVMNPYVATMRAVALRSCYEDVVSVSVENDLKLVVRWKAHTVINEEGKEERKVLYSAFSNTLSLQ
PLPRFVYQYFANGEKIIEDENIDTYRTNSIWAQNFTMHWANNYIVSCGAYYFAGMDDEKIVFSRNPDFYDPL
AALIDKRFVYFKESTDSLFQDFKTGKIDISYLPPNQRDNFYSFMKSSAYNKQVAKGGAVRETVSADRAYTYI
GWNCFSLFFQSRQVRCAMNMAIDRERIIEQCLDGQGYTISGPFASSSPSYNKQIEGWHYSPEEAARLLEEEGW
IDTDGDGIREKVIDGVIVPFRFRLCYYVKSVTAHTIADYVATACKEIGIECSLLGLDMADLSQAFDEKNFDALL

MGWCLGIPPEDPRALWHSEGAMEKGSANVVGFHNEEADKIIDRLSYEYDLKERNRLYHRFHEIIHEEAPYAF
LFSRHCSLLYKDYVKNIFVPTHRTDLIPEAQDETVNVTMVWLEKKEDPCLSTS

>core/85/5/Org5_Gene27

MKELRHESYNRALHKLSHQWVRYFLYTFVSCSFIVAIFTFAWLKVLYVPEYKAGEISRISLTAPMDFSLSWSA
HKFYKRTAHISEAFGKVYHLTLSPGSLLSKEGNADENTDYWFKKAADFLLSTNFVDSSTQKCLKDLCIYPPLL
GKEKKTLEININSNKGNVIAQCFCHLKIFLIQENCPQPCFDAIMDILKIANFEVAVDKEMSGCVKGELLGKRCI
EKITKGTPILEKYQRIDDRDAKILKQLRAQLLSVHTLFSCRSLWGAIFVVLLILLWGYGALKALCPEMLKSPQ
RFMLYIAILTLSLLWCRGTEIFCAYWVSYLSYPPILPFTAVLLGYFLGLPIAGFSCTFLALLYTLGSDLWNNSW
FLSINLLCSWRILVSLHRVSRLSSVFWACMKLGGVAMGSLLMFRIFTNTISREALYADGIESFVYSLITAISVVA
LIPVFEASFGASTNFSLLTYLSPENALLKRLFKEAPGTYQHSVLVGSLAEAAAQAIGADSLYCLVAAHYHDIG
KLINPGFFSENQKILQQSGHSLSPLECAKMIMRHIPEGVNLARQAGLPESFIQVIEEHHGTSVIRSAYYSHMVE
NPSTGSFDEELFRYSGNKPSSKETTIIMIADSFEAASRSLKNASLPDLQRLIDQIIQGKLQDGQFSCSPITLDELA
LISKSMVQTLYGALHSRMKYPEISYQISMDSCPKPSIGGT

>core/86/5/Org5_Gene859

MPEPLYTNKLITEKSPYLLLYAHTPVNWYPWGAEAFHIAAIENKPVFLSIGCKHSRWCQVMLQESYTNPEIAA
MLNEYFVNVKVDKEELPYVAKLYGDLAQMLAVSGDHQETVSWPLNVFLTPDLVPFFSVNYLGNEGKLGLP
SFPQIIDKLHFMWEDAEEREALVEQAMKVLEIASFLEGCVRKEILDESSLKRTVAALYQDIDPHYGGVKAFPK
RLPGLLLQFFLRYSLEYQESRGLFFVDRSLSMVALGGVRDHIGGGVYSYTIDDKWLIPAFEKRLIDNALMALN
YLEAWACLGKEEYRGIGKQILSYILSELYSPEVGAFYSSEQAENWGAGGQNFYTWSVEEISNALGEDAEIFCD
YYGISREGFFNGRNILHIPVHREIEELSEKYHRSIEAIEDIVDRSRDILKGIRAQRSHRSKDDLSLTFNNGWMIYT
FAYAGRLLGEVEYIEIGKKCGEFVRNSLYKHHELYRRWREGEAKYRASLEDYGALILGVLALYESGCGSFWL
SFAEELMQEVVLSFRSEEGGFYSVDGRDSTLLIKQSPLSDGETISGNALICQCLLSLHLITEKKHYLTYAEDILQ
IAQACAHTHKFSSLGLLIASQNYFSRKHVKVLIALGDQEDRSPVLKCLSGLFLPYLSLIWMTQENQEHLETVL
PEYEHCLIPKGDCTATTIYVLEVDQCKRFKDLELFRRYLISL

>core/88/5/Org5_Gene9

MKRCFLFLASFVLMGSSADALTHQEAVKKKNSYLSHFKSVSGIVTIEDGVLNIHNNLRIQANKVYVENTVGQ
SLKLVAHGNVMVNYRAKTLVCDYLEYYEDTDSCLLTNGRFAMYPWFLGGSMITLTPETIVIRKGYISTSEGP
KKDLCLSGDYLEYSSDSLLSIGKTTLRVCRIPILFLPPFSIMPMEIPKPPINFRGGTGGFLGSYLGMSYSPISRKHF
SSTFFLDSFFKHGVGMGFNLHCSQKQVPENVFNMKSYYAHRLAIDMAEAHDRYRLHGDFCFTHKHVNFSGE
YHLSDSWETVADIFPNNFMLKNTGPTRVDCTWNDNYFEGYLTSSVKVNSFQNANQELPYLTLRQYPISIYNT
GVYLENIVECGYLNFAFSDHIVGENFSSLRLAARPKLHKTVPLPIGTLSSTLGSSLIYYSDVPEISSRHSQLSAKL
QLDYRFLLHKSYIQRRHIIEPFVTFITETRPLAKNEDHYIFSIQDAFHSLNLLKAGIDTSVLSKTNPRFPRIHAKL
WTTHILSNTESKPTFPKTACELSLPFGKKNTVSLDAEWIWKKHCWDHMNIRWEWIGNDNVAMTLESLHRSK
YSLIKCDRENFILDVSRPIDQLLDSPLSDHRNLILGKLFVRPHPCWNYRLSLRYGWHRQDTPNYLEYQMILGT
KIFEHWQLYGVYERREADSRFFFFLKLDKPKKPPF

>core/89/5/Org5_Gene810

MNFQTISINLTEGKILVFETGKIARQANGAVLVRSGETCVFASACAVDLDDKVDFLPLRVDYQEKFSSTGKTL
GGFIKREGRPSEKEILVSRLIDRSLRPSFPYRLMQDVQVLSYVWSYDGQVLPDPLAICAASAALAISDIPQSNIV
AGVRIGCIDNQWVINPTKTELASSTLDLVLAGTENAILMIEGHCDFFTEEQVLDAIEFGHKHIVTICKRLQLWQ
EEVGKSKNLSAVYPLPAEVLTAVKECAQDKFTELFNIKDKKVHAATAHEIEENILEKLQREDDDLFSSFNIKA
ACKTLKSDTMRALIRDREIRADGRSLTTVRPITIETSYLPRTHGSCLFTRGETQTLAVCTLGSEAMAQRYEDLN
GEGLSKFYLQYFFPPFSVGEVGRIGSPGRREIGHGKLAEKALSHALPDSATFPYTIRIESNITESNGSSSMASVC
GGCLALMDAGVPISSPIAGIAMGLILDDQGAIILSDISGLEDHLGDMDFKIAGSGKGITAFQMDIKVEGITPAIM
KKALSQAKQGCNDILNIMNEALSAPKADLSQYAPRIETMQIKPTKIASVIGPGGKQIRQIIEETGVQIDVNDLG
VVSISASSASAINKAKEIIEGLVGEVEVGKTYRGRVTSVVAFGAFVEVLPGKEGLCHISECSRQRIENISDVVKE
GDIIDVKLLSINEKGQLKLSHKATLE

>core/90/5/Org5_Gene419

MSNQEFDLSAIRNIGIMAHIDAGKTTTTERILFYAGRTHKIGEVHEGGATMDWMAQEQERGITITSAATTVFW
LGAKINIIDTPGHVDFTIEVERSLRVLDGAVAVFDAVSGVEPQSETVWRQADKYGVPRIAFVNKMDRMGAD
YFAAVESMKEKLGANAFPVHCPIGSESQFVGMVDLISQKALYFLDDTLGAKWEEKEISEDLKERCAELRANL
LEELATIDESNEAFMMKVLEDPDSITEDEIHQVMRKGVIENKINPVLCGTAFKNKGVQQLLNVIVKWLPSPLD
RGNIRGINLKTDQEISLEPRRDGPLAALAFKIMTDPYVGRITFIRIYSGTLKKGSAILNSTKDKKERISRLLEMH
ANERTDRDEFTVGDIGACVGLKFSVTGDTLCDDNQEIVLERIEFPDPVIDMAIEPKSKGDREKLAQALSSLSEE
DPTFRVSTNEETGQTIISGMGELHLDILRDRMIREFKVEANVGKPQVSYKETITVSGNSETKYVKQSGGRGQY
AHVCLEIEPNEPGKGNEVVSKIVGGVIPKEYIPAVIKGIEEGLNTGVLAGYGLVDVKVSIVFGSYHEVDSSEM
AFKICGSMAVKDACRKAKPVILEPIMKVAVITPEDHLGDVIGDLNRRRGKILGQESSRGMAQVNAEVPLSEM
FGYTTSLRSLTSGRATSTMEPAFFAKVPQKIQEEIVKK

>core/92/5/Org5_Gene368

MYNLLHAHHDAASPDGRLVSHLKKLSPHIYEGEVLIENIPAYFLGFHLPQQCIQVNLKSSLAQLGVEAVLNHL
ELNKARKEARLHVLFMSQDPIATAMLELLEPGSFVCKLFAADDRRLVRSPCYLNRMFTHTDRTGSPLLRFGK
KLEHFITLEIINDRLVVFLPILPGTICYEETIYGFLPLMSKSLTRPHLKIRKFLPLYQMVTDRPPVPEDHKILLIKT
EPLHIRTVFARVVQDLLPQGLRHTAADILEPTTQESGDIYEFYGSTSEPIERIPLEFFTLEPYKEHSFFFYRDMLQ
ETLESPQEVFRVFESIPEGEDQAAMFISKGSELLELSQDSWIIKPRISPSDERHAREIQKHIEDQPCFPFLKAMET
DHITSQGVLFSRYFPSASLKGMFLSNYSRYYLQHIYFQIPSPTSGEFFSNRDRSFLLDLYFAGISVFWADLESKR
LLQYIKRRNKDVGMFVPKHQAEQFAQSYFIGIHGSCLIAGDYDEFLRELLTGMHTLSQQFTIPEFPPQTPLAIL
TGGGSGAMELANRVATELSILSCGNLISLDTTNAYVEAKMSYAIPDLLERQADFHVDLAVFVIGGMGTDFEL
LLELISLKTGKKALVPVFLIGPVDYWKSKITALYNSNHAVGTIRGSEWVHNCLFCLSSAKAGIAIFRRYLNHTL
PIGPEHPVPEDGFVIV

>core/93/5/Org5_Gene805

MKRSRRNFEQALENLEKLKEISLATSNDSYLNNPARFNQRKQTGSSVMEMKEALKNVENYLLEISCVSKSHA
DKALKESDFLIAGVQNVFSFLENQEDLYKSLLDEYSEVTKAYDEVKKNLKEVPTYDLSTDEETEEHKEPECFL
NNLVEVKRDRSYELFYMLDEQDKRFYNDALVQIIYKQNKLHETVNEGDPLTKTLLWNSEEVKNIASSLVIVN
DMPLRLFYQRALSHLDIEAVVKVHNAVMALFFSRYEATMVFKSPKKHNIWYFNDFLLFLREAWKDLNNNVI
DSQERKQTKLLASALSLGIFESKLVFEEASRYLYFNIQTKLENANGKKPLSPGQYLTDAYEELHRLISKYPNGP

LFKAMDRVLEHESRPYDPMILGILPSLEGTLKLHGKSIDIIRSPSPVTQSSILYANCNEEFLGFLNAKAHRSEVT
LVLNIQNRISRKERARSRVIEEALEQEEHAPYVHAFSFPEPEELLQNLESIHGDIETFADFFSILQEEFHKPLLAS
SFFLTKELKEFVGSFLKEKLTALKDIFFAKKKILFRNDKLLLLHLLSYLIVFKLIERTNPNSIVVVSKDGLDYVS
VFIAGFAFFSREAFWDEHSLKLLLTNVLSPTLVARDRLVFVSHIELLSKFVNCLKKNRQGFSSLKSFFKDDIEG
WEFTGYLHELTEVSHKHNL

>core/94/5/Org5_Gene641

MQPCLNMSIVRNSALPLPCLSRSETFKKVRSHMKFMKVLTPWIYRKDLWVTAFLLTAIPGSFAHTLVDIAGEP
RHAAQATGVSGDGKIVIGMKVPDDPFAITVGFQYIDGHLQPLEAVRPQCSVYPNGITPDGTVIVGTNYAIGM
GSVAVKWVNGKVSELPMLPDTLDSVASAVSADGRVIGGNRNINLGASVAVKWEDDVITQLPSLPDAMNAC
VNGISSDGSIIVGTMVDVSWRNTAVQWIGDQLSVIGTLGGTTSVASAISTDGTVIVGGSENADSQTHAYAYK
NGVMSDIGTLGGFYSLAHAVSSDGSVIVGVSTNSEHRYHAFQYADGQMVDLGTLGGPESYAQGVSGDGKVI
VGRAQVPSGDWHAFLCPFQAPSPAPVHGGSTVVTSQNPRGMVDINATYSSLKNSQQQLQRLLIQHSAKVESV
SSGAPSFTSVKGAISKQSPAVQNDVQKGTFLSYRSQVHGNVQNQQLLTGAFMDWKLASAPKCGFKVALHY
GSQDALVERAALPYTEQGLGSSVLSGFGGQVQGRYDFNLGETVVLQPFMGIQVLHLSREGYSEKNVRFPVSY
DSVAYSAATSFMGAHVFASLSPKMSTAATLGVERDLNSHIDEFKGSVSAMGNFVLENSTVSVLRPFASLAMY
YDVRQQQLVTLSVVMNQQPLTGTLSLVSQSSYNLSF

>core/95/5/Org5_Gene20

MGVVQNQVISSIRDVLKLVWELRFAEHKMLLLSRQSGSGGTFQLSCAGHELAGVLAGKSLIPGKDWSFPYY
RDQGFPIGLGCDLSEIFASFLARTTPNHSSARMMPYHYSHKKLRICCQSSVVGTQFLQAAGRAWAVKHSSAD
EVVYVSGGDGATSQGEFHEMLNFVALHQLPLITVIQNNHWAISVPFEDQCGADLASLGRCHQGLAVYEVDG
GNYTSLTETFSHAVDQARQHSVPALILIDVVRLSSHSNSDNQEKYRSALDLKLSMDKDPLILLEKEAINVFGLS
PFEIEEIKAEAQEEVRKSCEIAEALPFPSKGSTSHEVFSPYTETLIDYENSESAQNLRNSEPKVMRDAISEALVEE
MTRDSGVIVFGEDVAGDKGGVFGVTRNLTEKFGPQRCFNSPLAEATIIGTAIGMALDGIHKPVVEIQFADYIW
PGINQLFSEASSIYYRSAGEWEVPLVIRAPSGGYIQGGPYHSQSIEGFLAHCPGIKVAYPSNAADAKALLKAAI
RDPNPVVFLEHKALYQRRIFSACPVFSHDYVLPFGKAAIVHPGKDLTIVSWGMPLVLSLEVAQELASRGISIEV
IDLRTMVPCDFATVLKSLEKTGRLLVIHEASEFCGFGSELVATMSEQGYAYLDAPIRRLGGLHAPVPYSKVLE
NEVLPHKESILQAAKSLAEF

>core/96/5/Org5_Gene380

MLKKPKRKPGRRTYGKSLKIFIPGTLFVHARKGFGFVSPDNPEEYPFDIFVPARDLRGALDGDHVIVSVLPYPR
DGQKLKGTISEVLARGKTTLVGTITSLVSPTSALAYTSMSGSQSLIPVELLPGRTYKIGDRILLSTPPWVDKPQE
GASPALQMLEFIGHITNAKADFQAIQAEYNLAEEFPPEVIEEASLFSQKHITQVLHSRKDLRDLLCFTIDSSTAR
DFDDAISLTYDHNNNYILGVHIADVSHYVTPHSHLDKEAAKRCNSTYFPGKVIPMLPSALSDNLCSLKPNVDR
LAVSVFMTFTKSGHLSDYQIFRSVIRSKYRMTYDEVDNIIEKKHSHPLSKILNEMATLSKKFSDIREERGCIRFV
LPSVTMSLDNLQEPVALIENHQTFSHKLIEEFMLKANEVVAYHISHQGVSLPFRSHEPPNDENLLAFQELAKN
MGFDITFTPTQEPDYQYLLQTTSAGHPLEQVLHSQFVRSMKTASYSTENKGHYGLKLDYYTHFTSPIRRYIDL
IVHRLLFNPLSIDQTHLEIIVRACSTKERVSAKAENSFENLKKTRFINKFLQEQPKTTYHAYIITANHEGLSFVV
TEFCHEGFIAAAELPKEYSLKKNALPESIPDKMKPGASIKVTIDSVNLLTQKIVWSIATTTEDKPKKIKKTPSKK
KGTKKRAS

>core/98/5/Org5_Gene1003

MINKELDIGILGKIAGAIKQISIESIQKASSGHPGLPLGCAELAAYLYGYVLRQNPRDPHWINRDRFVLSAGHG
SXLLYSCLHLAGFDVSLEDLQEFRQLHSRTPGHPEYGETVGVEATTGPLGQGLGNAVGMALSMKMLESRFN
RPGHEIFNGKIYCLAGDGCFMEGVSHEVCSFAGSLNLNNLVVIYDYNNVVLDGYLNEISVEDTKKRFEAYG
WDVYEIDGYDFTHIHETFSSIKRGQERPVLVIAHTIIGHGSPKEGTNKAHGSPLGVEGTHETKQFWHLPEEKFF
VPPAVKNFFAHKIQEDRKAQEQWLDEVRVWSKQFPELHEEFVALTSHKLPKNLESLVQSVEMPDSIAGRAAS
NKLIQVLVQHIPYLIGGSADLSSSDGTWIANEKVIHTYDFSGRNIKYGVREFGMATIMNGLAYSQVFRPFGGT
FLVFSDYMRNAIRLAALSKLPVIYQFTHDSIFVGEDGPTHQPVEQLMSLRAIPGLYVIRPADANEVRGAWIAG
LKHTGPTVIVLSRQALPTLPAAHRPFKDGVGRGAYIVLKESGEKPDYTLFATGSEVSLALSVAKELEHLDKQV
RVVSFPCWELFEAQDVDYKQSIVGGDLGIRVSIEAGSALGWYKYIGSEGLAIAMDRFGYSGASDDVSEECGF
TTEQILQRILSQ

>core/99/5/Org5_Gene298

MEKVSSYPSVPLPLGASKISPNRYRFALYASQATEVILALTDENSEVIEVPLYPDTHRTGAIWHIEIEGISDQSS
YAFRVHGPKKHGMQYSFKEYLADPYAKNIHSPQSFGSRKKQGDYAFCYLKEEPFPWDGDQPLHLPKEEMIIY
EMHVRSFTQSSSSRVHAPGTFLGIIEKIDHLHKLGINAVELLPIFEFDETAHPFRNSKFPYLCNYWGYAPLNFFS
PCRRYAYASDPCAPSREFKTLVKTLHQEGIEVILDVVFNHTGLQGTTCSLPWIDTPSYYILDAQGHFTNYSGC
GNTLNTNRAPTTQWILDILRYWVEEMHVDGFRFDLASVFSRGPSGSPLQFAPVLEAISFDPLLASTKIIAEPWD
AGGLYQVGYFPTLSPRWSEWNGPYRDNVKAFLNGDQNLIGTFASRISGSQDIYPHGSPTNSINYVSCHDGFTL
CDTVTYNHKHNEANGEDNRDGTDANYSYNFGTEGKTEDPGILEVRERQLRNFFLTLMVSQGIPMIQSGDEY
AHTAEGNNNRWALDSNANYFLWDQLTAKPTLMHFLCDLIAFRKKYKTLFNRGFLSNKEISWVDAMGNPMT
WRPGNFLAFKIKSPKAHVYVAFHVGAQDQLATLPKASSNFLPYQIVAESQQGFVPQNVATPTVSLQPHTTLIA
ISHAKEVT

>core/100/5/Org5_Gene106

MKEENSQAHYLALCRELEDHDYSYYVLHRPRISDYEYDMKLRKLLEIERSHPEWKVLWSPSTRLGDRPSGTF
SVVSHKEPMLSIANSYSKEELSEFFSRVEKSLGTSPRYTVELKIDGIAVAIRYEDRVLVQALSRGNGKQGEDIT
SNIRTIRSLPLRLPEDAPEFIEVRGEVFFSYSTFQIINEKQQQLEKTIFANPRNAAGGTLKLLSPQEVAKRKLEISI
YNLIAPGDNDSHYENLQRCLEWGFPVSGKPRLCSTPEEVISVLKTIETERASLPMEIDGAVIKVDSLASQRVLG
ATGKHYRWALAYKYAPEEAETLLEDILVQVGRTGVLTPVAKLTPVLLSGSLVSRASLYNEDEIHRKDIRIGDT
VCVAKGGEVIPKVVRVCREKRPEGSEVWNMPEFCPVCHSHVVREEDRVSVRCVNPECVAGAIEKIRFFVGR
GALNIDHLGVKVITKLFELGLVHTCADLFQLTTEDLMQIPGIRERSARNILESIEQAKHVDLDRFLVALGIPLIG
IGVATVLAGHFETLDRVISATFEELLSLEGIGEKVAHAIAEYFSDSTHLNEIKKMQDLGVCISPYHKSGSTCFG
KAFVITGTLEGMSRLDAETAIRNCGGKVGSSVSKQTDYVVMGNNPGSKLEKARKLGVSILDQEAFTNLIHLE

>core/101/5/Org5_Gene965

MRLNIHKYLFIGRNKADFFSASRELGVVEFISKKCFITTEQGHRFVECLKVFDHLEAEYSLEALEFVKDESVSV
EDIVSEVLTLNKEIKGLLETVKALRKEIVRVKPLGAFSSSEIAELSRKTGISLRFFYRTHKDNEDLEEDSPNVFY
LSTAYNFDYYLVLGVVDLPRDRYTEIEAPRSVNELQVDLANLQREIRNRSDRLCDLYAYRREVLRGLCNYDN
EQRLHQAKECCEDLFDGKVFAVAGWVIVDRIKELQSLCNRYQIYMERVPVDPDETIPTYLENKGVGXMGED

LVQIYDTPAYSDKDPSTWVFFAFVLFFSMIVNDAGYGLLFLMSSLLFSWKFRRKMKFSKHLSRMLKMTAILG
LGCICWGTTTTSFFGMSFSKTSVFREYSMTHVLALKKAEYYLQMRPKAYKELTNEYPSLKAIRDPKAFLLAT
EIGSAGIESRYVVYDKFIDNILMELALFIGVVHLSLGMLRYLRYRYSGIGWILFMVSAYLYVPIYLGTVSLIHY
LFHVPYELGGQIGYYGMFGGIGLAVVLAMIQRSWRGVEEIISVIQVFSDVLSYLRIYALGLAGAMMGATFNQ
MGARLPMLLGSIVILLGHSVNIILSIMGGVIHGLRLNFIEWYHYSFDGGGRPLRPLRKIVCSEDAEASGIHLDN
NSIV

>core/102/5/Org5_Gene379

MSEHKKSSKIIGIDLGTTNSCVSVMEGGQAKVITSSEGTRTTPSIVAFKGNEKLVGIPAKRQAVTNPEKTLGST
KRFIGRKYSEVASEIQTVPYTVTSGSKGDAVFEVDGKQYTPEEIGAQILMKMKETAEAYLGETVTEAVITVPA
YFNDSQRASTKDAGRIAGLDVKRIIPEPTAAALAYGIDKVGDKKIAVFDLGGGTFDISILEIGDGVFEVLSTNG
DTLLGGDDFDEVIIKWMIEEFKKQEGIDLSKDNMALQRLKDAAEKAKIELSGVSSTEINQPFITMDAQGPKHL
ALTLTRAQFEKLAASLIERTKSPCIKALSDAKLSAKDIDDVLLVGGMSRMPAVQETVKELFGKEPNKGVNPD
EVVAIGAAIQGGVLGGEVKDVLLLDVIPLSLGIETLGGVMTTLVERNTTIPTQKKQIFSTAADNQPAVTIVVLQ
GERPMAKDNKEIGRFDLTDIPPAPRGHPQIEVSFDIDANGIFHVSAKDVASGKEQKIRIEASSGLQEDEIQRMV
RDAEINKEEDKKRREASDAKNEADSMIFRAEKAIKDYKEQIPETLVKEIEERIENVRNALKDDAPIEKIKEVTE
DLSKHMQKIGESMQSQSASAAASSAANAKGGPNINTEDLKKHSFSTKPPSNNGSSEDHIEEADVEIIDNDDK

>core/103/5/Org5_Gene919

MKLLLKAVLRHKNHLVILGCSLLAILGLTFSSQMEIFSLGMIAKTGPDAFLLFGRKESGKLVKVSELSQKDILE
NWQAISKDSETLKVSDATTYIAEHGKSTASLTSKLSKFVRNYIDVSRFRGLAIFLICVAIFKAVTLFFQRFLGQ
VVAIRVSRDLRQDYFKALQQLPMTFFHDHDIGNLSNRVMTDSASIALAVNSLMINYIQAPITFILTLGVCLSIS
WKFSILICVAFPIFILPIVVIARKIKNLAKRIQKSQDSFSSVLYDFLAGVMTVKVFRTEKFAFTKYCEHNNKISA
LEEKSAAYGLLPRPLLHTIASLFFAFVVVIGIYKFAIPPEELIVFCGLLYLIYDPIKKFGDENTSIMRGCAAAERF
YEVLNHPDLHSQKEREIEFLGLSNTITFENVSFGYQEDKHILKNLSFTLHKGEALGIVGPTGSGKTTLVKLLPR
LYEVSQGKILIDSLPITEYNKGSLRNHIACVLQNPFLFYDTVWNNLTCGKDMEEEAVLEALKRAYADEFILKL
PKGVHSVLEESGKNLSGGQQQRLAIARALLKNASILILDEATSALDAISENYIKNIIGELKGQCTQIIIAHKLTTL
EHVDRVLYIENGQKIAEGTKEELLQTCPEFLKMWELSGTKEYNRVFVPDHKLVANPTDMAITT

>core/104/5/Org5_Gene702

MFSRLFFTSFSAEVVNTFFESGMSEDTSPLLSKQNRKLSHNLPLKSAYLSLGTYLIALLSFWLHAKNLSNLFVV
FTFFLAGTPALIKSLDNICQKVVNIDILMTSAAFGSIFIGGALEGALLLVLFAISEALGQMVSGKAKSTLVSLKQ
LAPTTGWLVLEDGNLQKVAINKIEVGNILRIKSGEVVPLDGEILHGSSSINLMHLTGEKVPKSCHPGSIVPAGA
HNMEGSFDLRVLRTGSDSTIAHIINLVIQAQNSKPRLQQRLDKYSSVYALSIFAIACGIALLVPLFTSIPLLGPQS
AFYRALAFLIAASPCALIIAIPIAYLSAINACAKHGVLLKGGVILDRLVSCNSVVMDKTGTLTTGELTCIGCDYF
GSKNETFFPSVLALEQSSSHPIAEAIVSYLMEQKVSSLPADRYLTVPGEGVRGYFNEQEAFVGRVETGLGKVP
SEYLEDIEQKIYQAKQHGEICSLAYVGNSFALFYFRDIPRPQAKEIIQDLKDLGYPVSMLTGDHKVSAENTAEI
LGISEVFFDLTPEDKLAKIRELATQRQIMMVGDGINDAPALAQATVGIAMGEAGSATAIEAADIVLLHDSLSS
LPWIIQKAKQTKKVVSQNLALALAIILLVSWPASLGIIPLWLAVILHEGSTVIVGLNALRLLKS

>core/105/5/Org5_Gene644

MTFQLHAPFAPCGDQPEAIARLSAGVRNQVKSQVLLGTTGSGKTFTIANVVANVNLPTLVLAHNKTLAAQL
YQEFREFFPNNAVEYFISYYDYYQPEAYIARSDTYIEKSLLINDEIDKLRLSATRSILERRDTLIVSSVSCIYGIGS
PENYTSMALVLEVGKEYPRNILTAQLVKMHYQASPIPQRSAFRERGSVIDIFPAYESELALRLEFLNDTLTSIE
YSDPLTMIPKESVPSATLYPGSHYVIPEAIREQAIRTIQEELEERMAFFDDRPIEKDRIFHRTTHDIEMIKEIGFCK
GIENYSRHFTGAPPGAPPTCLLDYFPEDFLLIIDESHQTLPQIRAMYRGDQSRKQSLVEYGFRLPSAFDNRPLT
YEEAQKYFRKVIYVSATPGDTEVQESSGHIVQQIIRPTGIPDPMPEIRPATGQVDDLLEEIRLRLSQKHEKILVIS
ITKKLAEDMAGFLSELEIPAAYLHSGIETAERTQILTDLRSGVIDVLIGVNLLREGLDLPEVSLVAILDADKEGF
LRSTSSLIQFCGRAARNINGKVIFYADQKTRSIEETLRETERRRQIQLDYNKEHNIVPKPIIKAIFANPILQTSKDS
ESPKESQRPLSKEDLEEQIKKYEALMQRAAKEFRFNEAAKYRDAMQACKEQLLYLF

>core/106/5/Org5_Gene452

MATPAQKSPTFQDPSFVRELGSNHPVFSPLTLEERGEMAIARVQQCGWNHTIVKVSLIILALLTILGGGLLVGL
LPAVPMFIGTGLIALGAVIFALALILCLYDSQGLPEELPPVPEPQQIQIEDLRNETREVLEGTLLEVLLKDRDAK
DPAVPQVVVDCEKRLGMLDRKLRREEEILYRSTAHLKDEERYEFLLELLEMRSLVADRLEFNRRSYERFVQG
IMTVRSEEGEKEISRLQDLISLQQQTVQDLRSRIDDEQKRCWTALQRINQSQKDIQRAHDREASQRACEGTEM
DCAERQQLEKDLRRQLKSMQEWIEMRGTIHQQEKAWRKQNAKLERLQEDLRLTGIAFDEQSLFYREYKEKY
LSQKLDMQKILQEVNAEKSEKACLESLVHDYEKQLEQKDANLKKAAAVWEEELGKQQQEDYEQTQEIRRL
STFILEYQDSLREAEKVEKDFQELQQRYSRLQEEKQVKEKILEESMNHFADLFEKAQKENMAYKKKLADLE
GAAAPTEIGEDDDWVLTDSASLSQKKIRELVEENQELLKALAFKSNELTQLVADAVEAEKEISKLREHIEEQK
EGLRALDKMHAQAIKDCEAAQRKCCDLESLLSPVREDAGMRFELEVELQRLQEENAQLRAEVERLEQEQFQ
G

>core/107/5/Org5_Gene326

MSYRKRSTLIVLGVFALYALLVLRYYKIQICEGDHWAAEALGQHEFCVRDPFRRGTFFANTTVRKGDKDLQ
QPFAVDITKFHLCADPLAIPECHRDEIIQGILQFIEGQTYDDLSLKLDKKSRYCKLYPLLDVSVHDRLSLWWK
GYATKHRLPTNALFFITDYQRSYPFGKLLGQVLHTLREIKDEKTGKAFPTGGMEAYFNHILEGDVGERKLLRS
PLNRLDTNRVIKLPKDGSDIYLTINPVIQTIAEEELERGVLEAKAQGGRLILMNSQTGEILALAQYPFFDPTNYK
EYFNNKERIEHTKVSFVSDVFEPGSIMKPLTVAIALQANEEASLKSQKKIFDPEEPIDVTRTLFPGRKGSPLKDI
SRNSQLNMYMAIQKSSNVYVAQLADRIIQSLGVAWYQQKLLALGFGRKTGIELPSEASGLVPSPHRFHINGSL
EWSLSTPYSLAMGYNILATGIQMVQAYAILANGGYAVRPTLVKKIVSASGEEYHLPTKEKTRLFSEEITREVV
RAMRFTTLPGGSGFRASPKHHSSAGKTGTTEKMIHGKYDKRRHIASFIGFTPVESSEGNFPPLVMLVSIDDPEY
GLRADGTKNYMGGRCAAPIFSRVADRTLLYLGILPDKKLRNCDEEAAALKRLYEEWNRSPKQGGTR

>core/108/5/Org5_Gene584

MVNPIGPGPIDETERTPPADLSAQGLEASAANKSAEAQRIAGAEAKPKESKTDSVERWSILRSAVNALMSLAD
KLGIASSNSSSSTSRSADVDSTTATAPTPPPPTFDDYKTQAQTAYDTIFTSTSLADIQAALVSLQDAVTNIKDTA
ATDEETAIAAEWETKNADAVKVGAQITELAKYASDNQAILDSLGKLTSFDLLQAALLQSVANNNKAAELLK
EMQDNPVVPGKTPAIAQSLVDQTDATATQIEKDGNAIRDAYFAGQNASGAVENAKSNNSISNIDSAKAAIAT
AKTQIAEAQKKFPDSPILQEAEQMVIQAEKDLKNIKPADGSDVPNPGTTVGGSKQQGSSIGSIRVSMLLDDAE
NETASILMSGFRQMIHMFNTENPDSQAAQQELAAQARAAKAAGDDSAAAALADAQKALEAALGKAGQQQ
GILNALGQIASAAVVSAGVPPAAASSIGSSVKQLYKTSKSTGSDYKTQISAGYDAYKSINDAYGRARNDATR

DVINNVSTPALTRSVPRARTEARGPEKTDQALARVISGNSRTLGDVYSQVSALQSVMQIIQSNPQANNEEIRQ
KLTSAVTKPPQFGYPYVQLSNDSTQKFIAKLESLFAEGSRTAAEIKALSFETNSLFIQQVLVNIGSLYSGYLQ

>core/109/5/Org5_Gene424

MFVMKKLVRLCVVLLSLLPNVLFSSDLLREEGIKKMMDKLIEYHVDAQEVSTDILSRSLSSYIQSFDPHKSYL
SNQEVAVFLQSPETKKRLLKNYKAGNFAIYRNINQLIHESILRARQWRNEWVKNPKELVLEASSYQISKQPM
QWSKSLDEVKQRQRALLLSYLSLHLAGASSSRYEGKEEQLAALCLRQIENHENVYLGINDHGVAMDRDEEA
YQFHIRVVKALAHSLDAHTAYFSKDEALAMRIQLEKGMCGIGVVLKEDIDGVVVREIIPGGPAAKSGDLQLG
DIIYRVDGKDIEHLSFRGVLDCLRGGHGSTVVLDIHRGESDHTIALRREKILLEDRRVDVSYEPYGDGVIGKVT
LHSFYEGENQVSSEQDLRRAIQGLKEKNLLGLVLDIRENTGGFLSQAIKVSGLFMTNGVVVVSRYADGTMKC
YRTVSPKKFYDGPLAILVSKSSASAAEIVAQTLQDYGVALVVGDEQTYGKGTIQHQTITGDASQDDCFKVTV
GKYYSPSGKSTQLQGVKSDILIPSLYAEDRLGERFLEHPLPADCCDNVLHDPLTDLDTQTRPWFQKYYLPNLQ
KQETLWREMLPQLTKNSEQRLSENSNFQAFLSQIKSSEKTDLSYGSNDLQLEESINILKDMILLQQCRK

>core/110/5/Org5_Gene862

MTSSSCPLLDLILSPADLKKLSISQLPGLAEEIRYRIISVLSQTGGHLSSNLGIVELTIALHYVFSSPKDKFIFDVG
HQTYPHKLLTGRNNEGFDHIRNDNGLSGFTNPTESDHDLFFSGHAGTALSLALGMAQTTPLESRTHVIPILGD
AAFSCGLTLEALNNISTDLSKFVVILNDNNMSISKNVGAMSRIFSRWLHHPATNKLTKQVEKWLAKIPRYGD
SLAKHSRRLSQCVKNLFCPTPLFEQFGLAYVGPIDGHNVKKLIPILQSVRNLPFPILVHVCTTKGKGLDQAQN
NPAKYHGVRANFNKRESAKHLPAIKPKPSFPDIFGQTLCELGEVSSRLHVVTPAMSIGSRLEGFKQKFPERFFD
VGIAEGHAVTFSAGIAKAGNPVICSIYSTFLHRALDNVFHDVCMQDLPVIFAIDRAGLAYGDGRSHHGIYDMS
FLRAMPQMIICQPRSQVVFQQLLYSSLHWSSPSAIRYPNIPAPHGDPLTGDPNFLRSPGNAETLSQGEDVLIIAL
GTLCFTALSIKHQLLAYGISATVVDPIFIKPFDNDLFSLLLMSHSKVITIEEHSIRGGLASEFNNFVATFNFKVDI
LNFAIPDTFLSHGSKEALTKSIGLDESSMTNRILTHFNFRSKKQTVGDVRV

>core/111/5/Org5_Gene622

MTCISELNEAQRKAVTAPLNPVLVLAGAGAGKTRVVTYRILHLINQGIAPREILAVTFTNKAARELKERIVNQ
CASTNEFDVPMVCTFHSLGVFILRRSINLLNRENNFTIYDQSDAEKLIKHALQQHNLKPNLASKIQAHVSQAK
NRLLFPEDLDPNDYIDPVVSIYQEYQKKLIEANALDFDDLLFLTVRLLRESPEAQELYNQLWKALLIDEYQDT
NHAQYTLMQLLSKQHRNVFAVGDPDQSIYSWRGANIHNILNFENDYPNAKVLCLEENYRSYGNILNAANALI
KNNASRLEKELRSVKGPGEKIRLFLGSTDREEADFVAAEILQLHRVGNIKLRDICIFYRTNSQSRTFEDALLRR
RIPYEIIGGLSFYKRKEIQDILAFLRIFISKSDIVAFDRTVNLPKRGIGSTTIFALTQYAIAQGLPILKACQQALDT
KDVKLSKKQQEGLQEYLALFPQIEHAYNTLSLRDFIESVVRITGYLEILKEDADTFKDRKSNLEELYHKALESE
QQNPKTHLELFLDDLALKGSDDDLNLTADRVNLMTLHNGKGLEFRVSFLVGLEEQLLPHANSLGGTYENIEE
ERRLCYVGITRAQDLLYLTAAQVRSLWGTVRMMKPSRFLKEIPKDYMIQVR

>core/112/5/Org5_Gene75

MKQHYSLNKSRHILRSTYKLLKSKKLAHSPADKKQLQELLEQLEEAIFEHDQETASDLAQQALAFSNRYPNS
FGRKTYELIKALLFAGVVAFLVRQFWFELYEVPTGSMRPTILEQDRILVSKTTFGLHCPFAKKPLAFNPESVTR
GGLVVFTVGDLPIPDADTKYFGLIPGKKRYIKRCMGRPGDFLYFYGGKIYGLDDAGKRIEFPSVHGLENLYH
VPYISFDGTTSSHTEGQKTIIDFKQFNQSYGRLIFPQTSMYGQFFDHKEWHQDEPNKLKDPHLSPVSYADLFG

MGNYAMVRILTEHQARTSHLLPNPGSPTKVYLEICHTANLSYPKPLLRHYEHQLSPAIQPMKTLLPLRKEHLH
LIRNNLTTSRFIVAQGCAYKYHQFKINTSGIAKAYAILLPKVPDGCYEYSKGEAYQIGFGEIRYKLKSSHPLTQ
LNDKQVIELFNCGINFSSIYNPVNPLQAPLPNRYAFFNQGNLYIMDSPVFIKNDPTLQKFVTSETEKQEGSSET
QPYIAFVDKGLPPEDFKEFVEFIHNFGIQVPKGHVLVLGDNYPMSADSREFGFVPMENLLGSPLCTFWPIGRM
GRLTGVSAPTTLSGYLVSGIALATGLSLIGYVYYQKRRRLFPKKEEKNHKK

>core/113/5/Org5_Gene639

MIPFTKTIGFRLWLACAVAIIAPLGINIVWLNLDQYRTIVSAISTALKENAAFKANTLTQIVPLNVDVLSLFSDV
LDLDAGIPETPNVLLSNEMQKVFQGIYNEISLIKVFPNGDKIVVASSIPEHLGENYNHKIDIPKNTPFLAALKQS
PKNQEVFSVMQANVFDAKTQELQGILYTTFSAESLLKDLLINKQSYLTVKTAILSKYGVILKASDPALHLHTV
YPDMTKEKFCQVFLNDDPCPIDSELGPLTLSPLDIGENFYSFKIKDTEIWGCIENVPSIDIAVLSYAKKEESFAPL
WRRARMYTAYFFCILLGSLIAFIVARRLSLPIRKLATAMIESRKNKNCLYTDDSLGFEINRLGHIFNAMVENLH
KQQHLAKTNFEMKENAQNALHLGEQAQQRLLPNTLPSYPHIELAKAYIPAITVGGDFFDVFVVGEGSKARLF
LIVADASGKGVNACGYSLFLKNMLRTFLSRSSSLQQAIQETSRLFYNNTKNSGMFVTLCVYCYHQTSNTMEY
YSCGHPPACYLDPDGETSWLFHPGMALGFLPEVANITSKLFHPKPGSLFVLYSDGITEAHNNNNDMFGEERL
QAAIQGLTGKSAADAVHRLMLSVKTFVGNSHQHDDITLLILKVLES

>core/114/5/Org5_Gene649

MIQVTCDQKNYEVLEGTTAAELAKQLKNSHQFIGVLINERPRDLSTHLNEGDTLVFLTSEDPEGREIFLHTSA
HLLAQAVLRLWPDAIPTIGPVIDHGFYYDFANLSISESDFPLIEDTVKQIVDEKLAISRFTYGDKQQALAQFPQ
NPFKTELIRELPENEEISAYSQGEFFDLCRGPHLPSTAHVKAFKVLRTSAAYWRGDPSRESLVRIYGTSFPTSKE
LRAHLEQIEEAKKRDHRVLGAKLDLFSQQESSPGMPFFHPRGMIVWDALIRYWKQLHTAAGYKEILTPQLM
NRQLWEVSGHWDNYKANMYTLQIDDEDYAIKPMNCPGCMLYYKTRLHSYKEFPLRVAEVGHVHRQEASG
ALSGLMRVRAFHQDDAHVFLTPEQVEEETLNILQLVSTLYGTFGLEYHLELSTRPEKDTIGDDSLWELATDAL
NRALVQSGTPFIVRPGEGAFYGPKIDIHVKDAIQRTWQCGTIQLDMFLPERFELEYTTAQGTKSVPVMLHRAL
FGSIERFLGILIENFKGRFPLWLSPEQVRIITVADRHIPRAKELEEAWKRLGLVVTLDDSSESVSKKIRNAQNM
QVNYMITLGDHEINENVLAVRTRDNRVINDVSVERFLNTILEEKNSLSLTALL

>core/117/5/Org5_Gene636

MDENRKVIVVGGGLAGLSAAMQLANLGIIVELVSLTKVKRSHSVCAQGGINAALNLKPEEEDSPYVHAYDTI
KGGDFLADQPPVLEMCLAAPRIIKMLDNFGCPFNRGPSGNLDVRRFGGTLYHRTVFCGASTGQQLMYTLDE
QVRRREHAGRVIKRENHEFVRLVTDHSGRACGIILMNLFNNRLEILRGDAVIIATGGPGVIFKMSTNSTFCTGA
ANGRLFLQGMAYANPEFIQIHPTAIPGRDKLRLISESVRGEGGRVWVPGDSSKRIVFPDGSERPCGETGAPWY
FLEDMYPAYGNLVSRDVGARAILRVCEAGLGIDGRMEAYLDVTHLPEKTRHKLEVVLDIYKKFTGEDPNTV
PMRIFPAVHYSMGGAWVDWPAADDPDRDSRFRQMTNIPGCFNCGESDFQYHGANRLGANSLLSCLFAGLVS
GDEASRFIEAFGASQATSSDFDRALQQEKEENARLLSASGKENIFVLHEEIAKIMVRNVTVKRNNRDLQETM
DKLKEFRERLKNVSVLDSSPFANKSFHFVRQMGPMLELALAITKGALLRNEFRGSHYKPEFPERDDEHWLKT
TVAVYAPEEPEISYLPVDTRHVAPTLRDYTKSSTGKIELTNIPDNIRLPI

>core/118/5/Org5_Gene928

MTIIYFILAALALGILVLIHELGHLVVAKAVGMAVESFSIGFGPALFKKRIGGIEYRIGCIPFGGYVRIRGMERT
KEKGEKGKIDSVYDIPQGFFSKSPWKRILVLVAGPLANILLAVLAFSILYMNGGRSKNYSDCSKVVGWVHPV
LQAEGLLPGDEILTCNGKPYVGDKDMLTTSLLEGHLNLEIKRPGYLTVPSKEFAIDVEFDPTKFGVPCSGASY
LLYGNQVPLTKNSPMENSELRPNDRFVWMDGTLLFSMAQISQILNESYAFVKVARNDKIFFSRQPRVLASVL
HYTPYLRNELIDTQYEAGLKGKWSSLYTLPYVINSYGYIEGELTAIDPESPLPQPQERLQLGDRILAIDGTPVSG
SVDILRLVQNHRVSIIVQQMSPQELEEVNSRDADKRFIASYHSEDLLQILNHLGESHPVEVAGPYRLLDPVQPR
PWIDVYSSESLDKQLEVAKKIKNKDKQRYYLERLDAEKQKPSLGISLKDLKVRYNPSPVVMLSNITKESLITL
KALVTGHLSPQWLSGPVGIVQVLHTGWSVGFSEVLFWIGLISMNLAVLNLLPIPVLDGGYILLCLWEIVTRRR
LNMKIVERILVPFTFLLIIFFIFLTFQDLFRFFG

>core/119/5/Org5_Gene388

MRRSVCYVNPSIARAGQISTWKFLYSLATPLPAGTKCKFDLAGSGKPTDWEAPATDLSQTRNVIYAEMPEGE
IIEATAIPVKDNPVPQFEFTLPYELQVGETLTIVMGASPNHPQVDDAGNGAQLFAQRRKPFYLYIDPTGEGNY
DEPDVFSMDIRGNVLKKIEIFTPSYVVKNKRFDITVRFEDEFGNLTNFSPEETRIELSYEHLRENLNWQLFIPET
GFVILPNLYFNEPGIYRIQLKNLSTQEIFISAPIKCFADSAPNLMWGLLHGESERVDSEENIETCMRYFRDDRAL
NFYASSSFENQENLSPDIWKLINQTVSDFNEEDRFITLSGFQYSGEPHLEGVRHILHTKETKSHSKHKEYKHIPL
AKLYKSTVNHDMISIPSFTASKEHGFDFENFYPEFERVVEIYNAWGSSETTAALNNPFPIQGKDSEDPRGTVIE
GLKKNLRFGFVAGGLDDRGIYKDYFDSPQVQYSPGLTAIICNKYTRESLVEALFARHCYATTGPRIVLSFNITS
APMGSELSTGSKPGLNVNRHISGHVAGTALLKTVEIIRNGEVLHTFFPDSNNLDYEYDDMVPLSSVTLKDPNG
KAPFVFYYLRVTQADNAMAWSSPIWVDLN

>core/120/5/Org5_Gene955

MESEKDIGAKFLGDYRILYRKGQSLWSEDLLAEHRFIKKRYLIRLLLPDLGSSQPFMEAFHDVVVKLAKLNHP
GILSIENVSESEGRCFLVTQEQDIPILSLTQYLKSIPRKLTELEIVDIVSQLASLLDYVHSEGLAQEEWNLDSVYI
HILNGVPKVILPDLGFASLIKERILDGFISDEENRESKIKERVLLHTSEGKQGREDTYAFGAITYYLLFGFLPQGI
FPMPSKVFSDFIYDWDFLISSCLSCFMEERAKELFPLIRKKTLGEELQNVVTNCIESSLREVPDPLESSQNLPQA
VLKVGETKASHQQKESAEHLEFVLVEACSIDEAMDTAIESESSSGVEEEGYSLALQSLLVREPVVSRYVEAEK
EEPKPQPILTEMVLIEGGEFSRGSVEGQRDELPVHKVILHSFFLDVHPVTNEQFIRYLECCGSEQDKYYNELIRL
RDSRIQRRSGRLVIEPGYAKHPVVGVTWYGASGYAEWIGKRLPTEAEWEIAASGGVAALRYPCGEEIEKSRA
NFFTADTTTVMSYPPNPYGLYDMAGNVYEWCQDWYGYDFYEISAQEPESPQGPAQGVYRVLRGGCWKSLK
DDLRCAHRHRNNPGAVNSTYGFRCAKNIN

>core/121/5/Org5_Gene989

MKKGKLGAIVFGLLFTSSVAGFSKDLTKDNAYQDLNVIEHLISLKYAPLPWKELLFGWDLSQQTQQARLQLV
LEEKPTTNYCQKVLSNYVRSLNDYHAGITFYRTESAYIPYVLKLSEDGHVFVVDVQTSQGDIYLGDEILEVDG
MGIREAIESLRFGRGSATDYSAAVRSLTSRSAAFGDAVPSGIAMLKLRRPSGLIRSTPVRWRYTPEHIGDFSLV
APLIPEHKPQLPTQSCVLFRSGVNSQSSSSSLFSSYMVPYFWEELRVQNKQRFDSNHHIGSRNGFLPTFGPILW
EQDKGPYRSYIFKAKDSQGNPHRIGFLRISSYVWTDLEGLEEDHKDSPWELFGEIIDHLEKETDALIIDQTHNP
GGSVFYLYSLLSMLTDHPLDTPKHRMIFTQDEVSSALHWQDLLEDVFTDEQAVAVLGETMEGYCMDMHAV
ASLQNFSQSVLSSWVSGDINLSKPMPLLGFAQVRPHPKHQYTKPLFMLIDEDDFSCGDLAPAILKDNGRATLI

GKPTAGAGGFVFQVTFPNRSGIKGLSLTGSLAVRKDGEFIENLGVAPHIDLGFTSRDLQTSRFTDYVEAVKTIV
LTSLSENAKKSEEQTSPQETPEVIRVSYPTTTSAL

>core/122/5/Org5_Gene968

MFGTLVSTLCCPANSERDWEDHEVNCIYIASTSDTQLEAVQGGMHITELRGEPVRVLYETGHLYAFARENTC
HSRLEVSHTVRAMTYFWDRFFSRHWNVGRRFLVFYQGNGGAYVQAALDSSMHTQDIYVLGLSPTVYIRGN
YHVQHYRVRGFWPSCLDSLAACAENTSVLPYGESSDGIFYPSLFSHTFDNAIRYGERCLLVCSEGMGMLPET
QQQTSPLTSLEGGHEVALVLNPQQNPEALSIASRLMHEERGGRLESNYMPGRSSNPFMTSMYVLVRLNTLAQ
IYLMSPYYSFQSNDIVCLIFISSAAVETVSYIFLTVTDSTCGRRYLRVPRLVCTGLRNLALPTTLLELLILSYPRS
VEGVPFNVRFILGYMCTTRVVFFAWNLILHWPFRCLRHGIQLFVHRSIIGHTLGARITDLTLASMRYAIVFPSI
VSSCLLTALAHANTNILALDPYRLIESGDLRRPAFNDDEMQQADNPWDAYSIGLVINTCIYMLILFANLIFMV
YSVRRYHRSRR

>core/123/5/Org5_Gene284

MTLITPAINSSRRKTHTVRIGNLYIGSDHSIKTQSMTTTLTTDIDSTVEQIYALAEHNCDIVRVTVQGIKEAQAC
EKIKERLIALGLNIPLVADIHFFPQAAMLVADFADKVRINPGNYIDKRNMFKGTKIYTEASYAQSLLRLEEKFA
PLVEKCKRLGKAMRIGVNHGSLSERIMQKYGDTIEGMVASAIEYIAVCEKLNYRDVVFSMKSSNPKIMVTAY
RQLAKDLDARGWLYPLHLGVTEAGMGVDGIIKSAVGIGTLLAEGLGDTIRCSLTGCPTTEIPVCDSLLRHTKI
YLDLPEKKNPFSLQHSENFVSAAEKPAKTTLWGDVYGVFLKLYPHHLTDFTPEELLEHLGVNPVTKEKAFTT
PEGVVVPPELKDAPITDVLREHFLVFHHHQVPCLYEHNEEIWDSPAVHQAPFVHFHASDPFIHTSRDFFEKQG
HQGKPTKLVFSRDFDNKEEAAISIATEFGALLLDGLGEAVVLDLPNLPLQDVLKIAFGTLQNAGVRLVKTEYI
SCPMCGRTLFDLEEVTTRIRKRTQHLPGLKIAIMGCIVNGPGEMADADFGFVGSKTGMIDLYVKHTCVKAHIP
MEDAEEELIRLLQEHGVWKDPEETKLTV

>core/124/5/Org5_Gene481

MWTHPIAYDVIVVGAGHAGCEAAYCSAKMGVSVLMLTSNLDTIAKLSCNPAVGGIGKGHIVREIDALGGIM
AEVTDQSGIQFRILNQTKGPAVRAPRAQVDKQLYHIHMKRLLENTPGLHIMQATVESLLDKEGVISGVTTKE
GWMFSGKTVVLSSGTFMRGLIHIGDRNFSGGRLGDPSSQGLSEDLKKRGFPISRLKTGTPPRLLASSINFSCME
EQPGDLGVGFVHRTEPFQPPLPQLSCFITHTMEKTKAIISANLHRSALYGGCIEGVGPRYCPSIEDKIVKFSDKE
RHHVFLEPEGLHTQEIYANGLSTSMPFDVQYDMIRSVLGLENAIITRPAYAIEYDYIHGNVIHPTLESKLIEGLF
LCGQINGTTGYEEAAAQGLIAGINAVNKVFNRPPFIPSRQESYIGVMLDDLTTQILDEPYRMFTGRAEHRLLLR
QDNACARLSHYGYELGLLSEERYELVKKQNQLLEEEKVRLQKTFRQYGQSVVSLAKALSRPEVSYDMLREA
FPNDIRDLGAVLNASLEMEIKYSGYIDRQKILIQSLEKAESLLIPEDLDYKQITALSLEAQEKLAKFTPRTLGSA
SRISGIASADIQVLMIALKKHAHH

>core/125/5/Org5_Gene95

MTTELKTEALPTRTQVDPKHCWDTTLMYANREEWKKDFDLCSSGKDRSPIWPEFSPSHYQIDNPESLLELLS
KKFSVERKLDQLYIYAHLIHDQDITNPEGESDYQSIVYLYTLFSQEISWIQPALIALSEEKVAALLSSSVLAPYR
FYLEKIFRLSPHTGTANEEKILASSFAALNVSNKAFSSLSDAEIPFGIAKDSNGEEHPLSHALASLYMQSPDQEL
RRTAYLAQFQRYYDYRNTFANLLNGKVQAHLFEAKARNYPSCLEASLFQHNIPTTVYINLINETKKHTSLINR
YFNLKKEALNLKEFHFYDVYAPISQTTSKNYSYEEGVDLVCKSLLPLGTHYVEILRNGLLSNRWVDRYENKH

KRSGAYSSGCYDSAPYILLNYTNTLYDVSVIAHEAGHSMHSYFSREAQPYHDAQYPLFLAEIASTFNEMLLM
EALSKSDQSKEDKIVIITKTLDTIFATLFRQTFFAAFEYEIHSAAEQGTPLTEEFLSATYGNLQKEFYGGVVTSD
SLSALEWARIPHFYYNFYVYQYATGIIAALSFAEKILTQEPGALELYLKFLKSGRSDFPLNILKKSGLDMTTSA
PLDKAFAFITKKIDLLSSLLSED

>core/126/5/Org5_Gene1023

MFRCILFGIFLLTCFSSGGVLYYLFCSHDFSIGPKEKSRSVWIEEEKEFTDSVLHHLPSQHQHLHILCFQGFLLQ
KQQKFSQAEKIFSKVYDEAQDGPFLFKEEILGSRLINSFFLEKTDVMETILCLLNQRCPNSPYYHLFKALVCYK
QKLYREVIEQLAYWQEEKTRALAPLLNISIEQLLTDFLLDYISAHSLIEQKMFPEGRVILNRNINRLLKHECEW
NAKTYDRIAILLSRSYFLELVESKSADIYFDYYEMVLFYLKKIYILEQCPYAELLPEEELVSLIMEHVFILPKDK
LYPLIQLLEMWQKHYVHPNSSLVVQILVDRFSTHMEGAIRFCEALVSFSGLEELHQQIITTFEELLSNKVQQIK
TEEAKQCVALLHILDPSISISEKLALSSDTLQNIVSGDDEQHTKLRNYLDLWEAIQSYDIDRQQLVHHLVYGA
KDLWKKGGNDEKALNLLQLVLRFTSYDIECESVVFLFIKQAYKQALSSHAIARLLKLEKFISEANIPSIVISEAE
KANFLADAEYLFAHEDYDKCYLYSMWLTKVAPSPQSYRLAGLCLMENKRYDEALEFLCMLSPNNSINDYK
TQKALAFCQKHQSKDRAAS

>core/127/5/Org5_Gene994

MCGIFGYLGNQDGVSIVLEGLAKLEYRGYDSAGLAAVVEQELFIRKTVGRVQELSNLFQEREIPTASVIGHTR
WATHGVPTEINAHPHVDEGRSCAVVHNGIIENFKELRRELTAQGISFASDTDSEIIVQLFSLYYQESQDLVFSF
CQTLAQLRGSVACALIHKDHPHTILCASQESPLILGLGKEETFIASDSRAFFKYTRHSQALASGEFAIVSQGKEP
EVYNLELKKIHKDVRQITCSEDASDKSGYGYYMLKEIYDQPEVLEGLIQKHMDEEGHILSEFLSDVPIKSFKEI
TIVACGSSYHAGYLAKYIIESLVSIPVHIEVASEFRYRRPYIGKDTLGILISQSGETADTLAALKELRRRNIAYLL
GICNVPESAIALGVDHCLFLEAGVEIGVATTKAFTSQLLLLVFLGLKLANVHGALTHAEQCSFGQGLQSLPDL
CQKLLANESLHSWAQPYSYEDKFLFLGRRLMYPVVMEAALKLKEIAYIEANAYPGGEMKHGPIALISKGTPV
IAFCGDDIVYEKMIGNMMEVKARHAHVIAIAPESREDIAAVSDQQIFVPDCHFLAAPVLYTIVGQVMAYAMA
LAKGMEIDCPRNLAKSVTVE

>core/128/5/Org5_Gene783

MIPSGLVYLLYPLGFLASLFFGSAFSIQWWLSKKRKEVYAPRSFWILSSIGATLMIVHGTIQSQFPVTVLHVINL
IIYLRNLNITSSRPISFRATLVLMALSVVFVTLPFLYVNMEWMASPNIFHLPLPPAQLSWHLIGCLGLAIFSGRF
LIQWFYIESNNTKDFPLLFWKIGLLGGLLALVYFIRIGDPINILCYGCGLFPSIANLRLFYKEQRSTPYLDTHCFL
SAGEASGDILGGKLIQSIKSLYPNIRFWGVGGPAMRQEGLQPILNMEEFQVSGFAEVLGSLFRLYRNYRKILKT
ILKHKPATLIFIDFPDFHLLLIKKLRKHGYRGKIIHYVCPSIWAWRPKRKRILEQHLDMLLLILPFEEGLFKNTSL
ETVYLGHPLVEEISDYKEQASWKEKFLNSDRPIVAAFPGSRRGDISRNLRIQVQAFLNSSLSQTHQFVVSSSSA
KYDEIIEDTLKAEGCQHSQIIPMNFRYELMRSCDCALAKCGTIVLETALNQTPTIVMCRLRPFDTFLAKYIFKIL
LPAYSLPNIIMNSVIFPEFIGGKKDFHPEEIATALDLLNQHGSKEKQKEDCRKLCKVMTTGQIASEEFLKRIFDT
LPAV

>core/129/5/Org5_Gene638

MKHTFTKRVLFFFFLVIPIPLLLNLMVVGFFSFSAAKANLVQVLHTRATNLSIEFEKKLTIHKLFLDRLANTLA
LKSYASPSAEPYAQAYNEMMALSNTDFSLCLIDPFDGSVRTKNPGDPFIRYLKQHPEMKKKLSAAVGKAFLL

TIPGKPLLHYLILVEDVASWDSTTTSGLLVSFYPMSFLQKDLFQSLHITKGNICLVNKYGEVLFCAQDSESSFV
FSLDLPNLPQFQARSPSAIEIEKASGILGGENLITVSINKKRYLGLVLNKIPIQGTYTLSLVPVSDLIQSALKVPL
NICFFYVLAFLLMWWIFSKINTKLNKPLQELTFCMEAAWRGNHNVRFEPQPYGYEFNELGNIFNCTLLLLLNS
IEKADIDYHSGEKLQKELGILSSLQSALLSPDFPTFPKVTFSSQHLRRRQLSGHFNGWTVQDGGDTLLGIIGLA
GDIGLPSYLYALSARSLFLAYASSDVSLQKISKDTADSFSKTTEGNEAVVAMTFIKYVEKDRSLELLSLSEGAP
TMFLQRGESFVRLPLETHQALQPGDRLICLTGGEDILKYFSQLPIEELLKDPLNPLNTENLIDSLTMMLNNETE
HSADGTLTILSFS

>core/130/5/Org5_Gene764

MRIEDFSLKLIPSSPGVYLMKDVHDQVLYIGKAKNLKNRLASYFHEKGDSRERIPFLMKKTASIETIVVSNETE
ALLLENNLIKQHHPKYNVLLKDDKTFFCLAISLSHSWPKVEAIRTKAITSSQRQLIFGPYVSAEACHTLLEVISQ
WFPLRTCSDREFALRKRPCILYDMKRCLAPCVGYCTPEEYQGTLDKAILFLKGKIEEVVKDLEKVIQKASDNL
EFEQAANYYRTLSLIKQAMAKQQVEKFHFQNIDALGLYRHKQRTILTLLTVRSGKLLGARHFSFFENAQEDQ
DLLSSFILQYYVSQPYIPKEILTPLPLEFPTLSYVLNAESPPRLRSPKTGYGKELLDLAYRNAKAYAATTLPSST
LPYQDFQNILRMSQYPYRIECYDNAHMQGAHATGVYIVFENNGFDPKQYRTFSIDSEKTQNDLALLEEVLLR
RFHSLTTALPDMIVVDGGKTHYNKTKKIIQTLNLTGIQVVTIAKEKSNHSRGLNKEKIFCETFPEGFSLPPTSNL
LQFFQILRDEAHRFAISKHRKKRGKALFEQEKIPGIGEVKRKRLLQKFKSWKQVMLSSQEELEAIPGLTKKDIA
VLLARQKDFNKSD

>core/131/5/Org5_Gene272

MKEYKIENIRNFSIIAHIDHGKSTIADRLLESTSTVEEREMREQLLDSMDLERERGITIKAHPVTMTYLYEGEV
YQLNLIDTPGHVDFSYEVSRSLSACEGALLIVDAAQGVQAQSLANVYLALERDLEIIPVLNKIDLPAADPVRIA
QQIEDYIGLDTTNIIACSAKTGQGIPAILKAIIDLVPPPKAPAETELKALVFDSHYDPYVGIMVYVRIISGELKKG
DRITFMAAKGSSFEVLGIGAFLPKATFIEGSLRPGQVGFFIANLKKVKDVKIGDTVTKTKHPAKTPLEGFKEIN
PVVFAGIYPIDSSDFDTLKDALGRLQLNDSALTIEQESSHSLGFGFRCGFLGLLHLEIIFERIIREFDLDIIATAPS
VIYKVVLKNGKVLDIDNPSGYPDPAIIEHVEEPWVHVNIITPQEYLSNIMNLCLDKRGICVKTEMLDQHRLVL
AYELPLNEIVSDFNDKLKSVTKGYGSFDYRLGDYRKGSIIKLEVLINEEPIDAFSCLVHRDKAESRGRSICEKL
VDVIPQQLFKIPIQAAINKKVIARETIRALSKNVTAKCYGGDITRKRKLWEKQKKGKKRMKEFGKVSIPNTAFI
EVLKLD

>core/132/5/Org5_Gene688

MVWSTNIKHEGLKSWIDEVAKLTTPKDIRLCDGSDTEYDELCTLMESTGTMIRLNPEFHPNCFLVRSSADDV
ARVEQFTFICTSTEAEAGPTNNWRDPQEMRRELHQLFRGCMQGRTLYIVPFCMGPLDSPFSIVGVELTDSPYV
VCSMKIMTRMGDDVLRSLGTSGKFLKCLHSVGKPLSPGEADVSWPCNPKSMRIVHFQDDSSVMSFGSGYGG
NALLGKKCVALRLASYMAKSQGWLAEHMLIIGITNPEGKKKYFSASFPSACGKTNLAMLMPKLPGWKIECIG
DDIAWIRPGRDGRLYAVNPEYGFFGVAPGTSERTNPNALATCRSNSIFTNVALTADGDVWWEGLTEQPPEPL
TDWLGKPWKPGGSPAAHPNSRFTAPLRQCPSLDPEWNSPQGVPLDAIIFGGRRSETIPLVYEALSWEHGVTIG
AGMSSTTTAAIVGQLGKLRHDPFAMLPFCGYNMAYYFQHWLSFAENRSLKLPKIFGVNWFRKNNQGEFLW
PGFSENLRVLEWIFQRTDGLEDIAERTPIGYLPNIQKFNLNGLNLDLQTVQELFSVDAEGWLAEVENIGEYLKI
FGSDCPQQITDELLRIKSELKEK

>core/133/5/Org5_Gene572

MAAYTEASILSLASLDHIRLRAGMYIGRLGNGSQKEDGIYTLFKEVVDNGIDEFIMGHGKSLKISASDKQISIQ
DQGRGIPLGKLIDCVSKINTGAKYTQDVFHFSVGLNGVGLKAVNALSEIFSVRSVRKKKYHLATFHRGVLQE
SKQGSTKDPDGTFVSFTPDPSIFPEFTFNHDFLKDKIRQYTYLHSGLEIRFNDEVFISHNGLKDLFDAEITEPPLY
SPLFFQNEDLTFIFSHLEGNTERYFSFVNGQETLDGGTHLTAFKEAIVKGVNEFFGKTFVSNDIREGIVGCIAIKI
ASPIFESQTKNKLGNTQIRSSLIKDVKEAIVQALRKDKVAPELLLEKIKFNEKTRKNIQFIKQDLKSKQKKVHY
KIPKLRDCKFHYNDRSLYGEASSIFLTEGESASASILASRNPLTQAVFSLRGKPMNVFSLEETKMYKNDELFYL
ATALGITQNEIQHLRYNKVILATDADVDGMHIRNLLITFFLKTLLPLVENNHLFILETPLFKVRNKTTTLYYYS
EQEKMQALQQFGKKDSSLEITRFKGLGEISPKEFAAFIGPEIRLTPVTITSLESISSILQFYMGKNTKERKQFIMD
NLITDF

>core/134/5/Org5_Gene970

MKEVEQRIRSLYDAVTAENICRWLSNDCTQQDAKTILGWLDTDPAQLEDLFGATLTFGTGGLRSLMGIGTNR
INLFTIRRTTQGLVQVLRAHLPHPGDPMRVVVGCDTRHNSIEFAQETAKVLAGNGCEVFLFQYPEPLALVSFT
VRYERAIGGVMITASHNPPNYNGYKVYMASGGQVLPPLDQEIVAACSAVNEILSVPSIDHPNIHLIGKEYEAL
YRDTLKQLQLYPEANRISGRSLSISYSPLHGTGISLVPHVLKDWGFLSVHLVEKQAIGDGDFPTVQLPNPEDPE
ALTLGTEQMLANDDDLFIATDPDADRVGVVCLEDGQPYRFNGNQMASLLADHILGAWSKTRHLGEHDKLV
KSLVTTEMLSAIAKHYHVDLINVGTGFKYIGEKIESWRNSTNKFVFGAEESYGCLYGTHVEDKDAIIASALIA
EAALQQKLQGKTLCDALLSLYETYGYFANKTESVVFSAKTDEQEIRKKLSHLEEISSANFFSGKYQVEKFENY
KQGIGFNLLSKDSYALTLPKTSMLCYYFSGGGRVIIRPSGTEPKIKFYFEMSTHYPERVTDKEIQKQREAESFQ
HLDDFIFDFKEKFSNL

>core/135/5/Org5_Gene57

MVTVSEQTAQGHVIEAYGNLLRVRFDGYVRQGEVAYVNVDNTWLKAEVIEVADQEVKVQVFEDTQGACR
GALVTFSGHLLEAELGPGLLQGIFDGLQNRLEVLAEDSSFLQRGKHVNAISDHNLWNYTPVASVGDTLRRGD
LLGTVPEGRFTHKIMVPFSCFQEVTLTWVISEGTYNAHTVVAKARDAQGKECAFTMVQRWPIKQAFIEGEKI
PAHKIMDVGLRILDTQIPVLKGGTFCTPGPFGAGKTVLQHHLSKYAAVDIVILCACGERAGEVVEVLQEFPHL
IDPHTGKSLMHRTCIICNTSSMPVAARESSIYLGVTIAEYYRQMGLDILLLADSTSRWAQALREISGRLEEIPGE
EAFPAYLSSRIAAFYERGGAITTKDGSEGSLTICGAVSPAGGNFEEPVTQSTLAVVGAFCGLSKARADARRYP
SIDPLISWSKYLNQVGQILEEKVSGWGGAVKKAAQFLEKGSEIGKRMEVVGEEGVSMEDMEIYLKAELYDF
CYLQQNAFDPVDCYCPFERQIELFSLISRIFDAKFVFDSPDDARSFFLELQSKIKTLNGLKFLSEEYHESKEVIV
RLLEKTMVQMA

>core/136/5/Org5_Gene292

MTAPTESRSSPPTLLEETEPLSPNPIPADIQIPRITISPPSLDVSTVASSAEDISVFIAGGPRSSSSASVASDVYELV
CLCGGDEDPEPPDSEVRTLYVNGSWQTHQEAVQELLYISEVRGEAVRLLYNDGSGMSPWPISPCRTLPTLDH
PLCQALLTVWEQFFSAPENQNREFLVIFYGDASPYIQQALTQSRHSPRIVVVGISPTVFIQGDFRVHNYRVSGD
FFSSLDCRGTRAENTTILPYSSGLEGVFLPSIRCPSFTWAVRFGEQCLVANRGEDVEDRGGLSQDAERSQLPHS
ERDLAVVIDSTDPSSMSRLVEWLNQGSPSSDMEINPYPQRCPDVALSALYAISRVSGLAQEWILASVHEGLDL
QICYSLILMHTTFAVRYFFLLFTNYPQSRERFRTARIVAQSLYLPSILVLVFDCGNVLRKLWMPQEILRAIFISA

STISGSIVFVECTRWMGRGLRHRVQQFVQQRVIGSGLPVGTVRASYRDRAGFIIGFLQTVHGGLYLPVSIMVL
NQIAIQVPRILVRPNNTAVYDLHNKSAEENWSSGDVLAVGQTLNFILCAFVLFVNLWFFVKSVLRHSRRRRR

>core/137/5/Org5_Gene821

MKKKFIFYFVIVFSLLFLWEMTSRHRPTFSFFCPPPSSIASSTLQSLPLLLTSAWHTLKAILGGFFLAITLSIVLAT
IMLSYKSAKDLLQPLFILLQCTPMFALAPLIVLWFGWGIGAVIVPTALTIFFPLTLTIYQGILSTPEELIEQFVLC
GATKFQLLIKLRIPHALPHIFSGLKIAIGSAGFAAIAGEWVASQSGLGILMLESRRNYEMELAFAGLATLSILTL
SLFQITLLIEKLIFSLFRVKRMSLKHKSVAKKALSVLALIPIMLIPWKGNSKSPPDKKNLTSLTLLLDWTPNPNH
IPLYAGVAKGYFKQHGLDLQLQKNTDSSSAVPHVLFEQVDMALYHALGIMKTSIKGMPIQIVGRLIDSSLQGF
LYRSQDPIYKFEDLNGKVLGFCLNNSRDLNRLLETLNRNGVVPSEVKNVSSDLISPMLLNKIDFLYGAFYNIE
GVKLQTLGMPVKCFLSDTCDLPTGPQLIVFTKKGTKASEPEIVEAFQKALQESIIFSKDHPEDAFKLYAKETKS
IPKNLYQEYLQWEETFPLLAQSQDPLSKDLVDKLLETIIKRYPELASEVAKFSLNDLYNPSLPEEQSV

>core/138/5/Org5_Gene765

MYTEESLDNLRHSIDIVDVLSEHIHLKRSGATYKACCPFHTEKTPSFIVNPAGAHYHCFGCGAHGDAIGFLMQ
HLGYSFTEAILVLSKKFQVDLVLQPKDSGYTPPQGLKEELRHINSEAETFFRYCLYHLPEARHALQYLYHRGF
SPDTIDRFHLGYGPEQSLFLQAMEERKISQEQLHTAGFFGNKWFLFARRIIFPVHDALGHTIGFSARKFLENSQ
GGKYVNTPETPIFKKSRILFGLNFSRRRIAKEKKVILVEGQADCLQMIDSGFNCTVAAQGTAFTEEHVKELSK
LGVLKVFLLFDSDEAGNKAALRVGDLCQTAQMSVFVCKLPQGHDPDSFLMQRGSSGLIALLEQSQDYLTFLI
SEKMSSYPKFGPREKALLVEEAIRQIKHWGSPILVYEHLKQLASLMMVPEDMVLSLANPQVTAEPQNIPIKQK
VPKIHPHIVMETDILRCMLFCGSNTKILYTAQFYFVPEDFKHPECRKLFAFMISYYEKYRKNVPFDEACQVLS
DSQILQLLTKRRLNTEALDTIFVQSLQKMADRRWREQCKPLSLNQNIQDKKLEILEDYVQLRKDRTIITLLDPE
SELIP

>core/139/5/Org5_Gene358

MSTSPISNDPRYLSLSNATEKTSLLANSRSLSPVPNSLVPSNPEDTGLRKSIFTHSVTLFAGLVVLLVAVSVVV
VALTVLAPGVPQAILLGIAISGVGIGGFSIMKSLVYMVRDYMSPRMQESSRIKSALAVGTGFTVMGLVMKVG
ANFVPGGYGGLVGSLGSSAYSRGSQTTLASFSHYIYTKFFRSEKVAKGEKLTEAETIKEAKKLHYITLSIATIG
VGLAVLGILLAIAGTVLLGGAPATIAIILAPPLISIGLTTVLQTILHSSIGKWRAFLLTQEKKDLFVDTSLKDIRLE
KLPPSEVEESETSQSVIEVPDSEGIAETRISAEEIDTRLSLTTRQKVIFALATLLLLASIAAFIVTGFGGLTVMQVL
LVASVGSAVASVTLPMVSSGFSYVAYQLKARLNISKLRWKEAKNKKRVRQFLIESGVIASDREFNQMWKTV
YKKQIQKTDAAIREEVRNFEKGGEVNSALVGGILLGVGTGIMLLALVPAFAPIVPGILALGGSTLGIAGSILMR
KFVNWLYDELVKLYERRRNRRELLYGPESKMRSIATDLVVEALAASHDHLFDLDGPVDFIDVDVDIDGAA

>core/140/5/Org5_Gene431

MTNSDNASAAGLLWAHPKEDPAFLGMIIKEFHLPPTVAQIFISRGFQTIQEIHKFLYSHLSSLYDPGLFLDMSK
AVERLLLLARDRKEHVMIYGDSDVDGMTGVALLVEFLRDIDVHVSYFFLGAILKQHGETSTLIAKLKEEGITLL
ITVDCGITAGKEVSDITRQGIDVIITDHHMPTGKIPHCVATLNPKLRDHTYPNRELTGVGVAFKLARGVLNALI
SRNLVPKSQGSLKKLLDLVTLGTITDVGVLLGENRVMVRYGIKEIARGARPGLNKLCALCGVEKSEVTSTDIV
LKIAPKLNSLGRLDDPAKGVELLLTQDDERVDALIMELDNINRERQRIEAEVFQDVQEILNSNPEILKQAAIVL
SSTAWHARVIPIISARLAKTYNKPVVIAIQRGIGKGSARTIGSFPLLGVLKKCSSLLLSYGGHDFAAGVIMKED

KVEDFKKKFVHLVNSSLKKGDTLPHLEIDAYADFDAIDYDLLASMELFEPFGKGNLMPIFYSKVRQVRYPKV
LPGNHLKLYLSQKERNLEGVAFGLGRHADALKASWHYPLEIAYTPRLSQTSGSGVIHLLVRDFRISSEPRFSD

>core/141/5/Org5_Gene254

MSFLRRHISLFRSQKQLIDVFAPVSPNLELAEIHRRVIEDQGPALLFHNVIGSSFPVLTNLFGTKHRVDQLFSQA
PDNLIARVAHLISSTPKLSSLWKSRDLLKRISSLGLKKARFRRFPFVSMSSVNLDHLPLLTSWPEDGGAFLTLP
LVYTESPTLTTPNLGMYRVQRFNQNTMGLHFQIQKGGGMHLYEAEQKKQNLPVSVFLSGNPFLTLSAIAPLP
ENVSELLFATFLQGAKLLYKKTNDHPHPLLYDAEFILVGESPAGKRRPEGPFGDHFGYYSLQHDFPEFHCHKI
YHRKDAIYPATVVGKPYQEDFYIGNKLQEYLSPLFPLVMPGVRRLKSYGESGFHALTAAVVKERYWRESLTT
ALRILGEGQLSLTKFLMVTDQEVPLDRFSVVLETILERLQPDRDLIIFSETANDTLDYTGPSLNKGSKGIFMGIG
KAIRDLPHGYQGGKIHGVQDIAPFCRGCLVLETSLEDRCIKSLLHHPDLKSWPLIILADNLRETIQSEKDFLWR
TFTRCAPANDLHALHSHFATHRPNYNFPFVIDALMKPSYPKEVEVDPSTKQKVSERWHAYFPNKETFYI

>core/142/5/Org5_Gene522

MKYRTHRCNELTSNHIGENVQLAGWVHRYRNHGGVVFIDLRDRFGITQIVCREDEQPELHQRLDAVRSEWV
LSVRGKVCPRLAGMENPNLATGHIEVEVASFEVLSKSQNLPFSIADDHINVNEELRLEYRYLDMRRGDIIEKL
LCRHQVMLACRNFMDAQGFTEIVTPVLGKSTPEGARDYLVPSRIYPGKFYALPQSPQLFKQLLMVGGLDRYF
QIATCFRDEDLRADRQPEFAQIDIEMSFGDTQDLLPIIEQLVATLFATQGIEIPLPLAKMTYQEAKDSYGTDKP
DLRFDLKLKDCRDYAKRSSFSIFLDQLAHGGTIKGFCVPGGATMSRKQLDGYTEFVKRYGAMGLVWIKNQE
GKVASNIAKFMDEEVFHELFAYFDAKDQDILLLIAAPESVANQSLDHLRRLIAKERELYSDNQYNFVWITDFP
LFSLEDGKIVAEHHPFTAPLEEDIPLLETDPLAVRSSSYDLVLNGYEIASGSQRIHNPDLQSQIFTILKISPESIQE
KFGFFIKALSFGTPPHLGIALGLDRLVMVLTAAESIREVIAFPKTQKASDLMMNAPSEIMSSQLKELSIKVAF

>core/143/5/Org5_Gene275

MSGKKDGVRGMIFVPLSILVLIFLPLPQILLDFGLCISFALSLLTVCWVFTLNSSNSAKLFPPFFLYLCLLRLGLN
LASTRWIVSSGTASSLIVSLGSFFSLGSLWAATFACLLLFFVNFLMVSKGSERIAEVRSRFFLEALPAKQMALD
SDLVSGRASYKAVKKQKNALIEEGDFFSAMEGVFRFVKGDAIISCILLLVNVVSVTCLYYTSGYALEQMWFT
VLGDALVSQVPALLTSCAAATLISKIDKEESLLNYLFEYYKQLRQHFRVVSLLIFSLCCIPSSPKFPIVLLASLL
WLAYRKEEPASEDSCIERAFSYVEGACPKEQESQFYQVYRAASEEVFEDLGVRLPVLTSLRIEERPWLRVFGQ
NVYLDEMTPEAVLPFLRNIAHEALNAEVVQKYLEESERVFGIAVEDIVPKKISLSSLVVLSRLLVRERVSLKLF
PKILEAVAVYQNSGDSLEILAEKVRKSLGYWIGRSLWDQKQTLEVITIDFHVEELINSSYSKSNPVMQENVIRR
VDSLLERSVFKDFRAIVTSCETRFEMKKMLDPHFPDLLVLSHDELPKEIPISFLGIVSDEVLVP

>core/144/5/Org5_Gene243

MPKQAEYTWGSKKILDNIECLTEDVAEFKDLLYTAHRITSSEEESDNEIQPGAILKGTVVDINKDFVVVDVGL
KSEGVIPMSEFIDSSEGLVLGAEVEVYLDQAEDEEGKVVLSREKATRQRQWEYILAHCEEGSIVKGQITRKVK
GGLIVDIGMEAFLPGSQIDNKKIKNLDDYVGKVCEFKILKINVERRNIVVSRRELLEAERISKKAELIEQISIGEY
RKGVVKNITDFGVFLDLDGIDGLLHITDMTWKRIRHPSEMVELNQELEVIILSVDKEKGRVALGLKQKEHNP
WEDIEKKYPPGKRVLGKIVKLLPYGAFIEIEEGIEGLIHISEMSWVKNIVDPSEVVNKGDEVEAIVLSIQKDEGK
ISLGLKQTERNPWDNIEEKYPIGLHVNAEIKNLTNYGAFVELEPGIEGLIHISDMSWIKKVSHPSELFKKGNSV

EAVILSVDKESKKITLGVKQLSSNPWNEIEAMFPAGTVISGVVTKITAFGAFVELQNGIEGLIHVSELSDKPFA
KIEDIISIGENVSAKVIKLDPDHKKVSLSVKEYLADNAYDQDSRTELDFKDSQGPKERKKKGK

>core/145/5/Org5_Gene654

MSTRRPIQLLDPLTINQIAAGEVIENSVSVVKELIENSLDAGADEIEIETLGGGQGAIIIRDNGCGFRAEDIPIALQ
RHATSKIREFSDIFSLNSFGFRGEALPSIASISKMEIQSSIEGDEGVRTVIHGGDIVSCEPCARQLGTTVIVNSLFY
NVPVRRGFQKSMQSDRLGIRKLIENRILSTANIGWSWISEGHHEIQIAKQQGFQERVAYVMGDHFMQDALTI
DKEANGVRIVGVLGSPSFHRPTRQGQKIFINDRPIESLFISKKVGDAYALLLPLHRYPVFVLKLYLPSSWCDFN
VHPQKIEARILKEELVGDCIKEAIVETLACPPGILCRTHQEIEESDSVPLPMFRMLETSDVQEEESVEFDQNLFA
YSSEDVSLEKQEYTSRGPKSQMDWIYSSDVRFLTSLGRVVLAEDLEGVHIIFTAAARKHLFFLSLMQENSRM
YQSQALLIPLRLQVTPEEAFFFSHHGRTLCDLGIEISQVGPCVFSIESTPTVIGEEELKEWLLLLAARGSTDINSE
ALTALMKETLTQATFSKHQHVFDVSWLKLLWSVGKPEKGFDGARIRRLILDSDFMEG

>core/146/5/Org5_Gene256

MAVSGGGGVQPSSDPGKWNPALQGEQAEGPSPLKESIFSETKQASSAAKQESLVRSGSTGMYATESQINKAK
YRKAQDRSSTSPKSKLKGTFSKMRASVQGFMSGFGSRASRVSAKRASDSGEGTSLLPTEMDVALKKGNRISP
EMQGFFLDASGMGGSSSDISQLSLEALKSSAFSGARSLSLSSSESSSVASFGSFQKAIEPMSEEKVNAWTVARL
GGEMVSSLLDPNVETSSLVRRAMATGNEGMIDLSDLGQEEVSTAMTSPRAVEGKVKVSSSDSPEANPTGIPN
SNTLERAEKEAEKQESREQLSEDQMMLARAMAGLLTGAAPQEVLSNSVWSGPSTVFPPPKFSGTLPTQRSGD
KSKHKSPGIEKSTNHTNFSPLREGTVKSAEVKSLPHPESMYRFPKDSIVSREEPEAVVKESTAFKNPENSSQNF
LPIAVESVFPKESGTGGALGSDAVSSSYHFLAQRGVSLLAPLPRATDDYKEKLEAHKGPGGPPDPLIYQYRNV
AVEPPIVLRSPQPFSGSSRLSVQGKPEAASVHDDGGGGNSGGFSGDQRRGSSGQKASRQEKKGKKLSTDI

>core/147/5/Org5_Gene462

MQKHPSFYQRFLSAYYKNLLASLSWKFFISVALIGIYAPLFASSKPLLVTWHGEIFFPLLRYLFFPGYYTKPVD
LFFNVLMVTFPFFILSFKLTRGWLRRWLLGLCIISQCMIFAWAYSGKVQDPALAENLKKMRAEKVRENISKV
NSEMVMLLPKDTRTWEMERRYMSTYEQLGILIKAKYRKKQEASVKKYQVAFEEKRQSPMPTLRHLEMKNE
GICLKRLQQRVDKMQRPYEMAQQAWNRATDNYRPFLMALTRIEHELRLADYNNWGQPEDLCIAYANVEKR
AEPYKKSLLEIRQVLEDYAKLRSAISFIQDKRLWIEKESEDLRILINPFFSSFHWEDDAGGSREMNKYVPWWQ
LSRVTRKDLLAALVFGIRIALVVAGIGITIALAIGIMIGLVSGYFGGTVDMILSRFTEIWETMPVLFILMLVISIT
QQKSLLLNTVLLGCFSWTGFSRYVRIEVLKQRDRGYVLAATNLGYSHYYIMVHQILPNAIVPVISLVPFAMM
AMISCEAGLTFLGLGEESSASWGNLMREGVTGFPAESAVLWPPAIILTMLLIAIALIGDGVRDALDPRLQDS

>core/148/5/Org5_Gene1027

MTDFPTHFKGPKLNPIKVNPNFFERNPKVARVLQITAVVLGIIALLSGIVLIIGTPLGAPISMILGGCLLASGGAL
FVGGTIATILQARNSYKKAVNQKKLSEPLMERPELKALDYSLDLKEVWDLHHSVVKHLKKLDLNLSETQRE
VLNQIKIDDEGPSLGECAAMISENYDACLKMLAYREELLKEQTQYQETRFNQNLTHRNKVLLSILSRITDNIS
KAGGVFSLKFSTLSSRMSRIHTTTTVILALSAVVSVMVVAALIPGGILALPILLAVAISAGVIVTGLSYLVRQIL
SNTKRNRQDFYKDFVKNVDIELLNQTVTLQRFLFEMLKGVLKEEEEVSLEGQDWYTQYITNAPIEKRLIEEIR
VTYKEIDAQTKKMKTDLEFLENEVRSGRLSVASPSEDPSETPIFTQGKEFAKLRRQTSQNISTIYGPDNENIDPE

FSLPWMPKKEEEIDHSLEPVTKLEPGSREELLLVEGVNPTLRELNMRIALLQQQLSSVRKWRHPRGEHYGNVI
YSDTELDRIQMLEGAFYNHLREAQEEITQSLGDLVDIQNRILGIIVEGDSDSRTEEEPQE

>core/149/5/Org5_Gene41

MANPTQSRPPSPEISIEELELQELAGSSNTETISNTPPPSCAATAEEVSLFIEGGRRNSEDEEGPLGSCEVYDVVC
ITNQGDPEVRDHEVRVMYINGSGRTQHEGILDAMNICDLRGEPVRFIHNSGYGLGSCFLGIRNRIPPRDNVISQ
AIQARWNEFFIFAENANRDYIVLFSGNGGLYLQVALDNSIYSHHILCVGIGSSYYIQGNYRVHNYRVTGDWTT
LLDRRGATAVNTTTLPYADSAEGLFLPSVRCPSYQWALRCGEQCLIMDNNQQVGFRPQDSSSEIALVVNLNQ
DHSTWTRLIEWIDRGDSQAVLELNPQPSHCRDIALTALYATTRISSLLQECLMISVTYAPEVFVTYAIVTGYSI
MTLRYFILLLTNRPGCRRHFRVLRLAALGLQSLGFLTVLLDHINVTRRVNRRPPLISVIFCTASFATGSFIYVDL
TRMFFTSLRSRLQLFVQRRLTGRGLPLRRVFVNHLDSLRFSQNALITFHGGLFMPLIIGFFNQLVIQVPRVVIRP
NTTAVYDLNQTSQEAWDSGDVLAIGQTINFLLCMILLVINTFFFVRSVRRNLHRRPHR

>core/150/5/Org5_Gene607

MFMNTQNSQATEVSSEEESQKKLEELVALAKEQGFITYEEINEILPMSFDTPEQIDQVLIFLTGMDIQVLNQID
VERQKEKKKEAKELEGLARRTEGTPDDPVRMYLKEMGTVPLLTREEEVEISKRIEKAQVQIERIILRFRYSAK
EAISIAHYLISGKERFDKIISEKEVEDKTHFLKLLPKLITLLKEEDTYLENLLLSLKQPDLSKQEAAKLNDSLEK
CRIRTQAYLRCFHCRHNVTEDFGEVVFKAYDSFLHLEQQINDLKVRAERNKFAAAKLAAAKRKLYKREVAA
GRTLEEFKKDVRMLQRWMDKSQEAKKEMVESNLRLVISIAKKYTNRGLSFLDLIQEGNMGLMKAVEKFEY
RRGYKFSTYATWWIRQAVTRAIADQARTIRIPVHMIETINKVLRGAKKLMMETGKEPTPEELAEELGLTPDR
VREIYKIAQHPISLQAEVGEGSESSFGDFLEDTAVESPAEATGYSMLKDKMKEVLKTLTDRERFVLIHRFGLL
DGKPKTLEEVGSAFNVTRERIRQIEAKALRKMRHPIRSKQLRAFLDLLEEEKTGTSKVKSLKSK

>core/151/5/Org5_Gene975

MDTQSSIGNEEWRIAGTSVVSGMALGKVFFLGTSPLHVRELTLPQEEVEHEIHRYYKALNRSKSDIVALEQEV
TGQQGLQEVSSILQAHLEIMKDPLLTEEVVNTIRKDRKNAEYVFSSVMGKIEESLTAVRGMPSVVDRVQDIH
DISNRVIGHLCCQHKSSLGESDQNLIIFSEELTPSEVASANSAYIRGFVSLVGAATSHTAIVSRAKSIPYLANISE
ELWNIAKRYNGKLVLIDGYRGELIFNPKPATLQSCYKKELSVVAHTSQRLVRKSLHPIVSSHAGSDKDVEDLL
ENFPQTSIGLFRSEFLAVILGRLPTLREQVDLYEKLARFPGDSPSVLRLFDFGEDKPCPGIKNKKERSIRWLLDY
SVILEDQLQAIAKASLQGSIKVLIPGVSDVSEIIEVKKKWETIQTRFPKGHKVSWGTMIEFPSAVWMIEEILPEC
DFLSIGTNDLVQYTLGISRESALPKHLNVTLPPAVIRMIHHVLQAAKQNQVPVSICGEAAGQLSLTPLFIGLGV
QELSVAMPVINRLRNHIALLELNSCLEITEALLQAKTCSEVEELLNRNNKITS

>core/152/5/Org5_Gene823

MKVPWAFKNFIPKLYTSIKEGYSFNTFKKDFQAGITVGILAFPFAIAIAIGVGVSPIQGLLASIIGGLLASAMGG
SNVLISGPSSAFISILYCLSAKYGAEALFTVTLLAGVFLIAFGLTGLGTFIKYMPYPVVTGLTTGLAIIIFSSQIKD
FLGLQMGANIPADFLPKWIAYWDHLWTWDSKSFAVGLFTLLIMIYFRNYKPRYPGVMIAIVTATTLVWLLKI
DIPTIGSRYGTLPTAIPLPKIPQLSITKILQLMPDALTIAVLSGLETLLSAVVADGMTGWRHQSNCQLVAQGVA
NIGTSLFSGIPVTGSLSRTAASIKSEATTPIAGIVHSIFICFILLLLAPLTVKIPLTCLAAVLILIAWNMSEIHHFIHL
FTAPKKDIVVLLTVFILTVMTTITAAVQVGMMLAAFLFMKQMSDLSDVISTAKYFDKDSDFLSKAEVPQNTEI

YEINGPFFFGIADRLKNLLNDIEKPPKIFILCMTRVPTIDASAMHALEEFFLECDRQGTLLLLAGVKKTPLADLK
RYHLDELIGVDHIFSNIKSALLFAQALTNLESKTSTRHLV

>core/153/5/Org5_Gene10

MGLFHLTLFGLLLCSLPISLVAKFPESVGHKILYISTQSTQQALATYLEALDAYGDHDFFVLRKIGEDYLKQSI
HSSDPQTRKSTIIGAGLAGSSEALDVLSQAMETADPLQQLLVLSAVSGHLGKTSDDLLFKALASPYPVIRLEA
AYRLANLKNTKVIDHLHSFIHKLPEEIQCLSAAIFLRLETEESDAYIRDLLAAKKSAIRSATALQIGEYQQKRFL
PTLRNLLTSASPQDQEAILYALGKLKDGQSYYNIKKQLQKPDVDVTLAAAQALIALGKEEDALPVIKKQALE
ERPRALYALRHLPSEIGIPIALPIFLKTKNSEAKLNVALALLELGCDTPKLLEYITERLVQPHYNETLALSFSKG
RTLQNWKRVNIIVPQDPQERERLLSTTRGLEEQILTFLFRLPKEAYLPCIYKLLASQKTQLATTAISFLSHTSHQ
EALDLLFQAAKLPGEPIIRAYADLAIYNLTKDPEKKRSLHDYAKKLIQETLLFVDTENQRPHPSMPYLRYQVT
PESRTKLMLDILETLATSKSSEDIRLLIQLMTEGDAKNFPVLAGLLIKIVE

>core/154/5/Org5_Gene376

MKTSQLFYKTSKNANKSAAVLSNELLEKAGYLFKVSKGVYTYTPLLWRVVSKMMNIIREELNAIGGQELLLP
LLHNAELWQHTGRWEAFTSEGLLYTLKDREGKSHCLAPTHEEVICSFVAQWLSSKRQLPLHLYQIATKFRDE
IRPRFGLIRSRELLMEDSYTFSDSPEQMNEQYEKLRSAYSKIFDRLGLAYVIVTADGGKIGKGKSEEFQVLCSL
GEDTICVSGSYGANIEAAVSIPPQHAYDREFLPVEEVATPGITTIEALANFFSIPLHKILKTLVVKLSYSNEEKFI
AIGMRGDRQVNLVKVASKLNADDIALASDEEIERVLGTEKGFIGPLNCPIDFFADETTSPMTNFVCAGNAKD
KHYVNVNWDRDLLPPQYGDFLLAEEGDTCPENPGHPYRIYQGIEVAHIFNLGTRYTDSFEVNFQDEHGQTQQ
CWMGTYGIGVGRTLAACVEQLADDRGIVWPKALAPFSITIAFNGGDTVSQELAETIYHELQSQGYEPLLDDR
DERLGFKLKDSDLIGIPYKLILGKSYQSSGIFEIESRSGEKYTVSPEAFPTWCQNHLA

>core/155/5/Org5_Gene592

MTVSVKKKSFRALVTTHFLTIINDNLYKFLLAFFLLEGKTLTENAKILSCVSFFFALPFLLLAPLAGSLADRFQ
KRNIILATRFIEILCTILGTYFFFIQSVVGGYVVLILMACHTTIFGPAKLGILPEMLPSEQLSQANGIMTAATYTG
SILGSCLAPLLVDVTHRLGVNSYVWPTLMCVIVSIISTLISFCIRPSNVKNVKQKITLVSFKDLWKVLKDTRMI
HYLTVSIFLGSFFLLIGAYTQLEIIPFVEFTLKYPKHYGAYLFPIVALGVGTGSYITGKISGKDIKIGYVPLAAIGL
ALVFMGLYAFACSILFVLFFLLALGFLGGVYQVPLHAYVQYASPEHKRGQILAANNFLDFFGVLVAAGVIRV
LGSNLGLSPETSFFYIGWFVLAVSIWTLWIWREHVYRLLLGIILRRQLGYYLKIHQSSSPKCYFVAVQSYREIR
RVLAALTKTVRSRVIILDQKLVPGWRAWLLSWCVPTVVSSVRDNDSEAQDAWAVLQANHLKTSLKKFPDV
SVVCLGLPKNVERFTSILQEQGIDLHPIQLVQKEGKKRVIYTLVFPHA

>core/156/5/Org5_Gene438

MSTLLSILSVICSQAIAKAFPNLEDWAPEITPSTKEHFGHYQCNDAMKLARVLKKAPRAIAEAIVAELPQEPFS
LIEIAGAGFINFTFSPVFLNQQLEHFKDALKLGFQVSQPKKIIIDFSSPNIAKDMHVGHLRSTIIGDSLARIFSYVG
HDVLRLNHIGDWGTAFGMLITYLQENPCDYSDLEDLTSLYKKAYVCFTNDEEFKKRSQQNVVALQAKDPQA
IAIWEKICETSEKAFQKIYDILDIVVEKRGESFYNPFLPEIIEDLEKKGLLTVSNDAKCVFHEAFSIPFMVQKSDG
GYNYATTDLAAMRYRIEEDHADKIIIVTDLGQSLHFQLLEATAIAAGYLQPGIFSHVGFGLVLDPQGKKLKTR
SGENVKLRELLDTAIEKAEEALREHRPELTDEAIQERAPVIGINAIKYSDLSSHRTSDYVFSFEKMLRFEGNTA

MFLLYAYVRIQGIKRRLGISQLSLEGPPEIQEPAEELLALTLLRFPEALESTIKELCPHFLTDYLYNLTHKFNGFF
RDSHIQDSPYAKSRLFLCALAEQVLATGMHLLGLKTLERL

>core/157/5/Org5_Gene426

MSKLIRRVVTVLALTSMASCFASGGIEAAVAESLITKIVASAETKPAPVPMTAKKVRLVRRNKQPVEQKSRG
AFCDKEFYPCEEGRCQPVEAQQESCYGRLYSVKVNDDCNVEICQSVPEYATVGSPYPIEILAIGKKDCVDVVI
TQQLPCEAEFVSSDPETTPTSDGKLVWKIDRLGAGDKCKITVWVKPLKEGCCFTAATVCACPELRSYTKCGQ
PAICIKQEGPDCACLRCPVCYKIEVVNTGSAIARNVTVDNPVPDGYSHASGQRVLSFNLGDMRPGDKKVFTV
EFCPQRRGQITNVATVTYCGGHKCSANVTTVVNEPCVQVNISGADWSYVCKPVEYSISVSNPGDLVLHDVVI
QDTLPSGVTVLEAPGGEICCNKVVWRIKEMCPGETLQFKLVVKAQVPGRFTNQVAVTSESNCGTCTSCAETT
THWKGLAATHMCVLDTNDPICVGENTVYRICVTNRGSAEDTNVSLILKFSKELQPIASSGPTKGTISGNTVVF
DALPKLGSKESVEFSVTLKGIAPGDARGEAILSSDTLTSPVSDTENTHVY

>core/158/5/Org5_Gene951

MELLSLNKSYFEIQRLRYRPEILTLLETIRSKHIQETSSPPSPPPELQKHIPNLCRIPEVSIYTEQETSSKPLKIGVL
LSGGQAPGGHNVVIGLFDALRVFNPKTRLFGFIKGPLGLTRGLYKDLDISVIYDYYNMGGFDMLSSSREKIKT
EEQKKNILNTVKQLKLDGLLIIGGNNSNTDTAMLAEYFLAHNCKTSVIGVPKTIDGDLKNCWIETSLGFHTSC
RTYSEMIGNLAKDALSAKKYHHFIRLMGQQASYTTLECGLQTLPNIALISELIATRKISLKQLSEQLALGLVRR
YKSGKNYSTVLIPEGLIEHIFDTRKLIDELNVLLANGDSSIEKILSKLSPETLKTFHLFPKDIANQLLLARDSHGN
VRVSKIATEELLAVMVKKEIEKIKPHMEFHSVSHFFGYEARAGFPSNFDCNYGIALGIISALFLVRQKTGYMIT
INNLAQSYTEWQGGATPLYKMMHLENRCGTETPVIKTDSVDPKSPAVQHLLQQSDSCLVEDLYRFPGPLQYF
GKEELIDQRPLTLLWENQTHSPLL

>core/159/5/Org5_Gene864

MSSPPQAVASLTERIKTLLESNFCQIIVKGELSNVSLQPSGHLYFGIKDSQAFLNGAFFHFKSKYYDRKPKDGD
AVIIHGKLAVYAPRGQYQIVAHALVYAGEGDLLQKFEETKRRLTAEGYFATEKKKPLPFAPQCIGVITSPTGA
VIQDILRVLSRRARNYKILVYPVTVQGNSAAHEISKAIEVMNAENLADVLIIARGGGSIEDLWAFNEEILVKAI
HASTIPIVSAVGHETDYTLCDFASDVRAPTPSAAAEIVCKSSEEQVQVFEGYLRHLLSHSRQLLTSKKQQLLP
WRRFLDRAEFYTTAQQQLDSIEIAIQKGVQGKIHESKQRYDNISRWLQGDLVSRMTCRLQSLKKMLSQALSH
KALSLQVRCHQLKKSLTYPRQIQQASQKLSPWRQQLDTLISRRLHYQKEEYFHKHTRLKHAHNVLEQQLRS
HVQKLELLGRRLSRGCELNLQNQKIAYANVKETLATILERRYENSVARYSALKEQLHSLNPKNVLKRGYAM
LFDFNENSAMISVDSLQENARVRIQLQDGEAILTVTNIEICKLIKG

>core/160/5/Org5_Gene1000

MDNSDNSFHTLETEQGSFLNDELAVEEVAYTESTEISDATLCFADEIQELPSPEKKVAFILNKMREALTGSSQG
SDLRLFWDLRKQCLPLFNEIEDTAKRADHWRCYIELTKEGRHLKGLQDEEGSFVVGQIDLAITCLEKDILKFQ
EGTEDKIFKDREDNFLESQALDKHQAFYKQHHTSLLWLSSFSSKIIDLRKELINVGMRMRLKSKFFQRLSNLG
NQVFPKRKELIEKVSQTFAEDVDAFVAKYFIGSDKETLKKTVFFLRKEIKNLQHAAKRLFVSSHVFAETRLKL
SKCWDQLKGMEKEIRQEQGRLRVVSAENSKEVRQMLAEVSSLLIEGNDLSKVRKDLEGISKKIRALDLTHDD
VISLKKEMQQLFDQLREKQDAAEHSYQEQLAKDKQVKKEAARSLAERITTFSKTCSEGNITSESREEWQTLK

ELLGKMSFLPPPEKISLDNQLNLALQTIVNFFEEQLLSSPDSREKLVNMRQVLKQRRERRQELKDKLEQDKKL
LGSSGLDFDRAMQYSALVEEDKRALEELDASILELKQQIQQLL

>core/161/5/Org5_Gene960

MPQKVLITSALPYANGPLHFGHIAGVYLPADVYARFRRLLGDDVLYICGSDEFGIAITLNADREGLGYQEYV
DMYHKLHKDTFEKLGFALDFFSRTTNPFHAELVQDFYSQLKASGLIENRISEQLYSEQEQRFLADRYVEGTCP
RCGFDHARGDECQSCGADYEAIDLIDPKSKISGVELVKKETEHSYFLLDRMKDALLSFIQGCYLPDHVRKFV
VDYIEHVRSRAITRDLSWGIPVPDFPGKVFYVWFDAPIGYISGTMEWAASQGNPDEWKRFWLEDGVEYVQFI
GKDNLPFHSVVFPAMELGQKLDYKKVDALVVSEFYLLEGRQFSKSEGNYVDMDKFLSSYSLDKLRYVLAAT
APETSDSEFTFLDFKTRCNSELVGKFGNFINRVLAFAEKNHYDKLSYHSVVLEDSDRAFLEEVRQLVRDAEK
CYREYSLRKATSVIMSLAALGNVYFNQQAPWKLLKEGTRERVEAILFCACYCQKLLALISYPIIPESAVAIWE
MISPKSLENCNLDTMYARDLWKEEILDVINEEFHLKSPRLLFTTVE

>core/162/5/Org5_Gene151

MHPLYVDLDTIISSYSPPLPKEFQEAASLIAVPDTSHSKPVVPGVKTLFPQTYHLPYLKFVQGENVVHTPLKVG
VMFSGGPAPGGHNVIQGLFNSLKDFHPDSSLVGFVNNGDGLTNNKSIDITEEFLSKFRNSGGFNCIGTGRKKIV
TPEAKEACLKTAEALDLDGLVIIGGDGSNTATAILAEYFAKRRPKTSIVGVPKTIDGDLQHTFLDLTFGFDTAT
KFYSSIISNISRDALSCKAHYHFIKLMGRSASHIALECALQTHPNIALIGEEIAEKNLPLKTIIHKICSVIADRAAM
EKYYGVILIPEGIIEFIPEIINLITEIESLSEYEDKISRLSPESQRLLKSFPAPIIEQILNDRDAHGNVYVSKISVDKLL
IHLVSNHLQQYFPNVPFNAISHFLGYEGRSGLPTKFDNTYGYSLGYGAGILVRNHCNGYLSTIESLACPFMKW
KLRAIPVVKMFTVKQQADGTLQPKIKKYLVDIGSTAFRKFKLYRKIWALEDSYRFLGPLQIETPPEMHSDNFP
PLTLLLNHNFWQRHQGCIEIPDTTY

>core/163/5/Org5_Gene586

MSRKDNEVSLARSIFNILSGTFCSRITGIFREIAMATYFGADPIVAAFWLGFRTVFFLRKILGGLILEQAFIPHFE
FLRAQSLDRAAFFFRRFSRLIKGSTIIFTLLIEAVLWVVLQYVEEGTYDMILLTMILLPCGIFLMMYNVNGALL
HCENKFFGVGLAPVVVNIIWIFFVIAARHSDPRERIIGLSVALVIGFFFEWLITVPGVWKFLLEAKSPPQEHDSV
RALLAPLSLGILTSSIFQLNLLSDICLARYVHEIGPLYLMYSLKIYQLPIHLFGFGVFTVLLPAISRCVQREDHER
GLKLMKFVLTLTMSVMIIMTAGLLLLALPGVRVLYEHGLFPQSAVYAIVRVLRGYGASIIPMALAPLVSVLFY
AQRQYAVPLFIGIGTALANIVLSLVLGRWVLKDVSGISYATSITAWVQLYFLWYYSSKRLPMYSKLLWESIRR
SIKVMGTTMLACMITLGLNILTQTTYVIFLNPLTPLAWPLSSITAQAIAFLSESCIFLAFLFGFAKLLRVEDLINL
ASFEYWRGQRGLLQRQHVMQDTQN

>core/164/5/Org5_Gene93

MAAKNIKYNEEARKKIHKGVKTLAEAVKVTLGPKGRHVVIDKSFGSPQVTKDGVTVAKEIELEDKHENMGA
QMVKEVASKTADKAGDGTTTATVLAEAIYSEGLRNVTAGANPMDLKRGIDKAVKVVVDELKKISKPVQHH
KEIAQVATISANNDSEIGNLIAEAMEKVGKNGSITVEEAKGFETVLDVVEGMNFNRGYLSSYFSTNPETQECV
LEDALILIYDKKISGIKDFLPVLQQVAESGRPLLIIAEEIEGEALATLVVNRLRAGFRVCAVKAPGFGDRRKAM
LEDIAILTGGQLVSEELGMKLENTTLAMLGKAKKVIVTKEDTTIVEGLGNKPDIQARCDNIKKQIEDSTSDYD
KEKLQERLAKLSGGVAVIRVGAATEIEMKEKKDRVDDAQHATIAAVEEGILPGGGTALVRCIPTLEAFLPML

ANEDEAIGTRIILKALTAPLKQIASNAGKEGAIICQQVLARSANEGYDALRDAYTDMIDAGILDPTKVTRSALE
SAASIAGLLLTTEALIADIPEEKSSSAPAMPSAGMDY

>core/165/5/Org5_Gene366

MFGSESLRYQLLIQDFAKVSEEGIGLLESKEYSLLQAKLVLRALAQNSSFDDWFRSFKKCQISYPELAHDRDV
LEEFGIQVLREGIENPSVTVRAVSVLAIGLARDFRLVPLLLQSCNDDSAIVRSLALQVAVNYGSESLKKAIVEL
ARNDDSIHVRITAYQVVALLQIEELLPFLRERAENKLVDSVERREAWKACLELSSQFLETGVAKDDIDQALFT
CEVLRNGMLPETTEIFTELLSVEHPEVQESLLLSALAWSHQLQNHKEFLSKVRHVMCTSPFAKVRFQAAALL
HLHGDPLGRDSLVEGLRSPQPLVCEAASAALCSLGIHGVPLAKEHLESLSSRKAAANLSILLLVSREDIERAGD
VIARYLSNPEMCWAIEYFLWDAQWNLRGDTFPLYSDMIKREIGRKLIRLLAVARYSQAKAVTATFLSGQQA
QGWSFFSGMFWEEGDVKTSEDLVTDACFAAKLEGALASLCQKKDQASLQRVSQLYNDSRWQDKLAILESV
AFSENLDAVPFLLDCCHHEAPSLRSAAAGALFSIFK

>core/166/5/Org5_Gene513

MLRIFCFVISWCLIAFAQPDLSGFVSILGAACGYGFFWYSLEPLKKPSLPLRTLFVSCFFWIFTIEGIHFSWMLS
DQYIGKLIYLVWLTLITILSVLFSGFSCLLVAIVRQKRTAFLWSLPGVWVAIEMLRFYGIFSGMSFDYLGWPM
TASAYGRQFGGFLGWAGQSFAVIAVNMSFYCLLLKKPHAKMLWVLTLLLPYTFGAIHYEYLKHAFQQDKR
ALRVAVVQPAHPPIRPKLKSPIVVWEQLLQLVSPIQQPIDLLIFPEVVVPFGKHRQVYPYESCAHLLSSFAPLPE
GKAFLSNSDCATALSQHFQCPVIIGLERWVKKENVLYWYNSAEVISHKGISVGYDKRILVPGGEYIPGGKFGS
LICRQLFPKYALGCKRLPGRRSGVVQVRGLPRIGITICYEETFGYRLQSYKRQGAELLVNLTNDGWYPESRLP
KVHFLHGMLRNQEFGMPCVRACQTGVTAAVDSLGRILKILPYDTRETKAPSGVLETSLPLFNYKTLYGYCGD
YPMILIAFCAVSYLGGGFLGYRLLAKKEIR

>core/167/5/Org5_Gene479

MQSSEVKPFSRLRAYLCPIYKSEFSKFVPLFLLAFFVGFNYCLLKNMKDTLVIVGSDAGAEVIPFLKVWGIVP
GAVIVTMVYGWLGSRYPRDTVFYCFMAAFLGFFFLFAVIIYPVGDSLHLNSLADKLQELLPQGLRGFIVMVR
YWSYSIYYVMSELWSSVVLSMLFWGLANQITTITEAGRFYALINTGLNLSSICAGEISYWMGKQTFVAYSFA
CDSWHSVMLNLTMLITCSGLIMIWLYRRIHHLTIDTSIPPSRRVLAEEGAATANLKEKKKPKAKARNLFLHLI
QSRYLLGLAIIVLSYNLVIHLFEVVWKDQVSQIYSSHVEFNGYMSRITTLIGVVSVLAAVLLTGQCIRKWGWT
VGALVTPLVMLVSGLLFFGTIFAAKRDISIFGGVLGMTPLALAAWTGGMQNVLSRGTKFTFFDQTKEMAFIP
LSPEDKNHGKAAIDGVVSRIGKSGGSLIYQGLLVIFSSVAASLNVIALVLLIIMVVWIAVVAYIGKEYYSRAAD
AVATLKQPKEPSSSIVREAQESVEQEEMAVL

>core/168/5/Org5_Gene747

MHDQRNRGHNNHNLRLRPGSTLLEAFLILCSEHEEGIACFDEHLGSLSYRELRNAIIAVAIKVSKFSEDRVGV
MMPASIGAFIAYFGILLAGKTPVMMNWSQGLRELRACTKTVEVRRVLTSQQFIKHLTEVQGFVEYPFDLMY
MEDVRKRLSWWEKCRIGLYSKCSVPWLLRIFGVSGVESDDTAVILFTSGTEKLPKAVPLTHKNLMENQEACL
KFFDPNTQDVMLAFLPPFHAYGFNSCGLFPLLMGVHVVFASNPLNPKKLVEFIDDKKVTFFGSTPVFFDYILK
TAKKQNSCLESLRLVVIGGDALKDTLYEETKKLQPQIALYQGYGATECSPVISITTKESPRKSECVGMPIEGM
DVLIISKETHIPVSSGEQGLIVVRGNSVFSGYLGNHEHQSFVSLGGDQWYLTGDLGHIGPSGDLFLEGRLSRFV

KIGGEMVSLEALESILHEHFTENQNEDAGSLVVCGIPGDKVRLCLFTTLATTIHEVNDILKSAETSSIVKISYVHQVESIPILGIGKPDYVSLNALAVSLFG

>core/169/5/Org5_Gene941

MPFKCIFLTGGVVSSLGKGLTAASLALILERQRLNVAMLKLDPYLNVDPGTMNPFEHGEIYVTDDGVETDLDLGHYHRFSSAALSRHSSATSGQIYARVIKREREGDYLGSTVQVIPHITNEIIQVILDAAKEHSPDVLIVEIGGTIGDIESLPFLEAIRQFRYDHSEDCLNIHMTYVPYLQAADEVKSKPTQHSVQTLRGIGIIPDAILCRSEKPLTQEVKSKISLFCNVPNRAVFNVIDVKHTIYEMPLMLAQEKIANFIGEKLKLATVPENLDDWRVLVNQLSQDLPKVKIGVVGKYVQHRDAYKSIFEALTHAALRLGHAAEIIPIDAEDENLTMELSQCDACLVPGGFGVRGWEGKIAAAKFCREQGIPYFGICLGMQVLVVEYARNVLNLDQANSLEMDPNTPHPIVYVMEGQDPLVATGGTMRLGAYPCLLKPGSKAHKAYNESSLIQERHRHRYEVNPDYIQSLEDHGLRIVGTCPPQGLCEIIEVSDHPWMIGVQFHPEFVSKLISPHPLFIAFIEAALVYSKDASHV

>core/171/5/Org5_Gene140

MRKISVGICITILLSLSVVLQGCKESSHSSTSRGELAINIRDEPRSLDPRQVRLLSEISLVKHIYEGLVQENNLSGNIEPALAEDYSLSSDGLTYTFKLKSAFWSNGDPLTAEDFIESWKQVATQEVSGIYAFALNPIKNVRKIQEGHLSIDHFGVHSPNESTLVVTLESPTSHFLKLLALPVFFPVHKSQRTLQSKSLPIASGAFYPKNIKQKQWIKLSKNPHYYNQSQVETKTITIHFIPDANTAAKLFNQGKLNWQGPPWGERIPQETLSNLQSKGHLHSFDVAGTSWLTFNINKFPLNNMKLREALASALDKEALVSTIFLGRAKTADHLLPTNIHSYPEHQKQEMAQRQAYAKKLFKEALEELQITAKDLEHLNLIFPVSSSASSLLVQLIREQWKESLGFAIPIVGKEFALLQADLSSGNFSLATGGWFADFADPMAFLTIFAYPSGVPPYAINHKDFLEILQNIEQEQDHQKRSELVSQASLYLETFHIIEPIYHDAFQFAMNKKLSNLGVSPTGVVDFRYAKEN

>core/172/5/Org5_Gene600

MLGKEEEFTCKQKQCLSHFVTNLTSDVFALKNLPEVVKGALFSKYSRSVLGLRALLLKEFLSNEEDGDVCDEAYDFETDVQKAADFYQRVLDNFGDDSVGELGGAHLAMENVSILAAKVLEDARIGGSPLEKSTRYVYFDQKVRGEYLYYRDPILMTSAFKDMFLGTCDFLFDTYSALIPQVRAYFEKLYPKDSKTPASAYATSLRAKVLDCIRGLLPAATLTNLGFFGNGRFWQNLIHKLQGHNLAELRRLGDESLTELMKVIPSFVSRAEPHHHHHQAMMQYRRALKEQLKGLAEQATFSEEMSSSPSVQLVYGDPDGIYKVAAGFLFPYSNRSLTDLIDYCKKMPHEDLVQILESSVSARENRRHKSPRGLECVEFGFDILADFGAYRDLQRHRTLTQERQLLSTHHGYNFPVELLDTPMEKSYREAMERANETYNEIVQEFPEEAQYMVPMAYNIRWFFHVNARALQWICELRSQPQGHQNYRTIATGLVREVVKFNPMYELFFKFVDYSDIDLGRLNQEMRKEPTT

>core/173/5/Org5_Gene103

MFKSFIVRYMFVGGLVSFLLPIPDLECANNVTKTYDKKASVISRDLKLQEDCQKFWNLDPYKLESLCAYQVLYHDDYSSKRIRELFPQIQKDEVPIFATMILTLGKVDRGFSPEEISLIQKLSYPGLSLASLRGSTEIDPNTDLARALVVSEFSGDLGKNRADYYSNCLDILALRIHAERQRYLDQSPCVPGTSEFHKATIEAINTILFYEEAVRYPSKKEMFSDEFSFLSSVTDRKFGVCLGVSSLYFSLSQRLDLPLEAVTPPGHIYLRYQGGEVNIETTAGGRHLPTASYCDCLDLEDLQVRTPEEMIGLTFMNQGSFALQKKKYKEAEEAYKKAQEYLGDEELQELLGFVQILGGKKKEGKSLIGKSPRASQKGSVAYDYLKGRINIPTLALLFSYPGSNYEEIASYEEELKKAMKSSMPCCEGQRRLASVAFHLGK

TAEAVALLEKCVEDIPNDLSLHLRLCKILCDRHEYTKALKYFIIAERLMEDQGFLKKDNRSFALFYEVKKIISK
VAPQKANTLLLMESER

>core/174/5/Org5_Gene12

MSIVLDKIGKSLGTRILFDDVSVVFNPGNCYGLTGPNGAGKSTLLKIIMGMIEPTRGSISLPKKVGILRQNIDSF
HDTTVLDCVIMGNTRLWEALQRRDNLYLQEFTDAIGMELGEIEEIIGEENGYRADSEAEELLTGIGIPNEMFD
KKMAMIPIDLQFRVLLCQALFGHPEALLLDEPTNHLDLYSINWLGNFLKDYEGTVIVVSHDRHFLNTITTHIA
DIDYDTIIIYPGNYDDMVEMKTASREQEKADIKSKEKKISQLKEFVAKFGAGSRASQVQSRLREIKKLQPQEL
KKSNIQRPYIRFPLSDKSSGKVVLSLEAITKDYGDHQVIHPFSLEIYQGDKLGIIGNNGLGKTTLMKLLAGVEA
PSSGSIKLGHQAICSYFPQNHSDVLADCGQETLFEWLRNRKTGINDQEIRSVLGKMLFGGDDAFKQIQALSGG
ETARLLMAGMMLENHNVLILDEANNHLDLESVSALSWAINDYKGTAIFVSHDRGLIQDCATKLLIFDKDKIT
FFDGTMVDYTAGHKQLL

>core/175/5/Org5_Gene143

MHRLKPTLKSLIPNLLFLLLTLSSCSKQKQEPLGKHLVIAMSHDLADLDPRNAYLSRDASLAKALYEGLTRET
DQGIALALAESYTLSKDHKVYTFKLRPSVWSDGTPLTAYDFEKSIKQLYFEEFSPSIHTLLGVIKNSSAIHNAQ
KSLETLGIQAKDDLTLVITLEQPFPYFLTLIARPVFSPVHHTLRESYKKGTPPSTYISNGPFVLKKHEHQNYLIL
EKNPHYYDHESVKLDRVTLKIIPDASTATKLFKSKSIDWIGSPWSAPISNEDQKVLSQEKILTYSVSSTTLLIYN
LQKPLIQNKALRKAIAHAIDRKSILRLVPSGQEAVTLVPPNLSQLNLQKEISTEERQTKARAYFQEAKETLSEK
ELAELSILYPIDSSNSSIIAQEIQRQLKDTLGLKIKIQGMEYHCFLKKRRQGDFFIATGGWIAEYVSPVAFLSILG
NPRDLTQWRNSDYEKTLEKLYLPHAYKENLKRAEMIIEEETPIIPLYHGKYIYAIHPKIQNTFGSLLGHTDLKN
IDILS

>core/176/5/Org5_Gene986

MPGSVSSPPLSPVIVRERVPSSSGSDLIQPHAVLKISILIFALVTILGIVLVVSSALGALPSLVLTVSGCIAIAVGLI
GLGILVTRLILSTIRKVDAMGYDAAVKEEQYLSRIRELESENREIRDRNRAVEDQCAHLSEENKDLRDPEYLH
GMTERLIASLEIENQALVAENILLKDWNASLSRDFRAYKQKFPLGALEPWKEDIACIMEQNLFLKPECIAMVK
SLPLETQRLFLYPKGFQSLVNRFAPRSRFFQTPKYEYNSRNENEDGKVAAVCARLKKEFFSAVLGACSYEELG
GICERAVALKETLPLPEAVYDTLVQEFPNLLTAESLWKEWCFYSYPYLRPYLSVDYCKRLFVQLFEELCLKLF
TTGSPEDQALVRLFSYYRNHIPAVLASFGLPPPETGGSVFVLLPKQENLLWSQIEVLATRYLKDTFVRNSEWT
GSFEMMFSYNEMCKEISEGRIRFAEDYETRHSEEFPPSPLSEEGEGEEFLPPCSEEEVSVLERPDLDVDSMWV
WHPPVPKGPL

>core/177/5/Org5_Gene755

MTARAEYLDHEDFLYRSHKLQELSELGVVLYPYEFPGVFSCEDIKKTFASQELGNSEAAMSRSTPRVRFAGR
LVLFRAMGKNAFGQILDHNQTIQVMFNREFTSVHGLSEDAEITPIKFIEKKLDLGDILGIDGYLFFTHSGELTV
LVETVTLLCKSLLSLPDKHAGLSDKEVRYRKRWLDLISSREVSDTFVKRSYIIKLIRNYMDAHGFLEVETPILQ
NIYGGAEAKPFTTTMEALHSEMFLRISLEIALKKILVGGAPRIYELGKVFRNEGIDRTHNPEFTMIEAYAAYMD
YKEVMVFVENLVEHLVRAVNHDNTSLVYSYWKHGPQEVDFKAPWIRMTMKESIATYAGIDVDVHSDQKLK
EILKKKTTFPETAFATASRGMLIAALFDELVSDNLIAPHHITDHPVETTPLCKTLRSGDTAFVERFESFCLGKEL

CNAYSELNDPIRQRELLEQQHTKKELLPDSECHPIDEEFLEALCQGMPPAGGFGIGVDRLVMILTNAASIRDVL
YFPVMRRFDAEKTN

>core/178/5/Org5_Gene141

MLRFFAVFISTLWLITSGCSPSQSSKGIFVVNMKEMPRSLDPGKTRLIADQTLMRHLYEGLVEEHSQNGEIKPA
LAESYTISEDGTRYTFKIKNILWSNGDPLTAQDFVSSWKEILKEDASSVYLYAFLPIKNARAIFDDTESPENLG
VRALDKRHLEIQLETPCAHFLHFLTLPIFFPVHETLRNYSTSFEEMPITCGAFRPVSLEKGLRLHLEKNPMYHN
KSRVKLHKIIVQFISNANTAAILFKHKKLDWQGPPWGEPIPPEISASLHQDDQLFSLPGASTTWLLFNIQKKPW
NNAKLRKALSLAIDKDMLTKVVYQGLAEPTDHILHPRLYPGTYPERKRQNERILEAQQLFEEALDELQMTRE
DLEKETLTFSTFSFSYGRICQMLREQWKKVLKFTIPIVGQEFFTIQKNFLEGNYSLTVNQWTAAFIDPMSYLMI
FANPGGISPYHLQDSHFQTLLIKITQEHKKHLRNQLIIEALDYLEHCHILEPLCHPNLRIALNKNIKNFNLFVRR
TSDFRFIEKL

>core/179/5/Org5_Gene252

MNVLKYTKHSPSAHAWKLIGTSPKHGIYLPLFSIHTKNSCGIGEFLDLIPLISWCQKQGFSVIQLLPLNDTGEDT
SPYNSISSVALNPLFLSLSSLPNIDTIPEVAKKLQDMHELCSTPSVSYTQVKEKKWAFLREYYQKCCKSSLEGN
SNFSEFLESERYWLYPYGTFRAIKHHMHGEPINNWPKSLTDQENFPDLTKKFHDEVLFFSYLQFLCYQQLCEV
KAYADQHHVLLKGDLPILISKDSCDVWYFRDYFSSSRSVGAPPDLYNSEGQNWHLPIYNFSQLAKDDYIWW
KERLRYAQNFYSVYRLDHIIGFFRLWIWDSSGRGRFIPDNPKDYIKQGTEILSTMLGASSMLPIGEDLGIIPQDV
KTTLTHLGICGTRIPRWERNWESDSAFIPLKDYNPLSVTTLSTHDSDTFAQWWLNSPKEAKQFAKFLHLPFQK
TLTTETQIDILKLSHESASIFHINLFNDYLALCPDLVSKNLQRERINTPGTISKKNWSYRVRPSLEELAIHKKFN
GYIEKILTGL

>core/180/5/Org5_Gene987

MERKRFIDCDSTKILQELALNPLDLTAPGVLSAERIKKFSWLGGGFTFSFATERLDDAILAALISLAEERGLHES
MLAMQQGQVVNYIEGFPSEMRPALHTATRAWVTDSSFTGEAEDIAVRSRVEAQRLKDFLTKVRSQFTTIVQI
GIGGSELGPKALYRALRAYCPTDKHVHFISNIDPDNGAEVLDTIDCAKALVVVVSKSGTTIETAVNEAFFADY
FAKKGLSFKDHFIAVTCEGSPMDDTGKYLEVFHLWESIGGRFSSTSMVGGVVLGFAYGFEVFLQLLQGASA
MDQIALQPNARENLPMLSALISIWNRNFLGYPTEAVIPYSSGLEFFPAHLQQCCMESNGKSIVQDGRRVGFST
SPVIWGEPGTNGQHSFFQCLHQGTDIIPVEFIGFEKSQKGEDISFQGTTSSQKLFANMIAQAIALACGSENTNPN
KNFDGNRPSSVLVSSQLNPYSLGELLSYYENKIVFQGFCWGINSFDQEGVSLGKALANRVLELLEGADASNFP
EAASLLTLFNIKFR

>core/181/5/Org5_Gene267

MTKTEEKPFGKLRSFLWPIHTHELKKVLPMFLMFFCITFNYTVLRDTKDTLIVGAPGSGAEAIPFIKFWLVVPC
AIIFMLIYAKLSNILSKQALFYAVGTPFLIFFALFPTVIYPLRDVLHPTEFADRLQAILPPGLLGLVAILRNWTFA
AFYVLAELWGSVMLSLMFWGFANEITKIHEAKRFYALFGIGANISLLASGRAIVWASKLRASVSEGVDPWGI
SLRLLMAMTIVSGLVLMASYWWINKNVLTDPRFYNPEEMQKGKKGAKPKMNMKDSFLYLARSPYILLLAL
LVIAYGICINLIEVTWKSQLKLQYPNMNDYSEFMGNFSFWTGVVSVLIMLFVGGNVIRKFGWLTGALVTPVM
VLLTGIVFFALVIFRNQASGLVAMFGTTPLMLAVVVGAIQNILSKSTKYALFDSTKEMAYIPLDQEQKVKGK

AAIDVVAARFGKSGGALIQQGLLVICGSIGAMTPYLAVILLFIIAIWLVSATKLNKLFLAQSALKEQEVAQEDS
APASS

>core/182/5/Org5_Gene1019

MVWVFKSQFEGLSALKRGVHALTKAVTPAFGPRGYNVVIKKGKAPIVLTKNGIRIAKEIILQDAFESLGVKLA
KEALLKVVEQTGDGSTTALVVIDALFTQGLKGIAAGLDPQEIKAGILLSVEMVYQQLQRQAIELQSPKDVLH
VAMVAANHDVTLGTVVATVISQADLKGVFSSKDSGISKTRGLGKRVKSGYLSPYFVTRPETMDVVWEEALV
LILSHSLVSLSEELIRYLELISEQNTHPLVIIAEDFDQNVLRTLILNKLRNGLPVCAVKAPGSRELRQVVLEDLAI
LTGATLIGQESENCEIPVSLDVLGRVKQVMITKETFTFLEGGGDAEIIQARKQELCLAIAGSTSESECQELEERL
AIFIGSIPQVQITADTDTEQRERQFQLESALRATKAAMKGGIVPGGGVAFLRAAHAIEVPANLSSGMTFGFETL
LQAVRTPLKVLAQNCGRSSEEVIHTILSHENPRFGYNGMTDTFEDLVDAGICDPLIVTTSSLKCAVSVSCLLLT
SSFFISSRTKT

>core/183/5/Org5_Gene7

MYQENLRLLERLLYNSVQKSYADRLFSYEKTKMVHDTPLIPWEEDKEKCAEAEKAFLEQQKILLDYGKSIFW
LNENDEINLNDPWSWGLNTVRTRKVFQEVDDSERWNHKVLIQKLEDDYEKLLEESSKESTEANKKLLSDLV
DRLEDAKTKFFLKKQEEVETRVKDLRARYGGTVDPKQDTEAKKKVELEASLETFLDSIESELVQCLEDQDIY
WKEQDVKDLARTQELEEQDIEAKREEAAEDLRSLNERLKKSKTMLDRAKWHIENAEDSITWWTSQIEMKD
MKARLKILKEDITSVLPEIDEIETCLSLEELPLLTTRELLTKSYLKFKICSETLLKMTSVFENNIYVQEYEVQLQ
NLGFKLQGISQRFGKKQDDFANLEEQVALQKKRLRELTQNFEIQGFNFMKEDFKAAAKDLYIRSTAEQKMN
FDVPCMELFRRYHEEVNKPLLELMYNCADSYRDAKKKLCSLRLDEKELLQKEIKKEEFYQKKQQRHADRSR
HTTYQKLRIAEELALELKKKI

>core/184/5/Org5_Gene894

MVARGLCDFPTLVPNERLPIGPFFVPQHTSGAKGKEFAKRNFSIISGLDDILKLCILQRRPFALQWDNLSVKSD
YEEAGPAIGIRSLEPQVSQISPAHGRLCSTLVQWAPILGSEERLVWLEETMKRLKFPKSLGSKDAVIVDSEMVP
VNANPTQEIPAASETVESSPVAPGNTTDTMPAASGTTDTTSGVSEAAAAEATVDSTPGTEEEPSFSLRYALVV
QNVPYPEPPKEPEVMFTDEEKSLILEATRARRMELDLYNGYLADYELSKDEIQKHVPDLPENWRTNWRWSE
RLYKFFFKTKKEGLEEIFLNKELGNMILARGLAATQSQARIKVFNSLVAWLLQSFNVGRSCTAKPLPTSKLDL
FKSEFESKPKNNILTEFLVASDEEILFKGLRVLEPGIEGWYDHPDQAGEIRSVLEGLVQAGRISGYWENQPFGR
FVLRGVGERRTELVELLESLVASGEIMQFFESSDEEGAFIIDNEPSKTAMLKQRFKSCVRTKLVGSFADESLPR
GRFTILV

>core/185/5/Org5_Gene394

MLKVFEKFKKFAIVEIFTKVVAVVSLLHKFLENASGKKGQSLASTAYLAALDHLLNAFPSIGERIIDELKSQRS
HLKMIASENYSSLSVQLAMGNLLTDKYCEGSPFKRFYSCCENVDAIEWECVETAKELFAADCACVQPHSGA
DANLLAVMAILTHKVQGPAVSKLGYKTVNELTEEEYTLLKAEMSSCVCLGPSLNSGGHLTHGNVRLNVMSK
LMRCFPYDVNPDTECFDYAEISRLAKEYKPKVLIAGYSSYSRRLNFAVLKQIAEDCGSVLWVDMAHFAGLV
AGGVFVDEENPIPYADIVTTTTHKTLRGPRGGLVLATREYESTLNKACPLMMGGPLPHVIAAKTVALKEALS
VDFKKYAHQVVNNARRLAERFLSHGLRLLTGGTDNHMMVIDLGSLGISGKIAEDILSSVGIAVNRNSLPSDAI

GKWDTSGIRLGTPALTTLGMGIDEMEEVADIIVKVLRNIRLSCHVEGSSKKNKGELPEAIAQEARDRVRNLLL
RFPLYPEIDLEALV

>core/186/5/Org5_Gene727

MSEQEKLSNYNADKKLFSGIDKLFQIVKGSYGPKQSLSPTSFFKERGFYAISQTELSNSYENLGVDFAKAMVN
KIHKEHSDGATTGLILLHAILQESYAALEKGISTHKLIASLKLQGEKLQEALQQQSWPIKDALKVRNIIFSSLH
MPTIADHFYNAFSVVGPEGLISITKERENDKTSMDVFQGFKIPAGYASTYFVSDTASRLTRIAHPLILITDRKIS
MIHSLLPLLQEISEQNQHLIIFCEDIDPDVLATLVVNKLQGLLQVTVVTIPQLSTTNQELAEDIALFTGTHICPCQ
EASHVLAPEMVTLGSCLSIEISESQTTLIGGLHIPEVLTLKTRQLAEEIRTTSCLETKKRLIKSTNRLQSSVAILPT
DEDNEPLYTLALKIMESALSRGYVPGGGVALFYASLTLGTPKDDADENSIAISLLQKACCAPLKLLATNADLD
GDAVIAKLSSLGTTSLGISVFSREIEDLIAGGILDSLATTSTILAQALDTAILVLSSKILILENQYEISTL

>core/187/5/Org5_Gene778

MENEILLNIESKEIRYAHLKNGQLFDLTIERKKVRQLKGNIYRGRVTNILRNIQSAFINIDERENGFIHISDILENS
KKFEQMFDMDVDALPEEASEAPLLSSEEAPIEEFLKLDSPVLVQVVKEPIGSKGARLTSNISIPGRYLVLLPNSP
HRGVSRKIEDPHMREQLKQLIRSFEMPQDMGLICRTASTTASTEALINEAHDLLLTWKTILEKFYSTEQPCLLY
SETDILKKAVITCIDKNYKRLLIDDYATYQKCKHMLKKYSPDASIKIEYYRDSIPMFERFNIEKEIDKATRRKI
WLSSGGYLFFDKTEAMHTIDVNSGRSTQLESGVEETLVQINLEAAEEIARQLRLRNVGGLVIIDFIDMKSRKN
QRRVLERLKEHMKYDAARCTILSMSEFGLVEMTRQRNRESLMQTLFTLCPYCSGNAIIKTPESVVIEIERDLK
KVINHKEHSHLCLVVHPEIASYMKQENDDNEMINLAKQLKAKLQINTSDSVHLNHYQFFSLITGESIDL

>core/188/5/Org5_Gene218

MFNLFFFTANKETTASHELIYRKNQSFSLSPVTILCLLAISVLLLLGVVFALVGCHVLAAPLGLLVWGCAASV
CSMMAIVSLMCLYKGGKPLIEPSNEEKIDPTKDLEIKDPESLKPVPVEGQSLPKERKTVSFKAKIPSIVEDDFKP
YVIQSTFYHQNKVYSKPIAERMQSLEKEITTLIVDFPRALEESSKSSGSLLRGVISEIKNLFLPRFLSRKVKYSLT
ACLRRLGSIVEEYASSDLLILLLTKPEPLNMVTQQLIAHLNSLKTEKRKLTPHMQKLVLSINFWFYGWSLEEK
CIEKIVAYDPNLLTDELKAHLEAGNIVQFLLSFQSSEMQREFRALFPSDAQELPSAKDGSNYVPAINSSEYMY
DFKDLSVLKKSLSERLAFCEKIPSPSSWNFTSSVASHYKDFSLLFTFFSNQQSVILQNPFLLIELLHENPKCQTFL
KGLLEKAMPMSNWAALFRPMLMGMLCSGIARKKELKIIAEHLGVPFKEITQAIASGKILDLLLQHLFDF

>core/189/5/Org5_Gene349

MTILRNFLTCSALFLALPAAAQVVYLHESDGYNGAINNKSLEPKITCYPEGTSYIFLDDVRISNVKHDQEDAG
VFINRSGNLFFMGNRCNFTFHNLMTEGFGAAISNRVGDTTLTLSNFSYLAFTSAPLLPQGQGAIYSLGSVMIEN
SEEVTFCGNYSSWSGAAIYTPYLLGSKASRPSVNLSGNRYLVFRDNVSQGYGGAISTHNLTLTTRGPSCFENN
HAYHDVNSNGGAIAIAPGGSISISVKSGDLIFKGNTASQDGNTIHNSIHLQSGAQFKNLRAVSESGVYFYDPIS
HSESHKITDLVINAPEGKETYEGTISFSGLCLDDHEVCAENLTSTILQDVTLAGGTLSLSDGVTLQLHSFKQEA
SSTLTMSPGTTLLCSGDARVQNLHILIEDTDNFVPVRIRAEDKDALVSLEKLKVAFEAYWSVYDFPQFKEAFT
IPLLELLGPSFDSLLLGETTLERTQVTTENDAVRGFWSLSWEEYPPSLDKDRRITPTKKTVFLTWNPEITSTP

>core/190/5/Org5_Gene179

MTNVVQETIGGLNSPRTCPPCILVIFGATGDLTARKLLPALYHLTKEGRLSDQFVCVGFARREKSNELFRQEM
KQAVIQFSPSELDIKVWEDFQQRLFYHRSEFDNNMGYTSLKDSLEDLDKTYGTRGNRLFYLSTPPQYFSRIIEN
LNKHKLFYKNQDQGKPWSRVIIEKPFGRDLDSAKQLQQCINENLNENSVYHIDHYLGKETVQNILTTRFANTI
FESCWNSQYIDHVQISLSETIGIGSRGNFFEKSGMLRDMVQNHMMQLLCLLTMEPPTTFDADEIRKEKIKILQR
ISPFSEGSSIVRGQYGPGTVQGVSVLGYREEENVDKDSRVETYVALKTVINNPRWLGVPFYLRAGKRLAKKS
TDISIIFKKSPYNLFAAEECSRCPIENDLLIIRIQPDEGVALKFNCKVPGTNNIVRPVKMDFRYDSYFQTTTPEAY
ERLLCDCIIGDRTLFTGGDEVMASWKLFTPVLEEWDQDSSPSFPNYPAGSSGPKEADALIERDGRSWRPL

>core/191/5/Org5_Gene828

MSSWLSQASEVLLNQDPYIPDAPRSQESSVPKISYSITVAPQEAQKSLPKFFTQKFQSQCKSEPPITHHKTFIIAT
PRERILRFGSSFESQLHNTSQAQTSSPWNLFSQKNSTEASKALMQELTMPKSPEKTSEKALDKNLSSKQEGSC
KNFDTLHLQQHLKLFGTVDSLYSQSLDSEQQELLQSRREERSETYANQQSSEKKIETKVQIKDLCKDLFSQDQ
DSNQKQKKSPFQQDTSRKNRIAKAAQAVPVIPPPSIGVFTLSYLLTKQGILSDFSSYGCHKDSVESTQRELDAL
HEKRIETIKVSIEKEKRERLWGSLSDIIGWLAPFVSIGIGIVAILSGGGIFAFAGFFAGLISLVIKCLEKLKFWDW
LEKHLPIKNEELRRKIITIIQWVVYLTPVILSICTLKVENLGFSPIIEGAIKGIQPAIESTMAALRCAILFSQAEIYK
LKGKLTKIQLDIELKSFDRDDHYERSQELLDNMESSFEALSRILNYMRELDQVYLHSLRG

>core/192/5/Org5_Gene902

MAVGGVGGSRSPSPIPPNRRNSEDGKVSPKDNLGEHTVSSSDSSLASQGPTIEERKAQLGGTDKIPLPSVKEPG
DSPTSGRSGVLQRIWKGVKGVFKKTPQARPEVSSPRLPSHVQHGQRLPGLEGFRDRIQKRSENPEADLGKMK
RSYSDGDLDRVGHDSNEDSTEDSRSEGGEPSSKSSSFLSGVRGAVSKVHGALGDIKGKFQRSASEDDLTTQG
EDSAGDTVKERRSEEAEASSKSSSFLSGVRGATSTVQGALGDAKEKVSAFGEQAAGAIRSAPGNIRTRFQRSS
SEGDLSNVNKAAKHLRKALENLEKVAPEQVSPEVASRVQSLLARMEQLTHQEPPTVEDLITFVESNVGSDSV
EYASIVPQDGSQAPAETAEAPETGGVEGSAAQGAWKALRDFVVSIFQAVASFFRAIASRLSSARRESAVDDL
ASESNTQWFVEQEGVSNPSAAPSLSFAEEIARRAAEMSNRNAQSLEKLESGNVTDPVIQQGLGLARSFAPEGQ

>core/193/5/Org5_Gene107

MADILVIGANPTGLILANMLIQHGISVKVIDHRASPEDPSFLDCRKLPVILSCSSLELLHNSEMLGDFIQANHKI
FGARYHWKKRTLLFKFSQATDSPVPFSLSTTYQSLEQHLIDEFLKRGGVIDWSTRPVTLVDNSIFIESTKVSQN
FENREIYNPKWIIACEADNNLDIRDLVKSQLRARRINREVIFINCDEGEPFEEDHIHLLPITKNFLNFVFYNPQEK
TKQLCLPQGTHSISPKLKQKLLYTYNLVISDENFHIKTSHHAFPPEHGNVLFLGSLSNTLLLSYLNGINTNIHAA
FNLAWKLLPVLKKAALKHLVITKEQEDGNILPYISPTTEKRAKKLPFSRFYTPALMYYFLKGCRKFNTTGEEY
YYPPHQALKYRSSDIIKMSPQDKEIHGPGPGMRAIDARLENGSFLLDPLKSSKHLLIFFKDIPDLKEALQEEYG
EWIEICNVKEPRILNLYHANPNSLFIIRPDRYIGYRTHTFKLHELISYLLRIFASEKTS

>core/194/5/Org5_Gene429

MNWENVRVRVAPSPTGDPHVGTAYMALFNEIFAKRFKGKMILRIEDTDRTRSRQDYEENIFSALRWCGIQW
DEGPDVGGPYGPYRQSERTKIYQGYVETLLKTDCAYKCFATPQELAEMRAVASTLGYRGGYDRRYRYLSPE
EVASREAAGQPYTIRLKVPLSGECVFEDYSKGRVVFPWADVDDQVLVKSDGFPTYHFANVIDDHLMGITHV
LRGEEWLSSTPKHLLLYEAFGWEPPVFLHMPLLLNPDGTKLSKRKNPTSIFYYRDSGYVKEAFVNFLTLMGY
SMEGDEEVYSLERIIETFNPRRIGKSGAVFDIQKLDWMNKHYLNHEGSPECLLKELQGWLLNDEFFLKILPLC

QSRITTLAEFINLTSFFFSGLLEYRVEELLPQALSPEKAAILLYSYVKYLEKTDQWTKETCYLGSKWLAQAFN
VHHKKAIIPLLYVAITGKKQGLPLFDSIEILGKPRARARLVYAEKLLGGVPKKLAATVDKFMQREDFEEATFD
L

>core/195/5/Org5_Gene560

MDCRGGIPLPEPQVIGGYHVKKILSKKLRSRVVHGLHPETRHSTVIKVFSPSPSFTSRSVYNFLKEAQSLHQIT
HPNIVKFHRYGKWQDCLYIAMEYIEGISLREYILAQFISLPQAIDIIFDIAQALEHLHSRNILHKDIKPENILITPQ
GKIKLIDFGLADWDTEIQRAHPSVIGTPYYMSPEQRQGESHSPASDIYALGLLAYELILGHLSLGRVFLSLVPE
RISKILAKALQPSPNNRYSSTREFIQDIHHYRMSGDMQEDLRIKDHTVALYEQLQTQRFWLAPETLRFPDFISG
VLYHQGYPLYPHAYDTLLEGDVFNLWLGYSPISNATIALSVVKSLVCQQDLQRPLLDRVCEINECLIRMKIPI
DEMGISILCLEISKENKELSWIACGKTVFWIKRQGRVVQDFESFSPGLGKITSLQIRETKVAWEIGDEAVVCTL
ELEESVASLKTLSLAELQDRRQKAIFCPIESIHGGIQSRQHGSNSPSTLISLKRIR

>core/196/5/Org5_Gene334

MLKKFINSLWKLCQQDKYQRFTPIVDAIDTFCYEPIETPSKPPFIRDSVDVKRWMMLVVIALFPATFVAIWNS
GLQSIVYSSGNPVLMEQFLHISGFGSYLSFVYKEIHIVPILWEGLKIFIPLLTISYVVGGTCEVLFAVVRGHKIAE
GLLVTGILYPLTLPPTIPYWMAALGIAFGIVVSKELFGGTGMNILNPALSGRAFLFFTFPAKMSGDVWVGSNP
GVIKDSLMKMNSSTGKVLIDGFSQSTCLQTLNSTPPSVKRLHVDAIAANMLHIPHVPTQDVIHSQFSLWTETH
PGWVLDNLTLTQLQTFVTAPVAEGGLGLLPTQFDSAYAITDVIYGIGKFSAGNLFWGNIIGSLGETSTFACLLG
AIFLIVTGIASWRTMAAFGIGAFLTGWLFKFISVLIVGQNGAWAPARFFIPAYRQLFLGGLAFGLVFMATDPVS
SPTMKLGKWIYGFFIGFMTIVIRLINPAYPEGVMLAILLGNVFAPLIDYFAVRKYRKRGV

>core/197/5/Org5_Gene189

MKFEFSVALKYLIPGRGRLYSAIVSLFSVGIISLVVWLSIVFISVIHGLEQRWIEDLSQLHSPITILPSDTYYSSYY
YQIDKHSSLSNYTTKTLGEKIASPQVDPYDPESDYLLPETFPLKDCDLGGQQKDPVKMTLESLGPYLQSQHGK
VIEFEQGVGYLDIKTSLKLQKPQPRNLTHFLTYPSKLSYEDKVLPYDETDYTSAELNPFNRSPSGWQQDFHHL
EELYRGASIILPSTYKDSGYKVGDTGVFSTYSIENEKETQYTVHVIGFYNPGLSPLGGRTVFIDPDLARSIRSQS
EGLGMSNGFHLFFPNTKRIVFVKKQIENILTSLGVDDYWEISSLHDYDYFQPILDQLQSDQVLFLFVCILILIVA
CSNIVTMSMLLVNNKKKEIGILKAMGTSSRSLKIIFACCGAFSGACGVVIGTIFAIITLKNLQFIVKALNYLQGR
ETFNTAFFGQNLPNSVHPQAIYFLGLGTLLLAAVSGALPARKVAKMHVSEILKAD

>core/198/5/Org5_Gene295

MVLFHAQASGRNRVKADAIVLPFWHFKDAKNAASFEAEFEPSYLPALENFQGKTGEIELLYSSPKAKEKRIV
LLGLGKNEELTSDVVFQTYATLTRVLRKAKCSTVNIILPTISELRLSAEEFLVGLSSGILSLNYDYPRYNKVDR
NLETPLSKVTVIGIVPKMADAIFRKEAAIFEGVYLTRDLVNRNADEITPKKLAEVALNLGKEFPSIDTKVLGKD
AIAKEKMGLLLAVSKGSCVDPHFIVVRYQGRPKSKDHTVLIGKGVTFDSGGLDLKPGKSMLTMKEDMAGG
ATVLGILSALAVLELPINVTGIIPATENAIDGASYKMGDVYVGMSGLSVEICSTDAEGRLILADAITYALKYCK
PTRIIDFATLTGAMVVSLGEEVAGFFSNNDVLAEDLLEASAETSEPLWRLPLVKKYDKTLHSDIADMKNLGS
NRAGAITAALFLQRFLEESSVAWAHLDIAGTAYHEKEEDRYPKYASGFGVRSILYYLENSLSK

>core/199/5/Org5_Gene827

MTVSYQSISTPPPEGEFDIFVDGNATEEAVVAAEVQVALPAGEQYAMLRATSELCFGILTQSECALTQALPPK
EKPLQEEQFLVKNGILMRSTSLPNLKPGQSQQTSLASHRNPLAQQSTSSNSTGKASTETTSSSFPFFSCKAPEG
DSSVDKTFTVSVQTPKAQEQQEASASQSQAQFHVRSYSSSTIKEHSAKEKVSQSTKSAETQKHTQTKSDATLS
PMSLYSTLHKEVPQALSSTKSQQKDEEHRDQRQQEGYEQEQEQEEGKKKTPWCTVESLQQTSSSNQVYESY
TPIIPDPIVEFALSESQLSVLAGKRVTNLDVLRICTELMKLMLKSRANDTMTRLEERELMEREAHELAASYSR
QAKYARWLGIATATLGILGAIAPMVGEISGDSILGFVQRISGRFKDATAKTFFKGIGKVFTSLSQLTEAASKVH
ELSESAVRAVAEYRKEVFRMRQDEVTRTIEEVKDNWKSMDNFLLNILQTEHDAARSLYQ

>core/200/5/Org5_Gene657

MAASILSQELLDILPYTFLKKHCLLPIEESSEAITIAHATATSVIAQDEVKLLIKKPVRFVLKEESEILQRLQQLY
SNREGNVSDMLLTMKEEDGTTISEEEDLLETTDTIPVVRLLNWILKEAIEERASDIHFEPCEDSMRIRYRIDGVL
HDRHSPPSHLRSALTTRLKVLAKMDIAEHRLPQDGRIKIHIGGQEVDMRVSTVPVIYGERVVLRILDKRNVIL
DIAGLHMPKGTEILFKDTITAPEGILLVTGPTGSGKTTTLYSVLQELKGPLTNIMTIEDPPEYKLPGIAQIAVKP
KIGLTFARGLRHLLRQDPDILMVGEIRDQETAEIAIQAALTGHLVVSTLHTNDAISAIPRLLDMGIESYLLSATL
VGVVAQRLVRTICPYCKVAYTPENQEKSFLASLGKDTEMPLYRGQGCVHCFRSGYKGRQGIYEFLRPNTLFR
SEVASNRPYHILRETAEQNGFLPILEHGIALAVSGETTLAEVLRVTKRCD

>core/201/5/Org5_Gene937

MNKKHASFSSRLGFIFSMIGIAVGAGNIWRFPRVAAQNGGGAFLILWLCFLFLWSIPLIIIELSIGKLTKKAPIGA
LIKTAGKKFAWAGGFITLVTTCILAYYSTIVGWGLSYFYYAVSGKIHLGNDFAKLWTSHYQSSIPLWAHLTSL
GLAYLVIRKGIVHGIEKCNKILIPAFFLCTIALLLRAVTLPGAVQGIKQLFSCDKSCFSNYKVWIEALTQNAWD
TGAGWGLLLVYAGFASKKTGVVSNGALTAICNNLVSLIMGIIIFSTCASLDILGTTQLQDGAGASSIGITFIYLP
ELFTRLPGGIYLTTLFSSIFFLAFSMAALSSMISMLFLLSQTLAEFGIKPYISETLATIIAFVLGIPSALSLTFFSNQ
DTVWGVALIVNGLIFIYAALVYGFPKLKKEVINAAPGDLRLNKAFDYIIKYLLLIEGILLLGWYFYEGLFPENG
QWWNPISLYSLGSLVLQWSLGLIILWKFNKQLYLRFSRYNHEIL

>core/203/5/Org5_Gene463

MLKYILKRLVLIPLTLFAIVSINFVILNAAPGDVLEEKSRDALGEAGKSDKMRSYKGPDRYLQFREHYGLTLPI
FFNTRPKITHKKIQTALQELANANNTTPSAKNAAKSLVYWGDCAKFVMPALLFEADDASRDDKYRHIAADL
FIRGGVLQGFVGPNLSPEQRAQNKEIAESNAFLVRQLNEEDLDTKVEALKGWFQDHGGTEVFCYSSKQFWK
TFFLETRFARYMSRVLRLDFGTLRNDAHKTVISEVIKRLRCSLVLSILPMIVGFVLCQIFGMIMALKRNRWIDH
SLNFIFLILFSIPVFVAVPWILDNFVINKTIPFTTIPMPYSGLRSPPEVFNELSTLGRIFDLVSHGFLPFCAVSYGAL
AAQSRLSRSIFLEVLSQDFICAAKARGLRWFDILYKHVGKNAAVSIVTSLASSLGTLLGGALVVETLFNIDGFG
NFFYQAILNRDHNVVLFSVLVGSALSLVGYLLGDICYVLLDPRVQLEGRRI

>core/204/5/Org5_Gene1022

MHTEFAPFLEDLVHQQVISPLDIAFASKHISSDFEESFVFLAVSSALWRYGHPFLSLEENRIRPSLGGISETDLY
RGFHNLPKEVRDKLFVVVSGRLYLRSLYTIRSKLLDKLSLLCSATPNYFPPSIDSSILSEEQNFIFNKITQGCFSI
VSGGPGTGKTFLAAQLILSLVKQQPKLRIAIVSPTGKATSHIRQILMKYNIFDDMVLMQTVHHFLQEYAYRRY
NSIDVLLVDEGSMVTFDLLYSLVQTLQGYEKDKKLYTSSLIILGDTNQLPPIGIGVGNPLQDLIGYFHENTFFL
KTSHRAKTGVVDQLTQSVLRGEMISFSPLPSISSAIEVLKNRFVKSLRQSEARLCVLTPMRHGPWGVLNLNTM

IHQRLARSDPDLRIPIMVTSRYETWGLFNGDTGLLCLKTQKLHFPQHEPIDSRALSQYVYNYVMSVHKSQGSE
YDEVIVIIPKGSEVFGVSILYTAITRAKYRVSVWGDPETLHKIIKKSNY

>core/205/5/Org5_Gene792

MLNHAKKHAKPYVLIFFSTKDKLSYCDIIFNNCSGKPMNLDSKHFDINSANFLEEFAKFISFPSISADSDHLQD
CENCAHFLVDHVNKIFDVELWETPGHPPIIYASYKSEDPLSPTLMLYNHYDVQPAQLSDGWKGDPFILREEN
GNLYARGASDNKGQCFYTLKALQHYYESQGNFPLNIIWLIEGEEESGSLALFTWLEKKKEALRADYLLIVDG
GFLSEKHPYVSIGARGIVSMKISLEEGNKDMHSGVLGGIAYNTNRALSEILSSLHHPDNSIAIEGFYDDLALPS
DSDRPDLPKSDTLRECEENLGFRPQGYEASYSPEESALRPTVEINGISGGYTGPGFKTVIPYRATAYLSCRLVP
NQDPDKAAHQVIHHLKQQVPSSLKFSYEILPGGSRGWRSSANLPIVKVLQEIYSDLYNEECLRLVMPATIPIGP
LLGEAAQTSPIICGTSYLSDDIHAAEEHFSMDQLKKGFLSICQLLDKLPKIKE

>core/206/5/Org5_Gene1015

MSISSSSGPDNQKNIMSQVLTSTPQGVPQQDKLSGNETKQIQQTRQGKNTEMESDATIAGASGKDKTSSTTKT
ETAPQQGVAAGKESSESQKAGADTGVSGAAATTASNTATKIAMQTSIEEASKSMESTLESLQSLSAAQMKEV
EAVVVAALSGKSSGSAKLETPELPKPGVTPRSEVIEIGLALAKAIQTLGEATKSALSNYASTQAQADQTNKLG
LEKQAIKIDKEREEYQEMKAAEQKSKDLEGTMDTVNTVMIAVSVAITVISIVAAIFTCGAGLAGLAAGAAVG
AAAAGGAAGAAAATTVATQITVQAVVQAVKQAVITAVRQAITAAIKAAVKSGIKAFIKTLVKAIAKAISKGI
SKVFAKGTQMIAKNFPKLSKVISSLTSKWVTVGVGVVVAAPALGKGIMQMQLSEMQQNVAQFQKEVGKLQ
AAADMISMFTQFWQQASKIASKQTGESNEMTQKATKLGAQILKAYAAISGAIAGAA

>core/207/5/Org5_Gene3

MYRYSALELAKAVTLGELTATGVTQHFFHRIEEAEGQVGAFISLCKEQALEQAELIDKKRSRGEPLGKLAGV
PVGIKDNIHVTGLKTTCASRVLENYQPPFDATVVERIKKEDGIILGKLNMDEFAMGSTTLYSAFHPTHNPWDL
SRVPGGSSGGSAAAVSARFCPVALGSDTGGSIRQPAAFCGVVGFKPSYGAVSRYGLVAFASSLDQIGPLANT
VEDVALMMDVFSGRDPKDATSREFFRDSFMSKLSTEVPKVIGVPRTFLEGLRDDIRENFFSSLAIFEGEGTHLV
DVELDILSHAVSIYYILASAEAATNLARFDGVRYGYRSPQAHTISQLYDLSRGEGFGKEVMRRILLGNYVLSA
ERQNVYYKKATAVRAKIVKAFRTAFEKCEILAMPVCSSPAFEIGEILDPVTLYLQDIYTVAMNLAYLPAIAVP
SGFSKEGLPLGLQIIGQQGQDQQVCQVGYSFQEHAQIKQLFSKRYAKSVVLGGQS

>core/208/5/Org5_Gene1057

MNGCWEDLKQTIFWVGEHDCTDIETVRKSCMWLDRYADKFILREKEEKMERHELFHATMVRKASGHAYA
KAKAAFEKERSNENQRKVKDVEKWLSKGLAEFRNQESRRARERLRELQTLYPEVSVEERVLERQRTKKVNL
ENLYADIEKKYHHCVREQEHYWKEVENKEAEYRENGEKVLSAEEVSECLQRLEDCLETWSKKLTKAEESVF
EMKFDATEKLGNKVLSDVTNRLEILCEDAEEMIFRIEEIEMTLRMVELPLLFMKNTFEKASLQYNSCKEMLA
KVEPQCKESPTYRSSQERLERLNQDLQTAYTNCQERLQGFSDLESKVRTCRDHLREQMKHFEVQGLNFINEE
LLWVGAELFTQARLDLVATVPYMEFYLQYHNIKREKVRSQWMAKTERYREIRQAFQGVMKEDLLAEDTIL
KEEDYWLLRDDWLLRDERKNRQRRLICNKIAAAQQRVKGF

>core/209/5/Org5_Gene959

MLGILLIASGIIFLAVAIPGLSSAVALGLGCGMTALGTVLLITGLVLLIRSEKLALEQVEIKQARTRVNNELDQL
SQYVFYTENVLDNLKRWSYRDLGFVRQAQEEVTNLEQDIEEIFLTLRDIRNALDNEEFFMTHAKQCLAQVGE
SLFQDASIDEFINLAHLSEIRQHLDINDPRWSMITKKVKGTVVRFIYVSTMYKQIKSNFEKSDFGQLRKMLLN
NYKTIEEVLYQSFQRGYNRAALLSEKTRIIHTSSLLHWEKDEDKHLNIKNECASRLENFKKFRTLFLGLSEEDV
IDFTGASGWDCSKLPRKEVPLDGGKKKLRFKRTFADEQVGDWDRTTSLEHMTPQEEDPLDRLMDQVEQEAT
SVLKDQDRYWKEIETSEAKFRSLPREDDFEKQSQIDSYIRDLDDHLSVWANQLSAAEDALIEVTDVQEHGNR
EMLKNIQQGLELIEDAVKATLPRVDFIQELLEKEELPLVAARMSLENS

>core/210/5/Org5_Gene871

MTFPCGNCNCYYRETPPPNPGGEDIPLQEGGQSGSQGGRVITQQPGTGGREMGISLGSDNVLGMVEQAGSLL
NNLLDSARMQRLGHYCYRTGTPWCREHCPGFLQWIWGGCCACCLETVDDPDNPSAQFLQQLIQQYGPICVG
MSFQQLPHCTQKIEQGEPLGDGDKQEVENGCKLHRELLKAAQPRCMGESLVKLLQNNGLGEDMQQTPPWS
LILQAVSEGALSFVTSSDNPPTCWILQPEQQPCPPPPTDEEQLQGAVGGAPAPQQKKHPAQECRVTCKLNFRT
LLQKLSRLEVLSLESGYKGPLGQAAKQIVDLIKKSLKRLVASDLATFLGPGIGLSLESQVFEVLVLLCLLSKGY
LPLDPLHPEQTVLDPRVQGPWQRILRKVLVTTTAGENIWRQTQGEAPRQAPPPPDPWDDDEIERDGIVTGGG
FGIPCQCLRCWRKLPTEKRPNRWL

>core/211/5/Org5_Gene782

MHMSNPISLFSPAELIAKYNLIPKTSPIYPRRTELIILEENACQTRLTNVAQVLHPSSLFSMSKKILNPCGCSGGP
LCWVILNILAFIITSVLFIILLPVNLIVAGLRLFMPLPPKKIVEDLSEPTTEETNEVIQPFIFALQALLFEDNKLRSF
KIVEQSVGKAPLPNPFLNRLVAISPQESQEAMRKIPDLCSQLKKVLKSLGVLTPEWKHMLKYFEGLKNEHDS
NPDKKTFPILIKLLIEALTGKSSLPKTPSTKEKMQAALFIASSCKTCKPTWGEVITRSLNRLYSIANEGDNQLLI
WVQEFKERELMSIQDGDDAEEYRFAAQQHGERYTEAIEQVLRNESAAKLQWHVINTMKFFHGKNLGLVTE
HLQDTLGALTLRQTTVDTHQGREDADLSAALFLNKYLNSGNQLVNSVFKSMQKADPETKALIREFALDILYA
SLRLPQTSAHTEVFSTLLMDPETYEPNKACIAYLLYVLKIIEL

>core/212/5/Org5_Gene4

MSAVYADWESVIGLEVHVELNTASKLFSSALNRFGDEPNTNISTVCTGLPGSLPVLNQSAVEKAVLFGCAVE
GEISLLSRFDRKSYFYPDSPRNFQITQFEHPIIRGGRIKAIVQGEERYFELAQTHIEDDAGMLKHFGEFAGVDYN
RAGVPLIEIVSKPCMFCPEDAVAYATSLVSLLDYIGISDCNMEEGSIRFDVNVSVRPKGSPELRNKVEIKNMNS
FAFMAQALEAEKQRQIDEYLNQPNKDPKLVIPAATYRWDPEKKKTVLMRLKESAEDYKYFPEPDLPTLQLTE
SYIERIRKTLPELPYDKYHRYIQEYGLSEDIASILISDKNIATFFEVACKDCKNFRSLSNWVTVEFGGRCKTLGV
KLPSSGIFPEGVAQLVNAIDQGVITGKIAKEIADLMMESPGKNPEEILKEKPELLPMSDEGELQKIIAEVVLANP
ESIVDYKNGKTKALGFLVGQIMKRTAGKAPPKRVNELLLLELDKG

>core/213/5/Org5_Gene992

MITKQLRSWLAVLVGSXLLALPLSGQAVGKKESRVSELPQDVLLKEISGGFSKVATKATPAVVYIESFPKSQA
VTHPSPGRRGPYENPFDYFNDEFFNRFFGLPSQREKPQSKEAVRGTGFLVSPDGYIVTNNHVVEDTGKIHVTL
HDGQKYPATVIGLDPKTDLAVIKIKSQNLPYLSFGNSDHLKVGDWAIAIGNPFGLQATVTVGVISAKGRNQL
HIADFEDFIQTDAAINPGNSGGPLLNIDGQVIGVNTAIVSGSGGYIGIGFAIPSLMANRIIDQLIRDGQVTRGFLG
VTLQPIDAELAACYKLEKVYGALVTDVVKGSPADKAGLKQEDVIIAYNGKEVDSLSMFRNAVSLMNPDTRI

VLKVVREGKVIEIPVTVSQAPKEDGMSALQRVGIRVQNLTPETAKKLGIAPETKGILIISVEPGSVAASSGIAPG
QLILAVNRQKVSSIEDLNRTLKDSNNENILLMVSQGDVIRFIALKPEE

>core/214/5/Org5_Gene976

MWNRCQVFSSFFFRYPISSWLIRLRASCECFQQRHPIFLCGLYWLAGITSRGYPECSALILIFLGMFLPRNPKQ
WLPLASAWIISLMLTPAPFLHDGPISGTFVIHHAGGQGTYYGEALCIQTPCGKRAHHLSCQILSESRLELKKVY
ELEGTLHHTSQIVFKSNACYKEIPRSRFYIMKEKCRESSCHFLNHRFPSSEVGPFASSLLLGTPLPQNLRDLFRQ
KGLSHLFAISGWHFSLCATTLWMLCALLPLKIKKILSFIVLTSLACIFPMSLSVWRSWISVTLLCFSWCFSGSCS
GLNRLGAGFILCSIFFSPFSPTFVLSFLATLGILLFFPKIFSFLYTPWTQFLSPFWLYPIRYLAMTLAISLSAQLFIV
LPIMQYFGSLPLEGLLYNLIVPFTILPIIVFLIATIILPCCSPITEALIQGFLSHPWLHNPNILKTLSFAPVPPWMLTL
ASLILFFIGILRTNVSPYASTSATSYRFIETL

>core/215/5/Org5_Gene681

MLKIAILGRPNVGKSSLFNRLCKRSLAIVNSQEGTTRDRLYGELHAFGVPAQVIDTGGVDHNSEDYFQKHIYN
QALTGAKEADVLLLVIDIRCGITEEDAHLAKLLLPLKKPLILVANKADSRQEELQIHETYKLGIRDIVVTSTAH
DKHIDTLLQRIKLVANLPEPREEEEEGLEELSVDEHEESEAALPSNTFPDFSEVFTEGFSPEEPCTIPESPQQAPK
TLKIALIGRPNVGKSSIINGLLNEERCIIDNTPGTTRDNIDILYSHKDRQYLFIDTAGLRKMKSVKNSIEWISSSR
TEKAISRADICLLVIDATQKLSSYEKRILSLISKRKKPHIILINKWDLLEEVRMEHYCKDLRATDPYLGQAKML
CISATTKRNLKKIFSAIDELHHVVSNKVPTPIVNKTLASALHRNHPQVIQGRRLRIYYAIQKTTTPLQFLLFINA
KSLLTKHYEYYLKNTLKSSFNLYGIPFDLEFKEKPKRHN

>core/216/5/Org5_Gene988

MNMPVPSAVPSANITLKEDSSTVSTASGILKTATGEVLVSCTALEGSSSTDALISLALGQIILATQQELLLQSTN
VHQLLFLPPEVVELEIQVVDLLVQLEHAETITSEPQETQTQSRSEQTLPQQSSSKQSALSPRSLKPEISDSKQQQ
ALQTPKGSAVRKHSEAPSPETQARASLSQASSSSQRSLPPQESAPERTLLEQQKASSFSPLSQFSAEKQKEALT
TSKSHELYKERDQDRQQREQHDRKHDQEEDAESKKKKKKRGLGVEAVAEEPGENLDIAALIFSDQMRPPAE
ETSKKETTFKKKLPSPMSVFSRFIPSKNPLSVGSSIHGPIQTPKVENVFLRFMKLMARILGQAEAEANELYMRV
KQRTDDVDTLTVLISKINNEKKDIDWSENEEMKALLNRAKEIGVTIDKEKYTWTEEEKRLLKENVQMRKEN
MEKITQMERTDMQRHLQEISQCHQARSNVLKLLKELMDTFIYNLRP

>core/217/5/Org5_Gene985

MTSRTKSSKNLGTIALAGMVVSSIIGGGIFSLPQNMAATAGAGAVILSWILTGFGMFFIANTFRILSTIRPDLKE
GIYMYSREGFGPYIGFTIGWGYWLCQIFGNVGYAVITMDALNYFFPPYFQGGNTLPAILGGSILIWVFNFIVLK
GIRQASIINVIGTIFKIIPLIIFIILTAFFFKLAVFKTDFWGHAVTKAQPSLGSVSSQLKGTMLVTLWAFIGIEGAV
VMSGRAKNPLSVGQATVLGFLGCLTIYILFSLLPFGSLFQHQLANIPNPSTAGVLDILVGKWGEVLMNVGLIIA
VLSSWLSWTIIVAEIPFSAAKNGTFPEIFTIENKEKSPSVSLYITSSVMQLAMLLVYFSSNAWNTMLSITGVMV
LPAYLASAAFLFKLSKSKTYPKKGSIKAPLAMITGILGVVYSLWLIYAGGLKYLFMALVLLALGIPFYIDAGK
KKKNAKTFFAKKEIVGMTFIGLLALTAIFLFSTGRIKI

>core/218/5/Org5_Gene65

MITRTKIICTIGPATNSPEMLAKLLDAGMNVARLNFSHGSHETHGQAIGFLKELREQKRVPLAIMLDTKGPEIR
LGNIPQPISVSQGQKLRLVSSDIDGSAEGGVSLYPKGIFPFVPEGADVLIDDGYIHAVVVSSEADSLELEFMNS
GLLKSHKSLSIRGVDVALPFMTEKDIADLKFGVEQNMDVVAASFVRYGEDIETMRKCLADLGNPKMPIIAKI
ENRLGVENFSKIAKLADGIMIARGDLGIELSVVEVPNLQKMMAKVSRETGHFCVTATQMLESMIRNVLPTRA
EVSDIANAIYDGSSAVMLSGETASGAHPVAAVKIMRSVILETEKNLSHDSFLKLDDSNSALQVSPYLSAIGLA
GIQIAERADAKALIVYTESGSSPMFLSKYRPKFPIIAVTPSTSVYYRLALEWGVYPMLTQESDRAVWRHQACI
YGIEQGILSNYDRILVLSRGACMEETNNLTLTIVNDILTGSEFPET

>core/219/5/Org5_Gene551

MGESVKVFLEEREDYPYGFVTPIESQGLTRGLSEETIEEIAALRNEPQFIIDFRLQAYRYWKQLHEPAWARLH
YGPIAYDDIVYFSSPKQKKPLGRLEDADPEILDTFKKLGIPLDEQKRLLNVENVAVDLVFDSVSIGTTFKEALE
KAGVIFCSLGEAIQEHPNLVKKYLGSVVSHRDNFFAALNAAVFSDGSFVYVPKGVKCPMDISTYFRINNKEA
GQFERTLIVVEDGGYASYLEGCTAPAYSSNQLHAAVVELVAHEHAVIRYSTVQNWYAGDKKTGKGGIYNFV
TKRGLCAGYRSKISWSQVEVGAAITWKYPSCILKGDESVGEFYSVALTSGKMQADTGTKMLHVGKRTTSTV
ISKGISSDESKNTFRSLVSLGKKAEHSSNYTQCDSMLIGKASGAYTDPKIVVENSTSSIEHEATTSKLREDQLLY
LRSRGLSPEEAVSLVIHGFCREIIEQLPLEFAQEASKLLLIKLENSVG

>core/220/5/Org5_Gene325

MDLKELLHGVQAKIYGKVRPLEVRNLTRDSRCVSVGDIFIAHKGQRYDGNDFAVDALANGAIAIASSLYNPF
LSVVQIITPNLEELEAELSAKYYEYPSSKLHTIGVTGTNGKTTVTCLIKALLDSYQKPSGLLGTIEHILGEGVIK
DGFTTPTPALLQKYLATMVRQNRDAVVMEVSSIGLASGRVAYTNFDTAVLTNITLDHLDFHGTFETYVAAK
AKLFSLVPPSGMVVINTDSPYASQCIESAKAPVITYGIESAADYRATDIQLSSSGTKYTLVYGDQKIACSSSFIG
KYNVYNLLAAISTVHASLRCDLEDLLEKIGLCQPPPGRLDPVLMGPCPVYIDYAHTPDALDNVLTGLHELLPE
GGRLIVVFGCGGDRDRSKRKLMAQVVERYGFAVVTSDNPRSEPPEDIVNEICDGFYSKNYFIEIDRKQAITYA
LSIASDRDIVLIAGKGHEAYQIFKHQTVAFDDKQTVCEVLASYV

>core/222/5/Org5_Gene273

MQTNIGLIGLAVMGKNLVLNMIDHGFSVSVYNRTPEKTRDFLKEYPNHRELVGFESLEDFVNSLERPRKIML
MIQAGKPVDQSIHALLPFLEPGDVIIDGGNSYFKDSERRCKELQEKGILFLGVGISGGEEGARHGPSIMPGGNP
EAWPLVAPIFQSIAAKVQGRPCCSWVGTGGAGHYVKAVHNGIEYGDIQLICEAYGILRDFLKLSATAVATILK
EWNTLELESYLIRIASEVLALKDPEGIPVIDTILDVVGQKGTGKWTAIDALNSGVPLSLIIGAVLARFLSSWKEI
REQAARNYPGTPLIFEMPHDPSVFIQDVFHALYASKIISYAQGFMLLGEASKEYNWGLDLGEIALMWRGGCII
QSAFLDVIHKGFAANPENTSLIFQEYFRGALRHAEMGWRRTVVTAIGAGLPIPCLAAAITFYDGYRTASSSMS
LAQGLRDYFGAHTYERNDRPRGEFYHTDWVHTKTTERVK

>core/223/5/Org5_Gene573

MRDVSELFRTHFMHYASYVILERAIPHILDGLKPVQRRLLWTLFLMDDGKMHKVANIAGRTMALHPHGDAP
IVEALVVLANKGYLIDTQGNFGNPLTGDPHAAARYIEARLSPLARETLFNTDLIAFHDSYDGREKEPDILPAKL
PVLLLHGVDGIAVGMTTKIFPHNFAELLKAQIAILNDKKFTVFPDFPSGGLMDPSEYQDGLGSITLRASIDIIND
KTLVVKQICPQSTTETLIRSIENAAKRGTIKIDTIQDFSTDVPHIEIKLPKGSRAKEMLPLLFEHTECQVILYSKPT
VIYENKPVECSISEILKLHTTALQGYLEKELLLLQEQLTLDHYHKTLEYIFIKHKLYDSVREVLAINKKISADDL

HQAVLHALEPWLHELATPVTKQDTSQLASLTIKKILCFNEEACTKELLAIEKKQAAIQKDLGRIKEVTVKYLK
GLLERHGHLGERKTQITNFKTAKTSILKQQTLI

>core/224/5/Org5_Gene839

MLCATVSGPSFCEAKQQILKSLHLVDIIELRLDLINELDDQELHTLITTAQNPILTFRQHKEMSTALWIQKLYSL
AKLEPKWMDIDVSLPKTALQTIRKSHPKIKLILSYHTDKNEDLDAIYNEMLATPAEIYKIVLSPENSSEALNYI
KKARLLPKPSTVLCMGTHGLPSRVLSPLISNAMNYAAGISAPQVAPGQPKLEELLSYNYSKLSEKSHIYGLIG
DPVDRSISHLSHNFLLSKLSLNATYIKFPVTIGEVVTFFSAIRDLPFSGLSVTMPLKTAIFDHVDALDASAQLCE
SINTLVFRNQKILGYNTDGEGVAKLLKQKNISVNNKHIAIVGAGGAAKAIAATLAMQGANLHIFNRTLSSAA
ALATCCKGKAYPLGSLENFKTIDIIINCLPPEVTFPWRFPPIVMDINTKPHPSPYLERAQKHGSLIIHGYEMFIEQ
ALLQFALWFPDFLTPESCDSFRNYVKNFMAKV

>core/225/5/Org5_Gene342

MMSRLRFRLAALGIFFILLVPNSVSAKTIVASDKEKVGVLVYDNSVEAFQQILDCIDHANFYVELCPCMTGGR
TLKEMVDHLEARMDLVPELCSYIIIQPTFTDAEDQKLLKALKERHPNRFFYVFTGCPPSTSILAPNVIEMHIKLS
IIDGKYCILGGTNFEEFMCTPGDEVPEKVDNPRLFVSGVRRPLAFRDQDIMLRSTAFGLQLREEYHKQFAMW
DYYAHHMWFIDNPEQFAGACPPLTLEQAEETVFPGFDKHEDLVLVDSSKIRIVLGGPHDKQPNPVTQEYLKLI
QGARSSVKLAHMYFIPKDELLNALVDVSHNHGVHLSLITNGCHELSPAITGPYAWGNRINYFALLYGKRYPL
WKKWFCEKLKPYERVSIYEFAIWETQLHKKCMIIDDEIFVIGSYNFGKKSDAFDYESIVVIESPEVAAKANKV
FNKDIGLSIPVSHGDIFSWYFHSVHHTLGHLQLTYMPA

>core/226/5/Org5_Gene770

MRIVQVAVEFTPIVKVGGLGDAVASLSKELAKQNDVEVLLPHYPLISKFSSSQVLSERSFYYEFLGKQQASAIS
YSYEGLTLTIITLDSQIELFSTTSVYSENNVVRFSAFAAAAAYLQEADPADIVHLHDWHVGLLAGLLKNPLN
PVHSKIVFTIHNFGYRGYCSTQLLAASQIDDFHLSHYQLFRDPQTSVLMKGALYCSDYITTVSLTYVQEIINDY
SDYELHDAILARNSVFSGIINGIDEDVWNPKTDPALAVQYDASLLSEPDVLFTKKEENRAVLYEKLGISSDYFP
LICVISRIVEEKGPEFMKEIILHAMEHSYAFILIGTSQNEVLLNEFRNLQDCLASSPNIRLILDFNDPLARLTYAA
ADMICIPSHREACGLTQLIAMRYGTVPLVRKTGGLADTVIPGVNGFTFFDTNNFNEFRAMLSNAVTTYRQEP
DVWLNLIESGMLRASGLDAMAKHYVNLYQSLLS

>core/227/5/Org5_Gene756

MAFSHIEGLYFYNTASQKKELFFPNHTPVRLYTCGPTVYDYAHIGNFRTYVFEDILKRTLVFFGYSVTHVMNI
TDVEDKTIAGASKKNIPLQEYTQPYTEAFFEDLDTLNIARADFYPHATHYIPQMIQAITKLLEQGIAYIGQDAS
VYFSLNRFPNYGKLSHLDLSSLRCCSRISADEYDKENPSDFVLWKAYNPERDGVIYWESPFGKGRPGWHLEC
SIMAMELLGDSLDIHAGGVDNIFPHHENEIAQSEALSGKPFARYWLHSEHLLIDGKKMSKSLGNFLTLRDLLH
QEFTGQEVRYMLLQSHYRTQLNFTEEALLACRHALRRLKDFVSRLEGVDLPGESPLPRTLDSSSQFIEAFSRA
LANDLNVSTGFASLFDFVHEINTLIDQGHFSKADSLYILDTLKKVDTVLGVLPLTTSVCIPETVMQLVAEREEA
RKTKNWAMADTLRDEILAAGFLVEDSKSGPKVKPL

>core/228/5/Org5_Gene900

MDTIDTPGEQGSQSFGNSLGARFDLPRKEQDPSQALAVASYQNKTDSQVVEEHLDELISLADSCGISVLETRS
WILKTPSASTYINVGKLEEIEEILKEFPSIGTLIIDEEITPSQQRNLEKRLGLVVLDRTELILEIFSSRALTAEANIQ
VQLAQARYLLPRLKRLWGHLSRQKSGGGSGGFVKGEGEKQIELDRRMVRERIHKLSAQLKAVIKQRAERRK
VKSRRGIPTFALIGYTNSGKSTLLNLLTAADTYVEDKLFATLDPKTRKCVLPGGRHVLLTDTVGFIRKLPHTL
VAAFKSTLEAAFHEDVLLHVVDASHPLALEHVQTTYDLFQELKIEKPRIITVLNKVDRLPQGSIPMKLRLLSPL
PVLISAKTGEGIQNLLSLMTEIIQEKSLHVTLNFPYTEYGKFTELCDAGVVASSRYQEDFLVVXAYLPKELQK
KFRPFISYVFPEDCGDDEGRGPVLESSFGD

>core/229/5/Org5_Gene219

MDSRTSHLDDELSFKLEKAFTCLSTDIHSHDLSKIVIEYNPIDLAYAVSCLPSESRAILYKNLSCITAKVAFIINT
DSASRWAIFRRLSDSEVCALIEQMPPDEAVWVLDDIPDRRYRRILELIDSKKALKIRDLQKHGRNTAGRLMTN
EFFAFLMETTVKDVSACIRSNPGIDLTRLVFVLDFKGELQGVVTDRSLIINPPEMSLKQIMNQIEHKVLPDATR
EEVVDLVERYKIAALPVVDEENFLIGAITYEDVVEAIEDIADETIARMAGTTEDVGYQTCHVVQRFLLRAPWL
LVTLFAGLISASVMAYFQKISPALLALIIFFIPLINGMSGNVGVQCSTILVRSMATGTLSFGRRRETIFKEMSIGL
LTGVVLGILCGLVVYLMGFLGLNIFSGGGIQLGVTVATGVLGASLTATTLGVLSPFFFAKLGVDPALASGPIV
TALNDIMSMIIFFLIAGGINFLFFN

>core/230/5/Org5_Gene943

MNKKKRFLSLLFLTAVLLGIWFSPHPASINSNAWQLFAIFTTTIMGIIFQPVPMGAIAIIGISTLLLTQTLTLEQG
LSGFHNPIAWLVFLSFSIAKGIIKTGLGERIAYFFVSALGKSPLGLSYGLVITDFFLAPAIPSVTARAGGILYPVV
TSLSDSFGSSAEKGTQDLIGSFLIKVAYQSSVITSAMFLTAMAGNPLVAALAGHVGVSLSWVLWAKAAIIPGL
LSLFLMPIILYKLYPPKITSCEEAIRSAKLRLKEMGPLKKEEKTILMIFFLLVVLWTFGDLLGISATTAALIGLSL
LILTNILDWQKDVIANTTAWETFIWFGALIMMASFLNQLGFIPLVGDSAAALVSGLSWKIGFPLLFLIYFYSHY
LFASNTAHIGAMYPIFLAVSISLGTNPIFAALTLAFASNLFGGLTHYGSGPAPLYFGSHLVTVQEWWRSGFALS
IVNIVIWIGIGSLWWKALGLI

>core/231/5/Org5_Gene1049

MTTKSLGSFNSVISKNKIHFISLGCSRNLVDSEVMLGILLKAGYESTNEIEDADYLILNTCAFLKSARDEAKDY
LDHLIDVKKENAKIIVTGCMTSNHKDELKPWMSHIHYLLGSGDVENILSAIESRESGEKISAKSYIEMGEVPRQ
LSTPKHYAYLKVAEGCRKRCAFCIIPSIKGKLRSKPLDQILKEFRILVNKSVKEIILIAQDLGDYGKDLSTDRSS
QLESLLHELLKEPGDYWLRMLYLYPDEVSDGIIDLMQSNPKLLPYVDIPLQHINDRILKQMRRTTSREQILGFL
EKLRAKVPQVVYIRSSVIVGFPGETQEEFQELADFIGEGWIDNLGIFLYSQEANTPAAELPDQIPEKVKESRLKIL
SQIQKRNVDKHNQKLIGEKIEAVIDNYHPETNLLLTARFYGQAPEVDPCIIVNEAKLVSHFGERCFIEITGTAG
YDLVGRVVKKSQNQALLKTSKA

>core/232/5/Org5_Gene216

MHSHSKPTKPLGTFTVGMLSLAVVISLRNLPLTAKHGLSTLFFYGLAVICFMIPYALISAELASFKPQGIYIWA
RDALGKWWGFFAIWMQWFHNMTWYPAVLAFIASTIVYKINPELAHNKVYIATVILAGFWILTFFNFLGITSS
ALFSSICVIIGTLIPGVILVSLALFWIFSGNPIAISLSWGNLLPNFSNVSSLVLLAGMLLALCGLEANANLASDM
VNPRKNYPKAVFIGAIATLTILVLGSLSIAIVIPKEEISLVSGLVKTFTLFFDKYNLSWMTGIVVVMTIAGSLGE
LNAWMFAGTKGLFISTQNDCLPRLFKKVNSKNVPTNLMLFQGIVVTIFTLLFLCLDSADLVYWILTALSVQM

YLAMYICLFLAGPILRIKEPRAQRLYSVPGKFLGICTMSILGILSCAFALWVSFLPPRELAQISEGSKIGYTTFLL
LAFSLNCLIPFGIYFTHKRLSKKS

>core/233/5/Org5_Gene884

MDKSTGVPLPSPPHSKESEMIVLGCMLTGVHYLNLAANQLYEEDFYYLEHKIIFRVLQDAFKQDKPIDVHLA
GEELKRHNQITVIGGPSYLITLAEFAGTAAYLEEYVDIIRSKSILRKMISTAKEIEKRALEQPKNVAEALDEAQN
SFFKISQSTSVSQYTLVADKLRGLTTTTDKPYLVQLQERQELFLQNAQGDNKSFFTGIPTHFIDLDQLIHGFSPS
NLMILAARPAMGKTALALNIAENLCFQNRLPIGIFSLEMTVDQLIHRMICSRSEVDSKKISIGDLSGHDFQRIVS
VINEMQEHTLLIDDQPGLKVSDLRARARRMKESYDIQFLIIDYLQLLSGSGTLRATESRQTEISEISRMLKTLAR
ELNIPILCLSQLSRKVEDRANHRPMMSDLRESGSIEQDSDLVMFLLRREYYDPNDKPGTAELIIAKNRHGSIGS
VPLVFEKELARFRNYSAFECIS

>core/234/5/Org5_Gene597

MKITVNRGLDLSLQGSPKESGFYNKIDPEFVSIDLRPFQPLSLKLKVEQGDAVCSGAPIAEYKHFPNTYITSHV
SGVVTAIRRGNKRSLLDVIIKKTPGPTSTEYTYDLQTLSRSDLSEIFKENGLFALIKQRPFDIPAIPTQTPRDVFIN
LADNRPFTPSPEKHLALFSSREEGFYVFVVGVRAIAKLFGLRPHIVFRDRLTLPTQELKTIAHLHTVSGPFPSGS
PSIHIHSVAPITNEKEVVFTLSFQDVLTIGHLFLKGRILHEQVTALAGTALKSSLRRYVITTKGASFSSLINLNDI
SDNDTLISGDPLTGRLCKKEEEPFLGFRDHSISVLHNPTKRELFSFLRIGFNKPTFTKTYLSGFFKKKRTYTNPD
TNLHGETRPIIDTDIYDKVMPMRIPVVPLIKAVITKNFDLANELGFLEVCGEDFALPTLIDPSKTEMLTIVKESLI
EYAKESGILTPHQD

>core/236/5/Org5_Gene475

MKEERSSEILPRVKETKKHAYVSMQEKSCVGECAVVASESEEAESVTVTKIAKLQRMGIEELNILARQYGVK
NIGSLTKSQVVFEIVKAKSERPDELLIGEGVLEVLPDGFGFLRSPTYNYLPSAEDIYVSPAQIRRFDLKKGDTIIG
TIRSPKEKEKYFALLKVDKINGSTPDKAKERVLFENLTPLYPNQRIVMEMGKDHLAERVLDLTAPIGKGQRG
LIVAPPRSGKTVILQSIAHAIAVNNPDIVLIVLLIDERPEEVTDMIRQVRGEVVASTFDEQPERHIQVAEMVIEK
ARRLVEHGNDVVILLDSITRLARAYNTVQPHSGKILTGGVDASALHKPKRFFGAARNIEGGGSLTILATALIDT
GSRMDEVIFEEFKGTGNMELVLDRRLSDRRTYPAIDLIKSGTRKEELLYHPSELERVYLFRQAIADLTTIDAM
HLLLGRLKKTNSNAEFLLSLKE

>core/237/5/Org5_Gene616

MNFKLPVYHIGLTKAENNTIKIAILQKTCKGWIVCHCEQIPEGKTWSLPKKYFAAPTTFSLQGSDILVKSSSSS
LKNRKNILKVALTNLEASLALPWESLIVQPQLGKPTDRGETPLTLWIAQKNTLKKELSFLSQAQIFPDKLSCRA
ADIFFLAEQSPLKSLPAYLLIYGGSEEVTCIFVKNHAIAVARSFSNHSTKKSCDDIHATLQYIQETFPQTVLPAIH
VAQISPNLQKILEQKLSLPLVVCQSMTYGVEDEDWEIYGDTIAAAHHGASRRPLTFPYDATSVSPAAQKHWL
LRSSLLIGKYALMATVVVSLGSVLKLKSLSSSASNHFAFACPEEGVLPRSLKAAEKTVKAIGKKNSASNYPLL
PTIPTSEQTLKFLLALGKSSPSIKFSYFSYTMTSYPSKDNPSLPYSALVEVKGQGQPEDIPQFLKKISSHPKLQHV
SESLEDQRSFKLQFTLSS

>core/238/5/Org5_Gene693

MTESVYSPSAMHVNSLADKLKAINQEHILDIWPSLSPKQQQRLFQQLTSVDIDFFRKQQQLLSSPTAILKDFHP
ITSFASSGEDPERAHAGTTLLKEKKVACVVLAGGQGSRLKCDGPKGLFPVSPIKKKPLFQLVAEKVRAASKL
AGQPLPLAFMTSPLNTRQTRSFFESNDYFHLDPNQVDFFCQPLWPLLTLSGDLFLEDMDTLALGPNGNGCIAT
LLYTSGVWEKWKNAGIEMVSVIPIDNPLALPFDVELCGFHAMSNNEVTIKAALRQTAIEDVGILVKSHDSGK
TSVIEYSEIPQNERFALNEDGKLKYCLANIGLYCLSMDFIRHAAYQQLPLYKVHKHAKQLGHTSLNEKNAWK
FEEFIFDLFCYSDHCQTLVYPRQECFAPLKNLEGNHSPDTVRQALSDRERQLFHKVTGKKLSPNTTFELEADF
YYPSTSTSLHWENKAFFEEPFFEAS

>core/239/5/Org5_Gene673

MTQEFDCVVIGAGPSGYVAAITAAQSKLRTALIEEDQAGGTCLNRGCIPSKALIAGANVVSHIKHAEQFGIHV
DGYTIDYPAMAKRKNTVVQGIRQGLEGLIRSNKITVLKGTGSLVSSTEVKVIGQDTTIIKANHIILATGSEPRPF
PGVPFSSRILSSTGILELEVLPKKLAIIGGGVIGCEFASLFHTLGVEITVIEALDHILAVNNKEVSQTVTNKFTKQ
GIRILTKASISAIEESQNQVRITVNDQVEEFDYVLVAIGRQFNTASIGLDNAGVIRDDRGVIPVDETMRTNVPNI
YAIGDITGKWLLAHVASHQGVIAAKNISGHHEVMDYSAIPSVIFTHPEIAMVGLSLQEAEQQNLPAKLTKFPF
KAIGKAVALGASDGFAAIVSHEITQQILGAYVIGPHASSLIGEMTLAIRNELTLPCIYETVHAHPTLSEVWAEG
ALLATNHPLHFPPKS

>core/240/5/Org5_Gene38

MMSSKRTSKIAVLSILLTFTHSIGFANANSSVGLGTVYITSEVVKKPQKGSERKQAKKEPRARKGYLVPSSRT
LSARAQKMKNSSRKESSGGCNEISANSTPRSVKLRRNKRAEQKAAKQGFSAFSNLTLKSLLPKLPSKQKTSIH
EREKATSRFVNESQLSSARKRYCTPSSAAPSLFLETEIVRAPVERTKELQDNEIHIPVVQVQTNPKEQNTKTTK
QLASQASIQQSEGTEQSLRELAQGASLPVLVRSNPEVSVQRQKEELLKELVAERRQCKRKSVRQALEARSLT
KKVARGGSVTSTLRYDPEKAAEIKSRRNCKVSPEAREQKYSSCKRDARANGKQDKTTPSEDASQEEQQTGA
GLVRKTPKSQVASNAQNFYRNSKNTNIDSYLTANQYSCSSEETDWPCSSCVSKRRTHNSISVCTMVVTVIAMI
VGALIIANATESQTTSDPTPPTPTP

>core/241/5/Org5_Gene238

MRAWEEFLLLQEKEIGTNTVDKWLRSLKVLCFDACNLYLEAQDSFQITWFEEHIRHKVKSGLVNNNNKPIRV
HVTSVDKAAPFYKEKQMQQEKTAYFTMHYGSVNPEMTFSNFLVTPENDLPFRVLQEFTKSPDENGGVTFNPI
YLFGPEGSGKTHLMQSAISVLRESGGKILYVSSDLFTEHLVSAIRSGEMQKFRSFYRNIDALFIEDIEVFSGKSA
TQEEFFHTFNSLHSEGKLIVVSSSYAPVDLVAVEDRLISRFEWGVAIPIHPLVQEGLRSFLMRQVERLSIRIQET
ALDFLIYALSSNVKTLLHALNLLAKRVMYKKLSHQLLYEDDVKTLLKDVLEAAGSVRLTPLKIIRNVAQYYG
VSQESILGRSQSREYVLPRQVAMYFCRQKLSLSYVRIGDVFSRDHSTVISSIRLIEQKIEENSHDIHMAIQDISKN
LNSLHKSLEFFPSEEMII

>core/242/5/Org5_Gene822

MRQEKDSLGIVEVPEDKLYGAQTMRSRNFFSWGPELMPYEVIRALVWIKKCAAQANQDLGFLDSKHCDMIV
AAADEILEGGFEEHFPLKVWQTGSGTQSNMNVNEVIANLAIRHHGGVLGSKDPIHPNDHVNKSQSSNDVFPT
AMHIAAVISLKNKLIPALDHMIRVLDAKVEEFRHDVKIGRTHLMDAVPMTLGQEFSGYSSQLRHCLESIAFSL
AHLYELAIGATAVGTGLNVPEGFVEKIIHYLRKETDEPFIPASNYFSALSCHDALVDAHGSLATLACALTKIAT
DLSFLGSGPRCGLGELFFPENEPGSSIMPGKVNPTQCEALQMVCAQVLGNNQTVIIGGSRGNFELNVMKPVII

YNFLQSVDLLSEGMRAFSEFFVKGLKVNKARLQDNINNSLMLVTALAPVLGYDKCSKAALKAFHESISLKEA
CLALGYLSEKEFDRLVVPENMVGNH

>core/243/5/Org5_Gene365

MLKGNIFHLGCGVLYFMNFSLFLFFLIAIQGICLYVGRRGSKKVEDRESYFLAGRSLKIFPLMMTFIATQIGGG
VLLGAAEEAFCYGYGGILYPLGVALGLIFLGMGPGKRLAEGSLTTVVSIFEVFYGSKKLRKIAFLLSAGSLFFI
LVAQVIALDRLFSSFPFGKYVTVAFWIVLASYTSTGGFRGVVRTDVIQAGFLLIAVLVCGVSVWLSVPKSLSV
LDPFQSLPCAKLSNWIFMPMLFMLVEQDMVQRCVAASSPKRLQWAAVGAGLVLLLFNFIPLFLGSLGAKAG
LKAGCPLIDTIAYFCNPSLAAVMAAAIGVAILSTADSLMNAVSQLIAEEYPTLKAPYYRYLVLGLAVAAPLVA
IGFTNIVDVLILSYSLSVCCLSVPVGFYLLAPKGRRVSGAAAWAGVLVGALGYGWVQIVSLGMFGELLAWV
GSLVAFSFVGFIEITWKNKVKTQT

>core/244/5/Org5_Gene784

MQQSVRKLFGTDGVRGRANFEPMTVETTVLLGKAVARVLREGRSGKHRVVVGKDTRLSGYMFENALIAGL
NSMGIETLVLGPIPTPGVAFITRAYRADAGIMISASHNPYRDNGIKIFSLEGFKISDVLEQRIETMVSEADFGPLP
EDHAVGKNKRVIDAMGRYVEFVKATFPKGRTLKGLKIVLDCAHGASYKVAPSVFEELDAEVICYGCEPTGIN
INEHCGALFPQVIQKAVIEHQAHLGIALDGDGDRIIMVDEKGHIVDGDMILSICAGDLKKRSALPHNRVVATI
MTNFGVLKYLEGLGLQVFTSPVGDRHVLHAMLEHEVTLGGEQSGHMIFLDYNTTGDGIVSALQVLRIMIESE
SMLSDLTAPIVKSPQTLINVAVREKIPLETIPLIERTLRDVQDALGPSGRILLRYSGTENICRVMVEGHKKHQV
DCLAKALADVIDAELGTGSRE

>core/245/5/Org5_Gene439

MQIAQVFGCGRLNGEVKVSGAKNAATKLLVASLLSDQKCTLRNVPDIGDVSLTVELCKSLGAHVSWDKETE
VLEIYTPEIQCTRVPPTFSNVNRIPILLLGALLGRCPEGVYVPTVGGDAIGERTLNFHFEGLKQLGVQISSDSSG
YYAKAPRGLKGNYIHLPYPSVGATENLILAAIHAKGRTVIKNVALEAEILDLVLFLQKAGADITTDNDRTIDIF
GTGGLGSVDHTILPDKIEAASFGMAAVVSGGRVFVRNAKQELLIPFLKMLRSIGGGFLVSESGIEFFQERPLVG
GVVLETDVHPGFLTDWQQPFAVLLSQAQGSSVIHETVHENRLGYLHGLQHMGAECQLFHQCLSTKACRYAI
GNFPHSAVIHGATPLWASHLVIPDLRAGFAYVMAALIAEGGGSIIENTHLLDRGYTNWVGKLRSLGAKIQIFD
MEQEELTTSPKSLALRDASL

>core/246/5/Org5_Gene710

MLYFIEQLNKLSTSFCVFPMILLLGGFLTWKLRGLQFHGLKLGFNLMLQNKLDDSSSKANEVSSYEAVAGIL
AGNFGTGNIAGMAVALACGGPGALVWVWLAALLGAIVQYAGSYLGSKYRKPEGNTGEFIGGPIACLAFGM
RKKILAGFFALFTIMTAFCAGNCVQVSCIVPLCAEGTPGKLLVGILLALVVIPVLAGGNNRILRFSARVIPFIAG
FYCISCGIILFQHASAILPAIKLICSSAFGIKAGLAGIGGYTLSQVISTGINRAVMATDCGSGMVSILQANTKSKN
PVVDGLVTLVPPVIVMVVCSITMLVLIVSGAYSSGAQGTLMVMSAFKNSLGSLGSVIVILAMALFGYTTILTW
FACAEKSLQYMIPGRRANLWLKAIYVLIIPLGGVIDMRMIWALSDTGFSGMVILNCIALIALLKDVLSTNRDV
ALLKERECSVADPVRNLDA

>core/247/5/Org5_Gene719

MFNVNFKFLEGLHQPAPRYTSYPTALEWEPSDAAPALLAFQRIRENPQPLSLYFHIPFCQSMCLYCGCSVVLN RREDIVEAYINTLIQEMKLVVETIGFRPQVSRIHFGGGTPSRLSRELFTLLFDHIHKLFDLSHAEEIAIEVDPRSL RNDMEKADFFQNVGFNRVSLGVQDTQADVQEAVRRRQSHEESLKAYEKFKELAFQSINIDLIYGLPKQTKES FSKTIQDILAMYPDRLALFSFASVPWIKPHQKAMKASDMPSMEEKFAIYSQSRHLLTKAGYQAIGMDHFSLP HDPLTLAFKNKTLIRNFQGYSLPPEEDLLGLGMTSTSFIRGIYLQNAKTLEEYHNTVLRGTFATVKSKILTEDD RIRKWAIHKLMCTFTINKEEFFNLFGYEFDTYFIESRDRLISMETTGLIHNSPGSLKVTPLGELFVRVIATAFDH YFLNKVSKKECFSASI

>core/248/5/Org5_Gene492

MTTLRQFFLITELRQKLFYTFALLTACRVGVFIPVPGINGELAVAYFKQLLGSGQNLFQLADIFSGGAFAQMT VIALGVVPYISASIIVQLFLVFMPALQREMRESSDQGKRRIGRLTRLFTVALAVIQSLLFAKFALRMNLTIPGIV LPTLLSSKLFGVPWIFYITTVVVMTTGTLLLMWIGEQISDKGIGNGISLIIALGILSSFPSVLGSIVNKLNLGSQD SSDLGLISILILALVFVFVLITTILIIEGVRKIPVQYARRVIGRREVPGGGSYLPLKVNYAGVIPVIFASSLLMFPA TIGQFIASESSWMKRIAALLAPGSLVYSICYVLLIIFFTYFWTATQFHPEQIASEMKKNNAFIPGIRQGKPTQHY LEYTMNRVTLLGALFLAAIAILPSLLGCLLRVDSNVSYFLGGTAMLIVVGVVLDTMKQVDAFLLMRRYDSVL KTDRTKGRH

>core/250/5/Org5_Gene525

MNVWTKFFQPPKHIKEIEDQEVVKKKYKYWRIRIFYSMFIGYIFYYFTRKSFTFAMPTLIADLGFDKAQLGIIG STLYFSYGISKFVSGVMSDQSNPRYFMAIGLMITGLTNIFFGMSSSIVLFALWWGLNGWFQGWGWPPCARLL THWYAKSERGTWWSVWSTSHNIGGALIPILTGFIIDYSGWRGAMYVPGILCIGMGLVLINRLRDTPQSLGLPPI EKYKRDPHHAHHEGKSASEGTEEIERELSTREILFTYVLTNQWLWFLAAASFFIYIVRMAVNDWSALFLIETK HYAAVKANFCVSLFEIGGLFGMLVAGWLSDKISKGNRGPMNVLFSLGLLFAILGMWFSRSHNQWWVDGTL LFVIGFFLYGPQMMIGLAAAELSHKKAAGTASGFTGWFAYFGATFAGYPLGKVTDVWGWKGFFIALLACAS IALLLFLPTWNATEKNTRSKA

>core/251/5/Org5_Gene948

MKKVLIANRGEIAVRIIRACHDLGLSTVAVYSLADQEALHVLLADEAICIGEPQAAKSYLKISNILAACEITGA DAVHPGYGFLSENANFASICESCGLTFIGPSSESIAMMGDKIAAKSLAKKIKCPVIPGSEGIIEDESEGLKIAEKI GFPIVIKAVAGGGGRGIRIVKEKDEFYRAFSAARAEAEAGFNNPNVYIEKFIENPRHLEIQVIGDTHGNYVHLG ERDCTIQRRRQKLIEETPSPILNAEIRVKVGKIAVDLARSAGYFSVGTVEFLLDKDKKFYFMEMNTRIQVEHTI TEEVTGIDLVKEQIHVAMGNKLPWKQKNIEFSGHIIQCRINAEDPTNNFSPSPGRLDYYLPPAGPSIRVDGACY SGYAIPPYYDSMIAKVIAKGKNREEAIAIMKRALKEFHIGGVQSTIPFHQFMLDNPKFLESNYDINYIDNLLAQ GNSFFKEF

>core/252/5/Org5_Gene31

MATKTKTQWTCNQCGATAPKWLGQCPGCHNWNSLVEEYVPQARSGTSSRSSTSAIALSSIELENESRIFIDHA GWDRILGGGVVRGSLTLLGGDPGIGKSTLLLQTAERLASQKYKVLYVCGEESVTQTSLRAKRLNISSPLIYLF PETNLDNIKQQIATLEPDILIIDSIQIIFNPTLNSAPGSVAQVREVTYELMQIAKSAQITTFIIGHVTKSGEIAGPRV LEHLVDTVLYFEGNSHANYRMIRSVKNRFGPTNELLILSMHADGLKEVSNPSGLFLQEKTGPTTGSMIIPIIEG SGALLIELQALVSSSPFANPVRKTAGFDPNRFSLLLAVLEKRAQVKLFTMDVFLSITGGLKIIEPAADLGALLA

VASSLYNRLLPNNSIVIGEVGLGGEIRHVAHLERRIKEGKLMGFEGAILPEGQISSLPKEIRENFRLQGVKTIKD
AIRLLL

>core/253/5/Org5_Gene708

MLKILKIKVLVFPLALLMGCNSIGYAGPQGSLQTNSQTKVKIGSEVWIEQKLRQYPELLWLTESGGAPLLTST
PIDMAYSEKLFNKKVPALDIAIRSMIHLHLLIQGSRQSYMQLSQILPSEEGGMTFKQFQTAHKQLLFFLNSPKS
FDNTLRILETAIVLRHVGCSAKAVTTFKPYFTDSCPQSFYAKALHVLRTFPELCPSYARLSPEQQEVLLSLRRL
GNYDSLLNLTEVPSAQLLSAWRTRRSLAILDLYLYCLDTCGDKNCSQEFYINFAPLLSMLQQHATIEEAFSRY
FTYRANRLGFEGTSRTDMTLVRLATLMNLSPSEASTLAWSFKNLPSDEAENLVNSFYTVQGEHIPLTFRGLPS
LVAGLSVATHGSTVSPENRLRQLYSTMLSLLVKSLRSHREMLNKQLLPQGTVLDFSETTLSSGGLDVFAESIA
VRIHLNGAVSINL

>core/254/5/Org5_Gene945

MRKRHSFDSTSTKKEAVSKAIQKIIKIMETTDPSLNVETPNAEIESILQEIKEIKQKLSKQAEDLGLLEKYCSQE
TLSNLENTNASLKLSIGSVIEELASLKQLVEESIEESLGQQDQLIQSVLIEISDKFLSSIGETLSGNLDMNQNVIQ
GLLIKENPEKSEAASVGYVQTLLEPLSKRIGETHKKVATHDVNISSLQFHMMSVAGGRFRGHIDMNGYRVLG
LGEPKNGEDAVSKDYLERYVSSQLTIDKVEDKPITKPNKGKLLYSQGTSPKLEGPLPLGLLTSGISGFTWKSAS
KSNDGSFPFSALRHKETESDTDCFQITSTTLSGNQAGTYTWSLSLKVLVPSIFQIEKPEVQLSLVYSYEDWLPID
NIFNMSQPRTIPLALLGQTMLAGQKYDILELAAHQTNQTLMISPNCSRFSLQLKQTNQFENSPVDFYIVHAAH
SCHWSGF

>core/255/5/Org5_Gene331

MLTCNECTTWEQFLNYVKTRCSKTAFENWISPIQVLEETQEKIRLEVPNIFVQNYLLDNYKRDLCSFVPLDVH
GEPALEFVVAEHKKPSAPVASQKESNEGISEVFEETKDFELKLNLSYRFDNFIEGPSNQFVKSAAVGIAGKPGR
SYNPLFIHGGVGLGKTHLLHAVGHYVREHHKNLRIHCITTEAFINDLVYHLKSKSVDKMKNFYRSLDLLLVD
DIQFLQNRQNFEEEFCNTFETLINLSKQIVITSDKPPSQLKLSERIIARMEWGLVAHVGIPDLETRVAILQHKAE
QKGLLIPNEMAFYIADHIYGNVRQLEGAINKLTAYCRLFGKSLTETTVRETLKELFRSPTKQKISVETILKSVA
TVFQVKLNDLKGNSRSKDLVLARQIAMYLAKTLITDSLVAIGAAFGKTHSTVLYACKTIEHKLQNDETLKRQ
VNLCKNHIVG

>core/256/5/Org5_Gene775

MILPPYSYSLKIGAAVLFFCSILHTFLTPWLYTLCQSYEHKKLVFPECWKRYARLSELFRILSRVEIVFFLWAVP
LFFWFLYTEGYRISMAYFNSRNYGFAVFIMVILILLESRPIVYFAELVLSSIAKLGKTSPKSWWWTLMIAPPLL
SCLLKETGAMIIGATLLMRHFYVFSPSRRFAYATMGLLFSNISIGGLTSYVSSRALFLIFPALKWEHSFFLSHFA
WKAIVAILISTTIYYFIFRKEFKKFPDIPSDKDPSVEKVPWWIICVNIIFVGSIILSRSTPLFMGALLLFYLGFQKFT
IFYQDPINLSKVCYVGLFYAGLVVFGDLQEWWVLNLMQGLSDFGYMTVSYTLSIFLDNALVNYLVHNLSVA
TDCYHYLVVAGCMAAGGLTLVSNIPNIVGYLILRSAFPSSTIHMGWLFLGALGPSIISLGVFWLLKNVPEFLYC
FFR

>core/257/5/Org5_Gene609

MSEPRFVCLSLGSNLGNRFKNLQIARTLLGEQAVLGLRSSVILETEALLLPGSPPEWDLPYFNSVLVGETTLSL
RELLVTIKQIEKVVGRAEESPPWSPRTIDVDILLYGDESFCCDHTEITIPLSNLLSRPFLIALIASLCPYRRFCTQG
SPYHNFTFGELAHHLPSPPGMIRRSLSPDTMLMGVVNVTNDSMSDGGMFLDPEKAVAQAEKLFTEGAAVIDF
GAQATNPKVKQFLSVDQEWERLEPVLRLLKETWSNRKQYPIISLDTFYPEIILRAMDIYPIQWINDVSGGSQS
MAEVARDCELSLVMNHSSSLPVDPKNILSFSVPIGEQLLSWGEKQLKMFSDVGLNANQVIFDPGIGFGKGAA
QSLATLYEIAKFKRLGCPILIGHSRKSFLSLFGNHDPKDRDWETVGLSILLQQQGVDYLRVHNVAAHQKALS
VAACEACAPI

>core/258/5/Org5_Gene407

MNRLLSLLSVFDDFFWSYVAFILIIVLGVSFSWKSRFFQFTKFSQFCKLFRYYSQNPQERETKQGVHPLKVFFA
SAGGNIGIGNVVGIVTAACIGGPGALFWVWIAGIFGSIVKYSEVYLGIKFRKLDRDGVYQGGPMYFLIKAFKT
PVVSVIVAILLCIYGVEIYQFSVITDSLAHCWNLPKVYPMLGLLFLVFYAIRGGLQRIGKICSIVLPFFMLLYCA
LSLYILVKEFHTLPHLLSTVFSSAFKGQSALGGFAGCTVATTIHQGISRAAYSGDIGIGFDSIIQSESSAKDPSTQ
AQLSIVGIAIDNLICTLSLLMVLASGSWSLGLENASQVVEHTLASYFPMVKFFLPTFFFVTGYTTIISYFLVGKK
CAKFLYGNTGAKIYTLYGLLILPLFCFLSQNTALLIMSVSGALLLCFNLLGVFILRKEVIFPARAASLTETSLST
E

>core/259/5/Org5_Gene263

MLSCVFSDTIFLSSFLAVTLICMTTALWGTILLISKQPLLSESLSHASYPGLLVGALMAQYVFSLQASIFWIVLF
GCAASVFGYGIIVFLGKVCKLHKDSALCFVLVVFFAIGVILASYVKESSPTLYNRINAYLYGQAATLGFLEAT
LAAIVFCASLFALWWWYRQIVVTTFDKDFAVTCGLKTVLYEALSLIFISLVIVSGVRSVGIVLISAMFVAPSLG
ARQLSDRLSTILILSAFFGGISGALGSYISVAFTCRAIIGQQAVPVTLPTGPLVVICAGLLAGLCLLFSPKSGWVI
RFVRRKHFSFSKDQEHLLKVFWHISHNRLENISVRDFVCSYKYQEYFGPKPFPRWRVQILEWRGYVKKEQDY
YRLTKKGRSEALRLVRAHRLWESYLVNSLDFSKESVHELAEEIEHVLTEELDHTLTEILNDPCYDPHRQIIPNK
KKEV

>core/260/5/Org5_Gene961

MINSLSQKLSSIFSFLVSSRRINEENISESIREVRLALLDADVNYHVVKDFISKVKEKILGEEIWKHVSPGQQFIR
CLHEELVAFLSDGREEFTIQKTPSIILLCGLQGAGKTTTAAKLADYVIKNKKAKKVLVVPCDLKRFAAVDQL
KILVAQTKAEFYQSQENKPIDVVVKALAYAKENGHDFVILDTAGRLNIDNELMEELTAIQKVSQANERLFVM
NVAMGQDVLATVQAFDQSLDLTGVILSMTDGDARAGAVFSIKHVLGKPIKFEGCGERIQDLRSFDPQSMAER
ILGMGDTINFVKEMREYISEEEDAELGKKLVTAAFTYEDYYKQMKAFRRMGPLRKLLGMMPGFNNAKPSQ
KEIEDSEQQMKRTEAIILSMTPEERKELVELDMSRMKRIASGCGLTLGDVNQFRKQMSQSKKFFKGMSKGK
MEQVRKKMSGGNQWR

>core/261/5/Org5_Gene68

MDALILSRIQFGLFITFHYLFVPLSMGLSMMLVIMEGLYLVTKKQIYKQMTWFWVGIFALTFVLGVVTGIMQI
FSFGSNWANFSEYTGNIFGTLLGSEGVFAFFLESGFLGILLFGRHKVSKKMHFFSTCMVALGAHMSAFWIICA
NSWMQTPSGYEMVMHKGKLIPALTSFWGVVFSPTTIDRFIHAVLGTWLSGVFLVISVSAYYLWKKRHHEFA
KQGMKIGTICAVIVLVLQLWSADVTARGVAKNQPAKLAAFEGIFKTEEYTPIWAFGYVDMEKERVIGLPIPG
ALSFLVHRNIKTPVTGLDQIPRDEWPNVQAVFQLYHLMIMLWGVMVALTLISWSAYKGWRWALKPFFLVIL

TFSVLLPEICNECGWCAAEMGRQPWVVQGLLKTKDAVSPIVQANQIVQSLVIFSLVFIALLTLFITVLCKKIKH
GPEEENDLTEFEVK

>core/262/5/Org5_Gene1002

MRAMLLEDWVSLMLSDVSCPKCDKKITGFAIDSQKVQPGDLFFALPGNATDGHQFLKHAATAGAVAAVVS
HDYQGDSFGLELIRVDDTKSALQEAGSNQCNLFQGTLVGITGSVGKTTTKEFSKTILSSIYKTHASPKSYNSQL
TVPLSLLMAEGDEDVMILEMGVSEPGNMQDLLRIVQPEIAVITHINDQHAMHFPQGIQEILKEKSYILQKSKL
QLLPKDSPYYLDLRSCSPTAEKFSFSFNDPLADFCYKAISGDSVVIQTPEENYCLPIAFSYKPAYTNLLIAVALS
WILEVPEEGVIRSLPELKLPPMRFEHSMRNGMQVINDAYNACPEAMIAALDALPLPSDGGKIILILGHMAELG
RYSEEGHALVAEKAASRGDMIFFIGEKWIPVQSVLKSYSCEVSFFSSAQDVKDILKQVARYGDVILLKGSRAL
ALESLLACF

>core/263/5/Org5_Gene1052

MSNITSPVIQNNRSCNYYFELKNSTTIHIVISAILLCGALIAFLCVAAPVSYILSGALLGLGLLIALIGVILGIKKIT
PMISSKEQVFPQELVNRIRAHYPKFVSDFVSEAKPNLKDLISFIDLLNQLHSEVGSSTNYNVSEELQQKIDTFEG
IARLKNEVRTASLKRLESAASSRPLFPSLAKILQKVFPFFWLGEFISAGSKVVELHRVKKIGGSLEEDLSDYIKP
EMLPTYWLIPLDFRPTNSSILNLHTLVLARVLTRDVFQHLKYAALNGEWNLNHSDLNTMKQQLFAKYHAAY
QSYKHLSQPSLQEDXFYNLLLCIFKHRYSWKQMSLIKTVPADLWENLCCLTLDHTGRPQDMEFASLIGTLYT
QGLIHKESEAFLSSLTLLSLDQFKTIRRQSTNIAMFLENLATHNSTFRSLPPITVHPLKRSVFSQPEEDESSLLIG

>core/264/5/Org5_Gene1026

MVITNLSINADTIYGKNPINIVASAANKNITLTGTLALVNADGAFYENHTLQDSQDYSFVKLSPGAGGTIITQD
ASQKPLEVAPSRPHYGYQGHWNVQVIPGTGTQPSQANLEWVRTGYLPNPERQGSLVPNSLWGSFVDQRAIQ
EIMVNSSQILCQERGVWGAGIANFLHRDKINEHGYRHSGVGYLVGVGTHAFSDATINAAFCQLFSRDKDYV
VSKNHGTSYSGVVFLEDTLEFRSPQGFYTDSSSEACCNQVVTIDMQLSYSHRNNDMKTKYTTYPEAQGSWA
NDVFGLEFGATTYYYPNSTFLFDYYSPFLRLQCTYAHQEDFKETGGEVRHFTSGDLFNLAVPIGVKFERFSDC
KRGSYELTFAYVPDVIRKDPKSTATLASGATWSTHGNNLSRQGLQLRLGNHCLINPGIEVFSHGAIELRGSSR
NYNINLGGKYRF

>core/265/5/Org5_Gene843

MLTYKVSPSSVYGNAFIPSSKSHTLRAILWASVAEGKSIIYNYLDSPDTEAMICACKQMGASIKKFPQILEIVG
NPLAIFPKYTLIDAGNSGIVLRFMTALACVFSKEITVTGSSQLQRRPMAPLLQALRNFGASFHFSSDKSVLPFT
MSGPLRSAYSDVEGSDSQFASALAVACSLAEGPCSFTIIEPKERPWFDLSLWWLEKLHLPYSCSDTTYSFPGSS
HPQGFSYHVTGDFSSAAFIAAAALLSKSLQPIRLRNLDILDIQGDKIFFSLMQNLGASIQYDNEEILVFPSSFSGG
SIDMDGCIDALPILTVLCCFADSPSHLYNARSAKDKESDRILAITEELQKMGACIQPTHDGLLVNPSPLYGAVL
DSHDDHRIAMALTIAALYASGDSRIHNTACVRKTFPNFVQTLNIMEARIEECHDNYSMWSTHKRKVFARESF
G

>core/266/5/Org5_Gene651

MTSGVSGSSSQDPTLAAQLAQSSQKAGNAQSGHDTKNVTKQGAQAEVAAGGFEDLIQDASAQSTGKKEATS
STTKSSKGEKSEKSGKSKSSTSVASASETATAQAVQGPKGLRQNNYDSPSLPTPEAQTINGIVLKKGMGTLAL

LGLVMTLMANAAGESWKASFQSQNQAIRSQVESAPAIGEAIKRQANHQASATEAQAKQSLISGIVNIVGFTV
SVGAGIFSAAKGATSALKSASFAKETGASAAGGAASKALTSASSSVQQTMASTAKAATTAASSAGSAATKA
AANLTDDMAAAASKMASDGASKASGGLFGEVLNKPNWSEKVSRGMNVVKTQGARVASFAGNALSSSMQ
MSQLMHGLTAAVEGLSAGQTGIEVAHHQRLAGQAEAQAEVLKQMSSVYGQQAGQAGQLQEQAMQSFNTA
LQTLQNIADSQTQTTSAIFN

>core/267/5/Org5_Gene24

MTLQPYQASSRKYRPQIFREILGQSSVVAVLKNALVFNRAAHAYLFSGIRGTGKTTLARILAKALNCVHLSED
GEPCNQCFSCKEIASGSSLDVLEIDGASHRGIEDIRQINETVLFTPVKAKFKIYIIDEVHMLTKEAFNALLKTLE
EPPQHVKFFFATTEIHKIPGTILSRCQKMHLQRIPEKTILEKLSLMAQDDHIEASQEALAPIARAAQGSLRDAES
LYDYVISLFPKSLSPDTVAQALGFASQDSLRTLDNAILQRDYATALGIVTDFLNSGVAPVTFLHDLTLFYRNLL
LTNSTTSKFSSQYKTEQLLEIIDFLGESAKHLQNTIFEQTFLETVIIHIIRIYQRPVLSELISSIKSRQFEGLRNIKEP
TLTQQVSAPQPQPTYKEQSFLEKKNQPAAEGKIISVEVKSSASIKSAAVDTLLQFAVVEFSGILRQ

>core/268/5/Org5_Gene677

MLKHDTIAAIATPPGEGSIAVVRLSGPQAIVIADRIFSGSVASFASHTIHLGQVIFEETLIDQALLLLMRSPRSFT
GEDVVEFQCHGGFFACSQILDALIALGARPALPGEFSQRAFLNGKIDLVQAEAIQNLIVAENIDAFRIAQTHFQ
GNFSKKIQEIHTLIIEALAFLEVLADFPEEEQPDLLVPQEKIQNALHIVEDFISSFDEGQRLAQGTSLILAGKPNV
GKSSLLNALLQKNRAIVTHIPGTTRDILEEQWLLQGKRIRLLDTAGQRTTDNDIEKEGIERALSAMEEADGIL
WVIDATQPLEDLPKILFTKPSFLLWNKADLTPPPFLDTSLPQFAISAKTGEGLTQVKQALIQWMQKQEAGKTS
KVFLVSSRHHMILQEVARCLKEAQQNLYLQPPEIIALELREALHSIGMLSGKEVTESILGEIFSKFCIGK

>core/269/5/Org5_Gene685

MPRSLSNEQFSVDLEESPGCIVSALVKVSPEVLNKLNKQALKKIKKEITLPGFRKGKAPDDVIASRYPTNVRK
ELGELVTQDAYHALSTVGDRRPLSPKAVRSNSITQFDLQEGAKVEFSYEAFPAISDLPWENLSLPQEEAASEIS
DSDIEKGLTNIGMFFATKTPVERPSQEGDFISISLHVSKSNDENASSAAIFENKYFKLSEEEMTDAFKEKFLGIS
TGHRVVETITSPEIQSFLRGDTLTFTVNAVIEVSIPEIDDEKARQLQAESLDDLKAKLRIQLEKQAKDKQLQKR
FSEAEDALAMLVDFELPTSLLEERISLITREKLLNARLIQYCSDEELEKRKSELIKEAEEDATKALKLLFLTHKI
FSDEKLTISREELQYMMDVCSRERFGQQPPKDISNDTLQELVMSARDRLTYSKAIEHVLRKAELLASTPSA

>core/270/5/Org5_Gene564

MDQLTTDFDTLMSQLGDVNLTTVVGRITEVVGMLIKAVVPNVRVGEVCLVKRNGMEPLVTEVVGFTQSFAF
LSPLGELSGVSPSSEVIPTGLPLHIRAGNGLLGRVLNGLGEPIDVETKGPLQNVDQTFPIFRAPPDPLHRAKLRQ
ILSTGVRCIDGMLTVARGQRIGIFAGAGVGKSSLLGMIARNAEEADVNVIALIGERGREVREFIEGDLGEEGM
KRSVIVVSTSDQSSQLRLNAAYVGTAIAEYFRDQGKTVVLMMDSVTRFARALREVGLAAGEPPARAGYTPS
VFSTLPRLLERSGASDKGTITAFYTVLVAGDDMNEPVADEVKSILDGHIVLSNALAQAYHYPAIDVLASISRL
LTAIVPEEQRRIIGKAREVLAKYKANEMLIRIGEYRRGSDREIDFAIDHIDKLNRFLKQDIHEKTNYEEAAQQL
RAIFR

>core/271/5/Org5_Gene679

MWLVILWALAASLAIALVAKGYYRFVYFRRYAVQVIREVRLSMELKEWALAEQQLLPILKKRSYRRQCLFE
YMRILRKMQRFEESEKLLAEAKKLGLRGPYFFLEIAYKAYRFGAFKECAQAFASVPQDLFEEEDAAKYASAL
VRLGDLDAACSLIEPWISPLSHQETFVTMGHIYFTSKRYKDAIDFYNRANALGVCPVEVTYNLAQAYRITSSY
AKAGKLFRKLLSNPVYKEEALFNIGLCEQKLGRPGKALLIYQSSDLWSRGDALLMKYAAMAAMDQRDYVL
AEPCWELALRCSTFAKDYKCGLGYGFSLCRLRKYGDAERVYCNLIQNFPECLTACKALAWLCGVGYATLLG
SEEGLMYAKKAVELDHSCETLELLSACEARCGNFDAAYEIQSFLSSRDTSLQEKQRRSQILRILRKKLPLNDH
HIVEVDALLAA

>core/272/5/Org5_Gene472

MIENDFPEASNFESSHFYRDKVGVIILCGGEGKRLSPLTNCRCKPTVSFGGRYKLIDIPISHAISAGFSKIFVIGQ
YLTYTLQQHLFKTYFYHGVLQDQIHLLAPEARQGDQIWYQGTADAIRKNLLYFEDTEIEYFLILSGDQLYNM
DFRSIVDTAIRTHVDMVLVAQPIPEKDAYRMGVLDIDSEGKLIDFYEKPQEKEVLKRFQLSSEDRRIHKLTEDS
GDFLGSMGIYLFRRDSLFSLLREEEGNDFGKHLIQAQMKRGQVQTLLYNGYWADIGTIESYYEANIALTQKP
HAEKRGLNCYDDNGMIYSKNHHLPGAIITDSMISSSLLCEGCVINTSHVSRSVLGIRSKIGENSVVDQSIIMGN
ARYGSPSMPSLGIGKDCEIRKAIIDENCCIGNGVKLQNLKGYIKYDSPDKKLFVRDNIIIVPQGTHIPDNYIF

>core/273/5/Org5_Gene956

MLNCSNQKHTVTFEEACQVFPGGVNSPVRACRSVGVTPPIVSSAQGDIFLDTHGREFIDFCGGWGALIHGHSH
PKIVKAIQKTALKGTSYGLTSEEEILFATMLLSSLKLKEHKIRFVSSGTEATMTAVRLARGITNRSIIIKFIGGYH
GHADTLLGGISTTEETIDNLTSLIHTPSPHSLLISLPYNNSQILHHVMEALGPQVAGIIFEPICANMGIVLPKAEF
LDDIIELCKRFGSLSIMDEVVTGFRVAFQGAKDIFNLSPDITIYGKILGGGLPAAALVGHRSILDHLMPEGTIFQ
AGTMSGNFLAMATGHAAIQLCQSEGFYDHLSQLEALFYSPIEEEIRSQGFPVSLVHQGTMFSLFFTESAPTNFD
EAKNSDVEKFQTFYSEVFDNGVYLSPSPLEANFISSAHTEENLTYAQNIIIDSLIKIFDSSAQRFF

>core/274/5/Org5_Gene621

MFQQKQKLSLKYLPSLRMQQGLQMLQSPLTELSSYVVQEIIDNPFFDLSSLEEEEWSPCYRPTNSTFSYLNQTP
GPQESLYTRLLPQIEEAFSTAEERFIAHQIAGNLSDEGLFLRNPEDFAQELELPLEKIHKVWDTIQNLSPEGIASP
SLQSYWMKLLRNSSHQQAYSIVRDCYPLMTNCEFAPIMKKFSLSLSELRNILKKALGSIPWCPAAACTVKPM
VSTPLPDIYLFYSSGSWKIEVSTRGLPSIKLNKETFHFYEHLPKEEQKNLSQQILSAKWLIKNLRKREQTLLQV
METLLPKQEDFLLGKIPAPYPLGIKDLAEDLSFHESTIFRAIENKAVAAPIGIFPLKHLFPRGIHQDSSHSKENVL
QWIRQWIATEQTPLSDSVISDRITAKGIPCARRTVAKYRAQLKILPANKRKKLFYIRSSNSHFRDRQF

>core/275/5/Org5_Gene984

MSKIVYKFGGTSLATAENICLVCDIICKDKPSFVVVSAIAGVTDLLVDFCSSSLREREEVLRKIEGKHEEIVKNL
AIPFPVSTWTSRLLPYLQHLEISDLDFARILSLGEDISASLVRAVCSTRGWDLGFLEARSVILTDDSYRSASPNL
DLMKAHWHQLELNQPSYIIQGFIGSNGLGETVLLGRGGSDYSATLIAELARATEVRIYTDVNGIYTMDPKVIS
DAQRIPELSFEEMQNLASFGAKVLYPPMLFPCMRAGIPIFVTSTFDPEKGGTWVYAVDKSVSYEPRIKALSLS
QYQSFCSVDYTVLGCGGLEEILGILESHGIDPELMIAQNNVVGFVMDDDIISQEAQEHLVDVLSLSSVTRLHHS
VALITMIGDNLSSPKVVSTITEKLRGFQGPVFCFCQSSMALSFVVASELAEGIIEELHNDYVKQKAIVAT

>core/276/5/Org5_Gene58

MQTIYTKITDIKGNLITVEAEGARLGELATITRSDGRSSYASVLRFDLKKVTLQVFGGTSGLSTGDHVTFLGRP
MEVTFGSSLLGRRLNGIGKPIDNEGECFGEPIEIATPTFNPVCRIVPRSMVRTNIPMIDVFNCLVKSQKIPIFSSSG
EHHNALLMRIAAQTDADIVVIGGMGLTFVDYSFFVEESKKLGFADKCVMFIHKAVDAPVECVLVPDMALAC
AEKFAVEEKKNVLVLLTDMTAFADALKEISITMDQIPANRGYPGSLYSDLALRYEKAVEIADGGSITLITVTT
MPSDDITHPVPDNTGYITEGQFYLRNNRIDPFGSLSRLKQLVIGKVTREDHGDLANALIRLYADSRKATERMA
MGFKLSNWDKKLLAFSELFETRLMSLEVNIPLEEALDIGWKILAQSFTSEEVGIKAQLINKYWPKACLSK

>core/277/5/Org5_Gene837

MISFRFLLLSGLCALGISSYAETPKETTGHYHRYKARIQKKHPESIKESAPSETPHHNSLLSPVTNIFCSHPWKD
GISVSNLLTSVEKATNTQISLDFSILPQWFYPHKALGQTQALEIPSWQFYFSPSTTWTLYDSPTAGQGIVDFSYT
LIHYWQTNGVDANQAAGTASSMNDYSNRENNLAQLTFSQTFPGDFLTLAIGQYSLYAIDGTLYDNDQYSGFI
SYALSQNASATYSLGSTGAYLQFTPNSEIKVQLGFQDSYNIDGTNFSIYNLTKSKYNFYGYASWTPKPSCGDG
QYSVLLYSTRKVPEQNSQVTGWSLNAAQHIHEKLYLFGRINGATGTALPINRSYVLGLVSENPLNRHSQDLL
GIGFATNKVNAKAISNVNKLRRYESVMEAFATIGFGPYISLTPDFQLYIHPALRPERRTSQVYGLRANLSL

>core/278/5/Org5_Gene952

MMLRGVHRIFKCFYDVVLVCAFVIALPKLLYKMLVYGKYKKSLAVRFGLKKPHVPGEGPLVWFHGASVGE
VRLLLPVLEKFCEEFPGWRCLVTSCTELGVQVASQVFIPMGATVSILPLDFSIIIKSVVAKLRPSLAVFSEGDC
WLNFIEEAKRIGATTLVINGRISIDSSKRFKFLKRLGKNYFSPVDGFLLQDEVQKQRFLSLGIPEHKLQVTGNIK
TYVAAQTALHLERETWRDRLRLPTDSKLVILGSMHRSDAGKWLPVVQKLIKEGVSVLWVPRHVEKTKDVE
ESLHRLHIPYGLWSRGANFSYVPVVVVDEIGLLKQLYVAGDLAFVGGTFDPKIGGHNLLEPLQCEVPLIFGPH
ITSQSELAQRLLLSGAGLCLDEIEPIIDTVSFLLNNQEVREAYVQKGKVFVKAETASFDRTWRALKSYIPLYKN
S

>core/279/5/Org5_Gene531

MKRPFFTYLCIIFYGSCASLSLHAGLSFPEVRGATAAVVHADSGKVFYDKDIDAVIYPASMTKIATALFILKHY
PTVLDTLIKVKQDAIASITPQAKKQSGYRSPPHWLETDGSTIQLHLREELLGWDLFHALLVCSANDAANVLA
MACCGSVEKFMDKLNFFLKEEIGCTHTHFNNPHGLHHPNHYTTTRDLISIMRCALKEPPFRGVISTTSYKIGAT
NLHGERILSPTNKLLLPGSTYHYPPALGGKTGTTKTAGKNLIMAAEKNNRLLVTIATGYSGPVSDLYQDVIAL
CETVFNEPLLRKELVPPSDCLQLEIANLGKLSCPLPEGLYYDFYASEDREPLSVSFIAHADAFPIEQGDLLGHW
VFYDDEGKKISSQPFYAPCRFERTIKPWKLYMKRVFTSYRTYMSITMLLMYFRIRKHRKYKNLKHYSKI

>core/280/5/Org5_Gene1004

MKRAIIIGAGISGLAAGWWLHKKFPQAEILVLDKEAYAGGFVRTESPQGFSFDLGPKGFLTRGDGEYTLKLIH
ELGLQNSLIFSDRAAKNRFVYYRGKARKISTWTLLRKGLLPSLIKDFRAPCYTQDSSVQDFLKRHSSQNFTSYI
LDPLITAIRAGHSSILSTHMAFPELAKREASSGSLLRSYLKNRSPKKSKTDRYLASLSPSMGTLITTIQEKLPAT
WKFSTSVTHIDCSPKEACVTTPSETFFADMVIYTGPLQQLPVLLPNYGIENLSKRVLPWNLSSISLGWHHANFS
LPKGYGMLFADELPLLGIVWNSQIFPQATPGKTVLSLLIEGKWRESEAHAFAIAALSEYLNINQKPDAFALFSS
QDGMPQHAVGFLERKERILPHLPGNLKIVGQNIAGPGLNRCIASAYHAICDLHTEETLAQPQSSL

>core/281/5/Org5_Gene142

MFSRWITLFLLFISLTGCSSYSSKHKQSLIIPIHDDPVAFSPEQAKRAMDLSIAQLLFDGLTRETHRESNDLELAI
ASRYTVSEDFCSYTFFIKDSALWSDGTPITSEDIRNAWEYAQENSPHIQIFQGLNFSTPSSNAITIHLDSPNPDFP
KLLAFPAFAIFKPENPKLFSGPYTLVEYFPGHNIHLKKNPNYYDYHCVSINSIKLLIIPDIYTAIHLLNRGKVDW
VGQPWHQGIPWELHKQSQYHYYTYPVEGAFWLCLNTKSPHLNDLQNRHRLATCIDKRSIIEEALQGTQQPAE
TLSRGAPQPNQYKKQKPLTPQEKLVLTYPSDILRCQRIAEILKEQWKAAGIDLILEGLEYHLFVNKRKVQDYA
IATQTGVAYYPGANLISEEDKLLQNFEIIPIYYLSYDYLTQDFIEGVIYNASGAVDLKYTYFP

>core/282/5/Org5_Gene244

MNKNLVAIFDYMEKEKGIQRSTIIGAIESALKIAAKKTLRDDANISVNINSRTGDIEVFCEKEIVEICQNPSKEIP
LDKAREYDPDCQIGQYMDVPFVSDNFGRIAAHAARQIIGQKLRHAERDVIYEEYRHRVNETLSGVVKRFAKG
SNLIIDLGKVEAILPTRFYPKTEKHKIGDKIYALLYEVQESENGGAEVILSRSHAEFVKQLFIQEVPELEEGSVEI
VKIAREAGYRTKLAVRSSDPKTDPVGAFVGMRGSRVKNIIRELNDEKIDIVNYSPVSTELLQNLLYPIEIQKIAI
LEDDKVIAIVVNDADYATVIGKRGINARLISHILDYELEVQRMSEYNKLLEIQRLQLAEFDSPHLDQPLEMEGI
SKLVIQNLEHAGYDTIRRVLLASANDLASVPGISLELAYKILEQVSKYGESKVDEKPEIED

>core/283/5/Org5_Gene1051

MSMTIVPHALFKNHCECHSTFPLSSRTIVRIAIASLFCIGALAALGCLAPPVSYIVGSVLAFIAFVILSLVILALIF
GEKKLPPTPRIIPDRFTHVIDEAYGLSISAFVREQQVTLAEFRQFSTALLCNISPEEKIKQLPSELRSKVESFGISR
LAGDLEKNNWPIFEDLLSQTCPLYWLQKFISAGDPQVCRDLRVPRECYGYYWLGPLGYSTAKATIFCKETHH
ILQQLTKEDVLLLKNKALQEKWDTDEVKAIVERIYTTYTARGTLKTEAGGLTKETISKELLLLSLHGYSFDQL
QLITQLPRDAWDWLCFVDNSTAYNLQLCALVGALSSQNLLDESSIDFDVNLGLYVIQDLKEAVQAFSASDEP
KKELGKFLLRHLSSVSKRLESVLRQGLHRIALEHGNARARVYDVNFVTGARIHRKTSIFFKD

>core/284/5/Org5_Gene1010

MNHLNKEKLHIHNWQPYRACGLLSKVSGNLIEVDGLSACLGELCKISSTKDPNLLAEVIGFHNHTTLLMSLSP
LHSVALGTEVLPLRRPPSLHLSDHLLGRVLDAFGNPIDKKEDLPKTHRKPLLSLPPSPMMRQPIDQIFPTGIKAI
DAFLTLGKGQRIGVFSEPGSGKSSLLSAIALGSKSTINVIALIGERGREVREHIEKHSNALKQQRTIIIAAPAHET
APTKVIAGRAAMTIAEYFREQGHEVLFIMDSLSRWIAALQEVALARGETLSAHQYAASVFHHVSEFTERAGN
NDKGSITALYAILYYPKHPDIFTDYLKSLLDGHFFLTSQGKALASPPIDILSSLSRSAQALALPHHYAAAERLRS
LLKVYNEALDIIHLGAYTPGQDEELDKAVKLLPSIKAFLAQPLSSYCYLDNTLKQLEALADS

>core/285/5/Org5_Gene819

MLKPMYVLSKRLYRWVNQLIKLGDLVKNSRSFSVEWVFISALLLIFGCLGCASVVKVSLVPFLLLFSFLAFPLI
LCFRGKGYALLLGVFVTLYVAKYVVGETLYVSFWLSGLGVSFLLAFGLFLQGVWLAQEEEMVKGKEQLRL
SEDLDAQRSAYEDLLLTKSQEKEFLDARAQGLDRELTECQELLKAAYQKQEYLTIDLKILADQKNSWLEDY
AELHNKYIELVSKNGDVVFPWVAEPSVGESQGSERVDVSRWVSALQEKEESLERLRNEILVEKQRCSDYEHR
CQELGLLLQNFTALERRCEELQNLLNQKETQINELHQLVCKSEEKVSVEPSAHAETSCVEEKQYKGLYSQLQ
EQFLEKSETLSLVRKKLFAVQEKYLTLKKKEELTKQDISFDDISMIQGLLERIEILEEEVSHLEELVSRSLSL

>core/286/5/Org5_Gene630

MLRQLCFQVFFFCFASLVYAEELEVVVRSEHITLPIEVSCQTDTKDPKIQKYLSSLTEIFCKDIALGDCLQPTAA
SKESSSPLAISLRLHVPQLSVVLLQSSKTPQTLCSFTISQNLSVDRQKIHHAADTVHYALTGIPGISAGKIVFALS
SLGKDQKLKQGELWTTDYDGKNLAPLTTECSLSITPKWVGVGSNFPYLYVSYKYGVPKIFLGSLENTEGKKV
LPLKGNQLMPTFSPRKKLLAFVADTYGNPDLFIQPFSLTSGPMGRPRRLLNENFGTQGNPSFNPEGSQLVFISN
KDGRPRLYIMSLDPEPQAPRLLTKKYRNSSCPAWSPDGKKIAFCSVIKGVRQICIYDLSSGEDYQLTTSPTNKE
SPSWAIDSRHLVFSAGNAEESELYLISLVTKKTNKIAIGVGEKRFPSWGAFPQQPIKRTL

>core/287/5/Org5_Gene716

MTWLSGLYFICIASLIFCAIGVILAGVILLSRKLFIKVHPCKLKINDNEELTKTVESGQTLLVSLLSSGIPIPSPCG
GKATCKQCKVRVVKNADEPLETDRSTFSKRQLEEGWRLSCQCKVQHDMSLEIEERYLNASSWEGTVISNDN
VATFIKELVVAVDPNKPIPFKPGGYLQITVPSYKTNSSDWKQTMAPEYYSDWEHFHLFDQVIDNSQLPADSA
NKAYSLASYPAELPTIKFNIRIATPPFINGKPNSEIPWGVCSSYVFSLKPGDKITVSGPYGESFMKDDDRPLIFLI
GGAGSSFGRSHILDLLLNKHSKREIDLWYGARSLKENIYQEEYENLERQFPNFHYHLVLSEPLPEDIAAGWDK
DDPTKTNFLFRAFNLGQLSRLDNPEDYLYYVCGPPLHNSSILKLLGDYGVERSSIILDDFGS

>core/288/5/Org5_Gene523

MTVTLPKGVFDIFPYLADAKQLWRHTSLWHSVEKAIHTVCMLYGFCEIRTPIFEKSEVFLHVGEESDVVKKE
VYSFLDRKGRSMTLRPEGTAAVVRSFLEHGASHRSDNKFYYILPMFRYERQQAGRYRQHHQFGVEAIGVRH
PLRDAEVLALLWDFYSRVGLQHMQIQLNFLGGSETRFRYDKVLRAYLKESMGELSALSQQRFSTNVLRILDS
KEPEDQEIIRQAPPILDYVSDEDLKYFNEILDALRVLEIPYAINPRLVRGLDYYSDLVFEATTTFQEVSYALGGG
GRYDGLISAFGGASLPACGFGVGLERAIQTLLAQKRIEPQFPHKLRLIPMEPDADQFCLEWSQHLRRLGIPTEV
DWSHKKVKGALKAASTEQVSFVCLIGERELISQQLVIKNMSLRKEFFGTKEEVEQRLLYEIQNTPL

>core/289/5/Org5_Gene947

MFRKLFPFSKKKTGQKQRLRNNGLLQAIIQSIKVLLHNEAXKEACVLSYYGLLTCVPILVFFLRLSQHLFTNL
NWKEWLIIKFPDYKKPIVAIVEAAYHATESNIGLVLVGSFFVFCWAGILMLLSLEDGLNKIFRTSWTPISLKRL
VSYFVITLVSPMIFIIVCGSWIYITQIMPIQYAKLFSLSHSMTALYFISRFVPYLLLYLALFCCYAFLPRVAIQKTS
ALISTLIIGSVWIVFQKAFFSLQVSIFNYSFTYGALVALPSFLLLLYIYTMIYLFGGALTFIIQNRGCTFIFLGDKIL
PSCYLQLITSTYILALTTRQFNEGLSPLTAQFIAKQSKVPIGEVSQCLDVLEKEGFLFPYNNGYQPVFNFSELTI
KDIADKLLHREIFKKFNPDLGITFIENSFQNIFNQASKNKENLTLSEIARRIK

>core/290/5/Org5_Gene995

MVCENNILSGRGLELLKKKSNITLTPTIYSVSNHNIKLKDFSPHALSVIKTLRKAGYIAYIVGGCIRDLLLNTTP
KDFDISTSAKPEEIKAIFKNCILVGKRFRLAHIRFSKQIIEVSTFRSGSTDEDVLITKDNLWGTPEEDVLRRDFTI
NGLFYDPEHEEIIDYTGGVNDLRNRYLRTIGDPFTRFKQDPVRMLRLLKILSRSPFTVETQTQEALIACRQELIK
SSRARVFEELIKMLNSGAAKNFFQLLIENHLLEILFPYMDKALRLNRALEEQTATYLKALDDKILKKEAEYDR
HQLMAIFLFPLVNFNVRYKHQKHPYLSLTSVFDYIKNFLEQFFADSFTSCSKKNFILTALILQMQYRLTPLIPTK
KALFFNKKLLHHTRFLEALSLLEIRSIVYPKLDKVYVAWIRHHQTLKCKKDSHSQK

>core/291/5/Org5_Gene235

MISLLKMPKLSPTMEVGTIVKWHKKSNDQVSFGDVIVEISTDKAILEHTANEDGWIREILRHEGEKIVIGTPIA
VLSTEANEPFNLEELLPKTEPSNLEASPKGSSEEVSPATTPQAASATFTAVTFKPEPPLSSPLVFKHVGTTNNLS
PLARQLAKEKNIDVSSIQGSGPGGRIVKKDLEKAPPKSIAGFGYPESPEVPPGSYHEENLSPIREVIAARLQAAK
ISIPHFYVRQQVYASPLLNLLKELQAQGIKLSINDCIVRACALALKEFPSINSGFNSVDNKIVRFDTIDISIAVAIP
DGIITPIIRCADRKNLGMISAEIKSLALKARNQSLQDTEYKGGSFCVSNLGMTGITEFTAIVNPPQAAILAVGSV
TEQALVLDGEITIGSTCNLTLSVDHRVIDGYPAAMFMKRLQKILEAPAVLLLN

>core/293/5/Org5_Gene1016

MFEAVIADIQAREILDSRGYPTLHVKVTTSTGSVGEARVPSGASTGKKEALEFRDTDSPRYQGKGVLQAVKN
VKEILFPLVKGCSVYEQSLIDSLMMDSDGSPNKETLGANAILGVSLATAHAAAATLRRPLYRYLGGCFACSLP
CPMMNLINGGMHADNGLGFQEFMIRPIGASSIKEAVNMGADVFHTLKKLLHERGLSTGVGDEGGFAPNLAS
NEEALELLLLAIEKAGFTPGKDISLALDCAASSFYNVKTGTYDGRHYEEQIAILSNLCDRYPIDSIEDGLAEED
YDGWALLTEVLGEKVQIVGDDLFVTNPELILEGISNGLANSVLIKPNQIGTLTETVYAIKLAQMAGYTTIISHR
SGETTDTTIADLAVAFNAGQIKTGSLSRSERVAKYNRLMEIEEELGSEAIFTDSNVFSYEDSEE

>core/294/5/Org5_Gene614

MDIKKLFCLFLCSSLIAMSPIYGKTGDYEKLTLTGINIIDRNGLSETICSKEKLKKYTKVDFLAPQPYQKVMRM
YKNKRGDNVSCLTAYHTNGQIKQYLECLNNRAYGRYREWHVNGNIKIQAEVIGGIADLHPSAESGWLFDQT
TFAYNDEGILEAAIVYEKGLLEGSSVYYHTNGNIWKECPYHKGVPQGKFLTYTSSGKLLKEQNYQQGKRHG
LSIRYSEDSEEDVLAWEEYHEGRLLKAEYLDPQTHEIYATIHEGNGIQAIYGKYAVIETRAFYRGEPYGKVTR
FDNSGTQIVQTYNLLQGAKHGEEFFFYPETGKPKLLLNWHEGILNGIVKTWYPGGTLESCKELVNNKKSGLL
TIYYPEGQIMATEEYDNDLLIKGEYFRPGDRHPYSKIDRGCGTAVFFSSAGTITKKIPYQDGKPLLN

>core/295/5/Org5_Gene72

MKKTMVIDTSVFIYDPEALFSFENTRIIIPFPVIEELEAFGKFRDESAKNASRALSNIRLLLENAKTKVTDGVLLP
SGSELRIEVAPLSNDDRRGKLLTLELLKIIAKREPMVFVTKSLGRRVRAEALQIESRDYESKRFSFRSLYRGFRE
LQVSQEDIENFYKNGYLDLPLDVVSSPNEYFFMSAGENHFALGRYYVSEGKIIALKAMDKSVWGIKPLNTEQ
RCALDLLLRDDVKLVTLIGQAGSGKTILALAAAMHKVFDKETYNKVLVSRPIVPMGRDIGFLPGLKEDKLM
HWMQPIYDNMEVLFSINQMGNSSEALQALMDAKKLEMEALTYIRGRSLPKAFIIIDEAQNLTPHEIKTIISRAG
KGTKIVLTGDPTQIDSLYFDENSNGLTYLVGKFHHLALYGHMFMTRTERSELAAAAATIL

>core/296/5/Org5_Gene704

MLDIKIIRKTPEECETRLRKKDPKISLEPVLSLDKEVRQLKTDSETLQAQRRLLSQDIHKAKTQGVDATNLIQE
VETLAADLEKIEQHLDQKNAQLHELLSHLPNYPADDIPVSEDKAGNQVIKSVGDLPIFSFPPKHHLELNQELDI
LDFQAAAKTTGSGWPAYKNRGVLLEWALLTYMLQKQAAHGFQLWLPPLLVKKEILFGSGQIPKFDGQYYR
VEDGEQYLYLIPTAEVVLNGFRSQDILTEKELPLYYAACTPCFRREAGAAGAQERGLVRVHQFHKVEMFAFT
TPNQDDIAYEKMLSIVEEMLTELKLPYRLSLLSTGDMSFTASKTIDAEVWLPGQKAFYEVSSISQCTDFQSRRS
GTRYKDSQGKLQFVHTLNGSGLATPRLLVAILENNQQADGSVVIPEVLRPYLGGLEILLPKDQ

>core/297/5/Org5_Gene539

MLPLIIFVLLCGFYTSWNIGANDVANAVGPSVGSGVLTLRQAVVIAAIFEFFGALLLGDRVAGTIESSIVSVTN
PMIASGDYMYGMTAALLATGVWLQLASFFGWPVSTTHSIVGAVIGFGLVLGKGTIIYWNSVGIILISWILSPF
MGGCVAYLIFSFIRRHIFYKNDPVLAMVRVAPFLAALVIMTLGTVMISGGVILKVSSTPWAVSGVLVCGLLSY
IITFYYVHTKHCSYISDTPKKGSLTYRLKERGGNYGRKYLVVERIFAYLQIIVACFMAFAHGSNDVANAIAPV
AGVLRQAYPASYTSYTLIRLMAFGGIGLVIGLAIWGWRVIETVGCKITELTPSRGFSVGMGSALTIALASILGL
PISTTHVVVGAVLGIGLARGIRAINLNIIKDIVLSWFITLPAGALLSILFFFALRALFH

>core/298/5/Org5_Gene322

MTLIFVIIIVWCNAFLIKLCVIMGLQSRLQHCIEVSQNSNFDSQVKQFIYACQDKTLRQSVLKIFRYHPLLKIHD
IARAVYLLMALEEGEDLGLSFLNVQQYPSGAVELFSCGGFPWKGLPYPAEHAEFGLLLLQIAEFYEESQAYVS
KMSHFQQALFDHQGSVFPSLWSQENSRLLKEKTTLSQSFLFQLGMQIHPEYSLEDPALGFWMQRTRSSSAFV
AASGCQSSLGAYSSGDVGVIAYGPCSGDISDCYYFGCCGIAKEFVCQKSHQTTEISFLTSTGKPHPRNTGFSYL
RDSYVHLPIRCKITISDKQYRVHAALAEATSAMTFSIFCKGKNCQVVDGPRLRSCSLDSYKGPGNDIMILGEN
DAINIVSASPYMEIFALQGKEKFWNADFLINIPYKEEGVMLIFEKKVTSEKGRFFTKMN

>core/299/5/Org5_Gene845

MDKQSSGNSGCIWHPFTQSALDSTPIKIVRGEGAYLYAESGTRYLDAISSWWCNLHGHGHPYITKKLCEQAQ
KLEHVIFANFTHEPALELVSKLAPLLPEGLERFFFSDNGSTSIEIAMKIAVQYYYNQNKAKSHFVGLSNAYHG
DTFGAMSIAGTSPTTVPFHDLFLPSSTIAAPYYGKEELAIAQAKTVFSESNIAAFIYEPLLQGAGGMLMYNPEG
LKEILKLAKHYGVLCIADEILTGFGRTGPLFASEFTDIPPDIICLSKGLTGGYLPLALTVTTKEIHDAFVSQDRM
KALLHGHTFTGNPLGCSAALASLDLTLSPECLQQRQMIERCHQEFQEAHGSLWQRCEVLGTVLALDYPAEAT
GYFSQYRDHLNRFFLERGVLLRPLGNTLYVLPPYCIQEEDLRIIYSHLQDALCLQPQ

>core/300/5/Org5_Gene1054

MAEISTPSLPDSSIVSQKTPPVPDPDSSPDHIPTIPTQAPFKPQRKKETPSSIVNAIAFAILAFLSCLGGVFAICLGC
SLEITXPLFILTAVFIAFTLLYFIHYLEKPKIPEPLPTPPPSPTLRAPTLTPEIPAPAPGIPLPPTLPKVDRTKLTCNP
DIHYPSTYDPKACFSLLKQLFSLDPETRPEDRKYSNKLASILLRSKEKSGFRFHCFKGHFSHDKILNKKSGAVV
ISSHSSMDFSTTLGRAFAVTTCLQRSCWEKIKNNIPTPEKHLPIGSCVSGPWDVEEGAQLYTSHLIVINPPTLET
LIKEKMRRAITLKDFSMKEAFTNLVLAYLQCFDICIEHNLESVQLEVFGLNNLSADQEEFTTWESCCHLALLE
SVRILLASKEEYALSNVSVNSISQVPLQTACRALFLN

>core/301/5/Org5_Gene585

MKKQVYQWLASVVLLALTISGYAELPLSEQKVKSHTYTTLDEVKDYLSKRGFVETRKQDGVLRIAGDVRAR
WLYFREDIKNPSDKDKYNPLPVNRYRSEFYLYIDYRAERNWLSSKMNWTAIAGGENTAAGVDINRAFLGYR
FYKNPETRTDFFMEIGRSGLGDLFESEVQFQSNFDGLHIYWTRELSKDYPYQVIVHGGPFVVNMTKKHYAW
VVEGILNRLPKQFFVKCSVVDWNTFVPSETSTTEKAATNAMKYKYCVWQWLVGKHSQVPWINGQKKPLYL
YGAFLMNPLAKATKTTLNGKENLAWFIGGTLGGLRKAGDWSATVRYEYVEALSVPEIDVSGIGRGNLLKFW
FAQAIAANYDPKEANGFTNYKGFSALYMYGITDSLSFRAYGAYSKPANDKLGSDFTFRKFDLGIISAF

>core/302/5/Org5_Gene1043

MAILNYVRSLMQSWLQSLQERNILENFTAGLESVEGPIAAYLGFDPTAPALHIGHWIGICFLKRLAALGITPIA
LVGGATGMVGDPSGKQSERSLLQTSEVFDNSQKITACLQRYLPGVTLVNNADWLQEISLIDFLRDIGKHFRLG
QMLVKDTIKQRVHSDEGISYTEFSYLILQSYDFYHLFKNYGTILQCGGSDQWGNITSGIDFIRRKGLGQAYGL
TYPLLTNAQGKKIGKTESGTVWLDSDLTSPFELYQYLLRLPDDTIPKIARTLTLLSNEEIQDIDRRVQTDPVAV
KEFVAQDILSAIHGDLGLEEALSVTRSMHPGNLSSLSEKDFHELFAGGMGASLDKSEVLGKRWLDLFLVLGL
CKSKGEIRRLIEQKGVYINNVPIANEHSVCEEQDICYGHYVLLAQGKKRKLVLYLN

>core/303/5/Org5_Gene901

MTVAEVKGTFKLVCLGCRVNQYEVQAYRDQLTILGYQEVLDSEIPADLCIINTCAVTASAESSGRHAVRQLC
RQNPTAHIVVTGCLGESDKEFFASLDRQCTLVSNKEKSRLIEKIFSYDTTFPEFKIHSFEGKSRAFIKVQDGCNS
FCSYCIIPYLRGRSVSRPAEKILAEIAGVVDQGYREVVIAGINVGDYCDGERSLASLIEQVDQIPGIERIRISSIDP
DDITEDLHRAITSSRHTCPSSHLVLQSGSNSILKRMNRKYSRGDFLDCVEKFRASDPRYAFTTDVIVGFPGESD
QDFEDTLRIIEDVGFIKVHSFPFSARRRTKAYTFDNQIPNQVIYERKKYLAEVAKRVGQKEMMKRLGETTEVL
VEKVTGQVATGHSPYFEKVSFPVVGTVAINTLVSVRLDRVEEEGLIGEIV

>core/304/5/Org5_Gene683

MNKKNLTICSFCGRSEKDVEKLIAGPSVYICDYCIKLCSGILDKKPSSTISSAPVSETPSQPSDLRVLTPKEIKKH
IDEYVIGQERAKKTIAVAVYNHYKRIRALLHNKQVSYGKSNVLLLGPTGSGKTLIAKTLAKILDVPFTIADAT
TLTEAGYVGEDVENIVLRLLQAADYDVARAERGIIYIDEIDKIGRTTANVSITRDVSGEGVQQALLKIVEGTTA
NVPPKGGRKHPNQEYIRVNTENILFIVGGAFVNLDKIIAKRLGKTTIGFSDDQADLSQKTRDHLLAKVETEDLI
AFGMIPEFVGRFNCIVNCEELSLDELVAILTEPTNAIVKQYMELFAEENVKLVFKKEALYAIAKKAKQAKTGA
RALGMILENLLRDLMFEIPSDPTVEAIHIQEDTIAENKAPIIIRRTPEAIA

>core/305/5/Org5_Gene66

MIKFLSQLFIRHWPRKVVSLGFAIIIWILVGQSVTITRTLTNVPVRIVDLHPDQTVLGLQKSGFLNKKVSLTITG
NKNTVQDLRPSNLEVVISAANHTESWIATIDKHNLVSVDHEINIRKDIHSVDANDIFVRLTQYVTEDILLTITKP
IGSPPKGYEYLDVWPKYLNQKVSGPKEYINALKEQGLELTFNLNKISFEELERNRIAQGSHDEIIFPIPKEWKKI
LIPFENTFMDLNDPQADFLRLLFLKRECIPLNLNLPVFLFFPVTFIQTMNPLEYSLDPVPPIILNHGIHQINIPLYV
KDVSRQFLDVVKNNMVLTIVMPSPQDPSSINWAIEFLDEKTLENTFLQTIIAQEHGILHDIALIDEAGIRHRFRE
YLRKLALFTADGEPLNLIAEIKNNKVVIQTKTKETTKLYKKEW

>core/306/5/Org5_Gene824

MLKLQLCALFLFGYLAIVFEHIVRVNKSAIALAMGGLMWLVCFSHIPMADHMILVEEIADMSQVIFFLFSAM
AIVELIDAHKGFSVIVKFCRIQSRTLLLWALIGLSFFLSAALDNLTSIIIIISILKRLVKAREDRLLLGAICVIAVNA
GGAWTPLGDVTTTMLWINNKITSWGIIRALFVPSLVCVLVAGFCGQFFLRKRGSTLIAKDVELQSAPPKSLWII
FIGLGSLLMVPVWKACLGLPPFMGALLGLGLVWLTSDWIHSPHGEDRYHLRVPHILTKIDISSITFFIGILLAVN
ALSFANLLTDFSLWMDKIFSRNVVAIVIGLLSSVLDNVPLVAATMGMYTLPLDDTLWKLIAYAAGTGGSILII
GSAAGVAFMGLEKVDFLWYFKRISWIALASYFGGLFSYFVLESLNFFI

>core/307/5/Org5_Gene157

MSSAIARDCFPSPSPQPSSTLGVHPPKYKSLILSVSLIVLGVLLLCVGMLLLVNAIFSFSVLTVGLGGAGVFLGS
LLLILGLIFFVSYHRKLSEATRSLEQKITLEYQPWADLRKELNEVQEWSNFLLDEWEDFKEVVAQHKSQFATF
EGDLLLFGREVEKYETIWKELDGRDVALLTELKNIWGPLEFLRKKGDRLQCEIDKLRKEVMKVGKSGLKLA
CELTKFKSALKDVKIEQECYRDKRKVEKLEVFPEGYRRELLEVLKTRLSVEKEIQLFEEVVSAFEEKLASLHR
TVFSEEELQEALDKAKAELLDIQVRKSVVEDLSCEPTLIQYHLLRLYEVQCRIVEQFLTQTFSSEQEKVLEEYE
ALKARIRKTLRVKLDQVRANVAFVASTTDLLSESESLDGNDSVFEDAHDDFLD

>core/309/5/Org5_Gene706

MIETREEVGSANFVSLERAIEDLRAGKFVIVVDEASREDEGDLIIAGEKITVEKMTFLLQHTTGVVCAALSQER
LLSLDLPPMVKDNRCRFKTPFTVSVDAAHGVTTGVSAADRTKVVQLLADPKSKPEDFISPGHFFPLASSPGGV
LKRAGHTESTVDLMELAGLQPCGVLAELVNEDYSMMRLPQILEFARKHNIAVIPVTSIIAHRMLSDRLVSKIS
SARLPTIYGDFTIHVYESLLEGMQHLALVKGNVAGKSNVLVRVHSECVTGDILGSKRCDCGEQLSSAMSYIA
EKGTGVLVYLRGQEGRGIGLGHKVRAYALQDNGYDTVDANLAMGFPVDSREYGIGAQILVDLKLTTIKLIT
HNPQKYFGLQGFGLSITERVPLPVRISEDNEQYLRTKQERMGHWLDLPCCNNRVQ

>core/310/5/Org5_Gene1014

MLCYSPNYVTDLYRISLSAEESLGGIRAFPQAESLLGGACALNFPDLEERLPDLRKELLFLGSNDRPDACGGK
FSLQLASSKECYIAALKERVYLNVTNSSRGPVYSFSPKGVPTELWIECFSVSVDGRVEVKVRLQGLHKELISKP
RDCETLFLNPPANKLDCWEIAGFRVDASFPVKQKIRRIGVDKFLLMHGGAEYADKATKERVDFVSSDEENYS
RYLAVGDVLLWDGNCWQTCGEFQGASSRAPLFEVKRIDDKVMIADLWNVGGTQRQTISLVKGVPSPIEINE
VIREIEFTGMRSWSKPIVLVGGQRLILSPDDWILRTAKGWEKLSRADQIQDYVTGKVTGPLLVFEKLEKDLRG
FVLRGHMFNAQRTLVETISLPLKQGFEPAVASQEVSSNTRSAAAHPGATNRGGS

>core/311/5/Org5_Gene915

MSQPPINPLGQPQVPAAASPSGQPSVVKRLKTSSTGLFKRFITVPDKYPKMRYVYDTGIIALAAIAILSILLTAS
GNSLMLYALAPALALGALGVTLLISDILDSPKAKKIGEAITAIVVPIIVLAIAAGLIAGAFVASSGTMLVFANPM
FVMGLITVGLYFMSLNKLTLDYFRREHLLRMEKKTQETAEPILVTPSADDAKKIAVEKKKDLSASARMEEHE
ASQRQDARHRRIGREAQGSFFYSSRNPEHRRSFGSLSRFKTKPSDAASTRPASISPPFKDDFQPYHFKDLRSSSF
GSGASSAFTPIMPASSRSPNFSTGTVLHPEPVYPKGGKEPSIPRVSSSSRRSPRDRQDKQQQQQNQDEEQKQQS
KKKSGKSNQSLKTPPPDGKSTANLSPSNPFSDGYDEREKRKHRKNK

>core/312/5/Org5_Gene728

MCQRILILGTGITGKSVARFLYQQGHYLIGADNSLESLISVDHLHDRLLMGASEFPENIDLVIRSPGIKPYHPW
VEQAVSLKIPVVTDIQVALKTPEFQRYPSFGITGSNGKTTTTLFLTHLLNTLGIPAIAMGNIGLPILDHMGQPGV
RVVEISSFQLATQEEHIPALSGSVFLNFSRNHLDYHRNLDAYFDAKLRIQKCLRQDKTFWVWEECSLGNSYQI
YSEEIEEILDKGDALKPIYLHDRDNYCAAYALANEVGWVSPEGFLKAIRTFEKPAHRLEYLGKKDGVHYIND
SKATTVTAVEKALMAVGKDVIVILGGKDKGGDFPALASVLSQTTKHVIAMGECRQTIADALSEKIPLTLSKD
LQEAVSIAQTIAQEGDTVLLSPGCASFDQFQSFKERGAYFKLLIREMQAVR

>core/313/5/Org5_Gene741

MSKKRVVVTGFGVVSCLGNEVDTFYDNLLAGVSGVRPITSFPCEDYATRFAGWIPEFNPEPYVDKKQARRV
DPFITYAMVAAKKAIAMSRWDKDHLPSDPVRCGVIVGSGMGGLSTLDQGMERLLVIHKKLSPFFIPYIITNMA
PALIAMDFGLMGPNYSISTACATGNYCIDAAYQHLVSGRADMIICGGTEAAVNRIGLEGFIANRALSERNDAP
DQASRPWDRDRDGFVLGEGAGILVLETLESALRRDAPIFAEMLGSYVTCDAFHITAPRDDGEGITACVLGAL
NSAGIPKERVNYVNAHGTSTPLGDLSEVLAVKKAFGSHVRNLRMNSTKSLIGHCLGAAGGVEAVVAIQAILT
GKLHPTINLDNPIAEIEDFDVVANKAQDWDIDVAMSNSFGFGGHNSTILFSRYVP

>core/314/5/Org5_Gene549

MLVSIETFSSIASGSPVQKAAEACYTQYSKQPSSKEVLSSFSWIQELSLFPDRYNLATGASELIKQHWLHNNHS
LAFECILINGKYEPSLSQLPEGVIVCGIDEARGSLSSFMQGFDVNKHPLAFLNAVCSEDRGVVIYIPEEMQTSDP
IFVRHISFPTVSDHDVIFSPRIVVILGQRASAQIQISHDVDLEMVGSSKTIVNGVTELFVGEGADLTVFMVPGYS
EEDTLSWSTIATVEKDAICRMTQNLLESCQGFGWFDNTSYIVGKKGHAESLVLVQSPRKTWVNNLMSHDAE
ETVSRQNIKSILYSGHFLFEGTISISSQGDLSDANQKHDTLLLSSEARVSTFPRLEIETDEVKASHGATVGPLDP
QQIFYMRSRGMTEAEAQEKLIHGFLKQGLVSDTFLGSSFQLNQTS

>core/315/5/Org5_Gene701

MHIGFCHCVRGGNFFYFVINNFHILEIYSLLNSNTIMRYHKYFRYVNSWVFLVVLTLMLLSVVVISSMDPTAM
LVTSSKGLLTNKSIMQLRHFALGWVVFFICAYFDYHLFKRWAWVLYFFMICALVGLFFVPSVQNVHRWYRI
PFIHMSVQPSEYGKLVIVIMLSYILESRKADITSKTTAFLACLVVALPFFLILKEPDLGTALVLCPVTLTIFYLSN
VHSLLVKFCTVVATIGIIGSLLIFSGIVSHQKVKPYALKVIKEYQYERLSPSNHHQRASLISIGLGGIRGRGWKT
GEFAGRGWLPYGYTDSVFSALGEEFGLLGLLFTLGLFYCLICFGCRTVAVATDDFGKLLAAGITVYLAMHVL
INISMMCGLLPITGVPLILISYGGSSVISTMASLGVLQSIYSHRFAKY

>core/316/5/Org5_Gene399

MKLWMKIFIGLFVGVTLGLVLEDKAIFFKPIGDIFLNLLSMVVYPLVFCSMVLGIASISDMKKLGRIGIKSVGL
YLGTTALAIVIGLCFAWIFSPGNGCDFAQAQSMDSAVTVIDSNKTAAYFLSIIAQVFPSNPVRSFAEGNILQIIIF
AIFLGIALRLSGERGRPVERFIDGFSEIMLRMVNMIMSFAPYGVGASMAWISGNHGLGVLWQLGKFIIAYYLA
CLFHATLVFGGLVRFGCKMSFSKFLSSMMDAISCAVSTASSSATLPVTMRCVSKNLGVSAEVSGFVLPLGAT
VNMNGTAIFQGMAAVFIAQAYNCPLSLSSLLLLVVTATFSAVGSAGVPGGGMITLGSVLASVGLPIQGIAILA
GIDRLRDIVGTPMNILGDAVVATYVASGEGELSPYESIKQESVETT

>core/317/5/Org5_Gene625

MKKIFYSFVLLSCIFPYVGCAQVFVGLDRIFSEGEYTRCIQGKKIALISHSAAINSRGQDALSVFYSRKHDCTVE
ILCTLEHGYYGATPTETVGNQPSRYPNLRSVSLYGVKEVPKEVAEHCDVFVYDVQDIGVRSYSFVTVLMQIV
KASERYGKQLIVLDRPNPMGGRIVDGPLPNPTTSGSLAIPYCYGMTPGELALFFKKTYAPNANVVVIPMKGW
NRSMTFDETGLIWMPTSPQMPDPQSPFFYAATGILGALSVASIGVGYTLPFKVLGAPWMDGEKVADELNRM
KLPGVLFLPFFYEPFFGKYKMEMCSGVLLVLQDPKIFYPVETQCTIWGVLKALYPKQVEQTLKSIERIPARRSS
ICNLFGGDEFLSISHKERYIVWPLRRLCKESRESFHQLRSSCLLSEYAES

>core/318/5/Org5_Gene1013

MKKNASHKTNDKKSLSIWSIGGSIFAMFFGAGNIVFPLALGYHYNAHPWSAYFGMMLTAVCVPLLGLVSML
FYSGDYQKFFFSIGRIPGMIFITAIILLIGPFGGIPRAIAVSHATLISLSEHKSAFIPSLPIFSAICCVLIYIFSCKLSRL
IQWLGSVFFPIMLVTLLWVIIRSFMIPTHPMVQEFIPNARQAWLAGFIEGFNTMDLLAAFFFCSIVLISLRQLVA
EEKHPTEEEIPLSFQGISKKNKRSLALGFFLAAILLGMTYLGFVLSAARHAGLLVNVSKGHILGRISAIALGPNS
ILAGVSVFIACLTTEIALVGIVADFLARVVSFKKLNYASAVICTLIPTYLISILNFETISHLLLPLLQLSYPALIVLA
CGNIAYKLWNFRYSPVLFYLTLSLTIVLKLVN

>core/319/5/Org5_Gene172

MDKETLENIYRHFRYRFLKLNILPAFLGLLLLCSPNTLNYTQVDVIFSDRLCSCLLIFLAIASLTKRSLLWLGAP
LGIWVTLFACVAGRSPTIFANDTLIGFAILAVVCISPTRPEALEVGPTLPEGFSYNPSAGGRRAAVLFLSLLGWL
EARYLTASSLGITSSQSSNFLLLYSSIMTVYSLLVVLSLAGSERRWHTRPKIVIATALALTGVIILTLLPIILHQLR
YDCWLCLCLTIEPALAVVFAYDETRATLRYISQFLGDKRALTRASFFGSEYYKHTLSWEERTVLPLRKAYKQ
AFEGISFPINQLLAILVATVFVKVNSSMGLPTFPRNFLNICCWFIIVLFILAFAESLRHLRWMNLIFSAAILFSPVL
FHIPVESPMFLPIIVTGLILIILSIGKRRRTKRKL

>core/320/5/Org5_Gene303

MIPTMLMFFIICFTLCSGFISLSQIALFSLPTSLISHYKRSKSKKQQRVATLLLHPHHLLITLIFCDIGLNIAIQNCF
AILFGDAASWWFTVGLPLAITLILGEILPKAVALPFNTQIASSVAPLILCVTKIFKPLLHWGIVGINYVVQWILS
KQQIDIIQPQELKEVLQSCKDFGVVNQEESRLLYGYLSLSDCSVKERMQPRQDILFYDIQTPLENLYLLFSKQH
CSRVPICNDNLQNLLGICTARSLLLHDKPLQSSDDLLPLLKKPYYMPETISAKMALCQMAAEDETLGMIIDEY
GSIEGLITQEDLFEIVAGEIVDQRDNKILYTTSGADVIIASGTLELREFSEIFDINLPTNNNIATIGGWLIEQIGTIP
TTGMKLSWNNLLFQVLDAAPNRIRRVYIRKLYD

>core/321/5/Org5_Gene949

MDESDGEEASKDSAFSASFSYEFVKSSTRESKNTVTHSTASRTLYILRQDCSYDPRALKVDDEFRYWVEKRL
DAKNPDSLNAFVKEVGTHYVASVTYGGIGFQVLKMSYLQVEELEKEKISISVAAASSLLKSKTSNATEKGYS
SYQSESSAQTVFLGGTVLPDLQQDKLDFKDWSESIPNEPIPLAISVSSITDLIIPELFPSEDAQVLSQKKSALGQV
ILNYLESHKPKEEGPKPVQITSGFNSSSSVFTLQAAKAPKTVSFPYIDYWSTIPYLFPTLKETSGAQPLSFYLRF
DDIFEQQNLVHNTSYILASTSVRLGYFGDSYRDYDALSFYGSWPQAYFDWAGYKDRCTWTLEKLNTTGDLF
IRSGDEIRLKHNTSGKYLATTSMSDGYQTLTCTTQTSDSVFIITV

>core/322/5/Org5_Gene794

MNLPVSLACLLLSGCVFFLGVFVSSSLYARKKRAFLEKIQKLEHENQLLQTSLNLSRHQEQLIEDFSNRLALSS
HKLIKDMKEEAQNYFGDTSKSFQSILSPIQTTLTTFKQSLETFETKHAEDRGRLKEQISQLLAVEKKLEHETHV
LTDILKHPGSRGRWGEIQLERILELAGMLKYCDYDSQTTSAQGAFRADIIIRLPQDRCLIIDAKAPISDSYFSVE
EIDKGDLVDKIKEHIKTLKSKSYWEKFHQSPEYVILFLPGESLFNDAIRLAPELMEIGASSNVILSSPLTLLALLK
TIAYMWKQENLQKQIQEVSLLGKELHRRLQVVFTHFQKIGKNLNQTVQSYNDMTSSFQYRVLPTLRKFEGLE
TSSSHQIEEPTPIESLATSFPHTCDIDTNLAVIESLEKQD

>core/323/5/Org5_Gene369

MHFLDSRTQVVIEKNRAYLFSLPVDSSLSEAITNFVRDLPFICAVEICERPYGECITRSSAERPLLPKEKTLGMPI
FCGKEGVWLPQNTILFSPLIADPRQVTNSAGIRFNEKVVGNRVGATIFGGDFILLRLFDVSRFHVDCDFGIQGG
VFSVFDLDHPESCMVNSDFFVAGLWSGAIDKWSFRFRLWHLSSHLGDEFILTHPNFPRFNLSDEGVDLFISFR
YTPQIRLYGGCGYIVSRDLTFPERPFYCEWGAELRPFGLREGNLHAQPIFAMHFRCWEEQKFGLDQSYILGME
WAKFQEIGRKIRAVLEYHQGFSKEGQFIREPCNYYGFRLTYGF

>core/324/5/Org5_Gene682

MTTIAIEAAKKVLIKLRNAGYQAYFVGGCVRDMLMNRPLEDIDIATNASPTIVSTIFPDVISIGVAFGIIVVKQD
GRLFEVATFRSDGEYKDGRHPDRIIFSSMREDALRRDFTVNGMYYDPFEDKVFDFVEGTRDIEKKVIRAIGHP
RLRFSEDKLRILRAIRFSSSLGFTLDPTTERAIIKEAPALVNSVSPERIWQELKKMLKRQPYGALSLLLKLKVLIF
IFPELRDIPYSLLRTTIEFARKFNPTHFPEILFLLPLFQGVSEEAATVAFGRLRISNKELKLIESWYEALPHFQNQS
GNRVFWAHFLASPTAPLFLELFSALQKDPSRQQHFISRVQELESRLEQFILRIKTSSPVVSAPDLIAKGISPGRLL
GDLLREAEILSIENECLDKEKILLLLQEKGFWK

>core/325/5/Org5_Gene548

MKNLKEDFPIFAAKAKENEPFIYLDSAATTQKPQQVIDAVANFYTSSYATVNRAIYSSSRNVTEAYAAVREK
VRKWVSAASDSEIVFTRGTTAGLNLLAISVNDLWIPKGGVVLVSEAEHHANVLSWEIACRRRGSLVKKIRVH
DSGLIDLDDLEKLLNEGAQFVSIPHVSNVTGCVQPLQQVAELVHRYDAYLAVDGAQGAPHLPIDVQLWDVD
FYVFSSHKIYGPTGIGVLYGKKDLLDQLPPVEGGGDMVAIYDHQNPEYLPAPMKFEAGTPNIAGVLGLGAAL
DYLDGLSAKFIYDKEIALTTYLHKELLEIPGVEILGPSIEEPRGALIGMTIDGAHPLDLGFLLDLRGIAVRTGHQ
CAQPAMERWNVGHVLRVSLGIYNDEDDIDQFILVLQDSLDKIRR

>core/326/5/Org5_Gene156

MVVVALFILGIFFLSGSLAFLVHTSCGVLLGAALPILCIGLVLLAVALIVFLCHKHKTRQDLDYYDQDLDSLVI
HKKEIPNDISELRVTFEKLQNLFQFHTKDFSDLSQELQGKFINCMEKWLTLEDEVTKFLIVRDRFLETRRNFTT
FGEQVKGIQSNIFDLHEEKSSLYLELYRLRKDLQVLLNFFLLPPGILKVDYDEIEAIKGLFIRLTSRLDKLDVKA
QERKKFINEMSREFKEVEKAFDIVDRATKKLMDRAKKESPARLFMGRTESLLEMKKNEEALKNQGLDPENL
SHPELFSPYQQLLILNYLNSEIVLHHYEFLISGTVTSGLTLEECENRMRAASTGLNALLVRKLQFRGAIKSAYF
EKLTEIEKELRSLQDVIKSLELELIHKIKDIVTEET

>core/327/5/Org5_Gene280

MTDSNPLPSYTDASLYRTPAKHSYPIRLPLNRTDRIEKILKIVTLTLALACALGFSIAAGILAMPIFSAVVVITLA
IAAVSLYSLLKKPKLYEILPQIEPESEQSSLSPSPQPPEQQDLPLQIDPLPDPESLPEVSLADLTTPPEELTAITVTP
GYEALLEQNWDLLPSLAAVDPSFTTETPQQPCFIWKLKDSKLIFISTSGDIAVPRIKTQGRVMIVNAANENISR
EGGGTNKALSLATSLQCWNASRLPRAHSRSGSQLQPGECRSAKWENSDHTSNDHVPGKAHFLAQLLGPEAA
KCNNDPKQAFEVSKKAFHNLFQEAEIIGVDVIQLPLIGCNLFAPSRLLNLGKTRAEWIEAIKLALITSLQDFGW
EQDNQEEQKIIILTDKDQPPIIPPRFDLTTP

>core/328/5/Org5_Gene938

MKKQRSHYTKNNLLLLLSILVGLGLGSVQSPWIVYSAECIANTFLKFLRLLSIPLVFCALGSTITSIQNFNTMVT
LGKRILYYTLLTTVIAASIGLLLFFLLRPQMITQDALATTTKCNPLGYLDVLSDTLPENIFKPFLQGNVISAACL

AVLLGTASLFLQEKEKHFVNQFFNSFFSIFLNLARGGLKLLPIAMLGFSVILFKELKDQSNLTMFAEYLLCVIG
ANLAQGFIVLPILLKINKVSPLKVAKAMSPALVTAFFSKSSAATLPLTMELAEDDLKINKNLSRFSFPLCSVIN
MNGCAAFILITVLFVATSNGMIISPLMSLGWIFIATLAAIGNAGVPMGCYFLTLSLLTSMNVPLSILGLILPFYT
VIDMIETSLNVWSDCCVVSLAN

>core/329/5/Org5_Gene304

MTNSALFWIGVNIICIVLQGFYSMMEMACVSFNRVRLQYYLTKDHKKARYINFLIRRPYRLFGTVMLGVNIA
LQVGSESSRNCYRALGITPDYAPFTQIFIVVIFAELLPLTISRKIPEKLALWGAPILYYSHYIFYPLIQLIGSLTEGL
YYLLNIRKEKLNSTLSRDEFQKALETHHEEQDFNTIATNIFSLSATCADQVCQPLEQVTMLPSSANVKDFCRTI
KNTDINFIPVYHKARKNVIGIAHPKDFVNKALDEPLINNLHSPWFITAKSKLIRILKEFRDNRSSVAVVLNASG
EPIGILSLNAIFKILFNTTNIAHLKPKTISVIERTFPGNSRIKDLQKELDIQFPQYPVETLAQLVLQLLDSPAEVGT
SVIINNLLLEVKEMSLSGIKTVSIKNLLS

>core/330/5/Org5_Gene538

MDKLTVQDLSPEEKKVLVRVDFNVPMQDGKILDDIRIRSAMPTINYLLKKHAAVILMSHLGRPKGQGFQEEY
SLQPVVDVLEGYLGHHVPLAPDCVGEVARQAVAQLSPGRVLLLENLRFHIGEEHPEKDPTFAAELSSYGDFY
VNDAFGTSHRKHASVYVVPQAFPGRAAAGLLMEKELEFLGRHLLTSPKRPFTAILGGAKISSKIGVIEALLNQ
VDYLLLAGGMGFTFLQALGKSLGNSLVEKSALDLARNVLKIAKSRNVTIVLPSDVKAAENLQSKEYSVISIDQ
GIPPHLQGFDIGPRTTEEFIRIINQSATVFWNGPVGVYEVPPFDSGSIAIANALGNHPSAVTVVGGGDAAAVVA
LAGCSTKVSHVSTGGGASLEFLEQGFLPGTEVLSPSKS

>core/331/5/Org5_Gene1006

MSTMQNCPHFGVCGGCSFPQSNYSDSLKKKEELLHQLFAPLVPSDMIAPIIPCSPSLRGRNKMEFSFFQTYEGE
KSLGFISSTKPKKGIPVTTCLLIHEQTMDILKLTREWWDKHPELMAYFPPKNKGSLCTLTVRTGSPQQNFMVI
LTTSGTPEYRVNEACIDEWKEILLSSSLNIASIYWEEKVAARGISTYYETKLLYGAPSIQQKLSLPSDGNSASFS
LRPRSFFQPQITQAAKIIETAKEFINPEGSETLLDLYCGAGTIGIMLSPYVKNVIGVEIIPDAVASAQENIKANNK
EDCVEVYLEDAKAFCKRNENCKAPDVIIIDPPRCGMQSKVLKYILRIGSPKIVYISCNPKTQFQECADLISGGY
RIKKMQPIDQFPYSTHLENIILLEREIDL

>core/332/5/Org5_Gene606

MLLVRKWLHTCFKYWIYFLPVVTLLLPLVCYPFLSISQKIYGYFVFTTISSLGWFFALRRRENQLKTAAVQLL
QTKIRKLTENNEGLRQIRESLKEHQQESAQLQIQSQKLKNSLFHLQGLLVKTKGEGQKLETLLLHRTEENRCL
KMQVDSLIQECGEKTEEVQTLNRELAETLAYQQALNDEYQATFSEQRNMLDKRQIYIGKLENKVQDLMYEI
RNLLQLESDIAENIPSQESNAVTGNISLQLSSELKKIAFKAENIEAASSLTASRYLHTDTSVHNYSLECRQLFDS
LREENLGMLFVYARQSQRAVFANALFKTWTGYCAEDFLKFGSDIVISGGKQWMEDLHSSREECSGRLVIKT
KSRGHLPFRYCLMALNKGPLCYHVLGVLYPLHKEVLQS

>core/333/5/Org5_Gene70

MRMLQISMLLLALGTAINSPAIYAADSQSVSFPEQLPSSFTGEIKGNHVRMRLAPHTDGTIIREFSKGDLVAVI
GESKDYYVISAPPGITGYVFRSFVLDNVVEGEQVNVRLEPSTSAPVLVRLSRGTQIQPASQEPHGKWLEVVLP
SQCVFYVAKNFVANKGPIELYTQREGQKKIAMDLINSALNFAHIELEKSLNEIDLEAIYKKINLVQSEEFKDVP

GIQGLIQKALEEIQDAYLSKSLESQNTSIASSQCSTPKVSSSEVTTSLLSRHIRKQTALKTAPLTQGRENLEYSLF
RIWASMQQGNDHSEALTQEAFYRAEQKKKQVLAGVLEVYPHVVKNNPGDYLLKAQENTIAFLYGTSINLEQ
WLGKRVTVECLPRPNNHFAFPAYYVVGIKEAS

>core/334/5/Org5_Gene250

MAASGGTGGLGGTQGVNLAAVEAAAAKADAAEVVASQEGSEMNMIQQSQDLTNPAAATRTKKKEEKFQT
LESRKKGEAGKAEKKSESTEEKPDTDLADKYASGNSEISGQELRGLRDAIGDDASPEDILALVQEKIKDPALQ
STALDYLVQTTPPSQGKLKEALIQARNTHTEQFGRTAIGAKNILFASQEYADQLNVSPSGLRSLYLEVTGDTH
TCDQLLSMLQDRYTYQDMAIVSSFLMKGMATELKRQGPYVPSAQLQVLMTETRNLQAVLTSYDYFESRVPI
LLDSLKAEGIQTPSDLNFVKVAESYHKIINDKFPTASKVEREVRNLIGDDVDSVTGVLNLFFSALRQTSSRLFS
SADKRQQLGAMIANALDAVNINNEDYPKASDFPKPYPWS

>core/335/5/Org5_Gene940

MVYFMVFSPSSESVVKANSVVRSNFCYFLENKFVSPSESTEVMFSEIMKGRVPDIESLFDRPTDMMMTGFKA
AQNLGNLFNSFGILIMCFSQCKSCQTPEKETSAIVLGATLLFFVIALILGPTLGALVYCAYKVYTLGKMIYSLN
KAKAKVLRHPAQNVFHRAAGVATIRSSEEAVKACKLYKSAMIGSLVVSLIASLALIALTAGIVLVLFFVAPGA
APVITAAMMGCCAAGGGALLISLLGLWIAIVRKAKHQEACVGHLTNVVLHTAVSEALLHDPSHFQTNALAR
DLFLTDCLSHYGHLFSNEEVAQLVQGGAPGGGSRPSQHYGGSSDYQNRRGGNGNFGGSHFGGGGGFAGSHF
GAGYPTAPTMPSAPPPFPPPAYDTIYG

>core/336/5/Org5_Gene377

MLSVTIVLVGLEMARSKVSKRDSKILDILFATTELYLKTGQPVGSKTLKESFCSDLSTATIRNYFAELEAEGFL
KKNHTSGGRIPTDLALRHYVDHQEECPEAEISAPIFDKISQLPSESRNIIKDLQKATELLGEILDLPTFFSSPRFEN
DSVTNIQITQVDKQRAVTILSTEFGQIFTDTLWLPEACDTLSIKRIEKFLQNYIRKLPTNEELSKKEEHLSMSLY
NEVVVRYLTRYCNFSEEDLYQTGMSKLLKYEAFKDPEVLALGLSLFENRRQMCELLNIGMHKGRATAFIGK
ELSDILGTSNPGCSVITIPYYMNRSPLGALGILGPINLPYKEALPLLKLFANKINETLTQSFYKFKLSFRRPLTSN
CKLSNEPILRTEYSSIKLLPSKETL

>core/337/5/Org5_Gene373

MRRNPHFSLLKPQYLFSEISKKLAQFRKENPEISVIDLSIGDTTQPLCRSITQAIKEFCVSQEKQETYRGYGPET
GLEKLRTKIASEVYENRISPEEIFISDGAKPDIFRLFSFFGSEKTLGLQDPVYPAYRDIAHITGIRDIIPLACRKET
GFIPELPNQQSLDILCLCYPNNPTGTVLTFQQLQALVNYANQHGTVLIFDAAYSAFVSDPSLPKSIFEIPEAKYC
AIEINSFSKSLGFTGMRLAWNVIPKELTYDNNEPMINDWKRLFATTFNGASLLMQEAGYYGLDLFPTPPAISL
YLTNAQKLKKSLETAGFSVHGGDHAPYLWVELPEGISDEEAFDFFLHQYHIAVTPGHGFGSCGQGFVRFSAL
TQPQNIALACDRLCTASLKETMVLA

>core/338/5/Org5_Gene785

MSNKVLGGSLLIAGSAIGAGVLAVPVLTAKGGFFPATFLYIVSWLFSMASGLCLLEVMTWMKESKNPVNML
SMAESILGHVGKISICLVYLFLFYSLLIAYFCEGGNILCRVFNCQNLGISWIRHLGPLGFAILMGPIIMAGTKVID
YCNRFFMFGLTVAFGIFCALGFLKIQPSFLVRSSWLTTINAFPVFFLAFGFQSIIPTLYYYMDKKVGDVKKAILI
GTLIPLVLYVLWEVVVLGAVSLPILSQAKIGGYTAVEALKQAHRSWAFYIAGELFGFFALVSSFVGVALGVM

DFLADGLKWNKKSHPFSIFFLTFIIPLAWAVCYPEIVLTCLKYAGGFGAAVIIGVFPTLIVWKGRYGKQHHRE
KQLVPGGKFALFLMFLLIVINVVSIYHEL

>core/339/5/Org5_Gene270

MVNIQPVYRNTQVNYSQATQFSVCQPALSLIIVSVVAAVLAIVALVCSQSLLSIELGTALVLVSLILFASAMFM
IYKMRQEPKELLIPKKIMELIQEHYPSIVVDFIRDQEVSIYEIHHLISILNKTNVFDKAPVYLQEKLLQFGIEKFK
DVHPSKLPNFEEILLQHCPLHWLGRLVYPMVSDVTPGTYGYYWCGPLGLYENAPSLFERRSLLLLKKISFGEF
ALLEDGLKKNTWSSSELVQIRQNLFTRYYADKEEVDEAELNADYEQFDSLLHLIFSHKLS

>core/340/5/Org5_Gene594

MSFFNHIPTFSPDAILGLQNVFFADKRPEKVNLVIGVYEHPQKRYGGLSCIRKAQTVILEEEQNKSYLPISGLQI
FLDEMRELVFGAVDPSAIVGFQSLGGTGALHLGARLLSVAKGSGKVYYVPEQTWSNHIRIFSQEGLEVIRYPYY
SKEQKQLLFEPLIAFLKEVEKNSVILLHGCCHNPTGVDFTEDMWKELAILMKERELIPFFDTAYQGFAHGIEL
DRKPIEIFISEGNTVLVAASSSKNFALYGERVGYFAVHSTFTDELVKIHSFLEEKIRGEYSSPQRWGVEIVSTILS
NPYLKEEWQSELNFIRESLGKMRTRFVQALRKVAGHTFDFLLSQHGFFAYPGFSDKQVLFLREQHAVYTTAG
GRMNLNGITEKNIDHVVQSFIQAYEL

>core/341/5/Org5_Gene155

MVVSIYSEILSFSELTSCKHSLFPFGPIETASIRIHHVFNVVIVCLIILGTLFVCLGMVFLGVFSTYLLGMSSMILG
LLLISIGLALLKFKERYGLEPKELFGVEGGFDKKLPSEIIQMQDQIADLARELDLEQKKDTLIRGFSARLDVLE
GSKTEKKQILKIGVPRNLSEIQERAQEQNSILEQCKEALLFRRKSAQEIFKKLYDRKAAFWRSYREDLWCYSEI
HVSKKALSNLYIGDVFEGTAPHFLMEAYAMCRTAKNLRNYVKVCVEDMRVNEEKKRAKQLSVSELLCCCT
EIETDLENETNLFTSDSEDVLEEYQIHCIRVTMLHALWAIYNDEVVSRKPIDTLDRVRARMAVEDCIETFEELQ
MCVVHTKTLELEIAQLYVDILLEA

>core/343/5/Org5_Gene46

MSKETFQRNKPHINIGTIGHVDHGKTTLTAAITRALSGDGLASFRDYSSIDNTPEEKARGITINASHVEYETPN
RHYAHVDCPGHADYVKNMITGAAQMDGAILVVSATDGAMPQTKEHILLARQVGVPYIVVFLNKVDMISQE
DAELIDLVEMELSELLEEKGYKGCPIIRGSALKALEGDANYIEKVRELMQAVDDNIPTPEREIDKPFLMPIEDV
FSISGRGTVVTGRIERGIVKVSDKVQLVGLGETKETIVTGVEMFRKELPEGRAGENVGLLLRGIGKNDVERG
MVVCQPNSVKPHTKFKSAVYVLQKEEGGRHKPFFSGYRPQFFFRTTDVTGVVTLPEGTEMVMPGDNVELDV
ELIGTVALEEGMRFAIREGGRTIGAGTISKINA

>core/344/5/Org5_Gene891

MIFEFRFPKIGETSSGGSIVRWLKNLGDHVARDEPLIEVSTDKIATELPSPKAGRLVRFCVNEGDEVASGDVLG
LIELEEISEADDESTSCPLTSCETKSEAGSSSSSVWFSPAVLSLAQREGIGLDNLQKIAGTGKGGRVTRQDLEA
YISESQQVSIPEIFQGEVNRIPMSPLRRAIASSLSKSSDEVPHASLVVDVDVTDLMNLISGERQRFLDTHGVKLT
ITSFIVQCLAQTLRQFPLLNGSLDGTTIVMKKSVNVGVAVNLNKEGVVVPVIHNCQDRGLVSIAKALADLSSR
ARLNKLDPSEVQDGSVTVTNFGMTGALIGMPIIRYPEVAILGIGTIQKRVVVRDDDSLAIRKMVYVTLTFDHR
VLDGIYGSEFLTSLKNRLESVTMG

>core/345/5/Org5_Gene19

MDYYSILGISKTASAEEIKKAYRKLAVKYHPDKNPGDAAAEKRFKEVSEAYEVLSDPQKRDSYDRFGKDGPF
AGAGGFGGAGGMGNMEDALRTFMGAFGGEFGGGSFFDGLFGGLGEAFGMRSDPAGARQGASKKVHINLTF
EEAAHGVEKELVVSGYKSCETCSGQGAVNPQGIKSCERCKGSGQVVQSRGFFSMASTCPECGGEGRIITDPCS
SCRGQGRVKDKRSVHVHIPAGVDSGMRLKMEGYGDAGQNGAPSGDLYVFIDVESHPVFERRGDDLILELPIG
FVDAALGMKKEIPTLLKTEGSCRLTVPEGIQSGTILKVRNQGFPNVHGKGRGDLLVRISVETPQNLSEEQKEL
LRTFASTEKAENFPKKRSFLDKIKGFFSDFTV

>core/346/5/Org5_Gene134

MSSPVNNTPSAPNIPIPAPTTPGIPTTKPRSSFIEKVIIVAKYILFAIAATSGALGTILGLSGALTPGIGIALLVIFFV
SMVLLGLILKDSISGGEERRLREEVSRFTSENQRLTVITTTLETEVKDLKAAKDQLTLEIEAFRNENGNLKTTA
EDLEEQVSKLSEQLEALERINQLIQANAGDAQEISSELKKLISGWDSKVVEQINTSIQALKVLLGQEWVQEAQ
THVKAMQEQIQALQAEILGMHNQSTALQKSVENLLVQDQALTRVVGELLESENKLSQACSALRQEIEKLAQ
HETSLQQRIDAMLAQEQNLAEQVTALEKMKQEAQKAESEFIACVRDRTFGRRETPPPTTPVVEGDESQEEDE
GGTPPVSQPSSPVDRATGDGQ

>core/347/5/Org5_Gene658

MPRYRYTYLDPKERRKRGYLEALHIQEAREKLAQENIQVLDIREVALRRMSIKSTELIVFTKQLLLLLRSGLPL
YESLVSLRDQYHEQKMGLLLTSFMETLRSGGSLSQAMAAHPNIFDHFYCSGVAAGESVGNLEGCLQNIIVVL
EERAQITKKMVGALSYPCVLLVFSFAVMLFFLLGVIPSLKETFENMEVKGLTKIVFGVSDCLSAYRYLFLGFA
SALITVGILMRHRIPWKKILEKLLFALPGTKKFVVKVAVNRFCSVASAILKGGGTLIEGLDLGCDAIPYDRLKT
DMRDIVQAVIGGGSLSQELAQRSWVPKLAIGMIALGEESGDLADVLGYVAHIYNEDTQKTLASITSWCQPVI
LIFLGGLIGVIMLAILIPLTSNIQTL

>core/348/5/Org5_Gene690

MHPKIEKRNSLPLTAVAPVFEESYHPSVATTVDYVDATTLSRHLTVLKDVIKEARNLDLGKAFLTSMKQGFI
NTGTELAIIQASLADQSSRESRKKEEKIFHQHLGKAAPQAATATSGVQPTADPVADKMPLQSAFAYVLLDKYI
PAQEEALYALGRELNLSGYAQNLFSPLLDMIKSFNSAPINYNLGSYISQTSGTANFAYGYEMILSRYNNEVSQ
CRLDIASTVKAKAALANMSASVKANVSLTDAQKKQIEDIIASYTKSLDVIHTQLTDVMTNLASITFVPGLNKY
DPSYRIVGGDLSIIALQNDEKVLVDGKVDITTAVNEGGLLNFFTTVLTDVQNYGDLAQTQQLMLDLELKAM
QQQWSLVSASLKLLNGMYTTVISGFKN

>core/349/5/Org5_Gene553

MKKLLKSALLSAAFAGSVGSLQALPVGNPSDPSLLIDGTIWEGAAGDPCDPCATWCDAISLRAGFYGDYVFD
RILKVDAPKTFSMGAKPTGSAAANYTTAVDRPNPAYNKHLHDAEWFTNAGFIALNIWDRFDVFCTLGASNG
YIRGNSTAFNLVGLFGVKGTTVNANELPNVSLSNGVVELYTDTSFSWSVGARGALWECGCATLGAEFQYAQ
SKPKVEELNVICNVSQFSVNKPKGYKGVAFPLPTDAGVATATGTKSATINYHEWQVGASLSYRLNSLVPYIG
VQWSRATFDADNIRIAQPKLPTAVLNLTAWNPSLLGNATALSTTDSFSDFMQIVSCQINKFKSRKACGVTVG
ATLVDADKWSLTAEARLINERAAHVSGQFRF

>core/350/5/Org5_Gene353

MMKQGVGQDAKELYTFLSRGNEHYQPCLWFSLEEELGFLFDEKMLCAPLSEDHYCHSYLVDLVDQHLKDLI
LSMFLDPQNISAGELLKVSINVGDSFSPLQQKDFLSMVLRDETGKNVVVVFKGVLSLPATQVCKLVEELNSK
DYSYLNIFSCHGDSSPQLLFRKELEGTSGRYFTVICALYLGDTDMRSLQLASERIMVSREFDLVDAYAARCKL
LKIDHTNWRPGTFSRHADFADAVDVSAGFNSREFKLITQANQGILESGELPLPSKTFWEGFLAFCDRVTVTRH
FIPMLDAAIKQAVWTHKHPSLIDKECEALDLKTQCLPSIVSYLEYVTNSHEKTSKGPFIQKEIIADCSPLKEALF
PGSDEDVPSTSEDPSDDHPSDLEDS

>core/351/5/Org5_Gene535

MGTPISGNDGDRNTISDPLEESAAEEGDSDLEDRVSESATQVIETIADTGIPEATPSEGTNSDLNSDLVDRVEY
EARGSLLTTMLARIRKAVSQIWMHVKTKRHPKEQGVRSLGDIPCDLLKATRLPKETAEPPYFYALETALASC
RSFFFHVFLRLFTLLRRQHPEAPLDLCGTDPISPEAAVAFALILRSCCKWVATDAVQEGLPLEVIEEAGMYNA
FSLEATTTVEEVSKRLSELLYSDKRIDGLANVRGITKIITSPYLGAGQCVSVVDNLKTYDLGRNYTQVLACAS
QIDEFADKGENEALVMKDILYLVRQDRSKELGDFLMMWSEEHASEVNYDVVLAILEVNLPILEEDYRSHPLA
YQKKLNYVICQFFCSERLTSIEPKD

>core/352/5/Org5_Gene386

MLHILLAIFCILLFLAFGLTQPSCHGSSKFLKTLNQRFFKDKGREYPPFPSAPTILATLLCILYGALGTKLYTLLP
PKTAHKDLLFWPLYSLSALIAYGFLPPWISTKVPKETTAHLRFLASVFQLGLFPLQLLFYRRRPNQQVRSSTSF
QSQLSEALSAFDNLIVREVMIPKVDIFALPEETTLQEALVLVSEEGYSRVPVYKKNLDNITGILLVKDLLLLYT
SSHDLSQPISSVAKPPFYAPEIKKASSLLQEFRQKHRHLAIIVNEYGFTEGIATMEDIIEEIIGEIADEHDVQENTP
YKKIGSSWIVDGRMNISDAEEYFNLKIDHENSYDTLGGHVFHKVGAVPQKGMRIHHENFDIEIITCTERNVGK
LKITPRKRKFNIS

>core/353/5/Org5_Gene453

MAIKNILVVDDEPLLRDFLSELLTSQGFIPDTAENLRNALQMIRSRDYDLVISDMSMPDGSGLDLIKIIKQSSPH
TPVLVVTAYGSIENAVEAMHQGAFNYLTKPFSSEALFAFISKAEELKNLVHENLFLHSQTTPDSHPLIAESKA
MKDLLAIAKKAASSSANIFIHGESGCGKEVLSFFIHHNSPRANHPYIKVNCAAIPETLLESELFGHEKGAFTGA
TTKKAGRFELAHKGTLLLDEITEVPVNLQAKLLRAIQEKEIEHLGGTKTLSVDVRILATSNRKLKEAIDDKSFR
QDLYYRLNVIPLHLPPLRDRQDDILPLANYFLNKFCRMNNTPLKTLSPKAQELLLNYPWPGNIRELSNVLERV
VILENTSLLTEDMLALA

>core/354/5/Org5_Gene788

MHLHEYQAKDLLASYDVPIPPYWVVSSEEEGELLITKSGLDSAVVKVQVHAGGRGKHGGVIVAKSSAGILQ
AVAKLLGMHFTSNQTADGFLPVEKVLISPLVAIQREYYVAVIMDRKHRCPVLMLSKAGGMDIEEVAHSSPEQ
ILTLPLTSYGHIYSYQLRQATKFMEWEGEVMHQGVQLIKKLAKCFYENDVSLLEINPLVLTLEGELLVLDSKI
TIDDNALYRHPNLEVLYDPSQENVRDVLAKQIGLSYIALSGNIGCIVNGAGLAMSTLDILKLHGGNAANFLDV
GGGASQKQIQEAVSLVLSDESVKVLFINIFGGIMDCSVVASGLVAVMETRDQVVPTVIRLEGTNVELGKEIVQ
QSGIPCQFVSSMEEGARRAVELSM

>core/355/5/Org5_Gene730

MKWFVISCLLGIFSLGLIMVFDTSSAEVLDRSLECSTHKALIRQVTYLILGLGVASLLYMMEWRDFLKISPVLL
SGAALALICVFIPGLGICRNGARRWLGFGQLTIQPSEFVKYLVPIVALYFLTFSSLYQKQLKMFLKLTAILFIPIL
LIAIEPDNGSAAVISASLIPVFIMTSVRLRYWLLPLLCVLIAGGALAYRMPYVRYRLNVYLHPELDIKGRGHQP
YQAKIAAGSGKLLGKGPGASLQKLTYLPEAQNDYIAAIYAEEFGFLGMLVLILLYMCFVYGGYAIAIKASSLE
GAALAMVITLIISMQAFMNLGVVSGLLPSKGVNLPFFSQGGSSLIANMCGVTLLLKVYDEENSKSSLGCRRFR
RPHCPSSLGKGSFFS

>core/356/5/Org5_Gene897

MSSVNQSSGTPNPEEVTSPESTEENKNVVSSDEAQATHAVALPIVTQLSLPEGVGTSSEETASNPKVDEIVAEV
SSSRAVADQISSLVERVGELLDDLKGAQSLFTSFQSELKNCLPAWKSSTRRLETRGAGDNADIARLELFRSDY
EAVLGHANQFHGKAHLILSKLTDVHHKLQGLSREDLSLAFDNNDRVLEHLGSLGLDVDAEGNWSLSCERGI
PRLVLTADSMLVQIKKVNLPTVEELRTLQGTTESSSDPRVEESLSCCERLLNELRRLWANFVGFISSCYDNIVF
VLMWIVRRINLLPGLGCLPFHNPDASQEDQRSSSGERSTRRERLSRRSDLSEEEMIVRAEGESIHPESPHGDGR
NQPSRGDKQDSDSEEETEL

>core/357/5/Org5_Gene697

MEKPQNRKAPRIFWLNNQVAIPPSERVKESYALHSDIFSLPPGSALKLAEKTEESIRQLVGLKDSHIFRFVPHFP
HVVHIVLAALVENLSMFQGRNHIILPAHDQQLLINSLCRHQGLGTTYDWVTVNHEGRIVEEQLIETLSPRSLLF
SLSAAHGLTGVIQPLDPLLSLCKDRRILLHLDISDILGRAPLTPEILNADIITFSSAALGGMGSIGGIFIRKSLERV
FSSWFPPHTSASLCFSAVAAMQTACEERISALPLFTFHTSNLCKKLIQELQSVLPSIQLAFSEVQNRLPNIVVAA
IPDIPAESLAFHLHQQGIYPSLGYERFQPLAQVLQNCGISPFLCHSALHFSLTERSKDLEFSKLARAMHDAIKHL
TPLLGSSS

>core/358/5/Org5_Gene534

MILDFQFSIGYYLRVLELAIRDGTRILAYDRKRLLLDAWPVNDPLPTNYDTSVSTIRQVIHELFSWSAISYSISS
RLLAIIELRLHEEKPQTGWLYRLFFPSKYHIKKAIVDKLCMFKSLILFESKRPVDKIVQAANKVFSKGKSNFSS
WEDFTHEVTVSEVQTPLAGEVQRRLAADASLQMIIEALTTLLEGHTAYLPLSLELLNQFIGEKAQPLKTLSEK
SYVLLRELIQLFSLSAEDFQTIIMSIISDSLSEVLANSLIGNQPLTFHGKTFVGLWQETALASPEDSKLALGFLAE
VLRKVIVEKKLHVSKSDNTTPEEVGNIYSIRDQNPALWDKMITMLLMRWLLDYDRDIGIALRKAAEYYNPHP
SFWRQFLRLWQRRP

>core/360/5/Org5_Gene181

MSAMISLSSSHEASIASNTQVRDVLVSLAMDEFVEHNTEILPIKVFLARGTLSSTAIIDDLKDVVETEGEHHFQ
VYSNISLKMIYQRFFEKIFGIGCCPLLLVTDSHHTDPCGALITGIFAAVLFTVLAIVFGPTLGILCYSAYKIYQLT
KKISSLSRTHTEVINSVQKSDPFIHRSGAVAAAAASQSTIKACKVFRQSTLIFFVLGLIITISLAALIVGLVFALFF
LDPGAPAVMTAAMIGCCAAGGTGILLSVIGFLLASVYSVQKSQEGVHHMHTALLRCIVSNTIIQMPYLPITPG
TKKVLTQSIRRYQQFFSDDEYRDIESEVPLNRQTTPPPSYETLFHEEGSDGSSNVIPRESPPAYSTIDSSNSPFPSS
SPPPYYR

>core/361/5/Org5_Gene840

MLQTMMSETIITTPHVVKLISNFFQKKLFSSISTAYPLVIITDVSVQQHLLGPILDHIKMLGYQVIVLTFPPGEPN
KTWETFISLQYQLVDQNISPKSSIIGIGGGTVLDMTGFLAATYCRGLPLYLIPTTITAMVDTSIGGKNGINLRGI
KNRLGTFYLPKEVWMCPQFLSTLPREEWYHGIAEAIKHGFIADAYLWEFLNSHSKMLFSSSQILHEFIKRNCQI
KAAIVAEDPYDRSLRKILNFGHSIAHAIETLAKGTVNHGQAVSVGMMIETRISLAEGVMKTPQLIDQLERLLK
RFNLPSTLKDLQSIVPEHLHNSLYSPENIIYTLGYDKKNLSQHELKMIMIEHLGRAAPFNGTYCASPNMEILYD
ILWSECHVMRHC

>core/362/5/Org5_Gene847

MLCQQFLIEALARRKSKHTYRSLSLNSHLIDFTSNDYLGFASSPELRKEYITKLHAIESLGATGSRLLTGHSQL
CQRIEEQLAAYHNFESCLIFNTGYTANLGLLYALATDQDRILHDLYIHASIYDGIRLSKAQSFPFNHNDLNHLE
KRLASSHLGRTFVCVESVYSLHGSVAPLQAISELCERYSAYLIVDEAHAVGVFGDQGEGLVSALGLQDKVLA
TVYTFGKALGTHGAAIAGSSILKDYLINFCRPFIYTTAQPPHALTAIELAYEHNQRAFNQREHLSALIHHFREK
AQNLGLQLMKDNTTTPIQSICVSGSHRARQAALQIQNSGYDVRPIVSPTVKQREELLRICLHAFNTKNEIDHLL
HTLEQIFLCNVSSL

>core/363/5/Org5_Gene221

MSCFNLTSTNESLRPISPKASFPKQGWQSYFRSALRKHRSDTLSVSVCKVNKYDANLFVRLTVIALAVVGVLI
LFSIMLASIQGTLVITSWPLVTAAILIPTILLTGGMYILHRLGKKVDVISGVCIPPFSRRCWVPISSSHTLEKFDEK
HVSACSYLDISTLSADGSGIAAVYQCPPLLFRAFPCFGIPCAMPFVALLRMIYNLIRFLVVPFYIIFRMIYEHFFC
KHLPEDDRFIYKDVAREMGRSLAAFLKAPFYASACMIGAFYSLLDPLAGRVLMGSVERDWNDNVILARSVS
LANEAHSLFRFEGGGGRKGLGQHAFYLMLCCQPQSVFLFDKGEIVSGAHPSIQLPERRGLDTSGRYPHISVIPD
SGNDSAKNFIV

>core/364/5/Org5_Gene536

MGINPSGNRSPDDVWVRGAQGDSSSTQGTGATNSNLGAHNVTTSTSQPQVASKAKQLWQTVREFFLGKKSP
DSSQGASGPAMQSPSGPTIRPTRPAPPPPTTGGANAKRPATHGKGRAPQPPTAGSSSGSEQPTAMSSEVAKLV
SELKDAVHSHAESQKVLKKVSQELQTKWTDWENNRGPDYLLHGYRVIARALQQTYTEQSMLIEGTSSTGPV
PQAVTVAKDAVTQTVRGAIKNLENPKPGNDPDGVLMQVVISLGIEGPTLDPGESIQNFLETRVSDFGGDDSDI
DYTSDIARLGSALDRVRENHPNEMPRIWIALARELGAAVHSHATSVRIANAGKNHTRDVVRMANESSRLLQ
GMKVLSVGAWANTMTVLIGDLFE

>core/365/5/Org5_Gene752

MIPSPTPINFRDDTILETDPKPSLIMFSSKKTEIASERRKAHPTLFKVLGTIWNIVKFIISIILFLPLALLWVLKKTC
QFFILPSSIISQSMSKTAVAIRRMTFLSHIKQLLSLKEISAADRVVIQYDDLVVDSLAIKIPHALPHRWILYSQGN
SGLMENLFDRGDSSLHQLAKATGSNLLVFNYPGIMSSKGEAKRENLVKSYQACVRYLRDEETGPKANQIIAF
GYSLGTSVQAAALDREVTDGSDGTSWIVVKDRGPRSLADVANQICKPIASAIIKLVGWNIDSVKPSERLRCPEI
FIYNSNHDQELISDGLFERENCVATPFLELPEVKTSGTKIPIPERDLLHLNPLSPNVVDRLAAVISNYLDSENRK
SQQPD

>core/366/5/Org5_Gene927

MKHLAVLGSTGSIGRQTLEIVRRYPSEFKIISMASYGNNLRLFFQQLEEFAPLAAAVYNEEVYNEACQRFPHM
QFFLGQEGLTQLCIMDTVTTVVAASSGIEALPAILESMKKGKALALANKEILVCAGELVSKTAKENGIKVLPI
DSEHNALYQCLEGRTIEGIKKLILTASGGPLLNKSLEELSCVTKQDVLNHPIWNMGSKVTVDSSTLVNKGLEII
EAYWLFGLENXEILAVIHPQSLIHGMVEFLDGSVISIMNPPDMLFPIQYALTAPERFASPRDGMDFSKKQTLEF
FPVDEERFPSIRLAQQVLEKQGSSGSFFNAANEVLVRRFLCEEISWCDILRKLTTLMECHKVYACHSLEDILEV
DGEARALAQEI

>core/367/5/Org5_Gene1061

MIVGQAQTADKSVHAFQYYNGEMKDLGTLGGTSSTAKTVSPDGKVIMGRSQIADGSWHAFMCHTDFSSNN
VLFDLDNTYKTLRENGRQLNSIFNLQNMMLQRASDHEFTEFGRSNIALGAGLYVNALQNLPSNLAAQYFGIA
YKIRPKYRLGVFLDHNFSSHVPNNFNVSHNRLWMGAFIGWQDSDALGSSVKVSFGYGKQKATITREQLENT
EAGSGESHFEGVAAQIEGRYGKSLGGHVRVQPFLGLQFVHITRKEYTENAVQFPVHYDPIDYSTGVVYLGIGS
HIALVDSLHVGTRMGMEQNFAAHTDRFSGSIASIGNFVFEKLDVTHTRAFAEMRVNYELPYLQSLNLILRVN
QQPLQGVMGFSSDLRYALGF

>core/368/5/Org5_Gene705

MEDFSEQQLFFMRRAIEIGEKGRITAPPNPWVGCVVVQENRIIGEGFHAYAGGPHAEELAIQNASMPISGSDV
YVSLEPCSHFGSCPPCANLLIKHKVSRVFVALVDPDPKVAGQGIAMLRQAGIQVYVGIGESEAQASLQPYLY
QRTHNFPWTILKSAASVDGQVADSQGKSQWITCPEARHDVGKLRAESQAILVGSRTVLSDDPWLTARQPQG
MLYPKQPLRVVLDSRGSVPPTSKVFDKTSPTLYVTTERCPENYIKVLDSLDVPVLLTESTPSGVDLHKVYEYL
AQKKILQVLVEGGTTLHTSLLKERFVNSLVLYSGPMILGDQKRPLVGVLGNLLESASPLTLKSSQILGNSLKV
VWEISPQVFEPIRN

>core/369/5/Org5_Gene753

MNLSNRSDILSGIFSNPHPVSYFSSTHAKQLSDFSKKHPILTKIVTIIVKIFKLLIGLIIPPLGIYWLCQLVCSLALF
PRSSMLYSVLKTCFKKYRLEQEIQDYFVKNLDPSFKDPAVSESKRITIQQDHLTIDTLAIHFSTARPKRWLLISL
GSGDFLEDMIGLKDSLFLSWKELAKLLGANILIYNYPGVKSSTGKLNLENLATAHNLCAKYLQDKIQGPGAN
EIITYGYSLGGVVQSAALQKNPFTNSETSWVAVKDRAPHSLPAAANSFFGPIGKLIAVLARWKMDAEKNSRE
LPCPEILVYSADRFRPSEVGDDTALLPEFTLAHAIKRTPFARSKKFIGEVNLLHSSPLKHPTIQKLAEAILESLSR
KN

>core/370/5/Org5_Gene101

MNTSLKRPLKSHFDVVGSFLRPEHLKKTRESLKEGSISLDQLMQIEDIAIQDLIKKQKAAGLSFITDGEFRRAT
WHYDFMWGFHGVGHHRATEGVFFDGERAMIDDTYLTDKISVSHHPFVDHFKFVKALEDEFTTAKQTLPAPA
QFLKQMIFPNNIEVTRKFYPTNQELIEDIVAGYRKVIRDLYDAGCRYLQLDDCTRGGLVDPRVCSWYGIDEK
GLQDLIQQYLLINNLVIADRPDDLVVNLHVCRGNYHSKFFASGSYDFIAKPLFEQTNVDGYYLEFDHERSGDF
SPLTFISGEKTVCLGLVTSKTPTLENKDEVIARIHQAADYLPLERLSLSPQCGFASCEIGNKLTEEEQWAKVAL
VKEISEEVWK

>core/371/5/Org5_Gene1034

MSDNAHNLLYDKFELPEAVKMLPVEGLPIDKHARFIAEPLERGMGHTLGNALRRALLIGLEAPAIISFAMTGV
LHEYMAIEGVIEDVTNIILNLKGALLKKYPMQDSSLGRTTQVLKASISIDASDLAAANGQKEVTLQDLLQEGD
FEAVNPDQVIFTVTQPIQLEVVLRIAFGRGYTPSERIVLEDKGVYEIVLDAAFSPVTLVNYFVEDTRVGQDTDF
DRLVLIVETDGRVTPKEALAFSTQILTKHFSIFENMDEKKIVFEEAISIEKENKDDILHKLILGINEIELSVRSTNC
LSNANIETIGELVIMPEPRLLQFRNFGKKSLCEIKNKLKEMKLELGMDLTQFGVGLDNVKEKMKWYAEKIRA
KNIKG

>core/372/5/Org5_Gene291

MNGKAPLALYIHIPFCTKKCRYCSFYTIPYKSESVSLYCNAVIQEGLRKLAPIQETHFIETVFFGGGTPSLVSPL
DLKRILKELAPHAREITLEANPENLTVSYLRQLQETPINRISVGVQTFDDSILQLLGRTHSSSAAITALQECQNH
GFSNLSIDLIYGLPTQSLEIFLSDLHQALTLPITHISLYNLTIDPHTSFYKHRKILVPTIAQEEILAEMSLLAENLLL
SQGFQRYELASYAKPDYPAKHNLYYWTDRPFLGLGVSASQYLHGERSKNYSHISHYLRAVRKNLPTQETSEI
LPKKERIKEALALRLRLLEGADLAEFPSTLISMLTQDVKLQNLFSVHGQCLALNRQGRLFHDTIAEEIMGYSF

>core/373/5/Org5_Gene889

MSIAIAREQHAAILDMHPKPSIAMFSSEQARTSWEKRQAHPYLYRLLEIIWGVVKFLLGLIFFIPLGLFWVLQK
ICQNFILLGAGGWIFRPICRDSNLLRQAYAARLFSASFQDHVSSVRRVCLQYDEVFIDGLELRLPNAKPDRWM
LISNGNSDCLEYRTVLQGEKDWIFRIAEESQSNILIFNYPGVMKSQGNITRNNVVKSYQACVRYLRDEPAGPQ
ARQIVAYGYSLGASVQAEALSKEIADGSDSVRWFVVKDRGARSTGAVAKQFIGSLGVWLANLTHWNINSEK
RSKDLHCPELFIYGKDSQGNLIGDGLFKKETCFAAPFLDPKNLEECSGKKIPVAQTGLRHDHILSDDVIKEVAG
HIRRHFDN

>core/374/5/Org5_Gene161

MALKFHLIHQSKKSQARVGQIETSHGVIDTPAFVPVATHGALKGVIDHSDIPLLFCNTYHLLLHPGPEAVAKL
GGLHQFMGRQAPIITDSGGFQIFSLAYGSVAEEIKSCGKKKGMSSLVKITDEGAWFKSYRDGRKLFLSPELSV
QAQKDLGADIIIPLDELLPFHTDQEYFLTSCSRTYVWEKRSLEYHRKDPRHQSMYGVIHGGLDPEQRRIGVRF
VEDEPFDGSAIGGSLGRNLQEMSEVVKITTSFLSKERPVHLLGIGDLPSIYAMVGFGIDSFDSSYPTKAARHGLI
LSKAGPIKIGQQKYSQDSSTIDPSCSCLTCLSGISRAYLRHLFKVREPNAAIWASIHNLHHMQQVMKEIREAIL
KDEI

>core/375/5/Org5_Gene998

MAPIHGSNAFVEDILHSHPSPQATYFSSTRAQKLHEFKDRHPVLTRIASVIIKIFKVLIGLIILPLGIYWLCQTLCT
NSILPSKNLLKIFKKQPNTKTLKTNYLRALQDYSSKNRVASMRRVPILQDNVLIDTLEICLSQAPTNRWMLISL
GSDCSLEEIACKEIFDSWQRFAKLIGANILVYNYPGVMSSTGSSSLKDLASAHNICTRYLKDKEQGPGAKEIIT
YGYSLGGLIQAEALRDQKIVANDDTTWIAVKDRCPLFISPEGFHSCRRIGKLVARLFGWGTKAVERSQDLPCL
EIFLYPTDSLRRSTVRQNKLLAPELTLAHAIKNSPYVQNKEFIEVRLSSDIDPIDSKTRVALATPILKKLS

>core/376/5/Org5_Gene281

MSTTEPNLTNVNLTMLISSESMPTQLASHKLKGLDLVAFILIIGIAVSSGTAAIILGIPLLFILTALAVLAFSILLY
FLLREPKSPISVTHQPTPIIKDTDLPPVPPLALTPVPTEAVLEEPPLPSPRTHQTLLQENWDRIPDLQANTDMPFI
AADNQTGYAWHLKNSNLTLISTLGPIEKPRYKTQGIVMIVNAATPNMANNVKGTSLALAKATSVRCWENSK

KSPDPLRSKQPLQLGECRSAKWENLNGTTNAGKAGLPQFLGQLLGPKASDYNYNPNDAFTFCRQAYLNCLN
EAKRRKTTVVQLPLLSSHFPGSPKDEETTSLRLQWIDGVKLALIDALQTFGSEAENQNQPWVIILTTLARHPLI
TP

>core/377/5/Org5_Gene561

MAVAADSSASWLKSRNNFLSSLGKTEEQVAAPEFPKELCQHKIREKFRLEDVQVSIKFRGSITAVEATKEFGV
HLLIQPMVVQPWEVENLLFLTSEEDLQELMVAVFDDASLASYFYEKDKLLGFHYYFVAEACKLFEELQWVP
SLSAKVGGDAIFTATSLQGSFQVVDISLRLDGKNVRCRLLLPEDTFQSCQKFFSGLHDESDLHNIDQTQQISLS
VEVGYSQLTQEEWHQVVPGSFIMLDSCLYDPETEESGALLTVQKHQFFGGRFLTPSSGEFKITSYPNLTHEDP
PLPENPQASAAPLPGYSRLVVEVARYSLAVSEFIKLNLGSILSLGNHPAYGVDIILDGAKVGRGEIIALGDVLGI
RVLEV

>core/378/5/Org5_Gene305

MIYLDNNAMTPPERGLLEFLQKTFLIEGTYANPSSVHQLGKKSRQLVLEASHWMQKVLSFQGRVLYTSGATE
SLNLAIASLPKDSHVITSGSEHPAILEPLKHSSLSVSYLNPEEGRCVLTIEQIERAVTPKTSAIILGWVNSETGAK
ADIAAIAHFAQERQLQFIVDATANVGKERIVLPSGVTMAAFSGHKFHALSGIGALLVSPGVKLHPQLWGGGQ
QGGLRAGTENLWGIASLLYIFKYLDLHQERISQEILTHRNGFEKAIKARIPDVHIHCADQPRANNVSAIAFPPL
EGEVLQIALDIEGVACGYGSACSSGATAPFKSLVSMGVDEELTLATLRFSFSHLLLQEDVERAVGIIEKVVERL
KNS

>core/379/5/Org5_Gene748

MTTSDVIDFVTNDFLGFARSPTIYCEVSKRFQIHCQQFPHEKLGIRGSRLMVGPSSVIDDLESKIASYHGAPNA
FIVNSGYMANLGLCHHVSRSTDVLLWDEEVHMSVVHSLSAISGQHHTFHHNNLEHLESLLQCYRISSKGRIFI
FVSSVYSFRGTLAPLEQIIALSKKYHAHLIVDEAHAMGIFGDDGKGLCHALGYENFYAVLVTYGKALGTMG
ASLLTSSEVKYDLMQNSPPLRYSTSLSPHTLISIGTAYDFLASEGEIARKQVFKLKEHFHECFDSHAPGCVQPIF
LPHTCLEEAISVLETTGIHVGVVAFAKHPFLRVNLHAYNTVDEVNLLAQVMKPYLEKSSHRVHINHEFHLWR
ELCQH

>core/380/5/Org5_Gene1039

MMLVYCFDPSVPTSPEHRLMAALDRWFFLGGHRVRILTLEGNHYRAFQENMSISTVEKILKLISYLLIPIVLIA
LLIRCFLHSRFKCNWKCDSLSDARVPHDVQPFNDFQLFNNQERLNIWKNRRYVSGIDVLMVPVDYLRSQFPG
FKEIPEAIRCENYVSDGQFSEESKTSYLRAMLTDIVGYILSLDETYWTNVILKIRAMCITFESFPGKEADPNYSP
RVTHHYFDESWKALARHVLGEGNMVNRLDEALIRTEKPGKEGECITKQFLKDYCKKHLEVMSCPDFIESLV
DEKIREFRCPSILNSAVCDVIDRKCQEHLLKAIINEANRRLPGMKNSSFTMRGNQVLFYTIFSPPKLPPAASSVY
F

>core/381/5/Org5_Gene389

MTTCLPQPPKTSPLYSIFEKLDAQERLSSEDALHLLLLTNKEDQRTLWNFADQVRKQRVGDTVYYSSTLYLY
PTNFCDFSCKFCSFYAKPGDPKGWLYSPDDLLQQIQNIKTPITEVHIVGGCFPSCNLQYYSDLFTKIKEYDPQIH
IKALTAIEYAYLSDLDNLSIRDVLLTLKDAGLDSIPGGGAEILVDKIRNFLAPKRLSSSDFLNIHKMAHQLGIHS
NITMLCYHKEGPEDLVTHMVKVRDLQDETQGFKNFILLKFAQENNVLGKRLRKSGQGHAIPLKSLMAVARIF

LDNFSNMKALWNYLGIEAALDLLSCGANDLSSTHMGEKVFQMASSKEPIKMDAEGMAALITQQGRTPCLTN
SSHV

>core/382/5/Org5_Gene1059

MRRQVREIMQQTVIVAMSGGVDSSVVAYLFKKFTNYKVIGLFMKNWEEDSEGGLCSSTKDYEDVERVCLQ
LDIPYYTVSFAKEYRERVFARFLKEYSLGYTPNPDILCNREIKFDLLQKKVQELGGDYLATGHYCRLNTELQE
TQLLRGCDPQKDQSYFLSGTPKSALHNVLFPLGEMNKTEVRAIAAQAALPTAEKKDSTGICFIGKRPFKEFLE
KFLPNKTGNVIDWDTKEIVGQHQGAHYYTIGQRRGLDLGGSEKPCYVVGKNIEENSIYIVRGEDHPQLYLRE
LTARELNWFTPPKSGCHCSAKVRYRSPDEACTIDYSSGDEVKVRFSQPVKAVTPGQTIAFYQGDTCLGSGVID
VPMIPSEG

>core/383/5/Org5_Gene310

MTKIAFSEKAKNFPVEALKKWFEKNKRSLPWRDNPTPYSVWVSEVMLQQTRAEVVIDYFNQWMERFPTIES
LAAAKEEDVIKLWEGLGYYSRARHLLEGARMVMEEFHGKIPDDAISLAQIRGVGPYTVHAILAFAFKRRAAA
VDGNVLRVLSRIFLIETSIDLESTRTWVSRIAQALLPHKSPEVIAEALIELGACICKKVPQCHRCPVRQACGAW
RENKQFVLPVRHARKKVIFLHRLVAIVLYDGSLVVEKRRPKEMMAGLYEFPYIEVEPEEGLQDIEGFTKKME
LSLESPLEFLGNLKEQRHAFTNHKVHLCPIIFKATSLPQFGELHLLSDIDHLAFSSGHKKIKDALLIYLGDVRSR
ESIGV

>core/384/5/Org5_Gene444

MQENLDKRLEALRTEISLAARSLFDLDKKQKELQVLEEESSEENFWQDSVHAGKISEQIVSLRRQIQEYQELK
SKIDAIEFFLEDADALEDPAICEDLEKEFLFCEKKLAVWETQRLLSGEADKNSCFLTINAGAGGTESCDWVEM
LFRMYSRWATKHQWALEVVDRLDGEVVGIKHVTVKFSGMYAYGYAKAERGVHRLVRISPFDSNGKRHTSF
ASVDVFPEIDDQIKIEIRPNDLRIDTFRSSGAGGQHVNVTESAVRITHLPSGVVVSCQNERSQIQNRESCMKML
QAKLYQQVLQERLEKQSLDRKDKKEIAWGSQIRNYVFQPYTLVKDVRTGHETGNVQAMLDGELLDEFIKAY
LAEFGEVS

>core/385/5/Org5_Gene42

MAVEGRVNSSQALNQDCQEVLANKQSKGLLRCRILSIVVAVITFIAGVVLIALTLASILTSVPYLALGVFLLIV
TLGCIIFALCSEKIKKVPPTPISHKEEIIAWFEERKNIDMEKEKEDPEHFGRTATDIPMRSALDQFNHSCHHIHES
PALTETYRSHQDVLLFKDWCPVTLPDVTSEEEVLIRSVVGSYLLMEACVPKVSMLIDELHNKLKSPSERECLF
IDKKTLQRKASFLFTQKDLATFFLAYTRVNDGHLAPFRAGAKWILIHYVRLRRQHNQNDFFTPGHSCYYARL
AFNQTQRLYHQLFNVEKLRSIYANMDKDPLCHPWAFIPIYDLLKTEDHGDGFLEQQEDREYPSRAAQDQFW
G

>core/386/5/Org5_Gene687

MSPHRNLFKLKNFSNRLYNRALGRFDKVFNFFSGNVGIDLGTANTLVYVRGRGIVLSEPSVVAVDAQTHAVL
AVGHKAKAMLGKTPRKIMAVRPMKDGVIADFEIAEGMLKALIKRVTPSRSVFRPRILIAVPSGITGVEKRAVE
DSALHAGAQEVILIEEPMAAAIGVDLPVHEPAASMIIDIGGGTTEIAIISLGGIVESRSLRIAGDEFDECIINYMR
RTYNLMIGPRTAEEIKITIGSAYPLGDQELEMEVRGRDQVAGLPITKRINSVEIRECLAEPIQQIIECVRLTLEKC
PPELSADLVERGMVLAGGGALIKGLDKALSKNTGLSVITAPHPLLAVCLGTGKALEHLDQFKKRKGNLV

>core/387/5/Org5_Gene930

MKFVVSRNELGNLIKKIQSVVPQNTPIPVLTHVLIETYNDELVFTATDLTVSTRCVTKAKVYEKGAISIPSKRF
FQLVKELTEANLEISYSAGEMAQITSGSSCFRLLSMEKEDFPMLPDIQNALRFSLPAEQLKTMLQRTSFAVSRE
ESRYVLTGVLLAIANGVATIVGTDGKRLAKIDAEVTLDKSFSGEYIIPIKAVEEIIKMCSDEGEATIFLDQDKIA
VECDNTLLITKLLSGEFPDFSPVISTESNVKLDLHREELITLLKQVALFTNESSHSVKFSFLPGELTLTANCTKV
GEGKVSMAVNYSGELLEIAFNPFFFLDILKHSKDELVSLGISDSYNPGIITDSASGLFVIMPMRLHDD

>core/388/5/Org5_Gene806

MLIWKRHLLTRFWFALTSLLVLALIFYASIHHSLHTLKGASTAASGASVKLSILYYLAQISLKAEFLMPQLVA
VATTSTLFAMQNKREIILLQASGLSLKSLMHPLLLSGAVIMMVLYANFQWLHPICEKISITKENMDRGTTDKE
QGKIPALYLKDQTVLLYSSIEPKTLTLNNVFWIKDPKTIYTMEKLAFTTLSLPIGLNVTQFFANDSENLELKEFF
DMKEFPEIEFNFYENPFSKLFSAGNKNRLSEFFKAIPWNATGLGLSTQVPQRILSLLAQFYYVLISPLACMAAII
LSAYLCLRFSRTPTVTLAYLIPLGTVNIFFVFLKAGIVLASSSVLPTLPVMAFPLIVLFLLTNYAYAKLQ

>core/389/5/Org5_Gene433

MKFPRISISDLIPTQMVIWWRGGGNVHYVPNAQNLPKKILGGVLACFGLALLGCAAFAAGVCQTIFPCIGLMI
LGLVLLGFAYLQYSKGWSRFERPLFRETKVFEKPINWLGCLSLLQSWKKIRPGCYYHPGCPQVEICEGSQEIV
TKIFQKKSDRNTSIFLIQEMDQIALRQGIEKSSLSRKTFAIDPSVVSSLLSEIQREEQQYLDPKVISWSSEDQASD
RTHPKSAIYVNISDAAQEPQGRCYIDAYTKAFFTVLDQIGDPNIVKKHTIYVLTPILGVPDALPKEEQENLKLL
SQAAFLYSAEQVAKRMREEKQDSIRIKFIFTDPTSPTSLYFSPHHSSTPHSVTPISLSGFVGEQESYTFA

>core/390/5/Org5_Gene1017

MSKKIKVLGHLTLCTLFRGVLCAAALSNIGYASTSQESPYQKSIEDWKGYTFTDLELLSKEGWSEAHAISGNG
SRIVGASGAGQGSVTAVIWESHLIKHLGTLGGEASSAEGISKDGEVVVGWSDTREGYTHAFVFDGRDMKDL
GTLGATYSVARGVSGDGSIIVGVSATARGEDYGWQVGVKWEKGKIKQLKLLPQGLWSEANAISEDGTVIVG
RGEISRNHIVAVKWNKNAVYSLGTLGGSVASAEAISANGKVIVGWSTTNNGETHAFMHKDETMHDLGTLG
GGFSVATGVSADGRAIVGFSAVKTGEIHAFYYAEGEMEDLTTLGGEEARVFDISSEGNDIIGSIKTDAGAERA
YLFHIHK

>core/391/5/Org5_Gene400

MKKRFPSTLFLFYRRVTIAISLEGILGWGWLGSLLSKVFAFLVACWNRFSWSTPYRARSTVISVGNIVVGGAG
KTPTVLWLAEALRLRGYSCGVLSRGYKSQSSRQKKLTVVDSKVHSASYVGDEPLLMAEKLPEGSVWVHKD
RRISAARAAEKFGILLLDDGLQYRKLHKDVEIAVVNGQDPLGGRAFFPKGRLRDFPLRLKTVDAIIVNGGGKE
AGTVVKRVSNAPQIFVKPTIASVVWTHNGERIPKEALRELRVGVFCGLGFPQGFLNMLREEGIHILGKYLLPD
HAAITKKELNYFCQQMAMRQGQGLLCTEKDSVKLPRLSGEVSLLPIAKVEMRLSVNQDDTLSLLNMIEQIHK
NRGN

>core/392/5/Org5_Gene451

MNVPDSKNLHPPAYELLEIKARITQSYKEASAILTAIPDGILLLSETGHFLICNSQAREILGIDENLEILNRSFTD
VLPDTCLGFSIQEALESLKVPKTLRLSLCKESKEKEVELFIRKNEISGYLFIQIRDRSDYKQLENAIERYKNIAEL

GKMTATLAHEIRNPLSGIVGFASILKKEISSPRHQRMLSSIISGTRSLNNLVSSMLEYTKSQPLNLKIINLQDFFS
SLIPLLSVSFPNCKFVREGAQPLFRSIDPDRMNSVVWNLVKNAVETGNSPITLTLHTSGDISVTNPGTIPSEIMD
KLFTPFFTTKREGNGLGLAEAQKIIRLHGGDIQLKTSDSAVSFFIIIPELLAALPKERAAS

>core/393/5/Org5_Gene247

MSHTECGIVGLPNVGKSGLFNALTGAQVASCNYPFCTIDPNVGIVPVIDERLEALAKISNSQKIIYADMKFVDI
AGLVKGASDGAGLGNRFLSHIRETHAIAHVVRCFDDPDVTHVSGKVNPVEDIEVINLELIFSDFSSAKNIHSKL
EKLAKGKREVGALLPLFDTIIAHLEKGLPLRTLELTPEQIVALKPYPFLTMKPMFYIANVDESSLPDMDNDYV
AAVREVAAKENSKVVPICVRIEEEIVSLPIEERLEFLMSLGLEKSGLHRLVRAAYDTLGLISYFTTGPQESRAW
TVVRGSSAWEAAGEIHTDIQKGFIRAEVITFEDMIECQGRAAARELGKLHIEGRDYIVQDGDTMLFLHN

>core/394/5/Org5_Gene288

MTTEVRIPNIAESISEVTVASLLVTEGALIQENQGLLEIESDKVNQLIYAPVSGRIFWEVSEGDVVPVGGVVGKI
EPAGEGEELGDSQSKETIEAEIICFPQSGVRQSPPENKTFIPLRDQMDQGSQGLSAGDRGETRERMTSIRKTISR
RLLSALHESAMLTTFNEVYMTPLFHLRKEKQEEFLSRYGVKLGFMSFFVKAVLEALKAYPRVNAYIDGEEIV
YRHYYDISIAVGIDRGLVVPVIRDCDKLSNGEIEQKLADLALRAREGLLAIAELEGGGFTITNGGVYGSLLSTPI
INPPQVGILGMHKIEKRPVVLDNEIVIADMMYVALSYDHRLIDGKEAVGFLVKVKEGLENPASLLDL

>core/395/5/Org5_Gene533

MVPFRQHHAYQLLKQLHTSAISEADRVSYYFKQNRSLGSKDRQWIQNIIFNILRHRRLLETLILDSGEQVTPEA
LVAKVNEGVLENLDSYSAIPWPVRYSISDDLAHFLVQDYGEEQAEEIAKIWLTEAPITIRVNTDKISVKELQEK
LEYPSSPGELPEALHFSKRHPLQSTEAFRRGFFEIQDENSQRISQGISLTDKDIVLDFCAGAGGKSLIFAQKAKH
VVINDSRKAILQTAKHRLLRAGARNFSLADQLRLGSFSVVIVDAPCSGTGVFRRHPEHKWQFSKKLLLNYVR
VQKSILKQASAYVGPRGRLVYITCSLLKEENEAHVAYMHSLGWKEVHRKTLPLQVGKGDAFFTSHFQKI

>core/396/5/Org5_Gene860

MKLYQTLRGIVLVSTGCIFLGMHGGYAAEVPVTSSGYENLLESKEQDPSGLAIHDRILFKVDEENVVTALDVI
HKLNLLFYNSYPHLIDSFPARSQYYTAMWPVVLESVIDEFLMVADAKAKRIATDPTAVNQEIEEMFGRDLSP
LYAHFEMSPNDIFNVIDRTLTAQRVMGMMVRSKVMLKVTPGKIREYYRKLEEEASRKVIWKYRVLTIKANT
ESLASQIADKVRARLNEAKTWDKDRLTALVISQGGQLVCSEEFSRENSELSQSHKQELDLIGYPKELCGLPKA
HKSGYKLYMLLDKTSGSIEPLDVMESKIKQHLFALEAESVEKQYKDRLRKRYGYDASMIAKLLSEEAPPLFS
LL

>core/397/5/Org5_Gene887

MAFKRKTRWLWQVLILSVGLNMLFLLLFYSAIFRKDIYKLHLFSGPLIAKSSRKVYLSEDFLNEISQASLDDLI
SLFKDERYMYGRPIKLWALSVAIASHHIDITPVLSKPLTYTELKGSSVRWLLPNIDLKDFPVILDYLRCHKYPY
TSKGLFLLIEKMVQEGWVDEDCLYHFCSTPEFLYLRTLLVGADVQASSVASLARMVIRCGSERFFHFCNEESR
TSMISATQRQKVLKSYLDCEESLAALLLLVHDSDVVLHEFCDEDLEKVIRLMPQESPYSQNFFSRLQHSPRRE
LACMSTXRVEAPRVQEDQDEEYVVQDGDSLWLIAKRFGIPMDKIIQKNGLNHHRLFPGKVLKLPAKQS

>core/398/5/Org5_Gene850

MHYCERTLDPKYILKIALKLRQSLSLFFQNSQSLQRAYSTPYSYYRIILQKENKEKQALARHKCISILEFFKNLL
FVHLLSLSKNQREGCSTDMAVVSTPFFNRNLWYRLLSSRFSLWKSYCPRFFLDYLEAFGLLSDFLDHQAVIKF
FELETHFSYYPVSGFVAPHQYLSLLQDRYFPIASVMRTLDKDNFSLTPDLIHDLLGHVPWLLHPSFSEFFINMG
RLFTKVIEKVQALPSKKQRIQTLQSNLIAIVRCFWFTVESGLIENHEGRKAYGAVLISSPQELGHAFIDNVRVLP
LELDQIIRLPFNTSTPQETLFSIRHFDELVELTSKLEWMLDQGLLESIPLYNQEKYLSGFEVLCQ

>core/400/5/Org5_Gene81

MKIDILSLFPGYFDGPLQTSILGRAIKQRLLDVQLTNLRDFGLGKWKQVDDTPFSGGGMLLMAEPVTSAIRSV
RKENSKVIYLSPQGALLTAEKSRELAAASHLILLCGHYEGIDERAIESEVDEEISIGDYVLTNGGIAALVLIDAV
SRFIPGVLGNQESAERDSLENGLLEGPQYTRPREFEGKEVPEVLLQGDHKAISQWRLEQSERRTYERRPDLYL
NYLYKRSIDHKFDEETTTNRDHFKCDKISVVLEVNKLKRAKNFYCKVFGLDAMSCENKFCLPHEGKTIFWLR
EVQAEKKNIVTLSLSLDCACEEDFCYLLRRWELFGGKLLEKQADEHAVWALAQDLDGHAWIFSWHRMK

>core/401/5/Org5_Gene248

MGEKTEKATPKRLRDARKKGQVAKSQDFPSAVTFIVSMFTAFSLSTFFFKHLGGFLVSMLSQAPTRHDPVITL
FYLKNCLMLILTASLPLLGAVAVVGVIVGFLIVGPTFSTEVFKPDIKKFNPIENIKQKFKIKTLIELIKSILKIFGA
ALILYITLKSKVSLIIETAGVSPIITAQIFKEIFYKAVTSIGIFFLIVAILDLVYQRHNFAKELKMEKFEVKQEFKD
TEGNPEIKGRRRQIAQEIAYEDSSSQVKHASTVVSNPKDIAVAIGYMPEKYKAPWIIAMGINLRAKRILDEAEK
YGIPIMRNVPLAHQLLDEGKELKFIPESTYEAIGEILLYITSLNAQNPNNKNTNQPDHL

>core/402/5/Org5_Gene232

MSEAPVYTLKQLAELLQVEVQGNIETPISGVEDISQAQPHHIAFLDNEKYSSFLKNTKAGAIILSRSQAMQHA
HLKKNFLITNESPSLTFQKCIELFIEPVTSGFPGIHPTAVIHPTARIEKNVTIEPYVVISQHAHIGSDTYIGAGSVIG
AHSVLGANCLIHPKVVIRERVLMGNRVVVQPGAVLGSCGFGYITNAFGHHKPLKHLGYVIVGDDVEIGANTT
IDRGRFKNTVIHEGTKIDNQVQVAHHVEIGKHSIIVAQAGIAGSTKIGEHVIIGGQTGITGHISIADHVIMIAQTG
VTKSITSPGIYGGAPARPYQETHRLIAKIRNLPKTEERLSKLEKQVRDLSTPSLAEIPSEI

>core/403/5/Org5_Gene892

MSGPSRTESSQVSVLSYVPRDKEIAPKKQFTIAKISTLAILASLALGALVAGISLTIVLGNPVFLALLITTALFSV
VTFLVYHQMTSKVSSNWQKVLEQNFKPLGKAWQEKNVDCYSNEMQFYNNHLNPKFKVAIQTDASQPFQPT
FLTGLRVIEKNQSTGIIFNPVGPTNLIDNTATNLSTILYSTLKDKSVWDTCKQREGXPAKGEDPFSPTEVRVVK
LPNEALDQTFNLNLSSAEKKSILPTFLGHVCGPKSEELPNQQEYYRQALLAYENCLKAAIESHAAIVALPLFTS
VYEVPPEEILPKEGTFYWDNQTQAFCKRALLDAIQNTALRYPQRSLLVILQDPFNTIESQSRSEE

>core/404/5/Org5_Gene841

MKNSFGSLFSFTTWGESHGPSIGVVIDGCPAGLELHESDFVPAMKRRRPGNPGTSSRKENDIVQILSGVYKGK
TTGTPLSLQILNTDVDSSPYENSERLYRPGHSQYTYEKKFGIVDPNGGGRSSARETACRVAAGVVAEKFLAN
QNIFTLAYLSSLGSLTLPHYLKISPELIHKIHTSPFYSPLPNEKIQEILTSLHDDSDSLGGVISFITSPIHDFLGEPLF
GKVHALLASALMSIPAAKGFEIGKGFASAQMRGSQYTDPFVMEGENITLKSNNCGGTLGGITIGVPIEGRIAF
KPTSSIKRPCATVTKTKKETTYRTPQTGRHDPCVAIRAVPVVEAMINLVLADLVLYQRCSKL

>core/405/5/Org5_Gene877

MTLPNDLLETLVKRKESPQANKVWPVTTFSLARNLSVSKFLPCLSKEQKLEILQFITSHFNHIEGFGEFIVLPLK
DTPLWQKEFLLEHFLLPYDLVGNPEGEALVVSRSGDFLAAINFQDHLVLHGIDFQGNVEKTLDQLVQLDSYL
HSKLSFAFSSEFGFLTTNPKNCGTGLKSQCFLHIPALLYSKEFTNLIDEEVEIITSSLLLGVTGFPGNIVVLSNRC
SLGLTEELLLSSLRITASKLSVAEVAAKKRLSEENSGDLKNLILRSLGLLTHSCQLELKETLDALSWIQLGIDLG
LIKVTENHPLWNPLFWQIRRAHLALQKQAEBSRDLQKDTISHLRASVLKELTKGLSPESF

>core/406/5/Org5_Gene731

MMKKIRKVALAVGGSGGHIVPALSVKEAFSREGIDVLLLGKGLKNHPSLQQGISYREIPSGLPTVLNPIKIMSR
TLSLCSGYLKARKELKIFDPDLVIGFGSYHSLPVLLAGLSHKIPLFLHEQNLVPGKVNQLFSRYARGIGVNFSP
VTKHFRCPAEEVFLPKRSFSLGSPMMKRCTNHTPTICVVGGSQGAQILNTCVPQALVKLVNKYPNMYVHHIV
GPKSDVMKVQHVYNRGEVLCCVKPFEEQLLDVLLAADLVISRAGATILEEILWAKVPGILIPYPGAYGHQEV
NAKFFVDVLEGGTMILEKELTEKLLVEKVTFALDSHNREKQRNSLAAYSQQRSTKTFHAFICECL

>core/407/5/Org5_Gene78

MKKKVAEYLNRLAEVEIKISNPEIFSNSKEYSALSKEHSYLLELKNAYDKILNLEKVLADDKQALAIEKDPEM
VVMLEEGINENKVELEKLNKILESLLVPPDPDDDLNVIMELRAGTGGEEAALFVGDCVRMYHLYASSKGWK
YEVLSASESDLKGYKEYVMGISGTGVKRLLQYEAGTHRVQRVPETETQGRVHTSAITIAVLPEPSEEDTELLI
NEKDLKIDTFRASGAGGQHVNVTDSAVRITHLPTGVVVTCQDERSQHKNKDKAMRILKARIRDAEMQKRHN
EASAMRSAQVGSGDRSERIRTYNFSQNRVTDHRIGLTLYNLDKVMEGDLDPITTAMVSHAYHQLLEHGN

>core/408/5/Org5_Gene807

MPILWKVLIFRYLKTAAFCTLSLICISIISSLQEIVAYIAKDVPYDTVLRLMAYQIPYLLPFILPGSCFVSAFSLFR
KLSDNNHMTFLRASGASQSIIMFPVLMVSGAICCLNFYTCSELASICRYQTCKEIANMAMTSPALLLQTLQKK
ENNRIFIAVDHCAKSKFDNVIVALKGNNEISHVGIIKSIIPDTTKDTVKAKDVVFISKLPDSLTESSSPSSQRFYIE
TLDELLIPKITSTLFAGKSYLKTRTDYLPWKQLVKQSLKHSHLPETLRRVAIGFLCITLTYAGMILGIHKPRFRK
SIALYFIFPILDLILLIVGKNTKNLPLAFMLFVFPQLVSWVVFAARAYRESRGYA

>core/409/5/Org5_Gene312

MSLLSGHRLEGFPPIAEVMAACDRCSMDFCEILKSQSMDLWADAASCVDGLLQDPFWSTAIASGIAKSSLQE
TEFECESKVMVLSSWGEQGAQVCSPFNLERICMSFPSLKVFSLKKNGCENMGIQLSASCMNLLMSIFFVATN
GGSTPIWITKENLMALVALVLSHYQCYFVPATGDPQRGNILGNPEVNAILARGMGMRVDLERKRGGESSSSR
YLELAARCFENSLTKTSLLSDANNVQERDKCLLQMSTSLMHTAGLNLQRPPVPTPSGVTAHPQPQPDPVVTS
QPSLLGARERSPVSSRGRFPVVLPLSVISPRSHPGRVERRDLEDEEEEVMF

>core/410/5/Org5_Gene655

MSHDRILRAQRALSEHNLDAILVEKSEDLAYFLHDEAIAGILLIGQQEVMFFVYRMDKDLYSHIQRVPLTFLT
QDVVADLSLYVQKQRYQKIGFDSASTVYHKFAQRQVLPCLWEPLECFTEKIRSIKSEEEIRRMQEAAALGSA
GYDYVLTLLREGITEKEVVRQLRAFWAEAGAEGPSFPPIIAFGEHSAFPHSIPTDRPLKKGDIVLIDIGVLLNGY

CSDMTRMTALGTPHPKLLESYPVVVEAQKRAMALCKEGVLWGDIDAEAVRVLREHHLDTYFIHGIGHGVG
RHIHEYPCSPRGSQVKLESGMTITVEPGVYFPGIGGIRIEDTLCIDKNKNFSLTARPVISELVCL

>core/411/5/Org5_Gene931

MNKRQKDKLKICVIISTLILVGIFARAPRGDTFKTFLKSEEAIIYSNQCNXDMRKILCDAIEHADEEIFLRIYNLS
EPKIQQSLTRQAQAKNKVTIYYQKFKIPQILKQASNVTLVEQPPAGRKLMHQKALSIDKKDAWLGSANYTNL
SLRLDNNLILGMHSSELCDLIITNTSGDFSIKDQTGKYFVLPQDRKIAIQAVLEKIQTAQKTIQVAMFALTHSEII
QALHQAKQRGIHVDIIIDRSHSKLTFKQLRQLNINKDFVSINTAPCTLHHKFAVIDNKTLLAGSINWSKGRFSL
NDESLIILENLTKQQNQKLRMIWKDLAKHSEHPTVDDEEKEIIEKSLPVEEQEAA

>core/412/5/Org5_Gene612

MNLPDRKKALEAAVAYIEKQFGAGSIMSLGRHSATHEISTIKTGALSLDLALGIHGVPKGRVIEIFGPESSGKT
TLATHIVANAQKMGGVAAYIDAEHALDPSYASLIGVNIDDLMISQPDCGEDALSIAELLARSGAVDVIVIDSV
AALVPKSELEGDIGDVHVGLQARMMSQALRKLTATLSRSQTCAVFINQIREKIGVSFGNPETTTGGRALKFYS
SIRLDIRRIGSIKGSDNSDIGNRIKVKVAKNKLAPPFRIAEFDILFNEGISSAGCILDLAVEYNIIEKKGSWFNYQE
KKLGQGREFVREELKRNRKLFEEIEKRIYDVIAANKTPSVHANETPQEVPAQTVEA

>core/413/5/Org5_Gene738

MNLCKRISFEEGLELFVSSPIERLQERADAIRKERYPSNEVTYVLDANPNYTNICKIDCTFCAFYRKPKSPDAY
LLSFDEVRSLLQRYVSSGVKTVLLQGGVHPGLGIDYLEELVRITVQEFPSIHPHFFSAVEIEHACRVSGISIEQG
LQRLWDAGQRTIPGGGAEILSERVRKIISPKKMQPGGWINLHKLAHLMGFRTTATMMFGHVENPEDILIHLQ
TLRDAQDSCPGFYSFIPWSYKPGNTALRRNVPQQASIETYYRILALGRIFLDNFDHVAASWFGEGKSLGAKAL
HYGADDFGGVILDESVHKATGWSIQSSEEEICNIIRSEGFIPVERNTFYQHISCTVSSL

>core/414/5/Org5_Gene744

MKYSLNFKEIKIDDYERVIEVTCSKVRLHAIIAIHQTAVGPALGGVRASLYSSFEDACTDALRLARGMTYKAII
SNTGTGGGKSVIILPQDAPSLTEDMLRAFGQAVNALEGTYICAEDLGVSINDISIVAEETPYVCGIADVSGDPSI
YTAHGGFLCIKETAKYLWGSSSLRGKKIAIQGIGSVGRRLLQSLFFEGAELYVADVLERAVQDAARLYGATIV
PTEEIHALECDIFSPCARGNVIRKDNLADLNCKAIVGVANNQLEDSSAGMMLHERGILYGPDYLVNAGGLLN
VAAAIEGRVYAPKEVLLKVEELPIVLSKLYNQSKTTGKDLVALSDSFVEDKLLAYTS

>core/417/5/Org5_Gene215

MLNIHDILGNDDENLLSYQCKHITKDKLTLPSHDFVDKVFGLSDRNNRVLRSLQTMFSHGRLANSGYLSILPV
DQGIEHSAGASFAINPIYFDPENIVKLAIESGCSAVASTYGTLSLLSRKYAHKIPFMLKLNHNELLSYPTKYHQI
FFTQVEAAYSMGAVAVGATVYFGSETSNEEIVAVSNAFAKARSLGLATVLWCYLRNPAFVANGVDYHTAA
DLTGQADHLGATLGADIVKQKLPTCQGGFKAINFGKTDERVYSELSSNHPIDLCRYQVLNSYCGKVGLINSG
GPSGKNDFTEAARTAVINKRAGGMGLILGRKAFQRPLSEGIQLLNLVQDIYLDPNITIA

>core/418/5/Org5_Gene1001

MIPLIPMFLKQSLFFSLALTGMTTLVLTVSLGVPVMKWLKRKNYRDYIHKEYCEKLEMLHKDKAEVPTGGG
VLLFISLIASLLVWLPWGKFSTWFFIILLTCYAGLGWYDDRIKIKRKQGHGLKAKHKFMVQIAIAAFTLIALPY

IYGSTEPLWTLKIPFMEGMLSLPFWLGKVFCLGLALVAIIGTSNAVNLTDGLDGLAAGTMSFAALGFIFVALR
SSTIPIAQDVAYVLAALVGACIGFLWYNGFPAQLFMGDTGSLLLGGLLGSCAVMLRAECILVVIGGVFVAEA
GSVILQVLSCRLRKKRLFLCSPLHHHYEYQGLPETKIVMRFWIFSFVCAGLGIAAVLWR

>core/419/5/Org5_Gene835

MRIAVLGAGYAGLSVTWHLLLHSQGTATIDLFDPIPLGEGASGMSSGLLHAFTGKKALKPPLADQGINATHA
LITEASKALNVPIVISQGILRPAIDEDQAQLFTERVEEFPKEVEWWEKARCEISIPSMVIPPNLGALFIKSGVTLN
NDLYIQGLADACMKLGTQFYDELIEDLADIEEFYDHIIVTPGANASILPELKDMPVNKVKGQLLEISWPKDLA
MLSFSINAHKYMVANTQKNTCILGATFEHNQPEETPDPAIAYQEIMPPVLSLFPGLKDAQVLHCYAGMRSSS
KSRLPVISRIREKLWFLGGLGSKGLLYHGITGDMLAQAVLRKSTAYIAKEFLFTI

>core/420/5/Org5_Gene757

MILLQNIKRCSLKQLKVLATLLLSLSLPTLEAAENRDSDSIVWHLDYQEALQKSKEAELPLLVIFSGSDWNGP
CMKIRKEVLESPEFIKRVQGKFVCVEVEYLKHRPQVENIRQQNLALKSKFKINELPCMILLSHEEREIYRIGSFG
NETGSNLGDSLCHIVESDSLLRRAFPMMTSLSLSELQRYYRLAEELSHKEFLKHALELGVRSDDYFFLSEKFR
LLVEVGKMDSEECQRIKKRLLNKDPKNEKQTHFTVALIEFQELAKRSRAGVRQDASQVIAPLESYISQFGQQD
KDNLWRVEMMIAQFYLDSDQWHHALQHAEVAFEAAPNEVRSHISRSLEYIRHQS

>core/421/5/Org5_Gene417

MYLQEKFKAQQVPLVLRELLSCSDSINDSDPIYRMVFDSNDTTISYKVGDALGVLPENSKEVSEHVLQLLGYS
PTTLVNVKKTSEKVSAQKFIQGYVDLDKIPAKLNSFFPDKDPKITLYDAIQEYRPQIPIELFAESVFPLLPRFYSI
ASSPDLHPKSIELLVKHVSYPGKYQKRFGVCSSFLCSELQVNDSAYIFVQPTKHFTLSTQTEGKPLVMIGAGT
GIAPYKAFLEERLFNKDPGNNLLFFGERKEKVNFYYREFWNHAEEEGKLKLFLAFSRERDQKVYVQDLLRIQ
KDEVRKAYEEGGFFFVCGRKVLGIEVKHALEEILGKDTLASLRKEHRYVVDVY

>core/422/5/Org5_Gene935

MDVSRKINRHTQFYVDSIDGVIKNFDHKPSEDKSRDHEELEEKLLTITKRIVASAQEFQNRKTDSKNYYLKKT
QWLPFKNEELEQTKELFAMLTSMDKKIAQLFFYSPGCSSDWVEFTEVICHLNDSIGLGGVLLCCGLFEQQCEH
VVTVNKKLDLPLLLGTTVVNSLRYYLTYRNISLLNCQSMSELGKELGDVLKQHGVAFTLIFKEIVDIDLLNYV
KLIQGLKRSGNIQARIYDNDVPTLPSVSSSPIALRYGLANTIRGLALHVDFSSLKFISPSILSNTEHTAKALNSGG
ECFIFSNLDEFNLGMKIVMQLLRTGKISPEILNKNIMKILMIKRRVRSLYI

>core/423/5/Org5_Gene797

MEADILDGKLKRVEVSKKGLVNCNQVDVNQLVPIKYKWAWEHYLNGCANNWLPTEVPMARDIELWKSDE
LSEDERRVILLNLGFFSTAESLVGNNIVLAIFKHITNPEARQYLLRQAFEEAVHTHTFLYICESLGLDEGEVFNA
YNERASIRAKDDFQMTLTVDVLDPNFSVQSSEGLGQFIKNLVGYYIIMEGIFFYSGFVMILSFHRQNKMTGIGE
QYQYILRDETIHLNFGIDLINGIKEENPEVWTTELQEEIVALIEKAVELEIEYAKDCLPRGILGLRSSMFIDYVRH
IADRRLERIGLKPIYHSRNPFPWMSETMDLNKEKNFFETRVTEYQTAGNLSW

>core/426/5/Org5_Gene69

MELSLTSLLPLAWYVILGVAVFAYSFGDGFDLGLGAVYLKAKEDKERRILLNSIGPVWDGNEVWLVIIVGGL
FAGFPACYATLLSIFYMPIWTLVLLYIFRGCSLEFRSKSESVSWKIFWDIIFICSGTAISFFLGTIVGNLILGLPLSP
DTSYASLSWILFFRPYAALCGAVVASAFAIHGSCFALMKTSDSLNARIAQQFPYILSSFLVFYVLFLGASLISIP
KRFDAFPTYPLLILLIALTSCCCVAAKTSVSKKRYGYAFIYSTLNLLSLILSAATLTFPNILLSTVDPQYSYTIYN
SAVETKTLKSLLIIVLIGLPFIITYTCYIYRVFRGKTNFPSIY

>core/427/5/Org5_Gene691

MNSKMLKHLRLATLSFSMFFGIVSSPAVYALGAGNPAAPVLPGVNPEQTGWCAFQLCNSYDLFAALAGSLK
FGFYGDYVFSESAHITNVPVITSVTTSGTGTTPTITSTTKNVDFDLNNSSISSSCVFATIALQETSPAAIPLLDIAF
TARVGGLKQYYRLPLNAYRDFTSNPLNAESEVTDGLIEVQSDYGIVWGLSLQKVLWKDGVSFVGVSADYRH
GSSPINYIIVYNKANPEIYFDATDGNLSYKEWSASIGISTYLNDYVLPYASVSIGNTSRKAPSDSFTELEKQFTN
FKFKIRKITNFDRVNFCFGTTCCISNNFYYSVEGRWGYQRAINITSGLQF

>core/428/5/Org5_Gene790

MMEVYSFSPSVRTSFQHRVMAALDNWFFLGGRRLKVVSLDSCNSGQACEEYVPISTTEKVLKILSYLLIPIVII
ALLIRYLLHSNFTAKVSQKPWLKTLQLGIDIKSFILPGSHVNTMDSATLFKAIRLEGKRVDVEYHRLHSSDKV
VFYIPAQKLPDDLRLTHWLPEKETRKTEYVRHMLAHVMGYLTSQGKERLQQVVQDSRSSTSLGAEKVLQYR
FIDHPQSQGEFQRLLNENITTKGSEDKEVVQSDLFDMAFQCWWPQFISVIQSPTFSEELVHEMSQKLDLDCIYP
EDDEFEQKFLNTLLKAVLHHGFEGISVASMGVIFLICPDSLALQIPFLRNQK

>core/429/5/Org5_Gene645

MNKKKRVLTGDRPTGKLHLGHWVGSIKNRLELQNSPEYDCFFIIADLHTLTTKIRKEEVLDVDNHIYEVLAD
WLSVGIDPTKSIIYLQSAIPEIYELHLLFSMLISINRVMGIPSLKDMARNASIEEGSLSYGLIGYPILQSADILLAK
AQFVPVGKDNEAHVELTRDIARNFNRLYGQVFPEPEVLQGELTSLVGIDGQGKMSKSANNAIYLSDSDATITE
KVRKMYTDPNRIRATTPGRVEGNPLFIYHDIFNPHKDEVEEFKARYRQGCIKDIEVKARLAEELIHFLKPIKER
RSEFLSKPLALQNVLEDGTHKMREVAKVTMEEVHDKFGFSHKWRSLLK

>core/430/5/Org5_Gene139

MLTLGLESSCDETACAIVNEDKQILANIIASQDIHASYGGVVPELASRAHLHIFPQVINKALQQANLLIEDMDL
IAVTQTPGLIGSLSVGVHFGKGIAIGAKKSLIGVNHVEAHLYAAYMAAQNVQFPALGLVVSGAHTAAFFIENP
TSYKLIGKTRDDAIGETFDKVGRFLGLPYPAGPLIEKLALEGSEDSYPFSPAKVPNYDFSFSGLKTAVLYAIKG
NNSSPRSPAPEISLEKQRDIAASFQKAACTTIAQKLPTIIKEFSCRSILIGGGVAINEYFRSAIQTACNLPVYFPPA
KLCSDNAAMIAGLGGENFQKNSSIPEIRICARYQWESVSPFSLASP

>core/431/5/Org5_Gene91

MENAMSSSFVYNGPSWILKTSVAQEVFKKHGKGIQVLLSTSVMLFIGLGVCAFIFPQYLIVFVLTIALLMLAIS
LVLFLLIRSVRSSMVDRLWCSEKGYALHQHENGPFLDVKRVQQILLRSPYIKVRALWPSGDIPEDPSQAAVLL
LSPWTFFSSVDVEALLPSPQEKEGKYIDPVLPKLSRIERVSLLVFLSAFTLDDLNEQGVNPLMNNEEFLFFINK
KAREHGIQDLKHEIMSSLEKTGVPLDPSMSFQVSQAMFSVYRYLRQRDLTTSELRCFHLLSCFKGDVVHCLA
SFENPKDLADSDFLEACKNVEWGEFISACEKALLKNPQGISIKDLKQFLVR

>core/432/5/Org5_Gene233

MDSSAPYNIASQGTEKSTVERILDLYGPASCIKFLKQMVLIREFEARGEEAYLEGLVGGFYHSYAGQEAVATA
AIANTGLDPWVFSSYRCHALAILLNIPLQEIAAELLGKETGCALGRGGSMHMCGPNFPGGFGIVGGQIPLAAG
AAFTIKYQEQKNRVSLCFIGDGAVAQGVFHETLNFVSLHQLPLMLIIENNGWSMGTSLNRAVAKQPIAESQG
SSYDIRAVTVNGFDLFNSLLGFREAYRYMVDTESPVLVECLCSRFRGHSISDPNLYRSKEEMQCLFKKDPIVL
AKDWLIRLEVLTEEEFQNIRQECKTAVLEAFSNAKLSSDPSVTTLEEGVYA

>core/433/5/Org5_Gene115

MKLYSISSDVDTPWIFQLMSKVDSYLFLGGNRIKVVSIVMQEPNLIIGKVENVRISTIVKILKILSFLIFPLILIAL
ALHYFLHAKYANHLLVSKILERAPQYVPIPGRSGDTASHYKLTTLVPVSQKNLQAMGSNPLEVEAALRTTKP
SFFCVPAKYRQIIISSHGIRFSLDLEQLADDINLDSVSWPTEYLNSTMDFCSKADKRVIQNVQNLRTGTYINSV
GKRSLLKFMLQHLFIDGITQENPEALPNNTSGRLTLFPSVRYIYSHFTPQNPTIWPQVFFRQGPLDEDRGGGFEI
LEQLQELGVRFPICPSQGPDNPNFQGFQGIRIYWEDSYQPNKEV

>core/434/5/Org5_Gene737

MLPFEFEFNTTSSPECDVCLDPQKLFVKLFKRTIVLLSGPTGSGKTDVSLALAPMIDGEIVSVDSMQVYQGMD
IGTAKVSLKARQEIPHHLIDIRHVQEPFNVVDFYYEAIQACQNILSRNKVPILVGGSGFYFHAFLSGPPKGPAA
DPQIREQLEAIAEEHGVSALYEDLLLKDPEYAQTITKNDKNKIIRGLEIIQLTGKKVSDHEWDIVPKASREYCC
RAWFLSPETEFLKNNIQMRCEAMLQEGLLEEVRGLLNQGIRENPSAFKAIGYREWIEFLDNGEKLEEYEETKR
KFVSNSWHYTKKQKTWFKRYSIFRELPTLGLSSDAIAQKIAKDYLLYS

>core/435/5/Org5_Gene696

MFFQNLAKKLTALGISPLGCLLIGGVVSCAILFGRSSNPSLAPTQVKTEKTSGNWLKLTQMGNPKLIESLTKK
EQLEKDLTSFHPIASAKVAIALSTEDDVMSPLHLSVILTLRKEESLTPSLLFSITDYLCSSLPGLKREHISLSDNL
GNLYIPESITVNSLFIHTLENYLGKIFPKEHFALAYHAKAEKPTLQLTLNENYIAHLTKEESEKIVAHTKHYLY
QNYDDSYDIVIETLPFARLQNKKSFPAKVLIGSMILVISLMIVALASFYLARHAYERVSPEPRKIKRGINISKLLE
IIQKESPEKIALILSYLDPKKAEALLNRLPEDLKHQVLKYKL

>core/436/5/Org5_Gene214

MSEQHSPIISVQDVSKKLGDHILLSKVSFSVYPGEVFGIVGHSGSGKTTLLRCLDFLDMPTSGSISVAGFDNSLP
TQKFSRRNFSKKVAYISQNYGLFSSKTVFENIAYPLRIHHSEMSKSEVEEQVYDTLNFLNLYHRHDAYPGNLS
GGQKQKVAIARAIVCQPEVVLCDEITSALDPKSTENIIERLLQLNQERGITLVLVSHEIDVVKKICSHVLVMHQ
GAVEELGTTEELFLNSENSITNELFHEDINIAALSSCYFAEDREEVLRLNFSKELAIQGIISKVIQTGLVSINILSG
NINLFRKSPMGFLIIVLEGEVEQRKKAKELLIELGVVIKEFY

>core/437/5/Org5_Gene898

MTILRKLSQYLFFFSLFCSFIYVATCGSQPDSVSSPKIAIFLSFPHPLLEDCSKSCIETLKDFENLPEIVVLNAEDSI
VKARKIARSLHTDKNVVAIVTLGTIATKVMSHIETQKPVIYAAVPDRESLTPPKNTMNIYGVNDTLDINQYCF
AIQAVATNAQSIVYLKPSEPFPSDLQKEIVKKLHASGIEVIEISITSSTFKTRIRQAIDKRPSAIFIPLSPLSHKEGT

AFLQEILKEKIPIITDDTSLISEGACIACSVDYKKSGKQIAKIVHHLLYNNHDVDSLRKIIAQRLSPTTTFNEDIIK
YLGIKLHKTERNQFLSFKSKKLEKSEKGKNVAVS

>core/438/5/Org5_Gene804

MEMKEEIEAVKQQFHSELDQVNSSQALADLKVRYLGKKGIFRSFSEKLKQCTDKAKLGSLINDFKTYVEDLL
QEKSLVLLASEQAEAFSKEKIDSSLPGDSQPSGGRHILKSILDDVVDIFVHLGFCVREAPNIESEANNFTLLNFT
EDHPARQMHDTFYLNATTVLRTHTSNVQARELKKQQPPIKVVAPGLCFRNEDISARSHVLFHQVEAFYVDH
NVTFSDLTAILSAFYHSFFQRKTELRFRHSYFPFVEPGIEVDVSCECCGKGCALCKHTGWLEVAGAGMIHPQV
LRNGNVDPEIYSGYAVGMGIERLAMLKYGVSDIRLFSENDLRFLQQFS

>core/439/5/Org5_Gene277

MATISPISLTVDHPLVDTKKKSCSNFDKIQSRILLITAIFAVLVTIGTLLIGLLLNIPVIYFLTGISFIAVVLSNFILY
KRATTLLKPRACGKHKEIKPKRVSTNLQYSSISIAINRSKENWEHQPKDLQNLPAPSALLTDNPYEIWKAKHS
LFSLVSLLPGGNPEHLLISASENLGKTLLIEETSQNAPISSYVDTTPSPKSLLNEAIQETRVEINTELPAGDSGER
LYWQPDFRGRVFLPQIPTTPEAIYQYYYALYVTYIQTAINTNTQIIQIPLYSLREHLYSRELPPQSRMQQSLAMI
TAVKYMAELHPEYPLTIACVERSLAQLPQESIEDLS

>core/440/5/Org5_Gene552

MEEAAKHLAKEFLCSGINLFLSGEYEQAEKRLKETLELDSTAALAYCYLGIIALETGRVSEALNWCSKGLASE
PGDSYLRYCYGVALDRGNQYEAAIEQYSAYVALHPDDVECWFSLGSVYHRLKRLQEALDCFDKILALDPW
NPQSLYNKAVILSEMDDEAESIRLLEVAVAKNPLYWKAWVKLGFLLSRSKRWDKATEAYERVVQLRPDLSD
GHYNLGLCYLTLDKTRLALKAFQEALFLNAEDADAHFYVGLAHLDLKQMREAYEAFNSALSINLEHERAHY
LLGYLHHMQGETDKATKELLFLQKKDSTFAPLLQKTVVSDPSSMQFERRLDTIS

>core/441/5/Org5_Gene571

MVLGVVGISYREAALKERERAIQYLQSFEKNLFLAQRFLGKGGAFIPLLTCHRAELYYYSESPEIAQAALLSEL
TSQGIRPYRHRGLSCFTHLFQVTSGIDSLIFGETEIQGQVKRAYLKGSKERELPFDLHFLFQKALKEGKEYRSRI
GFPDHQVTIESVVQEILLSYDKSIYTNFLFVGYSDINRKVAAYLYQHGYHRITFCSRQQVTAPYRTLSRETLSF
RQPYDVIFFGSSESASQFSDLSCESLASIPKRIVFDFNVPRTFLWKETPTGFVYLDIDFISECVQKRLQCTKEGV
NKAKLLLTCAAKKQWEIYEKKSSHITQRQISSPRIPSVLSY

>core/442/5/Org5_Gene89

MFPSANQESRTRNVPLGIFHGLVACLYWGIVFVIPNFLGSFGDLDIVLTRYTIFGIFSLIACAIKNPSVIKKTPLYI
WRKSLLWTLLINPVYYFGITLGIRYVGSAITVVIASLAPTAVLYHSNTKQKELPYSLLFAISSVIITGVILTHLSA
LNLPTAASPLYSILGVIAVILSTSLWVIYVIRNQSLLEKHPNLTPDTWSYLIGISALIICLPMIIILDLCGITHVTHN
LISHTPGSERLLFLLLCSAMGIFSSAKALIAWNKASLNLSPALLGAILIFEPIFGLVLTYLYSQSLPSLQEGIGIFL
MLGGSLLCLVLFGRKVQKSLENSQVSSSNE

>core/443/5/Org5_Gene642

MKKTCCQNYRSIGVVFSVVLFVLTTQTLFAGHFIDIGTSGLYSWARGVSGDGRVVVGYEGGNAFKYVDGEK
FLLEGLVPRSEALVFKASYDGSVIIGISDQDPSCRAVKWVNGALVDLGIFSEGMQSFAEGVSSDGKTIVGCLY

SDDTETNFAVKWDETGMVVLPNLPEDRHSCAWDASEDGSVIVGDAMGSEEIAKAVYWKDGEQHLLSNIPG
AKRSSAHAVSKDGSFIVGEFISEENEVHAFVYHNGVIKDIGTLGGDYSVATGVSRDGKVIVGHSTRTDGEYR
AFKYVDGRMIDLGTLGGSASFAFGVSDDGKTIVGKFETELGECHAFIYLDD

>core/444/5/Org5_Gene922

MTHQVAVLHQDKKFDVSLRPKGLEEFYGQHHLKERLDLFLCAALQRGEVPGHCLFFGPPGLGKTSLAHIVA
YTVGKGLVLASGPQLIKPSDLLGLLTSLQEGDVFFIDEIHRMGKVAEEYLYSAMEDFKVDITIDSGPGARSVR
VDLAPFTLVGATTRSGMLSEPLRTRFAFSARLSYYSDQDLKEILVRSSHLLGIEADSSALLEIAKRSRGTPRLA
NHLLRWVRDFAQIREGNCINGDVAEKALAMLLIDDWGLNEIDIKLLTTIIDYYQGGPVGIKTLSVAVGEDIKT
LEDVYEPFLILKGFIKKTPRGRMVTQLAYDHLKRHAKNLLSLGEGQ

>core/445/5/Org5_Gene593

MSYSLRNKKTKICVYIIIALGILSFRSIPQEVYDKIRSSFVSLHVKFFPKIKQAPSSHLANLELENLVLKERVASL
EEKLKLYEVSNHTPPLFPEILTPYFHKLVEGKVVYRDYTHWSSSCWVNVGKTHGIKKNSPVLSGNVLVGLVD
YVGEHQSRIRLITDVGMKPSVVAMRGDIQSWWIKHSLRELIRQVEQISHAYILEKDKYEKISQLQELDSLIQGE
GENQALLRGILSGVGGALWKEGSLCLEGEGFYFSEGKTLLPGDILVTTGLDGVFPPGLLVARVTKVKAPRDG
ACTFKIEAQSLEEKLMELDQLFILPPLEFNPNDRPDIFGLLWD

>core/446/5/Org5_Gene488

MKVVINGFGRIGRLVLRQILKRNSSVEVLAINDLVPGDALTYLFKFDSTHGRFPEDVRCEADHLIVGKRKIQF
LSERNVQNLPWKDLGVDLVIECTGLFTKKEDAEKHIQAGAKRVLISAPGKGDIPTFVMGVNHKTFNPEKDFV
ISNASCTTNCLAPIAKVLLDNFGITEGLMTTVHAATATQLVVDGPSKKDWRGGRGCLQNIIPASTGAAKAVT
LCLPELKGKLTGMAFRVPIEDVSVVDLTVRLDKSTTYDDICKAMKQASETDLKGILDYTDEQVVSSDFIGSEY
SSIFDALAGIALNDRFFKLVAWYDNETGYATRIVDLLEYVEKNSK

>core/447/5/Org5_Gene413

MFVDQITLELRAGKGGNGVVAWRKEKYLPKGGPYGGNGGNGGSVIIEATTSVYSFEAYRNIRFLKAPDGQS
GATNNRTGRSGKDLIVSVPTGTLLRDAETGEILHDFTVDGERLLVSQGGKGGKGNTFFKTSVNRAPTKATPG
KPGEIRQVELELKLIADIGLVGFPNAGKSTLFNTLAHTEVKVGAYPFTTLAPSLGLVLCKDRLYQKPWIIADIP
GIIEGAHQNKGLGLDFLRHIERTLLLLFVIDVSKRERNSPEEDLETLIHELHSHQPDFEKKDMLVALNKIDDLL
PDEQEECLQSFQKRFPSYTFVLISGLTGEGVDGLYRFFTQRLAV

>core/448/5/Org5_Gene228

MWFSVNKNKKAAIWATGSYLPEKVLSNADLEKMVDTSDEWIVTRTGIKERRIAGPQEYTSLMGAIAAEKAI
ANAGLSKDQIDCIIFSTAAPDYIFPSSGALAQAHLGIEDVPTFDCQAACTGYLYGLSVAKAYVESGTYNHVLLI
AADKLSSFVDYTDRNTCVLFGDGGAACVIGESRPGSLEINRLSLGADGKLGELLSLPAGGSRCPASKETLQSG
KHFIAMEGKEVFKHAVRRMETAAKHSIALAGIQEEDIDWFVPHQANERIIDALAKRFEIDESRVFKSVHKYGN
TAASSVGIALDELVHTESIKLDDYLLLVAFGGGLSWGAVVLKQV

>core/449/5/Org5_Gene618

MAAPIFIKNILLRSSIVYAPLAGFSDYPYRCMSALYQPGLMFCEMVKVEGILYAPERTSKLLDYNENMRPIGA
QLCGSNPETSGEAAKILEGLGFDLIDLNCGCPTDKITKDGSGSGLLKTPELIGRILDKIINSVSIPVTVKIRSGWD
MEHINVEDTVRIIRDAGASAVFVHGRTRAQGYHGPSKQEYISRAKAAAGKEFPVFGNGDIFSPEAAQAMLTT
GCDGVLVARGTLGAPWIGKQIQDYLTTGSYEKIPFIKRKAAFLEHMRLVEDYYQSETKFLSETRKLCGHYLIS
AAKVRFLRSSLAKATSYQEVYQLVNDYEEADDSSLETFVKC

>core/450/5/Org5_Gene44

MSTPLSSGGISPSDQYVPQELFCDRLSSSRSNSPDSNASGDSPIVSPPISALVALTDLKLVPYNQNSFSWTTRLK
NAVEKIGLFLQRNWKYILLYILAWALILVCHHTVALTLTIWLGVGLGIGVVFGIFTATCLDKENKHRHVNSL
WNLINHGILQLDPNGTRQILLATMIASISALIYAVPQAVGLVIGFSIGNQLSINTVYGARLGDEATYAIDRKAH
KKRIENIEQAINQHQIIKHQMINQKQLNALIEINRNNQTDPATANLLASLKLNLNQPMPYCFSMPECGVTSSYL
DLNNNSPDDIIARADQCIMTLSQTLQQIKKEPDRIIESNH

>core/451/5/Org5_Gene669

MVRRSISFCLFFLMTLLCCTSCNSRSLIVHGLPGREANEIVVLLVSKGVAAQKLPQAAAATAGAATEQMWDI
AVPSAQITEALAILNQAGLPRMKGTSLLDLFAKQGLVPSELQEKIRYQEGLSEQMASTIRKMDGVVDASVQIS
FTTENEDNLPLTASVYIKHRGVLDNPNSIMVSKIKRLIASAVPGLVPENVSVVSDRAAYSDITINGPWGLTEEI
DYVSVWGIILAKSSLTKFRLIFYVLILILFVISCGLLWVIWKTHTLIMTMGGTKGFFNPTPYTKNALEAKKAEG
AAADKEKKEDADSQGESKNAETSDKDSSDKDAPEGSNEIEGA

>core/452/5/Org5_Gene692

MKQHIGYLGMGIWGFCLASLLANKGYPVVAWSRNPDLIKQLQEERRHPLAPNVVISPNLSFTTDMKEAIHNA
FMIVEGVTSAGIRPVAEQLKQITDLSVPFVITSKGIEQNTGLLLSEIMLEVLGDSVTPYLGYLSGPSIAKEVLNG
SPCSVVVSAYDSQTLKQIHEAFSLPTFRVYPNTDIKGAALGGALKNVIAIACGIAEGLSFGNNAKAGLVTRGL
HEMRKLAAIMDCKPETLNGLAGLGDLCVTCFSESSRNLRFGHLLAQGLTFEQAKAKIGMVVEGAYTALSAY
QVAKHHKIDMPITTGIYRVLYENLDLKEGIALLLQRNTKEEFL

>core/453/5/Org5_Gene467

MKPLGFQENLEALCNKTSRQLLKYLIKQILFVCGASLLIALEFSFFLYFFLFSGKTVIPAFCLACFFLTLFVCLV
TRLYLLSGKGDFFEDLASEYLQGAVPPNKRSQNIVEEQSHLAAAATKLSINLQNQEYSLLSEIFKFLPKHDLIR
KFSCFCFWKDYFLFRECLLQKAIEAYIKVVQAIPVDLSAHVSLADAYVALSGLYADPRKYPEFDANYWIPSG
RYSAEIQEKFFATARRAIEEFQILNEYAPGNAWVHAQLAYSYHDLQMPMEEIQEYEIVLKLKPNDVETMSKL
GILYFQQGMNAKGLRIYEEIKKRDYKKSQKLIKFYGVEYKY

>core/455/5/Org5_Gene662

MQPFIFTLLCLTSLVSLVAFDAANARKRCACAQTIERGENFFSIKRSACAEIEYQEKSRHASAIERISKDKGKV
TPKQIAKVATKKKQRYRLLQVPFSRPPNNSRYNLYALLSEPPECYSDTASWYAIFIRLLRRAYVDTGNVPPGS
EYAIANALISNKQEILERGAQLGPDVIETLTLPEEQAEIFYKMLKGSSNSQSLLNFLHYEEKSLGHCKLNLIFM
DPLLLEAVLDHPDAYRETSLLRDGIWEAVKRQEHAIQEHGQAAALELFKTRTDFRLELRDKMQLLLSRYDLL
PLLNKKMFDYTLGSAGDYLFLVDPDTKAISRCRCPSKSIKL

>core/456/5/Org5_Gene509

MATNAIRSAGSAASKMLLPVAKEPAAVSSFAQKGIYCIQQFFTNPGNKLAKFVGATKSLDKCFKLSKAVSDC
VVGSLEEAGCTGDALTSARNAQGMLKTTREVVALANVLNGAVPSIVNSTQRCYQYTRQAFELGSKTKERKT
PGEYSKMLLTRGDYLLAASREACTAVGATTYSATFGVLRPLMLINKLTAKPFLDKATVGNFGTAVAGIMTIN
HMAGVAGAVGGIALEQKLFKRAKESLYNERCALENQQSQLSGDVILSAERALRKEHVATLKRNVLTLLEKA
LELVVDGVKLIPLPITVACSAAISGALTAASAGIGLYSIWQKTKSGK

>core/457/5/Org5_Gene852

MRIAVLGVTGLVGQKFVALLHKWYRDWVIAEVVASNSKYGQSYGDACIWQEPIGPMPEMVRDLPIRKIEEV
QSDIVVSFLPSSAESMEAYCLSQGKVVFSNASTYRMHSSVPIIIPEVNSDHFQLLEEQPYPGKIITSPNCCVSGIT
LALAPLRKFSLDHVHIVTLQSASGAGYPGVPSLDLLANTVPHIVGEEEKILRETVKILGSSKQPLPCKLSVTVH
RVPVAYGHTLSLHVTFSKDVDLDEILYSYQEKNKEFPNTYQLYDNPWSPQARKHLSHDDMRVHLGPITYGG
DFRTIKMNVLIHNLVRGAAGTLLASMENYFFDYLKREMCLR

>core/458/5/Org5_Gene478

MKTLWHFVSKAFLSIVGLCCGVVLAFVVIFALIASSLGNGDATFVSLPDAQGEVKDLGKTAPIIAVIEMKDVI
ASSKNTAKTIQNILEGFEKAPLKDRVKGIVIDMDCPGGEVFEIDRIYSMLRFWKERKGFPIYIYVNGLCASGGY
YVSCAATKIYATSSSLIGSIGVRSGPFFNVKEGLNRYGVESDLLTAGKDKAPMNPYTPWTSHDREERQATLDF
LYGQFVDIVTQNRPLLTKEKLVHTLGARIFSPEKAKQEGYIDVVGATKEQVLQDIVAVCKIEDNYRVIGSGGD
GWWKRVASAAASSPLVTGMIKHDILPLSHDAAYIPPYLAL

>core/460/5/Org5_Gene598

MSSLTLSRRPRRNRKTAAIRDLLAETHLSPKDLIAPFFVKYGNNIKEEIPSLPGVFRWSLDLLLKEIERLCTYGL
RAVMLFPIIPDDLKDAYGSYSSNPKNILCHSIHEIKNAFPHLCLISDIALDPYTTHGHDGIFLNGEVLNDESVRIF
GNIATLHAEMGADIVAPSDMMDGRIGYIRSKLDQSGYSKTSIMSYSVKYASCLYSPFRDALSSHVTSGDKKQ
YQMNPKNVLEALLESSLDEEEGADILMVKPAGLYLDVIYRIRQNTCLPLAAYQVSGEYAMILSAFQQGWLD
KETLFHESLIAIKRAGADMIISYSAPFILELLHQGFEF

>core/461/5/Org5_Gene111

MNIYQFSPGASPNWQASLMAQLNSYFCLGGETVTRIISLRPSGLILAKKEKAVVSTAEKILKILSFILFPLVLIAL
AIRYLLYNKFNKDLDRAVFFIPTEITKAEELIIAKNPALVKEAALTVSPLFYSLPKKYQLMKVETP

>core/462/5/Org5_Gene112

MLLEDLDTDSIPWPKLYLSEDFDFAYYPESKAIIDTVAKLEKNNPGEEFCLESKKILARYLLEQLFKLETGLNF
PTSTIDGGRESFLIEFSHETKKPTVWAFIYFYYYHSNGPKLEKDFKQAGCEVHNRLLNLGLKYRPQAGAQND
GRNGGPYGPIGFLIVWEENYGSVLKDHGFIKDN

>core/463/5/Org5_Gene777

MQFSRYLRYAFDNQYLPEPLYQKFSVFHQNYIDAATKKAAADQAEVLCLQWVKVIIEDLKNPFIFPPYHKKI
RAPIDLFRLSIDFFSLVIDDKNSRILNLHRLKEIEEYIARGDNVVLLANHQTECDPQLMYYALGKTHPELMEN
MIFVAGDRVTSDPLARPFSMGCDLLCIYSKRHIATPPELREEKLLHNQKSMQILKTLLNEGGKFIYVAPAGGR

DRKNAEGRLYPSEFSPESIEVFRLLAKASNQTTHFYPFALKTYDILPPPPKIENAIGEQRAIFFAPVFFNFGAELF
FDALCSKEELIHCDKHAQRTLRAEKVFSIVKNLYEEL

>core/464/5/Org5_Gene848

MREETVSWSLEDIREIYHTPVFELIHKANAILRSNFLHSELQTCYLISIKTGGCVEDCAYCAQSSRYHTHVTPEP
MMKIVDVVERAKRAVELGATRVCLGAAWRNAKDDRYFDRVLAMVKSITDLGAEVCCALGMLSEEQAKKL
YDAGLYAYNHNLDSSPEFYETIITTRSYEDRLNTLDVVNKSGISTCCGGIVGMGESEEDRIKLLHVLATRDHIP
ESVPVNLLWPIDGTPLQDQPPISFWEVLRTIATARVVFPRSMVRLAAGRAFLTVEQQTLCFLAGANSIFYGDK
LLTVENNDIDEDAEMIKLLGLIPRPSFGIERGNPCYANNS

>core/465/5/Org5_Gene360

MIDIMQHFKPYTMVPGQKLPIPGSLLYAQVFPTLWRLFSSKHEILNEQTLQVQGPLKRFAVFQDLHRGGLAV
TSERYKYYLLPSGECTQSIKGKLPSAAQAGPLLSLGVHKHADWQKVRCRRDLKEILPLWFRFAAMAPKGSY
RDLETTAIGSLVKTAHQRVLHRETTEIAPALLSIALAGFSECFLPRSYDEEFQGILPQDGDPEGGVPFELLSYSF
GMIQDIFLRHQGQLVEILPALPPEFPCGRLIHVALPNLGTLSIVWTKKTIRQVELHAEYSGEVFLKFCSSLCSAR
LREWSERRLSGSKRLSLGETLEIKAGTTYLWDCFHK

>core/466/5/Org5_Gene720

MSAFFDLLKSQTASHPPIWLLRQVGRYMPPYQELKGSQSLKTFFHNTEAIVEATLLGPSLLHVDAAILFADILS
ILDGFAVTYDFAPGPRIQFSPEQPFTFTSDPQTIFSYLLDAIRTLKQKLPVPLIVFAASPFTLACYLIDGGASKDF
SKTMSFLYVYPEKFDQLISTIIEGTAIYLKTQMDAGAAAVQLFESSSLRLPSALFTRYVTEPNRRLIAKLKEQAI
PVSLFCRCFEENFYTLQATQADTLHPDYHVDLHRIQKNLMLSLQGNLDPAIFLLPQEKLLHYVEAFLVPLRTY
PNFIFNSGHGILPETPLENVQLVVSYVQRQL

>core/467/5/Org5_Gene398

MPSPMISTDVCQDILGKQKEAVDFFFQAFQPKEAMQLAEKILGHSGWVFFSGVGKSGCVARKLVATLQSLSE
RALFFSPVDLLHGDLGLVSPGDIVCLFSKSGETQELLDTVPHLKSRRAILVAITSMPYSNLAALSDLVVILPSV
AELDPFNLIPTNSTTCQMIFGDFLAMLLFHSRGVSLSTYGKNHPSGQVGMKANGKVKDFMFPKTEVPFCHLG
DKVSFSLEVFSAYGCGCVCIVDPQFRLMGIFTDGDLRRSLASYGGEVLSLSLEKVMTANPRCITEDSDIAIALQ
LMESSSPVAVLPVLDNEENRHVTGLLHMHTLAKAGLL

>core/468/5/Org5_Gene833

MAFKEVVRVAVTGGKGQIAYNFLFALAHGDVFGVDRGVDLRIYDVPGTERALSGVRMELDDGAYPLLHRL
RVTTSLNDAFDGIDAAFLIGAVPRGPGMERGDLLKQNGQIFSLQGAALNTAAKRDAKIFVVGNPVNTNCWIA
MKHAPRLHRKNFHAMLRLDQNRMHSMLAHRAEVPLEEVSRVVIWGNHSAKQVPDFTQARISGKPAAEVIG
DRDWLENILVHSVQNRGSAVIEARGKSSAASASRALAEAARSIFCPKSDEWFSSGVCSDHNPYGIPEDLIFGFP
CRMLPSGDYEIIPGLPWEPFIRNKIQISLDEIAQEKASVSSL

>core/469/5/Org5_Gene234

MPKHKTLEIREALREAIDEEMSRDPNVCILGEEVGDYNGAYKVTKGLLDKWGPKRVIDAPISEAAFSGIGIGA
ALSGLRPIIEFMSWNFSFVALDQIISHAAKMHFMTGGKFSVPIVFRGPNGAAAQVSCQHSHCVESLYANIPGLI

IIAPSNPYDAKGLLKSAIRNNNPVLFLENELEYNLKGEVPTEEYLVPIGKAHRVQEGNDLTIITYSRMVSITKEA
CSLAKKRWGLSIEIIDLRTIKPLDISTILSSVRKTSRCIVIEEGHYFAGISSEIIALITEHVFDSLDAPPLRVCQKETP
MPYSKILEQATLPNVNRILDTIEKVMR

>core/470/5/Org5_Gene53

MSNQFDQLKKLSTIVCDSGDPELVKASGSQDATTNPSLILKVAQEPKFQELLNEAVVWGIRQNGDDLQTLSFI
LDKIQVNFALEIIKNIPGRISLEIDARLSFNVEAMVQRAVFLSQLFEAMGGDKKRLLVKIPGTWEGIRAVEFLE
AKGIACNVTLIFNLVQAIAAAKAKATLISPFVGRIYDWWIAAYGDEGYSIDADPGVASVSNIYAYYKKFGIPT
QIMAASFRTKEQVLALAGCDLLTISPKLLDELKKSQHPVKKELDPAEAKKLDVQPIELTESFFRFLMNEDAMA
TEKLAEGIRIFAGDTQILETAITEFIKQIAAEGA

>core/471/5/Org5_Gene468

MTTPAYLLANFGGPRHAKDLQEFLISLLTDRDVTGTFLPRVLHRHLFTFIAKKRVPKVLPQYQSLQNWSPIYF
DTETLAKTLSEILRAPVIPFHRYLPSTHEKTLLALRTLHTRHVIGIPLFPHFTYSVTGSIVRFFMKHVPEIPISWIP
QFGSDSKFVSLITCHIRDFLQKLGILEKECCFLFSVHGLPVRYISQGDPYSKQCYESFSAITTNFKQSENFLCFQS
KFGPGKWLSPSTAQLCQNIDTDKPNVIVVPFGFISDHLETLYEIERDYLPLLRSRGYRALRIPAIYSSPLWVSTL
VDIVKENSTVVAEELIKSGKKHTGIR

>core/472/5/Org5_Gene265

MDAKMGYIFKVMRWIFCFVACGITFGCTNSGFQNANSRPCILSMNRMIHDCVERVVGNRLATAVLIKGSLDP
HAYEMVKGDKDKIAGSAVIFCNGLGLEHTLSLRKHLENNPNSVKLGERLIARGAFVPLEEDGICDPHIWMDL
SIWKEAVIEITEVLIEKFPEWSAEFKANSEELVCEMSILDSWAKQCLSTIPENLRYLVSGHNAFSYFTRRYLATP
EEVASGAWRSRCISPEGLSPEAQISVRDIMAVVDYINEHDVSVVFPEDTLNQDALKKIVSSLKKSHLVRLAQK
PLYSDNVDDNYFSTFKHNVCLITEELGGVALECQR

>core/473/5/Org5_Gene391

MMTYPVPQNPLLLRILRLMDAFSKSDDERDFYLDRVEGFILYIDLDKDQEDLNKIYQELEENAERYCLIPKLT
FYEVKKIMETFINEKIYDIDTKEKFLEILQSKNAREQFLEFIYDHEAELEKWQQFYVERSRIRIIEWLRNNKFHF
VFEEDLDFTKNVLEQLKIHLFDAKVGKEITQARQLLSNKAKIYYSNEALNPRPKRGRPPKQSAKVETETTISSD
IYTKVPQAARRFLFLPEITSPSSITFSEKFDTEEEFLANLRGSTRVEDQLNLTNLSERFASLKELSAKLGYDSLST
GDFFGDDDEKVVTKTKGSKRGRKKSS

>core/474/5/Org5_Gene745

MHSELPNYQNIVESVVTEITTQLLNYRSEHRLVPFWEKSDGSFITAADYGSQYYLKQQLAKAFPNIPFIGEETL
YPDQDNEKIPEILKFTRLLTSSVSRDDLISTLVPPPSPTSLFWLVDPIDGTAGFIRHRAFAVAISLIYEYRPILSVM
ACPAYNQTFKLYSAAKGHGLSIVHSQNLDRRFVYADRKQTKQFCEASLAALNQQHHATRKLSLGLPNTPSP
RRVESQYKYALVAEGAVDFFIRYPFIDSPARAWDHVPGAFLVEEAGGRVTDALGAPLEYRKESLVLNNHAVI
LASGDQETHETTLAALQNQLNVVPTDKLIAL

>core/475/5/Org5_Gene576

MKTVTSFTVCKENSGRLDKYLTEVHPKYSRAFYQEHILSGLVQINGQINTRVATRLNCGDIVTIDIQEKEELLE
LLPEAIPLDKVYEDGMILVINKPRDMVVHPAPGHFHGTLVHALLHEIGERLKEEFPEEPWRPGIVHRLDKDTS
GLIITAKTRQAKKVFSELFSTKRLKKSYLAVCIGKPRSTTIHTHISRHQNKRKEMTVSSQGKEAVTHCQVLAF
NGKLSFVALSPETGRTHQLRVHMKHLGTPILGDPVYGIPSMNSSYGLDKQQLHAYSVDFTHPETRQFCSLKA
GLPEDMRSLLIKEFRNETTILNKNLLESILKEQ

>core/476/5/Org5_Gene204

MTIDMHCDLLSHPHFCRKDPAVRCSPEQLLSGGVRQQVCAIFVPHSRGEPNCDKQNSLFFSLPNQYPDIGLLS
YEEEENGSSSQKKSLSLIRSIENASALGDDTAPLGTLLAKLIHLTKQGPLAYLGIVWKGDNRFGGGTEAPKRL
SNDGKVLLDIMYELGVPIDLSHCSDKLAEDILDYTADKLPNLAVIASHSNFRSVLDHRRNLVDAHAKEIVRRK
GVIGLNLVRSYVGDSLGDLEKHVLHAENLGILSSIVLGSDFFYANEDENFFFNECSSAEAHPVLNQLIHRIFSK
GKAESILSSRAEKFLKQVIVEQVNPKITDVKL

>core/478/5/Org5_Gene957

MIEFAFVPHTSVTADRIEDRMACRMNKLSTLAITSLCVLISSVCIMIGILCISGTVGTYAFVVGIIFSVLALVACV
FFLYFFYFSSEEFKCASSQEFRFLPIPAVVSALRSYEYISQDAINDVIKDTMQLSTLSSLLDPEAFFLEFPYFNSLI
VNHSMKEADRLSREAFLILLGEITWKDCETKILPWLKDPNITPDDFWKLLKDHFDLKDFKKRIATWIRKAYPE
IRLPKKHCLDKSIYKGCCKFLLLAENDVQYQRLLHKVCYFSGEFPAMVLGLGSEVPMVLGLPKVPKDLTWE
MFMENMPVLLQSKREGHWKISLEDVASL

>core/479/5/Org5_Gene321

MELLPHEKQVVEYEKAIAEFKEKNKKNSLLSSSEIQKLEKRLDKLKEKIYSDLTPWERVQICRHPSRPRTVNYI
EGMCEEFVELCGDRTFRDDPAVVGGFVKIQGQRFVLIGQEKGCDTASRLHRNFGMLCPEGFRKALRLGKLA
EKFGLPVVFLVDTPGAYPGLTAEERGQGWAIAKNLFELSRLATPVIIVVIGEGCSGGALGMAVGDSVAMLEH
SYYSVISPEGCASILWKDPKKNSEAASMLKMHGENLKQFGIIDTVIKEPIGGAHHDPALVYSNVREFIIQEWLR
LKDLAIEELLEKRYEKFRSIGLYETTSESGPEA

>core/480/5/Org5_Gene541

MASNPILQIEDLSITLAKQRQQYPIVQSLSFTINEGQTLAIIGESGSGKSVSAHAILRLLPCPPFSVSGQVNFQGH
NLLTASRSIQKKIIGTEISMIFQNPQASLNPVFTIEQQFREIIHTHLALTAEVAKEKMLYALEETGFHDPRLCLNL
YPHQLSGGMLQRICIAMALLCSPKLLIADEPTTALDVSVQYQILQLLKTLQKKTGMSLLIITHNMGVVAETAD
DVLVLYAGRMVECAPAVQMFHNPSHPYTRDLLASRPSLQPQQLGSFNPIPGQPPHYTAFPSGCRYHPRCSKIL
NRCSAEAPEIYPVREGHKVRCWLYDD

>core/481/5/Org5_Gene262

MALGPSPYYGVSFFQFFSVFFSRLFSGSLFTGSLYIDDIQIIVFLAISCSGAFAGTFLVLRKMAMYANAVSHTVL
FGLVCVCLFTHQLTTLSLGTLTLAAMATAMLTGFLIYFIRNTFKVSEESSTALVFSLLFSLSLVLLVFMTKNAH
IGTELVLGNADSLTKEDIFPVTIVILANAVITIFAFRSLVCSSFDSVFASSLGIPIRLVDYLIIFQLSACLVGAFKA
VGVLMALAFLIIPSLIAKVIAKSIRSLMAWSLVFSIGTAFLAPASSRAILSAYDLGLSTSGISVVFLTMMYIVVK
FISYFRGYFSKNFEKISEKSSQY

>core/482/5/Org5_Gene542

MTTNFPQPLIQATSLTKHYYKRSFWFQGKTIASRPVDDVSFSLYSRRAVGLIGESGSGKSTLALALAGLLPLTS
GFLTFNGTPIKLHSKHGRHQLRSQVRLVFQNPQASLNPRKTILDSLGHSLLYHKLVPKEKVLATVREYLELVG
LSEEYFYRYPHQLSGGQQQRVSIARALLGVPQLIICDEIVSALDLSIQAQILNMLAELQKKLSLTYLFISHDLAV
VRSFCTEVFIMYKGQIVEKGNTKRIFSDPQHPYTRMLLNAQLPETPDQRQSKPIFQEYHKDSEESCSTGCYFY
NRCPQKQEACKSEIIPNQGDAHHTYRCIH

>core/483/5/Org5_Gene590

MEKKYYALAYYYITRVDNPHEEIALHKKFLEDLDVSCRIYISEQGINGQFSGYEPHAELYMQWLKERPNFSKI
KFKIHHIKENIFPRITVKYRKELAALGCEVDLSKQAKHISPQEWHEKLQENRCLILDVRNNYEWKIGHFDNAT
LPDIQTFREFPEYAEKLAQECDPETTPVMMYCTGGIRCELYSPVLLEKGFKEVYQLDGGVIAYGQQVGTGKW
LGKLFVFDDRLAIPIDESDPDVAPIAECCHCQTPSDAYYNCANTDCNALFLCCDECIHQHQGCCGEECSQSPR
VRKFDSSRGNKPFRRAHLCEISENSESASCCLI

>core/484/5/Org5_Gene460

MKQLLFCVCVFAMSCSAYASPRRQDPSVMKETFRNNYGIIVSGQEWVKRGSDGTITKVLKNGATLHEVYSG
GLLHGEITLTFPHTTALDVVQIYDQGRLVSRKTFFVNGLPSQEELFNEDGTFVLTRWPDNNDSDTITKPYFIET
TYQGHVIEGSYTSFNGKYSSSIHNGEGVRSVFSSNNILLSEETFNEGVMVKYTTFYPNRDPESITHYQNGQPHG
LRLTYLQGGIPNTIEEWRYGFQDGTTIVFKNGCKTSEIAYVKGVKEGLELRYNEQEIVAEEVSWRNDFLHGER
KIYAGGIQKHEWYYRGRSVSKAKFERLNAAG

>core/486/5/Org5_Gene881

MNLKVVYFGTPTFAATVLQDLLHHKIQITAVVTRVDKPQKRSAQLIPSPVKTIALTHGLPLLQPSKASDPQFIE
ELRAFNADVFIVVAYGAILRQIVLDIPRYGCYNLHAGLLPAYRGAAPIQRCIMEGATESGNTVIRMDAGMDT
GDMANITRVPIGPDMTSGELADALASQGAEVLIKTLQQIESGQLQLVSQDAALATIAPKLSKEEGQVPWDKP
AKEAYAHIRGVTPAPGAWTLFSFSEKAPKRLMIRKASLLAEAGRYGAPGTVVVTDRQELAVACSEGAICLHE
VQVEGKGSTNSKSFLNGYPAKKLKIVFTLNN

>core/488/5/Org5_Gene335

MSKGSSKHTVRINQTWYIVSFILGLSLFAGVLLSTIYYVLSPIQEQAATFDRNKQMLLAAHILDFKGRFQIQEK
KEWVPATFDKKTQLLEVATKKVSEVSYPELELYAERFVRPLLTDAQGKVFSFEEKNLNPIEFFEKYQESPPCQ
QSPLPFYVILENTSRTENMSGADVAKDLSTVQALIFPISGFGLWGPIHGYLGVKNDGDTVLGTAWYQQGETP
GLGANITNPEWQEQFYGKKIFLQDSSGTTNFATTDLGLEVVKGSVRTTLGDSPKALSAIDGISGATLTCNGVT
EAYVQSLACYRQLLINFSNLTHEKKTGE

>core/489/5/Org5_Gene486

MRLLSILKLHLFSLRSSSSLSPHYYHSCSRSMLHLLCRWKDADIMEWQQICNILSGVCSRMSGKLVSLQKETQ
DSCHQEHERIHLQYREQLSALEEEYRRREEAKNQDLEKLQQENTWLQNRLAEKLQQIRHQSDIIDEIKKELLQ
SVQRTEISEGRRLCYEHKIKQLEEQLQRYVSQHGAPSIEIEEDKSSAAYAEINRLKKSLIDLQQEKDIYIKTYHS

EIAKLREKLQRQEGAQTSSEVCSIEKLTEVQTDLAEKKKAIALLQDIVEDQYCQLRDLHKEKGMAMPSNTKL
DHLKGLLGKEPESEVDVVFSESKSLGS

>core/490/5/Org5_Gene780

MEVQIGIDLMGGDHSPLVVWQVLVDVLKSQSSTIPFAFTLFASEEIRKQIQEEFISDLPQEKFPKIISAENFVAM
EDSPLAAIRKKSSSMALGLDYLQEDKLDAFISTGNTGALVTLARAKIPLFPAVSRPALLVCVPTMRGHAVILD
VGANISVKPEEMVGFARMGLAYRQCLGDSKIPTIGLLNIGSEERKGTEAHRQTFRMLRETFGEAFLGNIESGA
VFDGAADIVVTDGFTGNIFLKTAEGVFEFLQRILGDKLEADIQRRLDYTFYPGSVVCGLSKLVIKCHGKACGS
SLFHGILGSINLAQARLCKRILSNLI

>core/491/5/Org5_Gene446

MLISISLATLPILAFSWASFIEPNWLRTTAIPWRLPKKHAHLHGLRIAQISDLHFHKRVPEKFLNKVSKSIKNFSP
DLIVFCGDLLCRARLEDKERLETFLNTLEAPLGVFAILGNHDYSSYISRNTKGEITCIPEEKSRPIQRAIIAVMQG
LFSSPSYRYDPNLTPQEPHPDLLKLLKNTPLTLLHNTTHVIPNTLNIVGLGDLFARQFHPEQAFKNYDPSLPGL
LLSHNPDGITRLQQYPGDFVLSGHSHGPQVTLSWPKFARKFFERLSGLENPYLARGYFVTKEGKQLYVNRGL
GGLKRIRFCSPPEICYITCSYD

>core/492/5/Org5_Gene808

MVLSSDLLRDDKQLDLFFASLDVKKRYLLALSGGSDSLFLFYLLKERGVSFTAVHIDHGWRSTSAQEAKELE
ELCAREGVPFVLYTLTAEEQGDKDLENQARKKRYAFLYESYRQLDAGGIFLAHHANDQAETVLKRLLESAH
LTNLKAMAERSYVEDVLLLRPLLHIPKSSLKEALDARGISYLQDPSNEDERYLRARMRKKLFPWLEEVFGKNI
TFPLLTLGEESAELSEYLEKQAQPFFSAATHQDSQGELPCPDCLIQQAFLCKWVMKKFFNNAGIAVSRHFLQ
MVYDHLSRSSCATLRMRNKIVIIKPGVVVID

>core/493/5/Org5_Gene880

MKFLLYVPLLLVLVSTGCDAKPVSFEPFSGKLSTQRFEPQHSAEEYFSQGQEFLKKGNFRKALLCFGIITHHFP
RDILRNQAQYLIGVCYFTQDHPDLADKAFASYLQLPDAEYSEELFQMKYAIAQRFAQGKRKRICRLEGFPKL
MNADEDALRIYDEILTAFPSKDLGAQALYSKAALLIVKNDLTEATKTLKKLTLQFPLHILSSEAFVRLSEIYLQ
QAKKEPHNLQYLHFAKLNEEAMKKQHPNHPMNEVVSANVGAMREHYARGLYATGRFYEKKKKAEAANIY
YRTAITNYPDTLLVAKCQKRLDRISKHTS

>core/494/5/Org5_Gene375

MNRRKARWVVALFAMTALISVGCCPWSQAKSRCSIDKYIPVVNRLLEVCGLPEAENVEDLIESSSAWVLTPE
ERFSGELVSICQVKDEHAFYNDLSLLHMTQAVPSYSATYDCAVVFGGPLPALRQRLDFLVREWQRGVRFKKI
VFLCGERGRYQSIEEQEHFFDSRYNPFPTEENWESGNRVTPSSEEEIAKFVWMQMLLPRAWRDSTSGVRVTF
LLAKPEENRVVANRKDTLLLFRSYQEAFPGRVLFVSSQPFIGLDACRVGQFFKGESYDLAGPGFAQGVLKYH
WAPRICLHTLAEWLKETNGCLNISEGCFG

>core/495/5/Org5_Gene953

MRKVAFLVSCLFSVAIGASAAPVRVPGFPQIPEDLVQIKTEVCPKQEVCLAVTIKCDDHNLIGVLHLPNTPTPE
GGFPTVVLFHGFRGTKFGGLTGAYRKLGRKFAAAGIATLRVDMAGCGDSEGVAEEVPIETYLRDAQTILETV

QEHPDLNAYRLGISGFSLGCHIAFELAKIYNPRDLNIKALSVWAPIADGGILLKELYENFSKHGEGDIISVGKDF
GFGPPPIIVCSGDVDLLIRIQDHVTANSLPTKPYILHQQGIDDTLVSRTQQTLFKNTAPGRMTFISYPNTGHNLA
TAPDLDMILDQIVSHFQRTL

>core/496/5/Org5_Gene917

MFKLLFHIAAFAGHVLSTPIFIVQDACGIDEEACKNPPPRPFSAQVQYLKVNDAKFKKLPHQTIGYRQYDGTF
LCTLPITEHSGLLFSTGYIGADIQWKSSLPISETDPNGLGWATFQDTSFYNYVLLSLGAYTLSLKNWQWSIILS
GLVDPKNIEMGYGLYQGVLSGKYQATEKLSAIFGVINETGLHQEKAWPLVGVSYKATDQLTLNCIYPVNFSI
DYRSTSVCNLGLAYRLTRFRKKLYKNHLISSRGIFEYQGREIEANVKLTPWPGSFIKGFYGWSIGNDISIADDH
NNNKTSHTFKTSAFFGGSAVMNF

>core/497/5/Org5_Gene153

MLVELEALKREFAHLKDQKPTSDQEITSLYQCLDHLEFVLLGLGQDKFLKATEDEDVLFESQKAIDAWNALL
TKARDVLGLGDIGAIYQTIEFLGAYLSKVNRRAFCIASEIHFLKTAIRDLNAYYLLDFRWPLCKIEEFVDWGN
DCVEIAKRKLCTFEKETKELNESLLREEHAMEKCSIQDLQRKLSDIIIELHDVSLFCFSKTPSQEEYQKDCLYQS
RLRYLLLLYEYTLLCKTSTDFQEQARAKEEFIREKFSLLELEKGIKQTKELEFAIAKSKLERGCLVMRKYEAA
AKHSLDSMFEEETVKSPRKDTE

>core/498/5/Org5_Gene293

MTLPMQKSLTSFDDFSQAYAEKVPAIALIGSALEDDKDALIELLVSESFKELGGQGLMPATLMSWTETFALFQ
EHETLGIIHAEKFPLATKEFLSRYARNPQPHLTILIFTTKQECFRELSKALPSALSLSLFGEWPADRQKRIIRLLL
QRAERVGISCSQSLASLFLRALASTSLPDILSEFDKLLCSVGKKTSLDHSDIKELVVKKEKASLWKFRDSLLKR
DPVEGHQQLHFLLEDGEDPLGIITFLRTQCLYGLRSIEEGSKENKHRMFVLYGKERLHQALNSLFYAETLIKN
NVQDPIVAVETLVIRMVNL

>core/499/5/Org5_Gene144

MFSYIKNRILFNLLSLWIVLTLTFLVMKTIPGDPFNDEGCNVLSEEVLQTLKSRYGLDKPLYQQYTQYLHSIAK
LDFGNSLVYKDRKVTNIISTAFPISAILGLQSLFLSIGGGIALGTIAALKKKKQRRYILGASILQISIPAFIFATLLQ
YVFAVKIPLLPIACWGSFTHTILPTLALAVTPMAFIIQLTYSSVSAALNKDYVLLAYAKGLSPLKVVIKHILPYA
IFPTISYSAFLTTTVITGTFAIENIFCIPGLGKWFICSIKQRDYPVALGLSVFYGTLFMLSSLLSDLIQSIIDPQIRYA
HGKEKKRK

>core/500/5/Org5_Gene261

MAMLPKFFLVLLCLGLCSCSQKTTTIEGEQMTIFYRIVLGTSLSAKEKASLSQQIDRCFHKIDSIYNNWNPYSE
LSIINRAPADVPITLSVELSEFLDQVDTLYKLSEGRFDPTVGPLKTLWLLHLKSQTLPPKDVWEQHYKDMGW
QHLEFQSNTKTLIKKNPHVQIDLCGVVKGYAVDCLNEICNTFCPNNYVEWGGEIKTSGHHPSGRPWRIFSEA
AGTILDIDDMAIATSGNHIQKWCVEGKIYTHILDTRTGKPLELSSYPIQSVSVVHPSCAYADAIATVLMTFDSK
IEAKQWAEEHHILTYINDGASS

>core/501/5/Org5_Gene422

MWRVVLRFLIIFILGRAVFPLRASESFSWETSTCLTVLGIPFIDIILTTNEDFVAQCGLQIGTISSTNNAKIKEIFLI
YKEKFPEASISFKRKEPLNLSQSHLSDLGILCMRNGETYAEGMANKENGPALKQPKDLRLVLRCPNQPDTLL
YSEKEAEKGIETNTCLCNQGYTLLDGQLILYGDSIEKFLKETKRKNNHTLVDLCDSQVVTTFLGRFWSLLNY
VQVLFLSEDSAKILAGIPDLAQATQLLSHTVPLLFIYTNDSIHIIEQGKESSFTYNQDLTEPILGFLFGYINRGSM
EYCFNCAQSSLGET

>core/502/5/Org5_Gene13

MIASIYSFLDYLKMVKSASPHTLRNYCLDLNGLKIFLEERGNLAPSSPLQLATEKRKVSELPFSLFTKEHVRM
YIAKLIENGKAKRTIKRCLSSIKSFAHYCVIQKILLENPAETIHGPRLPKELPSPMTYAQVEVLMATPDISKYHG
LRDRCLMELFYSSGLRISEIVAVNKQDFDLSTHLIRIRGKGKKERIIPVTSNAIQWIQIYLNHPDRKRLEKDPQA
IFLNRFGRRISTRSIDRSFQEYLRRSGLSGHITPHTIRHTIATHWLESGMDLKTIQALLGHSSLETTTVYTQVSV
KLKKQTHQEAHPHA

>core/503/5/Org5_Gene242

MIHSRLIIIGSGPSGYTAAIYASRALLHPLLFEGFFSGISGGQLMTTTEVENFPGFPEGILGPKLMNNMKEQAVR
FGTKTLAQDIISVDFSVRPFILKSKEETYSCDACIIATGASAKRLEIPGAGNDEFWQKGVTACAVCDGASPIFK
NKDLYVIGGGDSALEEALYLTRYGSHVYVVHRRDKLRASKAMEARAQNNEKITFLWNSEIVKISGDSIVRSV
DIKNVQTQEITTREAAGVFFAIGHKPNTDFLGGQLTLDESGYIVTEKGTSKTSVPGVFAAGDVQDKYYRQAV
TSAGSGCIAALDAERFLG

>core/504/5/Org5_Gene825

MRKLILCNPRGFCSGVVRAIQVVEVALEKWGAPIYVKHEIVHNRHVVNALRAKGAIFVEELVDVPEGERVIY
SAHGIPPSVRAEAKARKLIDIDATCGLVTKVHSAAKLYASKGYKIILIGHKKHVEVIGIVGEVPEHITVVEKVA
DVEALPFSSDTPLFYITQTTLSLDDVQEISSALLKRYPSIITLPSSSICYATTNRQKALRSVLSRVNYVYVVGDV
NSSNSNRLREVALRRGVPADLINNPEDIDTNIVNHSGDIAMTAGASTPEDVVQACIRKLSSLIPGLQVENDIFA
VEDVVFQLPKELRCS

>core/505/5/Org5_Gene672

MKCRPTLNTDQPRVRKKLPERFPKWLQRPLPQGSAFHATDATIKRSGMPTVCEEALCPNRAECWSRKTATY
LALGDVCTRSCGFCNIGHSKTPPALDPTEPERIALSAKELGLKHVVITMVARDDLEDGGAQGLVDIIQKLREE
LPQATTEVLASDFQGNVSALHTLLDSGITIYNHNVETVARLSPLVRHKATYARSMFMLEQAANYLPDLKIKS
GIMVGLGEMEGEVKQTLQDLASIGVRIVTIGQYLRPSRKHLQVKSYVTPETFDYYRRVGEAMGLFVYAGPFV
RSSFNADMILASVQDKASA

>core/507/5/Org5_Gene227

MKKRYAFLFPGQGSQYVGMGQDLYMEYPEVRELFDFANERLGFSLTSIMFEGPEDLLMETVHSQLAIYLHS
MAVVKVLSQRSSIQPSLVSGLSLGEYTALVASDRISVLDGLELVRKRGQLMNEACNQSPGAMAALLGLPSEV
IEENITSLGQGIWIANYNAPKQLVVAGIAEKVDQAIELFRDLGCKKAVRLKVSGAFHTPLMQVAQDGLAPDI
YALCMKDSSLPLVSHVVGKSLVNTEEMRECLARQMTSPTLWYQSCYHIESEVDEFLELGPGKVLAGLNRSIG
ISKPITSLGTFAQIEKFLSEV

>core/508/5/Org5_Gene435

MLNSNKFKSKTGAYGDLFQRVVVHSLVLTFLVLLLYSSLFPLTSFALGFITATCGAVGTYEYSSMAKAKMHY
PLSTFSAIGSFLFLALSFLSIRWGHSLPGFFDALPWTLLIVWVVWSIFRVRKSTIGALQLSGVTLFSILYVGIPIRL
FLHVLYSFIHTQEPYLGIWWASFLIATTKGADIFGYFFGKAFGNKKIAPQISPNKTVVGFVAGCLGATLISFIFF
LQIPTRFASYFPMPAILIPLGLALGITGFFGDIIESIFKRDAHLKNSNKLKAVGGMLDTLDSLLLSTPIAYLFLLIT
QSKEFIG

>core/509/5/Org5_Gene35

MRLFSYDKPKIKVQKIKADGFSGWLKCNHCHEMIHANELGQNYNCCPKCSYHYRITAIERVKLLADKDSWR
PLYTDLKSQDPLEFIDTDTYANRLEKARKNTTESEGVIVGICTIGLHPVALAVMDFNFMAGSMGAVVGEKLT
RLIEEAIETRLPVIIVSASGGARMQESVFSLMQMVKTSAALAKLHEAGLPYISVLTNPTSGGVTASFAALGDIII
AEPKALICFAGPRVVAQVIGEDLPEGAQKSEFLLEHGMIDKIVERKELKTTLQTLLDYFLAQEYTGGKSKAPR
DLSKRLKEIFLLTDDSE

>core/510/5/Org5_Gene932

MPMEIAYSLTSSFSVDSVTVGFFDGCHLGHSNLLSILTSYSGSSGVITFDSHPQTVLSLNHTKLINTKEERLQLL
QTFPIDWLGVLTFDLNFANQSAEEFLTLLHRNLKCKRLILGYDSCIGKEQQSNTEALDTIGKPLGIEVIKIPPYR
MDNIVVSSKAIRQFLSAGNLECAHRFLGHPYAISGKITEGSGIGGSLGFATINLPREESLIPLGVYACEIRYDSTT
CQGVMNLGTAPTFERESLYAEAHIFSFAENLYGKEVSIIPRKFLREEKKFQSKETLIRAIEKDILDAQDWFAKG
SFNYEGTA

>core/511/5/Org5_Gene14

MSSRELIILGCSSQQPTRTRNQGAYLFRWNGEGLLFDPGEGTQRQFIFANIAPTTVNRIFVSHFHGDHCLGLGS
MLMRLNLDKVSHPIHCYYPASGKKYFDRLRYGTIYHETIQVVEHPISEEGIVEDFGSFRIEAQRLQHQVDTLG
WRITEPDTIKFLPKELESRGIRGLIIQDLIRDQEISIGGSTVYLSDVSYVRKGDSIAIIADTLPCQAAIDLAKNSCM
MLCESTYLEQHRHLAESHFHMTAKQAATLAKRAATQKLILTHFSARYLNLDDFYKEASAVFPNVSVAQEYR
SYPFPKNPLLNK

>core/512/5/Org5_Gene666

MRSIFRFSLCFFTLSVSCCFADASLYENSCPSRCQPTPPPSNSNPLNVVQQPVAASSVPSYMPPLNADDVLPRD
HLSDGSFSDTYPDITTQAIILIFLALSPFLVMLLTSYLKIIITLVLLRNALGVQQTPPSQVLNGIALILSIYVMFPT
GVAMYKDARKEIEANTIPQSLFTAEGAETVFVALNKSKEPLRSFLIRNTPKAQIQSFYKISQKTFPSEIRAHLTA
SDFVIIIPAFIMGQIKNAFEIGVLIYLPFFVIDLVTANVLVAMQMMMLSPLSISLPLKLLLIVMVDGWTLLLQGL
MISFK

>core/513/5/Org5_Gene991

MKEAAPMHFPFPVRRSVWLNRYSTFRIGGPANYFKAIHTIEEAREVIRFLHSINYPFLIIGKGSNCLFDDRGFD
GFVLYNAIYGKQFLEDARIKAYSGLSFAALGKATAYNGYSGLEFAAGIPGSVGGAIFMNAGTNESDISSVVRN
VETINSEGELCSYSVEELELSYRSSRFHRQQEFILSATLQLSKKQVSADHSKSILQHRLMTQPYTQPSAGCIFRN

PEGTSAGKLIDAAGLKGLAIGGAQISPLHANFIINTGKATSDEVKQLIAIIQSTLKTQGIDLEHEIRIIPYQPKIHS
PVSEK

>core/514/5/Org5_Gene650

MSLFLVFLTAFIWSSSFALSKLVMNASAPIFATGARMVIAGAILALAAWFRGGFVGISKKIFLYIVLLALTGFY
LTNIFEFIGLQSLSSSKTCFIYGLSPLMSALFSYIQLKEKVTLKKVLGLSLGLVSYICYLTFGGGGDDSQPWTW
QIGLPELLILGAASLASFGWTLLRQIEKQSTLSVTAINAYAMLIAGMLSIMHSAVVEPWRPLPVQDISQFLYAT
LALVVISNLICYNLYAKLLRKYSSTFLSFCNLVMPLYSGFYGWILLGEKGVSLGLVLAVAFMVAGCRLIYHEE
FRQGYIVS

>core/515/5/Org5_Gene678

MQKPQYIDRITKKKVIEPIFYEKTMLFLYNSKLGKKLSVFLSTHPIFSRIYGWLQRCSWTRRQIRPFMNRYKIS
EKELTKPVADFTSFNDFFTRKLKPEARPIVGGKEVFITPVDGRYLVYPNVSEFDKFIVKSKAFSLPKLLGDHEL
TKLYAHGSIVFARLAPFDYHRFHFPCDCLPQKTRCVNGALFSVHPLAVKDNFILFCENKRTVTVLETEQFGNV
LYLEVGAMNVGSIVQTFSPNQTYAKGDEKGFFAFGGSTVILLFLPNAIRFDNDLLKNSRMGFETRCLMGQSL
GRSQREEI

>core/516/5/Org5_Gene831

MTMPSTQFHTTILEQFSLFLSVDRGLCQQSIAAYRQDISSFLTISAISSPQDISQNSVYIFAEELYRRKEAETTLA
RRLIALKVFFLFLKDQQLLPYPPIIEHPKIWKRLPSVLTPQEVDALLAVPLQMEKNPRHLAFRDTAILHTLYST
GVRVSELCDLRLGHVSDDCIRVTGKGSKTRLVPLGSRAREAIDAYLCPFRDQYQKKNPHEDHLFLSTRGHKL
ERSCVWRRIHNYAKQVTSKPVSPHSLRHAFATHLLDNKADLRVIQEMLGHARIASTEVYTHVAADSLIEKFL
AHHPRNL

>core/517/5/Org5_Gene314

MLKIDLTGKVAFVAGIGDDQGYGWGIAKLLAEAGATIIVGTWVPIYKIFSQSWELGKFNESRKLSNGTLLEIA
KIYPMDASFDSPEDVPEDIAENKRYKGITGFTISEVAEQVKKDFGHIDILVHSLANSPEISKSLLETSRKGYLAA
LSASSYSFVSLLSHFGSIMNRGGSTISLTYLASMRAVPGYGGGMSSAKAALESDTKTLAWEAGRRWGIRVNT
ISAGPLASRAGKAIGFIERMVDYYQEWAPIPEAMNAEQVGAVAAFLASPLASAITGETLYVDHGANVMGIGP
EMFPKDS

>core/518/5/Org5_Gene869

MPPPFVVTLTTSAQNNLRDQLKEKNFIFSQPQNTVFQARSNTVTCTLYPSGKLVIQGKGSEEFIEFFLEPEILHT
FTHARVEQDLRPRLGVDESGKGDFFGPLCIAAVYASNAEILKKLYENKVQDSKNLKDTKIASLARIIRSLCVC
DVIILYPEKYNELYGKFQNLNTLLAWAHATVINNLAPKPAGDVFAISDQFAASEYTLLKALQKKETDITLIQK
PRAEQDVVVAAASILARDAFVQSIQKLEEQYQVQLPKGAGFNVKAAGREIAKQRGKELLAKISKTHFKTFDE
ICSGK

>core/519/5/Org5_Gene207

MHLEEENQGWEALLRKVYHQEVPPAILLHGFTLPVLQDKAEQLASEILLSSSPGSEHKVSQKIHPDIYQFFPEG
KGRLHSIDLPRGIKKQIYISPFEANYKIYIIHEADRMTLAAISAFLKVFEEPPKHAVIILTTAKVQRLPKTIISRSLS

IFIERGEKILCSKETFSYLFRYAQCEIPVTEVSQIIKESSETDKQVLRDKVQRFMEVLLELYRDRYTLNLGLKAS
ALNYPEHVKEILQLPLLPLDKVLLIVESACRSLNNSSSAASVLEWVAIQLVSLQYKEKELVSVSPGQDLSN

>core/520/5/Org5_Gene200

MRLNYFLNLVNFKYSIFSILFLSASTVFALSINEISQNLSFKEGFKISVFGAIAFVFARTTGIVVNQCIDRFIDKKN
TRTSKRVLPANLVSLNFAWVLSLFCSFLFLFLCKILRIFSLGIASLTLMIVYPYMKRVTFFCHWGLGLVYTVAI
LMNFCAFAESGLSMRLCFLALLWGGSVGMVIAANDIIYAIEDTEFDREEGLRSVPAHYGEKKAVEIAKVNLW
VSYLAYIFSGFVGSLDKEFYFTAIIPLVVILKVVRMYSNYSKKDQEGESKFFLANIAIALSFLVSMTLFWSLSR

>core/521/5/Org5_Gene328

MSERAHIPVLVEECLALFAQRPPQTFRDVTLGAGGHAYAFLEAYPSLTCYDGSDRDLQALAIAEKRLETFQD
RVSFSHASFEDLANQPTPRLYDGVLADLGVSSMQLDTLSRGFSFQGEKEELDMRMDQTQELSASDVLNSLKE
EELGRIFREYGEEPQWKSAAKAVVHFRKHKKILSIQDVKEALLGVFPHYRFHRKIHPLTLIFQALRVYVNGED
RQLKSLLTSAISWLAPQGRLVIISFCSSEDRPVKWFFKEAEASGLGKVITKKVIQPTYQEVRRNPRSRSAKLRC
FEKASQ

>core/522/5/Org5_Gene635

MSRHEICPEVSHKKGKYYSTFIFRCIHSLAGIAFTFFLCEHLFTNMLASSYFSQGKGFVAMVNGFHKIPGLKIIE
VAGLVLPFLCHAIIGIVYLFQGKSNCYSGDGSRPHLRYAKNYSYTWQRWTAWILLFGIAFHVVHLRFIRYPVH
VDIHGTTYYAVDIQPSRYDVIVRGTKGFLTLNLPNTEASSIEVSRHDLGGADAALLSERNSYLLTPSAGTAFLY
VVRDALGSLFIALLYTILVIAAAFHGFNGLWTFCCRWGVVVSLRMQGVLRIVCYLAMIVVTFMGVSAVWNL
YSVA

>core/524/5/Org5_Gene942

MRRFLFLILSSLPLVAFSADNFTILEEKQSPLSRVSIIFALPGVTPVSFDGNCSIPWFSHSKKTLEGQRIYYSGDSF
GKYFVVSALWPNKVSSAVVACNMILKHRVDLILIIGSCYSRSQDSRFGSVLVSKGYINYDADVRPFFERFEIPD
IKKSVFATSEVHREAILRGGEEFISTHKQEIEELLKTHGYLKSTTKTEHTLMEGLVATGESFAMSRNYFLSLQK
LYPEIHGFDSVSGAVSQVCYEYSIPCLGVNILLPHPLESRSNEDWKHLQSEASKIYMDTLLKSVLKELCSSH

>core/525/5/Org5_Gene198

MSHGPRPTKFSFPLYFSKTLSWFILGGFLAACGVQMVLVPNELIDGGIVGLSIIASHFLGHKALPFCLVLFNLPF
VFLAFKQIGKYFVIQMLTAVIIFSCSLWLIDQLPSWLGMSPFVFKGSEMETVVLGGAIIGVGCGLIIRHGGSTD
GTEILGIIINKKKGYTVGQIILFVNFFIFALSGIVYKNWHTAFVSFLTYGIATKVMDMVILGLEDTKSVTIITSSP
RKLGHILMETLGIGLTYIHAEGGYSGEPRNLLYVVVERLQLSQLKEIVHREDPSAFIAIENLHEVINGRRT

>core/526/5/Org5_Gene726

MQEKPRHVHRIIHISDVHFHVLPVNPVHCFNKRLKGLLRKVFGLVHFQATTIGQRFPKVVRSLGADSVCITGD
FSLTAMDGEFLLAKHFVETLAKHSSVYLLPGNHDVYTLKSLAQQTFYTHFPNDQLQQNKVSFHKITDHWWL
ILLDCSCLNGWFSANGVVHLAQISAIETFLLSLSPEENVIIANHYPLLSSQNPSHDLINNTHLQNVLKKYPKVRL
YLHGHEHQAAVYNCADTSPSYILNSGSISLPTNSRFHVIDLYPEKYQVHTMILKNLLDFDAPLEIANEATWDC
QKL

>core/527/5/Org5_Gene315

MEKLLVTDIDGTITHQSHHLDKKVYERLYALHQAGWKLFFLTGRYYKYAARLFSDFDAPYLLGCQNGASVWSSTSSNLLYSKSLPSDLLCILQDCMEGATALFSVESGAPYGDHYYRFSPTPIAQDLHEYVDPRYFPNAKEREILFETRSLKDDYAFPSFAAAKVFGLRDEVIRIQKELERQEALTSVATMTLMRWPFDFRYAILFLTDKSVSKGKALDRVVNILYDGKKPFVMASGDDANDLDLIERGDFKIVMSSAPEEMHVHADFLAPPADKNGILSAWEAGVRYYDDLMSL

>core/528/5/Org5_Gene527

MKKLILYFAAFVASLFCGVFLWDRVPCAQKIMRLAADHSSEVFSKSCRFVRKISGFEELQVFERHVSPEQALALFPEYRDGKSFVELAFIPHTLMHVRFSKEEPVKKHIISQEGEILWSLVNGEMVLHTGTWTCSKGFRECLLLHAGKQDMRVIQTLATLGGTTSRESLAQALALKNIRAERVIKECQKKKLIFASGNQIGTHFQQFQPIRGCTTTLNNNPVWLQKPRHAAVFPAQYSEDRVRHLVKMIFGDNFLIVRSSMVYVPVYKISLVSADNSVRVEYINAVTGKSFQDL

>core/529/5/Org5_Gene789

MFHSLSKNTPIITQGITGKAGSFHTEQCLAYGTNFVGGVTPGKGGTLWLDLPVYDSVLEAKQATGCRATMIFVPPPYAAEAILEAEEAGIELIVCITEGIPVRDMLEVARVMDNSTSQLIGPNCPGIIKPGECKIGIMPGYIHLPGNIGVVSRSGTLTYEAVWQLTQLKIGQSICVGIGGDPLNGTSFIDVLQALEEDPYTELILMIGEIGGSAEEEAAAWIQAHCTKPVVAFIAGVTAPKGKRMGHAGAIISGNSGDAKSKIQVLRESGVTVVESPAHIGKTVDAVLRAKEL

>core/530/5/Org5_Gene723

MSKHTSESRIAQDMLERYSGSSVKQFCPYLLLTNFSYYIQTFAKLHGVPVFEGSMFSAAHAPHLKTSILDFKLGSPGAALTIDLCSFLPDLKAALMLGMCGGLRSHYQVGDYFVPVASIRGEGTSDAYFPPEVPALANFVVQKATTEVLEDKKANYHIGITHTTNIRFWEFNKKFRKKLYETKAQSAEMECATLFAAGYRRNLPIGALLLISDLPLRKEGIKTKSSGNFIFNTYTEDHILTGQEVIENLEKVMLKRAASDHKKDQQYRGLPHMEVGEADDTMASGSETSDSDY

>core/531/5/Org5_Gene588

MKVLPPPSIPLLGAHTSTAGGLKNAIYEGRDIGASTVQIFTANQRQWQRRALKEEVIEDFKAALKETDLSYIMSHAGYLINPGAPDPVILEKSRIGIYQEILDCITLGISFVNFHPGAALKSSKEDCMNKIVSSFSQSAPLFDSSPPLVVLLETTAGQGTLIGSNFEELGYLVQNLKNQIPIGVCVDTCHIFAAGYDITSPQGWEDVLNEFDEYVGLSYLRAFHLNDSMFPLGANKDRHAPLGEGYIGKESFKFLMTDERTRKIPKYLETPGGPENWQKEIGELLKFSKNRDS

>core/532/5/Org5_Gene412

MLSSLIRDSFPLLILLPTFLAALGASVAGGVMGTYIVVKRIVSISGSISHAILGGIGLTLWIQYKLHLSFFPMYGAIVGAIFLALCIGKIHLKYQEREDSLIAMIWSVGMAIGIIFISRLPTFNGELINFLFGNILWVTPSDLYSLGIFDLLVLGIVVLCHTRFLALCFDERYTALNHCSVQLWYFLLLVLTAITIVMLIYVMGTILMLSMLVLPVAIACRFSYKMTRIMFISVLLNILCSFSGICIAYCLDFPVGPTISLLMGLGYTASLCVKKRYNPSTPSPVSPEINTNV

>core/533/5/Org5_Gene820

MKRNDPCWCGSGRKWKQCHYPQPPKMSPEALKQHYASQYNILLKTPEQKAKIYNACQITARILDELCKASQ
KGVTTNELDELSQELHKKYDAIAAPFHYGSPPFPKTICTSLNEVICHGIPNDIPLKDGDIMNIDVSCIVDGYYG
DCSRMVMIGEVPEIKKKICQAALECLNDSIAILKPGIPLCEIGEAIEARADTYGFSVVDQFVGHGVGIEFHENPY
VPHYRNRSMIPLAPGMIFTIEPMINVGKKEGVVDPKNQWEARTCDNQPSAQWEHTIAITETGYEILTLLND

>core/534/5/Org5_Gene853

MHLLTATVTPFFPNGTIDFASLERLLSFQDAVGNGVVLLGSTGEGLSLTKKEKQALICFACDLQLKVPLFVGT
SGTLLEEVLDWIHFCNDLPISGFLMTTPIYTKPKLCGQILWFEAVLNAAKHPAILYNIPSRAATPLYLDTVKAL
AHHPQFLGIKDSGGSVEEFQSYKSIAPHIQLYCGDDVFWSEMAACGAHGLISVLSNAWPEEAREYVLNPQEQ
DYRSLWMETCRWVYTTTNPIGIKAILAYKKAITHAQLRLPLSIEDFDLENVSPAVESMLAWPKLRTSVFSYS

>core/535/5/Org5_Gene787

MFKFFRNKLQSLFKKNISLDLIEDAESLFYEADFGTELTEELCARLRRTKKADASTIKDLITVLLRESLEGLPSQ
ASQSSQTRPIVSLLLGTNGSGKTTTAAKLAHYYKERSESVMLVATDTFRAAGMDQARLWANELGCGFVSGQ
PGGDAAAIAFDGIQSAIARGYSRVIIDTSGRLHVHGNLMKELSKIVSVCGKALEGAPHEIFMTVDSTLGNNAIE
QVRVFHDVVPLSGLIFTKVDGSAKGGTLFQIAKRLKIPTKFIGYGESLKDLNEFDLDLFLNKLFPEVEKI

>core/536/5/Org5_Gene194

MKRRNLQKILPNASTPSTNVAENTGIKDQNLFLDQATLNVDGNVDIENFLETRDLKVADTITSPCEFTVGGGL
SAESSQFKATTLSKGLEITSEDQDGRVPKFTNVSDPQSPRDALTYNYYRNTGCQALNLYTYYSSSQPTTVGKP
IETVCQNPNPETYRISASAKIYDAVTRFPYIQFKAPGIYQVTIQIRRESGQHSGLDNPNLYLNLMIGNNKTLLCA
SDTRGYSGGHRTSIAVTGTFTLTEIVATPPHDYPWLFLETTIGLDIKSMSTCVIWFPFQANFAEVD

>core/537/5/Org5_Gene1032

MQIPRSIGTHDGSFHADEVTACALLIIFDLVDENKIIRSRDPVVLSKCEYVCDVGGVYSIENKRFDHHQVSYDG
SWSSAGMILHYLKEFGYMDCEEYHFLNNTLVHGVDEQDNGRFFSKEGFCSFSDIIKIYNPREEEETNSDADFS
CALHFTIDFLCRLRKKFQYDRVCRGIVREAMETEDMCLYFDRPLAWQENFFFLGGEKHPAAFVCFPSCDQWI
LRGIPPNLDRRMEVRVPFPENWAGLLGKELSKVSGIPGAVFCHKGLFLSVWTNRESCQRALRLTLQDRGII

>core/538/5/Org5_Gene664

MGISLPELFSNLGSAYLDYIFQHPPAYVWSVFLLLLARLLPIFAVAPFLGAKLFPSPIKIGISLSWLAIIFPKVLA
DTQITNYMDNNLFYVLLVKEMIIGIVIGFVLAFPFYAAQSAGSFITNQQGIQGLEGATSLISIEQTSPHGILYHYF
VTIIFWLVGGHRIVISLLLQTLEVIPIHSFFPAEMMSLSAPIWITMIKMCQLCLVMTIQLSAPAALAMLMSDLFL
GIINRMAPQVQVIYLLSALKAFMGLLFLTLAWWFIIKQIDYFTLAWFKEVPIMLLGSNPQVL

>core/539/5/Org5_Gene601

MLHALDTYRPSIESAIEKALEGFGPIGHPIRSPVEYALQGGGKRLRPGLVCMMAQGLGLNHDVMDSALAVEF
VHTSTLIADDLPCMDNDDERRGRPTVHKAFDEATALLASYALIPAAYSHLRLNAKKLKEQGCDPREIDIAYNI
IGDITDKNIGCSGVLGGQYDDMFFSNRGQEHVQSIMIKKTGSLFEIACISGWLFGGGDPQFAPIITSFSNNFGLL
FQIKDDFSDLQKDSQQIGLNYALLFGEKAALELLARSQNNCLELLDRLSAGGLKNSSEFETIISSLGSF

>core/540/5/Org5_Gene193

MSNPTPKTKISIPTFVRFNIQSINLTEDQKKTTFTVGGKVTTENTVVRGDLTCTDGGLTCQSDLTIQKDINIRPT
STNSMVFDGRLNLSNSPLSYKNSQGQDITDYEKMSSGKPQEYVPFGYYKRTQIMMAQRAAHSSGYVGGGSV
PSGSYVPWNKFDQTSTQKTSGTEIYIDPNDSTKLVFEVNNKVPKLFRISVIMAKHGSWLDNGTGADILLAANE
YEQGGGRINVTDLAMTTSRGSSYYETRPLQVVCVTYYAQNNGYFTFQNRAGGGLRVSFFSWNIVALPYVE

>core/541/5/Org5_Gene873

MLKIQKKRMCVSVVITVGAIVGFFNSADAAPKKKKIPIQILYSFTKVSSYLKNEDASTIFCVDVDRGLLQHRY
LGSPGWQETRRRQLFKSLENQSYGNERLGEETLAIDIFRNKECLESEIPEQMEAILANSSALVLGISSFGITGIPA
TLHSLLRQNLSFQKRSIASESFLLKIDSAPSDASVFYKGVLFRGETAIVDALSQLFAQLDLSPKKIIFLGEDPEV
VQAVGSACIGWGMNFLGLVYYPAQESLFSYVHPYSTATELQEAQGLQVISDEVAQLTLNALPKMN

>core/542/5/Org5_Gene978

MSHLIPSLRNSVTSYFHKPQPIKQAAPSKSIRDICNIAYLIIICVLVVVVLVGAMLCMFIPSVGIPLCLSSLALLV
LLSIFNPCLINWISTKKTKEIAPKDASESQPTKSASRKGSPQLSPHHDHEPKNFIRTQLEKGVNYVTNKFKSGEE
SPHISDEHHSPRQSKRSSEIESSDESSPELHRKAKGKAPHTATTKGSKTSTTESSKKKKKTKHSLHRTTSSIHKR
SAPKPMVPSKKRKPVLLKKTVPLPIEDLEHQSSGNESSDSSSPPPVQRKAILPWFCKQPTDP

>core/543/5/Org5_Gene79

MEIKKAIQEGTAYLDYYGVPLSDCEALYILMDLLEVSSRAKLFDLVGISETMLMEYRKRLALRGQRCPTAYL
NGAVSFLGLRLRVDSRVLIPRTETELLAEYIINYLLSHSEIQTFYDICCGSGCLGLAIKKSCPHVEVVLSDVCPQ
AVAVANENAKSNGLDVKILLGDLSAPYTRPADAFVCNPPYLSFNEIIHIDPEVRCYEPWKALVGGSTGLEFYQ
RIAQELPKIVTSTGVGWLEIGSSQGESIKNIFSKHGIYGRLHQDLSGRDRIFFLEMDGRDPVSSGAYS

>core/544/5/Org5_Gene239

MAVIYWDRSKIVWSFEPWSLRLTWYGVFFTVGIFLACLSARYLALSYYGLKDHLSFSKSQLRVALENFFIYSI
LFIVPGARLAYVIFYGWSFYLQHPEEIIQIWHGGLSSHGGVLGFLLWAAIFSWIYKKKISKLTFLFLTDLCGSVF
GIAAFFIRLGNFWNQEIVGTPTSLPWGVVFSDPMQGVQGVPVHPVQLYEGISYLVVSGILYFLSYKRYLHLGK
GYVTSIACISVAFIRFFAEYVKSHQGKVLAEDCLLTIGQILSIPLFLFGVALLIICSLKARRHRSHI

>core/545/5/Org5_Gene260

MLLRGIPAAEKILQRLKEEISQSPTSPGLAVVLIGNDPASEVYVGMKVKKATEIGIISKAHKLPSDSTLSSVLKL
IERLNQDPSIHGILVQLPLPKHLDSEVILQAISPDKDVDGLHPVNMGKLLLGNFDGLLPCTPAGIIELLNYYEIP
LRGRHAAIVGRSNIVGKPLAALMMQKHPQTNCTVTVLHSQSENLPEILKTADIIIAALGAPLFIKETMVAPHA
VIVDVGTTRVPADNAKGYTLLGDVDFNNVVTKCAAITPVPGGVGPMTVAMLMSNTWRCYQNFS

>core/546/5/Org5_Gene543

MTEEISKDTIIEVAIDDIRVSPFQPRRVFSNEELQELIASIKAVGLIHPPVVREICTGDRVLYYELIAGERRWRAM
QLAGATTIPVILKHVIADGTAAEATLIENIQRVNLNPIEMAEAFKRLIHVFGLTQDKVAYKVGKKRSTVANYL
RLLALSKTIQESLLQGQITLGHAKVILTLEDPILREKLNEIIIQEHLAVREAELIAKQLISEEGSSIELKPTPLDMA
ESSKQHEELQQRLSDLCGYKVQIKTRGSKATVSFHLQNTQDLQKLEAWLSSHGTLSESLS

>core/547/5/Org5_Gene205

MPDKKAQITFSLPEVMSAIHQGKIVALPTDTVYGFVLSLYASEAEERLYALKDREPSKAFALYVNSIEDIENIS
GYPLSPTAKKLAQLFPGAITLVVKHRNPRFPKETLAFRIVDHSVVREIVDHCGTLIGTSANLSEFPSALTAQEIF
ADFADHDLCIFDGPCSHGLESTVVASDPLYIYREGLISRSVIENIAGTEAKIFHRTSHAFSKHIKIYTVKNQEQL
VSFLSGSLDFKGVVCEHPKPKNFYTRLREALKKKTPSIVFIYDINTSDYPELFPFLSPYYIE

>core/548/5/Org5_Gene192

MKNNINNNECYFKLDSTVDGDLLAANLKTFDTQAQGISSTETFSVQGNATFKDQVSATGLTSGTTYNLNAQ
NFTSSQISIDFKNNRLSNCALPKEDCDPVPANYVRSPEYFFCSKPLIGDFDFNSGESYLPLTGSEYTLYQSRNVN
SIFRFIGWKQSTRELTVGGNTAIQFLAAGTYIVSFTVGKRWGWNNGWGGAIYINNGLGQVQCESTIYSGGGY
ATIGTLGTSIYRASVDVAPNPNDPNASDRYRAGIFYLSNGGSSAGIGNYSFSLLYYPDDRG

>core/549/5/Org5_Gene363

MHEVLILTFTYPLPRTLKQHPDEVHTVPISPNLSFGEGSPILIAGPCTLESYEHTVSSALTVKEAGAQVFRGSIR
KPRTSPFSFQGWEKECVLWHKEAQSIHGLPTETEVLDVRDVEITAEHVDILRIGAKNMHNTPLLQEVSKSHRP
IILKRSPAATLEEWLCAAEYILASSPSCPGVILCERGIRTFEHSTRYTLDLNTVALLKEISSLPVIVDPSHAAGKR
SLVLPLASAGLSVGADGLMIEVHAHPEKALCDAKQQITPEELHLFAKKHFCPSESRAHAIS

>core/550/5/Org5_Gene185

MKHYLSFSPSADFFSKQGAIETQVLFGERVLVKGSTCYAYSQLFHNELLWKPYPGHSFRSTLVPCTPEFHIHP
NVSVVSVDAFLDPWGIPLPFGTLLHVNSQNTVIFPKDILNHMNTIWGSGTPQCDPRHLRRLNYNFFAELLIKD
ADLLLNFPYVWGGRSVHESLEKPGVDCSGFINILYQAQGYNVPRNAADQYADCHWISSFENLPSGGLIFLYP
KEEKRISHVMLKQDSSTLIHASGGGKKVEYFILEQDGKFLDSTYLFFRNNQRGRAFFGIPRKRKAFL

>core/551/5/Org5_Gene944

MDNYLLNIKDLTITSTNPKRTLIENLSLQLKENRNLALVGESGSGKTTITKAILGFLPENCLIKTGSILFEDIDIT
KLSPKELHKIRGQKIATILQNAMGSLTPSMRIGMQIIETLRQHHKMNKEEAYNKAMQLLTDVCIPNPKYSFSQ
YPFELSGGMRQRVVIAIALASQPKLILADEPTTALDSMSQAQVLRILRNXQQQKQATILLVTHNLSLVKELCN
DICIIKDGKLIETGTVEEIFLSPKHPYTLKLLNAVSKIPIKKTSSPILKNKFQPLMSMQGGL

>core/552/5/Org5_Gene145

MENLSSAPSRSIWKSIIQNKMLVLGLTTLIILMLGALLLPWFYQDYEQTSLKDILVSPCSRFPFGTDTLGRCMF
ARTLRGLRLSLLIATIATLIDVCVGLLWATVAISGGKKIDFLMMRTTEILFSLPRIPIIILLLVIFHHGLLPLILAM
TITGWIPISRIIYGQFLLLKNKPFVLSAKAMHASTFHILKKHLLPNTLAPIISTLIFTIPNAIYTEAFISFLGLGIQPP
QASLGTLVKEGINAIDYYPWLFFFPSLIMIALSISFNLIGEGAKTLCLEEGSHG

>core/553/5/Org5_Gene127

MKQPMSLIFSSVCLGLGLGSLSSCNQKPSWNYHNTSTSEEFFVHGNKSVSQLPHYPSAFRTTQIFSEEHNDPY
VVAKTDEESRKIWREIHKNLKIKGSYIPISTYGSLMHPKSAALTLKTYRPHPIWINGYERSFNIDTGKYLKNGS
RRRTSHDGPKNRAVLNLIKSSGRRCNAIGLEMTEEDFVIARRREGVYSLYPVEVCSYPQGNPFVIAYAWIADE
SACSKEVLPVKGYYSLVWESVSSSDSLNAFGDSFAEDYLRSTFLANGTSILCVHESYKKVPPQP

>core/554/5/Org5_Gene505

MFKKFKPVTPGTRQLVLPAFDELTTRGELRGTKSKRSLRPNKKLSFFKKSSGGRDNLGHISCRHRGGGAKQL
YRVVDFKRNKDGITAKVVTVEYDPNRSAYIALLSYEDGEKRYILAPKGIQRGDVVVSGEGSPFKPGCCMTLK
SIPLGLSVHNIEMRPSSGGKLVRSAGLAAQVIAKSPGYVTLKMPSGEFRMLNEGCRATIGEVSNADHNLRVD
GKAGRRRWMGVRPTVRGTAMNPVDHPHGGGEGRHNGYIPRTPWGKVTKGLKTRDKNKSNKWIVKDRRK

>core/556/5/Org5_Gene510

MASIHPTAIIEPGAKIGKDVVIEPYVVIKATVTLCDNVVVKSYAYIDGNTTIGKGTTIWPSAMIGNKPQDLKYQ
GEKTYVTIGENCEIREFAIITSSTFEGTTVSIGNNCLIMPWAHVAHNCTIGNNVVLSNHAQLAGHVQVGDYAIL
GGMVGVHQFVRIGAHAMVGALSGIRRDVPPYTIGSGNPYQLAGINKVGLQRRQVPFATRLALIKAFKKIYRA
DGCFFESLEETLEEYGDIPEVKNFIEFCQSPSKRGIERSIDKQALEEESADKEGVLIES

>core/557/5/Org5_Gene555

MSDFSMETLKTLRQQTGVGLTKCKEALEACGGNLEEAVVYLRKLGLASAGKKEHRETKEGIIAAKTDANGT
ALIEVNVETDFVANNAVFREFVSNLLNDILKYKVDTVEALSQAASSQDPSLSVDELRAVTMQTVGENIRISRV
AYFPKATNSTVGIYSHGNGKTVALTMLSGSSTADSLAKDIAMHVVAAQPQFLSKESVPAEAIAKEKEVIASQI
QGKPQEVIEKIVTGKLNTFFQEACLLEQPFIKNADLSIQSLIDDFSKTSGSSVAIEQFILWKIGA

>core/558/5/Org5_Gene512

MLERTQRTLKREVRYSGVGIHLGKSSTLHLQPAQTNTGIVFQRQSASGNYENVPALLDHVYTTGRSTTLSRG
SAVIATVEHLMAALRSNNIDNLIIQCSGEEIPIGDGSSNVFVELIDQAGICEQEDKVSIARLTRPVYYQHQDIFL
AAFPSDELKISYTLHYPQSSTIGTQYKSLVINEESFRQEIAPCRTFALYNELCFLMEKGLIGGGCLDNAVVFKD
DGIISRGQLRFADEPVRHKILDLIGDLSLVGRPFVAHVLAVGSGHSSNIAFGKKILEALEL

>core/559/5/Org5_Gene604

MATAHLGRQALLHLRSWTPAIRASGNLFRQQSMSLHNNVLFAGDIVGAIKNSTAISRHALGSSHYAHAALQK
TEGFLGAADGVNTAVAGAMLWGQLLNGSMIFETDEETGELRRCNEADAEGCMTQKLQRRSALTITGKVAR
LASKTLGTATFLHEMDVVSLGANANKIGCKVTSCLNLVATGCSLTESSISLYRILSTRPETISDPENRNKPSAEF
AARSKAIRNAFIAWLGDVVDLVCDALGTLSLFLPAILGVHAVLIMAILGLISCVINFVKDYAKIG

>core/560/5/Org5_Gene197

MVLMNKRLKIILTNDDGITAKGMSCLVSALLEANIGDIYIAAPQAEQSGKSMAISLNQVVCASPYAYPQPVKE
AWAVGGSPTDCVRLGLRTLFESVSPDLVISGINCGNNICKNAWYSGTIGAAKQALVDGIPSMALSQDNHISFF
QQDKAPEILKALVIYLLSQPFPCLTGLNINFPTSPGGSSWEGMRLVPPGDEFFYEEPQYLGSVNKNQYYVGKIS
GVRIGEHPSEELACMLENHISVSPIFSQNSPIGLMTLEEFQKTQENFNASLLSSELTTKIF

>core/561/5/Org5_Gene562

MELKKTAESLYSAKTDNHTVYQNSPEPRDSRDVKVFSLEGKQTRQEKTTSSKGNTRTESRKFADEEKRVDD
EIAEVGSKEEEQESQEFCLAENAFAGMSLIDIAAAGSAEAVVEVAPIAVSSIDTQWIENIILSTVESMVISEINGE
QLVELVLDASSSVPEAFVGANLTLVQSGQDLSVKFSSFVDATQMAEAADLVTNNPSQLSSLVSALKGHQLTL
KEFSVGNLLVQLPKIEEVQTPLHMIASTIRHREEKDQRDQNQKQKQDDKEQDSYKIEEARL

>core/563/5/Org5_Gene834

MLWGVSMRQSFDELSQNAFKNIFNKQRFCFIFCSLCCFGFVFALFLKLCSRLAPEISLSTLGLGAFFCAFSVICA
SAIIVQFLLHKESQGETSKLCCAIKNTWSSLWLSLLVSMPFFIAMVAVVTVAMLSSFLGSLPWVGKLFHTVLIF
IPYLSATALILLFLGSFSCLFFCIPVLHNQESIDYRKLLECFRGNILRQFIGVVIALVPLALCSWLALDSFYLMTH
LVEIADIHTWSFLAQMFVLIVPIALILTPAVSFFFNFSFSFYLAKQEEEKALVK

>core/564/5/Org5_Gene890

MHKVIVFIFLTLYSLKSYGNDVIDKPHVLVSIAPYKFLVEQIAEETCFVYAIVTNHYDPHTYELPPQQIKELRQ
GDLWFRIGEAFEKTCERNLTCQQVDLSQNVSLIQGKPCCNQHTTNYDTHTWLSPKNLKVQVETIVTTLSKKY
PQHATLYQSNGEKLLLALDQLNEEILTITSKAKQRHILVSHGAFGYFCRDYNFSQHTIEKSSHVEPSPKDVAR
VFRDIEQYKISSVILLEYSGRRSSAMLADRFHMHTVNLDPYAENXLVNLKTIATTFSSL

>core/565/5/Org5_Gene487

MYFTRDPVIETVITSREGYKLSVRNTKHFSQDPFMVEAIEVISLGNICFFRNCDHSKPFLVPAGDYEVMEVRDT
KINLKAVGLDRGVKIAGGREALIKLTKSTPLPVIDEKPLADSPEEGTEPTSPSKKEKKEARKDSFKGEKWKEK
KKLSRRRNHKEIAEVTGASQEILDTVKEELWEESQENEIVEQKKFSLLPPPAKLISEVISQTVVDPVVTSADLN
ESLQALVRESSDLINALLSADDAIHFPETEEEPTSASFEESSAMFFPETSSATEEE

>core/566/5/Org5_Gene861

MTRSSPAQLSRFLSEIQNKPKKSLSQNFLVDQNIVKKIVATSEVIPQDWVLEIGPGFGALTEELIAAGAQVIAIE
KDPMFAPSLEELPIRLEIIDACKYPLDQLQEYKTLGKGRVVANLPYHITTPLLTKLFLEAPDFWKTVTVMVQD
EVARRIVAQPGGRDYGSLTIFLQFFADIHYAFKVSASCFYPKPQVQSAVIHMKVKETLPLSDEEIPVFFTLTRT
AFQQRRKVLANTLKGLYPKEQVEQALKELGLLLNVRPEVLSLNDYLALFHKMQAG

>core/567/5/Org5_Gene554

MESQSCKLTIKDLMSAGAHFGHQTRRWNPKMKLYIFEEKNGLYIINLAKTLQQLRNALPHIRKVIQDNKTVL
FVGTKKQAKCVIREAAIEAGEFFIAERWLGGMLTNMTTIRNSIKTLDKIEKDLSRNQAYLTKKEAALLAKRH
QKLLRNLEGIRYMKKAPGLLVVVDPSYEKIAVAEAKKLGIPVLALVDTNCDPTPIDHVIPCNDDSLKSIRLIIN
VIKENIIEAKHKLGIEIVSPVKSLEVPDLSAFESSQDDESDEENREEDLLAKKFDGEAN

>core/569/5/Org5_Gene668

MTANTFGTLDILMKHSKEDDLSRFLPKNLLVESPHPEEIPLKSLSFTMSWLPTIHPSWITIAMKEFPPEIQGQLL
AWLPEPLVQEILPLLPGISIAPHRCAPFGAFYLLDMLSKKIRPCGITEEIFLPASSANAILYYTGPVKIALINCLGL
YSIAKELKHILDKVVIERVKNALSPTEKLFLTYCQSHPMKHLETTNFLSSWTTDAELRQFVHKQGLEFLGKAL
TKENASFLWYFLRRLDVGRAYIVEQTLKTWYDHPYVDYFKSRLEQCMKVLVK

>core/570/5/Org5_Gene402

MDYKLLDSGDGNKLECFGPVTLIRPSSIAVWPKSRPELWSQAQLQYVREGERGAWKNFKRLPEEWEVAFSD
VRCLLKRTPFGHLGVFPEHMGFWPALKQAIEKHKERQVLNLFAYTGAGSIFAAKCGARVTHVDASQAAVR
WAQRNVEKNAFPERRIFWVIEDVISFLKKEIRRNKKYQVILLDPPSYGRGPDGEVFKIDKDLFPLLSLCSKLLA
DDASYFLLTSHTPGHTPEFLRAIARRSVPTLVSEAWSCGESFCGEGVGALPSGSFVQWIA

>core/571/5/Org5_Gene114

MITGVVLEKHEQRTMFSLTLLNNFTTFGLLHTPLHYNPPYPIVILLHGLASDKTGSKRSHVRLAQELTRLGIAA
LRVDLLGHGDCEGELMDFSLENYKQNIREIIEYTHSLLHIDQERLAIFGSSLGGTLALQTLPFFNKIKALAVWA
PTISGELMAAEAQKNAPEVITMSQKGAITYAGMTLNPDFYTQFLKIDIVKELMPSARNLPPILYMQGEQDLLV
SINHRTLFTEAFANQDKPITILTYPDVDHAFPFAESSALSDLTQWLKRELTSGE

>core/572/5/Org5_Gene581

MLLGFLCDCPCASWQCAAVANCYDSVFMSRPEHKPNIPYITKATRRGLRMKTLAYLASLKDARQLAYDFLK
DPGSLARLAKALIAPKEALQEGNLFFYGCSNIEDILEEMRRPHRILLLGFSYCQKPKACPEGRFNDACRYDPSH
PTCASCSIGTMMRLNARRYTTVIIPTFIDIAKHLHTLKKRYPGYQILFAVTACELSLKMFGDYASVMNLKGVG
IRLTGRICNTFKAFKLAERGVKPGVTILEEDGFEVLARILTEYSSAPFPRDFCEIH

>core/573/5/Org5_Gene874

MRRYLFMVLALCLYRAAPLEAVVIKITDAQAVLKFAREKTLVCFNIEDTVVFPKQMVGQSAWLYNRELDLK
TTLSEEQAREQAFLEWMGISFLVDYELVSANLRNVLTGLSLKRSWVLGISQRPVHLIKNTLRILRSFNIDFTSC
PAICEDGWLSHPTKDTTFDQAMAIEKNILFVGSLKNGQPMDAALEVLLSGISSPPSQIIYVDQDAERLRSIGAF
CKKANIYFIGMLYTPAKQRVESYNPKLTAIQWSQIRKNLSDEYYESLLSYVKSKG

>core/574/5/Org5_Gene71

MLLIYCKKKEIHLQWPQTAKIRFTPKIAMKVKINDQLICIPPFISARWSQIAFIESQEGENKDQGTLRLHLIDGKI
ISIPNLDQSIIDIAFQEHLLYLETSQSGKEDSRDDDKLGVGVLMNVLQQITKGNDIQVLPKNLISPLFSGTNPIEA
ILQHTPEHKDHPDAPTDVLEKMADVIRVLSGNNATLLPRPEPHCNCMHCQIGRVMNEEDTLAVSDKDLTFRT
WDIMQSGDKLYIVTNPLNPSDQFSVYLGPPIGCTCGEPNCEHIKAVLYT

>core/575/5/Org5_Gene299

MKLLKNVLLGLFFSMSISGFSEVKVSDTFVKQDTVVEPKIRVLLSNESTTALIEAKGPYRIYGDNVLLDTAIQG
QRCVVHALYEGIRWGEFYPGLQCLKIEPVDDTASLFFNGIQYQGSLYVHRKDNHCIMVSNEVTIEDYLKSVL
SIKYLEELDKEALSACIILERTALYEKLLARNPQNFWHVKAEEEGYAGFGVTKQFYGVEEAIDWTARLVVDS
PQGLIIDAQGLLQSNVDRLAIEGFNARQILEKFYKDVDFVVIESWNEELDGEIR

>core/576/5/Org5_Gene212

MKKKLSLLVGLIFVLSSCHKEDAQNKIRIVASPTPHAELLESLQEEAKDLGIKLKILPVDDYRIPNRLLLDKQV
DANYFQHQAFLDDECERYDCKGELVVIAKVHLEPQAIYSKKHSSLERLKSQKKLTIAIPVDRTNAQRALHLL
EECGLIVCKGPANLNMTAKDVCGKENRSINILEVSAPLLVGSLPDVDAAVIPGNFAIAANLSPKKDSLCLEDL
SVSKYTNLVVIRSEDVGSPKMIKLQKLFQSPSVQHFFDTKYHGNILTMTQDNG

>core/577/5/Org5_Gene361

MVRDIQSESIGKLVFLGTGNPEGIPVPFCSCRVCQNTGIHRLRSSVLIQYQNKTLVIDAGPDFRTQMLVAGVSE
LDGVFLTHPHYDHIGGIDDLRAWYIVTQRSLPLVLSASTYRFLNKAKEYLFATPNVESSLPAVLEFTILNEDCG
QEEFQGIPYTYVSYYQKSCHVTGFRFGNLAYLTDLCSYDAKIFSYLDNVETLILSAGPSETPIPFQGHKSSHLT
VEEAKAFANHAGIKNLIITHISHCLEAERDQHPEVTFAYDGMEVLWTL

>core/578/5/Org5_Gene735

MIIIKNNELMIRRFFKTLFPPGPQYSLCYASILIVLSSLVCVPTFCWLFLPELSLSKFNPSPIRNLFLVSSTLSKVPP
TAIAEHLRLSADAPTYLHEFSIKEAESSLHALGIFSSLVIEKSPDNKGITIFYTLQTPIAYVGNRSNTLCNLEGSC
FLGQPYFPSLNLPQIFFSQEDLKMQKLPKEKMLFTKILLKELAMESPKIIDLSLSDAYPGEIIVTLSSGSLLRLPIK
TLDRALDLYKHMKKSPVIESEKQYVYDLRFPNFLLLKAL

>core/579/5/Org5_Gene578

MFNNKMILIAGPCVIEGEDITLEIAGKLQSILAPYSDRIQWFFKSSYDKANRSSLNSFRGPGLTEGLRILAKVKE
TFGVGILTDVHTPQDAYAAAEVCNILQVPAFLCRQTDLLVATAETGAIVNLKKGQFLSPWDMEGPINKVLST
GNNKILLTERGCSFGYNNLVSDMRSIPVLSRSGFPVIFDATHSVQLPGALSTESGGLTEFVPTLSRAALAAGAH
GLFIETHTNPKIAKSDAASMLSLEEFAALLPTWDQLFTCVSSFDMVSA

>core/580/5/Org5_Gene888

MTKVALLIAYQGTAYSGWQQQPNDLSIQEVIESSLKKITKTRTPLIASGRTDAGVHAYGQVAHFRAPDHPLF
ANANLTKKALNAILPKDIVIRDVALFDDNFHARYLTIAKEYRYSLSRLAKPLPWQRHFCYTPRHPFSTELMQE
GANLLIGTHDFASFANHGRDYNSTVRTIYTLDIVDKGDSLSIICRGNGFLYKMVRNLVGALLDVGKGAYPPE
HLLDILEQKNRREGPSAAPAYGLSLHHVCYSSPYNNFCCEQCSVSTSNEG

>core/582/5/Org5_Gene966

MTQYYFLSSFLPTQLPESVPLFSISDLDDLLYLNLSENDLCNYGLLKRFFDFENFAFFWAGKPIPFSFGEVTQE
NVERMLSSQQWSDDNDFEDFFKDFLMNHKSSQDRLNHFSDLFREFLSYHQTNSSKFLRDYFRFQQQLRVVL
AGFRARVLNMDVSYVLRDEDSSDPVVLEVLMQKDSPNYELPEEFSDLQGVLDDYGLLPHTLNRALALYQFH
KLEGFCSDSYFDGNVILARCATYMFAIRNSLASVEKGREIINHIEKAIKW

>core/583/5/Org5_Gene135

MHSKFLSRRKKNSSHKEETSWDCIASSYNKIVQDKGHYYHRETILPQLLPSLTLGSKSSVLDIGCGQGFLERA
LPKECRYLGIDISSRLIALAKKMRSVNSHQFKVADLSKRLEFVEPTLFSHAVAILSLQNMEFPGEAIRNTATLL
EPLGQFFIVLNHPCFRIPRASSWHYDENKKAISRHIDRYLSPMKIPIMAHPGQKDSPSTLSFHFPLSYWFKELSS
HGFLVSGLEEWTSSKTSTGKRAKAENLCRKEFPLFLMISCIKIK

>core/584/5/Org5_Gene1009

MTLPLEPMIFWSSLSAKVMKKFLTPHCAGTFSEEDAEAKEAHLVTGKQGHRLMGNCVTFYWLVDKKNGVI
LDAKFQYFGHPYLIPLAEAVCNLVCGKSYXEAYKMTLDDIDKSLRVHAHQPALPEDSISLYHFVIDALDTAV
EQCLEIPLEDGSLPLQNSPMNLDFEDANPYSQSDWEALTHEQKLYALRATIAEKIGPYIAMDGGEVTVESLEN
FIVTIAYSGNCSGCPSSLGSTLNSIGQLLRAYIYPELQVKVDESSLNLSHP

>core/585/5/Org5_Gene712

MQGFFPLASGSKGNSAYLGTDSCKILIDLGVSKQVVTRELLSMNIDPEDIQAIFVTHEHSDHISGIKSFVKAYN
TPIVCNLETARALCHLLDSHPEFKIFSTGSSFCFQDLEVQTFNVPHDAVDPVAFIFHYREEKLGFCTDLGWVTS
WITHELYDCDYLLIESNHSPELVRQSQRPDVYKKRVLSKLGHISNQECGQLLQKIITPKLKKLYLAHLSTECNT
AELALSTVSESIASITSIAPEIALAQGITSPIYFSRLEVACPR

>core/586/5/Org5_Gene795

MAGLDLEARGKRRVVTPNAITAFGLCCGLFIIFKSVLRTSSSVELFHRLQGLSLLLISAMIADFSDGAIARIMKA
ESAFGAQFDSLSDAVTFGIAPPLIAIKSLDGIYVGNFFSSLLLITSIIYSLCGVLRLVRYNLFSQKTVDVSKPYCFI
GLPIPAAAASIVSLALFLASDFFPDLPAQLRVGLLSFALLFIGGLMISPWKFPGVKHFRFNVSSFLLVVTIGLAA
CLFFSGLVDHFVEVFFLVSWLYTLVGFPIFSIIYRKKS

>core/587/5/Org5_Gene21

MKKPDNDSTFDVRSFFPFDVLCIEQLRKEMSWEVVSAKIPRLPRGWYELMGLSKEDRIDFCLDFWCSVLGIE
HKESPSICRFFSLLETIEVYIYRLEKEPYQLKMFYVFRDGRCGFQGEPPLLDFLGHHRLPPLGDRHYEKFFSIHN
GFGKWEDEGIFPMRSLAKVQQKLRQQLVVMNKMQAEDNCYSLGIFPFYGYEEPFAYQSFFFDPEIRRDLPSP
NVLLNEESLEHRSLETIELLHLSKSYYPSFLSWLENYLHSEEVYNE

>core/590/5/Org5_Gene401

MDCIGKHNPLVKEALALKRSRCRKSSWFLVEGAREIQKALRTGYLCQHVFCSTHLSEKEKEFLYELKRNSTK
ILYCLDSTLAQLSFKEHHDSFVAVIQKRVWNKEDFLIQRKNAQPFYLIIEQVEKPGNVGAILRIADGAGVDGVI
LCNPIVDLYNPNVVRSSLGAVFSLPILSISREEGKELFKQEGWTVFVTSPRAETMYFSKNYLGPTALVFGSEKD
GLTEDWFSEDFSEIALPMLGESDSLNLATSVAAVAYEVVRQRWVN

>core/591/5/Org5_Gene851

MGSSMHVGVIGCSGRTGKVIVSALEQSSEYTLGPGFSRSSALTLFQVIAHNDVLVDFSHPLLTKEVVAHLLISP
KPLIIGTTGFPGKCKEAHDSLEELTHIVPVVVCPNASLGAYIHKRLVMLLSQLCNPQFDIRIRETHHRYKKDSL
SGTAQDLLDTIQQVKQEDWGEEYEVGQRDSSKKTIEVQSSRVGDIPGEHEVAFISSGEQILVRHTVFSRNVFG
RGILSILDWLKTLNPQPGLYSLGDTLELVLRNEHCLLKKTTDH

>core/592/5/Org5_Gene620

MEKLEFVTSLSSPDDDLITFNKQGLIAGPEEEKVAFLVRSNAMLDAGPETPASFPESLREQFDIFPEYVEVLYS
NEGLDVWEAGCTWILNNEVTIQLRKHHRKASRWLGMYSRDEVLAHEAVHAVRMKFHEPVFEEVLAYQTSR
WGWRRFFGPLFRSPGESYLLLFFTILGLGISLWYPAGILIMLVLPMYFLMRLCMAQSYLYRAMKKIRKMLGV
PPLWVLLRLTDKEIKMFAKEPIPVLEHYARKRKLENVRWKQIYQSYFV

>core/593/5/Org5_Gene895

MSNQLQPCISLGCVSYINSFPLSLQLIKRNDIRCVLAPPADLLNLLIEGKLDVALTSSLGAISHNLGYVPGFGIA
ANQRILSANLYAAPTFFNSPQPRIAATLESRSSIGLLKVLCRHLWRIPTPHILRFITTKVLRQTPENYDGLLLIGD
AALQHPVLPGFVTYDLASGWYDLTKLPFVFALLLHSTSWKEHPLPNLAMEEALQQFESSPEEVLKEAHQHTG
LPPSLLQEYYALCQYRLGEEHYESFEKFREYYGTLYQQARL

>core/594/5/Org5_Gene324

MKLTKYLNTKQLRSMISRLFVRYSLPMSKQLSFFALCVLGSHPIFAQTPNPPQRVRRSEVIFIDPGHGGKDQGT
ASKELHYEEKSLTLSLALTVQSYLKRMGYKPQLTRSSDVYVDLGKRVALSNRGQGDVFISIHCNHSSNAAAF
GTEVYFYNGKVGSPTRNRMSEVLGKNILAAMEKNGILKSRGLKTANFVVIRDTSMPAVLVETGFLSNSRERA
ALQDARYRMHVAKGIAEGVHNFLSGPSFQKPKQNIAKIRKPQIQAN

>core/597/5/Org5_Gene67

MPFDITYYTTPLLEIILIWVMLNYLLKFFWGTRAMDVVFGLLAFLFLFVLADKLHLPIIRRLMLHVVNIAAIVV
FIIFQPEIRLALSRIRFHGKKFFIDTQEQFVEQLAASIYQLSERQIGALVVLENKDSFDEYLSFSSVKINATFSEEL
LETIFEPSSPLHDGAVILRGDILAYARVVLPLAHDTTQLSRSMGTRHRAALGASQRSDALIITVSEENGSVSLSR
DGLLTRGVKIDRFKAVLRSILSPKEHKRKPLFSWIWKR

>core/599/5/Org5_Gene146

MTTLLSIKDLSLTIRGKKILNHINLNLIKGSYLTIVGPSGSGKSSLALTILDLLKPTTGTITFHMDPKIPRARKVQ
VIWQDIDSSLNPCMSIKGIISEPLNIIGTYSKAEQNKEIYNVLDLVNLPKSVLHLKPYKLSGGQKQRIAIAKALV
SKPELLICDEPLSSLDTLNQSLILDLFQTIKKEYQNTLLFITHDMSAAYYIADTIAVMDQGSLVEHACREKIFST
PKHTTTQDLLDAIPIFSLISTEMEPSEEYELQVASK

>core/600/5/Org5_Gene838

MKTWLFFTFLFSCSSFYASCRYAEVRSIHEVAGDILYDEENFWLILDLDDTLLQGGEALSHSIWKSKAIQGLQ
KQGTPEQEAWEAVVPFWIEIQEMGTVQPIESAIFLLIEKIQKQGKTTFVYTERPKTAKDLTLKQLHMLNVSLE
DTAPQPQAPLPKNLLYTSGILFSGDYHKGPGLDLFLEICTPLPAKIIYIDNQKENVLRIGDLCQKYGIAYFGITY
KAQELHPPIYFDNIAQVQYNYSKKLLSNEAAALLLRHQMHE

>core/601/5/Org5_Gene1064

MHSELKATQEDELCELFRTHIALPSKGYVRIKLVLSPNGEIQECSFLSEVSAADKQLLTQRIHALPFQKFLEKY
KVSKNISFHIKLVSNES

>core/602/5/Org5_Gene634

MDLADAHVHLSDDAFEEDINSVLQRAQDSGVSLVVNVTTTEKELNRSFAYAERFPKIRFCHVGGTPPQDVDQ
DIEEDYRNFHAAAHSKKLAAIGEVGLDYCFATEEGIARQKEVLQRYLALSLECELPLVVHCRGAFNDFFRML
DQYYHNDPRSRPGMLHCFTGTLEEAQELISRGWFISISGIVTFKNAQDLRDLVVELPLEHLLIETDAPFLAPVP
YRGKKNEPAHVLHTINAVANVKGMFPQELAALAYKNVLRFLHG

>core/603/5/Org5_Gene16

MFLQFFHPIVFSDQSLSFLPYLGKSSGIIEKCSNIVEHYLHLGGDTSVIITGVSGATFLSVDHALPISKSEKIIKILS
YILILPLILALFIKIVLRIILFFKYRGLILDVKKEDLKKTLTPDQENLSLPLPSPTTLKKIHALHILVRSGKTYNELI
QEGFSFTKITDLGQAPSPKQDIGFSYNSLLPNFYFHSLVSVPNISGEERALNYHKEQQEEMAVKLKTMQACSF
VFRSLHLPSMQTKDKKAGFGLLTFFPWKIYPL

>core/605/5/Org5_Gene319

MILTAAFSPCPNDIFLFRSFLKDPQFRPLLNQVTIADIETLNTLALQRRLSLMKMSAALFPLVSDYYNLMDVG
NTLGYNSGPIVLSLDPECSLDTLATPGEMTTAHALCKLYYPKAKLIPMPYDKILSAILQGKVDGGALIHEERFS
YDLQLTLRADFGELWRRKTIFPLPLGCLAIAKYVPMATVDALTAALRKSLICSLKDPITAGAKAVEYSKNKN
VTVIHRFIGTYINKETFQLSKTGKKALHMLWKANECCQYT

>core/606/5/Org5_Gene899

MIKQIGRFFRAFIFIMPLSLTSCESKIDRNRIWIVGTNATYPPFEYVDAQGEVVGFDIDLAKAISEKLGKQLEVR
EFAFDALILNLKKHRIDAILAGMSITPSRQKEIALLPYYGDEVQELMVVSKRSLETPVLPLTQHSSVAVQTGTF
QEHYLLSQPGICVRSFDSTLEVIMEVRYGKSPVAVLEPSVGRVVLKDFPNLVATRLELPPECWVLGCGLGVA
KDRPEEIQTIQQAITDLKSEGVIQSLTKKWQLSEVAYE

>core/607/5/Org5_Gene90

MLRNQVLVYCSEGVSPYYLRHTIRFLKYYSTQEGAFDILRVDGNFLIKNPFWEETTRLLVFPGGADRPYHRV
LHGLGTARIFQYVSEGGNFLGICAGAYFGSKMIYFYEPEGAPLQGARDLGFFPGTAKGPAYRGNFSYVSPSGV
RVSPQLFSDFGLGYAMFNGGCFFEGSEGYPGVNIESRYDDLPGKPASIVSRIVSKGLAVLSGPHIEYLPHYCR
MVKENVQKTREFLQRERTTLDRYCQNLVQRLRQPAFSKADC

>core/608/5/Org5_Gene264

MNVKDETFWSVHNLCVNYEHAAVLYHISFSLGKGSLTAILGPNGAGKSTLLKASLGLIKPSSGTVYFFNQKF
KKVRQRIAYMPQRASVDWDFPMTVLDLALMGCYSYKGMWGRISSDDRREAFHILERVGLESVADRQIGQLS
GGQQQRAFLARALMQKADLYLMDELFSAIDMASFKTSVGVLQELRDQGKTIVVVHHDLSHVRQLFDHVVL
LNKRLICCGPTDECLNGDTIFQTYGCEIELLEQTLKLSRGKQFGSC

>core/609/5/Org5_Gene521

MNRRWNLVLATVALALSVASCDVRSKDKDKDQGSLVEYKDNKDTNDIELSDNQKLSRTFGHLLARQLRKS
EDMFFDIAEVAKGLQAELVCKSAPLTETEYEEKMAEVQKLVFEKKSKENLSLAEKFLKENSKNAGVVEVQP
SKLQYKIIKEGAGKAISGKPSALLHYKGSFINGQVFSSSEGNNEPILLPLGQTIPGFALGMQGMKEGETRVLYI
HPDLAYGTAGQLPPNSLLIFEINLIQASADEVAAVPQEGNQGE

>core/611/5/Org5_Gene1018

MMENLETFILKIYRGVPGKQYWESFELPLHPGENVISALMEIEKRPVNILGEKVNPVVWEQGCLEEVCGSCSI
LVNGVPRQACTALIQEYIDATQSREIVLAPLTKFPLIRDLIVDRSIMFDNLERIQGWVAADIEGETFGPQVTQE
QQELLYALSQCMTCGCCTEACPQIDNKSDFIGPAAISQARYFNTYPGDKQSKKRWRALMGKGGIEGCGQAH
NCVRVCPKKLPLTESISAVGREISKFSLRSLFSALFKKKK

>core/613/5/Org5_Gene308

MSKFILLLSLGVAALASKNFFIWPAPSGKTPLKLRQVLFGGALLVFSSLVALSVSSQTAELLSTMTGISLAFAF
LFYLLFLPKDITRAILFSGERPVKTSWRALGSAIRMWIIIIPVTQLIGIMMSKFLTLVLPTQEIHTQEVTQEVQNS
LPITGHYISMILNLGVLTPFGEEVFFRGILQTFLKNKMTRIAAVLCSSIIFSFIHIEHSLGSWVFVPVLFVFSLSAG
FLYEKDRHILSPIALHGLFNLTSLLFLGIK

>core/614/5/Org5_Gene392

MAFYSPSTISKYFIYSGAGNRFLLGETLPEVEDVRFLCQETRVDGFLYLKPSSCADAQLIIFNSDGSRPTMCGN
GLRCAIAHLASQKGKSDISVSTDSGLYSGYFYSWDRVLVDMTLADWRASVHRLESRPDPLPKEVVCIHTGVP
HAVVILPEISTLDLSILGPFLRYHQTFSPDGVNVNFVQILGHCQLRVRTYERGVEGETAACGTGALASALVVS
NSYGWKESIQIHTWGGELMTVSQNRGRVYLQGSVTRDL

>core/615/5/Org5_Gene92

MFKLLKNLFLIGCCIVGYFWMRKESIVEQWLSNRLHTQVTVGRVSIRTSGIKIRHICIHNPLASERFPYAAEIEY
ADVRFSSISMLLTKQLEISELIIHGANFTIFPYDSHGTKTNWSLVWKNFHPQKETPSNLWIDRAPVLIRRCLFLN
TRLYGLRANHKDIPHLSVPSLEFHSHTSSAKELPKLSEALPSLLYLALEESLYHLNLPGDIIKPLSQQAHKHFYS
SYPQFQDRLNDINTPGTPTEEIIGFIRGLFFH

>core/616/5/Org5_Gene73

MVSPLSLFHKMLLENWTPVEEPFPWPPAEKNQKIFAWALNQSKLIFVSTSGNIAQPRLVTDSMSMMIVNAAN
RTMSRDGAGTNQVLSAAVSVDSWGLSQRPLNPERQGTPLNEGECRAGMWRNADGSNHTGKQGKPHYLAQ
LLGPKAVDHHNKSQAAFDRCKNAYLNCFSLAQTLGVTFLQIPLISSGIYAPPENRKKPNSEENKVRMRWIHA
VKCALVAAMQEFGNEPGNTDRRMLIVLTDLKTPAITDPKKKSHL

>core/617/5/Org5_Gene274

MKTQQTQNIIEVWNFYWETQEIEYRDSLIEFYLPLVKSVVHRLISGMPSHVKTEDLYASGVEGLVRAVERYN
PERSRRFEGYAVFLIKAAIIDDLRKQDWVPRSVHQKANKLSGAMDSLRQSLGKEPTDLELCEYLNISQQELSG
WFVSARPALIVSLNEEWPSQSDEGAGMALEERIPDERAETGYDVVDKQEFSLCLANAIQELEEKERKVMALY
YYEELVLKEIGKVLGVSESRVSQIHSKALLKLRAALSAFR

>core/618/5/Org5_Gene337

MWLGAYTWLNVFGILLQAAFIQNILLANFLGMCSYLACSTRVSTANGLGMSVALVLTVTGSINWFVHAFITG
PKALTWISPSLASVNLGFLELIIFIVVIAAFTQILELLLEKVSRNLYLSLGIFLPLIAVNCAILGGVLFGITRSYPFIP
MMIFSLGAGCGWWLAIVILATIKEKLAYSDIPKNLQGMGISFITTGLIAMAFMSLTGIDISKPSAKIQRAPLETE
VVENTTNPLKESSSKHQPSISKARTQRRSL

>core/619/5/Org5_Gene550

MLKIKHLHASCNDVKILDDFNLNIQPGTMHVIMGPNGAGKSTLAKILAGDESVLVSSGEIALQEQNLLSMLPE
ERSRAGLFVGFQMPPEIPGVNNKMFLRDAYNARRRANQEGDISIDEFNTLLSTVLETYEYNATTDLFLDRNV
NEGFSGGERKRNEICQMLVLEPEMVLLDEPDSGLDVDALRLICRVLEKYRELHPTSSLCIVTHNPKLGNLIRP
DVVHLLLDGRVALSGDVSLMHELEAKSYQEVTKRVAWR

>core/621/5/Org5_Gene648

MKTIAVNSFKGGTAKTSTTLHLGAALAQYHQARVLLIDFDAQANLTSGLGLDPDCYDSLAVVLQGEKEIQE
VIRPIQDTQLDLIPADTWLERIEVSGNLAADRYSHERLKYVLGSVQDKYDYVIIDTPPSLCWLTESALIAADYA
LICATPEFYSVKGLERLAGFIQGISARHPLTILGVALSFWNCRGKNNSAFAELIHKTFPGKLLNTKIRRDITVSE
AAIHGKPVFATSPSARASEDYFNLTKELLILLRDI

>core/622/5/Org5_Gene982

MTRQSYVLGNWKMHKTIQEAKEYVQTLASLLQGEPLSCTIGIASPFTSLRAIHEMINTTGAFLWLGAQNVHP
ELSGAFTGEISLPMLKEVGVEFVLVGHSERRHIFGESDAFIASKVKSVAQAGLVPVLCVGESLEVREEGKAHQ
VIKKQLLLGLEQMDNGSEFLIAYEPVWAIGTGKVAEASDVQDIHMFCREVVAERFSEATAEEISILYGGSVKV
DNAQRFGQCSDVDGLLVGGASLEGQSFFEVAKNFNV

>core/623/5/Org5_Gene397

MHDALLSILAIQELDIKMIRLMRVKKEHQKELAKVQSLKSDIRRKVQEKELEMENLKTQIRDGENRIQEISEQI
NKLENQQAAVKKMDEFNALTQEMTTANKERRSLEHQLSDLMDKQAGGEDLIVSLKESLASTENSSSVIEKEI
FESIKKINEEGKALLEQRTELKHATNPELLSIYERLLNNKKDRVVVPIENRVCSGCHIVLTPQHENLVRKKDRL
IFCEHCSRILYWQESQVNAQENSTAKRRRRRAAV

>core/624/5/Org5_Gene177

MKPEESECLCIGVLPARWNSSRYPGKPLAKIHGKSLIQRTYENASQSSLLDKIVVATDDQHIIDHVTDFGGYA
VMTSPTCSNGTERTGEVARKYFPKAEIIVNIQGDEPCLNSEVVDALVQKLRSSPEAELVTPVALTTDREEILTE
KKVKCVFDSEGRALYFSRSPIPFILKKATPVYLHIGVYAFKREALFRYLQHSSTPLSDAEDLEQLRFLEHGGKI
HVCIVDAKSPSVDYPEDIAKVEQYITCLSNAYF

>core/625/5/Org5_Gene746

MLIKLWRATYEGMYTFLVGALLKLRYRMQVEGWDTLNINPKQGCLFLANHVAEVDPIILEYLFWSRFHVRP
MAVEYLFHSRVVQWFLNSVRSIPIPQLVPGKESKRSLERMNVCYEEASRALNRGESLLLYPSGRLSRTGKEEI
VNQYSAYVLLHRVMECNVVLVRVSGLWGSAFSRYKQNSTPKLGPAFKEAFRALLRRGIFFMPKRFVKITLC
QVDHLFLKQFPTKQDLNTFLASWFNQGDDNLPIEVPYA

>core/626/5/Org5_Gene456

MQICVTGVVLRSRPLGKNHTLTTLFTPEGLFTFFAKQGQTLQCDYRETLVPISLGKYTLHRNGSRLPKLTHGD
ILNAFEAIKQTYALLEASGKMIQALLASQWKEKPSHKLFSLFLNFLHRIPESSNPEFFAAIFVLKLLQYEGILDL
TPACSLCKASLPYACYRYQGHKLCKKHQHKQAISIEKEEEQILQAIIHAKQFSELLAIAEFPIAIAEKIFYLFDSL
QEEKKSERNSSEDPYHEILRLSKVVHPY

>core/627/5/Org5_Gene547

MLIVLAFRQVFFSHSRSQLDRLKNYLRLLKQNFAITLPKERTSKGHSLMLTFDFASFDFYTNIFPFLEEQKIPAV
VGVASRYIPSNAAQDLHPSHRLKPSETLAFQDEIFSNYMPFCCQNELIEMAKSPYIQLASSGFAIRNLMNNPPY
LTTEILLSRHHIETITGAKPLAFLFPFGKSDPTSRKLAADHYPYSFLLGNTINRKLKTHNIYRLDIKPMQYVCPS
LFQSSRYLKNWIKEKSKQLYLKKQLPKR

>core/628/5/Org5_Gene150

MLMDISHILEDLAYDEGILPREAIEAAIVKQMQITPYLLHILHDATQRVPEIVNDGSYQGHLYAMYLLAQFRE
SRALPLIIKLFAFEDDTPHAIAGDVLTEDLPRILASVCNDDSLIKELIETPKINPYVKAAAISGLVTLVGAGKIPR
DKVIRYFAELLNYRLEKQPSFAWDNLIAGICTLYPGELFYPISKAFDGGLVDTSFISMEDVENIIHEETVESCIH
TLCSSTELINDTLEEMEKWLEDFPIEP

>core/629/5/Org5_Gene96

MNVADLLSHLETLLSSKIFQDYGPNGLQVGDPQTPVKKIAVAVTADLETIKQAVAAEANVLIVHHGIFWKG
MPYPITGMIHKRIQLLIEHNIQLIAYHLPLDAHPTLGNNWRVALDLNWHDLKPFGSSLPYLGVQGSFSPIDIDS
FIDLLSQYYQAPLKGSALGGPSRVSSAALISGGAYRELSSAATSQVDCFITGNFDEPAWSTALESNINFLAFGH
TATEKVGPKSLAEHLKSEFPISTTFIDTANPF

>core/630/5/Org5_Gene76

MKKQGKTLFLFLFLSFLFSTAFSGLFASQTSSLRTIQENIFLAKTGDYTVLSRGSQRTFVLVKSTTPKTVWIEIIH
FPCIAHKERPSLEQASWKTVIHQLESPSQVFVVSLSSEGSQFFSLNTRTKSLEPVGKSTTVPAFLQIFDLPLSPAP
ANVIKTKGKENKPWSPKVSFEGAPLTSISVNAWQGLWPKDRGPLSETGILMYFTQPDISVFPLWVSIETPKGT
SIVRAVDIGHGATSPYVYSLPDSKTQ

>core/632/5/Org5_Gene434

MSLATNNAESKFPSLQRLPNHVAIIMDGNRRWYKKHREECGHTHTSGHYYGAKVLPNILNAVLDLGIKVLT
LYTFSTENFGRPKEEIQEIFNIFYTQLDKQLPYLMENEICLRCIGDLSKLPKGIQTKINHVSRMTASFSRLELVLA
VNYGGKDELVRAFKKLHVDILNKKISSDDLSESLISSYLDTSGLTDPDLLIRTGGEMRVSNFLLWQIAYTELYI
TDTLWPDFTPQDLFEAINVYQQRSRRGGK

>core/633/5/Org5_Gene647

MGNLKTLLESRFKKNTPTKMEALARKRMEGDPSPLAVRLSNPTLSSKEKEQLRHLLQHYNFREQIEEPDLTQ
LCTLSAEVKQIHHQSVLLHGERITKVRDLLKSYREGAFSSWLLLTYGNRQTPYNFLVYYELFTLLPEPLKIEM
EKMPRQAVYTLASRQGPQEKKEEIIRNYRGERKSELLDRIRKEFPLVETDCRKTSPVKQALAMLTKGSQILTK
CTSLSSDEQIILEKLIKKLEKVKSNLFPDTKV

>core/634/5/Org5_Gene469

MKIKFSWKVNFLICLLAVGLIFFGCSRVKREVLVGRDATWFPKQFGIYTSDTNAFLNDLVSEINYKENLNINIV
NQDWVHLFENLDDKKTQGAFTSVLPTLEMLEHYQFSDPILLTGPVLVVAQDSPYQSIEDLKGRLIGVYKFDSS
VLVAQNIPDAVISLYQHVPIALEALTSNCYDALLAPVIEVTALIETAYKGRLKIISKPLNADGLRLAILKGTNG
DLLEGFNAGLVKTRRSGKYDAIKQRYRLP

>core/635/5/Org5_Gene515

MSLLKDTVFTCLDCEMTGLDVKKDRIIEIAAVRFTFDSVISSIEFLINPERVVSAESQRVHHISNAMLRDQPKIA
EVFPQIKAFFKEGDYIVGHSVGFDLQVLAQEMERIGETFLSKYTIIDTLRLAKEYGDSPNNSLESLAVHFNVPY
DGNHRAMKDVEININIFKHLCKRFRTLEQLKQVLAKPIKMKYMPLGKHKGRCFSEIPLAYLQWASKMDFDS
DLLFSIRHEIKHRQKGTGFSQVNNPFMEL

>core/636/5/Org5_Gene226

MDITLVGKKVIVTGGSRGIGLGIVKLFLENGADVEIWGLNEERGQAVIESLTGLGGEVSFARVDVSHNGGVK
DCVQKFLDKHNKIDILVNNAGITRDNLLMRMSEDDWQSVISTNLTSLYYTCSSVIRHMIKARSGSIINVASIVA
KIGSAGQTNYAAAKAGIIAFTKSLAKEVAARNIRVNCLAPGFIETDMTSVLNDNLKAEWLKSIPLGRAGTPED
VARVALFLASQLSSYMTAQTLVVDGGLTY

>core/637/5/Org5_Gene703

MTQDPHDHFKSRTPEDHIKHVRDKHRVCKGEPHTTFKGFFYHLANNALSTGVFIFFIRTLFFLIPTNRALQVKS
LISLGVGWTFYHGCLKARKAWAYMELSHRSMLEEKNEIEENFEQEKIELRILFENQGFKDPLLQEMVEYVCS
DSTLLLDTMIREELYIRKEDLPHPLIQGGSRILGGLCGLAIFLPLVLCISYTLAGVFSALMVLVLSFLKAKILKN
DKISEMVWVLGIFITSASIISSLMKLL

>core/638/5/Org5_Gene285

MTLSFHTHPLNYWTFEEFDGLPIRHGVFSKQKDAEGTVFAAKNPEIASALQSPKYCDLHQRHGTSVRCVTPT
SPTYQPADGLCTQSPLLSLHIRHSDCQAAIFYDREHHAIANVHSGWRGLLGNIYAVTVGTMKKLFHTKPQDL
FVAIGPSIGPDYAIYPDYATLFPRSFLPFMNPKNHFDLRAIARKQLTNLGISKDRIFISDLCTYTEHDAFFSSRYL
AHHPDPNLTGQHSKNRNNVTAVLLLPRD

>core/639/5/Org5_Gene556

MAKQTRRVLFKISGEALSKDSSNRIDEMRLSRLVSELRAVRNNDIEIALVIGGGNILRGLAEQKELQINRVSAD
QMGMLATLINGMAVADALKAEDIPCLLTSTLSCPQLADLYTPQKSIEALDQGKILICTTGAGSPYLTTDTGAA
LRACELNVDVLIKATMHVDGVYDKDPRLFPDAVKYDFVSYKDFLSNQLGVMDASAISLCMDSHIPIRVFSFL
QHSLEKALFDPTIGTLVSEDVNHVCSPRH

>core/640/5/Org5_Gene55

MKFFILFILIVAQFPAFSAQPRTQVSASHSKQAKARRTSRIRSSAATNASVSRYKTRAAARKKIGKFEKKPSLSP
VQWVRYSGKNYSIQTPSLWQCIDDKTQLPEKLDVLLIGKGKGNLTPTINIAQEITSKSSKEYIEEILAYHKANE
MTLESGIFTQIQSPSGEFTIIKTEKNSSWGRVFCLQATTVIDHTAYIFTSTATLDDYAELSFTFLKVVSSFQIRGG
KEATSGDAILEKALEALQNENK

>core/641/5/Org5_Gene471

MHIYGLADLHLALGVPEKTMEVFGDPWIGYHQKICSEWQAVVHPEDIVLLPGDISWAMNLSEAHKDFAFIG
DLPGTKYMIRGNHDYWSSASTSKILQALPPSLYYLNQGFALLTPHLAVVGVRLWDSPTICVKKENFLTPSTQE
QSYTEQDEKIFLRELGRLKRAFAALPKEVTEVIVMTHYPPISSDGTPGPISEFLEADGRVSLCLFGHIHKVQRPI
DGFGNIRGIHYILVAADYVNFVPQEVM

>core/642/5/Org5_Gene1021

MKITTVKTPKIYPYDDLYSILESSLPKLNERSIVVITSKIVSLCEGAVVELEKVSKDELIKQEADAYVFVEKYGI
YLTKKWGILIPSAGIDESNVEGYFVLYPRDXLLSVNTLGDWLRNFYHLEHCGIIISDSHTTPLRRGTMGLGLC
WNGFFPLYNYVGKPDCFGRALKMTYSNLLDGLSAAAVLCMGEGDEQTPIAIIEEAPKITFHSSPTTLQDMSTL
AIAEDEDLYGPLLQSMAWETPAPTS

>core/643/5/Org5_Gene921

MDFDYFGLSDIGRVRARNEDFWQVNLMSQVVAIADGVGGRLGGDIASQEAVTSLMELIDEQQSKLMGYED
DQYKETLKKILLEVNGVVYEHGQMEEHLQGMGTTLSFIQFRKDRAWLFHVGDSRIYRIREGELRRLTEDHSL
ENQLKNRYGLPKQSDKVYSYRHILTNVLGSRPYVMPDIRNLPCEKEDLYCLCSDGLTNMVPDIDIRDILNQPA
TLEERGNALISLANTRGGDDNATVVLVRIQ

>core/644/5/Org5_Gene411

MTIRILAEGLAFRYGSKGPNIIHDVSFSVYDGDFIGIIGPNGGGKSTLTMLILGLLTPTFGSLKTFPSHSAGKQTH
SMIGWVPQHFSYDPCFPISVKDVVLSGRLSQLSWHGKYKKKDFEAVDHALDLVGLSDHHHHCFAHLSGGQI
QRVLLARALASYPEILILDEPTTNIDPDNQQRILSILKKLNRTCTILMVTHDLHHTTNYFNKVFYMNKTLTSLA
DTSTLTDQFCCHPYKNQEFSCSPH

>core/646/5/Org5_Gene826

MSYFNYQKNSVVLRSLGLLAKFFSRLLYRVFFSFREGIYLFSSLYLKYPRLFFYDLGKYVYSLRHCPYAKLGR
LPGASLLKEGNVYGETPWSVLAKICQAFDITSQDILYDLGCGLGKVCFWFSHVVRCQVIGIDNQPHFIRFSSN
MHRKLSSGFALFDTEEFKNVVLSQASYVYFYGSSFSRRLLNEIILKLSEMAPGSVVISISFPLDSFSRGKECFFT
EKSCSVRFPWGKTIAYKNIRKGS

>core/647/5/Org5_Gene196

MSTLLLNPPWMKAGKRIESLVRKALYTHTMLANHRKIVVALSGGKDSLTLLLMLKAISGRGFPDLDLHAVNI
GGKYSCGAEVNKPYLTRICDQLCIPFRTIPSPYAPETPECYPCSQARRRLLFQAAKEIGASAIAFGHHRDDLVQ
TALLNLLHKAEFAGMLPVLDMVHFGVTILRPLIFTPEFWIRKFAKENGFARVTCRCPVVSLRSKAEQSLKLLE
EVFPLARHNIALAIQEHGSSKSQKI

>core/648/5/Org5_Gene206

MTDYSFFRRKIGNIEAIECPGNPQDPIIILCHGYGSLADNLTFFPSICSFSKLRPTWIFPNGILPLENDFRGSRACF
PLNVLLLQELSRLYANGVGNLQEKYDELFDVDLETPKEALEELILNLNRPYNEIIIGGFSQGAILATHLVLTSQ
NPYAGALIFAGARLFNQGWEEGLKQCAQVPFLQSHGYEDEILPYHLGAHLNDLLLTKLNGQFVSFHGGHEIP
SVVFQKMQVTVPNWIDPARG

>core/650/5/Org5_Gene580

MPILSVCNLVKKYNKKPVTNDVSFQINPGEIVGLLGPNGAGKTTAFYLTVGLIRPDSGKIIFKNVDVTKKTMD
HRARLGIGYLAQEPTIFKELTVQDNLICILEIIYKARKQQSHLLNTLVDDLQLGSCLHKKAGTLSGGERRRLEI
ACVLALNPSVLLLDEPFANVDPLVIQNVKYLIKILAGRGIGILITDHNAKELLSIADRCYLIIDGKIFFEGSSSQM
ISNPMVKQHYLGDSFSY

>core/651/5/Org5_Gene343

MKVRIVDSGKSSAASHMAKDRDLLESLQDGELILHLYEWENPCSLTYGHFMRPEKFLLSNYADLGLDAAVR
PTGGGFVFHKGDYAFSVLMSATHPSYSSSVLENYHTVNSFVAKVLEKVFRIQGMLAPEDENSSSRDSGNFCM
AKTSKYDVLFGDKKIGGAAQRKVQQGFLHQGSLFLSGSSSEFYQRFLKPEVLEEIIEQIQIHAFFPLGLEAADE
VLQEARQQVKEAFIKLFCGEGL

>core/652/5/Org5_Gene923

MTLYLLPNTLGTRAVETLPSVIGELVHRLDGLIVESDRGGRAFLSLWKIPEVHKFPLAILSKHARLPKAWDFY
LEPIVKHGENWGLISDAGLPCIADPGASLVRRARALGIPVQAFSGPCSITLALMLSGLPSQSFTFLGYLPQSPKE
RVKSIKKAATSKEVSTSVCIETPYRNVYTFESLLDTLPSYAELCVASDLSGPSELVLTRQVQSWRTTEDLGSV
KQSITKVPTIFLFHIPN

>core/653/5/Org5_Gene441

MAGHSKWANTKHRKERADHKKGKIFSRIIKELISAVKLGGADPKSNARLRMVIQKAKENNIPNENIERNLKK
ATSAEQKNFEEVTYELYGHGGVGIIVEAMTDNKNRTASDMRIAINKRGGSLVEPGSVLYNFARKGACTVAK
SSIDEEVIFSYAIEAGAEDLDTEDEENFLVICAPSELASVKEKLISQGATCSEDRLIYLPLRLVDCDEKDGEANL
ALIDWLEQIEDVDDVYHNMS

>core/654/5/Org5_Gene518

MDISGAVKQKLLQFLGKQKKPELLATYLFYLEQALSLRPVVFVRDKIIFKTPEDAVRILEQDKKIWRETEIQIS
SEKPQVNENTKRIYICPFTGKVFADNVYANPQDAIYDWLSSCPQNMEKQGGVRIKRFLVSEDPDVIKEYAVPP
KEPIIKTVFASAITGKLFHSLPPLLEDFISSYLRPMTLEEVQNQTKFQLESSFLSLLQDALVEDKIAAFIESLADD
TAFHVYISQWVDTEE

>core/655/5/Org5_Gene786

MGLYDRDYIQDSRVQGTFASRVYGWMTAGLIVTSCVALGLYFSGLYRSLFSFWWVWCFATLGVSFFINSKI
QTLSVSAVGGLFLLYSTLEGMFFGTLLPVYAAQYGGGVIWAAFGSAALVFGLAAVYGAFTKSDLTKISKIMT
FALIGLLLVTLVFAVVSMFVSMPLIYLLICYLGLVIFVGLTAADAQAIRRISSTIGDNNTLSYKLSLMFALKMY
CNVIMVFWYLLQIFSSSGNRD

>core/656/5/Org5_Gene32

MHPPIDITAIEAKLNFTFTQPKLLEIALTHPSYKNESAVQIEDSERLEFLGDAVLGLIVTEHLFLLFPSMDEGTLS
TARASLVNAKACCRYTTMLGIGDYLLIGKGEKIQSERGRLSAYANLFESILGAVYLDGGLSPARKLTVPLLPP
REEILPLMSGNPKNLLQQFTQKQFRVLPVYQSTAVTDAQGNVSYQIQVLVNQEVWGEGNASSKKEAEKIAA
QQALDTYGNKNQNTMDV

>core/657/5/Org5_Gene390

MKNSGNIMEPSTNKPDCKKIFDSIASKYDRTNTILSLGMHHFWNRSLIQILGSGYSLLDLCAGTGKVAKRYIA
AHPQASVTLVDFSSAMLDIAKQHLPQGSCSFIHSDINQLPLENHSYPLAAMAYGLRNLSDPHKALQEISRVLM
PSGKLGILELTPPKKTHPTYSAHKLYLRAVVPWIGKSVSKDPDAYSYLSKSIQQLPKDHDLEDLFSKSGFYIAK
KKKLFLGAATIWLLEKQ

>core/658/5/Org5_Gene318

MSSQTMDVLIFYDTETTGTQIERDRIIEIAAYNSVTDESFLTYVNPEIPIPDEASKIHGITTDAVLSAPKFPEAYE
GFRKFCGEDSILVAHNNDGFDFPLLGKECRRHSLEPLTNRTIDSLKWAQKYRPDLPKHNLQYLRQVYGFAEN
QAHRALDDVVILHKVFTSLIGDLPPQQVLDLLQQSYHPKVFKMPFGKYKGQPLVDIPKSYFEWLENQGALDK
PENKDIKAAIALLHQPT

>core/659/5/Org5_Gene623

MQNATIDQLPVSWQEQLPLCWREQLKEEWSKPYMQQLLIFLKQEYKEHTVYPEENCVFSALRSTPFDQVRV
VILGQDPYPGKGQAHGLSFSVPEGQRLPPSLINIFRELKTDLGIENHKGCLQSWANQGILLLNTVLTVRAGEPF
SHAGKGWELFTDAIVTKLIQERTHIIFVLWGAAARKKCELLFNSKHQHAVLSSPHPSPLAAHRGFFGCSHFSKI
NYLLNKLNKPMINWKLP

>core/660/5/Org5_Gene246

MDLAVELKEGILLVDKPQGRTSFSLIRALTKLIGVKKIGHAGTLDPFATGVMVMLIGRKFTRLSDILLFEDKEY
EAIAHLGTTTDSYDCDGKVVGRSKKIPSLEEVLSAAEYFQGEIQQLPPMFSAKKVQGKKLYEYARKGLSIERH
HSTVQVHLQITKYEYPLLHFVVSCSKGTYIRSIAHELGTMLGCGAYLEQLRRLRSGRFSIDECIDGNLLDHPDF
DISPYLRDAHGNSL

>core/661/5/Org5_Gene603

MIGDKIILFVTEDLSLSSQLKDLASQRSDYQILVSPVFPTSFESVAIFCEYLLLPEQIFSPGIFPEEDLIVLFDTFQE
EAITKVLNQGATGYLLRPITAKVLDAVIRAFLRQHEVLEHSIPDTMTFGDHTFRVLNLVIESPEGSVYLTPSEA
GILKKLLINRGHLCLRKNLLAEIKGNTKEIIARNVDVHIASLRKKLGPYGSKIVTIRGVGYLFSDDDSIPLQNHD
NTAHPNEE

>core/662/5/Org5_Gene1008

MTKVRLNKFLASAGVASRRKCDEIIFSGSVTVNGRVAEGPFVLVDPEDKVQVGGTSVHLTKKVYFMVHKAI
GYLCSSEKKFPGTKLVIDLFAHLPYRVFTVGRLDKETSGLILVTNDGEFANKIIHPSSGITKEYLLKVSRDVSA
KDLGKLMEGTFIDGKHVRPVSVTKIRRGTVKIVVSEGKKHEIRLFADAAGLPILELKRIRIGSLVLGGLRYGEY
RELTDAELGTYMKLSD

>core/663/5/Org5_Gene174

MLQAHRLCYSCDNQVILKDASFQASPGTITIILGSSGVGKTTLFRLLAGFLPLQEGELLWNGSPLNRKDVAYM
QQKEALLPWRTALKNMTLSTELGINTSHNALSNERLEEIIHNFDLGQLLDRYPDELSGGQRQRIALAAQCLSL
KPILLLDEPFSSLDVLLKEQLYQDIVALAKKENKTVLLVTHDFHDVSCLGDVLYVIKNKTLTPVPLDPSMRPL
NNGLCFIKDLKKHLYT

>core/664/5/Org5_Gene814

MRVIFPDKHNNFPNLSKLLKKLPSVILVTSCIAPFFSYIINKFFGIPGLLEILALSVKGIQKHHFWQFLTYPLITAD
SLSLNKDQSFEITQRLLLRNVLDFFLFYKAIQHLIRKLGAFSVLVVISGQALIIGAVLWGFMALIHSSQSFFGPE
SIICGVLTVQIFLDPEKRFTIGPTPLSVSIKWGFLFVLGFYCCILIFSGAFLLLLASMLAIVLAILFCKKEKIPNPYT
TSLRF

>core/665/5/Org5_Gene482

MPTTNCIFLDLRGHSILHQLQIEEALLRVANQNFCIINSGAKDSIVLGISRNLNQDVHISRAQADHIPIIRRYSGG
GTVFIDSNTLMVSWIMNSSEASAQPQELLAWTYGIYSPLLPNTFSIRENDYVLGHKKIGGNAQYIQRHRWVH
HTTFLWDIDLDKLSYYLPIPQQQPTYRNQRSHEEFLTTLRPWFPSRDDFLERIKASGSLLFTWEEFLDNELEEIL
AQPHRKATTVLN

>core/666/5/Org5_Gene30

MLSVCYSDPCLSDFCQGKRPLRIASRNSNLAKAQVHECISLLRSWYPKLWFQLSTTETTGDREKKIPLHLVEN
SYFFTDGVDALVHKGVCDLAIHSAKDLPETPSLPVVAITRCLHPADLLVYADHYVHEPLPLSPRLGSSSLRRS
AVLKQLFPQGQILDIRGTIEERLDQLHRGHYDAIVLAKAASLRLHLHHAYSIELPPPYHALQGSLAITAKDHA
GKWKQLFTPIHCHSS

>core/667/5/Org5_Gene667

MKFFSLIFKDDDVSPNKKVLSPEAFSAFLDAKELLEKTKADSEAYVAETEQKCAQIRQEAKDQGFKEGSESW
SKQIAFLEEETKNLRIRVREALVPLAIASVRKIIGKELELHPETIVSIISQALKELTQNKHIIISVNPKDLPLVEKSR
PELKNIVEYADSLILTAKPDVTPGGCIIETEAGIINAQLDVQLDALEKAFSTILKAKNPVDEPSETSSSTDSSSLS
NDQDKKE

>core/668/5/Org5_Gene729

MNRRDMVITAVVVNAILLVALFVTSKRIGVKDYDEGFRNFASSKVTQAVVSEEKVIEKPVVAEVPSRPIAKET
LAAQFIESKPVIVTTPPVPVVSETPEVPTVAVPPQPVRETVKEEQAPYATVVVKKGDFLERIARANHTTVAKL
MQINDLTTTQLKIGQVIKVPTSQDVSNEKTPQTQTANPENYYIVQEGDSPWTIALRNHIRLDDLLKMNDLDEY
KARRLKPGDQLRIR

>core/669/5/Org5_Gene170

MNKKILVLCTAMFFIVCFGFLIHKKHTILPPKAHIPTNAKHFPTIGNPYAPINITVFEEPSCSACAEFTTEVFPLL
KKHYIDTGEISFTLIPVCFIRGSKPAAQALLCIYHHDPRQADIDAYMEYFHRILTYPKEEGSHWATPEVLTKLA
EGLKINSGRSVNPKGLEQCIASGQYNEQIKKNNLYGSQVLGGQLATPTAVVGDYLIEDPTFHEIERAIQHIRQL
QAVEGDHDD

>core/670/5/Org5_Gene632

MVHFSHNPIIQAYTEADFFGKSIFFCLLILSVCTWTVLHQKLAIQKNFLKAGKSLKDFLIKNRHAPLSLDIHPEL
SPFADLYFTIKRGTLELLDKNRQSAPDRGPILSSEDIQSLETLLGAIMPKYKALLHKNSFIPATTISLAPFLGLLG
TVWGILVAFTHISSGSSGNSAIMEGLATALGTTIIGLFVAIPSLIAFNYLKAHSSELISEIEQTAYLLLNSIEVKYR
NTNL

>core/671/5/Org5_Gene49

MTKHGKRIRGILKNYDFSKSYSLREAIDILKQCPPVRFDQTVDVSIKLGIDPKKSDQQIRGAVFLPNGTGKTLR
ILVFASGNKVKEAVEAGADFMGSDDLVEKIKSGWLEFDVAVATPDMMREVGKLGKVLGPRNLMPTPKTGT
VTTDVAKAISELRKGKIEFKADRAGVCNVGVGKLSFESSQIKENIEALSSALIKAKPPAAKGQYLVSFTISSTM
GPGISIDTRELMAS

>core/672/5/Org5_Gene99

MEKDLHLHEKKCLAHEAATQVTSGMILGLGSGSTAKEFIFALAHRIQTESLAVHAIASSQNSYALAKQLAIPL
LNPEKFSSLDLTVDGADEVDPQLRMIKGGGGAIFREKILLRAAKRSIILVDESKLVPVLGKFRVPLEISRFGRSA
IIEEIRHLGYEGEWRLQDTGDLFITDSSNYIYDIFSPNSYPNPEKDLLKLIQIHGVIEVGFVIEKVEVWSSNSQGL
ISKKYSV

>core/673/5/Org5_Gene176

MLQSCKKALLSIVVSILAFHPIPGMGVEAKSGFLGKVKGWFSKKEIQEEARILPVKDSLSWKRYDYTSSSGFS
VEFPGEPDHSGQIVEVPQSEITIRYDTYVTETHPDNTVYVVSVWEYPEKVDISRPELNLQEGFSGMMQALPES
QVLFMQARQIQGHKALEFWIVCEDVYFRGMLISVNHTLYQVFMVYKNKNPQALDKEYEAFSQSFKITKIREP
RTIPSSVKKKVSL

>core/674/5/Org5_Gene201

MALDEINNQNNPSQQIASSTSQTSKINQDRKTFACTVTLLVVATLMILSGIVLLFTIGSLGLSVPLSGILGTFAV
TVGAVLFITGLTILVRKSLGIEQKNEDLNFLKIKTPTPPARPLMSKFSVTCSTTSIVLGMALLIGAVVSVFFLTG
YLQLGLCAGLVGLGTALFVAGLARMSPRSLADQEGSGSADSQSNIVGIGEPKAAQEQKWYKMAVVRGEDGI
PTAIRLTPEK

>core/675/5/Org5_Gene653

MSKPSPRNANQPQKPSASFNKKTRSRLAELAAQKKAKADDLEQVHPVPTEEEIKKALGNIFEGLSNGLDLQQI
LGLSDYLLEEIYTVAYTFYSQGKYNEAVGLFQLLAAAQPQNYKYMLGLSSCYHQLHLYNEAAFGFFLAFDA
QPDNPIPPYYIADSLLKLQQPEESNNFLDVTMDICGNNPEFKILKERCQIMKQSIEKQMAGETKKAPTKKPAG
KSKTTTNKKSGKKR

>core/676/5/Org5_Gene448

MYLEDYDVFFFDLDGLLVDTEPCFYRAFLQACAEFSLEVHWDFSTYYSHTTLGTEIFSKKFIEQYPQAQEYM
AEIFAKRLQIYYKSLEHAGPALMPGVEAFIELVLSLNKTFGVVTNSPRDATHTLRTMYPILNKFLFWVTRENY
ARPKPYGDSYDYAYRTFAREGMKVIGFEDSVKGLRALSKIPATLVCINSMAEITPEDYPELKGKEFFSYPSFD
VLTEHCSQQKLL

>core/677/5/Org5_Gene849

MNTSHRKTLVFSYLSSTFTLLLVLSNLVLSSKLIPTTFFNFIIPGGLILYPLTFLISDVVNEIFGPKKARVMIFSAFI
ANLLASSIVQIFMFFPVASPEMQTAWHCLFDLSPLRFLASLLAFIVSQQLDIVLYTFFKNRTPNSSLWLRSNGS
TWISQIPDTFIVDTCILYFGMGLSFPQTLNIMFYSYIYKITFCVLTTPLFYLAVNTIRKFLGMPSTKIANTVPLIN
QP

>core/678/5/Org5_Gene133

MKKQESVLVGPSIMGADLTCLGVEAKKLEQAGSDFIHIDIMDGHFVPNLTFGPGIIAAINRSTDLFLEVHAMIY
NPFEFIESFVRSGADRIIVHFEASEDIKELLSYIKKCGVQAGLAFSPDTSIEFLPSFLPFCDVVVLMSVYPGFTGQ
SFLPNTIEKIAFARHAIKTLGLKDSCLIEVDGGIDQQSAPLCRDAGADILVTASYLFEADSLAMEDKILLLRGEN
YGVK

>core/679/5/Org5_Gene159

MKGFLSVNELIFGFQTFSVVVLGVFFASRGKAWLTGWLSLLSSIMNVFVLKQIHLWGFEVTSADVYVIGLLT
CLNYAREHYEKNDINDAMLCSWVISIAFLVLTQLHLFLIPSPNDSSQEHFLALFSSTPRIVVASLVTLIFVQIVDI
KLFTFLQRVFSKKYFAMRSTISLLFSQLIDTIIFSFLGLYGLVSNLCDVMIFAMLVKGIVITLAIPTLTVTKAVLD
RRSS

>core/680/5/Org5_Gene454

MRPHRKHVSSKSLALKQSASTHVEITTKAFRLSMPLKQLILEKSDHLPPMETIRVVLTSHKDKLGTEVHVVAS
HGKEILQTKVHNANPYTAVINAFKKIRTMANKHSNKRKDRTKHDLGLAAKEERIAIQEEQEDRLSNEWLPVE
GLDAWDSLKTLGYVPASAKKKISKKKMSIRMLSQDEAIRQLESAAENFLIFLNEQEHKIQCIYKKHDGNYVLI
EPSLKPGFCI

>core/681/5/Org5_Gene698

MALLILLRHGQSVWNEKNLFSGWVDIPLSQQGIEEAFSAGRAIQNLPIDCIFTSTLVRSLMTALLAMTNHHSK
KIPYIVHEDPKAKEMSRIYSAEEENNMIPLYQSSALNERMYGELQGKNKKQTAEQFGEERVKLWRRSYKTAP
PQGESLYDTKQRTLPYFEKNILPQLQNGKNVFVSAHGNSLRSLIMDLEKLSEEEVLSLELPTGKPVVYQWKN
HKIEKHPEFFG

>core/682/5/Org5_Gene173

MSSQPLVTTSSSLSRYVVLTGEEKVACYKKAFNHIWHGAPAIILAAALLMFCIFGFVLGSILLGAPLEGASILY
DVILPWLLPSILVFVLLVLPLNIYAYSHHKQVLALHERITQSNYKEIYDHCEKEKKTPNKKALSLYIESQVLVP
EYSKRFSSMILGKTLKIIPKKDSPESLKHDELIQKALERAKENIYMNKNQREKRDEREAKKEAKNASKTNPLW
EGLGT

>core/683/5/Org5_Gene188

MSLLIEAKNLSKTIQQQNQNISILTDVSLSLHAGETISITGASGNGKTTLLHLLGTLDVPSSGSLRFFDKDLKNQ
DLANFRNQHIGFVFQNFYLLEDDTVLKNVLMPALIARKNISKGSPVYTRALELLDLVNLEDKVRTRCSKLSG
GEKQRVAIARALINEPAILLADEPSGNLDEETSEQIHNLLLEQASALCGILIVTHNKHLASRCSREGVLSNGKLF
FHNS

>core/684/5/Org5_Gene136

MTIRVRNLAYSVNKKKILDGVTFSLERGHITLFVGKSGSGKTMILRALAGLVQPTQGDIWIEGEAPALVFQQP
ELFSHMTVLGNCTHPQIHIKGRSTEEAREKAFELLHLLDIEEVAKNYPDQLSGGQKQRVAIVRSLCMDKHTLL
FDEPTSALDPFATASFRHLLETLRDQELTVGLTTHDMQFVHSCLDRIYLIDQGTVAGVYDKRDGELDSGHPLS
KYIHSAQ

>core/685/5/Org5_Gene969

MDLKLDEVASLLDVSEHTVLQWLKEGAIPSYSMNNEYRFSREEIEDWLLHNQALMIQERGEDKEALKDLSL
KYSLYKAIHRGGVLCDVVVHSKEEALQYASKYIAQKFQLDESVLFEMLSHRENLMSTGIGEGIALPHAKDFLI
NAYYDIVVPMFLAEPIEYGALDGKPVGILFFLFACQDKSHLNLVNKIVHLGMSLNARSFFKNYPNKDQLLAY
VKEWESQTH

>core/686/5/Org5_Gene449

MILRISTVSLLTSCSFSKNSRTCFVTPERITSQKDCPVLLHPKSTTISPPLYDWISPNREVITAYSFYCRGQGNSII
TPEGVLYDCDGLHHSITKEEFRYIHPRLIEVVRLLQQDHPKVSIIEAFCCPKHFHFLEASGISLSQLHLQGTAAT
FALDPPLPMEKLLATIKKLYKKNSDPSLSNFIVTEATLTNPELRLTQQDLGSHTEITVEILDNLQNKEALSSA

>core/687/5/Org5_Gene750

MENSQNFHDTLCQLLDRYSEELYPTLASLLNVTLPNTAISASVSSIPEKAVEVPNAEPQPITPPPPTNLSQEKTK
PSDWKCVPLHPDLSQNAILKEKYPALKDCSLPAPKIPCSIFVYEENNEEVLFFNRLAKILTQQLFPTKLTLIHAK
TNIFVNNPNFFLALAPLNVIRYKIPTTDYHQSLTQNGCIFLPLYSSLEYEKDSQLKRNLWAILNRLPFAYTPKSS

>core/688/5/Org5_Gene798

MKPQDLSPPFLWKERRPCIQDGVLYVPRHYFEHQNFSTSYHQEFFQNHTSIACELCSGNGDWVVAQAQKDP
QVLWIAVEQRFDRVRKIWSKMINHQIQNLRIVCGTAETFFQYYVPDQFLQRLVVNFPDPWPKMRHRKHRLL
QPSFVQEISRSLQDSAVFALATDDKTYLLESIEALQTHLAPRMETPYYIKMTDTYGNSWFENLWRTKGQEIFY
TEFIKKAGI

>core/689/5/Org5_Gene565

MIDPVECFPNLDGDAEAQSITQNSGTPLASELKKDISPFALGSYAAPKDTTLVQGFKPNPMAMMQDQNSNLI
DPELQEALESEELQEQINNLKGRLWDFRSTFEDSQTTAQFADEHFQAVGVIIDLINEDLNTIAEHTQQDARKE
DKEEGSVTRKIIDWVSSGEEVLNRALLYFSDRDGNRESLANFLKVQYAVQRATQRAELFASIVGTSVSSVKTI
MTTQLG

>core/690/5/Org5_Gene611

MTSWIELLDKQIEDQHMLKHEFYQRWSEGKLEKQQLQAYAKDYYLHIKAFPCYLSALHARCDDLQIRRQIL
ENLMDEEAGNPNHIDLWRQFALSLGVSEEELANHEFSQAAQDMVATFRRLCDMPQLAVGLGALYTYEIQIP
QVCVEKIRGLKEYFGVSARGYAYFTVHQEADIKHASEEKEMLQTLVGRENPDAVLQGSQEVLDTLWNFLSS
FINSTEPCSCK

>core/691/5/Org5_Gene507

MVLLSKFDFSGNKIGEVEVADSLFADEGDGLQLIKDYIVAIRANKRQWSACTRNRSEVSHSTKKPFKQKGTG
NARQGCLASPQFRGGGIVFGPKPKFNQHVRINRKERKAAIRLLLAQKIQTNKLTVVDDTVFVDALTAPKTQS
ALRFLKDCNVECRSILFIDHLDHVEKNENLRLSLRNLTAVKGFVYGININGYDLASAHNIVISKKALQELVERL
VSETKD

>core/692/5/Org5_Gene540

MQTLARLFGQSPFAPLQAHLEMVVSCVEYMLPIFTALRDGRYEELLEMAKLVSDKEYQADCIKNDMRNHLP
AGLFMPISRAGILEIISIQDSIADTAEDVAILLTIRRLNFYPSMETLFFRFLEKNLEAFELTMTLLHEFNQLLESSF
GGRKADKARLLVGRVAKSEHESDVLQRELMQIFFSDDFIIPEKEFYLWLQVIRRTAGISDSSEKLAHRINMTLE
EK

>core/693/5/Org5_Gene844

MSTTTVKHFIHTASRWEPVLKEIVASNYWHAQWINTLSFLENSGAKKISASEHPTEVKEEVLKHAAEEFRHG
HYLKTQISRISETSLPDYTSKNLLGGLLTKYYLHLLDLRTCRVLENEYSLSGQTLKTAAYILVTYAIELRASEL
YPLYHDILKEAQSKITVKSIILEEQGHLQEMERELKDLPHGEELLGYACQFEGELCLQFVERLEQMIFDPSSTF
TKF

>core/694/5/Org5_Gene167

MTKNAINSQTTTPQPNLTDAEPIASRAQCKSIAVIISLFALGMLLLCLGIILISIPIPGLAAQVALGLGIVSLILGIA
LANIGFLCLLLRCKQVPQKPDTLPSESSKQPSEGSTPTALPWQAGEFLEKVQVSATPILLPKNKDEELSAKVM
KEGAEAASSIKQAVLESTEKLIDARKQEESRREARKKIVAEEAEASRKRIQQQMAADQEALRKRKEEVAKRK

>core/695/5/Org5_Gene382

MKKYFITGLVILLPLAITIAIVTMIMNFLTQPFVGLASEFFEKFSFYTKHRALLKFVLQIILLFGLFFATVLLGFL
TRIMIFKSLLSIYDKILHRIPIIKTVYKAAQQVMTTIFGSKSGSFKQVVMVPFPNANVQCIGLVAGDAPTVCCT
GEKEDDPLVTVFIPTTPNPTSGFLTLFRKSDIVFLDMKIEDAFKYIISCGVLSTPMACPSSPLPDELHQDQGS

>core/696/5/Org5_Gene883

MGQKGCPIGFRTGVTKKWRSLWYGNKQEFGKFLIEDVRIRQFLRKKPSCQGAAGFVVRRMSGKIEVTIQTAR
PGLVIGKKGAEVDLLKEELRALTGKEVWLEIAEIKRPELNAKLVADNIARQIERRVSFRRAMKKAMQSVMD
AGAVGVKIQVSGRLAGAEIARSEWYKNGRVPLHTLRADIDYATACAETTYGIIGIKVWINLGENSSSTTPNNP
AAPSAAA

>core/697/5/Org5_Gene395

MTLYLGLNQKTARKYQAHYLPILTLFPYAKSTPQNKRALQFLPQATHVILTSPSSTHLFLSRMTSLLSKATLK
TKTYLCIGESTKERLLSFLGQVKYVVATQEIAEGIFPLLQALPSSARILYPHSSLARPVIREFLYNRFTFFSYPHY
TVKPRKLKKNILSKYKKIIFTSPSTVRAFAKIFPRFPEKTYWCQGRMTLQEFQKFSSQKQVSLLETLGKSRTSP

>core/698/5/Org5_Gene591

MLMMLMMIIGITGGSGAGKTTLTQNIKEIFGEDVSVICQDNYYKDRSHYTPEERANLIWDHPDAFDNDLLISD
IKRLKNNEIVQAPVFDFVLGNRSKTEIETIYPSKVILVEGILVFENQELRDLMDIRIFVDTDADERILRRMVRDV
QEQGDSVDCIMSRYLSMVKPMHEKFIEPTRKYADIIVHGNYRQNVVTNILSQKIKNHLENALESDETYYMVN
SK

>core/699/5/Org5_Gene213

MQSDLIQILLKETVNTLYMVSTAFFFSCAIGGMLGLGLFCTSPKSLNPKKSLYATISMILSFLTAIPFAILIVILFP
ITRWIVGTSLGPTASIVPLTIGAIPFVVTIVVDAFRNSALNYLESAVALGIPKRNILFGILLPESYPQLIFSLKSLV
VHLISCSTLAGFVGGGGLGQLLLQYGYYRFEWSVTTSVLVITLVLIESVRILGDFWGRRVLKYRGIL

>core/700/5/Org5_Gene1007

MSTVTTEPCSSIHISLNNDWRDSQPYSLDRASELLHFRFLPSLVFSNWKVEQQIETLCHKSEKRRLISPLAKWL
GKLHKQDLLCPPAPPVSVCWINAHVGYGVFARDEIAPWTYIGEYTGILRHRQAIWMDENDYCFRYPMPLFTL
RYFTIDSGKQGNVTRFINHSEQPNAEAIGVFSEGLFHVIIRTIAPIYAGQEICYHYGPLYWKHRKKREEFIPEEE

>core/701/5/Org5_Gene508

MRSHISVMGKKEGMIHIFDKDGSLVACSVIRVEPNVVTQIKTKESDGYFSLQIGAEEMNAPAHTITKRVSKPK
LGHLRKAGGRVFRFLKEVRGSEEALNGVSLGDAFGLEVFEDVSSVDVRGISKGKGFQGVMKKFGFRGGPGS
HGSGFHRHAGSIGMRSTPGRCFPGSKRPSHMGAENVTVKNLEVIKVDLEKKVLLVKGAIPGARGSIVIVKHSS
RT

>core/702/5/Org5_Gene846

MQRIIIVGIDTGVGKTIVSAILARALNAEYWKPIQAGNLENSDSNIVHELSGAYCHPEAYRLHKPLSPHKAAQI
DNVSIEESHICAPKTTSNLIIETSGGFLSPCTSKRLQGDVFSSWSCSWILVSQAYLGSINHTCLTVEAMRSRNLN
ILGMVVNGYPEDEEHWLTQEIKLPIIGTLAKEKEITKTIISCYAEQWKEVWTSNHQGIQGVSGTPSLNLH

>core/703/5/Org5_Gene320

MNAVNTPKKILCIVADYREISPLIEQLDFTQINEHLYSYRCTDYHLDLYIVHVWGSTAVLNALQSYCQAYTDY
DLWINPGFVGACSPEIPLGQCYTIEKIANLTTDTPPVLSEDPPYIFDALPDSLPKSSLVTSPVLYHYGFHKTFKL
LDMEGYAIASQAAEHHIPCSFLKITSDYTVPGDCPFSRLEEVSQKLTQTLVELLPELMERAIPPKLLLPCP

>core/704/5/Org5_Gene362

MLGSLPCYPGAGNIEEYKNRYFYCQLCAEVVSPYVVPVIVVDVQGAPPTGILQVLRCKQHKFQGLPVHGPIT
SLWALEPVGKGAPQLESAMYELCSQVRNFDICSIVSWVFGGLCIFAGLIVGVMVEAPLIAGLSAWVIPCIIGGV
GAILCLFAILMAYLGRGRVREWLNLSHEYITQCHCRQIQAHSQNYSVITEYPATCALSQPITKLPNGSRRDN

>core/705/5/Org5_Gene137

MDHWLAIARLLLRGCGYTLCVSGIGILCGSILGLLIGTVTSLYFPSKLTKLLANSYVTVIRGTPLFIQILIIYFGLP
EVLPIEPTPLVAGIIALSMNSAAYLAENIRGGINSLSIGQWESAMVLGYKKYQIFVYIIYPQVFKNILPSLTNEFV
SLIKESSILMVVGVPELTKVTKDIVSRELNPMEMYLICAGLYFLMTTSFSCISRLSEKRRSYDN

>core/706/5/Org5_Gene709

MKRVIYKTIFCGLTLLTSLSSCSLDPKGYNLETKNSRDLNQESVILKENRETPSLVKRLSRRSRRLFARRDQTQ
KDTLQVQANFKTYAEKISEQDERDLSFVVSSAAEKSSISLALSQGEIKDALYRIREVHPLALIEALAENPALIEG
MKKMQGRDWIWNLFLTQLSEVFSQAWSQGVISEEDIAAFASTLGLDSGTVASIVQGERWPELVDIVIT

>core/707/5/Org5_Gene546

MQKLVHNIWKKFYSFSSAIAICIVLASFLSLKIVSNTYKHSQAKRNSILLLTRAAEVAVSQGFLPSKSALSSLEQ
AYHLGGESMKPYAGFLASCFYIHNEPLRGAYYAGLAYNNSQALQLPHPIQKLLKEISEAQADQLYDVALSKS
YQLLQTANSSPEYPTLSFLTLLRVIELKELLHQDVSQDFAALKSSPLFHQFERMYSDGEWTLSKRFGKKG

>core/708/5/Org5_Gene436

MIITIDGPSGTGKSTTAKALADHLHFNYCNTGKMYRTLAYARLQSPWATLPLTKFLEEPPFSFTFATGQPLESF
FNGHLLTSELTTQEVANAASELSQLPEVRAFMQDLQRRYAQLGNCVFEGRDMGSKVFPNADLKIFLTSSPEV
RAQRRLKDLPEGTLSPEQLQAELVKRDAADAQRAHDPLVIPENGIVIDSSDLTIRQVLEKILALLFRNEL

>core/709/5/Org5_Gene307

MRKMLVLLASLGLLSPTLSSCTHLGSSGSYHPKLYTSGSKTKGVIAMLPVFHRPGKSLEPLPWNLQGEFTEEI
SKRFYASEKVFLIKHNASPQTVSQFYAPIANRLPETIIEQFLPAEFIVATELLEQKTGKEAGVDSVTASVRVRVF
DIRHHKIALIYQEIIECSQPLTTLVNDYHRYGWNSKHFDSTPMGLMHSRLFREVVARVEGYVCANYS

>core/710/5/Org5_Gene743

MSKKPLYVAHPWHSPTLTQDNYESLCCYIEITPYDSVKFELDKATGLLKVDRPQKFSNFCPCLYGLLPQTYC
GTASGNYSGEQTRREGIQGDKDPLDVCVLTEKNIHHGNILLQARPIGGLRIIDSGEADDKIIAVLEDDLVFAEIE
DISDCPGTVLDMIQHYFLTYKATPNHLIKGSPAKIEIVGIYGKKEAQKVIQLAHEDYLSYIGDTAEVN

>core/711/5/Org5_Gene537

MSVNPSGNSKNDLWITGAHDQHPDVKESGVTSANLGSHRVTASGGRQGLLARIKEAVTGFFSRMSFFRSGAP
RGSQQPSAPSADTVRSPLPGGDARATEGAGRNLIKKGYQPGMKVTIPQVPGGGAQRSSGSTTLKPTRPAPPPP
KTGGTNAKRPATHGKGPAPQPPKTGGTNAKRAATHGKGPAPQPPKGILKQPGQSGTSGKKRVSWSDED

>core/712/5/Org5_Gene83

MNTSISEIQRFLSMIAFEKELVSEDFSVVAGIDEAGRGPLAGPVVASACILPKGKVFPGVNDSKKLSPKQRAQV
RDALMQDPEVCFGIGVISVERIDQVNILEATKEAMLQAISSLPISPDILLVDGLYLPHDIPCKKIIQGDAKSASIA
AASILAKEHRDDLMLQLHRLYPEYGFDRHKGYGTSLHVEAIRRYGPSPCHRKSFSPIKQMCAIV

>core/713/5/Org5_Gene184

MTKGSVFIIMGPPGSGKGTQSQYLANRIGLPHISTGDLLRAIIREGTPNGLKAKAYLDKGAFVPSDFVWEILKE
KLQSQACSKGCIIDGFPRTLDQAHLLDSFLMDVHSNYTVIFLEISEDEILKRVCSRFLCPSCSRIYNTSQGHTEC
PDCHVPLIRRSDDTPEIIKERLTKYQERTAPVIAYYDSLGKLCRVSSENKEDLVFEDILKCIYK

>core/714/5/Org5_Gene336

MTSKKSYKSYFFDPLWSNNQILIAILGICSALAVTTTVQTAITMGIAVSIVTGCSSFFVSLLRKFTPDSVRMITQ
LIIISLFVIVIDQFLKAFFFDISKTLSVFVGLIITNCIVMGRSESLARHVTPIPAFLDGFASGLGYGWVLLVIGVIRE
LFGFGTLMGFRIIPQFVYASETHPDGYQNLSLMVLAPSAFFLLGIMIWLVNIRDSKKRKR

>core/715/5/Org5_Gene17

MYFYKYVIIDTSGYYPFLACVDNQQVLEHWSLPVGPDLGIVLEFLFKSKNLSFQGVAVALGPGNFSATRIGIS
FAQGLAMAKNVPLLGYSSLEGYLLSKDEKKALMLPLGKRGGVLTLSSEIPEEGLNEKRRGVGPGALLSYEEA
SDYCVAHGYYHVISPNPQLFASSFSDKITVEEVAPSVEQIRRHVISQFMFVEYDKQLSPDYRSYSCIF

>core/716/5/Org5_Gene345

MATSVAPSPVPESSPLSHATEVLNLPNAYITQPHPIPAAPWETFRSKLSTKHTLCFALTLLLTLGGTISAGYAG
YTGNWIICGIGLGIIVLTLILALLLAIPLKNKQTGTKLIDEISQDISSIGSGFVQRYGLMFSTIKSVHLPELTTQNQ
EKTRILNEIEAKKESIQNLELKITECQNKLAQKQPKRKSSQKSFMRSIKHLSKNPVILFDC

>core/717/5/Org5_Gene437

MIFRICKFFTWVAFSLFYKLKVYGVKKNFIKGPAIIAVNHNSFLDPIALHMCVHECIYHLARASLFNIPWLWK
QWGCFPVRQDEGNSAAFKIASRLFNKRKKLVIYPEGARSPDGQLQPGKVGIGMMAAKSRVPIIPVYIRGTFEA
FNRHQKIPHVWKTITCVFGTPMYFDDIIQNPEIKNKETYQIITNQTMNKIAELKAWYESGCKGDVP

>core/718/5/Org5_Gene447

MIKSSLILLSGGQGTRFGSKIPKQYLPLNGTPLVLHSLKILSSLPQIAEVIVVCDPSYQETFQEYPVSFAIPGERR
QDSVFSGLQQVSYPWVIIHDGARPFIYPDEIHDLLETAEKIGATALASPIPYTIKQRNPVRTLDRDNLAIIHTPQ
CIKTEILREGLALAKEKQLTLVDDIEAAEIIGKPSQLVFNKHPQIKISYPEDLTIAQALL

>core/719/5/Org5_Gene473

MMNYEDAKLRGQAVAILYQIGAIKFGKHILASGEETPLYVDMRLVISSPEVLQTVATLIWRLRPSFNSSLLCG
VPYTALTLATSISLKYNIPMVLRRKELQNVDPSDAIKVEGLFTPGQTCLVINDMVSSGKSIIETAVALEENGLV
VREALVFLDRRKEACQPLGPQGIKVSSVFTVPTLIKALIAYGKLSSGDLTLANKISEILEIES

>core/720/5/Org5_Gene589

MARYCGPKNRVARRFGANIFGRSRNPLLKKPHPPGQHGMQRKKKSDYGLQLEEKQKLKACYGMIMEKQLV
KAFKEVIHKQGNVAQMFLERFECRLDNMVYRMGFAKTIFAAQQLVAHGHILVNGRRVDRRSFFLRPGMQIS
LKEKSKRLQSVKDALESKDESSLPSYISLDKTGFKGELLVSPEQDQIEAQLPLPINISVVCEFLSHRT

>core/721/5/Org5_Gene676

MKQFILRTLNALFPNPKPSLEGWSSPFQLLIAILLSGNSTDKAVNSVTPQLFAKAPDAQSILDLPPGKLYQLIAP
CGLGERKSAYIYQLSQILVRDFHGEPPNDMALLTQLPGVGRKTASVFLGIAYGKPTFPVDTHILRLAQRWKIS
EKKSPSAAEKDLARFFGHENTPKLHLQLIYYARQYCPALHHKIDNCPICSYLAKEANSTRT

>core/722/5/Org5_Gene59

MSVQVKLTKNSFRLEKQKLARLQTYLPTLKLKKALLQAEVQNAVKDAAECDKDYVQAYERIYAFAELFSIP
LCTDCVEKSFEIQSIDNDFENIAGVEVPIVREVTLFPASYSLLGTPIWLDTMLSASKELVVKKVMAEVSKERLK
ILEEELRAVSIRVNLFEKKLIPETTKILKKIAVFLSDRSITDVGQVKMAKKKIELRKARGDECV

>core/723/5/Org5_Gene56

MANLNADGKLKQICDALRLDTLKPAEDEAAALLHNAKEQAKRIIQEAQEEARKILETAEERAHQKIKQGEVA
LSQAGKRALEALKQAVENKIFRESLVEWLEHVTTDPEVSTKLIQALVQALEAQGVSGNLTAYIGKHVSPRAV
NELLGKAVTTKLRKKSVVVGSFVGGVQLKVEEKNWVLDLSSSALLEIFTRYLQKDFREMIFQGS

>core/724/5/Org5_Gene602

MTYLASSIFSPEDFLYPEIISKAHYTWDILDLMDQMLENHVFSGIHGTVESGVTLKNIEKIEIAEDAYVESGAYI
VGPCILGSQTEVRHGAYLRGNVITGSRCVVGHCTEIKNSYLGHHTKAAHFAYLGDSVLSSEVNLGAGVRCA
NFRLDGRNIYVRSTSDKSKKIDTGRRKLGAFLGKGVAIGCNVVINPGQHILPHTRIRPGQVI

>core/726/5/Org5_Gene34

MSFVPYSLPELPYDYDALEPVISSEIMILHHQKHHQIYINNLNAALKRLDAAETQQNLNELIALEPALRFNGGG
HINHSLFWETLAPIDQGGGQPPKHELLSLIERFWGTMDNFLKKLIEVAAGVQGSGWAWLGFCPAKQELVLQ
ATANQDPLEPLTGKLPLLGVDVWEHAYYLQYKNVRMDYLKAFPQIINWGHIENRFSEIISSK

>core/727/5/Org5_Gene484

MYDYIRGTLTYVHTGAIVIECQGIGYHIAITERWAIECIRALHQDFLVFTHVIFRETEHLLYGFHSREERECFRI
LISFSGIGPKLALAILNALPLKVLCSVVRSEDIRALASVSGIGKKTAEKLMVELKQKLPDLLPLDSRVETSQTHT
TSSCLEEGIQALAALGYSKIAAERMIAEAIKDLPEGSSLTDILPIALKKNFSGVNKD

>core/728/5/Org5_Gene1020

MKIVIASSHGYKIRETKTFLKRLGDFDIFSLSDFPDYKLPQEQEDSITANALTKGIHAANHLGCWVIADDTMLR
VPALNGLPGPLSANFAGVGAYDKDHRKKLLDLMSSLESLVDRSAYFECCVVLVSPNQEIFKTYGICEGYISHQ
EKGSSGFGYDPIFVKYDYKQTFAELSEDVKNQVSHRAKALQKLAPHLQSLFEKHLLTRD

>core/729/5/Org5_Gene769

MGLPNYITFSRLFITPIFMILYLKGKWFGITPVVLPYVLLALLAISELTDAIDGYVARKFSQVTDLGKLLDPMA
DSIYRISIYLTFTQPPVNLPLLLVFIFLARDSVISTLRTVCAFRGRVVAARASGKLKAILQGVSFFLILLVMIPHSL
GLLSQNGLEIFASVTVSIIAVYSIASGIEYFWMNKNFLSQRAKTKDSEKNHESKD

>core/730/5/Org5_Gene208

MFIVIEGGEGSGKSSLAKALGDQLVAQDRKVLLTREPGGCLIGERLRDLILEPPHLELSRCCELFLFLGSRAQH
IQEVIIPALRDGYIVICERFHDSTIVYQGIAEGLGADFVADLCSKVVGPTPFLPNFVLLLDIPADIGLQRKHRQK
VFDKFEKKPLSYHNRIREGFLSLASADPSRYLVLDARESLASLIDKVMLHTQLGLCT

>core/731/5/Org5_Gene84

MNKILVDSPFSPDHQKCCPKLFTISAPAGVGKTTLVRMLEQEFSSAFAETISVTTRKPREGEVPGKDYHFVSHE
EFQRLLDRQALLEWVFLFGECYGTSMLEIERIWSLGKHAVAVIDIQGALFIRSRMPSVSIFIAPPSQEELERRLA
SRGSEEGSQRKERLEHSLIELAAANQFDYVIINDDLNQAYRVLKSIFIAEEHRNIL

>core/732/5/Org5_Gene628

MHAKLSFFILLSLLFSGIDCSRLHAAGRSPSLQGVLAEIEDISAKLASHEVEIVMLSERLDEQDSKCQKWTAAK
PETLAQKIRELESDQKALAKTLAVLTTSVKDLQTNLQSKLQEIQKDHRALAQDLRLVRRSLLALVDSSSPGA
YADFSDPVPENIYIVREGDSLSKIAKKYKLSVTELKKINKLDSDAIYAGQRLCLQRNKQ

>core/733/5/Org5_Gene294

MIGAQKKQSGKKTASRAVRKPAKKVAAKRTVKKATVRKTAVKKPAVRKTAAKKTVAKKTTAKRTVRKTV
AKKPAVKKVAAKRVVKKTVAKKTTAKRAVRKTVAKKPVARKTTVAKGSPKKAAACALACHKNHKHTSSC
KRVCSSTATRKHGSKSRVRTAHGWRHQLIKMMSR

>core/734/5/Org5_Gene223

MTSPIPFQSSGDASFLAEQPQQLPSTSESQLVTQLLTMMKHTQALSETVLQQQRDRLPTASIILQVGGAPTGG
AGAPFQPGPADDHHHPIPPPVVPAQIETEITTIRSELQLMRSTLQQSTKGARTGVLVVTAILMTISLLAIIIIILAV
LGFTGVLPQVALLMQGETNLIWAMVSGSIICFIALIGTLGLILTNKNTPLPAS

>core/735/5/Org5_Gene684

MTLVPYVVEDTGRGERAMDIYSRLLKDRIVMIGQEITEPLANTVIAQLLFLMSEDPKKDIQIFINSPGGYITAG
LAIYDTIRFLGCDVNTYCIGQAASMGALLLSAGTKGKRHALPHSRMMIHQPSGGIIGTSADIQLQAAEILTLKK
HLANILSECTGQPVEKIIEDSERDFFMGAEEAISYGLIDKVVTSAKETNKDTSST

>core/736/5/Org5_Gene476

MLKLLKVSITGDLSSGKTEACQVFQELGAYVVSADEISHSFLIPHTRIGRRVIDLLGSDVVVDGAFDAQAIAA
KVFYNSVLLQGLEAILHPEVCRIIEEQYHQSIQDGNYPLFVAEVPLLYEIHYAKWFDSVILVMANEDIRRERFM
KKTGRSSEDFDQRCSRFLNVEEKLAQADVVVENNGTKKELHQKIEEYFYALKGAL

>core/737/5/Org5_Gene990

MLILLNLSLLFYVLFDSPGSIPVFVALLKNFSRKKQQRVILRECLFALGALILFVTFGRSFFQFLDISLYAFQIIG
GFLLFTVSIKMMLAPMPEKAKDDTSKTEPIFFPLAFPVITGPAVITALLSYMEEGIYSREIIFTAMIIAWAFSLFT
LLCSSFFDRLSGNFGLLALERLFGIALLLMSVNLMLKGISIAFNIGFYIG

>core/738/5/Org5_Gene403

MFSGIIQELGEVCFFEAQGNGLSLGIKSTPLFVTPLVTGDSVAVDGVCLTLTSCNESKIFFDVIPETLACTTLGE
KRCSDQVNLEAALKMGDSIGGHLLSGHVFGTAEIFLIKENRYYFRGSKELSQYLFEKGFIAIDGISLTLVSVDS
DTFSVGLIPETLQRTTLGKKREGERVNIEIDMSTKIQVDTVKRILASSGKD

>core/739/5/Org5_Gene799

MFAYRTLLTHNVVQVSHEIFKTTVVPGDTVIDATCGNGNDSLFLARLLQGEGRLVVYDIQKEALSNALLLFE
THLSEQERSVIEMKEQSHEHILEKDVKLIHYNLGYLPKGNKEITTLARTTEISLEYALNIVRPDGLITVVCYPGH
PEGEKETHSVESLAQRLHPKEWCVSSFYVANRCRAPRLFIFQRQGSESSVDKG

>core/740/5/Org5_Gene229

MTRYPDYLSKLIFFLRKLPGIGFKTAEKLAFELISWDSEQLKILGNAFHNVASERSHCPLCFTLKESKEADCHF
CREERDNQSLCIVASPKDVFFLERSKVFKGRYHVLGSLLSPITGKHIENERLSILKSRIETLCPKEIILAIDATLEG
DATALFLKQELQHFSVNISRLALGLPIGLSFDYVDSGTLARAFSGRHSY

>core/741/5/Org5_Gene457

MGFACRYLFFFIVLFASGSFGNQLLSVPCWLSEEESFYTHRFDFSKSYPDMENMEIQAQRKKRVEFNLTGEFP
KLETLNYQGSFGHLRAKCRGVYPVLYALNFSCSSCKMDMDFRGKWNRSSTITISNQKESINLKLPKDVGVIV
NTKTSLKGNVCPGSTFIKQGWGVWNKIYHNDLVGFSEVTLIFNVSSEGGTITFS

>core/742/5/Org5_Gene528

MRLFSLGTIYLFFSLALSSCCGYSILNSPYHLSSLGKSLLQERIFIAPIKEDPHGQLCSALTYELSKRSFAISGRSS
CAGYTLKVELLNGIDKNIGFTYAPNKLGDKTHRHFIVSNEGRLSLSAKVQLINNDTQEVLIDQCVARESVDFD
FEPDLGTANAHEFALGQFEMHSEAIKSARRILSIRLAETIAQQVYYDLF

>core/743/5/Org5_Gene117

MNWVPKTIDHVDPESEIDIRKVVSCYKLIKECQPEFRSLISELLGVIRCGLRLLKRSKYQEQARTVSDEDAPLF
CLTRSYYQDGYLTPLRAGPRDLINHYIHLRRRENPKHFFSPKHPCYYARLAFNESVCVYRELFDIERLTKMYV
EGDYSKEQEKNLQAILSFVKTLDEGKDFLIEHKDTDLIGRGFTDVFCT

>core/744/5/Org5_Gene768

MRIALSLLSLLMIFPIFGEESRPGSEDGNSNTQEIVGSQDTQVCLYHSYEQGLQASRIEGKPLVIVVLCNSGDD
GQACTIGLSETCEEVLSVLSGSIFSELANFVVLVPSGVNPLIYPPIEDPILAEIVKFKELFKDESFPTGLSIIVVGV
TPEGPGDIIEVSPVSLTVEEEETLPSEQTTEVESTSELQSEDPAIA

>core/745/5/Org5_Gene425

MSSNLHPVGGTGTGAAAPESVLNIVEEIAASGSVTAGLQAITSSPGMVNLLIGWAKTKFIQPIRESKLFQSRAC
QITLLVLGILLVVAGLACMFIFHSQLGANAFWLIIPAAIGLIKLLVTSLCFDEACTSEKLMVFQKWAGVLEDQL
DDGILNNSNKIFGHVKTEGNTSRATTPVLNDGRGTPVLSPLVSKIARV

>core/746/5/Org5_Gene11

MSLPLVLGSSSPRRKFILEKFRVPFTVIPSNFDESKVSYSGDPIAYTQELAAQKAYAVSELHSPCDCIILTGDTIV
SYDGRIFTKPQDKADAIQMLKTLRNQTHDVVTSIAVLHKGKLLTGSETSQISLTMIPDHRIESYIDTVGTLNNC
GAYDVCHGGLILKKVHGCVYNVQGLPIQTLKYLLEELNIDLWDYSI

>core/747/5/Org5_Gene381

MLQEHFFLSEDVITLAQQLLGHKLITTHEGLITSGYIVETEAYRGPDDKACHAYNYRKTQRNRAMYLKGGSA
YLYRCYGMHHLLNVVTGPEDIPHAVLIRAILPDQGKELMIQRRQWRDKPPHLLTNGPGKVCQALGISLENNR
QRLNTPALYISKEKISGTLTATARIGIDYAQEYRDVPWRFLLSPEDSGKVLS

>core/748/5/Org5_Gene626

MTLSLVGKEAPDFVAQAVVNGETCTVSLKDYLGKYVVLFFYPKDFTYVCPTELHAFQDALGEFHTRGAEVI
GCSVDDIATHQQWLATKKKQGGIEGITYPLLSDEDKVISRSYHVLKPEEELSFRGVFLIDKGGIIRHLVVNDLP
LGRSIEEELRTLDALIFFETNGLVCPANWHEGERAMAPNEEGLQNYFGTID

>core/749/5/Org5_Gene332

MFRRTGKGPFEDVQTLYEEETSSPSSYSPYSRSERPETPPSLFDNPKASEARPLNHNLTEESSLPQWSSTPRTES
LLPLEEPETTLGEGVTFKGELAFERLLRIDGTFEGILVSKGKIIIGPKGVVKADIQLQEAIIEGVVEGNITVSGKV
ELRGGAIIKGDIQANTLCVDEGVRILGYLAIAGITDHSERERDL

>core/750/5/Org5_Gene836

MAYGTRYPTLAFHTGGIGESDDGMPPQPFETFCYDSALLQAKIENFNIVPYTSVLPKELFGNIVPVDTCVKSFK
HGAVLEVIMAGRGAALSDGTHAIATGIGICWGKDKNGELIGGWAAEYVEFFPTWINDEIAETHAKMWLKKS
LQHELDLRSIAKHSEFQFFHNYINIKQKFGFCLTALGFLNFENAEPAKVN

>core/751/5/Org5_Gene615

MKKWISILILSFLSLLSILPVLAITINHVKISQRWSDLNSQILTLKVIRDHEDQVIKHNARISKDRNNLSIESLNAS
CKQLRPLSKERERLNKLNSNSLLAQSKEVWERKRALEKSNHQLVWNCEQMHNDFAFVRLEQATEMDNEDI
ESLFSLFNPENPVAPLVFFTCWKMTKQTTPLGNEVWLTHAEAISRWI

>core/752/5/Org5_Gene629

MNIHSLWKLCTLLALLALPACSLSPNYGWEDSCNTCHHTRRKKPSSFGFVPLYTEEDFNPNFTFGEYDSKEEK
QYKSSQVAAFRNITFATDSYTIKGEENLAILTNLVHYMKKNPKATLYIEGHTDERGAASYNLALGARRANAI
KEHLRKQGISADRLSTISYGKEHPLNSGHNELAWQQNRRTEFKIHAR

>core/753/5/Org5_Gene333

MVLFSLLFPKLCYGCQAPGAYFCSNCLEKLLVEDREGRCLHCFRYLGSSETRLCSQCSPSSQLQAFSLYLPSQ
TALSVYARACEGKRPALQFFSKSIAFELASLDETPSCIAYITSTISRKIVVEVAKLEKLLRIPLWPWLPKKRQIE
KLPKGEGICFLSAYPLSQKWMQTIVGGSASPLVSISLFLSQNDQ

>core/754/5/Org5_Gene199

MKRYVVGISGASGVILAVKLIKELVNAKHQVEVIISPSGRKTLYYELGCQSFDALFSEENLEYIHTHSIQAIESS
LASGSCPVEATIIIPCSMTTVAAISIGLADNLLRRVADVALKERRPLILVPRETPLHTIHLENLLKLSKSGATIFPP
MPMWYFKPQSVEDLENALVGKILAYLNIPSDLTKQWSNPE

>core/755/5/Org5_Gene700

MKVIYYEIEEIPSTNTMAKSYMHLWDPYALTVISTKCQTAGTGKFGKSWKSSKGDLLNTFCFFITDLHIDVSR
LFRLGTEAVVALCKDLGITEAKIKWPNDVLVHGEKLCGVLPETLPVEGLLGVVLGIGLNGNTTKQALKDVG
QPATSLQEILGHPIDLETTRELLIHHLLGVLQENLPDSLATKSNRGNI

>core/757/5/Org5_Gene393

MADGEVHKLRDIIEKELLEARRVFFSEPVTEKSASDAIKKLWYLELKDPGKPIVFVINSPGGSVDAGFAVWDQ
IKMLTSPVTTVVTGLAASMGSVLSLCAAPGRRFATPHSRIMIHQPSIGGPITGQATDLDIHAREILKTKARIIDV
YVEATNQPRDIIEKAIDRDMWMTANEAKDFGLLDGILFSFNDL

>core/758/5/Org5_Gene43

MIKKFFIYSLIFSCSFSAPLKGICNEDVSSQSRIEEDPEVLITQLNELIETPIEEGKEIRNELQAISDGQKSSEEIEES
CGTSDSEGLSEKTDKESSNEYVLDFFDSMVQRLEGISKMCQSGQVAQIIDCFNREFDIRNRELELKNRELELRE
KDLEFKKSILDWNKEKVSRELAFQREQDIKQTLMLLKK

>core/759/5/Org5_Gene301

MSIKEDKWIREMALNADMIHPFVNGQVNVNEETGEKLISYGLSSYGYDLRLSREFKVFTNVYNSVVDPKCFT
EDIFISITDDVCIVPPNSFALARSVEYFRIPRNVLTMCIGKSTYARCGIIVNVTPFEPEWEGHVTIEISNTTPLPAK
IYANEGIAQVLFFESSTTCEVSYADRKGKYQKQQGITVPCV

>core/760/5/Org5_Gene724

MVRVSTSEFRVGLRIEIDGQPYLILQNDFVKPGKGQAFNRIKVKNFLTGRVIERTYKSGESVETADIVERSMRL
LYTDQEGATFMDDETFEQEVVFWEKLENIRQWLLEDTIYTLVLYNGDVVAVEPPIFMELSIAETAPGVRGDT
ASGRVLKPAVTNTGAKIMVPIFIDEGELVKVDTRTGSYESRVSK

>core/762/5/Org5_Gene97

MKIPYARLEKGSLLVASPDINQGVFARSVILLCEHSLNGSFGLILNKTLGFEISDDIFTFEKVSNHNIRFCMGGP
LQANQMMLLHSCSEIPEQTLEICPSVYLGGDLPFLQEIASSESGPEINLCFGYSGWQAGQLEKEFLSNDWFLAP
GNKDYVFYSEPEDLWALVLKDLGGKYASLSTVPDNLLLN

>core/763/5/Org5_Gene868

MIRRLEYYGSPILRKKSSPIAEITDEIRNLVSDMCDTMEAHRGVGLAAPQVGKNVSLFVMCVDRETEDGELIF
SESPRVFINPVLSDPSETPIIGKEGCLSIPGLRGEVFRPQKITVTAMDLNGKIFTEHLEGFTARIIMHETDHLNGV
LYIDLMEEPKDPKKFKASLEKIKRRYNTHLSKEELVS

>core/764/5/Org5_Gene801

MALNFKINRQIRAPKVRLIGSAGEQLGILAIKDALDLAREAGLDLVEVASNSEPPVCKIMDYGKYRYGLTKKE
KDSKKAQHQVRIKEVKLKPNIDENDFSTKLKQARTFVEKGNKVKITCMFRGRELAYPEHGFKVVQKMSQGL
EDIGFVEAEPKLAGRSLICVVAPGTVKTKKKQEKSHAQDENQ

>core/766/5/Org5_Gene470

MRILAGKYKGKSLKTFSNPHIRPTSGLVKEAFFSICREDIEGAAFLDLFAGMGAIGFEALSRGAASVVFVDISIK
AIQLIHTNSALLGEQLPVVIFRQDAQSAIQRLIKQKRSFDLIYIDPPYELCNCYVETLLQKIVSGNILNPEGTLFL
ENASDEEIACEGLTLRRRRKLGKTYLAEYIVEKDP

>core/768/5/Org5_Gene996

MELVVTSRETGKKSFLKKIRQQGGIPAVVYSAGKSLANITVDALVFKKFLSNLESGALSSTVFSLSYEGRIIKA
LVKDIQYQITTYDVIHLDFEELVEDRPVKLNIPIRCINAVDCIGVKLGGSLRQVIRAVRVVCKPKDIVPFLELDV
RSVGLSQTRKLSDIKIPAGIETITPLKEVAITVSRR

>core/770/5/Org5_Gene132

MVLSSQLSVGMFISTKDGLYKVTSVSKVAGPKGESFIKVALQAADSDVVIERNFKATQEVKEAQFETRTLEY
LYLEDESYLFLDLGNYEKLFIPQEIMKDNFLFLKAGVTVSAMVYDNVVFSVELPHFLELMVSKTDFPGDSLSL
SGGVKKALLETGIEVMVPPFVEIGDVIKIDTRTCEYIQRV

>core/771/5/Org5_Gene378

MTDTPPENEEQHESNVQNENEVEHLQQEIVTLKTELKEKNDKYLMALAESENSRKRLQKERQELMQYALEN
TLIDFLNPIESMEKALGFATQMSDDVKNWALGFNMILNQFKQIFEEKGIIEYSSIGQKFNPFLHEAVQTEETSE
VPEGTILEEFAKGYKIGERPIRVAKVKVAKAPTPKENKE

>core/772/5/Org5_Gene646

MAAKTKTLELEDNVFLLLEGNLKRIFATPIGYTTFREFQNVVFNCANGQQEIANFFFEMLINGKLTQELAPQQ
KQAAHSLIAEFMMPIRVAKDIHERGEFINFITSDMLTQQERCIFLNRLARVDGQEFLLMTDVQNTCHLIRHLL
ARLLEAQKNPVGEKNLQEIQEEITSLKNHFDELTKALQ

>core/773/5/Org5_Gene442

MVETVLHNFQRYLSKYLYRVFRFPCRKKTFLSSHRVLARPSFPVDYCPGKIYDLQEIYEELNAQLFQGALRLQ
IGWFGRKATRKGKSVVLGLFHENEQLIRIHRSLDRQEIPRFFMEYLVYHEMVHSVVPREYSLSGRSIFHGKKF
KEYEQRFPLYDRAVAWEKANAYLLRGYKKRVGGGYGRA

>core/775/5/Org5_Gene856

MNFAKIDHNHLYLTCLGDLGVACPILSTDCLPNYSEKASHEVLVYSKFRCISGEPSRLATSGNDTYYSIVSLPI GLRYEVTSPSGRHDFNIDMHVAPKIGAVLSHGTREAKEIPGSSKDYAFFSLTARESLMISEKLAMTFQVSEVIQ NCYSQCTKVTKTNLKEQYRHLSHNTGFELSVKSAF

>core/776/5/Org5_Gene496

MSRKAREPILLPQGVEVSIQDDKIIVKGPKGSLTQKSVKEVEITLKDNSIFVHAAPHVVDRPSCMQGLYWALIS NMVQGVHLGFEKRLEMIGVGFRASVQGAFLDLSIGVSHPTKIPIPSTLQVSVEKNTLISVKGLDKQLVGEFAA SIRAKRPPEPYKGKGIRYENEYVRRKAGKAAKTGKK

>core/777/5/Org5_Gene967

MYKWYVVQVFTAQEKKVKKALEDFKESSGMTDFIQEIILPIENVMEVKKGEHKVVEKYIWPGYLLVKMHLT DESWLYVKSTAGIVEFLGGGVPVALSEDEVRSILTDIEEKKSGVVQKHQFEVGSRVKINDGVFVNFIGTVSEV FHDKGRLSVMVSIFGRETRVDDLEFWQVEEVAPGQESE

>core/778/5/Org5_Gene455

MSLLNLPSSQDSASEDSTSQSQIFDPIRNRELVSTPEEKVRQRLLSFLMHKLNYPKKLIIIEKELKTLFPLLMRK GTLIPKRRPDILIITPPTYTDAQGNTHNLGDPKPLLLIECKALAVNQNALKQLLSYNYSIGATCIAMAGKHSQV SALFNPKTQTLDFYPGLPEYSQLLNYFISLNL

>core/779/5/Org5_Gene329

MVEIFNYSTSIYEQHASNNRIVSDFRKEIQMEGISIRDVAKHAQILDMNPKPSALTSLLQTNQKSHWACFSPPN NFYKQRFSTPYLAPSLGSPDQQDEDIEKISSFLKVLTRGKFSYRSQITPFLSYKDKEEEEDEDPEEDDDDPRVQ QGKVLLKALDLGVKSTNVMIDYVISRIFQFVQG

>core/780/5/Org5_Gene162

MSKESIRSYSEISTPTPIFRETPSKEGVAYKLQLRSPAKDCILRNRVSLKGALLRSIPFYGSFLGAKRIHSAWSA KDAPCTTRVYHYLVGGLELLGLGVVVLACKVLATALKFLFSKASSKIKQMKWREKARNLAAKDTVQSIKEF CSVDLTSCFTRCFRLRNRVVEEGASENQTVREIIV

>core/781/5/Org5_Gene613

MTDPKIEKSALRKLFISIRRDLSEERKHEASSAVASFVRSFSKESVVLSFVSFNHEIDMQEANRILIQKCTLALP KIDQENLYPVLIPSIDDLISVVHPKDPFSKQTPISSDKITHVLVPGLAFDQQGYRLGYGHGFYDRWLAQHPYPS IRTIGIGYCEQKIDRLPQESHDIPLSQIYLC

>core/782/5/Org5_Gene498

MSRLKKFYTEEIRKSLFEKFGYANKMQIPVLKKIVLSMGLAEAAKDKNLFQAHLEELTMISGQKPLVTKARN SIAGFKLREGQGIGAKVTLRGIRMYDFMDRFCNIVSPRIRDFRGFSNKGDGRGCYSVGLDDQQIFPEINLDRV KRTQGLNITWVTTAQTDDECTTLLELMGLRFKKAQ

>core/784/5/Org5_Gene771

MAKLIVAIGNPRHGYANTRHNAGFLLADRLVEELQGPPFKPLSKCHALMTLVESSSGPLVFIKPTTFVNLSGK
AVVLAKKYFNVALSHILVLADDVNRSFGKLRLCFNGGSGGHNGLKSITASLGSNEYWQLRFGVGRPLEEGV
ELSNFVLGKFSEEENLQLGSIFVEASTLFTEWCSKF

>core/785/5/Org5_Gene557

MSVLQDTEKKMAAALDFFHKEVKSFRTGKAHPALVETVVVDVYGTTMRLSDIASISVADLRQLVISPYDGN
NASAIAKGIIAANLNLQPEVEGSIIRIKVPEPTADYRQEMIKQLRRKCEEAKINVRNIRREANDKLKKDSALTE
DVVKGNEKKIQELTDKFCKQLDELTKQKEAEIASI

>core/786/5/Org5_Gene695

MTTPQSPGSLSQSHLPHPHDPWDTEPTSLPEDPNDKASQELHSLVHLFRKLSIHLLSEVEKTVQQLKPDLLELA
LLICEKFLYKKLENPQELALLLSTALQRHTTLRSLTPIKVFLHPEDLKTLTDWISTHELPMIKHAEFFPDTSCRR
SGFKIETPNGILRQEISEELDHLLSVLTA

>core/787/5/Org5_Gene430

MACEQHEGCYELEEREEIEDIKDSDTKWVSITQAAKLHNVTRQAIYVAIKQKKLKASKETRWEIDIKDLEEY
KRNRYSRKKSLYQGELVFDNGKGCYSINQVAQILGIPVQKVYYATRTGTIRGERKGAAWVIHVSEIERYKNE
YLSKQAAKKLKGAEPKEHQAPNFEPPTEIFPESN

>core/789/5/Org5_Gene842

MTIILCGLPTSGKSSLGKALAKFLNLPFYDLDDLIVSNYSSALYSSSAEIYKAYGDQKFSECEARILETLPPEDA
LISLGGGTLMYEASYRAIQTRGALVFLSVELPLIYERLEKRGLPERLKEAMKTKPLSEILTERIDRMKEIADYIF
PVDHVDHSSKSSLEQASQDLITLLKS

>core/790/5/Org5_Gene222

MSAPIPTPQELSDQITCLNVQYQQVSELARENKGDIEGLKTLTAALTADAGIQPSADEIYSLQTAAALILSASE
KPGSGPSGSTEGSVTVQSPCKFKKVLAVVLTIIALIAIAVLIACIIAACGGFPLLLSALNLYTIGACVSLPIIASTS
VALICLCTFVANSLIKPVITVRTTR

>core/791/5/Org5_Gene416

MDRDNEVPLPKPKWIYRTGIGQDSHRFLPESSTKPCILGGIIFDHCPGFQANSDGDIIFHAICNAISSVTNKIILG
KVADELLQTRGITDSGIYLEEALKSLKPNQKISHVAITIEGSRPKFLCKLSALRQNIAQVMNLTPTDIGITATSG
EGLSDFGCGDGVQCFCVLTVMEYCD

>core/793/5/Org5_Gene699

MGYVFYVIAGSIFLGISLGAYCQLYYSVKSVLFSWYLLTVYALEKRHALLALSQLVGEEDAQSQKEIDFLSQC
DKLSWRAFLKNSYEIIPTFKEMEDLLSERVQGFLESIETIAEHDRAILCIENFWASKNLFDFEIAAYEEAVEKYL
KLRQRAPLRLASKLFRFLDVPSIRFSS

>core/794/5/Org5_Gene1063

MSLVSYLSNPQKALVLGSKGFSMDCVDNLKLYIFRLKLPGDTERISYSISPEYIREKGEEELLNSPIEVEGSLGR
IDSDQWILSLSLKTQLGLCCPVCNNFFSHSVCLPDLQRVISHDEVGSGVFDCRPLIRQELLLESDCFEECSGQG
CPERKNILKFLEDRKKHEGNNPFEYL

>core/795/5/Org5_Gene344

MSSTLNGVFPSSLPEESADLFITNKEIVALGEKGNVFLTHSIPMHIAAITILVIVALAGIAIICLGCYSQSILLIAVG
IVLTILTLLCLQALVGFIKFIRQLPQQLHTTVQFIREKIRPESSLQLVTNAQRKTTQDTLKLYEELCDLSQKEFK
LQSTLYQKRFELSHKNEKTNQN

>core/796/5/Org5_Gene815

MSIQPVSNTTTKADKVIPDSTKVISDSITINKQSAFYFCISVMLRLSESTTEYGKSILAVLEDNTIVQQQRVKELI
NLPLLKVPDLQKKDGSDDEYKNQNEIQAYQSSNQQISANRQMIQQELSSAQQRAQANQKSVNSTTIESMQIL
QATSSMLSTLKELTIKANLTNSPSD

>core/797/5/Org5_Gene816

MWIIDPLSAKKPLQAAINVPGTPITGGPNTATADDIIAKFSKDSNPLIVTVYYVYQSVLVAQDNLSIIAQELQA
NSSAQTYLNNQEALYQYVSIPKNKLNDNSSSYLQNIQSDNQAIGASRQAIQNQISSLGNAAQVISSNLNTNNNI
IQQSLQVGQALIQTFSQIVSLIANI

>core/798/5/Org5_Gene461

MADDTLIPKLMKNSLSQACSEGLLIAKYPPLQVIVHFDNNLVVKTHLSVAPVFSCLFLGPAAHKAMQEIVLW
CSRYANKEHPPFSSHFAKDLIPSQYLEILNCVAEIPFGEQQTYAEIAKKTDTHPRTVGAACKQNPFLLFFPCHR
VVGSHGERNYVLGPVIHEILLKFENSY

>core/799/5/Org5_Gene916

MGFKNICKQGSQLYLNGIFPERILARKLKNCAKSYPRTALTIEVLVSSVLGALKVILIPCASTYAALTLPLRALF
NAIKTKSCQHLASYAMAWLLNILTIAVIIGLVFSLVFIPPPVVFISLGLLMSVTTSVTLFQVHKNLFPPYEPPPSR
PHTPPPFADEYVPLISESYFD

>core/800/5/Org5_Gene829

MSHLNYLLEKIAASSKEDFPFPDDLESYLEGYVPDKNIALDTYQKIFKISSEDLEKVYKEGYHAYLDKDYAKS
ITVFRWLVFFNPFVSKFWFSLGASLHMSEQYSQALHAYGVTAVLRDKDPYPHYYAYICYTLTNEHEEAEKAL
EMAWVRAQHKPLYNELKEEILDIRKHK

>core/802/5/Org5_Gene259

MSVHITPRKCFILCILSMFTLPTLFPKAHLILFSPYIVLCFYCFSKDKGLVLALGCGVLSDLALGSRGVFLLLYP
LTALITHKAHLIFSKESKAALVIVNMIFYGVFLLLTIPMCALFGHEVRWSIDVLMIPLKCSFLDNLIFTSVIYILP
CAINSGIHKMISFFRRLVCY

>core/803/5/Org5_Gene558

MVHSPTHQCYHCQQPATICYTEIDKDKVIRSYVCATCPCPSHYYNNEHLSLSKGVGVLTLECGNCKTVWHS
KQDDEQLLGCHQCYTNFKNQITSKLKSERVVSSSFTMEKGQGSLHIGRAPGEASNTNPLLKLIALNEALQDTL
EREDYEQAAVIRDQINHLKTKNPDDPS

>core/804/5/Org5_Gene443

MTAEKQNTGILGLEIRYTLPSDATYMLKWLNDPKILRGFPIQTEAEIRETVNFWVGFYRYHSSLTAVYNGNV
AGVATLVLNPYVKVSHHALISIIVGEEFRNKGIGTALLNNLIHLAKTRFKLEVLYLEVYEGNPALHLYQRFGF
VEVGRQNRFYKDEIGYLAKTTMEKDL

>core/805/5/Org5_Gene231

MKKLLFSTFLLVLGSTSAAHANLGYVNLKRCLEESDLGKKETEELEAMKQQFVKNAEKIEEELTSIYNKLQD
EDYMESLSDSASEELRKKFEDLSGEYNAYQSQYYQSINQSNVKRIQKLIQEVKIAAESVRSKEKLEAILNEEA
VLAIAPGTDKTTEIIAILNESFKKQN

>core/806/5/Org5_Gene87

MSEVKPLFLKNDSFDLATQRFQNLINMLQEQAEIYNEYEEKNARVQNEIKEQKDFVKRCIEDFEARGLGVLK
EELASLTRDFHDKAKAETSMLIECPCIGFYYSIHQEEQRQRQERLQKMAERYRDCKQVLEAVQVEQKDMISS
RVVVDDSYFEEEKEEQKVDNRKKEQD

>core/807/5/Org5_Gene610

MCKNRGVRGIVACDPRGVIGLEGKLPWHYPEDLQFFSETIQKFPIVMGRKTWETLPRKYFVDRAVVVFSHEK
RQGVHGEIWVTSLEEFLLLDLSSPTFLIGGGELYSLFLENQIVRDFFISHIKKEYAGDTFFPLSLLETWTKTVLR
DTQKITTCYYENHHSQNTKNISL

>core/808/5/Org5_Gene50

MKQEKTLLLQEVEDKISAAQGFILLRYLRFTAAYSREFRNSLSGVSAEFEVLKKRIFFKAIEAAGLEVDCSDTD
GHLGVVFSCGDPVSAAKQVLDFNKQHKDSLVFLAGRMDNASLSGAEVEAVAKLPSLKELRQQVVGLFAAP
MSQVVGIMNSVLSGVISCVDQKAGKN

>core/809/5/Org5_Gene61

MQRLGLSNLFHCLLLFLRYYYSKLVFGLTVLLAAISVICLLGCSEPSLSSFTEYVGPEYSAAAQLSIEQSCHDE
VYGQQVVVTWSLPSRMRKCLPVTLYLWVYYGNGKVEKLTYEVNQSAGYRVYCLKGLEYKELQGIISYRVA
LCSGNQEIVSRRHHLWMEVISLDSP

>core/810/5/Org5_Gene774

MKQQLLLLEDVDGLGRSGDLITARPGYVRNYLIPKKKAVIAGAGTLRLQAKLKEQRLIQAAADKADSERIAQ
ALKDIVLEFQVRVDPDNNMYGSVTIADIIAEAAKKNIFLVRKNFPHAHYAIKNLGKKNIPLKLKEEVTATLLV
EVTSDNEYVTVLAQGKQTEENQEG

>core/811/5/Org5_Gene211

MIINVRAPAFGITSVQQFSTNFQAAIPILNIVIGCSRISSTYAEDIEEVAQEKLEKSTHSKSSTSVNLWAHRVRGV
VEILGGGIVILALEITALVLQVIIKLIKCLIDVLCVCLFGLGVCVVAIIGAIAFCVVVVVKYLGFCSQGEELEPIE
VKTLISPDKPYPTVVYV

>core/812/5/Org5_Gene485

MSELIIGVDPGTIVAGYAIIAVEQRYQLRPYSYGAIRLSSDMPLPMRYKTLFEQLSGVLDDTQPNAMVLETQF
VNKNPQSTMKLAMARGIVLLAAAQRDILIFEYAPNVAKKAVVGKGHASKRQVQVMVSKILNVPEVLHPSNE
DIADAFALAICHTHVARSPLCGVR

>core/813/5/Org5_Gene480

MRQFCNLLSLSRLWLALYFCQEKLHIRLLAIVGAMLSDVLDGYLARRYKATSRLGSILDPITDKVFVFVCITV
LYMEGSLSIAHLFFICARDLFLIIFVCYLSLVKGWKGYDYGSLFWGKIFTVVQFIILLGVTAGGEIPWTGLVPL
VALGFLYFLERIMDYKKQFLR

>core/814/5/Org5_Gene406

MATRFRSTLLVITLFVLIDWVTKLVVLLQYKDLQILTHPTLYTHSWGRFSFSIAPVFNEGAAFGLFSNYKYFLF
LLRIFVILGLLAYLFFKKKSIQSTTQTALVLLCAGAIGNVGDIIFYGHIVDFISFNYKQWAFPTFNVADVLISLGT
LLLVYKFYFPTKQTEKKR

>core/815/5/Org5_Gene563

MAKYPLEPVLAIKKDRVDRAEKVVKEKRRLLEIEQEKLREKEAERDKVKNHYMQKIQQLRDLLDEGTTSDA
VLQIKSYIKVVAVQLSEEEEKVNKQKEVVLAASKELEKAEVNLAKRRKEEEKTRLHKEEWMKEALKEEARA
EEKEQDEMGQLLFQLRQKKKRESGGS

>core/816/5/Org5_Gene266

MAVEQSHIKEEIEKLIGKAIKRVCGNKENDLCRYLPGPSGGYMHHFTLKKMKSAAPEQLLKMLKTFILESETP
RTINPKPRAPRGSKKRRDFINFTKTDIERVLELARQVGDKDLLARFSPKKPLTSLKRELIRSIRNGIVSVELWNA
YVEAVKAVSSPNLEVTSPFV

>core/817/5/Org5_Gene131

MDLKQIEKLMIAMGRNGMKRFAIKREGLELELERDTREGNRQEPVFYDSRLFSGFSQERPIPTDPKKDTIKET
TTENSETSTTTSSGDFISSPLVGTFYGSPAPDSPSFVKPGDIVSEDTIVCIVEAMKVMNEVKAGMSGRVLEVLIT
NGDPVQFGSKLFRIAKDAS

>core/819/5/Org5_Gene195

MSKKINRNDLCPCGSNKKYKQCCLKKEEQTARYTTEGKFKFSAEVLSASEQGEAGDNCTKLFQRLSQSLTSE
QKAAVGKFHQITKNKEVMSKKALKKAQAKEEKLVTEKLQQHNFEILNTGENLAPPMESTATLNQDTNFVCE
DFIPTQEDFRISENSQKPPVEED

>core/820/5/Org5_Gene297

MSRQNAEENLKNFAKELKLPDVAFDQNNTCILFVDGEFSLHLTYEEHSDRLYVYAPLLDGLPDNTQRKLALYEKLLEGSMLGGQMAGGGVGVATKEQLILMHCVLDMKYAETNLLKAFAQLFIETVVKWRTVCADICAGREPSVDTMPQMPQGGGGMQPPPTGIRA

>core/821/5/Org5_Gene751

MKFWLQGCAFVGCLLLTLPCCAARRRASGENLQQTRPIAAANLQWESYAEALEHSKQDHKPICLFFTGSDWCMWCIKMQDQILQSSEFKHFAGVHLHMVEVDFPQKNHQPEEQRQKNQELKAQYKVTGFPELVFIDAEGKQLARMGFEPGGGAAYVSKVKSALKLR

>core/822/5/Org5_Gene420

MSRRHSAEKRDIPGDPIYGSVILEKFINKVMMHGKKSVARKIVYSALERFGKKLNLENVLEGFGEALENAKPILEVRSRRVGGATYQVPVEVASERRNCLAMQWIIKHARSKPGKSMEVGLATELIDCFNKQGATIKKREDTHRMAEANKAFAHYKW

>core/823/5/Org5_Gene494

MSLSKNSHKEDQLEEKVLVVNRCSKVVKGGRKFSFSALILVGDGKGRLGYGFAKANELTDAIRKGGEAAKKNLMKIEALEDGSIPHEVLVHHDGAQLLLKPAKPGTGIVAGSRIRLILEMAGIKDIVAKSFGSNNPMNQVKAAFKALTGLSPRKDLLRRGAAIND

>core/826/5/Org5_Gene579

MTKFLYCGLFYSLGLLVLAFGTMVAIIQVDQICDVSCMNKHFQESPPFLKIKKVNVSKQICSPEERFFHCKIDKSCMELHFPQSSYSCKEYLTRISGHILTQNFEKQMQFRGNSGLLNYQDGSLHVYDCRFQVDPVPGYGSPDKEDSSSGGMKTLYLSLFRN

>core/827/5/Org5_Gene767

MMHRYFIPLLALLIFSPSLVRAELQPSENRKGGWPTQLSCAEGSQLFCKFEAAYNNAIEEGKPGILVFFSERPTPEFADLTNGSFSLSTPIAKGFNVVVLCPGLISPLDFFHKMDPVILYMGSFLEMFPEVEAVSGPRLCYILIDEQGGAQCQAVLPLETKN

>core/828/5/Org5_Gene817

MSITTLGTLPTVNTINSSRPPLEPLNTPKIGAVLFSIYELLLQAIEIRQQTVLTQSQQLNDNTNIQQQLNQETNQIKYAIVSAGAKEDEITRVQNQNQNYSAQRSNIQDELVTTRQNGQIILSHASTNINIIQQQSSQDSSFIKTTNSIGSTVNQLNKPLG

>core/829/5/Org5_Gene317

MTTWTLNQNNLTKFLKSSDEEPFLERESGLTYINIQANGNELPLFFVIRSEGEILQLICYLPYQLHESHKASTARLLHLLNRDIDIPGFGMDEEQGLIFYRLVLPCLNGEIHDTLLRIYIDTIKLVCDSFSHAIGLISSGNMNLDELRRQALQEQQEKRNE

>core/831/5/Org5_Gene800

MATLSPEKFSGSPISISKEFPQQKMREIILQMLYALDMAPSAEDSLVPLLMSQTAVSQKHVLVALNQTKSILEK
SQELDLIIGNALKNKSFDSLDLVEKNVLRLTLFEHFYSPPINKAILIAEAIRLVKKFSYSEACPFIQAILNDIFTDS
SLNENSLSI

>core/832/5/Org5_Gene296

MMFGHFAGYLGADPEERMTSKGKRVITLRLGVKTRVGMKDETVWCKCNIWHNRYDKMLPYLKKGSGVIV
AGDISVESYMSKDGSPQSSLVISVDSLKFSPFGRNEGSRSPSLEDNHQQVGYESVSVGFEGEALDAEAIKDKD
MYAGYGQEQQYVCEDVPF

>core/834/5/Org5_Gene160

MSKKVFFESYEDFANVASSWPKSLRALVQGRYFVDSELKETPYRIHDFKKTPIHHRLYRSLPIISTIGGIIRLIEA
HSGPIHPRDKMKYRFEVLQAVIEILGLGVLILVFDIIGCFLAFLVAIILSLLLYCNSTFTCVQNLSFTERMLEGIG
EAVNFLA

>core/835/5/Org5_Gene316

MFFNLFSLVFKLSDELALAETIQEPISVHEMFPGSMKLEMFKMLGSLILLLTIFGFGVWAFKKFVRSRSHGFGG
SSQIKILERRSLTPKTSIYLIRVVNKTLVIAETPEKITLLTEFPPDTDINHLLQENNKQSSSSATSDFLSKAIQKIQK
KQQTNQD

>core/837/5/Org5_Gene37

MPSYCQNQQDFSLFSLLSPRLVMFLGKHSRDEILQDLTDLVDAAGLLEDKQAFFDALVRRENIMSTGIGMGV
AIPHGKLESCSNFFIAIGIHTQGILWDAIDGALVRLVFLIGGPENAQAEYLKLLSTLTLSLREESRRQQLLQVNT
IEEVMNVFVGM

>core/838/5/Org5_Gene663

MADETPKENSSKESSSQFDSLKRKVKDLHSNPKVGKWKKFLSHRACEAIGGCLVLVGIIADFISWAGGLFIAC
GVVLGFHVEIRKMLSNLQSYSIANGPIKNAILCGLILFFVLNIPSFAVSFIVLCVILSFITTAPSCSTCSKDHCDKH
QDTSNKPS

>core/839/5/Org5_Gene385

MTQEKIKIHVSNEQTCIPIHLVSVEKLVLTLLEHLKVTTNEIFIYFLEDKALAELHDKVFADPSLTDTITLPIDAP
GDPAYPHVLGEAFISPQAALRFLENTSPNQEDIYEEISRYLVHSILHMLGYDDTSSEEKRKMRVKENQILCML
RKKHALLTA

>core/841/5/Org5_Gene762

MLPISILLFYVILGCLSAYIADKKKRNVIGWFFAGAFFGFIGLVVLLLLPSRRNALEKPQNDPFDNSDLFDDLK
KSLAGNDEIPSSGDLQEIVIDTEKWFYLNKDRENVGPISFEELVVLLKGKTYPEEIWVWKKGMKDWQRVKD
VPSLQQALKEASK

>core/842/5/Org5_Gene255

MRIIRFDPYGALSAQSIAKDSRQNSPLVEKISEEIATNEAIRLALLAIGDREQEEKKQRHRYKLLGQKQAKVLL
SQLRHVHLDFKKLYCDSKKKEDQEKDEKNKQKRSIKVTKKKKGISLGAAASQAIAAAAEAWVIARNKGVLE
TASTLFYQKDEEA

>core/843/5/Org5_Gene763

MEKQNLKLDVKEIEFPETVFSRDIETRVIQVIILHCLAKINGVSLLGGNLIDALFGRDIERMKGIYVEQDSKNHL
VKVRVEVNVDYGVSIPEKTEEIQGCIVSEISEYTGLHVAAVHVIIKGLTQPKDRIDEEIEEEVSVQDLPSPEDFL
LENSEG

>core/844/5/Org5_Gene125

MIMTTISNSPSPALNPELSLIPPPTLVSSGTQTSLAYTIPAQGRRSTLRIILDIFIIILGLATIISTFIVIFFLNGLNLLS
TPSIISSSCLIIVGLLFLIMGLYFMISSLDQGLVGLLQKELSQAEEREEEYIQEIEALRGAPRAESPTESPSTWL

>core/845/5/Org5_Gene520

MRVVLHCPDIPQNTGNIGRTCVALGAELILVRPLGFSLADKFVKRAGMDYWDKLQLTVVDSIEEALHDVPED
QIFCLSTKGSASYTEFSLPSSGTYVFGSESKGLPKEILKKYYKNCLRIPMQQDIRSLNLATSVGIVLYEVVRQKT
VALQKNPTV

>core/846/5/Org5_Gene812

MEKDIFFMQQAFKEARKAYDQDEVPVGCVIVKDDKIIARAHNSVEKLKDATAHAEILCIGSAAQDLDNWRL
LDTVLYCTLEPCLMCAGAIQLARIPRIVWAAPDVRLGAGGSWVNIFTEEHPFHTVSCTGGVCSEEAEHLMKK
FFVEKRREKSEK

>core/847/5/Org5_Gene278

MGYLPVSATDVLFESPAAPLINSANTQNQKLIELKGKQQAESSPRTITSVILEVLLVIGCCLIVLSLLAIRPALQF
TLETGHPAAIAVLAVSGTILLVAVIILFCFLAAVPFAAKKTYKYVKTVDDYASWHSHQQTPTLGTIFSGIVYAE
SQAQL

>core/848/5/Org5_Gene514

MLKKKPVSFSCIDGHIYKIFPNDLNANNTVFGGLLMSLLDRLALVVAERHTESVCVTAFVDALRFYAPAYMG
ENLICKAAVNRTWRTSLEVGVKVWAENIYKQERRHITSAYFTFVAVNEDNQPIPVHQIVPETPEEKRRYNEA
DRRRQARLELK

>core/849/5/Org5_Gene707

MKTLKGHLSAKNLRIAIVGSCFNQAMADALVSGTQETFLKFGGSEDGLMTIRVPGAFEIPCTIKKLLSSERKF
DAIVACGVLIQGETDHYNQIVNQVAAGIGALSLEFCLPITLSIVAAPSAEIAWQRSGIKGRHLGVSGMTTAIEM
ATLFTQI

>core/850/5/Org5_Gene674

MPFAKETEMQRTCWKCEGSVSMHVPQCPYCSAFLQDPPVASGGFSSCHISFPEGASKEEAEDLFAVSSEDWE AVLGDQNPTQETNKQVIPEWTWLQSWPLAALFLGIGLLAFAFLILLFSTDSGLVLTWPKNRAYFYGIIGAAVA YRGYRKLPL

>core/851/5/Org5_Gene33

MRSALHLQHLRHFHNHGSILFENLLTIKDCFLLETKLQNFIAKASKTIDTVRWRENIFRSMPEIYTVVRKRRLD FFAAELVHRPKLSLVRDLWVFPGEEILEGEEDCMLFLLLSGDRAGSGIFFTGPYPSDLYELEKGTTGLLLAFSS VGIPVI

>core/852/5/Org5_Gene511

MNQPSVIKLRELLDLLPHRYPFLLVDKVLSYDIEARSITAQKNVTINEPFFMGHFPNAPIMPGVLILEALAQAA GVLIGLVLENDRNKRIALFLGIQKAKFRQAVRPGDVLTLQADFSLISSKGGKAWAQARVDSQLVTEAELSFA LVDKESI

>core/853/5/Org5_Gene404

MQCPFCNHGELKVIDSRNAPEANAIKRRRECLKCSQRFTTFETVELTLQVLKRDGRYENFQESKLIHGLNAAS SHTRIGQDQVHAIASNVKSELLGKQNREISTKEIGELVMKYLKKADMIAYIRFACVYRRFKDVGELMEVLLS ATPDMEK

>core/854/5/Org5_Gene1056

MAQKEIVSNRKALRNYEVIETLEAGIVLTGTEIKSLRDHGGNLGDAYVIVSKGEGWLLNASIAPYRFGNIYNH EERRKRKLLLHRYELRKLEGKIAQKGMTLIPLGMXLSRGYVKVRLGCCRGKKAYDKRRTIIEREKEREVAAA MKRRHH

>core/855/5/Org5_Gene240

MARNIKYFLILFPGILWISAGMKLLLKATAIALDPLSSFFTYCLLSMVSWGLASLKHRYLLSKTIRKQLSLSSEF FSQKITWIAYIKQTFISRRFLIMVIMIAFSLVLRRYISNPQALFVIRATVGYALIKTAIAYFSKLQNALMENPEGN

>core/856/5/Org5_Gene178

MSKPSSCKAYLGIDYGKKRIGLAYAAEPLLLTLPIGNIEAGKNLKLSAEALHKIILSRNITCVVLGNPLPMQKG LYSSLQEEVSLLAEELKKLSTVEIILWDERLSSVQAERMLKQDCGLSRKDRKGKTDSLAATLILTSFLDSLPKK LTL

>core/857/5/Org5_Gene742

MMKTKYEYSFGVIPIKFFGTPDKNTLKACFICHTRGKHWGFPKGHSEDKEGPQEAARELVEETGLSVVNFF PKVLIEQYSFNNEEQVFVRKEVTYFLAEVRGDIHADPMEICDSQWLSLQEGLRLLSFPELRDLTVEADKFINN YLFSS

>core/858/5/Org5_Gene711

MQLLSPAFAYGAPIPKKYTCQGAGISPPLTFVDVPGAAQSLALIVEDPDVPKEIRSDGLWIHWIVYNLSTTITN
LAEGAEIFAVQGLNTSGKPVYEGPCPPDKQHRYFFTLFALDVVLPEEENVTRDQLYEAMEFHIIEQAELMGTY
EKS

>core/859/5/Org5_Gene187

MEKRKDTKTTIVKSSETTKSWYVVDAAGKTLGRLSSEVAKILRGKHKVTYTPHVAMGDGVIVINAEKVRLT
GAKKGQKIYRYYTGYISGMREIPFENMMARKPNYIIEHAIKGMMPRTRLGKKQLKSLRIVKGDSYETFESQK
PILLDI

>core/860/5/Org5_Gene1028

MEFICPLQHARCLKKQHKIIEELFPEPFQKDHLYLKLMENSSSRDAFDKKRVLKENLVVGCQSDLYLYEVYQ
DGILFFFTYTKALMSSGIASLFTEVYSGETPSTILTCKPIFFQRLTPYLSFGRLNGGESLYMRMKQIAVQYLKPP
QT

>core/861/5/Org5_Gene566

MADLEVFQADFALLFEAGLLAIKQGDEDSARKLFQSLHILNPNHYGHDLGLALISLHKMDLFDAEERLSALIK
GNEDNWSIKAFLSLTHMLIVLHQGSSFEVRRESLESCLKFADQVIANCKIESTRALAQSVLDWHDTLVAKSA
GPLG

>core/863/5/Org5_Gene290

MKSERLKKLESELHDLTQWMQLGLVPKKEISRHQEEIRILEHKIYEEKERLQLLKENGEIEEYVTPRRSPAKTV
YPDGPSMSDIEFVEPTETEIDIDPGETVELELTDEGREDGAVEVDYSHEDDEDPFSDRNRWRRGGIIDPDANE
W

>core/864/5/Org5_Gene287

MKTKMNSRKKAGQWAIFNSPTPGVSSTLVLAWTPWGYYDKDVQDILERKDPMSSSLSEKDSKEFLKNLFVD
LLENGFTSVHIHAEEAFTPLDHTGKPHFKRDNVYLPGKLLGALNEAAVQANVSADTQFTLFLTQDECNPFHD
KKRG

>core/865/5/Org5_Gene660

MSNIMGSRRKLKRSFLLIEVLMALSLVCAVLLPCIRFYYAIHRSFEEDIFNLQLPALIDHCFLSVEEKMRQQMA
EGTVLTSGKGQTVSLAYTSQGIGYRIPYGYNVDIRQEVRGDNLKMKVCLADVVVELFPDQKQAVSVQRCLC
VTL

>core/866/5/Org5_Gene138

MKKKVTIDEALKEILRLEGAATQEELCAKLLAQGFATTQSSVSRWLRKIQAVKVAGERGARYSLPSSTEKTT
TRHLVLSIRHNASLIVIRTVPGSASWIAALLDQGLKDEILGTLAGDDTIFVTPIDEGRLPLLMVSIANLLQVFLD

>core/867/5/Org5_Gene251

MQNQYEQLLESLAPLLNTTLAPDKNNSCLIRFSDTHVPVQIEEDGNSGDLAVSTLLGTLPENVFRERIFKAALS
VNGSFQSSIKGILGYGEVTQQLYLSDILSMNYLNGEKLFEYLKLFSLHAKIWMESLRTGNLPDLHVLGIYYVA

>core/868/5/Org5_Gene733

MEEFVAYIVKNLVTNPEAVEIRSIEDEDNESIKLEIRVAAEDIGKIIGRRGNTIHALRTILRRVCSRLKKKVQIDL
VQPENGTDVIADQDYICDNDSSNSTEDTFGESDTCCSGHCHYDEDLNQEEQEEGNMHHSCECSNHH

>core/869/5/Org5_Gene98

MSLEKELLEETPLVLLNFYKLVSFCNYAGMILGTEEKKFAIYGHVSMGQAFQGADTEGHSPQRPFAHDLLNF
VFSGFDIQVLRVVINDYKDNVFYTRLFLEQKDREFLYVVDVDARPSDSIPLALTHKIPILCVKSVFDAVVPYEE

>core/870/5/Org5_Gene158

MNPVTFDRIQVDFIPEDTSLRINSYIVAGGLLILGVVLSILSVICLDIGLVGLSAGAAFTLGLGCLIFALFLFSFSL
ILLLSQEKRVPDVLSLYLEKEVPQYETPLYKEDLESERDMSAISERLGIIEEKLRIAEKFRYSDSVFV

>core/871/5/Org5_Gene36

MTVFCELDSGGELPEYTTPGAAGADLRANIEEPIALLPGQRALIPTGIKAEIPEGYELQVRPRSGLALKHGITVL
NSPGTIDSDYRGEIRVILINFGDSTFIIEPKMRIAQVVLSPVVQATFVVKQESLAETARGSGGFGHTGAS

>core/872/5/Org5_Gene529

MTFFEGETVFPAVLSELHSMLDLIKRAGKQSKCPQEKLLKLELACEELLVNIISYAYQGENSPGTIAISCISHRG
DLEVVIKDHGPSFNPLAVSINIQEDLPLEQRKLGGLGIFLAKSSVDEFLYAREDHCNIVHLKMLNGQHS

>core/873/5/Org5_Gene483

MEQTLSIIKPDSVSKAHIGEILSIFEQSGLRIAAMKMMHLSQTEAEGFYFVHRERPFFQELVDFMVSGPVVVLV
LEGANAVSRNRELMGATNPAEAASGTIRAKFGESIGVNAVHGSDTLENAAVEIAYFFSKIEVVNASKPLV

>core/874/5/Org5_Gene493

MIKLESLFDISERKRRKKLLGRGPSSGHGKTSGRGHKGDGSRSGYKRRFGYEGGGVPLYRRVPTRGFSHKRF
DKCVEEITTGRLAELFQEGEAITLDALKAKKAIARQAVRVKVILKGDLEKTFVWQDTAVVLSQGVQNLLGIT

>core/875/5/Org5_Gene182

MACSIFFRMSQGDYDDEPLSKKTACLVVDTMLYPVIAVVCAVVSVVLLILKVLFLLLSFPFKLCSASSALPGE
RVSLGSHFKCLYGGGLPYLLACLLIVPVIGTAIHGFIISHRTSEDARLSSAIVFMQAPILQLAGMSGLIKP

>core/876/5/Org5_Gene870

MQEHIHKELLHLGEIFRSSRESQSLSLKDVEAATSIRYSCLEAIEQGCLGKLISPVYAQGFIKKYATYLGLDGD
SILQEHPYVMKIFKEFSDHNMEMLLDLESMGGRNSPERAIHSWSNLWWAGLIIIGGIMVWWLGSLFSIF

>core/877/5/Org5_Gene530

MSLDFFEEFYHQSILNTGTSFPEGYLNIAEILSYPHCTDANTDFLCSQSDNDFIIAESKDKLTLFNADFAIWLVP
ELVQGQAVTRGYIAVSQGEGNYEPEMAFEASGQYNQSSLILEALQLYLKDIKDTENALRSFRFNNDH

>core/878/5/Org5_Gene489

MQHARKKFRVGRTSSHNRCMLANMLKSLIHYERIETTLPKAKELRRHADKMITLAKKNSLAARRIAIGRLM
VRYNKLTSKEARQAKGGDTSVYNVDRLVVNKLFDELGNRFVERKGGYTRILKLQNRIGDNAQKCIIEFLAS

>core/879/5/Org5_Gene596

MNSKSAQKIIDSIKQILTIYNIDFDPSFGSSLSSDSDADYEYLITKTQEKIQELDKRAQEILTQTGMSKEQMEVF
ANNPDNFSPEEWLALEKVRSSCDEYRKETENLINEITLDLHPTKESKRPKQKLSSTKKNKKKNWIPL

>core/880/5/Org5_Gene330

MLDNEWKAILGWGDDELEELRISGYSFLRQGHYSKAILFFEALVILDPLSIYDHQTLGGLYLQIGENSQALAV
LDQALRMQGDHLPTLLNKTKALFCLGRIEEATAIATYLSSCPIPAIANDAEALLMSYSKATKKNAALVR

>core/881/5/Org5_Gene517

MGRYRRVSHSSQETLLLGTELGQVLVPGAVLLLFGDYGAGKTEFVRGIVSGYLGDTIAEEVASPSFSILHVYG
NEPKRLCHYDLYRIDQKNQEYIFQDAEEDDVLCIEWADRLPKPRFCDTINIYITMQTNMEREIIIEKR

>core/882/5/Org5_Gene48

MSVKKVIKIIKLQIPGGKANPAPPIGPALGAAGVNIMGFCKEFNAATQDKPGDLLPVVITVYADKTFTFITKQP
PVSSLIKKTLNLESGSKIPNRNKVGKLTQAQVEAIAEQKMKDMDIVLLESAKRMVEGTARSMGIDVE

>core/883/5/Org5_Gene60

MIDMSVVGPALVLGLAMIGSAIGCGMAGVASHAVMSRIDEGHGKLIGMSAMPSSQSIYGFILMLLMQAAIKN
GTLSPVGGIAIGLSVGAALLVSSVMQGKCCVSGIQAYARSSSIYGKCYAAIGIVESFSLFAVVFALLLL

>core/885/5/Org5_Gene758

MHPLTLPKQSRVLKRKQFLYITRSGFCCRGSQATFYVVPSRHPGTCRMGITVSKKFGKAHERNSFKRVVREV
FRHVRHQLPNCQIVVFPKGHKQRPVFSKLLQDFINQIPEGLHRLGKTKATTGGECTPKSEKCVTAPR

>core/886/5/Org5_Gene503

MLMPKRTKFRKQQKGQFAGLSKGATFVDFGEYAMQTLERGWVTSRQIEACRVAINRYLKRRGKVWIRIFPD
KSVTKKPAETRMGKGKGAPDHWVAVVRPGRILFEVANVSKEDAQDALRRAAAKLGIKTRFVKRVERV

>core/887/5/Org5_Gene224

MNLIDRAFLLKKTIIFQSLDMDLLLTIADKTETIIFKPGSNVFSIGQPGFSFYIIVEGYITISKEKLESPLNLKPLDC
FGEESLFNNKPREYNASANTQVRMLVLSKGQILNIVEECPSVALSFLELYAKQIKFREP

>core/888/5/Org5_Gene163

MVNRYKSSAEFSADHYYDDNLVRMGYKRNLRGLAPVENEVCLFEENNLLESVMASIPIMGSILGLGRLHSV
WSTQDPKDSKISIIFHTALGILETLGLGIIVLLIKITITILLILFTPCLLCYFMYSAAYSDFHPI

>core/889/5/Org5_Gene169

MINFIRSYALYFAWAISCAGTLISIFYSYILNVEPCILCYYQRICLFPLTVILGISAYREDSSIKLYILPQAVLGLGI
SIYQVFLQEIPGMQLDICGRVSCSTKIFLFSYVTIPMASVVAFGAIVCLLVLTKKYRG

>core/890/5/Org5_Gene631

MKYRFTEEIEEEPLVNLTPLIDIVFVILMAFIVAVPLIKLDSIALAPGTQEQEVLSSENDSIAVIKVFADHSLTLN
EHPITLQELTVRLTLLHKAYPEKTPLLLQDGETSFRTYQNVKNAIEAAGFHELHVALQN

>core/891/5/Org5_Gene186

MAKSTIQESVATGRRKQAVSSVRLRPGSGKIDVNGKSFEDYFPLEIQRTTILSPLKKITEDQSQYDLIIRVSGGG
IQGQVIATRLGLARALLKENEENRQDLKSCGFLTRDPRKKERKKYGHKKARKSFQFSKR

>core/892/5/Org5_Gene608

MIAIERYQLIISKFRMWLFLGCSVEERHFKQPVLISVTFSYNEVPSACLSDKLSDACCYLEVTSLIEEIANTKPY
ALIEHLANELFDSLVISFGDKASKIDLEVEKERPPVPNLLNPIKFTISKELCPSPVLSA

>core/893/5/Org5_Gene168

MPYYANTLEFIQGTQSLCPLFKYGFVRHHYKGQLEIEDASHDWDFLEPPSTWKRTLLAAIPILGSVIGLGRLFS
IWSIREPQDSQEYKSIFWHTLCAVLEILGLGIVALILKILATFIMAMPGLKRVATFLFYS

>core/894/5/Org5_Gene497

MGMTSDSIADLLTRIRNALMAEHLYVDVEHSKMREAIVKILKHKGFVAHYLVKEENRKRAMRVFLQYSDD
RKPVIHQLKRVSKPSRRVYVSAAKIPYVFGNMGISVLSTSQGVMEGSLARSKNIGGELLCLVW

>core/895/5/Org5_Gene490

MVKNQAQAKKSVKRKQLKNIPSGVVHVKATFNNTIVSITDPAGNVISWASAGKVGYSGSRKSSAFAATVAA
QDAAKTAMNSGLKEVEVCLKGTGAGRESAVRALISAGLVVSVIRDETPVPHNGCRPRKRRRV

>core/896/5/Org5_Gene999

MNFVSTLTGSDFYAPVLEKLEEAFADTTGQAILFSSSPDFIVHPIAQQLGISSWYASCYRDQSAEQTIYKKCLT
GDKKAQILSYIKKINQARSHTFSDHILDLPFLMLGEEKTVVRPQGRLKKMAKKYYWNIV

>core/898/5/Org5_Gene203

MSQCQSSSTSTWEWMKSFVPNWKNPTPPLSPIPSEDEFILAYEPFVLPKTDPENAQANPPGTSTPNVENGIDDL
NPLLGQPNEQNNANNPGTSGSNPTSLPAPERLPETEENSQEEEQGSQNNEDLIG

>core/899/5/Org5_Gene570

MLEKLIKNFATYMGITSTLELDADGAYVLPISEVVKVRAQQNADNEIVLSASLGALPPSADTAKLYLQMMIG
NLFGRETGGSALGLDSEGNVVMVRRFSGDTTYDDFVRHVESFMNFSETWLSDLGLGKQ

>core/900/5/Org5_Gene309

MRDHAFSKLIGTVRAMVVEGRCPWSLQQSLVSMVEHILGECQEFHEAVLQGKTVQEVGSEAGDVLTLVLIL
CFLLEREGVLASEDVANEAMEKLRRRAPYIFAEDYKPVSIEEADRLWELAKHREKNEST

>core/901/5/Org5_Gene54

MKNKMDYKSQLVFSCPCCCKGNVCFSVFNLDVILTCNVCSSTYTFDSVIRNEIRQFVALCKRIHDANSILGNA
TVSVSVEDNQMDIPFQLLFSRFPVVLNLSLDGKKIAIRFLFDALNTSILHQESDLIS

>core/902/5/Org5_Gene351

MREEGSVSFREYFRAYMCDKIVAQKNFLFTLDAVIKQAGWRSQEKLNLFYVESQALGREIKVSLEEYIQSMV
GILGSQRTKKSFKFSVDFTPLEQALQERCSSDDDEDATATSTATGATASPTDMHEDE

>core/903/5/Org5_Gene51

MTTESLETLVEKLSNLTVLELSQLKKLLEEKWDVTASAPVVAVAAGGGGEAPVAAEPTEFAVTLEDVPADK
KIGVLKVVREVTGLALKEAKEMTEGLPKTVKEKTSKSDAEDTVKKLQDAGAKASFKGL

>core/904/5/Org5_Gene627

MKFTVALFGEAEKGSYDTAYFCRSLVDLHNYLGDVSSPGITLAIKTLLSDYNVVYFRVREEGYCVDSYFFGL
HFLNTQTTLKNIIAIGLPGVGNQHIIEASRSLCQKHNSLLLFFDHDLYDLLTFNQPF

>core/905/5/Org5_Gene950

MRAGGSLVTTYPKEGQRLRSPEQLRVLDDLVQSYPNHLHAIELDCGAIPQDLIGATYIITFADFSXYILSLRSY
QANSPSDDTWGIWFGSIDDPVQAVISFLKDHGFALPSTLAQDPLLCTNK

>core/906/5/Org5_Gene165

MLIGRYSSDDQFTEATKNTPTIIKLGFVRDNLEGLTNPISEIVSETSSSIKDSVLRSLPILGSILGCARLYSTLSTN
DPLDETQEKIWHTIFGALETLGLGILILLFKIIFVILHCIFHLVIGFCK

>core/908/5/Org5_Gene405

MPLSDDEIEQFKKRLLEMKAKLSHTLEGNAQEVKKPNEATGYSQHQADQGTDTFDRTISLEVTTKEYELLRQ
INRALEKINESSYGICDVSGEEIPLARLIAIPYATMTVKAQEQFEKGLLSGN

>core/909/5/Org5_Gene495

MESSLCKKSLMKRRRALRVRKVLKGSPTKPRLSVVKTNKHIYVQLIDDSIGKTLASVSTLSKLNKSQGLTKK
NQEVAKVLGTQIAELGKNLQLDRVVFDRGPFKYHGIVSMVADGAREGGLQF

>core/910/5/Org5_Gene421

MPTINQLIRKRRKSSLARKKSPALQKCPQKRGVCLQVKTKTPKKPNSALRKVAWVRLSNGQEVIAYIGGEGH
NLQEHSIVLIQGGRVKDLPGVRYHIVRGTLDCAAVKNRKQSRSRYGAKRPK

>core/911/5/Org5_Gene718

MALKDTAKKMKDLLDSIQHDLAKAEKGNKAAAQRVRTDSIKLEKVAKLYRKESIKAEKSGLLKRKPSTKAP
AKVKKTAEKKAPKKSSAAAAKTSKAVKASKPASKKTAAKKVKKPSKARGFRK

>core/912/5/Org5_Gene659

MKRQKRKQSITLIEMMVVITLIGIIGGALAFNMRGSIHKGKVFQSEQNCAKVYDILMMEYATGGSSLKEIIAH
KETVVEEASWCKEGRKLLKDAWGEDLIVQLNDKGDDLVIFSKRVQSSNKK

>core/913/5/Org5_Gene694

MRDRLGSLSLILKVKIHKYLDTLHNQKRLALTVSRNIQATNKRIADLHLERYEHFISRDNIKHYDILLEYLKTL
QSSLYKQQSESLRFLEIHHQQLQELINRRKIIEKIKNNKYSKDQEIGT

>core/914/5/Org5_Gene491

MPRIIGIDIPAKKKLKISLTYIYGIGSARSDEIIKKLKLDPEARASELTEEEVGRLNSLLQSEYTVEGDLRRRVQS
DIKRLIAIHSYRGQRHRLSLPVRGQRTKTNSRTRKGKRKTVAGKKK

>core/915/5/Org5_Gene500

MIQQESQLKVADNTGAKKVKCFKVLGGSRRRYATVGDVIVCSVRDVEPNSSIKKGDVIKAVIVRTRRHITRK
DGSTLKFDTNSCVIIDDKGNPKGTRIFGPVAREIRDRGFIKISSLAPEVI

>core/917/5/Org5_Gene241

MEIIHIGTDIIEISRIREAIATHGNRLLNRIFTEAEQKYCLEKTDPIPSFAGRFAGKEAVAKALGTGIGSVVAWKD
IEVFKVSHGPEVLLPSHVYAKIGISKVILSISHCKEYATATAIALA

>core/918/5/Org5_Gene82

MVNLLKELEQEQCRNDLPEFHVGDTIRLATKISEGGKERVQVFQGTVMARRGGGSGETVSLHRVAYGEGME
KSFLLNSPRIVSIEIVKRGKVARARLYYLRGKTGKAAKVKEFVGPRSSKK

>core/919/5/Org5_Gene803

MVRATGSVASRRRRKRILKQAKGFWGDRKGHIRQSRSSVMRAMAFNYMHRKDRKGDFRSLWIARLNVASR
IHSLSYSRLINGLKCANISLNRKMLSEIAIHNPEGFAEIANQAKKALEATV

>core/920/5/Org5_Gene164

MRELNAFELTQPEEYRNRWVLMPCLKCRFCRTQHAKVWSYRCVHEASLYEKNCFLTLTYDDKHLPQYGSL
VKLHLQLFLKRLRKMISPHKIRYFECGAYGTKLQRPHYHLLLS

>core/921/5/Org5_Gene237

MATVAQTPQTTQPQPSVSHKATHRYCSWVFFKPILVSLGLLLASLTTLGLVIASGVTLSLGIGIVLAIQIVLAGI
ALVLAFNHIRQFKQARTAELNSMKMISAPAAATVQKQKLEDRYSSK

>core/922/5/Org5_Gene245

MTENRRIKRVNALLQEAIAKVILKDVKHPKISNLWITVTRVSLSKDLHSARVYVSVMPHENTKEEALEALKV
SAGFIAHRASKNVVLKYFPELHFYLDDIFSPQDYIENLLWQIQEKEKS

>core/923/5/Org5_Gene652

MNKKPKKTKKAVQSKAAPVKRVPEESQEAAIQQLELAVSDLYKELPLAQTFASLTDKNQINSIIAALSGTLES
LHLEELTQGLFPSAQEDANFAKELSSVVHGLKNLTTVVNKQMVKGAE

>core/924/5/Org5_Gene408

MDNYLLGSLIFCCVLLSIGMCTIFVMTICFLRQLNKILKNIHRVTTILNFEAKILAPLMLGKKLLCGWLKKRKN
RGSLSEDIDELLDEKKQRSWKKNLDQGIKWCAALVLIWKVFRNKD

>core/925/5/Org5_Gene740

MDSFCFDLLKVAAKAIDDKKGNNLVVLDVRTISEFTDYFVFVEGSVNVHVKALANTIVEELKKQKVSPLHVE
GITDGNWVVIDYGFIVVHVFVSEIRGKYRLEELWKDGFIVTSKLLAS

>core/926/5/Org5_Gene282

MPVSSAPLPTSHRPSSGNLGLMEPNSKALKAKHQDKTTKTIKLLVKILVAILVIEVLGIIAAFFIPGTPPICLIILG
GLILTTVLCVLLLVIKLALVNKTEGTTAEQQIKRKLSSKSIS

>core/927/5/Org5_Gene80

MALKIRLRQQGRRNHVVYRLVLADVESPRDGKYIELLGWYDPHSSINYQLKSERIFYWLERGAQLSSKAEAL
VKQGAPGVYSALLSKQEARKLVVRKKRRAYRQRRSTQREEAAKDATK

>core/928/5/Org5_Gene867

MSLNKEIGMTVLFYAFLFIFLFLCVILCGLILVQESKSMGLGSSFGVDSGDSVFGVSTPDILKKVTSWCAVAFC
IGCLLLSFSTNLLGKKLDAKEFLLPAAEESDTQASSESVEADES

>core/929/5/Org5_Gene717

MLSRIVTCFLFLLSSLPLFAEEEAAQSKNTFVQPAVMLAIAILFFYFILWRPEQKRRKAMEKRKNDLAKGDKV
TAMGIIGTVDDIREHTVILNIASGKVEVLKGAISEILKPNDNKS

>core/930/5/Org5_Gene387

MSDIQKEEHGSTTIFHLHGKLDGISSPEVQENISQSLAAGSKNIILDCAHLDYMSSAGIRVLLQSYHQVGQHSG
KIVLTTVPKTIEQTLYVTGFLSYFKIFNTVDEAIQTLNKDGD

>core/932/5/Org5_Gene210

MFLKRKKRGGSQVQNKRTASPIKHAKHYLHNYLQELQKIMAARPHDAIDAWNQVFRDKYKGMSQAIGFRD
HILLVKVYNSSLYALLKQTPQNDLIMSLYQVASHVQIREIQFLLG

>core/933/5/Org5_Gene302

MSKVSVRKKNWGFRLLEEVMIKSWWVIFSILIGGFVYDRAIQELRTEELRLQSKVSSLCQDILSAQEKQRQLQLHLQHWQDSAAIEAALIQRLGLIPKGYKKLCVSPKQQSENKD

>core/934/5/Org5_Gene340

MWYSDYHVWILPVHERVVRLGLTEKMQKNLGAILHVDLPSVGSLCKEGEVLVILESSKSAIEVLSPVSGEVIDINLDLVDNPQKINEAPEGEGWLAVVRLDQDWDPSNLSLMDEE

>core/936/5/Org5_Gene772

MGKKENQLYEGAYVFSVTLSEEARRKALDKVISGITNYGGEIHKIHDQGRKKLAYTIRGAREGYYYFIYFSVSPGAITELWKEYHLNEDLLRFMTLRADSVKEVLEFASLPE

>core/937/5/Org5_Gene734

MTAVLILTSFPSEESARSLARHLITERLASCVHVFPKGTSTYLWEGKLCESEEHHIQIKSIDIRFSEICLAIQEFSGYEVPEVLLFPIENGDPRYLNWLTILSYPEKPPLSD

>core/938/5/Org5_Gene504

MFKATARYIRVQPRKARLAAGLMRNLSVQEAEEQLGFSQLKAGRCLKKVLNSAVANAELHENIKRENLSVTEVRVDAGPVYKRSKSKSRGGRSPILKRTSHLTVIVGEKER

>core/939/5/Org5_Gene499

MKKQNIRVGDKVFILAGNDKGKEGKVLSLTEDKVVVEGVNVRIKNIKRSQQNPKGKRISIEAPIHISNVRLTIAGEPAKLSVKVTEQGRELWQRRPDGTSQLYRLVRGKKG

>core/940/5/Org5_Gene338

MTTLPKYVPRSRQNPDTLTFLKRYSSVLLHSENSLSYRIFAKVLAILLTSLAVAFAVTLFSCEGSQLRLCALYIGIALAICVLLTIVVYCIASKIATACKKPPSISRIEIV

>core/943/5/Org5_Gene506

MKDPYDVIKRHYVTEKAKMLEHLSAGTGEGKKKGSFCKDPKFVFIVSHDATKPLIAQALEAIYVDKNVKVKSVNTINVKPQPARMFRGRRKGKTSGFKKAIVTFYQGHSVG

>core/945/5/Org5_Gene367

MTVFKQIIDGLIDCEKVFENENFIAIKDRFPQAPVHLLIIPKKPIPRFQDIPGDEMILMAEAGKIVQELAAEFGIADGYRVVINNGAEGGQAVFHLHIHLLGGRPLGAIA

>core/946/5/Org5_Gene736

MNLSAKEYGDIIVIYLQGSLDAVSVPSVQEYLEQFIQKKHLKIALNFTDVSYISSAGIRLLLSNFKLVQSLGGKMCLCCVKESVTEVMRIAGLDQLILLCQSEQECLSKL

>core/947/5/Org5_Gene396

MASSATPGFDGTAPSLFPPATRPRYNFKLALFVTIAIALVWIALIATTIAIGLCIHPLCSFIFLTAIPLYFISRYICS
HYARNVYIALDVVPDHSKLQDMRSHSPIFSDR

>core/948/5/Org5_Gene77

MKKNTHPEYRQVLFVDSSTGYKFVCGSTYQSEKTEVFEGKEYPVCYVSVSSSSHPFFTGSKKFVDAEGRVDK
FLKRYSNVRQPAQQPQPEEDALPAAKGKKKVVTKKKK

>core/949/5/Org5_Gene22

MNEPTRTYLESEKDTQDQIEELQATCIVKNAAGIHVRPAGVIVRLFDGEPCDVHFTYAGKTINAKSIMSILML
GAPQGGEILVTIRSKEAHRILQKIQDAFSSGFGEL

>core/950/5/Org5_Gene925

MSTSPIGVPSMLNAATSLNATTSKAPLPTSTLAERIKEWLPRILLLIVGAIFTIAGCIVMALTKQILYGLLCVVG
GLLLALGLLLKPENCIYRNAESAARSLSNALE

>core/952/5/Org5_Gene466

MDEITPNYPLLRQDSLWNRVRVSWRADLSVSSRYEIASAIAILGLLVAFCASAAVSIIFTANPLAQVFIDGCLA
LGLLPIPLVIGLLIIGIIVLLYGIYLFPQQRE

>core/954/5/Org5_Gene415

MEPYAVIQTGSKQYQVRSGDVIDVELLGEVASDKEVIFQDVLFVFDGTKASLGSPTIANAQVKAEYLSHVKG
EKVVAYKYKKRKNYHRKHGHRQKYLRVKIREILI

>core/955/5/Org5_Gene575

MSFTYFLALPVDRLMQERFLCSPKRWAPFINSPLYLTLIADHDTPYLAKNLDKFPLPVEQWEKTVLHVSSLLK
SIFLCSDLSSLRLLACTKFEILTLNDLYCAQNI

>core/956/5/Org5_Gene450

MNERTLLLLLKKKKGLFLAILDLTQTESSLTTPELEKVLKQKKIFLSCIDRVDLQIKEFRHAFSSELPQDIQEEL
EEIRDVIIRILDTDKRNYAQKKKEFGIYERP

>core/957/5/Org5_Gene418

MKQQKQKIRIRLKGFDQGQLDRSTADIVETAKRTGARVVGPIPLPTKREVYTVLRSPHVDKKSREQFEIRTHK
RLVDILDPTGKTIDALKMLALPAGVDIKIKAA

>core/958/5/Org5_Gene283

MSSPVVTGTSSASPVEQTKLGEFLERLSGSGRCIKIAFAASTALLLLNTFVSGIVAIAMIFVATSVGAYFTVIGP
LFLLSLILLAIMLISMYKITHPSQNTPISN

>core/959/5/Org5_Gene459

MSFKRFLQQIPVRICLLIIYLYQWLISPLLGSCCRFFPSCSHYAEQALKSHGFLMGCWLSIKRIGKCGPWHPGGIDMVPKTALQEVLEPYQEIDGGDSSHFSE

>core/961/5/Org5_Gene519

MVKIISSENFDSFIASGLVLVDFFAEWCGPCRMLTPILENLAAELPHVTIGKINIDENSKPAETYEVSSIPTLILFKDGNEVARVVGLKDKEFLTNLINKHA

>core/962/5/Org5_Gene94

MSDQATTLRIKPLGDRILVKREEEEATARGGIILPDTAKKKQDRAEVLVLGTGKRTDDGTLLPFEVQVGDIILMDKYAGQEITIDDEEYVILQSSEIMAVLK

>core/963/5/Org5_Gene339

MDPASPVAPHVLQDHVQLSSEELSALSSGVSRVKKLTIAIMVLSLIAISLVACGLFLTGSAPLQLSIWIAASCITLSMLVCACWRYKISNALEKTKVAHES

>core/964/5/Org5_Gene761

MAKKSSVAREAKRRRLVEANFKKRSDLRKIVKSLSVSEEEKENARISLNKMKRDTSPTRLHNRCLLTGRPRGYLRKFAISRICFRQMASMGEIPGVIKASW

>core/965/5/Org5_Gene2

MEQFHLDREEILLLAKASALQLSEELIQEYQTSLSAVITSMKEALAIEIDDADSCESLFMHVVNVEDLREDSVTSDFNREEFLRNVPESLGGLVKVPAVIK

>core/967/5/Org5_Gene409

MFRNNHKPKKTKCKRFRWLRGVLFGGFIATLLTCLFTPKSGVQLRKKILKVKNSGAKKSRVFFKNSKQHTKSFVKQAKLLAKNISHELQDFKKGILDDKD

>core/968/5/Org5_Gene323

MATMTKKKLISTISQDHKIHPNHVRTVIQNFLDKMTDALVKGDRLEFRDFGVLQVVERKPKVGRNPKNAAVPIHIPARRAVKFTPGKRMKRLIETPNKHS

>core/969/5/Org5_Gene605

MAPKKPNKKNVIQRRPSAEKRILTAQKRELINHSFKSKVKTIVKKFEASLKLDDTQATLSNLQSVYSVVDKAVKRGIFKDNKAARIKSKATLKVNARAS

>core/970/5/Org5_Gene830

MKKVVTLSIIFFATYCASELSAVTVVAVPLSEAPGKIQVRPVVGLQFQEEQGSVPYSFYYPYDYGYYYPETYGYTKNTGQESRECYTRFEDGTIFYECD

>core/971/5/Org5_Gene574

MEPRHIYIRKPETPKAPDVEKPGVPEYMTMANTPTFEGPVKTLDQLRRALIEQRGAEEGQKMYDNFIQSILIST
FGLVHKDMDRAQKASKRMRSVYKEQ

>core/972/5/Org5_Gene963

MCLIDCLGQGFEAAINTVCCCSDSSESKANVATVSAGLLALTAIVSFILIILICTGVLGASGMTFGMSNVAAVL
VLLVTILLSMFLSGACFAAKREILR

>core/974/5/Org5_Gene617

MLSYLLRTAINVYSFLILAYIFASWVPDCQSARWYQLVSKCVDPFLNFFRRFVPRIGFIDPSPFVGLLCLGILPF
VILRVLRFIILNIFHSPWLLQYL

>core/975/5/Org5_Gene258

MGKPKKSRTDRALAQEIQKKSTEVLKKPARIKAKNRRKFLIAKEQKTLKHRAQEYDQLVRSLLDSQKKDTD
KVLIFNYENGFVFTDKDHFSKYSIRL

>core/976/5/Org5_Gene85

MIKKDRFTNEKLNKLFDSPFSLVNYAIKQAKIKIAKGDVRSSNVAIETLVLLDREGIQPEFTEEIVVTASPTVER
KRSEHTNSRKKDPSAYTWSDVK

>core/977/5/Org5_Gene423

MYEGKSRMASPTPGQLHLQQKVESKAYDYSRSLAMIATALLFFIVALILSGLSLLPQVFLPFSGAYFIIGSFLAF
IALGILLINCVCDLKQYLTSS

>core/978/5/Org5_Gene23

MGSGYAKKKKEAKIMEQQFLEMEASLLEKRYEGQAGNGLVSVVINGKCDLISVKVQPTCLDPEDPEVIEDLF
RAAFKLAKEQMDQEMSLMRSTMPF

>core/979/5/Org5_Gene327

MNKSRFLRLCCCLCFCGSLFYFYINKQNSLTKLRLEIPCLSVRLRQLEQQNISLRFLIDKIERPDHLMEIAALPE
YQYLEYPSEESISLLSYELP

>core/980/5/Org5_Gene665

MLAFFATSFKSVLFEYSYQSLLLILIVSAPPIILASIVGIMVAIFQAATQIQEQTFAFAVKLVVIFGTLMISGGWL
SNMILRFAGQIFQNFYKWK

>core/982/5/Org5_Gene474

MLIRLFLGISLPKGFPLYLEPPLVLATFQGTQFVGTYSEATNPLYIDNLNLNYHYTQELLYKAVPCNYKSIYRE
IPLIIFPEVLIGSTPTQSTE

>core/983/5/Org5_Gene1

MLGKIIRGLSSLIVILCALNVGLIGITHNKLNIIAKLCGGVSTPATQITYIIIGIAGVICLLSFCPFCSKKSRHSHGD
SCSSGGCHSHHSDKN

>core/988/5/Org5_Gene276

MAKLVITSDDEQQEFELEDNSEIAEPCESMGIPFACTEGVCGTCVIEVLEGRENLSEFTEPEYDFLGEPEDSNER
LACQCRIKGGCVKVTF

>core/989/5/Org5_Gene374

MDDSWILEVKVTPKAKENKIVGFDGQALKVRVTEPPEKGKANDAVISLLAKALSLPKRDVTLIAGETSRKKK
FLLPNRVQDIIFSLHIDV

>core/990/5/Org5_Gene427

MKKAVLIAAMFCGVVSLSSCCRIVDCCFEDPCAPSSCNPCEVIRKKERSCGGNACGSYVPSCSNPCGSTECNS
QSPQVKGCTSPDGRCKQ

>core/991/5/Org5_Gene811

MSLDKGTKEEITKKFQLHEKDTGSADVQIAILTEHIAELKEHLKRSPKDQNSRLALLKLVGQRRKLLEYLNST
DTERYKNLITRLNLRK

>core/992/5/Org5_Gene253

MSRKCPLTGKRPRRGYSYTLRGIAKKKKGIGLKVTGKTKRRFFPNMLTKRLWSTEENRFLKLKISASALRHID
KLGLEKVLERAKSKNF

>core/993/5/Org5_Gene882

MSRSLRKGPFVDHHLLKKVRAMNIEEKKTPIKTWSRRSMITPEMIGHTFEVHNGKKFLTVFVSETMVGHKLG
EFSPTRIFKSHPVKKG

>core/994/5/Org5_Gene128

MKEYLDFLVQRNVERDPQTKRHCTVSQKFGGESIDAKTTTGQLFHIAGKTEPGHGKLCLGESILKQLLALGII
TGYENREREVWVYLD

>core/996/5/Org5_Gene445

MSQKNKNSAFMHPVNISTDLAVIVGKGPMPRTEIVKKVWEYIKKHNCQDQKNKRNILPDANLAKVFGSSDPI
DMFQMTKALSKHIVK

>core/997/5/Org5_Gene766

MKSFKFLLPFLSVILCCGNLLSSPRSRAISVTESIGMSAVKTLVLSEKAHEFLEGIGYGVGASSILRDWQTQQW
LEIESLLAQNEVM

>core/998/5/Org5_Gene501

MASEPRGSRKVKIGVVVSAKMEKTVVVRVERIFSHPQYLKVVRSSKKYYAHTELKVSEGDKVKIQETRPLSKLKRWRVIEHVGVVS

>core/999/5/Org5_Gene863

MVEIHHKDPSLKKLFALQQSLETLNSLSDIVATYEAMFSLIYEGLNKALRKDQLCYLLSVNSKGELLKSPSGDPIVQTFPIHPHH

>core/1000/5/Org5_Gene567

MATNKSCTAFDFNKMLDGVCTYVKGVQQYLTELETSTQGTVDLGTMFNLQFRMQILSQYMESVSNILTAVNTEMITMARAVKGS

>core/1001/5/Org5_Gene414

MAHKKGQGASRNGRDSKSKRLGVKVGAGQKVSTGSILVRQRGTRWNPAQNVGRGRDDTLFALVDGIVVMKKTNRTYISVVPEQL

>core/1003/5/Org5_Gene47

MKQQHNRKALSRKIGTVKKQAKFAGSFLDEIKKIEWVSKHDLKKYIKVVLISIFGFGFAIYFVDLVLRKSITCLDGITTFLFG

>core/1004/5/Org5_Gene568

MFNMENTAKEEKNSQPLLDLEQDMQDHDRAQELKASVQDKVHKLHALLREGSDKESFGQQQSLLAGYVALQKVLGRINRKMI

>core/1005/5/Org5_Gene773

MNKPVHNNEHRRKRFNKKCPFVSAGWKTIDYKDVETLKKFITERGKVLPRRITGVSSRFQGVLSQAIKRARHLGLLPFVGED

>core/1007/5/Org5_Gene40

MTKIQCSAQYYRSRPAERAQTPPQPFLARDRADFWERHPRFSACCRVLLLVAWVVLALLFLFVMLLPLAAGSYLLAF

>core/1009/5/Org5_Gene225

MSLEDDVIAIIVEQLGVDPKEVNENSSFIEDLNADSLDLTELIMTLEEKFAFEISEEDAEKLRTVGDVFTYIKKRQAEQ

>core/1012/5/Org5_Gene577

MKEFLAYIIKNLVDRPEEVRIKEVQGTHTIIYELSVAKPDIGKIIGKEGRTIKAIRTLLVSVASRNNVRVSLEIMEEK

>core/1013/5/Org5_Gene896

MMTLFLVICCATVLLGLGMGILLVGSHLLGRPLSKGCQKPDCCQKKTCDKTEHCATKSRENSTSKCSSNDDVPPTAP

>core/1014/5/Org5_Gene624

MNEGIHSVCFQKTPRLTAKSVVSMEMLLTTQQLPSAEGMPSVANLEADFLRAEALLAEMREIRGCLEQSLRTLVPSE

>core/1015/5/Org5_Gene813

MKNKIVTLLDQLYEDQESRLQKLGEEIVPNLTPEDLLQPMDFPQLEGNPAFRFEEGVLSGIGEVRAAILAALSQEN

>core/1016/5/Org5_Gene725

MRNMEAKKIKELSKEAQLLKKLREKSRVLDEKNKRKAWVAKLVAMPESIREIEKEERVETPQLFQAIAEKILEEGV

>core/1018/5/Org5_Gene582

MFFAPLLYESLRRGLMHPTSHMQQQLARLEFINDQLTTELEHVNELLCSLGFPEGLTTIKAIAEEVLSDDEPLLD

>core/1019/5/Org5_Gene983

MEEVPFENAMQRLEEIVDLMNQPTTSLDASLALYEEADALMRICESRIRQVEQRVRELAEKRHESSLFEEQAVVR

>core/1021/5/Org5_Gene45

MAKKEDTLVLEGKVEELLPGMHFRVILENGMPVTAHLCGKMRMSNIRLLVGDRVTVEMSAYDLTKARVVYRHR

>core/1022/5/Org5_Gene818

MWYKSLAGEEKDVSGNECNDYPEVFKDDVSAYVLVTCGQMSSEGKIQVEMTYEGDPAVISYLLTKARDSLDES

>core/1023/5/Org5_Gene1048

MSYKITLPKADETTAKKVTKISEASTLIFSVLKEKASLGNVHGFCQAENSLSVEANKIISVAENTLAGCFCK

>core/1025/5/Org5_Gene502

MAAKKDLLTQLRGKSDDDLDAYVHENKKALFALRAENLLQNKVVKVHMFSTHKKNIARALTVKQERKGKVHG

>core/1027/5/Org5_Gene879

MMFDFMLARAFRAANTIVNTKQKPFHIHGMATLRPNATSILNIKEDALSLGTVGSFASTASIKIDSH

>core/1028/5/Org5_Gene524

MKKLIALIGIFLVPIKGNTNKEHDAHATVLKAARAKYNLFFVQDVFPVHEVIEPISPDCLVHYEGWV

>core/1031/5/Org5_Gene885

MKKLMLALLLALVLPVAGSAFGYESCSAPQDEAQGSEDEGRDESSDSDTQTEKDEEGGSEENTAE

>core/1033/5/Org5_Gene802

MPKMKTNKSVSARFKLTASGQLKRTRPGKRHKLSKKSSQEKRNLSKQPLVDKGQVGMYKRMMLV

>core/1034/5/Org5_Gene364

MIKQACKFYLLQCLLCALYWLLKYCRKLLKGTLHHSEETLYQALLSSLIDLLYQLKQLPAPTNE

>core/1037/5/Org5_Gene428

MKLFYFCNIIFLTSLFSVSCTHTPPPRSYILAQGRTPLVNKAHSLEFFLARSVFNTCYNTNL

>core/1045/5/Org5_Gene779

MAVPRNRHSNARKNIRRSHDAKKACHAAKCSNCKHALLPHTICPSCGFYNGKAVMTVEKK

>core/1046/5/Org5_Gene532

MSIALNREEVWDNPHHLMFILMQFQQFSGEQDRFGSFLEATIRDRVSFLVLQEKIATLK

>core/1049/5/Org5_Gene865

MRVMRFFCLFFLGFLGSFHCVAEDKGVDLFGVWDDNQITECDDSYMTEGREEVEKVVDA

>core/1050/5/Org5_Gene18

MPSVKVRVGEPVDRALRILKKKIDKEGILKAAKSHRFYDKPSVKKRAKSKAAAKYRSR

>core/1054/5/Org5_Gene306

MQIENSSILFAEVVMKWFIFSVISAPVVFLPGCTLIPKEKVTKVPSQLWSESLSQP

>core/1056/5/Org5_Gene1044

MIDAENQNPKDGGSFTSLHTDPKNLFDEEGMPSPSDTLQCDLNNVFIFIKSMFF

>core/1058/5/Org5_Gene384

MSRHRSYGKSVKGVTKRNVLKRFERVEVLRKLGRWNDSTAKKVTGLPKTPILK

>core/1059/5/Org5_Gene190

MASKNREIIKLKSSESSDMYWTVKNKRKTTGRLELKKYDRKLRRHVIFKEAR

>core/1067/5/Org5_Gene832

MFFIAVRSRGFLDIHGILAARKGKQVVKSTAGAWIGSRGAVFYSLVS

>core/1068/5/Org5_Gene1047

MSESLEIPELTEVLSEQPSLSTPDSPPKVITGTLTLYFQEDIDPAS

>core/1069/5/Org5_Gene760

MKVSSSVKADPSKGDKLVRRKGRLYVINKKDPNRKQRQAGPARKK

>core/1070/5/Org5_Gene759

MKRTYQPSKRKRRNSVGFRTRMATRNGRKLLNRRRRHGRHSLVDL

>core/1073/5/Org5_Gene1062

MSSVKKKRRLKIAKHKRKKRRRRDRHKNK
