# Supplementary material for: Potential Therapeutic Candidates against Chlamydia pneumonia Discovered and Developed In Silico Using Core Proteomics and Molecular Docking and Simulation-Based Approaches
Source: Int J Environ Res Public Health. 2022 Jun 15;19(12):7306. doi: 10.3390/ijerph19127306 (PMC9223490; doi:10.3390/ijerph19127306)
Supplement: Supplementary file 1 [file ijerph-19-07306-s001.zip › file S2.pdf]

Sequence of Cytoplasmic proteins

>core/3/1/Org1_Gene886

MASSSNNSTKQDGIPSWVNPNVQWNRASQVGDQEANSLTPEAQTSRSWFSDRKHFLEVLDVSLEEMENNDLKKYSRYKTIILIATLV
TVAITCIVPISMVFGIPMWVPCLILFGAGLSSAFLSHRLQSKCKEIHLRYRAYQIYRQQLLSQYPDLRKSTLYKYSITHVKPKKGFVGKLVE
NLRPDLHKNKDDGGAAADSRLDFAGYGVKHYQTDALLGVSGVNSVEWQRLASLIMSVKNDILNDVGSREPIDKAQRSALVVSGKDIG
GEIQPGGILDISRDILAICGYGMNVGVEAKKAIDQYKKWYLNSSTFIAWNPQLPAIAQSYLLEQQRHLDYAAKIFQDLSALTTAHGTGQ
ALEDLDSLLCYYDQLIESKGVGEKIIASIHQKHLDLAMQDSCDQEHLKKWSNLYHVFSITIKEFTEGKLEQNEVVSRIQRLRGKLEKSKCSI
LGNCRTNAEYATKSEKKLADYLLQIGDREPFLTGMHKAIATGKAIQGKVEGVISQHPEKQIMMLRCSIERLEGMLRREDWGAILQKNE
DEVLALKSTMEAQLQGFKDLVGTWEGKYQEFKKNKLSKVLVYDFTKSYSNLLNRLEVLHAESSTDDLVLHVDRMSEDLKKTIEEIDGNL
FQVTPEELSLLAREYQGLMNELPLIVQEGNRLQEAISSEGVSQGLMLLNSLLNRDEKINKNIESSRKNLVAIAKQARSDARNIDSQGLAPL
IQRNRASLDNILQNMYLFNGSIRNIHALDTETLVATSSNMFSAMHTFDWNIYTNLLDVLEIQSKPAPAPMENPDLPGALPEEVQDAVA
EDVSGTHRLHHQVLKRRCADLKNMISQLQKSINKWGMAKAIVLGIVAVLFCVLSAIFIGQNILSLLILSCVGLLLTQVCPLIFDRISKSKEFE
KQVLETAQSLIPATKILPSEFNNKDLNRLAKLQDNLNLEGFGPTWARNIVSDLEGIPTKEKSLKDLTKEFRKDSKNLNKRIKRRFKEGLGQ
EAPVVRPTIPQDIRGAEVFAELHRELEHLQKQKEEISIRGDALVQERMGLCLEKSKYDNEKAHAAAMTKKVGKLQNIDRLQKNNETYV
RIQNFFRTLIQEKLGRDTVQEIDVVKEAKELHELAAIIYGNTSGKSQKQRAKKQFKENVLHIAGKGQLELLEAYLNVTASQGLCRHQMQ
ASFRERILLNPDGAKHGEAERTLASREEMLKTLGLSYLTPFVRFSSPESTQSGYNQILKVREQLFDIEQRLQNQETVSPEDYAAVQAALA
AYVRKHESLIVSTYGLGAQEGQTSSKVTTLMRDLHAVEELVEMGVETYRLNRSDQILHRVHSVLHSHLRDSDSSGNGIIDVVKKLFELLN
NNGNNPNDPECQKYMQILLDAPVSLLYGAFKSFKNEFLLNFTELNIANSTKAAEEEAKRYVEEKGRGFETYWEEAKQRLEAIAAELDDL
RNQETLLEQEIRLANLKISIFSDLNLREKVSVEKAALEEEIQGIQEQYAEMQGIEDLELKQKFEDLQKKLEALEERLLQIGRRIDSSVDKQKE
LLGLLGREEAA

>core/8/1/Org1_Gene500

MTWIPLHCHSQYSVLDAMSSIKDFVAKGQEFGIPALALTDHGNLYGAVDFYKECTQKGIQPIIGCECYIAPGSRFDKKKEKRSRAAHHLI
LLCKNEQGYRNLCILTSLAFTEGFYYFPRIDKDLLRQYSEGLICLSGCLSSSVSDAALKSPEALLLELQWFQDLFKDDYFTEVQLHKMSEESI
AGFKEEWLKQEYYSLIEKQIKVNTAVLEASKRLGIPTVATNDIHYINANDWQAHEILLNVQSGETVRIAKQNTHIPNPKRKVYRSREYYF
KSPAQMAELFKDIPEVISNTLEVAKRCDFTFDFSKKHYPIYVPESLKTLNSYTEEDRYQASAVFLKQLAEEALPKKYSSEVLAHIAKKFPHR
DPIDIVKERMDMEMAIIIPKGMCDYLLIVWDIIHWAKANGIPVGPGRGSGAGSVLLFLLGITEIEPIRFDLFFERFINPERLSYPDIDIDICM
AGRERVINYAIERHGKDNVAQIITFGTMKAKMAVKDVGRTLDMALSKVNHIAKHIPDLNTTLSKALETDPDLHQLYINDAESAQVIDM
ALCLEGSIRNTGVHAAGVIICGDQLTNHIPICISKDSTMITTQYSMKPVESVGMLKVDLLGLKTLTSINIAMSAIEKKTGQSLAMATLPLD
DATTFSLLHQGKTMGIFQMESKGMQELAKNLRPDLFEEIIAMGALYRPGPMDMIPSFINRKHGKEIIEYDHPLMESILKETYGIMVYQE
QVMQIAGALASYSLGEGDVLRRAMGKKDFQQMEQEREKFCKRACNNGIDPELATVIFDKMEKFAAYGFNKSHAAAYGLITYTTAYLK
ANYPKEWLAALLTCDSDDIEKIGKLIREAQSMGIPILPPHINVSSNHFVATDEGIRFAMGAIKGIGRGLIESIVEERDHHGPYESIRDFIQRS
DLKKVSKKSIESLIDAGCFDCFDSNRDLLLASVEPLYEAIAKDKKEAASGVMTFFTLGAMDRKNEVPICLPKDIPTRSKKELLKKEKELLGIY
LTEHPMDTVRDHLSRLSVVLAGEFENLPHGSVVRTVFIIDKVTTKISSKAQKKFAVLRVSDGIDSYELPIWPDMYEEQQELLEEDRLIYAIL
VLDKRSDSLRISCRWMKDLSIVNENIIYECDQAFDRIKNQVQKMSFTMSTSGKETKAKGNKPNENGHTQALAPVTLSLDLNELRHSHL
CILKKIVQKHPGSRTLVLVFTQDNERVASMSPDDAYFVCEDIEELRQELVTADLPVRVITV

>core/12/1/Org1_Gene955

MKRPKKFPIYLSIAQKTNRLLSGIVIAFAVIALRLWYLAVVEHEQKLEEAYKPQIRVLPQYVERATICDRFGKTLAVNQLQYDVSVAYGAIR
DLPTRAWRVDEHGHKQLIPVRKHYIMCLSELLSQELHLDREAIEDAIHAKASVLGSVPYLVAANVSERTYLKLKMLSKDWPGLHVEAVV
RRHYPQESVASDILGYVGPISLQEYKRVTQELSQLRECVRAYEEGEDPKLPEGLASIDQVRALLESVESNAYSLNALVGKMGVEACWDS
KLRGKIGKKPILVDRRGNFIQEMEGAVPEAPGTKLQLTLSAELQAYADALLLEYEKTETFRSAKSLKKREKLPPLFPWIKGGAIIALDPNNG
EILAMASSPRYRNNDFVNAKVAEDSKAVRSSIYRWLENKEHIAEIYDRKVPLIRERRNPLTGLCYEEILPLTFDCFLDFLFPENSVIKLQLKR
NSFVGQAIEVQNLVTRLLSLFPYEEGTCPCSAIFDAVFPNEEGHILIQEVISLREQKWIMECLNQHKADIEELKEALDQVFNELPANYDKIL
YTDILRLIVDPERFSPVLPSEVHRLSLSEFTELQGRYVVLRSAFSTILEDAFIEVHFKSWRKSEFLQYLAAKRQEEALRKQRYPTPYVDYLEEE
KTRQYKMFCQEHLDTFLAYLFSKTPYKEGLEPYYDILDLWINELDNGAHRALSWHEHYLFLKERVSHLSEHLPALFSTFREFNELQRPLLG
KYPISIVRNKRQTEQDLAASFYPVYGYGYLRPHAYGQAATLGSIFKLVSAYSVLSQRILWGHNEEPANPLVIIDKNSFGYRSSKPHVGFFK
DGTPIPTFFRGGSLPGNDFMGRGFIDLVSALEMSSNPYFSLLVGEGLGDPEDLADAASLFGFGEKTGLGLPGEYAGRVPHDLAYNRSGL
YATAIGQHTLVVTPLQTAVMLASLVNGGVVYVPKLLLGEWEGEHVSYLSSKKKRTIFMPDAVVEVLKTGMRNVIWGQYGTARAIQSQ
FPPQLLSRIIGKTSTAESIMRVGLDREYGTMKMKDIWFAAVGFSDQDLSLPTIVVIVYLRLGEFGRDAAPMAVKMIDMWEKIQQRESF
LRG

>core/24/1/Org1_Gene643

MLGFLKRFFGSSQERILKKFQKLVDKVNIYDEMLTPLSDDELRNKTAELKQRYQNGESLDSMLPEAYGVVKNVCRRLAGTPVEVSGYH
QRWDMVPYDVQILGAIAMHKGFITEMQTGEGKTLTAVMPLYLNALTGKPVHLVTVNDYLAQRDCEWVGSVLRWLGLTTGVLVSGT
LLEKRKKIYQCDVVYGTASEFGFDYLRDNSIATRLEEQVGRGYYFAIIDEVDSILIDEARTPLIISGPGEKHNPVYFELKEKVASLVYLQKELC
SRIALEARRGLDSFLDVDILPKDKKVLEGISEFCRSLWLVSKGMPLNRVLRRVREHPDLRAMIDKWDVYYHAEQNKEESLERLSELYIIVD
EHNNDFELTDKGMQQWVEYAGGSTEEFVMMDMGHEYALIENDETLSPADKINKKIAISEEDTLRKARAHGLRQLLRAQLLMERDVD
YIVRDDQIVIIDEHTGRPQPGRRFSEGLHQAIEAKEHVTIRKESQTLATVTLQNFFRLYEKLAGMTGTAITESREFKEIYNLYVLQVPTFKP
CLRIDHNDEFYMTEREKYHAIVNEIATIHGKGNPILVGTESVEVSEKLSRILRQNRIEHTVLNAKNHAQEAEIIAGAGKLGAVTVATNMA
GRGTDIKLDNEAVIVGGLHVIGTTRHQSRRIDRQLRGRCARLGDPGAAKFFLSFEDRLMRLFASPKLNTLIRHFRPPEGEAMSDPMFNR
LIETAQKRVEGRNYTIRKHTLEYDDVMNKQRQAIYAFRHDVLHAESVFDLAKEILCHVSLMVASLVMSDRQFKGWTLPNLEEWITSSF
PIALNIEELRQLKDTDSIAEKIAAELIQEFQVRFDHMVEGLSKAGGEELDASAICRDVVRSVMVMHIDEQWRIHLVDMDLLRSEVGLRT
VGQKDPLLEFKHESFLLFESLIRDIRITIARHLFRLELTVEPNPRVNNVIPTVATSFHNNVNYGPLELTVVTDSEDQD

>core/58/1/Org1_Gene679

MKKLYHPTLFLRPLIRLSLIFALSLTLISGNFPQQKSFGHCCADMHSALISGKNCEELFADFIERVLADRETLTARDWGTVVVLVREYLLKCI
RKGDCDYGVKILQKLLALRLPKDARKDLQILWHRLNPEQAPLRDVVDQLFTIGCHESLQDHLLFELYTVTLHSGYENRKQDMLLAKEQG
DYKKAIELAKELVAALEKGSCSPHPEIVQIEKTFLQKTLLALQIKVAQEAQESCDALLTPYCLSEIAYTEAMDALVLRIARGEVSRTNEVDS
VLLSHALQHLPFAREKAIPELEVLIDHGAYLESTLLYYAYFSLLELYHQNKDFASLERLLEKGDAVFVPEHPYFPEYGFFLGAYFYAKGKYES

AEKVFLQIIDPAVKLGATFARAYEYLGCIAYVQNHYEKAEEYFLRAYKSWGREESGIGLFLAYAVQKKKTACEDMLYHPKFSFTYRHLLDS
LCSLSYPHGENKGSSAIQRVHRAVPELSEIYSRCIYDMIKYRNVTYTHPIIELAYNQVRNLEKRNLEEICRDAQDPEYDKALAFWGALQSG
ASVPRSLIESSDVDEARITIRCYEALYFHNPDAIAMLPQAFSEECNSWQTALRLVWTLVRPKGAPNHAKYWDHLVLRPHGDSLYFFGY
DLQEYLIGKEDALKHLSVFAELFPKSSLLSLVYYLQGYSESSALRKVGWFVKALEEFTEISWSGEHMKTWAYIYYMVKLDLADTYISLGNF
SQAVHILEEVKEDWQVASHPKLHFLKGEDCYLAMELRWVEGLAYAYFQLHETAHLSNHLLEHVEKNLISPRSYRDYYGESLQRTLGLCQ
RFLGV

>core/65/1/Org1_Gene438

MRIPITLLQTYFSEPLSTKEILEACDHIGIEAEIENTTLYSFASVITAKILHTIPHPNADKLRVATLTDGEKEHQVVCGAPNCEAGLIVALALP
GAKLFDSEGQAYTIKKSKLRGVESQGMCCGADELGLDELQIQERALLELPEATPLGEDLATVLGNTSLEISLTPNLGHCASFLGLAREICH
VTQANLVIPKEFSFENLPTTALDMGNDPDICPFFSYVVITGISAQPSPIKLQESLQALKQKPINAIVDITNYIMLSLGQPLHAYDASHVALD
SLRVEKLSTPESLTLLNGETVLLPSGVPVVRDDHSLLGLGGVMGAKAPSFQETTTTTVIKAAYFLPEALRASQKLLPIPSESAYRFTRGIDP
QNVVPALQAAIHYILEIFPEATISPIYSSGEICRELKEVALRPKTLQRILGKSFSIEILSQKLQSLGFSTTPQETSLLVKVPSYRHDINEEIDLVEE
ICRTESWNIETQNPVSCYTPIYKLKRETAGFLANAGLQEFFTPDLLDPETVALTRKEKEEISLQGSKHTTVLRSSLLPGLLKSAATNLNRQA
PSVQAFEIGTVYAKHGEQYQETQTLAILLTEDGESRSWLPKPSLSFYSLKGWVERLLYHHHLSIDALTLESSALCEFHPYQQGVLRIHKQS
FATLGQVHPELAKKAQIKHPVFFAELNLDLLCKMLKKTTKLYKPYAIYPSSFRDLTLTVPEDIPANLLRQKLLHEGSKWLESVTIISIYQDKS
LETRNKNVSLRLVFQDYERTLSNQDIEEEYCRLVALLNELLTDTKGTINS

>core/67/1/Org1_Gene856

MLLLISGALFLTLGIPGLTAGVSFGLGIGLSALGGVLVVSGLLCLLVKREVSKVCPEEIPAVQPEETPEGVPVTPFEKPALDEAQKEQKTQK
ILDQLPQELDQLDRYIQEVFACLGPLKDLKYEDQGFLQDVKEEFQVFDFVQKDMIAEFVELQQILCQEGRLLEFVINQTRYIGRDLFKRE
DSLYKLWEWLGYLPSGDVRGERLKKSAREVVDRFMRTTCNIRKIAMTFDRHVYSVAKTAFEKAFGALETCVYESMRESYREAFCEYEK
AKLLGDEEKSAHAEQRFQDIKNRWEDVKDAFFWVKEDGKIEIDDAIGNSCKWSERYEEHRITRARWYKVAEHQLFNATMRVKDSLRE
HNEARVAFEKERSKENQRQVQKKKEKRLRDLKELHDQELPRAQERLRELQALYPEIAVSVVEARREVASDLEKAHESIDKHYQSCVREQ
ELY

>core/69/1/Org1_Gene1003

MLLLISGALFLTLGIPGLSAAISFGLGIGLSALGGVLMISGLLCLLVKREIPTVRPEEIPEGVSLAPSEEPALQAAQKTLAQLPKELDQLDTDI
QEVFACLRKLKDSKYESRSFLNDAKKELRVFDFVVEDTLSEIFELRQIVAQEGWDLNFLINGGRSLMMTAESESLDLFHVSKRLGYLPSG
DVRGEGLKKSAKEIVARLMSLHCEIHKVAVAFDRNSYAMAEKAFAKALGALEESVYRSLTQSYRDKFLESERAKIPWNGHITWLRDDAK
SGCAEKKLRDAEERWKKFRKAVFWVEEDGGFDINNLLGDWGTVLDPYRQERMDEITFHELYEKTTFLKRLHRKCALAKTTFEKKRSKK
NLQAVEEANARRLKYVRDWYDQEFQKAGERLEKLHALYPEVSVSIRENKIQETRSNLEKAYEAIEENYRCCVREQEDYWKEEEKREAEF

RERGNKILSPEELESSLEQFDHGLKNFSEKLMELEGHILKLQKEATAEVENKILSDAESRLEIVFEDVKEMPCRIEEIEKTLRMAELPLLPTK
KAFEKACSQYNSCAEMLEKVKPYCKESLAYVTSKERLVSLDEDLRRAYTECQKRFQGDSGLESEVRACREQLRERIQEFETQGLDLVEKE
LLCVSSRLRNTECDCVSGVKKEAPPGKKFYAQYYDEIYRVRVQSRWMTMSERLREGVQACNKMLKAGLSEEDKVLKEEEYWLYREER
KNKEKRLVGTKIVATQQRVAAFESIEVPEIPEAPEEKPSLLDKARSLFTREDHT

>core/76/1/Org1_Gene708

MGYIESSTFRLYAEVIVGSNINKVLDYGVPENLEHITKGTAVTISLRGGKKVGVIYQIKTTTQCKKILPILGLSDSEIVLPQDLLDLLFWISQY
YFAPLGKTLKLFLPAISSNVIQPKQHYRVVLKQSKAKTKEILAKLEVLHPSQGAVLKILLQHASPPGLSSLMETAKVSQSPIHSLEKLGILDIV
DAAQLELQEDLLTFFPPAPKDLHPEQQSAIDKIFSSLKTSQFHTHLLFGITGSGKTEIYLRATSEALKQGKSTILLVPEIALTVQTVSLFKARF
GKDVGVLHHKLSDSDKSRTWRQASEGSLRILIGPRSALFCPMKNLGLIIVDEEHDPAYKQTESPPCYHARDVAVMRGKLAHATVVLGS
ATPSLESYTNALSGKYVLSRLSSRAAAAHPAKISLINMNLEREKSKTKILFSQPVLKKIAERLEVGEQVLIFFNRRGYHTNVSCTVCKHTLKC
PHCDMVLTFHKYANVLLCHLCNSSPKDLPQSCPKCLGTMTLQYRGSGTEKIEKILQQIFPQIRTIRIDSDTTKFKGSHETLLRQFATGKAD
VLIGTQMIAKGMNFSAVTLAVILNGDSGLYIPDFRASEQVFQLITQVAGRSGRSHLPGEILIQSFLPDHPTIHSAMRQDYSAFYSQEITGR
ELCEYPPFIRLIRCIFMGKCPKQTWEEAHRVHNILKEQLESTNPLMPVTPCGHFKIKDTFRYQFLIKSAYVIPVNKKLHHALMLAKLSPKV
KFMIDVDPMTTFF

>core/79/1/Org1_Gene564

MDYLEKLQVLIEEGQSANFLSLWEEYCFNDVVRGRELVEILEKVKSSSLASLFGKIVDTVVPLWEKIPEGKDKDRVLQLILDLQTSNSQM
FFDIATEYVNKKYSGEENFNEALRVVGLRDGRDFQFSLSRFDFLMHMHKGNFVFHQGGWGVGEVMGVSFLQQKVLIEFEGIMSAKD
ISFETAFKSLTPLSGDHFLSRRFGDPDGFEAFAKENPIEVVEILLRDLGPKTAKEIKDELVDLVIPEADWNRWWQSAKTKIKKGTRIISPDN
PKEPYVLSDAGCSHMGQLERKLGLSLNSAEKISLIYHFIRDLHSELKNIEIRKSLVKALQDLDVEEGNKSLILQRELLLSEYLGIKDASIDKEYI
TSLSEDDTSRLLENMPIVALQKSFLSLVRKYSSFWQQVFMQILLYTTSPTMRDFVYKTIKNDPSSVEVLKKRLLDSAHQPMMFPELFVW
FFLKLGNHEDGLFDPEDKEVLRLFLESALNFMYQVASTPHKELGKKLHHYLVGQRYLAVRQMIEGASLPFLKELLLLSTKCPQFSSSDLNV
LQSLAEVVQPTLKKHKSNVEEENVLWSTSESFSRMKAKLQSLVGKEMVDNAKEIEDARSLGDLRENSEYKFALEKRARLQEEIRVLSEEI
NRARILTKDLVFTDKVGVGCKVTLKGDAGEVVEYTILGPWDADPDSCILSLQSKLAQNMLGKKLNDVVILQGKEYKISRIQSIWEEHGA

>core/82/1/Org1_Gene205

MIRSPLPFISSKRALNMLGLQDEFSCPEDVVDFLFSEIELLAQQDEPSEGYLALSRSLLMMTHNHPKVVKRVIFYGVSYGLKHKSMSIFID
VLTYIDFLFEKLGISASDRLSLCSARTCINFELYSQTGEMKFLSEVVDNFRLIEQLLKMHPQLKNRLGWEHFRIGAKQEEVSLVASASVYQ
AVGRSFIELYHKHLELSDLACGMKCLALALDLSPNNAHIHADYAKGLVVLGTRQGKSLLIERGMEHFSKAIFLSFSRDGDTLAYQNYRYS
YALASVKLFDLTYKKEHFDQAMNILYQTVQAFPNLSGLWMVWGELLIRSGWLNSNMKYIEVGLEKLASLQKKTNDPIALSGLLATGIAI
LGLYLEEPNLFKDSRHRLISAMRTFPGNSALVHALGVVQLCSALYFNEDSHFASAISCFQSCLEWDLDATGMWQKLFDAYFSWGIKKK

SARLLRKAVDVASRLCSLRPEAFLFWSDRGLALKCLAEATIDEAYKEIFLSESLLHYQRAWDLSGRLEILELWGQSHYLLAELQQSLFHYD
EAYTLLTKVDLTLSSSRVKLILAAVLLGKGRLLQDTDPAEEAREILEPLVEVYLEDENFLLLLGKVYLFLFWKNKNVCLGKLARTYLEKATSL
GCPEAYYTLGKFYAVIKDVNKAWGMVIRSAQYGVRITEAKWLNDPYLANLREIHAFREVVENQKGRLWLGNKTEMKRN

>core/84/1/Org1_Gene997

MYKRCVLDKILKGIVAGSLILLYWSSDLLERDIKSIKGNVRDIQEDIREISRVVKQQQTSQAIPAAPGVMLAPKLVRDEAFALLFGDPSYP
NLLSLDPYKQQTLPELLGTNFHPHGILRTAHVGKPENLSPFNGFDYVVGFYDLCIPSLASPHVGKYEEFSPDLAVKIEEHLVEDGSGDKEF
HIYLRPNVFWRPIDPKALPKHVQLDEVFQRPHPVTAHDIKFFYDAVMNPYVATMRAVALRSCYEDVVSVSVENDLKLVVRWKAHTVI
NEEGKEERKVLYSAFSNTLSLQPLPRFVYQYFANGEKIIEDENIDTYRTNSIWAQNFTMHWANNYIVSCGAYYFAGMDDEKIVFSRNP
DFYDPLAALIDKRFVYFKESTDSLFQDFKTGKIDISYLPPNQRDNFYSFMKSSAYNKQVAKGGAVRETVSADRAYTYIGWNCFSLFFQSR
QVRCAMNMAIDRERIIEQCLDGQGYTISGPFASSSPSYNKQIEGWHYSPEEAARLLEEEGWIDTDGDGIREKVIDGVTVPFRFRLCYYV
KSVTAHTIADYVATACKEIGIECSLLGLDMADLSQAFDEKNFDALLMGWCLGIPPEDPRALWHSEGAMEKGSANVVGFHNEEADKIID
RLSYEYDLKERNRLYHRFHEIIHEEAPYAFLFSRHCSLLYKDYVKNIFVPTHRTDLIPEAQDETVNVTMVWLEKKEDPCLSTS

>core/92/1/Org1_Gene351

MYNLLHAHHDAASPDGRLVSHLKKLSPHIYEGEVLIENIPAYFLGFHLPQQCIQVNLKSSLAQLGVEAVLNHLELNKARKEARLHVLFMS
QDPIATAMLELLEPGSFVCKLFAADDRRLVRSPCYLNRMFTHTDRTGSPLLRFGKKLEHFITLEIINDRLVVFLPILPGTICYEETIYGFLPL
MSKSLTRPHLKIRKFLPLYQMVTDRPPVPEDHKILLIKTEPLHIRTVFARVVQDLLPQGLRHTAADILEPTTQESGDIYEFYGSTSEPIERIPL
EFFTLEPYKEHSFFFYRDMLQETLESPQEVFRVFESIPEGEDQAAMFISKGSELLELSQDSWIIKPRISPSDERHAREIQKHIEDQPCFPFLK
AMETDHITSQGVLFSRYFPSASLKGMFLSNYSRYYLQHIYFQIPSPTSGEFFSNRDRSFLLDLYFAGISVFWADLESKRLLQYIKRRNKDV
GMFVPKHQAEQFAQSYFIGIHGSCLIAGDYDEFLRELLTGMHTLSQQFTIPEFPPQTPLAILTGGGSGAMELANRVATELSILSCGNLISL
DTTNAYVEAKMSYAIPDLLERQADFHVDLAVFVIGGMGTDFELLLLELISLKTGKKALVPVFLIGPVDYWKSKITALYNSNHAVGTIRGSE
WVHNCLFCLSSAKAGIAIFRRYLNHTLPIGPEHPVPEDGFVIV

>core/93/1/Org1_Gene763

MKRSRRNFEQALENLEKLKEISLATSNDSYLNNPARFNQRKQTGSSVMEMKEALKNVENYLLEISCVSKSHADKALKESDFLIAGVQNV
FSFLENQEDLYKSLLDEYSEVTKAYDEVKKNLKEVPTYDLSTDEETEEHKEPECFLNNLVEVKRDRSYELFYMLDEQDKRFYNDALVQIIY
KQNKLHETVNEGDPLTKTLLWNSEEVKNIASSLVIVNDMPLRLFYQRALSHLDIEAVVKVHNAVMALFFSRYEATMVFKSPKKHNIWY
FNDFLLFLREAWKDLNNNVIDSQERKQTKLLASALSLGIFESKLVFEEASRYLYFNIQTKLENANGKKPLSPGQYLTDAYEELHRLISKYPN
GPLFKAMDRVLEHESRPYDPMILGILPSLEGTLKLHGKSIDIIRSPSPVTQSSILYANCNEEFLGFLNAKAHRSEVTLVLNIQNRISRKERAR
SRVIEEALEQEEHAPYVHAFSFPEPEELLQNLESIHGDIETFADFFSILQEEFHKPLLASSFFLTKELKEFVGSFLKEKLTALKDIFFAKKKILFR
NDKLLLLHLLSYLIVFKLIERTNPNSIVVVSKDGLDYVSVFIAGFAFFSREAFWDEHSLKLLLTNVLSPTLVARDRLVFVSHIELLSKFVNCLK
KNRQGFSSLKSFFKDDIEGWEFTGYLHELTEVSHKHNL

>core/105/1/Org1_Gene609

MTFQLHAPFAPCGDQPEAIARLSAGVRNQVKSQVLLGTTGSGKTFTIANVVANVNLPTLVLAHNKTLAAQLYQEFREFFPNNAVEYFIS
YYDYYQPEAYIARSDTYIEKSLLINDEIDKLRLSATRSILERRDTLIVSSVSCIYGIGSPENYTSMALVLEVGKEYPRNILTAQLVKMHYQASPI
PQRSAFRERGSVIDIFPAYESELALRLEFLNDTLTSIEYSDPLTMIPKESVPSATLYPGSHYVIPEAIREQAIRTIQEELEERMAFFDDRPIEKD
RIFHRTTHDIEMIKEIGFCKGIENYSRHFTGAPPGAPPTCLLDYFPEDFLLIIDESHQTLPQIRAMYRGDQSRKQSLVEYGFRLPSAFDNRP
LTYEEAQKYFRKVIYVSATPGDTEVQESSGHIVQQIIRPTGIPDPMPEIRPATGQVDDLLEEIRLRLSQKHEKILVISITKKLAEDMAGFLSE
LEIPAAYLHSGIETAERTQILTDLRSGVIDVLIGVNLLREGLDLPEVSLVAILDADKEGFLRSTSSLIQFCGRAARNINGKVIFYADQKTRSIEE
TLRETERRRQIQLDYNKEHNIVPKPIIKAIFANPILQTSKDSESPKESQRPLSKEDLEEQIKKYEALMQRAAKEFRFNEAAKYRDAMQACK
EQLLYLF

>core/107/1/Org1_Gene306

MSYRKRSTLIVLGVFALYALLVLRYYKIQICEGDHWAAEALGQHEFCVRDPFRRGTFFANTTVRKGDKDLQQPFAVDITKFHLCADPLAI
PECHRDEIIQGILQFIEGQTYDDLSLKLDKKSRYCKLYPLLDVSVHDRLSLWWKGYATKHRLPTNALFFITDYQRSYPFGKLLGQVLHTLR
EIKDEKTGKAFPTGGMEAYFNHILEGDVGERKLLRSPLNRLDTNRVIKLPKDGSDIYLTINPVIQTIAEEELERGVLEAKAQGGRLILMNS
QTGEILALAQYPFFDPTNYKEYFNNKERIEHTKVSFVSDVFEPGSIMKPLTVAIALQANEEASLKSQKKIFDPEEPIDVTRTLFPGRKGSPL
KDISRNSQLNMYMAIQKSSNVYVAQLADRIIQSLGVAWYQQKLLALGFGRKTGIELPSEASGLVPSPHRFHINGSLEWSLSTPYSLAMG
YNILATGIQMVQAYAILANGGYAVRPTLVKKIVSASGEEYHLPTKEKTRLFSEEITREVVRAMRFTTLPGGSGFRASPKHHSSAGKTGTTE
KMIHGKYDKRRHIASFIGFTPVESSEGNFPPLVMLVSIDDPEYGLRADGTKNYMGGRCAAPIFSRVADRTLLYLGILPDKKLRNCDEEAA
ALKRLYEEWNRSPKQGGTR

>core/123/1/Org1_Gene268

MTLITPAINSSRRKTHTVRIGNLYIGSDHSIKTQSMTTTLTTDIDSTVEQIYALAEHNCDIVRVTVQGIKEAQACEKIKERLIALGLNIPLVA
DIHFFPQAAMLVADFADKVRINPGNYIDKRNMFKGTKIYTEASYAQSLLRLEEKFAPLVEKCKRLGKAMRIGVNHGSLSERIMQKYGDT
IEGMVASAIEYIAVCEKLNYRDVVFSMKSSNPKIMVTAYRQLAKDLDARGWLYPLHLGVTEAGMGVDGIIKSAVGIGTLLAEGLGDTIR
CSLTGCPTTEIPVCDSLLRHTKIYLDLPEKKNPFSLQHSENFVSAAEKPAKTTLWGDVYGVFLKLYPHHLTDFTPEELLEHLGVNPVTKEK
AFTTPEGVVVPPELKDAPITDVLREHFLVFHHHQVPCLYEHNEEIWDSPAVHQAPFVHFHASDPFIHTSRDFFEKQGHQGKPTKLVFSR
DFDNKEEAAISIATEFGALLLDGLGEAVVLDLPNLPLQDVLKIAFGTLQNAGVRLVKTEYISCPMCGRTLFDLEEVTTRIRKRTQHLPGLKI
AIMGCIVNGPGEMADADFGFVGSKTGMIDLYVKHTCVKAHIPMEDAEEELIRLLQEHGVWKDPEETKLTV

>core/126/1/Org1_Gene572

MFRCILFGIFLLTCFSSGGVLYYLFCSHDFSIGPKEKSRSVWIEEEKEFTDSVLHHLPSQHQHLHILCFQGFLLQKQQKFSQAEKIFSKVYDE
AQDGPFLFKEEILGSRLINSFFLEKTDVMETILCLLNQRCPNSPYYHLFKALVCYKQKLYREVIEQLAYWQEEKTRALAPLLNISIEQLLTDF
LLDYISAHSLIEQKMFPEGRVILNRNINRLLKHECEWNAKTYDRIAILLSRSYFLELVESKSADIYFDYYEMVLFYLKKIYILEQCPYAELLPEE
ELVSLIMEHVFILPKDKLYPLIQLLEMWQKHYVHPNSSLVVQILVDRFSTHMEGAIRFCEALVSFSGLEELHQQIITTFEELLSNKVQQIKT
EEAKQCVALLHILDPSISISEKLALSSDTLQNIVSGDDEQHTKLRNYLDLWEAIQSYDIDRQQLVHHLVYGAKDLWKKGGNDEKALNLLQ
LVLRFTSYDIECESVVFLFIKQAYKQALSSHAIARLLKLEKFISEANIPSIVISEAEKANFLADAEYLFAHEDYDKCYLYSMWLTKVAPSPQSY
RLAGLCLMENKRYDEALEFLCMLSPNDSINDYKTQKALAFCQKHQSKDRAAS

>core/130/1/Org1_Gene722

MRIEDFSLKLIPSSPGVYLMKDVHDQVLYIGKAKNLKNRLASYFHEKGDSRERIPFLMKKTASIETIVVSNETEALLLENNLIKQHHPKYN
VLLKDDKTFFCLAISLSHSWPKVEAIRTKAITSSQRQLIFGPYVSAEACHTLLEVISQWFPLRTCSDREFALRKRPCILYDMKRCLAPCVGY
CTPEEYQGTLDKAILFLKGKIEEVVKDLEKVIQKASDNLEFEQAANYYRTLSLIKQAMAKQQVEKFHFQNIDALGLYRHKQRTILTLLTVR
SGKLLGARHFSFFENAQEDQDLLSSFILQYYVSQPYIPKEILTPLPLEFPTLSYVLNAESPPRLRSPKTGYGKELLDLAYRNAKAYAATTLPSS
TLPYQDFQNILRMSQYPYRIECYDNAHMQGAHATGVYIVFENNGFDPKQYRTFSIDSEKTQNDLALLEEVLLRRFHSLTTALPDMIVVD
GGKTHYNKTKKIIQTLNLTGIQVVTIAKEKSNHSRGLNKEKIFCETFPEGFSLPPTSNLLQFFQILRDEAHRFAISKHRKKRGKALFEQEKIP
GIGEVKRKRLLQKFKSWKQVMLSSQEELEAIPGLTKKDIAVLLARQKDFNKSD

>core/138/1/Org1_Gene723

MYTEESLDNLRHSIDIVDVLSEHIHLKRSGATYKACCPFHTEKTPSFIVNPAGAHYHCFGCGAHGDAIGFLMQHLGYSFTEAILVLSKKFQ
VDLVLQPKDSGYTPPQGLKEELRHINSEAETFFRYCLYHLPEARHALQYLYHRGFSPDTIDRFHLGYGPEQSLFLQAMEERKISQEQLHT
AGFFGNKWFLFARRIIFPVHDALGHTIGFSARKFLENSQGGKYVNTPETPIFKKSRILFGLNFSRRRIAKEKKVILVEGQADCLQMIDSGF
NCTVAAQGTAFTEEHVKELSKLGVLKVFLLFDSDEAGNKAALRVGDLCQTAQMSVFVCKLPQGHDPDSFLMQRGSSGLIALLEQSQD
YLTFLISEKMSSYPKFGPREKALLVEEAIRQIKHWGSPILVYEHLKQLASLMMVPEDMVLSLANPQVTAEPQNIPIKQKVPKIHPHIVME
TDILRCMLFCGSNTKILYTAQFYFVPEDFKHPECRKLFAFMISYYEKYRKNVPFDEACQVLSDSQILQLLTKRRLNTEALDTIFVQSLQKM
ADRRWREQCKPLSLNQNIQDKKLEILEDYVQLRKDRTIITLLDPESELIP

>core/140/1/Org1_Gene409

MTNSDNASAAGLLWAHPKEDPAFLGMIIKEFHLPPTVAQIFISRGFQTIQEIHKFLYSHLSSLYDPGLFLDMSKAVERLLLARDRKEHVM
IYGDSDVDGMTGVALLVEFLRDIDVHVSYFFLGAILKQHGETSTLIAKLKEEGITLLITVDCGITAGKEVSDITRQGIDVIITDHHMPTGKIP
HCVATLNPKLRDHTYPNRELTGVGVAFKLARGVLNALISRNLVPKSQGSLKKLLDLVTLGTITDVGVLLGENRVMVRYGIKEIARGARPG

LNKLCALCGVEKSEVTSTDIVLKIAPKLNSLGRLDDPAKGVELLLTQDDERVDALIMELDNINRERQRIEAEVFQDVQEILNSNPEILKQA
AIVLSSTAWHARVIPIISARLAKTYNKPVVIIAIQRGIGKGSARTIGSFPLLGVLKKCSSLLLSYGGHDFAAGVIMKEDKVEDFKKKFVHLVN
SSLKKGDTLPHLEIDAYADFDAIDYDLLASMELFEPFGKGNLMPIFYSKVRQVRYPKVLPGNHLKLYLSQKERNLEGVAFGLGRHADALK
ASWHYPLEIAYTPRLSQTSGSGVIHLLVRDFRISSEPRFSD

>core/141/1/Org1_Gene240

MSFLRRHISLFRSQKQLIDVFAPVSPNLELAEIHRRVIEDQGPALLFHNVIGSSFPVLTNLFGTKHRVDQLFSQAPDNLIARVAHLISSTPK
LSSLWKSRDLLKRISSLGLKKARFRRFPFVSMSSVNLDHLPLLTSWPEDGGAFLTLPLVYTESPTLTTPNLGMYRVQRFNQNTMGLHFQI
QKGGGMHLYEAEQKKQNLPVSVFLSGNPFLTLSAIAPLPENVSELLFATFLQGAKLLYKKTNDHPHPLLYDAEFILVGESPAGKRRPEGP
FGDHFGYYSLQHDFPEFHCHKIYHRKDAIYPATVVGKPYQEDFYIGNKLQEYLSPLFPLVMPGVRRLKSYGESGFHALTAAVVKERYWR
ESLTTALRILGEGQLSLTKFLMVTDQEVPLDRFSVVLETILERLQPDRDLIIFSETANDTLDYTGPSLNKGSKGIFMGIGKAIRDLPHGYQG
GKIHGVQDIAPFCRGCLVLETSLEDRCIKSLLHHPDLKSWPLIILADNLRETIQSEKDFLWRTFTRCAPANDLHALHSHFATHRPNYNFPF
VIDALMKPSYPKEVEVDPSTKQKVSERWHAYFPNKETFYI

>core/150/1/Org1_Gene576

MFMNTQNSQATEVSSEEESQKKLEELVALAKEQGFITYEEINEILPMSFDTPEQIDQVLIFLTGMDIQVLNQIDVERQKEKKKEAKELEG
LARRTEGTPDDPVRMYLKEMGTVPLLTREEEVEISKRIEKAQVQIERIILRFRYSAKEAISIAHYLISGKERFDKIISEKEVEDKTHFLKLLPKLI
TLLKEEDTYLENLLLSLKQPDLSKQEAAKLNDSLEKCRIRTQAYLRCFHCRHNVTEDFGEVVFKAYDSFLHLEQQINDLKVRAERNKFAAA
KLAAAKRKLYKREVAAGRTLEEFKKDVRMLQRWMDKSQEAKKEMVESNLRLVISIAKKYTNRGLSFLDLIQEGNMGLMKAVEKFEYR
RGYKFSTYATWWIRQAVTRAIADQARTIRIPVHMIETINKVLRGAKKLMMETGKEPTPEELAEELGLTPDRVREIYKIAQHPISLQAEVG
EGSESSFGDFLEDTAVESPAEATGYSMLKDKMKEVLKTLTDRERFVLIHRFGLLDGKPKTLEEVGSAFNVTRERIRQIEAKALRKMRHPIR
SKQLRAFLDLLEEEKTGTSKVKSLKSK

>core/151/1/Org1_Gene901

MDTQSSIGNEEWRIAGTSIVSGMALGKVFFLGTSPLHVRELTLPQEEVEHEIHRYYKALNRSKSDIVALEQEVTGQQGLQEVSSILQAHL
EIMKDPLLTEEVVNTIRKDRKNAEYVFSSVMGKIEESLTAVRGMPSVVDRVQDIHDISNRVIGHLCCQHKSSLGESDQNLIIFSEELTPSE
VASANSAYIRGFVSLVGAATSHTAIVSRAKSIPYLANISEELWNIAKRYNGKLVLIDGYRGELIFNPKPATLQSCYKKELSVVAHTSQRLVR
KSLHPIVSSHAGSDKDVEDLLENFPQTSIGLFRSEFLAVILGRLPTLREQVDLYEKLARFPGDSPSVLRLFDFGEDKPCPGIKNKKERSIRWL
LDYSVILEDQLQAIAKASLQGSIKVLIPGVSDVSEIIEVKKKWETIQTRFPKGHKVSWGTMIEFPSAVWMIEEILPECDFLSIGTNDLVQYT
LGISRESALPKHLNVTLPPAVIRMIHHVLQAAKQNQVPVSICGEAAGQLSLTPLFIGLGVQELSVAMPVINRLRNHIALLELNSCLEITEAL
LQAKTCSEVEELLNRNNKITS

>core/153/1/Org1_Gene899

MGLFHLTLFGLLLCSLPISLVAKFPESVGHKILYISTQSTQQALATYLEALDAYGDHDFFVLRKIGEDYLKQSIHSSDPQTRKSTVIGAGLAG
SSEALDVLSQAMETADPLQQLLVLSAVSGHLGKTSDDLLFKALASPYPVIRLEAAYRLANLKNTKVIDHLHSFIHKLPEEIQCLSAAIFLRLE
TEESDAYIRDLLAAKKSAIRSATALQIGEYQQKRFLPTLRNLLTSASPQDQEAILYALGKLKDGQSYYNIKKQLQKPDVDVTLAAAQALIAL
GKEEDALPVIKKQALEERPRALYALRHLPSEIGIPIALPIFLKTKNSEAKLNVALALLELGCDTPKLLEYITERLVQPHYNETLALSFSKGRTLQ
NWKRVNIIVPQDPQERERLLSTTRGLEEQILTFLFRLPKEAYLPCIYKLLASQKTQLATTAISFLSHTSHQEALDLLFQAAKLPGEPIIRAYAD
LAIYNLTKDPEKKRSLHDYAKKLIQETLLFVDTENQRPHPSMPYLRYQVTPESRTKLMLDILETLATSKSSEDIRLLIQLMTEGDAKNFPVL
AGLLIKIVE

>core/159/1/Org1_Gene813

MSSPPQAVASLTERIKTLLESNFCQIIVKGELSNVSLQPSGHLYFGIKDSQAFLNGAFFHFKSKYYDRKPKDGDAVIIHGKLAVYAPRGQY
QIVAHALVYAGEGDLLQKFEETKRRLTAEGYFATEKKKPLPFAPQCIGVITSPTGAVIQDILRVLSRRARNYKILVYPVTVQGNSAAHEISK
AIEVMNAENLADVLIIARGGGSIEDLWAFNEEILVKAIHASTIPIVSAVGHETDYTLCDFASDVRAPTPSAAAEIVCKSSEEQVQVFEGYL
RHLLSHSRQLLTSKKQQLLPWRRFLDRAEFYTTAQQQLDSIEIAIQKGVQGKIHESKQRYDNISRWLQGDLVSRMTCRLQSLKKMLSQ
ALSHKALSLQVRCHQLKKSLTYPRQIQQASQKLSPWRQQLDTLISRRLHYQKEEYFHKHTRLKHAHNVLEQQLRSHVQKLELLGRRLSR
GCELNLQNQKIAYANVKETLATILERRYENSVARYSALKEQLHSLNPKNVLKRGYAMLFDFNENSAMISVDSLQENARVRIQLQDGEAI
LTVTNIEICKLIKG

>core/160/1/Org1_Gene973

MDNSDNSFHTLETEQGSFLNDELAVEEVASTESTEISDATLCFADEIQELPSPEKKVAFILNKMREALTGSSQGSDLRLFWDLRKQCLPL
FNEIEDTAKRADHWRCYIELTKEGRHLKGLQDEEGSFVVGQIDLAITCLEKDILKFQEGTEDKIFKDREDNFLESQALDKHQAFYKQHHT
SLLWLSSFSSKIIDLRKELINVGMRMRLKSKFFQRLSNLGNQVFPKRKELIEKVSQTFAEDVDAFVAKYFIGSDKETLKKTVFFLRKEIKNL
QHAAKRLFVSSHVFAETRLKLSKCWDQLKGMEKEIRQEQGRLRVVSAENSKEVRQMLAEVSSLLIEGNDLSKVRKDLEGISKKIRALDLT
HDDVISLKKEMQQLFDQLREKQDAAEHSYQEQLAKDKQVKKEAARSLAERITTFSKTCSEGNITSESREEWQTLKELLGKMSFLPPPEKI
SLDNQLNLALQTIVNFFEEQLLSSPDSREKLVNMRQVLKQRRERRQELKDKLEQDKKLLGSSGLDFDRAMQYSALVEEDKRALEELDAS
ILELKQQIQQLL

>core/162/1/Org1_Gene140

MHPLYVDLDTIISSYSPPLPKEFQEAASLIAVPDTSHSKPVVPGVKTLFPQTYHLPYLKFVQGENVVHTPLKVGVMFSGGPAPGGHNVI
QGLFNSLKDFHPDSSLVGFVNNGDGLTNNKSIDITEEFLSKFRNSGGFNCIGTGRKKIVTPEAKEACLKTAEALDLDGLVIIGGDGSNTAT
AILAEYFAKRRPKTSIVGVPKTIDGDLQHTFLDLTFGFDTATKFYSSIISNISRDALSCKAHYHFIKLMGRSASHIALECALQTHPNIALIGEEI
AEKNLPLKTIIHKICSVIADRAAMEKYYGVILIPEGIIEFIPEIINLITEIESLSEYEDKISRLSPESQRLLKSFPAPIIEQILNDRDAHGNVYVSKIS
VDKLLIHLVSNHLQQYFPNVPFNAISHFLGYEGRSGLPTKFDNTYGYSLGYGAGILVRNHCNGYLSTIESLACPFMKWKLRAIPVVKMFT
VKQQADGTLQPKIKKYLVDIGSTAFRKFKLYRKIWALEDSYRFLGPLQIETPPEMHSDNFPPLTLLLNHNFWQRHQGCIEIPDTTY

>core/165/1/Org1_Gene349

MFGSESLRYQLLIQDFAKVSEEGIGLLESKEYSLLQAKLVLRALAQNSSFDDWFRSFKKCQISYPELAHDRDVLEEFGIQVLREGIENPSVT
VRAVSVLAIGLARDFRLVPLLLQSCNDDSAIVRSLALQVAVNYGSESLKKAIVELARNDDSIHVRITAYQVVALLQIEELLPFLRERAENKL
VDSVERREAWKACLELSSQFLETGVAKDDIDQALFTCEVLRNGMLPETTEIFTELLSVEHPEVQESLLLSALAWSHQLQNHKEFLSKVRH
VMCTSPFAKVRFQAAALLHLHGDPLGRDSLVEGLRSPQPLVCEAASAALCSLGIHGVPLAKEHLESLSSRKAAANLSILLLVSREDIERAG
DVIARYLSNPEMCWAIEYFLWDAQWNLRGDTFPLYSDMIKREIGRKLIRLLAVARYSQAKAVTATFLSGQQAQGWSFFSGMFWEEG
DVKTSEDLVTDACFAAKLEGALASLCQKKDQASLQRVSQLYNDSRWQDKLAILESVAFSENLDAVPFLLDCCHHEAPSLRSAAAGALFS
IFK

>core/172/1/Org1_Gene568

MLGKEEEFTCKQKQCLSHFVTNLTSDVFALKNLPEVVKGALFSKYSRSVLGLRALLLKEFLSNEEDGDVCDEAYDFETDVQKAADFYQR
VLDNFGDDSVGELGGAHLAMENVSILAAKVLEDARIGGSPLEKSTRYVYFDQKVRGEYLYYRDPILMTSAFKDMFLGTCDFLFDTYSALI
PQVRAYFEKLYPKDSKTPASAYATSLRAKVLDCIRGLLPAATLTNLGFFGNGRFWQNLIHKLQGHNLAELRRLGDESLTELMKVIPSFVS
RAEPHHHHHQAMMQYRRALKEQLKGLAEQATFSEEMSSSPSVQLVYGDPDGIYKVAAGFLFPYSNRSLTDLIDYCKKMPHEDLVQIL
ESSVSARENRRHKSPRGLECVEFGFDILADFGAYRDLQRHRTLTQERQLLSTHHGYNFPVELLDTPMEKSYREAMERANETYNEIVQEF
PEEAQYMVPMAYNIRWFFHVNARALQWICELRSQPQGHQNYRTIATGLVREVVKFNPMYELFFKFVDYSDIDLGRLNQEMRKEPTT

>core/178/1/Org1_Gene132

MLRFFAVFISTLWLITSGCSPSQSSKGIFVVNMKEMPRSLDPGKTRLIADQTLMRHLYEGLVEEHSQNGEIKPALAESYTISEDGTRYTFK
IKNILWSNGDPLTAQDFVSSWKEILKEDASSVYLYAFLPIKNARAIFDDTESPENLGVRALDKRHLEIQLETPCAHFLHFLTLPIFFPVHETL
RNYSTSFEEMPITCGAFRPVSLEKGLRLHLEKNPMYHNKSRVKLHKIIVQFISNANTAAILFKHKKLDWQGPPWGEPIPPEISASLHQDD
QLFSLPGASTTWLLFNIQKKPWNNAKLRKALSLAIDKDMLTKVVYQGLAEPTDHILHPRLYPGTYPERKRQNERILEAQQLFEEALDELQ
MTREDLEKETLTFSTFSFSYGRICQMLREQWKKVLKFTIPIVGQEFFTIQKNFLEGNYSLTVNQWTAAFIDPMSYLMIFANPGGISPYHL
QDSHFQTLLIKITQEHKKHLRNQLIIEALDYLEHCHILEPLCHPNLRIALNKNIKNFNLFVRRTSDFRFIEKL

>core/183/1/Org1_Gene5

MYQENLRLLERLLYNSVQKSYADRLFSYEKTKMVHDTPLIPWEEDKEKCAEAEKAFLEQQKILLDYGKSIFWLNENDEINLNDPWSWG
LNTVRTRKVFQEVDDSERWNHKVLIQKLEDDYEKLLEESSKESTEANKKLLSDLVDRLEDAKTKFFLKKQEEVETRVKDLRARYGGTVDP
KQDTEAKKKVELEASLETFLDSIESELVQCLEDQDIYWKEQDVKDLARTQELEEQDIEAKREEAAEDLRSLNERLKKSKTMLDRAKWHIE
NAEDSITWWTSQIEMKDMKARLKILKEDITSVLPEIDEIETCLSLEELPLLTTRELLTKSYLKFKICSETLLKMTSVFENNIYVQEYEVQLQNL
GFKLQGISQRFGKKQDDFANLEEQVALQKKRLRELTQNFEIQGFNFMKEDFKAAAKDLYIRSTAEQKMNFDVPCMELFRRYHEEVNK
PLLELMYNCADSYRDAKKKLCSLRLDEKELLQKEIKKEEFYQKKQQRHADRSRHTTYQKLRIAEELALELKKKI

>core/184/1/Org1_Gene996

MVARGLCDFPTLVPNERLPIGPFFVPQHTSGAKGKEFAKRNFSIISGLDDILKLCILQRRPFALQWDNLSVKSDYEEAGPAIGIRSLEPQV
SQISPAHGRLCSTLVQWAPILGSEEQLVWLEETMKRLKFPKSLGSKDAVIVDSEMVPVNANPTQEIPAASETVESSPVAPGNTTDTMP
AASGTTDTTSGVSEAAAAEATVDSTPGTEEEPSFSLRYALVVQNVPYPEPPKEPEVMFTDEEKSLILEATRARRMELDLYNGYLADYELS
KDEIQKHVPDLPENWRTNWRWSERLYKFFFKTKKEGLEEIFLNKELGNMILARGLAATQSQARIKVFNSLVAWLLQSFNVGRSCTAKP
LPTSKLDLFKSEFESKPKNNILTEFLVASDEEILFKGLRVLEPGIEGWYDHPDQAGEIRSVLEGLVQAGRISGYWENQPFGRFVLRGVGER
RTELVELLESLVASGEIMQFFESSDEEGAFIIDNEPSKTAMLKQRFKSCVRTKLVGSFADESLPRGRFTILV

>core/186/1/Org1_Gene686

MSEQEKLSNYNADKKLFSGIDKLFQIVKGSYGPKQSLSPTSFFKERGFYAISQTELSNSYENLGVDFAKAMVNKIHKEHSDGATTGLILLH
AILQESYAALEKGISTHKLIASLKLQGEKLQEALQQQSWPIKDALKVRNIIFSSLHMPTIADHFYNAFSVVGPEGLISITKERENDKTSMDV
FQGFKIPAGYASTYFVSDTASRLTRIAHPLILITDRKISMIHSLLPLLQEISEQNQHLIIFCEDIDPDVLATLVVNKLQGLLQVTVVTIPQLSTT
NQELAEDIALFTGTHICPCQEASHVLAPEMVTLGSCLSIEISESQTTLIGGLHIPEVLTLKTRQLAEEIRTTSCLETKKRLIKSTNRLQSSVAIL
PTDEDNEPLYTLALKIMESALSRGYVPGGGVALFYASLTLGTPKDDADENSIAISLLQKACCAPLKLLATNADLDGDAVIAKLSSLGTTSLG
ISVFSREIEDLIAGGILDSLATTSTILAQALDTAILVLSSKILILENQYEISTL

>core/187/1/Org1_Gene736

MENEILLNIESKEIRYAHLKNGQLFDLTIERKKVRQLKGNIYRGRVTNILRNIQSAFINIDERENGFIHISDILENSKKFEQMFDMDVDALP
EEASEAPLLSSEEAPIEEFLKLDSPVLVQVVKEPIGSKGARLTSNISIPGRYLVLLPNSPHRGVSRKIEDPHMREQLKQLIRSFEMPQDMGL
ICRTASTTASTEALINEAHDLLLTWKTILEKFYSTEQPCLLYSETDILKKAVITCIDKNYKRLLIDDYATYQKCKHMLKKYSPDASIKIEYYRDS
IPMFERFNIEKEIDKATRRKIWLSSGGYLFFDKTEAMHTIDVNSGRSTQLESGVEETLVQINLEAAEEIARQLRLRNVGGLVIIDFIDMKSR

KNQRRVLERLKEHMKYDAARCTILSMSEFGLVEMTRQRNRESLMQTLFTLCPYCSGNAIIKTPESVVIEIERDLKKVINHKEHSHLCLVV
HPEIASYMKQENDDNEMINLAKQLKAKLQINTSDSVHLNHYQFFSLITGESIDL

>core/193/1/Org1_Gene97

MADILVIGANPTGLILANMLIQHGISVKVIDHRASPEDPSFLDCRKLPVILSCSSLELLHNSEMLGDFIQANHKIFGARYHWKKRTLLFKFS
QATDSPVPFSLSTTYQSLEQHLIDEFLKRGGVIDWSTRPVTLVDNSIFIESTKVSQNFENREIYNPKWIIACEADNNLDIRDLVKSQLRAR
RINREVIFINCDEGEPFEEDHIHLLPITKNFLNFVFYNPQEKTKQLCLPQGTHSISPKLKQKLLYTYNLVISDENFHIKTSHHAFPPEHGNVL
FLGSLSNTLLLSYLNGINTNIHAAFNLAWKLLPVLKKAALKHLVITKEQEDGNILPYISPTTEKRAKKLPFSRFYTPALMYYFLKGCRKFNTT
GEEYYYPPHQALKYRSSDIIKMSPQDKEIHGPGPGMRAIDARLENGSFLLDPLKSSKHLLIFFKDIPDLKEALQEEYGEWIEICNVKEPRIL
NLYHANPNSLFIIRPDRYIGYRTHTFKLHELISYLLRIFASEKTS

>core/200/1/Org1_Gene621

MAASILSQELLDILPYTFLKKHCLLPIEESSEAITIAHATATSVIAQDEVKLLIKKPVRFVLKEESEILQRLQQLYSNREGNVSDMLLTMKEED
GTTISEEEDLLETTDTIPVVRLLNWILKEAIEERASDIHFEPCEDSMRIRYRIDGVLHDRHSPPSHLRSALTTRLKVLAKMDIAEHRLPQDG
RIKIHIGGQEVDMRVSTVPVIYGERVVLRILDKRNVILDIAGLHMPKGTEILFKDTITAPEGILLVTGPTGSGKTTTLYSVLQELKGPLTNIM
TIEDPPEYKLPGIAQIAVKPKIGLTFARGLRHLLRQDPDILMVGEIRDQETAEIAIQAALTGHLVVSTLHTNDAISAIPRLLDMGIESYLLSA
TLVGVVAQRLVRTICPYCKVAYTPENQEKSFLASLGKDTEMPLYRGQGCVHCFRSGYKGRQGIYEFLRPNTLFRSEVASNRPYHILRETA
EQNGFLPILEHGIALAVSGETTLAEVLRVTKRCD

>core/220/1/Org1_Gene305

MDLKELLHGVQAKIYGKVRPLEVRNLTRDSRCVSVGDIFIAHKGQRYDGNDFAVDALANGAIAIASSLYNPFLSVVQIITPNLEELEAELS
AKYYEYPSSKLHTIGVTGTNGKTTVTCLIKALLDSYQKPSGLLGTIEHILGEGVIKDGFTTPTPALLQKYLATMVRQNRDAVVMEVSSIGL
ASGRVAYTNFDTAVLTNITLDHLDFHGTFETYVAAKAKLFSLVPPSGMVVINTDSPYASQCIESAKAPVITYGIESAADYRATDIQLSSSG
TKYTLVYGDQKIACSSSFIGKYNVYNLLAAISTVHASLRCDLEDLLEKIGLCQPPPGRLDPVLMGPCPVYIDYAHTPDALDNVLTGLHELLP
EGGRLIVVFGCGGDRDRSKRKLMAQVVERYGFAVVTSDNPRSEPPEDIVNEICDGFYSKNYFIEIDRKQAITYALSIASDRDIVLIAGKGH
EAYQIFKHQTVAFDDKQTVCEVLASYV

>core/224/1/Org1_Gene792

MLCATVSGPSFCEAKQQILKSLHLVDIIELRLDLINELDDQELHTLITTAQNPILTFRQHKEMSTALWIQKLYSLAKLEPKWMDIDVSLPKT
ALQTIRKSHPKIKLILSYHTDKNEDLDAIYNEMLATPAEIYKIVLSPENSSEALNYIKKARLLPKPSTVLCMGTHGLPSRVLSPLISNAMNYA
AGISAPQVAPGQPKLEELLSYNYSKLSEKSHIYGLIGDPVDRSISHLSHNFLLSKLSLNATYIKFPVTIGEVVTFFSAIRDLPFSGLSVTMPLK
TAIFDHVDALDASAQLCESINTLVFRNQKILGYNTDGEGVAKLLKQKNISVNNKHIAIVGAGGAAKAIAATLAMQGANLHIFNRTLSSA
AALATCCKGKAYPLGSLENFKTIDIIINCLPPEVTFPWRFPPIVMDINTKPHPSPYLERAQKHGSLIIHGYEMFIEQALLQFALWFPDFLTP
ESCDSFRNYVKNFMAKV

>core/225/1/Org1_Gene321

MMSRLRFRLAALGIFFILLVPNSVSAKTIVASDKEKVGVLVYDNSVEAFQQILDCIDHANFYVELCPCMTGGRTLKEMVDHLEARMDLV
PELCSYIIIQPTFTDAEDQKLLKALKERHPNRFFYVFTGCPPSTSILAPNVIEMHIKLSIIDGKYCILGGTNFEEFMCTPGDEVPEKVDNPRL
FVSGVRRPLAFRDQDIMLRSTAFGLQLREEYHKQFAMWDYYAHHMWFIDNPEQFAGACPPLTLEQAEETVFPGFDKHEDLVLVDSS
KIRIVLGGPHDKQPNPVTQEYLKLIQGARSSVKLAHMYFIPKDELLNALVDVSHNHGVHLSLITNGCHELSPAITGPYAWGNRINYFALL
YGKRYPLWKKWFCEKLKPYERVSIYEFAIWETQLHKKCMIIDDEIFVIGSYNFGKKSDAFDYESIVVIESPEVAAKANKVFNKDIGLSIPVS
HGDIFSWYFHSVHHTLGHLQLTYMPA

>core/226/1/Org1_Gene728

MRIVQVAVEFTPIVKVGGLGDAVASLSKELAKQNDVEVLLPHYPLISKFSSSQVLSERSFYYEFLGKQQASAISYSYEGLTLTIITLDSQIELF
STTSVYSENNVVRFSAFAAAAAAYLQEADPADIVHLHDWHVGLLAGLLKNPLNPVHSKIVFTIHNFGYRGYCSTQLLAASQIDDFHLSH
YQLFRDPQTSVLMKGALYCSDYITTVSLTYVQEIINDYSDYELHDAILARNSVFSGIINGIDEDVWNPKTDPALAVQYDASLLSEPDVLFT
KKEENRAVLYEKLGISSDYFPLICVISRIVEEKGPEFMKEIILHAMEHSYAFILIGTSQNEVLLNEFRNLQDCLASSPNIRLILDFNDPLARLTY
AAADMICIPSHREACGLTQLIAMRYGTVPLVRKTGGLADTVIPGVNGFTFFDTNNFNEFRAMLSNAVTTYRQEPDVWLNLIESGMLR
ASGLDAMAKHYVNLYQSLLS

>core/233/1/Org1_Gene824

MDKSTGVPLPSPPHSKESEMIVLGCMLTGVHYLNLAANQLYEEDFYYLEHKIIFRVLQDAFKQDKPIDVHLAGEELKRHNQITVIGGPSY
LITLAEFAGTAAYLEEYVDIIRSKSILRKMISTAKEIEKRALEQPKNVAEALDEAQNSFFKISQSTSVSQYTLVADKLRGLTTTTDKPYLVQL
QERQELFLQNAQGDNKSFFTGIPTHFIDLDQLIHGFSPSNLMILAARPAMGKTALALNIAENLCFQNRLPIGIFSLEMTVDQLIHRMICS
RSEVDSKKISIGDLSGHDFQRIVSVINEMQEHTLLIDDQPGLKVSDLRARARRMKESYDIQFLIIDYLQLLSGSGTLRATESRQTEISEISRM
LKTLARELNIPILCLSQLSRKVEDRANHRPMMSDLRESGSIEQDSDLVMFLLRREYYDPNDKPGTAELIIAKNRHGSIGSVPLVFEKELAR
FRNYSAFECIS

>core/234/1/Org1_Gene566

MKITVNRGLDLSLQGSPKESGFYNKIDPEFVSIDLRPFQPLSLKLKVEQGDAVCSGAPIAEYKHFPNTYITSHVSGVVTAIRRGNKRSLLD
VIIKKTPGPTSTEYTYDLQTLSRSDLSEIFKENGLFALIKQRPFDIPAIPTQTPRDVFINLADNRPFTPSPEKHLALFSSREEGFYVFVVGVRAI
AKLFGLRPHIVFRDRLTLPTQELKTIAHLHTVSGPFPSGSPSIHIHSVAPITNEKEVVFTLSFQDVLTIGHLFLKGRILHEQVTALAGTALKSS
LRRYVITTKGASFSSLINLNDISDNDTLISGDPLTGRLCKKEEEPFLGFRDHSISVLHNPTKRELFSFLRIGFNKPTFTKTYLSGFFKKKRTYTN
PDTNLHGETRPIIDTDIYDKVMPMRIPVVPLIKAVITKNFDLANELGFLEVCGEDFALPTLIDPSKTEMLTIVKESLIEYAKESGILTPHQD

>core/236/1/Org1_Gene452

MKEERSSEILPRVKETKKHAYVSMQEKSCVGECAVVASESEEAESVTVTKIAKLQRMGIEELNILARQYGVKNIGSLTKSQVVFEIVKAKS
ERPDELLIGEGVLEVLPDGFGFLRSPTYNYLPSAEDIYVSPAQIRRFDLKKGDTIIGTIRSPKEKEKYFALLKVDKINGSTPDKAKERVLFENL
TPLYPNQRIVMEMGKDHLAERVLDLTAPIGKGQRGLIVAPPRSGKTVILQSIAHAIAVNNPDIVLIVLLIDERPEEVTDMIRQVRGEVVA
STFDEQPERHIQVAEMVIEKARRLVEHGNDVVILLDSITRLARAYNTVQPHSGKILTGGVDASALHKPKRFFGAARNIEGGGSLTILATAL
IDTGSRMDEVIFEEFKGTGNMELVLDRRLSDRRTYPAIDLIKSGTRKEELLYHPSELERVYLFRQAIADLTTIDAMHLLLGRLKKTNSNAEF
LLSLKE

>core/245/1/Org1_Gene417

MQIAQVFGCGRLNGEVKVSGAKNAATKLLVASLLSDQKCTLRNVPDIGDVSLTVELCKSLGAHVSWDKETEVLEIYTPEIQCTRVPPTFS
NVNRIPILLLGALLGRCPEGVYVPTVGGDAIGERTLNFHFEGLKQLGVQISSDSSGYYAKAPRGLKGNYIHLPYPSVGATENLILAAIHAK
GRTVIKNVALEAEILDLVLFLQKAGADITTDNDRTIDIFGTGGLGSVDHTILPDKIEAASFGMAAVVSGGRVFVRNAKQELLIPFLKMLRS
IGGGFLVSESGIEFFQERPLVGGVVLETDVHPGFLTDWQQPFAVLLSQAQGSSVIHETVHENRLGYLHGLQHMGAECQLFHQCLSTKA
CRYAIGNFPHSAVIHGATPLWASHLVIPDLRAGFAYVMAALIAEGGGSIIENTHLLDRGYTNWVGKLRSLGAKIQIFDMEQEELTTSPKS
LALRDASL

>core/255/1/Org1_Gene311

MLTCNECTTWEQFLNYVKTRCSKTAFENWISPIQVLEETQEKIRLEVPNIFVQNYLLDNYKRDLCSFVPLDVHGEPALEFVVAEHKKPSA
PVASQKESNEGISEVFEETKDFELKLNLSYRFDNFIEGPSNQFVKSAAVGIAGKPGRSYNPLFIHGGVGLGKTHLLHAVGHYVREHHKNL
RIHCITTEAFINDLVYHLKSKSVDKMKNFYRSLDLLLVDDIQFLQNRQNFEEEFCNTFETLINLSKQIVITSDKPPSQLKLSERIIARMEWGL
VAHVGIPDLETRVAILQHKAEQKGLLIPNEMAFYIADHIYGNVRQLEGAINKLTAYCRLFGKSLTETTVRETLKELFRSPTKQKISVETILKS
VATVFQVKLNDLKGNSRSKDLVLARQIAMYLAKTLITDSLVAIGAAFGKTHSTVLYACKTIEHKLQNDETLKRQVNLCKNHIVG

>core/257/1/Org1_Gene578

MSEPRFVCLSLGSNLGNRFKNLQIARTLLGEQAVLGLRSSVILETEALLLPGSPPEWDLPYFNSVLVGETTLSLRELLVTIKQIEKVVGRAE
ESPPWSPRTIDVDILLYGDESFCCDHTEITIPLSNLLSRPFLIALIASLCPYRRFCTQGSPYHNFTFGELAHHLPSPPGMIRRSLSPDTMLMG
VVNVTNDSMSDGGMFLDPEKAVAQAEKLFTEGAAVIDFGAQATNPKVKQFLSVDQEWERLEPVLRLLKETWSNRKQYPIISLDTFYP
EIILRAMDIYPIQWINDVSGGSQSMAEVARDCELSLVMNHSSSLPVDPKNILSFSVPIGEQLLSWGEKQLKMFSDVGLNANQVIFDPGI
GFGKGAAQSLATLYEIAKFKRLGCPILIGHSRKSFLSLFGNHDPKDRDWETVGLSILLQQQGVDYLRVHNVAAHQKALSVAACEACAPI

>core/262/1/Org1_Gene687

MRAMLLEDWVSLMLSDVSCPKCDKKITGFAIDSQQVQPGDLFFALPGNATDGHQFLKHAATAGAVAAVVSHDYQGDSFGLELIRVD
DTKSALQEAGSNQCNLFQGTLVGITGSVGKTTTKEFSKTILSSIYKTHASPKSYNSQLTVPLSLLMAEGDEDVMILEMGVSEPGNMQDL
LRIVQPEIAVITHINDQHAMHFPQGIQEILKEKSYILQKSKLQLLPKDSPYYLDLRSCSPTAEKFSFSFNDPLADFCYKAISGDSVVIQTPEE
NYCLPIAFSYKPAYTNLLIAVALSWILEVPEEGVIRSLPELKLPPMRFEHSMRNGMQVINDAYNACPEAMIAALDALPLPSDGGKIILILG
HMAELGRYSEEGHALVAEKAASRGDMIFFIGEKWIPVQSVLKSYSCEVSFFSSAQDVKDILKQVARYGDVILLKGSRALALESLLACF

>core/269/1/Org1_Gene648

MPRSLSNEQFSVDLEESPGCIVSALVKVSPEVLNKLNKQALKKIKKEITLPGFRKGKAPDDVIASRYPTNVRKELGELVTQDAYHALSTVG
DRRPLSPKAVRSNSITQFDLQEGAKVEFSYEAFPAISDLPWENLSLPQEEAASEISDSDIEKGLTNIGMFFATKTPVERPSQEGDFISISLH
VSKSNDENASSAAIFENKYFKLSEEEMTDAFKEKFLGISTGHRVVETITSPEIQSFLRGDTLTFTVNAVIEVSIPEIDDEKARQLQAESLDDL
KAKLRIQLEKQAKDKQLQKRFSEAEDALAMLVDFELPTSLLEERISLITREKLLNARLIQYCSDEELEKRKSELIKEAEEDATKALKLLFLTHKI
FSDEKLTISREELQYMMDVCSRERFGQQPPKDISNDTLQELVMSARDRLTYSKAIEHVLRKAELLASTPSA

>core/271/1/Org1_Gene642

MWLVILWALAASLAIALVAKGYYRFVYFRRYAVQVIREVRLSMELKEWALAEQQLLPILKKRSYRRQCLFEYMRILRKMQRFEESEKLLA
EAKKLGLRGPYFFLEIAYKAYRFGAFKECAQAFASVPQDLFEEEDAAKYASALVRLGDLDAACSLIEPWISPLSHQETFVTMGHIYFTSKR
YKDAIDFYNRANALGVCPVEVTYNLAQAYRITSSYAKAGKLFRKLLSNPVYKEEALFNIGLCEQKLGRPGKALLIYQSSDLWSRGDALLM
KYAAMAAMDQRDYVLAEPCWELALRCSTFAKDYKCGLGYGFSLCRLRKYGDAERVYCNLIQNFPECLTACKALAWLCGVGYATLLGS
EEGLMYAKKAVELDHSCETLELLSACEARCGNFDAAYEIQSFLSSRDTSLQEKQRRSQILRILRKKLPLNDHHIVEVDALLAA

>core/272/1/Org1_Gene449

MIENDFPEASNFESSHFYRDKVGVIILCGGEGKRLSPLTNCRCKPTVSFGGRYKLIDIPISHAISAGFSKIFVIGQYLTYTLQQHLFKTYFYH
GVLQDQIHLLAPEARQGDQIWYQGTADAIRKNLLYFEDTEIEYFLILSGDQLYNMDFRSIVDTAIRTHVDMVLVAQPIPEKDAYRMGVL
DIDSEGKLIDFYEKPQEKEVLKRFQLSSEDRRIHKLTEDSGDFLGSMGIYLFRRDSLFSLLREEEGNDFGKHLIQAQMKRGQVQTLLYNGY
WADIGTIESYYEANIALTQKPHAEKRGLNCYDDNGMIYSKNHHLPGAIITDSMISSSLLCEGCVINTSHVSRSVLGIRSKIGENSVVDQSII
MGNARYGSPSMPSLGIGKDCEIRKAIIDENCCIGNGVKLQNLKGYIKYDSPDKKLFVRDNIIIVPQGTHIPDNYIF

>core/274/1/Org1_Gene588

MFQQKQKLSLKYLPSLRMQQGLQMLQSPLTELSSYVVQEIIDNPFFDLSSLEEEEWSPCYRPTNSTFSYLNQTPGPQESLYTRLLPQIEE
AFSTAEERFIAHQIAGNLSDEGLFLRNPEDFAQELELPLEKIHKVWDTIQNLSPEGIASPSLQSYWMKLLRNSSHQQAYSIVRDCYPLMT
NCEFAPIMKKFSLSLSELRNILKKALGSIPWCPAAACTVKPMVSTPLPDIYLFYSSGSWKIEVSTRGLPSIKLNKETFHFYEHLPKEEQKNLS
QQILSAKWLIKNLRKREQTLLQVMETLLPKQEDFLLGKIPAPYPLGIKDLAEDLSFHESTIFRAIENKAVAAPIGIFPLKHLFPRGIHQDSSH
SKENVLQWIRQWIATEQTPLSDSVISDRITAKGIPCARRTVAKYRAQLKILPANKRKKLFYIRSSNSHFRDRQF

>core/279/1/Org1_Gene506

MKRPFFTYLCIIFYGSCASLSLHAGLSFPEVRGATAAVVHADSGKVFYDKDIDAVIYPASMTKIATALFILKHYPTVLDTLIKVKQDAIASIT
PQAKKQSGYRSPPHWLETDGSTIQLHLREELLGWDLFHALLVCSANDAANVLAMACCGSVEKFMDKLNFFLKEEIGCTHTHFNNPHG
LHHPNHYTTTRDLISIMRCALKEPPFRGVISTTSYKIGATNLHGERILSPTNKLLLPGSTYHYPPALGGKTGTTKTAGKNLIMAAEKNNRLL
VTIATGYSGPVSDLYQDVIALCETVFNEPLLRKELVPPSDCLQLEIANLGKLSCPLPEGLYYDFYASEDREPLSVSFIAHADAFPIEQGDLLG
HWVFYDDEGKKISSQPFYAPCRFERTIKPWKLYMKRVFTSYRTYMSITMLLMYFRIRKHRKYKNLKHYSKI

>core/281/1/Org1_Gene133

MFSRWITLFLLFISLTGCSSYSSKHKQSLIIPIHDDPVAFSPEQAKRAMDLSIAQLLFDGLTRETHRESNDLELAIASRYTVSEDFCSYTFFIK
DSALWSDGTPITSEDIRNAWEYAQENSPHIQIFQGLNFSTPSSNAITIHLDSPNPDFPKLLAFPAFAIFKPENPKLFSGPYTLVEYFPGHNI
HLKKNPNYYDYHCVSINSIKLLIIPDIYTAIHLLNRGKVDWVGQPWHQGIPWELHKQSQYHYYTYPVEGAFWLCLNTKSPHLNDLQNR
HRLATCIDKRSIIEEALQGTQQPAETLSRGAPQPNQYKKQKPLTPQEKLVLTYPSDILRCQRIAEILKEQWKAAGIDLILEGLEYHLFVNKR
KVQDYAIATQTGVAYYPGANLISEEDKLLQNFEIIPIYYLSYDYLTQDFIEGVIYNASGAVDLKYTYFP

>core/282/1/Org1_Gene229

MNKNLVAIFDYMEKEKGIQRSTIIGAIESALKIAAKKTLRDDANISVNINSRTGDIEVFCEKEIVEICQNPSKEIPLDKAREYDPDCQIGQY
MDVPFVSDNFGRIAAHAARQIIGQKLRHAERDVIYEEYRHRVNETLSGVVKRFAKGSNLIIDLGKVEAILPTRFYPKTEKHKIGDKIYALLY
EVQESENGGAEVILSRSHAEFVKQLFIQEVPELEEGSVEIVKIAREAGYRTKLAVRSSDPKTDPVGAFVGMRGSRVKNIIRELNDEKIDIV
NYSPVSTELLQNLLYPIEIQKIAILEDDKVIAIVVNDADYATVIGKRGINARLISHILDYELEVQRMSEYNKLLEIQRLQLAEFDSPHLDQPLE
MEGISKLVIQNLEHAGYDTIRRVLLASANDLASVPGISLELAYKILEQVSKYGESKVDEKPEIED

>core/290/1/Org1_Gene742

MVCENNILSGRGLELLKKKSNITLTPTIYSVSNHNIKLKDFSPHALSVIKTLRKAGYIAYIVGGCIRDLLLNTTPKDFDISTSAKPEEIKAIFKN
CILVGKRFRLAHIRFSKQIIEVSTFRSGSTDEDVLITKDNLWGTPEEDVLRRDFTINGLFYDPEHEEIIDYTGGVNDLRNRYLRTIGDPFTRF
KQDPVRMLRLLKILSRSPFTVETQTQEALIACRQELIKSSQARVFEELIKMLNSGAAKNFFQLLIENHLLEILFPYMDKAFRLNRALEEQTA
TYLKALDDKILKKEAEYDRHQLMAIFLFPLVNFNVRYKHQKHPYLSLTSVFDYIKNFLEQFFADSFTSCSKKNFILTALILQMQYRLTPLIPT
KKALFFNKKLLHHTRFLEALSLLEIRSIVYPKLDKVYVAWIRHHQTLKCKKDSHSQK

>core/295/1/Org1_Gene909

MKKTMVIDTSVFIYDPEALFSFENTRIIIPFPVIEELEAFGKFRDESAKNASRALSNIRLLLENAKTKVTDGVLLPSGSELRIEVAPLSNDDRR
GKLLTLELLKIIAKREPMVFVTKSLGRRVRAEALQIESRDYESKRFSFRSLYRGFRELQVSQEDIENFYKNGYLDLPLDVVPSPNEYFFMSA
GENHFALGRYYVSEGKIIALKAMDKSVWGIKPLNTEQRCALDLLLRDDVKLVTLIGQAGSGKTILALAAAMHKVFDKETYNKVLVSRPIV
PMGRDIGFLPGLKEDKLMHWMQPIYDNMEVLFSINQMGNSSEALQALMDAKKLEMEALTYIRGRSLPKAFIIIDEAQNLTPHEIKTIIS
RAGKGTKIVLTGDPTQIDSLYFDENSNGLTYLVGKFHHLALYGHMFMTRTERSELAAAAATIL

>core/305/1/Org1_Gene60

MIKFLSQLFIRHWPRKVVSLGFAIIIWILVGQSVTITRTLTNVPVRIVDLHPDQTVLGLQKSGFLNKKVSLTITGNKNTVQDLRPSNLEVVI
SAANHTESWIATIDKHNLVSVDHEINIRKDIHSVDANDIFVRLTQYVTEDILLTITKPIGSPPKGYEYLDVWPKYLNQKVSGPKEYINALKE
QGLELTFNLNKISFEELERNRIAQGSHDEIIFPIPKEWKKILIPFENTFMDLNDPQADFLRLLFLKRECIPLNLNLPVFLFFPVTFIQTMNPLE
YSLDPVPPIILNHGIHQINIPLYVKDVSRQFLDVVKNNMVLTIVMPSPQDPSSINWAIEFLDEKTLENTFLQTIIAQEHGILHDIALIDEAGI
RHRFREYLRKLALFTADGEPLNLIAEIKNNKVVIQTKTKETTKLYKKEW

>core/307/1/Org1_Gene146

MSSAIARDCFPSPSPQPSSTLGVHPPKYKSLILSVSLIVLGVLLLCVGMLLLVNAIFSFSVLTVGLGGAGVFLGSLLLILGLIFFVSYHRKLSEA
TRSLEQKITLEYQPWADLRKELNEVQEWSNFLLDEWEDFKEVVAQHKSQFATFEGDLLLFGREVEKYETIWKELDGRDVALLTELKNI
WGPLEFLRKKGDRLQCEIDKLRKEVMKVGKSGLKLACELTKFKSALKDVKIEQECYRDKRKVEKLEVFPEGYRRELLEVLKTRLSVEKEIQL
FEEVVSAFEEKLASLHRTVFSEEELQEALDKAKAELLDIQVRKSVVEDLSCEPTLIQYHLLRLYEVQCRIVEQFLTQTFSSEQEKVLEEYEAL
KARIRKTLRVKLDQVRANVAFVASTTDLLSESESLDGNDSVFEDAHDDFLD

>core/309/1/Org1_Gene668

MIETREEVGSANFVSLERAIEDLRAGKFVIVVDEASREDEGDLIIAGEKITVEKMTFLLQHTTGVVCAALSQERLLSLDLPPMVKDNRCRF
KTPFTVSVDAAHGVTTGVSAADRTKVVQLLADPKSKPEDFISPGHFFPLASSPGGVLKRAGHTESTVDLMELAGLQPCGVLAELVNED
YSMMRLPQILEFARKHNIAVIPVTSIIAHRMLSDRLVSKISSARLPTIYGDFTIHVYESLLEGMQHLALVKGNVAGKSNVLVRVHSECVTG
DILGSKRCDCGEQLSSAMSYIAEKGTGVLVYLRGQEGRGIGLGHKVRAYALQDNGYDTVDANLAMGFPVDSREYGIGAQILVDLKLTTI
KLITHNPQKYFGLQGFGLSITERVPLPVRISEDNEQYLRTKQERMGHWLDLPCCNNRVQ

>core/312/1/Org1_Gene689

MCQRILILGTGITGKSVARFLYQQGHYLIGADNSLESLISVDHLHDRLLMGASEFPENIDLVIRSPGIKPYHPWVEQAVSLKIPVVTDIQV
ALKTPEFQRYPSFGITGSNGKTTTTLFLTHLLNTLGIPAIAMGNIGLPILDHMGQPGVRVVEISSFQLATQEEHIPALSGSVFLNFSRNHLD
YHRNLDAYFDAKLRIQKCLRQDKTFWVWEECSLGNSYQIYSEEIEEILDKGDALKPIYLHDRDNYCAAYALANEVGWVSPEGFLKAIRTF
EKPAHRLEYLGKKDGVHYINDSKATTVTAVEKALMAVGKDVIVILGGKDKGGDFPALASVLSQTTKHVIAMGECRQTIADALSEKIPLTL
SKDLQEAVSIAQTIAQEGDTVLLSPGCASFDQFQSFKERGAYFKLLIREMQAVR

>core/318/1/Org1_Gene874

MKKNASHKTNDKKSLSIWSIGGSIFAMFFGAGNIVFPLALGYHYNAHPWSAYFGMMLTAVCVPLLGLVSMLFYSGDYQKFFFSIGRIP
GMIFITAIILLIGPFGGIPRAIAVSHATLISLSEHKSAFIPSLPIFSAICCVLIYIFSCKLSRLIQWLGSVFFPIMLVTLLWVIIRSFMIPTHPMVQ
EFIPNARQAWLAGFIEGFNTMDLLAAFFFCSIVLISLRQLVAEEKHPTEEEIPLSFQGISKKNKRSLALGFFLAAILLGMTYLGFVLSAARHA
GLLVNVSKGHILGRISAIALGPNSILAGVSVFIACLTTEIALVGIVADFLARVVSFKKLNYASAVICTLIPTYLISILNFETISHLLLPLLQLSYPAL
IVLACGNIAYKLWNFRYSPVLFYLTLSLTIVLKLVN

>core/322/1/Org1_Gene751

MNLPVSLACLLLSGCVFFLGVFVSSSLYARKKRAFLEKIQKLEHENQLLQTSLNLSRHQEQLIEDFSNRLALSSHKLIKDMKEEAQNYFGD
TSKSFQSILSPIQTTLTTFKQSLETFETKHAEDRGRLKEQISQLLAVEKKLEHETHVLTDILKHPGSRGRWGEIQLERILELAGMLKYCDYDS
QTTSAQGAFRADIIIRLPQDRCLIIDAKAPISDSYFSVEEIDKGDLVDKIKEHIKTLKSKSYWEKFHQSPEYVILFLPGESLFNDAIRLAPELM
EIGASSNVILSSPLTLLALLKTIAYMWKQENLQKQIQEVSLLGKELHRRLQVVFTHFQKIGKNLNQTVQSYNDMTSSFQYRVLPTLRKFE
GLETSSSHQIEEPTPIESLATSFPHTCDIDTNLAVIESLEKQD

>core/323/1/Org1_Gene828

MKTAFHSCYSWFCWLFSFLVLFVGGIAGGEPLCPDCKYETKSVLRSDQLPDHLWNYENDCYLTGYVQSLLDMHFLDSRTQVVIEKNRA
YLFSLPVDSSLSEAITNFVRDLPFICAVEICERPYGECITRSSAERPLLPKEKTLGMPIFCGKEGVWLPQNTILFSPLIADPRQVTNSAGIRFN
EKVVGNRVGATIFGGDFILLRLFDVSRFHVDCDFGIQGGVFSVFDLDHPESCMVNSDFFVAGLWSGAIDKWSFRFRLWHLSSHLGDEF
ILTHPNFPRFNLSDEGVDLFISFRYTPQIRLYGGCGYIVSRDLTFPERPFYCEWGAELRPFGLREGNLHAQPIFAMHFRCWEEQKFGLDQ
SYILGMEWAKFQEIGRKIRAVLEYHQGFSKEGQFIREPCNYYGFRLTYGF

>core/326/1/Org1_Gene145

MVVVALFILGIFFLSGSLAFLVHTSCGVLLGAALPILCIGLVLLAVALIVFLCHKHKTRQDLDYYDQDLDSLVIHKKEIPNDISELRVTFEKLQ
NLFQFHTKDFSDLSQELQGKFINCMEKWLTLEDEVTKFLIVRDRFLETRRNFTTFGEQVKGIQSNIFDLHEEKSSLYLELYRLRKDLQVLL
NFFLLPPGILKVDYDEIEAIKGLFIRLTSRLDKLDVKAQERKKFINEMSREFKEVEKAFDIVDRATKKLMDRAKKESPARLFMGRTESLLEM
KKNEEALKNQGLDPENLSHPELFSPYQQLLILNYLNSEIVLHHYEFLISGTVTSGLTLEECENRMRAASTGLNALLVRKLQFRGAIKSAYFE
KLTEIEKELRSLQDVIKSLELELIHKIKDIVTEET

>core/336/1/Org1_Gene359

MLSVTIVLVGLEMARSKVSKRDSKILDILFATTELYLKTGQPVGSKTLKESFCSDLSTATIRNYFAELEAEGFLKKNHTSGGRIPTDLALRHY
VDHQEECPEAEISAPIFDKISQLPSESRNIIKDLQKATELLGEILDLPTFFSSPRFENDSVTNIQITQVDKQRAVTILSTEFGQIFTDTLWLPE
ACDTLSIKRIEKFLQNYIRKLPTNEELSKKEEHLSMSLYNEVVVRYLTRYCNFSEEDLYQTGMSKLLKYEAFKDPEVLALGLSLFENRRQMC
ELLNIGMHKGRATAFIGKELSDILGTSNPGCSVITIPYYMNRSPLGALGILGPINLPYKEALPLLKLFANKINETLTQSFYKFKLSFRRPLTSN
CKLSNEPILRTEYSSIKLLPSKETL

>core/337/1/Org1_Gene354

MRRNPHFSLLKPQYLFSEISKKLAQFRKENPEISVIDLSIGDTTQPLCRSITQAIKEFCVSQEKQETYRGYGPETGLEKLRTKIASEVYENRIS
PEEIFISDGAKPDIFRLFSFFGSEKTLGLQDPVYPAYRDIAHITGIRDIIPLACRKETGFIPELPNQQSLDILCLCYPNNPTGTVLTFQQLQAL
VNYANQHGTVLIFDAAYSAFVSDPSLPKSIFEIPEAKYCAIEINSFSKSLGFTGMRLAWNVIPKELTYDNNEPMINDWKRLFATTFNGAS
LLMQEAGYYGLDLFPTPPAISLYLTNAQKLKKSLETAGFSVHGGDHAPYLWVELPEGISDEEAFDFFLHQYHIAVTPGHGFGSCGQGFV
RFSALTQPQNIALACDRLCTASLKETMVLA

>core/345/1/Org1_Gene16

MDYYSILGISKTASAEEIKKAYRKLAVKYHPDKNPGDAAAEKRFKEVSEAYEVLSDPQKRDSYDRFGKDGPFAGAGGFGGAGGMGNM
EDALRTFMGAFGGEFGGGSFFDGLFGGLGEAFGMRSDPAGARQGASKKVHINLTFEEAAHGVEKELVVSGYKSCETCSGQGAVNPQ
GIKSCERCKGSGQVVQSRGFFSMASTCPECGGEGRIITDPCSSCRGQGRVKDKRSVHVHIPAGVDSGMRLKMEGYGDAGQNGAPSG
DLYVFIDVESHPVFERRGDDLILELPIGFVDAALGMKKEIPTLLKTEGSCRLTVPEGIQSGTILKVRNQGFPNVHGKGRGDLLVRISVETPQ
NLSEEQKELLRTFASTEKAENFPKKRSFLDKIKGFFSDFTV

>core/350/1/Org1_Gene992

MMKQGVGQDAKELYTFLSRGNEHYQPCLWFSLEEELGFLFDAKMLCAPLSEDHYCHSYLVDLVDQHLKDLILSMFLDPQNISAGELLK
VSINVGDSFSPLQQKDFLSMVLRDETGKNVVVVFKGVLSLPATQVCKLVEELNSKDYSYLNIFSCHGDSSPQLLFRKELEGTSGRYFTVIC
ALYLGDTDMRSLQLASERIMVSREFDLVDAYAARCKLLKIDHTNWRPGTFSRHADFADAVDVSAGFNSREFKLITQANQGILESGELPL
PSKTFWEGFLAFCDRVTVTRHFIPMLDAAIKQAVWTHKHPSLIDKECEALDLKTQCLPSIVSYLEYVTNSHEKTSKGPFIQKEIIADCSPLK
EALFPGSDEDVPSTSEDPSDDHPSDLEDS

>core/351/1/Org1_Gene509

MGTPISGNDGDRNTISDPLEESAAEEGDSDLEDRVSESATQVIETIADTGIPEATPSEGTNSDLNSDLVDRVEYEARGSLLTTMLARIRKA
VSQIWMHVKTKRHPKEQGVRSLGDIPCDLLKATRLPKETAEPPYFYALETALASCRSFFFHVFLRLFTLLRRQHPEAPLDLCGTDPISPEA
AVAFALILRSCCKWVATDAVQEGLPLEVIEEAGMYNAFSLEATTTVEEVSKRLSELLYSDKRIDGLANVRGITKIITSPYLGAGQCVSVVD
NLKTYDLGRNYTQVLACASQIDEFADKGENEALVMKDILYLVRQDRSKELGDFLMMWSEEHASEVNYDVVLAILEVNLPILEEDYRSH
PLAYQKKLNYVICQFFCSERLTSIEPKD

>core/353/1/Org1_Gene431

MAIKNILVVDDEPLLRDFLSELLTSQGFIPDTAENLRNALQMIRSRDYDLVISDMSMPDGSGLDLIKIIKQSSPHTPVLVVTAYGSIENAV
EAMHQGAFNYLTKPFSSEALFAFISKAEELKNLVHENLFLHSQTTPDSHPLIAESKAMKDLLAIAKKAASSSANIFIHGESGCGKEVLSFFI
HHNSPRANHPYIKVNCAAIPETLLESELFGHEKGAFTGATTKKAGRFELAHKGTLLLDEITEVPVNLQAKLLRAIQEKEIEHLGGTKTLSVD
VRILATSNRKLKEAIDDKSFRQDLYYRLNVIPLHLPPLRDRQDDILPLANYFLNKFCRMNNTPLKTLSPKAQELLLNYPWPGNIRELSNVL
ERVVILENTSLLTEDMLALA

>core/357/1/Org1_Gene660

MEKPQNRKAPRIFWLNNQVAIPPSERVKESYALHSDIFSLPPGSALKLAEKTEESIRQLVGLKDSHIFRFVPHFPHVVHIVLAALVENLSM
FQGRNHIILPAHDQQLLINSLCRHQGLGTTYDWVTVNHEGRIVEEQLIETLSPRSLLFSLSAAHGLTGVIQPLDPLLSLCKDRRILLHLDIS
DILGRAPLTPEILNADIITFSSAALGGMGSIGGIFIRKSLERVFSSWFPPHTSASLCFSAVAAMQTACEERISALPLFTFHTSNLCKKLIQELQ
SVLPSIQLAFSEVQNRLPNIVVAAIPDIPAESLAFHLHQQGIYPSLGYERFQPLAQVLQNCGISPFLCHSALHFSLTERSKDLEFSKLARAM
HDAIKHLTPLLGSSS

>core/366/1/Org1_Gene924

MKHLAVLGSTGSIGRQTLEIVRRYPSEFKIISMASYGNNLRLFFQQLEEFAPLAAAVYNEEVYNEACQRFPHMQFFLGQEGLTQLCIMD
TVTTVVAASSGIEALPAILESMKKGKALALANKEILVCAGELVSKTAKENGIKVLPIDSEHNALYQCLEGRTIEGIKKLILTASGGPLLNKSLE
ELSCVTKQDVLNHPIWNMGSKVTVDSSTLVNKGLEIIEAYWLFGLENIEILAVIHPQSLIHGMVEFLDGSVISIMNPPDMLFPIQYALTAP
ERFASPRDGMDFSKKQTLEFFPVDEERFPSIRLAQQVLEKQGSSGSFFNAANEVLVRRFLCEEISWCDILRKLTTLMECHKVYACHSLED
ILEVDGEARALAQEI

>core/370/1/Org1_Gene92

MNTSLKRPLKSHFDVVGSFLRPEHLKKTRESLKEGSISLDQLMQIEDIAIQDLIKKQKAAGLSFITDGEFRRATWHYDFMWGFHGVGH
HRATEGVFFDGERAMIDDTYLTDKISVSHHPFVDHFKFVKALEDEFTTAKQTLPAPAQFLKQMIFPNNIEVTRKFYPTNQELIEDIVAGY
RKVIRDLYDAGCRYLQLDDCTRGGLVDPRVCSWYGIDEKGLQDLIQQYLLINNLVIADRPDDLVVNLHVCRGNYHSKFFASGSYDFIAK
PLFEQTNVDGYYLEFDHERSGDFSPLTFISGEKTVCLGLVTSKTPTLENKDEVIARIHQAADYLPLERLSLSPQCGFASCEIGNKLTEEEQW
AKVALVKEISEEVWK

>core/371/1/Org1_Gene465

MSDNAHNLLYDKFELPEAVKMLPVEGLPIDKHARFIAEPLERGMGHTLGNALRRALLIGLEAPAIISFAMTGVLHEYMAIEGVIEDVTNI
ILNLKGALLKKYPMQDSSLGRTTQVLKASISIDASDLAAANGQKEVTLQDLLQEGDFEAVNPDQVIFTVTQPIQLEVVLRIAFGRGYTPS
ERIVLEDKGVYEIVLDAAFSPVTLVNYFVEDTRVGQDTDFDRLVLIVETDGRVTPKEALAFSTQILTKHFSIFENMDEKKIVFEEAISIEKEN
KDDILHKLILGINEIELSVRSTNCLSNANIETIGELVIMPEPRLLQFRNFGKKSLCEIKNKLKEMKLELGMDLTQFGVGLDNVKEKMKWYA
EKIRAKNTKG

>core/377/1/Org1_Gene532

MAVAADSSASWLKSRNNFLSSLGKTEEQVAAPEFPKELCQHKIREKFRLEDVQVSIKFRGSITAVEATKEFGVHLLIQPMVVQPWEVEN
LLFLTSEEDLQELMVAVFDDASLASYFYEKDKLLGFHYYFVAEACKLFEELQWVPSLSAKVGGDAIFTATSLQGSFQVVDISLRLDGKNV
RCRLLLPEDTFQSCQKFFSGLHDESDLHNIDQTQQISLSVEVGYSQLTQEEWHQVVPGSFIMLDSCLYDPETEESGALLTVQKHQFFGG
RFLTPSSGEFKITSYPNLTHEDPPLPENPQASAAPLPGYSRLVVEVARYSLAVSEFIKLNLGSILSLGNHPAYGVDIILDGAKVGRGEIIALG
DVLGIRVLEV

>core/380/1/Org1_Gene891

MMLVYCFDPSVPTSPEHRLMAALDRWFFLGGHRVRILTLEGNHYRAFQENMSISTVEKILKLISYLLIPIVLIALLIRCFLHSRFKCNWKCD
SLSDARVPHDVQPFNDFQLFNNQERLNIWKNRRYVSGIDVLMVPVDYLRSQFPGFKEIPEAIRCENYVSDGQFSEESKTSYLRAMLTDI
VGYILSLDETYWTNVILKIRAMCITFESFPGKEADPNYSPRVTHHYFDESWKALARHVLGEGNMVNRLDEALIRTEKPGKEGECITKQFL
KDYCKKHLEVMSCPDFIESLVDEKIREFRCPSILNSAVCDVIDRKCQEHLLKAIINEANRRLPGMKNSSFTMRGNQVLFYTIFSPPKLPPA
ASSVYF

>core/381/1/Org1_Gene370

MTTCLPQPPKTSPLYSIFEKLDAQERLSSEDALHLLLLTNKEDQRTLWNFADQVRKQRVGDTVYYSSTLYLYPTNFCDFSCKFCSFYAKP
GDPKGWLYSPDDLLQQIQNIKTPITEVHIVGGCFPSCNLQYYSDLFTKIKEYDPQIHIKALTAIEYAYLSDLDNLSIRDVLLTLKDAGLDSIP
GGGAEILVDKIRNFLAPKRLSSSDFLNIHKMAHQLGIHSNITMLCYHKEGPEDLVTHMVKVRDLQDETQGFKNFILLKFAQENNVLGKR
LRKSGQGHAIPLKSLMAVARIFLDNFSNMKALWNYLGIEAALDLLSCGANDLSSTHMGEKVFQMASSKEPIKMDAEGMAALITQQG
RTPCLTNSSHV

>core/385/1/Org1_Gene34

MAVEGRVNSSQALNQDCQEVLANKQSKGLLRCRILSIVVAVITFIAGVVLIALTLASILTSVPYLALGVFLLIVTLGCIIFALCSEKIKKVPPTP
ISHKEEIIAWFEERKNIDMEKEKEDPEHFGRTATDIPMRSALDQFNHSCHHIHESPALTETYRSHQDVLLFKDWCPVTLPDVTSEEEVLI
RSVVGSYLLMEACVPKVSMLIDELHNKLKSPSERECLFIDKKTLQRKASFLFTQKDLATFFLAYTRVNDGHLAPFRAGAKWILIHYVRLRR
QHNQNDFFTPGHSCYYARLAFNQTQRLYHQLFNVEKLRSIYANMDKDPLCHPWAFIPIYDLLKTEDHGDGFLEQQEDREYPSRAAQD
QFWG

>core/387/1/Org1_Gene888

MKFVVSRNELGNLIKKIQSVVPQNTPIPVLTHVLIETYNDELVFTATDLTVSTRCVTKAKVYEKGAISIPSKRFFQLVKELTEANLEISSSAG
EMAQITSGSSCFRLLSMEKEDFPMLPDIQNALRFSLPAEQLKTMLQRTSFAVSREESRYVLTGVLLAIANGVATIVGTDGKRLAKIDAEV
TLDKSFSGEYIIPIKAVEEIIKMCSDEGEATIFLDQDKIAVECDNTLLITKLLSGEFPDFSPVISTESNVKLDLHREELITLLKQVALFTNESSHS
VKFSFLPGELTLTANCTKVGEGKVSMAVNYSGELLEIAFNPFFFLDILKHSKDELVSLGISDSYNPGIITDSASGLFVIMPMRLHDD

>core/396/1/Org1_Gene810

MKLYQTLRGIVLVSTGCIFLGMHGGYAAEVPVTSSGYENLLESKEQDPSGLAIHDRILFKVDEENVVTALDVIHKLNLLFYNSYPHLIDSF
PARSQYYTAMWPVVLESVIDEFLMVADAKAKRIATDPTAVNQEIEEMFGRDLSPLYAHFEMSPNDIFNVIDRTLTAQRVMGMMVRS
KVMLKVTPGKIREYYRKLEEEASRKVIWKYRVLTIKANTESLASQIADKVRARLNEAKTWDKDRLTALVISQGGQLVCSEEFSRENSELSQ
SHKQELDLIGYPKELCGLPKAHKSGYKLYMLLDKTSGSIEPLDVMESKIKQHLFALEAESVEKQYKDRLRKRYGYDASMIAKLLSEEAPPLF
SLL

>core/397/1/Org1_Gene437

MAFKRKTRWLWQVLILSVGLNMLFLLLFYSAIFRKDIYKLHLFSGPLIAKSSRKVYLSEDFLNEISQASLDDLISLFKDERYMYGRPIKLWA
LSVAIASHHIDITPVLSKPLTYTELKGSSVRWLLPNIDLKDFPVILDYLRCHKYPYTSKGLFLLIEKMVQEGWVDEDCLYHFCSTPEFLYLRT
LLVGADVQASSVASLARMVIRCGSERFFHFCNEESRTSMISATQRQKVLKSYLDCEESLAALLLLVHDSDVVLHEFCDEDLEKVIRLMPQ
ESPYSQNFFSRLQHSPRRELACMSTQRVEAPRVQEDQDEEYVVQDGDSLWLIAKRFGIPMDKIIQKNGLNHHRLFPGKVLKLPAKQS

>core/400/1/Org1_Gene73

MKIDILSLFPGYFDGPLQTSILGRAIKQRLLDVQLTNLRDFGLGKWKQVDDTPFSGGGMLLMAEPVTSAIRSVRKENSKVIYLSPQGALL
TAEKSRELAAASHLILLCGHYEGIDERAIESEVDEEISIGDYVLTNGGIAALVLIDAVSRFIPGVLGNQESAERDSLENGLLEGPQYTRPREF
EGKEVPEVLLQGDHKAISQWRLEQSERRTYERRPDLYLNYLYKRSIDHKFDEETTTNRDHFKCDKISVVLEVNKLKRAKNFYCKVFGLDA

MSCENKFCLPHEGKTIFWLREVQAEKKNIVTLSLSLDCACEEDFCYLLRRWELFGGKLLEKQADEHAVWALAQDLDGHAWIFSWHR
MK

>core/402/1/Org1_Gene217

MSEAPVYTLKQLAELLQVEVQGNIETPISGVEDISQAQPHHIAFLDNEKYSSFLKNTKAGAIILSRSQAMQHAHLKKNFLITNESPSLTFQ
KCIELFIEPVTSGFPGIHPTAVIHPTARIEKNVTIEPYVVISQHAHIGSDTYIGAGSVIGAHSVLGANCLIHPKVVIRERVLMGNRVVVQPG
AVLGSCGFGYITNAFGHHKPLKHLGYVIVGDDVEIGANTTIDRGRFKNTVIHEGTKIDNQVQVAHHVEIGKHSIIVAQAGIAGSTKIGEH
VIIGGQTGITGHISIADHVIMIAQTGVTKSITSPGIYGGAPARPYQETHRLIAKIRNLPKTEERLSKLEKQVRDLSTPSLAEIPSEI

>core/406/1/Org1_Gene692

MMKKIRKVALAVGGSGGHIVPALSVKEAFSREGIDVLLLGKGLKNHPSLQQGISYREIPSGLPTVLNPIKIMSRTLSLCSGYLKARKELKIF
DPDLVIGFGSYHSLPVLLAGLSHKIPLFLHEQNLVPGKVNQLFSRYARGIGVNFSPVTKHFRCPAEEVFLPKRSFSLGSPMMKRCTNHTP
TICVVGGSQGAQILNTCVPQALVKLVNKYPNMYVHHIVGPKSDVMKVQHVYNRGEVLCCVKPFEEQLLDVLLAADLVISRAGATILEEI
LWAKVPGILIPYPGAYGHQEVNAKFFVDVLEGGTMILEKELTEKLLVEKVTFALDSHNREKQRNSLAAYSQQRSTKTFHAFICECL

>core/412/1/Org1_Gene961

MNLPDRKKALEAAVAYIEKQFGAGSIMSLGRHSATHEISTIKTGALSLDLALGIHGVPKGRVIEIFGPESSGKTTLATHIVANAQKMGGV
AAYIDAEHALDPSYASLIGVNIDDLMISQPDCGEDALSIAELLARSGAVDVIVIDSVAALVPKSELEGDIGDVHVGLQARMMSQALRKLT
ATLSRSQTCAVFINQIREKIGVSFGNPETTTGGRALKFYSSIRLDIRRIGSIKGSDNSDIGNRIKVKVAKNKLAPPFRIAEFDILFNEGISSAG
CILDLAVEYNIIEKKGSWFNYQEKKLGQGREFVREELKRNRKLFKEIEKRIYDVIAANKTPSVHANETPQEVPAQTVEA

>core/413/1/Org1_Gene697

MNLCKRISFEEGLELFVSSPIERLQERADAIRKERYPSNEVTYVLDANPNYTNICKIDCTFCAFYRKPKSPDAYLLSFDEVRSLLQRYVSSGV
KTVLLQGGVHPGLGIDYLEELVRITVQEFPSIHPHFFSAVEIEHACRVSGISIEQGLQRLWDAGQRTIPGGGAEILSERVRKIISPKKMQPG
GWINLHKLAHLMGFRTTATMMFGHVENPEDILIHLQTLRDAQDSCPGFYSFIPWSYKPGNTALRRNVPQQASIETYYRILALGRIFLDN
FDHVAASWFGEGKSLGAKALHYGADDFGGVILDESVHKATGWSIQSSEEEICNIIRSEGFIPVERNTFYQHISCTVSSL

>core/414/1/Org1_Gene703

MKYSLNFKEIKIDDYERVIEVTCSKVRLHAIIAIHQTAVGPALGGVRASLYSSFEDACTDALRLARGMTYKAIISNTGTGGGKSVIILPQDA
PSLTEDMLRAFGQAVNALEGTYICAEDLGVSINDISIVAEETPYVCGIADVSGDPSIYTAHGGFLCIKETAKYLWGSSSLRGKKIAIQGIGS
VGRRLLQSLFFEGAELYVADVLERAVQDAARLYGATIVPTEEIHALECDIFSPCARGNVIRKDNLADLNCKAIVGVANNQLEDSSAGMM
LHERGILYGPDYLVNAGGLLNVAAAIEGRVYAPKEVLLKVEELPIVLSKLYNQSKTTGKDLVALSDSFVEDKLLAYTS

>core/419/1/Org1_Gene789

MRIAVLGAGYAGLSVTWHLLLHSQGTATIDLFDPIPLGEGASGMSSGLLHAFTGKKALKPPLADQGINATHALITEASKALNVPIVISQG
ILRPAIDEDQAQLFTERVEEFPKEVEWWEKARCEISIPSMVIPPNLGALFIKSGVTLNNDLYIQGLADACMKLGTQFYDELIEDLADIEEFY
DHIIVTPGANASILPELKDMPVNKVKGQLLEISWPKDLAMLSFSINAHKYMVANTQKNTCILGATFEHNQPEETPDPAIAYQEIMPPVL
SLFPGLKDAQVLHCYAGMRSSSKSRLPVISRIREKLWFLGGLGSKGLLYHGITGDMLAQAVLRKSTAYIAKEFLFTI

>core/420/1/Org1_Gene715

MILLQNIKRCSLKQLKVLATLLLSLSLPTLEAAENRDSDSIVWHLDYQEALQKSKEAELPLLVIFSGSDWNGPCMKIRKEVLESPEFIKRVQ
GKFVCVEVEYLKHRPQVENIRQQNLALKSKFKINELPCMILLSHEEREIYRIGSFGNETGSNLGDSLCHIVESDSLLRRAFPMMTSLSLSEL
QRYYRLAEELSHKEFLKHALELGVRSDDYFFLSEKFRLLVEVGKMDSEECQRIKKRLLNKDPKNEKQTHFTVALIEFQELAKRSRAGVRQ
DASQVIAPLESYISQFGQQDKDNLWRVEMMIAQFYLDSDQWHHALQHAEVAFEAAPNEVRSHISRSLEYIRHQS

>core/422/1/Org1_Gene218

MDVSRKINRHTQFYVDSIDGVIKNFDHKPSEDKSRDHEELEEKLLTITKRIVASAQEFQNRKTDSKNYYLKKTQWLPFKNEELEQTKELF
AMLTSMDKKIAQLFFYSPGCSSDWVEFTEVICHLNDSIGLGGVLLCCGLFEQQCEHVVTVNKKLDLPLLLGTTVVNSLRYYLTYRNISLLN
CQSMSELGKELGDVLKQHGVAFTLIFKEIVDIDLLNYVKLIQGLKRSGNIQARIYDNDVPTLPSVSSSPIALRYSLANTIRGLALHVDFSSLK
FISPSILSNTEHTAKALNSGGECFIFSNLDEFNLGMKIVMQLLRTGKISPEILNKNIMKILMIKRRVRSLYI

>core/428/1/Org1_Gene748

MMEVYSFSPSVRTSFQHRVMAALDNWFFLGGRRLKVVSLDSCNSGQACEEYVPISTTEKVLKILSYLLIPIVIIALLIRYLLHSNFTAKVSQ
KPWLKTLQLGIDIKSFILPGSHVNTMDSATLFKAIRLEGKRVDVEYHRLHSSDKVVFYIPAQKLPDDLRLTHWLPEKETRKTEYVRHMLA
HVMGYLTSQGKERLQQVVQDSRSSTSLGAEKVLQYRFIDHPQSQGEFQRLLNENITTKGSEDKEVVQSDLFDMAFQCWWPQFISVIQ
SPTFSEELVHEMSQKLDLDCIYPEDDEFEQKFLNTLLKAVLHHGFEGISVASMGVIFLICPDSLALQIPFLRNQK

>core/435/1/Org1_Gene659

MFFQNLAKKLTALGISPLGCLLIGGVVSCAILFGRSSNPSLAPTQVKTEKTSGNWLKLTQMGNPKLIESLTKKEQLEKDLTSFHPIASAKV
AIALSTEDDVMSPLHLSVILTLRKEESLTPSLLFSITDYLCSSLPGLKREHISLSDNLGNLYIPESITVNSLFIHTLENYLGKIFPKEHFALAYHAK
AEKPTLQLTLNENYIAHLTKEESEKIVAHTKHYLYQNYDDSYDIVIETLPFARLQNKKSFPAKVLIGSMILVISLMIVALASFYLARHAYERV
SPEPRKIKRGINISKLLEIIQKESPEKIALILSYLDPKKAEALLNRLPEDLKHQVLKYKL

>core/437/1/Org1_Gene355

MTILRKLSQYLFFFSLFCSFIYVATCGSQPDSVSSPKIAIFLSFPHPLLEDCSKSCIETLKDFENLPEIVVLNAEDSIVKARKIARSLHTDKNVV
AIVTLGTIATKVMSHIETQKPVIYAAVPDRESLTLPKNTMNIYGVNDTLDINQYCFAIQAVATNAQSIVYLKPSEPFPSDLQKEIVKKLHAS
GIEVIEISITSSTFKTRIRQAIDKRPSAIFIPLSPLSHKEGTAFLQEILKEKIPIITDDTSLISEGACIACSVDYKKSGKQIAKIVHHLLYNNHDVDS
LRKIIAQRLSPTTTFNEDIIKYLGIKLHKTERNQFLSFKSKKLEKSEKGKNVAVS

>core/445/1/Org1_Gene562

MSYSLRNKKTKICVYIIIALGILSFRSIPQEVYDKIRSSFVSLHVKFFPKIKQAPSSHLANLELENLVLKERVASLEEKLKLYEVSNHTPPLFPEIL
TPYFHKLVEGKVVYRDYTHWSSSCWVNVGKTHGIKKNSPVLSGNVLVGLVDYVGEHQSRIRLITDVGMKPSVVAMRGDIQSWWIKH
SLRELIRQVEQISHAYILEKDKYEKISQLQELDSLIQGEGENQALLRGILSGVGGALWKEGSLCLEGEGFYFSEGKTLLPGDILVTTGLDGVF
PPGLLVARVTKVKAPRDGACTFKIEAQSLEEKLMELDQLFILPPLEFNPNDRPDIFGLLWD

>core/448/1/Org1_Gene214

MWFSVNKNKKAAIWATGSYLPEKVLSNADLEKMVDTSDEWIVTRTGIKERRIAGPQEYTSLMGAIAAEKAIANAGLSKDQIDCIIFSTA
APDYIFPSSGALAQAHLGIEDVPTFDCQAACTGYLYGLSVAKAYVESGTYNHVLLIAADKLSSFVDYTDRNTCVLFGDGGAACVIGESRP
GSLEINRLSLGADGKLGELLSLPAGGSRCPASKETLQSGKHFIAMEGKEVFKHAVRRMETAAKHSIALAGIQEEDIDWFVPHQANERIID
ALAKRFEIDESRVFKSVHKYGNTAASSVGIALDELVHTESIKLDDYLLLVAFGGGLSWGAVVLKQV

>core/455/1/Org1_Gene625

MQPFIFTLLCLTSLVSLVAFDAANARKRCACAQTIERGENFFSIKRSACAEIEYQEKSRHASAIERISKDKGKVTPKQIAKVATKKKQRYRL
LQVPFSRPPNNSRYNLYALLSEPPECYSDTASWYAIFIRLLRRAYVDTGNVPPGSEYAIANALISNKQEILERGAQLGPDVIETLTLPEEQA
EIFYKMLKGSSNSQSLLNFLHYEEKSLGHCKLNLIFMDPLLLEAVLDHPDAYRETSLLRDGIWEAVKRQEHAIQEHGQAAALELFKTRTD
FRLELRDKMQLLLSRYDLLPLLNKKMFDYTLGSAGDYLFLVDPDTKAISRCRCPSKSIKL

>core/457/1/Org1_Gene803

MRIAVLGVTGLVGQKFVALLHKWYRDWVIAEVVASNSKYGQSYGDACIWQEPIGPMPEMVRDLPIRKIEEVQSDIVVSFLPSSAESM
EAYCLSQGKVVFSNASTYRMHSSVPIIIPEVNSDHFQLLEEQPYPGKIITSPNCCVSGITLALAPLRKFSLDHVHIVTLQSASGAGYPGVPS
LDLLANTVPHIVGEEEKILRETVKILGSSKQPLPCKLSVTVHRVPVAYGHTLSLHVTFSKDVDLDEILYSYQEKNKEFPNTYQLYDNPWSP
QARKHLSHDDMRVHLGPITYGGDFRTIKMNVLIHNLVRGAAGTLLASMENYFFDYLKREMCLR

>core/460/1/Org1_Gene567

MSSLTLSRRPRRNRKTAAIRDLLAETHLSPKDLIAPFFVKYGNNIKEEIPSLPGVFRWSLDLLLKEIERLCTYGLRAVMLFPIIPDDLKDAYG
SYSSNPKNILCHSIHEIKNAFPHLCLISDIALDPYTTHGHDGIFLNGEVLNDESVRIFGNIATLHAEMGADIVAPSDMMDGRIGYIRSKLD
QSGYSKTSIMSYSVKYASCLYSPFRDALSSHVTSGDKKQYQMNPKNVLEALLESSLDEEEGADILMVKPAGLYLDVIYRIRQNTCLPLAA
YQVSGEYAMILSAFQQGWLDKETLFHESLIAIKRAGADMIISYSAPFILELLHQGFEF

>core/463/1/Org1_Gene735

MQFSRYLRYAFDNQYLPEPLYQKFSVFHQNYIDAATKKAAADQAEVLCLQWVKVIIEDLKNPFIFPPYHKKIRAPIDLFRLSIDFFSLVIDD
KNSRILNLHRLKEIEEYIARGDNVVLLANHQTECDPQLMYYALGKTHPELMENMIFVAGDRVTSDPLARPFSMGCDLLCIYSKRHIATP
PELREEKLLHNQKSMQILKTLLNEGGKFIYVAPAGGRDRKNAEGRLYPSEFSPESIEVFRLLAKASNQTTHFYPFALKTYDILPPPPKIENAI
GEQRAIFFAPVFFNFGAELFFDALCSKEELIHCDKHAQRTLRAEKVFSIVKNLYEEL

>core/464/1/Org1_Gene800

MREETVSWSLEDIREIYHTPVFELIHKANAILRSNFLHSELQTCYLISIKTGGCVEDCAYCAQSSRYHTHVTPEPMMKIVDVVERAKRAVE
LGATRVCLGAAWRNAKDDRYFDRVLAMVKSITDLGAEVCCALGMLSEEQAKKLYDAGLYAYNHNLDSSPEFYETIITTRSYEDRLNTLD
VVNKSGISTCCGGIVGMGESEEDRIKLLHVLATRDHIPESVPVNLLWPIDGTPLQDQPPISFWEVLRTIATARVVFPRSMVRLAAGRAFL
TVEQQTLCFLAGANSIFYGDKLLTVENNDIDEDAEMIKLLGLIPRPSFGIERGNPCYANNS

>core/465/1/Org1_Gene343

MIDIMQHFKPYTMVPGQKLPIPGSLLYAQVFPTLWRLFSSKHEILNEQTLQVQGPLKRFAVFQDLHRGGLAVTSERYKYYLLPSGECTQ
SIKGKLPSAAQAGPLLSLGVHKHADWQKVRCRRDLKEILPLWFRFAAMAPKGSYRDLETTAIGSLVKTAHQRVLHRETTEIAPALLSIAL
AGFSECFLPRSYDEEFQGILPQDGDPEGGVPFELLSYSFGMIQDIFLRHQGQLVEILPALPPEFPCGRLIHVALPNLGTLSIVWTKKTIRQV
ELHAEYSGEVFLKFCSSLCSARLREWSERRLSGSKRLSLGETLEIKAGTTYLWDCFHK

>core/467/1/Org1_Gene376

MPSPMISTDVCQDILGKQKEAVDFFFQAFQPKEAMQLAEKILGHSGWVFFSGVGKSGCVARKLVATLQSLSERALFFSPVDLLHGDLG
LVSPGDIVCLFSKSGETQELLDTVPHLKSRRAILVAITSMPYSNLAALSDLVVILPSVAELDPFNLIPTNSTTCQMIFGDFLAMLLFHSRGV
SLSTYGKNHPSGQVGMKANGKVKDFMFPKTEVPFCHLGDKVSFSLEVFSAYGCGCVCIVDPQFRLMGIFTDGDLRRSLASYGGEVLSL
SLEKVMTANPRCITEDSDIAIALQLMESSSPVAVLPVLDNEENRHVTGLLHMHTLAKAGLL

>core/472/1/Org1_Gene250

MDAKMGYIFKVMRWIFCFVACGITFGCTNSGFQNANSRPCILSMNRMIHDCVERVVGNRLATAVLIKGSLDPHAYEMVKGDKDKIA
GSAVIFCNGLGLEHTLSLRKHLENNPNSVKLGERLIARGAFVPLEEDGICDPHIWMDLSIWKEAVIEITEVLIEKFPEWSAEFKANSEELV
CEMSILDSWAKQCLSTIPENLRYLVSGHNAFSYFTRRYLATPEEVASGAWRSRCISPEGLSPEAQISVRDIMAVVDYINEHDVSVVFPED
TLNQDALKKIVSSLKKSHLVRLAQKPLYSDNVDDNYFSTFKHNVCLITEELGGVALECQR

>core/476/1/Org1_Gene190

MTIDMHCDLLSHPHFCRKDPAVRCSPEQLLSGGVRQQVCAIFVPHSRGEPNCDKQNSLFFSLPNQYPDIGLLSYEEEENGSSSQKKSLS
LIRSIENASALGDDTAPLGTLLAKLIHLTKQGPLAYLGIVWKGDNRFGGGTEAPKRLSNDGKVLLDIMYELGVPIDLSHCSDKLAEDILDY
TADKLPNLAVIASHSNFRSVLDHRRNLVDAHAKEIVRRKGVIGLNLVRSYVGDSLGDLEKHVLHAENLGILSSIVLGSDFFYANEDENFFF
NECSSAEAHPVLNQLIHRIFSKGKAESILSSRAEKFLKQVIVEQVNPKITDVKL

>core/479/1/Org1_Gene302

MELLPHEKQVVEYEKAIAEFKEKNKKNSLLSSSEIQKLEKRLDKLKEKIYSDLTPWERVQICRHPSRPRTVNYIEGMCEEFVELCGDRTFR
DDPAVVGGFVKIQGQRFVLIGQEKGCDTASRLHRNFGMLCPEGFRKALRLGKLAEKFGLPVVFLVDTPGAYPGLTAEERGQGWAIAK

NLFELSRLATPVIIVVIGEGCSGGALGMAVGDSVAMLEHSYYSVISPEGCASILWKDPKKNSEAASMLKMHGENLKQFGIIDTVIKEPIG
GAHHDPALVYSNVREFIIQEWLRLKDLAIEELLEKRYEKFRSIGLYETTSESGPEA

>core/489/1/Org1_Gene462

MRLLSILKLHLFSLRSSSSLSPHYYHSCSRSMLHLLCRWKDADIMEWQQICNILSGVCSRMSGKLVSLQKETQDSCHQEHERIHLQYRE
QLSALEEEYRRREEAKNQDLEKLQQENTWLQNRLAEKLQQIRHQSDIIDEIKKELLQSVQRTEISEGRRLCYEHKIKQLEEQLQRYVSQH
GAPSIEIEEDKSSAAYAEINRLKKSLIDLQQEKDIYIKTYHSEIAKLREKLQRQEGAQTSSEVCSIEKLTEVQTDLAEKKKAIALLQDIVEDQY
CQLRDLHKEKGMAMPSNTKLDHLKGLLGKEPESEVDVVFSESKSLGS

>core/490/1/Org1_Gene738

MEVQIGIDLMGGDHSPLVVWQVLVDVLKSQSSTIPFAFTLFASEEIRKQIQEEFISDLPQEKFPKIISAENFVAMEDSPLAAIRKKSSSMA
LGLDYLQEDKLDAFISTGNTGALVTLARAKIPLFPAVSRPALLVCVPTMRGHAVILDVGANISVKPEEMVGFARMGLAYRQCLGDSKIP
TIGLLNIGSEERKGTEAHRQTFRMLRETFGEAFLGNIESGAVFDGAADIVVTDGFTGNIFLKTAEGVFEFLQRILGDKLEADIQRRLDYTF
YPGSVVCGLSKLVIKCHGKACGSSLFHGILGSINLAQARLCKRILSNLI

>core/491/1/Org1_Gene424

MLISISLATLPILAFSWASFIEPNWLRTTAIPWRLPKKHAHLHGLRIAQISDLHFHKRVPEKFLNKVSKSIKNFSPDLIVFCGDLLCRARLED
KERLETFLNTLEAPLGVFAILGNHDYSSYISRNTKGEITCIPEEKSRPIQRAIIAVMQGLFSSPSYRYDPNLTPQEPHPDLLKLLKNTPLTLLH
NTTHVIPNTLNIVGLGDLFARQFHPEQAFKNYDPSLPGLLLSHNPDGITRLQQYPGDFVLSGHSHGPQVTLSWPKFARKFFERLSGLEN
PYLARGYFVTKEGKQLYVNRGLGGLKRIRFCSPPEICYITCSYD

>core/492/1/Org1_Gene765

MVLSSDLLRDDKQLDLFFASLDVKKRYLLALSGGSDSLFLFYLLKERGVSFTAVHIDHGWRSTSAQEAKELEELCAREGVPFVLYTLTAEE
QGDKDLENQARKKRYAFLYESYRQLDAGGIFLAHHANDQAETVLKRLLESAHLTNLKAMAERSYVEDVLLLRPLLHIPKSSLKEALDARG
ISYLQDPSNEDERYLRARMRKKLFPWLEEVFGKNITFPLLTLGEESAELSEYLEKQAQPFFSAATHQDSQGELPCPDCLIQQAFLCKWV
MKKFFNNAGIAVSRHFLQMVYDHLSRSSCATLRMRNKIVIIKPGVVVID

>core/493/1/Org1_Gene502

MKFLLYVPLLLVLVSTGCDAKPVSFEPFSGKLSTQRFEPQHSAEEYFSQGQEFLKKGNFRKALLCFGIITHHFPRDILRNQAQYLIGVCYFT
QDHPDLADKAFASYLQLPDAEYSEELFQMKYAIAQRFAQGKRKRICRLEGFPKLMNADEDALRIYDEILTAFPSKDLGAQALYSKAALLI
VKNDLTEATKTLKKLTLQFPLHILSSEAFVRLSEIYLQQAKKEPHNLQYLHFAKLNEEAMKKQHPNHPLNEVVSANVGAMREHYARGLY
ATGRFYEKKKKAEAANIYYRTAITNYPDTLLVAKCQKRLDRISKHTS

>core/494/1/Org1_Gene357

MNRRKARWVVALFAMTALISVGCCPWSQAKSRCSIDKYIPVVNRLLEVCGLPEAENVEDLIESSSAWVLTPEERFSGELVSICQVKDEH
AFYNDLSLLHMTQAVPSYSATYDCAVVFGGPLPALRQRLDFLVREWQRGVRFKKIVFLCGERGRYQSIEEQEHFFDSRYNPFPTEENW
ESGNRVTPSSEEEIAKFVWMQMLLPRAWRDSTSGVRVTFLLAKPEENRVVANRKDTLLLFRSYQEAFPGRVLFVSSQPFIGLDACRVG
QFFKGESYDLAGPGFAQGVLKYHWAPRICLHTLAEWLKETNGCLNISEGCFG

>core/495/1/Org1_Gene848

MRKVAFLVSCLFSVAIGASAAPVRVPGFPQIPEDLVQIKTEVCPKQEVCLAVTIKCDDHNLIGVLHLPNTPTPEGGFPTVVLFHGFRGTK
FGGLTGAYRKLGRKFAAAGIATLRVDMAGCGDSEGVAEEVPIETYLRDAQTILETVQEHPDLNAYRLGISGFSLGCHIAFELAKIYNPRDL
NIKALSVWAPIADGGILLKELYENFSKHGEGDIISVGKDFGFGPPPIIVCSGDVDLLIRIQDHVTANSLPTKPYILHQQGIDDTLVSRTQQT
LFKNTAPGRMTFISYPNTGHNLATAPDLDMILDQIVSHFQRTL

>core/497/1/Org1_Gene142

MLVELEALKREFAHLKDQKPTSDQEITSLYQCLDHLEFVLLGLGQDKFLKATEDEDVLFESQKAIDAWNALLTKARDVLGLGDIGAIYQTI
EFLGAYLSKVNRRAFCIASEIHFLKTAIRDLNAYYLLDFRWPLCKIEEFVDWGNDCVEIAKRKLCTFEKETKELNESLLREEHAMEKCSIQD
LQRKLSDIIIELHDVSLFCFSKTPSQEEYQKDCLYQSRLRYLLLLYEYTLLCKTSTDFQEQARAKEEFIREKFSLLELEKGIKQTKELEFAIAKSK
LERGCLVMRKYEAAAKHSLDSMFEEETVKSPRKDTE

>core/498/1/Org1_Gene277

MTLPMQKSLTSFDDFSQAYAEKVPAIALIGSALEDDKDALIELLVSESFKELGGQGLMPATLMSWTETFALFQEHETLGIIHAEKFPLAT
KEFLSRYARNPQPHLTILIFTTKQECFRELSKALPSALSLSLFGEWPADRQKRIIRLLLQRAERVGISCSQSLASLFLRALASTSLPDILSEFDK
LLCSVGKKTSLDHSDIKELVVKKEKASLWKFRDSLLKRDPVEGHQQLHFLLEDGEDPLGIITFLRTQCLYGLRSIEEGSKENKHRMFVLYG
KERLHQALNSLFYAETLIKNNVQDPIVAVETLVIRMVNL

>core/500/1/Org1_Gene246

MAMLPKFFLVLLCLGLCSCSQKTTTIEGEQMTIFYRIVLGTSLSAKEKASLSQQIDRCFHKIDSIYNNWNPYSELSIINRAPADVPITLSVEL
SEFLDQVDTLYKLSEGRFDPTVGPLKTLWLLHLKSQTLPPKDVWEQHYKDMGWQHLEFQSNTKTLIKKNPHVQIDLCGVVKGYAVDC
LNEICNTFCPNNYVEWGGEIKTSGHHPSGRPWRIFSEAAGTILDIDDMAIATSGNHIQKWCVEGKIYTHILDTRTGKPLELSSYPIQSVS
VVHPSCAYADAIATVLMTFDSKIEAKQWAEEHHILTYINDGASS

>core/502/1/Org1_Gene9

MIASIYSFLDYLKMVKSASPHTLRNYCLDLNGLKIFLEERGNLAPSSPLQLATEKRKVSELPFSLFTKEHVRMYIAKLIENGKAKRTIKRCLS
SIKSFAHYCVIQKILLENPAETIHGPRLPKELPSPMTYAQVEVLMATPDISKYHGLRDRCLMELFYSSGLRISEIVAVNKQDFDLSTHLIRIR
GKGKKERIIPVTSNAIQWIQIYLNHPDRKRLEKDPQAIFLNRFGRRISTRSIDRSFQEYLRRSGLSGHITPHTIRHTIATHWLESGMDLKTI
QALLGHSSLETTTVYTQVSVKLKKQTHQEAHPHA

>core/504/1/Org1_Gene780

MRKLILCNPRGFCSGVVRAIQVVEVALEKWGAPIYVKHEIVHNRHVVNALRAKGAIFVEELVDVPEGERVIYSAHGIPPSVRAEAKARKL
IDIDATCGLVTKVHSAAKLYASKGYKIILIGHKKHVEVIGIVGEVPEHITVVEKVADVEALPFSSDTPLFYITQTTLSLDDVQEISSALLKRYPS
IITLPSSSICYATTNRQKALRSVLSRVNYVYVVGDVNSSNSNRLREVALRRGVPADLINNPEDIDTNIVNHSGDIAMTAGASTPEDVVQA
CIRKLSSLIPGLQVENDIFAVEDVVFQLPKELRCS

>core/509/1/Org1_Gene28

MRLFSYDKPKIKVQKIKADGFSGWLKCNHCHEMIHANELGQNYNCCPKCSYHYRITAIERVKLLADKDSWRPLYTDLKSQDPLEFIDTD
TYANRLEKARKNTTESEGVIVGICTIGLHPVALAVMDFNFMAGSMGAVVGEKLTRLIEEAIETRLPVIIVSASGGARMQESVFSLMQM
VKTSAALAKLHEAGLPYISVLTNPTSGGVTASFAALGDIIIAEPKALICFAGPRVVAQVIGEDLPEGAQKSEFLLEHGMIDKIVERKELKTTL
QTLLDYFLAQEYTGGKSKAPRDLSKRLKEIFLLTDDSE

>core/516/1/Org1_Gene786

MTMPSTQFHTTILEQFSLFLSVDRGLCQQSIAAYRQDISSFLTISAISSPQDISQNSVYIFAEELYRRKEAETTLARRLIALKVFFLFLKDQQL
LPYPPIIEHPKIWKRLPSVLTPQEVDALLAVPLQMEKNPRHLAFRDTAILHTLYSTGVRVSELCDLRLGHVSDDCIRVTGKGSKTRLVPLG
SRAREAIDAYLCPFRDQYQKKNPHEDHLFLSTRGHKLERSCVWRRIHNYAKQVTSKPVSPHSLRHAFATHLLDNKADLRVIQEMLGHA
RIASTEVYTHVAADSLIEKFLAHHPRNL

>core/519/1/Org1_Gene192

MHLEEENQGWEALLRKVYHQEVPPAILLHGFTLPVLQDKAEQLASEILLSSSPGSEHKVSQKIHPDIYQFFPEGKGRLHSIDLPRGIKKQI
YISPFEANYKIYIIHEADRMTLAAISAFLKVFEEPPKHAVIILTTAKVQRLPKTIISRSLSIFIERGEKILCSKETFSYLFRYAQCEIPVTEVSQIIKE
SSETDKQVLRDKVQRFMEVLLELYRDRYTLNLGLKASALNYPEHVKEILQLPLLPLDKVLLIVESACRSLNNSSSAASVLEWVAIQLVSLQY
KEKELVSVSPGQDLSN

>core/526/1/Org1_Gene685

MQEKPRHVHRIIHISDVHFHVLPVNPVHCFNKRLKGLLRKVFGLVHFQATTIGQRFPKVVRSLGADSVCITGDFSLTAMDGEFLLAKHF
VETLAKHSSVYLLPGNHDVYTLKSLAQQTFYTHFPNDQLQQNKVSFHKITDHWWLILLDCSCLNGWFSANGVVHLAQISAIETFLLSLS
PEENVIIANHYPLLSSQNPSHDLINNTHLQNVLKKYPKVRLYLHGHEHQAAVYNCADTSPSYILNSGSISLPTNSRFHVIDLYPEKYQVHT
MILKNLLDFDAPLEIANEATWDCQKL

>core/527/1/Org1_Gene297

MEKLLVTDIDGTITHQSHHLDKKVYERLYALHQAGWKLFFLTGRYYKYAARLFSDFDAPYLLGCQNGASVWSSTSSNLLYSKSLPSDLLC
ILQDCMEGATALFSVESGAPYGDHYYRFSPTPIAQDLHEYVDPRYFPNAKEREILFETRSLKDDYAFPSFAAAKVFGLRDEVIRIQKELER
QEALTSVATMTLMRWPFDFRYAILFLTDKSVSKGKALDRVVNILYDGKKPFVMASGDDANDLDLIERGDFKIVMSSAPEEMHVHADF
LAPPADKNGILSAWEAGVRYYDDLMSL

>core/530/1/Org1_Gene683

MSKHTSESRIAQDMLERYSGSSVKQFCPYLLLTNFSYYIQTFAKLHGVPVFEGSMFSAAHAPHLKTSILDFKLGSPGAALTIDLCSFLPDL
KAALMLGMCGGLRSHYQVGDYFVPVASIRGEGTSDAYFPPEVPALANFVVQKATTEVLEDKKANYHIGITHTTNIRFWEFNKKFRKKL
YETKAQSAEMECATLFAAGYRRNLPIGALLLISDLPLRKEGIKTKSSGNFIFNTYTEDHILTGQEVIENLEKVMLKRAASDHKKDQQYRGL
PHMEVGEADDTMASGSETSDSDY

>core/531/1/Org1_Gene556

MKVLPPPSIPLLGAHTSTAGGLKNAIYEGRDIGASTVQIFTANQRQWQRRALKEEVIEDFKAALKETDLSYIMSHAGYLINPGAPDPVIL
EKSRIGIYQEILDCITLGISFVNFHPGAALKSSKEDCMNKIVSSFSQSAPLFDSSPPLVVLLETTAGQGTLIGSNFEELGYLVQNLKNQIPIGV
CVDTCHIFAAGYDITSPQGWEDVLNEFDEYVGLSYLRAFHLNDSMFPLGANKDRHAPLGEGYIGKESFKFLMTDERTRKIPKYLETPGG
PENWQKEIGELLKFSKNRDS

>core/546/1/Org1_Gene516

MTEEISKDTIIEVAIDDIRVSPFQPRRVFSNEELQELIASIKAVGLIHPPVVREICTGDRVLYYELIAGERRWRAMQLAGATTIPVILKHVIA
DGTAAEATLIENIQRVNLNPIEMAEAFKRLIHVFGLTQDKVAYKVGKKRSTVANYLRLLALSKTIQESLLQGQITLGHAKVILTLEDPILREK
LNEIIIQEHLAVREAELIAKQLISEEGSSIELKPTPLDMAESSKQHEELQQRLSDLCGYKVQIKTRGSKATVSFHLQNTQDLQKLEAWLSSH
GTLSESLS

>core/549/1/Org1_Gene347

MHEVLILTFTYPLPRTLKQHPDEVHTVPISPNLSFGEGSPILIAGPCTLESYEHTVSSALTVKEAGAQVFRGSIRKPRTSPFSFQGWEKECV
LWHKEAQSIHGLPTETEVLDVRDVEITAEHVDILRIGAKNMHNTPLLQEVSKSHRPIILKRSPAATLEEWLCAAEYILASSPSCPGVILCER
GIRTFEHSTRYTLDLNTVALLKEISSLPVIVDPSHAAGKRSLVLPLASAGLSVGADGLMIEVHAHPEKALCDAKQQITPEELHLFAKKHFCP
SESRAHAIS

>core/558/1/Org1_Gene487

MLERTQRTLKREVRYSGVGIHLGKSSTLHLQPAQTNTGIVFQRQSASGNYENVPALLDHVYTTGRSTTLSRGSAVIATVEHLMAALRSN
NIDNLIIQCSGEEIPIGDGSSNVFVELIDQAGICEQEDKVSIARLTRPVYYQHQDIFLAAFPSDELKISYTLHYPQSSTIGTQYKSLVINEESFR
QEIAPCRTFALYNELCFLMEKGLIGGGCLDNAVVFKDDGIISRGQLRFADEPVRHKILDLIGDLSLVGRPFVAHVLAVGSGHSSNIAFGKK
ILEALEL

>core/564/1/Org1_Gene390

MHKVIVFIFLTLYSLKSYGNDVIDKPHVLVSIAPYKFLVEQIAEETCFVYAIVTNHYDPHTYELPPQQIKELRQGDLWFRIGEAFEKTCERN
LTCQQVDLSQNVSLIQGKPCCNQHTTNYDTHTWLSPKNLKVQVETIVTTLSKKYPQHATLYQSNGEKLLLALDQLNEEILTITSKAKQRH

ILVSHGAFGYFCRDYNFSQHTIEKSSHVEPSPKDVARVFRDIEQYKISSVILLEYSGRRSSAMLADRFHMHTVNLDPYAENVLVNLKTIAT
TFSSL

>core/565/1/Org1_Gene950

MYFTRDPIIETVITSREGYKLSVRNTKHFSQDPFMVEAIEVISLGNICFFRNCDHSKPFLVPAGDYEVMEVRDTKINLKAVGLDRGVKIAG
GREALIKLTKSTPLPVIDEKPLADSPEEGTEPTSPSKKEKKEARKDSFKGEKWKEKKKLSRRRNHKEIAEVTGASQEILDTVKEELWEESQE
NEIVEQKKFSLLPPPAKLISEVISQTVVDPVVTSADLNESLQALVRESSDLINALLSADDAIHFPETEEEPTSASFEESSAMFFPETSSATEEE

>core/569/1/Org1_Gene631

MTANTFGTLDILMKHSKEDDLSRFLPKNLLVESPHPEEIPLKSLSFTMSWLPTIHPSWITIAMKEFPPEIQGQLLAWLPEPLVQEILPLLP
GISIAPHRCAPFGAFYLLDMLSKKIRPCGITEEIFLPASSANAILYYTGPVKIALINCLGLYSIAKELKHILDKVVIERVKNALSPTEKLFLTYCQ
SHPMKHLETTNFLSSWTTDAELRQFVHKQGLEFLGKALTKENASFLWYFLRRLDVGRAYIVEQTLKTWYDHPYVDYFKSRLEQCMKVL
VK

>core/570/1/Org1_Gene380

MDYKLLDSGDGNKLECFGPVTLIRPSSIAVWPKSRPELWSQAQLQYVREGERGAWKNFKRLPEEWEVAFSDVRCLLKRTPFGHLGVF
PEHMGFWPALKQAIEKHKERQVLNLFAYTGAGSIFAAKCGARVTHVDASQAAVRWAQRNVEKNAFPERRIFWVIEDVISFLKKEIRR
NKKYQVILLDPPSYGRGPDGEVFKIDKDLFPLLSLCSKLLADDASYFLLTSHTPGHTPEFLRAIARRSVPTLVSEAWSCGESFCGEGVGALP
SGSFVQWIA

>core/571/1/Org1_Gene104

MITGVVLEKHEQRTMFSLTLLNNFTTFGLLHTPLHYNPPYPIVILLHGLASDKTGSKRSHVRLAQELTRLGIAALRVDLLGHGDCEGELM
DFSLENYKQNIREIIEYTHSLLHIDQERLAIFGSSLGGTLALQTLPFFNKIKALAVWAPTISGELMAAEAQKNAPEVITMSQKGAITYAGM
TLNPDFYTQFLKIDIVKELMPSARNLPPILYMQGEQDLLVSINHRTLFTEAFANQDKPITILTYPDVDHAFPFAESSALSDLTQWLKRELTS
GE

>core/572/1/Org1_Gene551

MLLGFLCDCPCASWQCAAVANCYDSVFMSRPEHKPNIPYITKATRRGLRMKTLAYLASLKDARQLAYDFLKDPGSLARLAKALIAPKEA
LQEGNLFFYGCSNIEDILEEMRRPHRILLLGFSYCQKPKACPEGRFNDACRYDPSHPTCASCSIGTMMRLNARRYTTVIIPTFIDIAKHLHT
LKKRYPGYQILFAVTACELSLKMFGDYASVMNLKGVGIRLTGRICNTFKAFKLAERGVKPGVTILEEDGFEVLARILTEYSSAPFPRDFCEI
H

>core/575/1/Org1_Gene283

MKLLKNVLLGLFFSMSISGFSEVKVSDTFVKQDTVVEPKIRVLLSNESTTALIEAKGPYRIYGDNVLLDTAIQGQRCVVHALYEGIRWGEF
YPGLQCLKIEPVDDTASLFFNGIQYQGSLYVHRKDNHCIMVSNEVTIEDYLKSVLSIKYLEELDKEALSACIILERTALYEKLLARNPQNFW
HVKAEEEGYAGFGVTKQFYGVEEAIDWTARLVVDSPQGLIIDAQGLLQSNVDRLAIEGFNARQILEKFYKDVDFVVIESWNEELDGEIR

>core/576/1/Org1_Gene197

MKKKLSLLVGLIFVLSSCHKEDAQNKIRIVASPTPHAELLESLQEEAKDLGIKLKILPVDDYRIPNRLLLDKQVDANYFQHQAFLDDECERY
DCKGELVVIAKVHLEPQAIYSKKHSSLERLKSQKKLTIAIPVDRTNAQRALHLLEECGLIVCKGPANLNMTAKDVCGKENRSINILEVSAPL
LVGSLPDVDAAVIPGNFAIAANLSPKKDSLCLEDLSVSKYTNLVVIRSEDVGSPKMIKLQKLFQSPSVQHFFDTKYHGNILTMTQDNG

>core/577/1/Org1_Gene344

MVRDIQSESIGKLVFLGTGNPEGIPVPFCSCRVCQNTGIHRLRSSVLIQYQNKTLVIDAGPDFRTQMLVAGVSELDGVFLTHPHYDHIG
GIDDLRAWYIVTQRSLPLVLSASTYRFLNKAKEYLFATPNVESSLPAVLEFTILNEDCGQEEFQGIPYTYVSYYQKSCHVTGFRFGNLAYLT
DLCSYDAKIFSYLDNVETLILSAGPSETPIPFQGHKSSHLTVEEAKAFANHAGIKNLIITHISHCLEAERDQHPEVTFAYDGMEVLWTL

>core/582/1/Org1_Gene906

MTQYYFLSSFLPTQLPESVPLFSISDLDDLLYLNLSENDLCNYGLLKRFFDFENFAFFWAGKPIPFSFGEVTQENIERMLSSQQWSDDND
FEDFFKDFLMNHKSSQDRLNHFSDLFREFLSYHQTNSSKFLQDYFRFQQQLRVVLAGFRARVLNMDVSYVLRDEDSSDPVVLEVLMQ
KDSPNYELPEEFSDLQGVLDDYGLLPHTLNRALALYQFHKLEGFCSDSYFDGNVILARCATYMFAIRNSLASVEKGREIINHIEKAIKW

>core/583/1/Org1_Gene124

MHSKFLSRRKKNSSHKEETSWDCIASSYNKIVQDKGHYYHRETILPQLLPSLTLGSKSSVLDIGCGQGFLERALPKECRYLGIDISSRLIALA
KKMRSVNSHQFKVADLSKRLEFVEPTLFSHAVAILSLQNMEFPGEAIRNTATLLEPLGQFFIVLNHPCFRIPRASSWHYDENKKAISRHID
RYLSPMKIPIMAHPGQKDSPSTLSFHFPLSYWFKELSSHGFLVSGLEEWTSSKTSTGKRAKAENLCRKEFPLFLMISCIKIK

>core/585/1/Org1_Gene674

MQGFFPLASGSKGNSAYLGTDSCKILIDLGVSKQVVTRELLSMNIDPEDIQAIFVTHEHSDHISGIKSFVKAYNTPIVCNLETARALCHLLD
SHPEFKIFSTGSSFCFQDLEVQTFNVPHDAVDPVAFIFHYREEKLGFCTDLGWVTSWITHELYDCDYLLIESNHSPELVRQSQRPDVYKK
RVLSKLGHISNQECGQLLQKIITPKLKKLYLAHLSTECNTAELALSTVSESIASITSIAPEIALAQGITSPIYFSRLEVACPR

>core/587/1/Org1_Gene19

MKKPDNDSTFDVRSFFPFDVLCIEQLRKEMSWEVVSAKIPRLPRGWYELMGLSKEDRIDFCLDFWCSVLGIEHKESPSICRFFSLLETIEV
YIYRLEKEPYQLKMFYVFRDGRCGFQGEPPLLDFLGHHRLPPLGDRHYEKFFSIHNGFGKWEDEGIFPMRSLAKVQQKLRQQLVVMN
KMQAEDNCYSLGIFPFYGYEEPFAYQSFFFDPEIRRDLPSPNVLLNEESLEHRSLETIELLHLSKSYYPSFLSWLENYLHSEEVYNE

>core/593/1/Org1_Gene371

MSNQLQPCISLGCVSYINSFPLSLQLIKRNDIRCVLAPPADLLNLLIEGKLDVALTSSLGAISHNLGYVPGFGIAANQRILSVNLYAAPTFFN
SPQPRIAATLESRSSIGLLKVLCRHLWRIPTPHILRFITTKVLRQTPENYDGLLLIGDAALQHPVLPGFVTYDLASGWYDLTKLPFVFALLLH
STSWKEHPLPNLAMEEALQQFESSPEEVLKEAHQHTGLPPSLLQEYYALCQYRLGEEHYESFEKFREYYGTLYQQARL

>core/600/1/Org1_Gene791

MKTWLFFTFLFSCSSFYASCRYAEVRSIHEVAGDILYDEENFWLILDLDDTLLQGGEALSHSIWKSKAIQGLQKQGTPEQEAWEAVVPF
WIEIQEMGTVQPIESAIFLLIEKIQKQGKTTFVYTERPKTAKDLTLKQLHMLNVSLEDTAPQPQAPLPKNLLYTSGILFSGDYHKGPGLDL
FLEICTPLPAKIIYIDNQKENVLRIGDLCQKYGIAYFGITYKAQELHPPIYFDNIAQVQYNYSKKLLSNEAAALLLRHQMHE

>core/606/1/Org1_Gene346

MIKQIGRFFRAFIFIMPLSLTSCESKIDRNRIWIVGTNATYPPFEYVDAQGEVVGFDIDLAKAISEKLGKQLEVREFAFDALILNLKKHRIDA
ILAGMSITPSRQKEIALLPYYGDEVQELMVVSKRSLETPVLPLTQYSSVAVQTGTFQEHYLLSQPGICVRSFDSTLEVIMEVRYGKSPVAV
LEPSVGRVVLKDFPNLVATRLELPPECWVLGCGLGVAKDRPEEIQTIQQAITDLKSEGVIQSLTKKWQLSEVAYE

>core/615/1/Org1_Gene84

MFKLLKNLFLIGCCIVGYFWMRKESIVEQWLSNRLHTQVTVGRVSIRTSGIKIRHICIHNPLASERFPYAAEIEYADVRFSSISMLLTKQLEI
SELIIHGANFTIFPYDSHGTKTNWSLVWKNFHPQKETPSNLWIDRAPVLIRRCLFLNTRLYGLRANHKDIPHLSVPSLEFHSHTSSAKELP
KLSEALPSLLYLALEESLYHLNLPGDIIKPLSQQAHKHFYSSYPQFQDRLNDINTPGTPTEEIIGFIRGLFFH

>core/617/1/Org1_Gene258

MKTQQTQNIIEVWNFYWETQEIEYRDSLIEFYLPLVKSVVHRLISGMPSHVKTEDLYASGVEGLVRAVERYNPERSRRFEGYAVFLIKAA
IIDDLRKQDWVPRSVHQKANKLSGAMDSLRQSLGKEPTDLELCEYLNISQQELSGWFVSARPALIVSLNEEWPSQSDEGAGMALEERI
PDERAETGYDVVDKQEFSLCLANAIQELEEKERKVMALYYYEELVLKEIGKVLGVSESRVSQIHSKALLKLRAALSAFR

>core/621/1/Org1_Gene613

MKTIAVNSFKGGTAKTSTTLHLGAALAQYHQARVLLIDFDAQANLTSGLGLDPDCYDSLAVVLQGEKEIQEVIRPIQDTQLDLIPADTW
LERIEVSGNLAADRYSHERLKYVLGSVQDKYDYVIIDTPPSLCWLTESALIAADYALICATPEFYSVKGLERLAGFIQGISARHPLTILGVALS
FWNCRGKNNSAFAELIHKTFPGKLLNTKIRRDITVSEAAIHGKPVFATSPSARASEDYFNLTKELLILLRDI

>core/623/1/Org1_Gene375

MHDALLSILAIQELDIKMIRLMRVKKEHQKELAKVQSLKSDIRRKVQEKELEMENLKTQIRDGENRIQEISEQINKLENQQAAVKKMDE
FNALTQEMTTANKERRSLEHQLSDLMDKQAGGEDLIVSLKESLASTENSSSVIEKEIFESIKKINEEGKALLEQRTELKHATNPELLSIYERL
LNNKKDRVVVPIENRVCSGCHIVLTPQHENLVRKKDRLIFCEHCSRILYWQESQVNAQENSTAKRRRRRAAV

>core/624/1/Org1_Gene165

MKPEESECLCIGVLPARWNSSRYPGKPLAKIHGKSLIQRTYENASQSSLLDKIVVATDDQHIIDHVTDFGGYAVMTSPTCSNGTERTGE
VARKYFPKAEIIVNIQGDEPCLNSEVVDALVQKLRSSPEAELVTPVALTTDREEILTEKKVKCVFDSEGRALYFSRSPIPFILKKATPVYLHIG
VYAFKREALFRYLQHSSTPLSDAEDLEQLRFLEHGGKIHVCIVDAKSPSVDYPEDIAKVEQYITCLSNAYF

>core/626/1/Org1_Gene434

MQICVTGVVLRSRPLGKNHTLTTLFTPEGLFTFFAKQGQTLQCDYRETLVPISLGKYTLHRNGSRLPKLTHGDILNAFEAIKQTYALLEAS
GKMIQALLASQWKEKPSHKLFSLFLNFLHRIPESSNPEFFAAIFVLKLLQYEGILDLTPACSLCKASLPYACYRYQGHKLCKKHQHKQAISI
EKEEEQILQAIIHAKQFSELLAIAEFPIAIAEKIFYLFDSLQEEKKSERNSSEDPYHEILRLSKVVHPY

>core/628/1/Org1_Gene139

MLMDISHILEDLAYDEGILPREAIEAAIVKQMQITPYLLHILHDATQRVPEIVNDGSYQGHLYAMYLLAQFRESRALPLIIKLFAFEDDTPH
AIAGDVLTEDLPRILASVCNDDSLIKELIETPKINPYVKAAAISGLVTLVGAGKIPRDKVIRYFAELLNYRLEKQPSFAWDNLIAGICTLYPGE
LFYPISKAFDGGLVDTSFISMEDVENIIHEETVESCIHTLCSSTELINDTLEEMEKWLEDFPIEP

>core/633/1/Org1_Gene612

MGNLKTLLESRFKKNTPTKMEALARKRMEGDPSPLAVRLSNPTLSSKEKEQLRHLLQHYNFREQIEEPDLTQLCTLSAEVKQIHHQSVLL
HGERITKVRDLLKSYREGAFSSWLLLTYGNRQTPYNFLVYYELFTLLPEPLKIEMEKMPRQAVYTLASRQGPQEKKEEIIRNYRGERKSELL
DRIRKEFPLVETDCRKTSPVKQALAMLTKGSQILTKCTSLSSDEQIILEKLIKKLEKVKSNLFPDTKV

>core/635/1/Org1_Gene490

MSLLKDTVFTCLDCEMTGLDVKKDRIIEIAAVRFTFDSVISSIEFLINPERVVSAESQRVHHISNAMLRDQPKIAEVFPQIKAFFKEGDYIV
GHSVGFDLQVLAQEMERIGETFLSKYTIIDTLRLAKEYGDSPNNSLESLAVHFNVPYDGNHRAMKDVEININIFKHLCKRFRTLEQLKQV
LAKPIKMKYMPLGKHKGRCFSEIPLAYLQWASKMDFDSDLLFSIRHEIKHRQKGTGFSQVNNPFMEL

>core/639/1/Org1_Gene527

MAKQTRRVLFKISGEALSKDSSNRIDEMRLSRLVSELRAVRNNDIEIALVIGGGNILRGLAEQKELQINRVSADQMGMLATLINGMAVA
DALKAEDIPCLLTSTLSCPQLADLYTPQKSIEALDQGKILICTTGAGSPYLTTDTGAALRACELNVDVLIKATMHVDGVYDKDPRLFPDAV
KYDFVSYKDFLSNQLGVMDASAISLCMDSHIPIRVFSFLQHSLEKALFDPTIGTLVSEDVNHVCSPRH

>core/641/1/Org1_Gene448

MHIYGLADLHLALGVPEKTMEVFGDPWIGYHQKICSEWQAVVHPEDIVLLPGDISWAMNLSEAHKDFAFIGDLPGTKYMIRGNHDY
WSSASTSKILQALPPSLYYLNQGFALLTPHLAVVGVRLWDSPTICVKKENFLTPSTQEQSYTEQDEKIFLRELGRLKRAFAALPKEVTEVIV
MTHYPPISSDGTPGPISEFLEADGRVSLCLFGHIHKVQRPIDGFGNIRGIHYILVAADYVNFVPQEVM

>core/642/1/Org1_Gene580

MKITTVKTPKIYPYDDLYSILESSLPKLNERSIVVITSKIVSLCEGAVVELEKVSKDELIKQEADAYVFVEKYGIYLTKKWGILIPSAGIDESNVE
GYFVLYPRDFLLSVNTLGDWLRNFYHLEHCGIIISDSHTTPLRRGTMGLGLCWNGFFPLYNYVGKPDCFGRALKMTYSNLLDGLSAAAV
LCMGEGDEQTPIAIIEEAPKITFHSSPTTLQDMSTLAIAEDEDLYGPLLQSMAWETPAPTS

>core/651/1/Org1_Gene322

MKVRIVDSGKSSAASHMAKDRDLLESLQDGELILHLYEWENPCSLTYGHFMRPEKFLLSNYADLGLDAAVRPTGGGFVFHKGDYAFSV
LMSATHPSYSSSVLENYHTVNSFVAKVLEKVFRIQGMLAPEDENSSSRDSGNFCMAKTSKYDVLFGDKKIGGAAQRKVQQGFLHQGS
LFLSGSSSEFYQRFLKPEVLEEIIEQIQIHAFFPLGLEAADEVLQEARQQVKEAFIKLFCGEGL

>core/652/1/Org1_Gene835

MTLYLLPNTLGTRAVETLPSVIGELVHRLDGLIVESDRGGRAFLSLWKIPEVHKFPLAILSKHARLPKAWDFYLEPIVKHGENWGLISDAG
LPCIADPGASLVRRARALGIPVQAFSGPCSITLALMLSGLPSQSFTFLGYLPQSPKERVKSIKKAATSKEVSTSVCIETPYRNVYTFESLLDTL
PSYAELCVASDLSGPSELVLTRQVQSWRTTEDLGSVKQSITKVPTIFLFHIPN

>core/654/1/Org1_Gene492

MDISGAVKQKLLQFLGKQKKPELLATYLFYLEQALSLRPVVFVRDKIIFKTPEDAVRILEQDKKIWRETEIQISSEKPQVNENTKRIYICPFT
GKVFADNVYANPQDAIYDWLSSCPQNMEKQGGVRIKRFLVSEDPDVIKEYAVPPKEPIIKTVFASAITGKLFHSLPPLLEDFISSYLRPMT
LEEVQNQTKFQLESSFLSLLQDALVEDKIAAFIESLADDTAFHVYISQWVDTEE

>core/661/1/Org1_Gene571

MIGDKIILFVTEDLSLSSQLKDLASQRSDYQILVSPVFPTSFESVAIFCEYLLLPEQIFSPGIFPEEDLIVLFDTFQEEAITKVLNQGATGYLLRP
ITAKVLDAVIRAFLRQHEVLEHSIPDTMTFGDHTFRVLNLVIESPEGSVYLTPSEAGILKKLLINRGHLCLRKNLLAEIKGNTKEIIARNVDV
HIASLRKKLGPYGSKIVTIRGVGYLFSDDDSIPLQNHDNTAHPNEE

>core/662/1/Org1_Gene873

MTKVRLNKFLASAGVASRRKCDEIIFSGSVTVNGRVAEGPFVLVDPEDKVQVGGTSVHLTKKVYFMVHKAIGYLCSSEKKFPGTKLVIDL
FAHLPYRVFTVGRLDKETSGLILVTNDGEFANKIIHPSSGITKEYLLKVSRDVSAKDLGKLMEGTFIDGKHVRPVSVTKIRRGTVKIVVSEG
KKHEIRLFADAAGLPILELKRIRIGSLVLGGLRYGEYRELTDAELGTYMKLSD

>core/665/1/Org1_Gene458

MPTTNCIFLDLRGHSILHQLQIEEALLRVANQNFCIINSGAKDSIVLGISRNLNQDVHISRAQADHIPIIRRYSGGGTVFIDSNTLMVSWI
MNSSEASAQPQELLAWTYGIYSPLLPNTFSIRENDYVLGHKKIGGNAQYIQRHRWVHHTTFLWDIDLDKLSYYLPIPQQQPTYRNQRS
HEEFLTTLRPWFPSRDDFLERIKASGSLLFTWEEFLDNELEEILAQPHRKATTVLN

>core/667/1/Org1_Gene630

MKFFSLIFKDDDVSPNKKVLSPEAFSAFLDAKELLEKTKADSEAYVAETEQKCAQIRQEAKDQGFKEGSESWSKQIAFLEEETKNLRIRVR
EALVPLAIASVRKIIGKELELHPETIVSIISQALKELTQNKHIIISVNPKDLPLVEKSRPELKNIVEYADSLILTAKPDVTPGGCIIETEAGIINAQ
LDVQLDALEKAFSTILKAKNPVDEPSETSSSTDSSSLSNDQDKKE

>core/668/1/Org1_Gene690

MNRRDMVITAVVVNAILLVALFVTSKRIGVKDYDEGFRNFASSKVTQAVVSEEKVIEKPVVAEVPSRPIAKETLAAQFIESKPVIVTTPPV
PVVSETPEVPTVAVPPQPVRETVKEEQAPYATVVVKKGDFLERIARANHTTVAKLMQINDLTTTQLKIGQVIKVPTSQDVSNEKTPQTQ
TANPENYYIVQEGDSPWTIALRNHIRLDDLLKMNDLDEYKARRLKPGDQLRIR

>core/671/1/Org1_Gene43

MTKHGKRIRGILKNYDFSKSYSLREAIDILKQCPPVRFDQTVDVSIKLGIDPKKSDQQIRGAVFLPNGTGKTLRILVFASGNKVKEAVEAG
ADFMGSDDLVEKIKSGWLEFDVAVATPDMMREVGKLGKVLGPRNLMPTPKTGTVTTDVAKAISELRKGKIEFKADRAGVCNVGVGK
LSFESSQIKENIEALSSALIKAKPPAAKGQYLVSFTISSTMGPGISIDTRELMAS

>core/673/1/Org1_Gene164

MLQSCKKALLSIVVSILAFHPIPGMGVEAKSGFLGKVKGWFSKKEIQEEARILPVKDSLSWKRYDYTSSSGFSVEFPGEPDHSGQIVEVP
QSEITIRYDTYVTETHPDNTVYVVSVWEYPEKVDISRPELNLQEGFSGMMQALPESQVLFMQARQIQGHKALEFWIVCEDVYFRGMLI
SVNHTLYQVFMVYKNKNPQALDKEYEAFSQSFKITKIREPRTIPSSVKKKVSL

>core/676/1/Org1_Gene427

MYLEDYDVFFFDLDGLLVDTEPCFYRAFLQACAEFSLEVHWDFSTYYSHTTLGTEIFSKKFIEQYPQAQEYMAEIFAKRLQIYYKSLEHAG
PALMPGVEAFIELVLSLNKTFGVVTNSPRDATHTLRTMYPILNKFLFWVTRENYARPKPYGDSYDYAYRTFAREGMKVIGFEDSVKGLR
ALSKIPATLVCINSMAEITPEDYPELKGKEFFSYPSFDVLTEHCSQQKLL

>core/680/1/Org1_Gene432

MRPHRKHVSSKSLALKQSASTHVEITTKAFRLSMPLKQLILEKSDHLPPMETIRVVLTSHKDKLGTEVHVVASHGKEILQTKVHNANPYT
AVINAFKKIRTMANKHSNKRKDRTKHDLGLAAKEERIAIQEEQEDRLSNEWLPVEGLDAWDSLKTLGYVPASAKKKISKKKMSIRMLSQ
DEAIRQLESAAENFLIFLNEQEHKIQCIYKKHDGNYVLIEPSLKPGFCI

>core/685/1/Org1_Gene854

MDLKLDEVASLLDVSEHTVLQWLKEGAIPSYSMNNEYRFSREEIEDWLLHNQALMIQERGEDKEALKDLSLKYSLYKAIHRGGVLCDVV
VHSKEEALQYASKYIAQKFQLDESVLFEMLSHRENLMSTGIGEGIALPHAKDFLINAYYDIVVPMFLAEPIEYGALDGKPVGILFFLFACQ
DKSHLNLVNKIVHLGMSLNARSFFKNYPNKDQLLAYVKEWESQTH

>core/686/1/Org1_Gene948

MILRISTVSLLTSCSFSKNSRTCFVTPERITSQKDCPVLLHPKSTTISPPLYDWIPPNREVITAYSFYCRGQGNSIITPEGVLYDCDGLHHSIT
KEEFRYIHPRLIEVVRLLQQDHPKVSIIEAFCCPKHFHFLEASGISLSQLHLQGTAATFALDPPLPMEKLLATIKKLYKKNSDPSLSNFIVTEA
TLTNPELRLTQQDLGSHTEITVEILDNLQNKETLSSA

>core/688/1/Org1_Gene755

MKPQDLSPPFLWKERRPCIQDGVLYVPRHYFEHQNFSTSYHQEFFQNHTSIACELCSGNGDWVVAQAQKDPQVLWIAVEQRFDRVR
KIWSKMINHQIQNLRIVCGTAETFFQYYVPDQFLQRLVVNFPDPWPKMRHRKHRLLQPSFVQEISRSLQDSAVFALATDDKTYLLESIE
ALQTHLAPRMETPYYIKMTDTYGNSWFENLWRTKGQEIFYTEFIKKAGI

>core/690/1/Org1_Gene581

MTSWIELLDKQIEDQHMLKHEFYQRWSEGKLEKQQLQAYAKDYYLHIKAFPCYLSALHARCDDLQIRRQILENLMDEEAGNPNHIDL
WRQFALSLGVSEEELANHEFSQAAQDMVATFRRLCDMPQLAVGLGALYTYEIQIPQVCVEKIRGLKEYFGVSARGYAYFTVHQEADIK
HASEEKEMLQTLVGRENPDAVLQGSQEVLDTLWNFLSSFINSTEPCSCK

>core/692/1/Org1_Gene513

MQTLARLFGQSPFAPLQAHLEMVVSCVEYMLPIFTALRDGRYEELLEMAKLVSDKEYQADCIKNDMRNHLPAGLFMPISRAGILEIISI
QDSIADTAEDVAILLTIRRLNFYPSMETLFFRFLEKNLEAFELTMTLLHEFNQLLESSFGGRKADKARLLVGRVAKSEHESDVLQRELMQI
FFSDDFIIPEKEFYLWLQVIRRTAGISDSSEKLAHRINMTLEEK

>core/693/1/Org1_Gene796

MSTTTVKHFIHTASRWEPVLKEIVASNYWHAQWINTLSFLENSGAKKISASEHPTEVKEEVLKHAAEEFRHGHYLKTQISRISETSLPDYT
SKNLLGGLLTKYYLHLLDLRTCRVLENEYSLSGQTLKTAAYILVTYAIELRASELYPLYHDILKEAQSKITVKSIILEEQGHLQEMERELKDLPH
GEELLGYACQFEGELCLQFVERLEQMIFDPSSTFTKF

>core/696/1/Org1_Gene823

MGQKGCPIGFRTGVTKKWRSLWYGNKQEFGKFLIEDVRIRQFLRKKPSCQGAAGFVVRRMSGKIEVTIQTARPGLVIGKKGAEVDLLK
EELRALTGKEVWLEIAEIKRPELNAKLVADNIARQIERRVSFRRAMKKAMQSVMDAGAVGVKIQVSGRLAGAEIARSEWYKNGRVPL
HTLRADIDYATACAETTYGIIGIKVWINLGENSSSTTPNNPAAPSAAA

>core/702/1/Org1_Gene798

MQRIIIVGIDTGVGKTIVSAILARALNAEYWKPIQAGNLENSDSNIVHELSGAYCHPEAYRLHKPLSPHKAAQIDNVSIEESHICAPKTTSN
LIIETSGGFLSPCTSKRLQGDVFSSWSCSWILVSQAYLGSINHTCLTVEAMRSRNLNILGMVVNGYPEDEEHWLTQEIKLPIIGTLAKEKEI
TKTIISCYAEQWKEVWTSNHQGIQGVSGTPSLNLH

>core/703/1/Org1_Gene301

MNAVNTPKKILCIVADYREISPLIEQLDFTQINEHLYSYRCTDYHLDLYIVHVWGSTAVLNALQSYCQAYTDYDLWINPGFVGACSPEIPL
GQCYTIEKIANLTTDTPPVLSEDPPYIFDALPDSLPKSSLVTSPVLYHYGFHKTFKLLDMEGYAIASQAAEHHIPCSFLKITSDYTVPGDCPF
SRLEEVSQKLTQTLVELLPELMERAIPPKLLLPCP

>core/708/1/Org1_Gene414

MIITIDGPSGTGKSTTAKALADHLHFNYCNTGKMYRTLAYARLQSPWATLPLTKFLEEPPFSFTFATGQPLESFFNGHLLTSELTTQEVA
NAASELSQLPEVRAFMQDLQRRYAQLGNCVFEGRDMGSKVFPNADLKIFLTSSPEVRAQRRLKDLPEGTLSPEQLQAELVKRDAADA
QRAHDPLVIPENGIVIDSSDLTIRQVLEKILALLFRNEL

>core/709/1/Org1_Gene291

MRKMLVLLASLGLLSPTLSSCTHLGSSGSYHPKLYTSGSKTKGVIAMLPVFHRPGKSLEPLPWNLQGEFTEEISKRFYASEKVFLIKHNAS
PQTVSQFYAPIANRLPETIIEQFLPAEFIVATELLEQKTGKEAGVDSVTASVRVRVFDIRHHKIALIYQEIIECSQPLTTLVNDYHRYGWNS
KHFDSTPMGLMHSRLFREVVARVEGYVCANYS

>core/710/1/Org1_Gene702

MSKKPLYVAHPWHSPTLTQDNYESLCCYIEITPYDSVKFELDKATGLLKVDRPQKFSNFCPCLYGLLPQTYCGTASGNYSGEQTRREGIQ
GDKDPLDVCVLTEKNIHHGNILLQARPIGGLRIIDSGEADDKIIAVLEDDLVFAEIEDISDCPGTVLDMIQHYFLTYKATPNHLIKGSPAKIE
IVGIYGKKEAQKVIQLAHEDYLSYIGDTAEVN

>core/720/1/Org1_Gene557

MARYCGPKNRVARRFGANIFGRSRNPLLKKPHPPGQHGMQRKKKSDYGLQLEEKQKLKACYGMIMEKQLVKAFKEVIHKQGNVAQ
MFLERFECRLDNMVYRMGFAKTIFAAQQLVAHGHILVNGRRVDRRSFFLRPGMQISLKEKSKRLQSVKDALESKDESSLPSYISLDKTG
FKGELLVSPEQDQIEAQLPLPINISVVCEFLSHRT

>core/722/1/Org1_Gene52

MSVQVKLTKNSFRLEKQKLARLQTYLPTLKLKKALLQAEVQNAVKDAAECDKDYVQAYERIYAFAELFSIPLCTDCVEKSFEIQSIDNDFE
NIAGVEVPIVREVTLFPASYSLLGTPIWLDTMLSASKELVVKKVMAEVSKERLKILEEELRAVSIRVNLFEKKLIPETTKILKKIAVFLSDRSIT
DVGQVKMAKKKIELRKARGDECV

>core/723/1/Org1_Gene49

MANLNADGKLKQICDALRLDTLKPAEDEAAALLHNAKEQAKRIIQEAQEEARKILETAEERAHQKIKQGEVALSQAGKRALEALKQAVE
NKIFRESLVEWLEHVTTDPEVSTKLIQALVQALEAQGVSGNLTAYIGKHVSPRAVNELLGKAVTTKLRKKSVVVGSFVGGVQLKVEEKN
WVLDLSSSALLEIFTRYLQKDFREMIFQGS

>core/724/1/Org1_Gene570

MTYLASSIFSPEDFLYPEIISKAHYTWDILDLMDQMLENHVFSGIHGTVESGVTLKNIEKIEIAEDAYVESGAYIVGPCILGSQTEVRHGAY
LRGNVITGSRCVVGHCTEIKNSYLGHHTKAAHFAYLGDSVLSSEVNLGAGVRCANFRLDGRNIYVRSTSDKSKKIDTGRRKLGAFLGKG
VAIGCNVVINPGQHILPHTRIRPGQVI

>core/727/1/Org1_Gene460

MYDYIRGTLTYVHTGAIVIECQGIGYHIAITERWAIECIRALHQDFLVFTHVIFRETEHLLYGFHSREERECFRILISFSGIGPKLALAILNALP
LKVLCSVVRSEDIRALASVSGIGKKTAEKLMVELKQKLPDLLPLDSRVETSQTHTTSSCLEEGIQALAALGYSKIAAERMIAEAIKDLPEGSS
LTDILPIALKKNFSGVNKD

>core/730/1/Org1_Gene193

MFIVIEGGEGSGKSSLAKALGDQLVAQDRKVLLTREPGGCLIGERLRDLILEPPHLELSRCCELFLFLGSRAQHIQEVIIPALRDGYIVICERF
HDSTIVYQGIAEGLGADFVADLCSKVVGPTPFLPNFVLLLDIPADIGLQRKHRQKVFDKFEKKPLSYHNRIREGFLSLASADPSRYLVLDA
RESLASLIDKVMLHTQLGLCT

>core/736/1/Org1_Gene453

MLKLLKVSITGDLSSGKTEACQVFQELGAYVVSADEISHSFLIPHTRIGRRVIDLLGSDVVVDGAFDAQAIAAKVFYNSVLLQGLEAILHPE
VCRIIEEQYHQSIQDGNYPLFVAEVPLLYEIHYAKWFDSVILVMANEDIRRERFMKKTGRSSEDFDQRCSRFLNVEEKLAQADVVVENN
GTKKELHQKIEEYFYALKGAL

>core/740/1/Org1_Gene215

MTRYPDYLSKLIFFLRKLPGIGFKTAEKLAFELISWDSEQLKILGNAFHNVASERSHCPLCFTLKESKEADCHFCREERDNQSLCIVASPKD
VFFLERSKVFKGRYHVLGSLLSPITGKHIENERLSILKSRIETLCPKEIILAIDATLEGDATALFLKQELQHFSVNISRLALGLPIGLSFDYVDSG
TLARAFSGRHSY

>core/742/1/Org1_Gene503

MRLFSLGTIYLFFSLALSSCCGYSILNSPYHLSSLGKSLLQERIFIAPIKEDPHGQLCSALTYELSKRSFAISGRSSCAGYTLKVELLNGIDKNIG
FTYAPNKLGDKTHRHFIVSNEGRLSLSAKVQLINNDTQEVLIDQCVARESVDFDFEPDLGTANAHEFALGQFEMHSEAIKSARRILSIRLA
ETIAQQVYYDLF

>core/743/1/Org1_Gene915

MNWVPKTIDHVDPESEIDIRKVVSCYKLIKECQPEFRSLISELLGVIRCGLRLLKRSKYKEQARTVSDEDAPLFCLTRSYYQDGYLTPLRAG
PRDLINHYIHLRRRENPKHFFSPKHPCYYARLAFNESVCVYRELFDIERLTKMYVEGDYSKEQEKNLQAILSFVKTLDEGKDFLIEHKDTDL
IGRGFTDVFCT

>core/744/1/Org1_Gene726

MRIALSLLSLLMIFPIFGEESRPGSEDGNSNTQEIVGSQDTQVCLYHSYEQGLQASRIEGKPLVIVVLCNSGDDGQACTIGLSETCEEVLS
VLSGSIFSELANFVVLVPSGVNPLIYPPIEDPILAEIVKFKELFKDESFPTGLSIIVVGVTPEGPGDIIEVSPVSLTVEEEETLPSEQTTEVESTSE
LQSEDPAIA

>core/749/1/Org1_Gene312

MFRRTGKGPFEDVQTLYEEETSSPSSYSPYSRSERPETPPSLFDNPKASEARPLNHNLTEESSLPQWSSTPRTESLLPLEEPETTLGEGVTF
KGELAFERLLRIDGTFEGILVSKGKIIIGPKGVVKADIQLQEAIIEGVVEGNITVSGKVELRGGAIIKGDIQANTLCVDEGVRILGYLAIAGIT
DHSERERDL

>core/750/1/Org1_Gene790

MAYGTRYPTLAFHTGGIGESDDGMPPQPFETFCYDSALLQAKIENFNIVPYTSVLPKELFGNIVPVDTCVKSFKHGAVLEVIMAGRGAA
LSDGTHAIATGIGICWGKDKNGELIGGWAAEYVEFFPTWINDEIAETHAKMWLKKSLQHELDLRSIAKHSEFQFFHNYINIKQKFGFCL
TALGFLNFENAEPAKVN

>core/754/1/Org1_Gene185

MKRYVVGISGASGVILAVKLIKELVNAKHQVEVIISPSGRKTLYYELGCQSFDALFSEENLEYIHTHSIQAIESSLASGSCPVEATIIIPCSMTT
VAAISIGLADNLLRRVADVALKERRPLILVPRETPLHTIHLENLLKLSKSGATIFPPMPMWYFKPQSVEDLENALVGKILAYLNIPSDLTKQ
WSNPE

>core/755/1/Org1_Gene663

MKVIYYEIEEIPSTNTMAKSYMHLWDPYALTVISTKCQTAGTGKFGKSWKSSKGDLLNTFCFFITDLHIDVSRLFRLGTEAVVALCKDLGI
TEAKIKWPNDVLVHGEKLCGVLPETLPVEGLLGVVLGIGLNGNTTKQALKDVGQPATSLQEILGHPIDLETTRELLIHHLLGVLQENLPDS
LATKSNRGNI

>core/758/1/Org1_Gene35

MIKKFFIYSLIFSCSFSAPLKGICNEDVSSQSRIEEDPEVLITQLNELIETPIEEGKEIRNELQAISDGQKSSEEIEESCGTSDSEGLSEKTDKESS
NEYVLDFFDSMVQRLEGISKMCQSGQVAQIIDCFNREFDIRNRELELKNRELELREKDLEFKKSILDWNKEKVSRELAFQREQDIKQTLM
LLKK

>core/759/1/Org1_Gene285

MSIKEDKWIREMALNADMIHPFVNGQVNVNEETGEKLISYGLSSYGYDLRLSREFKVFTNVYNSVVDPKCFTEDIFISITDDVCIVPPNS
FALARSVEYFRIPRNVLTMCIGKSTYARCGIIVNVTPFEPEWEGHVTIEISNTTPLPAKIYANEGIAQVLFFESSTTCEVSYADRKGKYQKQ
QGITVPCV

>core/760/1/Org1_Gene684

MVRVSTSEFRVGLRIEIDGQPYLILQNDFVKPGKGQAFNRIKVKNFLTGRVIERTYKSGESVETADIVERSMRLLYTDQEGATFMDDETF
EQEVVFWEKLENIRQWLLEDTIYTLVLYNGDVVAVEPPIFMELSIAETAPGVRGDTASGRVLKPAVTNTGAKIMVPIFIDEGELVKVDTR
TGSYESRVSK

>core/762/1/Org1_Gene987

MMKIPYARLEKGSLLVASPDINQGVFARSVILLCEHSLNGSFGLILNKTLGFEISDDIFTFEKVSNHNIRFCMGGPLQANQMMLLHSCSE
IPEQTLEICPSVYLGGDLPFLQEIASSESGPEINLCFGYSGWQAGQLEKEFLSNDWFLAPGNKDYVFYSEPEDLWALVLKDLGGKYASLS
TVPDNLLLN

>core/766/1/Org1_Gene447

MRILAGKYKGKSLKTFSNPHIRPTSGLVKEAFFSICREDIEGAAFLDLFAGMGAIGFEALSRGAASVVFVDISIKAIQLIHTNSALLGEQLPV
VIFRQDAQSAIQRLIKQKRSFDLIYIDPPYELCNCYVETLLQKIVSGNILNPEGTLFLENASDEEIACEGLTLRRRRKLGKTYLAEYIVEKDP

>core/768/1/Org1_Gene865

MELVVTSRETGKKSFLKKIRQQGGIPAVVYSAGKSLANITVDALVFKKFLSNLESGALSSTVFSLSYEGRIIKALVKDIQYQITTYDVIHLDFE
ELVEDRPVKLNIPIRCINAVDCIGVKLGGSLRQVIRAVRVVCKPKDIVPFLELDVRSVGLSQTRKLSDIKIPAGIETITPLKEVAITVSRR

>core/770/1/Org1_Gene121

MVLSSQLSVGMFISTKDGLYKVTSVSKVAGPKGESFIKVALQAADSDVVIERNFKATQEVKEAQFETRTLEYLYLEDESYLFLDLGNYEKL
FIPQEIMKDNFLFLKAGVTVSAMVYDNVVFSVELPHFLELMVSKTDFPGDSLSLSGGVKKALLETGIEVMVPPFVEIGDVIKIDTRTCEYI
QRV

>core/771/1/Org1_Gene360

MTDTPPENEEQHESNVQNENEVEHLQQEIVTLKTELKEKNDKYLMALAESENSRKRLQKERQELMQYALENTLIDFLNPIESMEKALG
FATQMSDDVKNWALGFNMILNQFKQIFEEKGIIEYSSIGQKFNPFLHEAVQTEETSEVPEGTILEEFAKGYKIGERPIRVAKVKVAKAPTP
KENKE

>core/772/1/Org1_Gene611

MAAKTKTLELEDNVFLLLEGNLKRIFATPIGYTTFREFQNVVFNCANGQQEIANFFFEMLINGKLTQELAPQQKQAAHSLIAEFMMPIR
VAKDIHERGEFINFITSDMLTQQERCIFLNRLARVDGQEFLLMTDVQNTCHLIRHLLARLLEAQKNPVGEKNLQEIQEEITSLKNHFDELT
KALQ

>core/773/1/Org1_Gene420

MVETVLHNFQRYLSKYLYRVFRFPCRKKTFLSSHRVLARPSFPVDYCPGKIYDLQEIYEELNAQLFQGALRLQIGWFGRKATRKGKSVVL
GLFHENEQLIRIHRSLDRQEIPRFFMEYLVYHEMVHSVVPREYSLSGRSIFHGKKFKEYEQRFPLYDRAVAWEKANAYLLRGYKKRVGG
GYGRA

>core/776/1/Org1_Gene472

MSRKAREPILLPQGVEVSIQDDKIIVKGPKGSLTQKSVKEVEITLKDNSIFVHAAPHVVDRPSCMQGLYWALISNMVQGVHLGFEKRLE
MIGVGFRASVQGAFLDLSIGVSHPTKIPIPSTLQVSVEKNTLISVKGLDKQLVGEFAASIRAKRPPEPYKGKGIRYENEYVRRKAGKAAKT
GKK

>core/777/1/Org1_Gene41

MYKWYVVQVFTAQEKKVKKALEDFKESSGMTDFIQEIILPIENVMEVKKGEHKVVEKYIWPGYLLVKMHLTDESWLYVKSTAGIVEFL
GGGVPVALSEDEVRSILTDIEEKKSGVVQKHQFEVGSRVKINDGVFVNFIGMVSEVFHDKGRLSVMVSIFGRETRVDDLEFWQVEEVA
PGQESE

>core/782/1/Org1_Gene474

MSRLKKFYTEEIRKSLFEKFGYANKMQIPVLKKIVLSMGLAEAAKDKNLFQAHLEELTMISGQKPLVTKARNSIAGFKLREGQGIGAKVT
LRGIRMYDFMDRFCNIVSPRIRDFRGFSNKGDGRGCYSVGLDDQQIFPEINLDRVKRTQGLNITWVTTAQTDDECTTLLELMGLRFKK
AQ

>core/786/1/Org1_Gene658

MTTPQSPGSLSQSHLPHPHDPWDTEPTSLPEDPNDKASQELHSLVHLFRKLSIHLLSEVEKTVQQLKPDLLELALLICEKFLYKKLENPQE
LALLLSTALQRHTTLRSLTPIKVFLHPEDLKTLTDWISTHELPMIKHAEFFPDTSCRRSGFKIETPNGILRQEISEELDHLLSVLTA

>core/787/1/Org1_Gene407

MACEQHEGCYELEEREEIEDIKDSDTKWVSITQAAKLHNVTRQAIYVAIKQKKLKASKETRWEIDIKDLEEYKRNRYSRKKSLYQGELVFD
NGKGCYSINQVAQILGIPVQKVYYATRTGTIRGERKGAAWVIHVSEIERYKNEYLSKQAAKKLKGAEPKEHQAPNFEPPTEIFPESN

>core/789/1/Org1_Gene794

MTIILCGLPTSGKSSLGKALAKFLNLPFYDLDDLIVSNYSSALYSSSAEIYKAYGDQKFSECEARILETLPPEDALISLGGGTLMYEASYRAIQ
TRGALVFLSVELPLIYERLEKRGLPERLKEAMKTKPLSEILTERIDRMKEIADYIFPVDHVDHSSKSSLEQASQDLITLLKS

>core/791/1/Org1_Gene396

MDRDNEVPLPKPKWIYRTGIGQDSHRFLPESSTKPCILGGIIFDHCPGFQANSDGDIIFHAICNAISSVTNKIILGKVADELLQTRGITDSGI
YLEEALKSLKPNQKISHVAITIEGSRPKFLCKLSALRQNIAQVMNLTPTDIGITATSGEGLSDFGCGDGVQCFCVLTVMEYCD

>core/793/1/Org1_Gene662

MGYVFYVIAGSIFLGISLGAYCQLYYSVKSVLFSWYLLTVYALEKRHALLALSQLVGEEDAQSQKEIDFLSQCDKLSWRAFLKNSYEIIPTFK
EMEDLLSERVQGFLESIETIAEHDRAILCIENFWASKNLFDFEIAAYEEAVEKYLKLRQRAPLRLASKLFRFLDVPSIRFSS

>core/795/1/Org1_Gene991

MSSTLNGVFPSSLPEESADLFITNKEIVALGEKGNVFLTHSIPMHIAAITILVIVAPAGIAIICLGCYSQSILLIAVGIVLTILTLLCLQALVGFIK
FIRQLPQQLHTTVQFIREKIRPESSLQLVTNAQRKTTQDTLKLYEELCDLSQKEFKLQSTLYQKRFELSHKNEKTNQN

>core/800/1/Org1_Gene783

MSHLNYLLEKIAASSKEDFPFPDDLESYLEGYVPDKNIALDTYQKIFKISSEDLEKVYKEGYHAYLDKDYAKSITVFRWLVFFNPFVSKFWF
SLGASLHMSEQYSQALHAYGVTAVLRDKDPYPHYYAYICYTLTNEHEEAEKALEMAWVRAQHKPLYNELKEEILDIRKHK

>core/805/1/Org1_Gene216

MKKLLFSTFLLVLGSTSAAHANLGYVNLKRCLEESDLGKKETEELEAMKQQFVKNAEKIEEELTSIYNKLQDEDYMESLSDSASEELRKKF
EDLSGEYNAYQSQYYQSINQSNVKRIQKLIQEVKIAAESVRSKEKLEAILNEEAVLAIAPGTDKTTEIIAILNESFKKQN

>core/806/1/Org1_Gene80

MSEVKPLFLKNDSFDLATQRFQNLINMLQEQAEIYNEYEEKNARVQNEIKEQKDFVKRCIEDFEARGLGVLKEELASLTRDFHDKAKAE
TSMLIECPCIGFYYSIHQEEQRQRQERLQKMAERYRDCKQVLEAVQVEQKDMISSRVVVDDSYFEEEKEEQKVDNRKKEQD

>core/810/1/Org1_Gene732

MKQQLLLLEDVDGLGRSGDLITARPGYVRNYLIPKKKAVIAGAGTLRLQAKLKEQRLIQAAADKADSERIAQALKDIVLEFQVRVDPDN
NMYGSVTIADIIAEAAKKNIFLVRKNFPHAHYAIKNLGKKNIPLKLKEEVTATLLVEVTSDNEYVTVLAQGKQTEENQEG

>core/815/1/Org1_Gene534

MAKYPLEPVLAIKKDRVDRAEKVVKEKRRLLEIEQEKLREKEAERDKVKNHYMQKIQQLRDLLDEGTTSDAVLQIKSYIKVVAVQLSEEE
EKVNKQKEVVLAASKELEKAEVNLAKRRKEEEKTRLHKEEWMKEALKEEARAEEKEQDEMGQLLFQLRQKKKRESGGS

>core/816/1/Org1_Gene251

MAVEQSHIKEEIEKLIGKAIKRVCGNKENDLCRYLPGPSGGYMHHFTLKKMKSAAPEQLLKMLKTFILESETPRTINPKPRAPRGSKKRR
DFINFTKTDIERVLELARQVGDKDLLARFSPKKPLTSLKRELIRSIRNGIVSVELWNAYVEAVKAVSSPNLEVTSPFV

>core/820/1/Org1_Gene281

MSRQNAEENLKNFAKELKLPDVAFDQNNTCILFVDGEFSLHLTYEEHSDRLYVVYAPLLDGLPDNTQRKLALYEKLLEGSMLGGQMAGG
GVGVATKEQLILMHCVLDMKYAETNLLKAFAQLFIETVVKWRTVCADICAGREPSVDTMPQMPQGGGGMQPPPTGIRA

>core/829/1/Org1_Gene299

MTTWTLNQNNLTKFLKSSDEEPFLERESGLTYINIQANGNELPLFFVIRSEGEILQLICYLPYQLHESHKASTARLLHLLNRDIDIPGFGMD
EEQGLIFYRLVLPCLNGEIHDTLLRIYIDTIKLVCDSFSHAIGLISSGNMNLDELRRQALQEQQEKRNE

>core/831/1/Org1_Gene758

MATLSPEKFSGSPISISKEFPQQKMREIILQMLYALDMAPSAEDSLVPLLMSQTAVSQKHVLVALNQTKSILEKSQELDLIIGNALKNKSF
DSLDLVEKNVLRLTLFEHFYSPPINKAILIAEAIRLVKKFSYSEACPFIQAILNDIFTDSSLNENSLSI

>core/839/1/Org1_Gene366

MTQEKIKIHVSNEQTCIPIHLVSVEKLVLTLLEHLKVTTNEIFIYFLEDKALAELHDKVFADPSLTDTITLPIDAPGDPAYPHVLGEAFISPQA
ALRFLENTSPNQEDIYEEISRYLVHSILHMLGYDDTSSEEKRKMRVKENQILCMLRKKHALLTA

>core/842/1/Org1_Gene241

MRIIRFDPYGALSAQSIAKDSRQNSPLVEKISEEIATNEAIRLALLAIGDREQEEKKQRHRYKLLGQKQAKVLLSQLRHVHLDFKKLYCDSK
KKEDQEKDEKNKQKRSIKVTKKKKGISLGAAASQAIAAAAEAWVIARNKGVLETASTLFYQKDEEA

>core/843/1/Org1_Gene721

MEKQNLKLDVKEIEFPETVFSRDIETRVIQVIILHCLAKINGVSLLGGNLIDALFGRDIERMKGIYVEQDSKNHLVKVRVEVNVDYGVSIPE
KTEEIQGCIVSEISEYTGLHVAAVHVIIKGLTQPKDRIDEEIEEEVSVQDLPSPEDFLLENSEG

>core/890/1/Org1_Gene599

MKYRFTEEIEEEPLVNLTPLIDIVFVILMAFIVAVPLIKLDSIALAPGTQEQEVLSSENDSIAVIKVFADHSLTLNEHPITLQELTVRLTLLHKA
YPEKTPLLLQDGETSFRTYQNVKNAIEAAGFHELHVALQN

>core/892/1/Org1_Gene577

MIAIERYQLIISKFRMWLFLGCSVEERHFKQPVLISVTFSYNEVPSACLSDKLSDACCYLEVTSLIEEIANTKPYALIEHLANELFDSLVISFG
DKASKIDLEVEKERPPVPNLLNPIKFTISKELCPSPVLSA

>core/894/1/Org1_Gene473

MGMTSDSIADLLTRIRNALMAEHLYVDVEHSKMREAIVKILKHKGFVAHYLVKEENRKRAMRVFLQYSDDRKPVIHQLKRVSKPSRRV
YVSAAKIPYVFGNMGISVLSTSQGVMEGSLARSKNIGGELLCLVW

>core/900/1/Org1_Gene292

MRDHAFSKLIGTVRAMVVEGRCPWSLQQSLVSMVEHILGECQEFHEAVLQGKTVQEVGSEAGDVLTLVLILCFLLEREGVLASEDVAN
EAMEKLRRRAPYIFAEDYKPVSIEEADRLWELAKHREKNEST

>core/903/1/Org1_Gene45

MTTESLETLVEKLSNLTVLELSQLKKLLEEKWDVTASAPVVAVAAGGGGEAPVAAEPTEFAVTLEDVPADKKIGVLKVVREVTGLALKEA
KEMTEGLPKTVKEKTSKSDAEDTVKKLQDAGAKASFKGL

>core/904/1/Org1_Gene594

MKFTVALFGEAEKGSYDTAYFCRSLVDLHNYLGDVSSPGITLAIKTLLSDYNVVYFRVREEGYCVDSYFFGLHFLNTQTTLKNIIAIGLPGV
GNQHIIEASRSLCQKHNSLLLFFDHDLYDLLTFNQPF

>core/906/1/Org1_Gene154

MLIGRYSSDDQFTEATKNTPTIIKLGFVRDNLEGLTNPISEIVSETSSSIKDSVLRSLPILGSILGCARLYSTLSTNDPLDETQEKIWHTIFGAL
ETLGLGILILLFKIIFVILHCIFHLVIGFCK

>core/908/1/Org1_Gene383

MPLSDDEIEQFKKRLLEMKAKLSHTLEGNAQEVKKPNEATGYSQHQADQGTDTFDRTISLEVTTKEYELLRQINRALEKINESSYGICDV
SGEEIPLARLIAIPYATMTVKAQEQFEKGLLSGN

>core/912/1/Org1_Gene623

MKRQKRKQSITLIEMMVVITLIGIIGGALAFNMRGSIHKGKVFQSEQNCAKVYDILMMEYATGGSSLKEIIAHKETVVEEASWCKEGRK
LLKDAWGEDLIVQLNDKGDDLVIFSKRVQSSNKK

>core/913/1/Org1_Gene656

MRDRLGSLSLILKVKIHKYLDTLHNQKRLALTVSRNIQATNKRIADLHLERYEHFISRDNIKHYDILLEYLKTLQSSLYKQQSESLRFLEIHHQ
QLQELINRRKIIEKIKNNKYSKDQEIGT

>core/917/1/Org1_Gene226

MEIIHIGTDIIEISRIREAIATHGNRLLNRIFTEAEQKYCLEKTDPIPSFAGRFAGKEAVAKALGTGIGSVVAWKDIEVFKVSHGPEVLLPSH
VYAKIGISKVILSISHCKEYATATAIALA

>core/918/1/Org1_Gene74

MVNLLKELEQEQCRNDLPEFHVGDTIRLATKISEGGKERVQVFQGTVMARRGGGSGETVSLHRVAYGEGMEKSFLLNSPRIVSIEIVKR
GKVARARLYYLRGKTGKAAKVKEFVGPRSSKK

>core/922/1/Org1_Gene231

MTENRRIKRVNALLQEAIAKVILKDVKHPKISNLWITVTRVSLSKDLHSARVYVSVMPHENTKEEALEALKVSAGFIAHRASKNVVLKYF
PELHFYLDDIFSPQDYIENLLWQIQEKEKS

>core/930/1/Org1_Gene368

MSDIQKEEHGSTTIFHLHGKLDGISSPEVQENISQSLAAGSKNIILDCAHLDYMSSAGIRVLLQSYHQVGQHSGKIVLTTVPKTIEQTLYVTGFLSYFKIFNTVDEAIQTLNKDGD

>core/933/1/Org1_Gene286

MSKVSVRKKNWGFRLLEEVMIKSWWVIFSILIGGFVYDRAIQELRTEELRLQSKVSSLCQDILSAQEKQRQLQLHLQHWQDSAAIEAALIQRLGLIPKGYKKLCVSPKQQSENKD

>core/936/1/Org1_Gene730

MGKKENQLYEGAYVFSVTLSEEARRKALDKVISGITNYGGEIHKIHDQGRKKLAYTIRGAREGYYYFIYFSVSPGAITELWKEYHLNEDLLRFMTLRADSVKEVLEFASLPE

>core/938/1/Org1_Gene479

MFKATARYIRVQPRKARLAAGLMRNLSVQEAEEQLGFSQLKAGRCLKKVLNSAVANAELHENIKRENLSVTEVRVDAGPVYKRSKSKSRGGRSPILKRTSHLTVIVGEKER

>core/946/1/Org1_Gene695

MNLSAKEYGDIIVIYLQGSLDAVSVPSVQEYLEQFIQKKHLKIALNFTDVSYISSAGIRLLLSNFKLVQSLGGKMCLCCVKESVTEVMRIAGLDQLILLCQSEQECLSKL

>core/949/1/Org1_Gene20

MNEPTRTYLESEKDTQDQIEELQATCIVKNAAGIHVRPAGVIVRLFDGEPCDVHFTYAGKTINAKSIMSILMLGAPQGGEILVTIRSKEAHRILQKIQDAFSSGFGEL

>core/954/1/Org1_Gene395

MEPYAVIQTGSKQYQVRSGDVIDVELLGEVASDKEVIFQDVLFVFDGTKASLGSPTIANAQVKAEYLSHVKGEKVVAYKYKKRKNYHRK
HGHRQKYLRVKIREILI

>core/955/1/Org1_Gene545

MSFTYFLALPVDRLMQERFLCSPKRWAPFINSPLYLTLIADHDTPYLAKNLDKFPLPVEQWEKTVLHVSSLLKSIFLCSDLSSLRLLACTKF
EILTLNDLYCAQNI

>core/956/1/Org1_Gene428

MNERTLLLLLKKKKGLFLAILDLTQTESSLTTPELEKVLKQKKIFLSCIDRVDLQIKEFRHAFSSELPQDIQEELEEIRDVIIRILDTDKRNYAQK
KKEFGIYERP

>core/959/1/Org1_Gene436

MSFKRFLQQIPVRICLLIIYLYQWLISPLLGSCCRFFPSCSHYAEQALKSHGFLMGCWLSIKRIGKCGPWHPGGIDMVPKTALQEVLEPY
QEIDGGDSSHFSE

>core/965/1/Org1_Gene2

MEQFHLDREEILLLAKASALQLSEELIQEYQTSLSAVITSMKEALAIEIDDADSCESLFMHVVNVEDLREDSVTSDFNREEFLRNVPESLG
GLVKVPAVIK

>core/968/1/Org1_Gene303

MATMTKKKLISTISQDHKIHPNHVRTVIQNFLDKMTDALVKGDRLEFRDFGVLQVVERKPKVGRNPKNAAVPIHIPARRAVKFTPGKR
MKRLIETPNKHS

>core/976/1/Org1_Gene77

MIKKDRFTNEKLNKLFDSPFSLVNYAIKQAKIKIAKGDVRSSNVAIETLVLLDREGIQPEFTEEIVVTASPTVERKRSEHTNSRKKDPSAYT
WSDVK

>core/978/1/Org1_Gene21

MGSGYAKKKKEAKIMEQQFLEMEASLLEKRYEGQAGNGLVSVVINGKCDLISVKVQPTCLDPEDPEVIEDLFRAAFKLAKEQMDQEM
SLMRSTMPF

>core/979/1/Org1_Gene307

MNKSRFLRLCCCLCFCGSLFYFYINKQNSLTKLRLEIPCLSVRLRQLEQQNISLRFLIDKIERPDHLMEIAALPEYQYLEYPSEESISLLSYELP

>core/982/1/Org1_Gene451

MLIRLFLGISLPKGFPLYLEPPLVLATFQGTQFVGTYSEATNPLYIDNLNLNYHYTQELLYKAVPCNYKSIYREIPLIIFPEVLIGSTPTQSTE

>core/988/1/Org1_Gene260

MAKLVITSDDEQQEFELEDNSEIAEPCESMGIPFACTEGVCGTCVIEVLEGRENLSEFTEPEYDFLGEPEDSNERLACQCRIKGGCVKVTF

>core/992/1/Org1_Gene239

MSRKCPLTGKRPRRGYSYTLRGIAKKKKGIGLKVTGKTKRRFFPNMLTKRLWSTEENRFLKLKISASALRHIDKLGLEKVLERAKSKNF

>core/994/1/Org1_Gene116

MKEYLDFLVQRNVERDPQTKRHCTVSQKFGGESIDAKTTTGQLFHIAGKTEPGHGKLCLGESILKQLLALGIITGYENREREVWVYLD

>core/997/1/Org1_Gene724

MKSFKFLLPFLSVILCCGNLLSSPRSRAISVTESIGMSAVKTLVLSEKAHEFLEGIGYGVGASSILRDWQTQQWLEIESLLAQNEVM

>core/998/1/Org1_Gene477

MASEPRGSRKVKIGVVVSAKMEKTVVVRVERIFSHPQYLKVVRSSKKYYAHTELKVSEGDKVKIQETRPLSKLKRWRVIEHVGVVS

>core/999/1/Org1_Gene812

MVEIHHKDPSLKKLFALQQSLETLNSLSDIVATYEAMFSLIYEGLNKALRKDQLCYLLSVNSKGELLKSPSGDPIVQTFPIHPHH

>core/1000/1/Org1_Gene538

MATNKSCTAFDFNKMLDGVCTYVKGVQQYLTELETSTQGTVDLGTMFNLQFRMQILSQYMESVSNILTAVNTEMITMARAVKGS

>core/1003/1/Org1_Gene40

MKQQHNRKALSRKIGTVKKQAKFAGSFLDEIKKIEWVSKHDLKKYIKVVLISIFGFGFAIYFVDLVLRKSITCLDGITTFLFG

>core/1004/1/Org1_Gene539

MFNMENTAKEEKNSQPLLDLEQDMQDHDRAQELKASVQDKVHKLHALLREGSDKESFGQQQSLLAGYVALQKVLGRINRKMI

>core/1009/1/Org1_Gene211

MSLEDDVIAIIVEQLGVDPKEVNENSSFIEDLNADSLDLTELIMTLEEKFAFEISEEDAEKLRTVGDVFTYIKKRQAEQ

>core/1012/1/Org1_Gene547

MKEFLAYIIKNLVDRPEEVRIKEVQGTHTIIYELSVAKPDIGKIIGKEGRTIKAIRTLLVSVASRNNVRVSLEIMEEK

>core/1014/1/Org1_Gene590

MNEGIHSVCFQKTPRLTAKSVVSMEMLLTTQQLPSAEGMPSVANLEADFLRAEALLAEMREIRGCLEQSLRTLVPSE

>core/1015/1/Org1_Gene769

MKNKIVTLLDQLYEDQESRLQKLGEEIVPNLTPEDLLQPMDFPQLEGNPAFRFEEGVLSGIGEVRAAILAALSQEN

>core/1018/1/Org1_Gene552

MFFAPLLYESLRRGLMHPTSHMQQQLARLEFINDQLTTELEHVNELLCSLGFPEGLTTIKAIAEEVLSDDEPLLD

>core/1019/1/Org1_Gene858

MEEVPFENAMQRLEEIVDLMNQPTTSLDASLALYEEADALMRICESRIRQVEQRVRELAEKRHESSLFEEQAVVR

>core/1021/1/Org1_Gene38

MAKKEDTLVLEGKVEELLPGMHFRVILENGMPVTAHLCGKMRMSNIRLLVGDRVTVEMSAYDLTKARVVYRHR

>core/1023/1/Org1_Gene985

MSYKITLPKADETTAKKVTKISEASTLIFSVLKEKASLGNVHGFCQAENSLSVEANKIISVAENTLAGCFCK

>core/1025/1/Org1_Gene951

MAAKKDLLTQLRGKGDDDLDAYVHENKKALFALRAENLLQNKVVKVHMFSTHKKNIARALTVKQERKGKVHG

>core/1028/1/Org1_Gene498

MKKLIALIGIFLVPIKGNTNKEHDAHATVLKAARAKYNLFFVQDVFPVHEVIEPISPDCLVHYEGWV

>core/1034/1/Org1_Gene348

MIKQACKFYLLQCLLCALYWLLKYCRKLLKGTLHHSEETLYQALLSSLIDLLYQLKQLPAPTNE

>core/1046/1/Org1_Gene953

MSIALNREEVWDNPRHLMFILMQFQQFSGEQDRFGSFLEATIRDRVSFLVLQEKIATLK

>core/1056/1/Org1_Gene932

MIDAENQNPKDGGSFTSLHTDPKNLFDEEGMPSPSDTLQCDLNNVFIFIKSMFF

>core/1058/1/Org1_Gene365

MSRHRSYGKSVKGVTKRNVLKRFERVEVLRKLGRWNDSTAKKVTGLPKTPILK

>core/1059/1/Org1_Gene177

MASKNREIIKLKSSESSDMYWTVKNKRKTTGRLELKKYDRKLRRHVIFKEAR

>core/1067/1/Org1_Gene787

MFFIAVRSRGFLDIHGILAARKGKQVVKSTAGAWIGSRGAVFYSLVS

>core/1068/1/Org1_Gene984

MSESLEIPELTEVLSEQPSLSTPDSPPKVITGTLTLYFQEDIDPAS

>core/1070/1/Org1_Gene717

MKRTYQPSKRKRRNSVGFRTRMATRNGRKLLNRRRRHGRHSLVDL

>core/1073/1/Org1_Gene1014

MSSVKKKRRLKIAKHKRKKRRRRDRHKNK

>core/158/2/Org2_Gene129

MELLSLNKSYFEIQRLRYRPEILTLLETIRSKHIQETSSPPSPPPELQKHIPNLCRIPEVSIYTEQETSSKPLKIGVLLSGGQAPGGHNVVIGLF
DALRVFNPKTRLFGFIKGPLGLTRGLYKDLDISVIYDYYNMGGFDMLSSSREKIKTEEQKKNILNTVKQLKLDGLLIIGGNNSNTDTAMLA
EYFLAHNCKTSVIGVPKTIDGDLKNCWIETSLGFHTSCRTYSEMIGNLAKDALSAKKYHHFIRLMGQQASYTTLECGLQTLPNIALISELIA
TRKISLKQLSEQLALGLVRRYKSGKNYSTVLIPEGLIEHIFDTRKLIDELNVLLANGDSSIEKILSKLSPETLKTFHLFPKDIANQLLLARDSHG
NVRVSKIATEELLAVMVKKEIEKIKPHMEFHSVSHFFGYEARAGFPSNFDCNYGIALGIISALFLVRQKTGYMITINNLAQSYTEWQGGA
TPLYKMMHLENRCGTETPVIKTDSVDPKSPAVQHLLQQSDSCLVEDLYRFPGPLQYFGKEELIDQRPLTLLWENQTHSPFQALYSTSGK
RSL

>core/176/2/Org2_Gene914

MPGSVSSPPLSPVIVRERVPSSSGSDLIQPHAVLKISILIFALVTILGIVLVVLSSALGALPSLVLTVSGCIAIAVGLIGLGILVTRLILSTIRKVDA
MGYDAAVKEEQYLSRIRELESENREIRDRNRAVEDQCAHLSEENKDLRDPEYLHGMTERLIASLEIENQALVAENILLKDWNASLSRDFR

AYKQKFPLGALEPWKEDIACIMEQNLFLKPECIAMVKSLPLETQRLFLYPKGFQSLVNRFAPRSRFFQTPKYEYNSRNENEDGKVAAVC
ARLKKEFFSAVLGACSYEELGGICERAVALKETLPLPEAVYDTLVQEFPNLLTAESLWKEWCFYSYPYLRPYLSVDYCKRLFVQLFEELCLK
LFTTGSPEDQALVRLFSYYRNHIPAVLASFGLPPPETGGSVFVLLPKQENLLWSQIEVLATRYLKDTFVRNSEWTGSFEMMFSYNEMCK
EISEGRIRFAEDYETRHSEEFPPSPLSEEGEGEEFLPPCSEEEVSVLERPDLDVDSMWVWHPPVPKGPL

>core/20/4/Org4_Gene980

MYSCYSKGISHNYLLHPMSRLDIFVFDSLIANQDQNLLEEIFCSEDTVLFKAYRTTALQSPLAAKNLNIARKVANYILADNGEIDTVKLVD
AIHHLSQCTYPLGPHRHNEAQDREHLLKMLKALKENPKLKESIKTLFVPSYSTIQNLIRHTLALNPQTILSTIHVRQAALTALFTYLRQDVG
SCFATAPAILIHQEYPERFLKDLNDLISSGKLSRIVNQREIAVPINLSGCIGELFKPLRILDLYPDPVVKLSSSPGLKKAFSAANLIETLGDSEA
QIQQLLSHQYLMQKLQNVHETLTANDIIKSTLLHYYQLQESTVRAIFFKEGLFSKEQVAFSTQHPRELSEIQRVYHYLHAYEEAKSAFIHD
TQNPLLKAWEYTLATLADASQPTISNHIRLALGWKSEDPHSLVSLVTHFVEEEVENIRILVQQCEQTYHEARSQLEYIEGRMRNPLNNQ
DSQILTMDHMRFRQELNKALYEWDSAQEKAKKFLHLPEFLLSFYTKQIPLYFRSSYDAFIQEFAHLYADAPAGFRILFTHGRTHPNTWSP
IYSINEFIRFLSEFFTSTESELLGKHAVINLEKETSRLVHNITAMLHTDVFQEALLTRILEAYQLPVPPSILNHLDQLSQTPWVYVSGGTVDT
LLLDYFESSEPLTLTEKHPENPHELAAFYADALKDLPTGIKSYLEEGSHSLLSSSPTHVFSIIAGSPLFREAWDNDWYSYTWLRDVWVKQH
QDFLQDTILPQPSIYAFIENFCNKYALQHVVHDFHDFCSDHSLTLPELYDKGSRFLSSLFTKDKTVALIYIRRLLYLMVREVPYVSEQQLPE
VLDNVSSYLGISSRITYEKFRSLIEETIPKMTLLSSADLRHIYKGLLMQSYQKIYTEEDMYLRLTTAMRHHNLAYPAPLLFADSNWPSIYFG
FILNPGTTEIDLWKFNYAGLQGQPLDNIQELFATSRPWTLYANPIDYGMPPPPGYRSRLPKEFF

>core/67/4/Org4_Gene970

MLLLISGALFLTLGISGLSAAISFGLGIGFSALGGVLVVSGLLCLLVKQEVSEERPEEIPAVQPEEIPEGVPVTPFEKPALDEAQKEQKAQKIL
EQLPKELDQLDRYIQEVVSCFRKLKDLKCEDQGLLKDAKEKLQVFDFVWKDMMTEFVELQQIIVQEGWYKELHIQNLDDLGPNLFNDS
RLSEHCVSLCYLEELCGYLPSGDARASRLKRSLREVVARFMKVVHDTRKVAITFERNNYGVAKKTFEETFRALEDCVCRSLSKSYRDAFYE
YEKAKILRSGHIEWLRDDRNSVLAEQRFQDARDRWEYLKETVFWVKEDGSIDIEDVANPSAWNDRFPVRMFFQDRQDKILGDESWE
WWGGCRKAYHTSHAVLQAAFEKDKSKDNLQKVKKHDERMFTFFQKNCDREYQKALERLRELQALYPEVSVSLVEAGREENLGSNLE
RAYENLEEKYQRCVRDQEDYWRKVEKEEAEFRANGTKIRSMEEVLGYIQILEDLIESWSLKINKFEQSVLGFNFEVTQELDNKILFHATN
RLNVLGEDIKDMLSRVEEIEMMLRVIELPLLPIKQALEKAFVQYNSCKAKLTKVEPCFRESPAYITSEERLQSLDQTLERAYKEYQKRFQEP
SRLESEVSGCREHLREQVKQFETQGLDLIKEELIFVSDVLFRKMVSRLVSTVHVPFMEFYYEYFELHRLRLRAQWMANAEIYSKVRKAFP
EMLKETLEKAKAPREEEYWLLCEERKSKEKRLILNKIEAAQQRVKDLEPPPIKETGKQKRK

>core/69/4/Org4_Gene683

MLLLISGALFLTLGIPGLSGAISFSLGIGLSALGGVLMISGLLCLLVKREVSEERPEEIPTVQPEGVPLAPSEEPALQAAQKTLAQLPKELDQL
DTDIQEVFACLRKLKDSKYESRSFLQDAKEKLRIFDFVVEDALSEIFELRQIVDQEGWDLNFLINGGRSLMVTAEPESLDLFHVWKRLGYL

PSGDVRGEELKKSVKEIVARLMRLYCEIHKVAVAFDRNSYAMAEKAFAKALGALEESVYRSLTQSYRDKFLESERAKILWNGHITWLRD
DAKSGCAEKKLRDAEERWKKFRKAVFWVEEDGRIEITYVIGDRGEVLEPYRQERMNEITFHELYDKITSVKDTHRKYALAKTTFEKKRSR
RNLQAVEEANACRLKYVRDWYDQEFQKAGERLEKLHALYPEVSVSIRENRIQATRSNLEKAYEAIEENYRCCVREQEDYWKEEEKREAE
FREKGTKVYSPEEVSEHLQILEDLLEVCSTQLTIAEVVVLGVGLEATEEIEYTILSDAANRLKVLCEDADDMTFRIKEIEMMLRMAELPLLPI
KQAFTNAFVQYNSCKENLAKVEPYCQESPAYVNSNKRLESLDQALQNVYQEYQKRFQGASGLESEVSAYREYLREQITEFETQGLDVIK
EELLFVSSTLKSKSSYDPLIAKVPCMKFYYQYYDGIDKARVQSRWLEKSERYRKAKKGFQEMLKEGLFKEDQALKEEDYRLYREERMNKE
SHLICNKIAAARRRVQEFESMEISEIPEEKAGFMDKARSLFTREDRS

>core/106/4/Org4_Gene361

MATPAQKSPTFQDPSFVRELGSNHPVFSPLTLEERGEMAIAPVQQCGWNHTIVKVSLFILALLTILGGGLLVGLLPAVPMFIGTGLIALG
AVIFALALILCLCDSQGLPEELPPVPEPQQIQIEYLRNETREVLEGTLLEVLLKDRDAKDPAVPQVVVDCEKRLGMLDRKLRREEEILYRST
AHLKDEARYEFLLELLEMRSLVADRLEFNRRSYERFVQGIMTVRSEEGEKEISRLQDLIGLQQQTVQDLRSQIDDEQKRCWTSLQRIHQ
SQKDIQRAHDREASQRACEGTEMDCAERHQLEKDLRRQLKSMREWIEMRGTIHQQEKAWRKQNAKLERLQEDLRLTKIAFDEQPLF
YREYKEKYLSQKLDMQKILQEVNAEKSEKARLKSLVRDYEKQLEQQDANLKKAKAIWEEELGKQQLKDHEQTQEIKRLNTFMLEYQDG
LREAEQVAEQVRQDLRQLEEKYSHLQEEKQEKEKILEQSVNHFAERFEALQKENVVYKKKLADLEGAAAPTEIREEDGWILAGSASLSQ
KKIRELNEENQELLKLLAFKTRELTQLAADVGEAEKEISKLREQIEEQKEELRVLDTMHSEAVKDCEAAQRKCRDLEGLLSPVREDAGMR
FELEIELQRLREENAQLRLEVERLEQEQLQG

>core/208/4/Org4_Gene991

MQRDGRFKWLQDEDTSAHAEQRFRDINGCWEDLKQTIFWVGEHDCMDIETVRKSYMMWLDRYADKFILREKEEKMKRHELFHAT
MVRKASGHAYAKAKAAFEKERSNENQRKVKDIEKWLSKGLAEFRNQESRRARERLRELQTLYPGVSVEERVLERQRTKKVNLENLYAD
IEKKYHHCVREQEHYWKEVENKEAEFRENREKVLSTEEVLECLQRLEDCLETWSKQLTKAEGSVFEMMFDATEELGNKVLSDVTNRLEI
LCEDAEEMIFRIEEIEMTLRMVELPLLFMKNTFEKASLQYNSCKEMLAKAEPHCKESAIYRSSEERLERLNRDLQTAYTNCHERLQGFSKL
ESEVRTCKDHLREQMKHFEVQGLNFINEELLWVGAELFTQARLDLVATVPYMEFYLQYHNIKREKVRSQWMAKTERYREIRQAFQGV
MKEDLLAEDTILKEEDYWLLRDDWLLRDERKNRQRRLICNKIAAAQQRVKGF

>core/209/4/Org4_Gene637

MLGILLIASGIIFLAVAIPGLSSAVALGLGCGMTALGTVLLITGLVSLIRSEQLALEQVEIKQARTRVNNELDQLSQYVFYTENVLDNLKRW
SYRDLGFVRQAQEEVTNLEQDIEEIFLTLRDIRNALDNEEFFMTHAKQCLAQVGESLFQDASIDEFINLAHLSEIRQHLDINDPRWSMIT
KKVKGTVVRFIYVSTMYKQIKSNFEKSDFGQLRKMLLNNYKTIEEVLYQSFQKGYNRAALLSEKTRIIHTSSLLHWEKDEDKHLNIKNECA
SRLENFKKFRTLFLGLSEEDVIDFTGASGWDCSKLPRKELPLDGGKKKLRFKRTFADEQVGDWDRTTSLEHMTPQEEDPLDKLMDQVE

QEATSVLKDQDRYWKEIETSEAKFRSLPQEDDFEKQSQIDSYIRDLDDHLSVWANQLSAAEDALIEVTDVQEHGNREMLKNIQQGLELI
EDAVKATLPRVDFIQELLEKEELPLVAARMSLENSYLKISS

>core/341/4/Org4_Gene932

MVVSIYSEILSFSELTSCKHSLFPFGPIETASIRIHHVFNVVIVCLIILGTLFVCLGMVFLGVFSTYLLGMSSMILGLLLISIGLALLKFKERYGLE
PKELFGVEGGFDKKLPSEIIQMQDQIADLARELDLEQKKDTLIRGFSARLDVLEGSKTEKKQILKIGVPRNLSEIQERAQELNSILEQCKEA
LLFRRKSAQEIFKKLYDRKAAFWRSYREDLWCYSEIHVSKKALSNLYIGDVFEGTAPHFLMEAYAMCRTAKNLRNYVKVCVEDMRMRI
DEEKKRAKQLSVSELLCCCTEIETDLENETNLFTSDSEDVLEEYQIHCIRVTMLHALWAIYNDEVVCRKPIDTLDRVRARMAVEDCIETFE
ELQMCVVHTKTLELEIAQLYVDILLEA

>core/380/4/Org4_Gene764

MEVYSFHPAVRTSFQHRVMAGLDNWFFLGGYRLKVVFLDKNNCGWACQKLVSISKTEKILKILSYLLVPIVLIALLIRCFLHARFKCTWN
RDSLIDSRVPSDVRPFHDFRLFNNQERLNIWKNRIYVSGVDVLVASVDYLKSQFSGFNHIPKAIRCENYVRNGQFSEEGKKSYLRGMLT
HIVGYILSVDETYWEDVILKIPAMNILSEAAYSRSVSTDPNYQHHGVYLFLDKSLQVPARHVLGEGNMLNTAKWMHIRAERPGKEGEC
IAKQFLKDYCKKHLEVMNCPDFIESLLDEKIREFHCPSVLNSAVCDIIDHKCQEHLLKAIINEANRRLPGMKNSSFTMCGDQVLFYTVFSP
PKLPPAASSAYF

>core/461/4/Org4_Gene615

MNIYQFSPGVSPNWQASLMARLDSYFCLGGETVTRIISLRPSGLILAKKEKAVVSIAAKILKILSFILFPLVLTALAIRYLLYNKFNKDLDRAV
FFIPTEITKTEELIIAKNPALVKEAALNVSPFFYSLPKKYQRMKVETPEGKFYKITFSVNLDLLVEDLHLETLDWPTELLLKNRPFDFTGHPEE
EKLIKDILLKEEGNEYFSLESKKLLARHMMHNIVVLSEEPGRSAFLGRTAFFPNKYPIAKGGVGIPSTTSNLFTIWYCFYFYRAATPQSDHP
DGCGFILLERLKELGAEFFYCDLRESNTTGFTLLFEGSNKGVLKNHLFIRDE

>core/462/4/Org4_Gene614

MSNIYSFSPGICPNWQAGLMSKLDSLCCFGGETATRIFSMTPSGFSLATEEKVHISTAEKVIKILALIFFPIILIALAIRYFLHRKFDRKCFVIP
QDTPKELELILAANPQLVEKAAREVHPGFFALPTKYQSMYIQTSKGQPPKITLSINIDLLLEDLDTDSIPWPKLYLDEDFDFAYYPESKAIID
TVTKLEKNNPGEEFCLESKKILAHYLLEQLFKLETGLNFPTSTIDGGRESFLIKFSHETKKPTVWAFIYFYYHHSNGPKLEKDFKQAGCEVH
NRLLNLGLKYRPQAGAQNDGCDGGPYGPRGFLIVWEENYGSVLKDHGFIKDN

>core/473/4/Org4_Gene406

MMTYPVPQNPLLLRILRLMDAFSKSDDERDFYLDRVEGFILYIDLDKDQEDLNKIYQELEENAERYCLIPKLTFYEVKKIMETFINEKIYDID
TKEKFLEILQSKNAREQFLEFIYDHEAELEKWQQFYVERSRIRIIEWLRNNKFHFVFEEDLDFTKNVLEQLKIHLFDAKVGKEITQARQLLS
NKAKIYYSNEALNPRPKRGRPPKQSAKVETETTISSDIYTKVPQAARRFLFLPEITSPSSITFSEKFDTEEEFLANLRGSTRVEDQLNLTNLSE
RFASLKELSAKLGYDSLSTGDFFGDDEEEEEVVTKTKGSKRGRKKSS

>core/556/4/Org4_Gene319

MASIHPTAIIEPGAKIGKDVVIEPYVVIKATVTLCDNVVVKSYAYIDGNTTIGKGTTIWPSAMIGNKPQDLKYQGEKTYVTIGENCEIREF
AIITSSTFEGTTVSIGNNCLIMPWAHVAHNCTIGNNVVLSNHAQLAGHVQVGDYAILGGMVGVHQFVRIGAHAMVGALSGIRRDVP
PYTIGSGNPYQLAGINKVGLQRRQVPFTTRLALIKAFKKIYRADGCFFESLEETLEETLEEYGDIPEVKNFIEFCQSPSKRGIERSIDKQALEE
ESADKEGVLIES

>core/743/4/Org4_Gene935

MNWAPKTMDHVDPKSETNIRLVISCYKLIKACQLEFPSLVDEVLLGMKCCAWESFKLVRQYQEQAKTVSAKNAPLFCLTRSYYRDGHL
TPLRAGPRAALSNYLDLRRRENSEKFFNPGHPCYYARLAFNETIQIYRTLFDISKLQIMFESGDYEKGQRENIAVILNFVKTLDDNEKVNFL
LRHNDTRIPGRESATVFCS

>core/865/4/Org4_Gene979

MSNIMGSRRKLKRSFLLIEVLMALSLVCAVLLPCIRFYYAIHRSFEEDIFNLQLPALIDHCFLSVEEKMRQQMAEGTVFTSGKGQTVSLAY
TSQGIGYRIPYGYNVDIRQEVRGDNLKMKVCLADVVVELFPDQKQAVSVQRCLCVTL

>core/868/4/Org4_Gene809

MEEFVAYIVKNLVTNPEAVEIRSIEDEDNESIKLEIRVAAEDIGKIIGRRGNTIHALRTILRRVCSRLKKKVQIDLIQPENGTDVIADQDYICD
NDSSNSTEDTFGESDTCCSGHCHYDEDLNQEELDQEEQEEDNMHHSCECSNHH

>core/905/4/Org4_Gene937

MRAGGSLVTTYPKEGRRLRSPEQLRVLDDLVQSYPNHLHAIELDCDAIPQDLIGATYIITFADFSTYILSLRSYQANSPSDDTWGIWFGSI
DDPVIADRFQAVISFLKDHGFALPSTLAQDPLLCTNK

>core/920/4/Org4_Gene578

MRELNAFELTQPEEYRNRWVLMPCLKCRFCRTQHAKVWSYRCVHEASLYEKNCFLTLTYDDKHLPQYGSLVKLHLQLFLKRLRDRISP
HKIRYFGCGEYGTKLQRPHYHLLIFNYDSLLDG

>core/1056/4/Org4_Gene448

MIDAENQNPKDGGSFTSLHTDPKNLFDEEGMPSHLILYSAISITYSSL

>core/19/5/Org5_Gene1037

MESFVSEHPLTLQSMIATILRFWSEQGCVIHQGYDLEVGAGTFNPATFLRALGPEPYKAAYVEPSRRPQDGRYGVHPNRLQNYHQLQ
VILKPVPENFLSLYTESLRAIGLDLRDHDIRFIHDDWENPTIGAWGLGWEVWLNGMEITQLTYFQAIGSKPLDTISGEITYGIERIAMYLQ
KKTSIYDVLWNDTLTYGQITQASEKAWSEYNFDYANTEMWFKHFEDFAEEALRTLKNGLSVPAYDFVIKASHAFNILDARGTISVTERT
RYIARIRQLTRLVADSYVEWRASLNYPLLSLSSTSEPKETSESVVPMISSTEDLLLEIGSEELPATFVPIGIQQLESLARQVLTDHNIVYEGLE
VLGSPRRLALLVKNVAPEVVQKAFEKKGPMLTSLFSPDGDVSPQGQQFFASQGVDISHYQDLSRHASLAIRTVNGSEYLFLLHPEIRLRT
ADILMQELPLLIQRMKFPKKMVWDNSGVEYARPIRWLVALYGEHILPITLGTIIASRNSFGHRQLDPRKISISSPQDYVETLRQACVVVS
QKERRMIIEQGLRAHSSDTISAIPLPRLIEEATFLSEHPFVSCGQFSEQFCALPKELLIAEMVNHQKYFPTHETSSGAISNFFIVVCDNSPN
DTIIEGNEKALTPRLTDGEFLFKQDLQTPLTTFIEKLKSVTYFEALGSLYDKVERLKAHQRVFSTFSSLAASEDLDIAIQYCKADLVSAVVNE
FPELQGIMGEYYLKHANLPTASAVAVGEHLRHITMGQKLSTIGTLLSLLDRLDNLLACFILGLKPTSSHDPYALRRQSLEVLTLVSASRLPI
DLASLLDRLADHFPSTIEEKVWDKSKTIHEILEFIWGRLKTFMGSLEFRKDEIAAVLIDSATKNPIEILDTAEALQLLKEEHTEKLAVITTTHN
RLKKILSSLKLSMTSSPIEVLGDRESNFKQVLDAFPGFPKETSAHAFLEYFLSLADLSNDIQDFLNTVHIANDDGAIRNLRISLLLTAMDKFS
LCHWESVAV

>core/78/5/Org5_Gene341

MRIYQQDLFCRLCRDPAWFFSLLSFTLRFYCLGRGWTLLSFFYKHQKKFIGIVIAVVCVSGIGVGWGRFSRKGSAESTSRRTVFTTASGK
RYVEKDFMAMKKFFAHEAYPFTGNPRAWNFINEGLLTDYFLTTRVGEKLFLKVYHPGEKIFSKEKAYQPYRRFDAPFISSEEVWKSSAP
QLLEILKVFQQIENPISKEGFLARAKLFLEERRFPHYVLRQMLEYRRQMFALPPDEALSRGKDLRLFGYQTIQDWFGDAYLSAAVELLIRFI

DEQKKVLPRPSKQEARDDFYDKAKHAYTKISKNKEFSLGFEEFVNSYFQFLEISESEFFNMYRDILLCKRALLLLQGGVSFDFQPLTTFFVQ
GKDSIQVEFFRLPKEYSFKTKQELKAFEVYLKLVSLPKSDSLDVPNEILPIATIKAKEPRLVGRRFSIDYKRVALQDLAATVPMVEVLHWQ
QNSEHFQEILQQFPDVETCQSYKDFQHLKPALRDKISLFTRKEILRARPERILQSLQQVPKQSQEVLLSAGKNSALPGISDGQQLAKVLLE
NEVLDLYSQDAETYYTIIVNSSFEKEEVLPYREVLKRDLASQLLTSHGHLVDMERLESALRTRYPGEEGASLWQRRLWKVVENHRLGRH
LEGSFSWSLDRSLKTFSRGDKELPQEFDRIFSMKVGDYSSVFMSPNEGPCYYQCLSHLLYDRPASVDKLFLAKSQLDEELLGSYMERFIE
QGVVR

>core/173/5/Org5_Gene103

MFKSFIVRYMFVGGLVSFLLPIPDLECANNVTKTYDKKASVISRDLKLQEDCQKFWNLDPYKLESLCAYQVLYHDDYSSKRIRELFPQIQK
DEVPIFATMILTLGKVDRGFSPEEISLIQKLSYPGLSLASLRGSTEIDPNTDLARALVVSEFSGDLGKNRADYYSNCLDILALRIHAERQRYL
DQSPCVPGTSEFHKATIEAINTILFYEEAVRYPSKKEMFSDEFSFLSSVTDRKFGVCLGVSSLYFSLSQRLDLPLEAVTPPGHIYLRYQGGEV
NIETTAGGRHLPTASYCDCLDLEDLQVRTPEEMIGLTFMNQGSFALQKKKYKEAEEAYKKAQEYLGDEELQELLGFVQILGGKKKEGKS
LIGKSPRASQKGSVAYDYLKGRINIPTLALLFSYPGSNYEEIASYEEELKKAMKSSMPCCEGQRRLASVAFHLGKTAEAVALLEKCVEDIPN
DLSLHLRLCKILCDRHEYTKALKYFIIAERLMEDQGFLKKDNRSFALFYEVKKIISKVAPQKANTLLLMESER

>core/574/5/Org5_Gene71

MLLIYCKKKEIHLQWPQTAKIRFTPKIAMKVKINDQLICIPPFISARWSQIAFIESQEGENKDQGTLRLHLIDGKIISIPNLDQSIIDIAFQEH
LLYLETSQSGKEDSRDDDKLGVGVLMNVLQQITKGNDIQVLPKNLISPLFSGTNPIEAILQHTPEHKDHPDAPTDVLEKMADVIRVLSG
NNATLLPRPEPHCNCMHCQIGRVMNEEDTLAVSDKDLTFRTWDIMQSGDKLYIVTNPLNPSDQFSVYLGPPIGCTCGEPNCEHIKAVL
YT

>core/669/5/Org5_Gene170

MNKKILVLCTAMFFIVCFGFLIHKKHTILPPKAHIPTNAKHFPTIGNPYAPINITVFEEPSCSACAEFTTEVFPLLKKHYIDTGEISFTLIPVCFI
RGSKPAAQALLCIYHHDPRQADIDAYMEYFHRILTYPKEEGSHWATPEVLTKLAEGLKINSGRSVNPKGLEQCIASGQYNEQIKKNNLY
GSQVLGGQLATPTAVVGDYLIEDPTFHEIERAIQHIRQLQAVEGDHDD

>core/794/5/Org5_Gene1063

MSLVSYLSNPQKALVLGSKGFSMDCVDNLKLYIFRLKLPGDTERISYSISPEYIREKGEEELLNSPIEVEGSLGRIDSDQWILSLSLKTQLGLC
CPVCNNFFSHSVCLPDLQRVISHDEVGSGVFDCRPLIRQELLLESDCFEECSGQGCPERKNILKFLEDRKKHEGNNPFEYL

>core/901/5/Org5_Gene54

MKNKMDYKSQLVFSCPCCCKGNVCFSVFNLDVILTCNVCSSTYTFDSVIRNEIRQFVALCKRIHDANSILGNATVSVSVEDNQMDIPFQ
LLFSRFPVVLNLSLDGKKIAIRFLFDALNTSILHQESDLIS

>core/1016/5/Org5_Gene725

MRNMEAKKIKELSKEAQLLKKLREKSRVLDEKNKRKAWVAKLVAMPESIREIEKEERVETPQLFQAIAEKILEEGV

>core/1022/5/Org5_Gene818

MWYKSLAGEEKDVSGNECNDYPEVFKDDVSAYVLVTCGQMSSEGKIQVEMTYEGDPAVISYLLTKARDSLDES

>core/1049/5/Org5_Gene865

MRVMRFFCLFFLGFLGSFHCVAEDKGVDLFGVWDDNQITECDDSYMTEGREEVEKVVDA
