# Supplementary material for: Potential Therapeutic Candidates against Chlamydia pneumonia Discovered and Developed In Silico Using Core Proteomics and Molecular Docking and Simulation-Based Approaches
Source: Int J Environ Res Public Health. 2022 Jun 15;19(12):7306. doi: 10.3390/ijerph19127306 (PMC9223490; doi:10.3390/ijerph19127306)
Supplement: Supplementary file 1 [file ijerph-19-07306-s001.zip › file S3.pdf]

Amino acid sequences of final proteins

>tr|Q9Z8C4|Q9Z8C4_CHLPN Penicillin-binding protein OS=Chlamydia pneumoniae OX=83558 GN=pbp3 PE=4 SV=1

MSYRKRSTLIVLGVFALYALLVLRYYKIQICEGDHWAAEALGQHEFCVRDPFRRGTFFAN
TTVRKGDKDLQQPFAVDITKFHLCADPLAIPECHRDEIIQGILQFIEGQTYDDLSLKLDK
KSRYCKLYPLLDVSVHDRLSLWWKGYATKHRLPTNALFFITDYQRSYPFGKLLGQVLHTL
REIKDEKTGKAFPTGGMEAYFNHILEGDVGERKLLRSPLNRLDTNRVIKLPKDGSDIYLT
INPVIQTIAEEELERGVLEAKAQGGRLILMNSQTGEILALAQYPFFDPTNYKEYFNNKER
IEHTKVSFVSDVFEPGSIMKPLTVAIALQANEEASLKSQKKIFDPEEPIDVTRTLFPGRK
GSPLKDISRNSQLNMYMAIQKSSNVYVAQLADRIIQSLGVAWYQQKLLALGFGRKTGIEL
PSEASGLVPSPHRFHINGSLEWSLSTPYSLAMGYNILATGIQMVQAYAILANGGYAVRPT
LVKKIVSASGEEYHLPTKEKTRLFSEEITREVVRAMRFTTLPGGSGFRASPKHHSSAGKT
GTTEKMIHGKYDKRRHIASFIGFTPVESSEGNFPPLVMLVSIDDPEYGLRADGTKNYMGG
RCAAPIFSRVADRTLLYLGILPDKKLRNCDEEAAALKRLYEEWNRSPKQGGTR

>tr|Q9Z8X6|Q9Z8X6_CHLPN Probable ATP-dependent 6-phosphofructokinase OS=Chlamydia pneumoniae OX=83558 GN=pfkA_2 PE=3 SV=1

MHPLYVDLDTIISSYSPPLPKEFQEAASLIAVPDTSHSKPVVPGVKTLFPQTYHLPYLKF
VQGENVVHTPLKVGVMFSGGPAPGGHNVIQGLFNSLKDFHPDSSLVGFVNNGDGLTNNKS
IDITEEFLSKFRNSGGFNCIGTGRKKIVTPEAKEACLKTAEALDLDGLVIIGGDGSNTAT
AILAEYFAKRRPKTSIVGVPKTIDGDLQHTFLDLTFGFDTATKFYSSIISNISRDALSCK
AHYHFIKLMGRSASHIALECALQTHPNIALIGEEIAEKNLPLKTIIHKICSVIADRAAME
KYYGVILIPEGIIEFIPEIINLITEIESLSEYEDKISRLSPESQRLLKSFPAPIIEQILN
DRDAHGNVYVSKISVDKLLIHLVSNHLQQYFPNVPFNAISHFLGYEGRSGLPTKFDNTYG
YSLGYGAGILVRNHCNGYLSTIESLACPFMKWKLRAIPVVKMFTVKQQADGTLQPKIKKY
LVDIGSTAFRKFKLYRKIWALEDSYRFLGPLQIETPPEMHSDNFPPLTLLLNHNFWQRHQ
GCIEIPDTTY

>sp|Q9Z7Y2|MURA_CHLPN UDP-N-acetylglucosamine 1-carboxyvinyltransferase OS=Chlamydia pneumoniae OX=83558 GN=murA PE=3 SV=1

MQIAQVFGCGRLNGEVKVSGAKNAATKLLVASLLSDQKCTLRNVPDIGDVSLTVELCKSL
GAHVSWDKETEVLEIYTPEIQCTRVPPTFSNVNRIPILLLGALLGRCPEGVYVPTVGGDA
IGERTLNFHFEGLKQLGVQISSDSSGYYAKAPRGLKGNYIHLPYPSVGATENLILAAIHA
KGRTVIKNVALEAEILDLVLFLQKAGADITTDNDRTIDIFGTGGLGSVDHTILPDKIEAA
SFGMAAVVSGGRVFVRNAKQELLIPFLKMLRSIGGGFLVSESGIEFFQERPLVGGVVLET
DVHPGFLTDWQQPFAVLLSQAQGSSVIHETVHENRLGYLHGLQHMGAECQLFHQCLSTKA
CRYAIGNFPHSAVIHGATPLWASHLVIPDLRAGFAYVMAALIAEGGGSIIENTHLLDRGY
TNWVGKLRSLGAKIQIFDMEQEELTTSPKSLALRDASL

>tr|Q9Z7N2|Q9Z7N2_CHLPN D-Ala-D-Ala Carboxypeptidase OS=Chlamydia pneumoniae OX=83558 GN=dacF PE=4 SV=1

MKRPFFTYLCIIFYGSCASLSLHAGLSFPEVRGATAAVVHADSGKVFYDKDIDAVIYPAS
MTKIATALFILKHYPTVLDTLIKVKQDAIASITPQAKKQSGYRSPPHWLETDGSTIQLHL
REELLGWDLFHALLVCSANDAANVLAMACCGSVEKFMDKLNFFLKEEIGCTHTHFNNPHG
LHHPNHYTTTRDLISIMRCALKEPPFRGVISTTSYKIGATNLHGERILSPTNKLLLPGST
YHYPPALGGKTGTTKTAGKNLIMAAEKNNRLLVTIATGYSGPVSDLYQDVIALCETVFNE
PLLRKELVPPSDCLQLEIANLGKLSCPLPEGLYYDFYASEDREPLSVSFIAHADAFPIEQ

GDLLGHWVFYDDEGKKISSQPFYAPCRFERTIKPWKLYMKRVFTSYRTYMSITMLLMYFR
IRKHRKYKNLKHYSKI
>tr|Q9Z744|Q9Z744_CHLPN NifS protein, putative OS=Chlamydia pneumoniae OX=83558 GN=yfhO_2 PE=4 SV=1
MEKPQNRKAPRIFWLNNQVAIPPSERVKESYALHSDIFSLPPGSALKLAEKTEESIRQLV
GLKDSHIFRFVPHFPHVVHIVLAALVENLSMFQGRNHIILPAHDQQLLINSLCRHQGLGT
TYDWVTVNHEGRIVEEQLIETLSPRSLLFSLSAAHGLTGVIQPLDPLLSLCKDRRILLHL
DISDILGRAPLTPEILNADIITFSSAALGGMGSIGGIFIRKSLERVFSSWFPPHTSASLC
FSAVAAMQTACEERISALPLFTFHTSNLCKKLIQELQSVLPSIQLAFSEVQNRLPNIVVA
AIPDIPAESLAFHLHQQGIYPSLGYERFQPLAQVLQNCGISPFLCHSALHFSLTERSKDL
EFSKLARAMHDAIKHLTPLLGSSS

>sp|Q9Z8P0|FABH_CHLPN 3-oxoacyl-[acyl-carrier-protein] synthase 3 OS=Chlamydia pneumoniae OX=83558 GN=fabH PE=3 SV=1
MWFSVNKNKKAAIWATGSYLPEKVLSNADLEKMVDTSDEWIVTRTGIKERRIAGPQEYTS
LMGAIAAEKAIANAGLSKDQIDCIIFSTAAPDYIFPSSGALAQAHLGIEDVPTFDCQAAC
TGYLYGLSVAKAYVESGTYNHVLLIAADKLSSFVDYTDRNTCVLFGDGGAACVIGESRPG
SLEINRLSLGADGKLGELLSLPAGGSRCPASKETLQSGKHFIAMEGKEVFKHAVRRMETA
AKHSIALAGIQEEDIDWFVPHQANERIIDALAKRFEIDESRVFKSVHKYGNTAASSVGIA
LDELVHTESIKLDDYLLLVAFGGGLSWGAVVLKQV

>sp|Q9Z7G1|HEM2_CHLPN Delta-aminolevulinic acid dehydratase OS=Chlamydia pneumoniae OX=83558 GN=hemB PE=3 SV=1
MSSLTLSRRPRRNRKTAAIRDLLAETHLSPKDLIAPFFVKYGNNIKEEIPSLPGVFRWSL
DLLLKEIERLCTYGLRAVMLFPIIPDDLKDAYGSYSSNPKNILCHSIHEIKNAFPHLCLI
SDIALDPYTTHGHDGIFLNGEVLNDESVRIFGNIATLHAEMGADIVAPSDMMDGRIGYIR
SKLDQSGYSKTSIMSYSVKYASCLYSPFRDALSSHVTSGDKKQYQMNPKNVLEALLESSL
DEEEGADILMVKPAGLYLDVIYRIRQNTCLPLAAYQVSGEYAMILSAFQQGWLDKETLFH
ESLIAIKRAGADMIISYSAPFILELLHQGFEF
>tr|Q9Z9C3|Q9Z9C3_CHLPN Acetyl-coenzyme A carboxylase carboxyl transferase subunit beta OS=Chlamydia pneumoniae OX=83558 GN=accD PE=3 SV=1
MRLFSYDKPKIKVQKIKADGFSGWLKCNHCHEMIHANELGQNYNCCPKCSYHYRITAIER
VKLLADKDSWRPLYTDLKSQDPLEFIDTDTYANRLEKARKNTTESEGVIVGICTIGLHPV
ALAVMDFNFMAGSMGAVVGEKLTRLIEEAIETRLPVIIVSASGGARMQESVFSLMQMVKT
SAALAKLHEAGLPYISVLTNPTSGGVTASFAALGDIIIAEPKALICFAGPRVVAQVIGED
LPEGAQKSEFLLEHGMIDKIVERKELKTTLQTLLDYFLAQEYTGGKSKAPRDLSKRLKEI
FLLTDDSE
>tr|Q9Z7T6|Q9Z7T6_CHLPN Lipoate--protein ligase A OS=Chlamydia pneumoniae OX=83558 GN=lplA_2 PE=4 SV=1
MPTTNCIFLDLRGHSILHQLQIEEALLRVANQNFCIINSGAKDSIVLGISRNLNQDVHIS
RAQADHIPIIRRYSGGGTVFIDSNTLMVSWIMNSSEASAQPQELLAWTYGIYSPLLPNTF
SIRENDYVLGHKKIGGNAQYIQRHRWVHHTTFLWDIDLDKLSYYLPIPQQQPTYRNQRSH
EEFLTTLRPWFPSRDDFLERIKASGSLLFTWEEFLDNELEEILAQPHRKATTVLN

>sp|Q9Z7H2|RS4_CHLPN 30S ribosomal protein S4 OS=Chlamydia pneumoniae OX=83558 GN=rpsD PE=3 SV=1

MARYCGPKNRVARRFGANIFGRSRNPLLKKPHPPGQHGMQRKKKSDYGLQLEEKQKLKAC
YGMIMEKQLVKAFKEVIHKQGNVAQMFLERFECRLDNMVYRMGFAKTIFAAQQLVAHGHI
LVNGRRVDRRSFFLRPGMQISLKEKSKRLQSVKDALESKDESSLPSYISLDKTGFKGELL
VSPEQDQIEAQLPLPINISVVCEFLSHRT

>sp|Q9Z8P3|ACP_CHLPN Acyl carrier protein OS=Chlamydia pneumoniae OX=83558 GN=acpP PE=3 SV=1

MSLEDDVIAIIVEQLGVDPKEVNENSSFIEDLNADSLDLTELIMTLEEKFAFEISEEDAE
KLRTVGDVFTYIKKRQAEQ

>sp|Q9Z8L0|BSDC_CHLPN 4-hydroxybenzoate decarboxylase subunit C OS=Chlamydia pneumoniae OX=83558 GN=CPn_0328 PE=1 SV=1

MSFLRRHISLFRSQKQLIDVFAPVSPNLELAEIHRRVIEDQGPALLFHNVIGSSFPVLTN
LFGTKHRVDQLFSQAPDNLIARVAHLISSTPKLSSLWKSRDLLKRISSLGLKKARFRRFP
FVSMSSVNLDHLPLLTSWPEDGGAFLTLPLVYTESPTLTTPNLGMYRVQRFNQNTMGLHF
QIQKGGGMHLYEAEQKKQNLPVSVFLSGNPFLTLSAIAPLPENVSELLFATFLQGAKLLY
KKTNDHPHPLLYDAEFILVGESPAGKRRPEGPFGDHFGYYSLQHDFPEFHCHKIYHRKDA
IYPATVVGKPYQEDFYIGNKLQEYLSPLFPLVMPGVRRLKSYGESGFHALTAAVVKERYW
RESLTTALRILGEGQLSLTKFLMVTDQEVPLDRFSVVLETILERLQPDRDLIIFSETAND
TLDYTGPSLNKGSKGIFMGIGKAIRDLPHGYQGGKIHGVQDIAPFCRGCLVLETSLEDRC
IKSLLHHPDLKSWPLIILADNLRETIQSEKDFLWRTFTRCAPANDLHALHSHFATHRPNY
NFPFVIDALMKPSYPKEVEVDPSTKQKVSERWHAYFPNKETFYI

>tr|Q9Z8Y8|Q9Z8Y8_CHLPN Oligopeptide Binding Protein OS=Chlamydia pneumoniae OX=83558 GN=oppA_2 PE=4 SV=1

MLRFFAVFISTLWLITSGCSPSQSSKGIFVVNMKEMPRSLDPGKTRLIADQTLMRHLYEG
LVEEHSQNGEIKPALAESYTISEDGTRYTFKIKNILWSNGDPLTAQDFVSSWKEILKEDA
SSVYLYAFLPIKNARAIFDDTESPENLGVRALDKRHLEIQLETPCAHFLHFLTLPIFFPV
HETLRNYSTSFEEMPITCGAFRPVSLEKGLRLHLEKNPMYHNKSRVKLHKIIVQFISNAN
TAAILFKHKKLDWQGPPWGEPIPPEISASLHQDDQLFSLPGASTTWLLFNIQKKPWNNAK
LRKALSLAIDKDMLTKVVYQGLAEPTDHILHPRLYPGTYPERKRQNERILEAQQLFEEAL
DELQMTREDLEKETLTFSTFSFSYGRICQMLREQWKKVLKFTIPIVGQEFFTIQKNFLEG
NYSLTVNQWTAAFIDPMSYLMIFANPGGISPYHLQDSHFQTLLIKITQEHKKHLRNQLII
EALDYLEHCHILEPLCHPNLRIALNKNIKNFNLFVRRTSDFRFIEKL
